# Whether Gametophytes Are Reduced or Unreduced in Angiosperms Might Be Determined Metabolically

**DOI:** 10.3390/genes11121449

**Published:** 2020-12-02

**Authors:** Mayelyn Mateo de Arias, Lei Gao, David A. Sherwood, Krishna K. Dwivedi, Bo J. Price, Michelle Jamison, Becky M. Kowallis, John G. Carman

**Affiliations:** 1 Plants, Soils, and Climate Department, Utah State University, Logan, UT 84322-4820, USA; mayelyn.mateo@intec.edu.do (M.M.d.A.); gao.lei@jju.edu.cn (L.G.); david@sherwoodpethealth.com (D.A.S.); bojprice57@gmail.com (B.J.P.); 2 Instituto Tecnológico de Santo Domingo, 10103 Santo Domingo, Dominican Republic; 3 College of Pharmacy and Life Science, Jiujiang University, Jiujiang 332000, China; 4 Sherwood Pet Health, Logan, UT 84321, USA; 5 Caisson Laboratories, Inc., Smithfield, UT 84335, USA; dwivedi1976@gmail.com (K.K.D.); m.jamison@elitechgroup.com (M.J.); becky.kowallis@cytiva.com (B.M.K.); 6 Crop Improvement Division, Indian Grassland and Fodder Research Institute, 284003 Jhansi, India; 7 Molecular Biology Program, University of Utah, Salt Lake City, UT 84112-5750, USA; 8 Wescor, Inc. An Elitech Company, Logan, UT 84321, USA; 9 Cytiva, Inc., Logan, UT 84321, USA

**Keywords:** 5-azacytidine, abscisic acid, apomixis, apospory, diplospory, expression profiling, fluridone, metabolic homeostasis, oxidative stress, sucrose non-fermenting-related protein kinase

## Abstract

In angiosperms, meiotic failure coupled with the formation of genetically unreduced gametophytes in ovules (apomeiosis) constitute major components of gametophytic apomixis. These aberrant developmental events are generally thought to be caused by mutation. However, efforts to locate the responsible mutations have failed. Herein, we tested a fundamentally different hypothesis: apomeiosis is a polyphenism of meiosis, with meiosis and apomeiosis being maintained by different states of metabolic homeostasis. Microarray analyses of ovules and pistils were used to differentiate meiotic from apomeiotic processes in *Boechera* (Brassicaceae). Genes associated with translation, cell division, epigenetic silencing, flowering, and meiosis characterized sexual *Boechera* (meiotic). In contrast, genes associated with stress responses, abscisic acid signaling, reactive oxygen species production, and stress attenuation mechanisms characterized apomictic *Boechera* (apomeiotic). We next tested whether these metabolic differences regulate reproductive mode. Apomeiosis switched to meiosis when premeiotic ovules of apomicts were cultured on media that increased oxidative stress. These treatments included drought, starvation, and H_2_O_2_ applications. In contrast, meiosis switched to apomeiosis when premeiotic pistils of sexual plants were cultured on media that relieved oxidative stress. These treatments included antioxidants, glucose, abscisic acid, fluridone, and 5-azacytidine. High-frequency apomeiosis was initiated in all sexual species tested: Brassicaceae, *Boechera stricta*, *Boechera exilis,* and *Arabidopsis thaliana*; Fabaceae, *Vigna unguiculata*; Asteraceae, *Antennaria dioica*. Unreduced gametophytes formed from ameiotic female and male sporocytes, first division restitution dyads, and nucellar cells. These results are consistent with modes of reproduction and types of apomixis, in natural apomicts, being regulated metabolically.

## 1. Introduction

Apomixis renews life cycles by producing unreduced, mitotically active (parthenogenetic) gametes or gamete like cells [1], and it occurs in all eukaryote kingdoms [2,3,4,5]. Once discovered in angiosperms [6], it was soon documented that the timing of apomixis induction during ovule development and the types of ovule cells involved vary among taxa. This temporal and spatial variation became the focal point for defining multiple types of gametophytic apomixis in angiosperms, and within the first decade of the 20th century, three morphologically distinct types had been described [4,7]. Subsequent Mendelian analyses suggested that these types, and their elements, i.e., apomeiosis (unreduced gametophyte formation), parthenogenesis (embryo formation without fertilization), and endosperm formation without the normal 2M:1P (maternal paternal) genome ratio, are under separate genetic controls [4,8,9,10,11]. Accordingly, it became widely viewed that the different apomixis types are caused by different mutations that in mysterious ways destabilize meiosis (megasporogenesis), gametophyte (embryo sac) formation, egg formation, and syngamy [8,9,10,11,12,13].

In the Antennaria and Eragrostis types of diplospory (gonial apospory), the unreduced gametophyte, with its parthenogenetic egg, forms from a meiosis-aborted megasporocyte (megaspore mother cell, MMC). In Taraxacum type diplospory, meiosis aborts slightly later, during the meiotic prophase stage of the MMC. The heterotypic division (reductional) then fails and a first division restitution replaces it. The equational mitotic-like second division then occurs producing two unreduced spores. The unreduced gametophyte, with its parthenogenetic egg, then forms from one of the unreduced spores. In Hieracium type apospory, sex aborts during meiosis, and the unreduced gametophyte, with its parthenogenetic egg, forms from a cell of the ovule wall (nucellus). In sporophytic apomixis (adventitious embryony), sex—if it aborts at all—aborts after a genetically reduced egg forms. The clonal embryo then develops adventitiously, from an unreduced egg-like cell that forms in the ovule wall, and this embryo is nourished by the sexually derived endosperm [8].

Based on numerous reports of apomixis switching to sex in response to stress, reviewed in [14,15], we hypothesized that (i) sexual development is induced and maintained by a stress-like state of metabolic homeostasis, and (ii) ovules in apomictic plants maintain a state of metabolic homeostasis that detoxifies oxidative stress and induces apomixis. To test this, we profiled the reproductive tissues of sexual and apomictic *Boechera* (Brassicaceae). Our goal was to identify differentially expressed genes (DEGs) indicative of differences in metabolic states. Upon observing such differences, we designed and conducted whole-plant stress physiology experiments that tested the effects of drought and heat on frequencies of sexual and apomictic development in facultatively apomictic *Boechera*. We also excised immature pistils, containing ovules in the MMC stage, of sexual and apomictic *Boechera*, sexual *Arabidopsis thaliana* (L.) Heynh. (Brassicaceae), and sexual *Vigna unguiculata* (L.) Walp. (Fabaceae). These were then cultured on media amended with chemicals selected to alter stress associated metabolic pathways. Additionally, we placed cut stems of male dioecious *Antennaria dioica* (L.) Gaertn. (Asteraceae), which contained premeiotic microsporocytes (pollen mother cells, PMCs), in chemically amended liquid media. Through these experiments, we demonstrated that apomeiotic spore and gametophyte formation, of various types (Taraxacum, Antennaria, and Hieracium), are inducible in sexual species of *Boechera*, *Arabidopsis*, *Vigna,* and *Antennaria* by altering homeostasis-based processes of stress perception and attenuation.

## 2. Materials and Methods

### 2.1. Plant Materials and Growth Conditions

Plants of the following taxa were grown from seed: diploid aposporous *Boechera microphylla* (Nuttall) Dorn from Millard County, UT USA (*Boechera imnahaensis* × *yellowstonensis*, UT10003), diploid diplosporous and aposporous *B. microphylla* from Cache County, UT USA (UT05001), diploid diplosporous and aposporous *Boechera retrofracta × stricta* (CO11010), diploid sexual *A. thaliana* (Col O), diploid sexual *Boechera exilis* (A. Nelson) Dorn (NV14003), diploid sexual *Boechera stricta* (Graham) Al-Shehbaz (UT10007), diploid sexual *V. unguiculata* (cowpea), diploid diplosporous *B. exilis* × *retrofracta* (UT11004), triploid diplosporous *Boechera* cf. *gunnisoniana* (Rollins) W.A. Weber (CO11005), and diploid diplosporous *Boechera lignifera* (A. Nelson) W. A. Weber (*B. exilis* × *thompsonii*, WY05001). A population of diplosporous hybrids of mixed parentage, which resemble sexual *B. formosa* (Greene) Windham and Al-Shehbaz (UT10006), are referred to herein as *B. × formosa* and were transplanted from native habitats, six from Duchesne County, UT USA (40.169 N, −110.328 W) and 58 from Carbon County, UT USA (39.547 N, −110.649 W). Male plants of diploid *A. dioica* were transplanted from landscape plantings at Utah State University, Logan, UT USA. Unless otherwise indicated, field collection information, ploidy, and voucher specimen numbers of *Boechera* taxa are from [16,17]. Seed germination, establishment, vernalization, and growing conditions were as previously reported [16].

### 2.2. Microarray Analyses

Based on the close phylogenic relationship between *Boechera* and *Arabidopsis* [18,19], Affymetrix (Santa Clara, CA, USA) ATH1 gene chips were chosen in 2005 for *Boechera* profiling, and this profiling system was retained for consistency through 2013. Ovules and pistils were collected from 9:00 A.M. to 12:00 noon, placed in Nuclease-Free water (Ambion, Life Technologies Corporation, Carlsbad, CA, USA), and measured. Needles from 1.0 mL syringes were used to excise ovules. Instruments and work surfaces were cleaned with RNAse ZAP (Ambion) prior to dissection and at 20–30 min intervals. Numbers of ovules per replicate were ca. 1600, 1400, 1200, and 800 for the MMC, active meiocyte (meiotic MMC, AM), young 1–4 nucleate gametophyte (YG), and mature gametophyte (MG) stages, respectively. Eighty pistils were obtained per replicate for *B. stricta*. Ovules and pistils were immediately placed in RNAlater (Ambion) and stored at −80 °C. RNA was extracted and purified from ovules using TRIzol reagent (Invitrogen, Life Technologies Corporation) and RNeasy columns (Qiagen, Germantown, MD, USA) and from pistils using PureLink^®^ RNA Mini Kits (Ambion, Austin, TX, USA) and TURBO DNA-free™ Kits (Ambion, Austin, TX, USA). NanoDrop (Thermo Scientific, Wilmington, DE, USA) and Bioanalyzer (Agilent, Santa Clara, CA, USA) instruments were used to determine RNA yield and integrity. Affymetrix two-cycle cDNA synthesis kits (P/N 900494) were used for cDNA synthesis and RNA amplification of ovule RNA. The MEGAscript T7 kit (Ambion) with unlabeled nucleotides was used for first-cycle cRNA amplifications. For in vitro transcription (IVT), 20 μL cDNA was mixed with 30 μL first-cycle IVT master mix, and reactions were incubated at 37 °C for 24 h. Second-cycle first and second strand cDNA syntheses were performed as above. The Affymetrix GeneChip IVT labeling kit (P/N 900449) was used for the second IVT. First and second cycle cRNA was purified using Affymetrix sample cleanup modules P/N 900371, and cRNA was fragmented at 94 °C for 35 min. Pistil RNA (500 ng) was amplified, labeled, and fragmented using GeneChip^®^ 3′ IVT Express Kits (Affymetrix). ATH1 arrays were hybridized for 16 h at 45 °C using 15 µg of labeled and fragmented cRNA (Affymetrix Technical Analysis Manual). Arrays were stained with streptavidin-phycoerythrin in an Affymetrix GeneChip^®^ Fluidics Workstation 400 and scanned with a GeneChip^®^ Scanner 3000.

### 2.3. Expression Profiling

Cel files (ncbi.nlm.nih.gov/geo/query/acc.cgi?acc=GSE156684) were deleted if RMAExpress (rmaexpress.bmbolstad.com/) values exceeded 99% of the RLE-NUSE T2 multivariate statistic. The remaining files were RMA normalized using BRB-ArrayTools (4.6.0-beta-2, Dr. Richard Simon, and the BRB-ArrayTools Development Team, linus.nci.nih.gov/BRB-ArrayTools.html). Low variability genes (<10% of expression values exceeding 1.5-fold change from the median) or genes missing >50% of their expression data were removed. The BRB-ArrayTools class comparison option (random variance model with a normalized significance level of 0.001 for univariate tests) was used to identify DEGs. Where multiple ATH1 probe sets identified the same gene (~0.8% of probe sets), probe sets producing the greatest fold change were retained. Each comparison used to identify DEGs involved detectable expression data from ca. 16,000 (59%) of 27,048 *Boechera* genes [19]. Tests for DEGs were performed within stages for all pairwise cross taxa comparisons and within taxa but across stages. Multi-stage comparisons, across taxa, were performed when comparisons of adjoining stages within taxa produced few or no DEGs.

### 2.4. Gene Ontology Analyses

Two overrepresentation gene ontology (OGO) analyses (Panther Version 15, pantherdb.org/, 2020–10-9 release) were performed for all comparisons that produced ≥10 DEGs, one for DEGs upregulated in one taxon and one for DEGs upregulated in the other. For Panther enrichment GO (EGO) analyses, gene trimming criteria were relaxed. Here, genes were removed if 0–40% of sample values differed from the median by ≥8%. The sample value threshold was varied so as to retain 10–16 thousand genes. Since EGO analyses are based on all expression data, rather than just DEGs, it was possible to perform these analyses for all within stage cross taxa comparisons. All OGO and EGO analyses were corrected for false discovery rate (FDR, *p* ≤ 0.05). GO terms (all GOs of DEGs, OGOs, and EGOs) were partitioned into higher-order groups, e.g., [20,21,22], that were defined by the broader biological concepts being investigated. Partitioning was performed manually by assigning each GO term to one of four groups and to one of 13 subgroups (Tables S1 and S2). Subgroup frequencies were then compared across taxa by chi-square tests for independence [23], and subgroups responsible for significant pooled chi-square values were identified based on *p* values of adjusted residuals [24] (Table S3). Molecular pathway components were identified by searching DEG narratives (Araport11, 1/1/2020 release) and additional literature as referenced for DEGs in TAIR (www.arabidopsis.org).

### 2.5. Expression Verification

Fourteen DEGs identified between the YG stages of *B. microphylla* and *B.* × *formosa* were selected for quantitative reverse transcription PCR (qRT-PCR). RNA was converted to cDNA using Superscript III First-Strand Synthesis kits (Invitrogen). Primers were designed from *A. thaliana* data using Primer3 (bioinfo.ut.ee/primer3/), quality checked by PrimerSelect (dnastar.com), and searched (blast.ncbi.nlm.nih.gov/Blast.cgi) for like sequences. Sequences predicted to form internal loops or dimers or to be homologous to other genes were discarded. Primers were tested using *Boechera* gDNA. qRT-PCR reactions (25 µL) contained QuantiTect SYBR Green (Qiagen), 200–250 nM forward and reverse primers and 3–5 µL of dilute cDNA. Reaction cycles were: 50 °C, 2 min; 95 °C, 15 min; 40 cycles of 94 °C for 15 s, 58 °C for 30 s, and 72 °C for 30 s. Melt curve analyses were performed from 65 °C to 95 °C in 5 sec 0.5 °C increments. A DNA Engine (Opticon 2, Continuous Fluorescence Detection System) with MicroAmp Optical 96-well plates (Bio-Rad, Hercules, CA, USA) was used. Three reps with two technical reps each were conducted. Expression values and standard deviations were calculated using the comparative C_T_ method using separate sets of housekeeping replicates for each gene. Delta Ct values were normalized (delta-delta Ct method) and subjected to Student’s *t*-tests (*p* ≤ 0.05).

### 2.6. Drought and Heat-Stress Experiments

Seedlings (ca. 60 days post-planting) were randomly assigned to three groups, well-watered, drought-stressed, and heat plus drought-stressed (8–12 plants per taxon per group) and vernalized (60 days, 4 °C). After vernalization, plants targeted for the well-watered and droughted treatments were grown in the greenhouse, and plants targeted for heat plus drought were grown in a growth chamber (32 °C, 500 µmol m^−2^ s^−1^ photosynthetic photon flux, 16/8 h day/night photoperiod). To minimize surface evaporation, perlite was added to the surface of pots, and field capacity weights were obtained (weighed 2 h after drenching). Enough water was added each day to the well-watered plants to bring them to field capacity (based on pot weight). Water was added to droughted plants each day to equal 50% of the transpiration rate of well-watered plants. Upon flowering, pistils were collected for cytology and expression profiling. Ovule parameters were compared by chi-square tests [25].

### 2.7. In Vitro Pistil Culture Experiments

Chemicals were from MilliporeSigma (St. Louis, MO, USA) unless otherwise indicated. The basal tissue culture medium was MS [26] salts and vitamins (Caisson Laboratories, Smithfield, UT, USA) with 20 g L^−1^ sucrose and solidified with 1.5 g L^−1^ Phytogel (pH 5.7 prior to autoclaving). For osmotic stress, basal media were amended with 0, 20, 30, 40, or 60 g L^−1^ polyethylene glycol (PEG) 6000. Water potentials (Ψ) were estimated by adding the water potential of basal MS medium (ca. −0.5 MPa [27]) to the water potential of PEG in water (ca. 0.0, −0.013, −0.022, −0.034, −0.065 MPa, respectively [28]) and multiplying the sums by 1.075 [28,29]. Brassinozole (BZR, dissolved in minimal DMSO), epibrassinolide (epiBL, dissolved in 80% ethanol), ABA (dissolved in ethanol), fluridone (dissolved in DMSO), 5-azacytidine (5-azaC, dissolved in water), and (S)-2-aminobutane-1,4-dithiol hydrochloride (DTBA, dissolved in water) were added by filter sterilization after media were autoclaved and before solidification. Two factorial experiments were conducted to determine the effects of multiple treatments on ovule development in *B. stricta* and *A. thaliana*. Variables tested were: sucrose (20 or 30 g L^−1^), glucose (20 g L^−1^); MS or B5 vitamins; 0.5 mmol L^−1^ 5-azaC; 0.5 or 1.0 μmol L^−1^ epiBL; 0.5 or 1.0 μmol L^−1^ DTBA; and 0.5 and 1.0 μmol L^−1^ epiBL and DTBA. Results were combined to show the main effects. A third factorial experiment was conducted to determine the effects of multiple treatments on ovule development in *V. unguiculata*. The basal medium here was amended with B5 vitamins, 0.5 μmol L^−1^ naphthalene acetic acid (NAA), and 5.0 μmol L^−1^ 6-benzylaminopurine (BAP). Variables tested were: 60 g L^−1^ sucrose or 30 g L^−1^ glucose; 1.0 μmol L^−1^ epiBL; 1.0 μmol L^−1^ DTBA; and 1.0 μmol L^−1^ epiBL and DTBA. Results were combined to show main effects. Pistils for individual and factorial experiments were staged by length (based on predetermined ovule development stages), aseptically excised, and immediately transferred without disinfestation to media (horizontally, ca. 30% of pistil bodies pressed into the medium). For experiments involving a pretreatment, excised pistils were soaked in liquid media followed by transfer to solidified media. Ovule parameters were tabulated 24–72 h after culture initiation and analyzed by chi-square tests [25].

### 2.8. Cytoembryological Analyses

Pistils and anthers were fixed, cleared, and observed by differential interference contrast microscopy as previously described [16]. The following characteristics were recorded: (i) MMCs (without a large vacuole), (ii) Antennaria type diplosporous 1–2 nucleate gametophytes (with one or more large vacuoles; ADGs), (iii) sexual or Taraxacum type diplosporous dyads, (iv) sexual tetrads (no large vacuoles), (v) sexual 1–2 nucleate gametophytes (with one or more large vacuoles and degenerating tetrad remnants still visible); (vi) Taraxacum type diplosporous 1–2 nucleate gametophytes (with one or more large vacuoles; TDGs), (vii) aposporous initials (enlarged non-vacuolate nucellar cells), (viii) aposporous gametophytes (nucellar cells with one or more large vacuoles and one or more nuclei), (ix) microspore dyads and tetrads, and (x) 1–3 nucleate unreduced male gametophytes. Developmental stages of ovules per pistil were recorded, and pistil lengths corresponding to the following *A. thaliana* stages [30] were recorded: 1-I, late pre-MMC; 1-II, ovule meristems protruding from placentae (precedes MMC formation); 2-II, enlarging MMC; 2-III, mature MMC; 2-IV, meiosis; 3-III, large vacuole present in immature gametophyte.

## 3. Results

### 3.1. Expression Profiling

We recently documented reproduction in 44 *Boechera* taxa. These ranged from 100% sexual in species to nearly 100% apomictic, by TDG formation, in hybrids. Between these extremes were facultative apomicts that expressed combinations of meiosis and TDG and/or Hieracium-type gametophyte (HAG) formation [16]. Here, we report transcriptome analyses for four of these taxa: sexual *B. stricta*, strongly diplosporous *B. × formosa*, facultatively aposporous, and diplosporous *B. microphylla* (Cache County, UT), and facultatively diplosporous *B. lignifera*. Pistil lengths, array normalization results, array vs. qRT-PCR comparisons, and primers used for qRT-PCR are shown in Figures S1–S3 and Table S4, respectively.

**Figure 1:**
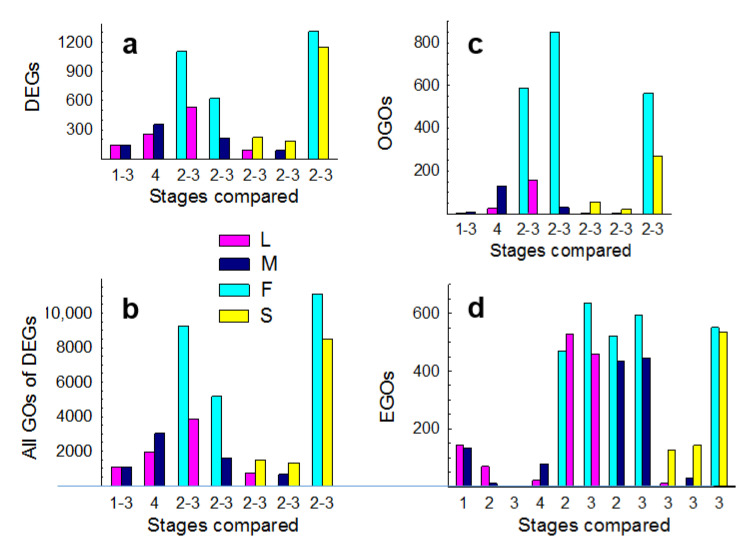
Differentially expressed genes (DEGs) and associated gene ontology categories (GOs) by comparison: (**a**) DEGs, (**b**) all GOs of all DEGs, (**c**) overrepresented GOs (OGOs), (**d**) enriched GOs (EGOs). L (*Boechera lignifera*), M (*Boechera microphylla*), F (*Boechera × formosa*), S (*Boechera stricta*), 1 (megasporocyte (MMC) stage), 2 (active meiocyte stage), 3 (young gametophyte stage), 4 (mature gametophyte stage).

Comparisons among the MMC (megasporocyte), AM (active meiocyte), and YG (young gametophyte) stages within diplosporous *B. lignifera* and aposporous *B. microphylla* produced few DEGs (Table S5). This near absence of DEGs may have occurred because pistil length intervals for the respective samples differed only minimally (Figure S1). However, few DEGs were detected at these stages even when comparisons were made across taxa. Hence, we combined the MMC, AM, and YG stages, within taxa, to make a single *B. lignifera* vs. *B. microphylla* comparison, which yielded 283 DEGs. At the MG stage (mature gametophyte), 609 DEGs were detected (Figure 1a, Table S5).

**Figure 2:**
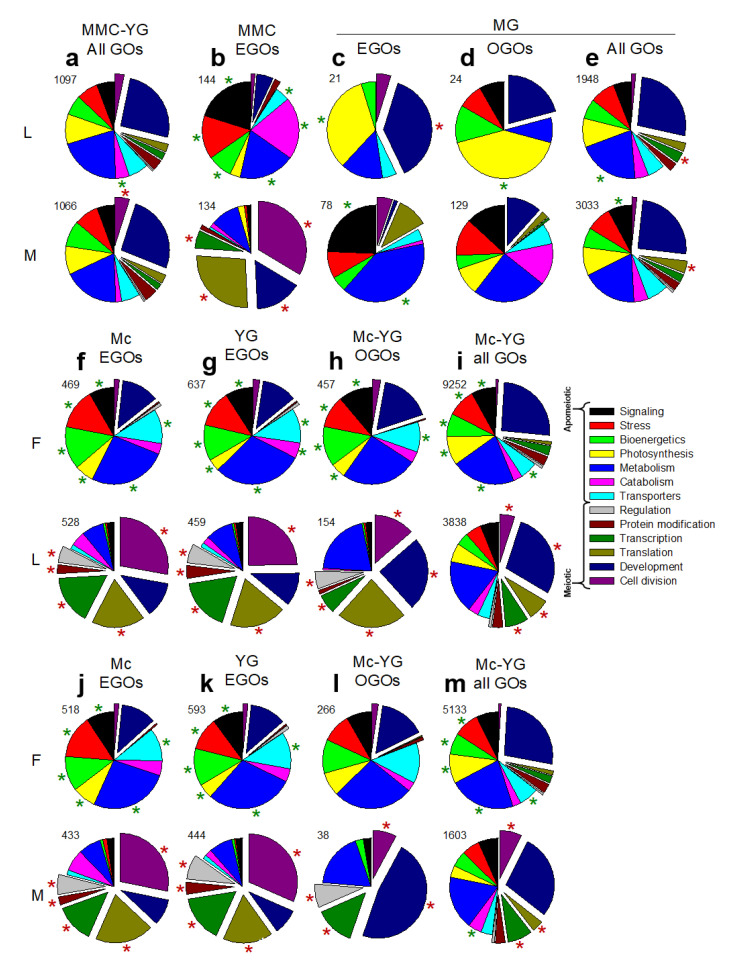
Pie charts of enriched gene ontology categories (EGOs), overexpressed GOs (OGOs), and GOs of differentially expressed genes (all GOs) by GO group and by comparison: (**a**–**e**), L vs. M; (**f**–**i**), F vs. L; (**j**–**m**), F vs. M. F (*Boechera × formosa*), L (*Boechera lignifera*), M (*Boechera microphylla*), MMC (megasporocyte stage), Mc (meiocyte stage), YG (young gametophyte stage), MG (mature gametophyte stage). Normal and exploded sections tended to be more frequently observed in less and more meiotic taxa, respectively. Starred GO groups differed significantly in frequency (*p* ≤ 0.05): green stars, normal; red stars, exploded; values next to pies are total numbers of GOs observed.

**Figure 3:**
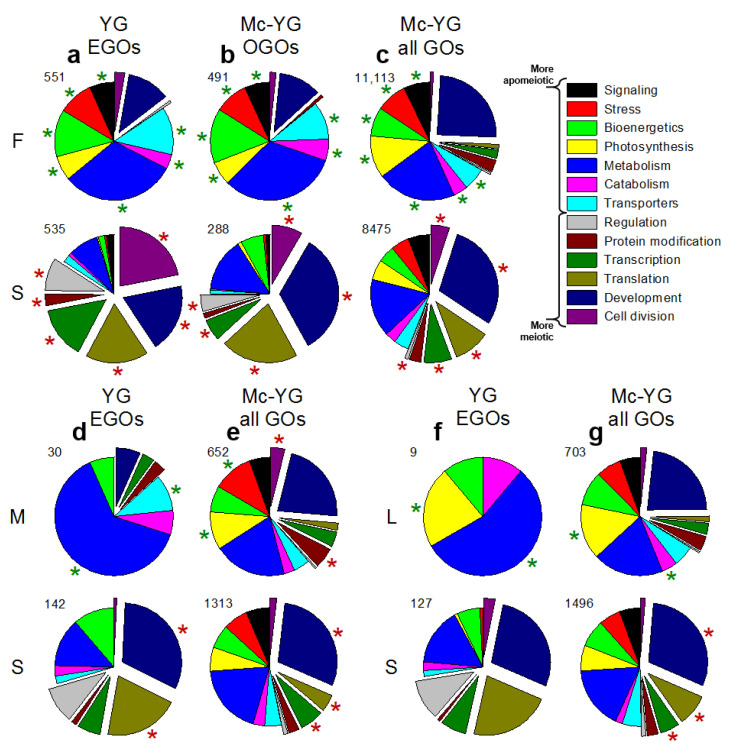
Pie charts of enriched gene ontology categories (EGOs), overexpressed GOs (OGOs), and GOs of differentially expressed genes (all GOs) by GO group and by comparison: (**a**–**c**), F vs. S; (**d**,**e**), M vs. S; (**f**,**g**), L vs. S. F (*Boechera × formosa*), L (*Boechera lignifera*), M (*Boechera microphylla*), S (*Boechera stricta*), Mc (meiocyte stage), YG (young gametophyte stage). Normal and exploded subgroups tended to be more frequently observed in less and more meiotic taxa, respectively. Starred subgroups differed significantly in frequency (*p* ≤ 0.05): green stars, normal; red stars, exploded; values next to pies are total numbers of GOs observed.

Across all comparisons, 3723 distinct DEGs were detected (Figure 1a; Table S5), which are linked to 3980 distinct GO terms (Figure 1b; Table S6). Overrepresented GOs (OGOs) occurs when more DEGs are linked to a GO term than expected by chance (pantherdb.org/). Among our OGO comparisons (Figure 1c), 930 distinct OGOs were detected (Table S7). EGOs occur when more genes of a GO term are up or down-regulated (significant or not) than expected by chance (pantherdb.org/). Since all expression data are used, EGO analyses can detect shifts in gene expression that may be missed by OGO analyses [31,32]. Among our EGO comparisons (Figure 1d), 1773 distinct EGOs were detected (Table S8). Collectively, 4822 distinct GO terms were identified. To more readily visualize large metabolism-related shifts in gene expression, we partitioned the 4822 GO terms into 13 user-defined subgroups (Tables S1 and S2). Subgroup frequencies per taxon were then compared among taxa by chi-square analyses (Figure 2 and Figure 3; Table S3; see Materials and Methods).

Only 5–20% of ovules of the TDG forming *B. lignifera* were facultatively meiotic [16]. Hence, we tested whether fewer meiosis related GO terms (all GOs of DEGs) occurred in *B. lignifera* compared to the TDG and HAG forming *B. microphylla*, where ca. 40% of ovules were meiotic [16]. For the combined MMC to YG comparison, meiosis and gametophyte related GOs occurred nearly exclusively in the more meiotic (and aposporous) *B. microphylla* (Table S6, lines 1–35 vs. 1098–1151, see also lines 2164–92 vs. 4112–62). These meiosis and gametophyte related GOs belong to the ‘cell division’ subgroup, which collectively represented a significantly larger percentage of GO terms in the aposporous *B. microphylla* compared to the diplosporous *B. lignifera* (Figure 2a, red star).

We used EGO analyses to compare *B. lignifera* to *B. microphylla* at each stage individually. The MMC stage produced the most EGOs (Figure 1d). Here, signaling, stress, bioenergetics, metabolism, catabolism, and transporter EGOs occurred more frequently in ovules of the TDG forming *B. lignifera* (least meiotic) (Figure 2b). These included carbohydrate, fatty acid, and protein metabolism processes, responses to organic and inorganic stimuli, activation of homeostasis processes, regulation of ROS and other stresses, and ABA signaling (Table S8, lines 11–79, 84–91, 95–144). In contrast, transcription, translation, development, and cell division EGOs were more frequent in ovules of the more meiotic HAG forming *B. microphylla* (Figure 2b). These included 13 meiosis associated terms and numerous ribosome-associated terms (Table S8, lines 145–210, 232–75). Fewer differences were observed at the AM, YG, and MG stages (Table S8, lines 290–471; Figure 2a,b). Collectively, these findings revealed major metabolism-related differences. Gene expression in the TDG forming *B. lignifera* favored signaling, stress, and catabolism. In the meiotic HAG forming *B. microphylla*, gene expression favored transcription, translation, development, and cell division.

Meiosis associated EGO subgroups at the MMC stage (exploded pie sections, Figure 2b) comprised ca. 10% and 80% of the diplosporous *B. lignifera* and the more meiotic but aposporous *B. microphylla*, respectively. Interestingly, nearly the same percentages were observed when diplosporous *B. lignifera* and aposporous *B. microphylla* were compared individually to the more diplosporous *B. × formosa* (Figure 2f,j). These results reveal a continuum where meiosis associated gene expression was highest in *B. microphylla* and lowest in *B. × formosa* with *B. lignifera* in between (compare Figure 2b,f,j). One of our objectives was to determine if these distinct shifts in gene expression are responsible for reproductive mode and apomixis type or are simply coincidental (discussed below). Analyses involving EGOs, OGOs, and all GOs of DEGs at the YG or AM/YG stages generally mirrored those at the AM stage for comparisons between the nearly obligate apomictic *B. × formosa* and the facultatively apomictic *B. lignifera* and *B. microphylla* (Figure 2f–i, j–m).

For this study, we collected *Boechera* species from native habitats in the Great Basin and Rocky Mountain Cordillera of North America [16]. However, embryological analyses revealed apomixis for taxa we thought, at the time of collection, were sexual. We eventually found sexual species, but resources for ovule dissections were no longer available. Instead, we created expression profiles from whole pistils of sexual *B. stricta*, each pistil of which contained ca. 160–180 ovules. We then compared ovule profiles of the apomicts to the same pistil profile from sexual *B. stricta* (Figure 3). We first considered differential gene expression between the nearly obligate TDG forming *B. × formosa* versus the completely sexual *B. stricta*. This comparison produced the most DEGs, OGOs, all GOs of DEGs and EGOs (Figure 1). Importantly, it produced highly similar frequency patterns for meiosis-associated vs. apomeiosis-associated EGO subgroups (Figure 3a) as had been observed for the more vs. the less meiotic apomicts (Figure 2b,f,j). Similar GO subgroup patterns were observed for OGOs and all GOs of DEGs (Figure 3a–c) and for the facultative apomicts compared to sexual *B. stricta* (Figure 3d–g).

In the three most meiotic taxa, *B. stricta* (100%), *B. microphylla* (40%), and *B. lignifera* (5–20%), 17, 14, and 10 meiosis associated EGOs per comparison were detected compared to the nearly obligate *B. × formosa*. Meiosis associated EGOs in these comparisons were not enriched in *B. × formosa* (Table S8: 458–65 vs. 927–1073, 1455–69 vs. 2092–205, 2551–8 vs. 3072–193, 3505–14 vs. 4098–237, 4851–65 vs. 5402–518). The more meiotic taxon of each comparison was also enriched for EGOs associated with transcriptional regulators involving DNA conformation changes, histone and DNA modifications, chromatin remodeling and silencing, epigenetic regulation, and posttranscriptional gene silencing by RNA (Table S8: 92–4 vs. 232–9; 816–23 vs. 1259–345; 1946–55 vs. 2369–449; 2942–8 vs. 3348–403; 3962–72 vs. 4386–4455; 5306–11 vs. 5756–831). In contrast, the more diplosporous taxon of each comparison tended to be enriched for EGOs associated with carbohydrate, fatty acid, and lipid metabolism; photosynthesis; responses to biotic and abiotic stimuli; kinase and phosphatase activities; calcium signaling; hormone metabolic pathways; second messenger signaling; ABA, salt, drought, hypoxia and ROS responses; regulation of ABA signaling; detoxification processes; homeostasis processes; pH regulation; peroxidase activities; and the biosynthesis of ascorbate, flavonoid and glutathione antioxidants (Table S8, 11–91 and 95–144 vs. 211–29 and 276–8; 306–42 vs. 348–57; 525–815 and 824–926 vs. 1137–213 and 1440–54; 1545–945 and 1956–2091 vs. 2257–319 and 2537–50; 2620–941 and 2949–3071 vs. 3232–3306 and 3489–504; 3586–961 and 3973–4097 vs. 4276–330 and 4530–4541; 4542–51 vs. 4592–625 and 4678; 4681–706 vs. 4755–96; 4931–5303 and 5312–401 vs. 5620–90 and 5923–36).

### 3.2. Molecular Pathways Upregulated in Meiotic Taxa

To better understand large scale shifts in gene expression between meiotic and apomeiotic taxa (Figure 2 and Figure 3), we searched gene narratives (Araport11 release, 1 January 2020) of our DEGs (Table S5) using the following terms: ABA, AGL, AGO, AOX, ARF, ascorbate, BR, Ca, calmodulin, catalase, chromatin modification, detoxification, epigenetic, ERF, ethylene, flavonol, gametophyte, glutathione, H_2_O_2_, H3K4, HDAC, histone, homeostasis, HSP, imprinting, LEA, MAPK, meiosis, miRNA, NaCl, oxidative damage, peroxidase, PP2C, RBOH, RDDM, ribosome, RNAi, ROS, salt responsive, silencing, siRNA, SnRK, SPO, stress, superoxide dismutase, thioredoxin, translation, and transport. After manual screening, 514 DEGs were identified for further analyses across all comparisons (Table S9).

**Figure 4:**
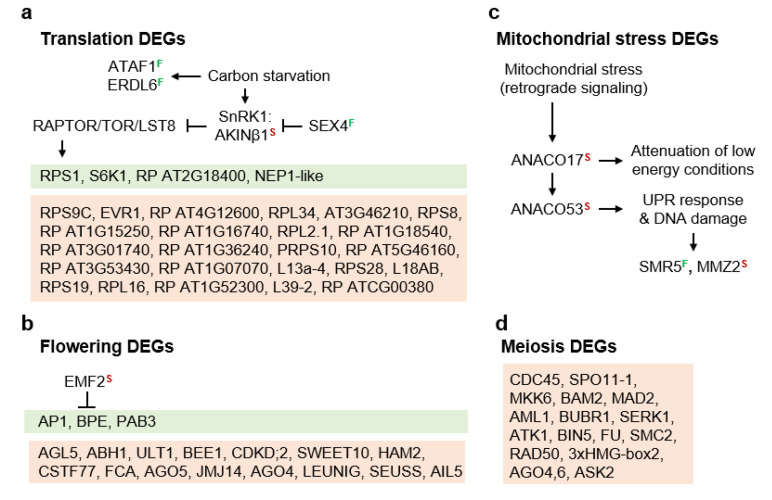
Differentially expressed genes (DEGs) observed between diplosporous *Boechera × formosa* and sexual *Boechera stricta* (Tables S5 and S9) as associated with: (**a**), translation; (**b**), flowering; (**c**), mitochondrial stress; and (**d**), meiosis. Superscripts identify taxa in which DEGs are upregulated; F, *B. × formosa*; S, *B. stricta*; red and green highlights, up or downregulation in *B. stricta*, respectively; arrows indicate upregulation or activation; blunt end lines represent downregulation or suppression; RP, ribosomal protein; UPR, unfolded protein response.

Carbon starvation downregulates translation by inactivating TARGET OF RAPAMYCIN (TOR) [33]. DEGs in the red and green boxes (Figure 4a) are translation machinery genes (Table S9). Most of these were upregulated in *B. stricta* (red box), which suggests that diplosporous *B. × formosa* was experiencing carbon starvation. Further evidence for this involved upregulated levels of ARABIDOPSIS NAC DOMAIN CONTAINING PROTEIN 2 (ATAF1), a transcription factor (TF) that upregulates TREHALASE1 (TRE1). TRE1 promotes the formation of a sugar starvation metabolome by catabolizing the signaling molecule trehalose-6-phosphate, thus further reducing TOR signaling by increasing SUCROSE-NON-FERMENTING-RELATED PROTEIN KINASE-1 (SnRK1) activity [34,35,36]. Carbon starvation also upregulates the vacuolar glucose exporter EARLY RESPONSIVE TO DEHYDRATION-LIKE 6 gene (*ERDL6*) [37] and the DUAL-SPECIFICITY PROTEIN PHOSPHATASE 4 gene (*SEX4*) [38], the products of which replenish monosaccharide levels to maintain primary metabolism. SEX4 might also inactivate SnRK1, thus reinitiating TOR activity in the presence of restored energy levels [39]. These three-carbon starvation genes were upregulated in diplosporous *B. × formosa* (Figure 4a). In contrast, SNF1-RELATED PROTEIN KINASE REGULATORY β SUBUNIT 1 (*AKIN*β*1*), which encodes an SnRK1 subunit that directs SnRK1 to the nucleus for gene regulation but also silences SnRK1 upon *N*-myristoylation [40], was downregulated in *B. × formosa* (Figure 4a), possibly as a feedback mechanism responding to TOR inactivation.

The early flowering gene EMBRYONIC FLOWER 2 (*EMF2*) encodes a polycomb repressive complex member that suppresses flowering [41]. It was downregulated in *B. × formosa* (Figure 4b), suggesting that this apomict may exhibit an early flowering phenotype, which is common for apomicts in nature [4]. Additionally, genes responsible for flower formation were mostly downregulated in *B. × formosa* (Figure 4b, red box). Taken together, these results suggest that the earliness of flowering was favored in apomictic *B. × formosa* but that several epigenetically regulated programs of reproduction were only weakly expressed in this taxon.

In apomictic strains of *Saccharomyces cerevisiae* (brewer’s yeast), heat stress switches apomeiotic divisions, which normally produce dyads of unreduced spores, to meiotic divisions, which produce tetrads of reduced spores. However, when mitochondrial transcription in apomictic yeast is interrupted by erythromycin, the heat-stress switch from apomeiosis to meiosis no longer occurs, i.e., the yeast remains apomictic. This finding, plus research in other organisms, suggests that meiosis depends on stress-induced nucleomitochondrial communication [3,42,43]. In the present study, genes for two nuclear TFs, NAC DOMAIN CONTAINING PROTEIN 17 (ANACO17) and 53 (ANACO53), which participate in drought-induced mitochondrial retrograde signaling, were upregulated in sexual *B. stricta* (downregulated in the TDG-forming 1st division restitution *B. × formosa*, Figure 4c). This is consistent with a mitochondrial role in the initiation and completion of meiosis I, a role that is downregulated during 1st division restitution apomeioses. ANAC017 normally induces senescence, suppresses auxin and chloroplast function, reprograms mitochondrial metabolism for lower energy conditions, and possibly activates SnRK1 [44]. It also upregulates ANACO53, which upregulates ROS production genes and initiates unfolded protein responses (UPRs) that attenuate proteotoxic conditions in the ER [45]. MMS ZWEI HOMOLOGUE 2 (MMZ2), which was strongly upregulated in *B. stricta*, is upregulated by DNA damage and UPRs, and it normally participates in post replicative DNA repair. Since these genes were upregulated in sexual *B. stricta*, their expression is consistent with the view that meiosis evolved as a DNA repair mechanism [15,46,47,48] at about the same time that early eukaryotes were domesticating their oxygen respiring endocytically obtained organelles [46]. The downregulation of meiosis genes and mitochondrial stress response genes in apomictic *B. × formosa* (Figure 4c,d) is further evidence that reproductive processes, from flower formation through meiosis, were only weakly supported at the molecular level in apomictic *B. × formosa*.

**Figure 5:**
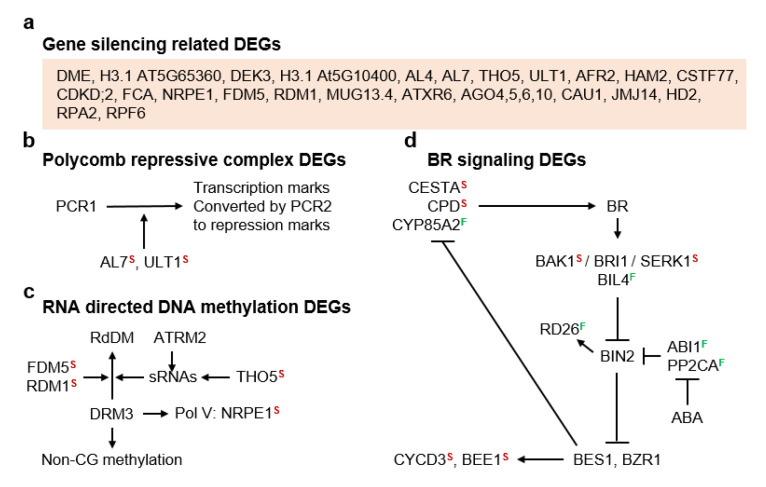
Differentially expressed genes (DEGs) observed between diplosporous *Boechera × formosa* and sexual *Boechera stricta* (see Tables S5 and S9) as associated with: (**a**), gene silencing; (**b**), polycomb repressive complex formation, (**c**), RNA directed DNA methylation, and (**d**), brassinosteroid (BR) signaling. Superscripts identify taxa in which DEGs are upregulated; F, *B. × formosa*; S, *B. stricta*; red highlights, upregulation in sexual *B. stricta*; arrows indicate upregulation or activation; blunt end lines represent downregulation or suppression.

DEGs of two additional signaling related categories, gene silencing and BR signaling, also provide clues concerning sex apomixis switching (Figure 5). Chromatin remodeling by methylation and demethylation occurs throughout plant development. In sexual cell lineages, RNA-directed DNA methylation (RdDM) is required to accomplish meiosis-specific gene splicing [49]. RdDM may also occur in tissues that produce sex cells because it silences potentially harmful transposons [49]. In the present study, silencing processes by RdDM and by polycomb repressive complex activity were upregulated in the ovules of sexual *B. stricta* vs. apomictic *B. × formosa* (Figure 5a–c). This again is consistent with other upregulated reproductive processes in *B. stricta* as described above.

ALFIN-LIKE 7 (AL7) is a nuclear-localized H3K4me3 binding protein that directs polycomb repressive complex (PcG) 1s (PCR1s) to transcriptionally active chromatin where the H3K4me3 expression marks are converted to H2Aub1 repression marks. These are then stabilized by PCR2 H3K27me3 silencing marks [50]. ULTRAPETALA1 (ULT1) is a trithorax factor that functions as an anti-repressor to counteract PcG silencing at 1000s of loci, including many genes where specific up or downregulation is required for normal reproductive development [51,52]. The upregulation in *B. stricta* of AL7 and ULT1 genes (Figure 5a–c) is further evidence that floral development at the molecular level was strongly supported in sexual *B. stricta* but weakly supported in apomictic *B. × formosa*. Overall, it appears that *B. stricta* ovules embarked on epigenetic paths of sexual development that apomeiotic ovules were reluctant to follow.

BR interacts with other processes to regulate growth and development as well as abiotic and biotic stresses [53,54]. The major hub of BR signaling is the kinase BRASSINOSTEROID-INSENSITIVE 2 (BIN2), which phosphorylation inactivates the major BR TFs BRASSINAZOLE-RESISTANT 1 (BZR1) and BRI1-EMS-SUPPRESSOR 1 (BES1). When BIN2 is inhibited, BZR1, and BES1 are activated, and this activation upregulates cascades of gene expression involving multiple downstream TFs and ca. 2000 downstream genes [55]. BR is a growth-promoting hormone. Hence, it is generally upregulated by TOR and downregulated by ABA. Consistent with this pattern, the two BR regulated signaling genes identified in our *B. × formosa* vs. *B. stricta* comparison (Table S9) were upregulated in *B. stricta* (Figure 5d): CYCLIN D3;1 (CYCD3) is a cyclin that initiates cell division [56], and BR-ENHANCED EXPRESSION 1 (BEE1) combines with a blue light photoreceptor, CRYPTOCHROME 2 (CRY2), to initiate photoperiod induced flowering [57]. Several additional BR associated genes were upregulated in *B. stricta*. These included two BR synthesis genes (Figure 5d), which, like TOR (Figure 4a), are upregulated under favorable conditions: HALF FILLED (CESTA) is a TF that upregulates multiple BR biosynthesis genes [58], including CONSTITUTIVE PHOTOMORPHOGENIC DWARF (*CPD*), which is critical for BR biosynthesis [59]. Additionally upregulated in *B. stricta* were two BR receptor-associated proteins, including BRI1-ASSOCIATED RECEPTOR KINASE (BAK1) [54] and SOMATIC EMBRYOGENESIS RECEPTOR-LIKE KINASE 1 (SERK1) [60]. Both of these proteins enhance BR signal transduction across membranes by forming protective complexes with the cell surface BR receptor kinase BR INSENSITIVE 1 (BRI1). One of these, SERK1, also binds EXCESS MICROSPOROCYTES 1 (EMS1) during reproductive development in processes that convert somatic cells to reproductive cells (microsporocytes) in anthers [61]. The downregulation of these BR genes in *B. × formosa* is consistent with other downregulated reproduction processes in *B. × formosa* (Figure 4 and Figure 5).

### 3.3. Molecular Pathways Upregulated in Apomeiotic Taxa

**Figure 6:**
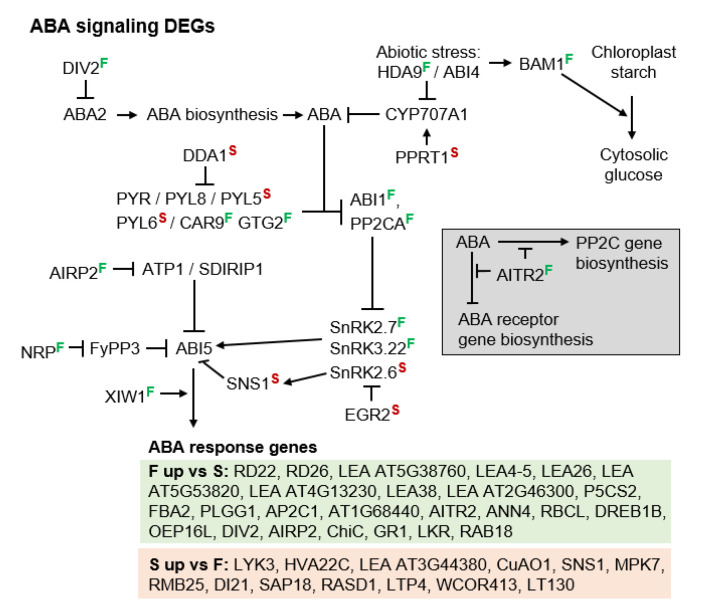
ABA signaling associated differentially expressed genes (DEGs) observed between diplosporous *Boechera × formosa* and sexual *Boechera stricta* (see Tables S5 and S9). Superscripts identify taxa in which DEGs are upregulated; F, *B. × formosa*; S, *B. stricta*; red and green highlights, up and down-regulation in *B. stricta*, respectively; arrows indicate upregulation or activation; blunt end lines represent downregulation or suppression.

Several BR associated genes were upregulated in *B. × formosa* (Figure 5d). One of these, BRASSINOSTEROID-6-OXIDASE 2 (*CYP85A2*), is a circadian rhythm regulated gene that encodes an enzyme that catalyzes the last step in BR biosynthesis. When upregulated, CYP85A2 increases BR but decreases ABA signaling [62]. BRZ-INSENSITIVE-LONG HYPOCOTYLS 4 (*BIL4*) was also upregulated in *B. × formosa*. It encodes a BRI1-interacting transmembrane protein that is involved in cell elongation. BIL4 suppresses BRI1 degradation and physically maintains BRI1 close to the plasma membrane [63]. Two protein phosphatase 2C (PP2C) genes, ABA INSENSITIVE 1 (*ABI1*) and PROTEIN PHOSPHATASE 2CA (*PP2CA*) were also upregulated in *B. × formosa*. These genes upregulate BZR1 and BES1 by phosphatase inactivating BIN2 (Figure 5d). They are also upregulated by ABA as part of a feedback inhibition mechanism where (i) stress induces ABA biosynthesis, (ii) ABA inactivates ABI1 and PP2CA, (iii) ABA signaling upregulates ABI1 and PP2CA biosynthesis, and (iv) additional accumulation of ABI1 and PP2CA suppresses ABA signaling (Figure 6). These variable findings are not surprising given the abundant crosstalk that occurs among tissues and cells during hormonal and environmental signaling [54].

A very different state of cellular homeostasis exists during stress than during favorable conditions. During stress, ABA-inactivated PP2Cs permit the self-activation by autophosphorylation of SnRK2,3s as well as BIN2. Activated BIN2 phosphorylation inactivates the growth-enhancing BR TFs but phosphorylation activates the NAC TF RESPONSIVE TO DESICCATION 26 (RD26), which upregulates multiple stress response genes [53,64]. *RD26* was upregulated 4.2 fold in *B. × formosa* vs. *B. stricta* (Figure 5d, Table S9). Activated SnRK2,3s upregulate ABA specific stress response genes [65] (Figure 6). These data suggest that apomictic ovules experienced states of cellular homeostasis that were maintained by enhanced stress perception with feedback suppression of ABA signaling occurring through *CYP85A2*, *ABI1,* and *PP2CA* [53,62,64,65].

**Figure 7:**
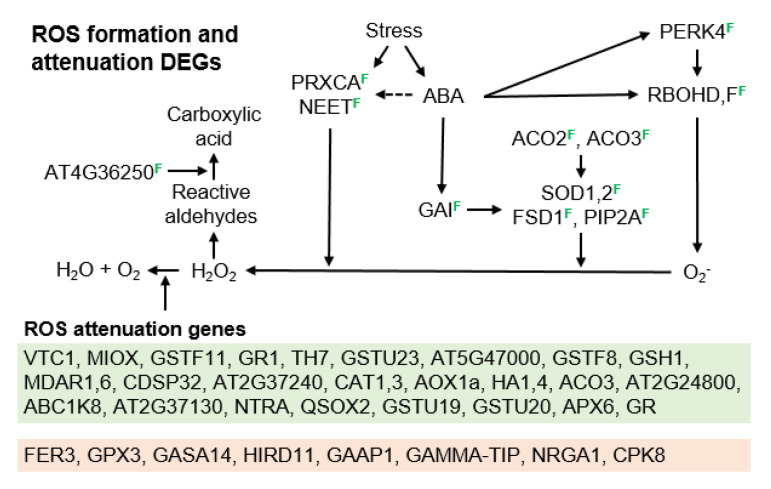
Reactive oxygen species (ROS) formation and attenuation pathways and associated differentially expressed genes (DEGs) as observed between diplosporous *Boechera × formosa* and sexual *Boechera stricta* (see Tables S5 and S9). Superscripts identify taxa in which DEGs were upregulated; F, *B. × formosa*; S, *B. stricta*; red and green highlights indicate up and down-regulation in *B. stricta*, respectively; arrows indicate upregulation or activation; blunt end lines represent downregulation or suppression.

The molecular pathways presented to this point suggest that apomeiosis is caused by carbon starvation (Figure 4a, low-level TOR activity), ABA (Figure 6), and upregulated drought response genes (Figure 5d). However, these pathways do not explain how a stress-like state of metabolic homeostasis induces apomixis, especially when stressed apomicts in many cases revert to sex [15,66]. Recognizing this paradox, we hypothesized that the stress-like state of metabolic homeostasis observed in ovules of apomicts is a chronic condition that is maintained by imbalances in wild type gene expression that occur as a result of hybridization, polyploidization, or other chromosome aberrations [13,67,68]. We further hypothesized that this chronic stress response state enhances stress tolerance, and it is this enhanced stress tolerance that simultaneously suppresses sex while inducing apomixis. Accordingly, we searched our DEGs for candidates that might be responsible for shifting metabolic homeostasis toward a chronic dual state of stress-response but even stronger stress-attenuation (Figure 6 and Figure 7).

We first considered the possibility that the biosynthesis of ABA itself, which is upregulated by stress [69], might be chronically upregulated in the apomicts. ABA DEFICIENT 2 (*ABA2*), an ABA biosynthesis gene, was upregulated in aposporous *B. microphylla* vs. sexual *B. stricta*. However, this gene was also upregulated in *B. microphylla* vs. *B. × formosa* (Table S9), which is inconsistent with ABA synthesis being a major contributor. Furthermore, the ABA and salt stress-induced TF DIVARICATA 2 (DIV2), which negatively regulates ABA biosynthesis by downregulating *ABA1,2* [70], was upregulated in *B. × formosa* vs. *B. stricta* (Table S9). Hence, a chronic stress-like phenotype being caused by upregulated ABA biosynthesis is unlikely.

Decreased catabolism of ABA is another possibility. Here, a drought-upregulated histone deacetylase gene, HISTONE DEACETYLASE 9 (*HDA9*), was upregulated 1.9 fold in *B. × formosa* vs. *B. stricta* (Figure 6). HDA9 forms a complex with ABA INSENSITIVE 4 (ABI4) that downregulates the major ABA catabolism genes CYTOCHROME P450, FAMILY 707, SUBFAMILY A, POLYPEPTIDE 1,2 (*CYP707A1,2*) [71]. Moreover, the salt stress-induced PROTEIN WITH THE RING DOMAIN AND TMEMB_185A 1 (*PPRT1*) gene [72], a C3HC4 zinc-finger ubiquitin E3 ligase that upregulates the ABA catabolism genes *CYP707A1,3* [73], was downregulated in *B. × formosa* vs. *B. stricta*. Hence, the up and downregulation of *HDA9* and *PPRT1*, respectively, in *B. × formosa* likely contributed to its stronger ABA signaling. Hence, from our expression profiles, suppressing ABA catabolism is a reasonable candidate for upregulating ABA signaling and inducing a homeostatic state of stress attenuation.

Upregulating the biosynthesis of ABA receptor genes is another way in which ABA signaling in *B. × formosa* might have been enhanced. Normally, stress-induced ABA feedback inhibits ABA receptor gene biosynthesis, and this may have occurred for the ABA receptor genes PYRABACTIN RESISTANCE 1-LIKE 5,6 (*PYL5,6*) (downregulated 67% in *B. × formosa* vs. *B. stricta*; PYL5 was also downregulated 67% in *B. lignifera* and *B. microphylla* vs. *B. stricta*; Table S9). However, the downregulation of ABA receptor genes by feedback mechanisms is suppressed by the ABA-INDUCED TRANSCRIPTION REPRESSOR 2 gene (*AITR2*) [74,75], which was upregulated 2.1-fold in *B. × formosa* vs. *B. stricta*. Additionally, the C2-DOMAIN ABA-RELATED 9 gene (*CAR9*), an ABA receptor (Figure 6), was upregulated 4.3-fold in *B. × formosa* vs. *B. stricta*. CAR9 positively regulates ABA signaling by interacting with ABA and various PYRABACTIN RESISTANCE (PYR) and PYL ABA receptors at the plasma membrane [76]. Furthermore, the ABA receptor gene GPCR-TYPE G PROTEIN 2 (*GTG2*), which encodes a G-protein with nine transmembrane domains [77], was also upregulated 2.1-fold in *B. × formosa* vs. *B. stricta*. Hence, ABA receptor upregulation in *B. × formosa* likely enhanced ABA signaling in this apomict.

Downregulating proteins that target ABA receptors for degradation, could also enhance ABA signaling. Here, the DET1 AND DDB1 ASSOCIATED 1 gene (*DDA1*), which downregulates ABA signaling by targeting *PYL4,8,9* for degradation [78], was downregulated in all three apomicts (Table S9). Hence, DDA1 is a reasonable candidate for shifting metabolic homeostasis toward ABA signaling. In general, the majority of ABA receptors were, for various reasons, upregulated in apomictic *B. × formosa* (Figure 6).

Under favorable conditions, PP2Cs promote growth by promoting BR signaling (Figure 5d, by inactivating BIN2), promoting TOR activity (Figure 4a, by inactivating SnRK1), and preventing ABA signaling (Figure 6, by inactivating SnRK2,3s). During stress, ABA synthesis is upregulated, ABA and ABA receptors bind, and the receptor complexes inactivate PP2Cs, thus suppressing growth. In addition to downregulating ABA receptors, stress-induced ABA signaling also upregulates PP2C biosynthesis (Figure 6), thus reducing ABA signaling by feedback inhibition. Hence, the upregulated PP2Cs (ABI1 and PP2CA) in *B. × formosa* (Table S9) is further evidence of upregulated ABA signaling. Interestingly, AITR2, which suppresses ABA-induced suppression of ABA receptor gene biosynthesis, also suppresses ABA-induced upregulation of PP2C biosynthesis [75] (Figure 6). AITR2 was upregulated in *B. lignifera* vs. *B. stricta* 29.5 fold and in *B. × formosa* vs. *B. stricta* 2.1 fold (Table S9). Hence, *AITR2* is an interesting candidate gene for upregulating ABA signaling.

Next in ABA signaling are certain SnRK2,3s that phosphorylation activate the major ABA TF ABA INSENSITIVE 5 (ABI5) and other ABA TFs (Figure 6). During favorable conditions, SnRK2,3s are inactivated by PP2Cs. Hence, by upregulating the biosynthesis of SnRK2,3s, ABA signaling might be upregulated simply by overwhelming PP2C suppression. Here, *SnRK2.6* is an interesting case. It was upregulated in the opposite direction, i.e., 3-fold in sexual *B. stricta* vs. *B. × formosa* (Table S9). However, an even greater upregulation of *SnRK2.6* was likely prevented by the EARLY GROWTH RESPONSE 2 gene *EGR2*, which also was upregulated in *B. stricta* (2.4-fold). In warm temperatures, the EGR2 phosphatase inactivates SnRK2.6. In cold weather, EGR2 is inactivated, and SnRK2.6 upregulates cold stress genes [79]. Furthermore, SnRK2.6 activates SNRK2-SUBSTRATE 1 (SNS1), which suppresses ABA signaling [80]. Compared to diplosporous *B. × formosa*, *SNS1* was upregulated 5.3, 3.4, and 3.2-fold in the more meiotic *B. stricta*, *B. microphylla,* and *B. lignifera*, respectively (Table S9). Hence, SnRK upregulation in *B. × formosa* may have also contributed to a stress-attenuation-based metabolic homeostasis.

Several SnRK2,3s upregulate stress response genes independently of ABA. SnRK2.7 is activated by salt and osmotic stress [81], and its synthesis was upregulated in *B. × formosa* vs. *B. stricta* 2.5-fold. The *SnRK3.22* gene (PROTEIN KINASE SOS2-LIKE 5, *PKS5*), which activates ABI5 [82], is also upregulated by salt stress, and it was upregulated 3-fold in *B. × formosa* vs. *B. stricta*. In the absence of stress, SnRK3.22 inactivates the H^+^ ATPase driven SALT OVERLY SENSITIVE 2 Na^+^/H^+^ antiporter gene (*SOS2*). During salt stress, this antiporter is activated, by Ca^2+^ signaling, whereupon it exports Na^+^ from cells [83].

Other targets for altering ABA signaling are the ABA TFs themselves (Figure 6). XPO1-INTERACTING WD40 PROTEIN 1 (*XIW1*) was upregulated in *B. × formosa* 3.5, 2.8, and 2.1-fold vs. *B. stricta*, *B. microphylla,* and *B. lignifera*, respectively. It upregulates ABA signaling by stabilizing ABI5 [84]. ASPARAGINE-RICH PROTEIN (*NRP*) was upregulated in *B. × formosa* 3.0, 5.6, and 4.1-fold vs. *B. stricta*, *B. microphylla,* and *B. lignifera*, respectively. It upregulates ABA signaling by targeting FLOWER-SPECIFIC, PHYTOCHROME-ASSOCIATED PROTEIN PHOSPHATASE 3 (FyPP3) for degradation [85]. FyPP3 decreases ABA signaling by inactivating ABI5. Likewise, ABA INSENSITIVE RING PROTEIN 2 (*AIRP2*) was upregulated in *B. × formosa* 2.2-fold vs. *B. stricta*. It encodes a cytosolic RING-type E3 ubiquitin ligase that upregulates ABA signaling, in response to ABA or salt stress, by targeting ATAIRP2 TARGET PROTEIN 1 (ATP1) for ubiquitination [86]. In the absence of salt stress, ATP1 decreases ABA signaling by silencing ABI5. Accordingly, enhancing ABI5 stability represents another means whereby ABA signaling was enhanced in *B. × formosa*. Other heat, salt, and osmotic stress induced DEGs that were upregulated in *B. × formosa* vs. sexual *B. stricta* included *SHE*, *HSP70 AT3G12580*, *HSP AT3G47940*, *AT1G49670*, *MYC2*, *CML9*, *CALS5*, *ZAT10*, *AKR4C8*, *ADC2*, *DOB1*, *ChlAKR*, *DREB A-2*, *HSP70 AT3G09440*, *FBP7*, *TPR4*, *DJC77*, *ALDA10A9*, *HMAD1*, *AT4G29780*, *RDUF1*, *CYS-3A*, *SRP3*, *HDA9*, *SnRK2.7*, *MPK4*, *AT4G37530*, *AT2G37130*, *AT2G24800*, *GSTU19*, *APG6*, *EGR3*, *DJA7*, *ERDL6*, *DNAJ*, *RD21*, *SAT32*, *MKK9*, *NHX1*, *PCaP1*, *TYDC*, *AT2G25940*, and *GSTF11* (Tables S5 and S9). In most of these cases, it is not known whether ABA alone or other stresses activate their expression.

Many of the ABA response DEGs (Figure 6) are also upregulated by heat, salt, or osmotic stress (Table S9), suggesting that hypersensitivity to these conditions may have also contributed to the chronic stress induction/tolerance homeostasis observed in *B. × formosa*. Many of these were late embryogenesis abundant (LEA) genes, the proteins of which protect cells and proteins from the deleterious effects of ROS, drought, and freezing [87]. Others included: DELTA 1-PYRROLINE-5-CARBOXYLATE SYNTHASE 2 (*P5CS2*), upregulated 9.9, 17.1 and 6.6-fold in *B. × formosa* vs. *B. stricta*, *B. microphylla* and *B. lignifera*, respectively; FRUCTOSE-BISPHOSPHATE ALDOLASE 2 (*FBA2*) [88], upregulated 8.8, 8.8 and 7.3-fold in *B. × formosa* vs. *B. stricta*, *B. microphylla* and *B. lignifera*, respectively; RAB GTPASE HOMOLOG B18 (*RAB18*), upregulated 4.7-fold in *B. × formosa* vs. *B. stricta*, *B. microphylla* and *B. lignifera*, which is a dehydrin that interacts with aquaporins in responses to drought stress [89]; RESPONSIVE TO DESICCATION 22 (*RD22*) [90], a NAC TF upregulated in *B. × formosa* 11.9, 9.4 and 6.3-fold vs. *B. stricta*, *B. microphylla* and *B. lignifera*, respectively; RD26 [64], a NAC TF upregulated 4.2-fold in *B. × formosa* vs. *B. stricta*, which upregulates drought response genes; GLUTATHIONE-DISULFIDE REDUCTASE (*GR1*), upregulated 1.9-fold in *B. × formosa* vs. *B. stricta*, which is a major peroxisomal glutathione reductase that functions in the H_2_O_2_ detoxification ascorbate glutathione pathway [91]; and the protein-serine/threonine phosphatase gene *AP2C1*, upregulated 12.1 and 6.1-fold in *B. × formosa* vs. *B. stricta* and *B. lignifera*, respectively. AP2C1 modulates K^+^ homeostasis by dephosphorylation inactivating CBL-INTERACTING PROTEIN KINASE 9 (CIPK9), which normally enhances K^+^ ion uptake through K^+^ channels, particularly under low K^+^ conditions [92]. It also moderates responses to pathogens by dephosphorylation inactivating MAP KINASE 4,6 (MPK4,6) in the cytoplasm and nucleus. These MAPKs affect the expression of hundreds of genes by modifying the activity of 88 TFs from 21 TF families [93].

ABA signaling upregulates ROS synthesis, ROS signaling, and large suites of peroxisomal and other antioxidant network genes [91,94]. The first step in ROS signaling is an upregulation of ROS biosynthesis by peroxidases or RBOHs. Five ROS biosynthesis genes were highly upregulated in the TDG forming *B. × formosa*. PEROXIDASE CA (*PRXCA*) (Figure 7) encodes a cell wall peroxidase that produces H_2_O_2_ in the apoplast [95]. It was upregulated in *B. × formosa* 11.4, 10.0, and 8.5-fold vs. the meiosis exhibiting *B. stricta*, *B. microphylla,* and *B. lignifera*, respectively (Table S9). NEET is a ROS homeostasis regulator that was upregulated in *B. × formosa* 7.8, 4.9, and 10.7-fold vs. *B. stricta*, *B. microphylla,* and *B. lignifera*. AT-NEET (*NEET*) encodes a protein that regulates Fe and ROS homeostasis by controlling the biogenesis of Fe-S clusters that function in electron transport. In the absence of NEET, excess Fe-S clusters form and react with oxygen to produce ROS [96]. PROLINE-RICH RECEPTOR-LIKE PROTEIN KINASE 4 (PERK4) enhances ROS accumulation by enabling ABA-induced expression of RBOH genes, including *RBOHC* [97]. PERK4 was upregulated in *B. × formosa* 6.7, 5.6, and 6.5-fold vs. *B. stricta*, *B. microphylla,* and *B. lignifera*. RBOHD produces superoxide in response to heat and wounding and its gene was upregulated in *B. × formosa* 2.9, 5.1, and 8.2-fold vs. *B. stricta*, *B. microphylla,* and *B. lignifera*. Both *RBOHD* and *RBOHF* are upregulated by ABA, and their expression, through ROS signaling, is critical to cell survival under ER stress [98]. *RBOHF* was upregulated in *B. × formosa* 2.9, 4.2, and 3.5-fold vs. *B. stricta*, *B. microphylla,* and *B. lignifera*.

Conversion of superoxides to H_2_O_2_ by superoxide dismutases (SODs) is the next step in ROS signaling. Five genes responsible for this conversion were upregulated in *B. × formosa*. ACONITASE 2,3 (ACO2,3) upregulate SUPEROXIDE DISMUTASE 2 (*SOD2*) (Figure 7). *ACO2* was upregulated 3.1, 4.1, and 5.0-fold in *B. × formosa* vs. *B. stricta*, *B. microphylla,* and *B. lignifera*, respectively, and *ACO3* was upregulated 2.5-fold in *B. × formosa* vs. *B. stricta* (Table S9). SOD1 is a cytosolic copper/zinc SOD. Under favorable conditions, its transcripts are cleaved by miR398, which decreases SOD1 levels. Under stress conditions, miR398 expression is suppressed, and SOD1 levels increase [99]. SOD1 was upregulated 3.2-fold in *B. × formosa* vs. *B. lignifera* and 2.9-fold in *B. microphylla* vs. *B. lignifera*. SOD2 is a chloroplastic copper/zinc SOD. Its transcripts are also cleaved by miR398 under favorable conditions. Under stress conditions, miR398 expression is suppressed, and SOD2 levels increase [99,100]. SOD2 was upregulated 1.8-fold in *B. × formosa* vs. *B. microphylla*, 2.8-fold in *B. × formosa* vs. *B. lignifera*, and 2.0-fold in *B. microphylla* vs. *B. lignifera*. FE SUPEROXIDE DISMUTASE 1 (FSD1) is a salt stress-induced chloroplastic SOD and is also an osmoprotectant [101]. It was upregulated 5.6-fold in *B. × formosa* vs. *B. stricta*, 12.2-fold in *B. × formosa* vs. *B. microphylla*, and 5.8-fold in *B. lignifera* vs. *B. microphylla*.

Several other DEGs increase H_2_O_2_ levels in cells. PLASMA MEMBRANE INTRINSIC PROTEIN 2A (PIP2A) is a water and H_2_O_2_ transmembrane aquaporin that is downregulated by salt stress. Apoplastic RBOHF-generated ROS cause PIP2A clustering, and subsequent endocytosis of the PIP2A clusters appears to regulate ROS levels [102]. *PIP2A* transcripts were upregulated 2.8-fold in *B. × formosa* vs. *B. stricta* and 5.3-fold in *B. × formosa* vs. *B. lignifera* (Table S9). GIBBERELLIC ACID INSENSITIVE (GAI) is a transcriptional regulator that interacts with TFs. It is upregulated by ABA, ethylene, and salt stress and increases drought tolerance by restraining cell proliferation and expansion. It possibly increases H_2_O_2_ levels by upregulating SODs [103]. GAI was upregulated 2.4-fold in *B. × formosa* vs. *B. stricta* and 2.2-fold in *B. × formosa* vs. *B. lignifera*.

Several H_2_O_2_ attenuation processes were upregulated in *B. × formosa*. The most prominent of these, based on numbers of DEGs, was the classic glutathione ascorbate H_2_O_2_ attenuation cycle. Here, VITAMIN C DEFECTIVE 1 (*VTC1*), which encodes an essential enzyme in ascorbate biosynthesis, the levels of which are correlated with ascorbate levels [104], was upregulated 2.1, 4.5, and 2.1-fold in *B. × formosa* vs. *B. stricta*, *B. microphylla* and *B. lignifera*, respectively (Table S9). MYO-INOSITOL OXYGENASE 2 (MIOX) also functions in ascorbate biosynthesis [105]. *MIOX* was upregulated 4.7, 3.7, and 3.3-fold in *B. × formosa* vs. *B. stricta*, *B. microphylla,* and *B. lignifera*, respectively. ALTERNATIVE OXIDASE 1A (AOX1a) is an ascorbate peroxidase that is highly suppressed at the transcriptional and post-transcriptional levels under favorable growing conditions. During stress, *AOX1a* is upregulated by multiple stress-related TFs including SnRK1 [106]. It was upregulated 1.7 and 2.1-fold in *B. × formosa* vs. *B. stricta* and *B. lignifera*. MONODEHYDROASCORBATE REDUCTASE 1 (MDAR1) is a peroxisomal monodehydroascorbate reductase that functions in the ascorbate-glutathione cycle. *MDAR1* was upregulated 6.2 and 2.6-fold in *B. × formosa* vs. *B. stricta* and *B. lignifera*. ASCORBATE PEROXIDASE 6 (APX6) is a major cytosolic ascorbate peroxidase [107]. *APX6* was upregulated 3.9 and 1.9-fold in *B. × formosa* vs. *B. stricta* and *B. microphylla*. It was also upregulated 2.1-fold in *B. microphylla* vs. *B. stricta* and 2.0-fold in *B. lignifera* vs. *B. microphylla*. GLUTAMATE-CYSTEINE LIGASE (GSH1) catalyzes the rate-limiting step in glutathione biosynthesis. *GSH1* was upregulated 2.0 and 4.3-fold in *B. × formosa* vs. *B. stricta* and *B. lignifera*. GLUTATHIONE REDUCTASE (GR) is a plastid glutathione reductase that maintains a highly reduced glutathione pool that balances ROS and enables the redox buffering required to maintain efficient photosynthesis [108]. *GR* was upregulated 2.9, 7.7, and 7.3-fold in *B. × formosa* vs. *B. stricta*, *B. microphylla,* and *B. lignifera*, respectively. It was also upregulated 2.9-fold in *B. stricta* vs. *B. microphylla*. GLUTATHIONE-DISULFIDE REDUCTASE (GR1) is a glutathione reductase that is upregulated by drought, dehydration, and ABA and is a major peroxisomal glutathione reductase in the glutathione ascorbate cycle [91]. *GR1* was upregulated in *B. × formosa* 1.9-fold vs. *B. stricta*. Five glutathione transferases that are involved in ROS attenuation were also upregulated. GLUTATHIONE S-TRANSFERASE U23 (GSTU23) detoxifies ROS under high oxidative stress conditions [109]. *GSTU23* was upregulated 3.8, 4.0, and 3.9-fold in *B. × formosa* vs. *B. stricta*, *B. microphylla,* and *B. lignifera*, respectively. GLUTATHIONE S-TRANSFERASE F11 (*GSTF11*) was upregulated 7.7, 12.3, and 20.9-fold in *B. × formosa* vs. *B. stricta*, *B. microphylla,* and *B. lignifera*, respectively. *GSTF11* was also upregulated 3.3-fold in *B. stricta* vs. *B. lignifera*. GSTF8 reduces oxidative damage by binding to heavy metals. *GSTF8* was upregulated 2.0 and 2.5-fold in *B. × formosa* vs. *B. stricta* and *B. microphylla*, respectively. *GSTU19* is plant-specific and is upregulated by salt, drought, and oxidative stress. Its protein functions redundantly with other GSTs to maintain redox homeostasis [110]. It was upregulated 1.7-fold in *B. × formosa* vs. *B. stricta*. GSTU20 is required for plant responses to far-red light [111]. *GSTU20* is upregulated by stress and was upregulated 10.6 and 3.2-fold in *B. × formosa* vs. *B. stricta* and *B. lignifera*, respectively. It was also upregulated 5.9-fold in *B. microphylla* vs. *B. stricta* and 3.6-fold in *B. lignifera* vs. *B. stricta*.

Several other classes of ROS attenuation genes were upregulated in *B. × formosa*, including catalases, peroxidases, and thioredoxins. CATALASE 1 (CAT1) catalyzes the reduction of H_2_O_2_. *CAT1* is upregulated by ROS and was upregulated 3.1-fold in *B. × formosa* vs. *B. stricta* (Table S9). CAT3 also catalyzes the reduction of H_2_O_2_ but also catalyzes the transnitrosylation of REPRESSOR OF GSNOR1 (GSNOR1) to activate H_2_O_2_ reduction in the presence of NO [112]. *CAT3* was upregulated 3.2 and 4.4-fold in *B. × formosa* vs. *B. stricta* and *B. lignifera*, respectively. Three peroxidases, AT5G47000, AT2G24800, AT2G37130, which are upregulated in response to oxidative stress, were upregulated 3.0, 2.1, and 1.9-fold in *B. × formosa* vs. *B. stricta*, respectively. The former was also upregulated 2.6-fold in *B. × formosa* vs. *B. microphylla* and *B. lignifera*. The respiratory burst oxidase homolog (RBOH) NADPH-DEPENDENT THIOREDOXIN REDUCTASE A (NTRA) is a major cytosolic isoform of NADPH-dependent thioredoxin reductases. It redundantly regulates enzymes involved in photorespiration, ATP synthesis, stress-related reactions, redox homeostasis processes, and TCA cycle enzymes [113]. *NTRA* was upregulated 1.9-fold in *B. × formosa* vs. *B. stricta*. CHLOROPLASTIC DROUGHT-INDUCED STRESS PROTEIN OF 32 KD (CDSP32), thioredoxin upregulated by oxidative stress and drought, participates in maintaining cell redox homeostasis. *CDSP32* was upregulated 1.8 and 2.2-fold in *B. × formosa* vs. *B. stricta* and *B. lignifera*, respectively. AT2G37240, thioredoxin that participates in maintaining cell redox homeostasis, was upregulated 1.9 and 1.6-fold in *B. × formosa* vs. *B. stricta* and *B. lignifera*, respectively. QUIESCIN-SULFHYDRYL OXIDASE 2 (QSOX2) is a thioredoxin involved in protein folding and in maintaining cell redox homeostasis. *QSOX2* was upregulated 1.8-fold in *B. × formosa* vs. *B. stricta*.

Three additional classes of ROS attenuation genes were upregulated in *B. × formosa*. THIOREDOXIN H-TYPE 7 (TH7) functions as a redox sensor/transmitter in the ER where it participates in protein folding and transport of NADPH to peroxisomes [114]. *TH7* was upregulated 5.0, 4.5, and 4.9-fold in *B. × formosa* vs. *B. stricta*, *B. microphylla,* and *B. lignifera*, respectively (Table S9). H(+)-ATPase 1,4 (HA1,4) are plasma membrane proton ATPases that acidify the apoplast while increasing cytosolic pH and decreasing ROS levels [115]. *HA1* and *HA4* were upregulated 3.7 and 2.4-fold in *B. × formosa* vs. *B. stricta*, respectively. ABC2 HOMOLOG 13 (ABC1K8) is a chloroplast localized protein kinase that is upregulated by heavy metals and mediates iron distribution and lipid membrane changes during oxidative stress [116]. It was upregulated 2-fold in *B. × formosa* vs. *B. stricta*.

### 3.4. Metabolically Induced Switching from Apomeiosis to Meiosis

**Figure 8:**
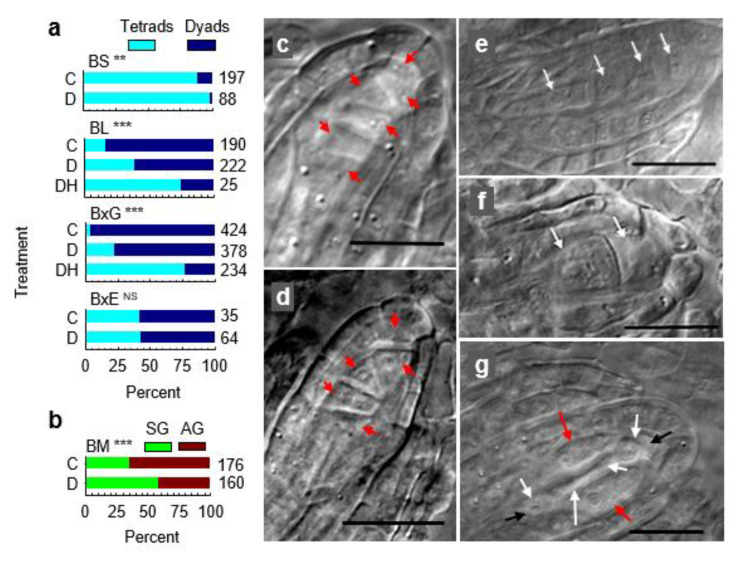
Effects of no stress (C), drought (D) and drought plus heat (DH) on (**a**) percentages of meiotic or apomeiotic dyads and meiotic tetrads in sexual *Boechera stricta* (BS), facultatively diplosporous *Boechera lignifera* (BL), triploid and nearly obligately diplosporous *Boechera* cf. *gunnisoniana* (BxG), and facultatively diplosporous *Boechera exilis* × *retrofracta* (BxE); (**b**) percentages of aposporous (AG) and sexual (SG) gametophyte formation in aposporous *Boechera microphylla* (BM); (**c**,**d**) meiotic hexad and pentad of megaspores from stress-induced meioses in triploid diplosporous *B.* cf. *gunnisoniana*; (**e**) meiotic tetrad of megaspores from sexual *B. stricta*; (**f**) diplosporous dyad from diplosporous *B. lignifera*; (**g**) degenerating tetrad (between black arrows) of Millard County *B. microphylla* with early vacuolate, 1-2 nucleate aposporous gametophytes on each side (red arrows). Numbers of ovules examined in (**a**) and (**b**) that contained scorable dyads, tetrads, SGs, and AGs per treatment are indicated at the right of the colored bars; cytology bars, 20 μm. Chi-square *p* values: **, *** are 0.01, 0.001; NS, not significant.

We hypothesized above that apomicts express chronic stress responses coupled with chronic, overcompensating stress attenuation mechanisms that suppress meiosis and induce apomeiosis. If this is correct, meiosis should be inducible in apomicts by applying stresses that exceed the apomict’s stress attenuation capabilities. In 1951, Bocher [117] reported shifts from 1st division restitutions in microsporocytes of apomictic *Boechera*, which produced unreduced pollen, to complete meioses in weak plants that flowered sporadically late in the season. He noted that the processes that produce unreduced male and female spores, 1st division restitution, occurred in anthers and ovules. He also suggested that stress had induced the observed shifts from apomeiosis to meiosis, late in the season, and he speculated that stress might affect ovules in the same way. We viewed Bocher’s speculations as an opportunity to gain further insights into these processes of sex/apomixis switching in facultative apomicts. Accordingly, we exposed randomly selected groups of *Boechera* plants to three growth conditions: well-watered, droughted, and droughted plus heat. Taxa studied were sexual *B. stricta*, facultative TDG forming *B. lignifera*, triploid, and highly TDG forming *B.* cf. *gunnisoniana*, HAG forming *B. microphylla* from Millard County Utah (meiotic and aposporous, but not diplosporous), and facultative TDG forming *B. exilis × retrofracta* [16]. The combination of drought and heat increased meiosis frequencies (tetrad formation) from 15 to 75% in *B. lignifera* and from 2 to 77% in *B.* cf. *gunnisoniana* (Figure 8a). Since most dyads in these droughted and heat-stressed plants (23 to 25% of ovules, Figure 8a) were possibly produced by meiosis, actual frequencies of sexual meioses in these stressed TDG-forming apomicts may have been closer to 90%. Drought alone in TDG forming *B. lignifera* and *B.* cf. *gunnisoniana* was less effective in switching apomeiosis to meiosis (Figure 8a). Disturbed meioses that produced pentads and hexads of imbalanced megaspores also occurred among the stress-induced meioses of triploid *B.* cf. *gunnisoniana* (Figure 8c,d). Likewise, Bocher also observed microspore pentads and hexads in the anthers of late-flowering *Boechera* triploids [117].

Due to lethality, data were not obtained for the double stressed (drought and heat) *B. stricta*, *B. exilis × retrofracta,* and *B. microphylla*. Drought alone had minor effects on the reproductive mode in sexual *B. stricta* and facultative *B. exilis × retrofracta*. The sexual dyad stage of meiosis is brief and difficult to catch cytologically. Accordingly, it is possible that drought accelerated the dyad stage (e.g., more rapid onset of M_II_) such that fewer dyads were observed in ovules of *B. stricta* (Figure 8a). Drought also shifted frequencies of gametophyte formation from aposporous to sexual in the Millard County *B. microphylla* (Figure 8b). Figure 8e–g provides examples of sexual tetrads, diplosporous dyads, and early onset stages of aposporous gametophyte formation as observed in this experiment.

Our greenhouse experiment exposed plants to chronic drought or drought plus heat for the 10–40 days required for flowering to occur following vernalization. Accordingly, we asked whether long-term chronic stress is required to switch apomeiosis to meiosis or whether this switch might occur rapidly, i.e., following acute stresses applied to pistils within days or even hours of apomeiosis. To answer this question, we developed a simple MS-based [26] pistil culture protocol that supported ovule development in cultured pistils from the late pre-MMC stage (1-I) through the enlarging MMC (2-II), mature MMC (2-III), meiosis (2-IV), late meiosis to functional megaspore (2-V), and early vacuolate multinucleate embryo sac (3-III) stages. Ovules of pistils cultured on basal medium (without pharmacological additives) progressed through these developmental stages within 48–72 h of culture initiation. However, development beyond the early gametophyte stage was irregular, in controls and treatments, which prevented us from evaluating in vitro the effects of metabolic modifications on later stages of gametophyte development and seed formation.

**Figure 9:**
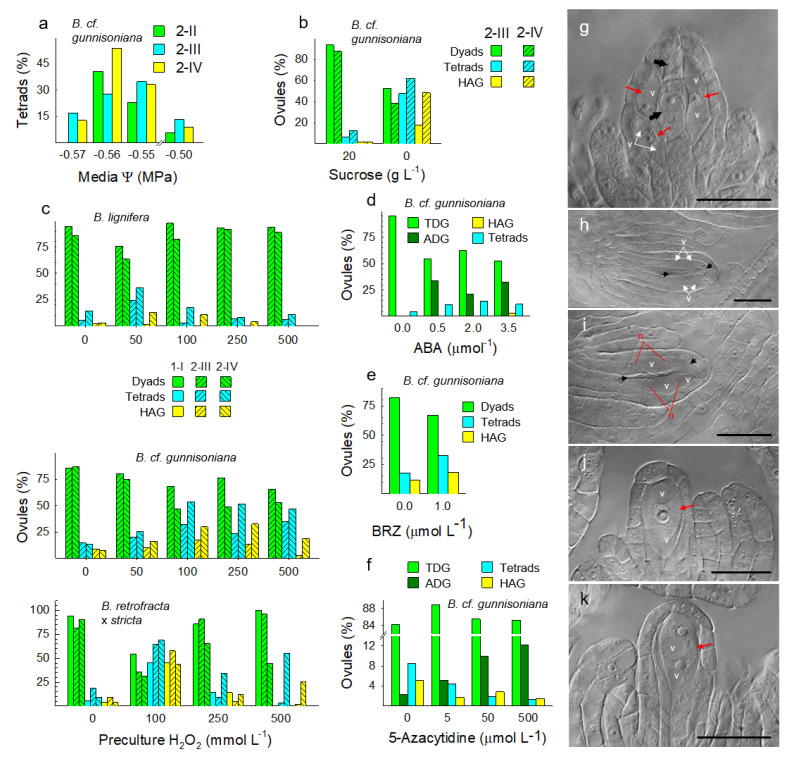
Effects of pharmacological treatments and pistil maturity on conversion from Taraxacum-type gametophyte formation (TDG) to (i) meiosis, (ii) Hieracium-type gametophyte formation (HAG), and (iii) Antennaria-type gametophyte formation (ADG): (**a**) drought (media water potential); (**b**) energy starvation; (**c**) reactive oxygen species stress (H_2_O_2_); (**d**) abscisic acid (ABA); (**e**) brassinazole (BRZ, brassinosteroid synthesis inhibitor); (**f**) 5-azacytidine (DNA methylation inhibitor); (**g**) sucrose starved *Boechera* cf. *gunnisoniana* ovule with a Taraxacum-type diplosporous dyad (black arrows) and three 1 nucleate vacuolate HAGs (red arrows; v, vacuoles); (**h**–**i**) H_2_O_2_ induced conversions from TDG formation to meiosis, but with all four megaspores degenerating (between black arrows), and from programmed cell death of nucellar cells to apospory (n, nuclei); (**j**,**k**) ABA induced conversions from TDGs to ADGs. Numbers of informative ovules per treatment (average, range): (**a**), 222, 103–289; (**b**), 81, 22-164; (**c**), *Boechera lignifera* (70, 29–168), *B.* cf. *gunnisoniana* (217, 78–465), *Boechera retrofracta × stricta* (77, 6–138); (**d**), 183, 160–201; (**e**), 102, 84–119; (**f**), 382, 178–487. Pooled chi-squares were significant (*p <* 0.001). 1-I, late pre-MMC; 2-II, enlarging MMC; 2-III, mature MMC; 2-IV, meiosis; cytology bars, 20 μm.

The TDG forming triploid *B.* cf. *gunnisoniana* responded strongly to chronic drought (Figure 8a). Hence, we chose it for acute drought experiments that involved culturing 1.5–1.9 mm long pistils, each containing 40–50 ovules at the early to late MMC stage, on low water potential media (Figure 9a). Even on control media (−0.50 MPa), frequencies of meiotic tetrads were elevated above the 2–3% background level. Apparently, excising and culturing these young desiccation sensitive pistils was itself stressful enough to switch apomeiosis to meiosis in some ovules, presumably by overwhelming stress attenuation processes in ovules of the cultured pistils. Meiotic tetrad frequencies were highest (~50%) in the −0.56 MPa treatment, and the highest tetrad frequencies occurred in pistils that contained late MMC staged ovules at the time of culture initiation. Hence, the duration of drought exposure may be less important than drought exposure occurring at the late MMC stage when meiosis or apomeiosis fates are determined. Ovules with less mature MMCs at the time of culture initiation possibly had more time in culture to restore metabolic homeostasis. Importantly, this experiment revealed that extended periods of stress are not required to reprogram genomes for meiosis. Instead, this reprogramming occurred within hours of stress induction.

Comparisons of the more apomeiotic vs. the more meiotic taxa revealed reduced molecular support for translation in ovules of apomicts (Figure 2b,f,j and Figure 4a). Moreover, these ovules were more metabolically active, were responding to ABA, and were more actively attenuating stress (Figure 6 and Figure 7). Hence, it appears that our chronic and acute drought stresses overwhelmed the stress attenuation capabilities of these apomicts and switched apomeiosis to meiosis. We next asked whether severe carbon starvation, which generates oxidative stress and terminates translation [118], might also increase apomeiosis to meiosis conversions. To address this question, we cultured pistils of *B.* cf. *gunnisoniana* (containing early to late-stage MMCs) on basal medium either with or without sucrose. Again, younger pistils on the control medium (with sucrose) produced fewer tetrads than more mature pistils (Figure 9b, compare 2-III to 2-IV results). Presumably, this occurred because cells of the younger 2-III staged pistils had more time to adjust their stress shocked metabolism to pre-excision levels prior to reaching the point of stress tolerance that defines facultativeness (apomeiosis/meiosis switching) in apomicts. In the no sucrose treatment, TDG (1st division restitution dyad) frequencies decreased by ca. 50%. This was matched by corresponding increases in meiotic tetrads, but it was also matched by HAG formation onset (Figure 9g). Frequencies of both phenomena approached 50% in ovules of the more mature pistils (Figure 9b). Hence, under severe carbon starvation, apomeiosis switched to meiosis, but the meioses in these ovules were often accompanied by HAG formation (apospory) with little or no net decrease in apomixis. We next tested whether oxidative stress (exogenously applying H_2_O_2_) would induce apomeiosis to meiosis conversions as well as diplospory to apospory conversions, i.e., switching nucellar cell programmed cell death (the norm) to HAG formation. For this experiment, we used pistils (with ovules at the MMC stage) from three apomicts, the facultative TDG forming *B. lignifera* (1.0–1.4 mm long pistils), the nearly obligate TDG forming *B.* cf. *gunnisoniana* (1.5–1.9 mm long pistils), and the facultative TDG forming *B. retrofracta × stricta* (1.6–2.1 mm long pistils). Pistils were excised and soaked for 5 min in H_2_O_2_ solutions prior to culture (Figure 9c). These H_2_O_2_ pretreatments induced up to 60% apomeiosis to meiosis conversions. However, all megaspores from many of the induced meiotic tetrads degenerated, and in many cases, this degeneration was accompanied by HAG formation (Figure 9h–i). These conversions were again most prominent in ovules of more mature pistils, suggesting that younger ovules had more time (ca. 12–24 h) to detoxify the exogenously added H_2_O_2_ before committing themselves to very different developmental paths.

The experiments reported so far illustrate the importance of plants maintaining highly stable states of metabolic homeostasis that restrict reproduction to a single sexual or apomictic type, and they suggest that apomixis occurs when oxidative stress is overly attenuated (Figure 7). Continuing with this theme, we asked whether upregulating stress attenuation by increasing ABA signaling (Figure 6 and Figure 7) might prevent meiotic prophase and shift TDG formation to ADG formation in *B.* cf. *gunnisoniana* (Figure 9d). Such treatments induced ADG formation in 25–35% of ovules (gametophyte formation directly from MMCs; Figure 9j–k). These findings place oxidative stress and oxidative stress attenuation at the center of reproductive decisions in *Boechera*, with strong and moderate stress attenuation causing ADG and TDG formation and oxidative stress causing nucellar cell destabilization followed by HAG formation.

Our expression profiling studies suggested that ABA signaling in the TDG forming *B. × formosa* reduces BR signaling at BIN2 (Figure 5d). To further evaluate this, we cultured TDG forming *B.* cf. *gunnisoniana* pistils, with ovules at the MMC stage, on media containing the BR biosynthesis inhibitor brassinazole (BRZ). This more thorough reduction in BR signaling increased frequencies of meiosis and apospory (Figure 9e), possibly by further suppressing mechanisms of oxidative stress attenuation.

Meiosis is a cell-lineage-specific process that requires a meiosis-specific transcriptome that includes meiosis-specific *de novo* RNA-directed DNA methylation (RdDM) [49]. Here, we asked whether disrupting RdDM using 5-azaC, a methyltransferase inhibitor that reduces DNA methylation [119], might prevent meiotic prophase in TDG forming *B.* cf. *gunnisoniana* but still permit gametophyte formation directly from MMCs (ADG formation). Though not as efficient as ABA, 5-azaC induced ADG formation at frequencies that were correlated with 5-azaC concentrations (Figure 9f). That both ABA and 5-azaC induce ADG formation (Figure 9d,f,j–k) is consistent with ABA-induced oxidative stress attenuation silencing meiosis-specific RdDM in germline cells (Figure 4, Figure 5, Figure 6 and Figure 7).

### 3.5. Metabolically Induced Switching from Meiosis to Apomeiosis

We demonstrated above that the apomicts studied herein chronically express stress attenuation mechanisms, but when these mechanisms are overwhelmed, apomeiosis reverts to meiosis. Here, we show that the reverse also occurs, i.e., when stress attenuation mechanisms are enhanced in sexual plants, meiosis reverts to apomeiosis. For these experiments, we chose four sexual diploid species: *A. thaliana*, *B. stricta,* and *B. exilis* of the Brassicaceae family and *V. unguiculata* of the Fabaceae family. Pistils containing MMC staged ovules were used. Pistil lengths were 0.5–1.2, 1.3–1.9, 1.5–2.3, and 1.5–1.9 mm, respectively. Dioecious *A. dioica* (Asteraceae) was used to study microsporogenesis.

**Figure 10:**
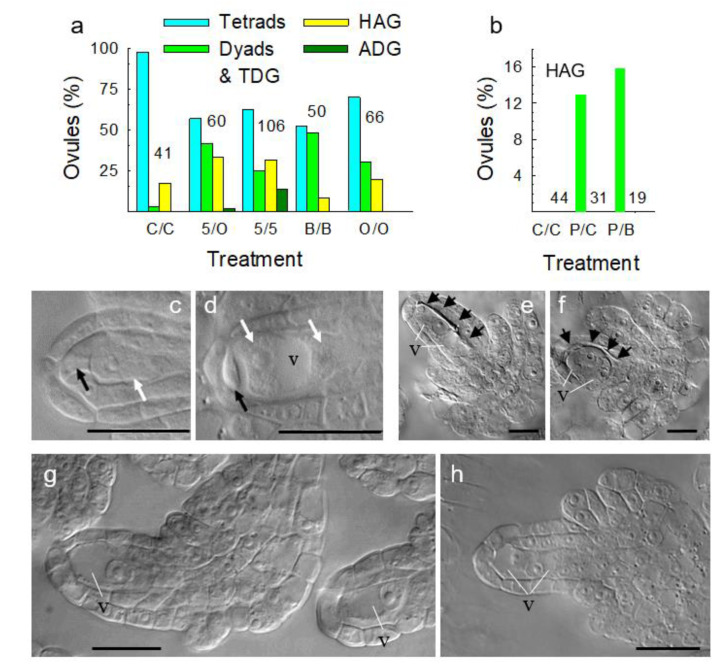
Taraxacum (TDG), Antennaria (ADG), and Hieracium (HAG) types of gametophyte formation induced in cultured *Arabidopsis thaliana* pistils: (**a**) percentages of ovules that produced sexual tetrads, sexual or TDG dyads or TDGs, HAGs and ADGs by treatment; (**b**) percentages of ovules that produced HAGs by treatment; (**c**–**d**) 1 and 2-nucleate TDGs induced by DTBA; (**e**–**f**) 1 and 2-nucleate HAGs induced by DTBA; (**g**–**h**) 1 and 2 nucleate ADGs induced by 5-azaC. 5, 5-azaC; B, brassinolide (BR); C, control; O, other (B or DTBA); P, 7 min H_2_O_2_ pretreatment. Numbers of informative ovules analyzed appear next to treatment bars in (**a**) and (**b**) (values are sums of four and two replicates, respectively). Pooled chi-squares were significant (*p <* 0.001). v, vacuole; cytology bars, 20 μm.

Treatments applied to excised *A. thaliana* pistils included a 60 min pretreatment emersion. In the controls (Figure 10a), percentages of tetrads to dyads were as expected. However, 17% of control ovules (presoaked for 60 min) contained vacuolate HAGs. This response may have been caused by anoxia induced ROS (e.g., Figure 9c). To test this, we pre-soaked sets of pistils for 7 min in water or 100 mmol L^−1^ H_2_O_2_ and then cultured them on basal medium or medium amended with 1.0 μmol L^−1^ epiBL. Water alone did not induce HAG formation, but such formation did occur in 12–15% of ovules pretreated with H_2_O_2_ (Figure 10b). Diplospory was absent in these treatments. Hence, exogenous H_2_O_2_ may have reinforced meiosis while destabilizing nucellar cells and either inducing or allowing aposporous development. The TF BZR1, which is activated by endogenous H_2_O_2_ [120], may have also been involved. These experiments indicate that nucellar cells in ovules of diplosporous and sexual plants respond similarly to H_2_O_2_ treatments in terms of inducing apospory.

To determine whether apomeiosis is inducible by reducing oxidative stress, we excised early MMC staged *A. thaliana* pistils, soaked them in the antioxidant (S)-2-aminobutane-1,4-dithiol hydrochloride (DTBA) for 60 min, and cultured them on DTBA containing medium for 4 days. In 15 of 41 scored ovules, a first division meiotic restitution occurred, which produced a dyad of megaspores (Figure 10a). The micropylar dyad member in some of these was degenerating and the chalazal dyad member was becoming vacuolate and undergoing endomitosis to form a multinucleate gametophyte (Figure 10c,d). An additional 7 of 41 scored ovules completed meiosis, but all four megaspores in these ovules were degenerating and being replaced by HAGs (Figure 10e–f).

We also asked whether BR alone would induce apomeiosis. In 24 of 50 ovules, BR induced a 1st division restitution and subsequent TDG formation. Low-level HAG formation (8%) also occurred. Adding an H_2_O_2_ pretreatment to BR-treated pistils prevented TDG formation possibly by reinforced meiosis (Figure 10b). Nevertheless, the combination of BR and H_2_O_2_ supported post-meiotic abortion coupled with HAG formation. We next tested whether 5-azaC would terminate meiosis and induce ADG formation in *A. thaliana* as it had done in *B.* cf. *gunnisoniana* (Figure 9d,j,k). Here, we soaked pre-MMC staged *A. thaliana* pistils in 5-azaC for 60 min and cultured them on control or 5-azaC medium. In 5-azaC treated pistils, 14 of 106 appropriately staged ovules were forming ADGs (Figure 10a,g,h). Of all treated pistils (Figure 10a), 64% (180 of 282) initiated either TDG, ADG, or HAG formation, i.e., all three major types of apomeiosis were induced in *A. thaliana* by metabolic modifications.

**Figure 11:**
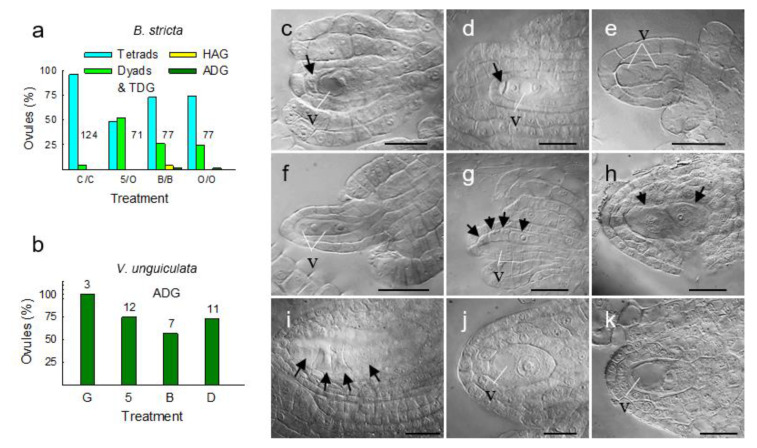
Taraxacum (TDG), Antennaria (ADG) and Hieracium (HAG) types of gametophyte formation induced in sexual *Boechera stricta* and ADG formation induced in sexual *Vigna unguiculata*: (**a**) percentages of ovules that produced sexual tetrads, TDGs, ADGs and HAGs in *B. stricta* by treatment; (**b**) percentages of ovules that produced ADGs in *V. unguiculata* by treatment; (**c**–**d**) 1 and 2 nucleate TDGs in *B. stricta*; (**e**–**f**) 1 and 2 nucleate ADG formation in *B. stricta*; (**g**) 1 nucleate HAG in *B. stricta*; (**h**–**i**) sexual dyad and tetrad formation in *V. unguiculata*; (**j**–**k**) vacuolate 1 nucleate ADG formation in *V. unguiculata*. Pretreatment/treatment acronyms: 5, 5-azaC; B, brassinolide (BR); C, control; D, DTBA; G, glucose; O, other (B or D). Numbers of informative ovules analyzed appear next to treatment bars in (**a**) and (**b**) (values are sums of two replicates). The *B. stricta* pooled chi-square was significant (*p <* 0.001). v, vacuole; cytology bars, 20 μm.

To determine if apomeiosis is inducible in other angiosperms, we exposed pistils of sexual *B. stricta* and sexual *V. unguiculata* to treatments similar to those used for *A. thaliana* (Figure 11a,b). The *A*. *thaliana* and *B*. *stricta* treatments were performed in duplicate, but with glucose and sucrose used individually as energy sources. The sugar main effect was not significant in *A. thaliana*, but TDG formation (Figure 11c–d) increased significantly in *B. stricta* when glucose was used (38 of 152 ovules vs. 26 of 164 ovules). ADG formation was only rarely observed in *B. stricta* but was induced by all treatments in *V. unguiculata* (Figure 11b,j–k). The *V. unguiculata* study included 74 pistils, but only 34 ovules were at the MMC stage at culture initiation. Of these 34, 33 had been exposed to apomeiosis-inducing treatments, of which 24 produced ADGs (73%). One MMC staged ovule had been exposed to the sucrose control, and it was meiotic.

**Figure 12:**
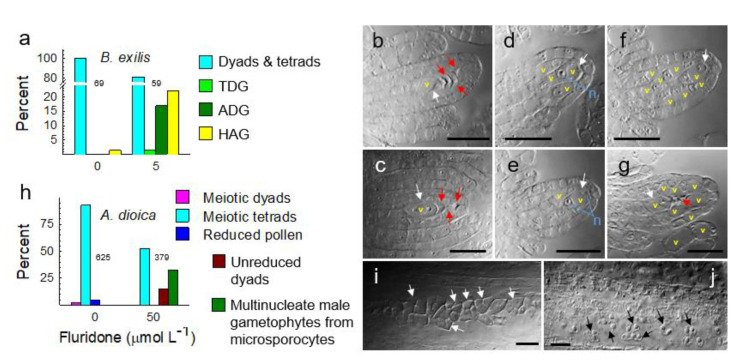
Fluridone induced unreduced gametophyte formation in ovules of *Boechera exilis* and anthers of *Antennaria dioica*: (**a**) percentages of ovules that produced Taraxacum (TDG), Antennaria (ADG) and Hieracium (HAG) types of gametophyte formation in ovules of sexual *B. exilis* when treated with fluridone; (**b**,**c**) tetrad and 2-nucleate sexual gametophyte in control pistils; (**d**,**e**) 1 and 2-nucleate ADGs in ovules of fluridone treated pistils (apospory is also occurring in these ovules); (**f**) 2-nucleate ADG and HAG formation in the same ovule of a fluridone treated pistil; (**g**) formation of multiple 1–2 nucleate HAGs in the same ovule of a fluridone treated pistil (this pistil had also successfully completed meiosis and was forming a vacuolate sexual gametophyte (white arrow)); (**h**) fluridone induced ameiotic male gametophyte formation from microsporocytes in young anthers of stem-cultured dioecious *A. dioica*; (**i**) normal microspore tetrads in anthers of control *A. dioica* plants; (**j**) multinucleate and vacuolate ameiotic 1–3 nucleate pollen grains that formed directly from microsporocytes in *A. dioica* anthers from stems that were cultured in fluridone amended medium. Numbers of informative ovules or anther meiocytes analyzed appear next to treatment bars in (**a**) and (**h**). Pooled chi-squares were significant (*p <* 0.001). red arrows, degenerating haploid megaspores; n, nucleus; v, vacuole; cytology bars, 20 μm.

ABA signaling includes oxidative stress induction by respiratory burst oxidase homologs and superoxide dismutases and subsequent oxidative stress attenuation by antioxidants. The evolution of this compensatory stress tolerance process facilitated the evolution of land plants. Here, we wondered if terminating ABA synthesis might induce apomeiosis by downregulating PP2C production, hence upregulating SnRK2,3 activities and the subsequent production of stress attenuation mechanisms (Figure 6 and Figure 7). To evaluate this, we exposed pistils of sexual *B. exilis* to 5.0 μmol L^−1^ fluridone treatments for 24–48 h before meiosis initiation (early to the mid-MMC stage). Fluridone, which inhibits ABA synthesis [121,122], induced 17%, 22%, and 2% of ovules to undergo ADG, HAG, and TDG formation, respectively (Figure 12a–g). In some ovules, ADG and HAG formation occurred simultaneously (Figure 12d–f). Evolutionarily, the nucellus is sporangial, like fern sporangia [5]. By altering ABA signaling, nucellar cells may have been released from programmed cell death, their normal fate, to pursue an ancestral fate, that of gametophyte formation.

We also tested whether chemicals that induce apomeiosis in megasporocytes might also induce apomeiosis in microsporocytes. Here we placed cut stems of male dioecious *A. dioica* (Asteraceae) in liquid MS media amended with fluridone. As was observed for MMCs, the ameiotic PMCs developed precociously into unreduced male gametophytes (Figure 12h–j). Note that the four-fold meiotically replicated and expanding tetrads of microspores crowded the anther locule in the controls (Figure 12i). In contrast, the meiotically skipped, precociously forming unreduced pollen grains of the fluridone treated plants were widely spaced throughout the locule (Figure 12j).

## 4. Discussion

### 4.1. Sucrose Non-Fermenting-Related Protein Kinases May Be Central to Meiosis Apomeiosis Switching

**Figure 13:**
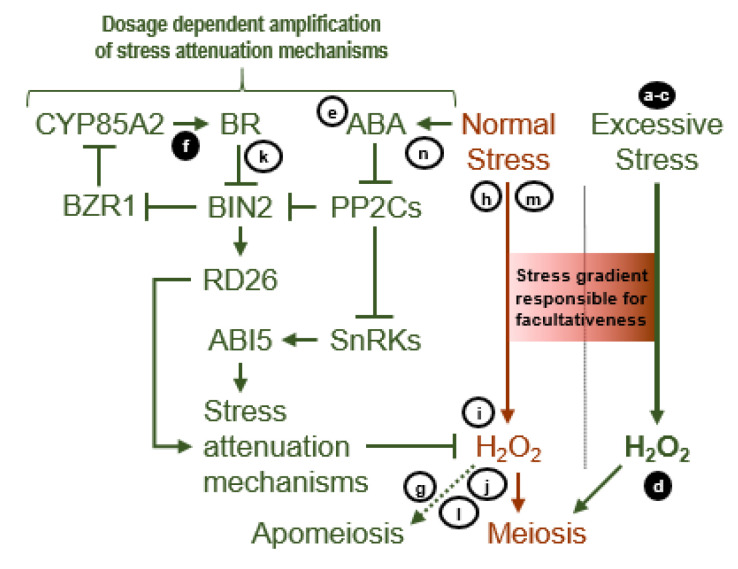
Simplified model of apomixis sex switching in plants. Green text to the left represents enhanced stress attenuation pathways in apomeiotic plants that suppress meiosis under non-stress conditions. The red text represents normal levels of stress attenuation in sexual plants, which are meiosis permissive. Green text to the right represents apomictic plants exposed to excessive stress, which overwhelms stress attenuation mechanisms and induces meiosis. White letters represent experiments that tested metabolic processes of apomeiosis to meiosis switching: **a**, heat and drought; **b**, osmotic stress; **c**, carbon starvation; **d**, H_2_O_2_; **f**, inhibition of brassinosteroid (BR) synthesis by brassinazole. Black numbers represent experiments that tested metabolic processes of meiosis to apomeiosis switching: **e**, abscisic acid (ABA); **g** and **l**, 5-azacytidine (DNA methylation suppression); **h**, anoxia (60 min emersion prior to pistil culture); **i**, H_2_O_2_; **j**, (S)-2-aminobutane-1,4-dithiol hydrochloride (antioxidant); **k**, BR; **m**, glucose; **n**, fluridone (ABA synthesis suppression). Arrows indicate upregulation or activation; blunt end lines represent downregulation or suppression; ABI5, ABA INSENSITIVE 5; BZR1, BRASSINAZOLE-RESISTANT 1; BIN2, BRASSINOSTEROID-INSENSITIVE 2; CYP85A2, BRASSINOSTEROID-6-OXIDASE 2; PP2Cs, protein phosphatase 2Cs; RD26, RESPONSIVE TO DESICCATION 26; SnRKs, sucrose non-fermenting related protein kinases.

The evolution of land plants required new water retaining and transporting morphologies, new stress detection and signaling pathways, and new processes for catabolizing toxins induced by terrestrial stresses [123,124,125]. Here, the evolution of ABA and SnRK2,3 stress response pathways was critical for increasing ROS attenuation capabilities [124]. ROS form constantly as byproducts of respiration, photosynthesis, and the activities of peroxidases and peroxisomes. However, in land plants, ROS are also produced to propagate waves of ROS signals from stressed cells to more distant cells [126]. Two ABA-induced pathways accomplish this: (i) propagation of ROS signaling through tissues via RBOHs and SODs (Figure 7) and (ii) upregulation of stress attenuation mechanisms through SnRK2,3 ABI5 signaling (Figure 6). During such signaling, H_2_O_2_ activates or inactivates redox-regulated protein phosphatases and other enzymes [127,128]. It is critical that excess H_2_O_2_ is catabolized by ROS attenuation mechanisms [94,129,130]. Metabolic processes that establish such redox homeostasis function by balancing ROS generation, from multiple sources, with ROS catabolism. We discovered, and report herein, that apomeiosis occurs when this redox balance is chronically tilted toward H_2_O_2_ catabolism, i.e., when SnRK2,3s and RD26 are activated (Figure 13). We further show that meiosis apomeiosis interconversions are inducible by applying chemicals that acutely disturb these taxa specific states of redox homeostasis.

In addition to the plant-specific SnRK2,3s (Figure 6), SnRK1 was also more active in the apomicts (Figure 4a). In eukaryotes, SnRK1 regulates growth no-growth decisions. When phosphorylated, SnRK1 dissociates the TORC1 complex [33,36,124,131,132,133,134,135], and this suppresses translation and BR regulated cell proliferation [33,120,124,136,137,138,139]. Translation downregulation in the more apomeiotic taxa (14 of 16 comparisons during early ovule development, Figure 2a,e–i and Figure 3a–g) indicates that TOR was less active in the apomicts due to increased SnRK1 activity. That stress or low energy conditions in the apomicts activated SnRK1, which suppresses TOR, is further shown by upregulated genes in the apomicts for fatty acid, amino acid, carbohydrate, and other macromolecule catabolism (Figure 2a,f,g,l, and Figure 3a–d,g).

In the apomicts, profiling revealed upregulated genes associated with signaling, stress responses, metabolism, photosynthesis, and bioenergetics (Figure 1a,e–j,l, and Figure 3a–g). Here, the *B. × formosa* population may have contained triploids, which is the common polyploid level among *Boechera* apomicts [17]. Polyploids often show distinct patterns of gene expression [140] as well as increased stress attenuation capabilities [15,141]. Increased metabolic activity seems counterintuitive given the low energy induced suppression of TOR in this taxon. However, the evolution of SnRK2,3s enabled land plants to avoid toxic levels of ROS, due to enhanced ROS attenuation processes, while maintaining growth and other metabolic processes [124,125]. Under favorable conditions, all three categories of SnRKs (SnRK1,2,3s) were largely phosphatase inactivated in the more meiotic plants by ABI1,2 and the HIGHLY ABA-INDUCED PP2C GENE 2 (AIP1). In contrast, these SnRKs appeared to remain partially active in the apomicts, which likely explains their greater ROS attenuation capacity. Switching from apomeiosis to meiosis (facultativeness) in response to stress (Figure 8 and Figure 9) suggests that ROS accumulation, due to stress treatments, exceeded ROS catabolism (Figure 13).

### 4.2. Metabolically Inducing Meiosis Apomeiosis Interconversions

Stress attenuation in plants involves the activities of ascorbic acid, ascorbate peroxidases, glutathiones, thioredoxins, catalases, superoxide dismutases (SODs), tocopherols, flavonoids, carotenoids, and ion homeostasis regulators. These are upregulated by oxidative stress, ABA-activated SnRKs, and ABA insensitive SnRKs (activated by salt, osmotic, or other stress) [124,128]. Genes encoding many of these were upregulated in ovules of the more apomeiotic taxa (Tables S2 and S5). In this respect, Shah et al. [142] provided evidence that chronic stress tolerances in apomictic *Boechera* might persist throughout the life cycle. They further suggested that this increased tolerance contributes to geographical parthenogenesis (superior stress tolerance in apomicts). We provide evidence herein that the chronic states of stress tolerance in apomicts suppress meiosis and induce apomeiosis. They might also suppress syngamy and induce parthenogenesis. This notion recently gained traction by the demonstration that parthenogenesis in rice is inducible by altering the location of expression of a wild type *APETALA2/ERF* gene [143], a member of a TF family that regulates development and stress by integrating redox, hormone, and environmental signals [144].

Using pharmacological approaches, we tested whether the differences in molecular pathways observed between apomeiotic and meiotic taxa (Figure 2, Figure 3, Figure 4, Figure 5, Figure 6 and Figure 7) are responsible for inducing meiosis or apomeiosis or are simply coincidental. To do this, we identified chemicals that perturb specific elements of metabolic homeostasis. Here, apomeiosis switched to meiosis when the chosen treatments increased oxidative stress. These included drought, energy starvation, topical H_2_O_2_ applications, and BR synthesis inhibition (Figure 9a–c,e; Figure 13a–d,f). In contrast, meiosis switched to apomeiosis when the chosen treatments relieved oxidative stress. These included glucose, BR, antioxidants, ABA, and the ABA synthesis inhibitor fluridone (Figure 10, Figure 11 and Figure 12 and Figure 13e,g–n). Additionally, 5-azaC terminated meiosis in sexual plants (Figure 10 and Figure 11), terminated meiotic prophase in TDG forming plants (Figure 9f), and permitted or induced ADG formation, directly from ameiotic MMCs, in sexual and TDG forming plants. This suggests that while RdDM may be required for meiosis [49,145], it is not required for the early stages of gametophyte formation.

When used as the sole carbon source, glucose blocked meiosis, and induced TDG formation in *B. stricta* and ADG formation in *V. unguiculata* (Figure 11b–d,j,k). In *S. cerevisiae*, glucose, which inhibits Snf1 kinase (SnRK1 in plants) and possibly activates TOR [146], also blocks meiosis [147]. Thus, in plants and yeast, meiosis is correlated with Snf1/SnRK1 inactivity and possibly TOR activity (Figure 4a). In plants, meiosis was further correlated with SnRK2,3 inactivity (reduced ABA signaling). In contrast, the more apomeiotic taxa expressed elevated levels of ABA signaling and correspondingly higher levels of antioxidant production (Figure 6 and Figure 7). Since glucose in plants also upregulates ABA synthesis [148], glucose upregulated apomeiosis in *B. stricta* and *V. unguiculata* (Figure 11) is consistent with upregulated ABA signaling followed by increased stress attenuation (Figure 13).

Surprisingly, carefully timed H_2_O_2_ treatments not only converted apomeiosis to meiosis in TDG forming *Boechera* (apomixis to sex) but also induced HAG formation (sex to apomixis) in many of the same ovules (Figure 9c). H_2_O_2_ also induced HAG formation in sexual *A. thaliana* (Figure 10b). We suggested that H_2_O_2_ induces HAG formation directly. However, treating pistils with the antioxidant DTBA also induced HAG formation (Figure 10a,e,f). Hence, it is also possible that the exogenous H_2_O_2_ treatments triggered an amplification of ROS attenuation mechanisms by upregulating ABA signaling (Figure 6 and Figure 7). Upregulated ROS catabolism may have then freed nucellar cells from redox induced programmed cell death, thus allowing them to pursue gametophyte formation.

In addition to inducing ADG and HAG formation in sexual *B. exilis* (Figure 12a–g), fluridone also induced gametophyte formation directly from PMCs (Figure 12h–j). Since ABA upregulates PP2C formation, the suppression of ABA synthesis may have indirectly increased antioxidant production downstream of SnRK2,3s (Figure 6). PMCs, MMCs, and nucellar cells share an evolutionary origin that includes homosporous meioses, e.g., as occur in sporangia of many ferns [12,149,150]. Hence, it is not surprising that metabolic disturbances that induce ADG formation from MMCs and HAG formation from nucellar cells (Figure 12a–g) can also induce gametophyte formation from PMCs (Figure 12h–j). The effects of fluridone persisted throughout the 3–4 days duration of the experiment. Accordingly, most nucellar cells and some integument cells initiated HAG formation (Figure 12d–g).

### 4.3. Apomixis Genes: Horses of a Different Color

From the early 1900s to the 1940s, common views concerning the inheritance of apomixis stemmed from Strasburger’s work, which suggested that certain sexual plants possess propensities for apomixis that are released by hybridization and polyploidization [4,151]. Starting in the late 1930s, genetic studies suggested that apomixis genes exist, which were later found to be isolated in nonrecombining genomic regions [10]. Aside from these more common views, a short-lived hypothesis (in the 1920s) was introduced by Ernst [152] who concluded that apomixis was caused by hybridization. Importantly, Ernst emphasized that apomixis propensities in parent plants are not required. In the 1990′s, Carman [13] comprehensively reviewed the genomics of known apomicts and reported highly significant correlations between apomixis and hybridization, polyploidization, and a relative absence of recent paleopolyploidization (low chromosome base numbers). Carman also concluded that hybridization was singularly important to apomixis evolution and that apomixis specific genes are not necessary. Ernst thought that phylogenetic distance between parental species determined apomixis [152]. In contrast, Carman cited physiological and biochemical examples of asynchronously expressed duplicate genes in hybrids and hypothesized that apomixis is caused by asynchronous expression of germline development genes in the ovules of certain hybrids.

The asynchrony hypothesis suggests that ecotype-driven divergences in wild type genes occurred during speciation along ecological gradients and that these new alleles modified ovule development timing. Following secondary contact hybridization, germline asynchronies were resolved in certain hybrids by eliminating meiosis and syngamy, thus producing apomixis [13]. The present paper updates this hypothesis. Here, asynchronies in germline development are replaced by dosage-dependent disparities in metabolic homeostasis (Figure 13). In terms of genetic mapping, this revision simply shifts the types of genes expected to be found in apomixis associated genomic regions from meiosis/syngamy genes to metabolic homeostasis genes. Interestingly, apomixis is much more common in land plants than in many other eukaryotes. The addition of a major ROS attenuation mechanism (ABA/SnRK2,3 signaling) possibly made apomixis-conducive states of redox homeostasis more readily attainable through hybridization and polyploidization.

It is important to address the question as to why “occasional apomixis” in sexual plants—sensu Asker and Jerling [4]—is rare. The simplest explanation is that the chronic states of stress attenuation responsible for apomixis (Figure 13, left side) are not environmentally inducible in sexual plants. Instead, such states of homeostasis are achieved only rarely through genomic aberrations associated with hybridization, recombination, or polyploidization. In contrast, states of stress attenuation in natural apomicts, induced by genomic aberrations, are variable (Figure 13, right side) such that some plants are weakly apomictic while others remain nearly obligately apomictic even when stressed.

Several interesting examples of homeostatic states of chronic stress attenuation, which possibly enforce apomixis, exist in apomicts of other kingdoms. Apomeiosis in bdelloid and monogonont rotifers (Metazoa) is mitotic-like, and the 2n eggs develop into embryos parthenogenetically. Bdelloid rotifers are obligately apomictic, and they exhibit constitutive anti-oxidant protection provided by extensively duplicated anti-oxidant genes [153,154]. Generally, in cyclical animal apomicts, it is the onset of overwhelming stress, from various factors, that cause apomictic females to apomictically produce clonal but sexually functional males and females (genders induced environmentally). These breed to produce resting eggs that hatch into apomictic females. This pattern is observed in monogonont rotifers, water fleas (*Daphnia*), aphids (Arthropoda, Metazoa) [155,156], diatoms (Chromista) [157], and green alga (Plantae) from the genera *Volvox* (Plantae). As in bdelloid rotifers, a major class of oxidative stress detoxifying enzymes, glutathione S-transferases, are upregulated during the apomictic female phase in the pea aphid *Acyrthosiphon pisum* [156]. Major oxidative stresses are also required to induce the switch from apomictic to sexual reproduction in *Daphnia* [155] and *Volvox* [158,159,160], and to induce apomixis to sex conversions in facultatively apomictic slime molds (Protozoa) [161,162,163] and brewer’s yeast (Fungi) [3]. In our experiments, attenuating oxidative stress in sexual sporocytes of *Boechera*, *Arabidopsis*, *Vigna,* and *Antennaria* caused meiosis to switch to apomeiosis. In contrast, when we overwhelmed ovules of facultatively to nearly obligately apomictic *Boechera* with oxidative stress, apomeiosis switched to meiosis. If our interpretations are correct, states of metabolic homeostasis that induce apomeiosis in extant eukaryotes are generally rare, are characterized by strong anti-oxidant properties, and are usually caused by genomic aberrations that grossly disturb the expression of wild type genes.

## 5. Conclusions

Switching from apomeiosis to meiosis in apomictic plants and from meiosis to apomeiosis in sexual plants was readily achieved through appropriate treatments. However, the treatment effects dissipated rapidly, with taxon-specific levels of cellular homeostasis apparently returning quickly to pretreatment levels. For example, the effectiveness of treatments applied 24–48 h before apomeiosis were diminished by up to 90% compared to treatments applied only a few hours prior to apomeiosis (Figure 9). Accordingly, achieving levels of cellular homeostasis that assure complete penetrance of apomeiosis (and parthenogenesis) will likely require genetic engineering modifications that acutely alter cellular levels of metabolic homeostasis throughout ovule and early seed development. Candidate genes and pathways for achieving such modifications are described herein.

Our studies shed light on two important barriers that have frustrated the development of apomixis as a technology for stabilizing hybrid vigor in crops: (i) a correct understanding of what apomixis is, and (ii) a correct understanding of what biological properties trigger meiosis/apomeiosis interconversions. Concerning the first, we have demonstrated that apomeiosis does not require mutations, meiotic or otherwise, but is instead a polyphenism of meiosis, a conserved polyphenism that may be as ancient as meiosis itself [5]. Concerning the second, we have demonstrated that the polyphenic shifts from apomeiosis to meiosis or vice versa are metabolically regulated. Here, MMCs of apomictic TDG forming taxa reverted to meiosis at high frequencies when exposed to meiosis promoting metabolic conditions. Likewise, MMCs, nucellar cells, and/or microsporocytes from five sexual taxa—representing three angiospermous families—reverted to apomeiosis at high frequencies when exposed to apomeiosis promoting metabolic conditions.

The experiments reported herein can be easily modified to further elucidate metabolic and molecular details of meiosis apomeiosis switching using florets from a single genotype, or even from a single plant. Through such studies, it may be possible to identify wild type genes that faithfully induce apomixis in crops when their levels and locations of expression are transgenically manipulated.

## Supplementary Materials

The following are available online at https://www.mdpi.com/2073-4425/11/12/1449/s1, Figure S1: Mean (±SD) lengths of pistils by ovule development stage, Figure S2: Microarrays that passed quality control analyses, Figure S3: qRT-PCR verification of microarray expression profiling, Table S1: Rules used to assign gene ontology (GO) terms to groups and subgroups, Table S2: Gene ontology (GO) groups and subgroups for all GO terms of differentially expressed genes, Table S3: Calculations for chi-square tests of independence and for the determination of standard and adjusted residuals, Table S4: Primers used for qRT-PCR verification of microarray data, Table S5: Differentially expressed genes for 17 microarray comparisons, Table S6: All gene ontology categories (GOs) of all differentially expressed genes (DEGs) by comparison, Table S7: GO terms by comparison using DEGs that were overrepresented or underrepresented, Table S8: Enriched gene ontology terms (EGOs) of comparisons, Table S9: Genes by comparison identified by keyword searches of DEG descriptions.
