# Supplementary material for: Whether Gametophytes Are Reduced or Unreduced in Angiosperms Might Be Determined Metabolically
Source: Genes (Basel). 2020 Dec 2;11(12):1449. doi: 10.3390/genes11121449 (PMC7761559; doi:10.3390/genes11121449)
Supplement: Supplementary file 1 [file genes-11-01449-s001.zip › Table S1 GO assignment rules.pdf]

Table S2. Rules used to assign gene ontology (GO) terms to groups and subgroups. Underlined terms in the excluded column indicate where the corresponding terms were assigned.

Group and subgroup	Included	Excluded
Metabolism: bioenergetics	glycolysis; TCA cycle; mitochondrial ETC; ATP synthase; mitochondrial components; glyoxysomes; fatty acid and carbohydrate synthesis, catabolism, modification and regulation processes; regulation of mitochondrial development and function; mitochondrial pyruvate transport; NAD(H) processes; starch and trehalose processes; TOR processes; ATPase activity	mitochondrial signaling; stress homeostasis; mitochondrial transcription or translation processes; mitochondrial targeting or localization (transporters); photosynthetic ferredoxin NADP processes; stress attenuating monodehydroascorbate reductase (NADH) processes; transcription regulating histone deacetylase associated NAD processes; ATPase-coupled transporter activities; fatty acid and sugar signaling, homeostasis and transport processes
Metabolism: catabolism	terms that include catabolic processes in their descriptions; autophagic processes; peroxisome and proteasome processes; ubiquitin processes; abscission and senescence processes	catabolic processes that participate actively in bioenergetics pathways; ubiquitin processes associated with cell division or transcription
Metabolism: metabolism	Metabolic processes except as noted below	metabolic processes associated with central metabolism (bioenergetics), cell cycle processes (cell division), photosynthesis, catabolism, transporter functions, protein modification, transcription, translation, signaling and stress
Metabolism: photosynthesis	photosynthesis related organelles and metabolic processes	
Metabolism: transporters	cytoplasmic and transmembrane molecule transport	transferase based molecular modifications
Regulation: protein modification	post translational modifications and processes	protein modifications associated with signaling and stress
Regulation: regulation	regulation of developmental and metabolic activities	regulation of bioenergetics, catabolism, cell division, photosynthesis, signaling, stress and transporter processes; post translational modifications and processes (protein modification)
Regulation: transcription	regulation of processes associated with transcription and gene silencing	
Regulation: translation	regulation of translation, post-transcriptional silencing, mRNA splicing and processing, and ribosome biosynthesis and function	post-translational modifications (protein modification)

Signaling and response: signaling	detection, regulation and responses to non-stress biotic and abiotic environmental factors	abscisic acid signaling, responses to stress conditions and molecules
Signaling and response: stress	detection, regulation and responses to abscisic acid, calcium, ethylene and jasmonic acid; callose synthesis; biosynthesis of antioxidants and related processes; homeostasis processes; catalase; detoxification processes; heat acclimation processes; responses to stress; defense responses	detection, regulation and responses to non-stress biotic and abiotic signaling
Development: cell division	meiotic and mitotic processes and regulation; interphase processes; chromosome repair mechanisms; gametophyte formation; reproductive processes and regulation	
Development: development	chromosomal, organellar, cellular, tissue and organ structures and developmental processes	mitochondrial (bioenergetics) and chloroplast (photosynthesis) structures and developmental processes
