# Supplementary material for: Whether Gametophytes Are Reduced or Unreduced in Angiosperms Might Be Determined Metabolically
Source: Genes (Basel). 2020 Dec 2;11(12):1449. doi: 10.3390/genes11121449 (PMC7761559; doi:10.3390/genes11121449)
Supplement: Supplementary file 1 [file genes-11-01449-s001.zip › Table S2 GO master list.pdf]

Table S2. Gene ontology (GO) groups and subgroups for all GO terms of differentially expressed genes identified by expression profiling and for GO terms identified by overrepresentation (OGO) and enrichment (EGO) analyses (see Table S1 for assignment rules used for partitioning).

Line no.	GO no.	GO group	GO subgroup	Type	GO description
1	GO:0005658	Development	Cell division	C	alpha DNA polymerase:primase complex
2	GO:0005680	Development	Cell division	C	anaphase-promoting complex
3	GO:0010997	Development	Cell division	F	anaphase-promoting complex binding
4	GO:0031145	Development	Cell division	P	anaphase-promoting complex-dependent catabolic process
5	GO:0009971	Development	Cell division	P	anastral spindle assembly involved in male meiosis
6	GO:0008356	Development	Cell division	P	asymmetric cell division
7	GO:1990939	Development	Cell division	F	ATP-dependent microtubule motor activity
8	GO:0006284	Development	Cell division	P	base-excision repair
9	GO:0097510	Development	Cell division	P	base-excision repair, AP site formation via deaminated base removal
10	GO:0090661	Development	Cell division	C	box H/ACA telomerase RNP complex
11	GO:0070531	Development	Cell division	C	BRCA1-A complex
12	GO:0070552	Development	Cell division	C	BRISC complex
13	GO:1990298	Development	Cell division	C	bub1-bub3 complex
14	GO:0015030	Development	Cell division	C	Cajal body
15	GO:0007049	Development	Cell division	P	cell cycle
16	GO:0007050	Development	Cell division	P	cell cycle arrest
17	GO:0000075	Development	Cell division	P	cell cycle checkpoint
18	GO:0044786	Development	Cell division	P	cell cycle DNA replication
19	GO:1902292	Development	Cell division	P	cell cycle DNA replication initiation
20	GO:0044843	Development	Cell division	P	cell cycle G1/S phase transition
21	GO:0044770	Development	Cell division	P	cell cycle phase transition
22	GO:0022402	Development	Cell division	P	cell cycle process
23	GO:0051301	Development	Cell division	P	cell division
24	GO:0008283	Development	Cell division	P	cell population proliferation
25	GO:0030896	Development	Cell division	C	checkpoint clamp complex
26	GO:0051026	Development	Cell division	P	chiasma assembly
27	GO:0031497	Development	Cell division	P	chromatin assembly
28	GO:0005694	Development	Cell division	C	chromosome
29	GO:0030261	Development	Cell division	P	chromosome condensation
30	GO:0070192	Development	Cell division	P	chromosome organization involved in meiotic cell cycle
31	GO:0032133	Development	Cell division	C	chromosome passenger complex
32	GO:0007059	Development	Cell division	P	chromosome segregation
33	GO:0051304	Development	Cell division	P	chromosome separation
34	GO:0000775	Development	Cell division	C	chromosome, centromeric region
35	GO:0000243	Development	Cell division	C	commitment complex
36	GO:0000793	Development	Cell division	C	condensed chromosome
37	GO:0000777	Development	Cell division	C	condensed chromosome kinetochore
38	GO:0000779	Development	Cell division	C	condensed chromosome, centromeric region
39	GO:0000794	Development	Cell division	C	condensed nuclear chromosome
40	GO:0000778	Development	Cell division	C	condensed nuclear chromosome kinetochore
41	GO:0000780	Development	Cell division	C	condensed nuclear chromosome, centromeric region
42	GO:0000796	Development	Cell division	C	condensin complex
43	GO:0030332	Development	Cell division	F	cyclin binding
44	GO:0000307	Development	Cell division	C	cyclin-dependent protein kinase holoenzyme complex
45	GO:0061575	Development	Cell division	F	cyclin-dependent protein serine/threonine kinase activator activity
46	GO:0004693	Development	Cell division	F	cyclin-dependent protein serine/threonine kinase activity
47	GO:0016538	Development	Cell division	F	cyclin-dependent protein serine/threonine kinase regulator activity
48	GO:0000910	Development	Cell division	P	cytokinesis
49	GO:0000911	Development	Cell division	P	cytokinesis by cell plate formation
50	GO:0061640	Development	Cell division	P	cytoskeleton-dependent cytokinesis
51	GO:0043625	Development	Cell division	C	delta DNA polymerase complex
52	GO:0071897	Development	Cell division	P	DNA biosynthetic process
53	GO:0003689	Development	Cell division	F	DNA clamp loader activity
54	GO:0000077	Development	Cell division	P	DNA damage checkpoint
55	GO:0080111	Development	Cell division	P	DNA demethylation
56	GO:0032508	Development	Cell division	P	DNA duplex unwinding
57	GO:0042023	Development	Cell division	P	DNA endoreduplication
58	GO:0003678	Development	Cell division	F	DNA helicase activity
59	GO:0033202	Development	Cell division	C	DNA helicase complex
60	GO:0031570	Development	Cell division	P	DNA integrity checkpoint
61	GO:0003910	Development	Cell division	F	DNA ligase (ATP) activity
62	GO:0006266	Development	Cell division	P	DNA ligation
63	GO:0051103	Development	Cell division	P	DNA ligation involved in DNA repair
64	GO:0009294	Development	Cell division	P	DNA mediated transformation
65	GO:0006323	Development	Cell division	P	DNA packaging
66	GO:0044815	Development	Cell division	C	DNA packaging complex
67	GO:0070182	Development	Cell division	F	DNA polymerase binding
68	GO:0042575	Development	Cell division	C	DNA polymerase complex
69	GO:0030337	Development	Cell division	F	DNA polymerase processivity factor activity
70	GO:0006310	Development	Cell division	P	DNA recombination
71	GO:0006281	Development	Cell division	P	DNA repair
72	GO:1990391	Development	Cell division	C	DNA repair complex
73	GO:0006260	Development	Cell division	P	DNA replication

74	GO:0000076	Development	Cell division	P	DNA replication checkpoint
75	GO:0005662	Development	Cell division	C	DNA replication factor A complex
76	GO:0005663	Development	Cell division	C	DNA replication factor C complex
77	GO:0006270	Development	Cell division	P	DNA replication initiation
78	GO:0003688	Development	Cell division	F	DNA replication origin binding
79	GO:0031261	Development	Cell division	C	DNA replication preinitiation complex
80	GO:0071163	Development	Cell division	P	DNA replication preinitiation complex assembly
81	GO:0006336	Development	Cell division	P	DNA replication-independent nucleosome assembly
82	GO:0022616	Development	Cell division	P	DNA strand elongation
83	GO:0006271	Development	Cell division	P	DNA strand elongation involved in DNA replication
84	GO:0003916	Development	Cell division	F	DNA topoisomerase activity
85	GO:0003918	Development	Cell division	F	DNA topoisomerase type II (double strand cut, ATP-hydrolyzing) activity
86	GO:0009330	Development	Cell division	C	DNA topoisomerase type II (double strand cut, ATP-hydrolyzing) complex
87	GO:0006265	Development	Cell division	P	DNA topological change
88	GO:0006268	Development	Cell division	P	DNA unwinding involved in DNA replication
89	GO:0003906	Development	Cell division	F	DNA-(apurinic or apyrimidinic site) endonuclease activity
90	GO:0006261	Development	Cell division	P	DNA-dependent DNA replication
91	GO:0003887	Development	Cell division	F	DNA-directed DNA polymerase activity
92	GO:0006302	Development	Cell division	P	double-strand break repair
93	GO:0000727	Development	Cell division	P	double-strand break repair via break-induced replication
94	GO:0000724	Development	Cell division	P	double-strand break repair via homologous recombination
95	GO:0006303	Development	Cell division	P	double-strand break repair via nonhomologous end joining
96	GO:0008309	Development	Cell division	F	double-stranded DNA exodeoxyribonuclease activity
97	GO:0010385	Development	Cell division	F	double-stranded methylated DNA binding
98	GO:0004519	Development	Cell division	F	endonuclease activity
99	GO:0001935	Development	Cell division	P	endothelial cell proliferation
100	GO:0010481	Development	Cell division	P	epidermal cell division
101	GO:0070522	Development	Cell division	C	ERCC4-ERCC1 complex
102	GO:0000132	Development	Cell division	P	establishment of mitotic spindle orientation
103	GO:0007292	Development	Cell division	P	female gamete generation
104	GO:0007143	Development	Cell division	P	female meiotic nuclear division
105	GO:0000400	Development	Cell division	F	four-way junction DNA binding
106	GO:0000082	Development	Cell division	P	G1/S transition of mitotic cell cycle
107	GO:0000086	Development	Cell division	P	G2/M transition of mitotic cell cycle
108	GO:0007276	Development	Cell division	P	gamete generation
109	GO:0048229	Development	Cell division	P	gametophyte development
110	GO:0000811	Development	Cell division	C	GINS complex
111	GO:0051880	Development	Cell division	F	G-quadruplex DNA binding
112	GO:0070652	Development	Cell division	C	HAUS complex
113	GO:0000417	Development	Cell division	C	HIR complex
114	GO:0045143	Development	Cell division	P	homologous chromosome segregation
115	GO:0035825	Development	Cell division	P	homologous recombination
116	GO:0036297	Development	Cell division	P	interstrand cross-link repair
117	GO:0000741	Development	Cell division	P	karyogamy
118	GO:0000776	Development	Cell division	C	kinetochore
119	GO:0006273	Development	Cell division	P	lagging strand elongation
120	GO:0006272	Development	Cell division	P	leading strand elongation
121	GO:0034090	Development	Cell division	P	maintenance of meiotic sister chromatid cohesion
122	GO:0034088	Development	Cell division	P	maintenance of mitotic sister chromatid cohesion
123	GO:0048232	Development	Cell division	P	male gamete generation
124	GO:0007112	Development	Cell division	P	male meiosis cytokinesis
125	GO:0007140	Development	Cell division	P	male meiotic nuclear division
126	GO:0042555	Development	Cell division	C	MCM complex
127	GO:0009561	Development	Cell division	P	megagametogenesis
128	GO:0009554	Development	Cell division	P	megasporogenesis
129	GO:0007127	Development	Cell division	P	meiosis I
130	GO:0061982	Development	Cell division	P	meiosis I cell cycle process
131	GO:0051321	Development	Cell division	P	meiotic cell cycle
132	GO:1903046	Development	Cell division	P	meiotic cell cycle process
133	GO:0010032	Development	Cell division	P	meiotic chromosome condensation
134	GO:0045132	Development	Cell division	P	meiotic chromosome segregation
135	GO:0051307	Development	Cell division	P	meiotic chromosome separation
136	GO:0033206	Development	Cell division	P	meiotic cytokinesis
137	GO:0042138	Development	Cell division	P	meiotic DNA double-strand break formation
138	GO:0000706	Development	Cell division	P	meiotic DNA double-strand break processing
139	GO:0000710	Development	Cell division	P	meiotic mismatch repair
140	GO:0140013	Development	Cell division	P	meiotic nuclear division
141	GO:0051754	Development	Cell division	P	meiotic sister chromatid cohesion, centromeric
142	GO:0055046	Development	Cell division	P	microgametogenesis
143	GO:0010480	Development	Cell division	P	microsporocyte differentiation
144	GO:0009556	Development	Cell division	P	microsporogenesis
145	GO:1902850	Development	Cell division	P	microtubule cytoskeleton organization involved in mitosis
146	GO:0003777	Development	Cell division	F	microtubule motor activity
147	GO:0008568	Development	Cell division	F	microtubule-severing ATPase activity
148	GO:0005872	Development	Cell division	C	minus-end kinesin complex
149	GO:0006298	Development	Cell division	P	mismatch repair
150	GO:0032300	Development	Cell division	C	mismatch repair complex
151	GO:0030983	Development	Cell division	F	mismatched DNA binding

152	GO:0000278	Development	Cell division	P	mitotic cell cycle
153	GO:0007093	Development	Cell division	P	mitotic cell cycle checkpoint
154	GO:0044772	Development	Cell division	P	mitotic cell cycle phase transition
155	GO:1903047	Development	Cell division	P	mitotic cell cycle process
156	GO:0033597	Development	Cell division	C	mitotic checkpoint complex
157	GO:0007076	Development	Cell division	P	mitotic chromosome condensation
158	GO:0000281	Development	Cell division	P	mitotic cytokinesis
159	GO:0044774	Development	Cell division	P	mitotic DNA integrity checkpoint
160	GO:1902969	Development	Cell division	P	mitotic DNA replication
161	GO:0033314	Development	Cell division	P	mitotic DNA replication checkpoint
162	GO:1902975	Development	Cell division	P	mitotic DNA replication initiation
163	GO:1902977	Development	Cell division	P	mitotic DNA replication preinitiation complex assembly
164	GO:0000080	Development	Cell division	P	mitotic G1 phase
165	GO:0000085	Development	Cell division	P	mitotic G2 phase
166	GO:0044818	Development	Cell division	P	mitotic G2/M transition checkpoint
167	GO:0140014	Development	Cell division	P	mitotic nuclear division
168	GO:0006312	Development	Cell division	P	mitotic recombination
169	GO:0000084	Development	Cell division	P	mitotic S phase
170	GO:0000070	Development	Cell division	P	mitotic sister chromatid segregation
171	GO:0072686	Development	Cell division	C	mitotic spindle
172	GO:0090307	Development	Cell division	P	mitotic spindle assembly
173	GO:0007094	Development	Cell division	P	mitotic spindle assembly checkpoint
174	GO:0071174	Development	Cell division	P	mitotic spindle checkpoint
175	GO:0007052	Development	Cell division	P	mitotic spindle organization
176	GO:0030870	Development	Cell division	C	Mre11 complex
177	GO:0032504	Development	Cell division	P	multicellular organism reproduction
178	GO:0032301	Development	Cell division	C	MutSalpha complex
179	GO:0045786	Development	Cell division	P	negative regulation of cell cycle
180	GO:1902750	Development	Cell division	P	negative regulation of cell cycle G2/M phase transition
181	GO:1901988	Development	Cell division	P	negative regulation of cell cycle phase transition
182	GO:0010948	Development	Cell division	P	negative regulation of cell cycle process
183	GO:0051782	Development	Cell division	P	negative regulation of cell division
184	GO:0008285	Development	Cell division	P	negative regulation of cell population proliferation
185	GO:2001251	Development	Cell division	P	negative regulation of chromosome organization
186	GO:0051985	Development	Cell division	P	negative regulation of chromosome segregation
187	GO:1905819	Development	Cell division	P	negative regulation of chromosome separation
188	GO:0045910	Development	Cell division	P	negative regulation of DNA recombination
189	GO:0010972	Development	Cell division	P	negative regulation of G2/M transition of mitotic cell cycle
190	GO:1902100	Development	Cell division	P	negative regulation of metaphase/anaphase transition of cell cycle
191	GO:0045930	Development	Cell division	P	negative regulation of mitotic cell cycle
192	GO:1901991	Development	Cell division	P	negative regulation of mitotic cell cycle phase transition
193	GO:0045841	Development	Cell division	P	negative regulation of mitotic metaphase/anaphase transition
194	GO:0045839	Development	Cell division	P	negative regulation of mitotic nuclear division
195	GO:0033048	Development	Cell division	P	negative regulation of mitotic sister chromatid segregation
196	GO:2000816	Development	Cell division	P	negative regulation of mitotic sister chromatid separation
197	GO:0051784	Development	Cell division	P	negative regulation of nuclear division
198	GO:0045128	Development	Cell division	P	negative regulation of reciprocal meiotic recombination
199	GO:2000242	Development	Cell division	P	negative regulation of reproductive process
200	GO:0033046	Development	Cell division	P	negative regulation of sister chromatid segregation
201	GO:0010213	Development	Cell division	P	non-photoreactive DNA repair
202	GO:1902315	Development	Cell division	P	nuclear cell cycle DNA replication initiation
203	GO:0098813	Development	Cell division	P	nuclear chromosome segregation
204	GO:0000280	Development	Cell division	P	nuclear division
205	GO:0033260	Development	Cell division	P	nuclear DNA replication
206	GO:0031468	Development	Cell division	P	nuclear envelope reassembly
207	GO:0007097	Development	Cell division	P	nuclear migration
208	GO:0031022	Development	Cell division	P	nuclear migration along microfilament
209	GO:0005664	Development	Cell division	C	nuclear origin of replication recognition complex
210	GO:0043596	Development	Cell division	C	nuclear replication fork
211	GO:0043224	Development	Cell division	C	nuclear SCF ubiquitin ligase complex
212	GO:0004518	Development	Cell division	F	nuclease activity
213	GO:0090304	Development	Cell division	P	nucleic acid metabolic process
214	GO:0009112	Development	Cell division	P	nucleobase metabolic process
215	GO:0006139	Development	Cell division	P	nucleobase-containing compound metabolic process
216	GO:0055086	Development	Cell division	P	nucleobase-containing small molecule metabolic process
217	GO:0033865	Development	Cell division	P	nucleoside bisphosphate metabolic process
218	GO:0009132	Development	Cell division	P	nucleoside diphosphate metabolic process
219	GO:0006165	Development	Cell division	P	nucleoside diphosphate phosphorylation
220	GO:0009116	Development	Cell division	P	nucleoside metabolic process
221	GO:1901265	Development	Cell division	F	nucleoside phosphate binding
222	GO:0006753	Development	Cell division	P	nucleoside phosphate metabolic process
223	GO:0009142	Development	Cell division	P	nucleoside triphosphate biosynthetic process
224	GO:0017110	Development	Cell division	F	nucleoside-diphosphatase activity
225	GO:0017111	Development	Cell division	F	nucleoside-triphosphatase activity
226	GO:0031492	Development	Cell division	F	nucleosomal DNA binding
227	GO:0000786	Development	Cell division	C	nucleosome
228	GO:0006334	Development	Cell division	P	nucleosome assembly
229	GO:0031491	Development	Cell division	F	nucleosome binding

230	GO:0016584	Development	Cell division	P	nucleosome positioning
231	GO:0008252	Development	Cell division	F	nucleotidase activity
232	GO:0000166	Development	Cell division	F	nucleotide binding
233	GO:0009165	Development	Cell division	P	nucleotide biosynthetic process
234	GO:0009117	Development	Cell division	P	nucleotide metabolic process
235	GO:0006289	Development	Cell division	P	nucleotide-excision repair
236	GO:0000110	Development	Cell division	C	nucleotide-excision repair factor 1 complex
237	GO:0000111	Development	Cell division	C	nucleotide-excision repair factor 2 complex
238	GO:0000717	Development	Cell division	P	nucleotide-excision repair, DNA duplex unwinding
239	GO:0033683	Development	Cell division	P	nucleotide-excision repair, DNA incision
240	GO:0006296	Development	Cell division	P	nucleotide-excision repair, DNA incision, 5'-to lesion
241	GO:0006294	Development	Cell division	P	nucleotide-excision repair, preincision complex assembly
242	GO:0009226	Development	Cell division	P	nucleotide-sugar biosynthetic process
243	GO:0009225	Development	Cell division	P	nucleotide-sugar metabolic process
244	GO:0016779	Development	Cell division	F	nucleotidyltransferase activity
245	GO:0000808	Development	Cell division	C	origin recognition complex
246	GO:0032357	Development	Cell division	F	oxidized purine DNA binding
247	GO:0043186	Development	Cell division	C	P granule
248	GO:0043626	Development	Cell division	C	PCNA complex
249	GO:0000914	Development	Cell division	P	phragmoplast assembly
250	GO:0080175	Development	Cell division	P	phragmoplast microtubule organization
251	GO:0010197	Development	Cell division	P	polar nucleus fusion
252	GO:0009846	Development	Cell division	P	pollen germination
253	GO:0048235	Development	Cell division	P	pollen sperm cell differentiation
254	GO:0048868	Development	Cell division	P	pollen tube development
255	GO:0009860	Development	Cell division	P	pollen tube growth
256	GO:0010183	Development	Cell division	P	pollen tube guidance
257	GO:0009856	Development	Cell division	P	pollination
258	GO:0045787	Development	Cell division	P	positive regulation of cell cycle
259	GO:0051781	Development	Cell division	P	positive regulation of cell division
260	GO:0008284	Development	Cell division	P	positive regulation of cell population proliferation
261	GO:1901972	Development	Cell division	P	positive regulation of DNA-5-methylcytosine glycosylase activity
262	GO:1900087	Development	Cell division	P	positive regulation of G1/S transition of mitotic cell cycle
263	GO:0045836	Development	Cell division	P	positive regulation of meiotic nuclear division
264	GO:0045931	Development	Cell division	P	positive regulation of mitotic cell cycle
265	GO:0045951	Development	Cell division	P	positive regulation of mitotic recombination
266	GO:1901969	Development	Cell division	P	positive regulation of polynucleotide 3'-phosphatase activity
267	GO:0006301	Development	Cell division	P	postreplication repair
268	GO:0006279	Development	Cell division	P	premeiotic DNA replication
269	GO:0009574	Development	Cell division	C	preprophase band
270	GO:0000913	Development	Cell division	P	preprophase band assembly
271	GO:0036388	Development	Cell division	P	pre-replicative complex assembly
272	GO:1902299	Development	Cell division	P	pre-replicative complex assembly involved in cell cycle DNA replication
273	GO:0006267	Development	Cell division	P	pre-replicative complex assembly involved in nuclear cell cycle DNA replication
274	GO:0006290	Development	Cell division	P	pyrimidine dimer repair
275	GO:0140527	Development	Cell division	F	reciprocal homologous recombination
276	GO:0007131	Development	Cell division	P	reciprocal meiotic recombination
277	GO:0048544	Development	Cell division	P	recognition of pollen
278	GO:0000725	Development	Cell division	P	recombinational repair
279	GO:0051726	Development	Cell division	P	regulation of cell cycle
280	GO:1902806	Development	Cell division	P	regulation of cell cycle G1/S phase transition
281	GO:1902749	Development	Cell division	P	regulation of cell cycle G2/M phase transition
282	GO:1901987	Development	Cell division	P	regulation of cell cycle phase transition
283	GO:0010564	Development	Cell division	P	regulation of cell cycle process
284	GO:0051302	Development	Cell division	P	regulation of cell division
285	GO:0042127	Development	Cell division	P	regulation of cell population proliferation
286	GO:1902275	Development	Cell division	P	regulation of chromatin organization
287	GO:0033044	Development	Cell division	P	regulation of chromosome organization
288	GO:0051983	Development	Cell division	P	regulation of chromosome segregation
289	GO:1905818	Development	Cell division	P	regulation of chromosome separation
290	GO:1904029	Development	Cell division	P	regulation of cyclin-dependent protein kinase activity
291	GO:0032465	Development	Cell division	P	regulation of cytokinesis
292	GO:0032875	Development	Cell division	P	regulation of DNA endoreduplication
293	GO:0051052	Development	Cell division	P	regulation of DNA metabolic process
294	GO:0000018	Development	Cell division	P	regulation of DNA recombination
295	GO:0006282	Development	Cell division	P	regulation of DNA repair
296	GO:0006275	Development	Cell division	P	regulation of DNA replication
297	GO:0090329	Development	Cell division	P	regulation of DNA-dependent DNA replication
298	GO:0030174	Development	Cell division	P	regulation of DNA-dependent DNA replication initiation
299	GO:0080155	Development	Cell division	P	regulation of double fertilization forming a zygote and endosperm
300	GO:2000779	Development	Cell division	P	regulation of double-strand break repair
301	GO:0010569	Development	Cell division	P	regulation of double-strand break repair via homologous recombination
302	GO:0010389	Development	Cell division	P	regulation of G2/M transition of mitotic cell cycle
303	GO:0051095	Development	Cell division	P	regulation of helicase activity
304	GO:0051445	Development	Cell division	P	regulation of meiotic cell cycle
305	GO:0040020	Development	Cell division	P	regulation of meiotic nuclear division
306	GO:1902099	Development	Cell division	P	regulation of metaphase/anaphase transition of cell cycle
307	GO:0007346	Development	Cell division	P	regulation of mitotic cell cycle

308	GO:1901990	Development	Cell division	P	regulation of mitotic cell cycle phase transition
309	GO:0090266	Development	Cell division	P	regulation of mitotic cell cycle spindle assembly checkpoint
310	GO:0030071	Development	Cell division	P	regulation of mitotic metaphase/anaphase transition
311	GO:0007088	Development	Cell division	P	regulation of mitotic nuclear division
312	GO:0000019	Development	Cell division	P	regulation of mitotic recombination
313	GO:0033047	Development	Cell division	P	regulation of mitotic sister chromatid segregation
314	GO:0010965	Development	Cell division	P	regulation of mitotic sister chromatid separation
315	GO:0060236	Development	Cell division	P	regulation of mitotic spindle organization
316	GO:0051783	Development	Cell division	P	regulation of nuclear division
317	GO:0033045	Development	Cell division	P	regulation of sister chromatid segregation
318	GO:0090224	Development	Cell division	P	regulation of spindle organization
319	GO:2000035	Development	Cell division	P	regulation of stem cell division
320	GO:0032204	Development	Cell division	P	regulation of telomere maintenance
321	GO:0005657	Development	Cell division	C	replication fork
322	GO:0048478	Development	Cell division	P	replication fork protection
323	GO:0031298	Development	Cell division	C	replication fork protection complex
324	GO:0030894	Development	Cell division	C	replisome
325	GO:0000003	Development	Cell division	P	reproduction
326	GO:0022414	Development	Cell division	P	reproductive process
327	GO:0000712	Development	Cell division	P	resolution of meiotic recombination intermediates
328	GO:0043021	Development	Cell division	F	ribonucleoprotein complex binding
329	GO:0032549	Development	Cell division	F	ribonucleoside binding
330	GO:0033875	Development	Cell division	P	ribonucleoside bisphosphate metabolic process
331	GO:0005971	Development	Cell division	C	ribonucleoside-diphosphate reductase complex
332	GO:0009259	Development	Cell division	P	ribonucleotide metabolic process
333	GO:0004749	Development	Cell division	F	ribose phosphate diphosphokinase activity
334	GO:0002189	Development	Cell division	C	ribose phosphate diphosphokinase complex
335	GO:0019693	Development	Cell division	P	ribose phosphate metabolic process
336	GO:0004751	Development	Cell division	F	ribose-5-phosphate isomerase activity
337	GO:0004750	Development	Cell division	F	ribulose-phosphate 3-epimerase activity
338	GO:0016070	Development	Cell division	P	RNA metabolic process
339	GO:0019005	Development	Cell division	C	SCF ubiquitin ligase complex
340	GO:0019953	Development	Cell division	P	sexual reproduction
341	GO:0007338	Development	Cell division	P	single fertilization
342	GO:0000012	Development	Cell division	P	single strand break repair
343	GO:1990518	Development	Cell division	F	single-stranded 3'-5' DNA helicase activity
344	GO:0003697	Development	Cell division	F	single-stranded DNA binding
345	GO:0000014	Development	Cell division	F	single-stranded DNA endodeoxyribonuclease activity
346	GO:0043047	Development	Cell division	F	single-stranded telomeric DNA binding
347	GO:0007062	Development	Cell division	P	sister chromatid cohesion
348	GO:0000819	Development	Cell division	P	sister chromatid segregation
349	GO:0035861	Development	Cell division	C	site of double-strand break
350	GO:0032116	Development	Cell division	C	SMC loading complex
351	GO:0005819	Development	Cell division	C	spindle
352	GO:0051225	Development	Cell division	P	spindle assembly
353	GO:0071173	Development	Cell division	P	spindle assembly checkpoint
354	GO:0031577	Development	Cell division	P	spindle checkpoint
355	GO:0005876	Development	Cell division	C	spindle microtubule
356	GO:0051233	Development	Cell division	C	spindle midzone
357	GO:0007051	Development	Cell division	P	spindle organization
358	GO:0000922	Development	Cell division	C	spindle pole
359	GO:0031616	Development	Cell division	C	spindle pole centrosome
360	GO:0098973	Development	Cell division	F	structural constituent of postsynaptic actin cytoskeleton
361	GO:0098725	Development	Cell division	P	symmetric cell division
362	GO:0007129	Development	Cell division	P	synapsis
363	GO:0007130	Development	Cell division	P	synaptonemal complex assembly
364	GO:0006949	Development	Cell division	P	syncytium formation
365	GO:0010198	Development	Cell division	P	synergid death
366	GO:0043765	Development	Cell division	F	T/G mismatch-specific endonuclease activity
367	GO:0005697	Development	Cell division	C	telomerase holoenzyme complex
368	GO:0016233	Development	Cell division	P	telomere capping
369	GO:0000723	Development	Cell division	P	telomere maintenance
370	GO:0000722	Development	Cell division	P	telomere maintenance via recombination
371	GO:0007004	Development	Cell division	P	telomere maintenance via telomerase
372	GO:0010833	Development	Cell division	P	telomere maintenance via telomere lengthening
373	GO:0032200	Development	Cell division	P	telomere organization
374	GO:0042162	Development	Cell division	F	telomeric DNA binding
375	GO:0019079	Development	Cell division	P	viral genome replication
376	GO:0071942	Development	Cell division	C	XPC complex
377	GO:0010158	Development	Development	P	abaxial cell fate specification
378	GO:0030479	Development	Development	C	actin cortical patch
379	GO:0015629	Development	Development	C	actin cytoskeleton
380	GO:0030036	Development	Development	P	actin cytoskeleton organization
381	GO:0005884	Development	Development	C	actin filament
382	GO:0032432	Development	Development	C	actin filament bundle
383	GO:0042643	Development	Development	C	actomyosin, actin portion
384	GO:0009943	Development	Development	P	adaxial/abaxial axis specification
385	GO:0009955	Development	Development	P	adaxial/abaxial pattern specification

386	GO:0048830	Development	Development	P	adventitious root development
387	GO:0009501	Development	Development	C	amyloplast
388	GO:0048856	Development	Development	P	anatomical structure development
389	GO:0048646	Development	Development	P	anatomical structure formation involved in morphogenesis
390	GO:0060249	Development	Development	P	anatomical structure homeostasis
391	GO:0009653	Development	Development	P	anatomical structure morphogenesis
392	GO:0031225	Development	Development	C	anchored component of membrane
393	GO:0046658	Development	Development	C	anchored component of plasma membrane
394	GO:0009887	Development	Development	P	animal organ morphogenesis
395	GO:0051211	Development	Development	P	anisotropic cell growth
396	GO:0009901	Development	Development	P	anther dehiscence
397	GO:0048653	Development	Development	P	anther development
398	GO:0048657	Development	Development	P	anther wall tapetum cell differentiation
399	GO:0048658	Development	Development	P	anther wall tapetum development
400	GO:0045177	Development	Development	C	apical part of cell
401	GO:0016324	Development	Development	C	apical plasma membrane
402	GO:0048046	Development	Development	C	apoplast
403	GO:0090506	Development	Development	P	axillary shoot meristem initiation
404	GO:0009925	Development	Development	C	basal plasma membrane
405	GO:0098588	Development	Development	C	bounding membrane of organelle
406	GO:0090610	Development	Development	P	bundle sheath cell fate specification
407	GO:0048440	Development	Development	P	carpel development
408	GO:0048226	Development	Development	C	Casparian strip
409	GO:0005901	Development	Development	C	caveola
410	GO:0005623	Development	Development	C	cell
411	GO:0007155	Development	Development	P	cell adhesion
412	GO:0005938	Development	Development	C	cell cortex
413	GO:0048468	Development	Development	P	cell development
414	GO:0030154	Development	Development	P	cell differentiation
415	GO:0045165	Development	Development	P	cell fate commitment
416	GO:0001709	Development	Development	P	cell fate determination
417	GO:0001708	Development	Development	P	cell fate specification
418	GO:0016049	Development	Development	P	cell growth
419	GO:0030054	Development	Development	C	cell junction
420	GO:0000902	Development	Development	P	cell morphogenesis
421	GO:0000904	Development	Development	P	cell morphogenesis involved in differentiation
422	GO:0071944	Development	Development	C	cell periphery
423	GO:0009504	Development	Development	C	cell plate
424	GO:0000919	Development	Development	P	cell plate assembly
425	GO:0009920	Development	Development	P	cell plate formation involved in plant-type cell wall biogenesis
426	GO:0060187	Development	Development	C	cell pole
427	GO:0042995	Development	Development	C	cell projection
428	GO:0009986	Development	Development	C	cell surface
429	GO:0051286	Development	Development	C	cell tip
430	GO:0009932	Development	Development	P	cell tip growth
431	GO:0005618	Development	Development	C	cell wall
432	GO:0042546	Development	Development	P	cell wall biogenesis
433	GO:0042545	Development	Development	P	cell wall modification
434	GO:0042547	Development	Development	P	cell wall modification involved in multidimensional cell growth
435	GO:0071555	Development	Development	P	cell wall organization
436	GO:0071554	Development	Development	P	cell wall organization or biogenesis
437	GO:0052546	Development	Development	P	cell wall pectin metabolic process
438	GO:0005911	Development	Development	C	cell-cell junction
439	GO:0007043	Development	Development	P	cell-cell junction assembly
440	GO:0110165	Development	Development	C	cellular anatomical entity
441	GO:0022607	Development	Development	P	cellular component assembly
442	GO:0010927	Development	Development	P	cellular component assembly involved in morphogenesis
443	GO:0044085	Development	Development	P	cellular component biogenesis
444	GO:0032989	Development	Development	P	cellular component morphogenesis
445	GO:0016043	Development	Development	P	cellular component organization
446	GO:0071840	Development	Development	P	cellular component organization or biogenesis
447	GO:0048869	Development	Development	P	cellular developmental process
448	GO:0051641	Development	Development	P	cellular localization
449	GO:0009987	Development	Development	P	cellular process
450	GO:0005575	Development	Development	C	cellular_component
451	GO:0042807	Development	Development	C	central vacuole
452	GO:0098653	Development	Development	P	centromere clustering
453	GO:0034508	Development	Development	P	centromere complex assembly
454	GO:0005813	Development	Development	C	centrosome
455	GO:0007098	Development	Development	P	centrosome cycle
456	GO:0000785	Development	Development	C	chromatin
457	GO:0006333	Development	Development	P	chromatin assembly or disassembly
458	GO:0006325	Development	Development	P	chromatin organization
459	GO:0010369	Development	Development	C	chromocenter
460	GO:0009661	Development	Development	P	chromoplast organization
461	GO:0098687	Development	Development	C	chromosomal region
462	GO:0051276	Development	Development	P	chromosome organization
463	GO:0000781	Development	Development	C	chromosome, telomeric region

464	GO:0003013	Development	Development	P	circulatory system process
465	GO:0005801	Development	Development	C	cis-Golgi network
466	GO:0048268	Development	Development	P	clathrin coat assembly
467	GO:0072318	Development	Development	P	clathrin coat disassembly
468	GO:0030132	Development	Development	C	clathrin coat of coated pit
469	GO:0030130	Development	Development	C	clathrin coat of trans-Golgi network vesicle
470	GO:0032050	Development	Development	F	clathrin heavy chain binding
471	GO:0030125	Development	Development	C	clathrin vesicle coat
472	GO:0045334	Development	Development	C	clathrin-coated endocytic vesicle
473	GO:0005905	Development	Development	C	clathrin-coated pit
474	GO:0030136	Development	Development	C	clathrin-coated vesicle
475	GO:0072583	Development	Development	P	clathrin-dependent endocytosis
476	GO:0043674	Development	Development	C	columella
477	GO:0030864	Development	Development	C	cortical actin cytoskeleton
478	GO:0030863	Development	Development	C	cortical cytoskeleton
479	GO:0030865	Development	Development	P	cortical cytoskeleton organization
480	GO:0055028	Development	Development	C	cortical microtubule
481	GO:0030981	Development	Development	C	cortical microtubule cytoskeleton
482	GO:0043622	Development	Development	P	cortical microtubule organization
483	GO:0010005	Development	Development	C	cortical microtubule, transverse to long axis
484	GO:0048825	Development	Development	P	cotyledon development
485	GO:0048826	Development	Development	P	cotyledon morphogenesis
486	GO:0010588	Development	Development	P	cotyledon vascular tissue pattern formation
487	GO:0042335	Development	Development	P	cuticle development
488	GO:0005737	Development	Development	C	cytoplasm
489	GO:0005881	Development	Development	C	cytoplasmic microtubule
490	GO:0031122	Development	Development	P	cytoplasmic microtubule organization
491	GO:0009898	Development	Development	C	cytoplasmic side of plasma membrane
492	GO:0008092	Development	Development	F	cytoskeletal protein binding
493	GO:0005856	Development	Development	C	cytoskeleton
494	GO:0007010	Development	Development	P	cytoskeleton organization
495	GO:0005829	Development	Development	C	cytosol
496	GO:0009855	Development	Development	P	determination of bilateral symmetry
497	GO:0048588	Development	Development	P	developmental cell growth
498	GO:0048589	Development	Development	P	developmental growth
499	GO:0060560	Development	Development	P	developmental growth involved in morphogenesis
500	GO:0032502	Development	Development	P	developmental process
501	GO:0003006	Development	Development	P	developmental process involved in reproduction
502	GO:0080186	Development	Development	P	developmental vegetative growth
503	GO:0009567	Development	Development	P	double fertilization forming a zygote and endosperm
504	GO:0009790	Development	Development	P	embryo development
505	GO:0009793	Development	Development	P	embryo development ending in seed dormancy
506	GO:0009558	Development	Development	P	embryo sac cellularization
507	GO:0009559	Development	Development	P	embryo sac central cell differentiation
508	GO:0009553	Development	Development	P	embryo sac development
509	GO:0000578	Development	Development	P	embryonic axis specification
510	GO:0048598	Development	Development	P	embryonic morphogenesis
511	GO:0012505	Development	Development	C	endomembrane system
512	GO:0010256	Development	Development	P	endomembrane system organization
513	GO:0005788	Development	Development	C	endoplasmic reticulum lumen
514	GO:0016320	Development	Development	P	endoplasmic reticulum membrane fusion
515	GO:0090158	Development	Development	P	endoplasmic reticulum membrane organization
516	GO:0007029	Development	Development	P	endoplasmic reticulum organization
517	GO:0044432	Development	Development	C	endoplasmic reticulum part
518	GO:0098827	Development	Development	C	endoplasmic reticulum subcompartment
519	GO:0071782	Development	Development	C	endoplasmic reticulum tubular network
520	GO:0071786	Development	Development	P	endoplasmic reticulum tubular network organization
521	GO:0005793	Development	Development	C	endoplasmic reticulum-Golgi intermediate compartment
522	GO:0061817	Development	Development	P	endoplasmic reticulum-plasma membrane tethering
523	GO:0009960	Development	Development	P	endosperm development
524	GO:0031975	Development	Development	C	envelope
525	GO:0009913	Development	Development	P	epidermal cell differentiation
526	GO:0009957	Development	Development	P	epidermal cell fate specification
527	GO:0000923	Development	Development	C	equatorial microtubule organizing center
528	GO:0010168	Development	Development	C	ER body
529	GO:0000815	Development	Development	C	ESCRT III complex
530	GO:0007164	Development	Development	P	establishment of tissue polarity
531	GO:0007163	Development	Development	P	establishment or maintenance of cell polarity
532	GO:0030951	Development	Development	P	establishment or maintenance of microtubule cytoskeleton polarity
533	GO:0009513	Development	Development	C	etioplast
534	GO:0034426	Development	Development	C	etioplast membrane
535	GO:0000145	Development	Development	C	exocyst
536	GO:0030312	Development	Development	C	external encapsulating structure
537	GO:0045229	Development	Development	P	external encapsulating structure organization
538	GO:0009897	Development	Development	C	external side of plasma membrane
539	GO:0070062	Development	Development	C	extracellular exosome
540	GO:0031012	Development	Development	C	extracellular matrix
541	GO:0030198	Development	Development	P	extracellular matrix organization

542	GO:0005576	Development	Development	C	extracellular region
543	GO:0005615	Development	Development	C	extracellular space
544	GO:0031234	Development	Development	C	extrinsic component of cytoplasmic side of plasma membrane
545	GO:0042406	Development	Development	C	extrinsic component of endoplasmic reticulum membrane
546	GO:0010582	Development	Development	P	floral meristem determinacy
547	GO:0048437	Development	Development	P	floral organ development
548	GO:0048449	Development	Development	P	floral organ formation
549	GO:0048444	Development	Development	P	floral organ morphogenesis
550	GO:0048438	Development	Development	P	floral whorl development
551	GO:0009908	Development	Development	P	flower development
552	GO:0048041	Development	Development	P	focal adhesion assembly
553	GO:0010154	Development	Development	P	fruit development
554	GO:1990058	Development	Development	P	fruit replum development
555	GO:0009835	Development	Development	P	fruit ripening
556	GO:0080127	Development	Development	P	fruit septum development
557	GO:0000930	Development	Development	C	gamma-tubulin complex
558	GO:0033566	Development	Development	P	gamma-tubulin complex localization
559	GO:0008274	Development	Development	C	gamma-tubulin ring complex
560	GO:0000137	Development	Development	C	Golgi cis cisterna
561	GO:0031985	Development	Development	C	Golgi cisterna
562	GO:0005796	Development	Development	C	Golgi lumen
563	GO:0005797	Development	Development	C	Golgi medial cisterna
564	GO:0007030	Development	Development	P	Golgi organization
565	GO:0005795	Development	Development	C	Golgi stack
566	GO:0098791	Development	Development	C	Golgi subcompartment
567	GO:0000138	Development	Development	C	Golgi trans cisterna
568	GO:0030660	Development	Development	C	Golgi-associated vesicle membrane
569	GO:0006505	Development	Development	P	GPI anchor metabolic process
570	GO:0090391	Development	Development	P	granum assembly
571	GO:0035838	Development	Development	C	growing cell tip
572	GO:0040007	Development	Development	P	growth
573	GO:0010052	Development	Development	P	guard cell differentiation
574	GO:0010442	Development	Development	P	guard cell morphogenesis
575	GO:0010444	Development	Development	P	guard mother cell differentiation
576	GO:0048467	Development	Development	P	gynoecium development
577	GO:0010410	Development	Development	P	hemicellulose metabolic process
578	GO:0000792	Development	Development	C	heterochromatin
579	GO:0031507	Development	Development	P	heterochromatin assembly
580	GO:0070828	Development	Development	P	heterochromatin organization
581	GO:0043657	Development	Development	C	host cell
582	GO:0042025	Development	Development	C	host cell nucleus
583	GO:0033643	Development	Development	C	host cell part
584	GO:0018995	Development	Development	C	host cellular component
585	GO:0033648	Development	Development	C	host intracellular membrane-bounded organelle
586	GO:0033647	Development	Development	C	host intracellular organelle
587	GO:0033646	Development	Development	C	host intracellular part
588	GO:0043656	Development	Development	C	host intracellular region
589	GO:0048283	Development	Development	P	indeterminate inflorescence morphogenesis
590	GO:0010229	Development	Development	P	inflorescence development
591	GO:0010450	Development	Development	P	inflorescence meristem growth
592	GO:0048281	Development	Development	P	inflorescence morphogenesis
593	GO:0071458	Development	Development	C	integral component of cytoplasmic side of endoplasmic reticulum membrane
594	GO:0030176	Development	Development	C	integral component of endoplasmic reticulum membrane
595	GO:0071556	Development	Development	C	integral component of lumenal side of endoplasmic reticulum membrane
596	GO:0016021	Development	Development	C	integral component of membrane
597	GO:0005887	Development	Development	C	integral component of plasma membrane
598	GO:0080060	Development	Development	P	integument development
599	GO:0031021	Development	Development	C	interphase microtubule organizing center
600	GO:0005622	Development	Development	C	intracellular
601	GO:0043231	Development	Development	C	intracellular membrane-bounded organelle
602	GO:0043232	Development	Development	C	intracellular non-membrane-bounded organelle
603	GO:0043229	Development	Development	C	intracellular organelle
604	GO:0070013	Development	Development	C	intracellular organelle lumen
605	GO:0097708	Development	Development	C	intracellular vesicle
606	GO:0070676	Development	Development	P	intralumenal vesicle formation
607	GO:0005828	Development	Development	C	kinetochore microtubule
608	GO:0005770	Development	Development	C	late endosome
609	GO:0031902	Development	Development	C	late endosome membrane
610	GO:0016328	Development	Development	C	lateral plasma membrane
611	GO:0048527	Development	Development	P	lateral root development
612	GO:0010311	Development	Development	P	lateral root formation
613	GO:0010102	Development	Development	P	lateral root morphogenesis
614	GO:0048366	Development	Development	P	leaf development
615	GO:0010338	Development	Development	P	leaf formation
616	GO:0009965	Development	Development	P	leaf morphogenesis
617	GO:0060772	Development	Development	P	leaf phyllotactic patterning
618	GO:0010358	Development	Development	P	leaf shaping
619	GO:0010305	Development	Development	P	leaf vascular tissue pattern formation

620	GO:0005811	Development	Development	C	lipid droplet
621	GO:0009942	Development	Development	P	longitudinal axis specification
622	GO:0005765	Development	Development	C	lysosomal membrane
623	GO:0000323	Development	Development	C	lytic vacuole
624	GO:0000327	Development	Development	C	lytic vacuole within protein storage vacuole
625	GO:0010076	Development	Development	P	maintenance of floral meristem identity
626	GO:0048497	Development	Development	P	maintenance of floral organ identity
627	GO:0010077	Development	Development	P	maintenance of inflorescence meristem identity
628	GO:0010074	Development	Development	P	maintenance of meristem identity
629	GO:0090700	Development	Development	P	maintenance of plant organ identity
630	GO:0010078	Development	Development	P	maintenance of root meristem identity
631	GO:0010492	Development	Development	P	maintenance of shoot apical meristem identity
632	GO:0080173	Development	Development	P	male-female gamete recognition during double fertilization forming a zygote and endosperm
633	GO:0016020	Development	Development	C	membrane
634	GO:0061025	Development	Development	P	membrane fusion
635	GO:0061024	Development	Development	P	membrane organization
636	GO:0098796	Development	Development	C	membrane protein complex
637	GO:0043227	Development	Development	C	membrane-bounded organelle
638	GO:0031974	Development	Development	C	membrane-enclosed lumen
639	GO:0010022	Development	Development	P	meristem determinacy
640	GO:0048507	Development	Development	P	meristem development
641	GO:0035266	Development	Development	P	meristem growth
642	GO:0010014	Development	Development	P	meristem initiation
643	GO:0010073	Development	Development	P	meristem maintenance
644	GO:0009933	Development	Development	P	meristem structural organization
645	GO:0042579	Development	Development	C	microbody
646	GO:0031903	Development	Development	C	microbody membrane
647	GO:0005874	Development	Development	C	microtubule
648	GO:0005875	Development	Development	C	microtubule associated complex
649	GO:0001578	Development	Development	P	microtubule bundle formation
650	GO:0015630	Development	Development	C	microtubule cytoskeleton
651	GO:0000226	Development	Development	P	microtubule cytoskeleton organization
652	GO:0007020	Development	Development	P	microtubule nucleation
653	GO:0051415	Development	Development	P	microtubule nucleation by interphase microtubule organizing center
654	GO:0051418	Development	Development	P	microtubule nucleation by microtubule organizing center
655	GO:0005815	Development	Development	C	microtubule organizing center
656	GO:0035371	Development	Development	C	microtubule plus-end
657	GO:0046785	Development	Development	P	microtubule polymerization
658	GO:0007018	Development	Development	P	microtubule-based movement
659	GO:0007017	Development	Development	P	microtubule-based process
660	GO:0012511	Development	Development	C	monolayer-surrounded lipid storage body
661	GO:0042814	Development	Development	P	monopolar cell growth
662	GO:0006928	Development	Development	P	movement of cell or subcellular component
663	GO:0080001	Development	Development	P	mucilage extrusion from seed coat
664	GO:0007275	Development	Development	P	multicellular organism development
665	GO:0035264	Development	Development	P	multicellular organism growth
666	GO:0009825	Development	Development	P	multidimensional cell growth
667	GO:0044706	Development	Development	P	multi-multicellular organism process
668	GO:0005771	Development	Development	C	multivesicular body
669	GO:0016459	Development	Development	C	myosin complex
670	GO:0071836	Development	Development	P	nectar secretion
671	GO:0043228	Development	Development	C	non-membrane-bounded organelle
672	GO:0016604	Development	Development	C	nuclear body
673	GO:0005846	Development	Development	C	nuclear cap binding complex
674	GO:0000790	Development	Development	C	nuclear chromatin
675	GO:0000228	Development	Development	C	nuclear chromosome
676	GO:0000784	Development	Development	C	nuclear chromosome, telomeric region
677	GO:0000799	Development	Development	C	nuclear condensin complex
678	GO:0010445	Development	Development	C	nuclear dicing body
679	GO:0005635	Development	Development	C	nuclear envelope
680	GO:0005719	Development	Development	C	nuclear euchromatin
681	GO:0000176	Development	Development	C	nuclear exosome (RNase complex)
682	GO:0005652	Development	Development	C	nuclear lamina
683	GO:0031981	Development	Development	C	nuclear lumen
684	GO:0016363	Development	Development	C	nuclear matrix
685	GO:0031965	Development	Development	C	nuclear membrane
686	GO:0000818	Development	Development	C	nuclear MIS12/MIND complex
687	GO:0005640	Development	Development	C	nuclear outer membrane
688	GO:0042175	Development	Development	C	nuclear outer membrane-endoplasmic reticulum membrane network
689	GO:0034399	Development	Development	C	nuclear periphery
690	GO:0005643	Development	Development	C	nuclear pore
691	GO:0044611	Development	Development	C	nuclear pore inner ring
692	GO:0006999	Development	Development	P	nuclear pore organization
693	GO:0031080	Development	Development	C	nuclear pore outer ring
694	GO:0005656	Development	Development	C	nuclear pre-replicative complex
695	GO:0043601	Development	Development	C	nuclear replisome
696	GO:0090305	Development	Development	P	nucleic acid phosphodiester bond hydrolysis

697	GO:0009295	Development	Development	C	nucleoid
698	GO:0007000	Development	Development	P	nucleolus organization
699	GO:0005654	Development	Development	C	nucleoplasm
700	GO:0005634	Development	Development	C	nucleus
701	GO:0006997	Development	Development	P	nucleus organization
702	GO:0035265	Development	Development	P	organ growth
703	GO:0043226	Development	Development	C	organelle
704	GO:0070925	Development	Development	P	organelle assembly
705	GO:0031967	Development	Development	C	organelle envelope
706	GO:0031970	Development	Development	C	organelle envelope lumen
707	GO:0048285	Development	Development	P	organelle fission
708	GO:0019866	Development	Development	C	organelle inner membrane
709	GO:0051640	Development	Development	P	organelle localization
710	GO:0043233	Development	Development	C	organelle lumen
711	GO:0031090	Development	Development	C	organelle membrane
712	GO:0006996	Development	Development	P	organelle organization
713	GO:0031984	Development	Development	C	organelle subcompartment
714	GO:0007389	Development	Development	P	pattern specification process
715	GO:0048471	Development	Development	C	perinuclear region of cytoplasm
716	GO:0048441	Development	Development	P	petal development
717	GO:0080172	Development	Development	P	petal epidermis patterning
718	GO:0048446	Development	Development	P	petal morphogenesis
719	GO:0045335	Development	Development	C	phagocytic vesicle
720	GO:0010088	Development	Development	P	phloem development
721	GO:0090449	Development	Development	P	phloem glucosinolate loading
722	GO:0110126	Development	Development	P	phloem loading
723	GO:0010087	Development	Development	P	phloem or xylem histogenesis
724	GO:0000719	Development	Development	P	photoreactive repair
725	GO:0009524	Development	Development	C	phragmoplast
726	GO:0048827	Development	Development	P	phyllome development
727	GO:0060771	Development	Development	P	phyllotactic patterning
728	GO:0090627	Development	Development	P	plant epidermal cell differentiation
729	GO:0090626	Development	Development	P	plant epidermis morphogenesis
730	GO:0099402	Development	Development	P	plant organ development
731	GO:1905392	Development	Development	P	plant organ morphogenesis
732	GO:0048481	Development	Development	P	plant ovule development
733	GO:0009505	Development	Development	C	plant-type cell wall
734	GO:0009832	Development	Development	P	plant-type cell wall biogenesis
735	GO:0009828	Development	Development	P	plant-type cell wall loosening
736	GO:0009827	Development	Development	P	plant-type cell wall modification
737	GO:0009831	Development	Development	P	plant-type cell wall modification involved in multidimensional cell growth
738	GO:0009664	Development	Development	P	plant-type cell wall organization
739	GO:0071669	Development	Development	P	plant-type cell wall organization or biogenesis
740	GO:0035670	Development	Development	P	plant-type ovary development
741	GO:0009833	Development	Development	P	plant-type primary cell wall biogenesis
742	GO:0009834	Development	Development	P	plant-type secondary cell wall biogenesis
743	GO:0000325	Development	Development	C	plant-type vacuole
744	GO:0009705	Development	Development	C	plant-type vacuole membrane
745	GO:0005886	Development	Development	C	plasma membrane
746	GO:0120025	Development	Development	C	plasma membrane bounded cell projection
747	GO:0007009	Development	Development	P	plasma membrane organization
748	GO:0098590	Development	Development	C	plasma membrane region
749	GO:0009663	Development	Development	P	plasmodesma organization
750	GO:0009511	Development	Development	C	plasmodesmatal endoplasmic reticulum
751	GO:0016605	Development	Development	C	PML body
752	GO:0043078	Development	Development	C	polar nucleus
753	GO:0009944	Development	Development	P	polarity specification of adaxial/abaxial axis
754	GO:0009555	Development	Development	P	pollen development
755	GO:0010584	Development	Development	P	pollen exine formation
756	GO:0010152	Development	Development	P	pollen maturation
757	GO:0090406	Development	Development	C	pollen tube
758	GO:0090404	Development	Development	C	pollen tube tip
759	GO:0010208	Development	Development	P	pollen wall assembly
760	GO:0099513	Development	Development	C	polymeric cytoskeletal fiber
761	GO:0009791	Development	Development	P	post-embryonic development
762	GO:0090698	Development	Development	P	post-embryonic plant morphogenesis
763	GO:0048528	Development	Development	P	post-embryonic root development
764	GO:0009530	Development	Development	C	primary cell wall
765	GO:0080022	Development	Development	P	primary root development
766	GO:0010072	Development	Development	P	primary shoot apical meristem specification
767	GO:0010067	Development	Development	P	procambium histogenesis
768	GO:0061919	Development	Development	P	process utilizing autophagic mechanism
769	GO:0000326	Development	Development	C	protein storage vacuole
770	GO:0032586	Development	Development	C	protein storage vacuole membrane
771	GO:1990019	Development	Development	P	protein storage vacuole organization
772	GO:0009946	Development	Development	P	proximal/distal axis specification
773	GO:1904961	Development	Development	P	quiescent center organization
774	GO:0010245	Development	Development	P	radial microtubular system formation

775	GO:0009956	Development	Development	P	radial pattern formation
776	GO:0055038	Development	Development	C	recycling endosome membrane
777	GO:0003002	Development	Development	P	regionalization
778	GO:0090567	Development	Development	P	reproductive shoot system development
779	GO:0048608	Development	Development	P	reproductive structure development
780	GO:0061458	Development	Development	P	reproductive system development
781	GO:0048829	Development	Development	P	root cap development
782	GO:0048364	Development	Development	P	root development
783	GO:0010053	Development	Development	P	root epidermal cell differentiation
784	GO:0035618	Development	Development	C	root hair
785	GO:0080147	Development	Development	P	root hair cell development
786	GO:0048768	Development	Development	P	root hair cell tip growth
787	GO:0048767	Development	Development	P	root hair elongation
788	GO:0048766	Development	Development	P	root hair initiation
789	GO:0010449	Development	Development	P	root meristem growth
790	GO:0010015	Development	Development	P	root morphogenesis
791	GO:0090057	Development	Development	P	root radial pattern formation
792	GO:0031209	Development	Development	C	SCAR complex
793	GO:0010223	Development	Development	P	secondary shoot formation
794	GO:0080191	Development	Development	P	secondary thickening
795	GO:0046903	Development	Development	P	secretion
796	GO:0032940	Development	Development	P	secretion by cell
797	GO:0010214	Development	Development	P	seed coat development
798	GO:0048316	Development	Development	P	seed development
799	GO:0010162	Development	Development	P	seed dormancy process
800	GO:0009845	Development	Development	P	seed germination
801	GO:0080112	Development	Development	P	seed growth
802	GO:0010431	Development	Development	P	seed maturation
803	GO:0010344	Development	Development	P	seed oilbody biogenesis
804	GO:0090351	Development	Development	P	seedling development
805	GO:1902182	Development	Development	P	shoot apical meristem development
806	GO:0048367	Development	Development	P	shoot system development
807	GO:0010016	Development	Development	P	shoot system morphogenesis
808	GO:0048756	Development	Development	P	sieve cell differentiation
809	GO:0060776	Development	Development	P	simple leaf morphogenesis
810	GO:0030427	Development	Development	C	site of polarized growth
811	GO:0009647	Development	Development	P	skotomorphogenesis
812	GO:0035019	Development	Development	P	somatic stem cell population maintenance
813	GO:0010093	Development	Development	P	specification of floral organ identity
814	GO:0048833	Development	Development	P	specification of floral organ number
815	GO:0090708	Development	Development	P	specification of plant organ axis polarity
816	GO:0090701	Development	Development	P	specification of plant organ identity
817	GO:0090706	Development	Development	P	specification of plant organ position
818	GO:0009799	Development	Development	P	specification of symmetry
819	GO:0048443	Development	Development	P	stamen development
820	GO:0080086	Development	Development	P	stamen filament development
821	GO:0010479	Development	Development	P	stele development
822	GO:0019827	Development	Development	P	stem cell population maintenance
823	GO:0010222	Development	Development	P	stem vascular tissue pattern formation
824	GO:0048480	Development	Development	P	stigma development
825	GO:0010374	Development	Development	P	stomatal complex development
826	GO:0010376	Development	Development	P	stomatal complex formation
827	GO:0010103	Development	Development	P	stomatal complex morphogenesis
828	GO:0010375	Development	Development	P	stomatal complex patterning
829	GO:0010440	Development	Development	P	stomatal lineage progression
830	GO:1990069	Development	Development	P	stomatal opening
831	GO:0080166	Development	Development	P	stomium development
832	GO:0010319	Development	Development	C	stromule
833	GO:0005199	Development	Development	F	structural constituent of cell wall
834	GO:0030527	Development	Development	F	structural constituent of chromatin
835	GO:0005200	Development	Development	F	structural constituent of cytoskeleton
836	GO:0017056	Development	Development	F	structural constituent of nuclear pore
837	GO:0005198	Development	Development	F	structural molecule activity
838	GO:0010098	Development	Development	P	suspensor development
839	GO:0055044	Development	Development	C	symplast
840	GO:0048731	Development	Development	P	system development
841	GO:0003008	Development	Development	P	system process
842	GO:0009888	Development	Development	P	tissue development
843	GO:0032588	Development	Development	C	trans-Golgi network membrane
844	GO:0010500	Development	Development	P	transmitting tissue development
845	GO:0010148	Development	Development	P	transpiration
846	GO:0010091	Development	Development	P	trichome branching
847	GO:0010026	Development	Development	P	trichome differentiation
848	GO:0010090	Development	Development	P	trichome morphogenesis
849	GO:1905499	Development	Development	P	trichome papilla formation
850	GO:0048629	Development	Development	P	trichome patterning
851	GO:0007021	Development	Development	P	tubulin complex assembly
852	GO:0009826	Development	Development	P	unidimensional cell growth

853	GO:0005775	Development	Development	C	vacuolar lumen
854	GO:0005774	Development	Development	C	vacuolar membrane
855	GO:0005773	Development	Development	C	vacuole
856	GO:0003018	Development	Development	P	vascular process in circulatory system
857	GO:0001944	Development	Development	P	vasculature development
858	GO:0010228	Development	Development	P	vegetative to reproductive phase transition of meristem
859	GO:0006900	Development	Development	P	vesicle budding from membrane
860	GO:0048278	Development	Development	P	vesicle docking
861	GO:0006906	Development	Development	P	vesicle fusion
862	GO:0048280	Development	Development	P	vesicle fusion with Golgi apparatus
863	GO:0012506	Development	Development	C	vesicle membrane
864	GO:0090522	Development	Development	P	vesicle tethering involved in exocytosis
865	GO:0019034	Development	Development	C	viral replication complex
866	GO:0010051	Development	Development	P	xylem and phloem pattern formation
867	GO:0035798	Metabolism	Bioenergetics	F	2-alkenal reductase (NADP+) activity
868	GO:0032440	Metabolism	Bioenergetics	F	2-alkenal reductase [NAD(P)+] activity
869	GO:0004316	Metabolism	Bioenergetics	F	3-oxoacyl-[acyl-carrier-protein] reductase (NADPH) activity
870	GO:0102339	Metabolism	Bioenergetics	F	3-oxo-arachidoyl-CoA reductase activity
871	GO:0102336	Metabolism	Bioenergetics	F	3-oxo-arachidoyl-CoA synthase activity
872	GO:0102340	Metabolism	Bioenergetics	F	3-oxo-behenoyl-CoA reductase activity
873	GO:0102342	Metabolism	Bioenergetics	F	3-oxo-cerotoyl-CoA reductase activity
874	GO:0102337	Metabolism	Bioenergetics	F	3-oxo-cerotoyl-CoA synthase activity
875	GO:0102131	Metabolism	Bioenergetics	F	3-oxo-glutaryl-[acp] methyl ester reductase activity
876	GO:0102338	Metabolism	Bioenergetics	F	3-oxo-lignoceronyl-CoA synthase activity
877	GO:0102341	Metabolism	Bioenergetics	F	3-oxo-lignoceroyl-CoA reductase activity
878	GO:0102132	Metabolism	Bioenergetics	F	3-oxo-pimeloyl-[acp] methyl ester reductase activity
879	GO:0003866	Metabolism	Bioenergetics	F	3-phosphoshikimate 1-carboxyvinyltransferase activity
880	GO:0016207	Metabolism	Bioenergetics	F	4-coumarate-CoA ligase activity
881	GO:0003872	Metabolism	Bioenergetics	F	6-phosphofructokinase activity
882	GO:0003987	Metabolism	Bioenergetics	F	acetate-CoA ligase activity
883	GO:0018454	Metabolism	Bioenergetics	F	acetoacetyl-CoA reductase activity
884	GO:0010327	Metabolism	Bioenergetics	F	acetyl CoA:(Z)-3-hexen-1-ol acetyltransferase activity
885	GO:0006085	Metabolism	Bioenergetics	P	acetyl-CoA biosynthetic process
886	GO:0019427	Metabolism	Bioenergetics	P	acetyl-CoA biosynthetic process from acetate
887	GO:0006086	Metabolism	Bioenergetics	P	acetyl-CoA biosynthetic process from pyruvate
888	GO:0003988	Metabolism	Bioenergetics	F	acetyl-CoA C-acyltransferase activity
889	GO:0003989	Metabolism	Bioenergetics	F	acetyl-CoA carboxylase activity
890	GO:0009317	Metabolism	Bioenergetics	C	acetyl-CoA carboxylase complex
891	GO:0006084	Metabolism	Bioenergetics	P	acetyl-CoA metabolic process
892	GO:0004042	Metabolism	Bioenergetics	F	acetyl-CoA:L-glutamate N-acetyltransferase activity
893	GO:0071616	Metabolism	Bioenergetics	P	acyl-CoA biosynthetic process
894	GO:0003995	Metabolism	Bioenergetics	F	acyl-CoA dehydrogenase activity
895	GO:0047617	Metabolism	Bioenergetics	F	acyl-CoA hydrolase activity
896	GO:0003996	Metabolism	Bioenergetics	F	acyl-CoA ligase activity
897	GO:0006637	Metabolism	Bioenergetics	P	acyl-CoA metabolic process
898	GO:0003997	Metabolism	Bioenergetics	F	acyl-CoA oxidase activity
899	GO:0043531	Metabolism	Bioenergetics	F	ADP binding
900	GO:0046031	Metabolism	Bioenergetics	P	ADP metabolic process
901	GO:0080042	Metabolism	Bioenergetics	F	ADP-glucose pyrophosphohydrolase activity
902	GO:0102502	Metabolism	Bioenergetics	F	ADP-glucose-starch glucosyltransferase activity
903	GO:0047631	Metabolism	Bioenergetics	F	ADP-ribose diphosphatase activity
904	GO:0080041	Metabolism	Bioenergetics	F	ADP-ribose pyrophosphohydrolase activity
905	GO:0009060	Metabolism	Bioenergetics	P	aerobic respiration
906	GO:0004022	Metabolism	Bioenergetics	F	alcohol dehydrogenase (NAD+) activity
907	GO:0008106	Metabolism	Bioenergetics	F	alcohol dehydrogenase (NADP+) activity
908	GO:0004029	Metabolism	Bioenergetics	F	aldehyde dehydrogenase (NAD+) activity
909	GO:0004030	Metabolism	Bioenergetics	F	aldehyde dehydrogenase [NAD(P)+] activity
910	GO:0004032	Metabolism	Bioenergetics	F	alditol:NADP+ 1-oxidoreductase activity
911	GO:0004033	Metabolism	Bioenergetics	F	aldo-keto reductase (NADP) activity
912	GO:0004555	Metabolism	Bioenergetics	F	alpha,alpha-trehalase activity
913	GO:0003825	Metabolism	Bioenergetics	F	alpha,alpha-trehalose-phosphate synthase (UDP-forming) activity
914	GO:0004556	Metabolism	Bioenergetics	F	alpha-amylase activity
915	GO:0103025	Metabolism	Bioenergetics	F	alpha-amylase activity (releasing maltohexaose)
916	GO:0102966	Metabolism	Bioenergetics	F	arachidoyl-CoA:1-dodecanol O-acyltransferase activity
917	GO:0047681	Metabolism	Bioenergetics	F	aryl-alcohol dehydrogenase (NADP+) activity
918	GO:0006754	Metabolism	Bioenergetics	P	ATP biosynthetic process
919	GO:0003878	Metabolism	Bioenergetics	F	ATP citrate synthase activity
920	GO:0046034	Metabolism	Bioenergetics	P	ATP metabolic process
921	GO:0003879	Metabolism	Bioenergetics	F	ATP phosphoribosyltransferase activity
922	GO:0042773	Metabolism	Bioenergetics	P	ATP synthesis coupled electron transport
923	GO:0015986	Metabolism	Bioenergetics	P	ATP synthesis coupled proton transport
924	GO:0001671	Metabolism	Bioenergetics	F	ATPase activator activity
925	GO:0016887	Metabolism	Bioenergetics	F	ATPase activity
926	GO:0042623	Metabolism	Bioenergetics	F	ATPase activity, coupled
927	GO:0044769	Metabolism	Bioenergetics	F	ATPase activity, coupled to transmembrane movement of ions, rotational mechanism
928	GO:0051117	Metabolism	Bioenergetics	F	ATPase binding
929	GO:1904949	Metabolism	Bioenergetics	C	ATPase complex

930	GO:0008569	Metabolism	Bioenergetics	F	ATP-dependent microtubule motor activity, minus-end-directed
931	GO:0008574	Metabolism	Bioenergetics	F	ATP-dependent microtubule motor activity, plus-end-directed
932	GO:0016161	Metabolism	Bioenergetics	F	beta-amylase activity
933	GO:0042409	Metabolism	Bioenergetics	F	caffeoyl-CoA O-methyltransferase activity
934	GO:0030246	Metabolism	Bioenergetics	F	carbohydrate binding
935	GO:0016051	Metabolism	Bioenergetics	P	carbohydrate biosynthetic process
936	GO:0016052	Metabolism	Bioenergetics	P	carbohydrate catabolic process
937	GO:0097367	Metabolism	Bioenergetics	F	carbohydrate derivative binding
938	GO:1901137	Metabolism	Bioenergetics	P	carbohydrate derivative biosynthetic process
939	GO:1901135	Metabolism	Bioenergetics	P	carbohydrate derivative metabolic process
940	GO:0019200	Metabolism	Bioenergetics	F	carbohydrate kinase activity
941	GO:0005975	Metabolism	Bioenergetics	P	carbohydrate metabolic process
942	GO:0019203	Metabolism	Bioenergetics	F	carbohydrate phosphatase activity
943	GO:0046835	Metabolism	Bioenergetics	P	carbohydrate phosphorylation
944	GO:0034637	Metabolism	Bioenergetics	P	cellular carbohydrate biosynthetic process
945	GO:0044275	Metabolism	Bioenergetics	P	cellular carbohydrate catabolic process
946	GO:0044262	Metabolism	Bioenergetics	P	cellular carbohydrate metabolic process
947	GO:0033692	Metabolism	Bioenergetics	P	cellular polysaccharide biosynthetic process
948	GO:0044264	Metabolism	Bioenergetics	P	cellular polysaccharide metabolic process
949	GO:0045333	Metabolism	Bioenergetics	P	cellular respiration
950	GO:0004108	Metabolism	Bioenergetics	F	citrate (Si)-synthase activity
951	GO:0009346	Metabolism	Bioenergetics	C	citrate lyase complex
952	GO:0006101	Metabolism	Bioenergetics	P	citrate metabolic process
953	GO:0016405	Metabolism	Bioenergetics	F	CoA-ligase activity
954	GO:0015937	Metabolism	Bioenergetics	P	coenzyme A biosynthetic process
955	GO:0015936	Metabolism	Bioenergetics	P	coenzyme A metabolic process
956	GO:0050662	Metabolism	Bioenergetics	F	coenzyme binding
957	GO:0070069	Metabolism	Bioenergetics	C	cytochrome complex
958	GO:0017004	Metabolism	Bioenergetics	P	cytochrome complex assembly
959	GO:0004129	Metabolism	Bioenergetics	F	cytochrome-c oxidase activity
960	GO:0006176	Metabolism	Bioenergetics	P	dATP biosynthetic process from ADP
961	GO:0102391	Metabolism	Bioenergetics	F	decanoate-CoA ligase activity
962	GO:0016720	Metabolism	Bioenergetics	F	delta12-fatty acid dehydrogenase activity
963	GO:0102985	Metabolism	Bioenergetics	F	Delta12-fatty-acid desaturase activity
964	GO:0017113	Metabolism	Bioenergetics	F	dihydropyrimidine dehydrogenase (NADP+) activity
965	GO:0046351	Metabolism	Bioenergetics	P	disaccharide biosynthetic process
966	GO:0005984	Metabolism	Bioenergetics	P	disaccharide metabolic process
967	GO:0008094	Metabolism	Bioenergetics	F	DNA-dependent ATPase activity
968	GO:0009055	Metabolism	Bioenergetics	F	electron transfer activity
969	GO:0022900	Metabolism	Bioenergetics	P	electron transport chain
970	GO:0015990	Metabolism	Bioenergetics	P	electron transport coupled proton transport
971	GO:0004174	Metabolism	Bioenergetics	F	electron-transferring-flavoprotein dehydrogenase activity
972	GO:0015980	Metabolism	Bioenergetics	P	energy derivation by oxidation of organic compounds
973	GO:0004300	Metabolism	Bioenergetics	F	enoyl-CoA hydratase activity
974	GO:0031314	Metabolism	Bioenergetics	C	extrinsic component of mitochondrial inner membrane
975	GO:0080132	Metabolism	Bioenergetics	F	fatty acid alpha-hydroxylase activity
976	GO:0001561	Metabolism	Bioenergetics	P	fatty acid alpha-oxidation
977	GO:0006635	Metabolism	Bioenergetics	P	fatty acid beta-oxidation
978	GO:0033539	Metabolism	Bioenergetics	P	fatty acid beta-oxidation using acyl-CoA dehydrogenase
979	GO:0033540	Metabolism	Bioenergetics	P	fatty acid beta-oxidation using acyl-CoA oxidase
980	GO:0005504	Metabolism	Bioenergetics	F	fatty acid binding
981	GO:0006633	Metabolism	Bioenergetics	P	fatty acid biosynthetic process
982	GO:0009062	Metabolism	Bioenergetics	P	fatty acid catabolic process
983	GO:1901570	Metabolism	Bioenergetics	P	fatty acid derivative biosynthetic process
984	GO:1901568	Metabolism	Bioenergetics	P	fatty acid derivative metabolic process
985	GO:0009922	Metabolism	Bioenergetics	F	fatty acid elongase activity
986	GO:0009923	Metabolism	Bioenergetics	C	fatty acid elongase complex
987	GO:0030497	Metabolism	Bioenergetics	P	fatty acid elongation
988	GO:0052722	Metabolism	Bioenergetics	F	fatty acid in-chain hydroxylase activity
989	GO:0015645	Metabolism	Bioenergetics	F	fatty acid ligase activity
990	GO:0006631	Metabolism	Bioenergetics	P	fatty acid metabolic process
991	GO:0019395	Metabolism	Bioenergetics	P	fatty acid oxidation
992	GO:0046949	Metabolism	Bioenergetics	P	fatty-acyl-CoA biosynthetic process
993	GO:0004321	Metabolism	Bioenergetics	F	fatty-acyl-CoA synthase activity
994	GO:0008865	Metabolism	Bioenergetics	F	fructokinase activity
995	GO:1902334	Metabolism	Bioenergetics	P	fructose export from vacuole to cytoplasm
996	GO:0006000	Metabolism	Bioenergetics	P	fructose metabolic process
997	GO:0006091	Metabolism	Bioenergetics	P	generation of precursor metabolites and energy
998	GO:0006094	Metabolism	Bioenergetics	P	gluconeogenesis
999	GO:0051156	Metabolism	Bioenergetics	P	glucose 6-phosphate metabolic process
1000	GO:0006007	Metabolism	Bioenergetics	P	glucose catabolic process
1001	GO:0006006	Metabolism	Bioenergetics	P	glucose metabolic process
1002	GO:0008878	Metabolism	Bioenergetics	F	glucose-1-phosphate adenylyltransferase activity
1003	GO:0047938	Metabolism	Bioenergetics	F	glucose-6-phosphate 1-epimerase activity
1004	GO:0004345	Metabolism	Bioenergetics	F	glucose-6-phosphate dehydrogenase activity
1005	GO:0004352	Metabolism	Bioenergetics	F	glutamate dehydrogenase (NAD+) activity
1006	GO:0004353	Metabolism	Bioenergetics	F	glutamate dehydrogenase [NAD(P)+] activity
1007	GO:0016040	Metabolism	Bioenergetics	F	glutamate synthase (NADH) activity

1008	GO:0043878	Metabolism	Bioenergetics	F	glyceraldehyde-3-phosphate dehydrogenase (NAD+) (non-phosphorylating) activity
1009	GO:0004365	Metabolism	Bioenergetics	F	glyceraldehyde-3-phosphate dehydrogenase (NAD+) (phosphorylating) activity
1010	GO:0008886	Metabolism	Bioenergetics	F	glyceraldehyde-3-phosphate dehydrogenase (NADP+) (non-phosphorylating) activity
1011	GO:0047100	Metabolism	Bioenergetics	F	glyceraldehyde-3-phosphate dehydrogenase (NADP+) (phosphorylating) activity
1012	GO:0004373	Metabolism	Bioenergetics	F	glycogen (starch) synthase activity
1013	GO:0008891	Metabolism	Bioenergetics	F	glycolate oxidase activity
1014	GO:0006096	Metabolism	Bioenergetics	P	glycolytic process
1015	GO:0006097	Metabolism	Bioenergetics	P	glyoxylate cycle
1016	GO:0030267	Metabolism	Bioenergetics	F	glyoxylate reductase (NADP) activity
1017	GO:0046861	Metabolism	Bioenergetics	C	glyoxysomal membrane
1018	GO:0009514	Metabolism	Bioenergetics	C	glyoxysome
1019	GO:0010111	Metabolism	Bioenergetics	P	glyoxysome organization
1020	GO:0030931	Metabolism	Bioenergetics	C	heterotetrameric ADPG pyrophosphorylase complex
1021	GO:0019319	Metabolism	Bioenergetics	P	hexose biosynthetic process
1022	GO:0019318	Metabolism	Bioenergetics	P	hexose metabolic process
1023	GO:0050505	Metabolism	Bioenergetics	F	hydroquinone glucosyltransferase activity
1024	GO:0052716	Metabolism	Bioenergetics	F	hydroquinone:oxygen oxidoreductase activity
1025	GO:0004419	Metabolism	Bioenergetics	F	hydroxymethylglutaryl-CoA lyase activity
1026	GO:0004420	Metabolism	Bioenergetics	F	hydroxymethylglutaryl-CoA reductase (NADPH) activity
1027	GO:0042282	Metabolism	Bioenergetics	F	hydroxymethylglutaryl-CoA reductase activity
1028	GO:0098800	Metabolism	Bioenergetics	C	inner mitochondrial membrane protein complex
1029	GO:0031305	Metabolism	Bioenergetics	C	integral component of mitochondrial inner membrane
1030	GO:0031307	Metabolism	Bioenergetics	C	integral component of mitochondrial outer membrane
1031	GO:0048312	Metabolism	Bioenergetics	P	intracellular distribution of mitochondria
1032	GO:0031304	Metabolism	Bioenergetics	C	intrinsic component of mitochondrial inner membrane
1033	GO:0004449	Metabolism	Bioenergetics	F	isocitrate dehydrogenase (NAD+) activity
1034	GO:0004450	Metabolism	Bioenergetics	F	isocitrate dehydrogenase (NADP+) activity
1035	GO:0004448	Metabolism	Bioenergetics	F	isocitrate dehydrogenase activity
1036	GO:0006102	Metabolism	Bioenergetics	P	isocitrate metabolic process
1037	GO:0034440	Metabolism	Bioenergetics	P	lipid oxidation
1038	GO:0030060	Metabolism	Bioenergetics	F	L-malate dehydrogenase activity
1039	GO:0001676	Metabolism	Bioenergetics	P	long-chain fatty acid metabolic process
1040	GO:0004467	Metabolism	Bioenergetics	F	long-chain fatty acid-CoA ligase activity
1041	GO:0052853	Metabolism	Bioenergetics	F	long-chain-(S)-2-hydroxy-long-chain-acid oxidase activity
1042	GO:0047196	Metabolism	Bioenergetics	F	long-chain-alcohol O-fatty-acyltransferase activity
1043	GO:0004471	Metabolism	Bioenergetics	F	malate dehydrogenase (decarboxylating) (NAD+) activity
1044	GO:0004473	Metabolism	Bioenergetics	F	malate dehydrogenase (decarboxylating) (NADP+) activity
1045	GO:0046554	Metabolism	Bioenergetics	F	malate dehydrogenase (NADP+) activity
1046	GO:0016615	Metabolism	Bioenergetics	F	malate dehydrogenase activity
1047	GO:0006108	Metabolism	Bioenergetics	P	malate metabolic process
1048	GO:0004470	Metabolism	Bioenergetics	F	malic enzyme activity
1049	GO:0090409	Metabolism	Bioenergetics	F	malonyl-CoA synthetase activity
1050	GO:0031956	Metabolism	Bioenergetics	F	medium-chain fatty acid-CoA ligase activity
1051	GO:0004485	Metabolism	Bioenergetics	F	methylcrotonoyl-CoA carboxylase activity
1052	GO:0004488	Metabolism	Bioenergetics	F	methylenetetrahydrofolate dehydrogenase (NADP+) activity
1053	GO:0004489	Metabolism	Bioenergetics	F	methylenetetrahydrofolate reductase (NAD(P)H) activity
1054	GO:0004490	Metabolism	Bioenergetics	F	methylglutaconyl-CoA hydratase activity
1055	GO:0005947	Metabolism	Bioenergetics	C	mitochondrial alpha-ketoglutarate dehydrogenase complex
1056	GO:0042775	Metabolism	Bioenergetics	P	mitochondrial ATP synthesis coupled electron transport
1057	GO:0042776	Metabolism	Bioenergetics	P	mitochondrial ATP synthesis coupled proton transport
1058	GO:0006123	Metabolism	Bioenergetics	P	mitochondrial electron transport, cytochrome c to oxygen
1059	GO:0006120	Metabolism	Bioenergetics	P	mitochondrial electron transport, NADH to ubiquinone
1060	GO:0006121	Metabolism	Bioenergetics	P	mitochondrial electron transport, succinate to ubiquinone
1061	GO:0006122	Metabolism	Bioenergetics	P	mitochondrial electron transport, ubiquinol to cytochrome c
1062	GO:0005740	Metabolism	Bioenergetics	C	mitochondrial envelope
1063	GO:0000266	Metabolism	Bioenergetics	P	mitochondrial fission
1064	GO:0005743	Metabolism	Bioenergetics	C	mitochondrial inner membrane
1065	GO:0005758	Metabolism	Bioenergetics	C	mitochondrial intermembrane space
1066	GO:0005759	Metabolism	Bioenergetics	C	mitochondrial matrix
1067	GO:0031966	Metabolism	Bioenergetics	C	mitochondrial membrane
1068	GO:0005741	Metabolism	Bioenergetics	C	mitochondrial outer membrane
1069	GO:0005742	Metabolism	Bioenergetics	C	mitochondrial outer membrane translocase complex
1070	GO:0017087	Metabolism	Bioenergetics	C	mitochondrial processing peptidase complex
1071	GO:0098798	Metabolism	Bioenergetics	C	mitochondrial protein complex
1072	GO:0034982	Metabolism	Bioenergetics	P	mitochondrial protein processing
1073	GO:0005753	Metabolism	Bioenergetics	C	mitochondrial proton-transporting ATP synthase complex
1074	GO:0033615	Metabolism	Bioenergetics	P	mitochondrial proton-transporting ATP synthase complex assembly
1075	GO:0000275	Metabolism	Bioenergetics	C	mitochondrial proton-transporting ATP synthase complex, catalytic core F(1)
1076	GO:0000276	Metabolism	Bioenergetics	C	mitochondrial proton-transporting ATP synthase complex, coupling factor F(o)
1077	GO:0005754	Metabolism	Bioenergetics	C	mitochondrial proton-transporting ATP synthase, catalytic core
1078	GO:0000274	Metabolism	Bioenergetics	C	mitochondrial proton-transporting ATP synthase, stator stalk
1079	GO:0006850	Metabolism	Bioenergetics	P	mitochondrial pyruvate transmembrane transport
1080	GO:0005746	Metabolism	Bioenergetics	C	mitochondrial respirasome
1081	GO:0005747	Metabolism	Bioenergetics	C	mitochondrial respiratory chain complex I
1082	GO:0032981	Metabolism	Bioenergetics	P	mitochondrial respiratory chain complex I assembly

1083	GO:0005749	Metabolism	Bioenergetics	C	mitochondrial respiratory chain complex II, succinate dehydrogenase complex (ubiquinone)
1084	GO:0005750	Metabolism	Bioenergetics	C	mitochondrial respiratory chain complex III
1085	GO:0034551	Metabolism	Bioenergetics	P	mitochondrial respiratory chain complex III assembly
1086	GO:0005751	Metabolism	Bioenergetics	C	mitochondrial respiratory chain complex IV
1087	GO:0033617	Metabolism	Bioenergetics	P	mitochondrial respiratory chain complex IV assembly
1088	GO:0005739	Metabolism	Bioenergetics	C	mitochondrion
1089	GO:0007005	Metabolism	Bioenergetics	P	mitochondrion organization
1090	GO:0046364	Metabolism	Bioenergetics	P	monosaccharide biosynthetic process
1091	GO:0051287	Metabolism	Bioenergetics	F	NAD binding
1092	GO:0034356	Metabolism	Bioenergetics	P	NAD biosynthesis via nicotinamide riboside salvage pathway
1093	GO:0009435	Metabolism	Bioenergetics	P	NAD biosynthetic process
1094	GO:0008746	Metabolism	Bioenergetics	F	NAD(P)+ transhydrogenase activity
1095	GO:0003955	Metabolism	Bioenergetics	F	NAD(P)H dehydrogenase (quinone) activity
1096	GO:0010598	Metabolism	Bioenergetics	C	NAD(P)H dehydrogenase complex (plastoquinone)
1097	GO:0010275	Metabolism	Bioenergetics	P	NAD(P)H dehydrogenase complex assembly
1098	GO:0016174	Metabolism	Bioenergetics	F	NAD(P)H oxidase activity
1099	GO:0003950	Metabolism	Bioenergetics	F	NAD+ ADP-ribosyltransferase activity
1100	GO:0070403	Metabolism	Bioenergetics	F	NAD+ binding
1101	GO:0000210	Metabolism	Bioenergetics	F	NAD+ diphosphatase activity
1102	GO:0008137	Metabolism	Bioenergetics	F	NADH dehydrogenase (ubiquinone) activity
1103	GO:0003954	Metabolism	Bioenergetics	F	NADH dehydrogenase activity
1104	GO:0030964	Metabolism	Bioenergetics	C	NADH dehydrogenase complex
1105	GO:0010258	Metabolism	Bioenergetics	P	NADH dehydrogenase complex (plastoquinone) assembly
1106	GO:0006734	Metabolism	Bioenergetics	P	NADH metabolic process
1107	GO:0006116	Metabolism	Bioenergetics	P	NADH oxidation
1108	GO:0035529	Metabolism	Bioenergetics	F	NADH pyrophosphatase activity
1109	GO:0052856	Metabolism	Bioenergetics	F	NADHX epimerase activity
1110	GO:0050661	Metabolism	Bioenergetics	F	NADP binding
1111	GO:0006739	Metabolism	Bioenergetics	P	NADP metabolic process
1112	GO:0070401	Metabolism	Bioenergetics	F	NADP+ binding
1113	GO:0070402	Metabolism	Bioenergetics	F	NADPH binding
1114	GO:0003959	Metabolism	Bioenergetics	F	NADPH dehydrogenase activity
1115	GO:0006740	Metabolism	Bioenergetics	P	NADPH regeneration
1116	GO:0003958	Metabolism	Bioenergetics	F	NADPH-hemoprotein reductase activity
1117	GO:0052857	Metabolism	Bioenergetics	F	NADPHX epimerase activity
1118	GO:0031999	Metabolism	Bioenergetics	P	negative regulation of fatty acid beta-oxidation
1119	GO:0008942	Metabolism	Bioenergetics	F	nitrite reductase [NAD(P)H] activity
1120	GO:0031588	Metabolism	Bioenergetics	C	nucleotide-activated protein kinase complex
1121	GO:0009312	Metabolism	Bioenergetics	P	oligosaccharide biosynthetic process
1122	GO:0009311	Metabolism	Bioenergetics	P	oligosaccharide metabolic process
1123	GO:0042389	Metabolism	Bioenergetics	F	omega-3 fatty acid desaturase activity
1124	GO:0045485	Metabolism	Bioenergetics	F	omega-6 fatty acid desaturase activity
1125	GO:0050203	Metabolism	Bioenergetics	F	oxalate-CoA ligase activity
1126	GO:0016491	Metabolism	Bioenergetics	F	oxidoreductase activity
1127	GO:0016667	Metabolism	Bioenergetics	F	oxidoreductase activity, acting on a sulfur group of donors
1128	GO:0016671	Metabolism	Bioenergetics	F	oxidoreductase activity, acting on a sulfur group of donors, disulfide as acceptor
1129	GO:0016614	Metabolism	Bioenergetics	F	oxidoreductase activity, acting on CH-OH group of donors
1130	GO:0016681	Metabolism	Bioenergetics	F	oxidoreductase activity, acting on diphenols and related substances as donors, cytochrome as acceptor
1131	GO:0016682	Metabolism	Bioenergetics	F	oxidoreductase activity, acting on diphenols and related substances as donors, oxygen as acceptor
1132	GO:0016730	Metabolism	Bioenergetics	F	oxidoreductase activity, acting on iron-sulfur proteins as donors
1133	GO:0016651	Metabolism	Bioenergetics	F	oxidoreductase activity, acting on NAD(P)H
1134	GO:0050664	Metabolism	Bioenergetics	F	oxidoreductase activity, acting on NAD(P)H, oxygen as acceptor
1135	GO:0016655	Metabolism	Bioenergetics	F	oxidoreductase activity, acting on NAD(P)H, quinone or similar compound as acceptor
1136	GO:0016661	Metabolism	Bioenergetics	F	oxidoreductase activity, acting on other nitrogenous compounds as donors
1137	GO:0016705	Metabolism	Bioenergetics	F	oxidoreductase activity, acting on paired donors, with incorporation or reduction of molecular oxygen
1138	GO:0016709	Metabolism	Bioenergetics	F	oxidoreductase activity, acting on paired donors, with incorporation or reduction of molecular oxygen, NAD(P)H as one donor, and incorporation of one atom of oxygen
1139	GO:0016712	Metabolism	Bioenergetics	F	oxidoreductase activity, acting on paired donors, with incorporation or reduction of molecular oxygen, reduced flavin or flavoprotein as one donor, and incorporation of one atom of oxygen
1140	GO:0016713	Metabolism	Bioenergetics	F	oxidoreductase activity, acting on paired donors, with incorporation or reduction of molecular oxygen, reduced iron-sulfur protein as one donor, and incorporation of one atom of oxygen
1141	GO:0016717	Metabolism	Bioenergetics	F	oxidoreductase activity, acting on paired donors, with oxidation of a pair of donors resulting in the reduction of molecular oxygen to two molecules of water
1142	GO:0016701	Metabolism	Bioenergetics	F	oxidoreductase activity, acting on single donors with incorporation of molecular oxygen
1143	GO:0016702	Metabolism	Bioenergetics	F	oxidoreductase activity, acting on single donors with incorporation of molecular oxygen, incorporation of two atoms of oxygen
1144	GO:0016903	Metabolism	Bioenergetics	F	oxidoreductase activity, acting on the aldehyde or oxo group of donors
1145	GO:0016620	Metabolism	Bioenergetics	F	oxidoreductase activity, acting on the aldehyde or oxo group of donors, NAD or NADP as acceptor

1146	GO:0016627	Metabolism	Bioenergetics	F	oxidoreductase activity, acting on the CH-CH group of donors
1147	GO:0016638	Metabolism	Bioenergetics	F	oxidoreductase activity, acting on the CH-NH2 group of donors
1148	GO:0016616	Metabolism	Bioenergetics	F	oxidoreductase activity, acting on the CH-OH group of donors, NAD or NADP as acceptor
1149	GO:0016899	Metabolism	Bioenergetics	F	oxidoreductase activity, acting on the CH-OH group of donors, oxygen as acceptor
1150	GO:0016722	Metabolism	Bioenergetics	F	oxidoreductase activity, oxidizing metal ions
1151	GO:0016724	Metabolism	Bioenergetics	F	oxidoreductase activity, oxidizing metal ions, oxygen as acceptor
1152	GO:1990204	Metabolism	Bioenergetics	C	oxidoreductase complex
1153	GO:0045252	Metabolism	Bioenergetics	C	oxoglutarate dehydrogenase complex
1154	GO:0001405	Metabolism	Bioenergetics	C	PAM complex, Tim23 associated import motor
1155	GO:0006098	Metabolism	Bioenergetics	P	pentose-phosphate shunt
1156	GO:0009052	Metabolism	Bioenergetics	P	pentose-phosphate shunt, non-oxidative branch
1157	GO:0009051	Metabolism	Bioenergetics	P	pentose-phosphate shunt, oxidative branch
1158	GO:0000015	Metabolism	Bioenergetics	C	phosphopyruvate hydratase complex
1159	GO:0009853	Metabolism	Bioenergetics	P	photorespiration
1160	GO:0048244	Metabolism	Bioenergetics	F	phytanoyl-CoA dioxygenase activity
1161	GO:0045272	Metabolism	Bioenergetics	C	plasma membrane respiratory chain complex I
1162	GO:0000271	Metabolism	Bioenergetics	P	polysaccharide biosynthetic process
1163	GO:0005976	Metabolism	Bioenergetics	P	polysaccharide metabolic process
1164	GO:0032781	Metabolism	Bioenergetics	P	positive regulation of ATPase activity
1165	GO:0045723	Metabolism	Bioenergetics	P	positive regulation of fatty acid biosynthetic process
1166	GO:0010636	Metabolism	Bioenergetics	P	positive regulation of mitochondrial fusion
1167	GO:1903862	Metabolism	Bioenergetics	P	positive regulation of oxidative phosphorylation
1168	GO:1903942	Metabolism	Bioenergetics	P	positive regulation of respiratory gaseous exchange
1169	GO:0044238	Metabolism	Bioenergetics	P	primary metabolic process
1170	GO:1902600	Metabolism	Bioenergetics	P	proton transmembrane transport
1171	GO:0008553	Metabolism	Bioenergetics	F	proton-exporting ATPase activity, phosphorylative mechanism
1172	GO:0046933	Metabolism	Bioenergetics	F	proton-transporting ATP synthase activity, rotational mechanism
1173	GO:0045259	Metabolism	Bioenergetics	C	proton-transporting ATP synthase complex
1174	GO:0043461	Metabolism	Bioenergetics	P	proton-transporting ATP synthase complex assembly
1175	GO:0045261	Metabolism	Bioenergetics	C	proton-transporting ATP synthase complex, catalytic core F(1)
1176	GO:0045263	Metabolism	Bioenergetics	C	proton-transporting ATP synthase complex, coupling factor F(o)
1177	GO:0046961	Metabolism	Bioenergetics	F	proton-transporting ATPase activity, rotational mechanism
1178	GO:0016469	Metabolism	Bioenergetics	C	proton-transporting two-sector ATPase complex
1179	GO:0033178	Metabolism	Bioenergetics	C	proton-transporting two-sector ATPase complex, catalytic domain
1180	GO:0033177	Metabolism	Bioenergetics	C	proton-transporting two-sector ATPase complex, proton-transporting domain
1181	GO:0033176	Metabolism	Bioenergetics	C	proton-transporting V-type ATPase complex
1182	GO:0070070	Metabolism	Bioenergetics	P	proton-transporting V-type ATPase complex assembly
1183	GO:0033179	Metabolism	Bioenergetics	C	proton-transporting V-type ATPase, V0 domain
1184	GO:0033180	Metabolism	Bioenergetics	C	proton-transporting V-type ATPase, V1 domain
1185	GO:0009678	Metabolism	Bioenergetics	F	pyrophosphate hydrolysis-driven proton transmembrane transporter activity
1186	GO:0010318	Metabolism	Bioenergetics	C	pyrophosphate-dependent phosphofructokinase complex, beta-subunit complex
1187	GO:0004739	Metabolism	Bioenergetics	F	pyruvate dehydrogenase (acetyl-transferring) activity
1188	GO:0045254	Metabolism	Bioenergetics	C	pyruvate dehydrogenase complex
1189	GO:0009078	Metabolism	Bioenergetics	P	pyruvate family amino acid metabolic process
1190	GO:0004743	Metabolism	Bioenergetics	F	pyruvate kinase activity
1191	GO:0006090	Metabolism	Bioenergetics	P	pyruvate metabolic process
1192	GO:0019253	Metabolism	Bioenergetics	P	reductive pentose-phosphate cycle
1193	GO:0043462	Metabolism	Bioenergetics	P	regulation of ATPase activity
1194	GO:0043255	Metabolism	Bioenergetics	P	regulation of carbohydrate biosynthetic process
1195	GO:0006109	Metabolism	Bioenergetics	P	regulation of carbohydrate metabolic process
1196	GO:0043610	Metabolism	Bioenergetics	P	regulation of carbohydrate utilization
1197	GO:0043457	Metabolism	Bioenergetics	P	regulation of cellular respiration
1198	GO:0031998	Metabolism	Bioenergetics	P	regulation of fatty acid beta-oxidation
1199	GO:0010821	Metabolism	Bioenergetics	P	regulation of mitochondrion organization
1200	GO:0080093	Metabolism	Bioenergetics	P	regulation of photorespiration
1201	GO:0010581	Metabolism	Bioenergetics	P	regulation of starch biosynthetic process
1202	GO:0070469	Metabolism	Bioenergetics	C	respirasome
1203	GO:0098803	Metabolism	Bioenergetics	C	respiratory chain complex
1204	GO:0045271	Metabolism	Bioenergetics	C	respiratory chain complex I
1205	GO:0045273	Metabolism	Bioenergetics	C	respiratory chain complex II
1206	GO:0045275	Metabolism	Bioenergetics	C	respiratory chain complex III
1207	GO:0022904	Metabolism	Bioenergetics	P	respiratory electron transport chain
1208	GO:0047131	Metabolism	Bioenergetics	F	saccharopine dehydrogenase (NAD+, L-glutamate-forming) activity
1209	GO:0047130	Metabolism	Bioenergetics	F	saccharopine dehydrogenase (NADP+, L-lysine-forming) activity
1210	GO:0001401	Metabolism	Bioenergetics	C	SAM complex
1211	GO:0033587	Metabolism	Bioenergetics	P	shikimate biosynthetic process
1212	GO:0046459	Metabolism	Bioenergetics	P	short-chain fatty acid metabolic process
1213	GO:0102420	Metabolism	Bioenergetics	F	sn-1-glycerol-3-phosphate C16:0-DCA-CoA acyl transferase activity
1214	GO:0102419	Metabolism	Bioenergetics	F	sn-2-glycerol-3-phosphate omega-OH-C22:0-CoA acyl transferase activity
1215	GO:2001070	Metabolism	Bioenergetics	F	starch binding
1216	GO:0019252	Metabolism	Bioenergetics	P	starch biosynthetic process
1217	GO:0005983	Metabolism	Bioenergetics	P	starch catabolic process
1218	GO:0043036	Metabolism	Bioenergetics	C	starch grain
1219	GO:0005982	Metabolism	Bioenergetics	P	starch metabolic process
1220	GO:0009011	Metabolism	Bioenergetics	F	starch synthase activity

1221	GO:0102218	Metabolism	Bioenergetics	F	starch, H2O dikinase activity
1222	GO:0008177	Metabolism	Bioenergetics	F	succinate dehydrogenase (ubiquinone) activity
1223	GO:0045281	Metabolism	Bioenergetics	C	succinate dehydrogenase complex
1224	GO:0004775	Metabolism	Bioenergetics	F	succinate-CoA ligase (ADP-forming) activity
1225	GO:0004777	Metabolism	Bioenergetics	F	succinate-semialdehyde dehydrogenase (NAD+) activity
1226	GO:0009013	Metabolism	Bioenergetics	F	succinate-semialdehyde dehydrogenase [NAD(P)+] activity
1227	GO:0006104	Metabolism	Bioenergetics	P	succinyl-CoA metabolic process
1228	GO:0005986	Metabolism	Bioenergetics	P	sucrose biosynthetic process
1229	GO:0005987	Metabolism	Bioenergetics	P	sucrose catabolic process
1230	GO:0005985	Metabolism	Bioenergetics	P	sucrose metabolic process
1231	GO:0016157	Metabolism	Bioenergetics	F	sucrose synthase activity
1232	GO:0046524	Metabolism	Bioenergetics	F	sucrose-phosphate synthase activity
1233	GO:0050308	Metabolism	Bioenergetics	C	sugar-phosphatase activity
1234	GO:0042721	Metabolism	Bioenergetics	C	TIM22 mitochondrial import inner membrane insertion complex
1235	GO:0005744	Metabolism	Bioenergetics	C	TIM23 mitochondrial import inner membrane translocase complex
1236	GO:0019166	Metabolism	Bioenergetics	F	trans-2-enoyl-CoA reductase (NADPH) activity
1237	GO:0005992	Metabolism	Bioenergetics	P	trehalose biosynthetic process
1238	GO:0005993	Metabolism	Bioenergetics	P	trehalose catabolic process
1239	GO:0005991	Metabolism	Bioenergetics	P	trehalose metabolic process
1240	GO:0004805	Metabolism	Bioenergetics	F	trehalose-phosphatase activity
1241	GO:0006099	Metabolism	Bioenergetics	P	tricarboxylic acid cycle
1242	GO:0045239	Metabolism	Bioenergetics	C	tricarboxylic acid cycle enzyme complex
1243	GO:0072350	Metabolism	Bioenergetics	P	tricarboxylic acid metabolic process
1244	GO:0035436	Metabolism	Bioenergetics	P	triose phosphate transmembrane transport
1245	GO:0102721	Metabolism	Bioenergetics	F	ubiquinol:oxygen oxidoreductase activity
1246	GO:0008121	Metabolism	Bioenergetics	F	ubiquinol-cytochrome-c reductase activity
1247	GO:0048039	Metabolism	Bioenergetics	F	ubiquinone binding
1248	GO:0006744	Metabolism	Bioenergetics	P	ubiquinone biosynthetic process
1249	GO:1901006	Metabolism	Bioenergetics	P	ubiquinone-6 biosynthetic process
1250	GO:0006636	Metabolism	Bioenergetics	P	unsaturated fatty acid biosynthetic process
1251	GO:0033559	Metabolism	Bioenergetics	P	unsaturated fatty acid metabolic process
1252	GO:0016471	Metabolism	Bioenergetics	C	vacuolar proton-transporting V-type ATPase complex
1253	GO:0000220	Metabolism	Bioenergetics	C	vacuolar proton-transporting V-type ATPase, V0 domain
1254	GO:0000221	Metabolism	Bioenergetics	C	vacuolar proton-transporting V-type ATPase, V1 domain
1255	GO:0042761	Metabolism	Bioenergetics	P	very long-chain fatty acid biosynthetic process
1256	GO:0000038	Metabolism	Bioenergetics	P	very long-chain fatty acid metabolic process
1257	GO:0102756	Metabolism	Bioenergetics	F	very-long-chain 3-ketoacyl-CoA synthase activity
1258	GO:0102758	Metabolism	Bioenergetics	F	very-long-chain enoyl-CoA reductase activity
1259	GO:0052852	Metabolism	Bioenergetics	F	very-long-chain-(S)-2-hydroxy-acid oxidase activity
1260	GO:0061158	Metabolism	Catabolism	P	3'-UTR-mediated mRNA destabilization
1261	GO:0009838	Metabolism	Catabolism	P	abscission
1262	GO:0006168	Metabolism	Catabolism	P	adenine salvage
1263	GO:0006524	Metabolism	Catabolism	P	alanine catabolic process
1264	GO:0046164	Metabolism	Catabolism	P	alcohol catabolic process
1265	GO:0046185	Metabolism	Catabolism	P	aldehyde catabolic process
1266	GO:0071771	Metabolism	Catabolism	F	aldehyde decarbonylase activity
1267	GO:0043447	Metabolism	Catabolism	P	alkane biosynthetic process
1268	GO:0043448	Metabolism	Catabolism	P	alkane catabolic process
1269	GO:1901606	Metabolism	Catabolism	P	alpha-amino acid catabolic process
1270	GO:0046556	Metabolism	Catabolism	F	alpha-L-arabinofuranosidase activity
1271	GO:0004177	Metabolism	Catabolism	F	aminopeptidase activity
1272	GO:0031222	Metabolism	Catabolism	P	arabinan catabolic process
1273	GO:0006527	Metabolism	Catabolism	P	arginine catabolic process
1274	GO:0009074	Metabolism	Catabolism	P	aromatic amino acid family catabolic process
1275	GO:0019439	Metabolism	Catabolism	P	aromatic compound catabolic process
1276	GO:0005776	Metabolism	Catabolism	C	autophagosome
1277	GO:0000045	Metabolism	Catabolism	P	autophagosome assembly
1278	GO:0000421	Metabolism	Catabolism	C	autophagosome membrane
1279	GO:0006914	Metabolism	Catabolism	P	autophagy
1280	GO:0009083	Metabolism	Catabolism	P	branched-chain amino acid catabolic process
1281	GO:0004089	Metabolism	Catabolism	F	carbonate dehydratase activity
1282	GO:0046395	Metabolism	Catabolism	P	carboxylic acid catabolic process
1283	GO:0009056	Metabolism	Catabolism	P	catabolic process
1284	GO:0008219	Metabolism	Catabolism	P	cell death
1285	GO:0016998	Metabolism	Catabolism	P	cell wall macromolecule catabolic process
1286	GO:0009063	Metabolism	Catabolism	P	cellular amino acid catabolic process
1287	GO:0044248	Metabolism	Catabolism	P	cellular catabolic process
1288	GO:0044242	Metabolism	Catabolism	P	cellular lipid catabolic process
1289	GO:0044265	Metabolism	Catabolism	P	cellular macromolecule catabolic process
1290	GO:0043094	Metabolism	Catabolism	P	cellular metabolic compound salvage
1291	GO:0044257	Metabolism	Catabolism	P	cellular protein catabolic process
1292	GO:0030245	Metabolism	Catabolism	P	cellulose catabolic process
1293	GO:0006032	Metabolism	Catabolism	P	chitin catabolic process
1294	GO:0015996	Metabolism	Catabolism	P	chlorophyll catabolic process
1295	GO:0030574	Metabolism	Catabolism	P	collagen catabolic process
1296	GO:0031462	Metabolism	Catabolism	C	Cul2-RING ubiquitin ligase complex
1297	GO:0031463	Metabolism	Catabolism	C	Cul3-RING ubiquitin ligase complex
1298	GO:0031464	Metabolism	Catabolism	C	Cul4A-RING E3 ubiquitin ligase complex

1299	GO:0080008	Metabolism	Catabolism	C	Cul4-RING E3 ubiquitin ligase complex
1300	GO:0031467	Metabolism	Catabolism	C	Cul7-RING ubiquitin ligase complex
1301	GO:0031461	Metabolism	Catabolism	C	cullin-RING ubiquitin ligase complex
1302	GO:0009440	Metabolism	Catabolism	P	cyanate catabolic process
1303	GO:0004197	Metabolism	Catabolism	F	cysteine-type endopeptidase activity
1304	GO:0006216	Metabolism	Catabolism	P	cytidine catabolic process
1305	GO:0009264	Metabolism	Catabolism	P	deoxyribonucleotide catabolic process
1306	GO:0046177	Metabolism	Catabolism	P	D-gluconate catabolic process
1307	GO:0043649	Metabolism	Catabolism	P	dicarboxylic acid catabolic process
1308	GO:0006308	Metabolism	Catabolism	P	DNA catabolic process
1309	GO:0000737	Metabolism	Catabolism	P	DNA catabolic process, endonucleolytic
1310	GO:0030139	Metabolism	Catabolism	C	endocytic vesicle
1311	GO:0004175	Metabolism	Catabolism	F	endopeptidase activity
1312	GO:1905369	Metabolism	Catabolism	C	endopeptidase complex
1313	GO:0004521	Metabolism	Catabolism	F	endoribonuclease activity
1314	GO:1902222	Metabolism	Catabolism	P	erythrose 4-phosphate/phosphoenolpyruvate family amino acid catabolic process
1315	GO:0004529	Metabolism	Catabolism	F	exodeoxyribonuclease activity
1316	GO:0016895	Metabolism	Catabolism	F	exodeoxyribonuclease activity, producing 5'-phosphomonoesters
1317	GO:0004527	Metabolism	Catabolism	F	exonuclease activity
1318	GO:0043928	Metabolism	Catabolism	P	exonucleolytic catabolism of deadenylated mRNA
1319	GO:1905354	Metabolism	Catabolism	C	exoribonuclease complex
1320	GO:0008859	Metabolism	Catabolism	F	exoribonuclease II activity
1321	GO:0000178	Metabolism	Catabolism	C	exosome (RNase complex)
1322	GO:0019898	Metabolism	Catabolism	C	extrinsic component of membrane
1323	GO:0010227	Metabolism	Catabolism	P	floral organ abscission
1324	GO:0080187	Metabolism	Catabolism	P	floral organ senescence
1325	GO:0046294	Metabolism	Catabolism	P	formaldehyde catabolic process
1326	GO:0010047	Metabolism	Catabolism	P	fruit dehiscence
1327	GO:0019388	Metabolism	Catabolism	P	galactose catabolic process
1328	GO:0033499	Metabolism	Catabolism	P	galactose catabolic process via UDP-galactose
1329	GO:0009450	Metabolism	Catabolism	P	gamma-aminobutyric acid catabolic process
1330	GO:0034722	Metabolism	Catabolism	F	gamma-glutamyl-peptidase activity
1331	GO:0045487	Metabolism	Catabolism	P	gibberellin catabolic process
1332	GO:0019762	Metabolism	Catabolism	P	glucosinolate catabolic process
1333	GO:0006538	Metabolism	Catabolism	P	glutamate catabolic process
1334	GO:0009065	Metabolism	Catabolism	P	glutamine family amino acid catabolic process
1335	GO:0006751	Metabolism	Catabolism	P	glutathione catabolic process
1336	GO:0019563	Metabolism	Catabolism	P	glycerol catabolic process
1337	GO:0046168	Metabolism	Catabolism	P	glycerol-3-phosphate catabolic process
1338	GO:0006546	Metabolism	Catabolism	P	glycine catabolic process
1339	GO:1902000	Metabolism	Catabolism	P	homogentisate catabolic process
1340	GO:0032264	Metabolism	Catabolism	P	IMP salvage
1341	GO:0042344	Metabolism	Catabolism	P	indole glucosinolate catabolic process
1342	GO:0019310	Metabolism	Catabolism	P	inositol catabolic process
1343	GO:0005779	Metabolism	Catabolism	C	integral component of peroxisomal membrane
1344	GO:0042853	Metabolism	Catabolism	P	L-alanine catabolic process
1345	GO:0060866	Metabolism	Catabolism	P	leaf abscission
1346	GO:0010150	Metabolism	Catabolism	P	leaf senescence
1347	GO:0006552	Metabolism	Catabolism	P	leucine catabolic process
1348	GO:0046274	Metabolism	Catabolism	P	lignin catabolic process
1349	GO:0016042	Metabolism	Catabolism	P	lipid catabolic process
1350	GO:0019477	Metabolism	Catabolism	P	L-lysine catabolic process
1351	GO:0033512	Metabolism	Catabolism	P	L-lysine catabolic process to acetyl-CoA via saccharopine
1352	GO:0019509	Metabolism	Catabolism	P	L-methionine salvage from methylthioadenosine
1353	GO:0006559	Metabolism	Catabolism	P	L-phenylalanine catabolic process
1354	GO:0006565	Metabolism	Catabolism	P	L-serine catabolic process
1355	GO:0005764	Metabolism	Catabolism	C	lysosome
1356	GO:0009057	Metabolism	Catabolism	P	macromolecule catabolic process
1357	GO:0090410	Metabolism	Catabolism	P	malonate catabolic process
1358	GO:0000025	Metabolism	Catabolism	P	maltose catabolic process
1359	GO:0033619	Metabolism	Catabolism	P	membrane protein proteolysis
1360	GO:0004222	Metabolism	Catabolism	F	metalloendopeptidase activity
1361	GO:1905202	Metabolism	Catabolism	C	methylcrotonoyl-CoA carboxylase complex
1362	GO:0043632	Metabolism	Catabolism	P	modification-dependent macromolecule catabolic process
1363	GO:0019941	Metabolism	Catabolism	P	modification-dependent protein catabolic process
1364	GO:0034053	Metabolism	Catabolism	P	modulation by symbiont of host defense-related programmed cell death
1365	GO:0072329	Metabolism	Catabolism	P	monocarboxylic acid catabolic process
1366	GO:0006402	Metabolism	Catabolism	P	mRNA catabolic process
1367	GO:0061157	Metabolism	Catabolism	P	mRNA destabilization
1368	GO:1903298	Metabolism	Catabolism	P	negative regulation of hypoxia-induced intrinsic apoptotic signaling pathway
1369	GO:1900056	Metabolism	Catabolism	P	negative regulation of leaf senescence
1370	GO:0043069	Metabolism	Catabolism	P	negative regulation of programmed cell death
1371	GO:0016054	Metabolism	Catabolism	P	organic acid catabolic process
1372	GO:1901361	Metabolism	Catabolism	P	organic cyclic compound catabolic process
1373	GO:1901616	Metabolism	Catabolism	P	organic hydroxy compound catabolic process
1374	GO:1901575	Metabolism	Catabolism	P	organic substance catabolic process
1375	GO:1901565	Metabolism	Catabolism	P	organonitrogen compound catabolic process

1376	GO:0046434	Metabolism	Catabolism	P	organophosphate catabolic process
1377	GO:0033611	Metabolism	Catabolism	P	oxalate catabolic process
1378	GO:0019822	Metabolism	Catabolism	C	P4 peroxisome
1379	GO:0045490	Metabolism	Catabolism	P	pectin catabolic process
1380	GO:0019323	Metabolism	Catabolism	P	pentose catabolic process
1381	GO:0008233	Metabolism	Catabolism	F	peptidase activity
1382	GO:1905368	Metabolism	Catabolism	C	peptidase complex
1383	GO:0043171	Metabolism	Catabolism	P	peptide catabolic process
1384	GO:0004601	Metabolism	Catabolism	F	peroxidase activity
1385	GO:0005782	Metabolism	Catabolism	C	peroxisomal matrix
1386	GO:0005778	Metabolism	Catabolism	C	peroxisomal membrane
1387	GO:0005777	Metabolism	Catabolism	C	peroxisome
1388	GO:0016559	Metabolism	Catabolism	P	peroxisome fission
1389	GO:0007031	Metabolism	Catabolism	P	peroxisome organization
1390	GO:0000407	Metabolism	Catabolism	C	phagophore assembly site
1391	GO:0019336	Metabolism	Catabolism	P	phenol-containing compound catabolic process
1392	GO:0010124	Metabolism	Catabolism	P	phenylacetate catabolic process
1393	GO:0009395	Metabolism	Catabolism	P	phospholipid catabolic process
1394	GO:0090693	Metabolism	Catabolism	P	plant organ senescence
1395	GO:1990137	Metabolism	Catabolism	F	plant seed peroxidase activity
1396	GO:0006598	Metabolism	Catabolism	P	polyamine catabolic process
1397	GO:0046174	Metabolism	Catabolism	P	polyol catabolic process
1398	GO:0000272	Metabolism	Catabolism	P	polysaccharide catabolic process
1399	GO:0031593	Metabolism	Catabolism	F	polyubiquitin modification-dependent protein binding
1400	GO:0009896	Metabolism	Catabolism	P	positive regulation of catabolic process
1401	GO:0043085	Metabolism	Catabolism	P	positive regulation of catalytic activity
1402	GO:0031331	Metabolism	Catabolism	P	positive regulation of cellular catabolic process
1403	GO:1903364	Metabolism	Catabolism	P	positive regulation of cellular protein catabolic process
1404	GO:1900064	Metabolism	Catabolism	P	positive regulation of peroxisome organization
1405	GO:1901800	Metabolism	Catabolism	P	positive regulation of proteasomal protein catabolic process
1406	GO:0032436	Metabolism	Catabolism	P	positive regulation of proteasomal ubiquitin-dependent protein catabolic process
1407	GO:0045732	Metabolism	Catabolism	P	positive regulation of protein catabolic process
1408	GO:0045862	Metabolism	Catabolism	P	positive regulation of proteolysis
1409	GO:1903052	Metabolism	Catabolism	P	positive regulation of proteolysis involved in cellular protein catabolic process
1410	GO:1904668	Metabolism	Catabolism	P	positive regulation of ubiquitin protein ligase activity
1411	GO:2000060	Metabolism	Catabolism	P	positive regulation of ubiquitin-dependent protein catabolic process
1412	GO:0051443	Metabolism	Catabolism	P	positive regulation of ubiquitin-protein transferase activity
1413	GO:0012501	Metabolism	Catabolism	P	programmed cell death
1414	GO:0010623	Metabolism	Catabolism	P	programmed cell death involved in cell development
1415	GO:0006562	Metabolism	Catabolism	P	proline catabolic process
1416	GO:0010133	Metabolism	Catabolism	P	proline catabolic process to glutamate
1417	GO:0010498	Metabolism	Catabolism	P	proteasomal protein catabolic process
1418	GO:0010499	Metabolism	Catabolism	P	proteasomal ubiquitin-independent protein catabolic process
1419	GO:0043248	Metabolism	Catabolism	P	proteasome assembly
1420	GO:0070628	Metabolism	Catabolism	F	proteasome binding
1421	GO:0000502	Metabolism	Catabolism	C	proteasome complex
1422	GO:0005839	Metabolism	Catabolism	C	proteasome core complex
1423	GO:0019773	Metabolism	Catabolism	C	proteasome core complex, alpha-subunit complex
1424	GO:0008540	Metabolism	Catabolism	C	proteasome regulatory particle, base subcomplex
1425	GO:0008541	Metabolism	Catabolism	C	proteasome regulatory particle, lid subcomplex
1426	GO:0036402	Metabolism	Catabolism	F	proteasome-activating ATPase activity
1427	GO:0043161	Metabolism	Catabolism	P	proteasome-mediated ubiquitin-dependent protein catabolic process
1428	GO:0051865	Metabolism	Catabolism	P	protein autoubiquitination
1429	GO:0030163	Metabolism	Catabolism	P	protein catabolic process
1430	GO:0031648	Metabolism	Catabolism	P	protein destabilization
1431	GO:0016558	Metabolism	Catabolism	P	protein import into peroxisome matrix
1432	GO:0070936	Metabolism	Catabolism	P	protein K48-linked ubiquitination
1433	GO:0070534	Metabolism	Catabolism	P	protein K63-linked ubiquitination
1434	GO:0006513	Metabolism	Catabolism	P	protein monoubiquitination
1435	GO:0000209	Metabolism	Catabolism	P	protein polyubiquitination
1436	GO:0043328	Metabolism	Catabolism	P	protein transport to vacuole involved in ubiquitin-dependent protein catabolic process via the multivesicular body sorting pathway
1437	GO:0016567	Metabolism	Catabolism	P	protein ubiquitination
1438	GO:0032991	Metabolism	Catabolism	C	protein-containing complex
1439	GO:0006508	Metabolism	Catabolism	P	proteolysis
1440	GO:0051603	Metabolism	Catabolism	P	proteolysis involved in cellular protein catabolic process
1441	GO:0010304	Metabolism	Catabolism	P	PSII associated light-harvesting complex II catabolic process
1442	GO:0006166	Metabolism	Catabolism	P	purine ribonucleoside salvage
1443	GO:0019365	Metabolism	Catabolism	P	pyridine nucleotide salvage
1444	GO:0009080	Metabolism	Catabolism	P	pyruvate family amino acid catabolic process
1445	GO:0034213	Metabolism	Catabolism	P	quinolinate catabolic process
1446	GO:0052692	Metabolism	Catabolism	F	raffinose alpha-galactosidase activity
1447	GO:0009894	Metabolism	Catabolism	P	regulation of catabolic process
1448	GO:1903362	Metabolism	Catabolism	P	regulation of cellular protein catabolic process
1449	GO:1900055	Metabolism	Catabolism	P	regulation of leaf senescence
1450	GO:0044375	Metabolism	Catabolism	P	regulation of peroxisome size
1451	GO:0043067	Metabolism	Catabolism	P	regulation of programmed cell death

1452	GO:0061136	Metabolism	Catabolism	P	regulation of proteasomal protein catabolic process
1453	GO:0032434	Metabolism	Catabolism	P	regulation of proteasomal ubiquitin-dependent protein catabolic process
1454	GO:0042176	Metabolism	Catabolism	P	regulation of protein catabolic process
1455	GO:0030162	Metabolism	Catabolism	P	regulation of proteolysis
1456	GO:1903050	Metabolism	Catabolism	P	regulation of proteolysis involved in cellular protein catabolic process
1457	GO:2000058	Metabolism	Catabolism	P	regulation of ubiquitin-dependent protein catabolic process
1458	GO:1990116	Metabolism	Catabolism	P	ribosome-associated ubiquitin-dependent protein catabolic process
1459	GO:0006401	Metabolism	Catabolism	P	RNA catabolic process
1460	GO:0090502	Metabolism	Catabolism	P	RNA phosphodiester bond hydrolysis, endonucleolytic
1461	GO:0016075	Metabolism	Catabolism	P	rRNA catabolic process
1462	GO:0046244	Metabolism	Catabolism	P	salicylic acid catabolic process
1463	GO:0010265	Metabolism	Catabolism	P	SCF complex assembly
1464	GO:0031146	Metabolism	Catabolism	P	SCF-dependent proteasomal ubiquitin-dependent protein catabolic process
1465	GO:0010282	Metabolism	Catabolism	C	senescence-associated vacuole
1466	GO:0009071	Metabolism	Catabolism	P	serine family amino acid catabolic process
1467	GO:0004185	Metabolism	Catabolism	F	serine-type carboxypeptidase activity
1468	GO:0004252	Metabolism	Catabolism	F	serine-type endopeptidase activity
1469	GO:0044282	Metabolism	Catabolism	P	small molecule catabolic process
1470	GO:0046203	Metabolism	Catabolism	P	spermidine catabolic process
1471	GO:0046208	Metabolism	Catabolism	P	spermine catabolic process
1472	GO:0046521	Metabolism	Catabolism	P	sphingoid catabolic process
1473	GO:0030149	Metabolism	Catabolism	P	sphingolipid catabolic process
1474	GO:0036459	Metabolism	Catabolism	F	thiol-dependent ubiquitinyl hydrolase activity
1475	GO:0006567	Metabolism	Catabolism	P	threonine catabolic process
1476	GO:0004298	Metabolism	Catabolism	F	threonine-type endopeptidase activity
1477	GO:0006210	Metabolism	Catabolism	P	thymine catabolic process
1478	GO:0006569	Metabolism	Catabolism	P	tryptophan catabolic process
1479	GO:0019441	Metabolism	Catabolism	P	tryptophan catabolic process to kynurenine
1480	GO:0006572	Metabolism	Catabolism	P	tyrosine catabolic process
1481	GO:0004839	Metabolism	Catabolism	F	ubiquitin activating enzyme activity
1482	GO:0043130	Metabolism	Catabolism	F	ubiquitin binding
1483	GO:0061631	Metabolism	Catabolism	F	ubiquitin conjugating enzyme activity
1484	GO:0031624	Metabolism	Catabolism	F	ubiquitin conjugating enzyme binding
1485	GO:0000151	Metabolism	Catabolism	C	ubiquitin ligase complex
1486	GO:0061630	Metabolism	Catabolism	F	ubiquitin protein ligase activity
1487	GO:0031625	Metabolism	Catabolism	F	ubiquitin protein ligase binding
1488	GO:0010992	Metabolism	Catabolism	P	ubiquitin recycling
1489	GO:0006511	Metabolism	Catabolism	P	ubiquitin-dependent protein catabolic process
1490	GO:0032182	Metabolism	Catabolism	F	ubiquitin-like protein binding
1491	GO:0061650	Metabolism	Catabolism	F	ubiquitin-like protein conjugating enzyme activity
1492	GO:0044389	Metabolism	Catabolism	F	ubiquitin-like protein ligase binding
1493	GO:0019787	Metabolism	Catabolism	F	ubiquitin-like protein transferase activity
1494	GO:0097027	Metabolism	Catabolism	F	ubiquitin-protein transferase activator activity
1495	GO:0004842	Metabolism	Catabolism	F	ubiquitin-protein transferase activity
1496	GO:1990381	Metabolism	Catabolism	F	ubiquitin-specific protease binding
1497	GO:0006212	Metabolism	Catabolism	P	uracil catabolic process
1498	GO:0019628	Metabolism	Catabolism	P	urate catabolic process
1499	GO:0030891	Metabolism	Catabolism	C	VCB complex
1500	GO:0042178	Metabolism	Catabolism	P	xenobiotic catabolic process
1501	GO:0009044	Metabolism	Catabolism	F	xylan 1,4-beta-xylosidase activity
1502	GO:0045493	Metabolism	Catabolism	P	xylan catabolic process
1503	GO:0047504	Metabolism	Metabolism	F	(-)-menthol dehydrogenase activity
1504	GO:0047501	Metabolism	Metabolism	F	(+)-neomenthol dehydrogenase activity
1505	GO:0097008	Metabolism	Metabolism	F	(3E)-4,8-dimethyl-1,3,7-nonatriene synthase activity
1506	GO:0034768	Metabolism	Metabolism	F	(E)-beta-ocimene synthase activity
1507	GO:1902609	Metabolism	Metabolism	P	(R)-2-hydroxy-alpha-linolenic acid biosynthetic process
1508	GO:0051990	Metabolism	Metabolism	F	(R)-2-hydroxyglutarate dehydrogenase activity
1509	GO:0102165	Metabolism	Metabolism	F	(Z)-3-hexen-1-ol acetyltransferase activity
1510	GO:0044571	Metabolism	Metabolism	P	[2Fe-2S] cluster assembly
1511	GO:0046509	Metabolism	Metabolism	F	1,2-diacylglycerol 3-beta-galactosyltransferase activity
1512	GO:0009257	Metabolism	Metabolism	P	10-formyltetrahydrofolate biosynthetic process
1513	GO:0016629	Metabolism	Metabolism	F	12-oxophytodienoate reductase activity
1514	GO:0009979	Metabolism	Metabolism	F	16:0 monogalactosyldiacylglycerol desaturase activity
1515	GO:0003841	Metabolism	Metabolism	F	1-acylglycerol-3-phosphate O-acyltransferase activity
1516	GO:0009815	Metabolism	Metabolism	F	1-aminocyclopropane-1-carboxylate oxidase activity
1517	GO:0016847	Metabolism	Metabolism	F	1-aminocyclopropane-1-carboxylate synthase activity
1518	GO:0052865	Metabolism	Metabolism	P	1-deoxy-D-xylulose 5-phosphate biosynthetic process
1519	GO:0030604	Metabolism	Metabolism	F	1-deoxy-D-xylulose-5-phosphate reductoisomerase activity
1520	GO:0008661	Metabolism	Metabolism	F	1-deoxy-D-xylulose-5-phosphate synthase activity
1521	GO:0080179	Metabolism	Metabolism	P	1-methylguanosine metabolic process
1522	GO:0005545	Metabolism	Metabolism	F	1-phosphatidylinositol binding
1523	GO:0051537	Metabolism	Metabolism	F	2 iron, 2 sulfur cluster binding
1524	GO:0046537	Metabolism	Metabolism	F	2,3-bisphosphoglycerate-independent phosphoglycerate mutase activity
1525	GO:0047545	Metabolism	Metabolism	F	2-hydroxyglutarate dehydrogenase activity
1526	GO:0051997	Metabolism	Metabolism	F	2-oxo-4-hydroxy-4-carboxy-5-ureidoimidazoline decarboxylase activity
1527	GO:0016706	Metabolism	Metabolism	F	2-oxoglutarate-dependent dioxygenase activity
1528	GO:0051538	Metabolism	Metabolism	F	3 iron, 4 sulfur cluster binding
1529	GO:0008686	Metabolism	Metabolism	F	3,4-dihydroxy-2-butanone-4-phosphate synthase activity

1530	GO:0008408	Metabolism	Metabolism	F	3'-5' exonuclease activity
1531	GO:0000175	Metabolism	Metabolism	F	3'-5'-exoribonuclease activity
1532	GO:0004028	Metabolism	Metabolism	F	3-chloroallyl aldehyde dehydrogenase activity
1533	GO:0003849	Metabolism	Metabolism	F	3-deoxy-7-phosphoheptulonate synthase activity
1534	GO:0008690	Metabolism	Metabolism	F	3-deoxy-manno-octulosonate cytidylyltransferase activity
1535	GO:0003860	Metabolism	Metabolism	F	3-hydroxyisobutyryl-CoA hydrolase activity
1536	GO:0003861	Metabolism	Metabolism	F	3-isopropylmalate dehydratase activity
1537	GO:0003862	Metabolism	Metabolism	F	3-isopropylmalate dehydrogenase activity
1538	GO:0003863	Metabolism	Metabolism	F	3-methyl-2-oxobutanoate dehydrogenase (2-methylpropanoyl-transferring) activity
1539	GO:0080102	Metabolism	Metabolism	F	3-methylthiopropyl glucosinolate S-oxygenase activity
1540	GO:0080066	Metabolism	Metabolism	F	3-methylthiopropyl-desulfoglucosinolate sulfotransferase activity
1541	GO:0051539	Metabolism	Metabolism	F	4 iron, 4 sulfur cluster binding
1542	GO:0097007	Metabolism	Metabolism	F	4,8,12-trimethyltrideca-1,3,7,11-tetraene synthase activity
1543	GO:0004134	Metabolism	Metabolism	F	4-alpha-glucanotransferase activity
1544	GO:0046429	Metabolism	Metabolism	F	4-hydroxy-3-methylbut-2-en-1-yl diphosphate synthase activity
1545	GO:0047443	Metabolism	Metabolism	F	4-hydroxy-4-methyl-2-oxoglutarate aldolase activity
1546	GO:0008839	Metabolism	Metabolism	F	4-hydroxy-tetrahydrodipicolinate reductase
1547	GO:0080067	Metabolism	Metabolism	F	4-methylthiobutyl-desulfoglucosinolate sulfotransferase activity
1548	GO:0080103	Metabolism	Metabolism	F	4-methylthiopropyl glucosinolate S-oxygenase activity
1549	GO:0080081	Metabolism	Metabolism	F	4-methylumbelliferyl-beta-D-glucopyranoside beta-glucosidase activity
1550	GO:0008409	Metabolism	Metabolism	F	5'-3' exonuclease activity
1551	GO:0017108	Metabolism	Metabolism	F	5'-flap endonuclease activity
1552	GO:0003871	Metabolism	Metabolism	F	5-methyltetrahydropteroyltriglutamate-homocysteine S-methyltransferase
1553	GO:0080068	Metabolism	Metabolism	F	5-methylthiopentyl-desulfoglucosinolate sulfotransferase activity
1554	GO:0080104	Metabolism	Metabolism	F	5-methylthiopropyl glucosinolate S-oxygenase activity
1555	GO:0008253	Metabolism	Metabolism	F	5'-nucleotidase activity
1556	GO:0006015	Metabolism	Metabolism	P	5-phosphoribose 1-diphosphate biosynthetic process
1557	GO:0102217	Metabolism	Metabolism	F	6-phosphoglucan, water dikinase activity
1558	GO:0017057	Metabolism	Metabolism	F	6-phosphogluconolactonase activity
1559	GO:0080069	Metabolism	Metabolism	F	7-methylthioheptyl-desulfoglucosinolate sulfotransferase activity
1560	GO:0080106	Metabolism	Metabolism	F	7-methylthiopropyl glucosinolate S-oxygenase activity
1561	GO:0080070	Metabolism	Metabolism	F	8-methylthiooctyl-desulfoglucosinolate sulfotransferase activity
1562	GO:0080107	Metabolism	Metabolism	F	8-methylthiopropyl glucosinolate S-oxygenase activity
1563	GO:0044715	Metabolism	Metabolism	F	8-oxo-dGDP phosphatase activity
1564	GO:0102490	Metabolism	Metabolism	F	8-oxo-dGTP phosphohydrolase activity
1565	GO:0006083	Metabolism	Metabolism	P	acetate metabolic process
1566	GO:0008126	Metabolism	Metabolism	F	acetylesterase activity
1567	GO:0008375	Metabolism	Metabolism	F	acetylglucosaminyltransferase activity
1568	GO:0017096	Metabolism	Metabolism	F	acetylserotonin O-methyltransferase activity
1569	GO:0016407	Metabolism	Metabolism	F	acetyltransferase activity
1570	GO:1902493	Metabolism	Metabolism	C	acetyltransferase complex
1571	GO:0016881	Metabolism	Metabolism	F	acid-amino acid ligase activity
1572	GO:0016880	Metabolism	Metabolism	F	acid-ammonia (or amide) ligase activity
1573	GO:0016878	Metabolism	Metabolism	F	acid-thiol ligase activity
1574	GO:0010309	Metabolism	Metabolism	F	acireductone dioxygenase [iron(II)-requiring] activity
1575	GO:0003994	Metabolism	Metabolism	F	aconitate hydratase activity
1576	GO:0003779	Metabolism	Metabolism	F	actin binding
1577	GO:0051015	Metabolism	Metabolism	F	actin filament binding
1578	GO:0051017	Metabolism	Metabolism	P	actin filament bundle assembly
1579	GO:0061572	Metabolism	Metabolism	P	actin filament bundle organization
1580	GO:0051693	Metabolism	Metabolism	P	actin filament capping
1581	GO:0030042	Metabolism	Metabolism	P	actin filament depolymerization
1582	GO:0051639	Metabolism	Metabolism	P	actin filament network formation
1583	GO:0007015	Metabolism	Metabolism	P	actin filament organization
1584	GO:0030041	Metabolism	Metabolism	P	actin filament polymerization
1585	GO:0030048	Metabolism	Metabolism	P	actin filament-based movement
1586	GO:0030029	Metabolism	Metabolism	P	actin filament-based process
1587	GO:0003785	Metabolism	Metabolism	F	actin monomer binding
1588	GO:0045010	Metabolism	Metabolism	P	actin nucleation
1589	GO:0008154	Metabolism	Metabolism	P	actin polymerization or depolymerization
1590	GO:0000035	Metabolism	Metabolism	F	acyl binding
1591	GO:0000036	Metabolism	Metabolism	F	acyl carrier activity
1592	GO:0045300	Metabolism	Metabolism	F	acyl-[acyl-carrier-protein] desaturase activity
1593	GO:0016297	Metabolism	Metabolism	F	acyl-[acyl-carrier-protein] hydrolase activity
1594	GO:0047372	Metabolism	Metabolism	F	acylglycerol lipase activity
1595	GO:0003999	Metabolism	Metabolism	F	adenine phosphoribosyltransferase activity
1596	GO:0004013	Metabolism	Metabolism	F	adenosylhomocysteinase activity
1597	GO:0004014	Metabolism	Metabolism	F	adenosylmethionine decarboxylase activity
1598	GO:0008179	Metabolism	Metabolism	F	adenylate cyclase binding
1599	GO:0004017	Metabolism	Metabolism	F	adenylate kinase activity
1600	GO:0047627	Metabolism	Metabolism	F	adenylylsulfatase activity
1601	GO:0004020	Metabolism	Metabolism	F	adenylylsulfate kinase activity
1602	GO:0009973	Metabolism	Metabolism	F	adenylyl-sulfate reductase activity
1603	GO:0006522	Metabolism	Metabolism	P	alanine metabolic process
1604	GO:0008453	Metabolism	Metabolism	F	alanine-glyoxylate transaminase activity
1605	GO:0046165	Metabolism	Metabolism	P	alcohol biosynthetic process
1606	GO:0004024	Metabolism	Metabolism	F	alcohol dehydrogenase activity, zinc-dependent

1607	GO:0006066	Metabolism	Metabolism	P	alcohol metabolic process
1608	GO:0004031	Metabolism	Metabolism	F	aldehyde oxidase activity
1609	GO:1990465	Metabolism	Metabolism	F	aldehyde oxygenase (deformylating) activity
1610	GO:0016832	Metabolism	Metabolism	F	aldehyde-lyase activity
1611	GO:0004034	Metabolism	Metabolism	F	aldose 1-epimerase activity
1612	GO:0052595	Metabolism	Metabolism	F	aliphatic-amine oxidase activity
1613	GO:0009820	Metabolism	Metabolism	P	alkaloid metabolic process
1614	GO:0018685	Metabolism	Metabolism	F	alkane 1-monooxygenase activity
1615	GO:0003905	Metabolism	Metabolism	F	alkylbase DNA N-glycosylase activity
1616	GO:0019428	Metabolism	Metabolism	P	allantoin biosynthetic process
1617	GO:0009978	Metabolism	Metabolism	F	allene oxide synthase activity
1618	GO:0046423	Metabolism	Metabolism	F	allene-oxide cyclase activity
1619	GO:0052924	Metabolism	Metabolism	F	all-trans-nonaprenyl-diphosphate synthase (geranylgeranyl-diphosphate specific) activity
1620	GO:0033201	Metabolism	Metabolism	F	alpha-1,4-glucan synthase activity
1621	GO:1901607	Metabolism	Metabolism	P	alpha-amino acid biosynthetic process
1622	GO:1901605	Metabolism	Metabolism	P	alpha-amino acid metabolic process
1623	GO:0004557	Metabolism	Metabolism	F	alpha-galactosidase activity
1624	GO:0003826	Metabolism	Metabolism	F	alpha-ketoacid dehydrogenase activity
1625	GO:0043604	Metabolism	Metabolism	P	amide biosynthetic process
1626	GO:0004044	Metabolism	Metabolism	F	amidophosphoribosyltransferase activity
1627	GO:0009309	Metabolism	Metabolism	P	amine biosynthetic process
1628	GO:0009308	Metabolism	Metabolism	P	amine metabolic process
1629	GO:0016843	Metabolism	Metabolism	F	amine-lyase activity
1630	GO:0016597	Metabolism	Metabolism	F	amino acid binding
1631	GO:0052594	Metabolism	Metabolism	F	aminoacetone:oxygen oxidoreductase(deaminating) activity
1632	GO:0004046	Metabolism	Metabolism	F	aminoacylase activity
1633	GO:0006023	Metabolism	Metabolism	P	aminoglycan biosynthetic process
1634	GO:0006022	Metabolism	Metabolism	P	aminoglycan metabolic process
1635	GO:0004047	Metabolism	Metabolism	F	aminomethyltransferase activity
1636	GO:0009940	Metabolism	Metabolism	F	amino-terminal vacuolar sorting propeptide binding
1637	GO:0019676	Metabolism	Metabolism	P	ammonia assimilation cycle
1638	GO:0016211	Metabolism	Metabolism	C	ammonia ligase activity
1639	GO:0016208	Metabolism	Metabolism	F	AMP binding
1640	GO:0003876	Metabolism	Metabolism	F	AMP deaminase activity
1641	GO:0046033	Metabolism	Metabolism	P	AMP metabolic process
1642	GO:0044209	Metabolism	Metabolism	P	AMP salvage
1643	GO:0047668	Metabolism	Metabolism	F	amygdalin beta-glucosidase activity
1644	GO:2001066	Metabolism	Metabolism	F	amylopectin binding
1645	GO:0010021	Metabolism	Metabolism	P	amylopectin biosynthetic process
1646	GO:0102229	Metabolism	Metabolism	F	amylopectin maltohydrolase activity
1647	GO:0070161	Metabolism	Metabolism	C	anchoring junction
1648	GO:0043168	Metabolism	Metabolism	F	anion binding
1649	GO:0009718	Metabolism	Metabolism	P	anthocyanin-containing compound biosynthetic process
1650	GO:0046283	Metabolism	Metabolism	P	anthocyanin-containing compound metabolic process
1651	GO:0004048	Metabolism	Metabolism	F	anthranilate phosphoribosyltransferase activity
1652	GO:0004049	Metabolism	Metabolism	F	anthranilate synthase activity
1653	GO:0005950	Metabolism	Metabolism	C	anthranilate synthase complex
1654	GO:0042868	Metabolism	Metabolism	P	antisense RNA metabolic process
1655	GO:0010405	Metabolism	Metabolism	P	arabinogalactan protein metabolic process
1656	GO:0019567	Metabolism	Metabolism	P	arabinose biosynthetic process
1657	GO:0019146	Metabolism	Metabolism	F	arabinose-5-phosphate isomerase activity
1658	GO:0006526	Metabolism	Metabolism	P	arginine biosynthetic process
1659	GO:0008792	Metabolism	Metabolism	F	arginine decarboxylase activity
1660	GO:0047769	Metabolism	Metabolism	F	arogenate dehydratase activity
1661	GO:0070330	Metabolism	Metabolism	F	aromatase activity
1662	GO:0009073	Metabolism	Metabolism	P	aromatic amino acid family biosynthetic process
1663	GO:0009072	Metabolism	Metabolism	P	aromatic amino acid family metabolic process
1664	GO:0019438	Metabolism	Metabolism	P	aromatic compound biosynthetic process
1665	GO:0004058	Metabolism	Metabolism	F	aromatic-L-amino-acid decarboxylase activity
1666	GO:0034314	Metabolism	Metabolism	P	Arp2/3 complex-mediated actin nucleation
1667	GO:0005885	Metabolism	Metabolism	C	Arp2/3 protein complex
1668	GO:0004067	Metabolism	Metabolism	F	asparaginase activity
1669	GO:0009067	Metabolism	Metabolism	P	aspartate family amino acid biosynthetic process
1670	GO:0009066	Metabolism	Metabolism	P	aspartate family amino acid metabolic process
1671	GO:0004072	Metabolism	Metabolism	F	aspartate kinase activity
1672	GO:0042500	Metabolism	Metabolism	F	aspartic endopeptidase activity, intramembrane cleaving
1673	GO:0004190	Metabolism	Metabolism	F	aspartic-type endopeptidase activity
1674	GO:0045330	Metabolism	Metabolism	F	aspartyl esterase activity
1675	GO:0005524	Metabolism	Metabolism	F	ATP binding
1676	GO:0004176	Metabolism	Metabolism	F	ATP-dependent peptidase activity
1677	GO:0042537	Metabolism	Metabolism	P	benzene-containing compound metabolic process
1678	GO:0018874	Metabolism	Metabolism	P	benzoate metabolic process
1679	GO:0052641	Metabolism	Metabolism	F	benzoic acid glucosyltransferase activity
1680	GO:0102996	Metabolism	Metabolism	F	beta,beta digalactosyldiacylglycerol galactosyltransferase activity
1681	GO:0019483	Metabolism	Metabolism	P	beta-alanine biosynthetic process
1682	GO:0033396	Metabolism	Metabolism	P	beta-alanine biosynthetic process via 3-ureidopropionate
1683	GO:0019482	Metabolism	Metabolism	P	beta-alanine metabolic process

1684	GO:0008798	Metabolism	Metabolism	F	beta-aspartyl-peptidase activity
1685	GO:0004564	Metabolism	Metabolism	F	beta-fructofuranosidase activity
1686	GO:0004565	Metabolism	Metabolism	F	beta-galactosidase activity
1687	GO:0080083	Metabolism	Metabolism	F	beta-gentiobiose beta-glucosidase activity
1688	GO:0051274	Metabolism	Metabolism	P	beta-glucan biosynthetic process
1689	GO:0051273	Metabolism	Metabolism	P	beta-glucan metabolic process
1690	GO:0008422	Metabolism	Metabolism	F	beta-glucosidase activity
1691	GO:0008802	Metabolism	Metabolism	F	betaine-aldehyde dehydrogenase activity
1692	GO:0102500	Metabolism	Metabolism	F	beta-maltose 4-alpha-glucanotransferase activity
1693	GO:0004567	Metabolism	Metabolism	F	beta-mannosidase activity
1694	GO:0048487	Metabolism	Metabolism	F	beta-tubulin binding
1695	GO:0003837	Metabolism	Metabolism	F	beta-ureidopropionase activity
1696	GO:0005488	Metabolism	Metabolism	F	binding
1697	GO:0008150	Metabolism	Metabolism	P	biological_process
1698	GO:0009058	Metabolism	Metabolism	P	biosynthetic process
1699	GO:0009374	Metabolism	Metabolism	F	biotin binding
1700	GO:0009082	Metabolism	Metabolism	P	branched-chain amino acid biosynthetic process
1701	GO:0009081	Metabolism	Metabolism	P	branched-chain amino acid metabolic process
1702	GO:0004084	Metabolism	Metabolism	F	branched-chain-amino-acid transaminase activity
1703	GO:0047763	Metabolism	Metabolism	F	caffeate O-methyltransferase activity
1704	GO:0010120	Metabolism	Metabolism	P	camalexin biosynthetic process
1705	GO:0052317	Metabolism	Metabolism	P	camalexin metabolic process
1706	GO:0030552	Metabolism	Metabolism	F	cAMP binding
1707	GO:0045230	Metabolism	Metabolism	P	capsule organization
1708	GO:0045227	Metabolism	Metabolism	P	capsule polysaccharide biosynthetic process
1709	GO:0015976	Metabolism	Metabolism	P	carbon utilization
1710	GO:0016830	Metabolism	Metabolism	F	carbon-carbon lyase activity
1711	GO:0016835	Metabolism	Metabolism	F	carbon-oxygen lyase activity
1712	GO:0016846	Metabolism	Metabolism	F	carbon-sulfur lyase activity
1713	GO:0046394	Metabolism	Metabolism	P	carboxylic acid biosynthetic process
1714	GO:0019752	Metabolism	Metabolism	P	carboxylic acid metabolic process
1715	GO:0052689	Metabolism	Metabolism	F	carboxylic ester hydrolase activity
1716	GO:0016831	Metabolism	Metabolism	F	carboxy-lyase activity
1717	GO:0004180	Metabolism	Metabolism	F	carboxypeptidase activity
1718	GO:0032806	Metabolism	Metabolism	C	carboxy-terminal domain protein kinase complex
1719	GO:0010291	Metabolism	Metabolism	F	carotene beta-ring hydroxylase activity
1720	GO:0016119	Metabolism	Metabolism	P	carotene metabolic process
1721	GO:0003824	Metabolism	Metabolism	F	catalytic activity
1722	GO:0140096	Metabolism	Metabolism	F	catalytic activity, acting on a protein
1723	GO:0140097	Metabolism	Metabolism	F	catalytic activity, acting on DNA
1724	GO:1902494	Metabolism	Metabolism	C	catalytic complex
1725	GO:0043169	Metabolism	Metabolism	F	cation binding
1726	GO:0016024	Metabolism	Metabolism	P	CDP-diacylglycerol biosynthetic process
1727	GO:0003881	Metabolism	Metabolism	F	CDP-diacylglycerol-inositol 3-phosphatidyltransferase activity
1728	GO:0044038	Metabolism	Metabolism	P	cell wall macromolecule biosynthetic process
1729	GO:0044036	Metabolism	Metabolism	P	cell wall macromolecule metabolic process
1730	GO:0000032	Metabolism	Metabolism	P	cell wall mannoprotein biosynthetic process
1731	GO:0052325	Metabolism	Metabolism	P	cell wall pectin biosynthetic process
1732	GO:0070592	Metabolism	Metabolism	P	cell wall polysaccharide biosynthetic process
1733	GO:0010383	Metabolism	Metabolism	P	cell wall polysaccharide metabolic process
1734	GO:0080079	Metabolism	Metabolism	F	cellobiose glucosidase activity
1735	GO:0006081	Metabolism	Metabolism	P	cellular aldehyde metabolic process
1736	GO:0043603	Metabolism	Metabolism	P	cellular amide metabolic process
1737	GO:0044106	Metabolism	Metabolism	P	cellular amine metabolic process
1738	GO:0008652	Metabolism	Metabolism	P	cellular amino acid biosynthetic process
1739	GO:0006520	Metabolism	Metabolism	P	cellular amino acid metabolic process
1740	GO:0006725	Metabolism	Metabolism	P	cellular aromatic compound metabolic process
1741	GO:0042401	Metabolism	Metabolism	P	cellular biogenic amine biosynthetic process
1742	GO:0006576	Metabolism	Metabolism	P	cellular biogenic amine metabolic process
1743	GO:0044249	Metabolism	Metabolism	P	cellular biosynthetic process
1744	GO:0070589	Metabolism	Metabolism	P	cellular component macromolecule biosynthetic process
1745	GO:0006073	Metabolism	Metabolism	P	cellular glucan metabolic process
1746	GO:0042180	Metabolism	Metabolism	P	cellular ketone metabolic process
1747	GO:0044255	Metabolism	Metabolism	P	cellular lipid metabolic process
1748	GO:0034645	Metabolism	Metabolism	P	cellular macromolecule biosynthetic process
1749	GO:0044260	Metabolism	Metabolism	P	cellular macromolecule metabolic process
1750	GO:0044237	Metabolism	Metabolism	P	cellular metabolic process
1751	GO:0006575	Metabolism	Metabolism	P	cellular modified amino acid metabolic process
1752	GO:0044271	Metabolism	Metabolism	P	cellular nitrogen compound biosynthetic process
1753	GO:0034641	Metabolism	Metabolism	P	cellular nitrogen compound metabolic process
1754	GO:0034613	Metabolism	Metabolism	P	cellular protein localization
1755	GO:0044267	Metabolism	Metabolism	P	cellular protein metabolic process
1756	GO:0006464	Metabolism	Metabolism	P	cellular protein modification process
1757	GO:0034622	Metabolism	Metabolism	P	cellular protein-containing complex assembly
1758	GO:0008810	Metabolism	Metabolism	F	cellulase activity
1759	GO:0030244	Metabolism	Metabolism	P	cellulose biosynthetic process
1760	GO:0030243	Metabolism	Metabolism	P	cellulose metabolic process
1761	GO:0010215	Metabolism	Metabolism	P	cellulose microfibril organization

1762	GO:0016760	Metabolism	Metabolism	F	cellulose synthase (UDP-forming) activity
1763	GO:0016759	Metabolism	Metabolism	F	cellulose synthase activity
1764	GO:0010330	Metabolism	Metabolism	C	cellulose synthase complex
1765	GO:0034080	Metabolism	Metabolism	P	CENP-A containing nucleosome assembly
1766	GO:1902387	Metabolism	Metabolism	F	ceramide 1-phosphate binding
1767	GO:1902388	Metabolism	Metabolism	F	ceramide 1-phosphate transfer activity
1768	GO:0046513	Metabolism	Metabolism	P	ceramide biosynthetic process
1769	GO:0047493	Metabolism	Metabolism	F	ceramide cholinephosphotransferase activity
1770	GO:0006672	Metabolism	Metabolism	P	ceramide metabolic process
1771	GO:0030553	Metabolism	Metabolism	F	cGMP binding
1772	GO:0009715	Metabolism	Metabolism	P	chalcone biosynthetic process
1773	GO:0008061	Metabolism	Metabolism	F	chitin binding
1774	GO:0004568	Metabolism	Metabolism	F	chitinase activity
1775	GO:0042425	Metabolism	Metabolism	P	choline biosynthetic process
1776	GO:0004103	Metabolism	Metabolism	F	choline kinase activity
1777	GO:0004105	Metabolism	Metabolism	F	choline-phosphate cytidylyltransferase activity
1778	GO:0009423	Metabolism	Metabolism	P	chorismate biosynthetic process
1779	GO:0003682	Metabolism	Metabolism	F	chromatin binding
1780	GO:0050412	Metabolism	Metabolism	F	cinnamate beta-D-glucosyltransferase activity
1781	GO:0009800	Metabolism	Metabolism	P	cinnamic acid biosynthetic process
1782	GO:0009801	Metabolism	Metabolism	P	cinnamic acid ester metabolic process
1783	GO:0009803	Metabolism	Metabolism	P	cinnamic acid metabolic process
1784	GO:0045551	Metabolism	Metabolism	F	cinnamyl-alcohol dehydrogenase activity
1785	GO:0033961	Metabolism	Metabolism	F	cis-stilbene-oxide hydrolase activity
1786	GO:0033468	Metabolism	Metabolism	P	CMP-keto-3-deoxy-D-manno-octulosonic acid biosynthetic process
1787	GO:0009236	Metabolism	Metabolism	P	cobalamin biosynthetic process
1788	GO:0048037	Metabolism	Metabolism	F	cofactor binding
1789	GO:0047782	Metabolism	Metabolism	F	coniferin beta-glucosidase activity
1790	GO:0050268	Metabolism	Metabolism	F	coniferyl-alcohol dehydrogenase activity
1791	GO:0050269	Metabolism	Metabolism	F	coniferyl-aldehyde dehydrogenase activity
1792	GO:0016531	Metabolism	Metabolism	F	copper chaperone activity
1793	GO:0005507	Metabolism	Metabolism	F	copper ion binding
1794	GO:0009805	Metabolism	Metabolism	P	coumarin biosynthetic process
1795	GO:0009804	Metabolism	Metabolism	P	coumarin metabolic process
1796	GO:0006241	Metabolism	Metabolism	P	CTP biosynthetic process
1797	GO:0003883	Metabolism	Metabolism	F	CTP synthase activity
1798	GO:0097602	Metabolism	Metabolism	F	cullin family protein binding
1799	GO:0006723	Metabolism	Metabolism	P	cuticle hydrocarbon biosynthetic process
1800	GO:0010143	Metabolism	Metabolism	P	cutin biosynthetic process
1801	GO:0008824	Metabolism	Metabolism	F	cyanate hydratase activity
1802	GO:0102580	Metabolism	Metabolism	F	cyanidin 3-O-glucoside 2-O''-xylosyltransferase activity
1803	GO:1901038	Metabolism	Metabolism	P	cyanidin 3-O-glucoside metabolic process
1804	GO:0047793	Metabolism	Metabolism	F	cycloeucalenol cycloisomerase activity
1805	GO:0016018	Metabolism	Metabolism	F	cyclosporin A binding
1806	GO:0004121	Metabolism	Metabolism	F	cystathionine beta-lyase activity
1807	GO:0004123	Metabolism	Metabolism	F	cystathionine gamma-lyase activity
1808	GO:0019344	Metabolism	Metabolism	P	cysteine biosynthetic process
1809	GO:0006535	Metabolism	Metabolism	P	cysteine biosynthetic process from serine
1810	GO:0019343	Metabolism	Metabolism	P	cysteine biosynthetic process via cystathionine
1811	GO:0031071	Metabolism	Metabolism	F	cysteine desulfurase activity
1812	GO:0017172	Metabolism	Metabolism	F	cysteine dioxygenase activity
1813	GO:0006534	Metabolism	Metabolism	P	cysteine metabolic process
1814	GO:0004124	Metabolism	Metabolism	F	cysteine synthase activity
1815	GO:0047804	Metabolism	Metabolism	F	cysteine-S-conjugate beta-lyase activity
1816	GO:0008234	Metabolism	Metabolism	F	cysteine-type peptidase activity
1817	GO:0004126	Metabolism	Metabolism	F	cytidine deaminase activity
1818	GO:0009972	Metabolism	Metabolism	P	cytidine deamination
1819	GO:0016554	Metabolism	Metabolism	P	cytidine to uridine editing
1820	GO:0102485	Metabolism	Metabolism	F	dATP phosphohydrolase activity
1821	GO:0102486	Metabolism	Metabolism	F	dCTP phosphohydrolase activity
1822	GO:0044210	Metabolism	Metabolism	P	'de novo' CTP biosynthetic process
1823	GO:0042351	Metabolism	Metabolism	P	'de novo' GDP-L-fucose biosynthetic process
1824	GO:0006189	Metabolism	Metabolism	P	'de novo' IMP biosynthetic process
1825	GO:0006207	Metabolism	Metabolism	P	'de novo' pyrimidine nucleobase biosynthetic process
1826	GO:0044205	Metabolism	Metabolism	P	'de novo' UMP biosynthetic process
1827	GO:0019239	Metabolism	Metabolism	F	deaminase activity
1828	GO:0009704	Metabolism	Metabolism	P	de-etiolation
1829	GO:0045547	Metabolism	Metabolism	F	dehydrodolichyl diphosphate synthase activity
1830	GO:0052637	Metabolism	Metabolism	F	delta 3-trans-hexadecenoic acid phosphatidylglycerol desaturase activity
1831	GO:0050613	Metabolism	Metabolism	F	delta14-sterol reductase activity
1832	GO:0047844	Metabolism	Metabolism	F	deoxycytidine deaminase activity
1833	GO:0019136	Metabolism	Metabolism	F	deoxynucleoside kinase activity
1834	GO:0004536	Metabolism	Metabolism	F	deoxyribonuclease activity
1835	GO:0009157	Metabolism	Metabolism	P	deoxyribonucleoside monophosphate biosynthetic process
1836	GO:0009263	Metabolism	Metabolism	P	deoxyribonucleotide biosynthetic process
1837	GO:0047364	Metabolism	Metabolism	F	desulfoglucosinolate sulfotransferase activity
1838	GO:0046396	Metabolism	Metabolism	P	D-galacturonate metabolic process
1839	GO:0102491	Metabolism	Metabolism	F	dGTP phosphohydrolase activity

1840	GO:0004144	Metabolism	Metabolism	F	diacylglycerol O-acyltransferase activity
1841	GO:0019877	Metabolism	Metabolism	P	diaminopimelate biosynthetic process
1842	GO:0008837	Metabolism	Metabolism	F	diaminopimelate epimerase activity
1843	GO:0043648	Metabolism	Metabolism	P	dicarboxylic acid metabolic process
1844	GO:0072548	Metabolism	Metabolism	F	dicoumaroyl monocaffeoyl spermidine meta-hydroxylase activity
1845	GO:0072551	Metabolism	Metabolism	F	diferuloyl mono-(hydroxyferuloyl) spermidine meta-hydroxylase activity
1846	GO:0045552	Metabolism	Metabolism	F	dihydrokaempferol 4-reductase activity
1847	GO:0004148	Metabolism	Metabolism	F	dihydrolipoyl dehydrogenase activity
1848	GO:0004742	Metabolism	Metabolism	F	dihydrolipoyllysine-residue acetyltransferase activity
1849	GO:0004149	Metabolism	Metabolism	F	dihydrolipoyllysine-residue succinyltransferase activity
1850	GO:0004151	Metabolism	Metabolism	F	dihydroorotase activity
1851	GO:0050992	Metabolism	Metabolism	P	dimethylallyl diphosphate biosynthetic process
1852	GO:0004161	Metabolism	Metabolism	F	dimethylallyltranstransferase activity
1853	GO:0051213	Metabolism	Metabolism	F	dioxygenase activity
1854	GO:0016805	Metabolism	Metabolism	F	dipeptidase activity
1855	GO:0008239	Metabolism	Metabolism	F	dipeptidyl-peptidase activity
1856	GO:0047334	Metabolism	Metabolism	F	diphosphate-fructose-6-phosphate 1-phosphotransferase activity
1857	GO:0004163	Metabolism	Metabolism	F	diphosphomevalonate decarboxylase activity
1858	GO:0003677	Metabolism	Metabolism	F	DNA binding
1859	GO:0006259	Metabolism	Metabolism	P	DNA metabolic process
1860	GO:0019104	Metabolism	Metabolism	F	DNA N-glycosylase activity
1861	GO:0003913	Metabolism	Metabolism	F	DNA photolyase activity
1862	GO:0000217	Metabolism	Metabolism	F	DNA secondary structure binding
1863	GO:0008725	Metabolism	Metabolism	F	DNA-3-methyladenine glycosylase activity
1864	GO:0052822	Metabolism	Metabolism	F	DNA-3-methylguanine glycosylase activity
1865	GO:0052821	Metabolism	Metabolism	F	DNA-7-methyladenine glycosylase activity
1866	GO:0043916	Metabolism	Metabolism	F	DNA-7-methylguanine glycosylase activity
1867	GO:0019408	Metabolism	Metabolism	P	dolichol biosynthetic process
1868	GO:0019348	Metabolism	Metabolism	P	dolichol metabolic process
1869	GO:0006488	Metabolism	Metabolism	P	dolichol-linked oligosaccharide biosynthetic process
1870	GO:0106073	Metabolism	Metabolism	F	dolichyl pyrophosphate Glc2Man9GlcNAc2 alpha-1,2-glucosyltransferase
1871	GO:0042281	Metabolism	Metabolism	F	dolichyl pyrophosphate Man9GlcNAc2 alpha-1,3-glucosyltransferase activity
1872	GO:0004579	Metabolism	Metabolism	F	dolichyl-diphosphooligosaccharide-protein glycotransferase activity
1873	GO:0004582	Metabolism	Metabolism	F	dolichyl-phosphate beta-D-mannosyltransferase activity
1874	GO:0004583	Metabolism	Metabolism	F	dolichyl-phosphate-glucose-glycolipid alpha-glucosyltransferase activity
1875	GO:0003690	Metabolism	Metabolism	F	double-stranded DNA binding
1876	GO:0003691	Metabolism	Metabolism	F	double-stranded telomeric DNA binding
1877	GO:0008830	Metabolism	Metabolism	F	dTDP-4-dehydrorhamnose 3,5-epimerase activity
1878	GO:0008831	Metabolism	Metabolism	F	dTDP-4-dehydrorhamnose reductase activity
1879	GO:0008460	Metabolism	Metabolism	F	dTDP-glucose 4,6-dehydratase activity
1880	GO:0019305	Metabolism	Metabolism	P	dTDP-rhamnose biosynthetic process
1881	GO:0102488	Metabolism	Metabolism	F	dTTP phosphohydrolase activity
1882	GO:0102487	Metabolism	Metabolism	F	dUTP phosphohydrolase activity
1883	GO:0042732	Metabolism	Metabolism	P	D-xylose metabolic process
1884	GO:0046526	Metabolism	Metabolism	F	D-xylulose reductase activity
1885	GO:0008843	Metabolism	Metabolism	F	endochitinase activity
1886	GO:0006897	Metabolism	Metabolism	P	endocytosis
1887	GO:0016889	Metabolism	Metabolism	F	endodeoxyribonuclease activity, producing 3'-phosphomonoesters
1888	GO:0004866	Metabolism	Metabolism	F	endopeptidase inhibitor activity
1889	GO:0016891	Metabolism	Metabolism	F	endoribonuclease activity, producing 5'-phosphomonoesters
1890	GO:0035671	Metabolism	Metabolism	F	enone reductase activity
1891	GO:0008047	Metabolism	Metabolism	F	enzyme activator activity
1892	GO:0019899	Metabolism	Metabolism	F	enzyme binding
1893	GO:0004857	Metabolism	Metabolism	F	enzyme inhibitor activity
1894	GO:0004301	Metabolism	Metabolism	F	epoxide hydrolase activity
1895	GO:0019373	Metabolism	Metabolism	P	epoxygenase P450 pathway
1896	GO:0006696	Metabolism	Metabolism	P	ergosterol biosynthetic process
1897	GO:1902221	Metabolism	Metabolism	P	erythrose 4-phosphate/phosphoenolpyruvate family amino acid metabolic process
1898	GO:0080082	Metabolism	Metabolism	F	esculin beta-glucosidase activity
1899	GO:0051234	Metabolism	Metabolism	P	establishment of localization
1900	GO:0004306	Metabolism	Metabolism	F	ethanolamine-phosphate cytidylyltransferase activity
1901	GO:0035885	Metabolism	Metabolism	F	exochitinase activity
1902	GO:0006887	Metabolism	Metabolism	P	exocytosis
1903	GO:0010409	Metabolism	Metabolism	P	extensin metabolic process
1904	GO:0045226	Metabolism	Metabolism	P	extracellular polysaccharide biosynthetic process
1905	GO:0046379	Metabolism	Metabolism	P	extracellular polysaccharide metabolic process
1906	GO:0071949	Metabolism	Metabolism	F	FAD binding
1907	GO:0006747	Metabolism	Metabolism	P	FAD biosynthetic process
1908	GO:0045337	Metabolism	Metabolism	P	farnesyl diphosphate biosynthetic process
1909	GO:0004310	Metabolism	Metabolism	F	farnesyl-diphosphate farnesyltransferase activity
1910	GO:0004311	Metabolism	Metabolism	F	farnesyltranstransferase activity
1911	GO:0008199	Metabolism	Metabolism	F	ferric iron binding
1912	GO:0000293	Metabolism	Metabolism	F	ferric-chelate reductase activity
1913	GO:0004325	Metabolism	Metabolism	F	ferrochelatase activity
1914	GO:0008198	Metabolism	Metabolism	F	ferrous iron binding
1915	GO:0004322	Metabolism	Metabolism	F	ferroxidase activity
1916	GO:0047890	Metabolism	Metabolism	F	flavanone 4-reductase activity

1917	GO:0050660	Metabolism	Metabolism	F	flavin adenine dinucleotide binding
1918	GO:0072387	Metabolism	Metabolism	P	flavin adenine dinucleotide metabolic process
1919	GO:0016971	Metabolism	Metabolism	F	flavin-linked sulfhydryl oxidase activity
1920	GO:0033759	Metabolism	Metabolism	F	flavone synthase activity
1921	GO:0047893	Metabolism	Metabolism	F	flavonol 3-O-glucosyltransferase activity
1922	GO:0033836	Metabolism	Metabolism	F	flavonol 7-O-beta-glucosyltransferase activity
1923	GO:0045431	Metabolism	Metabolism	F	flavonol synthase activity
1924	GO:0003919	Metabolism	Metabolism	F	FMN adenylyltransferase activity
1925	GO:0010181	Metabolism	Metabolism	F	FMN binding
1926	GO:0009398	Metabolism	Metabolism	P	FMN biosynthetic process
1927	GO:0046655	Metabolism	Metabolism	P	folic acid metabolic process
1928	GO:0004328	Metabolism	Metabolism	F	formamidase activity
1929	GO:0004329	Metabolism	Metabolism	F	formate-tetrahydrofolate ligase activity
1930	GO:0006004	Metabolism	Metabolism	P	fucose metabolic process
1931	GO:0004333	Metabolism	Metabolism	F	fumarate hydratase activity
1932	GO:0006106	Metabolism	Metabolism	P	fumarate metabolic process
1933	GO:0047274	Metabolism	Metabolism	F	galactinol-sucrose galactosyltransferase activity
1934	GO:0010392	Metabolism	Metabolism	P	galactoglucomannan metabolic process
1935	GO:0004335	Metabolism	Metabolism	F	galactokinase activity
1936	GO:0019375	Metabolism	Metabolism	P	galactolipid biosynthetic process
1937	GO:0046480	Metabolism	Metabolism	F	galactolipid galactosyltransferase activity
1938	GO:0019374	Metabolism	Metabolism	P	galactolipid metabolic process
1939	GO:0051070	Metabolism	Metabolism	P	galactomannan biosynthetic process
1940	GO:0005534	Metabolism	Metabolism	F	galactose binding
1941	GO:0046369	Metabolism	Metabolism	P	galactose biosynthetic process
1942	GO:0006012	Metabolism	Metabolism	P	galactose metabolic process
1943	GO:0015925	Metabolism	Metabolism	F	galactosidase activity
1944	GO:0008107	Metabolism	Metabolism	F	galactoside 2-alpha-L-fucosyltransferase activity
1945	GO:0008378	Metabolism	Metabolism	F	galactosyltransferase activity
1946	GO:0047911	Metabolism	Metabolism	F	galacturan 1,4-alpha-galacturonidase activity
1947	GO:0010393	Metabolism	Metabolism	P	galacturonan metabolic process
1948	GO:0033481	Metabolism	Metabolism	P	galacturonate biosynthetic process
1949	GO:0019586	Metabolism	Metabolism	P	galacturonate metabolic process
1950	GO:0047912	Metabolism	Metabolism	F	galacturonokinase activity
1951	GO:0003839	Metabolism	Metabolism	F	gamma-glutamylcyclotransferase activity
1952	GO:0019003	Metabolism	Metabolism	F	GDP binding
1953	GO:0008446	Metabolism	Metabolism	F	GDP-mannose 4,6-dehydratase activity
1954	GO:0009298	Metabolism	Metabolism	P	GDP-mannose biosynthetic process
1955	GO:0019673	Metabolism	Metabolism	P	GDP-mannose metabolic process
1956	GO:0102797	Metabolism	Metabolism	F	geranial:oxygen oxidoreductase activity
1957	GO:0033384	Metabolism	Metabolism	P	geranyl diphosphate biosynthetic process
1958	GO:0033386	Metabolism	Metabolism	P	geranylgeranyl diphosphate biosynthetic process
1959	GO:0016767	Metabolism	Metabolism	F	geranylgeranyl-diphosphate geranylgeranyltransferase activity
1960	GO:0004337	Metabolism	Metabolism	F	geranyltranstransferase activity
1961	GO:0009250	Metabolism	Metabolism	P	glucan biosynthetic process
1962	GO:0044042	Metabolism	Metabolism	P	glucan metabolic process
1963	GO:0047259	Metabolism	Metabolism	F	glucomannan 4-beta-mannosyltransferase activity
1964	GO:0019761	Metabolism	Metabolism	P	glucosinolate biosynthetic process
1965	GO:0102799	Metabolism	Metabolism	F	glucosinolate glucohydrolase activity
1966	GO:0019760	Metabolism	Metabolism	P	glucosinolate metabolic process
1967	GO:0046527	Metabolism	Metabolism	F	glucosyltransferase activity
1968	GO:0015020	Metabolism	Metabolism	F	glucuronosyltransferase activity
1969	GO:0030775	Metabolism	Metabolism	F	glucuronoxylan 4-O-methyltransferase activity
1970	GO:0010417	Metabolism	Metabolism	P	glucuronoxylan biosynthetic process
1971	GO:0004349	Metabolism	Metabolism	F	glutamate 5-kinase activity
1972	GO:0016595	Metabolism	Metabolism	F	glutamate binding
1973	GO:0006537	Metabolism	Metabolism	P	glutamate biosynthetic process
1974	GO:0004351	Metabolism	Metabolism	F	glutamate decarboxylase activity
1975	GO:0006540	Metabolism	Metabolism	P	glutamate decarboxylation to succinate
1976	GO:0006536	Metabolism	Metabolism	P	glutamate metabolic process
1977	GO:0016041	Metabolism	Metabolism	F	glutamate synthase (ferredoxin) activity
1978	GO:0004350	Metabolism	Metabolism	F	glutamate-5-semialdehyde dehydrogenase activity
1979	GO:0004356	Metabolism	Metabolism	F	glutamate-ammonia ligase activity
1980	GO:0004357	Metabolism	Metabolism	F	glutamate-cysteine ligase activity
1981	GO:0004359	Metabolism	Metabolism	F	glutaminase activity
1982	GO:0006542	Metabolism	Metabolism	P	glutamine biosynthetic process
1983	GO:0009084	Metabolism	Metabolism	P	glutamine family amino acid biosynthetic process
1984	GO:0009064	Metabolism	Metabolism	P	glutamine family amino acid metabolic process
1985	GO:0006541	Metabolism	Metabolism	P	glutamine metabolic process
1986	GO:0016603	Metabolism	Metabolism	F	glutaminyl-peptide cyclotransferase activity
1987	GO:0046166	Metabolism	Metabolism	P	glyceraldehyde-3-phosphate biosynthetic process
1988	GO:0008465	Metabolism	Metabolism	F	glycerate dehydrogenase activity
1989	GO:0008887	Metabolism	Metabolism	F	glycerate kinase activity
1990	GO:0006662	Metabolism	Metabolism	P	glycerol ether metabolic process
1991	GO:0004370	Metabolism	Metabolism	F	glycerol kinase activity
1992	GO:0006071	Metabolism	Metabolism	P	glycerol metabolic process
1993	GO:0090447	Metabolism	Metabolism	F	glycerol-3-phosphate 2-O-acyltransferase activity
1994	GO:0046167	Metabolism	Metabolism	P	glycerol-3-phosphate biosynthetic process

1995	GO:0004367	Metabolism	Metabolism	F	glycerol-3-phosphate dehydrogenase [NAD+] activity
1996	GO:0009331	Metabolism	Metabolism	C	glycerol-3-phosphate dehydrogenase complex
1997	GO:0006072	Metabolism	Metabolism	P	glycerol-3-phosphate metabolic process
1998	GO:0004366	Metabolism	Metabolism	F	glycerol-3-phosphate O-acyltransferase activity
1999	GO:0046486	Metabolism	Metabolism	P	glycerolipid metabolic process
2000	GO:0004371	Metabolism	Metabolism	F	glycerone kinase activity
2001	GO:0008889	Metabolism	Metabolism	F	glycerophosphodiester phosphodiesterase activity
2002	GO:0006650	Metabolism	Metabolism	P	glycerophospholipid metabolic process
2003	GO:0016594	Metabolism	Metabolism	F	glycine binding
2004	GO:0006545	Metabolism	Metabolism	P	glycine biosynthetic process
2005	GO:0019264	Metabolism	Metabolism	P	glycine biosynthetic process from serine
2006	GO:0005960	Metabolism	Metabolism	C	glycine cleavage complex
2007	GO:0019464	Metabolism	Metabolism	P	glycine decarboxylation via glycine cleavage system
2008	GO:0004375	Metabolism	Metabolism	F	glycine dehydrogenase (decarboxylating) activity
2009	GO:0004372	Metabolism	Metabolism	F	glycine hydroxymethyltransferase activity
2010	GO:0006544	Metabolism	Metabolism	P	glycine metabolic process
2011	GO:0047958	Metabolism	Metabolism	F	glycine:2-oxoglutarate aminotransferase activity
2012	GO:0005978	Metabolism	Metabolism	P	glycogen biosynthetic process
2013	GO:0005977	Metabolism	Metabolism	P	glycogen metabolic process
2014	GO:0009247	Metabolism	Metabolism	P	glycolipid biosynthetic process
2015	GO:0034203	Metabolism	Metabolism	P	glycolipid translocation
2016	GO:0033926	Metabolism	Metabolism	F	glycopeptide alpha-N-acetylgalactosaminidase activity
2017	GO:0009100	Metabolism	Metabolism	P	glycoprotein metabolic process
2018	GO:0006024	Metabolism	Metabolism	P	glycosaminoglycan biosynthetic process
2019	GO:0030203	Metabolism	Metabolism	P	glycosaminoglycan metabolic process
2020	GO:0019758	Metabolism	Metabolism	P	glycosinolate biosynthetic process
2021	GO:0019757	Metabolism	Metabolism	P	glycosinolate metabolic process
2022	GO:1901659	Metabolism	Metabolism	P	glycosyl compound biosynthetic process
2023	GO:1901657	Metabolism	Metabolism	P	glycosyl compound metabolic process
2024	GO:0019172	Metabolism	Metabolism	F	glyoxalase III activity
2025	GO:0018920	Metabolism	Metabolism	P	glyphosate metabolic process
2026	GO:0032580	Metabolism	Metabolism	C	Golgi cisterna membrane
2027	GO:0006506	Metabolism	Metabolism	P	GPI anchor biosynthetic process
2028	GO:0003923	Metabolism	Metabolism	F	GPI-anchor transamidase activity
2029	GO:0042765	Metabolism	Metabolism	C	GPI-anchor transamidase complex
2030	GO:0010597	Metabolism	Metabolism	P	green leaf volatile biosynthetic process
2031	GO:0005525	Metabolism	Metabolism	F	GTP binding
2032	GO:0006183	Metabolism	Metabolism	P	GTP biosynthetic process
2033	GO:0003935	Metabolism	Metabolism	F	GTP cyclohydrolase II activity
2034	GO:0005096	Metabolism	Metabolism	F	GTPase activator activity
2035	GO:0003924	Metabolism	Metabolism	F	GTPase activity
2036	GO:0106046	Metabolism	Metabolism	P	guanine deglycation, glyoxal removal
2037	GO:0015969	Metabolism	Metabolism	P	guanosine tetraphosphate metabolic process
2038	GO:0004383	Metabolism	Metabolism	F	guanylate cyclase activity
2039	GO:0005085	Metabolism	Metabolism	F	guanyl-nucleotide exchange factor activity
2040	GO:0020037	Metabolism	Metabolism	F	heme binding
2041	GO:0102798	Metabolism	Metabolism	F	heptaldehyde:oxygen oxidoreductase activity
2042	GO:0018130	Metabolism	Metabolism	P	heterocycle biosynthetic process
2043	GO:0046483	Metabolism	Metabolism	P	heterocycle metabolic process
2044	GO:1901363	Metabolism	Metabolism	F	heterocyclic compound binding
2045	GO:0010297	Metabolism	Metabolism	F	heteropolysaccharide binding
2046	GO:0000105	Metabolism	Metabolism	P	histidine biosynthetic process
2047	GO:0009927	Metabolism	Metabolism	F	histidine phosphotransfer kinase activity
2048	GO:0010289	Metabolism	Metabolism	P	homogalacturonan biosynthetic process
2049	GO:0010394	Metabolism	Metabolism	P	homogalacturonan metabolic process
2050	GO:0004411	Metabolism	Metabolism	F	homogentisate 1,2-dioxygenase activity
2051	GO:0009090	Metabolism	Metabolism	P	homoserine biosynthetic process
2052	GO:0004412	Metabolism	Metabolism	F	homoserine dehydrogenase activity
2053	GO:0004413	Metabolism	Metabolism	F	homoserine kinase activity
2054	GO:0120251	Metabolism	Metabolism	P	hydrocarbon biosynthetic process
2055	GO:0120252	Metabolism	Metabolism	P	hydrocarbon metabolic process
2056	GO:0070814	Metabolism	Metabolism	P	hydrogen sulfide biosynthetic process
2057	GO:0070813	Metabolism	Metabolism	P	hydrogen sulfide metabolic process
2058	GO:0016787	Metabolism	Metabolism	F	hydrolase activity
2059	GO:0016817	Metabolism	Metabolism	F	hydrolase activity, acting on acid anhydrides
2060	GO:0016818	Metabolism	Metabolism	F	hydrolase activity, acting on acid anhydrides, in phosphorus-containing anhydrides
2061	GO:0016811	Metabolism	Metabolism	F	hydrolase activity, acting on carbon-nitrogen (but not peptide) bonds, in linear amides
2062	GO:0016788	Metabolism	Metabolism	F	hydrolase activity, acting on ester bonds
2063	GO:0016798	Metabolism	Metabolism	F	hydrolase activity, acting on glycosyl bonds
2064	GO:0016799	Metabolism	Metabolism	F	hydrolase activity, hydrolyzing N-glycosyl compounds
2065	GO:0004553	Metabolism	Metabolism	F	hydrolase activity, hydrolyzing O-glycosyl compounds
2066	GO:0016836	Metabolism	Metabolism	F	hydro-lyase activity
2067	GO:0033971	Metabolism	Metabolism	F	hydroxyisourate hydrolase activity
2068	GO:1990714	Metabolism	Metabolism	F	hydroxyproline O-galactosyltransferase activity
2069	GO:0016618	Metabolism	Metabolism	F	hydroxypyruvate reductase activity
2070	GO:0102953	Metabolism	Metabolism	F	hypoglycin A gamma-glutamyl transpeptidase activity

2071	GO:0042802	Metabolism	Metabolism	F	identical protein binding
2072	GO:0006188	Metabolism	Metabolism	P	IMP biosynthetic process
2073	GO:0003937	Metabolism	Metabolism	F	IMP cyclohydrolase activity
2074	GO:0080071	Metabolism	Metabolism	F	indol-3-yl-methyl-desulfoglucosinolate sulfotransferase activity
2075	GO:0009759	Metabolism	Metabolism	P	indole glucosinolate biosynthetic process
2076	GO:0042343	Metabolism	Metabolism	P	indole glucosinolate metabolic process
2077	GO:0009700	Metabolism	Metabolism	P	indole phytoalexin biosynthetic process
2078	GO:0103007	Metabolism	Metabolism	F	indole-3-acetate carboxyl methyltransferase activity
2079	GO:0052638	Metabolism	Metabolism	F	indole-3-butyrate beta-glucosyltransferase activity
2080	GO:0004425	Metabolism	Metabolism	F	indole-3-glycerol-phosphate synthase activity
2081	GO:0103075	Metabolism	Metabolism	F	indole-3-pyruvate monooxygenase activity
2082	GO:0042435	Metabolism	Metabolism	P	indole-containing compound biosynthetic process
2083	GO:0042430	Metabolism	Metabolism	P	indole-containing compound metabolic process
2084	GO:0004427	Metabolism	Metabolism	F	inorganic diphosphatase activity
2085	GO:0006021	Metabolism	Metabolism	P	inositol biosynthetic process
2086	GO:0000822	Metabolism	Metabolism	F	inositol hexakisphosphate binding
2087	GO:0006020	Metabolism	Metabolism	P	inositol metabolic process
2088	GO:0008934	Metabolism	Metabolism	F	inositol monophosphate 1-phosphatase activity
2089	GO:0052832	Metabolism	Metabolism	F	inositol monophosphate 3-phosphatase activity
2090	GO:0052833	Metabolism	Metabolism	F	inositol monophosphate 4-phosphatase activity
2091	GO:0043647	Metabolism	Metabolism	P	inositol phosphate metabolic process
2092	GO:0045140	Metabolism	Metabolism	F	inositol phosphoceramide synthase activity
2093	GO:0004512	Metabolism	Metabolism	F	inositol-3-phosphate synthase activity
2094	GO:0006880	Metabolism	Metabolism	P	intracellular sequestering of iron ion
2095	GO:0016860	Metabolism	Metabolism	F	intramolecular oxidoreductase activity
2096	GO:0016861	Metabolism	Metabolism	F	intramolecular oxidoreductase activity, interconverting aldoses and ketoses
2097	GO:0016866	Metabolism	Metabolism	F	intramolecular transferase activity
2098	GO:0005242	Metabolism	Metabolism	F	inward rectifier potassium channel activity
2099	GO:0043167	Metabolism	Metabolism	F	ion binding
2100	GO:0005506	Metabolism	Metabolism	F	iron ion binding
2101	GO:0016226	Metabolism	Metabolism	P	iron-sulfur cluster assembly
2102	GO:0051536	Metabolism	Metabolism	F	iron-sulfur cluster binding
2103	GO:0008909	Metabolism	Metabolism	F	isochorismate synthase activity
2104	GO:0009097	Metabolism	Metabolism	P	isoleucine biosynthetic process
2105	GO:0016853	Metabolism	Metabolism	F	isomerase activity
2106	GO:0009240	Metabolism	Metabolism	P	isopentenyl diphosphate biosynthetic process
2107	GO:0019288	Metabolism	Metabolism	P	isopentenyl diphosphate biosynthetic process, methylerythritol 4-phosphate pathway
2108	GO:0051484	Metabolism	Metabolism	P	isopentenyl diphosphate biosynthetic process, methylerythritol 4-phosphate pathway involved in terpenoid biosynthetic process
2109	GO:0019287	Metabolism	Metabolism	P	isopentenyl diphosphate biosynthetic process, mevalonate pathway
2110	GO:0004452	Metabolism	Metabolism	F	isopentenyl-diphosphate delta-isomerase activity
2111	GO:0008299	Metabolism	Metabolism	P	isoprenoid biosynthetic process
2112	GO:0006720	Metabolism	Metabolism	P	isoprenoid metabolic process
2113	GO:0052694	Metabolism	Metabolism	F	jasmonoyl-isoleucine-12-hydroxylase activity
2114	GO:0046951	Metabolism	Metabolism	P	ketone body biosynthetic process
2115	GO:0045703	Metabolism	Metabolism	F	ketoreductase activity
2116	GO:0019894	Metabolism	Metabolism	F	kinesin binding
2117	GO:0016212	Metabolism	Metabolism	F	kynurenine-oxoglutarate transaminase activity
2118	GO:0008911	Metabolism	Metabolism	F	lactaldehyde dehydrogenase activity
2119	GO:0019516	Metabolism	Metabolism	P	lactate oxidation
2120	GO:0042851	Metabolism	Metabolism	P	L-alanine metabolic process
2121	GO:0004021	Metabolism	Metabolism	F	L-alanine:2-oxoglutarate aminotransferase activity
2122	GO:0008732	Metabolism	Metabolism	F	L-allo-threonine aldolase activity
2123	GO:0046373	Metabolism	Metabolism	P	L-arabinose metabolic process
2124	GO:0004069	Metabolism	Metabolism	F	L-aspartate:2-oxoglutarate aminotransferase activity
2125	GO:0009098	Metabolism	Metabolism	P	leucine biosynthetic process
2126	GO:0006551	Metabolism	Metabolism	P	leucine metabolic process
2127	GO:0019370	Metabolism	Metabolism	P	leukotriene biosynthetic process
2128	GO:0103068	Metabolism	Metabolism	F	leukotriene C4 gamma-glutamyl transferase activity
2129	GO:0097054	Metabolism	Metabolism	P	L-glutamate biosynthetic process
2130	GO:0016874	Metabolism	Metabolism	F	ligase activity
2131	GO:0016877	Metabolism	Metabolism	F	ligase activity, forming carbon-sulfur bonds
2132	GO:0009807	Metabolism	Metabolism	P	lignan biosynthetic process
2133	GO:0009809	Metabolism	Metabolism	P	lignin biosynthetic process
2134	GO:0009808	Metabolism	Metabolism	P	lignin metabolic process
2135	GO:0016165	Metabolism	Metabolism	F	linoleate 13S-lipoxygenase activity
2136	GO:1990136	Metabolism	Metabolism	F	linoleate 9S-lipoxygenase activity
2137	GO:0016298	Metabolism	Metabolism	F	lipase activity
2138	GO:0008289	Metabolism	Metabolism	F	lipid binding
2139	GO:0008610	Metabolism	Metabolism	P	lipid biosynthetic process
2140	GO:0140042	Metabolism	Metabolism	P	lipid droplet formation
2141	GO:0034389	Metabolism	Metabolism	P	lipid droplet organization
2142	GO:0030259	Metabolism	Metabolism	P	lipid glycosylation
2143	GO:0010876	Metabolism	Metabolism	P	lipid localization
2144	GO:0006629	Metabolism	Metabolism	P	lipid metabolic process
2145	GO:0030258	Metabolism	Metabolism	P	lipid modification
2146	GO:0019915	Metabolism	Metabolism	P	lipid storage

2147	GO:0009107	Metabolism	Metabolism	P	lipoate biosynthetic process
2148	GO:0016992	Metabolism	Metabolism	F	lipoate synthase activity
2149	GO:0031405	Metabolism	Metabolism	F	lipoic acid binding
2150	GO:0052656	Metabolism	Metabolism	F	L-isoleucine transaminase activity
2151	GO:0004459	Metabolism	Metabolism	F	L-lactate dehydrogenase activity
2152	GO:0052654	Metabolism	Metabolism	F	L-leucine transaminase activity
2153	GO:0062045	Metabolism	Metabolism	F	L-lysine alpha-aminotransferase
2154	GO:0019279	Metabolism	Metabolism	P	L-methionine biosynthetic process from L-homoserine via cystathionine
2155	GO:0036456	Metabolism	Metabolism	F	L-methionine-(S)-S-oxide reductase activity
2156	GO:0033744	Metabolism	Metabolism	F	L-methionine:thioredoxin-disulfide S-oxidoreductase activity
2157	GO:0051179	Metabolism	Metabolism	P	localization
2158	GO:0046577	Metabolism	Metabolism	F	long-chain-alcohol oxidase activity
2159	GO:0009094	Metabolism	Metabolism	P	L-phenylalanine biosynthetic process
2160	GO:0006558	Metabolism	Metabolism	P	L-phenylalanine metabolic process
2161	GO:0062034	Metabolism	Metabolism	P	L-pipecolic acid biosynthetic process
2162	GO:0055129	Metabolism	Metabolism	P	L-proline biosynthetic process
2163	GO:0006564	Metabolism	Metabolism	P	L-serine biosynthetic process
2164	GO:0006563	Metabolism	Metabolism	P	L-serine metabolic process
2165	GO:0050362	Metabolism	Metabolism	F	L-tryptophan:2-oxoglutarate aminotransferase activity
2166	GO:0004838	Metabolism	Metabolism	F	L-tyrosine:2-oxoglutarate aminotransferase activity
2167	GO:0030744	Metabolism	Metabolism	F	luteolin O-methyltransferase activity
2168	GO:0052655	Metabolism	Metabolism	F	L-valine transaminase activity
2169	GO:0016829	Metabolism	Metabolism	F	lyase activity
2170	GO:0019878	Metabolism	Metabolism	P	lysine biosynthetic process via aminoadipic acid
2171	GO:0009089	Metabolism	Metabolism	P	lysine biosynthetic process via diaminopimelate
2172	GO:0016278	Metabolism	Metabolism	F	lysine N-methyltransferase activity
2173	GO:0042171	Metabolism	Metabolism	F	lysophosphatidic acid acyltransferase activity
2174	GO:0080171	Metabolism	Metabolism	P	lytic vacuole organization
2175	GO:0009059	Metabolism	Metabolism	P	macromolecule biosynthetic process
2176	GO:0043170	Metabolism	Metabolism	P	macromolecule metabolic process
2177	GO:0016851	Metabolism	Metabolism	F	magnesium chelatase activity
2178	GO:0000287	Metabolism	Metabolism	F	magnesium ion binding
2179	GO:0048529	Metabolism	Metabolism	F	magnesium-protoporphyrin IX monomethyl ester (oxidative) cyclase activity
2180	GO:0016034	Metabolism	Metabolism	F	maleylacetoacetate isomerase activity
2181	GO:0102216	Metabolism	Metabolism	F	maltodextrin water dikinase
2182	GO:1901982	Metabolism	Metabolism	F	maltose binding
2183	GO:0000024	Metabolism	Metabolism	P	maltose biosynthetic process
2184	GO:0030145	Metabolism	Metabolism	F	manganese ion binding
2185	GO:0010412	Metabolism	Metabolism	P	mannan metabolic process
2186	GO:0051753	Metabolism	Metabolism	F	mannan synthase activity
2187	GO:0004475	Metabolism	Metabolism	F	mannose-1-phosphate guanylyltransferase activity
2188	GO:0004476	Metabolism	Metabolism	F	mannose-6-phosphate isomerase activity
2189	GO:0097502	Metabolism	Metabolism	P	mannosylation
2190	GO:0000030	Metabolism	Metabolism	F	mannosyltransferase activity
2191	GO:0008381	Metabolism	Metabolism	F	mechanosensitive ion channel activity
2192	GO:0052854	Metabolism	Metabolism	F	medium-chain-(S)-2-hydroxy-acid oxidase activity
2193	GO:1904408	Metabolism	Metabolism	F	melatonin binding
2194	GO:0030187	Metabolism	Metabolism	P	melatonin biosynthetic process
2195	GO:0008502	Metabolism	Metabolism	F	melatonin receptor activity
2196	GO:0046467	Metabolism	Metabolism	P	membrane lipid biosynthetic process
2197	GO:0006643	Metabolism	Metabolism	P	membrane lipid metabolic process
2198	GO:0008152	Metabolism	Metabolism	P	metabolic process
2199	GO:0046872	Metabolism	Metabolism	F	metal ion binding
2200	GO:0070006	Metabolism	Metabolism	F	metalloaminopeptidase activity
2201	GO:0008235	Metabolism	Metabolism	F	metalloexopeptidase activity
2202	GO:0008237	Metabolism	Metabolism	F	metallopeptidase activity
2203	GO:0004477	Metabolism	Metabolism	F	methenyltetrahydrofolate cyclohydrolase activity
2204	GO:0103045	Metabolism	Metabolism	F	methione N-acyltransferase activity
2205	GO:0004478	Metabolism	Metabolism	F	methionine adenosyltransferase activity
2206	GO:0009086	Metabolism	Metabolism	P	methionine biosynthetic process
2207	GO:0006555	Metabolism	Metabolism	P	methionine metabolic process
2208	GO:0030732	Metabolism	Metabolism	F	methionine S-methyltransferase activity
2209	GO:0008705	Metabolism	Metabolism	F	methionine synthase activity
2210	GO:0010326	Metabolism	Metabolism	F	methionine-oxo-acid transaminase activity
2211	GO:0102078	Metabolism	Metabolism	F	methyl jasmonate methylesterase activity
2212	GO:0008327	Metabolism	Metabolism	F	methyl-CpG binding
2213	GO:0008168	Metabolism	Metabolism	F	methyltransferase activity
2214	GO:0000146	Metabolism	Metabolism	F	microfilament motor activity
2215	GO:0008017	Metabolism	Metabolism	F	microtubule binding
2216	GO:0007019	Metabolism	Metabolism	P	microtubule depolymerization
2217	GO:0051011	Metabolism	Metabolism	F	microtubule minus-end binding
2218	GO:0051010	Metabolism	Metabolism	F	microtubule plus-end binding
2219	GO:0031109	Metabolism	Metabolism	P	microtubule polymerization or depolymerization
2220	GO:0019048	Metabolism	Metabolism	P	modulation by virus of host morphology or physiology
2221	GO:0060090	Metabolism	Metabolism	F	molecular adaptor activity
2222	GO:0003674	Metabolism	Metabolism	F	molecular_function
2223	GO:0030151	Metabolism	Metabolism	F	molybdenum ion binding
2224	GO:0030366	Metabolism	Metabolism	F	molybdopterin synthase activity

2225	GO:0006777	Metabolism	Metabolism	P	Mo-molybdopterin cofactor biosynthetic process
2226	GO:0072330	Metabolism	Metabolism	P	monocarboxylic acid biosynthetic process
2227	GO:0032787	Metabolism	Metabolism	P	monocarboxylic acid metabolic process
2228	GO:0072549	Metabolism	Metabolism	F	monocoumaroyl dicaffeoyl spermidine meta-hydroxylase activity
2229	GO:0072552	Metabolism	Metabolism	F	monoferuloyl di-(hydroxyferuloyl) spermidine meta-hydroxylase activity
2230	GO:0004497	Metabolism	Metabolism	F	monooxygenase activity
2231	GO:0005996	Metabolism	Metabolism	P	monosaccharide metabolic process
2232	GO:0043693	Metabolism	Metabolism	P	monoterpene biosynthetic process
2233	GO:0016099	Metabolism	Metabolism	P	monoterpenoid biosynthetic process
2234	GO:0005451	Metabolism	Metabolism	F	monovalent cation:proton antiporter activity
2235	GO:0003774	Metabolism	Metabolism	F	motor activity
2236	GO:0010192	Metabolism	Metabolism	P	mucilage biosynthetic process
2237	GO:0048354	Metabolism	Metabolism	P	mucilage biosynthetic process involved in seed coat development
2238	GO:0010191	Metabolism	Metabolism	P	mucilage metabolic process
2239	GO:0048359	Metabolism	Metabolism	P	mucilage metabolic process involved in seed coat development
2240	GO:0048358	Metabolism	Metabolism	P	mucilage pectin biosynthetic process
2241	GO:0032405	Metabolism	Metabolism	F	MutLalpha complex binding
2242	GO:0010264	Metabolism	Metabolism	P	myo-inositol hexakisphosphate biosynthetic process
2243	GO:0017022	Metabolism	Metabolism	F	myosin binding
2244	GO:0045159	Metabolism	Metabolism	F	myosin II binding
2245	GO:0080115	Metabolism	Metabolism	F	myosin XI tail binding
2246	GO:0050551	Metabolism	Metabolism	F	myrcene synthase activity
2247	GO:0033799	Metabolism	Metabolism	F	myricetin 3'-O-methyltransferase activity
2248	GO:0061711	Metabolism	Metabolism	F	N(6)-L-threonylcarbamoyladenine synthase activity
2249	GO:0004499	Metabolism	Metabolism	F	N,N-dimethylaniline monooxygenase activity
2250	GO:0052895	Metabolism	Metabolism	F	N1-acetylspermine:oxygen oxidoreductase (N1-acetylspermidine-forming) activity
2251	GO:0010013	Metabolism	Metabolism	F	N-1-naphthylphthalamic acid binding
2252	GO:0003992	Metabolism	Metabolism	F	N2-acetyl-L-ornithine:2-oxoglutarate 5-aminotransferase activity
2253	GO:0004577	Metabolism	Metabolism	F	N-acetylglucosaminyldiphosphodolichol N-acetylglucosaminyltransferase activity
2254	GO:0008080	Metabolism	Metabolism	F	N-acetyltransferase activity
2255	GO:0045486	Metabolism	Metabolism	F	naringenin 3-dioxygenase activity
2256	GO:0016210	Metabolism	Metabolism	F	naringenin-chalcone synthase activity
2257	GO:0050126	Metabolism	Metabolism	F	N-carbamoylputrescine amidase activity
2258	GO:0019788	Metabolism	Metabolism	F	NEDD8 transferase activity
2259	GO:0016151	Metabolism	Metabolism	F	nickel cation binding
2260	GO:0030418	Metabolism	Metabolism	P	nicotianamine biosynthetic process
2261	GO:0030410	Metabolism	Metabolism	F	nicotianamine synthase activity
2262	GO:0008936	Metabolism	Metabolism	F	nicotinamidase activity
2263	GO:0000309	Metabolism	Metabolism	F	nicotinamide-nucleotide adenylyltransferase activity
2264	GO:0004514	Metabolism	Metabolism	F	nicotinate-nucleotide diphosphorylase (carboxylating) activity
2265	GO:0090704	Metabolism	Metabolism	F	nicotinate-O-glucosyltransferase activity
2266	GO:0042128	Metabolism	Metabolism	P	nitrate assimilation
2267	GO:0042126	Metabolism	Metabolism	P	nitrate metabolic process
2268	GO:0009671	Metabolism	Metabolism	F	nitrate:proton symporter activity
2269	GO:0080028	Metabolism	Metabolism	P	nitrile biosynthetic process
2270	GO:0006807	Metabolism	Metabolism	P	nitrogen compound metabolic process
2271	GO:0071941	Metabolism	Metabolism	P	nitrogen cycle metabolic process
2272	GO:0019740	Metabolism	Metabolism	P	nitrogen utilization
2273	GO:0018580	Metabolism	Metabolism	F	nitronate monooxygenase activity
2274	GO:0006607	Metabolism	Metabolism	P	NLS-bearing protein import into nucleus
2275	GO:0008170	Metabolism	Metabolism	F	N-methyltransferase activity
2276	GO:0052894	Metabolism	Metabolism	F	norspermine:oxygen oxidoreductase activity
2277	GO:0018008	Metabolism	Metabolism	P	N-terminal peptidyl-glycine N-myristoylation
2278	GO:0017196	Metabolism	Metabolism	P	N-terminal peptidyl-methionine acetylation
2279	GO:0003676	Metabolism	Metabolism	F	nucleic acid binding
2280	GO:0045735	Metabolism	Metabolism	F	nutrient reservoir activity
2281	GO:0016413	Metabolism	Metabolism	F	O-acetyltransferase activity
2282	GO:0009924	Metabolism	Metabolism	F	octadecanal decarbonylase activity
2283	GO:0120254	Metabolism	Metabolism	P	olefinic compound metabolic process
2284	GO:0070012	Metabolism	Metabolism	F	oligopeptidase activity
2285	GO:1900750	Metabolism	Metabolism	F	oligopeptide binding
2286	GO:0070492	Metabolism	Metabolism	F	oligosaccharide binding
2287	GO:0006490	Metabolism	Metabolism	P	oligosaccharide-lipid intermediate biosynthetic process
2288	GO:0004576	Metabolism	Metabolism	F	oligosaccharyl transferase activity
2289	GO:0008171	Metabolism	Metabolism	F	O-methyltransferase activity
2290	GO:0006730	Metabolism	Metabolism	P	one-carbon metabolic process
2291	GO:0004648	Metabolism	Metabolism	F	O-phospho-L-serine:2-oxoglutarate aminotransferase activity
2292	GO:0016053	Metabolism	Metabolism	P	organic acid biosynthetic process
2293	GO:0006082	Metabolism	Metabolism	P	organic acid metabolic process
2294	GO:0097159	Metabolism	Metabolism	F	organic cyclic compound binding
2295	GO:1901362	Metabolism	Metabolism	P	organic cyclic compound biosynthetic process
2296	GO:1901360	Metabolism	Metabolism	P	organic cyclic compound metabolic process
2297	GO:1901617	Metabolism	Metabolism	P	organic hydroxy compound biosynthetic process
2298	GO:1901615	Metabolism	Metabolism	P	organic hydroxy compound metabolic process
2299	GO:1901576	Metabolism	Metabolism	P	organic substance biosynthetic process
2300	GO:0071704	Metabolism	Metabolism	P	organic substance metabolic process

2301	GO:1901566	Metabolism	Metabolism	P	organonitrogen compound biosynthetic process
2302	GO:1901564	Metabolism	Metabolism	P	organonitrogen compound metabolic process
2303	GO:0090407	Metabolism	Metabolism	P	organophosphate biosynthetic process
2304	GO:0019637	Metabolism	Metabolism	P	organophosphate metabolic process
2305	GO:0008948	Metabolism	Metabolism	F	oxaloacetate decarboxylase activity
2306	GO:0006107	Metabolism	Metabolism	P	oxaloacetate metabolic process
2307	GO:0018131	Metabolism	Metabolism	P	oxazole or thiazole biosynthetic process
2308	GO:0055114	Metabolism	Metabolism	P	oxidation-reduction process
2309	GO:0017077	Metabolism	Metabolism	F	oxidative phosphorylation uncoupler activity
2310	GO:0043436	Metabolism	Metabolism	P	oxoacid metabolic process
2311	GO:0019825	Metabolism	Metabolism	F	oxygen binding
2312	GO:0004729	Metabolism	Metabolism	F	oxygen-dependent protoporphyrinogen oxidase activity
2313	GO:0031408	Metabolism	Metabolism	P	oxylipin biosynthetic process
2314	GO:0031407	Metabolism	Metabolism	P	oxylipin metabolic process
2315	GO:0004594	Metabolism	Metabolism	F	pantothenate kinase activity
2316	GO:0046482	Metabolism	Metabolism	P	para-aminobenzoic acid metabolic process
2317	GO:0046409	Metabolism	Metabolism	F	p-coumarate 3-hydroxylase activity
2318	GO:0030570	Metabolism	Metabolism	F	pectate lyase activity
2319	GO:0010401	Metabolism	Metabolism	P	pectic galactan metabolic process
2320	GO:0052793	Metabolism	Metabolism	F	pectin acetylesterase activity
2321	GO:0045489	Metabolism	Metabolism	P	pectin biosynthetic process
2322	GO:0045488	Metabolism	Metabolism	P	pectin metabolic process
2323	GO:0030599	Metabolism	Metabolism	F	pectinesterase activity
2324	GO:0046910	Metabolism	Metabolism	F	pectinesterase inhibitor activity
2325	GO:0019321	Metabolism	Metabolism	P	pentose metabolic process
2326	GO:0042277	Metabolism	Metabolism	F	peptide binding
2327	GO:0006518	Metabolism	Metabolism	P	peptide metabolic process
2328	GO:0017183	Metabolism	Metabolism	P	peptidyl-diphthamide biosynthetic process from peptidyl-histidine
2329	GO:0000048	Metabolism	Metabolism	F	peptidyltransferase activity
2330	GO:0052596	Metabolism	Metabolism	F	phenethylamine:oxygen oxidoreductase (deaminating) activity
2331	GO:0045548	Metabolism	Metabolism	F	phenylalanine ammonia-lyase activity
2332	GO:0032442	Metabolism	Metabolism	F	phenylcoumaran benzylic ether reductase activity
2333	GO:0009699	Metabolism	Metabolism	P	phenylpropanoid biosynthetic process
2334	GO:0009698	Metabolism	Metabolism	P	phenylpropanoid metabolic process
2335	GO:0080124	Metabolism	Metabolism	F	pheophytinase activity
2336	GO:0006796	Metabolism	Metabolism	P	phosphate-containing compound metabolic process
2337	GO:0070300	Metabolism	Metabolism	F	phosphatidic acid binding
2338	GO:0006656	Metabolism	Metabolism	P	phosphatidylcholine biosynthetic process
2339	GO:0006646	Metabolism	Metabolism	P	phosphatidylethanolamine biosynthetic process
2340	GO:0046471	Metabolism	Metabolism	P	phosphatidylglycerol metabolic process
2341	GO:0035091	Metabolism	Metabolism	F	phosphatidylinositol binding
2342	GO:0006661	Metabolism	Metabolism	P	phosphatidylinositol biosynthetic process
2343	GO:0034593	Metabolism	Metabolism	P	phosphatidylinositol bisphosphate phosphatase activity
2344	GO:0046488	Metabolism	Metabolism	P	phosphatidylinositol metabolic process
2345	GO:0017176	Metabolism	Metabolism	F	phosphatidylinositol N-acetylglucosaminyltransferase activity
2346	GO:0008526	Metabolism	Metabolism	F	phosphatidylinositol transfer activity
2347	GO:0043325	Metabolism	Metabolism	F	phosphatidylinositol-3,4-bisphosphate binding
2348	GO:0080025	Metabolism	Metabolism	F	phosphatidylinositol-3,5-bisphosphate binding
2349	GO:0005546	Metabolism	Metabolism	F	phosphatidylinositol-4,5-bisphosphate binding
2350	GO:0006659	Metabolism	Metabolism	P	phosphatidylserine biosynthetic process
2351	GO:0004609	Metabolism	Metabolism	F	phosphatidylserine decarboxylase activity
2352	GO:0004610	Metabolism	Metabolism	F	phosphoacetylglucosamine mutase activity
2353	GO:0004612	Metabolism	Metabolism	F	phosphoenolpyruvate carboxykinase (ATP) activity
2354	GO:0008964	Metabolism	Metabolism	F	phosphoenolpyruvate carboxylase activity
2355	GO:0000234	Metabolism	Metabolism	F	phosphoethanolamine N-methyltransferase activity
2356	GO:0051752	Metabolism	Metabolism	F	phosphoglucan, water dikinase activity
2357	GO:0102219	Metabolism	Metabolism	F	phosphogluco-amylopectin water dikinase activity
2358	GO:0004614	Metabolism	Metabolism	F	phosphoglucomutase activity
2359	GO:0008114	Metabolism	Metabolism	F	phosphogluconate 2-dehydrogenase activity
2360	GO:0004616	Metabolism	Metabolism	F	phosphogluconate dehydrogenase (decarboxylating) activity
2361	GO:0004617	Metabolism	Metabolism	F	phosphoglycerate dehydrogenase activity
2362	GO:0005543	Metabolism	Metabolism	F	phospholipid binding
2363	GO:0008654	Metabolism	Metabolism	P	phospholipid biosynthetic process
2364	GO:0006644	Metabolism	Metabolism	P	phospholipid metabolic process
2365	GO:0052667	Metabolism	Metabolism	F	phosphomethylethanolamine N-methyltransferase activity
2366	GO:0004633	Metabolism	Metabolism	F	phosphopantothenoylcysteine decarboxylase activity
2367	GO:0004634	Metabolism	Metabolism	F	phosphopyruvate hydratase activity
2368	GO:0004637	Metabolism	Metabolism	F	phosphoribosylamine-glycine ligase activity
2369	GO:0004643	Metabolism	Metabolism	F	phosphoribosylaminoimidazolecarboxamide formyltransferase activity
2370	GO:0004640	Metabolism	Metabolism	F	phosphoribosylanthranilate isomerase activity
2371	GO:0004641	Metabolism	Metabolism	F	phosphoribosylformylglycinamidine cyclo-ligase activity
2372	GO:0008081	Metabolism	Metabolism	F	phosphoric diester hydrolase activity
2373	GO:0042578	Metabolism	Metabolism	F	phosphoric ester hydrolase activity
2374	GO:0006793	Metabolism	Metabolism	P	phosphorus metabolic process
2375	GO:0046838	Metabolism	Metabolism	P	phosphorylated carbohydrate dephosphorylation
2376	GO:0016310	Metabolism	Metabolism	P	phosphorylation
2377	GO:0016773	Metabolism	Metabolism	F	phosphotransferase activity, alcohol group as acceptor
2378	GO:0042372	Metabolism	Metabolism	P	phylloquinone biosynthetic process

2379	GO:0010024	Metabolism	Metabolism	P	phytochromobilin biosynthetic process
2380	GO:0046905	Metabolism	Metabolism	F	phytoene synthase activity
2381	GO:0033306	Metabolism	Metabolism	P	phytol metabolic process
2382	GO:0031409	Metabolism	Metabolism	F	pigment binding
2383	GO:0046148	Metabolism	Metabolism	P	pigment biosynthetic process
2384	GO:0042440	Metabolism	Metabolism	P	pigment metabolic process
2385	GO:0006907	Metabolism	Metabolism	P	pinocytosis
2386	GO:0052324	Metabolism	Metabolism	P	plant-type cell wall cellulose biosynthetic process
2387	GO:0052541	Metabolism	Metabolism	P	plant-type cell wall cellulose metabolic process
2388	GO:0010236	Metabolism	Metabolism	P	plastoquinone biosynthetic process
2389	GO:0006596	Metabolism	Metabolism	P	polyamine biosynthetic process
2390	GO:0006595	Metabolism	Metabolism	P	polyamine metabolic process
2391	GO:0046592	Metabolism	Metabolism	F	polyamine oxidase activity
2392	GO:0004650	Metabolism	Metabolism	F	polygalacturonase activity
2393	GO:0090353	Metabolism	Metabolism	F	polygalacturonase inhibitor activity
2394	GO:0047262	Metabolism	Metabolism	F	polygalacturonate 4-alpha-galacturonosyltransferase activity
2395	GO:0019751	Metabolism	Metabolism	P	polyol metabolic process
2396	GO:0016094	Metabolism	Metabolism	P	polyprenol biosynthetic process
2397	GO:0002094	Metabolism	Metabolism	F	polyprenyltransferase activity
2398	GO:0008187	Metabolism	Metabolism	F	poly-pyrimidine tract binding
2399	GO:0030247	Metabolism	Metabolism	F	polysaccharide binding
2400	GO:0015288	Metabolism	Metabolism	F	porin activity
2401	GO:0006778	Metabolism	Metabolism	P	porphyrin-containing compound metabolic process
2402	GO:0090358	Metabolism	Metabolism	P	positive regulation of tryptophan metabolic process
2403	GO:0030955	Metabolism	Metabolism	F	potassium ion binding
2404	GO:0004664	Metabolism	Metabolism	F	prephenate dehydratase activity
2405	GO:0034308	Metabolism	Metabolism	P	primary alcohol metabolic process
2406	GO:0008131	Metabolism	Metabolism	F	primary amine oxidase activity
2407	GO:0006561	Metabolism	Metabolism	P	proline biosynthetic process
2408	GO:0004657	Metabolism	Metabolism	F	proline dehydrogenase activity
2409	GO:1990404	Metabolism	Metabolism	F	protein ADP-ribosylase activity
2410	GO:0005515	Metabolism	Metabolism	F	protein binding
2411	GO:0030674	Metabolism	Metabolism	F	protein binding, bridging
2412	GO:0008022	Metabolism	Metabolism	F	protein C-terminus binding
2413	GO:0004673	Metabolism	Metabolism	F	protein histidine kinase activity
2414	GO:0043424	Metabolism	Metabolism	F	protein histidine kinase binding
2415	GO:0019538	Metabolism	Metabolism	P	protein metabolic process
2416	GO:0016262	Metabolism	Metabolism	F	protein N-acetylglucosaminyltransferase activity
2417	GO:0008318	Metabolism	Metabolism	F	protein prenyltransferase activity
2418	GO:0043394	Metabolism	Metabolism	F	proteoglycan binding
2419	GO:0015252	Metabolism	Metabolism	F	proton channel activity
2420	GO:0120029	Metabolism	Metabolism	P	proton export across plasma membrane
2421	GO:0009982	Metabolism	Metabolism	F	pseudouridine synthase activity
2422	GO:0001522	Metabolism	Metabolism	P	pseudouridine synthesis
2423	GO:0009113	Metabolism	Metabolism	P	purine nucleobase biosynthetic process
2424	GO:0006144	Metabolism	Metabolism	P	purine nucleobase metabolic process
2425	GO:0034032	Metabolism	Metabolism	P	purine nucleoside bisphosphate metabolic process
2426	GO:0009135	Metabolism	Metabolism	P	purine nucleoside diphosphate metabolic process
2427	GO:0009145	Metabolism	Metabolism	P	purine nucleoside triphosphate biosynthetic process
2428	GO:0009144	Metabolism	Metabolism	P	purine nucleoside triphosphate metabolic process
2429	GO:0017076	Metabolism	Metabolism	F	purine nucleotide binding
2430	GO:0006164	Metabolism	Metabolism	P	purine nucleotide biosynthetic process
2431	GO:0006163	Metabolism	Metabolism	P	purine nucleotide metabolic process
2432	GO:0009179	Metabolism	Metabolism	P	purine ribonucleoside diphosphate metabolic process
2433	GO:0035639	Metabolism	Metabolism	F	purine ribonucleoside triphosphate binding
2434	GO:0009206	Metabolism	Metabolism	P	purine ribonucleoside triphosphate biosynthetic process
2435	GO:0009205	Metabolism	Metabolism	P	purine ribonucleoside triphosphate metabolic process
2436	GO:0032555	Metabolism	Metabolism	F	purine ribonucleotide binding
2437	GO:0009150	Metabolism	Metabolism	P	purine ribonucleotide metabolic process
2438	GO:0072522	Metabolism	Metabolism	P	purine-containing compound biosynthetic process
2439	GO:0072521	Metabolism	Metabolism	P	purine-containing compound metabolic process
2440	GO:0009446	Metabolism	Metabolism	P	putrescine biosynthetic process
2441	GO:0033388	Metabolism	Metabolism	P	putrescine biosynthetic process from arginine
2442	GO:0036381	Metabolism	Metabolism	F	pyridoxal 5'-phosphate synthase (glutamine hydrolysing) activity
2443	GO:0042817	Metabolism	Metabolism	P	pyridoxal metabolic process
2444	GO:0030170	Metabolism	Metabolism	F	pyridoxal phosphate binding
2445	GO:0042823	Metabolism	Metabolism	P	pyridoxal phosphate biosynthetic process
2446	GO:0004733	Metabolism	Metabolism	F	pyridoxamine-phosphate oxidase activity
2447	GO:0008615	Metabolism	Metabolism	P	pyridoxine biosynthetic process
2448	GO:0019856	Metabolism	Metabolism	P	pyrimidine nucleobase biosynthetic process
2449	GO:0006221	Metabolism	Metabolism	P	pyrimidine nucleotide biosynthetic process
2450	GO:0006220	Metabolism	Metabolism	P	pyrimidine nucleotide metabolic process
2451	GO:0016462	Metabolism	Metabolism	F	pyrophosphatase activity
2452	GO:0050242	Metabolism	Metabolism	F	pyruvate, phosphate dikinase activity
2453	GO:0080043	Metabolism	Metabolism	F	quercetin 3-O-glucosyltransferase activity
2454	GO:0102822	Metabolism	Metabolism	F	quercetin 3'-O-methyltransferase activity
2455	GO:0030755	Metabolism	Metabolism	F	quercetin 3-O-methyltransferase activity
2456	GO:0080046	Metabolism	Metabolism	F	quercetin 4'-O-glucosyltransferase activity

2457	GO:0080044	Metabolism	Metabolism	F	quercetin 7-O-glucosyltransferase activity
2458	GO:0048038	Metabolism	Metabolism	F	quinone binding
2459	GO:0016854	Metabolism	Metabolism	F	racemase and epimerase activity
2460	GO:0016857	Metabolism	Metabolism	F	racemase and epimerase activity, acting on carbohydrates and derivatives
2461	GO:0010325	Metabolism	Metabolism	P	raffinose family oligosaccharide biosynthetic process
2462	GO:0030904	Metabolism	Metabolism	C	retromer complex
2463	GO:0010395	Metabolism	Metabolism	P	rhamnogalacturonan I metabolic process
2464	GO:0050255	Metabolism	Metabolism	F	ribitol 2-dehydrogenase activity
2465	GO:0009231	Metabolism	Metabolism	P	riboflavin biosynthetic process
2466	GO:0008531	Metabolism	Metabolism	F	riboflavin kinase activity
2467	GO:0004753	Metabolism	Metabolism	F	saccharopine dehydrogenase activity
2468	GO:0006557	Metabolism	Metabolism	P	S-adenosylmethioninamine biosynthetic process
2469	GO:0006556	Metabolism	Metabolism	P	S-adenosylmethionine biosynthetic process
2470	GO:0033353	Metabolism	Metabolism	P	S-adenosylmethionine cycle
2471	GO:0046500	Metabolism	Metabolism	P	S-adenosylmethionine metabolic process
2472	GO:0008757	Metabolism	Metabolism	F	S-adenosylmethionine-dependent methyltransferase activity
2473	GO:0034785	Metabolism	Metabolism	F	salicylate 5-hydroxylase activity
2474	GO:0005090	Metabolism	Metabolism	F	Sar guanyl-nucleotide exchange factor activity
2475	GO:0102483	Metabolism	Metabolism	F	scopolin beta-glucosidase activity
2476	GO:1902652	Metabolism	Metabolism	P	secondary alcohol metabolic process
2477	GO:0019748	Metabolism	Metabolism	P	secondary metabolic process
2478	GO:0044550	Metabolism	Metabolism	P	secondary metabolite biosynthetic process
2479	GO:0004801	Metabolism	Metabolism	F	sedoheptulose-7-phosphate:D-glyceraldehyde-3-phosphate glyceronetransferase activity
2480	GO:0050278	Metabolism	Metabolism	F	sedoheptulose-bisphosphatase activity
2481	GO:0008430	Metabolism	Metabolism	F	selenium binding
2482	GO:0001887	Metabolism	Metabolism	P	selenium compound metabolic process
2483	GO:0042989	Metabolism	Metabolism	P	sequestering of actin monomers
2484	GO:0070905	Metabolism	Metabolism	F	serine binding
2485	GO:0004758	Metabolism	Metabolism	F	serine C-palmitoyltransferase activity
2486	GO:0009070	Metabolism	Metabolism	P	serine family amino acid biosynthetic process
2487	GO:0009069	Metabolism	Metabolism	P	serine family amino acid metabolic process
2488	GO:0009001	Metabolism	Metabolism	F	serine O-acetyltransferase activity
2489	GO:0008236	Metabolism	Metabolism	F	serine-type peptidase activity
2490	GO:0016144	Metabolism	Metabolism	P	S-glycoside biosynthetic process
2491	GO:0016143	Metabolism	Metabolism	P	S-glycoside metabolic process
2492	GO:0016987	Metabolism	Metabolism	F	sigma factor activity
2493	GO:0050284	Metabolism	Metabolism	F	sinapate 1-glucosyltransferase activity
2494	GO:0047158	Metabolism	Metabolism	F	sinapoylglucose-sinapoylglucose O-sinapoyltransferase activity
2495	GO:0052747	Metabolism	Metabolism	F	sinapyl alcohol dehydrogenase activity
2496	GO:0032143	Metabolism	Metabolism	F	single thymine insertion binding
2497	GO:0019354	Metabolism	Metabolism	P	siroheme biosynthetic process
2498	GO:0016852	Metabolism	Metabolism	F	sirohydrochlorin cobaltochelatase activity
2499	GO:0051266	Metabolism	Metabolism	F	sirohydrochlorin ferrochelatase activity
2500	GO:0036094	Metabolism	Metabolism	F	small molecule binding
2501	GO:0044283	Metabolism	Metabolism	P	small molecule biosynthetic process
2502	GO:0044281	Metabolism	Metabolism	P	small molecule metabolic process
2503	GO:0046523	Metabolism	Metabolism	F	S-methyl-5-thioribose-1-phosphate isomerase activity
2504	GO:0120114	Metabolism	Metabolism	C	Sm-like protein family complex
2505	GO:0005483	Metabolism	Metabolism	F	soluble NSF attachment protein activity
2506	GO:0008295	Metabolism	Metabolism	P	spermidine biosynthetic process
2507	GO:0080088	Metabolism	Metabolism	P	spermidine hydroxycinnamate conjugate biosynthetic process
2508	GO:0008216	Metabolism	Metabolism	P	spermidine metabolic process
2509	GO:0004766	Metabolism	Metabolism	F	spermidine synthase activity
2510	GO:0006597	Metabolism	Metabolism	P	spermine biosynthetic process
2511	GO:0008215	Metabolism	Metabolism	P	spermine metabolic process
2512	GO:0016768	Metabolism	Metabolism	F	spermine synthase activity
2513	GO:0052901	Metabolism	Metabolism	F	spermine:oxygen oxidoreductase (spermidine-forming) activity
2514	GO:0008117	Metabolism	Metabolism	F	sphinganine-1-phosphate aldolase activity
2515	GO:0046520	Metabolism	Metabolism	P	sphingoid biosynthetic process
2516	GO:0030148	Metabolism	Metabolism	P	sphingolipid biosynthetic process
2517	GO:0052631	Metabolism	Metabolism	F	sphingolipid delta-8 desaturase activity
2518	GO:0006665	Metabolism	Metabolism	P	sphingolipid metabolic process
2519	GO:0033188	Metabolism	Metabolism	F	sphingomyelin synthase activity
2520	GO:0000170	Metabolism	Metabolism	F	sphingosine hydroxylase activity
2521	GO:0080110	Metabolism	Metabolism	P	sporopollenin biosynthetic process
2522	GO:0035339	Metabolism	Metabolism	C	SPOTS complex
2523	GO:0004506	Metabolism	Metabolism	F	squalene monooxygenase activity
2524	GO:0051996	Metabolism	Metabolism	F	squalene synthase activity
2525	GO:0102786	Metabolism	Metabolism	F	stearoyl-[acp] desaturase activity
2526	GO:0000104	Metabolism	Metabolism	F	succinate dehydrogenase activity
2527	GO:0006105	Metabolism	Metabolism	P	succinate metabolic process
2528	GO:0004575	Metabolism	Metabolism	F	sucrose alpha-glucosidase activity
2529	GO:0050307	Metabolism	Metabolism	F	sucrose-phosphate phosphatase activity
2530	GO:0004781	Metabolism	Metabolism	F	sulfate adenylyltransferase (ATP) activity
2531	GO:0019419	Metabolism	Metabolism	P	sulfate reduction
2532	GO:0046506	Metabolism	Metabolism	P	sulfolipid biosynthetic process
2533	GO:0008146	Metabolism	Metabolism	F	sulfotransferase activity

2534	GO:0000097	Metabolism	Metabolism	P	sulfur amino acid biosynthetic process
2535	GO:0000096	Metabolism	Metabolism	P	sulfur amino acid metabolic process
2536	GO:1901681	Metabolism	Metabolism	F	sulfur compound binding
2537	GO:0044272	Metabolism	Metabolism	P	sulfur compound biosynthetic process
2538	GO:0006790	Metabolism	Metabolism	P	sulfur compound metabolic process
2539	GO:0050313	Metabolism	Metabolism	F	sulfur dioxygenase activity
2540	GO:0016783	Metabolism	Metabolism	F	sulfurtransferase activity
2541	GO:0099080	Metabolism	Metabolism	C	supramolecular complex
2542	GO:0099512	Metabolism	Metabolism	C	supramolecular fiber
2543	GO:0099081	Metabolism	Metabolism	C	supramolecular polymer
2544	GO:0048488	Metabolism	Metabolism	P	synaptic vesicle endocytosis
2545	GO:0019905	Metabolism	Metabolism	F	syntaxin binding
2546	GO:0017025	Metabolism	Metabolism	F	TBP-class protein binding
2547	GO:0046246	Metabolism	Metabolism	P	terpene biosynthetic process
2548	GO:0006721	Metabolism	Metabolism	P	terpenoid metabolic process
2549	GO:0035999	Metabolism	Metabolism	P	tetrahydrofolate interconversion
2550	GO:0046653	Metabolism	Metabolism	P	tetrahydrofolate metabolic process
2551	GO:0046906	Metabolism	Metabolism	F	tetrapyrrole binding
2552	GO:0033014	Metabolism	Metabolism	P	tetrapyrrole biosynthetic process
2553	GO:0033013	Metabolism	Metabolism	P	tetrapyrrole metabolic process
2554	GO:0009228	Metabolism	Metabolism	P	thiamine biosynthetic process
2555	GO:0009229	Metabolism	Metabolism	P	thiamine diphosphate biosynthetic process
2556	GO:0030976	Metabolism	Metabolism	F	thiamine pyrophosphate binding
2557	GO:0035384	Metabolism	Metabolism	P	thioester biosynthetic process
2558	GO:0035383	Metabolism	Metabolism	P	thioester metabolic process
2559	GO:0019137	Metabolism	Metabolism	F	thioglucosidase activity
2560	GO:0010180	Metabolism	Metabolism	F	thioglucosidase binding
2561	GO:0010169	Metabolism	Metabolism	C	thioglucosidase complex
2562	GO:0018708	Metabolism	Metabolism	F	thiol S-methyltransferase activity
2563	GO:0004791	Metabolism	Metabolism	F	thioredoxin-disulfide reductase activity
2564	GO:0004793	Metabolism	Metabolism	F	threonine aldolase activity
2565	GO:0009088	Metabolism	Metabolism	P	threonine biosynthetic process
2566	GO:0004797	Metabolism	Metabolism	F	thymidine kinase activity
2567	GO:0046104	Metabolism	Metabolism	P	thymidine metabolic process
2568	GO:0008483	Metabolism	Metabolism	F	transaminase activity
2569	GO:0016710	Metabolism	Metabolism	F	trans-cinnamate 4-monooxygenase activity
2570	GO:0016740	Metabolism	Metabolism	F	transferase activity
2571	GO:0016746	Metabolism	Metabolism	F	transferase activity, transferring acyl groups
2572	GO:0016747	Metabolism	Metabolism	F	transferase activity, transferring acyl groups other than amino-acyl groups
2573	GO:0046912	Metabolism	Metabolism	F	transferase activity, transferring acyl groups, acyl groups converted into alkyl on transfer
2574	GO:0016765	Metabolism	Metabolism	F	transferase activity, transferring alkyl or aryl (other than methyl) groups
2575	GO:0016757	Metabolism	Metabolism	F	transferase activity, transferring glycosyl groups
2576	GO:0016758	Metabolism	Metabolism	F	transferase activity, transferring hexosyl groups
2577	GO:0016769	Metabolism	Metabolism	F	transferase activity, transferring nitrogenous groups
2578	GO:0016741	Metabolism	Metabolism	F	transferase activity, transferring one-carbon groups
2579	GO:1990234	Metabolism	Metabolism	C	transferase complex
2580	GO:0061695	Metabolism	Metabolism	C	transferase complex, transferring phosphorus-containing groups
2581	GO:0046914	Metabolism	Metabolism	F	transition metal ion binding
2582	GO:0019985	Metabolism	Metabolism	P	translesion synthesis
2583	GO:0050347	Metabolism	Metabolism	F	trans-octaprenyltranstransferase activity
2584	GO:0019346	Metabolism	Metabolism	P	transsulfuration
2585	GO:0072532	Metabolism	Metabolism	F	tri-(feruloyl or hydroxyferuloyl) spermidine meta-hydroxylase activity
2586	GO:0072547	Metabolism	Metabolism	F	tricoumaroylspermidine meta-hydroxylase activity
2587	GO:0102701	Metabolism	Metabolism	F	tricyclene synthase activity
2588	GO:0072550	Metabolism	Metabolism	F	triferuloylspermidine meta-hydroxylase activity
2589	GO:0019432	Metabolism	Metabolism	P	triglyceride biosynthetic process
2590	GO:0004806	Metabolism	Metabolism	F	triglyceride lipase activity
2591	GO:0006641	Metabolism	Metabolism	P	triglyceride metabolic process
2592	GO:0004807	Metabolism	Metabolism	F	triose-phosphate isomerase activity
2593	GO:0016104	Metabolism	Metabolism	P	triterpenoid biosynthetic process
2594	GO:0052593	Metabolism	Metabolism	F	tryptamine:oxygen oxidoreductase (deaminating) activity
2595	GO:0000162	Metabolism	Metabolism	P	tryptophan biosynthetic process
2596	GO:0006568	Metabolism	Metabolism	P	tryptophan metabolic process
2597	GO:0004834	Metabolism	Metabolism	F	tryptophan synthase activity
2598	GO:0015631	Metabolism	Metabolism	F	tubulin binding
2599	GO:0045298	Metabolism	Metabolism	C	tubulin complex
2600	GO:1901695	Metabolism	Metabolism	P	tyramine biosynthetic process
2601	GO:0004837	Metabolism	Metabolism	F	tyrosine decarboxylase activity
2602	GO:0006570	Metabolism	Metabolism	P	tyrosine metabolic process
2603	GO:0010489	Metabolism	Metabolism	F	UDP-4-keto-6-deoxy-glucose-3,5-epimerase activity
2604	GO:0010490	Metabolism	Metabolism	F	UDP-4-keto-rhamnose-4-keto-reductase activity
2605	GO:0052691	Metabolism	Metabolism	F	UDP-arabinopyranose mutase activity
2606	GO:0050373	Metabolism	Metabolism	F	UDP-arabinose 4-epimerase activity
2607	GO:0033320	Metabolism	Metabolism	P	UDP-D-xylose biosynthetic process
2608	GO:0035250	Metabolism	Metabolism	F	UDP-galactosyltransferase activity
2609	GO:0050377	Metabolism	Metabolism	F	UDP-glucose 4,6-dehydratase activity
2610	GO:0003978	Metabolism	Metabolism	F	UDP-glucose 4-epimerase activity

2611	GO:0003979	Metabolism	Metabolism	F	UDP-glucose 6-dehydrogenase activity
2612	GO:0006011	Metabolism	Metabolism	P	UDP-glucose metabolic process
2613	GO:0080002	Metabolism	Metabolism	F	UDP-glucose:4-aminobenzoate acylglucosyltransferase activity
2614	GO:0035251	Metabolism	Metabolism	F	UDP-glucosyltransferase activity
2615	GO:0050378	Metabolism	Metabolism	F	UDP-glucuronate 4-epimerase activity
2616	GO:0006065	Metabolism	Metabolism	P	UDP-glucuronate biosynthetic process
2617	GO:0048040	Metabolism	Metabolism	F	UDP-glucuronate decarboxylase activity
2618	GO:0046398	Metabolism	Metabolism	P	UDP-glucuronate metabolic process
2619	GO:0008194	Metabolism	Metabolism	F	UDP-glycosyltransferase activity
2620	GO:0033358	Metabolism	Metabolism	P	UDP-L-arabinose biosynthetic process
2621	GO:0033356	Metabolism	Metabolism	P	UDP-L-arabinose metabolic process
2622	GO:0010280	Metabolism	Metabolism	F	UDP-L-rhamnose synthase activity
2623	GO:0006048	Metabolism	Metabolism	P	UDP-N-acetylglucosamine biosynthetic process
2624	GO:0010253	Metabolism	Metabolism	P	UDP-rhamnose biosynthetic process
2625	GO:0033478	Metabolism	Metabolism	P	UDP-rhamnose metabolic process
2626	GO:0046510	Metabolism	Metabolism	F	UDP-sulfoquinovose:DAG sulfoquinovosyltransferase activity
2627	GO:0035252	Metabolism	Metabolism	F	UDP-xylosyltransferase activity
2628	GO:0071568	Metabolism	Metabolism	F	UFM1 transferase activity
2629	GO:0002058	Metabolism	Metabolism	F	uracil binding
2630	GO:0006063	Metabolism	Metabolism	P	uronic acid metabolic process
2631	GO:0006228	Metabolism	Metabolism	P	UTP biosynthetic process
2632	GO:0003983	Metabolism	Metabolism	F	UTP:glucose-1-phosphate uridylyltransferase activity
2633	GO:0007035	Metabolism	Metabolism	P	vacuolar acidification
2634	GO:0006624	Metabolism	Metabolism	P	vacuolar protein processing
2635	GO:0010209	Metabolism	Metabolism	F	vacuolar sorting signal binding
2636	GO:0009099	Metabolism	Metabolism	P	valine biosynthetic process
2637	GO:0070279	Metabolism	Metabolism	F	vitamin B6 binding
2638	GO:0042819	Metabolism	Metabolism	P	vitamin B6 biosynthetic process
2639	GO:0019842	Metabolism	Metabolism	F	vitamin binding
2640	GO:0010025	Metabolism	Metabolism	P	wax biosynthetic process
2641	GO:0010166	Metabolism	Metabolism	P	wax metabolic process
2642	GO:0016123	Metabolism	Metabolism	P	xanthophyll biosynthetic process
2643	GO:0010301	Metabolism	Metabolism	F	xanthoxin dehydrogenase activity
2644	GO:0006805	Metabolism	Metabolism	P	xenobiotic metabolic process
2645	GO:0045492	Metabolism	Metabolism	P	xylan biosynthetic process
2646	GO:0045491	Metabolism	Metabolism	P	xylan metabolic process
2647	GO:1990538	Metabolism	Metabolism	F	xylan O-acetyltransferase activity
2648	GO:0010089	Metabolism	Metabolism	P	xylem development
2649	GO:0033843	Metabolism	Metabolism	F	xyloglucan 6-xylosyltransferase activity
2650	GO:0009969	Metabolism	Metabolism	P	xyloglucan biosynthetic process
2651	GO:0010411	Metabolism	Metabolism	P	xyloglucan metabolic process
2652	GO:0016762	Metabolism	Metabolism	F	xyloglucan:xyloglucosyl transferase activity
2653	GO:0033946	Metabolism	Metabolism	F	xyloglucan-specific endo-beta-1,4-glucanase activity
2654	GO:0009045	Metabolism	Metabolism	F	xylose isomerase activity
2655	GO:0009974	Metabolism	Metabolism	F	zeinoxanthin epsilon hydroxylase activity
2656	GO:0008270	Metabolism	Metabolism	F	zinc ion binding
2657	GO:0009882	Metabolism	Photosynthesis	F	blue light photoreceptor activity
2658	GO:0015977	Metabolism	Photosynthesis	P	carbon fixation
2659	GO:0016120	Metabolism	Photosynthesis	P	carotene biosynthetic process
2660	GO:0016117	Metabolism	Photosynthesis	P	carotenoid biosynthetic process
2661	GO:0016168	Metabolism	Photosynthesis	F	chlorophyll binding
2662	GO:0015995	Metabolism	Photosynthesis	P	chlorophyll biosynthetic process
2663	GO:0015994	Metabolism	Photosynthesis	P	chlorophyll metabolic process
2664	GO:0046408	Metabolism	Photosynthesis	F	chlorophyll synthetase activity
2665	GO:0010277	Metabolism	Photosynthesis	F	chlorophyllide a oxygenase [overall] activity
2666	GO:0009507	Metabolism	Photosynthesis	C	chloroplast
2667	GO:0009904	Metabolism	Photosynthesis	P	chloroplast accumulation movement
2668	GO:0009544	Metabolism	Photosynthesis	C	chloroplast ATP synthase complex
2669	GO:0009903	Metabolism	Photosynthesis	P	chloroplast avoidance movement
2670	GO:0010151	Metabolism	Photosynthesis	P	chloroplast elongation
2671	GO:0009941	Metabolism	Photosynthesis	C	chloroplast envelope
2672	GO:0010020	Metabolism	Photosynthesis	P	chloroplast fission
2673	GO:0009706	Metabolism	Photosynthesis	C	chloroplast inner membrane
2674	GO:0031972	Metabolism	Photosynthesis	C	chloroplast intermembrane space
2675	GO:0019750	Metabolism	Photosynthesis	P	chloroplast localization
2676	GO:0031969	Metabolism	Photosynthesis	C	chloroplast membrane
2677	GO:0042644	Metabolism	Photosynthesis	C	chloroplast nucleoid
2678	GO:0009658	Metabolism	Photosynthesis	P	chloroplast organization
2679	GO:0009707	Metabolism	Photosynthesis	C	chloroplast outer membrane
2680	GO:0030093	Metabolism	Photosynthesis	C	chloroplast photosystem I
2681	GO:0009902	Metabolism	Photosynthesis	P	chloroplast relocation
2682	GO:0043253	Metabolism	Photosynthesis	C	chloroplast ribosome
2683	GO:0031425	Metabolism	Photosynthesis	P	chloroplast RNA processing
2684	GO:1901259	Metabolism	Photosynthesis	P	chloroplast rRNA processing
2685	GO:0009569	Metabolism	Photosynthesis	C	chloroplast starch grain
2686	GO:0009570	Metabolism	Photosynthesis	C	chloroplast stroma
2687	GO:0009533	Metabolism	Photosynthesis	C	chloroplast stromal thylakoid
2688	GO:0009534	Metabolism	Photosynthesis	C	chloroplast thylakoid

2689	GO:0009543	Metabolism	Photosynthesis	C	chloroplast thylakoid lumen
2690	GO:0009535	Metabolism	Photosynthesis	C	chloroplast thylakoid membrane
2691	GO:0098807	Metabolism	Photosynthesis	C	chloroplast thylakoid membrane protein complex
2692	GO:0009840	Metabolism	Photosynthesis	C	chloroplastic endopeptidase Clp complex
2693	GO:0010019	Metabolism	Photosynthesis	P	chloroplast-nucleus signaling pathway
2694	GO:0009575	Metabolism	Photosynthesis	C	chromoplast stroma
2695	GO:0009512	Metabolism	Photosynthesis	C	cytochrome b6f complex
2696	GO:0010190	Metabolism	Photosynthesis	P	cytochrome b6f complex assembly
2697	GO:0046028	Metabolism	Photosynthesis	F	electron transporter, transferring electrons from cytochrome b6/f complex of photosystem II activity
2698	GO:0045158	Metabolism	Photosynthesis	F	electron transporter, transferring electrons within cytochrome b6/f complex of photosystem II activity
2699	GO:0045156	Metabolism	Photosynthesis	F	electron transporter, transferring electrons within the cyclic electron transport pathway of photosynthesis activity
2700	GO:0045157	Metabolism	Photosynthesis	F	electron transporter, transferring electrons within the noncyclic electron transport pathway of photosynthesis activity
2701	GO:1990066	Metabolism	Photosynthesis	P	energy quenching
2702	GO:0051667	Metabolism	Photosynthesis	P	establishment of plastid localization
2703	GO:0035448	Metabolism	Photosynthesis	C	extrinsic component of thylakoid membrane
2704	GO:0004324	Metabolism	Photosynthesis	F	ferredoxin-NADP+ reductase activity
2705	GO:0042132	Metabolism	Photosynthesis	F	fructose 1,6-bisphosphate 1-phosphatase activity
2706	GO:0030388	Metabolism	Photosynthesis	P	fructose 1,6-bisphosphate metabolic process
2707	GO:0006002	Metabolism	Photosynthesis	P	fructose 6-phosphate metabolic process
2708	GO:0004332	Metabolism	Photosynthesis	F	fructose-bisphosphate aldolase activity
2709	GO:0009515	Metabolism	Photosynthesis	C	granal stacked thylakoid
2710	GO:0006783	Metabolism	Photosynthesis	P	heme biosynthetic process
2711	GO:0006788	Metabolism	Photosynthesis	P	heme oxidation
2712	GO:0004392	Metabolism	Photosynthesis	F	heme oxygenase (decyclizing) activity
2713	GO:0031357	Metabolism	Photosynthesis	C	integral component of chloroplast inner membrane
2714	GO:0031361	Metabolism	Photosynthesis	C	integral component of thylakoid membrane
2715	GO:0010007	Metabolism	Photosynthesis	C	magnesium chelatase complex
2716	GO:1902325	Metabolism	Photosynthesis	P	negative regulation of chlorophyll biosynthetic process
2717	GO:1905156	Metabolism	Photosynthesis	P	negative regulation of photosynthesis
2718	GO:0080153	Metabolism	Photosynthesis	P	negative regulation of reductive pentose-phosphate cycle
2719	GO:0009854	Metabolism	Photosynthesis	P	oxidative photosynthetic carbon pathway
2720	GO:0010242	Metabolism	Photosynthesis	F	oxygen evolving activity
2721	GO:0009915	Metabolism	Photosynthesis	P	phloem sucrose loading
2722	GO:0110128	Metabolism	Photosynthesis	P	phloem sucrose unloading
2723	GO:0015979	Metabolism	Photosynthesis	P	photosynthesis
2724	GO:0019685	Metabolism	Photosynthesis	P	photosynthesis, dark reaction
2725	GO:0009765	Metabolism	Photosynthesis	P	photosynthesis, light harvesting
2726	GO:0009768	Metabolism	Photosynthesis	P	photosynthesis, light harvesting in photosystem I
2727	GO:0009769	Metabolism	Photosynthesis	P	photosynthesis, light harvesting in photosystem II
2728	GO:0019684	Metabolism	Photosynthesis	P	photosynthesis, light reaction
2729	GO:0009643	Metabolism	Photosynthesis	P	photosynthetic acclimation
2730	GO:0009767	Metabolism	Photosynthesis	P	photosynthetic electron transport chain
2731	GO:0009773	Metabolism	Photosynthesis	P	photosynthetic electron transport in photosystem I
2732	GO:0009772	Metabolism	Photosynthesis	P	photosynthetic electron transport in photosystem II
2733	GO:0034357	Metabolism	Photosynthesis	C	photosynthetic membrane
2734	GO:0009521	Metabolism	Photosynthesis	C	photosystem
2735	GO:0009522	Metabolism	Photosynthesis	C	photosystem I
2736	GO:0009782	Metabolism	Photosynthesis	C	photosystem I antenna complex
2737	GO:0048564	Metabolism	Photosynthesis	P	photosystem I assembly
2738	GO:0009538	Metabolism	Photosynthesis	C	photosystem I reaction center
2739	GO:0009523	Metabolism	Photosynthesis	C	photosystem II
2740	GO:0009783	Metabolism	Photosynthesis	C	photosystem II antenna complex
2741	GO:0010207	Metabolism	Photosynthesis	P	photosystem II assembly
2742	GO:0009654	Metabolism	Photosynthesis	C	photosystem II oxygen evolving complex
2743	GO:0010270	Metabolism	Photosynthesis	P	photosystem II oxygen evolving complex assembly
2744	GO:0009539	Metabolism	Photosynthesis	C	photosystem II reaction center
2745	GO:0010206	Metabolism	Photosynthesis	P	photosystem II repair
2746	GO:0042549	Metabolism	Photosynthesis	P	photosystem II stabilization
2747	GO:0080005	Metabolism	Photosynthesis	P	photosystem stoichiometry adjustment
2748	GO:0009536	Metabolism	Photosynthesis	C	plastid
2749	GO:0009508	Metabolism	Photosynthesis	C	plastid chromosome
2750	GO:0009526	Metabolism	Photosynthesis	C	plastid envelope
2751	GO:0009528	Metabolism	Photosynthesis	C	plastid inner membrane
2752	GO:0000311	Metabolism	Photosynthesis	C	plastid large ribosomal subunit
2753	GO:0051644	Metabolism	Photosynthesis	P	plastid localization
2754	GO:0042170	Metabolism	Photosynthesis	C	plastid membrane
2755	GO:0009668	Metabolism	Photosynthesis	P	plastid membrane organization
2756	GO:0042646	Metabolism	Photosynthesis	C	plastid nucleoid
2757	GO:0009657	Metabolism	Photosynthesis	P	plastid organization
2758	GO:0009527	Metabolism	Photosynthesis	C	plastid outer membrane
2759	GO:0009547	Metabolism	Photosynthesis	C	plastid ribosome
2760	GO:0000312	Metabolism	Photosynthesis	C	plastid small ribosomal subunit
2761	GO:0009532	Metabolism	Photosynthesis	C	plastid stroma
2762	GO:0031976	Metabolism	Photosynthesis	C	plastid thylakoid

2763	GO:0031978	Metabolism	Photosynthesis	C	plastid thylakoid lumen
2764	GO:0055035	Metabolism	Photosynthesis	C	plastid thylakoid membrane
2765	GO:0042793	Metabolism	Photosynthesis	P	plastid transcription
2766	GO:0032544	Metabolism	Photosynthesis	P	plastid translation
2767	GO:0010287	Metabolism	Photosynthesis	C	plastoglobule
2768	GO:0080177	Metabolism	Photosynthesis	P	plastoglobule organization
2769	GO:0009496	Metabolism	Photosynthesis	F	plastoquinol--plastocyanin reductase activity
2770	GO:0006779	Metabolism	Photosynthesis	P	porphyrin-containing compound biosynthetic process
2771	GO:1902326	Metabolism	Photosynthesis	P	positive regulation of chlorophyll biosynthetic process
2772	GO:1905157	Metabolism	Photosynthesis	P	positive regulation of photosynthesis
2773	GO:0045037	Metabolism	Photosynthesis	P	protein import into chloroplast stroma
2774	GO:0045038	Metabolism	Photosynthesis	P	protein import into chloroplast thylakoid membrane
2775	GO:0072598	Metabolism	Photosynthesis	P	protein localization to chloroplast
2776	GO:0045036	Metabolism	Photosynthesis	P	protein targeting to chloroplast
2777	GO:0018298	Metabolism	Photosynthesis	P	protein-chromophore linkage
2778	GO:0016630	Metabolism	Photosynthesis	F	protochlorophyllide reductase activity
2779	GO:0006782	Metabolism	Photosynthesis	P	protoporphyrinogen IX biosynthetic process
2780	GO:0009517	Metabolism	Photosynthesis	C	PSII associated light-harvesting complex II
2781	GO:0031540	Metabolism	Photosynthesis	P	regulation of anthocyanin biosynthetic process
2782	GO:0010380	Metabolism	Photosynthesis	P	regulation of chlorophyll biosynthetic process
2783	GO:0043467	Metabolism	Photosynthesis	P	regulation of generation of precursor metabolites and energy
2784	GO:0010109	Metabolism	Photosynthesis	P	regulation of photosynthesis
2785	GO:0042548	Metabolism	Photosynthesis	P	regulation of photosynthesis, light reaction
2786	GO:1904215	Metabolism	Photosynthesis	P	regulation of protein import into chloroplast stroma
2787	GO:0046863	Metabolism	Photosynthesis	F	ribulose-1,5-bisphosphate carboxylase/oxygenase activator activity
2788	GO:0016984	Metabolism	Photosynthesis	F	ribulose-bisphosphate carboxylase activity
2789	GO:0098572	Metabolism	Photosynthesis	C	stromal side of plastid thylakoid membrane
2790	GO:0016114	Metabolism	Photosynthesis	P	terpenoid biosynthetic process
2791	GO:0009579	Metabolism	Photosynthesis	C	thylakoid
2792	GO:0031977	Metabolism	Photosynthesis	C	thylakoid lumen
2793	GO:0042651	Metabolism	Photosynthesis	C	thylakoid membrane
2794	GO:0010027	Metabolism	Photosynthesis	P	thylakoid membrane organization
2795	GO:0031897	Metabolism	Photosynthesis	C	Tic complex
2796	GO:0010006	Metabolism	Photosynthesis	C	Toc complex
2797	GO:0061927	Metabolism	Photosynthesis	C	TOC-TIC supercomplex I
2798	GO:0051738	Metabolism	Photosynthesis	F	xanthophyll binding
2799	GO:0046964	Metabolism	Transporters	F	3'-phosphoadenosine 5'-phosphosulfate transmembrane transporter activity
2800	GO:0015172	Metabolism	Transporters	F	acidic amino acid transmembrane transporter activity
2801	GO:0015800	Metabolism	Transporters	P	acidic amino acid transport
2802	GO:0046715	Metabolism	Transporters	F	active borate transmembrane transporter activity
2803	GO:0022853	Metabolism	Transporters	F	active ion transmembrane transporter activity
2804	GO:0022804	Metabolism	Transporters	F	active transmembrane transporter activity
2805	GO:0034196	Metabolism	Transporters	P	acylglycerol transport
2806	GO:0051503	Metabolism	Transporters	P	adenine nucleotide transport
2807	GO:0015720	Metabolism	Transporters	P	allantoin transport
2808	GO:0005274	Metabolism	Transporters	F	allantoin:proton symporter activity
2809	GO:0043090	Metabolism	Transporters	P	amino acid import
2810	GO:0003333	Metabolism	Transporters	P	amino acid transmembrane transport
2811	GO:0015171	Metabolism	Transporters	F	amino acid transmembrane transporter activity
2812	GO:0006865	Metabolism	Transporters	P	amino acid transport
2813	GO:0008519	Metabolism	Transporters	F	ammonium transmembrane transporter activity
2814	GO:0005253	Metabolism	Transporters	F	anion channel activity
2815	GO:0098656	Metabolism	Transporters	P	anion transmembrane transport
2816	GO:0008509	Metabolism	Transporters	F	anion transmembrane transporter activity
2817	GO:0006820	Metabolism	Transporters	P	anion transport
2818	GO:0015301	Metabolism	Transporters	F	anion:anion antiporter activity
2819	GO:0015297	Metabolism	Transporters	F	antiporter activity
2820	GO:0030121	Metabolism	Transporters	C	AP-1 adaptor complex
2821	GO:0015181	Metabolism	Transporters	F	arginine transmembrane transporter activity
2822	GO:0015173	Metabolism	Transporters	F	aromatic amino acid transmembrane transporter activity
2823	GO:1901684	Metabolism	Transporters	P	arsenate ion transmembrane transport
2824	GO:0005471	Metabolism	Transporters	F	ATP:ADP antiporter activity
2825	GO:0019829	Metabolism	Transporters	F	ATPase-coupled cation transmembrane transporter activity
2826	GO:0140326	Metabolism	Transporters	F	ATPase-coupled intramembrane lipid transporter activity
2827	GO:0042625	Metabolism	Transporters	F	ATPase-coupled ion transmembrane transporter activity
2828	GO:0042626	Metabolism	Transporters	F	ATPase-coupled transmembrane transporter activity
2829	GO:0008559	Metabolism	Transporters	F	ATPase-coupled xenobiotic transmembrane transporter activity
2830	GO:0015174	Metabolism	Transporters	F	basic amino acid transmembrane transporter activity
2831	GO:0080139	Metabolism	Transporters	F	borate efflux transmembrane transporter activity
2832	GO:0035445	Metabolism	Transporters	P	borate transmembrane transport
2833	GO:0046713	Metabolism	Transporters	P	borate transport
2834	GO:0015556	Metabolism	Transporters	F	C4-dicarboxylate transmembrane transporter activity
2835	GO:0015740	Metabolism	Transporters	P	C4-dicarboxylate transport
2836	GO:0015839	Metabolism	Transporters	P	cadaverine transport
2837	GO:0015086	Metabolism	Transporters	F	cadmium ion transmembrane transporter activity
2838	GO:0015691	Metabolism	Transporters	P	cadmium ion transport
2839	GO:0070509	Metabolism	Transporters	P	calcium ion import
2840	GO:0070588	Metabolism	Transporters	P	calcium ion transmembrane transport

2841	GO:0015085	Metabolism	Transporters	F	calcium ion transmembrane transporter activity
2842	GO:0006816	Metabolism	Transporters	P	calcium ion transport
2843	GO:0005388	Metabolism	Transporters	F	calcium transmembrane transporter activity, phosphorylative mechanism
2844	GO:1901264	Metabolism	Transporters	P	carbohydrate derivative transport
2845	GO:0033231	Metabolism	Transporters	P	carbohydrate export
2846	GO:0034219	Metabolism	Transporters	P	carbohydrate transmembrane transport
2847	GO:0015144	Metabolism	Transporters	F	carbohydrate transmembrane transporter activity
2848	GO:0008643	Metabolism	Transporters	P	carbohydrate transport
2849	GO:0005351	Metabolism	Transporters	F	carbohydrate:proton symporter activity
2850	GO:1905039	Metabolism	Transporters	P	carboxylic acid transmembrane transport
2851	GO:0046943	Metabolism	Transporters	F	carboxylic acid transmembrane transporter activity
2852	GO:0046942	Metabolism	Transporters	P	carboxylic acid transport
2853	GO:0015226	Metabolism	Transporters	F	carnitine transmembrane transporter activity
2854	GO:0005261	Metabolism	Transporters	F	cation channel activity
2855	GO:0098655	Metabolism	Transporters	P	cation transmembrane transport
2856	GO:0008324	Metabolism	Transporters	F	cation transmembrane transporter activity
2857	GO:0006812	Metabolism	Transporters	P	cation transport
2858	GO:0015491	Metabolism	Transporters	F	cation:cation antiporter activity
2859	GO:0070727	Metabolism	Transporters	P	cellular macromolecule localization
2860	GO:0035627	Metabolism	Transporters	P	ceramide transport
2861	GO:0015267	Metabolism	Transporters	F	channel activity
2862	GO:0072321	Metabolism	Transporters	P	chaperone-mediated protein transport
2863	GO:0006935	Metabolism	Transporters	P	chemotaxis
2864	GO:0034707	Metabolism	Transporters	C	chloride channel complex
2865	GO:0015108	Metabolism	Transporters	F	chloride transmembrane transporter activity
2866	GO:0006821	Metabolism	Transporters	P	chloride transport
2867	GO:0015137	Metabolism	Transporters	F	citrate transmembrane transporter activity
2868	GO:0030276	Metabolism	Transporters	F	clathrin binding
2869	GO:0030665	Metabolism	Transporters	C	clathrin-coated vesicle membrane
2870	GO:0006824	Metabolism	Transporters	P	cobalt ion transport
2871	GO:0015228	Metabolism	Transporters	F	coenzyme A transmembrane transporter activity
2872	GO:0030126	Metabolism	Transporters	C	COPI vesicle coat
2873	GO:0030127	Metabolism	Transporters	C	COPII vesicle coat
2874	GO:0030134	Metabolism	Transporters	C	COPII-coated ER to Golgi transport vesicle
2875	GO:0005375	Metabolism	Transporters	F	copper ion transmembrane transporter activity
2876	GO:0006825	Metabolism	Transporters	P	copper ion transport
2877	GO:0080051	Metabolism	Transporters	P	cutin transport
2878	GO:0031410	Metabolism	Transporters	C	cytoplasmic vesicle
2879	GO:0005310	Metabolism	Transporters	F	dicarboxylic acid transmembrane transporter activity
2880	GO:0006835	Metabolism	Transporters	P	dicarboxylic acid transport
2881	GO:0042938	Metabolism	Transporters	P	dipeptide transport
2882	GO:0015766	Metabolism	Transporters	P	disaccharide transport
2883	GO:0072509	Metabolism	Transporters	F	divalent inorganic cation transmembrane transporter activity
2884	GO:0072511	Metabolism	Transporters	P	divalent inorganic cation transport
2885	GO:0070838	Metabolism	Transporters	P	divalent metal ion transport
2886	GO:0005769	Metabolism	Transporters	C	early endosome
2887	GO:0031901	Metabolism	Transporters	C	early endosome membrane
2888	GO:0006888	Metabolism	Transporters	P	endoplasmic reticulum to Golgi vesicle-mediated transport
2889	GO:0005768	Metabolism	Transporters	C	endosome
2890	GO:0010008	Metabolism	Transporters	C	endosome membrane
2891	GO:0008333	Metabolism	Transporters	P	endosome to lysosome transport
2892	GO:0032509	Metabolism	Transporters	P	endosome transport via multivesicular body sorting pathway
2893	GO:1990052	Metabolism	Transporters	P	ER to chloroplast lipid transport
2894	GO:0012507	Metabolism	Transporters	C	ER to Golgi transport vesicle membrane
2895	GO:0072663	Metabolism	Transporters	P	establishment of protein localization to peroxisome
2896	GO:0140115	Metabolism	Transporters	P	export across plasma membrane
2897	GO:0140352	Metabolism	Transporters	P	export from cell
2898	GO:0015230	Metabolism	Transporters	F	FAD transmembrane transporter activity
2899	GO:0015245	Metabolism	Transporters	F	fatty acid transmembrane transporter activity
2900	GO:0015908	Metabolism	Transporters	P	fatty acid transport
2901	GO:0015916	Metabolism	Transporters	P	fatty-acyl-CoA transport
2902	GO:0015093	Metabolism	Transporters	F	ferrous iron transmembrane transporter activity
2903	GO:0042044	Metabolism	Transporters	P	fluid transport
2904	GO:1903424	Metabolism	Transporters	P	fluoride transmembrane transport
2905	GO:1903425	Metabolism	Transporters	F	fluoride transmembrane transporter activity
2906	GO:0008517	Metabolism	Transporters	F	folic acid transmembrane transporter activity
2907	GO:0015884	Metabolism	Transporters	P	folic acid transport
2908	GO:0015755	Metabolism	Transporters	P	fructose transmembrane transport
2909	GO:0005353	Metabolism	Transporters	F	fructose transmembrane transporter activity
2910	GO:0015284	Metabolism	Transporters	F	fructose uniporter activity
2911	GO:0005354	Metabolism	Transporters	F	galactose transmembrane transporter activity
2912	GO:0015185	Metabolism	Transporters	F	gamma-aminobutyric acid transmembrane transporter activity
2913	GO:0015812	Metabolism	Transporters	P	gamma-aminobutyric acid transport
2914	GO:0015783	Metabolism	Transporters	P	GDP-fucose transmembrane transport
2915	GO:0005457	Metabolism	Transporters	F	GDP-fucose transmembrane transporter activity
2916	GO:1904659	Metabolism	Transporters	P	glucose transmembrane transport
2917	GO:0005355	Metabolism	Transporters	F	glucose transmembrane transporter activity
2918	GO:0015152	Metabolism	Transporters	F	glucose-6-phosphate transmembrane transporter activity

2919	GO:0015760	Metabolism	Transporters	P	glucose-6-phosphate transport
2920	GO:1901349	Metabolism	Transporters	P	glucosinolate transport
2921	GO:0090448	Metabolism	Transporters	F	glucosinolate:proton symporter activity
2922	GO:0015168	Metabolism	Transporters	F	glycerol transmembrane transporter activity
2923	GO:0006895	Metabolism	Transporters	P	Golgi to endosome transport
2924	GO:0006893	Metabolism	Transporters	P	Golgi to plasma membrane transport
2925	GO:0006896	Metabolism	Transporters	P	Golgi to vacuole transport
2926	GO:0017119	Metabolism	Transporters	C	Golgi transport complex
2927	GO:0048193	Metabolism	Transporters	P	Golgi vesicle transport
2928	GO:0015886	Metabolism	Transporters	P	heme transport
2929	GO:0015232	Metabolism	Transporters	F	heme transporter activity
2930	GO:0051170	Metabolism	Transporters	P	import into nucleus
2931	GO:0005452	Metabolism	Transporters	F	inorganic anion exchanger activity
2932	GO:0098661	Metabolism	Transporters	P	inorganic anion transmembrane transport
2933	GO:0015103	Metabolism	Transporters	F	inorganic anion transmembrane transporter activity
2934	GO:0015698	Metabolism	Transporters	P	inorganic anion transport
2935	GO:0098662	Metabolism	Transporters	P	inorganic cation transmembrane transport
2936	GO:0022890	Metabolism	Transporters	F	inorganic cation transmembrane transporter activity
2937	GO:0098660	Metabolism	Transporters	P	inorganic ion transmembrane transport
2938	GO:0015318	Metabolism	Transporters	F	inorganic molecular entity transmembrane transporter activity
2939	GO:0005315	Metabolism	Transporters	F	inorganic phosphate transmembrane transporter activity
2940	GO:0031359	Metabolism	Transporters	C	integral component of chloroplast outer membrane
2941	GO:0120009	Metabolism	Transporters	P	intermembrane lipid transfer
2942	GO:0005222	Metabolism	Transporters	F	intracellular cAMP-activated cation channel activity
2943	GO:0005221	Metabolism	Transporters	F	intracellular cyclic nucleotide activated cation channel activity
2944	GO:0065002	Metabolism	Transporters	P	intracellular protein transmembrane transport
2945	GO:0006886	Metabolism	Transporters	P	intracellular protein transport
2946	GO:0046907	Metabolism	Transporters	P	intracellular transport
2947	GO:0075733	Metabolism	Transporters	P	intracellular transport of virus
2948	GO:0006891	Metabolism	Transporters	P	intra-Golgi vesicle-mediated transport
2949	GO:0005216	Metabolism	Transporters	F	ion channel activity
2950	GO:0034220	Metabolism	Transporters	P	ion transmembrane transport
2951	GO:0015075	Metabolism	Transporters	F	ion transmembrane transporter activity
2952	GO:0015662	Metabolism	Transporters	F	ion transmembrane transporter activity, phosphorylative mechanism
2953	GO:0006811	Metabolism	Transporters	P	ion transport
2954	GO:0034755	Metabolism	Transporters	P	iron ion transmembrane transport
2955	GO:0005381	Metabolism	Transporters	F	iron ion transmembrane transporter activity
2956	GO:0006826	Metabolism	Transporters	P	iron ion transport
2957	GO:0051980	Metabolism	Transporters	F	iron-nicotianamine transmembrane transporter activity
2958	GO:0005871	Metabolism	Transporters	C	kinesin complex
2959	GO:0015180	Metabolism	Transporters	F	L-alanine transmembrane transporter activity
2960	GO:0015808	Metabolism	Transporters	P	L-alanine transport
2961	GO:1902475	Metabolism	Transporters	P	L-alpha-amino acid transmembrane transport
2962	GO:0015179	Metabolism	Transporters	F	L-amino acid transmembrane transporter activity
2963	GO:0045324	Metabolism	Transporters	P	late endosome to vacuole transport
2964	GO:0015184	Metabolism	Transporters	F	L-cystine transmembrane transporter activity
2965	GO:0015811	Metabolism	Transporters	P	L-cystine transport
2966	GO:0098712	Metabolism	Transporters	P	L-glutamate import across plasma membrane
2967	GO:0015813	Metabolism	Transporters	P	L-glutamate transmembrane transport
2968	GO:0005313	Metabolism	Transporters	F	L-glutamate transmembrane transporter activity
2969	GO:0015186	Metabolism	Transporters	F	L-glutamine transmembrane transporter activity
2970	GO:0006869	Metabolism	Transporters	P	lipid transport
2971	GO:0005319	Metabolism	Transporters	F	lipid transporter activity
2972	GO:0010351	Metabolism	Transporters	P	lithium ion transport
2973	GO:0015189	Metabolism	Transporters	F	L-lysine transmembrane transporter activity
2974	GO:0040011	Metabolism	Transporters	P	locomotion
2975	GO:0000064	Metabolism	Transporters	F	L-ornithine transmembrane transporter activity
2976	GO:0080054	Metabolism	Transporters	F	low-affinity nitrate transmembrane transporter activity
2977	GO:0009673	Metabolism	Transporters	F	low-affinity phosphate transmembrane transporter activity
2978	GO:0015193	Metabolism	Transporters	F	L-proline transmembrane transporter activity
2979	GO:0015194	Metabolism	Transporters	F	L-serine transmembrane transporter activity
2980	GO:0005302	Metabolism	Transporters	F	L-tyrosine transmembrane transporter activity
2981	GO:0033036	Metabolism	Transporters	P	macromolecule localization
2982	GO:0015095	Metabolism	Transporters	F	magnesium ion transmembrane transporter activity
2983	GO:0015693	Metabolism	Transporters	P	magnesium ion transport
2984	GO:0071423	Metabolism	Transporters	P	malate transmembrane transport
2985	GO:0015140	Metabolism	Transporters	F	malate transmembrane transporter activity
2986	GO:0005384	Metabolism	Transporters	F	manganese ion transmembrane transporter activity
2987	GO:0006828	Metabolism	Transporters	P	manganese ion transport
2988	GO:0015410	Metabolism	Transporters	F	manganese transmembrane transporter activity, phosphorylative mechanism
2989	GO:0046873	Metabolism	Transporters	F	metal ion transmembrane transporter activity
2990	GO:0030001	Metabolism	Transporters	P	metal ion transport
2991	GO:0015200	Metabolism	Transporters	F	methylammonium transmembrane transporter activity
2992	GO:1990575	Metabolism	Transporters	P	mitochondrial L-ornithine transmembrane transport
2993	GO:0006839	Metabolism	Transporters	P	mitochondrial transport
2994	GO:0030943	Metabolism	Transporters	F	mitochondrion targeting sequence binding
2995	GO:0015098	Metabolism	Transporters	F	molybdate ion transmembrane transporter activity
2996	GO:0015689	Metabolism	Transporters	P	molybdate ion transport

2997	GO:0008028	Metabolism	Transporters	F	monocarboxylic acid transmembrane transporter activity
2998	GO:0015749	Metabolism	Transporters	P	monosaccharide transmembrane transport
2999	GO:0015145	Metabolism	Transporters	F	monosaccharide transmembrane transporter activity
3000	GO:0015077	Metabolism	Transporters	F	monovalent inorganic cation transmembrane transporter activity
3001	GO:0015672	Metabolism	Transporters	P	monovalent inorganic cation transport
3002	GO:0005366	Metabolism	Transporters	F	myo-inositol:proton symporter activity
3003	GO:0010360	Metabolism	Transporters	P	negative regulation of anion channel activity
3004	GO:0010362	Metabolism	Transporters	P	negative regulation of anion channel activity by blue light
3005	GO:1901017	Metabolism	Transporters	P	negative regulation of potassium ion transmembrane transporter activity
3006	GO:0051051	Metabolism	Transporters	P	negative regulation of transport
3007	GO:0015175	Metabolism	Transporters	F	neutral amino acid transmembrane transporter activity
3008	GO:0015804	Metabolism	Transporters	P	neutral amino acid transport
3009	GO:1902025	Metabolism	Transporters	P	nitrate import
3010	GO:0015112	Metabolism	Transporters	F	nitrate transmembrane transporter activity
3011	GO:0015706	Metabolism	Transporters	P	nitrate transport
3012	GO:0071705	Metabolism	Transporters	P	nitrogen compound transport
3013	GO:0051168	Metabolism	Transporters	P	nuclear export
3014	GO:0008139	Metabolism	Transporters	F	nuclear localization sequence binding
3015	GO:0005880	Metabolism	Transporters	C	nuclear microtubule
3016	GO:0044613	Metabolism	Transporters	C	nuclear pore central transport channel
3017	GO:0051169	Metabolism	Transporters	P	nuclear transport
3018	GO:0050657	Metabolism	Transporters	P	nucleic acid transport
3019	GO:0006913	Metabolism	Transporters	P	nucleocytoplasmic transport
3020	GO:0005337	Metabolism	Transporters	F	nucleoside transmembrane transporter activity
3021	GO:0015780	Metabolism	Transporters	P	nucleotide-sugar transmembrane transport
3022	GO:0005338	Metabolism	Transporters	F	nucleotide-sugar transmembrane transporter activity
3023	GO:0035673	Metabolism	Transporters	F	oligopeptide transmembrane transporter activity
3024	GO:0006857	Metabolism	Transporters	P	oligopeptide transport
3025	GO:0019755	Metabolism	Transporters	P	one-carbon compound transport
3026	GO:1903825	Metabolism	Transporters	P	organic acid transmembrane transport
3027	GO:0005342	Metabolism	Transporters	F	organic acid transmembrane transporter activity
3028	GO:0015849	Metabolism	Transporters	P	organic acid transport
3029	GO:0008514	Metabolism	Transporters	F	organic anion transmembrane transporter activity
3030	GO:0015711	Metabolism	Transporters	P	organic anion transport
3031	GO:0015850	Metabolism	Transporters	P	organic hydroxy compound transport
3032	GO:0071702	Metabolism	Transporters	P	organic substance transport
3033	GO:0015822	Metabolism	Transporters	P	ornithine transport
3034	GO:0015131	Metabolism	Transporters	F	oxaloacetate transmembrane transporter activity
3035	GO:0015729	Metabolism	Transporters	P	oxaloacetate transport
3036	GO:0015367	Metabolism	Transporters	F	oxoglutarate:malate antiporter activity
3037	GO:1901140	Metabolism	Transporters	P	p-coumaryl alcohol transport
3038	GO:1904680	Metabolism	Transporters	F	peptide transmembrane transporter activity
3039	GO:0015333	Metabolism	Transporters	F	peptide:proton symporter activity
3040	GO:1990429	Metabolism	Transporters	C	peroxisomal importomer complex
3041	GO:0010233	Metabolism	Transporters	P	phloem transport
3042	GO:0035435	Metabolism	Transporters	P	phosphate ion transmembrane transport
3043	GO:0015114	Metabolism	Transporters	F	phosphate ion transmembrane transporter activity
3044	GO:0006817	Metabolism	Transporters	P	phosphate ion transport
3045	GO:0015714	Metabolism	Transporters	P	phosphoenolpyruvate transport
3046	GO:0015713	Metabolism	Transporters	P	phosphoglycerate transmembrane transport
3047	GO:0015120	Metabolism	Transporters	F	phosphoglycerate transmembrane transporter activity
3048	GO:0045332	Metabolism	Transporters	P	phospholipid translocation
3049	GO:0015914	Metabolism	Transporters	P	phospholipid transport
3050	GO:0009506	Metabolism	Transporters	C	plasmodesma
3051	GO:0010497	Metabolism	Transporters	P	plasmodesmata-mediated intercellular transport
3052	GO:0015203	Metabolism	Transporters	F	polyamine transmembrane transporter activity
3053	GO:0015846	Metabolism	Transporters	P	polyamine transport
3054	GO:0015774	Metabolism	Transporters	P	polysaccharide transport
3055	GO:0046930	Metabolism	Transporters	C	pore complex
3056	GO:0050918	Metabolism	Transporters	P	positive chemotaxis
3057	GO:0031204	Metabolism	Transporters	P	posttranslational protein targeting to membrane, translocation
3058	GO:0071805	Metabolism	Transporters	P	potassium ion transmembrane transport
3059	GO:0015079	Metabolism	Transporters	F	potassium ion transmembrane transporter activity
3060	GO:0006813	Metabolism	Transporters	P	potassium ion transport
3061	GO:0015386	Metabolism	Transporters	F	potassium:proton antiporter activity
3062	GO:0015450	Metabolism	Transporters	F	P-P-bond-hydrolysis-driven protein transmembrane transporter activity
3063	GO:0015399	Metabolism	Transporters	F	primary active transmembrane transporter activity
3064	GO:0015824	Metabolism	Transporters	P	proline transport
3065	GO:0032527	Metabolism	Transporters	P	protein exit from endoplasmic reticulum
3066	GO:0006611	Metabolism	Transporters	P	protein export from nucleus
3067	GO:0045041	Metabolism	Transporters	P	protein import into mitochondrial intermembrane space
3068	GO:0030150	Metabolism	Transporters	P	protein import into mitochondrial matrix
3069	GO:0006606	Metabolism	Transporters	P	protein import into nucleus
3070	GO:0016560	Metabolism	Transporters	P	protein import into peroxisome matrix, docking
3071	GO:0045039	Metabolism	Transporters	P	protein insertion into mitochondrial inner membrane
3072	GO:0045040	Metabolism	Transporters	P	protein insertion into mitochondrial outer membrane
3073	GO:0008104	Metabolism	Transporters	P	protein localization
3074	GO:1901703	Metabolism	Transporters	P	protein localization involved in auxin polar transport

3075	GO:0070973	Metabolism	Transporters	P	protein localization to endoplasmic reticulum exit site
3076	GO:1903292	Metabolism	Transporters	P	protein localization to Golgi membrane
3077	GO:0035372	Metabolism	Transporters	P	protein localization to microtubule
3078	GO:1904825	Metabolism	Transporters	P	protein localization to microtubule plus-end
3079	GO:0033365	Metabolism	Transporters	P	protein localization to organelle
3080	GO:0072662	Metabolism	Transporters	P	protein localization to peroxisome
3081	GO:0072659	Metabolism	Transporters	P	protein localization to plasma membrane
3082	GO:0043495	Metabolism	Transporters	F	protein membrane anchor
3083	GO:0006627	Metabolism	Transporters	P	protein processing involved in protein targeting to mitochondrion
3084	GO:0009306	Metabolism	Transporters	P	protein secretion
3085	GO:0006626	Metabolism	Transporters	P	protein targeting to mitochondrion
3086	GO:0006625	Metabolism	Transporters	P	protein targeting to peroxisome
3087	GO:0006623	Metabolism	Transporters	P	protein targeting to vacuole
3088	GO:0008320	Metabolism	Transporters	F	protein transmembrane transporter activity
3089	GO:0015031	Metabolism	Transporters	P	protein transport
3090	GO:0043953	Metabolism	Transporters	P	protein transport by the Tat complex
3091	GO:0009977	Metabolism	Transporters	F	proton motive force dependent protein transmembrane transporter activity
3092	GO:0015078	Metabolism	Transporters	F	proton transmembrane transporter activity
3093	GO:0005345	Metabolism	Transporters	F	purine nucleobase transmembrane transporter activity
3094	GO:0006863	Metabolism	Transporters	P	purine nucleobase transport
3095	GO:0015211	Metabolism	Transporters	F	purine nucleoside transmembrane transporter activity
3096	GO:0015865	Metabolism	Transporters	P	purine nucleotide transport
3097	GO:0090481	Metabolism	Transporters	P	pyrimidine nucleotide-sugar transmembrane transport
3098	GO:0015165	Metabolism	Transporters	F	pyrimidine nucleotide-sugar transmembrane transporter activity
3099	GO:0050833	Metabolism	Transporters	F	pyruvate transmembrane transporter activity
3100	GO:0003400	Metabolism	Transporters	P	regulation of COPII vesicle coating
3101	GO:1901979	Metabolism	Transporters	P	regulation of inward rectifier potassium channel activity
3102	GO:0034765	Metabolism	Transporters	P	regulation of ion transmembrane transport
3103	GO:0032412	Metabolism	Transporters	P	regulation of ion transmembrane transporter activity
3104	GO:0010966	Metabolism	Transporters	P	regulation of phosphate transport
3105	GO:0042306	Metabolism	Transporters	P	regulation of protein import into nucleus
3106	GO:0010155	Metabolism	Transporters	P	regulation of proton transport
3107	GO:0022898	Metabolism	Transporters	P	regulation of transmembrane transporter activity
3108	GO:0032409	Metabolism	Transporters	P	regulation of transporter activity
3109	GO:0048209	Metabolism	Transporters	P	regulation of vesicle targeting, to, from or within Golgi
3110	GO:0060627	Metabolism	Transporters	P	regulation of vesicle-mediated transport
3111	GO:0050658	Metabolism	Transporters	P	RNA transport
3112	GO:0008271	Metabolism	Transporters	F	secondary active sulfate transmembrane transporter activity
3113	GO:0015291	Metabolism	Transporters	F	secondary active transmembrane transporter activity
3114	GO:0099503	Metabolism	Transporters	C	secretory vesicle
3115	GO:0005484	Metabolism	Transporters	F	SNAP receptor activity
3116	GO:0000149	Metabolism	Transporters	F	SNARE binding
3117	GO:0031201	Metabolism	Transporters	C	SNARE complex
3118	GO:0035493	Metabolism	Transporters	P	SNARE complex assembly
3119	GO:0035494	Metabolism	Transporters	P	SNARE complex disassembly
3120	GO:0098719	Metabolism	Transporters	P	sodium ion import across plasma membrane
3121	GO:0035725	Metabolism	Transporters	P	sodium ion transmembrane transport
3122	GO:0015081	Metabolism	Transporters	F	sodium ion transmembrane transporter activity
3123	GO:0006814	Metabolism	Transporters	P	sodium ion transport
3124	GO:0015385	Metabolism	Transporters	F	sodium:proton antiporter activity
3125	GO:0140323	Metabolism	Transporters	F	solute:anion antiporter activity
3126	GO:0015298	Metabolism	Transporters	F	solute:cation antiporter activity
3127	GO:0015294	Metabolism	Transporters	F	solute:cation symporter activity
3128	GO:0015299	Metabolism	Transporters	F	solute:proton antiporter activity
3129	GO:0015295	Metabolism	Transporters	F	solute:proton symporter activity
3130	GO:0006616	Metabolism	Transporters	P	SRP-dependent cotranslational protein targeting to membrane, translocation
3131	GO:0000322	Metabolism	Transporters	C	storage vacuole
3132	GO:0071422	Metabolism	Transporters	P	succinate transmembrane transport
3133	GO:0015141	Metabolism	Transporters	F	succinate transmembrane transporter activity
3134	GO:0008515	Metabolism	Transporters	F	sucrose transmembrane transporter activity
3135	GO:0015770	Metabolism	Transporters	P	sucrose transport
3136	GO:0008506	Metabolism	Transporters	F	sucrose:proton symporter activity
3137	GO:0051119	Metabolism	Transporters	F	sugar transmembrane transporter activity
3138	GO:0000103	Metabolism	Transporters	P	sulfate assimilation
3139	GO:0015116	Metabolism	Transporters	F	sulfate transmembrane transporter activity
3140	GO:0008272	Metabolism	Transporters	P	sulfate transport
3141	GO:0030581	Metabolism	Transporters	P	symbiont intracellular protein transport in host
3142	GO:0015293	Metabolism	Transporters	F	symporter activity
3143	GO:0033281	Metabolism	Transporters	C	TAT protein transport complex
3144	GO:0015117	Metabolism	Transporters	F	thiosulfate transmembrane transporter activity
3145	GO:0015709	Metabolism	Transporters	P	thiosulfate transport
3146	GO:0005802	Metabolism	Transporters	C	trans-Golgi network
3147	GO:0012510	Metabolism	Transporters	C	trans-Golgi network transport vesicle membrane
3148	GO:0046915	Metabolism	Transporters	F	transition metal ion transmembrane transporter activity
3149	GO:0000041	Metabolism	Transporters	P	transition metal ion transport
3150	GO:1905011	Metabolism	Transporters	P	transmembrane phosphate ion transport from cytosol to vacuole
3151	GO:0055085	Metabolism	Transporters	P	transmembrane transport
3152	GO:0022857	Metabolism	Transporters	F	transmembrane transporter activity

3153	GO:0006810	Metabolism	Transporters	P	transport
3154	GO:0046739	Metabolism	Transporters	P	transport of virus in multicellular host
3155	GO:0030133	Metabolism	Transporters	C	transport vesicle
3156	GO:0030658	Metabolism	Transporters	C	transport vesicle membrane
3157	GO:0005215	Metabolism	Transporters	F	transporter activity
3158	GO:0030008	Metabolism	Transporters	C	TRAPP complex
3159	GO:0035674	Metabolism	Transporters	P	tricarboxylic acid transmembrane transport
3160	GO:0015142	Metabolism	Transporters	F	tricarboxylic acid transmembrane transporter activity
3161	GO:0071917	Metabolism	Transporters	F	triose-phosphate transmembrane transporter activity
3162	GO:0042937	Metabolism	Transporters	F	tripeptide transmembrane transporter activity
3163	GO:0042939	Metabolism	Transporters	P	tripeptide transport
3164	GO:0072334	Metabolism	Transporters	P	UDP-galactose transmembrane transport
3165	GO:0005459	Metabolism	Transporters	F	UDP-galactose transmembrane transporter activity
3166	GO:0015786	Metabolism	Transporters	P	UDP-glucose transmembrane transport
3167	GO:0005460	Metabolism	Transporters	F	UDP-glucose transmembrane transporter activity
3168	GO:0015790	Metabolism	Transporters	P	UDP-xylose transmembrane transport
3169	GO:0005464	Metabolism	Transporters	F	UDP-xylose transmembrane transporter activity
3170	GO:0015292	Metabolism	Transporters	F	uniporter activity
3171	GO:0015210	Metabolism	Transporters	F	uracil transmembrane transporter activity
3172	GO:0015857	Metabolism	Transporters	P	uracil transport
3173	GO:0015505	Metabolism	Transporters	F	uracil:cation symporter activity
3174	GO:0015204	Metabolism	Transporters	F	urea transmembrane transporter activity
3175	GO:0015840	Metabolism	Transporters	P	urea transport
3176	GO:0043182	Metabolism	Transporters	P	vacuolar sequestering of sodium ion
3177	GO:0007034	Metabolism	Transporters	P	vacuolar transport
3178	GO:0010232	Metabolism	Transporters	P	vascular transport
3179	GO:0031982	Metabolism	Transporters	C	vesicle
3180	GO:0006904	Metabolism	Transporters	P	vesicle docking involved in exocytosis
3181	GO:0016050	Metabolism	Transporters	P	vesicle organization
3182	GO:0016192	Metabolism	Transporters	P	vesicle-mediated transport
3183	GO:0008308	Metabolism	Transporters	F	voltage-gated anion channel activity
3184	GO:0005245	Metabolism	Transporters	F	voltage-gated calcium channel activity
3185	GO:0005247	Metabolism	Transporters	F	voltage-gated chloride channel activity
3186	GO:0015250	Metabolism	Transporters	F	water channel activity
3187	GO:0005372	Metabolism	Transporters	F	water transmembrane transporter activity
3188	GO:0006833	Metabolism	Transporters	P	water transport
3189	GO:0042907	Metabolism	Transporters	F	xanthine transmembrane transporter activity
3190	GO:0042906	Metabolism	Transporters	P	xanthine transport
3191	GO:0042910	Metabolism	Transporters	F	xenobiotic transmembrane transporter activity
3192	GO:0042908	Metabolism	Transporters	P	xenobiotic transport
3193	GO:1990388	Metabolism	Transporters	P	xylem-to-phloem iron transport
3194	GO:0015341	Metabolism	Transporters	F	zinc efflux active transmembrane transporter activity
3195	GO:0022883	Metabolism	Transporters	F	zinc efflux transmembrane transporter activity
3196	GO:0071577	Metabolism	Transporters	P	zinc ion transmembrane transport
3197	GO:0005385	Metabolism	Transporters	F	zinc ion transmembrane transporter activity
3198	GO:0006829	Metabolism	Transporters	P	zinc ion transport
3199	GO:0016255	Regulation	Protein modification	P	attachment of GPI anchor to protein
3200	GO:0051087	Regulation	Protein modification	F	chaperone binding
3201	GO:0061077	Regulation	Protein modification	P	chaperone-mediated protein folding
3202	GO:0097361	Regulation	Protein modification	C	CIA complex
3203	GO:0043686	Regulation	Protein modification	P	co-translational protein modification
3204	GO:0006458	Regulation	Protein modification	P	'de novo' protein folding
3205	GO:0033185	Regulation	Protein modification	C	dolichol-phosphate-mannose synthase complex
3206	GO:0005783	Regulation	Protein modification	C	endoplasmic reticulum
3207	GO:0034663	Regulation	Protein modification	C	endoplasmic reticulum chaperone complex
3208	GO:0070971	Regulation	Protein modification	C	endoplasmic reticulum exit site
3209	GO:0005789	Regulation	Protein modification	C	endoplasmic reticulum membrane
3210	GO:0030234	Regulation	Protein modification	F	enzyme regulator activity
3211	GO:0072546	Regulation	Protein modification	C	ER membrane protein complex
3212	GO:0005794	Regulation	Protein modification	C	Golgi apparatus
3213	GO:0000139	Regulation	Protein modification	C	Golgi membrane
3214	GO:0030173	Regulation	Protein modification	C	integral component of Golgi membrane
3215	GO:0018393	Regulation	Protein modification	P	internal peptidyl-lysine acetylation
3216	GO:0006475	Regulation	Protein modification	P	internal protein amino acid acetylation
3217	GO:0042157	Regulation	Protein modification	P	lipoprotein metabolic process
3218	GO:0051651	Regulation	Protein modification	P	maintenance of location in cell
3219	GO:0051457	Regulation	Protein modification	P	maintenance of protein location in nucleus
3220	GO:0032259	Regulation	Protein modification	P	methylation
3221	GO:0031416	Regulation	Protein modification	C	NatB complex
3222	GO:0031365	Regulation	Protein modification	P	N-terminal protein amino acid modification
3223	GO:0006499	Regulation	Protein modification	P	N-terminal protein myristoylation
3224	GO:0008250	Regulation	Protein modification	C	oligosaccharyltransferase complex
3225	GO:0018119	Regulation	Protein modification	P	peptidyl-cysteine S-nitrosylation
3226	GO:0018230	Regulation	Protein modification	P	peptidyl-L-cysteine S-palmitoylation
3227	GO:0018394	Regulation	Protein modification	P	peptidyl-lysine acetylation
3228	GO:0003755	Regulation	Protein modification	F	peptidyl-prolyl cis-trans isomerase activity
3229	GO:0017186	Regulation	Protein modification	P	peptidyl-pyroglutamic acid biosynthetic process, using glutaminyl-peptide cyclotransferase

3230	GO:0018209	Regulation	Protein modification	P	peptidyl-serine modification
3231	GO:0035335	Regulation	Protein modification	P	peptidyl-tyrosine dephosphorylation
3232	GO:0061087	Regulation	Protein modification	P	positive regulation of histone H3-K27 methylation
3233	GO:0033674	Regulation	Protein modification	P	positive regulation of kinase activity
3234	GO:0007023	Regulation	Protein modification	P	post-chaperonin tubulin folding pathway
3235	GO:0016272	Regulation	Protein modification	C	prefoldin complex
3236	GO:0008213	Regulation	Protein modification	P	protein alkylation
3237	GO:0016540	Regulation	Protein modification	P	protein autoprocessing
3238	GO:0051259	Regulation	Protein modification	P	protein complex oligomerization
3239	GO:0008214	Regulation	Protein modification	P	protein dealkylation
3240	GO:0036524	Regulation	Protein modification	F	protein deglycase activity
3241	GO:0036529	Regulation	Protein modification	P	protein deglycation, glyoxal removal
3242	GO:0000338	Regulation	Protein modification	P	protein deneddylation
3243	GO:0016579	Regulation	Protein modification	P	protein deubiquitination
3244	GO:0046983	Regulation	Protein modification	F	protein dimerization activity
3245	GO:0003756	Regulation	Protein modification	F	protein disulfide isomerase activity
3246	GO:0015035	Regulation	Protein modification	F	protein disulfide oxidoreductase activity
3247	GO:0019904	Regulation	Protein modification	F	protein domain specific binding
3248	GO:0018343	Regulation	Protein modification	P	protein farnesylation
3249	GO:0006457	Regulation	Protein modification	P	protein folding
3250	GO:0018344	Regulation	Protein modification	P	protein geranylgeranylation
3251	GO:0010731	Regulation	Protein modification	P	protein glutathionylation
3252	GO:0006486	Regulation	Protein modification	P	protein glycosylation
3253	GO:0046982	Regulation	Protein modification	F	protein heterodimerization activity
3254	GO:0042803	Regulation	Protein modification	F	protein homodimerization activity
3255	GO:0051260	Regulation	Protein modification	P	protein homooligomerization
3256	GO:0051289	Regulation	Protein modification	P	protein homotetramerization
3257	GO:1990592	Regulation	Protein modification	P	protein K69-linked ufmylation
3258	GO:0005956	Regulation	Protein modification	C	protein kinase CK2 complex
3259	GO:0072657	Regulation	Protein modification	P	protein localization to membrane
3260	GO:0006479	Regulation	Protein modification	P	protein methylation
3261	GO:0008276	Regulation	Protein modification	F	protein methyltransferase activity
3262	GO:0032446	Regulation	Protein modification	P	protein modification by small protein conjugation
3263	GO:0018377	Regulation	Protein modification	P	protein myristoylation
3264	GO:0045116	Regulation	Protein modification	P	protein neddylation
3265	GO:0006487	Regulation	Protein modification	P	protein N-linked glycosylation
3266	GO:0018279	Regulation	Protein modification	P	protein N-linked glycosylation via asparagine
3267	GO:0018258	Regulation	Protein modification	P	protein O-linked glycosylation via hydroxyproline
3268	GO:0018345	Regulation	Protein modification	P	protein palmitoylation
3269	GO:0000413	Regulation	Protein modification	P	protein peptidyl-prolyl isomerization
3270	GO:0070212	Regulation	Protein modification	P	protein poly-ADP-ribosylation
3271	GO:0018342	Regulation	Protein modification	P	protein prenylation
3272	GO:0043621	Regulation	Protein modification	F	protein self-association
3273	GO:0050821	Regulation	Protein modification	P	protein stabilization
3274	GO:0016925	Regulation	Protein modification	P	protein sumoylation
3275	GO:0031386	Regulation	Protein modification	F	protein tag
3276	GO:0006605	Regulation	Protein modification	P	protein targeting
3277	GO:0045047	Regulation	Protein modification	P	protein targeting to ER
3278	GO:0006612	Regulation	Protein modification	P	protein targeting to membrane
3279	GO:0051262	Regulation	Protein modification	P	protein tetramerization
3280	GO:0070206	Regulation	Protein modification	P	protein trimerization
3281	GO:0044877	Regulation	Protein modification	F	protein-containing complex binding
3282	GO:0019707	Regulation	Protein modification	F	protein-cysteine S-acyltransferase activity
3283	GO:0019706	Regulation	Protein modification	F	protein-cysteine S-palmitoyltransferase activity
3284	GO:0016279	Regulation	Protein modification	F	protein-lysine N-methyltransferase activity
3285	GO:0050790	Regulation	Protein modification	P	regulation of catalytic activity
3286	GO:0031060	Regulation	Protein modification	F	regulation of histone methylation
3287	GO:0043496	Regulation	Protein modification	P	regulation of protein homodimerization activity
3288	GO:0051246	Regulation	Protein modification	P	regulation of protein metabolic process
3289	GO:0031647	Regulation	Protein modification	P	regulation of protein stability
3290	GO:0006614	Regulation	Protein modification	P	SRP-dependent cotranslational protein targeting to membrane
3291	GO:0043541	Regulation	Protein modification	C	UDP-N-acetylglucosamine transferase complex
3292	GO:0072341	Regulation	Protein modification	F	modified amino acid binding
3293	GO:0032272	Regulation	Protein modification	P	negative regulation of protein polymerization
3294	GO:0031333	Regulation	Protein modification	P	negative regulation of protein-containing complex assembly
3295	GO:0004596	Regulation	Protein modification	F	peptide alpha-N-acetyltransferase activity
3296	GO:0018316	Regulation	Protein modification	P	peptide cross-linking via L-cystine
3297	GO:0042586	Regulation	Protein modification	F	peptide deformylase activity
3298	GO:0033743	Regulation	Protein modification	F	peptide-methionine (R)-S-oxide reductase activity
3299	GO:0008113	Regulation	Protein modification	F	peptide-methionine (S)-S-oxide reductase activity
3300	GO:0018193	Regulation	Protein modification	P	peptidyl-amino acid modification
3301	GO:0018205	Regulation	Protein modification	P	peptidyl-lysine modification
3302	GO:0031401	Regulation	Protein modification	P	positive regulation of protein modification process
3303	GO:0070647	Regulation	Protein modification	P	protein modification by small protein conjugation or removal
3304	GO:0070646	Regulation	Protein modification	P	protein modification by small protein removal
3305	GO:0036211	Regulation	Protein modification	P	protein modification process
3306	GO:0065003	Regulation	Protein modification	P	protein-containing complex assembly
3307	GO:0031503	Regulation	Protein modification	P	protein-containing complex localization

3308	GO:0043933	Regulation	Protein modification	P	protein-containing complex subunit organization
3309	GO:0031399	Regulation	Protein modification	P	regulation of protein modification process
3310	GO:0065007	Regulation	Regulation	P	biological regulation
3311	GO:0098772	Regulation	Regulation	F	molecular function regulator
3312	GO:0030835	Regulation	Regulation	P	negative regulation of actin filament depolymerization
3313	GO:0030837	Regulation	Regulation	P	negative regulation of actin filament polymerization
3314	GO:0048519	Regulation	Regulation	P	negative regulation of biological process
3315	GO:0009890	Regulation	Regulation	P	negative regulation of biosynthetic process
3316	GO:0043086	Regulation	Regulation	P	negative regulation of catalytic activity
3317	GO:0045596	Regulation	Regulation	P	negative regulation of cell differentiation
3318	GO:0030308	Regulation	Regulation	P	negative regulation of cell growth
3319	GO:0031327	Regulation	Regulation	P	negative regulation of cellular biosynthetic process
3320	GO:2000113	Regulation	Regulation	P	negative regulation of cellular macromolecule biosynthetic process
3321	GO:0031324	Regulation	Regulation	P	negative regulation of cellular metabolic process
3322	GO:0048523	Regulation	Regulation	P	negative regulation of cellular process
3323	GO:1900060	Regulation	Regulation	P	negative regulation of ceramide biosynthetic process
3324	GO:0051494	Regulation	Regulation	P	negative regulation of cytoskeleton organization
3325	GO:0051053	Regulation	Regulation	P	negative regulation of DNA metabolic process
3326	GO:1901918	Regulation	Regulation	P	negative regulation of exoribonuclease activity
3327	GO:0009910	Regulation	Regulation	P	negative regulation of flower development
3328	GO:0045926	Regulation	Regulation	P	negative regulation of growth
3329	GO:1901332	Regulation	Regulation	P	negative regulation of lateral root development
3330	GO:0010558	Regulation	Regulation	P	negative regulation of macromolecule biosynthetic process
3331	GO:0010605	Regulation	Regulation	P	negative regulation of macromolecule metabolic process
3332	GO:0009892	Regulation	Regulation	P	negative regulation of metabolic process
3333	GO:0007026	Regulation	Regulation	P	negative regulation of microtubule depolymerization
3334	GO:0031115	Regulation	Regulation	P	negative regulation of microtubule polymerization
3335	GO:0051172	Regulation	Regulation	P	negative regulation of nitrogen compound metabolic process
3336	GO:0045934	Regulation	Regulation	P	negative regulation of nucleobase-containing compound metabolic process
3337	GO:0035308	Regulation	Regulation	P	negative regulation of protein dephosphorylation
3338	GO:2000604	Regulation	Regulation	P	negative regulation of secondary growth
3339	GO:1902184	Regulation	Regulation	P	negative regulation of shoot apical meristem development
3340	GO:2000122	Regulation	Regulation	P	negative regulation of stomatal complex development
3341	GO:1902904	Regulation	Regulation	P	negative regulation of supramolecular fiber organization
3342	GO:0032211	Regulation	Regulation	P	negative regulation of telomere maintenance via telomerase
3343	GO:0051511	Regulation	Regulation	P	negative regulation of unidimensional cell growth
3344	GO:1904277	Regulation	Regulation	P	negative regulation of wax biosynthetic process
3345	GO:0030836	Regulation	Regulation	P	positive regulation of actin filament depolymerization
3346	GO:0048518	Regulation	Regulation	P	positive regulation of biological process
3347	GO:0009891	Regulation	Regulation	P	positive regulation of biosynthetic process
3348	GO:1904143	Regulation	Regulation	P	positive regulation of carotenoid biosynthetic process
3349	GO:0045597	Regulation	Regulation	P	positive regulation of cell differentiation
3350	GO:0031328	Regulation	Regulation	P	positive regulation of cellular biosynthetic process
3351	GO:0031325	Regulation	Regulation	P	positive regulation of cellular metabolic process
3352	GO:0048522	Regulation	Regulation	P	positive regulation of cellular process
3353	GO:0032270	Regulation	Regulation	P	positive regulation of cellular protein metabolic process
3354	GO:1901959	Regulation	Regulation	P	positive regulation of cutin biosynthetic process
3355	GO:0045962	Regulation	Regulation	P	positive regulation of development, heterochronic
3356	GO:0048639	Regulation	Regulation	P	positive regulation of developmental growth
3357	GO:0040019	Regulation	Regulation	P	positive regulation of embryonic development
3358	GO:0009911	Regulation	Regulation	P	positive regulation of flower development
3359	GO:0080114	Regulation	Regulation	P	positive regulation of glycine hydroxymethyltransferase activity
3360	GO:0045927	Regulation	Regulation	P	positive regulation of growth
3361	GO:1901333	Regulation	Regulation	P	positive regulation of lateral root development
3362	GO:0010557	Regulation	Regulation	P	positive regulation of macromolecule biosynthetic process
3363	GO:0010604	Regulation	Regulation	P	positive regulation of macromolecule metabolic process
3364	GO:0009893	Regulation	Regulation	P	positive regulation of metabolic process
3365	GO:0031117	Regulation	Regulation	P	positive regulation of microtubule depolymerization
3366	GO:0031116	Regulation	Regulation	P	positive regulation of microtubule polymerization
3367	GO:0051173	Regulation	Regulation	P	positive regulation of nitrogen compound metabolic process
3368	GO:0045935	Regulation	Regulation	P	positive regulation of nucleobase-containing compound metabolic process
3369	GO:0046622	Regulation	Regulation	P	positive regulation of organ growth
3370	GO:1903288	Regulation	Regulation	P	positive regulation of potassium ion import across plasma membrane
3371	GO:0031334	Regulation	Regulation	P	positive regulation of protein complex assembly
3372	GO:0070863	Regulation	Regulation	P	positive regulation of protein exit from endoplasmic reticulum
3373	GO:0051247	Regulation	Regulation	P	positive regulation of protein metabolic process
3374	GO:0010030	Regulation	Regulation	P	positive regulation of seed germination
3375	GO:1902458	Regulation	Regulation	P	positive regulation of stomatal opening
3376	GO:0010063	Regulation	Regulation	P	positive regulation of trichoblast fate specification
3377	GO:0051512	Regulation	Regulation	P	positive regulation of unidimensional cell growth
3378	GO:1905182	Regulation	Regulation	P	positive regulation of urease activity
3379	GO:1904278	Regulation	Regulation	P	positive regulation of wax biosynthetic process
3380	GO:0032956	Regulation	Regulation	P	regulation of actin cytoskeleton organization
3381	GO:0032970	Regulation	Regulation	P	regulation of actin filament-based process
3382	GO:2000011	Regulation	Regulation	P	regulation of adaxial/abaxial pattern formation
3383	GO:0031537	Regulation	Regulation	P	regulation of anthocyanin metabolic process
3384	GO:0050789	Regulation	Regulation	P	regulation of biological process
3385	GO:0065008	Regulation	Regulation	P	regulation of biological quality

3386	GO:0009889	Regulation	Regulation	P	regulation of biosynthetic process
3387	GO:0030155	Regulation	Regulation	P	regulation of cell adhesion
3388	GO:0001558	Regulation	Regulation	P	regulation of cell growth
3389	GO:0001560	Regulation	Regulation	P	regulation of cell growth by extracellular stimulus
3390	GO:0010769	Regulation	Regulation	P	regulation of cell morphogenesis involved in differentiation
3391	GO:0008360	Regulation	Regulation	P	regulation of cell shape
3392	GO:1903338	Regulation	Regulation	P	regulation of cell wall organization or biogenesis
3393	GO:0034248	Regulation	Regulation	P	regulation of cellular amide metabolic process
3394	GO:0031326	Regulation	Regulation	P	regulation of cellular biosynthetic process
3395	GO:0051128	Regulation	Regulation	F	regulation of cellular component organization
3396	GO:0010565	Regulation	Regulation	P	regulation of cellular ketone metabolic process
3397	GO:2000112	Regulation	Regulation	P	regulation of cellular macromolecule biosynthetic process
3398	GO:0031323	Regulation	Regulation	P	regulation of cellular metabolic process
3399	GO:0050794	Regulation	Regulation	P	regulation of cellular process
3400	GO:0032268	Regulation	Regulation	P	regulation of cellular protein metabolic process
3401	GO:1901957	Regulation	Regulation	P	regulation of cutin biosynthetic process
3402	GO:0035303	Regulation	Regulation	P	regulation of dephosphorylation
3403	GO:0040034	Regulation	Regulation	P	regulation of development, heterochronic
3404	GO:0045995	Regulation	Regulation	P	regulation of embryonic development
3405	GO:0045604	Regulation	Regulation	P	regulation of epidermal cell differentiation
3406	GO:2000769	Regulation	Regulation	P	regulation of establishment or maintenance of cell polarity regulating cell shape
3407	GO:0017157	Regulation	Regulation	P	regulation of exocytosis
3408	GO:0010080	Regulation	Regulation	P	regulation of floral meristem growth
3409	GO:0009909	Regulation	Regulation	P	regulation of flower development
3410	GO:0010439	Regulation	Regulation	P	regulation of glucosinolate biosynthetic process
3411	GO:0040008	Regulation	Regulation	P	regulation of growth
3412	GO:0010081	Regulation	Regulation	P	regulation of inflorescence meristem growth
3413	GO:2000023	Regulation	Regulation	P	regulation of lateral root development
3414	GO:2000024	Regulation	Regulation	P	regulation of leaf development
3415	GO:2000025	Regulation	Regulation	P	regulation of leaf formation
3416	GO:1901371	Regulation	Regulation	P	regulation of leaf morphogenesis
3417	GO:1901141	Regulation	Regulation	P	regulation of lignin biosynthetic process
3418	GO:0010556	Regulation	Regulation	P	regulation of macromolecule biosynthetic process
3419	GO:0060255	Regulation	Regulation	P	regulation of macromolecule metabolic process
3420	GO:1905038	Regulation	Regulation	P	regulation of membrane lipid metabolic process
3421	GO:0048509	Regulation	Regulation	P	regulation of meristem development
3422	GO:0010075	Regulation	Regulation	P	regulation of meristem growth
3423	GO:0009934	Regulation	Regulation	P	regulation of meristem structural organization
3424	GO:0019222	Regulation	Regulation	P	regulation of metabolic process
3425	GO:0070507	Regulation	Regulation	P	regulation of microtubule cytoskeleton organization
3426	GO:0031110	Regulation	Regulation	P	regulation of microtubule polymerization or depolymerization
3427	GO:0032886	Regulation	Regulation	P	regulation of microtubule-based process
3428	GO:0065009	Regulation	Regulation	P	regulation of molecular function
3429	GO:0040014	Regulation	Regulation	P	regulation of multicellular organism growth
3430	GO:2000026	Regulation	Regulation	P	regulation of multicellular organismal development
3431	GO:0061062	Regulation	Regulation	P	regulation of nematode larval development
3432	GO:0051171	Regulation	Regulation	P	regulation of nitrogen compound metabolic process
3433	GO:0006808	Regulation	Regulation	P	regulation of nitrogen utilization
3434	GO:0019219	Regulation	Regulation	P	regulation of nucleobase-containing compound metabolic process
3435	GO:0033043	Regulation	Regulation	P	regulation of organelle organization
3436	GO:1900030	Regulation	Regulation	P	regulation of pectin biosynthetic process
3437	GO:0019220	Regulation	Regulation	P	regulation of phosphate metabolic process
3438	GO:0051174	Regulation	Regulation	P	regulation of phosphorus metabolic process
3439	GO:1905421	Regulation	Regulation	P	regulation of plant organ morphogenesis
3440	GO:2001009	Regulation	Regulation	P	regulation of plant-type cell wall cellulose biosynthetic process
3441	GO:0080092	Regulation	Regulation	P	regulation of pollen tube growth
3442	GO:0048580	Regulation	Regulation	P	regulation of post-embryonic development
3443	GO:2000069	Regulation	Regulation	P	regulation of post-embryonic root development
3444	GO:0080090	Regulation	Regulation	P	regulation of primary metabolic process
3445	GO:2000214	Regulation	Regulation	P	regulation of proline metabolic process
3446	GO:0010082	Regulation	Regulation	P	regulation of root meristem growth
3447	GO:0010337	Regulation	Regulation	P	regulation of salicylic acid metabolic process
3448	GO:2000652	Regulation	Regulation	P	regulation of secondary cell wall biogenesis
3449	GO:1900376	Regulation	Regulation	P	regulation of secondary metabolite biosynthetic process
3450	GO:2000032	Regulation	Regulation	P	regulation of secondary shoot formation
3451	GO:0080050	Regulation	Regulation	P	regulation of seed development
3452	GO:0080113	Regulation	Regulation	P	regulation of seed growth
3453	GO:1900140	Regulation	Regulation	P	regulation of seedling development
3454	GO:1902183	Regulation	Regulation	P	regulation of shoot apical meristem development
3455	GO:0048831	Regulation	Regulation	P	regulation of shoot system development
3456	GO:0062012	Regulation	Regulation	P	regulation of small molecule metabolic process
3457	GO:0090153	Regulation	Regulation	P	regulation of sphingolipid biosynthetic process
3458	GO:2000038	Regulation	Regulation	P	regulation of stomatal complex development
3459	GO:2000037	Regulation	Regulation	P	regulation of stomatal complex patterning
3460	GO:1901401	Regulation	Regulation	P	regulation of tetrapyrrole metabolic process
3461	GO:0051338	Regulation	Regulation	P	regulation of transferase activity
3462	GO:0051510	Regulation	Regulation	P	regulation of unidimensional cell growth
3463	GO:0043138	Regulation	Transcription	F	3'-5' DNA helicase activity

3464	GO:0034062	Regulation	Transcription	F	5'-3' RNA polymerase activity
3465	GO:0006370	Regulation	Transcription	P	7-methylguanosine mRNA capping
3466	GO:0033613	Regulation	Transcription	F	activating transcription factor binding
3467	GO:0003680	Regulation	Transcription	F	AT DNA binding
3468	GO:0043044	Regulation	Transcription	P	ATP-dependent chromatin remodeling
3469	GO:0045014	Regulation	Transcription	P	carbon catabolite repression of transcription by glucose
3470	GO:0016593	Regulation	Transcription	C	Cdc73/Paf1 complex
3471	GO:0031490	Regulation	Transcription	F	chromatin DNA binding
3472	GO:0097549	Regulation	Transcription	P	chromatin organization involved in negative regulation of transcription
3473	GO:0034401	Regulation	Transcription	P	chromatin organization involved in regulation of transcription
3474	GO:0006338	Regulation	Transcription	P	chromatin remodeling
3475	GO:0006342	Regulation	Transcription	P	chromatin silencing
3476	GO:0030702	Regulation	Transcription	P	chromatin silencing at centromere
3477	GO:0031048	Regulation	Transcription	P	chromatin silencing by small RNA
3478	GO:0070847	Regulation	Transcription	C	core mediator complex
3479	GO:0016569	Regulation	Transcription	P	covalent chromatin modification
3480	GO:0070988	Regulation	Transcription	P	demethylation
3481	GO:0070940	Regulation	Transcription	P	dephosphorylation of RNA polymerase II C-terminal domain
3482	GO:0071103	Regulation	Transcription	P	DNA conformation change
3483	GO:0032392	Regulation	Transcription	P	DNA geometric change
3484	GO:0006306	Regulation	Transcription	P	DNA methylation
3485	GO:0006304	Regulation	Transcription	P	DNA modification
3486	GO:0001216	Regulation	Transcription	F	DNA-binding transcription activator activity
3487	GO:0001228	Regulation	Transcription	F	DNA-binding transcription activator activity, RNA polymerase II-specific
3488	GO:0003700	Regulation	Transcription	F	DNA-binding transcription factor activity
3489	GO:0000981	Regulation	Transcription	F	DNA-binding transcription factor activity, RNA polymerase II-specific
3490	GO:0003899	Regulation	Transcription	F	DNA-directed 5'-3' RNA polymerase activity
3491	GO:0000428	Regulation	Transcription	C	DNA-directed RNA polymerase complex
3492	GO:0006354	Regulation	Transcription	P	DNA-templated transcription, elongation
3493	GO:0006352	Regulation	Transcription	P	DNA-templated transcription, initiation
3494	GO:0006353	Regulation	Transcription	P	DNA-templated transcription, termination
3495	GO:0032044	Regulation	Transcription	C	DSIF complex
3496	GO:0031050	Regulation	Transcription	P	dsRNA processing
3497	GO:0000408	Regulation	Transcription	C	EKC/KEOPS complex
3498	GO:0032783	Regulation	Transcription	C	ELL-EAF complex
3499	GO:0033588	Regulation	Transcription	C	Elongator holoenzyme complex
3500	GO:0070449	Regulation	Transcription	C	elongin complex
3501	GO:0001158	Regulation	Transcription	F	enhancer sequence-specific DNA binding
3502	GO:0010467	Regulation	Transcription	P	gene expression
3503	GO:0016458	Regulation	Transcription	P	gene silencing
3504	GO:0035195	Regulation	Transcription	P	gene silencing by miRNA
3505	GO:0031047	Regulation	Transcription	P	gene silencing by RNA
3506	GO:0004818	Regulation	Transcription	F	glutamate-tRNA ligase activity
3507	GO:0008883	Regulation	Transcription	F	glutamyl-tRNA reductase activity
3508	GO:1902562	Regulation	Transcription	C	H4 histone acetyltransferase complex
3509	GO:0043189	Regulation	Transcription	C	H4/H2A histone acetyltransferase complex
3510	GO:0004386	Regulation	Transcription	F	helicase activity
3511	GO:0016573	Regulation	Transcription	P	histone acetylation
3512	GO:0004402	Regulation	Transcription	F	histone acetyltransferase activity
3513	GO:0043995	Regulation	Transcription	F	histone acetyltransferase activity (H4-K5 specific)
3514	GO:0035035	Regulation	Transcription	F	histone acetyltransferase binding
3515	GO:0000123	Regulation	Transcription	C	histone acetyltransferase complex
3516	GO:0034969	Regulation	Transcription	P	histone arginine methylation
3517	GO:0042393	Regulation	Transcription	F	histone binding
3518	GO:0004407	Regulation	Transcription	F	histone deacetylase activity
3519	GO:0000118	Regulation	Transcription	C	histone deacetylase complex
3520	GO:0032452	Regulation	Transcription	F	histone demethylase activity
3521	GO:0032453	Regulation	Transcription	F	histone demethylase activity (H3-K4 specific)
3522	GO:0032454	Regulation	Transcription	F	histone demethylase activity (H3-K9 specific)
3523	GO:0034647	Regulation	Transcription	F	histone demethylase activity (H3-trimethyl-K4 specific)
3524	GO:0016577	Regulation	Transcription	P	histone demethylation
3525	GO:1990258	Regulation	Transcription	P	histone glutamine methylation
3526	GO:1990164	Regulation	Transcription	P	histone H2A phosphorylation
3527	GO:0033523	Regulation	Transcription	P	histone H2B ubiquitination
3528	GO:0070932	Regulation	Transcription	P	histone H3 deacetylation
3529	GO:0070734	Regulation	Transcription	P	histone H3-K27 methylation
3530	GO:0070544	Regulation	Transcription	P	histone H3-K36 demethylation
3531	GO:0010452	Regulation	Transcription	P	histone H3-K36 methylation
3532	GO:0034720	Regulation	Transcription	P	histone H3-K4 demethylation
3533	GO:0051568	Regulation	Transcription	P	histone H3-K4 methylation
3534	GO:0043970	Regulation	Transcription	P	histone H3-K9 acetylation
3535	GO:1990619	Regulation	Transcription	P	histone H3-K9 deacetylation
3536	GO:0033169	Regulation	Transcription	P	histone H3-K9 demethylation
3537	GO:0051567	Regulation	Transcription	P	histone H3-K9 methylation
3538	GO:0035407	Regulation	Transcription	P	histone H3-T11 phosphorylation
3539	GO:0072355	Regulation	Transcription	P	histone H3-T3 phosphorylation
3540	GO:0070933	Regulation	Transcription	P	histone H4 deacetylation
3541	GO:0043985	Regulation	Transcription	P	histone H4-R3 methylation

3542	GO:0035175	Regulation	Transcription	F	histone kinase activity (H3-S10 specific)
3543	GO:0035402	Regulation	Transcription	F	histone kinase activity (H3-T11 specific)
3544	GO:0072354	Regulation	Transcription	F	histone kinase activity (H3-T3 specific)
3545	GO:0070076	Regulation	Transcription	P	histone lysine demethylation
3546	GO:0034968	Regulation	Transcription	P	histone lysine methylation
3547	GO:0016571	Regulation	Transcription	P	histone methylation
3548	GO:0042054	Regulation	Transcription	F	histone methyltransferase activity
3549	GO:0046976	Regulation	Transcription	F	histone methyltransferase activity (H3-K27 specific)
3550	GO:0046975	Regulation	Transcription	F	histone methyltransferase activity (H3-K36 specific)
3551	GO:0035097	Regulation	Transcription	C	histone methyltransferase complex
3552	GO:0016570	Regulation	Transcription	P	histone modification
3553	GO:0010390	Regulation	Transcription	P	histone monoubiquitination
3554	GO:0016572	Regulation	Transcription	P	histone phosphorylation
3555	GO:0035174	Regulation	Transcription	F	histone serine kinase activity
3556	GO:0008469	Regulation	Transcription	F	histone-arginine N-methyltransferase activity
3557	GO:1990259	Regulation	Transcription	F	histone-glutamine methyltransferase activity
3558	GO:0018024	Regulation	Transcription	F	histone-lysine N-methyltransferase activity
3559	GO:0031011	Regulation	Transcription	C	Ino80 complex
3560	GO:0097346	Regulation	Transcription	C	INO80-type complex
3561	GO:0098732	Regulation	Transcription	P	macromolecule deacylation
3562	GO:0043414	Regulation	Transcription	P	macromolecule methylation
3563	GO:0043412	Regulation	Transcription	P	macromolecule modification
3564	GO:0006344	Regulation	Transcription	P	maintenance of chromatin silencing
3565	GO:0010216	Regulation	Transcription	P	maintenance of DNA methylation
3566	GO:0043570	Regulation	Transcription	P	maintenance of DNA repeat elements
3567	GO:0001193	Regulation	Transcription	P	maintenance of transcriptional fidelity during DNA-templated transcription elongation from RNA polymerase II promoter
3568	GO:0016592	Regulation	Transcription	C	mediator complex
3569	GO:0035064	Regulation	Transcription	F	methylated histone binding
3570	GO:0140034	Regulation	Transcription	F	methylation-dependent protein binding
3571	GO:0034708	Regulation	Transcription	C	methyltransferase complex
3572	GO:0048027	Regulation	Transcription	F	mRNA 5'-UTR binding
3573	GO:0006378	Regulation	Transcription	P	mRNA polyadenylation
3574	GO:0006397	Regulation	Transcription	P	mRNA processing
3575	GO:0032041	Regulation	Transcription	F	NAD-dependent histone deacetylase activity (H3-K14 specific)
3576	GO:0043433	Regulation	Transcription	P	negative regulation of DNA-binding transcription factor activity
3577	GO:2000143	Regulation	Transcription	P	negative regulation of DNA-templated transcription, initiation
3578	GO:0010629	Regulation	Transcription	P	negative regulation of gene expression
3579	GO:0045814	Regulation	Transcription	P	negative regulation of gene expression, epigenetic
3580	GO:0035067	Regulation	Transcription	P	negative regulation of histone acetylation
3581	GO:1900364	Regulation	Transcription	P	negative regulation of mRNA polyadenylation
3582	GO:1903507	Regulation	Transcription	P	negative regulation of nucleic acid-templated transcription
3583	GO:1902679	Regulation	Transcription	P	negative regulation of RNA biosynthetic process
3584	GO:1900369	Regulation	Transcription	P	negative regulation of RNA interference
3585	GO:0000122	Regulation	Transcription	P	negative regulation of transcription by RNA polymerase II
3586	GO:0045892	Regulation	Transcription	P	negative regulation of transcription, DNA-templated
3587	GO:0010529	Regulation	Transcription	P	negative regulation of transposition
3588	GO:0035267	Regulation	Transcription	C	NuA4 histone acetyltransferase complex
3589	GO:0055029	Regulation	Transcription	C	nuclear DNA-directed RNA polymerase complex
3590	GO:0071048	Regulation	Transcription	P	nuclear retention of unspliced pre-mRNA at the site of transcription
3591	GO:0031380	Regulation	Transcription	C	nuclear RNA-directed RNA polymerase complex
3592	GO:0016607	Regulation	Transcription	C	nuclear speck
3593	GO:0000289	Regulation	Transcription	P	nuclear-transcribed mRNA poly(A) tail shortening
3594	GO:0097659	Regulation	Transcription	P	nucleic acid-templated transcription
3595	GO:0005730	Regulation	Transcription	C	nucleolus
3596	GO:0034728	Regulation	Transcription	P	nucleosome organization
3597	GO:0000932	Regulation	Transcription	C	P-body
3598	GO:0035246	Regulation	Transcription	P	peptidyl-arginine N-methylation
3599	GO:0018027	Regulation	Transcription	P	peptidyl-lysine dimethylation
3600	GO:0018022	Regulation	Transcription	P	peptidyl-lysine methylation
3601	GO:0018026	Regulation	Transcription	P	peptidyl-lysine monomethylation
3602	GO:0070816	Regulation	Transcription	P	phosphorylation of RNA polymerase II C-terminal domain
3603	GO:0000427	Regulation	Transcription	C	plastid-encoded plastid RNA polymerase complex
3604	GO:0008143	Regulation	Transcription	F	poly(A) binding
3605	GO:1905269	Regulation	Transcription	P	positive regulation of chromatin organization
3606	GO:2001252	Regulation	Transcription	P	positive regulation of chromosome organization
3607	GO:0032786	Regulation	Transcription	P	positive regulation of DNA-templated transcription, elongation
3608	GO:0010628	Regulation	Transcription	P	positive regulation of gene expression
3609	GO:1900111	Regulation	Transcription	P	positive regulation of histone H3-K9 dimethylation
3610	GO:0031062	Regulation	Transcription	P	positive regulation of histone methylation
3611	GO:0031058	Regulation	Transcription	P	positive regulation of histone modification
3612	GO:0060213	Regulation	Transcription	P	positive regulation of nuclear-transcribed mRNA poly(A) tail shortening
3613	GO:1903508	Regulation	Transcription	P	positive regulation of nucleic acid-templated transcription
3614	GO:0060148	Regulation	Transcription	P	positive regulation of posttranscriptional gene silencing
3615	GO:1903705	Regulation	Transcription	P	positive regulation of production of siRNA involved in RNA interference
3616	GO:1902680	Regulation	Transcription	P	positive regulation of RNA biosynthetic process
3617	GO:1900370	Regulation	Transcription	P	positive regulation of RNA interference
3618	GO:0045943	Regulation	Transcription	P	positive regulation of transcription by RNA polymerase I

3619	GO:0045944	Regulation	Transcription	P	positive regulation of transcription by RNA polymerase II
3620	GO:0032968	Regulation	Transcription	P	positive regulation of transcription elongation from RNA polymerase II promoter
3621	GO:0061408	Regulation	Transcription	P	positive regulation of transcription from RNA polymerase II promoter in response to heat stress
3622	GO:0006990	Regulation	Transcription	P	positive regulation of transcription from RNA polymerase II promoter involved in unfolded protein response
3623	GO:0045893	Regulation	Transcription	P	positive regulation of transcription, DNA-templated
3624	GO:0035194	Regulation	Transcription	P	posttranscriptional gene silencing by RNA
3625	GO:0140458	Regulation	Transcription	P	pre-transcriptional gene silencing by RNA
3626	GO:0070919	Regulation	Transcription	P	production of siRNA involved in chromatin silencing by small RNA
3627	GO:0030422	Regulation	Transcription	P	production of siRNA involved in RNA interference
3628	GO:0070918	Regulation	Transcription	P	production of small RNA involved in gene silencing by RNA
3629	GO:0010267	Regulation	Transcription	P	production of ta-siRNAs involved in RNA interference
3630	GO:0006473	Regulation	Transcription	P	protein acetylation
3631	GO:0031248	Regulation	Transcription	C	protein acetyltransferase complex
3632	GO:0043543	Regulation	Transcription	P	protein acylation
3633	GO:0035601	Regulation	Transcription	P	protein deacylation
3634	GO:0006482	Regulation	Transcription	P	protein demethylation
3635	GO:0032993	Regulation	Transcription	C	protein-DNA complex
3636	GO:0065004	Regulation	Transcription	P	protein-DNA complex assembly
3637	GO:0071824	Regulation	Transcription	P	protein-DNA complex subunit organization
3638	GO:0034402	Regulation	Transcription	P	recruitment of 3'-end processing factors to RNA polymerase II holoenzyme complex
3639	GO:0031935	Regulation	Transcription	P	regulation of chromatin silencing
3640	GO:0031938	Regulation	Transcription	P	regulation of chromatin silencing at telomere
3641	GO:0044030	Regulation	Transcription	P	regulation of DNA methylation
3642	GO:0051090	Regulation	Transcription	P	regulation of DNA-binding transcription factor activity
3643	GO:0032784	Regulation	Transcription	P	regulation of DNA-templated transcription, elongation
3644	GO:0010468	Regulation	Transcription	P	regulation of gene expression
3645	GO:0006349	Regulation	Transcription	P	regulation of gene expression by genetic imprinting
3646	GO:0040029	Regulation	Transcription	P	regulation of gene expression, epigenetic
3647	GO:0060968	Regulation	Transcription	P	regulation of gene silencing
3648	GO:0060964	Regulation	Transcription	P	regulation of gene silencing by miRNA
3649	GO:0060966	Regulation	Transcription	P	regulation of gene silencing by RNA
3650	GO:1903506	Regulation	Transcription	P	regulation of nucleic acid-templated transcription
3651	GO:2001141	Regulation	Transcription	P	regulation of RNA biosynthetic process
3652	GO:1900368	Regulation	Transcription	P	regulation of RNA interference
3653	GO:0006357	Regulation	Transcription	P	regulation of transcription by RNA polymerase II
3654	GO:0043619	Regulation	Transcription	P	regulation of transcription from RNA polymerase II promoter in response to oxidative stress
3655	GO:0010767	Regulation	Transcription	P	regulation of transcription from RNA polymerase II promoter in response to UV-induced DNA damage
3656	GO:0006355	Regulation	Transcription	P	regulation of transcription, DNA-templated
3657	GO:0001067	Regulation	Transcription	F	regulatory region nucleic acid binding
3658	GO:0071076	Regulation	Transcription	P	RNA 3' uridylation
3659	GO:0031123	Regulation	Transcription	P	RNA 3'-end processing
3660	GO:0003723	Regulation	Transcription	F	RNA binding
3661	GO:0032774	Regulation	Transcription	P	RNA biosynthetic process
3662	GO:0034337	Regulation	Transcription	P	RNA folding
3663	GO:0001510	Regulation	Transcription	P	RNA methylation
3664	GO:0009451	Regulation	Transcription	P	RNA modification
3665	GO:0097747	Regulation	Transcription	F	RNA polymerase activity
3666	GO:0030880	Regulation	Transcription	C	RNA polymerase complex
3667	GO:0001054	Regulation	Transcription	F	RNA polymerase I activity
3668	GO:0005736	Regulation	Transcription	C	RNA polymerase I complex
3669	GO:0001102	Regulation	Transcription	F	RNA polymerase II activating transcription factor binding
3670	GO:0001055	Regulation	Transcription	F	RNA polymerase II activity
3671	GO:0000993	Regulation	Transcription	F	RNA polymerase II complex binding
3672	GO:0001139	Regulation	Transcription	F	RNA polymerase II complex recruiting activity
3673	GO:0000979	Regulation	Transcription	F	RNA polymerase II core promoter sequence-specific DNA binding
3674	GO:0008353	Regulation	Transcription	F	RNA polymerase II CTD heptapeptide repeat kinase activity
3675	GO:0008420	Regulation	Transcription	F	RNA polymerase II CTD heptapeptide repeat phosphatase activity
3676	GO:0051123	Regulation	Transcription	P	RNA polymerase II preinitiation complex assembly
3677	GO:0000978	Regulation	Transcription	F	RNA polymerase II proximal promoter sequence-specific DNA binding
3678	GO:0000977	Regulation	Transcription	F	RNA polymerase II regulatory region sequence-specific DNA binding
3679	GO:0090575	Regulation	Transcription	C	RNA polymerase II transcription factor complex
3680	GO:0005665	Regulation	Transcription	C	RNA polymerase II, core complex
3681	GO:0016591	Regulation	Transcription	C	RNA polymerase II, holoenzyme
3682	GO:0001056	Regulation	Transcription	F	RNA polymerase III activity
3683	GO:0005666	Regulation	Transcription	C	RNA polymerase III complex
3684	GO:0000995	Regulation	Transcription	F	RNA polymerase III general transcription initiation factor activity
3685	GO:0000418	Regulation	Transcription	C	RNA polymerase IV complex
3686	GO:0000419	Regulation	Transcription	C	RNA polymerase V complex
3687	GO:0050265	Regulation	Transcription	F	RNA uridylyltransferase activity
3688	GO:0003968	Regulation	Transcription	F	RNA-directed 5'-3' RNA polymerase activity
3689	GO:0080188	Regulation	Transcription	P	RNA-directed DNA methylation
3690	GO:0000124	Regulation	Transcription	C	SAGA complex
3691	GO:0043565	Regulation	Transcription	F	sequence-specific DNA binding

3692	GO:1990837	Regulation	Transcription	F	sequence-specific double-stranded DNA binding
3693	GO:0035197	Regulation	Transcription	F	siRNA binding
3694	GO:0046695	Regulation	Transcription	C	SLIK (SAGA-like) complex
3695	GO:0070603	Regulation	Transcription	C	SWI/SNF superfamily-type complex
3696	GO:0070034	Regulation	Transcription	F	telomerase RNA binding
3697	GO:0000347	Regulation	Transcription	C	THO complex
3698	GO:0000445	Regulation	Transcription	C	THO complex part of transcription export complex
3699	GO:0006366	Regulation	Transcription	P	transcription by RNA polymerase II
3700	GO:0006383	Regulation	Transcription	P	transcription by RNA polymerase III
3701	GO:0003713	Regulation	Transcription	F	transcription coactivator activity
3702	GO:0003712	Regulation	Transcription	F	transcription coregulator activity
3703	GO:0003714	Regulation	Transcription	F	transcription corepressor activity
3704	GO:0008134	Regulation	Transcription	F	transcription factor binding
3705	GO:0005667	Regulation	Transcription	C	transcription factor complex
3706	GO:0005669	Regulation	Transcription	C	transcription factor TFIID complex
3707	GO:0005674	Regulation	Transcription	C	transcription factor TFIIF complex
3708	GO:0000439	Regulation	Transcription	C	transcription factor TFIIH core complex
3709	GO:0005675	Regulation	Transcription	C	transcription factor TFIIH holo complex
3710	GO:0000127	Regulation	Transcription	C	transcription factor TFIIIC complex
3711	GO:0070985	Regulation	Transcription	C	transcription factor TFIIK complex
3712	GO:0006367	Regulation	Transcription	P	transcription initiation from RNA polymerase II promoter
3713	GO:0006384	Regulation	Transcription	P	transcription initiation from RNA polymerase III promoter
3714	GO:0070897	Regulation	Transcription	P	transcription preinitiation complex assembly
3715	GO:0140110	Regulation	Transcription	F	transcription regulator activity
3716	GO:0044212	Regulation	Transcription	F	transcription regulatory region DNA binding
3717	GO:0000976	Regulation	Transcription	F	transcription regulatory region sequence-specific DNA binding
3718	GO:0006351	Regulation	Transcription	P	transcription, DNA-templated
3719	GO:0097550	Regulation	Transcription	C	transcriptional preinitiation complex
3720	GO:0001174	Regulation	Transcription	P	transcriptional start site selection at RNA polymerase II promoter
3721	GO:0006283	Regulation	Transcription	P	transcription-coupled nucleotide-excision repair
3722	GO:0003746	Regulation	Transcription	F	translation elongation factor activity
3723	GO:0003743	Regulation	Transcription	F	translation initiation factor activity
3724	GO:0031372	Regulation	Transcription	C	UBC13-MMS2 complex
3725	GO:0004844	Regulation	Transcription	F	uracil DNA N-glycosylase activity
3726	GO:0060145	Regulation	Transcription	P	viral gene silencing in virus induced gene silencing
3727	GO:0042781	Regulation	Translation	F	3'-tRNA processing endoribonuclease activity
3728	GO:0008097	Regulation	Translation	F	5S rRNA binding
3729	GO:0030686	Regulation	Translation	C	90S preribosome
3730	GO:0000380	Regulation	Translation	P	alternative mRNA splicing, via spliceosome
3731	GO:0002161	Regulation	Translation	F	aminoacyl-tRNA editing activity
3732	GO:0004812	Regulation	Translation	F	aminoacyl-tRNA ligase activity
3733	GO:1902626	Regulation	Translation	P	assembly of large subunit precursor of preribosome
3734	GO:0000494	Regulation	Translation	P	box C/D snoRNA 3'-end processing
3735	GO:0031428	Regulation	Translation	C	box C/D snoRNP complex
3736	GO:0034513	Regulation	Translation	F	box H/ACA snoRNA binding
3737	GO:0031429	Regulation	Translation	C	box H/ACA snoRNP complex
3738	GO:0140101	Regulation	Translation	F	catalytic activity, acting on a tRNA
3739	GO:0140098	Regulation	Translation	F	catalytic activity, acting on RNA
3740	GO:0071013	Regulation	Translation	C	catalytic step 2 spliceosome
3741	GO:0030014	Regulation	Translation	C	CCR4-NOT complex
3742	GO:0030015	Regulation	Translation	C	CCR4-NOT core complex
3743	GO:0000354	Regulation	Translation	P	cis assembly of pre-catalytic spliceosome
3744	GO:0000469	Regulation	Translation	P	cleavage involved in rRNA processing
3745	GO:0000177	Regulation	Translation	C	cytoplasmic exosome (RNase complex)
3746	GO:0036464	Regulation	Translation	C	cytoplasmic ribonucleoprotein granule
3747	GO:0002181	Regulation	Translation	P	cytoplasmic translation
3748	GO:0002183	Regulation	Translation	P	cytoplasmic translational initiation
3749	GO:0002184	Regulation	Translation	P	cytoplasmic translational termination
3750	GO:0022625	Regulation	Translation	C	cytosolic large ribosomal subunit
3751	GO:0022626	Regulation	Translation	C	cytosolic ribosome
3752	GO:0022627	Regulation	Translation	C	cytosolic small ribosomal subunit
3753	GO:0000290	Regulation	Translation	P	deadenylation-dependent decapping of nuclear-transcribed mRNA
3754	GO:0003725	Regulation	Translation	F	double-stranded RNA binding
3755	GO:0000447	Regulation	Translation	P	endonucleolytic cleavage in ITS1 to separate SSU-rRNA from 5.8S rRNA and LSU-rRNA from tricistronic rRNA transcript (SSU-rRNA, 5.8S rRNA, LSU-rRNA)
3756	GO:0000478	Regulation	Translation	P	endonucleolytic cleavage involved in rRNA processing
3757	GO:0000461	Regulation	Translation	P	endonucleolytic cleavage to generate mature 3'-end of SSU-rRNA from (SSU-rRNA, 5.8S rRNA, LSU-rRNA)
3758	GO:0071784	Regulation	Translation	P	endoplasmic reticulum cisternal network assembly
3759	GO:0051236	Regulation	Translation	P	establishment of RNA localization
3760	GO:0033290	Regulation	Translation	C	eukaryotic 48S preinitiation complex
3761	GO:0005850	Regulation	Translation	C	eukaryotic translation initiation factor 2 complex
3762	GO:0005851	Regulation	Translation	C	eukaryotic translation initiation factor 2B complex
3763	GO:0005852	Regulation	Translation	C	eukaryotic translation initiation factor 3 complex
3764	GO:0016281	Regulation	Translation	C	eukaryotic translation initiation factor 4F complex
3765	GO:0000467	Regulation	Translation	P	exonucleolytic trimming to generate mature 3'-end of 5.8S rRNA from tricistronic rRNA transcript (SSU-rRNA, 5.8S rRNA, LSU-rRNA)
3766	GO:0004532	Regulation	Translation	F	exoribonuclease activity

3767	GO:0001731	Regulation	Translation	P	formation of translation preinitiation complex
3768	GO:0000350	Regulation	Translation	P	generation of catalytic spliceosome for second transesterification step
3769	GO:0004819	Regulation	Translation	F	glutamine-tRNA ligase activity
3770	GO:0006425	Regulation	Translation	P	glutaminyl-tRNA aminoacylation
3771	GO:0006424	Regulation	Translation	P	glutamyl-tRNA aminoacylation
3772	GO:0000373	Regulation	Translation	P	Group II intron splicing
3773	GO:0015934	Regulation	Translation	C	large ribosomal subunit
3774	GO:0070180	Regulation	Translation	F	large ribosomal subunit rRNA binding
3775	GO:1990726	Regulation	Translation	C	Lsm1-7-Pat1 complex
3776	GO:0000460	Regulation	Translation	P	maturation of 5.8S rRNA
3777	GO:0000466	Regulation	Translation	P	maturation of 5.8S rRNA from tricistronic rRNA transcript (SSU-rRNA, 5.8S rRNA, LSU-rRNA)
3778	GO:0000481	Regulation	Translation	P	maturation of 5S rRNA
3779	GO:0000470	Regulation	Translation	P	maturation of LSU-rRNA
3780	GO:0000463	Regulation	Translation	P	maturation of LSU-rRNA from tricistronic rRNA transcript (SSU-rRNA, 5.8S rRNA, LSU-rRNA)
3781	GO:0030490	Regulation	Translation	P	maturation of SSU-rRNA
3782	GO:0000462	Regulation	Translation	P	maturation of SSU-rRNA from tricistronic rRNA transcript (SSU-rRNA, 5.8S rRNA, LSU-rRNA)
3783	GO:0042256	Regulation	Translation	P	mature ribosome assembly
3784	GO:0034709	Regulation	Translation	C	methylosome
3785	GO:0035198	Regulation	Translation	F	miRNA binding
3786	GO:0035278	Regulation	Translation	P	miRNA mediated inhibition of translation
3787	GO:0010586	Regulation	Translation	P	miRNA metabolic process
3788	GO:0005762	Regulation	Translation	C	mitochondrial large ribosomal subunit
3789	GO:0080156	Regulation	Translation	P	mitochondrial mRNA modification
3790	GO:0005761	Regulation	Translation	C	mitochondrial ribosome
3791	GO:1900864	Regulation	Translation	P	mitochondrial RNA modification
3792	GO:0005763	Regulation	Translation	C	mitochondrial small ribosomal subunit
3793	GO:0032543	Regulation	Translation	P	mitochondrial translation
3794	GO:0090646	Regulation	Translation	P	mitochondrial tRNA processing
3795	GO:0031124	Regulation	Translation	P	mRNA 3'-end processing
3796	GO:0003730	Regulation	Translation	F	mRNA 3'-UTR binding
3797	GO:0003729	Regulation	Translation	F	mRNA binding
3798	GO:0005845	Regulation	Translation	C	mRNA cap binding complex
3799	GO:0045292	Regulation	Translation	P	mRNA cis splicing, via spliceosome
3800	GO:0006379	Regulation	Translation	P	mRNA cleavage
3801	GO:0005849	Regulation	Translation	C	mRNA cleavage factor complex
3802	GO:0005848	Regulation	Translation	C	mRNA cleavage stimulating factor complex
3803	GO:0006406	Regulation	Translation	P	mRNA export from nucleus
3804	GO:0016071	Regulation	Translation	P	mRNA metabolic process
3805	GO:0016556	Regulation	Translation	P	mRNA modification
3806	GO:1990481	Regulation	Translation	P	mRNA pseudouridine synthesis
3807	GO:0006376	Regulation	Translation	P	mRNA splice site selection
3808	GO:0000398	Regulation	Translation	P	mRNA splicing, via spliceosome
3809	GO:0051028	Regulation	Translation	P	mRNA transport
3810	GO:0071427	Regulation	Translation	P	mRNA-containing ribonucleoprotein complex export from nucleus
3811	GO:0043614	Regulation	Translation	C	multi-eIF complex
3812	GO:0005854	Regulation	Translation	C	nascent polypeptide-associated complex
3813	GO:0043628	Regulation	Translation	P	ncRNA 3'-end processing
3814	GO:0034660	Regulation	Translation	P	ncRNA metabolic process
3815	GO:0034470	Regulation	Translation	P	ncRNA processing
3816	GO:0051253	Regulation	Translation	P	negative regulation of RNA metabolic process
3817	GO:0045900	Regulation	Translation	P	negative regulation of translational elongation
3818	GO:0030690	Regulation	Translation	C	Noc1p-Noc2p complex
3819	GO:0030691	Regulation	Translation	C	Noc2p-Noc3p complex
3820	GO:0071028	Regulation	Translation	P	nuclear mRNA surveillance
3821	GO:0071042	Regulation	Translation	P	nuclear polyadenylation-dependent mRNA catabolic process
3822	GO:0071035	Regulation	Translation	P	nuclear polyadenylation-dependent rRNA catabolic process
3823	GO:0071038	Regulation	Translation	P	nuclear polyadenylation-dependent tRNA catabolic process
3824	GO:0000956	Regulation	Translation	P	nuclear-transcribed mRNA catabolic process
3825	GO:0000288	Regulation	Translation	P	nuclear-transcribed mRNA catabolic process, deadenylation-dependent decay
3826	GO:0000291	Regulation	Translation	P	nuclear-transcribed mRNA catabolic process, exonucleolytic
3827	GO:0034427	Regulation	Translation	P	nuclear-transcribed mRNA catabolic process, exonucleolytic, 3'-5'
3828	GO:0000184	Regulation	Translation	P	nuclear-transcribed mRNA catabolic process, nonsense-mediated decay
3829	GO:0000315	Regulation	Translation	C	organellar large ribosomal subunit
3830	GO:0000313	Regulation	Translation	C	organellar ribosome
3831	GO:0000314	Regulation	Translation	C	organellar small ribosomal subunit
3832	GO:0070545	Regulation	Translation	C	PeBoW complex
3833	GO:0043043	Regulation	Translation	P	peptide biosynthetic process
3834	GO:0004826	Regulation	Translation	F	phenylalanine-tRNA ligase activity
3835	GO:0009328	Regulation	Translation	C	phenylalanine-tRNA ligase complex
3836	GO:0006432	Regulation	Translation	P	phenylalanyl-tRNA aminoacylation
3837	GO:0034715	Regulation	Translation	C	pICln-Sm protein complex
3838	GO:0004535	Regulation	Translation	F	poly(A)-specific ribonuclease activity
3839	GO:0008266	Regulation	Translation	F	poly(U) RNA binding
3840	GO:0071051	Regulation	Translation	P	polyadenylation-dependent snoRNA 3'-end processing
3841	GO:0031426	Regulation	Translation	P	polycistronic mRNA processing

3842	GO:0042788	Regulation	Translation	C	polysomal ribosome
3843	GO:0048026	Regulation	Translation	P	positive regulation of mRNA splicing, via spliceosome
3844	GO:0051254	Regulation	Translation	P	positive regulation of RNA metabolic process
3845	GO:0045727	Regulation	Translation	P	positive regulation of translation
3846	GO:0045901	Regulation	Translation	P	positive regulation of translational elongation
3847	GO:0045903	Regulation	Translation	P	positive regulation of translational fidelity
3848	GO:0045948	Regulation	Translation	P	positive regulation of translational initiation
3849	GO:0045905	Regulation	Translation	P	positive regulation of translational termination
3850	GO:0016441	Regulation	Translation	P	posttranscriptional gene silencing
3851	GO:0010608	Regulation	Translation	P	posttranscriptional regulation of gene expression
3852	GO:0071011	Regulation	Translation	C	precatalytic spliceosome
3853	GO:0031054	Regulation	Translation	P	pre-miRNA processing
3854	GO:0030628	Regulation	Translation	F	pre-mRNA 3'-splice site binding
3855	GO:0097157	Regulation	Translation	F	pre-mRNA intronic binding
3856	GO:0030684	Regulation	Translation	C	preribosome
3857	GO:0030687	Regulation	Translation	C	preribosome, large subunit precursor
3858	GO:0071010	Regulation	Translation	C	prespliceosome
3859	GO:0031053	Regulation	Translation	P	primary miRNA processing
3860	GO:0010599	Regulation	Translation	P	production of lsiRNA involved in RNA interference
3861	GO:0035196	Regulation	Translation	P	production of miRNAs involved in gene silencing by miRNA
3862	GO:0000974	Regulation	Translation	C	Prp19 complex
3863	GO:0034388	Regulation	Translation	C	Pwp2p-containing subcomplex of 90S preribosome
3864	GO:0000182	Regulation	Translation	F	rDNA binding
3865	GO:0030875	Regulation	Translation	C	rDNA protrusion
3866	GO:0000381	Regulation	Translation	P	regulation of alternative mRNA splicing, via spliceosome
3867	GO:0010603	Regulation	Translation	P	regulation of cytoplasmic mRNA processing body assembly
3868	GO:0043488	Regulation	Translation	P	regulation of mRNA stability
3869	GO:0060211	Regulation	Translation	P	regulation of nuclear-transcribed mRNA poly(A) tail shortening
3870	GO:0051252	Regulation	Translation	P	regulation of RNA metabolic process
3871	GO:0043484	Regulation	Translation	P	regulation of RNA splicing
3872	GO:0006417	Regulation	Translation	P	regulation of translation
3873	GO:0006446	Regulation	Translation	P	regulation of translational initiation
3874	GO:0061980	Regulation	Translation	F	regulatory RNA binding
3875	GO:0070274	Regulation	Translation	C	RES complex
3876	GO:0072344	Regulation	Translation	P	rescue of stalled ribosome
3877	GO:0004540	Regulation	Translation	F	ribonuclease activity
3878	GO:0004525	Regulation	Translation	F	ribonuclease III activity
3879	GO:0008428	Regulation	Translation	F	ribonuclease inhibitor activity
3880	GO:0004526	Regulation	Translation	F	ribonuclease P activity
3881	GO:0033897	Regulation	Translation	F	ribonuclease T2 activity
3882	GO:1990904	Regulation	Translation	C	ribonucleoprotein complex
3883	GO:0022618	Regulation	Translation	P	ribonucleoprotein complex assembly
3884	GO:0022613	Regulation	Translation	P	ribonucleoprotein complex biogenesis
3885	GO:0071426	Regulation	Translation	P	ribonucleoprotein complex export from nucleus
3886	GO:0071166	Regulation	Translation	P	ribonucleoprotein complex localization
3887	GO:0071826	Regulation	Translation	P	ribonucleoprotein complex subunit organization
3888	GO:0035770	Regulation	Translation	C	ribonucleoprotein granule
3889	GO:0004748	Regulation	Translation	F	ribonucleoside-diphosphate reductase activity, thioredoxin disulfide as acceptor
3890	GO:0032553	Regulation	Translation	F	ribonucleotide binding
3891	GO:0000027	Regulation	Translation	P	ribosomal large subunit assembly
3892	GO:0043023	Regulation	Translation	F	ribosomal large subunit binding
3893	GO:0042273	Regulation	Translation	P	ribosomal large subunit biogenesis
3894	GO:0000028	Regulation	Translation	P	ribosomal small subunit assembly
3895	GO:0043024	Regulation	Translation	F	ribosomal small subunit binding
3896	GO:0042274	Regulation	Translation	P	ribosomal small subunit biogenesis
3897	GO:0044391	Regulation	Translation	C	ribosomal subunit
3898	GO:0005840	Regulation	Translation	C	ribosome
3899	GO:0042255	Regulation	translation	P	ribosome assembly
3900	GO:0043022	Regulation	Translation	F	ribosome binding
3901	GO:0042254	Regulation	Translation	P	ribosome biogenesis
3902	GO:0032790	Regulation	Translation	P	ribosome disassembly
3903	GO:0097344	Regulation	Translation	C	Rix1 complex
3904	GO:0000340	Regulation	Translation	F	RNA 7-methylguanosine cap binding
3905	GO:0000339	Regulation	Translation	F	RNA cap binding
3906	GO:0006405	Regulation	Translation	P	RNA export from nucleus
3907	GO:0003724	Regulation	Translation	F	RNA helicase activity
3908	GO:0016246	Regulation	Translation	P	RNA interference
3909	GO:0006403	Regulation	Translation	P	RNA localization
3910	GO:0008173	Regulation	Translation	F	RNA methyltransferase activity
3911	GO:0090501	Regulation	Translation	P	RNA phosphodiester bond hydrolysis
3912	GO:0043631	Regulation	Translation	P	RNA polyadenylation
3913	GO:0006396	Regulation	Translation	P	RNA processing
3914	GO:0010501	Regulation	Translation	P	RNA secondary structure unwinding
3915	GO:0008380	Regulation	Translation	P	RNA splicing
3916	GO:0000394	Regulation	Translation	P	RNA splicing, via endonucleolytic cleavage and ligation
3917	GO:0000375	Regulation	Translation	P	RNA splicing, via transesterification reactions
3918	GO:0000377	Regulation	Translation	P	RNA splicing, via transesterification reactions with bulged adenosine as nucleophile

3919	GO:0043489	Regulation	Translation	P	RNA stabilization
3920	GO:0070042	Regulation	Translation	F	rRNA (uridine-N3-)-methyltransferase activity
3921	GO:1904812	Regulation	Translation	P	rRNA acetylation involved in maturation of SSU-rRNA
3922	GO:0070475	Regulation	Translation	P	rRNA base methylation
3923	GO:0019843	Regulation	Translation	F	rRNA binding
3924	GO:1990883	Regulation	Translation	F	rRNA cytidine N-acetyltransferase activity
3925	GO:0016072	Regulation	Translation	P	rRNA metabolic process
3926	GO:0031167	Regulation	Translation	P	rRNA methylation
3927	GO:0008649	Regulation	Translation	F	rRNA methyltransferase activity
3928	GO:0000154	Regulation	Translation	P	rRNA modification
3929	GO:0006364	Regulation	Translation	P	rRNA processing
3930	GO:0031118	Regulation	Translation	P	rRNA pseudouridine synthesis
3931	GO:0000386	Regulation	Translation	F	second spliceosomal transesterification activity
3932	GO:1990825	Regulation	Translation	F	sequence-specific mRNA binding
3933	GO:0003727	Regulation	Translation	F	single-stranded RNA binding
3934	GO:0030532	Regulation	Translation	C	small nuclear ribonucleoprotein complex
3935	GO:0005732	Regulation	Translation	C	small nucleolar ribonucleoprotein complex
3936	GO:0015935	Regulation	Translation	C	small ribosomal subunit
3937	GO:0070181	Regulation	Translation	F	small ribosomal subunit rRNA binding
3938	GO:0032040	Regulation	Translation	C	small-subunit processome
3939	GO:0034719	Regulation	Translation	C	SMN-Sm protein complex
3940	GO:0030515	Regulation	Translation	F	snoRNA binding
3941	GO:0043144	Regulation	Translation	P	snoRNA processing
3942	GO:0034247	Regulation	Translation	P	snoRNA splicing
3943	GO:0017069	Regulation	Translation	F	snRNA binding
3944	GO:0061015	Regulation	Translation	P	snRNA import into nucleus
3945	GO:0016073	Regulation	Translation	P	snRNA metabolic process
3946	GO:0040031	Regulation	Translation	P	snRNA modification
3947	GO:0016180	Regulation	Translation	P	snRNA processing
3948	GO:0031120	Regulation	Translation	P	snRNA pseudouridine synthesis
3949	GO:0005681	Regulation	Translation	C	spliceosomal complex
3950	GO:0000245	Regulation	Translation	P	spliceosomal complex assembly
3951	GO:0000387	Regulation	Translation	P	spliceosomal snRNP assembly
3952	GO:0097525	Regulation	Translation	C	spliceosomal snRNP complex
3953	GO:0097526	Regulation	Translation	C	spliceosomal tri-snRNP complex
3954	GO:0000244	Regulation	Translation	P	spliceosomal tri-snRNP complex assembly
3955	GO:0003735	Regulation	Translation	F	structural constituent of ribosome
3956	GO:0006412	Regulation	Translation	P	translation
3957	GO:0031369	Regulation	Translation	F	translation initiation factor binding
3958	GO:0002188	Regulation	Translation	P	translation reinitiation
3959	GO:0003747	Regulation	Translation	F	translation release factor activity
3960	GO:0016149	Regulation	Translation	F	translation release factor activity, codon specific
3961	GO:0018444	Regulation	Translation	C	translation release factor complex
3962	GO:0006414	Regulation	Translation	P	translational elongation
3963	GO:0006452	Regulation	Translation	P	translational frameshifting
3964	GO:0006413	Regulation	Translation	P	translational initiation
3965	GO:0006415	Regulation	Translation	P	translational termination
3966	GO:0016429	Regulation	Translation	F	tRNA (adenine-N1-)-methyltransferase activity
3967	GO:0016423	Regulation	Translation	F	tRNA (guanine) methyltransferase activity
3968	GO:0009019	Regulation	Translation	F	tRNA (guanine-N1-)-methyltransferase activity
3969	GO:0009020	Regulation	Translation	F	tRNA (guanosine-2'-O-)-methyltransferase activity
3970	GO:0031515	Regulation	Translation	C	tRNA (m1A) methyltransferase complex
3971	GO:0042780	Regulation	Translation	P	tRNA 3'-end processing
3972	GO:0034414	Regulation	Translation	P	tRNA 3'-trailer cleavage, endonucleolytic
3973	GO:0001682	Regulation	Translation	P	tRNA 5'-leader removal
3974	GO:0043039	Regulation	Translation	P	tRNA aminoacylation
3975	GO:0000049	Regulation	Translation	F	tRNA binding
3976	GO:0017150	Regulation	Translation	F	tRNA dihydrouridine synthase activity
3977	GO:0002938	Regulation	Translation	P	tRNA guanine ribose methylation
3978	GO:0016031	Regulation	Translation	P	tRNA import into mitochondrion
3979	GO:0006399	Regulation	Translation	P	tRNA metabolic process
3980	GO:0030488	Regulation	Translation	P	tRNA methylation
3981	GO:0008175	Regulation	Translation	F	tRNA methyltransferase activity
3982	GO:0006400	Regulation	Translation	P	tRNA modification
3983	GO:0008033	Regulation	Translation	P	tRNA processing
3984	GO:0106029	Regulation	Translation	F	tRNA pseudouridine synthase activity
3985	GO:0031119	Regulation	Translation	P	tRNA pseudouridine synthesis
3986	GO:0034227	Regulation	Translation	P	tRNA thio-modification
3987	GO:0002926	Regulation	Translation	P	tRNA wobble base 5-methoxycarbonylmethyl-2-thiouridinylation
3988	GO:0002143	Regulation	Translation	P	tRNA wobble position uridine thiolation
3989	GO:0002098	Regulation	Translation	P	tRNA wobble uridine modification
3990	GO:0034473	Regulation	Translation	P	U1 snRNA 3'-end processing
3991	GO:0030619	Regulation	Translation	F	U1 snRNA binding
3992	GO:0005685	Regulation	Translation	C	U1 snRNP
3993	GO:0005689	Regulation	Translation	C	U12-type spliceosomal complex
3994	GO:0030620	Regulation	Translation	F	U2 snRNA binding
3995	GO:0005686	Regulation	Translation	C	U2 snRNP
3996	GO:0089701	Regulation	Translation	C	U2AF

3997	GO:0071021	Regulation	Translation	C	U2-type post-spliceosomal complex
3998	GO:0071004	Regulation	Translation	C	U2-type prespliceosome
3999	GO:0005684	Regulation	Translation	C	U2-type spliceosomal complex
4000	GO:0034511	Regulation	Translation	F	U3 snoRNA binding
4001	GO:0034475	Regulation	Translation	P	U4 snRNA 3'-end processing
4002	GO:0005687	Regulation	Translation	C	U4 snRNP
4003	GO:0046540	Regulation	Translation	C	U4/U6 x U5 tri-snRNP complex
4004	GO:0034476	Regulation	Translation	P	U5 snRNA 3'-end processing
4005	GO:0030623	Regulation	Translation	F	U5 snRNA binding
4006	GO:0005682	Regulation	translation	C	U5 snRNP
4007	GO:0017070	Regulation	Translation	F	U6 snRNA binding
4008	GO:0005688	Regulation	Translation	C	U6 snRNP
4009	GO:0004832	Regulation	Translation	F	valine-tRNA ligase activity
4010	GO:0006438	Regulation	Translation	P	valyl-tRNA aminoacylation
4011	GO:0004430	Signaling and response	Signaling	F	1-phosphatidylinositol 4-kinase activity
4012	GO:0016308	Signaling and response	Signaling	F	1-phosphatidylinositol-4-phosphate 5-kinase activity
4013	GO:0030797	Signaling and response	Signaling	F	24-methylenesterol C-methyltransferase activity
4014	GO:0060321	Signaling and response	Signaling	P	acceptance of pollen
4015	GO:0003993	Signaling and response	Signaling	F	acid phosphatase activity
4016	GO:0010541	Signaling and response	Signaling	P	acropetal auxin transport
4017	GO:0090630	Signaling and response	Signaling	P	activation of GTPase activity
4018	GO:0032147	Signaling and response	Signaling	P	activation of protein kinase activity
4019	GO:0007188	Signaling and response	Signaling	P	adenylate cyclase-modulating G protein-coupled receptor signaling pathway
4020	GO:0010011	Signaling and response	Signaling	F	auxin binding
4021	GO:0009851	Signaling and response	Signaling	P	auxin biosynthetic process
4022	GO:0010315	Signaling and response	Signaling	P	auxin efflux
4023	GO:0010329	Signaling and response	Signaling	F	auxin efflux transmembrane transporter activity
4024	GO:0060919	Signaling and response	Signaling	P	auxin influx
4025	GO:0010328	Signaling and response	Signaling	F	auxin influx transmembrane transporter activity
4026	GO:0060774	Signaling and response	Signaling	P	auxin mediated signaling pathway involved in phyllotactic patterning
4027	GO:0009850	Signaling and response	Signaling	P	auxin metabolic process
4028	GO:0009926	Signaling and response	Signaling	P	auxin polar transport
4029	GO:0060918	Signaling and response	Signaling	P	auxin transport
4030	GO:0009734	Signaling and response	Signaling	P	auxin-activated signaling pathway
4031	GO:0010540	Signaling and response	Signaling	P	basipetal auxin transport
4032	GO:0009785	Signaling and response	Signaling	P	blue light signaling pathway
4033	GO:0016132	Signaling and response	Signaling	P	brassinosteroid biosynthetic process
4034	GO:0009742	Signaling and response	Signaling	P	brassinosteroid mediated signaling pathway
4035	GO:0016131	Signaling and response	Signaling	P	brassinosteroid metabolic process
4036	GO:0052634	Signaling and response	Signaling	F	C-19 gibberellin 2-beta-dioxygenase activity
4037	GO:0000248	Signaling and response	Signaling	F	C-5 sterol desaturase activity
4038	GO:0005509	Signaling and response	Signaling	F	calcium ion binding
4039	GO:0009931	Signaling and response	Signaling	F	calcium-dependent protein serine/threonine kinase activity
4040	GO:0019722	Signaling and response	Signaling	P	calcium-mediated signaling
4041	GO:0005516	Signaling and response	Signaling	F	calmodulin binding
4042	GO:0004683	Signaling and response	Signaling	F	calmodulin-dependent protein kinase activity
4043	GO:0007154	Signaling and response	Signaling	P	cell communication
4044	GO:0007166	Signaling and response	Signaling	P	cell surface receptor signaling pathway
4045	GO:0007267	Signaling and response	Signaling	P	cell-cell signaling
4046	GO:0071214	Signaling and response	Signaling	P	cellular response to abiotic stimulus
4047	GO:0071229	Signaling and response	Signaling	P	cellular response to acid chemical
4048	GO:0071318	Signaling and response	Signaling	P	cellular response to ATP
4049	GO:0071365	Signaling and response	Signaling	P	cellular response to auxin stimulus
4050	GO:0071483	Signaling and response	Signaling	P	cellular response to blue light
4051	GO:0080029	Signaling and response	Signaling	P	cellular response to boron-containing substance levels
4052	GO:0071367	Signaling and response	Signaling	P	cellular response to brassinosteroid stimulus
4053	GO:0071277	Signaling and response	Signaling	P	cellular response to calcium ion
4054	GO:0071244	Signaling and response	Signaling	P	cellular response to carbon dioxide
4055	GO:0070887	Signaling and response	Signaling	P	cellular response to chemical stimulus
4056	GO:0070417	Signaling and response	Signaling	P	cellular response to cold
4057	GO:0071280	Signaling and response	Signaling	P	cellular response to copper ion
4058	GO:0071368	Signaling and response	Signaling	P	cellular response to cytokinin stimulus
4059	GO:0104004	Signaling and response	Signaling	P	cellular response to environmental stimulus
4060	GO:0071496	Signaling and response	Signaling	P	cellular response to external stimulus
4061	GO:0031668	Signaling and response	Signaling	P	cellular response to extracellular stimulus
4062	GO:0071490	Signaling and response	Signaling	P	cellular response to far red light
4063	GO:0071398	Signaling and response	Signaling	P	cellular response to fatty acid
4064	GO:0071370	Signaling and response	Signaling	P	cellular response to gibberellin stimulus
4065	GO:0071366	Signaling and response	Signaling	P	cellular response to indolebutyric acid stimulus
4066	GO:0071281	Signaling and response	Signaling	P	cellular response to iron ion
4067	GO:0010106	Signaling and response	Signaling	P	cellular response to iron ion starvation
4068	GO:0071395	Signaling and response	Signaling	P	cellular response to jasmonic acid stimulus
4069	GO:0071482	Signaling and response	Signaling	P	cellular response to light stimulus
4070	GO:0071396	Signaling and response	Signaling	P	cellular response to lipid
4071	GO:0071286	Signaling and response	Signaling	P	cellular response to magnesium ion
4072	GO:0010350	Signaling and response	Signaling	P	cellular response to magnesium starvation
4073	GO:0071325	Signaling and response	Signaling	P	cellular response to mannitol stimulus
4074	GO:0071249	Signaling and response	Signaling	P	cellular response to nitrate

4075	GO:0043562	Signaling and response	Signaling	P	cellular response to nitrogen levels
4076	GO:0031669	Signaling and response	Signaling	P	cellular response to nutrient levels
4077	GO:0071453	Signaling and response	Signaling	P	cellular response to oxygen levels
4078	GO:1901701	Signaling and response	Signaling	P	cellular response to oxygen-containing compound
4079	GO:0035865	Signaling and response	Signaling	P	cellular response to potassium ion
4080	GO:0071478	Signaling and response	Signaling	P	cellular response to radiation
4081	GO:0071491	Signaling and response	Signaling	P	cellular response to red light
4082	GO:0071446	Signaling and response	Signaling	P	cellular response to salicylic acid stimulus
4083	GO:0072709	Signaling and response	Signaling	P	cellular response to sorbitol
4084	GO:0051716	Signaling and response	Signaling	P	cellular response to stimulus
4085	GO:0071329	Signaling and response	Signaling	P	cellular response to sucrose stimulus
4086	GO:1904482	Signaling and response	Signaling	P	cellular response to tetrahydrofolate
4087	GO:0071462	Signaling and response	Signaling	P	cellular response to water stimulus
4088	GO:0010617	Signaling and response	Signaling	P	circadian regulation of calcium ion oscillation
4089	GO:0007623	Signaling and response	Signaling	P	circadian rhythm
4090	GO:0010031	Signaling and response	Signaling	P	circumnutation
4091	GO:0050897	Signaling and response	Signaling	F	cobalt ion binding
4092	GO:0008180	Signaling and response	Signaling	C	COP9 signalosome
4093	GO:0010387	Signaling and response	Signaling	P	COP9 signalosome assembly
4094	GO:0009691	Signaling and response	Signaling	P	cytokinin biosynthetic process
4095	GO:0009690	Signaling and response	Signaling	P	cytokinin metabolic process
4096	GO:0009736	Signaling and response	Signaling	P	cytokinin-activated signaling pathway
4097	GO:0016311	Signaling and response	Signaling	P	dephosphorylation
4098	GO:0009729	Signaling and response	Signaling	P	detection of brassinosteroid stimulus
4099	GO:0005513	Signaling and response	Signaling	P	detection of calcium ion
4100	GO:0050982	Signaling and response	Signaling	P	detection of mechanical stimulus
4101	GO:0015036	Signaling and response	Signaling	F	disulfide oxidoreductase activity
4102	GO:0009649	Signaling and response	Signaling	P	entrainment of circadian clock
4103	GO:0106167	Signaling and response	Signaling	P	extracellular ATP signaling
4104	GO:0010018	Signaling and response	Signaling	P	far-red light signaling pathway
4105	GO:0001664	Signaling and response	Signaling	F	G protein-coupled receptor binding
4106	GO:0007186	Signaling and response	Signaling	P	G protein-coupled receptor signaling pathway
4107	GO:0009740	Signaling and response	Signaling	P	gibberellic acid mediated signaling pathway
4108	GO:1905200	Signaling and response	Signaling	P	gibberellic acid transmembrane transport
4109	GO:0045544	Signaling and response	Signaling	F	gibberellin 20-oxidase activity
4110	GO:0102118	Signaling and response	Signaling	F	gibberellin A4 carboxyl methyltransferase activity
4111	GO:0010331	Signaling and response	Signaling	F	gibberellin binding
4112	GO:0009686	Signaling and response	Signaling	P	gibberellin biosynthetic process
4113	GO:0010341	Signaling and response	Signaling	F	gibberellin carboxyl-O-methyltransferase activity
4114	GO:0010476	Signaling and response	Signaling	P	gibberellin mediated signaling pathway
4115	GO:1905201	Signaling and response	Signaling	F	gibberellin transmembrane transporter activity
4116	GO:0010255	Signaling and response	Signaling	P	glucose mediated signaling pathway
4117	GO:0031683	Signaling and response	Signaling	F	G-protein beta/gamma-subunit complex binding
4118	GO:0009630	Signaling and response	Signaling	P	gravitropism
4119	GO:0008083	Signaling and response	Signaling	F	growth factor activity
4120	GO:0008728	Signaling and response	Signaling	F	GTP diphosphokinase activity
4121	GO:0102489	Signaling and response	Signaling	F	GTP phosphohydrolase activity
4122	GO:0051020	Signaling and response	Signaling	F	GTPase binding
4123	GO:0030742	Signaling and response	Signaling	F	GTP-dependent protein binding
4124	GO:0005834	Signaling and response	Signaling	C	heterotrimeric G-protein complex
4125	GO:0009755	Signaling and response	Signaling	P	hormone-mediated signaling pathway
4126	GO:0010274	Signaling and response	Signaling	P	hydrotropism
4127	GO:0010179	Signaling and response	Signaling	F	IAA-Ala conjugate hydrolase activity
4128	GO:0010211	Signaling and response	Signaling	F	IAA-Leu conjugate hydrolase activity
4129	GO:0010210	Signaling and response	Signaling	F	IAA-Phe conjugate hydrolase activity
4130	GO:0051749	Signaling and response	Signaling	F	indole acetic acid carboxyl methyltransferase activity
4131	GO:0047215	Signaling and response	Signaling	F	indole-3-acetate beta-glucosyltransferase activity
4132	GO:0010279	Signaling and response	Signaling	F	indole-3-acetic acid amido synthetase activity
4133	GO:0009684	Signaling and response	Signaling	P	indoleacetic acid biosynthetic process
4134	GO:0046855	Signaling and response	Signaling	P	inositol phosphate dephosphorylation
4135	GO:0007229	Signaling and response	Signaling	P	integrin-mediated signaling pathway
4136	GO:0035556	Signaling and response	Signaling	P	intracellular signal transduction
4137	GO:0032366	Signaling and response	Signaling	P	intracellular sterol transport
4138	GO:0030795	Signaling and response	Signaling	F	jasmonate O-methyltransferase activity
4139	GO:0009695	Signaling and response	Signaling	P	jasmonic acid biosynthetic process
4140	GO:0120091	Signaling and response	Signaling	F	jasmonic acid hydrolase
4141	GO:0009867	Signaling and response	Signaling	P	jasmonic acid mediated signaling pathway
4142	GO:0009694	Signaling and response	Signaling	P	jasmonic acid metabolic process
4143	GO:0016301	Signaling and response	Signaling	F	kinase activity
4144	GO:0019900	Signaling and response	Signaling	F	kinase binding
4145	GO:0019210	Signaling and response	Signaling	F	kinase inhibitor activity
4146	GO:0019207	Signaling and response	Signaling	F	kinase regulator activity
4147	GO:0048574	Signaling and response	Signaling	P	long-day photoperiodism, flowering
4148	GO:0004724	Signaling and response	Signaling	F	magnesium-dependent protein serine/threonine phosphatase activity
4149	GO:0004707	Signaling and response	Signaling	F	MAP kinase activity
4150	GO:0004708	Signaling and response	Signaling	F	MAP kinase kinase activity
4151	GO:0004709	Signaling and response	Signaling	F	MAP kinase kinase kinase activity
4152	GO:0033549	Signaling and response	Signaling	F	MAP kinase phosphatase activity

4153	GO:0000165	Signaling and response	Signaling	P	MAPK cascade
4154	GO:0005078	Signaling and response	Signaling	F	MAP-kinase scaffold activity
4155	GO:0080030	Signaling and response	Signaling	F	methyl indole-3-acetate esterase activity
4156	GO:0080032	Signaling and response	Signaling	F	methyl jasmonate esterase activity
4157	GO:0080031	Signaling and response	Signaling	F	methyl salicylate esterase activity
4158	GO:0031930	Signaling and response	Signaling	P	mitochondria-nucleus signaling pathway
4159	GO:0051019	Signaling and response	Signaling	F	mitogen-activated protein kinase binding
4160	GO:0031435	Signaling and response	Signaling	F	mitogen-activated protein kinase kinase kinase binding
4161	GO:0032501	Signaling and response	Signaling	P	multicellular organismal process
4162	GO:0051704	Signaling and response	Signaling	P	multi-organism process
4163	GO:0009959	Signaling and response	Signaling	P	negative gravitropism
4164	GO:0042754	Signaling and response	Signaling	P	negative regulation of circadian rhythm
4165	GO:0009938	Signaling and response	Signaling	P	negative regulation of gibberellic acid mediated signaling pathway
4166	GO:1901997	Signaling and response	Signaling	P	negative regulation of indoleacetic acid biosynthetic process via tryptophan
4167	GO:0048579	Signaling and response	Signaling	P	negative regulation of long-day photoperiodism, flowering
4168	GO:0043407	Signaling and response	Signaling	P	negative regulation of MAP kinase activity
4169	GO:0010100	Signaling and response	Signaling	P	negative regulation of photomorphogenesis
4170	GO:0048577	Signaling and response	Signaling	P	negative regulation of short-day photoperiodism, flowering
4171	GO:1902457	Signaling and response	Signaling	P	negative regulation of stomatal opening
4172	GO:0004715	Signaling and response	Signaling	F	non-membrane spanning protein tyrosine kinase activity
4173	GO:0061608	Signaling and response	Signaling	F	nuclear import signal receptor activity
4174	GO:0004550	Signaling and response	Signaling	F	nucleoside diphosphate kinase activity
4175	GO:0005034	Signaling and response	Signaling	F	osmosensor activity
4176	GO:0007231	Signaling and response	Signaling	P	osmosensory signaling pathway
4177	GO:0006119	Signaling and response	Signaling	P	oxidative phosphorylation
4178	GO:0002221	Signaling and response	Signaling	P	pattern recognition receptor signaling pathway
4179	GO:0017046	Signaling and response	Signaling	F	peptide hormone binding
4180	GO:0051428	Signaling and response	Signaling	F	peptide hormone receptor binding
4181	GO:0001653	Signaling and response	Signaling	F	peptide receptor activity
4182	GO:0018105	Signaling and response	Signaling	P	peptidyl-serine phosphorylation
4183	GO:0018107	Signaling and response	Signaling	P	peptidyl-threonine phosphorylation
4184	GO:0016791	Signaling and response	Signaling	F	phosphatase activity
4185	GO:0019902	Signaling and response	Signaling	F	phosphatase binding
4186	GO:0036149	Signaling and response	Signaling	P	phosphatidylinositol acyl-chain remodeling
4187	GO:0046856	Signaling and response	Signaling	P	phosphatidylinositol dephosphorylation
4188	GO:0016307	Signaling and response	Signaling	F	phosphatidylinositol phosphate kinase activity
4189	GO:0052866	Signaling and response	Signaling	F	phosphatidylinositol phosphate phosphatase activity
4190	GO:0004435	Signaling and response	Signaling	F	phosphatidylinositol phospholipase C activity
4191	GO:0016314	Signaling and response	Signaling	F	phosphatidylinositol-3,4,5-trisphosphate 3-phosphatase activity
4192	GO:0034485	Signaling and response	Signaling	F	phosphatidylinositol-3,4,5-trisphosphate 5-phosphatase activity
4193	GO:0005547	Signaling and response	Signaling	F	phosphatidylinositol-3,4,5-trisphosphate binding
4194	GO:0004439	Signaling and response	Signaling	F	phosphatidylinositol-4,5-bisphosphate 5-phosphatase activity
4195	GO:0004618	Signaling and response	Signaling	F	phosphoglycerate kinase activity
4196	GO:0008967	Signaling and response	Signaling	F	phosphoglycolate phosphatase activity
4197	GO:0008970	Signaling and response	Signaling	F	phospholipase A1 activity
4198	GO:0004623	Signaling and response	Signaling	F	phospholipase A2 activity
4199	GO:0016004	Signaling and response	Signaling	F	phospholipase activator activity
4200	GO:0004620	Signaling and response	Signaling	F	phospholipase activity
4201	GO:0004629	Signaling and response	Signaling	F	phospholipase C activity
4202	GO:0046839	Signaling and response	Signaling	P	phospholipid dephosphorylation
4203	GO:0004721	Signaling and response	Signaling	F	phosphoprotein phosphatase activity
4204	GO:0000156	Signaling and response	Signaling	F	phosphorelay response regulator activity
4205	GO:0000155	Signaling and response	Signaling	F	phosphorelay sensor kinase activity
4206	GO:0000160	Signaling and response	Signaling	P	phosphorelay signal transduction system
4207	GO:0010205	Signaling and response	Signaling	P	photoinhibition
4208	GO:0009640	Signaling and response	Signaling	P	photomorphogenesis
4209	GO:0009648	Signaling and response	Signaling	P	photoperiodism
4210	GO:0048573	Signaling and response	Signaling	P	photoperiodism, flowering
4211	GO:0009881	Signaling and response	Signaling	F	photoreceptor activity
4212	GO:0009638	Signaling and response	Signaling	P	phototropism
4213	GO:0009958	Signaling and response	Signaling	P	positive gravitropism
4214	GO:0010601	Signaling and response	Signaling	P	positive regulation of auxin biosynthetic process
4215	GO:0010929	Signaling and response	Signaling	P	positive regulation of auxin mediated signaling pathway
4216	GO:0090355	Signaling and response	Signaling	P	positive regulation of auxin metabolic process
4217	GO:2000488	Signaling and response	Signaling	P	positive regulation of brassinosteroid biosynthetic process
4218	GO:0042753	Signaling and response	Signaling	P	positive regulation of circadian rhythm
4219	GO:0009939	Signaling and response	Signaling	P	positive regulation of gibberellic acid mediated signaling pathway
4220	GO:0048578	Signaling and response	Signaling	P	positive regulation of long-day photoperiodism, flowering
4221	GO:2000306	Signaling and response	Signaling	P	positive regulation of photomorphogenesis
4222	GO:0001934	Signaling and response	Signaling	P	positive regulation of protein phosphorylation
4223	GO:1902448	Signaling and response	Signaling	P	positive regulation of shade avoidance
4224	GO:0009967	Signaling and response	Signaling	P	positive regulation of signal transduction
4225	GO:0010220	Signaling and response	Signaling	P	positive regulation of vernalization response
4226	GO:0046777	Signaling and response	Signaling	P	protein autophosphorylation
4227	GO:0006470	Signaling and response	Signaling	P	protein dephosphorylation
4228	GO:0004660	Signaling and response	Signaling	F	protein farnesyltransferase activity
4229	GO:0005965	Signaling and response	Signaling	C	protein farnesyltransferase complex
4230	GO:0030295	Signaling and response	Signaling	F	protein kinase activator activity

4231	GO:0004672	Signaling and response	Signaling	F	protein kinase activity
4232	GO:0019901	Signaling and response	Signaling	F	protein kinase binding
4233	GO:0005080	Signaling and response	Signaling	F	protein kinase C binding
4234	GO:1902911	Signaling and response	Signaling	C	protein kinase complex
4235	GO:0004860	Signaling and response	Signaling	F	protein kinase inhibitor activity
4236	GO:0019887	Signaling and response	Signaling	F	protein kinase regulator activity
4237	GO:0034394	Signaling and response	Signaling	P	protein localization to cell surface
4238	GO:0072542	Signaling and response	Signaling	F	protein phosphatase activator activity
4239	GO:0004864	Signaling and response	Signaling	F	protein phosphatase inhibitor activity
4240	GO:0019888	Signaling and response	Signaling	F	protein phosphatase regulator activity
4241	GO:0000164	Signaling and response	Signaling	C	protein phosphatase type 1 complex
4242	GO:0000159	Signaling and response	Signaling	C	protein phosphatase type 2A complex
4243	GO:0045309	Signaling and response	Signaling	F	protein phosphorylated amino acid binding
4244	GO:0006468	Signaling and response	Signaling	P	protein phosphorylation
4245	GO:0004674	Signaling and response	Signaling	F	protein serine/threonine kinase activity
4246	GO:0004722	Signaling and response	Signaling	F	protein serine/threonine phosphatase activity
4247	GO:0008287	Signaling and response	Signaling	C	protein serine/threonine phosphatase complex
4248	GO:0004712	Signaling and response	Signaling	F	protein serine/threonine/tyrosine kinase activity
4249	GO:0004713	Signaling and response	Signaling	F	protein tyrosine kinase activity
4250	GO:0004725	Signaling and response	Signaling	F	protein tyrosine phosphatase activity
4251	GO:0008138	Signaling and response	Signaling	F	protein tyrosine/serine/threonine phosphatase activity
4252	GO:0047134	Signaling and response	Signaling	F	protein-disulfide reductase activity
4253	GO:0005093	Signaling and response	Signaling	F	Rab GDP-dissociation inhibitor activity
4254	GO:0004663	Signaling and response	Signaling	F	Rab geranylgeranyltransferase activity
4255	GO:0017137	Signaling and response	Signaling	F	Rab GTPase binding
4256	GO:0017112	Signaling and response	Signaling	F	Rab guanyl-nucleotide exchange factor activity
4257	GO:0032482	Signaling and response	Signaling	P	Rab protein signal transduction
4258	GO:0005968	Signaling and response	Signaling	C	Rab-protein geranylgeranyltransferase complex
4259	GO:0048365	Signaling and response	Signaling	F	Rac GTPase binding
4260	GO:0008536	Signaling and response	Signaling	F	Ran GTPase binding
4261	GO:0043235	Signaling and response	Signaling	C	receptor complex
4262	GO:0033612	Signaling and response	Signaling	F	receptor serine/threonine kinase binding
4263	GO:0010161	Signaling and response	Signaling	P	red light signaling pathway
4264	GO:0010017	Signaling and response	Signaling	P	red or far-red light signaling pathway
4265	GO:0009585	Signaling and response	Signaling	P	red, far-red light phototransduction
4266	GO:0010600	Signaling and response	Signaling	P	regulation of auxin biosynthetic process
4267	GO:0010928	Signaling and response	Signaling	P	regulation of auxin mediated signaling pathway
4268	GO:0090354	Signaling and response	Signaling	P	regulation of auxin metabolic process
4269	GO:2000012	Signaling and response	Signaling	P	regulation of auxin polar transport
4270	GO:1900457	Signaling and response	Signaling	P	regulation of brassinosteroid mediated signaling pathway
4271	GO:0042752	Signaling and response	Signaling	P	regulation of circadian rhythm
4272	GO:0000079	Signaling and response	Signaling	P	regulation of cyclin-dependent protein serine/threonine kinase activity
4273	GO:0080036	Signaling and response	Signaling	P	regulation of cytokinin-activated signaling pathway
4274	GO:0009937	Signaling and response	Signaling	P	regulation of gibberellic acid mediated signaling pathway
4275	GO:0010371	Signaling and response	Signaling	P	regulation of gibberellin biosynthetic process
4276	GO:0043087	Signaling and response	Signaling	P	regulation of GTPase activity
4277	GO:0080141	Signaling and response	Signaling	P	regulation of jasmonic acid biosynthetic process
4278	GO:2000022	Signaling and response	Signaling	P	regulation of jasmonic acid mediated signaling pathway
4279	GO:0043549	Signaling and response	Signaling	P	regulation of kinase activity
4280	GO:0048586	Signaling and response	Signaling	P	regulation of long-day photoperiodism, flowering
4281	GO:0010921	Signaling and response	Signaling	P	regulation of phosphatase activity
4282	GO:0042325	Signaling and response	Signaling	P	regulation of phosphorylation
4283	GO:0010099	Signaling and response	Signaling	P	regulation of photomorphogenesis
4284	GO:2000028	Signaling and response	Signaling	P	regulation of photoperiodism, flowering
4285	GO:0031952	Signaling and response	Signaling	P	regulation of protein autophosphorylation
4286	GO:0035304	Signaling and response	Signaling	P	regulation of protein dephosphorylation
4287	GO:0045859	Signaling and response	Signaling	P	regulation of protein kinase activity
4288	GO:0001932	Signaling and response	Signaling	P	regulation of protein phosphorylation
4289	GO:0071900	Signaling and response	Signaling	P	regulation of protein serine/threonine kinase activity
4290	GO:0080163	Signaling and response	Signaling	P	regulation of protein serine/threonine phosphatase activity
4291	GO:2000030	Signaling and response	Signaling	P	regulation of response to red or far red light
4292	GO:2000280	Signaling and response	Signaling	P	regulation of root development
4293	GO:2000067	Signaling and response	Signaling	P	regulation of root morphogenesis
4294	GO:0080142	Signaling and response	Signaling	P	regulation of salicylic acid biosynthetic process
4295	GO:2000031	Signaling and response	Signaling	P	regulation of salicylic acid mediated signaling pathway
4296	GO:0010029	Signaling and response	Signaling	P	regulation of seed germination
4297	GO:0048510	Signaling and response	Signaling	P	regulation of timing of transition from vegetative to reproductive phase
4298	GO:0010321	Signaling and response	Signaling	P	regulation of vegetative phase change
4299	GO:0060320	Signaling and response	Signaling	P	rejection of self pollen
4300	GO:0048838	Signaling and response	Signaling	P	release of seed from dormancy
4301	GO:0009961	Signaling and response	Signaling	P	response to 1-aminocyclopropane-1-carboxylic acid
4302	GO:0009628	Signaling and response	Signaling	P	response to abiotic stimulus
4303	GO:0001101	Signaling and response	Signaling	P	response to acid chemical
4304	GO:0010044	Signaling and response	Signaling	P	response to aluminum ion
4305	GO:0072722	Signaling and response	Signaling	P	response to amitrole
4306	GO:0060359	Signaling and response	Signaling	P	response to ammonium ion
4307	GO:0009733	Signaling and response	Signaling	P	response to auxin
4308	GO:0009617	Signaling and response	Signaling	P	response to bacterium

4309	GO:0009607	Signaling and response	Signaling	P	response to biotic stimulus
4310	GO:0009637	Signaling and response	Signaling	P	response to blue light
4311	GO:0010036	Signaling and response	Signaling	P	response to boron-containing substance
4312	GO:0009741	Signaling and response	Signaling	P	response to brassinosteroid
4313	GO:0046686	Signaling and response	Signaling	P	response to cadmium ion
4314	GO:0051592	Signaling and response	Signaling	P	response to calcium ion
4315	GO:0009743	Signaling and response	Signaling	P	response to carbohydrate
4316	GO:0010037	Signaling and response	Signaling	P	response to carbon dioxide
4317	GO:0042221	Signaling and response	Signaling	P	response to chemical
4318	GO:0010200	Signaling and response	Signaling	P	response to chitin
4319	GO:0032025	Signaling and response	Signaling	P	response to cobalt ion
4320	GO:0046688	Signaling and response	Signaling	P	response to copper ion
4321	GO:0009735	Signaling and response	Signaling	P	response to cytokinin
4322	GO:0034285	Signaling and response	Signaling	P	response to disaccharide
4323	GO:0009719	Signaling and response	Signaling	P	response to endogenous stimulus
4324	GO:0043207	Signaling and response	Signaling	P	response to external biotic stimulus
4325	GO:0009605	Signaling and response	Signaling	P	response to external stimulus
4326	GO:0009991	Signaling and response	Signaling	P	response to extracellular stimulus
4327	GO:0010218	Signaling and response	Signaling	P	response to far red light
4328	GO:0009750	Signaling and response	Signaling	P	response to fructose
4329	GO:0009739	Signaling and response	Signaling	P	response to gibberellin
4330	GO:0009749	Signaling and response	Signaling	P	response to glucose
4331	GO:0009629	Signaling and response	Signaling	P	response to gravity
4332	GO:0060416	Signaling and response	Signaling	P	response to growth hormone
4333	GO:0009725	Signaling and response	Signaling	P	response to hormone
4334	GO:0010035	Signaling and response	Signaling	P	response to inorganic substance
4335	GO:0010212	Signaling and response	Signaling	P	response to ionizing radiation
4336	GO:0010039	Signaling and response	Signaling	P	response to iron ion
4337	GO:0009753	Signaling and response	Signaling	P	response to jasmonic acid
4338	GO:0080167	Signaling and response	Signaling	P	response to karrikin
4339	GO:1902065	Signaling and response	Signaling	P	response to L-glutamate
4340	GO:0009642	Signaling and response	Signaling	P	response to light intensity
4341	GO:0009416	Signaling and response	Signaling	P	response to light stimulus
4342	GO:0033993	Signaling and response	Signaling	P	response to lipid
4343	GO:0010555	Signaling and response	Signaling	P	response to mannitol
4344	GO:1905582	Signaling and response	Signaling	P	response to mannose
4345	GO:0010038	Signaling and response	Signaling	P	response to metal ion
4346	GO:0010167	Signaling and response	Signaling	P	response to nitrate
4347	GO:1901698	Signaling and response	Signaling	P	response to nitrogen compound
4348	GO:0007584	Signaling and response	Signaling	P	response to nutrient
4349	GO:0031667	Signaling and response	Signaling	P	response to nutrient levels
4350	GO:0014070	Signaling and response	Signaling	P	response to organic cyclic compound
4351	GO:0010033	Signaling and response	Signaling	P	response to organic substance
4352	GO:0010243	Signaling and response	Signaling	P	response to organonitrogen compound
4353	GO:0070482	Signaling and response	Signaling	P	response to oxygen levels
4354	GO:1901700	Signaling and response	Signaling	P	response to oxygen-containing compound
4355	GO:0080184	Signaling and response	Signaling	P	response to phenylpropanoid
4356	GO:0019236	Signaling and response	Signaling	P	response to pheromone
4357	GO:0010114	Signaling and response	Signaling	P	response to red light
4358	GO:0009639	Signaling and response	Signaling	P	response to red or far red light
4359	GO:0009751	Signaling and response	Signaling	P	response to salicylic acid
4360	GO:0010272	Signaling and response	Signaling	P	response to silver ion
4361	GO:0072708	Signaling and response	Signaling	P	response to sorbitol
4362	GO:0050896	Signaling and response	Signaling	P	response to stimulus
4363	GO:1902347	Signaling and response	Signaling	P	response to strigolactone
4364	GO:0009744	Signaling and response	Signaling	P	response to sucrose
4365	GO:0009266	Signaling and response	Signaling	P	response to temperature stimulus
4366	GO:0009411	Signaling and response	Signaling	P	response to UV
4367	GO:0070141	Signaling and response	Signaling	P	response to UV-A
4368	GO:0010225	Signaling and response	Signaling	P	response to UV-C
4369	GO:0010266	Signaling and response	Signaling	P	response to vitamin B1
4370	GO:0009415	Signaling and response	Signaling	P	response to water
4371	GO:0010043	Signaling and response	Signaling	P	response to zinc ion
4372	GO:0030970	Signaling and response	Signaling	P	retrograde protein transport, ER to cytosol
4373	GO:0042147	Signaling and response	Signaling	P	retrograde transport, endosome to Golgi
4374	GO:0006890	Signaling and response	Signaling	P	retrograde vesicle-mediated transport, Golgi to endoplasmic reticulum
4375	GO:0007266	Signaling and response	Signaling	P	Rho protein signal transduction
4376	GO:0048511	Signaling and response	Signaling	P	rhythmic process
4377	GO:0009697	Signaling and response	Signaling	P	salicylic acid biosynthetic process
4378	GO:0052639	Signaling and response	Signaling	F	salicylic acid glucosyltransferase (ester-forming) activity
4379	GO:0052640	Signaling and response	Signaling	F	salicylic acid glucosyltransferase (glucoside-forming) activity
4380	GO:0009863	Signaling and response	Signaling	P	salicylic acid mediated signaling pathway
4381	GO:0009696	Signaling and response	Signaling	P	salicylic acid metabolic process
4382	GO:1902554	Signaling and response	Signaling	C	serine/threonine protein kinase complex
4383	GO:0009641	Signaling and response	Signaling	P	shade avoidance
4384	GO:0048575	Signaling and response	Signaling	P	short-day photoperiodism, flowering
4385	GO:0005787	Signaling and response	Signaling	C	signal peptidase complex
4386	GO:0006465	Signaling and response	Signaling	P	signal peptide processing

4387	GO:0005047	Signaling and response	Signaling	F	signal recognition particle binding
4388	GO:0005785	Signaling and response	Signaling	C	signal recognition particle receptor complex
4389	GO:0005048	Signaling and response	Signaling	F	signal sequence binding
4390	GO:0007165	Signaling and response	Signaling	P	signal transduction
4391	GO:0023014	Signaling and response	Signaling	P	signal transduction by protein phosphorylation
4392	GO:0038023	Signaling and response	Signaling	F	signaling receptor activity
4393	GO:0005102	Signaling and response	Signaling	F	signaling receptor binding
4394	GO:0007264	Signaling and response	Signaling	P	small GTPase mediated signal transduction
4395	GO:0010012	Signaling and response	Signaling	F	steroid 22-alpha hydroxylase activity
4396	GO:0005496	Signaling and response	Signaling	F	steroid binding
4397	GO:0016229	Signaling and response	Signaling	F	steroid dehydrogenase activity
4398	GO:0008395	Signaling and response	Signaling	F	steroid hydroxylase activity
4399	GO:0003838	Signaling and response	Signaling	F	sterol 24-C-methyltransferase activity
4400	GO:0032934	Signaling and response	Signaling	F	sterol binding
4401	GO:0016126	Signaling and response	Signaling	P	sterol biosynthetic process
4402	GO:0016125	Signaling and response	Signaling	P	sterol metabolic process
4403	GO:0015918	Signaling and response	Signaling	P	sterol transport
4404	GO:0047012	Signaling and response	Signaling	F	sterol-4-alpha-carboxylate 3-dehydrogenase (decarboxylating) activity
4405	GO:0010118	Signaling and response	Signaling	P	stomatal movement
4406	GO:0010182	Signaling and response	Signaling	P	sugar mediated signaling pathway
4407	GO:0044403	Signaling and response	Signaling	P	symbiotic process
4408	GO:0042330	Signaling and response	Signaling	P	taxis
4409	GO:0009652	Signaling and response	Signaling	P	thigmotropism
4410	GO:0019199	Signaling and response	Signaling	F	transmembrane receptor protein kinase activity
4411	GO:0004714	Signaling and response	Signaling	F	transmembrane receptor protein tyrosine kinase activity
4412	GO:0009606	Signaling and response	Signaling	P	tropism
4413	GO:0010050	Signaling and response	Signaling	P	vegetative phase change
4414	GO:0010295	Signaling and response	Stress	F	(+)-abscisic acid 8'-hydroxylase activity
4415	GO:1902418	Signaling and response	Stress	P	(+)-abscisic acid D-glucopyranosyl ester transmembrane transport
4416	GO:1902417	Signaling and response	Stress	F	(+)-abscisic acid D-glucopyranosyl ester transmembrane transporter activity
4417	GO:0001872	Signaling and response	Stress	F	(1->3)-beta-D-glucan binding
4418	GO:0006075	Signaling and response	Stress	P	(1->3)-beta-D-glucan biosynthetic process
4419	GO:0003843	Signaling and response	Stress	F	1,3-beta-D-glucan synthase activity
4420	GO:0000148	Signaling and response	Stress	C	1,3-beta-D-glucan synthase complex
4421	GO:0010427	Signaling and response	Stress	F	abscisic acid binding
4422	GO:0009688	Signaling and response	Stress	P	abscisic acid biosynthetic process
4423	GO:0046345	Signaling and response	Stress	P	abscisic acid catabolic process
4424	GO:0010294	Signaling and response	Stress	F	abscisic acid glucosyltransferase activity
4425	GO:0009687	Signaling and response	Stress	P	abscisic acid metabolic process
4426	GO:0080168	Signaling and response	Stress	P	abscisic acid transport
4427	GO:0009738	Signaling and response	Stress	P	abscisic acid-activated signaling pathway
4428	GO:0033741	Signaling and response	Stress	F	adenylyl-sulfate reductase (glutathione) activity
4429	GO:0007568	Signaling and response	Stress	P	aging
4430	GO:0009916	Signaling and response	Stress	F	alternative oxidase activity
4431	GO:0010230	Signaling and response	Stress	P	alternative respiration
4432	GO:0080144	Signaling and response	Stress	P	amino acid homeostasis
4433	GO:0055081	Signaling and response	Stress	P	anion homeostasis
4434	GO:0016209	Signaling and response	Stress	F	antioxidant activity
4435	GO:1901683	Signaling and response	Stress	F	arsenate ion transmembrane transporter activity
4436	GO:0015105	Signaling and response	Stress	F	arsenite transmembrane transporter activity
4437	GO:0015700	Signaling and response	Stress	P	arsenite transport
4438	GO:0061574	Signaling and response	Stress	C	ASAP complex
4439	GO:0010252	Signaling and response	Stress	P	auxin homeostasis
4440	GO:0010268	Signaling and response	Stress	P	brassinosteroid homeostasis
4441	GO:0080120	Signaling and response	Stress	P	CAAX-box protein maturation
4442	GO:0071586	Signaling and response	Stress	P	CAAX-box protein processing
4443	GO:0004662	Signaling and response	Stress	F	CAAX-protein geranylgeranyltransferase activity
4444	GO:0005953	Signaling and response	Stress	C	CAAX-protein geranylgeranyltransferase complex
4445	GO:0055073	Signaling and response	Stress	P	cadmium ion homeostasis
4446	GO:0005227	Signaling and response	Stress	F	calcium activated cation channel activity
4447	GO:0005262	Signaling and response	Stress	F	calcium channel activity
4448	GO:0015368	Signaling and response	Stress	F	calcium:cation antiporter activity
4449	GO:0015369	Signaling and response	Stress	F	calcium:proton antiporter activity
4450	GO:0005432	Signaling and response	Stress	F	calcium:sodium antiporter activity
4451	GO:0005544	Signaling and response	Stress	F	calcium-dependent phospholipid binding
4452	GO:0052543	Signaling and response	Stress	P	callose deposition in cell wall
4453	GO:0033500	Signaling and response	Stress	P	carbohydrate homeostasis
4454	GO:0004096	Signaling and response	Stress	F	catalase activity
4455	GO:0055080	Signaling and response	Stress	P	cation homeostasis
4456	GO:0045454	Signaling and response	Stress	P	cell redox homeostasis
4457	GO:0006884	Signaling and response	Stress	P	cell volume homeostasis
4458	GO:0006876	Signaling and response	Stress	P	cellular cadmium ion homeostasis
4459	GO:0006874	Signaling and response	Stress	P	cellular calcium ion homeostasis
4460	GO:0030003	Signaling and response	Stress	P	cellular cation homeostasis
4461	GO:0055082	Signaling and response	Stress	P	cellular chemical homeostasis
4462	GO:0006878	Signaling and response	Stress	P	cellular copper ion homeostasis
4463	GO:1990748	Signaling and response	Stress	P	cellular detoxification
4464	GO:0070370	Signaling and response	Stress	P	cellular heat acclimation

4465	GO:0019725	Signaling and response	Stress	P	cellular homeostasis
4466	GO:0006873	Signaling and response	Stress	P	cellular ion homeostasis
4467	GO:0006879	Signaling and response	Stress	P	cellular iron ion homeostasis
4468	GO:0030026	Signaling and response	Stress	P	cellular manganese ion homeostasis
4469	GO:0006875	Signaling and response	Stress	P	cellular metal ion homeostasis
4470	GO:0030004	Signaling and response	Stress	P	cellular monovalent inorganic cation homeostasis
4471	GO:0098869	Signaling and response	Stress	P	cellular oxidant detoxification
4472	GO:0030643	Signaling and response	Stress	P	cellular phosphate ion homeostasis
4473	GO:0071215	Signaling and response	Stress	P	cellular response to abscisic acid stimulus
4474	GO:0071485	Signaling and response	Stress	P	cellular response to absence of light
4475	GO:0071454	Signaling and response	Stress	P	cellular response to anoxia
4476	GO:0062197	Signaling and response	Stress	P	cellular response to chemical stress
4477	GO:0036294	Signaling and response	Stress	P	cellular response to decreased oxygen levels
4478	GO:0006974	Signaling and response	Stress	P	cellular response to DNA damage stimulus
4479	GO:0071369	Signaling and response	Stress	P	cellular response to ethylene stimulus
4480	GO:0071217	Signaling and response	Stress	P	cellular response to external biotic stimulus
4481	GO:0034605	Signaling and response	Stress	P	cellular response to heat
4482	GO:0071486	Signaling and response	Stress	P	cellular response to high light intensity
4483	GO:0070301	Signaling and response	Stress	P	cellular response to hydrogen peroxide
4484	GO:0071456	Signaling and response	Stress	P	cellular response to hypoxia
4485	GO:0071484	Signaling and response	Stress	P	cellular response to light intensity
4486	GO:0071219	Signaling and response	Stress	P	cellular response to molecule of bacterial origin
4487	GO:0071732	Signaling and response	Stress	P	cellular response to nitric oxide
4488	GO:0006995	Signaling and response	Stress	P	cellular response to nitrogen starvation
4489	GO:0071470	Signaling and response	Stress	P	cellular response to osmotic stress
4490	GO:0034599	Signaling and response	Stress	P	cellular response to oxidative stress
4491	GO:0071450	Signaling and response	Stress	P	cellular response to oxygen radical
4492	GO:0071457	Signaling and response	Stress	P	cellular response to ozone
4493	GO:0016036	Signaling and response	Stress	P	cellular response to phosphate starvation
4494	GO:0051365	Signaling and response	Stress	P	cellular response to potassium ion starvation
4495	GO:0034614	Signaling and response	Stress	P	cellular response to reactive oxygen species
4496	GO:0071461	Signaling and response	Stress	P	cellular response to redox state
4497	GO:0071472	Signaling and response	Stress	P	cellular response to salt stress
4498	GO:0009267	Signaling and response	Stress	P	cellular response to starvation
4499	GO:0033554	Signaling and response	Stress	P	cellular response to stress
4500	GO:0009970	Signaling and response	Stress	P	cellular response to sulfate starvation
4501	GO:0071451	Signaling and response	Stress	P	cellular response to superoxide
4502	GO:0097237	Signaling and response	Stress	P	cellular response to toxic substance
4503	GO:0034620	Signaling and response	Stress	P	cellular response to unfolded protein
4504	GO:0071492	Signaling and response	Stress	P	cellular response to UV-A
4505	GO:0071493	Signaling and response	Stress	P	cellular response to UV-B
4506	GO:0042631	Signaling and response	Stress	P	cellular response to water deprivation
4507	GO:0090156	Signaling and response	Stress	P	cellular sphingolipid homeostasis
4508	GO:0006882	Signaling and response	Stress	P	cellular zinc ion homeostasis
4509	GO:0051085	Signaling and response	Stress	P	chaperone cofactor-dependent protein refolding
4510	GO:0048878	Signaling and response	Stress	P	chemical homeostasis
4511	GO:0009631	Signaling and response	Stress	P	cold acclimation
4512	GO:0055070	Signaling and response	Stress	P	copper ion homeostasis
4513	GO:0004869	Signaling and response	Stress	F	cysteine-type endopeptidase inhibitor activity
4514	GO:0010494	Signaling and response	Stress	C	cytoplasmic stress granule
4515	GO:0003684	Signaling and response	Stress	F	damaged DNA binding
4516	GO:0006952	Signaling and response	Stress	P	defense response
4517	GO:0052542	Signaling and response	Stress	P	defense response by callose deposition
4518	GO:0052544	Signaling and response	Stress	P	defense response by callose deposition in cell wall
4519	GO:0009870	Signaling and response	Stress	P	defense response signaling pathway, resistance gene-dependent
4520	GO:0010204	Signaling and response	Stress	P	defense response signaling pathway, resistance gene-independent
4521	GO:0042742	Signaling and response	Stress	P	defense response to bacterium
4522	GO:0009816	Signaling and response	Stress	P	defense response to bacterium, incompatible interaction
4523	GO:0050832	Signaling and response	Stress	P	defense response to fungus
4524	GO:0009817	Signaling and response	Stress	P	defense response to fungus, incompatible interaction
4525	GO:0050829	Signaling and response	Stress	P	defense response to Gram-negative bacterium
4526	GO:0002213	Signaling and response	Stress	P	defense response to insect
4527	GO:0002229	Signaling and response	Stress	P	defense response to oomycetes
4528	GO:0098542	Signaling and response	Stress	P	defense response to other organism
4529	GO:0051607	Signaling and response	Stress	P	defense response to virus
4530	GO:0009814	Signaling and response	Stress	P	defense response, incompatible interaction
4531	GO:0036513	Signaling and response	Stress	C	Derlin-1 retrotranslocation complex
4532	GO:0016045	Signaling and response	Stress	P	detection of bacterium
4533	GO:0009727	Signaling and response	Stress	P	detection of ethylene stimulus
4534	GO:0098754	Signaling and response	Stress	P	detoxification
4535	GO:0071585	Signaling and response	Stress	P	detoxification of cadmium ion
4536	GO:0010273	Signaling and response	Stress	P	detoxification of copper ion
4537	GO:0072505	Signaling and response	Stress	P	divalent inorganic anion homeostasis
4538	GO:0009819	Signaling and response	Stress	P	drought recovery
4539	GO:0009368	Signaling and response	Stress	C	endopeptidase Clp complex
4540	GO:0030968	Signaling and response	Stress	P	endoplasmic reticulum unfolded protein response
4541	GO:0006983	Signaling and response	Stress	P	ER overload response
4542	GO:0051740	Signaling and response	Stress	F	ethylene binding

4543	GO:0009693	Signaling and response	Stress	P	ethylene biosynthetic process
4544	GO:0038199	Signaling and response	Stress	F	ethylene receptor activity
4545	GO:0009873	Signaling and response	Stress	P	ethylene-activated signaling pathway
4546	GO:0055089	Signaling and response	Stress	P	fatty acid homeostasis
4547	GO:0030385	Signaling and response	Stress	F	ferredoxin:thioredoxin reductase activity
4548	GO:0048307	Signaling and response	Stress	F	ferredoxin-nitrite reductase activity
4549	GO:0103012	Signaling and response	Stress	F	ferredoxin-thioredoxin reductase activity
4550	GO:0009813	Signaling and response	Stress	P	flavonoid biosynthetic process
4551	GO:0009812	Signaling and response	Stress	P	flavonoid metabolic process
4552	GO:0051555	Signaling and response	Stress	P	flavonol biosynthetic process
4553	GO:0052096	Signaling and response	Stress	P	formation by symbiont of syncytium involving giant cell for nutrient acquisition from host
4554	GO:0010336	Signaling and response	Stress	P	gibberellic acid homeostasis
4555	GO:0042973	Signaling and response	Stress	F	glucan endo-1,3-beta-D-glucosidase activity
4556	GO:0042593	Signaling and response	Stress	P	glucose homeostasis
4557	GO:0043295	Signaling and response	Stress	F	glutathione binding
4558	GO:0006750	Signaling and response	Stress	P	glutathione biosynthetic process
4559	GO:0045174	Signaling and response	Stress	F	glutathione dehydrogenase (ascorbate) activity
4560	GO:0016756	Signaling and response	Stress	F	glutathione gamma-glutamylcysteinyltransferase activity
4561	GO:0036374	Signaling and response	Stress	F	glutathione hydrolase activity
4562	GO:0006749	Signaling and response	Stress	P	glutathione metabolic process
4563	GO:0004602	Signaling and response	Stress	F	glutathione peroxidase activity
4564	GO:0004363	Signaling and response	Stress	F	glutathione synthase activity
4565	GO:0004364	Signaling and response	Stress	F	glutathione transferase activity
4566	GO:0034635	Signaling and response	Stress	P	glutathione transport
4567	GO:0004362	Signaling and response	Stress	F	glutathione-disulfide reductase activity
4568	GO:0010235	Signaling and response	Stress	P	guard mother cell cytokinesis
4569	GO:0010286	Signaling and response	Stress	P	heat acclimation
4570	GO:0031072	Signaling and response	Stress	F	heat shock protein binding
4571	GO:0042592	Signaling and response	Stress	P	homeostatic process
4572	GO:0080034	Signaling and response	Stress	P	host response to induction by symbiont of tumor, nodule or growth in host
4573	GO:0000839	Signaling and response	Stress	C	Hrd1p ubiquitin ligase ERAD-L complex
4574	GO:0051879	Signaling and response	Stress	F	Hsp90 protein binding
4575	GO:0050665	Signaling and response	Stress	P	hydrogen peroxide biosynthetic process
4576	GO:0042744	Signaling and response	Stress	P	hydrogen peroxide catabolic process
4577	GO:0042743	Signaling and response	Stress	P	hydrogen peroxide metabolic process
4578	GO:0080170	Signaling and response	Stress	P	hydrogen peroxide transmembrane transport
4579	GO:0047987	Signaling and response	Stress	F	hydroperoxide dehydratase activity
4580	GO:0004416	Signaling and response	Stress	F	hydroxyacylglutathione hydrolase activity
4581	GO:0006972	Signaling and response	Stress	P	hyperosmotic response
4582	GO:0042538	Signaling and response	Stress	P	hyperosmotic salinity response
4583	GO:0042539	Signaling and response	Stress	P	hypotonic salinity response
4584	GO:0006955	Signaling and response	Stress	P	immune response
4585	GO:0002376	Signaling and response	Stress	P	immune system process
4586	GO:0046217	Signaling and response	Stress	P	indole phytoalexin metabolic process
4587	GO:0009682	Signaling and response	Stress	P	induced systemic resistance
4588	GO:0045087	Signaling and response	Stress	P	innate immune response
4589	GO:0098771	Signaling and response	Stress	P	inorganic ion homeostasis
4590	GO:0050113	Signaling and response	Stress	F	inositol oxygenase activity
4591	GO:0052658	Signaling and response	Stress	F	inositol-1,4,5-trisphosphate 5-phosphatase activity
4592	GO:0051701	Signaling and response	Stress	P	interaction with host
4593	GO:0044419	Signaling and response	Stress	P	interspecies interaction between organisms
4594	GO:0050801	Signaling and response	Stress	P	ion homeostasis
4595	GO:0055072	Signaling and response	Stress	P	iron ion homeostasis
4596	GO:0009861	Signaling and response	Stress	P	jasmonic acid and ethylene-dependent systemic resistance
4597	GO:0009871	Signaling and response	Stress	P	jasmonic acid and ethylene-dependent systemic resistance, ethylene mediated signaling pathway
4598	GO:0009868	Signaling and response	Stress	P	jasmonic acid and ethylene-dependent systemic resistance, jasmonic acid mediated signaling pathway
4599	GO:0031640	Signaling and response	Stress	P	killing of cells of other organism
4600	GO:0004462	Signaling and response	Stress	F	lactoylglutathione lyase activity
4601	GO:0016688	Signaling and response	Stress	F	L-ascorbate peroxidase activity
4602	GO:0031418	Signaling and response	Stress	F	L-ascorbic acid binding
4603	GO:0019853	Signaling and response	Stress	P	L-ascorbic acid biosynthetic process
4604	GO:0055088	Signaling and response	Stress	P	lipid homeostasis
4605	GO:0055071	Signaling and response	Stress	P	manganese ion homeostasis
4606	GO:0055065	Signaling and response	Stress	P	metal ion homeostasis
4607	GO:0019243	Signaling and response	Stress	P	methylglyoxal catabolic process to D-lactate via S-lactoyl-glutathione
4608	GO:0010587	Signaling and response	Stress	P	miRNA catabolic process
4609	GO:0051787	Signaling and response	Stress	F	misfolded protein binding
4610	GO:0051560	Signaling and response	Stress	P	mitochondrial calcium ion homeostasis
4611	GO:0044003	Signaling and response	Stress	P	modulation by symbiont of host process
4612	GO:0035821	Signaling and response	Stress	P	modulation of process of other organism
4613	GO:0051817	Signaling and response	Stress	P	modulation of process of other organism involved in symbiotic interaction
4614	GO:0016656	Signaling and response	Stress	F	monodehydroascorbate reductase (NADH) activity
4615	GO:0055083	Signaling and response	Stress	P	monovalent inorganic anion homeostasis
4616	GO:0055067	Signaling and response	Stress	P	monovalent inorganic cation homeostasis
4617	GO:0031990	Signaling and response	Stress	P	mRNA export from nucleus in response to heat stress

4618	GO:0060586	Signaling and response	Stress	P	multicellular organismal iron ion homeostasis
4619	GO:0090359	Signaling and response	Stress	P	negative regulation of abscisic acid biosynthetic process
4620	GO:0009788	Signaling and response	Stress	P	negative regulation of abscisic acid-activated signaling pathway
4621	GO:0043066	Signaling and response	Stress	P	negative regulation of apoptotic process
4622	GO:0010507	Signaling and response	Stress	P	negative regulation of autophagy
4623	GO:0060548	Signaling and response	Stress	P	negative regulation of cell death
4624	GO:1901181	Signaling and response	Stress	P	negative regulation of cellular response to caffeine
4625	GO:0031936	Signaling and response	Stress	P	negative regulation of chromatin silencing
4626	GO:0031348	Signaling and response	Stress	P	negative regulation of defense response
4627	GO:1900425	Signaling and response	Stress	P	negative regulation of defense response to bacterium
4628	GO:1902478	Signaling and response	Stress	P	negative regulation of defense response to bacterium, incompatible interaction
4629	GO:1900366	Signaling and response	Stress	P	negative regulation of defense response to insect
4630	GO:0010951	Signaling and response	Stress	P	negative regulation of endopeptidase activity
4631	GO:0010366	Signaling and response	Stress	P	negative regulation of ethylene biosynthetic process
4632	GO:0010105	Signaling and response	Stress	P	negative regulation of ethylene-activated signaling pathway
4633	GO:0010730	Signaling and response	Stress	P	negative regulation of hydrogen peroxide biosynthetic process
4634	GO:0034051	Signaling and response	Stress	P	negative regulation of plant-type hypersensitive response
4635	GO:1901001	Signaling and response	Stress	P	negative regulation of response to salt stress
4636	GO:0080148	Signaling and response	Stress	P	negative regulation of response to water deprivation
4637	GO:0010187	Signaling and response	Stress	P	negative regulation of seed germination
4638	GO:0010113	Signaling and response	Stress	P	negative regulation of systemic acquired resistance
4639	GO:0017148	Signaling and response	Stress	P	negative regulation of translation
4640	GO:0006809	Signaling and response	Stress	P	nitric oxide biosynthetic process
4641	GO:0007263	Signaling and response	Stress	P	nitric oxide mediated signal transduction
4642	GO:0050421	Signaling and response	Stress	F	nitrite reductase (NO-forming) activity
4643	GO:0010196	Signaling and response	Stress	P	nonphotochemical quenching
4644	GO:0016684	Signaling and response	Stress	F	oxidoreductase activity, acting on peroxide as acceptor
4645	GO:0009405	Signaling and response	Stress	P	pathogenesis
4646	GO:0018401	Signaling and response	Stress	P	peptidyl-proline hydroxylation to 4-hydroxy-L-proline
4647	GO:0051920	Signaling and response	Stress	F	peroxiredoxin activity
4648	GO:0055062	Signaling and response	Stress	P	phosphate ion homeostasis
4649	GO:0031210	Signaling and response	Stress	F	phosphatidylcholine binding
4650	GO:0046854	Signaling and response	Stress	P	phosphatidylinositol phosphorylation
4651	GO:0048015	Signaling and response	Stress	P	phosphatidylinositol-mediated signaling
4652	GO:0055091	Signaling and response	Stress	P	phospholipid homeostasis
4653	GO:0047066	Signaling and response	Stress	F	phospholipid-hydroperoxide glutathione peroxidase activity
4654	GO:0010117	Signaling and response	Stress	P	photoprotection
4655	GO:0052315	Signaling and response	Stress	P	phytoalexin biosynthetic process
4656	GO:0052314	Signaling and response	Stress	P	phytoalexin metabolic process
4657	GO:0009626	Signaling and response	Stress	P	plant-type hypersensitive response
4658	GO:0009789	Signaling and response	Stress	P	positive regulation of abscisic acid-activated signaling pathway
4659	GO:0010186	Signaling and response	Stress	P	positive regulation of cellular defense response
4660	GO:1900103	Signaling and response	Stress	P	positive regulation of endoplasmic reticulum unfolded protein response
4661	GO:0010365	Signaling and response	Stress	P	positive regulation of ethylene biosynthetic process
4662	GO:0009963	Signaling and response	Stress	P	positive regulation of flavonoid biosynthetic process
4663	GO:1900057	Signaling and response	Stress	P	positive regulation of leaf senescence
4664	GO:0034052	Signaling and response	Stress	P	positive regulation of plant-type hypersensitive response
4665	GO:0043068	Signaling and response	Stress	P	positive regulation of programmed cell death
4666	GO:2000379	Signaling and response	Stress	P	positive regulation of reactive oxygen species metabolic process
4667	GO:1902584	Signaling and response	Stress	P	positive regulation of response to water deprivation
4668	GO:1901672	Signaling and response	Stress	P	positive regulation of systemic acquired resistance
4669	GO:0055075	Signaling and response	Stress	P	potassium ion homeostasis
4670	GO:0080136	Signaling and response	Stress	P	priming of cellular response to stress
4671	GO:0004656	Signaling and response	Stress	F	procollagen-proline 4-dioxygenase activity
4672	GO:0097468	Signaling and response	Stress	P	programmed cell death in response to reactive oxygen species
4673	GO:0002020	Signaling and response	Stress	F	protease binding
4674	GO:0044183	Signaling and response	Stress	F	protein folding chaperone
4675	GO:0006515	Signaling and response	Stress	P	protein quality control for misfolded or incompletely synthesized proteins
4676	GO:0042026	Signaling and response	Stress	P	protein refolding
4677	GO:0030091	Signaling and response	Stress	P	protein repair
4678	GO:0006621	Signaling and response	Stress	P	protein retention in ER lumen
4679	GO:2001057	Signaling and response	Stress	P	reactive nitrogen species metabolic process
4680	GO:0072593	Signaling and response	Stress	P	reactive oxygen species metabolic process
4681	GO:0006898	Signaling and response	Stress	P	receptor-mediated endocytosis
4682	GO:0010115	Signaling and response	Stress	P	regulation of abscisic acid biosynthetic process
4683	GO:0009787	Signaling and response	Stress	P	regulation of abscisic acid-activated signaling pathway
4684	GO:0010359	Signaling and response	Stress	P	regulation of anion channel activity
4685	GO:0030641	Signaling and response	Stress	P	regulation of cellular pH
4686	GO:1905957	Signaling and response	Stress	P	regulation of cellular response to alcohol
4687	GO:1900067	Signaling and response	Stress	P	regulation of cellular response to alkaline pH
4688	GO:0031347	Signaling and response	Stress	P	regulation of defense response
4689	GO:1900424	Signaling and response	Stress	P	regulation of defense response to bacterium
4690	GO:1900150	Signaling and response	Stress	P	regulation of defense response to fungus
4691	GO:2000068	Signaling and response	Stress	P	regulation of defense response to insect
4692	GO:0050691	Signaling and response	Stress	P	regulation of defense response to virus by host
4693	GO:0010104	Signaling and response	Stress	P	regulation of ethylene-activated signaling pathway
4694	GO:0045088	Signaling and response	Stress	P	regulation of innate immune response
4695	GO:0051453	Signaling and response	Stress	P	regulation of intracellular pH

4696	GO:2000082	Signaling and response	Stress	P	regulation of L-ascorbic acid biosynthetic process
4697	GO:0006885	Signaling and response	Stress	P	regulation of pH
4698	GO:0010363	Signaling and response	Stress	P	regulation of plant-type hypersensitive response
4699	GO:1903426	Signaling and response	Stress	P	regulation of reactive oxygen species biosynthetic process
4700	GO:2000377	Signaling and response	Stress	P	regulation of reactive oxygen species metabolic process
4701	GO:2000121	Signaling and response	Stress	P	regulation of removal of superoxide radicals
4702	GO:1901419	Signaling and response	Stress	P	regulation of response to alcohol
4703	GO:2001020	Signaling and response	Stress	P	regulation of response to DNA damage stimulus
4704	GO:0047484	Signaling and response	Stress	P	regulation of response to osmotic stress
4705	GO:1901031	Signaling and response	Stress	P	regulation of response to reactive oxygen species
4706	GO:1901000	Signaling and response	Stress	P	regulation of response to salt stress
4707	GO:2000070	Signaling and response	Stress	P	regulation of response to water deprivation
4708	GO:2000033	Signaling and response	Stress	P	regulation of seed dormancy process
4709	GO:0090333	Signaling and response	Stress	P	regulation of stomatal closure
4710	GO:0010119	Signaling and response	Stress	P	regulation of stomatal movement
4711	GO:1902456	Signaling and response	Stress	P	regulation of stomatal opening
4712	GO:0043903	Signaling and response	Stress	P	regulation of symbiosis, encompassing mutualism through parasitism
4713	GO:0010112	Signaling and response	Stress	P	regulation of systemic acquired resistance
4714	GO:0051209	Signaling and response	Stress	P	release of sequestered calcium ion into cytosol
4715	GO:0019430	Signaling and response	Stress	P	removal of superoxide radicals
4716	GO:0002679	Signaling and response	Stress	P	respiratory burst involved in defense response
4717	GO:0009737	Signaling and response	Stress	P	response to abscisic acid
4718	GO:0009646	Signaling and response	Stress	P	response to absence of light
4719	GO:0097305	Signaling and response	Stress	P	response to alcohol
4720	GO:0046685	Signaling and response	Stress	P	response to arsenic-containing substance
4721	GO:0090549	Signaling and response	Stress	P	response to carbon starvation
4722	GO:0009409	Signaling and response	Stress	P	response to cold
4723	GO:0010201	Signaling and response	Stress	P	response to continuous far red light stimulus by the high-irradiance response system
4724	GO:0046898	Signaling and response	Stress	P	response to cycloheximide
4725	GO:0010583	Signaling and response	Stress	P	response to cyclopentenone
4726	GO:0036293	Signaling and response	Stress	P	response to decreased oxygen levels
4727	GO:0009269	Signaling and response	Stress	P	response to desiccation
4728	GO:0042493	Signaling and response	Stress	P	response to drug
4729	GO:0034976	Signaling and response	Stress	P	response to endoplasmic reticulum stress
4730	GO:0045471	Signaling and response	Stress	P	response to ethanol
4731	GO:0009723	Signaling and response	Stress	P	response to ethylene
4732	GO:0009413	Signaling and response	Stress	P	response to flooding
4733	GO:0050826	Signaling and response	Stress	P	response to freezing
4734	GO:0009620	Signaling and response	Stress	P	response to fungus
4735	GO:0010332	Signaling and response	Stress	P	response to gamma radiation
4736	GO:0009408	Signaling and response	Stress	P	response to heat
4737	GO:0009635	Signaling and response	Stress	P	response to herbicide
4738	GO:0080027	Signaling and response	Stress	P	response to herbivore
4739	GO:0009644	Signaling and response	Stress	P	response to high light intensity
4740	GO:0042542	Signaling and response	Stress	P	response to hydrogen peroxide
4741	GO:0001666	Signaling and response	Stress	P	response to hypoxia
4742	GO:0009625	Signaling and response	Stress	P	response to insect
4743	GO:1990641	Signaling and response	Stress	P	response to iron ion starvation
4744	GO:0033591	Signaling and response	Stress	P	response to L-ascorbic acid
4745	GO:0010288	Signaling and response	Stress	P	response to lead ion
4746	GO:0006982	Signaling and response	Stress	P	response to lipid hydroperoxide
4747	GO:0010244	Signaling and response	Stress	P	response to low fluence blue light stimulus by blue low-fluence system
4748	GO:0009645	Signaling and response	Stress	P	response to low light intensity stimulus
4749	GO:0009612	Signaling and response	Stress	P	response to mechanical stimulus
4750	GO:0010188	Signaling and response	Stress	P	response to microbial phytotoxin
4751	GO:0051788	Signaling and response	Stress	P	response to misfolded protein
4752	GO:0002237	Signaling and response	Stress	P	response to molecule of bacterial origin
4753	GO:0002238	Signaling and response	Stress	P	response to molecule of fungal origin
4754	GO:0009624	Signaling and response	Stress	P	response to nematode
4755	GO:0090548	Signaling and response	Stress	P	response to nitrate starvation
4756	GO:0071731	Signaling and response	Stress	P	response to nitric oxide
4757	GO:0010335	Signaling and response	Stress	P	response to non-ionic osmotic stress
4758	GO:0002239	Signaling and response	Stress	P	response to oomycetes
4759	GO:0006970	Signaling and response	Stress	P	response to osmotic stress
4760	GO:0051707	Signaling and response	Stress	P	response to other organism
4761	GO:0006979	Signaling and response	Stress	P	response to oxidative stress
4762	GO:0000305	Signaling and response	Stress	P	response to oxygen radical
4763	GO:0010193	Signaling and response	Stress	P	response to ozone
4764	GO:0009623	Signaling and response	Stress	P	response to parasitic fungus
4765	GO:0009268	Signaling and response	Stress	P	response to pH
4766	GO:0080183	Signaling and response	Stress	P	response to photooxidative stress
4767	GO:0009314	Signaling and response	Stress	P	response to radiation
4768	GO:0000302	Signaling and response	Stress	P	response to reactive oxygen species
4769	GO:0051775	Signaling and response	Stress	P	response to redox state
4770	GO:1902074	Signaling and response	Stress	P	response to salt
4771	GO:0009651	Signaling and response	Stress	P	response to salt stress
4772	GO:0042594	Signaling and response	Stress	P	response to starvation

4773	GO:0006950	Signaling and response	Stress	P	response to stress
4774	GO:0000303	Signaling and response	Stress	P	response to superoxide
4775	GO:0009610	Signaling and response	Stress	P	response to symbiotic fungus
4776	GO:0009636	Signaling and response	Stress	P	response to toxic substance
4777	GO:0010353	Signaling and response	Stress	P	response to trehalose
4778	GO:0006986	Signaling and response	Stress	P	response to unfolded protein
4779	GO:0010224	Signaling and response	Stress	P	response to UV-B
4780	GO:0009615	Signaling and response	Stress	P	response to virus
4781	GO:0009414	Signaling and response	Stress	P	response to water deprivation
4782	GO:0009611	Signaling and response	Stress	P	response to wounding
4783	GO:0005094	Signaling and response	Stress	F	Rho GDP-dissociation inhibitor activity
4784	GO:0005089	Signaling and response	Stress	F	Rho guanyl-nucleotide exchange factor activity
4785	GO:0006278	Signaling and response	Stress	P	RNA-dependent DNA biosynthetic process
4786	GO:1990112	Signaling and response	Stress	C	RQC complex
4787	GO:0009303	Signaling and response	Stress	P	rRNA transcription
4788	GO:0051903	Signaling and response	Stress	F	S-(hydroxymethyl)glutathione dehydrogenase activity
4789	GO:0004867	Signaling and response	Stress	F	serine-type endopeptidase inhibitor activity
4790	GO:0090734	Signaling and response	Stress	C	site of DNA damage
4791	GO:0016444	Signaling and response	Stress	P	somatic cell DNA recombination
4792	GO:0090332	Signaling and response	Stress	P	stomatal closure
4793	GO:0034063	Signaling and response	Stress	P	stress granule assembly
4794	GO:0004784	Signaling and response	Stress	F	superoxide dismutase activity
4795	GO:0006801	Signaling and response	Stress	P	superoxide metabolic process
4796	GO:0009627	Signaling and response	Stress	P	systemic acquired resistance
4797	GO:0009862	Signaling and response	Stress	P	systemic acquired resistance, salicylic acid mediated signaling pathway
4798	GO:0043247	Signaling and response	Stress	P	telomere maintenance in response to DNA damage
4799	GO:0004843	Signaling and response	Stress	F	thiol-dependent ubiquitin-specific protease activity
4800	GO:0008379	Signaling and response	Stress	F	thioredoxin peroxidase activity
4801	GO:0042964	Signaling and response	Stress	P	thioredoxin reduction
4802	GO:0090729	Signaling and response	Stress	F	toxin activity
4803	GO:0009403	Signaling and response	Stress	P	toxin biosynthetic process
4804	GO:0009407	Signaling and response	Stress	P	toxin catabolic process
4805	GO:0009404	Signaling and response	Stress	P	toxin metabolic process
4806	GO:0055076	Signaling and response	Stress	P	transition metal ion homeostasis
4807	GO:0004675	Signaling and response	Stress	F	transmembrane receptor protein serine/threonine kinase activity
4808	GO:0070413	Signaling and response	Stress	P	trehalose metabolism in response to stress
4809	GO:0070328	Signaling and response	Stress	P	triglyceride homeostasis
4810	GO:0072506	Signaling and response	Stress	P	trivalent inorganic anion homeostasis
4811	GO:0030433	Signaling and response	Stress	P	ubiquitin-dependent ERAD pathway
4812	GO:0051082	Signaling and response	Stress	F	unfolded protein binding
4813	GO:0009650	Signaling and response	Stress	P	UV protection
4814	GO:0070914	Signaling and response	Stress	P	UV-damage excision repair
4815	GO:0016032	Signaling and response	Stress	P	viral process
4816	GO:0039694	Signaling and response	Stress	P	viral RNA genome replication
4817	GO:0009110	Signaling and response	Stress	P	vitamin biosynthetic process
4818	GO:0010189	Signaling and response	Stress	P	vitamin E biosynthetic process
4819	GO:0006766	Signaling and response	Stress	P	vitamin metabolic process
4820	GO:0030104	Signaling and response	Stress	P	water homeostasis
4821	GO:0042364	Signaling and response	Stress	P	water-soluble vitamin biosynthetic process
4822	GO:0006767	Signaling and response	Stress	P	water-soluble vitamin metabolic process
