# Supplementary material for: Whether Gametophytes Are Reduced or Unreduced in Angiosperms Might Be Determined Metabolically
Source: Genes (Basel). 2020 Dec 2;11(12):1449. doi: 10.3390/genes11121449 (PMC7761559; doi:10.3390/genes11121449)
Supplement: Supplementary file 1 [file genes-11-01449-s001.zip › Table S3 Adjusted residuals.pdf]

Table S3. Calculations for chi square tests of independence and for the determination of standard and adjusted residuals. Yellow highlighted values are significant at P < 0.05. Orange highlights indicate minimal absolule value adjustments made to accomodate overrepresentation gene ontology (OGO) analyses where underrepresented (-) GO categories were present.

Type	Test	Up + -	Group	Subgroup	Obs	Obs	Total	Exp	Exp	Chi square	1st %	2nd %	standard residuals		p value		adjusted residuals		p value
EGO	L1M1	+	Sig & Res	Sig	29	2	31	16.06	14.94	21.64	20.1	1.5	3.23	-3.35	0.0012	0.0008	4.9354	-4.9354	0.0000
EGO	L1M1	+	Sig & Res	Stress	21	1	22	11.40	10.60	16.79	14.6	0.7	2.85	-2.95	0.0044	0.0032	4.2704	-4.2704	0.0000
EGO	L1M1	+	Metab	Bioen	12	0	12	6.22	5.78	11.17	8.3	0.0	2.32	-2.41	0.0203	0.0162	3.4162	-3.4162	0.0006
EGO	L1M1	+	Metab	Photosyn	5	3	8	4.14	3.86	0.37	3.5	2.2	0.42	-0.44	0.6741	0.6629	0.6147	-0.6147	0.5388
EGO	L1M1	+	Metab	Metab	27	13	40	20.72	19.28	3.95	18.8	9.7	1.38	-1.43	0.1677	0.1526	2.1479	-2.1479	0.0317
EGO	L1M1	+	Metab	Catab	30	2	32	16.58	15.42	22.56	20.8	1.5	3.30	-3.42	0.0010	0.0006	5.0488	-5.0488	0.0000
EGO	L1M1	+	Metab	Transp	7	1	8	4.14	3.86	4.08	4.9	0.7	1.40	-1.45	0.1606	0.1458	2.0506	-2.0506	0.0403
EGO	L1M1	+	Reg	Reg							0.0	0.0							
EGO	L1M1	+	Reg	Prot mod	3	2	5	2.59	2.41	0.13	2.1	1.5	0.25	-0.26	0.7989	0.7917	0.3704	-0.3704	0.7111
EGO	L1M1	+	Reg	Transcri	0	8	8	4.14	3.86	8.60	0.0	6.0	-2.04	2.11	0.0418	0.0348	-2.9752	2.9752	0.0029
EGO	L1M1	+	Reg	Transla	0	36	36	18.65	17.35	38.69	0.0	26.9	-4.32	4.48	0.0000	0.0000	-6.6665	6.6665	0.0000
EGO	L1M1	+	Devel	Devel	8	21	29	15.02	13.98	6.81	5.6	15.7	-1.81	1.88	0.0700	0.0604	-2.7572	2.7572	0.0058
EGO	L1M1	+	Devel	Cell div	2	45	47	24.35	22.65	42.55	1.4	33.6	-4.53	4.69	0.0000	0.0000	-7.1559	7.1559	0.0000
					144	134	278	144.00	134.00	177.34	100.0	100.0	SS	177.34					
EGO	L2M2	+	Sig & Res	Sig															
EGO	L2M2	+	Sig & Res	Stress															
EGO	L2M2	+	Metab	Bioen	6	0	6	5.24	0.76	0.87	8.7	0.0	0.33	-0.87	0.7401	0.3835	0.9701	-0.9701	0.3320
EGO	L2M2	+	Metab	Photosyn	6	0	6	5.24	0.76	0.87	8.7	0.0	0.33	-0.87	0.7401	0.3835	0.9701	-0.9701	0.3320
EGO	L2M2	+	Metab	Metab	20	9	29	25.33	3.67	8.86	29.0	90.0	-1.06	2.78	0.2897	0.0054	-3.7410	3.7410	0.0002
EGO	L2M2	+	Metab	Catab	4	0	4	3.49	0.51	0.58	5.8	0.0	0.27	-0.71	0.7865	0.4767	0.7814	-0.7814	0.4346
EGO	L2M2	+	Metab	Transp	1	1	2	1.75	0.25	2.52	1.4	10.0	-0.57	1.48	0.5720	0.1377	-1.6087	1.6087	0.1077
EGO	L2M2	+	Reg	Reg															
EGO	L2M2	+	Reg	Prot mod															
EGO	L2M2	+	Reg	Transcri	2	0	2	1.75	0.25	0.29	2.9	0.0	0.19	-0.50	0.8481	0.6149	0.5453	-0.5453	0.5855
EGO	L2M2	+	Reg	Transla	3	0	3	2.62	0.38	0.43	4.3	0.0	0.23	-0.62	0.8145	0.5377	0.6723	-0.6723	0.5014
EGO	L2M2	+	Devel	Devel	22	0	22	19.22	2.78	3.19	31.9	0.0	0.64	-1.67	0.5252	0.0952	2.1021	-2.1021	0.0355
EGO	L2M2	+	Devel	Cell div	5	0	5	4.37	0.63	0.72	7.2	0.0	0.30	-0.80	0.7620	0.4263	0.8795	-0.8795	0.3791
					69	10	79	69.00	10.00	18.34	100.0	100.0	SS	18.34					
EGO	L4M4	+	Sig & Res	Sig	0	19	19	4.03	14.97	5.12	0.0	24.4	-2.01	1.04	0.0447	0.2976	-2.5160	2.5160	0.0119
EGO	L4M4	+	Sig & Res	Stress	0	7	7	1.48	5.52	1.88	0.0	9.0	-1.22	0.63	0.2230	0.5272	-1.4241	1.4241	0.1544
EGO	L4M4	+	Metab	Bioen	1	4	5	1.06	3.94	0.00	4.8	5.1	-0.06	0.03	0.9531	0.9756	-0.0680	0.0680	0.9458
EGO	L4M4	+	Metab	Photosyn	7	0	7	1.48	5.52	26.00	33.3	0.0	4.53	-2.35	0.0000	0.0189	5.2894	-5.2894	0.0000
EGO	L4M4	+	Metab	Metab	3	31	34	7.21	26.79	3.12	14.3	39.7	-1.57	0.81	0.1168	0.4157	-2.1807	2.1807	0.0292
EGO	L4M4	+	Metab	Catab	0	1	1	0.21	0.79	0.27	0.0	1.3	-0.46	0.24	0.6451	0.8111	-0.5215	0.5215	0.6020
EGO	L4M4	+	Metab	Transp	1	3	4	0.85	3.15	0.03	4.8	3.8	0.16	-0.09	0.8693	0.9320	0.1892	-0.1892	0.8500
EGO	L4M4	+	Reg	Reg															
EGO	L4M4	+	Reg	Prot mod															
EGO	L4M4	+	Reg	Transcri															
EGO	L4M4	+	Reg	Transla	0	8	8	1.70	6.30	2.15	0.0	10.3	-1.30	0.68	0.1927	0.4991	-1.5307	1.5307	0.1258
EGO	L4M4	+	Devel	Devel	8	1	9	1.91	7.09	24.66	38.1	1.3	4.41	-2.29	0.0000	0.0222	5.2088	-5.2088	0.0000
EGO	L4M4	+	Devel	Cell div	1	4	5	1.06	3.94	0.00	4.8	5.1	-0.06	0.03	0.9531	0.9756	-0.0680	0.0680	0.9458
					21	78	99	21.00	78.00	63.25	#######	#######	SS	63.25					
EGO	F2L2	+	Sig & Res	Sig	39	12	51	23.99	27.01	17.73	8.3	2.3	3.06	-2.89	0.0022	0.0039	4.3228	-4.3228	0.0000
EGO	F2L2	+	Sig & Res	Stress	64	3	67	31.52	35.48	63.21	13.6	0.6	5.79	-5.45	0.0000	0.0000	8.2321	-8.2321	0.0000
EGO	F2L2	+	Metab	Bioen	67	4	71	33.40	37.60	63.83	14.3	0.8	5.81	-5.48	0.0000	0.0000	8.2900	-8.2900	0.0000
EGO	F2L2	+	Metab	Photosyn	30	0	30	14.11	15.89	33.77	6.4	0.0	4.23	-3.99	0.0000	0.0001	5.9010	-5.9010	0.0000
EGO	F2L2	+	Metab	Metab	123	39	162	76.21	85.79	54.25	26.2	7.4	5.36	-5.05	0.0000	0.0000	8.0486	-8.0486	0.0000
EGO	F2L2	+	Metab	Catab	17	26	43	20.23	22.77	0.97	3.6	4.9	-0.72	0.68	0.4730	0.4988	-1.0081	1.0081	0.3134
EGO	F2L2	+	Metab	Transp	54	8	62	29.17	32.83	39.93	11.5	1.5	4.60	-4.33	0.0000	0.0000	6.5252	-6.5252	0.0000
EGO	F2L2	+	Reg	Reg	5	29	34	15.99	18.01	14.27	1.1	5.5	-2.75	2.59	0.0060	0.0096	-3.8436	3.8436	0.0001
EGO	F2L2	+	Reg	Prot mod	2	16	18	8.47	9.53	9.33	0.4	3.0	-2.22	2.09	0.0262	0.0362	-3.0821	3.0821	0.0021
EGO	F2L2	+	Reg	Transcri	0	87	87	40.93	46.07	77.28	0.0	16.5	-6.40	6.03	0.0000	0.0000	-9.2014	9.2014	0.0000
EGO	F2L2	+	Reg	Transla	1	94	95	44.69	50.31	80.65	0.2	17.8	-6.54	6.16	0.0000	0.0000	-9.4416	9.4416	0.0000
EGO	F2L2	+	Devel	Devel	59	63	122	57.39	64.61	0.09	12.6	11.9	0.21	-0.20	0.8317	0.8413	0.3117	-0.3117	0.7553
EGO	F2L2	+	Devel	Cell div	8	147	155	72.91	82.09	109.13	1.7	27.8	-7.60	7.16	0.0000	0.0000	-11.3672	11.3672	0.0000
					469	528	997	469.00	528.00	564.44	#######	#######	SS	564.44					
EGO	F3L3	+	Sig & Res	Sig	58	11	69	40.10	28.90	19.07	9.1	2.4	2.83	-3.33	0.0047	0.0009	4.5114	-4.5114	0.0000

EGO	F3L3	+	Sig & Res	Stress	78	3	81	47.08	33.92	48.50	12.2	0.7	4.51	-5.31	0.0000	0.0000	7.2367	-7.2367	0.0000
EGO	F3L3	+	Metab	Bioen	76	3	79	45.92	33.08	47.07	11.9	0.7	4.44	-5.23	0.0000	0.0000	7.1222	-7.1222	0.0000
EGO	F3L3	+	Metab	Photosyn	26	0	26	15.11	10.89	18.73	4.1	0.0	2.80	-3.30	0.0051	0.0010	4.3806	-4.3806	0.0000
EGO	F3L3	+	Metab	Metab	192	42	234	136.00	98.00	55.06	30.1	9.2	4.80	-5.66	0.0000	0.0000	8.3667	-8.3667	0.0000
EGO	F3L3	+	Metab	Catab	33	11	44	25.57	18.43	5.15	5.2	2.4	1.47	-1.73	0.1419	0.0836	2.3164	-2.3164	0.0205
EGO	F3L3	+	Metab	Transp	74	7	81	47.08	33.92	36.76	11.6	1.5	3.92	-4.62	0.0001	0.0000	6.3006	-6.3006	0.0000
EGO	F3L3	+	Reg	Reg	6	29	35	20.34	14.66	24.15	0.9	6.3	-3.18	3.75	0.0015	0.0002	-4.9942	4.9942	0.0000
EGO	F3L3	+	Reg	Prot mod	3	20	23	13.37	9.63	19.20	0.5	4.4	-2.84	3.34	0.0046	0.0008	-4.4285	4.4285	0.0000
EGO	F3L3	+	Reg	Transcri	0	81	81	47.08	33.92	112.41	0.0	17.6	-6.86	8.08	0.0000	0.0000	-11.0174	11.0174	0.0000
EGO	F3L3	+	Reg	Transla	1	87	88	51.15	36.85	117.40	0.2	19.0	-7.01	8.26	0.0000	0.0000	-11.2981	11.2981	0.0000
EGO	F3L3	+	Devel	Devel	75	51	126	73.23	52.77	0.10	11.8	11.1	0.21	-0.24	0.8363	0.8077	0.3394	-0.3394	0.7343
EGO	F3L3	+	Devel	Cell div	15	114	129	74.98	54.02	114.56	2.4	24.8	-6.93	8.16	0.0000	0.0000	-11.3947	11.3947	0.0000
					637	459	1096	637.00	459.00	618.16	#######	#######	SS	618.16					
EGO	F2M2	+	Sig & Res	Sig	48	10	58	31.59	26.41	18.72	9.3	2.3	2.92	-3.19	0.0035	0.0014	4.4645	-4.4645	0.0000
EGO	F2M2	+	Sig & Res	Stress	75	6	81	44.12	36.88	47.47	14.5	1.4	4.65	-5.08	0.0000	0.0000	7.2034	-7.2034	0.0000
EGO	F2M2	+	Metab	Bioen	60	4	64	34.86	29.14	39.82	11.6	0.9	4.26	-4.66	0.0000	0.0000	6.5339	-6.5339	0.0000
EGO	F2M2	+	Metab	Photosyn	41	0	41	22.33	18.67	34.27	7.9	0.0	3.95	-4.32	0.0001	0.0000	5.9847	-5.9847	0.0000
EGO	F2M2	+	Metab	Metab	138	33	171	93.14	77.86	47.45	26.6	7.6	4.65	-5.08	0.0000	0.0000	7.6060	-7.6060	0.0000
EGO	F2M2	+	Metab	Catab	26	31	57	31.05	25.95	1.80	5.0	7.2	-0.91	0.99	0.3650	0.3218	-1.3846	1.3846	0.1662
EGO	F2M2	+	Metab	Transp	57	7	64	34.86	29.14	30.88	11.0	1.6	3.75	-4.10	0.0002	0.0000	5.7542	-5.7542	0.0000
EGO	F2M2	+	Reg	Reg	0	29	29	15.80	13.20	34.69	0.0	6.7	-3.97	4.35	0.0001	0.0000	-5.9820	5.9820	0.0000
EGO	F2M2	+	Reg	Prot mod	3	12	15	8.17	6.83	7.19	0.6	2.8	-1.81	1.98	0.0705	0.0479	-2.7021	2.7021	0.0069
EGO	F2M2	+	Reg	Transcri	0	56	56	30.50	25.50	66.99	0.0	12.9	-5.52	6.04	0.0000	0.0000	-8.4371	8.4371	0.0000
EGO	F2M2	+	Reg	Transla	1	85	86	46.84	39.16	98.54	0.2	19.6	-6.70	7.33	0.0000	0.0000	-10.4083	10.4083	0.0000
EGO	F2M2	+	Devel	Devel	61	38	99	53.92	45.08	2.04	11.8	8.8	0.96	-1.05	0.3353	0.2919	1.5087	-1.5087	0.1314
EGO	F2M2	+	Devel	Cell div	8	122	130	70.81	59.19	122.36	1.5	28.2	-7.46	8.16	0.0000	0.0000	-11.9054	11.9054	0.0000
					518	433	951	518.00	433.00	552.22	#######	#######	SS	552.22					
EGO	F3M3	+	Sig & Res	Sig	60	10	70	40.03	29.97	23.27	10.1	2.3	3.16	-3.65	0.0016	0.0003	4.9956	-4.9956	0.0000
EGO	F3M3	+	Sig & Res	Stress	65	2	67	38.31	28.69	43.41	11.0	0.5	4.31	-4.98	0.0000	0.0000	6.8127	-6.8127	0.0000
EGO	F3M3	+	Metab	Bioen	74	4	78	44.60	33.40	45.25	12.5	0.9	4.40	-5.09	0.0000	0.0000	6.9950	-6.9950	0.0000
EGO	F3M3	+	Metab	Photosyn	29	0	29	16.58	12.42	21.71	4.9	0.0	3.05	-3.52	0.0023	0.0004	4.7263	-4.7263	0.0000
EGO	F3M3	+	Metab	Metab	174	35	209	119.51	89.49	58.01	29.3	7.9	4.98	-5.76	0.0000	0.0000	8.5239	-8.5239	0.0000
EGO	F3M3	+	Metab	Catab	26	10	36	20.59	15.41	3.33	4.4	2.3	1.19	-1.38	0.2328	0.1679	1.8560	-1.8560	0.0635
EGO	F3M3	+	Metab	Transp	73	6	79	45.18	33.82	40.03	12.3	1.4	4.14	-4.78	0.0000	0.0000	6.5823	-6.5823	0.0000
EGO	F3M3	+	Reg	Reg	7	37	44	25.16	18.84	30.62	1.2	8.3	-3.62	4.18	0.0003	0.0000	-5.6544	5.6544	0.0000
EGO	F3M3	+	Reg	Prot mod	3	18	21	12.01	8.99	15.78	0.5	4.1	-2.60	3.00	0.0093	0.0027	-4.0138	4.0138	0.0001
EGO	F3M3	+	Reg	Transcri	0	70	70	40.03	29.97	93.49	0.0	15.8	-6.33	7.31	0.0000	0.0000	-10.0129	10.0129	0.0000
EGO	F3M3	+	Reg	Transla	1	74	75	42.89	32.11	95.55	0.2	16.7	-6.40	7.39	0.0000	0.0000	-10.1490	10.1490	0.0000
EGO	F3M3	+	Devel	Devel	71	38	109	62.33	46.67	2.82	12.0	8.6	1.10	-1.27	0.2722	0.2044	1.7740	-1.7740	0.0761
EGO	F3M3	+	Devel	Cell div	10	140	150	85.78	64.22	156.35	1.7	31.5	-8.18	9.46	0.0000	0.0000	-13.5200	13.5200	0.0000
					593	444	1037	593.00	444.00	629.62	#######	#######	SS	629.62					
EGO	L3S3	+	Sig & Res	Sig	0	0	0	0.00	0.00										
EGO	L3S3	+	Sig & Res	Stress	0	1	1	0.07	0.93	0.07	0.0	0.8	-0.26	0.07	0.7970	0.9454	-0.2672	0.2672	0.7893
EGO	L3S3	+	Metab	Bioen	1	8	9	0.60	8.40	0.29	11.1	6.3	0.52	-0.14	0.6003	0.8891	0.5612	-0.5612	0.5747
EGO	L3S3	+	Metab	Photosyn	2	1	3	0.20	2.80	17.51	22.2	0.8	4.04	-1.08	0.0001	0.2818	4.2308	-4.2308	0.0000
EGO	L3S3	+	Metab	Metab	5	20	25	1.65	23.35	7.24	55.6	15.7	2.60	-0.69	0.0093	0.4887	2.9794	-2.9794	0.0029
EGO	L3S3	+	Metab	Catab	1	3	4	0.26	3.74	2.19	11.1	2.4	1.43	-0.38	0.1530	0.7036	1.5012	-1.5012	0.1333
EGO	L3S3	+	Metab	Transp	0	2	2	0.13	1.87	0.14	0.0	1.6	-0.36	0.10	0.7160	0.9228	-0.3793	0.3793	0.7045
EGO	L3S3	+	Reg	Reg	0	14	14	0.93	13.07	0.99	0.0	11.0	-0.96	0.26	0.3358	0.7978	-1.0517	1.0517	0.2930
EGO	L3S3	+	Reg	Prot mod	0	1	1	0.07	0.93	0.07	0.0	0.8	-0.26	0.07	0.7970	0.9454	-0.2672	0.2672	0.7893
EGO	L3S3	+	Reg	Transcri	0	9	9	0.60	8.40	0.64	0.0	7.1	-0.77	0.21	0.4403	0.8372	-0.8264	0.8264	0.4086
EGO	L3S3	+	Reg	Transla	0	28	28	1.85	26.15	1.98	0.0	22.0	-1.36	0.36	0.1734	0.7171	-1.5807	1.5807	0.1139
EGO	L3S3	+	Devel	Devel	0	36	36	2.38	33.62	2.55	0.0	28.3	-1.54	0.41	0.1227	0.6812	-1.8627	1.8627	0.0625
EGO	L3S3	+	Devel	Cell div	0	4	4	0.26	3.74	0.28	0.0	3.1	-0.51	0.14	0.6069	0.8911	-0.5404	0.5404	0.5889
					9	127	136	9.00	127.00	33.96	#######	#######	SS	33.96					
EGO	M3S3	+	Sig & Res	Sig															
EGO	M3S3	+	Sig & Res	Stress															
EGO	M3S3	+	Metab	Bioen	2	16	18	3.14	14.86	0.50	6.7	11.3	-0.64	0.30	0.5201	0.7675	-0.7480	0.7480	0.4544
EGO	M3S3	+	Metab	Photosyn															
EGO	M3S3	+	Metab	Metab	19	19	38	6.63	31.37	27.97	63.3	13.4	4.81	-2.21	0.0000	0.0272	5.9922	-5.9922	0.0000
EGO	M3S3	+	Metab	Catab	2	4	6	1.05	4.95	1.05	6.7	2.8	0.93	-0.43	0.3513	0.6684	1.0442	-1.0442	0.2964

EGO	M3S3	+	Metab	Transp	3	3	6	1.05	4.95	4.42	10.0	2.1	1.91	-0.88	0.0562	0.3801	2.1393	-2.1393	0.0324
EGO	M3S3	+	Reg	Reg	0	14	14	2.44	11.56	2.96	0.0	9.9	-1.56	0.72	0.1181	0.4726	-1.7944	1.7944	0.0728
EGO	M3S3	+	Reg	Prot mod	1	2	3	0.52	2.48	0.53	3.3	1.4	0.66	-0.30	0.5099	0.7619	0.7318	-0.7318	0.4643
EGO	M3S3	+	Reg	Transcri	1	9	10	1.74	8.26	0.38	3.3	6.3	-0.56	0.26	0.5731	0.7956	-0.6390	0.6390	0.5228
EGO	M3S3	+	Reg	Transla	0	29	29	5.06	23.94	6.13	0.0	20.4	-2.25	1.03	0.0245	0.3013	-2.7146	2.7146	0.0066
EGO	M3S3	+	Devel	Devel	2	45	47	8.20	38.80	5.68	6.7	31.7	-2.16	0.99	0.0304	0.3198	-2.7946	2.7946	0.0052
EGO	M3S3	+	Devel	Cell div	0	1	1	0.17	0.83	0.21	0.0	0.7	-0.42	0.19	0.6762	0.8478	-0.4610	0.4610	0.6448
					30	142	172	30.00	142.00	49.83	#######	#######	SS	49.83					
EGO	F3S3	+	Sig & Res	Sig	37	11	48	24.35	23.65	13.33	6.7	2.1	2.56	-2.60	0.0104	0.0093	3.7346	-3.7346	0.0002
EGO	F3S3	+	Sig & Res	Stress	53	3	56	28.41	27.59	43.19	9.6	0.6	4.61	-4.68	0.0000	0.0000	6.7483	-6.7483	0.0000
EGO	F3S3	+	Metab	Bioen	71	9	80	40.59	39.41	46.25	12.9	1.7	4.77	-4.84	0.0000	0.0000	7.0660	-7.0660	0.0000
EGO	F3S3	+	Metab	Photosyn	37	2	39	19.79	19.21	30.39	6.7	0.4	3.87	-3.93	0.0001	0.0001	5.6148	-5.6148	0.0000
EGO	F3S3	+	Metab	Metab	173	44	217	110.10	106.90	72.95	31.4	8.2	5.99	-6.08	0.0000	0.0000	9.5480	-9.5480	0.0000
EGO	F3S3	+	Metab	Catab	22	5	27	13.70	13.30	10.21	4.0	0.9	2.24	-2.28	0.0249	0.0228	3.2359	-3.2359	0.0012
EGO	F3S3	+	Metab	Transp	72	11	83	42.11	40.89	43.06	13.1	2.1	4.61	-4.67	0.0000	0.0000	6.8282	-6.8282	0.0000
EGO	F3S3	+	Reg	Reg	4	48	52	26.38	25.62	38.55	0.7	9.0	-4.36	4.42	0.0000	0.0000	-6.3628	6.3628	0.0000
EGO	F3S3	+	Reg	Prot mod	1	17	18	9.13	8.87	14.70	0.2	3.2	-2.69	2.73	0.0071	0.0063	-3.8663	3.8663	0.0001
EGO	F3S3	+	Reg	Transcri	0	76	76	38.56	37.44	78.27	0.0	14.2	-6.21	6.30	0.0000	0.0000	-9.1740	9.1740	0.0000
EGO	F3S3	+	Reg	Transla	1	91	92	46.68	45.32	90.74	0.2	17.0	-6.69	6.78	0.0000	0.0000	-9.9566	9.9566	0.0000
EGO	F3S3	+	Devel	Devel	65	101	166	84.22	81.78	8.91	11.8	18.9	-2.09	2.13	0.0362	0.0335	-3.2424	3.2424	0.0012
EGO	F3S3	+	Devel	Cell div	15	117	132	66.97	65.03	81.87	2.7	21.9	-6.35	6.45	0.0000	0.0000	-9.6539	9.6539	0.0000
					551	535	1086	551.00	535.00	572.42	#######	#######	SS	572.42					
OGO	L4M4		Sig & Res	Sig	2	17	19	2.98	16.02	0.38	8.3	13.2	-0.57	0.24	0.5701	0.8065	-0.6609	0.6609	0.5087
OGO	L4M4		Sig & Res	Stress	2	16	18	2.82	15.18	0.28	8.3	12.4	-0.49	0.21	0.6241	0.8326	-0.5682	0.5682	0.5699
OGO	L4M4		Metab	Bioen	3	6	9	1.41	7.59	2.12	12.5	4.7	1.34	-0.58	0.1813	0.5642	1.5005	-1.5005	0.1335
OGO	L4M4		Metab	Photosyn	10	12	22	3.45	18.55	14.74	41.7	9.3	3.53	-1.52	0.0004	0.1284	4.1492	-4.1492	0.0000
OGO	L4M4		Metab	Metab	2	32	34	5.33	28.67	2.47	8.3	24.8	-1.44	0.62	0.1489	0.5336	-1.7824	1.7824	0.0747
OGO	L4M4		Metab	Catab	0	18	18	2.82	15.18	3.35	0.0	14.0	-1.68	0.72	0.0929	0.4686	-1.9482	1.9482	0.0514
OGO	L4M4		Metab	Transp	0	9	9	1.41	7.59	1.67	0.0	7.0	-1.19	0.51	0.2348	0.6083	-1.3338	1.3338	0.1823
OGO	L4M4		Reg	Reg															
OGO	L4M4		Reg	Prot mod															
OGO	L4M4		Reg	Transcri	0	1	1	0.16	0.84	0.19	0.0	0.8	-0.40	0.17	0.6921	0.8644	-0.4327	0.4327	0.6652
OGO	L4M4		Reg	Transla	0	3	3	0.47	2.53	0.56	0.0	2.3	-0.69	0.30	0.4927	0.7673	-0.7545	0.7545	0.4505
OGO	L4M4		Devel	Devel	5	15	20	3.14	16.86	1.31	20.8	11.6	1.05	-0.45	0.2930	0.6501	1.2284	-1.2284	0.2193
OGO	L4M4		Devel	Cell div															
					24	129	153	24.00	129.00	27.08	100.00	100.00	SS	27.08					
OGO	F23L23		Sig & Res	Sig	51	3	51												
OGO	F23L23		Sig & Res	Stress	49	1	49												
OGO	F23L23		Metab	Bioen	61	1	61												
OGO	F23L23		Metab	Photosyn	23	0	23												
OGO	F23L23		Metab	Metab	117	32	117												
OGO	F23L23		Metab	Catab	17	1	17												
OGO	F23L23		Metab	Transp	46	0	46												
OGO	F23L23		Reg	Reg	-1	8	-1												
OGO	F23L23		Reg	Prot mod	2	2	2												
OGO	F23L23		Reg	Transcri	-6	4	-6												
OGO	F23L23		Reg	Transla	-3	33	-3												
OGO	F23L23		Devel	Devel	78	38	78												
OGO	F23L23		Devel	Cell div	13	21	13												
					447	144	591												
						0.3221													
	**Adjusted**																		
OGO	F23L23		Sig & Res	Sig	51	3	54	40.39	13.61	11.06	11.2	1.9	1.67	-2.88	0.0950	0.0040	3.4830	-3.4830	0.0005
OGO	F23L23		Sig & Res	Stress	49	1	50	37.40	12.60	14.28	10.7	0.6	1.90	-3.27	0.0578	0.0011	3.9439	-3.9439	0.0001
OGO	F23L23		Metab	Bioen	61	1	62	46.37	15.63	18.30	13.3	0.6	2.15	-3.70	0.0317	0.0002	4.5135	-4.5135	0.0000
OGO	F23L23		Metab	Photosyn	23	0	23	17.20	5.80	7.75	5.0	0.0	1.40	-2.41	0.1622	0.0161	2.8379	-2.8379	0.0045
OGO	F23L23		Metab	Metab	117	32	149	111.45	37.55	1.10	25.6	20.8	0.53	-0.91	0.5988	0.3647	1.2053	-1.2053	0.2281
OGO	F23L23		Metab	Catab	17	1	18	13.46	4.54	3.69	3.7	0.6	0.96	-1.66	0.3351	0.0968	1.9489	-1.9489	0.0513
OGO	F23L23		Metab	Transp	46	0	46	34.41	11.59	15.50	10.1	0.0	1.98	-3.41	0.0481	0.0007	4.0943	-4.0943	0.0000
OGO	F23L23		Reg	Reg	0	9	9	6.73	2.27	26.71	0.0	5.8	-2.59	4.47	0.0095	0.0000	-5.2064	5.2064	0.0000
OGO	F23L23		Reg	Prot mod	2	2	4	2.99	1.01	1.30	0.4	1.3	-0.57	0.99	0.5664	0.3233	-1.1459	1.1459	0.2518
OGO	F23L23		Reg	Transcri	0	10	10	7.48	2.52	29.68	0.0	6.5	-2.73	4.71	0.0062	0.0000	-5.4926	5.4926	0.0000

OGO	F23L23	Reg	Transla	0	36	36	26.93	9.07	106.83	0.0	23.4	-5.19	8.94	0.0000	0.0000	-10.6546	10.6546	0.0000
OGO	F23L23	Devel	Devel	78	38	116	86.76	29.24	3.51	17.1	24.7	-0.94	1.62	0.3468	0.1051	-2.0818	2.0818	0.0374
OGO	F23L23	Devel	Cell div	13	21	34	25.43	8.57	24.11	2.8	13.6	-2.46	4.25	0.0137	0.0000	-5.0525	5.0525	0.0000
				457	154	611	457.00	154.00	263.82	100.00	100.00	SS	263.82					
					0.337													
OGO	F23M22	Sig & Res	Sig	22	1	23												
OGO	F23M23	Sig & Res	Stress	26	0	26												
OGO	F23M23	Metab	Bioen	30	1	31												
OGO	F23M23	Metab	Photosyn	21	0	21												
OGO	F23M23	Metab	Metab	72	7	79												
OGO	F23M23	Metab	Catab	8	0	8												
OGO	F23M23	Metab	Transp	36	0	36												
OGO	F23M23	Reg	Reg	-3	0	-3												
OGO	F23M23	Reg	Prot mod	3	0	3												
OGO	F23M23	Reg	Transcri	-5	0	-5												
OGO	F23M23	Reg	Transla	1	0	1												
OGO	F23M23	Devel	Devel	41	18	59												
OGO	F23M23	Devel	Cell div	6	3	9												
				236	29	265 0												
					0.1229													
	**Adjusted**																	
OGO	F23M22	Sig & Res	Sig	22	1	23	21.77	3.11	1.43	8.3	2.6	0.05	-1.20	0.9611	0.2315	0.2205	-1.3429	0.8255
OGO	F23M23	Sig & Res	Stress	26	0	26	24.61	3.52	3.59	9.8	0.0	0.28	-1.88	0.7797	0.0608	1.2711	-2.1167	0.2037
OGO	F23M23	Metab	Bioen	30	1	31	29.35	4.19	2.45	11.3	2.6	0.12	-1.56	0.9038	0.1190	0.5547	-1.7775	0.5791
OGO	F23M23	Metab	Photosyn	21	0	21	19.88	2.84	2.90	7.9	0.0	0.25	-1.69	0.8015	0.0920	1.1313	-1.8839	0.2579
OGO	F23M23	Metab	Metab	72	7	79	74.78	10.68	1.37	27.1	18.4	-0.32	-1.13	0.7476	0.2598	-1.6428	-1.4293	0.1004
OGO	F23M23	Metab	Catab	8	0	8	7.57	1.08	1.11	3.0	0.0	0.16	-1.04	0.8767	0.2983	0.6814	-1.1348	0.4956
OGO	F23M23	Metab	Transp	36	0	36	34.08	4.87	4.98	13.5	0.0	0.33	-2.21	0.7420	0.0274	1.5259	-2.5410	0.1270
OGO	F23M23	Reg	Reg	0	3	3	2.84	0.41	19.43	0.0	7.9	-1.69	4.07	0.0920	0.0000	-7.3331	4.4035	0.0000
OGO	F23M23	Reg	Prot mod	3	0	3	2.84	0.41	0.41	1.1	0.0	0.10	-0.64	0.9243	0.5242	0.4135	-0.6886	0.6792
OGO	F23M23	Reg	Transcri	0	5	5	4.73	0.68	32.38	0.0	13.2	-2.18	5.26	0.0296	0.0000	-9.5012	5.7055	0.0000
OGO	F23M23	Reg	Transla	1	0	1	0.95	0.14	0.14	0.4	0.0	0.05	-0.37	0.9562	0.7131	0.2379	-0.3962	0.8120
OGO	F23M23	Devel	Devel	41	18	59	55.85	7.98	16.54	15.4	47.4	-1.99	3.55	0.0469	0.0004	-9.6764	4.2923	0.0000
OGO	F23M23	Devel	Cell div	6	3	9	8.52	1.22	3.36	2.3	7.9	-0.86	1.62	0.3880	0.1061	-3.7975	1.7664	0.0001
				266	38	281 0	266.00	38.00	90.09	100.00	100.00	SS	90.09					
OGO	L23S23	Sig & Res	Sig															
OGO	L23S23	Sig & Res	Stress															
OGO	L23S23	Metab	Bioen	0	14	14	0.49	13.51	0.51	0.0	25.5	-0.70	0.13	0.4834	0.8937	-0.8264	0.8264	0.4086
OGO	L23S23	Metab	Photosyn															
OGO	L23S23	Metab	Metab	1	11	11	0.39	10.61	0.99	50.0	20.0	0.99	0.12	0.3230	0.9057	1.1249	0.7071	0.2606
OGO	L23S23	Metab	Catab	0	1	1	0.04	0.96	0.04	0.0	1.8	-0.19	0.04	0.8514	0.9715	-0.1925	0.1925	0.8474
OGO	L23S23	Metab	Transp	0	18	18	0.63	17.37	0.65	0.0	32.7	-0.79	0.15	0.4268	0.8795	-0.9864	0.9864	0.3239
OGO	L23S23	Reg	Reg															
OGO	L23S23	Reg	Prot mod															
OGO	L23S23	Reg	Transcri	0	1	1	0.04	0.96	0.04	0.0	1.8	-0.19	0.04	0.8514	0.9715	-0.1925	0.1925	0.8474
OGO	L23S23	Reg	Transla															
OGO	L23S23	Devel	Devel	1	10	10	0.35	9.65	1.21	50.0	18.2	1.10	0.11	0.2731	0.9101	1.2333	0.6667	0.2175
OGO	L23S23	Devel	Cell div	0	0		0.00	0.00										
				2	55	57	1.93	53.07	3.44	100.00	100.00	SS	3.44					
OGO	M23S23	Sig & Res	Sig															
OGO	M23S23	Sig & Res	Stress															
OGO	M23S23	Metab	Bioen	0	1	1	0.17	0.83	0.21	0.0	5.3	-0.42	0.19	0.6767	0.8483	-0.4691	0.4691	0.6390
OGO	M23S23	Metab	Photosyn															
OGO	M23S23	Metab	Metab	3	5	8	1.39	6.61	2.25	75.0	26.3	1.36	-0.63	0.1726	0.5315	1.8581	-1.8581	0.0632
OGO	M23S23	Metab	Catab															
OGO	M23S23	Metab	Transp															
OGO	M23S23	Reg	Reg															
OGO	M23S23	Reg	Prot mod															
OGO	M23S23	Reg	Transcri															
OGO	M23S23	Reg	Transla	0	7	7	1.22	5.78	1.47	0.0	36.8	-1.10	0.51	0.2699	0.6127	-1.4555	1.4555	0.1455
OGO	M23S23	Devel	Devel	1	6	7	1.22	5.78	0.05	25.0	31.6	-0.20	0.09	0.8438	0.9280	-0.2599	0.2599	0.7949

OGO	M23S23	Devel	Cell div															
				4	19	23	4.00	19.00	3.98	100.00	100.00	SS	3.98					
OGO	F23S23	Sig & Res	Sig	34	3	37												
OGO	F23S23	Sig & Res	Stress	45	2	47												
OGO	F23S23	Metab	Bioen	73	18	91												
OGO	F23S23	Metab	Photosyn	32	3	35												
OGO	F23S23	Metab	Metab	158	42	200												
OGO	F23S23	Metab	Catab	29	1	30												
OGO	F23S23	Metab	Transp	51	3	54												
OGO	F23S23	Reg	Reg	-5	8	3												
OGO	F23S23	Reg	Prot mod	4	4	8												
OGO	F23S23	Reg	Transcri	-9	8	-1												
OGO	F23S23	Reg	Transla	-4	57	53												
OGO	F23S23	Devel	Devel	57	97	154												
OGO	F23S23	Devel	Cell div	8	24	32												
				473	270	743												
	Adjusted																	
OGO	F23S23	Sig & Res	Sig	34	3	37	23.32	13.68	13.23	6.9	1.0	2.21	-2.89	0.0270	0.0039	3.7265	-3.7265	0.0002
OGO	F23S23	Sig & Res	Stress	45	2	47	29.62	17.38	21.59	9.2	0.7	2.83	-3.69	0.0047	0.0002	4.7930	-4.7930	0.0000
OGO	F23S23	Metab	Bioen	73	18	91	57.36	33.64	11.54	14.9	6.3	2.07	-2.70	0.0389	0.0070	3.6147	-3.6147	0.0003
OGO	F23S23	Metab	Photosyn	32	3	35	22.06	12.94	12.11	6.5	1.0	2.12	-2.76	0.0343	0.0057	3.5614	-3.5614	0.0004
OGO	F23S23	Metab	Metab	158	42	200	126.06	73.94	21.89	32.2	14.6	2.84	-3.71	0.0044	0.0002	5.4270	-5.4270	0.0000
OGO	F23S23	Metab	Catab	29	1	30	18.91	11.09	14.57	5.9	0.3	2.32	-3.03	0.0203	0.0024	3.8923	-3.8923	0.0001
OGO	F23S23	Metab	Transp	51	3	54	34.04	19.96	22.87	10.4	1.0	2.91	-3.80	0.0036	0.0001	4.9572	-4.9572	0.0000
OGO	F23S23	Reg	Reg	0	13	13	8.19	4.81	22.16	0.0	4.5	-2.86	3.74	0.0042	0.0002	-4.7476	4.7476	0.0000
OGO	F23S23	Reg	Prot mod	4	4	8	5.04	2.96	0.58	0.8	1.4	-0.46	0.61	0.6425	0.5444	-0.7674	0.7674	0.4429
OGO	F23S23	Reg	Transcri	0	17	17	10.72	6.28	28.98	0.0	5.9	-3.27	4.27	0.0011	0.0000	-5.4433	5.4433	0.0000
OGO	F23S23	Reg	Transla	0	61	61	38.45	22.55	104.00	0.0	21.2	-6.20	8.10	0.0000	0.0000	-10.6222	10.6222	0.0000
OGO	F23S23	Devel	Devel	57	97	154	97.07	56.93	44.73	11.6	33.7	-4.07	5.31	0.0000	0.0000	-7.4669	7.4669	0.0000
OGO	F23S23	Devel	Cell div	8	24	32	20.17	11.83	19.86	1.6	8.3	-2.71	3.54	0.0067	0.0004	-4.5510	4.5510	0.0000
				491	288	779	491.00	288.00	338.11	100.00	100.00	SS	338.11					
All GO	L123M123	Sig & Res	Sig	63	60	123	62.38	60.62	0.01	5.7	5.6	0.08	-0.08	0.9376	0.9367	0.1149	-0.1149	0.9085
All GO	L123M123	Sig & Res	Stress	79	88	167	84.70	82.30	0.78	7.2	8.3	-0.62	0.63	0.5359	0.5300	-0.9179	0.9179	0.3587
All GO	L123M123	Metab	Bioen	73	92	165	83.68	81.32	2.77	6.7	8.6	-1.17	1.18	0.2429	0.2362	-1.7307	1.7307	0.0835
All GO	L123M123	Metab	Photosyn	110	101	211	107.01	103.99	0.17	10.0	9.5	0.29	-0.29	0.7727	0.7695	0.4331	-0.4331	0.6649
All GO	L123M123	Metab	Metab	230	199	429	217.57	211.43	1.44	21.0	18.7	0.84	-0.85	0.3996	0.3928	1.3402	-1.3402	0.1802
All GO	L123M123	Metab	Catab	51	21	72	36.52	35.48	11.66	4.6	2.0	2.40	-2.43	0.0165	0.0150	3.4726	-3.4726	0.0005
All GO	L123M123	Metab	Transp	74	66	140	71.00	69.00	0.26	6.7	6.2	0.36	-0.36	0.7221	0.7183	0.5238	-0.5238	0.6004
All GO	L123M123	Reg	Reg	5	6	11	5.58	5.42	0.12	0.5	0.6	-0.25	0.25	0.8064	0.8037	-0.3500	0.3500	0.7264
All GO	L123M123	Reg	Prot mod	40	47	87	44.12	42.88	0.78	3.6	4.4	-0.62	0.63	0.5348	0.5289	-0.9026	0.9026	0.3667
All GO	L123M123	Reg	Transcri	26	23	49	24.85	24.15	0.11	2.4	2.2	0.23	-0.23	0.8177	0.8151	0.3321	-0.3321	0.7398
All GO	L123M123	Reg	Transla	30	32	62	31.44	30.56	0.13	2.7	3.0	-0.26	0.26	0.7967	0.7939	-0.3723	0.3723	0.7097
All GO	L123M123	Devel	Devel	281	279	560	284.01	275.99	0.06	25.6	26.2	-0.18	0.18	0.8581	0.8561	-0.2958	0.2958	0.7674
All GO	L123M123	Devel	Cell div	35	52	87	44.12	42.88	3.83	3.2	4.9	-1.37	1.39	0.1696	0.1635	-1.9970	1.9970	0.0458
				1097	1066	2163	1097.00	1066.00	22.12	100.00	100.00	SS	22.12					
All GO	L4M4	Sig & Res	Sig	116	239	355	138.84	216.16	6.17	6.0	7.9	-1.94	1.55	0.0526	0.1204	-2.5771	2.5771	0.0100
All GO	L4M4	Sig & Res	Stress	165	256	421	164.65	256.35	0.00	8.5	8.4	0.03	-0.02	0.9781	0.9824	0.0368	-0.0368	0.9706
All GO	L4M4	Metab	Bioen	129	199	328	128.28	199.72	0.01	6.6	6.6	0.06	-0.05	0.9490	0.9592	0.0847	-0.0847	0.9325
All GO	L4M4	Metab	Photosyn	188	287	475	185.77	289.23	0.04	9.7	9.5	0.16	-0.13	0.8698	0.8955	0.2209	-0.2209	0.8252
All GO	L4M4	Metab	Metab	402	560	962	376.22	585.78	2.90	20.6	18.5	1.33	-1.06	0.1839	0.2869	1.8958	-1.8958	0.0580
All GO	L4M4	Metab	Catab	89	147	236	92.30	143.70	0.19	4.6	4.8	-0.34	0.27	0.7315	0.7833	-0.4505	0.4505	0.6523
All GO	L4M4	Metab	Transp	112	218	330	129.06	200.94	3.70	5.7	7.2	-1.50	1.20	0.1332	0.2288	-1.9914	1.9914	0.0464
All GO	L4M4	Reg	Reg	8	23	31	12.12	18.88	2.30	0.4	0.8	-1.18	0.95	0.2363	0.3426	-1.5225	1.5225	0.1279
All GO	L4M4	Reg	Prot mod	57	85	142	55.53	86.47	0.06	2.9	2.8	0.20	-0.16	0.8441	0.8747	0.2557	-0.2557	0.7982
All GO	L4M4	Reg	Transcri	71	80	151	59.05	91.95	3.97	3.6	2.6	1.55	-1.25	0.1201	0.2128	2.0230	-2.0230	0.0431
All GO	L4M4	Reg	Transla	56	126	182	71.18	110.82	5.32	2.9	4.2	-1.80	1.44	0.0720	0.1494	-2.3488	2.3488	0.0188
All GO	L4M4	Devel	Devel	526	762	1288	503.72	784.28	1.62	27.0	25.1	0.99	-0.80	0.3208	0.4263	1.4775	-1.4775	0.1395
All GO	L4M4	Devel	Cell div	29	51	80	31.29	48.71	0.27	1.5	1.7	-0.41	0.33	0.6826	0.7432	-0.5282	0.5282	0.5974
				1948	3033	4981	1948.00	3033.00	26.56	100.00	100.00	SS	26.56					

All GO	F23L23	Sig & Res	Sig	746	234	980	692.66	287.34	14.01	8.1	6.1	2.03	-3.15	0.0427	0.0017	3.8912	-3.8912	0.0001
All GO	F23L23	Sig & Res	Stress	872	207	1079	762.64	316.36	53.49	9.4	5.4	3.96	-6.15	0.0001	0.0000	7.6351	-7.6351	0.0000
All GO	F23L23	Metab	Bioen	701	162	863	609.97	253.03	46.34	7.6	4.2	3.69	-5.72	0.0002	0.0000	7.0432	-7.0432	0.0000
All GO	F23L23	Metab	Photosyn	908	236	1144	808.58	335.42	41.69	9.8	6.1	3.50	-5.43	0.0005	0.0000	6.7592	-6.7592	0.0000
All GO	F23L23	Metab	Metab	1974	678	2652	1874.43	777.57	18.04	21.3	17.7	2.30	-3.57	0.0215	0.0004	4.7563	-4.7563	0.0000
All GO	F23L23	Metab	Catab	288	132	420	296.86	123.14	0.90	3.1	3.4	-0.51	0.80	0.6073	0.4249	-0.9648	0.9648	0.3346
All GO	F23L23	Metab	Transp	545	155	700	494.76	205.24	17.40	5.9	4.0	2.26	-3.51	0.0239	0.0005	4.2876	-4.2876	0.0000
All GO	F23L23	Reg	Reg	80	39	119	84.11	34.89	0.68	0.9	1.0	-0.45	0.70	0.6541	0.4866	-0.8312	0.8312	0.4058
All GO	F23L23	Reg	Prot mod	291	132	423	298.98	124.02	0.73	3.1	3.4	-0.46	0.72	0.6446	0.4739	-0.8660	0.8660	0.3865
All GO	F23L23	Reg	Transcri	287	298	585	413.48	171.52	131.95	3.1	7.8	-6.22	9.66	0.0000	0.0000	-11.7526	11.7526	0.0000
All GO	F23L23	Reg	Transla	105	279	384	271.41	112.59	347.99	1.1	7.3	-10.10	15.68	0.0000	0.0000	-18.9344	18.9344	0.0000
All GO	F23L23	Devel	Devel	2368	1094	3462	2446.94	########	8.69	25.6	28.5	-1.60	2.48	0.1105	0.0132	-3.4363	3.4363	0.0006
All GO	F23L23	Devel	Cell div	87	192	279	197.20	81.80	210.03	0.9	5.0	-7.85	12.18	0.0000	0.0000	-14.6492	14.6492	0.0000
				9252	3838	13090	9252.00	3838.00	891.93	100.00	100.00	SS	891.93					

All GO	F23M23	Sig & Res	Sig	375	106	481	366.53	114.47	0.82	7.3	6.6	0.44	-0.79	0.6583	0.4288	0.9407	-0.9407	0.3469
All GO	F23M23	Sig & Res	Stress	439	96	535	407.68	127.32	10.11	8.6	6.0	1.55	-2.78	0.1209	0.0055	3.3137	-3.3137	0.0009
All GO	F23M23	Metab	Bioen	363	85	448	341.39	106.61	5.75	7.1	5.3	1.17	-2.09	0.2421	0.0363	2.4818	-2.4818	0.0131
All GO	F23M23	Metab	Photosyn	503	63	566	431.31	134.69	50.08	9.8	3.9	3.45	-6.18	0.0006	0.0000	7.3940	-7.3940	0.0000
All GO	F23M23	Metab	Metab	1142	287	1429	1088.93	340.07	10.87	22.2	17.9	1.61	-2.88	0.1078	0.0040	3.7139	-3.7139	0.0002
All GO	F23M23	Metab	Catab	145	73	218	166.12	51.88	11.28	2.8	4.6	-1.64	2.93	0.1013	0.0034	-3.4150	3.4150	0.0006
All GO	F23M23	Metab	Transp	340	61	401	305.57	95.43	16.30	6.6	3.8	1.97	-3.52	0.0489	0.0004	4.1631	-4.1631	0.0000
All GO	F23M23	Reg	Reg	31	15	46	35.05	10.95	1.97	0.6	0.9	-0.68	1.23	0.4936	0.2206	-1.4082	1.4082	0.1591
All GO	F23M23	Reg	Prot mod	162	52	214	163.07	50.93	0.03	3.2	3.2	-0.08	0.15	0.9330	0.8804	-0.1751	0.1751	0.8610
All GO	F23M23	Reg	Transcri	128	133	261	198.89	62.11	106.17	2.5	8.3	-5.03	8.99	0.0000	0.0000	-10.5096	10.5096	0.0000
All GO	F23M23	Reg	Transla	72	62	134	102.11	31.89	37.31	1.4	3.9	-2.98	5.33	0.0029	0.0000	-6.1701	6.1701	0.0000
All GO	F23M23	Devel	Devel	1386	452	1838	1400.60	437.40	0.64	27.0	28.2	-0.39	0.70	0.6964	0.4851	-0.9379	0.9379	0.3483
All GO	F23M23	Devel	Cell div	47	118	165	125.73	39.27	207.18	0.9	7.4	-7.02	12.56	0.0000	0.0000	-14.5732	14.5732	0.0000
				5133	1603	6736	5133.00	1603.00	458.51	100.00	100.00	SS	458.51					

All GO	L23S23	Sig & Res	Sig	39	85	124	39.64	84.36	0.02	5.5	5.7	-0.10	0.07	0.9188	0.9443	-0.1272	0.1272	0.8988
All GO	L23S23	Sig & Res	Stress	48	89	137	43.80	93.20	0.59	6.8	5.9	0.63	-0.44	0.5254	0.6634	0.7950	-0.7950	0.4266
All GO	L23S23	Metab	Bioen	66	111	177	56.59	120.41	2.30	9.4	7.4	1.25	-0.86	0.2107	0.3909	1.5824	-1.5824	0.1136
All GO	L23S23	Metab	Photosyn	106	105	211	67.45	143.55	32.38	15.1	7.0	4.69	-3.22	0.0000	0.0013	5.9843	-5.9843	0.0000
All GO	L23S23	Metab	Metab	136	256	392	125.32	266.68	1.34	19.3	17.1	0.95	-0.65	0.3400	0.5131	1.2761	-1.2761	0.2019
All GO	L23S23	Metab	Catab	30	29	59	18.86	40.14	9.67	4.3	1.9	2.56	-1.76	0.0103	0.0787	3.1519	-3.1519	0.0016
All GO	L23S23	Metab	Transp	40	77	117	37.40	79.60	0.26	5.7	5.1	0.42	-0.29	0.6712	0.7711	0.5289	-0.5289	0.5969
All GO	L23S23	Reg	Reg	3	18	21	6.71	14.29	3.02	0.4	1.2	-1.43	0.98	0.1518	0.3259	-1.7460	1.7460	0.0808
All GO	L23S23	Reg	Prot mod	28	45	73	23.34	49.66	1.37	4.0	3.0	0.97	-0.66	0.3345	0.5082	1.1901	-1.1901	0.2340
All GO	L23S23	Reg	Transcri	22	79	101	32.29	68.71	4.82	3.1	5.3	-1.81	1.24	0.0702	0.2145	-2.2475	2.2475	0.0246
All GO	L23S23	Reg	Transla	10	130	140	44.76	95.24	39.67	1.4	8.7	-5.20	3.56	0.0000	0.0004	-6.5094	6.5094	0.0000
All GO	L23S23	Devel	Devel	163	452	615	196.61	418.39	8.45	23.2	30.2	-2.40	1.64	0.0165	0.1004	-3.4241	3.4241	0.0006
All GO	L23S23	Devel	Cell div	12	20	32	10.23	21.77	0.45	1.7	1.3	0.55	-0.38	0.5800	0.7044	0.6758	-0.6758	0.4991
				703	1496	2199	703.00	1496.00	104.34	100.00	100.00	SS	104.34					

All GO	M23S23	Sig & Res	Sig	36	85	121	40.15	80.85	0.64	5.5	6.5	-0.65	0.46	0.5126	0.6445	-0.8268	0.8268	0.4083
All GO	M23S23	Sig & Res	Stress	72	89	161	53.42	107.58	9.67	11.0	6.8	2.54	-1.79	0.0110	0.0732	3.2455	-3.2455	0.0012
All GO	M23S23	Metab	Bioen	47	84	131	43.47	87.53	0.43	7.2	6.4	0.54	-0.38	0.5920	0.7057	0.6786	-0.6786	0.4974
All GO	M23S23	Metab	Photosyn	67	86	153	50.77	102.23	7.77	10.3	6.5	2.28	-1.61	0.0227	0.1084	2.9025	-2.9025	0.0037
All GO	M23S23	Metab	Metab	130	254	384	127.41	256.59	0.08	19.9	19.3	0.23	-0.16	0.8188	0.8717	0.3125	-0.3125	0.7547
All GO	M23S23	Metab	Catab	19	41	60	19.91	40.09	0.06	2.9	3.1	-0.20	0.14	0.8387	0.8859	-0.2530	0.2530	0.8003
All GO	M23S23	Metab	Transp	29	60	89	29.53	59.47	0.01	4.4	4.6	-0.10	0.07	0.9222	0.9451	-0.1223	0.1223	0.9027
All GO	M23S23	Reg	Reg	3	15	18	5.97	12.03	2.21	0.5	1.1	-1.22	0.86	0.2239	0.3914	-1.4948	1.4948	0.1350
All GO	M23S23	Reg	Prot mod	37	38	75	24.89	50.11	8.83	5.7	2.9	2.43	-1.71	0.0152	0.0870	3.0292	-3.0292	0.0025
All GO	M23S23	Reg	Transcri	27	86	113	37.49	75.51	4.40	4.1	6.5	-1.71	1.21	0.0866	0.2272	-2.1596	2.1596	0.0308
All GO	M23S23	Reg	Transla	13	61	74	24.55	49.45	8.14	2.0	4.6	-2.33	1.64	0.0197	0.1004	-2.9077	2.9077	0.0036
All GO	M23S23	Devel	Devel	147	390	537	178.18	358.82	8.17	22.5	29.7	-2.34	1.65	0.0195	0.0998	-3.3521	3.3521	0.0008
All GO	M23S23	Devel	Cell div	25	24	49	16.26	32.74	7.03	3.8	1.8	2.17	-1.53	0.0302	0.1266	2.6858	-2.6858	0.0072
				652	1313	1965	652.00	1313.00	57.44	100.00	100.00	SS	57.44					

All GO	F23S23	Sig & Res	Sig	798	506	1304	739.81	564.19	10.58	7.2	6.0	2.14	-2.45	0.0324	0.0143	3.3666	-3.3666	0.0008
All GO	F23S23	Sig & Res	Stress	943	425	1368	776.12	591.88	82.94	8.5	5.0	5.99	-6.86	0.0000	0.0000	9.4427	-9.4427	0.0000
All GO	F23S23	Metab	Bioen	881	387	1268	719.38	548.62	83.92	7.9	4.6	6.03	-6.90	0.0000	0.0000	9.4725	-9.4725	0.0000
All GO	F23S23	Metab	Photosyn	1277	471	1748	991.71	756.29	189.69	11.5	5.6	9.06	-10.37	0.0000	0.0000	14.4320	-14.4320	0.0000
All GO	F23S23	Metab	Metab	2405	1343	3748	2126.38	########	84.38	21.6	15.8	6.04	-6.92	0.0000	0.0000	10.2149	-10.2149	0.0000

All GO	F23S23	Metab	Catab	440	254	694	393.73	300.27	12.57	4.0	3.0	2.33	-2.67	0.0197	0.0076	3.6094	-3.6094	0.0003
All GO	F23S23	Metab	Transp	688	345	1033	586.06	446.94	40.98	6.2	4.1	4.21	-4.82	0.0000	0.0000	6.5776	-6.5776	0.0000
All GO	F23S23	Reg	Reg	59	82	141	79.99	61.01	12.74	0.5	1.0	-2.35	2.69	0.0189	0.0072	-3.5815	3.5815	0.0003
All GO	F23S23	Reg	Prot mod	373	263	636	360.83	275.17	0.95	3.4	3.1	0.64	-0.73	0.5216	0.4630	0.9905	-0.9905	0.3219
All GO	F23S23	Reg	Transcri	262	643	905	513.44	391.56	284.60	2.4	7.6	-11.10	12.71	0.0000	0.0000	-17.2737	17.2737	0.0000
All GO	F23S23	Reg	Transla	134	846	980	555.99	424.01	740.27	1.2	10.0	-17.90	20.49	0.0000	0.0000	-27.9151	27.9151	0.0000
All GO	F23S23	Devel	Devel	2750	2489	5239	2972.28	########	38.42	24.7	29.4	-4.08	4.67	0.0000	0.0000	-7.2421	7.2421	0.0000
All GO	F23S23	Devel	Cell div	103	421	524	297.28	226.72	293.46	0.9	5.0	-11.27	12.90	0.0000	0.0000	-17.3646	17.3646	0.0000
				11113	8475	19588	11113.00	8475.00	1875.49	100.00	100.00	SS	1875.49					
