# Supplementary material for: Whether Gametophytes Are Reduced or Unreduced in Angiosperms Might Be Determined Metabolically
Source: Genes (Basel). 2020 Dec 2;11(12):1449. doi: 10.3390/genes11121449 (PMC7761559; doi:10.3390/genes11121449)
Supplement: Supplementary file 1 [file genes-11-01449-s001.zip › Table S4 qPCR primers.pdf]

**Table S4.** Primers used for qRT-PCR verification of microarray data.

| Gene | Name | Dir. | Primer | Tm |
|---|---|---|---|---|
| AT1G32640 | MYC2 | F | CGACGACAACGCTTCTATGA | 60.4 |
| | | R | CCAACCTTCGTGTGTTCCTT | 62.4 |
| AT5G20240 | PI | F | CTTCCATGGATCTTGGTGCT | 60.4 |
| | | R | ATCTCCATCTGGTGGTCTCG | 60.4 |
| AT1G69120 | AP1 | F | GCAAGCAATGAGCCCTAAAG | 60.4 |
| | | R | ACTGCTCCTGTTGAGCCCTA | 62.4 |
| AT5G53250 | AGP22 | F | CCTGAAATTTCCGTTGGAGA | 58.4 |
| | | R | GCCAAAGCCACCATCATTAG | 60.4 |
| AT4G09960 | STK | F | TGCCGCGTACTATCAACAAG | 60.4 |
| | | R | TGCTTCTTGGACCTGATCCT | 60.4 |
| AT1G20930 | CDKB2 | F | ATCTTTGCGGGAGACTCTGA | 60.4 |
| | | R | GTTTTGCTGGCTCGTACTCC | 62.4 |
| AT5G51230 | EMF2 | F | CGTTTCTCCTAAGCCTGTCG | 62.4 |
| | | R | GATCCTGATTTTGCCTCCAA | 58.4 |
| AT3G52940 | FK | F | TGTTACTGGGCGAAGTTCCT | 60.4 |
| | | R | GGAACCATCCTGCACACTTT | 60.4 |
| AT3G13160 | PPR | F | GTTCAAGAAAGCCTGCCAAG | 60.4 |
| | | R | GGCATTTCGTCGAACACTTT | 58.4 |
| AT5G61380 | TOC1 | F | GATCTCCCAATGGCTAAGG | 60.4 |
| | | R | CATGCGTCTTCTTCTCCACA | 60.4 |
| AT4G30270 | MERI5B | F | CAACGGCCAGCTTCTTACTC | 62.4 |
| | | R | TCATCCCAAGTGGATCCTTC | 60.4 |
| AT4G25100 | FSD1 | F | TGGAGGAAAACCATCAGGAG | 60.4 |
| | | R | GGATTCACAGCATTGGGAGT | 60.4 |
| AT3G13580 | RPL7D | F | TCATCTGCGTTGAGGATCTG | 60.4 |
| | | R | GATGAAGTTCTCGCGGTTTC | 60.4 |
| AT2G35940 | BLH1 | F | AACATGGGATCCATGGAAAA | 56.3 |
| | | R | ATTGTCTCGTCGCTGGTTCT | 60.4 |
