# Supplementary material for: Whether Gametophytes Are Reduced or Unreduced in Angiosperms Might Be Determined Metabolically
Source: Genes (Basel). 2020 Dec 2;11(12):1449. doi: 10.3390/genes11121449 (PMC7761559; doi:10.3390/genes11121449)
Supplement: Supplementary file 1 [file genes-11-01449-s001.zip › Table S5 DEGs.pdf]

Table S5. Differentially expressed genes for 17 microarray comparisons involving ovules from facultatively aposporous B. microphylla Cache (M), facultatively diplosporous B. lignifera (L), diplosporous B. x formosa (FH) population, and pistils from sexual B. stricta (SP). Ovule stages 1-4: MMC, active meiocyte, young gametophyte, and mature gametophyte, respectively. Pistil stage 3: young gametophyte.

Line no.		Comparison	ProbeSet	*A. thaliana* ortholog	*A. thaliana* gene description	L1-3	M1-3	Fold	p-value	FDR
1	1	L1-3 vs. M1-3	245008_at	ATCG00360	Encodes a protein required for photosystem I assembly and stability. In *Chlamydomonas reinhardtii* , this protein seems to act as a PSI specific chaperone facilitating the assembly of the complex by interacting with PsaA and PsaD. A loss of function mutation in tobacco leads to a loss of photosystem I.	4538.94	169.74	26.74	< 1e-07	< 1e-07
2	1	L1-3 vs. M1-3	257890_s_a	AT3G42570	peroxidase family protein;(source:Araport11)	329.68	32.03	10.29	4.40E-05	1.17E-03
3	1	L1-3 vs. M1-3	250508_at	AT5G09950	Encodes a DYW-class PPR protein required for RNA editing at four sites in mitochondria of A. thaliana.	118.54	12.21	9.71	3.00E-07	4.26E-05
4	1	L1-3 vs. M1-3	253487_at	AT4G31700	Encodes a putative ribosomal protein S6 (rps6a). RPS6A and RPS6B are fully redundant and essential during gametogenesis.	319.97	44.72	7.15	< 1e-07	< 1e-07
5	1	L1-3 vs. M1-3	256243_at	AT3G12500	encodes a basic chitinase involved in ethylene/jasmonic acid mediated signalling pathway during systemic acquired resistance based on expression analyses.	146.13	21.55	6.78	8.00E-07	8.73E-05
6	1	L1-3 vs. M1-3	254098_at	AT4G25100	Fe-superoxide dismutase	208.70	35.72	5.84	3.76E-04	5.18E-03
7	1	L1-3 vs. M1-3	247163_at	AT5G65685	UDP-Glycosyltransferase superfamily protein;(source:Araport11)	61.04	10.94	5.58	5.00E-07	5.91E-05
8	1	L1-3 vs. M1-3	252372_at	AT3G48000	Encodes a putative (NAD+) aldehyde dehydrogenase.	149.09	33.39	4.47	2.87E-05	8.40E-04
9	1	L1-3 vs. M1-3	245330_at	AT4G14930	Survival protein SurE-like phosphatase/nucleotidase;(source:Araport11)	253.57	61.49	4.12	5.27E-05	1.36E-03
10	1	L1-3 vs. M1-3	244971_at	ATCG00670	Encodes the only ClpP (caseinolytic protease) encoded within the plastid genome. Contains a highly conserved catalytic triad of Ser-type proteases (Ser-His-Asp). Part of the 350 kDa chloroplast Clp complex. The name reflects nomenclature described in Adam et. al (2001).	5528.25	1420.42	3.89	5.00E-06	2.73E-04
11	1	L1-3 vs. M1-3	252095_at	AT3G51000	alpha/beta-Hydrolases superfamily protein;(source:Araport11)	138.94	36.90	3.76	6.42E-05	1.56E-03
12	1	L1-3 vs. M1-3	256091_at	AT1G20693	Encodes a protein belonging to the subgroup of HMGB (high mobility group B) proteins that have a distinctive DNA-binding motif, the HMG-box domain. The motif confers non-sequence specific interaction with linear DNA and structure-specific binding to distorted DNA sites. The HMGB proteins are involved in the assembly of nucleoprotein complexes. Can be phosphorylated by CK2alpha. The mRNA is cell-to-cell mobile.	93.85	29.07	3.23	1.10E-06	1.01E-04
13	1	L1-3 vs. M1-3	246619_at	AT5G36290	Member of the UPF0016 family of membrane proteins, belongs to the conserved group of Mn/Ca transporters.	107.14	33.47	3.20	4.80E-06	2.72E-04
14	1	L1-3 vs. M1-3	252267_at	AT3G49640	Aldolase-type TIM barrel family protein;(source:Araport11)	66.74	21.41	3.12	9.00E-07	9.46E-05
15	1	L1-3 vs. M1-3	257004_s_a	AT3G14130	Aldolase-type TIM barrel family protein;(source:Araport11)	72.75	23.64	3.08	2.70E-06	1.78E-04
16	1	L1-3 vs. M1-3	264223_s_a	AT1G67520	lectin protein kinase family protein;(source:Araport11)	28.54	9.26	3.08	4.01E-04	5.44E-03
17	1	L1-3 vs. M1-3	261819_at	AT1G11410	S-locus lectin protein kinase family protein;(source:Araport11)	64.12	21.22	3.02	5.70E-06	2.89E-04
18	1	L1-3 vs. M1-3	258054_at	AT3G16240	Delta tonoplast intrinsic protein, functions as a water channel and ammonium (NH3) transporter. Highly expressed in flower, shoot, and stem. Expression shows diurnal regulation and is induced by ammonium (NH3). Protein localized to vacuolar membrane. The mRNA is cell-to-cell mobile.	379.24	127.47	2.98	2.67E-05	7.98E-04
19	1	L1-3 vs. M1-3	260201_at	AT1G67600	Acid phosphatase/vanadium-dependent haloperoxidase-related protein;(source:Araport11)	111.06	38.26	2.90	3.00E-07	4.26E-05
20	1	L1-3 vs. M1-3	251304_at	AT3G61990	Encodes a protein methyltransferase. Involved in the methylation of plant transmembrane proteins.	41.61	14.45	2.88	2.06E-04	3.40E-03
21	1	L1-3 vs. M1-3	266352_at	AT2G01610	Plant invertase/pectin methylesterase inhibitor superfamily protein;(source:Araport11)	65.54	23.65	2.77	4.05E-05	1.10E-03
22	1	L1-3 vs. M1-3	244912_at	ATMG00830	cytochrome c biogenesis orf382	360.31	132.68	2.72	5.28E-05	1.36E-03
23	1	L1-3 vs. M1-3	253041_at	AT4G37870	Encodes a phosphoenolpyruvate carboxykinase that localizes to the cytosol.	610.79	224.95	2.72	2.13E-04	3.43E-03
24	1	L1-3 vs. M1-3	251265_at	AT3G62310	RNA helicase family protein;(source:Araport11)	114.48	42.38	2.70	6.98E-04	7.56E-03

25	1	L1-3 vs. M1-3	252950_at	AT4G38690	PLC-like phosphodiesterases superfamily protein;(source:Araport11)	27.28	10.32	2.64	2.16E-04	3.46E-03
26	1	L1-3 vs. M1-3	258846_at	AT3G03070	NADH-ubiquinone oxidoreductase-like protein;(source:Araport11)	22.34	8.49	2.63	2.50E-06	1.76E-04
27	1	L1-3 vs. M1-3	245292_at	AT4G15093	catalytic LigB subunit of aromatic ring-opening dioxygenase family;(source:Araport11)	106.67	40.57	2.63	5.30E-06	2.79E-04
28	1	L1-3 vs. M1-3	253684_at	AT4G29690	Alkaline-phosphatase-like family protein;(source:Araport11)	82.33	31.67	2.60	9.85E-05	2.09E-03
29	1	L1-3 vs. M1-3	250736_s_a	AT1G01350	Zinc finger (CCCH-type/C3HC4-type RING finger) family protein;(source:Araport11)	424.15	164.69	2.58	1.28E-05	5.02E-04
30	1	L1-3 vs. M1-3	262885_at	AT1G64740	alpha-tubulin expressed primarily in stamens and mature pollen	162.90	63.42	2.57	9.30E-06	4.00E-04
31	1	L1-3 vs. M1-3	254824_s_a t	AT4G12620	Origin Recognition Complex subunit 1b. Involved in the initiation of DNA replication. Regulated transcriptionally during cell cycle, peaking at G1/S-phase. Target of E2F/DF family of transcription factors. Interacts with ORC2 and ORC5. Highly expressed in proliferating cells. Expression levels are independent of light regime.	307.26	120.51	2.55	1.69E-05	5.78E-04
32	1	L1-3 vs. M1-3	258401_at	AT3G15430	Regulator of chromosome condensation (RCC1) family protein;(source:Araport11)	165.03	64.99	2.54	1.06E-05	4.24E-04
33	1	L1-3 vs. M1-3	248200_at	AT5G54160	A caffeic acid/5-hydroxyferulic acid O-methyltransferase. Interacts with 14-4-3 proteins in yeast 2 hybrid assay. AtOMT1 (At5g54160) encodes a flavonol 3?-O-methyltransferase that is highly active towards quercetin and myricetin. The substrate specificity identifies the enzyme as flavonol 3?-methyltransferase which replaces the former annotation of the gene to encode a caffeic acid/5-hydroxyferulic acid O-methyltransferase The mRNA is cell-to-cell mobile.	542.75	215.97	2.51	1.00E-06	9.79E-05
34	1	L1-3 vs. M1-3	259498_at	AT1G15880	Golgi SNARE 11 protein (GOS11)	356.52	148.57	2.40	1.60E-04	2.96E-03
35	1	L1-3 vs. M1-3	251099_at	AT5G01660	influenza virus NS1A-binding protein;(source:Araport11)	119.78	50.32	2.38	3.47E-05	9.65E-04
36	1	L1-3 vs. M1-3	255513_at	AT4G02060	Member of the minichromosome maintenance complex, involved in DNA replication initiation. Abundant in proliferating and endocycling tissues. Localized in the nucleus during G1, S and G2 phases of the cell cycle, and are released into the cytoplasmic compartment during mitosis. Binds chromatin.	24.21	10.22	2.37	1.64E-04	3.00E-03
37	1	L1-3 vs. M1-3	259029_at	AT3G09300	OSBP(oxysterol binding protein)-related protein 3B;(source:Araport11)	223.00	94.57	2.36	5.00E-07	5.91E-05
38	1	L1-3 vs. M1-3	248793_at	AT5G47240	nudix hydrolase homolog 8;(source:Araport11)	62.64	26.50	2.36	4.77E-04	5.88E-03
39	1	L1-3 vs. M1-3	254851_at	AT4G12010	Leucine-rich repeat domain (NLR) receptor. Dominant negative alleles suppress catma3 autoimmunity. Co-regulates with WRKY19 basal levels of immunity to root-knot nematodes.	24.59	10.60	2.32	4.30E-06	2.60E-04
40	1	L1-3 vs. M1-3	258448_at	AT3G22290	Endoplasmic reticulum vesicle transporter protein;(source:Araport11)	78.40	34.09	2.30	2.24E-05	6.84E-04
41	1	L1-3 vs. M1-3	265387_at	AT2G20670	sugar phosphate exchanger, putative (DUF506);(source:Araport11)	61.49	26.71	2.30	1.68E-04	3.04E-03
42	1	L1-3 vs. M1-3	248580_at	AT5G49890	member of Anion channel protein family	114.28	49.93	2.29	6.00E-05	1.51E-03
43	1	L1-3 vs. M1-3	267492_at	AT2G30620	winged-helix DNA-binding transcription factor family protein;(source:Araport11)	226.77	100.00	2.27	1.63E-05	5.71E-04
44	1	L1-3 vs. M1-3	246508_at	AT5G16150	Encodes a putative plastidic glucose transporter.	419.83	188.86	2.22	3.07E-04	4.40E-03
45	1	L1-3 vs. M1-3	267082_at	AT2G41140	Encodes CDPK-related kinase 1 (CRK1).	116.47	52.97	2.20	1.00E-06	9.79E-05
46	1	L1-3 vs. M1-3	247918_at	AT5G57610	kinase superfamily with octicosapeptide/Phox/Bem1p domain-containing protein;(source:Araport11)	104.25	48.19	2.16	3.17E-05	9.09E-04
47	1	L1-3 vs. M1-3	265195_at	AT2G36730	Pentatricopeptide repeat (PPR) superfamily protein;(source:Araport11)	31.18	14.41	2.16	7.52E-04	8.11E-03
48	1	L1-3 vs. M1-3	257892_at	AT3G17020	Adenine nucleotide alpha hydrolases-like superfamily protein;(source:Araport11)	266.70	124.58	2.14	2.60E-06	1.76E-04
49	1	L1-3 vs. M1-3	260042_at	AT1G68820	Putative C3HC4 zinc-finger ubiquitin E3 ligase, negative regulator in ABA and drought stress response.	42.86	20.27	2.11	3.28E-04	4.63E-03
50	1	L1-3 vs. M1-3	255997_s_a t	AT1G29930	Subunit of light-harvesting complex II (LHCII),which absorbs light and transfers energy to the photosynthetic reaction center. The mRNA is cell-to-cell mobile.	1106.95	525.73	2.11	3.57E-04	4.94E-03
51	1	L1-3 vs. M1-3	263924_at	AT2G36530	Involved in light-dependent cold tolerance and encodes an enolase. Protein is tyrosine-phosphorylated and its phosphorylation state is modulated in response to ABA in Arabidopsis thaliana seeds.	439.72	209.39	2.10	4.05E-05	1.10E-03
52	1	L1-3 vs. M1-3	259911_at	AT1G72680	cinnamyl-alcohol dehydrogenase;(source:Araport11)	93.69	44.88	2.09	1.00E-04	2.11E-03
53	1	L1-3 vs. M1-3	250062_at	AT5G17760	P-loop containing nucleoside triphosphate hydrolases superfamily protein;(source:Araport11)	34.17	16.32	2.09	8.13E-04	8.54E-03
54	1	L1-3 vs. M1-3	251383_at	AT3G60740	Encodes tubulin-folding cofactor D. Mutants arrest during embryogenesis with embryos that are small, mushroom-shaped (Primepilz Prime) and consist of only one or few large cells each containing one or more variably enlarged nuclei and often cell wall stubs. Gene product necessary for continuous microtubule organization.	16.98	8.17	2.08	1.50E-06	1.22E-04
55	1	L1-3 vs. M1-3	262537_s_a t	AT1G17280	Group XIV ubiquitin-conjugating enzyme that functions negative regulation of drought stress.	241.70	116.15	2.08	4.34E-04	5.68E-03
56	1	L1-3 vs. M1-3	258314_at	AT3G16100	RAB GTPase homolog G3C;(source:Araport11)	31.99	15.51	2.06	5.60E-06	2.89E-04
57	1	L1-3 vs. M1-3	247650_at	AT5G59960	K-stimulated pyrophosphate-energized sodium pump protein;(source:Araport11)	68.44	33.26	2.06	6.08E-04	6.91E-03

58	1	L1-3 vs. M1-3	260579_at	AT2G47380	Cytochrome c oxidase subunit Vc family protein;(source:Araport11)	218.43	106.57	2.05	< 1e-07	2.58E-05
59	1	L1-3 vs. M1-3	257784_at	AT3G26980	membrane-anchored ubiquitin-fold protein 4 precursor;(source:Araport11)	60.20	29.53	2.04	2.80E-04	4.20E-03
60	1	L1-3 vs. M1-3	266358_at	AT2G32280	Encodes a member of a plant-specific gene family that is required for embryo provasculature development. The gene product regulates vascular network complexity and connectivity in cotyledons.	108.04	53.06	2.04	3.07E-04	4.40E-03
61	1	L1-3 vs. M1-3	245436_at	AT4G16620	nodulin MtN21-like transporter family protein	26.84	13.21	2.03	2.13E-04	3.43E-03
62	1	L1-3 vs. M1-3	245308_at	AT4G17486	PPPDE putative thiol peptidase family protein;(source:Araport11)	218.18	107.79	2.02	1.82E-05	6.01E-04
63	1	L1-3 vs. M1-3	253225_at	AT4G35020	A member of ROP GTPase gene family; Encodes a Rho-like GTP binding protein.	279.14	138.70	2.01	4.17E-04	5.56E-03
64	1	L1-3 vs. M1-3	259928_at	AT1G34380	5-3 exonuclease family protein;(source:Araport11)	96.47	47.95	2.01	5.45E-04	6.36E-03
65	1	L1-3 vs. M1-3	253203_at	AT4G34710	Encodes a arginine decarboxylase (ADC), a rate-limiting enzyme that catalyzes the first step of polyamine (PA) biosynthesis via ADC pathway in Arabidopsis thaliana. Arabidopsis genome has two ADC paralogs, ADC1 and ADC2. ADC2 is stress-inducible (osmotic stress). Double mutant analysis showed that ADC genes are essential for the production of PA, and are required for normal seed development. Overexpression causes phenotypes similar to GA-deficient plants and these plants show reduced levels of GA due to lower expression levels of AtGA20ox1, AtGA3ox3 and AtGA3ox1.	231.72	116.53	1.99	6.34E-04	7.14E-03
66	1	L1-3 vs. M1-3	265187_at	AT1G23490	Gene encoding ADP-ribosylation factor and similar to other ARFs and ARF-like proteins. A member of ARF GTPase family. Arabidopsis has 21 known members, known to be essential for vesicle coating and uncoating and functions in GTP-binding. The gene is shown to play a role in cell division, cell expansion and cellulose production using antisense construct.	236.39	119.68	1.98	1.16E-04	2.35E-03
67	1	L1-3 vs. M1-3	253477_at	AT4G32320	Encodes a cytosolic ascorbate peroxidase APX6. Ascorbate peroxidases are enzymes that scavenge hydrogen peroxide in plant cells. Eight types of APX have been described for Arabidopsis: three cytosolic (APX1, APX2, APX6), two chloroplastic types (stromal sAPX, thylakoid tAPX), and three microsomal (APX3, APX4, APX5) isoforms.	84.33	42.85	1.97	4.10E-06	2.53E-04
68	1	L1-3 vs. M1-3	248967_at	AT5G45350	proline-rich family protein;(source:Araport11)	213.37	109.75	1.94	1.30E-06	1.09E-04
69	1	L1-3 vs. M1-3	266925_at	AT2G45740	member of the peroxin11 (PEX11) gene family, integral to peroxisome membrane, controls peroxisome proliferation. The mRNA is cell-to-cell mobile.	502.15	259.01	1.94	1.00E-05	4.09E-04
70	1	L1-3 vs. M1-3	251834_at	AT3G55170	Ribosomal L29 family protein;(source:Araport11)	12.97	6.68	1.94	7.88E-04	8.34E-03
71	1	L1-3 vs. M1-3	256926_at	AT3G22540	hypothetical protein (DUF1677);(source:Araport11)	81.75	42.27	1.93	4.97E-04	6.08E-03
72	1	L1-3 vs. M1-3	267586_at	AT2G41950	DNA-directed RNA polymerase subunit beta;(source:Araport11)	25.07	13.08	1.92	2.56E-04	3.94E-03
73	1	L1-3 vs. M1-3	257029_at	AT3G19240	Vacuolar import/degradation, Vid27-related protein;(source:Araport11)	144.29	75.51	1.91	2.00E-07	3.78E-05
74	1	L1-3 vs. M1-3	257771_at	AT3G23000	Encodes a serine/threonine protein kinase with similarities to CBL-interacting protein kinases, SNF1 and SOS2. The mRNA is cell-to-cell mobile.	28.41	14.85	1.91	1.40E-05	5.30E-04
75	1	L1-3 vs. M1-3	252414_at	AT3G47420	Encodes a Pi starvation-responsive protein AtPS3. A member of the phosphate starvation-induced glycerol-3-phosphate permease gene family: AT3G47420(G3Pp1), AT4G25220(G3Pp2), AT1G30560(G3Pp3), AT4G17550(G3Pp4) and AT2G13100(G3Pp5). Its expression is responsive to phosphate (Pi) and not phosphite (Phi) in roots and shoots.	23.61	12.39	1.91	4.18E-04	5.56E-03
76	1	L1-3 vs. M1-3	261149_s_a	AT1G19550	Glutathione S-transferase family protein;(source:Araport11)	443.19	232.71	1.90	9.29E-04	9.38E-03
77	1	L1-3 vs. M1-3	261852_at	AT1G50440	RING/FYVE/PHD zinc finger superfamily protein;(source:Araport11)	65.27	34.68	1.88	9.16E-05	1.98E-03
78	1	L1-3 vs. M1-3	253670_at	AT4G30010	ATP-dependent RNA helicase;(source:Araport11)	137.25	72.81	1.88	3.38E-04	4.73E-03
79	1	L1-3 vs. M1-3	254577_at	AT4G19450	Major facilitator superfamily protein;(source:Araport11)	294.77	156.87	1.88	4.21E-04	5.58E-03
80	1	L1-3 vs. M1-3	259750_at	AT1G71130	encodes a member of the ERF (ethylene response factor) subfamily B-5 of ERF/AP2 transcription factor family. The protein contains one AP2 domain. There are 7 members in this subfamily.	35.68	19.13	1.87	6.22E-05	1.53E-03
81	1	L1-3 vs. M1-3	246154_at	AT5G19940	Enables plants to cope with moderate light stress and affects cadmium tolerance.	70.68	37.78	1.87	2.45E-04	3.84E-03
82	1	L1-3 vs. M1-3	267534_at	AT2G41900	AtOXS2 specifcally entered the nuclear under salt stress. Te specifc nuclear localization of AtOXS2 could play a role in salt tolerance at the molecular level. Tese results implied that AtOXS2 might target some downstream cis-elements which are required for salt stress responses	62.27	33.90	1.84	8.00E-06	3.60E-04
83	1	L1-3 vs. M1-3	253304_at	AT4G33640	costars family protein;(source:Araport11)	95.26	52.13	1.83	6.80E-04	7.45E-03
84	1	L1-3 vs. M1-3	261601_at	AT1G49670	molecular function has not been defined. Was shown involved in oxidative stress tolerance.	50.41	27.71	1.82	3.00E-07	4.26E-05
85	1	L1-3 vs. M1-3	264256_at	AT1G09270	Protein interacts with Agrobacterium proteins VirD2 and VirE2.	213.76	117.30	1.82	8.35E-04	8.65E-03

86	1	L1-3 vs. M1-3	258507_at	AT3G06500	Encodes an alkaline/neutral invertase which localizes in mitochondria. It may be modulating hormone balance in relation to the radicle emergence. Mutants display severely reduced shoot growth and reduced oxygen consumption. Mutant root development is not affected as reported for A/N-InvA mutant (inva) plants.The mRNA is cell-to-cell mobile.	138.08	76.31	1.81	2.00E-07	3.78E-05
87	1	L1-3 vs. M1-3	252127_at	AT3G50960	Encodes a protein that functions in microtubule assembly. PLP3a can bind to several different tubulin family members in Y2H assays. Plants with reduced levels of both PLP3a and PLP3b (At5g66410) show defects in cytokinesis, cortical microtubule array formation, oriented cell growth, and maintenance of proper ploidy.	46.68	25.79	1.81	1.30E-06	1.09E-04
88	1	L1-3 vs. M1-3	249876_at	AT5G23060	Encodes a chloroplast-localized protein that modulates cytoplasmic Ca2+ concentration and is crucial for proper stomatal regulation in response to elevations of external Ca2+. Phosphorylation of this protein is dependent on calcium.	35.19	19.49	1.81	1.02E-04	2.12E-03
89	1	L1-3 vs. M1-3	259727_at	AT1G60950	encodes a major leaf ferredoxin	2741.94	1515.30	1.81	1.49E-04	2.84E-03
90	1	L1-3 vs. M1-3	261793_at	AT1G16080	nuclear protein;(source:Araport11)	107.63	60.08	1.79	1.52E-04	2.88E-03
91	1	L1-3 vs. M1-3	252190_at	AT3G50170	transmembrane protein, putative (DUF247);(source:Araport11)	14.30	8.01	1.78	7.54E-05	1.73E-03
92	1	L1-3 vs. M1-3	266906_at	AT2G34585	transmembrane protein;(source:Araport11)	81.59	45.71	1.78	4.76E-04	5.88E-03
93	1	L1-3 vs. M1-3	265012_at	AT1G24470	Encodes one of the two Arabidopsis homologues to YBR159w encoding a S. cerevisiae beta-ketoacyl reductase (KCR), which catalyzes the first reduction during VLCFA (very long chain fatty acids, >18 carbon) elongation: KCR1 (At1g67730), KCR2 (At1g24470). Complementation of the yeast ybr159Delta mutant demonstrated that the two KCR proteins are divergent and that only AtKCR1 can restore heterologous elongase activity similar to the native yeast KCR gene.	30.06	16.92	1.78	5.23E-04	6.22E-03
94	1	L1-3 vs. M1-3	263113_at	AT1G03150	Acyl-CoA N-acyltransferases (NAT) superfamily protein;(source:Araport11)	96.60	54.72	1.77	8.38E-04	8.65E-03
95	1	L1-3 vs. M1-3	266275_at	AT2G29370	NAD(P)-binding Rossmann-fold superfamily protein;(source:Araport11)	30.27	17.22	1.76	6.72E-04	7.42E-03
96	1	L1-3 vs. M1-3	267027_at	AT2G38330	MATE efflux family protein;(source:Araport11)	35.63	20.31	1.75	2.50E-05	7.55E-04
97	1	L1-3 vs. M1-3	260986_at	AT1G53580	Mononuclear Fe(II)-containing member of the b-lactamase fold superfamily. ETHE1 is homodimeric in solution, exhibits low-level esterase activity, and specifically binds a single Fe(II) atom in the active site.	76.79	43.94	1.75	2.91E-04	4.27E-03
98	1	L1-3 vs. M1-3	245985_at	AT5G13120	Encodes a lumenal cyclophilin with peptidyl-prolyl isomerase activity that is associated with the NAD(P)H dehydrogenase complex in stromal regions of the thylakoid membrane. It is likely to be important for the accumulation of the hydrophobic domain of the NAD(P)H dehydrogenase complex. This complex is associated with PSI and is responsible for the reduction of plastoquinone.	625.06	359.13	1.74	3.12E-04	4.45E-03
99	1	L1-3 vs. M1-3	251117_at	AT3G63390	hypothetical protein;(source:Araport11)	121.71	70.15	1.73	1.73E-05	5.78E-04
100	1	L1-3 vs. M1-3	265872_at	AT2G01670	nudix hydrolase homolog 17;(source:Araport11)	104.76	60.64	1.73	3.36E-04	4.72E-03
101	1	L1-3 vs. M1-3	260789_s_a	AT3G06690	putative acyl-coenzyme A oxidase;(source:Araport11)	22.88	13.21	1.73	5.24E-04	6.22E-03
102	1	L1-3 vs. M1-3	255785_at	AT1G19920	encodes a chloroplast form of ATP sulfurylase.	67.41	39.19	1.72	6.90E-06	3.44E-04
103	1	L1-3 vs. M1-3	253967_at	AT4G26550	Got1/Sft2-like vescicle transport protein family;(source:Araport11)	99.06	57.53	1.72	7.57E-04	8.12E-03
104	1	L1-3 vs. M1-3	247192_at	AT5G65360	Histone superfamily protein;(source:Araport11)	27.06	15.74	1.72	7.84E-04	8.34E-03
105	1	L1-3 vs. M1-3	265479_at	AT2G15760	calmodulin-binding protein (DUF1645);(source:Araport11)	33.90	19.79	1.71	4.07E-05	1.10E-03
106	1	L1-3 vs. M1-3	245809_at	AT1G58440	Encodes a putative protein that has been speculated, based on sequence similarities, to have squalene monooxygenase activity.	73.81	43.12	1.71	9.57E-05	2.06E-03
107	1	L1-3 vs. M1-3	254658_at	AT4G18230	UDP-N-acetylglucosamine transferase subunit ALG14-like protein;(source:Araport11)	51.15	29.86	1.71	4.43E-04	5.72E-03
108	1	L1-3 vs. M1-3	245442_at	AT4G16710	glycosyltransferase family protein 28;(source:Araport11)	197.32	116.28	1.70	1.75E-04	3.10E-03
109	1	L1-3 vs. M1-3	248655_at	AT5G48760	Ribosomal protein L13 family protein;(source:Araport11)	346.40	203.91	1.70	2.31E-04	3.69E-03
110	1	L1-3 vs. M1-3	247609_at	AT5G60940	Member of CstF complex.	37.87	22.41	1.69	1.93E-04	3.29E-03
111	1	L1-3 vs. M1-3	248462_at	AT5G50960	Highly similar to Saccharomyces cerevisiae NBP35, locus YGL091C. Cytosolic protein that homodimerizes and can assemble both 4Fe-4S - type and 2Fe-2S - type clusters on its amino terminal and carboxy therminal respectively. Null mutants are embryo lethal.	158.70	93.71	1.69	3.43E-04	4.77E-03
112	1	L1-3 vs. M1-3	264526_at	AT1G10130	Encodes a golgi localized P2A-type Ca2+ ATPase involved in Mn nutrition and homeostasis.	138.00	81.59	1.69	5.33E-04	6.28E-03
113	1	L1-3 vs. M1-3	245885_at	AT5G09440	EXORDIUM like 4;(source:Araport11)	714.96	423.78	1.69	9.10E-04	9.26E-03
114	1	L1-3 vs. M1-3	261239_at	AT1G32930	Galactosyltransferase family protein;(source:Araport11)	264.56	157.05	1.68	8.33E-05	1.85E-03

115	1	L1-3 vs. M1-3	262844_at	AT1G14890	Plant invertase/pectin methylesterase inhibitor superfamily protein;(source:Araport11)	25.99	15.56	1.67	5.81E-05	1.47E-03
116	1	L1-3 vs. M1-3	265244_at	AT2G43020	Encodes a polyamine oxidase.	98.00	58.52	1.67	2.39E-04	3.77E-03
117	1	L1-3 vs. M1-3	248820_at	AT5G47060	hypothetical protein (DUF581);(source:Araport11)	84.59	50.79	1.67	4.56E-04	5.81E-03
118	1	L1-3 vs. M1-3	247986_at	AT5G56880	hypothetical protein;(source:Araport11)	148.75	90.95	1.64	2.13E-04	3.43E-03
119	1	L1-3 vs. M1-3	250465_at	AT5G10070	RNase L inhibitor protein-like protein;(source:Araport11)	70.60	43.18	1.64	6.70E-04	7.42E-03
120	1	L1-3 vs. M1-3	256112_at	AT1G16920	small GTP-binding protein (Rab11)similar to YPT3/RAB11 proteins in yeast and mammals, respectively. YPT3/RAB11 is involved in intracellular protein trafficking.	809.12	491.98	1.64	1.00E-03	9.91E-03
121	1	L1-3 vs. M1-3	253776_at	AT4G28390	Encodes a mitochondrial ADP/ATP carrier protein. Shown in heterologous systems to be located in the plasma membrane. Has comparable affinity for ADP and ATP (in E.coli).	423.21	259.43	1.63	2.64E-04	4.03E-03
122	1	L1-3 vs. M1-3	250033_at	AT5G18110	Putative cap-binding protein;(source:Araport11)	110.21	67.96	1.62	5.30E-04	6.27E-03
123	1	L1-3 vs. M1-3	265738_at	AT2G01350	At2g01350 encodes quinolinate phosphoribosyl transferase involved in NAD biosynthesis as shown by heterologous expression in E. coli.	21.62	13.43	1.61	5.22E-04	6.22E-03
124	1	L1-3 vs. M1-3	253272_at	AT4G34190	Encodes a stress enhanced protein that localizes to the thylakoid membrane and whose mRNA is upregulated in response to high light intensity. It may be involved in chlorophyll binding.	48.02	30.00	1.60	1.03E-04	2.13E-03
125	1	L1-3 vs. M1-3	262716_at	AT1G16470	Encodes 20S proteasome subunit PAB1 (PAB1).	394.45	245.81	1.60	1.35E-04	2.62E-03
126	1	L1-3 vs. M1-3	248693_at	AT5G48330	Regulator of chromosome condensation (RCC1) family protein;(source:Araport11)	30.51	19.07	1.60	2.02E-04	3.39E-03
127	1	L1-3 vs. M1-3	249792_at	AT5G23720	Encodes a protein tyrosine phosphatase Propyzamide-Hypersensitive 1 (PHS1). One of the mutant alleles, phs1-1, is hypersensitive to the microtubule-destabilizing drug propyzamide, suggesting that PHS1 may be involved in phosphorylation cascades that control the dynamics of cortical microtubules in plant cells. A second allele, phs1-3, is hypersensitive to abscisic acid, indicating a possible involvement of PHS1 in ABA signalling.	14.44	9.01	1.60	4.45E-04	5.72E-03
128	1	L1-3 vs. M1-3	264970_at	AT1G67280	Encodes a Ni+ dependent glyoxylase.	271.38	170.34	1.59	8.22E-04	8.55E-03
129	1	L1-3 vs. M1-3	261483_at	AT1G14270	CAAX amino terminal protease family protein;(source:Araport11)	35.36	22.54	1.57	1.29E-04	2.52E-03
130	1	L1-3 vs. M1-3	252941_at	AT4G39280	phenylalanyl-tRNA synthetase, putative / phenylalanine-tRNA ligase;(source:Araport11)	476.51	303.98	1.57	2.76E-04	4.16E-03
131	1	L1-3 vs. M1-3	263987_at	AT2G42690	alpha/beta-Hydrolases superfamily protein;(source:Araport11)	49.36	31.44	1.57	5.11E-04	6.18E-03
132	1	L1-3 vs. M1-3	266459_at	AT2G47970	Nuclear pore localization protein NPL4;(source:Araport11)	45.55	28.97	1.57	7.58E-04	8.12E-03
133	1	L1-3 vs. M1-3	262823_at	AT1G11750	One of several nuclear-encoded ClpPs (caseinolytic protease). Contains a highly conserved catalytic triad of Ser-type proteases (Ser-His-Asp). The name reflects nomenclature described in Adam et. al (2001).	172.11	110.20	1.56	4.32E-04	5.68E-03
134	1	L1-3 vs. M1-3	254596_at	AT4G18975	Pentatricopeptide repeat (PPR) superfamily protein;(source:Araport11)	29.88	19.33	1.55	5.77E-04	6.60E-03
135	1	L1-3 vs. M1-3	264120_at	AT1G79340	Encodes MCP2d, the predominant and constitutively expressed member of type II metacaspases (MCPs). MCP2d plays a positive regulatory role in biotic and abiotic stress-induced programmed cell death (PCD). Arabidopsis contains three type I MCP genes (MCP1a-c) and six type II MCP genes (MCP2a?f): AtMCP1a/At5g64240, AtMCP1b/At1g02170, AtMCP1c/At4g25110, AtMCP2a/At1g79310, AtMCP2b/At1g79330, AtMCP2c/At1g79320, AtMCP2d/At1g79340, AtMCP2e/At1g16420, AtMCP2f/At5g04200. The mRNA is cell-to-cell mobile.	40.04	26.06	1.54	1.90E-04	3.28E-03
136	1	L1-3 vs. M1-3	260496_at	AT2G41700	ATP-binding cassette A1;(source:Araport11)	107.18	69.45	1.54	4.74E-04	5.88E-03
137	1	L1-3 vs. M1-3	251961_at	AT3G53620	Encodes a soluble protein with inorganic pyrophosphatase activity that is highly specific for Mg-inorganic pyrophosphate. The mRNA is cell-to-cell mobile.	127.28	82.61	1.54	8.85E-04	9.07E-03
138	1	L1-3 vs. M1-3	263705_at	AT1G31190	Encodes a myo-inositol monophosphatase IMPL1 (myo-Inositol monophosphatase like 1).	164.28	107.24	1.53	1.25E-04	2.47E-03
139	1	L1-3 vs. M1-3	246027_at	AT5G21060	Glyceraldehyde-3-phosphate dehydrogenase-like family protein;(source:Araport11)	184.44	121.53	1.52	4.85E-04	5.95E-03
140	1	L1-3 vs. M1-3	259801_at	AT1G72230	Cupredoxin superfamily protein;(source:Araport11)	29.73	19.73	1.51	1.69E-04	3.04E-03
141	1	L1-3 vs. M1-3	257587_at	AT1G56310	DEDDy-type 3′ -> 5′ exoribonuclease involved in miRNA degradation.	260.47	172.21	1.51	6.63E-04	7.41E-03

142	1	L1-3 vs. M1-3	258675_at	AT3G08770	Predicted to encode a PR (pathogenesis-related) protein. Belongs to the lipid transfer protein (PR-14) family with the following members: At2g38540/LTP1, At2g38530/LTP2, At5g59320/LTP3, At5g59310/LTP4, At3g51600/LTP5, At3g08770/LTP6, At2g15050/LTP7, At2g18370/LTP8, At2g15325/LTP9, At5g01870/LTP10, At4g33355/LTP11, At3g51590/LTP12, At5g44265/LTP13, At5g62065/LTP14, At4g08530/LTP15.	44.97	29.90	1.50	9.72E-04	9.75E-03
143	1	L1-3 vs. M1-3	255443_at	AT4G02700	sulfate transporter 3;(source:Araport11)	17.73	11.89	1.49	2.03E-04	3.39E-03
144	1	L1-3 vs. M1-3	245352_at	AT4G15490	Encodes a protein that might have sinapic acid:UDP-glucose glucosyltransferase activity.	26.06	17.66	1.48	8.19E-04	8.55E-03
145	1	L1-3 vs. M1-3	248575_at	AT5G49840	ATP-dependent Clp protease;(source:Araport11)	119.71	81.20	1.47	5.16E-04	6.21E-03
146	1	L1-3 vs. M1-3	263930_at	AT2G36300	Integral membrane Yip1 family protein;(source:Araport11)	81.98	124.35	0.66	6.98E-04	7.56E-03
147	1	L1-3 vs. M1-3	260822_at	AT1G06790	Encodes a subunit of RNA polymerase III involved in maintaining global RNA homeostasis, not just that of genes transcribed by RNA pol III.	130.88	199.83	0.65	7.30E-06	3.51E-04
148	1	L1-3 vs. M1-3	247210_at	AT5G65020	Annexins are calcium binding proteins that are localized in the cytoplasm. When cytosolic Ca2+ increases, they relocate to the plasma membrane. They may be involved in the Golgi-mediated secretion of polysaccharides.	600.28	928.32	0.65	3.28E-05	9.22E-04
149	1	L1-3 vs. M1-3	257179_at	AT3G13170	Encodes AtSPO11-1, one of the three Arabidopsis homologues of the archaeal DNA topoisomerase VIA subunit (topo VIA). Required for meiotic recombination. AtSPO11-1 and AtSPO11-2 have overlapping functions (i.e. both required for meiotic recombination) whereas AtSPO11-3 functions in DNA replication. AtSPO11-1 accumulates in foci in early G2. At 1 h post-S phase, no foci are observed, but by 3 h a majority (80%) of meiocytes at this time point contain >50 foci. However, by 5 h, AtSPO11-1 foci are no longer detectable. This suggests that the protein undergoes a rapid cycle of accumulation and disappearance in meiocytes over a period of between 1 and 5 h post-S phase.	10.67	16.29	0.65	1.94E-04	3.29E-03
150	1	L1-3 vs. M1-3	251323_at	AT3G61580	Fatty acid/sphingolipid desaturase;(source:Araport11)	64.87	100.43	0.65	4.74E-04	5.88E-03
151	1	L1-3 vs. M1-3	263101_s_a	AT2G05250	DNAJ heat shock N-terminal domain-containing protein;(source:Araport11)	58.83	90.28	0.65	5.96E-04	6.79E-03
152	1	L1-3 vs. M1-3	254789_at	AT4G12880	early nodulin-like protein 19;(source:Araport11)	82.39	128.57	0.64	1.88E-04	3.26E-03
153	1	L1-3 vs. M1-3	262842_at	AT1G14720	member of Glycoside Hydrolase Family 16	12.78	19.85	0.64	5.00E-04	6.09E-03
154	1	L1-3 vs. M1-3	247636_at	AT5G60570	Galactose oxidase/kelch repeat superfamily protein;(source:Araport11)	23.24	36.47	0.64	5.67E-04	6.54E-03
155	1	L1-3 vs. M1-3	254111_at	AT4G24890	purple acid phosphatase 24;(source:Araport11)	11.27	17.98	0.63	7.90E-06	3.60E-04
156	1	L1-3 vs. M1-3	264060_at	AT2G27980	Acyl-CoA N-acyltransferase with RING/FYVE/PHD-type zinc finger domain-containing protein;(source:Araport11)	27.37	43.77	0.63	7.98E-04	8.42E-03
157	1	L1-3 vs. M1-3	250545_at	AT5G08160	Encodes a serine/threonine protein kinase.	119.61	194.81	0.61	5.00E-07	5.91E-05
158	1	L1-3 vs. M1-3	259617_at	AT1G47970	nucleolin;(source:Araport11)	42.75	70.46	0.61	4.40E-06	2.60E-04
159	1	L1-3 vs. M1-3	245522_at	AT4G15890	CAP-3D is a subunit of condensin. It a target of MMD1 regulation and also involved in meiotic chromosome condensation. Mutants have reduced fertility. Required for the correct spatial relationship between centromeres and rDNA arrays.	21.36	34.93	0.61	3.00E-05	8.69E-04
160	1	L1-3 vs. M1-3	259523_at	AT1G12500	Nucleotide-sugar transporter family protein;(source:Araport11)	22.32	36.55	0.61	4.75E-04	5.88E-03
161	1	L1-3 vs. M1-3	249889_at	AT5G22540	Associated with a QTL for quantitative disease resistance.	24.43	40.08	0.61	5.72E-04	6.57E-03
162	1	L1-3 vs. M1-3	261722_at	AT1G08510	Encodes an acyl-acyl carrier protein thioesterase. Hydrolyzes primarily saturated acyl-ACPs with chain lengths that vary between 8 and 18 carbons. Involved in saturated fatty acid synthesis. Nuclear-encoded, plastid-targeted globular protein that is functional as dimer.	52.41	85.71	0.61	6.66E-04	7.41E-03
163	1	L1-3 vs. M1-3	254810_at	AT4G12390	pectin methylesterase inhibitor 1;(source:Araport11)	17.56	28.61	0.61	6.77E-04	7.44E-03
164	1	L1-3 vs. M1-3	264996_at	AT1G67230	Encodes a nuclear coiled-coil protein related to the carrot peripheral nuclear protein NMCP1 that is involved in the determination of plant nuclear structure. Member of a small gene family in Arabidopsis containing 4 proteins (LNC1-4 or CRWN 1-4) with redundant functions in protection from oxidative damage, control of nuclear morphology and degradation of ABI5.	44.78	74.58	0.60	4.46E-04	5.72E-03
165	1	L1-3 vs. M1-3	249334_at	AT5G41000	Arabidopsis thaliana metal-nicotianamine transporter YSL4	16.13	27.36	0.59	4.76E-05	1.25E-03
166	1	L1-3 vs. M1-3	252524_at	AT3G46430	Mitochondrial F1F0-ATP synthase.	166.72	284.43	0.59	6.86E-05	1.60E-03
167	1	L1-3 vs. M1-3	249284_at	AT5G41810	Avr9/Cf-9 rapidly elicited protein;(source:Araport11)	18.27	30.96	0.59	1.77E-04	3.12E-03
168	1	L1-3 vs. M1-3	248962_at	AT5G45680	Peptidyl-Prolyl Isomerase located in chloroplast thylakoid lumen The mRNA is cell-to-cell mobile.	68.88	117.19	0.59	2.90E-04	4.27E-03

169	1	L1-3 vs. M1-3	247281_at	AT5G64350	Encodes FK506-binding protein 12 (FKBP12 or FKP12). FKP12 overexpression dramatically enhances rapamycin sensitivity, whereas rapamycin inhibition is relieved in transgenic plants deficient in FKP12.	343.56	578.74	0.59	3.27E-04	4.63E-03
170	1	L1-3 vs. M1-3	255704_at	AT4G00170	Plant VAMP (vesicle-associated membrane protein) family protein;(source:Araport11)	32.32	55.16	0.59	4.23E-04	5.58E-03
171	1	L1-3 vs. M1-3	264243_at	AT1G54650	Methyltransferase family protein;(source:Araport11)	57.82	100.27	0.58	6.00E-07	6.81E-05
172	1	L1-3 vs. M1-3	263728_at	AT1G60070	Adaptor protein complex AP-1, gamma subunit;(source:Araport11)	60.93	104.93	0.58	2.87E-05	8.40E-04
173	1	L1-3 vs. M1-3	256677_at	AT3G52190	Encodes a plant specific protein structurally related to the SEC12 proteins of the early secretory pathway. Mutation of PHF1 impairs Pi transport. Expression was detected in all tissues, and was induced by Pi starvation. Localized in endoplasmic reticulum (ER), and mutation of PHF1 resulted in ER retention and reduced accumulation of the plasma membrane PHT1;1 transporter. Its expression is responsive to both phosphate (Pi) and phosphite (Phi) in shoots.	65.88	113.94	0.58	8.15E-05	1.82E-03
174	1	L1-3 vs. M1-3	261354_at	AT1G79690	nudix hydrolase homolog 3;(source:Araport11)	34.60	59.38	0.58	4.04E-04	5.45E-03
175	1	L1-3 vs. M1-3	256725_at	AT2G34070	Encodes a member of the TBL (TRICHOME BIREFRINGENCE-LIKE) gene family containing a plant-specific DUF231 (domain of unknown function) domain. TBL gene family has 46 members, two of which (TBR/AT5G06700 and TBL3/AT5G01360) have been shown to be involved in the synthesis and deposition of secondary wall cellulose, presumably by influencing the esterification state of pectic polymers. A nomenclature for this gene family has been proposed (Volker Bischoff & Wolf Scheible, 2010, personal communication).	26.57	45.80	0.58	4.49E-04	5.74E-03
176	1	L1-3 vs. M1-3	252870_at	AT4G39940	adenosine-5'-phosphosulfate-kinase (akn2) mRNA, complete The mRNA is cell-to-cell mobile.	12.10	20.89	0.58	8.21E-04	8.55E-03
177	1	L1-3 vs. M1-3	255430_at	AT4G03320	Encodes a component of the TIC (translocon at the inner envelope membrane of chloroplasts) protein translocation machinery mediating the protein translocation across the inner envelope of plastids. The Arabidopsis genome encodes four Tic20 homologous proteins, AT1G04940(Tic20-I), AT2G47840(Tic20-II), AT4G03320(Tic20-IV) and AT5G55710(Tic20-V).	70.45	122.12	0.58	9.07E-04	9.26E-03
178	1	L1-3 vs. M1-3	245351_at	AT4G17640	Encodes casein kinase II beta (regulatory) subunit.	143.87	247.51	0.58	9.15E-04	9.27E-03
179	1	L1-3 vs. M1-3	266075_s_a	AT2G40710	hemolysin-III related integral membrane protein;(source:Araport11)	75.88	132.09	0.57	8.40E-06	3.72E-04
180	1	L1-3 vs. M1-3	261537_at	AT1G01800	NAD(P)-binding Rossmann-fold superfamily protein;(source:Araport11)	14.19	24.71	0.57	2.05E-05	6.46E-04
181	1	L1-3 vs. M1-3	248746_at	AT5G47890	NADH-ubiquinone oxidoreductase B8 subunit;(source:Araport11)	133.04	231.95	0.57	6.99E-05	1.61E-03
182	1	L1-3 vs. M1-3	249751_at	AT5G24650	HP30/Tric1 is a component of the mitochondrial protein translocation complex and is involved in tRNA transport along with HP30-2/Tric2.It interacts with several members of the TOM complex such as TOM40 and this interaction is mediated by the SAM domain.	68.26	118.95	0.57	7.62E-05	1.73E-03
183	1	L1-3 vs. M1-3	258851_at	AT3G03190	Encodes glutathione transferase belonging to the phi class of GSTs. Naming convention according to Wagner et al. (2002).	11.60	20.43	0.57	7.86E-05	1.77E-03
184	1	L1-3 vs. M1-3	247338_at	AT5G63680	Pyruvate kinase family protein;(source:Araport11)	32.47	56.82	0.57	1.23E-04	2.45E-03
185	1	L1-3 vs. M1-3	260993_at	AT1G12140	belongs to the flavin-monooxygenase (FMO) family, encodes a glucosinolate S-oxygenase that catalyzes the conversion of methylthioalkyl glucosinolates to methylsulfinylalkyl glucosinolates	86.23	150.00	0.57	1.37E-04	2.64E-03
186	1	L1-3 vs. M1-3	262558_at	AT1G31335	transmembrane protein;(source:Araport11)	51.25	90.06	0.57	2.82E-04	4.21E-03
187	1	L1-3 vs. M1-3	262536_at	AT1G17100	SOUL heme-binding family protein;(source:Araport11)	50.07	87.19	0.57	2.93E-04	4.28E-03
188	1	L1-3 vs. M1-3	253987_at	AT4G26270	phosphofructokinase 3;(source:Araport11)	120.69	211.64	0.57	5.61E-04	6.50E-03
189	1	L1-3 vs. M1-3	259085_at	AT3G05000	Part of multi-protein complex, acting as guanine nucleotide exchange factors (GEFs) and possibly as tethers, regulating intracellular trafficking.	66.60	116.84	0.57	9.90E-04	9.89E-03
190	1	L1-3 vs. M1-3	244961_at	ATCG01040	Cytochrome C assembly protein;(source:Araport11)	645.34	1152.46	0.56	1.80E-06	1.42E-04
191	1	L1-3 vs. M1-3	256038_at	AT1G19170	Pectin lyase-like superfamily protein;(source:Araport11)	11.75	20.92	0.56	3.20E-06	2.06E-04
192	1	L1-3 vs. M1-3	252363_at	AT3G48460	GDSL-motif esterase/acyltransferase/lipase. Enzyme group with broad substrate specificity that may catalyze acyltransfer or hydrolase reactions with lipid and non-lipid substrates.	13.94	25.06	0.56	2.89E-04	4.27E-03
193	1	L1-3 vs. M1-3	244972_at	ATCG00680	encodes for CP47, subunit of the photosystem II reaction center.	72.97	129.61	0.56	7.77E-04	8.29E-03
194	1	L1-3 vs. M1-3	246463_at	AT5G16970	encodes a 2-alkenal reductase (EC 1.3.1.74), plays a key role in the detoxification of reactive carbonyls	50.18	91.25	0.55	2.50E-04	3.87E-03
195	1	L1-3 vs. M1-3	262786_at	AT1G10740	alpha/beta-Hydrolases superfamily protein;(source:Araport11)	13.37	24.34	0.55	4.68E-04	5.88E-03
196	1	L1-3 vs. M1-3	264355_at	AT1G03210	Phenazine biosynthesis PhzC/PhzF protein;(source:Araport11)	70.00	130.60	0.54	2.08E-05	6.49E-04

197	1	L1-3 vs. M1-3	247429_at	AT5G62620	Encodes a Golgi-localized hydroxyproline-O-galactosyltransferase. Mutants display multiple phenotypes including reduced seed coat mucilage and accelerated leaf senescence.	114.17	211.24	0.54	6.81E-05	1.60E-03
198	1	L1-3 vs. M1-3	245316_at	AT4G14000	Putative methyltransferase family protein;(source:Araport11)	163.74	305.19	0.54	1.81E-04	3.17E-03
199	1	L1-3 vs. M1-3	264646_at	AT1G08860	Encodes a copine-like protein, which is a member of a newly identified class of calcium-dependent, phospholipid binding proteins that are present in a wide range of organisms. Overexpression of this gene suppresses bon1-1 phenotypes. Double mutant analyses with bon1-1 suggest that BON1 and BON3 have overlapping functions in maintaining cellular homeostasis and inhibiting cell death.	34.26	63.55	0.54	2.36E-04	3.74E-03
200	1	L1-3 vs. M1-3	262509_at	AT1G11290	Pentatricopeptide Repeat Protein containing the DYW motif. Required for editing of multiple plastid transcripts. Endonuclease activity.	56.97	108.13	0.53	2.20E-06	1.64E-04
201	1	L1-3 vs. M1-3	266167_at	AT2G38860	Encodes protease I (pfpI)-like protein YLS5.	79.50	149.14	0.53	6.96E-04	7.56E-03
202	1	L1-3 vs. M1-3	266038_at	AT2G07680	Encodes ABCC13/MRP11, a member of the multidrug resistance associated protein MRP/ABCC subfamily. Its expression is induced by gibberellic acid and downregulated by naphthalene acetic acid, abscisic acid, and zeatin.	22.24	42.58	0.52	7.90E-06	3.60E-04
203	1	L1-3 vs. M1-3	266613_at	AT2G14900	Gibberellin-regulated family protein;(source:Araport11)	197.31	376.69	0.52	1.45E-05	5.34E-04
204	1	L1-3 vs. M1-3	252163_at	AT3G50610	DNA-directed RNA polymerase II subunit RPB1-like protein;(source:Araport11)	12.72	24.69	0.52	1.70E-05	5.78E-04
205	1	L1-3 vs. M1-3	252883_at	AT4G39520	Encodes a member of the DRG (developmentally regulated G-protein) family. Has GTPase activity.	45.85	87.54	0.52	1.39E-04	2.66E-03
206	1	L1-3 vs. M1-3	258875_at	AT3G03160	B-cell receptor-associated-like protein;(source:Araport11)	146.34	279.08	0.52	6.24E-04	7.06E-03
207	1	L1-3 vs. M1-3	258864_at	AT3G03210	Pmr5/Cas1p GDSL/SGNH-like acyl-esterase family protein;(source:Araport11)	52.55	102.39	0.51	7.60E-06	3.59E-04
208	1	L1-3 vs. M1-3	254953_at	AT4G10925	Nuclear transport factor 2 (NTF2) family protein;(source:Araport11)	101.95	200.02	0.51	1.62E-05	5.71E-04
209	1	L1-3 vs. M1-3	262725_at	AT1G43580	Sphingomyelin synthetase family protein;(source:Araport11)	28.95	57.17	0.51	8.45E-05	1.86E-03
210	1	L1-3 vs. M1-3	259625_at	AT1G42970	Encodes chloroplast localized glyceraldehyde-3-phosphate dehydrogenase that can use both NADH and NADPH to reduce 1,3-diphosphate glycerate. It forms A2B2 heterotetramers with GapA forms of the GADPH enzyme. These complexes are active in the light under reducing conditions, but show reduced NADPH-dependent activity in response to oxidized thioredoxins and increased NAD(H)/NADP(H) ratios due to the formation of inactive A8B8 hexadecamers. The mRNA is cell-to-cell mobile.	129.54	255.19	0.51	3.98E-04	5.44E-03
211	1	L1-3 vs. M1-3	259003_at	AT3G02010	Pentatricopeptide repeat (PPR) superfamily protein;(source:Araport11)	88.44	177.42	0.50	3.26E-05	9.22E-04
212	1	L1-3 vs. M1-3	251277_at	AT3G61760	DYNAMIN-like 1B;(source:Araport11)	10.72	21.53	0.50	1.61E-04	2.97E-03
213	1	L1-3 vs. M1-3	263108_at	AT1G65240	Eukaryotic aspartyl protease family protein;(source:Araport11)	17.65	35.16	0.50	5.37E-04	6.29E-03
214	1	L1-3 vs. M1-3	259130_at	AT3G02190	Ribosomal protein L39 family protein;(source:Araport11)	120.22	243.45	0.49	1.31E-05	5.02E-04
215	1	L1-3 vs. M1-3	256629_at	AT3G19980	Encodes catalytic subunit of serine/threonine protein phosphatase 2A. It can associate with phytochromes A and B in vitro. Mutant plants display an accelerated flowering phenotype.Acts antagonistically to SnRK2 to regulate ABI5 phosphorylation. It inteacts with NRP which results in tethering to endosomes leading to its degradation.	54.76	112.23	0.49	6.54E-05	1.57E-03
216	1	L1-3 vs. M1-3	248377_at	AT5G51720	Encodes a protein with biochemical, structural, and biophysical characteristics of a NEET protein. It plays a key role in plant development, senescence, reactive oxygen homeostasis, and Fe metabolism.	40.68	82.85	0.49	6.77E-05	1.60E-03
217	1	L1-3 vs. M1-3	251796_at	AT3G55360	Enoyl-CoA reductase is involved in all very long chain fatty acids (VLCFA) elongation reactions that are required for cuticular wax, storage lipid and sphingolipid metabolism. The protein is located in the ER, but in contrast to its yeast homolog TSC13 is not particularly enriched in the nuclear envelope-vacuole junction. Mutants in this gene show abnormal organ morphology and stem glossiness. Cells in all tissues are only about 1/3 of the size of wild type cells. The morphological changes are most likely to result from the reduction in the VLCFA content of sphingolipids. Mutants also show abnormalities in the endocytic membrane organization and transport as well as reduced trichome papillae.	117.51	240.80	0.49	1.94E-04	3.29E-03
218	1	L1-3 vs. M1-3	249373_at	AT5G40670	PQ-loop repeat family protein / transmembrane family protein;(source:Araport11)	20.29	41.24	0.49	4.12E-04	5.54E-03
219	1	L1-3 vs. M1-3	248697_at	AT5G48370	Thioesterase/thiol ester dehydrase-isomerase superfamily protein;(source:Araport11)	9.90	20.12	0.49	4.63E-04	5.86E-03
220	1	L1-3 vs. M1-3	257141_at	AT3G28900	Ribosomal protein L34e superfamily protein;(source:Araport11)	88.86	181.50	0.49	5.49E-04	6.39E-03

221	1	L1-3 vs. M1-3	266165_at	AT2G28190	Encodes a chloroplastic copper/zinc superoxide dismutase CSD2 that can detoxify superoxide radicals. Its expression is affected by miR398-directed mRNA cleavage. Activation depends totally on CCS. Overexpression of a miR398-resistant form of CSD2 leads to more dramatic improvements in stress (hight light, Cu2+ and methyl viologen) tolerance than overexpression of wild-type CSD2. The mRNA is cell-to-cell mobile.	320.92	651.23	0.49	6.50E-04	7.29E-03
222	1	L1-3 vs. M1-3	264262_at	AT1G09200	Histone superfamily protein;(source:Araport11)	187.34	393.32	0.48	1.24E-04	2.45E-03
223	1	L1-3 vs. M1-3	248114_at	AT5G55370	MBOAT (membrane bound O-acyl transferase) family protein;(source:Araport11)	11.20	23.47	0.48	1.95E-04	3.30E-03
224	1	L1-3 vs. M1-3	245631_at	AT1G25350	glutamine-tRNA ligase, putative / glutaminyl-tRNA synthetase, putative / GlnRS;(source:Araport11)	27.21	56.72	0.48	2.69E-04	4.08E-03
225	1	L1-3 vs. M1-3	247267_at	AT5G64140	Encodes a putative ribosomal protein S28.	20.04	42.33	0.47	9.79E-05	2.09E-03
226	1	L1-3 vs. M1-3	248526_at	AT5G50740	Heavy metal transport/detoxification superfamily protein;(source:Araport11)	177.53	373.78	0.47	4.00E-04	5.44E-03
227	1	L1-3 vs. M1-3	261655_at	AT1G01940	Cyclophilin-like peptidyl-prolyl cis-trans isomerase family protein;(source:Araport11)	367.16	794.05	0.46	1.20E-06	1.06E-04
228	1	L1-3 vs. M1-3	252946_at	AT4G39235	hypothetical protein;(source:Araport11)	13.33	28.75	0.46	4.36E-05	1.17E-03
229	1	L1-3 vs. M1-3	267618_at	AT2G26760	Cyclin B1;(source:Araport11)	31.30	67.58	0.46	1.69E-04	3.04E-03
230	1	L1-3 vs. M1-3	251089_at	AT5G01390	DNAJ heat shock family protein;(source:Araport11)	19.80	44.26	0.45	2.12E-04	3.43E-03
231	1	L1-3 vs. M1-3	251021_at	AT5G02140	Pathogenesis-related thaumatin superfamily protein;(source:Araport11)	19.08	43.71	0.44	1.01E-05	4.09E-04
232	1	L1-3 vs. M1-3	247185_at	AT5G65460	Kinesin that binds cyclin-dependent kinase CDKA;1 as homodimer or as heterodimer with KCA1	19.29	44.17	0.44	2.05E-05	6.46E-04
233	1	L1-3 vs. M1-3	252337_at	AT3G48750	A-type cyclin-dependent kinase. Together with its specific inhibitor, the Kip-related protein, KRP2 they regulate the mitosis-to-endocycle transition during leaf development. Dominant negative mutations abolish cell division. Loss of function phenotype has reduced fertility with failure to transmit via pollen. Pollen development is arrested at the second mitotic division. Expression is regulated by environmental and chemical signals. Part of the promoter is responsible for expression in trichomes. Functions as a positive regulator of cell proliferation during development of the male gametophyte, embryo and endosperm. Phosphorylation of threonine 161 is required for activation of its associated kinase.	82.00	186.47	0.44	1.59E-04	2.96E-03
234	1	L1-3 vs. M1-3	260943_at	AT1G45145	encodes a cytosolic thioredoxin that reduces disulfide bridges of target proteins by the reversible formation of a disulfide bridge between two neighboring Cys residues present in the active site. Thioredoxins have been found to regulate a variety of biological reactions in prokaryotic and eukaryotic cells.	23.15	53.24	0.43	1.11E-04	2.27E-03
235	1	L1-3 vs. M1-3	267095_at	AT2G38280	Encodes a protein with in vitro AMP deaminase activity that is involved in embryogenesis. Homozygous mutant embryos fail to develop past the zygote stage.	115.73	268.79	0.43	8.66E-04	8.90E-03
236	1	L1-3 vs. M1-3	264443_at	AT1G27385	phosphoribosylformylglycinamidine synthase;(source:Araport11)	74.92	180.16	0.42	1.10E-06	1.01E-04
237	1	L1-3 vs. M1-3	251945_at	AT3G53520	Encodes an isoform of UDP-glucuronic acid decarboxylase, which is predicted to be membrane-bound by PSORT analysis. This enzyme produces UDP-xylose, which is a substrate for many cell wall carbohydrates including hemicellulose and pectin. UDP-xylose is also known to feedback regulate several cell wall biosynthetic enzymes.	26.62	63.64	0.42	1.44E-05	5.34E-04
238	1	L1-3 vs. M1-3	247983_at	AT5G56630	phosphofructokinase 7;(source:Araport11)	212.89	502.41	0.42	5.55E-05	1.42E-03
239	1	L1-3 vs. M1-3	245446_at	AT4G16800	3-methylglutaconyl-CoA hydratase localized to mitochondria. Knockout displays accelerated senescence when subjected to extended dark conditions;knockout senescing leaves and knockout seeds accumulate leu, ile, and val.	49.32	121.38	0.41	2.49E-04	3.87E-03
240	1	L1-3 vs. M1-3	254480_at	AT4G20360	Nuclear transcribed, plastid localized EF-Tu translation elongation factor. Referred to as AtRabE1b in DOI:10.1104/pp.013052. However, wider community usage and more publications assign the symbol RabE1b to At5g59840.	42.25	106.75	0.40	2.00E-07	3.78E-05
241	1	L1-3 vs. M1-3	246443_at	AT5G17620	nuclear matrix protein;(source:Araport11)	83.46	209.64	0.40	5.00E-06	2.73E-04
242	1	L1-3 vs. M1-3	258976_at	AT3G01980	NAD(P)-binding Rossmann-fold superfamily protein;(source:Araport11)	61.67	153.20	0.40	1.01E-05	4.09E-04
243	1	L1-3 vs. M1-3	266070_at	AT2G18660	Encodes PNP-A (Plant Natriuretic Peptide A). PNPs are a class of systemically mobile molecules distantly related to expansins; their biological role has remained elusive. PNP-A contains a signal peptide domain and is secreted into the extracellular space. Co-expression analyses using microarray data suggest that PNP-A may function as a component of plant defence response and SAR in particular, and could be classified as a newly identified PR protein. It is stress responsive and can enhance its own expression.	31.23	77.37	0.40	1.31E-05	5.02E-04

244	1	L1-3 vs. M1-3	264052_at	AT2G22330	Encodes a cytochrome P450. Involved in tryptophan metabolism. Converts Trp to indole-3-acetaldoxime (IAOx), a precursor to IAA and indole glucosinolates.The mRNA is cell-to-cell mobile.	21.11	52.44	0.40	1.95E-05	6.36E-04
245	1	L1-3 vs. M1-3	246408_at	AT1G57680	plasminogen activator inhibitor;(source:Araport11)	199.77	497.84	0.40	6.14E-05	1.53E-03
246	1	L1-3 vs. M1-3	264972_at	AT1G67370	meiotic asynaptic mutant 1 (ASY1). ASY1 protein is initially distributed as numerous foci throughout the chromatin. During early G2, the foci are juxtaposed to the nascent chromosome axes to form a continuous axis associated signal.	18.31	45.69	0.40	1.14E-04	2.33E-03
247	1	L1-3 vs. M1-3	256916_at	AT3G24050	Encodes a member of the GATA factor family of zinc finger transcription factors.	12.60	31.91	0.40	3.01E-04	4.35E-03
248	1	L1-3 vs. M1-3	260976_at	AT1G53650	RNA-binding protein, putative, similar to RNA-binding protein GB:AAA86641 GI:1174153 from (Arabidopsis thaliana).Contains PAB2 domain which facilitates binding to PABC proteins.	57.53	150.07	0.38	2.58E-04	3.96E-03
249	1	L1-3 vs. M1-3	252272_at	AT3G49670	Encodes a CLAVATA1-related receptor kinase-like protein required for both shoot and flower meristem function. Very similar to BAM1,with more than 85% a.a. identity. It has a broad expression pattern and is involved in vascular strand development in the leaf, control of leaf shape, size and symmetry, male gametophyte development and ovule specification and function. Anthers of double mutants (bam1bam2) appeared abnormal at a very early stage and lack the endothecium, middle, and tapetum layers. Further analyses revealed that cells interior to the epidermis (in anther tissue) acquire some characteristics of pollen mother cells (PMCs), suggesting defects in cell fate specification. The pollen mother-like cells degenerate before the completion of meiosis, suggesting that these cells are defective. In addition, the BAM2 expression pattern supports both an early role in promoting somatic cell fates and a subsequent function in the PMCs. The mRNA is cell-to-cell mobile.	24.76	66.78	0.37	1.63E-05	5.71E-04
250	1	L1-3 vs. M1-3	263679_at	AT1G59990	DEA(D/H)-box RNA helicase family protein;(source:Araport11)	19.44	53.05	0.37	1.56E-04	2.94E-03
251	1	L1-3 vs. M1-3	260368_at	AT1G69700	Part of the AtHVA22 family. Protein expression is ABA- and stress-inducible. The mRNA is cell-to-cell mobile.	24.32	65.28	0.37	5.08E-04	6.16E-03
252	1	L1-3 vs. M1-3	258140_at	AT3G24503	Arabidopsis thaliana aldehyde dehydrogenase AtALDH1a mRNA. a sinapaldehyde dehydrogenase catalyzes both the oxidation of coniferylaldehyde and sinapaldehyde forming ferulic acid and sinapic acid, respectively	60.80	168.09	0.36	1.00E-05	4.09E-04
253	1	L1-3 vs. M1-3	261023_at	AT1G12200	Putative flavin monooxygenase.	10.51	29.00	0.36	1.71E-05	5.78E-04
254	1	L1-3 vs. M1-3	266732_at	AT2G03240	EXS (ERD1/XPR1/SYG1) family protein;(source:Araport11)	35.66	102.25	0.35	9.10E-06	3.97E-04
255	1	L1-3 vs. M1-3	258716_at	AT3G09700	Chaperone DnaJ-domain superfamily protein;(source:Araport11)	33.69	96.44	0.35	2.01E-05	6.46E-04
256	1	L1-3 vs. M1-3	254578_at	AT4G19410	Pectinacetylesterase family protein;(source:Araport11)	122.96	355.69	0.35	1.18E-04	2.37E-03
257	1	L1-3 vs. M1-3	264809_at	AT1G08830	Encodes a cytosolic copper/zinc superoxide dismutase CSD1 that can detoxify superoxide radicals. Its expression is affected by miR398-directed mRNA cleavage. Regulated by biotic and abiotic stress. Activation of CSD1 in the cytoplasm involves both a CCS-dependent and -independent pathway.	314.80	890.86	0.35	2.97E-04	4.32E-03
258	1	L1-3 vs. M1-3	260297_at	AT1G80280	alpha/beta-Hydrolases superfamily protein;(source:Araport11)	15.51	44.89	0.35	4.42E-04	5.72E-03
259	1	L1-3 vs. M1-3	244970_at	ATCG00660	encodes a chloroplast ribosomal protein L20, a constituent of the large subunit of the ribosomal complex	106.58	317.21	0.34	1.74E-04	3.10E-03
260	1	L1-3 vs. M1-3	248658_at	AT5G48600	member of SMC subfamily	23.56	74.50	0.32	2.06E-04	3.40E-03
261	1	L1-3 vs. M1-3	263151_at	AT1G54120	hypothetical protein;(source:Araport11)	39.34	135.44	0.29	< 1e-07	< 1e-07
262	1	L1-3 vs. M1-3	249242_at	AT5G42250	Zinc-binding alcohol dehydrogenase family protein;(source:Araport11)	52.66	183.35	0.29	3.00E-07	4.26E-05
263	1	L1-3 vs. M1-3	267153_at	AT2G30860	Encodes glutathione transferase belonging to the phi class of GSTs. Naming convention according to Wagner et al. (2002).	400.89	1364.81	0.29	4.00E-07	5.41E-05
264	1	L1-3 vs. M1-3	257224_at	AT3G27870	ATPase E1-E2 type family protein / haloacid dehalogenase-like hydrolase family protein;(source:Araport11)	34.01	123.00	0.28	2.40E-06	1.75E-04
265	1	L1-3 vs. M1-3	259381_s_at	AT3G16410	Encodes a nitrile-specifier protein NSP4. NSP4 is one out of five (At3g16400/NSP1, At2g33070/NSP2, At3g16390/NSP3, At3g16410/NSP4 and At5g48180/NSP5) A. thaliana epithiospecifier protein (ESP) homologues that promote simple nitrile, but not epithionitrile or thiocyanate formation. The mRNA is cell-to-cell mobile.	23.38	83.88	0.28	1.59E-05	5.71E-04
266	1	L1-3 vs. M1-3	245293_at	AT4G16660	heat shock protein 70 (Hsp 70) family protein;(source:Araport11)	22.79	81.12	0.28	6.27E-05	1.53E-03
267	1	L1-3 vs. M1-3	256899_at	AT3G24660	member of Receptor kinase-like protein family	17.48	62.10	0.28	8.67E-05	1.89E-03
268	1	L1-3 vs. M1-3	256322_at	AT1G54990	auxin response mutant (AXR4) The mRNA is cell-to-cell mobile.	40.47	155.98	0.26	< 1e-07	2.58E-05
269	1	L1-3 vs. M1-3	259391_s_a	AT1G06350	Fatty acid desaturase family protein;(source:Araport11)	200.64	782.34	0.26	3.00E-07	4.26E-05
270	1	L1-3 vs. M1-3	257334_at	ATMG01370	transmembrane protein;(source:Araport11)	135.26	515.02	0.26	2.12E-05	6.54E-04

271	1	L1-3 vs. M1-3	266262_at	AT2G27590	S-adenosyl-L-methionine-dependent methyltransferases superfamily protein;(source:Araport11)	26.71	102.48	0.26	6.67E-05	1.59E-03
272	1	L1-3 vs. M1-3	245275_at	AT4G15210	cytosolic beta-amylase expressed in rosette leaves and inducible by sugar. RAM1 mutants have reduced beta amylase in leaves and stems.	115.80	463.78	0.25	9.45E-04	9.51E-03
273	1	L1-3 vs. M1-3	266012_s_a	ATMG00410	ATPase subunit 6	176.74	756.56	0.23	3.60E-06	2.27E-04
274	1	L1-3 vs. M1-3	255459_at	AT4G02890	Polyubiquitin gene containing 4 ubiquitin repeats.	746.67	3334.43	0.22	< 1e-07	< 1e-07
275	1	L1-3 vs. M1-3	245009_at	ATCG00380	Chloroplast encoded ribosomal protein S4	57.10	260.15	0.22	5.10E-06	2.73E-04
276	1	L1-3 vs. M1-3	261650_at	AT1G27770	Encodes a chloroplast envelope Ca2+-ATPase with an N-terminal autoinhibitor.	21.57	102.31	0.21	< 1e-07	< 1e-07
277	1	L1-3 vs. M1-3	263734_at	AT1G60030	nucleobase-ascorbate transporter 7;(source:Araport11)	20.75	103.50	0.20	< 1e-07	< 1e-07
278	1	L1-3 vs. M1-3	264577_at	AT1G05260	Encodes a cold-inducible cationic peroxidase that is involved in the stress response. In response to low temperature, RCI3 transcripts accumulate in the aerial part and in roots of etiolated seedlings but only in roots of light-grown seedlings. The mRNA is cell-to-cell mobile.	40.00	204.24	0.20	2.20E-06	1.64E-04
279	1	L1-3 vs. M1-3	253534_at	AT4G31500	Encodes an oxime-metabolizing enzyme in the biosynthetic pathway of glucosinolates. Is required for phytochrome signal transduction in red light. Mutation confers auxin overproduction.	188.35	938.18	0.20	7.20E-06	3.51E-04
280	1	L1-3 vs. M1-3	244964_at	ATCG00560	PSII L protein	620.80	3316.70	0.19	2.00E-07	3.78E-05
281	1	L1-3 vs. M1-3	265230_s_a	ATMG00480	Encodes subunit 8 of the mitochondrial F(O) ATP synthase complex.	62.40	385.96	0.16	< 1e-07	< 1e-07
282	1	L1-3 vs. M1-3	261178_at	AT1G04760	member of Synaptobrevin -like protein family	14.11	117.85	0.12	< 1e-07	< 1e-07
283	1	L1-3 vs. M1-3	245928_s_a t	AT5G24780	encodes an acid phosphatase similar to soybean vegetative storage proteins. Gene expression is induced by wounding and jasmonic acid.	30.83	709.89	0.04	< 1e-07	< 1e-07
284	2	L4 vs. M4	248227_at	AT5G53820	Late embryogenesis abundant protein (LEA) family protein;(source:Araport11)	98.69	15.29	6.45	1.00E-06	1.34E-05
285	2	L4 vs. M4	264223_s_a	AT1G67520	lectin protein kinase family protein;(source:Araport11)	137.09	21.97	6.24	< 1e-07	< 1e-07
286	2	L4 vs. M4	254098_at	AT4G25100	Fe-superoxide dismutase	265.10	44.19	6.00	< 1e-07	< 1e-07
287	2	L4 vs. M4	267256_s_a	AT2G23010	serine carboxypeptidase-like 9;(source:Araport11)	198.59	38.00	5.23	1.36E-05	5.88E-05
288	2	L4 vs. M4	259063_at	AT3G07450	Bifunctional inhibitor/lipid-transfer protein/seed storage 2S albumin superfamily protein;(source:Araport11)	150.71	28.98	5.20	3.00E-07	6.20E-06
289	2	L4 vs. M4	249536_at	AT5G38760	Late embryogenesis abundant protein (LEA) family protein;(source:Araport11)	97.79	18.91	5.17	< 1e-07	< 1e-07
290	2	L4 vs. M4	246028_at	AT5G21170	Encodes AKINbeta1, a subunit of the SnRK1 kinase (Sucrose non-fermenting-1-related protein kinase). Involved in regulation of nitrogen and sugar metabolism. As part of the regulatory subunit, it binds maltose which promotes kinase activity. Acts as a global regulator of genes involved in carbon, lipid and nitrogen metabolism.	191.15	38.03	5.03	4.80E-06	3.41E-05
291	2	L4 vs. M4	251590_at	AT3G57690	Encodes a putative arabinogalactan-protein (AGP23).	2528.19	505.48	5.00	< 1e-07	< 1e-07
292	2	L4 vs. M4	247762_at	AT5G59170	Proline-rich extensin-like family protein;(source:Araport11)	888.02	178.29	4.98	< 1e-07	3.36E-06
293	2	L4 vs. M4	262566_at	AT1G34310	auxin response factor 12;(source:Araport11)	2402.10	501.18	4.79	< 1e-07	< 1e-07
294	2	L4 vs. M4	251109_at	AT5G01600	Encodes a ferretin protein that is targeted to the chloroplast. Member of a Ferritin gene family. Gene expression is induced in response to iron overload and by nitric oxide. Expression of the gene is downregulated in the presence of paraquat, an inducer of photoxidative stress.	249.40	53.76	4.64	< 1e-07	3.36E-06
295	2	L4 vs. M4	257041_at	AT3G28550	Proline-rich extensin-like family protein;(source:Araport11)	249.65	56.70	4.40	1.40E-06	1.63E-05
296	2	L4 vs. M4	258686_at	AT3G07840	Pectin lyase-like superfamily protein;(source:Araport11)	223.75	52.12	4.29	1.80E-06	1.90E-05
297	2	L4 vs. M4	248367_at	AT5G52360	ADF10 is an actin-depolymerizing factor that preferentially binds ADP-G-actin and inhibits G-actin nucleotide exchange. ADF10 promotes actin turnover in pollen , regulating organization of actin filaments and vesicle trafficking during pollen tube growth.	290.03	71.11	4.08	< 1e-07	3.36E-06
298	2	L4 vs. M4	259727_at	AT1G60950	encodes a major leaf ferredoxin	4882.75	1261.89	3.87	5.00E-06	3.46E-05
299	2	L4 vs. M4	248467_at	AT5G50800	Encodes a member of the SWEET sucrose efflux transporter family proteins, together with RPG1, it is involved in pollen development. Together with SWEET14, it is likely involved in modulating the GA response and is required for proper development of anthers, seeds and seedlings.	378.23	97.72	3.87	9.00E-07	1.29E-05
300	2	L4 vs. M4	249527_at	AT5G38710	Methylenetetrahydrofolate reductase family protein;(source:Araport11)	248.82	67.33	3.70	8.00E-07	1.21E-05
301	2	L4 vs. M4	257816_at	AT3G25140	Quasimodo1, encodes a glycosyltransferase, involved in homogalacturonan biosynthesis; mutant shows cell adhesion defect and lower wall uronic acid content. The mRNA is cell-to-cell mobile.	316.61	87.57	3.62	8.00E-07	1.21E-05

302	2	L4 vs. M4	253161_at	AT4G35770	Senescence-associated gene that is strongly induced by phosphate starvation. Transcripts are differentially regulated at the level of mRNA stability at different times of day. mRNAs are targets of the mRNA degradation pathway mediated by the downstream (DST) instability determinant.	150.55	42.05	3.58	2.00E-07	5.51E-06
303	2	L4 vs. M4	258751_at	AT3G05890	Low temperature and salt responsive protein family;(source:Araport11)	72.51	21.00	3.45	1.38E-05	5.88E-05
304	2	L4 vs. M4	245232_at	AT4G25590	actin depolymerizing factor 7;(source:Araport11)	109.01	32.51	3.35	3.00E-07	6.20E-06
305	2	L4 vs. M4	245946_at	AT5G19580	glyoxal oxidase-related protein;(source:Araport11)	835.90	249.30	3.35	1.10E-06	1.44E-05
306	2	L4 vs. M4	265002_at	AT1G24400	High-affinity transporter for neutral and acidic amino acids, expressed in tapetum tissue of anthers. Transport of 1-Aminocyclopropane-1-carboxylic acid (ACC).	144.51	43.22	3.34	7.60E-06	4.10E-05
307	2	L4 vs. M4	249375_at	AT5G40730	Encodes an arabinogalactan-protein (AGP24).	845.68	256.53	3.30	2.20E-06	2.09E-05
308	2	L4 vs. M4	265552_at	AT2G07560	H[+]-ATPase 6;(source:Araport11)	194.22	59.01	3.29	1.00E-07	3.36E-06
309	2	L4 vs. M4	257986_at	AT3G20865	Encodes a putative arabinogalactan-protein (AGP40) that is expressed in pollen.	196.35	60.63	3.24	5.40E-06	3.52E-05
310	2	L4 vs. M4	251770_at	AT3G55970	One of 4 paralogs encoding a 2-oxoglutarate/Fe(II)-dependent oxygenases that hydroxylates JA to 12-OH-JA.	369.60	115.00	3.21	2.30E-06	2.09E-05
311	2	L4 vs. M4	262912_at	AT1G59740	Major facilitator superfamily protein;(source:Araport11)	121.91	38.49	3.17	1.20E-06	1.50E-05
312	2	L4 vs. M4	253125_at	AT4G36040	Chaperone DnaJ-domain superfamily protein;(source:Araport11)	317.60	101.92	3.12	6.60E-04	1.18E-03
313	2	L4 vs. M4	254104_at	AT4G25040	Uncharacterized protein family (UPF0497);(source:Araport11)	106.08	34.24	3.10	3.00E-07	6.20E-06
314	2	L4 vs. M4	256955_at	AT3G13390	SKU5 similar 11;(source:Araport11)	123.65	40.08	3.09	4.50E-06	3.27E-05
315	2	L4 vs. M4	248926_at	AT5G45880	Pollen Ole e 1 allergen and extensin family protein;(source:Araport11)	304.10	99.56	3.05	9.00E-07	1.29E-05
316	2	L4 vs. M4	263113_at	AT1G03150	Acyl-CoA N-acyltransferases (NAT) superfamily protein;(source:Araport11)	178.26	59.09	3.02	3.00E-07	6.20E-06
317	2	L4 vs. M4	255997_s_at	AT1G29930	Subunit of light-harvesting complex II (LHCII),which absorbs light and transfers energy to the photosynthetic reaction center. The mRNA is cell-to-cell mobile.	2759.91	921.84	2.99	5.50E-06	3.52E-05
318	2	L4 vs. M4	255515_at	AT4G02250	Plant invertase/pectin methylesterase inhibitor superfamily protein;(source:Araport11)	107.07	35.83	2.99	3.00E-07	6.20E-06
319	2	L4 vs. M4	254716_at	AT4G13560	Late embryogenesis abundant protein (LEA) family protein;(source:Araport11)	209.52	70.37	2.98	5.50E-06	3.52E-05
320	2	L4 vs. M4	253627_at	AT4G30650	Low temperature and salt responsive protein family;(source:Araport11)	222.44	74.75	2.98	1.88E-04	4.37E-04
321	2	L4 vs. M4	253812_at	AT4G28240	Member of the wound-induced polypeptide (WIP) family. Positively regulates plant resistance against Pst DC3000 by enhancing PTI responses.	228.98	77.48	2.96	4.00E-07	7.54E-06
322	2	L4 vs. M4	252267_at	AT3G49640	Aldolase-type TIM barrel family protein;(source:Araport11)	172.63	59.03	2.92	1.71E-05	6.90E-05
323	2	L4 vs. M4	261395_at	AT1G79700	WRI4 encodes an AP2/ERF-type transcriptional activator that specifically controls cuticular wax biosynthesis in Arabidopsis stems. It also functions to activate transcription of genes involved fatty acid biosynthesis during seed and flower development as well as stem wax biosynthesis. Targets identified by ChIP-seq include: LACS1, KCR1, PAS2, ECR, and WSD1.	169.01	58.01	2.91	1.00E-06	1.34E-05
324	2	L4 vs. M4	250610_at	AT5G07550	member of Oleosin-like protein family	1801.91	637.49	2.83	7.00E-07	1.12E-05
325	2	L4 vs. M4	260042_at	AT1G68820	Putative C3HC4 zinc-finger ubiquitin E3 ligase, negative regulator in ABA and drought stress response.	133.51	47.51	2.81	7.10E-06	4.03E-05
326	2	L4 vs. M4	256772_at	AT3G13750	beta-galactosidase, glycosyl hydrolase family 35 The mRNA is cell-to-cell mobile.	872.14	314.28	2.77	1.04E-05	4.96E-05
327	2	L4 vs. M4	250514_at	AT5G09550	GDP dissociation inhibitor family protein / Rab GTPase activator family protein;(source:Araport11)	69.90	25.22	2.77	2.80E-06	2.34E-05
328	2	L4 vs. M4	250073_at	AT5G17170	rubredoxin family protein;(source:Araport11)	1226.89	442.98	2.77	4.00E-07	7.54E-06
329	2	L4 vs. M4	251222_at	AT3G62580	Late embryogenesis abundant protein (LEA) family protein;(source:Araport11)	120.75	43.98	2.75	3.50E-06	2.70E-05
330	2	L4 vs. M4	255257_at	AT4G05050	polyubiquitin gene, belongs to a subtype group with UBQ10 and UBQ14. Various ecotypes of Arabidopsis have different numbers of ubiquitin repeats within this gene.	140.89	51.64	2.73	1.42E-04	3.48E-04
331	2	L4 vs. M4	252063_at	AT3G51590	Encodes a member of the lipid transfer protein family. Proteins of this family are generally small (~9 kD), basic, expressed abundantly and contain eight Cys residues. The proteins can bind fatty acids and acylCoA esters and can transfer several different phospholipids. They are localized to the cell wall. The LTP12 promoter is active exclusively in the tapetum during the uninucleate microspore and bicellular pollen stages. Predicted to be a member of PR-14 pathogenesis-related protein family with the following members: At2g38540/LTP1, At2g38530/LTP2, At5g59320/LTP3, At5g59310/LTP4, At3g51600/LTP5, At3g08770/LTP6, At2g15050/LTP7, At2g18370/LTP8, At2g15325/LTP9, At5g01870/LTP10, At4g33355/LTP11, At3g51590/LTP12, At5g44265/LTP13, At5g62065/LTP14, At4g08530/LTP15.	190.10	70.70	2.69	6.30E-06	3.82E-05
332	2	L4 vs. M4	246545_at	AT5G15110	Pectate lyase family protein;(source:Araport11)	208.65	77.47	2.69	8.27E-05	2.23E-04
333	2	L4 vs. M4	251258_at	AT3G62170	VANGUARD-like protein;(source:Araport11)	472.01	177.07	2.67	1.38E-05	5.88E-05

334	2	L4 vs. M4	264140_at	AT1G79210	N-terminal nucleophile aminohydrolases (Ntn hydrolases) superfamily protein;(source:Araport11)	632.72	237.80	2.66	5.70E-06	3.60E-05
335	2	L4 vs. M4	266154_at	AT2G12190	Cytochrome P450 superfamily protein;(source:Araport11)	155.16	58.30	2.66	1.13E-05	5.19E-05
336	2	L4 vs. M4	263156_at	AT1G54030	Encodes a vacuolar protein. Mutation causes organizational defects in the endoplasmic reticulum and aberrant protein trafficking in the plant secretory pathway.The mRNA is cell-to-cell mobile.	139.48	52.79	2.64	2.60E-06	2.25E-05
337	2	L4 vs. M4	254085_at	AT4G24960	Homologous to a eukaryote specific ABA- and stress-inducible gene first isolated from barley. Groups in one subfamily with ATHVA22E. Along with other members of the ATHVA22 family, it may be involved in regulation of autophagy during development. The mRNA is cell-to-cell mobile.	402.29	152.46	2.64	6.00E-07	9.91E-06
338	2	L4 vs. M4	265387_at	AT2G20670	sugar phosphate exchanger, putative (DUF506);(source:Araport11)	103.22	39.25	2.63	1.21E-05	5.44E-05
339	2	L4 vs. M4	256416_at	AT3G11050	ferritin 2;(source:Araport11)	150.98	57.42	2.63	8.90E-06	4.49E-05
340	2	L4 vs. M4	253061_at	AT4G37610	BTB and TAZ domain protein. Located in cytoplasm and expressed in fruit, flower and leaves.	108.58	41.28	2.63	7.00E-07	1.12E-05
341	2	L4 vs. M4	262707_at	AT1G16290	transglycosylase;(source:Araport11)	112.41	43.14	2.61	1.00E-06	1.34E-05
342	2	L4 vs. M4	261096_at	AT1G62940	encodes an acyl-CoA synthetase, has in vitro activity towards medium- to long-chain fatty acids and their hydroxylated derivatives. Expressed in the tapetum. Involved in pollen wall exine formation. Null mutants were devoid of pollen grains at anther maturity and were completely male sterile.	131.74	51.15	2.58	6.80E-06	3.98E-05
343	2	L4 vs. M4	251037_at	AT5G02100	Encodes a protein that binds to beta-sitosterol and localizes to the ER. The WFDE motif in ORP3a appears to be important for a direct interaction with PVA12 [Plant VAMP-Associated protein 12]. Mutation of this motif causes ORP3a to relocalize to the Golgi and cytosol. The interaction between PVA12 and ORP3a does not appear to be sterol-dependent.	295.11	114.47	2.58	1.60E-06	1.74E-05
344	2	L4 vs. M4	250545_at	AT5G08160	Encodes a serine/threonine protein kinase.	443.47	172.03	2.58	1.36E-05	5.88E-05
345	2	L4 vs. M4	248638_at	AT5G49070	Encodes KCS21, a member of the 3-ketoacyl-CoA synthase family involved in the biosynthesis of VLCFA (very long chain fatty acids).	120.49	46.82	2.57	1.20E-06	1.50E-05
346	2	L4 vs. M4	263548_at	AT2G21660	Encodes a small glycine-rich RNA binding protein that is part of a negative-feedback loop through which AtGRP7 regulates the circadian oscillations of its own transcript. Gene expression is induced by cold. GRP7 appears to promote stomatal opening and reduce tolerance under salt and dehydration stress conditions, but, promotes stomatal closing and thereby increases stress tolerance under conditions of cold tolerance. Loss of function mutations have increased susceptibility to pathogens suggesting a role in mediating innate immune response. Mutants are also late flowering in a non-photoperiodic manner and are responsive to vernalization suggesting an interaction with the autonomous flowering pathway. There is a reduction of mRNA export from the nucleus in grp7 mutants. GRP7:GFP fusion proteins can be found in the cytosol and nucleus. A substrate of the type III effector HopU1 (mono-ADP-ribosyltransferase).	293.91	114.60	2.56	8.20E-06	4.21E-05
347	2	L4 vs. M4	266592_at	AT2G46210	Fatty acid/sphingolipid desaturase;(source:Araport11)	91.96	36.09	2.55	9.10E-06	4.52E-05
348	2	L4 vs. M4	264993_at	AT1G67290	glyoxal oxidase-related protein;(source:Araport11)	261.67	103.11	2.54	1.69E-05	6.90E-05
349	2	L4 vs. M4	248001_at	AT5G55990	Encodes a member of the Arabidopsis CBL (Calcineurin B-like Calcium Sensor) protein family.	179.62	70.62	2.54	5.70E-06	3.60E-05
350	2	L4 vs. M4	252140_at	AT3G51070	S-adenosyl-L-methionine-dependent methyltransferases superfamily protein;(source:Araport11)	63.40	25.11	2.53	5.00E-06	3.46E-05
351	2	L4 vs. M4	258645_s_a	AT3G07850	Pectin lyase-like superfamily protein;(source:Araport11)	97.02	38.54	2.52	1.55E-05	6.43E-05
352	2	L4 vs. M4	251735_at	AT3G56090	Encodes FERRITIN 3, AtFER3. Ferritins are a class of 24-mer multi-meric proteins found in all kingdoms of life. Function as the main iron store in mammals. Evidence suggests that Arabidopsis ferritins are essential to protect cells against oxidative damage, but they do not constitute the major iron pool.	423.87	168.53	2.52	1.50E-06	1.71E-05
353	2	L4 vs. M4	265080_at	AT1G55570	SKU5 similar 12;(source:Araport11)	56.03	22.51	2.49	1.40E-06	1.63E-05
354	2	L4 vs. M4	253226_at	AT4G35010	putative beta-galactosidase (BGAL11 gene)	70.67	28.39	2.49	1.90E-06	1.94E-05
355	2	L4 vs. M4	261518_at	AT1G71695	Peroxidase superfamily protein;(source:Araport11)	97.58	39.40	2.48	8.00E-06	4.15E-05
356	2	L4 vs. M4	258637_at	AT3G07880	RhoGTPase GDP dissociation inhibitor (RhoGDI) that spatially restricts the sites of growth to a single point on the trichoblast. It regulates the NADPH oxidase RHD2/AtrbohC, which is required for hair growth.	734.80	296.40	2.48	3.10E-06	2.47E-05

357	2	L4 vs. M4	260201_at	AT1G67600	Acid phosphatase/vanadium-dependent haloperoxidase-related protein;(source:Araport11)	215.08	87.53	2.46	9.00E-07	1.29E-05
358	2	L4 vs. M4	252430_at	AT3G47470	Encodes a chlorophyll a/b-binding protein that is more similar to the PSI Cab proteins than the PSII cab proteins. The predicted protein is about 20 amino acids shorter than most known Cab proteins.	1376.47	562.83	2.45	4.80E-06	3.41E-05
359	2	L4 vs. M4	266123_at	AT2G45180	Bifunctional inhibitor/lipid-transfer protein/seed storage 2S albumin superfamily protein;(source:Araport11)	130.59	53.48	2.44	1.40E-06	1.63E-05
360	2	L4 vs. M4	250814_at	AT5G05080	ubiquitin-conjugating enzyme 22;(source:Araport11)	582.78	239.18	2.44	5.30E-06	3.52E-05
361	2	L4 vs. M4	245658_at	AT1G28270	Member of a diversely expressed predicted peptide family showing sequence similarity to tobacco Rapid Alkalinization Factor (RALF), and is believed to play an essential role in the physiology of Arabidopsis. Consists of a single exon and is characterized by a conserved C-terminal motif and N-terminal signal peptide. RALF4 and RALF19 act redundantly in the pollen tube to regulate pollen tube growth.	87.30	35.97	2.43	1.75E-04	4.16E-04
362	2	L4 vs. M4	255331_at	AT4G04330	Encodes a chloroplast thylakoid localized RbcX protein that acts as a chaperone in the folding of Rubisco.	161.72	67.19	2.41	7.60E-06	4.10E-05
363	2	L4 vs. M4	259802_at	AT1G72260	Encodes a thionin which is a cysteine rich protein having antimicrobial properties. Thi2.1 is expressed in response to a variety of pathogens and induced by ethylene and jasmonic acid. Belongs to the plant thionin (PR-13) family with the following members: At1g66100, At5g36910, At1g72260, At2g15010, At1g12663, At1g12660.	36.95	15.42	2.40	3.80E-06	2.87E-05
364	2	L4 vs. M4	252161_at	AT3G50580	transmembrane protein;(source:Araport11)	97.76	40.75	2.40	6.10E-06	3.74E-05
365	2	L4 vs. M4	245700_at	AT5G04180	alpha carbonic anhydrase 3;(source:Araport11)	58.92	24.57	2.40	1.40E-06	1.63E-05
366	2	L4 vs. M4	248402_at	AT5G52100	Is essential for chloroplast NAD(P)H dehydrogenase activity, which is involved in electron transfer between PSII and PSI. Likely functions in biogenesis or stabilization of the NAD(P)H dehydrogenase complex. The mRNA is cell-to-cell mobile.	95.81	39.94	2.40	3.80E-06	2.87E-05
367	2	L4 vs. M4	267443_at	AT2G19000	hypothetical protein;(source:Araport11)	54.63	22.84	2.39	2.70E-04	5.75E-04
368	2	L4 vs. M4	254024_at	AT4G25780	CAP (Cysteine-rich secretory proteins, Antigen 5, and Pathogenesis-related 1 protein) superfamily protein;(source:Araport11)	157.00	65.56	2.39	1.73E-05	6.96E-05
369	2	L4 vs. M4	260430_at	AT1G68200	Zinc finger C-x8-C-x5-C-x3-H type family protein;(source:Araport11)	143.61	60.35	2.38	1.50E-06	1.71E-05
370	2	L4 vs. M4	253725_at	AT4G29340	Profilin is a low-molecular weight, actin monomer-binding protein that regulates the organization of actin cytoskeleton in eukaryotes, including higher plants. PRF4 and PRF5 are late pollen-specific and are not detectable in other cell types of the plant body including microspores and root hairs. Immunocytochemical studies at the subcellular level reveal that both the constitutive and pollen-specific profilins are abundant in the cytoplasm. In vegetative cell types, such as root apical cells, profilins showed localization to nuclei in addition to the cytoplasmic staining.	118.78	49.81	2.38	2.30E-06	2.09E-05
371	2	L4 vs. M4	265633_at	AT2G25490	Encodes an F-box protein involved in the ubiquitin/proteasome-dependent proteolysis of EIN3. The mRNA is cell-to-cell mobile.	182.90	77.31	2.37	4.90E-06	3.46E-05
372	2	L4 vs. M4	249800_at	AT5G23660	Encodes a member of the SWEET sucrose efflux transporter family proteins.	217.93	91.87	2.37	2.75E-05	9.91E-05
373	2	L4 vs. M4	251082_at	AT5G01530	light harvesting complex photosystem II;(source:Araport11)	2064.28	877.08	2.35	1.09E-04	2.82E-04
374	2	L4 vs. M4	262832_s_a	AT1G14880	PLANT CADMIUM RESISTANCE 1;(source:Araport11)	126.83	54.10	2.34	6.50E-06	3.88E-05
375	2	L4 vs. M4	264882_at	AT1G61110	NAC transcription regulator. Regulates endosperm cell expansion during germination.	397.95	170.02	2.34	2.00E-06	1.99E-05
376	2	L4 vs. M4	262202_at	AT2G01110	mutant is Albino and pale green; Chloroplast Protein Translocation (tatC). Core subunit of the chloroplast Tat translocase. Integral chloroplast thylakoid membrane protein.	231.55	99.56	2.33	2.50E-06	2.20E-05
377	2	L4 vs. M4	252649_at	AT3G44680	Encodes HDA9 (a RPD3-like histone deacetylase). Functions in promoting the onset of leaf senescence.The hda9 mutant shows enhanced H3K9 acetylation levels,based on immunodetection using H3K9ac antibodies.	140.97	60.48	2.33	1.60E-06	1.74E-05
378	2	L4 vs. M4	261901_at	AT1G80920	A nuclear encoded soluble protein found in the chloroplast stroma. Negatively regulated by light and has rapid turnover in darkness.	868.14	373.84	2.32	2.05E-05	7.89E-05
379	2	L4 vs. M4	253195_at	AT4G35420	Encodes DRL1 (Dihydroflavonol 4-reductase-like1), a closely related homolog of the rice anther-specific gene OsDFR2. DRL1 may be involved in a metabolic pathway essential for pollen wall development and male fertility. Mutant plants have impaired pollen formation and seed production.	40.03	17.25	2.32	1.92E-05	7.53E-05

380	2	L4 vs. M4	261412_at	AT1G07890	Encodes a cytosolic ascorbate peroxidase APX1. Ascorbate peroxidases are enzymes that scavenge hydrogen peroxide in plant cells. Eight types of APX have been described for Arabidopsis: three cytosolic (APX1, APX2, APX6), two chloroplastic types (stromal sAPX, thylakoid tAPX), and three microsomal (APX3, APX4, APX5) isoforms. At least part of the induction of heat shock proteins during light stress in Arabidopsis is mediated by H2O2 that is scavenged by APX1. Expression of the gene is downregulated in the presence of paraquat, an inducer of photoxidative stress. The mRNA is cell-to-cell mobile.	287.07	124.18	2.31	5.65E-04	1.05E-03
381	2	L4 vs. M4	256398_at	AT3G06100	Encodes NIP7;1, an anther-specific boric acid transporter of the aquaporin superfamily regulated by an unusual tyrosine in helix 2 of the transport pore.	512.04	222.41	2.30	3.16E-05	1.07E-04
382	2	L4 vs. M4	251013_at	AT5G02540	NAD(P)-binding Rossmann-fold superfamily protein;(source:Araport11)	126.54	54.96	2.30	9.20E-06	4.53E-05
383	2	L4 vs. M4	265111_at	AT1G62510	Expressed in the root cortex.	61.50	27.01	2.28	9.10E-06	4.52E-05
384	2	L4 vs. M4	256381_at	AT1G66850	Bifunctional inhibitor/lipid-transfer protein/seed storage 2S albumin superfamily protein;(source:Araport11)	129.33	56.62	2.28	5.50E-06	3.52E-05
385	2	L4 vs. M4	248714_at	AT5G48140	Pectin lyase-like superfamily protein;(source:Araport11)	714.94	314.17	2.28	2.20E-06	2.09E-05
386	2	L4 vs. M4	255795_at	AT2G33380	Encodes a calcium binding protein whose mRNA is induced upon treatment with NaCl, ABA and in response to desiccation. mRNA expression under drought conditions is apparent particularly in leaves and flowers. Isoform of caleosin with a role as a peroxygenase involved in oxylipin metabolism during biotic and abiotic stress. Involved in the production of 2-hydroxy-octadecatrienoic acid. The peroxygenase has a narrow substrate specificity thus acting as a fatty acid hydroperoxide reductase in vivo.	150.65	66.41	2.27	7.78E-05	2.14E-04
387	2	L4 vs. M4	264327_at	AT1G04170	protein synthesis initiation factor eIF2 gamma The mRNA is cell-to-cell mobile.	177.97	78.92	2.26	2.50E-06	2.20E-05
388	2	L4 vs. M4	249251_at	AT5G42000	ORMDL family protein;(source:Araport11)	83.87	37.34	2.25	2.94E-05	1.02E-04
389	2	L4 vs. M4	262417_at	AT1G50170	encodes sirohydrochlorin ferrochelatase catalyzing the last step of the siroheme biosynthesis	235.34	104.99	2.24	7.80E-06	4.12E-05
390	2	L4 vs. M4	247763_at	AT5G59180	Non-catalytic subunit specific to DNA-directed RNA polymerase II; the ortholog of budding yeast RPB7	145.47	64.94	2.24	9.80E-06	4.78E-05
391	2	L4 vs. M4	264007_at	AT2G21140	Proline-rich protein expressed in expanding leaves, stems, flowers, and siliques.	485.71	217.57	2.23	4.00E-06	2.98E-05
392	2	L4 vs. M4	252402_s_at	AT3G48050	Encodes a large protein with N-terminal bromo-adjacent homology (BAH) and transcription elongation factor S-II (TFS2N) domains and two C-terminal GW (glycine and tryptophan) repeats. It is nuclear and colocalizes with the processing-body component DCP1 in the cytoplasm. SOU is a component of the miRNA pathway and is involved in translational repression.	419.46	188.17	2.23	2.80E-06	2.34E-05
393	2	L4 vs. M4	259161_at	AT3G01500	Encodes a putative beta-carbonic anhydrase betaCA1. Together with betaCA4 (At1g70410) regulates CO2-controlled stomatal movements in guard cells, as well as attenuates immunity. Differential CA gene expression in response to changing atmospheric CO2 conditions contribute to altered disease resistance levels. Activated by OXS2 under the treatment of salt.	584.64	263.91	2.22	4.50E-06	3.27E-05
394	2	L4 vs. M4	251996_at	AT3G52840	beta-galactosidase 2;(source:Araport11)	303.60	137.05	2.22	1.20E-04	3.06E-04
395	2	L4 vs. M4	254658_at	AT4G18230	UDP-N-acetylglucosamine transferase subunit ALG14-like protein;(source:Araport11)	82.89	37.42	2.21	1.21E-04	3.07E-04
396	2	L4 vs. M4	261955_at	AT1G64520	regulatory particle non-ATPase 12A;(source:Araport11)	249.41	113.55	2.20	1.89E-04	4.37E-04
397	2	L4 vs. M4	250079_at	AT5G16650	Chaperone DnaJ-domain superfamily protein;(source:Araport11)	872.72	398.31	2.19	6.30E-06	3.82E-05
398	2	L4 vs. M4	246114_at	AT5G20250	encodes a member of glycosyl hydrolase family 36. Expression is induced within 3 hours of dark treatment, in senescing leaves and treatment with exogenous photosynthesis inhibitor. Induction of gene expression was suppressed in excised leaves supplied with sugar. The authors suggest that the gene's expression pattern is responding to the level of sugar in the cell. The mRNA is cell-to-cell mobile.	533.17	243.73	2.19	3.31E-05	1.11E-04
399	2	L4 vs. M4	247981_at	AT5G56640	Myo-Inositol Oxygenase gene family	110.88	50.54	2.19	3.50E-06	2.70E-05
400	2	L4 vs. M4	255248_at	AT4G05180	Encodes the PsbQ subunit of the oxygen evolving complex of photosystem II.	227.40	104.48	2.18	2.41E-05	8.99E-05
401	2	L4 vs. M4	267002_s_at	AT2G34430	Photosystem II type I chlorophyll a/b-binding protein The mRNA is cell-to-cell mobile.	1508.44	695.13	2.17	1.05E-04	2.75E-04
402	2	L4 vs. M4	245330_at	AT4G14930	Survival protein SurE-like phosphatase/nucleotidase;(source:Araport11)	102.15	47.15	2.17	8.74E-04	1.52E-03
403	2	L4 vs. M4	253329_at	AT4G33480	BTB/POZ domain protein TNFAIP protein;(source:Araport11)	110.65	51.08	2.17	1.48E-04	3.59E-04
404	2	L4 vs. M4	248245_at	AT5G53190	Nodulin MtN3 family protein;(source:Araport11)	180.37	83.21	2.17	4.93E-05	1.52E-04
405	2	L4 vs. M4	262341_at	AT1G64230	ubiquitin-conjugating enzyme 28;(source:Araport11)	989.01	457.70	2.16	1.04E-04	2.75E-04

406	2	L4 vs. M4	258402_at	AT3G15450	aluminum induced protein with YGL and LRDR motifs;(source:Araport11)	96.72	44.76	2.16	4.84E-05	1.50E-04
407	2	L4 vs. M4	254267_at	AT4G22930	Encodes dihydroorotase (PYR4).	257.28	119.39	2.16	9.74E-04	1.66E-03
408	2	L4 vs. M4	260536_at	AT2G43400	Encodes a unique electron-transfer flavoprotein:ubiquinone oxidoreductase that is localized to the mitochondrion. Mutants are more sensitive to sugar starvation when plants are kept in the dark for long periods.	141.95	66.44	2.14	1.03E-05	4.96E-05
409	2	L4 vs. M4	253042_at	AT4G37550	Acetamidase/Formamidase family protein;(source:Araport11)	190.31	88.78	2.14	1.52E-05	6.33E-05
410	2	L4 vs. M4	247374_at	AT5G63190	Encodes a member of the MRF (MA3 DOMAIN-CONTAINING TRANSLATION REGULATORY FACTOR) gene family under TOR control that is transcriptionally induced by dark and starvation. MRF1 can be phosphorylated in vitro by S6K1 and S6K2.	424.66	198.61	2.14	7.40E-06	4.10E-05
411	2	L4 vs. M4	256966_at	AT3G13400	SKU5 similar 13;(source:Araport11)	778.68	364.78	2.13	1.71E-05	6.90E-05
412	2	L4 vs. M4	264439_at	AT1G27450	Adenosine phosphoribosyl transferase(E.C:2.4.2.7), involved in the one-step salvage of adenine to AMP.	1149.30	542.51	2.12	4.49E-05	1.41E-04
413	2	L4 vs. M4	264931_at	AT1G60590	Pectin lyase-like superfamily protein;(source:Araport11)	143.99	67.78	2.12	1.07E-05	5.00E-05
414	2	L4 vs. M4	263064_at	AT2G18160	Encodes a b-ZIP transcription factor.	258.10	121.96	2.12	1.97E-04	4.53E-04
415	2	L4 vs. M4	266764_at	AT2G47050	Plant invertase/pectin methylesterase inhibitor superfamily protein;(source:Araport11)	44.54	21.03	2.12	1.98E-04	4.54E-04
416	2	L4 vs. M4	254824_s_at	AT4G12620	Origin Recognition Complex subunit 1b. Involved in the initiation of DNA replication. Regulated transcriptionally during cell cycle, peaking at G1/S-phase. Target of E2F/DF family of transcription factors. Interacts with ORC2 and ORC5. Highly expressed in proliferating cells. Expression levels are independent of light regime.	762.46	360.48	2.12	1.83E-05	7.25E-05
417	2	L4 vs. M4	250159_at	AT5G15200	Ribosomal protein S4;(source:Araport11)	1078.00	508.90	2.12	3.74E-05	1.23E-04
418	2	L4 vs. M4	256836_at	AT3G22960	encodes a chloroplast pyruvate kinase alpha subunit. Important for seed oil biosynthesis. Ubiquitously expressed, with significantly increased expression in maturing seeds. The mRNA is cell-to-cell mobile.	245.00	116.13	2.11	9.20E-06	4.53E-05
419	2	L4 vs. M4	254561_at	AT4G19160	transglutaminase family protein;(source:Araport11)	1711.76	810.20	2.11	5.50E-06	3.52E-05
420	2	L4 vs. M4	259593_at	AT1G27970	Encodes an ortholog of yeast NTF2, a nuclear envelop transport protein that functions as the nuclear import receptor for RanGDP, an essential player in nucleocytoplasmic transport. The mRNA is cell-to-cell mobile.	112.54	53.48	2.10	6.96E-05	2.00E-04
421	2	L4 vs. M4	245060_at	AT2G39770	Encodes a GDP-mannose pyrophosphorylase/ mannose-1-pyrophosphatase. This enzyme provides GDP-mannose, which is used for cell wall carbohydrate biosynthesis and protein glycosylation as well as for ascorbate (vitamin C) biosynthesis. Mutations in this gene confer hypersensitivity to NH4+.	494.65	235.13	2.10	5.10E-06	3.51E-05
422	2	L4 vs. M4	265263_at	AT2G42940	Encodes a nuclear matrix protein with AT-hook DNA binding motifs that acts in the maintenance of genomic integrity by silencing TEs and repeat-containing genes through epigenetic machinery. It interacts with FVE and MSI5 which are components of HDAC corepressor complexes. It is expressed in tapetum during the tetrad stage.	60.45	28.76	2.10	2.71E-04	5.75E-04
423	2	L4 vs. M4	252055_at	AT3G52580	Ribosomal protein S11 family protein;(source:Araport11)	615.36	293.26	2.10	1.78E-05	7.11E-05
424	2	L4 vs. M4	255013_at	AT4G10000	Thioredoxin family protein;(source:Araport11)	142.23	67.57	2.10	9.89E-05	2.62E-04
425	2	L4 vs. M4	253743_at	AT4G28940	Phosphorylase superfamily protein;(source:Araport11)	2605.35	1240.37	2.10	1.47E-05	6.14E-05
426	2	L4 vs. M4	258646_at	AT3G08040	Encodes a member of the MATE (multidrug and toxin efflux family), expressed in roots but not shoots. Mutants accumulate excess iron, manganese and zinc, and express root Fe(III) chelatase activity even under iron sufficiency conditions. FRD3 is likely to function in root xylem loading of an iron chelator or other factor necessary for efficient iron uptake out of the xylem or apoplastic space and into leaf cells.	162.87	77.97	2.09	6.40E-06	3.86E-05
427	2	L4 vs. M4	261525_at	AT1G14330	Galactose oxidase/kelch repeat superfamily protein;(source:Araport11)	740.17	355.81	2.08	3.80E-05	1.23E-04
428	2	L4 vs. M4	254999_at	AT4G09830	nuclear receptor family 2 group C protein;(source:Araport11)	133.16	63.93	2.08	1.29E-05	5.63E-05
429	2	L4 vs. M4	266656_at	AT2G25900	Encodes a protein with two tandem-arrayed CCCH-type zinc fingers that binds RNA and is involved in RNA turnover. The mRNA is cell-to-cell mobile.	129.21	62.48	2.07	3.69E-04	7.26E-04
430	2	L4 vs. M4	266766_at	AT2G46880	purple acid phosphatase 14;(source:Araport11)	54.15	26.21	2.07	6.74E-05	1.96E-04
431	2	L4 vs. M4	266952_at	AT2G34555	Encodes a gibberellin 2-oxidase that acts on C19 gibberellins to deactivate them.	71.09	34.49	2.06	5.15E-05	1.58E-04
432	2	L4 vs. M4	262716_at	AT1G16470	Encodes 20S proteasome subunit PAB1 (PAB1).	433.30	211.29	2.05	7.00E-06	4.00E-05
433	2	L4 vs. M4	264529_at	AT1G30820	Cytidine triphosphate synthase.	52.44	25.64	2.05	2.81E-05	9.99E-05
434	2	L4 vs. M4	267582_at	AT2G41970	Encodes MRI, a plasma membrane-localized member of the RLCK-VIII subfamily. Preferentially expressed in both pollen tubes and root hairs. mri-knockout mutants display spontaneous pollen tube and root-hair bursting.	49.76	24.31	2.05	7.68E-05	2.13E-04

435	2	L4 vs. M4	252090_at	AT3G52130	Bifunctional inhibitor/lipid-transfer protein/seed storage 2S albumin superfamily protein;(source:Araport11)	97.01	47.37	2.05	3.26E-04	6.62E-04
436	2	L4 vs. M4	250576_at	AT5G08250	Cytochrome P450 superfamily protein;(source:Araport11)	74.90	36.52	2.05	3.78E-05	1.23E-04
437	2	L4 vs. M4	259928_at	AT1G34380	5-3 exonuclease family protein;(source:Araport11)	252.95	123.96	2.04	1.71E-05	6.90E-05
438	2	L4 vs. M4	267027_at	AT2G38330	MATE efflux family protein;(source:Araport11)	112.84	55.20	2.04	3.75E-04	7.33E-04
439	2	L4 vs. M4	246917_at	AT5G25280	serine-rich protein-like protein;(source:Araport11)	61.00	29.96	2.04	5.99E-04	1.10E-03
440	2	L4 vs. M4	260997_at	AT1G26610	C2H2-like zinc finger protein;(source:Araport11)	170.43	84.14	2.03	1.90E-05	7.47E-05
441	2	L4 vs. M4	266662_at	AT2G25830	YebC-like protein;(source:Araport11)	251.36	123.83	2.03	3.64E-04	7.18E-04
442	2	L4 vs. M4	250334_at	AT5G11770	NADH-ubiquinone oxidoreductase 20 kDa subunit;(source:Araport11)	1794.75	885.87	2.03	2.88E-05	1.02E-04
443	2	L4 vs. M4	260769_at	AT1G49010	Duplicated homeodomain-like superfamily protein;(source:Araport11)	122.75	60.65	2.02	1.21E-05	5.44E-05
444	2	L4 vs. M4	266319_s_at	AT2G46720	Encodes KCS13, a member of the 3-ketoacyl-CoA synthase family involved in the biosynthesis of VLCFA (very long chain fatty acids).	139.60	69.23	2.02	8.16E-05	2.22E-04
445	2	L4 vs. M4	250606_s_at	AT5G07410	Encodes a pectin methylesterase implicated in the remodeling of pectins in pollen. Its expression is restricted to the male gametophyte (dry pollen, imbibed pollen and pollen tube). Mutants do not display any phenotype in the vegetative tissues. Pollen germination of KO mutant is strongly delayed in vivo and in vitro. Extra calcium restores the wild type phenotype in vitro.	767.48	379.03	2.02	1.15E-04	2.97E-04
446	2	L4 vs. M4	257333_at	ATMG01360	cytochrome c oxidase subunit 1	523.99	259.33	2.02	9.72E-04	1.66E-03
447	2	L4 vs. M4	245968_at	AT5G19810	Proline-rich extensin-like family protein;(source:Araport11)	67.17	33.44	2.01	8.83E-04	1.53E-03
448	2	L4 vs. M4	254830_at	AT4G12590	ER membrane protein complex subunit-like protein (Protein of unknown function DUF106, transmembrane);(source:Araport11)	212.56	106.55	1.99	2.94E-05	1.02E-04
449	2	L4 vs. M4	248961_at	AT5G45650	subtilase family protein;(source:Araport11)	81.72	41.14	1.99	1.25E-04	3.15E-04
450	2	L4 vs. M4	256469_at	AT1G32540	Encodes a protein with 3 plant-specific zinc finger domains that acts as a positive regulator of cell death.	113.56	57.25	1.98	1.16E-05	5.28E-05
451	2	L4 vs. M4	263160_at	AT1G54080	oligouridylate-binding protein 1A;(source:Araport11)	290.14	146.64	1.98	2.82E-05	1.00E-04
452	2	L4 vs. M4	262313_at	AT1G70900	hypothetical protein;(source:Araport11)	318.68	161.26	1.98	7.18E-04	1.27E-03
453	2	L4 vs. M4	258685_at	AT3G07830	Pectin lyase-like superfamily protein;(source:Araport11)	235.78	119.05	1.98	1.92E-04	4.43E-04
454	2	L4 vs. M4	262399_at	AT1G49500	transcription initiation factor TFIID subunit 1b-like protein;(source:Araport11)	216.91	110.24	1.97	6.27E-05	1.83E-04
455	2	L4 vs. M4	264025_at	AT2G21050	Encodes LAX2 (LIKE AUXIN RESISTANT), a member of the AUX1 LAX family of auxin influx carriers. Required for the establishment of embryonic root cell organization.	59.07	29.99	1.97	1.66E-04	3.99E-04
456	2	L4 vs. M4	250993_at	AT5G02280	Part of multi-protein complex, acting as guanine nucleotide exchange factors (GEFs) and possibly as tethers, regulating intracellular trafficking.	539.01	274.05	1.97	1.13E-04	2.91E-04
457	2	L4 vs. M4	261559_at	AT1G01780	Encodes a member of the Arabidopsis LIM proteins: a family of actin bundlers with distinct expression patterns. WLIM1, WLIM2a, and WLIM2b are widely expressed, whereas PLIM2a, PLIM2b, and PLIM2c are predominantly expressed in pollen. Regulates actin cytoskeleton organization. The mRNA is cell-to-cell mobile.	53.92	27.58	1.96	3.63E-04	7.18E-04
458	2	L4 vs. M4	250565_at	AT5G08000	Encodes a member of the X8-GPI family of proteins. It localizes to the plasmodesmata and binds callose.	497.27	253.41	1.96	1.17E-04	3.00E-04
459	2	L4 vs. M4	250243_at	AT5G13630	Encodes magnesium chelatase involved in plastid-to-nucleus signal transduction.	394.15	201.15	1.96	2.48E-04	5.40E-04
460	2	L4 vs. M4	264373_at	AT1G11890	member of SEC22 Gene Family	799.59	409.86	1.95	1.90E-04	4.41E-04
461	2	L4 vs. M4	262922_at	AT1G79420	C-type mannose receptor (DUF620);(source:Araport11)	59.19	30.31	1.95	1.25E-04	3.14E-04
462	2	L4 vs. M4	258035_at	AT3G21180	one of the type IIB calcium pump isoforms. encodes an autoinhibited Ca(2+)-ATPase that contains an N-terminal calmodulin binding autoinhibitory domain.	75.23	38.67	1.95	5.15E-05	1.58E-04
463	2	L4 vs. M4	250206_at	AT5G14040	Encodes a mitochondrial phosphate transporter. Modulates plant responses to salt stress.	1582.72	809.65	1.95	7.25E-05	2.05E-04
464	2	L4 vs. M4	248200_at	AT5G54160	A caffeic acid/5-hydroxyferulic acid O-methyltransferase. Interacts with 14-4-3 proteins in yeast 2 hybrid assay. AtOMT1 (At5g54160) encodes a flavonol 3?-O-methyltransferase that is highly active towards quercetin and myricetin. The substrate specificity identifies the enzyme as flavonol 3?-methyltransferase which replaces the former annotation of the gene to encode a caffeic acid/5-hydroxyferulic acid O-methyltransferase The mRNA is cell-to-cell mobile.	1285.72	658.70	1.95	4.31E-05	1.37E-04
465	2	L4 vs. M4	260903_at	AT1G02460	Pectin lyase-like superfamily protein;(source:Araport11)	340.40	175.70	1.94	1.39E-04	3.43E-04
466	2	L4 vs. M4	248663_at	AT5G48590	phosphoserine aminotransferase, putative (DUF760);(source:Araport11)	150.60	78.11	1.93	6.75E-05	1.96E-04
467	2	L4 vs. M4	260352_at	AT1G69295	Encodes a member of the X8-GPI family of proteins. It localizes to the plasmodesmata and is predicted to bind callose.	145.71	75.97	1.92	2.53E-04	5.46E-04

468	2	L4 vs. M4	267034_at	AT2G38310	Encodes a member of the PYR (pyrabactin resistance)/PYL(PYR1-like)/RCAR (regulatory components of ABA receptor) family proteins with 14 members. PYR/PYL/RCAR family proteins function as abscisic acid sensors. Mediate ABA-dependent regulation of protein phosphatase 2Cs ABI1 and ABI2. The mRNA is cell-to-cell mobile.	54.78	28.48	1.92	5.41E-05	1.62E-04
469	2	L4 vs. M4	262038_at	AT1G35580	CINV1 / A/N-InvG is an alkaline/neutral invertase that breaks sucrose down into fructose and glucose (GH100). The exact localization of CINV1 remains under investigation but there is evidence that fluorescently-tagged CINV1 localizes to the cytoplasm. atinvg mutants have reduced root growth, reduced invertase activity, and increased expression of antioxidant genes under basal conditions. The levels of CINV1 / A/N-InvG transcripts rise in response to a hydrogen peroxide treatment. The protein has been shown to interact with PIP5K9.	143.76	75.46	1.91	3.51E-05	1.17E-04
470	2	L4 vs. M4	258392_at	AT3G15400	Encodes a protein with novel repeat sequences and a glycine-rich domain which has a 53% identity to GRP1, a petunia glycine-rich cell wall protein.	599.36	314.48	1.91	1.28E-04	3.19E-04
471	2	L4 vs. M4	255479_at	AT4G02380	Encodes AtLEA5 (late embryogenesis abundant like protein). Also known as SENESCENCE-ASSOCIATED GENE 21 (SAG21). Has a role on oxidative stress tolerance. mRNA levels are elevated in response to various stresses.	95.74	50.72	1.89	1.22E-04	3.10E-04
472	2	L4 vs. M4	253880_at	AT4G27590	Heavy metal transport/detoxification superfamily protein;(source:Araport11)	70.31	37.23	1.89	4.14E-05	1.33E-04
473	2	L4 vs. M4	267489_s_at	AT4G30100	P-loop containing nucleoside triphosphate hydrolases superfamily protein;(source:Araport11)	55.37	29.32	1.89	1.97E-04	4.53E-04
474	2	L4 vs. M4	250972_at	AT5G02840	Encodes RVE4, a homolog of the circadian rhythm regulator RVE8. rve4 rve6 rve8 triple mutants display an extremely long circadian period, with delayed and reduced expression of evening-phased clock genes. Involved in heat shock response.	52.42	27.73	1.89	2.58E-05	9.36E-05
475	2	L4 vs. M4	247794_at	AT5G58670	phosphatidylinositol-specific phospholipase C is induced to a significant extent under various environmental stresses, such as dehydration, salinity, and low temperature. May play a role in secondary ABA response. There are two genes called ATPLC1, one corresponding to AT4g38530 and one corresponding ot AT5g58670 (this one).	72.19	38.12	1.89	7.18E-05	2.04E-04
476	2	L4 vs. M4	259892_at	AT1G72610	germin-like protein (GLP1)	434.98	231.41	1.88	1.04E-04	2.75E-04
477	2	L4 vs. M4	252871_at	AT4G40000	S-adenosyl-L-methionine-dependent methyltransferases superfamily protein;(source:Araport11)	138.32	73.67	1.88	3.39E-04	6.84E-04
478	2	L4 vs. M4	244974_at	ATCG00700	PSII low MW protein	104.04	55.42	1.88	6.38E-04	1.16E-03
479	2	L4 vs. M4	246463_at	AT5G16970	encodes a 2-alkenal reductase (EC 1.3.1.74), plays a key role in the detoxification of reactive carbonyls	61.21	32.78	1.87	7.57E-04	1.33E-03
480	2	L4 vs. M4	249427_at	AT5G39850	Ribosomal protein S4;(source:Araport11)	305.81	163.29	1.87	5.25E-05	1.59E-04
481	2	L4 vs. M4	257082_at	AT3G20580	COBRA-like protein 10 precursor;(source:Araport11)	34.37	18.62	1.85	1.79E-04	4.23E-04
482	2	L4 vs. M4	260238_at	AT1G74520	Part of the AtHVA22a family. Protein expression is ABA- and stress-inducible.	184.40	100.04	1.84	6.05E-05	1.78E-04
483	2	L4 vs. M4	263876_at	AT2G21880	RAB GTPase homolog 7A;(source:Araport11)	143.20	77.88	1.84	3.89E-04	7.56E-04
484	2	L4 vs. M4	258054_at	AT3G16240	Delta tonoplast intrinsic protein, functions as a water channel and ammonium (NH3) transporter. Highly expressed in flower, shoot, and stem. Expression shows diurnal regulation and is induced by ammonium (NH3). Protein localized to vacuolar membrane. The mRNA is cell-to-cell mobile.	367.84	199.98	1.84	8.07E-05	2.21E-04
485	2	L4 vs. M4	257315_at	AT3G30775	Encodes a proline oxidase that is predicted to localize to the inner mitochondrial membrane, its mRNA expression induced by high levels of Al and by osmotic stress. The promoter contains an L-proline-inducible element.	362.16	196.69	1.84	3.30E-04	6.70E-04
486	2	L4 vs. M4	251127_at	AT5G01080	Beta-galactosidase related protein;(source:Araport11)	109.32	59.38	1.84	5.42E-05	1.62E-04
487	2	L4 vs. M4	250293_s_a	AT5G13360	Auxin-responsive GH3 family protein;(source:Araport11)	245.41	133.05	1.84	2.11E-04	4.75E-04
488	2	L4 vs. M4	247163_at	AT5G65685	UDP-Glycosyltransferase superfamily protein;(source:Araport11)	321.23	174.41	1.84	3.08E-04	6.41E-04
489	2	L4 vs. M4	265459_at	AT2G46540	fiber;(source:Araport11)	246.75	134.66	1.83	5.63E-05	1.67E-04
490	2	L4 vs. M4	254448_at	AT4G20870	encodes a fatty acid hydroxylase, required for the AtBI-1-mediated suppression of programmed cell death.	259.27	142.04	1.83	6.48E-04	1.17E-03
491	2	L4 vs. M4	262765_at	AT1G13150	member of CYP86C	178.67	98.19	1.82	6.48E-04	1.17E-03
492	2	L4 vs. M4	261296_at	AT1G48460	tRNA-processing ribonuclease BN;(source:Araport11)	235.29	129.04	1.82	9.64E-05	2.58E-04
493	2	L4 vs. M4	260493_at	AT2G41830	Uncharacterized protein;(source:Araport11)	291.03	160.12	1.82	6.84E-04	1.22E-03
494	2	L4 vs. M4	250194_at	AT5G14550	Core-2/I-branching beta-1,6-N-acetylglucosaminyltransferase family protein;(source:Araport11)	53.74	29.58	1.82	4.04E-04	7.79E-04
495	2	L4 vs. M4	246841_at	AT5G26700	RmlC-like cupins superfamily protein;(source:Araport11)	97.09	53.40	1.82	8.86E-04	1.53E-03

496	2	L4 vs. M4	267239_at	AT2G02510	NADH dehydrogenase (ubiquinone)s;(source:Araport11)	165.83	91.81	1.81	3.61E-04	7.18E-04
497	2	L4 vs. M4	250288_at	AT5G13350	Auxin-responsive GH3 family protein;(source:Araport11)	261.25	144.39	1.81	1.99E-04	4.55E-04
498	2	L4 vs. M4	265576_at	AT2G20190	Encodes a microtubule-associated protein that is involved in both cell division and cell expansion. It likely promotes microtubule stability.	108.90	60.52	1.80	1.37E-04	3.40E-04
499	2	L4 vs. M4	266636_at	AT2G35370	Encodes glycine decarboxylase complex H protein. Involved in photorespiration. The mRNA is cell-to-cell mobile.	91.42	50.86	1.80	5.76E-04	1.07E-03
500	2	L4 vs. M4	266981_at	AT2G39460	Encodes a 60S ribosomal protein L23aA (AtrpL23aA). Paralog of RLPL23aB.	150.22	83.54	1.80	6.42E-04	1.16E-03
501	2	L4 vs. M4	266750_s_at	AT2G47040	Share high homologies with a group of pectin methylesterases (PME), pollen specific, and is required for enhancing the growth of pollen tube in style and transmitting tract tissues.	845.19	470.75	1.80	1.45E-04	3.54E-04
502	2	L4 vs. M4	258636_at	AT3G07890	Ypt/Rab-GAP domain of gyp1p superfamily protein;(source:Araport11)	141.71	78.88	1.80	3.02E-04	6.29E-04
503	2	L4 vs. M4	250862_s_at	AT5G04800	Ribosomal S17 family protein;(source:Araport11)	747.71	415.58	1.80	2.19E-04	4.88E-04
504	2	L4 vs. M4	248957_at	AT5G45620	Proteasome component (PCI) domain protein;(source:Araport11)	202.14	112.39	1.80	3.13E-04	6.46E-04
505	2	L4 vs. M4	259416_at	AT1G02305	Encodes a capase involved in stress induced cell death.	1483.27	830.12	1.79	4.03E-04	7.79E-04
506	2	L4 vs. M4	260228_at	AT1G74540	Encodes a tricoumaroylspermidine / triferuloylspermidine meta-hydroxylase that participates in the formation of N¹,N⁵-di(hydroxyferuloyol)- N¹⁰-sinapoyl spermidine, an important constituent of pollen. This gene appears to be expressed in young flower buds and inflorescence tips with notably high levels of expression in the tapetum and pollen.	439.36	245.72	1.79	9.85E-05	2.62E-04
507	2	L4 vs. M4	259192_at	AT3G01740	Mitochondrial ribosomal protein L37;(source:Araport11)	75.45	42.37	1.78	1.99E-04	4.55E-04
508	2	L4 vs. M4	246004_at	AT5G20630	Encodes a germin-like protein. Its transcripts are more abundant in RNA from leaves collected in the evening, suggesting some kind of circadian regulation.	122.70	68.81	1.78	6.53E-04	1.17E-03
509	2	L4 vs. M4	267383_at	AT2G44360	ecotropic viral integration site protein;(source:Araport11)	241.69	136.47	1.77	2.20E-04	4.88E-04
510	2	L4 vs. M4	262121_at	AT1G02800	Encodes a protein with similarity to endo-1,4-b-glucanases and is a member of Glycoside Hydrolase Family 9. CEL2 is induced by nemotodes and is expressed in syncitia induced by Heterodera schachtii.May be involved in the development and function of syncitia.	313.49	177.83	1.76	5.46E-04	1.02E-03
511	2	L4 vs. M4	259498_at	AT1G15880	Golgi SNARE 11 protein (GOS11)	933.98	531.05	1.76	3.64E-04	7.18E-04
512	2	L4 vs. M4	259635_at	AT1G56360	purple acid phosphatase 6;(source:Araport11)	26.61	15.13	1.76	3.36E-04	6.79E-04
513	2	L4 vs. M4	264040_at	AT2G03730	Member of a small family of ACT domain containing proteins. ACT domains are thought to be involved in amino acid binding.	101.27	57.44	1.76	3.52E-04	7.05E-04
514	2	L4 vs. M4	264246_at	AT1G60140	Encodes an enzyme putatively involved in trehalose biosynthesis. The protein has a trehalose synthase (TPS)-like domain that may or may not be active as well as a trehalose phosphatase (TPP)-like domain.	91.12	52.18	1.75	3.17E-04	6.51E-04
515	2	L4 vs. M4	263950_at	AT2G36020	HVA22-like protein J;(source:Araport11)	46.26	26.41	1.75	4.73E-04	8.97E-04
516	2	L4 vs. M4	254001_at	AT4G26260	Encodes a myo-inositol oxygenase, which is the first enzyme in the inositol route to ascorbate (L‐ascorbic acid, AsA, vitamin C). Overexpression results in enhanced biomass and abiotic stress tolerance.	48.73	27.84	1.75	3.26E-04	6.62E-04
517	2	L4 vs. M4	248127_at	AT5G54750	Part of multi-protein complex, acting as guanine nucleotide exchange factors (GEFs) and possibly as tethers, regulating intracellular trafficking.	380.86	218.88	1.74	3.52E-04	7.05E-04
518	2	L4 vs. M4	259838_at	AT1G52220	Thylakoid membrane localized protein that interacts with other CURT family proteins. Oligomerization is associated with grana thylakoid curavature.	73.90	42.76	1.73	4.13E-04	7.92E-04
519	2	L4 vs. M4	262162_at	AT1G78020	FCS like zinc finger 6 is induced during energy starvation through SnRK1 signaling. Mutants accumulate more SnRK1alpha1 which results in the inhibition of seedling growth under favorable growth conditions. Increased SnRK1 activity in the mutant also results in the downregulation of TOR signaling (DOI:10.1111/tpj.13854).	131.62	75.93	1.73	1.87E-04	4.35E-04
520	2	L4 vs. M4	253950_at	AT4G26910	Dihydrolipoamide succinyltransferase;(source:Araport11)	375.57	216.87	1.73	6.77E-04	1.21E-03
521	2	L4 vs. M4	266591_at	AT2G46225	Encodes a subunit of the WAVE complex. The WAVE complex is required for activation of ARP2/3 complex which functions in actin microfilament nucleation and branching. One of four ABI-like proteins.	93.54	54.51	1.72	2.96E-04	6.19E-04
522	2	L4 vs. M4	250739_at	AT5G05740	S2P-like putative metalloprotease, also contain transmembrane helices near their C-termini and many of them, five of seven, contain a conserved zinc-binding motif HEXXH. Homolog of EGY1. Each of the EGY1 and EGY-like proteins share two additional highly conserved motifs, the previously reported NPDG motif (aa 442?454 in EGY1, Rudner et al., 1999) and a newly defined GNLR motif (aa 171?179 in EGY1). The GNLR motif is a novel signature motif unique to EGY1 and EGY-like proteins as well as other EGY1 orthologs found in cyanobacteria.	300.60	174.57	1.72	2.23E-04	4.94E-04

523	2	L4 vs. M4	249645_at	AT5G36910	Encodes a thionin that is expressed at a low basal level in seedlings and shows circadian variation. Predicted to encode a PR (pathogenesis-related) protein. Belongs to the plant thionin (PR-13) family with the following members: At1g66100, At5g36910, At1g72260, At2g15010, At1g12663, At1g12660.	114.46	66.71	1.72	2.90E-04	6.09E-04
524	2	L4 vs. M4	261560_at	AT1G01710	acyl-CoA thioesterase II;(source:Araport11)	147.11	86.49	1.70	3.05E-04	6.35E-04
525	2	L4 vs. M4	261126_at	AT1G04950	Encodes one of two Arabidopsis proteins with significant similarity to the histone fold TBP-associated factor TAF6. Mutants are embryo lethal and transmission of the mutant allele through the male gametophyte is significantly reduced. This is due to reduced pollen tube growth of the mutant.	195.50	115.02	1.70	2.88E-04	6.06E-04
526	2	L4 vs. M4	265587_at	AT2G19980	CAP (Cysteine-rich secretory proteins, Antigen 5, and Pathogenesis-related 1 protein) superfamily protein;(source:Araport11)	41.74	24.61	1.70	7.27E-04	1.28E-03
527	2	L4 vs. M4	254083_at	AT4G24920	secE/sec61-gamma protein transport protein;(source:Araport11)	79.12	46.49	1.70	3.16E-04	6.50E-04
528	2	L4 vs. M4	256243_at	AT3G12500	encodes a basic chitinase involved in ethylene/jasmonic acid mediated signalling pathway during systemic acquired resistance based on expression analyses.	384.14	228.22	1.68	3.72E-04	7.29E-04
529	2	L4 vs. M4	260264_at	AT1G68500	hypothetical protein;(source:Araport11)	216.12	129.53	1.67	6.22E-04	1.14E-03
530	2	L4 vs. M4	260775_at	AT1G78300	G-box binding factor GF14 omega encoding a 14-3-3 protein The mRNA is cell-to-cell mobile.	386.54	230.94	1.67	6.52E-04	1.17E-03
531	2	L4 vs. M4	259349_at	AT3G03860	Encodes a protein disulfide isomerase-like (PDIL) protein, a member of a multigene family within the thioredoxin (TRX) superfamily. This protein also belongs to the adenosine 5'-phosphosulfate reductase-like (APRL) group. The mRNA is cell-to-cell mobile.	210.51	126.39	1.67	5.96E-04	1.10E-03
532	2	L4 vs. M4	255456_at	AT4G02920	hypothetical protein;(source:Araport11)	24.29	14.52	1.67	8.29E-04	1.44E-03
533	2	L4 vs. M4	251842_at	AT3G54580	Proline-rich extensin-like family protein;(source:Araport11)	40.54	24.35	1.66	5.97E-04	1.10E-03
534	2	L4 vs. M4	256664_at	AT3G12040	Encodes a 3-methyladenine-DNA glycosylase. Arabdiopsis cDNA complements the methyl methanesulfonate-sensitive phenotype of an Escherichia coli double mutant deficient in 3-methyladenine glycosylases (DNA-3-methyladenine glycosidases I and II, EC 3.2.2.20 and 3.2.2.21, respectively, encoded by tag and alkA).	60.28	36.79	1.64	9.39E-04	1.61E-03
535	2	L4 vs. M4	257808_at	AT3G26935	DHHC-type zinc finger family protein;(source:Araport11)	74.61	45.48	1.64	4.77E-04	9.02E-04
536	2	L4 vs. M4	262609_at	AT1G13930	Involved in response to salt stress. Knockout mutants are hypersensitive to salt stress. The mRNA is cell-to-cell mobile.	821.29	507.25	1.62	8.94E-04	1.54E-03
537	2	L4 vs. M4	245015_at	ATCG00490	large subunit of RUBISCO. Protein is tyrosine-phosphorylated and its phosphorylation state is modulated in response to ABA in Arabidopsis thaliana seeds.	7402.27	4670.41	1.58	9.88E-04	1.68E-03
538	2	L4 vs. M4	263250_at	AT2G31390	Encodes a member of the fructokinase gene family. Nomenclature according to Riggs 2017 has been adopted for the family by the community (personal communication, Boernke, Callis, Granot, Boernke, and Smeekens).	127.94	205.80	0.62	7.49E-04	1.32E-03
539	2	L4 vs. M4	256276_at	AT3G12070	RAB geranylgeranyl transferase beta subunit 2;(source:Araport11)	345.06	554.83	0.62	9.22E-04	1.58E-03
540	2	L4 vs. M4	253805_at	AT4G28260	acyl-UDP-N-acetylglucosamine O-acyltransferase;(source:Araport11)	78.85	126.90	0.62	7.88E-04	1.38E-03
541	2	L4 vs. M4	249126_at	AT5G43380	encodes a type I serine/threonine protein phosphatase expressed in expressed in roots, rosettes and flowers.	62.62	100.82	0.62	9.76E-04	1.66E-03
542	2	L4 vs. M4	248751_at	AT5G47540	Mo25 family protein;(source:Araport11)	115.67	187.77	0.62	7.17E-04	1.27E-03
543	2	L4 vs. M4	262691_at	AT1G62740	Encodes one of the 36 carboxylate clamp (CC)-tetratricopeptide repeat (TPR) proteins (Prasad 2010, Pubmed ID: 20856808) with potential to interact with Hsp90/Hsp70 as co-chaperones. The mRNA is cell-to-cell mobile.	184.72	301.74	0.61	4.50E-04	8.57E-04
544	2	L4 vs. M4	265772_at	AT2G48010	receptor-like serine/threonine kinase (RKF3) The mRNA is cell-to-cell mobile.	46.80	76.51	0.61	7.85E-04	1.37E-03
545	2	L4 vs. M4	245987_at	AT5G13180	Encodes a NAC domain transcription factor that interacts with VND7 and negatively regulates xylem vessel formation.	37.63	61.43	0.61	5.91E-04	1.09E-03
546	2	L4 vs. M4	247449_at	AT5G62290	nucleotide-sensitive chloride conductance regulator (ICln) family protein;(source:Araport11)	142.99	236.15	0.61	3.92E-04	7.60E-04
547	2	L4 vs. M4	264590_at	AT2G17710	Big1;(source:Araport11)	88.38	147.25	0.60	7.81E-04	1.37E-03
548	2	L4 vs. M4	251584_at	AT3G58620	Encodes one of the 36 carboxylate clamp (CC)-tetratricopeptide repeat (TPR) proteins (Prasad 2010, Pubmed ID: 20856808) with potential to interact with Hsp90/Hsp70 as co-chaperones. The TTL family is required for osmotic stress tolerance and male sporogenesis.	20.26	33.78	0.60	3.60E-04	7.18E-04
549	2	L4 vs. M4	245254_at	AT4G14680	Encodes one of three A. thaliana ATP-sulfurylases. APS is the first enzyme of sulfate assimilation that catalyzes the formation of adenosine-5'-phosphosulfate from ATP and sulfate.	68.26	114.64	0.60	4.27E-04	8.17E-04

550	2	L4 vs. M4	254200_at	AT4G24110	NADP-specific glutamate dehydrogenase;(source:Araport11)	58.22	96.37	0.60	5.88E-04	1.09E-03
551	2	L4 vs. M4	260410_at	AT1G69870	Encodes a low affinity nitrate transporter NRT1.7. Expressed in phloem. Responsible for source-to-sink remobilization of nitrate. The mRNA is cell-to-cell mobile.	18.92	31.88	0.59	6.51E-04	1.17E-03
552	2	L4 vs. M4	263881_at	AT2G21820	seed maturation protein;(source:Araport11)	51.13	87.30	0.59	3.40E-04	6.84E-04
553	2	L4 vs. M4	256900_at	AT3G24670	Pectin lyase-like superfamily protein;(source:Araport11)	33.93	57.82	0.59	2.19E-04	4.88E-04
554	2	L4 vs. M4	253362_s_at	AT4G33120	S-adenosyl-L-methionine-dependent methyltransferases superfamily protein;(source:Araport11)	44.21	75.30	0.59	2.36E-04	5.16E-04
555	2	L4 vs. M4	253341_at	AT4G33410	SIGNAL PEPTIDE PEPTIDASE-LIKE 1;(source:Araport11)	185.15	311.58	0.59	6.24E-04	1.14E-03
556	2	L4 vs. M4	246785_at	AT5G27380	Encodes a protein with similarity to glutathione synthetases, which catalyzes one of the early steps in glutathione biosynthesis. Two transcripts have been detected; the longer transcript is less abundant and the protein is localized to the chloroplast. The smaller transcript, in which the transit peptide is truncated, is localized to the cytosol. Increased glutathione accumulation in response to cesium stress.	28.38	48.10	0.59	8.30E-04	1.44E-03
557	2	L4 vs. M4	264356_at	AT1G03190	UV damage and heat induce a common stress response in plants that leads to tissue death and reduced chloroplast function. The UVH6 product is suggested to be a negative regulator of this response.	42.69	73.72	0.58	6.42E-04	1.16E-03
558	2	L4 vs. M4	261689_at	AT1G50140	P-loop containing nucleoside triphosphate hydrolases superfamily protein;(source:Araport11)	143.97	247.25	0.58	6.39E-04	1.16E-03
559	2	L4 vs. M4	245188_at	AT1G67660	Restriction endonuclease, type II-like superfamily protein;(source:Araport11)	101.47	173.98	0.58	2.00E-04	4.56E-04
560	2	L4 vs. M4	265375_at	AT2G06530	SNF7 family protein;(source:Araport11)	66.82	115.94	0.58	8.56E-04	1.49E-03
561	2	L4 vs. M4	266864_at	AT2G26800	Mutant has increased seed ile, leu and val as well as his and arg.	186.49	323.54	0.58	3.26E-04	6.62E-04
562	2	L4 vs. M4	263824_at	AT2G40360	Encodes BOP1, an ortholog of Block of cell proliferation (BOP) protein. A T-DNA null allele of the BOP1 gene is lethal, and a 50% decrease in transcript accumulation is sufficient to cause severe developmental defects linked to defective cell division.	158.53	274.12	0.58	6.09E-04	1.12E-03
563	2	L4 vs. M4	250398_at	AT5G11000	hypothetical protein (DUF868);(source:Araport11)	49.76	85.83	0.58	8.80E-04	1.52E-03
564	2	L4 vs. M4	244938_at	ATCG01110	Encodes the 49KDa plastid NAD(P)H dehydrogenase subunit H protein. Its transcription is regulated by an ndhF-specific plastid sigma factor, SIG4.	55.08	94.30	0.58	6.34E-04	1.15E-03
565	2	L4 vs. M4	265092_at	AT1G03910	Encodes the Arabidopsis homolog of a conserved eukaryotic protein without known functional domains. The protein that localizes to nuclear speckles and colocalizes with known splicing proteins.	190.63	334.78	0.57	2.56E-04	5.49E-04
566	2	L4 vs. M4	261164_at	AT1G34470	magnesium transporter, putative (DUF803);(source:Araport11)	51.60	91.02	0.57	2.59E-04	5.53E-04
567	2	L4 vs. M4	260943_at	AT1G45145	encodes a cytosolic thioredoxin that reduces disulfide bridges of target proteins by the reversible formation of a disulfide bridge between two neighboring Cys residues present in the active site. Thioredoxins have been found to regulate a variety of biological reactions in prokaryotic and eukaryotic cells.	36.51	63.91	0.57	1.67E-04	4.01E-04
568	2	L4 vs. M4	264741_at	AT1G62290	Saposin-like aspartyl protease family protein;(source:Araport11)	52.54	92.94	0.57	1.38E-04	3.43E-04
569	2	L4 vs. M4	264313_at	AT1G70410	Encodes a putative beta-carbonic anhydrase betaCA4. Together with betaCA1 (At3g01500) regulates CO2-controlled stomatal movements in guard cells, as well as attenuates immunity. Differential CA gene expression in response to changing atmospheric CO2 conditions contribute to altered disease resistance levels.	155.07	271.62	0.57	2.29E-04	5.04E-04
570	2	L4 vs. M4	AFFX-Athal-GAPDH_M_s_at	AT3G04120	encodes cytosolic GADPH (C subunit) involved in the glycolytic pathway but also interacts with H2O2 potentially placing it in a signalling cascade induced by ROS. The mRNA is cell-to-cell mobile.	672.84	1190.39	0.57	3.24E-04	6.62E-04
571	2	L4 vs. M4	257703_at	AT3G12640	RNA binding (RRM/RBD/RNP motifs) family protein;(source:Araport11)	91.24	159.49	0.57	3.40E-04	6.84E-04
572	2	L4 vs. M4	254167_at	AT4G24400	Encodes a CBL (calcineurin B-like calcium sensor proteins) -interacting serine/threonine protein kinase. Regulates the low-affinity phase of the primary nitrate response. The mRNA is cell-to-cell mobile.	86.90	151.74	0.57	7.80E-04	1.37E-03
573	2	L4 vs. M4	245913_at	AT5G19860	transmembrane protein, putative (Protein of unknown function, DUF538);(source:Araport11)	103.75	181.24	0.57	7.14E-04	1.26E-03
574	2	L4 vs. M4	247082_at	AT5G66030	Involved in golgi protein trafficking. AtARL1 binds directly to the GRIP domain of AtGRIP in a GTP-dependent manner. Localized to the golgi apparatus, tyrosine 717 in AtGRIP is crucial for Golgi localization.	96.74	171.13	0.57	2.35E-04	5.16E-04
575	2	L4 vs. M4	256038_at	AT1G19170	Pectin lyase-like superfamily protein;(source:Araport11)	25.48	45.11	0.56	2.52E-04	5.45E-04
576	2	L4 vs. M4	260715_at	AT1G48200	hypothetical protein;(source:Araport11)	34.24	61.33	0.56	5.35E-04	1.00E-03
577	2	L4 vs. M4	267560_at	AT2G45580	cytochrome P450, family 76, subfamily C, polypeptide 3;(source:Araport11)	49.64	88.86	0.56	4.80E-04	9.06E-04

578	2	L4 vs. M4	258774_at	AT3G10740	Encodes a bifunctional alpha-l-arabinofuranosidase/beta-d-xylosidase that belongs to family 51 of glycoside hydrolases. It may be involved in cell wall modification.	160.97	287.34	0.56	2.19E-04	4.88E-04
579	2	L4 vs. M4	258207_at	AT3G14050	RELA/SPOT homolog 2;(source:Araport11)	480.64	863.53	0.56	8.25E-05	2.23E-04
580	2	L4 vs. M4	258416_at	AT3G17310	Encodes DRM3 (Domains Rearranged Methyltransferase3), a catalytically mutated paralog of the cytosine methyltransferase DRM2. Despite being catalytically mutated, DRM3 is required for normal maintenance of non-CG DNA methylation, establishment of RNA-directed DNA methylation triggered by repeat sequences and accumulation of repeat-associated small RNAs.	32.28	58.04	0.56	1.08E-04	2.82E-04
581	2	L4 vs. M4	251668_at	AT3G57010	Calcium-dependent phosphotriesterase superfamily protein;(source:Araport11)	112.39	199.07	0.56	2.50E-04	5.43E-04
582	2	L4 vs. M4	246201_at	AT4G36750	Quinone reductase family protein;(source:Araport11)	43.51	77.67	0.56	3.18E-04	6.52E-04
583	2	L4 vs. M4	246253_at	AT4G37260	Member of the R2R3 factor gene family. The mRNA is cell-to-cell mobile.	162.30	288.08	0.56	2.44E-04	5.32E-04
584	2	L4 vs. M4	246055_at	AT5G08380	alpha-galactosidase 1;(source:Araport11)	212.87	383.53	0.56	2.99E-04	6.24E-04
585	2	L4 vs. M4	250527_at	AT5G08590	Encodes a member of SNF1-related protein kinases (SnRK2) whose activity is activated by ionic (salt) and non-ionic (mannitol) osmotic stress. Similar to the calcium/calmodulin-dependent protein kinase subfamily and the SNF1 kinase subfamily.	127.92	230.11	0.56	1.24E-04	3.12E-04
586	2	L4 vs. M4	246447_at	AT5G16780	Encodes a protein belonging to SART-1 family. The gene is expressed in the basal region of the developing embryo during heart stage. Phenotypic analyses of dot2 mutants suggest that this protein plays a role in root, shoot, and flower development. dot2 mutants are dwarved plants that display an aberrant spurred leaf venation pattern and fail to flower.	77.11	138.61	0.56	9.44E-04	1.61E-03
587	2	L4 vs. M4	247806_at	AT5G58270	Encodes a mitochondrial half-molecule ABC transporter, a member of ATM subfamily. Mutants are dwarfed, chlorotic plants with altered leaf morphology. ATM3 transcription is induced by Cd(II) or Pb(II). Involved in heavy metal resistance. Arabidopsis thaliana has three ATM genes, namely ATM1, ATM2 and ATM3. Only ATM3 has an important function for plant growth. Role in Moco biosynthesis.	93.07	166.37	0.56	4.89E-04	9.20E-04
588	2	L4 vs. M4	247575_at	AT5G61030	Encodes a glycine-rich RNA binding protein that is involved in C-> U RNA editing in mitochondria. Gene expression is induced by cold. The mRNA is cell-to-cell mobile.	180.68	324.37	0.56	4.77E-04	9.02E-04
589	2	L4 vs. M4	259391_s_a	AT1G06350	Fatty acid desaturase family protein;(source:Araport11)	75.17	136.01	0.55	7.79E-05	2.14E-04
590	2	L4 vs. M4	256061_at	AT1G07040	plant/protein;(source:Araport11)	71.58	130.32	0.55	2.85E-04	6.02E-04
591	2	L4 vs. M4	261424_at	AT1G18700	DNAJ heat shock N-terminal domain-containing protein;(source:Araport11)	20.07	36.34	0.55	9.27E-04	1.59E-03
592	2	L4 vs. M4	259632_at	AT1G56430	Encodes a protein with nicotianamine synthase activity.	36.10	65.24	0.55	2.36E-04	5.16E-04
593	2	L4 vs. M4	266098_at	AT2G37870	Bifunctional inhibitor/lipid-transfer protein/seed storage 2S albumin superfamily protein;(source:Araport11)	260.25	469.23	0.55	1.40E-04	3.45E-04
594	2	L4 vs. M4	258270_at	AT3G15650	alpha/beta-Hydrolases superfamily protein;(source:Araport11)	61.82	112.34	0.55	1.80E-04	4.24E-04
595	2	L4 vs. M4	251948_at	AT3G53580	diaminopimelate epimerase family protein;(source:Araport11)	686.58	1240.35	0.55	3.26E-04	6.62E-04
596	2	L4 vs. M4	251721_s_a	AT3G56190	Encodes one of two alpha-SNAPs (soluble NSF attachment protein) in Arabidopsis	145.36	264.19	0.55	1.55E-04	3.75E-04
597	2	L4 vs. M4	253551_at	AT4G30996	NKS1, a plant-specific gene, encodes a 19 kDa endomembrane-localized protein. It is part of SOS independent ion homeostasis regulation pathway.	373.53	683.76	0.55	3.41E-04	6.85E-04
598	2	L4 vs. M4	246257_at	AT4G36690	Regulates flowering time and displays a redundant role in pollen tube growth together with AtU2AF65b.	259.24	468.33	0.55	7.35E-05	2.07E-04
599	2	L4 vs. M4	250911_at	AT5G03730	Homologous to the RAF family of serine/threonine protein kinases. Negative regulator in the ethylene signal transduction pathway. Interacts with the putative ethylene receptors ETR1 and ERS. Constitutively expressed.	62.82	114.23	0.55	1.07E-04	2.81E-04
600	2	L4 vs. M4	245904_at	AT5G11110	Encodes a protein with putative sucrose-phosphate synthase activity.Involved in pollen exine formation.	67.73	122.73	0.55	7.04E-04	1.25E-03
601	2	L4 vs. M4	246499_at	AT5G16260	Encodes a RNA binding protein ELF9 (EARLY FLOWERING9). Loss of ELF9 function in the Wassilewskija ecotype causes early flowering in short days. ELF9 reduces SOC1 (SUPPRESSOR OF OVEREXPRESSION OF CO1) transcript levels, possibly via nonsense-mediated mRNA decay. The mRNA is cell-to-cell mobile.	76.74	139.34	0.55	9.30E-05	2.50E-04
602	2	L4 vs. M4	246733_at	AT5G27660	Encodes a protein with similarity to human PARK13, a mitochondrial protease implicated in Parkinson disease. DEG14 is induced by heat stress and involved in degradation of misfolded proteins.	78.55	143.74	0.55	2.15E-04	4.81E-04
603	2	L4 vs. M4	247811_at	AT5G58430	A member of EXO70 gene family, putative exocyst subunits, conserved in land plants. Arabidopsis thaliana contains 23 putative EXO70 genes, which can be classified into eight clusters on the phylogenetic tree. Targeted by AvrPtoB to manipulate the defense molecule secretion machinery.	31.71	57.35	0.55	5.77E-04	1.07E-03

604	2	L4 vs. M4	261537_at	AT1G01800	NAD(P)-binding Rossmann-fold superfamily protein;(source:Araport11)	45.89	84.23	0.54	4.81E-04	9.07E-04
605	2	L4 vs. M4	256022_at	AT1G58360	Encodes AAP1 (amino acid permease 1), a neutral amino acid transporter expressed in seeds. Functions in amino acid uptake into embryos. The transporter also functions in acquisition of glutamate and neutral amino acids by the root.	392.79	723.23	0.54	9.79E-05	2.61E-04
606	2	L4 vs. M4	265661_at	AT2G24360	STYK serine threonine kinase that phosphorylates several oil body proteins including OLE1 and CLO4/CAL4.	84.93	158.26	0.54	1.03E-04	2.73E-04
607	2	L4 vs. M4	263425_at	AT2G31530	Encodes a component of the thylakoid-localized Sec system involved in the translocation of cytoplasmic proteins into plastid. Loss-of-function mutations result in arrest at the globular stage and embryo lethality.	76.68	140.71	0.54	1.72E-04	4.09E-04
608	2	L4 vs. M4	251924_at	AT3G53730	Histone superfamily protein;(source:Araport11)	345.72	640.92	0.54	7.90E-05	2.16E-04
609	2	L4 vs. M4	251431_at	AT3G60240	protein synthesis initiation factor 4G (EIF4G). A mutation in this gene (cum2-1) results in decreased accumulation of CMV coat protein in upper, uninoculated leaves. Likely affects cell-to-cell movement of the virus, also affects TCV multiplication.	574.52	1058.97	0.54	7.47E-05	2.09E-04
610	2	L4 vs. M4	254080_at	AT4G25630	encodes a fibrillarin, a key nucleolar protein in eukaryotes which associates with box C/D small nucleolar RNAs (snoRNAs) directing 2'-O-ribose methylation of the rRNA. This gene also encodes a novel box C/D snoRNA, U60.2f in its fifth intron that accumulates in seedlings and that their targeted residue on the 25 S rRNA is methylated. The mRNA is cell-to-cell mobile.	384.65	710.45	0.54	1.19E-04	3.05E-04
611	2	L4 vs. M4	253755_at	AT4G29040	RPT2a encodes the 26S proteasome subunit. It is required for root meristem maintenance, and regulates gametogenesis. RPT2a is also shown to regulate gene silencing via DNA methylation.	579.63	1078.79	0.54	7.16E-05	2.04E-04
612	2	L4 vs. M4	253083_at	AT4G36250	Encodes a putative aldehyde dehydrogenase. The gene is not responsive to osmotic stress and is expressed constitutively at a low level in plantlets and root cultures.	455.81	846.87	0.54	1.09E-04	2.82E-04
613	2	L4 vs. M4	250793_at	AT5G05600	Encodes a protein with similarity to flavonol synthases that is involved in the detoxifcation polycyclic aromatic hydrocarbons.One of 4 paralogs encoding a 2-oxoglutarate/Fe(II)-dependent oxygenases that hydroxylates JA to 12-OH-JA.	234.13	429.62	0.54	7.76E-05	2.14E-04
614	2	L4 vs. M4	250094_at	AT5G17380	Thiamine pyrophosphate dependent pyruvate decarboxylase family protein;(source:Araport11)	291.34	540.93	0.54	2.29E-04	5.04E-04
615	2	L4 vs. M4	253898_s_a	AT5G54810	A.thaliana tryptophan synthase beta subunit (trpB)	1611.07	2991.81	0.54	7.51E-05	2.10E-04
616	2	L4 vs. M4	247077_at	AT5G66420	TIM-barrel signal transduction protein;(source:Araport11)	131.64	242.66	0.54	9.35E-05	2.51E-04
617	2	L4 vs. M4	262105_at	AT1G02810	Plant invertase/pectin methylesterase inhibitor superfamily;(source:Araport11)	38.28	72.34	0.53	5.02E-05	1.54E-04
618	2	L4 vs. M4	263183_at	AT1G05570	Encodes a callose synthase 1 catalytic subunit . Member of Glycosyltransferase Family- 48.	667.49	1249.36	0.53	5.70E-05	1.69E-04
619	2	L4 vs. M4	264992_at	AT1G67300	Major facilitator superfamily protein;(source:Araport11)	76.45	144.30	0.53	1.85E-04	4.33E-04
620	2	L4 vs. M4	260445_at	AT1G68370	DnaJ-like protein with homology to coiled coils found in cytoskeleton-interacting proteins.	23.63	44.71	0.53	6.97E-05	2.00E-04
621	2	L4 vs. M4	257715_at	AT3G12750	A member of Zrt- and Irt-related protein (ZIP) family. transcript is induced in response to zinc deficiency in the root.	22.13	41.49	0.53	2.76E-04	5.85E-04
622	2	L4 vs. M4	252277_at	AT3G49470	nascent polypeptide-associated complex subunit alpha-like protein 2;(source:Araport11)	69.17	130.25	0.53	3.50E-05	1.17E-04
623	2	L4 vs. M4	245007_at	ATCG00350	Encodes psaA protein comprising the reaction center for photosystem I along with psaB protein; hydrophobic protein encoded by the chloroplast genome.	43.31	81.46	0.53	6.36E-04	1.16E-03
624	2	L4 vs. M4	263184_at	AT1G05560	A UDP-glucose transferase localized in the phragmoplast. It has been co-purified with the callose synthase complex and may transfer UDP-glucose from sucrose synthase to the callose synthase and thus help form a substrate channel for the synthesis of callose at the forming cell plate. Induced by salicylic acid. Independent of NPR1 for their induction by salicylic acid. UGT1 encodes a protein with glucosyltransferase activity with high sequence homology to UGT2 (AT1G05530). It belongs to an UGT subfamily that binds UDP-glucose but not UDP-glucuronate, UDP-galactose, or UDP-rhamnose as the glycosyl donor. UGT1 was shown to be able to use abscisic acid as glycosylation substrate in the presence of UDP-glucose.	17.76	33.91	0.52	3.63E-05	1.20E-04
625	2	L4 vs. M4	261150_at	AT1G19640	Encodes a S-adenosyl-L-methionine:jasmonic acid carboxyl methyltransferase that catalyzes the formation of methyljasmonate from jasmonic acid. Its expression is induced in response to wounding or methyljasmonate treatment.	21.07	40.40	0.52	3.79E-05	1.23E-04

626	2	L4 vs. M4	262744_at	AT1G28680	Catalyses trans-cis isomerization and lactonization in the biosynthesis of coumarins in roots.	67.91	129.70	0.52	3.61E-05	1.20E-04
627	2	L4 vs. M4	260232_at	AT1G74640	alpha/beta-Hydrolases superfamily protein;(source:Araport11)	66.16	127.90	0.52	1.56E-04	3.77E-04
628	2	L4 vs. M4	261881_at	AT1G80760	Encodes a protein with boron transporter activity. It helps to preferentially direct boron to young developing tissues in the shoot, such as immature leaves, under low boron conditions. This boron channel appears to be impermeable to water, unlike the closely related NIP5;1 boron transporter. This protein also allows the transport of glycerol, urea, and formimide but not larger uncharged solutes such as arabitol and sucrose when it is expressed heterologously.	41.81	80.98	0.52	5.36E-04	1.00E-03
629	2	L4 vs. M4	266732_at	AT2G03240	EXS (ERD1/XPR1/SYG1) family protein;(source:Araport11)	57.58	110.52	0.52	5.47E-04	1.02E-03
630	2	L4 vs. M4	263807_at	AT2G04400	Acts during tryptophan biosynthesis controlled by ERF109.	287.26	550.95	0.52	3.84E-04	7.48E-04
631	2	L4 vs. M4	265938_at	AT2G19620	Plays a role in dehydration stress response.	77.97	149.82	0.52	2.47E-05	9.15E-05
632	2	L4 vs. M4	264002_at	AT2G22360	DNAJ heat shock family protein;(source:Araport11)	26.55	50.75	0.52	4.50E-04	8.57E-04
633	2	L4 vs. M4	265911_at	AT2G25670	hypothetical protein;(source:Araport11)	227.59	441.38	0.52	1.80E-04	4.24E-04
634	2	L4 vs. M4	263986_at	AT2G42790	Encodes a peroxisomal citrate synthase that is expressed throughout seedling and shoot development.	307.40	592.92	0.52	2.77E-05	9.92E-05
635	2	L4 vs. M4	266749_at	AT2G47060	Encodes Pto-interacting 1-4 (PTI1-4), a member of the PTI1-like serine/threonine protein kinases that share strong sequence identity to the tomato PTI1 kinase.	34.74	66.18	0.52	1.69E-04	4.04E-04
636	2	L4 vs. M4	258485_at	AT3G02630	One of seven acyl acyl carrier proteins. Expressed primarily in developing seeds.Involved in fatty acid metabolism.	135.84	261.73	0.52	3.56E-05	1.19E-04
637	2	L4 vs. M4	258679_at	AT3G08590	Encodes a 2,3-biphosphoglycerate-independent phosphoglycerate mutase that is involved in pollen development and stomatal movement.	325.13	620.20	0.52	4.28E-05	1.36E-04
638	2	L4 vs. M4	257781_at	AT3G27120	Encodes a conserved AAA-ATPase that acts as a negative regulator of meiotic CO formation.	22.50	43.10	0.52	2.00E-04	4.55E-04
639	2	L4 vs. M4	246307_at	AT3G51800	putative nuclear DNA-binding protein G2p (AtG2) mRNA,	204.56	392.33	0.52	7.58E-05	2.11E-04
640	2	L4 vs. M4	251221_at	AT3G62550	Adenine nucleotide alpha hydrolases-like superfamily protein;(source:Araport11)	58.92	112.64	0.52	2.77E-05	9.92E-05
641	2	L4 vs. M4	254915_s_a	AT4G11310	cysteine proteinase precursor-like protein	59.62	115.59	0.52	4.06E-04	7.82E-04
642	2	L4 vs. M4	254020_at	AT4G25700	Converts beta-carotene to zeaxanthin via cryptoxanthin.	128.91	247.29	0.52	4.42E-05	1.40E-04
643	2	L4 vs. M4	246050_s_a	AT5G28900	Calcium-binding EF-hand family protein;(source:Araport11)	49.02	94.46	0.52	1.97E-05	7.67E-05
644	2	L4 vs. M4	249513_at	AT5G38460	ALG6, ALG8 glycosyltransferase family;(source:Araport11)	31.58	60.50	0.52	1.65E-04	3.98E-04
645	2	L4 vs. M4	249211_at	AT5G42680	MIZU-KUSSEI-like protein (Protein of unknown function, DUF617);(source:Araport11)	55.54	107.24	0.52	5.36E-05	1.62E-04
646	2	L4 vs. M4	248747_at	AT5G47930	Zinc-binding ribosomal protein family protein;(source:Araport11)	39.57	75.98	0.52	4.02E-05	1.30E-04
647	2	L4 vs. M4	248499_at	AT5G50400	purple acid phosphatase 27;(source:Araport11)	44.23	84.96	0.52	1.31E-04	3.27E-04
648	2	L4 vs. M4	247261_at	AT5G64460	Phosphoglycerate mutase family protein;(source:Araport11)	230.91	447.80	0.52	3.72E-05	1.23E-04
649	2	L4 vs. M4	244968_at	ATCG00640	encodes a chloroplast ribosomal protein L33, a constituent of the large subunit of the ribosomal complex	38.74	73.86	0.52	1.24E-04	3.12E-04
650	2	L4 vs. M4	261570_at	AT1G01120	Encodes a condensing enzyme KCS1 (3-ketoacyl-CoA synthase 1) which is involved in the critical fatty acid elongation process in wax biosynthesis.	54.49	106.11	0.51	3.64E-04	7.18E-04
651	2	L4 vs. M4	265700_at	AT2G03470	ELM2 domain-containing protein;(source:Araport11)	21.52	42.48	0.51	4.53E-05	1.41E-04
652	2	L4 vs. M4	266849_at	AT2G25940	Encodes a vacuolar processing enzyme belonging to a novel group of cysteine proteinases that is expressed in vegetative organs and is upregulated in association with various types of cell death and under stressed conditions.	321.92	629.17	0.51	3.15E-05	1.07E-04
653	2	L4 vs. M4	267380_at	AT2G26170	Encodes a protein with similarity to thromboxane-A synthase, member of the CYP711A cytochrome P450 family. MAX1 is a specific repressor of vegetative axillary buds generated by the axillary meristem. Expressed in vascular traces in the rosette stem and axillary buds throughout plant development. Mutants have increased axillary branches. Along with MAX3,4 thought to mediate control of shoot branching via synthesis of a signal molecule which is transported over long distance mediated by MAX2. cDNA supports the existence of the longer transcript predicted for this locus, no cDNA isolated for shorter transcript. MAX1 downregulates 11 genes involved in flavonoid pathway (CHS, CHI, F3H, F3'H, FLS, DFR, ANS, UFGT, RT, AAC and GST).	193.32	376.43	0.51	4.65E-05	1.44E-04
654	2	L4 vs. M4	267381_at	AT2G26190	IQM4 is a plastid localized, Ca2+ independent calmodulin binding protein that is involved in promoting seed dormancy.	86.42	170.40	0.51	2.66E-05	9.62E-05

655	2	L4 vs. M4	258697_at	AT3G09660	Encodes a minichromosome maintenance protein that is involved with RAD51 in a backup pathway that repairs meiotic double strand breaks without giving meiotic crossovers when the major pathway, which relies on DMC1, fails.	34.58	68.04	0.51	2.49E-05	9.16E-05
656	2	L4 vs. M4	259383_at	AT3G16470	Encodes a JA-responsive gene that coordinates with GRP7 in shaping plant development through the regulation of RNA processing in Arabidopsis. AtJAC1 interacts with RNA binding protein GRP7 specifically in the cytoplasm to regulate its nucleocytoplasmic distribution.	60.49	119.34	0.51	2.14E-05	8.12E-05
657	2	L4 vs. M4	251929_at	AT3G53920	Encodes a sigma-like transcription factor, Sigma 3 (SIG3 or SIGC). As a subunit of chloroplast RNA polymerase, SIG3 confers the ability to recognize promoter sequences on the core enzyme. SIG3 transcribes specifically the psbN gene in plastids.	25.97	51.24	0.51	9.21E-04	1.58E-03
658	2	L4 vs. M4	251594_at	AT3G57630	Encodes a glycoprotein glycosyl transferase ExAD. Knockout mutants show truncated root hair phenotype.	146.19	289.25	0.51	2.55E-04	5.47E-04
659	2	L4 vs. M4	253184_at	AT4G35230	Encodes BR-signaling kinase 1 (BSK1), one of the three homologous BR-signaling kinases (BSK1, AT4G35230; BSK2, AT5G46570; BSK3, AT4G00710). Mediates signal transduction from receptor kinase BRI1 by functioning as the substrate of BRI1. Plasma membrane localized.	31.64	62.17	0.51	3.64E-04	7.18E-04
660	2	L4 vs. M4	250897_at	AT5G03430	phosphoadenosine phosphosulfate (PAPS) reductase family protein;(source:Araport11)	63.16	123.71	0.51	1.10E-04	2.85E-04
661	2	L4 vs. M4	246829_at	AT5G26570	chloroplastidic phosphoglucan, water dikinase (PWD) which is required for normal degradation of leaf starch in Arabidopsis. NMR analysis of the mutants, suggests that the gene is specifically involved in the phosphorylation of the glucosyl residues of starch at the C3 position.	430.66	850.08	0.51	3.81E-05	1.23E-04
662	2	L4 vs. M4	249237_at	AT5G42050	Stress responsive asparagine-rich protein. Binds to PevD (Verticillium dahliae) fungal effector protein. NRP interacts with CRY2, leading to increased cytoplasmic accumulation of CRY2 in a blue light-independent manner (PMID:28633330).NRP also binds FyPP3 and recruits it to endosomes and thus targets it for degradation.	94.20	183.37	0.51	2.24E-05	8.41E-05
663	2	L4 vs. M4	247267_at	AT5G64140	Encodes a putative ribosomal protein S28.	74.35	147.11	0.51	2.89E-05	1.02E-04
664	2	L4 vs. M4	264614_s_at	AT1G02590	Aldehyde oxidase/xanthine dehydrogenase, molybdopterin binding protein;(source:Araport11)	70.43	139.97	0.50	7.43E-05	2.09E-04
665	2	L4 vs. M4	261490_at	AT1G14320	Encodes a ribosomal protein L10 and may be involved in translation regulation. Semi-dominant mutations in SAC552 can suppress defects in acaulis5, which encodes a thermospermine synthase, by enhancing translation of acl5 and itself.	1528.54	3029.30	0.50	1.26E-05	5.52E-05
666	2	L4 vs. M4	264160_at	AT1G65450	Contains dual transcription units and alternative splicing that could rescue the sterility defect of glc mutants. Shares homology to BAHD (for BEAT, AHCT, HCBT, and DAT) acyl-transferases. Functions in double fertilization.	158.97	316.81	0.50	1.23E-05	5.46E-05
667	2	L4 vs. M4	259975_at	AT1G76470	NAD(P)-binding Rossmann-fold superfamily protein;(source:Araport11)	25.22	50.87	0.50	8.60E-06	4.38E-05
668	2	L4 vs. M4	255872_at	AT2G30360	Encodes a SOS2-like protein kinase that is a member of the CBL-interacting protein kinase family.Loss of function mutants show a decrease in sensitivity to high pH.Phosphorylates AHA2, a plasma membrane H+ ATPase.This phosphorylation appears to regulate the activity of the proton transporter.	36.68	72.82	0.50	2.01E-05	7.77E-05
669	2	L4 vs. M4	263954_at	AT2G35840	Sucrose-6F-phosphate phosphohydrolase family protein;(source:Araport11)	367.10	738.65	0.50	2.13E-05	8.11E-05
670	2	L4 vs. M4	267368_at	AT2G44350	encodes a mitochrondrion targeted citrate synthase, the first enzyme of the tricarboxylic acid cycle, catalyzing the condensation of acetyl-CoA and oxaloacetate, finally yielding citrate and CoA.	657.11	1314.31	0.50	2.24E-05	8.41E-05
671	2	L4 vs. M4	257932_at	AT3G17040	It is a RNA tetratricopeptide repeat-containing protein required for normal processing of transcripts from the polycistronic chloroplast psbB-psbT-psbH-petB-petD operon coding for proteins of the photosystem II and cytochrome b6/f complexes. Localizes to the chloroplast membrane. Involved in regulating plastidial gene expression and biogenesis. It binds in the psbT?psbH intercistronic region and blocks the progression of 5′ → 3′ exoribonucleases, which defines the 5′ end of processed psbH transcripts and also stabilizes the downstream RNA segment. In addition, HCF107 binding remodels the structure of the psbH 5′ UTR in a way that can account for its ability to enhance psbH translation.	256.78	516.75	0.50	1.37E-05	5.88E-05

672	2	L4 vs. M4	258452_at	AT3G22370	Encodes AOX1a, an isoform of alternative oxidase that is expressed in rosettes, flowers, and root. The alternative oxidase of plant mitochondria transfers electrons from the ubiquinone pool to oxygen without energy conservations. It is regulated through transcriptional control and by pyruvate. Plays a role in shoot acclimation to low temperature. Also is capable of ameliorating reactive oxygen species production when the cytochrome pathway is inhibited. AOX1a also functions as a marker for mitochondrial retrograde response. The mRNA is cell-to-cell mobile.	110.23	220.81	0.50	3.70E-04	7.26E-04
673	2	L4 vs. M4	252287_at	AT3G49080	Mitochondrial ribosomal protein, similar to RPS9 from E.coli. Loss of function results in gametophyte lethality, particularly the megagametophyte.	138.75	280.24	0.50	2.96E-05	1.02E-04
674	2	L4 vs. M4	252308_at	AT3G49310	Major facilitator superfamily protein;(source:Araport11)	37.78	75.96	0.50	2.85E-04	6.02E-04
675	2	L4 vs. M4	251193_at	AT3G62910	Encodes a plastid-localized ribosome release factor 1 that is essential in chloroplast development. Pale green, albino mutant seedlings arrest early in seedling development.	121.53	242.75	0.50	1.47E-04	3.59E-04
676	2	L4 vs. M4	252896_at	AT4G39480	member of CYP96A	19.15	38.57	0.50	6.92E-05	2.00E-04
677	2	L4 vs. M4	252830_at	AT4G39850	Encodes a peroxisomal protein of the ATP binding cassette (ABC) transporter class (PMP subfamily) with significant identity to the human X-linked adrenoleukodystrophy protein (ALDP). The gene product promotes germination and represses embryo dormancy. ABI3, ABA1, FUS3 and LEC1 are epistatic to this gene. Mutants accumulate fatty acyl CoA suggesting a defect in uptake of fatty acyl CoA into the peroxisome.	408.43	812.89	0.50	1.85E-04	4.33E-04
678	2	L4 vs. M4	250828_at	AT5G05250	hypothetical protein;(source:Araport11)	71.45	143.39	0.50	2.30E-05	8.61E-05
679	2	L4 vs. M4	250326_at	AT5G12080	Encodes a mechanosensitive (or stretch-activated) ion channel in the plasma membrane with a moderate preference for anions.Cell death activity is negatively regulated by phosphorylation while mechanosensitive properties are unaffected.	55.22	110.38	0.50	4.19E-05	1.34E-04
680	2	L4 vs. M4	248921_at	AT5G45950	GDSL-motif esterase/acyltransferase/lipase. Enzyme group with broad substrate specificity that may catalyze acyltransfer or hydrolase reactions with lipid and non-lipid substrates.	258.62	518.41	0.50	7.21E-05	2.04E-04
681	2	L4 vs. M4	246987_at	AT5G67300	Member of the R2R3 factor MYB gene family involved in mediating plant responses to a variety of abiotic stimiuli. The mRNA is cell-to-cell mobile.	192.22	382.82	0.50	2.90E-04	6.09E-04
682	2	L4 vs. M4	260005_at	AT1G67920	hypothetical protein;(source:Araport11)	60.29	122.47	0.49	3.10E-04	6.44E-04
683	2	L4 vs. M4	263136_at	AT1G78580	Encodes an enzyme putatively involved in trehalose biosynthesis. The protein has a trehalose synthase (TPS)-like domain but no trehalose phosphatase (TPP)-like domain. ATTPS1 is able to complement yeast tps1 mutants in vivo. The gene product modulates cell growth but not cell differentiation by determining cell wall deposition and cell division.	38.64	78.17	0.49	3.05E-05	1.05E-04
684	2	L4 vs. M4	265415_at	AT2G20890	Chloroplast-localized Thylakoid formation1 gene product involved in vesicle-mediated formation of thylakoid membranes. Thf1 antisense lines contain abnormal chloroplasts early in leaf development (chloroplasts have loosely stacked thylakoid membranes). Expression was induced in the light and decreased under dark conditions. G-alpha interaction partner that functions downstream of the plasma membrane?delimited heterotrimeric G-protein (GPA1) in a D-glucose signaling pathway. Localized to both the outer plastid membrane and the stroma. Probably involved in the metabolic pathway that controls the assembly of the PS II complex. The mRNA is cell-to-cell mobile.	297.94	606.80	0.49	1.45E-05	6.11E-05
685	2	L4 vs. M4	263845_at	AT2G37040	Encodes PAL1, a phenylalanine ammonia-lyase. Arabidopsis has four PALs: AT2G37040 (PAL1), AT3G53260 (PAL2), AT5G04230 (PAL3) and AT3G10340 (PAL4).	46.77	95.14	0.49	4.64E-05	1.44E-04
686	2	L4 vs. M4	260580_at	AT2G47390	Prolyl oligopeptidase family protein;(source:Araport11)	106.65	219.10	0.49	1.77E-05	7.09E-05
687	2	L4 vs. M4	251248_at	AT3G62150	Encodes a facultative transporter controlling auxin concentrations in plant cells.	102.46	211.25	0.49	3.70E-05	1.22E-04
688	2	L4 vs. M4	255590_at	AT4G01610	Encodes a capase involved in stress induced cell death. Activity detected in leaf and cell culture.	713.76	1444.64	0.49	7.90E-06	4.12E-05
689	2	L4 vs. M4	245293_at	AT4G16660	heat shock protein 70 (Hsp 70) family protein;(source:Araport11)	59.58	122.44	0.49	2.79E-05	9.96E-05
690	2	L4 vs. M4	253421_at	AT4G32340	Tetratricopeptide repeat (TPR)-like superfamily protein;(source:Araport11)	34.16	69.82	0.49	1.69E-04	4.04E-04
691	2	L4 vs. M4	253266_s_a	AT4G34080	myosin-4 protein (DUF641);(source:Araport11)	116.51	239.13	0.49	5.36E-04	1.00E-03
692	2	L4 vs. M4	246162_at	AT4G36400	Encodes a (D)-2-hydroxyglutarate dehydrogenase.	20.71	42.39	0.49	2.94E-05	1.02E-04

693	2	L4 vs. M4	248622_at	AT5G49360	Encodes a bifunctional {beta}-D-xylosidase/{alpha}-L-arabinofuranosidase required for pectic arabinan modification. Located in the extracellular matrix. Gene is expressed specifically in tissues undergoing secondary wall thickening. This is a member of glycosyl hydrolase family 3 and has six other closely related members.	950.79	1956.75	0.49	5.22E-05	1.59E-04
694	2	L4 vs. M4	244998_at	ATCG00180	RNA polymerase beta' subunit-1	36.94	74.80	0.49	1.47E-05	6.14E-05
695	2	L4 vs. M4	260932_s_at	AT1G02520	Encodes an ATP-binding cassette (ABC) transporter. Expressed in the vascular tissue of primary stem.The mRNA is cell-to-cell mobile.	16.88	35.28	0.48	1.43E-04	3.50E-04
696	2	L4 vs. M4	260944_at	AT1G45130	beta-galactosidase 5;(source:Araport11)	138.57	289.16	0.48	1.24E-05	5.48E-05
697	2	L4 vs. M4	264916_at	AT1G60810	One of the three genes encoding subunit A of the trimeric enzyme ATP Citrate lyase	1021.43	2114.78	0.48	1.37E-05	5.88E-05
698	2	L4 vs. M4	267115_s_at	AT2G32530	encodes a gene similar to cellulose synthase	166.51	345.11	0.48	2.50E-05	9.16E-05
699	2	L4 vs. M4	267388_at	AT2G44450	beta glucosidase 15;(source:Araport11)	56.63	118.82	0.48	1.76E-04	4.17E-04
700	2	L4 vs. M4	259134_at	AT3G05390	S-adenosyl-L-methionine-dependent methyltransferase;(source:Araport11)	54.88	115.30	0.48	2.46E-05	9.14E-05
701	2	L4 vs. M4	259227_at	AT3G07750	3-5-exoribonuclease family protein;(source:Araport11)	113.67	238.56	0.48	7.40E-06	4.10E-05
702	2	L4 vs. M4	258375_at	AT3G17470	Ca2+-activated RelA/spot-like protein;(source:Araport11)	93.89	196.79	0.48	1.39E-05	5.90E-05
703	2	L4 vs. M4	252272_at	AT3G49670	Encodes a CLAVATA1-related receptor kinase-like protein required for both shoot and flower meristem function. Very similar to BAM1,with more than 85% a.a. identity. It has a broad expression pattern and is involved in vascular strand development in the leaf, control of leaf shape, size and symmetry, male gametophyte development and ovule specification and function. Anthers of double mutants (bam1bam2) appeared abnormal at a very early stage and lack the endothecium, middle, and tapetum layers. Further analyses revealed that cells interior to the epidermis (in anther tissue) acquire some characteristics of pollen mother cells (PMCs), suggesting defects in cell fate specification. The pollen mother-like cells degenerate before the completion of meiosis, suggesting that these cells are defective. In addition, the BAM2 expression pattern supports both an early role in promoting somatic cell fates and a subsequent function in the PMCs. The mRNA is cell-to-cell mobile.	35.38	73.41	0.48	1.27E-04	3.18E-04
704	2	L4 vs. M4	251554_at	AT3G58670	2-aminoethanethiol dioxygenase, putative (DUF1637);(source:Araport11)	31.05	64.49	0.48	2.54E-05	9.25E-05
705	2	L4 vs. M4	253358_at	AT4G32940	Encodes a vacuolar processing enzyme belonging to a novel group of cysteine proteinases that is expressed in vegetative organs and is upregulated in association with various types of cell death and under stressed conditions. They are essential in processing seed storage proteins and for mediating the susceptible response of toxin-induced cell death.	857.97	1781.17	0.48	6.10E-06	3.74E-05
706	2	L4 vs. M4	253268_s_at	AT4G34135	The At4g34135 gene encodes a flavonol 7-O-glucosyltransferase (EC 2.4.1.237) that glucosylates also with a 20 fold lower activity flavonols (kaempferol and quercetin) at the 3-O-position.	102.53	213.45	0.48	3.78E-04	7.39E-04
707	2	L4 vs. M4	249691_at	AT5G36170	Required for normal processing of polycistronic plastidial transcripts	134.72	282.93	0.48	5.20E-05	1.59E-04
708	2	L4 vs. M4	247903_at	AT5G57340	ras guanine nucleotide exchange factor Q-like protein;(source:Araport11)	19.10	40.04	0.48	1.13E-05	5.19E-05
709	2	L4 vs. M4	260780_at	AT1G14610	Required for proper proliferation of basal cells.	51.52	110.09	0.47	2.62E-04	5.60E-04
710	2	L4 vs. M4	265056_at	AT1G51980	Insulinase (Peptidase family M16) protein;(source:Araport11)	118.88	253.69	0.47	2.42E-04	5.28E-04
711	2	L4 vs. M4	260297_at	AT1G80280	alpha/beta-Hydrolases superfamily protein;(source:Araport11)	42.03	88.59	0.47	6.90E-06	3.98E-05
712	2	L4 vs. M4	245164_at	AT2G33210	Involved in the RNA splicing of rpl2 and ccmFC introns in mitochondria.	287.64	615.72	0.47	5.37E-05	1.62E-04
713	2	L4 vs. M4	263957_at	AT2G35880	TPX2 (targeting protein for Xklp2) protein family;(source:Araport11)	37.11	78.29	0.47	9.60E-06	4.71E-05
714	2	L4 vs. M4	267623_at	AT2G39650	cruciferin (DUF506);(source:Araport11)	51.78	110.13	0.47	6.70E-06	3.95E-05
715	2	L4 vs. M4	258565_at	AT3G04350	vacuolar sorting-associated protein (DUF946);(source:Araport11)	105.43	224.33	0.47	6.03E-05	1.77E-04
716	2	L4 vs. M4	257694_at	AT3G12860	NOP56-like pre RNA processing ribonucleoprotein;(source:Araport11)	80.98	171.84	0.47	2.25E-04	4.98E-04
717	2	L4 vs. M4	256633_at	AT3G28340	Encodes a protein with putative galacturonosyltransferase activity.	37.19	78.70	0.47	2.54E-05	9.25E-05
718	2	L4 vs. M4	250100_at	AT5G16570	Encodes a cytosolic glutamine synthetase, the enzyme has high affinity with substrate ammonium	45.29	96.12	0.47	1.80E-04	4.24E-04
719	2	L4 vs. M4	248249_at	AT5G53140	Protein phosphatase 2C family protein;(source:Araport11)	80.94	172.14	0.47	5.33E-05	1.61E-04
720	2	L4 vs. M4	247624_at	AT5G60160	Vacuolar aspartyl aminopeptidase which also functions as a molecular chaperone.	378.82	807.22	0.47	8.10E-06	4.18E-05
721	2	L4 vs. M4	247627_at	AT5G60360	Encodes a senescence-associated thiol protease. The mRNA is cell-to-cell mobile.	467.48	984.70	0.47	1.14E-05	5.21E-05
722	2	L4 vs. M4	262769_at	AT1G13180	Mutant has defect in trichome cell expansion and actin organization resulting in a distorted trichome phenotype.	77.48	169.04	0.46	1.86E-04	4.35E-04

723	2	L4 vs. M4	260690_at	AT1G32340	Encodes a protein whose sequence is similar to tobacco hairpin-induced gene (HIN1) and Arabidopsis non-race specific disease resistance gene (NDR1). Expression is not detected under normal conditions and in response to cucumber mosaic virus or spermine.	27.58	60.22	0.46	4.03E-04	7.79E-04
724	2	L4 vs. M4	260072_at	AT1G73650	3-oxo-5-alpha-steroid 4-dehydrogenase (DUF1295);(source:Araport11)	77.79	168.54	0.46	2.93E-05	1.02E-04
725	2	L4 vs. M4	260331_at	AT1G80270	PENTATRICOPEPTIDE REPEAT 596;(source:Araport11)	404.84	871.16	0.46	9.10E-06	4.52E-05
726	2	L4 vs. M4	266743_at	AT2G02990	Encodes a member of the ribonuclease T2 family that responds to inorganic phosphate starvation, and inhibits production of anthocyanin. Also involved in wound-induced signaling independent of jasmonic acid. Its expression is responsive to both phosphate (Pi) and phosphite (Phi) in roots.	270.16	588.96	0.46	1.04E-05	4.96E-05
727	2	L4 vs. M4	267421_at	AT2G35040	AICARFT/IMPCHase bienzyme family protein;(source:Araport11)	223.05	485.11	0.46	8.80E-06	4.46E-05
728	2	L4 vs. M4	266366_at	AT2G41420	proline-rich family protein;(source:Araport11)	64.95	142.35	0.46	6.90E-06	3.98E-05
729	2	L4 vs. M4	259190_at	AT3G01780	Encodes TPLATE, a cytokinesis protein targeted to the cell plate. Functions in vesicle-trafficking events required for site-specific cell wall modifications during pollen germination and for anchoring of the cell plate to the mother wall at the correct cortical position.	232.22	506.23	0.46	1.33E-04	3.32E-04
730	2	L4 vs. M4	257062_at	AT3G18290	Encodes BRUTUS (BTS), a putative E3 ligase protein with metal ion binding and DNA binding domains, which negatively regulates the response to iron deficiency. The mRNA is cell-to-cell mobile.	212.98	463.10	0.46	2.26E-04	5.00E-04
731	2	L4 vs. M4	258005_at	AT3G19390	Granulin repeat cysteine protease family protein;(source:Araport11)	627.45	1360.89	0.46	1.83E-05	7.25E-05
732	2	L4 vs. M4	251840_at	AT3G54960	Encodes a protein disulfide isomerase-like (PDIL) protein, a member of a multigene family within the thioredoxin (TRX) superfamily. Transcript levels for this gene are up-regulated in response to three different chemical inducers of ER stress (dithiothreitol, beta-mercaptoethanol, and tunicamycin). Neither AtIRE1-2 nor AtbZIP60 appear to be required for this response.	410.31	898.78	0.46	2.90E-06	2.34E-05
733	2	L4 vs. M4	255226_at	AT4G05420	Structurally similar to damaged DNA binding proteins. DDB1a is part of a 350 KDa nuclear localized DET1 protein complex. This complex may physically interact with histone tails and while bound to chromatin- repress transcription of genes involved in photomorphogenesis. DDB1a is shown to be RUB-modified.	188.65	406.49	0.46	5.40E-06	3.52E-05
734	2	L4 vs. M4	254201_at	AT4G24130	DUF538 family protein (Protein of unknown function, DUF538);(source:Araport11)	64.97	142.67	0.46	3.08E-05	1.05E-04
735	2	L4 vs. M4	254053_s_at	AT4G25310	2-oxoglutarate (2OG) and Fe(II)-dependent oxygenase superfamily protein;(source:Araport11)	41.60	91.04	0.46	1.41E-04	3.46E-04
736	2	L4 vs. M4	253735_at	AT4G29160	SNF7 family protein;(source:Araport11)	83.38	183.22	0.46	2.08E-04	4.72E-04
737	2	L4 vs. M4	246516_at	AT5G15740	RRT1 is a member of a novel glycosyltransferase famly in plants. It functions as a rhamnosyltransferase, elongating the RG-1 backbone. It functions during seed coat mucilage development.	52.63	114.57	0.46	2.09E-04	4.72E-04
738	2	L4 vs. M4	249346_at	AT5G40780	Encodes LHT1 (lysine histidine transporter), a high-affinity transporter for cellular amino acid uptake in both root epidermis and leaf mesophyll.	120.27	261.06	0.46	1.17E-05	5.30E-05
739	2	L4 vs. M4	249215_at	AT5G42800	dihydroflavonol reductase. Catalyzes the conversion of dihydroquercetin to leucocyanidin in the biosynthesis of anthocyanins. Not expressed in roots (qRT-PCR). The mRNA is cell-to-cell mobile.	44.03	94.90	0.46	5.30E-06	3.52E-05
740	2	L4 vs. M4	247281_at	AT5G64350	Encodes FK506-binding protein 12 (FKBP12 or FKP12). FKP12 overexpression dramatically enhances rapamycin sensitivity, whereas rapamycin inhibition is relieved in transgenic plants deficient in FKP12.	282.99	615.34	0.46	1.12E-05	5.18E-05
741	2	L4 vs. M4	247864_s_a	AT1G25155	Glutamine amidotransferase type 1 family protein;(source:Araport11)	105.83	232.87	0.45	2.60E-06	2.25E-05
742	2	L4 vs. M4	256008_s_at	AT1G34040	Pyridoxal phosphate (PLP)-dependent transferases superfamily protein;(source:Araport11)	45.65	102.53	0.45	2.40E-06	2.17E-05
743	2	L4 vs. M4	264400_at	AT1G61800	glucose6-Phosphate/phosphate transporter 2. Expression is upregulated in the shoot of cax1/cax3 mutant. The mRNA is cell-to-cell mobile.	368.65	827.97	0.45	5.40E-06	3.52E-05
744	2	L4 vs. M4	259913_at	AT1G72660	P-loop containing nucleoside triphosphate hydrolases superfamily protein;(source:Araport11)	22.97	51.00	0.45	4.53E-05	1.41E-04
745	2	L4 vs. M4	264000_at	AT2G22500	Encodes one of the mitochondrial dicarboxylate carriers (DIC): DIC1 (AT2G22500), DIC2 (AT4G24570), DIC3 (AT5G09470).	108.14	239.24	0.45	6.98E-04	1.24E-03
746	2	L4 vs. M4	267040_at	AT2G34300	S-adenosyl-L-methionine-dependent methyltransferases superfamily protein;(source:Araport11)	140.36	308.74	0.45	3.12E-04	6.45E-04
747	2	L4 vs. M4	267358_at	AT2G39890	Encodes a proline transporter with affinity for gly betaine, proline and GABA. Protein is expressed in the vascular tissue, specifically the phloem.	391.65	867.56	0.45	5.00E-06	3.46E-05

748	2	L4 vs. M4	258162_at	AT3G17810	Encodes a protein predicted to have dihydropyrimidine dehydrogenase activity. Its activity has not been demonstrated in vivo, but, it is required for efficient uracil catabolism in Arabidopsis. It localizes to the plastid.	146.12	328.16	0.45	1.95E-05	7.62E-05
749	2	L4 vs. M4	252158_at	AT3G50530	CDPK-related kinase	960.60	2132.88	0.45	2.70E-06	2.32E-05
750	2	L4 vs. M4	253360_at	AT4G33090	encodes an aminopeptidase, a ortholog of mouse microsomal AP (EC 3.4.11.2).	513.33	1151.38	0.45	7.12E-05	2.03E-04
751	2	L4 vs. M4	250259_at	AT5G13800	Encodes a pheophytinase that is involved in chlorophyll breakdown. Its transcript levels increase during senescence and pph-1 mutants have a stay-green phenotype.	94.34	211.64	0.45	4.50E-06	3.27E-05
752	2	L4 vs. M4	249242_at	AT5G42250	Zinc-binding alcohol dehydrogenase family protein;(source:Araport11)	72.43	160.51	0.45	6.00E-04	1.10E-03
753	2	L4 vs. M4	249153_s_a	AT5G43390	plant/protein;(source:Araport11)	17.75	39.09	0.45	5.40E-06	3.52E-05
754	2	L4 vs. M4	248583_at	AT5G49930	zinc knuckle (CCHC-type) family protein;(source:Araport11)	51.64	114.64	0.45	8.23E-05	2.23E-04
755	2	L4 vs. M4	248545_at	AT5G50260	Encodes a papain-like cysteine protease involved in tapetal programmed cell death and pollen development.CEP1 is expressed specifically in the tapetum from stages 5 to 11 of anther development. The CEP1 protein first appears as a proenzyme in precursor protease vesicles, and is then transported to the vacuole and transformed into the mature enzyme before rupture of the vacuole. CEP1 was also released to the tapetal cell wall during late stage 6 and stage 7. After the tapetal cell wall degenerated, the CEP1 enzyme entered the callose wall from the degenerated tapetal cell wall and was probably involved in degeneration of the callose wall.	109.99	244.47	0.45	1.08E-05	5.02E-05
756	2	L4 vs. M4	247929_at	AT5G57330	Galactose mutarotase-like superfamily protein;(source:Araport11)	90.62	202.92	0.45	7.70E-06	4.11E-05
757	2	L4 vs. M4	247902_at	AT5G57350	member of Plasma membrane H+-ATPase family	593.54	1317.49	0.45	4.00E-06	2.98E-05
758	2	L4 vs. M4	247771_at	AT5G58590	Encodes a Ran-binding protein 1 homolog (RanBP1).	48.87	109.63	0.45	1.23E-05	5.46E-05
759	2	L4 vs. M4	263709_at	AT1G09310	plant/protein (Protein of unknown function, DUF538);(source:Araport11)	247.41	560.79	0.44	2.50E-06	2.20E-05
760	2	L4 vs. M4	262454_at	AT1G11190	Encodes a bifunctional nuclease that acts on both RNA and DNA involved in nucleic acid degradation to facilitate nucleotide and phosphate recovery during senescence. It has mismatch-specific endonuclease activity with wide recognition of single base mismatches as well as the ability to cleave indel types of mismatches (heteroduplexes with loops).	95.78	215.31	0.44	7.70E-06	4.11E-05
761	2	L4 vs. M4	266033_at	AT2G05830	Encodes a 5-methylthioribose-1-phosphate isomerase.	215.44	484.20	0.44	8.60E-06	4.38E-05
762	2	L4 vs. M4	266038_at	AT2G07680	Encodes ABCC13/MRP11, a member of the multidrug resistance associated protein MRP/ABCC subfamily. Its expression is induced by gibberellic acid and downregulated by naphthalene acetic acid, abscisic acid, and zeatin.	58.89	135.00	0.44	4.80E-06	3.41E-05
763	2	L4 vs. M4	252190_at	AT3G50170	transmembrane protein, putative (DUF247);(source:Araport11)	18.31	41.88	0.44	3.81E-04	7.42E-04
764	2	L4 vs. M4	252184_at	AT3G50660	Encodes a 22α hydroxylase whose reaction is a rate-limiting step in brassinosteroid biosynthetic pathway. The protein is a member of CYP90B gene family. CLM is an epi-allele with small, compressed rosette, reduced internode length, and reduced fertility, appears in selfed ddm mutant plants possibly due to loss of cytosine methylation. Transcripts accumulate in actively growing tissues, and GUS expression is negatively regulated by brassinosteroids. Localized in the endoplasmic reticulum. The in vitro expressed protein can perform the C-22 hydroxylation of a variety of C27-, C28- and C29-sterols. Cholesterol was the best substrate, followed by campesterol. Sitosterol was a poor substrate.	361.68	820.19	0.44	2.90E-06	2.34E-05
765	2	L4 vs. M4	251945_at	AT3G53520	Encodes an isoform of UDP-glucuronic acid decarboxylase, which is predicted to be membrane-bound by PSORT analysis. This enzyme produces UDP-xylose, which is a substrate for many cell wall carbohydrates including hemicellulose and pectin. UDP-xylose is also known to feedback regulate several cell wall biosynthetic enzymes.	46.93	107.55	0.44	4.07E-04	7.83E-04
766	2	L4 vs. M4	254101_at	AT4G25000	Predicted to be secreted protein based on signalP prediction. Involved in starch mobilization. Mutants are defective in alpha-amylase activity. (Note: AMY1 has been found in the literature to be referred to as AMY3, which is not to be confused with AMY3/At1g69830).	352.80	810.04	0.44	5.82E-05	1.72E-04
767	2	L4 vs. M4	253373_at	AT4G33150	This is a splice variant of the LKR/SDH locus. It encodes a bifunctional polypeptide lysine-ketoglutarate reductase and saccharopine dehydrogenase involved in lysine degradation. There is another splice variant that encodes a mono saccharopine dehydrogenase protein. Gene expression is induced by abscisic acid, jasmonate, and under sucrose starvation.	38.98	88.41	0.44	7.50E-06	4.10E-05

768	2	L4 vs. M4	245701_at	AT5G04140	Encodes a gene whose sequence is similar to ferredoxin dependent glutamate synthase (Fd-GOGAT). Expression in leaves is induced by light and sucrose. Proposed to be involved in photorespiration and nitrogen assimilation. The mRNA is cell-to-cell mobile.	585.94	1326.25	0.44	1.63E-05	6.71E-05
769	2	L4 vs. M4	246566_at	AT5G14940	Major facilitator superfamily protein;(source:Araport11)	61.91	140.38	0.44	1.69E-05	6.90E-05
770	2	L4 vs. M4	246510_at	AT5G15410	'defense, no death' gene (DND1) encodes a mutated cyclic nucleotide-gated cation channel; Same as CNGC2 (article ID 229): Cyclic nucleotide gated channel, activated by cAMP, conducts K+ and other monovalent cations but excludes Na+, does not contain the GYG amino acid sequence found in other channels with this conductivity profile. Conducts Ca2+ into cells which is linked to the generation of NO and the NO signaling pathway involved in the innate immune response to pathogens. CNGC2 could be the key step mediating bulk Ca2+ influx into leaf cells after unloading from the vascular and have no direct roles in the leaf development and HR.	65.67	148.80	0.44	2.53E-04	5.46E-04
771	2	L4 vs. M4	264550_at	AT1G09450	Encodes a protein kinase that phosphorylates histone H3 at Thr3 and Thr11 and plays a role in mitotic cell division.	32.53	76.08	0.43	2.20E-06	2.09E-05
772	2	L4 vs. M4	261032_at	AT1G17430	alpha/beta-Hydrolases superfamily protein;(source:Araport11)	88.55	203.65	0.43	2.30E-06	2.09E-05
773	2	L4 vs. M4	265940_at	AT2G19480	This gene is predicted to encode a nucleosome assembly protein. Plant lines expressing an RNAi construct directed against this gene show a reduction in agrobacterium-mediated root transformation. The mRNA is cell-to-cell mobile. Plants mutated in three ubiquitously expressed NAP1 genes (NAP1;1~NAP1;3) and organ-specifically expressed NAP1;4 gene show hypersensitivity to genotoxic stresses including UV and DSB-inducing agent Bleomycin. The NAP1 genes act synergistically with NRP genes in promoting somatic homologous recombination.	74.20	171.97	0.43	7.90E-06	4.12E-05
774	2	L4 vs. M4	267293_at	AT2G23810	Member of TETRASPANIN family	67.43	155.54	0.43	3.20E-06	2.53E-05
775	2	L4 vs. M4	266989_at	AT2G39330	jacalin-related lectin 23;(source:Araport11)	939.85	2208.50	0.43	2.00E-06	1.99E-05
776	2	L4 vs. M4	253872_at	AT4G27410	Encodes a NAC transcription factor induced in response to desiccation. It is localized to the nucleus and acts as a transcriptional activator in ABA-mediated dehydration response.	66.67	156.13	0.43	1.80E-06	1.90E-05
777	2	L4 vs. M4	251055_at	AT5G01710	methyltransferase;(source:Araport11)	62.01	144.46	0.43	1.90E-06	1.94E-05
778	2	L4 vs. M4	250676_at	AT5G06320	encodes a protein whose sequence is similar to tobacco hairpin-induced gene (HIN1) and Arabidopsis non-race specific disease resistance gene (NDR1). Expression of this gene is induced by cucumber mosaic virus, spermine and Pseudomonas syringae pv. tomato DC3000. The gene product is localized to the plasma membrane.	40.37	94.59	0.43	1.84E-05	7.27E-05
779	2	L4 vs. M4	248968_at	AT5G45280	Pectinacetylesterase family protein;(source:Araport11)	357.16	822.73	0.43	5.50E-06	3.52E-05
780	2	L4 vs. M4	261405_at	AT1G18740	DUF793 domain containing protein. Expression is induced by cold. Loss of function mutations are more sensitive to freezing and have reduced levels of CBFs. May act by preventing degradation of CBFs.	200.74	474.64	0.42	1.05E-05	4.97E-05
781	2	L4 vs. M4	261650_at	AT1G27770	Encodes a chloroplast envelope Ca2+-ATPase with an N-terminal autoinhibitor.	81.26	192.62	0.42	5.90E-06	3.71E-05
782	2	L4 vs. M4	263157_at	AT1G54100	Aldehyde dehydrogenase	849.10	2028.09	0.42	2.90E-06	2.34E-05
783	2	L4 vs. M4	265929_s_a	AT2G18570	UDP-Glycosyltransferase superfamily protein;(source:Araport11)	295.01	694.61	0.42	1.04E-05	4.96E-05
784	2	L4 vs. M4	267319_at	AT2G34660	encodes a multidrug resistance-associated protein that is MgATP-energized glutathione S-conjugate pump. An ABCC-type arsenite-phytochelatin transporter. The expression of this gene is upregulated by herbicide safeners such as benoxacor and fenclorim. The mRNA is cell-to-cell mobile.	171.85	413.22	0.42	6.50E-06	3.88E-05
785	2	L4 vs. M4	252622_at	AT3G45310	Cysteine proteinases superfamily protein;(source:Araport11)	148.11	349.66	0.42	7.40E-06	4.10E-05
786	2	L4 vs. M4	249618_at	AT5G37490	ARM repeat superfamily protein;(source:Araport11)	18.44	44.19	0.42	2.06E-05	7.90E-05
787	2	L4 vs. M4	249581_at	AT5G37600	encodes a cytosolic glutamine synthetase, the enzyme has high affinity with substrate ammonium	628.87	1496.74	0.42	1.60E-06	1.74E-05
788	2	L4 vs. M4	265741_at	AT2G01320	ABC-2 type transporter family protein;(source:Araport11)	48.42	117.36	0.41	3.70E-06	2.84E-05
789	2	L4 vs. M4	266613_at	AT2G14900	Gibberellin-regulated family protein;(source:Araport11)	118.28	285.97	0.41	1.60E-06	1.74E-05
790	2	L4 vs. M4	259116_at	AT3G01350	Major facilitator superfamily protein;(source:Araport11)	22.98	56.29	0.41	7.90E-06	4.12E-05
791	2	L4 vs. M4	252123_at	AT3G51240	Encodes flavanone 3-hydroxylase that is coordinately expressed with chalcone synthase and chalcone isomerases. Regulates flavonoid biosynthesis. Not responsive to auxin or ethylene stimulus (qRT-PCR).	68.31	165.83	0.41	1.60E-06	1.74E-05
792	2	L4 vs. M4	251753_at	AT3G55760	hypothetical protein;(source:Araport11)	77.17	189.35	0.41	9.57E-05	2.56E-04
793	2	L4 vs. M4	251189_at	AT3G62650	hypothetical protein;(source:Araport11)	45.35	109.54	0.41	4.40E-06	3.26E-05

794	2	L4 vs. M4	255447_at	AT4G02790	Encodes a GTPase that is targeted to chloroplasts and co-fractionated with chloroplast ribosomes. Mutants are embryo lethal due to this essential function being lost.	126.97	307.29	0.41	7.69E-05	2.13E-04
795	2	L4 vs. M4	254283_s_at	AT4G22870	2-oxoglutarate (2OG) and Fe(II)-dependent oxygenase superfamily protein;(source:Araport11)	183.27	448.25	0.41	2.90E-06	2.34E-05
796	2	L4 vs. M4	250273_at	AT5G13010	Encodes a nuclear localized DEAH-box containing protein that is involved in miRNA biogenesis. Loss of function mutants are embryo lethal. Gene silencing experiments demonstrated its role in the localization of DCL-1 and HYL1 to the nuclear D-body. In silenced lines, miRNA production is suppressed and plants have developmental abnormalities and are hypersensitive to fungal pathogens.	67.92	166.31	0.41	8.22E-05	2.23E-04
797	2	L4 vs. M4	245981_at	AT5G13100	Gap junction beta-4 protein;(source:Araport11)	166.82	409.90	0.41	1.40E-06	1.63E-05
798	2	L4 vs. M4	259393_at	AT1G06410	Encodes an enzyme putatively involved in trehalose biosynthesis. Though the protein has both trehalose-6-phosphate synthase (TPS)-like and trehalose-6-phosphate phosphatase (TPP)-like domains, neither activity has been detected in enzymatic assays nor has the protein been able to complement yeast TPS or TPP mutants.	239.74	604.01	0.40	3.08E-05	1.05E-04
799	2	L4 vs. M4	255940_at	AT1G20380	Prolyl oligopeptidase family protein;(source:Araport11)	115.37	290.31	0.40	1.05E-05	4.97E-05
800	2	L4 vs. M4	261294_at	AT1G48430	Dihydroxyacetone kinase;(source:Araport11)	60.78	150.54	0.40	6.00E-06	3.72E-05
801	2	L4 vs. M4	262360_at	AT1G73080	Encodes a leucine-rich repeat receptor kinase. Functions as a receptor for AtPep1 to amplify innate immunity response to pathogen attacks. The mRNA is cell-to-cell mobile.	102.80	258.48	0.40	7.50E-06	4.10E-05
802	2	L4 vs. M4	267357_at	AT2G40000	ortholog of sugar beet HS1 PRO-1 2;(source:Araport11)	54.68	138.40	0.40	7.74E-05	2.14E-04
803	2	L4 vs. M4	260567_at	AT2G43820	Encodes a nicotinate-O-glycosyltransferase. Induced by Salicylic acid, virus, fungus and bacteria. Also involved in the tryptophan synthesis pathway. Independent of NPR1 for their induction by salicylic acid. UGT74F1 transfers UDP:glucose to salicylic acid (forming a glucoside (SAG) and a glucose ester (SGE)), benzoic acid, and anthranilate in vitro. UGT74F2 shows a weak ability to catalyze the formation of the p-aminobenzoate-glucose ester in vitro. But, UGT75B1 appears to be the dominant pABA acylglucosyltransferase in vivo based on assays in leaves, flowers, and siliques.	170.05	420.91	0.40	2.30E-06	2.09E-05
804	2	L4 vs. M4	263776_s_at	AT2G46440	Member of Cyclic nucleotide gated channel family. Positive regulator of resistance against avirulent fungal pathogen.The mRNA is cell-to-cell mobile.	21.52	53.91	0.40	2.08E-04	4.72E-04
805	2	L4 vs. M4	258299_at	AT3G23410	Encodes a fatty alcohol oxidase.	37.62	93.27	0.40	7.60E-06	4.10E-05
806	2	L4 vs. M4	254630_at	AT4G18360	Encodes a glycolate oxidase that modulates reactive oxygen species-mediated signal transduction during nonhost resistance.	26.99	68.24	0.40	2.95E-05	1.02E-04
807	2	L4 vs. M4	248309_at	AT5G52540	keratin-associated protein, putative (DUF819);(source:Araport11)	481.86	1190.12	0.40	7.80E-06	4.12E-05
808	2	L4 vs. M4	248255_at	AT5G53350	CLP protease regulatory subunit CLPX mRNA, nuclear gene	33.39	83.68	0.40	4.44E-05	1.40E-04
809	2	L4 vs. M4	244936_at	ATCG01100	NADH dehydrogenase ND1	52.25	130.66	0.40	2.54E-04	5.46E-04
810	2	L4 vs. M4	252592_at	AT3G45640	Encodes a mitogen-activated kinase whose mRNA levels increase in response to touch, cold, salinity stress and chitin oligomers.Also functions in ovule development. Heterozygous MPK3 mutants in a homozygous MPK6 background are female sterile due to defects in integument development. MPK3 can be dephosphorylated by MKP2 in vitro. The mRNA is cell-to-cell mobile.	51.84	131.54	0.39	7.60E-06	4.10E-05
811	2	L4 vs. M4	251962_at	AT3G53420	a member of the plasma membrane intrinsic protein subfamily PIP2. localizes to the plasma membrane and exhibits water transport activity in Xenopus oocyte. expressed specifically in the vascular bundles and protein level increases slightly during leaf dev. When expressed in yeast cells can conduct hydrogen peroxide into those cells.	790.85	2005.21	0.39	7.00E-06	4.00E-05
812	2	L4 vs. M4	253343_at	AT4G33540	metallo-beta-lactamase family protein;(source:Araport11)	172.97	442.35	0.39	8.00E-07	1.21E-05
813	2	L4 vs. M4	245692_at	AT5G04150	Encodes a member of the basic helix-loop-helix transcription factor family protein. Functions as a key regulator of iron-deficiency responses independent of the master regulator FIT. Likely regulates genes involved in the distribution of iron within the plant.	30.20	78.04	0.39	2.11E-04	4.75E-04
814	2	L4 vs. M4	246002_at	AT5G20740	Plant invertase/pectin methylesterase inhibitor superfamily protein;(source:Araport11)	179.80	460.68	0.39	8.00E-07	1.21E-05
815	2	L4 vs. M4	247156_at	AT5G65760	Serine carboxypeptidase S28 family protein;(source:Araport11)	86.47	223.60	0.39	6.00E-06	3.72E-05

816	2	L4 vs. M4	262238_at	AT1G48300	Cytosolic iron-sulfur protein with a [2Fe-2S] cluster which synthesizes triacylglycerol (DGAT activity).	75.57	200.07	0.38	1.23E-05	5.46E-05
817	2	L4 vs. M4	257641_s_at	AT3G25760	encodes allene oxide cyclase. One of four genes in Arabidopsis that encode this enzyme, which catalyzes an essential step in jasmonic acid biosynthesis. Gene expression is induced during senescence, a process that involves jasmonic acid signalling pathway. The mRNA is cell-to-cell mobile.	242.05	640.23	0.38	4.38E-05	1.39E-04
818	2	L4 vs. M4	257629_at	AT3G26140	Cellulase (glycosyl hydrolase family 5) protein;(source:Araport11)	1381.91	3610.53	0.38	1.90E-06	1.94E-05
819	2	L4 vs. M4	255016_at	AT4G10120	Encodes a protein with putative sucrose-phosphate synthase activity.	162.03	430.41	0.38	6.90E-06	3.98E-05
820	2	L4 vs. M4	246216_at	AT4G36380	Encodes a cytochrome P-450 gene that is involved in leaf blade expansion by controlling polar cell expansion in the leaf length direction. Member of the CYP90C CYP450 family. ROT3 was shown to be involved in brassinosteroid biosynthesis, most likely in the conversion step of typhasterol (TY) to castasterone (CS). As 6-deoxo-CS was unable to restore the phenotype of rot3-1, it has been postulated that ROT3 might be specifically involved in the conversion of TY to CS in the C6-oxidation pathway of brassinolide. Recently, CYP90C1 was shown to catalyse the C-23 hydroxylation of several brassinosteroids (the enzyme has a broad specificity for 22-hydroxylated substrates).	75.15	196.32	0.38	6.00E-07	9.91E-06
821	2	L4 vs. M4	249690_at	AT5G36210	alpha/beta-Hydrolases superfamily protein;(source:Araport11)	82.51	219.15	0.38	2.20E-06	2.09E-05
822	2	L4 vs. M4	264751_at	AT1G23020	Encodes a ferric chelate reductase whose transcription is regulated by FIT1. Expressed in the root, shoot, flower and cotyledon.	26.71	71.53	0.37	3.12E-04	6.45E-04
823	2	L4 vs. M4	265184_at	AT1G23710	hypothetical protein (DUF1645);(source:Araport11)	26.95	72.49	0.37	6.60E-06	3.92E-05
824	2	L4 vs. M4	264186_at	AT1G54570	Encodes a protein with phytyl ester synthesis and diacylglycerol acyltransferase activities that is involved in the deposition of free phytol and free fatty acids in the form of phytyl esters in chloroplasts, a process involved in maintaining the integrity of the photosynthetic membrane during abiotic stress and senescence.	127.92	344.82	0.37	4.00E-07	7.54E-06
825	2	L4 vs. M4	262094_at	AT1G56110	NOP56-like protein	183.86	501.10	0.37	3.81E-05	1.23E-04
826	2	L4 vs. M4	257701_at	AT3G12710	DNA glycosylase superfamily protein;(source:Araport11)	39.51	106.18	0.37	7.20E-06	4.07E-05
827	2	L4 vs. M4	258033_at	AT3G21250	member of MRP subfamily	61.00	165.85	0.37	5.00E-07	9.10E-06
828	2	L4 vs. M4	249719_at	AT5G35735	Auxin-responsive family protein;(source:Araport11)	22.27	60.84	0.37	1.80E-06	1.90E-05
829	2	L4 vs. M4	247013_at	AT5G67480	BTB and TAZ domain protein. Located in cytoplasm and expressed in fruit, flower and leaves.	52.03	142.17	0.37	2.30E-06	2.09E-05
830	2	L4 vs. M4	245002_at	ATCG00270	PSII D2 protein	109.45	298.84	0.37	1.06E-05	4.97E-05
831	2	L4 vs. M4	261226_at	AT1G20190	member of Alpha-Expansin Gene Family. Naming convention from the Expansin Working Group (Kende et al, 2004. Plant Mol Bio)	76.73	213.55	0.36	6.00E-07	9.91E-06
832	2	L4 vs. M4	261864_s_at	AT1G50480	10-formyltetrahydrofolate synthetase (THFS) mRNA, complete The mRNA is cell-to-cell mobile.	1081.71	2974.66	0.36	1.00E-06	1.34E-05
833	2	L4 vs. M4	267644_s_a	AT2G32880	TRAF-like family protein;(source:Araport11)	27.72	77.83	0.36	1.66E-04	3.99E-04
834	2	L4 vs. M4	258979_at	AT3G09440	Heat shock protein 70 (Hsp 70) family protein;(source:Araport11)	67.06	187.43	0.36	2.20E-06	2.09E-05
835	2	L4 vs. M4	251882_at	AT3G54140	Encodes a di- and tri-peptide transporter that recognizes a variety of different amino acid combinations. GFP-tagged PTR1 localizes to the plasma membrane and has 8 to 11 predicted transmembrane domains. PTR1 is expressed in a number of different vascular tissues throughout the plant based on promoter:GUS expression analysis. ptr1 mutants have a lower dry weight than wild type plants when both are grown with Pro-Ala or Ala-Ala dipeptides as their nitrogen source, suggesting that PTR1 plays a role in dipeptide uptake in the roots. Furthermore N content of ptr1 mutants is lower than that of wild type plants when grown with Pro-Ala or a mixture of dipeptides as nitrogen source	129.51	364.78	0.36	1.45E-05	6.11E-05
836	2	L4 vs. M4	259826_at	AT1G29340	Encodes a protein containing a UND, a U-box, and an ARM domain. This protein has E3 ubiquitin ligase activity. It is required for cell death and full resistance specified by Arabidopsis RPM1 and RPS4 resistance proteins against Pseudomonas syringae pv tomato. The mRNA is cell-to-cell mobile.	69.90	202.59	0.35	1.40E-06	1.63E-05
837	2	L4 vs. M4	253404_at	AT4G32840	phosphofructokinase 6;(source:Araport11)	145.95	414.20	0.35	3.50E-06	2.70E-05
838	2	L4 vs. M4	250788_at	AT5G05570	transducin family protein / WD-40 repeat family protein;(source:Araport11)	95.40	272.48	0.35	5.75E-05	1.70E-04
839	2	L4 vs. M4	260462_at	AT1G10970	A member of Zrt- and Irt-related protein (ZIP) family. transcript is induced in response to zinc deficiency in the root and shoot. Expression is regulated by copper, but response to copper deficiency is detected only after three weeks of deficiency.	74.01	219.63	0.34	3.10E-06	2.47E-05
840	2	L4 vs. M4	261767_s_a	AT1G15500	TLC ATP/ADP transporter;(source:Araport11)	114.74	336.42	0.34	3.00E-07	6.20E-06

841	2	L4 vs. M4	262073_at	AT1G59640	A basic helix-loop-helix encoding gene (BIGPETAL, BPE) involved in the control of petal size. BPE is expressed via two mRNAs derived from an alternative splicing event. The BPEub (AT1G59640.1)transcript is expressed ubiquitously, whereas the BPEp (AT1G59640.2) transcript is preferentially expressed in petals. Plants that lack the petal-expressed variant BPEp have larger petals as a result of increased cell size. BPEp is positively regulated downstream of APETALA3, PISTILLATA, APETALA1 and PISTILLATA3 and is negatively regulated downstream of AGAMOUS.	48.44	140.91	0.34	2.00E-07	5.51E-06
842	2	L4 vs. M4	245006_at	ATCG00340	Encodes the D1 subunit of photosystem I reaction center.	4541.33	13400.47	0.34	7.03E-05	2.01E-04
843	2	L4 vs. M4	264595_at	AT1G04750	Encodes vesicle-associated membrane protein 7B (VAMP7B, or VAMP721). Required for cell plate formation.VAMP721 interacts with KAT1 and KC1 K+ channels, affects channel gating and suppresses the K+ current within the physiological voltage range. Post-transcriptionally regulated by CRT1/2 under ER stress.	168.67	516.18	0.33	2.50E-05	9.16E-05
844	2	L4 vs. M4	246627_s_at	AT1G48860	5-enolpyruvylshikimate-3-phosphate synthase involved in shikimic acid biosynthesis.	449.09	1358.16	0.33	3.00E-07	6.20E-06
845	2	L4 vs. M4	257895_at	AT3G16950	encodes a plastid lipoamide dehydrogenase, subunit of the pyruvate dehydrogenase complex which provides acetyl-CoA for de novo fatty acid biosynthesis. The gene is highly expressed in developing seeds.	281.30	858.54	0.33	1.06E-05	4.97E-05
846	2	L4 vs. M4	256617_at	AT3G22240	cysteine-rich/transmembrane domain PCC1-like protein;(source:Araport11)	142.79	432.98	0.33	3.00E-07	6.20E-06
847	2	L4 vs. M4	246018_at	AT5G10695	methionyl-tRNA synthetase;(source:Araport11)	88.29	267.67	0.33	1.10E-06	1.44E-05
848	2	L4 vs. M4	244972_at	ATCG00680	encodes for CP47, subunit of the photosystem II reaction center.	93.73	281.31	0.33	5.43E-05	1.62E-04
849	2	L4 vs. M4	257122_at	AT3G20250	Encodes a member of the Arabidopsis Pumilio (APUM) proteins containing PUF domain (eight repeats of approximately 36 amino acids each). PUF proteins regulate both mRNA stability and translation through sequence-specific binding to the 3 Prime UTR of target mRNA transcripts. APUM5 is involved in susceptibility to CMV and is not required for bacterial or fungal pathogen resistance although its expression is induced upon bacterial and fungal infection. It is involved in the osmotic, salt, and drought stress responses.	38.32	121.19	0.32	1.00E-06	1.34E-05
850	2	L4 vs. M4	254740_s_at	AT4G13930	Encodes a serine hydroxymethyltransferase maximally expressed in root	464.06	1429.53	0.32	6.00E-07	9.91E-06
851	2	L4 vs. M4	255926_at	AT1G22190	The gene encodes a putative transcription factor belongings to the abiotic stress-associated DREB A-6 clade. The mRNA is cell-to-cell mobile.	35.13	111.70	0.31	2.00E-05	7.75E-05
852	2	L4 vs. M4	252606_at	AT3G45010	serine carboxypeptidase-like 48;(source:Araport11)	479.17	1568.18	0.31	5.00E-07	9.10E-06
853	2	L4 vs. M4	247060_at	AT5G66760	One of two genes in Arabidopsis that encode a flavoprotein subunit of the mitochondrial succinate dehydrogenase complex. The mRNA is cell-to-cell mobile.	446.32	1439.11	0.31	1.26E-05	5.52E-05
854	2	L4 vs. M4	262660_at	AT1G14000	Encodes a protein with similarity to members of the C1 subgroup of MAP kinase kinase kinases. Interacts physically with the receptor kinase BRL2/VH1 and appears to be involved in auxin and brassinosteriod signaling. The mRNA is cell-to-cell mobile.	178.64	597.71	0.30	3.00E-07	6.20E-06
855	2	L4 vs. M4	245803_at	AT1G47128	cysteine proteinase precursor-like protein/ dehydration stress-responsive gene (RD21). Has been shown to have peptide ligase activity and protease activity in vitro. RD21 is involved in immunity to the necrotrophic fungal pathogen Botrytis cinerea.Activity detected in root, leaf, flower and cell culture.	1957.37	6632.95	0.30	1.00E-07	3.36E-06

856	2	L4 vs. M4	264851_at	AT2G17290	Encodes calcium dependent protein kinase 6 (CPK6), a member of the Arabidopsis CDPK gene family. CDPKs contain an intrinsic Ca2+-activation domain with four EF hand Ca2+-binding sites. CDPKs protein kinases have been proposed to function in multiple plant signal transduction pathways downstream of [Ca2+]cyt elevations, thus transducing various physiological responses. CPK6 is expressed in both guard cells and mesophyll cells. Functions in guard cell ion channel regulation. ABA and Ca(2+) activation of slow-type anion channels and, interestingly, ABA activation of plasma membrane Ca(2+)-permeable channels were impaired in independent alleles of single and double cpk3cpk6 mutant guard cells. Furthermore, ABA- and Ca(2+)-induced stomatal closing were partially impaired in these cpk3cpk6 mutant alleles. The protein kinase CPK6 is shown in biochemical assays to be directly activated by elevations in calcium concentrations in the physiological range (Laanements et al., 2013 PlantPhys.; PMID: 23766366). These data correlate with the in vivo function of CPK6 in Ca2+ and ABA activation of S-type anion channels (Mori et al., 2006 PLoS Biol.; PMID: 17032064) and the ability of CPK6 to mediate ABA activation of SLAC1 (Brandt et al., 2012 PNAS; PMID: 22689970).The mRNA is cell-to-cell mobile.	44.24	149.60	0.30	6.00E-07	9.91E-06
857	2	L4 vs. M4	258610_at	AT3G02875	Hydrolyzes amino acid conjugates of the plant growth regulator indole-3-acetic acid (IAA), including IAA-Leu and IAA-Phe. Uses Mg and Co ions as cofactors.	84.28	281.31	0.30	2.17E-05	8.21E-05
858	2	L4 vs. M4	245296_at	AT4G16370	Encodes a phloem-specific iron transporter that is essential for systemic iron signaling and redistribution of iron and cadmium. It loads iron into the phloem, facilitates iron recirculation from the xylem to the phloem, and regulates both shoot-to-root iron signaling and iron redistribution from mature to developing tissues.	760.65	2528.09	0.30	6.00E-07	9.91E-06
859	2	L4 vs. M4	254833_s_a	AT4G12280	copper amine oxidase family protein;(source:Araport11)	356.18	1219.51	0.29	1.00E-07	3.36E-06
860	2	L4 vs. M4	254657_s_a	AT4G18220	Drug/metabolite transporter superfamily protein;(source:Araport11)	240.35	831.91	0.29	1.00E-07	3.36E-06
861	2	L4 vs. M4	254543_at	AT4G19810	ChiC encodes a Class V chitinase that is a part of glycoside hydrolase family 18 based on CAZy groupings. It appears to primarily act as an exochitinase in vitro where it predominantly cleaves a chitobiose (GlcNAc)2 residue from the non-reducing end of a chitin oligosaccharide. However, it shows some minor endochitinase activity in vitro, as well. A putative 24 amino-acid signal peptide may direct this protein to the secretory system and it has been detected in cell wall apoplastic fluid. RT-PCR experiments demonstrate that ChiC transcript levels are increased in response to abscisisc acid, jasmonic acid, and NaCl stress. Microarray results also suggest that transcript levels rise in response to osmotic stress, two fungal pathogens, a bacterial pathogen, and the elicitor flagellin. The mRNA is cell-to-cell mobile.	43.16	151.10	0.29	2.90E-06	2.34E-05
862	2	L4 vs. M4	265530_at	AT2G06050	Encodes a 12-oxophytodienoate reductase that is required for jasmonate biosynthesis. Mutants are male sterile and defective in pollen dehiscence. Shows activity towards 2,4,6-trinitrotoluene. CFA-Ile, CFA-Leu, CFA-Val, CFA-Met and CFA-Ala can restore the fertility of opr3 plants by inducing filament elongation and anther dehiscence.	327.67	1152.25	0.28	3.00E-07	6.20E-06
863	2	L4 vs. M4	249688_at	AT5G36160	Encodes a cytosolic L-tyrosine aminotransferase. AtTAT2 exhibits much broader amino donor specificity than AtTAT1 and can use not only Tyr but also Phe, Trp, His, Met, Leu, Ala, Ser, Cys, Asp, Asn, Gln, and Arg as amino donors.	26.60	95.02	0.28	3.00E-07	6.20E-06
864	2	L4 vs. M4	258599_at	AT3G04520	Encodes a threonine aldolase, involved in threonine degradation to glycine. Expressed in vascular tissue through out the plant.	188.00	698.03	0.27	2.00E-07	5.51E-06
865	2	L4 vs. M4	252278_at	AT3G49530	Transcription factor that serves as a molecular link between cold signals and pathogen resistance responses. Undergoes proteolytic processing triggered by cold-induced changes in membrane fluidity.It relocates from the plasma membrane to the nucleus in response to ER stress. NAC062 is phosphorylated by SnRK2.8 at Thr-142.	17.24	66.17	0.26	3.23E-05	1.09E-04
866	2	L4 vs. M4	245757_at	AT1G35140	EXL1 is involved in the C-starvation response. Phenotypic changes of an exl1 loss of function mutant became evident only under corresponding experimental conditions. For example, the mutant showed diminished biomass production in a short-day/low light growth regime, impaired survival during extended night, and impaired survival of anoxia stress.	30.63	123.61	0.25	2.00E-06	1.99E-05
867	2	L4 vs. M4	260101_at	AT1G73260	Encodes a trypsin inhibitor involved in modulating programmed cell death in plant-pathogen interactions.	79.59	315.35	0.25	4.00E-07	7.54E-06

868	2	L4 vs. M4	251503_at	AT3G59140	member of MRP subfamily	172.80	680.62	0.25	2.00E-07	5.51E-06
869	2	L4 vs. M4	251937_at	AT3G53400	peptide upstream protein;(source:Araport11)	56.45	233.47	0.24	2.00E-07	5.51E-06
870	2	L4 vs. M4	244983_at	ATCG00790	chloroplast gene encoding a ribosomal protein L16, which is a constituent of 50S large ribosomal subunit	318.33	1337.84	0.24	1.00E-03	1.69E-03
871	2	L4 vs. M4	264574_at	AT1G05300	member of Fe(II) transporter isolog family	87.65	381.66	0.23	1.00E-07	3.36E-06
872	2	L4 vs. M4	261892_at	AT1G80840	Pathogen-induced transcription factor. Binds W-box sequences in vitro. Forms protein complexes with itself and with WRKY40 and WRKY60. Coexpression with WRKY18 or WRKY60 made plants more susceptible to both P. syringae and B. cinerea. WRKY18, WRKY40, and WRKY60 have partially redundant roles in response to the hemibiotrophic bacterial pathogen Pseudomonas syringae and the necrotrophic fungal pathogen Botrytis cinerea, with WRKY18 playing a more important role than the other two. The mRNA is cell-to-cell mobile.	80.84	348.20	0.23	3.00E-07	6.20E-06
873	2	L4 vs. M4	265460_at	AT2G46600	Calcium-binding EF-hand family protein;(source:Araport11)	281.39	1219.42	0.23	< 1e-07	< 1e-07
874	2	L4 vs. M4	246998_at	AT5G67370	DUF1230 family protein (DUF1230);(source:Araport11)	38.70	164.96	0.23	< 1e-07	< 1e-07
875	2	L4 vs. M4	245009_at	ATCG00380	Chloroplast encoded ribosomal protein S4	88.04	386.72	0.23	2.90E-06	2.34E-05
876	2	L4 vs. M4	261754_at	AT1G76130	alpha-amylase, putative / 1,4-alpha-D-glucan glucanohydrolase, putative, strong similarity to alpha-amylase GI:7532799 from (Malus x domestica);contains Pfam profile PF00128: Alpha amylase, catalytic domain. Predicted to be secreted based on SignalP analysis.	136.98	635.85	0.22	< 1e-07	< 1e-07
877	2	L4 vs. M4	244973_at	ATCG00690	Encodes photosystem II 5 kD protein subunit PSII-T. This is a plastid-encoded gene (PsbTc) which also has a nuclear-encoded paralog (PsbTn).	34.80	155.84	0.22	5.50E-06	3.52E-05
878	2	L4 vs. M4	266262_at	AT2G27590	S-adenosyl-L-methionine-dependent methyltransferases superfamily protein;(source:Araport11)	28.79	140.21	0.21	< 1e-07	< 1e-07
879	2	L4 vs. M4	249438_at	AT5G40010	Encodes a mitochondrial ATPase involved in seed and silique development.	28.04	135.92	0.21	1.20E-06	1.50E-05
880	2	L4 vs. M4	245021_at	ATCG00560	PSII L protein	672.90	3450.89	0.19	< 1e-07	< 1e-07
881	2	L4 vs. M4	255459_at	AT4G02890	Polyubiquitin gene containing 4 ubiquitin repeats.	907.11	4734.62	0.19	2.00E-07	5.51E-06
882	2	L4 vs. M4	262680_at	AT1G75880	SGNH hydrolase-type esterase superfamily protein;(source:Araport11)	81.67	444.08	0.18	< 1e-07	< 1e-07
883	2	L4 vs. M4	245088_at	AT2G39850	Subtilisin-like serine endopeptidase family protein;(source:Araport11)	74.99	462.22	0.16	< 1e-07	< 1e-07
884	2	L4 vs. M4	248918_at	AT5G45890	Senescence-associated gene 12 (SAG12) encoding a cysteine protease influenced by cytokinin, auxin, and sugars.Localized to special vacuole found during senescence called senescence associated vacuoles which are different from central vacuole in the tonoplast composition and pH.	25.88	158.86	0.16	< 1e-07	3.36E-06
885	2	L4 vs. M4	253643_at	AT4G29780	Expression of the gene is affected by multiple stresses. Knockout and overexpression lines show no obvious phenotypes.	28.38	216.22	0.13	< 1e-07	< 1e-07
886	2	L4 vs. M4	248270_at	AT5G53450	OBP3-responsive protein 1;(source:Araport11)	140.09	1305.69	0.11	< 1e-07	< 1e-07
887	2	L4 vs. M4	266834_s_at	AT2G30020	Encodes AP2C1. Belongs to the clade B of the PP2C-superfamily. Acts as a MAPK phosphatase that negatively regulates MPK4 and MPK6.	144.50	1428.33	0.10	< 1e-07	< 1e-07
888	2	L4 vs. M4	258063_at	AT3G14620	putative cytochrome P450 The mRNA is cell-to-cell mobile.	39.83	435.46	0.09	< 1e-07	< 1e-07
889	2	L4 vs. M4	263972_at	AT2G42760	DUF1685 family protein;(source:Araport11)	34.72	389.41	0.09	< 1e-07	< 1e-07
890	2	L4 vs. M4	259381_s_at	AT3G16410	Encodes a nitrile-specifier protein NSP4. NSP4 is one out of five (At3g16400/NSP1, At2g33070/NSP2, At3g16390/NSP3, At3g16410/NSP4 and At5g48180/NSP5) A. thaliana epithiospecifier protein (ESP) homologues that promote simple nitrile, but not epithionitrile or thiocyanate formation. The mRNA is cell-to-cell mobile.	57.54	838.08	0.07	< 1e-07	3.36E-06
891	2	L4 vs. M4	244970_at	ATCG00660	encodes a chloroplast ribosomal protein L20, a constituent of the large subunit of the ribosomal complex	50.44	775.54	0.07	< 1e-07	3.36E-06
892	2	L4 vs. M4	244986_at	ATCG00820	Encodes a 6.8-kDa protein of the small ribosomal subunit.	102.96	1702.98	0.06	2.00E-07	5.51E-06
893	3	F2-3 vs. L2-3	246880_s_at	AT5G25980	Myrosinase (thioglucoside glucohydrolase) gene involved in glucosinoloate metabolism. The mRNA is cell-to-cell mobile.	5041.14	20.91	241.10	< 1e-07	< 1e-07
894	3	F2-3 vs. L2-3	248227_at	AT5G53820	Late embryogenesis abundant protein (LEA) family protein;(source:Araport11)	1371.93	15.02	91.34	< 1e-07	< 1e-07
895	3	F2-3 vs. L2-3	246687_at	AT5G33370	GDSL-motif esterase/acyltransferase/lipase. Enzyme group with broad substrate specificity that may catalyze acyltransfer or hydrolase reactions with lipid and non-lipid substrates. Mutants are defective in cuticle formation with reduced sepal cuticle ridge formation.	1648.20	22.61	72.89	< 1e-07	< 1e-07
896	3	F2-3 vs. L2-3	265552_at	AT2G07560	H[+]-ATPase 6;(source:Araport11)	1117.09	17.18	65.02	< 1e-07	< 1e-07
897	3	F2-3 vs. L2-3	266353_at	AT2G01520	Encodes a cis-cinnamic acid responsive gene that is a member of the major latex protein-like gene family and plays a role in promoting vegetative growth and delaying ﬂowering. The mRNA is cell-to-cell mobile.	2765.39	44.57	62.05	3.60E-06	5.76E-05

898	3	F2-3 vs. L2-3	261943_at	AT1G80660	H[+]-ATPase 9;(source:Araport11)	920.91	16.24	56.71	< 1e-07	< 1e-07
899	3	F2-3 vs. L2-3	245928_s_at	AT5G24780	encodes an acid phosphatase similar to soybean vegetative storage proteins. Gene expression is induced by wounding and jasmonic acid.	2992.69	69.62	42.98	< 1e-07	< 1e-07
900	3	F2-3 vs. L2-3	245010_at	ATCG00420	Encodes NADH dehydrogenase subunit J. Its transcription is increased upon sulfur depletion.	9425.33	220.06	42.83	< 1e-07	< 1e-07
901	3	F2-3 vs. L2-3	246072_at	AT5G20240	Floral homeotic gene encoding a MADS domain transcription factor. Required for the specification of petal and stamen identities.	596.07	15.84	37.63	< 1e-07	< 1e-07
902	3	F2-3 vs. L2-3	254687_at	AT4G13770	Encodes a cytochrome p450 enzyme that catalyzes the initial conversion of aldoximes to thiohydroximates in the synthesis of glucosinolates not derived from tryptophan. Also has a role in auxin homeostasis.	1178.82	31.78	37.10	< 1e-07	< 1e-07
903	3	F2-3 vs. L2-3	251258_at	AT3G62170	VANGUARD-like protein;(source:Araport11)	2579.65	88.77	29.06	9.35E-04	3.04E-03
904	3	F2-3 vs. L2-3	252820_at	AT3G42640	H[+]-ATPase 8;(source:Araport11)	3041.01	107.96	28.17	3.22E-05	2.62E-04
905	3	F2-3 vs. L2-3	252123_at	AT3G51240	Encodes flavanone 3-hydroxylase that is coordinately expressed with chalcone synthase and chalcone isomerases. Regulates flavonoid biosynthesis. Not responsive to auxin or ethylene stimulus (qRT-PCR).	656.75	23.77	27.63	5.00E-07	1.44E-05
906	3	F2-3 vs. L2-3	253226_at	AT4G35010	putative beta-galactosidase (BGAL11 gene)	604.49	22.01	27.47	2.70E-06	4.78E-05
907	3	F2-3 vs. L2-3	252618_at	AT3G45140	Chloroplast lipoxygenase required for wound-induced jasmonic acid accumulation in Arabidopsis.Mutants are resistant to Staphylococcus aureus and accumulate salicylic acid upon infection.CFA-Leu, CFA-Val, CFA-Met and CFA-Ala can induce the expression of LOX2.The mRNA is cell-to-cell mobile.	535.73	19.96	26.84	< 1e-07	< 1e-07
908	3	F2-3 vs. L2-3	262463_at	AT1G50310	Sucrose transporter, expressed in pollen tubes.	585.53	24.43	23.97	< 1e-07	< 1e-07
909	3	F2-3 vs. L2-3	260888_at	AT1G29140	Pollen Ole e 1 allergen and extensin family protein;(source:Araport11)	791.17	34.86	22.70	2.60E-06	4.67E-05
910	3	F2-3 vs. L2-3	249939_at	AT5G22430	Pollen Ole e 1 allergen and extensin family protein;(source:Araport11)	326.74	14.49	22.54	< 1e-07	< 1e-07
911	3	F2-3 vs. L2-3	265080_at	AT1G55570	SKU5 similar 12;(source:Araport11)	420.13	19.97	21.04	4.86E-05	3.55E-04
912	3	F2-3 vs. L2-3	254104_at	AT4G25040	Uncharacterized protein family (UPF0497);(source:Araport11)	416.28	19.84	20.98	< 1e-07	< 1e-07
913	3	F2-3 vs. L2-3	245842_at	AT1G58430	Encodes an anther-specific proline-rich protein.	264.00	12.60	20.96	< 1e-07	< 1e-07
914	3	F2-3 vs. L2-3	258851_at	AT3G03190	Encodes glutathione transferase belonging to the phi class of GSTs. Naming convention according to Wagner et al. (2002).	421.92	20.21	20.87	< 1e-07	< 1e-07
915	3	F2-3 vs. L2-3	257173_at	AT3G23810	S-adenosyl-l-homocysteine (SAH) hydrolase 2;(source:Araport11)	3763.75	181.62	20.72	< 1e-07	4.57E-06
916	3	F2-3 vs. L2-3	256638_at	AT3G19090	RNA-binding protein;(source:Araport11)	804.07	39.30	20.46	< 1e-07	< 1e-07
917	3	F2-3 vs. L2-3	259161_at	AT3G01500	Encodes a putative beta-carbonic anhydrase betaCA1. Together with betaCA4 (At1g70410) regulates CO2-controlled stomatal movements in guard cells, as well as attenuates immunity. Differential CA gene expression in response to changing atmospheric CO2 conditions contribute to altered disease resistance levels. Activated by OXS2 under the treatment of salt.	563.57	28.45	19.81	< 1e-07	< 1e-07
918	3	F2-3 vs. L2-3	266743_at	AT2G02990	Encodes a member of the ribonuclease T2 family that responds to inorganic phosphate starvation, and inhibits production of anthocyanin. Also involved in wound-induced signaling independent of jasmonic acid. Its expression is responsive to both phosphate (Pi) and phosphite (Phi) in roots.	1968.32	100.15	19.65	7.80E-06	1.01E-04
919	3	F2-3 vs. L2-3	256955_at	AT3G13390	SKU5 similar 11;(source:Araport11)	706.01	36.93	19.12	1.17E-05	1.31E-04
920	3	F2-3 vs. L2-3	249584_s_at	AT5G37810	NOD26-like intrinsic protein 4;(source:Araport11)	216.14	11.53	18.74	< 1e-07	< 1e-07
921	3	F2-3 vs. L2-3	254716_at	AT4G13560	Late embryogenesis abundant protein (LEA) family protein;(source:Araport11)	666.23	40.53	16.44	< 1e-07	< 1e-07
922	3	F2-3 vs. L2-3	249614_at	AT5G37300	Encodes a bifunctional enzyme, wax ester synthase (WS) and diacylglycerol acyltransferase (DGAT). In vitro assay indicated a ratio of 10.9 between its WS and DGAT activities. Both mutant and in vivo expression/analysis in yeast studies indicated a role in wax biosynthesis.	191.24	11.72	16.32	< 1e-07	< 1e-07
923	3	F2-3 vs. L2-3	249996_at	AT5G18600	Encodes a member of the CC-type glutaredoxin (ROXY) family that has been shown to interact with the transcription factor TGA2 and suppress ORA59 promoter activity.	305.23	19.17	15.92	< 1e-07	< 1e-07
924	3	F2-3 vs. L2-3	258639_at	AT3G07820	Pectin lyase-like superfamily protein;(source:Araport11)	449.66	29.77	15.10	1.69E-04	8.51E-04
925	3	F2-3 vs. L2-3	245061_at	AT2G39730	Rubisco activase, a nuclear-encoded chloroplast protein that consists of two isoforms arising from alternative splicing in most plants. Required for the light activation of rubisco. Involved in jasmonate-induced leaf senescence.	822.31	54.83	15.00	< 1e-07	< 1e-07
926	3	F2-3 vs. L2-3	248367_at	AT5G52360	ADF10 is an actin-depolymerizing factor that preferentially binds ADP-G-actin and inhibits G-actin nucleotide exchange. ADF10 promotes actin turnover in pollen , regulating organization of actin filaments and vesicle trafficking during pollen tube growth.	550.59	38.21	14.41	3.10E-06	5.20E-05

927	3	F2-3 vs. L2-3	245465_at	AT4G16590	encodes a gene similar to cellulose synthase	231.55	16.25	14.25	< 1e-07	< 1e-07
928	3	F2-3 vs. L2-3	253083_at	AT4G36250	Encodes a putative aldehyde dehydrogenase. The gene is not responsive to osmotic stress and is expressed constitutively at a low level in plantlets and root cultures.	421.58	29.60	14.24	< 1e-07	< 1e-07
929	3	F2-3 vs. L2-3	252607_at	AT3G44990	Encodes a xyloglucan endotransglycosylase/hydrolase. Protein sequence and phylogenetic analysis indicates that this enzyme resides in Group III-A of the XTH family, with high similarity to Tropaeolum majus (nasturtium) xyloglucanase 1 (TmNXG1).Enzyme kinetic analysis indicates predominant xyloglucan endo-hydrolase activity (EC 3.2.1.151) with only limited potential to act as a xyloglucan endo-transglycosylase (EC 2.4.1.207).	176.74	12.53	14.10	< 1e-07	< 1e-07
930	3	F2-3 vs. L2-3	267213_at	AT2G44120	Ribosomal protein L30/L7 family protein;(source:Araport11)	259.80	18.74	13.86	< 1e-07	< 1e-07
931	3	F2-3 vs. L2-3	252863_at	AT4G39800	** Referred to as MIPS2 in Mitsuhashi et al 2008. myo-inositol-1-phosphate synthase isoform 1.Expressed in leaf, root and silique. Immunolocalization experiments with an antibody recognizing MIPS1, MIPS2, and MIPS3 showed endosperm localization.	1119.49	81.13	13.80	< 1e-07	4.57E-06
932	3	F2-3 vs. L2-3	248714_at	AT5G48140	Pectin lyase-like superfamily protein;(source:Araport11)	2621.64	190.85	13.74	2.43E-05	2.16E-04
933	3	F2-3 vs. L2-3	265405_at	AT2G16750	kinase with adenine nucleotide alpha hydrolases-like domain-containing protein;(source:Araport11)	213.83	15.83	13.51	4.00E-07	1.25E-05
934	3	F2-3 vs. L2-3	247538_at	AT5G61700	ABC2 homolog 16;(source:Araport11)	544.15	40.90	13.30	< 1e-07	< 1e-07
935	3	F2-3 vs. L2-3	258003_at	AT3G29030	Encodes an expansin. Naming convention from the Expansin Working Group (Kende et al, 2004. Plant Mol Bio)	553.31	42.15	13.13	< 1e-07	< 1e-07
936	3	F2-3 vs. L2-3	247537_s_a	AT5G61690	ABC2 homolog 15;(source:Araport11)	360.15	27.94	12.89	1.10E-06	2.61E-05
937	3	F2-3 vs. L2-3	258591_at	AT3G04360	Calcium-dependent lipid-binding (CaLB domain) family protein;(source:Araport11)	427.74	33.37	12.82	< 1e-07	< 1e-07
938	3	F2-3 vs. L2-3	253725_at	AT4G29340	Profilin is a low-molecular weight, actin monomer-binding protein that regulates the organization of actin cytoskeleton in eukaryotes, including higher plants. PRF4 and PRF5 are late pollen-specific and are not detectable in other cell types of the plant body including microspores and root hairs. Immunocytochemical studies at the subcellular level reveal that both the constitutive and pollen-specific profilins are abundant in the cytoplasm. In vegetative cell types, such as root apical cells, profilins showed localization to nuclei in addition to the cytoplasmic staining.	705.97	55.39	12.74	3.00E-07	1.05E-05
939	3	F2-3 vs. L2-3	261892_at	AT1G80840	Pathogen-induced transcription factor. Binds W-box sequences in vitro. Forms protein complexes with itself and with WRKY40 and WRKY60. Coexpression with WRKY18 or WRKY60 made plants more susceptible to both P. syringae and B. cinerea. WRKY18, WRKY40, and WRKY60 have partially redundant roles in response to the hemibiotrophic bacterial pathogen Pseudomonas syringae and the necrotrophic fungal pathogen Botrytis cinerea, with WRKY18 playing a more important role than the other two. The mRNA is cell-to-cell mobile.	474.48	37.38	12.70	< 1e-07	< 1e-07
940	3	F2-3 vs. L2-3	266697_at	AT2G19770	Encodes profilin 5, originally named profilin 4 (PRO4/PFN4). Low-molecular weight, actin monomer-binding protein that regulates the organization of actin cytoskeleton. Pollen-specific plant profilin present predominantly in mature pollen and growing pollen tubes.	260.22	20.63	12.62	5.00E-07	1.44E-05
941	3	F2-3 vs. L2-3	258686_at	AT3G07840	Pectin lyase-like superfamily protein;(source:Araport11)	847.44	67.34	12.59	2.00E-07	7.75E-06
942	3	F2-3 vs. L2-3	245011_at	ATCG00430	Encodes a protein which was originally thought to be part of photosystem II but its wheat homolog was later shown to encode for subunit K of NADH dehydrogenase.	453.54	36.11	12.56	< 1e-07	< 1e-07
943	3	F2-3 vs. L2-3	264208_at	AT1G22760	Putative poly(A) binding protein May there fore function in posttranscriptional regulation, including mRNA turnover and translational initiation. Expression detected only in floral organs.	242.70	19.49	12.45	< 1e-07	< 1e-07
944	3	F2-3 vs. L2-3	265547_at	AT2G28305	Putative lysine decarboxylase family protein;(source:Araport11)	553.71	45.27	12.23	< 1e-07	< 1e-07
945	3	F2-3 vs. L2-3	244964_at	ATCG00560	PSII L protein	10690.31	877.67	12.18	1.14E-05	1.29E-04
946	3	F2-3 vs. L2-3	245701_at	AT5G04140	Encodes a gene whose sequence is similar to ferredoxin dependent glutamate synthase (Fd-GOGAT). Expression in leaves is induced by light and sucrose. Proposed to be involved in photorespiration and nitrogen assimilation. The mRNA is cell-to-cell mobile.	218.40	17.98	12.15	< 1e-07	< 1e-07
947	3	F2-3 vs. L2-3	259372_at	AT1G69120	Floral homeotic gene encoding a MADS domain protein homologous to SRF transcription factors. Specifies floral meristem and sepal identity. Required for the transcriptional activation of AGAMOUS. Interacts with LEAFY.Binds to promoter and regulates the expression of flowering time genes SVP, SOC1 and AGL24.	200.77	16.59	12.10	< 1e-07	< 1e-07

948	3	F2-3 vs. L2-3	262717_s_a	AT1G16410	member of CYP79F The mRNA is cell-to-cell mobile.	153.91	12.74	12.08	< 1e-07	< 1e-07
949	3	F2-3 vs. L2-3	250793_at	AT5G05600	Encodes a protein with similarity to flavonol synthases that is involved in the detoxifcation polycyclic aromatic hydrocarbons.One of 4 paralogs encoding a 2-oxoglutarate/Fe(II)-dependent oxygenases that hydroxylates JA to 12-OH-JA.	947.71	82.74	11.45	1.00E-06	2.42E-05
950	3	F2-3 vs. L2-3	251733_at	AT3G56240	CCH protein belongs to a family of eukaryotic proteins that participate in intracellular copper homeostasis by delivering this metal to the secretory pathway; mainly located along the vascular bundles of senescing leaves and petioles as well as in stem sieve elements; hypothesized to have a role in copper mobilization from decaying organs towards reproductive structures, as a result of metalloprotein breakdown. The plant-specific C-terminal domain of the CCH protein forms amyloid-like fibrils in vitro.	1467.82	130.92	11.21	5.09E-04	1.91E-03
951	3	F2-3 vs. L2-3	254122_at	AT4G24510	Encodes a component of the fatty acid elongation machinery required for C28 to C30 fatty acid elongation. It does not require the acyltransferase catalytic site for biological function.	182.55	16.37	11.15	< 1e-07	< 1e-07
952	3	F2-3 vs. L2-3	251916_at	AT3G53960	Major facilitator superfamily protein;(source:Araport11)	161.99	14.55	11.13	< 1e-07	< 1e-07
953	3	F2-3 vs. L2-3	252140_at	AT3G51070	S-adenosyl-L-methionine-dependent methyltransferases superfamily protein;(source:Araport11)	163.66	14.71	11.12	< 1e-07	< 1e-07
954	3	F2-3 vs. L2-3	261648_at	AT1G27730	Related to Cys2/His2-type zinc-finger proteins found in higher plants. Compensated for a subset of calcineurin deficiency in yeast. Salt tolerance produced by ZAT10 appeared to be partially dependent on ENA1/PMR2, a P-type ATPase required for Li+ and Na+ efflux in yeast. The protein is localized to the nucleus, acts as a transcriptional repressor and is responsive to chitin oligomers. Also involved in response to photooxidative stress.	293.67	26.53	11.07	1.00E-06	2.42E-05
955	3	F2-3 vs. L2-3	247162_at	AT5G65730	xyloglucan endotransglucosylase/hydrolase 6;(source:Araport11)	165.90	15.30	10.84	< 1e-07	< 1e-07
956	3	F2-3 vs. L2-3	258445_at	AT3G22400	Encodes lipoxygenase5 (LOX5). LOX5 activity in roots facilitates green peach aphid colonization of Arabidopsis foliage by promoting green peach aphid feeding from sieve element and water consumption from xylem.	159.72	14.78	10.81	< 1e-07	< 1e-07
957	3	F2-3 vs. L2-3	245749_at	AT1G51090	Heavy metal associated domain containing protein involved in plant immunity.Mutants show an increase in root length under NO stress and reduction in root length under H2O2 stress conditions. Mutants show increases in defense responses to pathogens including hypersensitive lesions, increased resistance and induction of SAR genes.	182.84	16.95	10.79	< 1e-07	< 1e-07
958	3	F2-3 vs. L2-3	248377_at	AT5G51720	Encodes a protein with biochemical, structural, and biophysical characteristics of a NEET protein. It plays a key role in plant development, senescence, reactive oxygen homeostasis, and Fe metabolism.	489.84	45.88	10.68	< 1e-07	< 1e-07
959	3	F2-3 vs. L2-3	247902_at	AT5G57350	member of Plasma membrane H+-ATPase family	385.97	36.42	10.60	3.00E-07	1.05E-05
960	3	F2-3 vs. L2-3	263845_at	AT2G37040	Encodes PAL1, a phenylalanine ammonia-lyase. Arabidopsis has four PALs: AT2G37040 (PAL1), AT3G53260 (PAL2), AT5G04230 (PAL3) and AT3G10340 (PAL4).	170.52	16.34	10.43	4.00E-07	1.25E-05
961	3	F2-3 vs. L2-3	250498_at	AT5G09660	encodes a microbody NAD-dependent malate dehydrogenase encodes an peroxisomal NAD-malate dehydrogenase that is involved in fatty acid beta-oxidation through providing NAD to the process of converting fatty acyl CoA to acetyl CoA.	339.34	32.52	10.43	< 1e-07	4.57E-06
962	3	F2-3 vs. L2-3	265768_at	AT2G48020	Encodes a zinc transporter ZIF2. Expression of ZIF2 is regulated by alternative splicing.	807.09	77.67	10.39	< 1e-07	< 1e-07
963	3	F2-3 vs. L2-3	249048_at	AT5G44300	Dormancy/auxin associated family protein;(source:Araport11)	301.98	29.33	10.30	2.00E-07	7.75E-06
964	3	F2-3 vs. L2-3	250631_at	AT5G07430	Pectin lyase-like superfamily protein;(source:Araport11)	231.28	22.48	10.29	6.94E-04	2.41E-03
965	3	F2-3 vs. L2-3	257641_s_a t	AT3G25760	encodes allene oxide cyclase. One of four genes in Arabidopsis that encode this enzyme, which catalyzes an essential step in jasmonic acid biosynthesis. Gene expression is induced during senescence, a process that involves jasmonic acid signalling pathway. The mRNA is cell-to-cell mobile.	2696.20	277.14	9.73	< 1e-07	< 1e-07
966	3	F2-3 vs. L2-3	266918_at	AT2G45800	Encodes a member of the Arabidopsis LIM proteins: a family of actin bundlers with distinct expression patterns. WLIM1, WLIM2a, and WLIM2b are widely expressed, whereas PLIM2a, PLIM2b, and PLIM2c are predominantly expressed in pollen. Regulates actin cytoskeleton organization.	187.59	19.39	9.68	3.00E-07	1.05E-05
967	3	F2-3 vs. L2-3	258979_at	AT3G09440	Heat shock protein 70 (Hsp 70) family protein;(source:Araport11)	1105.44	115.69	9.56	4.00E-07	1.25E-05
968	3	F2-3 vs. L2-3	262122_at	AT1G02790	encodes a exopolygalacturonase.	159.19	16.79	9.48	5.00E-07	1.44E-05
969	3	F2-3 vs. L2-3	253619_at	AT4G30460	glycine-rich protein;(source:Araport11)	261.99	28.13	9.31	2.00E-07	7.75E-06

970	3	F2-3 vs. L2-3	250349_at	AT5G12000	kinase with adenine nucleotide alpha hydrolases-like domain-containing protein;(source:Araport11)	435.53	47.53	9.16	1.53E-04	7.92E-04
971	3	F2-3 vs. L2-3	254283_s_at	AT4G22870	2-oxoglutarate (2OG) and Fe(II)-dependent oxygenase superfamily protein;(source:Araport11)	751.44	82.82	9.07	8.25E-05	5.15E-04
972	3	F2-3 vs. L2-3	253177_s_a	AT4G35150	O-methyltransferase family protein;(source:Araport11)	473.33	52.50	9.02	6.40E-06	8.73E-05
973	3	F2-3 vs. L2-3	258158_at	AT3G17790	Expression is upregulated in the shoot of cax1/cax3 mutant and is responsive to phosphate (Pi) and not phosphite (Phi) in roots and shoots.	255.07	29.03	8.79	< 1e-07	< 1e-07
974	3	F2-3 vs. L2-3	261970_at	AT1G65960	glutamate decarboxylase (GAD2)The mRNA is cell-to-cell mobile.	1657.23	191.20	8.67	8.00E-07	2.05E-05
975	3	F2-3 vs. L2-3	267322_at	AT2G19330	Encodes PIRL6, a member of the Plant Intracellular Ras-group-related LRRs (Leucine rich repeat proteins). PIRLs are a distinct, plant-specific class of intracellular LRRs that likely mediate protein interactions, possibly in the context of signal transduction.	154.09	18.18	8.48	2.00E-07	7.75E-06
976	3	F2-3 vs. L2-3	252291_s_at	AT3G49110	Class III peroxidase Perx33. Expressed in roots. Located in the cell wall. Involved in cell elongation. Expression activated by light. May play a role in generating H2O2 during defense response. The mRNA is cell-to-cell mobile.	105.47	12.45	8.47	6.00E-07	1.65E-05
977	3	F2-3 vs. L2-3	260241_at	AT1G63710	Encodes a member of the CYP86A subfamily of cytochrome p450 genes. Expressed at highest level in mature stems and flowers.	158.44	18.78	8.44	< 1e-07	4.57E-06
978	3	F2-3 vs. L2-3	264204_at	AT1G22710	Encodes for a high-affinity transporter essential for phloem loading and long-distance transport. A major sucrose transporter, AtSUC2 can also transport a wide range of physiological and synthetic glucose conjugates with both α- or β-linkage.	631.63	75.82	8.33	1.60E-06	3.46E-05
979	3	F2-3 vs. L2-3	264400_at	AT1G61800	glucose6-Phosphate/phosphate transporter 2. Expression is upregulated in the shoot of cax1/cax3 mutant. The mRNA is cell-to-cell mobile.	3412.12	413.67	8.25	2.15E-05	1.98E-04
980	3	F2-3 vs. L2-3	267500_s_a	AT2G44890	member of CYP704A	141.21	17.18	8.22	< 1e-07	< 1e-07
981	3	F2-3 vs. L2-3	248719_at	AT5G47910	NADPH/respiratory burst oxidase protein D (RbohD).Interacts with AtrbohF gene to fine tune the spatial control of ROI production and hypersensitive response to cell in and around infection site. The mRNA is cell-to-cell mobile.	163.34	19.99	8.17	< 1e-07	< 1e-07
982	3	F2-3 vs. L2-3	252733_at	AT3G43120	SAUR-like auxin-responsive protein family;(source:Araport11)	113.95	13.97	8.16	1.00E-06	2.42E-05
983	3	F2-3 vs. L2-3	262664_at	AT1G13970	beta-hexosaminidase (DUF1336);(source:Araport11)	222.53	27.35	8.14	9.50E-06	1.14E-04
984	3	F2-3 vs. L2-3	261881_at	AT1G80760	Encodes a protein with boron transporter activity. It helps to preferentially direct boron to young developing tissues in the shoot, such as immature leaves, under low boron conditions. This boron channel appears to be impermeable to water, unlike the closely related NIP5;1 boron transporter. This protein also allows the transport of glycerol, urea, and formimide but not larger uncharged solutes such as arabitol and sucrose when it is expressed heterologously.	175.63	21.80	8.06	< 1e-07	< 1e-07
985	3	F2-3 vs. L2-3	252377_at	AT3G47960	Encodes a high-affinity, proton-dependent glucosinolate-specific transporter that is crucial for the transport of both methionine- and tryptophan-derived glucosinolates to seeds.	336.65	41.75	8.06	< 1e-07	< 1e-07
986	3	F2-3 vs. L2-3	261266_at	AT1G26770	Encodes an expansin. Naming convention from the Expansin Working Group (Kende et al, Plant Mol Bio). Involved in the formation of nematode-induced syncytia in roots of Arabidopsis thaliana.	161.12	20.33	7.93	2.00E-06	3.93E-05
987	3	F2-3 vs. L2-3	262281_at	AT1G68570	NPF3.1 is a membrane localized GA transporter that is expressed in the root endodermis.	138.82	17.51	7.93	< 1e-07	< 1e-07
988	3	F2-3 vs. L2-3	259625_at	AT1G42970	Encodes chloroplast localized glyceraldehyde-3-phosphate dehydrogenase that can use both NADH and NADPH to reduce 1,3-diphosphate glycerate. It forms A2B2 heterotetramers with GapA forms of the GADPH enzyme. These complexes are active in the light under reducing conditions, but show reduced NADPH-dependent activity in response to oxidized thioredoxins and increased NAD(H)/NADP(H) ratios due to the formation of inactive A8B8 hexadecamers. The mRNA is cell-to-cell mobile.	1520.22	194.10	7.83	4.00E-07	1.25E-05
989	3	F2-3 vs. L2-3	264139_at	AT1G78940	kinase with adenine nucleotide alpha hydrolases-like domain-containing protein;(source:Araport11)	135.93	17.45	7.79	1.01E-05	1.19E-04
990	3	F2-3 vs. L2-3	263676_at	AT1G09340	Encodes CHLOROPLAST RNA BINDING (CRB), a putative RNA-binding protein. CRB is important for the proper functioning of the chloroplast. Mutations in CRB also affects the circadian system, altering the expression of both oscillator and output genes. The mRNA is cell-to-cell mobile.	580.39	74.61	7.78	< 1e-07	4.57E-06

991	3	F2-3 vs. L2-3	256745_at	AT3G29360	Encodes one of four UDP-glucose dehydrogenase UGD) genes. Mutation of this gene in combination with UGD3 leads to swollen plant cell walls and severe developmental defects associated with changes in pectic polysaccharides.	281.49	36.17	7.78	< 1e-07	< 1e-07
992	3	F2-3 vs. L2-3	248073_at	AT5G55720	Pectin lyase-like superfamily protein;(source:Araport11)	78.85	10.20	7.73	< 1e-07	< 1e-07
993	3	F2-3 vs. L2-3	259451_at	AT1G13890	Encodes a member of a gene family homologous to mammalian SNAP25, a type of SNARE proteins with two chains. There are three members in Arabidopsis: SNAP30, SNAP29, and SNAP33.	106.97	14.18	7.54	1.70E-06	3.64E-05
994	3	F2-3 vs. L2-3	253643_at	AT4G29780	Expression of the gene is affected by multiple stresses. Knockout and overexpression lines show no obvious phenotypes.	888.57	118.22	7.52	2.80E-06	4.91E-05
995	3	F2-3 vs. L2-3	265002_at	AT1G24400	High-affinity transporter for neutral and acidic amino acids, expressed in tapetum tissue of anthers. Transport of 1-Aminocyclopropane-1-carboxylic acid (ACC).	133.97	17.99	7.45	< 1e-07	< 1e-07
996	3	F2-3 vs. L2-3	267298_at	AT2G23760	Encodes a member of the BEL family of homeodomain proteins. Plants doubly mutant for saw1/saw2 (blh2/blh4) have serrated leaves. BP is expressed in the serrated leaves, therefore saw2 and saw1 may act redundantly to repress BP in leaves. Regulates together with BLH2 demethylesterification of homogalacturonan in seed mucilage.	90.22	12.12	7.44	2.00E-07	7.75E-06
997	3	F2-3 vs. L2-3	260205_at	AT1G70700	JAZ9 is a protein presumed to be involved in jasmonate signaling. JAZ9 transcript levels rise in response to a jasmonate stimulus. JAZ9 can interact with the COI1 F-box subunit of an SCF E3 ubiquitin ligase in a yeast-two-hybrid assay only in the presence of jasmonate-isoleucine (JA-ILE) or coronatine. The Jas domain appears to be important for JAZ9-COI1 interactions in the presence of coronatine. Two positive residues (R205 and R206) in the Jas domain shown to be important for coronatine -dependent COI1 binding are not required for binding AtMYC2. The mRNA is cell-to-cell mobile.	189.67	25.55	7.42	1.16E-05	1.31E-04
998	3	F2-3 vs. L2-3	251697_at	AT3G56600	phosphatidylinositol 4-kinase gamma-like protein;(source:Araport11)	174.30	23.51	7.41	6.25E-05	4.25E-04
999	3	F2-3 vs. L2-3	253606_at	AT4G30530	Encodes a gamma-glutamyl peptidase, outside the GGT family, that can hydrolyze gamma-glutamyl peptide bonds. The mRNA is cell-to-cell mobile.	366.36	49.44	7.41	1.60E-06	3.46E-05
1000	3	F2-3 vs. L2-3	260399_at	AT1G72520	PLAT/LH2 domain-containing lipoxygenase family protein;(source:Araport11)	140.76	19.28	7.30	4.00E-07	1.25E-05
1001	3	F2-3 vs. L2-3	252929_at	AT4G38970	Protein is tyrosine-phosphorylated and its phosphorylation state is modulated in response to ABA in Arabidopsis thaliana seeds.	3206.21	440.63	7.28	2.00E-06	3.93E-05
1002	3	F2-3 vs. L2-3	248918_at	AT5G45890	Senescence-associated gene 12 (SAG12) encoding a cysteine protease influenced by cytokinin, auxin, and sugars.Localized to special vacuole found during senescence called senescence associated vacuoles which are different from central vacuole in the tonoplast composition and pH.	229.71	31.61	7.27	< 1e-07	4.57E-06
1003	3	F2-3 vs. L2-3	251860_at	AT3G54660	Encodes glutathione reductase that is most likely localized in the chloroplast.	491.09	67.63	7.26	< 1e-07	< 1e-07
1004	3	F2-3 vs. L2-3	251898_at	AT3G54340	Floral homeotic gene encoding a MADS domain protein homologous to SRF transcription factors. Specifies petal and stamen identities. Associates with PISTILLATA.	334.23	46.16	7.24	4.00E-07	1.25E-05
1005	3	F2-3 vs. L2-3	248335_at	AT5G52450	MATE efflux family protein;(source:Araport11)	236.39	32.78	7.21	< 1e-07	< 1e-07
1006	3	F2-3 vs. L2-3	249008_at	AT5G44680	DNA glycosylase superfamily protein;(source:Araport11)	704.16	98.63	7.14	1.77E-04	8.81E-04
1007	3	F2-3 vs. L2-3	266115_at	AT2G02140	Predicted to encode a PR (pathogenesis-related) protein. Belongs to the plant defensin (PDF) family with the following members: At1g75830/PDF1.1, At5g44420/PDF1.2a, At2g26020/PDF1.2b, At5g44430/PDF1.2c, At2g26010/PDF1.3, At1g19610/PDF1.4, At1g55010/PDF1.5, At2g02120/PDF2.1, At2g02100/PDF2.2, At2g02130/PDF2.3, At1g61070/PDF2.4, At5g63660/PDF2.5, At2g02140/PDF2.6, At5g38330/PDF3.1 and At4g30070/PDF3.2.	166.52	23.47	7.09	4.10E-06	6.26E-05
1008	3	F2-3 vs. L2-3	263450_at	AT2G31500	member of Calcium Dependent Protein Kinase	99.12	14.10	7.03	3.90E-06	6.01E-05
1009	3	F2-3 vs. L2-3	254607_at	AT4G18920	histone acetyltransferase (DUF1264);(source:Araport11)	115.46	16.43	7.03	1.00E-07	4.57E-06
1010	3	F2-3 vs. L2-3	252606_at	AT3G45010	serine carboxypeptidase-like 48;(source:Araport11)	725.95	104.14	6.97	2.34E-04	1.07E-03
1011	3	F2-3 vs. L2-3	255008_at	AT4G10060	Glucosylceramidase that preferentially hydrolyzes long acyl chain glucosylceramides.	159.67	22.89	6.97	< 1e-07	< 1e-07
1012	3	F2-3 vs. L2-3	265091_s_a	AT1G03495	HXXXD-type acyl-transferase family protein;(source:Araport11)	109.47	15.72	6.96	< 1e-07	< 1e-07
1013	3	F2-3 vs. L2-3	267639_at	AT2G42200	Encodes a putative transcriptional regulator that is involved in the vegetative to reproductive phase transition. Expression is regulated by MIR156b. SPL activity nonautonomously inhibits initiation of new leaves at the shoot apical meristem.	126.36	18.52	6.82	< 1e-07	< 1e-07
1014	3	F2-3 vs. L2-3	256008_s_a t	AT1G34040	Pyridoxal phosphate (PLP)-dependent transferases superfamily protein;(source:Araport11)	96.79	14.23	6.80	3.00E-07	1.05E-05

1015	3	F2-3 vs. L2-3	245232_at	AT4G25590	actin depolymerizing factor 7;(source:Araport11)	220.66	32.53	6.78	5.00E-06	7.29E-05
1016	3	F2-3 vs. L2-3	250514_at	AT5G09550	GDP dissociation inhibitor family protein / Rab GTPase activator family protein;(source:Araport11)	219.43	32.45	6.76	6.50E-06	8.82E-05
1017	3	F2-3 vs. L2-3	260829_s_at	AT2G30440	Encodes a thylakoidal processing peptidase that removes signal sequences from proteins synthesized in the cytoplasm and transported into the thylakoid lumen. The mRNA is cell-to-cell mobile.	277.18	41.46	6.69	2.17E-05	1.99E-04
1018	3	F2-3 vs. L2-3	246208_at	AT4G36490	SEC14-like 12;(source:Araport11)	166.76	25.03	6.66	2.60E-06	4.67E-05
1019	3	F2-3 vs. L2-3	249894_at	AT5G22580	Stress responsive A/B Barrel Domain-containing protein;(source:Araport11)	887.96	133.54	6.65	4.36E-04	1.70E-03
1020	3	F2-3 vs. L2-3	257951_at	AT3G21700	Monomeric G protein. Expressed in root epidermal cells that are destined to become atrichoblasts. Also expressed during pollen development and in the pollen tube tip.	291.11	43.83	6.64	9.29E-05	5.58E-04
1021	3	F2-3 vs. L2-3	251775_s_at	AT3G55610	encodes delta 1-pyrroline-5-carboxylate synthetase B. Gene expression is induced by dehydration, high salt and ABA. Knock-out mutations in P5CS2 are embryo-lethal. P5CS2 appears to be present in different cells and/or different subcellular locations from P5CS1 in a tissue-dependent manner. Mutants are defective in pollen development.	898.52	135.61	6.63	7.36E-04	2.52E-03
1022	3	F2-3 vs. L2-3	258561_at	AT3G05960	Encodes a hexose sugar transporter that is expressed in pollen. STP6 may play a role in providing sugars during late pollen maturation or pollen tube germination.	115.49	17.50	6.60	6.00E-07	1.65E-05
1023	3	F2-3 vs. L2-3	254578_at	AT4G19410	Pectinacetylesterase family protein;(source:Araport11)	821.27	125.65	6.54	1.70E-06	3.64E-05
1024	3	F2-3 vs. L2-3	265923_at	AT2G18470	Proline-rich extensin-like receptor kinase 4. Functions at an early stage of ABA signalling inhibiting primary root cell elongation by perturbing Ca2+ homeostasis.	96.25	14.76	6.52	1.72E-05	1.75E-04
1025	3	F2-3 vs. L2-3	263956_at	AT2G35940	Encodes a member of the BEL-like homeodomain protein family. Ecotopic expression in the embryo sac leads to defects in nuclear migration and cellularization and embryo sacs with multiple egg cells. Loss of function alleles have no female gametophyte defects. The ecotopic expression phenotype requires KNAT3 because it can be suppressed by loss of KNAT3 function alleles. Localized to the nucleus but interaction with OFP1 relocates it to the cytoplasm.	139.55	21.44	6.51	< 1e-07	4.57E-06
1026	3	F2-3 vs. L2-3	259848_at	AT1G72180	Encodes a leucine-rich repeat receptor kinase that functions as a receptor for CEP1 peptide. Mediates nitrate uptake signaling.	634.81	97.93	6.48	1.60E-06	3.46E-05
1027	3	F2-3 vs. L2-3	252343_at	AT3G48610	Non-specific phospholipase C6 involved in gametophyte development.	132.14	20.59	6.42	< 1e-07	< 1e-07
1028	3	F2-3 vs. L2-3	264474_s_at	AT5G38420	Encodes a member of the Rubisco small subunit (RBCS) multigene family: RBCS1A (At1g67090), RBCS1B (At5g38430), RBCS2B (At5g38420), and RBCS3B (At5g38410). Activated by OXS2 under the treatment of salt.	14651.90	2297.97	6.38	< 1e-07	< 1e-07
1029	3	F2-3 vs. L2-3	246308_at	AT3G51820	Encodes a protein with chlorophyll synthase activity. This enzyme has been shown to perform the esterification of chlorophyllide (a and b), the last step of chlorophyll biosynthesis. Although it can use either geranylgeranyl pyrophosphate (GGPP) or phytyl pyrophosphate (PhyPP) as substrates, the esterification reaction was faster with GGPP than with PhyPP.	1050.08	165.00	6.36	1.00E-07	4.57E-06
1030	3	F2-3 vs. L2-3	245528_at	AT4G15530	Encodes a dual-targeted protein believed to act as a pyruvate, orthophosphate dikinase. These enzymes are normally associated with C4 photosynthesis which does not occur in Arabidopsis. However, PPDK may play a role in remobilizing nitrogen during leaf senescence in Arabidopsis. The product of the long transcript (.1 gene model) was shown to be targeted to the chloroplast, whereas the shorter transcript (no targeting sequence) accumulates in the cytosol. The two proteins were also found to be expressed in slightly different tissues.	174.27	27.42	6.36	7.00E-07	1.83E-05
1031	3	F2-3 vs. L2-3	245806_at	AT1G45474	Encodes a component of the light harvesting complex of photosystem I.	213.05	33.53	6.35	< 1e-07	< 1e-07
1032	3	F2-3 vs. L2-3	258751_at	AT3G05890	Low temperature and salt responsive protein family;(source:Araport11)	203.24	32.02	6.35	3.05E-04	1.31E-03
1033	3	F2-3 vs. L2-3	246908_at	AT5G25610	responsive to dehydration 22 (RD22) mediated by ABA	2061.09	325.33	6.34	1.00E-07	4.57E-06
1034	3	F2-3 vs. L2-3	258415_at	AT3G17390	S-adenosylmethionine synthetase	3327.54	531.09	6.27	4.00E-07	1.25E-05
1035	3	F2-3 vs. L2-3	258140_at	AT3G24503	Arabidopsis thaliana aldehyde dehydrogenase AtALDH1a mRNA. a sinapaldehyde dehydrogenase catalyzes both the oxidation of coniferylaldehyde and sinapaldehyde forming ferulic acid and sinapic acid, respectively	297.04	47.93	6.20	3.99E-05	3.09E-04
1036	3	F2-3 vs. L2-3	253042_at	AT4G37550	Acetamidase/Formamidase family protein;(source:Araport11)	184.73	30.18	6.12	6.60E-06	8.91E-05
1037	3	F2-3 vs. L2-3	266834_s_at	AT2G30020	Encodes AP2C1. Belongs to the clade B of the PP2C-superfamily. Acts as a MAPK phosphatase that negatively regulates MPK4 and MPK6.	3998.62	657.73	6.08	2.12E-04	9.99E-04
1038	3	F2-3 vs. L2-3	254123_at	AT4G24640	Encodes AppB protein (AppB1).	159.76	26.33	6.07	6.00E-07	1.65E-05

1039	3	F2-3 vs. L2-3	248622_at	AT5G49360	Encodes a bifunctional {beta}-D-xylosidase/{alpha}-L-arabinofuranosidase required for pectic arabinan modification. Located in the extracellular matrix. Gene is expressed specifically in tissues undergoing secondary wall thickening. This is a member of glycosyl hydrolase family 3 and has six other closely related members.	685.37	112.91	6.07	5.00E-07	1.44E-05
1040	3	F2-3 vs. L2-3	250610_at	AT5G07550	member of Oleosin-like protein family	84.59	14.06	6.02	3.00E-07	1.05E-05
1041	3	F2-3 vs. L2-3	265984_at	AT2G24210	terpene synthase 10;(source:Araport11)	90.26	15.05	6.00	1.00E-07	4.57E-06
1042	3	F2-3 vs. L2-3	264207_at	AT1G22750	transmembrane protein;(source:Araport11)	142.23	23.84	5.97	4.00E-07	1.25E-05
1043	3	F2-3 vs. L2-3	267590_at	AT2G39700	putative expansin. Naming convention from the Expansin Working Group (Kende et al, 2004. Plant Mol Bio). Involved in the formation of nematode-induced syncytia in roots of Arabidopsis thaliana.	131.99	22.10	5.97	< 1e-07	4.57E-06
1044	3	F2-3 vs. L2-3	261015_at	AT1G26480	14-3-3 protein GF14iota (grf12)	126.50	21.34	5.93	2.00E-07	7.75E-06
1045	3	F2-3 vs. L2-3	267144_at	AT2G38110	bifunctional sn-glycerol-3-phosphate 2-O-acyltransferase/phosphatase. Involved in cutin assembly.	427.42	72.16	5.92	1.36E-04	7.30E-04
1046	3	F2-3 vs. L2-3	265884_at	AT2G42320	nucleolar protein gar2-like protein;(source:Araport11)	315.43	53.26	5.92	7.00E-07	1.83E-05
1047	3	F2-3 vs. L2-3	257144_at	AT3G27300	glucose-6-phosphate dehydrogenase 5;(source:Araport11)	191.42	32.53	5.88	2.62E-05	2.27E-04
1048	3	F2-3 vs. L2-3	253172_at	AT4G35060	Heavy metal transport/detoxification superfamily protein;(source:Araport11)	154.21	26.28	5.87	1.94E-05	1.88E-04
1049	3	F2-3 vs. L2-3	252101_at	AT3G51290	pyridoxal-phosphate-dependent serine hydroxymethyltransferase, putative (DUF632);(source:Araport11)	1018.93	175.56	5.80	4.00E-07	1.25E-05
1050	3	F2-3 vs. L2-3	262796_at	AT1G20850	Cysteine peptidase. Enzyme activity detected in leaf.	521.80	90.18	5.79	< 1e-07	< 1e-07
1051	3	F2-3 vs. L2-3	257313_at	AT3G26520	gamma tonoplast intrinsic protein 2 (TIP2). expressed throughout the plant and transcript level is increased upon NaCl or ABA treatments. NaCl stress-sensitive yeast mutant strains exhibit more resistance to salt when expressing this protein.	233.12	40.95	5.69	3.00E-07	1.05E-05
1052	3	F2-3 vs. L2-3	263838_at	AT2G36880	methionine adenosyltransferase 3;(source:Araport11)	2125.42	374.08	5.68	7.63E-05	4.85E-04
1053	3	F2-3 vs. L2-3	245658_at	AT1G28270	Member of a diversely expressed predicted peptide family showing sequence similarity to tobacco Rapid Alkalinization Factor (RALF), and is believed to play an essential role in the physiology of Arabidopsis. Consists of a single exon and is characterized by a conserved C-terminal motif and N-terminal signal peptide. RALF4 and RALF19 act redundantly in the pollen tube to regulate pollen tube growth.	149.87	26.41	5.67	< 1e-07	4.57E-06
1054	3	F2-3 vs. L2-3	247776_at	AT5G58700	phosphatidylinositol-speciwc phospholipase C4;(source:Araport11)	222.78	39.69	5.61	< 1e-07	< 1e-07
1055	3	F2-3 vs. L2-3	263433_at	AT2G22240	** Referred to as MIPS1 in Mitsuhashi et al 2008. Myo-inositol-1-phosphate synthase isoform 2. Expressed in leaf, root and silique. Immunolocalization experiments with an antibody recognizing MIPS1, MIPS2, and MIPS3 showed endosperm localization.	792.73	141.89	5.59	3.70E-06	5.84E-05
1056	3	F2-3 vs. L2-3	261754_at	AT1G76130	alpha-amylase, putative / 1,4-alpha-D-glucan glucanohydrolase, putative, strong similarity to alpha-amylase GI:7532799 from (Malus x domestica);contains Pfam profile PF00128: Alpha amylase, catalytic domain. Predicted to be secreted based on SignalP analysis.	380.39	68.26	5.57	5.00E-07	1.44E-05
1057	3	F2-3 vs. L2-3	266802_at	AT2G22900	Encodes MUCI10, a galactomannan-1,6-galactosyltransferase. MUCI10 likely decorates glucomannan, synthesized by CSLA2, with galactose residues in vivo. The degree of galactosylation is essential for the synthesis of the GGM backbone, the structure of cellulose, mucilage density, as well as the adherence of pectin.	315.60	56.63	5.57	< 1e-07	< 1e-07
1058	3	F2-3 vs. L2-3	257106_at	AT3G29060	EXS (ERD1/XPR1/SYG1) family protein;(source:Araport11)	157.39	28.27	5.57	1.40E-05	1.51E-04
1059	3	F2-3 vs. L2-3	254551_at	AT4G19840	encodes a phloem lectin, similar to phloem lectin in cucumber and celery. Gene is expressed in the phloem, predominantly in the companion cells. The mRNA is cell-to-cell mobile.	226.49	40.81	5.55	2.00E-07	7.75E-06
1060	3	F2-3 vs. L2-3	245275_at	AT4G15210	cytosolic beta-amylase expressed in rosette leaves and inducible by sugar. RAM1 mutants have reduced beta amylase in leaves and stems.	1508.57	273.09	5.52	2.00E-06	3.93E-05
1061	3	F2-3 vs. L2-3	251358_at	AT3G61160	Protein kinase superfamily protein;(source:Araport11)	307.46	55.81	5.51	3.50E-06	5.69E-05
1062	3	F2-3 vs. L2-3	267181_at	AT2G37760	Encodes an NADPH-dependent aldo-keto reductase that can act on a wide variety of substrates in vitro including aliphatic and aromatic aldehydes and steroids. Transcript levels for this gene are up-regulated in response to cold, salt, and drought stress.	481.46	87.71	5.49	1.07E-05	1.23E-04
1063	3	F2-3 vs. L2-3	259429_at	AT1G01600	Encodes a member of the CYP86A subfamily of cytochrome p450 genes. Expressed significantly at highest level in mature stems and flowers.	122.76	22.44	5.47	3.90E-06	6.01E-05
1064	3	F2-3 vs. L2-3	264835_at	AT1G03550	Secretory carrier membrane protein (SCAMP) family protein;(source:Araport11)	73.37	13.51	5.43	7.00E-07	1.83E-05

1065	3	F2-3 vs. L2-3	261559_at	AT1G01780	Encodes a member of the Arabidopsis LIM proteins: a family of actin bundlers with distinct expression patterns. WLIM1, WLIM2a, and WLIM2b are widely expressed, whereas PLIM2a, PLIM2b, and PLIM2c are predominantly expressed in pollen. Regulates actin cytoskeleton organization. The mRNA is cell-to-cell mobile.	142.63	26.50	5.38	2.00E-07	7.75E-06
1066	3	F2-3 vs. L2-3	253344_at	AT4G33550	Bifunctional inhibitor/lipid-transfer protein/seed storage 2S albumin superfamily protein;(source:Araport11)	151.83	28.20	5.38	1.01E-05	1.19E-04
1067	3	F2-3 vs. L2-3	246878_at	AT5G26060	Plant self-incompatibility protein S1 family;(source:Araport11)	170.31	31.68	5.38	3.00E-07	1.05E-05
1068	3	F2-3 vs. L2-3	244959_s_a	AT2G07708	hypothetical protein;(source:Araport11)	2004.15	377.65	5.31	7.69E-05	4.86E-04
1069	3	F2-3 vs. L2-3	258369_at	AT3G14310	encodes a pectin methylesterase, targeted by a cellulose binding protein (CBP) from the parasitic nematode Heterodera schachtii during parasitism.	136.63	25.84	5.29	2.00E-07	7.75E-06
1070	3	F2-3 vs. L2-3	251962_at	AT3G53420	a member of the plasma membrane intrinsic protein subfamily PIP2. localizes to the plasma membrane and exhibits water transport activity in Xenopus oocyte. expressed specifically in the vascular bundles and protein level increases slightly during leaf dev. When expressed in yeast cells can conduct hydrogen peroxide into those cells.	2221.49	420.25	5.29	6.60E-06	8.91E-05
1071	3	F2-3 vs. L2-3	247377_at	AT5G63180	Pectin lyase-like superfamily protein;(source:Araport11)	118.01	22.32	5.29	7.49E-05	4.77E-04
1072	3	F2-3 vs. L2-3	255016_at	AT4G10120	Encodes a protein with putative sucrose-phosphate synthase activity.	135.49	25.75	5.26	9.85E-04	3.18E-03
1073	3	F2-3 vs. L2-3	249536_at	AT5G38760	Late embryogenesis abundant protein (LEA) family protein;(source:Araport11)	60.70	11.56	5.25	< 1e-07	< 1e-07
1074	3	F2-3 vs. L2-3	255459_at	AT4G02890	Polyubiquitin gene containing 4 ubiquitin repeats.	7096.89	1353.78	5.24	7.30E-06	9.59E-05
1075	3	F2-3 vs. L2-3	260693_at	AT1G32450	Transmembrane nitrate transporter. Involved in xylem transport of nitrate from root to shoot. Induced in response to high and low concentrations of nitrate. Not involved in nitrate uptake. Expressed in root pericycle cells under the control of MYB59. Also functions as a proton-coupled H+/K+ antiporter for K+ loading into the xylem.	486.13	93.23	5.21	2.26E-04	1.05E-03
1076	3	F2-3 vs. L2-3	254926_at	AT4G11280	encodes a a member of the 1-aminocyclopropane-1-carboxylate (ACC) synthase (S-adenosyl-L-methionine methylthioadenosine-lyase, EC 4.4.1.14) gene family The mRNA is cell-to-cell mobile.	505.25	96.95	5.21	1.02E-05	1.20E-04
1077	3	F2-3 vs. L2-3	267595_at	AT2G32990	glycosyl hydrolase 9B8;(source:Araport11)	113.29	21.81	5.20	2.00E-06	3.93E-05
1078	3	F2-3 vs. L2-3	263867_at	AT2G36830	Encodes a tonoplast intrinsic protein, which functions as water channel. It has also been shown to be able to facilitate the transport of urea and hydrogen peroxide. Highly expressed in vascular tissues of the root, stem, cauline leaves and flowers but not in the apical meristems. The mRNA is cell-to-cell mobile.	626.20	120.56	5.19	9.00E-07	2.22E-05
1079	3	F2-3 vs. L2-3	253191_at	AT4G35350	tracheary element vacuolar protein	245.23	47.22	5.19	1.00E-07	4.57E-06
1080	3	F2-3 vs. L2-3	263761_at	AT2G21330	fructose-bisphosphate aldolase 1;(source:Araport11)	1051.90	203.22	5.18	7.70E-06	9.97E-05
1081	3	F2-3 vs. L2-3	262951_at	AT1G75500	An Arabidopsis thaliana homolog of Medicago truncatula NODULIN21 (MtN21). The gene encodes a plant-specific, predicted integral membrane protein and is a member of the Plant-Drug/Metabolite Exporter (P-DME) family (Transporter Classification number: TC 2.A.7.3) and the nodulin MtN21-like transporter family.	388.40	75.21	5.16	< 1e-07	4.57E-06
1082	3	F2-3 vs. L2-3	258005_at	AT3G19390	Granulin repeat cysteine protease family protein;(source:Araport11)	1054.12	204.41	5.16	3.69E-04	1.50E-03
1083	3	F2-3 vs. L2-3	252592_at	AT3G45640	Encodes a mitogen-activated kinase whose mRNA levels increase in response to touch, cold, salinity stress and chitin oligomers.Also functions in ovule development. Heterozygous MPK3 mutants in a homozygous MPK6 background are female sterile due to defects in integument development. MPK3 can be dephosphorylated by MKP2 in vitro. The mRNA is cell-to-cell mobile.	189.72	36.74	5.16	1.06E-05	1.22E-04
1084	3	F2-3 vs. L2-3	258685_at	AT3G07830	Pectin lyase-like superfamily protein;(source:Araport11)	527.66	102.53	5.15	1.40E-06	3.16E-05
1085	3	F2-3 vs. L2-3	256279_at	AT3G12620	Protein phosphatase 2C family protein;(source:Araport11)	304.51	59.57	5.11	2.62E-05	2.27E-04
1086	3	F2-3 vs. L2-3	256754_at	AT3G25690	actin binding protein required for normal chloroplast positioningThe mRNA is cell-to-cell mobile.	324.67	64.10	5.07	< 1e-07	< 1e-07
1087	3	F2-3 vs. L2-3	248916_at	AT5G45840	Encodes a leucine-rich-repeat RLK that is localized to the plasma membrane of pollen tubes and functions with MIK1/2 as the male receptor of the pollen tube chemo-attractant LURE1.MDIS1 forms a complex with MIK1/2 and binds LURE1.	65.44	12.96	5.05	2.80E-06	4.91E-05
1088	3	F2-3 vs. L2-3	265337_at	AT2G18390	Encodes a member of ARF-like GTPase family. A thaliana has 21 members, in two subfamilies, ARF and ARF-like (ARL) GTPases. Mutant has abnormal mitosis and cell cycle control during seed development.	356.08	70.65	5.04	2.00E-07	7.75E-06
1089	3	F2-3 vs. L2-3	253638_at	AT4G30470	NAD(P)-binding Rossmann-fold superfamily protein;(source:Araport11)	624.68	123.85	5.04	9.00E-07	2.22E-05
1090	3	F2-3 vs. L2-3	251405_at	AT3G60330	H[+]-ATPase 7;(source:Araport11)	193.16	38.49	5.02	2.00E-07	7.75E-06

1091	3	F2-3 vs. L2-3	256017_at	AT1G19180	JAZ1 is a nuclear-localized protein involved in jasmonate signaling. JAZ1 transcript levels rise in response to a jasmonate stimulus. JAZ1 can interact with the COI1 F-box subunit of an SCF E3 ubiquitin ligase in a yeast-two-hybrid assay only in the presence of jasmonate-isoleucine (JA-ILE) or coronatine. Application of jasmonate methyl ester to Arabidopsis roots reduces the levels of a JAZ1:GUS fusion protein, presumably by stimulating ubiquitin-proteasome-mediated degradation. The Jas domain appears to be important for JAZ1-COI1 interactions in the presence of coronatine. Two positive residues (R205 and R206) in the Jas domain shown to be important for coronatine -dependent COI1 binding are not required for binding AtMYC2. The mRNA is cell-to-cell mobile.	464.88	92.76	5.01	3.00E-06	5.09E-05
1092	3	F2-3 vs. L2-3	251591_at	AT3G57680	Peptidase S41 family protein;(source:Araport11)	373.61	74.71	5.00	< 1e-07	< 1e-07
1093	3	F2-3 vs. L2-3	253954_at	AT4G26970	Encodes an aconitase that can catalyze the conversion of citrate to isocitrate through a cis-aconitate intermediate, indicating that it may participate in the TCA cycle and other primary metabolic pathways. The protein is believed to accumulate in the mitochondria and the cytosol. It affects CSD2 (At2g28190 - a superoxide dismutase) transcript levels and may play a role in the response to oxidative stress. One member of the family (ACO1 - At35830) was shown to specifically bind to the 5' UTR of CSD2 in vitro. The mRNA is cell-to-cell mobile.	1665.80	334.03	4.99	3.20E-06	5.35E-05
1094	3	F2-3 vs. L2-3	255149_at	AT4G08150	A member of class I knotted1-like homeobox gene family (together with KNAT2). Similar to the knotted1 (kn1) homeobox gene of maize. Normally expressed in the peripheral and rib zone of shoot apical meristem but not in the leaf primordia. It is also expressed in the fourth floral whorl, in the region that would become style, particularly in the cell surrounding the transmitting tissue. No expression was detected in the first three floral whorls. Expression is repressed by auxin and AS1 which results in the promotion of leaf fate.	84.83	17.04	4.98	6.00E-07	1.65E-05
1095	3	F2-3 vs. L2-3	254563_at	AT4G19120	S-adenosyl-L-methionine-dependent methyltransferases superfamily protein;(source:Araport11)	232.25	46.59	4.98	1.46E-05	1.56E-04
1096	3	F2-3 vs. L2-3	267112_at	AT2G14750	Encodes adenosine-5'-phosphosulfate kinase. Provides activated sulfate for sulfation of secondary metabolites, including the glucosinolates. Essential for pollen viability. The mRNA is cell-to-cell mobile.	159.79	32.14	4.97	< 1e-07	4.57E-06
1097	3	F2-3 vs. L2-3	248199_at	AT5G54170	Polyketide cyclase/dehydrase and lipid transport superfamily protein;(source:Araport11)	285.96	57.68	4.96	< 1e-07	4.57E-06
1098	3	F2-3 vs. L2-3	257986_at	AT3G20865	Encodes a putative arabinogalactan-protein (AGP40) that is expressed in pollen.	378.40	76.42	4.95	6.84E-04	2.38E-03
1099	3	F2-3 vs. L2-3	266708_at	AT2G03200	Atypical aspartic protease which modulates lateral root development.	87.00	17.60	4.94	9.00E-07	2.22E-05
1100	3	F2-3 vs. L2-3	266764_at	AT2G47050	Plant invertase/pectin methylesterase inhibitor superfamily protein;(source:Araport11)	119.77	24.23	4.94	3.75E-04	1.52E-03
1101	3	F2-3 vs. L2-3	259226_at	AT3G07700	ABC1K7 is a member of an atypical protein kinase family that is induced by salt stress. Loss of function mutations affect the metabolic profile of chloroplast lipids. It appears to function along with ABC1K8 in mediating lipid membrane changes in response to stress.	376.98	76.87	4.90	1.05E-04	6.09E-04
1102	3	F2-3 vs. L2-3	262905_at	AT1G59730	Thioredoxin H-type 7 , oxidoreductase located in cytosol and ER. Interacts with GPT1.	67.10	13.81	4.86	2.90E-06	5.02E-05
1103	3	F2-3 vs. L2-3	259736_at	AT1G64390	glycosyl hydrolase 9C2;(source:Araport11)	351.18	72.92	4.82	5.17E-05	3.71E-04
1104	3	F2-3 vs. L2-3	265115_at	AT1G62450	Immunoglobulin E-set superfamily protein;(source:Araport11)	96.59	20.10	4.81	2.00E-06	3.93E-05
1105	3	F2-3 vs. L2-3	256627_at	AT3G19970	alpha/beta-Hydrolases superfamily protein;(source:Araport11)	285.38	59.35	4.81	1.64E-04	8.34E-04
1106	3	F2-3 vs. L2-3	245101_at	AT2G40890	encodes coumarate 3-hydroxylase (C3H), a P450-dependent monooxygenase. Involved in lignin biosynthesis and flavonoid biosynthesis. Also affects the biosynthesis of coumarins such as scopoletin and scopolin as a branching-out-pathway from the phenylpropanoid acid level.	283.16	59.02	4.80	< 1e-07	4.57E-06
1107	3	F2-3 vs. L2-3	267255_at	AT2G22950	Encodes a putative auto-regulated Ca2+-ATPase located in the plasma membrane involved in transporting Ca2+ outside developing pollen grains. This activity is important to support normal pollen development, particularly the progression to uninucleated microspores to bicellular pollen grains.	268.37	56.28	4.77	4.20E-06	6.33E-05
1108	3	F2-3 vs. L2-3	258645_s_a	AT3G07850	Pectin lyase-like superfamily protein;(source:Araport11)	78.03	16.34	4.77	3.81E-05	2.98E-04
1109	3	F2-3 vs. L2-3	258035_at	AT3G21180	one of the type IIB calcium pump isoforms. encodes an autoinhibited Ca(2+)-ATPase that contains an N-terminal calmodulin binding autoinhibitory domain.	114.94	24.10	4.77	5.45E-05	3.87E-04
1110	3	F2-3 vs. L2-3	263718_at	AT2G13570	nuclear factor Y, subunit B7;(source:Araport11)	70.59	14.86	4.75	6.00E-07	1.65E-05

1111	3	F2-3 vs. L2-3	263706_s_at	AT5G14200	The AtIMD1 is one out of 3 genes encoding the enzyme 3-isopropylmalate dehydrogenase involved in leucine biosynthesis in Arabidopsis. Its subcellular location has been targeted to plastids. Encodes methylthioalkylmalate dehydrogenase. Involved in glucosinolate biosynthesis, in methionine chain elongation. The mRNA is cell-to-cell mobile.	57.46	12.13	4.74	1.50E-05	1.58E-04
1112	3	F2-3 vs. L2-3	265475_at	AT2G15620	Involved in the second step of nitrate assimilation. Its expression is induced by nitrate. The mRNA is cell-to-cell mobile.	154.90	32.98	4.70	< 1e-07	< 1e-07
1113	3	F2-3 vs. L2-3	263709_at	AT1G09310	plant/protein (Protein of unknown function, DUF538);(source:Araport11)	149.29	32.10	4.65	5.02E-05	3.63E-04
1114	3	F2-3 vs. L2-3	262146_at	AT1G52580	RHOMBOID-like protein 5;(source:Araport11)	446.77	96.84	4.61	2.50E-06	4.58E-05
1115	3	F2-3 vs. L2-3	257054_at	AT3G15353	metallothionein, binds to and detoxifies excess copper and other metals, limiting oxidative damage	65.31	14.21	4.60	8.40E-06	1.05E-04
1116	3	F2-3 vs. L2-3	AFFX-r2-At-GAPDH-5_s_at	AT3G04120	encodes cytosolic GADPH (C subunit) involved in the glycolytic pathway but also interacts with H2O2 potentially placing it in a signalling cascade induced by ROS. The mRNA is cell-to-cell mobile.	602.35	131.25	4.59	3.00E-06	5.09E-05
1117	3	F2-3 vs. L2-3	263379_at	AT2G40140	zinc finger (CCCH-type) family protein;(source:Araport11)	236.54	51.50	4.59	5.60E-06	7.97E-05
1118	3	F2-3 vs. L2-3	265368_at	AT2G13350	Calcium-dependent lipid-binding (CaLB domain) family protein;(source:Araport11)	342.43	74.92	4.57	3.00E-07	1.05E-05
1119	3	F2-3 vs. L2-3	254762_at	AT4G13230	Late embryogenesis abundant protein (LEA) family protein;(source:Araport11)	84.68	18.59	4.56	2.40E-06	4.54E-05
1120	3	F2-3 vs. L2-3	267168_at	AT2G37770	Encodes an NADPH-dependent aldo-keto reductase that can act on a wide variety of substrates in vitro including saturated and unsaturated aldehydes, steroids, and sugars. GFP-tagged AKR4C9 localizes to the chloroplast where it may play a role in detoxifying reactive carbonyl compounds that threaten to impair the photosynthetic process. Transcript levels for this gene are up-regulated in response to cold, salt, and drought stress.	62.27	13.74	4.53	2.00E-07	7.75E-06
1121	3	F2-3 vs. L2-3	244935_at	ATCG01090	Encodes subunit of the chloroplast NAD(P)H dehydrogenase complex	142.22	31.66	4.49	3.92E-05	3.05E-04
1122	3	F2-3 vs. L2-3	265444_s_at	AT2G37180	a member of the plasma membrane intrinsic protein PIP2. functions as aquaporin and is involved in desiccation.	1361.75	303.89	4.48	1.20E-06	2.80E-05
1123	3	F2-3 vs. L2-3	257119_at	AT3G20190	Leucine-rich repeat protein kinase family protein;(source:Araport11)	61.77	13.81	4.47	< 1e-07	< 1e-07
1124	3	F2-3 vs. L2-3	259544_at	AT1G20620	Catalase, catalyzes the breakdown of hydrogen peroxide (H2O2) into water and oxygen. The mRNA is cell-to-cell mobile.	1678.52	382.28	4.39	1.81E-04	8.86E-04
1125	3	F2-3 vs. L2-3	260221_at	AT1G74670	Gibberellin-regulated family protein;(source:Araport11)	44.06	10.04	4.39	7.00E-07	1.83E-05
1126	3	F2-3 vs. L2-3	251304_at	AT3G61990	Encodes a protein methyltransferase. Involved in the methylation of plant transmembrane proteins.	521.52	119.54	4.36	3.32E-04	1.39E-03
1127	3	F2-3 vs. L2-3	247643_at	AT5G60450	Encodes a member of the ARF family of transcription factors which mediate auxin responses. ARF4 appears to have redundant function with ETT(ARF3) in specifying abaxial cell identity.	139.30	32.09	4.34	1.95E-05	1.88E-04
1128	3	F2-3 vs. L2-3	254270_at	AT4G23100	Encodes the enzyme glutamate-cysteine ligase catalyzing the first, and rate-limiting, step of glutathione biosynthesis. Required for cell proliferation at the root tip. Involved in susceptibility to the bacterial pathogen Pseudomonas syringae. Mutants are phytoalexin defective.	102.52	23.88	4.29	3.00E-07	1.05E-05
1129	3	F2-3 vs. L2-3	246998_at	AT5G67370	DUF1230 family protein (DUF1230);(source:Araport11)	140.64	32.91	4.27	2.50E-06	4.58E-05
1130	3	F2-3 vs. L2-3	245463_at	AT4G17030	Encodes EXLB1 (expansin-like B1), a member of the expansin family.	89.78	21.09	4.26	3.30E-06	5.45E-05
1131	3	F2-3 vs. L2-3	262637_at	AT1G06640	encodes a protein whose sequence is similar to a 2-oxoglutarate-dependent dioxygenase The mRNA is cell-to-cell mobile.	311.41	73.90	4.21	1.65E-05	1.69E-04
1132	3	F2-3 vs. L2-3	260385_at	AT1G74090	encodes a desulfoglucosinolate sulfotransferase, involved in the final step of glucosinolate core structure biosynthesis. Has a broad-substrate specificity with preference with methionine-derived desulfoglucosinolates.	185.37	44.23	4.19	1.00E-07	4.57E-06
1133	3	F2-3 vs. L2-3	250129_at	AT5G16450	Ribonuclease E inhibitor RraA/Dimethylmenaquinone methyltransferase;(source:Araport11)	464.79	111.27	4.18	1.31E-04	7.11E-04
1134	3	F2-3 vs. L2-3	266865_at	AT2G29980	Endoplasmic reticulum enzyme responsible for the synthesis of 18:3 fatty acids from phospholipids. Uses cytochrome b5 as electron donor.	341.10	82.18	4.15	1.38E-05	1.50E-04
1135	3	F2-3 vs. L2-3	253387_at	AT4G33010	glycine decarboxylase P-protein 1;(source:Araport11)	702.81	169.38	4.15	7.12E-04	2.46E-03
1136	3	F2-3 vs. L2-3	256075_at	AT1G18150	Encodes mitogen-activated protein kinase 8 (MPK8). MPK8 connects protein phosphorylation, Ca2+, and ROS in the wound-signaling pathway.	163.99	39.62	4.14	8.48E-05	5.24E-04
1137	3	F2-3 vs. L2-3	245745_at	AT1G51110	localized to chloroplasts	46.80	11.37	4.12	1.50E-06	3.34E-05

1138	3	F2-3 vs. L2-3	249237_at	AT5G42050	Stress responsive asparagine-rich protein. Binds to PevD (Verticillium dahliae) fungal effector protein. NRP interacts with CRY2, leading to increased cytoplasmic accumulation of CRY2 in a blue light-independent manner (PMID:28633330).NRP also binds FyPP3 and recruits it to endosomes and thus targets it for degradation.	330.93	80.23	4.12	6.88E-05	4.51E-04
1139	3	F2-3 vs. L2-3	261713_at	AT1G32640	Encodes a MYC-related transcriptional activator with a typical DNA binding domain of a basic helix-loop-helix leucine zipper motif. Binds to an extended G-Box promoter motif and interacts with Jasmonate ZIM-domain proteins. MYC2 interacts with EIN3 and EIL1 to repress hook curvature and resistance to Botrytis cinera.Its transcription is induced by dehydration stress, ABA treatment and blue light via CRY1. Negative regulator of blue light-mediated photomorphogenic growth and blue and far-red-light-regulated gene expression. Positive regulator of lateral root formation. Regulates diverse JA-dependent functions. Negatively regulates Trp metabolism and biosynthesis of Trp-derived secondary metabolites. Positively regulates flavonoid biosynthesis, resistance to insects, and response to oxidative stress. Regulates other transcription factors, and negatively regulates its own expression. For example it binds to and regulates the expression of NST1. Its stability is modulated by PUB10 through polyubiquitination.	294.50	71.58	4.11	1.70E-06	3.64E-05
1140	3	F2-3 vs. L2-3	263477_at	AT2G31790	UDP-Glycosyltransferase superfamily protein;(source:Araport11)	176.84	43.05	4.11	< 1e-07	4.57E-06
1141	3	F2-3 vs. L2-3	254050_s_a	AT4G25670	stress response NST1-like protein;(source:Araport11)	265.80	64.72	4.11	6.00E-07	1.65E-05
1142	3	F2-3 vs. L2-3	254972_at	AT4G10440	S-adenosyl-L-methionine-dependent methyltransferases superfamily protein;(source:Araport11)	63.13	15.46	4.08	7.00E-07	1.83E-05
1143	3	F2-3 vs. L2-3	261053_at	AT1G01320	Encodes REDUCED CHLOROPLAST COVERAGE 1 (REC1). Located in the nucleus and cytosol. Contributes to establishing the size of the chloroplast compartment.	121.34	29.82	4.07	1.41E-05	1.52E-04
1144	3	F2-3 vs. L2-3	262680_at	AT1G75880	SGNH hydrolase-type esterase superfamily protein;(source:Araport11)	42.31	10.38	4.07	3.00E-07	1.05E-05
1145	3	F2-3 vs. L2-3	263533_at	AT2G24820	translocon at the inner envelope membrane of chloroplasts 55-II;(source:Araport11)	171.96	42.26	4.07	< 1e-07	4.57E-06
1146	3	F2-3 vs. L2-3	266262_at	AT2G27590	S-adenosyl-L-methionine-dependent methyltransferases superfamily protein;(source:Araport11)	128.09	31.74	4.04	4.18E-04	1.64E-03
1147	3	F2-3 vs. L2-3	249438_at	AT5G40010	Encodes a mitochondrial ATPase involved in seed and silique development.	50.20	12.43	4.04	2.29E-05	2.08E-04
1148	3	F2-3 vs. L2-3	259068_at	AT3G07560	Encodes peroxin 13 (PEX13) involved in protein transport into peroxisomes, for example, peroxisomal import of nitric oxide synthase.	400.61	99.34	4.03	5.00E-07	1.44E-05
1149	3	F2-3 vs. L2-3	253743_at	AT4G28940	Phosphorylase superfamily protein;(source:Araport11)	696.51	172.67	4.03	5.35E-05	3.82E-04
1150	3	F2-3 vs. L2-3	263662_at	AT1G04430	S-adenosyl-L-methionine-dependent methyltransferases superfamily protein;(source:Araport11)	202.47	50.32	4.02	4.20E-06	6.33E-05
1151	3	F2-3 vs. L2-3	261363_at	AT1G41830	SKU5-similar 6;(source:Araport11)	399.06	99.35	4.02	2.21E-04	1.03E-03
1152	3	F2-3 vs. L2-3	252971_at	AT4G38770	Encodes one of four proline-rich proteins in Arabidopsis which are predicted to localize to the cell wall. Transcripts are most abundant in aerial organs of the plant.	239.16	59.51	4.02	6.80E-05	4.47E-04
1153	3	F2-3 vs. L2-3	254862_at	AT4G12030	Required for the biosynthesis of methionine-derived glucosinolates. Involved in the transport of 2-keto acids between chloroplasts and the cytosol.	100.66	25.11	4.01	1.45E-04	7.62E-04
1154	3	F2-3 vs. L2-3	247882_at	AT5G57785	hypothetical protein;(source:Araport11)	355.02	88.67	4.00	7.40E-06	9.65E-05
1155	3	F2-3 vs. L2-3	265230_s_a	ATMG00480	Encodes subunit 8 of the mitochondrial F(O) ATP synthase complex.	396.85	99.55	3.99	5.76E-05	4.02E-04
1156	3	F2-3 vs. L2-3	265200_s_at	AT2G36790	The At2g36790 gene encodes a UDP-glucose:flavonol-3-O-glycoside-7-O-glucosyltransferase (UGT73C6)attaching a glucosyl residue to the 7-O-position of the flavonols kaempferol, quercetin and their 3-O-glycoside derivatives.	84.18	21.14	3.98	6.40E-06	8.73E-05
1157	3	F2-3 vs. L2-3	252921_at	AT4G39030	Encodes an orphan multidrug and toxin extrusion transporter. Essential component of salicylic acid-dependent signaling for disease resistance. Member of the MATE-transporter family. Expression induced by salicylic acid. Mutants are salicylic acid-deficient.	154.29	38.79	3.98	3.10E-06	5.20E-05

1158	3	F2-3 vs. L2-3	266673_at	AT2G29630	Encodes a protein involved in thiamin biosynthesis. The protein is an iron-sulfur cluster protein predicted to catalyze the conversion of 5-aminoimidazole ribonucleotide (AIR) to hydroxymethylpyrimidine (HMP) or hydroxymethylpyrimidine phosphate (HMP-P). A severe reduction of THIC levels in plants decreases vitamin B1 (thiamin diphosphate (TPP)) levels and also leads to changes in the levels of numerous other metabolites since so many primary metabolic enzymes require a TPP co-factor. thiC mutants are chlorotic and arrest in their development at the cotyledon stage. A N-terminal targeting sequence directs the THIC protein to the chloroplast stroma. A conserved TPP-binding site is located in the 3' UTR of the At2g29630.2 gene model, and is predicted to function as a riboswitch. The riboswitch controls the formation of transcripts with alternative 3' UTR lengths, which affect mRNA accumulation and protein production. THIC transcripts are observed in seedlings 5 or more days after germination, and light promotes the expression of this gene. Recessive mutant isolated by Redei. Leaves but not cotyledons white, lethal; restored to normal by thiamine or 2,5-dimethyl-4-aminopyrimidine.	332.60	83.71	3.97	4.77E-05	3.50E-04
1159	3	F2-3 vs. L2-3	245346_at	AT4G17090	Encodes a beta-amylase targeted to the chloroplast. Transgenic BMY8 RNAi lines fail to accumulate maltose during cold shock suggesting that maltose accumulation coincides with BMY8 expression. Apart from maltose, the sugar content of the RNAi lines were similar to wildtype (glucose and sucrose unaffected).BAM3 activity declines 2 and 4 days after start of cold stress despite an increase in transcript levels. BAM3 activity has a lower temperature optimum than BAM1 (PMID:25293962).	154.00	38.95	3.95	2.31E-05	2.08E-04
1160	3	F2-3 vs. L2-3	256156_at	AT3G08510	Phosphoinositide-specific phospholipase C (PI-PLC), catalyzes hydrolysis of phosphatidylinositol 4,5-bisphosphate into inositol 1,4,5-trisphosphate and diacylglycerol. It is involved in auxin biosynthesis and signaling, modulating development of both male and female gametophytes. It also regulates MAMP-triggered immunity by modulating ROS production.	203.12	51.55	3.94	7.70E-06	9.97E-05
1161	3	F2-3 vs. L2-3	257121_at	AT3G20220	SAUR-like auxin-responsive protein family;(source:Araport11)	119.57	30.47	3.92	5.83E-05	4.05E-04
1162	3	F2-3 vs. L2-3	252134_at	AT3G50910	netrin receptor DCC;(source:Araport11)	362.22	92.52	3.91	2.31E-04	1.06E-03
1163	3	F2-3 vs. L2-3	249215_at	AT5G42800	dihydroflavonol reductase. Catalyzes the conversion of dihydroquercetin to leucocyanidin in the biosynthesis of anthocyanins. Not expressed in roots (qRT-PCR). The mRNA is cell-to-cell mobile.	60.05	15.34	3.91	9.00E-06	1.11E-04
1164	3	F2-3 vs. L2-3	245306_at	AT4G14690	Encodes an early light-induced protein. ELIPs are thought not to be directly involved in the synthesis and assembly of specific photosynthetic complexes, but rather affect the biogenesis of all chlorophyll-binding complexes. A study (PMID 17553115) has shown that the chlorophyll synthesis pathway was downregulated as a result of constitutive ELIP2 expression, leading to decreased chlorophyll availability for the assembly of pigment-binding proteins for photosynthesis.	304.76	78.44	3.89	2.00E-07	7.75E-06
1165	3	F2-3 vs. L2-3	266184_s_at	AT3G54700	Encodes Pht1;7, a member of the Pht1 family of phosphate transporters which include: Pht1;1/At5g43350, Pht1;2/At5g43370, Pht1;3/At5g43360, Pht1;4/At2g38940, Pht1;5/At2g32830, Pht1;6/At5g43340, Pht1;7/At3g54700, Pht1;8/At1g20860, Pht1;9/At1g76430 (Plant Journal 2002, 31:341).	74.51	19.20	3.88	1.05E-05	1.22E-04
1166	3	F2-3 vs. L2-3	264603_at	AT1G04670	hypothetical protein;(source:Araport11)	39.66	10.26	3.86	1.00E-07	4.57E-06
1167	3	F2-3 vs. L2-3	251649_at	AT3G57330	Lesion mimic phenotype when mutation in the gene is combined with a mutation in ACA4. Lesion mimic phenotype of double knockout can be suppressed by nutritional supplements that increase anion levels (e.g. 15 mM Nitrate, Chloride, or Phosphate)	140.27	36.36	3.86	2.50E-06	4.58E-05
1168	3	F2-3 vs. L2-3	248573_at	AT5G49720	Encodes a membrane-bound endo-1,4-beta-D-glucanase, involved in cellulose biosynthesis. Loss-of-function mutants have severe cellulose-deficient phenotypes. During cell elongation, KOR1 is associated with Golgi apparatus and early endosome. Inhibition of cellulose biosynthesis promoted a redistribution of KOR1 in subcellular locations. These observations suggest that deposition of cellulose involves the intracellular cycling of KOR1.	682.03	176.46	3.86	< 1e-07	4.57E-06
1169	3	F2-3 vs. L2-3	260805_at	AT1G78320	Encodes glutathione transferase belonging to the tau class of GSTs. Naming convention according to Wagner et al. (2002).	116.96	30.38	3.85	2.38E-04	1.08E-03

1170	3	F2-3 vs. L2-3	257769_at	AT3G23050	Transcription regulator acting as repressor of auxin-inducible gene expression. Plays role in the control of gravitropic growth and development in light-grown seedlings. Auxin induces the degradation of the protein in a dosage-dependent manner in a process mediated by AtRac1. Auxin induced the relocalization of the protein within the nucleus from a diffused nucleoplasmic pattern to a discrete particulated pattern named nuclear protein bodies or NPB in a process also mediated by Rac1. Colocalizes with SCF, CSN and 26S proteasome components. Pseudomonas syringae type III effector AvrRpt2 stimulates AXR2 protein turnover.	82.07	21.47	3.82	1.00E-07	4.57E-06
1171	3	F2-3 vs. L2-3	255552_at	AT4G01850	S-adenosylmethionine synthetase 2;(source:Araport11)	986.13	258.42	3.82	1.47E-04	7.67E-04
1172	3	F2-3 vs. L2-3	262917_at	AT1G64800	DNA binding / transcription factor;(source:Araport11)	50.26	13.18	3.81	3.13E-05	2.58E-04
1173	3	F2-3 vs. L2-3	259044_at	AT3G03430	Calcium-binding EF-hand family protein;(source:Araport11)	47.83	12.58	3.80	6.11E-05	4.19E-04
1174	3	F2-3 vs. L2-3	256865_at	AT3G23820	Encodes a UDP-D-glucuronate 4-epimerase involved in pectin biosynthesis in the cell wall and affects cell wall integrity and immunity to fungi and bacteria. The mRNA is cell-to-cell mobile.	75.83	20.05	3.78	1.60E-06	3.46E-05
1175	3	F2-3 vs. L2-3	254480_at	AT4G20360	Nuclear transcribed, plastid localized EF-Tu translation elongation factor. Referred to as AtRabE1b in DOI:10.1104/pp.013052. However, wider community usage and more publications assign the symbol RabE1b to At5g59840.	267.88	70.99	3.77	< 1e-07	< 1e-07
1176	3	F2-3 vs. L2-3	262432_at	AT1G47530	MATE efflux family protein;(source:Araport11)	187.62	49.84	3.76	2.50E-06	4.58E-05
1177	3	F2-3 vs. L2-3	258151_at	AT3G18080	B-S glucosidase 44;(source:Araport11)	59.69	15.92	3.75	3.60E-06	5.76E-05
1178	3	F2-3 vs. L2-3	258500_at	AT3G02470	Encodes a S-adenosylmethionine decarboxylase involved in polyamine biosynthesis.	646.92	172.84	3.74	5.00E-07	1.44E-05
1179	3	F2-3 vs. L2-3	253048_at	AT4G37560	Acetamidase/Formamidase family protein;(source:Araport11)	51.36	13.77	3.73	2.80E-06	4.91E-05
1180	3	F2-3 vs. L2-3	265715_s_at	AT1G13860	Encodes QUASIMODO2 LIKE1 (QUL1), a paralog of QUASIMODO2 (QUA2). AT1G78240 (QUA2), AT1G13860 (QUL1) and AT2G03480 (QUL2) form a clade with a possible role in plant vasculature development.	43.77	11.78	3.72	2.70E-06	4.78E-05
1181	3	F2-3 vs. L2-3	256861_at	AT3G23920	Encodes a chloroplast beta-amylase. Is necessary for leaf starch breakdown in the absence of BAM3.Activity of BAM1 increases 4 days after osmotic stress. BAM1 has a higher temperature optimum than BAM3 (PMID:25293962).	513.13	137.99	3.72	1.40E-06	3.16E-05
1182	3	F2-3 vs. L2-3	248968_at	AT5G45280	Pectinacetylesterase family protein;(source:Araport11)	324.02	87.17	3.72	1.50E-05	1.58E-04
1183	3	F2-3 vs. L2-3	262282_at	AT1G68610	Target promoter of the male germline-specific transcription factor DUO1.	52.88	14.25	3.71	1.10E-06	2.61E-05
1184	3	F2-3 vs. L2-3	257262_at	AT3G21890	B-box type zinc finger family protein;(source:Araport11)	481.69	130.43	3.69	5.41E-05	3.85E-04
1185	3	F2-3 vs. L2-3	257886_at	AT3G17060	Pectin lyase-like superfamily protein;(source:Araport11)	164.25	44.59	3.68	9.80E-06	1.17E-04
1186	3	F2-3 vs. L2-3	248643_at	AT5G49130	MATE efflux family protein;(source:Araport11)	248.54	67.60	3.68	3.05E-05	2.54E-04
1187	3	F2-3 vs. L2-3	261593_at	AT1G33170	S-adenosyl-L-methionine-dependent methyltransferases superfamily protein;(source:Araport11)	78.24	21.31	3.67	4.20E-06	6.33E-05
1188	3	F2-3 vs. L2-3	255877_at	AT2G40460	Major facilitator superfamily protein;(source:Araport11)	80.09	21.81	3.67	1.09E-05	1.25E-04
1189	3	F2-3 vs. L2-3	254740_s_a	AT4G13930	Encodes a serine hydroxymethyltransferase maximally expressed in root	2185.38	595.02	3.67	1.00E-07	4.57E-06
1190	3	F2-3 vs. L2-3	259615_at	AT1G47980	desiccation-like protein;(source:Araport11)	99.38	27.23	3.65	2.90E-06	5.02E-05
1191	3	F2-3 vs. L2-3	267357_at	AT2G40000	ortholog of sugar beet HS1 PRO-1 2;(source:Araport11)	431.13	118.36	3.64	2.10E-06	4.08E-05
1192	3	F2-3 vs. L2-3	266075_s_a	AT2G40710	hemolysin-III related integral membrane protein;(source:Araport11)	382.99	105.43	3.63	1.03E-05	1.21E-04
1193	3	F2-3 vs. L2-3	250676_at	AT5G06320	encodes a protein whose sequence is similar to tobacco hairpin-induced gene (HIN1) and Arabidopsis non-race specific disease resistance gene (NDR1). Expression of this gene is induced by cucumber mosaic virus, spermine and Pseudomonas syringae pv. tomato DC3000. The gene product is localized to the plasma membrane.	379.50	104.41	3.63	6.53E-04	2.30E-03
1194	3	F2-3 vs. L2-3	252474_at	AT3G46620	Encodes an ABA- and drought-induced RING-DUF1117 gene whose mutation results in hyposensitive phenotypes toward ABA in terms of germination rate and stomatal closure and markedly reduced tolerance to drought stress relative to wild-type plants.	551.92	152.53	3.62	3.04E-04	1.31E-03
1195	3	F2-3 vs. L2-3	253522_at	AT4G31290	ChaC-like family protein;(source:Araport11)	373.58	104.57	3.57	1.30E-06	3.01E-05
1196	3	F2-3 vs. L2-3	258977_s_a	AT5G14060	lysine-sensitive aspartate kinase	632.68	177.36	3.57	1.51E-04	7.85E-04
1197	3	F2-3 vs. L2-3	251658_at	AT3G57020	Calcium-dependent phosphotriesterase superfamily protein;(source:Araport11)	155.98	43.77	3.56	1.34E-04	7.21E-04
1198	3	F2-3 vs. L2-3	247736_at	AT5G59370	Encodes one of eight Arabidopsis actins. ACT4 belongs to the reproductive actin subclass which is predominantly expressed in developing and reproductive tissues, such as pollen, pollen tubes, ovules, and developing seeds. Expression of the ACT4/GUS fusion was restricted to young vascular tissues, tapetum, and developing and mature pollen.	50.78	14.27	3.56	1.49E-05	1.58E-04

1199	3	F2-3 vs. L2-3	267470_at	AT2G30490	Encodes a cinnamate-4-hydroxylase. Mutations in this gene impact phenylpropanoid metabolism, growth and development.	174.73	49.17	3.55	2.20E-06	4.25E-05
1200	3	F2-3 vs. L2-3	262344_at	AT1G64060	Interacts with AtrbohD gene to fine tune the spatial control of ROI production and hypersensitive response to cell in and around infection site.	189.87	53.66	3.54	1.90E-06	3.89E-05
1201	3	F2-3 vs. L2-3	255580_at	AT4G01470	Encodes AtTIP1;3, functions as water and urea channels in pollen.	236.80	66.92	3.54	1.41E-04	7.49E-04
1202	3	F2-3 vs. L2-3	262998_at	AT1G54280	Encodes a member of the P4 subfamily of P-type ATPases expressed in the pollen plasma membrane. Double mutants with ALA7 display pollen and pollen tube defects.	50.20	14.23	3.53	4.63E-05	3.43E-04
1203	3	F2-3 vs. L2-3	262707_at	AT1G16290	transglycosylase;(source:Araport11)	137.26	39.10	3.51	3.26E-04	1.37E-03
1204	3	F2-3 vs. L2-3	264112_at	AT2G13680	Responsible for the synthesis of callose deposited at the primary cell wall of meiocytes, tetrads and microspores. Required for exine formation during microgametogenesis and for pollen viability. Highest expression in meiocytes, tetrads, microspores and mature pollen.	192.07	54.75	3.51	6.27E-04	2.23E-03
1205	3	F2-3 vs. L2-3	261722_at	AT1G08510	Encodes an acyl-acyl carrier protein thioesterase. Hydrolyzes primarily saturated acyl-ACPs with chain lengths that vary between 8 and 18 carbons. Involved in saturated fatty acid synthesis. Nuclear-encoded, plastid-targeted globular protein that is functional as dimer.	244.82	69.89	3.50	1.00E-07	4.57E-06
1206	3	F2-3 vs. L2-3	264394_at	AT1G11860	Glycine cleavage T-protein family;(source:Araport11)	1043.89	298.26	3.50	3.83E-04	1.54E-03
1207	3	F2-3 vs. L2-3	252870_at	AT4G39940	adenosine-5'-phosphosulfate-kinase (akn2) mRNA, complete The mRNA is cell-to-cell mobile.	64.39	18.42	3.50	2.00E-07	7.75E-06
1208	3	F2-3 vs. L2-3	265587_at	AT2G19980	CAP (Cysteine-rich secretory proteins, Antigen 5, and Pathogenesis-related 1 protein) superfamily protein;(source:Araport11)	63.60	18.22	3.49	1.62E-04	8.26E-04
1209	3	F2-3 vs. L2-3	260568_at	AT2G43570	chitinase;(source:Araport11)	50.12	14.35	3.49	1.48E-05	1.57E-04
1210	3	F2-3 vs. L2-3	252543_at	AT3G45780	Blue-light photoreceptor. Contains a light activated serine-threonine kinase domain and LOV1 and LOV2 repeats. Mutants are defective in blue-light response. Mediates blue light-induced growth enhancements. PHOT1 and PHOT2 mediate blue light-dependent activation of the plasma membrane H+-ATPase in guard cell protoplasts. PHOT1 undergoes blue-light-dependent autophosphorylation. At least eight phosphorylation sites have been identified in PHOT1. Phosphorylation of serine851 in the activation loop of PHOT1 appears to be required for stomatal opening, chloroplast accumulation, leaf flattening, and phototropism, and phosphorylation of serine849 may also contribute to the regulation of these responses. Phosphorylation-dependent binding of 14-3-3 proteins to the Hinge1 region of PHOT1 appears to require serine350 and serine376.	62.55	17.91	3.49	3.30E-05	2.67E-04
1211	3	F2-3 vs. L2-3	265073_at	AT1G55480	Encodes a member of a novel plant protein family containing a PDZ, a K-box, and a TPR motif. mRNA but not protein levels decrease after wounding. ZKT is phosphorylated at Thr and Ser residues after wounding. The mRNA is cell-to-cell mobile.	352.19	101.18	3.48	3.00E-06	5.09E-05
1212	3	F2-3 vs. L2-3	247161_at	AT5G65810	Encodes CGR3 (cotton Golgi-related 3). CGR3 and a close homologue CGR2 have overlapping roles in pectin methylesterification and plant growth.	582.57	168.10	3.47	9.00E-07	2.22E-05
1213	3	F2-3 vs. L2-3	259520_at	AT1G12320	ankyrin repeat/KH domain protein (DUF1442);(source:Araport11)	48.39	14.02	3.45	3.10E-06	5.20E-05
1214	3	F2-3 vs. L2-3	260632_at	AT1G62360	Class I knotted-like homeodomain protein that is required for shoot apical meristem (SAM) formation during embryogenesis and for SAM function throughout the lifetime of the plant. Functions by preventing incorporation of cells in the meristem center into differentiating organ primordia. It has also been shown to have a role in the specification of flower meristem identity.	95.79	27.90	3.43	2.40E-06	4.54E-05
1215	3	F2-3 vs. L2-3	260309_at	AT1G70580	Encodes a protein with glyoxylate aminotransferase activity. It can act on a number of different small substrates and amino acids in vitro.	398.17	116.02	3.43	1.73E-05	1.75E-04
1216	3	F2-3 vs. L2-3	248153_at	AT5G54250	member of Cyclic nucleotide gated channel family, downstream component of the signaling pathways leading to HR resistance. mutant plants exhibit gene-for-gene disease resistance against avirulent Pseudomonas syringae despite the near-complete absence of the hypersensitive response (HR). Salicylic acid accumulation in dnd2 mutants is completely PAD4-independent.	84.97	24.76	3.43	2.30E-06	4.39E-05
1217	3	F2-3 vs. L2-3	265022_at	AT1G24520	Male fertility gene acting on tapetum and microspore	263.93	77.32	3.41	2.08E-04	9.86E-04

1218	3	F2-3 vs. L2-3	245253_at	AT4G15440	Encodes a hydroperoxide lyase. Also a member of the CYP74B cytochrome p450 family. In the ecotype Columbia (Col) the gene contains a 10-nucleotide deletion in its first exon that causes it to code for a truncated protein that results in a non-functional hydroperoxide lyase.	136.07	39.95	3.41	1.90E-06	3.89E-05
1219	3	F2-3 vs. L2-3	247884_at	AT5G57800	encodes a transmembrane protein with similarity to the sterol desaturase family at the N-terminus and to the short-chain dehydrogenase/reductase family at the C-terminus. Mutant analyses indicate this protein is involved in cuticle membrane and wax biosynthesis. The mRNA is cell-to-cell mobile.	104.26	30.77	3.39	4.98E-04	1.87E-03
1220	3	F2-3 vs. L2-3	255719_at	AT1G32080	Encodes a plant LrgAB/CidAB protein localized to the chloroplast envelope that is involved in chloroplast development, carbon partitioning, ABA/drought response, and leaf senescence. The gene may have evolved from gene fusion of bacterial lrgA and lrgB.	77.64	22.95	3.38	3.00E-06	5.09E-05
1221	3	F2-3 vs. L2-3	265441_at	AT2G20870	cell wall protein precursor;(source:Araport11)	70.86	20.95	3.38	6.00E-07	1.65E-05
1222	3	F2-3 vs. L2-3	257749_at	AT3G18780	Encodes an actin that is constitutively expressed in vegetative structures but not pollen. ACT2 is involved in tip growth of root hairs.	1619.99	478.86	3.38	3.00E-06	5.09E-05
1223	3	F2-3 vs. L2-3	263553_at	AT2G16430	Encodes an acid phosphatase involved plant acclimation to Pi deprivation.	165.83	49.21	3.37	1.21E-04	6.71E-04
1224	3	F2-3 vs. L2-3	263012_at	AT1G23380	homeodomain transcription factor KNAT6, belonging to class I of KN transcription factor family (which also includes KNAT1 and KNAT2). Expression is increased in as and bop1 leaf mutants.	48.40	14.45	3.35	1.98E-05	1.89E-04
1225	3	F2-3 vs. L2-3	264851_at	AT2G17290	Encodes calcium dependent protein kinase 6 (CPK6), a member of the Arabidopsis CDPK gene family. CDPKs contain an intrinsic Ca2+-activation domain with four EF hand Ca2+-binding sites. CDPKs protein kinases have been proposed to function in multiple plant signal transduction pathways downstream of [Ca2+]cyt elevations, thus transducing various physiological responses. CPK6 is expressed in both guard cells and mesophyll cells. Functions in guard cell ion channel regulation. ABA and Ca(2+) activation of slow-type anion channels and, interestingly, ABA activation of plasma membrane Ca(2+)-permeable channels were impaired in independent alleles of single and double cpk3cpk6 mutant guard cells. Furthermore, ABA- and Ca(2+)-induced stomatal closing were partially impaired in these cpk3cpk6 mutant alleles. The protein kinase CPK6 is shown in biochemical assays to be directly activated by elevations in calcium concentrations in the physiological range (Laanements et al., 2013 PlantPhys.; PMID: 23766366). These data correlate with the in vivo function of CPK6 in Ca2+ and ABA activation of S-type anion channels (Mori et al., 2006 PLoS Biol.; PMID: 17032064) and the ability of CPK6 to mediate ABA activation of SLAC1 (Brandt et al., 2012 PNAS; PMID: 22689970).The mRNA is cell-to-cell mobile.	145.31	43.57	3.34	3.91E-04	1.56E-03
1226	3	F2-3 vs. L2-3	257650_at	AT3G16800	EGR3 functions as a negative regulator of plant growth with prominent effect on plant growth during drought stress, EGR3 regulates microtubule organization and likely affects additional cytoskeleton and trafficking processes along the plasma membrane.	78.84	23.62	3.34	6.00E-06	8.44E-05
1227	3	F2-3 vs. L2-3	250824_at	AT5G05200	Protein kinase superfamily protein;(source:Araport11)	216.48	64.88	3.34	2.00E-07	7.75E-06
1228	3	F2-3 vs. L2-3	246596_at	AT5G14740	Encodes a beta carbonic anhydrase likely to be localized in the cytoplasm. Expression of its mRNA is seen in etiolated seedlings and points to a possible nonphotosynthetic role for this isoform.	59.24	17.81	3.33	4.00E-07	1.25E-05
1229	3	F2-3 vs. L2-3	245629_at	AT1G56580	Encodes SMALLER WITH VARIABLE BRANCHES (SVB), a protein with a conserved domain of unknown function (DUF538). The trichomes of the SVB mutants are smaller and exhibit branches of variable length and number.	319.73	96.18	3.32	2.00E-07	7.75E-06
1230	3	F2-3 vs. L2-3	267153_at	AT2G30860	Encodes glutathione transferase belonging to the phi class of GSTs. Naming convention according to Wagner et al. (2002).	1797.23	541.52	3.32	3.07E-05	2.55E-04
1231	3	F2-3 vs. L2-3	263954_at	AT2G35840	Sucrose-6F-phosphate phosphohydrolase family protein;(source:Araport11)	1001.90	301.98	3.32	3.00E-07	1.05E-05
1232	3	F2-3 vs. L2-3	259275_at	AT3G01060	lysine-tRNA ligase;(source:Araport11)	208.47	62.73	3.32	5.00E-07	1.44E-05
1233	3	F2-3 vs. L2-3	260997_at	AT1G26610	C2H2-like zinc finger protein;(source:Araport11)	171.15	51.77	3.31	8.00E-07	2.05E-05
1234	3	F2-3 vs. L2-3	265119_at	AT1G62570	belongs to the flavin-monooxygenase (FMO) family, encodes a glucosinolate S-oxygenase that catalyzes the conversion of methylthioalkyl glucosinolates to methylsulfinylalkyl glucosinolates The mRNA is cell-to-cell mobile.	86.67	26.26	3.30	1.90E-05	1.85E-04
1235	3	F2-3 vs. L2-3	252740_at	AT3G43270	Plant invertase/pectin methylesterase inhibitor superfamily;(source:Araport11)	73.01	22.13	3.30	6.77E-05	4.47E-04
1236	3	F2-3 vs. L2-3	252482_at	AT3G46670	UDP-glucosyl transferase 76E11;(source:Araport11)	80.80	24.48	3.30	1.20E-06	2.80E-05
1237	3	F2-3 vs. L2-3	251855_at	AT3G54690	Sugar isomerase (SIS) family protein;(source:Araport11)	422.21	128.05	3.30	1.11E-04	6.34E-04

1238	3	F2-3 vs. L2-3	247440_at	AT5G62680	Encodes a high-affinity, proton-dependent glucosinolate-specific transporter that is crucial for the transport of both methionine- and tryptophan-derived glucosinolates to seeds.	152.73	46.29	3.30	2.50E-06	4.58E-05
1239	3	F2-3 vs. L2-3	260267_at	AT1G68530	Encodes KCS6, a member of the 3-ketoacyl-CoA synthase family involved in the biosynthesis of VLCFA (very long chain fatty acids).	124.33	37.94	3.28	2.26E-04	1.05E-03
1240	3	F2-3 vs. L2-3	254001_at	AT4G26260	Encodes a myo-inositol oxygenase, which is the first enzyme in the inositol route to ascorbate (L‐ascorbic acid, AsA, vitamin C). Overexpression results in enhanced biomass and abiotic stress tolerance.	75.07	22.88	3.28	2.73E-05	2.34E-04
1241	3	F2-3 vs. L2-3	262073_at	AT1G59640	A basic helix-loop-helix encoding gene (BIGPETAL, BPE) involved in the control of petal size. BPE is expressed via two mRNAs derived from an alternative splicing event. The BPEub (AT1G59640.1)transcript is expressed ubiquitously, whereas the BPEp (AT1G59640.2) transcript is preferentially expressed in petals. Plants that lack the petal-expressed variant BPEp have larger petals as a result of increased cell size. BPEp is positively regulated downstream of APETALA3, PISTILLATA, APETALA1 and PISTILLATA3 and is negatively regulated downstream of AGAMOUS.	129.73	39.71	3.27	3.90E-06	6.01E-05
1242	3	F2-3 vs. L2-3	249754_at	AT5G24530	Encodes a putative 2OG-Fe(II) oxygenase that is defense-associated but required for susceptibility to downy mildew. The mRNA is cell-to-cell mobile.	128.50	39.31	3.27	6.90E-06	9.18E-05
1243	3	F2-3 vs. L2-3	264016_at	AT2G21220	SAUR-like auxin-responsive protein family;(source:Araport11)	39.45	12.09	3.26	2.00E-07	7.75E-06
1244	3	F2-3 vs. L2-3	258976_at	AT3G01980	NAD(P)-binding Rossmann-fold superfamily protein;(source:Araport11)	348.78	106.90	3.26	< 1e-07	4.57E-06
1245	3	F2-3 vs. L2-3	246511_at	AT5G15490	Encodes one of four UDP-glucose dehydrogenase UGD) genes. Mutation of this gene in combination with UGD2 leads to swollen plant cell walls and severe developmental defects associated with changes in pectic polysaccharides.	355.18	108.93	3.26	4.69E-04	1.78E-03
1246	3	F2-3 vs. L2-3	261443_at	AT1G28480	Encodes GRX480, a member of the glutaredoxin family that regulates protein redox state. GRX480 interacts with TGA factors and suppresses JA-responsive PDF1.2 transcription. GRX480 transcription is SA-inducible and requires NPR1. Maybe involved in SA/JA cross-talk. It has also been shown to interact with the transcription factor TGA2 and suppress ORA59 promoter activity.	65.56	20.19	3.25	9.20E-06	1.13E-04
1247	3	F2-3 vs. L2-3	256311_at	AT1G30330	Encodes a member of the auxin response factor family. Mediates auxin response via expression of auxin regulated genes. Acts redundantly with ARF8 to control stamen elongation and flower maturation. Expression of ARF6 is controlled by miR167.	1132.38	348.21	3.25	7.00E-07	1.83E-05
1248	3	F2-3 vs. L2-3	255675_at	AT4G00480	MYC-related protein with a basic helix-loop-helix motif at the C-terminus and a region similar to the maize B/R family at the N-terminus	46.07	14.16	3.25	3.30E-06	5.45E-05
1249	3	F2-3 vs. L2-3	244936_at	ATCG01100	NADH dehydrogenase ND1	214.84	66.14	3.25	6.48E-05	4.33E-04
1250	3	F2-3 vs. L2-3	256299_at	AT1G69530	Member of Alpha-Expansin Gene Family. Naming convention from the Expansin Working Group (Kende et al, Plant Mol Bio). Involved in the formation of nematode-induced syncytia in roots of Arabidopsis thaliana.	88.85	27.45	3.24	5.98E-05	4.12E-04
1251	3	F2-3 vs. L2-3	265634_at	AT2G25530	AFG1-like ATPase family protein;(source:Araport11)	124.27	38.50	3.23	5.00E-07	1.44E-05
1252	3	F2-3 vs. L2-3	256417_s_at	AT3G11170	Chloroplastic enzyme responsible for the synthesis of 16:3 and 18:3 fatty acids from galactolipids, sulpholipids and phosphatidylglycerol. Uses ferredoxin as electron donor. Gene expression is induced by wounding in shoot and root. The wound-response in shoot is independent of jasmonic acid mediated pathway whereas the root response is mediated by jasmonic acid. The mRNA is cell-to-cell mobile.	320.70	99.25	3.23	1.06E-05	1.22E-04
1253	3	F2-3 vs. L2-3	264809_at	AT1G08830	Encodes a cytosolic copper/zinc superoxide dismutase CSD1 that can detoxify superoxide radicals. Its expression is affected by miR398-directed mRNA cleavage. Regulated by biotic and abiotic stress. Activation of CSD1 in the cytoplasm involves both a CCS-dependent and -independent pathway.	1279.68	397.53	3.22	6.27E-04	2.23E-03
1254	3	F2-3 vs. L2-3	255872_at	AT2G30360	Encodes a SOS2-like protein kinase that is a member of the CBL-interacting protein kinase family.Loss of function mutants show a decrease in sensitivity to high pH.Phosphorylates AHA2, a plasma membrane H+ ATPase.This phosphorylation appears to regulate the activity of the proton transporter.	88.05	27.33	3.22	1.29E-04	7.05E-04
1255	3	F2-3 vs. L2-3	250505_at	AT5G09870	Encodes a cellulose synthase CESA5 that produces seed mucilage cellulose.Mutants are defective in seed coat mucilage.Involved in the regulation of mucilage composition and/or mucilage synthesis.	672.67	208.78	3.22	2.71E-05	2.33E-04
1256	3	F2-3 vs. L2-3	264279_s_at	AT1G78820	D-mannose binding lectin protein with Apple-like carbohydrate-binding domain-containing protein;(source:Araport11)	100.53	31.27	3.21	1.60E-06	3.46E-05

1257	3	F2-3 vs. L2-3	265998_at	AT2G24270	Encodes a protein with non-phosphorylating NADP-dependent glyceraldehyde-3-phosphate dehydrogenase activity. The activity of the enzyme was determined from leaf extracts; the enzyme has not been purified to confirm activity.	647.36	201.44	3.21	3.66E-05	2.88E-04
1258	3	F2-3 vs. L2-3	250690_at	AT5G06530	Encodes ABCG22, an ABC transporter gene. Mutation results in increased water transpiration and drought susceptibility.	77.32	24.11	3.21	1.40E-05	1.51E-04
1259	3	F2-3 vs. L2-3	247450_at	AT5G62350	Plant invertase/pectin methylesterase inhibitor superfamily protein;(source:Araport11)	410.43	127.97	3.21	2.34E-05	2.10E-04
1260	3	F2-3 vs. L2-3	255622_at	AT4G01070	the glycosyltransferase (UGT72B1) is involved in metabolizing xenobiotica (chloroaniline and chlorophenole). Comparison between wild type and knock-out mutant demonstrates the central role of this gene for metabolizing chloroaniline but significantly less for chlorophenole. The glucosyltransferase preferred UDP-xylose over UDP-glucose indicating its (additional) functioning as a xylosyltransferase in planta	75.69	23.66	3.20	1.88E-05	1.83E-04
1261	3	F2-3 vs. L2-3	260745_at	AT1G78370	Encodes glutathione transferase belonging to the tau class of GSTs. Naming convention according to Wagner et al. (2002).	228.99	71.74	3.19	5.00E-07	1.44E-05
1262	3	F2-3 vs. L2-3	245293_at	AT4G16660	heat shock protein 70 (Hsp 70) family protein;(source:Araport11)	97.08	30.45	3.19	7.20E-06	9.51E-05
1263	3	F2-3 vs. L2-3	265182_at	AT1G23740	AOR is an alkenal/one oxidoreductase that acts on compounds with unsaturated alpha,beta-carbonyls. The activity of this enzyme with a number of substrates, including acrolein and 3-buten-2-one, was demonstrated in vitro using a truncated form of the protein that lacked approximately 80 of the first amino acids. This protein appears to localize to the chloroplast where it likely helps to maintain the photosynthetic process by detoxifying reactive carbonyls formed during lipid peroxidation.	425.44	134.25	3.17	7.00E-07	1.83E-05
1264	3	F2-3 vs. L2-3	264760_at	AT1G61290	member of SYP12 Gene Family	116.48	36.78	3.17	8.70E-06	1.08E-04
1265	3	F2-3 vs. L2-3	245854_at	AT5G13490	Encodes mitochondrial ADP/ATP carrier	1076.07	340.52	3.16	2.04E-05	1.92E-04
1266	3	F2-3 vs. L2-3	261933_at	AT1G22410	Class-II DAHP synthetase family protein;(source:Araport11)	713.21	226.80	3.14	2.60E-06	4.67E-05
1267	3	F2-3 vs. L2-3	253833_at	AT4G27790	Calcium-binding EF hand family protein;(source:Araport11)	71.59	22.81	3.14	2.27E-05	2.07E-04
1268	3	F2-3 vs. L2-3	246488_at	AT5G16010	3-oxo-5-alpha-steroid 4-dehydrogenase family protein;(source:Araport11)	207.87	66.27	3.14	6.70E-06	8.98E-05
1269	3	F2-3 vs. L2-3	258619_at	AT3G02780	Encodes a protein with isopentenyl diphosphate:dimethylallyl diphosphate isomerase activity. There is genetic evidence that it functions in the mevalonate, but not the MEP biosynthetic pathway.	446.09	142.38	3.13	6.95E-05	4.53E-04
1270	3	F2-3 vs. L2-3	251361_at	AT3G61230	Encodes a member of the Arabidopsis LIM proteins: a family of actin bundlers with distinct expression patterns. WLIM1, WLIM2a, and WLIM2b are widely expressed, whereas PLIM2a, PLIM2b, and PLIM2c are predominantly expressed in pollen. Regulates actin cytoskeleton organization.	44.96	14.38	3.13	9.40E-06	1.14E-04
1271	3	F2-3 vs. L2-3	261376_at	AT1G18660	Membrane localized protein of unknown function. Involved in negative regulation of immune response. Mutants have increased resistance to pathogens.	127.50	40.80	3.12	8.32E-05	5.18E-04
1272	3	F2-3 vs. L2-3	252213_at	AT3G50210	2-oxoglutarate (2OG) and Fe(II)-dependent oxygenase superfamily protein;(source:Araport11)	313.00	100.24	3.12	6.20E-06	8.57E-05
1273	3	F2-3 vs. L2-3	267095_at	AT2G38280	Encodes a protein with in vitro AMP deaminase activity that is involved in embryogenesis. Homozygous mutant embryos fail to develop past the zygote stage.	384.41	123.71	3.11	6.23E-05	4.24E-04
1274	3	F2-3 vs. L2-3	251091_at	AT5G01410	Encodes a protein predicted to function in tandem with PDX2 to form glutamine amidotransferase complex with involved in vitamin B6 biosynthesis.	678.21	218.19	3.11	3.40E-06	5.58E-05
1275	3	F2-3 vs. L2-3	248263_at	AT5G53370	pectin methylesterase PCR fragment F;(source:Araport11)	96.65	31.05	3.11	4.30E-06	6.44E-05
1276	3	F2-3 vs. L2-3	245001_at	ATCG00220	PSII low MW protein	2612.77	839.23	3.11	6.91E-04	2.40E-03
1277	3	F2-3 vs. L2-3	246036_at	AT5G08370	Member of Glycoside Hydrolase Family 27 (GH27)that functions as an α-galactosidase.	47.27	15.25	3.10	2.50E-06	4.58E-05
1278	3	F2-3 vs. L2-3	261218_at	AT1G20020	Encodes a leaf-type ferredoxin:NADP(H) oxidoreductase. It is present in both chloroplast stroma and thylakoid membranes but is more abundant in the stroma The mRNA is cell-to-cell mobile.	230.64	74.66	3.09	1.10E-04	6.29E-04
1279	3	F2-3 vs. L2-3	251433_at	AT3G59830	Integrin-linked protein kinase family;(source:Araport11)	53.30	17.22	3.09	4.50E-06	6.65E-05
1280	3	F2-3 vs. L2-3	253276_at	AT4G34050	Methyltransferase in the lignin biosynthetic pathway.	1049.65	339.37	3.09	6.20E-06	8.57E-05
1281	3	F2-3 vs. L2-3	262801_at	AT1G21010	poly polymerase;(source:Araport11)	62.40	20.28	3.08	5.09E-05	3.66E-04
1282	3	F2-3 vs. L2-3	251598_at	AT3G57600	encodes a member of the DREB subfamily A-2 of ERF/AP2 transcription factor family. The protein contains one AP2 domain. There are eight members in this subfamily including DREB2A AND DREB2B that are involved in response to drought.	49.63	16.11	3.08	7.00E-07	1.83E-05

1283	3	F2-3 vs. L2-3	250547_at	AT5G08100	Encodes an asparaginase that catalyzes the degradation of L-asparagine to L-aspartic acid and ammonia.	135.73	44.08	3.08	2.00E-06	3.93E-05
1284	3	F2-3 vs. L2-3	257217_at	AT3G14940	Encodes a cytosolic phosphoenolpyruvate carboxylase (PEPC) that has activity when expressed in E.coli. Its mRNA is most abundantly expressed in roots and siliques. PPC3 belongs to the plant-type PEPC family. It can form an enzymatically active complex with a castor bean ortholog of PPC4, which encodes a bacterial-type PEPC.The mRNA is cell-to-cell mobile.	64.30	20.97	3.07	7.40E-06	9.65E-05
1285	3	F2-3 vs. L2-3	246460_at	AT5G16910	encodes a gene similar to cellulose synthase. Located in Golgi membranes. The mRNA is cell-to-cell mobile.	479.52	156.24	3.07	3.45E-04	1.43E-03
1286	3	F2-3 vs. L2-3	249710_at	AT5G35630	chloroplastic glutamine synthetase The mRNA is cell-to-cell mobile.	1035.03	337.32	3.07	4.17E-05	3.20E-04
1287	3	F2-3 vs. L2-3	247251_at	AT5G64740	Encodes a cellulose synthase isomer. CESA6 mutants have cellulose defect in the primary cell wall. Multiple lines of evidence suggest that CESA6, along with CESA1 and CESA3 are present in the same plasma membrane complex for cellulose biosynthesis. CESA2 and CESA5 are related to CESA6, having partially redundant roles. As inferred from the null role of secondary wall-type CesAs, included in a set of five primary wall-type CesAs that may support trichome cell wall thickening. The mRNA is cell-to-cell mobile.	518.82	169.16	3.07	2.40E-06	4.54E-05
1288	3	F2-3 vs. L2-3	249688_at	AT5G36160	Encodes a cytosolic L-tyrosine aminotransferase. AtTAT2 exhibits much broader amino donor specificity than AtTAT1 and can use not only Tyr but also Phe, Trp, His, Met, Leu, Ala, Ser, Cys, Asp, Asn, Gln, and Arg as amino donors.	61.15	20.00	3.06	3.70E-04	1.50E-03
1289	3	F2-3 vs. L2-3	259277_at	AT3G01180	starch synthase 2;(source:Araport11)	516.77	169.35	3.05	9.86E-05	5.81E-04
1290	3	F2-3 vs. L2-3	252052_at	AT3G52600	cell wall invertase 2;(source:Araport11)	81.51	26.73	3.05	4.75E-05	3.49E-04
1291	3	F2-3 vs. L2-3	252011_at	AT3G52720	Encodes an alpha carbonic anhydrase (CAH1) located in the chloroplast stroma. Most chloroplast proteins are encoded by the nuclear genome and imported with the help of sorting signals that are intrinsic parts of the polypeptides. CAH1 takes an alternative route through the secretory pathway, and becomes N-glycosylated before entering the chloroplast. Glycosylation and intra-molecular disulfide bridge fromation are necessary for the correct folding, ER export, trafficking and activity of the protein.	79.69	26.13	3.05	1.08E-05	1.24E-04
1292	3	F2-3 vs. L2-3	265559_at	AT2G05530	Glycine-rich protein family;(source:Araport11)	154.54	50.81	3.04	4.38E-04	1.70E-03
1293	3	F2-3 vs. L2-3	245159_at	AT2G33100	encodes a gene similar to cellulose synthase	78.84	25.93	3.04	1.52E-05	1.59E-04
1294	3	F2-3 vs. L2-3	251109_at	AT5G01600	Encodes a ferretin protein that is targeted to the chloroplast. Member of a Ferritin gene family. Gene expression is induced in response to iron overload and by nitric oxide. Expression of the gene is downregulated in the presence of paraquat, an inducer of photoxidative stress.	133.47	43.93	3.04	2.60E-04	1.16E-03
1295	3	F2-3 vs. L2-3	263950_at	AT2G36020	HVA22-like protein J;(source:Araport11)	100.90	33.25	3.03	4.90E-06	7.16E-05
1296	3	F2-3 vs. L2-3	252363_at	AT3G48460	GDSL-motif esterase/acyltransferase/lipase. Enzyme group with broad substrate specificity that may catalyze acyltransfer or hydrolase reactions with lipid and non-lipid substrates.	56.00	18.48	3.03	1.66E-05	1.70E-04
1297	3	F2-3 vs. L2-3	256891_at	AT3G19030	transcription initiation factor TFIID subunit 1b-like protein;(source:Araport11)	143.03	47.41	3.02	6.46E-05	4.33E-04
1298	3	F2-3 vs. L2-3	248758_at	AT5G47620	RNA-binding (RRM/RBD/RNP motifs) family protein;(source:Araport11)	415.50	137.77	3.02	5.01E-05	3.63E-04
1299	3	F2-3 vs. L2-3	253891_at	AT4G27720	Major facilitator superfamily protein;(source:Araport11)	368.56	122.28	3.01	8.00E-07	2.05E-05
1300	3	F2-3 vs. L2-3	248382_at	AT5G51890	encodes peroxidase involved in the lignification of tracheary elements (TE) in roots	61.80	20.70	2.99	4.20E-05	3.21E-04
1301	3	F2-3 vs. L2-3	248129_at	AT5G54780	Ypt/Rab-GAP domain of gyp1p superfamily protein;(source:Araport11)	286.45	95.69	2.99	7.74E-05	4.89E-04
1302	3	F2-3 vs. L2-3	264348_at	AT1G12110	Encodes NRT1.1 (CHL1), a dual-affinity nitrate transporter. The protein is expressed in guard cells and function in stomatal opening. Mutants have less transpiration and are more tolerant to drought. Expressed in lateral roots. Involved in nitrate signaling which enables the plant root system to detect and exploit nitrate-rich soil patches. Comparing to the wild type, the mutant displays a strongly decreased lateral root proliferation phenotype in nitrate rich patches on growth medium. Affects flowering time via interaction with the FLC dependent flowering pathway to influence its target gene FT.	102.53	34.40	2.98	7.20E-06	9.51E-05
1303	3	F2-3 vs. L2-3	262988_at	AT1G23310	Identified by cloning the gene that corresponded to a purified protein having glyoxylate aminotransferase activity. Localized to the peroxisome and thought to be involved in photorespiration/ metabolic salvage pathway.	343.21	115.16	2.98	2.50E-06	4.58E-05
1304	3	F2-3 vs. L2-3	248243_at	AT5G53590	SAUR-like auxin-responsive protein family;(source:Araport11)	59.27	19.89	2.98	1.62E-05	1.67E-04
1305	3	F2-3 vs. L2-3	260944_at	AT1G45130	beta-galactosidase 5;(source:Araport11)	249.05	84.26	2.96	1.46E-04	7.65E-04

1306	3	F2-3 vs. L2-3	267612_at	AT2G26690	Major facilitator superfamily protein;(source:Araport11)	58.60	19.78	2.96	2.70E-06	4.78E-05
1307	3	F2-3 vs. L2-3	258614_at	AT3G02770	Ribonuclease E inhibitor RraA/Dimethylmenaquinone methyltransferase;(source:Araport11)	146.33	49.62	2.95	3.64E-05	2.88E-04
1308	3	F2-3 vs. L2-3	251157_at	AT3G63140	Encodes a protein with ribonuclease activity that is involved in plastid rRNA maturation.	106.06	36.00	2.95	1.06E-05	1.22E-04
1309	3	F2-3 vs. L2-3	260985_at	AT1G53500	encodes a putative NDP-L-rhamnose synthase, an enzyme required for the synthesis of the pectin rhamnogalacturonan I, the major component of Arabidopsis mucilage. Gene is involved in seed coat mucilage cell development. Mutant analyses suggest that MUM4 is required for complete mucilage synthesis, cytoplasmic rearrangement and seed coat development.	225.48	76.70	2.94	1.18E-04	6.63E-04
1310	3	F2-3 vs. L2-3	252998_at	AT4G38510	One of three genes encoding the vacuolar ATP synthase subunit B1. The protein binds to and co-localizes with F-actin, bundles F-actin to form higher-order structure, and stabilizes actin filaments in vitro.	213.87	72.71	2.94	1.37E-04	7.31E-04
1311	3	F2-3 vs. L2-3	249327_at	AT5G40890	Encodes a member of the voltage-dependent chloride channel. Also functions as a NO3-/H+ exchanger that serves to accumulate nitrate nutrient in vacuoles. Mutants homozygous for the T-DNA insertion mutation have reduced nitrate uptake capacity in high nitrate environment and exhibit hypersensitivity to chlorate.	105.82	36.02	2.94	1.56E-04	8.04E-04
1312	3	F2-3 vs. L2-3	262473_at	AT1G50250	encodes an FTSH protease that is localized to the chloroplast. Involved in the D1 repair cycle of Photosystem II. FtsH1 and FtsH5 are interchangeable in thylakoid membranes.	357.02	121.98	2.93	3.44E-05	2.75E-04
1313	3	F2-3 vs. L2-3	258750_at	AT3G05910	Pectinacetylesterase family protein;(source:Araport11)	159.41	54.35	2.93	1.84E-04	8.97E-04
1314	3	F2-3 vs. L2-3	249126_at	AT5G43380	encodes a type I serine/threonine protein phosphatase expressed in expressed in roots, rosettes and flowers.	89.23	30.45	2.93	3.21E-04	1.35E-03
1315	3	F2-3 vs. L2-3	260014_at	AT1G68010	Encodes hydroxypyruvate reductase.	400.00	137.17	2.92	6.10E-06	8.52E-05
1316	3	F2-3 vs. L2-3	266790_at	AT2G28950	Encodes an expansin. Naming convention from the Expansin Working Group (Kende et al, 2004. Plant Mol Bio). Involved in the formation of nematode-induced syncytia in roots of Arabidopsis thaliana.	349.33	119.79	2.92	5.54E-05	3.91E-04
1317	3	F2-3 vs. L2-3	266097_at	AT2G37970	SOUL heme-binding family protein;(source:Araport11)	175.23	60.02	2.92	6.36E-05	4.30E-04
1318	3	F2-3 vs. L2-3	267035_at	AT2G38400	alanine:glyoxylate aminotransferase 2 homolog (AGT3) mRNA,	398.92	136.78	2.92	4.42E-04	1.71E-03
1319	3	F2-3 vs. L2-3	249343_at	AT5G40650	One of three isoforms of the iron-sulfur component of the succinate dehydrogenase complex, a component of the mitochondrial respiratory chain complex II. The product of the nuclear encoded gene is imported into the mitochondrion. Expressed during germination and post-germinative growth.	1251.26	428.06	2.92	1.60E-06	3.46E-05
1320	3	F2-3 vs. L2-3	257309_at	AT3G28150	Encodes a member of the TBL (TRICHOME BIREFRINGENCE-LIKE) gene family containing a plant-specific DUF231 (domain of unknown function) domain. A putative xyloglucan O-acetyltransferase. TBL gene family has 46 members, two of which (TBR/AT5G06700 and TBL3/AT5G01360) have been shown to be involved in the synthesis and deposition of secondary wall cellulose, presumably by influencing the esterification state of pectic polymers. A nomenclature for this gene family has been proposed (Volker Bischoff & Wolf Scheible, 2010, personal communication).	36.98	12.70	2.91	1.20E-06	2.80E-05
1321	3	F2-3 vs. L2-3	245993_at	AT5G20700	senescence-associated family protein, putative (DUF581);(source:Araport11)	475.25	163.21	2.91	< 1e-07	4.57E-06
1322	3	F2-3 vs. L2-3	249773_at	AT5G24140	Encodes a protein with similarity to squalene monoxygenases.	35.39	12.15	2.91	1.56E-05	1.62E-04
1323	3	F2-3 vs. L2-3	265419_at	AT2G20840	Secretory carrier membrane protein (SCAMP) family protein;(source:Araport11)	580.36	199.87	2.90	4.41E-05	3.32E-04
1324	3	F2-3 vs. L2-3	267093_at	AT2G38170	Encodes a high affinity vacuolar calcium antiporter. The residue His 338 is critical to Ca2+ transport activity. Disruption of CAX1 reduces manganese and zinc of shoot tissue and results in a decrease in the activity of vacuolar V-type proton ATPase.	143.29	49.47	2.90	1.10E-06	2.61E-05
1325	3	F2-3 vs. L2-3	264264_at	AT1G09250	basic helix-loop-helix (bHLH) DNA-binding superfamily protein;(source:Araport11)	117.36	40.66	2.89	6.70E-06	8.98E-05
1326	3	F2-3 vs. L2-3	260560_at	AT2G43590	Chitinase family protein;(source:Araport11)	38.26	13.24	2.89	8.00E-06	1.03E-04
1327	3	F2-3 vs. L2-3	264916_at	AT1G60810	One of the three genes encoding subunit A of the trimeric enzyme ATP Citrate lyase	2606.23	905.68	2.88	3.06E-04	1.31E-03
1328	3	F2-3 vs. L2-3	266732_at	AT2G03240	EXS (ERD1/XPR1/SYG1) family protein;(source:Araport11)	130.47	45.32	2.88	2.70E-06	4.78E-05
1329	3	F2-3 vs. L2-3	258298_at	AT3G23300	S-adenosyl-L-methionine-dependent methyltransferases superfamily protein;(source:Araport11)	43.70	15.19	2.88	1.06E-04	6.14E-04
1330	3	F2-3 vs. L2-3	255435_at	AT4G03280	Encodes the Rieske FeS center of cytochrome b6f complex. Gene is expressed in shoot but not in root. Mutant has reduced electron transport at saturating light intensities and Q-cycle activity is hypersensitive to acidification of the thylakoid lumen. The mRNA is cell-to-cell mobile.	2971.62	1032.30	2.88	1.64E-05	1.69E-04

1331	3	F2-3 vs. L2-3	255011_at	AT4G10040	Encodes cytochrome c. Promoter directs preferential expression in vascular tissues of cotyledons, leaves, roots, and hypocotyls, and in anthers. Double mutants with CYTC-1 accumulate starch during the day, have delayed growth and development and reduced GA and DELLA proteins linking cellular metabolism and GA homeostasis.	204.87	71.22	2.88	4.72E-05	3.48E-04
1332	3	F2-3 vs. L2-3	250533_at	AT5G08640	Encodes a flavonol synthase that catalyzes formation of flavonols from dihydroflavonols. Co-expressed with CHI and CHS (qRT-PCR).	59.24	20.55	2.88	8.60E-05	5.29E-04
1333	3	F2-3 vs. L2-3	262264_at	AT1G42470	Patched family protein;(source:Araport11)	353.89	123.82	2.86	2.27E-05	2.07E-04
1334	3	F2-3 vs. L2-3	263419_at	AT2G17220	Encodes a putative serine/threonine-specific protein kinase kin3. Protein is N-myristoylated.	89.81	31.45	2.86	1.81E-05	1.80E-04
1335	3	F2-3 vs. L2-3	251711_at	AT3G56960	Encodes a protein with phosphatidylinositol-4-phosphate 5-kinase activity that plays a role in pollen tip growth. The enzyme localizes to the apical plasma membrane and adjacent cytosolic region of pollen tubes. Overexpression of this gene leads to increased deposition of pectin in the cell wall at the tip of the pollen tube and causes altered pollen tube morphology.	44.64	15.60	2.86	2.71E-05	2.33E-04
1336	3	F2-3 vs. L2-3	256015_at	AT1G19150	PSI type II chlorophyll a/b-binding protein (Lhca2*1) mRNA, The mRNA is cell-to-cell mobile.	242.13	84.90	2.85	3.50E-06	5.69E-05
1337	3	F2-3 vs. L2-3	257392_at	AT2G24450	Fasciclin-like arabinogalactan protein. Possibly involved in embryogenesis and seed development.	123.54	43.29	2.85	2.04E-04	9.72E-04
1338	3	F2-3 vs. L2-3	245057_at	AT2G26490	JGB contains seven WD40 repeats and is highly conserved in flowering plants. Overexpression inhibits pollen germination. suggesting JGB is a negative regulator of pollen germination	75.03	26.29	2.85	5.20E-06	7.50E-05
1339	3	F2-3 vs. L2-3	266363_at	AT2G41250	Haloacid dehalogenase-like hydrolase (HAD) superfamily protein;(source:Araport11)	45.33	15.90	2.85	2.60E-06	4.67E-05
1340	3	F2-3 vs. L2-3	250034_at	AT5G18280	Encodes an enzyme with ATPase and ADPase activity (an apyrase) that when mutated in combination with ATAPY1 causes a complete inhibition of pollen germination.	133.99	46.98	2.85	4.00E-07	1.25E-05
1341	3	F2-3 vs. L2-3	259109_at	AT3G05580	Encodes a Type One Protein Phosphatase that acts as a nucleocytoplasmic negative regulator of tip growth. Mutants affect pollen germination, pollen tube growth, and root hair growth. It acts genetically downstream of ANX1 (AT3G04690) and ANX2 (AT5G28680) and is functionally redundant with TOPP8 (AT5G27840).	748.15	263.48	2.84	2.56E-05	2.24E-04
1342	3	F2-3 vs. L2-3	264984_at	AT1G27000	GRIP/coiled-coil protein, putative (DUF1664);(source:Araport11)	278.10	98.27	2.83	1.21E-04	6.71E-04
1343	3	F2-3 vs. L2-3	259839_at	AT1G52190	Encodes a low affinity nitrate transporter that is expressed in the plasma membrane and found in the phloem of the major veins of leaves. It is responsible for nitrate redistribution to young leaves.	889.79	314.19	2.83	1.86E-05	1.83E-04
1344	3	F2-3 vs. L2-3	264956_at	AT1G76990	ACT domain repeat 3;(source:Araport11)	309.99	109.43	2.83	1.94E-04	9.32E-04
1345	3	F2-3 vs. L2-3	245431_at	AT4G17080	Histone H3 K4-specific methyltransferase SET7/9 family protein;(source:Araport11)	77.18	27.39	2.82	5.40E-06	7.72E-05
1346	3	F2-3 vs. L2-3	249090_at	AT5G43745	ion channel POLLUX-like protein, putative (DUF1012);(source:Araport11)	51.97	18.41	2.82	7.27E-04	2.49E-03
1347	3	F2-3 vs. L2-3	248267_at	AT5G53460	NADH-dependent glutamate synthase The mRNA is cell-to-cell mobile.	117.75	41.69	2.82	2.07E-04	9.85E-04
1348	3	F2-3 vs. L2-3	266165_at	AT2G28190	Encodes a chloroplastic copper/zinc superoxide dismutase CSD2 that can detoxify superoxide radicals. Its expression is affected by miR398-directed mRNA cleavage. Activation depends totally on CCS. Overexpression of a miR398-resistant form of CSD2 leads to more dramatic improvements in stress (hight light, Cu2+ and methyl viologen) tolerance than overexpression of wild-type CSD2. The mRNA is cell-to-cell mobile.	1009.84	358.88	2.81	2.72E-04	1.20E-03
1349	3	F2-3 vs. L2-3	267425_at	AT2G34810	FAD-binding Berberine family protein;(source:Araport11)	81.34	28.91	2.81	9.18E-05	5.55E-04
1350	3	F2-3 vs. L2-3	254848_at	AT4G11960	Encodes PGRL1B, a transmembrane protein present in thylakoids. PGRL1B has a highly homologous isoform PGRL1A encoded by At4g22890. Plants lacking PGRL1 show perturbation of cyclic electron flow, similar to PGR5-deficient plants. PGRL1 and PGR5 interact physically and associate with PSI (photosystem I).	91.43	32.52	2.81	3.30E-06	5.45E-05
1351	3	F2-3 vs. L2-3	260421_at	AT1G69640	Encodes one of the two redundant sphingoid base hydroxylases (SBH). Involved in sphingolipid trihydroxy long-chain base (4-hydroxysphinganine) biosynthesis. Double mutants of SBHs were dwarfed and not able to progress from vegetative to reproductive growth.	67.95	24.26	2.80	8.64E-05	5.29E-04
1352	3	F2-3 vs. L2-3	250738_at	AT5G05730	ASA1 encodes the alpha subunit of anthranilate synthase, which catalyzes the rate-limiting step of tryptophan synthesis. ASA1 is induced by ethylene, and forms a link between ethylene signalling and auxin synthesis in roots.	542.49	193.73	2.80	2.97E-04	1.28E-03
1353	3	F2-3 vs. L2-3	251668_at	AT3G57010	Calcium-dependent phosphotriesterase superfamily protein;(source:Araport11)	272.02	97.88	2.78	3.48E-04	1.44E-03

1354	3	F2-3 vs. L2-3	255645_at	AT4G00880	SAUR-like auxin-responsive protein family;(source:Araport11)	47.61	17.15	2.78	1.34E-04	7.21E-04
1355	3	F2-3 vs. L2-3	244995_at	ATCG00150	Encodes a subunit of ATPase complex CF0, which is a proton channel that supplies the proton motive force to drive ATP synthesis by CF1 portion of the complex.	199.50	71.71	2.78	1.10E-05	1.26E-04
1356	3	F2-3 vs. L2-3	254809_at	AT4G12410	SAUR-like auxin-responsive protein family;(source:Araport11)	122.02	43.97	2.77	6.58E-05	4.38E-04
1357	3	F2-3 vs. L2-3	261149_s_a	AT1G19550	Glutathione S-transferase family protein;(source:Araport11)	2243.08	813.15	2.76	4.10E-06	6.26E-05
1358	3	F2-3 vs. L2-3	264301_at	AT1G78780	pathogenesis-related family protein;(source:Araport11)	259.80	94.00	2.76	3.78E-04	1.52E-03
1359	3	F2-3 vs. L2-3	258359_s_a t	AT3G14420	Encodes a glycolate oxidase that modulates reactive oxygen species-mediated signal transduction during nonhost resistance. The mRNA is cell-to-cell mobile.	106.44	38.62	2.76	6.85E-05	4.49E-04
1360	3	F2-3 vs. L2-3	256835_at	AT3G22890	encodes ATP sulfurylase, the first enzyme in the sulfate assimilation pathway of Arabidopsis. It may also participate in selenium metabolism. The mRNA is cell-to-cell mobile.	1303.87	471.99	2.76	2.00E-06	3.93E-05
1361	3	F2-3 vs. L2-3	252531_at	AT3G46520	Member of actin subclass composed of ACT12 and ACT4. RNA is expressed at very low levels in vegetative organs, low levels in flowers and very high levels in pollen. Expression of an ACT12/GUS fusion was found in vascular tissues, tapetum, developing and mature pollen, the root cap and in a ring of pericycle tissues during lateral root initiation and early development.	39.90	14.48	2.76	1.33E-05	1.47E-04
1362	3	F2-3 vs. L2-3	247994_at	AT5G56140	RNA-binding KH domain-containing protein;(source:Araport11)	50.69	18.35	2.76	4.70E-06	6.91E-05
1363	3	F2-3 vs. L2-3	247038_at	AT5G67160	Encodes a member of the BAHD acyltransferase superfamily. Mutants have enhanced susceptibility to virulent and avirulent pathogens and are defective in pathogen induced SA biosynthesis. EPS1 may act upstream of SA biosynthesis as application of SA can rescue the mutant phenotype.	312.21	113.15	2.76	1.89E-05	1.84E-04
1364	3	F2-3 vs. L2-3	267101_at	AT2G41480	Encodes a cationic cell-wall-bound peroxidase homolog that is involved in the lignification of cell walls. Regulated by COG1, involved in seed longevity.	63.03	22.91	2.75	1.78E-05	1.78E-04
1365	3	F2-3 vs. L2-3	256020_at	AT1G58290	Encodes a protein with glutamyl-tRNA reductase (GluTR) activity, catalyzing the NADPH-dependent reduction of Glu-tRNA(Glu) to glutamate 1-semialdehyde (GSA) with the release of free tRNA(Glu). It is involved in the early steps of chlorophyll biosynthesis.	198.58	72.58	2.74	3.00E-06	5.09E-05
1366	3	F2-3 vs. L2-3	265929_s_a	AT2G18570	UDP-Glycosyltransferase superfamily protein;(source:Araport11)	286.91	104.65	2.74	5.07E-05	3.65E-04
1367	3	F2-3 vs. L2-3	266607_at	AT2G46300	Late embryogenesis abundant (LEA) hydroxyproline-rich glycoprotein family;(source:Araport11)	53.18	19.42	2.74	1.96E-04	9.42E-04
1368	3	F2-3 vs. L2-3	247402_at	AT5G62750	hypothetical protein;(source:Araport11)	24.92	9.09	2.74	2.97E-04	1.28E-03
1369	3	F2-3 vs. L2-3	244986_at	ATCG00820	Encodes a 6.8-kDa protein of the small ribosomal subunit.	532.78	194.27	2.74	2.05E-05	1.92E-04
1370	3	F2-3 vs. L2-3	264787_at	AT2G17840	Identified as drought-inducible gene by differential hybridization. Upregulated by high light, drought, cold and salt stress determined by microarray analysis.	533.79	197.13	2.71	2.72E-05	2.33E-04
1371	3	F2-3 vs. L2-3	256433_at	AT3G10985	A senescence-associated gene whose expression is induced in response to treatment with Nep1, a fungal protein that causes necrosis. The mRNA is cell-to-cell mobile.	64.18	23.68	2.71	1.17E-05	1.31E-04
1372	3	F2-3 vs. L2-3	257066_at	AT3G18280	Bifunctional inhibitor/lipid-transfer protein/seed storage 2S albumin superfamily protein;(source:Araport11)	55.80	20.57	2.71	7.27E-04	2.49E-03
1373	3	F2-3 vs. L2-3	258037_at	AT3G21230	The gene encodes a 4-coumarate coenzyme A ligase being able to use sinapate as substrate. The catalytic efficiency was in the following (descending) order: p-coumaric acid, caffeic acid, 5-OH-ferulic acid, ferulic acid and sinapic acid. At4CL5 was unable to use cinnamic acid as substrate. Knockout of At4CL5 (4cl5) revealed no effect on syringyl lignin content indicating that the activity observed does probably not occur in vivo.	83.50	30.83	2.71	8.00E-05	5.02E-04
1374	3	F2-3 vs. L2-3	246431_at	AT5G17480	pollen calcium-binding protein 1;(source:Araport11)	25.68	9.49	2.71	< 1e-07	4.57E-06
1375	3	F2-3 vs. L2-3	248193_at	AT5G54080	Encodes a homogentisate 1,2-dioxygenase that can convert homogentisate to malylacetoacetate and is likely to be involved in tyrosine catabolism.	251.60	92.98	2.71	3.50E-04	1.44E-03
1376	3	F2-3 vs. L2-3	265658_at	AT2G13810	ALD1 is a L-lysine alpha-aminotransferase. It is part of the pipecolic acid biosynthetic pathway, where it catalyzes the biochemical conversion of lysine to epsilon-amino-alpha-ketocaproic acid (KAC) which is subject to subsequent transamination, cyclization and isomerization to form 2,3-dehydropipecolic acid.	43.54	16.13	2.70	2.50E-06	4.58E-05
1377	3	F2-3 vs. L2-3	259598_at	AT1G27980	dihydrosphingosine phosphate lyase;(source:Araport11)	125.68	46.71	2.69	4.50E-06	6.65E-05
1378	3	F2-3 vs. L2-3	258398_at	AT3G15360	encodes a prokaryotic thioredoxin The mRNA is cell-to-cell mobile.	66.20	24.65	2.69	1.81E-04	8.86E-04

1379	3	F2-3 vs. L2-3	261285_at	AT1G35720	Encodes a member of the annexin gene family, a diverse, multigene family of calcium-dependent, membrane-binding proteins. The protein was determined to have peroxidase activity. This activity is thought to be dependent on the presence of post-translational modifications (most likely phosphorylation). The protein was shown to be present as a mixture of monomer and homodimer. The homodimerization seems to be dependent on the presence of Ca2+ or H2O2. The dimerization was prevented by the addition of DTT, β-mercaptoethanol and TCEP. Annat1 mRNA is expressed in flowers, roots,leaves and stems and is most abundant in stems. mRNA levels are increased in response to oxidative stress. Developmental expression patterns suggest a role in Golgi-mediated polysaccharide secretion. It is a Ca 2+-permeable transporter providing a molecular link between reactive oxygen species and cytosolic Ca 2+ in plants. The mRNA is cell-to-cell mobile.	52.55	19.64	2.68	2.34E-04	1.07E-03
1380	3	F2-3 vs. L2-3	245213_at	AT1G44575	Encoding PSII-S (CP22), a ubiquitous pigment-binding protein associated with photosystem II (PSII) of higher plants. Involved in nonphotochemical quenching rather than in photosynthesis. Mutant has a normal violaxanthin cycle but has a limited capacity of quenching singlet excited chlorophylls and is tolerant to lipid peroxidation.	1591.51	594.30	2.68	1.87E-05	1.83E-04
1381	3	F2-3 vs. L2-3	257522_at	AT3G08990	Yippee family putative zinc-binding protein;(source:Araport11)	53.26	19.84	2.68	1.10E-06	2.61E-05
1382	3	F2-3 vs. L2-3	249638_at	AT5G36880	Encodes a plastidic acetyl-coA synthetase. This enzyme plays a role in converting acetate to acetyl-coA in the plastids. It does not appear to be a major contributor to fatty acid biosynthesis based on mutant phenotypes. The enzyme seems to act as a monomer and may play an important role in preventing the toxic accumulation of fermentation products including acetaldehyde, acetate, and ethanol. It participates in the pyruvate dehydrogenase bypass pathway	1063.18	396.39	2.68	4.95E-04	1.86E-03
1383	3	F2-3 vs. L2-3	266070_at	AT2G18660	Encodes PNP-A (Plant Natriuretic Peptide A). PNPs are a class of systemically mobile molecules distantly related to expansins; their biological role has remained elusive. PNP-A contains a signal peptide domain and is secreted into the extracellular space. Co-expression analyses using microarray data suggest that PNP-A may function as a component of plant defence response and SAR in particular, and could be classified as a newly identified PR protein. It is stress responsive and can enhance its own expression.	78.29	29.32	2.67	4.35E-05	3.29E-04
1384	3	F2-3 vs. L2-3	264317_at	AT1G70310	Spermidine synthase.	76.34	28.75	2.66	9.00E-07	2.22E-05
1385	3	F2-3 vs. L2-3	258063_at	AT3G14620	putative cytochrome P450 The mRNA is cell-to-cell mobile.	51.23	19.26	2.66	6.10E-06	8.52E-05
1386	3	F2-3 vs. L2-3	249769_at	AT5G24120	Encodes a specialized sigma factor that functions in regulation of plastid genes and is responsible for the light-dependent transcription at the psbD LRP. Activation of SIG5 is dependent upon blue light and mediated by cryptochromes.	66.25	24.93	2.66	5.50E-05	3.89E-04
1387	3	F2-3 vs. L2-3	248880_at	AT5G46200	carboxyl-terminal proteinase-like protein (DUF239);(source:Araport11)	82.89	31.16	2.66	2.93E-05	2.46E-04
1388	3	F2-3 vs. L2-3	248163_at	AT5G54510	Encodes an IAA-amido synthase that conjugates Ala, Asp, Phe, and Trp to auxin. Lines overexpressing this gene accumulate IAA-ASP and are hypersensitive to several auxins. Identified as a dominant mutation that displays shorter hypocotyls in light grown plants when compared to wild type siblings. Protein is similar to auxin inducible gene from pea (GH3).	110.91	41.68	2.66	9.00E-06	1.11E-04
1389	3	F2-3 vs. L2-3	245024_at	ATCG00120	Encodes the ATPase alpha subunit, which is a subunit of ATP synthase and part of the CF1 portion which catalyzes the conversion of ADP to ATP using the proton motive force. This complex is located in the thylakoid membrane of the chloroplast.	1986.55	747.42	2.66	3.60E-06	5.76E-05
1390	3	F2-3 vs. L2-3	256057_at	AT1G07180	Internal NAD(P)H dehydrogenase in mitochondria. The predicted protein sequence has high homology with other designated NAD(P)H DHs from microorganisms; the capacity for matrix NAD(P)H oxidation via the rotenone-insensitive pathway is significantly reduced in the Atndi1 mutant plant line; the in vitro translation product of AtNDI1 is imported into isolated mitochondria and located on the inside of the inner membrane.	37.32	14.08	2.65	3.80E-06	5.94E-05
1391	3	F2-3 vs. L2-3	265184_at	AT1G23710	hypothetical protein (DUF1645);(source:Araport11)	47.52	17.93	2.65	1.50E-06	3.34E-05
1392	3	F2-3 vs. L2-3	261506_at	AT1G71697	Encodes choline kinase. mRNA levels are increased in response to wounding. The mRNA is cell-to-cell mobile.	59.95	22.84	2.62	1.40E-06	3.16E-05
1393	3	F2-3 vs. L2-3	259096_at	AT3G04840	Ribosomal protein S3Ae;(source:Araport11)	628.55	240.32	2.62	1.09E-04	6.27E-04
1394	3	F2-3 vs. L2-3	260656_at	AT1G19380	sugar, putative (DUF1195);(source:Araport11)	51.32	19.65	2.61	3.72E-04	1.51E-03
1395	3	F2-3 vs. L2-3	262428_at	AT1G47570	RING/U-box superfamily protein;(source:Araport11)	181.54	69.57	2.61	1.79E-04	8.84E-04

1396	3	F2-3 vs. L2-3	259896_at	AT1G71500	Encodes PSB33, a protein conserved in the plastid lineage. PSB33 is associated with the chloroplast thylakoid membrane and provides stability to Photosystem II. The mRNA is cell-to-cell mobile.	70.55	27.07	2.61	1.42E-05	1.53E-04
1397	3	F2-3 vs. L2-3	253428_at	AT4G32410	Encodes a cellulose synthase isomer. CESA1 mutants have cellulose defect in the primary cell wall. Multiple lines of evidence suggest that CESA1, along with CESA3 and CESA6 are present in the same plasma membrane complex for cellulose biosynthesis. lasma membrane complex for cellulose biosynthesis. As inferred from the null role of secondary wall-type CesAs, included in a set of five primary wall-type CesAs that may support trichome cell wall thickening.	269.45	103.19	2.61	1.70E-05	1.73E-04
1398	3	F2-3 vs. L2-3	250204_at	AT5G13990	A member of EXO70 gene family, putative exocyst subunits, conserved in land plants. Arabidopsis thaliana contains 23 putative EXO70 genes, which can be classified into eight clusters on the phylogenetic tree. This particular member is expressed in pollen and is involved in pollen tube elongation. Found in the cytoplasm and surprisingly, not found in the plasma membrane and is not found to colocalize with or interact with core exocyst subunits.	45.04	17.24	2.61	8.83E-05	5.37E-04
1399	3	F2-3 vs. L2-3	248467_at	AT5G50800	Encodes a member of the SWEET sucrose efflux transporter family proteins, together with RPG1, it is involved in pollen development. Together with SWEET14, it is likely involved in modulating the GA response and is required for proper development of anthers, seeds and seedlings.	69.34	26.53	2.61	7.65E-05	4.85E-04
1400	3	F2-3 vs. L2-3	257816_at	AT3G25140	Quasimodo1, encodes a glycosyltransferase, involved in homogalacturonan biosynthesis; mutant shows cell adhesion defect and lower wall uronic acid content. The mRNA is cell-to-cell mobile.	435.53	168.01	2.59	2.60E-06	4.67E-05
1401	3	F2-3 vs. L2-3	252368_at	AT3G48520	CYP94B3 is a jasmonoyl-isoleucine-12-hydroxylase that catalyzes the formation of 12-OH-JA-Ile from JA-Ile. By reducing the levels of this the biologically active phytohormone, CYP94B3 attenuates the jasmonic acid signaling cascade. CYP94B3 transcript levels rise in response to wounding.	90.13	34.86	2.59	2.18E-04	1.02E-03
1402	3	F2-3 vs. L2-3	251309_at	AT3G61220	CytADR/SDR1 is an aldehyde reductase that catalyzes the reduction of the aldehyde carbonyl groups on alpha,beta-unsaturated aldehydes with more than 5 carbons in vitro. It can also act on menthone and neomenthol in vitro, but these do not represent likely endogenous activities of this enzyme in planta. GFP-tagged CytADR appears to localize to the cytosol where it likely plays a role in detoxifying reactive carbonyls. sdr1 mutants have altered responses to pathogens.The mRNA is cell-to-cell mobile.	53.83	20.78	2.59	2.10E-06	4.08E-05
1403	3	F2-3 vs. L2-3	247013_at	AT5G67480	BTB and TAZ domain protein. Located in cytoplasm and expressed in fruit, flower and leaves.	55.48	21.43	2.59	2.91E-05	2.45E-04
1404	3	F2-3 vs. L2-3	252024_at	AT3G52880	Encodes a peroxisomal monodehydroascorbate reductase, involved in the ascorbate-glutathione cycle which removes toxic H2O2	1411.56	546.46	2.58	2.25E-05	2.06E-04
1405	3	F2-3 vs. L2-3	262751_at	AT1G16310	Cation efflux family protein which affects ABA-JA crosstalk and susceptibility to Mamestra brassicae herbivory.	42.66	16.63	2.57	2.47E-05	2.17E-04
1406	3	F2-3 vs. L2-3	266716_at	AT2G46820	Encodes the P subunit of Photosystem I. About 25% of the TMP14 pool appeared to be phosphorylated, and this ratio is not affected by light. Contains seven phosphorylation sites on threonine residue and chloroplast targeting signal. Located in the proximity of PSI-L, -H and -O subunits. Forms oligomers with other members of CURT1 family to modulate grana structure.	444.56	172.97	2.57	6.04E-05	4.15E-04
1407	3	F2-3 vs. L2-3	255690_at	AT4G00360	Encodes a member of the CYP86A subfamily of cytochrome p450 genes. Expressed at moderate levels in flowers, leaves, roots and stems.	39.07	15.17	2.57	1.33E-05	1.47E-04
1408	3	F2-3 vs. L2-3	254559_at	AT4G19200	proline-rich family protein;(source:Araport11)	123.30	47.98	2.57	3.70E-06	5.84E-05
1409	3	F2-3 vs. L2-3	264372_at	AT1G11840	Encodes Ni+ dependent glyoxalase I homolog ATGLX1.	1990.64	778.32	2.56	2.83E-05	2.41E-04
1410	3	F2-3 vs. L2-3	258875_at	AT3G03160	B-cell receptor-associated-like protein;(source:Araport11)	603.96	235.92	2.56	3.13E-05	2.58E-04
1411	3	F2-3 vs. L2-3	249481_at	AT5G38900	Thioredoxin superfamily protein;(source:Araport11)	38.57	15.07	2.56	8.30E-04	2.77E-03
1412	3	F2-3 vs. L2-3	263166_at	AT1G03050	Phosphatidylinositol binding clathrin assembly protein 5A/B are recent paralogs with overlapping functions in recycling ANXUR proteins to the pollen tube membrane.	51.36	20.16	2.55	8.40E-06	1.05E-04
1413	3	F2-3 vs. L2-3	266613_at	AT2G14900	Gibberellin-regulated family protein;(source:Araport11)	902.14	353.55	2.55	4.60E-05	3.42E-04
1414	3	F2-3 vs. L2-3	266592_at	AT2G46210	Fatty acid/sphingolipid desaturase;(source:Araport11)	275.26	108.08	2.55	8.10E-04	2.72E-03
1415	3	F2-3 vs. L2-3	248822_at	AT5G47000	Peroxidase superfamily protein;(source:Araport11)	33.63	13.21	2.55	6.30E-06	8.66E-05
1416	3	F2-3 vs. L2-3	258188_at	AT3G17800	mRNA level of the MEB5.2 gene (At3g17800) remains unchanged after cutting the inflorescence stem	241.60	94.94	2.54	7.00E-07	1.83E-05

1417	3	F2-3 vs. L2-3	246490_at	AT5G15950	Adenosylmethionine decarboxylase family protein;(source:Araport11)	140.19	55.22	2.54	2.24E-04	1.04E-03
1418	3	F2-3 vs. L2-3	247627_at	AT5G60360	Encodes a senescence-associated thiol protease. The mRNA is cell-to-cell mobile.	971.22	382.18	2.54	3.89E-04	1.56E-03
1419	3	F2-3 vs. L2-3	246995_at	AT5G67470	formin homolog 6;(source:Araport11)	36.79	14.50	2.54	1.76E-05	1.77E-04
1420	3	F2-3 vs. L2-3	263734_at	AT1G60030	nucleobase-ascorbate transporter 7;(source:Araport11)	92.49	36.58	2.53	2.00E-05	1.90E-04
1421	3	F2-3 vs. L2-3	252366_at	AT3G48420	Haloacid dehalogenase-like hydrolase (HAD) superfamily protein;(source:Araport11)	341.18	134.67	2.53	1.78E-04	8.82E-04
1422	3	F2-3 vs. L2-3	263108_at	AT1G65240	Eukaryotic aspartyl protease family protein;(source:Araport11)	55.47	22.05	2.52	9.06E-05	5.49E-04
1423	3	F2-3 vs. L2-3	265306_at	AT2G20320	DENN (AEX-3) domain-containing protein;(source:Araport11)	56.34	22.40	2.52	9.58E-05	5.69E-04
1424	3	F2-3 vs. L2-3	256629_at	AT3G19980	Encodes catalytic subunit of serine/threonine protein phosphatase 2A. It can associate with phytochromes A and B in vitro. Mutant plants display an accelerated flowering phenotype.Acts antagonistically to SnRK2 to regulate ABI5 phosphorylation. It inteacts with NRP which results in tethering to endosomes leading to its degradation.	162.81	64.57	2.52	3.11E-04	1.33E-03
1425	3	F2-3 vs. L2-3	258321_at	AT3G22840	Encodes an early light-inducible protein.	586.85	232.93	2.52	3.60E-06	5.76E-05
1426	3	F2-3 vs. L2-3	251840_at	AT3G54960	Encodes a protein disulfide isomerase-like (PDIL) protein, a member of a multigene family within the thioredoxin (TRX) superfamily. Transcript levels for this gene are up-regulated in response to three different chemical inducers of ER stress (dithiothreitol, beta-mercaptoethanol, and tunicamycin). Neither AtIRE1-2 nor AtbZIP60 appear to be required for this response.	643.16	255.06	2.52	8.81E-04	2.91E-03
1427	3	F2-3 vs. L2-3	262885_at	AT1G64740	alpha-tubulin expressed primarily in stamens and mature pollen	426.00	169.85	2.51	1.48E-04	7.71E-04
1428	3	F2-3 vs. L2-3	266979_at	AT2G39470	PsbP-like protein 2;(source:Araport11)	60.86	24.25	2.51	2.94E-05	2.46E-04
1429	3	F2-3 vs. L2-3	251234_s_at	AT2G47650	encodes a protein similar to UDP-glucuronic acid decarboxylase. UDP-glucuronic acid decarboxylase produces UDP-xylose, which is a substrate for many cell wall carbohydrates including hemicellulose and pectin. UDP-xylose is also known to feedback regulate several cell wall biosynthetic enzymes.	1058.50	422.50	2.51	3.99E-04	1.59E-03
1430	3	F2-3 vs. L2-3	252058_at	AT3G52470	Late embryogenesis abundant (LEA) hydroxyproline-rich glycoprotein family;(source:Araport11)	328.25	130.76	2.51	2.03E-04	9.66E-04
1431	3	F2-3 vs. L2-3	252984_at	AT4G37990	Encodes an aromatic alcohol:NADP+ oxidoreductase whose mRNA levels are increased in response to treatment with a variety of phytopathogenic bacteria. Though similar to mannitol dehydrogenases, this enzyme does not have mannitol dehydrogenase activity.	83.70	33.30	2.51	5.80E-05	4.03E-04
1432	3	F2-3 vs. L2-3	251020_at	AT5G02270	member of NAP subfamily	87.33	34.75	2.51	2.00E-07	7.75E-06
1433	3	F2-3 vs. L2-3	262262_at	AT1G70780	hypothetical protein;(source:Araport11)	584.91	233.79	2.50	1.78E-05	1.78E-04
1434	3	F2-3 vs. L2-3	248534_at	AT5G50030	Plant invertase/pectin methylesterase inhibitor superfamily protein;(source:Araport11)	34.98	14.02	2.50	4.53E-04	1.74E-03
1435	3	F2-3 vs. L2-3	254573_at	AT4G19420	Pectinacetylesterase family protein;(source:Araport11)	147.41	59.26	2.49	1.95E-04	9.39E-04
1436	3	F2-3 vs. L2-3	250366_at	AT5G11420	Encodes a DUF642 cell wall protein.	111.42	44.80	2.49	2.60E-06	4.67E-05
1437	3	F2-3 vs. L2-3	262357_at	AT1G73040	Mannose-binding lectin superfamily protein;(source:Araport11)	71.98	29.00	2.48	8.64E-05	5.29E-04
1438	3	F2-3 vs. L2-3	265338_at	AT2G18400	ribosomal protein L6 family protein;(source:Araport11)	1900.34	765.93	2.48	1.20E-06	2.80E-05
1439	3	F2-3 vs. L2-3	267367_at	AT2G44210	carboxyl-terminal peptidase (DUF239);(source:Araport11)	247.64	99.92	2.48	7.22E-04	2.48E-03
1440	3	F2-3 vs. L2-3	258167_at	AT3G21560	Encodes a protein with sinapic acid:UDP-glucose glucosyltransferase activity. Mutants defective in this gene are hyper-fluorescent (which accumulate in their trichomes a compound that is likely to be 3',5'-dimethoxynaringenin chalcone or sinapoyltriacetic acid lactone, potential products of the concerted action of 4-coumarate CoA ligase and chalcone synthase on sinapic acid). Also shown to be required for Arabidopsis nonhost resistance to the Asian soybean rust pathogen Phakopsora pachyrhizi.	188.06	75.70	2.48	3.65E-05	2.88E-04
1441	3	F2-3 vs. L2-3	255590_at	AT4G01610	Encodes a capase involved in stress induced cell death. Activity detected in leaf and cell culture.	2149.37	867.86	2.48	2.85E-04	1.24E-03
1442	3	F2-3 vs. L2-3	250991_at	AT5G02380	cysteine-rich protein with copper-binding activity	8182.97	3303.85	2.48	1.86E-05	1.83E-04
1443	3	F2-3 vs. L2-3	261059_at	AT1G01250	encodes a member of the DREB subfamily A-4 of ERF/AP2 transcription factor family. The protein contains one AP2 domain. There are 17 members in this subfamily including TINY.	50.41	20.44	2.47	8.30E-06	1.05E-04
1444	3	F2-3 vs. L2-3	262326_at	AT1G64140	WRKY transcription factor;(source:Araport11)	174.40	70.68	2.47	1.26E-05	1.40E-04

1445	3	F2-3 vs. L2-3	263319_at	AT2G47160	Boron transporter. Protein accumulates in shoots and roots under conditions of boron deficiency and is degraded within several hours of restoring boron supply. Localized to the plasma membrane under B limitation, and to the cytoplasm after B application before degradation. Protein is transferred via the endosomes to the vacuole for degradation. Localized to the inner plasma membrane domain in the columella, lateral root cap, epidermis, and endodermis in the root tip region, and in the epidermis and endodermis in the elongation zone. Under high-boron is transported to the vacuole for degradation.	70.66	28.62	2.47	3.85E-05	3.01E-04
1446	3	F2-3 vs. L2-3	250967_at	AT5G02790	Glutathione S-transferase family protein;(source:Araport11)	205.66	83.18	2.47	9.30E-06	1.13E-04
1447	3	F2-3 vs. L2-3	261084_at	AT1G07440	NAD(P)-binding Rossmann-fold superfamily protein;(source:Araport11)	103.82	42.26	2.46	5.51E-05	3.89E-04
1448	3	F2-3 vs. L2-3	256209_at	AT1G50940	Encodes the electron transfer flavoprotein ETF alpha, a putative subunit of the mitochondrial electron transfer flavoprotein complex (ETF beta is At5g43430.1) in Arabidopsis. Mutations of the ETF beta gene results in accelerated senescence and early death compared to wild-type during extended darkness.	103.11	41.99	2.46	2.44E-05	2.16E-04
1449	3	F2-3 vs. L2-3	255587_at	AT4G01480	Encodes a protein that might have inorganic pyrophosphatase activity.	58.00	23.59	2.46	3.20E-05	2.61E-04
1450	3	F2-3 vs. L2-3	254158_at	AT4G24380	dihydrofolate reductase;(source:Araport11)	135.01	54.85	2.46	3.60E-06	5.76E-05
1451	3	F2-3 vs. L2-3	254077_at	AT4G25640	Encodes a multidrug and toxin efflux family transporter. Involved in flavonoid metabolism, affecting Root growth, seed development and germination, and pollen development, release and viability.	228.69	92.79	2.46	2.90E-05	2.44E-04
1452	3	F2-3 vs. L2-3	246947_at	AT5G25120	putative cytochrome P450 The mRNA is cell-to-cell mobile.	59.77	24.26	2.46	4.34E-05	3.28E-04
1453	3	F2-3 vs. L2-3	247593_at	AT5G60790	member of GCN subfamily	404.49	164.65	2.46	4.40E-06	6.55E-05
1454	3	F2-3 vs. L2-3	261105_at	AT1G63000	nucleotide-rhamnose synthase/epimerase-reductase;(source:Araport11)	161.50	65.90	2.45	1.65E-05	1.69E-04
1455	3	F2-3 vs. L2-3	258748_at	AT3G05930	germin-like protein (GLP8)	58.59	23.93	2.45	3.01E-05	2.52E-04
1456	3	F2-3 vs. L2-3	249771_at	AT5G24080	Protein kinase superfamily protein;(source:Araport11)	37.09	15.13	2.45	1.95E-05	1.88E-04
1457	3	F2-3 vs. L2-3	246802_at	AT5G27000	Encodes a kinesin-like protein that binds microtubules in an ATP-dependent manner.	42.61	17.41	2.45	2.63E-05	2.28E-04
1458	3	F2-3 vs. L2-3	248531_at	AT5G49950	alpha/beta-Hydrolases superfamily protein;(source:Araport11)	376.53	153.58	2.45	2.13E-04	1.00E-03
1459	3	F2-3 vs. L2-3	259460_at	AT1G44000	STAY-GREEN-like protein;(source:Araport11)	151.05	62.00	2.44	3.11E-04	1.33E-03
1460	3	F2-3 vs. L2-3	265741_at	AT2G01320	ABC-2 type transporter family protein;(source:Araport11)	57.14	23.43	2.44	4.75E-05	3.49E-04
1461	3	F2-3 vs. L2-3	251218_at	AT3G62410	CP12-2 encodes a small peptide found in the chloroplast stroma. It belongs to the CP12 gene family thought to be involved in the formation of a supramolecular complex with glyceraldehyde-3-phosphate dehydrogenase (GAPDH) and phosphoribulokinase (PRK) embedded in the Calvin cycle. CP12-2 is coordinately regulated by light with the photosynthetic GAPDH and PRK. The annotation of this gene is based on article 32494. The mRNA is cell-to-cell mobile.	101.48	41.62	2.44	1.08E-04	6.22E-04
1462	3	F2-3 vs. L2-3	253343_at	AT4G33540	metallo-beta-lactamase family protein;(source:Araport11)	69.63	28.52	2.44	1.93E-05	1.87E-04
1463	3	F2-3 vs. L2-3	265400_at	AT2G10940	Bifunctional inhibitor/lipid-transfer protein/seed storage 2S albumin superfamily protein;(source:Araport11)	85.14	35.08	2.43	4.20E-05	3.21E-04
1464	3	F2-3 vs. L2-3	267517_at	AT2G30520	light inducible root phototropism 2 encoding a signal transducer of the phototropic response in Arabidopsis	34.39	14.18	2.43	1.62E-04	8.26E-04
1465	3	F2-3 vs. L2-3	254491_at	AT4G20300	Serine/Threonine-kinase, putative (DUF1639);(source:Araport11)	235.89	96.90	2.43	1.00E-05	1.18E-04
1466	3	F2-3 vs. L2-3	253846_at	AT4G28000	P-loop containing nucleoside triphosphate hydrolases superfamily protein;(source:Araport11)	38.55	15.90	2.43	1.18E-04	6.62E-04
1467	3	F2-3 vs. L2-3	253551_at	AT4G30996	NKS1, a plant-specific gene, encodes a 19 kDa endomembrane-localized protein. It is part of SOS independent ion homeostasis regulation pathway.	675.67	278.11	2.43	4.50E-06	6.65E-05
1468	3	F2-3 vs. L2-3	249928_at	AT5G22250	Encodes one of the homologs of the yeast CCR4-associated factor 1: AT3G44260 (CAF1a), AT5G22250 (CAF1b). Has mRNA deadenylation activity. Also plays a role in plant defense responses.	93.92	38.63	2.43	2.90E-06	5.02E-05
1469	3	F2-3 vs. L2-3	259054_at	AT3G03480	acetyl CoA:(Z)-3-hexen-1-ol acetyltransferase;(source:Araport11)	44.10	18.25	2.42	6.90E-06	9.18E-05
1470	3	F2-3 vs. L2-3	258399_at	AT3G15540	Primary auxin-responsive gene. Involved in the regulation stamen filaments development.	323.07	133.73	2.42	1.37E-05	1.50E-04
1471	3	F2-3 vs. L2-3	251928_at	AT3G53980	Bifunctional inhibitor/lipid-transfer protein/seed storage 2S albumin superfamily protein;(source:Araport11)	104.89	43.32	2.42	1.78E-05	1.78E-04
1472	3	F2-3 vs. L2-3	245501_at	AT4G15620	Uncharacterized protein family (UPF0497);(source:Araport11)	69.04	28.48	2.42	1.40E-06	3.16E-05
1473	3	F2-3 vs. L2-3	264528_at	AT1G30810	JMJ18 encodes a novel JmjC domain- containing histone H3K4 demethylase.	50.80	21.05	2.41	2.67E-05	2.30E-04
1474	3	F2-3 vs. L2-3	260297_at	AT1G80280	alpha/beta-Hydrolases superfamily protein;(source:Araport11)	71.65	29.77	2.41	1.72E-04	8.59E-04
1475	3	F2-3 vs. L2-3	266765_at	AT2G46860	Encodes a protein that might have inorganic pyrophosphatase activity.	54.10	22.48	2.41	1.07E-04	6.21E-04

1476	3	F2-3 vs. L2-3	259003_at	AT3G02010	Pentatricopeptide repeat (PPR) superfamily protein;(source:Araport11)	271.07	112.60	2.41	4.30E-05	3.26E-04
1477	3	F2-3 vs. L2-3	258729_at	AT3G11900	encodes an amino acid transporter that transports aromatic and neutral amino acids, IAA, and 2,4-D. Expressed in all tissues with highest abundance in flowers and cauline leaves. a member of a small gene family in Arabidopsis and represents a new class of amino acid transporters.	498.45	206.99	2.41	1.50E-04	7.83E-04
1478	3	F2-3 vs. L2-3	253525_at	AT4G31330	transmembrane protein, putative (Protein of unknown function, DUF599);(source:Araport11)	39.27	16.29	2.41	8.90E-06	1.10E-04
1479	3	F2-3 vs. L2-3	261135_at	AT1G19610	Predicted to encode a PR (pathogenesis-related) protein. Belongs to the plant defensin (PDF) family with the following members: At1g75830/PDF1.1, At5g44420/PDF1.2a, At2g26020/PDF1.2b, At5g44430/PDF1.2c, At2g26010/PDF1.3, At1g19610/PDF1.4, At1g55010/PDF1.5, At2g02120/PDF2.1, At2g02100/PDF2.2, At2g02130/PDF2.3, At1g61070/PDF2.4, At5g63660/PDF2.5, At2g02140/PDF2.6, At5g38330/PDF3.1 and At4g30070/PDF3.2.	47.38	19.78	2.40	1.48E-05	1.57E-04
1480	3	F2-3 vs. L2-3	258416_at	AT3G17310	Encodes DRM3 (Domains Rearranged Methyltransferase3), a catalytically mutated paralog of the cytosine methyltransferase DRM2. Despite being catalytically mutated, DRM3 is required for normal maintenance of non-CG DNA methylation, establishment of RNA-directed DNA methylation triggered by repeat sequences and accumulation of repeat-associated small RNAs.	66.10	27.56	2.40	6.80E-05	4.47E-04
1481	3	F2-3 vs. L2-3	257021_at	AT3G19710	Belongs to the branched-chain amino acid aminotransferase gene family. Encodes a methionine-oxo-acid transaminase. Involved in the methionine chain elongation pathway that leads to the ultimate biosynthesis of methionine-derived glucosinolates.	35.05	14.59	2.40	6.38E-05	4.30E-04
1482	3	F2-3 vs. L2-3	251762_at	AT3G55800	Encodes the chloroplast enzyme sedoheptulose-1,7-bisphosphatase (SBPase), involved in the carbon reduction of the Calvin cycle. Increase in SBPase activity in transgenic lines accumulate up to 50% more sucrose and starch than wild-type. The mRNA is cell-to-cell mobile.	817.23	340.02	2.40	2.05E-05	1.92E-04
1483	3	F2-3 vs. L2-3	253137_at	AT4G35500	Protein kinase superfamily protein;(source:Araport11)	148.65	61.88	2.40	4.00E-07	1.25E-05
1484	3	F2-3 vs. L2-3	248746_at	AT5G47890	NADH-ubiquinone oxidoreductase B8 subunit;(source:Araport11)	579.63	242.57	2.39	5.63E-05	3.95E-04
1485	3	F2-3 vs. L2-3	260730_at	AT1G48030	Encodes a mitochondrial lipoamide dehydrogenase whose expression is induced by light.	386.51	162.39	2.38	9.22E-05	5.56E-04
1486	3	F2-3 vs. L2-3	245724_at	AT1G73390	Endosomal targeting BRO1-like domain-containing protein;(source:Araport11)	69.52	29.25	2.38	3.06E-05	2.54E-04
1487	3	F2-3 vs. L2-3	252617_at	AT3G45100	encodes Arabidopsis homolog of a conserved protein involved in the first step of the GPI biosynthetic pathway.	342.92	144.25	2.38	8.38E-05	5.21E-04
1488	3	F2-3 vs. L2-3	253963_at	AT4G26470	Calcium-binding EF-hand family protein;(source:Araport11)	45.13	18.96	2.38	1.68E-05	1.72E-04
1489	3	F2-3 vs. L2-3	246429_at	AT5G17450	Heavy metal transport/detoxification superfamily protein;(source:Araport11)	34.99	14.72	2.38	1.05E-05	1.22E-04
1490	3	F2-3 vs. L2-3	264365_s_a	AT1G03230	Eukaryotic aspartyl protease family protein;(source:Araport11)	195.72	82.62	2.37	4.00E-07	1.25E-05
1491	3	F2-3 vs. L2-3	267157_at	AT2G37630	Encodes a MYB-domain protein involved in specification of the leaf proximodistal axis. Mutation results in lobed and dissected leaves with a characteristic asymmetry. Homologous to the Antirrhinum PHANTASTICA (PHAN) and maize ROUGH SHEATH2 (RS2) genes Asymmetric placement of auxin response at the distal leaf tip precedes visible asymmetric leaf growth. Acts alongside AXR1 to exclude BP expression in leaves and with PIN1 to repress BP and promote lateral organ growth. Interacts physically with AS2 to form a complex that binds to the BP promoter and silences BP. Also functions as a regulator of the plant immune response.	221.32	93.51	2.37	3.70E-06	5.84E-05
1492	3	F2-3 vs. L2-3	247156_at	AT5G65760	Serine carboxypeptidase S28 family protein;(source:Araport11)	247.13	104.38	2.37	2.14E-05	1.97E-04
1493	3	F2-3 vs. L2-3	261033_at	AT1G17380	jasmonate-zim-domain protein 5;(source:Araport11)	243.88	103.23	2.36	1.43E-05	1.53E-04
1494	3	F2-3 vs. L2-3	258677_at	AT3G08730	Encodes a protein-serine kinase that phosphorylates ribosomal protein in vitro. Activation of AtS6k is regulated by 1-naphthylacetic acid and kinetin, at least in part, via a lipid kinase-dependent pathway. Involved in translational up-regulation of ribosomal proteins. Phosphorylated by PDK1. Interacts with RAPTOR1, which in turn interacts with TOR. SPK6 activity is affected by osmotic stress, and plants overexpressing S6k1 are hypersensitive to osmotic stress. The gene is expressed in all tissues examined, with highest expression level detected in metabolically active tissues.	30.73	13.01	2.36	9.34E-05	5.59E-04

1495	3	F2-3 vs. L2-3	245286_at	AT4G14880	Encodes a cytosolic isoform of cytosolic O-acetylserine(thiol)lyase, a key enzyme in cysteine biosynthesis and for the fixation of inorganic sulfide. It catalyzes the formation of cysteine from O-acetylserine and inorganic sulfide. Gene expression is predominant in the root cortex and the xylem parenchyma. Gene expression is induced in leave, stems and roots by high salt and heavy metal stresses, mediated by ABA. Lines carrying semi-dominant mutations exhibit early senescence. Required for pollen tube growth and/or fertilization.	842.26	357.33	2.36	1.58E-04	8.13E-04
1496	3	F2-3 vs. L2-3	262179_at	AT1G77980	Encodes a member of the MIKC (MADS box, Keratin binding domain, and C terminal domain containing)family of transcriptional regulators. AGL66 is expressed in pollen.It forms heterodimers with other MICK family members (AGL104). Involved in late stages of pollen development and pollen tube growth.	53.90	22.92	2.35	2.13E-04	1.00E-03
1497	3	F2-3 vs. L2-3	266181_at	AT2G02390	Encodes glutathione transferase belonging to the zeta class of GSTs. Naming convention according to Wagner et al. (2002). The protein undergoes spontaneous thiolation following treatment with the oxidant tert-butylhydroperoxide. It functions in vitro as a maleylacetoacetate isomerase and is likely to be involved in tyrosine catabolism.	129.67	55.14	2.35	1.14E-04	6.47E-04
1498	3	F2-3 vs. L2-3	257253_at	AT3G24190	Protein kinase superfamily protein;(source:Araport11)	100.96	43.00	2.35	1.13E-05	1.28E-04
1499	3	F2-3 vs. L2-3	251854_at	AT3G54800	Pleckstrin homology (PH) and lipid-binding START domains-containing protein;(source:Araport11)	66.92	28.48	2.35	9.22E-05	5.56E-04
1500	3	F2-3 vs. L2-3	267187_s_a	AT3G59970	methylenetetrahydrofolate reductase MTHFR1 mRNA, complete	635.88	270.04	2.35	4.55E-05	3.40E-04
1501	3	F2-3 vs. L2-3	245619_at	AT4G13990	Exostosin family protein;(source:Araport11)	56.99	24.23	2.35	4.25E-05	3.24E-04
1502	3	F2-3 vs. L2-3	253730_at	AT4G29480	Mitochondrial ATP synthase subunit G protein;(source:Araport11)	97.57	41.54	2.35	8.30E-06	1.05E-04
1503	3	F2-3 vs. L2-3	252880_at	AT4G39730	PLAT1 domain stress protein family member. Involved in mediating response to stresses such as pathogen infection. It is found in endoplasmic reticulum bodies. PLAT1 is induced by pathogenic fungi and induces the production of scopolin.	1176.17	499.88	2.35	3.23E-04	1.36E-03
1504	3	F2-3 vs. L2-3	245683_at	AT5G22030	ubiquitin-specific protease 8;(source:Araport11)	97.79	41.68	2.35	3.15E-04	1.33E-03
1505	3	F2-3 vs. L2-3	266000_at	AT2G24180	Encodes a cytochrome P450 monooxygenase that converts indole-3-acetonitrile to indole-3-aldehyde / indole-3-carboxylic acid and cyanide. The mRNA is cell-to-cell mobile.	52.59	22.52	2.34	1.67E-04	8.43E-04
1506	3	F2-3 vs. L2-3	263249_at	AT2G31360	Encodes a protein homologous to delta 9 acyl-lipid desaturases of cyanobacteria and acyl-CoA desaturases of yeast and mammals. expression up-regulated by cold temperature. It is involved in the synthesis of the 24:1n-9 and 26:1n-9 components of seed lipids, sphingolipids and the membrane phospholipids phosphatidylserine (PS), and phosphatidylethanolamine (PE).	326.42	139.74	2.34	8.20E-06	1.05E-04
1507	3	F2-3 vs. L2-3	252009_at	AT3G52800	A20/AN1-like zinc finger family protein;(source:Araport11)	255.63	109.30	2.34	4.72E-04	1.79E-03
1508	3	F2-3 vs. L2-3	264558_at	AT1G09600	Protein kinase superfamily protein;(source:Araport11)	63.47	27.29	2.33	2.10E-06	4.08E-05
1509	3	F2-3 vs. L2-3	262803_at	AT1G21000	PLATZ transcription factor family protein;(source:Araport11)	56.89	24.40	2.33	4.53E-04	1.74E-03
1510	3	F2-3 vs. L2-3	255345_at	AT4G04460	Saposin-like aspartyl protease family protein;(source:Araport11)	50.52	21.68	2.33	9.17E-04	3.00E-03
1511	3	F2-3 vs. L2-3	262634_at	AT1G06690	NAD(P)-linked oxidoreductase superfamily protein;(source:Araport11)	117.26	50.57	2.32	1.60E-05	1.66E-04
1512	3	F2-3 vs. L2-3	251524_at	AT3G58990	isopropylmalate isomerase 1;(source:Araport11)	51.74	22.33	2.32	2.46E-04	1.11E-03
1513	3	F2-3 vs. L2-3	255259_at	AT4G05020	Miitochondrial alternative NADH dehydrogenase.	148.91	64.49	2.31	5.50E-06	7.84E-05
1514	3	F2-3 vs. L2-3	267005_at	AT2G34460	NAD(P)-binding Rossmann-fold superfamily protein;(source:Araport11)	171.64	74.78	2.30	5.90E-05	4.08E-04
1515	3	F2-3 vs. L2-3	251738_at	AT3G56150	member of eIF3c - eukaryotic initiation factor 3c	64.44	28.10	2.29	8.76E-05	5.35E-04
1516	3	F2-3 vs. L2-3	249469_at	AT5G39320	UDP-glucose 6-dehydrogenase family protein;(source:Araport11)	348.57	152.15	2.29	4.46E-04	1.72E-03
1517	3	F2-3 vs. L2-3	260007_at	AT1G67870	glycine-rich protein;(source:Araport11)	41.96	18.39	2.28	5.72E-05	4.00E-04
1518	3	F2-3 vs. L2-3	252107_at	AT3G51490	tonoplast monosaccharide transporter3;(source:Araport11)	58.30	25.54	2.28	1.99E-05	1.89E-04
1519	3	F2-3 vs. L2-3	251277_at	AT3G61760	DYNAMIN-like 1B;(source:Araport11)	33.96	14.91	2.28	4.20E-06	6.33E-05
1520	3	F2-3 vs. L2-3	253736_at	AT4G28780	GDSL-motif esterase/acyltransferase/lipase. Enzyme group with broad substrate specificity that may catalyze acyltransfer or hydrolase reactions with lipid and non-lipid substrates.	52.56	23.10	2.28	2.09E-04	9.88E-04
1521	3	F2-3 vs. L2-3	249472_at	AT5G39210	Encodes a protein of the chloroplastic NAD(P)H dehydrogenase complex (NDH Complex) involved in respiration, photosystem I (PSI) cyclic electron transport and CO2 uptake. The product of this gene appears to be essential for the stable formation of the NDH Complex. The mRNA is cell-to-cell mobile.	25.16	11.03	2.28	5.10E-04	1.91E-03
1522	3	F2-3 vs. L2-3	249284_at	AT5G41810	Avr9/Cf-9 rapidly elicited protein;(source:Araport11)	58.01	25.46	2.28	2.75E-05	2.35E-04
1523	3	F2-3 vs. L2-3	265194_at	AT1G05010	Encodes 1-aminocyclopropane-1-carboxylate oxidase	70.78	31.11	2.27	6.29E-05	4.26E-04
1524	3	F2-3 vs. L2-3	267486_at	AT2G02800	Encodes protein kinase APK2b.	263.11	115.99	2.27	8.11E-05	5.07E-04

1525	3	F2-3 vs. L2-3	258957_at	AT3G01420	Encodes an alpha-dioxygenase involved in protection against oxidative stress and cell death. Induced in response to Salicylic acid and oxidative stress. Independent of NPR1 in induction by salicylic acid. The mRNA is cell-to-cell mobile.	36.53	16.06	2.27	2.34E-04	1.07E-03
1526	3	F2-3 vs. L2-3	259348_at	AT3G03770	Leucine-rich repeat protein kinase family protein;(source:Araport11)	413.63	182.59	2.27	1.80E-06	3.80E-05
1527	3	F2-3 vs. L2-3	258689_at	AT3G07940	Calcium-dependent ARF-type GTPase activating protein family;(source:Araport11)	252.61	111.43	2.27	5.38E-04	1.99E-03
1528	3	F2-3 vs. L2-3	258392_at	AT3G15400	Encodes a protein with novel repeat sequences and a glycine-rich domain which has a 53% identity to GRP1, a petunia glycine-rich cell wall protein.	53.07	23.34	2.27	1.19E-05	1.33E-04
1529	3	F2-3 vs. L2-3	263410_at	AT2G04039	NdhV is loosely associated with the NDH complex and is required for stabilizing NDH subcomplexes A and E.	34.76	15.35	2.26	6.12E-05	4.19E-04
1530	3	F2-3 vs. L2-3	265174_s_a	AT1G23460	Pectin lyase-like superfamily protein;(source:Araport11)	33.02	14.68	2.25	2.59E-04	1.16E-03
1531	3	F2-3 vs. L2-3	265203_at	AT2G36630	Sulfite exporter TauE/SafE family protein;(source:Araport11)	102.54	45.59	2.25	2.01E-04	9.60E-04
1532	3	F2-3 vs. L2-3	255479_at	AT4G02380	Encodes AtLEA5 (late embryogenesis abundant like protein). Also known as SENESCENCE-ASSOCIATED GENE 21 (SAG21). Has a role on oxidative stress tolerance. mRNA levels are elevated in response to various stresses.	106.65	47.34	2.25	8.82E-04	2.91E-03
1533	3	F2-3 vs. L2-3	245318_at	AT4G16980	arabinogalactan-protein family;(source:Araport11)	30.29	13.44	2.25	8.86E-05	5.38E-04
1534	3	F2-3 vs. L2-3	261040_at	AT1G17370	Encodes an RNA?binding protein involved in stress granule formation. Regulated by a transposable element small RNA.	343.46	153.34	2.24	7.82E-04	2.64E-03
1535	3	F2-3 vs. L2-3	261751_at	AT1G76080	Encodes a thioredoxin localized in chloroplast stroma. Known as CDSP32 (CHLOROPLASTIC DROUGHT-INDUCED STRESS PROTEIN OF 32 KD).	136.79	61.15	2.24	8.42E-04	2.81E-03
1536	3	F2-3 vs. L2-3	257954_at	AT3G21760	Encodes HYR1, a UDP glycosyltransferase (UGT). HYR1 glucosylates hypostatin, an inhibitor of cell expansion in vivo to form a bioactive glucoside.	97.36	43.55	2.24	2.31E-05	2.08E-04
1537	3	F2-3 vs. L2-3	248146_at	AT5G54940	Translation initiation factor SUI1 family protein;(source:Araport11)	65.39	29.18	2.24	1.19E-04	6.65E-04
1538	3	F2-3 vs. L2-3	260308_at	AT1G70610	member of TAP subfamily	220.37	98.73	2.23	2.57E-05	2.25E-04
1539	3	F2-3 vs. L2-3	266636_at	AT2G35370	Encodes glycine decarboxylase complex H protein. Involved in photorespiration. The mRNA is cell-to-cell mobile.	43.96	19.71	2.23	4.80E-06	7.03E-05
1540	3	F2-3 vs. L2-3	256673_at	AT3G52370	Fasciclin-like arabinogalactan protein. Possibly involved in embryogenesis and seed development.	146.21	65.57	2.23	1.70E-04	8.51E-04
1541	3	F2-3 vs. L2-3	248967_at	AT5G45350	proline-rich family protein;(source:Araport11)	651.96	291.93	2.23	3.55E-05	2.81E-04
1542	3	F2-3 vs. L2-3	247222_at	AT5G64840	member of GCN subfamily	589.49	264.10	2.23	6.41E-04	2.27E-03
1543	3	F2-3 vs. L2-3	259983_at	AT1G76490	Encodes a 3-hydroxy-3-methylglutaryl coenzyme A reductase, which is involved in melavonate biosynthesis and performs the first committed step in isoprenoid biosynthesis. Expression is activated in dark in leaf tissue but not controlled by light in the root (confine The mRNA is cell-to-cell mobile.	67.70	30.54	2.22	9.22E-04	3.01E-03
1544	3	F2-3 vs. L2-3	245247_at	AT4G17230	Encodes a scarecrow-like protein (SCL13). Member of GRAS gene family. Regulated by heat shock.	146.91	66.05	2.22	2.07E-04	9.84E-04
1545	3	F2-3 vs. L2-3	253203_at	AT4G34710	Encodes a arginine decarboxylase (ADC), a rate-limiting enzyme that catalyzes the first step of polyamine (PA) biosynthesis via ADC pathway in Arabidopsis thaliana. Arabidopsis genome has two ADC paralogs, ADC1 and ADC2. ADC2 is stress-inducible (osmotic stress). Double mutant analysis showed that ADC genes are essential for the production of PA, and are required for normal seed development. Overexpression causes phenotypes similar to GA-deficient plants and these plants show reduced levels of GA due to lower expression levels of AtGA20ox1, AtGA3ox3 and AtGA3ox1.	548.60	247.54	2.22	6.99E-05	4.55E-04
1546	3	F2-3 vs. L2-3	250254_at	AT5G13710	SMT1 controls the level of cholesterol in plants	1223.04	551.22	2.22	4.76E-04	1.80E-03
1547	3	F2-3 vs. L2-3	245088_at	AT2G39850	Subtilisin-like serine endopeptidase family protein;(source:Araport11)	27.08	12.26	2.21	4.26E-05	3.24E-04
1548	3	F2-3 vs. L2-3	258512_at	AT3G06510	Encodes a protein with beta-glucosidase and galactosyltransferase activity, mutants show increased sensitivity to freezing. Though it is classified as a family I glycosyl hydrolase, it has no hydrolase activity in vitro.	799.26	361.49	2.21	7.20E-04	2.48E-03
1549	3	F2-3 vs. L2-3	256237_at	AT3G12610	Plays role in DNA-damage repair/toleration. Partially complements RecA- phenotypes.	165.67	74.94	2.21	9.20E-06	1.13E-04
1550	3	F2-3 vs. L2-3	253420_at	AT4G32260	ATPase, F0 complex, subunit B/B, bacterial/chloroplast;(source:Araport11)	655.28	296.63	2.21	3.20E-05	2.61E-04
1551	3	F2-3 vs. L2-3	247989_at	AT5G56350	Pyruvate kinase family protein;(source:Araport11)	255.57	115.88	2.21	2.52E-04	1.13E-03
1552	3	F2-3 vs. L2-3	266867_at	AT2G45770	chloroplast SRP receptor homolog, alpha subunit CPFTSY. Required for LHCP integration into isolated thylakoids.	77.36	35.10	2.20	9.26E-05	5.57E-04

1553	3	F2-3 vs. L2-3	250207_at	AT5G13930	Encodes chalcone synthase (CHS), a key enzyme involved in the biosynthesis of flavonoids. Required for the accumulation of purple anthocyanins in leaves and stems. Also involved in the regulation of auxin transport and the modulation of root gravitropism. The mRNA is cell-to-cell mobile.	69.31	31.52	2.20	7.53E-04	2.56E-03
1554	3	F2-3 vs. L2-3	246620_at	AT5G36220	This gene is a member of the CYP81D family of cytochrome p450s. It was originally called CYP91A1, but was later renamed to CYP81D1.	26.60	12.07	2.20	3.03E-05	2.53E-04
1555	3	F2-3 vs. L2-3	255702_at	AT4G00230	xylem serine peptidase 1;(source:Araport11)	106.06	48.52	2.19	2.65E-04	1.18E-03
1556	3	F2-3 vs. L2-3	253101_at	AT4G37430	Encodes a member of the CYP81F cytochrome P450 monooxygenase subfamily.	84.38	38.48	2.19	2.45E-05	2.17E-04
1557	3	F2-3 vs. L2-3	249927_at	AT5G19220	Encodes the large subunit of ADP-glucose pyrophosphorylase which catalyzes the first, rate limiting step in starch biosynthesis. The large subunit plays a regulatory role whereas the small subunit (ApS) is the catalytic isoform. Four isoforms (ApL1-4) have been identified. ApL1 is the major large subunit isoform present in leaves. Mutational analysis of APS1 suggests that APL1 and APL2 can compensate for loss of APS1 catalytic activity,suggesting both have catalytic as well as regulatory functions.	27.47	12.57	2.19	1.19E-04	6.64E-04
1558	3	F2-3 vs. L2-3	247706_at	AT5G59480	Haloacid dehalogenase-like hydrolase (HAD) superfamily protein;(source:Araport11)	112.77	51.43	2.19	3.19E-05	2.61E-04
1559	3	F2-3 vs. L2-3	262850_at	AT1G14920	Similar to a putative transcription factor and transcriptional coactivators. Repressor of GA responses and involved in gibberellic acid mediated signaling. Member of the DELLA proteins that restrain the cell proliferation and expansion that drives plant growth. The protein undergoes degradation in response to GA via the 26S proteasome. GAI may be involved in reducing ROS accumulation in response to stress by up-regulating the transcription of superoxide dismutases. Represses GA-induced vegetative growth and floral initiation. Rapidly degraded in response to GA.	694.70	318.43	2.18	3.58E-05	2.83E-04
1560	3	F2-3 vs. L2-3	264007_at	AT2G21140	Proline-rich protein expressed in expanding leaves, stems, flowers, and siliques.	104.29	47.76	2.18	1.80E-04	8.86E-04
1561	3	F2-3 vs. L2-3	252192_at	AT3G50000	Encodes the casein kinase II (CK2) catalytic subunit (alpha).	1100.57	506.68	2.17	3.92E-04	1.57E-03
1562	3	F2-3 vs. L2-3	245333_at	AT4G14615	sporulation-specific protein;(source:Araport11)	956.04	439.93	2.17	4.14E-04	1.63E-03
1563	3	F2-3 vs. L2-3	253555_at	AT4G31080	Encodes one of two LUNAPARK proteins in Arabidopsis. Both LNPA and LNPB are predominantly distributed throughout the ER, but not preferentially localized at the three-way junctions. Mutation of both LNPA and LNPB together caused the cortical ER to develop poor ER cisternae and a less dense tubular network.	99.45	45.77	2.17	3.94E-04	1.57E-03
1564	3	F2-3 vs. L2-3	245986_at	AT5G13160	Mutant is defective in perception of Pseudomonas syringae avirulence gene avrPphB. Encodes a putative serine-threonine kinase.	109.18	50.40	2.17	9.19E-04	3.00E-03
1565	3	F2-3 vs. L2-3	260227_at	AT1G74450	Plants overexpressing At1g74450 are stunted in height and have reduced male fertility.	165.87	76.95	2.16	7.13E-04	2.46E-03
1566	3	F2-3 vs. L2-3	267019_at	AT2G39130	Transmembrane amino acid transporter family protein;(source:Araport11)	25.75	11.94	2.16	2.88E-04	1.25E-03
1567	3	F2-3 vs. L2-3	254120_at	AT4G24570	Encodes one of the mitochondrial dicarboxylate carriers (DIC): DIC1 (AT2G22500), DIC2 (AT4G24570), DIC3 (AT5G09470). The mRNA is cell-to-cell mobile.	123.87	57.26	2.16	1.35E-04	7.28E-04
1568	3	F2-3 vs. L2-3	266581_at	AT2G46140	Late embryogenesis abundant protein;(source:Araport11)	110.91	51.47	2.15	4.92E-04	1.85E-03
1569	3	F2-3 vs. L2-3	257699_at	AT3G12780	PGK1 was localized exclusively in the chloroplasts of photosynthetic tissues and is the photosynthetic isoform. The pgk1.1 knock-down mutant displayed reduced growth, lower photosynthetic capacity and starch content. Expression studies in PGK mutants showed that PGK1 and PGK3 were down-regulated in pgk3.2 and pgk1.1, respectively. These results indicate that the down-regulation of photosynthetic activity could be a plant strategy when glycolysis is impaired to achieve metabolic adjustment and optimize growth (DOI:10.1104/pp.17.01227).Functions redundantly with AT1G56190 in the chloroplast in the biosynthesis of thylakoid membrane galactolipids. Double mutants are photosynthetically incompetent, plants are albino and seedling lethal	925.91	431.23	2.15	1.81E-04	8.86E-04
1570	3	F2-3 vs. L2-3	251825_at	AT3G55100	ABC-2 type transporter family protein;(source:Araport11)	35.46	16.48	2.15	1.83E-05	1.81E-04
1571	3	F2-3 vs. L2-3	251557_at	AT3G58730	vacuolar ATP synthase subunit D (VATD) / V-ATPase D subunit / vacuolar proton pump D subunit (VATPD);(source:Araport11)	137.96	64.21	2.15	1.14E-05	1.29E-04
1572	3	F2-3 vs. L2-3	249271_at	AT5G41790	CIP1 physically interacts specifically with the putative coiled-coil region of COP1 in vitro. In hypocotyl and cotyledon protoplasts, it is associated to the cytoskeleton, but not in the root. IN the root the protein is found around epidermal cells. Expression is induced by ABA and osmotic stress but is not regulated by light. Loss of function mutants are also sensitive to osmotic stress and are somewhat ABA insensitive. The mRNA is cell-to-cell mobile.	28.86	13.44	2.15	7.09E-04	2.45E-03

1573	3	F2-3 vs. L2-3	247338_at	AT5G63680	Pyruvate kinase family protein;(source:Araport11)	112.83	52.44	2.15	6.65E-05	4.41E-04
1574	3	F2-3 vs. L2-3	247240_at	AT5G64660	CYS, MET, PRO, and GLY protein 2;(source:Araport11)	40.73	18.97	2.15	6.82E-04	2.38E-03
1575	3	F2-3 vs. L2-3	265183_at	AT1G23750	Nucleic acid-binding, OB-fold-like protein;(source:Araport11)	152.54	71.40	2.14	2.71E-04	1.19E-03
1576	3	F2-3 vs. L2-3	252973_s_a	AT4G38740	Encodes cytosolic cyclophilin ROC1.	744.96	347.97	2.14	4.95E-05	3.59E-04
1577	3	F2-3 vs. L2-3	246673_at	AT5G30510	ribosomal protein S1;(source:Araport11)	1803.55	840.82	2.14	2.00E-06	3.93E-05
1578	3	F2-3 vs. L2-3	259603_at	AT1G56500	Encodes a thylakoid membrane protein with thioredoxin-like and beta-propeller domains located in the lumen and a haloacid-dehalogenase domain exposed to the chloroplast stroma. The protein Primes role may be to prevent formation of a slowly reversible form of antenna quenching, thereby maintaining the efficiency of light harvesting.The mRNA is cell-to-cell mobile.	99.15	46.58	2.13	3.08E-05	2.55E-04
1579	3	F2-3 vs. L2-3	266578_at	AT2G23910	NAD(P)-binding Rossmann-fold superfamily protein;(source:Araport11)	45.44	21.30	2.13	2.61E-05	2.27E-04
1580	3	F2-3 vs. L2-3	266901_at	AT2G34600	jasmonate-zim-domain protein 7;(source:Araport11)	29.92	14.05	2.13	4.00E-07	1.25E-05
1581	3	F2-3 vs. L2-3	267623_at	AT2G39650	cruciferin (DUF506);(source:Araport11)	122.65	57.65	2.13	9.00E-07	2.22E-05
1582	3	F2-3 vs. L2-3	257090_at	AT3G20530	Protein kinase superfamily protein, expressed in the peroxisome.	89.75	42.17	2.13	3.77E-04	1.52E-03
1583	3	F2-3 vs. L2-3	253200_at	AT4G34720	vacuolar H+-pumping ATPase 16 kDa proteolipid (ava-p1)	1450.20	679.48	2.13	2.87E-05	2.42E-04
1584	3	F2-3 vs. L2-3	250054_at	AT5G17860	calcium exchanger 7;(source:Araport11)	41.05	19.29	2.13	1.20E-04	6.65E-04
1585	3	F2-3 vs. L2-3	247285_at	AT5G64400	CHCH domain protein;(source:Araport11) involved in mechanotransduction. Loss of both At12cys-1 and At12cys-2 lead to enhanced tolerance to drought and light stress and increased anti-oxidant capacity.	3199.18	1504.90	2.13	5.52E-04	2.02E-03
1586	3	F2-3 vs. L2-3	258690_at	AT3G07960	Encodes phosphatidylinositol-4-phosphate 5-kinase 6 (PIP5K6). Regulates clathrin-dependent endocytosis in pollen tubes.	37.85	17.86	2.12	9.44E-05	5.62E-04
1587	3	F2-3 vs. L2-3	258962_at	AT3G10570	member of CYP77A	152.41	71.76	2.12	5.18E-04	1.93E-03
1588	3	F2-3 vs. L2-3	245299_at	AT4G17530	AtRabD2c encodes a Rab GTPase, which plays important roles in pollen development, germination and tube elongation.	188.83	89.21	2.12	2.38E-04	1.08E-03
1589	3	F2-3 vs. L2-3	254657_s_a	AT4G18220	Drug/metabolite transporter superfamily protein;(source:Araport11)	579.53	272.92	2.12	2.00E-04	9.59E-04
1590	3	F2-3 vs. L2-3	254562_at	AT4G19230	Encodes a protein with ABA 8'-hydroxylase activity, involved in ABA catabolism. Member of the CYP707A gene family. CYP707A1 appears to play an important role in determining the ABA levels in dry seeds. Gene involved in postgermination growth. Overexpression of CYP707A1 leads to a decrease in ABA levels and a reduction in after-ripening period to break dormancy.	44.73	21.06	2.12	2.65E-05	2.29E-04
1591	3	F2-3 vs. L2-3	253877_at	AT4G27435	fiber (DUF1218);(source:Araport11)	30.71	14.49	2.12	1.68E-04	8.46E-04
1592	3	F2-3 vs. L2-3	250347_at	AT5G11960	magnesium transporter, putative (DUF803);(source:Araport11)	128.10	60.48	2.12	1.79E-04	8.84E-04
1593	3	F2-3 vs. L2-3	264235_at	AT1G54560	Encodes a class XI myosin that is involved in organelle motility, actin organization, and optimal growth of pollen tubes.	39.61	18.74	2.11	7.34E-05	4.71E-04
1594	3	F2-3 vs. L2-3	250803_at	AT5G04980	DNAse I-like superfamily protein;(source:Araport11)	43.07	20.40	2.11	6.31E-04	2.24E-03
1595	3	F2-3 vs. L2-3	248474_at	AT5G50850	Transketolase family protein;(source:Araport11)	1065.50	505.28	2.11	7.40E-06	9.65E-05
1596	3	F2-3 vs. L2-3	247780_at	AT5G58770	AtCPT7 synthesizes medium-chain polyprenols of approximately 55 carbons in length. The enzyme utlizes geranylgeranyl pyrophosphate (GGPP) and isopentenyl pyrophosphate (IPP) as substrates. The enzymatic product accumulates into plastdial membranes (DOI:10.1105/tpc.16.00796).	36.70	17.42	2.11	3.08E-04	1.32E-03
1597	3	F2-3 vs. L2-3	264609_at	AT1G04530	Encodes one of the 36 carboxylate clamp (CC)-tetratricopeptide repeat (TPR) proteins (Prasad 2010, Pubmed ID: 20856808) with potential to interact with Hsp90/Hsp70 as co-chaperones.	255.16	121.58	2.10	1.90E-06	3.89E-05
1598	3	F2-3 vs. L2-3	260950_s_a	AT1G06090	Fatty acid desaturase family protein;(source:Araport11)	72.15	34.36	2.10	1.70E-04	8.53E-04
1599	3	F2-3 vs. L2-3	246582_at	AT1G31750	proline-rich family protein;(source:Araport11)	59.24	28.23	2.10	3.65E-04	1.49E-03
1600	3	F2-3 vs. L2-3	259105_at	AT3G05500	Encodes a protein that associates with lipid droplet surfaces and shares sequence homology with family of small rubber particle proteins. Plays dual roles as positive factors for tissue growth and development and in drought stress responses. The mRNA is cell-to-cell mobile.	258.89	123.27	2.10	1.59E-04	8.16E-04
1601	3	F2-3 vs. L2-3	247380_at	AT5G63380	Encodes a peroxisomal protein involved in the activation of fatty acids through esterification with CoA. At5g63380 preferentially activates fatty acids with increased chain length (C9:0 to C8:0) and thus shares characteristics with long-chain fatty acyl-CoA synthases. Also able to catalyze the conversion of OPDA to its CoA ester and is therefore thought to be involved in the peroxisomal β-oxidation steps of jasmonic acid biosynthesis.	473.40	225.80	2.10	3.30E-06	5.45E-05
1602	3	F2-3 vs. L2-3	260481_at	AT1G10960	ferredoxin 1;(source:Araport11)	1127.02	540.41	2.09	3.15E-04	1.33E-03
1603	3	F2-3 vs. L2-3	258681_at	AT3G08610	NADH dehydrogenase ubiquinone 1 alpha subcomplex subunit;(source:Araport11)	1415.84	676.14	2.09	4.99E-04	1.87E-03

1604	3	F2-3 vs. L2-3	252933_at	AT4G39110	bups1 and bups1/2 double mutants have reduced feritlity due to premature rupture of pollen tubes before they reach the ovule. BUSP1 interacts with RALF4/19 peptide ligands and ANX1/2 receptors. BUPS/ANX signaling may regulate and promote pollen tube growth.	51.62	24.71	2.09	4.22E-05	3.22E-04
1605	3	F2-3 vs. L2-3	256093_at	AT1G20823	Encodes a RING E3 ubiquitin ligase ATL80. Involved in phosphate mobilization and cold stress response in sufficient phosphate growth conditions. The mRNA is cell-to-cell mobile.	39.78	19.14	2.08	5.69E-04	2.07E-03
1606	3	F2-3 vs. L2-3	245756_at	AT1G35190	2-oxoglutarate (2OG) and Fe(II)-dependent oxygenase superfamily protein;(source:Araport11)	67.71	32.58	2.08	2.74E-04	1.21E-03
1607	3	F2-3 vs. L2-3	262022_at	AT1G35490	bZIP family transcription factor;(source:Araport11)	24.16	11.59	2.08	1.08E-04	6.22E-04
1608	3	F2-3 vs. L2-3	263134_at	AT1G78570	Encodes a UDP-L-Rhamnose synthase involved in the biosynthesis of rhamnose, a major monosaccharide component of pectin. Catalyzes the conversion of UDP-D-Glc to UDP-L-Rha. The dehydrogenase domain of RHM1 was shown to catalyze the conversion of UDP-D-Glc to the reaction intermediate UDP-4-keto-6-deoxy-D-Glc using recombinant protein assay but the activity of the full-length protein was not determined as it could not be expressed in <i>E. coli</i>.	239.24	115.24	2.08	6.66E-04	2.33E-03
1609	3	F2-3 vs. L2-3	264102_at	AT1G79270	evolutionarily conserved C-terminal region 8;(source:Araport11)	22.76	10.93	2.08	1.57E-05	1.63E-04
1610	3	F2-3 vs. L2-3	263985_at	AT2G42750	DNAJ heat shock N-terminal domain-containing protein;(source:Araport11)	434.31	208.56	2.08	2.92E-04	1.26E-03
1611	3	F2-3 vs. L2-3	266719_at	AT2G46830	Encodes a transcriptional repressor that performs overlapping functions with LHY in a regulatory feedback loop that is closely associated with the circadian oscillator of Arabidopsis. Binds to the evening element in the promoter of TOC1 and represses TOC1 transcription. CCA1 and LHY colocalize in the nucleus and form heterodimers in vivo. CCA1 and LHY function synergistically in regulating circadian rhythms of Arabidopsis. CCA1 binds the GI promoter.	87.45	42.14	2.08	3.52E-04	1.45E-03
1612	3	F2-3 vs. L2-3	257609_at	AT3G13845	transmembrane protein;(source:Araport11)	207.55	99.55	2.08	4.04E-04	1.60E-03
1613	3	F2-3 vs. L2-3	250854_at	AT5G04710	Plastid localized metalloaminopeptidase.	91.73	44.20	2.08	4.15E-04	1.63E-03
1614	3	F2-3 vs. L2-3	247150_at	AT5G65650	sugar transporter, putative (DUF1195);(source:Araport11)	67.34	32.44	2.08	7.01E-04	2.43E-03
1615	3	F2-3 vs. L2-3	261226_at	AT1G20190	member of Alpha-Expansin Gene Family. Naming convention from the Expansin Working Group (Kende et al, 2004. Plant Mol Bio)	42.33	20.43	2.07	1.13E-04	6.40E-04
1616	3	F2-3 vs. L2-3	265761_at	AT2G01330	nucleotide binding protein;(source:Araport11)	61.28	29.58	2.07	2.46E-05	2.17E-04
1617	3	F2-3 vs. L2-3	245060_at	AT2G39770	Encodes a GDP-mannose pyrophosphorylase/ mannose-1-pyrophosphatase. This enzyme provides GDP-mannose, which is used for cell wall carbohydrate biosynthesis and protein glycosylation as well as for ascorbate (vitamin C) biosynthesis. Mutations in this gene confer hypersensitivity to NH4+.	315.42	152.17	2.07	1.06E-05	1.22E-04
1618	3	F2-3 vs. L2-3	259338_at	AT3G03800	member of SYP13 Gene Family	36.58	17.69	2.07	4.60E-04	1.76E-03
1619	3	F2-3 vs. L2-3	247816_at	AT5G58260	Encodes subunit NDH-N of NAD(P)H:plastoquinone dehydrogenase complex (Ndh complex) present in the thylakoid membrane of chloroplasts. This subunit is thought to be required for Ndh complex assembly.	86.05	41.58	2.07	2.55E-05	2.24E-04
1620	3	F2-3 vs. L2-3	245138_at	AT2G45190	Encodes a member of the YABBY family of transcriptional regulators that is involved in abaxial cell type specification in leaves and fruits. YAB1 acts in a non-cell autonomous fashion within the meristem to affect phyllotactic patterning. The non-autonomous effect on the central region of the meristem is mediated through the activity if Lateral Suppressor (LAS).	43.86	21.27	2.06	1.08E-04	6.22E-04
1621	3	F2-3 vs. L2-3	258682_at	AT3G08720	Encodes a ribosomal-protein S6 kinase. Gene expression is induced by cold and salt (NaCl). Activation of AtS6k is regulated by 1-naphthylacetic acid and kinetin, at least in part, via a lipid kinase-dependent pathway. Phosphorylates specifically mammalian and plant S6 at 25 degrees C but not at 37 degrees C. Involved in translational up-regulation of ribosomal proteins.	92.47	44.82	2.06	4.07E-04	1.61E-03
1622	3	F2-3 vs. L2-3	257807_at	AT3G26650	Encodes one of the two subunits forming the photosynthetic glyceraldehyde-3-phosphate dehydrogenase (GAPDH) and as such a constituent of the supramolecular complex with phosphoribulokinase (PRK) thought to be linked by a small peptide encoded by CP12-2. GapA-1 is coordinately expressed by light with PRK and CP12-2. The enzyme activity, tested in leaf protein extracts dropped significantly after external sucrose treatment for the photosynthetic GAPDH (NADPH-dependent) but not for the cytosolic GAPDH (NADH-dependent).	708.76	344.14	2.06	7.68E-05	4.86E-04
1623	3	F2-3 vs. L2-3	252016_at	AT3G52950	CBS / octicosapeptide/Phox/Bemp1 (PB1) domains-containing protein;(source:Araport11)	159.05	77.29	2.06	1.70E-05	1.73E-04

1624	3	F2-3 vs. L2-3	253039_at	AT4G37760	squalene epoxidase 3;(source:Araport11)	109.57	53.13	2.06	3.15E-04	1.33E-03
1625	3	F2-3 vs. L2-3	252896_at	AT4G39480	member of CYP96A	49.39	23.95	2.06	5.96E-04	2.14E-03
1626	3	F2-3 vs. L2-3	260968_at	AT1G12250	Pentapeptide repeat-containing protein;(source:Araport11)	135.20	65.79	2.05	2.29E-04	1.06E-03
1627	3	F2-3 vs. L2-3	262597_at	AT1G15470	WD40 nucleoplasmic shuttling protein that positively regulates the Abscisic acid (ABA) response by interacting with and maintaining the stability of ABI5 in the nucleus. Nuclear export of XIW1 is XPO1-dependent. Involved in regulating seed germination, primary root growth, and drought stress resistance.	46.09	22.49	2.05	6.60E-05	4.38E-04
1628	3	F2-3 vs. L2-3	262868_at	AT1G64980	Encodes a putative nucleotide-diphospho-sugar transferase required for pollen germination and tube growth.	295.75	144.46	2.05	3.39E-05	2.72E-04
1629	3	F2-3 vs. L2-3	260800_at	AT1G78240	Encodes TSD2 (TUMOROUS SHOOT DEVELOPMENT2), a putative methyltransferase with an essential role in cell adhesion and coordinated plant development.	728.19	355.08	2.05	6.67E-05	4.42E-04
1630	3	F2-3 vs. L2-3	258038_at	AT3G21260	Glycolipid transfer protein (GLTP) family protein;(source:Araport11)	24.10	11.77	2.05	3.39E-05	2.72E-04
1631	3	F2-3 vs. L2-3	258452_at	AT3G22370	Encodes AOX1a, an isoform of alternative oxidase that is expressed in rosettes, flowers, and root. The alternative oxidase of plant mitochondria transfers electrons from the ubiquinone pool to oxygen without energy conservations. It is regulated through transcriptional control and by pyruvate. Plays a role in shoot acclimation to low temperature. Also is capable of ameliorating reactive oxygen species production when the cytochrome pathway is inhibited. AOX1a also functions as a marker for mitochondrial retrograde response. The mRNA is cell-to-cell mobile.	120.68	58.84	2.05	6.57E-05	4.38E-04
1632	3	F2-3 vs. L2-3	251197_at	AT3G62960	Encodes a member of the CC-type glutaredoxin (ROXY) family that has been shown to interact with the transcription factor TGA2.	39.08	19.05	2.05	5.71E-04	2.07E-03
1633	3	F2-3 vs. L2-3	261178_at	AT1G04760	member of Synaptobrevin -like protein family	46.21	22.68	2.04	1.52E-04	7.91E-04
1634	3	F2-3 vs. L2-3	266653_at	AT2G25740	ATP-dependent protease La (LON) domain protein;(source:Araport11)	101.92	49.91	2.04	3.86E-04	1.55E-03
1635	3	F2-3 vs. L2-3	258181_at	AT3G21670	Major facilitator superfamily protein;(source:Araport11)	66.36	32.49	2.04	3.88E-04	1.55E-03
1636	3	F2-3 vs. L2-3	252327_at	AT3G48740	Encodes a member of the SWEET sucrose efflux transporter family proteins.	148.81	72.97	2.04	6.73E-04	2.35E-03
1637	3	F2-3 vs. L2-3	253943_at	AT4G27030	Encodes an unusual palmitate desaturase that is highly substrate specific. It introduces a delta-3 trans double bond at palmitate at the sn-2 position of phosphatidylglycerol. The mRNA is cell-to-cell mobile.	32.30	15.81	2.04	1.30E-06	3.01E-05
1638	3	F2-3 vs. L2-3	253533_at	AT4G31590	encodes a gene similar to cellulose synthase	182.99	89.66	2.04	3.60E-04	1.47E-03
1639	3	F2-3 vs. L2-3	247320_at	AT5G64040	Encodes the only subunit of photosystem I located entirely in the thylakoid lumen. May be involved in the interaction between plastocyanin and the photosystem I complex. Phosphorylation of this protein is dependent on calcium.	1683.49	825.42	2.04	8.22E-04	2.75E-03
1640	3	F2-3 vs. L2-3	258408_at	AT3G17630	member of Putative Na+/H+ antiporter family	28.53	14.08	2.03	4.57E-05	3.40E-04
1641	3	F2-3 vs. L2-3	251301_at	AT3G61880	Encodes a cytochrome p450 monooxygenase. Overexpression of this gene allows fruit growth independently of fertilization. The gene is normally expressed only in floral organs(during the Arabidopsis stage 14 flower) and in the funiculus at anthesis.	55.15	27.12	2.03	4.80E-05	3.52E-04
1642	3	F2-3 vs. L2-3	253197_at	AT4G35250	HCF244 is a member of the atypical short-chain dehydrogenase/reductase superfamily, a modified group, which has lost enzyme activity.HCF244 interacts with unknown partners in a 200-400 kD membrane associated complex.	404.42	198.99	2.03	7.31E-04	2.51E-03
1643	3	F2-3 vs. L2-3	255764_at	AT1G16720	Encodes HCF173, a protein with weak similiarities to the superfamily of the short-chain dehydrogenases/reductases. HCF173 is involved in the initiation of translation of the psbA mRNA and binds a specific site in the 5' UTR of psbA mRNA. . Mutants shows a high chlorophyll fluorescence phenotype (hcf) and are severely affected in the accumulation of PSII subunits. The protein HCF173 is localized in the chloroplast, where it is mainly associated with the membrane system and is part of a higher molecular weight complex with psbA mRNA as a component of this complex.	397.21	196.20	2.02	9.20E-04	3.00E-03
1644	3	F2-3 vs. L2-3	264280_at	AT1G61820	beta glucosidase 46;(source:Araport11)	24.42	12.11	2.02	4.51E-04	1.73E-03
1645	3	F2-3 vs. L2-3	245148_at	AT2G45220	Plant invertase/pectin methylesterase inhibitor superfamily;(source:Araport11)	61.54	30.44	2.02	9.04E-04	2.97E-03
1646	3	F2-3 vs. L2-3	257194_at	AT3G13110	Encodes a mitochondrial serine O-acetyltransferase involved in sulfur assimilation and cysteine biosynthesis. Expressed in the vascular system.	138.85	68.90	2.02	6.50E-06	8.82E-05
1647	3	F2-3 vs. L2-3	253567_at	AT4G31230	kinase with adenine nucleotide alpha hydrolases-like domain-containing protein;(source:Araport11)	27.85	13.78	2.02	1.38E-05	1.50E-04
1648	3	F2-3 vs. L2-3	246230_at	AT4G36710	GRAS family transcription factor;(source:Araport11)	46.11	22.86	2.02	1.06E-04	6.14E-04
1649	3	F2-3 vs. L2-3	247281_at	AT5G64350	Encodes FK506-binding protein 12 (FKBP12 or FKP12). FKP12 overexpression dramatically enhances rapamycin sensitivity, whereas rapamycin inhibition is relieved in transgenic plants deficient in FKP12.	925.46	457.97	2.02	9.02E-04	2.97E-03

1650	3	F2-3 vs. L2-3	256475_s_at	AT1G42680	P-loop containing nucleoside triphosphate hydrolases superfamily protein;(source:Araport11)	23.19	11.56	2.01	2.88E-04	1.25E-03
1651	3	F2-3 vs. L2-3	261875_at	AT1G50610	Leucine-rich repeat protein kinase family protein;(source:Araport11)	31.24	15.53	2.01	1.08E-04	6.23E-04
1652	3	F2-3 vs. L2-3	259856_at	AT1G68440	Transmembrane protein;(source:Araport11). Expression induced by abiotic stressors such as ABA, drought, heat, light, NaCl, osmotic stress and wounding.	83.74	41.61	2.01	6.21E-05	4.24E-04
1653	3	F2-3 vs. L2-3	263297_at	AT2G15310	A member of ARF GTPase family. A thaliana has 21 members of this family, known to be essential for vesicle coating and uncoating and functions in GTP-binding. Gene encoding ADP-ribosylation factor and similar to ADP-ribosylation factor (GI:861205) (Chlamydomonas reinhardtii), other ARFs and ARF-like proteins.	33.73	16.75	2.01	2.05E-05	1.92E-04
1654	3	F2-3 vs. L2-3	266835_at	AT2G29990	alternative NAD(P)H dehydrogenase 2;(source:Araport11)	35.81	17.86	2.01	1.03E-04	6.00E-04
1655	3	F2-3 vs. L2-3	266993_at	AT2G39210	Major facilitator superfamily protein;(source:Araport11)	77.77	38.78	2.01	2.80E-05	2.39E-04
1656	3	F2-3 vs. L2-3	258941_at	AT3G09940	Encodes a member of the monodehydroascorbate reductase gene family. Critical for a mutualistic symbiosis between the host Arabidopsis and the root colonizing fungus Piriformospora indica.	40.80	20.33	2.01	7.70E-06	9.97E-05
1657	3	F2-3 vs. L2-3	254605_at	AT4G18950	BHP1 is a Raf-like protein kinase involved in mediating blue light dependent stomatal opening.	145.26	72.29	2.01	9.30E-06	1.13E-04
1658	3	F2-3 vs. L2-3	255720_at	AT1G32060	phosphoribulokinase;(source:Araport11)	81.99	41.02	2.00	3.44E-04	1.43E-03
1659	3	F2-3 vs. L2-3	260517_at	AT1G51420	sucrose-phosphatase 1;(source:Araport11)	41.74	20.88	2.00	2.71E-05	2.33E-04
1660	3	F2-3 vs. L2-3	263382_at	AT2G40230	HXXXD-type acyl-transferase family protein;(source:Araport11)	70.98	35.47	2.00	1.99E-04	9.54E-04
1661	3	F2-3 vs. L2-3	258208_at	AT3G13930	Encodes a subunit of the mitochondrial pyruvate dehydrogenase complex.	186.93	93.63	2.00	9.30E-06	1.13E-04
1662	3	F2-3 vs. L2-3	253995_at	AT4G26100	Encodes a member of the casein kinase 1 protein family that is expressed in punctate particles at the cell periphery suggesting possible plasmodesmatal localization (member of CKL-B group).	142.37	71.06	2.00	3.14E-05	2.59E-04
1663	3	F2-3 vs. L2-3	251102_at	AT5G01690	member of Putative Na+/H+ antiporter family	58.43	29.20	2.00	2.49E-04	1.12E-03
1664	3	F2-3 vs. L2-3	250545_at	AT5G08160	Encodes a serine/threonine protein kinase.	332.94	166.60	2.00	1.80E-06	3.80E-05
1665	3	F2-3 vs. L2-3	263473_at	AT2G31750	Encodes an auxin glycosyltransferase that is likely to be involved in regulation of auxin by glycosylation.	33.73	16.91	1.99	1.86E-05	1.83E-04
1666	3	F2-3 vs. L2-3	258791_at	AT3G04720	Encodes a protein similar to the antifungal chitin-binding protein hevein from rubber tree latex. mRNA levels increase in response to ethylene and turnip crinkle virus infection. The mRNA is cell-to-cell mobile.	150.05	75.32	1.99	2.59E-04	1.16E-03
1667	3	F2-3 vs. L2-3	251455_at	AT3G60100	citrate synthase 5;(source:Araport11)	39.76	19.94	1.99	1.90E-06	3.89E-05
1668	3	F2-3 vs. L2-3	245304_at	AT4G15630	Uncharacterized protein family (UPF0497);(source:Araport11)	233.90	117.59	1.99	1.02E-04	5.96E-04
1669	3	F2-3 vs. L2-3	265034_at	AT1G61660	Encodes a transcriptional activator that regulates the expression of genes by binding to their GCG- or E-boxes to mediate physiological responses, including proline biosynthesis and ROS scavenging pathways, to enhance stress tolerance.	39.41	19.95	1.98	2.64E-04	1.17E-03
1670	3	F2-3 vs. L2-3	260222_at	AT1G74380	xyloglucan xylosyltransferase 5;(source:Araport11)	127.40	64.23	1.98	1.87E-05	1.83E-04
1671	3	F2-3 vs. L2-3	249876_at	AT5G23060	Encodes a chloroplast-localized protein that modulates cytoplasmic Ca2+ concentration and is crucial for proper stomatal regulation in response to elevations of external Ca2+. Phosphorylation of this protein is dependent on calcium.	141.63	71.77	1.97	3.68E-05	2.89E-04
1672	3	F2-3 vs. L2-3	247431_at	AT5G62520	Encodes a protein with similarity to RCD1 but without the WWE domain. The protein does have a PARP signature upstream of the C-terminal protein interaction domain. The PARP signature may bind NAD+ and attach the ADP-ribose-moiety from NAD+ to the target molecule. Its presence suggests a role for the protein in ADP ribosylation. Up-regulated by NaCl. SRO5 and P5CDH (an overlapping gene in the antisense orientation) generate 24-nt and 21-nt siRNAs, which together are components of a regulatory loop controlling reactive oxygen species (ROS) production and stress response.	44.79	22.79	1.97	2.09E-05	1.95E-04
1673	3	F2-3 vs. L2-3	264146_at	AT1G02205	Expression of the CER1 gene associated with production of stem epicuticular wax and pollen fertility. Biochemical studies showed that cer1 mutants are blocked in the conversion of stem wax C30 aldehydes to C29 alkanes, and they also lack the secondary alcohols and ketones. These suggested the CER1 protein is an aldehyde decarbonylase, but the exact molecular function of this protein remains to be determined.	167.90	85.83	1.96	4.51E-04	1.73E-03

1674	3	F2-3 vs. L2-3	262899_at	AT1G59870	ATP binding cassette transporter. Localized to the plasma membrane in uninfected cells. In infected leaves, the protein concentrated at infection sites. Contributes to nonhost resistance to inappropriate pathogens that enter by direct penetration in a salicylic acid?dependent manner. Required for mlo resistance. Has Cd transporter activity (Cd2+ extrusion pump) and contributes to heavy metal resistance. The mRNA is cell-to-cell mobile.	29.93	15.30	1.96	5.50E-05	3.89E-04
1675	3	F2-3 vs. L2-3	260774_at	AT1G78290	encodes a member of SNF1-related protein kinase (SnRK2) family whose activity is activated by ionic (salt) and non-ionic (mannitol) osmotic stress and dehydration.	38.50	19.61	1.96	3.39E-05	2.72E-04
1676	3	F2-3 vs. L2-3	259237_at	AT3G11630	Encodes a 2-Cys peroxiredoxin (2-Cys PrxA) that contains two catalytic Cys residues. Functions in redox cascade with TrxL2 via the ferredoxin-thioredoxin reductase (FTR)/thioredoxin (Trx) pathway to mediate the light-responsive reductive control of target proteins. Continuously transfers reducing power from TrxL2 to H2O2.	640.35	327.36	1.96	6.29E-05	4.26E-04
1677	3	F2-3 vs. L2-3	250307_at	AT5G12170	Encodes one of the CRT-Like transporters (CLT1/AT5G19380, CLT2/AT4G24460, CLT3/AT5G12170). Required for glutathione homeostasis and stress responses. Mutants lacking these transporters are heavy metal-sensitive, glutathione(GSH)-deficient, and hypersensitive to Phytophthora infection. The mRNA is cell-to-cell mobile.	29.35	14.94	1.96	2.05E-04	9.75E-04
1678	3	F2-3 vs. L2-3	248164_at	AT5G54490	Encodes a PINOID (PID)-binding protein containing putative EF-hand calcium-binding motifs. The interaction is dependent on the presence of calcium. mRNA expression is up-regulated by auxin. Not a phosphorylation target of PID, likely acts upstream of PID to regulate the activity of this protein in response to changes in calcium levels.	96.02	49.03	1.96	8.54E-05	5.27E-04
1679	3	F2-3 vs. L2-3	259363_at	AT1G13270	Encodes a methionine aminopeptidase formerly called MAP1B, renamed to MAP1C.	519.97	267.01	1.95	1.71E-05	1.74E-04
1680	3	F2-3 vs. L2-3	267607_s_at	AT2G26740	Encodes a soluble epoxide hydrolase whose expression is induced by auxin and water stress.	330.89	169.68	1.95	2.30E-04	1.06E-03
1681	3	F2-3 vs. L2-3	267429_at	AT2G34850	NAD(P)-binding Rossmann-fold superfamily protein;(source:Araport11)	208.75	107.09	1.95	7.66E-05	4.85E-04
1682	3	F2-3 vs. L2-3	251469_at	AT3G59530	Calcium-dependent phosphotriesterase superfamily protein;(source:Araport11)	44.42	22.74	1.95	2.49E-04	1.12E-03
1683	3	F2-3 vs. L2-3	254683_at	AT4G13800	magnesium transporter NIPA (DUF803);(source:Araport11)	30.99	15.90	1.95	6.90E-06	9.18E-05
1684	3	F2-3 vs. L2-3	246555_at	AT5G15470	Encodes a protein with putative galacturonosyltransferase activity.	35.60	18.25	1.95	1.50E-05	1.58E-04
1685	3	F2-3 vs. L2-3	260320_at	AT1G63930	from the Czech roh meaning corner;(source:Araport11)	129.35	66.55	1.94	4.51E-04	1.73E-03
1686	3	F2-3 vs. L2-3	260207_at	AT1G70730	Encodes a cytosolic phosphoglucomutase (PGM). Two Arabidopsis PGM proteins (AT1G70730/PGM2 and AT1G23190/PGM3) have high sequence similarities and redundant functions. Mature plants possessing a single cPGM allele had a major reduction in cPGM activity. Whereas pgm2 and pgm3 single mutants are undistinguishable from the wild type, loss of both PGM2 and PGM3 severely impairs male and female gametophyte development.	225.45	116.45	1.94	1.30E-04	7.07E-04
1687	3	F2-3 vs. L2-3	258671_at	AT3G08560	vacuolar H+-ATPase subunit E isoform 2;(source:Araport11)	33.14	17.13	1.94	8.40E-06	1.05E-04
1688	3	F2-3 vs. L2-3	256416_at	AT3G11050	ferritin 2;(source:Araport11)	171.18	88.03	1.94	5.10E-05	3.67E-04
1689	3	F2-3 vs. L2-3	251968_at	AT3G53100	GDSL-motif esterase/acyltransferase/lipase. Enzyme group with broad substrate specificity that may catalyze acyltransfer or hydrolase reactions with lipid and non-lipid substrates.	45.31	23.39	1.94	3.11E-04	1.33E-03
1690	3	F2-3 vs. L2-3	245216_at	AT4G16143	Protein interacts with Agrobacterium proteins VirD2 and VirE2. Is not individually essential for Agrobacterium-mediated root transformation, but when overexpressed can rescue the impa-4 decreased transformation susceptibility phenotype.	79.23	40.85	1.94	6.30E-06	8.66E-05
1691	3	F2-3 vs. L2-3	254187_at	AT4G23890	NAD(P)H-quinone oxidoreductase subunit S;(source:Araport11)	301.54	155.73	1.94	5.20E-04	1.94E-03
1692	3	F2-3 vs. L2-3	247925_at	AT5G57560	Encodes a cell wall-modifying enzyme, rapidly upregulated in response to environmental stimuli.	126.31	65.13	1.94	4.03E-05	3.11E-04
1693	3	F2-3 vs. L2-3	261279_at	AT1G05850	Encodes an endo chitinase-like protein AtCTL1. Essential for tolerance to heat, salt and drought stresses. Also involved in root hair development, cell expansion and response to cytokinin. Allelic to erh2. 11 alleles described in Hauser (1995). Mutant is defective in acquired thermotolerance, appears semidwarf throughout its life cycle and has extra lateral branches. There are two EMS alleles. Expression of AtHSP101 is not affected in the mutants.	409.55	211.95	1.93	5.12E-05	3.68E-04
1694	3	F2-3 vs. L2-3	261210_at	AT1G12840	Encodes subunit C of the vacuolar H(+)-ATPase (V-ATPase). Bound and phosphorylated by AtWNK8. The mRNA is cell-to-cell mobile.	1155.78	600.32	1.93	9.03E-05	5.48E-04

1695	3	F2-3 vs. L2-3	262660_at	AT1G14000	Encodes a protein with similarity to members of the C1 subgroup of MAP kinase kinase kinases. Interacts physically with the receptor kinase BRL2/VH1 and appears to be involved in auxin and brassinosteriod signaling. The mRNA is cell-to-cell mobile.	182.08	94.49	1.93	4.40E-06	6.55E-05
1696	3	F2-3 vs. L2-3	260777_at	AT1G14560	Encodes a mitochondrial CoA transporter.	83.85	43.41	1.93	1.19E-04	6.65E-04
1697	3	F2-3 vs. L2-3	262546_at	AT1G31260	member of Fe(II) transporter isolog family	30.86	15.98	1.93	8.24E-04	2.76E-03
1698	3	F2-3 vs. L2-3	256019_at	AT1G58265	Cytochrome P450 superfamily protein;(source:Araport11)	24.33	12.60	1.93	3.34E-05	2.69E-04
1699	3	F2-3 vs. L2-3	264959_at	AT1G77090	thylakoid lumenal protein (Mog1/PsbP/DUF1795-like photosystem II reaction center PsbP family protein);(source:Araport11)	119.14	61.77	1.93	7.84E-05	4.94E-04
1700	3	F2-3 vs. L2-3	266300_at	AT2G01420	Encodes a putative auxin efflux carrier that is localized in developing and mature root meristems. It is involved in the maintenance of embryonic auxin gradients. A role for AtPIN4 in generating a sink for auxin below the quiescent center of the root meristem that is essential for auxin distribution and patterning is proposed. In the root, PIN4 is detected around the quiescent center and cells surrounding it, and localizes basally in provascular cells. PIN4 expression is upregulated in brassinosteroid-insensitive mutant (PMID 16141452).	33.72	17.49	1.93	1.67E-04	8.43E-04
1701	3	F2-3 vs. L2-3	245044_at	AT2G26500	cytochrome b6f complex subunit (petM);(source:Araport11)	1006.31	521.49	1.93	9.44E-05	5.62E-04
1702	3	F2-3 vs. L2-3	259180_at	AT3G01680	Encodes a protein localized to phloem filaments that is required for phloem filament formation. The mRNA is cell-to-cell mobile.	34.17	17.72	1.93	1.44E-05	1.54E-04
1703	3	F2-3 vs. L2-3	257713_at	AT3G27380	One of three isoforms of the iron-sulfur component of the succinate dehydrogenase complex, a component of the mitochondrial respiratory chain complex II. The product of the nuclear encoded gene is imported into the mitochondrion. Expressed during germination and post-germinative growth.	720.34	373.75	1.93	2.54E-04	1.14E-03
1704	3	F2-3 vs. L2-3	255243_at	AT4G05590	Encodes NRGA1, a putative mitochondrial pyruvate carrier that mediates ABA regulation of guard cell ion channels and drought stress responses.	24.30	12.59	1.93	6.59E-04	2.31E-03
1705	3	F2-3 vs. L2-3	254961_at	AT4G11030	AMP-dependent synthetase and ligase family protein;(source:Araport11)	22.69	11.74	1.93	3.80E-06	5.94E-05
1706	3	F2-3 vs. L2-3	254419_at	AT4G21490	NAD(P)H dehydrogenase B3;(source:Araport11)	35.68	18.50	1.93	2.08E-04	9.86E-04
1707	3	F2-3 vs. L2-3	249875_at	AT5G23120	encodes a stability and/or assembly factor of photosystem II The mRNA is cell-to-cell mobile.	732.10	379.29	1.93	5.59E-04	2.04E-03
1708	3	F2-3 vs. L2-3	246411_at	AT1G57770	FAD/NAD(P)-binding oxidoreductase family protein;(source:Araport11)	29.45	15.36	1.92	1.97E-05	1.89E-04
1709	3	F2-3 vs. L2-3	265705_at	AT2G03410	Mo25 family protein;(source:Araport11)	28.68	14.93	1.92	7.37E-04	2.52E-03
1710	3	F2-3 vs. L2-3	248191_at	AT5G54130	Calcium-binding endonuclease/exonuclease/phosphatase family;(source:Araport11)	32.35	16.85	1.92	8.07E-04	2.72E-03
1711	3	F2-3 vs. L2-3	264395_at	AT1G12070	Immunoglobulin E-set superfamily protein;(source:Araport11)	44.99	23.59	1.91	3.25E-04	1.37E-03
1712	3	F2-3 vs. L2-3	260304_at	AT1G70480	OBP32pep, putative (DUF220);(source:Araport11)	163.91	85.64	1.91	4.38E-05	3.30E-04
1713	3	F2-3 vs. L2-3	259892_at	AT1G72610	germin-like protein (GLP1)	65.26	34.17	1.91	1.69E-04	8.50E-04
1714	3	F2-3 vs. L2-3	252115_at	AT3G51600	Predicted to encode a PR (pathogenesis-related) protein. Belongs to the lipid transfer protein (PR-14) family with the following members: At2g38540/LTP1, At2g38530/LTP2, At5g59320/LTP3, At5g59310/LTP4, At3g51600/LTP5, At3g08770/LTP6, At2g15050/LTP7, At2g18370/LTP8, At2g15325/LTP9, At5g01870/LTP10, At4g33355/LTP11, At3g51590/LTP12, At5g44265/LTP13, At5g62065/LTP14, At4g08530/LTP15.	35.92	18.81	1.91	9.80E-06	1.17E-04
1715	3	F2-3 vs. L2-3	250889_at	AT5G04500	Encodes a member of the CAZy Glycosyltransferase Family 64 that is involved in glycosylinositolphosphorylceramide and sphingolipid glycosylation. In mutants, seed germination was less sensitive to salt stress than in wild-type plants. [The protein was expected to be Golgi-localized based on function as well as the Golgi localization of its homolog GMT1. However, GFP-fusion proteins localized both to the ER and Golgi, and especially to ER when co-expressed with Golgi markers. Therefore, localization cannot confidently be defined. (pers. communication, J. Mortimer)]	75.77	39.68	1.91	5.01E-04	1.88E-03
1716	3	F2-3 vs. L2-3	246440_at	AT5G17650	glycine/proline-rich protein;(source:Araport11)	44.27	23.12	1.91	3.04E-04	1.31E-03
1717	3	F2-3 vs. L2-3	247775_at	AT5G58690	phosphatidylinositol-speciwc phospholipase C5;(source:Araport11)	22.69	11.89	1.91	1.83E-04	8.93E-04

1718	3	F2-3 vs. L2-3	260242_at	AT1G63650	Mutant has reduced trichomes, anthocyanin, and seed coat mucilage and abnormally patterned stomates. Mutants are defective in jasmonate-induced anthocyanin accumulation. Encodes a bHLH Transcription Factor 1. The protein is functionally redundant with GL3 and TT8 and interacts with TTG1, the myb proteins GL1, PAP1 and 2, CPC and TRY, and it will form heterodimers with GL3. Expression in N (non-hair cell forming) cell layers is negatively regulated by WER. Expression in H cells (hair cell forming) is promoted by CPC/TRY.	34.37	18.06	1.90	2.76E-04	1.21E-03
1719	3	F2-3 vs. L2-3	256548_at	AT3G14770	Nodulin MtN3 family protein;(source:Araport11)	34.19	17.96	1.90	2.65E-04	1.18E-03
1720	3	F2-3 vs. L2-3	254033_at	AT4G25950	V-ATPase G-subunit like protein	16.90	8.90	1.90	6.32E-04	2.24E-03
1721	3	F2-3 vs. L2-3	246616_at	AT5G36260	Eukaryotic aspartyl protease family protein;(source:Araport11)	67.79	35.65	1.90	1.98E-05	1.89E-04
1722	3	F2-3 vs. L2-3	262879_at	AT1G64860	Subunit of chloroplast RNA polymerase, confers the ability to recognize promoter sequences on the core enzyme	212.96	112.51	1.89	9.04E-04	2.97E-03
1723	3	F2-3 vs. L2-3	263796_at	AT2G24540	Galactose oxidase/kelch repeat superfamily protein;(source:Araport11)	40.32	21.36	1.89	1.85E-04	8.99E-04
1724	3	F2-3 vs. L2-3	257966_at	AT3G19800	Encodes the DUF177B version of the two DUF177 proteins in Arabidopsis. This version differs from DUF177A in containing a 23 aa insertion compared to the DUF177A sequence.	88.13	46.75	1.89	4.40E-04	1.71E-03
1725	3	F2-3 vs. L2-3	252354_at	AT3G48170	ALDH10A9 encodes a protein that can function as a betaine aldehyde dehydrogenase in vitro. The C-terminal amino acids of this protein direct GFP to the peroxisome suggesting that ALDH10A9 accumulates in this organelle. ALDH10A9 transcript levels rise in response to ABA, NaCl, chilling, methyl viologen, and dehydration stress. The enzyme can catalyze the formation of glycine betaine in vitro, but there are still questions about whether Arabidopsis makes this protective compound under natural conditions. This enzyme may be involved in oxidizing aminoaldehydes formed through polyamine metabolism.	1476.17	780.64	1.89	9.83E-04	3.18E-03
1726	3	F2-3 vs. L2-3	254343_at	AT4G21990	Encodes a protein disulfide isomerase-like (PDIL) protein, a member of a multigene family within the thioredoxin (TRX) superfamily. This protein also belongs to the adenosine 5'-phosphosulfate reductase-like (APRL) group.	326.49	172.35	1.89	2.87E-05	2.42E-04
1727	3	F2-3 vs. L2-3	245906_at	AT5G11070	hypothetical protein;(source:Araport11)	106.83	56.50	1.89	5.44E-04	2.00E-03
1728	3	F2-3 vs. L2-3	246566_at	AT5G14940	Major facilitator superfamily protein;(source:Araport11)	45.60	24.17	1.89	9.42E-05	5.62E-04
1729	3	F2-3 vs. L2-3	247544_at	AT5G61670	Encodes a close homolog of the Cauliflower OR (Orange) protein that is located in the chloroplast of light grown organs but in the nucleus of etiolated cotyledons. The function of OR is to induce the differentiation of proplastids or other noncolored plastids into chromoplasts for carotenoid accumulation. Both proteins contain a Cysteine-rich zinc finger domain that is highly specific to DnaJ-like molecular chaperons. The AtOR protein interacts directly with the PSY (phytoene synthase) protein and acts as a positive posttranscriptional regulator of its expression, thereby affecting carotenoid biosynthesis.	102.10	53.94	1.89	4.42E-05	3.32E-04
1730	3	F2-3 vs. L2-3	264815_at	AT1G03620	ELMO/CED-12 family protein;(source:Araport11)	28.24	15.00	1.88	1.04E-04	6.07E-04
1731	3	F2-3 vs. L2-3	262959_at	AT1G54290	Translation initiation factor SUI1 family protein;(source:Araport11)	539.91	286.83	1.88	8.55E-04	2.84E-03
1732	3	F2-3 vs. L2-3	264186_at	AT1G54570	Encodes a protein with phytyl ester synthesis and diacylglycerol acyltransferase activities that is involved in the deposition of free phytol and free fatty acids in the form of phytyl esters in chloroplasts, a process involved in maintaining the integrity of the photosynthetic membrane during abiotic stress and senescence.	65.25	34.67	1.88	1.38E-05	1.50E-04
1733	3	F2-3 vs. L2-3	264741_at	AT1G62290	Saposin-like aspartyl protease family protein;(source:Araport11)	36.75	19.53	1.88	7.68E-04	2.60E-03
1734	3	F2-3 vs. L2-3	258805_at	AT3G04010	O-Glycosyl hydrolases family 17 protein;(source:Araport11)	40.43	21.53	1.88	3.42E-05	2.74E-04
1735	3	F2-3 vs. L2-3	259380_at	AT3G16340	Encodes a p-coumaryl alcohol exporter involved in lignin biosynthesis.	31.84	16.93	1.88	1.37E-04	7.31E-04
1736	3	F2-3 vs. L2-3	257172_at	AT3G23700	Encodes a chloroplast-localized S1 domain-containing protein with RNA chaperone activity that affects the splicing and processing of chloroplast transcripts and plays a role in Arabidopsis seedling growth in the presence of ABA. Binds the chloroplast psbA RNA and some other chloroplast RNAs. Required for the stability of the chloroplast ndhC RNA. Inhibits ribosome association with psbA RNA and ycf1 RNA. Not required for the splicing of chloroplast trnL, as had been reported previously.	111.83	59.64	1.88	9.05E-04	2.97E-03
1737	3	F2-3 vs. L2-3	252671_at	AT3G44190	FAD/NAD(P)-binding oxidoreductase family protein;(source:Araport11)	221.11	117.75	1.88	6.24E-04	2.23E-03
1738	3	F2-3 vs. L2-3	254103_at	AT4G25030	Plastid localized protein of unknown function. Mutants are more susceptible to P. syringae and produce less callose upon infection.	133.61	70.91	1.88	5.20E-06	7.50E-05
1739	3	F2-3 vs. L2-3	253330_at	AT4G33530	potassium transporter	100.50	53.43	1.88	3.61E-04	1.47E-03

1740	3	F2-3 vs. L2-3	250517_at	AT5G08260	serine carboxypeptidase-like 35;(source:Araport11)	61.17	32.51	1.88	1.45E-04	7.63E-04
1741	3	F2-3 vs. L2-3	247478_at	AT5G62360	Pectin methylesterase inhibitor expressed throughout the plant.	32.70	17.37	1.88	2.38E-05	2.13E-04
1742	3	F2-3 vs. L2-3	261583_at	AT1G01090	pyruvate dehydrogenase E1 alpha subunit	269.57	143.92	1.87	1.42E-04	7.51E-04
1743	3	F2-3 vs. L2-3	264727_at	AT1G22840	Encodes cytochrome c. Contains two site II (TGGGCC/T) elements, which interact with a TCP-domain transcription factor, and a downstream internal telomeric repeat, and are required for expression of the Cytc-1 gene. Promoter directs preferential expression in root and shoot meristems and in anthers. Double mutants with CYTC-2 accumulate starch during the day, have delayed growth and development and reduced GA and DELLA proteins linking cellular metabolism and GA homeostasis.	1183.42	634.21	1.87	5.47E-04	2.01E-03
1744	3	F2-3 vs. L2-3	256213_at	AT1G50990	kinase with tetratricopeptide repeat domain-containing protein;(source:Araport11)	23.82	12.73	1.87	4.02E-05	3.10E-04
1745	3	F2-3 vs. L2-3	260002_at	AT1G67940	member of NAP subfamily The mRNA is cell-to-cell mobile.	98.16	52.62	1.87	3.87E-04	1.55E-03
1746	3	F2-3 vs. L2-3	260214_at	AT1G74510	Galactose oxidase/kelch repeat superfamily protein;(source:Araport11)	78.75	42.22	1.87	4.58E-04	1.75E-03
1747	3	F2-3 vs. L2-3	266977_at	AT2G39420	alpha/beta-Hydrolases superfamily protein;(source:Araport11)	23.40	12.49	1.87	3.50E-05	2.78E-04
1748	3	F2-3 vs. L2-3	259056_at	AT3G03420	Ku70-binding family protein;(source:Araport11)	51.35	27.47	1.87	2.35E-04	1.07E-03
1749	3	F2-3 vs. L2-3	258312_at	AT3G16170	Encodes a malonyl-CoA synthetase that is localized to the cytosol and mitochondrion. AAE13 produces two transcripts one of which includes an N terminal mitochondrial targeting motif. Loss of function of the mtAAE13 product results in growth arrest and lethality.	655.37	349.73	1.87	3.04E-04	1.31E-03
1750	3	F2-3 vs. L2-3	255418_at	AT4G03200	catalytics;(source:Araport11)	147.66	79.04	1.87	5.72E-04	2.07E-03
1751	3	F2-3 vs. L2-3	246215_at	AT4G37180	UIF1 is a nuclear and cytoplasmically localized myb-domain containing member of the GARP G2-like subfamily of transcription factors. Interacts with ULT1 and binds to the WUS promoter. UIF1 binding domains are also found in CUC and AG promoters suggesting they are also direct targets. This locus was also identified as a putative cytoskeletal protein in a yeast screen.	32.00	17.13	1.87	2.70E-04	1.19E-03
1752	3	F2-3 vs. L2-3	250633_at	AT5G07460	ubiquitous enzyme that repairs oxidatively damaged proteins. Methionine sulfoxide reductase activity. Mutant lacking reductase activity showed increased protein oxidation, nitration and glycation of specific amino acid residues during darkness.	26.62	14.21	1.87	5.69E-04	2.07E-03
1753	3	F2-3 vs. L2-3	264504_at	AT1G09430	Encodes subunit A of the heteromeric enzyme ATP citrate lyase (ACL). In animals, ACL is encoded by a single gene; ACL in Arabidopsis is composed of two polypeptides, ACLA (encoded by 3 genes) and ACLB (encoded by 2 genes). The holoenzyme has an A(4)B(4)stoichiometry. Expression of both ACLA and ACLB but not of either of the subunits alone results in ACL activity.	84.86	45.50	1.86	1.77E-04	8.79E-04
1754	3	F2-3 vs. L2-3	260969_at	AT1G12240	Encodes a vacuolar invertase betaFruct4. betaFruct4 is transported from the endoplasmic reticulum through the intermediate compartments as a membrane protein. The N-terminal cytoplasmic domain contains multiple sequence motifs that are involved at various stages in the trafficking of betaFruct4 from the ER to the central vacuole. The mRNA is cell-to-cell mobile.	24.64	13.24	1.86	6.74E-04	2.35E-03
1755	3	F2-3 vs. L2-3	261031_at	AT1G17360	LOW protein: protein phosphatase 1 regulatory subunit-like protein;(source:Araport11)	108.88	58.63	1.86	5.86E-04	2.11E-03
1756	3	F2-3 vs. L2-3	261405_at	AT1G18740	DUF793 domain containing protein. Expression is induced by cold. Loss of function mutations are more sensitive to freezing and have reduced levels of CBFs. May act by preventing degradation of CBFs.	719.07	386.65	1.86	1.55E-04	8.02E-04
1757	3	F2-3 vs. L2-3	259826_at	AT1G29340	Encodes a protein containing a UND, a U-box, and an ARM domain. This protein has E3 ubiquitin ligase activity. It is required for cell death and full resistance specified by Arabidopsis RPM1 and RPS4 resistance proteins against Pseudomonas syringae pv tomato. The mRNA is cell-to-cell mobile.	392.88	210.94	1.86	5.54E-04	2.03E-03
1758	3	F2-3 vs. L2-3	261511_at	AT1G71770	Encodes a Class I polyA-binding protein. Expressed in floral organs. Binds polyA sepharose in vitro.	51.11	27.47	1.86	2.63E-04	1.17E-03
1759	3	F2-3 vs. L2-3	258483_at	AT3G02570	Encodes a protein with phosphomannose isomerase activity.	79.00	42.44	1.86	5.01E-05	3.63E-04
1760	3	F2-3 vs. L2-3	258161_at	AT3G17930	Encodes a thylakoid membrane protein involved in the accumulation of the cytochrome b6/f complex.	101.27	54.35	1.86	1.51E-05	1.59E-04
1761	3	F2-3 vs. L2-3	250146_at	AT5G14660	Encodes a peptide deformylase PDF1B. The crystal structure has been determined at a resolution of 0.24 nm (Biochem J, 2008, vol 413:417-427).	146.43	78.66	1.86	7.50E-04	2.55E-03
1762	3	F2-3 vs. L2-3	249719_at	AT5G35735	Auxin-responsive family protein;(source:Araport11)	31.19	16.74	1.86	1.08E-04	6.22E-04
1763	3	F2-3 vs. L2-3	261037_at	AT1G17420	LOX3 encode a Lipoxygenase. Lipoxygenases (LOXs) catalyze the oxygenation of fatty acids (FAs).	29.93	16.21	1.85	9.95E-05	5.85E-04

1764	3	F2-3 vs. L2-3	265943_at	AT2G19570	Encodes a cytidine deaminase that deaminates cytidine and deoxycytidine and is competitively inhibited by cytosine-containing compounds.	95.52	51.57	1.85	1.60E-04	8.18E-04
1765	3	F2-3 vs. L2-3	263252_at	AT2G31380	a B-box zinc finger protein that interacts with COP1. contains a novel 11 amino acid motif at the C-terminus (also found at the N-terminus of HY5) that is involved in the COP1 interaction.	51.05	27.53	1.85	5.62E-05	3.95E-04
1766	3	F2-3 vs. L2-3	258849_at	AT3G03250	Is thought to encode a cytosolic UDP-glucose pyrophosphorylase with strong similarity to potato UTP--glucose-1-phosphate uridylyltransferase. Downregulated by flooding.	795.97	429.13	1.85	1.08E-04	6.22E-04
1767	3	F2-3 vs. L2-3	252164_at	AT3G50620	P-loop containing nucleoside triphosphate hydrolases superfamily protein;(source:Araport11)	28.79	15.52	1.85	7.07E-04	2.45E-03
1768	3	F2-3 vs. L2-3	254890_at	AT4G11600	Encodes glutathione peroxidase.	63.65	34.35	1.85	4.65E-04	1.77E-03
1769	3	F2-3 vs. L2-3	250800_at	AT5G05370	Cytochrome b-c1 complex, subunit 8 protein;(source:Araport11)	181.37	98.03	1.85	2.57E-04	1.15E-03
1770	3	F2-3 vs. L2-3	249063_at	AT5G44110	Encodes a member of the NAP subfamily of ABC transporters whose expression pattern is regulated by light and sucrose.	188.43	101.66	1.85	2.02E-05	1.91E-04
1771	3	F2-3 vs. L2-3	244902_at	ATMG00650	Encodes NADH dehydrogenase subunit 4L.	152.04	82.34	1.85	7.34E-05	4.71E-04
1772	3	F2-3 vs. L2-3	265680_at	AT2G32150	Haloacid dehalogenase-like hydrolase (HAD) superfamily protein;(source:Araport11)	81.15	44.01	1.84	2.44E-04	1.10E-03
1773	3	F2-3 vs. L2-3	262323_at	AT1G64190	6-phosphogluconate dehydrogenase family protein;(source:Araport11)	477.36	261.52	1.83	1.32E-04	7.19E-04
1774	3	F2-3 vs. L2-3	259850_at	AT1G72240	hypothetical protein;(source:Araport11)	27.24	14.90	1.83	8.63E-04	2.86E-03
1775	3	F2-3 vs. L2-3	265742_at	AT2G01290	Cytosolic ribose-5-phosphate isomerase. Knockout mutation causes chloroplast dysfunction, late flowering and premature cell death.	53.61	29.37	1.83	1.36E-04	7.28E-04
1776	3	F2-3 vs. L2-3	258602_at	AT3G02750	Protein phosphatase 2C family protein;(source:Araport11)	58.51	31.91	1.83	1.99E-04	9.56E-04
1777	3	F2-3 vs. L2-3	257701_at	AT3G12710	DNA glycosylase superfamily protein;(source:Araport11)	48.31	26.37	1.83	1.45E-04	7.63E-04
1778	3	F2-3 vs. L2-3	256711_at	AT3G30390	Encodes a putative amino acid transporter.	968.53	529.80	1.83	4.46E-04	1.72E-03
1779	3	F2-3 vs. L2-3	251666_at	AT3G57050	Encodes cystathionine beta-lyase, the second enzyme in the methionine biosynthetic pathway. Mutants show defects in root development, reduced methylation and maintenance of the quiescent center.	811.75	444.63	1.83	7.57E-05	4.82E-04
1780	3	F2-3 vs. L2-3	248466_at	AT5G50720	Encodes one of five HVA22 homologs in Arabidopsis. HVA22 is an ABA- and stress-inducible gene first isolated from barley. Members of this gene family have only been found in eukaryotes. AtHVA22e mRNA is upregulated to varying degrees in response to cold stress, salt stress, ABA treatment or dehydration.	44.22	24.16	1.83	1.18E-04	6.62E-04
1781	3	F2-3 vs. L2-3	246396_at	AT1G58180	beta carbonic anhydrase 6;(source:Araport11)	27.38	15.05	1.82	1.33E-04	7.21E-04
1782	3	F2-3 vs. L2-3	259882_at	AT1G76670	Nucleotide-sugar transporter family protein;(source:Araport11)	323.86	178.01	1.82	1.49E-04	7.78E-04
1783	3	F2-3 vs. L2-3	251257_at	AT3G62290	A member of ARF GTPase family. A thaliana has 21 members of this family, known to be essential for vesicle coating and uncoating and functions in GTP-binding. The gene is shown to play a role in cell division, cell expansion and cellulose production using antisense construct. The mRNA is cell-to-cell mobile.	2912.21	1601.77	1.82	4.94E-05	3.59E-04
1784	3	F2-3 vs. L2-3	263669_at	AT1G04400	Blue light receptor mediating blue-light regulated cotyledon expansion and flowering time. Positive regulator of the flowering-time gene CONSTANS. This gene possesses a light-induced CNT2 N-terminal homodimerisation domain.Involved in blue-light induced stomatal opening. Involved in triggering chromatin decondensation. An 80-residue motif (NC80) is sufficient to confer CRY2's physiological function. It is proposed that the PHR domain and the C-terminal tail of the unphosphorylated CRY2 form a "closed" conformation to suppress the NC80 motif in the absence of light. In response to blue light, the C-terminal tail of CRY2 is phosphorylated and electrostatically repelled from the surface of the PHR domain to form an "open" conformation, resulting in derepression of the NC80 motif and signal transduction to trigger photomorphogenic responses. Cry2 phosphorylation and degradation both occur in the nucleus.The life-time of cry2 signaling state in situ (in planta) is about 16 min.	191.65	106.13	1.81	1.90E-04	9.16E-04
1785	3	F2-3 vs. L2-3	261455_at	AT1G21070	Nucleotide-sugar transporter family protein;(source:Araport11)	38.76	21.39	1.81	4.11E-04	1.62E-03
1786	3	F2-3 vs. L2-3	262500_at	AT1G21760	This gene is predicted to encode an F-box protein that is evolutionarily conserved between Arabidopsis and other eukaryotes including S.cerevisiae and humans. It may play a role in regulating translation under conditions of temperature stress. FBP7 transcript levels are increased at high and low temperatures. The mRNA is cell-to-cell mobile.	77.90	42.98	1.81	6.47E-05	4.33E-04

1787	3	F2-3 vs. L2-3	262263_at	AT1G70940	A regulator of auxin efflux and involved in differential growth. PIN3 is expressed in gravity-sensing tissues, with PIN3 protein accumulating predominantly at the lateral cell surface. PIN3 localizes to the plasma membrane and to vesicles. In roots, PIN3 is expressed without pronounced polarity in tiers two and three of the columella cells, at the basal side of vascular cells, and to the lateral side of pericycle cells of the elongation zone. PIN3 overexpression inhibits root cell growth. Protein phosphorylation plays a role in regulating PIN3 trafficking to the plasma membrane. The mRNA is cell-to-cell mobile.	46.84	25.94	1.81	2.91E-04	1.26E-03
1788	3	F2-3 vs. L2-3	260080_at	AT1G78160	Encodes a member of the Arabidopsis Pumilio (APUM) proteins containing PUF domain (eight repeats of approximately 36 amino acids each). PUF proteins regulate both mRNA stability and translation through sequence-specific binding to the 3' UTR of target mRNA transcripts.	36.82	20.36	1.81	1.04E-04	6.08E-04
1789	3	F2-3 vs. L2-3	267029_at	AT2G38460	Encodes IRON REGULATED1 (IREG1/FPN1), one of the Arabidopsis orthologs (AT2G38460/IREG1/FPN1 and AT5G03570/IREG2/FPN2) the iron efflux transporter ferroportin (FPN) identified in animals.	114.56	63.17	1.81	1.20E-04	6.65E-04
1790	3	F2-3 vs. L2-3	266078_at	AT2G40670	response regulator 16	29.99	16.59	1.81	9.90E-05	5.82E-04
1791	3	F2-3 vs. L2-3	258040_at	AT3G21190	O-fucosyltransferase family protein;(source:Araport11)	450.61	248.76	1.81	2.63E-04	1.17E-03
1792	3	F2-3 vs. L2-3	257150_at	AT3G27230	S-adenosyl-L-methionine-dependent methyltransferases superfamily protein;(source:Araport11)	119.38	66.07	1.81	3.33E-05	2.69E-04
1793	3	F2-3 vs. L2-3	252906_at	AT4G39640	The gene encodes a gamma-glutamyltransferase (AKA gamma-glutamyl transpeptidase, EC 2.3.2.2) that is located in vascular tissues (predominantly phloem) of leaves and is involved in the degradation of glutathione. The encoded enzyme also mitigates oxidative stress by metabolizing GSSG (oxidized form of GSH - glutathione) in the apoplast.	57.86	31.95	1.81	6.11E-05	4.19E-04
1794	3	F2-3 vs. L2-3	248140_at	AT5G54980	Uncharacterized protein family (UPF0497);(source:Araport11)	98.36	54.26	1.81	1.36E-04	7.28E-04
1795	3	F2-3 vs. L2-3	247030_at	AT5G67210	Encode a DUF579 (domain of unknown function 579) containing protein essential for normal xylan synthesis and deposition in the secondary cell wall.	27.21	15.07	1.81	6.51E-04	2.30E-03
1796	3	F2-3 vs. L2-3	262756_at	AT1G16370	organic cation/carnitine transporter 6;(source:Araport11)	28.42	15.79	1.80	4.74E-04	1.80E-03
1797	3	F2-3 vs. L2-3	260518_at	AT1G51410	similar to Eucalyptus gunnii alcohol dehydrogenase of unknown physiological function (GI:1143445), apple tree, PIR:T16995; NOT a cinnamyl-alcohol dehydrogenase	41.89	23.34	1.80	1.86E-05	1.83E-04
1798	3	F2-3 vs. L2-3	260274_at	AT1G80460	Encodes a protein similar to glycerol kinase, which converts glycerol to glycerol 3-phosphate and performs a rate-limiting step in glycerol metabolism. This gene is required for both general and specific resistance against bacteria and fungi. Arabidopsis thaliana glycerol kinase (GLR1) mRNA.Involved in flagellin-induced non-host resistance to Pseudomonas. Coronatine partially suppresses flagellin-induced expression of NHO1.	706.70	391.77	1.80	4.66E-04	1.78E-03
1799	3	F2-3 vs. L2-3	267151_at	AT2G30970	ASPARTATE AMINOTRANSFERASE 1	138.54	76.94	1.80	5.41E-04	1.99E-03
1800	3	F2-3 vs. L2-3	258545_at	AT3G07050	Arabidopsis NSN1 encodes a nucleolar GTP- binding protein and is required for maintenance of inflorescence meristem identity and floral organ development.	247.88	137.97	1.80	1.02E-05	1.20E-04
1801	3	F2-3 vs. L2-3	257881_at	AT3G17180	serine carboxypeptidase-like 33;(source:Araport11)	46.18	25.59	1.80	1.81E-04	8.86E-04
1802	3	F2-3 vs. L2-3	257082_at	AT3G20580	COBRA-like protein 10 precursor;(source:Araport11)	45.39	25.23	1.80	8.55E-04	2.84E-03
1803	3	F2-3 vs. L2-3	257824_at	AT3G25290	Auxin-responsive family protein;(source:Araport11)	23.17	12.85	1.80	1.75E-04	8.74E-04
1804	3	F2-3 vs. L2-3	252736_at	AT3G43210	Encodes a kinesin TETRASPORE. Required for cytokinesis in pollen. In mutants, all four microspore nuclei remain within the same cytoplasm after meiosis.	46.18	25.64	1.80	7.90E-06	1.02E-04
1805	3	F2-3 vs. L2-3	251757_at	AT3G55640	Mitochondrial substrate carrier family protein;(source:Araport11)	199.68	111.02	1.80	4.68E-05	3.45E-04
1806	3	F2-3 vs. L2-3	253779_at	AT4G28490	Member of Receptor kinase-like protein family. Controls the separation step of floral organ abscission. The mRNA is cell-to-cell mobile.	35.10	19.49	1.80	5.90E-06	8.35E-05
1807	3	F2-3 vs. L2-3	250337_at	AT5G11790	Plays a role in dehydration stress response.	121.21	67.26	1.80	1.36E-04	7.28E-04
1808	3	F2-3 vs. L2-3	262342_at	AT1G64150	PAM71 encodes an integral thylakoid membrane protein that is required for normal operation of oxygen-evolving complex (as evidenced by oxygen evolution rates) and for manganese incorporation. PAM71 belongs to a small gene family in Arabidopsis comprising five members. PAM71 is well conserved in the green lineage and shares homology with putative Ca2+/H+ exchangers from yeast (Saccharomyces cerevisiae) (GDT1) and human (Homo sapiens) (TMEM165).	94.54	52.95	1.79	5.21E-04	1.94E-03
1809	3	F2-3 vs. L2-3	264856_at	AT2G17370	Encodes a 3-hydroxy-3-methylglutaryl-CoA reductase (HMGR) that is involved in the synthesis of sterol and triterpenoid compounds.	41.73	23.34	1.79	6.41E-04	2.27E-03

1810	3	F2-3 vs. L2-3	267087_at	AT2G32460	Member of the R2R3 factor gene family.	50.06	28.04	1.79	7.25E-05	4.66E-04
1811	3	F2-3 vs. L2-3	258288_at	AT3G23290	LIGHT-DEPENDENT SHORT HYPOCOTYLS-like protein (DUF640);(source:Araport11)	40.53	22.64	1.79	4.94E-04	1.86E-03
1812	3	F2-3 vs. L2-3	250075_at	AT5G17670	alpha/beta-Hydrolases superfamily protein;(source:Araport11)	172.33	96.27	1.79	2.59E-04	1.16E-03
1813	3	F2-3 vs. L2-3	265093_at	AT1G03905	P-loop containing nucleoside triphosphate hydrolases superfamily protein;(source:Araport11)	67.30	37.78	1.78	3.73E-04	1.51E-03
1814	3	F2-3 vs. L2-3	256530_at	AT1G33290	P-loop containing nucleoside triphosphate hydrolases superfamily protein;(source:Araport11)	64.08	35.97	1.78	1.78E-04	8.81E-04
1815	3	F2-3 vs. L2-3	261326_s_a	AT1G44820	Peptidase M20/M25/M40 family protein;(source:Araport11)	58.19	32.68	1.78	4.20E-06	6.33E-05
1816	3	F2-3 vs. L2-3	260616_at	AT1G53280	Encodes a homolog of animal DJ-1 superfamily protein. In the A. thaliana genome, three genes encoding close homologs of human DJ-1 were identified AT3G14990 (DJ1A), AT1G53280 (DJ1B) and AT4G34020 (DJ1C). Among the three homologs, DJ1C is essential for chloroplast development and viability. It exhibits glyoxalase activity towards glyoxal and methylglyoxal.	792.17	446.20	1.78	1.11E-04	6.34E-04
1817	3	F2-3 vs. L2-3	261958_at	AT1G64500	A member of a protein family found in plants and animals that contain conserved C-terminal glutaredoxin-like and putative zinc-binding cysteine-rich domains. It is involved in light stimulated actin bundling and chloroplast movement. The mRNA is cell-to-cell mobile.	43.51	24.38	1.78	6.28E-04	2.23E-03
1818	3	F2-3 vs. L2-3	263876_at	AT2G21880	RAB GTPase homolog 7A;(source:Araport11)	53.17	29.95	1.78	5.84E-05	4.05E-04
1819	3	F2-3 vs. L2-3	266168_at	AT2G38870	Predicted to encode a PR (pathogenesis-related) peptide that belongs to the PR-6 proteinase inhibitor family. Six putative PR-6-type protein encoding genes are found in Arabidopsis: At2g38900, At2g38870, At5g43570, At5g43580, At3g50020 and At3g46860.	38.90	21.91	1.78	7.35E-04	2.52E-03
1820	3	F2-3 vs. L2-3	247109_at	AT5G65870	Probable phytosulfokines 5 precursor, coding for a unique plant peptide growth factor.	17.63	9.92	1.78	2.81E-04	1.23E-03
1821	3	F2-3 vs. L2-3	264440_at	AT1G27340	Encodes a putative F-box protein that is involved in the regulation of leaf morphology.	117.04	66.31	1.77	3.90E-05	3.04E-04
1822	3	F2-3 vs. L2-3	245114_at	AT2G41630	Encodes a transcription factor, TFIIB1, that plays important roles in pollen tube growth, guidance, and reception as well as endosperm development and is partially functionally different from AtTFIIB2 and AtTFIIB3/AtpBRP2.	26.64	15.05	1.77	1.99E-05	1.89E-04
1823	3	F2-3 vs. L2-3	253512_at	AT4G31750	Encodes HopW1-1-Interacting protein 2 (WIN2). Interacts with the P. syringae effector HopW1-1. WIN2 has protein phosphatase activity. Modulates plant defenses against bacteria. Three WIN proteins are identified so far (WIN1: AT1G80600; WIN2: AT4G31750; WIN3: AT5G13320).	502.12	284.43	1.77	3.01E-04	1.29E-03
1824	3	F2-3 vs. L2-3	249397_at	AT5G40230	nodulin MtN21-like transporter family protein	53.13	29.94	1.77	3.19E-05	2.61E-04
1825	3	F2-3 vs. L2-3	248838_at	AT5G46800	Seedling lethal mutation; Mitochondrial Carnitine Acyl Carrier-Like Protein	346.20	196.03	1.77	2.08E-04	9.86E-04
1826	3	F2-3 vs. L2-3	252293_at	AT3G48990	Encodes an oxalyl-CoA synthetase and is required for oxalate degradation, for normal seed development, and for defense against an oxalate-producing fungal pathogen.	380.97	217.01	1.76	8.63E-04	2.86E-03
1827	3	F2-3 vs. L2-3	254710_at	AT4G18050	P-glycoprotein 9;(source:Araport11)	23.49	13.35	1.76	6.91E-04	2.40E-03
1828	3	F2-3 vs. L2-3	245891_at	AT5G09220	member of AAAP family The mRNA is cell-to-cell mobile.	47.70	27.16	1.76	5.18E-04	1.93E-03
1829	3	F2-3 vs. L2-3	245895_at	AT5G09230	Encodes SRT2, a member of the SIR2 (sirtuin) family HDAC (histone deacetylase) (SRT1/AT5g55760, SRT2/AT5G09230).	103.70	59.06	1.76	3.04E-04	1.31E-03
1830	3	F2-3 vs. L2-3	248237_at	AT5G53890	Encodes a leucine-rich repeat receptor kinase (LRR-RK) involved in the perception of phytosulfokine (PSK), which is a 5-aa tyrosine-sulfated peptide that primarily promotes cellular proliferation.	35.33	20.04	1.76	3.19E-04	1.35E-03
1831	3	F2-3 vs. L2-3	247693_at	AT5G59730	A member of EXO70 gene family, putative exocyst subunits, conserved in land plants. Arabidopsis thaliana contains 23 putative EXO70 genes, which can be classified into eight clusters on the phylogenetic tree. The mRNA is cell-to-cell mobile.	78.73	44.69	1.76	1.79E-04	8.84E-04
1832	3	F2-3 vs. L2-3	AFFX-r2-At-Actin-	AT5G09810	Member of Actin gene family.Mutants are defective in germination and root growth. The mRNA is cell-to-cell mobile.	702.19	400.13	1.75	2.23E-04	1.04E-03
1833	3	F2-3 vs. L2-3	264436_at	AT1G10370	Encodes GSTU17 (Glutathione S-Transferase U17). Functions as a negative component of stress-mediated signal transduction pathways in drought and salt stress responses.	130.28	74.33	1.75	9.50E-06	1.14E-04

1834	3	F2-3 vs. L2-3	256115_at	AT1G16880	Encodes a ACT domain-containing protein. The ACT domain, named after bacterial aspartate kinase, chorismate mutase and TyrA (prephenate dehydrogenase), is a regulatory domain that serves as an amino acid-binding site in feedback-regulated amino acid metabolic enzymes. The mRNA is cell-to-cell mobile.	261.08	149.07	1.75	8.81E-05	5.37E-04
1835	3	F2-3 vs. L2-3	259588_at	AT1G27930	Arabinogalactan methylesterase,involved in arabinogalactan glucuronic acid methylation. Interacts with eIF3.	59.22	33.88	1.75	5.84E-04	2.11E-03
1836	3	F2-3 vs. L2-3	245742_at	AT1G44170	Encodes an aldehyde dehydrogenase induced by ABA and dehydration that can oxidize saturated aliphatic aldehydes. It is also able to oxidize beta-unsaturated aldehydes, but not aromatic aldehydes. Activity of ALDH3H1 is NAD +-dependent.	51.60	29.56	1.75	2.31E-05	2.08E-04
1837	3	F2-3 vs. L2-3	265122_at	AT1G62540	belongs to the flavin-monooxygenase (FMO) family, encodes a glucosinolate S-oxygenase that catalyzes the conversion of methylthioalkyl glucosinolates to methylsulfinylalkyl glucosinolatesThe mRNA is cell-to-cell mobile.	21.15	12.10	1.75	1.64E-04	8.34E-04
1838	3	F2-3 vs. L2-3	263489_at	AT2G31830	Encodes a 5-inositol-polyphosphate phosphatase, that, in vitro, shows some activity against Ins(1,4,5)P3 and PI(3,4,5)P3, but even higher activity against PI(4,5)P2	27.73	15.87	1.75	9.09E-04	2.98E-03
1839	3	F2-3 vs. L2-3	263775_at	AT2G46410	Nuclear-localized R3-type MYB transcription factor. Positive regulator of hair-cell differentiation. Preferentially transcribed in hairless cells. Moves from atrichoblasts into trichoblast via plasmodesmata in a tissue-specific mode. N-terminus and part of the Myb domain are required for this movement, with W76 playing a crucial role. Capability to increase the size-exclusion limit of plasmodesmata. Regulated by WEREWOLF.	31.40	17.94	1.75	2.90E-04	1.26E-03
1840	3	F2-3 vs. L2-3	252483_at	AT3G46600	GRAS family transcription factor;(source:Araport11)	32.65	18.69	1.75	3.16E-05	2.60E-04
1841	3	F2-3 vs. L2-3	254279_at	AT4G22750	Encodes a protein S-acyltransferase that, together with PAT14, cooperatively regulates leaf senescence.	49.88	28.58	1.75	2.95E-04	1.27E-03
1842	3	F2-3 vs. L2-3	247426_at	AT5G62570	Calmodulin binding protein-like protein;(source:Araport11)	54.12	31.00	1.75	6.68E-04	2.34E-03
1843	3	F2-3 vs. L2-3	259693_at	AT1G63060	ribosome biogenesis NEP1-like protein;(source:Araport11)	25.31	14.53	1.74	8.03E-05	5.03E-04
1844	3	F2-3 vs. L2-3	262727_at	AT1G75800	Pathogenesis-related thaumatin superfamily protein;(source:Araport11)	49.19	28.34	1.74	4.06E-04	1.61E-03
1845	3	F2-3 vs. L2-3	264045_at	AT2G22450	riboflavin biosynthesis protein;(source:Araport11)	65.92	37.78	1.74	5.37E-04	1.99E-03
1846	3	F2-3 vs. L2-3	258470_at	AT3G06035	Glycoprotein membrane precursor GPI-anchored;(source:Araport11)	21.28	12.22	1.74	1.12E-05	1.27E-04
1847	3	F2-3 vs. L2-3	246486_at	AT5G15910	NAD(P)-binding Rossmann-fold superfamily protein;(source:Araport11)	42.02	24.18	1.74	4.87E-04	1.84E-03
1848	3	F2-3 vs. L2-3	249658_s_a	AT5G36700	2-phosphoglycolate phosphatase 1;(source:Araport11)	316.49	181.78	1.74	3.14E-04	1.33E-03
1849	3	F2-3 vs. L2-3	247185_at	AT5G65460	Kinesin that binds cyclin-dependent kinase CDKA;1 as homodimer or as heterodimer with KCA1	49.55	28.53	1.74	2.40E-04	1.09E-03
1850	3	F2-3 vs. L2-3	247125_at	AT5G66070	E3 ubiquitin ligase that functions in negative regulation of ABA signaling.	144.16	82.86	1.74	1.23E-04	6.80E-04
1851	3	F2-3 vs. L2-3	246288_at	AT1G31850	S-adenosyl-L-methionine-dependent methyltransferases superfamily protein;(source:Araport11)	37.07	21.42	1.73	4.08E-04	1.61E-03
1852	3	F2-3 vs. L2-3	263368_at	AT2G20470	AGC (cAMP-dependent, cGMP-dependent and protein kinase C) kinase family protein;(source:Araport11)	70.75	40.86	1.73	8.36E-04	2.79E-03
1853	3	F2-3 vs. L2-3	258925_at	AT3G10420	P-loop containing nucleoside triphosphate hydrolases superfamily protein;(source:Araport11)	299.86	173.70	1.73	2.55E-04	1.14E-03
1854	3	F2-3 vs. L2-3	258017_at	AT3G19370	filament-like protein (DUF869);(source:Araport11)	19.27	11.13	1.73	6.16E-05	4.21E-04
1855	3	F2-3 vs. L2-3	255305_at	AT4G04770	Encodes an iron-stimulated ATPase. A member of the NAP subfamily of ABC transporters. Involved in Fe-S cluster assembly. Similar to SufB. Involved in the regulation of iron homeostasis. Able to form homodimers. Interacts with AtNAP7 inside the chloroplast.	58.46	33.86	1.73	5.18E-04	1.93E-03
1856	3	F2-3 vs. L2-3	254485_at	AT4G20760	NAD(P)-binding Rossmann-fold superfamily protein;(source:Araport11)	71.32	41.29	1.73	1.52E-04	7.88E-04
1857	3	F2-3 vs. L2-3	250217_at	AT5G14120	Major facilitator superfamily protein;(source:Araport11)	175.56	101.62	1.73	9.77E-05	5.77E-04
1858	3	F2-3 vs. L2-3	248402_at	AT5G52100	Is essential for chloroplast NAD(P)H dehydrogenase activity, which is involved in electron transfer between PSII and PSI. Likely functions in biogenesis or stabilization of the NAD(P)H dehydrogenase complex. The mRNA is cell-to-cell mobile.	36.10	20.92	1.73	1.06E-05	1.22E-04
1859	3	F2-3 vs. L2-3	257375_at	AT2G38640	LURP-one-like protein (DUF567);(source:Araport11)	20.85	12.15	1.72	3.04E-05	2.53E-04
1860	3	F2-3 vs. L2-3	259231_at	AT3G11410	Encodes protein phosphatase 2C. Negative regulator of ABA signalling. Expressed in seeds during germination. mRNA up-regulated by drought and ABA.	459.42	266.43	1.72	7.20E-06	9.51E-05
1861	3	F2-3 vs. L2-3	254886_at	AT4G11760	Encodes a member of a family of small,secreted, cysteine rich protein with sequence similarity to the PCP (pollen coat protein) gene family.	26.86	15.62	1.72	8.46E-05	5.24E-04
1862	3	F2-3 vs. L2-3	254137_at	AT4G24930	thylakoid lumenal 17.9 kDa protein, chloroplast;(source:Araport11)	68.90	39.97	1.72	4.96E-04	1.87E-03
1863	3	F2-3 vs. L2-3	248126_at	AT5G54760	Translation initiation factor SUI1 family protein;(source:Araport11)	1807.25	1050.46	1.72	2.05E-04	9.74E-04

1864	3	F2-3 vs. L2-3	247613_at	AT5G60740	ABC transporter family protein;(source:Araport11)	29.76	17.34	1.72	3.31E-04	1.39E-03
1865	3	F2-3 vs. L2-3	261924_at	AT1G22550	Tonoplast localized pH dependent, low affinity nitrogen transporter.In shoots, expressed in leaf veins and mesophyll. In roots, GUS activity was detected in root vascular stele. More highly expressed in roots.	38.91	22.78	1.71	5.93E-04	2.13E-03
1866	3	F2-3 vs. L2-3	252822_at	AT4G39955	alpha/beta-Hydrolases superfamily protein;(source:Araport11)	33.69	19.65	1.71	1.40E-04	7.43E-04
1867	3	F2-3 vs. L2-3	258516_at	AT3G06490	Encodes a MYB transcription factor involved in regulating anther dehiscence as well as regulating cell death, and cuticle-related Botrytis immunity.	20.65	12.14	1.70	1.16E-04	6.55E-04
1868	3	F2-3 vs. L2-3	257267_at	AT3G15030	Arabidopsis thaliana TCP family transcription factor. Regulated by miR319. Involved in heterchronic regulation of leaf differentiation.	98.05	57.64	1.70	6.35E-04	2.25E-03
1869	3	F2-3 vs. L2-3	252037_at	AT3G51920	encodes a divergent member of calmodulin, which is an EF-hand family of Ca2+-binding proteins. This gene is expressed in leaves, flowers and siliques. The gene functionally complements yeast calmodulin 1 (CAM1) but only when selected against the plasmid harboring wild-type yeast sequences. Also the protein does not form formed a complex with a basic amphiphilic helical peptide in the presence of Ca2+ in vitro. Authors suggest that this gene may represent a Ca2+-binding sensor protein that interacts with a more limited set of target proteins than do more conventional CaM isoforms. Mutations in this gene alter plant responses to abiotic stress and abscisic acid.	19.95	11.73	1.70	5.84E-05	4.05E-04
1870	3	F2-3 vs. L2-3	251839_at	AT3G54950	Encodes pPLAIIIbeta, a member of the Group 3 patatin-related phospholipases. pPLAIIIbeta hydrolyzes phospholipids and galactolipids and additionally has acyl-CoA thioesterase activity. Alterations of pPLAIIIβ result in changes in lipid levels and composition.	137.06	80.79	1.70	2.09E-05	1.95E-04
1871	3	F2-3 vs. L2-3	255365_at	AT4G04040	Phosphofructokinase family protein;(source:Araport11)	681.89	401.12	1.70	9.64E-04	3.12E-03
1872	3	F2-3 vs. L2-3	247813_at	AT5G58330	lactate/malate dehydrogenase family protein;(source:Araport11)	580.97	342.54	1.70	7.97E-04	2.68E-03
1873	3	F2-3 vs. L2-3	246987_at	AT5G67300	Member of the R2R3 factor MYB gene family involved in mediating plant responses to a variety of abiotic stimiuli. The mRNA is cell-to-cell mobile.	463.63	273.15	1.70	1.73E-04	8.64E-04
1874	3	F2-3 vs. L2-3	259098_at	AT3G04790	Ribose 5-phosphate isomerase, type A protein;(source:Araport11)	229.17	135.59	1.69	2.37E-04	1.08E-03
1875	3	F2-3 vs. L2-3	245407_at	AT4G17170	member of RAB gene family	169.90	100.35	1.69	3.81E-04	1.54E-03
1876	3	F2-3 vs. L2-3	249211_at	AT5G42680	MIZU-KUSSEI-like protein (Protein of unknown function, DUF617);(source:Araport11)	34.74	20.55	1.69	4.14E-04	1.63E-03
1877	3	F2-3 vs. L2-3	245150_at	AT2G47590	photolyase/blue light photoreceptor PHR2 (PHR2) mRNA,	57.50	34.28	1.68	3.84E-04	1.54E-03
1878	3	F2-3 vs. L2-3	258135_at	AT3G24506	Participates in the late stages of the biogenesis of 50S ribosomal subunits in plastids.	51.21	30.42	1.68	3.14E-04	1.33E-03
1879	3	F2-3 vs. L2-3	253455_at	AT4G32020	serine/arginine repetitive matrix-like protein;(source:Araport11)	135.91	80.92	1.68	2.13E-04	1.00E-03
1880	3	F2-3 vs. L2-3	255959_at	AT1G21980	Type I phosphatidylinositol-4-phosphate 5-kinase. Preferentially phosphorylates PtdIns4P. Induced by water stress and abscisic acid in Arabidopsis thaliana. Expressed in procambial cells of leaves, flowers and roots. A N-terminal Membrane Occupation and Recognition Nexus (MORN)affects enzyme activity and distribution.	28.93	17.28	1.67	3.42E-04	1.42E-03
1881	3	F2-3 vs. L2-3	265188_at	AT1G23800	Encodes a mitochondrial aldehyde dehydrogenase; nuclear gene for mitochondrial product.	25.69	15.36	1.67	3.40E-04	1.41E-03
1882	3	F2-3 vs. L2-3	261954_at	AT1G64510	Translation elongation factor EF1B/ribosomal protein S6 family protein;(source:Araport11)	29.11	17.47	1.67	1.23E-04	6.78E-04
1883	3	F2-3 vs. L2-3	258766_at	AT3G10700	Encodes a GHMP kinase family protein that acts as a galacturonic acid-1-phosphate kinase that catalyzes the production of galacturonic acid-1-phosphate. This is a precursor of the important cell wall building block UDP-galacturonic acid. Based on gene trap line GT8007, the gene appears to be expressed in a petal and stamen-specific manner, between flower stages 8 to 11, however, later RT-qPCR analysis demonstrates that the transcript is present throughout the plant in all tissues tested.	31.18	18.63	1.67	7.46E-05	4.76E-04
1884	3	F2-3 vs. L2-3	245373_s_a	AT4G14710	RmlC-like cupins superfamily protein;(source:Araport11)	182.90	109.78	1.67	1.43E-04	7.56E-04
1885	3	F2-3 vs. L2-3	253405_at	AT4G32800	encodes a member of the DREB subfamily A-4 of ERF/AP2 transcription factor family. The protein contains one AP2 domain. There are 17 members in this subfamily including TINY.	32.41	19.45	1.67	1.65E-04	8.35E-04
1886	3	F2-3 vs. L2-3	248061_at	AT5G55340	MBOAT (membrane bound O-acyl transferase) family protein;(source:Araport11)	47.72	28.64	1.67	6.81E-05	4.47E-04
1887	3	F2-3 vs. L2-3	247539_at	AT5G61710	cotton fiber protein;(source:Araport11)	38.18	22.90	1.67	9.44E-05	5.62E-04

1888	3	F2-3 vs. L2-3	261788_at	AT1G15980	encodes a novel subunit of the chloroplast NAD(P)H dehydrogenase complex, involved in cyclic electron flow around photosystem I to produce ATP.	55.33	33.43	1.66	2.86E-04	1.25E-03
1889	3	F2-3 vs. L2-3	261309_at	AT1G48600	Encodes a phosphoethanolamine N-methyltransferase that catalyses the last two methylation steps of the three sequential methylations of phosphoethanolamine (PEA) that are required for the synthesis of phosphocholine (PCho) in plants.	148.22	89.04	1.66	6.51E-05	4.35E-04
1890	3	F2-3 vs. L2-3	267206_at	AT2G30830	encodes a protein whose sequence is similar to 2-oxoglutarate-dependent dioxygenase	26.68	16.04	1.66	1.72E-04	8.60E-04
1891	3	F2-3 vs. L2-3	266395_at	AT2G43100	isopropylmalate isomerase 2;(source:Araport11)	34.55	20.79	1.66	3.54E-04	1.45E-03
1892	3	F2-3 vs. L2-3	252271_s_a	AT5G01430	Got1/Sft2-like vescicle transport protein family;(source:Araport11)	60.57	36.41	1.66	1.29E-04	7.05E-04
1893	3	F2-3 vs. L2-3	247509_at	AT5G62020	member of Heat Stress Transcription Factor (Hsf) family The mRNA is cell-to-cell mobile.	93.69	56.56	1.66	7.73E-04	2.62E-03
1894	3	F2-3 vs. L2-3	247275_at	AT5G64370	PYD3 encodes a beta-ureidopropionase which, when expressed in E. coli, has been shown to convert beta-ureidopropionate into beta-alanine. It localizes to the cytosol and plays an important role in uracil degradation.	152.49	91.76	1.66	8.41E-04	2.81E-03
1895	3	F2-3 vs. L2-3	265821_at	AT2G17950	Homeobox gene controlling the stem cell pool. Expressed in the stem cell organizing center of meristems. Required to keep the stem cells in an undifferentiated state. Regulation of WUS transcription is a central checkpoint in stem cell control. The size of the WUS expression domain controls the size of the stem cell population through WUS indirectly activating the expression of CLAVATA3 (CLV3) in the stem cells and CLV3 repressing WUS transcription through the CLV1 receptor kinase signaling pathway. Repression of WUS transcription through AGAMOUS (AG) activity controls stem cell activity in the determinate floral meristem. Binds to TAAT element core motif. WUS is also involved in cell differentiation during anther development.	18.03	10.94	1.65	2.59E-05	2.26E-04
1896	3	F2-3 vs. L2-3	252085_s_a	AT3G52000	serine carboxypeptidase-like 36;(source:Araport11)	16.63	10.11	1.65	1.57E-04	8.09E-04
1897	3	F2-3 vs. L2-3	251704_at	AT3G56360	hypothetical protein;(source:Araport11)	61.25	37.17	1.65	4.95E-05	3.59E-04
1898	3	F2-3 vs. L2-3	253005_at	AT4G38100	CURVATURE THYLAKOID 1D-like protein; involved in thylakoid membrane organization.	33.28	20.17	1.65	2.38E-04	1.08E-03
1899	3	F2-3 vs. L2-3	252911_at	AT4G39510	member of CYP96A	62.29	37.84	1.65	3.53E-04	1.45E-03
1900	3	F2-3 vs. L2-3	247553_at	AT5G60910	MADS box gene negatively regulated by APETALA1	22.09	13.42	1.65	5.68E-05	3.98E-04
1901	3	F2-3 vs. L2-3	265569_at	AT2G05620	Involved in electron flow in Photosystem I. Essential for photoprotection.	45.62	27.77	1.64	7.57E-04	2.57E-03
1902	3	F2-3 vs. L2-3	251465_at	AT3G59360	UDP-galactose transporter 6;(source:Araport11)	67.54	41.21	1.64	1.16E-04	6.55E-04
1903	3	F2-3 vs. L2-3	250137_at	AT5G15390	tRNA/rRNA methyltransferase (SpoU) family protein;(source:Araport11)	170.64	104.00	1.64	7.24E-05	4.66E-04
1904	3	F2-3 vs. L2-3	249910_at	AT5G22630	Encodes a plastid-localized arogenate dehydratase involved in phenylalanine biosynthesis. Not less than six genes encoding ADT were identiﬁed in the Arabidopsis genome: ADT1 [At1g11790]; ADT2 [At3g07630]; ADT3 [At2g27820]; ADT4 [At3g44720]; ADT5 [At5g22630]; and ADT6 [At1g08250]. The mRNA is cell-to-cell mobile.	49.79	30.39	1.64	4.89E-05	3.56E-04
1905	3	F2-3 vs. L2-3	248028_at	AT5G55620	hypothetical protein;(source:Araport11)	20.57	12.57	1.64	9.29E-04	3.03E-03
1906	3	F2-3 vs. L2-3	261462_at	AT1G07850	transferring glycosyl group transferase (DUF604);(source:Araport11)	37.92	23.30	1.63	2.38E-04	1.08E-03
1907	3	F2-3 vs. L2-3	260637_at	AT1G62380	Encodes a protein similar to 1-aminocyclopropane-1-carboxylic oxidase (ACC oxidase). Expression of the AtACO2 transcripts is affected by ethylene.	217.44	133.30	1.63	7.20E-04	2.48E-03
1908	3	F2-3 vs. L2-3	261727_at	AT1G76090	Encodes S-adenosyl-methionine-sterol-C-methyltransferase, an enzyme in the sterol biosynthetic pathway.	58.61	35.96	1.63	5.00E-04	1.88E-03
1909	3	F2-3 vs. L2-3	265494_at	AT2G15680	Encodes a calmodulin-like protein.	20.60	12.64	1.63	3.30E-05	2.67E-04
1910	3	F2-3 vs. L2-3	257821_at	AT3G25170	Member of a diversely expressed predicted peptide family showing sequence similarity to tobacco Rapid Alkalinization Factor (RALF), and is believed to play an essential role in the physiology of Arabidopsis. Consists of a single exon and is characterized by a conserved C-terminal motif and N-terminal signal peptide.	37.51	23.07	1.63	4.56E-04	1.75E-03
1911	3	F2-3 vs. L2-3	257790_at	AT3G27090	DCD (Development and Cell Death) domain protein;(source:Araport11)	450.54	276.82	1.63	4.57E-04	1.75E-03
1912	3	F2-3 vs. L2-3	254153_at	AT4G24450	phosphoglucan, water dikinase;(source:Araport11)	35.87	22.06	1.63	1.22E-04	6.74E-04
1913	3	F2-3 vs. L2-3	252886_at	AT4G39350	Encodes a cellulose synthase isomer, related to CESA6. As inferred from the null role of secondary wall-type CesAs, included in a set of five primary wall-type CesAs that may support trichome cell wall thickening. The mRNA is cell-to-cell mobile.	97.14	59.59	1.63	7.46E-04	2.54E-03
1914	3	F2-3 vs. L2-3	249519_at	AT5G38660	mutant has Altered acclimation responses;	157.13	96.43	1.63	2.63E-04	1.17E-03
1915	3	F2-3 vs. L2-3	259891_at	AT1G72730	DEA(D/H)-box RNA helicase family protein;(source:Araport11)	106.71	66.04	1.62	8.73E-04	2.89E-03

1916	3	F2-3 vs. L2-3	267147_at	AT2G38240	One of 4 paralogs encoding a 2-oxoglutarate/Fe(II)-dependent oxygenases that hydroxylates JA to 12-OH-JA.	42.91	26.53	1.62	5.66E-04	2.06E-03
1917	3	F2-3 vs. L2-3	260572_at	AT2G43760	molybdopterin biosynthesis MoaE family protein;(source:Araport11)	117.60	72.65	1.62	3.66E-05	2.88E-04
1918	3	F2-3 vs. L2-3	258655_at	AT3G09810	Encodes a catalytic subunit of the mitochondrially-localized NAD+- dependent isocitrate dehydrogenase	67.92	42.05	1.62	4.94E-04	1.86E-03
1919	3	F2-3 vs. L2-3	251870_at	AT3G54510	Early-responsive to dehydration stress protein (ERD4);(source:Araport11)	26.71	16.51	1.62	4.45E-04	1.72E-03
1920	3	F2-3 vs. L2-3	253402_at	AT4G32880	member of homeodomain-leucine zipper family, acting as a differentiation-promoting transcription factor of the vascular meristems.	42.56	26.25	1.62	9.96E-05	5.85E-04
1921	3	F2-3 vs. L2-3	248687_at	AT5G48300	Encodes the small subunit of ADP-glucose pyrophosphorylase. The small subunit is the catalytic isoform responsible for ADP-glucose pyrophosphorylase activity. The presence of the small subunit is required for large subunit stability. Two isoforms of the small subunit (ApS1 and ApS2) have been described. ApS1 is the major small subunit isoform present in all plant tissues tested. The mRNA is cell-to-cell mobile.	94.16	58.05	1.62	2.45E-05	2.17E-04
1922	3	F2-3 vs. L2-3	264645_at	AT1G08940	Phosphoglycerate mutase family protein;(source:Araport11)	22.84	14.15	1.61	2.58E-05	2.25E-04
1923	3	F2-3 vs. L2-3	256506_at	AT1G75160	DUF620 family protein (DUF620);(source:Araport11)	19.28	12.01	1.61	7.46E-04	2.54E-03
1924	3	F2-3 vs. L2-3	267458_at	AT2G33670	A member of a large family of seven-transmembrane domain proteins specific to plants, homologs of the barley mildew resistance locus o (MLO) protein. The Arabidopsis genome contains 15 genes encoding MLO proteins, with localization in plasma membrane. Phylogenetic analysis revealed four clades of closely-related AtMLO genes. ATMLO5 belongs to the clade III, with AtMLO7, AtMLO8, AtMLO9, and AtMLO10. The gene is expressed during seedling growth, in cotyledon vascular system, and in stigma, anther and pollen grains; it was not expressed in rosette leaves, as shown by GUS activity patterns. The expression of several phylogenetically closely-related AtMLO genes showed similar or overlapping tissue specificity and analogous responsiveness to external stimuli, suggesting functional redundancy, co-function, or antagonistic function(s).	24.07	14.96	1.61	5.09E-04	1.91E-03
1925	3	F2-3 vs. L2-3	266965_at	AT2G39510	Encodes a plasma membrane-localized amino acid transporter likely involved in amino acid export in the developing seed.	78.95	49.04	1.61	1.77E-04	8.80E-04
1926	3	F2-3 vs. L2-3	265721_at	AT2G40090	member of ATH subfamily	30.97	19.22	1.61	1.54E-04	7.98E-04
1927	3	F2-3 vs. L2-3	259173_at	AT3G03640	Encodes beta-glucosidase (GLUC).	27.85	17.28	1.61	1.82E-04	8.87E-04
1928	3	F2-3 vs. L2-3	256900_at	AT3G24670	Pectin lyase-like superfamily protein;(source:Araport11)	59.96	37.22	1.61	6.34E-04	2.25E-03
1929	3	F2-3 vs. L2-3	255417_at	AT4G03190	Encodes an F box protein belonging to the TIR1 subfamily. This protein forms SCF complexes with ASK1 and CUL1 and interacts with Aux/IAA proteins in an auxin-dependent manner. It also has sequence similarity to the yeast protein GRR1, which is involved in glucose repression.	43.63	27.10	1.61	1.43E-04	7.56E-04
1930	3	F2-3 vs. L2-3	253485_at	AT4G31800	Pathogen-induced transcription factor. Binds W-box sequences in vitro. Forms protein complexes with itself and with WRKY40 and WRKY60. Constitutive expression of WRKY18 enhanced resistance to P. syringae, but its coexpression with WRKY40 or WRKY60 made plants more susceptible to both P. syringae and B. cinerea. WRKY18, WRKY40, and WRKY60 have partially redundant roles in response to the hemibiotrophic bacterial pathogen Pseudomonas syringae and the necrotrophic fungal pathogen Botrytis cinerea, with WRKY18 playing a more important role than the other two.The mRNA is cell-to-cell mobile.	42.00	26.07	1.61	5.56E-04	2.04E-03
1931	3	F2-3 vs. L2-3	250829_at	AT5G04720	Encodes a member of the ADR1 family nucleotide-binding leucine-rich repeat (NB-LRR) immune receptors. The mRNA is cell-to-cell mobile.	24.52	15.23	1.61	5.80E-04	2.10E-03
1932	3	F2-3 vs. L2-3	250580_at	AT5G07440	Encodes the alpha-subunit of the glutamate dehydrogenase. The enzyme is almost exclusively found in the mitochondria of stem and leaf companion cells.	17.40	10.84	1.61	7.81E-05	4.93E-04
1933	3	F2-3 vs. L2-3	246539_at	AT5G15460	membrane-anchored ubiquitin-fold protein 2;(source:Araport11)	130.18	80.85	1.61	1.89E-04	9.14E-04
1934	3	F2-3 vs. L2-3	249751_at	AT5G24650	HP30/Tric1 is a component of the mitochondrial protein translocation complex and is involved in tRNA transport along with HP30-2/Tric2.It interacts with several members of the TOM complex such as TOM40 and this interaction is mediated by the SAM domain.	227.28	141.15	1.61	3.46E-05	2.76E-04

1935	3	F2-3 vs. L2-3	247182_at	AT5G65410	Encodes ZFHD2, a member of the zinc finger homeodomain transcriptional factor family.Gain of function of ATHB25 (35S and UBQ10 proomoters) and double loss of function of ATHB25 and ATHB22 increases and decreases, respectively, seed longevity. This phenotype is maternal and related to seed coat alterations. Gain of function increases expression of GA3OX2 and GA4 and GA1 levels.	67.45	41.98	1.61	4.70E-04	1.79E-03
1936	3	F2-3 vs. L2-3	262418_at	AT1G50320	encodes a prokaryotic thioredoxin	136.08	84.97	1.60	6.45E-05	4.33E-04
1937	3	F2-3 vs. L2-3	266368_at	AT2G41380	S-adenosyl-L-methionine-dependent methyltransferases superfamily protein;(source:Araport11)	19.86	12.38	1.60	1.48E-05	1.57E-04
1938	3	F2-3 vs. L2-3	257696_at	AT3G12690	Encodes a putative serine/threonine kinase It is expressed specifically in pollen and appears to function redundantly with AGC1.7 to regulate polarized growth of pollen tubes.	133.94	83.89	1.60	1.56E-04	8.05E-04
1939	3	F2-3 vs. L2-3	247648_at	AT5G60020	LAC17 appears to have laccase activity based on enzyme assays performed using lac17 mutants. Notably, these mutants appear to have a reduced deposition of G lignin units. LAC17 is expressed in interfascicular fibers and likely contributes to lignin biosynthesis, and hence, cell wall biosynthesis, there.	48.54	30.38	1.60	1.01E-04	5.92E-04
1940	3	F2-3 vs. L2-3	247523_at	AT5G61410	Arabidopsis thaliana ribulose-5-phosphate-3-epimerase mRNA	391.94	245.44	1.60	7.93E-05	4.98E-04
1941	3	F2-3 vs. L2-3	262744_at	AT1G28680	Catalyses trans-cis isomerization and lactonization in the biosynthesis of coumarins in roots.	83.45	52.44	1.59	9.89E-04	3.19E-03
1942	3	F2-3 vs. L2-3	262165_at	AT1G75020	lysophosphatidyl acyltransferase 4;(source:Araport11)	25.22	15.88	1.59	4.57E-04	1.75E-03
1943	3	F2-3 vs. L2-3	262970_at	AT1G75690	Thylakoid Thiol/Disulfide-Modulating Protein.	123.93	77.79	1.59	4.05E-04	1.61E-03
1944	3	F2-3 vs. L2-3	245076_at	AT2G23170	encodes an IAA-amido synthase that conjugates Asp and other amino acids to auxin in vitro.	31.31	19.73	1.59	1.29E-04	7.07E-04
1945	3	F2-3 vs. L2-3	265959_at	AT2G37240	Thioredoxin superfamily protein;(source:Araport11)	105.98	66.65	1.59	8.71E-04	2.88E-03
1946	3	F2-3 vs. L2-3	257892_at	AT3G17020	Adenine nucleotide alpha hydrolases-like superfamily protein;(source:Araport11)	930.20	584.24	1.59	3.55E-04	1.46E-03
1947	3	F2-3 vs. L2-3	248754_at	AT5G47670	Encodes LEC1-Like (L1L), closely related to LEC1 (Leafy Cotyledon1). Functions as a regulator of embryo development.	29.64	18.59	1.59	2.74E-04	1.21E-03
1948	3	F2-3 vs. L2-3	259753_at	AT1G71050	Heavy metal transport/detoxification superfamily protein;(source:Araport11)	60.46	38.29	1.58	8.45E-05	5.24E-04
1949	3	F2-3 vs. L2-3	262892_at	AT1G79440	Encodes a mitochondrial succinic semialdehyde dehydrogenase (SSADH). Nomenclature according to Kirch, et al (2004).	173.96	110.31	1.58	5.40E-04	1.99E-03
1950	3	F2-3 vs. L2-3	265530_at	AT2G06050	Encodes a 12-oxophytodienoate reductase that is required for jasmonate biosynthesis. Mutants are male sterile and defective in pollen dehiscence. Shows activity towards 2,4,6-trinitrotoluene. CFA-Ile, CFA-Leu, CFA-Val, CFA-Met and CFA-Ala can restore the fertility of opr3 plants by inducing filament elongation and anther dehiscence.	348.70	220.56	1.58	1.16E-04	6.55E-04
1951	3	F2-3 vs. L2-3	266428_at	AT2G07180	Protein kinase superfamily protein;(source:Araport11)	37.27	23.56	1.58	2.18E-04	1.02E-03
1952	3	F2-3 vs. L2-3	266805_at	AT2G30010	Encodes a member of the TBL (TRICHOME BIREFRINGENCE-LIKE) gene family containing a plant-specific DUF231 (domain of unknown function) domain. TBL gene family has 46 members, two of which (TBR/AT5G06700 and TBL3/AT5G01360) have been shown to be involved in the synthesis and deposition of secondary wall cellulose, presumably by influencing the esterification state of pectic polymers. A nomenclature for this gene family has been proposed (Volker Bischoff & Wolf Scheible, 2010, personal communication).	38.35	24.21	1.58	5.24E-04	1.95E-03
1953	3	F2-3 vs. L2-3	267527_at	AT2G45610	alpha/beta-Hydrolases superfamily protein;(source:Araport11)	29.62	18.70	1.58	3.15E-04	1.33E-03
1954	3	F2-3 vs. L2-3	258888_at	AT3G05620	Plant invertase/pectin methylesterase inhibitor superfamily;(source:Araport11)	22.96	14.49	1.58	1.34E-04	7.21E-04
1955	3	F2-3 vs. L2-3	250385_at	AT5G11520	Encodes the chloroplastic isozyme of aspartate aminotransferase. Involved in aspartate biosynthesis and nitrogen metabolism. mRNA is expressed in senescing leaves.	133.46	84.46	1.58	5.97E-05	4.12E-04
1956	3	F2-3 vs. L2-3	250103_at	AT5G16600	Encodes a putative transcription factor (MYB43).	19.07	12.05	1.58	5.07E-05	3.65E-04
1957	3	F2-3 vs. L2-3	247980_at	AT5G56860	Encodes a member of the GATA factor family of zinc finger transcription factors. Modulate chlorophyll biosynthesis and glutamate synthase (GLU1/Fd-GOGAT) expression.	21.83	13.79	1.58	3.63E-04	1.48E-03
1958	3	F2-3 vs. L2-3	258501_at	AT3G06780	glycine-rich protein;(source:Araport11)	152.32	96.81	1.57	1.05E-04	6.09E-04
1959	3	F2-3 vs. L2-3	253372_at	AT4G33220	pectin methylesterase 44;(source:Araport11)	265.61	169.63	1.57	1.90E-04	9.18E-04
1960	3	F2-3 vs. L2-3	248042_at	AT5G55960	transmembrane protein C9orf5 protein;(source:Araport11)	48.91	31.10	1.57	2.48E-04	1.12E-03
1961	3	F2-3 vs. L2-3	247286_at	AT5G64280	dicarboxylate transporter 2.2;(source:Araport11)	89.53	56.89	1.57	2.94E-04	1.27E-03

1962	3	F2-3 vs. L2-3	261564_at	AT1G01720	Belongs to a large family of putative transcriptional activators with NAC domain. Transcript level increases in response to wounding and abscisic acid. ATAF1 attentuates ABA signaling and sythesis. Mutants are hyposensitive to ABA. The mRNA is cell-to-cell mobile.	134.17	86.21	1.56	2.15E-04	1.00E-03
1963	3	F2-3 vs. L2-3	259418_at	AT1G02390	putative sn-glycerol-3-phosphate 2-O-acyltransferase	28.67	18.36	1.56	5.51E-04	2.02E-03
1964	3	F2-3 vs. L2-3	252167_at	AT3G50560	NAD(P)-binding Rossmann-fold superfamily protein;(source:Araport11)	39.65	25.44	1.56	7.75E-04	2.62E-03
1965	3	F2-3 vs. L2-3	264390_at	AT1G11950	Transcription factor jumonji (jmjC) domain-containing protein;(source:Araport11)	38.80	25.04	1.55	9.84E-04	3.18E-03
1966	3	F2-3 vs. L2-3	263426_at	AT2G31570	glutathione peroxidase GPx	101.87	65.57	1.55	3.19E-04	1.35E-03
1967	3	F2-3 vs. L2-3	258954_at	AT3G01400	ARM repeat superfamily protein;(source:Araport11)	104.95	67.61	1.55	1.24E-04	6.83E-04
1968	3	F2-3 vs. L2-3	258406_at	AT3G17611	RHOMBOID-like protein 14;(source:Araport11)	138.89	89.68	1.55	4.62E-04	1.76E-03
1969	3	F2-3 vs. L2-3	252394_at	AT3G47940	DNAJ heat shock family protein;(source:Araport11)	82.91	53.36	1.55	2.94E-04	1.27E-03
1970	3	F2-3 vs. L2-3	253215_at	AT4G34950	Major facilitator superfamily protein;(source:Araport11)	48.70	31.46	1.55	3.27E-04	1.37E-03
1971	3	F2-3 vs. L2-3	252997_at	AT4G38400	member of EXPANSIN-LIKE. Naming convention from the Expansin Working Group (Kende et al, 2004. Plant Mol Bio)	92.25	59.41	1.55	8.93E-04	2.94E-03
1972	3	F2-3 vs. L2-3	259809_at	AT1G49800	Homolog of PIP1.	28.00	18.21	1.54	4.34E-04	1.69E-03
1973	3	F2-3 vs. L2-3	266989_at	AT2G39330	jacalin-related lectin 23;(source:Araport11)	1941.91	1260.78	1.54	6.24E-04	2.23E-03
1974	3	F2-3 vs. L2-3	267081_at	AT2G41210	Encodes a protein with phosphatidylinositol-4-phosphate 5-kinase activity that plays a role in pollen tip growth. The enzyme localizes to the apical plasma membrane and adjacent cytosolic region of pollen tubes. Overexpression of this gene leads to increased deposition of pectin in the cell wall at the tip of the pollen tube and causes altered pollen tube morphology.	29.02	18.88	1.54	1.19E-04	6.64E-04
1975	3	F2-3 vs. L2-3	256492_at	AT1G31490	HXXXD-type acyl-transferase family protein;(source:Araport11)	28.15	18.38	1.53	5.25E-04	1.95E-03
1976	3	F2-3 vs. L2-3	264424_at	AT1G61740	Sulfite exporter TauE/SafE family protein;(source:Araport11)	64.32	41.91	1.53	9.41E-05	5.62E-04
1977	3	F2-3 vs. L2-3	251661_at	AT3G56950	One of the Major Intrinsic Proteins(MIPs) which facilitate the passive transport of small molecules across membranes.Belongs to a family of plant aquaporins.Similar to yeast and radish aquaporins. Located on ER. Probably involved in the alleviation of ER stress; the lack of SIP2;1 reduces both pollen germination and pollen tube elongation.	107.55	70.40	1.53	5.33E-04	1.97E-03
1978	3	F2-3 vs. L2-3	245631_at	AT1G25350	glutamine-tRNA ligase, putative / glutaminyl-tRNA synthetase, putative / GlnRS;(source:Araport11)	66.18	43.41	1.52	7.44E-04	2.54E-03
1979	3	F2-3 vs. L2-3	262283_at	AT1G68590	Ribosomal protein PSRP-3/Ycf65;(source:Araport11)	25.41	16.74	1.52	7.11E-04	2.46E-03
1980	3	F2-3 vs. L2-3	263880_at	AT2G21960	transmembrane protein;(source:Araport11)	69.25	45.53	1.52	1.16E-04	6.55E-04
1981	3	F2-3 vs. L2-3	259375_at	AT3G16370	GDSL-motif esterase/acyltransferase/lipase. Enzyme group with broad substrate specificity that may catalyze acyltransfer or hydrolase reactions with lipid and non-lipid substrates. The mRNA is cell-to-cell mobile.	222.12	146.19	1.52	1.48E-04	7.71E-04
1982	3	F2-3 vs. L2-3	254468_at	AT4G20460	NAD(P)-binding Rossmann-fold superfamily protein;(source:Araport11)	36.38	24.01	1.52	7.11E-04	2.46E-03
1983	3	F2-3 vs. L2-3	254111_at	AT4G24890	purple acid phosphatase 24;(source:Araport11)	29.35	19.25	1.52	6.16E-05	4.21E-04
1984	3	F2-3 vs. L2-3	250650_at	AT5G06850	Encodes an endoplasmic reticulum protein that is involved in the transport of the florigen FT from companion cells to sieve elements, thus affecting FT transport through the phloem to the SAM.	46.46	30.59	1.52	5.59E-04	2.04E-03
1985	3	F2-3 vs. L2-3	247843_at	AT5G58050	Encodes a member of the glycerophosphodiester phosphodiesterase like (GDPD-like) family.	70.57	46.57	1.52	2.08E-04	9.86E-04
1986	3	F2-3 vs. L2-3	247637_at	AT5G60600	Encodes a chloroplast-localized hydroxy-2-methyl-2-(E)-butenyl 4-diphosphate (HMBPP) synthase (HDS), catalyzes the formation of HMBPP from 2-C-methyl-D-erythrytol 2,4-cyclodiphosphate (MEcPP). The HDS enzyme controls the penultimate steps of the biosynthesis of IPP and dimethylallyl diphosphate (DMAPP) via the MEP pathway and may serve as a metabolic control point for SA-mediated disease resistance. In the light, the electrons required for the reaction catalyzed by HDS are directly provided by the electron flow from photosynthesis via ferredoxin. In the dark however, the enzyme requires an electron shuttle: ferredoxin-NADP⁺ reductase. The mRNA is cell-to-cell mobile.	22.93	15.13	1.52	9.66E-04	3.13E-03
1987	3	F2-3 vs. L2-3	259886_at	AT1G76370	Protein kinase superfamily protein;(source:Araport11)	33.09	21.94	1.51	4.79E-04	1.81E-03
1988	3	F2-3 vs. L2-3	245506_at	AT4G15700	Encodes a member of the CC-type glutaredoxin (ROXY) family.	14.71	9.71	1.51	1.18E-04	6.62E-04
1989	3	F2-3 vs. L2-3	253727_at	AT4G29350	Encodes profilin2, a low-molecular weight, actin monomer-binding protein that regulates the organization of actin cytoskeleton. Expressed in vegetative organs. The first intron of PRF2 enhances gene expression. The mRNA is cell-to-cell mobile.	627.72	415.11	1.51	4.38E-04	1.70E-03

1990	3	F2-3 vs. L2-3	250496_at	AT5G09650	Encodes a protein with inorganic pyrophosphatase activity.	262.11	173.87	1.51	3.12E-04	1.33E-03
1991	3	F2-3 vs. L2-3	261563_at	AT1G01630	Sec14p-like phosphatidylinositol transfer family protein;(source:Araport11)	49.65	33.07	1.50	3.96E-04	1.58E-03
1992	3	F2-3 vs. L2-3	245763_at	AT1G27850	Encodes a microtubule-associated protein involved in cortical microtubule organization during leaf development.	41.81	28.09	1.49	1.34E-04	7.21E-04
1993	3	F2-3 vs. L2-3	245737_at	AT1G44160	HSP40/DnaJ peptide-binding protein;(source:Araport11)	20.27	13.62	1.49	6.38E-05	4.30E-04
1994	3	F2-3 vs. L2-3	265401_at	AT2G10970	Plant invertase/pectin methylesterase inhibitor superfamily protein;(source:Araport11)	24.67	16.59	1.49	9.02E-04	2.97E-03
1995	3	F2-3 vs. L2-3	263456_at	AT2G22125	Encodes a protein involved in cell elongation in root and anther filaments. Mutants have greater cell volumes in root tissues and have additive phenotypes with other cell expansion mutants such as those carrying mutations in COB, QUI and POM1 loci. POM2/CSI1 promotes Cellulose Synthase and microtubule co-alignment. The mRNA is cell-to-cell mobile.	65.32	43.87	1.49	8.13E-04	2.73E-03
1996	3	F2-3 vs. L2-3	258087_at	AT3G26060	encodes periredoxin Q which decomposes peroxides and plays a role in the protection of the photosynthetic apparatus	170.54	114.53	1.49	9.72E-04	3.15E-03
1997	3	F2-3 vs. L2-3	257850_at	AT3G13065	STRUBBELIG-receptor family 4;(source:Araport11)	20.94	14.11	1.48	7.81E-04	2.63E-03
1998	3	F2-3 vs. L2-3	248765_at	AT5G47650	Encodes an ADP-ribose pyrophosphatase that confers enhanced tolerance to oxidative stress.	59.61	40.20	1.48	6.64E-04	2.33E-03
1999	3	F2-3 vs. L2-3	261451_at	AT1G21060	Serine/Threonine-kinase, putative (Protein of unknown function, DUF547);(source:Araport11)	37.48	25.53	1.47	6.70E-04	2.35E-03
2000	3	F2-3 vs. L2-3	255251_at	AT4G04980	hypothetical protein;(source:Araport11)	15.14	10.67	1.42	9.51E-04	3.09E-03
2001	3	F2-3 vs. L2-3	247092_at	AT5G66380	Encodes a folate transporter that is located in the chloroplast envelope and is able to mediate exogenous folate uptake when expressed in E. coli. However, this is not the sole folate transporter for chloroplasts as null mutants of this gene have no discernible phenotype when grown under folate-sufficient conditions and contained wild-type levels of folates in leaves.	67.09	95.02	0.71	7.73E-04	2.62E-03
2002	3	F2-3 vs. L2-3	260868_at	AT1G43860	sequence-specific DNA binding transcription factor;(source:Araport11)	36.45	52.24	0.70	7.01E-04	2.43E-03
2003	3	F2-3 vs. L2-3	265676_at	AT2G32070	Deadenylase.	25.85	37.06	0.70	2.43E-04	1.10E-03
2004	3	F2-3 vs. L2-3	259406_at	AT1G17690	U3 small nucleolar RNA-associated protein;(source:Araport11)	98.98	142.50	0.69	9.15E-04	2.99E-03
2005	3	F2-3 vs. L2-3	260081_at	AT1G78170	E3 ubiquitin-protein ligase;(source:Araport11)	12.08	17.62	0.69	8.66E-04	2.87E-03
2006	3	F2-3 vs. L2-3	250282_at	AT5G13260	myosin;(source:Araport11)	42.19	61.52	0.69	1.37E-04	7.31E-04
2007	3	F2-3 vs. L2-3	245793_at	AT1G32220	NAD(P)-binding Rossmann-fold superfamily protein;(source:Araport11)	93.19	137.13	0.68	7.65E-05	4.85E-04
2008	3	F2-3 vs. L2-3	251464_at	AT3G59380	Encodes the alpha-subunit shared between protein farnesyltransferase and protein geranylgeranyltransferase-I. Involved in protein prenylation: covalent attachment of the C-15 isoprene farnesyl or the C-20 isoprene geranylgeranyl groups to the C-terminal end of some proteins. Involved in shoot and flower meristem homeostasis, and response to ABA and drought. Also regulates leaf cell shape. Mutant is epistatic to era1.	209.01	306.12	0.68	5.43E-04	2.00E-03
2009	3	F2-3 vs. L2-3	259126_at	AT3G02280	Flavodoxin family protein;(source:Araport11)	28.50	42.30	0.67	1.95E-04	9.37E-04
2010	3	F2-3 vs. L2-3	258390_at	AT3G15410	Leucine-rich repeat (LRR) family protein;(source:Araport11)	38.82	58.14	0.67	4.09E-04	1.61E-03
2011	3	F2-3 vs. L2-3	246255_at	AT4G36650	Encodes a protein with similarity to the general transcription factor TFIIB. pBRP binds rDNA sequences in vitro. pBRP has been localized to the outer face of the plastid membrane with GFP fusion however, under conditions of proteosome inhibition it is found in the nucleus.	17.45	26.18	0.67	6.13E-04	2.19E-03
2012	3	F2-3 vs. L2-3	262774_at	AT1G13230	Encodes a leucine-rich repeat protein pii-2. Located in the endoplasmic reticulum/plasma membrane continuum in Arabidopsis roots. Required for growth promotion and enhanced seed production mediated by the endophytic fungus Piriformospora indica in Arabidopsis.	15.73	23.99	0.66	3.46E-04	1.43E-03
2013	3	F2-3 vs. L2-3	265798_at	AT2G35790	transmembrane protein;(source:Araport11)	94.44	143.31	0.66	4.09E-04	1.61E-03
2014	3	F2-3 vs. L2-3	252927_at	AT4G39090	Similar to cysteine proteinases, induced by desiccation but not abscisic acid. Required for RRS1-R mediated resistance against Ralstonia solanacearum. Interacts with the R. solanacearum type III effector PopP2. RD19 associates with PopP2 to form a nuclear complex that is required for activation of the RRS1-R?mediated resistance response.	1111.56	1682.73	0.66	1.81E-04	8.86E-04
2015	3	F2-3 vs. L2-3	250981_at	AT5G03140	Concanavalin A-like lectin protein kinase family protein;(source:Araport11)	71.92	109.06	0.66	1.18E-04	6.62E-04

2016	3	F2-3 vs. L2-3	249193_at	AT5G42480	Shows homology to the cyanobacterial cell division protein Ftn2, mutant only has two mesophyll cell chloroplasts. Protein was localized to a ring at the center of the chloroplasts. Probably involved in functions in the assembly and/or stabilization of the plastid-dividing FtsZ ring, inhibiting FtsZ filament formation in the chloroplast.	383.36	582.21		0.66	4.23E-04	1.66E-03
2017	3	F2-3 vs. L2-3	248655_at	AT5G48760	Ribosomal protein L13 family protein;(source:Araport11)	295.07	449.96		0.66	8.81E-04	2.91E-03
2018	3	F2-3 vs. L2-3	259920_at	AT1G72550	tRNA synthetase beta subunit family protein;(source:Araport11)	26.01	40.31		0.65	3.11E-04	1.33E-03
2019	3	F2-3 vs. L2-3	253645_at	AT4G29830	VIP3 protein is composed of repeats of WD motif which is involved in protein complex formation. The gene is involved in flower timing and flower development. This gene is predicted to encode a protein with a DWD motif. It can bind to DDB1a in Y2H assays, and DDB1b in co-IP assays, and may be involved in the formation of a CUL4-based E3 ubiquitin ligase. Loss of gene function leads to a redistribution of H3K4me3 and K3K36me2 modifications within genes but not a change in the overall abundance of these modifications within chromatin. Also known as SKI8, a component of the SKI complex involved in exosome mediated RNA degredation. Member of PAF-C complex.	300.80	464.17		0.65	3.29E-04	1.38E-03
2020	3	F2-3 vs. L2-3	246621_at	AT5G36230	ARM repeat superfamily protein;(source:Araport11)	559.01	855.89		0.65	9.30E-05	5.58E-04
2021	3	F2-3 vs. L2-3	247336_at	AT5G63640	ENTH/VHS/GAT family protein;(source:Araport11)	47.52	73.25		0.65	6.53E-04	2.30E-03
2022	3	F2-3 vs. L2-3	247249_at	AT5G64670	Ribosomal protein L18e/L15 superfamily protein;(source:Araport11)	206.03	316.31		0.65	1.34E-04	7.22E-04
2023	3	F2-3 vs. L2-3	264179_at	AT1G02180	ferredoxin-like protein;(source:Araport11)	42.94	66.77		0.64	3.49E-04	1.44E-03
2024	3	F2-3 vs. L2-3	265858_at	AT2G01720	Ribophorin I;(source:Araport11)	232.43	360.93		0.64	1.84E-04	8.96E-04
2025	3	F2-3 vs. L2-3	266579_at	AT2G23930	Putative small nuclear ribonucleoprotein G;(source:Araport11)	256.70	398.15		0.64	8.76E-05	5.35E-04
2026	3	F2-3 vs. L2-3	252124_at	AT3G51010	protein translocase subunit;(source:Araport11)	171.62	268.56		0.64	1.46E-04	7.67E-04
2027	3	F2-3 vs. L2-3	250758_at	AT5G06000	One of the 2 genes that code for the G subunit of eukaryotic initiation factor 3 (EIF3).	33.13	51.45		0.64	5.57E-04	2.04E-03
2028	3	F2-3 vs. L2-3	248148_at	AT5G54930	AT hook motif-containing protein;(source:Araport11)	33.78	52.58		0.64	4.24E-04	1.66E-03
2029	3	F2-3 vs. L2-3	247260_at	AT5G64500	Major facilitator superfamily protein;(source:Araport11)	242.85	380.72		0.64	1.65E-04	8.35E-04
2030	3	F2-3 vs. L2-3	256065_at	AT1G07070	Ribosomal protein L35Ae family protein;(source:Araport11)	73.15	116.97		0.63	8.77E-04	2.90E-03
2031	3	F2-3 vs. L2-3	264872_at	AT1G24260	Member of the MADs box transcription factor family. SEP3 is redundant with SEP1 and 2. Flowers of SEP1/2/3 triple mutants show a conversion of petals and stamens to sepals.SEP3 forms heterotetrameric complexes with other MADS box family members and binds to the CArG box motif.	180.47	284.42		0.63	4.36E-05	3.29E-04
2032	3	F2-3 vs. L2-3	262440_at	AT1G47710	Serine protease inhibitor (SERPIN) family protein;(source:Araport11)	108.22	173.07		0.63	2.74E-04	1.21E-03
2033	3	F2-3 vs. L2-3	266932_s_a	AT1G63250	DEA(D/H)-box RNA helicase family protein;(source:Araport11)	26.30	41.74		0.63	2.10E-04	9.94E-04
2034	3	F2-3 vs. L2-3	245730_at	AT1G73470	hypothetical protein;(source:Araport11)	49.27	78.65		0.63	4.49E-04	1.73E-03
2035	3	F2-3 vs. L2-3	266506_at	AT2G47850	Zinc finger C-x8-C-x5-C-x3-H type family protein;(source:Araport11)	53.02	84.34		0.63	4.20E-04	1.65E-03
2036	3	F2-3 vs. L2-3	258138_at	AT3G24495	encodes a DNA mismatch repair homolog of human MutS gene, MSH6. There are four MutS genes in Arabidopsis, MSH2, MSH3, MSH6, and MSH7, which all act as heterodimers and bind to 51-mer duplexes. MSH2*MSH7 exhibit moderate affinity for a (T/G) substrate and weak binding of (+T), suggesting MSH2*MSH7 may be specialized for lesions/base mispairs not tested or for (T/G) mispairs in special contexts.	18.95	30.16		0.63	6.59E-04	2.31E-03
2037	3	F2-3 vs. L2-3	256613_at	AT3G29290	Pentatricopeptide repeat (PPR) superfamily protein;(source:Araport11)	14.51	22.87		0.63	4.56E-04	1.75E-03
2038	3	F2-3 vs. L2-3	253078_at	AT4G36180	Leucine-rich receptor-like protein kinase family protein;(source:Araport11)	13.35	21.28		0.63	1.71E-04	8.58E-04
2039	3	F2-3 vs. L2-3	246190_at	AT5G21010	Encodes a member of the MATH-BTB domain proteins (BPMs) that directly interact with and target for proteasomal degradation the class I homeobox-leucine zipper (HD-ZIP) transcription factor ATHB6. Known members include AT5G19000 (BPM1), AT3G06190 (BPM2), AT2G39760 (BPM3), AT3G03740 (BPM4), AT5G21010 (BPM5) and AT3G43700 (BPM6).	79.76	127.26		0.63	9.57E-04	3.10E-03
2040	3	F2-3 vs. L2-3	248710_at	AT5G48480	Lactoylglutathione lyase / glyoxalase I family protein;(source:Araport11)	38.39	61.25		0.63	1.10E-04	6.29E-04
2041	3	F2-3 vs. L2-3	261671_at	AT1G18340	basal transcription factor complex subunit-like protein;(source:Araport11)	67.36	108.88		0.62	3.31E-04	1.38E-03
2042	3	F2-3 vs. L2-3	265918_at	AT2G15090	Encodes KCS8, a member of the 3-ketoacyl-CoA synthase family involved in the biosynthesis of VLCFA (very long chain fatty acids). The mRNA is cell-to-cell mobile.	237.39	382.04		0.62	5.71E-04	2.07E-03
2043	3	F2-3 vs. L2-3	251574_at	AT3G58100	Encodes a member of the X8-GPI family of proteins. It localizes to the plasmodesmata and is predicted to bind callose.	32.60	52.23		0.62	6.34E-05	4.29E-04
2044	3	F2-3 vs. L2-3	253495_at	AT4G31850	encodes a protein containing 27 pentatrico-peptide repeat (PPR) motifs. Functions in the stabilization of petL operon RNA and also in the translation of petL.	42.64	68.71		0.62	9.96E-04	3.21E-03

2045	3	F2-3 vs. L2-3	253115_at	AT4G35910	Encodes a cytoplasmic thiouridylase that is essential for tRNA thiolation. Its activity appears to be important in root development.	32.69	52.47	0.62	2.43E-05	2.16E-04
2046	3	F2-3 vs. L2-3	250860_at	AT5G04770	Encodes a member of the cationic amino acid transporter (CAT) subfamily of amino acid polyamine choline transporters. Does not mediate efficient uptake of basic amino acids in yeast or Xenopus systems but can transport neutral and acidic amino acid analogs. Expressed in sink tissues. Induced during infestation of roots by the plant parasitic root-knot nematode, Meloidogyne incognita. Localized in the plasma membrane.	18.76	30.17	0.62	4.43E-04	1.72E-03
2047	3	F2-3 vs. L2-3	250042_at	AT5G18420	CCR4-NOT transcription complex subunit;(source:Araport11)	282.95	457.94	0.62	3.45E-04	1.43E-03
2048	3	F2-3 vs. L2-3	249801_at	AT5G23580	Member of a unique family of enzymes containing a single polypeptide chain with a kinase domain at the amino terminus and a putative calcium-binding EF hands structure at the carboxyl terminus; recombinant protein is fully active and induced by Ca2+	96.32	156.03	0.62	3.58E-04	1.47E-03
2049	3	F2-3 vs. L2-3	246735_at	AT5G27670	Encodes HTA7, a histone H2A protein.	113.34	184.16	0.62	8.30E-05	5.17E-04
2050	3	F2-3 vs. L2-3	248565_at	AT5G49710	RING finger protein;(source:Araport11)	64.73	103.66	0.62	1.59E-04	8.16E-04
2051	3	F2-3 vs. L2-3	247210_at	AT5G65020	Annexins are calcium binding proteins that are localized in the cytoplasm. When cytosolic Ca2+ increases, they relocate to the plasma membrane. They may be involved in the Golgi-mediated secretion of polysaccharides.	654.82	1058.74	0.62	2.67E-04	1.18E-03
2052	3	F2-3 vs. L2-3	260390_at	AT1G73940	tumor necrosis factor receptor family protein;(source:Araport11)	53.72	87.84	0.61	2.35E-04	1.08E-03
2053	3	F2-3 vs. L2-3	263239_at	AT2G16570	Amidophosphoribosyltransferase (ATase: EC 2.4.2.14) is a key enzyme in the pathway of purine nucleotide biosynthesis	138.92	227.52	0.61	9.04E-04	2.97E-03
2054	3	F2-3 vs. L2-3	245142_at	AT2G45270	Mitochondrial protein essential for embryo development.	49.96	81.98	0.61	7.82E-04	2.64E-03
2055	3	F2-3 vs. L2-3	258793_at	AT3G04780	Encodes a protein with little sequence identity with any other protein of known structure or function. Part of this protein shows a 42% sequence identity with the C-terminal domain of the 32-kD human thioredoxin-like protein.	79.37	130.08	0.61	2.40E-04	1.09E-03
2056	3	F2-3 vs. L2-3	258332_at	AT3G16180	Encodes a low affinity nitrate transporter that is expressed in the plasma membrane and found in the phloem of the major veins of leaves. It is responsible for nitrate redistribution to young leaves.	12.71	20.83	0.61	4.30E-04	1.68E-03
2057	3	F2-3 vs. L2-3	256939_at	AT3G22630	Encodes 20S proteasome beta subunit PBD1 (PBD1).	209.20	344.48	0.61	1.30E-04	7.10E-04
2058	3	F2-3 vs. L2-3	252562_s_a	AT3G45930	Histone superfamily protein;(source:Araport11)	39.63	64.53	0.61	8.61E-04	2.86E-03
2059	3	F2-3 vs. L2-3	251938_at	AT3G53430	Ribosomal protein L11 family protein;(source:Araport11)	146.39	240.03	0.61	1.80E-04	8.86E-04
2060	3	F2-3 vs. L2-3	251852_at	AT3G54750	downstream neighbor of Son;(source:Araport11)	19.68	32.41	0.61	1.11E-04	6.34E-04
2061	3	F2-3 vs. L2-3	251233_at	AT3G62800	Encodes a nuclear dsRNA-binding protein DRB4 that interacts specifically with DCL4. May regulate DCL4 function and thereby affect miRNA biogenesis. Also has an impact on polymerase IV-dependent siRNA levels. DRB4 interacts with the P6 viral protein from Cauliflower mosaic virus and may be a target of viral silencing suppression.	27.86	45.55	0.61	1.26E-04	6.94E-04
2062	3	F2-3 vs. L2-3	248965_at	AT5G45370	nodulin MtN21-like transporter family protein	39.80	65.18	0.61	4.23E-05	3.23E-04
2063	3	F2-3 vs. L2-3	261089_at	AT1G07570	Protein kinase capable of phosphorylating tyrosine, serine, and threonine residues	214.21	357.49	0.60	1.29E-04	7.05E-04
2064	3	F2-3 vs. L2-3	256533_at	AT1G33250	beta-1,3-n-acetylglucosaminyltransferase radical fringe protein, putative (DUF604);(source:Araport11)	30.92	51.75	0.60	6.81E-05	4.47E-04
2065	3	F2-3 vs. L2-3	260008_at	AT1G68070	Zinc finger, C3HC4 type (RING finger) family protein;(source:Araport11)	166.33	276.18	0.60	1.58E-04	8.14E-04
2066	3	F2-3 vs. L2-3	261887_at	AT1G80780	Deadenylase.	61.05	102.42	0.60	5.63E-05	3.95E-04
2067	3	F2-3 vs. L2-3	265332_at	AT2G18410	elongator complex protein;(source:Araport11)	14.94	24.79	0.60	1.20E-04	6.66E-04
2068	3	F2-3 vs. L2-3	266831_at	AT2G22830	squalene epoxidase 2;(source:Araport11)	20.48	33.94	0.60	1.41E-04	7.47E-04
2069	3	F2-3 vs. L2-3	266887_at	AT2G44650	Encodes a chloroplast-localized chaperonin 10 whose mRNA is expressed in leaves and stems but not roots.	82.06	137.75	0.60	8.16E-04	2.74E-03
2070	3	F2-3 vs. L2-3	266459_at	AT2G47970	Nuclear pore localization protein NPL4;(source:Araport11)	37.91	62.96	0.60	9.13E-04	2.99E-03
2071	3	F2-3 vs. L2-3	252603_at	AT3G45050	transmembrane protein;(source:Araport11)	28.14	47.25	0.60	5.30E-04	1.96E-03
2072	3	F2-3 vs. L2-3	252189_at	AT3G50070	Encode CYCD3;3, a CYCD3 D-type cyclin. Important for determining cell number in developing lateral organs. Mediating cytokinin effects in apical growth and development.	79.29	132.92	0.60	5.45E-04	2.01E-03
2073	3	F2-3 vs. L2-3	251921_at	AT3G53890	Cytosolic ribosomal protein. Mutants enhance the varigation effect of var2 mutations suggesting a link between cytosolic translation and chloroplast development.	13.03	21.86	0.60	5.85E-05	4.05E-04

2074	3	F2-3 vs. L2-3	251200_at	AT3G63010	Encodes a gibberellin (GA) receptor ortholog of the rice GA receptor gene (OsGID1). Has GA-binding activity, showing higher affinity to GA4. Interacts with DELLA proteins in vivo in the presence of GA4. The mRNA is cell-to-cell mobile.	21.56	36.23	0.60	3.98E-04	1.58E-03
2075	3	F2-3 vs. L2-3	254188_at	AT4G23920	Encodes a protein with UDP-D-glucose 4-epimerase activity. Involved in growth and cell wall carbohydrate biosynthesis.	74.59	124.77	0.60	6.37E-05	4.30E-04
2076	3	F2-3 vs. L2-3	253976_at	AT4G26610	D6PK family kinase involved in pulse-induced phototropism but also for time-dependent second positive phototropism, and continuous light-induced hypocotyl phototropism.	52.55	88.14	0.60	7.79E-04	2.63E-03
2077	3	F2-3 vs. L2-3	252883_at	AT4G39520	Encodes a member of the DRG (developmentally regulated G-protein) family. Has GTPase activity.	21.62	36.07	0.60	7.32E-04	2.51E-03
2078	3	F2-3 vs. L2-3	250434_at	AT5G10390	Histone superfamily protein;(source:Araport11)	124.52	207.95	0.60	6.02E-04	2.16E-03
2079	3	F2-3 vs. L2-3	250382_at	AT5G11580	Regulator of chromosome condensation (RCC1) family protein;(source:Araport11)	95.32	157.95	0.60	6.29E-05	4.26E-04
2080	3	F2-3 vs. L2-3	249074_at	AT5G44080	Basic-leucine zipper (bZIP) transcription factor family protein;(source:Araport11)	77.23	129.41	0.60	2.47E-04	1.12E-03
2081	3	F2-3 vs. L2-3	248878_at	AT5G46160	Ribosomal protein L14p/L23e family protein;(source:Araport11)	145.70	244.32	0.60	1.17E-04	6.58E-04
2082	3	F2-3 vs. L2-3	247862_at	AT5G58250	Involved in tetrapyrrole biosynthesis. May function as a scaffold protein to stabilize CHL27.	64.35	107.16	0.60	3.19E-04	1.35E-03
2083	3	F2-3 vs. L2-3	255883_at	AT1G20270	2-oxoglutarate (2OG) and Fe(II)-dependent oxygenase superfamily protein;(source:Araport11)	68.22	115.85	0.59	3.66E-04	1.49E-03
2084	3	F2-3 vs. L2-3	265101_at	AT1G30880	hypothetical protein;(source:Araport11)	74.14	125.06	0.59	4.41E-04	1.71E-03
2085	3	F2-3 vs. L2-3	255985_at	AT1G34150	Pseudouridine synthase family protein;(source:Araport11)	56.25	94.84	0.59	4.00E-04	1.59E-03
2086	3	F2-3 vs. L2-3	262245_at	AT1G48240	member of NPSN Gene Family	13.42	22.69	0.59	3.04E-04	1.31E-03
2087	3	F2-3 vs. L2-3	265426_at	AT2G20740	Tetraspanin family protein;(source:Araport11)	363.83	617.70	0.59	1.87E-05	1.83E-04
2088	3	F2-3 vs. L2-3	264044_at	AT2G22480	phosphofructokinase 5;(source:Araport11)	181.88	306.39	0.59	2.21E-04	1.03E-03
2089	3	F2-3 vs. L2-3	267284_at	AT2G23700	Itga6 (Protein of unknown function, DUF547);(source:Araport11)	54.69	92.39	0.59	1.85E-04	8.99E-04
2090	3	F2-3 vs. L2-3	266789_at	AT2G29020	Rab5-interacting family protein;(source:Araport11)	401.53	680.35	0.59	8.58E-04	2.85E-03
2091	3	F2-3 vs. L2-3	256278_at	AT3G12130	KHZ1 is a CCCH zinc-finger and KH domain protein belonging to the VII subfamily. It is expressed throughout the plant. Highly similar to KHZ2. khz1 mutants are late flowering and double mutants with khz2 are even more late flowering. Overexpression leads to increased rates of leaf senescence.	33.27	56.56	0.59	4.27E-05	3.24E-04
2092	3	F2-3 vs. L2-3	245442_at	AT4G16710	glycosyltransferase family protein 28;(source:Araport11)	176.51	297.60	0.59	5.54E-04	2.03E-03
2093	3	F2-3 vs. L2-3	254476_at	AT4G20410	Encodes a member of the gamma-soluble NSF attachment protein (gSNAP) gene family.	117.61	199.89	0.59	6.34E-04	2.25E-03
2094	3	F2-3 vs. L2-3	245998_at	AT5G20830	Encodes a protein with sucrose synthase activity (SUS1).	261.01	439.90	0.59	9.34E-04	3.04E-03
2095	3	F2-3 vs. L2-3	247053_at	AT5G66710	Protein kinase superfamily protein;(source:Araport11)	16.45	27.82	0.59	6.17E-04	2.21E-03
2096	3	F2-3 vs. L2-3	261030_at	AT1G17410	Nucleoside diphosphate kinase family protein;(source:Araport11)	16.30	27.87	0.58	8.25E-04	2.76E-03
2097	3	F2-3 vs. L2-3	245644_at	AT1G25320	Leucine-rich repeat protein kinase family protein;(source:Araport11)	87.94	150.99	0.58	6.71E-04	2.35E-03
2098	3	F2-3 vs. L2-3	261294_at	AT1G48430	Dihydroxyacetone kinase;(source:Araport11)	28.03	48.38	0.58	5.74E-05	4.01E-04
2099	3	F2-3 vs. L2-3	259612_at	AT1G52300	Zinc-binding ribosomal protein family protein;(source:Araport11)	675.52	1168.38	0.58	4.76E-04	1.80E-03
2100	3	F2-3 vs. L2-3	256293_at	AT1G69440	Encodes ARGONAUTE7, a member of the ARGONAUTE family, characterised by the presence of PAZ and PIWI domains. Involved in the regulation of developmental timing. Required for the accumulation of TAS3 ta-siRNAs but not for accumulation of miR171, miR173, miR390 or mi391. Localized in mature rosette leaves and floral buds.	22.98	39.36	0.58	1.10E-04	6.29E-04
2101	3	F2-3 vs. L2-3	260373_at	AT1G73970	obscurin-like protein;(source:Araport11)	25.52	43.84	0.58	4.46E-04	1.72E-03
2102	3	F2-3 vs. L2-3	264114_at	AT2G31270	Encodes a cyclin-dependent protein kinase. Involved in nuclear DNA replication and plastid division. Located in nucleus and chloroplast.	14.61	25.34	0.58	6.86E-04	2.39E-03
2103	3	F2-3 vs. L2-3	266929_at	AT2G45850	AT hook motif DNA-binding family protein;(source:Araport11)	35.19	60.73	0.58	9.29E-04	3.03E-03
2104	3	F2-3 vs. L2-3	259099_at	AT3G05010	Encodes a candidate G-protein Coupled Receptor that is involved in the regulation of root growth by bacterial N-acyl-homoserine lactones (AHLs) and plays a role in mediating interactions between plants and microbes.	302.52	521.01	0.58	9.24E-05	5.56E-04
2105	3	F2-3 vs. L2-3	259255_at	AT3G07690	6-phosphogluconate dehydrogenase family protein;(source:Araport11)	68.79	119.01	0.58	2.13E-04	1.00E-03
2106	3	F2-3 vs. L2-3	256961_at	AT3G13445	TBP (TATA binding protein) associates with TAF(II)s (TBP-associated factors) to form the TFIID general transcription factor complex	71.17	122.03	0.58	2.10E-05	1.95E-04
2107	3	F2-3 vs. L2-3	256854_at	AT3G15180	ARM repeat superfamily protein;(source:Araport11)	95.40	164.68	0.58	7.03E-04	2.44E-03

2108	3	F2-3 vs. L2-3	258155_at	AT3G18130	Encodes a protein with similarity to mammalian RACKs. RACKs function to shuttle activated protein kinase C to different subcellular sites and may also function as a scaffold through physical interactions with other proteins. RACK1C has no phenotype on its own and probably acts redundantly with RACK1A and RACK1B.	641.34	1102.92	0.58	2.53E-04	1.13E-03
2109	3	F2-3 vs. L2-3	257199_at	AT3G23710	Tic22-like family protein;(source:Araport11)	67.41	116.23	0.58	3.23E-04	1.36E-03
2110	3	F2-3 vs. L2-3	255128_at	AT4G08310	DNA ligase;(source:Araport11)	14.57	25.33	0.58	5.57E-04	2.04E-03
2111	3	F2-3 vs. L2-3	255025_at	AT4G09900	Encodes a protein predicted to act as a carboxylesterase. It has similarity to the SABP2 methyl salicylate esterase from tobacco. This protein does not act on methyl IAA, methyl JA, MeSA, MeGA4, or MEGA9 in vitro.	22.04	37.85	0.58	2.78E-04	1.22E-03
2112	3	F2-3 vs. L2-3	245602_at	AT4G14270	Protein containing PAM2 motif which mediates interaction with the PABC domain of polyadenyl binding proteins.	56.20	97.27	0.58	4.77E-04	1.80E-03
2113	3	F2-3 vs. L2-3	254370_at	AT4G21750	Encodes a homeobox protein similar to GL2. It is expressed in both the apical and basal daughter cells of the zygote as well as their progeny. Expression is detected starting the two-celled stage of embryo development and is later restricted to the outermost, epidermal cell layer from its inception. Its promoter is highly modular with each region contributing to specific aspects of the gene Primes spatial and temporal expression. Double mutant analysis with PDF2, another L1-specific gene, suggests that their functions are partially redundant and the absence of both of the genes result in abnormal shoot development.	102.41	176.64	0.58	2.01E-05	1.90E-04
2114	3	F2-3 vs. L2-3	253368_at	AT4G33200	member of Myosin-like proteins	79.66	136.95	0.58	5.40E-04	1.99E-03
2115	3	F2-3 vs. L2-3	252935_at	AT4G39150	DNAJ heat shock N-terminal domain-containing protein;(source:Araport11)	80.74	139.49	0.58	8.66E-05	5.30E-04
2116	3	F2-3 vs. L2-3	245930_at	AT5G09240	ssDNA-binding transcriptional regulator;(source:Araport11)	39.91	68.76	0.58	5.76E-04	2.08E-03
2117	3	F2-3 vs. L2-3	245898_at	AT5G11020	Protein kinase superfamily protein;(source:Araport11)	165.76	285.29	0.58	9.33E-04	3.04E-03
2118	3	F2-3 vs. L2-3	248820_at	AT5G47060	hypothetical protein (DUF581);(source:Araport11)	78.12	134.46	0.58	2.87E-04	1.25E-03
2119	3	F2-3 vs. L2-3	247626_at	AT5G60300	Encodes a legume-type lectin receptor kinase that is structurally distinct from the mammalian extracellular ATP receptors and acts as an extracellular ATP receptor in Arabidopsis. Extracellular ATP acts as a damage-associated molecular pattern in plants, and its signaling through P2K1 is important for mounting an effective defense response against various pathogenic microorganisms. It also plays a role in cell wall-plasma membrane adhesion.	89.89	156.25	0.58	6.83E-04	2.38E-03
2120	3	F2-3 vs. L2-3	264364_at	AT1G03330	Small nuclear ribonucleoprotein family protein;(source:Araport11)	155.85	273.98	0.57	8.60E-06	1.07E-04
2121	3	F2-3 vs. L2-3	262238_at	AT1G48300	Cytosolic iron-sulfur protein with a [2Fe-2S] cluster which synthesizes triacylglycerol (DGAT activity).	170.82	298.28	0.57	5.00E-04	1.87E-03
2122	3	F2-3 vs. L2-3	260604_at	AT1G55840	Sec14p-like phosphatidylinositol transfer family protein;(source:Araport11)	355.62	625.28	0.57	2.24E-04	1.04E-03
2123	3	F2-3 vs. L2-3	257460_at	AT1G75580	SAUR-like auxin-responsive protein family;(source:Araport11)	9.10	16.08	0.57	8.37E-04	2.79E-03
2124	3	F2-3 vs. L2-3	263332_at	AT2G03870	Small nuclear ribonucleoprotein family protein;(source:Araport11)	242.82	424.81	0.57	8.40E-06	1.05E-04
2125	3	F2-3 vs. L2-3	258799_at	AT3G04770	40s ribosomal protein SA B;(source:Araport11)	162.51	286.74	0.57	9.75E-05	5.77E-04
2126	3	F2-3 vs. L2-3	256282_at	AT3G12550	Belongs to a subgroup of SGS3-like proteins that act redundantly in RNA-directed DNA methylation: AT1G15910 (FDM1), AT4G00380 (FDM2), AT3G12550 (FDM3), AT1G13790 (FDM4), AT1G80790 (FDM5).	25.80	45.17	0.57	3.88E-05	3.03E-04
2127	3	F2-3 vs. L2-3	257190_at	AT3G13120	Ribosomal protein S10p/S20e family protein;(source:Araport11)	36.04	62.98	0.57	6.52E-04	2.30E-03
2128	3	F2-3 vs. L2-3	252637_at	AT3G44530	Encodes a nuclear localized WD-repeat containing protein involved in negative regulation of knox gene expression via epigenetic mechanism of chromatin re-organization. It is a part of the HISTONE REGULATOR complex that deposits histones in a DNA synthesis-independent manner and affects both nucleosome occupancy and the maintenance of transcriptional silencing. Interacts physically and genetically with AS1. Expressed in meristem and leaf primordia. Homozygous mutants are embryo lethal. Phenotype of cosuppressed lines is variable but show effects on leaf development similar to as1/as2.	80.19	141.11	0.57	8.40E-05	5.21E-04
2129	3	F2-3 vs. L2-3	251922_at	AT3G54030	kinase with tetratricopeptide repeat domain-containing protein;(source:Araport11)	18.23	32.23	0.57	7.07E-04	2.45E-03
2130	3	F2-3 vs. L2-3	255478_at	AT4G02440	EID1 is an F-box protein that functions as a negative regulator in phytochrome A (phyA)-specific light signalling. Expressed at all stages of plant development independently of light conditions, localizes to the nucleus, and forms nuclear speckles under continuous far-red light. Forms stable dimeric and trimeric complexes with several ASK proteins and Cullin1 in yeast and in planta.	54.05	95.02	0.57	8.53E-04	2.84E-03
2131	3	F2-3 vs. L2-3	250386_at	AT5G11510	Arabidopsis thaliana putative c-myb-like transcription factor MYB3R-4. Functions in powdery mildew induced host endoreduplication at the site of infection.	38.06	66.64	0.57	5.93E-04	2.13E-03

2132	3	F2-3 vs. L2-3	248421_at	AT5G51510	jagunal-like protein;(source:Araport11)	150.21	261.43	0.57	6.93E-05	4.53E-04
2133	3	F2-3 vs. L2-3	264857_at	AT1G24170	Encodes a protein with putative galacturonosyltransferase activity.	74.57	132.29	0.56	2.14E-05	1.97E-04
2134	3	F2-3 vs. L2-3	260842_at	AT1G29150	specifically interacts with FUS6/COP11 via the C-terminal domain of FUS6/COP11 and associates with an ATPase subunit of the 19S proteasome regulatory complex, AtS6A. The mRNA is cell-to-cell mobile.	64.07	115.26	0.56	6.11E-04	2.19E-03
2135	3	F2-3 vs. L2-3	264497_at	AT1G30840	Member of a family of proteins related to PUP1, a purine transporter. May be involved in the transport of purine and purine derivatives such as cytokinins, across the plasma membrane.	27.36	48.45	0.56	3.25E-04	1.37E-03
2136	3	F2-3 vs. L2-3	260229_at	AT1G74370	RING/U-box superfamily protein;(source:Araport11)	48.67	86.42	0.56	7.16E-05	4.63E-04
2137	3	F2-3 vs. L2-3	262699_at	AT1G75980	Single hybrid motif superfamily protein;(source:Araport11)	86.41	154.02	0.56	6.98E-05	4.55E-04
2138	3	F2-3 vs. L2-3	259732_at	AT1G77470	Encodes a protein with high homology to the Replication Factor C, Subunit 3 (RFC3) of yeast and other eukaryotes. rfc3 mutants are hypersensitive to salicylic acid and exhibit enhanced induction of PR genes and resistance against virulent oomycete Hyaloperonospora arabidopsidis Noco2. The enhanced pathogen resistance in the mutant is NPR1-independent.	104.86	187.93	0.56	2.20E-05	2.02E-04
2139	3	F2-3 vs. L2-3	267309_at	AT2G19385	zinc ion binding protein;(source:Araport11)	31.90	56.95	0.56	1.55E-04	8.02E-04
2140	3	F2-3 vs. L2-3	267462_at	AT2G33735	Chaperone DnaJ-domain superfamily protein;(source:Araport11)	77.70	137.98	0.56	7.32E-04	2.51E-03
2141	3	F2-3 vs. L2-3	258801_at	AT3G04560	nucleolar/coiled-body phosphoprotein;(source:Araport11)	235.52	421.34	0.56	1.79E-04	8.84E-04
2142	3	F2-3 vs. L2-3	259250_at	AT3G07580	hypothetical protein;(source:Araport11)	21.12	37.55	0.56	3.83E-04	1.54E-03
2143	3	F2-3 vs. L2-3	258410_at	AT3G16780	Ribosomal protein L19e family protein;(source:Araport11)	402.54	720.08	0.56	1.44E-04	7.62E-04
2144	3	F2-3 vs. L2-3	257601_at	AT3G24820	BSD domain-containing protein;(source:Araport11)	12.71	22.90	0.56	8.22E-04	2.75E-03
2145	3	F2-3 vs. L2-3	257809_at	AT3G27060	Encodes one of the 3 ribonucleotide reductase (RNR) small subunit genes. TSO2 transcription occurs predominantly at the S-phase of the cell cycle and its expression pattern is consistent with its role in dNDP biosynthesis during DNA replication in actively dividing cells. Critical for cell cycle progression, DNA damage repair and plant development.	76.21	135.77	0.56	6.22E-04	2.22E-03
2146	3	F2-3 vs. L2-3	257714_at	AT3G27360	Histone superfamily protein;(source:Araport11)	35.46	63.59	0.56	9.52E-05	5.66E-04
2147	3	F2-3 vs. L2-3	251251_at	AT3G62220	Protein kinase superfamily protein;(source:Araport11)	117.77	209.23	0.56	9.64E-05	5.71E-04
2148	3	F2-3 vs. L2-3	255553_at	AT4G01960	transmembrane protein;(source:Araport11)	86.96	155.26	0.56	3.43E-04	1.42E-03
2149	3	F2-3 vs. L2-3	254952_at	AT4G10960	Encodes a protein with UDP-D-glucose 4-epimerase activity.	47.86	85.14	0.56	1.75E-04	8.74E-04
2150	3	F2-3 vs. L2-3	254576_at	AT4G19350	embryo defective 3006;(source:Araport11)	105.15	186.25	0.56	2.10E-05	1.95E-04
2151	3	F2-3 vs. L2-3	254035_at	AT4G25970	Encodes the major form of the two non-mitochondrail phosphatidylserine decarboxylase. Located at the ER. The mRNA is cell-to-cell mobile.	98.73	174.77	0.56	5.58E-04	2.04E-03
2152	3	F2-3 vs. L2-3	252890_at	AT4G39400	Encodes a plasma membrane localized leucine-rich repeat receptor kinase involved in brassinosteroid signal transduction. BRI1 ligand is brassinolide which binds at the extracellular domain. Binding results in phosphorylation of the kinase domain which activates the BRI1 protein leading to BR responses. Residue T-1049 and either S-1044 or T-1045 were essential for kinase function in vitro and normal BRI1 signaling in planta. The structure of BRI1 ligand-binding domain has been determined at 2.5A resolution. Although BAK1 and BRI1 alone localize in the plasma membrane, when BAK1 and BRI1 are coexpressed, the heterodimer BAK1/BRI1 they form is localized in the endosome. BRI1 appears to be involved in the autonomous pathway that regulates the transition to flowering, primarily through its effects on FLC expression levels, as uncovered by double mutant analyses. This most likely occurs as a result of BRI1-dependent effects on histone acetylation, but not histone triMeH3K4 methylation, at the FLC locus. The mRNA is cell-to-cell mobile.	142.96	253.33	0.56	4.05E-04	1.61E-03
2153	3	F2-3 vs. L2-3	251139_at	AT5G01230	Encodes a tRNA methyltransferase that mediates the 2'-O-ribose methylation of selected tRNA species in the anticodon loop. Mutation of this gene compromises plant immunity against bacteria.	106.12	188.15	0.56	4.88E-05	3.56E-04
2154	3	F2-3 vs. L2-3	250584_at	AT5G07590	Transducin/WD40 repeat-like superfamily protein;(source:Araport11)	63.69	114.68	0.56	7.35E-05	4.71E-04
2155	3	F2-3 vs. L2-3	249181_at	AT5G42920	Encodes a component of the putative Arabidopsis THO/TREX complex: THO1 or HPR1 (At5g09860), THO2 (At1g24706), THO3 or TEX1 (At5g56130), THO5 (At5g42920, At1g45233), THO6 (At2g19430), and THO7 (At5g16790, At3g02950). THO/TREX complexes in animals have been implicated in the transport of mRNA precursors. Mutants of THO3/TEX1, THO1, THO6 accumulate reduced amount of small interfering (si)RNA, suggesting a role of the putative Arabidopsis THO/TREX in siRNA biosynthesis.	41.27	74.02	0.56	1.54E-05	1.61E-04

2156	3	F2-3 vs. L2-3	248694_at	AT5G48340	hypothetical protein;(source:Araport11)	98.08	175.76	0.56	6.34E-04	2.25E-03
2157	3	F2-3 vs. L2-3	248419_at	AT5G51550	EXORDIUM like 3;(source:Araport11)	369.78	661.43	0.56	9.36E-04	3.04E-03
2158	3	F2-3 vs. L2-3	248250_at	AT5G53130	member of Cyclic nucleotide gated channel family	199.41	355.79	0.56	6.95E-05	4.53E-04
2159	3	F2-3 vs. L2-3	247054_at	AT5G66730	C2H2-like zinc finger protein;(source:Araport11)	28.62	51.07	0.56	5.26E-04	1.95E-03
2160	3	F2-3 vs. L2-3	263113_at	AT1G03150	Acyl-CoA N-acyltransferases (NAT) superfamily protein;(source:Araport11)	71.05	129.89	0.55	3.71E-04	1.51E-03
2161	3	F2-3 vs. L2-3	265027_at	AT1G24450	Ribonuclease III family protein;(source:Araport11)	27.14	49.08	0.55	1.16E-04	6.55E-04
2162	3	F2-3 vs. L2-3	265012_at	AT1G24470	Encodes one of the two Arabidopsis homologues to YBR159w encoding a S. cerevisiae beta-ketoacyl reductase (KCR), which catalyzes the first reduction during VLCFA (very long chain fatty acids, >18 carbon) elongation: KCR1 (At1g67730), KCR2 (At1g24470). Complementation of the yeast ybr159Delta mutant demonstrated that the two KCR proteins are divergent and that only AtKCR1 can restore heterologous elongase activity similar to the native yeast KCR gene.	20.13	36.55	0.55	1.25E-04	6.86E-04
2163	3	F2-3 vs. L2-3	259928_at	AT1G34380	5-3 exonuclease family protein;(source:Araport11)	74.53	134.61	0.55	6.02E-04	2.16E-03
2164	3	F2-3 vs. L2-3	262257_at	AT1G53860	Encodes a protein that is highly methylated in a WT DML background.	28.84	52.43	0.55	9.53E-04	3.09E-03
2165	3	F2-3 vs. L2-3	264701_at	AT1G70160	zinc finger MYND domain protein;(source:Araport11)	157.88	287.83	0.55	3.01E-04	1.30E-03
2166	3	F2-3 vs. L2-3	262201_at	AT2G01120	Origin Recognition Complex subunit 4. Involved in the initiation of DNA replication. Regulated transcriptionally during cell cycle, peaking at G1/S-phase. Target of E2F/DF family of transcription factors. Interacts with all ORC subunits except ORC1b.	22.19	40.50	0.55	5.27E-04	1.95E-03
2167	3	F2-3 vs. L2-3	267484_at	AT2G02760	ubiquitin conjugating enzyme UBC2. Homolog of the yeast RAD6 gene.	329.61	597.49	0.55	2.75E-04	1.21E-03
2168	3	F2-3 vs. L2-3	263074_at	AT2G17560	Encodes a protein belonging to the subgroup of HMGB (high mobility group B) proteins that have a distinctive DNA-binding motif, the HMG-box domain. The motif confers non-sequence specific interaction with linear DNA and structure-specific binding to distorted DNA sites. The HMGB proteins are involved in the assembly of nucleoprotein complexes. Can be phosphorylated by CK2alpha.	130.75	236.35	0.55	8.62E-05	5.29E-04
2169	3	F2-3 vs. L2-3	266225_at	AT2G28900	Encodes AtOEP16, a 16-KDa plastid outer membrane protein involved in plastid import of protochlorophyllide oxidoreductase A. Predominantly expressed in leaves and is also inducible by cold treatment.	322.09	583.49	0.55	9.76E-05	5.77E-04
2170	3	F2-3 vs. L2-3	267591_at	AT2G39705	ROTUNDIFOLIA like 8;(source:Araport11)	26.81	48.60	0.55	3.68E-04	1.50E-03
2171	3	F2-3 vs. L2-3	259005_at	AT3G01930	Major facilitator superfamily protein;(source:Araport11)	52.40	95.32	0.55	5.67E-04	2.06E-03
2172	3	F2-3 vs. L2-3	258853_at	AT3G06440	Encodes a Golgi-localized hydroxyproline-O-galactosyltransferase. Mutants display multiple phenotypes including reduced root hair growth and reduced seed coat mucilage.	39.23	70.79	0.55	1.87E-04	9.06E-04
2173	3	F2-3 vs. L2-3	258666_at	AT3G08550	mutant is Dwarfed and shows defects in cell elongation; Cellulose deficient; Plasma Membrane Protein	135.96	248.26	0.55	8.55E-04	2.84E-03
2174	3	F2-3 vs. L2-3	257897_at	AT3G18030	flavin mononucleotide flavoprotein involved in salt and osmotic tolerance HAL3A encodes for phosphopantothenoylcysteine decarboxylase being involved in Coenzyme A biosynthesis. HAL3A is predominant over another gene with the presumably same function (HAL3B).	73.61	134.38	0.55	4.29E-04	1.68E-03
2175	3	F2-3 vs. L2-3	252469_at	AT3G46920	kinase superfamily with octicosapeptide/Phox/Bem1p domain-containing protein;(source:Araport11)	12.04	21.77	0.55	1.93E-04	9.30E-04
2176	3	F2-3 vs. L2-3	252349_at	AT3G48150	anaphase-promoting complex or cyclosome subunit	76.72	139.05	0.55	3.94E-05	3.06E-04
2177	3	F2-3 vs. L2-3	251623_at	AT3G57390	encodes a MADS-box containing protein likely to be a transcription factor that is expressed in endosperm and developing gametophytes. The protein sequence is most similar to that of AGL15, which is expressed in developing embryos.	93.77	169.43	0.55	1.57E-04	8.09E-04
2178	3	F2-3 vs. L2-3	245255_at	AT4G14110	Represses photomorphogenesis and induces skotomorphogenesis in the dark. A component of the COP9 signalosome complex.	113.18	205.70	0.55	1.39E-04	7.42E-04
2179	3	F2-3 vs. L2-3	253703_at	AT4G29910	Origin Recognition Complex subunit 5. Involved in the initiation of DNA replication. Interacts strongly with all ORC subunits.	50.76	91.55	0.55	2.35E-05	2.11E-04
2180	3	F2-3 vs. L2-3	253668_at	AT4G30220	small nuclear ribonucleoprotein F;(source:Araport11)	292.75	533.79	0.55	6.43E-04	2.27E-03
2181	3	F2-3 vs. L2-3	253633_at	AT4G30480	Encodes one of the 36 carboxylate clamp (CC)-tetratricopeptide repeat (TPR) proteins (Prasad 2010, Pubmed ID: 20856808). Interacts with Hsp90/Hsp70 as co-chaperone.	167.93	307.76	0.55	2.33E-04	1.07E-03
2182	3	F2-3 vs. L2-3	247826_at	AT5G58480	O-Glycosyl hydrolases family 17 protein;(source:Araport11)	39.97	73.06	0.55	5.19E-04	1.93E-03
2183	3	F2-3 vs. L2-3	261493_at	AT1G14410	Encodes a homolog of the potato p24 protein. Binds single strand telomeric repeats. Negatively regulates telomerase activity and telomere length.	79.95	147.52	0.54	2.12E-04	9.99E-04
2184	3	F2-3 vs. L2-3	260665_at	AT1G19360	Encodes an arabinosyltransferase that modifies extensin proteins in root hair cells.	26.24	48.25	0.54	2.33E-04	1.07E-03

2185	3	F2-3 vs. L2-3	260257_at	AT1G74340	Encodes a subunit of the dolichol phosphate mannase synthase (DPMS) complex that may serve as membrane anchors for the catalytic core, DPMS1, or provide catalytic assistance. It is localized in the ER and mediates isoprenyl-linked glycan biogenesis.	41.00	76.10	0.54	3.74E-04	1.52E-03
2186	3	F2-3 vs. L2-3	263624_at	AT2G04700	Encodes catalytic subunit of ferredoxin/thioredoxin reductase.	253.47	473.38	0.54	4.82E-04	1.82E-03
2187	3	F2-3 vs. L2-3	266037_at	AT2G05940	Encodes a receptor-like cytoplasmic kinase that phosphorylates the host target RIN4, leading to the activation of a plant innate immune receptor RPM1.	73.12	134.88	0.54	9.88E-04	3.19E-03
2188	3	F2-3 vs. L2-3	263564_s_at	AT2G15400	Non-catalytic subunit of Nuclear DNA-dependent RNA polymerase V; homologous to budding yeast RPB3 and the E. coli RNA polymerase alpha subunit. A closely related paralog, At2g15430 can substitute for At2g15400 in the context of Pol V and encodes the equivalent subunit of Pol II and Pol IV.	43.63	80.73	0.54	7.89E-05	4.96E-04
2189	3	F2-3 vs. L2-3	267012_at	AT2G39220	PATATIN-like protein 6;(source:Araport11)	438.40	815.96	0.54	2.47E-05	2.17E-04
2190	3	F2-3 vs. L2-3	263990_at	AT2G42810	Encodes a phytochrome-specific type 5 serine/threonine protein phosphatase. It dephosphorylates active Pfr-phytochromes. Controls light signal flux by enhancing phytochrome stability and affinity for a signal transducer. The gene is alternately spliced. This variant is an integral membrane protein localized to the ER and nuclear envelope. Belongs to one of the 36 carboxylate clamp (CC)-tetratricopeptide repeat (TPR) proteins (Prasad 2010, Pubmed ID: 20856808) with potential to interact with Hsp90/Hsp70 as co-chaperones. It also regulates tetrapyrrole biosynthesis through the accumulation of Mg-ProtoIX and acts as a negative regulator of photosynthesis associated nuclear gene expression during chloroplast biogenesis and development.	68.86	126.88	0.54	9.34E-05	5.59E-04
2191	3	F2-3 vs. L2-3	258206_at	AT3G14010	hydroxyproline-rich glycoprotein family protein, similar to Mrs16p (GI:2737884) (Saccharomyces cerevisiae); weak similarity to ataxin-2 related protein (GI:1679686) (Homo sapiens). Included in a family of CTC interacting domain proteins found to interact with PAB2.	26.74	49.60	0.54	7.20E-05	4.64E-04
2192	3	F2-3 vs. L2-3	252297_at	AT3G48930	Nucleic acid-binding, OB-fold-like protein;(source:Araport11)	219.50	408.46	0.54	6.24E-04	2.23E-03
2193	3	F2-3 vs. L2-3	253687_at	AT4G29520	SES1 is an ER localized chaperone involved in salt and heat stress response.	132.58	244.42	0.54	7.19E-04	2.48E-03
2194	3	F2-3 vs. L2-3	247743_at	AT5G59010	kinase with tetratricopeptide repeat domain-containing protein;(source:Araport11)	65.36	120.83	0.54	5.78E-04	2.09E-03
2195	3	F2-3 vs. L2-3	247583_at	AT5G60750	Encodes a chloroplast endoproteinase, SNOWY COTYLEDON4 (SCO4), required for photosynthetic acclimation to higher light intensities.	19.96	37.24	0.54	3.99E-04	1.59E-03
2196	3	F2-3 vs. L2-3	247609_at	AT5G60940	Member of CstF complex.	36.31	66.75	0.54	5.91E-04	2.13E-03
2197	3	F2-3 vs. L2-3	261277_at	AT1G20230	Pentatricopeptide repeat (PPR) superfamily protein;(source:Araport11)	90.70	172.75	0.53	2.32E-04	1.07E-03
2198	3	F2-3 vs. L2-3	261802_at	AT1G30545	S-adenosyl-L-methionine-dependent methyltransferase superfamily protein;(source:Araport11)	24.94	47.29	0.53	3.11E-04	1.33E-03
2199	3	F2-3 vs. L2-3	256460_at	AT1G36240	Ribosomal protein L7Ae/L30e/S12e/Gadd45 family protein;(source:Araport11)	1064.22	1999.55	0.53	1.46E-04	7.65E-04
2200	3	F2-3 vs. L2-3	260644_at	AT1G53290	Galactosyltransferase family protein;(source:Araport11)	72.68	138.12	0.53	3.53E-04	1.45E-03
2201	3	F2-3 vs. L2-3	265760_at	AT2G01220	Nucleotidylyl transferase superfamily protein;(source:Araport11)	28.16	53.34	0.53	8.06E-04	2.71E-03
2202	3	F2-3 vs. L2-3	266182_at	AT2G02370	SNARE associated Golgi protein family;(source:Araport11)	92.28	174.94	0.53	5.24E-05	3.75E-04
2203	3	F2-3 vs. L2-3	266226_at	AT2G28740	histone 4	21.23	40.27	0.53	5.18E-04	1.93E-03
2204	3	F2-3 vs. L2-3	266922_s_a	AT2G45950	SKP1-like 20;(source:Araport11)	96.97	184.33	0.53	2.06E-05	1.93E-04
2205	3	F2-3 vs. L2-3	259029_at	AT3G09300	OSBP(oxysterol binding protein)-related protein 3B;(source:Araport11)	163.48	308.95	0.53	5.26E-04	1.95E-03
2206	3	F2-3 vs. L2-3	252335_at	AT3G48860	coiled-coil protein;(source:Araport11)	32.97	61.87	0.53	2.70E-04	1.19E-03
2207	3	F2-3 vs. L2-3	251802_at	AT3G55380	ubiquitin-conjugating enzyme 14;(source:Araport11)	83.41	156.37	0.53	8.10E-06	1.04E-04
2208	3	F2-3 vs. L2-3	251446_at	AT3G59840	allyl alcohol dehydrogenase-like protein;(source:Araport11)	34.54	65.26	0.53	7.01E-04	2.43E-03
2209	3	F2-3 vs. L2-3	251271_at	AT3G62050	Putative endonuclease or glycosyl hydrolase;(source:Araport11)	17.36	32.99	0.53	7.19E-05	4.64E-04
2210	3	F2-3 vs. L2-3	245254_at	AT4G14680	Encodes one of three A. thaliana ATP-sulfurylases. APS is the first enzyme of sulfate assimilation that catalyzes the formation of adenosine-5'-phosphosulfate from ATP and sulfate.	30.96	58.24	0.53	2.31E-04	1.06E-03
2211	3	F2-3 vs. L2-3	254359_at	AT4G22360	SWIB complex BAF60b domain-containing protein;(source:Araport11)	76.13	144.49	0.53	9.70E-06	1.16E-04
2212	3	F2-3 vs. L2-3	253984_at	AT4G26590	oligopeptide transporter	82.31	156.10	0.53	1.67E-04	8.43E-04
2213	3	F2-3 vs. L2-3	252871_at	AT4G40000	S-adenosyl-L-methionine-dependent methyltransferases superfamily protein;(source:Araport11)	89.74	170.82	0.53	1.79E-04	8.84E-04
2214	3	F2-3 vs. L2-3	251040_at	AT5G02250	Encodes a exoribonuclease involved in rRNA processing in mitochondria and chloroplasts.Loss of function mutations are pale green and require supplementation with sucrose for germination and early development. Plants are pale green due to defects in chloroplast biogenesis.	34.45	65.61	0.53	3.58E-04	1.47E-03

2215	3	F2-3 vs. L2-3	250284_at	AT5G13290	Encodes a protein with predicted Ser/Thr kinase activity and membrane localization that is involved in the CLV3 signaling pathway that represses WUS expression in the meristem. Loss of function of CRN can suppress the phenotype caused by overexpression of CLV3. SOL2 isolated as a suppressor of root- specific overexpression of CLE19, a clavata3 like gene. sol2 partially suppresses the short root phenotype caused by CLE19 overexpression. Mutant flowers have extra carpels.	88.81	166.90	0.53	5.51E-05	3.89E-04
2216	3	F2-3 vs. L2-3	250051_at	AT5G17800	Member of the R2R3 factor gene family that acts as a cell-specific repressor of quiescent center (QC) divisions in the primary root, acting through the BR signaling pathway. Works with BES1 to regulate QC division in the root.	46.92	88.55	0.53	3.68E-04	1.50E-03
2217	3	F2-3 vs. L2-3	245914_at	AT5G19620	AtOEP80 is paralog to the chloroplastic protein translocation channel Toc75. Mutations in this locus result in embryo lethality.	66.59	126.74	0.53	6.46E-04	2.28E-03
2218	3	F2-3 vs. L2-3	249742_at	AT5G24490	30S ribosomal protein;(source:Araport11)	115.47	218.62	0.53	9.11E-04	2.99E-03
2219	3	F2-3 vs. L2-3	262630_at	AT1G06520	sn-glycerol-3-phosphate 2-O-acyltransferase. Expressed in flower buds and siliques. Homozygous mutant plants are male sterile.	19.53	37.29	0.52	3.85E-04	1.55E-03
2220	3	F2-3 vs. L2-3	260782_at	AT1G14570	Encodes a nuclear UBX-containing protein that can bridge ubiquitin to AtCDC48A.	181.00	346.81	0.52	9.62E-04	3.12E-03
2221	3	F2-3 vs. L2-3	263160_at	AT1G54080	oligouridylate-binding protein 1A;(source:Araport11)	77.34	149.07	0.52	1.96E-05	1.89E-04
2222	3	F2-3 vs. L2-3	257547_at	AT3G13000	ubiquinone biosynthesis protein (Protein of unknown function, DUF547);(source:Araport11)	16.03	31.07	0.52	5.25E-04	1.95E-03
2223	3	F2-3 vs. L2-3	256889_at	AT3G24010	ING1 encodes a member of the Inhibitor of Growth family of nuclear-localized PhD domain containing homeodomain proteins. Binds to H3K4 di or trimethylated DNA.	93.07	180.46	0.52	6.16E-04	2.20E-03
2224	3	F2-3 vs. L2-3	245404_at	AT4G17610	tRNA/rRNA methyltransferase (SpoU) family protein;(source:Araport11)	79.34	152.41	0.52	1.20E-04	6.65E-04
2225	3	F2-3 vs. L2-3	253946_at	AT4G26790	GDSL-motif esterase/acyltransferase/lipase. Enzyme group with broad substrate specificity that may catalyze acyltransfer or hydrolase reactions with lipid and non-lipid substrates.	20.26	39.13	0.52	9.60E-05	5.69E-04
2226	3	F2-3 vs. L2-3	250150_at	AT5G14710	proteasome assembly chaperone-like protein;(source:Araport11)	69.99	133.50	0.52	7.64E-04	2.59E-03
2227	3	F2-3 vs. L2-3	249398_at	AT5G40250	RING/U-box superfamily protein;(source:Araport11)	67.13	129.69	0.52	8.30E-06	1.05E-04
2228	3	F2-3 vs. L2-3	248861_at	AT5G46700	Encodes a transmembrane protein of the tetraspanin (TET) family, one of 17 members found in Arabidopsis. Double mutant analysis showed that TRN1 and TRN2 act in the same pathway. Required for the maintenance of both the radial pattern of tissue differentiation in the root and for the subsequent circumferential pattern within the epidermis.	20.16	38.48	0.52	1.65E-04	8.35E-04
2229	3	F2-3 vs. L2-3	248684_at	AT5G48485	Encodes a putative apoplastic lipid transfer protein that is involved in systemic acquired resistance. Mutants in this gene exhibit wild-type local resistance to avirulent and virulent Pseudomonas syringae, but pathogenesis-related gene expression is abolished in uninoculated distant leaves and fail to develop SAR to virulent Pseudomonas or Peronospora parasitica. DIR1 protein is cell-to-cell mobile and is transported via phloem sap.	23.57	45.71	0.52	6.92E-05	4.53E-04
2230	3	F2-3 vs. L2-3	260949_at	AT1G06110	SKP1/ASK-interacting protein 16;(source:Araport11)	191.66	375.11	0.51	7.49E-04	2.55E-03
2231	3	F2-3 vs. L2-3	264674_at	AT1G09815	polymerase delta 4;(source:Araport11)	55.33	107.71	0.51	1.74E-05	1.76E-04
2232	3	F2-3 vs. L2-3	264441_at	AT1G27435	hypothetical protein;(source:Araport11)	232.42	452.20	0.51	1.64E-04	8.34E-04
2233	3	F2-3 vs. L2-3	262514_at	AT1G34190	Encodes a NAC domain transcription factor that regulates the mitochondrial retrograde response and coordinates organellar functions and stress responses.	150.40	297.77	0.51	1.40E-06	3.16E-05
2234	3	F2-3 vs. L2-3	264418_at	AT1G43190	polypyrimidine tract-binding protein 3;(source:Araport11)	223.50	439.37	0.51	8.78E-05	5.35E-04
2235	3	F2-3 vs. L2-3	261628_at	AT1G50000	methyltransferase;(source:Araport11)	34.28	66.62	0.51	2.71E-04	1.19E-03
2236	3	F2-3 vs. L2-3	263590_at	AT2G01820	Leucine-rich repeat protein kinase family protein;(source:Araport11)	370.32	729.78	0.51	1.67E-04	8.43E-04
2237	3	F2-3 vs. L2-3	264001_at	AT2G22420	Encodes a cell wall-localized class III peroxidase that is directly regulated by the MADS-box transcription factor AGL15 and is involved in lignified tissue formation.	39.51	77.15	0.51	4.33E-04	1.69E-03
2238	3	F2-3 vs. L2-3	259192_at	AT3G01740	Mitochondrial ribosomal protein L37;(source:Araport11)	42.64	83.06	0.51	8.00E-06	1.03E-04
2239	3	F2-3 vs. L2-3	258921_at	AT3G10500	Encodes a transcriptional activator that is associated with the plasma membrane in a dormant form and is proteolytically cleaved to create a form that can enter the nucleus. It is thought to promote ROS production by binding directly to the promoters of genes encoding ROS biosynthetic enzymes during drought-induced leaf senescence.The mRNA is cell-to-cell mobile.	144.60	282.29	0.51	1.35E-05	1.48E-04
2240	3	F2-3 vs. L2-3	256626_at	AT3G20015	Eukaryotic aspartyl protease family protein;(source:Araport11)	41.42	81.51	0.51	8.95E-04	2.95E-03
2241	3	F2-3 vs. L2-3	251425_at	AT3G60210	GroES-like family protein;(source:Araport11)	222.18	438.83	0.51	3.34E-05	2.69E-04
2242	3	F2-3 vs. L2-3	255807_at	AT4G10270	Member of the wound-induced polypeptide (WIP) family.	17.80	34.99	0.51	3.77E-04	1.52E-03

2243	3	F2-3 vs. L2-3	254991_at	AT4G10620	P-loop containing nucleoside triphosphate hydrolases superfamily protein;(source:Araport11)	93.61	182.16	0.51	1.81E-04	8.86E-04
2244	3	F2-3 vs. L2-3	254529_at	AT4G19540	Encodes a iron-suflur protein required for NADH dehydrogenase.	64.36	127.26	0.51	1.64E-04	8.35E-04
2245	3	F2-3 vs. L2-3	253324_at	AT4G33940	RING/U-box superfamily protein;(source:Araport11)	55.49	109.82	0.51	5.28E-05	3.78E-04
2246	3	F2-3 vs. L2-3	252968_at	AT4G38890	FMN-linked oxidoreductases superfamily protein;(source:Araport11)	206.61	407.02	0.51	7.01E-05	4.56E-04
2247	3	F2-3 vs. L2-3	245885_at	AT5G09440	EXORDIUM like 4;(source:Araport11)	471.46	922.32	0.51	4.20E-05	3.21E-04
2248	3	F2-3 vs. L2-3	245965_at	AT5G19730	Pectin lyase-like superfamily protein;(source:Araport11)	70.37	137.69	0.51	1.05E-04	6.09E-04
2249	3	F2-3 vs. L2-3	247606_at	AT5G61000	Replication factor-A protein 1-like protein;(source:Araport11)	84.54	166.02	0.51	3.38E-04	1.41E-03
2250	3	F2-3 vs. L2-3	262501_at	AT1G21690	ATPase family associated with various cellular activities (AAA);(source:Araport11)	85.67	169.65	0.50	1.61E-04	8.23E-04
2251	3	F2-3 vs. L2-3	257403_at	AT1G62520	sulfated surface-like glycoprotein;(source:Araport11)	14.32	28.40	0.50	9.90E-05	5.82E-04
2252	3	F2-3 vs. L2-3	262919_at	AT1G79380	Encodes a ubiquitin ligase that is an essential upstream modulator of JA signaling in response to various stimuli.	156.39	314.91	0.50	6.71E-04	2.35E-03
2253	3	F2-3 vs. L2-3	263439_at	AT2G28650	A member of EXO70 gene family, putative exocyst subunits, conserved in land plants. Arabidopsis thaliana contains 23 putative EXO70 genes, which can be classified into eight clusters on the phylogenetic tree.	44.98	90.87	0.50	6.71E-04	2.35E-03
2254	3	F2-3 vs. L2-3	265960_at	AT2G37470	Histone superfamily protein;(source:Araport11)	23.75	47.49	0.50	3.15E-05	2.59E-04
2255	3	F2-3 vs. L2-3	258846_at	AT3G03070	NADH-ubiquinone oxidoreductase-like protein;(source:Araport11)	11.67	23.11	0.50	3.69E-04	1.50E-03
2256	3	F2-3 vs. L2-3	259093_at	AT3G04860	hypothetical protein (DUF868);(source:Araport11)	49.67	98.95	0.50	6.28E-04	2.23E-03
2257	3	F2-3 vs. L2-3	259225_at	AT3G07590	SmD1a is one of two Yeast SmD1 orthologs, lower levels than SmD1b.It is localized to the nucleus and may play a minor role in RNA splicing and indirectly facilitating PTGS.	31.27	62.48	0.50	1.33E-04	7.21E-04
2258	3	F2-3 vs. L2-3	254791_at	AT4G12910	serine carboxypeptidase-like 20;(source:Araport11)	47.96	95.25	0.50	4.57E-04	1.75E-03
2259	3	F2-3 vs. L2-3	254425_at	AT4G21450	PapD-like superfamily protein;(source:Araport11)	264.78	534.24	0.50	7.11E-05	4.61E-04
2260	3	F2-3 vs. L2-3	253753_at	AT4G29030	Putative membrane lipoprotein;(source:Araport11)	157.00	313.41	0.50	5.61E-05	3.95E-04
2261	3	F2-3 vs. L2-3	253163_at	AT4G35750	SEC14 cytosolic factor family protein / phosphoglyceride transfer family protein;(source:Araport11)	40.98	81.44	0.50	1.44E-04	7.62E-04
2262	3	F2-3 vs. L2-3	251048_at	AT5G02410	Encodes ALG10, an ER-resident alpha1,2-glucosyltransferase that is required for lipid-linked oligosaccharide biosynthesis and subsequently for normal leaf development and abiotic stress response.	26.32	52.61	0.50	1.10E-04	6.29E-04
2263	3	F2-3 vs. L2-3	245183_at	AT5G12440	CCCH-type zinc fingerfamily protein with RNA-binding domain-containing protein;(source:Araport11)	92.72	185.36	0.50	4.07E-04	1.61E-03
2264	3	F2-3 vs. L2-3	246026_at	AT5G21070	Fe(3+) dicitrate transport system permease;(source:Araport11)	43.08	86.33	0.50	3.48E-05	2.77E-04
2265	3	F2-3 vs. L2-3	249434_at	AT5G39960	GTP-binding protein;(source:Araport11)	98.30	198.59	0.50	7.93E-05	4.98E-04
2266	3	F2-3 vs. L2-3	248103_at	AT5G55160	Encodes a small ubiquitin-like modifier (SUMO) polypeptide that becomes covalently attached to various intracellular protein targets, much like ubiquitination, leading to post-translational modification of those targets. SUMO2 can form SUMO chains through lysine residue 10 during in vitro assays.	366.31	738.66	0.50	2.34E-04	1.07E-03
2267	3	F2-3 vs. L2-3	247271_at	AT5G64270	splicing factor;(source:Araport11)	231.79	464.79	0.50	4.23E-04	1.66E-03
2268	3	F2-3 vs. L2-3	264252_at	AT1G09180	A member of ARF-like GTPase family. A thaliana has 21 members, in two subfamilies, ARF and ARF-like (ARL) GTPases.	112.67	230.81	0.49	2.77E-04	1.21E-03
2269	3	F2-3 vs. L2-3	255776_at	AT1G18540	Ribosomal protein L6 family protein;(source:Araport11)	149.19	302.65	0.49	3.59E-04	1.47E-03
2270	3	F2-3 vs. L2-3	259477_at	AT1G19120	Small nuclear ribonucleoprotein family protein;(source:Araport11)	509.65	1030.17	0.49	1.61E-04	8.24E-04
2271	3	F2-3 vs. L2-3	260028_at	AT1G29980	choice-of-anchor C domain protein, putative (Protein of unknown function, DUF642);(source:Araport11)	253.47	521.91	0.49	1.43E-04	7.55E-04
2272	3	F2-3 vs. L2-3	262558_at	AT1G31335	transmembrane protein;(source:Araport11)	48.40	99.35	0.49	7.72E-04	2.62E-03
2273	3	F2-3 vs. L2-3	265629_at	AT2G27230	Encodes a nuclear-localized transcriptional activator with weak sequence similarity to basic helix-loop-helix(bHLH)-domain proteins. It promotes the production of stele cells in root meristems and is required to establish and maintain the normal vascular cell number and pattern in primary and lateral roots.	113.21	230.39	0.49	4.47E-04	1.73E-03
2274	3	F2-3 vs. L2-3	263787_at	AT2G46420	helicase with zinc finger protein;(source:Araport11)	92.94	190.71	0.49	4.01E-05	3.10E-04
2275	3	F2-3 vs. L2-3	260530_at	AT2G47320	Cyclophilin-like peptidyl-prolyl cis-trans isomerase family protein;(source:Araport11)	191.55	388.81	0.49	8.50E-06	1.06E-04
2276	3	F2-3 vs. L2-3	257662_at	AT3G13320	low affinity calcium antiporter CAX2 The mRNA is cell-to-cell mobile.	60.02	122.38	0.49	9.05E-05	5.49E-04
2277	3	F2-3 vs. L2-3	257203_at	AT3G23730	xyloglucan endotransglucosylase/hydrolase 16;(source:Araport11)	470.02	954.48	0.49	9.55E-04	3.10E-03
2278	3	F2-3 vs. L2-3	252495_at	AT3G46770	AP2/B3-like transcriptional factor family protein;(source:Araport11)	35.47	73.00	0.49	4.64E-04	1.77E-03
2279	3	F2-3 vs. L2-3	252008_at	AT3G52610	GATA zinc finger protein;(source:Araport11)	53.41	108.10	0.49	8.80E-06	1.09E-04
2280	3	F2-3 vs. L2-3	251982_at	AT3G53190	Pectin lyase-like superfamily protein;(source:Araport11)	98.58	199.54	0.49	2.04E-05	1.92E-04
2281	3	F2-3 vs. L2-3	253424_at	AT4G32330	WDL5 is an target of EIN3 that co-localizes with cortical microtubles. It its thought to function to stabilize microtubles during ethylene induced hypocotyl elongation.	33.49	68.96	0.49	1.14E-04	6.46E-04

2282	3	F2-3 vs. L2-3	250221_at	AT5G14220	Encodes PPO2, a putative protoporphyrinogen oxidase based on sequence homology. Also known as MEE61 (maternal effect embryo arrest 61). mee61 mutant shows arrested endosperm development.	124.73	253.37	0.49	3.25E-05	2.64E-04
2283	3	F2-3 vs. L2-3	249456_at	AT5G39410	Saccharopine dehydrogenase;(source:Araport11)	71.88	146.08	0.49	9.30E-06	1.13E-04
2284	3	F2-3 vs. L2-3	248418_at	AT5G51660	cleavage and polyadenylation specificity factor 160;(source:Araport11)	155.24	319.08	0.49	2.41E-04	1.09E-03
2285	3	F2-3 vs. L2-3	246983_at	AT5G67200	Leucine-rich repeat protein kinase family protein;(source:Araport11)	27.39	55.76	0.49	5.83E-04	2.11E-03
2286	3	F2-3 vs. L2-3	264408_at	AT1G10240	FAR1-related sequence 11;(source:Araport11)	69.66	144.08	0.48	2.88E-05	2.43E-04
2287	3	F2-3 vs. L2-3	259767_s_a	AT1G29370	RNA polymerase II degradation factor-like protein (DUF1296);(source:Araport11)	125.28	263.10	0.48	2.81E-04	1.23E-03
2288	3	F2-3 vs. L2-3	257587_at	AT1G56310	DEDDy-type 3′ -> 5′ exoribonuclease involved in miRNA degradation.	172.53	357.10	0.48	4.48E-05	3.35E-04
2289	3	F2-3 vs. L2-3	262355_at	AT1G72820	Mitochondrial substrate carrier family protein;(source:Araport11)	140.85	292.48	0.48	7.78E-04	2.63E-03
2290	3	F2-3 vs. L2-3	261886_s_a	AT1G80700	stress response NST1-like protein;(source:Araport11)	62.51	130.65	0.48	5.39E-05	3.84E-04
2291	3	F2-3 vs. L2-3	258553_at	AT3G06960	TGD4 is an integral dimeric beta-barrel lipid transfer protein that binds PtdOH with its N-terminus and contains dimerization domains at its C-terminus. Mutations in this gene suppress the low temperature-induced phenotype of Arabidopsis tocopherol-deficient mutant vte2.	21.82	45.09	0.48	2.84E-04	1.24E-03
2292	3	F2-3 vs. L2-3	252561_at	AT3G45980	Encodes a histone 2B (H2B) protein. This protein can be ubiquitinated in planta, and this modification depends on the HUB1 and HUB2 E3 ubiquitin ligases as well as the UBC1 and UBC2 E2 ubiquitin conjugating enzymes. Lysine 146 appears to be the site of the ubiquitin addition.	28.95	60.24	0.48	8.60E-06	1.07E-04
2293	3	F2-3 vs. L2-3	255509_at	AT4G02210	Myb/SANT-like DNA-binding domain protein;(source:Araport11)	40.53	84.05	0.48	6.71E-05	4.44E-04
2294	3	F2-3 vs. L2-3	254682_at	AT4G13640	Homeodomain-like superfamily protein;(source:Araport11)	50.17	105.05	0.48	1.16E-04	6.55E-04
2295	3	F2-3 vs. L2-3	253688_at	AT4G29590	S-adenosyl-L-methionine-dependent methyltransferases superfamily protein;(source:Araport11)	36.26	76.25	0.48	1.12E-05	1.27E-04
2296	3	F2-3 vs. L2-3	266535_s_a	AT5G15220	Ribosomal protein L27 family protein;(source:Araport11)	661.72	1372.19	0.48	5.63E-05	3.95E-04
2297	3	F2-3 vs. L2-3	247725_at	AT5G59410	Rab5-interacting family protein;(source:Araport11)	85.85	178.78	0.48	4.18E-04	1.64E-03
2298	3	F2-3 vs. L2-3	244987_s_at	ATCG00830	encodes a chloroplast ribosomal protein L2, a constituent of the large subunit of the ribosomal complex	1969.34	4089.75	0.48	1.46E-04	7.65E-04
2299	3	F2-3 vs. L2-3	262594_at	AT1G15250	Zinc-binding ribosomal protein family protein;(source:Araport11)	44.45	95.50	0.47	2.23E-05	2.04E-04
2300	3	F2-3 vs. L2-3	255767_at	AT1G16740	Ribosomal protein L20;(source:Araport11)	277.31	589.91	0.47	9.80E-06	1.17E-04
2301	3	F2-3 vs. L2-3	261603_at	AT1G49600	RNA-binding protein 47A;(source:Araport11)	19.59	41.27	0.47	1.20E-05	1.34E-04
2302	3	F2-3 vs. L2-3	262941_at	AT1G79490	Pentatricopeptide repeat (PPR) superfamily protein;(source:Araport11)	48.33	102.49	0.47	4.63E-05	3.43E-04
2303	3	F2-3 vs. L2-3	263591_at	AT2G01910	Binds microtubules. Induces a crisscross mesh of microtubules, not bundles. Not involved in microtubule polymerization nor nucleation. Localizes to mitochondria. The mRNA is cell-to-cell mobile.	89.56	188.58	0.47	2.70E-04	1.19E-03
2304	3	F2-3 vs. L2-3	266984_at	AT2G39570	Encodes a ACT domain-containing protein. The ACT domain, named after bacterial aspartate kinase, chorismate mutase and TyrA (prephenate dehydrogenase), is a regulatory domain that serves as an amino acid-binding site in feedback-regulated amino acid metabolic enzymes.	50.77	107.90	0.47	1.35E-05	1.48E-04
2305	3	F2-3 vs. L2-3	256798_at	AT3G18630	Encodes a uracil-DNA glycosylase (UDG) involved in a base excision DNA repair pathway in mitochondria.	33.05	70.81	0.47	2.01E-04	9.61E-04
2306	3	F2-3 vs. L2-3	257615_at	AT3G26510	Octicosapeptide/Phox/Bem1p family protein;(source:Araport11)	89.40	189.11	0.47	2.21E-04	1.03E-03
2307	3	F2-3 vs. L2-3	255569_at	AT4G01320	CAAX protease with broad substrate specificity. Localized exclusively to the endoplasmic reticulum.	254.66	547.48	0.47	9.20E-05	5.56E-04
2308	3	F2-3 vs. L2-3	254957_at	AT4G10970	UAP56-interacting factor1, binds single stranded RNA and, along with UIEF2,,appears to play a role in nuclear export of RNA.	40.87	87.54	0.47	1.75E-05	1.76E-04
2309	3	F2-3 vs. L2-3	252789_s_a	AT1G21930	transmembrane protein;(source:Araport11)	153.86	332.95	0.46	2.81E-05	2.39E-04
2310	3	F2-3 vs. L2-3	261859_at	AT1G50490	Encodes one of two ubiquitin-conjugating enzymes belonging to the E2-C gene family (the other being UBC19). Transcript is always found in diving cells, but also in other non-dividing cells.	168.61	366.57	0.46	3.93E-04	1.57E-03
2311	3	F2-3 vs. L2-3	261549_at	AT1G63470	AT hook motif DNA-binding family protein;(source:Araport11)	156.39	337.66	0.46	1.38E-05	1.50E-04
2312	3	F2-3 vs. L2-3	264111_at	AT2G13690	PRLI-interacting factor;(source:Araport11)	333.06	716.72	0.46	1.83E-04	8.93E-04
2313	3	F2-3 vs. L2-3	266303_at	AT2G27060	Leucine-rich repeat protein kinase family protein;(source:Araport11)	39.06	84.39	0.46	5.52E-04	2.02E-03
2314	3	F2-3 vs. L2-3	267383_at	AT2G44360	ecotropic viral integration site protein;(source:Araport11)	107.83	232.68	0.46	8.57E-05	5.28E-04
2315	3	F2-3 vs. L2-3	267561_at	AT2G45590	Protein kinase superfamily protein;(source:Araport11)	174.62	378.35	0.46	1.33E-04	7.19E-04
2316	3	F2-3 vs. L2-3	258803_at	AT3G04670	member of WRKY Transcription Factor; Group II-d	83.53	180.27	0.46	5.39E-05	3.84E-04
2317	3	F2-3 vs. L2-3	256607_at	AT3G32930	Protein of unknown function. Locus is correlated with bacterial hypersensitive response, expression is reduced after injection with avrRpm1.	191.33	411.74	0.46	3.96E-05	3.07E-04

2318	3	F2-3 vs. L2-3	248726_at	AT5G47960	Encodes a small molecular weight g-protein.	14.76	32.06	0.46	7.17E-05	4.63E-04
2319	3	F2-3 vs. L2-3	247986_at	AT5G56880	hypothetical protein;(source:Araport11)	106.34	228.85	0.46	2.13E-04	1.00E-03
2320	3	F2-3 vs. L2-3	247525_at	AT5G61380	Pseudo response regulator involved in the generation of circadian rhythms. TOC1 appears to shorten the period of circumnutation speed. TOC1 contributes to the plant fitness (carbon fixation, biomass) by influencing the circadian clock period. PRR3 may increase the stability of TOC1 by preventing interactions between TOC1 and the F-box protein ZTL. Expression of TOC1 is correlated with rhythmic changes in chromatin organization. The mRNA is cell-to-cell mobile.	66.67	145.86	0.46	2.12E-04	9.99E-04
2321	3	F2-3 vs. L2-3	244981_at	ATCG00770	chloroplast 30S ribosomal protein S8	415.32	900.15	0.46	3.20E-05	2.61E-04
2322	3	F2-3 vs. L2-3	244982_at	ATCG00790	chloroplast gene encoding a ribosomal protein L16, which is a constituent of 50S large ribosomal subunit	472.33	1019.63	0.46	5.90E-06	8.35E-05
2323	3	F2-3 vs. L2-3	263116_s_at	AT1G03140	PRP18a is one of two paralogs (the other being PRP18b) which are highly similar to the step II splicing factors in yeast. Loss of function mutations show defects in alternative splicing, mostly intron retention events.	177.63	390.41	0.45	2.96E-04	1.28E-03
2324	3	F2-3 vs. L2-3	266110_at	AT2G02080	C2H2 BIRD transcription factor family.	36.94	82.93	0.45	6.08E-04	2.18E-03
2325	3	F2-3 vs. L2-3	266001_at	AT2G24150	heptahelical transmembrane protein HHP3	43.92	97.13	0.45	3.50E-05	2.78E-04
2326	3	F2-3 vs. L2-3	267586_at	AT2G41950	DNA-directed RNA polymerase subunit beta;(source:Araport11)	20.08	45.12	0.45	6.10E-06	8.52E-05
2327	3	F2-3 vs. L2-3	259235_at	AT3G11600	One of two plant specific paralogs of unknown function. Interacts with GL2. GIR1/GIR2 loss of function resembles gl2 lof mutations.	20.99	46.44	0.45	8.42E-04	2.81E-03
2328	3	F2-3 vs. L2-3	251995_at	AT3G52940	Encodes a sterol C-14 reductase required for cell division and expansion and is involved in proper organization of the embryo.	90.41	201.66	0.45	1.90E-06	3.89E-05
2329	3	F2-3 vs. L2-3	246317_at	AT3G56900	Encodes ALADIN, a component of the nuclear pore complex.	45.84	102.36	0.45	8.61E-05	5.29E-04
2330	3	F2-3 vs. L2-3	245367_at	AT4G16265	One of two highly similar, non-catalytic subunits common to nuclear DNA-directed RNA polymerases II, IV and V; homologous to budding yeast RPB9. Appears to be redundant with At3g16980	105.45	233.65	0.45	1.37E-05	1.50E-04
2331	3	F2-3 vs. L2-3	254041_at	AT4G25830	Uncharacterized protein family (UPF0497);(source:Araport11)	49.40	109.14	0.45	4.49E-04	1.73E-03
2332	3	F2-3 vs. L2-3	253981_at	AT4G26670	Mitochondrial import inner membrane translocase subunit Tim17/Tim22/Tim23 family protein;(source:Araport11)	45.62	101.25	0.45	9.00E-07	2.22E-05
2333	3	F2-3 vs. L2-3	253148_at	AT4G35620	Cyclin B2;(source:Araport11)	22.95	50.58	0.45	1.81E-04	8.86E-04
2334	3	F2-3 vs. L2-3	250814_at	AT5G05080	ubiquitin-conjugating enzyme 22;(source:Araport11)	248.17	552.91	0.45	2.02E-04	9.64E-04
2335	3	F2-3 vs. L2-3	249238_at	AT5G41970	Metal-dependent protein hydrolase;(source:Araport11)	150.85	337.20	0.45	2.00E-06	3.93E-05
2336	3	F2-3 vs. L2-3	247238_at	AT5G64610	Encodes an enzyme with histone acetyltransferase activity. HAM1 primarily acetylate histone H4, but also display some ability to acetylate H3. Prior acetylation of lysine 5 on histone H4 reduces radioactive acetylation by either HAM1. HAM1 acetylates histone H4 lysine 5.	81.04	178.57	0.45	2.12E-05	1.96E-04
2337	3	F2-3 vs. L2-3	259528_at	AT1G12330	cyclin-dependent kinase-like protein;(source:Araport11)	97.10	218.37	0.44	2.49E-04	1.12E-03
2338	3	F2-3 vs. L2-3	256112_at	AT1G16920	small GTP-binding protein (Rab11)similar to YPT3/RAB11 proteins in yeast and mammals, respectively. YPT3/RAB11 is involved in intracellular protein trafficking.	508.96	1157.70	0.44	2.80E-04	1.23E-03
2339	3	F2-3 vs. L2-3	245821_at	AT1G26270	Phosphatidylinositol 3- and 4-kinase family protein;(source:Araport11)	103.47	237.35	0.44	2.86E-05	2.42E-04
2340	3	F2-3 vs. L2-3	259942_at	AT1G71260	Encodes WHY2, a homolog of the potato p24 protein. It shares the conserved KGKAAL domain, a putative DNA-binding domain, with potato p24 and is localized to mitochondria and not the nucleus. WHY2 is a member of the Whirly family proteins present mainly in the plant kingdom performing various activities related to DNA metabolism. Crystal structure of Solanum tuberosum WHY2, a close homolog of Arabidopsis WHY2, reveal that Whirly proteins bind to single strand DNA to promote accurate repair of DNA double-strand breaks over an error-prone repair pathway.	28.65	64.41	0.44	2.63E-04	1.17E-03
2341	3	F2-3 vs. L2-3	256341_at	AT1G72040	Encodes a multisubstrate deoxyribonucleoside kinase that salvages DNA precursors.	41.12	93.55	0.44	1.97E-05	1.89E-04
2342	3	F2-3 vs. L2-3	259912_at	AT1G72670	Member of IQ67 (CaM binding) domain containing family.	42.88	97.45	0.44	1.34E-04	7.21E-04
2343	3	F2-3 vs. L2-3	259103_at	AT3G11690	hypothetical protein;(source:Araport11)	68.98	158.10	0.44	1.59E-04	8.15E-04
2344	3	F2-3 vs. L2-3	256320_at	AT3G12170	Chaperone DnaJ-domain superfamily protein;(source:Araport11)	46.73	105.47	0.44	1.82E-05	1.81E-04
2345	3	F2-3 vs. L2-3	256595_x_a	AT3G28530	UDP-glucose 4-epimerase;(source:Araport11)	237.21	536.54	0.44	4.67E-05	3.45E-04
2346	3	F2-3 vs. L2-3	252508_at	AT3G46210	Ribosomal protein S5 domain 2-like superfamily protein;(source:Araport11)	41.29	94.61	0.44	2.00E-06	3.93E-05
2347	3	F2-3 vs. L2-3	255021_at	AT4G10330	glycine-rich protein;(source:Araport11)	74.91	170.95	0.44	1.23E-04	6.80E-04

2348	3	F2-3 vs. L2-3	252830_at	AT4G39850	Encodes a peroxisomal protein of the ATP binding cassette (ABC) transporter class (PMP subfamily) with significant identity to the human X-linked adrenoleukodystrophy protein (ALDP). The gene product promotes germination and represses embryo dormancy. ABI3, ABA1, FUS3 and LEC1 are epistatic to this gene. Mutants accumulate fatty acyl CoA suggesting a defect in uptake of fatty acyl CoA into the peroxisome.	147.95	338.12	0.44	2.30E-04	1.06E-03
2349	3	F2-3 vs. L2-3	252824_at	AT4G40030	Histone superfamily protein;(source:Araport11)	1050.94	2401.53	0.44	3.40E-06	5.58E-05
2350	3	F2-3 vs. L2-3	248403_at	AT5G51410	LUC7 N terminus domain-containing protein;(source:Araport11)	73.67	166.70	0.44	3.13E-04	1.33E-03
2351	3	F2-3 vs. L2-3	248385_at	AT5G51910	TCP family transcription factor;(source:Araport11)	26.19	59.49	0.44	5.98E-04	2.15E-03
2352	3	F2-3 vs. L2-3	247284_at	AT5G64410	oligopeptide transporter	39.56	89.70	0.44	9.28E-04	3.03E-03
2353	3	F2-3 vs. L2-3	244966_at	ATCG00590	electron carrier;(source:Araport11)	22.21	50.80	0.44	8.52E-05	5.26E-04
2354	3	F2-3 vs. L2-3	260369_at	AT1G69620	putative 60S ribosomal protein L34 The mRNA is cell-to-cell mobile.	441.98	1033.10	0.43	2.35E-05	2.11E-04
2355	3	F2-3 vs. L2-3	263064_at	AT2G18160	Encodes a b-ZIP transcription factor.	51.09	118.29	0.43	9.39E-05	5.62E-04
2356	3	F2-3 vs. L2-3	252184_at	AT3G50660	Encodes a 22α hydroxylase whose reaction is a rate-limiting step in brassinosteroid biosynthetic pathway. The protein is a member of CYP90B gene family. CLM is an epi-allele with small, compressed rosette, reduced internode length, and reduced fertility, appears in selfed ddm mutant plants possibly due to loss of cytosine methylation. Transcripts accumulate in actively growing tissues, and GUS expression is negatively regulated by brassinosteroids. Localized in the endoplasmic reticulum. The in vitro expressed protein can perform the C-22 hydroxylation of a variety of C27-, C28- and C29-sterols. Cholesterol was the best substrate, followed by campesterol. Sitosterol was a poor substrate.	239.54	560.60	0.43	3.50E-06	5.69E-05
2357	3	F2-3 vs. L2-3	252128_at	AT3G50870	Encodes a GATA transcriptional regulator required to position the proembryo boundary in the early embryo. Regulates shoot apical meristem and flower development.	48.24	111.75	0.43	1.81E-04	8.86E-04
2358	3	F2-3 vs. L2-3	254784_at	AT4G12720	Encodes a protein with ADP-ribose hydrolase activity. Negatively regulates EDS1-conditioned plant defense and programmed cell death.	204.75	472.25	0.43	9.60E-06	1.15E-04
2359	3	F2-3 vs. L2-3	254391_at	AT4G21590	Encodes a putative endonuclease but no demonstrable endonuclease activity, either towards single stranded DNA or mismatches, has been seen in vitro. Activated by AGAMOUS in a cal-1, ap1-1 background. Expressed in the floral meristem and during stamen development.	195.59	457.42	0.43	2.30E-05	2.08E-04
2360	3	F2-3 vs. L2-3	248438_at	AT5G51230	Polycomb group protein with zinc finger domain involved in negative regulation of reproductive development. Forms a complex with FIE, CLF, and MSI1. This complex modulates the expression of target genes including AG, PI and AP3.	214.53	499.25	0.43	3.47E-04	1.43E-03
2361	3	F2-3 vs. L2-3	247702_at	AT5G59500	protein C-terminal S-isoprenylcysteine carboxyl O-methyltransferase;(source:Araport11)	222.02	512.58	0.43	4.56E-05	3.40E-04
2362	3	F2-3 vs. L2-3	247112_at	AT5G65950	TRAPPIII complex protein which regulates TGN integrity, by altered TGN/EE association of several residents, including SYNTAXIN OF PLANTS 61 (SYP61), and altered vesicle morphology. Involved in regulation of endosomal function and salt stress response.	212.60	493.63	0.43	7.17E-05	4.63E-04
2363	3	F2-3 vs. L2-3	244991_s_a	ATCG01250	NADH dehydrogenase ND2	20.54	47.51	0.43	4.56E-04	1.75E-03
2364	3	F2-3 vs. L2-3	255889_at	AT1G17840	Encodes a plasma membrane-localized ATP-binding cassette transporter, that is required for cutin transport to the extracellular matrix. The mRNA is cell-to-cell mobile.	436.29	1035.50	0.42	8.06E-05	5.04E-04
2365	3	F2-3 vs. L2-3	262400_at	AT1G49480	Encodes a nuclear-localized DNA-binding protein that interacts with ITN1 at the PM and nuclei in vivo and may regulate ITN Primes subcellular localization.	171.61	409.94	0.42	6.45E-04	2.28E-03
2366	3	F2-3 vs. L2-3	262203_at	AT2G01060	myb-like HTH transcriptional regulator family protein;(source:Araport11)	38.42	92.35	0.42	1.84E-04	8.94E-04
2367	3	F2-3 vs. L2-3	257985_at	AT3G20810	JMJD5 encodes a protein which contains a jumonji-C (jmjC) domain. jmjd5 mutant plants have a short-period circadian phenotype. JMJD5 has histone demethylase activity and interacts with EFM to repress FT.	27.42	64.62	0.42	4.69E-04	1.78E-03
2368	3	F2-3 vs. L2-3	256761_at	AT3G25670	Leucine-rich repeat (LRR) family protein;(source:Araport11)	9.59	22.62	0.42	3.76E-04	1.52E-03
2369	3	F2-3 vs. L2-3	251319_at	AT3G61610	Galactose mutarotase-like superfamily protein;(source:Araport11)	49.39	116.57	0.42	3.70E-06	5.84E-05
2370	3	F2-3 vs. L2-3	254986_at	AT4G10640	Overexpression of IQD16 in transgenic Arabidopsis Pro35:IQD16 lines alters microtubule organization, cell shape, and plant growth. Phenotypes are reminiscent of lng1-1D plants, which overexpress LNG1/TRM2. IQD16 induces elongation of aerial tissues.	24.95	59.14	0.42	3.90E-06	6.01E-05

2371	3	F2-3 vs. L2-3	250269_at	AT5G12970	Calcium-dependent lipid-binding (CaLB domain) plant phosphoribosyltransferase family protein;(source:Araport11)	45.50	107.84	0.42	4.48E-05	3.35E-04
2372	3	F2-3 vs. L2-3	249922_at	AT5G19140	aluminum induced protein with YGL and LRDR motifs;(source:Araport11)	1815.65	4363.45	0.42	2.42E-05	2.16E-04
2373	3	F2-3 vs. L2-3	263727_at	AT2G13540	Encodes a nuclear cap-binding protein that forms a heterodimeric complex with CBP20 and is involved in ABA signaling and flowering. Mutants are early flowering and exhibit hypersensitive response to ABA in germination inhibition.Loss of ABH1 function results in abnormal processing of mRNAs for several important floral regulators (FLC, CO, FLM). Analysis of loss of function mutations suggests a role in pri-miRNA processing and mRNA splicing. Note that two different mutant alleles were given the same name abh1-7 (Kuhn et al 2007; Kim et al 2008). To avoid confusion, abh1-7 described in Kim et al (2008) has been renamed abh1-107 (other names: ensalada-1, ens-1).	82.44	201.84	0.41	1.58E-05	1.64E-04
2374	3	F2-3 vs. L2-3	266758_at	AT2G46920	Pol mutations are recessive, partial suppressors of meristem defects in strong clv1 and clv3 mutants, and nearly complete suppressors of weak clv1 mutants. Single mutants appear normal. Acts downstream of the CLV signaling pathway in meristem development and is required together with PLL1 for stem-cell maintenance through the regulation of WUS.	86.16	209.64	0.41	4.74E-04	1.80E-03
2375	3	F2-3 vs. L2-3	259154_at	AT3G10260	Reticulon family protein;(source:Araport11)	223.24	549.06	0.41	1.27E-04	6.98E-04
2376	3	F2-3 vs. L2-3	252267_at	AT3G49640	Aldolase-type TIM barrel family protein;(source:Araport11)	70.17	170.85	0.41	2.14E-04	1.00E-03
2377	3	F2-3 vs. L2-3	254990_at	AT4G10610	RNA-binding protein, putative. Member of a family of proteins having an PABC binding domain (PAM motif).	167.71	408.06	0.41	5.78E-05	4.02E-04
2378	3	F2-3 vs. L2-3	253871_at	AT4G27440	light-dependent NADPH:protochlorophyllide oxidoreductase B The mRNA is cell-to-cell mobile.	811.40	1968.09	0.41	8.11E-04	2.73E-03
2379	3	F2-3 vs. L2-3	253403_at	AT4G32830	Encodes a member of a family of Ser/Thr kinases whose activities peak during cell division. Transcripts are abundant in tissues rich in dividing cells like roots and flowers but are low or absent in fully expanded leaves and stems. In interphase cells, the protein is predominantly nuclear. During mitosis, the protein associates with plant-specific cytoskeletal structures (preprophase band, phragmoplast, nascent cell plate) that are necessary for cytokinesis as well as with the microtubule spindle. It specifically phosphorylates Ser10 of histone H3 and colocalizes with phosphorylated histone H3 during mitosis.	183.89	453.01	0.41	3.91E-05	3.04E-04
2380	3	F2-3 vs. L2-3	250141_at	AT5G14640	shaggy-like kinase 13;(source:Araport11)	278.79	686.11	0.41	3.90E-06	6.01E-05
2381	3	F2-3 vs. L2-3	248277_at	AT5G52860	ABC-2 type transporter family protein;(source:Araport11)	40.45	99.13	0.41	5.74E-04	2.08E-03
2382	3	F2-3 vs. L2-3	264582_at	AT1G05230	Encodes a homeobox-leucine zipper family protein belonging to the HD-ZIP IV family. Mutants have trichomes that appear glass-like under a dissecting microscope as compared to the wild-type trichomes. The mutations do not affect trichome growth or branch number.	80.68	201.42	0.40	6.53E-05	4.35E-04
2383	3	F2-3 vs. L2-3	261486_at	AT1G14510	Encodes a member of the Alfin1-like family of nuclear-localized PHD (plant homeodomain) domain containing proteins. All AL proteins except AL3 bind to di- or trimethylated histone H3 (H3K4me3/2). Members of this family include: AT5G05610 (AL1), AT3G11200 (AL2), AT3G42790 (AL3), AT5G26210 (AL4), AT5G20510 (AL5), AT2G02470 (AL6), AT1G14510 (AL7).	195.43	485.75	0.40	2.00E-06	3.93E-05
2384	3	F2-3 vs. L2-3	263924_at	AT2G36530	Involved in light-dependent cold tolerance and encodes an enolase. Protein is tyrosine-phosphorylated and its phosphorylation state is modulated in response to ABA in Arabidopsis thaliana seeds.	327.20	814.48	0.40	3.37E-04	1.41E-03
2385	3	F2-3 vs. L2-3	257858_at	AT3G12920	Encodes one of the BRGs (BOI-related gene) involved in resistance to Botrytis cinerea.	177.03	440.44	0.40	2.60E-04	1.16E-03
2386	3	F2-3 vs. L2-3	254596_at	AT4G18975	Pentatricopeptide repeat (PPR) superfamily protein;(source:Araport11)	25.57	64.45	0.40	2.45E-04	1.11E-03
2387	3	F2-3 vs. L2-3	253811_at	AT4G28190	Encodes a novel Cys-rich protein with a B-box like domain that acts as a negative regulator of meristem cell accumulation in inflorescence and floral meristems as loss-of-function ult1 mutations cause inflorescence meristem enlargement, the production of extra flowers and floral organs, and a decrease in floral meristem determinacy. Acts opposite to CLF which represses AG, but preventing deposition of CLF repressive methylation marks.ULT1 acts as an anti-repressor that counteracts EMF1 action through modulation of histone marks on target genes. Regulates developmental as well as biotic and abiotic stress response genes.	29.54	74.35	0.40	4.57E-05	3.40E-04
2388	3	F2-3 vs. L2-3	250768_at	AT5G05670	signal recognition particle binding protein;(source:Araport11)	145.78	368.98	0.40	3.86E-04	1.55E-03

2389	3	F2-3 vs. L2-3	250433_at	AT5G10400	Histone superfamily protein;(source:Araport11)	93.40	231.58	0.40	2.92E-05	2.45E-04
2390	3	F2-3 vs. L2-3	246489_at	AT5G16050	Encodes GF14 upsilon chain, a 14-3-3 gene family member.	301.27	761.10	0.40	1.05E-04	6.09E-04
2391	3	F2-3 vs. L2-3	249484_at	AT5G38970	Encodes a polypeptide involved in the C-6 oxidation of brassinosteroids. Heterologous expression of the protein in yeast conferred the ability to catalyze multiple reactions in which the C-6 position of 6-deoxocastasterone, 6-deoxotyphasterol, 3-dehydro-6-deoxoteasterone and 6-deoxoteasterone are oxidized.	11.80	29.48	0.40	4.28E-04	1.67E-03
2392	3	F2-3 vs. L2-3	247692_s_a	AT5G59970	Histone superfamily protein;(source:Araport11)	429.58	1079.90	0.40	1.92E-04	9.24E-04
2393	3	F2-3 vs. L2-3	263258_at	AT1G10540	nucleobase-ascorbate transporter 8;(source:Araport11)	56.72	145.42	0.39	3.67E-05	2.88E-04
2394	3	F2-3 vs. L2-3	261207_at	AT1G12830	nucleolin;(source:Araport11)	44.17	114.61	0.39	1.20E-04	6.65E-04
2395	3	F2-3 vs. L2-3	262417_at	AT1G50170	encodes sirohydrochlorin ferrochelatase catalyzing the last step of the siroheme biosynthesis	49.43	125.99	0.39	6.40E-06	8.73E-05
2396	3	F2-3 vs. L2-3	263156_at	AT1G54030	Encodes a vacuolar protein. Mutation causes organizational defects in the endoplasmic reticulum and aberrant protein trafficking in the plant secretory pathway.The mRNA is cell-to-cell mobile.	141.21	361.63	0.39	1.27E-04	6.99E-04
2397	3	F2-3 vs. L2-3	260401_at	AT1G69840	SPFH/Band 7/PHB domain-containing membrane-associated protein family;(source:Araport11)	115.04	294.15	0.39	1.38E-04	7.38E-04
2398	3	F2-3 vs. L2-3	259978_at	AT1G76540	Encodes a cyclin-dependent protein kinase involved in regulation of the G2/M transition of the mitotic cell cycle. Specifically binds to the cyclin CYCD4;1, expressed in shoot meristem, young leaves and vascular tissue during the G2/M phase. Required for proper organization of the shoot apical meristem and for hormone signaling.	61.37	159.14	0.39	2.14E-04	1.00E-03
2399	3	F2-3 vs. L2-3	264026_at	AT2G21060	Glycine-rich protein (AtGRP2b). Also named as CSP4 (cold shock domain protein 4) containing a well conserved cold shock domain (CSD) and glycine-rich motifs interspersed by two retroviral-like CCHC zinc fingers. AtCSP4 is expressed in all tissues but accumulates in reproductive tissues and those undergoing cell divisions. Overexpression of AtCSP4 reduces silique length and induces embryo lethality.	22.15	57.49	0.39	1.76E-05	1.77E-04
2400	3	F2-3 vs. L2-3	266358_at	AT2G32280	Encodes a member of a plant-specific gene family that is required for embryo provasculature development. The gene product regulates vascular network complexity and connectivity in cotyledons.	57.21	146.13	0.39	1.54E-05	1.61E-04
2401	3	F2-3 vs. L2-3	266899_at	AT2G34620	Mitochondrial transcription termination factor family member.	73.85	191.31	0.39	2.85E-04	1.24E-03
2402	3	F2-3 vs. L2-3	256664_at	AT3G12040	Encodes a 3-methyladenine-DNA glycosylase. Arabdiopsis cDNA complements the methyl methanesulfonate-sensitive phenotype of an Escherichia coli double mutant deficient in 3-methyladenine glycosylases (DNA-3-methyladenine glycosidases I and II, EC 3.2.2.20 and 3.2.2.21, respectively, encoded by tag and alkA).	22.99	58.33	0.39	3.86E-04	1.55E-03
2403	3	F2-3 vs. L2-3	258324_at	AT3G22780	CHC protein involved in cell cycle progression (positive regulator).	78.53	203.90	0.39	1.04E-04	6.05E-04
2404	3	F2-3 vs. L2-3	254119_at	AT4G24780	Encodes a pectate lyase involved in response to nematodes.	51.75	133.43	0.39	7.59E-04	2.58E-03
2405	3	F2-3 vs. L2-3	246508_at	AT5G16150	Encodes a putative plastidic glucose transporter.	210.32	545.06	0.39	1.15E-05	1.30E-04
2406	3	F2-3 vs. L2-3	250015_at	AT5G18070	encodes a novel protein involved in DNA repair from UV damage. Isolated by functional complementation of E. coli UV-sensitive mutants (UVR genes).	40.69	103.28	0.39	2.14E-05	1.97E-04
2407	3	F2-3 vs. L2-3	247892_at	AT5G57970	DNA glycosylase superfamily protein;(source:Araport11)	85.87	219.41	0.39	5.62E-04	2.05E-03
2408	3	F2-3 vs. L2-3	247325_at	AT5G64200	encodes an SC35-like splicing factor of 35 kD localized to the nuclear specks.	122.19	313.72	0.39	5.00E-07	1.44E-05
2409	3	F2-3 vs. L2-3	262109_at	AT1G02730	Encodes a gene similar to cellulose synthase. Knock-out mutant has reduced growth, reduced xylan level and reduced xylan synthase activity in stems.It's expression is cell cycle dependent and it appears to function in cell plate formation.	97.34	257.45	0.38	1.54E-04	7.97E-04
2410	3	F2-3 vs. L2-3	266072_at	AT2G18700	Encodes an enzyme putatively involved in trehalose biosynthesis. The protein has a trehalose synthase (TPS)-like domain that may or may not be active as well as a trehalose phosphatase (TPP)-like domain.	336.90	885.74	0.38	8.17E-04	2.74E-03
2411	3	F2-3 vs. L2-3	256970_at	AT3G21090	ABC-2 type transporter family protein;(source:Araport11)	47.41	123.49	0.38	5.89E-04	2.12E-03
2412	3	F2-3 vs. L2-3	252230_at	AT3G49810	Encodes a protein with E3 ubiquitin ligase activity that is involved in negative regulation of salt stress tolerance during germination.	34.10	89.10	0.38	1.90E-06	3.89E-05
2413	3	F2-3 vs. L2-3	254628_at	AT4G18593	dual specificity protein phosphatase-like protein;(source:Araport11)	34.55	90.49	0.38	8.61E-04	2.86E-03
2414	3	F2-3 vs. L2-3	253388_at	AT4G32910	SBB1 is a putative nucleoporin that is localized to the nuclear envelope. SBB1 was identified in a screen for supressors of BAK1, BKK1 mediated cell death.	96.65	253.97	0.38	1.78E-04	8.82E-04
2415	3	F2-3 vs. L2-3	248914_at	AT5G45750	RAB GTPase homolog A1C;(source:Araport11)	101.78	269.70	0.38	3.40E-05	2.73E-04

2416	3	F2-3 vs. L2-3	247506_at	AT5G61960	A member of mei2-like gene family, predominantly plant-based family of genes encoding RNA binding proteins with characteristic presence of a highly conserved RNA binding motif first described in the mei2 gene of the fission yeast S. pombe. In silico analyses reveal nine mei2 -like genes in A. thaliana. They were grouped into four distinct clades, based on overall sequence similarity and subfamily-specific sequence elements. AML1 is a member of two sister clades of mei2-like gene family, AML1 through AML5 and belongs to the clade named ALM14. AML1 is expressed during early embryo development, particularly along embryonic axis at torpedo stage, in shoot apex (weaker expression) and in the organogenic regions of floral apices.	68.00	180.74	0.38	3.00E-07	1.05E-05
2417	3	F2-3 vs. L2-3	244974_at	ATCG00700	PSII low MW protein	49.80	130.99	0.38	4.85E-04	1.83E-03
2418	3	F2-3 vs. L2-3	262504_at	AT1G21750	Encodes a protein disulfide isomerase-like (PDIL) protein, a member of a multigene family within the thioredoxin (TRX) superfamily; isoform contains non-consensus GA donor splice site at intron 9. Transcript levels for this gene are up-regulated in response to three different chemical inducers of ER stress (dithiothreitol, beta-mercaptoethanol, and tunicamycin). Neither AtIRE1-2 nor AtbZIP60 appear to be required for this response. The mRNA is cell-to-cell mobile.	47.03	126.01	0.37	3.43E-05	2.74E-04
2419	3	F2-3 vs. L2-3	255499_at	AT4G02730	Transducin/WD40 repeat-like superfamily protein;(source:Araport11)	93.50	249.85	0.37	6.43E-05	4.32E-04
2420	3	F2-3 vs. L2-3	254831_at	AT4G12600	Ribosomal protein L7Ae/L30e/S12e/Gadd45 family protein;(source:Araport11)	52.97	142.73	0.37	3.08E-04	1.32E-03
2421	3	F2-3 vs. L2-3	259273_s_a	AT5G15080	Protein kinase superfamily protein;(source:Araport11)	38.66	104.36	0.37	9.78E-04	3.16E-03
2422	3	F2-3 vs. L2-3	257333_at	ATMG01360	cytochrome c oxidase subunit 1	367.64	992.87	0.37	3.26E-04	1.37E-03
2423	3	F2-3 vs. L2-3	262924_s_a t	AT1G75630	vacuolar H+-pumping ATPase 16 kD proteolipid (ava-p) mRNA, The mRNA is cell-to-cell mobile.	626.36	1742.68	0.36	1.25E-05	1.39E-04
2424	3	F2-3 vs. L2-3	263631_at	AT2G04900	hypothetical protein;(source:Araport11)	47.82	132.82	0.36	4.47E-05	3.35E-04
2425	3	F2-3 vs. L2-3	266294_at	AT2G29500	HSP20-like chaperones superfamily protein;(source:Araport11)	15.84	43.50	0.36	2.66E-05	2.30E-04
2426	3	F2-3 vs. L2-3	263286_at	AT2G36160	Ribosomal protein S11 family protein;(source:Araport11)	75.34	209.30	0.36	1.01E-04	5.93E-04
2427	3	F2-3 vs. L2-3	251778_at	AT3G55660	Encodes a member of KPP-like gene family, homolog of KPP (kinase partner protein) gene in tomato. Also a member of the RopGEF (guanine nucleotide exchange factor) family, containing the novel PRONE domain (plant-specific Rop nucleotide exchanger), which is exclusively active towards members of the Rop subfamily.	70.38	197.88	0.36	5.30E-06	7.62E-05
2428	3	F2-3 vs. L2-3	249060_at	AT5G44560	SNF7 family protein;(source:Araport11)	77.67	217.91	0.36	2.41E-05	2.15E-04
2429	3	F2-3 vs. L2-3	263373_at	AT2G20515	pollen Ole e I family allergen protein;(source:Araport11)	21.59	61.00	0.35	4.33E-04	1.69E-03
2430	3	F2-3 vs. L2-3	257967_at	AT3G19910	RING/U-box superfamily protein;(source:Araport11)	351.36	1013.35	0.35	1.80E-06	3.80E-05
2431	3	F2-3 vs. L2-3	248462_at	AT5G50960	Highly similar to Saccharomyces cerevisiae NBP35, locus YGL091C. Cytosolic protein that homodimerizes and can assemble both 4Fe-4S - type and 2Fe-2S - type clusters on its amino terminal and carboxy therminal respectively. Null mutants are embryo lethal.	90.33	260.94	0.35	1.94E-05	1.88E-04
2432	3	F2-3 vs. L2-3	257474_at	AT1G80850	DNA glycosylase superfamily protein;(source:Araport11)	71.82	212.17	0.34	6.04E-05	4.15E-04
2433	3	F2-3 vs. L2-3	259349_at	AT3G03860	Encodes a protein disulfide isomerase-like (PDIL) protein, a member of a multigene family within the thioredoxin (TRX) superfamily. This protein also belongs to the adenosine 5'-phosphosulfate reductase-like (APRL) group. The mRNA is cell-to-cell mobile.	30.96	90.85	0.34	2.84E-05	2.41E-04
2434	3	F2-3 vs. L2-3	257167_at	AT3G24340	Involved in gene silencing. Locus-specific regulator of 24nt-siRNA expression, works together with CLSY1-3 as the master regulators of essentially all Pol-IV-dependent 24nt-siRNAs.	25.01	73.94	0.34	6.57E-04	2.31E-03
2435	3	F2-3 vs. L2-3	252127_at	AT3G50960	Encodes a protein that functions in microtubule assembly. PLP3a can bind to several different tubulin family members in Y2H assays. Plants with reduced levels of both PLP3a and PLP3b (At5g66410) show defects in cytokinesis, cortical microtubule array formation, oriented cell growth, and maintenance of proper ploidy.	35.00	103.22	0.34	1.00E-07	4.57E-06
2436	3	F2-3 vs. L2-3	246205_at	AT4G36970	Remorin family protein;(source:Araport11)	126.05	367.33	0.34	2.30E-06	4.39E-05
2437	3	F2-3 vs. L2-3	247963_at	AT5G56590	O-Glycosyl hydrolases family 17 protein;(source:Araport11)	164.63	479.73	0.34	5.00E-07	1.44E-05
2438	3	F2-3 vs. L2-3	262609_at	AT1G13930	Involved in response to salt stress. Knockout mutants are hypersensitive to salt stress. The mRNA is cell-to-cell mobile.	67.87	203.41	0.33	6.39E-05	4.30E-04
2439	3	F2-3 vs. L2-3	256426_at	AT1G33420	RING/FYVE/PHD zinc finger superfamily protein;(source:Araport11)	90.62	276.69	0.33	3.51E-04	1.45E-03
2440	3	F2-3 vs. L2-3	265387_at	AT2G20670	sugar phosphate exchanger, putative (DUF506);(source:Araport11)	39.49	120.55	0.33	6.20E-06	8.57E-05
2441	3	F2-3 vs. L2-3	252995_at	AT4G38370	Phosphoglycerate mutase family protein;(source:Araport11)	73.30	220.70	0.33	7.23E-04	2.48E-03

2442	3	F2-3 vs. L2-3	246856_at	AT5G26210	Encodes a member of the Alfin1-like family of nuclear-localized PHD (plant homeodomain) domain containing proteins. All AL proteins except AL3 bind to di- or trimethylated histone H3 (H3K4me3/2). Members of this family include: AT5G05610 (AL1), AT3G11200 (AL2), AT3G42790 (AL3), AT5G26210 (AL4), AT5G20510 (AL5), AT2G02470 (AL6), AT1G14510 (AL7).	128.80	394.58	0.33	1.98E-05	1.89E-04
2443	3	F2-3 vs. L2-3	255782_at	AT1G19850	Encodes a transcription factor (IAA24) mediating embryo axis formation and vascular development. Similar to AUXIN RESPONSIVE FACTOR 1 (ARF1) shown to bind to auxin responsive elements (AREs), and to the maize transcriptional activator VIVIPAROUS 1(VP1). In situ hybridization shows expression in provascular tissue of embryos, the emerging shoot primordia, then is restricted to provascular tissue, and in the root central vascular cylinder.	65.28	203.31	0.32	6.06E-04	2.18E-03
2444	3	F2-3 vs. L2-3	259541_at	AT1G20650	Protein kinase superfamily protein;(source:Araport11)	20.41	63.81	0.32	7.65E-04	2.59E-03
2445	3	F2-3 vs. L2-3	261729_s_a	AT1G47845	hexokinase;(source:Araport11)	19.80	61.25	0.32	2.52E-04	1.13E-03
2446	3	F2-3 vs. L2-3	266106_at	AT2G45170	Involved in autophagy. Under nutrient starvation the protein localizes to autophagosomes.	14.27	44.33	0.32	4.64E-05	3.43E-04
2447	3	F2-3 vs. L2-3	257879_at	AT3G17160	hypothetical protein;(source:Araport11)	144.72	453.33	0.32	6.00E-07	1.65E-05
2448	3	F2-3 vs. L2-3	258041_at	AT3G21175	member of a novel family of plant-specific GATA-type transcription factors.	32.38	99.97	0.32	4.60E-06	6.78E-05
2449	3	F2-3 vs. L2-3	253597_at	AT4G30690	SVR9-LIKE1 (SVR9L1)	48.91	152.91	0.32	1.00E-07	4.57E-06
2450	3	F2-3 vs. L2-3	261019_at	AT1G26470	chromatin modification-like protein;(source:Araport11)	28.14	90.68	0.31	1.90E-06	3.89E-05
2451	3	F2-3 vs. L2-3	245814_at	AT1G49910	Encodes a homolog of the yeast and human BUB3 (BUDDING UNINHIBITED BY BENZYMIDAZOL 3) protein. Yeast and human BUB3s function in spindle assembly checkpoint control.	17.92	58.27	0.31	3.35E-04	1.40E-03
2452	3	F2-3 vs. L2-3	267472_at	AT2G02850	Encodes plantacyanin one of blue copper proteins. Involved in anther development and pollination. Expressed in the transmitting tract of the pistil.	256.96	830.09	0.31	3.00E-07	1.05E-05
2453	3	F2-3 vs. L2-3	255257_at	AT4G05050	polyubiquitin gene, belongs to a subtype group with UBQ10 and UBQ14. Various ecotypes of Arabidopsis have different numbers of ubiquitin repeats within this gene.	82.71	265.79	0.31	1.31E-04	7.13E-04
2454	3	F2-3 vs. L2-3	254853_at	AT4G12080	AT-hook motif nuclear-localized protein 1;(source:Araport11)	41.02	132.56	0.31	2.30E-05	2.08E-04
2455	3	F2-3 vs. L2-3	246090_at	AT5G20520	Encodes a Bem46-like protein. WAV2 negatively regulates root bending when roots alter their growth direction. It's not involved in sensing environmental stimuli (e.g. gravity, light, water, touch).	83.16	267.73	0.31	9.00E-07	2.22E-05
2456	3	F2-3 vs. L2-3	248842_at	AT5G46850	phosphatidylinositol-glycan biosynthesis class X-like protein;(source:Araport11)	96.10	305.56	0.31	4.00E-07	1.25E-05
2457	3	F2-3 vs. L2-3	248427_at	AT5G51750	subtilase 1.3;(source:Araport11)	498.84	1592.99	0.31	7.37E-05	4.71E-04
2458	3	F2-3 vs. L2-3	247192_at	AT5G65360	Histone superfamily protein;(source:Araport11)	30.17	96.58	0.31	3.41E-04	1.42E-03
2459	3	F2-3 vs. L2-3	262785_at	AT1G10750	carboxyl-terminal peptidase, putative (DUF239);(source:Araport11)	44.59	149.46	0.30	1.50E-06	3.34E-05
2460	3	F2-3 vs. L2-3	255775_at	AT1G18600	Mitochondrion-located rhomboid-like protein	45.72	154.56	0.30	1.90E-06	3.89E-05
2461	3	F2-3 vs. L2-3	260673_at	AT1G19330	Evening-expressed key component of Sin3-HDAC complex, which bind directly to the CIRCADIAN CLOCK ASSOCIATED 1 (CCA1) and PSEUDO-RESPONSE REGULATOR 9 (PRR9) promoters and catalyze histone 3 (H3) deacetylation at the cognate regions to repress expression, allowing the declining phase of their expression at dusk.	77.96	255.70	0.30	4.84E-05	3.54E-04
2462	3	F2-3 vs. L2-3	266591_at	AT2G46225	Encodes a subunit of the WAVE complex. The WAVE complex is required for activation of ARP2/3 complex which functions in actin microfilament nucleation and branching. One of four ABI-like proteins.	78.73	260.66	0.30	6.00E-06	8.44E-05
2463	3	F2-3 vs. L2-3	257702_at	AT3G12670	Cytidine triphosphate synthase.	54.01	177.21	0.30	9.40E-06	1.14E-04
2464	3	F2-3 vs. L2-3	251638_at	AT3G57490	Ribosomal protein S5 family protein;(source:Araport11)	38.82	130.47	0.30	1.53E-05	1.60E-04
2465	3	F2-3 vs. L2-3	245324_at	AT4G17260	Lactate/malate dehydrogenase family protein;(source:Araport11)	68.57	231.38	0.30	< 1e-07	4.57E-06
2466	3	F2-3 vs. L2-3	253225_at	AT4G35020	A member of ROP GTPase gene family; Encodes a Rho-like GTP binding protein.	96.47	317.75	0.30	4.10E-06	6.26E-05
2467	3	F2-3 vs. L2-3	246917_at	AT5G25280	serine-rich protein-like protein;(source:Araport11)	27.26	90.99	0.30	2.29E-05	2.08E-04
2468	3	F2-3 vs. L2-3	248767_at	AT5G47710	Calcium-dependent lipid-binding (CaLB domain) family protein;(source:Araport11)	153.01	517.82	0.30	5.00E-07	1.44E-05
2469	3	F2-3 vs. L2-3	264978_at	AT1G27120	Encodes a Golgi-localized hydroxyproline-O-galactosyltransferase.	96.02	326.51	0.29	8.00E-07	2.05E-05
2470	3	F2-3 vs. L2-3	261239_at	AT1G32930	Galactosyltransferase family protein;(source:Araport11)	114.94	397.22	0.29	< 1e-07	< 1e-07
2471	3	F2-3 vs. L2-3	253051_at	AT4G37490	Cyclin-dependent protein kinase CYCB1;1. Functions as an effector of growth control at G2/M. Regulated by TCP20.	40.45	141.07	0.29	2.33E-04	1.07E-03
2472	3	F2-3 vs. L2-3	247770_at	AT5G58930	hypothetical protein (DUF740);(source:Araport11)	112.03	379.88	0.29	5.40E-06	7.72E-05

2473	3	F2-3 vs. L2-3	262989_at	AT1G23420	Essential for formation and asymmetric growth of the ovule outer integument. Member of the YABBY protein family of putative transcription factors that contain apparent Cys(2)-Cys(2) zinc-finger domains and regions of similarity to the high mobility group (HMG) transcription factors. INO may be required for polarity determination in the central part of the ovule.	22.05	79.76	0.28	3.33E-04	1.39E-03
2474	3	F2-3 vs. L2-3	262969_at	AT1G75710	C2H2-like zinc finger protein;(source:Araport11)	351.06	1259.85	0.28	4.00E-07	1.25E-05
2475	3	F2-3 vs. L2-3	265985_at	AT2G24220	Member of a family of proteins related to PUP1, a purine transporter. May be involved in the transport of purine and purine derivatives such as cytokinins, across the plasma membrane.	56.49	203.32	0.28	3.00E-07	1.05E-05
2476	3	F2-3 vs. L2-3	256621_at	AT3G24450	Heavy metal transport/detoxification superfamily protein;(source:Araport11)	48.77	172.52	0.28	1.60E-06	3.46E-05
2477	3	F2-3 vs. L2-3	263726_at	AT2G13610	ABC-2 type transporter family protein;(source:Araport11)	56.84	208.73	0.27	7.39E-05	4.72E-04
2478	3	F2-3 vs. L2-3	263979_at	AT2G42840	Encodes a putative extracellular proline-rich protein is exclusively expressed in the L1 layer of vegetative, inflorescence and floral meristems and the protoderm of organ primordia.	367.33	1347.40	0.27	7.19E-04	2.48E-03
2479	3	F2-3 vs. L2-3	245228_at	AT3G29810	During the course of seed coat epidermal cell differentiation, COBRA-LIKE 2 plays a role in cellulose deposition into mucilage secretory cells of Arabidopsis seeds. COBRA-LIKE 2 affects mucilage solubility and cellulosic ray formation.	25.74	94.46	0.27	2.76E-04	1.21E-03
2480	3	F2-3 vs. L2-3	253812_at	AT4G28240	Member of the wound-induced polypeptide (WIP) family. Positively regulates plant resistance against Pst DC3000 by enhancing PTI responses.	78.51	295.04	0.27	4.15E-04	1.63E-03
2481	3	F2-3 vs. L2-3	250780_at	AT5G05290	Encodes an expansin. Naming convention from the Expansin Working Group (Kende et al, 2004. Plant Mol Bio)	60.50	221.43	0.27	8.87E-04	2.92E-03
2482	3	F2-3 vs. L2-3	249427_at	AT5G39850	Ribosomal protein S4;(source:Araport11)	241.14	897.53	0.27	2.00E-07	7.75E-06
2483	3	F2-3 vs. L2-3	247355_at	AT5G63670	SPT4 homolog 2;(source:Araport11)	66.65	244.42	0.27	1.40E-06	3.16E-05
2484	3	F2-3 vs. L2-3	266123_at	AT2G45180	Bifunctional inhibitor/lipid-transfer protein/seed storage 2S albumin superfamily protein;(source:Araport11)	21.50	81.42	0.26	1.79E-05	1.78E-04
2485	3	F2-3 vs. L2-3	258480_at	AT3G02640	transmembrane protein;(source:Araport11)	70.75	271.04	0.26	1.73E-05	1.75E-04
2486	3	F2-3 vs. L2-3	245512_at	AT4G15770	RNA binding protein;(source:Araport11)	162.00	622.76	0.26	< 1e-07	4.57E-06
2487	3	F2-3 vs. L2-3	254609_at	AT4G18970	GDSL-motif esterase/acyltransferase/lipase. Enzyme group with broad substrate specificity that may catalyze acyltransfer or hydrolase reactions with lipid and non-lipid substrates.	113.02	435.70	0.26	6.82E-04	2.38E-03
2488	3	F2-3 vs. L2-3	264377_at	AT2G25060	early nodulin-like protein 14;(source:Araport11)	88.65	355.27	0.25	1.00E-06	2.42E-05
2489	3	F2-3 vs. L2-3	263640_at	AT2G25270	transmembrane protein;(source:Araport11)	25.94	105.49	0.25	4.29E-04	1.68E-03
2490	3	F2-3 vs. L2-3	259171_at	AT3G03590	SWIB/MDM2 domain superfamily protein;(source:Araport11)	84.99	346.17	0.25	5.00E-07	1.44E-05
2491	3	F2-3 vs. L2-3	251365_at	AT3G61310	AT hook motif DNA-binding family protein;(source:Araport11)	79.87	319.45	0.25	1.69E-04	8.50E-04
2492	3	F2-3 vs. L2-3	245008_at	ATCG00360	Encodes a protein required for photosystem I assembly and stability. In Chlamydomonas reinhardtii, this protein seems to act as a PSI specific chaperone facilitating the assembly of the complex by interacting with PsaA and PsaD. A loss of function mutation in tobacco leads to a loss of photosystem I.	1165.10	4618.82	0.25	7.02E-05	4.56E-04
2493	3	F2-3 vs. L2-3	266656_at	AT2G25900	Encodes a protein with two tandem-arrayed CCCH-type zinc fingers that binds RNA and is involved in RNA turnover. The mRNA is cell-to-cell mobile.	66.67	280.73	0.24	3.70E-06	5.84E-05
2494	3	F2-3 vs. L2-3	264061_at	AT2G27970	CDK-subunit 2;(source:Araport11)	83.52	341.38	0.24	5.10E-06	7.41E-05
2495	3	F2-3 vs. L2-3	266401_s_a	AT2G38620	Encodes a member of a plant specific family of cyclin dependent kinases.	142.35	596.64	0.24	2.20E-06	4.25E-05
2496	3	F2-3 vs. L2-3	255433_at	AT4G03210	encodes a member of xyloglucan endotransglucosylase/hydrolases (XTHs) that catalyze the cleavage and molecular grafting of xyloglucan chains function in loosening and rearrangement of the cell wall. Gene is expressed in shoot apex region, flower buds, flower stalks and internodes bearing flowers.	71.43	303.70	0.24	4.28E-05	3.25E-04
2497	3	F2-3 vs. L2-3	246203_at	AT4G36610	alpha/beta-Hydrolases superfamily protein;(source:Araport11)	47.06	198.61	0.24	7.46E-04	2.54E-03
2498	3	F2-3 vs. L2-3	247962_at	AT5G56580	Encodes a member of the MAP Kinase Kinase family of proteins. It can phosphorylate MPK12 in vitro and it can be dephosphorylated by MKP2 in vitro.	50.63	213.05	0.24	7.00E-07	1.83E-05
2499	3	F2-3 vs. L2-3	256125_at	AT1G18250	encodes a thaumatin-like protein	46.70	201.76	0.23	1.35E-05	1.48E-04
2500	3	F2-3 vs. L2-3	262367_at	AT1G73030	Encodes an ESCRT-related protein: CHMP1A/AT1G73030; CHMP1B/AT1G17730. CHMP1A and B mediate multivesicular body sorting of auxin carriers and are required for plant development. ESCRT: Endosomal Sorting Complexes Required For Transport machinery; CHMP: Charged Multivesicular Body Protein/Chromatin Modifying Protein.	105.65	451.48	0.23	5.20E-06	7.50E-05
2501	3	F2-3 vs. L2-3	263488_at	AT2G31840	Thioredoxin superfamily protein;(source:Araport11)	35.25	151.06	0.23	< 1e-07	4.57E-06

2502	3	F2-3 vs. L2-3	258067_at	AT3G25980	Encodes MAD2 (MITOTIC ARREST-DEFICIENT 2). May have the spindle assembly checkpoint protein functions conserved from yeast to humans.	33.10	145.08	0.23	6.20E-06	8.57E-05
2503	3	F2-3 vs. L2-3	263988_at	AT2G42830	AGAMOUS [AG]-like MADS box protein (AGL5) involved in fruit development (valve margin and dehiscence zone differentiation). A putative direct target of AG. SHP2 has been shown to be a downstream gene of the complex formed by AG and SEP proteins (SEP4 alone does not form a functional complex with AG).	121.79	555.07	0.22	7.30E-06	9.59E-05
2504	3	F2-3 vs. L2-3	257334_at	ATMG01370	transmembrane protein;(source:Araport11)	40.84	192.48	0.21	2.36E-04	1.08E-03
2505	3	F2-3 vs. L2-3	262667_at	AT1G62810	Encodes COPPER AMINE OXIDASE1 (CuAO1). Contributes to abscisic acid- and polyamine-induced nitric oxide biosynthesis and abscisic acid signal transduction.	23.98	119.14	0.20	5.33E-04	1.97E-03
2506	3	F2-3 vs. L2-3	260181_at	AT1G70710	endo-1,4-beta-glucanase. Involved in cell elongation.	128.94	639.18	0.20	1.34E-05	1.48E-04
2507	3	F2-3 vs. L2-3	255727_at	AT1G25510	Eukaryotic aspartyl protease family protein;(source:Araport11)	54.86	287.83	0.19	2.30E-06	4.39E-05
2508	3	F2-3 vs. L2-3	260391_at	AT1G74020	Encodes AtSS-2 strictosidine synthase.	90.21	464.14	0.19	2.11E-04	9.94E-04
2509	3	F2-3 vs. L2-3	255277_at	AT4G04890	Encodes a homeodomain protein that is expressed in the LI layer of the vegetative, floral and inflorescence meristems. Binds to the L1 box promoter element which is required in some proteins for L1 specific expression.	150.17	777.38	0.19	1.72E-04	8.60E-04
2510	3	F2-3 vs. L2-3	248011_at	AT5G56300	A member of the Arabidopsis SABATH methyltransferase gene family. Encodes GAMT2, a methyltransferase that uses S-adenosine-L-methionine (SAM) as a methyl donor to methylate the carboxyl group of GAs, resulting in the methyl esters of GAs (MeGAs). Expressed most highly in the siliques during seed development.	30.81	161.44	0.19	3.54E-04	1.46E-03
2511	3	F2-3 vs. L2-3	264327_at	AT1G04170	protein synthesis initiation factor eIF2 gamma The mRNA is cell-to-cell mobile.	15.18	85.03	0.18	4.06E-05	3.12E-04
2512	3	F2-3 vs. L2-3	252320_at	AT3G48580	xyloglucan endotransglucosylase/hydrolase 11;(source:Araport11)	21.66	122.71	0.18	5.66E-04	2.06E-03
2513	3	F2-3 vs. L2-3	246923_at	AT5G25100	Endomembrane protein 70 protein family;(source:Araport11)	25.41	137.94	0.18	2.50E-06	4.58E-05
2514	3	F2-3 vs. L2-3	265058_s_at	AT1G52030	Similar to myrosinase binding proteins which may be involved in metabolizing glucosinolates and forming defense compounds to protect against herbivory. Also similar to lectins and other agglutinating factors. Expressed only in flowers.	139.70	809.17	0.17	1.00E-07	4.57E-06
2515	3	F2-3 vs. L2-3	253684_at	AT4G29690	Alkaline-phosphatase-like family protein;(source:Araport11)	23.03	136.65	0.17	< 1e-07	< 1e-07
2516	3	F2-3 vs. L2-3	262802_at	AT1G20930	Cyclin-dependent kinase, expressed in flowers and suspension cell culture, expression peaks during M phase in synchronized cultures. Required for proper organization of the shoot apical meristem and for hormone signaling. Expressed in the shoot apical meristem. Involved in regulation of the G2/M transition of the mitotic cell cycle.	39.56	247.26	0.16	3.00E-07	1.05E-05
2517	3	F2-3 vs. L2-3	265117_at	AT1G62500	Bifunctional inhibitor/lipid-transfer protein/seed storage 2S albumin superfamily protein;(source:Araport11)	134.35	857.43	0.16	3.83E-04	1.54E-03
2518	3	F2-3 vs. L2-3	256243_at	AT3G12500	encodes a basic chitinase involved in ethylene/jasmonic acid mediated signalling pathway during systemic acquired resistance based on expression analyses.	21.56	136.72	0.16	< 1e-07	< 1e-07
2519	3	F2-3 vs. L2-3	251340_at	AT3G60830	Encodes an actin-related protein required for normal embryogenesis, plant architecture and floral organ abscission.	64.77	404.91	0.16	2.90E-06	5.02E-05
2520	3	F2-3 vs. L2-3	267614_at	AT2G26710	Encodes a member of the cytochrome p450 family that serves as a control point between multiple photoreceptor systems and brassinosteroid signal transduction. Involved in brassinolide metabolism. Mediates response to a variety of light signals including hypocotyl elongation and cotyledon expansion.	20.15	135.45	0.15	2.00E-07	7.75E-06
2521	3	F2-3 vs. L2-3	254824_s_at	AT4G12620	Origin Recognition Complex subunit 1b. Involved in the initiation of DNA replication. Regulated transcriptionally during cell cycle, peaking at G1/S-phase. Target of E2F/DF family of transcription factors. Interacts with ORC2 and ORC5. Highly expressed in proliferating cells. Expression levels are independent of light regime.	90.62	595.34	0.15	< 1e-07	< 1e-07
2522	3	F2-3 vs. L2-3	250508_at	AT5G09950	Encodes a DYW-class PPR protein required for RNA editing at four sites in mitochondria of A. thaliana.	20.47	162.00	0.13	2.00E-07	7.75E-06
2523	3	F2-3 vs. L2-3	252357_at	AT3G48410	alpha/beta-Hydrolases superfamily protein;(source:Araport11)	22.96	194.04	0.12	5.00E-07	1.44E-05
2524	3	F2-3 vs. L2-3	251555_at	AT3G58780	One of two genes (SHP1 and SHP2) that are required for fruit dehiscence. The two genes control dehiscence zone differentiation and promote the lignification of adjacent cells.	98.71	885.62	0.11	< 1e-07	< 1e-07
2525	3	F2-3 vs. L2-3	262121_at	AT1G02800	Encodes a protein with similarity to endo-1,4-b-glucanases and is a member of Glycoside Hydrolase Family 9. CEL2 is induced by nemotodes and is expressed in syncitia induced by Heterodera schachtii.May be involved in the development and function of syncitia.	63.28	664.82	0.10	6.81E-05	4.47E-04

2526	3	F2-3 vs. L2-3	255014_at	AT4G09960	Encodes a MADS box transcription factor expressed in the carpel and ovules. Plays a maternal role in fertilization and seed development. Controls the structure and mechanical properties of the seed coat. Controls fruit size by regulating cytokinin levels and FRUITFULL.	35.87	422.68	0.09	< 1e-07	< 1e-07
2527	3	F2-3 vs. L2-3	253874_at	AT4G27450	aluminum induced protein with YGL and LRDR motifs;(source:Araport11)	43.82	545.71	0.08	< 1e-07	< 1e-07
2528	3	F2-3 vs. L2-3	246550_at	AT5G14920	Encodes a GASA domain containing protein that regulates increases in plant growth through GA-induced and DELLA-dependent signal transduction and that can increase abiotic stress resistance by reducing ROS accumulation.	44.19	577.26	0.08	3.80E-06	5.94E-05
2529	3	F2-3 vs. L2-3	252321_at	AT3G48510	ABA‐induced transcription repressor that acts as feedback regulator in ABA signalling.	71.35	1004.16	0.07	< 1e-07	< 1e-07
2530	3	F2-3 vs. L2-3	244971_at	ATCG00670	Encodes the only ClpP (caseinolytic protease) encoded within the plastid genome. Contains a highly conserved catalytic triad of Ser-type proteases (Ser-His-Asp). Part of the 350 kDa chloroplast Clp complex. The name reflects nomenclature described in Adam et. al (2001).	549.91	7769.24	0.07	< 1e-07	< 1e-07
2531	3	F2-3 vs. L2-3	247208_at	AT5G64870	Belongs to the group of plant flotillins, which are plasma membrane proteins. Flot3 is found in membrane nanodomains.	62.04	1192.56	0.05	< 1e-07	< 1e-07
2532	3	F2-3 vs. L2-3	257629_at	AT3G26140	Cellulase (glycosyl hydrolase family 5) protein;(source:Araport11)	50.43	1082.65	0.05	4.30E-06	6.44E-05
2533	3	F2-3 vs. L2-3	257890_s_at	AT3G42570	peroxidase family protein;(source:Araport11)	29.40	700.58	0.04	7.00E-07	1.83E-05
2534	3	F2-3 vs. L2-3	256648_at	AT3G13580	Ribosomal protein L30/L7 family protein;(source:Araport11)	14.85	1396.97	0.01	< 1e-07	< 1e-07
2535	4	F2-3 vs. M2-3	250606_s_at	AT5G07410	Encodes a pectin methylesterase implicated in the remodeling of pectins in pollen. Its expression is restricted to the male gametophyte (dry pollen, imbibed pollen and pollen tube). Mutants do not display any phenotype in the vegetative tissues. Pollen germination of KO mutant is strongly delayed in vivo and in vitro. Extra calcium restores the wild type phenotype in vitro.	5737.01	9.23	621.32	< 1e-07	< 1e-07
2536	4	F2-3 vs. M2-3	266750_s_at	AT2G47040	Share high homologies with a group of pectin methylesterases (PME), pollen specific, and is required for enhancing the growth of pollen tube in style and transmitting tract tissues.	7716.04	14.79	521.72	< 1e-07	< 1e-07
2537	4	F2-3 vs. M2-3	246880_s_at	AT5G25980	Myrosinase (thioglucoside glucohydrolase) gene involved in glucosinoloate metabolism. The mRNA is cell-to-cell mobile.	8914.54	21.32	418.06	< 1e-07	< 1e-07
2538	4	F2-3 vs. M2-3	256966_at	AT3G13400	SKU5 similar 13;(source:Araport11)	2870.82	16.95	169.37	5.00E-07	6.51E-05
2539	4	F2-3 vs. M2-3	255515_at	AT4G02250	Plant invertase/pectin methylesterase inhibitor superfamily protein;(source:Araport11)	1545.08	10.60	145.82	3.00E-07	4.88E-05
2540	4	F2-3 vs. M2-3	246687_at	AT5G33370	GDSL-motif esterase/acyltransferase/lipase. Enzyme group with broad substrate specificity that may catalyze acyltransfer or hydrolase reactions with lipid and non-lipid substrates. Mutants are defective in cuticle formation with reduced sepal cuticle ridge formation.	2352.70	19.78	118.97	< 1e-07	< 1e-07
2541	4	F2-3 vs. M2-3	259269_at	AT3G01270	Pectate lyase family protein;(source:Araport11)	2423.12	20.52	118.06	< 1e-07	< 1e-07
2542	4	F2-3 vs. M2-3	251258_at	AT3G62170	VANGUARD-like protein;(source:Araport11)	3054.47	26.97	113.24	1.00E-07	2.17E-05
2543	4	F2-3 vs. M2-3	248227_at	AT5G53820	Late embryogenesis abundant protein (LEA) family protein;(source:Araport11)	1220.54	11.78	103.63	3.00E-07	4.88E-05
2544	4	F2-3 vs. M2-3	265127_at	AT1G55560	SKU5 similar 14;(source:Araport11)	1235.75	12.25	100.90	1.00E-07	2.17E-05
2545	4	F2-3 vs. M2-3	252820_at	AT3G42640	H[+]-ATPase 8;(source:Araport11)	3179.09	38.79	81.97	1.00E-07	2.17E-05
2546	4	F2-3 vs. M2-3	248926_at	AT5G45880	Pollen Ole e 1 allergen and extensin family protein;(source:Araport11)	1332.48	16.31	81.69	< 1e-07	< 1e-07
2547	4	F2-3 vs. M2-3	251590_at	AT3G57690	Encodes a putative arabinogalactan-protein (AGP23).	9925.07	131.86	75.27	4.00E-07	5.48E-05
2548	4	F2-3 vs. M2-3	261943_at	AT1G80660	H[+]-ATPase 9;(source:Araport11)	812.70	11.66	69.69	< 1e-07	2.17E-05
2549	4	F2-3 vs. M2-3	245946_at	AT5G19580	glyoxal oxidase-related protein;(source:Araport11)	1498.35	21.85	68.59	1.20E-06	1.14E-04
2550	4	F2-3 vs. M2-3	248714_at	AT5G48140	Pectin lyase-like superfamily protein;(source:Araport11)	686.89	10.17	67.57	2.00E-07	3.72E-05
2551	4	F2-3 vs. M2-3	265552_at	AT2G07560	H[+]-ATPase 6;(source:Araport11)	819.78	12.23	67.04	< 1e-07	< 1e-07
2552	4	F2-3 vs. M2-3	245010_at	ATCG00420	Encodes NADH dehydrogenase subunit J. Its transcription is increased upon sulfur depletion.	7175.90	114.05	62.92	8.00E-07	9.26E-05
2553	4	F2-3 vs. M2-3	253226_at	AT4G35010	putative beta-galactosidase (BGAL11 gene)	652.31	11.46	56.93	< 1e-07	< 1e-07
2554	4	F2-3 vs. M2-3	265080_at	AT1G55570	SKU5 similar 12;(source:Araport11)	626.28	12.29	50.96	2.00E-07	3.72E-05

2555	4	F2-3 vs. M2-3	251733_at	AT3G56240	CCH protein belongs to a family of eukaryotic proteins that participate in intracellular copper homeostasis by delivering this metal to the secretory pathway; mainly located along the vascular bundles of senescing leaves and petioles as well as in stem sieve elements; hypothesized to have a role in copper mobilization from decaying organs towards reproductive structures, as a result of metalloprotein breakdown. The plant-specific C-terminal domain of the CCH protein forms amyloid-like fibrils in vitro.	1361.88	29.11	46.79	4.00E-07	5.48E-05
2556	4	F2-3 vs. M2-3	248367_at	AT5G52360	ADF10 is an actin-depolymerizing factor that preferentially binds ADP-G-actin and inhibits G-actin nucleotide exchange. ADF10 promotes actin turnover in pollen , regulating organization of actin filaments and vesicle trafficking during pollen tube growth.	775.69	19.55	39.69	4.00E-07	5.48E-05
2557	4	F2-3 vs. M2-3	260888_at	AT1G29140	Pollen Ole e 1 allergen and extensin family protein;(source:Araport11)	426.02	13.43	31.71	1.10E-06	1.08E-04
2558	4	F2-3 vs. M2-3	246072_at	AT5G20240	Floral homeotic gene encoding a MADS domain transcription factor. Required for the specification of petal and stamen identities.	525.58	16.76	31.36	2.00E-07	3.72E-05
2559	4	F2-3 vs. M2-3	266743_at	AT2G02990	Encodes a member of the ribonuclease T2 family that responds to inorganic phosphate starvation, and inhibits production of anthocyanin. Also involved in wound-induced signaling independent of jasmonic acid. Its expression is responsive to both phosphate (Pi) and phosphite (Phi) in roots.	964.31	31.65	30.47	7.70E-06	3.29E-04
2560	4	F2-3 vs. M2-3	256955_at	AT3G13390	SKU5 similar 11;(source:Araport11)	464.22	15.93	29.14	5.80E-06	2.82E-04
2561	4	F2-3 vs. M2-3	262760_at	AT1G10770	Encodes a putative pectin methylesterase/invertase inhibitor. Anti-sense reduction of this gene's transcript results in pollen tube growth retardation and then partial male sterility and reduced seed set.	494.95	17.50	28.29	2.30E-06	1.62E-04
2562	4	F2-3 vs. M2-3	258639_at	AT3G07820	Pectin lyase-like superfamily protein;(source:Araport11)	243.11	9.36	25.98	< 1e-07	2.17E-05
2563	4	F2-3 vs. M2-3	251304_at	AT3G61990	Encodes a protein methyltransferase. Involved in the methylation of plant transmembrane proteins.	510.37	21.38	23.88	2.90E-06	1.91E-04
2564	4	F2-3 vs. M2-3	257173_at	AT3G23810	S-adenosyl-l-homocysteine (SAH) hydrolase 2;(source:Araport11)	4626.75	194.32	23.81	1.46E-05	5.10E-04
2565	4	F2-3 vs. M2-3	254104_at	AT4G25040	Uncharacterized protein family (UPF0497);(source:Araport11)	616.86	26.16	23.58	< 1e-07	< 1e-07
2566	4	F2-3 vs. M2-3	245842_at	AT1G58430	Encodes an anther-specific proline-rich protein.	323.61	13.77	23.51	6.00E-07	7.44E-05
2567	4	F2-3 vs. M2-3	266353_at	AT2G01520	Encodes a cis-cinnamic acid responsive gene that is a member of the major latex protein-like gene family and plays a role in promoting vegetative growth and delaying ﬂowering. The mRNA is cell-to-cell mobile.	2556.56	112.64	22.70	1.40E-06	1.22E-04
2568	4	F2-3 vs. M2-3	254716_at	AT4G13560	Late embryogenesis abundant protein (LEA) family protein;(source:Araport11)	631.25	27.81	22.70	5.00E-07	6.51E-05
2569	4	F2-3 vs. M2-3	252606_at	AT3G45010	serine carboxypeptidase-like 48;(source:Araport11)	622.45	27.54	22.60	1.85E-05	6.16E-04
2570	4	F2-3 vs. M2-3	267213_at	AT2G44120	Ribosomal protein L30/L7 family protein;(source:Araport11)	358.00	15.96	22.44	3.20E-06	2.06E-04
2571	4	F2-3 vs. M2-3	249584_s_a	AT5G37810	NOD26-like intrinsic protein 4;(source:Araport11)	214.36	9.84	21.79	1.40E-06	1.22E-04
2572	4	F2-3 vs. M2-3	262463_at	AT1G50310	Sucrose transporter, expressed in pollen tubes.	347.34	16.24	21.39	6.90E-06	3.02E-04
2573	4	F2-3 vs. M2-3	259161_at	AT3G01500	Encodes a putative beta-carbonic anhydrase betaCA1. Together with betaCA4 (At1g70410) regulates CO2-controlled stomatal movements in guard cells, as well as attenuates immunity. Differential CA gene expression in response to changing atmospheric CO2 conditions contribute to altered disease resistance levels. Activated by OXS2 under the treatment of salt.	380.65	18.38	20.72	1.90E-06	1.48E-04
2574	4	F2-3 vs. M2-3	258003_at	AT3G29030	Encodes an expansin. Naming convention from the Expansin Working Group (Kende et al, 2004. Plant Mol Bio)	397.73	19.39	20.51	< 1e-07	< 1e-07
2575	4	F2-3 vs. M2-3	258686_at	AT3G07840	Pectin lyase-like superfamily protein;(source:Araport11)	552.39	26.95	20.50	5.40E-06	2.78E-04
2576	4	F2-3 vs. M2-3	248252_at	AT5G53250	arabinogalactan protein 22;(source:Araport11)	1087.93	53.66	20.27	1.31E-04	1.88E-03
2577	4	F2-3 vs. M2-3	252607_at	AT3G44990	Encodes a xyloglucan endotransglycosylase/hydrolase. Protein sequence and phylogenetic analysis indicates that this enzyme resides in Group III-A of the XTH family, with high similarity to Tropaeolum majus (nasturtium) xyloglucanase 1 (TmNXG1).Enzyme kinetic analysis indicates predominant xyloglucan endo-hydrolase activity (EC 3.2.1.151) with only limited potential to act as a xyloglucan endo-transglycosylase (EC 2.4.1.207).	200.56	10.20	19.65	1.80E-06	1.42E-04
2578	4	F2-3 vs. M2-3	266918_at	AT2G45800	Encodes a member of the Arabidopsis LIM proteins: a family of actin bundlers with distinct expression patterns. WLIM1, WLIM2a, and WLIM2b are widely expressed, whereas PLIM2a, PLIM2b, and PLIM2c are predominantly expressed in pollen. Regulates actin cytoskeleton organization.	256.04	13.43	19.06	1.00E-06	1.06E-04
2579	4	F2-3 vs. M2-3	246545_at	AT5G15110	Pectate lyase family protein;(source:Araport11)	207.74	11.18	18.59	3.00E-07	4.88E-05

2580	4	F2-3 vs. M2-3	249614_at	AT5G37300	Encodes a bifunctional enzyme, wax ester synthase (WS) and diacylglycerol acyltransferase (DGAT). In vitro assay indicated a ratio of 10.9 between its WS and DGAT activities. Both mutant and in vivo expression/analysis in yeast studies indicated a role in wax biosynthesis.	233.55	12.61	18.52	3.50E-06	2.20E-04
2581	4	F2-3 vs. M2-3	250631_at	AT5G07430	Pectin lyase-like superfamily protein;(source:Araport11)	195.35	11.02	17.73	2.00E-07	3.72E-05
2582	4	F2-3 vs. M2-3	249375_at	AT5G40730	Encodes an arabinogalactan-protein (AGP24).	1324.88	76.88	17.23	1.94E-04	2.36E-03
2583	4	F2-3 vs. M2-3	251775_s_at	AT3G55610	encodes delta 1-pyrroline-5-carboxylate synthetase B. Gene expression is induced by dehydration, high salt and ABA. Knock-out mutations in P5CS2 are embryo-lethal. P5CS2 appears to be present in different cells and/or different subcellular locations from P5CS1 in a tissue-dependent manner. Mutants are defective in pollen development.	834.52	48.78	17.11	2.19E-05	6.79E-04
2584	4	F2-3 vs. M2-3	252140_at	AT3G51070	S-adenosyl-L-methionine-dependent methyltransferases superfamily protein;(source:Araport11)	208.13	12.19	17.07	1.20E-06	1.14E-04
2585	4	F2-3 vs. M2-3	256638_at	AT3G19090	RNA-binding protein;(source:Araport11)	739.58	47.04	15.72	2.50E-06	1.69E-04
2586	4	F2-3 vs. M2-3	261266_at	AT1G26770	Encodes an expansin. Naming convention from the Expansin Working Group (Kende et al, Plant Mol Bio). Involved in the formation of nematode-induced syncytia in roots of Arabidopsis thaliana.	226.92	14.74	15.40	2.10E-05	6.59E-04
2587	4	F2-3 vs. M2-3	261970_at	AT1G65960	glutamate decarboxylase (GAD2)The mRNA is cell-to-cell mobile.	2324.83	152.61	15.23	1.40E-06	1.22E-04
2588	4	F2-3 vs. M2-3	265768_at	AT2G48020	Encodes a zinc transporter ZIF2. Expression of ZIF2 is regulated by alternative splicing.	1326.82	90.52	14.66	< 1e-07	2.17E-05
2589	4	F2-3 vs. M2-3	252863_at	AT4G39800	** Referred to as MIPS2 in Mitsuhashi et al 2008. myo-inositol-1-phosphate synthase isoform 1.Expressed in leaf, root and silique. Immunolocalization experiments with an antibody recognizing MIPS1, MIPS2, and MIPS3 showed endosperm localization.	795.03	54.56	14.57	1.10E-04	1.70E-03
2590	4	F2-3 vs. M2-3	247902_at	AT5G57350	member of Plasma membrane H+-ATPase family	399.01	28.08	14.21	1.85E-04	2.30E-03
2591	4	F2-3 vs. M2-3	252123_at	AT3G51240	Encodes flavanone 3-hydroxylase that is coordinately expressed with chalcone synthase and chalcone isomerases. Regulates flavonoid biosynthesis. Not responsive to auxin or ethylene stimulus (qRT-PCR).	562.77	40.26	13.98	2.88E-05	7.94E-04
2592	4	F2-3 vs. M2-3	253083_at	AT4G36250	Encodes a putative aldehyde dehydrogenase. The gene is not responsive to osmotic stress and is expressed constitutively at a low level in plantlets and root cultures.	466.83	34.28	13.62	1.60E-06	1.30E-04
2593	4	F2-3 vs. M2-3	255016_at	AT4G10120	Encodes a protein with putative sucrose-phosphate synthase activity.	133.95	9.95	13.47	8.00E-07	9.26E-05
2594	4	F2-3 vs. M2-3	249939_at	AT5G22430	Pollen Ole e 1 allergen and extensin family protein;(source:Araport11)	296.77	22.35	13.28	5.37E-05	1.14E-03
2595	4	F2-3 vs. M2-3	245232_at	AT4G25590	actin depolymerizing factor 7;(source:Araport11)	178.24	13.64	13.07	4.00E-07	5.48E-05
2596	4	F2-3 vs. M2-3	258751_at	AT3G05890	Low temperature and salt responsive protein family;(source:Araport11)	168.98	12.98	13.02	5.36E-05	1.14E-03
2597	4	F2-3 vs. M2-3	247538_at	AT5G61700	ABC2 homolog 16;(source:Araport11)	250.74	19.48	12.87	6.50E-06	2.99E-04
2598	4	F2-3 vs. M2-3	262717_s_a	AT1G16410	member of CYP79F The mRNA is cell-to-cell mobile.	143.94	11.23	12.81	4.00E-07	5.48E-05
2599	4	F2-3 vs. M2-3	258591_at	AT3G04360	Calcium-dependent lipid-binding (CaLB domain) family protein;(source:Araport11)	229.97	18.08	12.72	3.00E-06	1.95E-04
2600	4	F2-3 vs. M2-3	253725_at	AT4G29340	Profilin is a low-molecular weight, actin monomer-binding protein that regulates the organization of actin cytoskeleton in eukaryotes, including higher plants. PRF4 and PRF5 are late pollen-specific and are not detectable in other cell types of the plant body including microspores and root hairs. Immunocytochemical studies at the subcellular level reveal that both the constitutive and pollen-specific profilins are abundant in the cytoplasm. In vegetative cell types, such as root apical cells, profilins showed localization to nuclei in addition to the cytoplasmic staining.	319.33	25.14	12.70	< 1e-07	2.17E-05
2601	4	F2-3 vs. M2-3	258158_at	AT3G17790	Expression is upregulated in the shoot of cax1/cax3 mutant and is responsive to phosphate (Pi) and not phosphite (Phi) in roots and shoots.	235.19	18.56	12.67	1.12E-05	4.26E-04
2602	4	F2-3 vs. M2-3	250349_at	AT5G12000	kinase with adenine nucleotide alpha hydrolases-like domain-containing protein;(source:Araport11)	165.88	13.12	12.64	1.00E-06	1.06E-04
2603	4	F2-3 vs. M2-3	265547_at	AT2G28305	Putative lysine decarboxylase family protein;(source:Araport11)	349.95	27.95	12.52	1.07E-04	1.68E-03
2604	4	F2-3 vs. M2-3	245011_at	ATCG00430	Encodes a protein which was originally thought to be part of photosystem II but its wheat homolog was later shown to encode for subunit K of NADH dehydrogenase.	405.07	32.49	12.47	5.50E-06	2.81E-04
2605	4	F2-3 vs. M2-3	263450_at	AT2G31500	member of Calcium Dependent Protein Kinase	129.20	10.44	12.37	2.49E-05	7.09E-04

2606	4	F2-3 vs. M2-3	261648_at	AT1G27730	Related to Cys2/His2-type zinc-finger proteins found in higher plants. Compensated for a subset of calcineurin deficiency in yeast. Salt tolerance produced by ZAT10 appeared to be partially dependent on ENA1/PMR2, a P-type ATPase required for Li+ and Na+ efflux in yeast. The protein is localized to the nucleus, acts as a transcriptional repressor and is responsive to chitin oligomers. Also involved in response to photooxidative stress.	310.78	25.20	12.33	1.23E-04	1.82E-03
2607	4	F2-3 vs. M2-3	258851_at	AT3G03190	Encodes glutathione transferase belonging to the phi class of GSTs. Naming convention according to Wagner et al. (2002).	318.07	25.90	12.28	9.00E-06	3.69E-04
2608	4	F2-3 vs. M2-3	254098_at	AT4G25100	Fe-superoxide dismutase	1218.52	99.62	12.23	5.02E-04	4.10E-03
2609	4	F2-3 vs. M2-3	252377_at	AT3G47960	Encodes a high-affinity, proton-dependent glucosinolate-specific transporter that is crucial for the transport of both methionine- and tryptophan-derived glucosinolates to seeds.	402.40	33.95	11.85	1.40E-06	1.22E-04
2610	4	F2-3 vs. M2-3	264208_at	AT1G22760	Putative poly(A) binding protein May there fore function in posttranscriptional regulation, including mRNA turnover and translational initiation. Expression detected only in floral organs.	204.29	17.28	11.82	< 1e-07	< 1e-07
2611	4	F2-3 vs. M2-3	252733_at	AT3G43120	SAUR-like auxin-responsive protein family;(source:Araport11)	119.86	10.16	11.80	5.80E-06	2.82E-04
2612	4	F2-3 vs. M2-3	259372_at	AT1G69120	Floral homeotic gene encoding a MADS domain protein homologous to SRF transcription factors. Specifies floral meristem and sepal identity. Required for the transcriptional activation of AGAMOUS. Interacts with LEAFY.Binds to promoter and regulates the expression of flowering time genes SVP, SOC1 and AGL24.	125.32	10.69	11.72	3.00E-07	4.88E-05
2613	4	F2-3 vs. M2-3	267144_at	AT2G38110	bifunctional sn-glycerol-3-phosphate 2-O-acyltransferase/phosphatase. Involved in cutin assembly.	262.08	23.32	11.24	2.83E-04	3.01E-03
2614	4	F2-3 vs. M2-3	266115_at	AT2G02140	Predicted to encode a PR (pathogenesis-related) protein. Belongs to the plant defensin (PDF) family with the following members: At1g75830/PDF1.1, At5g44420/PDF1.2a, At2g26020/PDF1.2b, At5g44430/PDF1.2c, At2g26010/PDF1.3, At1g19610/PDF1.4, At1g55010/PDF1.5, At2g02120/PDF2.1, At2g02100/PDF2.2, At2g02130/PDF2.3, At1g61070/PDF2.4, At5g63660/PDF2.5, At2g02140/PDF2.6, At5g38330/PDF3.1 and At4g30070/PDF3.2.	216.49	19.38	11.17	1.62E-05	5.55E-04
2615	4	F2-3 vs. M2-3	267476_at	AT2G02720	Pectate lyase family protein;(source:Araport11)	186.23	16.72	11.14	3.04E-05	8.25E-04
2616	4	F2-3 vs. M2-3	266697_at	AT2G19770	Encodes profilin 5, originally named profilin 4 (PRO4/PFN4). Low-molecular weight, actin monomer-binding protein that regulates the organization of actin cytoskeleton. Pollen-specific plant profilin present predominantly in mature pollen and growing pollen tubes.	202.21	18.15	11.14	9.38E-05	1.54E-03
2617	4	F2-3 vs. M2-3	247162_at	AT5G65730	xyloglucan endotransglucosylase/hydrolase 6;(source:Araport11)	136.75	12.32	11.10	2.50E-06	1.69E-04
2618	4	F2-3 vs. M2-3	245701_at	AT5G04140	Encodes a gene whose sequence is similar to ferredoxin dependent glutamate synthase (Fd-GOGAT). Expression in leaves is induced by light and sucrose. Proposed to be involved in photorespiration and nitrogen assimilation. The mRNA is cell-to-cell mobile.	252.82	23.17	10.91	3.62E-05	9.26E-04
2619	4	F2-3 vs. M2-3	245749_at	AT1G51090	Heavy metal associated domain containing protein involved in plant immunity.Mutants show an increase in root length under NO stress and reduction in root length under H2O2 stress conditions. Mutants show increases in defense responses to pathogens including hypersensitive lesions, increased resistance and induction of SAR genes.	168.81	16.09	10.49	1.00E-07	2.17E-05
2620	4	F2-3 vs. M2-3	260997_at	AT1G26610	C2H2-like zinc finger protein;(source:Araport11)	507.50	49.13	10.33	1.17E-04	1.76E-03
2621	4	F2-3 vs. M2-3	259451_at	AT1G13890	Encodes a member of a gene family homologous to mammalian SNAP25, a type of SNARE proteins with two chains. There are three members in Arabidopsis: SNAP30, SNAP29, and SNAP33.	100.74	9.77	10.31	7.17E-05	1.33E-03
2622	4	F2-3 vs. M2-3	245061_at	AT2G39730	Rubisco activase, a nuclear-encoded chloroplast protein that consists of two isoforms arising from alternative splicing in most plants. Required for the light activation of rubisco. Involved in jasmonate-induced leaf senescence.	675.54	65.64	10.29	9.50E-05	1.55E-03
2623	4	F2-3 vs. M2-3	249996_at	AT5G18600	Encodes a member of the CC-type glutaredoxin (ROXY) family that has been shown to interact with the transcription factor TGA2 and suppress ORA59 promoter activity.	224.51	22.15	10.14	2.89E-04	3.03E-03
2624	4	F2-3 vs. M2-3	250498_at	AT5G09660	encodes a microbody NAD-dependent malate dehydrogenase encodes an peroxisomal NAD-malate dehydrogenase that is involved in fatty acid beta-oxidation through providing NAD to the process of converting fatty acyl CoA to acetyl CoA.	352.14	34.81	10.11	3.90E-06	2.31E-04

2625	4	F2-3 vs. M2-3	252291_s_at	AT3G49110	Class III peroxidase Perx33. Expressed in roots. Located in the cell wall. Involved in cell elongation. Expression activated by light. May play a role in generating H2O2 during defense response. The mRNA is cell-to-cell mobile.	116.67	11.63	10.04	1.03E-04	1.65E-03
2626	4	F2-3 vs. M2-3	250514_at	AT5G09550	GDP dissociation inhibitor family protein / Rab GTPase activator family protein;(source:Araport11)	139.97	13.96	10.03	4.20E-06	2.40E-04
2627	4	F2-3 vs. M2-3	251916_at	AT3G53960	Major facilitator superfamily protein;(source:Araport11)	239.96	24.11	9.95	2.82E-05	7.85E-04
2628	4	F2-3 vs. M2-3	245629_at	AT1G56580	Encodes SMALLER WITH VARIABLE BRANCHES (SVB), a protein with a conserved domain of unknown function (DUF538). The trichomes of the SVB mutants are smaller and exhibit branches of variable length and number.	331.64	33.42	9.92	7.68E-05	1.36E-03
2629	4	F2-3 vs. M2-3	262664_at	AT1G13970	beta-hexosaminidase (DUF1336);(source:Araport11)	231.87	23.55	9.85	7.92E-05	1.39E-03
2630	4	F2-3 vs. M2-3	258561_at	AT3G05960	Encodes a hexose sugar transporter that is expressed in pollen. STP6 may play a role in providing sugars during late pollen maturation or pollen tube germination.	128.51	13.42	9.57	5.07E-05	1.13E-03
2631	4	F2-3 vs. M2-3	252618_at	AT3G45140	Chloroplast lipoxygenase required for wound-induced jasmonic acid accumulation in Arabidopsis.Mutants are resistant to Staphylococcus aureus and accumulate salicylic acid upon infection.CFA-Leu, CFA-Val, CFA-Met and CFA-Ala can induce the expression of LOX2.The mRNA is cell-to-cell mobile.	578.39	60.43	9.57	3.71E-05	9.26E-04
2632	4	F2-3 vs. M2-3	257951_at	AT3G21700	Monomeric G protein. Expressed in root epidermal cells that are destined to become atrichoblasts. Also expressed during pollen development and in the pollen tube tip.	192.77	20.26	9.51	5.20E-06	2.71E-04
2633	4	F2-3 vs. M2-3	251358_at	AT3G61160	Protein kinase superfamily protein;(source:Araport11)	188.33	19.85	9.49	3.41E-05	8.97E-04
2634	4	F2-3 vs. M2-3	246908_at	AT5G25610	responsive to dehydration 22 (RD22) mediated by ABA	2057.19	218.35	9.42	8.73E-05	1.46E-03
2635	4	F2-3 vs. M2-3	264139_at	AT1G78940	kinase with adenine nucleotide alpha hydrolases-like domain-containing protein;(source:Araport11)	124.44	13.57	9.17	4.31E-05	1.02E-03
2636	4	F2-3 vs. M2-3	245465_at	AT4G16590	encodes a gene similar to cellulose synthase	188.55	20.59	9.16	4.43E-05	1.04E-03
2637	4	F2-3 vs. M2-3	261363_at	AT1G41830	SKU5-similar 6;(source:Araport11)	250.30	27.43	9.12	8.40E-06	3.50E-04
2638	4	F2-3 vs. M2-3	244959_s_a	AT2G07708	hypothetical protein;(source:Araport11)	1798.49	202.96	8.86	2.73E-04	2.96E-03
2639	4	F2-3 vs. M2-3	252929_at	AT4G38970	Protein is tyrosine-phosphorylated and its phosphorylation state is modulated in response to ABA in Arabidopsis thaliana seeds.	2617.69	298.20	8.78	2.25E-05	6.85E-04
2640	4	F2-3 vs. M2-3	267443_at	AT2G19000	hypothetical protein;(source:Araport11)	101.61	11.63	8.74	1.20E-05	4.43E-04
2641	4	F2-3 vs. M2-3	262281_at	AT1G68570	NPF3.1 is a membrane localized GA transporter that is expressed in the root endodermis.	154.91	17.78	8.71	1.50E-06	1.24E-04
2642	4	F2-3 vs. M2-3	266764_at	AT2G47050	Plant invertase/pectin methylesterase inhibitor superfamily protein;(source:Araport11)	102.85	11.84	8.68	5.24E-05	1.13E-03
2643	4	F2-3 vs. M2-3	245658_at	AT1G28270	Member of a diversely expressed predicted peptide family showing sequence similarity to tobacco Rapid Alkalinization Factor (RALF), and is believed to play an essential role in the physiology of Arabidopsis. Consists of a single exon and is characterized by a conserved C-terminal motif and N-terminal signal peptide. RALF4 and RALF19 act redundantly in the pollen tube to regulate pollen tube growth.	109.41	12.71	8.61	2.52E-05	7.13E-04
2644	4	F2-3 vs. M2-3	252101_at	AT3G51290	pyridoxal-phosphate-dependent serine hydroxymethyltransferase, putative (DUF632);(source:Araport11)	1103.91	132.31	8.34	2.74E-05	7.67E-04
2645	4	F2-3 vs. M2-3	245008_at	ATCG00360	Encodes a protein required for photosystem I assembly and stability. In Chlamydomonas reinhardtii, this protein seems to act as a PSI specific chaperone facilitating the assembly of the complex by interacting with PsaA and PsaD. A loss of function mutation in tobacco leads to a loss of photosystem I.	1268.77	152.55	8.32	9.40E-04	5.94E-03
2646	4	F2-3 vs. M2-3	245528_at	AT4G15530	Encodes a dual-targeted protein believed to act as a pyruvate, orthophosphate dikinase. These enzymes are normally associated with C4 photosynthesis which does not occur in Arabidopsis. However, PPDK may play a role in remobilizing nitrogen during leaf senescence in Arabidopsis. The product of the long transcript (.1 gene model) was shown to be targeted to the chloroplast, whereas the shorter transcript (no targeting sequence) accumulates in the cytosol. The two proteins were also found to be expressed in slightly different tissues.	163.62	19.84	8.25	2.98E-04	3.05E-03
2647	4	F2-3 vs. M2-3	263845_at	AT2G37040	Encodes PAL1, a phenylalanine ammonia-lyase. Arabidopsis has four PALs: AT2G37040 (PAL1), AT3G53260 (PAL2), AT5G04230 (PAL3) and AT3G10340 (PAL4).	119.28	14.54	8.20	9.50E-06	3.81E-04
2648	4	F2-3 vs. M2-3	254122_at	AT4G24510	Encodes a component of the fatty acid elongation machinery required for C28 to C30 fatty acid elongation. It does not require the acyltransferase catalytic site for biological function.	103.37	12.66	8.16	4.36E-05	1.03E-03

2649	4	F2-3 vs. M2-3	259343_s_at	AT3G03780	Encodes a cytosolic methionine synthase, involved in methionine regeneration via the activated methyl cycle (or SAM cycle)	2170.15	267.25	8.12	7.52E-04	5.17E-03
2650	4	F2-3 vs. M2-3	247537_s_at	AT5G61690	ABC2 homolog 15;(source:Araport11)	287.98	35.51	8.11	6.16E-05	1.21E-03
2651	4	F2-3 vs. M2-3	247377_at	AT5G63180	Pectin lyase-like superfamily protein;(source:Araport11)	217.85	26.94	8.09	3.80E-06	2.30E-04
2652	4	F2-3 vs. M2-3	253487_at	AT4G31700	Encodes a putative ribosomal protein S6 (rps6a). RPS6A and RPS6B are fully redundant and essential during gametogenesis.	486.83	60.35	8.07	5.76E-05	1.19E-03
2653	4	F2-3 vs. M2-3	262122_at	AT1G02790	encodes a exopolygalacturonase.	119.53	14.85	8.05	1.05E-04	1.67E-03
2654	4	F2-3 vs. M2-3	267181_at	AT2G37760	Encodes an NADPH-dependent aldo-keto reductase that can act on a wide variety of substrates in vitro including aliphatic and aromatic aldehydes and steroids. Transcript levels for this gene are up-regulated in response to cold, salt, and drought stress.	180.16	22.50	8.01	1.13E-04	1.73E-03
2655	4	F2-3 vs. M2-3	262684_s_at	AT1G76030	One of three genes encoding the vacuolar ATP synthase subunit B1. This subunit was shown to interact with the gene product of hexokinase1 (ATHXK1). This interaction, however, is solely restricted to the nucleus. The protein binds to and co-localizes with F-actin, bundles F-actin to form higher-order structure, and stabilizes actin filaments in vitro. The mRNA is cell-to-cell mobile.	469.85	59.20	7.94	3.83E-04	3.51E-03
2656	4	F2-3 vs. M2-3	256627_at	AT3G19970	alpha/beta-Hydrolases superfamily protein;(source:Araport11)	322.22	40.58	7.94	3.59E-05	9.26E-04
2657	4	F2-3 vs. M2-3	256745_at	AT3G29360	Encodes one of four UDP-glucose dehydrogenase UGD) genes. Mutation of this gene in combination with UGD3 leads to swollen plant cell walls and severe developmental defects associated with changes in pectic polysaccharides.	245.96	31.02	7.93	1.06E-04	1.68E-03
2658	4	F2-3 vs. M2-3	253177_s_at	AT4G35150	O-methyltransferase family protein;(source:Araport11)	292.23	37.03	7.89	2.85E-04	3.03E-03
2659	4	F2-3 vs. M2-3	263761_at	AT2G21330	fructose-bisphosphate aldolase 1;(source:Araport11)	653.50	83.39	7.84	1.00E-07	2.17E-05
2660	4	F2-3 vs. M2-3	251860_at	AT3G54660	Encodes glutathione reductase that is most likely localized in the chloroplast.	428.38	55.60	7.70	1.10E-06	1.08E-04
2661	4	F2-3 vs. M2-3	247981_at	AT5G56640	Myo-Inositol Oxygenase gene family	197.13	25.84	7.63	3.60E-06	2.23E-04
2662	4	F2-3 vs. M2-3	251697_at	AT3G56600	phosphatidylinositol 4-kinase gamma-like protein;(source:Araport11)	129.08	16.96	7.61	< 1e-07	< 1e-07
2663	4	F2-3 vs. M2-3	260829_s_at	AT2G30440	Encodes a thylakoidal processing peptidase that removes signal sequences from proteins synthesized in the cytoplasm and transported into the thylakoid lumen. The mRNA is cell-to-cell mobile.	123.46	16.31	7.57	1.54E-04	2.07E-03
2664	4	F2-3 vs. M2-3	254687_at	AT4G13770	Encodes a cytochrome p450 enzyme that catalyzes the initial conversion of aldoximes to thiohydroximates in the synthesis of glucosinolates not derived from tryptophan. Also has a role in auxin homeostasis.	557.46	74.23	7.51	1.35E-04	1.92E-03
2665	4	F2-3 vs. M2-3	258500_at	AT3G02470	Encodes a S-adenosylmethionine decarboxylase involved in polyamine biosynthesis.	410.99	56.05	7.33	6.88E-05	1.29E-03
2666	4	F2-3 vs. M2-3	267590_at	AT2G39700	putative expansin. Naming convention from the Expansin Working Group (Kende et al, 2004. Plant Mol Bio). Involved in the formation of nematode-induced syncytia in roots of Arabidopsis thaliana.	110.77	15.26	7.26	4.00E-07	5.48E-05
2667	4	F2-3 vs. M2-3	261754_at	AT1G76130	alpha-amylase, putative / 1,4-alpha-D-glucan glucanohydrolase, putative, strong similarity to alpha-amylase GI:7532799 from (Malus x domestica);contains Pfam profile PF00128: Alpha amylase, catalytic domain. Predicted to be secreted based on SignalP analysis.	259.11	35.72	7.25	5.70E-06	2.82E-04
2668	4	F2-3 vs. M2-3	262146_at	AT1G52580	RHOMBOID-like protein 5;(source:Araport11)	176.87	24.45	7.23	1.80E-06	1.42E-04
2669	4	F2-3 vs. M2-3	254024_at	AT4G25780	CAP (Cysteine-rich secretory proteins, Antigen 5, and Pathogenesis-related 1 protein) superfamily protein;(source:Araport11)	344.77	48.00	7.18	6.26E-05	1.21E-03
2670	4	F2-3 vs. M2-3	257986_at	AT3G20865	Encodes a putative arabinogalactan-protein (AGP40) that is expressed in pollen.	174.69	24.41	7.16	8.24E-04	5.50E-03
2671	4	F2-3 vs. M2-3	254607_at	AT4G18920	histone acetyltransferase (DUF1264);(source:Araport11)	117.33	16.51	7.11	1.10E-06	1.08E-04
2672	4	F2-3 vs. M2-3	264204_at	AT1G22710	Encodes for a high-affinity transporter essential for phloem loading and long-distance transport. A major sucrose transporter, AtSUC2 can also transport a wide range of physiological and synthetic glucose conjugates with both α- or β-linkage.	568.29	80.20	7.09	3.42E-04	3.28E-03
2673	4	F2-3 vs. M2-3	263867_at	AT2G36830	Encodes a tonoplast intrinsic protein, which functions as water channel. It has also been shown to be able to facilitate the transport of urea and hydrogen peroxide. Highly expressed in vascular tissues of the root, stem, cauline leaves and flowers but not in the apical meristems. The mRNA is cell-to-cell mobile.	538.49	75.97	7.09	2.78E-04	2.99E-03
2674	4	F2-3 vs. M2-3	258645_s_at	AT3G07850	Pectin lyase-like superfamily protein;(source:Araport11)	116.47	16.46	7.08	4.37E-04	3.79E-03

2675	4	F2-3 vs. M2-3	267322_at	AT2G19330	Encodes PIRL6, a member of the Plant Intracellular Ras-group-related LRRs (Leucine rich repeat proteins). PIRLs are a distinct, plant-specific class of intracellular LRRs that likely mediate protein interactions, possibly in the context of signal transduction.	128.21	18.30	7.01	6.54E-05	1.24E-03
2676	4	F2-3 vs. M2-3	252414_at	AT3G47420	Encodes a Pi starvation-responsive protein AtPS3. A member of the phosphate starvation-induced glycerol-3-phosphate permease gene family: AT3G47420(G3Pp1), AT4G25220(G3Pp2), AT1G30560(G3Pp3), AT4G17550(G3Pp4) and AT2G13100(G3Pp5). Its expression is responsive to phosphate (Pi) and not phosphite (Phi) in roots and shoots.	125.76	18.02	6.98	3.14E-05	8.40E-04
2677	4	F2-3 vs. M2-3	254563_at	AT4G19120	S-adenosyl-L-methionine-dependent methyltransferases superfamily protein;(source:Araport11)	263.57	37.90	6.95	1.18E-04	1.77E-03
2678	4	F2-3 vs. M2-3	265002_at	AT1G24400	High-affinity transporter for neutral and acidic amino acids, expressed in tapetum tissue of anthers. Transport of 1-Aminocyclopropane-1-carboxylic acid (ACC).	141.66	20.52	6.90	5.20E-06	2.71E-04
2679	4	F2-3 vs. M2-3	259736_at	AT1G64390	glycosyl hydrolase 9C2;(source:Araport11)	279.67	40.60	6.89	2.74E-04	2.96E-03
2680	4	F2-3 vs. M2-3	246208_at	AT4G36490	SEC14-like 12;(source:Araport11)	87.18	12.64	6.89	2.30E-06	1.62E-04
2681	4	F2-3 vs. M2-3	254123_at	AT4G24640	Encodes AppB protein (AppB1).	79.96	11.64	6.87	2.45E-05	7.01E-04
2682	4	F2-3 vs. M2-3	265405_at	AT2G16750	kinase with adenine nucleotide alpha hydrolases-like domain-containing protein;(source:Araport11)	159.66	23.30	6.85	9.48E-04	5.97E-03
2683	4	F2-3 vs. M2-3	265368_at	AT2G13350	Calcium-dependent lipid-binding (CaLB domain) family protein;(source:Araport11)	160.80	23.54	6.83	2.39E-05	6.95E-04
2684	4	F2-3 vs. M2-3	261015_at	AT1G26480	14-3-3 protein GF14iota (grf12)	133.07	19.56	6.80	1.71E-04	2.21E-03
2685	4	F2-3 vs. M2-3	261559_at	AT1G01780	Encodes a member of the Arabidopsis LIM proteins: a family of actin bundlers with distinct expression patterns. WLIM1, WLIM2a, and WLIM2b are widely expressed, whereas PLIM2a, PLIM2b, and PLIM2c are predominantly expressed in pollen. Regulates actin cytoskeleton organization. The mRNA is cell-to-cell mobile.	115.32	17.07	6.75	1.88E-05	6.16E-04
2686	4	F2-3 vs. M2-3	246878_at	AT5G26060	Plant self-incompatibility protein S1 family;(source:Araport11)	181.18	26.83	6.75	7.20E-06	3.12E-04
2687	4	F2-3 vs. M2-3	253619_at	AT4G30460	glycine-rich protein;(source:Araport11)	199.31	29.68	6.71	7.18E-05	1.33E-03
2688	4	F2-3 vs. M2-3	253042_at	AT4G37550	Acetamidase/Formamidase family protein;(source:Araport11)	110.43	16.50	6.69	1.64E-04	2.16E-03
2689	4	F2-3 vs. M2-3	262885_at	AT1G64740	alpha-tubulin expressed primarily in stamens and mature pollen	427.75	65.87	6.49	4.90E-06	2.60E-04
2690	4	F2-3 vs. M2-3	265115_at	AT1G62450	Immunoglobulin E-set superfamily protein;(source:Araport11)	96.12	14.96	6.43	2.87E-05	7.94E-04
2691	4	F2-3 vs. M2-3	251898_at	AT3G54340	Floral homeotic gene encoding a MADS domain protein homologous to SRF transcription factors. Specifies petal and stamen identities. Associates with PISTILLATA.	340.64	52.97	6.43	1.16E-04	1.76E-03
2692	4	F2-3 vs. M2-3	265091_s_a	AT1G03495	HXXXD-type acyl-transferase family protein;(source:Araport11)	83.95	13.10	6.41	9.70E-06	3.86E-04
2693	4	F2-3 vs. M2-3	253344_at	AT4G33550	Bifunctional inhibitor/lipid-transfer protein/seed storage 2S albumin superfamily protein;(source:Araport11)	128.41	20.19	6.36	4.27E-04	3.72E-03
2694	4	F2-3 vs. M2-3	261933_at	AT1G22410	Class-II DAHP synthetase family protein;(source:Araport11)	592.86	94.06	6.30	5.20E-04	4.18E-03
2695	4	F2-3 vs. M2-3	247882_at	AT5G57785	hypothetical protein;(source:Araport11)	172.38	27.39	6.29	3.43E-04	3.28E-03
2696	4	F2-3 vs. M2-3	248335_at	AT5G52450	MATE efflux family protein;(source:Araport11)	242.51	38.76	6.26	1.58E-05	5.45E-04
2697	4	F2-3 vs. M2-3	267298_at	AT2G23760	Encodes a member of the BEL family of homeodomain proteins. Plants doubly mutant for saw1/saw2 (blh2/blh4) have serrated leaves. BP is expressed in the serrated leaves, therefore saw2 and saw1 may act redundantly to repress BP in leaves. Regulates together with BLH2 demethylesterification of homogalacturonan in seed mucilage.	82.38	13.23	6.23	1.19E-05	4.43E-04
2698	4	F2-3 vs. M2-3	261881_at	AT1G80760	Encodes a protein with boron transporter activity. It helps to preferentially direct boron to young developing tissues in the shoot, such as immature leaves, under low boron conditions. This boron channel appears to be impermeable to water, unlike the closely related NIP5;1 boron transporter. This protein also allows the transport of glycerol, urea, and formimide but not larger uncharged solutes such as arabitol and sucrose when it is expressed heterologously.	165.55	26.64	6.22	5.68E-05	1.19E-03
2699	4	F2-3 vs. M2-3	249048_at	AT5G44300	Dormancy/auxin associated family protein;(source:Araport11)	120.44	19.38	6.22	2.33E-05	6.86E-04
2700	4	F2-3 vs. M2-3	251591_at	AT3G57680	Peptidase S41 family protein;(source:Araport11)	272.81	43.92	6.21	3.90E-06	2.31E-04
2701	4	F2-3 vs. M2-3	248580_at	AT5G49890	member of Anion channel protein family	317.19	51.25	6.19	4.60E-06	2.55E-04
2702	4	F2-3 vs. M2-3	255008_at	AT4G10060	Glucosylceramidase that preferentially hydrolyzes long acyl chain glucosylceramides.	150.03	24.34	6.16	5.60E-06	2.82E-04

2703	4	F2-3 vs. M2-3	263676_at	AT1G09340	Encodes CHLOROPLAST RNA BINDING (CRB), a putative RNA-binding protein. CRB is important for the proper functioning of the chloroplast. Mutations in CRB also affects the circadian system, altering the expression of both oscillator and output genes. The mRNA is cell-to-cell mobile.	443.63	72.18	6.15	1.29E-05	4.70E-04
2704	4	F2-3 vs. M2-3	258979_at	AT3G09440	Heat shock protein 70 (Hsp 70) family protein;(source:Araport11)	823.92	134.89	6.11	3.15E-05	8.40E-04
2705	4	F2-3 vs. M2-3	258369_at	AT3G14310	encodes a pectin methylesterase, targeted by a cellulose binding protein (CBP) from the parasitic nematode Heterodera schachtii during parasitism.	134.38	21.99	6.11	3.75E-05	9.26E-04
2706	4	F2-3 vs. M2-3	251405_at	AT3G60330	H[+]-ATPase 7;(source:Araport11)	136.02	22.43	6.06	1.82E-05	6.12E-04
2707	4	F2-3 vs. M2-3	261713_at	AT1G32640	Encodes a MYC-related transcriptional activator with a typical DNA binding domain of a basic helix-loop-helix leucine zipper motif. Binds to an extended G-Box promoter motif and interacts with Jasmonate ZIM-domain proteins. MYC2 interacts with EIN3 and EIL1 to repress hook curvature and resistance to Botrytis cinera.Its transcription is induced by dehydration stress, ABA treatment and blue light via CRY1. Negative regulator of blue light-mediated photomorphogenic growth and blue and far-red-light-regulated gene expression. Positive regulator of lateral root formation. Regulates diverse JA-dependent functions. Negatively regulates Trp metabolism and biosynthesis of Trp-derived secondary metabolites. Positively regulates flavonoid biosynthesis, resistance to insects, and response to oxidative stress. Regulates other transcription factors, and negatively regulates its own expression. For example it binds to and regulates the expression of NST1. Its stability is modulated by PUB10 through polyubiquitination.	217.07	35.98	6.03	1.49E-04	2.02E-03
2708	4	F2-3 vs. M2-3	260241_at	AT1G63710	Encodes a member of the CYP86A subfamily of cytochrome p450 genes. Expressed at highest level in mature stems and flowers.	72.47	12.15	5.96	6.91E-05	1.29E-03
2709	4	F2-3 vs. M2-3	251770_at	AT3G55970	One of 4 paralogs encoding a 2-oxoglutarate/Fe(II)-dependent oxygenases that hydroxylates JA to 12-OH-JA.	101.56	17.03	5.96	1.12E-05	4.26E-04
2710	4	F2-3 vs. M2-3	248199_at	AT5G54170	Polyketide cyclase/dehydrase and lipid transport superfamily protein;(source:Araport11)	129.98	21.80	5.96	4.40E-06	2.49E-04
2711	4	F2-3 vs. M2-3	263533_at	AT2G24820	translocon at the inner envelope membrane of chloroplasts 55-II;(source:Araport11)	472.46	80.23	5.89	1.40E-06	1.22E-04
2712	4	F2-3 vs. M2-3	262291_at	AT1G70790	C2-domain ABA-related (CAR) protein, involved in the recruitment of ABA receptors to the plasma membrane to facilitate ABA signaling. Its stability and dynamic localization is regulated by LOT1.	191.42	33.02	5.80	8.40E-06	3.50E-04
2713	4	F2-3 vs. M2-3	253276_at	AT4G34050	Methyltransferase in the lignin biosynthetic pathway.	923.89	159.19	5.80	2.30E-06	1.62E-04
2714	4	F2-3 vs. M2-3	260267_at	AT1G68530	Encodes KCS6, a member of the 3-ketoacyl-CoA synthase family involved in the biosynthesis of VLCFA (very long chain fatty acids).	119.36	20.80	5.74	< 1e-07	< 1e-07
2715	4	F2-3 vs. M2-3	263838_at	AT2G36880	methionine adenosyltransferase 3;(source:Araport11)	1834.13	319.58	5.74	2.37E-05	6.93E-04
2716	4	F2-3 vs. M2-3	261149_s_a	AT1G19550	Glutathione S-transferase family protein;(source:Araport11)	1932.50	337.90	5.72	< 1e-07	< 1e-07
2717	4	F2-3 vs. M2-3	250793_at	AT5G05600	Encodes a protein with similarity to flavonol synthases that is involved in the detoxifcation polycyclic aromatic hydrocarbons.One of 4 paralogs encoding a 2-oxoglutarate/Fe(II)-dependent oxygenases that hydroxylates JA to 12-OH-JA.	539.28	94.36	5.71	4.95E-04	4.10E-03
2718	4	F2-3 vs. M2-3	265444_s_a t	AT2G37180	a member of the plasma membrane intrinsic protein PIP2. functions as aquaporin and is involved in desiccation.	1863.44	327.06	5.70	3.68E-04	3.43E-03
2719	4	F2-3 vs. M2-3	267255_at	AT2G22950	Encodes a putative auto-regulated Ca2+-ATPase located in the plasma membrane involved in transporting Ca2+ outside developing pollen grains. This activity is important to support normal pollen development, particularly the progression to uninucleated microspores to bicellular pollen grains.	110.21	19.52	5.65	8.00E-06	3.39E-04
2720	4	F2-3 vs. M2-3	259226_at	AT3G07700	ABC1K7 is a member of an atypical protein kinase family that is induced by salt stress. Loss of function mutations affect the metabolic profile of chloroplast lipids. It appears to function along with ABC1K8 in mediating lipid membrane changes in response to stress.	296.20	52.43	5.65	3.48E-04	3.31E-03
2721	4	F2-3 vs. M2-3	250610_at	AT5G07550	member of Oleosin-like protein family	78.96	14.04	5.62	1.29E-04	1.88E-03
2722	4	F2-3 vs. M2-3	249237_at	AT5G42050	Stress responsive asparagine-rich protein. Binds to PevD (Verticillium dahliae) fungal effector protein. NRP interacts with CRY2, leading to increased cytoplasmic accumulation of CRY2 in a blue light-independent manner (PMID:28633330).NRP also binds FyPP3 and recruits it to endosomes and thus targets it for degradation.	278.59	49.56	5.62	3.02E-05	8.23E-04
2723	4	F2-3 vs. M2-3	259068_at	AT3G07560	Encodes peroxin 13 (PEX13) involved in protein transport into peroxisomes, for example, peroxisomal import of nitric oxide synthase.	333.44	59.80	5.58	1.28E-04	1.88E-03
2724	4	F2-3 vs. M2-3	264835_at	AT1G03550	Secretory carrier membrane protein (SCAMP) family protein;(source:Araport11)	68.36	12.29	5.56	4.79E-05	1.08E-03

2725	4	F2-3 vs. M2-3	265923_at	AT2G18470	Proline-rich extensin-like receptor kinase 4. Functions at an early stage of ABA signalling inhibiting primary root cell elongation by perturbing Ca2+ homeostasis.	114.45	20.63	5.55	1.87E-05	6.16E-04
2726	4	F2-3 vs. M2-3	262432_at	AT1G47530	MATE efflux family protein;(source:Araport11)	203.26	36.82	5.52	1.20E-05	4.43E-04
2727	4	F2-3 vs. M2-3	262796_at	AT1G20850	Cysteine peptidase. Enzyme activity detected in leaf.	420.52	77.10	5.45	3.74E-04	3.46E-03
2728	4	F2-3 vs. M2-3	264112_at	AT2G13680	Responsible for the synthesis of callose deposited at the primary cell wall of meiocytes, tetrads and microspores. Required for exine formation during microgametogenesis and for pollen viability. Highest expression in meiocytes, tetrads, microspores and mature pollen.	84.93	15.58	5.45	7.19E-04	5.05E-03
2729	4	F2-3 vs. M2-3	266716_at	AT2G46820	Encodes the P subunit of Photosystem I. About 25% of the TMP14 pool appeared to be phosphorylated, and this ratio is not affected by light. Contains seven phosphorylation sites on threonine residue and chloroplast targeting signal. Located in the proximity of PSI-L, -H and -O subunits. Forms oligomers with other members of CURT1 family to modulate grana structure.	491.87	90.59	5.43	4.10E-06	2.37E-04
2730	4	F2-3 vs. M2-3	253203_at	AT4G34710	Encodes a arginine decarboxylase (ADC), a rate-limiting enzyme that catalyzes the first step of polyamine (PA) biosynthesis via ADC pathway in Arabidopsis thaliana. Arabidopsis genome has two ADC paralogs, ADC1 and ADC2. ADC2 is stress-inducible (osmotic stress). Double mutant analysis showed that ADC genes are essential for the production of PA, and are required for normal seed development. Overexpression causes phenotypes similar to GA-deficient plants and these plants show reduced levels of GA due to lower expression levels of AtGA20ox1, AtGA3ox3 and AtGA3ox1.	568.70	104.82	5.43	2.14E-04	2.52E-03
2731	4	F2-3 vs. M2-3	266673_at	AT2G29630	Encodes a protein involved in thiamin biosynthesis. The protein is an iron-sulfur cluster protein predicted to catalyze the conversion of 5-aminoimidazole ribonucleotide (AIR) to hydroxymethylpyrimidine (HMP) or hydroxymethylpyrimidine phosphate (HMP-P). A severe reduction of THIC levels in plants decreases vitamin B1 (thiamin diphosphate (TPP)) levels and also leads to changes in the levels of numerous other metabolites since so many primary metabolic enzymes require a TPP co-factor. thiC mutants are chlorotic and arrest in their development at the cotyledon stage. A N-terminal targeting sequence directs the THIC protein to the chloroplast stroma. A conserved TPP-binding site is located in the 3' UTR of the At2g29630.2 gene model, and is predicted to function as a riboswitch. The riboswitch controls the formation of transcripts with alternative 3' UTR lengths, which affect mRNA accumulation and protein production. THIC transcripts are observed in seedlings 5 or more days after germination, and light promotes the expression of this gene. Recessive mutant isolated by Redei. Leaves but not cotyledons white, lethal; restored to normal by thiamine or 2,5-dimethyl-4-aminopyrimidine.	290.74	53.67	5.42	2.30E-06	1.62E-04
2732	4	F2-3 vs. M2-3	255877_at	AT2G40460	Major facilitator superfamily protein;(source:Araport11)	63.93	12.05	5.31	1.96E-05	6.30E-04
2733	4	F2-3 vs. M2-3	253172_at	AT4G35060	Heavy metal transport/detoxification superfamily protein;(source:Araport11)	131.29	24.91	5.27	5.61E-04	4.37E-03
2734	4	F2-3 vs. M2-3	248073_at	AT5G55720	Pectin lyase-like superfamily protein;(source:Araport11)	59.34	11.30	5.25	2.43E-05	7.01E-04
2735	4	F2-3 vs. M2-3	265984_at	AT2G24210	terpene synthase 10;(source:Araport11)	67.78	12.98	5.22	5.93E-05	1.20E-03
2736	4	F2-3 vs. M2-3	259429_at	AT1G01600	Encodes a member of the CYP86A subfamily of cytochrome p450 genes. Expressed significantly at highest level in mature stems and flowers.	83.99	16.17	5.19	2.31E-05	6.86E-04
2737	4	F2-3 vs. M2-3	258685_at	AT3G07830	Pectin lyase-like superfamily protein;(source:Araport11)	241.75	46.80	5.17	2.04E-04	2.45E-03
2738	4	F2-3 vs. M2-3	267425_at	AT2G34810	FAD-binding Berberine family protein;(source:Araport11)	91.55	17.75	5.16	1.76E-04	2.25E-03
2739	4	F2-3 vs. M2-3	257641_s_at	AT3G25760	encodes allene oxide cyclase. One of four genes in Arabidopsis that encode this enzyme, which catalyzes an essential step in jasmonic acid biosynthesis. Gene expression is induced during senescence, a process that involves jasmonic acid signalling pathway. The mRNA is cell-to-cell mobile.	2093.61	405.88	5.16	4.80E-06	2.60E-04
2740	4	F2-3 vs. M2-3	256754_at	AT3G25690	actin binding protein required for normal chloroplast positioningThe mRNA is cell-to-cell mobile.	171.65	33.36	5.15	5.19E-05	1.13E-03
2741	4	F2-3 vs. M2-3	260221_at	AT1G74670	Gibberellin-regulated family protein;(source:Araport11)	52.25	10.16	5.14	4.06E-05	9.74E-04
2742	4	F2-3 vs. M2-3	263718_at	AT2G13570	nuclear factor Y, subunit B7;(source:Araport11)	62.41	12.16	5.13	2.22E-05	6.84E-04
2743	4	F2-3 vs. M2-3	248719_at	AT5G47910	NADPH/respiratory burst oxidase protein D (RbohD).Interacts with AtrbohF gene to fine tune the spatial control of ROI production and hypersensitive response to cell in and around infection site. The mRNA is cell-to-cell mobile.	153.69	30.11	5.10	8.90E-06	3.68E-04

2744	4	F2-3 vs. M2-3	245306_at	AT4G14690	Encodes an early light-induced protein. ELIPs are thought not to be directly involved in the synthesis and assembly of specific photosynthetic complexes, but rather affect the biogenesis of all chlorophyll-binding complexes. A study (PMID 17553115) has shown that the chlorophyll synthesis pathway was downregulated as a result of constitutive ELIP2 expression, leading to decreased chlorophyll availability for the assembly of pigment-binding proteins for photosynthesis.	410.65	81.01	5.07	5.27E-05	1.13E-03
2745	4	F2-3 vs. M2-3	256075_at	AT1G18150	Encodes mitogen-activated protein kinase 8 (MPK8). MPK8 connects protein phosphorylation, Ca2+, and ROS in the wound-signaling pathway.	141.09	28.51	4.95	6.54E-05	1.24E-03
2746	4	F2-3 vs. M2-3	245636_at	AT1G25240	ENTH/VHS/GAT family protein;(source:Araport11)	72.84	14.77	4.93	1.38E-04	1.95E-03
2747	4	F2-3 vs. M2-3	258415_at	AT3G17390	S-adenosylmethionine synthetase	2974.07	610.00	4.88	6.60E-06	2.99E-04
2748	4	F2-3 vs. M2-3	248377_at	AT5G51720	Encodes a protein with biochemical, structural, and biophysical characteristics of a NEET protein. It plays a key role in plant development, senescence, reactive oxygen homeostasis, and Fe metabolism.	395.40	81.32	4.86	6.24E-05	1.21E-03
2749	4	F2-3 vs. M2-3	253387_at	AT4G33010	glycine decarboxylase P-protein 1;(source:Araport11)	597.91	123.63	4.84	8.69E-04	5.68E-03
2750	4	F2-3 vs. M2-3	255580_at	AT4G01470	Encodes AtTIP1;3, functions as water and urea channels in pollen.	87.64	18.38	4.77	4.65E-05	1.07E-03
2751	4	F2-3 vs. M2-3	267639_at	AT2G42200	Encodes a putative transcriptional regulator that is involved in the vegetative to reproductive phase transition. Expression is regulated by MIR156b. SPL activity nonautonomously inhibits initiation of new leaves at the shoot apical meristem.	108.70	22.85	4.76	6.07E-05	1.21E-03
2752	4	F2-3 vs. M2-3	266592_at	AT2G46210	Fatty acid/sphingolipid desaturase;(source:Araport11)	154.14	32.48	4.74	2.12E-04	2.52E-03
2753	4	F2-3 vs. M2-3	249876_at	AT5G23060	Encodes a chloroplast-localized protein that modulates cytoplasmic Ca2+ concentration and is crucial for proper stomatal regulation in response to elevations of external Ca2+. Phosphorylation of this protein is dependent on calcium.	109.71	23.27	4.71	3.29E-05	8.70E-04
2754	4	F2-3 vs. M2-3	259615_at	AT1G47980	desiccation-like protein;(source:Araport11)	172.63	36.98	4.67	1.96E-04	2.38E-03
2755	4	F2-3 vs. M2-3	256861_at	AT3G23920	Encodes a chloroplast beta-amylase. Is necessary for leaf starch breakdown in the absence of BAM3.Activity of BAM1 increases 4 days after osmotic stress. BAM1 has a higher temperature optimum than BAM3 (PMID:25293962).	775.47	165.97	4.67	6.76E-04	4.88E-03
2756	4	F2-3 vs. M2-3	257106_at	AT3G29060	EXS (ERD1/XPR1/SYG1) family protein;(source:Araport11)	90.46	19.50	4.64	3.54E-05	9.20E-04
2757	4	F2-3 vs. M2-3	258035_at	AT3G21180	one of the type IIB calcium pump isoforms. encodes an autoinhibited Ca(2+)-ATPase that contains an N-terminal calmodulin binding autoinhibitory domain.	60.75	13.27	4.58	1.06E-04	1.68E-03
2758	4	F2-3 vs. M2-3	254050_s_a	AT4G25670	stress response NST1-like protein;(source:Araport11)	115.17	25.17	4.58	1.30E-04	1.88E-03
2759	4	F2-3 vs. M2-3	248153_at	AT5G54250	member of Cyclic nucleotide gated channel family, downstream component of the signaling pathways leading to HR resistance. mutant plants exhibit gene-for-gene disease resistance against avirulent Pseudomonas syringae despite the near-complete absence of the hypersensitive response (HR). Salicylic acid accumulation in dnd2 mutants is completely PAD4-independent.	63.76	13.92	4.58	7.96E-04	5.37E-03
2760	4	F2-3 vs. M2-3	262905_at	AT1G59730	Thioredoxin H-type 7 , oxidoreductase located in cytosol and ER. Interacts with GPT1.	71.76	15.80	4.54	2.10E-04	2.49E-03
2761	4	F2-3 vs. M2-3	258151_at	AT3G18080	B-S glucosidase 44;(source:Araport11)	59.84	13.20	4.53	4.10E-06	2.37E-04
2762	4	F2-3 vs. M2-3	260385_at	AT1G74090	encodes a desulfoglucosinolate sulfotransferase, involved in the final step of glucosinolate core structure biosynthesis. Has a broad-substrate specificity with preference with methionine-derived desulfoglucosinolates.	129.03	28.53	4.52	8.00E-07	9.26E-05
2763	4	F2-3 vs. M2-3	267168_at	AT2G37770	Encodes an NADPH-dependent aldo-keto reductase that can act on a wide variety of substrates in vitro including saturated and unsaturated aldehydes, steroids, and sugars. GFP-tagged AKR4C9 localizes to the chloroplast where it may play a role in detoxifying reactive carbonyl compounds that threaten to impair the photosynthetic process. Transcript levels for this gene are up-regulated in response to cold, salt, and drought stress.	59.97	13.29	4.51	1.24E-05	4.55E-04
2764	4	F2-3 vs. M2-3	245060_at	AT2G39770	Encodes a GDP-mannose pyrophosphorylase/ mannose-1-pyrophosphatase. This enzyme provides GDP-mannose, which is used for cell wall carbohydrate biosynthesis and protein glycosylation as well as for ascorbate (vitamin C) biosynthesis. Mutations in this gene confer hypersensitivity to NH4+.	168.92	37.47	4.51	7.59E-04	5.21E-03
2765	4	F2-3 vs. M2-3	251109_at	AT5G01600	Encodes a ferretin protein that is targeted to the chloroplast. Member of a Ferritin gene family. Gene expression is induced in response to iron overload and by nitric oxide. Expression of the gene is downregulated in the presence of paraquat, an inducer of photoxidative stress.	139.95	31.00	4.51	5.08E-04	4.13E-03
2766	4	F2-3 vs. M2-3	259044_at	AT3G03430	Calcium-binding EF-hand family protein;(source:Araport11)	41.21	9.15	4.50	4.99E-04	4.10E-03

2767	4	F2-3 vs. M2-3	256524_at	AT1G66200	encodes a cytosolic glutamate synthetase, this enzyme has low affinity with substrate ammonium	171.66	38.26	4.49	3.46E-04	3.30E-03
2768	4	F2-3 vs. M2-3	265022_at	AT1G24520	Male fertility gene acting on tapetum and microspore	232.60	51.86	4.48	9.10E-06	3.70E-04
2769	4	F2-3 vs. M2-3	266708_at	AT2G03200	Atypical aspartic protease which modulates lateral root development.	53.03	12.03	4.41	1.09E-04	1.70E-03
2770	4	F2-3 vs. M2-3	260309_at	AT1G70580	Encodes a protein with glyoxylate aminotransferase activity. It can act on a number of different small substrates and amino acids in vitro.	387.10	88.11	4.39	3.78E-04	3.48E-03
2771	4	F2-3 vs. M2-3	247161_at	AT5G65810	Encodes CGR3 (cotton Golgi-related 3). CGR3 and a close homologue CGR2 have overlapping roles in pectin methylesterification and plant growth.	373.72	85.08	4.39	6.90E-06	3.02E-04
2772	4	F2-3 vs. M2-3	246511_at	AT5G15490	Encodes one of four UDP-glucose dehydrogenase UGD) genes. Mutation of this gene in combination with UGD2 leads to swollen plant cell walls and severe developmental defects associated with changes in pectic polysaccharides.	297.30	67.88	4.38	5.06E-04	4.12E-03
2773	4	F2-3 vs. M2-3	265884_at	AT2G42320	nucleolar protein gar2-like protein;(source:Araport11)	280.30	64.16	4.37	2.40E-06	1.67E-04
2774	4	F2-3 vs. M2-3	250608_at	AT5G07420	Pectin lyase-like superfamily protein;(source:Araport11)	47.42	10.95	4.33	1.40E-04	1.95E-03
2775	4	F2-3 vs. M2-3	245806_at	AT1G45474	Encodes a component of the light harvesting complex of photosystem I.	203.06	47.01	4.32	3.70E-06	2.27E-04
2776	4	F2-3 vs. M2-3	263379_at	AT2G40140	zinc finger (CCCH-type) family protein;(source:Araport11)	220.01	50.96	4.32	2.97E-04	3.05E-03
2777	4	F2-3 vs. M2-3	AFFX-r2-At-GAPDH-5_s_at	AT3G04120	encodes cytosolic GADPH (C subunit) involved in the glycolytic pathway but also interacts with H2O2 potentially placing it in a signalling cascade induced by ROS. The mRNA is cell-to-cell mobile.	979.30	227.30	4.31	1.39E-04	1.95E-03
2778	4	F2-3 vs. M2-3	264146_at	AT1G02205	Expression of the CER1 gene associated with production of stem epicuticular wax and pollen fertility. Biochemical studies showed that cer1 mutants are blocked in the conversion of stem wax C30 aldehydes to C29 alkanes, and they also lack the secondary alcohols and ketones. These suggested the CER1 protein is an aldehyde decarbonylase, but the exact molecular function of this protein remains to be determined.	163.73	37.96	4.31	1.71E-05	5.78E-04
2779	4	F2-3 vs. M2-3	256017_at	AT1G19180	JAZ1 is a nuclear-localized protein involved in jasmonate signaling. JAZ1 transcript levels rise in response to a jasmonate stimulus. JAZ1 can interact with the COI1 F-box subunit of an SCF E3 ubiquitin ligase in a yeast-two-hybrid assay only in the presence of jasmonate-isoleucine (JA-ILE) or coronatine. Application of jasmonate methyl ester to Arabidopsis roots reduces the levels of a JAZ1:GUS fusion protein, presumably by stimulating ubiquitin-proteasome-mediated degradation. The Jas domain appears to be important for JAZ1-COI1 interactions in the presence of coronatine. Two positive residues (R205 and R206) in the Jas domain shown to be important for coronatine -dependent COI1 binding are not required for binding AtMYC2. The mRNA is cell-to-cell mobile.	311.23	72.35	4.30	1.73E-04	2.23E-03
2780	4	F2-3 vs. M2-3	265475_at	AT2G15620	Involved in the second step of nitrate assimilation. Its expression is induced by nitrate. The mRNA is cell-to-cell mobile.	153.29	35.86	4.28	1.75E-04	2.23E-03
2781	4	F2-3 vs. M2-3	259848_at	AT1G72180	Encodes a leucine-rich repeat receptor kinase that functions as a receptor for CEP1 peptide. Mediates nitrate uptake signaling.	498.96	117.69	4.24	2.95E-04	3.05E-03
2782	4	F2-3 vs. M2-3	245253_at	AT4G15440	Encodes a hydroperoxide lyase. Also a member of the CYP74B cytochrome p450 family. In the ecotype Columbia (Col) the gene contains a 10-nucleotide deletion in its first exon that causes it to code for a truncated protein that results in a non-functional hydroperoxide lyase.	94.63	22.32	4.24	3.40E-04	3.28E-03
2783	4	F2-3 vs. M2-3	254762_at	AT4G13230	Late embryogenesis abundant protein (LEA) family protein;(source:Araport11)	80.21	18.94	4.23	2.16E-04	2.54E-03
2784	4	F2-3 vs. M2-3	256015_at	AT1G19150	PSI type II chlorophyll a/b-binding protein (Lhca2*1) mRNA, The mRNA is cell-to-cell mobile.	247.25	58.79	4.21	3.89E-05	9.38E-04
2785	4	F2-3 vs. M2-3	262344_at	AT1G64060	Interacts with AtrbohD gene to fine tune the spatial control of ROI production and hypersensitive response to cell in and around infection site.	111.00	26.44	4.20	8.97E-04	5.79E-03
2786	4	F2-3 vs. M2-3	246308_at	AT3G51820	Encodes a protein with chlorophyll synthase activity. This enzyme has been shown to perform the esterification of chlorophyllide (a and b), the last step of chlorophyll biosynthesis. Although it can use either geranylgeranyl pyrophosphate (GGPP) or phytyl pyrophosphate (PhyPP) as substrates, the esterification reaction was faster with GGPP than with PhyPP.	810.47	193.33	4.19	3.41E-04	3.28E-03
2787	4	F2-3 vs. M2-3	265441_at	AT2G20870	cell wall protein precursor;(source:Araport11)	96.63	23.30	4.15	7.36E-05	1.34E-03
2788	4	F2-3 vs. M2-3	254551_at	AT4G19840	encodes a phloem lectin, similar to phloem lectin in cucumber and celery. Gene is expressed in the phloem, predominantly in the companion cells. The mRNA is cell-to-cell mobile.	226.95	54.70	4.15	5.38E-05	1.14E-03
2789	4	F2-3 vs. M2-3	255552_at	AT4G01850	S-adenosylmethionine synthetase 2;(source:Araport11)	789.50	192.16	4.11	7.32E-04	5.10E-03

2790	4	F2-3 vs. M2-3	253191_at	AT4G35350	tracheary element vacuolar protein	142.92	34.74	4.11	3.72E-04	3.45E-03
2791	4	F2-3 vs. M2-3	245057_at	AT2G26490	JGB contains seven WD40 repeats and is highly conserved in flowering plants. Overexpression inhibits pollen germination. suggesting JGB is a negative regulator of pollen germination	87.01	21.33	4.08	1.34E-05	4.81E-04
2792	4	F2-3 vs. M2-3	253833_at	AT4G27790	Calcium-binding EF hand family protein;(source:Araport11)	64.50	15.83	4.07	3.30E-04	3.22E-03
2793	4	F2-3 vs. M2-3	249536_at	AT5G38760	Late embryogenesis abundant protein (LEA) family protein;(source:Araport11)	44.98	11.06	4.07	4.74E-05	1.07E-03
2794	4	F2-3 vs. M2-3	253954_at	AT4G26970	Encodes an aconitase that can catalyze the conversion of citrate to isocitrate through a cis-aconitate intermediate, indicating that it may participate in the TCA cycle and other primary metabolic pathways. The protein is believed to accumulate in the mitochondria and the cytosol. It affects CSD2 (At2g28190 - a superoxide dismutase) transcript levels and may play a role in the response to oxidative stress. One member of the family (ACO1 - At35830) was shown to specifically bind to the 5' UTR of CSD2 in vitro. The mRNA is cell-to-cell mobile.	1368.90	337.27	4.06	7.05E-04	4.98E-03
2795	4	F2-3 vs. M2-3	247884_at	AT5G57800	encodes a transmembrane protein with similarity to the sterol desaturase family at the N-terminus and to the short-chain dehydrogenase/reductase family at the C-terminus. Mutant analyses indicate this protein is involved in cuticle membrane and wax biosynthesis. The mRNA is cell-to-cell mobile.	76.35	18.82	4.06	6.99E-04	4.97E-03
2796	4	F2-3 vs. M2-3	265587_at	AT2G19980	CAP (Cysteine-rich secretory proteins, Antigen 5, and Pathogenesis-related 1 protein) superfamily protein;(source:Araport11)	48.87	12.07	4.05	1.54E-04	2.07E-03
2797	4	F2-3 vs. M2-3	260985_at	AT1G53500	encodes a putative NDP-L-rhamnose synthase, an enzyme required for the synthesis of the pectin rhamnogalacturonan I, the major component of Arabidopsis mucilage. Gene is involved in seed coat mucilage cell development. Mutant analyses suggest that MUM4 is required for complete mucilage synthesis, cytoplasmic rearrangement and seed coat development.	159.28	39.44	4.04	1.25E-04	1.84E-03
2798	4	F2-3 vs. M2-3	260805_at	AT1G78320	Encodes glutathione transferase belonging to the tau class of GSTs. Naming convention according to Wagner et al. (2002).	81.10	20.09	4.04	3.29E-04	3.22E-03
2799	4	F2-3 vs. M2-3	265337_at	AT2G18390	Encodes a member of ARF-like GTPase family. A thaliana has 21 members, in two subfamilies, ARF and ARF-like (ARL) GTPases. Mutant has abnormal mitosis and cell cycle control during seed development.	411.48	101.75	4.04	1.07E-04	1.68E-03
2800	4	F2-3 vs. M2-3	263954_at	AT2G35840	Sucrose-6F-phosphate phosphohydrolase family protein;(source:Araport11)	865.81	214.63	4.03	1.50E-06	1.24E-04
2801	4	F2-3 vs. M2-3	246075_at	AT5G20410	Encodes a type B monogalactosyldiacylglycerol (MGDG) synthase. Strongly induced by phosphate deprivation, and in non-photosynthetic tissues. Does not contribute to galactolipid synthesis under Pi-sufficient conditions but does under Pi starvation.	119.37	29.63	4.03	1.59E-04	2.12E-03
2802	4	F2-3 vs. M2-3	248967_at	AT5G45350	proline-rich family protein;(source:Araport11)	607.93	151.04	4.02	2.74E-05	7.67E-04
2803	4	F2-3 vs. M2-3	251091_at	AT5G01410	Encodes a protein predicted to function in tandem with PDX2 to form glutamine amidotransferase complex with involved in vitamin B6 biosynthesis.	343.04	85.76	4.00	7.56E-05	1.36E-03
2804	4	F2-3 vs. M2-3	265338_at	AT2G18400	ribosomal protein L6 family protein;(source:Araport11)	1713.93	430.42	3.98	1.00E-05	3.95E-04
2805	4	F2-3 vs. M2-3	247163_at	AT5G65685	UDP-Glycosyltransferase superfamily protein;(source:Araport11)	56.23	14.13	3.98	3.77E-05	9.26E-04
2806	4	F2-3 vs. M2-3	262073_at	AT1G59640	A basic helix-loop-helix encoding gene (BIGPETAL, BPE) involved in the control of petal size. BPE is expressed via two mRNAs derived from an alternative splicing event. The BPEub (AT1G59640.1)transcript is expressed ubiquitously, whereas the BPEp (AT1G59640.2) transcript is preferentially expressed in petals. Plants that lack the petal-expressed variant BPEp have larger petals as a result of increased cell size. BPEp is positively regulated downstream of APETALA3, PISTILLATA, APETALA1 and PISTILLATA3 and is negatively regulated downstream of AGAMOUS.	81.44	20.51	3.97	1.02E-05	3.99E-04
2807	4	F2-3 vs. M2-3	253048_at	AT4G37560	Acetamidase/Formamidase family protein;(source:Araport11)	42.69	10.79	3.96	1.30E-04	1.88E-03
2808	4	F2-3 vs. M2-3	267367_at	AT2G44210	carboxyl-terminal peptidase (DUF239);(source:Araport11)	236.26	60.54	3.90	5.54E-04	4.36E-03
2809	4	F2-3 vs. M2-3	245333_at	AT4G14615	sporulation-specific protein;(source:Araport11)	498.50	128.10	3.89	1.86E-04	2.31E-03
2810	4	F2-3 vs. M2-3	252973_s_a	AT4G38740	Encodes cytosolic cyclophilin ROC1.	788.19	202.75	3.89	9.66E-05	1.57E-03
2811	4	F2-3 vs. M2-3	250547_at	AT5G08100	Encodes an asparaginase that catalyzes the degradation of L-asparagine to L-aspartic acid and ammonia.	121.32	31.20	3.89	6.72E-05	1.27E-03
2812	4	F2-3 vs. M2-3	252372_at	AT3G48000	Encodes a putative (NAD+) aldehyde dehydrogenase.	196.61	50.99	3.86	5.64E-04	4.37E-03
2813	4	F2-3 vs. M2-3	262680_at	AT1G75880	SGNH hydrolase-type esterase superfamily protein;(source:Araport11)	45.44	11.80	3.85	6.17E-05	1.21E-03
2814	4	F2-3 vs. M2-3	263477_at	AT2G31790	UDP-Glycosyltransferase superfamily protein;(source:Araport11)	275.54	71.48	3.85	3.67E-05	9.26E-04
2815	4	F2-3 vs. M2-3	251433_at	AT3G59830	Integrin-linked protein kinase family;(source:Araport11)	48.98	12.72	3.85	2.90E-04	3.04E-03

2816	4	F2-3 vs. M2-3	248880_at	AT5G46200	carboxyl-terminal proteinase-like protein (DUF239);(source:Araport11)	84.70	22.41	3.78	1.66E-04	2.17E-03
2817	4	F2-3 vs. M2-3	256156_at	AT3G08510	Phosphoinositide-specific phospholipase C (PI-PLC), catalyzes hydrolysis of phosphatidylinositol 4,5-bisphosphate into inositol 1,4,5-trisphosphate and diacylglycerol. It is involved in auxin biosynthesis and signaling, modulating development of both male and female gametophytes. It also regulates MAMP-triggered immunity by modulating ROS production.	175.98	46.73	3.77	5.80E-06	2.82E-04
2818	4	F2-3 vs. M2-3	248534_at	AT5G50030	Plant invertase/pectin methylesterase inhibitor superfamily protein;(source:Araport11)	53.63	14.24	3.77	7.30E-05	1.34E-03
2819	4	F2-3 vs. M2-3	255149_at	AT4G08150	A member of class I knotted1-like homeobox gene family (together with KNAT2). Similar to the knotted1 (kn1) homeobox gene of maize. Normally expressed in the peripheral and rib zone of shoot apical meristem but not in the leaf primordia. It is also expressed in the fourth floral whorl, in the region that would become style, particularly in the cell surrounding the transmitting tissue. No expression was detected in the first three floral whorls. Expression is repressed by auxin and AS1 which results in the promotion of leaf fate.	45.01	11.99	3.75	4.22E-04	3.70E-03
2820	4	F2-3 vs. M2-3	251228_at	AT3G62710	Glycosyl hydrolase family protein;(source:Araport11)	89.68	23.96	3.74	1.83E-04	2.30E-03
2821	4	F2-3 vs. M2-3	254972_at	AT4G10440	S-adenosyl-L-methionine-dependent methyltransferases superfamily protein;(source:Araport11)	52.29	13.96	3.74	5.18E-05	1.13E-03
2822	4	F2-3 vs. M2-3	261593_at	AT1G33170	S-adenosyl-L-methionine-dependent methyltransferases superfamily protein;(source:Araport11)	81.44	21.89	3.72	2.30E-05	6.86E-04
2823	4	F2-3 vs. M2-3	257037_at	AT3G19130	RBP47B, is a component of the stress granule proteome and interacts with 2',3'-cAMP.	93.31	25.12	3.72	5.99E-04	4.51E-03
2824	4	F2-3 vs. M2-3	259520_at	AT1G12320	ankyrin repeat/KH domain protein (DUF1442);(source:Araport11)	56.05	15.20	3.69	6.74E-05	1.27E-03
2825	4	F2-3 vs. M2-3	264207_at	AT1G22750	transmembrane protein;(source:Araport11)	133.33	36.14	3.69	7.09E-04	4.99E-03
2826	4	F2-3 vs. M2-3	259275_at	AT3G01060	lysine-tRNA ligase;(source:Araport11)	275.56	74.59	3.69	1.87E-04	2.31E-03
2827	4	F2-3 vs. M2-3	253891_at	AT4G27720	Major facilitator superfamily protein;(source:Araport11)	323.22	87.70	3.69	6.25E-05	1.21E-03
2828	4	F2-3 vs. M2-3	251361_at	AT3G61230	Encodes a member of the Arabidopsis LIM proteins: a family of actin bundlers with distinct expression patterns. WLIM1, WLIM2a, and WLIM2b are widely expressed, whereas PLIM2a, PLIM2b, and PLIM2c are predominantly expressed in pollen. Regulates actin cytoskeleton organization.	37.73	10.24	3.68	7.62E-04	5.21E-03
2829	4	F2-3 vs. M2-3	254001_at	AT4G26260	Encodes a myo-inositol oxygenase, which is the first enzyme in the inositol route to ascorbate (L‐ascorbic acid, AsA, vitamin C). Overexpression results in enhanced biomass and abiotic stress tolerance.	56.90	15.53	3.67	4.25E-04	3.72E-03
2830	4	F2-3 vs. M2-3	246183_at	AT5G20940	Glycosyl hydrolase family protein;(source:Araport11)	104.13	28.39	3.67	6.03E-04	4.52E-03
2831	4	F2-3 vs. M2-3	257119_at	AT3G20190	Leucine-rich repeat protein kinase family protein;(source:Araport11)	41.94	11.46	3.66	1.12E-05	4.26E-04
2832	4	F2-3 vs. M2-3	251855_at	AT3G54690	Sugar isomerase (SIS) family protein;(source:Araport11)	265.51	72.60	3.66	6.30E-06	2.98E-04
2833	4	F2-3 vs. M2-3	255675_at	AT4G00480	MYC-related protein with a basic helix-loop-helix motif at the C-terminus and a region similar to the maize B/R family at the N-terminus	44.11	12.05	3.66	9.22E-04	5.88E-03
2834	4	F2-3 vs. M2-3	246998_at	AT5G67370	DUF1230 family protein (DUF1230);(source:Araport11)	114.04	31.20	3.66	1.40E-04	1.95E-03
2835	4	F2-3 vs. M2-3	266802_at	AT2G22900	Encodes MUCI10, a galactomannan-1,6-galactosyltransferase. MUCI10 likely decorates glucomannan, synthesized by CSLA2, with galactose residues in vivo. The degree of galactosylation is essential for the synthesis of the GGM backbone, the structure of cellulose, mucilage density, as well as the adherence of pectin.	351.89	97.04	3.63	6.45E-05	1.23E-03
2836	4	F2-3 vs. M2-3	256279_at	AT3G12620	Protein phosphatase 2C family protein;(source:Araport11)	228.46	63.00	3.63	< 1e-07	2.17E-05
2837	4	F2-3 vs. M2-3	265741_at	AT2G01320	ABC-2 type transporter family protein;(source:Araport11)	53.02	14.68	3.61	5.89E-05	1.20E-03
2838	4	F2-3 vs. M2-3	255702_at	AT4G00230	xylem serine peptidase 1;(source:Araport11)	77.04	21.42	3.60	6.06E-05	1.21E-03
2839	4	F2-3 vs. M2-3	257262_at	AT3G21890	B-box type zinc finger family protein;(source:Araport11)	382.71	106.76	3.58	7.70E-06	3.29E-04
2840	4	F2-3 vs. M2-3	258314_at	AT3G16100	RAB GTPase homolog G3C;(source:Araport11)	86.00	24.09	3.57	9.64E-05	1.57E-03
2841	4	F2-3 vs. M2-3	262751_at	AT1G16310	Cation efflux family protein which affects ABA-JA crosstalk and susceptibility to Mamestra brassicae herbivory.	47.94	13.46	3.56	4.90E-06	2.60E-04
2842	4	F2-3 vs. M2-3	248758_at	AT5G47620	RNA-binding (RRM/RBD/RNP motifs) family protein;(source:Araport11)	443.06	124.40	3.56	6.22E-05	1.21E-03
2843	4	F2-3 vs. M2-3	261144_s_a	AT1G19660	Wound-responsive family protein;(source:Araport11)	1116.48	314.39	3.55	6.35E-04	4.70E-03
2844	4	F2-3 vs. M2-3	266578_at	AT2G23910	NAD(P)-binding Rossmann-fold superfamily protein;(source:Araport11)	43.18	12.18	3.55	3.16E-05	8.40E-04
2845	4	F2-3 vs. M2-3	248916_at	AT5G45840	Encodes a leucine-rich-repeat RLK that is localized to the plasma membrane of pollen tubes and functions with MIK1/2 as the male receptor of the pollen tube chemo-attractant LURE1.MDIS1 forms a complex with MIK1/2 and binds LURE1.	32.27	9.19	3.51	2.85E-04	3.03E-03

2846	4	F2-3 vs. M2-3	259983_at	AT1G76490	Encodes a 3-hydroxy-3-methylglutaryl coenzyme A reductase, which is involved in melavonate biosynthesis and performs the first committed step in isoprenoid biosynthesis. Expression is activated in dark in leaf tissue but not controlled by light in the root (confine The mRNA is cell-to-cell mobile.	52.31	15.12	3.46	1.43E-04	1.97E-03
2847	4	F2-3 vs. M2-3	245463_at	AT4G17030	Encodes EXLB1 (expansin-like B1), a member of the expansin family.	101.48	29.67	3.42	1.39E-04	1.95E-03
2848	4	F2-3 vs. M2-3	252011_at	AT3G52720	Encodes an alpha carbonic anhydrase (CAH1) located in the chloroplast stroma. Most chloroplast proteins are encoded by the nuclear genome and imported with the help of sorting signals that are intrinsic parts of the polypeptides. CAH1 takes an alternative route through the secretory pathway, and becomes N-glycosylated before entering the chloroplast. Glycosylation and intra-molecular disulfide bridge fromation are necessary for the correct folding, ER export, trafficking and activity of the protein.	63.96	18.74	3.41	2.14E-05	6.67E-04
2849	4	F2-3 vs. M2-3	254862_at	AT4G12030	Required for the biosynthesis of methionine-derived glucosinolates. Involved in the transport of 2-keto acids between chloroplasts and the cytosol.	82.71	24.26	3.41	6.29E-04	4.67E-03
2850	4	F2-3 vs. M2-3	258321_at	AT3G22840	Encodes an early light-inducible protein.	317.82	93.38	3.40	1.07E-04	1.68E-03
2851	4	F2-3 vs. M2-3	256311_at	AT1G30330	Encodes a member of the auxin response factor family. Mediates auxin response via expression of auxin regulated genes. Acts redundantly with ARF8 to control stamen elongation and flower maturation. Expression of ARF6 is controlled by miR167.	471.29	139.76	3.37	2.00E-05	6.35E-04
2852	4	F2-3 vs. M2-3	255719_at	AT1G32080	Encodes a plant LrgAB/CidAB protein localized to the chloroplast envelope that is involved in chloroplast development, carbon partitioning, ABA/drought response, and leaf senescence. The gene may have evolved from gene fusion of bacterial lrgA and lrgB.	68.36	20.36	3.36	5.66E-05	1.19E-03
2853	4	F2-3 vs. M2-3	251762_at	AT3G55800	Encodes the chloroplast enzyme sedoheptulose-1,7-bisphosphatase (SBPase), involved in the carbon reduction of the Calvin cycle. Increase in SBPase activity in transgenic lines accumulate up to 50% more sucrose and starch than wild-type. The mRNA is cell-to-cell mobile.	595.29	177.29	3.36	4.27E-04	3.72E-03
2854	4	F2-3 vs. M2-3	246488_at	AT5G16010	3-oxo-5-alpha-steroid 4-dehydrogenase family protein;(source:Araport11)	161.70	48.27	3.35	7.72E-05	1.36E-03
2855	4	F2-3 vs. M2-3	249688_at	AT5G36160	Encodes a cytosolic L-tyrosine aminotransferase. AtTAT2 exhibits much broader amino donor specificity than AtTAT1 and can use not only Tyr but also Phe, Trp, His, Met, Leu, Ala, Ser, Cys, Asp, Asn, Gln, and Arg as amino donors.	46.56	13.91	3.35	1.22E-04	1.81E-03
2856	4	F2-3 vs. M2-3	264970_at	AT1G67280	Encodes a Ni+ dependent glyoxylase.	755.77	226.86	3.33	7.96E-05	1.39E-03
2857	4	F2-3 vs. M2-3	266184_s_at	AT3G54700	Encodes Pht1;7, a member of the Pht1 family of phosphate transporters which include: Pht1;1/At5g43350, Pht1;2/At5g43370, Pht1;3/At5g43360, Pht1;4/At2g38940, Pht1;5/At2g32830, Pht1;6/At5g43340, Pht1;7/At3g54700, Pht1;8/At1g20860, Pht1;9/At1g76430 (Plant Journal 2002, 31:341).	49.97	15.02	3.33	8.17E-05	1.41E-03
2858	4	F2-3 vs. M2-3	252550_at	AT3G45870	nodulin MtN21-like transporter family protein	75.67	22.83	3.31	1.95E-04	2.36E-03
2859	4	F2-3 vs. M2-3	253638_at	AT4G30470	NAD(P)-binding Rossmann-fold superfamily protein;(source:Araport11)	692.57	210.42	3.29	9.01E-04	5.81E-03
2860	4	F2-3 vs. M2-3	255645_at	AT4G00880	SAUR-like auxin-responsive protein family;(source:Araport11)	42.84	13.05	3.28	8.44E-04	5.55E-03
2861	4	F2-3 vs. M2-3	246431_at	AT5G17480	pollen calcium-binding protein 1;(source:Araport11)	27.48	8.38	3.28	1.10E-06	1.08E-04
2862	4	F2-3 vs. M2-3	264558_at	AT1G09600	Protein kinase superfamily protein;(source:Araport11)	57.94	17.70	3.27	5.66E-05	1.19E-03
2863	4	F2-3 vs. M2-3	256310_at	AT1G30360	Early-responsive to dehydration stress protein (ERD4);(source:Araport11)	136.21	41.62	3.27	5.37E-04	4.27E-03
2864	4	F2-3 vs. M2-3	245501_at	AT4G15620	Uncharacterized protein family (UPF0497);(source:Araport11)	61.97	18.93	3.27	4.11E-04	3.65E-03
2865	4	F2-3 vs. M2-3	256865_at	AT3G23820	Encodes a UDP-D-glucuronate 4-epimerase involved in pectin biosynthesis in the cell wall and affects cell wall integrity and immunity to fungi and bacteria. The mRNA is cell-to-cell mobile.	64.04	19.72	3.25	1.43E-04	1.97E-03
2866	4	F2-3 vs. M2-3	247994_at	AT5G56140	RNA-binding KH domain-containing protein;(source:Araport11)	61.06	18.99	3.22	5.71E-05	1.19E-03
2867	4	F2-3 vs. M2-3	260014_at	AT1G68010	Encodes hydroxypyruvate reductase.	270.89	84.83	3.19	8.40E-04	5.55E-03
2868	4	F2-3 vs. M2-3	264984_at	AT1G27000	GRIP/coiled-coil protein, putative (DUF1664);(source:Araport11)	163.52	51.44	3.18	1.37E-04	1.94E-03
2869	4	F2-3 vs. M2-3	267019_at	AT2G39130	Transmembrane amino acid transporter family protein;(source:Araport11)	49.98	15.72	3.18	5.97E-04	4.51E-03
2870	4	F2-3 vs. M2-3	247440_at	AT5G62680	Encodes a high-affinity, proton-dependent glucosinolate-specific transporter that is crucial for the transport of both methionine- and tryptophan-derived glucosinolates to seeds.	101.68	32.01	3.18	5.65E-04	4.37E-03
2871	4	F2-3 vs. M2-3	262264_at	AT1G42470	Patched family protein;(source:Araport11)	313.35	98.87	3.17	2.02E-04	2.43E-03

2872	4	F2-3 vs. M2-3	247736_at	AT5G59370	Encodes one of eight Arabidopsis actins. ACT4 belongs to the reproductive actin subclass which is predominantly expressed in developing and reproductive tissues, such as pollen, pollen tubes, ovules, and developing seeds. Expression of the ACT4/GUS fusion was restricted to young vascular tissues, tapetum, and developing and mature pollen.	43.81	13.99	3.13	1.22E-04	1.81E-03
2873	4	F2-3 vs. M2-3	247450_at	AT5G62350	Plant invertase/pectin methylesterase inhibitor superfamily protein;(source:Araport11)	532.94	170.10	3.13	1.30E-04	1.88E-03
2874	4	F2-3 vs. M2-3	251157_at	AT3G63140	Encodes a protein with ribonuclease activity that is involved in plastid rRNA maturation.	63.75	20.42	3.12	4.80E-04	4.01E-03
2875	4	F2-3 vs. M2-3	245745_at	AT1G51110	localized to chloroplasts	44.12	14.23	3.10	1.84E-04	2.30E-03
2876	4	F2-3 vs. M2-3	252984_at	AT4G37990	Encodes an aromatic alcohol:NADP+ oxidoreductase whose mRNA levels are increased in response to treatment with a variety of phytopathogenic bacteria. Though similar to mannitol dehydrogenases, this enzyme does not have mannitol dehydrogenase activity.	83.07	27.02	3.07	7.93E-04	5.36E-03
2877	4	F2-3 vs. M2-3	264317_at	AT1G70310	Spermidine synthase.	96.18	31.43	3.06	2.64E-04	2.88E-03
2878	4	F2-3 vs. M2-3	259892_at	AT1G72610	germin-like protein (GLP1)	199.61	65.63	3.04	7.31E-04	5.10E-03
2879	4	F2-3 vs. M2-3	245989_s_a	AT5G20620	encodes a ubiquitin polyprotein.	100.18	32.96	3.04	5.81E-04	4.44E-03
2880	4	F2-3 vs. M2-3	259277_at	AT3G01180	starch synthase 2;(source:Araport11)	422.34	140.04	3.02	2.73E-04	2.96E-03
2881	4	F2-3 vs. M2-3	248129_at	AT5G54780	Ypt/Rab-GAP domain of gyp1p superfamily protein;(source:Araport11)	74.78	24.82	3.01	5.80E-04	4.44E-03
2882	4	F2-3 vs. M2-3	248146_at	AT5G54940	Translation initiation factor SUI1 family protein;(source:Araport11)	37.70	12.52	3.01	8.44E-04	5.55E-03
2883	4	F2-3 vs. M2-3	250366_at	AT5G11420	Encodes a DUF642 cell wall protein.	106.67	35.55	3.00	3.43E-04	3.28E-03
2884	4	F2-3 vs. M2-3	255622_at	AT4G01070	the glycosyltransferase (UGT72B1) is involved in metabolizing xenobiotica (chloroaniline and chlorophenole). Comparison between wild type and knock-out mutant demonstrates the central role of this gene for metabolizing chloroaniline but significantly less for chlorophenole. The glucosyltransferase preferred UDP-xylose over UDP-glucose indicating its (additional) functioning as a xylosyltransferase in planta	40.76	13.67	2.98	2.13E-04	2.52E-03
2885	4	F2-3 vs. M2-3	257886_at	AT3G17060	Pectin lyase-like superfamily protein;(source:Araport11)	87.76	29.50	2.97	2.98E-04	3.05E-03
2886	4	F2-3 vs. M2-3	245281_at	AT4G15560	Encodes a protein with 1-deoxyxylulose 5-phosphate synthase activity involved in the MEP pathway. It is essential for chloroplast development in Arabidopsis	1763.46	593.19	2.97	2.56E-04	2.85E-03
2887	4	F2-3 vs. M2-3	AFFX-r2-At-Actin-5_s_at	AT5G09810	Member of Actin gene family.Mutants are defective in germination and root growth. The mRNA is cell-to-cell mobile.	60.23	20.37	2.96	8.35E-04	5.53E-03
2888	4	F2-3 vs. M2-3	260579_at	AT2G47380	Cytochrome c oxidase subunit Vc family protein;(source:Araport11)	386.28	131.58	2.94	4.25E-04	3.72E-03
2889	4	F2-3 vs. M2-3	259109_at	AT3G05580	Encodes a Type One Protein Phosphatase that acts as a nucleocytoplasmic negative regulator of tip growth. Mutants affect pollen germination, pollen tube growth, and root hair growth. It acts genetically downstream of ANX1 (AT3G04690) and ANX2 (AT5G28680) and is functionally redundant with TOPP8 (AT5G27840).	332.79	113.02	2.94	5.46E-05	1.16E-03
2890	4	F2-3 vs. M2-3	255690_at	AT4G00360	Encodes a member of the CYP86A subfamily of cytochrome p450 genes. Expressed at moderate levels in flowers, leaves, roots and stems.	47.44	16.11	2.94	7.79E-05	1.37E-03
2891	4	F2-3 vs. M2-3	246044_at	AT5G19450	calcium-dependent protein kinase (CDPK19) mRNA, complete	61.68	20.98	2.94	7.24E-04	5.07E-03
2892	4	F2-3 vs. M2-3	265715_s_at	AT1G13860	Encodes QUASIMODO2 LIKE1 (QUL1), a paralog of QUASIMODO2 (QUA2). AT1G78240 (QUA2), AT1G13860 (QUL1) and AT2G03480 (QUL2) form a clade with a possible role in plant vasculature development.	38.63	13.20	2.93	7.14E-04	5.02E-03
2893	4	F2-3 vs. M2-3	257071_at	AT3G28180	encodes a gene similar to cellulose synthase The mRNA is cell-to-cell mobile.	81.49	27.94	2.92	2.29E-05	6.86E-04
2894	4	F2-3 vs. M2-3	251649_at	AT3G57330	Lesion mimic phenotype when mutation in the gene is combined with a mutation in ACA4. Lesion mimic phenotype of double knockout can be suppressed by nutritional supplements that increase anion levels (e.g. 15 mM Nitrate, Chloride, or Phosphate)	60.27	20.82	2.90	1.63E-05	5.55E-04
2895	4	F2-3 vs. M2-3	253736_at	AT4G28780	GDSL-motif esterase/acyltransferase/lipase. Enzyme group with broad substrate specificity that may catalyze acyltransfer or hydrolase reactions with lipid and non-lipid substrates.	73.77	25.41	2.90	4.68E-04	3.94E-03
2896	4	F2-3 vs. M2-3	267429_at	AT2G34850	NAD(P)-binding Rossmann-fold superfamily protein;(source:Araport11)	170.78	59.10	2.89	2.41E-04	2.74E-03
2897	4	F2-3 vs. M2-3	267093_at	AT2G38170	Encodes a high affinity vacuolar calcium antiporter. The residue His 338 is critical to Ca2+ transport activity. Disruption of CAX1 reduces manganese and zinc of shoot tissue and results in a decrease in the activity of vacuolar V-type proton ATPase.	129.56	44.89	2.89	2.99E-04	3.05E-03
2898	4	F2-3 vs. M2-3	251020_at	AT5G02270	member of NAP subfamily	133.99	46.46	2.88	3.39E-04	3.28E-03

2899	4	F2-3 vs. M2-3	265658_at	AT2G13810	ALD1 is a L-lysine alpha-aminotransferase. It is part of the pipecolic acid biosynthetic pathway, where it catalyzes the biochemical conversion of lysine to epsilon-amino-alpha-ketocaproic acid (KAC) which is subject to subsequent transamination, cyclization and isomerization to form 2,3-dehydropipecolic acid.	68.53	23.98	2.86	9.32E-04	5.90E-03
2900	4	F2-3 vs. M2-3	247251_at	AT5G64740	Encodes a cellulose synthase isomer. CESA6 mutants have cellulose defect in the primary cell wall. Multiple lines of evidence suggest that CESA6, along with CESA1 and CESA3 are present in the same plasma membrane complex for cellulose biosynthesis. CESA2 and CESA5 are related to CESA6, having partially redundant roles. As inferred from the null role of secondary wall-type CesAs, included in a set of five primary wall-type CesAs that may support trichome cell wall thickening. The mRNA is cell-to-cell mobile.	474.52	165.92	2.86	2.60E-04	2.87E-03
2901	4	F2-3 vs. M2-3	262988_at	AT1G23310	Identified by cloning the gene that corresponded to a purified protein having glyoxylate aminotransferase activity. Localized to the peroxisome and thought to be involved in photorespiration/ metabolic salvage pathway.	319.54	112.18	2.85	2.73E-04	2.96E-03
2902	4	F2-3 vs. M2-3	259771_at	AT1G29470	S-adenosyl-L-methionine-dependent methyltransferases superfamily protein;(source:Araport11)	299.55	105.09	2.85	8.02E-04	5.39E-03
2903	4	F2-3 vs. M2-3	262868_at	AT1G64980	Encodes a putative nucleotide-diphospho-sugar transferase required for pollen germination and tube growth.	167.16	58.56	2.85	2.94E-04	3.05E-03
2904	4	F2-3 vs. M2-3	252482_at	AT3G46670	UDP-glucosyl transferase 76E11;(source:Araport11)	53.77	18.87	2.85	4.14E-04	3.66E-03
2905	4	F2-3 vs. M2-3	253420_at	AT4G32260	ATPase, F0 complex, subunit B/B, bacterial/chloroplast;(source:Araport11)	365.40	128.22	2.85	2.45E-05	7.01E-04
2906	4	F2-3 vs. M2-3	260207_at	AT1G70730	Encodes a cytosolic phosphoglucomutase (PGM). Two Arabidopsis PGM proteins (AT1G70730/PGM2 and AT1G23190/PGM3) have high sequence similarities and redundant functions. Mature plants possessing a single cPGM allele had a major reduction in cPGM activity. Whereas pgm2 and pgm3 single mutants are undistinguishable from the wild type, loss of both PGM2 and PGM3 severely impairs male and female gametophyte development.	130.11	45.73	2.84	4.62E-05	1.06E-03
2907	4	F2-3 vs. M2-3	257121_at	AT3G20220	SAUR-like auxin-responsive protein family;(source:Araport11)	49.70	17.52	2.84	1.51E-04	2.04E-03
2908	4	F2-3 vs. M2-3	252543_at	AT3G45780	Blue-light photoreceptor. Contains a light activated serine-threonine kinase domain and LOV1 and LOV2 repeats. Mutants are defective in blue-light response. Mediates blue light-induced growth enhancements. PHOT1 and PHOT2 mediate blue light-dependent activation of the plasma membrane H+-ATPase in guard cell protoplasts. PHOT1 undergoes blue-light-dependent autophosphorylation. At least eight phosphorylation sites have been identified in PHOT1. Phosphorylation of serine851 in the activation loop of PHOT1 appears to be required for stomatal opening, chloroplast accumulation, leaf flattening, and phototropism, and phosphorylation of serine849 may also contribute to the regulation of these responses. Phosphorylation-dependent binding of 14-3-3 proteins to the Hinge1 region of PHOT1 appears to require serine350 and serine376.	41.03	14.44	2.84	5.51E-04	4.35E-03
2909	4	F2-3 vs. M2-3	262357_at	AT1G73040	Mannose-binding lectin superfamily protein;(source:Araport11)	59.47	20.99	2.83	5.93E-04	4.49E-03
2910	4	F2-3 vs. M2-3	260560_at	AT2G43590	Chitinase family protein;(source:Araport11)	79.51	28.16	2.82	4.17E-04	3.68E-03
2911	4	F2-3 vs. M2-3	253963_at	AT4G26470	Calcium-binding EF-hand family protein;(source:Araport11)	36.72	13.01	2.82	1.51E-04	2.04E-03
2912	4	F2-3 vs. M2-3	259498_at	AT1G15880	Golgi SNARE 11 protein (GOS11)	557.87	198.42	2.81	3.52E-04	3.33E-03
2913	4	F2-3 vs. M2-3	250533_at	AT5G08640	Encodes a flavonol synthase that catalyzes formation of flavonols from dihydroflavonols. Co-expressed with CHI and CHS (qRT-PCR).	42.95	15.31	2.81	7.31E-05	1.34E-03
2914	4	F2-3 vs. M2-3	259593_at	AT1G27970	Encodes an ortholog of yeast NTF2, a nuclear envelop transport protein that functions as the nuclear import receptor for RanGDP, an essential player in nucleocytoplasmic transport. The mRNA is cell-to-cell mobile.	273.25	98.69	2.77	3.42E-04	3.28E-03
2915	4	F2-3 vs. M2-3	245088_at	AT2G39850	Subtilisin-like serine endopeptidase family protein;(source:Araport11)	31.90	11.52	2.77	1.89E-04	2.33E-03
2916	4	F2-3 vs. M2-3	245993_at	AT5G20700	senescence-associated family protein, putative (DUF581);(source:Araport11)	686.71	247.62	2.77	1.37E-04	1.94E-03
2917	4	F2-3 vs. M2-3	262597_at	AT1G15470	WD40 nucleoplasmic shuttling protein that positively regulates the Abscisic acid (ABA) response by interacting with and maintaining the stability of ABI5 in the nucleus. Nuclear export of XIW1 is XPO1-dependent. Involved in regulating seed germination, primary root growth, and drought stress resistance.	51.45	18.64	2.76	2.30E-06	1.62E-04
2918	4	F2-3 vs. M2-3	252107_at	AT3G51490	tonoplast monosaccharide transporter3;(source:Araport11)	53.55	19.41	2.76	2.70E-06	1.80E-04

2919	4	F2-3 vs. M2-3	251598_at	AT3G57600	encodes a member of the DREB subfamily A-2 of ERF/AP2 transcription factor family. The protein contains one AP2 domain. There are eight members in this subfamily including DREB2A AND DREB2B that are involved in response to drought.	51.10	18.55	2.75	1.47E-04	2.00E-03
2920	4	F2-3 vs. M2-3	254446_at	AT4G20890	tubulin 9 The mRNA is cell-to-cell mobile.	220.36	80.44	2.74	4.50E-06	2.52E-04
2921	4	F2-3 vs. M2-3	266097_at	AT2G37970	SOUL heme-binding family protein;(source:Araport11)	144.83	53.06	2.73	4.55E-05	1.05E-03
2922	4	F2-3 vs. M2-3	253522_at	AT4G31290	ChaC-like family protein;(source:Araport11)	296.21	108.65	2.73	5.59E-04	4.37E-03
2923	4	F2-3 vs. M2-3	264394_at	AT1G11860	Glycine cleavage T-protein family;(source:Araport11)	1332.93	489.69	2.72	2.33E-04	2.68E-03
2924	4	F2-3 vs. M2-3	263950_at	AT2G36020	HVA22-like protein J;(source:Araport11)	41.48	15.24	2.72	1.66E-04	2.17E-03
2925	4	F2-3 vs. M2-3	266558_at	AT2G23900	Pectin lyase-like superfamily protein;(source:Araport11)	42.05	15.52	2.71	8.18E-04	5.47E-03
2926	4	F2-3 vs. M2-3	249910_at	AT5G22630	Encodes a plastid-localized arogenate dehydratase involved in phenylalanine biosynthesis. Not less than six genes encoding ADT were identiﬁed in the Arabidopsis genome: ADT1 [At1g11790]; ADT2 [At3g07630]; ADT3 [At2g27820]; ADT4 [At3g44720]; ADT5 [At5g22630]; and ADT6 [At1g08250]. The mRNA is cell-to-cell mobile.	42.31	15.63	2.71	2.32E-05	6.86E-04
2927	4	F2-3 vs. M2-3	258512_at	AT3G06510	Encodes a protein with beta-glucosidase and galactosyltransferase activity, mutants show increased sensitivity to freezing. Though it is classified as a family I glycosyl hydrolase, it has no hydrolase activity in vitro.	635.18	237.33	2.68	5.76E-04	4.42E-03
2928	4	F2-3 vs. M2-3	245854_at	AT5G13490	Encodes mitochondrial ADP/ATP carrier	792.90	296.40	2.68	9.57E-04	6.01E-03
2929	4	F2-3 vs. M2-3	261601_at	AT1G49670	molecular function has not been defined. Was shown involved in oxidative stress tolerance.	74.57	27.94	2.67	2.99E-04	3.05E-03
2930	4	F2-3 vs. M2-3	258690_at	AT3G07960	Encodes phosphatidylinositol-4-phosphate 5-kinase 6 (PIP5K6). Regulates clathrin-dependent endocytosis in pollen tubes.	54.23	20.33	2.67	2.88E-04	3.03E-03
2931	4	F2-3 vs. M2-3	245292_at	AT4G15093	catalytic LigB subunit of aromatic ring-opening dioxygenase family;(source:Araport11)	102.31	38.45	2.66	2.61E-04	2.88E-03
2932	4	F2-3 vs. M2-3	252921_at	AT4G39030	Encodes an orphan multidrug and toxin extrusion transporter. Essential component of salicylic acid-dependent signaling for disease resistance. Member of the MATE-transporter family. Expression induced by salicylic acid. Mutants are salicylic acid-deficient.	133.36	50.11	2.66	6.07E-04	4.54E-03
2933	4	F2-3 vs. M2-3	260288_at	AT1G80530	Major facilitator superfamily protein;(source:Araport11)	569.48	215.09	2.65	5.60E-04	4.37E-03
2934	4	F2-3 vs. M2-3	250174_at	AT5G14380	Encodes an arabinogalactan protein that is expressed in pollen, pollen sac and pollen tube. Loss of AGP6 function results in decreased fertility due to defects in pollen tube growth.	45.62	17.23	2.65	4.91E-04	4.07E-03
2935	4	F2-3 vs. M2-3	263012_at	AT1G23380	homeodomain transcription factor KNAT6, belonging to class I of KN transcription factor family (which also includes KNAT1 and KNAT2). Expression is increased in as and bop1 leaf mutants.	40.26	15.32	2.63	8.86E-04	5.74E-03
2936	4	F2-3 vs. M2-3	258160_at	AT3G17820	encodes a cytosolic glutamine synthetase, the enzyme has low affinity with substrate ammonium The mRNA is cell-to-cell mobile.	320.10	122.30	2.62	1.16E-04	1.76E-03
2937	4	F2-3 vs. M2-3	250129_at	AT5G16450	Ribonuclease E inhibitor RraA/Dimethylmenaquinone methyltransferase;(source:Araport11)	518.33	197.60	2.62	3.79E-04	3.48E-03
2938	4	F2-3 vs. M2-3	264931_at	AT1G60590	Pectin lyase-like superfamily protein;(source:Araport11)	45.43	17.39	2.61	3.10E-04	3.09E-03
2939	4	F2-3 vs. M2-3	259056_at	AT3G03420	Ku70-binding family protein;(source:Araport11)	68.87	26.41	2.61	5.89E-04	4.47E-03
2940	4	F2-3 vs. M2-3	250633_at	AT5G07460	ubiquitous enzyme that repairs oxidatively damaged proteins. Methionine sulfoxide reductase activity. Mutant lacking reductase activity showed increased protein oxidation, nitration and glycation of specific amino acid residues during darkness.	33.90	12.97	2.61	2.26E-04	2.63E-03
2941	4	F2-3 vs. M2-3	249090_at	AT5G43745	ion channel POLLUX-like protein, putative (DUF1012);(source:Araport11)	46.18	17.73	2.61	4.84E-04	4.03E-03
2942	4	F2-3 vs. M2-3	264760_at	AT1G61290	member of SYP12 Gene Family	69.41	26.70	2.60	4.42E-04	3.81E-03
2943	4	F2-3 vs. M2-3	250565_at	AT5G08000	Encodes a member of the X8-GPI family of proteins. It localizes to the plasmodesmata and binds callose.	153.12	58.88	2.60	8.00E-04	5.38E-03
2944	4	F2-3 vs. M2-3	248822_at	AT5G47000	Peroxidase superfamily protein;(source:Araport11)	27.13	10.45	2.60	3.71E-04	3.44E-03
2945	4	F2-3 vs. M2-3	262179_at	AT1G77980	Encodes a member of the MIKC (MADS box, Keratin binding domain, and C terminal domain containing)family of transcriptional regulators. AGL66 is expressed in pollen.It forms heterodimers with other MICK family members (AGL104). Involved in late stages of pollen development and pollen tube growth.	46.35	17.90	2.59	3.59E-04	3.36E-03
2946	4	F2-3 vs. M2-3	259348_at	AT3G03770	Leucine-rich repeat protein kinase family protein;(source:Araport11)	315.60	121.91	2.59	6.62E-04	4.83E-03

2947	4	F2-3 vs. M2-3	261059_at	AT1G01250	encodes a member of the DREB subfamily A-4 of ERF/AP2 transcription factor family. The protein contains one AP2 domain. There are 17 members in this subfamily including TINY.	39.14	15.16	2.58	2.09E-04	2.49E-03
2948	4	F2-3 vs. M2-3	246215_at	AT4G37180	UIF1 is a nuclear and cytoplasmically localized myb-domain containing member of the GARP G2-like subfamily of transcription factors. Interacts with ULT1 and binds to the WUS promoter. UIF1 binding domains are also found in CUC and AG promoters suggesting they are also direct targets. This locus was also identified as a putative cytoskeletal protein in a yeast screen.	26.46	10.30	2.57	4.70E-05	1.07E-03
2949	4	F2-3 vs. M2-3	264603_at	AT1G04670	hypothetical protein;(source:Araport11)	22.63	8.87	2.55	1.17E-05	4.42E-04
2950	4	F2-3 vs. M2-3	267470_at	AT2G30490	Encodes a cinnamate-4-hydroxylase. Mutations in this gene impact phenylpropanoid metabolism, growth and development.	155.23	61.19	2.54	5.38E-04	4.27E-03
2951	4	F2-3 vs. M2-3	255997_s_at	AT1G29930	Subunit of light-harvesting complex II (LHCII),which absorbs light and transfers energy to the photosynthetic reaction center. The mRNA is cell-to-cell mobile.	1896.68	751.47	2.52	1.77E-04	2.25E-03
2952	4	F2-3 vs. M2-3	256020_at	AT1G58290	Encodes a protein with glutamyl-tRNA reductase (GluTR) activity, catalyzing the NADPH-dependent reduction of Glu-tRNA(Glu) to glutamate 1-semialdehyde (GSA) with the release of free tRNA(Glu). It is involved in the early steps of chlorophyll biosynthesis.	114.85	45.57	2.52	4.00E-04	3.59E-03
2953	4	F2-3 vs. M2-3	254491_at	AT4G20300	Serine/Threonine-kinase, putative (DUF1639);(source:Araport11)	161.87	64.55	2.51	5.00E-04	4.10E-03
2954	4	F2-3 vs. M2-3	253551_at	AT4G30996	NKS1, a plant-specific gene, encodes a 19 kDa endomembrane-localized protein. It is part of SOS independent ion homeostasis regulation pathway.	818.57	325.60	2.51	4.49E-05	1.05E-03
2955	4	F2-3 vs. M2-3	249773_at	AT5G24140	Encodes a protein with similarity to squalene monoxygenases.	29.38	11.72	2.51	5.65E-04	4.37E-03
2956	4	F2-3 vs. M2-3	249754_at	AT5G24530	Encodes a putative 2OG-Fe(II) oxygenase that is defense-associated but required for susceptibility to downy mildew. The mRNA is cell-to-cell mobile.	82.43	32.85	2.51	3.64E-05	9.26E-04
2957	4	F2-3 vs. M2-3	263166_at	AT1G03050	Phosphatidylinositol binding clathrin assembly protein 5A/B are recent paralogs with overlapping functions in recycling ANXUR proteins to the pollen tube membrane.	42.99	17.19	2.50	1.10E-04	1.70E-03
2958	4	F2-3 vs. M2-3	260968_at	AT1G12250	Pentapeptide repeat-containing protein;(source:Araport11)	118.35	47.57	2.49	3.20E-04	3.16E-03
2959	4	F2-3 vs. M2-3	260777_at	AT1G14560	Encodes a mitochondrial CoA transporter.	74.05	29.79	2.49	4.84E-04	4.03E-03
2960	4	F2-3 vs. M2-3	266275_at	AT2G29370	NAD(P)-binding Rossmann-fold superfamily protein;(source:Araport11)	61.87	24.89	2.49	1.70E-04	2.21E-03
2961	4	F2-3 vs. M2-3	266867_at	AT2G45770	chloroplast SRP receptor homolog, alpha subunit CPFTSY. Required for LHCP integration into isolated thylakoids.	72.55	29.17	2.49	7.91E-04	5.36E-03
2962	4	F2-3 vs. M2-3	254605_at	AT4G18950	BHP1 is a Raf-like protein kinase involved in mediating blue light dependent stomatal opening.	45.52	18.25	2.49	1.02E-04	1.65E-03
2963	4	F2-3 vs. M2-3	257816_at	AT3G25140	Quasimodo1, encodes a glycosyltransferase, involved in homogalacturonan biosynthesis; mutant shows cell adhesion defect and lower wall uronic acid content. The mRNA is cell-to-cell mobile.	397.28	160.01	2.48	8.11E-04	5.43E-03
2964	4	F2-3 vs. M2-3	252271_s_a	AT5G01430	Got1/Sft2-like vescicle transport protein family;(source:Araport11)	112.91	45.55	2.48	2.51E-04	2.82E-03
2965	4	F2-3 vs. M2-3	261376_at	AT1G18660	Membrane localized protein of unknown function. Involved in negative regulation of immune response. Mutants have increased resistance to pathogens.	108.92	44.15	2.47	9.83E-04	6.11E-03
2966	4	F2-3 vs. M2-3	257749_at	AT3G18780	Encodes an actin that is constitutively expressed in vegetative structures but not pollen. ACT2 is involved in tip growth of root hairs.	1106.73	447.72	2.47	9.69E-05	1.57E-03
2967	4	F2-3 vs. M2-3	251928_at	AT3G53980	Bifunctional inhibitor/lipid-transfer protein/seed storage 2S albumin superfamily protein;(source:Araport11)	82.08	33.26	2.47	2.09E-04	2.49E-03
2968	4	F2-3 vs. M2-3	245939_at	AT5G19760	Encodes a novel mitochondrial carrier capable of transporting both dicarboxylates (such as malate, oxaloacetate, oxoglutarate, and maleate) and tricarboxylates (such as citrate, isocitrate, cis-aconitate, and trans-aconitate).	1006.70	407.79	2.47	7.84E-04	5.32E-03
2969	4	F2-3 vs. M2-3	247979_at	AT5G56750	AGB1/AGG dimmer interacting protein, response to water deficit.	46.29	18.77	2.47	6.11E-05	1.21E-03
2970	4	F2-3 vs. M2-3	248200_at	AT5G54160	A caffeic acid/5-hydroxyferulic acid O-methyltransferase. Interacts with 14-4-3 proteins in yeast 2 hybrid assay. AtOMT1 (At5g54160) encodes a flavonol 3?-O-methyltransferase that is highly active towards quercetin and myricetin. The substrate specificity identifies the enzyme as flavonol 3?-methyltransferase which replaces the former annotation of the gene to encode a caffeic acid/5-hydroxyferulic acid O-methyltransferase The mRNA is cell-to-cell mobile.	572.46	232.47	2.46	6.08E-05	1.21E-03
2971	4	F2-3 vs. M2-3	261506_at	AT1G71697	Encodes choline kinase. mRNA levels are increased in response to wounding. The mRNA is cell-to-cell mobile.	39.45	16.11	2.45	2.59E-04	2.87E-03
2972	4	F2-3 vs. M2-3	266461_at	AT2G47730	Encodes glutathione transferase belonging to the phi class of GSTs. Naming convention according to Wagner et al. (2002).	137.67	56.15	2.45	3.41E-04	3.28E-03

2973	4	F2-3 vs. M2-3	263410_at	AT2G04039	NdhV is loosely associated with the NDH complex and is required for stabilizing NDH subcomplexes A and E.	31.43	12.87	2.44	4.80E-04	4.01E-03
2974	4	F2-3 vs. M2-3	263841_at	AT2G36870	Encodes a xyloglucan endotransglycosylase/hydrolase. Protein sequence and phylogenetic analysis indicates that this enzyme resides in Group III-A of the XTH family, with high similarity to Tropaeolum majus (nasturtium) xyloglucanase 1 (TmNXG1). By sequence similarity to XTH31 (At3g44990) and in vivo analysis, likely to exhibit predominant xyloglucan endo-hydrolase activity (EC 3.2.1.151) with only limited potential to act as a xyloglucan endo-transglycosylase (EC 2.4.1.207).	99.95	41.05	2.43	2.34E-04	2.68E-03
2975	4	F2-3 vs. M2-3	250967_at	AT5G02790	Glutathione S-transferase family protein;(source:Araport11)	68.14	28.05	2.43	1.57E-04	2.10E-03
2976	4	F2-3 vs. M2-3	248140_at	AT5G54980	Uncharacterized protein family (UPF0497);(source:Araport11)	115.92	47.79	2.43	2.83E-04	3.01E-03
2977	4	F2-3 vs. M2-3	265174_s_a	AT1G23460	Pectin lyase-like superfamily protein;(source:Araport11)	24.73	10.21	2.42	3.18E-04	3.16E-03
2978	4	F2-3 vs. M2-3	253512_at	AT4G31750	Encodes HopW1-1-Interacting protein 2 (WIN2). Interacts with the P. syringae effector HopW1-1. WIN2 has protein phosphatase activity. Modulates plant defenses against bacteria. Three WIN proteins are identified so far (WIN1: AT1G80600; WIN2: AT4G31750; WIN3: AT5G13320).	391.84	161.97	2.42	8.31E-04	5.52E-03
2979	4	F2-3 vs. M2-3	264738_at	AT1G62250	orotidine 5-phosphate decarboxylase;(source:Araport11)	79.02	32.81	2.41	6.72E-04	4.87E-03
2980	4	F2-3 vs. M2-3	263419_at	AT2G17220	Encodes a putative serine/threonine-specific protein kinase kin3. Protein is N-myristoylated.	38.49	16.03	2.40	4.38E-04	3.79E-03
2981	4	F2-3 vs. M2-3	245299_at	AT4G17530	AtRabD2c encodes a Rab GTPase, which plays important roles in pollen development, germination and tube elongation.	254.56	105.86	2.40	1.11E-04	1.71E-03
2982	4	F2-3 vs. M2-3	245724_at	AT1G73390	Endosomal targeting BRO1-like domain-containing protein;(source:Araport11)	62.40	26.15	2.39	6.59E-04	4.82E-03
2983	4	F2-3 vs. M2-3	261728_at	AT1G76160	SKU5 similar 5;(source:Araport11)	103.19	43.35	2.38	5.70E-04	4.39E-03
2984	4	F2-3 vs. M2-3	249769_at	AT5G24120	Encodes a specialized sigma factor that functions in regulation of plastid genes and is responsible for the light-dependent transcription at the psbD LRP. Activation of SIG5 is dependent upon blue light and mediated by cryptochromes.	42.55	17.89	2.38	9.74E-04	6.09E-03
2985	4	F2-3 vs. M2-3	261326_s_a	AT1G44820	Peptidase M20/M25/M40 family protein;(source:Araport11)	53.21	22.53	2.36	1.04E-04	1.67E-03
2986	4	F2-3 vs. M2-3	258677_at	AT3G08730	Encodes a protein-serine kinase that phosphorylates ribosomal protein in vitro. Activation of AtS6k is regulated by 1-naphthylacetic acid and kinetin, at least in part, via a lipid kinase-dependent pathway. Involved in translational up-regulation of ribosomal proteins. Phosphorylated by PDK1. Interacts with RAPTOR1, which in turn interacts with TOR. SPK6 activity is affected by osmotic stress, and plants overexpressing S6k1 are hypersensitive to osmotic stress. The gene is expressed in all tissues examined, with highest expression level detected in metabolically active tissues.	20.46	8.67	2.36	3.21E-04	3.16E-03
2987	4	F2-3 vs. M2-3	259054_at	AT3G03480	acetyl CoA:(Z)-3-hexen-1-ol acetyltransferase;(source:Araport11)	41.76	17.81	2.35	5.69E-04	4.39E-03
2988	4	F2-3 vs. M2-3	258416_at	AT3G17310	Encodes DRM3 (Domains Rearranged Methyltransferase3), a catalytically mutated paralog of the cytosine methyltransferase DRM2. Despite being catalytically mutated, DRM3 is required for normal maintenance of non-CG DNA methylation, establishment of RNA-directed DNA methylation triggered by repeat sequences and accumulation of repeat-associated small RNAs.	55.65	23.77	2.34	5.09E-04	4.13E-03
2989	4	F2-3 vs. M2-3	254158_at	AT4G24380	dihydrofolate reductase;(source:Araport11)	118.61	50.82	2.33	7.74E-04	5.28E-03
2990	4	F2-3 vs. M2-3	245619_at	AT4G13990	Exostosin family protein;(source:Araport11)	55.03	23.75	2.32	1.41E-05	4.96E-04
2991	4	F2-3 vs. M2-3	252906_at	AT4G39640	The gene encodes a gamma-glutamyltransferase (AKA gamma-glutamyl transpeptidase, EC 2.3.2.2) that is located in vascular tissues (predominantly phloem) of leaves and is involved in the degradation of glutathione. The encoded enzyme also mitigates oxidative stress by metabolizing GSSG (oxidized form of GSH - glutathione) in the apoplast.	56.70	24.46	2.32	5.73E-05	1.19E-03
2992	4	F2-3 vs. M2-3	258401_at	AT3G15430	Regulator of chromosome condensation (RCC1) family protein;(source:Araport11)	198.54	85.95	2.31	6.19E-04	4.61E-03
2993	4	F2-3 vs. M2-3	255587_at	AT4G01480	Encodes a protein that might have inorganic pyrophosphatase activity.	49.68	21.61	2.30	3.02E-04	3.07E-03
2994	4	F2-3 vs. M2-3	255345_at	AT4G04460	Saposin-like aspartyl protease family protein;(source:Araport11)	30.87	13.42	2.30	2.14E-04	2.53E-03
2995	4	F2-3 vs. M2-3	262832_s_a	AT1G14880	PLANT CADMIUM RESISTANCE 1;(source:Araport11)	28.80	12.60	2.29	6.73E-04	4.87E-03
2996	4	F2-3 vs. M2-3	252115_at	AT3G51600	Predicted to encode a PR (pathogenesis-related) protein. Belongs to the lipid transfer protein (PR-14) family with the following members: At2g38540/LTP1, At2g38530/LTP2, At5g59320/LTP3, At5g59310/LTP4, At3g51600/LTP5, At3g08770/LTP6, At2g15050/LTP7, At2g18370/LTP8, At2g15325/LTP9, At5g01870/LTP10, At4g33355/LTP11, At3g51590/LTP12, At5g44265/LTP13, At5g62065/LTP14, At4g08530/LTP15.	36.56	15.94	2.29	2.25E-05	6.85E-04

2997	4	F2-3 vs. M2-3	250259_at	AT5G13800	Encodes a pheophytinase that is involved in chlorophyll breakdown. Its transcript levels increase during senescence and pph-1 mutants have a stay-green phenotype.	127.21	55.53	2.29	4.74E-05	1.07E-03
2998	4	F2-3 vs. M2-3	256548_at	AT3G14770	Nodulin MtN3 family protein;(source:Araport11)	29.82	13.14	2.27	3.18E-04	3.16E-03
2999	4	F2-3 vs. M2-3	256584_at	AT3G28750	hypothetical protein;(source:Araport11)	28.11	12.37	2.27	3.93E-04	3.54E-03
3000	4	F2-3 vs. M2-3	245742_at	AT1G44170	Encodes an aldehyde dehydrogenase induced by ABA and dehydration that can oxidize saturated aliphatic aldehydes. It is also able to oxidize beta-unsaturated aldehydes, but not aromatic aldehydes. Activity of ALDH3H1 is NAD +-dependent.	54.92	24.35	2.26	1.61E-04	2.13E-03
3001	4	F2-3 vs. M2-3	247852_at	AT5G58060	member of YKT6 Gene Family	155.81	68.82	2.26	3.93E-04	3.54E-03
3002	4	F2-3 vs. M2-3	251885_at	AT3G54050	Encodes a chloroplastic fructose 1,6-bisphosphate phosphatase. also known as HCEF1 (High Cyclic Electron Flow 1). hcef1 mutants have constitutively elevated electron flow (CEFI) and plants with antisense suppression of this enzyme have higher levels of net leaf photosynthesis and increased sucrose biosynthesis. The mRNA is cell-to-cell mobile.	458.26	203.70	2.25	1.47E-04	2.00E-03
3003	4	F2-3 vs. M2-3	267027_at	AT2G38330	MATE efflux family protein;(source:Araport11)	73.04	32.91	2.22	4.67E-04	3.94E-03
3004	4	F2-3 vs. M2-3	257021_at	AT3G19710	Belongs to the branched-chain amino acid aminotransferase gene family. Encodes a methionine-oxo-acid transaminase. Involved in the methionine chain elongation pathway that leads to the ultimate biosynthesis of methionine-derived glucosinolates.	30.90	13.94	2.22	6.85E-04	4.92E-03
3005	4	F2-3 vs. M2-3	251839_at	AT3G54950	Encodes pPLAIIIbeta, a member of the Group 3 patatin-related phospholipases. pPLAIIIbeta hydrolyzes phospholipids and galactolipids and additionally has acyl-CoA thioesterase activity. Alterations of pPLAIIIβ result in changes in lipid levels and composition.	69.88	31.52	2.22	5.91E-05	1.20E-03
3006	4	F2-3 vs. M2-3	265415_at	AT2G20890	Chloroplast-localized Thylakoid formation1 gene product involved in vesicle-mediated formation of thylakoid membranes. Thf1 antisense lines contain abnormal chloroplasts early in leaf development (chloroplasts have loosely stacked thylakoid membranes). Expression was induced in the light and decreased under dark conditions. G-alpha interaction partner that functions downstream of the plasma membrane?delimited heterotrimeric G-protein (GPA1) in a D-glucose signaling pathway. Localized to both the outer plastid membrane and the stroma. Probably involved in the metabolic pathway that controls the assembly of the PS II complex. The mRNA is cell-to-cell mobile.	506.71	229.39	2.21	1.10E-04	1.70E-03
3007	4	F2-3 vs. M2-3	251197_at	AT3G62960	Encodes a member of the CC-type glutaredoxin (ROXY) family that has been shown to interact with the transcription factor TGA2.	53.66	24.32	2.21	3.70E-04	3.44E-03
3008	4	F2-3 vs. M2-3	254559_at	AT4G19200	proline-rich family protein;(source:Araport11)	131.18	59.32	2.21	8.34E-04	5.53E-03
3009	4	F2-3 vs. M2-3	248126_at	AT5G54760	Translation initiation factor SUI1 family protein;(source:Araport11)	1909.80	862.74	2.21	1.56E-04	2.09E-03
3010	4	F2-3 vs. M2-3	261623_at	AT1G01980	Encodes an oligogalacturonide oxidase that inactivates the elicitor-active oligogalacturonides (OGs).	24.57	11.18	2.20	2.37E-04	2.71E-03
3011	4	F2-3 vs. M2-3	267531_at	AT2G41860	member of Calcium Dependent Protein Kinase	21.30	9.67	2.20	1.19E-04	1.78E-03
3012	4	F2-3 vs. M2-3	267214_at	AT2G43970	RNA-binding protein;(source:Araport11)	123.01	55.95	2.20	9.16E-04	5.86E-03
3013	4	F2-3 vs. M2-3	258188_at	AT3G17800	mRNA level of the MEB5.2 gene (At3g17800) remains unchanged after cutting the inflorescence stem	189.66	86.18	2.20	4.61E-04	3.92E-03
3014	4	F2-3 vs. M2-3	258087_at	AT3G26060	encodes periredoxin Q which decomposes peroxides and plays a role in the protection of the photosynthetic apparatus	135.40	61.68	2.20	2.36E-04	2.70E-03
3015	4	F2-3 vs. M2-3	252213_at	AT3G50210	2-oxoglutarate (2OG) and Fe(II)-dependent oxygenase superfamily protein;(source:Araport11)	208.81	94.73	2.20	3.79E-04	3.48E-03
3016	4	F2-3 vs. M2-3	254137_at	AT4G24930	thylakoid lumenal 17.9 kDa protein, chloroplast;(source:Araport11)	34.82	15.80	2.20	7.46E-04	5.16E-03
3017	4	F2-3 vs. M2-3	254103_at	AT4G25030	Plastid localized protein of unknown function. Mutants are more susceptible to P. syringae and produce less callose upon infection.	129.84	59.19	2.19	3.73E-04	3.45E-03
3018	4	F2-3 vs. M2-3	252933_at	AT4G39110	bups1 and bups1/2 double mutants have reduced feritlity due to premature rupture of pollen tubes before they reach the ovule. BUSP1 interacts with RALF4/19 peptide ligands and ANX1/2 receptors. BUPS/ANX signaling may regulate and promote pollen tube growth.	32.77	15.07	2.17	2.29E-05	6.86E-04
3019	4	F2-3 vs. M2-3	246673_at	AT5G30510	ribosomal protein S1;(source:Araport11)	1638.25	758.85	2.16	6.02E-05	1.21E-03
3020	4	F2-3 vs. M2-3	258359_s_at	AT3G14420	Encodes a glycolate oxidase that modulates reactive oxygen species-mediated signal transduction during nonhost resistance. The mRNA is cell-to-cell mobile.	60.47	28.22	2.14	3.57E-04	3.36E-03
3021	4	F2-3 vs. M2-3	247741_at	AT5G58960	Mutant plants display impaired light-regulation of the hypocotyl randomization response.	934.66	436.02	2.14	6.89E-04	4.93E-03

3022	4	F2-3 vs. M2-3	252531_at	AT3G46520	Member of actin subclass composed of ACT12 and ACT4. RNA is expressed at very low levels in vegetative organs, low levels in flowers and very high levels in pollen. Expression of an ACT12/GUS fusion was found in vascular tissues, tapetum, developing and mature pollen, the root cap and in a ring of pericycle tissues during lateral root initiation and early development.	31.19	14.64	2.13	1.77E-04	2.25E-03
3023	4	F2-3 vs. M2-3	261226_at	AT1G20190	member of Alpha-Expansin Gene Family. Naming convention from the Expansin Working Group (Kende et al, 2004. Plant Mol Bio)	44.05	20.80	2.12	7.06E-04	4.99E-03
3024	4	F2-3 vs. M2-3	256209_at	AT1G50940	Encodes the electron transfer flavoprotein ETF alpha, a putative subunit of the mitochondrial electron transfer flavoprotein complex (ETF beta is At5g43430.1) in Arabidopsis. Mutations of the ETF beta gene results in accelerated senescence and early death compared to wild-type during extended darkness.	52.66	24.84	2.12	3.34E-04	3.25E-03
3025	4	F2-3 vs. M2-3	266636_at	AT2G35370	Encodes glycine decarboxylase complex H protein. Involved in photorespiration. The mRNA is cell-to-cell mobile.	45.37	21.40	2.12	4.19E-04	3.69E-03
3026	4	F2-3 vs. M2-3	251301_at	AT3G61880	Encodes a cytochrome p450 monooxygenase. Overexpression of this gene allows fruit growth independently of fertilization. The gene is normally expressed only in floral organs(during the Arabidopsis stage 14 flower) and in the funiculus at anthesis.	43.81	20.64	2.12	3.88E-04	3.54E-03
3027	4	F2-3 vs. M2-3	250347_at	AT5G11960	magnesium transporter, putative (DUF803);(source:Araport11)	125.11	58.93	2.12	2.47E-04	2.79E-03
3028	4	F2-3 vs. M2-3	260517_at	AT1G51420	sucrose-phosphatase 1;(source:Araport11)	54.29	25.89	2.10	5.94E-04	4.49E-03
3029	4	F2-3 vs. M2-3	264102_at	AT1G79270	evolutionarily conserved C-terminal region 8;(source:Araport11)	20.37	9.68	2.10	6.39E-05	1.23E-03
3030	4	F2-3 vs. M2-3	256416_at	AT3G11050	ferritin 2;(source:Araport11)	145.03	69.23	2.10	5.53E-04	4.35E-03
3031	4	F2-3 vs. M2-3	258208_at	AT3G13930	Encodes a subunit of the mitochondrial pyruvate dehydrogenase complex.	202.71	96.44	2.10	4.11E-04	3.65E-03
3032	4	F2-3 vs. M2-3	264348_at	AT1G12110	Encodes NRT1.1 (CHL1), a dual-affinity nitrate transporter. The protein is expressed in guard cells and function in stomatal opening. Mutants have less transpiration and are more tolerant to drought. Expressed in lateral roots. Involved in nitrate signaling which enables the plant root system to detect and exploit nitrate-rich soil patches. Comparing to the wild type, the mutant displays a strongly decreased lateral root proliferation phenotype in nitrate rich patches on growth medium. Affects flowering time via interaction with the FLC dependent flowering pathway to influence its target gene FT.	84.38	40.55	2.08	1.88E-05	6.16E-04
3033	4	F2-3 vs. M2-3	258140_at	AT3G24503	Arabidopsis thaliana aldehyde dehydrogenase AtALDH1a mRNA. a sinapaldehyde dehydrogenase catalyzes both the oxidation of coniferylaldehyde and sinapaldehyde forming ferulic acid and sinapic acid, respectively	385.20	185.30	2.08	6.98E-04	4.97E-03
3034	4	F2-3 vs. M2-3	255243_at	AT4G05590	Encodes NRGA1, a putative mitochondrial pyruvate carrier that mediates ABA regulation of guard cell ion channels and drought stress responses.	26.22	12.59	2.08	1.74E-04	2.23E-03
3035	4	F2-3 vs. M2-3	263705_at	AT1G31190	Encodes a myo-inositol monophosphatase IMPL1 (myo-Inositol monophosphatase like 1).	281.55	135.78	2.07	1.91E-04	2.34E-03
3036	4	F2-3 vs. M2-3	255855_at	AT1G66950	Encodes a plasma membrane-localized ABC transporter. Confers tolerance to herbicide paraquat.	27.74	13.49	2.06	8.05E-04	5.40E-03
3037	4	F2-3 vs. M2-3	254683_at	AT4G13800	magnesium transporter NIPA (DUF803);(source:Araport11)	24.33	11.80	2.06	2.42E-04	2.74E-03
3038	4	F2-3 vs. M2-3	247523_at	AT5G61410	Arabidopsis thaliana ribulose-5-phosphate-3-epimerase mRNA	323.40	156.68	2.06	3.00E-05	8.22E-04
3039	4	F2-3 vs. M2-3	257375_at	AT2G38640	LURP-one-like protein (DUF567);(source:Araport11)	19.33	9.44	2.05	3.72E-05	9.26E-04
3040	4	F2-3 vs. M2-3	257701_at	AT3G12710	DNA glycosylase superfamily protein;(source:Araport11)	40.44	19.74	2.05	5.24E-04	4.20E-03
3041	4	F2-3 vs. M2-3	249244_at	AT5G42270	VAR1 contains a conserved motif for ATPase and a metalloprotease characteristic to FtsH proteins, and is targeted into chloroplasts. A VAR1-fusion protein synthesized in vitro exhibited ATPase activity and partial metalloprotease activity. This protein is located to the thylakoid membrane and forms a complex with VAR2. FtsH1 (VAR1) and FtsH5 are interchangeable in thylakoid membranes. Phosphorylation of this protein is dependent on calcium. The mRNA is cell-to-cell mobile.	365.62	178.87	2.04	1.68E-04	2.20E-03
3042	4	F2-3 vs. M2-3	260304_at	AT1G70480	OBP32pep, putative (DUF220);(source:Araport11)	162.78	80.18	2.03	1.87E-04	2.31E-03
3043	4	F2-3 vs. M2-3	257606_at	AT3G13870	required for regulated cell expansion and normal root hair development. Encodes an evolutionarily conserved protein with putative GTP-binding motifs that is implicated in the control of vesicle trafficking between the endoplasmic reticulum and the Golgi compartments.	127.56	62.79	2.03	1.69E-04	2.20E-03

3044	4	F2-3 vs. M2-3	258054_at	AT3G16240	Delta tonoplast intrinsic protein, functions as a water channel and ammonium (NH3) transporter. Highly expressed in flower, shoot, and stem. Expression shows diurnal regulation and is induced by ammonium (NH3). Protein localized to vacuolar membrane. The mRNA is cell-to-cell mobile.	164.44	81.06	2.03	4.95E-04	4.10E-03
3045	4	F2-3 vs. M2-3	264395_at	AT1G12070	Immunoglobulin E-set superfamily protein;(source:Araport11)	23.10	11.45	2.02	2.27E-04	2.64E-03
3046	4	F2-3 vs. M2-3	261373_at	AT1G53000	Encodes a mitochondrial-localized CMP-KDO (3-deoxy-D-manno-octulosonate) synthetase. This is the enzyme activating KDO as a nucleotide sugar prior to its incorporation into rhamnogalacturonan-II. Heterozygous mutants are defective in pollen development and in pollen tube elongation.	100.67	49.77	2.02	2.33E-04	2.68E-03
3047	4	F2-3 vs. M2-3	260080_at	AT1G78160	Encodes a member of the Arabidopsis Pumilio (APUM) proteins containing PUF domain (eight repeats of approximately 36 amino acids each). PUF proteins regulate both mRNA stability and translation through sequence-specific binding to the 3' UTR of target mRNA transcripts.	29.06	14.42	2.02	9.35E-05	1.54E-03
3048	4	F2-3 vs. M2-3	252004_at	AT3G52780	Purple acid phosphatases superfamily protein;(source:Araport11)	121.24	60.02	2.02	1.81E-04	2.29E-03
3049	4	F2-3 vs. M2-3	252331_s_at	AT3G48780	Encodes one of the two LCB2 subunits (LCB2a and LCB2b) of serine palmitoyltransferase, an enzyme involved in sphingolipid biosynthesis. LCB2a and LCB2b are functional redundant. Double mutants are gametophytic lethal. The mRNA is cell-to-cell mobile.	104.72	52.09	2.01	7.10E-04	4.99E-03
3050	4	F2-3 vs. M2-3	252058_at	AT3G52470	Late embryogenesis abundant (LEA) hydroxyproline-rich glycoprotein family;(source:Araport11)	174.04	86.39	2.01	4.08E-04	3.64E-03
3051	4	F2-3 vs. M2-3	265043_at	AT1G03900	member of NAP family, an heterogeneous subfamily of the ATP-binding Cassette (ABC) superfamily of membrane transporters. The NAPs proteins are characterized by having only one nucleotide-binding folds (NBFs) domain.	280.38	140.48	2.00	9.09E-04	5.84E-03
3052	4	F2-3 vs. M2-3	255959_at	AT1G21980	Type I phosphatidylinositol-4-phosphate 5-kinase. Preferentially phosphorylates PtdIns4P. Induced by water stress and abscisic acid in Arabidopsis thaliana. Expressed in procambial cells of leaves, flowers and roots. A N-terminal Membrane Occupation and Recognition Nexus (MORN)affects enzyme activity and distribution.	30.26	15.12	2.00	6.55E-04	4.80E-03
3053	4	F2-3 vs. M2-3	259727_at	AT1G60950	encodes a major leaf ferredoxin	3805.93	1907.38	2.00	9.15E-05	1.52E-03
3054	4	F2-3 vs. M2-3	251961_at	AT3G53620	Encodes a soluble protein with inorganic pyrophosphatase activity that is highly specific for Mg-inorganic pyrophosphate. The mRNA is cell-to-cell mobile.	222.57	111.11	2.00	3.89E-04	3.54E-03
3055	4	F2-3 vs. M2-3	253372_at	AT4G33220	pectin methylesterase 44;(source:Araport11)	245.25	122.80	2.00	6.95E-04	4.96E-03
3056	4	F2-3 vs. M2-3	250137_at	AT5G15390	tRNA/rRNA methyltransferase (SpoU) family protein;(source:Araport11)	103.60	51.74	2.00	8.08E-05	1.40E-03
3057	4	F2-3 vs. M2-3	257407_at	AT1G27100	Actin cross-linking protein;(source:Araport11)	183.50	92.40	1.99	5.59E-04	4.37E-03
3058	4	F2-3 vs. M2-3	257790_at	AT3G27090	DCD (Development and Cell Death) domain protein;(source:Araport11)	398.82	200.57	1.99	7.42E-04	5.15E-03
3059	4	F2-3 vs. M2-3	247539_at	AT5G61710	cotton fiber protein;(source:Araport11)	28.49	14.36	1.98	9.08E-04	5.84E-03
3060	4	F2-3 vs. M2-3	257253_at	AT3G24190	Protein kinase superfamily protein;(source:Araport11)	63.28	32.11	1.97	5.56E-04	4.36E-03
3061	4	F2-3 vs. M2-3	250827_at	AT5G05170	Encodes a cellulose synthase isomer. CESA3 mutants have cellulose defect in the primary cell wall. Multiple lines of evidence suggest that CESA3, along with CESA1 and CESA6 are present in the same plasma membrane complex for cellulose biosynthesis. As inferred from the null role of secondary wall-type CesAs, included in a set of five primary wall-type CesAs that may support trichome cell wall thickening. The xylem cells in primary root have reduced cell expansion and higher than normal lignification.	1253.06	635.76	1.97	9.76E-04	6.09E-03
3062	4	F2-3 vs. M2-3	256093_at	AT1G20823	Encodes a RING E3 ubiquitin ligase ATL80. Involved in phosphate mobilization and cold stress response in sufficient phosphate growth conditions. The mRNA is cell-to-cell mobile.	38.21	19.48	1.96	3.03E-04	3.07E-03
3063	4	F2-3 vs. M2-3	256213_at	AT1G50990	kinase with tetratricopeptide repeat domain-containing protein;(source:Araport11)	22.62	11.62	1.95	5.15E-04	4.16E-03
3064	4	F2-3 vs. M2-3	249343_at	AT5G40650	One of three isoforms of the iron-sulfur component of the succinate dehydrogenase complex, a component of the mitochondrial respiratory chain complex II. The product of the nuclear encoded gene is imported into the mitochondrion. Expressed during germination and post-germinative growth.	1204.09	617.88	1.95	1.79E-04	2.27E-03
3065	4	F2-3 vs. M2-3	265400_at	AT2G10940	Bifunctional inhibitor/lipid-transfer protein/seed storage 2S albumin superfamily protein;(source:Araport11)	144.90	74.70	1.94	4.05E-04	3.62E-03
3066	4	F2-3 vs. M2-3	260789_s_a	AT3G06690	putative acyl-coenzyme A oxidase;(source:Araport11)	32.73	16.92	1.93	1.94E-04	2.36E-03
3067	4	F2-3 vs. M2-3	249447_at	AT5G39400	Calcium/lipid-binding (CaLB) phosphatase;(source:Araport11)	24.19	12.54	1.93	2.54E-04	2.84E-03

3068	4	F2-3 vs. M2-3	253477_at	AT4G32320	Encodes a cytosolic ascorbate peroxidase APX6. Ascorbate peroxidases are enzymes that scavenge hydrogen peroxide in plant cells. Eight types of APX have been described for Arabidopsis: three cytosolic (APX1, APX2, APX6), two chloroplastic types (stromal sAPX, thylakoid tAPX), and three microsomal (APX3, APX4, APX5) isoforms.	117.62	61.11	1.92	6.30E-06	2.98E-04
3069	4	F2-3 vs. M2-3	250621_at	AT5G07250	RHOMBOID-like protein 3;(source:Araport11)	164.77	85.67	1.92	1.52E-05	5.28E-04
3070	4	F2-3 vs. M2-3	260305_at	AT1G70490	A member of ARF GTPase family. A thaliana has 21 members of this family, known to be essential for vesicle coating and uncoating and functions in GTP-binding. Gene encoding ADP-ribosylation factor and similar to other ARFs and ARF-like proteins. The gene is shown to play a role in cell division, cell expansion and cellulose production using antisense construct.	107.12	56.10	1.91	6.17E-04	4.60E-03
3071	4	F2-3 vs. M2-3	251465_at	AT3G59360	UDP-galactose transporter 6;(source:Araport11)	66.09	34.56	1.91	8.45E-05	1.44E-03
3072	4	F2-3 vs. M2-3	254419_at	AT4G21490	NAD(P)H dehydrogenase B3;(source:Araport11)	40.37	21.12	1.91	8.27E-04	5.50E-03
3073	4	F2-3 vs. M2-3	250517_at	AT5G08260	serine carboxypeptidase-like 35;(source:Araport11)	48.43	25.41	1.91	5.33E-04	4.25E-03
3074	4	F2-3 vs. M2-3	260320_at	AT1G63930	from the Czech roh meaning corner;(source:Araport11)	73.76	38.92	1.90	1.32E-04	1.89E-03
3075	4	F2-3 vs. M2-3	251854_at	AT3G54800	Pleckstrin homology (PH) and lipid-binding START domains-containing protein;(source:Araport11)	21.09	11.09	1.90	8.93E-04	5.77E-03
3076	4	F2-3 vs. M2-3	259464_at	AT1G18990	myosin-binding protein, putative (Protein of unknown function, DUF593);(source:Araport11)	18.41	9.75	1.89	9.80E-04	6.11E-03
3077	4	F2-3 vs. M2-3	264120_at	AT1G79340	Encodes MCP2d, the predominant and constitutively expressed member of type II metacaspases (MCPs). MCP2d plays a positive regulatory role in biotic and abiotic stress-induced programmed cell death (PCD). Arabidopsis contains three type I MCP genes (MCP1a-c) and six type II MCP genes (MCP2a?f): AtMCP1a/At5g64240, AtMCP1b/At1g02170, AtMCP1c/At4g25110, AtMCP2a/At1g79310, AtMCP2b/At1g79330, AtMCP2c/At1g79320, AtMCP2d/At1g79340, AtMCP2e/At1g16420, AtMCP2f/At5g04200. The mRNA is cell-to-cell mobile.	62.58	33.05	1.89	9.17E-04	5.86E-03
3078	4	F2-3 vs. M2-3	254033_at	AT4G25950	V-ATPase G-subunit like protein	18.23	9.65	1.89	9.11E-04	5.84E-03
3079	4	F2-3 vs. M2-3	250330_at	AT5G11810	rhomboid family protein;(source:Araport11)	181.36	95.98	1.89	1.07E-05	4.16E-04
3080	4	F2-3 vs. M2-3	250155_at	AT5G15160	BNQ2 belongs to a family of atypical non-DNA binding basic helix-loop-helix (bHLH) proteins that heterodimerize with and negatively regulate bHLH transcription factors. Directly and negatively regulated by AP3 and PI in petals.Required for appropriate regulation of flowering time.	19.49	10.30	1.89	8.80E-04	5.72E-03
3081	4	F2-3 vs. M2-3	264235_at	AT1G54560	Encodes a class XI myosin that is involved in organelle motility, actin organization, and optimal growth of pollen tubes.	35.33	18.76	1.88	5.03E-04	4.10E-03
3082	4	F2-3 vs. M2-3	251557_at	AT3G58730	vacuolar ATP synthase subunit D (VATD) / V-ATPase D subunit / vacuolar proton pump D subunit (VATPD);(source:Araport11)	160.14	84.97	1.88	6.00E-07	7.44E-05
3083	4	F2-3 vs. M2-3	262727_at	AT1G75800	Pathogenesis-related thaumatin superfamily protein;(source:Araport11)	34.12	18.27	1.87	3.06E-04	3.07E-03
3084	4	F2-3 vs. M2-3	263368_at	AT2G20470	AGC (cAMP-dependent, cGMP-dependent and protein kinase C) kinase family protein;(source:Araport11)	54.12	29.04	1.86	1.18E-04	1.77E-03
3085	4	F2-3 vs. M2-3	267212_at	AT2G44060	Late embryogenesis abundant protein, group 2;(source:Araport11)	456.51	244.99	1.86	5.35E-04	4.26E-03
3086	4	F2-3 vs. M2-3	251825_at	AT3G55100	ABC-2 type transporter family protein;(source:Araport11)	25.92	13.91	1.86	1.74E-04	2.23E-03
3087	4	F2-3 vs. M2-3	253525_at	AT4G31330	transmembrane protein, putative (Protein of unknown function, DUF599);(source:Araport11)	27.53	14.87	1.85	2.00E-06	1.53E-04
3088	4	F2-3 vs. M2-3	264855_at	AT2G17265	Encodes a homoserine kinase (HSK) which produces O-phospho-L-homoserine (HserP), a compound at the branching point of methionine and threonine biosynthesis. HSK is found in the stromal fraction of chloroplasts. Mutation of this gene results in higher level of the amino acid homoserine and resistance to downy mildew pathogen Hyaloperonospora arabidopsidis.	122.42	66.43	1.84	2.55E-04	2.84E-03
3089	4	F2-3 vs. M2-3	247586_at	AT5G60660	A member of the plasma membrane intrinsic protein subfamily PIP2.When expressed in yeast cells can conduct hydrogen peroxide into those cells. Mutants exhibit longer root hairs.	75.91	41.17	1.84	2.71E-04	2.95E-03
3090	4	F2-3 vs. M2-3	264645_at	AT1G08940	Phosphoglycerate mutase family protein;(source:Araport11)	35.62	19.47	1.83	3.46E-04	3.30E-03
3091	4	F2-3 vs. M2-3	245867_at	AT1G58080	ATP phosphoribosyl transferase, catalyses first step of histidine biosynthesis	108.14	58.98	1.83	2.51E-04	2.82E-03

3092	4	F2-3 vs. M2-3	266165_at	AT2G28190	Encodes a chloroplastic copper/zinc superoxide dismutase CSD2 that can detoxify superoxide radicals. Its expression is affected by miR398-directed mRNA cleavage. Activation depends totally on CCS. Overexpression of a miR398-resistant form of CSD2 leads to more dramatic improvements in stress (hight light, Cu2+ and methyl viologen) tolerance than overexpression of wild-type CSD2. The mRNA is cell-to-cell mobile.	1340.29	731.95	1.83	4.14E-04	3.66E-03
3093	4	F2-3 vs. M2-3	251383_at	AT3G60740	Encodes tubulin-folding cofactor D. Mutants arrest during embryogenesis with embryos that are small, mushroom-shaped (Primepilz Prime) and consist of only one or few large cells each containing one or more variably enlarged nuclei and often cell wall stubs. Gene product necessary for continuous microtubule organization.	21.34	11.67	1.83	3.57E-04	3.36E-03
3094	4	F2-3 vs. M2-3	249472_at	AT5G39210	Encodes a protein of the chloroplastic NAD(P)H dehydrogenase complex (NDH Complex) involved in respiration, photosystem I (PSI) cyclic electron transport and CO2 uptake. The product of this gene appears to be essential for the stable formation of the NDH Complex. The mRNA is cell-to-cell mobile.	23.95	13.10	1.83	5.70E-04	4.39E-03
3095	4	F2-3 vs. M2-3	259839_at	AT1G52190	Encodes a low affinity nitrate transporter that is expressed in the plasma membrane and found in the phloem of the major veins of leaves. It is responsible for nitrate redistribution to young leaves.	762.78	419.45	1.82	3.22E-04	3.17E-03
3096	4	F2-3 vs. M2-3	263876_at	AT2G21880	RAB GTPase homolog 7A;(source:Araport11)	37.80	20.73	1.82	5.40E-04	4.28E-03
3097	4	F2-3 vs. M2-3	258038_at	AT3G21260	Glycolipid transfer protein (GLTP) family protein;(source:Araport11)	21.51	11.84	1.82	8.60E-05	1.45E-03
3098	4	F2-3 vs. M2-3	247307_at	AT5G63860	UV-B-specific signaling component that orchestrates expression of a range of genes with vital UV-protective functions. Located in the nucleus and the cytosol. Associates with chromatin via histones. UV-B light promotes URV8 protein accumulation in the nucleus. UVR8 interaction with COP1 is negatively regulated by RUP1 and RUP2.	45.02	24.72	1.82	5.03E-04	4.10E-03
3099	4	F2-3 vs. M2-3	264815_at	AT1G03620	ELMO/CED-12 family protein;(source:Araport11)	23.98	13.25	1.81	1.33E-04	1.89E-03
3100	4	F2-3 vs. M2-3	254848_at	AT4G11960	Encodes PGRL1B, a transmembrane protein present in thylakoids. PGRL1B has a highly homologous isoform PGRL1A encoded by At4g22890. Plants lacking PGRL1 show perturbation of cyclic electron flow, similar to PGR5-deficient plants. PGRL1 and PGR5 interact physically and associate with PSI (photosystem I).	68.36	37.77	1.81	6.86E-04	4.92E-03
3101	4	F2-3 vs. M2-3	259338_at	AT3G03800	member of SYP13 Gene Family	29.48	16.50	1.79	9.29E-04	5.90E-03
3102	4	F2-3 vs. M2-3	254562_at	AT4G19230	Encodes a protein with ABA 8'-hydroxylase activity, involved in ABA catabolism. Member of the CYP707A gene family. CYP707A1 appears to play an important role in determining the ABA levels in dry seeds. Gene involved in postgermination growth. Overexpression of CYP707A1 leads to a decrease in ABA levels and a reduction in after-ripening period to break dormancy.	28.69	15.99	1.79	1.24E-04	1.82E-03
3103	4	F2-3 vs. M2-3	246620_at	AT5G36220	This gene is a member of the CYP81D family of cytochrome p450s. It was originally called CYP91A1, but was later renamed to CYP81D1.	23.30	13.03	1.79	6.00E-04	4.51E-03
3104	4	F2-3 vs. M2-3	260818_at	AT1G06890	UXT3 is a member of the NST-KT subfamily of nucleotide/sugar transporters. It is localized to the golgi and functions as a UDP-Xyl transporter.	193.02	108.73	1.78	9.99E-04	6.17E-03
3105	4	F2-3 vs. M2-3	246427_at	AT5G17400	This gene is predicted to encode an ER-localized adenine nucleotide transporter with six putative transmembrane helices. It appears to act as a ATP:ADP antiporter when expressed in E.coli plasma membranes. Transcript levels for several ER-localized chaperones (e.g. BIP1/2) and other ATP-requiring ER proteins (e.g. CPK2) are reduced in er-ant1 knock-out lines, suggesting a lack of adequate ATP transport into the ER in these mutants. They also have reduced seed oil and seed protein levels.	38.49	21.60	1.78	6.51E-04	4.79E-03
3106	4	F2-3 vs. M2-3	261687_at	AT1G47280	hypothetical protein;(source:Araport11)	21.37	12.08	1.77	7.40E-04	5.14E-03
3107	4	F2-3 vs. M2-3	259603_at	AT1G56500	Encodes a thylakoid membrane protein with thioredoxin-like and beta-propeller domains located in the lumen and a haloacid-dehalogenase domain exposed to the chloroplast stroma. The protein Primes role may be to prevent formation of a slowly reversible form of antenna quenching, thereby maintaining the efficiency of light harvesting.The mRNA is cell-to-cell mobile.	91.65	51.71	1.77	6.39E-04	4.72E-03
3108	4	F2-3 vs. M2-3	245352_at	AT4G15490	Encodes a protein that might have sinapic acid:UDP-glucose glucosyltransferase activity.	40.79	23.01	1.77	4.42E-04	3.81E-03

3109	4	F2-3 vs. M2-3	267157_at	AT2G37630	Encodes a MYB-domain protein involved in specification of the leaf proximodistal axis. Mutation results in lobed and dissected leaves with a characteristic asymmetry. Homologous to the Antirrhinum PHANTASTICA (PHAN) and maize ROUGH SHEATH2 (RS2) genes Asymmetric placement of auxin response at the distal leaf tip precedes visible asymmetric leaf growth. Acts alongside AXR1 to exclude BP expression in leaves and with PIN1 to repress BP and promote lateral organ growth. Interacts physically with AS2 to form a complex that binds to the BP promoter and silences BP. Also functions as a regulator of the plant immune response.	209.09	118.82	1.76	7.50E-04	5.17E-03
3110	4	F2-3 vs. M2-3	254961_at	AT4G11030	AMP-dependent synthetase and ligase family protein;(source:Araport11)	20.58	11.73	1.75	7.22E-05	1.33E-03
3111	4	F2-3 vs. M2-3	247125_at	AT5G66070	E3 ubiquitin ligase that functions in negative regulation of ABA signaling.	110.55	63.19	1.75	4.51E-04	3.88E-03
3112	4	F2-3 vs. M2-3	263881_at	AT2G21820	seed maturation protein;(source:Araport11)	32.67	18.79	1.74	1.15E-04	1.76E-03
3113	4	F2-3 vs. M2-3	249527_at	AT5G38710	Methylenetetrahydrofolate reductase family protein;(source:Araport11)	27.32	15.72	1.74	5.26E-04	4.20E-03
3114	4	F2-3 vs. M2-3	265872_at	AT2G01670	nudix hydrolase homolog 17;(source:Araport11)	132.28	76.36	1.73	7.40E-04	5.14E-03
3115	4	F2-3 vs. M2-3	258671_at	AT3G08560	vacuolar H+-ATPase subunit E isoform 2;(source:Araport11)	37.07	21.42	1.73	4.72E-04	3.96E-03
3116	4	F2-3 vs. M2-3	259071_at	AT3G11650	Encodes a protein whose sequence is similar to tobacco hairpin-induced gene (HIN1) and Arabidopsis non-race specific disease resistance gene (NDR1). Expression of this gene is induced by cucumber mosaic virus and spermine. Overexpression of the gene induces the expression of PR-1 gene and shows light-dependent 'speck disease-like' symptom on leaves. The gene product is localized to the chloroplast	35.51	20.50	1.73	5.19E-04	4.18E-03
3117	4	F2-3 vs. M2-3	251494_at	AT3G59350	Protein kinase superfamily protein;(source:Araport11)	68.32	39.53	1.73	8.89E-04	5.75E-03
3118	4	F2-3 vs. M2-3	248007_at	AT5G56260	Ribonuclease E inhibitor RraA/Dimethylmenaquinone methyltransferase;(source:Araport11)	22.09	12.76	1.73	9.98E-04	6.17E-03
3119	4	F2-3 vs. M2-3	252822_at	AT4G39955	alpha/beta-Hydrolases superfamily protein;(source:Araport11)	25.93	15.11	1.72	3.16E-04	3.14E-03
3120	4	F2-3 vs. M2-3	259756_at	AT1G71080	RNA polymerase II transcription elongation factor;(source:Araport11)	77.53	45.26	1.71	9.30E-04	5.90E-03
3121	4	F2-3 vs. M2-3	259180_at	AT3G01680	Encodes a protein localized to phloem filaments that is required for phloem filament formation. The mRNA is cell-to-cell mobile.	34.90	20.54	1.70	8.09E-04	5.42E-03
3122	4	F2-3 vs. M2-3	247320_at	AT5G64040	Encodes the only subunit of photosystem I located entirely in the thylakoid lumen. May be involved in the interaction between plastocyanin and the photosystem I complex. Phosphorylation of this protein is dependent on calcium.	2121.52	1244.50	1.70	9.72E-04	6.09E-03
3123	4	F2-3 vs. M2-3	263034_at	AT1G24020	MLP-like protein 423;(source:Araport11)	2241.08	1329.37	1.69	6.32E-04	4.69E-03
3124	4	F2-3 vs. M2-3	264186_at	AT1G54570	Encodes a protein with phytyl ester synthesis and diacylglycerol acyltransferase activities that is involved in the deposition of free phytol and free fatty acids in the form of phytyl esters in chloroplasts, a process involved in maintaining the integrity of the photosynthetic membrane during abiotic stress and senescence.	55.07	32.61	1.69	7.35E-04	5.12E-03
3125	4	F2-3 vs. M2-3	266924_at	AT2G45730	eukaryotic initiation factor 3 gamma subunit family protein;(source:Araport11)	61.33	36.31	1.69	6.99E-04	4.97E-03
3126	4	F2-3 vs. M2-3	252037_at	AT3G51920	encodes a divergent member of calmodulin, which is an EF-hand family of Ca2+-binding proteins. This gene is expressed in leaves, flowers and siliques. The gene functionally complements yeast calmodulin 1 (CAM1) but only when selected against the plasmid harboring wild-type yeast sequences. Also the protein does not form formed a complex with a basic amphiphilic helical peptide in the presence of Ca2+ in vitro. Authors suggest that this gene may represent a Ca2+-binding sensor protein that interacts with a more limited set of target proteins than do more conventional CaM isoforms. Mutations in this gene alter plant responses to abiotic stress and abscisic acid.	18.62	10.99	1.69	1.14E-04	1.74E-03
3127	4	F2-3 vs. M2-3	263228_at	AT1G30700	FAD-binding Berberine family protein;(source:Araport11)	17.26	10.29	1.68	6.69E-04	4.86E-03
3128	4	F2-3 vs. M2-3	267029_at	AT2G38460	Encodes IRON REGULATED1 (IREG1/FPN1), one of the Arabidopsis orthologs (AT2G38460/IREG1/FPN1 and AT5G03570/IREG2/FPN2) the iron efflux transporter ferroportin (FPN) identified in animals.	105.26	62.83	1.68	5.85E-04	4.45E-03
3129	4	F2-3 vs. M2-3	266977_at	AT2G39420	alpha/beta-Hydrolases superfamily protein;(source:Araport11)	24.82	14.74	1.68	5.40E-04	4.28E-03
3130	4	F2-3 vs. M2-3	259232_at	AT3G11420	beta-1,3-N-acetylglucosaminyltransferase lunatic protein, putative (DUF604);(source:Araport11)	29.69	17.70	1.68	9.28E-04	5.90E-03
3131	4	F2-3 vs. M2-3	256115_at	AT1G16880	Encodes a ACT domain-containing protein. The ACT domain, named after bacterial aspartate kinase, chorismate mutase and TyrA (prephenate dehydrogenase), is a regulatory domain that serves as an amino acid-binding site in feedback-regulated amino acid metabolic enzymes. The mRNA is cell-to-cell mobile.	249.78	149.83	1.67	5.23E-05	1.13E-03

3132	4	F2-3 vs. M2-3	262174_at	AT1G74910	KONJAC1 is imilar to sugar pyrophosphorylases but has an insertion of 2 AA in the pyrophosphorylase consensus motif that is highly conserved in GMPPs. It lacks GDP-mannose pyrophosphorylase activity but can simulate the GDP-mannose pyrophosphorylase activity of VTC1.	35.17	21.11	1.67	9.76E-04	6.09E-03
3133	4	F2-3 vs. M2-3	252996_s_at	AT4G38460	Encodes a type II small subunit of the heteromeric geranyl(geranyl) diphosphate synthase that is localized to the chloroplast, expressed in petals and sepals and is involved in monoterpene biosynthesis. The mRNA is cell-to-cell mobile.	158.70	95.28	1.67	6.79E-04	4.89E-03
3134	4	F2-3 vs. M2-3	247240_at	AT5G64660	CYS, MET, PRO, and GLY protein 2;(source:Araport11)	30.95	18.49	1.67	7.73E-04	5.28E-03
3135	4	F2-3 vs. M2-3	253727_at	AT4G29350	Encodes profilin2, a low-molecular weight, actin monomer-binding protein that regulates the organization of actin cytoskeleton. Expressed in vegetative organs. The first intron of PRF2 enhances gene expression. The mRNA is cell-to-cell mobile.	641.83	386.36	1.66	6.52E-04	4.79E-03
3136	4	F2-3 vs. M2-3	245252_at	AT4G17500	Encodes a member of the ERF (ethylene response factor) subfamily B-3 of ERF/AP2 transcription factor family (ATERF-1). The protein contains one AP2 domain. There are 18 members in this subfamily including ATERF-1, ATERF-2, AND ATERF-5. The mRNA is cell-to-cell mobile.	24.51	14.85	1.65	5.14E-04	4.16E-03
3137	4	F2-3 vs. M2-3	266562_at	AT2G23970	Class I glutamine amidotransferase-like superfamily protein;(source:Araport11)	19.12	11.63	1.64	3.20E-04	3.16E-03
3138	4	F2-3 vs. M2-3	264080_at	AT2G28520	Vacuolar proton ATPase subunit VHA-a isoform 1. Localized in the trans-Golgi network. The mRNA is cell-to-cell mobile.	163.67	99.65	1.64	4.90E-06	2.60E-04
3139	4	F2-3 vs. M2-3	259098_at	AT3G04790	Ribose 5-phosphate isomerase, type A protein;(source:Araport11)	148.00	90.22	1.64	6.10E-04	4.56E-03
3140	4	F2-3 vs. M2-3	256673_at	AT3G52370	Fasciclin-like arabinogalactan protein. Possibly involved in embryogenesis and seed development.	155.48	94.67	1.64	9.52E-04	5.98E-03
3141	4	F2-3 vs. M2-3	259598_at	AT1G27980	dihydrosphingosine phosphate lyase;(source:Araport11)	75.45	46.96	1.61	2.60E-04	2.87E-03
3142	4	F2-3 vs. M2-3	263748_at	AT2G21480	BUSP2 plays a smaller role than BUSP1 in pollen tube growth. bups1/2 double mutants have reduced feritlity due to premature rupture of pollen tubes before they reach the ovule but single busp2 mutants are fertile. BUSP2 interacts with RALF4/19 peptide ligands and ANX1/2 receptors. BUPS/ANX signaling may regulate and promote pollen tube growth.	16.22	10.06	1.61	5.99E-04	4.51E-03
3143	4	F2-3 vs. M2-3	251834_at	AT3G55170	Ribosomal L29 family protein;(source:Araport11)	12.17	7.54	1.61	5.86E-04	4.45E-03
3144	4	F2-3 vs. M2-3	245150_at	AT2G47590	photolyase/blue light photoreceptor PHR2 (PHR2) mRNA,	47.35	29.54	1.60	2.89E-04	3.03E-03
3145	4	F2-3 vs. M2-3	261948_at	AT1G64680	beta-carotene isomerase D27;(source:Araport11)	148.49	94.08	1.58	5.91E-05	1.20E-03
3146	4	F2-3 vs. M2-3	254398_at	AT4G21280	Encodes the PsbQ subunit of the oxygen evolving complex of photosystem II.	185.07	117.94	1.57	1.14E-04	1.75E-03
3147	4	F2-3 vs. M2-3	245076_at	AT2G23170	encodes an IAA-amido synthase that conjugates Asp and other amino acids to auxin in vitro.	20.33	13.04	1.56	3.31E-04	3.23E-03
3148	4	F2-3 vs. M2-3	266483_at	AT2G47910	Encodes a chloroplast thylakoid membrane protein. Required for the assembly/accumulation of the NAD(P)H dehydrogenase complex of the photosynthetic electron transport chain.	24.88	16.05	1.55	5.24E-04	4.20E-03
3149	4	F2-3 vs. M2-3	258815_at	AT3G04000	ChlADR is an aldehyde reductase that catalyzes the reduction of the aldehyde carbonyl groups on saturated and alpha,beta-unsaturated aldehydes with more than 5 carbons in vitro. The N-terminal region of this protein directs GFP to the chloroplast where where ChlADR likely helps to maintain the photosynthetic process by detoxifying reactive carbonyls formed during lipid peroxidation. In addition, this enzyme can also reduce cis-3-hexenal, a major plant volatile compound that contributes to green leaf odor, as well as methylglyoxal in vitro.	65.37	42.27	1.55	1.93E-04	2.36E-03
3150	4	F2-3 vs. M2-3	245506_at	AT4G15700	Encodes a member of the CC-type glutaredoxin (ROXY) family.	14.32	9.37	1.53	3.51E-04	3.33E-03
3151	4	F2-3 vs. M2-3	266118_at	AT2G02130	Predicted to encode a PR (pathogenesis-related) protein. Belongs to the plant defensin (PDF) family with the following members: At1g75830/PDF1.1, At5g44420/PDF1.2a, At2g26020/PDF1.2b, At5g44430/PDF1.2c, At2g26010/PDF1.3, At1g19610/PDF1.4, At1g55010/PDF1.5, At2g02120/PDF2.1, At2g02100/PDF2.2, At2g02130/PDF2.3, At1g61070/PDF2.4, At5g63660/PDF2.5, At2g02140/PDF2.6, At5g38330/PDF3.1 and At4g30070/PDF3.2. The mRNA is cell-to-cell mobile.	48.42	31.94	1.52	9.37E-04	5.92E-03
3152	4	F2-3 vs. M2-3	261248_at	AT1G20030	Pathogenesis-related thaumatin superfamily protein;(source:Araport11)	17.73	11.73	1.51	8.58E-05	1.45E-03
3153	4	F2-3 vs. M2-3	250335_at	AT5G11650	alpha/beta-Hydrolases superfamily protein;(source:Araport11)	134.99	90.00	1.50	8.60E-04	5.63E-03
3154	4	F2-3 vs. M2-3	254040_at	AT4G25900	Galactose mutarotase-like superfamily protein;(source:Araport11)	14.60	9.94	1.47	8.80E-04	5.72E-03
3155	4	F2-3 vs. M2-3	262118_at	AT1G02850	beta glucosidase 11;(source:Araport11)	18.97	13.26	1.43	6.88E-04	4.93E-03
3156	4	F2-3 vs. M2-3	255605_at	AT4G01090	Hypothetical protein; participates in wound-induced lateral root development.	10.60	16.01	0.66	1.66E-04	2.17E-03
3157	4	F2-3 vs. M2-3	259224_at	AT3G07800	Encodes a thymidine kinase that salvages DNA precursors.	66.20	101.96	0.65	8.86E-04	5.74E-03

3158	4	F2-3 vs. M2-3	262711_at	AT1G16500	filamentous hemagglutinin transporter;(source:Araport11)	31.70	50.54	0.63	7.03E-04	4.98E-03
3159	4	F2-3 vs. M2-3	249703_at	AT5G35560	DENN (AEX-3) domain-containing protein;(source:Araport11)	46.58	75.70	0.62	2.98E-04	3.05E-03
3160	4	F2-3 vs. M2-3	258283_at	AT3G26750	HECT-like ubiquitin-conjugating enzyme (E2)-binding protein;(source:Araport11)	16.62	27.41	0.61	1.94E-04	2.36E-03
3161	4	F2-3 vs. M2-3	265426_at	AT2G20740	Tetraspanin family protein;(source:Araport11)	313.86	524.98	0.60	3.05E-04	3.07E-03
3162	4	F2-3 vs. M2-3	256722_at	AT2G34050	ATP synthase F1 complex assembly factor;(source:Araport11)	35.29	59.28	0.60	7.60E-04	5.21E-03
3163	4	F2-3 vs. M2-3	266102_at	AT2G40720	Tetratricopeptide repeat (TPR)-like superfamily protein;(source:Araport11)	20.62	34.39	0.60	3.98E-04	3.58E-03
3164	4	F2-3 vs. M2-3	247964_at	AT5G56600	Encodes profilin3, a low-molecular weight, actin monomer-binding protein that regulates the organization of actin cytoskeleton. Originally known as profilin5, and later named profilin3. Expressed in vegetative organs. Mutants have slightly elongated petioles.	135.31	227.34	0.60	6.67E-04	4.85E-03
3165	4	F2-3 vs. M2-3	263359_at	AT2G15230	Lipase active on medium and short chain triacylglycerols, but not on phospho- or galactolipids. Active between pH4 and 7 with an optimum at pH6. Knock-out mutant has not obvious phenotype. Predicted to be extracellular.	36.45	61.81	0.59	7.39E-05	1.35E-03
3166	4	F2-3 vs. M2-3	251251_at	AT3G62220	Protein kinase superfamily protein;(source:Araport11)	101.18	171.83	0.59	3.06E-04	3.07E-03
3167	4	F2-3 vs. M2-3	260515_at	AT1G51460	ABCG13 encodes a member of the ATP-binding cassette (ABC) transporter family protein. Mutants show defects in petal elongation resulting in a folded petal phenotype.	44.85	77.31	0.58	2.89E-04	3.03E-03
3168	4	F2-3 vs. M2-3	245066_at	AT2G39840	Encodes the catalytic subunit of a Type 1 phosphoprotein Ser/Thr phosphatase, expressed in roots, shoots and flowers. The mRNA is cell-to-cell mobile.	187.68	323.93	0.58	2.82E-04	3.01E-03
3169	4	F2-3 vs. M2-3	266929_at	AT2G45850	AT hook motif DNA-binding family protein;(source:Araport11)	26.29	45.12	0.58	6.75E-04	4.88E-03
3170	4	F2-3 vs. M2-3	252506_at	AT3G46180	UDP-galactose transporter 5;(source:Araport11)	19.65	34.01	0.58	3.44E-04	3.29E-03
3171	4	F2-3 vs. M2-3	254348_at	AT4G22150	Arabidopsis thaliana CDC48-interacting UBX-domain protein (PUX3)	329.00	567.57	0.58	5.78E-05	1.19E-03
3172	4	F2-3 vs. M2-3	251048_at	AT5G02410	Encodes ALG10, an ER-resident alpha1,2-glucosyltransferase that is required for lipid-linked oligosaccharide biosynthesis and subsequently for normal leaf development and abiotic stress response.	26.59	45.63	0.58	6.74E-04	4.87E-03
3173	4	F2-3 vs. M2-3	249344_at	AT5G40770	prohibitin 3	184.52	315.59	0.58	3.06E-04	3.07E-03
3174	4	F2-3 vs. M2-3	249129_at	AT5G43080	Cyclin A3;(source:Araport11)	62.22	106.90	0.58	8.14E-05	1.41E-03
3175	4	F2-3 vs. M2-3	266515_at	AT2G47900	Member of plant TLP family which differs in having an F box domain. Plasma membrane tethering is mediated by PIP2 binding domain. Under abiotic stress TLP3 detaches from the PM and translocates to the nucleus. Mutants are insensitive to ABA.	179.11	314.85	0.57	2.95E-04	3.05E-03
3176	4	F2-3 vs. M2-3	252935_at	AT4G39150	DNAJ heat shock N-terminal domain-containing protein;(source:Araport11)	57.58	100.60	0.57	2.87E-04	3.03E-03
3177	4	F2-3 vs. M2-3	246089_at	AT5G20570	Encodes a ring-box 1 like protein and component of the SCF ubiquitinization complex mediating auxin responses. Forms a E3 ubiquitin ligase complex with CUL3A and At1g21780.1 a BTB domain protein.	205.39	359.46	0.57	3.30E-04	3.22E-03
3178	4	F2-3 vs. M2-3	247112_at	AT5G65950	TRAPPIII complex protein which regulates TGN integrity, by altered TGN/EE association of several residents, including SYNTAXIN OF PLANTS 61 (SYP61), and altered vesicle morphology. Involved in regulation of endosomal function and salt stress response.	281.21	491.18	0.57	2.30E-04	2.67E-03
3179	4	F2-3 vs. M2-3	259477_at	AT1G19120	Small nuclear ribonucleoprotein family protein;(source:Araport11)	351.88	626.64	0.56	2.62E-04	2.88E-03
3180	4	F2-3 vs. M2-3	245757_at	AT1G35140	EXL1 is involved in the C-starvation response. Phenotypic changes of an exl1 loss of function mutant became evident only under corresponding experimental conditions. For example, the mutant showed diminished biomass production in a short-day/low light growth regime, impaired survival during extended night, and impaired survival of anoxia stress.	13.00	23.01	0.56	5.26E-04	4.20E-03
3181	4	F2-3 vs. M2-3	260503_at	AT1G47250	Encodes 20S proteasome subunit PAF2 (PAF2).	75.54	134.85	0.56	6.11E-04	4.56E-03
3182	4	F2-3 vs. M2-3	260545_at	AT2G43350	Glutathione peroxidase. Functions as both a redox transducer and a scavenger in abscisic acid and drought stress responses. Interacts with ABI2 and ABI1.	21.55	38.35	0.56	4.24E-05	1.01E-03
3183	4	F2-3 vs. M2-3	254957_at	AT4G10970	UAP56-interacting factor1, binds single stranded RNA and, along with UIEF2,,appears to play a role in nuclear export of RNA.	61.96	111.40	0.56	3.76E-05	9.26E-04
3184	4	F2-3 vs. M2-3	253100_at	AT4G37400	member of CYP81F	31.30	55.88	0.56	8.78E-04	5.72E-03
3185	4	F2-3 vs. M2-3	246621_at	AT5G36230	ARM repeat superfamily protein;(source:Araport11)	805.10	1450.55	0.56	8.38E-04	5.54E-03
3186	4	F2-3 vs. M2-3	248117_at	AT5G55000	FH protein interacting protein FIP2	239.00	424.33	0.56	7.02E-04	4.98E-03
3187	4	F2-3 vs. M2-3	266890_at	AT2G44620	Encodes a member of the mitochondrial acyl carrier protein (ACP) family. As part of the mitochondrial matrix, it is likely to be involved in fatty acid or lipoic acid biogenesis. Its acylated form is predominantly present in the mitochondrial membrane while the non-acylated form is soluble. The mRNA is cell-to-cell mobile.	79.77	145.11	0.55	4.11E-04	3.65E-03

3188	4	F2-3 vs. M2-3	252464_at	AT3G47160	RING/U-box superfamily protein;(source:Araport11)	54.31	99.09	0.55	1.39E-04	1.95E-03
3189	4	F2-3 vs. M2-3	253854_at	AT4G27900	CCT motif family protein;(source:Araport11)	186.27	340.83	0.55	2.30E-04	2.67E-03
3190	4	F2-3 vs. M2-3	261239_at	AT1G32930	Galactosyltransferase family protein;(source:Araport11)	79.42	148.17	0.54	3.57E-04	3.36E-03
3191	4	F2-3 vs. M2-3	256143_at	AT1G48830	Ribosomal protein S7e family protein;(source:Araport11)	277.88	512.63	0.54	1.42E-04	1.97E-03
3192	4	F2-3 vs. M2-3	263738_at	AT1G60060	Serine/threonine-protein kinase WNK (With No Lysine)-like protein;(source:Araport11)	21.02	38.82	0.54	1.82E-04	2.29E-03
3193	4	F2-3 vs. M2-3	267071_at	AT2G40980	Protein kinase superfamily protein;(source:Araport11)	34.46	63.23	0.54	4.61E-04	3.92E-03
3194	4	F2-3 vs. M2-3	258206_at	AT3G14010	hydroxyproline-rich glycoprotein family protein, similar to Mrs16p (GI:2737884) (Saccharomyces cerevisiae); weak similarity to ataxin-2 related protein (GI:1679686) (Homo sapiens). Included in a family of CTC interacting domain proteins found to interact with PAB2.	20.89	38.78	0.54	9.92E-04	6.15E-03
3195	4	F2-3 vs. M2-3	245255_at	AT4G14110	Represses photomorphogenesis and induces skotomorphogenesis in the dark. A component of the COP9 signalosome complex.	64.21	119.49	0.54	8.50E-05	1.44E-03
3196	4	F2-3 vs. M2-3	254670_at	AT4G18390	TEOSINTE BRANCHED 1, cycloidea and PCF transcription factor 2;(source:Araport11)	50.62	93.18	0.54	6.64E-04	4.84E-03
3197	4	F2-3 vs. M2-3	253805_at	AT4G28260	acyl-UDP-N-acetylglucosamine O-acyltransferase;(source:Araport11)	74.07	136.13	0.54	8.45E-05	1.44E-03
3198	4	F2-3 vs. M2-3	253766_at	AT4G28770	Tetraspanin family protein;(source:Araport11)	29.00	53.59	0.54	3.85E-05	9.37E-04
3199	4	F2-3 vs. M2-3	250051_at	AT5G17800	Member of the R2R3 factor gene family that acts as a cell-specific repressor of quiescent center (QC) divisions in the primary root, acting through the BR signaling pathway. Works with BES1 to regulate QC division in the root.	51.25	94.57	0.54	7.50E-04	5.17E-03
3200	4	F2-3 vs. M2-3	263633_at	AT2G04890	Encodes a scarecrow-like protein (SCL21). Member of GRAS gene family.	83.34	155.95	0.53	4.12E-04	3.66E-03
3201	4	F2-3 vs. M2-3	267462_at	AT2G33735	Chaperone DnaJ-domain superfamily protein;(source:Araport11)	86.64	164.83	0.53	7.30E-04	5.10E-03
3202	4	F2-3 vs. M2-3	258665_at	AT3G08710	Associated to plasma membrane. Moves cell to cell, suggesting a role in intercellular communication.	51.46	96.45	0.53	3.06E-04	3.07E-03
3203	4	F2-3 vs. M2-3	257131_at	AT3G20240	Mitochondrial substrate carrier family protein;(source:Araport11)	54.15	102.68	0.53	9.96E-04	6.17E-03
3204	4	F2-3 vs. M2-3	256889_at	AT3G24010	ING1 encodes a member of the Inhibitor of Growth family of nuclear-localized PhD domain containing homeodomain proteins. Binds to H3K4 di or trimethylated DNA.	119.48	226.37	0.53	6.65E-04	4.84E-03
3205	4	F2-3 vs. M2-3	251352_at	AT3G61070	member of the peroxin11 (PEX11) gene family, integral to peroxisome membrane, controls peroxisome proliferation.	42.09	79.17	0.53	2.78E-04	2.99E-03
3206	4	F2-3 vs. M2-3	253633_at	AT4G30480	Encodes one of the 36 carboxylate clamp (CC)-tetratricopeptide repeat (TPR) proteins (Prasad 2010, Pubmed ID: 20856808). Interacts with Hsp90/Hsp70 as co-chaperone.	110.40	209.44	0.53	4.34E-04	3.77E-03
3207	4	F2-3 vs. M2-3	248694_at	AT5G48340	hypothetical protein;(source:Araport11)	111.75	211.10	0.53	2.89E-04	3.03E-03
3208	4	F2-3 vs. M2-3	259669_at	AT1G52340	Encodes a cytosolic short-chain dehydrogenase/reductase involved in the conversion of xanthoxin to ABA-aldehyde during ABA biosynthesis. Mutants are insensitive to sucrose and glucose.	24.56	47.22	0.52	2.79E-04	2.99E-03
3209	4	F2-3 vs. M2-3	262355_at	AT1G72820	Mitochondrial substrate carrier family protein;(source:Araport11)	119.01	226.68	0.52	4.66E-04	3.94E-03
3210	4	F2-3 vs. M2-3	263332_at	AT2G03870	Small nuclear ribonucleoprotein family protein;(source:Araport11)	208.33	398.12	0.52	1.96E-05	6.30E-04
3211	4	F2-3 vs. M2-3	263564_s_at	AT2G15400	Non-catalytic subunit of Nuclear DNA-dependent RNA polymerase V; homologous to budding yeast RPB3 and the E. coli RNA polymerase alpha subunit. A closely related paralog, At2g15430 can substitute for At2g15400 in the context of Pol V and encodes the equivalent subunit of Pol II and Pol IV.	55.29	107.36	0.52	6.60E-04	4.83E-03
3212	4	F2-3 vs. M2-3	258484_at	AT3G02580	Brassinosteroid biosynthetic enzyme, catalyzes delta7 sterol C-5 desaturation step. Mutant has dwarf phenotype.	106.32	205.18	0.52	4.97E-04	4.10E-03
3213	4	F2-3 vs. M2-3	258921_at	AT3G10500	Encodes a transcriptional activator that is associated with the plasma membrane in a dormant form and is proteolytically cleaved to create a form that can enter the nucleus. It is thought to promote ROS production by binding directly to the promoters of genes encoding ROS biosynthetic enzymes during drought-induced leaf senescence.The mRNA is cell-to-cell mobile.	108.38	209.48	0.52	3.58E-04	3.36E-03
3214	4	F2-3 vs. M2-3	257967_at	AT3G19910	RING/U-box superfamily protein;(source:Araport11)	267.06	517.12	0.52	4.54E-04	3.88E-03
3215	4	F2-3 vs. M2-3	251781_at	AT3G55320	P-glycoprotein 20;(source:Araport11)	27.33	52.13	0.52	4.68E-04	3.94E-03
3216	4	F2-3 vs. M2-3	255500_at	AT4G02390	Encodes a DNA dependent nuclear poly (ADP-ribose) polymerase (E.C.2.4.2.30), thought to be involved in post-translational modification .	26.23	50.79	0.52	3.98E-04	3.58E-03

3217	4	F2-3 vs. M2-3	263727_at	AT2G13540	Encodes a nuclear cap-binding protein that forms a heterodimeric complex with CBP20 and is involved in ABA signaling and flowering. Mutants are early flowering and exhibit hypersensitive response to ABA in germination inhibition.Loss of ABH1 function results in abnormal processing of mRNAs for several important floral regulators (FLC, CO, FLM). Analysis of loss of function mutations suggests a role in pri-miRNA processing and mRNA splicing. Note that two different mutant alleles were given the same name abh1-7 (Kuhn et al 2007; Kim et al 2008). To avoid confusion, abh1-7 described in Kim et al (2008) has been renamed abh1-107 (other names: ensalada-1, ens-1).	148.36	289.39	0.51	9.93E-04	6.15E-03
3218	4	F2-3 vs. M2-3	257236_at	AT3G15095	Encodes HCF243 (high chlorophyll fluorescence), a chloroplast-localized protein involved in the D1 protein stability of the photosystem II complex1.	71.89	141.24	0.51	4.58E-04	3.91E-03
3219	4	F2-3 vs. M2-3	249275_at	AT5G41870	Pectin lyase-like superfamily protein;(source:Araport11)	52.47	103.46	0.51	8.85E-04	5.74E-03
3220	4	F2-3 vs. M2-3	258612_at	AT3G02920	Replication protein A, subunit RPA32;(source:Araport11)	37.96	75.86	0.50	7.52E-04	5.17E-03
3221	4	F2-3 vs. M2-3	258155_at	AT3G18130	Encodes a protein with similarity to mammalian RACKs. RACKs function to shuttle activated protein kinase C to different subcellular sites and may also function as a scaffold through physical interactions with other proteins. RACK1C has no phenotype on its own and probably acts redundantly with RACK1A and RACK1B.	891.57	1767.71	0.50	9.35E-04	5.92E-03
3222	4	F2-3 vs. M2-3	266789_at	AT2G29020	Rab5-interacting family protein;(source:Araport11)	353.47	716.96	0.49	6.06E-04	4.54E-03
3223	4	F2-3 vs. M2-3	245362_at	AT4G17460	Encodes a class II HD-ZIP protein that regulates meristematic activity in different tissues, and that it is necessary for the correct formation of the gynoecium.	56.42	115.91	0.49	8.00E-04	5.38E-03
3224	4	F2-3 vs. M2-3	254359_at	AT4G22360	SWIB complex BAF60b domain-containing protein;(source:Araport11)	55.85	114.97	0.49	1.44E-04	1.97E-03
3225	4	F2-3 vs. M2-3	261207_at	AT1G12830	nucleolin;(source:Araport11)	26.02	54.36	0.48	6.94E-04	4.96E-03
3226	4	F2-3 vs. M2-3	261486_at	AT1G14510	Encodes a member of the Alfin1-like family of nuclear-localized PHD (plant homeodomain) domain containing proteins. All AL proteins except AL3 bind to di- or trimethylated histone H3 (H3K4me3/2). Members of this family include: AT5G05610 (AL1), AT3G11200 (AL2), AT3G42790 (AL3), AT5G26210 (AL4), AT5G20510 (AL5), AT2G02470 (AL6), AT1G14510 (AL7).	201.56	416.20	0.48	6.60E-06	2.99E-04
3227	4	F2-3 vs. M2-3	262514_at	AT1G34190	Encodes a NAC domain transcription factor that regulates the mitochondrial retrograde response and coordinates organellar functions and stress responses.	114.05	240.04	0.48	3.66E-05	9.26E-04
3228	4	F2-3 vs. M2-3	261364_at	AT1G53140	Encodes DRP5A, a dynamin protein involved in cytokinesis in Arabidopsis.	42.32	87.45	0.48	5.48E-04	4.33E-03
3229	4	F2-3 vs. M2-3	252148_at	AT3G51280	Tetratricopeptide repeat (TPR)-like superfamily protein;(source:Araport11)	41.22	86.05	0.48	1.74E-04	2.23E-03
3230	4	F2-3 vs. M2-3	251319_at	AT3G61610	Galactose mutarotase-like superfamily protein;(source:Araport11)	65.37	135.56	0.48	5.15E-04	4.16E-03
3231	4	F2-3 vs. M2-3	264872_at	AT1G24260	Member of the MADs box transcription factor family. SEP3 is redundant with SEP1 and 2. Flowers of SEP1/2/3 triple mutants show a conversion of petals and stamens to sepals.SEP3 forms heterotetrameric complexes with other MADS box family members and binds to the CArG box motif.	125.54	267.26	0.47	1.00E-06	1.06E-04
3232	4	F2-3 vs. M2-3	262417_at	AT1G50170	encodes sirohydrochlorin ferrochelatase catalyzing the last step of the siroheme biosynthesis	42.53	89.57	0.47	8.41E-04	5.55E-03
3233	4	F2-3 vs. M2-3	266038_at	AT2G07680	Encodes ABCC13/MRP11, a member of the multidrug resistance associated protein MRP/ABCC subfamily. Its expression is induced by gibberellic acid and downregulated by naphthalene acetic acid, abscisic acid, and zeatin.	25.49	54.38	0.47	8.27E-04	5.50E-03
3234	4	F2-3 vs. M2-3	259349_at	AT3G03860	Encodes a protein disulfide isomerase-like (PDIL) protein, a member of a multigene family within the thioredoxin (TRX) superfamily. This protein also belongs to the adenosine 5'-phosphosulfate reductase-like (APRL) group. The mRNA is cell-to-cell mobile.	20.90	44.34	0.47	5.61E-04	4.37E-03
3235	4	F2-3 vs. M2-3	252323_at	AT3G48530	SNF1-related protein kinase regulatory subunit gamma 1;(source:Araport11)	96.66	204.78	0.47	1.84E-04	2.30E-03
3236	4	F2-3 vs. M2-3	252230_at	AT3G49810	Encodes a protein with E3 ubiquitin ligase activity that is involved in negative regulation of salt stress tolerance during germination.	44.86	95.44	0.47	7.81E-04	5.31E-03
3237	4	F2-3 vs. M2-3	254953_at	AT4G10925	Nuclear transport factor 2 (NTF2) family protein;(source:Araport11)	110.34	233.99	0.47	5.65E-04	4.37E-03
3238	4	F2-3 vs. M2-3	254451_at	AT4G21090	MITOCHONDRIAL FERREDOXIN 2;(source:Araport11)	214.57	458.18	0.47	2.97E-04	3.05E-03
3239	4	F2-3 vs. M2-3	245965_at	AT5G19730	Pectin lyase-like superfamily protein;(source:Araport11)	69.13	148.18	0.47	7.48E-04	5.17E-03
3240	4	F2-3 vs. M2-3	246090_at	AT5G20520	Encodes a Bem46-like protein. WAV2 negatively regulates root bending when roots alter their growth direction. It's not involved in sensing environmental stimuli (e.g. gravity, light, water, touch).	61.86	130.40	0.47	9.11E-04	5.84E-03

3241	4	F2-3 vs. M2-3	252637_at	AT3G44530	Encodes a nuclear localized WD-repeat containing protein involved in negative regulation of knox gene expression via epigenetic mechanism of chromatin re-organization. It is a part of the HISTONE REGULATOR complex that deposits histones in a DNA synthesis-independent manner and affects both nucleosome occupancy and the maintenance of transcriptional silencing. Interacts physically and genetically with AS1. Expressed in meristem and leaf primordia. Homozygous mutants are embryo lethal. Phenotype of cosuppressed lines is variable but show effects on leaf development similar to as1/as2.	68.11	148.72	0.46	6.80E-06	3.02E-04
3242	4	F2-3 vs. M2-3	253946_at	AT4G26790	GDSL-motif esterase/acyltransfer/lipase. Enzyme group with broad substrate specificity that may catalyze acyltransfer or hydrolase reactions with lipid and non-lipid substrates.	24.29	53.10	0.46	8.70E-04	5.68E-03
3243	4	F2-3 vs. M2-3	253338_at	AT4G33430	Leu-rich receptor Serine/threonine protein kinase. Component of BR signaling that interacts with BRI1 in vitro and in vivo to form a heterodimer. Brassinolide-dependent association of BRI1 and BAK1 in vivo. Phosphorylation of both BRI1 and BAK1 on Thr residues was BR dependent. Although BAK1 and BRI1 alone localize in the plasma membrane, when BAK1 and BRI1 are coexpressed, the heterodimer BAK1/BRI1 they form is localized in the endosome.	25.42	54.71	0.46	8.50E-04	5.58E-03
3244	4	F2-3 vs. M2-3	250563_at	AT5G08050	Encodes a grana core localized protein. Mutant plants have reduced NPQ, affected organization of light-havesting complex II and an enhanced grana stacking.	81.58	178.25	0.46	2.98E-04	3.05E-03
3245	4	F2-3 vs. M2-3	247238_at	AT5G64610	Encodes an enzyme with histone acetyltransferase activity. HAM1 primarily acetylate histone H4, but also display some ability to acetylate H3. Prior acetylation of lysine 5 on histone H4 reduces radioactive acetylation by either HAM1. HAM1 acetylates histone H4 lysine 5.	76.48	166.44	0.46	5.85E-04	4.45E-03
3246	4	F2-3 vs. M2-3	261810_at	AT1G08130	Encodes the Arabidopsis DNA ligase 1 that provides the major DNA ligase activity in cells and plays a key role in both DNA replication and excision repair pathways. In addition, it is an important component of the active DNA demethylation machinery and is indispensable for cell viability. AtLIG1 expresses one major and two minor mRNA transcripts differing only in the length of the 5' untranslated leader sequences preceding a common ORF. Translation from the first in-frame start codon produces an AtLIG1 isoform that is targeted exclusively to the mitochondria. Translation initiation from the second in-frame start codon produces an AtLIG1 isoform targeted only to the nucleus.	109.70	242.76	0.45	7.65E-05	1.36E-03
3247	4	F2-3 vs. M2-3	262313_at	AT1G70900	hypothetical protein;(source:Araport11)	40.40	90.60	0.45	4.99E-04	4.10E-03
3248	4	F2-3 vs. M2-3	260331_at	AT1G80270	PENTATRICOPEPTIDE REPEAT 596;(source:Araport11)	312.13	687.92	0.45	1.31E-04	1.89E-03
3249	4	F2-3 vs. M2-3	258487_at	AT3G02550	LOB domain-containing protein 41;(source:Araport11)	30.25	67.12	0.45	5.18E-04	4.18E-03
3250	4	F2-3 vs. M2-3	256939_at	AT3G22630	Encodes 20S proteasome beta subunit PBD1 (PBD1).	196.38	445.34	0.44	3.42E-04	3.28E-03
3251	4	F2-3 vs. M2-3	253115_at	AT4G35910	Encodes a cytoplasmic thiouridylase that is essential for tRNA thiolation. Its activity appears to be important in root development.	23.36	53.60	0.44	2.63E-04	2.88E-03
3252	4	F2-3 vs. M2-3	247606_at	AT5G61000	Replication factor-A protein 1-like protein;(source:Araport11)	42.03	95.59	0.44	4.78E-05	1.08E-03
3253	4	F2-3 vs. M2-3	260943_at	AT1G45145	encodes a cytosolic thioredoxin that reduces disulfide bridges of target proteins by the reversible formation of a disulfide bridge between two neighboring Cys residues present in the active site. Thioredoxins have been found to regulate a variety of biological reactions in prokaryotic and eukaryotic cells.	34.55	81.15	0.43	9.50E-04	5.97E-03
3254	4	F2-3 vs. M2-3	261859_at	AT1G50490	Encodes one of two ubiquitin-conjugating enzymes belonging to the E2-C gene family (the other being UBC19). Transcript is always found in diving cells, but also in other non-dividing cells.	118.87	275.69	0.43	6.43E-04	4.74E-03
3255	4	F2-3 vs. M2-3	264243_at	AT1G54650	Methyltransferase family protein;(source:Araport11)	66.98	156.84	0.43	1.99E-05	6.35E-04
3256	4	F2-3 vs. M2-3	256341_at	AT1G72040	Encodes a multisubstrate deoxyribonucleoside kinase that salvages DNA precursors.	62.87	147.68	0.43	6.62E-05	1.25E-03
3257	4	F2-3 vs. M2-3	263239_at	AT2G16570	Amidophosphoribosyltransferase (ATase: EC 2.4.2.14) is a key enzyme in the pathway of purine nucleotide biosynthesis	79.80	187.20	0.43	3.02E-04	3.07E-03
3258	4	F2-3 vs. M2-3	258332_at	AT3G16180	Encodes a low affinity nitrate transporter that is expressed in the plasma membrane and found in the phloem of the major veins of leaves. It is responsible for nitrate redistribution to young leaves.	15.52	36.38	0.43	2.91E-04	3.04E-03
3259	4	F2-3 vs. M2-3	252008_at	AT3G52610	GATA zinc finger protein;(source:Araport11)	42.58	99.35	0.43	3.19E-04	3.16E-03
3260	4	F2-3 vs. M2-3	254853_at	AT4G12080	AT-hook motif nuclear-localized protein 1;(source:Araport11)	37.27	87.31	0.43	4.52E-04	3.88E-03

3261	4	F2-3 vs. M2-3	253976_at	AT4G26610	D6PK family kinase involved in pulse-induced phototropism but also for time-dependent second positive phototropism, and continuous light-induced hypocotyl phototropism.	43.64	100.97	0.43	4.71E-04	3.96E-03
3262	4	F2-3 vs. M2-3	252832_at	AT4G39910	Encodes a nuclear ubiquitin-specific protease.	29.94	69.91	0.43	4.00E-04	3.59E-03
3263	4	F2-3 vs. M2-3	249206_at	AT5G42630	Encodes a member of the KANADI family of putative transcription factors. Involved in integument formation during ovule development and expressed at the boundary between the inner and outer integuments. It is essential for directing laminar growth of the inner integument.Along with KAN1 and KAN2, KAN4 is involved in proper localization of PIN1 in early embryogenesis.	22.20	52.06	0.43	6.36E-04	4.71E-03
3264	4	F2-3 vs. M2-3	247210_at	AT5G65020	Annexins are calcium binding proteins that are localized in the cytoplasm. When cytosolic Ca2+ increases, they relocate to the plasma membrane. They may be involved in the Golgi-mediated secretion of polysaccharides.	587.73	1355.75	0.43	4.37E-05	1.03E-03
3265	4	F2-3 vs. M2-3	260401_at	AT1G69840	SPFH/Band 7/PHB domain-containing membrane-associated protein family;(source:Araport11)	103.65	248.53	0.42	1.32E-04	1.89E-03
3266	4	F2-3 vs. M2-3	253668_at	AT4G30220	small nuclear ribonucleoprotein F;(source:Araport11)	130.90	314.26	0.42	2.00E-04	2.41E-03
3267	4	F2-3 vs. M2-3	249242_at	AT5G42250	Zinc-binding alcohol dehydrogenase family protein;(source:Araport11)	80.69	194.40	0.42	7.95E-05	1.39E-03
3268	4	F2-3 vs. M2-3	265097_at	AT1G04020	Encodes a protein containing two tandem BRCA1 C-Terminal (BRCT) domains, which function in phosphorylation-dependent protein-protein interactions. Loss of function mutations cause defects in meristem organization due to failure to repress WUS. BARD1 binds to WUS promoter and over expression of BARD reduces the extent of WUS expression.	22.32	54.96	0.41	9.24E-04	5.88E-03
3269	4	F2-3 vs. M2-3	262509_at	AT1G11290	Pentatricopeptide Repeat Protein containing the DYW motif. Required for editing of multiple plastid transcripts. Endonuclease activity.	42.48	103.80	0.41	2.64E-04	2.88E-03
3270	4	F2-3 vs. M2-3	261277_at	AT1G20230	Pentatricopeptide repeat (PPR) superfamily protein;(source:Araport11)	99.11	243.47	0.41	7.82E-04	5.31E-03
3271	4	F2-3 vs. M2-3	256320_at	AT3G12170	Chaperone DnaJ-domain superfamily protein;(source:Araport11)	41.00	100.65	0.41	5.73E-04	4.40E-03
3272	4	F2-3 vs. M2-3	257203_at	AT3G23730	xyloglucan endotransglucosylase/hydrolase 16;(source:Araport11)	432.52	1058.78	0.41	9.42E-04	5.94E-03
3273	4	F2-3 vs. M2-3	254991_at	AT4G10620	P-loop containing nucleoside triphosphate hydrolases superfamily protein;(source:Araport11)	65.72	158.95	0.41	1.44E-04	1.97E-03
3274	4	F2-3 vs. M2-3	264978_at	AT1G27120	Encodes a Golgi-localized hydroxyproline-O-galactosyltransferase.	70.63	176.37	0.40	5.01E-04	4.10E-03
3275	4	F2-3 vs. M2-3	263488_at	AT2G31840	Thioredoxin superfamily protein;(source:Araport11)	28.72	71.08	0.40	1.94E-05	6.30E-04
3276	4	F2-3 vs. M2-3	258553_at	AT3G06960	TGD4 is an integral dimeric beta-barrel lipid transfer protein that binds PtdOH with its N-terminus and contains dimerization domains at its C-terminus. Mutations in this gene suppress the low temperature-induced phenotype of Arabidopsis tocopherol-deficient mutant vte2.	33.95	84.76	0.40	1.88E-04	2.32E-03
3277	4	F2-3 vs. M2-3	252359_at	AT3G48440	Zinc finger C-x8-C-x5-C-x3-H type family protein;(source:Araport11)	25.70	63.87	0.40	6.44E-04	4.75E-03
3278	4	F2-3 vs. M2-3	255025_at	AT4G09900	Encodes a protein predicted to act as a carboxylesterase. It has similarity to the SABP2 methyl salicylate esterase from tobacco. This protein does not act on methyl IAA, methyl JA, MeSA, MeGA4, or MEGA9 in vitro.	23.04	57.09	0.40	7.66E-05	1.36E-03
3279	4	F2-3 vs. M2-3	245343_at	AT4G15830	ARM repeat superfamily protein;(source:Araport11)	37.69	94.72	0.40	4.63E-04	3.93E-03
3280	4	F2-3 vs. M2-3	254035_at	AT4G25970	Encodes the major form of the two non-mitochondrail phosphatidylserine decarboxylase. Located at the ER. The mRNA is cell-to-cell mobile.	61.94	155.36	0.40	4.62E-04	3.92E-03
3281	4	F2-3 vs. M2-3	264327_at	AT1G04170	protein synthesis initiation factor eIF2 gamma The mRNA is cell-to-cell mobile.	13.29	33.92	0.39	3.90E-04	3.54E-03
3282	4	F2-3 vs. M2-3	266303_at	AT2G27060	Leucine-rich repeat protein kinase family protein;(source:Araport11)	42.23	108.48	0.39	7.94E-04	5.36E-03
3283	4	F2-3 vs. M2-3	256961_at	AT3G13445	TBP (TATA binding protein) associates with TAF(II)s (TBP-associated factors) to form the TFIID general transcription factor complex	68.66	178.24	0.39	5.99E-05	1.21E-03
3284	4	F2-3 vs. M2-3	255499_at	AT4G02730	Transducin/WD40 repeat-like superfamily protein;(source:Araport11)	100.98	260.05	0.39	2.42E-04	2.74E-03
3285	4	F2-3 vs. M2-3	255807_at	AT4G10270	Member of the wound-induced polypeptide (WIP) family.	16.68	42.45	0.39	9.18E-04	5.86E-03
3286	4	F2-3 vs. M2-3	254784_at	AT4G12720	Encodes a protein with ADP-ribose hydrolase activity. Negatively regulates EDS1-conditioned plant defense and programmed cell death.	188.99	487.93	0.39	5.20E-05	1.13E-03
3287	4	F2-3 vs. M2-3	251021_at	AT5G02140	Pathogenesis-related thaumatin superfamily protein;(source:Araport11)	21.50	54.91	0.39	8.74E-05	1.46E-03
3288	4	F2-3 vs. M2-3	248418_at	AT5G51660	cleavage and polyadenylation specificity factor 160;(source:Araport11)	125.53	320.99	0.39	4.45E-04	3.83E-03
3289	4	F2-3 vs. M2-3	248148_at	AT5G54930	AT hook motif-containing protein;(source:Araport11)	35.00	88.72	0.39	7.53E-05	1.36E-03
3290	4	F2-3 vs. M2-3	247963_at	AT5G56590	O-Glycosyl hydrolases family 17 protein;(source:Araport11)	143.60	366.99	0.39	9.65E-04	6.05E-03
3291	4	F2-3 vs. M2-3	261178_at	AT1G04760	member of Synaptobrevin -like protein family	57.94	153.15	0.38	3.05E-04	3.07E-03
3292	4	F2-3 vs. M2-3	261080_at	AT1G07370	Encodes putative proliferating cell nuclear antigen involved in cell cycle regulation. May be sumoylated.	100.72	264.03	0.38	6.11E-05	1.21E-03
3293	4	F2-3 vs. M2-3	259103_at	AT3G11690	hypothetical protein;(source:Araport11)	60.09	158.01	0.38	9.25E-05	1.53E-03

3294	4	F2-3 vs. M2-3	245898_at	AT5G11020	Protein kinase superfamily protein;(source:Araport11)	150.50	397.71	0.38	4.38E-04	3.79E-03
3295	4	F2-3 vs. M2-3	250150_at	AT5G14710	proteasome assembly chaperone-like protein;(source:Araport11)	69.38	184.57	0.38	3.84E-04	3.51E-03
3296	4	F2-3 vs. M2-3	246443_at	AT5G17620	nuclear matrix protein;(source:Araport11)	94.96	249.75	0.38	7.04E-04	4.98E-03
3297	4	F2-3 vs. M2-3	249427_at	AT5G39850	Ribosomal protein S4;(source:Araport11)	159.06	416.55	0.38	2.10E-06	1.59E-04
3298	4	F2-3 vs. M2-3	264443_at	AT1G27385	phosphoribosylformylglycinamidine synthase;(source:Araport11)	92.70	249.89	0.37	9.98E-04	6.17E-03
3299	4	F2-3 vs. M2-3	266182_at	AT2G02370	SNARE associated Golgi protein family;(source:Araport11)	42.29	113.11	0.37	6.50E-06	2.99E-04
3300	4	F2-3 vs. M2-3	267561_at	AT2G45590	Protein kinase superfamily protein;(source:Araport11)	123.48	336.30	0.37	1.98E-04	2.39E-03
3301	4	F2-3 vs. M2-3	257702_at	AT3G12670	Cytidine triphosphate synthase.	107.27	293.35	0.37	3.61E-04	3.37E-03
3302	4	F2-3 vs. M2-3	258410_at	AT3G16780	Ribosomal protein L19e family protein;(source:Araport11)	228.77	616.16	0.37	3.89E-05	9.38E-04
3303	4	F2-3 vs. M2-3	257879_at	AT3G17160	hypothetical protein;(source:Araport11)	158.14	430.61	0.37	2.52E-04	2.82E-03
3304	4	F2-3 vs. M2-3	249328_at	AT5G40820	Encodes a Arabidopsis ortholog of the ATR protein kinase that is involved in a wide range of responses to DNA damage and plays a central role in cell-cycle regulation. Dominant loss of function alleles identified as suppressors of ALS also exhibit increased tolerance to aluminum. This may be due to the inhibition of terminal differentiation of the root apex upon exposure to Al.	74.82	201.92	0.37	5.17E-05	1.13E-03
3305	4	F2-3 vs. M2-3	248438_at	AT5G51230	Polycomb group protein with zinc finger domain involved in negative regulation of reproductive development. Forms a complex with FIE, CLF, and MSI1. This complex modulates the expression of target genes including AG, PI and AP3.	180.72	489.55	0.37	3.10E-04	3.09E-03
3306	4	F2-3 vs. M2-3	255727_at	AT1G25510	Eukaryotic aspartyl protease family protein;(source:Araport11)	68.77	190.35	0.36	1.46E-04	2.00E-03
3307	4	F2-3 vs. M2-3	266229_at	AT2G28840	hypothetical protein;(source:Araport11)	29.53	81.89	0.36	7.69E-05	1.36E-03
3308	4	F2-3 vs. M2-3	245512_at	AT4G15770	RNA binding protein;(source:Araport11)	77.72	214.49	0.36	1.66E-04	2.17E-03
3309	4	F2-3 vs. M2-3	263631_at	AT2G04900	hypothetical protein;(source:Araport11)	33.30	94.60	0.35	3.81E-05	9.32E-04
3310	4	F2-3 vs. M2-3	256621_at	AT3G24450	Heavy metal transport/detoxification superfamily protein;(source:Araport11)	45.38	128.79	0.35	5.64E-04	4.37E-03
3311	4	F2-3 vs. M2-3	257813_at	AT3G25100	Required for normal meiosis, may act in the last round of DNA replication prior to meiosis, sequence similar to yeast CDC45	78.62	227.72	0.35	4.53E-04	3.88E-03
3312	4	F2-3 vs. M2-3	264646_at	AT1G08860	Encodes a copine-like protein, which is a member of a newly identified class of calcium-dependent, phospholipid binding proteins that are present in a wide range of organisms. Overexpression of this gene suppresses bon1-1 phenotypes. Double mutant analyses with bon1-1 suggest that BON1 and BON3 have overlapping functions in maintaining cellular homeostasis and inhibiting cell death.	26.22	78.21	0.34	4.90E-04	4.07E-03
3313	4	F2-3 vs. M2-3	262367_at	AT1G73030	Encodes an ESCRT-related protein: CHMP1A/AT1G73030; CHMP1B/AT1G17730. CHMP1A and B mediate multivesicular body sorting of auxin carriers and are required for plant development. ESCRT: Endosomal Sorting Complexes Required For Transport machinery; CHMP: Charged Multivesicular Body Protein/Chromatin Modifying Protein.	122.32	364.19	0.34	1.34E-05	4.81E-04
3314	4	F2-3 vs. M2-3	262941_at	AT1G79490	Pentatricopeptide repeat (PPR) superfamily protein;(source:Araport11)	36.12	106.45	0.34	3.72E-05	9.26E-04
3315	4	F2-3 vs. M2-3	260530_at	AT2G47320	Cyclophilin-like peptidyl-prolyl cis-trans isomerase family protein;(source:Araport11)	134.12	394.17	0.34	9.30E-06	3.75E-04
3316	4	F2-3 vs. M2-3	258041_at	AT3G21175	member of a novel family of plant-specific GATA-type transcription factors.	38.18	113.96	0.34	5.23E-05	1.13E-03
3317	4	F2-3 vs. M2-3	246463_at	AT5G16970	encodes a 2-alkenal reductase (EC 1.3.1.74), plays a key role in the detoxification of reactive carbonyls	39.35	117.35	0.34	3.00E-04	3.06E-03
3318	4	F2-3 vs. M2-3	259942_at	AT1G71260	Encodes WHY2, a homolog of the potato p24 protein. It shares the conserved KGKAAL domain, a putative DNA-binding domain, with potato p24 and is localized to mitochondria and not the nucleus. WHY2 is a member of the Whirly family proteins present mainly in the plant kingdom performing various activities related to DNA metabolism. Crystal structure of Solanum tuberosum WHY2, a close homolog of Arabidopsis WHY2, reveal that Whirly proteins bind to single strand DNA to promote accurate repair of DNA double-strand breaks over an error-prone repair pathway.	20.23	61.58	0.33	7.27E-04	5.09E-03
3319	4	F2-3 vs. M2-3	259154_at	AT3G10260	Reticulon family protein;(source:Araport11)	245.86	755.59	0.33	1.17E-04	1.77E-03
3320	4	F2-3 vs. M2-3	253753_at	AT4G29030	Putative membrane lipoprotein;(source:Araport11)	132.48	406.80	0.33	3.58E-04	3.36E-03
3321	4	F2-3 vs. M2-3	267472_at	AT2G02850	Encodes plantacyanin one of blue copper proteins. Involved in anther development and pollination. Expressed in the transmitting tract of the pistil.	204.20	638.85	0.32	5.55E-04	4.36E-03
3322	4	F2-3 vs. M2-3	255478_at	AT4G02440	EID1 is an F-box protein that functions as a negative regulator in phytochrome A (phyA)-specific light signalling. Expressed at all stages of plant development independently of light conditions, localizes to the nucleus, and forms nuclear speckles under continuous far-red light. Forms stable dimeric and trimeric complexes with several ASK proteins and Cullin1 in yeast and in planta.	67.07	209.71	0.32	8.20E-05	1.41E-03

3323	4	F2-3 vs. M2-3	250433_at	AT5G10400	Histone superfamily protein;(source:Araport11)	64.20	200.23	0.32	4.81E-04	4.01E-03
3324	4	F2-3 vs. M2-3	249342_at	AT5G40660	ATP12 protein-like protein;(source:Araport11)	60.86	190.68	0.32	3.90E-04	3.54E-03
3325	4	F2-3 vs. M2-3	262924_s_at	AT1G75630	vacuolar H+-pumping ATPase 16 kD proteolipid (ava-p) mRNA, The mRNA is cell-to-cell mobile.	553.36	1801.11	0.31	4.27E-04	3.72E-03
3326	4	F2-3 vs. M2-3	262969_at	AT1G75710	C2H2-like zinc finger protein;(source:Araport11)	265.09	844.38	0.31	9.46E-05	1.55E-03
3327	4	F2-3 vs. M2-3	263988_at	AT2G42830	AGAMOUS [AG]-like MADS box protein (AGL5) involved in fruit development (valve margin and dehiscence zone differentiation). A putative direct target of AG. SHP2 has been shown to be a downstream gene of the complex formed by AG and SEP proteins (SEP4 alone does not form a functional complex with AG).	95.58	311.90	0.31	1.09E-04	1.70E-03
3328	4	F2-3 vs. M2-3	252184_at	AT3G50660	Encodes a 22α hydroxylase whose reaction is a rate-limiting step in brassinosteroid biosynthetic pathway. The protein is a member of CYP90B gene family. CLM is an epi-allele with small, compressed rosette, reduced internode length, and reduced fertility, appears in selfed ddm mutant plants possibly due to loss of cytosine methylation. Transcripts accumulate in actively growing tissues, and GUS expression is negatively regulated by brassinosteroids. Localized in the endoplasmic reticulum. The in vitro expressed protein can perform the C-22 hydroxylation of a variety of C27-, C28- and C29-sterols. Cholesterol was the best substrate, followed by campesterol. Sitosterol was a poor substrate.	155.25	493.83	0.31	6.90E-04	4.94E-03
3329	4	F2-3 vs. M2-3	251735_at	AT3G56090	Encodes FERRITIN 3, AtFER3. Ferritins are a class of 24-mer multi-meric proteins found in all kingdoms of life. Function as the main iron store in mammals. Evidence suggests that Arabidopsis ferritins are essential to protect cells against oxidative damage, but they do not constitute the major iron pool.	99.64	323.43	0.31	8.68E-04	5.68E-03
3330	4	F2-3 vs. M2-3	264674_at	AT1G09815	polymerase delta 4;(source:Araport11)	39.37	136.10	0.29	7.55E-05	1.36E-03
3331	4	F2-3 vs. M2-3	261023_at	AT1G12200	Putative flavin monooxygenase.	13.84	47.34	0.29	3.91E-04	3.54E-03
3332	4	F2-3 vs. M2-3	261019_at	AT1G26470	chromatin modification-like protein;(source:Araport11)	29.70	101.18	0.29	4.98E-04	4.10E-03
3333	4	F2-3 vs. M2-3	263151_at	AT1G54120	hypothetical protein;(source:Araport11)	44.20	152.36	0.29	2.42E-04	2.74E-03
3334	4	F2-3 vs. M2-3	266391_at	AT2G41290	Although this enzyme is predicted to encode a strictosidine synthase (SS), it lacks a conserved catalytic glutamate residue found in active SS enzymes and it is not expected to have SS activity.	184.11	652.49	0.28	6.70E-06	3.01E-04
3335	4	F2-3 vs. M2-3	251995_at	AT3G52940	Encodes a sterol C-14 reductase required for cell division and expansion and is involved in proper organization of the embryo.	57.31	204.33	0.28	2.16E-04	2.53E-03
3336	4	F2-3 vs. M2-3	246002_at	AT5G20740	Plant invertase/pectin methylesterase inhibitor superfamily protein;(source:Araport11)	279.45	980.59	0.28	8.22E-04	5.49E-03
3337	4	F2-3 vs. M2-3	247962_at	AT5G56580	Encodes a member of the MAP Kinase Kinase family of proteins. It can phosphorylate MPK12 in vitro and it can be dephosphorylated by MKP2 in vitro.	57.14	213.94	0.27	3.90E-04	3.54E-03
3338	4	F2-3 vs. M2-3	247355_at	AT5G63670	SPT4 homolog 2;(source:Araport11)	53.62	198.76	0.27	1.33E-04	1.89E-03
3339	4	F2-3 vs. M2-3	259381_s_at	AT3G16410	Encodes a nitrile-specifier protein NSP4. NSP4 is one out of five (At3g16400/NSP1, At2g33070/NSP2, At3g16390/NSP3, At3g16410/NSP4 and At5g48180/NSP5) A. thaliana epithiospecifier protein (ESP) homologues that promote simple nitrile, but not epithionitrile or thiocyanate formation. The mRNA is cell-to-cell mobile.	27.97	108.52	0.26	3.14E-05	8.40E-04
3340	4	F2-3 vs. M2-3	264061_at	AT2G27970	CDK-subunit 2;(source:Araport11)	102.69	408.45	0.25	8.42E-04	5.55E-03
3341	4	F2-3 vs. M2-3	265005_at	AT1G61667	serine protease, putative (Protein of unknown function, DUF538);(source:Araport11)	33.43	138.15	0.24	7.81E-04	5.31E-03
3342	4	F2-3 vs. M2-3	266401_s_a	AT2G38620	Encodes a member of a plant specific family of cyclin dependent kinases.	115.91	487.01	0.24	2.77E-04	2.98E-03
3343	4	F2-3 vs. M2-3	257224_at	AT3G27870	ATPase E1-E2 type family protein / haloacid dehalogenase-like hydrolase family protein;(source:Araport11)	30.56	126.98	0.24	1.23E-04	1.82E-03
3344	4	F2-3 vs. M2-3	245228_at	AT3G29810	During the course of seed coat epidermal cell differentiation, COBRA-LIKE 2 plays a role in cellulose deposition into mucilage secretory cells of Arabidopsis seeds. COBRA-LIKE 2 affects mucilage solubility and cellulosic ray formation.	21.14	89.67	0.24	1.64E-04	2.16E-03
3345	4	F2-3 vs. M2-3	266123_at	AT2G45180	Bifunctional inhibitor/lipid-transfer protein/seed storage 2S albumin superfamily protein;(source:Araport11)	22.14	94.26	0.23	2.18E-04	2.55E-03
3346	4	F2-3 vs. M2-3	251778_at	AT3G55660	Encodes a member of KPP-like gene family, homolog of KPP (kinase partner protein) gene in tomato. Also a member of the RopGEF (guanine nucleotide exchange factor) family, containing the novel PRONE domain (plant-specific Rop nucleotide exchanger), which is exclusively active towards members of the Rop subfamily.	50.54	215.93	0.23	3.50E-05	9.16E-04
3347	4	F2-3 vs. M2-3	253357_at	AT4G33400	Vacuolar import/degradation, Vid27-related protein;(source:Araport11)	209.61	953.72	0.22	7.53E-04	5.17E-03
3348	4	F2-3 vs. M2-3	249832_at	AT5G23400	Leucine-rich repeat (LRR) family protein;(source:Araport11)	17.30	85.02	0.20	2.34E-04	2.68E-03

3349	4	F2-3 vs. M2-3	260181_at	AT1G70710	endo-1,4-beta-glucanase. Involved in cell elongation.	120.00	647.99	0.19	5.97E-04	4.51E-03
3350	4	F2-3 vs. M2-3	246923_at	AT5G25100	Endomembrane protein 70 protein family;(source:Araport11)	20.83	109.57	0.19	8.56E-04	5.62E-03
3351	4	F2-3 vs. M2-3	261650_at	AT1G27770	Encodes a chloroplast envelope Ca2+-ATPase with an N-terminal autoinhibitor.	21.30	123.76	0.17	1.36E-05	4.85E-04
3352	4	F2-3 vs. M2-3	255433_at	AT4G03210	encodes a member of xyloglucan endotransglucosylase/hydrolases (XTHs) that catalyze the cleavage and molecular grafting of xyloglucan chains function in loosening and rearrangement of the cell wall. Gene is expressed in shoot apex region, flower buds, flower stalks and internodes bearing flowers.	129.27	778.46	0.17	6.30E-06	2.98E-04
3353	4	F2-3 vs. M2-3	262802_at	AT1G20930	Cyclin-dependent kinase, expressed in flowers and suspension cell culture, expression peaks during M phase in synchronized cultures. Required for proper organization of the shoot apical meristem and for hormone signaling. Expressed in the shoot apical meristem. Involved in regulation of the G2/M transition of the mitotic cell cycle.	36.06	243.14	0.15	1.05E-04	1.67E-03
3354	4	F2-3 vs. M2-3	264577_at	AT1G05260	Encodes a cold-inducible cationic peroxidase that is involved in the stress response. In response to low temperature, RCI3 transcripts accumulate in the aerial part and in roots of etiolated seedlings but only in roots of light-grown seedlings. The mRNA is cell-to-cell mobile.	51.40	389.20	0.13	8.63E-05	1.45E-03
3355	4	F2-3 vs. M2-3	259541_at	AT1G20650	Protein kinase superfamily protein;(source:Araport11)	24.87	201.38	0.12	4.15E-04	3.66E-03
3356	4	F2-3 vs. M2-3	253874_at	AT4G27450	aluminum induced protein with YGL and LRDR motifs;(source:Araport11)	50.18	411.65	0.12	6.40E-06	2.99E-04
3357	4	F2-3 vs. M2-3	246203_at	AT4G36610	alpha/beta-Hydrolases superfamily protein;(source:Araport11)	32.31	267.90	0.12	2.61E-04	2.88E-03
3358	4	F2-3 vs. M2-3	267614_at	AT2G26710	Encodes a member of the cytochrome p450 family that serves as a control point between multiple photoreceptor systems and brassinosteroid signal transduction. Involved in brassinolide metabolism. Mediates response to a variety of light signals including hypocotyl elongation and cotyledon expansion.	12.67	115.06	0.11	7.70E-05	1.36E-03
3359	4	F2-3 vs. M2-3	256125_at	AT1G18250	encodes a thaumatin-like protein	38.92	381.77	0.10	2.03E-05	6.41E-04
3360	4	F2-3 vs. M2-3	251555_at	AT3G58780	One of two genes (SHP1 and SHP2) that are required for fruit dehiscence. The two genes control dehiscence zone differentiation and promote the lignification of adjacent cells.	82.37	833.16	0.10	1.38E-05	4.89E-04
3361	4	F2-3 vs. M2-3	247208_at	AT5G64870	Belongs to the group of plant flotillins, which are plasma membrane proteins. Flot3 is found in membrane nanodomains.	79.98	866.65	0.09	1.50E-06	1.24E-04
3362	4	F2-3 vs. M2-3	266012_s_a	ATMG00410	ATPase subunit 6	69.79	771.71	0.09	4.53E-05	1.05E-03
3363	4	F2-3 vs. M2-3	255014_at	AT4G09960	Encodes a MADS box transcription factor expressed in the carpel and ovules. Plays a maternal role in fertilization and seed development. Controls the structure and mechanical properties of the seed coat. Controls fruit size by regulating cytokinin levels and FRUITFULL.	32.02	372.80	0.09	9.00E-07	1.02E-04
3364	4	F2-3 vs. M2-3	265058_s_at	AT1G52030	Similar to myrosinase binding proteins which may be involved in metabolizing glucosinolates and forming defense compounds to protect against herbivory. Also similar to lectins and other agglutinating factors. Expressed only in flowers.	104.12	1365.63	0.08	2.49E-04	2.80E-03
3365	4	F2-3 vs. M2-3	257334_at	ATMG01370	transmembrane protein;(source:Araport11)	34.23	611.61	0.06	3.55E-05	9.20E-04
3366	4	F2-3 vs. M2-3	256648_at	AT3G13580	Ribosomal protein L30/L7 family protein;(source:Araport11)	20.65	1432.78	0.01	3.40E-06	2.16E-04
3367	5	L2-3 vs. S2-3	256648_at	AT3G13580	Ribosomal protein L30/L7 family protein;(source:Araport11)	1287.46	19.48	66.1	6.40E-06	5.27E-03
3368	5	L2-3 vs. S2-3	245026_at	ATCG00140	ATPase III subunit	2472.88	54.19	45.63	1.99E-05	5.48E-03
3369	5	L2-3 vs. S2-3	252321_at	AT3G48510	ABA‐induced transcription repressor that acts as feedback regulator in ABA signalling.	919.76	31.2	29.48	1.18E-04	9.20E-03
3370	5	L2-3 vs. S2-3	244959_s_at	AT2G07708	hypothetical protein;(source:Araport11)	300.84	21.09	14.26	3.66E-04	1.19E-02
3371	5	L2-3 vs. S2-3	245499_at	AT4G16480	Encodes a high affinity H+:<i>myo</i>-inositol symporter. The only other compound shown to be transported was pinitol, a methylated derivative of <i>myo</i>-inositol. The mRNA is cell-to-cell mobile.	493.5	49.91	9.89	2.87E-05	6.15E-03
3372	5	L2-3 vs. S2-3	267607_s_at	AT2G26740	Encodes a soluble epoxide hydrolase whose expression is induced by auxin and water stress.	102.92	13.14	7.83	1.26E-04	9.27E-03
3373	5	L2-3 vs. S2-3	245010_at	ATCG00420	Encodes NADH dehydrogenase subunit J. Its transcription is increased upon sulfur depletion.	188.77	26.58	7.1	5.58E-04	1.45E-02
3374	5	L2-3 vs. S2-3	252095_at	AT3G51000	alpha/beta-Hydrolases superfamily protein;(source:Araport11)	179.01	27.07	6.61	1.55E-04	1.02E-02
3375	5	L2-3 vs. S2-3	265929_s_at	AT2G18570	UDP-Glycosyltransferase superfamily protein;(source:Araport11)	85.08	13.22	6.43	9.73E-04	1.79E-02
3376	5	L2-3 vs. S2-3	244972_at	ATCG00680	encodes for CP47, subunit of the photosystem II reaction center.	94.71	16.45	5.76	3.67E-04	1.19E-02

3377	5	L2-3 vs. S2-3	254824_s_at	AT4G12620	Origin Recognition Complex subunit 1b. Involved in the initiation of DNA replication. Regulated transcriptionally during cell cycle, peaking at G1/S-phase. Target of E2F/DF family of transcription factors. Interacts with ORC2 and ORC5. Highly expressed in proliferating cells. Expression levels are independent of light regime.	428.47	75.7	5.66	3.10E-05	6.15E-03
3378	5	L2-3 vs. S2-3	247208_at	AT5G64870	Belongs to the group of plant flotillins, which are plasma membrane proteins. Flot3 is found in membrane nanodomains.	1033.03	214.23	4.82	2.00E-05	5.48E-03
3379	5	L2-3 vs. S2-3	258321_at	AT3G22840	Encodes an early light-inducible protein.	125.75	26.88	4.68	3.93E-05	6.43E-03
3380	5	L2-3 vs. S2-3	266849_at	AT2G25940	Encodes a vacuolar processing enzyme belonging to a novel group of cysteine proteinases that is expressed in vegetative organs and is upregulated in association with various types of cell death and under stressed conditions.	132.96	28.81	4.62	2.12E-04	1.07E-02
3381	5	L2-3 vs. S2-3	258041_at	AT3G21175	member of a novel family of plant-specific GATA-type transcription factors.	92.27	20.28	4.55	2.76E-04	1.13E-02
3382	5	L2-3 vs. S2-3	247033_at	AT5G67250	Encodes an SKP1 interacting partner (SKIP2).Encodes an F-box protein. Based on genetic analysis appears to be functionally redundant with VFB1,2, and 3. When expression of all 4 genes is reduced plants show defects in growth and reduced expression of auxin response genes.	481.1	120.27	4	1.23E-04	9.20E-03
3383	5	L2-3 vs. S2-3	244966_at	ATCG00590	electron carrier;(source:Araport11)	54.87	15.16	3.62	2.12E-04	1.07E-02
3384	5	L2-3 vs. S2-3	260745_at	AT1G78370	Encodes glutathione transferase belonging to the tau class of GSTs. Naming convention according to Wagner et al. (2002).	65.43	18.08	3.62	3.97E-04	1.24E-02
3385	5	L2-3 vs. S2-3	260967_at	AT1G12230	Aldolase superfamily protein;(source:Araport11)	368.48	103.3	3.57	2.33E-04	1.08E-02
3386	5	L2-3 vs. S2-3	267012_at	AT2G39220	PATATIN-like protein 6;(source:Araport11)	656.18	190.71	3.44	7.66E-05	7.98E-03
3387	5	L2-3 vs. S2-3	248250_at	AT5G53130	member of Cyclic nucleotide gated channel family	196.02	57.19	3.43	8.89E-05	8.40E-03
3388	5	L2-3 vs. S2-3	252184_at	AT3G50660	Encodes a 22α hydroxylase whose reaction is a rate-limiting step in brassinosteroid biosynthetic pathway. The protein is a member of CYP90B gene family. CLM is an epi-allele with small, compressed rosette, reduced internode length, and reduced fertility, appears in selfed ddm mutant plants possibly due to loss of cytosine methylation. Transcripts accumulate in actively growing tissues, and GUS expression is negatively regulated by brassinosteroids. Localized in the endoplasmic reticulum. The in vitro expressed protein can perform the C-22 hydroxylation of a variety of C27-, C28- and C29-sterols. Cholesterol was the best substrate, followed by campesterol. Sitosterol was a poor substrate.	425.54	125.19	3.4	3.04E-05	6.15E-03
3389	5	L2-3 vs. S2-3	244963_at	ATCG00560	PSII L protein	4392.72	1319.79	3.33	9.33E-04	1.77E-02
3390	5	L2-3 vs. S2-3	257816_at	AT3G25140	Quasimodo1, encodes a glycosyltransferase, involved in homogalacturonan biosynthesis; mutant shows cell adhesion defect and lower wall uronic acid content. The mRNA is cell-to-cell mobile.	127.11	38.69	3.29	8.55E-04	1.75E-02
3391	5	L2-3 vs. S2-3	262892_at	AT1G79440	Encodes a mitochondrial succinic semialdehyde dehydrogenase (SSADH). Nomenclature according to Kirch, et al (2004).	84.52	26.05	3.24	6.15E-04	1.51E-02
3392	5	L2-3 vs. S2-3	260997_at	AT1G26610	C2H2-like zinc finger protein;(source:Araport11)	77.32	24.1	3.21	5.28E-04	1.42E-02
3393	5	L2-3 vs. S2-3	251885_at	AT3G54050	Encodes a chloroplastic fructose 1,6-bisphosphate phosphatase. also known as HCEF1 (High Cyclic Electron Flow 1). hcef1 mutants have constitutively elevated electron flow (CEFI) and plants with antisense suppression of this enzyme have higher levels of net leaf photosynthesis and increased sucrose biosynthesis. The mRNA is cell-to-cell mobile.	268.95	88.98	3.02	8.90E-04	1.75E-02
3394	5	L2-3 vs. S2-3	251386_at	AT3G60800	Encodes a protein S-acyltransferase that, together with PAT13, cooperatively regulates leaf senescence.	68.64	22.79	3.01	6.15E-04	1.51E-02
3395	5	L2-3 vs. S2-3	244962_at	ATCG01050	Represents a plastid-encoded subunit of a NAD(P)H dehydrogenase complex. Its mRNA is edited at four positions. Translation data is not available for this gene.	45.69	15.22	3	8.31E-04	1.74E-02
3396	5	L2-3 vs. S2-3	245002_at	ATCG00270	PSII D2 protein	99.21	33.37	2.97	9.90E-04	1.79E-02
3397	5	L2-3 vs. S2-3	246017_at	AT5G10730	NAD(P)-binding Rossmann-fold superfamily protein;(source:Araport11)	164.45	58.74	2.8	6.08E-04	1.51E-02
3398	5	L2-3 vs. S2-3	262785_at	AT1G10750	carboxyl-terminal peptidase, putative (DUF239);(source:Araport11)	159.53	57.57	2.77	3.56E-04	1.19E-02
3399	5	L2-3 vs. S2-3	254188_at	AT4G23920	Encodes a protein with UDP-D-glucose 4-epimerase activity. Involved in growth and cell wall carbohydrate biosynthesis.	140.26	50.78	2.76	2.22E-04	1.07E-02

3400	5	L2-3 vs. S2-3	265182_at	AT1G23740	AOR is an alkenal/one oxidoreductase that acts on compounds with unsaturated alpha,beta-carbonyls. The activity of this enzyme with a number of substrates, including acrolein and 3-buten-2-one, was demonstrated in vitro using a truncated form of the protein that lacked approximately 80 of the first amino acids. This protein appears to localize to the chloroplast where it likely helps to maintain the photosynthetic process by detoxifying reactive carbonyls formed during lipid peroxidation.	120.09	43.57	2.76	2.43E-04	1.09E-02
3401	5	L2-3 vs. S2-3	256161_at	AT1G30090	Galactose oxidase/kelch repeat superfamily protein;(source:Araport11)	115	41.89	2.75	5.33E-04	1.43E-02
3402	5	L2-3 vs. S2-3	256246_at	AT3G66658	Encodes a putative aldehyde dehydrogenase. The gene is not responsive to osmotic stress and is expressed constitutively at a low level in plantlets and root cultures.	130.62	47.68	2.74	2.76E-04	1.13E-02
3403	5	L2-3 vs. S2-3	263047_at	AT2G17630	Pyridoxal phosphate (PLP)-dependent transferases superfamily protein;(source:Araport11)	202.18	73.9	2.74	8.38E-04	1.74E-02
3404	5	L2-3 vs. S2-3	245993_at	AT5G20700	senescence-associated family protein, putative (DUF581);(source:Araport11)	150.85	55.33	2.73	9.04E-05	8.40E-03
3405	5	L2-3 vs. S2-3	261601_at	AT1G49670	molecular function has not been defined. Was shown involved in oxidative stress tolerance.	77.32	28.37	2.72	4.36E-04	1.30E-02
3406	5	L2-3 vs. S2-3	265985_at	AT2G24220	Member of a family of proteins related to PUP1, a purine transporter. May be involved in the transport of purine and purine derivatives such as cytokinins, across the plasma membrane.	139.12	52.17	2.67	6.25E-05	7.20E-03
3407	5	L2-3 vs. S2-3	263954_at	AT2G35840	Sucrose-6F-phosphate phosphohydrolase family protein;(source:Araport11)	218.79	81.95	2.67	1.22E-04	9.20E-03
3408	5	L2-3 vs. S2-3	248838_at	AT5G46800	Seedling lethal mutation; Mitochondrial Carnitine Acyl Carrier-Like Protein	172.48	67.37	2.56	9.30E-04	1.77E-02
3409	5	L2-3 vs. S2-3	262819_at	AT1G11600	member of CYP77B	412.34	167.42	2.46	8.66E-04	1.75E-02
3410	5	L2-3 vs. S2-3	246198_at	AT4G36810	Encodes a protein with geranylgeranyl pyrophosphate synthase activity involved in isoprenoid biosynthesis. The enzyme appears to be targeted to the chloroplast in epidermal cells and guard cells of leaves, and in etioplasts in roots. The mRNA is cell-to-cell mobile.	91.92	37.75	2.44	4.05E-05	6.43E-03
3411	5	L2-3 vs. S2-3	265763_at	AT2G48060	piezo-type mechanosensitive ion channel component;(source:Araport11)	279.95	114.59	2.44	2.09E-04	1.07E-02
3412	5	L2-3 vs. S2-3	247238_at	AT5G64610	Encodes an enzyme with histone acetyltransferase activity. HAM1 primarily acetylate histone H4, but also display some ability to acetylate H3. Prior acetylation of lysine 5 on histone H4 reduces radioactive acetylation by either HAM1. HAM1 acetylates histone H4 lysine 5.	168.05	68.93	2.44	9.15E-04	1.77E-02
3413	5	L2-3 vs. S2-3	255834_at	AT2G33410	Belongs to a member of the RNA-binding glycine-rich (RBG) gene superfamily. The mRNA is cell-to-cell mobile.	580.81	240.01	2.42	8.56E-05	8.36E-03
3414	5	L2-3 vs. S2-3	245868_at	AT1G58030	Encodes a member of the cationic amino acid transporter (CAT) subfamily of amino acid polyamine choline transporters. Localized to the tonoplast.	56.63	23.72	2.39	3.35E-05	6.23E-03
3415	5	L2-3 vs. S2-3	266225_at	AT2G28900	Encodes AtOEP16, a 16-KDa plastid outer membrane protein involved in plastid import of protochlorophyllide oxidoreductase A. Predominantly expressed in leaves and is also inducible by cold treatment.	443.23	189.34	2.34	2.68E-04	1.12E-02
3416	5	L2-3 vs. S2-3	247228_at	AT5G65140	Haloacid dehalogenase-like hydrolase (HAD) superfamily protein;(source:Araport11)	113.61	49.06	2.32	1.74E-04	1.02E-02
3417	5	L2-3 vs. S2-3	254874_at	AT4G11570	Encodes plastid localized protein involved in riboflavin biosynthesis. It dephosphorylates 5-amino-6-ribitylamino- 2,4(1H,3H) pyrimidinedione 5′-phosphate (ARPP) .	300.28	132.47	2.27	1.04E-04	9.04E-03
3418	5	L2-3 vs. S2-3	258044_at	AT3G21270	Encodes Dof zinc finger protein adof2.	112.09	50.11	2.24	4.34E-04	1.30E-02
3419	5	L2-3 vs. S2-3	248965_at	AT5G45370	nodulin MtN21-like transporter family protein	53.55	24.2	2.21	7.04E-05	7.69E-03
3420	5	L2-3 vs. S2-3	246183_at	AT5G20940	Glycosyl hydrolase family protein;(source:Araport11)	46.49	21.07	2.21	6.27E-04	1.51E-02
3421	5	L2-3 vs. S2-3	247811_at	AT5G58430	A member of EXO70 gene family, putative exocyst subunits, conserved in land plants. Arabidopsis thaliana contains 23 putative EXO70 genes, which can be classified into eight clusters on the phylogenetic tree. Targeted by AvrPtoB to manipulate the defense molecule secretion machinery.	31.8	14.62	2.18	8.85E-04	1.75E-02
3422	5	L2-3 vs. S2-3	247986_at	AT5G56880	hypothetical protein;(source:Araport11)	217.83	100.76	2.16	3.40E-04	1.16E-02
3423	5	L2-3 vs. S2-3	244971_at	ATCG00670	Encodes the only ClpP (caseinolytic protease) encoded within the plastid genome. Contains a highly conserved catalytic triad of Ser-type proteases (Ser-His-Asp). Part of the 350 kDa chloroplast Clp complex. The name reflects nomenclature described in Adam et. al (2001).	7253.01	3420.12	2.12	3.28E-04	1.15E-02
3424	5	L2-3 vs. S2-3	266351_at	AT2G01490	Encodes a phytanoyl-CoA 2-hydroxylase (PAHX). The mRNA is cell-to-cell mobile.	246.85	119.49	2.07	3.63E-04	1.19E-02
3425	5	L2-3 vs. S2-3	257295_at	AT3G17420	Serine/threonine protein kinase-like protein expressed in etiolated cotyledons and found in glyoxysomes.	50.65	24.76	2.05	5.15E-04	1.40E-02

3426	5	L2-3 vs. S2-3	248454_at	AT5G51350	Encodes a receptor-like kinase that represses secondary growth, the production of secondary vascular tissues.	89.32	43.75	2.04	6.95E-05	7.69E-03
3427	5	L2-3 vs. S2-3	247260_at	AT5G64500	Major facilitator superfamily protein;(source:Araport11)	268.68	131.86	2.04	2.25E-04	1.07E-02
3428	5	L2-3 vs. S2-3	248983_at	AT5G45130	small GTP binding protein. The mRNA is cell-to-cell mobile.	2326.81	1145.93	2.03	2.52E-05	6.15E-03
3429	5	L2-3 vs. S2-3	264903_at	AT1G23190	Encodes a cytosolic phosphoglucomutase (PGM). Two Arabidopsis PGM proteins (AT1G70730/PGM2 and AT1G23190/PGM3) have high sequence similarities and redundant functions. Mature plants possessing a single cPGM allele had a major reduction in cPGM activity. Whereas pgm2 and pgm3 single mutants are undistinguishable from the wild type, loss of both PGM2 and PGM3 severely impairs male and female gametophyte development. The mRNA is cell-to-cell mobile.	40.57	20.25	2	2.12E-04	1.07E-02
3430	5	L2-3 vs. S2-3	245308_at	AT4G17486	PPPDE putative thiol peptidase family protein;(source:Araport11)	241.03	120.84	1.99	6.72E-04	1.58E-02
3431	5	L2-3 vs. S2-3	256663_at	AT3G12050	Aha1 domain-containing protein;(source:Araport11)	285.81	146.49	1.95	2.21E-04	1.07E-02
3432	5	L2-3 vs. S2-3	254081_at	AT4G25660	PPPDE putative thiol peptidase family protein;(source:Araport11)	215.96	110.75	1.95	9.32E-04	1.77E-02
3433	5	L2-3 vs. S2-3	250981_at	AT5G03140	Concanavalin A-like lectin protein kinase family protein;(source:Araport11)	98.31	50.82	1.93	6.14E-05	7.20E-03
3434	5	L2-3 vs. S2-3	263799_at	AT2G24550	major centromere autoantigen B-like protein;(source:Araport11)	30.16	15.61	1.93	1.90E-04	1.07E-02
3435	5	L2-3 vs. S2-3	261324_at	AT1G44770	elongation factor;(source:Araport11)	594.68	310.12	1.92	2.47E-04	1.10E-02
3436	5	L2-3 vs. S2-3	253279_at	AT4G34030	MCC-B is involved in leucine degradation in mitochondria. The active protein is a dimer of MCC-A and MCC-B. MCC-A is biotinylated whereas MCC-B is not. The mRNA is cell-to-cell mobile.	136.84	71.54	1.91	8.91E-04	1.75E-02
3437	5	L2-3 vs. S2-3	246832_at	AT5G26600	Pyridoxal phosphate (PLP)-dependent transferases superfamily protein;(source:Araport11)	403	214.02	1.88	3.27E-04	1.15E-02
3438	5	L2-3 vs. S2-3	248842_at	AT5G46850	phosphatidylinositol-glycan biosynthesis class X-like protein;(source:Araport11)	226.89	123.61	1.84	1.60E-04	1.02E-02
3439	5	L2-3 vs. S2-3	260530_at	AT2G47320	Cyclophilin-like peptidyl-prolyl cis-trans isomerase family protein;(source:Araport11)	381.35	208.33	1.83	5.52E-04	1.45E-02
3440	5	L2-3 vs. S2-3	253137_at	AT4G35500	Protein kinase superfamily protein;(source:Araport11)	53.27	29.12	1.83	8.37E-04	1.74E-02
3441	5	L2-3 vs. S2-3	261085_at	AT1G17480	Transient expression of Pro35S:GFP-IQD7 in leaves of N. benthamiana alters microtubule organization, in patterns similar to Pro35S:GFP-IQD8 and Pro35S:GFP-IQD6.Member of IQ67 (CaM binding) domain containing family.	50.38	27.98	1.8	6.26E-04	1.51E-02
3442	5	L2-3 vs. S2-3	256380_at	AT1G66680	unknown function	93.99	52.58	1.79	9.10E-04	1.77E-02
3443	5	L2-3 vs. S2-3	265858_at	AT2G01720	Ribophorin I;(source:Araport11)	243.85	136.94	1.78	1.50E-05	5.44E-03
3444	5	L2-3 vs. S2-3	247380_at	AT5G63380	Encodes a peroxisomal protein involved in the activation of fatty acids through esterification with CoA. At5g63380 preferentially activates fatty acids with increased chain length (C9:0 to C8:0) and thus shares characteristics with long-chain fatty acyl-CoA synthases. Also able to catalyze the conversion of OPDA to its CoA ester and is therefore thought to be involved in the peroxisomal β-oxidation steps of jasmonic acid biosynthesis.	158.65	88.89	1.78	8.42E-04	1.75E-02
3445	5	L2-3 vs. S2-3	253871_at	AT4G27440	light-dependent NADPH:protochlorophyllide oxidoreductase B The mRNA is cell-to-cell mobile.	846.24	489.56	1.73	4.26E-04	1.30E-02
3446	5	L2-3 vs. S2-3	255841_at	AT2G33260	Tryptophan/tyrosine permease;(source:Araport11)	64.25	37.56	1.71	2.76E-04	1.13E-02
3447	5	L2-3 vs. S2-3	254359_at	AT4G22360	SWIB complex BAF60b domain-containing protein;(source:Araport11)	112.04	65.86	1.7	1.95E-04	1.07E-02
3448	5	L2-3 vs. S2-3	255303_at	AT4G04860	DERLIN-2.2;(source:Araport11)	219.48	130.91	1.68	2.89E-04	1.13E-02
3449	5	L2-3 vs. S2-3	258332_at	AT3G16180	Encodes a low affinity nitrate transporter that is expressed in the plasma membrane and found in the phloem of the major veins of leaves. It is responsible for nitrate redistribution to young leaves.	25.54	15.26	1.67	5.91E-05	7.20E-03
3450	5	L2-3 vs. S2-3	262571_at	AT1G15430	hypothetical protein (DUF1644);(source:Araport11)	39.17	23.45	1.67	4.91E-04	1.37E-02
3451	5	L2-3 vs. S2-3	258502_at	AT3G02490	Pentatricopeptide repeat (PPR) superfamily protein;(source:Araport11)	35.05	21.07	1.66	3.32E-04	1.15E-02
3452	5	L2-3 vs. S2-3	247859_at	AT5G58410	HEAT/U-box domain-containing protein;(source:Araport11)	65.41	39.45	1.66	9.37E-04	1.77E-02
3453	5	L2-3 vs. S2-3	250130_at	AT5G16510	RGP5 is a member of the reversably-glycosylated family of proteins. Analyses using tagged RGP5 suggest that it is present in the cytosol and in association with the Golgi apparatus. Recombinant RGP5 does not have UDP-arabinose mutase activity based on an in vitro assay even though the related RGP1, RGP2, and RGP3 proteins do have activity in the same assay. RGP5 can form complexes with RGP1 and RGP2.	179.46	109.73	1.64	2.83E-04	1.13E-02
3454	5	L2-3 vs. S2-3	248726_at	AT5G47960	Encodes a small molecular weight g-protein.	27.59	17.12	1.61	5.14E-04	1.40E-02
3455	5	L2-3 vs. S2-3	245159_at	AT2G33100	encodes a gene similar to cellulose synthase	19.07	11.95	1.6	4.86E-04	1.37E-02
3456	5	L2-3 vs. S2-3	266826_at	AT2G22910	N-acetyl-l-glutamate synthase 1;(source:Araport11)	24.82	15.58	1.59	8.50E-04	1.75E-02
3457	5	L2-3 vs. S2-3	254197_at	AT4G24040	Encodes a trehalase, member of Glycoside Hydrolase Family 37.	36.93	23.78	1.55	3.37E-04	1.16E-02
3458	5	L2-3 vs. S2-3	257004_s_a	AT3G14130	Aldolase-type TIM barrel family protein;(source:Araport11)	96.72	65.87	1.47	7.38E-04	1.67E-02

3459	5	L2-3 vs. S2-3	258545_at	AT3G07050	Arabidopsis NSN1 encodes a nucleolar GTP- binding protein and is required for maintenance of inflorescence meristem identity and floral organ development.	111.06	146.41	0.76	9.83E-04	1.79E-02
3460	5	L2-3 vs. S2-3	249910_at	AT5G22630	Encodes a plastid-localized arogenate dehydratase involved in phenylalanine biosynthesis. Not less than six genes encoding ADT were identiﬁed in the Arabidopsis genome: ADT1 [At1g11790]; ADT2 [At3g07630]; ADT3 [At2g27820]; ADT4 [At3g44720]; ADT5 [At5g22630]; and ADT6 [At1g08250]. The mRNA is cell-to-cell mobile.	20.45	27.6	0.74	1.94E-04	1.07E-02
3461	5	L2-3 vs. S2-3	258035_at	AT3G21180	one of the type IIB calcium pump isoforms. encodes an autoinhibited Ca(2+)-ATPase that contains an N-terminal calmodulin binding autoinhibitory domain.	15.41	21.3	0.72	7.86E-04	1.70E-02
3462	5	L2-3 vs. S2-3	253790_at	AT4G28660	Similar to PsbW subunit of photosystem II.	39.23	58.82	0.67	6.26E-04	1.51E-02
3463	5	L2-3 vs. S2-3	246936_at	AT5G25360	hypothetical protein;(source:Araport11)	62.63	95.55	0.66	1.55E-04	1.02E-02
3464	5	L2-3 vs. S2-3	250694_at	AT5G06710	Homeobox-leucine zipper protein.	13	20.21	0.64	1.57E-05	5.44E-03
3465	5	L2-3 vs. S2-3	251935_at	AT3G54090	Encodes a fructokinase-like protein (AT3G54090/FLN1, AT1G69200/FLN2), a member of the pfkB-carbohydrate kinase family. FLN1 and FLN2 are potential plastidial thioredoxin z (TRX z) targets. Mutants display mutant chloroplast development, general plant growth and development defects and defects in PEP-dependent transcription.	181.83	285.16	0.64	2.92E-05	6.15E-03
3466	5	L2-3 vs. S2-3	265691_at	AT2G24330	Encodes one of two LUNAPARK proteins in Arabidopsis. Both LNPA and LNPB are predominantly distributed throughout the ER, but not preferentially localized at the three-way junctions. Mutation of both LNPA and LNPB together caused the cortical ER to develop poor ER cisternae and a less dense tubular network.	29.07	45.39	0.64	4.74E-05	7.12E-03
3467	5	L2-3 vs. S2-3	256057_at	AT1G07180	Internal NAD(P)H dehydrogenase in mitochondria. The predicted protein sequence has high homology with other designated NAD(P)H DHs from microorganisms; the capacity for matrix NAD(P)H oxidation via the rotenone-insensitive pathway is significantly reduced in the Atndi1 mutant plant line; the in vitro translation product of AtNDI1 is imported into isolated mitochondria and located on the inside of the inner membrane.	13.41	21.01	0.64	5.48E-04	1.45E-02
3468	5	L2-3 vs. S2-3	253808_at	AT4G28300	Encodes a protein with 13.6% proline amino acids that is predicted to localize to the cell wall. The mRNA is cell-to-cell mobile.	22.11	34.67	0.64	7.40E-04	1.67E-02
3469	5	L2-3 vs. S2-3	248817_at	AT5G47020	MraZ;(source:Araport11)	37.95	60.01	0.63	5.63E-04	1.45E-02
3470	5	L2-3 vs. S2-3	254855_s_a	AT4G12140	RING/U-box superfamily protein;(source:Araport11)	8.94	14.75	0.61	3.48E-04	1.18E-02
3471	5	L2-3 vs. S2-3	249815_at	AT5G23900	Ribosomal protein L13e family protein;(source:Araport11)	61.74	100.96	0.61	4.48E-04	1.30E-02
3472	5	L2-3 vs. S2-3	264028_at	AT2G03680	The SPR1 gene encodes a plant-specific 12-kD protein which has a repeated motif at both ends, separated by a predicted rod-like domain, suggesting that it may act as an intermolecular linker. Ubiquitously expressed and belongs to a six-member gene family in Arabidopsis; expressed in transgenic seedlings localized to microtubules within the cortical array, preprophase band, phragmoplast, and mitotic spindle.	60.15	100.91	0.6	3.89E-05	6.43E-03
3473	5	L2-3 vs. S2-3	250836_at	AT5G04560	Encodes a DNA glycosylase DEMETER (DME). Responsible for endosperm maternal-allele-specific hypomethylation at the MEDEA (MEA) gene. DME can excise 5-methylcytosine in vitro and when expressed in E. coli. DME establishes MEA imprinting by removing 5-methylcytosine to activate the maternal allele.	17.69	29.72	0.6	7.58E-04	1.68E-02
3474	5	L2-3 vs. S2-3	253900_at	AT4G27130	Translation initiation factor SUI1 family protein;(source:Araport11)	368.73	625.26	0.59	8.90E-05	8.40E-03
3475	5	L2-3 vs. S2-3	266698_at	AT2G19830	SNF7 family protein;(source:Araport11)	47.62	80.69	0.59	4.44E-04	1.30E-02
3476	5	L2-3 vs. S2-3	261013_at	AT1G26440	Encodes a ureide permease, uptake assays in yeast mutants indicated the longer splice variant is a cellular importer for allantoin, uracil and xanthine. Encodes 2 splice variants, UPS5L and UPS5S, which under nonstress conditions may function in allantoin degradation for nutrient recycling, whereas under stress, both genes may be involved in vesicular export allowing allantoin translocation from roots to shoots. Expression of this variant is enhanced in roots under salt stress. The central loop of the protein faces the cytosol (ReTA).	14.25	23.97	0.59	6.95E-04	1.61E-02
3477	5	L2-3 vs. S2-3	264490_at	AT1G27390	Form of TOM20, which is a component of the TOM complex, involved in transport of nuclear-encoded mitochondrial proteins	33.87	57.42	0.59	7.79E-04	1.70E-02
3478	5	L2-3 vs. S2-3	260151_at	AT1G52910	fiber (DUF1218);(source:Araport11)	89.13	151.82	0.59	9.51E-04	1.77E-02
3479	5	L2-3 vs. S2-3	254800_at	AT4G13070	RNA-binding CRS1 / YhbY (CRM) domain protein;(source:Araport11)	12.2	21.2	0.58	2.06E-04	1.07E-02
3480	5	L2-3 vs. S2-3	260718_at	AT1G48110	evolutionarily conserved C-terminal region 7;(source:Araport11)	109.13	186.66	0.58	2.85E-04	1.13E-02
3481	5	L2-3 vs. S2-3	264099_at	AT1G79050	recA DNA recombination family protein;(source:Araport11)	38.84	66.71	0.58	3.14E-04	1.15E-02

3482	5	L2-3 vs. S2-3	252683_at	AT3G44380	Late embryogenesis abundant (LEA) hydroxyproline-rich glycoprotein family;(source:Araport11)	16.97	29.36	0.58	8.03E-04	1.73E-02
3483	5	L2-3 vs. S2-3	256980_at	AT3G26932	dsRNA-binding protein 3;(source:Araport11)	11.22	19.66	0.57	1.01E-04	8.94E-03
3484	5	L2-3 vs. S2-3	246730_at	AT5G28060	Ribosomal protein S24e family protein;(source:Araport11)	34.78	61.31	0.57	2.18E-04	1.07E-02
3485	5	L2-3 vs. S2-3	245524_at	AT4G15920	Encodes a vacuolar fructose transporter expressed in parenchyma and xylem that controls leaf fructose content. When its expression is reduced, fructose accumulates in leaves.	9.98	17.6	0.57	5.45E-04	1.45E-02
3486	5	L2-3 vs. S2-3	247721_at	AT5G59140	BTB/POZ domain-containing protein;(source:Araport11)	43.78	76.68	0.57	5.58E-04	1.45E-02
3487	5	L2-3 vs. S2-3	263847_at	AT2G36970	UDP-Glycosyltransferase superfamily protein;(source:Araport11)	20.51	36.29	0.57	6.86E-04	1.60E-02
3488	5	L2-3 vs. S2-3	252669_at	AT3G44100	MD-2-related lipid recognition domain-containing protein;(source:Araport11)	102.03	177.55	0.57	8.63E-04	1.75E-02
3489	5	L2-3 vs. S2-3	254355_at	AT4G22380	Ribosomal protein L7Ae/L30e/S12e/Gadd45 family protein;(source:Araport11)	117.52	204.5	0.57	9.79E-04	1.79E-02
3490	5	L2-3 vs. S2-3	251695_at	AT3G56590	hydroxyproline-rich glycoprotein family protein;(source:Araport11)	13.05	23.74	0.55	8.38E-05	8.33E-03
3491	5	L2-3 vs. S2-3	255285_at	AT4G04630	senescence regulator (Protein of unknown function, DUF584);(source:Araport11)	13	23.64	0.55	2.42E-04	1.09E-02
3492	5	L2-3 vs. S2-3	260865_at	AT1G43760	DNAse I-like superfamily protein;(source:Araport11)	13.06	23.92	0.55	2.86E-04	1.13E-02
3493	5	L2-3 vs. S2-3	259735_at	AT1G64405	hypothetical protein;(source:Araport11)	15.42	28.34	0.54	1.59E-04	1.02E-02
3494	5	L2-3 vs. S2-3	249228_at	AT5G42190	Similar to SKP1 in yeast and humans which are involved in mitotic cell cycle control and ubiquitin mediated proteolysis.	25.5	46.87	0.54	1.73E-04	1.02E-02
3495	5	L2-3 vs. S2-3	259612_at	AT1G52300	Zinc-binding ribosomal protein family protein;(source:Araport11)	1178.8	2196.12	0.54	2.57E-04	1.11E-02
3496	5	L2-3 vs. S2-3	259391_s_a	AT1G06350	Fatty acid desaturase family protein;(source:Araport11)	249.25	464.14	0.54	6.99E-04	1.62E-02
3497	5	L2-3 vs. S2-3	261481_at	AT1G14260	RING/FYVE/PHD zinc finger superfamily protein;(source:Araport11)	13.39	25.06	0.53	3.09E-04	1.14E-02
3498	5	L2-3 vs. S2-3	246517_at	AT5G15760	Ribosomal protein PSRP-3/Ycf65;(source:Araport11)	13.46	25.17	0.53	5.20E-04	1.41E-02
3499	5	L2-3 vs. S2-3	257894_at	AT3G17100	sequence-specific DNA binding transcription factor;(source:Araport11)	17.27	32.48	0.53	7.41E-04	1.67E-02
3500	5	L2-3 vs. S2-3	252136_at	AT3G50770	calmodulin-like 41;(source:Araport11)	12.51	23.47	0.53	9.75E-04	1.79E-02
3501	5	L2-3 vs. S2-3	248139_at	AT5G54970	hypothetical protein;(source:Araport11)	14.91	28.64	0.52	3.05E-04	1.14E-02
3502	5	L2-3 vs. S2-3	266306_at	AT2G26970	Polynucleotidyl transferase, ribonuclease H-like superfamily protein;(source:Araport11)	30.15	58.81	0.51	6.17E-05	7.20E-03
3503	5	L2-3 vs. S2-3	259096_at	AT3G04840	Ribosomal protein S3Ae;(source:Araport11)	241.35	473.09	0.51	2.36E-04	1.09E-02
3504	5	L2-3 vs. S2-3	258772_at	AT3G10860	Cytochrome b-c1 complex, subunit 8 protein;(source:Araport11)	569.28	1108.84	0.51	3.00E-04	1.13E-02
3505	5	L2-3 vs. S2-3	246371_at	AT1G51940	Encodes a LysM-containing receptor-like kinase. Induction of chitin-responsive genes by chitin treatment is not blocked in the mutant. Based on protein sequence alignment analysis, it has a typical RD signaling domain in its catalytic loop and possesses autophosphorylation activity.It is required for the suppression of defense responses in absence of pathogen infection or upon abscisic acid treatment. Loss-of-function mutants display enhanced resistance to Botrytis cinerea and Pectobacterium carotovorum. Its expression is repressed by pathogen infection and biological elicitors and is induced abscisic acid.Expression is strongly repressed by elicitors and fungal infection, and is induced by the hormone abscisic acid (ABA). Insertional mutants show increased expression of PHYTOALEXIN-DEFICIENT 3 (PAD3), enhanced resistance to Botrytis cinerea and Pectobacterium carotovorum infection and reduced physiological responses to ABA, suggesting that LYK3 is important for the cross-talk between signaling pathways activated by ABA and pathogens (PMID:24639336).	63.37	127.93	0.5	1.21E-04	9.20E-03
3506	5	L2-3 vs. S2-3	255899_at	AT1G17970	RING/U-box superfamily protein;(source:Araport11)	19.97	39.7	0.5	1.49E-04	1.02E-02
3507	5	L2-3 vs. S2-3	249107_at	AT5G43680	Encodes a TatB--like component of the mitochondrial Twin arginine translocation (Tat) pathway. The protein is localized to the inner mitochondrial membrane that is nuclear-encoded and is essential for plant growth and development. Mutants are embryo lethal.	12.12	24.23	0.5	3.61E-04	1.19E-02
3508	5	L2-3 vs. S2-3	247382_at	AT5G63400	encodes a protein similar to adenylate kinase.	99.96	200.26	0.5	5.13E-04	1.40E-02
3509	5	L2-3 vs. S2-3	253138_at	AT4G35490	mitochondrial ribosomal protein L11;(source:Araport11)	185.5	370.83	0.5	6.11E-04	1.51E-02
3510	5	L2-3 vs. S2-3	248587_at	AT5G49550	Putative homolog of mammalian BLOC-1 Subunit 2. Protein - protein interaction with BLOS1.	21.09	42.52	0.5	7.23E-04	1.65E-02
3511	5	L2-3 vs. S2-3	258855_at	AT3G02070	Cysteine proteinases superfamily protein;(source:Araport11)	46.69	96.04	0.49	1.35E-05	5.44E-03
3512	5	L2-3 vs. S2-3	256927_at	AT3G22550	NAD(P)H-quinone oxidoreductase subunit, putative (DUF581);(source:Araport11)	121.83	246.84	0.49	1.74E-04	1.02E-02
3513	5	L2-3 vs. S2-3	265443_at	AT2G20750	member of BETA-EXPANSINS. Naming convention from the Expansin Working Group (Kende et al, 2004. Plant Mol Bio)	23.71	48.78	0.49	3.24E-04	1.15E-02
3514	5	L2-3 vs. S2-3	265459_at	AT2G46540	fiber;(source:Araport11)	191.62	401.53	0.48	6.38E-04	1.52E-02
3515	5	L2-3 vs. S2-3	250881_at	AT5G04080	cysteine-rich TM module stress tolerance protein;(source:Araport11)	9.52	20.08	0.47	1.75E-04	1.02E-02

3516	5	L2-3 vs. S2-3	247049_at	AT5G66440	tRNA-methyltransferase non-catalytic subunit trm6MTase subunit;(source:Araport11)	12.2	26.11	0.47	2.66E-04	1.12E-02
3517	5	L2-3 vs. S2-3	267349_at	AT2G40010	Ribosomal protein L10 family protein;(source:Araport11)	21.82	46.41	0.47	7.09E-04	1.63E-02
3518	5	L2-3 vs. S2-3	263410_at	AT2G04039	NdhV is loosely associated with the NDH complex and is required for stabilizing NDH subcomplexes A and E.	13.13	28.06	0.47	8.75E-04	1.75E-02
3519	5	L2-3 vs. S2-3	249750_at	AT5G24570	hypothetical protein;(source:Araport11)	13.92	30.54	0.46	4.07E-04	1.26E-02
3520	5	L2-3 vs. S2-3	252425_at	AT3G47620	Encodes a transcription factor AtTCP14 that regulates seed germination. AtTCP14 shows elevated expression level just prior to germination. AtTCP14 is predominantly expressed in the vascular tissue of the embryo, and affects gene expression in radicles in a non-cell-autonomous manner. Modulates GA-dependent stamen filament elongation by direct activation of SAUR63 subfamily genes through conserved target sites in their promoters.	21.67	47	0.46	6.11E-04	1.51E-02
3521	5	L2-3 vs. S2-3	261354_at	AT1G79690	nudix hydrolase homolog 3;(source:Araport11)	46.95	102.3	0.46	9.36E-04	1.77E-02
3522	5	L2-3 vs. S2-3	246379_s_a	AT1G57860	Translation protein SH3-like family protein;(source:Araport11)	270.76	600.68	0.45	1.22E-04	9.20E-03
3523	5	L2-3 vs. S2-3	253097_at	AT4G37320	member of CYP81D	19.1	42.34	0.45	3.17E-04	1.15E-02
3524	5	L2-3 vs. S2-3	253891_at	AT4G27720	Major facilitator superfamily protein;(source:Araport11)	156.03	350.35	0.45	3.76E-04	1.20E-02
3525	5	L2-3 vs. S2-3	263489_at	AT2G31830	Encodes a 5-inositol-polyphosphate phosphatase, that, in vitro, shows some activity against Ins(1,4,5)P3 and PI(3,4,5)P3, but even higher activity against PI(4,5)P2	18.47	41.42	0.45	4.47E-04	1.30E-02
3526	5	L2-3 vs. S2-3	259166_at	AT3G01670	Encodes a protein localized to phloem filaments that is required for phloem filament formation.The mRNA is cell-to-cell mobile.	15.98	36.3	0.44	3.86E-04	1.21E-02
3527	5	L2-3 vs. S2-3	250233_at	AT5G13460	Member of IQ67 (CaM binding) domain containing family.	31.02	70.97	0.44	4.90E-04	1.37E-02
3528	5	L2-3 vs. S2-3	245061_at	AT2G39730	Rubisco activase, a nuclear-encoded chloroplast protein that consists of two isoforms arising from alternative splicing in most plants. Required for the light activation of rubisco. Involved in jasmonate-induced leaf senescence.	56.37	127.75	0.44	6.49E-04	1.53E-02
3529	5	L2-3 vs. S2-3	252601_s_a	AT3G45030	Ribosomal protein S10p/S20e family protein;(source:Araport11)	453.19	1037.19	0.44	7.44E-04	1.67E-02
3530	5	L2-3 vs. S2-3	258858_at	AT3G02080	Ribosomal protein S19e family protein;(source:Araport11)	287.43	658.7	0.44	7.56E-04	1.68E-02
3531	5	L2-3 vs. S2-3	247328_at	AT5G64130	cAMP-regulated phosphoprotein 19-related protein;(source:Araport11)	434.57	990.95	0.44	9.06E-04	1.76E-02
3532	5	L2-3 vs. S2-3	258630_at	AT3G02820	zinc knuckle (CCHC-type) family protein;(source:Araport11)	13.27	30.04	0.44	9.85E-04	1.79E-02
3533	5	L2-3 vs. S2-3	245861_at	AT5G28300	Encodes a Ca(2+)-dependent CaM-binding protein. AtGT2L specifically targets the nucleus and possesses both transcriptional activation and DNA-binding abilities, implicating its function as a nuclear transcription factor.	13.22	31.02	0.43	1.70E-05	5.44E-03
3534	5	L2-3 vs. S2-3	261886_s_a	AT1G80700	stress response NST1-like protein;(source:Araport11)	110.5	256.65	0.43	1.44E-04	1.02E-02
3535	5	L2-3 vs. S2-3	249614_at	AT5G37300	Encodes a bifunctional enzyme, wax ester synthase (WS) and diacylglycerol acyltransferase (DGAT). In vitro assay indicated a ratio of 10.9 between its WS and DGAT activities. Both mutant and in vivo expression/analysis in yeast studies indicated a role in wax biosynthesis.	12.29	28.61	0.43	2.57E-04	1.11E-02
3536	5	L2-3 vs. S2-3	267431_at	AT2G34870	hydroxyproline-rich glycoprotein family protein;(source:Araport11)	14.25	32.76	0.43	3.80E-04	1.20E-02
3537	5	L2-3 vs. S2-3	254491_at	AT4G20300	Serine/Threonine-kinase, putative (DUF1639);(source:Araport11)	58.63	136.83	0.43	5.66E-04	1.46E-02
3538	5	L2-3 vs. S2-3	257134_at	AT3G12870	transmembrane protein;(source:Araport11)	25.37	58.61	0.43	6.26E-04	1.51E-02
3539	5	L2-3 vs. S2-3	246289_at	AT3G56880	VQ motif-containing protein;(source:Araport11)	106.6	246.12	0.43	7.40E-04	1.67E-02
3540	5	L2-3 vs. S2-3	266481_at	AT2G31070	TCP family protein involved in heterchronic regulation of leaf differentiation.	28	66.37	0.42	5.55E-05	7.12E-03
3541	5	L2-3 vs. S2-3	265730_at	AT2G32220	Ribosomal L27e protein family;(source:Araport11)	25.53	61.46	0.42	1.82E-04	1.05E-02
3542	5	L2-3 vs. S2-3	252906_at	AT4G39640	The gene encodes a gamma-glutamyltransferase (AKA gamma-glutamyl transpeptidase, EC 2.3.2.2) that is located in vascular tissues (predominantly phloem) of leaves and is involved in the degradation of glutathione. The encoded enzyme also mitigates oxidative stress by metabolizing GSSG (oxidized form of GSH - glutathione) in the apoplast.	27.75	66.22	0.42	4.17E-04	1.29E-02
3543	5	L2-3 vs. S2-3	253436_at	AT4G32470	Cytochrome bd ubiquinol oxidase, 14kDa subunit;(source:Araport11)	521.9	1232.67	0.42	4.20E-04	1.29E-02
3544	5	L2-3 vs. S2-3	247427_at	AT5G62580	ARM repeat superfamily protein;(source:Araport11)	27.24	65.35	0.42	8.22E-04	1.74E-02
3545	5	L2-3 vs. S2-3	259371_at	AT1G69080	Adenine nucleotide alpha hydrolases-like superfamily protein;(source:Araport11)	16.62	39.38	0.42	9.03E-04	1.76E-02
3546	5	L2-3 vs. S2-3	265386_at	AT2G20930	Part of multi-protein complex, acting as guanine nucleotide exchange factors (GEFs) and possibly as tethers, regulating intracellular trafficking.	41.87	100.83	0.42	9.58E-04	1.78E-02
3547	5	L2-3 vs. S2-3	259588_at	AT1G27930	Arabinogalactan methylesterase,involved in arabinogalactan glucuronic acid methylation. Interacts with eIF3.	26.81	64.68	0.41	4.80E-04	1.36E-02
3548	5	L2-3 vs. S2-3	260974_at	AT1G53440	Leucine-rich repeat transmembrane protein kinase;(source:Araport11)	11	27	0.41	5.63E-04	1.45E-02
3549	5	L2-3 vs. S2-3	258038_at	AT3G21260	Glycolipid transfer protein (GLTP) family protein;(source:Araport11)	10.83	26.39	0.41	7.68E-04	1.69E-02
3550	5	L2-3 vs. S2-3	257115_at	AT3G20150	Kinesin motor family protein;(source:Araport11)	10.18	24.64	0.41	9.41E-04	1.77E-02

3551	5	L2-3 vs. S2-3	250800_at	AT5G05370	Cytochrome b-c1 complex, subunit 8 protein;(source:Araport11)	84.22	208.83	0.4	3.20E-05	6.15E-03
3552	5	L2-3 vs. S2-3	266636_at	AT2G35370	Encodes glycine decarboxylase complex H protein. Involved in photorespiration. The mRNA is cell-to-cell mobile.	19.76	49.82	0.4	8.33E-05	8.33E-03
3553	5	L2-3 vs. S2-3	244993_s_a	ATCG01130	Ycf1 protein;(source:Araport11)	942.44	2349.95	0.4	2.66E-04	1.12E-02
3554	5	L2-3 vs. S2-3	258709_at	AT3G09500	Ribosomal L29 family protein;(source:Araport11)	344.12	867.28	0.4	3.64E-04	1.19E-02
3555	5	L2-3 vs. S2-3	250797_at	AT5G05310	TLC ATP/ADP transporter;(source:Araport11)	48.57	121.61	0.4	6.23E-04	1.51E-02
3556	5	L2-3 vs. S2-3	246975_at	AT5G24890	stress response NST1-like protein;(source:Araport11)	28.64	71.26	0.4	8.21E-04	1.74E-02
3557	5	L2-3 vs. S2-3	258237_at	AT3G27810	Encodes a member of the R2R3-MYB transcription factor gene family. Induced by jasmonate. Involved in jasmonate response during stamen development. MYB21 interacts with JAZ proteins, and functions redundantly with MYB24 and MYB57 to regulate stamen development.	18.11	46.43	0.39	2.31E-04	1.08E-02
3558	5	L2-3 vs. S2-3	256062_at	AT1G07090	LIGHT-DEPENDENT SHORT HYPOCOTYLS-like protein (DUF640);(source:Araport11)	12.9	33.23	0.39	2.94E-04	1.13E-02
3559	5	L2-3 vs. S2-3	266693_at	AT2G19800	Encodes a myo-inositol oxygenase family gene.	38.31	97.47	0.39	4.48E-04	1.30E-02
3560	5	L2-3 vs. S2-3	258976_at	AT3G01980	NAD(P)-binding Rossmann-fold superfamily protein;(source:Araport11)	72.21	184.28	0.39	5.79E-04	1.48E-02
3561	5	L2-3 vs. S2-3	255221_at	AT4G05150	Octicosapeptide/Phox/Bem1p family protein;(source:Araport11)	172.95	446.11	0.39	9.37E-04	1.77E-02
3562	5	L2-3 vs. S2-3	256342_at	AT1G72020	TonB-dependent heme receptor A;(source:Araport11)	227.96	604.56	0.38	5.30E-05	7.12E-03
3563	5	L2-3 vs. S2-3	257570_at	AT3G13662	Disease resistance-responsive (dirigent-like protein) family protein;(source:Araport11)	21.52	57.14	0.38	3.27E-04	1.15E-02
3564	5	L2-3 vs. S2-3	246905_at	AT5G25570	polyamine-modulated factor 1-binding protein;(source:Araport11)	10.8	28.53	0.38	5.40E-04	1.44E-02
3565	5	L2-3 vs. S2-3	252880_at	AT4G39730	PLAT1 domain stress protein family member. Involved in mediating response to stresses such as pathogen infection. It is found in endoplasmic reticulum bodies. PLAT1 is induced by pathogenic fungi and induces the production of scopolin.	506.34	1337.79	0.38	7.66E-04	1.69E-02
3566	5	L2-3 vs. S2-3	256629_at	AT3G19980	Encodes catalytic subunit of serine/threonine protein phosphatase 2A. It can associate with phytochromes A and B in vitro. Mutant plants display an accelerated flowering phenotype.Acts antagonistically to SnRK2 to regulate ABI5 phosphorylation. It inteacts with NRP which results in tethering to endosomes leading to its degradation.	72.53	192.82	0.38	8.24E-04	1.74E-02
3567	5	L2-3 vs. S2-3	247457_at	AT5G62170	LOW protein: M-phase inducer phosphatase-like protein;(source:Araport11)	12.94	33.87	0.38	8.82E-04	1.75E-02
3568	5	L2-3 vs. S2-3	251662_at	AT3G57080	Non-catalytic subunit unique to Nuclear DNA-dependent RNA polymerase V; homologous to budding yeast RPB5.	17.85	48.61	0.37	3.77E-05	6.43E-03
3569	5	L2-3 vs. S2-3	262281_at	AT1G68570	NPF3.1 is a membrane localized GA transporter that is expressed in the root endodermis.	14.34	38.69	0.37	2.09E-04	1.07E-02
3570	5	L2-3 vs. S2-3	249268_at	AT5G41685	Mitochondrial outer membrane translocase complex, subunit Tom7;(source:Araport11)	11.41	30.91	0.37	5.16E-04	1.40E-02
3571	5	L2-3 vs. S2-3	262112_at	AT1G02870	nucleolar-like protein;(source:Araport11)	19.17	52.35	0.37	6.45E-04	1.53E-02
3572	5	L2-3 vs. S2-3	252676_at	AT3G44280	peptidyl-prolyl cis-trans isomerase G;(source:Araport11)	30.45	83.38	0.37	8.12E-04	1.74E-02
3573	5	L2-3 vs. S2-3	264579_at	AT1G05205	hexokinase-1 protein;(source:Araport11)	16.8	46.06	0.36	1.63E-05	5.44E-03
3574	5	L2-3 vs. S2-3	266579_at	AT2G23930	Putative small nuclear ribonucleoprotein G;(source:Araport11)	289.39	813.38	0.36	9.65E-05	8.72E-03
3575	5	L2-3 vs. S2-3	249651_at	AT5G37020	Encodes a member of the auxin response factor family. Mediates auxin response via expression of auxin regulated genes. Acts redundantly with ARF6 to control stamen elongation and flower maturation. Expression of ARF8 is controlled by miR167.	114.19	318.63	0.36	2.01E-04	1.07E-02
3576	5	L2-3 vs. S2-3	262593_at	AT1G15120	Ubiquinol-cytochrome C reductase hinge protein;(source:Araport11)	101.34	280.81	0.36	2.44E-04	1.09E-02
3577	5	L2-3 vs. S2-3	265210_at	AT2G36620	RPL24A encodes ribosomal protein L24, homolog of cytosolic RPL24, found in archaea and higher eukaryotes. Arabidopsis has two RPL24 homologs, RPL24A (AT2G36620) and RPL24B (AT3G53020).	268.23	742.84	0.36	3.00E-04	1.13E-02
3578	5	L2-3 vs. S2-3	252271_s_a	AT5G01430	Got1/Sft2-like vescicle transport protein family;(source:Araport11)	31.78	87.42	0.36	3.20E-04	1.15E-02
3579	5	L2-3 vs. S2-3	259130_at	AT3G02190	Ribosomal protein L39 family protein;(source:Araport11)	150.11	411.65	0.36	4.93E-04	1.37E-02
3580	5	L2-3 vs. S2-3	246135_at	AT5G20885	RING/U-box superfamily protein;(source:Araport11)	19.64	55.31	0.36	1.00E-03	1.81E-02
3581	5	L2-3 vs. S2-3	261572_at	AT1G01170	ozone-responsive stress-like protein (DUF1138);(source:Araport11)	183.33	528.54	0.35	5.22E-05	7.12E-03
3582	5	L2-3 vs. S2-3	246870_at	AT5G26030	encodes ferrochelatase I located in plastids. Involved in heme biosynthesis in non-photosynthetic tissues and induced by oxidative stress in photosynthetic tissues to supply heme for defensive hemoproteins The mRNA is cell-to-cell mobile.	102.29	290.54	0.35	3.51E-04	1.18E-02
3583	5	L2-3 vs. S2-3	AFFX-r2-At-GAPDH-3_s_at	AT3G04120	encodes cytosolic GADPH (C subunit) involved in the glycolytic pathway but also interacts with H2O2 potentially placing it in a signalling cascade induced by ROS. The mRNA is cell-to-cell mobile.	635.5	1837.5	0.35	6.29E-04	1.51E-02

3584	5	L2-3 vs. S2-3	264872_at	AT1G24260	Member of the MADs box transcription factor family. SEP3 is redundant with SEP1 and 2. Flowers of SEP1/2/3 triple mutants show a conversion of petals and stamens to sepals.SEP3 forms heterotetrameric complexes with other MADS box family members and binds to the CArG box motif.	220.21	656.26	0.34	7.20E-05	7.69E-03
3585	5	L2-3 vs. S2-3	266845_at	AT2G26110	bromodomain protein (DUF761);(source:Araport11)	17.39	50.86	0.34	1.52E-04	1.02E-02
3586	5	L2-3 vs. S2-3	255149_at	AT4G08150	A member of class I knotted1-like homeobox gene family (together with KNAT2). Similar to the knotted1 (kn1) homeobox gene of maize. Normally expressed in the peripheral and rib zone of shoot apical meristem but not in the leaf primordia. It is also expressed in the fourth floral whorl, in the region that would become style, particularly in the cell surrounding the transmitting tissue. No expression was detected in the first three floral whorls. Expression is repressed by auxin and AS1 which results in the promotion of leaf fate.	13.8	41.02	0.34	2.22E-04	1.07E-02
3587	5	L2-3 vs. S2-3	265400_at	AT2G10940	Bifunctional inhibitor/lipid-transfer protein/seed storage 2S albumin superfamily protein;(source:Araport11)	49.78	146.78	0.34	4.50E-04	1.30E-02
3588	5	L2-3 vs. S2-3	263552_x_a	AT2G24980	Proline-rich extensin-like family protein;(source:Araport11)	15.6	46.23	0.34	7.74E-04	1.70E-02
3589	5	L2-3 vs. S2-3	262000_at	AT1G33810	zinc finger/BTB domain protein;(source:Araport11)	67.03	205.75	0.33	1.50E-05	5.44E-03
3590	5	L2-3 vs. S2-3	249313_at	AT5G41560	Encodes a substrate receptor for CRL4-CDD complexes that provides substrate specificity for CRL4 by interacting with ubiquitination targets. By its interaction and regulation of levels of PYL8 through proteasomal degradation, it negatively regulates ABA-mediated developmental responses, including inhibition of seed germination, seedling establishment, and root growth	16.05	49.33	0.33	7.75E-05	7.98E-03
3591	5	L2-3 vs. S2-3	252524_at	AT3G46430	Mitochondrial F1F0-ATP synthase.	210.97	642.92	0.33	1.09E-04	9.20E-03
3592	5	L2-3 vs. S2-3	254111_at	AT4G24890	purple acid phosphatase 24;(source:Araport11)	22.39	68.87	0.33	2.37E-04	1.09E-02
3593	5	L2-3 vs. S2-3	262490_at	AT1G21840	Encodes a urease accessory protein which is essential for the activation of plant urease.	23.56	71.05	0.33	2.88E-04	1.13E-02
3594	5	L2-3 vs. S2-3	250777_at	AT5G05440	Encodes a member of the PYR (pyrabactin resistance)/PYL(PYR1-like)/RCAR (regulatory components of ABA receptor) family proteins with 14 members. PYR/PYL/RCAR family proteins function as abscisic acid sensors. Mediate ABA-dependent regulation of protein phosphatase 2Cs ABI1 and ABI2.	28.2	86.21	0.33	3.27E-04	1.15E-02
3595	5	L2-3 vs. S2-3	266044_s_a	AT2G07725	Ribosomal L5P family protein;(source:Araport11)	21.65	65.68	0.33	4.33E-04	1.30E-02
3596	5	L2-3 vs. S2-3	254694_at	AT4G17900	PLATZ transcription factor family protein;(source:Araport11)	80.34	252.31	0.32	9.20E-04	1.77E-02
3597	5	L2-3 vs. S2-3	257051_at	AT3G15270	Encodes a member of the SPL (squamosa-promoter binding protein-like)gene family, a novel gene family encoding DNA binding proteins and putative transcription factors. Contains the SBP-box, which encodes the SBP-domain, required and sufficient for interaction with DNA. It is involved in regulation of flowering and vegetative phase change. Its temporal expression is regulated by the microRNA miR156. The target site for the microRNA is in the 3'UTR.	20.22	62.69	0.32	9.31E-04	1.77E-02
3598	5	L2-3 vs. S2-3	244982_at	ATCG00790	chloroplast gene encoding a ribosomal protein L16, which is a constituent of 50S large ribosomal subunit	1039.48	3301.03	0.31	2.14E-04	1.07E-02
3599	5	L2-3 vs. S2-3	265058_s_at	AT1G52030	Similar to myrosinase binding proteins which may be involved in metabolizing glucosinolates and forming defense compounds to protect against herbivory. Also similar to lectins and other agglutinating factors. Expressed only in flowers.	830.18	2710.8	0.31	3.51E-04	1.18E-02
3600	5	L2-3 vs. S2-3	256794_at	AT3G22230	Ribosomal L27e protein family;(source:Araport11)	28.31	90.53	0.31	3.83E-04	1.21E-02
3601	5	L2-3 vs. S2-3	250207_at	AT5G13930	Encodes chalcone synthase (CHS), a key enzyme involved in the biosynthesis of flavonoids. Required for the accumulation of purple anthocyanins in leaves and stems. Also involved in the regulation of auxin transport and the modulation of root gravitropism. The mRNA is cell-to-cell mobile.	31.24	101.54	0.31	7.81E-04	1.70E-02
3602	5	L2-3 vs. S2-3	252789_s_a	AT1G21930	transmembrane protein;(source:Araport11)	303.58	968.98	0.31	8.33E-04	1.74E-02
3603	5	L2-3 vs. S2-3	266119_at	AT2G02100	Predicted to encode a PR (pathogenesis-related) protein. Belongs to the plant defensin (PDF) family with the following members: At1g75830/PDF1.1, At5g44420/PDF1.2a, At2g26020/PDF1.2b, At5g44430/PDF1.2c, At2g26010/PDF1.3, At1g19610/PDF1.4, At1g55010/PDF1.5, At2g02120/PDF2.1, At2g02100/PDF2.2, At2g02130/PDF2.3, At1g61070/PDF2.4, At5g63660/PDF2.5, At2g02140/PDF2.6, At5g38330/PDF3.1 and At4g30070/PDF3.2. The mRNA is cell-to-cell mobile.	474.83	1567.69	0.3	2.84E-05	6.15E-03
3604	5	L2-3 vs. S2-3	254687_at	AT4G13770	Encodes a cytochrome p450 enzyme that catalyzes the initial conversion of aldoximes to thiohydroximates in the synthesis of glucosinolates not derived from tryptophan. Also has a role in auxin homeostasis.	34.15	114.14	0.3	9.68E-05	8.72E-03

3605	5	L2-3 vs. S2-3	258851_at	AT3G03190	Encodes glutathione transferase belonging to the phi class of GSTs. Naming convention according to Wagner et al. (2002).	14.27	46.86	0.3	1.67E-04	1.02E-02
3606	5	L2-3 vs. S2-3	250517_at	AT5G08260	serine carboxypeptidase-like 35;(source:Araport11)	30.93	104.69	0.3	5.94E-04	1.51E-02
3607	5	L2-3 vs. S2-3	261053_at	AT1G01320	Encodes REDUCED CHLOROPLAST COVERAGE 1 (REC1). Located in the nucleus and cytosol. Contributes to establishing the size of the chloroplast compartment.	31.89	111.07	0.29	1.67E-04	1.02E-02
3608	5	L2-3 vs. S2-3	250565_at	AT5G08000	Encodes a member of the X8-GPI family of proteins. It localizes to the plasmodesmata and binds callose.	163.97	586.64	0.28	3.00E-05	6.15E-03
3609	5	L2-3 vs. S2-3	259617_at	AT1G47970	nucleolin;(source:Araport11)	65.42	231.29	0.28	1.66E-04	1.02E-02
3610	5	L2-3 vs. S2-3	253482_at	AT4G31985	Expressed protein	719.61	2564.96	0.28	2.01E-04	1.07E-02
3611	5	L2-3 vs. S2-3	253202_at	AT4G34555	Ribosomal protein S25 family protein;(source:Araport11)	312.4	1103.02	0.28	3.28E-04	1.15E-02
3612	5	L2-3 vs. S2-3	261738_s_a	AT1G47813	hypothetical protein;(source:Araport11)	55.95	202.83	0.28	8.81E-04	1.75E-02
3613	5	L2-3 vs. S2-3	249492_at	AT5G39160	RmlC-like cupins superfamily protein;(source:Araport11)	9.23	34.02	0.27	1.12E-04	9.20E-03
3614	5	L2-3 vs. S2-3	260538_at	AT2G43460	Ribosomal L38e protein family;(source:Araport11)	917.33	3393.19	0.27	1.21E-04	9.20E-03
3615	5	L2-3 vs. S2-3	264173_at	AT1G02160	Cox19 family protein (CHCH motif);(source:Araport11)	143.99	532.06	0.27	4.38E-04	1.30E-02
3616	5	L2-3 vs. S2-3	265844_at	AT2G35620	Encodes a plasma membrane localized leucine-rich repeat receptor kinase that is involved in cell wall elongation. Loss of function mutations of FEI1 and FEI2 exhibit defects in root and hypocotyl cell elongation. Double mutants are defective in cell wall biosynthesis and have thick hypocotyls, and short, thick roots.Mucilage is easily detached from fei2 mutants seeds, and forms a capsule that is >50% smaller relative to wild-type.	17.18	62.6	0.27	8.61E-04	1.75E-02
3617	5	L2-3 vs. S2-3	265741_at	AT2G01320	ABC-2 type transporter family protein;(source:Araport11)	20.29	78.51	0.26	6.34E-04	1.52E-02
3618	5	L2-3 vs. S2-3	266906_at	AT2G34585	transmembrane protein;(source:Araport11)	102.74	409.72	0.25	1.85E-05	5.48E-03
3619	5	L2-3 vs. S2-3	255140_x_a	AT4G08410	Proline-rich extensin-like family protein;(source:Araport11)	22.02	89.21	0.25	3.77E-04	1.20E-02
3620	5	L2-3 vs. S2-3	253627_at	AT4G30650	Low temperature and salt responsive protein family;(source:Araport11)	53.96	216.57	0.25	8.53E-04	1.75E-02
3621	5	L2-3 vs. S2-3	259090_at	AT3G04920	Ribosomal protein S24e family protein;(source:Araport11)	93.91	396.14	0.24	1.62E-04	1.02E-02
3622	5	L2-3 vs. S2-3	256475_s_a t	AT1G42680	P-loop containing nucleoside triphosphate hydrolases superfamily protein;(source:Araport11)	9.74	40.42	0.24	2.54E-04	1.11E-02
3623	5	L2-3 vs. S2-3	260257_at	AT1G74340	Encodes a subunit of the dolichol phosphate mannase synthase (DPMS) complex that may serve as membrane anchors for the catalytic core, DPMS1, or provide catalytic assistance. It is localized in the ER and mediates isoprenyl-linked glycan biogenesis.	86.12	360.13	0.24	2.64E-04	1.12E-02
3624	5	L2-3 vs. S2-3	254137_at	AT4G24930	thylakoid lumenal 17.9 kDa protein, chloroplast;(source:Araport11)	26.08	106.68	0.24	2.91E-04	1.13E-02
3625	5	L2-3 vs. S2-3	258003_at	AT3G29030	Encodes an expansin. Naming convention from the Expansin Working Group (Kende et al, 2004. Plant Mol Bio)	35.24	147.73	0.24	7.51E-04	1.68E-02
3626	5	L2-3 vs. S2-3	261598_at	AT1G49750	Leucine-rich repeat (LRR) family protein;(source:Araport11)	20.45	86.92	0.24	8.94E-04	1.75E-02
3627	5	L2-3 vs. S2-3	266118_at	AT2G02130	Predicted to encode a PR (pathogenesis-related) protein. Belongs to the plant defensin (PDF) family with the following members: At1g75830/PDF1.1, At5g44420/PDF1.2a, At2g26020/PDF1.2b, At5g44430/PDF1.2c, At2g26010/PDF1.3, At1g19610/PDF1.4, At1g55010/PDF1.5, At2g02120/PDF2.1, At2g02100/PDF2.2, At2g02130/PDF2.3, At1g61070/PDF2.4, At5g63660/PDF2.5, At2g02140/PDF2.6, At5g38330/PDF3.1 and At4g30070/PDF3.2. The mRNA is cell-to-cell mobile.	38.81	166.58	0.23	1.52E-05	5.44E-03
3628	5	L2-3 vs. S2-3	256311_at	AT1G30330	Encodes a member of the auxin response factor family. Mediates auxin response via expression of auxin regulated genes. Acts redundantly with ARF8 to control stamen elongation and flower maturation. Expression of ARF6 is controlled by miR167.	277.8	1207.57	0.23	7.18E-05	7.69E-03
3629	5	L2-3 vs. S2-3	254559_at	AT4G19200	proline-rich family protein;(source:Araport11)	46.93	201.11	0.23	1.56E-04	1.02E-02
3630	5	L2-3 vs. S2-3	260632_at	AT1G62360	Class I knotted-like homeodomain protein that is required for shoot apical meristem (SAM) formation during embryogenesis and for SAM function throughout the lifetime of the plant. Functions by preventing incorporation of cells in the meristem center into differentiating organ primordia. It has also been shown to have a role in the specification of flower meristem identity.	26.58	118.12	0.23	4.42E-04	1.30E-02
3631	5	L2-3 vs. S2-3	260421_at	AT1G69640	Encodes one of the two redundant sphingoid base hydroxylases (SBH). Involved in sphingolipid trihydroxy long-chain base (4-hydroxysphinganine) biosynthesis. Double mutants of SBHs were dwarfed and not able to progress from vegetative to reproductive growth.	22.66	96.88	0.23	6.28E-04	1.51E-02
3632	5	L2-3 vs. S2-3	266460_at	AT2G47930	arabinogalactan protein 26;(source:Araport11)	14.77	67.03	0.22	1.52E-04	1.02E-02

3633	5	L2-3 vs. S2-3	245842_at	AT1G58430	Encodes an anther-specific proline-rich protein.	12.89	58.56	0.22	1.97E-04	1.07E-02
3634	5	L2-3 vs. S2-3	263420_at	AT2G17240	Participates in the late stages of the biogenesis of 50S ribosomal subunits in plastids.	80.15	363.8	0.22	2.91E-04	1.13E-02
3635	5	L2-3 vs. S2-3	262602_at	AT1G15270	Translation machinery associated TMA7;(source:Araport11)	91.33	406.35	0.22	4.69E-04	1.34E-02
3636	5	L2-3 vs. S2-3	251007_at	AT5G02610	Ribosomal L29 family protein;(source:Araport11)	95.83	462.04	0.21	1.05E-05	5.44E-03
3637	5	L2-3 vs. S2-3	265768_at	AT2G48020	Encodes a zinc transporter ZIF2. Expression of ZIF2 is regulated by alternative splicing.	67.38	322.79	0.21	1.16E-05	5.44E-03
3638	5	L2-3 vs. S2-3	250682_x_a	AT5G06630	proline-rich extensin-like family protein;(source:Araport11)	13.41	64.2	0.21	2.98E-04	1.13E-02
3639	5	L2-3 vs. S2-3	253382_at	AT4G33040	Encodes a member of the CC-type glutaredoxin (ROXY) family that has been shown to interact with the transcription factor TGA2.	131.96	625.42	0.21	8.38E-04	1.74E-02
3640	5	L2-3 vs. S2-3	261864_s_a t	AT1G50480	10-formyltetrahydrofolate synthetase (THFS) mRNA, complete The mRNA is cell-to-cell mobile.	268.01	1361.47	0.2	1.86E-04	1.06E-02
3641	5	L2-3 vs. S2-3	259331_at	AT3G03840	SAUR-like auxin-responsive protein family;(source:Araport11)	8.78	44.62	0.2	2.88E-04	1.13E-02
3642	5	L2-3 vs. S2-3	247900_at	AT5G57290	60S acidic ribosomal protein family;(source:Araport11)	205.64	1070.45	0.19	1.05E-04	9.04E-03
3643	5	L2-3 vs. S2-3	257701_at	AT3G12710	DNA glycosylase superfamily protein;(source:Araport11)	23.36	125.8	0.19	4.99E-04	1.38E-02
3644	5	L2-3 vs. S2-3	250007_at	AT5G18670	putative beta-amylase BMY3 (BMY3)	657.13	3474.2	0.19	8.85E-04	1.75E-02
3645	5	L2-3 vs. S2-3	255243_at	AT4G05590	Encodes NRGA1, a putative mitochondrial pyruvate carrier that mediates ABA regulation of guard cell ion channels and drought stress responses.	11.07	62.45	0.18	7.01E-04	1.62E-02
3646	5	L2-3 vs. S2-3	251738_at	AT3G56150	member of eIF3c - eukaryotic initiation factor 3c	26.59	146.45	0.18	8.82E-04	1.75E-02
3647	5	L2-3 vs. S2-3	254083_at	AT4G24920	secE/sec61-gamma protein transport protein;(source:Araport11)	141.54	783.15	0.18	9.64E-04	1.78E-02
3648	5	L2-3 vs. S2-3	247450_at	AT5G62350	Plant invertase/pectin methylesterase inhibitor superfamily protein;(source:Araport11)	122.54	740.75	0.17	3.62E-04	1.19E-02
3649	5	L2-3 vs. S2-3	247148_at	AT5G65670	auxin (indole-3-acetic acid) induced gene The mRNA is cell-to-cell mobile.	215.33	1258.07	0.17	9.49E-04	1.77E-02
3650	5	L2-3 vs. S2-3	265984_at	AT2G24210	terpene synthase 10;(source:Araport11)	12.61	78.07	0.16	6.73E-04	1.58E-02
3651	5	L2-3 vs. S2-3	259156_at	AT3G10380	Subunit of the Putative Arabidopsis Exocyst Complex	38.31	243.56	0.16	7.91E-04	1.71E-02
3652	5	L2-3 vs. S2-3	246747_at	AT5G27700	Cytosolic ribosomal protein. Similar to EVR1 and redundant with EVR1. Also enhances VAR2 mutant varigation, but to a lesser extent than evr1.	289.89	1984.55	0.15	1.33E-04	9.58E-03
3653	5	L2-3 vs. S2-3	249754_at	AT5G24530	Encodes a putative 2OG-Fe(II) oxygenase that is defense-associated but required for susceptibility to downy mildew. The mRNA is cell-to-cell mobile.	37.61	254.74	0.15	2.22E-04	1.07E-02
3654	5	L2-3 vs. S2-3	266865_at	AT2G29980	Endoplasmic reticulum enzyme responsible for the synthesis of 18:3 fatty acids from phospholipids. Uses cytochrome b5 as electron donor.	58.59	402.93	0.15	3.08E-04	1.14E-02
3655	5	L2-3 vs. S2-3	246422_at	AT5G17060	A member of ARF GTPase family. A thaliana has 21 members of this family, known to be essential for vesicle coating and uncoating and functions in GTP-binding. Gene encoding ADP-ribosylation factor and similar to ADP-ribosylation factor 1; ARF 1 (GP:385340) {Drosophila melanogaster}, other ARFs and ARF-like proteins.	56.01	372.73	0.15	9.54E-04	1.77E-02
3656	5	L2-3 vs. S2-3	254915_s_a	AT4G11310	cysteine proteinase precursor-like protein	12.51	88.99	0.14	9.00E-07	2.59E-03
3657	5	L2-3 vs. S2-3	244986_at	ATCG00820	Encodes a 6.8-kDa protein of the small ribosomal subunit.	183.08	1292.8	0.14	2.30E-06	3.31E-03
3658	5	L2-3 vs. S2-3	261489_at	AT1G14450	NADH dehydrogenase (ubiquinone)s;(source:Araport11)	43.62	315.96	0.14	5.33E-05	7.12E-03
3659	5	L2-3 vs. S2-3	254163_s_a	AT4G24350	Phosphorylase superfamily protein;(source:Araport11)	23.53	165.3	0.14	1.16E-04	9.20E-03
3660	5	L2-3 vs. S2-3	255298_at	AT4G04840	methionine sulfoxide reductase B6;(source:Araport11)	17.45	124.41	0.14	2.03E-04	1.07E-02
3661	5	L2-3 vs. S2-3	247776_at	AT5G58700	phosphatidylinositol-speciwc phospholipase C4;(source:Araport11)	40.84	321.71	0.13	5.46E-05	7.12E-03
3662	5	L2-3 vs. S2-3	267298_at	AT2G23760	Encodes a member of the BEL family of homeodomain proteins. Plants doubly mutant for saw1/saw2 (blh2/blh4) have serrated leaves. BP is expressed in the serrated leaves, therefore saw2 and saw1 may act redundantly to repress BP in leaves. Regulates together with BLH2 demethylesterification of homogalacturonan in seed mucilage.	10.66	82.17	0.13	1.63E-04	1.02E-02
3663	5	L2-3 vs. S2-3	267239_at	AT2G02510	NADH dehydrogenase (ubiquinone)s;(source:Araport11)	121.3	984.95	0.12	1.14E-05	5.44E-03
3664	5	L2-3 vs. S2-3	260297_at	AT1G80280	alpha/beta-Hydrolases superfamily protein;(source:Araport11)	23.14	196.05	0.12	1.32E-04	9.58E-03
3665	5	L2-3 vs. S2-3	247162_at	AT5G65730	xyloglucan endotransglucosylase/hydrolase 6;(source:Araport11)	12.82	102.75	0.12	2.26E-04	1.07E-02
3666	5	L2-3 vs. S2-3	262951_at	AT1G75500	An Arabidopsis thaliana homolog of Medicago truncatula NODULIN21 (MtN21). The gene encodes a plant-specific, predicted integral membrane protein and is a member of the Plant-Drug/Metabolite Exporter (P-DME) family (Transporter Classification number: TC 2.A.7.3) and the nodulin MtN21-like transporter family.	52.15	464.06	0.11	4.13E-05	6.43E-03
3667	5	L2-3 vs. S2-3	260368_at	AT1G69700	Part of the AtHVA22 family. Protein expression is ABA- and stress-inducible. The mRNA is cell-to-cell mobile.	41.84	406.99	0.1	1.70E-04	1.02E-02
3668	5	L2-3 vs. S2-3	266123_at	AT2G45180	Bifunctional inhibitor/lipid-transfer protein/seed storage 2S albumin superfamily protein;(source:Araport11)	68.23	665.55	0.1	2.50E-04	1.10E-02

3669	5	L2-3 vs. S2-3	263867_at	AT2G36830	Encodes a tonoplast intrinsic protein, which functions as water channel. It has also been shown to be able to facilitate the transport of urea and hydrogen peroxide. Highly expressed in vascular tissues of the root, stem, cauline leaves and flowers but not in the apical meristems. The mRNA is cell-to-cell mobile.	96.44	1068.2	0.09	5.56E-05	7.12E-03
3670	5	L2-3 vs. S2-3	245009_at	ATCG00380	Chloroplast encoded ribosomal protein S4	77.02	1159.86	0.066	6.13E-04	1.51E-02
3671	5	L2-3 vs. S2-3	245928_s_at	AT5G24780	encodes an acid phosphatase similar to soybean vegetative storage proteins. Gene expression is induced by wounding and jasmonic acid.	40.49	710.31	0.057	4.67E-04	1.34E-02
3672	5	L2-3 vs. S2-3	247494_at	AT5G61790	calnexin 1;(source:Araport11)	57.21	1105.77	0.052	3.47E-05	6.25E-03
3673	5	L2-3 vs. S2-3	247478_at	AT5G62360	Pectin methylesterase inhibitor expressed throughout the plant.	9.78	186.62	0.052	5.90E-05	7.20E-03
3674	5	L2-3 vs. S2-3	249732_at	AT5G24420	Encodes a cytosolic 6-phosphogluconolactonase (PGL) thought to be involved in the oxidative pentose-phosphate pathway (OPPP).	9.96	195.99	0.051	2.09E-05	5.48E-03
3675	5	L2-3 vs. S2-3	245138_at	AT2G45190	Encodes a member of the YABBY family of transcriptional regulators that is involved in abaxial cell type specification in leaves and fruits. YAB1 acts in a non-cell autonomous fashion within the meristem to affect phyllotactic patterning. The non-autonomous effect on the central region of the meristem is mediated through the activity if Lateral Suppressor (LAS).	19.81	455.16	0.044	4.80E-06	4.61E-03
3676	5	L2-3 vs. S2-3	267213_at	AT2G44120	Ribosomal protein L30/L7 family protein;(source:Araport11)	14.9	349.33	0.043	8.60E-06	5.44E-03
3677	5	L2-3 vs. S2-3	247553_at	AT5G60910	MADS box gene negatively regulated by APETALA1	13.82	398.67	0.035	7.00E-07	2.59E-03
3678	5	L2-3 vs. S2-3	267639_at	AT2G42200	Encodes a putative transcriptional regulator that is involved in the vegetative to reproductive phase transition. Expression is regulated by MIR156b. SPL activity nonautonomously inhibits initiation of new leaves at the shoot apical meristem.	18.65	575.57	0.032	1.70E-06	3.27E-03
3679	5	L2-3 vs. S2-3	259381_s_at	AT3G16410	Encodes a nitrile-specifier protein NSP4. NSP4 is one out of five (At3g16400/NSP1, At2g33070/NSP2, At3g16390/NSP3, At3g16410/NSP4 and At5g48180/NSP5) A. thaliana epithiospecifier protein (ESP) homologues that promote simple nitrile, but not epithionitrile or thiocyanate formation. The mRNA is cell-to-cell mobile.	17.51	764.71	0.023	4.50E-06	4.61E-03
3680	6	M2-3 vs. S2-3	256648_at	AT3G13580	Ribosomal protein L30/L7 family protein;(source:Araport11)	1400.94	19.48	71.92	4.84E-05	7.54E-03
3681	6	M2-3 vs. S2-3	245026_at	ATCG00140	ATPase III subunit	3590.3	54.19	66.25	4.12E-05	6.79E-03
3682	6	M2-3 vs. S2-3	245275_at	AT4G15210	cytosolic beta-amylase expressed in rosette leaves and inducible by sugar. RAM1 mutants have reduced beta amylase in leaves and stems.	665.13	32.89	20.23	8.71E-04	1.98E-02
3683	6	M2-3 vs. S2-3	244964_at	ATCG00560	PSII L protein	4123.17	250.76	16.44	1.59E-04	1.29E-02
3684	6	M2-3 vs. S2-3	266012_s_a	ATMG00410	ATPase subunit 6	923.9	79.17	11.67	3.34E-05	6.43E-03
3685	6	M2-3 vs. S2-3	261080_at	AT1G07370	Encodes putative proliferating cell nuclear antigen involved in cell cycle regulation. May be sumoylated.	226.35	20.93	10.81	3.30E-05	6.43E-03
3686	6	M2-3 vs. S2-3	245499_at	AT4G16480	Encodes a high affinity H+:<i>myo</i>-inositol symporter. The only other compound shown to be transported was pinitol, a methylated derivative of <i>myo</i>-inositol. The mRNA is cell-to-cell mobile.	516.84	49.91	10.35	5.40E-04	1.70E-02
3687	6	M2-3 vs. S2-3	244959_s_a	AT2G07708	hypothetical protein;(source:Araport11)	197.24	21.09	9.35	2.40E-04	1.35E-02
3688	6	M2-3 vs. S2-3	266391_at	AT2G41290	Although this enzyme is predicted to encode a strictosidine synthase (SS), it lacks a conserved catalytic glutamate residue found in active SS enzymes and it is not expected to have SS activity.	684.05	75.37	9.08	2.45E-05	5.65E-03
3689	6	M2-3 vs. S2-3	244972_at	ATCG00680	encodes for CP47, subunit of the photosystem II reaction center.	148.49	16.45	9.03	4.15E-04	1.50E-02
3690	6	M2-3 vs. S2-3	267607_s_at	AT2G26740	Encodes a soluble epoxide hydrolase whose expression is induced by auxin and water stress.	107.22	13.14	8.16	3.47E-04	1.42E-02
3691	6	M2-3 vs. S2-3	256245_at	AT3G12580	heat shock protein 70;(source:Araport11)	179.77	30.34	5.93	8.64E-04	1.98E-02
3692	6	M2-3 vs. S2-3	260745_at	AT1G78370	Encodes glutathione transferase belonging to the tau class of GSTs. Naming convention according to Wagner et al. (2002).	106.89	18.08	5.91	7.41E-04	1.94E-02
3693	6	M2-3 vs. S2-3	258041_at	AT3G21175	member of a novel family of plant-specific GATA-type transcription factors.	102.67	20.28	5.06	3.40E-05	6.43E-03
3694	6	M2-3 vs. S2-3	244920_s_a	AT2G07674	transmembrane protein;(source:Araport11)	648.72	136.5	4.75	8.12E-04	1.97E-02
3695	6	M2-3 vs. S2-3	251658_at	AT3G57020	Calcium-dependent phosphotriesterase superfamily protein;(source:Araport11)	78.98	17.69	4.47	8.62E-04	1.98E-02
3696	6	M2-3 vs. S2-3	258336_at	AT3G16050	Encodes a protein with pyridoxal phosphate synthase activity whose transcripts were detected mostly in roots and accumulate during senescence. The protein was found in very low abundance, which prevented a specific localisation.	665.09	155.09	4.29	9.96E-04	2.10E-02
3697	6	M2-3 vs. S2-3	247033_at	AT5G67250	Encodes an SKP1 interacting partner (SKIP2).Encodes an F-box protein. Based on genetic analysis appears to be functionally redundant with VFB1,2, and 3. When expression of all 4 genes is reduced plants show defects in growth and reduced expression of auxin response genes.	490.86	120.27	4.08	2.46E-04	1.35E-02

3698	6	M2-3 vs. S2-3	247208_at	AT5G64870	Belongs to the group of plant flotillins, which are plasma membrane proteins. Flot3 is found in membrane nanodomains.	846.39	214.23	3.95	1.73E-04	1.29E-02
3699	6	M2-3 vs. S2-3	249242_at	AT5G42250	Zinc-binding alcohol dehydrogenase family protein;(source:Araport11)	203.33	52.92	3.84	7.95E-05	1.02E-02
3700	6	M2-3 vs. S2-3	254630_at	AT4G18360	Encodes a glycolate oxidase that modulates reactive oxygen species-mediated signal transduction during nonhost resistance.	106.97	28.2	3.79	8.09E-04	1.97E-02
3701	6	M2-3 vs. S2-3	262969_at	AT1G75710	C2H2-like zinc finger protein;(source:Araport11)	864.8	230.02	3.76	8.56E-04	1.98E-02
3702	6	M2-3 vs. S2-3	248250_at	AT5G53130	member of Cyclic nucleotide gated channel family	213.78	57.19	3.74	7.91E-05	1.02E-02
3703	6	M2-3 vs. S2-3	261023_at	AT1G12200	Putative flavin monooxygenase.	83.3	23.19	3.59	2.70E-04	1.37E-02
3704	6	M2-3 vs. S2-3	248248_at	AT5G53120	encodes a novel spermine synthase and is a paralog of previously characterized spermidine synthases, SPDS1 and SPDS2. SPDS3 forms heterodimers with SDPS2, which in turn forms heterodimers with SDPS1 in vivo. The gene does not complement speDelta3 deficiency of spermidine synthase in yeast but DOES complement speDelta4 deficiency.	1354.21	405.03	3.34	8.40E-04	1.98E-02
3705	6	M2-3 vs. S2-3	258321_at	AT3G22840	Encodes an early light-inducible protein.	89.76	26.88	3.34	9.66E-04	2.06E-02
3706	6	M2-3 vs. S2-3	245993_at	AT5G20700	senescence-associated family protein, putative (DUF581);(source:Araport11)	172.17	55.33	3.11	4.60E-04	1.57E-02
3707	6	M2-3 vs. S2-3	252004_at	AT3G52780	Purple acid phosphatases superfamily protein;(source:Araport11)	37.47	12.1	3.1	1.61E-04	1.29E-02
3708	6	M2-3 vs. S2-3	253277_at	AT4G34230	Encodes a catalytically active cinnamyl alcohol dehydrogenase which uses p-coumaryl aldehyde as a preferred substrate. It can also use sinapyl, caffeyl, coniferyl and d-hydroxyconiferyl aldehydes as substrates.	193.74	62.53	3.1	7.37E-04	1.94E-02
3709	6	M2-3 vs. S2-3	254784_at	AT4G12720	Encodes a protein with ADP-ribose hydrolase activity. Negatively regulates EDS1-conditioned plant defense and programmed cell death.	520.44	173.07	3.01	3.80E-04	1.48E-02
3710	6	M2-3 vs. S2-3	244966_at	ATCG00590	electron carrier;(source:Araport11)	44.64	15.16	2.94	1.35E-04	1.22E-02
3711	6	M2-3 vs. S2-3	260967_at	AT1G12230	Aldolase superfamily protein;(source:Araport11)	297.05	103.3	2.88	9.04E-04	2.03E-02
3712	6	M2-3 vs. S2-3	246017_at	AT5G10730	NAD(P)-binding Rossmann-fold superfamily protein;(source:Araport11)	168.65	58.74	2.87	1.49E-04	1.26E-02
3713	6	M2-3 vs. S2-3	265182_at	AT1G23740	AOR is an alkenal/one oxidoreductase that acts on compounds with unsaturated alpha,beta-carbonyls. The activity of this enzyme with a number of substrates, including acrolein and 3-buten-2-one, was demonstrated in vitro using a truncated form of the protein that lacked approximately 80 of the first amino acids. This protein appears to localize to the chloroplast where it likely helps to maintain the photosynthetic process by detoxifying reactive carbonyls formed during lipid peroxidation.	124.39	43.57	2.85	7.96E-04	1.97E-02
3714	6	M2-3 vs. S2-3	249328_at	AT5G40820	Encodes a Arabidopsis ortholog of the ATR protein kinase that is involved in a wide range of responses to DNA damage and plays a central role in cell-cycle regulation. Dominant loss of function alleles identified as suppressors of ALS also exhibit increased tolerance to aluminum. This may be due to the inhibition of terminal differentiation of the root apex upon exposure to Al.	198.63	71.05	2.8	1.47E-05	3.85E-03
3715	6	M2-3 vs. S2-3	262509_at	AT1G11290	Pentatricopeptide Repeat Protein containing the DYW motif. Required for editing of multiple plastid transcripts. Endonuclease activity.	105.71	38.39	2.75	6.85E-04	1.92E-02
3716	6	M2-3 vs. S2-3	249701_at	AT5G35460	membrane protein;(source:Araport11)	260.97	95.34	2.74	9.14E-04	2.03E-02
3717	6	M2-3 vs. S2-3	253625_at	AT4G30600	signal recognition particle receptor alpha subunit family protein;(source:Araport11)	983.05	362.1	2.71	2.17E-04	1.34E-02
3718	6	M2-3 vs. S2-3	254727_at	AT4G13670	plastid transcriptionally active 5;(source:Araport11)	128.97	47.86	2.69	6.39E-05	8.70E-03
3719	6	M2-3 vs. S2-3	252472_at	AT3G46830	RAB GTPase homolog A2C;(source:Araport11)	199.37	75.93	2.63	8.13E-05	1.02E-02
3720	6	M2-3 vs. S2-3	248148_at	AT5G54930	AT hook motif-containing protein;(source:Araport11)	75.43	29.01	2.6	4.70E-04	1.57E-02
3721	6	M2-3 vs. S2-3	248543_at	AT5G50230	autophagy-related (ATG) gene	118.82	45.67	2.6	7.59E-04	1.97E-02
3722	6	M2-3 vs. S2-3	255497_at	AT4G02590	basic helix-loop-helix (bHLH) DNA-binding superfamily protein;(source:Araport11)	111.33	42.99	2.59	4.56E-04	1.57E-02
3723	6	M2-3 vs. S2-3	252181_at	AT3G50685	anti-muellerian hormone type-2 receptor;(source:Araport11)	346.54	136.88	2.53	2.77E-04	1.37E-02
3724	6	M2-3 vs. S2-3	267561_at	AT2G45590	Protein kinase superfamily protein;(source:Araport11)	356.45	141.75	2.51	8.47E-05	1.02E-02
3725	6	M2-3 vs. S2-3	261685_at	AT1G47290	Encodes an enzyme with 3β-hydroxysteroid dehydrogenase/C4-decarboxylase activity <i>in vitro</i>. The activity of the enzyme was determined using microsomal extracts of yeast overexpressing the <i>Arabidopsis</i> gene. Cytosolic fractions failed to be associated to the activity, leading to the speculation that the enzyme is membrane-bound.	150.94	62.02	2.43	9.48E-04	2.05E-02
3726	6	M2-3 vs. S2-3	263954_at	AT2G35840	Sucrose-6F-phosphate phosphohydrolase family protein;(source:Araport11)	197.61	81.95	2.41	5.41E-05	7.94E-03
3727	6	M2-3 vs. S2-3	245929_at	AT5G24760	GroES-like zinc-binding dehydrogenase family protein;(source:Araport11)	190.29	78.93	2.41	4.86E-04	1.59E-02
3728	6	M2-3 vs. S2-3	264243_at	AT1G54650	Methyltransferase family protein;(source:Araport11)	152.02	63.11	2.41	8.38E-04	1.98E-02
3729	6	M2-3 vs. S2-3	260530_at	AT2G47320	Cyclophilin-like peptidyl-prolyl cis-trans isomerase family protein;(source:Araport11)	498.53	208.33	2.39	7.00E-07	1.25E-03

3730	6	M2-3 vs. S2-3	259669_at	AT1G52340	Encodes a cytosolic short-chain dehydrogenase/reductase involved in the conversion of xanthoxin to ABA-aldehyde during ABA biosynthesis. Mutants are insensitive to sucrose and glucose.	67.95	29	2.34	2.54E-04	1.37E-02
3731	6	M2-3 vs. S2-3	247260_at	AT5G64500	Major facilitator superfamily protein;(source:Araport11)	306.77	131.86	2.33	7.62E-04	1.97E-02
3732	6	M2-3 vs. S2-3	258044_at	AT3G21270	Encodes Dof zinc finger protein adof2.	114.34	50.11	2.28	7.41E-04	1.94E-02
3733	6	M2-3 vs. S2-3	262355_at	AT1G72820	Mitochondrial substrate carrier family protein;(source:Araport11)	255.11	112.08	2.28	8.73E-04	1.98E-02
3734	6	M2-3 vs. S2-3	245066_at	AT2G39840	Encodes the catalytic subunit of a Type 1 phosphoprotein Ser/Thr phosphatase, expressed in roots, shoots and flowers. The mRNA is cell-to-cell mobile.	352.87	155.05	2.28	9.13E-04	2.03E-02
3735	6	M2-3 vs. S2-3	265763_at	AT2G48060	piezo-type mechanosensitive ion channel component;(source:Araport11)	260.55	114.59	2.27	1.70E-06	1.40E-03
3736	6	M2-3 vs. S2-3	255303_at	AT4G04860	DERLIN-2.2;(source:Araport11)	294.31	130.91	2.25	6.95E-04	1.94E-02
3737	6	M2-3 vs. S2-3	251616_at	AT3G57990	Encodes a ?-barrel protein, named OEP40, locates in in the outer envelope of chloroplasts, and functions as a solute channel which is selectively permeable for glucose.	93.29	41.81	2.23	2.30E-04	1.34E-02
3738	6	M2-3 vs. S2-3	253839_at	AT4G27890	HSP20-like chaperones superfamily protein;(source:Araport11)	43.43	19.49	2.23	3.30E-04	1.42E-02
3739	6	M2-3 vs. S2-3	247894_at	AT5G58003	Encodes a polypeptide that contains FCPH and BRCT domains. RNAi suppression mutant lines were generated, which displayed a range of phenotypic abnormalities, including: incomplete to no cotyledon expansion, slow growth, epinastic leaves or small inflorescences.	96.32	44.53	2.16	8.49E-04	1.98E-02
3740	6	M2-3 vs. S2-3	267012_at	AT2G39220	PATATIN-like protein 6;(source:Araport11)	408.38	190.71	2.14	3.01E-04	1.41E-02
3741	6	M2-3 vs. S2-3	261065_at	AT1G07500	SMR5 is a member of the SIAMESE-RELATED Cyclin-Dependent Kinase Inhibitor family. It is induced by ROS/oxidative stress.	28.97	13.68	2.12	5.33E-05	7.94E-03
3742	6	M2-3 vs. S2-3	265413_s_at	AT2G16650	Encodes a proteinaceous RNase P that supports RNase P activity in vivo in both organelles and the nucleus. It is also involved in the maturation of small nucleolar RNA (snoRNA) and mRNA.	28.23	13.3	2.12	2.16E-04	1.34E-02
3743	6	M2-3 vs. S2-3	253477_at	AT4G32320	Encodes a cytosolic ascorbate peroxidase APX6. Ascorbate peroxidases are enzymes that scavenge hydrogen peroxide in plant cells. Eight types of APX have been described for Arabidopsis: three cytosolic (APX1, APX2, APX6), two chloroplastic types (stromal sAPX, thylakoid tAPX), and three microsomal (APX3, APX4, APX5) isoforms.	69.47	33.36	2.08	1.19E-04	1.16E-02
3744	6	M2-3 vs. S2-3	247040_at	AT5G67150	HXXXD-type acyl-transferase family protein;(source:Araport11)	31.76	15.54	2.04	3.00E-04	1.41E-02
3745	6	M2-3 vs. S2-3	246038_s_at	AT5G19460	nudix hydrolase homolog 20;(source:Araport11)	107.88	55.95	1.93	3.17E-04	1.42E-02
3746	6	M2-3 vs. S2-3	250889_at	AT5G04500	Encodes a member of the CAZy Glycosyltransferase Family 64 that is involved in glycosylinositolphosphorylceramide and sphingolipid glycosylation. In mutants, seed germination was less sensitive to salt stress than in wild-type plants. [The protein was expected to be Golgi-localized based on function as well as the Golgi localization of its homolog GMT1. However, GFP-fusion proteins localized both to the ER and Golgi, and especially to ER when co-expressed with Golgi markers. Therefore, localization cannot confidently be defined. (pers. communication, J. Mortimer)]	72.55	37.64	1.93	3.44E-04	1.42E-02
3747	6	M2-3 vs. S2-3	262950_at	AT1G75510	Transcription initiation factor IIF, beta subunit;(source:Araport11)	91.9	48.3	1.9	1.31E-04	1.22E-02
3748	6	M2-3 vs. S2-3	255364_s_at	AT4G04020	Fibrillin precursor protein. The fibrillin preprotein, but not the mature protein interacts with ABI2. Regulated by abscisic acid response regulators. Involved in abscisic acid-mediated photoprotection. The mRNA is cell-to-cell mobile.	19.93	10.61	1.88	3.67E-04	1.47E-02
3749	6	M2-3 vs. S2-3	254035_at	AT4G25970	Encodes the major form of the two non-mitochondrail phosphatidylserine decarboxylase. Located at the ER. The mRNA is cell-to-cell mobile.	281.08	149.8	1.88	8.65E-04	1.98E-02
3750	6	M2-3 vs. S2-3	261324_at	AT1G44770	elongation factor;(source:Araport11)	565	310.12	1.82	2.40E-04	1.35E-02
3751	6	M2-3 vs. S2-3	250130_at	AT5G16510	RGP5 is a member of the reversably-glycosylated family of proteins. Analyses using tagged RGP5 suggest that it is present in the cytosol and in association with the Golgi apparatus. Recombinant RGP5 does not have UDP-arabinose mutase activity based on an in vitro assay even though the related RGP1, RGP2, and RGP3 proteins do have activity in the same assay. RGP5 can form complexes with RGP1 and RGP2.	197.74	109.73	1.8	1.54E-04	1.29E-02

3752	6	M2-3 vs. S2-3	263241_at	AT2G16500	Encodes a arginine decarboxylase (ADC), a rate-limiting enzyme that catalyzes the first step of polyamine (PA) biosynthesis via ADC pathway in Arabidopsis thaliana. Arabidopsis genome has two ADC paralogs, ADC1 and ADC2. Double mutant analysis showed that ADC genes are essential for the production of PA, and are required for normal seed development. Promoter region of ADC1 contains 742-bp AT-rich transposable element, called AtATE, that belongs to the MITE families of repetitive elements.	43.65	24.71	1.77	4.54E-04	1.57E-02
3753	6	M2-3 vs. S2-3	251881_at	AT3G54250	Encodes mevalonate diphosphate decarboxylase, the enzyme that catalyzes the synthesis of isopentenyl diphosphate, used in sterol and isoprenoid biosynthesis.	304.44	175.95	1.73	2.00E-07	5.76E-04
3754	6	M2-3 vs. S2-3	261579_at	AT1G01050	Encodes a soluble protein with inorganic pyrophosphatase activity that is highly specific for Mg-inorganic pyrophosphate.	239.07	138.93	1.72	2.23E-04	1.34E-02
3755	6	M2-3 vs. S2-3	254030_at	AT4G25890	60S acidic ribosomal protein family;(source:Araport11)	111.72	66.59	1.68	9.93E-04	2.10E-02
3756	6	M2-3 vs. S2-3	249140_at	AT5G43190	Galactose oxidase/kelch repeat superfamily protein;(source:Araport11)	42	25.21	1.67	1.27E-05	3.66E-03
3757	6	M2-3 vs. S2-3	265858_at	AT2G01720	Ribophorin I;(source:Araport11)	227.35	136.94	1.66	1.33E-04	1.22E-02
3758	6	M2-3 vs. S2-3	260648_at	AT1G08050	Zinc finger (C3HC4-type RING finger) family protein;(source:Araport11)	40.79	24.57	1.66	8.07E-04	1.97E-02
3759	6	M2-3 vs. S2-3	255994_at	AT1G29760	Membrane protein involved in lipid droplet biogenesis primarily in pollen.	100.53	60.78	1.65	4.08E-04	1.49E-02
3760	6	M2-3 vs. S2-3	252394_at	AT3G47940	DNAJ heat shock family protein;(source:Araport11)	41.32	25.25	1.64	2.51E-04	1.36E-02
3761	6	M2-3 vs. S2-3	253216_at	AT4G34960	Cyclophilin-like peptidyl-prolyl cis-trans isomerase family protein;(source:Araport11)	42.75	26.18	1.63	9.51E-04	2.05E-02
3762	6	M2-3 vs. S2-3	254911_at	AT4G11100	gelsolin protein;(source:Araport11)	14.53	9.25	1.57	3.11E-04	1.41E-02
3763	6	M2-3 vs. S2-3	256396_at	AT3G06150	cytochrome P450 family protein;(source:Araport11)	41.4	26.43	1.57	8.09E-04	1.97E-02
3764	6	M2-3 vs. S2-3	266930_at	AT2G45930	hypothetical protein;(source:Araport11)	24.67	16.41	1.5	1.94E-04	1.34E-02
3765	6	M2-3 vs. S2-3	252230_at	AT3G49810	Encodes a protein with E3 ubiquitin ligase activity that is involved in negative regulation of salt stress tolerance during germination.	63.51	45.17	1.41	1.86E-04	1.33E-02
3766	6	M2-3 vs. S2-3	261455_at	AT1G21070	Nucleotide-sugar transporter family protein;(source:Araport11)	19.84	16.49	1.2	2.23E-04	1.34E-02
3767	6	M2-3 vs. S2-3	256381_at	AT1G66850	Bifunctional inhibitor/lipid-transfer protein/seed storage 2S albumin superfamily protein;(source:Araport11)	14.54	18.31	0.79	< 1e-07	< 1e-07
3768	6	M2-3 vs. S2-3	264524_at	AT1G10070	Encodes a chloroplast branched-chain amino acid aminotransferase. Complements the yeast leu/iso-leu/val auxotrophy mutant. Involved in cell wall development.	20.84	29.73	0.7	5.90E-04	1.75E-02
3769	6	M2-3 vs. S2-3	258098_at	AT3G23670	Microtubule motor kinesin PAKRP1L/Kinesin-12B. Together with PAKRP1/Kinesin-12A, serve as linkers of the plus ends of antiparallel microtubules in the phragmoplast.	16.82	26.46	0.64	8.68E-04	1.98E-02
3770	6	M2-3 vs. S2-3	254562_at	AT4G19230	Encodes a protein with ABA 8'-hydroxylase activity, involved in ABA catabolism. Member of the CYP707A gene family. CYP707A1 appears to play an important role in determining the ABA levels in dry seeds. Gene involved in postgermination growth. Overexpression of CYP707A1 leads to a decrease in ABA levels and a reduction in after-ripening period to break dormancy.	16.12	25.67	0.63	7.40E-04	1.94E-02
3771	6	M2-3 vs. S2-3	260625_at	AT1G08060	Encodes a transcriptional silencer that is required for proper expression of PRR/NLR immune receptor genes.	34.6	55.23	0.63	9.55E-04	2.05E-02
3772	6	M2-3 vs. S2-3	245573_at	AT4G14730	Stress induced membrane protein. Mutants show enhanced cell death under stress.	22.4	36.09	0.62	4.93E-04	1.61E-02
3773	6	M2-3 vs. S2-3	254446_at	AT4G20890	tubulin 9 The mRNA is cell-to-cell mobile.	73.82	119	0.62	6.45E-04	1.83E-02
3774	6	M2-3 vs. S2-3	264028_at	AT2G03680	The SPR1 gene encodes a plant-specific 12-kD protein which has a repeated motif at both ends, separated by a predicted rod-like domain, suggesting that it may act as an intermolecular linker. Ubiquitously expressed and belongs to a six-member gene family in Arabidopsis; expressed in transgenic seedlings localized to microtubules within the cortical array, preprophase band, phragmoplast, and mitotic spindle.	61.1	100.91	0.61	1.21E-05	3.66E-03
3775	6	M2-3 vs. S2-3	254855_s_a	AT4G12140	RING/U-box superfamily protein;(source:Araport11)	9.02	14.75	0.61	1.70E-04	1.29E-02
3776	6	M2-3 vs. S2-3	252210_at	AT3G50410	Arabidopsis Dof protein containing a single 51-amino acid zinc finger DNA-binding domain, which may play an important roles in plant growth and development.	25.5	41.91	0.61	3.52E-04	1.42E-02
3777	6	M2-3 vs. S2-3	250694_at	AT5G06710	Homeobox-leucine zipper protein.	12.39	20.21	0.61	7.77E-04	1.97E-02
3778	6	M2-3 vs. S2-3	257826_at	AT3G26730	RING/U-box superfamily protein;(source:Araport11)	97.88	163.17	0.6	9.46E-04	2.05E-02
3779	6	M2-3 vs. S2-3	248868_at	AT5G46780	VQ motif-containing protein;(source:Araport11)	13.05	22.57	0.58	5.41E-04	1.70E-02
3780	6	M2-3 vs. S2-3	254683_at	AT4G13800	magnesium transporter NIPA (DUF803);(source:Araport11)	12.14	20.84	0.58	8.07E-04	1.97E-02
3781	6	M2-3 vs. S2-3	254398_at	AT4G21280	Encodes the PsbQ subunit of the oxygen evolving complex of photosystem II.	126.17	221.46	0.57	7.20E-06	2.81E-03
3782	6	M2-3 vs. S2-3	251321_at	AT3G61460	Encodes a novel ring finger protein and forms an N-terminal hydrophobic domain and a C-terminal RING-H2 signature. Expression is down regulated by brassinolide.	40.6	71.62	0.57	2.34E-04	1.35E-02

3783	6	M2-3 vs. S2-3	246900_at	AT5G25620	Encodes a member of a family of flavin monooxygenases with an important role in auxin biosynthesis. YUC6 possesses an additional thiol-reductase activity that confers drought resistance independently of auxin biosynthesis.	15.21	26.71	0.57	7.16E-04	1.94E-02
3784	6	M2-3 vs. S2-3	262911_s_at	AT1G07400	HSP20-like chaperones superfamily protein;(source:Araport11)	8.32	14.98	0.56	2.78E-04	1.37E-02
3785	6	M2-3 vs. S2-3	266735_at	AT2G46930	Encodes a pectin acetylesterase that removes cell wall acetate associated with pectin formation in Arabidopsis leaves.	16.55	29.88	0.55	3.09E-04	1.41E-02
3786	6	M2-3 vs. S2-3	250439_at	AT5G10450	Encodes a member of the 14-3-3 gene family that is a lambda isoform (14-3-3λ). Interacts with APX3 (ascorbate peroxidase) and AKR2 , suggesting a role in mediating oxidative metabolism in stress response. This protein was shown to colocalize and interact with SERK1 by which it is phosphorylated. This protein is also reported to interact with the phosphorylated form of the BZR1 transcription factor involved in brassinosteroid signaling and may affect the nucleocytoplasmic shuttling of BZR1. Interacts with JAZ10.4 which lacks the Jas motif. It is also phosphorylated by CRPK1 as part of the response to cold and translocates to the nucleus after phosphorylation.	334.27	602.41	0.55	8.51E-04	1.98E-02
3787	6	M2-3 vs. S2-3	249750_at	AT5G24570	hypothetical protein;(source:Araport11)	16.48	30.54	0.54	2.75E-04	1.37E-02
3788	6	M2-3 vs. S2-3	254800_at	AT4G13070	RNA-binding CRS1 / YhbY (CRM) domain protein;(source:Araport11)	11.21	21.2	0.53	1.15E-04	1.16E-02
3789	6	M2-3 vs. S2-3	267405_at	AT2G33740	encodes a copper binding protein that forms tetramers in vitro. Gene is expressed in all tissues examined and protein is localized to the chloroplast. The mRNA is cell-to-cell mobile.	104.09	196.52	0.53	2.83E-04	1.37E-02
3790	6	M2-3 vs. S2-3	263477_at	AT2G31790	UDP-Glycosyltransferase superfamily protein;(source:Araport11)	58.56	109.8	0.53	3.73E-04	1.48E-02
3791	6	M2-3 vs. S2-3	256491_at	AT1G31500	HESP identified based on similarity to nocturnins and presence circadian regulatory elements in the promoter. It functions as a Mg(II) dependent poly(A) exoribonuclease.It is under circadian regulation and expressed at night. Knockdowns affect the regulation of circadian genes CCA1 and TOC1.	14.03	26.77	0.52	4.48E-04	1.57E-02
3792	6	M2-3 vs. S2-3	259257_at	AT3G07760	Ortholog of Peach WEEP gene containing a sterile alpha motif. In peach, WEEP is responsible for pendulous branching phenotype. However in Arabidopsis no morphological branching defect has been observed in mutant lines.	42.67	82.12	0.52	8.11E-04	1.97E-02
3793	6	M2-3 vs. S2-3	262341_at	AT1G64230	ubiquitin-conjugating enzyme 28;(source:Araport11)	696.36	1368.53	0.51	9.87E-04	2.10E-02
3794	6	M2-3 vs. S2-3	248587_at	AT5G49550	Putative homolog of mammalian BLOC-1 Subunit 2. Protein - protein interaction with BLOS1.	21.3	42.52	0.5	1.02E-04	1.09E-02
3795	6	M2-3 vs. S2-3	266684_at	AT2G19720	ribosomal protein S15A B;(source:Araport11)	42.09	84.9	0.5	3.86E-04	1.48E-02
3796	6	M2-3 vs. S2-3	259181_at	AT3G01690	alpha/beta-Hydrolases superfamily protein;(source:Araport11)	163.87	327.18	0.5	6.47E-04	1.83E-02
3797	6	M2-3 vs. S2-3	248139_at	AT5G54970	hypothetical protein;(source:Araport11)	13.94	28.64	0.49	1.71E-04	1.29E-02
3798	6	M2-3 vs. S2-3	255285_at	AT4G04630	senescence regulator (Protein of unknown function, DUF584);(source:Araport11)	11.64	23.64	0.49	3.52E-04	1.42E-02
3799	6	M2-3 vs. S2-3	265034_at	AT1G61660	Encodes a transcriptional activator that regulates the expression of genes by binding to their GCG- or E-boxes to mediate physiological responses, including proline biosynthesis and ROS scavenging pathways, to enhance stress tolerance.	19.73	40.59	0.49	9.35E-04	2.04E-02
3800	6	M2-3 vs. S2-3	257232_at	AT3G16500	phytochrome-associated protein 1 (PAP1)	14.66	30.41	0.48	1.45E-04	1.26E-02
3801	6	M2-3 vs. S2-3	260385_at	AT1G74090	encodes a desulfoglucosinolate sulfotransferase, involved in the final step of glucosinolate core structure biosynthesis. Has a broad-substrate specificity with preference with methionine-derived desulfoglucosinolates.	30.3	62.88	0.48	5.42E-04	1.70E-02
3802	6	M2-3 vs. S2-3	260718_at	AT1G48110	evolutionarily conserved C-terminal region 7;(source:Araport11)	89.42	186.66	0.48	7.71E-04	1.97E-02
3803	6	M2-3 vs. S2-3	261352_at	AT1G79650	Encodes a member of the RADIATION SENSITIVE23 (RAD23) family: AT1G16190(RAD23A), AT1G79650(RAD23B), AT3G02540(RAD23C), AT5G38470(RAD23D). RAD23 proteins play an essential role in the cell cycle, morphology, and fertility of plants through their delivery of UPS (ubiquitin/26S proteasome system) substrates to the 26S proteasome.	21.97	46.36	0.47	2.44E-04	1.35E-02
3804	6	M2-3 vs. S2-3	246730_at	AT5G28060	Ribosomal protein S24e family protein;(source:Araport11)	28.51	61.31	0.47	3.18E-04	1.42E-02
3805	6	M2-3 vs. S2-3	253038_at	AT4G37790	Encodes homeobox protein HAT22, member of the HD-Zip II family. The mRNA is cell-to-cell mobile.	15.13	32.47	0.47	5.95E-04	1.75E-02
3806	6	M2-3 vs. S2-3	254111_at	AT4G24890	purple acid phosphatase 24;(source:Araport11)	31.88	68.87	0.46	9.64E-05	1.07E-02
3807	6	M2-3 vs. S2-3	247427_at	AT5G62580	ARM repeat superfamily protein;(source:Araport11)	30	65.35	0.46	1.47E-04	1.26E-02
3808	6	M2-3 vs. S2-3	252931_at	AT4G38930	Ubiquitin fusion degradation UFD1 family protein;(source:Araport11)	11.84	25.94	0.46	1.66E-04	1.29E-02
3809	6	M2-3 vs. S2-3	255675_at	AT4G00480	MYC-related protein with a basic helix-loop-helix motif at the C-terminus and a region similar to the maize B/R family at the N-terminus	10	21.66	0.46	1.99E-04	1.34E-02
3810	6	M2-3 vs. S2-3	257115_at	AT3G20150	Kinesin motor family protein;(source:Araport11)	11.33	24.64	0.46	2.19E-04	1.34E-02

3811	6	M2-3 vs. S2-3	253964_at	AT4G26480	RNA-binding KH domain-containing protein;(source:Araport11)	58.22	127.45	0.46	5.78E-04	1.75E-02
3812	6	M2-3 vs. S2-3	258151_at	AT3G18080	B-S glucosidase 44;(source:Araport11)	13.67	29.88	0.46	6.17E-04	1.78E-02
3813	6	M2-3 vs. S2-3	253420_at	AT4G32260	ATPase, F0 complex, subunit B/B, bacterial/chloroplast;(source:Araport11)	193.13	425.45	0.45	3.64E-05	6.43E-03
3814	6	M2-3 vs. S2-3	259617_at	AT1G47970	nucleolin;(source:Araport11)	103.45	231.29	0.45	2.96E-04	1.41E-02
3815	6	M2-3 vs. S2-3	262100_s_at	AT4G14250	This locus is annotated as a protein-coding gene in TAIR10. Based on communication with Jean-Luc GALLOIS (April 2013), this gene is split into two UBX domain-containing pseudogenes: one retains the original name: AT4G14250 (Chr4:8213237..8211984), one given a new locus identifier AT4G14245 (Chr4:8210231..8208985). Note that the Map Detail Image on the locus detial page and in GBrowse will not be updated until after the next genome release.	17.75	39.09	0.45	3.29E-04	1.42E-02
3816	6	M2-3 vs. S2-3	258038_at	AT3G21260	Glycolipid transfer protein (GLTP) family protein;(source:Araport11)	11.82	26.39	0.45	4.29E-04	1.54E-02
3817	6	M2-3 vs. S2-3	266636_at	AT2G35370	Encodes glycine decarboxylase complex H protein. Involved in photorespiration. The mRNA is cell-to-cell mobile.	21.97	49.82	0.44	1.08E-04	1.11E-02
3818	6	M2-3 vs. S2-3	261593_at	AT1G33170	S-adenosyl-L-methionine-dependent methyltransferases superfamily protein;(source:Araport11)	26.58	60.64	0.44	5.19E-04	1.67E-02
3819	6	M2-3 vs. S2-3	264579_at	AT1G05205	hexokinase-1 protein;(source:Araport11)	20.02	46.06	0.43	6.49E-05	8.70E-03
3820	6	M2-3 vs. S2-3	253977_at	AT4G26630	Encodes a chromatin-associated protein that specifically binds histones H3 and H4 and contributes to modulation of Arabidopsis chromatin structure and function.	27.81	65.03	0.43	3.22E-04	1.42E-02
3821	6	M2-3 vs. S2-3	266481_at	AT2G31070	TCP family protein involved in heterchronic regulation of leaf differentiation.	28.38	66.37	0.43	4.79E-04	1.58E-02
3822	6	M2-3 vs. S2-3	249613_at	AT5G37380	Chaperone DnaJ-domain superfamily protein;(source:Araport11)	29.26	68.81	0.43	5.78E-04	1.75E-02
3823	6	M2-3 vs. S2-3	251707_at	AT3G56490	Encodes a protein that has adenylylsulfate sulfohydrolase activity (E.C. 3.6.2.1) in vitro.	453.14	1063.56	0.43	7.93E-04	1.97E-02
3824	6	M2-3 vs. S2-3	256062_at	AT1G07090	LIGHT-DEPENDENT SHORT HYPOCOTYLS-like protein (DUF640);(source:Araport11)	14.13	33.23	0.43	7.98E-04	1.97E-02
3825	6	M2-3 vs. S2-3	264872_at	AT1G24260	Member of the MADs box transcription factor family. SEP3 is redundant with SEP1 and 2. Flowers of SEP1/2/3 triple mutants show a conversion of petals and stamens to sepals.SEP3 forms heterotetrameric complexes with other MADS box family members and binds to the CArG box motif.	274.89	656.26	0.42	1.73E-04	1.29E-02
3826	6	M2-3 vs. S2-3	AFFX-Athal-Actin_5_r_at	AT5G09810	Member of Actin gene family.Mutants are defective in germination and root growth. The mRNA is cell-to-cell mobile.	31.23	74.39	0.42	2.18E-04	1.34E-02
3827	6	M2-3 vs. S2-3	254328_at	AT4G22570	Encodes an adenine phosphoribosyltransferase (APT; EC 2.4.2.7), which is a constitutively expressed enzyme involved in the one-step salvage of adenine to AMP. APT3 has higher affinity for zeatin, isopentenyladenine and benzyladenine than APT1 but lower Vmax than APT1.	85.22	205.21	0.42	2.21E-04	1.34E-02
3828	6	M2-3 vs. S2-3	262432_at	AT1G47530	MATE efflux family protein;(source:Araport11)	26.24	62.32	0.42	5.54E-04	1.70E-02
3829	6	M2-3 vs. S2-3	259971_at	AT1G76580	Squamosa promoter-binding protein-like (SBP domain) transcription factor family protein;(source:Araport11)	77.81	184.47	0.42	5.95E-04	1.75E-02
3830	6	M2-3 vs. S2-3	247787_at	AT5G58640	Selenoprotein, Rdx type;(source:Araport11)	91.42	215.14	0.42	6.00E-04	1.75E-02
3831	6	M2-3 vs. S2-3	251753_at	AT3G55760	hypothetical protein;(source:Araport11)	39.56	94.03	0.42	7.79E-04	1.97E-02
3832	6	M2-3 vs. S2-3	248349_at	AT5G52240	Encodes a protein with similarity to progesterone-binding proteins in animals. Has been shown to bind steroids in vitro. Expressed in aerial portions of the plant excluding mature flowers and siliques. Antisense experiments suggest a role in inhibition of hypocotyl cell elongation. Expression is suppressed light grown seedlings transferred to the dark. The mRNA is cell-to-cell mobile.	259.07	612.95	0.42	9.17E-04	2.03E-02
3833	6	M2-3 vs. S2-3	257177_at	AT3G23490	Encodes a cyanase that catalyzes the bicarbonate-dependent breakdown of cyanate to ammonia and bicarbonate. CYN forms a hexadecamer and is believed to be a cytosolic protein. Long-term exposure to NaCl increases CYN transcript levels. It is also expressed at higher levels in flowers relative to stems, roots, and seedlings.	13.96	34.22	0.41	7.30E-06	2.81E-03
3834	6	M2-3 vs. S2-3	259166_at	AT3G01670	Encodes a protein localized to phloem filaments that is required for phloem filament formation.The mRNA is cell-to-cell mobile.	14.84	36.3	0.41	3.88E-04	1.48E-02
3835	6	M2-3 vs. S2-3	249313_at	AT5G41560	Encodes a substrate receptor for CRL4-CDD complexes that provides substrate specificity for CRL4 by interacting with ubiquitination targets. By its interaction and regulation of levels of PYL8 through proteasomal degradation, it negatively regulates ABA-mediated developmental responses, including inhibition of seed germination, seedling establishment, and root growth	20.19	49.33	0.41	9.26E-04	2.04E-02
3836	6	M2-3 vs. S2-3	258709_at	AT3G09500	Ribosomal L29 family protein;(source:Araport11)	349.15	867.28	0.4	2.85E-04	1.37E-02
3837	6	M2-3 vs. S2-3	251333_at	AT3G61690	Putative TNAase	39.71	98.23	0.4	4.59E-04	1.57E-02

3838	6	M2-3 vs. S2-3	267431_at	AT2G34870	hydroxyproline-rich glycoprotein family protein;(source:Araport11)	13.14	32.76	0.4	5.11E-04	1.65E-02
3839	6	M2-3 vs. S2-3	251413_at	AT3G60320	bZIP domain class transcription factor (DUF630 and DUF632);(source:Araport11)	67.13	169.28	0.4	5.55E-04	1.70E-02
3840	6	M2-3 vs. S2-3	257570_at	AT3G13662	Disease resistance-responsive (dirigent-like protein) family protein;(source:Araport11)	22.67	57.14	0.4	5.97E-04	1.75E-02
3841	6	M2-3 vs. S2-3	262331_at	AT1G64050	hypothetical protein;(source:Araport11)	11.17	28.84	0.39	4.80E-04	1.58E-02
3842	6	M2-3 vs. S2-3	246154_at	AT5G19940	Enables plants to cope with moderate light stress and affects cadmium tolerance.	37.46	98.39	0.38	7.42E-04	1.94E-02
3843	6	M2-3 vs. S2-3	258466_at	AT3G06040	Ribosomal protein L12/ ATP-dependent Clp protease adaptor protein ClpS family protein;(source:Araport11)	21.7	56.53	0.38	7.92E-04	1.97E-02
3844	6	M2-3 vs. S2-3	253049_at	AT4G37300	maternal effect embryo arrest 59;(source:Araport11)	24.11	64.79	0.37	2.06E-04	1.34E-02
3845	6	M2-3 vs. S2-3	246905_at	AT5G25570	polyamine-modulated factor 1-binding protein;(source:Araport11)	10.59	28.53	0.37	3.91E-04	1.48E-02
3846	6	M2-3 vs. S2-3	261886_s_a	AT1G80700	stress response NST1-like protein;(source:Araport11)	94.55	256.65	0.37	5.93E-04	1.75E-02
3847	6	M2-3 vs. S2-3	253891_at	AT4G27720	Major facilitator superfamily protein;(source:Araport11)	128.16	350.35	0.37	6.23E-04	1.79E-02
3848	6	M2-3 vs. S2-3	262281_at	AT1G68570	NPF3.1 is a membrane localized GA transporter that is expressed in the root endodermis.	14.21	38.69	0.37	7.32E-04	1.94E-02
3849	6	M2-3 vs. S2-3	252601_s_a	AT3G45030	Ribosomal protein S10p/S20e family protein;(source:Araport11)	378.29	1037.19	0.36	2.96E-05	6.32E-03
3850	6	M2-3 vs. S2-3	258237_at	AT3G27810	Encodes a member of the R2R3-MYB transcription factor gene family. Induced by jasmonate. Involved in jasmonate response during stamen development. MYB21 interacts with JAZ proteins, and functions redundantly with MYB24 and MYB57 to regulate stamen development.	16.67	46.43	0.36	8.51E-05	1.02E-02
3851	6	M2-3 vs. S2-3	252906_at	AT4G39640	The gene encodes a gamma-glutamyltransferase (AKA gamma-glutamyl transpeptidase, EC 2.3.2.2) that is located in vascular tissues (predominantly phloem) of leaves and is involved in the degradation of glutathione. The encoded enzyme also mitigates oxidative stress by metabolizing GSSG (oxidized form of GSH - glutathione) in the apoplast.	23.91	66.22	0.36	1.04E-04	1.09E-02
3852	6	M2-3 vs. S2-3	264056_at	AT2G28510	DOF transcription factor with a conserved zinc finger (ZF) DNA-binding domain.	23.24	64.3	0.36	2.28E-04	1.34E-02
3853	6	M2-3 vs. S2-3	246125_at	AT5G19875	transmembrane protein;(source:Araport11)	19.41	53.53	0.36	2.86E-04	1.37E-02
3854	6	M2-3 vs. S2-3	249651_at	AT5G37020	Encodes a member of the auxin response factor family. Mediates auxin response via expression of auxin regulated genes. Acts redundantly with ARF6 to control stamen elongation and flower maturation. Expression of ARF8 is controlled by miR167.	113.59	318.63	0.36	7.24E-04	1.94E-02
3855	6	M2-3 vs. S2-3	252676_at	AT3G44280	peptidyl-prolyl cis-trans isomerase G;(source:Araport11)	29.23	83.38	0.35	1.63E-05	4.08E-03
3856	6	M2-3 vs. S2-3	258471_at	AT3G06030	NPK1-related protein kinase 3	17.56	50.73	0.35	3.82E-04	1.48E-02
3857	6	M2-3 vs. S2-3	251860_at	AT3G54660	Encodes glutathione reductase that is most likely localized in the chloroplast.	75.57	213.82	0.35	5.87E-04	1.75E-02
3858	6	M2-3 vs. S2-3	260579_at	AT2G47380	Cytochrome c oxidase subunit Vc family protein;(source:Araport11)	165.68	471.51	0.35	7.24E-04	1.94E-02
3859	6	M2-3 vs. S2-3	248200_at	AT5G54160	A caffeic acid/5-hydroxyferulic acid O-methyltransferase. Interacts with 14-4-3 proteins in yeast 2 hybrid assay. AtOMT1 (At5g54160) encodes a flavonol 3?-O-methyltransferase that is highly active towards quercetin and myricetin. The substrate specificity identifies the enzyme as flavonol 3?-methyltransferase which replaces the former annotation of the gene to encode a caffeic acid/5-hydroxyferulic acid O-methyltransferase The mRNA is cell-to-cell mobile.	294.6	848.2	0.35	7.39E-04	1.94E-02
3860	6	M2-3 vs. S2-3	266845_at	AT2G26110	bromodomain protein (DUF761);(source:Araport11)	17.72	50.86	0.35	9.67E-04	2.06E-02
3861	6	M2-3 vs. S2-3	249268_at	AT5G41685	Mitochondrial outer membrane translocase complex, subunit Tom7;(source:Araport11)	10.4	30.91	0.34	1.01E-04	1.09E-02
3862	6	M2-3 vs. S2-3	250777_at	AT5G05440	Encodes a member of the PYR (pyrabactin resistance)/PYL(PYR1-like)/RCAR (regulatory components of ABA receptor) family proteins with 14 members. PYR/PYL/RCAR family proteins function as abscisic acid sensors. Mediate ABA-dependent regulation of protein phosphatase 2Cs ABI1 and ABI2.	29.62	86.21	0.34	3.36E-04	1.42E-02
3863	6	M2-3 vs. S2-3	250384_at	AT5G11500	coiled-coil protein;(source:Araport11)	201.99	589.52	0.34	8.41E-04	1.98E-02
3864	6	M2-3 vs. S2-3	262206_at	AT2G01090	Ubiquinol-cytochrome C reductase hinge protein;(source:Araport11)	622.83	1887.53	0.33	4.47E-04	1.57E-02
3865	6	M2-3 vs. S2-3	248387_at	AT5G51960	complex 1 protein, LYR family protein;(source:Araport11)	41.32	124.75	0.33	7.53E-04	1.96E-02
3866	6	M2-3 vs. S2-3	255443_at	AT4G02700	sulfate transporter 3;(source:Araport11)	15.72	48.89	0.32	1.12E-05	3.59E-03
3867	6	M2-3 vs. S2-3	251662_at	AT3G57080	Non-catalytic subunit unique to Nuclear DNA-dependent RNA polymerase V; homologous to budding yeast RPB5.	15.54	48.61	0.32	3.79E-05	6.43E-03

3868	6	M2-3 vs. S2-3	266119_at	AT2G02100	Predicted to encode a PR (pathogenesis-related) protein. Belongs to the plant defensin (PDF) family with the following members: At1g75830/PDF1.1, At5g44420/PDF1.2a, At2g26020/PDF1.2b, At5g44430/PDF1.2c, At2g26010/PDF1.3, At1g19610/PDF1.4, At1g55010/PDF1.5, At2g02120/PDF2.1, At2g02100/PDF2.2, At2g02130/PDF2.3, At1g61070/PDF2.4, At5g63660/PDF2.5, At2g02140/PDF2.6, At5g38330/PDF3.1 and At4g30070/PDF3.2. The mRNA is cell-to-cell mobile.	496.59	1567.69	0.32	2.65E-04	1.37E-02
3869	6	M2-3 vs. S2-3	250511_at	AT5G09960	sorbin/SH3 domain protein;(source:Araport11)	56.63	175.93	0.32	8.34E-04	1.98E-02
3870	6	M2-3 vs. S2-3	249755_at	AT5G24580	Heavy metal transport/detoxification superfamily protein;(source:Araport11)	23.64	75.35	0.31	1.74E-04	1.29E-02
3871	6	M2-3 vs. S2-3	248041_at	AT5G55940	Uncharacterized protein family (UPF0172);(source:Araport11)	96.46	307.58	0.31	2.28E-04	1.34E-02
3872	6	M2-3 vs. S2-3	257311_at	AT3G26570	low affinity phosphate transporter	68.68	219.2	0.31	3.39E-04	1.42E-02
3873	6	M2-3 vs. S2-3	258846_at	AT3G03070	NADH-ubiquinone oxidoreductase-like protein;(source:Araport11)	14.39	46.55	0.31	5.28E-04	1.69E-02
3874	6	M2-3 vs. S2-3	256979_at	AT3G21055	Encodes photosystem II 5 kD protein subunit PSII-T. This is a nuclear-encoded gene (PsbTn) which also has a plastid-encoded paralog (PsbTc).	145.86	472.39	0.31	5.51E-04	1.70E-02
3875	6	M2-3 vs. S2-3	259308_at	AT3G05180	GDSL-motif esterase/acyltransferase/lipase. Enzyme group with broad substrate specificity that may catalyze acyltransfer or hydrolase reactions with lipid and non-lipid substrates.	11.93	38.79	0.31	8.73E-04	1.98E-02
3876	6	M2-3 vs. S2-3	255035_at	AT4G09550	Encodes a gamma-tubulin complex protein that plays a role in gamma-tubulin complex localization, spindle stability and chromosomal segregation.	55.89	186.96	0.3	4.78E-04	1.58E-02
3877	6	M2-3 vs. S2-3	247566_at	AT5G61170	Ribosomal protein S19e family protein;(source:Araport11)	659.9	2396.68	0.28	2.20E-04	1.34E-02
3878	6	M2-3 vs. S2-3	266150_s_a	AT2G12290	hypothetical protein;(source:Araport11)	24.34	88.43	0.28	6.46E-04	1.83E-02
3879	6	M2-3 vs. S2-3	254694_at	AT4G17900	PLATZ transcription factor family protein;(source:Araport11)	68.34	252.31	0.27	2.82E-04	1.37E-02
3880	6	M2-3 vs. S2-3	246506_at	AT5G16110	hypothetical protein;(source:Araport11)	45.1	166.49	0.27	3.38E-04	1.42E-02
3881	6	M2-3 vs. S2-3	251997_at	AT3G53020	RPL24B encodes ribosomal protein L24, homolog of cytosolic RPL24, found in archaea and higher eukaryotes. Arabidopsis has two RPL24 homologs, RPL24A (AT2G36620) and RPL24B. Mutants showed defects in apical-basal gynoecium patterning similar to previously described ett and mp mutants. Transformation of stv1-1 mutant with a uORF-eliminated ETT construct partially suppressed the stv1 gynoecium phenotype, implying that STV1 could influence ETT translation through its uORFs. Regulated by TCP20.	547.79	2018.22	0.27	4.08E-04	1.49E-02
3882	6	M2-3 vs. S2-3	250517_at	AT5G08260	serine carboxypeptidase-like 35;(source:Araport11)	28.71	104.69	0.27	5.48E-04	1.70E-02
3883	6	M2-3 vs. S2-3	252789_s_a	AT1G21930	transmembrane protein;(source:Araport11)	260.96	968.98	0.27	6.61E-04	1.86E-02
3884	6	M2-3 vs. S2-3	266352_at	AT2G01610	Plant invertase/pectin methylesterase inhibitor superfamily protein;(source:Araport11)	34.72	126.91	0.27	7.36E-04	1.94E-02
3885	6	M2-3 vs. S2-3	253264_at	AT4G33950	Encodes calcium-independent ABA-activated protein kinase, a member of SNF1-related protein kinases (SnRK2) whose activity is activated by ionic (salt) and non-ionic (mannitol) osmotic stress. Mutations disrupted ABA induction of stomatal closure as well as ABA inhibition of light-induced stomatal opening. However, regulation of stomatal opening/closing by light or CO(2) is not affected in these mutants. May act in the interval between ABA perception and reactive oxygen species production in the ABA signalling network.	41.65	151.79	0.27	9.94E-04	2.10E-02
3886	6	M2-3 vs. S2-3	259538_at	AT1G12310	Calcium-binding EF-hand family protein;(source:Araport11)	45.78	174.78	0.26	4.80E-06	2.31E-03
3887	6	M2-3 vs. S2-3	255140_x_a	AT4G08410	Proline-rich extensin-like family protein;(source:Araport11)	23.01	89.21	0.26	9.23E-05	1.04E-02
3888	6	M2-3 vs. S2-3	265115_at	AT1G62450	Immunoglobulin E-set superfamily protein;(source:Araport11)	12.97	49.9	0.26	2.30E-04	1.34E-02
3889	6	M2-3 vs. S2-3	266460_at	AT2G47930	arabinogalactan protein 26;(source:Araport11)	16.47	67.03	0.25	2.10E-06	1.51E-03
3890	6	M2-3 vs. S2-3	261572_at	AT1G01170	ozone-responsive stress-like protein (DUF1138);(source:Araport11)	134.16	528.54	0.25	2.61E-04	1.37E-02
3891	6	M2-3 vs. S2-3	247148_at	AT5G65670	auxin (indole-3-acetic acid) induced gene The mRNA is cell-to-cell mobile.	310.98	1258.07	0.25	7.78E-04	1.97E-02
3892	6	M2-3 vs. S2-3	256311_at	AT1G30330	Encodes a member of the auxin response factor family. Mediates auxin response via expression of auxin regulated genes. Acts redundantly with ARF8 to control stamen elongation and flower maturation. Expression of ARF6 is controlled by miR167.	288.46	1207.57	0.24	1.79E-04	1.30E-02
3893	6	M2-3 vs. S2-3	266768_s_a	AT2G47110	polyubiquitin gene The mRNA is cell-to-cell mobile.	1145.59	4720.05	0.24	3.05E-04	1.41E-02
3894	6	M2-3 vs. S2-3	258699_at	AT3G09455	dolichyl-diphosphooligosaccharide-protein glycosyltransferase subunit;(source:Araport11)	131.25	548.13	0.24	3.96E-04	1.48E-02
3895	6	M2-3 vs. S2-3	247192_at	AT5G65360	Histone superfamily protein;(source:Araport11)	20.45	84.99	0.24	4.41E-04	1.57E-02
3896	6	M2-3 vs. S2-3	253382_at	AT4G33040	Encodes a member of the CC-type glutaredoxin (ROXY) family that has been shown to interact with the transcription factor TGA2.	149.92	625.42	0.24	4.66E-04	1.57E-02

3897	6	M2-3 vs. S2-3	245253_at	AT4G15440	Encodes a hydroperoxide lyase. Also a member of the CYP74B cytochrome p450 family. In the ecotype Columbia (Col) the gene contains a 10-nucleotide deletion in its first exon that causes it to code for a truncated protein that results in a non-functional hydroperoxide lyase.	27.2	120.39	0.23	8.80E-06	3.17E-03
3898	6	M2-3 vs. S2-3	264173_at	AT1G02160	Cox19 family protein (CHCH motif);(source:Araport11)	117.82	532.06	0.22	4.36E-04	1.56E-02
3899	6	M2-3 vs. S2-3	248747_at	AT5G47930	Zinc-binding ribosomal protein family protein;(source:Araport11)	212.68	976.3	0.22	6.07E-04	1.76E-02
3900	6	M2-3 vs. S2-3	245842_at	AT1G58430	Encodes an anther-specific proline-rich protein.	12.4	58.56	0.21	1.62E-04	1.29E-02
3901	6	M2-3 vs. S2-3	254083_at	AT4G24920	secE/sec61-gamma protein transport protein;(source:Araport11)	162.85	783.15	0.21	8.61E-04	1.98E-02
3902	6	M2-3 vs. S2-3	252235_at	AT3G49910	Translation protein SH3-like family protein;(source:Araport11)	850.63	4021.99	0.21	9.31E-04	2.04E-02
3903	6	M2-3 vs. S2-3	261149_s_a	AT1G19550	Glutathione S-transferase family protein;(source:Araport11)	354.43	1817.27	0.2	2.90E-06	1.67E-03
3904	6	M2-3 vs. S2-3	260257_at	AT1G74340	Encodes a subunit of the dolichol phosphate mannase synthase (DPMS) complex that may serve as membrane anchors for the catalytic core, DPMS1, or provide catalytic assistance. It is localized in the ER and mediates isoprenyl-linked glycan biogenesis.	73.42	360.13	0.2	1.28E-04	1.21E-02
3905	6	M2-3 vs. S2-3	251007_at	AT5G02610	Ribosomal L29 family protein;(source:Araport11)	90.82	462.04	0.2	1.47E-04	1.26E-02
3906	6	M2-3 vs. S2-3	255243_at	AT4G05590	Encodes NRGA1, a putative mitochondrial pyruvate carrier that mediates ABA regulation of guard cell ion channels and drought stress responses.	12.24	62.45	0.2	2.42E-04	1.35E-02
3907	6	M2-3 vs. S2-3	246028_at	AT5G21170	Encodes AKINbeta1, a subunit of the SnRK1 kinase (Sucrose non-fermenting-1-related protein kinase). Involved in regulation of nitrogen and sugar metabolism. As part of the regulatory subunit, it binds maltose which promotes kinase activity. Acts as a global regulator of genes involved in carbon, lipid and nitrogen metabolism.	38.46	189.08	0.2	2.85E-04	1.37E-02
3908	6	M2-3 vs. S2-3	259331_at	AT3G03840	SAUR-like auxin-responsive protein family;(source:Araport11)	9.06	44.62	0.2	3.28E-04	1.42E-02
3909	6	M2-3 vs. S2-3	249754_at	AT5G24530	Encodes a putative 2OG-Fe(II) oxygenase that is defense-associated but required for susceptibility to downy mildew. The mRNA is cell-to-cell mobile.	50.35	254.74	0.2	4.06E-04	1.49E-02
3910	6	M2-3 vs. S2-3	266118_at	AT2G02130	Predicted to encode a PR (pathogenesis-related) protein. Belongs to the plant defensin (PDF) family with the following members: At1g75830/PDF1.1, At5g44420/PDF1.2a, At2g26020/PDF1.2b, At5g44430/PDF1.2c, At2g26010/PDF1.3, At1g19610/PDF1.4, At1g55010/PDF1.5, At2g02120/PDF2.1, At2g02100/PDF2.2, At2g02130/PDF2.3, At1g61070/PDF2.4, At5g63660/PDF2.5, At2g02140/PDF2.6, At5g38330/PDF3.1 and At4g30070/PDF3.2. The mRNA is cell-to-cell mobile.	31.78	166.58	0.19	2.60E-06	1.67E-03
3911	6	M2-3 vs. S2-3	265768_at	AT2G48020	Encodes a zinc transporter ZIF2. Expression of ZIF2 is regulated by alternative splicing.	61.95	322.79	0.19	3.75E-05	6.43E-03
3912	6	M2-3 vs. S2-3	254137_at	AT4G24930	thylakoid lumenal 17.9 kDa protein, chloroplast;(source:Araport11)	19.94	106.68	0.19	1.18E-04	1.16E-02
3913	6	M2-3 vs. S2-3	265741_at	AT2G01320	ABC-2 type transporter family protein;(source:Araport11)	15.06	78.51	0.19	1.27E-04	1.21E-02
3914	6	M2-3 vs. S2-3	254915_s_a	AT4G11310	cysteine proteinase precursor-like protein	15.93	88.99	0.18	2.88E-05	6.32E-03
3915	6	M2-3 vs. S2-3	258003_at	AT3G29030	Encodes an expansin. Naming convention from the Expansin Working Group (Kende et al, 2004. Plant Mol Bio)	26.13	147.73	0.18	1.85E-04	1.33E-02
3916	6	M2-3 vs. S2-3	262951_at	AT1G75500	An Arabidopsis thaliana homolog of Medicago truncatula NODULIN21 (MtN21). The gene encodes a plant-specific, predicted integral membrane protein and is a member of the Plant-Drug/Metabolite Exporter (P-DME) family (Transporter Classification number: TC 2.A.7.3) and the nodulin MtN21-like transporter family.	81.53	464.06	0.18	7.36E-04	1.94E-02
3917	6	M2-3 vs. S2-3	253284_at	AT4G34150	Calcium-dependent lipid-binding (CaLB domain) family protein;(source:Araport11)	19.46	113.66	0.17	1.85E-05	4.44E-03
3918	6	M2-3 vs. S2-3	247163_at	AT5G65685	UDP-Glycosyltransferase superfamily protein;(source:Araport11)	25.64	148.65	0.17	3.75E-04	1.48E-02
3919	6	M2-3 vs. S2-3	263420_at	AT2G17240	Participates in the late stages of the biogenesis of 50S ribosomal subunits in plastids.	62.32	363.8	0.17	3.99E-04	1.49E-02
3920	6	M2-3 vs. S2-3	265984_at	AT2G24210	terpene synthase 10;(source:Araport11)	13.2	78.07	0.17	7.88E-04	1.97E-02
3921	6	M2-3 vs. S2-3	257701_at	AT3G12710	DNA glycosylase superfamily protein;(source:Araport11)	20.62	125.8	0.16	8.67E-05	1.02E-02
3922	6	M2-3 vs. S2-3	247468_at	AT5G62000	Encodes an auxin response factor. Mutants have many defects including enlarged rosette leaves, reduced fertility, later senescence, hypocotyl elongation defects, enlarged seeds and enlarged cotyledons. May not mediate auxin effects. Increase in seed size due to increased cell proliferation. The mRNA is cell-to-cell mobile.	202.38	1235.81	0.16	9.02E-05	1.04E-02
3923	6	M2-3 vs. S2-3	246044_at	AT5G19450	calcium-dependent protein kinase (CDPK19) mRNA, complete	60.45	372.68	0.16	1.91E-04	1.34E-02
3924	6	M2-3 vs. S2-3	267298_at	AT2G23760	Encodes a member of the BEL family of homeodomain proteins. Plants doubly mutant for saw1/saw2 (blh2/blh4) have serrated leaves. BP is expressed in the serrated leaves, therefore saw2 and saw1 may act redundantly to repress BP in leaves. Regulates together with BLH2 demethylesterification of homogalacturonan in seed mucilage.	12.95	82.17	0.16	3.96E-04	1.48E-02

3925	6	M2-3 vs. S2-3	247450_at	AT5G62350	Plant invertase/pectin methylesterase inhibitor superfamily protein;(source:Araport11)	117.38	740.75	0.16	4.67E-04	1.57E-02
3926	6	M2-3 vs. S2-3	266123_at	AT2G45180	Bifunctional inhibitor/lipid-transfer protein/seed storage 2S albumin superfamily protein;(source:Araport11)	101.7	665.55	0.15	8.85E-04	2.00E-02
3927	6	M2-3 vs. S2-3	246747_at	AT5G27700	Cytosolic ribosomal protein. Similar to EVR1 and redundant with EVR1. Also enhances VAR2 mutant varigation, but to a lesser extent than evr1.	276.44	1984.55	0.14	2.86E-04	1.37E-02
3928	6	M2-3 vs. S2-3	261489_at	AT1G14450	NADH dehydrogenase (ubiquinone)s;(source:Araport11)	42.93	315.96	0.14	8.91E-04	2.01E-02
3929	6	M2-3 vs. S2-3	255298_at	AT4G04840	methionine sulfoxide reductase B6;(source:Araport11)	15.67	124.41	0.13	3.63E-05	6.43E-03
3930	6	M2-3 vs. S2-3	256091_at	AT1G20693	Encodes a protein belonging to the subgroup of HMGB (high mobility group B) proteins that have a distinctive DNA-binding motif, the HMG-box domain. The motif confers non-sequence specific interaction with linear DNA and structure-specific binding to distorted DNA sites. The HMGB proteins are involved in the assembly of nucleoprotein complexes. Can be phosphorylated by CK2alpha. The mRNA is cell-to-cell mobile.	36.39	275.53	0.13	5.87E-05	8.25E-03
3931	6	M2-3 vs. S2-3	247162_at	AT5G65730	xyloglucan endotransglucosylase/hydrolase 6;(source:Araport11)	13.71	102.75	0.13	2.58E-04	1.37E-02
3932	6	M2-3 vs. S2-3	267239_at	AT2G02510	NADH dehydrogenase (ubiquinone)s;(source:Araport11)	125.26	984.95	0.13	8.45E-04	1.98E-02
3933	6	M2-3 vs. S2-3	244933_at	ATCG01070	NADH dehydrogenase ND4L	123.7	1004.57	0.12	3.38E-04	1.42E-02
3934	6	M2-3 vs. S2-3	250565_at	AT5G08000	Encodes a member of the X8-GPI family of proteins. It localizes to the plasmodesmata and binds callose.	70.68	586.64	0.12	4.54E-04	1.57E-02
3935	6	M2-3 vs. S2-3	266906_at	AT2G34585	transmembrane protein;(source:Araport11)	50.17	409.72	0.12	5.71E-04	1.74E-02
3936	6	M2-3 vs. S2-3	254163_s_a	AT4G24350	Phosphorylase superfamily protein;(source:Araport11)	17.75	165.3	0.11	1.30E-06	1.25E-03
3937	6	M2-3 vs. S2-3	266882_at	AT2G44670	senescence-associated family protein (DUF581);(source:Araport11)	36.99	355.08	0.1	4.61E-05	7.38E-03
3938	6	M2-3 vs. S2-3	259381_s_at	AT3G16410	Encodes a nitrile-specifier protein NSP4. NSP4 is one out of five (At3g16400/NSP1, At2g33070/NSP2, At3g16390/NSP3, At3g16410/NSP4 and At5g48180/NSP5) A. thaliana epithiospecifier protein (ESP) homologues that promote simple nitrile, but not epithionitrile or thiocyanate formation. The mRNA is cell-to-cell mobile.	77.01	764.71	0.1	2.28E-04	1.34E-02
3939	6	M2-3 vs. S2-3	249732_at	AT5G24420	Encodes a cytosolic 6-phosphogluconolactonase (PGL) thought to be involved in the oxidative pentose-phosphate pathway (OPPP).	15.11	195.99	0.077	1.30E-06	1.25E-03
3940	6	M2-3 vs. S2-3	263867_at	AT2G36830	Encodes a tonoplast intrinsic protein, which functions as water channel. It has also been shown to be able to facilitate the transport of urea and hydrogen peroxide. Highly expressed in vascular tissues of the root, stem, cauline leaves and flowers but not in the apical meristems. The mRNA is cell-to-cell mobile.	74.45	1068.2	0.07	1.96E-04	1.34E-02
3941	6	M2-3 vs. S2-3	247478_at	AT5G62360	Pectin methylesterase inhibitor expressed throughout the plant.	11.48	186.62	0.062	5.51E-05	7.94E-03
3942	6	M2-3 vs. S2-3	245138_at	AT2G45190	Encodes a member of the YABBY family of transcriptional regulators that is involved in abaxial cell type specification in leaves and fruits. YAB1 acts in a non-cell autonomous fashion within the meristem to affect phyllotactic patterning. The non-autonomous effect on the central region of the meristem is mediated through the activity if Lateral Suppressor (LAS).	20.4	455.16	0.045	6.30E-06	2.79E-03
3943	6	M2-3 vs. S2-3	247553_at	AT5G60910	MADS box gene negatively regulated by APETALA1	15.98	398.67	0.04	1.00E-06	1.25E-03
3944	6	M2-3 vs. S2-3	267213_at	AT2G44120	Ribosomal protein L30/L7 family protein;(source:Araport11)	13.66	349.33	0.039	4.40E-06	2.31E-03
3945	6	M2-3 vs. S2-3	266353_at	AT2G01520	Encodes a cis-cinnamic acid responsive gene that is a member of the major latex protein-like gene family and plays a role in promoting vegetative growth and delaying ﬂowering. The mRNA is cell-to-cell mobile.	110.68	2827.8	0.039	9.50E-06	3.22E-03
3946	6	M2-3 vs. S2-3	267639_at	AT2G42200	Encodes a putative transcriptional regulator that is involved in the vegetative to reproductive phase transition. Expression is regulated by MIR156b. SPL activity nonautonomously inhibits initiation of new leaves at the shoot apical meristem.	22.49	575.57	0.039	1.44E-05	3.85E-03
3947	6	M2-3 vs. S2-3	252282_at	AT3G49360	Encodes a cytosolic 6-phosphogluconolactonase (PGL) thought to be involved in the oxidative pentose-phosphate pathway (OPPP).	63.18	2524.14	0.025	7.00E-04	1.94E-02
3948	7	F2-3 vs. S2-3	250606_s_at	AT5G07410	Encodes a pectin methylesterase implicated in the remodeling of pectins in pollen. Its expression is restricted to the male gametophyte (dry pollen, imbibed pollen and pollen tube). Mutants do not display any phenotype in the vegetative tissues. Pollen germination of KO mutant is strongly delayed in vivo and in vitro. Extra calcium restores the wild type phenotype in vitro.	6125.4	7.83	782.2	< 1e-07	< 1e-07
3949	7	F2-3 vs. S2-3	266750_s_at	AT2G47040	Share high homologies with a group of pectin methylesterases (PME), pollen specific, and is required for enhancing the growth of pollen tube in style and transmitting tract tissues.	7363.02	11.31	651.2	< 1e-07	< 1e-07

3950	7	F2-3 vs. S2-3	245010_at	ATCG00420	Encodes NADH dehydrogenase subunit J. Its transcription is increased upon sulfur depletion.	8804.59	26.58	331.21	< 1e-07	< 1e-07
3951	7	F2-3 vs. S2-3	246880_s_at	AT5G25980	Myrosinase (thioglucoside glucohydrolase) gene involved in glucosinoloate metabolism. The mRNA is cell-to-cell mobile.	8698.56	26.96	322.63	< 1e-07	< 1e-07
3952	7	F2-3 vs. S2-3	251258_at	AT3G62170	VANGUARD-like protein;(source:Araport11)	2375.01	11.16	212.81	< 1e-07	< 1e-07
3953	7	F2-3 vs. S2-3	256966_at	AT3G13400	SKU5 similar 13;(source:Araport11)	2543.16	13.46	188.88	< 1e-07	< 1e-07
3954	7	F2-3 vs. S2-3	259269_at	AT3G01270	Pectate lyase family protein;(source:Araport11)	2755.66	15.63	176.27	< 1e-07	< 1e-07
3955	7	F2-3 vs. S2-3	261438_at	AT1G07600	metallothionein, binds to and detoxifies excess copper and other metals, limiting oxidative damage.	6953.4	42.95	161.9	< 1e-07	< 1e-07
3956	7	F2-3 vs. S2-3	255515_at	AT4G02250	Plant invertase/pectin methylesterase inhibitor superfamily protein;(source:Araport11)	1484.66	11	134.97	< 1e-07	< 1e-07
3957	7	F2-3 vs. S2-3	248227_at	AT5G53820	Late embryogenesis abundant protein (LEA) family protein;(source:Araport11)	1194.71	9.19	130.05	< 1e-07	< 1e-07
3958	7	F2-3 vs. S2-3	265127_at	AT1G55560	SKU5 similar 14;(source:Araport11)	1191.5	9.72	122.61	< 1e-07	< 1e-07
3959	7	F2-3 vs. S2-3	248926_at	AT5G45880	Pollen Ole e 1 allergen and extensin family protein;(source:Araport11)	1889.99	20.94	90.27	< 1e-07	< 1e-07
3960	7	F2-3 vs. S2-3	245946_at	AT5G19580	glyoxal oxidase-related protein;(source:Araport11)	1367.93	16.74	81.71	< 1e-07	< 1e-07
3961	7	F2-3 vs. S2-3	261410_at	AT1G07610	one of the five metallothioneins (MTs) genes identified in Arabidopsis. MTs are cysteine-rich proteins required for heavy metal tolerance. The mRNA is cell-to-cell mobile.	1496.85	19.23	77.82	< 1e-07	< 1e-07
3962	7	F2-3 vs. S2-3	248714_at	AT5G48140	Pectin lyase-like superfamily protein;(source:Araport11)	721.95	9.31	77.52	< 1e-07	< 1e-07
3963	7	F2-3 vs. S2-3	244959_s_at	AT2G07708	hypothetical protein;(source:Araport11)	1620.53	21.09	76.82	< 1e-07	2.96E-06
3964	7	F2-3 vs. S2-3	253226_at	AT4G35010	putative beta-galactosidase (BGAL11 gene)	922.82	12.75	72.39	< 1e-07	< 1e-07
3965	7	F2-3 vs. S2-3	246687_at	AT5G33370	GDSL-motif esterase/acyltransferase/lipase. Enzyme group with broad substrate specificity that may catalyze acyltransfer or hydrolase reactions with lipid and non-lipid substrates. Mutants are defective in cuticle formation with reduced sepal cuticle ridge formation.	1549.18	22.67	68.32	< 1e-07	< 1e-07
3966	7	F2-3 vs. S2-3	261943_at	AT1G80660	H[+]-ATPase 9;(source:Araport11)	960.07	15.64	61.37	< 1e-07	< 1e-07
3967	7	F2-3 vs. S2-3	265552_at	AT2G07560	H[+]-ATPase 6;(source:Araport11)	820.33	14.08	58.24	< 1e-07	< 1e-07
3968	7	F2-3 vs. S2-3	252820_at	AT3G42640	H[+]-ATPase 8;(source:Araport11)	3348.06	61.95	54.05	< 1e-07	< 1e-07
3969	7	F2-3 vs. S2-3	256955_at	AT3G13390	SKU5 similar 11;(source:Araport11)	578.38	11.11	52.04	< 1e-07	< 1e-07
3970	7	F2-3 vs. S2-3	260888_at	AT1G29140	Pollen Ole e 1 allergen and extensin family protein;(source:Araport11)	496.37	9.91	50.07	< 1e-07	< 1e-07
3971	7	F2-3 vs. S2-3	266743_at	AT2G02990	Encodes a member of the ribonuclease T2 family that responds to inorganic phosphate starvation, and inhibits production of anthocyanin. Also involved in wound-induced signaling independent of jasmonic acid. Its expression is responsive to both phosphate (Pi) and phosphite (Phi) in roots.	966.2	19.82	48.75	< 1e-07	< 1e-07
3972	7	F2-3 vs. S2-3	248367_at	AT5G52360	ADF10 is an actin-depolymerizing factor that preferentially binds ADP-G-actin and inhibits G-actin nucleotide exchange. ADF10 promotes actin turnover in pollen , regulating organization of actin filaments and vesicle trafficking during pollen tube growth.	696.87	14.66	47.55	< 1e-07	< 1e-07
3973	7	F2-3 vs. S2-3	265080_at	AT1G55570	SKU5 similar 12;(source:Araport11)	649.82	14.37	45.21	< 1e-07	< 1e-07
3974	7	F2-3 vs. S2-3	244964_at	ATCG00560	PSII L protein	10363.9	250.76	41.33	6.00E-07	1.10E-05
3975	7	F2-3 vs. S2-3	245026_at	ATCG00140	ATPase III subunit	2135.9	54.19	39.41	< 1e-07	< 1e-07
3976	7	F2-3 vs. S2-3	254716_at	AT4G13560	Late embryogenesis abundant protein (LEA) family protein;(source:Araport11)	450.49	12.34	36.51	< 1e-07	< 1e-07
3977	7	F2-3 vs. S2-3	246545_at	AT5G15110	Pectate lyase family protein;(source:Araport11)	414.89	11.45	36.23	< 1e-07	< 1e-07
3978	7	F2-3 vs. S2-3	245275_at	AT4G15210	cytosolic beta-amylase expressed in rosette leaves and inducible by sugar. RAM1 mutants have reduced beta amylase in leaves and stems.	1023.96	32.89	31.14	< 1e-07	< 1e-07
3979	7	F2-3 vs. S2-3	258639_at	AT3G07820	Pectin lyase-like superfamily protein;(source:Araport11)	262.04	8.95	29.26	< 1e-07	< 1e-07
3980	7	F2-3 vs. S2-3	246072_at	AT5G20240	Floral homeotic gene encoding a MADS domain transcription factor. Required for the specification of petal and stamen identities.	329.85	11.32	29.13	< 1e-07	< 1e-07
3981	7	F2-3 vs. S2-3	257641_s_at	AT3G25760	encodes allene oxide cyclase. One of four genes in Arabidopsis that encode this enzyme, which catalyzes an essential step in jasmonic acid biosynthesis. Gene expression is induced during senescence, a process that involves jasmonic acid signalling pathway. The mRNA is cell-to-cell mobile.	2445.4	87.9	27.82	8.00E-07	1.32E-05
3982	7	F2-3 vs. S2-3	247537_s_at	AT5G61690	ABC2 homolog 15;(source:Araport11)	481.74	17.57	27.41	< 1e-07	< 1e-07
3983	7	F2-3 vs. S2-3	254104_at	AT4G25040	Uncharacterized protein family (UPF0497);(source:Araport11)	526	19.38	27.14	< 1e-07	< 1e-07
3984	7	F2-3 vs. S2-3	262760_at	AT1G10770	Encodes a putative pectin methylesterase/invertase inhibitor. Anti-sense reduction of this gene's transcript results in pollen tube growth retardation and then partial male sterility and reduced seed set.	568.18	21.2	26.81	< 1e-07	< 1e-07
3985	7	F2-3 vs. S2-3	258686_at	AT3G07840	Pectin lyase-like superfamily protein;(source:Araport11)	395.79	15.78	25.08	< 1e-07	< 1e-07

3986	7	F2-3 vs. S2-3	248622_at	AT5G49360	Encodes a bifunctional {beta}-D-xylosidase/{alpha}-L-arabinofuranosidase required for pectic arabinan modification. Located in the extracellular matrix. Gene is expressed specifically in tissues undergoing secondary wall thickening. This is a member of glycosyl hydrolase family 3 and has six other closely related members.	891.76	38.51	23.16	< 1e-07	< 1e-07
3987	7	F2-3 vs. S2-3	258005_at	AT3G19390	Granulin repeat cysteine protease family protein;(source:Araport11)	850.16	37.88	22.44	< 1e-07	< 1e-07
3988	7	F2-3 vs. S2-3	253725_at	AT4G29340	Profilin is a low-molecular weight, actin monomer-binding protein that regulates the organization of actin cytoskeleton in eukaryotes, including higher plants. PRF4 and PRF5 are late pollen-specific and are not detectable in other cell types of the plant body including microspores and root hairs. Immunocytochemical studies at the subcellular level reveal that both the constitutive and pollen-specific profilins are abundant in the cytoplasm. In vegetative cell types, such as root apical cells, profilins showed localization to nuclei in addition to the cytoplasmic staining.	423.88	19.48	21.76	< 1e-07	< 1e-07
3989	7	F2-3 vs. S2-3	245011_at	ATCG00430	Encodes a protein which was originally thought to be part of photosystem II but its wheat homolog was later shown to encode for subunit K of NADH dehydrogenase.	416.5	19.76	21.07	< 1e-07	< 1e-07
3990	7	F2-3 vs. S2-3	256638_at	AT3G19090	RNA-binding protein;(source:Araport11)	823.49	39.11	21.05	< 1e-07	< 1e-07
3991	7	F2-3 vs. S2-3	251733_at	AT3G56240	CCH protein belongs to a family of eukaryotic proteins that participate in intracellular copper homeostasis by delivering this metal to the secretory pathway; mainly located along the vascular bundles of senescing leaves and petioles as well as in stem sieve elements; hypothesized to have a role in copper mobilization from decaying organs towards reproductive structures, as a result of metalloprotein breakdown. The plant-specific C-terminal domain of the CCH protein forms amyloid-like fibrils in vitro.	1952.99	93.94	20.79	< 1e-07	2.96E-06
3992	7	F2-3 vs. S2-3	252607_at	AT3G44990	Encodes a xyloglucan endotransglycosylase/hydrolase. Protein sequence and phylogenetic analysis indicates that this enzyme resides in Group III-A of the XTH family, with high similarity to Tropaeolum majus (nasturtium) xyloglucanase 1 (TmNXG1).Enzyme kinetic analysis indicates predominant xyloglucan endo-hydrolase activity (EC 3.2.1.151) with only limited potential to act as a xyloglucan endo-transglycosylase (EC 2.4.1.207).	216.47	10.49	20.64	< 1e-07	< 1e-07
3993	7	F2-3 vs. S2-3	252606_at	AT3G45010	serine carboxypeptidase-like 48;(source:Araport11)	695.23	35.26	19.72	< 1e-07	< 1e-07
3994	7	F2-3 vs. S2-3	267476_at	AT2G02720	Pectate lyase family protein;(source:Araport11)	202.12	10.45	19.34	< 1e-07	< 1e-07
3995	7	F2-3 vs. S2-3	249939_at	AT5G22430	Pollen Ole e 1 allergen and extensin family protein;(source:Araport11)	240.85	12.64	19.06	< 1e-07	< 1e-07
3996	7	F2-3 vs. S2-3	262463_at	AT1G50310	Sucrose transporter, expressed in pollen tubes.	394.22	20.78	18.97	< 1e-07	< 1e-07
3997	7	F2-3 vs. S2-3	265929_s_a	AT2G18570	UDP-Glycosyltransferase superfamily protein;(source:Araport11)	247.83	13.22	18.74	< 1e-07	< 1e-07
3998	7	F2-3 vs. S2-3	251590_at	AT3G57690	Encodes a putative arabinogalactan-protein (AGP23).	5965.4	318.7	18.72	< 1e-07	< 1e-07
3999	7	F2-3 vs. S2-3	245232_at	AT4G25590	actin depolymerizing factor 7;(source:Araport11)	188.13	10.07	18.67	< 1e-07	< 1e-07
4000	7	F2-3 vs. S2-3	249584_s_a	AT5G37810	NOD26-like intrinsic protein 4;(source:Araport11)	215.85	11.74	18.38	< 1e-07	< 1e-07
4001	7	F2-3 vs. S2-3	248252_at	AT5G53250	arabinogalactan protein 22;(source:Araport11)	972.32	53.11	18.31	< 1e-07	< 1e-07
4002	7	F2-3 vs. S2-3	262664_at	AT1G13970	beta-hexosaminidase (DUF1336);(source:Araport11)	174.18	9.72	17.92	< 1e-07	< 1e-07
4003	7	F2-3 vs. S2-3	250349_at	AT5G12000	kinase with adenine nucleotide alpha hydrolases-like domain-containing protein;(source:Araport11)	193.02	10.78	17.9	< 1e-07	< 1e-07
4004	7	F2-3 vs. S2-3	250631_at	AT5G07430	Pectin lyase-like superfamily protein;(source:Araport11)	232.23	13.22	17.56	< 1e-07	2.96E-06
4005	7	F2-3 vs. S2-3	258591_at	AT3G04360	Calcium-dependent lipid-binding (CaLB domain) family protein;(source:Araport11)	258.45	14.74	17.54	< 1e-07	2.96E-06
4006	7	F2-3 vs. S2-3	266697_at	AT2G19770	Encodes profilin 5, originally named profilin 4 (PRO4/PFN4). Low-molecular weight, actin monomer-binding protein that regulates the organization of actin cytoskeleton. Pollen-specific plant profilin present predominantly in mature pollen and growing pollen tubes.	302.64	17.88	16.93	1.10E-06	1.64E-05
4007	7	F2-3 vs. S2-3	267607_s_a t	AT2G26740	Encodes a soluble epoxide hydrolase whose expression is induced by auxin and water stress.	221.61	13.14	16.87	< 1e-07	< 1e-07
4008	7	F2-3 vs. S2-3	247538_at	AT5G61700	ABC2 homolog 16;(source:Araport11)	286.37	17.13	16.71	< 1e-07	< 1e-07
4009	7	F2-3 vs. S2-3	260241_at	AT1G63710	Encodes a member of the CYP86A subfamily of cytochrome p450 genes. Expressed at highest level in mature stems and flowers.	158.73	9.59	16.55	< 1e-07	< 1e-07
4010	7	F2-3 vs. S2-3	254283_s_a t	AT4G22870	2-oxoglutarate (2OG) and Fe(II)-dependent oxygenase superfamily protein;(source:Araport11)	903.2	55.45	16.29	3.00E-07	6.68E-06
4011	7	F2-3 vs. S2-3	255016_at	AT4G10120	Encodes a protein with putative sucrose-phosphate synthase activity.	160.68	9.98	16.11	< 1e-07	< 1e-07
4012	7	F2-3 vs. S2-3	260997_at	AT1G26610	C2H2-like zinc finger protein;(source:Araport11)	379.73	24.1	15.76	< 1e-07	< 1e-07

4013	7	F2-3 vs. S2-3	266918_at	AT2G45800	Encodes a member of the Arabidopsis LIM proteins: a family of actin bundlers with distinct expression patterns. WLIM1, WLIM2a, and WLIM2b are widely expressed, whereas PLIM2a, PLIM2b, and PLIM2c are predominantly expressed in pollen. Regulates actin cytoskeleton organization.	200.98	12.78	15.72	< 1e-07	< 1e-07
4014	7	F2-3 vs. S2-3	254024_at	AT4G25780	CAP (Cysteine-rich secretory proteins, Antigen 5, and Pathogenesis-related 1 protein) superfamily protein;(source:Araport11)	482.46	31.04	15.54	3.00E-07	6.68E-06
4015	7	F2-3 vs. S2-3	249996_at	AT5G18600	Encodes a member of the CC-type glutaredoxin (ROXY) family that has been shown to interact with the transcription factor TGA2 and suppress ORA59 promoter activity.	205.11	13.23	15.5	4.00E-07	8.26E-06
4016	7	F2-3 vs. S2-3	259161_at	AT3G01500	Encodes a putative beta-carbonic anhydrase betaCA1. Together with betaCA4 (At1g70410) regulates CO2-controlled stomatal movements in guard cells, as well as attenuates immunity. Differential CA gene expression in response to changing atmospheric CO2 conditions contribute to altered disease resistance levels. Activated by OXS2 under the treatment of salt.	312.24	20.57	15.18	< 1e-07	< 1e-07
4017	7	F2-3 vs. S2-3	245701_at	AT5G04140	Encodes a gene whose sequence is similar to ferredoxin dependent glutamate synthase (Fd-GOGAT). Expression in leaves is induced by light and sucrose. Proposed to be involved in photorespiration and nitrogen assimilation. The mRNA is cell-to-cell mobile.	209.67	14.05	14.93	< 1e-07	< 1e-07
4018	7	F2-3 vs. S2-3	257173_at	AT3G23810	S-adenosyl-l-homocysteine (SAH) hydrolase 2;(source:Araport11)	4516.75	310.93	14.53	< 1e-07	< 1e-07
4019	7	F2-3 vs. S2-3	264208_at	AT1G22760	Putative poly(A) binding protein May there fore function in posttranscriptional regulation, including mRNA turnover and translational initiation. Expression detected only in floral organs.	239.94	16.94	14.17	< 1e-07	< 1e-07
4020	7	F2-3 vs. S2-3	252140_at	AT3G51070	S-adenosyl-L-methionine-dependent methyltransferases superfamily protein;(source:Araport11)	178	12.78	13.93	< 1e-07	< 1e-07
4021	7	F2-3 vs. S2-3	262717_s_a	AT1G16410	member of CYP79F The mRNA is cell-to-cell mobile.	141.39	10.21	13.85	< 1e-07	< 1e-07
4022	7	F2-3 vs. S2-3	250498_at	AT5G09660	encodes a microbody NAD-dependent malate dehydrogenase encodes an peroxisomal NAD-malate dehydrogenase that is involved in fatty acid beta-oxidation through providing NAD to the process of converting fatty acyl CoA to acetyl CoA.	301.81	21.91	13.77	1.30E-06	1.81E-05
4023	7	F2-3 vs. S2-3	261648_at	AT1G27730	Related to Cys2/His2-type zinc-finger proteins found in higher plants. Compensated for a subset of calcineurin deficiency in yeast. Salt tolerance produced by ZAT10 appeared to be partially dependent on ENA1/PMR2, a P-type ATPase required for Li+ and Na+ efflux in yeast. The protein is localized to the nucleus, acts as a transcriptional repressor and is responsive to chitin oligomers. Also involved in response to photooxidative stress.	388.48	28.8	13.49	2.00E-07	4.97E-06
4024	7	F2-3 vs. S2-3	245465_at	AT4G16590	encodes a gene similar to cellulose synthase	248.69	19.01	13.08	< 1e-07	< 1e-07
4025	7	F2-3 vs. S2-3	249375_at	AT5G40730	Encodes an arabinogalactan-protein (AGP24).	1114.91	85.52	13.04	1.15E-05	8.34E-05
4026	7	F2-3 vs. S2-3	255590_at	AT4G01610	Encodes a capase involved in stress induced cell death. Activity detected in leaf and cell culture.	5358.72	412.15	13	< 1e-07	< 1e-07
4027	7	F2-3 vs. S2-3	261970_at	AT1G65960	glutamate decarboxylase (GAD2)The mRNA is cell-to-cell mobile.	3140.23	246.14	12.76	1.70E-06	2.21E-05
4028	7	F2-3 vs. S2-3	260693_at	AT1G32450	Transmembrane nitrate transporter. Involved in xylem transport of nitrate from root to shoot. Induced in response to high and low concentrations of nitrate. Not involved in nitrate uptake. Expressed in root pericycle cells under the control of MYB59. Also functions as a proton-coupled H+/K+ antiporter for K+ loading into the xylem.	460.65	36.25	12.71	< 1e-07	2.96E-06
4029	7	F2-3 vs. S2-3	259372_at	AT1G69120	Floral homeotic gene encoding a MADS domain protein homologous to SRF transcription factors. Specifies floral meristem and sepal identity. Required for the transcriptional activation of AGAMOUS. Interacts with LEAFY.Binds to promoter and regulates the expression of flowering time genes SVP, SOC1 and AGL24.	165.34	13.1	12.62	< 1e-07	< 1e-07
4030	7	F2-3 vs. S2-3	245047_at	ATCG00020	Encodes chlorophyll binding protein D1, a part of the photosystem II reaction center core	498.19	39.89	12.49	5.79E-05	2.63E-04
4031	7	F2-3 vs. S2-3	253099_s_a	AT4G37530	Peroxidase superfamily protein;(source:Araport11)	163.68	13.34	12.27	< 1e-07	< 1e-07
4032	7	F2-3 vs. S2-3	252863_at	AT4G39800	** Referred to as MIPS2 in Mitsuhashi et al 2008. myo-inositol-1-phosphate synthase isoform 1.Expressed in leaf, root and silique. Immunolocalization experiments with an antibody recognizing MIPS1, MIPS2, and MIPS3 showed endosperm localization.	877.18	71.75	12.23	< 1e-07	< 1e-07
4033	7	F2-3 vs. S2-3	258158_at	AT3G17790	Expression is upregulated in the shoot of cax1/cax3 mutant and is responsive to phosphate (Pi) and not phosphite (Phi) in roots and shoots.	254.33	21.01	12.1	< 1e-07	< 1e-07

4034	7	F2-3 vs. S2-3	266834_s_at	AT2G30020	Encodes AP2C1. Belongs to the clade B of the PP2C-superfamily. Acts as a MAPK phosphatase that negatively regulates MPK4 and MPK6.	3797.12	314.03	12.09	1.82E-05	1.13E-04
4035	7	F2-3 vs. S2-3	251658_at	AT3G57020	Calcium-dependent phosphotriesterase superfamily protein;(source:Araport11)	212.7	17.69	12.03	< 1e-07	< 1e-07
4036	7	F2-3 vs. S2-3	246908_at	AT5G25610	responsive to dehydration 22 (RD22) mediated by ABA	1650.56	138.69	11.9	< 1e-07	< 1e-07
4037	7	F2-3 vs. S2-3	258321_at	AT3G22840	Encodes an early light-inducible protein.	313.75	26.88	11.67	< 1e-07	< 1e-07
4038	7	F2-3 vs. S2-3	252291_s_at	AT3G49110	Class III peroxidase Perx33. Expressed in roots. Located in the cell wall. Involved in cell elongation. Expression activated by light. May play a role in generating H2O2 during defense response. The mRNA is cell-to-cell mobile.	103.45	9.07	11.41	1.30E-06	1.81E-05
4039	7	F2-3 vs. S2-3	245658_at	AT1G28270	Member of a diversely expressed predicted peptide family showing sequence similarity to tobacco Rapid Alkalinization Factor (RALF), and is believed to play an essential role in the physiology of Arabidopsis. Consists of a single exon and is characterized by a conserved C-terminal motif and N-terminal signal peptide. RALF4 and RALF19 act redundantly in the pollen tube to regulate pollen tube growth.	111.12	9.79	11.35	2.00E-07	4.97E-06
4040	7	F2-3 vs. S2-3	259451_at	AT1G13890	Encodes a member of a gene family homologous to mammalian SNAP25, a type of SNARE proteins with two chains. There are three members in Arabidopsis: SNAP30, SNAP29, and SNAP33.	104.68	9.59	10.92	1.30E-06	1.81E-05
4041	7	F2-3 vs. S2-3	266764_at	AT2G47050	Plant invertase/pectin methylesterase inhibitor superfamily protein;(source:Araport11)	134.01	12.27	10.92	6.00E-07	1.10E-05
4042	7	F2-3 vs. S2-3	255008_at	AT4G10060	Glucosylceramidase that preferentially hydrolyzes long acyl chain glucosylceramides.	179.24	16.83	10.65	< 1e-07	< 1e-07
4043	7	F2-3 vs. S2-3	260745_at	AT1G78370	Encodes glutathione transferase belonging to the tau class of GSTs. Naming convention according to Wagner et al. (2002).	192.04	18.08	10.62	3.00E-07	6.68E-06
4044	7	F2-3 vs. S2-3	245993_at	AT5G20700	senescence-associated family protein, putative (DUF581);(source:Araport11)	580.58	55.33	10.49	< 1e-07	< 1e-07
4045	7	F2-3 vs. S2-3	262146_at	AT1G52580	RHOMBOID-like protein 5;(source:Araport11)	141.47	13.65	10.36	< 1e-07	< 1e-07
4046	7	F2-3 vs. S2-3	252733_at	AT3G43120	SAUR-like auxin-responsive protein family;(source:Araport11)	118.8	11.51	10.32	1.00E-07	2.96E-06
4047	7	F2-3 vs. S2-3	246878_at	AT5G26060	Plant self-incompatibility protein S1 family;(source:Araport11)	153.75	14.9	10.32	< 1e-07	< 1e-07
4048	7	F2-3 vs. S2-3	247882_at	AT5G57785	hypothetical protein;(source:Araport11)	283.06	27.46	10.31	1.60E-06	2.10E-05
4049	7	F2-3 vs. S2-3	262122_at	AT1G02790	encodes a exopolygalacturonase.	121.69	11.85	10.27	2.50E-06	2.85E-05
4050	7	F2-3 vs. S2-3	265405_at	AT2G16750	kinase with adenine nucleotide alpha hydrolases-like domain-containing protein;(source:Araport11)	244.53	23.82	10.27	2.00E-07	4.97E-06
4051	7	F2-3 vs. S2-3	258162_at	AT3G17810	Encodes a protein predicted to have dihydropyrimidine dehydrogenase activity. Its activity has not been demonstrated in vivo, but, it is required for efficient uracil catabolism in Arabidopsis. It localizes to the plastid.	577.71	56.31	10.26	< 1e-07	< 1e-07
4052	7	F2-3 vs. S2-3	263450_at	AT2G31500	member of Calcium Dependent Protein Kinase	124.91	12.19	10.25	< 1e-07	< 1e-07
4053	7	F2-3 vs. S2-3	267144_at	AT2G38110	bifunctional sn-glycerol-3-phosphate 2-O-acyltransferase/phosphatase. Involved in cutin assembly.	285.16	27.98	10.19	< 1e-07	< 1e-07
4054	7	F2-3 vs. S2-3	257951_at	AT3G21700	Monomeric G protein. Expressed in root epidermal cells that are destined to become atrichoblasts. Also expressed during pollen development and in the pollen tube tip.	244.43	24.06	10.16	< 1e-07	< 1e-07
4055	7	F2-3 vs. S2-3	257106_at	AT3G29060	EXS (ERD1/XPR1/SYG1) family protein;(source:Araport11)	170.44	16.86	10.11	< 1e-07	< 1e-07
4056	7	F2-3 vs. S2-3	263954_at	AT2G35840	Sucrose-6F-phosphate phosphohydrolase family protein;(source:Araport11)	825.38	81.95	10.07	< 1e-07	< 1e-07
4057	7	F2-3 vs. S2-3	244976_at	ATCG00720	Encodes the cytochrome b(6) subunit of the cytochrome b6f complex.	717.56	71.27	10.07	1.00E-07	2.96E-06
4058	7	F2-3 vs. S2-3	248918_at	AT5G45890	Senescence-associated gene 12 (SAG12) encoding a cysteine protease influenced by cytokinin, auxin, and sugars.Localized to special vacuole found during senescence called senescence associated vacuoles which are different from central vacuole in the tonoplast composition and pH.	133.13	13.33	9.99	7.00E-07	1.20E-05
4059	7	F2-3 vs. S2-3	251775_s_at	AT3G55610	encodes delta 1-pyrroline-5-carboxylate synthetase B. Gene expression is induced by dehydration, high salt and ABA. Knock-out mutations in P5CS2 are embryo-lethal. P5CS2 appears to be present in different cells and/or different subcellular locations from P5CS1 in a tissue-dependent manner. Mutants are defective in pollen development.	722.75	72.52	9.97	1.65E-05	1.06E-04
4060	7	F2-3 vs. S2-3	252101_at	AT3G51290	pyridoxal-phosphate-dependent serine hydroxymethyltransferase, putative (DUF632);(source:Araport11)	920.65	96.36	9.55	< 1e-07	< 1e-07

4061	7	F2-3 vs. S2-3	265182_at	AT1G23740	AOR is an alkenal/one oxidoreductase that acts on compounds with unsaturated alpha,beta-carbonyls. The activity of this enzyme with a number of substrates, including acrolein and 3-buten-2-one, was demonstrated in vitro using a truncated form of the protein that lacked approximately 80 of the first amino acids. This protein appears to localize to the chloroplast where it likely helps to maintain the photosynthetic process by detoxifying reactive carbonyls formed during lipid peroxidation.	414.63	43.57	9.52	< 1e-07	< 1e-07
4062	7	F2-3 vs. S2-3	257816_at	AT3G25140	Quasimodo1, encodes a glycosyltransferase, involved in homogalacturonan biosynthesis; mutant shows cell adhesion defect and lower wall uronic acid content. The mRNA is cell-to-cell mobile.	364.7	38.69	9.43	< 1e-07	2.96E-06
4063	7	F2-3 vs. S2-3	245499_at	AT4G16480	Encodes a high affinity H+:<i>myo</i>-inositol symporter. The only other compound shown to be transported was pinitol, a methylated derivative of <i>myo</i>-inositol. The mRNA is cell-to-cell mobile.	469.36	49.91	9.4	< 1e-07	< 1e-07
4064	7	F2-3 vs. S2-3	251697_at	AT3G56600	phosphatidylinositol 4-kinase gamma-like protein;(source:Araport11)	145.19	15.85	9.16	< 1e-07	< 1e-07
4065	7	F2-3 vs. S2-3	264139_at	AT1G78940	kinase with adenine nucleotide alpha hydrolases-like domain-containing protein;(source:Araport11)	121.31	13.34	9.09	1.40E-06	1.89E-05
4066	7	F2-3 vs. S2-3	250514_at	AT5G09550	GDP dissociation inhibitor family protein / Rab GTPase activator family protein;(source:Araport11)	161.55	17.83	9.06	< 1e-07	< 1e-07
4067	7	F2-3 vs. S2-3	267181_at	AT2G37760	Encodes an NADPH-dependent aldo-keto reductase that can act on a wide variety of substrates in vitro including aliphatic and aromatic aldehydes and steroids. Transcript levels for this gene are up-regulated in response to cold, salt, and drought stress.	213.55	23.86	8.95	< 1e-07	2.96E-06
4068	7	F2-3 vs. S2-3	267357_at	AT2G40000	ortholog of sugar beet HS1 PRO-1 2;(source:Araport11)	200.57	22.44	8.94	< 1e-07	< 1e-07
4069	7	F2-3 vs. S2-3	247377_at	AT5G63180	Pectin lyase-like superfamily protein;(source:Araport11)	197.4	22.25	8.87	< 1e-07	< 1e-07
4070	7	F2-3 vs. S2-3	252929_at	AT4G38970	Protein is tyrosine-phosphorylated and its phosphorylation state is modulated in response to ABA in Arabidopsis thaliana seeds.	2992.59	339.55	8.81	< 1e-07	< 1e-07
4071	7	F2-3 vs. S2-3	267322_at	AT2G19330	Encodes PIRL6, a member of the Plant Intracellular Ras-group-related LRRs (Leucine rich repeat proteins). PIRLs are a distinct, plant-specific class of intracellular LRRs that likely mediate protein interactions, possibly in the context of signal transduction.	150.97	17.43	8.66	5.00E-07	9.67E-06
4072	7	F2-3 vs. S2-3	258685_at	AT3G07830	Pectin lyase-like superfamily protein;(source:Araport11)	264.41	30.52	8.66	5.00E-07	9.67E-06
4073	7	F2-3 vs. S2-3	267443_at	AT2G19000	hypothetical protein;(source:Araport11)	110.98	12.87	8.63	3.00E-07	6.68E-06
4074	7	F2-3 vs. S2-3	266115_at	AT2G02140	Predicted to encode a PR (pathogenesis-related) protein. Belongs to the plant defensin (PDF) family with the following members: At1g75830/PDF1.1, At5g44420/PDF1.2a, At2g26020/PDF1.2b, At5g44430/PDF1.2c, At2g26010/PDF1.3, At1g19610/PDF1.4, At1g55010/PDF1.5, At2g02120/PDF2.1, At2g02100/PDF2.2, At2g02130/PDF2.3, At1g61070/PDF2.4, At5g63660/PDF2.5, At2g02140/PDF2.6, At5g38330/PDF3.1 and At4g30070/PDF3.2.	154.75	18	8.6	3.00E-07	6.68E-06
4075	7	F2-3 vs. S2-3	249614_at	AT5G37300	Encodes a bifunctional enzyme, wax ester synthase (WS) and diacylglycerol acyltransferase (DGAT). In vitro assay indicated a ratio of 10.9 between its WS and DGAT activities. Both mutant and in vivo expression/analysis in yeast studies indicated a role in wax biosynthesis.	244.53	28.61	8.55	< 1e-07	< 1e-07
4076	7	F2-3 vs. S2-3	245024_at	ATCG00120	Encodes the ATPase alpha subunit, which is a subunit of ATP synthase and part of the CF1 portion which catalyzes the conversion of ADP to ATP using the proton motive force. This complex is located in the thylakoid membrane of the chloroplast.	1892.86	221.57	8.54	4.00E-07	8.26E-06
4077	7	F2-3 vs. S2-3	252004_at	AT3G52780	Purple acid phosphatases superfamily protein;(source:Araport11)	102.39	12.1	8.46	< 1e-07	< 1e-07
4078	7	F2-3 vs. S2-3	245061_at	AT2G39730	Rubisco activase, a nuclear-encoded chloroplast protein that consists of two isoforms arising from alternative splicing in most plants. Required for the light activation of rubisco. Involved in jasmonate-induced leaf senescence.	1074.59	127.75	8.41	< 1e-07	< 1e-07
4079	7	F2-3 vs. S2-3	254122_at	AT4G24510	Encodes a component of the fatty acid elongation machinery required for C28 to C30 fatty acid elongation. It does not require the acyltransferase catalytic site for biological function.	146.65	17.5	8.38	< 1e-07	< 1e-07
4080	7	F2-3 vs. S2-3	265368_at	AT2G13350	Calcium-dependent lipid-binding (CaLB domain) family protein;(source:Araport11)	143.6	17.16	8.37	3.00E-07	6.68E-06
4081	7	F2-3 vs. S2-3	258561_at	AT3G05960	Encodes a hexose sugar transporter that is expressed in pollen. STP6 may play a role in providing sugars during late pollen maturation or pollen tube germination.	130.3	15.69	8.3	1.80E-06	2.29E-05
4082	7	F2-3 vs. S2-3	248335_at	AT5G52450	MATE efflux family protein;(source:Araport11)	240.88	29.37	8.2	< 1e-07	< 1e-07

4083	7	F2-3 vs. S2-3	265002_at	AT1G24400	High-affinity transporter for neutral and acidic amino acids, expressed in tapetum tissue of anthers. Transport of 1-Aminocyclopropane-1-carboxylic acid (ACC).	106.1	13.19	8.04	< 1e-07	< 1e-07
4084	7	F2-3 vs. S2-3	254123_at	AT4G24640	Encodes AppB protein (AppB1).	90.84	11.31	8.03	7.00E-07	1.20E-05
4085	7	F2-3 vs. S2-3	254607_at	AT4G18920	histone acetyltransferase (DUF1264);(source:Araport11)	112.15	13.99	8.02	1.00E-07	2.96E-06
4086	7	F2-3 vs. S2-3	256008_s_at	AT1G34040	Pyridoxal phosphate (PLP)-dependent transferases superfamily protein;(source:Araport11)	104.34	13.08	7.97	3.30E-06	3.44E-05
4087	7	F2-3 vs. S2-3	245749_at	AT1G51090	Heavy metal associated domain containing protein involved in plant immunity.Mutants show an increase in root length under NO stress and reduction in root length under H2O2 stress conditions. Mutants show increases in defense responses to pathogens including hypersensitive lesions, increased resistance and induction of SAR genes.	167.92	21.27	7.9	< 1e-07	2.96E-06
4088	7	F2-3 vs. S2-3	256627_at	AT3G19970	alpha/beta-Hydrolases superfamily protein;(source:Araport11)	295.7	37.49	7.89	2.30E-06	2.69E-05
4089	7	F2-3 vs. S2-3	248377_at	AT5G51720	Encodes a protein with biochemical, structural, and biophysical characteristics of a NEET protein. It plays a key role in plant development, senescence, reactive oxygen homeostasis, and Fe metabolism.	408.68	52.11	7.84	1.80E-06	2.29E-05
4090	7	F2-3 vs. S2-3	261713_at	AT1G32640	Encodes a MYC-related transcriptional activator with a typical DNA binding domain of a basic helix-loop-helix leucine zipper motif. Binds to an extended G-Box promoter motif and interacts with Jasmonate ZIM-domain proteins. MYC2 interacts with EIN3 and EIL1 to repress hook curvature and resistance to Botrytis cinera.Its transcription is induced by dehydration stress, ABA treatment and blue light via CRY1. Negative regulator of blue light-mediated photomorphogenic growth and blue and far-red-light-regulated gene expression. Positive regulator of lateral root formation. Regulates diverse JA-dependent functions. Negatively regulates Trp metabolism and biosynthesis of Trp-derived secondary metabolites. Positively regulates flavonoid biosynthesis, resistance to insects, and response to oxidative stress. Regulates other transcription factors, and negatively regulates its own expression. For example it binds to and regulates the expression of NST1. Its stability is modulated by PUB10 through polyubiquitination.	373.08	47.84	7.8	< 1e-07	2.96E-06
4091	7	F2-3 vs. S2-3	259343_s_at	AT3G03780	Encodes a cytosolic methionine synthase, involved in methionine regeneration via the activated methyl cycle (or SAM cycle)	1883.09	242.47	7.77	2.00E-07	4.97E-06
4092	7	F2-3 vs. S2-3	266592_at	AT2G46210	Fatty acid/sphingolipid desaturase;(source:Araport11)	176.4	22.75	7.75	1.00E-07	2.96E-06
4093	7	F2-3 vs. S2-3	251916_at	AT3G53960	Major facilitator superfamily protein;(source:Araport11)	231.48	29.94	7.73	< 1e-07	< 1e-07
4094	7	F2-3 vs. S2-3	261559_at	AT1G01780	Encodes a member of the Arabidopsis LIM proteins: a family of actin bundlers with distinct expression patterns. WLIM1, WLIM2a, and WLIM2b are widely expressed, whereas PLIM2a, PLIM2b, and PLIM2c are predominantly expressed in pollen. Regulates actin cytoskeleton organization. The mRNA is cell-to-cell mobile.	98.65	12.98	7.6	6.00E-07	1.10E-05
4095	7	F2-3 vs. S2-3	266075_s_a	AT2G40710	hemolysin-III related integral membrane protein;(source:Araport11)	365.66	48.15	7.59	3.40E-06	3.52E-05
4096	7	F2-3 vs. S2-3	265444_s_at	AT2G37180	a member of the plasma membrane intrinsic protein PIP2. functions as aquaporin and is involved in desiccation.	1236.36	163.55	7.56	< 1e-07	< 1e-07
4097	7	F2-3 vs. S2-3	264207_at	AT1G22750	transmembrane protein;(source:Araport11)	122.47	16.26	7.53	< 1e-07	< 1e-07
4098	7	F2-3 vs. S2-3	258851_at	AT3G03190	Encodes glutathione transferase belonging to the phi class of GSTs. Naming convention according to Wagner et al. (2002).	351.25	46.86	7.5	< 1e-07	< 1e-07
4099	7	F2-3 vs. S2-3	252006_at	AT3G52820	purple acid phosphatase 22;(source:Araport11)	160.63	21.42	7.5	< 1e-07	2.96E-06
4100	7	F2-3 vs. S2-3	254174_at	AT4G24120	Member of a small family of oligopeptide transporters similar to the yellow stripe locus of maize (ZmYS1).	351.5	47.18	7.45	3.00E-07	6.68E-06
4101	7	F2-3 vs. S2-3	249048_at	AT5G44300	Dormancy/auxin associated family protein;(source:Araport11)	170.17	22.86	7.44	1.00E-07	2.96E-06
4102	7	F2-3 vs. S2-3	261015_at	AT1G26480	14-3-3 protein GF14iota (grf12)	133.82	18.17	7.36	2.00E-07	4.97E-06
4103	7	F2-3 vs. S2-3	257986_at	AT3G20865	Encodes a putative arabinogalactan-protein (AGP40) that is expressed in pollen.	274.8	37.79	7.27	5.93E-05	2.67E-04
4104	7	F2-3 vs. S2-3	248073_at	AT5G55720	Pectin lyase-like superfamily protein;(source:Araport11)	81.9	11.27	7.27	2.00E-07	4.97E-06
4105	7	F2-3 vs. S2-3	251898_at	AT3G54340	Floral homeotic gene encoding a MADS domain protein homologous to SRF transcription factors. Specifies petal and stamen identities. Associates with PISTILLATA.	277.62	38.23	7.26	5.00E-07	9.67E-06
4106	7	F2-3 vs. S2-3	251304_at	AT3G61990	Encodes a protein methyltransferase. Involved in the methylation of plant transmembrane proteins.	299.38	42.43	7.06	< 1e-07	< 1e-07
4107	7	F2-3 vs. S2-3	251405_at	AT3G60330	H[+]-ATPase 7;(source:Araport11)	144.82	20.77	6.97	1.00E-07	2.96E-06
4108	7	F2-3 vs. S2-3	253177_s_a	AT4G35150	O-methyltransferase family protein;(source:Araport11)	413.91	59.41	6.97	2.00E-07	4.97E-06

4109	7	F2-3 vs. S2-3	248713_at	AT5G48180	Encodes a nitrile-specifier protein NSP5. NSP5 is one out of five (At3g16400/NSP1, At2g33070/NSP2, At3g16390/NSP3, At3g16410/NSP4 and At5g48180/NSP5) A. thaliana epithiospecifier protein (ESP) homologues that promote simple nitrile, but not epithionitrile or thiocyanate formation.	342.72	49.2	6.97	4.30E-06	4.17E-05
4110	7	F2-3 vs. S2-3	255997_s_at	AT1G29930	Subunit of light-harvesting complex II (LHCII),which absorbs light and transfers energy to the photosynthetic reaction center. The mRNA is cell-to-cell mobile.	2058.1	295.67	6.96	< 1e-07	2.96E-06
4111	7	F2-3 vs. S2-3	262396_at	AT1G49470	transmembrane epididymal protein (DUF716);(source:Araport11)	131.83	19.12	6.9	< 1e-07	< 1e-07
4112	7	F2-3 vs. S2-3	253344_at	AT4G33550	Bifunctional inhibitor/lipid-transfer protein/seed storage 2S albumin superfamily protein;(source:Araport11)	168.85	24.49	6.9	3.80E-06	3.79E-05
4113	7	F2-3 vs. S2-3	247902_at	AT5G57350	member of Plasma membrane H+-ATPase family	409.62	59.33	6.9	4.90E-06	4.55E-05
4114	7	F2-3 vs. S2-3	254740_s_at	AT4G13930	Encodes a serine hydroxymethyltransferase maximally expressed in root	1901.59	276.83	6.87	2.00E-07	4.97E-06
4115	7	F2-3 vs. S2-3	265547_at	AT2G28305	Putative lysine decarboxylase family protein;(source:Araport11)	266.53	39.15	6.81	1.00E-07	2.96E-06
4116	7	F2-3 vs. S2-3	263379_at	AT2G40140	zinc finger (CCCH-type) family protein;(source:Araport11)	363.05	53.57	6.78	< 1e-07	2.96E-06
4117	7	F2-3 vs. S2-3	252973_s_at	AT4G38740	Encodes cytosolic cyclophilin ROC1.	881.5	130.47	6.76	1.80E-06	2.29E-05
4118	7	F2-3 vs. S2-3	263533_at	AT2G24820	translocon at the inner envelope membrane of chloroplasts 55-II;(source:Araport11)	207.73	30.88	6.73	< 1e-07	< 1e-07
4119	7	F2-3 vs. S2-3	251591_at	AT3G57680	Peptidase S41 family protein;(source:Araport11)	364.16	54.21	6.72	< 1e-07	< 1e-07
4120	7	F2-3 vs. S2-3	265923_at	AT2G18470	Proline-rich extensin-like receptor kinase 4. Functions at an early stage of ABA signalling inhibiting primary root cell elongation by perturbing Ca2+ homeostasis.	110.01	16.54	6.65	2.65E-05	1.49E-04
4121	7	F2-3 vs. S2-3	261881_at	AT1G80760	Encodes a protein with boron transporter activity. It helps to preferentially direct boron to young developing tissues in the shoot, such as immature leaves, under low boron conditions. This boron channel appears to be impermeable to water, unlike the closely related NIP5;1 boron transporter. This protein also allows the transport of glycerol, urea, and formimide but not larger uncharged solutes such as arabitol and sucrose when it is expressed heterologously.	151.95	22.96	6.62	< 1e-07	2.96E-06
4122	7	F2-3 vs. S2-3	253643_at	AT4G29780	Expression of the gene is affected by multiple stresses. Knockout and overexpression lines show no obvious phenotypes.	816.98	123.83	6.6	< 1e-07	< 1e-07
4123	7	F2-3 vs. S2-3	265475_at	AT2G15620	Involved in the second step of nitrate assimilation. Its expression is induced by nitrate. The mRNA is cell-to-cell mobile.	119.95	18.29	6.56	< 1e-07	< 1e-07
4124	7	F2-3 vs. S2-3	262454_at	AT1G11190	Encodes a bifunctional nuclease that acts on both RNA and DNA involved in nucleic acid degradation to facilitate nucleotide and phosphate recovery during senescence. It has mismatch-specific endonuclease activity with wide recognition of single base mismatches as well as the ability to cleave indel types of mismatches (heteroduplexes with loops).	170.32	26.16	6.51	< 1e-07	< 1e-07
4125	7	F2-3 vs. S2-3	264204_at	AT1G22710	Encodes for a high-affinity transporter essential for phloem loading and long-distance transport. A major sucrose transporter, AtSUC2 can also transport a wide range of physiological and synthetic glucose conjugates with both α- or β-linkage.	466.32	71.74	6.5	2.20E-06	2.63E-05
4126	7	F2-3 vs. S2-3	244936_at	ATCG01100	NADH dehydrogenase ND1	121.74	18.77	6.49	1.20E-06	1.75E-05
4127	7	F2-3 vs. S2-3	258614_at	AT3G02770	Ribonuclease E inhibitor RraA/Dimethylmenaquinone methyltransferase;(source:Araport11)	141.56	21.84	6.48	3.00E-07	6.68E-06
4128	7	F2-3 vs. S2-3	259429_at	AT1G01600	Encodes a member of the CYP86A subfamily of cytochrome p450 genes. Expressed significantly at highest level in mature stems and flowers.	101.17	15.63	6.47	2.00E-07	4.97E-06
4129	7	F2-3 vs. S2-3	248643_at	AT5G49130	MATE efflux family protein;(source:Araport11)	183.55	28.42	6.46	3.73E-05	1.92E-04
4130	7	F2-3 vs. S2-3	247033_at	AT5G67250	Encodes an SKP1 interacting partner (SKIP2).Encodes an F-box protein. Based on genetic analysis appears to be functionally redundant with VFB1,2, and 3. When expression of all 4 genes is reduced plants show defects in growth and reduced expression of auxin response genes.	774.08	120.27	6.44	< 1e-07	2.96E-06
4131	7	F2-3 vs. S2-3	261754_at	AT1G76130	alpha-amylase, putative / 1,4-alpha-D-glucan glucanohydrolase, putative, strong similarity to alpha-amylase GI:7532799 from (Malus x domestica);contains Pfam profile PF00128: Alpha amylase, catalytic domain. Predicted to be secreted based on SignalP analysis.	431.53	67.09	6.43	1.80E-06	2.29E-05
4132	7	F2-3 vs. S2-3	265091_s_at	AT1G03495	HXXXD-type acyl-transferase family protein;(source:Araport11)	82.99	13.01	6.38	3.00E-07	6.68E-06
4133	7	F2-3 vs. S2-3	251358_at	AT3G61160	Protein kinase superfamily protein;(source:Araport11)	276.31	44.1	6.27	5.00E-07	9.67E-06
4134	7	F2-3 vs. S2-3	263838_at	AT2G36880	methionine adenosyltransferase 3;(source:Araport11)	1902.39	303.69	6.26	< 1e-07	< 1e-07
4135	7	F2-3 vs. S2-3	256245_at	AT3G12580	heat shock protein 70;(source:Araport11)	189.71	30.34	6.25	< 1e-07	2.96E-06

4136	7	F2-3 vs. S2-3	266184_s_at	AT3G54700	Encodes Pht1;7, a member of the Pht1 family of phosphate transporters which include: Pht1;1/At5g43350, Pht1;2/At5g43370, Pht1;3/At5g43360, Pht1;4/At2g38940, Pht1;5/At2g32830, Pht1;6/At5g43340, Pht1;7/At3g54700, Pht1;8/At1g20860, Pht1;9/At1g76430 (Plant Journal 2002, 31:341).	59.73	9.56	6.25	6.00E-07	1.10E-05
4137	7	F2-3 vs. S2-3	260399_at	AT1G72520	PLAT/LH2 domain-containing lipoxygenase family protein;(source:Araport11)	123.44	19.78	6.24	3.00E-07	6.68E-06
4138	7	F2-3 vs. S2-3	247954_at	AT5G56870	beta-galactosidase 4;(source:Araport11)	244.54	39.24	6.23	5.00E-07	9.67E-06
4139	7	F2-3 vs. S2-3	250610_at	AT5G07550	member of Oleosin-like protein family	75.1	12.12	6.2	7.00E-07	1.20E-05
4140	7	F2-3 vs. S2-3	252024_at	AT3G52880	Encodes a peroxisomal monodehydroascorbate reductase, involved in the ascorbate-glutathione cycle which removes toxic H2O2	1201.57	194.01	6.19	4.00E-07	8.26E-06
4141	7	F2-3 vs. S2-3	258338_at	AT3G16150	Encodes an asparaginase that catalyzes the degradation of L-asparagine to L-aspartic acid and ammonia. The mRNA is cell-to-cell mobile.	280.83	45.49	6.17	< 1e-07	< 1e-07
4142	7	F2-3 vs. S2-3	267233_s_at	AT2G43920	S-adenosyl-L-methionine-dependent methyltransferases superfamily protein;(source:Araport11)	177.96	29.36	6.06	7.40E-06	6.07E-05
4143	7	F2-3 vs. S2-3	246208_at	AT4G36490	SEC14-like 12;(source:Araport11)	81.11	13.39	6.06	< 1e-07	2.96E-06
4144	7	F2-3 vs. S2-3	246998_at	AT5G67370	DUF1230 family protein (DUF1230);(source:Araport11)	106.05	17.51	6.06	1.50E-06	2.00E-05
4145	7	F2-3 vs. S2-3	247627_at	AT5G60360	Encodes a senescence-associated thiol protease. The mRNA is cell-to-cell mobile.	1289.51	214.33	6.02	2.00E-07	4.97E-06
4146	7	F2-3 vs. S2-3	252618_at	AT3G45140	Chloroplast lipoxygenase required for wound-induced jasmonic acid accumulation in Arabidopsis.Mutants are resistant to Staphylococcus aureus and accumulate salicylic acid upon infection.CFA-Leu, CFA-Val, CFA-Met and CFA-Ala can induce the expression of LOX2.The mRNA is cell-to-cell mobile.	513.48	85.45	6.01	1.10E-06	1.64E-05
4147	7	F2-3 vs. S2-3	244972_at	ATCG00680	encodes for CP47, subunit of the photosystem II reaction center.	98.43	16.45	5.98	2.00E-07	4.97E-06
4148	7	F2-3 vs. S2-3	261266_at	AT1G26770	Encodes an expansin. Naming convention from the Expansin Working Group (Kende et al, Plant Mol Bio). Involved in the formation of nematode-induced syncytia in roots of Arabidopsis thaliana.	182.06	30.63	5.94	2.24E-05	1.31E-04
4149	7	F2-3 vs. S2-3	267255_at	AT2G22950	Encodes a putative auto-regulated Ca2+-ATPase located in the plasma membrane involved in transporting Ca2+ outside developing pollen grains. This activity is important to support normal pollen development, particularly the progression to uninucleated microspores to bicellular pollen grains.	117.21	19.92	5.89	< 1e-07	< 1e-07
4150	7	F2-3 vs. S2-3	245057_at	AT2G26490	JGB contains seven WD40 repeats and is highly conserved in flowering plants. Overexpression inhibits pollen germination. suggesting JGB is a negative regulator of pollen germination	86.9	14.79	5.88	< 1e-07	2.96E-06
4151	7	F2-3 vs. S2-3	253083_at	AT4G36250	Encodes a putative aldehyde dehydrogenase. The gene is not responsive to osmotic stress and is expressed constitutively at a low level in plantlets and root cultures.	434.46	73.86	5.88	3.00E-07	6.68E-06
4152	7	F2-3 vs. S2-3	248199_at	AT5G54170	Polyketide cyclase/dehydrase and lipid transport superfamily protein;(source:Araport11)	220.85	37.65	5.87	2.80E-06	3.09E-05
4153	7	F2-3 vs. S2-3	260221_at	AT1G74670	Gibberellin-regulated family protein;(source:Araport11)	55.47	9.5	5.84	3.90E-06	3.86E-05
4154	7	F2-3 vs. S2-3	257130_at	AT3G20210	Encodes a vacuolar processing enzyme with caspase-1-like activity that is specifically expressed in inner integument of developing seeds. Mutants display abnormal seed coat development. It is speculated to be involved in cell death of limited cell layers, the purpose of which is to form a seed coat.	104.24	17.89	5.83	1.30E-06	1.81E-05
4155	7	F2-3 vs. S2-3	249536_at	AT5G38760	Late embryogenesis abundant protein (LEA) family protein;(source:Araport11)	56.62	9.73	5.82	< 1e-07	< 1e-07
4156	7	F2-3 vs. S2-3	252474_at	AT3G46620	Encodes an ABA- and drought-induced RING-DUF1117 gene whose mutation results in hyposensitive phenotypes toward ABA in terms of germination rate and stomatal closure and markedly reduced tolerance to drought stress relative to wild-type plants.	627.08	108.31	5.79	< 1e-07	< 1e-07
4157	7	F2-3 vs. S2-3	254687_at	AT4G13770	Encodes a cytochrome p450 enzyme that catalyzes the initial conversion of aldoximes to thiohydroximates in the synthesis of glucosinolates not derived from tryptophan. Also has a role in auxin homeostasis.	658.33	114.14	5.77	< 1e-07	< 1e-07
4158	7	F2-3 vs. S2-3	245304_at	AT4G15630	Uncharacterized protein family (UPF0497);(source:Araport11)	104.82	18.23	5.75	2.00E-07	4.97E-06
4159	7	F2-3 vs. S2-3	262796_at	AT1G20850	Cysteine peptidase. Enzyme activity detected in leaf.	429.98	75.1	5.73	9.00E-07	1.44E-05
4160	7	F2-3 vs. S2-3	262473_at	AT1G50250	encodes an FTSH protease that is localized to the chloroplast. Involved in the D1 repair cycle of Photosystem II. FtsH1 and FtsH5 are interchangeable in thylakoid membranes.	479.18	83.59	5.73	1.40E-06	1.89E-05
4161	7	F2-3 vs. S2-3	263758_s_a	AT2G21250	NAD(P)-linked oxidoreductase superfamily protein;(source:Araport11)	726.31	126.65	5.73	2.90E-06	3.14E-05
4162	7	F2-3 vs. S2-3	251091_at	AT5G01410	Encodes a protein predicted to function in tandem with PDX2 to form glutamine amidotransferase complex with involved in vitamin B6 biosynthesis.	518.96	91.44	5.68	1.00E-07	2.96E-06

4163	7	F2-3 vs. S2-3	252095_at	AT3G51000	alpha/beta-Hydrolases superfamily protein;(source:Araport11)	152.96	27.07	5.65	1.40E-06	1.89E-05
4164	7	F2-3 vs. S2-3	266708_at	AT2G03200	Atypical aspartic protease which modulates lateral root development.	99.37	17.67	5.62	2.00E-07	4.97E-06
4165	7	F2-3 vs. S2-3	259615_at	AT1G47980	desiccation-like protein;(source:Araport11)	136.58	24.35	5.61	4.00E-07	8.26E-06
4166	7	F2-3 vs. S2-3	254098_at	AT4G25100	Fe-superoxide dismutase	820.89	146.94	5.59	2.00E-06	2.46E-05
4167	7	F2-3 vs. S2-3	245003_at	ATCG00280	chloroplast gene encoding a CP43 subunit of the photosystem II reaction center. promoter contains a blue-light responsive element.	456.19	81.97	5.57	3.80E-06	3.79E-05
4168	7	F2-3 vs. S2-3	264112_at	AT2G13680	Responsible for the synthesis of callose deposited at the primary cell wall of meiocytes, tetrads and microspores. Required for exine formation during microgametogenesis and for pollen viability. Highest expression in meiocytes, tetrads, microspores and mature pollen.	88.7	16.01	5.54	1.21E-04	4.48E-04
4169	7	F2-3 vs. S2-3	251762_at	AT3G55800	Encodes the chloroplast enzyme sedoheptulose-1,7-bisphosphatase (SBPase), involved in the carbon reduction of the Calvin cycle. Increase in SBPase activity in transgenic lines accumulate up to 50% more sucrose and starch than wild-type. The mRNA is cell-to-cell mobile.	647.97	117.04	5.54	6.00E-07	1.10E-05
4170	7	F2-3 vs. S2-3	252377_at	AT3G47960	Encodes a high-affinity, proton-dependent glucosinolate-specific transporter that is crucial for the transport of both methionine- and tryptophan-derived glucosinolates to seeds.	288.18	52.37	5.5	7.00E-07	1.20E-05
4171	7	F2-3 vs. S2-3	254573_at	AT4G19420	Pectinacetylesterase family protein;(source:Araport11)	259.19	47.29	5.48	2.90E-06	3.14E-05
4172	7	F2-3 vs. S2-3	248545_at	AT5G50260	Encodes a papain-like cysteine protease involved in tapetal programmed cell death and pollen development.CEP1 is expressed specifically in the tapetum from stages 5 to 11 of anther development. The CEP1 protein first appears as a proenzyme in precursor protease vesicles, and is then transported to the vacuole and transformed into the mature enzyme before rupture of the vacuole. CEP1 was also released to the tapetal cell wall during late stage 6 and stage 7. After the tapetal cell wall degenerated, the CEP1 enzyme entered the callose wall from the degenerated tapetal cell wall and was probably involved in degeneration of the callose wall.	58.03	10.61	5.47	< 1e-07	2.96E-06
4173	7	F2-3 vs. S2-3	259625_at	AT1G42970	Encodes chloroplast localized glyceraldehyde-3-phosphate dehydrogenase that can use both NADH and NADPH to reduce 1,3-diphosphate glycerate. It forms A2B2 heterotetramers with GapA forms of the GADPH enzyme. These complexes are active in the light under reducing conditions, but show reduced NADPH-dependent activity in response to oxidized thioredoxins and increased NAD(H)/NADP(H) ratios due to the formation of inactive A8B8 hexadecamers. The mRNA is cell-to-cell mobile.	1308.44	239.81	5.46	8.00E-07	1.32E-05
4174	7	F2-3 vs. S2-3	257262_at	AT3G21890	B-box type zinc finger family protein;(source:Araport11)	362.09	66.44	5.45	1.00E-07	2.96E-06
4175	7	F2-3 vs. S2-3	261892_at	AT1G80840	Pathogen-induced transcription factor. Binds W-box sequences in vitro. Forms protein complexes with itself and with WRKY40 and WRKY60. Coexpression with WRKY18 or WRKY60 made plants more susceptible to both P. syringae and B. cinerea. WRKY18, WRKY40, and WRKY60 have partially redundant roles in response to the hemibiotrophic bacterial pathogen Pseudomonas syringae and the necrotrophic fungal pathogen Botrytis cinerea, with WRKY18 playing a more important role than the other two. The mRNA is cell-to-cell mobile.	332.58	61.13	5.44	1.00E-07	2.96E-06
4176	7	F2-3 vs. S2-3	248129_at	AT5G54780	Ypt/Rab-GAP domain of gyp1p superfamily protein;(source:Araport11)	81.78	15.08	5.42	6.00E-06	5.18E-05
4177	7	F2-3 vs. S2-3	265022_at	AT1G24520	Male fertility gene acting on tapetum and microspore	225.56	41.85	5.39	< 1e-07	2.96E-06
4178	7	F2-3 vs. S2-3	247736_at	AT5G59370	Encodes one of eight Arabidopsis actins. ACT4 belongs to the reproductive actin subclass which is predominantly expressed in developing and reproductive tissues, such as pollen, pollen tubes, ovules, and developing seeds. Expression of the ACT4/GUS fusion was restricted to young vascular tissues, tapetum, and developing and mature pollen.	48.83	9.06	5.39	5.00E-07	9.67E-06
4179	7	F2-3 vs. S2-3	253619_at	AT4G30460	glycine-rich protein;(source:Araport11)	242.09	44.96	5.38	2.19E-05	1.30E-04
4180	7	F2-3 vs. S2-3	256279_at	AT3G12620	Protein phosphatase 2C family protein;(source:Araport11)	213.57	39.81	5.37	1.00E-07	2.96E-06
4181	7	F2-3 vs. S2-3	258140_at	AT3G24503	Arabidopsis thaliana aldehyde dehydrogenase AtALDH1a mRNA. a sinapaldehyde dehydrogenase catalyzes both the oxidation of coniferylaldehyde and sinapaldehyde forming ferulic acid and sinapic acid, respectively	460.82	86.46	5.33	3.00E-07	6.68E-06
4182	7	F2-3 vs. S2-3	267500_s_a	AT2G44890	member of CYP704A	122.46	23.14	5.29	2.30E-06	2.69E-05
4183	7	F2-3 vs. S2-3	263718_at	AT2G13570	nuclear factor Y, subunit B7;(source:Araport11)	73.1	13.9	5.26	1.70E-06	2.21E-05
4184	7	F2-3 vs. S2-3	245806_at	AT1G45474	Encodes a component of the light harvesting complex of photosystem I.	207.45	39.53	5.25	1.00E-07	2.96E-06

4185	7	F2-3 vs. S2-3	265587_at	AT2G19980	CAP (Cysteine-rich secretory proteins, Antigen 5, and Pathogenesis-related 1 protein) superfamily protein;(source:Araport11)	48.11	9.16	5.25	5.00E-07	9.67E-06
4186	7	F2-3 vs. S2-3	253638_at	AT4G30470	NAD(P)-binding Rossmann-fold superfamily protein;(source:Araport11)	981.15	187.43	5.23	2.40E-06	2.77E-05
4187	7	F2-3 vs. S2-3	246183_at	AT5G20940	Glycosyl hydrolase family protein;(source:Araport11)	110.01	21.07	5.22	1.00E-06	1.54E-05
4188	7	F2-3 vs. S2-3	245636_at	AT1G25240	ENTH/VHS/GAT family protein;(source:Araport11)	70.87	13.61	5.21	2.40E-06	2.77E-05
4189	7	F2-3 vs. S2-3	252482_at	AT3G46670	UDP-glucosyl transferase 76E11;(source:Araport11)	127.63	24.49	5.21	1.20E-06	1.75E-05
4190	7	F2-3 vs. S2-3	253172_at	AT4G35060	Heavy metal transport/detoxification superfamily protein;(source:Araport11)	143.12	27.59	5.19	3.00E-07	6.68E-06
4191	7	F2-3 vs. S2-3	258186_s_at	AT1G48490	Protein kinase which together with IREH1 plays an important role in controlling root skewing and maintaining the microtubule network.	361.2	69.77	5.18	2.21E-05	1.30E-04
4192	7	F2-3 vs. S2-3	256617_at	AT3G22240	cysteine-rich/transmembrane domain PCC1-like protein;(source:Araport11)	358.05	69.21	5.17	1.82E-05	1.13E-04
4193	7	F2-3 vs. S2-3	260847_s_a	AT1G72330	Encodes for alanine aminotransferase ALAAT2.	449.09	87.27	5.15	4.40E-06	4.21E-05
4194	7	F2-3 vs. S2-3	258729_at	AT3G11900	encodes an amino acid transporter that transports aromatic and neutral amino acids, IAA, and 2,4-D. Expressed in all tissues with highest abundance in flowers and cauline leaves. a member of a small gene family in Arabidopsis and represents a new class of amino acid transporters.	418.38	81.22	5.15	1.40E-06	1.89E-05
4195	7	F2-3 vs. S2-3	260829_s_at	AT2G30440	Encodes a thylakoidal processing peptidase that removes signal sequences from proteins synthesized in the cytoplasm and transported into the thylakoid lumen. The mRNA is cell-to-cell mobile.	235.59	45.82	5.14	5.63E-04	1.45E-03
4196	7	F2-3 vs. S2-3	245842_at	AT1G58430	Encodes an anther-specific proline-rich protein.	300.27	58.56	5.13	1.00E-06	1.54E-05
4197	7	F2-3 vs. S2-3	252679_at	AT3G44260	Encodes one of the homologs of the yeast CCR4-associated factor 1: AT3G44260 (CAF1a), AT5G22250 (CAF1b). Has mRNA deadenylation activity. Also plays a role in plant defense responses.	110.58	21.63	5.11	2.00E-07	4.97E-06
4198	7	F2-3 vs. S2-3	250256_at	AT5G13650	Encodes SVR3, a putative chloroplast TypA translation elongation GTPase. Loss of SVR3 suppresses variegation mediated by var2. SVR3 is essential for plants? ability to develop functional chloroplasts under chilling stress (8C), but not at normal temperature (22C).	377.65	74.06	5.1	3.68E-05	1.90E-04
4199	7	F2-3 vs. S2-3	248207_at	AT5G53970	Encodes a cytosolic tyrosine aminotransferase which is strongly induced upon aging and coronatine treatment. AtTAT1 prefers Tyr as an amino donor but can also use Phe, Trp, His, Met, and Leu. The mRNA is cell-to-cell mobile.	133.56	26.2	5.1	< 1e-07	2.96E-06
4200	7	F2-3 vs. S2-3	266319_s_at	AT2G46720	Encodes KCS13, a member of the 3-ketoacyl-CoA synthase family involved in the biosynthesis of VLCFA (very long chain fatty acids).	182.4	36.1	5.05	2.05E-05	1.24E-04
4201	7	F2-3 vs. S2-3	260944_at	AT1G45130	beta-galactosidase 5;(source:Araport11)	218.82	43.42	5.04	8.00E-07	1.32E-05
4202	7	F2-3 vs. S2-3	262905_at	AT1G59730	Thioredoxin H-type 7 , oxidoreductase located in cytosol and ER. Interacts with GPT1.	63.17	12.52	5.04	9.10E-06	7.07E-05
4203	7	F2-3 vs. S2-3	251885_at	AT3G54050	Encodes a chloroplastic fructose 1,6-bisphosphate phosphatase. also known as HCEF1 (High Cyclic Electron Flow 1). hcef1 mutants have constitutively elevated electron flow (CEFI) and plants with antisense suppression of this enzyme have higher levels of net leaf photosynthesis and increased sucrose biosynthesis. The mRNA is cell-to-cell mobile.	447.25	88.98	5.03	4.00E-07	8.26E-06
4204	7	F2-3 vs. S2-3	251109_at	AT5G01600	Encodes a ferretin protein that is targeted to the chloroplast. Member of a Ferritin gene family. Gene expression is induced in response to iron overload and by nitric oxide. Expression of the gene is downregulated in the presence of paraquat, an inducer of photoxidative stress.	168.45	33.55	5.02	1.00E-07	2.96E-06
4205	7	F2-3 vs. S2-3	254563_at	AT4G19120	S-adenosyl-L-methionine-dependent methyltransferases superfamily protein;(source:Araport11)	320.95	64.02	5.01	7.00E-07	1.20E-05
4206	7	F2-3 vs. S2-3	245159_at	AT2G33100	encodes a gene similar to cellulose synthase	59.72	11.95	5	< 1e-07	2.96E-06
4207	7	F2-3 vs. S2-3	262707_at	AT1G16290	transglycosylase;(source:Araport11)	131.98	26.55	4.97	< 1e-07	2.96E-06
4208	7	F2-3 vs. S2-3	254926_at	AT4G11280	encodes a a member of the 1-aminocyclopropane-1-carboxylate (ACC) synthase (S-adenosyl-L-methionine methylthioadenosine-lyase, EC 4.4.1.14) gene family The mRNA is cell-to-cell mobile.	330.86	66.56	4.97	5.50E-06	4.90E-05
4209	7	F2-3 vs. S2-3	263676_at	AT1G09340	Encodes CHLOROPLAST RNA BINDING (CRB), a putative RNA-binding protein. CRB is important for the proper functioning of the chloroplast. Mutations in CRB also affects the circadian system, altering the expression of both oscillator and output genes. The mRNA is cell-to-cell mobile.	458.7	92.54	4.96	2.00E-07	4.97E-06
4210	7	F2-3 vs. S2-3	258645_s_a	AT3G07850	Pectin lyase-like superfamily protein;(source:Araport11)	85.12	17.15	4.96	9.23E-05	3.68E-04
4211	7	F2-3 vs. S2-3	264005_at	AT2G22470	Encodes arabinogalactan-protein (AGP2).	117.02	23.65	4.95	7.00E-07	1.20E-05
4212	7	F2-3 vs. S2-3	267115_s_a	AT2G32530	encodes a gene similar to cellulose synthase	96.25	19.58	4.92	2.00E-07	4.97E-06

4213	7	F2-3 vs. S2-3	248916_at	AT5G45840	Encodes a leucine-rich-repeat RLK that is localized to the plasma membrane of pollen tubes and functions with MIK1/2 as the male receptor of the pollen tube chemo-attractant LURE1.MDIS1 forms a complex with MIK1/2 and binds LURE1.	54.64	11.1	4.92	1.33E-05	9.14E-05
4214	7	F2-3 vs. S2-3	266849_at	AT2G25940	Encodes a vacuolar processing enzyme belonging to a novel group of cysteine proteinases that is expressed in vegetative organs and is upregulated in association with various types of cell death and under stressed conditions.	140.14	28.81	4.87	1.10E-06	1.64E-05
4215	7	F2-3 vs. S2-3	245306_at	AT4G14690	Encodes an early light-induced protein. ELIPs are thought not to be directly involved in the synthesis and assembly of specific photosynthetic complexes, but rather affect the biogenesis of all chlorophyll-binding complexes. A study (PMID 17553115) has shown that the chlorophyll synthesis pathway was downregulated as a result of constitutive ELIP2 expression, leading to decreased chlorophyll availability for the assembly of pigment-binding proteins for photosynthesis.	241.21	49.58	4.86	2.10E-06	2.53E-05
4216	7	F2-3 vs. S2-3	250824_at	AT5G05200	Protein kinase superfamily protein;(source:Araport11)	203.66	42.06	4.84	9.00E-07	1.44E-05
4217	7	F2-3 vs. S2-3	265998_at	AT2G24270	Encodes a protein with non-phosphorylating NADP-dependent glyceraldehyde-3-phosphate dehydrogenase activity. The activity of the enzyme was determined from leaf extracts; the enzyme has not been purified to confirm activity.	656.59	135.96	4.83	< 1e-07	2.96E-06
4218	7	F2-3 vs. S2-3	246302_at	AT3G51860	cation exchanger 3;(source:Araport11)	209.33	43.3	4.83	3.00E-07	6.68E-06
4219	7	F2-3 vs. S2-3	250608_at	AT5G07420	Pectin lyase-like superfamily protein;(source:Araport11)	48.37	10.02	4.83	2.40E-06	2.77E-05
4220	7	F2-3 vs. S2-3	262892_at	AT1G79440	Encodes a mitochondrial succinic semialdehyde dehydrogenase (SSADH). Nomenclature according to Kirch, et al (2004).	125.02	26.05	4.8	1.40E-06	1.89E-05
4221	7	F2-3 vs. S2-3	263706_s_at	AT5G14200	The AtIMD1 is one out of 3 genes encoding the enzyme 3-isopropylmalate dehydrogenase involved in leucine biosynthesis in Arabidopsis. Its subcellular location has been targeted to plastids. Encodes methylthioalkylmalate dehydrogenase. Involved in glucosinolate biosynthesis, in methionine chain elongation. The mRNA is cell-to-cell mobile.	42.82	8.94	4.79	2.34E-04	7.39E-04
4222	7	F2-3 vs. S2-3	259694_at	AT1G63180	Encodes a protein with UDP-D-glucose 4-epimerase activity. Involved in pollen development.	123.3	25.86	4.77	1.08E-04	4.15E-04
4223	7	F2-3 vs. S2-3	260014_at	AT1G68010	Encodes hydroxypyruvate reductase.	211.91	44.67	4.74	4.00E-07	8.26E-06
4224	7	F2-3 vs. S2-3	264835_at	AT1G03550	Secretory carrier membrane protein (SCAMP) family protein;(source:Araport11)	72.48	15.31	4.73	8.30E-06	6.60E-05
4225	7	F2-3 vs. S2-3	249438_at	AT5G40010	Encodes a mitochondrial ATPase involved in seed and silique development.	40.8	8.66	4.71	1.03E-05	7.67E-05
4226	7	F2-3 vs. S2-3	247095_at	AT5G66400	Belongs to the dehydrin protein family, which contains highly conserved stretches of 7-17 residues that are repetitively scattered in their sequences, the K-, S-, Y- and lysine rich segments. ABA- and drought-induced glycine-rice dehydrin protein. The ABA-induced expression of RAB18 was reduced following ACC application, indicating that ethylene inhibits the ABA signaling pathway. RAB18 is also expressed in response to the formation of the phospholipid diacylglycerol pyrophosphate. COR47 and RAB18 double overexpressor plants are cold tolerant. Expressed in guard cells.	72.93	15.49	4.71	3.00E-07	6.68E-06
4227	7	F2-3 vs. S2-3	256754_at	AT3G25690	actin binding protein required for normal chloroplast positioningThe mRNA is cell-to-cell mobile.	184.55	39.31	4.69	2.50E-06	2.85E-05
4228	7	F2-3 vs. S2-3	254001_at	AT4G26260	Encodes a myo-inositol oxygenase, which is the first enzyme in the inositol route to ascorbate (L‐ascorbic acid, AsA, vitamin C). Overexpression results in enhanced biomass and abiotic stress tolerance.	61.56	13.16	4.68	1.12E-05	8.21E-05
4229	7	F2-3 vs. S2-3	255690_at	AT4G00360	Encodes a member of the CYP86A subfamily of cytochrome p450 genes. Expressed at moderate levels in flowers, leaves, roots and stems.	53.4	11.47	4.65	2.00E-07	4.97E-06
4230	7	F2-3 vs. S2-3	259044_at	AT3G03430	Calcium-binding EF-hand family protein;(source:Araport11)	43.57	9.38	4.64	2.16E-05	1.28E-04
4231	7	F2-3 vs. S2-3	263689_at	AT1G26820	Encodes ribonuclease RNS3.	150.28	32.67	4.6	3.85E-05	1.96E-04
4232	7	F2-3 vs. S2-3	253137_at	AT4G35500	Protein kinase superfamily protein;(source:Araport11)	133.96	29.12	4.6	2.00E-07	4.97E-06
4233	7	F2-3 vs. S2-3	257392_at	AT2G24450	Fasciclin-like arabinogalactan protein. Possibly involved in embryogenesis and seed development.	68.59	14.93	4.59	2.76E-05	1.53E-04
4234	7	F2-3 vs. S2-3	246431_at	AT5G17480	pollen calcium-binding protein 1;(source:Araport11)	32.03	6.99	4.58	1.00E-06	1.54E-05
4235	7	F2-3 vs. S2-3	262282_at	AT1G68610	Target promoter of the male germline-specific transcription factor DUO1.	54.05	11.9	4.54	1.80E-06	2.29E-05
4236	7	F2-3 vs. S2-3	248880_at	AT5G46200	carboxyl-terminal proteinase-like protein (DUF239);(source:Araport11)	76.52	16.91	4.52	6.50E-06	5.49E-05
4237	7	F2-3 vs. S2-3	259856_at	AT1G68440	Transmembrane protein;(source:Araport11). Expression induced by abiotic stressors such as ABA, drought, heat, light, NaCl, osmotic stress and wounding.	74.05	16.43	4.51	5.00E-07	9.67E-06
4238	7	F2-3 vs. S2-3	253833_at	AT4G27790	Calcium-binding EF hand family protein;(source:Araport11)	53.67	11.91	4.51	1.71E-05	1.09E-04
4239	7	F2-3 vs. S2-3	255877_at	AT2G40460	Major facilitator superfamily protein;(source:Araport11)	70.69	15.76	4.48	2.70E-06	3.02E-05

4240	7	F2-3 vs. S2-3	267293_at	AT2G23810	Member of TETRASPANIN family	387.42	86.74	4.47	< 1e-07	2.96E-06
4241	7	F2-3 vs. S2-3	248838_at	AT5G46800	Seedling lethal mutation; Mitochondrial Carnitine Acyl Carrier-Like Protein	300.87	67.37	4.47	< 1e-07	2.96E-06
4242	7	F2-3 vs. S2-3	256861_at	AT3G23920	Encodes a chloroplast beta-amylase. Is necessary for leaf starch breakdown in the absence of BAM3.Activity of BAM1 increases 4 days after osmotic stress. BAM1 has a higher temperature optimum than BAM3 (PMID:25293962).	652.22	146.29	4.46	1.78E-04	6.03E-04
4243	7	F2-3 vs. S2-3	250129_at	AT5G16450	Ribonuclease E inhibitor RraA/Dimethylmenaquinone methyltransferase;(source:Araport11)	713.49	161.31	4.42	3.00E-07	6.68E-06
4244	7	F2-3 vs. S2-3	257119_at	AT3G20190	Leucine-rich repeat protein kinase family protein;(source:Araport11)	50.37	11.47	4.39	7.00E-07	1.20E-05
4245	7	F2-3 vs. S2-3	259277_at	AT3G01180	starch synthase 2;(source:Araport11)	432.8	99.31	4.36	3.00E-07	6.68E-06
4246	7	F2-3 vs. S2-3	245018_at	ATCG00520	Encodes a protein required for photosystem I assembly and stability. In cyanobacteria, loss of function mutation in this gene increases PSII/PSI ratio without any influence on photoautotrophic growth.	279.39	64.14	4.36	6.00E-07	1.10E-05
4247	7	F2-3 vs. S2-3	253373_at	AT4G33150	This is a splice variant of the LKR/SDH locus. It encodes a bifunctional polypeptide lysine-ketoglutarate reductase and saccharopine dehydrogenase involved in lysine degradation. There is another splice variant that encodes a mono saccharopine dehydrogenase protein. Gene expression is induced by abscisic acid, jasmonate, and under sucrose starvation.	40.76	9.4	4.34	8.40E-06	6.65E-05
4248	7	F2-3 vs. S2-3	252921_at	AT4G39030	Encodes an orphan multidrug and toxin extrusion transporter. Essential component of salicylic acid-dependent signaling for disease resistance. Member of the MATE-transporter family. Expression induced by salicylic acid. Mutants are salicylic acid-deficient.	142.78	32.94	4.33	1.50E-06	2.00E-05
4249	7	F2-3 vs. S2-3	249134_at	AT5G43150	elongation factor;(source:Araport11)	65.16	15.06	4.33	1.64E-04	5.67E-04
4250	7	F2-3 vs. S2-3	259275_at	AT3G01060	lysine-tRNA ligase;(source:Araport11)	311.97	72.29	4.32	1.00E-06	1.54E-05
4251	7	F2-3 vs. S2-3	264264_at	AT1G09250	basic helix-loop-helix (bHLH) DNA-binding superfamily protein;(source:Araport11)	72.96	16.93	4.31	4.17E-05	2.09E-04
4252	7	F2-3 vs. S2-3	259826_at	AT1G29340	Encodes a protein containing a UND, a U-box, and an ARM domain. This protein has E3 ubiquitin ligase activity. It is required for cell death and full resistance specified by Arabidopsis RPM1 and RPS4 resistance proteins against Pseudomonas syringae pv tomato. The mRNA is cell-to-cell mobile.	336.84	78.35	4.3	1.17E-05	8.37E-05
4253	7	F2-3 vs. S2-3	262291_at	AT1G70790	C2-domain ABA-related (CAR) protein, involved in the recruitment of ABA receptors to the plasma membrane to facilitate ABA signaling. Its stability and dynamic localization is regulated by LOT1.	252.15	58.71	4.3	3.80E-06	3.79E-05
4254	7	F2-3 vs. S2-3	262680_at	AT1G75880	SGNH hydrolase-type esterase superfamily protein;(source:Araport11)	43.26	10.06	4.3	8.00E-07	1.32E-05
4255	7	F2-3 vs. S2-3	266615_s_at	AT2G35660	Encodes a member of a novel gene family with homology to known proteins involved in hydroxylation and oxidation of an aromatic ring.	247.73	57.67	4.3	4.50E-06	4.27E-05
4256	7	F2-3 vs. S2-3	259109_at	AT3G05580	Encodes a Type One Protein Phosphatase that acts as a nucleocytoplasmic negative regulator of tip growth. Mutants affect pollen germination, pollen tube growth, and root hair growth. It acts genetically downstream of ANX1 (AT3G04690) and ANX2 (AT5G28680) and is functionally redundant with TOPP8 (AT5G27840).	351.4	81.87	4.29	1.00E-06	1.54E-05
4257	7	F2-3 vs. S2-3	253276_at	AT4G34050	Methyltransferase in the lignin biosynthetic pathway.	1028.07	242.18	4.24	4.10E-06	4.02E-05
4258	7	F2-3 vs. S2-3	263433_at	AT2G22240	** Referred to as MIPS1 in Mitsuhashi et al 2008. Myo-inositol-1-phosphate synthase isoform 2. Expressed in leaf, root and silique. Immunolocalization experiments with an antibody recognizing MIPS1, MIPS2, and MIPS3 showed endosperm localization.	852.65	201.66	4.23	7.00E-07	1.20E-05
4259	7	F2-3 vs. S2-3	254762_at	AT4G13230	Late embryogenesis abundant protein (LEA) family protein;(source:Araport11)	70.15	16.58	4.23	4.27E-05	2.12E-04
4260	7	F2-3 vs. S2-3	250676_at	AT5G06320	encodes a protein whose sequence is similar to tobacco hairpin-induced gene (HIN1) and Arabidopsis non-race specific disease resistance gene (NDR1). Expression of this gene is induced by cucumber mosaic virus, spermine and Pseudomonas syringae pv. tomato DC3000. The gene product is localized to the plasma membrane.	300.4	70.95	4.23	5.60E-06	4.97E-05
4261	7	F2-3 vs. S2-3	251668_at	AT3G57010	Calcium-dependent phosphotriesterase superfamily protein;(source:Araport11)	189.57	44.92	4.22	1.02E-05	7.64E-05
4262	7	F2-3 vs. S2-3	253872_at	AT4G27410	Encodes a NAC transcription factor induced in response to desiccation. It is localized to the nucleus and acts as a transcriptional activator in ABA-mediated dehydration response.	117.06	27.76	4.22	4.80E-06	4.48E-05
4263	7	F2-3 vs. S2-3	261080_at	AT1G07370	Encodes putative proliferating cell nuclear antigen involved in cell cycle regulation. May be sumoylated.	88.17	20.93	4.21	3.20E-06	3.37E-05
4264	7	F2-3 vs. S2-3	245501_at	AT4G15620	Uncharacterized protein family (UPF0497);(source:Araport11)	93.76	22.32	4.2	6.00E-07	1.10E-05
4265	7	F2-3 vs. S2-3	253666_at	AT4G30270	encodes a protein similar to endo xyloglucan transferase in sequence. It is also very similar to BRU1 in soybean, which is involved in brassinosteroid response.	172.8	41.17	4.2	5.54E-05	2.55E-04

4266	7	F2-3 vs. S2-3	AFFX-Athal-GAPDH_5_s_at	AT3G04120	encodes cytosolic GADPH (C subunit) involved in the glycolytic pathway but also interacts with H2O2 potentially placing it in a signalling cascade induced by ROS. The mRNA is cell-to-cell mobile.	220.76	52.68	4.19	1.60E-06	2.10E-05
4267	7	F2-3 vs. S2-3	246304_at	AT3G51840	Encodes a short-chain acyl-CoA oxidase, which catalyzes the first step of peroxisomal fatty acid beta-oxidation during early, post-germinative growth in oilseed species. Null mutants virtually lack short-chain acyl-CoA and are resistant to 2,4-dichlorophenoxybutyric acid, which is converted to the herbicide and auxin analogue 2,4-dichlorophenoxyacetic acid by beta-oxidation. Despite the almost complete loss of short-chain activity, lipid catabolism and seedling growth and establishment was unaltered in the acx4 mutant. However, double mutants in acx3acx4 (acx3 encodes medium chain acyl CoA oxidase) were not viable and arrested during embryogenesis.	686.83	163.74	4.19	1.00E-06	1.54E-05
4268	7	F2-3 vs. S2-3	256835_at	AT3G22890	encodes ATP sulfurylase, the first enzyme in the sulfate assimilation pathway of Arabidopsis. It may also participate in selenium metabolism. The mRNA is cell-to-cell mobile.	1442.88	345.86	4.17	8.00E-07	1.32E-05
4269	7	F2-3 vs. S2-3	245700_at	AT5G04180	alpha carbonic anhydrase 3;(source:Araport11)	49.12	11.81	4.16	4.21E-05	2.10E-04
4270	7	F2-3 vs. S2-3	255365_at	AT4G04040	Phosphofructokinase family protein;(source:Araport11)	478.12	115.99	4.12	3.70E-06	3.73E-05
4271	7	F2-3 vs. S2-3	256524_at	AT1G66200	encodes a cytosolic glutamate synthetase, this enzyme has low affinity with substrate ammonium	206.68	50.5	4.09	2.30E-06	2.69E-05
4272	7	F2-3 vs. S2-3	267319_at	AT2G34660	encodes a multidrug resistance-associated protein that is MgATP-energized glutathione S-conjugate pump. An ABCC-type arsenite-phytochelatin transporter. The expression of this gene is upregulated by herbicide safeners such as benoxacor and fenclorim. The mRNA is cell-to-cell mobile.	338.22	82.7	4.09	8.70E-06	6.82E-05
4273	7	F2-3 vs. S2-3	266418_at	AT2G38750	Annexins are a family of calcium dependent membrane binding proteins though to be involved in Golgi mediated secretion. This is one of four annexins identified in Arabidopsis.	276	67.52	4.09	6.09E-05	2.71E-04
4274	7	F2-3 vs. S2-3	257886_at	AT3G17060	Pectin lyase-like superfamily protein;(source:Araport11)	75.86	18.55	4.09	9.00E-07	1.44E-05
4275	7	F2-3 vs. S2-3	257121_at	AT3G20220	SAUR-like auxin-responsive protein family;(source:Araport11)	77.42	18.95	4.09	3.00E-07	6.68E-06
4276	7	F2-3 vs. S2-3	266558_at	AT2G23900	Pectin lyase-like superfamily protein;(source:Araport11)	60.53	14.82	4.08	3.22E-05	1.71E-04
4277	7	F2-3 vs. S2-3	266673_at	AT2G29630	Encodes a protein involved in thiamin biosynthesis. The protein is an iron-sulfur cluster protein predicted to catalyze the conversion of 5-aminoimidazole ribonucleotide (AIR) to hydroxymethylpyrimidine (HMP) or hydroxymethylpyrimidine phosphate (HMP-P). A severe reduction of THIC levels in plants decreases vitamin B1 (thiamin diphosphate (TPP)) levels and also leads to changes in the levels of numerous other metabolites since so many primary metabolic enzymes require a TPP co-factor. thiC mutants are chlorotic and arrest in their development at the cotyledon stage. A N-terminal targeting sequence directs the THIC protein to the chloroplast stroma. A conserved TPP-binding site is located in the 3' UTR of the At2g29630.2 gene model, and is predicted to function as a riboswitch. The riboswitch controls the formation of transcripts with alternative 3' UTR lengths, which affect mRNA accumulation and protein production. THIC transcripts are observed in seedlings 5 or more days after germination, and light promotes the expression of this gene. Recessive mutant isolated by Redei. Leaves but not cotyledons white, lethal; restored to normal by thiamine or 2,5-dimethyl-4-aminopyrimidine.	285.16	70.06	4.07	1.00E-07	2.96E-06
4278	7	F2-3 vs. S2-3	254862_at	AT4G12030	Required for the biosynthesis of methionine-derived glucosinolates. Involved in the transport of 2-keto acids between chloroplasts and the cytosol.	135.47	33.34	4.06	7.50E-06	6.08E-05
4279	7	F2-3 vs. S2-3	254630_at	AT4G18360	Encodes a glycolate oxidase that modulates reactive oxygen species-mediated signal transduction during nonhost resistance.	114.5	28.2	4.06	5.70E-06	5.00E-05
4280	7	F2-3 vs. S2-3	251433_at	AT3G59830	Integrin-linked protein kinase family;(source:Araport11)	57.96	14.32	4.05	1.20E-06	1.75E-05
4281	7	F2-3 vs. S2-3	244902_at	ATMG00650	Encodes NADH dehydrogenase subunit 4L.	113.2	27.96	4.05	1.90E-06	2.37E-05
4282	7	F2-3 vs. S2-3	245213_at	AT1G44575	Encoding PSII-S (CP22), a ubiquitous pigment-binding protein associated with photosystem II (PSII) of higher plants. Involved in nonphotochemical quenching rather than in photosynthesis. Mutant has a normal violaxanthin cycle but has a limited capacity of quenching singlet excited chlorophylls and is tolerant to lipid peroxidation.	1276.8	316	4.04	1.40E-06	1.89E-05
4283	7	F2-3 vs. S2-3	265441_at	AT2G20870	cell wall protein precursor;(source:Araport11)	93.85	23.22	4.04	2.10E-06	2.53E-05

4284	7	F2-3 vs. S2-3	254874_at	AT4G11570	Encodes plastid localized protein involved in riboflavin biosynthesis. It dephosphorylates 5-amino-6-ribitylamino- 2,4(1H,3H) pyrimidinedione 5′-phosphate (ARPP) .	535.13	132.47	4.04	2.00E-07	4.97E-06
4285	7	F2-3 vs. S2-3	258312_at	AT3G16170	Encodes a malonyl-CoA synthetase that is localized to the cytosol and mitochondrion. AAE13 produces two transcripts one of which includes an N terminal mitochondrial targeting motif. Loss of function of the mtAAE13 product results in growth arrest and lethality.	549.09	136.68	4.02	4.20E-05	2.10E-04
4286	7	F2-3 vs. S2-3	263709_at	AT1G09310	plant/protein (Protein of unknown function, DUF538);(source:Araport11)	136.95	34.16	4.01	8.00E-07	1.32E-05
4287	7	F2-3 vs. S2-3	259098_at	AT3G04790	Ribose 5-phosphate isomerase, type A protein;(source:Araport11)	148.98	37.14	4.01	7.60E-06	6.14E-05
4288	7	F2-3 vs. S2-3	245528_at	AT4G15530	Encodes a dual-targeted protein believed to act as a pyruvate, orthophosphate dikinase. These enzymes are normally associated with C4 photosynthesis which does not occur in Arabidopsis. However, PPDK may play a role in remobilizing nitrogen during leaf senescence in Arabidopsis. The product of the long transcript (.1 gene model) was shown to be targeted to the chloroplast, whereas the shorter transcript (no targeting sequence) accumulates in the cytosol. The two proteins were also found to be expressed in slightly different tissues.	208.44	51.98	4.01	4.74E-05	2.28E-04
4289	7	F2-3 vs. S2-3	249638_at	AT5G36880	Encodes a plastidic acetyl-coA synthetase. This enzyme plays a role in converting acetate to acetyl-coA in the plastids. It does not appear to be a major contributor to fatty acid biosynthesis based on mutant phenotypes. The enzyme seems to act as a monomer and may play an important role in preventing the toxic accumulation of fermentation products including acetaldehyde, acetate, and ethanol. It participates in the pyruvate dehydrogenase bypass pathway	940.02	235.26	4	1.30E-06	1.81E-05
4290	7	F2-3 vs. S2-3	253898_s_a	AT5G54810	A.thaliana tryptophan synthase beta subunit (trpB)	1536.04	384.4	4	1.79E-05	1.11E-04
4291	7	F2-3 vs. S2-3	258415_at	AT3G17390	S-adenosylmethionine synthetase	2487.45	623.67	3.99	1.10E-06	1.64E-05
4292	7	F2-3 vs. S2-3	245699_at	AT5G04250	Cysteine proteinases superfamily protein;(source:Araport11)	192.38	48.29	3.98	7.30E-06	6.00E-05
4293	7	F2-3 vs. S2-3	267168_at	AT2G37770	Encodes an NADPH-dependent aldo-keto reductase that can act on a wide variety of substrates in vitro including saturated and unsaturated aldehydes, steroids, and sugars. GFP-tagged AKR4C9 localizes to the chloroplast where it may play a role in detoxifying reactive carbonyl compounds that threaten to impair the photosynthetic process. Transcript levels for this gene are up-regulated in response to cold, salt, and drought stress.	66.61	16.86	3.95	2.90E-06	3.14E-05
4294	7	F2-3 vs. S2-3	267035_at	AT2G38400	alanine:glyoxylate aminotransferase 2 homolog (AGT3) mRNA,	332.63	84.31	3.95	2.26E-05	1.32E-04
4295	7	F2-3 vs. S2-3	248185_at	AT5G54060	Encodes a anthocyanin 3-O-glucoside: 2''-O-xylosyl-transferase involved in anthocyanin modification that converts cyanidin 3-O-glucoside to cyanidin 3-O-xylosyl(1->2)glucoside. Its preferred sugar donor is UDP-xylose.	173.07	44.03	3.93	1.62E-05	1.04E-04
4296	7	F2-3 vs. S2-3	261144_s_a	AT1G19660	Wound-responsive family protein;(source:Araport11)	1123.69	287.53	3.91	4.00E-07	8.26E-06
4297	7	F2-3 vs. S2-3	258979_at	AT3G09440	Heat shock protein 70 (Hsp 70) family protein;(source:Araport11)	956.98	244.86	3.91	3.10E-06	3.28E-05
4298	7	F2-3 vs. S2-3	254551_at	AT4G19840	encodes a phloem lectin, similar to phloem lectin in cucumber and celery. Gene is expressed in the phloem, predominantly in the companion cells. The mRNA is cell-to-cell mobile.	150.73	38.52	3.91	2.60E-06	2.94E-05
4299	7	F2-3 vs. S2-3	253477_at	AT4G32320	Encodes a cytosolic ascorbate peroxidase APX6. Ascorbate peroxidases are enzymes that scavenge hydrogen peroxide in plant cells. Eight types of APX have been described for Arabidopsis: three cytosolic (APX1, APX2, APX6), two chloroplastic types (stromal sAPX, thylakoid tAPX), and three microsomal (APX3, APX4, APX5) isoforms.	130.25	33.36	3.9	2.00E-07	4.97E-06
4300	7	F2-3 vs. S2-3	264344_at	AT1G11910	Encodes an aspartic proteinase that forms a heterodimer and is stable over a broad pH range (ph 3-8).	4334.17	1119.48	3.87	1.10E-06	1.64E-05
4301	7	F2-3 vs. S2-3	258791_at	AT3G04720	Encodes a protein similar to the antifungal chitin-binding protein hevein from rubber tree latex. mRNA levels increase in response to ethylene and turnip crinkle virus infection. The mRNA is cell-to-cell mobile.	127.46	32.94	3.87	5.00E-07	9.67E-06
4302	7	F2-3 vs. S2-3	263761_at	AT2G21330	fructose-bisphosphate aldolase 1;(source:Araport11)	912.65	236.24	3.86	6.00E-07	1.10E-05
4303	7	F2-3 vs. S2-3	247380_at	AT5G63380	Encodes a peroxisomal protein involved in the activation of fatty acids through esterification with CoA. At5g63380 preferentially activates fatty acids with increased chain length (C9:0 to C8:0) and thus shares characteristics with long-chain fatty acyl-CoA synthases. Also able to catalyze the conversion of OPDA to its CoA ester and is therefore thought to be involved in the peroxisomal β-oxidation steps of jasmonic acid biosynthesis.	342.76	88.89	3.86	2.00E-07	4.97E-06

4304	7	F2-3 vs. S2-3	264400_at	AT1G61800	glucose6-Phosphate/phosphate transporter 2. Expression is upregulated in the shoot of cax1/cax3 mutant. The mRNA is cell-to-cell mobile.	3591.61	936.05	3.84	1.00E-06	1.54E-05
4305	7	F2-3 vs. S2-3	246279_at	AT4G36740	Encodes a homeodomain leucine zipper class I (HD-Zip I) protein.	51.48	13.42	3.84	1.40E-06	1.89E-05
4306	7	F2-3 vs. S2-3	250350_at	AT5G12010	nuclease;(source:Araport11)	208.23	54.2	3.84	8.00E-07	1.32E-05
4307	7	F2-3 vs. S2-3	259520_at	AT1G12320	ankyrin repeat/KH domain protein (DUF1442);(source:Araport11)	52.02	13.57	3.83	6.00E-07	1.10E-05
4308	7	F2-3 vs. S2-3	252123_at	AT3G51240	Encodes flavanone 3-hydroxylase that is coordinately expressed with chalcone synthase and chalcone isomerases. Regulates flavonoid biosynthesis. Not responsive to auxin or ethylene stimulus (qRT-PCR).	813.52	212.2	3.83	2.30E-06	2.69E-05
4309	7	F2-3 vs. S2-3	246554_at	AT5G15450	Encodes a chloroplast-targeted Hsp101 homologue. Functions as a molecular chaperone involved in plastid differentiation mediating internal thylakoid membrane formation and conferring thermotolerance to chloroplasts during heat stress. APG6 is constitutively expressed in the root tips, the organ boundary region, the reproductive tissues of mature plants where plastids exist as proplastids, and slightly in the stems and leaves. APG6 expression is upregulated in response to heat shock in various organs, but not in response to other abiotic stresses. Apg6 mutants have a pale-green phenotype.	361.96	94.6	3.83	1.13E-04	4.30E-04
4310	7	F2-3 vs. S2-3	247440_at	AT5G62680	Encodes a high-affinity, proton-dependent glucosinolate-specific transporter that is crucial for the transport of both methionine- and tryptophan-derived glucosinolates to seeds.	92.79	24.25	3.83	1.50E-06	2.00E-05
4311	7	F2-3 vs. S2-3	260805_at	AT1G78320	Encodes glutathione transferase belonging to the tau class of GSTs. Naming convention according to Wagner et al. (2002).	82.84	21.78	3.8	2.00E-07	4.97E-06
4312	7	F2-3 vs. S2-3	245463_at	AT4G17030	Encodes EXLB1 (expansin-like B1), a member of the expansin family.	86.44	22.78	3.79	1.03E-05	7.67E-05
4313	7	F2-3 vs. S2-3	257071_at	AT3G28180	encodes a gene similar to cellulose synthase The mRNA is cell-to-cell mobile.	63.26	16.75	3.78	3.00E-07	6.68E-06
4314	7	F2-3 vs. S2-3	252363_at	AT3G48460	GDSL-motif esterase/acyltransferase/lipase. Enzyme group with broad substrate specificity that may catalyze acyltransfer or hydrolase reactions with lipid and non-lipid substrates.	48.07	12.71	3.78	1.00E-06	1.54E-05
4315	7	F2-3 vs. S2-3	253196_at	AT4G35260	Encodes a regulatory subunit of the mitochondrially-localized NAD+- dependent isocitrate dehydrogenase.	757.91	200.51	3.78	1.61E-05	1.04E-04
4316	7	F2-3 vs. S2-3	249928_at	AT5G22250	Encodes one of the homologs of the yeast CCR4-associated factor 1: AT3G44260 (CAF1a), AT5G22250 (CAF1b). Has mRNA deadenylation activity. Also plays a role in plant defense responses.	124.98	33.25	3.76	8.00E-07	1.32E-05
4317	7	F2-3 vs. S2-3	245982_at	AT5G13170	Encodes a member of the SWEET sucrose efflux transporter family proteins.	135.64	36.13	3.75	1.30E-06	1.81E-05
4318	7	F2-3 vs. S2-3	247164_at	AT5G65720	Encodes a cysteine desulfurase whose activity is dependent on AtSufE activation. It requires pyridoxal phosphate (PLP) for proper folding. Its catalytic efficiency is increase three-fold in the presence of AtFH (frataxin).	278.89	74.4	3.75	2.58E-05	1.46E-04
4319	7	F2-3 vs. S2-3	265073_at	AT1G55480	Encodes a member of a novel plant protein family containing a PDZ, a K-box, and a TPR motif. mRNA but not protein levels decrease after wounding. ZKT is phosphorylated at Thr and Ser residues after wounding. The mRNA is cell-to-cell mobile.	300.62	80.65	3.73	5.40E-06	4.85E-05
4320	7	F2-3 vs. S2-3	260309_at	AT1G70580	Encodes a protein with glyoxylate aminotransferase activity. It can act on a number of different small substrates and amino acids in vitro.	350.2	93.96	3.73	5.00E-07	9.67E-06
4321	7	F2-3 vs. S2-3	252671_at	AT3G44190	FAD/NAD(P)-binding oxidoreductase family protein;(source:Araport11)	196.79	52.75	3.73	2.10E-06	2.53E-05
4322	7	F2-3 vs. S2-3	250648_at	AT5G06760	Encodes LEA4-5, a member of the Late Embryogenesis Abundant (LEA) proteins which typically accumulate in response to low water availability conditions imposed during development or by the environment. Most of the diverse set of LEA proteins can be grouped according to properties such as high hydrophilicity and high content of glycine or other small amino acids in what has been termed hydrophilins. LEA4-5 protects enzyme activities from the adverse effects induced by freeze-thaw cycles in vitro.	144.78	38.94	3.72	9.00E-07	1.44E-05
4323	7	F2-3 vs. S2-3	263986_at	AT2G42790	Encodes a peroxisomal citrate synthase that is expressed throughout seedling and shoot development.	431.14	116.15	3.71	5.47E-05	2.53E-04
4324	7	F2-3 vs. S2-3	258003_at	AT3G29030	Encodes an expansin. Naming convention from the Expansin Working Group (Kende et al, 2004. Plant Mol Bio)	548.66	147.73	3.71	5.90E-06	5.12E-05
4325	7	F2-3 vs. S2-3	251228_at	AT3G62710	Glycosyl hydrolase family protein;(source:Araport11)	69.85	18.84	3.71	3.12E-05	1.67E-04

4326	7	F2-3 vs. S2-3	266939_at	AT2G18960	Encodes a plasma membrane proton ATPase. Mutants have a reduced ability to close their stomata in response to drought and blue light, and are affected in stomatal but not seed responsiveness to ABA.It is the major isoform involved in the blue light mediated stomatal respsonse.The mRNA is cell-to-cell mobile.	2057.14	557.1	3.69	1.40E-06	1.89E-05
4327	7	F2-3 vs. S2-3	267429_at	AT2G34850	NAD(P)-binding Rossmann-fold superfamily protein;(source:Araport11)	180.99	49.2	3.68	2.46E-05	1.41E-04
4328	7	F2-3 vs. S2-3	250174_at	AT5G14380	Encodes an arabinogalactan protein that is expressed in pollen, pollen sac and pollen tube. Loss of AGP6 function results in decreased fertility due to defects in pollen tube growth.	64.77	17.59	3.68	1.07E-04	4.12E-04
4329	7	F2-3 vs. S2-3	264289_at	AT1G61890	MATE efflux family protein;(source:Araport11)	224.89	61.2	3.67	2.51E-04	7.75E-04
4330	7	F2-3 vs. S2-3	245745_at	AT1G51110	localized to chloroplasts	48.69	13.32	3.66	1.31E-05	9.05E-05
4331	7	F2-3 vs. S2-3	267112_at	AT2G14750	Encodes adenosine-5'-phosphosulfate kinase. Provides activated sulfate for sulfation of secondary metabolites, including the glucosinolates. Essential for pollen viability. The mRNA is cell-to-cell mobile.	135.03	36.98	3.65	1.22E-05	8.58E-05
4332	7	F2-3 vs. S2-3	247709_at	AT5G59250	Encodes a chloroplast localized H+/glucose antiporter.	261.04	71.55	3.65	2.40E-06	2.77E-05
4333	7	F2-3 vs. S2-3	261032_at	AT1G17430	alpha/beta-Hydrolases superfamily protein;(source:Araport11)	250.41	68.95	3.63	3.10E-06	3.28E-05
4334	7	F2-3 vs. S2-3	257217_at	AT3G14940	Encodes a cytosolic phosphoenolpyruvate carboxylase (PEPC) that has activity when expressed in E.coli. Its mRNA is most abundantly expressed in roots and siliques. PPC3 belongs to the plant-type PEPC family. It can form an enzymatically active complex with a castor bean ortholog of PPC4, which encodes a bacterial-type PEPC.The mRNA is cell-to-cell mobile.	49.8	13.7	3.63	1.00E-06	1.54E-05
4335	7	F2-3 vs. S2-3	252130_at	AT3G50820	Encodes a protein which is an extrinsic subunit of photosystem II and which has been proposed to play a central role in stabilization of the catalytic manganese cluster. In <i>Arabidopsis thaliana</i> the PsbO proteins are encoded by two genes: <i>psbO1</i> and <i>psbO2</i>. PsbO2 is the minor isoform in the wild-type. Mutants defective in this gene have been shown to be affected in the dephosphorylation of the D1 protein of PSII.	875.3	241.03	3.63	5.12E-05	2.42E-04
4336	7	F2-3 vs. S2-3	267489_s_at	AT4G30100	P-loop containing nucleoside triphosphate hydrolases superfamily protein;(source:Araport11)	47.91	13.22	3.62	3.57E-04	1.03E-03
4337	7	F2-3 vs. S2-3	262022_at	AT1G35490	bZIP family transcription factor;(source:Araport11)	33.7	9.33	3.61	1.08E-04	4.16E-04
4338	7	F2-3 vs. S2-3	245803_at	AT1G47128	cysteine proteinase precursor-like protein/ dehydration stress-responsive gene (RD21). Has been shown to have peptide ligase activity and protease activity in vitro. RD21 is involved in immunity to the necrotrophic fungal pathogen Botrytis cinerea.Activity detected in root, leaf, flower and cell culture.	1654.89	457.83	3.61	4.00E-07	8.26E-06
4339	7	F2-3 vs. S2-3	255622_at	AT4G01070	the glycosyltransferase (UGT72B1) is involved in metabolizing xenobiotica (chloroaniline and chlorophenole). Comparison between wild type and knock-out mutant demonstrates the central role of this gene for metabolizing chloroaniline but significantly less for chlorophenole. The glucosyltransferase preferred UDP-xylose over UDP-glucose indicating its (additional) functioning as a xylosyltransferase in planta	74.74	20.7	3.61	1.06E-05	7.84E-05
4340	7	F2-3 vs. S2-3	252740_at	AT3G43270	Plant invertase/pectin methylesterase inhibitor superfamily;(source:Araport11)	55.5	15.4	3.6	1.80E-06	2.29E-05
4341	7	F2-3 vs. S2-3	246596_at	AT5G14740	Encodes a beta carbonic anhydrase likely to be localized in the cytoplasm. Expression of its mRNA is seen in etiolated seedlings and points to a possible nonphotosynthetic role for this isoform.	58.79	16.45	3.57	4.50E-06	4.27E-05
4342	7	F2-3 vs. S2-3	258962_at	AT3G10570	member of CYP77A	124.66	35.2	3.54	1.00E-06	1.54E-05
4343	7	F2-3 vs. S2-3	264558_at	AT1G09600	Protein kinase superfamily protein;(source:Araport11)	54.58	15.46	3.53	2.30E-06	2.69E-05
4344	7	F2-3 vs. S2-3	266021_at	AT2G05910	LURP-one-like protein (DUF567);(source:Araport11)	220.88	62.6	3.53	3.00E-06	3.23E-05
4345	7	F2-3 vs. S2-3	258599_at	AT3G04520	Encodes a threonine aldolase, involved in threonine degradation to glycine. Expressed in vascular tissue through out the plant.	222.7	63.01	3.53	3.00E-07	6.68E-06
4346	7	F2-3 vs. S2-3	259231_at	AT3G11410	Encodes protein phosphatase 2C. Negative regulator of ABA signalling. Expressed in seeds during germination. mRNA up-regulated by drought and ABA.	428.26	121.31	3.53	5.50E-06	4.90E-05
4347	7	F2-3 vs. S2-3	248534_at	AT5G50030	Plant invertase/pectin methylesterase inhibitor superfamily protein;(source:Araport11)	54.82	15.52	3.53	1.00E-05	7.51E-05
4348	7	F2-3 vs. S2-3	256075_at	AT1G18150	Encodes mitogen-activated protein kinase 8 (MPK8). MPK8 connects protein phosphorylation, Ca2+, and ROS in the wound-signaling pathway.	135.98	38.7	3.51	6.40E-06	5.42E-05

4349	7	F2-3 vs. S2-3	262597_at	AT1G15470	WD40 nucleoplasmic shuttling protein that positively regulates the Abscisic acid (ABA) response by interacting with and maintaining the stability of ABI5 in the nucleus. Nuclear export of XIW1 is XPO1-dependent. Involved in regulating seed germination, primary root growth, and drought stress resistance.	55.75	15.98	3.49	4.60E-06	4.33E-05
4350	7	F2-3 vs. S2-3	245629_at	AT1G56580	Encodes SMALLER WITH VARIABLE BRANCHES (SVB), a protein with a conserved domain of unknown function (DUF538). The trichomes of the SVB mutants are smaller and exhibit branches of variable length and number.	272.56	78.09	3.49	1.10E-06	1.64E-05
4351	7	F2-3 vs. S2-3	256891_at	AT3G19030	transcription initiation factor TFIID subunit 1b-like protein;(source:Araport11)	124.33	35.62	3.49	4.97E-05	2.36E-04
4352	7	F2-3 vs. S2-3	263845_at	AT2G37040	Encodes PAL1, a phenylalanine ammonia-lyase. Arabidopsis has four PALs: AT2G37040 (PAL1), AT3G53260 (PAL2), AT5G04230 (PAL3) and AT3G10340 (PAL4).	115.41	33.29	3.47	6.96E-05	2.98E-04
4353	7	F2-3 vs. S2-3	251361_at	AT3G61230	Encodes a member of the Arabidopsis LIM proteins: a family of actin bundlers with distinct expression patterns. WLIM1, WLIM2a, and WLIM2b are widely expressed, whereas PLIM2a, PLIM2b, and PLIM2c are predominantly expressed in pollen. Regulates actin cytoskeleton organization.	55.48	15.99	3.47	2.01E-04	6.64E-04
4354	7	F2-3 vs. S2-3	260727_at	AT1G48100	Pectin lyase-like superfamily protein;(source:Araport11)	134.01	38.8	3.45	9.00E-06	7.00E-05
4355	7	F2-3 vs. S2-3	255580_at	AT4G01470	Encodes AtTIP1;3, functions as water and urea channels in pollen.	88.27	25.59	3.45	1.54E-05	1.01E-04
4356	7	F2-3 vs. S2-3	245019_at	ATCG00530	CemA-like proton extrusion protein-like protein;(source:Araport11)	210.58	60.98	3.45	9.59E-04	2.21E-03
4357	7	F2-3 vs. S2-3	260568_at	AT2G43570	chitinase;(source:Araport11)	55.05	16.03	3.43	3.30E-06	3.44E-05
4358	7	F2-3 vs. S2-3	256583_at	AT3G28850	Glutaredoxin family protein;(source:Araport11)	116.96	34.16	3.42	5.20E-06	4.73E-05
4359	7	F2-3 vs. S2-3	247161_at	AT5G65810	Encodes CGR3 (cotton Golgi-related 3). CGR3 and a close homologue CGR2 have overlapping roles in pectin methylesterification and plant growth.	503.45	147.08	3.42	4.00E-07	8.26E-06
4360	7	F2-3 vs. S2-3	265200_s_at	AT2G36790	The At2g36790 gene encodes a UDP-glucose:flavonol-3-O-glycoside-7-O-glucosyltransferase (UGT73C6)attaching a glucosyl residue to the 7-O-position of the flavonols kaempferol, quercetin and their 3-O-glycoside derivatives.	50.49	14.86	3.4	1.15E-05	8.34E-05
4361	7	F2-3 vs. S2-3	252366_at	AT3G48420	Haloacid dehalogenase-like hydrolase (HAD) superfamily protein;(source:Araport11)	293.84	86.34	3.4	2.81E-05	1.54E-04
4362	7	F2-3 vs. S2-3	245002_at	ATCG00270	PSII D2 protein	113.57	33.37	3.4	2.17E-05	1.29E-04
4363	7	F2-3 vs. S2-3	264984_at	AT1G27000	GRIP/coiled-coil protein, putative (DUF1664);(source:Araport11)	199.74	58.88	3.39	1.50E-06	2.00E-05
4364	7	F2-3 vs. S2-3	259056_at	AT3G03420	Ku70-binding family protein;(source:Araport11)	63.14	18.6	3.39	7.00E-07	1.20E-05
4365	7	F2-3 vs. S2-3	249769_at	AT5G24120	Encodes a specialized sigma factor that functions in regulation of plastid genes and is responsible for the light-dependent transcription at the psbD LRP. Activation of SIG5 is dependent upon blue light and mediated by cryptochromes.	79.54	23.46	3.39	1.30E-05	9.01E-05
4366	7	F2-3 vs. S2-3	264916_at	AT1G60810	One of the three genes encoding subunit A of the trimeric enzyme ATP Citrate lyase	2401.12	709.39	3.38	5.00E-07	9.67E-06
4367	7	F2-3 vs. S2-3	261528_at	AT1G14420	Pectate lyase family protein;(source:Araport11)	32.38	9.6	3.37	7.94E-05	3.30E-04
4368	7	F2-3 vs. S2-3	251711_at	AT3G56960	Encodes a protein with phosphatidylinositol-4-phosphate 5-kinase activity that plays a role in pollen tip growth. The enzyme localizes to the apical plasma membrane and adjacent cytosolic region of pollen tubes. Overexpression of this gene leads to increased deposition of pectin in the cell wall at the tip of the pollen tube and causes altered pollen tube morphology.	49.95	14.82	3.37	1.27E-05	8.84E-05
4369	7	F2-3 vs. S2-3	245014_at	ATCG00480	chloroplast-encoded gene for beta subunit of ATP synthase	5587.61	1659.36	3.37	1.90E-06	2.37E-05
4370	7	F2-3 vs. S2-3	253197_at	AT4G35250	HCF244 is a member of the atypical short-chain dehydrogenase/reductase superfamily, a modified group, which has lost enzyme activity.HCF244 interacts with unknown partners in a 200-400 kD membrane associated complex.	275.82	82.07	3.36	2.33E-05	1.35E-04
4371	7	F2-3 vs. S2-3	264760_at	AT1G61290	member of SYP12 Gene Family	79.35	23.71	3.35	5.00E-07	9.67E-06
4372	7	F2-3 vs. S2-3	263779_at	AT2G46340	Encodes a member of the SPA (suppressor of phyA-105) protein family (SPA1-SPA4). SPA proteins contain an N-terminal serine/threonine kinase-like motif followed by a coiled-coil structure and a C-terminal WD-repeat domain. SPA1 is a PHYA signaling intermediate, putative regulator of PHYA signaling pathway. Light responsive repressor of photomorphogenesis. Involved in regulating circadian rhythms and flowering time in plants. Under constant light, the abundance of SPA1 protein exhibited circadian regulation, whereas under constant darkness, SPA1 protein levels remained unchanged. In addition, the spa1-3 mutation slightly shortened circadian period of CCA1, TOC1/PRR1 and SPA1 transcript accumulation under constant light.	199.18	59.58	3.34	4.40E-06	4.21E-05
4373	7	F2-3 vs. S2-3	257939_at	AT3G19930	Encodes a sucrose hydrogen symporter that is induced by wounding.The mRNA is cell-to-cell mobile.	175.46	52.57	3.34	5.50E-06	4.90E-05

4374	7	F2-3 vs. S2-3	256745_at	AT3G29360	Encodes one of four UDP-glucose dehydrogenase UGD) genes. Mutation of this gene in combination with UGD3 leads to swollen plant cell walls and severe developmental defects associated with changes in pectic polysaccharides.	246.38	73.71	3.34	1.54E-04	5.41E-04
4375	7	F2-3 vs. S2-3	246017_at	AT5G10730	NAD(P)-binding Rossmann-fold superfamily protein;(source:Araport11)	196.02	58.74	3.34	1.35E-05	9.22E-05
4376	7	F2-3 vs. S2-3	249710_at	AT5G35630	chloroplastic glutamine synthetase The mRNA is cell-to-cell mobile.	642.43	192.29	3.34	1.43E-04	5.09E-04
4377	7	F2-3 vs. S2-3	250854_at	AT5G04710	Plastid localized metalloaminopeptidase.	76.04	22.86	3.33	7.42E-04	1.79E-03
4378	7	F2-3 vs. S2-3	249244_at	AT5G42270	VAR1 contains a conserved motif for ATPase and a metalloprotease characteristic to FtsH proteins, and is targeted into chloroplasts. A VAR1-fusion protein synthesized in vitro exhibited ATPase activity and partial metalloprotease activity. This protein is located to the thylakoid membrane and forms a complex with VAR2. FtsH1 (VAR1) and FtsH5 are interchangeable in thylakoid membranes. Phosphorylation of this protein is dependent on calcium. The mRNA is cell-to-cell mobile.	385.52	115.95	3.33	3.70E-06	3.73E-05
4379	7	F2-3 vs. S2-3	262682_at	AT1G75900	GDSL-motif esterase/acyltransferase/lipase. Enzyme group with broad substrate specificity that may catalyze acyltransfer or hydrolase reactions with lipid and non-lipid substrates.	243.28	73.32	3.32	4.22E-05	2.10E-04
4380	7	F2-3 vs. S2-3	267590_at	AT2G39700	putative expansin. Naming convention from the Expansin Working Group (Kende et al, 2004. Plant Mol Bio). Involved in the formation of nematode-induced syncytia in roots of Arabidopsis thaliana.	96.75	29.12	3.32	3.10E-06	3.28E-05
4381	7	F2-3 vs. S2-3	263972_at	AT2G42760	DUF1685 family protein;(source:Araport11)	254.33	76.57	3.32	6.70E-06	5.61E-05
4382	7	F2-3 vs. S2-3	262988_at	AT1G23310	Identified by cloning the gene that corresponded to a purified protein having glyoxylate aminotransferase activity. Localized to the peroxisome and thought to be involved in photorespiration/ metabolic salvage pathway.	314.57	95.1	3.31	3.60E-06	3.67E-05
4383	7	F2-3 vs. S2-3	262998_at	AT1G54280	Encodes a member of the P4 subfamily of P-type ATPases expressed in the pollen plasma membrane. Double mutants with ALA7 display pollen and pollen tube defects.	43.11	13.03	3.31	3.41E-04	9.84E-04
4384	7	F2-3 vs. S2-3	260453_s_a	AT2G09970	DUF1677 family protein (DUF1677);(source:Araport11)	62.68	18.94	3.31	6.38E-05	2.80E-04
4385	7	F2-3 vs. S2-3	267358_at	AT2G39890	Encodes a proline transporter with affinity for gly betaine, proline and GABA. Protein is expressed in the vascular tissue, specifically the phloem.	172.1	51.94	3.31	1.27E-05	8.84E-05
4386	7	F2-3 vs. S2-3	247925_at	AT5G57560	Encodes a cell wall-modifying enzyme, rapidly upregulated in response to environmental stimuli.	117.52	35.48	3.31	2.10E-06	2.53E-05
4387	7	F2-3 vs. S2-3	264016_at	AT2G21220	SAUR-like auxin-responsive protein family;(source:Araport11)	31.63	9.58	3.3	7.00E-07	1.20E-05
4388	7	F2-3 vs. S2-3	260274_at	AT1G80460	Encodes a protein similar to glycerol kinase, which converts glycerol to glycerol 3-phosphate and performs a rate-limiting step in glycerol metabolism. This gene is required for both general and specific resistance against bacteria and fungi. Arabidopsis thaliana glycerol kinase (GLR1) mRNA.Involved in flagellin-induced non-host resistance to Pseudomonas. Coronatine partially suppresses flagellin-induced expression of NHO1.	419.72	127.59	3.29	1.30E-05	9.01E-05
4389	7	F2-3 vs. S2-3	250690_at	AT5G06530	Encodes ABCG22, an ABC transporter gene. Mutation results in increased water transpiration and drought susceptibility.	81.85	25.01	3.27	2.74E-05	1.52E-04
4390	7	F2-3 vs. S2-3	261218_at	AT1G20020	Encodes a leaf-type ferredoxin:NADP(H) oxidoreductase. It is present in both chloroplast stroma and thylakoid membranes but is more abundant in the stroma The mRNA is cell-to-cell mobile.	166.82	51.23	3.26	1.92E-04	6.38E-04
4391	7	F2-3 vs. S2-3	263595_at	AT2G01890	Encodes a purple acid phosphatase (PAP) belonging to the low molecular weight plant PAP group.	72.72	22.28	3.26	1.60E-06	2.10E-05
4392	7	F2-3 vs. S2-3	245168_at	AT2G33150	Encodes an organellar (peroxisome, glyoxysome) 3-ketoacyl-CoA thiolase, involved in fatty acid b-oxidation during germination and subsequent seedling growth. Mutants have defects in glyoxysomal fatty acid beta-oxidation. EC2.3.1.16 thiolase.	1236.58	379.79	3.26	2.90E-06	3.14E-05
4393	7	F2-3 vs. S2-3	254809_at	AT4G12410	SAUR-like auxin-responsive protein family;(source:Araport11)	63.14	19.38	3.26	4.28E-04	1.17E-03
4394	7	F2-3 vs. S2-3	253343_at	AT4G33540	metallo-beta-lactamase family protein;(source:Araport11)	75.05	23	3.26	1.06E-04	4.08E-04
4395	7	F2-3 vs. S2-3	265194_at	AT1G05010	Encodes 1-aminocyclopropane-1-carboxylate oxidase	36.3	11.19	3.25	7.00E-07	1.20E-05
4396	7	F2-3 vs. S2-3	260985_at	AT1G53500	encodes a putative NDP-L-rhamnose synthase, an enzyme required for the synthesis of the pectin rhamnogalacturonan I, the major component of Arabidopsis mucilage. Gene is involved in seed coat mucilage cell development. Mutant analyses suggest that MUM4 is required for complete mucilage synthesis, cytoplasmic rearrangement and seed coat development.	194.86	59.9	3.25	2.10E-06	2.53E-05

4397	7	F2-3 vs. S2-3	265119_at	AT1G62570	belongs to the flavin-monooxygenase (FMO) family, encodes a glucosinolate S-oxygenase that catalyzes the conversion of methylthioalkyl glucosinolates to methylsulfinylalkyl glucosinolates The mRNA is cell-to-cell mobile.	81.45	25.1	3.25	1.19E-05	8.49E-05
4398	7	F2-3 vs. S2-3	262357_at	AT1G73040	Mannose-binding lectin superfamily protein;(source:Araport11)	61.68	18.97	3.25	2.07E-05	1.24E-04
4399	7	F2-3 vs. S2-3	245483_at	AT4G16190	Papain family cysteine protease;(source:Araport11)	500.96	154.47	3.24	2.80E-05	1.54E-04
4400	7	F2-3 vs. S2-3	248163_at	AT5G54510	Encodes an IAA-amido synthase that conjugates Ala, Asp, Phe, and Trp to auxin. Lines overexpressing this gene accumulate IAA-ASP and are hypersensitive to several auxins. Identified as a dominant mutation that displays shorter hypocotyls in light grown plants when compared to wild type siblings. Protein is similar to auxin inducible gene from pea (GH3).	120.93	37.49	3.23	1.30E-06	1.81E-05
4401	7	F2-3 vs. S2-3	259544_at	AT1G20620	Catalase, catalyzes the breakdown of hydrogen peroxide (H2O2) into water and oxygen. The mRNA is cell-to-cell mobile.	1432.02	444.45	3.22	5.00E-06	4.58E-05
4402	7	F2-3 vs. S2-3	261601_at	AT1G49670	molecular function has not been defined. Was shown involved in oxidative stress tolerance.	91.46	28.37	3.22	1.20E-06	1.75E-05
4403	7	F2-3 vs. S2-3	266359_at	AT2G32260	phosphorylcholine cytidylyltransferase;(source:Araport11)	252.38	78.34	3.22	6.00E-07	1.10E-05
4404	7	F2-3 vs. S2-3	262885_at	AT1G64740	alpha-tubulin expressed primarily in stamens and mature pollen	370.87	115.66	3.21	1.81E-05	1.12E-04
4405	7	F2-3 vs. S2-3	263241_at	AT2G16500	Encodes a arginine decarboxylase (ADC), a rate-limiting enzyme that catalyzes the first step of polyamine (PA) biosynthesis via ADC pathway in Arabidopsis thaliana. Arabidopsis genome has two ADC paralogs, ADC1 and ADC2. Double mutant analysis showed that ADC genes are essential for the production of PA, and are required for normal seed development. Promoter region of ADC1 contains 742-bp AT-rich transposable element, called AtATE, that belongs to the MITE families of repetitive elements.	79.39	24.71	3.21	5.69E-05	2.60E-04
4406	7	F2-3 vs. S2-3	248751_at	AT5G47540	Mo25 family protein;(source:Araport11)	223.7	69.78	3.21	6.09E-05	2.71E-04
4407	7	F2-3 vs. S2-3	262917_at	AT1G64800	DNA binding / transcription factor;(source:Araport11)	37.96	11.86	3.2	9.45E-05	3.75E-04
4408	7	F2-3 vs. S2-3	264970_at	AT1G67280	Encodes a Ni+ dependent glyoxylase.	773.68	241.99	3.2	1.30E-06	1.81E-05
4409	7	F2-3 vs. S2-3	245904_at	AT5G11110	Encodes a protein with putative sucrose-phosphate synthase activity.Involved in pollen exine formation.	106.94	33.41	3.2	1.26E-04	4.64E-04
4410	7	F2-3 vs. S2-3	248758_at	AT5G47620	RNA-binding (RRM/RBD/RNP motifs) family protein;(source:Araport11)	423.91	132.65	3.2	1.40E-06	1.89E-05
4411	7	F2-3 vs. S2-3	256015_at	AT1G19150	PSI type II chlorophyll a/b-binding protein (Lhca2*1) mRNA, The mRNA is cell-to-cell mobile.	231.45	72.64	3.19	5.40E-06	4.85E-05
4412	7	F2-3 vs. S2-3	252327_at	AT3G48740	Encodes a member of the SWEET sucrose efflux transporter family proteins.	152.97	47.9	3.19	4.84E-05	2.31E-04
4413	7	F2-3 vs. S2-3	252134_at	AT3G50910	netrin receptor DCC;(source:Araport11)	429.07	134.99	3.18	4.86E-05	2.32E-04
4414	7	F2-3 vs. S2-3	245431_at	AT4G17080	Histone H3 K4-specific methyltransferase SET7/9 family protein;(source:Araport11)	61.81	19.46	3.18	1.22E-05	8.58E-05
4415	7	F2-3 vs. S2-3	245928_s_at	AT5G24780	encodes an acid phosphatase similar to soybean vegetative storage proteins. Gene expression is induced by wounding and jasmonic acid.	2260.39	710.31	3.18	2.70E-06	3.02E-05
4416	7	F2-3 vs. S2-3	252011_at	AT3G52720	Encodes an alpha carbonic anhydrase (CAH1) located in the chloroplast stroma. Most chloroplast proteins are encoded by the nuclear genome and imported with the help of sorting signals that are intrinsic parts of the polypeptides. CAH1 takes an alternative route through the secretory pathway, and becomes N-glycosylated before entering the chloroplast. Glycosylation and intra-molecular disulfide bridge fromation are necessary for the correct folding, ER export, trafficking and activity of the protein.	54.28	17.12	3.17	5.00E-06	4.58E-05
4417	7	F2-3 vs. S2-3	251309_at	AT3G61220	CytADR/SDR1 is an aldehyde reductase that catalyzes the reduction of the aldehyde carbonyl groups on alpha,beta-unsaturated aldehydes with more than 5 carbons in vitro. It can also act on menthone and neomenthol in vitro, but these do not represent likely endogenous activities of this enzyme in planta. GFP-tagged CytADR appears to localize to the cytosol where it likely plays a role in detoxifying reactive carbonyls. sdr1 mutants have altered responses to pathogens.The mRNA is cell-to-cell mobile.	45.88	14.46	3.17	8.59E-05	3.48E-04
4418	7	F2-3 vs. S2-3	254050_s_a	AT4G25670	stress response NST1-like protein;(source:Araport11)	119.63	37.78	3.17	5.33E-05	2.49E-04
4419	7	F2-3 vs. S2-3	249875_at	AT5G23120	encodes a stability and/or assembly factor of photosystem II The mRNA is cell-to-cell mobile.	695.13	218.98	3.17	1.50E-05	9.94E-05
4420	7	F2-3 vs. S2-3	248243_at	AT5G53590	SAUR-like auxin-responsive protein family;(source:Araport11)	66.55	21.09	3.15	1.16E-04	4.36E-04
4421	7	F2-3 vs. S2-3	259517_at	AT1G20630	Catalyzes the reduction of hydrogen peroxide using heme group as cofactor. Protects cells from toxicity by H2O2.	335.99	107.04	3.14	1.30E-06	1.81E-05
4422	7	F2-3 vs. S2-3	258554_at	AT3G06980	DEA(D/H)-box RNA helicase family protein;(source:Araport11)	73.44	23.38	3.14	7.00E-07	1.20E-05

4423	7	F2-3 vs. S2-3	266098_at	AT2G37870	Bifunctional inhibitor/lipid-transfer protein/seed storage 2S albumin superfamily protein;(source:Araport11)	409.91	130.9	3.13	9.09E-05	3.64E-04
4424	7	F2-3 vs. S2-3	249688_at	AT5G36160	Encodes a cytosolic L-tyrosine aminotransferase. AtTAT2 exhibits much broader amino donor specificity than AtTAT1 and can use not only Tyr but also Phe, Trp, His, Met, Leu, Ala, Ser, Cys, Asp, Asn, Gln, and Arg as amino donors.	46.3	14.86	3.12	4.80E-06	4.48E-05
4425	7	F2-3 vs. S2-3	261564_at	AT1G01720	Belongs to a large family of putative transcriptional activators with NAC domain. Transcript level increases in response to wounding and abscisic acid. ATAF1 attentuates ABA signaling and sythesis. Mutants are hyposensitive to ABA. The mRNA is cell-to-cell mobile.	95.9	30.83	3.11	1.24E-04	4.59E-04
4426	7	F2-3 vs. S2-3	260286_at	AT1G80600	Encodes HopW1-1-Interacting protein 1 (WIN1). Interacts with the P. syringae effector HopW1-1. WIN1 is a putative acetylornithine transaminase. Modulates plant defenses against bacteria. Three WIN proteins are identified so far (WIN1: AT1G80600; WIN2: AT4G31750; WIN3: AT5G13320). Mediates red-light inhibition of seed germination.	192.36	61.75	3.11	7.02E-05	2.99E-04
4427	7	F2-3 vs. S2-3	253954_at	AT4G26970	Encodes an aconitase that can catalyze the conversion of citrate to isocitrate through a cis-aconitate intermediate, indicating that it may participate in the TCA cycle and other primary metabolic pathways. The protein is believed to accumulate in the mitochondria and the cytosol. It affects CSD2 (At2g28190 - a superoxide dismutase) transcript levels and may play a role in the response to oxidative stress. One member of the family (ACO1 - At35830) was shown to specifically bind to the 5' UTR of CSD2 in vitro. The mRNA is cell-to-cell mobile.	1600.17	515.29	3.11	1.34E-04	4.85E-04
4428	7	F2-3 vs. S2-3	256156_at	AT3G08510	Phosphoinositide-specific phospholipase C (PI-PLC), catalyzes hydrolysis of phosphatidylinositol 4,5-bisphosphate into inositol 1,4,5-trisphosphate and diacylglycerol. It is involved in auxin biosynthesis and signaling, modulating development of both male and female gametophytes. It also regulates MAMP-triggered immunity by modulating ROS production.	192.79	62.21	3.1	1.80E-06	2.29E-05
4429	7	F2-3 vs. S2-3	254972_at	AT4G10440	S-adenosyl-L-methionine-dependent methyltransferases superfamily protein;(source:Araport11)	48.16	15.53	3.1	2.39E-05	1.38E-04
4430	7	F2-3 vs. S2-3	264603_at	AT1G04670	hypothetical protein;(source:Araport11)	29.13	9.42	3.09	2.70E-06	3.02E-05
4431	7	F2-3 vs. S2-3	258167_at	AT3G21560	Encodes a protein with sinapic acid:UDP-glucose glucosyltransferase activity. Mutants defective in this gene are hyper-fluorescent (which accumulate in their trichomes a compound that is likely to be 3',5'-dimethoxynaringenin chalcone or sinapoyltriacetic acid lactone, potential products of the concerted action of 4-coumarate CoA ligase and chalcone synthase on sinapic acid). Also shown to be required for Arabidopsis nonhost resistance to the Asian soybean rust pathogen Phakopsora pachyrhizi.	158.2	51.15	3.09	5.29E-05	2.47E-04
4432	7	F2-3 vs. S2-3	252331_s_at	AT3G48780	Encodes one of the two LCB2 subunits (LCB2a and LCB2b) of serine palmitoyltransferase, an enzyme involved in sphingolipid biosynthesis. LCB2a and LCB2b are functional redundant. Double mutants are gametophytic lethal. The mRNA is cell-to-cell mobile.	89.75	29.02	3.09	2.50E-06	2.85E-05
4433	7	F2-3 vs. S2-3	247593_at	AT5G60790	member of GCN subfamily	333.28	108.11	3.08	2.20E-06	2.63E-05
4434	7	F2-3 vs. S2-3	259420_at	AT1G13900	Encodes a dual-localized acid phosphatase (mitochondria and chloroplast) that modulates carbon metabolism.	207.16	67.46	3.07	9.80E-06	7.45E-05
4435	7	F2-3 vs. S2-3	262281_at	AT1G68570	NPF3.1 is a membrane localized GA transporter that is expressed in the root endodermis.	118.74	38.69	3.07	4.60E-06	4.33E-05
4436	7	F2-3 vs. S2-3	263950_at	AT2G36020	HVA22-like protein J;(source:Araport11)	45.31	14.76	3.07	4.20E-06	4.10E-05
4437	7	F2-3 vs. S2-3	263157_at	AT1G54100	Aldehyde dehydrogenase	852.78	278.59	3.06	5.87E-04	1.50E-03
4438	7	F2-3 vs. S2-3	255345_at	AT4G04460	Saposin-like aspartyl protease family protein;(source:Araport11)	38.57	12.61	3.06	1.30E-06	1.81E-05
4439	7	F2-3 vs. S2-3	249187_at	AT5G43060	Peptidase, activity detected in extracts of root, leaf and cell culture.	168.45	55.04	3.06	3.80E-06	3.79E-05
4440	7	F2-3 vs. S2-3	260325_at	AT1G63940	monodehydroascorbate reductase 6;(source:Araport11)	93.83	30.8	3.05	8.00E-06	6.40E-05
4441	7	F2-3 vs. S2-3	258445_at	AT3G22400	Encodes lipoxygenase5 (LOX5). LOX5 activity in roots facilitates green peach aphid colonization of Arabidopsis foliage by promoting green peach aphid feeding from sieve element and water consumption from xylem.	124.43	40.77	3.05	1.05E-05	7.79E-05
4442	7	F2-3 vs. S2-3	247470_at	AT5G62220	Encodes a Golgi apparatus-localized galactosyltransferase involved in galactosyl-substitution of xyloglucan at position 2.	106.97	35.07	3.05	1.17E-04	4.39E-04

4443	7	F2-3 vs. S2-3	261920_at	AT1G65930	Encodes a NADP+-isocitrate dehydrogenase that is believed to function in the cytosol. It appears to contribute to NADPH production under oxidative stress, and thereby to participate in redox signalling linked to defense responses. The mRNA is cell-to-cell mobile.	981.78	322.44	3.04	3.64E-05	1.89E-04
4444	7	F2-3 vs. S2-3	266205_s_at	AT2G27860	Encodes UDP-d-apiose/UDP-d-xylose synthase that requires NAD+ for enzymatic activity and is strongly inhibited by UDP-d-galacturonate.	1112.08	366.78	3.03	1.94E-05	1.18E-04
4445	7	F2-3 vs. S2-3	257309_at	AT3G28150	Encodes a member of the TBL (TRICHOME BIREFRINGENCE-LIKE) gene family containing a plant-specific DUF231 (domain of unknown function) domain. A putative xyloglucan O-acetyltransferase. TBL gene family has 46 members, two of which (TBR/AT5G06700 and TBL3/AT5G01360) have been shown to be involved in the synthesis and deposition of secondary wall cellulose, presumably by influencing the esterification state of pectic polymers. A nomenclature for this gene family has been proposed (Volker Bischoff & Wolf Scheible, 2010, personal communication).	32.94	10.88	3.03	1.50E-06	2.00E-05
4446	7	F2-3 vs. S2-3	261443_at	AT1G28480	Encodes GRX480, a member of the glutaredoxin family that regulates protein redox state. GRX480 interacts with TGA factors and suppresses JA-responsive PDF1.2 transcription. GRX480 transcription is SA-inducible and requires NPR1. Maybe involved in SA/JA cross-talk. It has also been shown to interact with the transcription factor TGA2 and suppress ORA59 promoter activity.	67.07	22.28	3.01	7.50E-06	6.08E-05
4447	7	F2-3 vs. S2-3	265634_at	AT2G25530	AFG1-like ATPase family protein;(source:Araport11)	119.72	39.92	3	7.50E-06	6.08E-05
4448	7	F2-3 vs. S2-3	266302_at	AT2G27050	ethylene-insensitive3-like1 (EIL1) The mRNA is cell-to-cell mobile.	100.55	33.47	3	1.70E-04	5.83E-04
4449	7	F2-3 vs. S2-3	255872_at	AT2G30360	Encodes a SOS2-like protein kinase that is a member of the CBL-interacting protein kinase family.Loss of function mutants show a decrease in sensitivity to high pH.Phosphorylates AHA2, a plasma membrane H+ ATPase.This phosphorylation appears to regulate the activity of the proton transporter.	85.67	28.53	3	7.14E-04	1.74E-03
4450	7	F2-3 vs. S2-3	247811_at	AT5G58430	A member of EXO70 gene family, putative exocyst subunits, conserved in land plants. Arabidopsis thaliana contains 23 putative EXO70 genes, which can be classified into eight clusters on the phylogenetic tree. Targeted by AvrPtoB to manipulate the defense molecule secretion machinery.	43.86	14.62	3	9.90E-06	7.47E-05
4451	7	F2-3 vs. S2-3	249237_at	AT5G42050	Stress responsive asparagine-rich protein. Binds to PevD (Verticillium dahliae) fungal effector protein. NRP interacts with CRY2, leading to increased cytoplasmic accumulation of CRY2 in a blue light-independent manner (PMID:28633330).NRP also binds FyPP3 and recruits it to endosomes and thus targets it for degradation.	214.93	71.83	2.99	3.42E-05	1.79E-04
4452	7	F2-3 vs. S2-3	263166_at	AT1G03050	Phosphatidylinositol binding clathrin assembly protein 5A/B are recent paralogs with overlapping functions in recycling ANXUR proteins to the pollen tube membrane.	32.12	10.77	2.98	4.50E-06	4.27E-05
4453	7	F2-3 vs. S2-3	266553_at	AT2G46170	Reticulon family protein;(source:Araport11)	338.37	113.65	2.98	7.70E-06	6.20E-05
4454	7	F2-3 vs. S2-3	250738_at	AT5G05730	ASA1 encodes the alpha subunit of anthranilate synthase, which catalyzes the rate-limiting step of tryptophan synthesis. ASA1 is induced by ethylene, and forms a link between ethylene signalling and auxin synthesis in roots.	437.42	147.02	2.98	4.85E-05	2.31E-04
4455	7	F2-3 vs. S2-3	246488_at	AT5G16010	3-oxo-5-alpha-steroid 4-dehydrogenase family protein;(source:Araport11)	169.45	56.93	2.98	8.23E-05	3.38E-04
4456	7	F2-3 vs. S2-3	248822_at	AT5G47000	Peroxidase superfamily protein;(source:Araport11)	30.89	10.35	2.98	3.40E-06	3.52E-05
4457	7	F2-3 vs. S2-3	247401_at	AT5G62790	1-Deoxy-d-xylulose 5-phosphate reductoisomerase (DXR) catalyzes the first committed step of the 2-C-methyl-d-erythritol 4-phosphate pathway for isoprenoid biosynthesis. In Arabidopsis, DXR is encoded by a single-copy gene. Arabidopsis DXR is targeted to plastids and localizes into chloroplasts of leaf cells. DXR knockout or strongly silenced lines have a seedling lethal, albino phenotype. Transgenic, partially silenced lines expressing 35S:DXR have a variegated phenotype.	1214.71	407.45	2.98	5.52E-05	2.55E-04
4458	7	F2-3 vs. S2-3	246987_at	AT5G67300	Member of the R2R3 factor MYB gene family involved in mediating plant responses to a variety of abiotic stimiuli. The mRNA is cell-to-cell mobile.	406.64	136.68	2.98	7.10E-06	5.88E-05
4459	7	F2-3 vs. S2-3	249215_at	AT5G42800	dihydroflavonol reductase. Catalyzes the conversion of dihydroquercetin to leucocyanidin in the biosynthesis of anthocyanins. Not expressed in roots (qRT-PCR). The mRNA is cell-to-cell mobile.	50.26	16.94	2.97	1.19E-04	4.46E-04
4460	7	F2-3 vs. S2-3	249126_at	AT5G43380	encodes a type I serine/threonine protein phosphatase expressed in expressed in roots, rosettes and flowers.	72.33	24.34	2.97	1.00E-06	1.54E-05
4461	7	F2-3 vs. S2-3	247981_at	AT5G56640	Myo-Inositol Oxygenase gene family	189.82	63.84	2.97	1.30E-06	1.81E-05
4462	7	F2-3 vs. S2-3	261767_s_at	AT1G15500	TLC ATP/ADP transporter;(source:Araport11)	248.22	83.92	2.96	2.84E-04	8.54E-04

4463	7	F2-3 vs. S2-3	245868_at	AT1G58030	Encodes a member of the cationic amino acid transporter (CAT) subfamily of amino acid polyamine choline transporters. Localized to the tonoplast.	70.1	23.72	2.96	1.50E-05	9.94E-05
4464	7	F2-3 vs. S2-3	262263_at	AT1G70940	A regulator of auxin efflux and involved in differential growth. PIN3 is expressed in gravity-sensing tissues, with PIN3 protein accumulating predominantly at the lateral cell surface. PIN3 localizes to the plasma membrane and to vesicles. In roots, PIN3 is expressed without pronounced polarity in tiers two and three of the columella cells, at the basal side of vascular cells, and to the lateral side of pericycle cells of the elongation zone. PIN3 overexpression inhibits root cell growth. Protein phosphorylation plays a role in regulating PIN3 trafficking to the plasma membrane. The mRNA is cell-to-cell mobile.	66.39	22.4	2.96	8.70E-06	6.82E-05
4465	7	F2-3 vs. S2-3	263881_at	AT2G21820	seed maturation protein;(source:Araport11)	39.82	13.44	2.96	5.99E-05	2.69E-04
4466	7	F2-3 vs. S2-3	267175_s_a	AT2G37620	Member of the actin gene family. Expressed in mature pollen.	1173.76	396.15	2.96	3.10E-06	3.28E-05
4467	7	F2-3 vs. S2-3	246308_at	AT3G51820	Encodes a protein with chlorophyll synthase activity. This enzyme has been shown to perform the esterification of chlorophyllide (a and b), the last step of chlorophyll biosynthesis. Although it can use either geranylgeranyl pyrophosphate (GGPP) or phytyl pyrophosphate (PhyPP) as substrates, the esterification reaction was faster with GGPP than with PhyPP.	1047.23	354.13	2.96	1.32E-04	4.81E-04
4468	7	F2-3 vs. S2-3	253522_at	AT4G31290	ChaC-like family protein;(source:Araport11)	322.23	108.85	2.96	3.10E-06	3.28E-05
4469	7	F2-3 vs. S2-3	263084_at	AT2G27180	hypothetical protein;(source:Araport11)	41.38	14.05	2.95	6.48E-04	1.62E-03
4470	7	F2-3 vs. S2-3	262344_at	AT1G64060	Interacts with AtrbohD gene to fine tune the spatial control of ROI production and hypersensitive response to cell in and around infection site.	165.73	56.38	2.94	2.12E-04	6.88E-04
4471	7	F2-3 vs. S2-3	267623_at	AT2G39650	cruciferin (DUF506);(source:Araport11)	109.64	37.25	2.94	8.90E-06	6.93E-05
4472	7	F2-3 vs. S2-3	258849_at	AT3G03250	Is thought to encode a cytosolic UDP-glucose pyrophosphorylase with strong similarity to potato UTP--glucose-1-phosphate uridylyltransferase. Downregulated by flooding.	685.49	233.45	2.94	1.60E-06	2.10E-05
4473	7	F2-3 vs. S2-3	258398_at	AT3G15360	encodes a prokaryotic thioredoxin The mRNA is cell-to-cell mobile.	53.58	18.25	2.94	3.25E-04	9.50E-04
4474	7	F2-3 vs. S2-3	246947_at	AT5G25120	putative cytochrome P450 The mRNA is cell-to-cell mobile.	54.79	18.61	2.94	1.81E-05	1.12E-04
4475	7	F2-3 vs. S2-3	251860_at	AT3G54660	Encodes glutathione reductase that is most likely localized in the chloroplast.	625.36	213.82	2.92	2.18E-05	1.29E-04
4476	7	F2-3 vs. S2-3	248719_at	AT5G47910	NADPH/respiratory burst oxidase protein D (RbohD).Interacts with AtrbohF gene to fine tune the spatial control of ROI production and hypersensitive response to cell in and around infection site. The mRNA is cell-to-cell mobile.	153	52.38	2.92	2.70E-05	1.51E-04
4477	7	F2-3 vs. S2-3	255764_at	AT1G16720	Encodes HCF173, a protein with weak similarities to the superfamily of the short-chain dehydrogenases/reductases. HCF173 is involved in the initiation of translation of the psbA mRNA and binds a specific site in the 5' UTR of psbA mRNA. . Mutants shows a high chlorophyll fluorescence phenotype (hcf) and are severely affected in the accumulation of PSII subunits. The protein HCF173 is localized in the chloroplast, where it is mainly associated with the membrane system and is part of a higher molecular weight complex with psbA mRNA as a component of this complex.	382.58	131.32	2.91	1.60E-06	2.10E-05
4478	7	F2-3 vs. S2-3	265338_at	AT2G18400	ribosomal protein L6 family protein;(source:Araport11)	1981.37	681.15	2.91	2.30E-06	2.69E-05
4479	7	F2-3 vs. S2-3	266765_at	AT2G46860	Encodes a protein that might have inorganic pyrophosphatase activity.	39.99	13.76	2.91	1.51E-04	5.33E-04
4480	7	F2-3 vs. S2-3	257212_at	AT3G15090	GroES-like zinc-binding alcohol dehydrogenase family protein;(source:Araport11)	206.92	71.19	2.91	9.60E-06	7.35E-05
4481	7	F2-3 vs. S2-3	254492_at	AT4G20260	Encodes a Ca2+ and Cu2+ binding protein. N-terminal myristylation on glycine 2 appears to enable it to associate tightly with the plasma membrane. Recombinant PCaP1 interacts strongly with phosphatidylinositol 3,5-bisphosphate (PtdIns(3,5)P2) and PtdIns (3,4,5)P3, and weakly with PtdIns(3,5)P2 and PtdIns(4,5). It also interacts with calmodulin (CaM) in a calcium-dependent manner. CaM does not interfere with PCaP1 membrane localization but does weaken interactions between it and the PtdInsPs. PCaP1 has an apparent Kd of 10 uM for Cu2+ and can bind six ions per protein. Transcript levels for PCaP1 first fall and then rise following exposure to CuCl2. Mannitol, sorbitol, and the flg22 oligopeptide also increase expression levels.The mRNA is cell-to-cell mobile.	38.56	13.25	2.91	3.35E-04	9.71E-04
4482	7	F2-3 vs. S2-3	250347_at	AT5G11960	magnesium transporter, putative (DUF803);(source:Araport11)	135.41	46.52	2.91	6.30E-06	5.36E-05
4483	7	F2-3 vs. S2-3	245683_at	AT5G22030	ubiquitin-specific protease 8;(source:Araport11)	77.17	26.54	2.91	3.50E-06	3.60E-05
4484	7	F2-3 vs. S2-3	264244_at	AT1G60440	The gene AT1G60440 encodes pantothenate kinase 1. Its molecular function was shown to phosphorylate pantothenate to form 4?-phosphopantothenate.	514.43	177.12	2.9	1.72E-05	1.09E-04
4485	7	F2-3 vs. S2-3	260304_at	AT1G70480	OBP32pep, putative (DUF220);(source:Araport11)	157.87	54.44	2.9	9.90E-06	7.47E-05

4486	7	F2-3 vs. S2-3	257650_at	AT3G16800	EGR3 functions as a negative regulator of plant growth with prominent effect on plant growth during drought stress, EGR3 regulates microtubule organization and likely affects additional cytoskeleton and trafficking processes along the plasma membrane.	59.11	20.36	2.9	3.50E-06	3.60E-05
4487	7	F2-3 vs. S2-3	251757_at	AT3G55640	Mitochondrial substrate carrier family protein;(source:Araport11)	160.32	55.3	2.9	4.35E-05	2.14E-04
4488	7	F2-3 vs. S2-3	253387_at	AT4G33010	glycine decarboxylase P-protein 1;(source:Araport11)	596.03	205.47	2.9	1.24E-05	8.66E-05
4489	7	F2-3 vs. S2-3	258207_at	AT3G14050	RELA/SPOT homolog 2;(source:Araport11)	184.02	63.65	2.89	1.80E-06	2.29E-05
4490	7	F2-3 vs. S2-3	252550_at	AT3G45870	nodulin MtN21-like transporter family protein	58.79	20.34	2.89	7.20E-06	5.94E-05
4491	7	F2-3 vs. S2-3	266892_at	AT2G26080	glycine decarboxylase P-protein 2;(source:Araport11)	240.55	83.41	2.88	1.24E-04	4.58E-04
4492	7	F2-3 vs. S2-3	252372_at	AT3G48000	Encodes a putative (NAD+) aldehyde dehydrogenase.	205.02	71.23	2.88	6.70E-06	5.61E-05
4493	7	F2-3 vs. S2-3	264903_at	AT1G23190	Encodes a cytosolic phosphoglucomutase (PGM). Two Arabidopsis PGM proteins (AT1G70730/PGM2 and AT1G23190/PGM3) have high sequence similarities and redundant functions. Mature plants possessing a single cPGM allele had a major reduction in cPGM activity. Whereas pgm2 and pgm3 single mutants are undistinguishable from the wild type, loss of both PGM2 and PGM3 severely impairs male and female gametophyte development. The mRNA is cell-to-cell mobile.	58.14	20.25	2.87	2.08E-04	6.81E-04
4494	7	F2-3 vs. S2-3	262878_at	AT1G64770	encodes a novel subunit of the chloroplast NAD(P)H dehydrogenase complex, involved in cyclic electron flow around photosystem I to produce ATP.	135.4	47.16	2.87	1.92E-04	6.39E-04
4495	7	F2-3 vs. S2-3	267051_at	AT2G38500	2-oxoglutarate (2OG) and Fe(II)-dependent oxygenase superfamily protein;(source:Araport11)	171.76	59.77	2.87	3.76E-04	1.07E-03
4496	7	F2-3 vs. S2-3	252472_at	AT3G46830	RAB GTPase homolog A2C;(source:Araport11)	217.81	75.93	2.87	1.10E-06	1.64E-05
4497	7	F2-3 vs. S2-3	251839_at	AT3G54950	Encodes pPLAIIIbeta, a member of the Group 3 patatin-related phospholipases. pPLAIIIbeta hydrolyzes phospholipids and galactolipids and additionally has acyl-CoA thioesterase activity. Alterations of pPLAIIIβ result in changes in lipid levels and composition.	94.58	32.93	2.87	5.70E-06	5.00E-05
4498	7	F2-3 vs. S2-3	251020_at	AT5G02270	member of NAP subfamily	97.74	34.04	2.87	5.70E-06	5.00E-05
4499	7	F2-3 vs. S2-3	250858_at	AT5G04760	R-R-type MYB protein which plays negative roles in salt stress and is required for ABA signaling in Arabidopsis.	56.03	19.51	2.87	2.58E-05	1.46E-04
4500	7	F2-3 vs. S2-3	253039_at	AT4G37760	squalene epoxidase 3;(source:Araport11)	137.47	48.12	2.86	6.05E-05	2.71E-04
4501	7	F2-3 vs. S2-3	254270_at	AT4G23100	Encodes the enzyme glutamate-cysteine ligase catalyzing the first, and rate-limiting, step of glutathione biosynthesis. Required for cell proliferation at the root tip. Involved in susceptibility to the bacterial pathogen Pseudomonas syringae. Mutants are phytoalexin defective.	140.36	49.31	2.85	8.07E-05	3.34E-04
4502	7	F2-3 vs. S2-3	248128_at	AT5G54770	Encodes a thiamine biosynthetic gene that has a dual function in thiamine biosynthesis and mitochondrial DNA damage tolerance. It appears to be involved in producing the thiazole portion of thiamine (vitamin B1). A crystal structure of the protein reveals that it forms a 2-ring homo-octamer. The mRNA is cell-to-cell mobile.	867.58	304.07	2.85	2.00E-06	2.46E-05
4503	7	F2-3 vs. S2-3	265935_at	AT2G19580	Member of TETRASPANIN family	83.56	29.47	2.84	1.70E-06	2.21E-05
4504	7	F2-3 vs. S2-3	245088_at	AT2G39850	Subtilisin-like serine endopeptidase family protein;(source:Araport11)	28.77	10.15	2.84	4.66E-05	2.26E-04
4505	7	F2-3 vs. S2-3	245987_at	AT5G13180	Encodes a NAC domain transcription factor that interacts with VND7 and negatively regulates xylem vessel formation.	44.38	15.65	2.84	1.20E-05	8.55E-05
4506	7	F2-3 vs. S2-3	256161_at	AT1G30090	Galactose oxidase/kelch repeat superfamily protein;(source:Araport11)	118.61	41.89	2.83	2.70E-06	3.02E-05
4507	7	F2-3 vs. S2-3	260002_at	AT1G67940	member of NAP subfamily The mRNA is cell-to-cell mobile.	61.72	21.82	2.83	1.20E-04	4.48E-04
4508	7	F2-3 vs. S2-3	262262_at	AT1G70780	hypothetical protein;(source:Araport11)	655.37	231.45	2.83	2.83E-04	8.51E-04
4509	7	F2-3 vs. S2-3	252971_at	AT4G38770	Encodes one of four proline-rich proteins in Arabidopsis which are predicted to localize to the cell wall. Transcripts are most abundant in aerial organs of the plant.	403.06	142.62	2.83	1.43E-04	5.11E-04
4510	7	F2-3 vs. S2-3	265184_at	AT1G23710	hypothetical protein (DUF1645);(source:Araport11)	75.2	26.64	2.82	2.60E-04	7.98E-04
4511	7	F2-3 vs. S2-3	251704_at	AT3G56360	hypothetical protein;(source:Araport11)	55.24	19.61	2.82	1.92E-04	6.38E-04
4512	7	F2-3 vs. S2-3	265174_s_a	AT1G23460	Pectin lyase-like superfamily protein;(source:Araport11)	28.77	10.25	2.81	3.87E-05	1.96E-04
4513	7	F2-3 vs. S2-3	266181_at	AT2G02390	Encodes glutathione transferase belonging to the zeta class of GSTs. Naming convention according to Wagner et al. (2002). The protein undergoes spontaneous thiolation following treatment with the oxidant tert-butylhydroperoxide. It functions in vitro as a maleylacetoacetate isomerase and is likely to be involved in tyrosine catabolism.	216.25	76.85	2.81	2.46E-05	1.41E-04
4514	7	F2-3 vs. S2-3	263047_at	AT2G17630	Pyridoxal phosphate (PLP)-dependent transferases superfamily protein;(source:Araport11)	207.86	73.9	2.81	9.90E-06	7.47E-05

4515	7	F2-3 vs. S2-3	264590_at	AT2G17710	Big1;(source:Araport11)	72.19	25.65	2.81	1.04E-05	7.72E-05
4516	7	F2-3 vs. S2-3	252475_s_at	AT3G46640	Encodes a myb family transcription factor with a single Myb DNA-binding domain (type SHAQKYF) that is unique to plants and is essential for circadian rhythms, specifically for transcriptional regulation within the circadian clock. LUX is required for normal rhythmic expression of multiple clock outputs in both constant light and darkness. It is coregulated with TOC1 and seems to be repressed by CCA1 and LHY by direct binding of these proteins to the evening element in the LUX promoter. The mRNA is cell-to-cell mobile.	38.95	13.86	2.81	1.00E-04	3.94E-04
4517	7	F2-3 vs. S2-3	252354_at	AT3G48170	ALDH10A9 encodes a protein that can function as a betaine aldehyde dehydrogenase in vitro. The C-terminal amino acids of this protein direct GFP to the peroxisome suggesting that ALDH10A9 accumulates in this organelle. ALDH10A9 transcript levels rise in response to ABA, NaCl, chilling, methyl viologen, and dehydration stress. The enzyme can catalyze the formation of glycine betaine in vitro, but there are still questions about whether Arabidopsis makes this protective compound under natural conditions. This enzyme may be involved in oxidizing aminoaldehydes formed through polyamine metabolism.	1859.43	661.64	2.81	3.66E-05	1.89E-04
4518	7	F2-3 vs. S2-3	245619_at	AT4G13990	Exostosin family protein;(source:Araport11)	55.3	19.65	2.81	5.00E-06	4.58E-05
4519	7	F2-3 vs. S2-3	253268_s_at	AT4G34135	The At4g34135 gene encodes a flavonol 7-O-glucosyltransferase (EC 2.4.1.237) that glucosylates also with a 20 fold lower activity flavonols (kaempferol and quercetin) at the 3-O-position.	81.03	28.82	2.81	1.02E-04	3.97E-04
4520	7	F2-3 vs. S2-3	260023_at	AT1G30040	Encodes a gibberellin 2-oxidase that acts on C-19 gibberellins. AtGA2OX2 expression is responsive to cytokinin and KNOX activities.	114.01	40.78	2.8	6.30E-06	5.36E-05
4521	7	F2-3 vs. S2-3	266653_at	AT2G25740	ATP-dependent protease La (LON) domain protein;(source:Araport11)	80.36	28.73	2.8	1.03E-04	4.01E-04
4522	7	F2-3 vs. S2-3	251218_at	AT3G62410	CP12-2 encodes a small peptide found in the chloroplast stroma. It belongs to the CP12 gene family thought to be involved in the formation of a supramolecular complex with glyceraldehyde-3-phosphate dehydrogenase (GAPDH) and phosphoribulokinase (PRK) embedded in the Calvin cycle. CP12-2 is coordinately regulated by light with the photosynthetic GAPDH and PRK. The annotation of this gene is based on article 32494. The mRNA is cell-to-cell mobile.	75.77	27.08	2.8	4.28E-05	2.12E-04
4523	7	F2-3 vs. S2-3	255479_at	AT4G02380	Encodes AtLEA5 (late embryogenesis abundant like protein). Also known as SENESCENCE-ASSOCIATED GENE 21 (SAG21). Has a role on oxidative stress tolerance. mRNA levels are elevated in response to various stresses.	61.46	21.96	2.8	2.80E-06	3.09E-05
4524	7	F2-3 vs. S2-3	263012_at	AT1G23380	homeodomain transcription factor KNAT6, belonging to class I of KN transcription factor family (which also includes KNAT1 and KNAT2). Expression is increased in as and bop1 leaf mutants.	43.68	15.64	2.79	2.73E-05	1.52E-04
4525	7	F2-3 vs. S2-3	260267_at	AT1G68530	Encodes KCS6, a member of the 3-ketoacyl-CoA synthase family involved in the biosynthesis of VLCFA (very long chain fatty acids).	73.56	26.36	2.79	3.74E-05	1.92E-04
4526	7	F2-3 vs. S2-3	256416_at	AT3G11050	ferritin 2;(source:Araport11)	137.98	49.49	2.79	9.34E-05	3.72E-04
4527	7	F2-3 vs. S2-3	258359_s_at	AT3G14420	Encodes a glycolate oxidase that modulates reactive oxygen species-mediated signal transduction during nonhost resistance. The mRNA is cell-to-cell mobile.	72.16	25.89	2.79	5.80E-06	5.07E-05
4528	7	F2-3 vs. S2-3	246075_at	AT5G20410	Encodes a type B monogalactosyldiacylglycerol (MGDG) synthase. Strongly induced by phosphate deprivation, and in non-photosynthetic tissues. Does not contribute to galactolipid synthesis under Pi-sufficient conditions but does under Pi starvation.	153.54	55.06	2.79	2.85E-04	8.56E-04
4529	7	F2-3 vs. S2-3	262868_at	AT1G64980	Encodes a putative nucleotide-diphospho-sugar transferase required for pollen germination and tube growth.	249.98	89.94	2.78	5.14E-05	2.42E-04
4530	7	F2-3 vs. S2-3	267503_at	AT2G45600	alpha/beta-Hydrolases superfamily protein;(source:Araport11)	48.97	17.61	2.78	8.83E-04	2.06E-03
4531	7	F2-3 vs. S2-3	252531_at	AT3G46520	Member of actin subclass composed of ACT12 and ACT4. RNA is expressed at very low levels in vegetative organs, low levels in flowers and very high levels in pollen. Expression of an ACT12/GUS fusion was found in vascular tissues, tapetum, developing and mature pollen, the root cap and in a ring of pericycle tissues during lateral root initiation and early development.	34.53	12.44	2.78	3.51E-05	1.83E-04
4532	7	F2-3 vs. S2-3	251962_at	AT3G53420	a member of the plasma membrane intrinsic protein subfamily PIP2. localizes to the plasma membrane and exhibits water transport activity in Xenopus oocyte. expressed specifically in the vascular bundles and protein level increases slightly during leaf dev. When expressed in yeast cells can conduct hydrogen peroxide into those cells.	2458.28	884.35	2.78	5.00E-06	4.58E-05

4533	7	F2-3 vs. S2-3	245906_at	AT5G11070	hypothetical protein;(source:Araport11)	103.63	37.24	2.78	2.16E-05	1.28E-04
4534	7	F2-3 vs. S2-3	248461_s_at	AT5G50950	Encodes a fumarase enzyme initially shown to be in the mitochondria through proteomic studies but later shown to be present in the cytosol using an RFP fluorescent protein tag. It appears to be important for the accumulation of fumarate from malate in leaves in the light, and helps to promote nitrogen assimilation under high nitrogen conditions. It does not appear to be necessary for lipid metabolism and seedling growth. Inhibition of fumarate accumulation results in an overall shift in the cold response of leaves, with a complete inhibition of cold acclimation of photosynthesis.	839.33	301.76	2.78	4.10E-06	4.02E-05
4535	7	F2-3 vs. S2-3	252441_at	AT3G46780	plastid transcriptionally active 16;(source:Araport11)	38.51	13.89	2.77	1.44E-04	5.12E-04
4536	7	F2-3 vs. S2-3	264317_at	AT1G70310	Spermidine synthase.	77.51	28.09	2.76	2.65E-05	1.49E-04
4537	7	F2-3 vs. S2-3	245724_at	AT1G73390	Endosomal targeting BRO1-like domain-containing protein;(source:Araport11)	60.21	21.8	2.76	1.75E-04	5.95E-04
4538	7	F2-3 vs. S2-3	257271_at	AT3G28007	Nodulin MtN3 family protein;(source:Araport11)	314.43	113.92	2.76	4.80E-06	4.48E-05
4539	7	F2-3 vs. S2-3	255435_at	AT4G03280	Encodes the Rieske FeS center of cytochrome b6f complex. Gene is expressed in shoot but not in root. Mutant has reduced electron transport at saturating light intensities and Q-cycle activity is hypersensitive to acidification of the thylakoid lumen. The mRNA is cell-to-cell mobile.	2655.11	960.35	2.76	3.90E-06	3.86E-05
4540	7	F2-3 vs. S2-3	253963_at	AT4G26470	Calcium-binding EF-hand family protein;(source:Araport11)	29.15	10.55	2.76	5.17E-05	2.43E-04
4541	7	F2-3 vs. S2-3	247780_at	AT5G58770	AtCPT7 synthesizes medium-chain polyprenols of approximately 55 carbons in length. The enzyme utlizes geranylgeranyl pyrophosphate (GGPP) and isopentenyl pyrophosphate (IPP) as substrates. The enzymatic product accumulates into plastdial membranes (DOI:10.1105/tpc.16.00796).	36.42	13.18	2.76	7.20E-06	5.94E-05
4542	7	F2-3 vs. S2-3	265642_at	AT2G27450	Encodes N-carbamoylputrescine amidohydrolase that is involved in putrescine and polyamine biosynthesis.	111.35	40.42	2.75	7.50E-06	6.08E-05
4543	7	F2-3 vs. S2-3	258809_at	AT3G04070	NAC domain containing protein 47;(source:Araport11)	45.75	16.64	2.75	1.52E-05	1.00E-04
4544	7	F2-3 vs. S2-3	249469_at	AT5G39320	UDP-glucose 6-dehydrogenase family protein;(source:Araport11)	210.26	76.34	2.75	3.70E-06	3.73E-05
4545	7	F2-3 vs. S2-3	247047_at	AT5G66650	Chloroplast localized mitochondrial calcium uniporter.	91.28	33.19	2.75	4.11E-04	1.14E-03
4546	7	F2-3 vs. S2-3	256020_at	AT1G58290	Encodes a protein with glutamyl-tRNA reductase (GluTR) activity, catalyzing the NADPH-dependent reduction of Glu-tRNA(Glu) to glutamate 1-semialdehyde (GSA) with the release of free tRNA(Glu). It is involved in the early steps of chlorophyll biosynthesis.	138.46	50.61	2.74	5.40E-06	4.85E-05
4547	7	F2-3 vs. S2-3	259054_at	AT3G03480	acetyl CoA:(Z)-3-hexen-1-ol acetyltransferase;(source:Araport11)	36.64	13.37	2.74	5.80E-06	5.07E-05
4548	7	F2-3 vs. S2-3	258408_at	AT3G17630	member of Putative Na+/H+ antiporter family	33.47	12.21	2.74	8.30E-05	3.40E-04
4549	7	F2-3 vs. S2-3	253625_at	AT4G30600	signal recognition particle receptor alpha subunit family protein;(source:Araport11)	991.77	362.1	2.74	5.50E-06	4.90E-05
4550	7	F2-3 vs. S2-3	246841_at	AT5G26700	RmlC-like cupins superfamily protein;(source:Araport11)	31.19	11.38	2.74	1.73E-04	5.90E-04
4551	7	F2-3 vs. S2-3	248749_at	AT5G47880	Encodes a eukaryotic release factor 1 homolog. Cosuppression of the gene's expression results affects cell elongation of the inflorescence stem, specifically the internodes, and radial cell division. Expression of the protein is primarily observed in the vascular system and in actively growing and elongating zones.	95.29	34.82	2.74	5.32E-05	2.48E-04
4552	7	F2-3 vs. S2-3	247038_at	AT5G67160	Encodes a member of the BAHD acyltransferase superfamily. Mutants have enhanced susceptibility to virulent and avirulent pathogens and are defective in pathogen induced SA biosynthesis. EPS1 may act upstream of SA biosynthesis as application of SA can rescue the mutant phenotype.	309.27	112.82	2.74	2.10E-06	2.53E-05
4553	7	F2-3 vs. S2-3	266983_at	AT2G39400	alpha/beta-Hydrolases superfamily protein;(source:Araport11)	32.78	12	2.73	1.64E-05	1.05E-04
4554	7	F2-3 vs. S2-3	267393_at	AT2G44500	O-fucosyltransferase family protein;(source:Araport11)	40.93	15.02	2.73	1.68E-05	1.07E-04
4555	7	F2-3 vs. S2-3	252414_at	AT3G47420	Encodes a Pi starvation-responsive protein AtPS3. A member of the phosphate starvation-induced glycerol-3-phosphate permease gene family: AT3G47420(G3Pp1), AT4G25220(G3Pp2), AT1G30560(G3Pp3), AT4G17550(G3Pp4) and AT2G13100(G3Pp5). Its expression is responsive to phosphate (Pi) and not phosphite (Phi) in roots and shoots.	119.24	43.65	2.73	8.51E-04	2.00E-03
4556	7	F2-3 vs. S2-3	255552_at	AT4G01850	S-adenosylmethionine synthetase 2;(source:Araport11)	891.95	326.45	2.73	4.54E-05	2.21E-04
4557	7	F2-3 vs. S2-3	254848_at	AT4G11960	Encodes PGRL1B, a transmembrane protein present in thylakoids. PGRL1B has a highly homologous isoform PGRL1A encoded by At4g22890. Plants lacking PGRL1 show perturbation of cyclic electron flow, similar to PGR5-deficient plants. PGRL1 and PGR5 interact physically and associate with PSI (photosystem I).	64.95	23.77	2.73	6.29E-05	2.77E-04
4558	7	F2-3 vs. S2-3	254187_at	AT4G23890	NAD(P)H-quinone oxidoreductase subunit S;(source:Araport11)	342.07	125.33	2.73	2.92E-05	1.59E-04
4559	7	F2-3 vs. S2-3	247222_at	AT5G64840	member of GCN subfamily	835.61	305.88	2.73	6.31E-04	1.58E-03

4560	7	F2-3 vs. S2-3	264394_at	AT1G11860	Glycine cleavage T-protein family;(source:Araport11)	899.59	331.17	2.72	1.16E-05	8.35E-05
4561	7	F2-3 vs. S2-3	263678_at	AT1G04420	NAD(P)-linked oxidoreductase superfamily protein;(source:Araport11)	107.85	39.86	2.71	2.15E-04	6.94E-04
4562	7	F2-3 vs. S2-3	256299_at	AT1G69530	Member of Alpha-Expansin Gene Family. Naming convention from the Expansin Working Group (Kende et al, Plant Mol Bio). Involved in the formation of nematode-induced syncytia in roots of Arabidopsis thaliana.	60.66	22.35	2.71	1.85E-05	1.14E-04
4563	7	F2-3 vs. S2-3	245060_at	AT2G39770	Encodes a GDP-mannose pyrophosphorylase/ mannose-1-pyrophosphatase. This enzyme provides GDP-mannose, which is used for cell wall carbohydrate biosynthesis and protein glycosylation as well as for ascorbate (vitamin C) biosynthesis. Mutations in this gene confer hypersensitivity to NH4+.	178.48	65.8	2.71	1.72E-05	1.09E-04
4564	7	F2-3 vs. S2-3	259189_at	AT3G01700	Encodes an arabinogalactan protein that is expressed in pollen, pollen sac and pollen tube. Loss of AGP11 function results in decreased fertility due to defects in pollen tube growth.	31.57	11.64	2.71	4.96E-05	2.35E-04
4565	7	F2-3 vs. S2-3	251598_at	AT3G57600	encodes a member of the DREB subfamily A-2 of ERF/AP2 transcription factor family. The protein contains one AP2 domain. There are eight members in this subfamily including DREB2A AND DREB2B that are involved in response to drought.	36.04	13.28	2.71	6.00E-06	5.18E-05
4566	7	F2-3 vs. S2-3	253048_at	AT4G37560	Acetamidase/Formamidase family protein;(source:Araport11)	49.1	18.11	2.71	3.46E-04	9.97E-04
4567	7	F2-3 vs. S2-3	260950_s_a	AT1G06090	Fatty acid desaturase family protein;(source:Araport11)	71.31	26.37	2.7	7.50E-06	6.08E-05
4568	7	F2-3 vs. S2-3	256017_at	AT1G19180	JAZ1 is a nuclear-localized protein involved in jasmonate signaling. JAZ1 transcript levels rise in response to a jasmonate stimulus. JAZ1 can interact with the COI1 F-box subunit of an SCF E3 ubiquitin ligase in a yeast-two-hybrid assay only in the presence of jasmonate-isoleucine (JA-ILE) or coronatine. Application of jasmonate methyl ester to Arabidopsis roots reduces the levels of a JAZ1:GUS fusion protein, presumably by stimulating ubiquitin-proteasome-mediated degradation. The Jas domain appears to be important for JAZ1-COI1 interactions in the presence of coronatine. Two positive residues (R205 and R206) in the Jas domain shown to be important for coronatine -dependent COI1 binding are not required for binding AtMYC2. The mRNA is cell-to-cell mobile.	355.15	131.4	2.7	9.59E-04	2.21E-03
4569	7	F2-3 vs. S2-3	264280_at	AT1G61820	beta glucosidase 46;(source:Araport11)	24.18	8.95	2.7	4.11E-04	1.14E-03
4570	7	F2-3 vs. S2-3	250356_at	AT5G11710	EPSIN1 plays an important role in the vacuolar trafficking of soluble proteins at the trans-Golgi network via its interaction with gamma-ADR, VTI11, VSR1, and clathrin. Associated with actin filaments and with the Golgi complex. Expressed in most tissues.The mRNA is cell-to-cell mobile.	165.1	61.29	2.69	5.57E-04	1.44E-03
4571	7	F2-3 vs. S2-3	262645_at	AT1G62750	Nuclear encoded protein consists of the five domains conserved in EF-G proteins, with two GTP-binding sites in the first domain, and an additional transit peptide at the N-terminus. Localized in chloroplasts. Point mutation results in a delay in the onset of germination. At early developmental stage embryos still contain undifferentiated proplastids. The greening of cotyledons is severely impaired in light-grown mutant sco1 seedlings, whereas the following true leaves develop normally as in wild-type plants.	506.23	188.58	2.68	1.22E-05	8.58E-05
4572	7	F2-3 vs. S2-3	263956_at	AT2G35940	Encodes a member of the BEL-like homeodomain protein family. Ecotopic expression in the embryo sac leads to defects in nuclear migration and cellularization and embryo sacs with multiple egg cells. Loss of function alleles have no female gametophyte defects. The ecotopic expression phenotype requires KNAT3 because it can be suppressed by loss of KNAT3 function alleles. Localized to the nucleus but interaction with OFP1 relocates it to the cytoplasm.	99.5	37.11	2.68	1.35E-05	9.22E-05
4573	7	F2-3 vs. S2-3	249208_at	AT5G42650	Encodes a member of the cytochrome p450 CYP74 gene family that functions as an allene oxide synthase. This enzyme catalyzes dehydration of the hydroperoxide to an unstable allene oxide in the JA biosynthetic pathway. It shows a dual catalytic activity, the major one being a 13-AOS but also expressing a 9-AOS activity. CFA-Leu, CFA-Val, CFA-Met and CFA-Ala can induce the expression of AOS.	66.92	25	2.68	1.14E-05	8.32E-05
4574	7	F2-3 vs. S2-3	248576_at	AT5G49810	Arabidopsis thaliana methionine S-methyltransferase, an enzyme that catalyzes S -methylmethionine formation. The mRNA is cell-to-cell mobile.	108.99	40.68	2.68	4.40E-06	4.21E-05
4575	7	F2-3 vs. S2-3	248404_at	AT5G51460	homologous to the C-terminal part of microbial trehalose-6-phosphate phosphatases	49.92	18.63	2.68	3.58E-04	1.03E-03

4576	7	F2-3 vs. S2-3	252984_at	AT4G37990	Encodes an aromatic alcohol:NADP+ oxidoreductase whose mRNA levels are increased in response to treatment with a variety of phytopathogenic bacteria. Though similar to mannitol dehydrogenases, this enzyme does not have mannitol dehydrogenase activity.	72.42	27.08	2.67	2.12E-04	6.88E-04
4577	7	F2-3 vs. S2-3	264652_at	AT1G08920	Encodes ESL1, a transporter for monosaccharides.	100.28	37.68	2.66	8.49E-05	3.45E-04
4578	7	F2-3 vs. S2-3	257004_s_a	AT3G14130	Aldolase-type TIM barrel family protein;(source:Araport11)	175.5	65.87	2.66	4.10E-06	4.02E-05
4579	7	F2-3 vs. S2-3	256711_at	AT3G30390	Encodes a putative amino acid transporter.	839.51	315.04	2.66	5.62E-05	2.58E-04
4580	7	F2-3 vs. S2-3	263250_at	AT2G31390	Encodes a member of the fructokinase gene family. Nomenclature according to Riggs 2017 has been adopted for the family by the community (personal communication, Boernke, Callis, Granot, Boernke, and Smeekens).	105.66	39.87	2.65	3.20E-06	3.37E-05
4581	7	F2-3 vs. S2-3	266607_at	AT2G46300	Late embryogenesis abundant (LEA) hydroxyproline-rich glycoprotein family;(source:Araport11)	38.53	14.56	2.65	7.61E-04	1.82E-03
4582	7	F2-3 vs. S2-3	251930_at	AT3G53780	RHOMBOID-like protein 4;(source:Araport11)	426.04	161.03	2.65	4.80E-06	4.48E-05
4583	7	F2-3 vs. S2-3	255259_at	AT4G05020	Miitochondrial alternative NADH dehydrogenase.	100.75	38.23	2.64	1.70E-04	5.83E-04
4584	7	F2-3 vs. S2-3	259430_at	AT1G01610	bifunctional sn-glycerol-3-phosphate 2-O-acyltransferase/phosphatase. Involved in cutin assembly and is functionally redundant with GPAT8.	593.99	226.2	2.63	1.39E-05	9.43E-05
4585	7	F2-3 vs. S2-3	262801_at	AT1G21010	poly polymerase;(source:Araport11)	56.1	21.34	2.63	1.67E-05	1.07E-04
4586	7	F2-3 vs. S2-3	258264_at	AT3G15790	Protein containing methyl-CpG-binding domain.Has sequence similarity to human MBD proteins.	50.95	19.38	2.63	9.54E-05	3.77E-04
4587	7	F2-3 vs. S2-3	256061_at	AT1G07040	plant/protein;(source:Araport11)	106.17	40.47	2.62	5.85E-05	2.64E-04
4588	7	F2-3 vs. S2-3	262879_at	AT1G64860	Subunit of chloroplast RNA polymerase, confers the ability to recognize promoter sequences on the core enzyme	212.39	81.09	2.62	4.50E-05	2.19E-04
4589	7	F2-3 vs. S2-3	260227_at	AT1G74450	Plants overexpressing At1g74450 are stunted in height and have reduced male fertility.	150.6	57.52	2.62	5.49E-05	2.54E-04
4590	7	F2-3 vs. S2-3	252430_at	AT3G47470	Encodes a chlorophyll a/b-binding protein that is more similar to the PSI Cab proteins than the PSII cab proteins. The predicted protein is about 20 amino acids shorter than most known Cab proteins.	2019.43	770.84	2.62	7.90E-06	6.34E-05
4591	7	F2-3 vs. S2-3	246968_at	AT5G24870	RING/U-box superfamily protein;(source:Araport11)	47.05	17.96	2.62	3.23E-04	9.45E-04
4592	7	F2-3 vs. S2-3	248474_at	AT5G50850	Transketolase family protein;(source:Araport11)	845.18	322.02	2.62	1.79E-05	1.11E-04
4593	7	F2-3 vs. S2-3	247694_at	AT5G59750	monofunctional riboflavin biosynthesis protein RIBA 3;(source:Araport11)	169.75	64.76	2.62	4.40E-06	4.21E-05
4594	7	F2-3 vs. S2-3	262505_at	AT1G21680	DPP6 N-terminal domain-like protein;(source:Araport11)	102.55	39.33	2.61	2.68E-04	8.19E-04
4595	7	F2-3 vs. S2-3	260616_at	AT1G53280	Encodes a homolog of animal DJ-1 superfamily protein. In the A. thaliana genome, three genes encoding close homologs of human DJ-1 were identified AT3G14990 (DJ1A), AT1G53280 (DJ1B) and AT4G34020 (DJ1C). Among the three homologs, DJ1C is essential for chloroplast development and viability. It exhibits glyoxalase activity towards glyoxal and methylglyoxal.	738.49	283.03	2.61	1.75E-05	1.10E-04
4596	7	F2-3 vs. S2-3	267005_at	AT2G34460	NAD(P)-binding Rossmann-fold superfamily protein;(source:Araport11)	175.34	67.12	2.61	1.50E-05	9.94E-05
4597	7	F2-3 vs. S2-3	251301_at	AT3G61880	Encodes a cytochrome p450 monooxygenase. Overexpression of this gene allows fruit growth independently of fertilization. The gene is normally expressed only in floral organs(during the Arabidopsis stage 14 flower) and in the funiculus at anthesis.	45.41	17.43	2.61	1.03E-04	4.00E-04
4598	7	F2-3 vs. S2-3	260968_at	AT1G12250	Pentapeptide repeat-containing protein;(source:Araport11)	118.39	45.58	2.6	1.08E-04	4.16E-04
4599	7	F2-3 vs. S2-3	261363_at	AT1G41830	SKU5-similar 6;(source:Araport11)	300.71	115.74	2.6	8.18E-05	3.36E-04
4600	7	F2-3 vs. S2-3	255702_at	AT4G00230	xylem serine peptidase 1;(source:Araport11)	81.07	31.23	2.6	1.39E-05	9.43E-05
4601	7	F2-3 vs. S2-3	254485_at	AT4G20760	NAD(P)-binding Rossmann-fold superfamily protein;(source:Araport11)	62.25	23.96	2.6	3.84E-05	1.95E-04
4602	7	F2-3 vs. S2-3	248590_at	AT5G49660	The gene encodes receptorlike kinase (RLK). Involved in the maintenance organization of cell files or cell morphology in conductive elements. Functions as a receptor for CEP1 peptide. Mediates nitrate uptake signaling.	94.87	36.55	2.6	4.01E-05	2.02E-04
4603	7	F2-3 vs. S2-3	246392_at	AT1G58120	hypothetical protein;(source:Araport11)	40.05	15.45	2.59	2.08E-04	6.81E-04
4604	7	F2-3 vs. S2-3	266391_at	AT2G41290	Although this enzyme is predicted to encode a strictosidine synthase (SS), it lacks a conserved catalytic glutamate residue found in active SS enzymes and it is not expected to have SS activity.	195.01	75.37	2.59	6.20E-06	5.30E-05
4605	7	F2-3 vs. S2-3	250339_at	AT5G11670	The malic enzyme (EC 1.1.1.40) encoded by AtNADP-ME2 is presumably a cytosolic enzyme involved in malate metabolism and possibly assisting the oxidative pentose phosphate pathway. AtNADP-ME2 counts for the major part of NADP-ME activity in mature tissues of Arabidopsis.	623.7	240.92	2.59	6.50E-06	5.49E-05
4606	7	F2-3 vs. S2-3	249701_at	AT5G35460	membrane protein;(source:Araport11)	247.25	95.34	2.59	2.30E-06	2.69E-05
4607	7	F2-3 vs. S2-3	248531_at	AT5G49950	alpha/beta-Hydrolases superfamily protein;(source:Araport11)	247.75	95.51	2.59	4.40E-06	4.21E-05

4608	7	F2-3 vs. S2-3	260222_at	AT1G74380	xyloglucan xylosyltransferase 5;(source:Araport11)	112.52	43.57	2.58	2.75E-04	8.35E-04
4609	7	F2-3 vs. S2-3	267612_at	AT2G26690	Major facilitator superfamily protein;(source:Araport11)	54.65	21.19	2.58	1.45E-05	9.72E-05
4610	7	F2-3 vs. S2-3	266363_at	AT2G41250	Haloacid dehalogenase-like hydrolase (HAD) superfamily protein;(source:Araport11)	51.81	20.09	2.58	2.03E-05	1.23E-04
4611	7	F2-3 vs. S2-3	254343_at	AT4G21990	Encodes a protein disulfide isomerase-like (PDIL) protein, a member of a multigene family within the thioredoxin (TRX) superfamily. This protein also belongs to the adenosine 5'-phosphosulfate reductase-like (APRL) group.	348.55	134.93	2.58	6.89E-05	2.95E-04
4612	7	F2-3 vs. S2-3	256387_at	AT3G06170	Serinc-domain containing serine and sphingolipid biosynthesis protein;(source:Araport11)	414.01	160.93	2.57	1.64E-05	1.05E-04
4613	7	F2-3 vs. S2-3	258063_at	AT3G14620	putative cytochrome P450 The mRNA is cell-to-cell mobile.	38.71	15.05	2.57	5.60E-05	2.57E-04
4614	7	F2-3 vs. S2-3	255011_at	AT4G10040	Encodes cytochrome c. Promoter directs preferential expression in vascular tissues of cotyledons, leaves, roots, and hypocotyls, and in anthers. Double mutants with CYTC-1 accumulate starch during the day, have delayed growth and development and reduced GA and DELLA proteins linking cellular metabolism and GA homeostasis.	149.87	58.3	2.57	9.90E-06	7.47E-05
4615	7	F2-3 vs. S2-3	253009_at	AT4G37930	Encodes a protein with mitochondrial serine hydroxymethyltransferase activity, which functions in the photorespiratory pathway, catalyzes the conversion of serine and tetrahydrofolate to glycine and 5,10-methylene tetrahydrofolate. Involved in controlling cell damage caused by abiotic stress, such as high light and salt and the hypersensitive defense response of plants.	2404.87	933.96	2.57	4.50E-06	4.27E-05
4616	7	F2-3 vs. S2-3	248580_at	AT5G49890	member of Anion channel protein family	327.11	127.29	2.57	3.30E-06	3.44E-05
4617	7	F2-3 vs. S2-3	262073_at	AT1G59640	A basic helix-loop-helix encoding gene (BIGPETAL, BPE) involved in the control of petal size. BPE is expressed via two mRNAs derived from an alternative splicing event. The BPEub (AT1G59640.1)transcript is expressed ubiquitously, whereas the BPEp (AT1G59640.2) transcript is preferentially expressed in petals. Plants that lack the petal-expressed variant BPEp have larger petals as a result of increased cell size. BPEp is positively regulated downstream of APETALA3, PISTILLATA, APETALA1 and PISTILLATA3 and is negatively regulated downstream of AGAMOUS.	80.24	31.29	2.56	2.15E-04	6.93E-04
4618	7	F2-3 vs. S2-3	260385_at	AT1G74090	encodes a desulfoglucosinolate sulfotransferase, involved in the final step of glucosinolate core structure biosynthesis. Has a broad-substrate specificity with preference with methionine-derived desulfoglucosinolates.	160.7	62.88	2.56	8.50E-06	6.72E-05
4619	7	F2-3 vs. S2-3	258619_at	AT3G02780	Encodes a protein with isopentenyl diphosphate:dimethylallyl diphosphate isomerase activity. There is genetic evidence that it functions in the mevalonate, but not the MEP biosynthetic pathway.	444.96	173.65	2.56	1.42E-05	9.57E-05
4620	7	F2-3 vs. S2-3	258449_s_at	AT3G22310	Sequence similarity ot DEAD-box RNA helicases. Binds RNA and DNA. Involved in drought, salt and cold stress responses.The mRNA is cell-to-cell mobile. Functions as a host factor to promote TuMV replication.	551.04	215.66	2.56	5.39E-05	2.50E-04
4621	7	F2-3 vs. S2-3	AFFX-r2-At-Actin-5_s_at	AT5G09810	Member of Actin gene family.Mutants are defective in germination and root growth. The mRNA is cell-to-cell mobile.	75.32	29.58	2.55	1.22E-04	4.53E-04
4622	7	F2-3 vs. S2-3	265742_at	AT2G01290	Cytosolic ribose-5-phosphate isomerase. Knockout mutation causes chloroplast dysfunction, late flowering and premature cell death.	40.06	15.71	2.55	5.60E-05	2.57E-04
4623	7	F2-3 vs. S2-3	258727_at	AT3G11930	Adenine nucleotide alpha hydrolases-like superfamily protein;(source:Araport11)	209.21	81.95	2.55	2.10E-06	2.53E-05
4624	7	F2-3 vs. S2-3	251455_at	AT3G60100	citrate synthase 5;(source:Araport11)	34.94	13.7	2.55	4.40E-06	4.21E-05
4625	7	F2-3 vs. S2-3	247320_at	AT5G64040	Encodes the only subunit of photosystem I located entirely in the thylakoid lumen. May be involved in the interaction between plastocyanin and the photosystem I complex. Phosphorylation of this protein is dependent on calcium.	1676.28	656.51	2.55	5.10E-06	4.67E-05
4626	7	F2-3 vs. S2-3	253358_at	AT4G32940	Encodes a vacuolar processing enzyme belonging to a novel group of cysteine proteinases that is expressed in vegetative organs and is upregulated in association with various types of cell death and under stressed conditions. They are essential in processing seed storage proteins and for mediating the susceptible response of toxin-induced cell death.	260.19	102.63	2.54	4.00E-06	3.95E-05
4627	7	F2-3 vs. S2-3	262323_at	AT1G64190	6-phosphogluconate dehydrogenase family protein;(source:Araport11)	486.23	192.47	2.53	7.59E-05	3.18E-04
4628	7	F2-3 vs. S2-3	252394_at	AT3G47940	DNAJ heat shock family protein;(source:Araport11)	63.93	25.25	2.53	7.10E-06	5.88E-05
4629	7	F2-3 vs. S2-3	245296_at	AT4G16370	Encodes a phloem-specific iron transporter that is essential for systemic iron signaling and redistribution of iron and cadmium. It loads iron into the phloem, facilitates iron recirculation from the xylem to the phloem, and regulates both shoot-to-root iron signaling and iron redistribution from mature to developing tissues.	477.8	189.12	2.53	2.11E-04	6.85E-04

4630	7	F2-3 vs. S2-3	246516_at	AT5G15740	RRT1 is a member of a novel glycosyltransferase famly in plants. It functions as a rhamnosyltransferase, elongating the RG-1 backbone. It functions during seed coat mucilage development.	132.36	52.37	2.53	4.30E-04	1.18E-03
4631	7	F2-3 vs. S2-3	247979_at	AT5G56750	AGB1/AGG dimmer interacting protein, response to water deficit.	38.19	15.07	2.53	5.90E-06	5.12E-05
4632	7	F2-3 vs. S2-3	260730_at	AT1G48030	Encodes a mitochondrial lipoamide dehydrogenase whose expression is induced by light.	393.19	156.28	2.52	1.17E-05	8.37E-05
4633	7	F2-3 vs. S2-3	265658_at	AT2G13810	ALD1 is a L-lysine alpha-aminotransferase. It is part of the pipecolic acid biosynthetic pathway, where it catalyzes the biochemical conversion of lysine to epsilon-amino-alpha-ketocaproic acid (KAC) which is subject to subsequent transamination, cyclization and isomerization to form 2,3-dehydropipecolic acid.	50.61	20.07	2.52	7.36E-05	3.09E-04
4634	7	F2-3 vs. S2-3	267040_at	AT2G34300	S-adenosyl-L-methionine-dependent methyltransferases superfamily protein;(source:Araport11)	268.85	106.5	2.52	1.12E-04	4.25E-04
4635	7	F2-3 vs. S2-3	257253_at	AT3G24190	Protein kinase superfamily protein;(source:Araport11)	80.48	31.99	2.52	8.86E-05	3.58E-04
4636	7	F2-3 vs. S2-3	253162_at	AT4G35630	Encodes a phosphoserine aminotransferase which is involved in serine biosynthesis in the chloroplast which operates via the phosphorylated pathway. The mRNA is cell-to-cell mobile.	1401.74	558.02	2.51	6.90E-06	5.76E-05
4637	7	F2-3 vs. S2-3	259363_at	AT1G13270	Encodes a methionine aminopeptidase formerly called MAP1B, renamed to MAP1C.	471.97	189.1	2.5	5.50E-06	4.90E-05
4638	7	F2-3 vs. S2-3	260328_at	AT1G80360	Encodes a methionine-specific aminotransferase that uses the ethylene biosynthetic intermediate methionine as an amino donor and the auxin biosynthetic intermediate indole-3-pyruvic acid as an amino acceptor to produce L-tryptophan and 2-oxo-4-methylthiobutyric acid. These actions allow VAS1 to coordinate both auxin and ethylene biosynthesis. It functions downstream of TAA1/SAV3 but upstream of YUCs to negatively modulate IAA biosynthesis directly by altering the 3-IPA pool.	168.14	67.37	2.5	1.38E-05	9.39E-05
4639	7	F2-3 vs. S2-3	266097_at	AT2G37970	SOUL heme-binding family protein;(source:Araport11)	168.75	67.41	2.5	1.17E-05	8.37E-05
4640	7	F2-3 vs. S2-3	256624_at	AT3G19990	E3 ubiquitin-protein ligase;(source:Araport11)	175.53	70.15	2.5	6.59E-05	2.86E-04
4641	7	F2-3 vs. S2-3	251854_at	AT3G54800	Pleckstrin homology (PH) and lipid-binding START domains-containing protein;(source:Araport11)	24.86	9.96	2.5	3.37E-05	1.78E-04
4642	7	F2-3 vs. S2-3	251649_at	AT3G57330	Lesion mimic phenotype when mutation in the gene is combined with a mutation in ACA4. Lesion mimic phenotype of double knockout can be suppressed by nutritional supplements that increase anion levels (e.g. 15 mM Nitrate, Chloride, or Phosphate)	99.18	39.64	2.5	1.80E-05	1.12E-04
4643	7	F2-3 vs. S2-3	252886_at	AT4G39350	Encodes a cellulose synthase isomer, related to CESA6. As inferred from the null role of secondary wall-type CesAs, included in a set of five primary wall-type CesAs that may support trichome cell wall thickening. The mRNA is cell-to-cell mobile.	64.45	25.8	2.5	1.22E-05	8.58E-05
4644	7	F2-3 vs. S2-3	246038_s_a	AT5G19460	nudix hydrolase homolog 20;(source:Araport11)	139.65	55.95	2.5	8.90E-06	6.93E-05
4645	7	F2-3 vs. S2-3	265768_at	AT2G48020	Encodes a zinc transporter ZIF2. Expression of ZIF2 is regulated by alternative splicing.	802.32	322.79	2.49	3.10E-06	3.28E-05
4646	7	F2-3 vs. S2-3	252872_at	AT4G40010	encodes a member of SNF1-related protein kinases (SnRK2) whose activity is activated by ionic (salt) and non-ionic (mannitol) osmotic stress.	177.68	71.33	2.49	2.37E-04	7.45E-04
4647	7	F2-3 vs. S2-3	255720_at	AT1G32060	phosphoribulokinase;(source:Araport11)	59.43	24.01	2.48	5.82E-04	1.49E-03
4648	7	F2-3 vs. S2-3	262179_at	AT1G77980	Encodes a member of the MIKC (MADS box, Keratin binding domain, and C terminal domain containing)family of transcriptional regulators. AGL66 is expressed in pollen.It forms heterodimers with other MICK family members (AGL104). Involved in late stages of pollen development and pollen tube growth.	41.99	16.9	2.48	1.76E-05	1.10E-04
4649	7	F2-3 vs. S2-3	263348_at	AT2G05710	Encodes an aconitase that can catalyze the conversion of citrate to isocitrate through a cis-aconitate intermediate, indicating that it may participate in the TCA cycle and other primary metabolic pathways. The protein is believed to accumulate in the mitochondria and the cytosol. It affects CSD2 (At2g28190 - a superoxide dismutase) transcript levels and may play a role in the response to oxidative stress. One member of the family (ACO1 - At35830) was shown to specifically bind to the 5' UTR of CSD2 in vitro. ACO3 is tyrosine-phosphorylated and its phosphorylation state is modulated in response to ABA in Arabidopsis thaliana seeds. The mRNA is cell-to-cell mobile.	437.87	176.86	2.48	2.17E-05	1.29E-04

4650	7	F2-3 vs. S2-3	261211_at	AT1G12780	Encodes a UDP-glucose epimerase that catalyzes the interconversion of the sugar nucleotides UDP-glucose UDP-galactose via a UDP-4-keto-hexose intermediate. Responsive to stress.	638.77	258.59	2.47	5.96E-04	1.52E-03
4651	7	F2-3 vs. S2-3	264284_at	AT1G61860	Protein kinase superfamily protein;(source:Araport11)	29.53	11.95	2.47	4.59E-05	2.23E-04
4652	7	F2-3 vs. S2-3	257954_at	AT3G21760	Encodes HYR1, a UDP glycosyltransferase (UGT). HYR1 glucosylates hypostatin, an inhibitor of cell expansion in vivo to form a bioactive glucoside.	60.56	24.53	2.47	5.26E-04	1.38E-03
4653	7	F2-3 vs. S2-3	247741_at	AT5G58960	Mutant plants display impaired light-regulation of the hypocotyl randomization response.	905.89	366.12	2.47	1.91E-04	6.36E-04
4654	7	F2-3 vs. S2-3	259527_at	AT1G12600	UDP-N-acetylglucosamine (UAA) transporter family;(source:Araport11)	79.05	32.19	2.46	1.81E-04	6.11E-04
4655	7	F2-3 vs. S2-3	261135_at	AT1G19610	Predicted to encode a PR (pathogenesis-related) protein. Belongs to the plant defensin (PDF) family with the following members: At1g75830/PDF1.1, At5g44420/PDF1.2a, At2g26020/PDF1.2b, At5g44430/PDF1.2c, At2g26010/PDF1.3, At1g19610/PDF1.4, At1g55010/PDF1.5, At2g02120/PDF2.1, At2g02100/PDF2.2, At2g02130/PDF2.3, At1g61070/PDF2.4, At5g63660/PDF2.5, At2g02140/PDF2.6, At5g38330/PDF3.1 and At4g30070/PDF3.2.	39.74	16.13	2.46	2.80E-06	3.09E-05
4656	7	F2-3 vs. S2-3	245981_at	AT5G13100	Gap junction beta-4 protein;(source:Araport11)	401.14	163.02	2.46	7.50E-06	6.08E-05
4657	7	F2-3 vs. S2-3	266156_at	AT2G28110	Homolog to AT5G22940, a member of glycosyltransferase family 47 that is involved in secondary cell wall biosynthesis. It exhibits high sequence similarity to tobacco (Nicotiana plumbaginifolia) pectin glucuronyltransferase. Protein has a domain that shares significant similarity with the pfam03016 domain. It is expressed specifically in developing vessels and fiber cells, and FRA8 is targeted to Golgi. Mutants have irregular xylem formation, reduced cellulose levels and plants are smaller than normal siblings.	41.14	16.77	2.45	4.23E-05	2.11E-04
4658	7	F2-3 vs. S2-3	260967_at	AT1G12230	Aldolase superfamily protein;(source:Araport11)	252.2	103.3	2.44	3.64E-05	1.89E-04
4659	7	F2-3 vs. S2-3	263598_at	AT2G01850	EXGT-A3 has homology to xyloglucan endotransglucosylases/hydrolases (XTHs). Mutants in this gene show a lesion mimic phenotype associated with leaf maturation and a reduction in the number of tertiary veins. Individual tracheary elements in the mutants are shorter, but phloem transport activity is not severely affected. EXGT-A3 plays a role in xyloglucan degradation in the differentiating tracheary elements of rosette leaves. The mRNA is cell-to-cell mobile.	221.23	90.52	2.44	4.16E-05	2.08E-04
4660	7	F2-3 vs. S2-3	255587_at	AT4G01480	Encodes a protein that might have inorganic pyrophosphatase activity.	49.19	20.12	2.44	5.60E-06	4.97E-05
4661	7	F2-3 vs. S2-3	254608_at	AT4G18910	Encodes an aquaporin homolog. Functions in arsenite transport and tolerance.When expressed in yeast cells can conduct hydrogen peroxide into those cells.	36.98	15.18	2.44	2.95E-05	1.60E-04
4662	7	F2-3 vs. S2-3	249343_at	AT5G40650	One of three isoforms of the iron-sulfur component of the succinate dehydrogenase complex, a component of the mitochondrial respiratory chain complex II. The product of the nuclear encoded gene is imported into the mitochondrion. Expressed during germination and post-germinative growth.	1215.82	499.17	2.44	5.03E-05	2.38E-04
4663	7	F2-3 vs. S2-3	248164_at	AT5G54490	Encodes a PINOID (PID)-binding protein containing putative EF-hand calcium-binding motifs. The interaction is dependent on the presence of calcium. mRNA expression is up-regulated by auxin. Not a phosphorylation target of PID, likely acts upstream of PID to regulate the activity of this protein in response to changes in calcium levels.	103.06	42.27	2.44	1.07E-04	4.10E-04
4664	7	F2-3 vs. S2-3	264609_at	AT1G04530	Encodes one of the 36 carboxylate clamp (CC)-tetratricopeptide repeat (TPR) proteins (Prasad 2010, Pubmed ID: 20856808) with potential to interact with Hsp90/Hsp70 as co-chaperones.	151.25	62.35	2.43	2.90E-05	1.58E-04
4665	7	F2-3 vs. S2-3	262637_at	AT1G06640	encodes a protein whose sequence is similar to a 2-oxoglutarate-dependent dioxygenase The mRNA is cell-to-cell mobile.	283.29	116.72	2.43	1.16E-05	8.35E-05
4666	7	F2-3 vs. S2-3	262850_at	AT1G14920	Similar to a putative transcription factor and transcriptional coactivators. Repressor of GA responses and involved in gibberellic acid mediated signaling. Member of the DELLA proteins that restrain the cell proliferation and expansion that drives plant growth. The protein undergoes degradation in response to GA via the 26S proteasome. GAI may be involved in reducing ROS accumulation in response to stress by up-regulating the transcription of superoxide dismutases. Represses GA-induced vegetative growth and floral initiation. Rapidly degraded in response to GA.	635.66	261.32	2.43	4.79E-04	1.28E-03
4667	7	F2-3 vs. S2-3	264000_at	AT2G22500	Encodes one of the mitochondrial dicarboxylate carriers (DIC): DIC1 (AT2G22500), DIC2 (AT4G24570), DIC3 (AT5G09470).	488.86	200.9	2.43	3.04E-04	9.04E-04

4668	7	F2-3 vs. S2-3	245053_at	AT2G26450	Plant invertase/pectin methylesterase inhibitor superfamily;(source:Araport11)	26.21	10.79	2.43	4.28E-04	1.17E-03
4669	7	F2-3 vs. S2-3	267423_at	AT2G35060	potassium transporter	62.76	25.8	2.43	5.52E-04	1.43E-03
4670	7	F2-3 vs. S2-3	253512_at	AT4G31750	Encodes HopW1-1-Interacting protein 2 (WIN2). Interacts with the P. syringae effector HopW1-1. WIN2 has protein phosphatase activity. Modulates plant defenses against bacteria. Three WIN proteins are identified so far (WIN1: AT1G80600; WIN2: AT4G31750; WIN3: AT5G13320).	405.99	167.24	2.43	1.49E-05	9.94E-05
4671	7	F2-3 vs. S2-3	247304_at	AT5G63850	Amino acid transporter whose expression is downregulated by dehydration.	44.27	18.25	2.43	1.96E-04	6.48E-04
4672	7	F2-3 vs. S2-3	245015_at	ATCG00490	large subunit of RUBISCO. Protein is tyrosine-phosphorylated and its phosphorylation state is modulated in response to ABA in Arabidopsis thaliana seeds.	2820.07	1158.41	2.43	4.50E-05	2.19E-04
4673	7	F2-3 vs. S2-3	262428_at	AT1G47570	RING/U-box superfamily protein;(source:Araport11)	152.58	63.14	2.42	1.55E-04	5.45E-04
4674	7	F2-3 vs. S2-3	263368_at	AT2G20470	AGC (cAMP-dependent, cGMP-dependent and protein kinase C) kinase family protein;(source:Araport11)	50.09	20.66	2.42	1.63E-04	5.64E-04
4675	7	F2-3 vs. S2-3	245216_at	AT4G16143	Protein interacts with Agrobacterium proteins VirD2 and VirE2. Is not individually essential for Agrobacterium-mediated root transformation, but when overexpressed can rescue the impa-4 decreased transformation susceptibility phenotype.	68.97	28.49	2.42	2.23E-05	1.31E-04
4676	7	F2-3 vs. S2-3	248467_at	AT5G50800	Encodes a member of the SWEET sucrose efflux transporter family proteins, together with RPG1, it is involved in pollen development. Together with SWEET14, it is likely involved in modulating the GA response and is required for proper development of anthers, seeds and seedlings.	59.76	24.68	2.42	2.67E-05	1.50E-04
4677	7	F2-3 vs. S2-3	264738_at	AT1G62250	orotidine 5-phosphate decarboxylase;(source:Araport11)	79.86	33.2	2.41	6.62E-05	2.87E-04
4678	7	F2-3 vs. S2-3	264848_at	AT2G17350	beta-mannosyltransferase-like protein;(source:Araport11)	225.79	93.85	2.41	3.02E-04	9.00E-04
4679	7	F2-3 vs. S2-3	263985_at	AT2G42750	DNAJ heat shock N-terminal domain-containing protein;(source:Araport11)	312.89	129.66	2.41	2.03E-05	1.23E-04
4680	7	F2-3 vs. S2-3	258188_at	AT3G17800	mRNA level of the MEB5.2 gene (At3g17800) remains unchanged after cutting the inflorescence stem	183.79	76.26	2.41	6.28E-05	2.77E-04
4681	7	F2-3 vs. S2-3	251557_at	AT3G58730	vacuolar ATP synthase subunit D (VATD) / V-ATPase D subunit / vacuolar proton pump D subunit (VATPD);(source:Araport11)	153.06	63.43	2.41	8.60E-06	6.77E-05
4682	7	F2-3 vs. S2-3	253788_at	AT4G28680	Encodes a stress-induced tyrosine decarboxylase (TyrDC). Recombinant (His)6-TyrDC expressed in E. coli catalyzes the conversion of L-tyrosine to tyramine. Recombinant TyrDC forms tetramers.	46.09	19.11	2.41	1.78E-05	1.11E-04
4683	7	F2-3 vs. S2-3	253191_at	AT4G35350	tracheary element vacuolar protein	178.47	74.15	2.41	6.66E-05	2.88E-04
4684	7	F2-3 vs. S2-3	250204_at	AT5G13990	A member of EXO70 gene family, putative exocyst subunits, conserved in land plants. Arabidopsis thaliana contains 23 putative EXO70 genes, which can be classified into eight clusters on the phylogenetic tree. This particular member is expressed in pollen and is involved in pollen tube elongation. Found in the cytoplasm and surprisingly, not found in the plasma membrane and is not found to colocalize with or interact with core exocyst subunits.	36.75	15.23	2.41	3.21E-05	1.71E-04
4685	7	F2-3 vs. S2-3	259736_at	AT1G64390	glycosyl hydrolase 9C2;(source:Araport11)	277.94	115.91	2.4	2.52E-04	7.76E-04
4686	7	F2-3 vs. S2-3	264102_at	AT1G79270	evolutionarily conserved C-terminal region 8;(source:Araport11)	24.05	10.03	2.4	7.60E-06	6.14E-05
4687	7	F2-3 vs. S2-3	266898_at	AT2G45990	ribosomal RNA small subunit methyltransferase G;(source:Araport11)	375.56	156.46	2.4	4.47E-04	1.21E-03
4688	7	F2-3 vs. S2-3	256328_at	AT3G02360	6-phosphogluconate dehydrogenase family protein;(source:Araport11)	607.09	253.18	2.4	3.80E-05	1.94E-04
4689	7	F2-3 vs. S2-3	258605_at	AT3G02970	EXORDIUM like 6;(source:Araport11)	23.58	9.82	2.4	4.41E-05	2.17E-04
4690	7	F2-3 vs. S2-3	258647_at	AT3G07870	FBX92 is an F-box containing protein. Overexpression produces plants with smaller leaves while reduced expression is correlated with increased leaf size and increased rates of cell proliferation.	35.21	14.69	2.4	8.90E-06	6.93E-05
4691	7	F2-3 vs. S2-3	258406_at	AT3G17611	RHOMBOID-like protein 14;(source:Araport11)	139.54	58.1	2.4	3.09E-04	9.14E-04
4692	7	F2-3 vs. S2-3	252395_at	AT3G47950	mutant has Slight reduction in root and shoot growth; Exaggerated defects in salt stress; Plasma Membrane H+ ATPase	106.82	44.44	2.4	6.41E-05	2.81E-04
4693	7	F2-3 vs. S2-3	253277_at	AT4G34230	Encodes a catalytically active cinnamyl alcohol dehydrogenase which uses p-coumaryl aldehyde as a preferred substrate. It can also use sinapyl, caffeyl, coniferyl and d-hydroxyconiferyl aldehydes as substrates.	149.85	62.53	2.4	1.49E-05	9.94E-05
4694	7	F2-3 vs. S2-3	262526_at	AT1G17050	Encodes one of the two paralogous solanesyl diphosphate synthases - SPS1 (At1g78510) and SPS2 (At1g17050) - that assemble the side-chain of plastoquinone-9 in plastids.	89.17	37.35	2.39	1.75E-05	1.10E-04
4695	7	F2-3 vs. S2-3	260308_at	AT1G70610	member of TAP subfamily	124.23	51.98	2.39	7.00E-04	1.72E-03

4696	7	F2-3 vs. S2-3	258336_at	AT3G16050	Encodes a protein with pyridoxal phosphate synthase activity whose transcripts were detected mostly in roots and accumulate during senescence. The protein was found in very low abundance, which prevented a specific localisation.	369.9	155.09	2.39	1.55E-04	5.44E-04
4697	7	F2-3 vs. S2-3	257021_at	AT3G19710	Belongs to the branched-chain amino acid aminotransferase gene family. Encodes a methionine-oxo-acid transaminase. Involved in the methionine chain elongation pathway that leads to the ultimate biosynthesis of methionine-derived glucosinolates.	31.15	13.05	2.39	4.85E-04	1.29E-03
4698	7	F2-3 vs. S2-3	245286_at	AT4G14880	Encodes a cytosolic isoform of cytosolic O-acetylserine(thiol)lyase, a key enzyme in cysteine biosynthesis and for the fixation of inorganic sulfide. It catalyzes the formation of cysteine from O-acetylserine and inorganic sulfide. Gene expression is predominant in the root cortex and the xylem parenchyma. Gene expression is induced in leave, stems and roots by high salt and heavy metal stresses, mediated by ABA. Lines carrying semi-dominant mutations exhibit early senescence. Required for pollen tube growth and/or fertilization.	552.4	231.56	2.39	1.58E-05	1.03E-04
4699	7	F2-3 vs. S2-3	252911_at	AT4G39510	member of CYP96A	88.97	37.3	2.39	2.42E-05	1.39E-04
4700	7	F2-3 vs. S2-3	247720_at	AT5G59290	Encodes an isoform of UDP-glucuronic acid decarboxylase, which is predicted to be cytosolic by PSORT. This enzyme produces UDP-xylose, which is a substrate for many cell wall carbohydrates including hemicellulose and pectin. UDP-xylose is also known to feedback regulate several cell wall biosynthetic enzymes.	573.81	240.48	2.39	6.22E-05	2.75E-04
4701	7	F2-3 vs. S2-3	257194_at	AT3G13110	Encodes a mitochondrial serine O-acetyltransferase involved in sulfur assimilation and cysteine biosynthesis. Expressed in the vascular system.	133.02	55.82	2.38	7.00E-06	5.84E-05
4702	7	F2-3 vs. S2-3	245285_s_a	AT4G14040	selenium-binding protein 2;(source:Araport11)	39.05	16.44	2.38	1.60E-04	5.56E-04
4703	7	F2-3 vs. S2-3	245292_at	AT4G15093	catalytic LigB subunit of aromatic ring-opening dioxygenase family;(source:Araport11)	147.83	62.03	2.38	4.20E-06	4.10E-05
4704	7	F2-3 vs. S2-3	254462_at	AT4G20150	excitatory amino acid transporter;(source:Araport11)	401.91	169.08	2.38	2.71E-04	8.26E-04
4705	7	F2-3 vs. S2-3	247289_at	AT5G64290	dicarboxylate transport 2.1;(source:Araport11)	213.29	89.45	2.38	1.03E-05	7.67E-05
4706	7	F2-3 vs. S2-3	265244_at	AT2G43020	Encodes a polyamine oxidase.	123.61	52.11	2.37	8.04E-05	3.33E-04
4707	7	F2-3 vs. S2-3	258181_at	AT3G21670	Major facilitator superfamily protein;(source:Araport11)	52.7	22.28	2.37	2.49E-04	7.73E-04
4708	7	F2-3 vs. S2-3	255483_at	AT4G02500	Encodes a protein with xylosyltransferase activity, which is specific for UDP-xylose as donor substrate and for oligosaccharides with a degree of polymerization >4. Although the enzyme utilizes either cellopentaose or cellohexaose, its activity is four-fold higher with cellohexaose as an acceptor compared to cellopentaose. The enzyme is able to add several xylosyl residues to the acceptor forming mono-, di- and trixylosylated polysaccharides. The mRNA is cell-to-cell mobile.	558.93	236.12	2.37	1.20E-04	4.48E-04
4709	7	F2-3 vs. S2-3	248237_at	AT5G53890	Encodes a leucine-rich repeat receptor kinase (LRR-RK) involved in the perception of phytosulfokine (PSK), which is a 5-aa tyrosine-sulfated peptide that primarily promotes cellular proliferation.	34.38	14.5	2.37	1.75E-04	5.95E-04
4710	7	F2-3 vs. S2-3	262781_s_a	AT1G13060	Encodes 20S proteasome beta subunit PBE1 (PBE1).	1411.54	597.64	2.36	4.06E-05	2.05E-04
4711	7	F2-3 vs. S2-3	263799_at	AT2G24550	major centromere autoantigen B-like protein;(source:Araport11)	36.89	15.61	2.36	7.68E-05	3.21E-04
4712	7	F2-3 vs. S2-3	246036_at	AT5G08370	Member of Glycoside Hydrolase Family 27 (GH27)that functions as an α-galactosidase.	50.94	21.58	2.36	3.59E-04	1.03E-03
4713	7	F2-3 vs. S2-3	255380_at	AT4G03560	Encodes a depolarization-activated Ca(2+) channel. Anti-sense experiments with this gene as well as Sucrose-H(+) symporters and complementation of yeast sucrose uptake mutant cch1 suggest that this protein mediates a voltage-activated Ca(2+)influx. Mutants lack detectable SV channel activity suggesting TPC1 is essential component of the SV channel. Patch clamp analysis of loss of function mutation indicates TPC1 does not affect Ca2+ signaling in response to abiotic and biotic stress.	170.61	72.66	2.35	1.54E-05	1.01E-04
4714	7	F2-3 vs. S2-3	253567_at	AT4G31230	kinase with adenine nucleotide alpha hydrolases-like domain-containing protein;(source:Araport11)	26.8	11.4	2.35	3.83E-05	1.95E-04
4715	7	F2-3 vs. S2-3	248153_at	AT5G54250	member of Cyclic nucleotide gated channel family, downstream component of the signaling pathways leading to HR resistance. mutant plants exhibit gene-for-gene disease resistance against avirulent Pseudomonas syringae despite the near-complete absence of the hypersensitive response (HR). Salicylic acid accumulation in dnd2 mutants is completely PAD4-independent.	67.1	28.6	2.35	6.81E-04	1.68E-03
4716	7	F2-3 vs. S2-3	262571_at	AT1G15430	hypothetical protein (DUF1644);(source:Araport11)	54.92	23.45	2.34	6.01E-05	2.69E-04

4717	7	F2-3 vs. S2-3	258500_at	AT3G02470	Encodes a S-adenosylmethionine decarboxylase involved in polyamine biosynthesis.	361.72	154.62	2.34	1.10E-05	8.08E-05
4718	7	F2-3 vs. S2-3	252027_at	AT3G52850	Encodes the Vacuolar Sorting Receptor-1 (VSR-1)/Epidermal Growth Factor Receptor-like protein1(VSR-1/ATELP1). Binds vacuolar targeting signals. Involved in sorting seed storage proteins into vacuoles. The mRNA is cell-to-cell mobile.	365.81	156.61	2.34	5.88E-05	2.65E-04
4719	7	F2-3 vs. S2-3	252896_at	AT4G39480	member of CYP96A	40.35	17.24	2.34	1.58E-04	5.52E-04
4720	7	F2-3 vs. S2-3	246555_at	AT5G15470	Encodes a protein with putative galacturonosyltransferase activity.	30.94	13.2	2.34	4.41E-05	2.17E-04
4721	7	F2-3 vs. S2-3	264668_at	AT1G09780	Encodes a 2,3-biphosphoglycerate-independent phosphoglycerate mutase that is involved in pollen development and stomatal movement.	822.02	352.15	2.33	4.16E-05	2.08E-04
4722	7	F2-3 vs. S2-3	259545_at	AT1G20560	acyl activating enzyme 1;(source:Araport11)	125.41	53.84	2.33	1.52E-05	1.00E-04
4723	7	F2-3 vs. S2-3	262744_at	AT1G28680	Catalyses trans-cis isomerization and lactonization in the biosynthesis of coumarins in roots.	110.43	47.3	2.33	6.01E-05	2.69E-04
4724	7	F2-3 vs. S2-3	256144_at	AT1G48630	Encodes a protein with similarity to mammalian RACKs. RACKs function to shuttle activated protein kinase C to different subcellular sites and may also function as a scaffold through physical interactions with other proteins. RACK1B has no phenotype on its own and probably acts redundantly with RACK1A and RACK1C.	527.53	226.78	2.33	9.45E-04	2.18E-03
4725	7	F2-3 vs. S2-3	260592_at	AT1G55850	encodes a protein similar to cellulose synthase The mRNA is cell-to-cell mobile.	46.82	20.05	2.33	9.55E-05	3.77E-04
4726	7	F2-3 vs. S2-3	260284_at	AT1G80380	encodes a glycerate kinase which catalyzes the last step of photorespiration C2 cycle.	118.81	51.06	2.33	3.87E-05	1.96E-04
4727	7	F2-3 vs. S2-3	266979_at	AT2G39470	PsbP-like protein 2;(source:Araport11)	61.64	26.41	2.33	8.54E-05	3.47E-04
4728	7	F2-3 vs. S2-3	249863_at	AT5G22950	SNF7 family protein;(source:Araport11)	189.82	81.62	2.33	8.40E-06	6.65E-05
4729	7	F2-3 vs. S2-3	248375_at	AT5G51710	member of Putative potassium proton antiporter family	189.01	81.13	2.33	1.25E-05	8.72E-05
4730	7	F2-3 vs. S2-3	245000_at	ATCG00210	electron transporter, transferring electrons within cytochrome b6/f complex of photosystem II;(source:Araport11)	157.52	67.49	2.33	6.07E-05	2.71E-04
4731	7	F2-3 vs. S2-3	260481_at	AT1G10960	ferredoxin 1;(source:Araport11)	1105.15	477.21	2.32	2.98E-04	8.88E-04
4732	7	F2-3 vs. S2-3	259603_at	AT1G56500	Encodes a thylakoid membrane protein with thioredoxin-like and beta-propeller domains located in the lumen and a haloacid-dehalogenase domain exposed to the chloroplast stroma. The protein Primes role may be to prevent formation of a slowly reversible form of antenna quenching, thereby maintaining the efficiency of light harvesting.The mRNA is cell-to-cell mobile.	122.66	52.83	2.32	3.41E-05	1.79E-04
4733	7	F2-3 vs. S2-3	265763_at	AT2G48060	piezo-type mechanosensitive ion channel component;(source:Araport11)	265.71	114.59	2.32	4.60E-06	4.33E-05
4734	7	F2-3 vs. S2-3	251386_at	AT3G60800	Encodes a protein S-acyltransferase that, together with PAT13, cooperatively regulates leaf senescence.	52.88	22.79	2.32	2.44E-04	7.58E-04
4735	7	F2-3 vs. S2-3	250254_at	AT5G13710	SMT1 controls the level of cholesterol in plants	1402.73	603.56	2.32	3.77E-05	1.93E-04
4736	7	F2-3 vs. S2-3	244962_at	ATCG01050	Represents a plastid-encoded subunit of a NAD(P)H dehydrogenase complex. Its mRNA is edited at four positions. Translation data is not available for this gene.	35.37	15.22	2.32	2.15E-04	6.94E-04
4737	7	F2-3 vs. S2-3	264302_at	AT1G78900	Encodes catalytic subunit A of the vacuolar ATP synthase. Mutants are devoid of vacuolar ATPase activity as subunit A is encoded only by this gene and show strong defects in male gametophyte development and in Golgi stack morphology. The mRNA is cell-to-cell mobile.	897.59	389.36	2.31	2.48E-05	1.42E-04
4738	7	F2-3 vs. S2-3	258298_at	AT3G23300	S-adenosyl-L-methionine-dependent methyltransferases superfamily protein;(source:Araport11)	38.32	16.6	2.31	2.12E-05	1.27E-04
4739	7	F2-3 vs. S2-3	254657_s_a	AT4G18220	Drug/metabolite transporter superfamily protein;(source:Araport11)	511.62	221.85	2.31	2.61E-04	8.01E-04
4740	7	F2-3 vs. S2-3	254417_at	AT4G21470	Bifunctional enzyme that catalyzes hydrolysis of FMN to riboflavin, and phosphorylation of riboflavin to FMN. The mRNA is cell-to-cell mobile.	165.2	71.47	2.31	4.78E-05	2.29E-04
4741	7	F2-3 vs. S2-3	253079_s_a	AT4G36190	Serine carboxypeptidase S28 family protein;(source:Araport11)	148.73	64.27	2.31	5.19E-04	1.36E-03
4742	7	F2-3 vs. S2-3	248248_at	AT5G53120	encodes a novel spermine synthase and is a paralog of previously characterized spermidine synthases, SPDS1 and SPDS2. SPDS3 forms heterodimers with SDPS2, which in turn forms heterodimers with SDPS1 in vivo. The gene does not complement speDelta3 deficiency of spermidine synthase in yeast but DOES complement speDelta4 deficiency.	934.25	405.03	2.31	3.64E-05	1.89E-04
4743	7	F2-3 vs. S2-3	248193_at	AT5G54080	Encodes a homogentisate 1,2-dioxygenase that can convert homogentisate to malylacetoacetate and is likely to be involved in tyrosine catabolism.	319.08	137.97	2.31	4.04E-04	1.12E-03
4744	7	F2-3 vs. S2-3	260288_at	AT1G80530	Major facilitator superfamily protein;(source:Araport11)	544.51	236.95	2.3	4.43E-05	2.17E-04
4745	7	F2-3 vs. S2-3	256246_at	AT3G66658	Encodes a putative aldehyde dehydrogenase. The gene is not responsive to osmotic stress and is expressed constitutively at a low level in plantlets and root cultures.	109.72	47.68	2.3	3.02E-05	1.63E-04

4746	7	F2-3 vs. S2-3	253994_at	AT4G26080	Involved in abscisic acid (ABA) signal transduction. Negative regulator of ABA promotion of stomatal closure.	231.31	100.6	2.3	1.21E-05	8.58E-05
4747	7	F2-3 vs. S2-3	253200_at	AT4G34720	vacuolar H+-pumping ATPase 16 kDa proteolipid (ava-p1)	1320.05	573.7	2.3	8.42E-05	3.43E-04
4748	7	F2-3 vs. S2-3	263704_at	AT1G31130	polyadenylate-binding protein 1-B-binding protein;(source:Araport11)	118.33	51.6	2.29	9.21E-04	2.14E-03
4749	7	F2-3 vs. S2-3	245759_at	AT1G66900	alpha/beta-Hydrolases superfamily protein;(source:Araport11)	59.95	26.26	2.28	5.69E-04	1.47E-03
4750	7	F2-3 vs. S2-3	260101_at	AT1G73260	Encodes a trypsin inhibitor involved in modulating programmed cell death in plant-pathogen interactions.	47.2	20.71	2.28	2.62E-05	1.48E-04
4751	7	F2-3 vs. S2-3	266716_at	AT2G46820	Encodes the P subunit of Photosystem I. About 25% of the TMP14 pool appeared to be phosphorylated, and this ratio is not affected by light. Contains seven phosphorylation sites on threonine residue and chloroplast targeting signal. Located in the proximity of PSI-L, -H and -O subunits. Forms oligomers with other members of CURT1 family to modulate grana structure.	314.98	138.39	2.28	2.42E-05	1.39E-04
4752	7	F2-3 vs. S2-3	258690_at	AT3G07960	Encodes phosphatidylinositol-4-phosphate 5-kinase 6 (PIP5K6). Regulates clathrin-dependent endocytosis in pollen tubes.	46.13	20.19	2.28	1.63E-04	5.64E-04
4753	7	F2-3 vs. S2-3	253779_at	AT4G28490	Member of Receptor kinase-like protein family. Controls the separation step of floral organ abscission. The mRNA is cell-to-cell mobile.	24.81	10.89	2.28	1.23E-05	8.61E-05
4754	7	F2-3 vs. S2-3	265680_at	AT2G32150	Haloacid dehalogenase-like hydrolase (HAD) superfamily protein;(source:Araport11)	82.07	36.17	2.27	4.08E-04	1.13E-03
4755	7	F2-3 vs. S2-3	251086_at	AT5G01450	RING/U-box superfamily protein;(source:Araport11)	86.37	38.11	2.27	3.61E-04	1.03E-03
4756	7	F2-3 vs. S2-3	245686_at	AT5G22060	Co-chaperonin similar to E. coli DnaJ	580.64	256.22	2.27	3.84E-05	1.95E-04
4757	7	F2-3 vs. S2-3	248263_at	AT5G53370	pectin methylesterase PCR fragment F;(source:Araport11)	50.09	22.04	2.27	4.57E-05	2.22E-04
4758	7	F2-3 vs. S2-3	247523_at	AT5G61410	Arabidopsis thaliana ribulose-5-phosphate-3-epimerase mRNA	330.79	145.74	2.27	1.22E-05	8.58E-05
4759	7	F2-3 vs. S2-3	264365_s_a	AT1G03230	Eukaryotic aspartyl protease family protein;(source:Araport11)	102.74	45.56	2.26	6.46E-05	2.82E-04
4760	7	F2-3 vs. S2-3	261651_at	AT1G27760	Encodes a protein with similarity to human interferon-related developmental regulator (IFRD)that is involved in salt tolerance. Loss of function mutations are hypersensitive to salt stress and have reduced fertility. SAT32 is found in the cytoplasm but appears to translocate to the nucleus when plants are subject to salt stress.	75.36	33.35	2.26	7.47E-04	1.80E-03
4761	7	F2-3 vs. S2-3	263297_at	AT2G15310	A member of ARF GTPase family. A thaliana has 21 members of this family, known to be essential for vesicle coating and uncoating and functions in GTP-binding. Gene encoding ADP-ribosylation factor and similar to ADP-ribosylation factor (GI:861205) (Chlamydomonas reinhardtii), other ARFs and ARF-like proteins.	29.65	13.15	2.26	1.03E-04	4.01E-04
4762	7	F2-3 vs. S2-3	265628_at	AT2G27290	FAM210B-like protein, putative (DUF1279);(source:Araport11)	72.11	31.85	2.26	5.70E-04	1.47E-03
4763	7	F2-3 vs. S2-3	254446_at	AT4G20890	tubulin 9 The mRNA is cell-to-cell mobile.	268.97	119	2.26	1.00E-05	7.51E-05
4764	7	F2-3 vs. S2-3	254158_at	AT4G24380	dihydrofolate reductase;(source:Araport11)	133.31	58.96	2.26	2.37E-05	1.37E-04
4765	7	F2-3 vs. S2-3	254096_at	AT4G25150	HAD superfamily, subfamily IIIB acid phosphatase;(source:Araport11)	32.07	14.2	2.26	1.17E-05	8.37E-05
4766	7	F2-3 vs. S2-3	253455_at	AT4G32020	serine/arginine repetitive matrix-like protein;(source:Araport11)	82	36.34	2.26	4.63E-04	1.24E-03
4767	7	F2-3 vs. S2-3	250034_at	AT5G18280	Encodes an enzyme with ATPase and ADPase activity (an apyrase) that when mutated in combination with ATAPY1 causes a complete inhibition of pollen germination.	95.79	42.44	2.26	1.35E-05	9.22E-05
4768	7	F2-3 vs. S2-3	249025_at	AT5G44720	Molybdenum cofactor sulfurase family protein;(source:Araport11)	242.3	107.32	2.26	9.43E-05	3.75E-04
4769	7	F2-3 vs. S2-3	248804_at	AT5G47470	nodulin MtN21-like transporter family protein	49.08	21.71	2.26	7.34E-04	1.78E-03
4770	7	F2-3 vs. S2-3	248276_at	AT5G53550	YELLOW STRIPE like 3;(source:Araport11)	36.02	15.94	2.26	2.24E-05	1.31E-04
4771	7	F2-3 vs. S2-3	248104_at	AT5G55250	Encodes an enzyme which specifically converts IAA to its methyl ester form MeIAA. This gene belongs to the family of carboxyl methyltransferases whose members catalyze the transfer of the methyl group from S-adenosyl-L-methionine to carboxylic acid-containing substrates to form small molecule methyl esters. Expression of TCP genes is downregulated in mutant iamt1-D. SABATH methyltransferase.	79.53	35.2	2.26	9.62E-05	3.79E-04
4772	7	F2-3 vs. S2-3	263921_at	AT2G36460	Aldolase superfamily protein;(source:Araport11)	169.3	75.26	2.25	1.03E-04	4.00E-04
4773	7	F2-3 vs. S2-3	259105_at	AT3G05500	Encodes a protein that associates with lipid droplet surfaces and shares sequence homology with family of small rubber particle proteins. Plays dual roles as positive factors for tissue growth and development and in drought stress responses. The mRNA is cell-to-cell mobile.	268.08	119.13	2.25	6.93E-04	1.70E-03
4774	7	F2-3 vs. S2-3	257082_at	AT3G20580	COBRA-like protein 10 precursor;(source:Araport11)	24.29	10.81	2.25	1.25E-04	4.61E-04
4775	7	F2-3 vs. S2-3	253943_at	AT4G27030	Encodes an unusual palmitate desaturase that is highly substrate specific. It introduces a delta-3 trans double bond at palmitate at the sn-2 position of phosphatidylglycerol. The mRNA is cell-to-cell mobile.	33.03	14.7	2.25	1.59E-04	5.56E-04

4776	7	F2-3 vs. S2-3	248983_at	AT5G45130	small GTP binding protein. The mRNA is cell-to-cell mobile.	2583.06	1145.93	2.25	7.10E-06	5.88E-05
4777	7	F2-3 vs. S2-3	248796_at	AT5G47180	Plant VAMP (vesicle-associated membrane protein) family protein;(source:Araport11)	77.23	34.28	2.25	1.45E-05	9.72E-05
4778	7	F2-3 vs. S2-3	261191_at	AT1G32900	UDP-Glycosyltransferase superfamily protein;(source:Araport11)	1382.61	616.58	2.24	2.80E-04	8.45E-04
4779	7	F2-3 vs. S2-3	245731_at	AT1G73500	member of MAP Kinase Kinase family. Autophosphorylates and also phosphorylates MPK3 and MPK6. Independently involved in ethylene and calmalexin biosynthesis. Induces transcription of ACS2, ACS6, ERF1, ERF2, ERF5, ERF6, CYP79B2, CYP79B3, CYP71A13 and PAD3.	126.09	56.24	2.24	4.45E-05	2.18E-04
4780	7	F2-3 vs. S2-3	255834_at	AT2G33410	Belongs to a member of the RNA-binding glycine-rich (RBG) gene superfamily. The mRNA is cell-to-cell mobile.	537.55	240.01	2.24	4.31E-05	2.13E-04
4781	7	F2-3 vs. S2-3	251855_at	AT3G54690	Sugar isomerase (SIS) family protein;(source:Araport11)	295.1	131.62	2.24	1.75E-05	1.10E-04
4782	7	F2-3 vs. S2-3	247283_at	AT5G64250	Aldolase-type TIM barrel family protein;(source:Araport11)	119.53	53.43	2.24	1.39E-05	9.43E-05
4783	7	F2-3 vs. S2-3	262888_at	AT1G14790	Encodes RNA-dependent RNA polymerase. While not required for virus-induced post-transcriptional gene silencing (PTGS), it can promote turnover of viral RNAs in infected plants. Nomenclature according to Xie, et al. (2004). Involved in the production of Cucumber Mosaic Virus siRNAs.	126.82	56.94	2.23	1.19E-04	4.44E-04
4784	7	F2-3 vs. S2-3	261727_at	AT1G76090	Encodes S-adenosyl-methionine-sterol-C-methyltransferase, an enzyme in the sterol biosynthetic pathway.	57.66	25.83	2.23	2.20E-05	1.30E-04
4785	7	F2-3 vs. S2-3	259077_s_at	AT3G02230	RGP1 is a UDP-arabinose mutase that catalyzes the interconversion between the pyranose and furanose forms of UDP-L-arabinose. It appears to be required for proper cell wall formation. rgp1/rgp2 (at5g15650) double mutants have a male gametophyte lethal phenotype. RGP1 fusion proteins can be found in the cytosol and peripherally associated with the Golgi apparatus. The mRNA is cell-to-cell mobile.	4734.64	2126.31	2.23	5.40E-04	1.41E-03
4786	7	F2-3 vs. S2-3	256838_at	AT3G22950	A member of ARF GTPase family. A thaliana has 21 members of this family, known to be essential for vesicle coating and uncoating and functions in GTP-binding. Gene encoding ADP-ribosylation factor and similar to ADP-ribosylation factor GB:P91924 (Dugesia japonica), other ARFs and ARF-like proteins.	91.4	40.91	2.23	9.46E-05	3.75E-04
4787	7	F2-3 vs. S2-3	252368_at	AT3G48520	CYP94B3 is a jasmonoyl-isoleucine-12-hydroxylase that catalyzes the formation of 12-OH-JA-Ile from JA-Ile. By reducing the levels of this the biologically active phytohormone, CYP94B3 attenuates the jasmonic acid signaling cascade. CYP94B3 transcript levels rise in response to wounding.	96.98	43.41	2.23	6.61E-04	1.64E-03
4788	7	F2-3 vs. S2-3	253602_s_a	AT4G30910	Cytosol aminopeptidase family protein;(source:Araport11)	167.06	74.8	2.23	3.90E-04	1.09E-03
4789	7	F2-3 vs. S2-3	253274_at	AT4G34200	Encodes a 3-phosphoglycerate dehydrogenase that is essential for embryo and pollen development.	159.2	71.3	2.23	2.20E-04	7.06E-04
4790	7	F2-3 vs. S2-3	246429_at	AT5G17450	Heavy metal transport/detoxification superfamily protein;(source:Araport11)	28.2	12.64	2.23	3.09E-05	1.66E-04
4791	7	F2-3 vs. S2-3	247586_at	AT5G60660	A member of the plasma membrane intrinsic protein subfamily PIP2.When expressed in yeast cells can conduct hydrogen peroxide into those cells. Mutants exhibit longer root hairs.	100.07	44.94	2.23	6.22E-05	2.75E-04
4792	7	F2-3 vs. S2-3	247545_at	AT5G61530	small G protein family protein / RhoGAP family protein;(source:Araport11)	223.57	100.47	2.23	2.87E-05	1.57E-04
4793	7	F2-3 vs. S2-3	264346_at	AT1G12010	Encodes a protein that appears to have 1-amino-cyclopropane-1-carboxylic acid oxidase activity based on mutant analyses. The mRNA is cell-to-cell mobile.	81.77	36.85	2.22	8.47E-04	1.99E-03
4794	7	F2-3 vs. S2-3	258251_at	AT3G15810	LURP-one-like protein (DUF567);(source:Araport11)	87.12	39.33	2.22	1.99E-04	6.57E-04
4795	7	F2-3 vs. S2-3	245352_at	AT4G15490	Encodes a protein that might have sinapic acid:UDP-glucose glucosyltransferase activity.	47.1	21.22	2.22	1.13E-05	8.27E-05
4796	7	F2-3 vs. S2-3	253360_at	AT4G33090	encodes an aminopeptidase, a ortholog of mouse microsomal AP (EC 3.4.11.2).	661.59	298.25	2.22	2.44E-04	7.58E-04
4797	7	F2-3 vs. S2-3	247693_at	AT5G59730	A member of EXO70 gene family, putative exocyst subunits, conserved in land plants. Arabidopsis thaliana contains 23 putative EXO70 genes, which can be classified into eight clusters on the phylogenetic tree. The mRNA is cell-to-cell mobile.	100.88	45.36	2.22	9.20E-04	2.14E-03
4798	7	F2-3 vs. S2-3	247539_at	AT5G61710	cotton fiber protein;(source:Araport11)	28.92	13.03	2.22	3.79E-04	1.07E-03
4799	7	F2-3 vs. S2-3	260214_at	AT1G74510	Galactose oxidase/kelch repeat superfamily protein;(source:Araport11)	87.34	39.58	2.21	8.45E-05	3.44E-04
4800	7	F2-3 vs. S2-3	251397_at	AT3G60570	member of BETA-EXPANSINS. Naming convention from the Expansin Working Group (Kende et al, 2004. Plant Mol Bio)	69.15	31.29	2.21	1.25E-04	4.62E-04
4801	7	F2-3 vs. S2-3	251127_at	AT5G01080	Beta-galactosidase related protein;(source:Araport11)	36.36	16.44	2.21	4.84E-04	1.29E-03
4802	7	F2-3 vs. S2-3	260777_at	AT1G14560	Encodes a mitochondrial CoA transporter.	79.59	36.12	2.2	4.84E-05	2.31E-04
4803	7	F2-3 vs. S2-3	265190_at	AT1G23780	F-box family protein;(source:Araport11)	57.66	26.22	2.2	5.25E-04	1.38E-03

4804	7	F2-3 vs. S2-3	262175_at	AT1G74880	Encodes subunit NDH-O of NAD(P)H:plastoquinone dehydrogenase complex (Ndh complex) present in the thylakoid membrane of chloroplasts. This subunit is thought to be required for Ndh complex assembly.	37.62	17.12	2.2	4.56E-04	1.23E-03
4805	7	F2-3 vs. S2-3	245391_at	AT4G16520	Autophagy protein.	1720.01	781.24	2.2	1.05E-04	4.05E-04
4806	7	F2-3 vs. S2-3	252933_at	AT4G39110	bups1 and bups1/2 double mutants have reduced feritlity due to premature rupture of pollen tubes before they reach the ovule. BUSP1 interacts with RALF4/19 peptide ligands and ANX1/2 receptors. BUPS/ANX signaling may regulate and promote pollen tube growth.	29.02	13.21	2.2	2.11E-04	6.86E-04
4807	7	F2-3 vs. S2-3	250929_at	AT5G03290	Encodes a catalytic subunit of the mitochondrially-localized NAD+- dependent isocitrate dehydrogenase. The mRNA is cell-to-cell mobile.	1590.54	721.78	2.2	4.44E-05	2.17E-04
4808	7	F2-3 vs. S2-3	246958_at	AT5G24690	plant/protein, putative (DUF3411);(source:Araport11)	451.86	205.27	2.2	2.04E-04	6.71E-04
4809	7	F2-3 vs. S2-3	246755_at	AT5G27920	F-box family protein;(source:Araport11)	164.08	74.7	2.2	9.50E-06	7.29E-05
4810	7	F2-3 vs. S2-3	261788_at	AT1G15980	encodes a novel subunit of the chloroplast NAD(P)H dehydrogenase complex, involved in cyclic electron flow around photosystem I to produce ATP.	48.46	22.18	2.19	3.04E-04	9.04E-04
4811	7	F2-3 vs. S2-3	264931_at	AT1G60590	Pectin lyase-like superfamily protein;(source:Araport11)	46.12	21.02	2.19	2.38E-04	7.46E-04
4812	7	F2-3 vs. S2-3	258085_at	AT3G26100	Regulator of chromosome condensation (RCC1) family protein;(source:Araport11)	77.84	35.51	2.19	1.39E-04	4.98E-04
4813	7	F2-3 vs. S2-3	257150_at	AT3G27230	S-adenosyl-L-methionine-dependent methyltransferases superfamily protein;(source:Araport11)	122.9	56.1	2.19	6.64E-05	2.87E-04
4814	7	F2-3 vs. S2-3	256598_at	AT3G30180	Encodes a cytochrome p450 enzyme that catalyzes the last reaction in the production of brassinolide. It is capable of converting 6-deoxocastasterone into castasterone, a C-6 oxidation, as well as the further conversion of castasterone into brassinolide by a Baeyer-Villinger oxidation reaction at C-6, resulting in the formation of an unusual seven-membered lactone ring. The enzyme possesses high affinity for both C28- and C27-Brassinosteroids. The expression of the gene using a CYP85A2 promoter:LUC fusion construct was shown to be under circadian and light control.	44.12	20.17	2.19	3.50E-04	1.01E-03
4815	7	F2-3 vs. S2-3	252099_at	AT3G51250	Senescence/dehydration-associated protein-like protein;(source:Araport11)	192.2	87.61	2.19	2.10E-05	1.26E-04
4816	7	F2-3 vs. S2-3	251084_at	AT5G01520	Encodes a cytosolic RING-type E3 ubiquitin (Ub) ligase that is critical for ABA and high salinity responses during germination. AtAIRP2 and SDIR1 likely play a combinatory role in ABA signaling and the response to high salt in Arabidopsis.	30.95	14.12	2.19	9.20E-06	7.13E-05
4817	7	F2-3 vs. S2-3	248560_at	AT5G49970	encodes the bifunctional pyridoxine (pyridoxamine) 5?-phosphate oxidase (PPOX)(EC 1.4.3.5) that is involved in the formation of pyridoxal 5'-phosphate (member of the vitamin B6 group). NAD(P)HX epimerase (AT5G49970) interconverts the two epimers of NAD(P)HX.	172.21	78.75	2.19	1.25E-04	4.63E-04
4818	7	F2-3 vs. S2-3	264856_at	AT2G17370	Encodes a 3-hydroxy-3-methylglutaryl-CoA reductase (HMGR) that is involved in the synthesis of sterol and triterpenoid compounds.	35.87	16.43	2.18	5.07E-05	2.40E-04
4819	7	F2-3 vs. S2-3	245101_at	AT2G40890	encodes coumarate 3-hydroxylase (C3H), a P450-dependent monooxygenase. Involved in lignin biosynthesis and flavonoid biosynthesis. Also affects the biosynthesis of coumarins such as scopoletin and scopolin as a branching-out-pathway from the phenylpropanoid acid level.	259.6	119.21	2.18	8.00E-04	1.90E-03
4820	7	F2-3 vs. S2-3	258830_at	AT3G07090	PPPDE putative thiol peptidase family protein;(source:Araport11)	85.15	39.12	2.18	4.60E-04	1.24E-03
4821	7	F2-3 vs. S2-3	247488_at	AT5G61820	stress up-regulated Nod 19 protein;(source:Araport11)	89.75	41.21	2.18	2.29E-04	7.27E-04
4822	7	F2-3 vs. S2-3	264146_at	AT1G02205	Expression of the CER1 gene associated with production of stem epicuticular wax and pollen fertility. Biochemical studies showed that cer1 mutants are blocked in the conversion of stem wax C30 aldehydes to C29 alkanes, and they also lack the secondary alcohols and ketones. These suggested the CER1 protein is an aldehyde decarbonylase, but the exact molecular function of this protein remains to be determined.	297.36	136.78	2.17	2.29E-04	7.27E-04
4823	7	F2-3 vs. S2-3	261420_at	AT1G07720	Encodes KCS3, a member of the 3-ketoacyl-CoA synthase family involved in the biosynthesis of VLCFA (very long chain fatty acids).	22.72	10.49	2.17	1.71E-05	1.09E-04
4824	7	F2-3 vs. S2-3	261722_at	AT1G08510	Encodes an acyl-acyl carrier protein thioesterase. Hydrolyzes primarily saturated acyl-ACPs with chain lengths that vary between 8 and 18 carbons. Involved in saturated fatty acid synthesis. Nuclear-encoded, plastid-targeted globular protein that is functional as dimer.	321.83	148.21	2.17	2.90E-05	1.58E-04
4825	7	F2-3 vs. S2-3	260517_at	AT1G51420	sucrose-phosphatase 1;(source:Araport11)	50.31	23.2	2.17	2.91E-04	8.73E-04
4826	7	F2-3 vs. S2-3	258774_at	AT3G10740	Encodes a bifunctional alpha-l-arabinofuranosidase/beta-d-xylosidase that belongs to family 51 of glycoside hydrolases. It may be involved in cell wall modification.	35.76	16.46	2.17	4.33E-04	1.18E-03

4827	7	F2-3 vs. S2-3	253374_at	AT4G33140	Haloacid dehalogenase-like hydrolase (HAD) superfamily protein;(source:Araport11)	73.64	33.96	2.17	1.14E-05	8.32E-05
4828	7	F2-3 vs. S2-3	250404_at	AT5G10960	Polynucleotidyl transferase, ribonuclease H-like superfamily protein;(source:Araport11)	58.13	26.78	2.17	9.09E-05	3.64E-04
4829	7	F2-3 vs. S2-3	245979_at	AT5G13150	A member of EXO70 gene family, putative exocyst subunits, conserved in land plants. Arabidopsis thaliana contains 23 putative EXO70 genes, which can be classified into eight clusters on the phylogenetic tree. This particular member is expressed in pollen and, together with EXO70C2, is involved in pollen tube elongation. Found in the cytoplasm and surprisingly, not found in the plasma membrane.	28.55	13.16	2.17	5.26E-04	1.38E-03
4830	7	F2-3 vs. S2-3	263760_at	AT2G21280	A nuclear-encoded, plastid-targeted protein (AtSulA) whose overexpression causes severe yet stochastic plastid (shown in chloroplasts and leucoplasts) division defects. The protein does not appear to interact with either AtFtsZ proteins when studied in a yeast two-hybrid system.	100.38	46.42	2.16	2.15E-04	6.94E-04
4831	7	F2-3 vs. S2-3	256277_at	AT3G12120	Major enzyme responsible for the synthesis of 18:2 fatty acids in the endoplasmic reticulum. Contains His-rich motifs, which contribute to the interaction with the electron donor cytochrome b5. Mutations in this gene suppress the low temperature-induced phenotype of Arabidopsis tocopherol-deficient mutant vte2.	363.61	168.39	2.16	2.04E-04	6.71E-04
4832	7	F2-3 vs. S2-3	258151_at	AT3G18080	B-S glucosidase 44;(source:Araport11)	64.69	29.88	2.16	1.44E-05	9.67E-05
4833	7	F2-3 vs. S2-3	261623_at	AT1G01980	Encodes an oligogalacturonide oxidase that inactivates the elicitor-active oligogalacturonides (OGs).	30.02	13.94	2.15	1.31E-04	4.79E-04
4834	7	F2-3 vs. S2-3	262483_at	AT1G17220	Encodes a chloroplast localized protein with similarity to translation initiation factor 2. Can complement loss of INFB in E.coli suggesting FUG1 does function as a translation initiation factor in vivo. Identified as a suppressor of the leaf variegation mutant var2-6. Suppression is only seen in hypomorphs as complete loss of function alleles are embryo lethal. The mRNA is cell-to-cell mobile.	547.53	254.12	2.15	1.66E-05	1.06E-04
4835	7	F2-3 vs. S2-3	253203_at	AT4G34710	Encodes a arginine decarboxylase (ADC), a rate-limiting enzyme that catalyzes the first step of polyamine (PA) biosynthesis via ADC pathway in Arabidopsis thaliana. Arabidopsis genome has two ADC paralogs, ADC1 and ADC2. ADC2 is stress-inducible (osmotic stress). Double mutant analysis showed that ADC genes are essential for the production of PA, and are required for normal seed development. Overexpression causes phenotypes similar to GA-deficient plants and these plants show reduced levels of GA due to lower expression levels of AtGA20ox1, AtGA3ox3 and AtGA3ox1.	554.78	258.38	2.15	3.23E-04	9.45E-04
4836	7	F2-3 vs. S2-3	249377_at	AT5G40690	histone-lysine N-methyltransferase trithorax-like protein;(source:Araport11)	28.61	13.32	2.15	5.25E-05	2.46E-04
4837	7	F2-3 vs. S2-3	247286_at	AT5G64280	dicarboxylate transporter 2.2;(source:Araport11)	80.43	37.48	2.15	2.44E-04	7.59E-04
4838	7	F2-3 vs. S2-3	261226_at	AT1G20190	member of Alpha-Expansin Gene Family. Naming convention from the Expansin Working Group (Kende et al, 2004. Plant Mol Bio)	34.63	16.16	2.14	8.17E-04	1.94E-03
4839	7	F2-3 vs. S2-3	261373_at	AT1G53000	Encodes a mitochondrial-localized CMP-KDO (3-deoxy-D-manno-octulosonate) synthetase. This is the enzyme activating KDO as a nucleotide sugar prior to its incorporation into rhamnogalacturonan-II. Heterozygous mutants are defective in pollen development and in pollen tube elongation.	102.85	48.03	2.14	7.00E-04	1.72E-03
4840	7	F2-3 vs. S2-3	264959_at	AT1G77090	thylakoid lumenal protein (Mog1/PsbP/DUF1795-like photosystem II reaction center PsbP family protein);(source:Araport11)	102.64	47.97	2.14	3.63E-04	1.04E-03
4841	7	F2-3 vs. S2-3	254468_at	AT4G20460	NAD(P)-binding Rossmann-fold superfamily protein;(source:Araport11)	32.39	15.12	2.14	1.22E-05	8.58E-05
4842	7	F2-3 vs. S2-3	250206_at	AT5G14040	Encodes a mitochondrial phosphate transporter. Modulates plant responses to salt stress.	1843.2	859.62	2.14	5.90E-05	2.66E-04
4843	7	F2-3 vs. S2-3	245929_at	AT5G24760	GroES-like zinc-binding dehydrogenase family protein;(source:Araport11)	168.55	78.93	2.14	1.10E-04	4.19E-04
4844	7	F2-3 vs. S2-3	262626_at	AT1G06430	encodes a FtsH protease that is localized to the chloroplast	51.86	24.4	2.13	9.09E-05	3.64E-04
4845	7	F2-3 vs. S2-3	263419_at	AT2G17220	Encodes a putative serine/threonine-specific protein kinase kin3. Protein is N-myristoylated.	97.02	45.63	2.13	3.26E-04	9.51E-04
4846	7	F2-3 vs. S2-3	263528_at	AT2G24800	Peroxidase superfamily protein;(source:Araport11)	24.4	11.43	2.13	8.17E-05	3.36E-04

4847	7	F2-3 vs. S2-3	258677_at	AT3G08730	Encodes a protein-serine kinase that phosphorylates ribosomal protein in vitro. Activation of AtS6k is regulated by 1-naphthylacetic acid and kinetin, at least in part, via a lipid kinase-dependent pathway. Involved in translational up-regulation of ribosomal proteins. Phosphorylated by PDK1. Interacts with RAPTOR1, which in turn interacts with TOR. SPK6 activity is affected by osmotic stress, and plants overexpressing S6k1 are hypersensitive to osmotic stress. The gene is expressed in all tissues examined, with highest expression level detected in metabolically active tissues.	29.3	13.76	2.13	2.78E-04	8.40E-04
4848	7	F2-3 vs. S2-3	257745_at	AT3G29240	PPR containing protein (DUF179);(source:Araport11)	385.1	180.77	2.13	6.67E-04	1.65E-03
4849	7	F2-3 vs. S2-3	261560_at	AT1G01710	acyl-CoA thioesterase II;(source:Araport11)	118.34	55.77	2.12	3.12E-04	9.20E-04
4850	7	F2-3 vs. S2-3	261379_at	AT1G18720	ER membrane protein, putative (DUF962);(source:Araport11)	183.38	86.53	2.12	1.28E-04	4.70E-04
4851	7	F2-3 vs. S2-3	261107_at	AT1G63010	Encodes an SPX domain protein that transports Pi into the vacuole and is essential for phosphate homeostasis.	235.52	111.29	2.12	1.14E-04	4.32E-04
4852	7	F2-3 vs. S2-3	256673_at	AT3G52370	Fasciclin-like arabinogalactan protein. Possibly involved in embryogenesis and seed development.	89.63	42.36	2.12	2.41E-05	1.39E-04
4853	7	F2-3 vs. S2-3	254744_at	AT4G13345	Serinc-domain containing serine and sphingolipid biosynthesis protein;(source:Araport11)	30.96	14.6	2.12	2.30E-04	7.28E-04
4854	7	F2-3 vs. S2-3	262869_s_at	AT4G27630	Encodes a GPCR-type G protein receptor with nine predicted transmembrane domains. The protein binds abscisic acid (ABA) and is predicted to function as an ABA receptor. It has GTP-binding and GTPase activity and binds to ABA more effectively in the presence of GDP. GTG2 binds to GPA1, the alpha subunit of the heterotrimeric G protein. GPA1 (in its GTP-bound state) affects the GTP binding and GTPase activity of GTG2 and may act to down-regulate GTG2 binding to ABA. GTG2 is widely expressed throughout the plant and appears to be involved in the regulation of several ABA-dependent responses including seed germination, plant development, and promotion of stomatal closure. GTG2 transcript levels do not appear to change in response to ABA or abiotic stresses.	86.05	40.58	2.12	9.94E-04	2.28E-03
4855	7	F2-3 vs. S2-3	252939_at	AT4G39230	encodes a protein whose sequence is similar to phenylcoumaran benzylic ether reductase (PCBER), which catalyzes NADPH-dependent reduction of 8-5' linked lignans such as dehydrodiconiferyl alcohol to give isodihydrodehydrodiconiferyl alcohol.	38.73	18.29	2.12	3.78E-04	1.07E-03
4856	7	F2-3 vs. S2-3	249828_at	AT5G23250	Succinyl-CoA ligase, alpha subunit;(source:Araport11)	132.12	62.18	2.12	1.41E-05	9.54E-05
4857	7	F2-3 vs. S2-3	249346_at	AT5G40780	Encodes LHT1 (lysine histidine transporter), a high-affinity transporter for cellular amino acid uptake in both root epidermis and leaf mesophyll.	103.99	48.99	2.12	9.90E-06	7.47E-05
4858	7	F2-3 vs. S2-3	262813_at	AT1G11670	MATE efflux family protein;(source:Araport11)	30.51	14.44	2.11	4.67E-04	1.25E-03
4859	7	F2-3 vs. S2-3	264395_at	AT1G12070	Immunoglobulin E-set superfamily protein;(source:Araport11)	29.94	14.2	2.11	5.35E-05	2.49E-04
4860	7	F2-3 vs. S2-3	264223_s_a	AT1G67520	lectin protein kinase family protein;(source:Araport11)	23.27	11.05	2.11	1.45E-04	5.18E-04
4861	7	F2-3 vs. S2-3	249063_at	AT5G44110	Encodes a member of the NAP subfamily of ABC transporters whose expression pattern is regulated by light and sucrose.	186.19	88.15	2.11	5.50E-05	2.54E-04
4862	7	F2-3 vs. S2-3	245006_at	ATCG00340	Encodes the D1 subunit of photosystem I reaction center.	4204.96	1992.45	2.11	2.46E-04	7.64E-04
4863	7	F2-3 vs. S2-3	259896_at	AT1G71500	Encodes PSB33, a protein conserved in the plastid lineage. PSB33 is associated with the chloroplast thylakoid membrane and provides stability to Photosystem II. The mRNA is cell-to-cell mobile.	66.66	31.71	2.1	3.03E-04	9.02E-04
4864	7	F2-3 vs. S2-3	258748_at	AT3G05930	germin-like protein (GLP8)	39.33	18.71	2.1	1.32E-05	9.10E-05
4865	7	F2-3 vs. S2-3	256392_at	AT3G06260	Encodes a protein with putative galacturonosyltransferase activity.	27.08	12.89	2.1	2.51E-04	7.75E-04
4866	7	F2-3 vs. S2-3	257238_at	AT3G17430	Nucleotide/sugar transporter family protein The mRNA is cell-to-cell mobile.	123.75	58.81	2.1	5.84E-05	2.64E-04
4867	7	F2-3 vs. S2-3	256676_at	AT3G52180	Encodes a plant-specific glucan phosphatase that contains a noncatalytic carbohydrate-binding module as well as a dual specificity protein phosphatase domain. SEX4 can dephosphorylate C6- and C3-glucosyl residues on native starch grains and related maltodextrin compounds in vitro. This protein interacts with the plant SnRK AKIN11, binds starch, and is localized in the chloroplast. sex4 mutants have elevated levels of starch.	188.55	89.58	2.1	6.63E-04	1.65E-03
4868	7	F2-3 vs. S2-3	254165_at	AT4G24330	hypothetical protein (DUF1682);(source:Araport11)	89.32	42.62	2.1	3.71E-05	1.91E-04
4869	7	F2-3 vs. S2-3	254077_at	AT4G25640	Encodes a multidrug and toxin efflux family transporter. Involved in flavonoid metabolism, affecting Root growth, seed development and germination, and pollen development, release and viability.	202.53	96.53	2.1	2.50E-05	1.43E-04

4870	7	F2-3 vs. S2-3	249581_at	AT5G37600	encodes a cytosolic glutamine synthetase, the enzyme has high affinity with substrate ammonium	430.69	204.74	2.1	2.64E-04	8.07E-04
4871	7	F2-3 vs. S2-3	264602_at	AT1G04700	PB1 domain-containing protein tyrosine kinase;(source:Araport11)	25.72	12.33	2.09	4.59E-04	1.24E-03
4872	7	F2-3 vs. S2-3	261455_at	AT1G21070	Nucleotide-sugar transporter family protein;(source:Araport11)	34.4	16.49	2.09	1.53E-05	1.00E-04
4873	7	F2-3 vs. S2-3	262500_at	AT1G21760	This gene is predicted to encode an F-box protein that is evolutionarily conserved between Arabidopsis and other eukaryotes including S.cerevisiae and humans. It may play a role in regulating translation under conditions of temperature stress. FBP7 transcript levels are increased at high and low temperatures. The mRNA is cell-to-cell mobile.	66.87	31.94	2.09	2.69E-05	1.50E-04
4874	7	F2-3 vs. S2-3	264752_at	AT1G23010	Encodes a protein with multicopper oxidase activity. Located in ER. Function together with LPR2 (AT1G71040) and a P5-type ATPase (At5g23630/PDR2) in a common pathway that adjusts root meristem activity to Pi (inorganic phosphate) availability.	32.74	15.65	2.09	7.35E-05	3.09E-04
4875	7	F2-3 vs. S2-3	256209_at	AT1G50940	Encodes the electron transfer flavoprotein ETF alpha, a putative subunit of the mitochondrial electron transfer flavoprotein complex (ETF beta is At5g43430.1) in Arabidopsis. Mutations of the ETF beta gene results in accelerated senescence and early death compared to wild-type during extended darkness.	95.79	45.84	2.09	7.02E-05	2.99E-04
4876	7	F2-3 vs. S2-3	265807_at	AT2G17990	Calcium-dependent protein kinase 1 adaptor protein involved in vacuolar transport and lytic vacuole biogenesis.	29.79	14.27	2.09	1.16E-04	4.36E-04
4877	7	F2-3 vs. S2-3	265419_at	AT2G20840	Secretory carrier membrane protein (SCAMP) family protein;(source:Araport11)	509	244.03	2.09	3.42E-05	1.79E-04
4878	7	F2-3 vs. S2-3	267304_at	AT2G30080	member of Fe(II) transporter isolog family. Gene expression is not regulated by iron, copper, or zinc deficiency or excess.	36.91	17.64	2.09	9.23E-05	3.68E-04
4879	7	F2-3 vs. S2-3	266101_at	AT2G37940	Inositol phosphorylceramide synthase 2;(source:Araport11)	99.98	47.86	2.09	7.19E-04	1.75E-03
4880	7	F2-3 vs. S2-3	260567_at	AT2G43820	Encodes a nicotinate-O-glycosyltransferase. Induced by Salicylic acid, virus, fungus and bacteria. Also involved in the tryptophan synthesis pathway. Independent of NPR1 for their induction by salicylic acid. UGT74F1 transfers UDP:glucose to salicylic acid (forming a glucoside (SAG) and a glucose ester (SGE)), benzoic acid, and anthranilate in vitro. UGT74F2 shows a weak ability to catalyze the formation of the p-aminobenzoate-glucose ester in vitro. But, UGT75B1 appears to be the dominant pABA acylglucosyltransferase in vivo based on assays in leaves, flowers, and siliques.	138.27	66.06	2.09	5.68E-04	1.46E-03
4881	7	F2-3 vs. S2-3	252496_at	AT3G46790	Encodes a member of a PCMP (plant combinatorial and modular protein) family (PCMP-H subfamily) with 9 pentatricopeptide (PPR) repeats. The protein is involved the intergenic processing of chloroplast RNA between rps7 and ndhB, which is essential for ndhB translation.	136.39	65.33	2.09	3.65E-04	1.04E-03
4882	7	F2-3 vs. S2-3	254776_at	AT4G13360	ATP-dependent caseinolytic (Clp) protease/crotonase family protein;(source:Araport11)	134.82	64.49	2.09	9.42E-04	2.18E-03
4883	7	F2-3 vs. S2-3	253719_at	AT4G29490	Metallopeptidase M24 family protein;(source:Araport11)	71.85	34.31	2.09	4.63E-04	1.25E-03
4884	7	F2-3 vs. S2-3	250533_at	AT5G08640	Encodes a flavonol synthase that catalyzes formation of flavonols from dihydroflavonols. Co-expressed with CHI and CHS (qRT-PCR).	37.58	18	2.09	1.78E-04	6.02E-04
4885	7	F2-3 vs. S2-3	250335_at	AT5G11650	alpha/beta-Hydrolases superfamily protein;(source:Araport11)	128.81	61.58	2.09	2.37E-04	7.44E-04
4886	7	F2-3 vs. S2-3	250014_at	AT5G17990	Encodes the tryptophan biosynthetic enzyme phosphoribosylanthranilate transferase (PAT1, called trpD in bacteria). Converts anthranilate and phosphoribosylpyrophosphate into phosphoribosylanthranilate and inorganic pyrophosphate. The mRNA is cell-to-cell mobile.	279.86	134	2.09	2.10E-04	6.85E-04
4887	7	F2-3 vs. S2-3	247813_at	AT5G58330	lactate/malate dehydrogenase family protein;(source:Araport11)	656.01	314.23	2.09	4.73E-05	2.28E-04
4888	7	F2-3 vs. S2-3	247156_at	AT5G65760	Serine carboxypeptidase S28 family protein;(source:Araport11)	256.77	123.08	2.09	2.31E-04	7.32E-04
4889	7	F2-3 vs. S2-3	264506_at	AT1G09560	Encodes a plasodesmata-located protein involved in regulating primary root growth by controlling phloem-mediated allocation of resources between the primary and lateral root meristems. The mRNA is cell-to-cell mobile.	138.54	66.62	2.08	3.37E-04	9.77E-04
4890	7	F2-3 vs. S2-3	265026_at	AT1G24360	NAD(P)-binding Rossmann-fold superfamily protein;(source:Araport11)	1381.36	664.28	2.08	7.10E-05	3.01E-04
4891	7	F2-3 vs. S2-3	261296_at	AT1G48460	tRNA-processing ribonuclease BN;(source:Araport11)	300.3	144.41	2.08	2.20E-05	1.30E-04
4892	7	F2-3 vs. S2-3	262959_at	AT1G54290	Translation initiation factor SUI1 family protein;(source:Araport11)	479.26	230.08	2.08	4.09E-05	2.06E-04
4893	7	F2-3 vs. S2-3	266856_at	AT2G26910	Encodes a member of the PLEIOTROPIC DRUG RESISTANCE family of ATP binding cassette transporters. Required for the formation of a functional cuticle.	115.71	55.6	2.08	1.60E-04	5.58E-04

4894	7	F2-3 vs. S2-3	255624_at	AT4G01370	Encodes a nuclear and cytoplasmically localized MAP kinase involved in mediating responses to pathogens. Its substrates include MKS1 and probably MAP65-1.The MAP65-1 interaction is involved in mediating cortical microtuble organization. Required for male-specific meiotic cytokinesis. The mRNA is cell-to-cell mobile.	41.32	19.82	2.08	2.74E-05	1.52E-04
4895	7	F2-3 vs. S2-3	254033_at	AT4G25950	V-ATPase G-subunit like protein	17.45	8.4	2.08	9.69E-04	2.23E-03
4896	7	F2-3 vs. S2-3	265134_at	AT1G51260	ACYL-COA:1-ACYLGLYCEROL-3-PHOSPHATE ACYLTRANSFERASE, PUTATIVE SIMILAR TO ACYL-COA:1-ACYLGLYCEROL-3-PHOSPHATE ACYLTRANSFERASE GI:4583544 FROM [BRASSICA NAPUS]	35.67	17.25	2.07	6.00E-04	1.53E-03
4897	7	F2-3 vs. S2-3	266613_at	AT2G14900	Gibberellin-regulated family protein;(source:Araport11)	794.33	384.49	2.07	2.06E-04	6.77E-04
4898	7	F2-3 vs. S2-3	258288_at	AT3G23290	LIGHT-DEPENDENT SHORT HYPOCOTYLS-like protein (DUF640);(source:Araport11)	34.12	16.46	2.07	1.72E-04	5.88E-04
4899	7	F2-3 vs. S2-3	249148_at	AT5G43260	chaperone protein dnaJ-like protein;(source:Araport11)	117.41	56.59	2.07	3.79E-04	1.07E-03
4900	7	F2-3 vs. S2-3	264993_at	AT1G67290	glyoxal oxidase-related protein;(source:Araport11)	44.2	21.47	2.06	3.89E-05	1.97E-04
4901	7	F2-3 vs. S2-3	257699_at	AT3G12780	PGK1 was localized exclusively in the chloroplasts of photosynthetic tissues and is the photosynthetic isoform. The pgk1.1 knock-down mutant displayed reduced growth, lower photosynthetic capacity and starch content. Expression studies in PGK mutants showed that PGK1 and PGK3 were down-regulated in pgk3.2 and pgk1.1, respectively. These results indicate that the down-regulation of photosynthetic activity could be a plant strategy when glycolysis is impaired to achieve metabolic adjustment and optimize growth (DOI:10.1104/pp.17.01227).Functions redundantly with AT1G56190 in the chloroplast in the biosynthesis of thylakoid membrane galactolipids. Double mutants are photosynthetically incompetent, plants are albino and seedling lethal	665.88	322.56	2.06	8.09E-05	3.34E-04
4902	7	F2-3 vs. S2-3	258399_at	AT3G15540	Primary auxin-responsive gene. Involved in the regulation stamen filaments development.	269.66	130.7	2.06	1.85E-05	1.14E-04
4903	7	F2-3 vs. S2-3	252321_at	AT3G48510	ABA‐induced transcription repressor that acts as feedback regulator in ABA signalling.	64.34	31.2	2.06	9.05E-05	3.64E-04
4904	7	F2-3 vs. S2-3	251616_at	AT3G57990	Encodes a ?-barrel protein, named OEP40, locates in in the outer envelope of chloroplasts, and functions as a solute channel which is selectively permeable for glucose.	86.21	41.81	2.06	2.72E-05	1.51E-04
4905	7	F2-3 vs. S2-3	254577_at	AT4G19450	Major facilitator superfamily protein;(source:Araport11)	333.2	161.67	2.06	3.84E-04	1.08E-03
4906	7	F2-3 vs. S2-3	254543_at	AT4G19810	ChiC encodes a Class V chitinase that is a part of glycoside hydrolase family 18 based on CAZy groupings. It appears to primarily act as an exochitinase in vitro where it predominantly cleaves a chitobiose (GlcNAc)2 residue from the non-reducing end of a chitin oligosaccharide. However, it shows some minor endochitinase activity in vitro, as well. A putative 24 amino-acid signal peptide may direct this protein to the secretory system and it has been detected in cell wall apoplastic fluid. RT-PCR experiments demonstrate that ChiC transcript levels are increased in response to abscisisc acid, jasmonic acid, and NaCl stress. Microarray results also suggest that transcript levels rise in response to osmotic stress, two fungal pathogens, a bacterial pathogen, and the elicitor flagellin. The mRNA is cell-to-cell mobile.	39.31	19.1	2.06	1.03E-04	4.00E-04
4907	7	F2-3 vs. S2-3	254082_at	AT4G25720	Encodes a protein with similarity to gamma-glutamylcyclotransferase that is involved in catalyzing the formation of pyroglutate residue on proteins that have been post-translationally processed to reveal a glutamine at their N-terminus. Some pathogenesis related proteins may be modified in this matter. In vitro the enzyme (using a protein that does not match AT4G25720.1, AT4G25720.2, or AT4G25720.3) works on a broad range of synthetic peptides, but shows some preference for substrates that have a hydrophobic residues adjacent to the glutamine. It can also act on a N-terminal glutamic residue in a synthetic substrate (Schilling 2007). It does not appear to have the ability to transform GSH or gamma-glutamyl cysteine to 5-oxoproline (Okhama-Ohtsu 2008). This protein is predicted to enter the secretory pathway. When it is expressed in a heterologous system, two glycosylated forms of the protein can be detected.	71.43	34.71	2.06	5.25E-04	1.38E-03
4908	7	F2-3 vs. S2-3	248711_at	AT5G48270	DUF868 family protein (DUF868);(source:Araport11)	19.56	9.5	2.06	1.69E-04	5.79E-04
4909	7	F2-3 vs. S2-3	259416_at	AT1G02305	Encodes a capase involved in stress induced cell death.	707.16	344.78	2.05	6.47E-04	1.62E-03
4910	7	F2-3 vs. S2-3	261312_at	AT1G05790	lipase class 3 family protein;(source:Araport11)	68.83	33.61	2.05	3.94E-04	1.10E-03

4911	7	F2-3 vs. S2-3	259693_at	AT1G63060	ribosome biogenesis NEP1-like protein;(source:Araport11)	21.12	10.29	2.05	1.58E-05	1.03E-04
4912	7	F2-3 vs. S2-3	245199_at	AT1G67730	Encodes one of the two Arabidopsis homologues to YBR159w encoding a S. cerevisiae beta-ketoacyl reductase (KCR), which catalyzes the first reduction during VLCFA (very long chain fatty acids, >18 carbon) elongation: KCR1 (At1g67730), KCR2 (At1g24470). Complementation of the yeast ybr159Delta mutant demonstrated that the two KCR proteins are divergent and that only AtKCR1 can restore heterologous elongase activity similar to the native yeast KCR gene. The mRNA is cell-to-cell mobile.	1373.6	668.96	2.05	1.77E-05	1.11E-04
4913	7	F2-3 vs. S2-3	260290_at	AT1G80510	Encodes a close relative of the amino acid transporter ANT1 (AT3G11900).	87.62	42.82	2.05	1.23E-04	4.57E-04
4914	7	F2-3 vs. S2-3	258512_at	AT3G06510	Encodes a protein with beta-glucosidase and galactosyltransferase activity, mutants show increased sensitivity to freezing. Though it is classified as a family I glycosyl hydrolase, it has no hydrolase activity in vitro.	610.79	297.93	2.05	1.33E-04	4.83E-04
4915	7	F2-3 vs. S2-3	258006_at	AT3G19400	Cysteine proteinases superfamily protein;(source:Araport11)	215.29	104.97	2.05	6.15E-04	1.55E-03
4916	7	F2-3 vs. S2-3	253639_at	AT4G30550	Class I glutamine amidotransferase-like superfamily protein;(source:Araport11)	37.34	18.21	2.05	9.47E-05	3.75E-04
4917	7	F2-3 vs. S2-3	247077_at	AT5G66420	TIM-barrel signal transduction protein;(source:Araport11)	189.93	92.64	2.05	3.80E-04	1.07E-03
4918	7	F2-3 vs. S2-3	266774_at	AT2G29050	RHOMBOID-like 1;(source:Araport11)	100.17	49.09	2.04	6.38E-04	1.60E-03
4919	7	F2-3 vs. S2-3	267414_at	AT2G34790	Encodes a BBE-like enzyme that acts in monolignol metabolism by catalyzing the oxidation of aromatic allylic alcohols, such as coumaryl-, sinapyl-, and coniferyl alcohol, to the corresponding aldehydes.	20.04	9.85	2.04	4.28E-04	1.17E-03
4920	7	F2-3 vs. S2-3	265882_at	AT2G42490	Peroxisome-localized copper amine oxidase involved in lateral root formation.	1190.4	584.11	2.04	5.38E-04	1.40E-03
4921	7	F2-3 vs. S2-3	258602_at	AT3G02750	Protein phosphatase 2C family protein;(source:Araport11)	56.4	27.61	2.04	9.09E-05	3.64E-04
4922	7	F2-3 vs. S2-3	256584_at	AT3G28750	hypothetical protein;(source:Araport11)	23.38	11.48	2.04	6.02E-04	1.53E-03
4923	7	F2-3 vs. S2-3	253113_at	AT4G35985	Senescence/dehydration-associated protein-like protein;(source:Araport11)	44.7	21.92	2.04	3.84E-04	1.08E-03
4924	7	F2-3 vs. S2-3	246763_at	AT5G27150	Encodes a vacuolar sodium/proton antiporter involved in salt tolerance, ion homeostasis, and leaf development. The mRNA is cell-to-cell mobile.	77.92	38.29	2.04	6.72E-05	2.89E-04
4925	7	F2-3 vs. S2-3	247775_at	AT5G58690	phosphatidylinositol-speciwc phospholipase C5;(source:Araport11)	22.72	11.14	2.04	2.18E-04	7.01E-04
4926	7	F2-3 vs. S2-3	263167_at	AT1G03030	P-loop containing nucleoside triphosphate hydrolases superfamily protein;(source:Araport11)	54.03	26.65	2.03	3.63E-04	1.04E-03
4927	7	F2-3 vs. S2-3	261309_at	AT1G48600	Encodes a phosphoethanolamine N-methyltransferase that catalyses the last two methylation steps of the three sequential methylations of phosphoethanolamine (PEA) that are required for the synthesis of phosphocholine (PCho) in plants.	114.46	56.38	2.03	4.25E-05	2.11E-04
4928	7	F2-3 vs. S2-3	246396_at	AT1G58180	beta carbonic anhydrase 6;(source:Araport11)	23.83	11.72	2.03	6.90E-04	1.70E-03
4929	7	F2-3 vs. S2-3	266457_at	AT2G22780	encodes an peroxisomal NAD-malate dehydrogenase that is involved in fatty acid beta-oxidation through providing NAD to the process of converting fatty acyl CoA to acetyl CoA.	198.42	97.66	2.03	3.13E-05	1.67E-04
4930	7	F2-3 vs. S2-3	254727_at	AT4G13670	plastid transcriptionally active 5;(source:Araport11)	97.02	47.86	2.03	1.03E-04	4.00E-04
4931	7	F2-3 vs. S2-3	245330_at	AT4G14930	Survival protein SurE-like phosphatase/nucleotidase;(source:Araport11)	155.14	76.46	2.03	2.12E-04	6.87E-04
4932	7	F2-3 vs. S2-3	250480_at	AT5G10290	leucine-rich repeat transmembrane protein kinase family protein;(source:Araport11)	30.55	15.07	2.03	5.13E-05	2.42E-04
4933	7	F2-3 vs. S2-3	248543_at	AT5G50230	autophagy-related (ATG) gene	92.54	45.67	2.03	3.49E-05	1.83E-04
4934	7	F2-3 vs. S2-3	247544_at	AT5G61670	Encodes a close homolog of the Cauliflower OR (Orange) protein that is located in the chloroplast of light grown organs but in the nucleus of etiolated cotyledons. The function of OR is to induce the differentiation of proplastids or other noncolored plastids into chromoplasts for carotenoid accumulation. Both proteins contain a Cysteine-rich zinc finger domain that is highly specific to DnaJ-like molecular chaperons. The AtOR protein interacts directly with the PSY (phytoene synthase) protein and acts as a positive posttranscriptional regulator of its expression, thereby affecting carotenoid biosynthesis.	105.07	51.69	2.03	2.98E-05	1.61E-04
4935	7	F2-3 vs. S2-3	260870_at	AT1G43890	ras-related small GTPase	65.64	32.42	2.02	4.68E-04	1.26E-03
4936	7	F2-3 vs. S2-3	256458_at	AT1G75220	Encodes a vacuolar glucose exporter that is induced in response to factors that activate vacuolar glucose pools like darkness, heat stress and wounding and repressed during conditions that trigger glucose accumulation in the vacuole like cold stress and external sugar supply.	124.09	61.55	2.02	1.16E-04	4.38E-04
4937	7	F2-3 vs. S2-3	266351_at	AT2G01490	Encodes a phytanoyl-CoA 2-hydroxylase (PAHX). The mRNA is cell-to-cell mobile.	241.63	119.49	2.02	1.19E-04	4.45E-04
4938	7	F2-3 vs. S2-3	255497_at	AT4G02590	basic helix-loop-helix (bHLH) DNA-binding superfamily protein;(source:Araport11)	86.93	42.99	2.02	4.73E-04	1.27E-03

4939	7	F2-3 vs. S2-3	254204_at	AT4G24160	Encodes a soluble lysophosphatidic acid acyltransferase with additional triacylglycerol lipase and phosphatidylcholine hydrolyzing enzymatic activities. Plays a pivotal role in maintaining the lipid homeostasis by regulating both phospholipid and neutral lipid levels.	296.87	147.29	2.02	6.52E-05	2.84E-04
4940	7	F2-3 vs. S2-3	254065_at	AT4G25420	Encodes gibberellin 20-oxidase that is involved in the later steps of the gibberellin biosynthetic pathway. Regulated by a circadian clock. Weak expression response to far red light.	42.05	20.86	2.02	8.28E-05	3.40E-04
4941	7	F2-3 vs. S2-3	253698_at	AT4G29680	Alkaline-phosphatase-like family protein;(source:Araport11)	24.08	11.94	2.02	3.68E-05	1.90E-04
4942	7	F2-3 vs. S2-3	253481_at	AT4G31990	Encodes a plastid-localized aspartate aminotransferase. Does not display any PAT (glutamate/aspartate-prephenate aminotransferase) activity even in the presence of a high concentration of prephenate.	649.81	322.22	2.02	2.20E-04	7.07E-04
4943	7	F2-3 vs. S2-3	250967_at	AT5G02790	Glutathione S-transferase family protein;(source:Araport11)	117.92	58.43	2.02	4.71E-05	2.27E-04
4944	7	F2-3 vs. S2-3	247228_at	AT5G65140	Haloacid dehalogenase-like hydrolase (HAD) superfamily protein;(source:Araport11)	99.14	49.06	2.02	2.48E-05	1.42E-04
4945	7	F2-3 vs. S2-3	261326_s_a	AT1G44820	Peptidase M20/M25/M40 family protein;(source:Araport11)	32.22	16	2.01	2.99E-04	8.93E-04
4946	7	F2-3 vs. S2-3	262684_s_a t	AT1G76030	One of three genes encoding the vacuolar ATP synthase subunit B1. This subunit was shown to interact with the gene product of hexokinase1 (ATHXK1). This interaction, however, is solely restricted to the nucleus. The protein binds to and co-localizes with F-actin, bundles F-actin to form higher-order structure, and stabilizes actin filaments in vitro. The mRNA is cell-to-cell mobile.	405.15	201.12	2.01	5.20E-05	2.44E-04
4947	7	F2-3 vs. S2-3	266993_at	AT2G39210	Major facilitator superfamily protein;(source:Araport11)	61.37	30.56	2.01	4.26E-04	1.17E-03
4948	7	F2-3 vs. S2-3	251117_at	AT3G63390	hypothetical protein;(source:Araport11)	250.96	124.76	2.01	5.49E-05	2.54E-04
4949	7	F2-3 vs. S2-3	260835_at	AT1G06700	Protein kinase superfamily protein;(source:Araport11)	514.57	257.46	2	1.16E-04	4.36E-04
4950	7	F2-3 vs. S2-3	262456_at	AT1G11260	Encodes a H+/hexose cotransporter. The mRNA is cell-to-cell mobile.	101.97	50.89	2	3.77E-04	1.07E-03
4951	7	F2-3 vs. S2-3	263382_at	AT2G40230	HXXXD-type acyl-transferase family protein;(source:Araport11)	61.37	30.73	2	2.10E-04	6.84E-04
4952	7	F2-3 vs. S2-3	252192_at	AT3G50000	Encodes the casein kinase II (CK2) catalytic subunit (alpha).	1040.38	521	2	1.28E-04	4.71E-04
4953	7	F2-3 vs. S2-3	252075_at	AT3G51730	saposin B domain-containing protein;(source:Araport11)	302.41	150.97	2	2.98E-05	1.61E-04
4954	7	F2-3 vs. S2-3	254804_at	AT4G13010	Oxidoreductase, zinc-binding dehydrogenase family protein;(source:Araport11)	173.64	86.72	2	6.82E-05	2.93E-04
4955	7	F2-3 vs. S2-3	247994_at	AT5G56140	RNA-binding KH domain-containing protein;(source:Araport11)	55.5	27.8	2	4.10E-04	1.14E-03
4956	7	F2-3 vs. S2-3	247608_at	AT5G60990	DEA(D/H)-box RNA helicase family protein;(source:Araport11)	119.48	59.69	2	1.91E-05	1.17E-04
4957	7	F2-3 vs. S2-3	261551_at	AT1G63440	The Arabidopsis P-type ATPase HMA5 is involved in Cu detoxification. hma5 mutant plants exhibit Cu hypersensitivity, which is especially dramatic in roots where HMA5 is mostly expressed.	85.3	42.82	1.99	1.92E-04	6.38E-04
4958	7	F2-3 vs. S2-3	262059_at	AT1G80030	Molecular chaperone Hsp40/DnaJ family protein;(source:Araport11)	97.46	49.07	1.99	1.05E-04	4.05E-04
4959	7	F2-3 vs. S2-3	263473_at	AT2G31750	Encodes an auxin glycosyltransferase that is likely to be involved in regulation of auxin by glycosylation.	34.46	17.29	1.99	1.66E-04	5.72E-04
4960	7	F2-3 vs. S2-3	266461_at	AT2G47730	Encodes glutathione transferase belonging to the phi class of GSTs. Naming convention according to Wagner et al. (2002).	142.55	71.48	1.99	7.27E-05	3.07E-04
4961	7	F2-3 vs. S2-3	257749_at	AT3G18780	Encodes an actin that is constitutively expressed in vegetative structures but not pollen. ACT2 is involved in tip growth of root hairs.	1215.07	610.16	1.99	3.34E-05	1.77E-04
4962	7	F2-3 vs. S2-3	245427_at	AT4G17550	Encodes a member of the phosphate starvation-induced glycerol-3-phosphate permease gene family: AT3G47420(G3Pp1), AT4G25220(G3Pp2), AT1G30560(G3Pp3), AT4G17550(G3Pp4) and AT2G13100(G3Pp5).	91.43	45.92	1.99	7.08E-04	1.73E-03
4963	7	F2-3 vs. S2-3	254103_at	AT4G25030	Plastid localized protein of unknown function. Mutants are more susceptible to P. syringae and produce less callose upon infection.	117.05	58.92	1.99	8.94E-05	3.60E-04
4964	7	F2-3 vs. S2-3	247924_at	AT5G57655	xylose isomerase family protein;(source:Araport11)	44.7	22.45	1.99	8.65E-04	2.03E-03
4965	7	F2-3 vs. S2-3	260590_at	AT1G53310	Encodes one of four Arabidopsis phosphoenolpyruvate carboxylase proteins.Plays an important role in carbon and nitrogen metabolism.	1140.2	574.44	1.98	2.61E-04	8.01E-04
4966	7	F2-3 vs. S2-3	261958_at	AT1G64500	A member of a protein family found in plants and animals that contain conserved C-terminal glutaredoxin-like and putative zinc-binding cysteine-rich domains. It is involved in light stimulated actin bundling and chloroplast movement. The mRNA is cell-to-cell mobile.	26.61	13.44	1.98	2.40E-05	1.39E-04
4967	7	F2-3 vs. S2-3	263282_at	AT2G14095	hypothetical protein;(source:Araport11)	23.36	11.81	1.98	3.24E-05	1.72E-04
4968	7	F2-3 vs. S2-3	264007_at	AT2G21140	Proline-rich protein expressed in expanding leaves, stems, flowers, and siliques.	85.14	43.05	1.98	4.76E-04	1.27E-03
4969	7	F2-3 vs. S2-3	267367_at	AT2G44210	carboxyl-terminal peptidase (DUF239);(source:Araport11)	234.42	118.15	1.98	5.98E-04	1.52E-03

4970	7	F2-3 vs. S2-3	258037_at	AT3G21230	The gene encodes a 4-coumarate coenzyme A ligase being able to use sinapate as substrate. The catalytic efficiency was in the following (descending) order: p-coumaric acid, caffeic acid, 5-OH-ferulic acid, ferulic acid and sinapic acid. At4CL5 was unable to use cinnamic acid as substrate. Knockout of At4CL5 (4cl5) revealed no effect on syringyl lignin content indicating that the activity observed does probably not occur in vivo.	90.51	45.69	1.98	5.08E-04	1.34E-03
4971	7	F2-3 vs. S2-3	258183_at	AT3G21550	transmembrane protein, putative (DUF679 domain membrane protein 2);(source:Araport11)	59.11	29.88	1.98	1.43E-04	5.11E-04
4972	7	F2-3 vs. S2-3	253215_at	AT4G34950	Major facilitator superfamily protein;(source:Araport11)	48.61	24.58	1.98	6.60E-05	2.86E-04
4973	7	F2-3 vs. S2-3	247232_at	AT5G64940	ABC1K8 is a member of an atypical protein kinase family that is induced by heavy metals. Loss of function mutations affect the metabolic profile of chloroplast lipids. It appears to function along with ABC1K7 in mediating lipid membrane changes in response to stress. The mRNA is cell-to-cell mobile.	578.23	292.56	1.98	3.27E-04	9.53E-04
4974	7	F2-3 vs. S2-3	260813_at	AT1G43700	Encodes a VirE2-interacting protein. VIP1 mediates nuclear translocation of VirE2 via its amino half, and interacts with histone H2A via it carboxyl half. Involved in osmosensory response. The mRNA is cell-to-cell mobile.	377.17	191.56	1.97	6.10E-05	2.71E-04
4975	7	F2-3 vs. S2-3	261687_at	AT1G47280	hypothetical protein;(source:Araport11)	18.87	9.59	1.97	3.44E-04	9.92E-04
4976	7	F2-3 vs. S2-3	263477_at	AT2G31790	UDP-Glycosyltransferase superfamily protein;(source:Araport11)	216.19	109.8	1.97	3.90E-05	1.97E-04
4977	7	F2-3 vs. S2-3	258161_at	AT3G17930	Encodes a thylakoid membrane protein involved in the accumulation of the cytochrome b6/f complex.	104.65	53.18	1.97	1.85E-04	6.23E-04
4978	7	F2-3 vs. S2-3	252107_at	AT3G51490	tonoplast monosaccharide transporter3;(source:Araport11)	58.61	29.82	1.97	2.36E-04	7.43E-04
4979	7	F2-3 vs. S2-3	251825_at	AT3G55100	ABC-2 type transporter family protein;(source:Araport11)	28.38	14.41	1.97	3.07E-05	1.66E-04
4980	7	F2-3 vs. S2-3	254775_at	AT4G13450	Adenine nucleotide alpha hydrolases-like superfamily protein;(source:Araport11)	23.46	11.92	1.97	8.08E-04	1.92E-03
4981	7	F2-3 vs. S2-3	248529_at	AT5G50000	Belongs to the Raf-like kinase subfamily of the mitogen-activated protein kinase kinase kinase (MAPKKK) family. Negatively regulates stomatal opening by negatively regulating plasma membrane H+-ATPase phosphorylation.	164.08	83.37	1.97	1.50E-04	5.31E-04
4982	7	F2-3 vs. S2-3	247894_at	AT5G58003	Encodes a polypeptide that contains FCPH and BRCT domains. RNAi suppression mutant lines were generated, which displayed a range of phenotypic abnormalities, including: incomplete to no cotyledon expansion, slow growth, epinastic leaves or small inflorescences.	87.61	44.53	1.97	7.34E-05	3.09E-04
4983	7	F2-3 vs. S2-3	261525_at	AT1G14330	Galactose oxidase/kelch repeat superfamily protein;(source:Araport11)	193.32	98.7	1.96	2.38E-04	7.46E-04
4984	7	F2-3 vs. S2-3	259638_at	AT1G52360	Coatomer, beta subunit;(source:Araport11)	212.82	108.8	1.96	5.71E-04	1.47E-03
4985	7	F2-3 vs. S2-3	263731_at	AT1G59970	Matrixin family protein;(source:Araport11)	66.65	34.03	1.96	9.94E-05	3.91E-04
4986	7	F2-3 vs. S2-3	267012_at	AT2G39220	PATATIN-like protein 6;(source:Araport11)	373.89	190.71	1.96	6.28E-05	2.77E-04
4987	7	F2-3 vs. S2-3	258134_at	AT3G24530	AAA-type ATPase family protein / ankyrin repeat family protein;(source:Araport11)	63.51	32.39	1.96	4.79E-04	1.28E-03
4988	7	F2-3 vs. S2-3	246198_at	AT4G36810	Encodes a protein with geranylgeranyl pyrophosphate synthase activity involved in isoprenoid biosynthesis. The enzyme appears to be targeted to the chloroplast in epidermal cells and guard cells of leaves, and in etioplasts in roots. The mRNA is cell-to-cell mobile.	73.99	37.75	1.96	4.46E-05	2.18E-04
4989	7	F2-3 vs. S2-3	250547_at	AT5G08100	Encodes an asparaginase that catalyzes the degradation of L-asparagine to L-aspartic acid and ammonia.	124	63.36	1.96	7.85E-05	3.27E-04
4990	7	F2-3 vs. S2-3	249447_at	AT5G39400	Calcium/lipid-binding (CaLB) phosphatase;(source:Araport11)	24.29	12.38	1.96	5.38E-04	1.40E-03
4991	7	F2-3 vs. S2-3	247944_at	AT5G57100	Nucleotide/sugar transporter family protein;(source:Araport11)	75.3	38.51	1.96	2.78E-04	8.40E-04
4992	7	F2-3 vs. S2-3	247060_at	AT5G66760	One of two genes in Arabidopsis that encode a flavoprotein subunit of the mitochondrial succinate dehydrogenase complex. The mRNA is cell-to-cell mobile.	943.73	481.74	1.96	8.80E-04	2.06E-03
4993	7	F2-3 vs. S2-3	265067_at	AT1G03850	Encodes glutaredoxin ATGRXS13, required to facilitate Botrytis cinerea infection of Arabidopsis thaliana plants. Sylvain La Camera et al (2011, PMID:21756272) reported a third splice variant in addition to the two annotated in TAIR10. It is a member of the CC-type glutaredoxin (ROXY) family that has been shown to interact with the transcription factor TGA2 and suppress ORA59 promoter activity.	21.31	10.91	1.95	2.35E-04	7.40E-04
4994	7	F2-3 vs. S2-3	261206_at	AT1G12800	SDP is a chloroplast localized RNA binding protein that is required for plastid rRNA processing. Plants harboring a mutation in SDP have numerous defects including reduced chlorophyll content, poor growth, yellow leaves and abnormal chloroplasts.	389.18	199.24	1.95	2.79E-04	8.42E-04
4995	7	F2-3 vs. S2-3	259491_at	AT1G15820	Lhcb6 protein (Lhcb6), light harvesting complex of photosystem II.	636.25	326.88	1.95	3.54E-05	1.84E-04

4996	7	F2-3 vs. S2-3	262342_at	AT1G64150	PAM71 encodes an integral thylakoid membrane protein that is required for normal operation of oxygen-evolving complex (as evidenced by oxygen evolution rates) and for manganese incorporation. PAM71 belongs to a small gene family in Arabidopsis comprising five members. PAM71 is well conserved in the green lineage and shares homology with putative Ca2+/H+ exchangers from yeast (Saccharomyces cerevisiae) (GDT1) and human (Homo sapiens) (TMEM165).	56.24	28.89	1.95	6.68E-05	2.88E-04
4997	7	F2-3 vs. S2-3	263553_at	AT2G16430	Encodes an acid phosphatase involved plant acclimation to Pi deprivation.	111.03	56.79	1.95	4.57E-04	1.23E-03
4998	7	F2-3 vs. S2-3	257267_at	AT3G15030	Arabidopsis thaliana TCP family transcription factor. Regulated by miR319. Involved in heterchronic regulation of leaf differentiation.	64	32.81	1.95	7.32E-04	1.77E-03
4999	7	F2-3 vs. S2-3	258033_at	AT3G21250	member of MRP subfamily	168.68	86.69	1.95	9.35E-04	2.17E-03
5000	7	F2-3 vs. S2-3	254074_at	AT4G25490	Transcriptional activator that binds to the DRE/CRT regulatory element and induces COR (cold-regulated) gene expression increasing plant freezing tolerance. It encodes a member of the DREB subfamily A-1 of ERF/AP2 transcription factor family (CBF1). The protein contains one AP2 domain. There are six members in this subfamily, including CBF1, CBF2, and CBF3. This gene is involved in response to low temperature and abscisic acid.	69.77	35.76	1.95	6.31E-04	1.58E-03
5001	7	F2-3 vs. S2-3	267483_at	AT2G02810	Encodes a multitransmembrane hydrophobic protein that functions as transporter of UDP-galactose and UDP-glucose into the Golgi. Localized in the ER. Involved in the unfolded protein response, a mechanism that controls proper protein folding in the ER.	116.85	60.19	1.94	6.29E-05	2.77E-04
5002	7	F2-3 vs. S2-3	265959_at	AT2G37240	Thioredoxin superfamily protein;(source:Araport11)	88.45	45.66	1.94	3.90E-04	1.10E-03
5003	7	F2-3 vs. S2-3	267582_at	AT2G41970	Encodes MRI, a plasma membrane-localized member of the RLCK-VIII subfamily. Preferentially expressed in both pollen tubes and root hairs. mri-knockout mutants display spontaneous pollen tube and root-hair bursting.	34.56	17.8	1.94	4.06E-04	1.13E-03
5004	7	F2-3 vs. S2-3	258057_at	AT3G28970	Identified in a screen for mutants resistant to an anti-auxin. Encodes a protein with unknown function that shares homology with DCN protein family.	92.19	47.48	1.94	9.18E-05	3.67E-04
5005	7	F2-3 vs. S2-3	252649_at	AT3G44680	Encodes HDA9 (a RPD3-like histone deacetylase). Functions in promoting the onset of leaf senescence.The hda9 mutant shows enhanced H3K9 acetylation levels,based on immunodetection using H3K9ac antibodies.	56.04	28.87	1.94	1.67E-04	5.75E-04
5006	7	F2-3 vs. S2-3	245299_at	AT4G17530	AtRabD2c encodes a Rab GTPase, which plays important roles in pollen development, germination and tube elongation.	221.04	114.1	1.94	2.62E-05	1.48E-04
5007	7	F2-3 vs. S2-3	249876_at	AT5G23060	Encodes a chloroplast-localized protein that modulates cytoplasmic Ca2+ concentration and is crucial for proper stomatal regulation in response to elevations of external Ca2+. Phosphorylation of this protein is dependent on calcium.	117.1	60.41	1.94	4.15E-04	1.15E-03
5008	7	F2-3 vs. S2-3	247240_at	AT5G64660	CYS, MET, PRO, and GLY protein 2;(source:Araport11)	31.63	16.33	1.94	2.66E-05	1.49E-04
5009	7	F2-3 vs. S2-3	262960_at	AT1G54320	LEM3 (ligand-effect modulator 3) family protein / CDC50 family protein;(source:Araport11)	531.94	275.76	1.93	6.58E-05	2.86E-04
5010	7	F2-3 vs. S2-3	265392_at	AT2G20860	LIP1,Lipoic acid synthase,	101.26	52.43	1.93	1.53E-04	5.38E-04
5011	7	F2-3 vs. S2-3	252115_at	AT3G51600	Predicted to encode a PR (pathogenesis-related) protein. Belongs to the lipid transfer protein (PR-14) family with the following members: At2g38540/LTP1, At2g38530/LTP2, At5g59320/LTP3, At5g59310/LTP4, At3g51600/LTP5, At3g08770/LTP6, At2g15050/LTP7, At2g18370/LTP8, At2g15325/LTP9, At5g01870/LTP10, At4g33355/LTP11, At3g51590/LTP12, At5g44265/LTP13, At5g62065/LTP14, At4g08530/LTP15.	35.59	18.45	1.93	5.43E-05	2.52E-04
5012	7	F2-3 vs. S2-3	253101_at	AT4G37430	Encodes a member of the CYP81F cytochrome P450 monooxygenase subfamily.	78.18	40.56	1.93	3.81E-04	1.07E-03
5013	7	F2-3 vs. S2-3	249229_at	AT5G42090	Lung seven transmembrane receptor family protein;(source:Araport11)	192.28	99.57	1.93	1.09E-04	4.16E-04
5014	7	F2-3 vs. S2-3	259498_at	AT1G15880	Golgi SNARE 11 protein (GOS11)	559.98	291.7	1.92	1.17E-04	4.39E-04
5015	7	F2-3 vs. S2-3	264229_at	AT1G67480	Galactose oxidase/kelch repeat superfamily protein;(source:Araport11)	77.8	40.58	1.92	8.33E-05	3.41E-04
5016	7	F2-3 vs. S2-3	265705_at	AT2G03410	Mo25 family protein;(source:Araport11)	19.94	10.38	1.92	2.12E-04	6.88E-04
5017	7	F2-3 vs. S2-3	266562_at	AT2G23970	Class I glutamine amidotransferase-like superfamily protein;(source:Araport11)	23.74	12.36	1.92	4.80E-04	1.28E-03
5018	7	F2-3 vs. S2-3	263796_at	AT2G24540	Galactose oxidase/kelch repeat superfamily protein;(source:Araport11)	33.19	17.27	1.92	3.31E-04	9.63E-04
5019	7	F2-3 vs. S2-3	265884_at	AT2G42320	nucleolar protein gar2-like protein;(source:Araport11)	287.72	150.14	1.92	3.08E-04	9.12E-04

5020	7	F2-3 vs. S2-3	245335_at	AT4G16160	Homologous to pea OEP16 and barley pPORA (OEP16), a member of Arabidopsis OEP16 family. Two OEP16 genes are closely related to each other and are conserved in all land plants, OEP16-2, also named OEP16-S, and OEP16-1 (renamed OEP16-L) are result of the gene duplication event that occurred prior to divergence of bryophytes and seed plants. Predominantly expressed in seed and is not inducible by cold treatment. atOEP16-S gained an additional exon. The promoter region of atOEP16-S (but not atOEP16-L) contains multiple G-box ABA-responsive elements. The atOEP16-S promoter conferred developmentally regulated seed- and pollen-specific GUS expression in tobacco.	24.27	12.67	1.92	4.38E-04	1.19E-03
5021	7	F2-3 vs. S2-3	254299_at	AT4G22920	Similar to the tomato senescence-inducible chloroplast stay-green protein 1. It is upregulated during maximal senescence in the Arabidopsis life cycle, especially in senescent leaves. Acts antagonistically with SGR2 to balance chlorophyll catabolism in chloroplasts with the dismantling and remobilizing of other cellular components in senescing leaf cells.	68.54	35.65	1.92	5.36E-05	2.49E-04
5022	7	F2-3 vs. S2-3	255940_at	AT1G20380	Prolyl oligopeptidase family protein;(source:Araport11)	158.33	82.8	1.91	3.32E-04	9.65E-04
5023	7	F2-3 vs. S2-3	260322_at	AT1G63900	Encodes a RING-type ubiquitin E3 ligase of the chloroplast outer membrane that associates with TOC complexes and mediates ubiquitination of TOC components, promoting their degradation. It not only regulates chloroplast protein import but also targets components of the peroxisome protein import apparatus, PEX13 in particular. Several studies have been done to examine the peroxisomal localization of this protein, with varying interpretations.	130.4	68.35	1.91	1.75E-04	5.95E-04
5024	7	F2-3 vs. S2-3	255855_at	AT1G66950	Encodes a plasma membrane-localized ABC transporter. Confers tolerance to herbicide paraquat.	53.77	28.16	1.91	3.13E-04	9.23E-04
5025	7	F2-3 vs. S2-3	263835_at	AT2G40290	Encodes an eIF2alpha homolog that can be phosphorylated by GCN2 in vitro.	225.32	117.87	1.91	7.04E-05	2.99E-04
5026	7	F2-3 vs. S2-3	267212_at	AT2G44060	Late embryogenesis abundant protein, group 2;(source:Araport11)	546.74	286.81	1.91	1.53E-04	5.41E-04
5027	7	F2-3 vs. S2-3	257252_at	AT3G24170	Encodes a cytosolic glutathione reductase.	304.97	159.61	1.91	3.73E-04	1.06E-03
5028	7	F2-3 vs. S2-3	251961_at	AT3G53620	Encodes a soluble protein with inorganic pyrophosphatase activity that is highly specific for Mg-inorganic pyrophosphate. The mRNA is cell-to-cell mobile.	224.88	117.46	1.91	7.17E-05	3.04E-04
5029	7	F2-3 vs. S2-3	253525_at	AT4G31330	transmembrane protein, putative (Protein of unknown function, DUF599);(source:Araport11)	37.77	19.76	1.91	5.79E-05	2.63E-04
5030	7	F2-3 vs. S2-3	250889_at	AT5G04500	Encodes a member of the CAZy Glycosyltransferase Family 64 that is involved in glycosylinositolphosphorylceramide and sphingolipid glycosylation. In mutants, seed germination was less sensitive to salt stress than in wild-type plants. [The protein was expected to be Golgi-localized based on function as well as the Golgi localization of its homolog GMT1. However, GFP-fusion proteins localized both to the ER and Golgi, and especially to ER when co-expressed with Golgi markers. Therefore, localization cannot confidently be defined. (pers. communication, J. Mortimer)]	71.76	37.64	1.91	1.55E-04	5.45E-04
5031	7	F2-3 vs. S2-3	245854_at	AT5G13490	Encodes mitochondrial ADP/ATP carrier	1048.03	547.92	1.91	1.66E-04	5.73E-04
5032	7	F2-3 vs. S2-3	264355_at	AT1G03210	Phenazine biosynthesis PhzC/PhzF protein;(source:Araport11)	92.57	48.69	1.9	5.40E-05	2.51E-04
5033	7	F2-3 vs. S2-3	259626_at	AT1G42990	bZIP60 consists of a bZIP DNA binding domain followed by a putative transmembrane domain. bZIP60 mRNA is upregulated by the addition of ER stress inducers, tunicamycin (inhibitor of N-linked glycosylation), DTT (inhibitor of disulfide bond formation) and azetin-2-carboxylate (proline analog perturbing protein structure). Upon ER stress, bZIP60 mRNA is spliced by IRE1A and IRE1B to produce bZIP60-S, an active transcription factor without the transmembrane domain. bZIP60-U, a product of unspliced form of bZIP60 mRNA, is localized at the ER membrane and bZIP60-S is localized in the nucleus.	73.52	38.77	1.9	8.40E-04	1.98E-03
5034	7	F2-3 vs. S2-3	260518_at	AT1G51410	similar to Eucalyptus gunnii alcohol dehydrogenase of unknown physiological function (GI:1143445), apple tree, PIR:T16995; NOT a cinnamyl-alcohol dehydrogenase	37.49	19.68	1.9	3.91E-05	1.98E-04
5035	7	F2-3 vs. S2-3	265446_at	AT2G37110	PLAC8 family protein;(source:Araport11)	1525.52	801.47	1.9	5.73E-05	2.61E-04
5036	7	F2-3 vs. S2-3	265471_at	AT2G37130	Peroxidase superfamily protein;(source:Araport11)	23.1	12.16	1.9	5.03E-04	1.33E-03
5037	7	F2-3 vs. S2-3	257713_at	AT3G27380	One of three isoforms of the iron-sulfur component of the succinate dehydrogenase complex, a component of the mitochondrial respiratory chain complex II. The product of the nuclear encoded gene is imported into the mitochondrion. Expressed during germination and post-germinative growth.	588.9	310.53	1.9	2.02E-04	6.66E-04

5038	7	F2-3 vs. S2-3	252105_at	AT3G51470	Protein phosphatase 2C family protein;(source:Araport11)	30.59	16.07	1.9	1.74E-04	5.94E-04
5039	7	F2-3 vs. S2-3	255717_at	AT4G00350	MATE efflux family protein;(source:Araport11)	29.23	15.38	1.9	2.31E-04	7.33E-04
5040	7	F2-3 vs. S2-3	253736_at	AT4G28780	GDSL-motif esterase/acyltransferase/lipase. Enzyme group with broad substrate specificity that may catalyze acyltransfer or hydrolase reactions with lipid and non-lipid substrates.	49.87	26.29	1.9	6.17E-05	2.74E-04
5041	7	F2-3 vs. S2-3	253321_at	AT4G33910	2-oxoglutarate (2OG) and Fe(II)-dependent oxygenase superfamily protein;(source:Araport11)	149.68	78.63	1.9	2.24E-04	7.16E-04
5042	7	F2-3 vs. S2-3	245895_at	AT5G09230	Encodes SRT2, a member of the SIR2 (sirtuin) family HDAC (histone deacetylase) (SRT1/AT5g55760, SRT2/AT5G09230).	95.59	50.22	1.9	7.17E-04	1.75E-03
5043	7	F2-3 vs. S2-3	246832_at	AT5G26600	Pyridoxal phosphate (PLP)-dependent transferases superfamily protein;(source:Araport11)	407.58	214.02	1.9	4.49E-05	2.19E-04
5044	7	F2-3 vs. S2-3	246660_at	AT5G35180	ENHANCED DISEASE RESISTANCE protein (DUF1336);(source:Araport11)	210.74	110.94	1.9	1.65E-04	5.71E-04
5045	7	F2-3 vs. S2-3	257090_at	AT3G20530	Protein kinase superfamily protein, expressed in the peroxisome.	55.98	29.69	1.89	4.67E-05	2.26E-04
5046	7	F2-3 vs. S2-3	251197_at	AT3G62960	Encodes a member of the CC-type glutaredoxin (ROXY) family that has been shown to interact with the transcription factor TGA2.	37.31	19.7	1.89	5.36E-04	1.40E-03
5047	7	F2-3 vs. S2-3	253330_at	AT4G33530	potassium transporter	73.79	39.13	1.89	4.16E-04	1.15E-03
5048	7	F2-3 vs. S2-3	250827_at	AT5G05170	Encodes a cellulose synthase isomer. CESA3 mutants have cellulose defect in the primary cell wall. Multiple lines of evidence suggest that CESA3, along with CESA1 and CESA6 are present in the same plasma membrane complex for cellulose biosynthesis. As inferred from the null role of secondary wall-type CesAs, included in a set of five primary wall-type CesAs that may support trichome cell wall thickening. The xylem cells in primary root have reduced cell expansion and higher than normal lignification.	1204.63	636.25	1.89	1.82E-04	6.14E-04
5049	7	F2-3 vs. S2-3	246490_at	AT5G15950	Adenosylmethionine decarboxylase family protein;(source:Araport11)	338.42	178.61	1.89	2.51E-04	7.75E-04
5050	7	F2-3 vs. S2-3	264474_s_at	AT5G38420	Encodes a member of the Rubisco small subunit (RBCS) multigene family: RBCS1A (At1g67090), RBCS1B (At5g38430), RBCS2B (At5g38420), and RBCS3B (At5g38410). Activated by OXS2 under the treatment of salt.	16542.4	8760.31	1.89	5.76E-04	1.48E-03
5051	7	F2-3 vs. S2-3	261875_at	AT1G50610	Leucine-rich repeat protein kinase family protein;(source:Araport11)	23.16	12.32	1.88	6.89E-04	1.70E-03
5052	7	F2-3 vs. S2-3	260144_at	AT1G71960	Encodes a plasma membrane localized ABC transporter involved in abscisic acid transport and responses.	59.52	31.68	1.88	2.83E-04	8.51E-04
5053	7	F2-3 vs. S2-3	253372_at	AT4G33220	pectin methylesterase 44;(source:Araport11)	268.14	142.77	1.88	6.66E-04	1.65E-03
5054	7	F2-3 vs. S2-3	261405_at	AT1G18740	DUF793 domain containing protein. Expression is induced by cold. Loss of function mutations are more sensitive to freezing and have reduced levels of CBFs. May act by preventing degradation of CBFs.	713.93	381.46	1.87	3.21E-04	9.42E-04
5055	7	F2-3 vs. S2-3	260840_at	AT1G29050	Encodes a member of the TBL (TRICHOME BIREFRINGENCE-LIKE) gene family containing a plant-specific DUF231 (domain of unknown function) domain. TBL gene family has 46 members, two of which (TBR/AT5G06700 and TBL3/AT5G01360) have been shown to be involved in the synthesis and deposition of secondary wall cellulose, presumably by influencing the esterification state of pectic polymers. A nomenclature for this gene family has been proposed (Volker Bischoff & Wolf Scheible, 2010, personal communication).	57.68	30.77	1.87	5.15E-04	1.36E-03
5056	7	F2-3 vs. S2-3	264165_at	AT1G65410	Encodes a member of NAP subfamily of transporters. Mutations in this gene suppress the low temperature-induced phenotype of Arabidopsis tocopherol-deficient mutant vte2.	155.05	83	1.87	8.34E-04	1.97E-03
5057	7	F2-3 vs. S2-3	266371_at	AT2G41410	Calcium-binding EF-hand family protein;(source:Araport11)	62.46	33.32	1.87	9.82E-04	2.26E-03
5058	7	F2-3 vs. S2-3	265461_at	AT2G46500	Phosphoinositide kinase which undergo autophosphorylation and phosphorylate serine/threonine residues of protein substrates. Contains phosphoinositide 3/4-kinase and ubiquitin-like domains. Phosphorylates PUFD1 and RPN10 in vitro.	72.08	38.54	1.87	9.44E-04	2.18E-03
5059	7	F2-3 vs. S2-3	258889_at	AT3G05610	Plant invertase/pectin methylesterase inhibitor superfamily;(source:Araport11)	18.94	10.11	1.87	4.26E-04	1.17E-03
5060	7	F2-3 vs. S2-3	256266_at	AT3G12320	hypothetical protein;(source:Araport11)	64.27	34.45	1.87	1.71E-04	5.84E-04
5061	7	F2-3 vs. S2-3	250130_at	AT5G16510	RGP5 is a member of the reversably-glycosylated family of proteins. Analyses using tagged RGP5 suggest that it is present in the cytosol and in association with the Golgi apparatus. Recombinant RGP5 does not have UDP-arabinose mutase activity based on an in vitro assay even though the related RGP1, RGP2, and RGP3 proteins do have activity in the same assay. RGP5 can form complexes with RGP1 and RGP2.	205.13	109.73	1.87	3.79E-05	1.94E-04
5062	7	F2-3 vs. S2-3	249397_at	AT5G40230	nodulin MtN21-like transporter family protein	44.04	23.59	1.87	1.06E-04	4.09E-04

5063	7	F2-3 vs. S2-3	249327_at	AT5G40890	Encodes a member of the voltage-dependent chloride channel. Also functions as a NO3-/H+ exchanger that serves to accumulate nitrate nutrient in vacuoles. Mutants homozygous for the T-DNA insertion mutation have reduced nitrate uptake capacity in high nitrate environment and exhibit hypersensitivity to chlorate.	99.27	53.03	1.87	7.99E-04	1.90E-03
5064	7	F2-3 vs. S2-3	255719_at	AT1G32080	Encodes a plant LrgAB/CidAB protein localized to the chloroplast envelope that is involved in chloroplast development, carbon partitioning, ABA/drought response, and leaf senescence. The gene may have evolved from gene fusion of bacterial lrgA and lrgB.	78.83	42.43	1.86	9.07E-04	2.11E-03
5065	7	F2-3 vs. S2-3	263108_at	AT1G65240	Eukaryotic aspartyl protease family protein;(source:Araport11)	53.26	28.64	1.86	3.63E-04	1.04E-03
5066	7	F2-3 vs. S2-3	262933_at	AT1G65840	encodes a peroxisomal polyamine oxidase, involved in the back-conversion polyamine degradation pathway. Among the five polyamine oxidases in the Arabidopsis genome, PAO4 is the major isoform in root peroxisomes. The mRNA is cell-to-cell mobile.	57.07	30.65	1.86	2.35E-04	7.40E-04
5067	7	F2-3 vs. S2-3	266901_at	AT2G34600	jasmonate-zim-domain protein 7;(source:Araport11)	25.87	13.9	1.86	4.77E-04	1.27E-03
5068	7	F2-3 vs. S2-3	267388_at	AT2G44450	beta glucosidase 15;(source:Araport11)	67.55	36.23	1.86	8.39E-05	3.42E-04
5069	7	F2-3 vs. S2-3	257696_at	AT3G12690	Encodes a putative serine/threonine kinase It is expressed specifically in pollen and appears to function redundantly with AGC1.7 to regulate polarized growth of pollen tubes.	109.05	58.6	1.86	5.34E-04	1.39E-03
5070	7	F2-3 vs. S2-3	257672_at	AT3G20300	extracellular ligand-gated ion channel protein (DUF3537);(source:Araport11)	25.43	13.64	1.86	2.07E-04	6.78E-04
5071	7	F2-3 vs. S2-3	258044_at	AT3G21270	Encodes Dof zinc finger protein adof2.	93.46	50.11	1.86	6.15E-05	2.73E-04
5072	7	F2-3 vs. S2-3	251257_at	AT3G62290	A member of ARF GTPase family. A thaliana has 21 members of this family, known to be essential for vesicle coating and uncoating and functions in GTP-binding. The gene is shown to play a role in cell division, cell expansion and cellulose production using antisense construct. The mRNA is cell-to-cell mobile.	2842.36	1524.14	1.86	8.32E-04	1.97E-03
5073	7	F2-3 vs. S2-3	251082_at	AT5G01530	light harvesting complex photosystem II;(source:Araport11)	1217.76	654.21	1.86	1.32E-04	4.80E-04
5074	7	F2-3 vs. S2-3	250859_at	AT5G04660	encodes a protein with cytochrome P450 domain	71.96	38.73	1.86	7.91E-05	3.30E-04
5075	7	F2-3 vs. S2-3	249927_at	AT5G19220	Encodes the large subunit of ADP-glucose pyrophosphorylase which catalyzes the first, rate limiting step in starch biosynthesis. The large subunit plays a regulatory role whereas the small subunit (ApS) is the catalytic isoform. Four isoforms (ApL1-4) have been identified. ApL1 is the major large subunit isoform present in leaves. Mutational analysis of APS1 suggests that APL1 and APL2 can compensate for loss of APS1 catalytic activity,suggesting both have catalytic as well as regulatory functions.	20.38	10.97	1.86	5.64E-04	1.46E-03
5076	7	F2-3 vs. S2-3	248586_at	AT5G49610	F-box family protein;(source:Araport11)	79.53	42.87	1.86	7.20E-04	1.75E-03
5077	7	F2-3 vs. S2-3	248398_at	AT5G51970	Encodes a putative sorbitol dehydrogenase that can be thiolated in vitro.	249.98	134.18	1.86	5.34E-05	2.49E-04
5078	7	F2-3 vs. S2-3	261444_at	AT1G28490	Encodes SYP61, one of 24 Arabidopsis syntaxins. Its mRNA has been shown to be expressed. SYP61 and SYP121 coordinate the trafficking of plasma membrane aquaporin PIP2;7 to modulate the cell membrane water permeability.	137.14	74.33	1.85	5.74E-04	1.48E-03
5079	7	F2-3 vs. S2-3	260397_at	AT1G69680	ran guanine nucleotide release factor, putative (Mog1/PsbP/DUF1795-like photosystem II reaction center PsbP family protein);(source:Araport11)	140.21	75.94	1.85	3.56E-04	1.02E-03
5080	7	F2-3 vs. S2-3	262936_at	AT1G79400	member of Putative Na+/H+ antiporter family	24.03	13.01	1.85	5.22E-04	1.37E-03
5081	7	F2-3 vs. S2-3	264904_s_at	AT2G17420	NADPH-dependent thioredoxin reductase, major cytosolic isoformThe mRNA is cell-to-cell mobile.	507.59	273.65	1.85	2.78E-04	8.40E-04
5082	7	F2-3 vs. S2-3	267179_at	AT2G37550	A member of ARF GAP domain (AGD), A thaliana has 15 members, grouped into four classes.	38.4	20.8	1.85	3.06E-04	9.08E-04
5083	7	F2-3 vs. S2-3	266087_at	AT2G37790	NAD(P)-linked oxidoreductase superfamily protein;(source:Araport11)	45.07	24.34	1.85	1.38E-04	4.95E-04
5084	7	F2-3 vs. S2-3	266399_at	AT2G38670	Encodes a mitochondrial ethanolamine-phosphate cytidylyltransferase, involved in phosphatidylethanolamine (PE) biosynthesis.	223.42	120.87	1.85	7.35E-05	3.09E-04

5085	7	F2-3 vs. S2-3	252543_at	AT3G45780	Blue-light photoreceptor. Contains a light activated serine-threonine kinase domain and LOV1 and LOV2 repeats. Mutants are defective in blue-light response. Mediates blue light-induced growth enhancements. PHOT1 and PHOT2 mediate blue light-dependent activation of the plasma membrane H+-ATPase in guard cell protoplasts. PHOT1 undergoes blue-light-dependent autophosphorylation. At least eight phosphorylation sites have been identified in PHOT1. Phosphorylation of serine851 in the activation loop of PHOT1 appears to be required for stomatal opening, chloroplast accumulation, leaf flattening, and phototropism, and phosphorylation of serine849 may also contribute to the regulation of these responses. Phosphorylation-dependent binding of 14-3-3 proteins to the Hinge1 region of PHOT1 appears to require serine350 and serine376.	49.85	26.88	1.85	3.68E-04	1.05E-03
5086	7	F2-3 vs. S2-3	253052_at	AT4G37310	member of CYP81H	49.5	26.73	1.85	6.24E-04	1.57E-03
5087	7	F2-3 vs. S2-3	251133_at	AT5G01240	Encodes LAX1 (LIKE AUXIN RESISTANT), a member of the AUX1 LAX family of auxin influx carriers. Required for the establishment of embryonic root cell organization.	113.15	61.12	1.85	3.90E-04	1.09E-03
5088	7	F2-3 vs. S2-3	250613_at	AT5G07240	Member of IQ67 (CaM binding) domain containing family.	29.68	16.07	1.85	3.06E-04	9.07E-04
5089	7	F2-3 vs. S2-3	259173_at	AT3G03640	Encodes beta-glucosidase (GLUC).	27.09	14.72	1.84	6.70E-05	2.89E-04
5090	7	F2-3 vs. S2-3	250075_at	AT5G17670	alpha/beta-Hydrolases superfamily protein;(source:Araport11)	123.04	66.83	1.84	5.86E-05	2.65E-04
5091	7	F2-3 vs. S2-3	246153_s_at	AT5G20010	A member of RAN GTPase gene family. Encodes a small soluble GTP-binding protein. Likely to be involved in nuclear translocation of proteins. May also be involved in cell cycle progression. The mRNA is cell-to-cell mobile.	1437.37	779.32	1.84	2.73E-04	8.30E-04
5092	7	F2-3 vs. S2-3	245989_s_a	AT5G20620	encodes a ubiquitin polyprotein.	79.57	43.21	1.84	5.22E-04	1.37E-03
5093	7	F2-3 vs. S2-3	249284_at	AT5G41810	Avr9/Cf-9 rapidly elicited protein;(source:Araport11)	60.8	33.06	1.84	6.38E-04	1.60E-03
5094	7	F2-3 vs. S2-3	248508_at	AT5G50320	A subunit of Elongator, a histone acetyl transferase complex, consisting of six subunits (ELP1?ELP6), that copurifies with the elongating RNAPII in yeast and humans. Three Arabidopsis thaliana genes, encoding homologs of the yeast Elongator subunits ELP1, ELP3 (histone acetyl transferase), and ELP4 are responsible for the narrow leaf phenotype in elongata mutants and for reduced root growth that results from a decreased cell division rate. Two lines with RNAi constructs directed against HAG3 grow normally and can produce root calli, but have defects in agrobacterium-mediated transformation.	364.1	197.73	1.84	1.10E-04	4.20E-04
5095	7	F2-3 vs. S2-3	248082_at	AT5G55400	Encodes a member of the fimbrin family. Different members of the fimbrin/plastin family have diverged biochemically during evolution to generate either tight actin bundles or loose networks with distinct biochemical and biophysical properties. FIM4 generates both actin bundles and branched actin filaments whereas FIM5 only generates actin bundles.	38.66	21.02	1.84	8.91E-05	3.59E-04
5096	7	F2-3 vs. S2-3	259460_at	AT1G44000	STAY-GREEN-like protein;(source:Araport11)	92.27	50.47	1.83	8.53E-05	3.46E-04
5097	7	F2-3 vs. S2-3	265414_at	AT2G16660	Major facilitator superfamily protein;(source:Araport11)	503.59	275.61	1.83	1.42E-04	5.09E-04
5098	7	F2-3 vs. S2-3	258483_at	AT3G02570	Encodes a protein with phosphomannose isomerase activity.	86.31	47.17	1.83	1.67E-04	5.74E-04
5099	7	F2-3 vs. S2-3	257536_at	AT3G02800	Encodes an atypical dual-speciﬁcity phosphatase.	39.24	21.45	1.83	1.94E-04	6.42E-04
5100	7	F2-3 vs. S2-3	256548_at	AT3G14770	Nodulin MtN3 family protein;(source:Araport11)	30.61	16.68	1.83	1.24E-04	4.59E-04
5101	7	F2-3 vs. S2-3	245952_at	AT5G28500	rubisco accumulation factor-like protein;(source:Araport11)	62.74	34.22	1.83	9.65E-04	2.22E-03
5102	7	F2-3 vs. S2-3	261751_at	AT1G76080	Encodes a thioredoxin localized in chloroplast stroma. Known as CDSP32 (CHLOROPLASTIC DROUGHT-INDUCED STRESS PROTEIN OF 32 KD).	157.61	86.44	1.82	3.21E-04	9.42E-04
5103	7	F2-3 vs. S2-3	265913_at	AT2G25625	Histone deacetylase-like protein;(source:Araport11). Induced by senescence and abiotic stresses.	42.51	23.34	1.82	1.60E-04	5.56E-04
5104	7	F2-3 vs. S2-3	266483_at	AT2G47910	Encodes a chloroplast thylakoid membrane protein. Required for the assembly/accumulation of the NAD(P)H dehydrogenase complex of the photosynthetic electron transport chain.	24.41	13.42	1.82	2.13E-04	6.89E-04
5105	7	F2-3 vs. S2-3	257611_at	AT3G26580	Tetratricopeptide repeat (TPR)-like superfamily protein;(source:Araport11)	281.31	154.59	1.82	4.54E-04	1.23E-03
5106	7	F2-3 vs. S2-3	244996_at	ATCG00160	Chloroplast ribosomal protein S2	160.18	88.03	1.82	2.23E-04	7.13E-04
5107	7	F2-3 vs. S2-3	261570_at	AT1G01120	Encodes a condensing enzyme KCS1 (3-ketoacyl-CoA synthase 1) which is involved in the critical fatty acid elongation process in wax biosynthesis.	100.6	55.59	1.81	7.12E-04	1.74E-03
5108	7	F2-3 vs. S2-3	254108_at	AT4G25230	RPM1 interacting protein 2, has a CUE domain which is sufficient for the interaction with RPM1.Positive regulator of RPM1 and PRS2 mediated hypersensitive response.Functions as ubiquitin ligase and binds to RPM1.	90.63	50.17	1.81	8.82E-04	2.06E-03
5109	7	F2-3 vs. S2-3	253720_at	AT4G29270	HAD superfamily, subfamily IIIB acid phosphatase;(source:Araport11)	126.08	69.76	1.81	4.27E-04	1.17E-03

5110	7	F2-3 vs. S2-3	246230_at	AT4G36710	GRAS family transcription factor;(source:Araport11)	44.91	24.81	1.81	1.61E-04	5.59E-04
5111	7	F2-3 vs. S2-3	251143_at	AT5G01220	Encodes a UDP-sulfoquinovose:DAG sulfoquinovosyltransferase that is involved in sulfolipid biosynthesis and whose expression is responsive to both phosphate (Pi) and phosphite (Phi) in both roots and shoots.	16.64	9.21	1.81	3.11E-04	9.17E-04
5112	7	F2-3 vs. S2-3	246287_at	AT1G31870	Ortholog of yeast BUD13. Functions in pre mRNA processing of RNAs expressed in embryos.	307.98	171.2	1.8	8.48E-04	2.00E-03
5113	7	F2-3 vs. S2-3	264957_at	AT1G77000	AtSKP2;2 is a homolog of human SKP2, the human F-box protein that recruits E2F1. Contains an F-box motif at the N-terminal region and a C-terminal Leu-rich repeat domain. Forms part of an E3-ubiquitin-ligase SCF (Skp1, cullin, F-box) complex and recruits phosphorylated AtE2Fc, a transcriptional factor that might play a role in cell division and during the transition from skotomorphogenesis to photomorphogenesis. AtSKP2;1 (At1g21410) and AtSKP2;2 (At1g77000) may be duplicated genes. AtSKP2b may also be involved in the degradation of KRP1/ICK1, a CDK inhibitor.	30.62	16.99	1.8	1.90E-04	6.33E-04
5114	7	F2-3 vs. S2-3	266192_at	AT2G39080	NAD(P)-binding Rossmann-fold superfamily protein;(source:Araport11)	151.59	84.19	1.8	1.17E-04	4.40E-04
5115	7	F2-3 vs. S2-3	250234_at	AT5G13420	Aldolase-type TIM barrel family protein;(source:Araport11)	434.68	241.33	1.8	2.02E-04	6.65E-04
5116	7	F2-3 vs. S2-3	249059_at	AT5G44530	Subtilase family protein;(source:Araport11)	53.35	29.57	1.8	6.27E-04	1.58E-03
5117	7	F2-3 vs. S2-3	261462_at	AT1G07850	transferring glycosyl group transferase (DUF604);(source:Araport11)	23.98	13.39	1.79	4.17E-04	1.15E-03
5118	7	F2-3 vs. S2-3	261708_at	AT1G32740	SBP (S-ribonuclease binding protein) family protein;(source:Araport11)	26.66	14.92	1.79	1.03E-04	4.00E-04
5119	7	F2-3 vs. S2-3	266835_at	AT2G29990	alternative NAD(P)H dehydrogenase 2;(source:Araport11)	38.39	21.42	1.79	9.94E-04	2.28E-03
5120	7	F2-3 vs. S2-3	258193_at	AT3G29090	Encodes an atypical pectin methylesterase that does not require salt for its activity and has a blockwise mode of pectin demethylesterification.	92.23	51.42	1.79	2.56E-04	7.88E-04
5121	7	F2-3 vs. S2-3	246486_at	AT5G15910	NAD(P)-binding Rossmann-fold superfamily protein;(source:Araport11)	39.64	22.15	1.79	6.78E-04	1.68E-03
5122	7	F2-3 vs. S2-3	256119_at	AT1G18070	Translation elongation factor EF1A/initiation factor IF2gamma family protein;(source:Araport11)	112.97	63.35	1.78	1.26E-04	4.63E-04
5123	7	F2-3 vs. S2-3	256022_at	AT1G58360	Encodes AAP1 (amino acid permease 1), a neutral amino acid transporter expressed in seeds. Functions in amino acid uptake into embryos. The transporter also functions in acquisition of glutamate and neutral amino acids by the root.	219.87	123.72	1.78	6.84E-05	2.94E-04
5124	7	F2-3 vs. S2-3	265739_at	AT2G01270	Encodes a protein disulfide isomerase-like (PDIL) protein, a member of a multigene family within the thioredoxin (TRX) superfamily. This protein also belongs to the quiescin-sulfhydryl oxidase (QSOX) family, which possess an Erv1-like domain at the COOH terminus in addition to a TRX domain.	243.56	136.78	1.78	1.69E-04	5.79E-04
5125	7	F2-3 vs. S2-3	245151_at	AT2G47550	Plant invertase/pectin methylesterase inhibitor superfamily;(source:Araport11)	21.72	12.17	1.78	1.32E-04	4.80E-04
5126	7	F2-3 vs. S2-3	258655_at	AT3G09810	Encodes a catalytic subunit of the mitochondrially-localized NAD+- dependent isocitrate dehydrogenase	47.01	26.39	1.78	2.24E-04	7.17E-04
5127	7	F2-3 vs. S2-3	258314_at	AT3G16100	RAB GTPase homolog G3C;(source:Araport11)	91.86	51.72	1.78	5.88E-04	1.51E-03
5128	7	F2-3 vs. S2-3	246673_at	AT5G30510	ribosomal protein S1;(source:Araport11)	1464.9	823.67	1.78	1.15E-04	4.33E-04
5129	7	F2-3 vs. S2-3	248250_at	AT5G53130	member of Cyclic nucleotide gated channel family	101.9	57.19	1.78	5.76E-04	1.48E-03
5130	7	F2-3 vs. S2-3	261065_at	AT1G07500	SMR5 is a member of the SIAMESE-RELATED Cyclin-Dependent Kinase Inhibitor family. It is induced by ROS/oxidative stress.	24.23	13.68	1.77	8.33E-05	3.41E-04
5131	7	F2-3 vs. S2-3	264372_at	AT1G11840	Encodes Ni+ dependent glyoxalase I homolog ATGLX1.	1518.38	859.37	1.77	9.68E-04	2.23E-03
5132	7	F2-3 vs. S2-3	245756_at	AT1G35190	2-oxoglutarate (2OG) and Fe(II)-dependent oxygenase superfamily protein;(source:Araport11)	56.93	32.11	1.77	6.57E-04	1.64E-03
5133	7	F2-3 vs. S2-3	263748_at	AT2G21480	BUSP2 plays a smaller role than BUSP1 in pollen tube growth. bups1/2 double mutants have reduced feritlity due to premature rupture of pollen tubes before they reach the ovule but single busp2 mutants are fertile. BUSP2 interacts with RALF4/19 peptide ligands and ANX1/2 receptors. BUPS/ANX signaling may regulate and promote pollen tube growth.	17.92	10.13	1.77	8.08E-04	1.92E-03
5134	7	F2-3 vs. S2-3	256396_at	AT3G06150	cytochrome P450 family protein;(source:Araport11)	46.71	26.43	1.77	7.09E-04	1.73E-03
5135	7	F2-3 vs. S2-3	254886_at	AT4G11760	Encodes a member of a family of small,secreted, cysteine rich protein with sequence similarity to the PCP (pollen coat protein) gene family.	24.12	13.65	1.77	8.10E-04	1.92E-03
5136	7	F2-3 vs. S2-3	246253_at	AT4G37260	Member of the R2R3 factor gene family. The mRNA is cell-to-cell mobile.	221.38	125.4	1.77	6.10E-04	1.55E-03
5137	7	F2-3 vs. S2-3	252997_at	AT4G38400	member of EXPANSIN-LIKE. Naming convention from the Expansin Working Group (Kende et al, 2004. Plant Mol Bio)	75.81	42.8	1.77	6.55E-04	1.63E-03
5138	7	F2-3 vs. S2-3	250408_at	AT5G10930	Encodes CBL-interacting protein kinase 5 (CIPK5).	36.09	20.34	1.77	1.76E-04	5.98E-04
5139	7	F2-3 vs. S2-3	245207_at	AT5G12310	RING/U-box superfamily protein;(source:Araport11)	69.6	39.35	1.77	6.66E-04	1.65E-03
5140	7	F2-3 vs. S2-3	259403_at	AT1G17745	encodes a 3-Phosphoglycerate dehydrogenase The mRNA is cell-to-cell mobile.	290.89	165.52	1.76	5.02E-04	1.33E-03

5141	7	F2-3 vs. S2-3	262546_at	AT1G31260	member of Fe(II) transporter isolog family	29.4	16.73	1.76	1.24E-04	4.57E-04
5142	7	F2-3 vs. S2-3	263594_at	AT2G01880	purple acid phosphatase 7;(source:Araport11)	40.07	22.82	1.76	2.54E-04	7.83E-04
5143	7	F2-3 vs. S2-3	266079_at	AT2G37860	Encodes a protein involved in differential development of bundle sheath and mesophyll cell chloroplasts.	57.5	32.75	1.76	5.02E-04	1.33E-03
5144	7	F2-3 vs. S2-3	267080_at	AT2G41190	Transmembrane amino acid transporter family protein;(source:Araport11)	86.69	49.21	1.76	2.09E-04	6.83E-04
5145	7	F2-3 vs. S2-3	254578_at	AT4G19410	Pectinacetylesterase family protein;(source:Araport11)	996.56	566.57	1.76	5.87E-04	1.50E-03
5146	7	F2-3 vs. S2-3	261579_at	AT1G01050	Encodes a soluble protein with inorganic pyrophosphatase activity that is highly specific for Mg-inorganic pyrophosphate.	242.8	138.93	1.75	2.14E-04	6.91E-04
5147	7	F2-3 vs. S2-3	259771_at	AT1G29470	S-adenosyl-L-methionine-dependent methyltransferases superfamily protein;(source:Araport11)	347.13	198.06	1.75	1.02E-04	3.98E-04
5148	7	F2-3 vs. S2-3	267477_at	AT2G02710	Encodes a putative blue light receptor protein.	93.07	53.25	1.75	5.17E-04	1.36E-03
5149	7	F2-3 vs. S2-3	257337_at	ATMG00060	Mitochondrial NADH dehydrogenase subunit 5. The gene is trans-spliced from the three different pre-cursors, NAD5a, NAD5b and NAD5c.	28.98	16.6	1.75	4.24E-04	1.17E-03
5150	7	F2-3 vs. S2-3	264235_at	AT1G54560	Encodes a class XI myosin that is involved in organelle motility, actin organization, and optimal growth of pollen tubes.	36.47	20.94	1.74	3.81E-04	1.07E-03
5151	7	F2-3 vs. S2-3	258452_at	AT3G22370	Encodes AOX1a, an isoform of alternative oxidase that is expressed in rosettes, flowers, and root. The alternative oxidase of plant mitochondria transfers electrons from the ubiquinone pool to oxygen without energy conservations. It is regulated through transcriptional control and by pyruvate. Plays a role in shoot acclimation to low temperature. Also is capable of ameliorating reactive oxygen species production when the cytochrome pathway is inhibited. AOX1a also functions as a marker for mitochondrial retrograde response. The mRNA is cell-to-cell mobile.	110.07	63.34	1.74	2.94E-04	8.81E-04
5152	7	F2-3 vs. S2-3	251163_at	AT3G63310	Mediates cell elongation in brassinosteroid signaling.	138.12	79.3	1.74	7.10E-04	1.73E-03
5153	7	F2-3 vs. S2-3	254961_at	AT4G11030	AMP-dependent synthetase and ligase family protein;(source:Araport11)	18.6	10.71	1.74	1.77E-04	5.99E-04
5154	7	F2-3 vs. S2-3	245308_at	AT4G17486	PPPDE putative thiol peptidase family protein;(source:Araport11)	210.72	120.84	1.74	3.34E-04	9.69E-04
5155	7	F2-3 vs. S2-3	250262_at	AT5G13410	FKBP-like peptidyl-prolyl cis-trans isomerase family protein;(source:Araport11)	321.2	184.29	1.74	4.26E-04	1.17E-03
5156	7	F2-3 vs. S2-3	247319_at	AT5G64050	Glutamate-tRNA ligase. Targeted to mitochondria and chloroplast. Its inactivation causes developmental arrest of chloroplasts and mitochondria in Nicotiana benthamiana.	193.55	111.51	1.74	8.69E-04	2.03E-03
5157	7	F2-3 vs. S2-3	262638_at	AT1G06650	encodes a protein whose sequence is similar to 2-oxoglutarate-dependent dioxygenase The mRNA is cell-to-cell mobile.	20.13	11.63	1.73	1.36E-04	4.90E-04
5158	7	F2-3 vs. S2-3	256019_at	AT1G58265	Cytochrome P450 superfamily protein;(source:Araport11)	21.34	12.35	1.73	3.33E-04	9.66E-04
5159	7	F2-3 vs. S2-3	262950_at	AT1G75510	Transcription initiation factor IIF, beta subunit;(source:Araport11)	83.57	48.3	1.73	8.10E-04	1.92E-03
5160	7	F2-3 vs. S2-3	257807_at	AT3G26650	Encodes one of the two subunits forming the photosynthetic glyceraldehyde-3-phosphate dehydrogenase (GAPDH) and as such a constituent of the supramolecular complex with phosphoribulokinase (PRK) thought to be linked by a small peptide encoded by CP12-2. GapA-1 is coordinately expressed by light with PRK and CP12-2. The enzyme activity, tested in leaf protein extracts dropped significantly after external sucrose treatment for the photosynthetic GAPDH (NADPH-dependent) but not for the cytosolic GAPDH (NADH-dependent).	614.7	354.7	1.73	1.37E-04	4.93E-04
5161	7	F2-3 vs. S2-3	251664_at	AT3G56940	Encodes a putative ZIP protein with varying mRNA accumulation in leaves, stems and roots. Has a consensus carboxylate-bridged di-iron binding site. The mRNA is cell-to-cell mobile.	476.3	275.88	1.73	1.67E-04	5.75E-04
5162	7	F2-3 vs. S2-3	253428_at	AT4G32410	Encodes a cellulose synthase isomer. CESA1 mutants have cellulose defect in the primary cell wall. Multiple lines of evidence suggest that CESA1, along with CESA3 and CESA6 are present in the same plasma membrane complex for cellulose biosynthesis. lasma membrane complex for cellulose biosynthesis. As inferred from the null role of secondary wall-type CesAs, included in a set of five primary wall-type CesAs that may support trichome cell wall thickening.	265.56	153.72	1.73	2.71E-04	8.26E-04
5163	7	F2-3 vs. S2-3	250867_at	AT5G03880	Thioredoxin family protein;(source:Araport11)	389.45	224.59	1.73	4.43E-04	1.20E-03
5164	7	F2-3 vs. S2-3	248693_at	AT5G48330	Regulator of chromosome condensation (RCC1) family protein;(source:Araport11)	44.32	25.68	1.73	1.37E-04	4.93E-04
5165	7	F2-3 vs. S2-3	247763_at	AT5G59180	Non-catalytic subunit specific to DNA-directed RNA polymerase II; the ortholog of budding yeast RPB7	116.13	67.23	1.73	9.97E-04	2.28E-03
5166	7	F2-3 vs. S2-3	259774_at	AT1G29520	AWPM-19-like family protein;(source:Araport11)	36.38	21.1	1.72	2.26E-04	7.20E-04
5167	7	F2-3 vs. S2-3	260097_at	AT1G73220	Encodes Organic Cation Transporter 1 (OCT1), likely to be involved in polyamine transport.	62.19	36.06	1.72	1.64E-04	5.67E-04

5168	7	F2-3 vs. S2-3	245076_at	AT2G23170	encodes an IAA-amido synthase that conjugates Asp and other amino acids to auxin in vitro.	21.49	12.53	1.72	3.96E-04	1.11E-03
5169	7	F2-3 vs. S2-3	265722_at	AT2G40100	Lhcb4:3 protein (Lhcb4.3, light harvesting complex of photosystem II The mRNA is cell-to-cell mobile.	209.8	122.01	1.72	9.45E-04	2.18E-03
5170	7	F2-3 vs. S2-3	251928_at	AT3G53980	Bifunctional inhibitor/lipid-transfer protein/seed storage 2S albumin superfamily protein;(source:Araport11)	95.14	55.36	1.72	2.48E-04	7.69E-04
5171	7	F2-3 vs. S2-3	254737_at	AT4G13840	HXXXD-type acyl-transferase family protein;(source:Araport11)	31.68	18.39	1.72	2.81E-04	8.46E-04
5172	7	F2-3 vs. S2-3	252998_at	AT4G38510	One of three genes encoding the vacuolar ATP synthase subunit B1. The protein binds to and co-localizes with F-actin, bundles F-actin to form higher-order structure, and stabilizes actin filaments in vitro.	226	131.28	1.72	1.96E-04	6.49E-04
5173	7	F2-3 vs. S2-3	245119_at	AT2G41640	Glycosyltransferase family 61 protein;(source:Araport11)	18.17	10.66	1.7	2.48E-04	7.69E-04
5174	7	F2-3 vs. S2-3	253216_at	AT4G34960	Cyclophilin-like peptidyl-prolyl cis-trans isomerase family protein;(source:Araport11)	44.54	26.18	1.7	4.46E-04	1.21E-03
5175	7	F2-3 vs. S2-3	252861_at	AT4G39820	Part of multi-protein complex, acting as guanine nucleotide exchange factors (GEFs) and possibly as tethers, regulating intracellular trafficking.	183.5	107.86	1.7	1.50E-04	5.32E-04
5176	7	F2-3 vs. S2-3	261709_at	AT1G32790	RNA-binding protein, putative, similar to RNA-binding protein GB:CAB40027 GI:4539439 from (Arabidopsis thaliana).Member of a family of PAB2 binding domain proteins.	220.63	130.58	1.69	3.27E-04	9.53E-04
5177	7	F2-3 vs. S2-3	259756_at	AT1G71080	RNA polymerase II transcription elongation factor;(source:Araport11)	90.98	53.82	1.69	4.32E-04	1.18E-03
5178	7	F2-3 vs. S2-3	266638_at	AT2G35490	Involved in photoprotection of photosystem II.	576.61	341.07	1.69	2.40E-04	7.50E-04
5179	7	F2-3 vs. S2-3	251801_at	AT3G55440	Encodes triosephosphate isomerase.	4378.55	2597.6	1.69	2.62E-04	8.02E-04
5180	7	F2-3 vs. S2-3	251666_at	AT3G57050	Encodes cystathionine beta-lyase, the second enzyme in the methionine biosynthetic pathway. Mutants show defects in root development, reduced methylation and maintenance of the quiescent center.	578.97	342.89	1.69	2.35E-04	7.40E-04
5181	7	F2-3 vs. S2-3	255364_s_at	AT4G04020	Fibrillin precursor protein. The fibrillin preprotein, but not the mature protein interacts with ABI2. Regulated by abscisic acid response regulators. Involved in abscisic acid-mediated photoprotection. The mRNA is cell-to-cell mobile.	17.95	10.61	1.69	2.71E-04	8.26E-04
5182	7	F2-3 vs. S2-3	255305_at	AT4G04770	Encodes an iron-stimulated ATPase. A member of the NAP subfamily of ABC transporters. Involved in Fe-S cluster assembly. Similar to SufB. Involved in the regulation of iron homeostasis. Able to form homodimers. Interacts with AtNAP7 inside the chloroplast.	51.11	30.18	1.69	7.26E-04	1.76E-03
5183	7	F2-3 vs. S2-3	249835_s_at	AT5G23490	hypothetical protein;(source:Araport11)	26.27	15.54	1.69	8.60E-04	2.02E-03
5184	7	F2-3 vs. S2-3	247843_at	AT5G58050	Encodes a member of the glycerophosphodiester phosphodiesterase like (GDPD-like) family.	57.5	33.94	1.69	9.94E-04	2.28E-03
5185	7	F2-3 vs. S2-3	256053_at	AT1G07260	Encodes a uridine diphosphate-glycosyltransferase that acts on methyl salicylate (MeSA) to form MeSA glucosides in vitro and in vivo and facilitates negative regulation of the SAR response by modulating homeostasis of MeSA and SA.	17.74	10.58	1.68	4.00E-04	1.11E-03
5186	7	F2-3 vs. S2-3	264504_at	AT1G09430	Encodes subunit A of the heteromeric enzyme ATP citrate lyase (ACL). In animals, ACL is encoded by a single gene; ACL in Arabidopsis is composed of two polypeptides, ACLA (encoded by 3 genes) and ACLB (encoded by 2 genes). The holoenzyme has an A(4)B(4)stoichiometry. Expression of both ACLA and ACLB but not of either of the subunits alone results in ACL activity.	71.33	42.52	1.68	3.06E-04	9.07E-04
5187	7	F2-3 vs. S2-3	255756_at	AT1G19940	glycosyl hydrolase 9B5;(source:Araport11)	23.81	14.18	1.68	3.85E-04	1.08E-03
5188	7	F2-3 vs. S2-3	256213_at	AT1G50990	kinase with tetratricopeptide repeat domain-containing protein;(source:Araport11)	29.51	17.59	1.68	7.56E-04	1.81E-03
5189	7	F2-3 vs. S2-3	260746_at	AT1G78380	Encodes a glutathione transferase that is a member of Tau GST gene family. Expression is induced by drought stress, oxidative stress, and high doses of auxin and cytokinin. naming convention according to Wagner et al. (2002) The expression of this gene is upregulated by herbicide safeners such as benoxacor and fenclorim.	268.65	160.12	1.68	8.92E-04	2.08E-03
5190	7	F2-3 vs. S2-3	256956_at	AT3G13410	2-C-methyl-D-erythritol 4-phosphate cytidylyltransferase;(source:Araport11)	676.06	402.5	1.68	2.94E-04	8.81E-04
5191	7	F2-3 vs. S2-3	258218_at	AT3G18000	Encodes a N-methyltransferase-like protein. Double mutants of NMT1 and NMT3 are defective in leaf, root, flower, seed, and pollen development.	39.69	23.61	1.68	8.46E-04	1.99E-03
5192	7	F2-3 vs. S2-3	253279_at	AT4G34030	MCC-B is involved in leucine degradation in mitochondria. The active protein is a dimer of MCC-A and MCC-B. MCC-A is biotinylated whereas MCC-B is not. The mRNA is cell-to-cell mobile.	119.83	71.54	1.68	5.99E-04	1.53E-03
5193	7	F2-3 vs. S2-3	250095_at	AT5G17230	Encodes phytoene synthase that is the rate-limiting enzyme in the carotenoid biosynthetic pathway and that interacts with the ORANGE (OR) protein. PSY's expression is posttransciptionally regulated by OR.	61.5	36.59	1.68	2.61E-04	8.00E-04
5194	7	F2-3 vs. S2-3	260474_at	AT1G11090	alpha/beta-Hydrolases superfamily protein;(source:Araport11)	76.4	45.74	1.67	6.10E-04	1.55E-03

5195	7	F2-3 vs. S2-3	256115_at	AT1G16880	Encodes a ACT domain-containing protein. The ACT domain, named after bacterial aspartate kinase, chorismate mutase and TyrA (prephenate dehydrogenase), is a regulatory domain that serves as an amino acid-binding site in feedback-regulated amino acid metabolic enzymes. The mRNA is cell-to-cell mobile.	236.89	141.64	1.67	2.17E-04	6.99E-04
5196	7	F2-3 vs. S2-3	261324_at	AT1G44770	elongation factor;(source:Araport11)	518.45	310.12	1.67	3.97E-04	1.11E-03
5197	7	F2-3 vs. S2-3	263922_s_a	AT2G36580	Pyruvate kinase family protein;(source:Araport11)	191.59	114.83	1.67	3.65E-04	1.04E-03
5198	7	F2-3 vs. S2-3	258369_at	AT3G14310	encodes a pectin methylesterase, targeted by a cellulose binding protein (CBP) from the parasitic nematode Heterodera schachtii during parasitism.	121.86	73.05	1.67	4.02E-04	1.12E-03
5199	7	F2-3 vs. S2-3	257824_at	AT3G25290	Auxin-responsive family protein;(source:Araport11)	24.19	14.45	1.67	4.44E-04	1.21E-03
5200	7	F2-3 vs. S2-3	258060_at	AT3G26030	protein phosphatase 2A regulatory subunit isoform B' delta The mRNA is cell-to-cell mobile.	142.02	85.16	1.67	1.96E-04	6.49E-04
5201	7	F2-3 vs. S2-3	254197_at	AT4G24040	Encodes a trehalase, member of Glycoside Hydrolase Family 37.	39.7	23.78	1.67	6.16E-04	1.56E-03
5202	7	F2-3 vs. S2-3	250948_at	AT5G03490	Encodes a dihydroxybenzoic acid (DHBA) glycosyltransferase. The Col-0 enzyme is responsible for biosynthesis of 2,3-DHBA xyloside and 2,5-DHBA xyloside. The Col-0 enzyme is specific for UDP-xylose and the C24 enzyme uses both UDP-glucose and UDP-xylose. This difference in sugar donor specificity was shown to be largely due to a single amino acid change between the two isoforms.	46.77	28.01	1.67	2.90E-04	8.71E-04
5203	7	F2-3 vs. S2-3	248833_at	AT5G47120	Encodes BI-1, a homolog of mammalian Bax inhibitor 1. Functions as an attenuator of biotic and abiotic types of cell death. Bax-induced cell death can be downregulated by ectopically expressing AtBI in planta. The mRNA is cell-to-cell mobile.	1154.44	690.28	1.67	4.74E-04	1.27E-03
5204	7	F2-3 vs. S2-3	258925_at	AT3G10420	P-loop containing nucleoside triphosphate hydrolases superfamily protein;(source:Araport11)	230.92	139.24	1.66	2.80E-04	8.45E-04
5205	7	F2-3 vs. S2-3	257120_at	AT3G20200	kinase with adenine nucleotide alpha hydrolases-like domain-containing protein;(source:Araport11)	27.5	16.54	1.66	3.47E-04	9.99E-04
5206	7	F2-3 vs. S2-3	252037_at	AT3G51920	encodes a divergent member of calmodulin, which is an EF-hand family of Ca2+-binding proteins. This gene is expressed in leaves, flowers and siliques. The gene functionally complements yeast calmodulin 1 (CAM1) but only when selected against the plasmid harboring wild-type yeast sequences. Also the protein does not form formed a complex with a basic amphiphilic helical peptide in the presence of Ca2+ in vitro. Authors suggest that this gene may represent a Ca2+-binding sensor protein that interacts with a more limited set of target proteins than do more conventional CaM isoforms. Mutations in this gene alter plant responses to abiotic stress and abscisic acid.	17.67	10.63	1.66	4.95E-04	1.31E-03
5207	7	F2-3 vs. S2-3	255453_at	AT4G02900	ERD (early-responsive to dehydration stress) family protein;(source:Araport11)	23.06	13.92	1.66	2.48E-04	7.69E-04
5208	7	F2-3 vs. S2-3	250137_at	AT5G15390	tRNA/rRNA methyltransferase (SpoU) family protein;(source:Araport11)	137.46	82.79	1.66	2.28E-04	7.25E-04
5209	7	F2-3 vs. S2-3	248221_at	AT5G53530	Homolog of yeast retromer subunit VPS26. Part of a retromer-like protein complex involved in endosome to lysosome protein transport.	278.28	167.28	1.66	8.69E-04	2.03E-03
5210	7	F2-3 vs. S2-3	259850_at	AT1G72240	hypothetical protein;(source:Araport11)	24.01	14.52	1.65	5.24E-04	1.38E-03
5211	7	F2-3 vs. S2-3	265415_at	AT2G20890	Chloroplast-localized Thylakoid formation1 gene product involved in vesicle-mediated formation of thylakoid membranes. Thf1 antisense lines contain abnormal chloroplasts early in leaf development (chloroplasts have loosely stacked thylakoid membranes). Expression was induced in the light and decreased under dark conditions. G-alpha interaction partner that functions downstream of the plasma membrane?delimited heterotrimeric G-protein (GPA1) in a D-glucose signaling pathway. Localized to both the outer plastid membrane and the stroma. Probably involved in the metabolic pathway that controls the assembly of the PS II complex. The mRNA is cell-to-cell mobile.	495.51	299.53	1.65	1.84E-04	6.19E-04
5212	7	F2-3 vs. S2-3	257382_at	AT2G40750	member of WRKY Transcription Factor; Group III	21.07	12.78	1.65	6.07E-04	1.54E-03
5213	7	F2-3 vs. S2-3	260500_at	AT2G41705	Encodes a fluoride export protein.	41.11	24.88	1.65	3.66E-04	1.04E-03
5214	7	F2-3 vs. S2-3	256804_at	AT3G20920	Encodes an endoplasmic reticulum localized protein with similarity to yeast Sec62p. Mutants display growth defects and significantly reduced fertility. AtSec62 does not complement the thermosensitive phenotype of yeast Sec62 mutants.	431.92	262.19	1.65	6.65E-04	1.65E-03
5215	7	F2-3 vs. S2-3	252483_at	AT3G46600	GRAS family transcription factor;(source:Araport11)	49.9	30.16	1.65	9.98E-04	2.28E-03
5216	7	F2-3 vs. S2-3	256679_at	AT3G52300	ATP synthase D chain;(source:Araport11)	259.6	157.62	1.65	5.62E-04	1.45E-03
5217	7	F2-3 vs. S2-3	254188_at	AT4G23920	Encodes a protein with UDP-D-glucose 4-epimerase activity. Involved in growth and cell wall carbohydrate biosynthesis.	83.88	50.78	1.65	9.84E-04	2.26E-03

5218	7	F2-3 vs. S2-3	246427_at	AT5G17400	This gene is predicted to encode an ER-localized adenine nucleotide transporter with six putative transmembrane helices. It appears to act as a ATP:ADP antiporter when expressed in E.coli plasma membranes. Transcript levels for several ER-localized chaperones (e.g. BIP1/2) and other ATP-requiring ER proteins (e.g. CPK2) are reduced in er-ant1 knock-out lines, suggesting a lack of adequate ATP transport into the ER in these mutants. They also have reduced seed oil and seed protein levels.	39.96	24.23	1.65	2.66E-04	8.12E-04
5219	7	F2-3 vs. S2-3	248061_at	AT5G55340	MBOAT (membrane bound O-acyl transferase) family protein;(source:Araport11)	36.31	21.97	1.65	1.89E-04	6.32E-04
5220	7	F2-3 vs. S2-3	264455_at	AT1G10330	Tetratricopeptide repeat (TPR)-like superfamily protein;(source:Araport11)	77.19	46.98	1.64	9.06E-04	2.11E-03
5221	7	F2-3 vs. S2-3	261933_at	AT1G22410	Class-II DAHP synthetase family protein;(source:Araport11)	592.59	361.84	1.64	2.66E-04	8.13E-04
5222	7	F2-3 vs. S2-3	263374_at	AT2G20560	DNAJ heat shock family protein;(source:Araport11)	23.96	14.65	1.64	5.58E-04	1.44E-03
5223	7	F2-3 vs. S2-3	245252_at	AT4G17500	Encodes a member of the ERF (ethylene response factor) subfamily B-3 of ERF/AP2 transcription factor family (ATERF-1). The protein contains one AP2 domain. There are 18 members in this subfamily including ATERF-1, ATERF-2, AND ATERF-5. The mRNA is cell-to-cell mobile.	26.52	16.2	1.64	9.42E-04	2.18E-03
5224	7	F2-3 vs. S2-3	250829_at	AT5G04720	Encodes a member of the ADR1 family nucleotide-binding leucine-rich repeat (NB-LRR) immune receptors. The mRNA is cell-to-cell mobile.	23.59	14.37	1.64	3.16E-04	9.29E-04
5225	7	F2-3 vs. S2-3	249917_at	AT5G22460	alpha/beta-Hydrolases superfamily protein;(source:Araport11)	115.46	70.41	1.64	3.22E-04	9.44E-04
5226	7	F2-3 vs. S2-3	266000_at	AT2G24180	Encodes a cytochrome P450 monooxygenase that converts indole-3-acetonitrile to indole-3-aldehyde / indole-3-carboxylic acid and cyanide. The mRNA is cell-to-cell mobile.	30.6	18.77	1.63	3.45E-04	9.95E-04
5227	7	F2-3 vs. S2-3	253521_at	AT4G31300	Encodes 20S proteasome subunit PBA1 (PBA1). PBA1 acts as a plant caspase-3-like enzyme.	1238.92	759.91	1.63	6.85E-04	1.69E-03
5228	7	F2-3 vs. S2-3	250621_at	AT5G07250	RHOMBOID-like protein 3;(source:Araport11)	170.59	104.51	1.63	3.84E-04	1.08E-03
5229	7	F2-3 vs. S2-3	249677_at	AT5G35970	P-loop containing nucleoside triphosphate hydrolases superfamily protein;(source:Araport11)	173.69	106.63	1.63	5.56E-04	1.44E-03
5230	7	F2-3 vs. S2-3	245164_at	AT2G33210	Involved in the RNA splicing of rpl2 and ccmFC introns in mitochondria.	660.87	407	1.62	7.79E-04	1.86E-03
5231	7	F2-3 vs. S2-3	246201_at	AT4G36750	Quinone reductase family protein;(source:Araport11)	26.66	16.46	1.62	7.55E-04	1.81E-03
5232	7	F2-3 vs. S2-3	265943_at	AT2G19570	Encodes a cytidine deaminase that deaminates cytidine and deoxycytidine and is competitively inhibited by cytosine-containing compounds.	85.78	53.35	1.61	4.41E-04	1.20E-03
5233	7	F2-3 vs. S2-3	252622_at	AT3G45310	Cysteine proteinases superfamily protein;(source:Araport11)	249.13	155.13	1.61	7.23E-04	1.76E-03
5234	7	F2-3 vs. S2-3	253475_at	AT4G32290	Core-2/I-branching beta-1,6-N-acetylglucosaminyltransferase family protein;(source:Araport11)	54.05	33.63	1.61	9.99E-04	2.29E-03
5235	7	F2-3 vs. S2-3	251578_at	AT3G58180	ARM repeat superfamily protein;(source:Araport11)	68.26	42.76	1.6	3.82E-04	1.08E-03
5236	7	F2-3 vs. S2-3	253333_at	AT4G33510	Enzyme catalyzing the first committed step in aromatic amino acid biosynthesis The mRNA is cell-to-cell mobile.	66.38	41.55	1.6	6.93E-04	1.70E-03
5237	7	F2-3 vs. S2-3	249519_at	AT5G38660	mutant has Altered acclimation responses;	135.43	84.44	1.6	9.46E-04	2.18E-03
5238	7	F2-3 vs. S2-3	256506_at	AT1G75160	DUF620 family protein (DUF620);(source:Araport11)	20.6	12.98	1.59	3.94E-04	1.10E-03
5239	7	F2-3 vs. S2-3	259180_at	AT3G01680	Encodes a protein localized to phloem filaments that is required for phloem filament formation. The mRNA is cell-to-cell mobile.	29.01	18.26	1.59	7.52E-04	1.81E-03
5240	7	F2-3 vs. S2-3	257606_at	AT3G13870	required for regulated cell expansion and normal root hair development. Encodes an evolutionarily conserved protein with putative GTP-binding motifs that is implicated in the control of vesicle trafficking between the endoplasmic reticulum and the Golgi compartments.	276.34	173.61	1.59	4.26E-04	1.17E-03
5241	7	F2-3 vs. S2-3	258126_at	AT3G24490	Alcohol dehydrogenase transcription factor Myb/SANT-like family protein;(source:Araport11)	239.54	150.56	1.59	7.23E-04	1.76E-03
5242	7	F2-3 vs. S2-3	245715_s_at	AT5G08690	Encodes the mitochondrial ATP synthase beta-subunit. This subunit is encoded by a multigene family of three members (At5g08670, At5g08680, At5g08690) that shared 98% sequence identity at the amino acid level. The mRNA is cell-to-cell mobile.	1349.84	847.8	1.59	8.44E-04	1.99E-03
5243	7	F2-3 vs. S2-3	248497_at	AT5G50380	A member of EXO70 gene family, putative exocyst subunits, conserved in land plants. Arabidopsis thaliana contains 23 putative EXO70 genes, which can be classified into eight clusters on the phylogenetic tree.	27.12	17.01	1.59	9.05E-04	2.11E-03
5244	7	F2-3 vs. S2-3	260800_at	AT1G78240	Encodes TSD2 (TUMOROUS SHOOT DEVELOPMENT2), a putative methyltransferase with an essential role in cell adhesion and coordinated plant development.	633.36	401.38	1.58	5.06E-04	1.33E-03

5245	7	F2-3 vs. S2-3	258722_at	AT3G09590	CAP (Cysteine-rich secretory proteins, Antigen 5, and Pathogenesis-related 1 protein) superfamily protein;(source:Araport11)	21.35	13.48	1.58	8.22E-04	1.94E-03
5246	7	F2-3 vs. S2-3	252371_at	AT3G48070	RING/U-box superfamily protein;(source:Araport11)	91.46	58.11	1.57	5.65E-04	1.46E-03
5247	7	F2-3 vs. S2-3	247040_at	AT5G67150	HXXXD-type acyl-transferase family protein;(source:Araport11)	24.42	15.54	1.57	6.04E-04	1.54E-03
5248	7	F2-3 vs. S2-3	252193_at	AT3G50060	Encodes a member of the R2R3 transcription factor gene family. Expressed in response to potassium deprivation and auxin. Involved in lateral root development. Interacts with ARF7 and regulates the expression of some auxin responsive genes.	111.63	71.57	1.56	6.30E-04	1.58E-03
5249	7	F2-3 vs. S2-3	265033_at	AT1G61520	PSI type III chlorophyll a/b-binding protein (Lhca3*1) The mRNA is cell-to-cell mobile.	2488.07	1618.51	1.54	6.83E-04	1.69E-03
5250	7	F2-3 vs. S2-3	265583_at	AT2G20010	Gls protein (DUF810);(source:Araport11)	83.42	54.22	1.54	6.99E-04	1.72E-03
5251	7	F2-3 vs. S2-3	267504_at	AT2G45530	RING/U-box superfamily protein;(source:Araport11)	65.73	42.81	1.54	6.36E-04	1.59E-03
5252	7	F2-3 vs. S2-3	265460_at	AT2G46600	Calcium-binding EF-hand family protein;(source:Araport11)	832.88	543.93	1.53	5.41E-04	1.41E-03
5253	7	F2-3 vs. S2-3	252870_at	AT4G39940	adenosine-5'-phosphosulfate-kinase (akn2) mRNA, complete The mRNA is cell-to-cell mobile.	61.72	40.41	1.53	7.07E-04	1.73E-03
5254	7	F2-3 vs. S2-3	263345_s_at	AT2G05100	Lhcb2.1 protein encoding a subunit of the light harvesting complex II. Member of a gene family with high degree of sequence similarity. Initially LHCB2.3 was considered as a separate gene but appears to be an allele of LHCB2.1.	4136.88	2713.37	1.52	6.17E-04	1.56E-03
5255	7	F2-3 vs. S2-3	265246_at	AT2G43050	Plant invertase/pectin methylesterase inhibitor superfamily;(source:Araport11)	18.59	12.25	1.52	9.82E-04	2.26E-03
5256	7	F2-3 vs. S2-3	254081_at	AT4G25660	PPPDE putative thiol peptidase family protein;(source:Araport11)	168.54	110.75	1.52	8.47E-04	1.99E-03
5257	7	F2-3 vs. S2-3	253205_at	AT4G34490	CYCLASE ASSOCIATED PROTEIN	1093.53	721.18	1.52	9.37E-04	2.17E-03
5258	7	F2-3 vs. S2-3	246185_at	AT5G20980	Encodes a plastidic methionine synthase, involved in methionine de novo synthesis in the chloroplast	75.74	49.81	1.52	7.09E-04	1.73E-03
5259	7	F2-3 vs. S2-3	247307_at	AT5G63860	UV-B-specific signaling component that orchestrates expression of a range of genes with vital UV-protective functions. Located in the nucleus and the cytosol. Associates with chromatin via histones. UV-B light promotes URV8 protein accumulation in the nucleus. UVR8 interaction with COP1 is negatively regulated by RUP1 and RUP2.	48.95	32.26	1.52	9.89E-04	2.27E-03
5260	7	F2-3 vs. S2-3	263221_at	AT1G30620	encodes a type-II membrane protein that catalyzes 4-epimerization of UDP-D-Xylose to UDP-L-Arabinose in vitro, the nucleotide sugar used by glycosyltransferases in the arabinosylation of cell wall polysaccharides and wall-resident proteoglycans.	342.32	229.04	1.49	9.08E-04	2.11E-03
5261	7	F2-3 vs. S2-3	266411_at	AT2G38730	Cyclophilin-like peptidyl-prolyl cis-trans isomerase family protein;(source:Araport11)	82.68	123.8	0.67	7.28E-04	1.76E-03
5262	7	F2-3 vs. S2-3	262911_s_a	AT1G07400	HSP20-like chaperones superfamily protein;(source:Araport11)	9.84	14.98	0.66	9.04E-04	2.11E-03
5263	7	F2-3 vs. S2-3	254171_at	AT4G24530	O-fucosyltransferase family protein;(source:Araport11)	13	19.73	0.66	8.75E-04	2.05E-03
5264	7	F2-3 vs. S2-3	259655_at	AT1G55210	Disease resistance-responsive (dirigent-like protein) family protein;(source:Araport11)	37.17	57.35	0.65	8.16E-04	1.93E-03
5265	7	F2-3 vs. S2-3	263461_at	AT2G31800	Integrin-linked protein kinase family;(source:Araport11)	31.57	48.35	0.65	6.03E-04	1.53E-03
5266	7	F2-3 vs. S2-3	247718_at	AT5G59310	Encodes a member of the lipid transfer protein family. Proteins of this family are generally small (~9 kD), basic, expressed abundantly and contain eight Cys residues. The proteins can bind fatty acids and acylCoA esters and can transfer several different phospholipids. They are localized to the cell wall. The mRNA is present in flowers and siliques, and is strongly up-regulated by abscisic acid. Predicted to be a member of PR-14 pathogenesis-related protein family with the following members: At2g38540/LTP1, At2g38530/LTP2, At5g59320/LTP3, At5g59310/LTP4, At3g51600/LTP5, At3g08770/LTP6, At2g15050/LTP7, At2g18370/LTP8, At2g15325/LTP9, At5g01870/LTP10, At4g33355/LTP11, At3g51590/LTP12, At5g44265/LTP13, At5g62065/LTP14, At4g08530/LTP15.	324.03	496.88	0.65	5.85E-04	1.50E-03
5267	7	F2-3 vs. S2-3	247378_at	AT5G63120	P-loop containing nucleoside triphosphate hydrolases superfamily protein;(source:Araport11)	26.67	40.88	0.65	9.16E-04	2.13E-03
5268	7	F2-3 vs. S2-3	260724_at	AT1G48140	Encodes a subunit of the dolichol phosphate mannase synthase (DPMS) complex that may serve as membrane anchors for the catalytic core, DPMS1, or provide catalytic assistance. It is localized in the ER and mediates isoprenyl-linked glycan biogenesis.	91.24	142.63	0.64	6.67E-04	1.65E-03
5269	7	F2-3 vs. S2-3	263864_at	AT2G04530	Encodes a protein with RNAse Z activity suggesting a role in tRNA processing. Protein contains a signal sequence for import into the chloroplast.	29.85	46.57	0.64	3.72E-04	1.06E-03
5270	7	F2-3 vs. S2-3	264907_at	AT2G17280	Phosphoglycerate mutase family protein;(source:Araport11)	82.01	127.8	0.64	9.58E-04	2.21E-03

5271	7	F2-3 vs. S2-3	263990_at	AT2G42810	Encodes a phytochrome-specific type 5 serine/threonine protein phosphatase. It dephosphorylates active Pfr-phytochromes. Controls light signal flux by enhancing phytochrome stability and affinity for a signal transducer. The gene is alternately spliced. This variant is an integral membrane protein localized to the ER and nuclear envelope. Belongs to one of the 36 carboxylate clamp (CC)-tetratricopeptide repeat (TPR) proteins (Prasad 2010, Pubmed ID: 20856808) with potential to interact with Hsp90/Hsp70 as co-chaperones. It also regulates tetrapyrrole biosynthesis through the accumulation of Mg-ProtoIX and acts as a negative regulator of photosynthesis associated nuclear gene expression during chloroplast biogenesis and development.	67.05	105.13	0.64	6.54E-04	1.63E-03
5272	7	F2-3 vs. S2-3	245442_at	AT4G16710	glycosyltransferase family protein 28;(source:Araport11)	148.56	233.2	0.64	4.93E-04	1.31E-03
5273	7	F2-3 vs. S2-3	245404_at	AT4G17610	tRNA/rRNA methyltransferase (SpoU) family protein;(source:Araport11)	75.12	117.89	0.64	6.97E-04	1.71E-03
5274	7	F2-3 vs. S2-3	249344_at	AT5G40770	prohibitin 3	198.7	309.39	0.64	5.64E-04	1.46E-03
5275	7	F2-3 vs. S2-3	248463_at	AT5G51130	S-adenosyl-L-methionine-dependent methyltransferases superfamily protein;(source:Araport11)	32.86	50.98	0.64	5.05E-04	1.33E-03
5276	7	F2-3 vs. S2-3	261524_at	AT1G14300	ARM repeat superfamily protein;(source:Araport11)	45.05	71.96	0.63	6.66E-04	1.65E-03
5277	7	F2-3 vs. S2-3	264028_at	AT2G03680	The SPR1 gene encodes a plant-specific 12-kD protein which has a repeated motif at both ends, separated by a predicted rod-like domain, suggesting that it may act as an intermolecular linker. Ubiquitously expressed and belongs to a six-member gene family in Arabidopsis; expressed in transgenic seedlings localized to microtubules within the cortical array, preprophase band, phragmoplast, and mitotic spindle.	63.98	100.91	0.63	3.98E-04	1.11E-03
5278	7	F2-3 vs. S2-3	263332_at	AT2G03870	Small nuclear ribonucleoprotein family protein;(source:Araport11)	212.17	338.86	0.63	9.53E-04	2.20E-03
5279	7	F2-3 vs. S2-3	257179_at	AT3G13170	Encodes AtSPO11-1, one of the three Arabidopsis homologues of the archaeal DNA topoisomerase VIA subunit (topo VIA). Required for meiotic recombination. AtSPO11-1 and AtSPO11-2 have overlapping functions (i.e. both required for meiotic recombination) whereas AtSPO11-3 functions in DNA replication. AtSPO11-1 accumulates in foci in early G2. At 1 h post-S phase, no foci are observed, but by 3 h a majority (80%) of meiocytes at this time point contain >50 foci. However, by 5 h, AtSPO11-1 foci are no longer detectable. This suggests that the protein undergoes a rapid cycle of accumulation and disappearance in meiocytes over a period of between 1 and 5 h post-S phase.	25.78	41.08	0.63	5.51E-04	1.43E-03
5280	7	F2-3 vs. S2-3	256961_at	AT3G13445	TBP (TATA binding protein) associates with TAF(II)s (TBP-associated factors) to form the TFIID general transcription factor complex	76.3	121.93	0.63	4.02E-04	1.12E-03
5281	7	F2-3 vs. S2-3	253808_at	AT4G28300	Encodes a protein with 13.6% proline amino acids that is predicted to localize to the cell wall. The mRNA is cell-to-cell mobile.	21.87	34.67	0.63	6.40E-04	1.60E-03
5282	7	F2-3 vs. S2-3	253221_at	AT4G34870	belongs to cyclophilin family	53	84.75	0.63	3.99E-04	1.11E-03
5283	7	F2-3 vs. S2-3	253097_at	AT4G37320	member of CYP81D	26.56	42.34	0.63	6.67E-04	1.65E-03
5284	7	F2-3 vs. S2-3	259431_at	AT1G01620	a member of the plasma membrane intrinsic protein subfamily PIP1. localizes to the plasma membrane and exhibits water transport activity in Xenopus oocyte. expressed ubiquitously and protein level decreases slightly during leaf development.	240.98	391.81	0.62	9.40E-04	2.18E-03
5285	7	F2-3 vs. S2-3	265735_at	AT2G01140	Aldolase superfamily protein;(source:Araport11)	166.97	269.23	0.62	4.22E-04	1.16E-03
5286	7	F2-3 vs. S2-3	266887_at	AT2G44650	Encodes a chloroplast-localized chaperonin 10 whose mRNA is expressed in leaves and stems but not roots.	85.04	137.62	0.62	5.82E-04	1.49E-03
5287	7	F2-3 vs. S2-3	266735_at	AT2G46930	Encodes a pectin acetylesterase that removes cell wall acetate associated with pectin formation in Arabidopsis leaves.	18.48	29.88	0.62	8.47E-04	1.99E-03
5288	7	F2-3 vs. S2-3	256567_at	AT3G19553	Encodes POLYAMINE UPTAKE TRANSPORTER 5, an amino acid permease family protein.	24.04	38.85	0.62	2.69E-04	8.22E-04
5289	7	F2-3 vs. S2-3	257826_at	AT3G26730	RING/U-box superfamily protein;(source:Araport11)	101.57	163.17	0.62	4.49E-04	1.21E-03
5290	7	F2-3 vs. S2-3	251857_at	AT3G54770	Encodes a putative RNA binding protein that is localized in the nucleus and affects ABA-regulated seed germination of Arabidopsis.	13.57	21.76	0.62	9.12E-04	2.12E-03
5291	7	F2-3 vs. S2-3	254267_at	AT4G22930	Encodes dihydroorotase (PYR4).	72.47	116.38	0.62	3.46E-04	9.98E-04
5292	7	F2-3 vs. S2-3	251122_at	AT5G01020	Protein kinase superfamily protein;(source:Araport11)	131.61	210.8	0.62	3.96E-04	1.11E-03
5293	7	F2-3 vs. S2-3	250694_at	AT5G06710	Homeobox-leucine zipper protein.	12.52	20.21	0.62	4.84E-04	1.29E-03
5294	7	F2-3 vs. S2-3	248403_at	AT5G51410	LUC7 N terminus domain-containing protein;(source:Araport11)	56.95	92.18	0.62	8.50E-04	2.00E-03
5295	7	F2-3 vs. S2-3	247039_at	AT5G67270	encodes a homolog of animal microtubule-end-binding protein. There are two other members of this family. EB1 forms foci at regions where the minus ends of microtubules are gathered during mitosis and early cytokinesis.	14.19	22.88	0.62	8.76E-04	2.05E-03

5296	7	F2-3 vs. S2-3	260930_at	AT1G02620	Ras-related small GTP-binding family protein;(source:Araport11)	538.11	879.41	0.61	8.24E-04	1.95E-03
5297	7	F2-3 vs. S2-3	260782_at	AT1G14570	Encodes a nuclear UBX-containing protein that can bridge ubiquitin to AtCDC48A.	130.04	214.53	0.61	2.42E-04	7.55E-04
5298	7	F2-3 vs. S2-3	256482_at	AT1G31420	Encodes a plasma membrane localized leucine-rich repeat receptor kinase that is involved in cell wall elongation. Loss of function mutations of FEI1 and FEI2 exhibit defects in root and hypocotyl cell elongation. Double mutants are defective in cell wall biosynthesis and have thick hypocotyls, and short, thick roots.	36.25	59.34	0.61	4.13E-04	1.14E-03
5299	7	F2-3 vs. S2-3	260865_at	AT1G43760	DNAse I-like superfamily protein;(source:Araport11)	14.62	23.92	0.61	8.69E-04	2.03E-03
5300	7	F2-3 vs. S2-3	260718_at	AT1G48110	evolutionarily conserved C-terminal region 7;(source:Araport11)	113.67	186.66	0.61	3.38E-04	9.78E-04
5301	7	F2-3 vs. S2-3	263101_s_a	AT2G05250	DNAJ heat shock N-terminal domain-containing protein;(source:Araport11)	73.21	120.8	0.61	2.97E-04	8.88E-04
5302	7	F2-3 vs. S2-3	264617_at	AT2G17660	RPM1-interacting protein 4 (RIN4) family protein;(source:Araport11)	10.53	17.41	0.61	3.32E-04	9.65E-04
5303	7	F2-3 vs. S2-3	266401_s_a	AT2G38620	Encodes a member of a plant specific family of cyclin dependent kinases.	121.87	200.17	0.61	8.14E-04	1.93E-03
5304	7	F2-3 vs. S2-3	252669_at	AT3G44100	MD-2-related lipid recognition domain-containing protein;(source:Araport11)	108.58	177.55	0.61	2.26E-04	7.20E-04
5305	7	F2-3 vs. S2-3	252508_at	AT3G46210	Ribosomal protein S5 domain 2-like superfamily protein;(source:Araport11)	46.52	76.73	0.61	8.51E-04	2.00E-03
5306	7	F2-3 vs. S2-3	254855_s_a	AT4G12140	RING/U-box superfamily protein;(source:Araport11)	9.05	14.75	0.61	2.81E-04	8.45E-04
5307	7	F2-3 vs. S2-3	245524_at	AT4G15920	Encodes a vacuolar fructose transporter expressed in parenchyma and xylem that controls leaf fructose content. When its expression is reduced, fructose accumulates in leaves.	10.78	17.6	0.61	2.73E-04	8.30E-04
5308	7	F2-3 vs. S2-3	250825_at	AT5G05210	Surfeit locus protein 6;(source:Araport11)	30.26	49.48	0.61	7.07E-04	1.73E-03
5309	7	F2-3 vs. S2-3	250462_at	AT5G10020	Leucine-rich receptor-like protein kinase family protein;(source:Araport11)	35.92	58.75	0.61	2.44E-04	7.59E-04
5310	7	F2-3 vs. S2-3	246508_at	AT5G16150	Encodes a putative plastidic glucose transporter.	186.7	307.78	0.61	7.51E-04	1.81E-03
5311	7	F2-3 vs. S2-3	246184_at	AT5G20950	Encodes a beta-glucosidase involved in xyloglucan metabolism.	118.39	194.72	0.61	7.11E-04	1.74E-03
5312	7	F2-3 vs. S2-3	248842_at	AT5G46850	phosphatidylinositol-glycan biosynthesis class X-like protein;(source:Araport11)	75.77	123.61	0.61	5.77E-04	1.48E-03
5313	7	F2-3 vs. S2-3	247721_at	AT5G59140	BTB/POZ domain-containing protein;(source:Araport11)	47.06	76.68	0.61	2.21E-04	7.11E-04
5314	7	F2-3 vs. S2-3	247584_at	AT5G60670	Ribosomal protein L11 family protein;(source:Araport11)	244.87	400.44	0.61	6.25E-04	1.57E-03
5315	7	F2-3 vs. S2-3	246983_at	AT5G67200	Leucine-rich repeat protein kinase family protein;(source:Araport11)	24.71	40.26	0.61	9.69E-04	2.23E-03
5316	7	F2-3 vs. S2-3	261428_at	AT1G18870	Encodes a protein with isochorismate synthase activity involved in phylloquinone biosynthesis. Mutant studies of this gene's function suggest that its function is redundant with that of ICS1 (AT1G7410).	13.8	22.95	0.6	3.80E-04	1.07E-03
5317	7	F2-3 vs. S2-3	259477_at	AT1G19120	Small nuclear ribonucleoprotein family protein;(source:Araport11)	433.34	722.84	0.6	4.04E-04	1.12E-03
5318	7	F2-3 vs. S2-3	263679_at	AT1G59990	DEA(D/H)-box RNA helicase family protein;(source:Araport11)	30.78	51.46	0.6	9.90E-04	2.27E-03
5319	7	F2-3 vs. S2-3	260264_at	AT1G68500	hypothetical protein;(source:Araport11)	36.74	61.59	0.6	6.15E-04	1.55E-03
5320	7	F2-3 vs. S2-3	260383_s_a	AT1G74050	Ribosomal protein L6 family protein;(source:Araport11)	463.24	776.93	0.6	3.26E-04	9.52E-04
5321	7	F2-3 vs. S2-3	262057_at	AT1G80040	ubiquitin system component Cue;(source:Araport11)	15.48	25.77	0.6	5.10E-04	1.34E-03
5322	7	F2-3 vs. S2-3	266045_s_a	AT2G07727	cytochrome b;(source:Araport11)	225.5	375.62	0.6	3.14E-04	9.25E-04
5323	7	F2-3 vs. S2-3	267618_at	AT2G26760	Cyclin B1;(source:Araport11)	35.73	59.79	0.6	8.98E-04	2.09E-03
5324	7	F2-3 vs. S2-3	267350_at	AT2G40030	Encodes the unique largest subunit of nuclear DNA-dependent RNA polymerase V; homologous to budding yeast RPB1 and the E. coli RNA polymerase beta prime subunit. Required for normal RNA-directed DNA methylation at non-CG methylation sites and transgene silencing. The nrpe1 mutant is more resistant to biotrophic pathogens and is primed to activate salicylic acid-dependent defence genes.	21.49	35.69	0.6	1.97E-04	6.51E-04
5325	7	F2-3 vs. S2-3	266102_at	AT2G40720	Tetratricopeptide repeat (TPR)-like superfamily protein;(source:Araport11)	22.69	37.92	0.6	2.44E-04	7.59E-04
5326	7	F2-3 vs. S2-3	259192_at	AT3G01740	Mitochondrial ribosomal protein L37;(source:Araport11)	40.42	67.58	0.6	7.41E-04	1.79E-03
5327	7	F2-3 vs. S2-3	258921_at	AT3G10500	Encodes a transcriptional activator that is associated with the plasma membrane in a dormant form and is proteolytically cleaved to create a form that can enter the nucleus. It is thought to promote ROS production by binding directly to the promoters of genes encoding ROS biosynthetic enzymes during drought-induced leaf senescence.The mRNA is cell-to-cell mobile.	109.16	183	0.6	1.89E-04	6.33E-04
5328	7	F2-3 vs. S2-3	258730_at	AT3G11730	Encodes a member of the Rab GTPase family of proteins. This protein interacts with the tail region of a myosin XI protein (AT5G43900) in a GTP-dependent manner. It has also been identified as an isoprenylated protein.	175.82	290.95	0.6	2.09E-04	6.83E-04
5329	7	F2-3 vs. S2-3	257894_at	AT3G17100	sequence-specific DNA binding transcription factor;(source:Araport11)	19.42	32.48	0.6	4.75E-04	1.27E-03
5330	7	F2-3 vs. S2-3	256828_at	AT3G22970	hypothetical protein (DUF506);(source:Araport11)	54.07	89.89	0.6	7.27E-04	1.76E-03
5331	7	F2-3 vs. S2-3	254719_at	AT4G13600	Carbohydrate-binding X8 domain superfamily protein;(source:Araport11)	12.52	20.8	0.6	2.95E-04	8.82E-04
5332	7	F2-3 vs. S2-3	250440_at	AT5G10360	RPS6A and RPS6B are fully redundant and essential during gametogenesis.	3676.3	6130.79	0.6	3.38E-04	9.79E-04
5333	7	F2-3 vs. S2-3	248885_at	AT5G46150	LEM3 (ligand-effect modulator 3) family protein / CDC50 family protein;(source:Araport11)	14.84	24.56	0.6	2.50E-04	7.74E-04

5334	7	F2-3 vs. S2-3	259402_at	AT1G17730	Encodes an ESCRT-related protein: CHMP1A/AT1G73030; CHMP1B/AT1G17730. CHMP1A and B mediate multivesicular body sorting of auxin carriers and are required for plant development. ESCRT: Endosomal Sorting Complexes Required For Transport machinery; CHMP: Charged Multivesicular Body Protein/Chromatin Modifying Protein.	19.19	32.32	0.59	2.73E-04	8.30E-04
5335	7	F2-3 vs. S2-3	264978_at	AT1G27120	Encodes a Golgi-localized hydroxyproline-O-galactosyltransferase.	61.56	103.95	0.59	3.17E-04	9.31E-04
5336	7	F2-3 vs. S2-3	264444_at	AT1G27360	In conjunction with SPL10 and SPL2, SPL11 redundantly controls proper development of lateral organs in association with shoot maturation in the reproductive phase. SPL2, SPL10, and SPL11, suppress root regeneration with age by inhibiting wound-induced auxin biosynthesis.	20.74	35.24	0.59	7.81E-04	1.86E-03
5337	7	F2-3 vs. S2-3	261327_at	AT1G44830	Encodes a nuclear-localized member of the DREB subfamily A-5 of ERF/AP2 transcription factor family. The protein contains one AP2 domain. There are 15 members in this subfamily including RAP2.1, RAP2.9 and RAP2.10. Overexpression in cultured cells results in an increase in pectin deposition.ERF014 differentially regulates responses to bacterial and fungal pathogens.	17.34	29.29	0.59	6.09E-04	1.55E-03
5338	7	F2-3 vs. S2-3	265150_at	AT1G51200	A20/AN1-like zinc finger family protein;(source:Araport11)	512.68	863.63	0.59	6.03E-04	1.53E-03
5339	7	F2-3 vs. S2-3	259609_at	AT1G52410	Contains a novel calcium-binding repeat sequence. Binds TSK in vitro. Localizes to small cytoplasmic vesicles in interphase cells. In cells synchronized for cell division, TSA1 and TSK relocalize to ends of spindle microtubules that are ahead of separating chromatids during metaphase and anaphase of mitosis. May be involved in mitosis together with TSK. Expressed preferentially in the flower and shoot apex. Can form multimers. The mRNA is cell-to-cell mobile.	83.96	142.62	0.59	1.55E-04	5.44E-04
5340	7	F2-3 vs. S2-3	260401_at	AT1G69840	SPFH/Band 7/PHB domain-containing membrane-associated protein family;(source:Araport11)	108.23	184.9	0.59	8.33E-04	1.97E-03
5341	7	F2-3 vs. S2-3	267146_at	AT2G38160	hypothetical protein;(source:Araport11)	11.87	19.98	0.59	8.56E-04	2.01E-03
5342	7	F2-3 vs. S2-3	265270_at	AT2G42910	Phosphoribosyltransferase family protein;(source:Araport11)	155.32	263.42	0.59	8.54E-04	2.00E-03
5343	7	F2-3 vs. S2-3	252252_at	AT3G49180	Transducin/WD40 repeat-like superfamily protein;(source:Araport11)	60.3	102.52	0.59	6.57E-04	1.64E-03
5344	7	F2-3 vs. S2-3	252126_at	AT3G50950	Encodes a canonical CC-type NLR protein that is required for the recognition of the T3SE HopZ1a as well as several other Hop effectors from the pathogenic bacteria P. syringae.	19.86	33.86	0.59	7.57E-04	1.82E-03
5345	7	F2-3 vs. S2-3	254800_at	AT4G13070	RNA-binding CRS1 / YhbY (CRM) domain protein;(source:Araport11)	12.45	21.2	0.59	1.87E-04	6.27E-04
5346	7	F2-3 vs. S2-3	253065_at	AT4G37740	Growth regulating factor encoding transcription activator. One of the nine members of a GRF gene family, containing nuclear targeting domain. Mutants result in smaller leaves indicating the role of the gene in leaf development. Expressed in root, shoot and flower	13.99	23.63	0.59	9.11E-04	2.12E-03
5347	7	F2-3 vs. S2-3	252871_at	AT4G40000	S-adenosyl-L-methionine-dependent methyltransferases superfamily protein;(source:Araport11)	73.25	123.58	0.59	2.59E-04	7.97E-04
5348	7	F2-3 vs. S2-3	251098_at	AT5G01650	Tautomerase/MIF superfamily protein;(source:Araport11)	190.23	323.65	0.59	2.27E-04	7.23E-04
5349	7	F2-3 vs. S2-3	250517_at	AT5G08260	serine carboxypeptidase-like 35;(source:Araport11)	61.88	104.69	0.59	2.41E-04	7.51E-04
5350	7	F2-3 vs. S2-3	250494_at	AT5G09740	Encodes an enzyme with histone acetyltransferase activity. HAM2 primarily acetylate histone H4, but also display some ability to acetylate H3. Prior acetylation of lysine 5 on histone H4 reduces radioactive acetylation by either HAM2.	54.06	91.55	0.59	7.80E-04	1.86E-03
5351	7	F2-3 vs. S2-3	246856_at	AT5G26210	Encodes a member of the Alfin1-like family of nuclear-localized PHD (plant homeodomain) domain containing proteins. All AL proteins except AL3 bind to di- or trimethylated histone H3 (H3K4me3/2). Members of this family include: AT5G05610 (AL1), AT3G11200 (AL2), AT3G42790 (AL3), AT5G26210 (AL4), AT5G20510 (AL5), AT2G02470 (AL6), AT1G14510 (AL7).	141.57	239.14	0.59	4.47E-04	1.21E-03
5352	7	F2-3 vs. S2-3	264254_at	AT1G09150	pseudouridine synthase and archaeosine transglycosylase (PUA) domain-containing protein;(source:Araport11)	43.72	75.24	0.58	4.41E-04	1.20E-03
5353	7	F2-3 vs. S2-3	260704_at	AT1G32470	Single hybrid motif superfamily protein;(source:Araport11)	338.78	580.81	0.58	6.80E-04	1.68E-03
5354	7	F2-3 vs. S2-3	260515_at	AT1G51460	ABCG13 encodes a member of the ATP-binding cassette (ABC) transporter family protein. Mutants show defects in petal elongation resulting in a folded petal phenotype.	40.56	69.66	0.58	6.20E-04	1.56E-03
5355	7	F2-3 vs. S2-3	259649_at	AT1G55300	TBP-associated factor 7;(source:Araport11)	80.06	138.84	0.58	2.76E-04	8.37E-04
5356	7	F2-3 vs. S2-3	259735_at	AT1G64405	hypothetical protein;(source:Araport11)	16.4	28.34	0.58	1.60E-04	5.58E-04

5357	7	F2-3 vs. S2-3	262951_at	AT1G75500	An Arabidopsis thaliana homolog of Medicago truncatula NODULIN21 (MtN21). The gene encodes a plant-specific, predicted integral membrane protein and is a member of the Plant-Drug/Metabolite Exporter (P-DME) family (Transporter Classification number: TC 2.A.7.3) and the nodulin MtN21-like transporter family.	267.59	464.06	0.58	4.22E-04	1.16E-03
5358	7	F2-3 vs. S2-3	262922_at	AT1G79420	C-type mannose receptor (DUF620);(source:Araport11)	23.71	41.13	0.58	5.97E-04	1.52E-03
5359	7	F2-3 vs. S2-3	265531_at	AT2G06200	Growth regulating factor encoding transcription activator. One of the nine members of a GRF gene family, containing nuclear targeting domain. Involved in leaf development and expressed in root, shoot and flower	17.95	30.89	0.58	2.16E-04	6.95E-04
5360	7	F2-3 vs. S2-3	266847_at	AT2G26060	Encodes a homolog of the yeast Cytosolic Iron-sulfur protein Assembly protein CIA1.	49.38	85.8	0.58	7.75E-04	1.85E-03
5361	7	F2-3 vs. S2-3	267492_at	AT2G30620	winged-helix DNA-binding transcription factor family protein;(source:Araport11)	585.79	1003	0.58	2.28E-04	7.24E-04
5362	7	F2-3 vs. S2-3	255790_at	AT2G33560	Encodes BUBR1. May have the spindle assembly checkpoint protein functions conserved from yeast to humans.	14.34	24.76	0.58	7.50E-04	1.81E-03
5363	7	F2-3 vs. S2-3	263847_at	AT2G36970	UDP-Glycosyltransferase superfamily protein;(source:Araport11)	21.15	36.29	0.58	4.51E-04	1.22E-03
5364	7	F2-3 vs. S2-3	265466_at	AT2G37060	nuclear factor Y, subunit B8;(source:Araport11)	16.09	27.93	0.58	2.22E-04	7.11E-04
5365	7	F2-3 vs. S2-3	258569_at	AT3G04400	Ribosomal protein L14p/L23e family protein;(source:Araport11)	3145.5	5407.88	0.58	2.29E-04	7.26E-04
5366	7	F2-3 vs. S2-3	256971_at	AT3G21100	RNA-binding (RRM/RBD/RNP motifs) family protein;(source:Araport11)	17.44	29.83	0.58	5.66E-04	1.46E-03
5367	7	F2-3 vs. S2-3	258098_at	AT3G23670	Microtubule motor kinesin PAKRP1L/Kinesin-12B. Together with PAKRP1/Kinesin-12A, serve as linkers of the plus ends of antiparallel microtubules in the phragmoplast.	15.3	26.46	0.58	5.25E-04	1.38E-03
5368	7	F2-3 vs. S2-3	257203_at	AT3G23730	xyloglucan endotransglucosylase/hydrolase 16;(source:Araport11)	407.07	701.32	0.58	5.05E-04	1.33E-03
5369	7	F2-3 vs. S2-3	251969_at	AT3G53130	Lutein-deficient 1 (LUT1) required for lutein biosynthesis, member of the xanthophyll class of carotenoids. Involved in epsilon ring hydroxylation. Maps at 67.3 cM on chromosome 3.	57.93	99.68	0.58	4.24E-04	1.17E-03
5370	7	F2-3 vs. S2-3	251830_at	AT3G55010	encoding phosphoribosylformylglycinamidine cyclo-ligase (syn. AIR synthetase)that phosphorylates 5-phosphoribosyl-N-formylglycinamidine (FGAM) to form 5-aminoimidazole ribonucleotide (AIR)	37.23	64.36	0.58	8.93E-04	2.08E-03
5371	7	F2-3 vs. S2-3	251687_at	AT3G56460	GroES-like zinc-binding alcohol dehydrogenase family protein;(source:Araport11)	45.88	79.5	0.58	4.86E-04	1.29E-03
5372	7	F2-3 vs. S2-3	251425_at	AT3G60210	GroES-like family protein;(source:Araport11)	191.68	331.64	0.58	6.58E-04	1.64E-03
5373	7	F2-3 vs. S2-3	251121_at	AT3G63420	heterotrimeric G protein gamma-subunit (AGG1) mRNA,	26.11	44.91	0.58	2.13E-04	6.89E-04
5374	7	F2-3 vs. S2-3	255553_at	AT4G01960	transmembrane protein;(source:Araport11)	80.19	137.76	0.58	4.87E-04	1.29E-03
5375	7	F2-3 vs. S2-3	254191_at	AT4G23900	Nucleoside diphosphate kinase family protein;(source:Araport11)	72.3	125.11	0.58	4.35E-04	1.19E-03
5376	7	F2-3 vs. S2-3	249275_at	AT5G41870	Pectin lyase-like superfamily protein;(source:Araport11)	40.56	70.34	0.58	6.72E-04	1.66E-03
5377	7	F2-3 vs. S2-3	261481_at	AT1G14260	RING/FYVE/PHD zinc finger superfamily protein;(source:Araport11)	14.28	25.06	0.57	1.99E-04	6.57E-04
5378	7	F2-3 vs. S2-3	255775_at	AT1G18600	Mitochondrion-located rhomboid-like protein	31.81	55.42	0.57	5.29E-04	1.38E-03
5379	7	F2-3 vs. S2-3	261364_at	AT1G53140	Encodes DRP5A, a dynamin protein involved in cytokinesis in Arabidopsis.	31.73	55.21	0.57	4.73E-04	1.27E-03
5380	7	F2-3 vs. S2-3	260618_at	AT1G53230	Encodes a member of a recently identified plant transcription factor family that includes Teosinte branched 1, Cycloidea 1, and proliferating cell nuclear antigen (PCNA) factors, PCF1 and 2. Regulated by miR319. Involved in heterochronic regulation of leaf differentiation.	12.62	21.97	0.57	5.90E-04	1.51E-03
5381	7	F2-3 vs. S2-3	259689_x_at	AT1G63130	Transacting siRNA generating locus. Its derived siR9as targets AT1G62930 for cleavage. Itself is targeted by TAS2-derived ta-siR2140 for cleavage.	73.71	130.41	0.57	1.14E-04	4.33E-04
5382	7	F2-3 vs. S2-3	260291_at	AT1G63700	Member of MEKK subfamily, a component of the stomatal development regulatory pathway. Mutations in this locus result in embryo lethality.	22.61	39.92	0.57	1.17E-04	4.39E-04
5383	7	F2-3 vs. S2-3	266534_at	AT2G16940	Splicing factor, CC1-like protein;(source:Araport11)	202.54	357.33	0.57	7.89E-04	1.88E-03
5384	7	F2-3 vs. S2-3	263065_at	AT2G18170	MAP kinase 7;(source:Araport11)	39.75	69.48	0.57	3.04E-04	9.04E-04
5385	7	F2-3 vs. S2-3	265695_at	AT2G24490	Encodes a component of Replication Protein A. Component of transcriptional gene silencing which does not affect endogenous small RNA accumulation nor DNA methylation. Localized in the nucleus. Involved in DNA repair. Interacts physically with ROS1.	57.31	100.34	0.57	9.28E-04	2.15E-03
5386	7	F2-3 vs. S2-3	267462_at	AT2G33735	Chaperone DnaJ-domain superfamily protein;(source:Araport11)	68.74	121.03	0.57	6.82E-04	1.68E-03
5387	7	F2-3 vs. S2-3	256915_at	AT3G23900	RNA recognition motif (RRM)-containing protein;(source:Araport11)	11.67	20.56	0.57	2.54E-04	7.83E-04

5388	7	F2-3 vs. S2-3	251890_at	AT3G54220	Encodes a member of a novel family having similarity to DNA binding proteins containing basic-leucine zipper regions; scr is expressed in cortex/endodermal initial cells and in the endodermal cell lineage. Regulates the radial organization of the root. Is required cell-autonomously for distal specification of the quiescent center, which in turn regulates stem cell fate of immediately surrounding cells. SCR appears to be a direct target of SHR. SCR and SCR-LIKE 23 act redundantly in bundle sheath cell fate specification.	37.37	65.56	0.57	1.54E-04	5.43E-04
5389	7	F2-3 vs. S2-3	251856_at	AT3G54720	Encodes glutamate carboxypeptidase. Various alleles show-increased cotyledon number and rate of leaf initiation, show transformation of leaves to cotyledons, altered flowering time and photomorphogenesis and an increased level of cytokinin biosynthesis. Involved in ethylene enhanced hypocotyl elongation in the light. Strong genetic interaction between TGH and AMP1.	37.01	64.56	0.57	4.50E-04	1.22E-03
5390	7	F2-3 vs. S2-3	251758_at	AT3G55770	Encodes a member of the Arabidopsis LIM proteins: a family of actin bundlers with distinct expression patterns. WLIM1, WLIM2a, and WLIM2b are widely expressed, whereas PLIM2a, PLIM2b, and PLIM2c are predominantly expressed in pollen. Regulates actin cytoskeleton organization.	136.04	240.21	0.57	5.52E-04	1.43E-03
5391	7	F2-3 vs. S2-3	251764_at	AT3G55830	A member of the Glycosyltransferase Family 64, homologous to Poplar cambium-expressed GT64 gene. The EPC1 protein plays a critical role during plant development in maintaining the integrity of organs via cell-cell adhesion, thereby providing mechanical strength and facilitating the movement of metabolites throughout the plant.Loss of function specifically affects glycosylinositolphosphorylceramide (GIPC) mannosylation.	90.86	160.15	0.57	3.56E-04	1.02E-03
5392	7	F2-3 vs. S2-3	251695_at	AT3G56590	hydroxyproline-rich glycoprotein family protein;(source:Araport11)	13.52	23.74	0.57	1.92E-04	6.38E-04
5393	7	F2-3 vs. S2-3	251352_at	AT3G61070	member of the peroxin11 (PEX11) gene family, integral to peroxisome membrane, controls peroxisome proliferation.	43.67	76.52	0.57	2.56E-04	7.87E-04
5394	7	F2-3 vs. S2-3	254991_at	AT4G10620	P-loop containing nucleoside triphosphate hydrolases superfamily protein;(source:Araport11)	67.65	118.99	0.57	8.17E-04	1.94E-03
5395	7	F2-3 vs. S2-3	245357_at	AT4G17560	Ribosomal protein L19 family protein;(source:Araport11)	18.51	32.76	0.57	3.24E-04	9.47E-04
5396	7	F2-3 vs. S2-3	254368_at	AT4G21710	Encodes the unique second-largest subunit of DNA-dependent RNA polymerase II; the ortholog of budding yeast RPB2 and a homolog of the E. coli RNA polymerase beta subunit.	68.9	121.06	0.57	5.49E-04	1.43E-03
5397	7	F2-3 vs. S2-3	245234_at	AT4G25560	LAF1 is a R2R3-MYB transcription factor and positive regulator of the phyA photoresponse. Interaction of LAF1 with HFR1 stabilize the proteins against ubiquitination by COP1(AT2G32950) and subsequent degrations. Mutants have an elongated hypocotyl specifically under far-red light but retain wild-type responses to other light wavelengths.	11.17	19.49	0.57	1.85E-04	6.23E-04
5398	7	F2-3 vs. S2-3	253753_at	AT4G29030	Putative membrane lipoprotein;(source:Araport11)	140.1	244.9	0.57	4.00E-04	1.11E-03
5399	7	F2-3 vs. S2-3	253717_at	AT4G29440	Regulator of Vps4 activity in the MVB pathway protein;(source:Araport11)	32.86	57.24	0.57	5.41E-04	1.41E-03
5400	7	F2-3 vs. S2-3	253474_at	AT4G32270	Ubiquitin-like superfamily protein;(source:Araport11)	18.3	32.12	0.57	2.16E-04	6.96E-04
5401	7	F2-3 vs. S2-3	251089_at	AT5G01390	DNAJ heat shock family protein;(source:Araport11)	39.28	69.18	0.57	1.96E-04	6.49E-04
5402	7	F2-3 vs. S2-3	250426_at	AT5G10510	Encodes an AP2-domain transcription factor involved in root stem cell identity and root development. It is also required to maintain high levels of PIN1 expression at the periphery of the meristem and modulate local auxin production in the central region of the SAM which underlies phyllotactic transitions. Intronic sequences are required for its expression in flowers.Acts redundantly with PLT5 and 7 in lateral root pattern formation.	12.73	22.38	0.57	1.93E-04	6.42E-04
5403	7	F2-3 vs. S2-3	245849_at	AT5G13520	peptidase M1 family protein;(source:Araport11)	70.91	125.44	0.57	1.85E-04	6.23E-04
5404	7	F2-3 vs. S2-3	249888_s_a	AT5G37340	ZPR1 zinc-finger domain protein;(source:Araport11)	680.49	1203.22	0.57	7.48E-04	1.80E-03
5405	7	F2-3 vs. S2-3	248817_at	AT5G47020	MraZ;(source:Araport11)	34	60.01	0.57	4.74E-04	1.27E-03
5406	7	F2-3 vs. S2-3	248655_at	AT5G48760	Ribosomal protein L13 family protein;(source:Araport11)	266.36	467.91	0.57	1.18E-04	4.41E-04
5407	7	F2-3 vs. S2-3	248304_at	AT5G53180	Encodes one of the two polypyrimidine tract-binding (PTB) protein homologs in the Arabidopsis genome. Double mutants have defects in pollen germination.	29.54	51.59	0.57	3.76E-04	1.07E-03
5408	7	F2-3 vs. S2-3	247929_at	AT5G57330	Galactose mutarotase-like superfamily protein;(source:Araport11)	357.83	624.18	0.57	2.40E-04	7.50E-04
5409	7	F2-3 vs. S2-3	247493_at	AT5G61910	DCD (Development and Cell Death) domain protein;(source:Araport11)	26.21	46.08	0.57	2.11E-04	6.85E-04
5410	7	F2-3 vs. S2-3	247068_at	AT5G66800	membrane-associated kinase regulator-like protein;(source:Araport11)	52.12	90.97	0.57	4.60E-04	1.24E-03
5411	7	F2-3 vs. S2-3	265042_at	AT1G04040	HAD superfamily, subfamily IIIB acid phosphatase;(source:Araport11)	26.74	48.11	0.56	4.86E-04	1.29E-03
5412	7	F2-3 vs. S2-3	264408_at	AT1G10240	FAR1-related sequence 11;(source:Araport11)	73.68	131.82	0.56	1.06E-04	4.07E-04

5413	7	F2-3 vs. S2-3	264490_at	AT1G27390	Form of TOM20, which is a component of the TOM complex, involved in transport of nuclear-encoded mitochondrial proteins	31.89	57.42	0.56	7.94E-05	3.30E-04
5414	7	F2-3 vs. S2-3	262954_at	AT1G54500	RBD1 is a thylakoid membrane-bound iron-binding protein that is required for the proper assembly of photosystem II in Arabidopsis. It is found in all oxygenic photoautotrophic organisms (plants, algae and cyanobacteria).	23.7	42.23	0.56	6.98E-04	1.71E-03
5415	7	F2-3 vs. S2-3	262699_at	AT1G75980	Single hybrid motif superfamily protein;(source:Araport11)	72.25	128.35	0.56	9.31E-05	3.71E-04
5416	7	F2-3 vs. S2-3	263726_at	AT2G13610	ABC-2 type transporter family protein;(source:Araport11)	34.31	60.86	0.56	6.39E-04	1.60E-03
5417	7	F2-3 vs. S2-3	266264_at	AT2G27775	PPR containing protein;(source:Araport11)	13.45	23.97	0.56	1.23E-04	4.57E-04
5418	7	F2-3 vs. S2-3	267412_at	AT2G34940	VACUOLAR SORTING RECEPTOR 5;(source:Araport11)	24.17	43.06	0.56	9.41E-04	2.18E-03
5419	7	F2-3 vs. S2-3	267052_at	AT2G38410	ENTH/VHS/GAT family protein;(source:Araport11)	15.46	27.55	0.56	2.31E-04	7.32E-04
5420	7	F2-3 vs. S2-3	245142_at	AT2G45270	Mitochondrial protein essential for embryo development.	40.79	72.19	0.56	1.34E-04	4.84E-04
5421	7	F2-3 vs. S2-3	257674_at	AT3G20390	Encodes a plastidial RidA (Reactive Intermediate Deaminase A) homolog that hydrolyzes the enamines/imines formed by Thr dehydratase from Ser or Thr. RidA accelerates the deamination of reactive enamine/imine intermediates produced by threonine dehydratase (At3g10050) with threonine or serine as substrates. In the absence of RidA, the serine-derived imine inactivates BCAT3 (At3g49680). RidA thus pre-empts damage to BCAT3 by hydrolyzing the reactive imine before it does damage.	148.45	262.82	0.56	1.67E-04	5.74E-04
5422	7	F2-3 vs. S2-3	245573_at	AT4G14730	Stress induced membrane protein. Mutants show enhanced cell death under stress.	20.25	36.09	0.56	1.93E-04	6.42E-04
5423	7	F2-3 vs. S2-3	245361_at	AT4G17790	SNARE associated Golgi protein family;(source:Araport11)	198.23	355.31	0.56	4.87E-04	1.29E-03
5424	7	F2-3 vs. S2-3	254643_at	AT4G18820	AAA-type ATPase family protein;(source:Araport11)	24.38	43.15	0.56	9.86E-04	2.26E-03
5425	7	F2-3 vs. S2-3	254328_at	AT4G22570	Encodes an adenine phosphoribosyltransferase (APT; EC 2.4.2.7), which is a constitutively expressed enzyme involved in the one-step salvage of adenine to AMP. APT3 has higher affinity for zeatin, isopentenyladenine and benzyladenine than APT1 but lower Vmax than APT1.	114.43	205.21	0.56	6.67E-04	1.65E-03
5426	7	F2-3 vs. S2-3	253266_s_a	AT4G34080	myosin-4 protein (DUF641);(source:Araport11)	110.64	196.28	0.56	4.28E-04	1.17E-03
5427	7	F2-3 vs. S2-3	246196_at	AT4G37090	UDP-N-acetylmuramoyl-L-alanyl-D-glutamate-2, 6-diaminopimelate ligase;(source:Araport11)	17.44	31.25	0.56	6.96E-04	1.71E-03
5428	7	F2-3 vs. S2-3	253053_at	AT4G37470	HTL belonging to the alpha/beta fold hydrolase superfamily. Mutant and over-expression studies indicates its involvement in seedling de-etiolation process. Involved in the perception of karrikins. Interacts with MAX2. Important for cotyledon expansion.	31.78	56.31	0.56	3.27E-04	9.52E-04
5429	7	F2-3 vs. S2-3	251050_at	AT5G02440	60S ribosomal protein L36;(source:Araport11)	13.16	23.46	0.56	4.70E-04	1.26E-03
5430	7	F2-3 vs. S2-3	250193_at	AT5G14540	basic salivary proline-rich-like protein (DUF1421);(source:Araport11)	19.02	34.19	0.56	1.66E-04	5.71E-04
5431	7	F2-3 vs. S2-3	246509_at	AT5G16715	protein EMBRYO DEFECTIVE 2247;(source:Araport11)	18.33	32.7	0.56	3.92E-04	1.10E-03
5432	7	F2-3 vs. S2-3	249815_at	AT5G23900	Ribosomal protein L13e family protein;(source:Araport11)	56.09	100.96	0.56	1.18E-04	4.43E-04
5433	7	F2-3 vs. S2-3	246900_at	AT5G25620	Encodes a member of a family of flavin monooxygenases with an important role in auxin biosynthesis. YUC6 possesses an additional thiol-reductase activity that confers drought resistance independently of auxin biosynthesis.	14.93	26.71	0.56	1.63E-04	5.66E-04
5434	7	F2-3 vs. S2-3	249425_at	AT5G39790	Encodes a chloroplast localized protein that is involved in protein translocation and starch metabolism. PTST helps localize GBSS to the starch granules where GBSS functions in amylose biosynthesis.	30.16	53.6	0.56	4.38E-04	1.19E-03
5435	7	F2-3 vs. S2-3	248801_at	AT5G47370	homeobox-leucine zipper genes induced by auxin, but not by other phytohormones. Plays opposite roles in the shoot and root tissues in regulating auxin-mediated morphogenesis.	33.5	59.88	0.56	4.44E-04	1.21E-03
5436	7	F2-3 vs. S2-3	248684_at	AT5G48485	Encodes a putative apoplastic lipid transfer protein that is involved in systemic acquired resistance. Mutants in this gene exhibit wild-type local resistance to avirulent and virulent Pseudomonas syringae, but pathogenesis-related gene expression is abolished in uninoculated distant leaves and fail to develop SAR to virulent Pseudomonas or Peronospora parasitica. DIR1 protein is cell-to-cell mobile and is transported via phloem sap.	30.48	54.73	0.56	2.23E-04	7.13E-04
5437	7	F2-3 vs. S2-3	247715_at	AT5G59360	hypothetical protein;(source:Araport11)	28.42	50.8	0.56	1.20E-04	4.47E-04
5438	7	F2-3 vs. S2-3	247585_at	AT5G60680	transcription initiation factor TFIID subunit (Protein of unknown function, DUF584);(source:Araport11)	113.09	200.94	0.56	4.14E-04	1.14E-03
5439	7	F2-3 vs. S2-3	247449_at	AT5G62290	nucleotide-sensitive chloride conductance regulator (ICln) family protein;(source:Araport11)	106.57	190.97	0.56	6.34E-04	1.59E-03
5440	7	F2-3 vs. S2-3	247427_at	AT5G62580	ARM repeat superfamily protein;(source:Araport11)	36.51	65.35	0.56	1.30E-04	4.75E-04

5441	7	F2-3 vs. S2-3	260625_at	AT1G08060	Encodes a transcriptional silencer that is required for proper expression of PRR/NLR immune receptor genes.	30.36	55.23	0.55	5.69E-05	2.60E-04
5442	7	F2-3 vs. S2-3	257463_at	AT1G14820	Sec14p-like phosphatidylinositol transfer family protein;(source:Araport11)	207.14	379.21	0.55	8.90E-05	3.59E-04
5443	7	F2-3 vs. S2-3	256491_at	AT1G31500	HESP identified based on similarity to nocturnins and presence circadian regulatory elements in the promoter. It functions as a Mg(II) dependent poly(A) exoribonuclease.It is under circadian regulation and expressed at night. Knockdowns affect the regulation of circadian genes CCA1 and TOC1.	14.64	26.77	0.55	2.83E-04	8.51E-04
5444	7	F2-3 vs. S2-3	245791_at	AT1G32210	Encodes protein involved in suppression of apoptosis. Complements a mammalian apoptosis suppressor mutation.	162.38	293.27	0.55	5.43E-04	1.41E-03
5445	7	F2-3 vs. S2-3	265139_at	AT1G51310	tRNA (5-methylaminomethyl-2-thiouridylate)-methyltransferase;(source:Araport11)	47.84	87.06	0.55	3.15E-04	9.27E-04
5446	7	F2-3 vs. S2-3	260042_at	AT1G68820	Putative C3HC4 zinc-finger ubiquitin E3 ligase, negative regulator in ABA and drought stress response.	53.28	96.21	0.55	2.33E-04	7.37E-04
5447	7	F2-3 vs. S2-3	267444_at	AT2G19180	hypothetical protein;(source:Araport11)	17.39	31.63	0.55	2.64E-04	8.07E-04
5448	7	F2-3 vs. S2-3	267104_at	AT2G41430	Encodes hydrophilic protein lacking Cys residues that is expressed in response to drought stress, light stress and treatment with plant-growth-promoting rhizobacteria (Paenibacillus polymyxa), possibly revealing a connection between responses to biotic and abiotic stress. Also identified as a CTC Interacting Domain (CID) protein in a yeast two hybrid screen using the PAB2 protein as bait. Contains PAM2 like domain which mediates interaction with PABC domain in PAB2.	282.27	513.71	0.55	8.47E-04	1.99E-03
5449	7	F2-3 vs. S2-3	256649_at	AT3G13570	encodes an SC35-like splicing factor of 30 kD that is localized to the nuclear specks. Barta et al (2010) have proposed a nomenclature for Serine/Arginine-Rich Protein Splicing Factors (SR proteins): Plant Cell. 2010, 22:2926.	181.92	332.51	0.55	1.28E-04	4.68E-04
5450	7	F2-3 vs. S2-3	256927_at	AT3G22550	NAD(P)H-quinone oxidoreductase subunit, putative (DUF581);(source:Araport11)	135.53	246.84	0.55	8.23E-04	1.95E-03
5451	7	F2-3 vs. S2-3	256980_at	AT3G26932	dsRNA-binding protein 3;(source:Araport11)	10.78	19.66	0.55	9.27E-05	3.70E-04
5452	7	F2-3 vs. S2-3	257315_at	AT3G30775	Encodes a proline oxidase that is predicted to localize to the inner mitochondrial membrane, its mRNA expression induced by high levels of Al and by osmotic stress. The promoter contains an L-proline-inducible element.	89.31	162.54	0.55	6.65E-05	2.88E-04
5453	7	F2-3 vs. S2-3	252450_s_a	AT3G47580	Leucine-rich repeat protein kinase family protein;(source:Araport11)	9.35	16.98	0.55	6.25E-04	1.57E-03
5454	7	F2-3 vs. S2-3	251527_at	AT3G58650	Encodes a member of the TRM superfamily, that plays a role in preprophase band formation during plant cell division and controls the robustness of the orientation of that cell division. Its transcription is regulated by the cell cycle and peaks at the G2/M transition.	28.03	50.8	0.55	4.00E-04	1.11E-03
5455	7	F2-3 vs. S2-3	245358_at	AT4G15900	Mutations confer hypersensitivity to glucose and sucrose and augments sensitivity to cytokinin, ethylene, ABA and auxin. Encodes a nuclear WD40 protein that is imported into the nucleus. Essential for plant innate immunity. Interacts with MOS4 and AtCDC5. It is also predicted to have two DWD motifs. It can bind to DDB1a in Y2H assays, and DDB1b in co-IP assays, and may be involved in the formation of a CUL4-based E3 ubiquitin ligase, and may affect the stability of AKIN10.	27.21	49.35	0.55	6.93E-04	1.70E-03
5456	7	F2-3 vs. S2-3	254268_at	AT4G23040	Ubiquitin-like superfamily protein;(source:Araport11)	18.72	33.81	0.55	2.08E-04	6.81E-04
5457	7	F2-3 vs. S2-3	253028_at	AT4G38160	Encodes a member of the mitochondrial transcription termination factor family. MTERF6 is localized to both the mitochondria and chloroplast. It is required for ILE tRNA maturation during chloroplast development. Loss of function of MTERF6 results in plants with defective chloroplasts or reduced rate of photosynthesis when MTERF6 expression is low.	31.56	57.41	0.55	3.08E-04	9.11E-04
5458	7	F2-3 vs. S2-3	246501_at	AT5G16280	Part of multi-protein complex, acting as guanine nucleotide exchange factors (GEFs) and possibly as tethers, regulating intracellular trafficking.	16.98	30.71	0.55	7.98E-05	3.31E-04
5459	7	F2-3 vs. S2-3	250027_at	AT5G18250	transmembrane protein;(source:Araport11)	14.94	26.97	0.55	8.77E-04	2.05E-03
5460	7	F2-3 vs. S2-3	246135_at	AT5G20885	RING/U-box superfamily protein;(source:Araport11)	30.21	55.31	0.55	6.90E-04	1.70E-03
5461	7	F2-3 vs. S2-3	246026_at	AT5G21070	Fe(3+) dicitrate transport system permease;(source:Araport11)	40.15	72.63	0.55	2.99E-04	8.93E-04
5462	7	F2-3 vs. S2-3	246845_at	AT5G26710	Glutamyl/glutaminyl-tRNA synthetase, class Ic;(source:Araport11)	133.33	242.41	0.55	8.57E-04	2.01E-03
5463	7	F2-3 vs. S2-3	249723_at	AT5G35430	Tetratricopeptide repeat (TPR)-like superfamily protein;(source:Araport11)	13.26	24.06	0.55	1.44E-04	5.12E-04
5464	7	F2-3 vs. S2-3	264796_at	AT1G08680	A member of ARF GAP domain (AGD), A thaliana has 15 members, grouped into four classes. AGD14 belongs to the class 4, together with AGD15.	33.69	62	0.54	8.18E-04	1.94E-03
5465	7	F2-3 vs. S2-3	257422_at	AT1G11940	Core-2/I-branching beta-1,6-N-acetylglucosaminyltransferase family protein;(source:Araport11)	25.23	46.41	0.54	6.34E-04	1.59E-03

5466	7	F2-3 vs. S2-3	261486_at	AT1G14510	Encodes a member of the Alfin1-like family of nuclear-localized PHD (plant homeodomain) domain containing proteins. All AL proteins except AL3 bind to di- or trimethylated histone H3 (H3K4me3/2). Members of this family include: AT5G05610 (AL1), AT3G11200 (AL2), AT3G42790 (AL3), AT5G26210 (AL4), AT5G20510 (AL5), AT2G02470 (AL6), AT1G14510 (AL7).	174.06	324	0.54	1.15E-04	4.35E-04
5467	7	F2-3 vs. S2-3	259406_at	AT1G17690	U3 small nucleolar RNA-associated protein;(source:Araport11)	93.83	175.1	0.54	7.51E-04	1.81E-03
5468	7	F2-3 vs. S2-3	259599_at	AT1G28110	serine carboxypeptidase-like 45;(source:Araport11)	44.68	82.6	0.54	6.26E-04	1.58E-03
5469	7	F2-3 vs. S2-3	262958_at	AT1G54410	Encodes a KS-type dehydrin can reduce the formation of reactive oxygen species (ROS) from Cu.	309.66	577.43	0.54	3.98E-04	1.11E-03
5470	7	F2-3 vs. S2-3	256217_at	AT1G56320	hypothetical protein;(source:Araport11)	23.1	42.5	0.54	6.07E-04	1.54E-03
5471	7	F2-3 vs. S2-3	264218_at	AT1G60200	RMB25 is an alternative splicing factor involved in mediation of abiotic stress response and ABA response. Its expression is modulated by a variety of stressors and it in turn appears to affect the ratio of splice variants of stress responsive genes such as HAB1.2/HAB1.1.	28.22	51.89	0.54	1.03E-04	4.00E-04
5472	7	F2-3 vs. S2-3	262667_at	AT1G62810	Encodes COPPER AMINE OXIDASE1 (CuAO1). Contributes to abscisic acid- and polyamine-induced nitric oxide biosynthesis and abscisic acid signal transduction.	22.91	42.65	0.54	8.92E-04	2.08E-03
5473	7	F2-3 vs. S2-3	262338_at	AT1G64185	Vicinal oxygen chelate (VOC) superfamily member.	23.05	42.5	0.54	1.82E-04	6.14E-04
5474	7	F2-3 vs. S2-3	261521_at	AT1G71830	Plasma membrane LRR receptor-like serine threonine kinase expressed during embryogenesis in locules until stage 6 anthers, with higher expression in the tapetal cell layer. SERK1 and SERK2 receptor kinases function redundantly as an important control point for sporophytic development controlling male gametophyte production. SERK1 interacts with and transphosphorylates EMS1	16.84	31.11	0.54	9.18E-04	2.13E-03
5475	7	F2-3 vs. S2-3	259978_at	AT1G76540	Encodes a cyclin-dependent protein kinase involved in regulation of the G2/M transition of the mitotic cell cycle. Specifically binds to the cyclin CYCD4;1, expressed in shoot meristem, young leaves and vascular tissue during the G2/M phase. Required for proper organization of the shoot apical meristem and for hormone signaling.	53.01	98.24	0.54	3.10E-04	9.14E-04
5476	7	F2-3 vs. S2-3	255874_at	AT2G40550	Encodes a nuclear localized target of E2Fa-DPa, transcription factors controlling cell cycle progression. Required for sister chromatid cohesion and DNA repair.	50.62	94.4	0.54	5.49E-04	1.43E-03
5477	7	F2-3 vs. S2-3	245124_at	AT2G47580	encodes spliceosomal protein U1A	247.48	460.53	0.54	1.11E-04	4.24E-04
5478	7	F2-3 vs. S2-3	259128_at	AT3G02260	Calossin-like protein required for polar auxin transport. Involved in regulating sugar response and C/N balance.	195.09	364.3	0.54	6.61E-05	2.87E-04
5479	7	F2-3 vs. S2-3	258793_at	AT3G04780	Encodes a protein with little sequence identity with any other protein of known structure or function. Part of this protein shows a 42% sequence identity with the C-terminal domain of the 32-kD human thioredoxin-like protein.	78.43	146.28	0.54	7.73E-04	1.85E-03
5480	7	F2-3 vs. S2-3	256397_at	AT3G06110	Encodes a nuclear-localized MAP kinase phosphatase. Plants with reduced levels of MKP2 transcripts are hypersensitive to ozone and ozone-mediated activation of MPK3 and MPK6 is prolonged in these plants.	42.86	79.55	0.54	4.80E-05	2.30E-04
5481	7	F2-3 vs. S2-3	258636_at	AT3G07890	Ypt/Rab-GAP domain of gyp1p superfamily protein;(source:Araport11)	45.31	84.09	0.54	4.37E-04	1.19E-03
5482	7	F2-3 vs. S2-3	252464_at	AT3G47160	RING/U-box superfamily protein;(source:Araport11)	49.58	91.16	0.54	8.15E-05	3.36E-04
5483	7	F2-3 vs. S2-3	245479_at	AT4G16140	proline-rich family protein;(source:Araport11)	15.3	28.31	0.54	3.95E-04	1.10E-03
5484	7	F2-3 vs. S2-3	254675_at	AT4G18465	RNA helicase family protein;(source:Araport11)	46.42	85.44	0.54	6.85E-04	1.69E-03
5485	7	F2-3 vs. S2-3	254314_at	AT4G22470	Encodes a hybrid proline-rich protein that contains two tandem PRD-8CMs (proline-rich domain-eight cysteine motif) that is involved in systemic acquired resistance.	16.27	30.15	0.54	6.21E-05	2.75E-04
5486	7	F2-3 vs. S2-3	253967_at	AT4G26550	Got1/Sft2-like vescicle transport protein family;(source:Araport11)	80.86	149.72	0.54	7.50E-05	3.15E-04
5487	7	F2-3 vs. S2-3	253868_at	AT4G27500	interacts with H+-ATPase, and regulates its activity The mRNA is cell-to-cell mobile.	237.09	437.71	0.54	9.35E-05	3.72E-04
5488	7	F2-3 vs. S2-3	253357_at	AT4G33400	Vacuolar import/degradation, Vid27-related protein;(source:Araport11)	210.89	394.04	0.54	6.44E-04	1.61E-03
5489	7	F2-3 vs. S2-3	253010_at	AT4G37750	ANT is required for control of cell proliferation and encodes a putative transcriptional regulator similar to AP2. Loss of function alleles have reduced fertility, abnormal ovules and abnormal lateral organs. Expressed in the chalaza, floral organ primordia, and lateral shoot organ primordia. Regulates growth and cell numbers during organogenesis.	35.77	65.94	0.54	5.75E-05	2.62E-04
5490	7	F2-3 vs. S2-3	250586_at	AT5G07630	lipid transporter;(source:Araport11)	21.01	39.21	0.54	2.72E-04	8.27E-04
5491	7	F2-3 vs. S2-3	245984_at	AT5G13090	hypothetical protein;(source:Araport11)	40.95	75.75	0.54	2.22E-04	7.12E-04

5492	7	F2-3 vs. S2-3	246546_at	AT5G15090	Encodes a voltage-dependent anion channel (VDAC: AT3G01280/VDAC1, AT5G67500/VDAC2, AT5G15090/VDAC3, AT5G57490/VDAC4, AT5G15090/VDAC5). VDACs are reported to be porin-type, beta-barrel diffusion pores. They are prominently localized in the outer mitochondrial membrane and are involved in metabolite exchange between the organelle and the cytosol. Purified VDAC3 is shown to have voltage-dependent anion channel activity.	30.92	57.21	0.54	1.55E-04	5.44E-04
5493	7	F2-3 vs. S2-3	246443_at	AT5G17620	nuclear matrix protein;(source:Araport11)	103.83	192.17	0.54	1.69E-04	5.79E-04
5494	7	F2-3 vs. S2-3	249954_at	AT5G18920	Cox19-like CHCH family protein;(source:Araport11)	10.51	19.3	0.54	7.25E-04	1.76E-03
5495	7	F2-3 vs. S2-3	249936_at	AT5G22450	spectrin beta chain, brain;(source:Araport11)	37.79	69.43	0.54	6.37E-04	1.60E-03
5496	7	F2-3 vs. S2-3	249127_at	AT5G43500	encodes a protein whose sequence is similar to actin-related proteins (ARPs) in other organisms. Member of nuclear ARP family of genes.	13.77	25.44	0.54	3.87E-04	1.09E-03
5497	7	F2-3 vs. S2-3	248933_at	AT5G46070	Guanylate-binding family protein;(source:Araport11)	22.12	41	0.54	6.17E-04	1.56E-03
5498	7	F2-3 vs. S2-3	247853_at	AT5G58140	Membrane-bound protein serine/threonine kinase that functions as blue light photoreceptor in redundancy with PHO1. Involved in stomatal opening, chloroplast movement and phototropism. Mediates blue light-induced growth enhancements. PHOT1 and PHOT2 mediate blue light-dependent activation of the plasma membrane H+-ATPase in guard cell protoplasts. PHOT2 possesses two LOV (LOV1 and LOV2, for light-oxygen-voltage-sensing) domains involved in FMN-binding and a C-terminus forming a serine/threonine kinase domain. LOV2 acts as an inhibitor of phototropin kinase in the dark, and light cancels the inhibition through cysteine-FMN adduct formation. LOV1 in contrast acts as an attenuator of photoactivation. Localized to the Golgi apparatus under the induction of blue light. The mRNA is cell-to-cell mobile.	41.99	77.2	0.54	5.15E-04	1.35E-03
5499	7	F2-3 vs. S2-3	247158_at	AT5G65780	Encodes a chloroplast branched-chain amino acid aminotransferase, can complement the yeast leu/iso-leu/val auxotrophy mutant. Note that the AT5G65780.2 gene model (TAIR10) has been obsoleted due to the lack of experimental support. The mRNA is cell-to-cell mobile.	16.05	29.59	0.54	7.74E-04	1.85E-03
5500	7	F2-3 vs. S2-3	247112_at	AT5G65950	TRAPPIII complex protein which regulates TGN integrity, by altered TGN/EE association of several residents, including SYNTAXIN OF PLANTS 61 (SYP61), and altered vesicle morphology. Involved in regulation of endosomal function and salt stress response.	286.3	533.85	0.54	8.04E-05	3.33E-04
5501	7	F2-3 vs. S2-3	247049_at	AT5G66440	tRNA-methyltransferase non-catalytic subunit trm6MTase subunit;(source:Araport11)	14	26.11	0.54	8.73E-05	3.53E-04
5502	7	F2-3 vs. S2-3	259401_at	AT1G17760	Encodes a homolog of the mammalian protein CstF77, a polyadenylation factor subunit. RNA 3′-end?processing factor of antisense FLC transcript. Mediates silencing of the floral repressor gene FLC. Member of CstF complex.	33.41	63	0.53	8.30E-05	3.40E-04
5503	7	F2-3 vs. S2-3	262989_at	AT1G23420	Essential for formation and asymmetric growth of the ovule outer integument. Member of the YABBY protein family of putative transcription factors that contain apparent Cys(2)-Cys(2) zinc-finger domains and regions of similarity to the high mobility group (HMG) transcription factors. INO may be required for polarity determination in the central part of the ovule.	22.49	42.76	0.53	4.78E-04	1.28E-03
5504	7	F2-3 vs. S2-3	259996_at	AT1G67910	hypothetical protein;(source:Araport11)	18.82	35.34	0.53	8.69E-05	3.52E-04
5505	7	F2-3 vs. S2-3	260279_at	AT1G80420	Encodes a component of plant break excision repair and functions at several stages during active DNA demethylation in Arabidopsis.	13.07	24.76	0.53	6.46E-05	2.82E-04
5506	7	F2-3 vs. S2-3	265343_at	AT2G22570	encodes a nicotinamidase that converts nicotinamide into nicotinic acid. As such the encoded enzyme is involved in the pyridine nucleotide salvage pathway which may be connected to the de novo NAD biosynthesis through the ABA signaling pathway.	32.77	61.36	0.53	4.00E-04	1.11E-03
5507	7	F2-3 vs. S2-3	267606_at	AT2G26640	Encodes KCS11, a member of the 3-ketoacyl-CoA synthase family involved in the biosynthesis of VLCFA (very long chain fatty acids).	19.87	37.46	0.53	5.63E-05	2.58E-04
5508	7	F2-3 vs. S2-3	258855_at	AT3G02070	Cysteine proteinases superfamily protein;(source:Araport11)	50.47	96.04	0.53	2.25E-04	7.17E-04
5509	7	F2-3 vs. S2-3	258665_at	AT3G08710	Associated to plasma membrane. Moves cell to cell, suggesting a role in intercellular communication.	82.59	155.17	0.53	1.01E-04	3.95E-04

5510	7	F2-3 vs. S2-3	259259_at	AT3G11540	Encodes a N-acetyl glucosamine transferase that may glycosylate other molecules involved in GA signaling. Contains a tetratricopeptide repeat region, and a novel carboxy-terminal region. SPY acts as both a repressor of GA responses and as a positive regulation of cytokinin signalling. SPY may be involved in reducing ROS accumulation in response to stress.	78.93	150.28	0.53	5.84E-05	2.64E-04
5511	7	F2-3 vs. S2-3	256570_at	AT3G19540	glutamyl-tRNA (Gln) amidotransferase subunit A (DUF620);(source:Araport11)	97.89	185.36	0.53	1.57E-04	5.51E-04
5512	7	F2-3 vs. S2-3	257177_at	AT3G23490	Encodes a cyanase that catalyzes the bicarbonate-dependent breakdown of cyanate to ammonia and bicarbonate. CYN forms a hexadecamer and is believed to be a cytosolic protein. Long-term exposure to NaCl increases CYN transcript levels. It is also expressed at higher levels in flowers relative to stems, roots, and seedlings.	18.17	34.22	0.53	5.95E-05	2.67E-04
5513	7	F2-3 vs. S2-3	258132_at	AT3G24550	Encodes a member of the proline-rich extensin-like receptor kinase (PERK) family. This family consists of 15 predicted receptor kinases (PMID: 15653807). The mRNA is cell-to-cell mobile.	80.99	151.74	0.53	1.90E-04	6.33E-04
5514	7	F2-3 vs. S2-3	257835_at	AT3G25180	Encodes a cytochrome P450 monooxygenase (CYP82G1) that catalyzes the production of two volatile homoterpenes, TMTT and DMNT, although it is only likely to produce TMTT in planta. TMTT can be involved in attracting predatory insects to protect Arabidopsis plants from herbivorous pests. Homoterpene synthesis is also stimulated by fungal elicitors which increase the transcript levels of CYP82G1.	41.6	78.22	0.53	7.67E-04	1.84E-03
5515	7	F2-3 vs. S2-3	252683_at	AT3G44380	Late embryogenesis abundant (LEA) hydroxyproline-rich glycoprotein family;(source:Araport11)	15.5	29.36	0.53	1.05E-04	4.05E-04
5516	7	F2-3 vs. S2-3	252603_at	AT3G45050	transmembrane protein;(source:Araport11)	31.21	58.81	0.53	1.62E-04	5.62E-04
5517	7	F2-3 vs. S2-3	252328_at	AT3G48570	secE/sec61-gamma protein transport protein;(source:Araport11)	146.08	276.9	0.53	2.27E-04	7.23E-04
5518	7	F2-3 vs. S2-3	251990_at	AT3G53320	Encodes a microtubule-associated protein track growing microtubule plus ends.	15.38	29.2	0.53	7.61E-05	3.18E-04
5519	7	F2-3 vs. S2-3	251795_at	AT3G55390	Uncharacterized protein family (UPF0497);(source:Araport11)	28.61	54.33	0.53	3.40E-04	9.84E-04
5520	7	F2-3 vs. S2-3	245380_at	AT4G17760	PCNA domain-containing protein;(source:Araport11)	22.12	42.03	0.53	3.40E-04	9.84E-04
5521	7	F2-3 vs. S2-3	253900_at	AT4G27130	Translation initiation factor SUI1 family protein;(source:Araport11)	331.59	625.26	0.53	1.37E-04	4.93E-04
5522	7	F2-3 vs. S2-3	252824_at	AT4G40030	Histone superfamily protein;(source:Araport11)	1287	2438.77	0.53	5.14E-04	1.35E-03
5523	7	F2-3 vs. S2-3	250881_at	AT5G04080	cysteine-rich TM module stress tolerance protein;(source:Araport11)	10.55	20.08	0.53	2.34E-04	7.38E-04
5524	7	F2-3 vs. S2-3	250836_at	AT5G04560	Encodes a DNA glycosylase DEMETER (DME). Responsible for endosperm maternal-allele-specific hypomethylation at the MEDEA (MEA) gene. DME can excise 5-methylcytosine in vitro and when expressed in E. coli. DME establishes MEA imprinting by removing 5-methylcytosine to activate the maternal allele.	15.71	29.72	0.53	7.47E-04	1.80E-03
5525	7	F2-3 vs. S2-3	246487_at	AT5G16030	mental retardation GTPase activating protein;(source:Araport11)	43.22	81.24	0.53	4.51E-04	1.22E-03
5526	7	F2-3 vs. S2-3	249841_at	AT5G23520	smr (Small MutS Related) domain-containing protein;(source:Araport11)	16.27	30.69	0.53	6.79E-04	1.68E-03
5527	7	F2-3 vs. S2-3	264364_at	AT1G03330	Small nuclear ribonucleoprotein family protein;(source:Araport11)	135.16	260.9	0.52	1.35E-04	4.87E-04
5528	7	F2-3 vs. S2-3	264811_at	AT1G08640	Encodes a choloroplast membrane protein CJD1 (Chloroplast J-like Domain 1). Predicted to contain a transit peptide, three transmembrane domains and an N-terminal J-like domain. Influences fatty acid composition of chloroplast lipids.	74.47	143.1	0.52	1.23E-04	4.57E-04
5529	7	F2-3 vs. S2-3	261209_at	AT1G12810	proline-rich family protein;(source:Araport11)	62.2	120.19	0.52	5.43E-04	1.41E-03
5530	7	F2-3 vs. S2-3	261439_at	AT1G28395	hypothetical protein;(source:Araport11)	34.59	66.99	0.52	1.59E-04	5.54E-04
5531	7	F2-3 vs. S2-3	261239_at	AT1G32930	Galactosyltransferase family protein;(source:Araport11)	93.49	180.67	0.52	7.98E-05	3.31E-04
5532	7	F2-3 vs. S2-3	260767_s_at	AT1G49140	NADH dehydrogenase ubiquinone 1 beta subcomplex subunit 10-B-like protein (Complex I subunit NDUFS6);(source:Araport11)	376.77	727.4	0.52	2.34E-04	7.38E-04
5533	7	F2-3 vs. S2-3	264099_at	AT1G79050	recA DNA recombination family protein;(source:Araport11)	35	66.71	0.52	1.31E-04	4.79E-04
5534	7	F2-3 vs. S2-3	262941_at	AT1G79490	Pentatricopeptide repeat (PPR) superfamily protein;(source:Araport11)	38.71	74.39	0.52	7.54E-04	1.81E-03
5535	7	F2-3 vs. S2-3	260331_at	AT1G80270	PENTATRICOPEPTIDE REPEAT 596;(source:Araport11)	335.09	645.53	0.52	9.56E-05	3.77E-04
5536	7	F2-3 vs. S2-3	261891_at	AT1G80790	Belongs to a subgroup of SGS3-like proteins that act redundantly in RNA-directed DNA methylation: AT1G15910 (FDM1), AT4G00380 (FDM2), AT3G12550 (FDM3), AT1G13790 (FDM4), AT1G80790 (FDM5).The mRNA is cell-to-cell mobile.	10.66	20.65	0.52	2.53E-04	7.80E-04
5537	7	F2-3 vs. S2-3	266698_at	AT2G19830	SNF7 family protein;(source:Araport11)	42.32	80.69	0.52	8.51E-05	3.46E-04
5538	7	F2-3 vs. S2-3	267301_at	AT2G30110	Encodes a ubiquitin-activating enzyme (E1), involved in the first step in conjugating multiple ubiquitins to proteins targeted for degradation. Gene is expressed in most tissues examined. Mutant is able to revert the constitutive defense responses phenotype of snc1, which indicates the gene is involved in defense response. It also indicates that ubiquitination plays a role in plant defense signalling.	155.76	301.28	0.52	1.13E-04	4.30E-04

5539	7	F2-3 vs. S2-3	255817_at	AT2G33330	Encodes a plasmodesmal protein that affects the intercellular movement of molecules through the plasmodesmata. The protein has two DUF26 domains and a single transmembrane domain.	48.46	92.54	0.52	4.20E-05	2.10E-04
5540	7	F2-3 vs. S2-3	255848_at	AT2G33640	DHHC-type zinc finger family protein that encodes a functional s-acyl transferase.	19.91	38.51	0.52	7.84E-04	1.87E-03
5541	7	F2-3 vs. S2-3	267171_at	AT2G37590	PEAR protein involved in the formation of a short-range concentration gradient that peaks at protophloem sieve elements, and activates gene expression that promotes radial growth. Locally promotes transcription of inhibitory HD-ZIP III genes, and thereby establishes a negative-feedback loop that forms a robust boundary that demarks the zone of cell division.	43.79	84.61	0.52	8.18E-05	3.36E-04
5542	7	F2-3 vs. S2-3	267513_at	AT2G45620	Nucleotidyltransferase family protein involved in transcript polyadenylation.	161.56	310.62	0.52	6.98E-05	2.98E-04
5543	7	F2-3 vs. S2-3	256400_at	AT3G06140	Paralog of LOG2 (At3g09770), a ubiquitin ligase that regulates amino acid export.	11.88	22.96	0.52	2.02E-04	6.66E-04
5544	7	F2-3 vs. S2-3	256286_at	AT3G12180	Cornichon family protein;(source:Araport11)	50.47	97.5	0.52	7.04E-05	2.99E-04
5545	7	F2-3 vs. S2-3	257051_at	AT3G15270	Encodes a member of the SPL (squamosa-promoter binding protein-like)gene family, a novel gene family encoding DNA binding proteins and putative transcription factors. Contains the SBP-box, which encodes the SBP-domain, required and sufficient for interaction with DNA. It is involved in regulation of flowering and vegetative phase change. Its temporal expression is regulated by the microRNA miR156. The target site for the microRNA is in the 3'UTR.	32.65	62.69	0.52	3.42E-05	1.79E-04
5546	7	F2-3 vs. S2-3	252190_at	AT3G50170	transmembrane protein, putative (DUF247);(source:Araport11)	10.16	19.64	0.52	1.72E-04	5.87E-04
5547	7	F2-3 vs. S2-3	252136_at	AT3G50770	calmodulin-like 41;(source:Araport11)	12.12	23.47	0.52	1.32E-04	4.81E-04
5548	7	F2-3 vs. S2-3	251753_at	AT3G55760	hypothetical protein;(source:Araport11)	48.81	94.03	0.52	4.91E-04	1.30E-03
5549	7	F2-3 vs. S2-3	246317_at	AT3G56900	Encodes ALADIN, a component of the nuclear pore complex.	28.09	54.19	0.52	7.56E-04	1.82E-03
5550	7	F2-3 vs. S2-3	251338_at	AT3G60600	Encodes VAP27 (for Vesicle-Associated Protein). VAP27 has high homology to the VAP33 family of SNARE-like proteins from animals. May be involved in vesicular transport to or from the ER. Located exclusively in limiting membrane of protein storage vacuoles. Binds SRC2.	400.03	773.13	0.52	1.05E-04	4.06E-04
5551	7	F2-3 vs. S2-3	254953_at	AT4G10925	Nuclear transport factor 2 (NTF2) family protein;(source:Araport11)	90.52	173.24	0.52	9.53E-05	3.77E-04
5552	7	F2-3 vs. S2-3	254865_s_a	AT4G12190	RING/U-box superfamily protein;(source:Araport11)	12.63	24.35	0.52	6.20E-04	1.56E-03
5553	7	F2-3 vs. S2-3	254340_at	AT4G22120	Calcium-permeable stretch activated cation channel.	23.99	45.93	0.52	6.48E-04	1.62E-03
5554	7	F2-3 vs. S2-3	254088_at	AT4G24880	snurportin-1 protein;(source:Araport11)	84.23	160.49	0.52	9.60E-05	3.78E-04
5555	7	F2-3 vs. S2-3	254111_at	AT4G24890	purple acid phosphatase 24;(source:Araport11)	35.5	68.87	0.52	1.01E-04	3.95E-04
5556	7	F2-3 vs. S2-3	253138_at	AT4G35490	mitochondrial ribosomal protein L11;(source:Araport11)	193.8	370.83	0.52	1.06E-04	4.08E-04
5557	7	F2-3 vs. S2-3	246517_at	AT5G15760	Ribosomal protein PSRP-3/Ycf65;(source:Araport11)	13.06	25.17	0.52	6.67E-05	2.88E-04
5558	7	F2-3 vs. S2-3	250125_at	AT5G16390	Encodes for the biotin carboxyl-carrier subunit of the multi-enzyme plastidial acetyl-coenzyme A carboxylase complex.	18.54	35.45	0.52	7.07E-05	3.01E-04
5559	7	F2-3 vs. S2-3	248975_at	AT5G45040	Encodes a Class I cytochrome c family member possessing a high structural homology with photosynthetic cytochrome c(6) from cyanobacteria, but structurally and functionally distinct through the presence of a disulfide bond.	19.16	36.66	0.52	2.39E-04	7.47E-04
5560	7	F2-3 vs. S2-3	244939_at	ATCG00065	chloroplast gene encoding ribosomal protein s12. The gene is located in three distinct loci on the chloroplast genome and is transpliced to make one transcript.	20.28	39.23	0.52	3.24E-04	9.48E-04
5561	7	F2-3 vs. S2-3	244940_at	ATCG01230	chloroplast gene encoding ribosomal protein s12. The gene is located in three distinct loci on the chloroplast genome and is transpliced to make one transcript.	670.39	1298.13	0.52	6.27E-05	2.77E-04
5562	7	F2-3 vs. S2-3	264810_at	AT1G08750	GPI8/PIG-K homolog involved in stomata development. Loss of function alleles do not transmit through the pollen.	49.41	96.34	0.51	1.33E-04	4.83E-04
5563	7	F2-3 vs. S2-3	255899_at	AT1G17970	RING/U-box superfamily protein;(source:Araport11)	20.12	39.7	0.51	3.46E-05	1.81E-04
5564	7	F2-3 vs. S2-3	261717_at	AT1G18400	Encodes the brassinosteroid signaling component BEE1 (BR-ENHANCED EXPRESSION 1). Positively modulates the shade avoidance syndrome in Arabidopsis seedlings.	13.98	27.41	0.51	8.51E-04	2.00E-03
5565	7	F2-3 vs. S2-3	261131_at	AT1G19835	TCS1 encodes a coiled-coil domain protein that binds to microtubules and co-localizes with the cortical microtubules. Mutants have defects in trichome branching and hypocotyl elongation. TCS1 interacts with ZWI and appears to be involved in microtubule assembly.	145.12	282.52	0.51	2.11E-04	6.85E-04
5566	7	F2-3 vs. S2-3	260974_at	AT1G53440	Leucine-rich repeat transmembrane protein kinase;(source:Araport11)	13.79	27	0.51	4.61E-04	1.24E-03
5567	7	F2-3 vs. S2-3	261354_at	AT1G79690	nudix hydrolase homolog 3;(source:Araport11)	51.69	102.3	0.51	5.97E-05	2.68E-04
5568	7	F2-3 vs. S2-3	261348_at	AT1G79810	Dominant suppressor of det1 phenotypes. Encodes a peroxisomal protein essential for Arabidopsis growth. Inserted directly from the cytosol into peroxisomes.	26.18	51.55	0.51	1.14E-04	4.32E-04

5569	7	F2-3 vs. S2-3	266120_at	AT2G02070	RAVEN is part of the network regulated by BLJUEJAY, JACKDAW, SACRECROW and SHORT-ROOT to regulate root tissue patterning through cell lineage specification and asymmetric cell division. RAVEN is directly activated by SHORT-ROOT and directly repressed by JACKDAW.	72.49	142.04	0.51	3.35E-05	1.77E-04
5570	7	F2-3 vs. S2-3	266110_at	AT2G02080	C2H2 BIRD transcription factor family.	33.04	65.25	0.51	7.08E-04	1.73E-03
5571	7	F2-3 vs. S2-3	266306_at	AT2G26970	Polynucleotidyl transferase, ribonuclease H-like superfamily protein;(source:Araport11)	30.22	58.81	0.51	2.78E-05	1.53E-04
5572	7	F2-3 vs. S2-3	265629_at	AT2G27230	Encodes a nuclear-localized transcriptional activator with weak sequence similarity to basic helix-loop-helix(bHLH)-domain proteins. It promotes the production of stele cells in root meristems and is required to establish and maintain the normal vascular cell number and pattern in primary and lateral roots.	118.66	234.23	0.51	1.19E-04	4.44E-04
5573	7	F2-3 vs. S2-3	260545_at	AT2G43350	Glutathione peroxidase. Functions as both a redox transducer and a scavenger in abscisic acid and drought stress responses. Interacts with ABI2 and ABI1.	22.4	44.2	0.51	1.33E-04	4.83E-04
5574	7	F2-3 vs. S2-3	259166_at	AT3G01670	Encodes a protein localized to phloem filaments that is required for phloem filament formation.The mRNA is cell-to-cell mobile.	18.63	36.3	0.51	5.65E-05	2.58E-04
5575	7	F2-3 vs. S2-3	258529_at	AT3G06740	Encodes a member of the GATA factor family of zinc finger transcription factors.	12.79	25.23	0.51	3.69E-04	1.05E-03
5576	7	F2-3 vs. S2-3	258286_at	AT3G16060	ATP binding microtubule motor family protein;(source:Araport11)	13.99	27.59	0.51	7.19E-04	1.75E-03
5577	7	F2-3 vs. S2-3	257974_at	AT3G20820	Leucine-rich repeat (LRR) family protein;(source:Araport11)	257.51	506.22	0.51	2.27E-04	7.23E-04
5578	7	F2-3 vs. S2-3	258184_at	AT3G21510	Encodes AHP1, one of the six Arabidopsis thaliana histidine phosphotransfer proteins (AHPs). AHPs function as redundant positive regulators of cytokinin signaling. Members of the AHP gene family include: AT3G21510 (AHP1), AT3G29350 (AHP2), AT5G39340 (AHP3), AT3G16360 (AHP4), AT1G03430 (AHP5) and AT1G80100 (AHP6).	20.98	41.16	0.51	4.24E-04	1.17E-03
5579	7	F2-3 vs. S2-3	251943_at	AT3G53500	Barta et al (2010) have proposed a nomenclature for Serine/Arginine-Rich Protein Splicing Factors (SR proteins): Plant Cell. 2010, 22:2926.	107.31	211.02	0.51	1.13E-04	4.28E-04
5580	7	F2-3 vs. S2-3	251714_at	AT3G56370	LRR-RLK with distinct polar localization within the plasma membrane in different cell types of the root. Mutants show defects in cell divisions within the root ground tissue.	76.1	148.82	0.51	4.11E-04	1.14E-03
5581	7	F2-3 vs. S2-3	251413_at	AT3G60320	bZIP domain class transcription factor (DUF630 and DUF632);(source:Araport11)	86.09	169.28	0.51	3.67E-04	1.05E-03
5582	7	F2-3 vs. S2-3	251391_at	AT3G60910	S-adenosyl-L-methionine-dependent methyltransferases superfamily protein;(source:Araport11)	46.82	92.37	0.51	9.00E-05	3.62E-04
5583	7	F2-3 vs. S2-3	254747_at	AT4G13020	Encodes a member of the cdc2+ family of protein kinases MHK. Similar to the mak genes of rats. mak encodes a protein kinase that may play a role in spermatogenesis.	29.93	59.1	0.51	5.29E-05	2.47E-04
5584	7	F2-3 vs. S2-3	245605_at	AT4G14300	Belongs to a member of the RNA-binding glycine-rich (RBG) gene superfamily.	166.91	324.39	0.51	1.19E-04	4.45E-04
5585	7	F2-3 vs. S2-3	245254_at	AT4G14680	Encodes one of three A. thaliana ATP-sulfurylases. APS is the first enzyme of sulfate assimilation that catalyzes the formation of adenosine-5'-phosphosulfate from ATP and sulfate.	23.91	47.04	0.51	3.18E-05	1.70E-04
5586	7	F2-3 vs. S2-3	245571_at	AT4G14695	Uncharacterized protein family (UPF0041);(source:Araport11)	12.46	24.62	0.51	2.81E-04	8.45E-04
5587	7	F2-3 vs. S2-3	245489_at	AT4G16280	Involved in the promotion of the transition of the vegetative meristem to reproductive development. Four forms of the protein (alpha, beta, delta and gamma) are produced by alternative splicing. Involved in RNA-mediated chromatin silencing. At one point it was believed to act as an abscisic acid receptor but the paper describing that function was retracted.	35.04	68.3	0.51	1.05E-04	4.05E-04
5588	7	F2-3 vs. S2-3	254545_at	AT4G19830	FKBP-like peptidyl-prolyl cis-trans isomerase family protein;(source:Araport11)	33.61	66.47	0.51	4.92E-04	1.30E-03
5589	7	F2-3 vs. S2-3	250467_at	AT5G10100	Trehalose-6-phosphate phosphatase which enhances drought tolerance by regulating stomatal apertures.	13.75	27.09	0.51	6.10E-04	1.55E-03
5590	7	F2-3 vs. S2-3	246840_at	AT5G26610	D111/G-patch domain-containing protein;(source:Araport11)	37.17	73.13	0.51	3.53E-04	1.01E-03
5591	7	F2-3 vs. S2-3	248757_at	AT5G47680	Encodes a protein involved in modification of nucleosides in tRNA. Mutants have 50% less 1-methylguanosine than wt counterparts.	157.03	309.86	0.51	4.96E-04	1.31E-03
5592	7	F2-3 vs. S2-3	248768_at	AT5G47700	Co-orthologous gene of large ribosomal subunit protein RPP1.	177.97	349.48	0.51	2.37E-04	7.45E-04
5593	7	F2-3 vs. S2-3	248683_at	AT5G48490	Encodes a protein with similarity to a lipid transfer protein that may contribute to systemic acquired resistance (SAR).	12.16	23.97	0.51	5.38E-04	1.40E-03

5594	7	F2-3 vs. S2-3	248595_at	AT5G49230	Identified in a screen for mutations hypersensitive to red and blue light. Mutants have shorter hypocotyls. Encodes a nuclear localized protein with similarity to drought induced proteins. Contains a ZZ zinc finger domain which is thought to mediate protein-protein interactions.May be involved in red and blue light signal transduction.	28.44	55.6	0.51	6.92E-05	2.97E-04
5595	7	F2-3 vs. S2-3	248142_at	AT5G55040	DNA-binding bromodomain-containing protein;(source:Araport11)	34.54	67.54	0.51	1.11E-04	4.24E-04
5596	7	F2-3 vs. S2-3	247434_at	AT5G62575	Encodes subunit 7 of mitochondrial complex II (succinate dehydrogenase complex) and participates in the respiratory chain. It contributes to anchoring succinate dehydrogenase to the inner mitochondrial membrane. The mRNA is cell-to-cell mobile.	34.48	67.23	0.51	9.93E-05	3.90E-04
5597	7	F2-3 vs. S2-3	265228_s_a	ATMG01190	ATPase subunit 1	107.22	210.75	0.51	7.53E-04	1.81E-03
5598	7	F2-3 vs. S2-3	261572_at	AT1G01170	ozone-responsive stress-like protein (DUF1138);(source:Araport11)	263.28	528.54	0.5	3.76E-04	1.07E-03
5599	7	F2-3 vs. S2-3	263660_at	AT1G04260	Encodes protein that interacts with CaMV movement protein. Colocalizes in the cytoplasm with the movement protein. Has similarity to mammalian proteins (such as the rat PRA1) which have been described as rab acceptors.	54.89	109.95	0.5	7.22E-04	1.75E-03
5600	7	F2-3 vs. S2-3	261416_at	AT1G07770	Encodes cytoplasmic ribosomal protein S15a.	540.94	1082.75	0.5	1.95E-05	1.19E-04
5601	7	F2-3 vs. S2-3	263255_at	AT1G10490	GNAT acetyltransferase (DUF699);(source:Araport11)	36.83	72.95	0.5	9.31E-04	2.16E-03
5602	7	F2-3 vs. S2-3	264489_at	AT1G27370	In conjunction with SPL11 and SPL2, SPL10 redundantly controls proper development of lateral organs in association with shoot maturation in the reproductive phase. SPL2, SPL10, and SPL11, suppress root regeneration with age by inhibiting wound-induced auxin biosynthesis. SPL10 also controls lamina shape during vegetative development.	23.59	47.11	0.5	1.28E-04	4.71E-04
5603	7	F2-3 vs. S2-3	261336_at	AT1G44790	ChaC-like family protein;(source:Araport11)	136.05	273.36	0.5	2.60E-04	7.98E-04
5604	7	F2-3 vs. S2-3	256215_at	AT1G50900	Encodes GDC1 (Grana Deficient Chloroplast 1), an ankyrin domain containing protein required fro chloroplast thylakoid grana formation. The mRNA is cell-to-cell mobile.	34.04	67.81	0.5	7.73E-05	3.23E-04
5605	7	F2-3 vs. S2-3	259875_s_at	AT1G76680	Encodes a member of an alpha/beta barrel fold family of FMN-containing oxidoreductases. One of the closely related 12-oxophytodienoic acid reductases. This enzyme is not expected to participate in jasmonic acid biosynthesis because during in vitro assays, it shows very little activity with the naturally occurring OPDA isomer. Shows activity towards 2,4,6-trinitrotoluene. Expressed predominately in root. Up-regulated by senescence and jasmonic acid. Induced by salicylic acid. Independent of NPR1 for their induction by salicylic acid. Predicted to be a cytosolic protein.	151.46	300.96	0.5	3.27E-04	9.53E-04
5606	7	F2-3 vs. S2-3	256329_at	AT1G76850	Encodes a member of the exocyst complex gene family. The exocyst is a protein complex involved in tethering vesicles to the plasma membrane during regulated or polarized secretion. The mRNA is cell-to-cell mobile.	59.24	118.83	0.5	6.15E-04	1.56E-03
5607	7	F2-3 vs. S2-3	264961_at	AT1G76950	Regulator of chromosome condensation (RCC1) family with FYVE zinc finger domain-containing protein;(source:Araport11)	23.83	48.05	0.5	9.47E-05	3.75E-04
5608	7	F2-3 vs. S2-3	263049_at	AT2G05260	alpha/beta-Hydrolases superfamily protein;(source:Araport11)	58.08	115.98	0.5	7.29E-05	3.08E-04
5609	7	F2-3 vs. S2-3	263821_s_a	AT2G09990	Ribosomal protein S5 domain 2-like superfamily protein;(source:Araport11)	2163.65	4321.51	0.5	1.80E-05	1.12E-04
5610	7	F2-3 vs. S2-3	266699_at	AT2G19730	Ribosomal L28e protein family;(source:Araport11)	181.61	364.59	0.5	1.89E-04	6.32E-04
5611	7	F2-3 vs. S2-3	264020_at	AT2G21160	Translocon-associated protein (TRAP), alpha subunit;(source:Araport11)	251.9	500.77	0.5	4.19E-04	1.16E-03

5612	7	F2-3 vs. S2-3	267376_at	AT2G26330	Homologous to receptor protein kinases. Involved in specification of organs originating from the shoot apical meristem. Contains a cytoplasmic protein kinase catalytic domain, a transmembrane region, and an extracellular leucine-rich repeat. ER has been identified as a quantitative trait locus for transpiration efficiency by influencing epidermal and mesophyll development, stomatal density and porosity of leaves. It has been implicated in resistance to the bacterium Ralstonia solanacearum and to the necrotrophic fungus Plectosphaerella cucumerina. Together with ERL1 and ERL2, ER governs the initial decision of protodermal cells to either divide proliferatively to produce pavement cells or divide asymmetrically to generate stomatal complexes. ER binds to the peptides STOMAGEN and EPF2 which compete for the same binding site. The ER-EFP2 complex activates MAPK signaling that inhibits stomatal development. ER-STOMAGEN does not activate MAPK signaling. Plants harboring loss of function alleles of er are more susceptible to heat stress than wild type. In Arabidopsis and other organisms, overexpression of ER confers thermotolerance via as yet undefined mechanisms.	44.08	87.76	0.5	1.26E-04	4.64E-04
5613	7	F2-3 vs. S2-3	266216_at	AT2G28810	Dof-type zinc finger DNA-binding family protein;(source:Araport11)	19.41	38.56	0.5	2.00E-05	1.21E-04
5614	7	F2-3 vs. S2-3	267523_at	AT2G30600	BTB/POZ domain-containing protein;(source:Araport11)	71.88	143.16	0.5	7.94E-04	1.89E-03
5615	7	F2-3 vs. S2-3	267555_at	AT2G32765	Encodes a small ubiquitin-like modifier (SUMO) protein that becomes covalently attached to various intracellular protein targets through an isopeptide bond. SUMOylation typically has a post-translational effect on the behavior of the target protein.	11.32	22.47	0.5	7.93E-05	3.30E-04
5616	7	F2-3 vs. S2-3	263867_at	AT2G36830	Encodes a tonoplast intrinsic protein, which functions as water channel. It has also been shown to be able to facilitate the transport of urea and hydrogen peroxide. Highly expressed in vascular tissues of the root, stem, cauline leaves and flowers but not in the apical meristems. The mRNA is cell-to-cell mobile.	532.63	1068.2	0.5	8.10E-04	1.92E-03
5617	7	F2-3 vs. S2-3	259131_at	AT3G02180	SPIRAL1-LIKE3 belongs to a six-member gene family in Arabidopsis; all members share high sequence similarity in amino- and carboxy-terminal regions. Regulates cortical microtubule organization. Mutant plants exhibit altered patterns of root, leaf and petal growth as a result of defective anisotropic cell expansion.	717.79	1439.92	0.5	7.24E-05	3.06E-04
5618	7	F2-3 vs. S2-3	258616_at	AT3G02880	Leucine-rich repeat protein kinase family protein;(source:Araport11)	92.32	183.73	0.5	1.36E-04	4.91E-04
5619	7	F2-3 vs. S2-3	257879_at	AT3G17160	hypothetical protein;(source:Araport11)	145.63	293.61	0.5	2.08E-04	6.80E-04
5620	7	F2-3 vs. S2-3	256657_at	AT3G18860	transducin family protein / WD-40 repeat family protein;(source:Araport11)	159.78	318.72	0.5	4.58E-04	1.24E-03
5621	7	F2-3 vs. S2-3	252560_at	AT3G46030	Histone superfamily protein;(source:Araport11)	12.45	24.92	0.5	2.76E-04	8.37E-04
5622	7	F2-3 vs. S2-3	255683_at	AT4G00570	Encodes an NAD-dependent malic enzyme (NAD-ME) that does not act on oxaloacetate, indicating that it belongs to EC 1.1.1.39. It is a member of the beta family of NAD-MEs in plants. It appears to function as a homodimer or as a heterodimer with the alpha-type NAD-ME2 (At2g13560). NAD-ME2 transcript and protein levels are higher during the night than during the day.	98.32	198.5	0.5	3.07E-04	9.10E-04
5623	7	F2-3 vs. S2-3	255603_at	AT4G01040	Glycosyl hydrolase superfamily protein;(source:Araport11)	104.24	207.41	0.5	6.31E-05	2.77E-04
5624	7	F2-3 vs. S2-3	254788_at	AT4G12790	GPN GTPase involved in selective nuclear import of RNA polymerase II.	349.89	705.63	0.5	5.54E-05	2.55E-04
5625	7	F2-3 vs. S2-3	254119_at	AT4G24780	Encodes a pectate lyase involved in response to nematodes.	52.83	105.38	0.5	4.27E-04	1.17E-03
5626	7	F2-3 vs. S2-3	253811_at	AT4G28190	Encodes a novel Cys-rich protein with a B-box like domain that acts as a negative regulator of meristem cell accumulation in inflorescence and floral meristems as loss-of-function ult1 mutations cause inflorescence meristem enlargement, the production of extra flowers and floral organs, and a decrease in floral meristem determinacy. Acts opposite to CLF which represses AG, but preventing deposition of CLF repressive methylation marks.ULT1 acts as an anti-repressor that counteracts EMF1 action through modulation of histone marks on target genes. Regulates developmental as well as biotic and abiotic stress response genes.	21.22	42.67	0.5	1.15E-04	4.34E-04

5627	7	F2-3 vs. S2-3	246216_at	AT4G36380	Encodes a cytochrome P-450 gene that is involved in leaf blade expansion by controlling polar cell expansion in the leaf length direction. Member of the CYP90C CYP450 family. ROT3 was shown to be involved in brassinosteroid biosynthesis, most likely in the conversion step of typhasterol (TY) to castasterone (CS). As 6-deoxo-CS was unable to restore the phenotype of rot3-1, it has been postulated that ROT3 might be specifically involved in the conversion of TY to CS in the C6-oxidation pathway of brassinolide. Recently, CYP90C1 was shown to catalyse the C-23 hydroxylation of several brassinosteroids (the enzyme has a broad specificity for 22-hydroxylated substrates).	52.47	104.04	0.5	5.83E-04	1.49E-03
5628	7	F2-3 vs. S2-3	250734_at	AT5G06270	One of two plant specific paralogs of unknown function. Interacts with GL2. GIR1/GIR2 loss of function resembles gl2 lof mutations	23.34	46.75	0.5	8.08E-05	3.34E-04
5629	7	F2-3 vs. S2-3	250670_at	AT5G06860	Encodes a polygalacturonase inhibiting protein involved in defense response. PGIPs inhibit the function of cell wall pectin degrading enzymes such as those produced by fungal pathogens. PGIP1 is induced by fungal infection. Suppressed in the proton sensitive stop1-mutant, but the transcription level was recovered by transformation of STOP2. Knockout mutant showed severe damage in the root tip in low Ca and low pH medium.	12.5	24.9	0.5	1.30E-04	4.74E-04
5630	7	F2-3 vs. S2-3	245852_at	AT5G13510	Ribosomal protein L10 family protein;(source:Araport11)	102.35	205.57	0.5	7.95E-04	1.89E-03
5631	7	F2-3 vs. S2-3	249290_at	AT5G41060	DHHC-type zinc finger family protein;(source:Araport11)	58.66	116.78	0.5	2.01E-04	6.63E-04
5632	7	F2-3 vs. S2-3	248472_at	AT5G50860	Protein kinase superfamily protein;(source:Araport11)	59.79	119.64	0.5	1.84E-04	6.21E-04
5633	7	F2-3 vs. S2-3	248412_at	AT5G51590	AT hook motif DNA-binding family protein;(source:Araport11)	137.29	272.11	0.5	4.01E-04	1.11E-03
5634	7	F2-3 vs. S2-3	248223_at	AT5G53620	RNA polymerase II degradation factor;(source:Araport11)	88.79	176.15	0.5	3.32E-04	9.65E-04
5635	7	F2-3 vs. S2-3	248173_at	AT5G54580	Encodes an RNA recognition motif (RRM) and is involved in C-> U RNA editing in mitochondria.	169.54	337.01	0.5	1.05E-04	4.05E-04
5636	7	F2-3 vs. S2-3	247964_at	AT5G56600	Encodes profilin3, a low-molecular weight, actin monomer-binding protein that regulates the organization of actin cytoskeleton. Originally known as profilin5, and later named profilin3. Expressed in vegetative organs. Mutants have slightly elongated petioles.	150.5	303.72	0.5	1.78E-05	1.11E-04
5637	7	F2-3 vs. S2-3	247115_at	AT5G65930	encodes a novel member of the kinesin superfamily of motor proteins. recessive mutations have reduced number of trichome branches.	77.08	153.48	0.5	8.47E-04	1.99E-03
5638	7	F2-3 vs. S2-3	263113_at	AT1G03150	Acyl-CoA N-acyltransferases (NAT) superfamily protein;(source:Araport11)	63.65	129.38	0.49	2.25E-04	7.18E-04
5639	7	F2-3 vs. S2-3	262501_at	AT1G21690	ATPase family associated with various cellular activities (AAA);(source:Araport11)	89.32	183.48	0.49	1.51E-04	5.34E-04
5640	7	F2-3 vs. S2-3	255977_at	AT1G34030	Ribosomal protein S13/S18 family;(source:Araport11)	283.01	574.32	0.49	7.47E-04	1.80E-03
5641	7	F2-3 vs. S2-3	262543_at	AT1G34245	Encodes a secretory peptide EPF2 expressed in proliferating cells of the stomatal lineage, known as meristemoids, and in guard mother cells, the progenitors of stomata. Controls asymmetric cell divisions during stomatal development. EPF2 is related to EPF1, also involved in stomatal development. Its transcript levels change after inducing MUTE expression in a mute background. EPF2 binds to the ER receptor triggering MAPK activation that in turn inhibits stomatal development. EPF2 competes with STOMAGEN for binding to receptor protein kinases ER, and TMM.	12.06	24.66	0.49	2.09E-05	1.25E-04
5642	7	F2-3 vs. S2-3	261634_at	AT1G49970	Encodes a ClpP-related sequence. Though similar to ClpP proteins, this does not contains the highly conserved catalytic triad of Ser-type proteases (Ser-His-Asp). The name reflects nomenclature described in Adam et. al (2001).	47.54	96.68	0.49	5.74E-05	2.61E-04
5643	7	F2-3 vs. S2-3	262095_at	AT1G56090	Tetratricopeptide repeat (TPR)-like superfamily protein;(source:Araport11)	28.16	58.01	0.49	4.56E-04	1.23E-03
5644	7	F2-3 vs. S2-3	256372_at	AT1G66750	Encodes a CDK-activating kinase that interacts with SPT5, a regulator of transcription and histone methylation.	37.31	75.59	0.49	3.63E-05	1.88E-04
5645	7	F2-3 vs. S2-3	264337_at	AT1G70350	hypothetical protein;(source:Araport11)	15.56	31.82	0.49	6.99E-05	2.98E-04
5646	7	F2-3 vs. S2-3	261352_at	AT1G79650	Encodes a member of the RADIATION SENSITIVE23 (RAD23) family: AT1G16190(RAD23A), AT1G79650(RAD23B), AT3G02540(RAD23C), AT5G38470(RAD23D). RAD23 proteins play an essential role in the cell cycle, morphology, and fertility of plants through their delivery of UPS (ubiquitin/26S proteasome system) substrates to the 26S proteasome.	22.83	46.36	0.49	1.92E-05	1.17E-04
5647	7	F2-3 vs. S2-3	266684_at	AT2G19720	ribosomal protein S15A B;(source:Araport11)	41.67	84.9	0.49	2.68E-05	1.50E-04
5648	7	F2-3 vs. S2-3	266317_at	AT2G27030	encodes a calmodulin that has higher affinity to kinesin-like calmodulin binding motor protein than CAM4 or CAM6. The mRNA is cell-to-cell mobile.	58.35	118.33	0.49	3.04E-05	1.64E-04
5649	7	F2-3 vs. S2-3	266259_at	AT2G27830	hypothetical protein;(source:Araport11)	14.58	29.85	0.49	2.05E-04	6.74E-04

5650	7	F2-3 vs. S2-3	264070_at	AT2G27960	catalytic subunit of cyclin dependent kinase 1. physically interact with cyclin-dependent kinases (CDKs) and play an essential, but yet not entirely resolved, role in the regulation of the cell cycle	15.66	31.99	0.49	4.71E-05	2.27E-04
5651	7	F2-3 vs. S2-3	263399_at	AT2G31490	neuronal acetylcholine receptor subunit alpha-5;(source:Araport11)	92.85	191.16	0.49	2.22E-05	1.31E-04
5652	7	F2-3 vs. S2-3	266886_at	AT2G44745	WRKY family transcription factor;(source:Araport11)	15.59	32.05	0.49	3.22E-04	9.45E-04
5653	7	F2-3 vs. S2-3	265459_at	AT2G46540	fiber;(source:Araport11)	195.7	401.53	0.49	2.38E-05	1.38E-04
5654	7	F2-3 vs. S2-3	259016_at	AT3G07480	2Fe-2S ferredoxin-like superfamily protein;(source:Araport11)	23.49	48.43	0.49	2.54E-05	1.44E-04
5655	7	F2-3 vs. S2-3	258684_at	AT3G08680	Leucine-rich repeat protein kinase family protein;(source:Araport11)	128.93	263.7	0.49	4.27E-05	2.12E-04
5656	7	F2-3 vs. S2-3	259215_at	AT3G09090	Encodes DEX1 (defective in exine formation). Required for exine pattern formation during pollen development.	197.23	398.91	0.49	6.10E-04	1.55E-03
5657	7	F2-3 vs. S2-3	258775_at	AT3G10850	glyoxalase II cytoplasmic isozyme (Glx2-2) mRNA, complete	77.53	159	0.49	3.00E-04	8.95E-04
5658	7	F2-3 vs. S2-3	256434_at	AT3G10950	Zinc-binding ribosomal protein family protein;(source:Araport11)	62.11	126.77	0.49	3.26E-04	9.51E-04
5659	7	F2-3 vs. S2-3	258007_at	AT3G19420	Encodes a phosphatase with low in vitro tyrosine phosphatase activity that is capable of dephosphorylating in vitro the 3'phosphate group of PI3P, PI(3,4)P2, and PI(3,5)P2 and may be an effector of lipid signaling. The mRNA is cell-to-cell mobile.	154.83	313.46	0.49	2.55E-04	7.86E-04
5660	7	F2-3 vs. S2-3	257906_at	AT3G25520	Encodes ribosomal protein L5 that binds to 5S ribosomal RNA and in involved in its export from the nucleus to the cytoplasm. Identified in a screen for enhancers of as1. as1/pgy double mutants show defects in leaf vascular patterning and adaxial cell fate. Double mutant analysis indicates pgy genes function in the same pathway as REV, KAN1 and KAN2.	352.97	725.18	0.49	5.22E-05	2.45E-04
5661	7	F2-3 vs. S2-3	257224_at	AT3G27870	ATPase E1-E2 type family protein / haloacid dehalogenase-like hydrolase family protein;(source:Araport11)	32.44	66.56	0.49	3.28E-04	9.54E-04
5662	7	F2-3 vs. S2-3	256607_at	AT3G32930	Protein of unknown function. Locus is correlated with bacterial hypersensitive response, expression is reduced after injection with avrRpm1.	112.18	230.53	0.49	2.78E-04	8.40E-04
5663	7	F2-3 vs. S2-3	252492_at	AT3G46740	Component of the translocon outer membrane (TOC) complex. Forms the outer envelope translocation channel (beta-barrel). Plays a role in preprotein conductance. Imported into chloroplast. Expressed in young dividing photosynthetic tissues. Knockout mutants are embryo lethal with arrested development at the two-cell stage. Knockout mutants have abnormal etioplasts.	113.5	229.5	0.49	2.72E-05	1.51E-04
5664	7	F2-3 vs. S2-3	252425_at	AT3G47620	Encodes a transcription factor AtTCP14 that regulates seed germination. AtTCP14 shows elevated expression level just prior to germination. AtTCP14 is predominantly expressed in the vascular tissue of the embryo, and affects gene expression in radicles in a non-cell-autonomous manner. Modulates GA-dependent stamen filament elongation by direct activation of SAUR63 subfamily genes through conserved target sites in their promoters.	23.16	47	0.49	1.29E-04	4.71E-04
5665	7	F2-3 vs. S2-3	252031_at	AT3G52140	Involved in regulating mitochondrial quality control. Regulates mitochondrial association time and thereby is involved in mitochondrial fusion. Mutants show unregulated autophagy and display transcriptomic markers of mitochondrial stress. Its activity can be modulated by Lys acetylation.	43.73	88.59	0.49	7.21E-04	1.75E-03
5666	7	F2-3 vs. S2-3	245367_at	AT4G16265	One of two highly similar, non-catalytic subunits common to nuclear DNA-directed RNA polymerases II, IV and V; homologous to budding yeast RPB9. Appears to be redundant with At3g16980	79.67	162.08	0.49	1.41E-04	5.05E-04
5667	7	F2-3 vs. S2-3	253976_at	AT4G26610	D6PK family kinase involved in pulse-induced phototropism but also for time-dependent second positive phototropism, and continuous light-induced hypocotyl phototropism.	49.77	100.61	0.49	8.34E-05	3.41E-04
5668	7	F2-3 vs. S2-3	253403_at	AT4G32830	Encodes a member of a family of Ser/Thr kinases whose activities peak during cell division. Transcripts are abundant in tissues rich in dividing cells like roots and flowers but are low or absent in fully expanded leaves and stems. In interphase cells, the protein is predominantly nuclear. During mitosis, the protein associates with plant-specific cytoskeletal structures (preprophase band, phragmoplast, nascent cell plate) that are necessary for cytokinesis as well as with the microtubule spindle. It specifically phosphorylates Ser10 of histone H3 and colocalizes with phosphorylated histone H3 during mitosis.	179.82	368.71	0.49	5.02E-04	1.33E-03
5669	7	F2-3 vs. S2-3	253213_at	AT4G34910	P-loop containing nucleoside triphosphate hydrolases superfamily protein;(source:Araport11)	35.34	72.36	0.49	3.66E-05	1.89E-04
5670	7	F2-3 vs. S2-3	253156_at	AT4G35730	Regulator of Vps4 activity in the MVB pathway protein;(source:Araport11)	20.42	41.51	0.49	1.64E-04	5.66E-04
5671	7	F2-3 vs. S2-3	252832_at	AT4G39910	Encodes a nuclear ubiquitin-specific protease.	52.68	108.31	0.49	3.91E-04	1.10E-03

5672	7	F2-3 vs. S2-3	250642_at	AT5G07180	Encodes a receptor-like kinase that, together with ER and ERL1 governs the initial decision of protodermal cells to either divide proliferatively to produce pavement cells or divide asymmetrically to generate stomatal complexes. It is also important for maintaining stomatal stem cell activity and preventing terminal differentiation of the meristemoid into the guard mother cell. When heterozygous in an er/erl1 null background, plants are female sterile due to cell division defect in the integuments.	80.94	166.32	0.49	3.95E-04	1.10E-03
5673	7	F2-3 vs. S2-3	250233_at	AT5G13460	Member of IQ67 (CaM binding) domain containing family.	34.97	70.97	0.49	7.76E-05	3.24E-04
5674	7	F2-3 vs. S2-3	250237_at	AT5G13560	structural maintenance of chromosomes protein;(source:Araport11)	97.4	199.12	0.49	6.06E-05	2.71E-04
5675	7	F2-3 vs. S2-3	246957_at	AT5G24670	A protein coding gene with unknown function. The 5?UTR of this gene overlaps with a RNA coding gene TER2. TER2 (GenBank accession no. HQ401285) encodes a putative template sequence corresponding to 1.5 copies of the Arabidopsis telomere repeat (PNAS 2011, 108:73-78). Natural epiallele in Nok-1, transmission of the epiallele over generations depends only on the selfreinforcing loop between CHROMOMETHYLASE 3 and KRYPTONITE, involving DNA methylated in the CHG context and histone H3 lysine 9 methylation.	151.46	308.45	0.49	5.63E-04	1.46E-03
5676	7	F2-3 vs. S2-3	248914_at	AT5G45750	RAB GTPase homolog A1C;(source:Araport11)	90.87	184.24	0.49	1.33E-04	4.83E-04
5677	7	F2-3 vs. S2-3	248588_at	AT5G49540	Rab5-interacting family protein;(source:Araport11)	213.59	439.82	0.49	5.53E-05	2.55E-04
5678	7	F2-3 vs. S2-3	248438_at	AT5G51230	Polycomb group protein with zinc finger domain involved in negative regulation of reproductive development. Forms a complex with FIE, CLF, and MSI1. This complex modulates the expression of target genes including AG, PI and AP3.	180.33	364.68	0.49	5.97E-04	1.52E-03
5679	7	F2-3 vs. S2-3	248070_at	AT5G55660	DEK domain-containing chromatin associated protein;(source:Araport11)	118.31	242.06	0.49	2.08E-04	6.81E-04
5680	7	F2-3 vs. S2-3	247910_at	AT5G57410	Encodes a microtubule-associated protein.	70.6	143.07	0.49	3.65E-04	1.04E-03
5681	7	F2-3 vs. S2-3	247847_at	AT5G58110	chaperone binding / ATPase activator;(source:Araport11)	546.12	1114.16	0.49	1.58E-04	5.54E-04
5682	7	F2-3 vs. S2-3	247815_at	AT5G58420	Ribosomal protein S4 (RPS4A) family protein;(source:Araport11)	383.39	777.77	0.49	8.12E-05	3.35E-04
5683	7	F2-3 vs. S2-3	244992_s_a	ATCG01240	30S chloroplast ribosomal protein S7	3164.58	6456	0.49	2.21E-05	1.30E-04
5684	7	F2-3 vs. S2-3	261694_at	AT1G08360	Ribosomal protein L1p/L10e family;(source:Araport11)	1445.73	3042.76	0.48	2.65E-04	8.11E-04
5685	7	F2-3 vs. S2-3	262823_at	AT1G11750	One of several nuclear-encoded ClpPs (caseinolytic protease). Contains a highly conserved catalytic triad of Ser-type proteases (Ser-His-Asp). The name reflects nomenclature described in Adam et. al (2001).	132.88	279.34	0.48	1.96E-04	6.49E-04
5686	7	F2-3 vs. S2-3	261137_at	AT1G19830	SAUR-like auxin-responsive protein family;(source:Araport11)	13.21	27.72	0.48	2.79E-05	1.54E-04
5687	7	F2-3 vs. S2-3	260898_at	AT1G29070	Ribosomal protein L34;(source:Araport11)	43.12	88.97	0.48	1.91E-04	6.37E-04
5688	7	F2-3 vs. S2-3	263156_at	AT1G54030	Encodes a vacuolar protein. Mutation causes organizational defects in the endoplasmic reticulum and aberrant protein trafficking in the plant secretory pathway.The mRNA is cell-to-cell mobile.	96.19	198.75	0.48	1.31E-04	4.78E-04
5689	7	F2-3 vs. S2-3	246379_s_a	AT1G57860	Translation protein SH3-like family protein;(source:Araport11)	289.91	600.68	0.48	1.42E-04	5.06E-04
5690	7	F2-3 vs. S2-3	256342_at	AT1G72020	TonB-dependent heme receptor A;(source:Araport11)	289.87	604.56	0.48	2.51E-05	1.43E-04
5691	7	F2-3 vs. S2-3	261117_at	AT1G75310	auxin-like 1 protein;(source:Araport11)	13.8	28.79	0.48	5.34E-05	2.49E-04
5692	7	F2-3 vs. S2-3	262068_at	AT1G80070	Encodes a factor that influences pre-mRNA splicing and is required for embryonic development. Mutations result in an abnormal suspensor and embryo lethality. The mRNA is cell-to-cell mobile.	274.57	571.56	0.48	2.13E-05	1.27E-04
5693	7	F2-3 vs. S2-3	265594_at	AT2G20060	Ribosomal protein L4/L1 family;(source:Araport11)	166.76	347.97	0.48	6.57E-05	2.86E-04
5694	7	F2-3 vs. S2-3	255871_at	AT2G30260	encodes U2B", which is a component of the U2 snRNP complex. Its precise role in pre-mRNA splicing is still unknown. It has been suggested that U2B0 may not be required for the splicing reaction itself but may have a role in U2 snRNP biogenesis. Deletion analysis of the U2B0 gene fusion has identified the N-terminal RNP-80 motif as sufficient for localization to the coiled body and the nucleus.	35.06	72.57	0.48	1.88E-04	6.32E-04
5695	7	F2-3 vs. S2-3	267461_at	AT2G33830	Dormancy/auxin associated family protein;(source:Araport11)	28.71	60.36	0.48	2.16E-04	6.96E-04
5696	7	F2-3 vs. S2-3	257690_at	AT3G12830	SAUR-like auxin-responsive protein family;(source:Araport11)	23.11	48.55	0.48	2.10E-05	1.26E-04
5697	7	F2-3 vs. S2-3	257190_at	AT3G13120	Ribosomal protein S10p/S20e family protein;(source:Araport11)	44.93	94.22	0.48	7.56E-05	3.17E-04
5698	7	F2-3 vs. S2-3	257967_at	AT3G19910	RING/U-box superfamily protein;(source:Araport11)	305.13	635.67	0.48	2.11E-04	6.85E-04
5699	7	F2-3 vs. S2-3	258311_at	AT3G26810	Auxin F box protein, the dominant auxin receptor in roots.	27.22	57.28	0.48	1.02E-04	3.99E-04
5700	7	F2-3 vs. S2-3	252127_at	AT3G50960	Encodes a protein that functions in microtubule assembly. PLP3a can bind to several different tubulin family members in Y2H assays. Plants with reduced levels of both PLP3a and PLP3b (At5g66410) show defects in cytokinesis, cortical microtubule array formation, oriented cell growth, and maintenance of proper ploidy.	31.47	66.1	0.48	4.43E-05	2.17E-04
5701	7	F2-3 vs. S2-3	251982_at	AT3G53190	Pectin lyase-like superfamily protein;(source:Araport11)	88.96	186.96	0.48	7.21E-05	3.05E-04
5702	7	F2-3 vs. S2-3	251271_at	AT3G62050	Putative endonuclease or glycosyl hydrolase;(source:Araport11)	11.47	23.94	0.48	1.33E-05	9.14E-05

5703	7	F2-3 vs. S2-3	255378_at	AT4G03550	Encodes a callose synthase that is required for wound and papillary callose formation in response to fungal pathogens Erysiphe and Blumeria. Mutants are resistant to P. parasitica and exhibit an exaggerated PR1 response.Contributes to PAMP-induced basal defense. The mRNA is cell-to-cell mobile.	117.06	241.73	0.48	3.72E-05	1.91E-04
5704	7	F2-3 vs. S2-3	254999_at	AT4G09830	nuclear receptor family 2 group C protein;(source:Araport11)	78.55	164.16	0.48	8.38E-05	3.42E-04
5705	7	F2-3 vs. S2-3	253284_at	AT4G34150	Calcium-dependent lipid-binding (CaLB domain) family protein;(source:Araport11)	54.3	113.66	0.48	3.68E-04	1.05E-03
5706	7	F2-3 vs. S2-3	250895_at	AT5G03850	Nucleic acid-binding, OB-fold-like protein;(source:Araport11)	319.76	663.62	0.48	2.39E-04	7.49E-04
5707	7	F2-3 vs. S2-3	250590_at	AT5G07710	Polynucleotidyl transferase, ribonuclease H-like superfamily protein;(source:Araport11)	27.98	58.26	0.48	4.15E-05	2.08E-04
5708	7	F2-3 vs. S2-3	250144_at	AT5G14680	Adenine nucleotide alpha hydrolases-like superfamily protein;(source:Araport11)	51.85	107.59	0.48	1.38E-04	4.96E-04
5709	7	F2-3 vs. S2-3	249998_at	AT5G18580	fass mutants have aberrant cell shapes due to defects in arrangement of cortical microtubules. Encodes a protein highly conserved in higher plants and similar in its C-terminal part to B' regulatory subunits of type 2A protein phosphatases. Interacts with an Arabidopsis type A subunit of PP2A in the yeast two-hybrid system.	53.49	112.46	0.48	1.11E-04	4.24E-04
5710	7	F2-3 vs. S2-3	246089_at	AT5G20570	Encodes a ring-box 1 like protein and component of the SCF ubiquitinization complex mediating auxin responses. Forms a E3 ubiquitin ligase complex with CUL3A and At1g21780.1 a BTB domain protein.	239.49	499.55	0.48	5.27E-05	2.47E-04
5711	7	F2-3 vs. S2-3	249750_at	AT5G24570	hypothetical protein;(source:Araport11)	14.56	30.54	0.48	3.83E-05	1.95E-04
5712	7	F2-3 vs. S2-3	248427_at	AT5G51750	subtilase 1.3;(source:Araport11)	391.64	823.91	0.48	2.05E-05	1.24E-04
5713	7	F2-3 vs. S2-3	248107_at	AT5G55190	A member of RAN GTPase gene family. Encodes a small soluble GTP-binding protein. Likely to be involved in nuclear translocation of proteins. May also be involved in cell cycle progression.	435.89	912.31	0.48	1.05E-04	4.05E-04
5714	7	F2-3 vs. S2-3	248095_at	AT5G55230	Binds and bundles microtubules. Plays a role in stabilizing anti-parallel microtubules in the central spindle at anaphase to early cytokinesis but is not essential at the midline of the phragmoplast at later stages. The timing with which the MAP65-1 was targeted to the spindle appears to be regulated by a phosphorylation sensitive switch. Enhances microtubule polymerization, promotes nucleation and stabilizes microtubules against cold treatment and dilution.	83.71	176.23	0.48	7.43E-04	1.79E-03
5715	7	F2-3 vs. S2-3	261025_at	AT1G01225	NC domain-containing protein-like protein;(source:Araport11)	23.12	49.28	0.47	4.64E-05	2.25E-04
5716	7	F2-3 vs. S2-3	260895_at	AT1G29250	Alba DNA/RNA-binding protein;(source:Araport11)	13.71	29.25	0.47	4.21E-04	1.16E-03
5717	7	F2-3 vs. S2-3	262514_at	AT1G34190	Encodes a NAC domain transcription factor that regulates the mitochondrial retrograde response and coordinates organellar functions and stress responses.	120.64	258.06	0.47	1.79E-04	6.05E-04
5718	7	F2-3 vs. S2-3	257510_at	AT1G55360	tRNA-splicing ligase (DUF239);(source:Araport11)	104.83	222.36	0.47	4.64E-05	2.25E-04
5719	7	F2-3 vs. S2-3	260806_at	AT1G78260	RNA-binding (RRM/RBD/RNP motifs) family protein;(source:Araport11)	13.18	27.99	0.47	2.40E-04	7.50E-04
5720	7	F2-3 vs. S2-3	264301_at	AT1G78780	pathogenesis-related family protein;(source:Araport11)	225.28	483.36	0.47	3.51E-05	1.83E-04
5721	7	F2-3 vs. S2-3	266922_s_a	AT2G45950	SKP1-like 20;(source:Araport11)	76.62	162.49	0.47	1.68E-05	1.07E-04
5722	7	F2-3 vs. S2-3	266325_at	AT2G46630	serine/arginine repetitive matrix protein;(source:Araport11)	14.61	31.02	0.47	6.70E-04	1.66E-03
5723	7	F2-3 vs. S2-3	258553_at	AT3G06960	TGD4 is an integral dimeric beta-barrel lipid transfer protein that binds PtdOH with its N-terminus and contains dimerization domains at its C-terminus. Mutations in this gene suppress the low temperature-induced phenotype of Arabidopsis tocopherol-deficient mutant vte2.	33.43	71.82	0.47	1.00E-05	7.51E-05
5724	7	F2-3 vs. S2-3	265963_s_a t	AT3G11940	One of two genes encoding the ribosomal protein S5. Mutants have semi-dominant developmental phenotypes. Most cell-division processes are delayed or disturbed in the heterozygous mutant, and development is completely arrested at an early embryonic stage in the homozygous mutant.	3912.93	8342.67	0.47	4.46E-05	2.18E-04
5725	7	F2-3 vs. S2-3	256926_at	AT3G22540	hypothetical protein (DUF1677);(source:Araport11)	70.25	150.76	0.47	1.96E-05	1.19E-04
5726	7	F2-3 vs. S2-3	258318_at	AT3G22680	Encodes RNA-DIRECTED DNA METHYLATION 1 (RDM1), forming a complex with DMS3 (AT3G49250) and DRD1 (AT2G16390). This complex is termed DDR. The DDR complex is required for polymerase V transcripts and RNA-directed DNA methylation.	13.04	27.57	0.47	6.79E-04	1.68E-03
5727	7	F2-3 vs. S2-3	256890_at	AT3G23830	encodes a glycine-rich RNA binding protein. Gene expression is induced by cold and reduced by ionic (salt) and non-ionic (mannitol) osmotic stress. Lines overexpressing the gene are slightly more tolerant to osmotic stress during germination.	97.94	206.95	0.47	2.28E-05	1.33E-04
5728	7	F2-3 vs. S2-3	256864_at	AT3G23890	Encodes a topoisomerase II that is highly expressed in young seedlings. The protein is localized in the nucleus and gene expression levels are increased in proliferative tissues.	38.49	81.11	0.47	3.15E-05	1.68E-04

5729	7	F2-3 vs. S2-3	252348_at	AT3G48140	B12D protein;(source:Araport11)	861.85	1823.29	0.47	2.80E-04	8.45E-04
5730	7	F2-3 vs. S2-3	252205_at	AT3G50350	membrane insertase, putative (DUF1685);(source:Araport11)	55.56	118.38	0.47	6.51E-04	1.63E-03
5731	7	F2-3 vs. S2-3	251938_at	AT3G53430	Ribosomal protein L11 family protein;(source:Araport11)	216.12	455.81	0.47	7.27E-04	1.76E-03
5732	7	F2-3 vs. S2-3	251800_at	AT3G55510	Encodes a regulator of floral determinacy in that interacts with both nucleolar and nucleoplasmic proteins.	30.27	64.65	0.47	1.03E-05	7.67E-05
5733	7	F2-3 vs. S2-3	255285_at	AT4G04630	senescence regulator (Protein of unknown function, DUF584);(source:Araport11)	11.12	23.64	0.47	1.09E-05	8.03E-05
5734	7	F2-3 vs. S2-3	262100_s_at	AT4G14250	This locus is annotated as a protein-coding gene in TAIR10. Based on communication with Jean-Luc GALLOIS (April 2013), this gene is split into two UBX domain-containing pseudogenes: one retains the original name: AT4G14250 (Chr4:8213237..8211984), one given a new locus identifier AT4G14245 (Chr4:8210231..8208985). Note that the Map Detail Image on the locus detial page and in GBrowse will not be updated until after the next genome release.	18.26	39.09	0.47	2.77E-05	1.53E-04
5735	7	F2-3 vs. S2-3	245576_at	AT4G14770	Regulates fate transition and cell Divisions in the stomatal lineage.	35.4	75.9	0.47	2.42E-05	1.39E-04
5736	7	F2-3 vs. S2-3	245276_at	AT4G16780	Encodes a homeodomain-leucine zipper protein that is rapidly and strongly induced by changes in the ratio of red to far-red light. It is also involved in cell expansion and cell proliferation and in the response to auxin. The mRNA is cell-to-cell mobile.	49.42	105.91	0.47	1.33E-05	9.14E-05
5737	7	F2-3 vs. S2-3	253644_at	AT4G29870	Oligosaccharyltransferase complex/magnesium transporter family protein;(source:Araport11)	51.2	108.18	0.47	1.08E-05	7.97E-05
5738	7	F2-3 vs. S2-3	246205_at	AT4G36970	Remorin family protein;(source:Araport11)	109.39	234.13	0.47	2.42E-05	1.39E-04
5739	7	F2-3 vs. S2-3	245930_at	AT5G09240	ssDNA-binding transcriptional regulator;(source:Araport11)	30.05	64.61	0.47	6.67E-04	1.65E-03
5740	7	F2-3 vs. S2-3	246423_at	AT5G16950	krueppel-like factor;(source:Araport11)	21.3	44.84	0.47	1.34E-04	4.84E-04
5741	7	F2-3 vs. S2-3	246415_at	AT5G17160	aspartic/glutamic acid-rich protein;(source:Araport11)	29.08	62.06	0.47	2.81E-04	8.46E-04
5742	7	F2-3 vs. S2-3	246650_at	AT5G35160	Endomembrane protein 70 protein family;(source:Araport11)	27.19	57.96	0.47	1.83E-05	1.13E-04
5743	7	F2-3 vs. S2-3	249231_at	AT5G42030	ABL interactor-like protein 4;(source:Araport11)	17.69	37.55	0.47	1.32E-04	4.80E-04
5744	7	F2-3 vs. S2-3	249192_at	AT5G42470	BRCA1-A complex subunit BRE-like protein;(source:Araport11)	28.16	59.88	0.47	1.17E-04	4.38E-04
5745	7	F2-3 vs. S2-3	248811_at	AT5G47310	PPPDE putative thiol peptidase family protein;(source:Araport11)	21.67	46.02	0.47	2.80E-05	1.54E-04
5746	7	F2-3 vs. S2-3	248473_at	AT5G50810	Encodes a small zinc finger-like protein that is a component of the mitochondrial protein import apparatus.	86.03	182.97	0.47	9.91E-05	3.90E-04
5747	7	F2-3 vs. S2-3	248378_at	AT5G51840	junctophilin-like protein;(source:Araport11)	25.9	54.86	0.47	7.83E-04	1.87E-03
5748	7	F2-3 vs. S2-3	248139_at	AT5G54970	hypothetical protein;(source:Araport11)	13.47	28.64	0.47	1.89E-05	1.16E-04
5749	7	F2-3 vs. S2-3	248098_at	AT5G55290	ATPase, V0 complex, subunit E;(source:Araport11)	43.9	93.02	0.47	3.31E-04	9.63E-04
5750	7	F2-3 vs. S2-3	247407_at	AT5G62900	basic-leucine zipper transcription factor K;(source:Araport11)	18.09	38.12	0.47	3.05E-04	9.06E-04
5751	7	F2-3 vs. S2-3	260725_at	AT1G48170	proteasome assembly chaperone;(source:Araport11)	58.4	127.98	0.46	6.82E-04	1.68E-03
5752	7	F2-3 vs. S2-3	265070_at	AT1G55510	branched-chain alpha-keto acid decarboxylase E1 beta	240.11	520.99	0.46	6.41E-05	2.81E-04
5753	7	F2-3 vs. S2-3	262341_at	AT1G64230	ubiquitin-conjugating enzyme 28;(source:Araport11)	634.18	1368.53	0.46	2.51E-05	1.43E-04
5754	7	F2-3 vs. S2-3	264878_at	AT2G17410	AT Rich domain protein.	104.88	229.28	0.46	1.89E-04	6.32E-04
5755	7	F2-3 vs. S2-3	265443_at	AT2G20750	member of BETA-EXPANSINS. Naming convention from the Expansin Working Group (Kende et al, 2004. Plant Mol Bio)	22.64	48.78	0.46	2.64E-05	1.49E-04
5756	7	F2-3 vs. S2-3	266314_at	AT2G27040	AGO4 is a member of a class of PAZ/PIWI domain containing proteins involved in siRNA mediated gene silencing.Loss of function mutations have reduced site specific CpNpG and CpHpH methylation and increased susceptibility to bacterial pathogens including Tobacco rattle virus.	164.82	357.79	0.46	8.30E-06	6.60E-05
5757	7	F2-3 vs. S2-3	266481_at	AT2G31070	TCP family protein involved in heterchronic regulation of leaf differentiation.	30.56	66.37	0.46	1.32E-05	9.10E-05
5758	7	F2-3 vs. S2-3	267405_at	AT2G33740	encodes a copper binding protein that forms tetramers in vitro. Gene is expressed in all tissues examined and protein is localized to the chloroplast. The mRNA is cell-to-cell mobile.	91.23	196.52	0.46	4.34E-05	2.14E-04
5759	7	F2-3 vs. S2-3	266094_at	AT2G37975	Yos1-like protein;(source:Araport11)	70.8	155.24	0.46	2.11E-04	6.85E-04
5760	7	F2-3 vs. S2-3	267510_at	AT2G45640	Involved in the regulation of salt stress. Expression of AtSAP18 is induced by NaCl, cold, drought, ABA, and ethylene treatment. AtSAP18 and HDA19 associate with ERF3 and ERF4 both in vitro and in vivo.	30.63	66.38	0.46	8.60E-06	6.77E-05
5761	7	F2-3 vs. S2-3	258630_at	AT3G02820	zinc knuckle (CCHC-type) family protein;(source:Araport11)	13.93	30.04	0.46	4.28E-05	2.12E-04
5762	7	F2-3 vs. S2-3	259083_at	AT3G04810	Encodes AtNek2, a member of the NIMA-related serine/threonine kinases (Neks) that have been linked to cell-cycle regulation in fungi and mammals. Plant Neks might be involved in plant development processes.	12.92	27.89	0.46	2.04E-04	6.71E-04
5763	7	F2-3 vs. S2-3	258780_at	AT3G11910	Ubiquitin-specific protease, which together with UBP12 deubiquitinates DA1, DAR1 and DAR2, hence reducing their peptidase activity. Works upstream of DA1, DAR1 and DAR2 to restrict their protease activity and hence fine-tune plant growth and development.	44.82	97.17	0.46	6.03E-04	1.53E-03

5764	7	F2-3 vs. S2-3	252056_at	AT3G52590	Ubiquitin extension protein The mRNA is cell-to-cell mobile.	55.65	120.9	0.46	7.98E-04	1.90E-03
5765	7	F2-3 vs. S2-3	251935_at	AT3G54090	Encodes a fructokinase-like protein (AT3G54090/FLN1, AT1G69200/FLN2), a member of the pfkB-carbohydrate kinase family. FLN1 and FLN2 are potential plastidial thioredoxin z (TRX z) targets. Mutants display mutant chloroplast development, general plant growth and development defects and defects in PEP-dependent transcription.	132.42	285.16	0.46	3.85E-04	1.08E-03
5766	7	F2-3 vs. S2-3	255517_at	AT4G02290	glycosyl hydrolase 9B13;(source:Araport11)	577.69	1268.08	0.46	9.18E-05	3.67E-04
5767	7	F2-3 vs. S2-3	255130_at	AT4G08240	histone-lysine N-methyltransferase;(source:Araport11)	80.35	175	0.46	7.51E-05	3.15E-04
5768	7	F2-3 vs. S2-3	245523_at	AT4G15910	encodes a gene whose transcript level in root and leaves increases to progressive drought stress. The transcript level is also affected by changes of endogenous or exogenous abscisic acid level. It appears to be a member of plant-specific gene family that includes late embryo-abundant and zinc-IAA-induced proteins in other plants. The mRNA is cell-to-cell mobile.	55.04	118.61	0.46	3.13E-04	9.23E-04
5769	7	F2-3 vs. S2-3	254291_at	AT4G23010	UDP-galactose transporter 2;(source:Araport11)	237.35	520.03	0.46	5.30E-04	1.38E-03
5770	7	F2-3 vs. S2-3	253977_at	AT4G26630	Encodes a chromatin-associated protein that specifically binds histones H3 and H4 and contributes to modulation of Arabidopsis chromatin structure and function.	29.94	65.03	0.46	5.55E-05	2.55E-04
5771	7	F2-3 vs. S2-3	263375_s_a	AT4G28510	prohibitin 1 (Atphb1)	431.08	940.64	0.46	7.04E-04	1.72E-03
5772	7	F2-3 vs. S2-3	253761_at	AT4G28820	HIT-type Zinc finger family protein;(source:Araport11)	33.46	73.17	0.46	4.31E-05	2.13E-04
5773	7	F2-3 vs. S2-3	253078_at	AT4G36180	Leucine-rich receptor-like protein kinase family protein;(source:Araport11)	10.67	23.4	0.46	1.13E-04	4.30E-04
5774	7	F2-3 vs. S2-3	252931_at	AT4G38930	Ubiquitin fusion degradation UFD1 family protein;(source:Araport11)	11.97	25.94	0.46	1.66E-05	1.06E-04
5775	7	F2-3 vs. S2-3	246060_at	AT5G08420	RNA-binding KH domain-containing protein;(source:Araport11)	46.66	100.98	0.46	1.46E-04	5.19E-04
5776	7	F2-3 vs. S2-3	250437_at	AT5G10430	Encodes arabinogalactan-protein (AGP4) that is expressed in female reproductive tissues. It is involved in promoting degeneration of the persistent synergid after fertilization. In mutant ovules, the persistent synergid does not degrade resulting in polytuby.	13.22	28.66	0.46	7.05E-04	1.73E-03
5777	7	F2-3 vs. S2-3	246124_at	AT5G20060	alpha/beta-Hydrolases superfamily protein;(source:Araport11)	76.46	164.44	0.46	1.50E-05	9.94E-05
5778	7	F2-3 vs. S2-3	249873_at	AT5G23140	One of several nuclear-encoded ClpPs (caseinolytic protease). Contains a highly conserved catalytic triad of Ser-type proteases (Ser-His-Asp). The name reflects nomenclature described in Adam et. al (2001).	34.59	75.93	0.46	6.94E-05	2.97E-04
5779	7	F2-3 vs. S2-3	246905_at	AT5G25570	polyamine-modulated factor 1-binding protein;(source:Araport11)	13.04	28.53	0.46	5.50E-05	2.54E-04
5780	7	F2-3 vs. S2-3	249228_at	AT5G42190	Similar to SKP1 in yeast and humans which are involved in mitotic cell cycle control and ubiquitin mediated proteolysis.	21.79	46.87	0.46	1.51E-05	9.98E-05
5781	7	F2-3 vs. S2-3	248587_at	AT5G49550	Putative homolog of mammalian BLOC-1 Subunit 2. Protein - protein interaction with BLOS1.	19.56	42.52	0.46	1.91E-05	1.17E-04
5782	7	F2-3 vs. S2-3	247963_at	AT5G56590	O-Glycosyl hydrolases family 17 protein;(source:Araport11)	152.03	332.19	0.46	8.70E-06	6.82E-05
5783	7	F2-3 vs. S2-3	263116_s_at	AT1G03140	PRP18a is one of two paralogs (the other being PRP18b) which are highly similar to the step II splicing factors in yeast. Loss of function mutations show defects in alternative splicing, mostly intron retention events.	159.27	357.03	0.45	8.07E-05	3.34E-04
5784	7	F2-3 vs. S2-3	264579_at	AT1G05205	hexokinase-1 protein;(source:Araport11)	20.68	46.06	0.45	1.58E-05	1.03E-04
5785	7	F2-3 vs. S2-3	264674_at	AT1G09815	polymerase delta 4;(source:Araport11)	39.59	88.76	0.45	5.64E-05	2.58E-04
5786	7	F2-3 vs. S2-3	263226_at	AT1G30690	Sec14p-like phosphatidylinositol transfer family protein;(source:Araport11)	35.32	79.18	0.45	1.80E-04	6.07E-04
5787	7	F2-3 vs. S2-3	264222_at	AT1G60230	Radical SAM superfamily protein;(source:Araport11)	26.12	57.81	0.45	4.13E-04	1.14E-03
5788	7	F2-3 vs. S2-3	261548_at	AT1G63480	AT hook motif DNA-binding family protein;(source:Araport11)	30.66	68.71	0.45	2.21E-05	1.30E-04
5789	7	F2-3 vs. S2-3	260352_at	AT1G69295	Encodes a member of the X8-GPI family of proteins. It localizes to the plasmodesmata and is predicted to bind callose.	105.96	233.27	0.45	2.67E-04	8.15E-04
5790	7	F2-3 vs. S2-3	260395_at	AT1G69780	Encodes a homeodomain leucine zipper class I (HD-Zip I) protein which is expressed during the seed-to-seedling transition, regulates some of the network nodes, and affects late seedling establishment. Knock-out mutants for athb13 showed increased primary root length as compared with wild type (Col-0) seedlings, suggesting that this transcription factor is a negative regulator of early root growth, possibly repressing cell division and/or cell elongation or the length of time cells elongate.	38.51	86.34	0.45	9.41E-04	2.18E-03
5791	7	F2-3 vs. S2-3	265895_at	AT2G15000	caspase-6 protein;(source:Araport11)	31.46	69.35	0.45	4.19E-04	1.15E-03
5792	7	F2-3 vs. S2-3	265383_at	AT2G16780	Encodes a WD-40 repeat protein similar to yeast MSI1.	36	80.91	0.45	5.42E-04	1.41E-03
5793	7	F2-3 vs. S2-3	264790_at	AT2G17820	Encodes a member of the histidine kinase family.	17.22	38.08	0.45	4.99E-04	1.32E-03
5794	7	F2-3 vs. S2-3	267465_at	AT2G19170	Encodes a novel subtilisin-like serine protease.	71.74	159.16	0.45	7.28E-04	1.76E-03
5795	7	F2-3 vs. S2-3	263534_at	AT2G24940	membrane-associated progesterone binding protein 2;(source:Araport11)	175.61	391.94	0.45	9.67E-05	3.81E-04

5796	7	F2-3 vs. S2-3	266315_at	AT2G27100	Identified as a leaf form mutant by Redei having serrated leaves. Further analysis of the single loss of function allele indicated pleiotropic effects extending to many aspects of shoot development such as taller meristems, alterations in phase transition, phyllotaxy and branching. Encodes a single zinc finger containing protein that is expressed in meristems and organ primordia and forms a complex with both AtCBC (cap binding complex) proteins to control alternative splicing.	57.39	127.29	0.45	5.61E-04	1.45E-03
5797	7	F2-3 vs. S2-3	267349_at	AT2G40010	Ribosomal protein L10 family protein;(source:Araport11)	20.97	46.41	0.45	1.92E-05	1.17E-04
5798	7	F2-3 vs. S2-3	260610_at	AT2G43680	Member of IQ67 (CaM binding) domain containing family.	15.55	34.51	0.45	5.27E-04	1.38E-03
5799	7	F2-3 vs. S2-3	267230_at	AT2G44080	Encodes ARL, a gene similar to ARGOS involved in cell expansion-dependent organ growth. Upregulated by brassinosteroid. Acts downstream of BRI1. The mRNA is cell-to-cell mobile.	112.89	253.16	0.45	3.09E-05	1.66E-04
5800	7	F2-3 vs. S2-3	266106_at	AT2G45170	Involved in autophagy. Under nutrient starvation the protein localizes to autophagosomes.	13.1	29.19	0.45	3.22E-05	1.71E-04
5801	7	F2-3 vs. S2-3	258466_at	AT3G06040	Ribosomal protein L12/ ATP-dependent Clp protease adaptor protein ClpS family protein;(source:Araport11)	25.5	56.53	0.45	1.70E-05	1.08E-04
5802	7	F2-3 vs. S2-3	258821_at	AT3G07230	wound-responsive protein-like protein;(source:Araport11)	756.95	1684.29	0.45	7.13E-04	1.74E-03
5803	7	F2-3 vs. S2-3	259250_at	AT3G07580	hypothetical protein;(source:Araport11)	22.2	49.42	0.45	1.16E-04	4.36E-04
5804	7	F2-3 vs. S2-3	258713_at	AT3G09735	S1FA-like DNA-binding protein;(source:Araport11)	75.45	166.78	0.45	2.39E-04	7.47E-04
5805	7	F2-3 vs. S2-3	256783_at	AT3G13670	MUT9-like protein kinase. Contributes to phosphorylation of photoexcited CRY2. Interaction with CRY2 occurs via the non catalytic PPKC domain.MLK4 phosphorylates the conserved H2A serine 95 residue. Synthetic mutants that cannot phosphorylate H2AS95 fail to complement the late flowering phenotype suggesting that MLK4 promotes long day flowering via phosphorylation.MLK4 is required for H2A295 phosphorylation of GI.	127.07	280.08	0.45	2.29E-04	7.27E-04
5806	7	F2-3 vs. S2-3	257922_at	AT3G23150	Involved in ethylene perception in ArabidopsisThe mRNA is cell-to-cell mobile.	35.81	79.38	0.45	1.52E-04	5.36E-04
5807	7	F2-3 vs. S2-3	257615_at	AT3G26510	Octicosapeptide/Phox/Bem1p family protein;(source:Araport11)	77.72	173.77	0.45	2.33E-05	1.35E-04
5808	7	F2-3 vs. S2-3	252121_at	AT3G51160	Catalyzes the first step in the de novo synthesis of GDP-L-fucose. Loss of function mutations result in reduced levels of fucosylation and decreased freezing tolerance.	17.62	39.05	0.45	6.10E-04	1.55E-03
5809	7	F2-3 vs. S2-3	251454_at	AT3G60080	RING/U-box superfamily protein;(source:Araport11)	20.26	45.51	0.45	6.95E-04	1.71E-03
5810	7	F2-3 vs. S2-3	251429_at	AT3G60190	At3g60190 encodes Arabidopsis dynamin-related protein 1E, DRP1E, also known as EDR3, ADL4 and ADL1E, which is 624 amino acid residues long, has a predicted mass of 69.8 kDa and a pI of 7.5. Dynamin-related protein 1E belongs to a plant-specific subclass of dynamin-related proteins (DRP1), consisting of five members in Arabidopsis (A, B, C, D, E). This class is characterized by having an N-terminal GTPase domain, a central `dynamin 2` domain and a C-terminal GTPase effector domain (GED), a typical structure for plant dynamin-related proteins. However, this class lacks a PH domain and a proline-rich domain, which are found in classical animal dynamin-like proteins. Based on work on animal dynamins, the plant DRP1 proteins should be able to form polymeric structures that wrap around membranes to facilitate membrane tubulation and pinching off of vesicles, processes that are essential to vesicle trafficking and membrane compartmentalization. The edr3 mutation causes a P77L substitution in the G2 motif of the GTPase domain of DRP1E. edr3 mutant Arabidopsis plants display enhanced cell death in response to powdery mildew infection.	17.81	39.21	0.45	4.94E-05	2.35E-04
5811	7	F2-3 vs. S2-3	251341_at	AT3G60770	Ribosomal protein S13/S15;(source:Araport11)	1069.3	2356.15	0.45	1.54E-04	5.42E-04
5812	7	F2-3 vs. S2-3	251337_at	AT3G60820	Encodes 20S proteasome beta subunit PBF1 (PBF1).	80.12	177.41	0.45	1.68E-04	5.75E-04
5813	7	F2-3 vs. S2-3	251357_at	AT3G61110	Arabidopsis ribosomal protein	2466.25	5513.18	0.45	1.96E-05	1.19E-04
5814	7	F2-3 vs. S2-3	251261_at	AT3G62110	Pectin lyase-like superfamily protein;(source:Araport11)	11.38	25.02	0.45	3.13E-05	1.67E-04
5815	7	F2-3 vs. S2-3	255221_at	AT4G05150	Octicosapeptide/Phox/Bem1p family protein;(source:Araport11)	200.45	446.11	0.45	4.30E-05	2.13E-04
5816	7	F2-3 vs. S2-3	245340_at	AT4G14420	HR-like lesion-inducing protein-like protein;(source:Araport11)	79.85	177.5	0.45	5.71E-05	2.60E-04
5817	7	F2-3 vs. S2-3	245415_at	AT4G17330	gene of unknown function expressed in seedlings, flower buds and stems	128.84	284.78	0.45	5.53E-05	2.55E-04
5818	7	F2-3 vs. S2-3	254355_at	AT4G22380	Ribosomal protein L7Ae/L30e/S12e/Gadd45 family protein;(source:Araport11)	92.4	204.5	0.45	1.29E-05	8.96E-05
5819	7	F2-3 vs. S2-3	254137_at	AT4G24930	thylakoid lumenal 17.9 kDa protein, chloroplast;(source:Araport11)	48.34	106.68	0.45	1.01E-04	3.95E-04
5820	7	F2-3 vs. S2-3	253368_at	AT4G33200	member of Myosin-like proteins	74.08	165.3	0.45	1.57E-04	5.50E-04
5821	7	F2-3 vs. S2-3	250819_at	AT5G05100	R3H RNA binding protein that interacts with AGO2 and miRNA.	67.17	147.82	0.45	9.74E-04	2.24E-03
5822	7	F2-3 vs. S2-3	250768_at	AT5G05670	signal recognition particle binding protein;(source:Araport11)	102.04	225.34	0.45	6.27E-04	1.58E-03
5823	7	F2-3 vs. S2-3	250584_at	AT5G07590	Transducin/WD40 repeat-like superfamily protein;(source:Araport11)	34.6	76.54	0.45	5.06E-04	1.33E-03

5824	7	F2-3 vs. S2-3	246513_at	AT5G15680	ARM repeat superfamily protein;(source:Araport11)	73.91	164.94	0.45	5.79E-04	1.49E-03
5825	7	F2-3 vs. S2-3	249181_at	AT5G42920	Encodes a component of the putative Arabidopsis THO/TREX complex: THO1 or HPR1 (At5g09860), THO2 (At1g24706), THO3 or TEX1 (At5g56130), THO5 (At5g42920, At1g45233), THO6 (At2g19430), and THO7 (At5g16790, At3g02950). THO/TREX complexes in animals have been implicated in the transport of mRNA precursors. Mutants of THO3/TEX1, THO1, THO6 accumulate reduced amount of small interfering (si)RNA, suggesting a role of the putative Arabidopsis THO/TREX in siRNA biosynthesis.	15.09	33.87	0.45	1.50E-04	5.32E-04
5826	7	F2-3 vs. S2-3	247978_at	AT5G56710	Ribosomal protein L31e family protein;(source:Araport11)	1384.92	3104.47	0.45	3.74E-05	1.92E-04
5827	7	F2-3 vs. S2-3	244978_at	ATCG00740	RNA polymerase alpha subunit	334.59	749.02	0.45	5.76E-04	1.48E-03
5828	7	F2-3 vs. S2-3	263667_at	AT1G04270	Encodes cytosolic ribosomal protein S15.	185.32	419.03	0.44	1.43E-05	9.63E-05
5829	7	F2-3 vs. S2-3	260781_at	AT1G14620	decoy;(source:Araport11)	57.21	131.08	0.44	7.36E-04	1.78E-03
5830	7	F2-3 vs. S2-3	264203_at	AT1G22780	S18 ribosomal protein involved in the binding of f-Met tRNA during initiation of mRNA translation. Expression restricted to meristems. Mutant phenotype-pointed first leaves,reduced fresh weight, growth retardation.	464.56	1066.5	0.44	1.44E-05	9.67E-05
5831	7	F2-3 vs. S2-3	261650_at	AT1G27770	Encodes a chloroplast envelope Ca2+-ATPase with an N-terminal autoinhibitor.	22.27	51.15	0.44	1.15E-05	8.34E-05
5832	7	F2-3 vs. S2-3	261741_at	AT1G47870	Member of the E2F transcription factors, (cell cycle genes), key components of the cyclin D/retinoblastoma/E2F pathway. AtE2Fc is regulated by a balance between gene expression and ubiquitin-proteasome proteolysis. AtE2Fc might play a role in cell division and during the transition from skotomorphogenesis to photomorphogenesis. E2Fc has been shown to interact with DPB in its nonphosphorylated form; when E2Fc is phosphorylated, the formation of the E2Fc/DPB heterodimer is lost. E2Fc is required for miR396 activity on cell proliferation under UV-B. Its role is independent of E2Fe, probably modulating DNA damage responses through the regulation of SOG1 and ATR transcript levels.	70.86	159.47	0.44	1.63E-04	5.66E-04
5833	7	F2-3 vs. S2-3	259617_at	AT1G47970	nucleolin;(source:Araport11)	102.86	231.29	0.44	8.38E-05	3.42E-04
5834	7	F2-3 vs. S2-3	246371_at	AT1G51940	Encodes a LysM-containing receptor-like kinase. Induction of chitin-responsive genes by chitin treatment is not blocked in the mutant. Based on protein sequence alignment analysis, it has a typical RD signaling domain in its catalytic loop and possesses autophosphorylation activity.It is required for the suppression of defense responses in absence of pathogen infection or upon abscisic acid treatment. Loss-of-function mutants display enhanced resistance to Botrytis cinerea and Pectobacterium carotovorum. Its expression is repressed by pathogen infection and biological elicitors and is induced abscisic acid.Expression is strongly repressed by elicitors and fungal infection, and is induced by the hormone abscisic acid (ABA). Insertional mutants show increased expression of PHYTOALEXIN-DEFICIENT 3 (PAD3), enhanced resistance to Botrytis cinerea and Pectobacterium carotovorum infection and reduced physiological responses to ABA, suggesting that LYK3 is important for the cross-talk between signaling pathways activated by ABA and pathogens (PMID:24639336).	56.27	127.93	0.44	6.00E-06	5.18E-05
5835	7	F2-3 vs. S2-3	246400_at	AT1G57540	40S ribosomal protein;(source:Araport11)	104.79	240.62	0.44	1.13E-05	8.27E-05
5836	7	F2-3 vs. S2-3	265790_at	AT2G01170	Encodes a bidirectional amino acid transporter that can transport ala, arg, glu and lys, GABA but not pro with both export and import activity. Its expression is localized in the vascular tissues suggesting a function in amino acids export from the phloem into sink tissue.	56.53	129.73	0.44	4.93E-05	2.34E-04
5837	7	F2-3 vs. S2-3	266797_at	AT2G22840	Growth regulating factor encoding transcription activator. One of the nine members of a GRF gene family, containing nuclear targeting domain. Mutants result in smaller leaves indicating the role of the gene in leaf development. Expressed in root, shoot and flower	51.31	115.73	0.44	3.37E-05	1.78E-04
5838	7	F2-3 vs. S2-3	266226_at	AT2G28740	histone 4	24.04	55	0.44	1.89E-04	6.33E-04
5839	7	F2-3 vs. S2-3	267004_at	AT2G34260	Encodes a WDxR motif-containing protein that is required for gametogenesis, seed and endosperm development.	47.84	108.1	0.44	4.62E-04	1.24E-03

5840	7	F2-3 vs. S2-3	252272_at	AT3G49670	Encodes a CLAVATA1-related receptor kinase-like protein required for both shoot and flower meristem function. Very similar to BAM1,with more than 85% a.a. identity. It has a broad expression pattern and is involved in vascular strand development in the leaf, control of leaf shape, size and symmetry, male gametophyte development and ovule specification and function. Anthers of double mutants (bam1bam2) appeared abnormal at a very early stage and lack the endothecium, middle, and tapetum layers. Further analyses revealed that cells interior to the epidermis (in anther tissue) acquire some characteristics of pollen mother cells (PMCs), suggesting defects in cell fate specification. The pollen mother-like cells degenerate before the completion of meiosis, suggesting that these cells are defective. In addition, the BAM2 expression pattern supports both an early role in promoting somatic cell fates and a subsequent function in the PMCs. The mRNA is cell-to-cell mobile.	15.82	36.26	0.44	1.26E-04	4.63E-04
5841	7	F2-3 vs. S2-3	251985_at	AT3G53220	Thioredoxin superfamily protein;(source:Araport11)	10.5	23.79	0.44	1.27E-04	4.67E-04
5842	7	F2-3 vs. S2-3	251802_at	AT3G55380	ubiquitin-conjugating enzyme 14;(source:Araport11)	83.21	189.29	0.44	1.59E-05	1.03E-04
5843	7	F2-3 vs. S2-3	251333_at	AT3G61690	Putative TNAase	43.27	98.23	0.44	9.50E-06	7.29E-05
5844	7	F2-3 vs. S2-3	254950_at	AT4G10810	hypothetical protein;(source:Araport11)	18.85	43.01	0.44	7.16E-04	1.75E-03
5845	7	F2-3 vs. S2-3	254815_at	AT4G12420	Encodes a protein of unknown function involved in directed root tip growth. It is a member of 19-member gene family and is distantly related structurally to the multiple-copper oxidases ascorbate oxidase and laccase, though it lacks the copper-binding domains. The protein is glycosylated and GPI-anchored. It is localized to the plasma membrane and the cell wall. The gene is expressed most strongly in expanding tissues.	23.46	52.96	0.44	1.73E-05	1.09E-04
5846	7	F2-3 vs. S2-3	254238_at	AT4G23540	ARM repeat superfamily protein;(source:Araport11)	12.35	27.92	0.44	2.34E-04	7.38E-04
5847	7	F2-3 vs. S2-3	253812_at	AT4G28240	Member of the wound-induced polypeptide (WIP) family. Positively regulates plant resistance against Pst DC3000 by enhancing PTI responses.	63.66	144.55	0.44	7.74E-04	1.85E-03
5848	7	F2-3 vs. S2-3	253443_at	AT4G32551	LEUNIG regulates floral organ identity,gynoecium and ovule development. Negatively regulates AGAMOUS . Encodes a glutamine-rich protein with seven WD repeats similar to transcriptional corepressors.	78.3	179.31	0.44	2.54E-04	7.82E-04
5849	7	F2-3 vs. S2-3	253336_at	AT4G33250	Encodes initiation factor 3k (EIF3k).	380.55	861.39	0.44	3.70E-04	1.05E-03
5850	7	F2-3 vs. S2-3	253338_at	AT4G33430	Leu-rich receptor Serine/threonine protein kinase. Component of BR signaling that interacts with BRI1 in vitro and in vivo to form a heterodimer. Brassinolide-dependent association of BRI1 and BAK1 in vivo. Phosphorylation of both BRI1 and BAK1 on Thr residues was BR dependent. Although BAK1 and BRI1 alone localize in the plasma membrane, when BAK1 and BRI1 are coexpressed, the heterodimer BAK1/BRI1 they form is localized in the endosome.	27.11	61.56	0.44	2.93E-05	1.59E-04
5851	7	F2-3 vs. S2-3	250888_at	AT5G04460	RING/U-box superfamily protein;(source:Araport11)	31.73	71.42	0.44	4.14E-05	2.08E-04
5852	7	F2-3 vs. S2-3	250581_at	AT5G07300	Encodes a copine-like protein, which is a member of a newly identified class of calcium-dependent, phospholipid binding proteins that are present in a wide range of organisms.	13.81	31.09	0.44	6.50E-05	2.84E-04
5853	7	F2-3 vs. S2-3	250141_at	AT5G14640	shaggy-like kinase 13;(source:Araport11)	291.5	655.25	0.44	4.87E-04	1.29E-03
5854	7	F2-3 vs. S2-3	246975_at	AT5G24890	stress response NST1-like protein;(source:Araport11)	31.08	71.26	0.44	3.14E-05	1.68E-04
5855	7	F2-3 vs. S2-3	246103_at	AT5G28640	Encodes a protein with similarity to mammalian transcriptional coactivator that is involved in cell proliferation during leaf and flower development. Loss of function mutations have narrow, pointed leaves and narrow floral organs. AN3 interacts with members of the growth regulating factor (GRF) family of transcription factors.	126.88	289.13	0.44	5.88E-05	2.65E-04
5856	7	F2-3 vs. S2-3	249443_at	AT5G39600	39S ribosomal protein;(source:Araport11)	50.07	114.08	0.44	8.19E-05	3.36E-04
5857	7	F2-3 vs. S2-3	249183_at	AT5G42990	ubiquitin-conjugating enzyme 18;(source:Araport11)	80.13	182.34	0.44	2.78E-05	1.53E-04
5858	7	F2-3 vs. S2-3	244903_at	ATMG00660	hypothetical protein;(source:Araport11)	20.41	45.9	0.44	7.10E-06	5.88E-05
5859	7	F2-3 vs. S2-3	264803_at	AT1G08580	hypothetical protein;(source:Araport11)	19.4	44.81	0.43	1.41E-04	5.05E-04
5860	7	F2-3 vs. S2-3	264883_s_a	AT1G11180	Secretory carrier membrane protein (SCAMP) family protein;(source:Araport11)	161.14	374.25	0.43	1.50E-05	9.94E-05
5861	7	F2-3 vs. S2-3	260673_at	AT1G19330	Evening-expressed key component of Sin3-HDAC complex, which bind directly to the CIRCADIAN CLOCK ASSOCIATED 1 (CCA1) and PSEUDO-RESPONSE REGULATOR 9 (PRR9) promoters and catalyze histone 3 (H3) deacetylation at the cognate regions to repress expression, allowing the declining phase of their expression at dusk.	82.16	191.48	0.43	4.21E-05	2.10E-04

5862	7	F2-3 vs. S2-3	245642_at	AT1G25275	Thionin-like gene involved in resistance against the beet cyst nematode (Heterodera schachtii).	39.22	91.9	0.43	8.59E-05	3.48E-04
5863	7	F2-3 vs. S2-3	245688_at	AT1G28290	Encodes an atypical arabinogalactan protein that is localized to the plasma membrange. AGP31 is highly expressed in flowers and vascular tissue and is repressed by jasmonic acid. AGP31 may play a role in vascular tissue function during defense and development.	33.27	78.02	0.43	1.36E-04	4.90E-04
5864	7	F2-3 vs. S2-3	262411_at	AT1G34640	peptidase;(source:Araport11)	102.87	238.01	0.43	3.98E-05	2.01E-04
5865	7	F2-3 vs. S2-3	261557_at	AT1G63640	P-loop nucleoside triphosphate hydrolases superfamily protein with CH (Calponin Homology) domain-containing protein;(source:Araport11)	53.5	123.82	0.43	3.83E-04	1.08E-03
5866	7	F2-3 vs. S2-3	262967_at	AT1G75730	hypothetical protein;(source:Araport11)	16.9	39.18	0.43	1.00E-05	7.51E-05
5867	7	F2-3 vs. S2-3	264279_s_at	AT1G78820	D-mannose binding lectin protein with Apple-like carbohydrate-binding domain-containing protein;(source:Araport11)	317.27	741.9	0.43	2.88E-04	8.63E-04
5868	7	F2-3 vs. S2-3	266588_at	AT2G14890	putative proline-rich protein (At2g14890) mRNA, complete The mRNA is cell-to-cell mobile.	63.41	147.93	0.43	5.82E-04	1.49E-03
5869	7	F2-3 vs. S2-3	265445_at	AT2G37190	Ribosomal protein L11 family protein;(source:Araport11)	76.77	180.09	0.43	7.34E-05	3.09E-04
5870	7	F2-3 vs. S2-3	265883_at	AT2G42310	ESSS subunit of NADH:ubiquinone oxidoreductase (complex I) protein;(source:Araport11)	150.16	350.28	0.43	8.03E-04	1.91E-03
5871	7	F2-3 vs. S2-3	258576_at	AT3G04230	Ribosomal protein S5 domain 2-like superfamily protein;(source:Araport11)	130.84	306.53	0.43	3.40E-05	1.79E-04
5872	7	F2-3 vs. S2-3	257855_at	AT3G13040	myb-like HTH transcriptional regulator family protein;(source:Araport11)	139.74	324.64	0.43	2.82E-05	1.55E-04
5873	7	F2-3 vs. S2-3	252390_at	AT3G47836	transmembrane protein;(source:Araport11)	195.35	456.41	0.43	3.10E-04	9.16E-04
5874	7	F2-3 vs. S2-3	252297_at	AT3G48930	Nucleic acid-binding, OB-fold-like protein;(source:Araport11)	257.75	592.7	0.43	1.77E-05	1.11E-04
5875	7	F2-3 vs. S2-3	245364_at	AT4G15790	uveal autoantigen with coiled-coil/ankyrin;(source:Araport11)	52.42	121.78	0.43	3.48E-05	1.82E-04
5876	7	F2-3 vs. S2-3	253952_at	AT4G26840	Encodes a small ubiquitin-like modifier (SUMO) polypeptide that becomes covalently attached to various intracellular protein targets, much like ubiquitination, leading to post-translational modification of those targets.	205.57	479.28	0.43	1.32E-04	4.81E-04
5877	7	F2-3 vs. S2-3	253436_at	AT4G32470	Cytochrome bd ubiquinol oxidase, 14kDa subunit;(source:Araport11)	532.95	1232.67	0.43	4.83E-05	2.31E-04
5878	7	F2-3 vs. S2-3	252913_at	AT4G39220	Key player of retrieval of ER membrane proteins	102.05	237.01	0.43	1.51E-05	9.98E-05
5879	7	F2-3 vs. S2-3	251023_at	AT5G02170	Transmembrane amino acid transporter family protein;(source:Araport11)	19.75	46.22	0.43	4.07E-04	1.13E-03
5880	7	F2-3 vs. S2-3	246598_at	AT5G14850	Encodes a putative mannosyltransferase homolog to human PIG-B and yeast GPI10, both of which are involved in the biosynthesis of glycosylphosphatidylinositol (GPI) anchors. Disruption of the gene affects COBRA-LIKE10 localization, a GPI-anchored protein (GPI-AP) important for pollen tube growth and guidance.	149.97	345.63	0.43	5.81E-05	2.63E-04
5881	7	F2-3 vs. S2-3	246044_at	AT5G19450	calcium-dependent protein kinase (CDPK19) mRNA, complete	161.24	372.68	0.43	1.61E-04	5.59E-04
5882	7	F2-3 vs. S2-3	248277_at	AT5G52860	ABC-2 type transporter family protein;(source:Araport11)	38.34	88.79	0.43	1.56E-04	5.46E-04
5883	7	F2-3 vs. S2-3	247923_at	AT5G57490	Encodes a voltage-dependent anion channel (VDAC: AT3G01280/VDAC1, AT5G67500/VDAC2, AT5G15090/VDAC3, AT5G57490/VDAC4, AT5G15090/VDAC5). VDACs are reported to be porin-type, beta-barrel diffusion pores. They are prominently localized in the outer mitochondrial membrane and are involved in metabolite exchange between the organelle and the cytosol.	47.69	109.69	0.43	6.27E-05	2.77E-04
5884	7	F2-3 vs. S2-3	247325_at	AT5G64200	encodes an SC35-like splicing factor of 35 kD localized to the nuclear specks.	101.26	235.19	0.43	3.76E-05	1.93E-04
5885	7	F2-3 vs. S2-3	260932_s_at	AT1G02520	Encodes an ATP-binding cassette (ABC) transporter. Expressed in the vascular tissue of primary stem.The mRNA is cell-to-cell mobile.	9.69	22.93	0.42	2.31E-05	1.35E-04
5886	7	F2-3 vs. S2-3	264371_at	AT1G12090	extensin-like protein (ELP)	237.01	558.5	0.42	7.71E-04	1.85E-03
5887	7	F2-3 vs. S2-3	262651_at	AT1G14100	member of Glycosyltransferase Family- 37. FUT8 was previously associated to AT1G14110	12.28	29.28	0.42	5.79E-05	2.63E-04
5888	7	F2-3 vs. S2-3	246324_at	AT1G16610	Encodes SR45, a member of the highly conserved family of serine/arginine-rich (SR) proteins, which play key roles in pre-mRNA splicing and other aspects of RNA metabolism. SR45 is a spliceosome protein, interacts with SR33 and the U1-70K protein of the U1 snRNP. Also involved in plant sugar response. sr45-1 mutation confers hypersensitivity to glucose during early seedling growth.	89.51	211.68	0.42	1.16E-05	8.35E-05
5889	7	F2-3 vs. S2-3	261926_at	AT1G22530	PATLs belong to a family of proteins having a Golgi dynamics (GOLD) domain in tandem with the Sec14p-like domain. PATLs are auxin regulated. Quadruple mutants (patl2456) show altered PIN1 lateralization in root endodermis cells.	56.54	135.28	0.42	5.90E-06	5.12E-05

5890	7	F2-3 vs. S2-3	259592_at	AT1G27950	Encodes LTPG1, a lipid transfer protein with a predicted GPI (glycosylphosphatidylinositol)-anchor domain. Localized in the plasma membrane. Disruption of the LTPG1 gene causes alterations of cuticular lipid composition, but no significant changes on total wax and cutin monomer loads are seen. The mRNA is cell-to-cell mobile.	43.01	101.92	0.42	6.24E-05	2.76E-04
5891	7	F2-3 vs. S2-3	256536_at	AT1G33330	Class I peptide chain release factor;(source:Araport11)	69.81	165.89	0.42	5.17E-04	1.36E-03
5892	7	F2-3 vs. S2-3	259942_at	AT1G71260	Encodes WHY2, a homolog of the potato p24 protein. It shares the conserved KGKAAL domain, a putative DNA-binding domain, with potato p24 and is localized to mitochondria and not the nucleus. WHY2 is a member of the Whirly family proteins present mainly in the plant kingdom performing various activities related to DNA metabolism. Crystal structure of Solanum tuberosum WHY2, a close homolog of Arabidopsis WHY2, reveal that Whirly proteins bind to single strand DNA to promote accurate repair of DNA double-strand breaks over an error-prone repair pathway.	18.22	43.06	0.42	2.07E-04	6.77E-04
5893	7	F2-3 vs. S2-3	259710_at	AT1G77670	Pyridoxal phosphate (PLP)-dependent transferases superfamily protein;(source:Araport11)	83.28	199.76	0.42	2.51E-04	7.75E-04
5894	7	F2-3 vs. S2-3	262206_at	AT2G01090	Ubiquinol-cytochrome C reductase hinge protein;(source:Araport11)	800.04	1887.53	0.42	1.15E-05	8.34E-05
5895	7	F2-3 vs. S2-3	263017_at	AT2G17620	Cyclin B2;(source:Araport11)	63.82	152.82	0.42	1.59E-05	1.03E-04
5896	7	F2-3 vs. S2-3	267088_at	AT2G38140	plastid-specific ribosomal protein 4 (PSRP4) mRNA, complete The mRNA is cell-to-cell mobile.	25.27	60.55	0.42	2.43E-04	7.58E-04
5897	7	F2-3 vs. S2-3	265451_at	AT2G46490	hypothetical protein;(source:Araport11)	65.69	157.42	0.42	1.40E-05	9.48E-05
5898	7	F2-3 vs. S2-3	266715_at	AT2G46780	RNA-binding (RRM/RBD/RNP motifs) family protein;(source:Araport11)	14.19	33.52	0.42	2.74E-04	8.31E-04
5899	7	F2-3 vs. S2-3	259181_at	AT3G01690	alpha/beta-Hydrolases superfamily protein;(source:Araport11)	136.34	327.18	0.42	4.12E-05	2.07E-04
5900	7	F2-3 vs. S2-3	258486_at	AT3G02560	Ribosomal protein S7e family protein;(source:Araport11)	458.65	1086.32	0.42	3.83E-05	1.95E-04
5901	7	F2-3 vs. S2-3	259304_at	AT3G05210	encodes a homolog of human ERCC1 protein (yeast RAD10), which is a DNA repair endonuclease. Mutants are sensitive to UV-B and gamma radiation (G2 cell cycle phase arrest) and are defective in dark-repair of pyrimidine pyrimidone dimers. This protein incises the 5 Prime end of damaged DNA, similar to ERCC1/RAD10.	40.92	97.12	0.42	3.87E-04	1.09E-03
5902	7	F2-3 vs. S2-3	257553_at	AT3G16830	TOPLESS-related 2;(source:Araport11)	193.51	461.53	0.42	2.73E-04	8.30E-04
5903	7	F2-3 vs. S2-3	257115_at	AT3G20150	Kinesin motor family protein;(source:Araport11)	10.36	24.64	0.42	6.20E-06	5.30E-05
5904	7	F2-3 vs. S2-3	252148_at	AT3G51280	Tetratricopeptide repeat (TPR)-like superfamily protein;(source:Araport11)	42.14	100.21	0.42	5.93E-04	1.51E-03
5905	7	F2-3 vs. S2-3	255706_at	AT4G00100	Encodes a cytoplasmic ribosomal protein S13 homologue involved in early leaf development The mRNA is cell-to-cell mobile.	312.89	742	0.42	6.07E-05	2.71E-04
5906	7	F2-3 vs. S2-3	255043_at	AT4G09640	magnesium transporter, putative (DUF803);(source:Araport11)	29.77	71.08	0.42	3.01E-04	8.96E-04
5907	7	F2-3 vs. S2-3	245274_at	AT4G14360	S-adenosyl-L-methionine-dependent methyltransferases superfamily protein;(source:Araport11)	48.23	114.48	0.42	2.93E-05	1.59E-04
5908	7	F2-3 vs. S2-3	245512_at	AT4G15770	RNA binding protein;(source:Araport11)	105.27	251.83	0.42	4.40E-04	1.20E-03
5909	7	F2-3 vs. S2-3	245351_at	AT4G17640	Encodes casein kinase II beta (regulatory) subunit.	124.54	294.38	0.42	2.50E-04	7.74E-04
5910	7	F2-3 vs. S2-3	254400_at	AT4G21270	Encodes a kinesin-like motor protein heavy chain. Loss of function mutations have reduced fertility and are defective in spindle formation in male meiosis.	20.74	48.97	0.42	2.61E-04	8.01E-04
5911	7	F2-3 vs. S2-3	253670_at	AT4G30010	ATP-dependent RNA helicase;(source:Araport11)	185.65	437.44	0.42	4.32E-05	2.13E-04
5912	7	F2-3 vs. S2-3	253291_at	AT4G33865	Ribosomal protein S14p/S29e family protein;(source:Araport11)	356.12	847.61	0.42	1.63E-04	5.66E-04
5913	7	F2-3 vs. S2-3	250969_at	AT5G02740	Ribosomal protein S24e family protein;(source:Araport11)	114.47	274.8	0.42	2.61E-05	1.47E-04
5914	7	F2-3 vs. S2-3	250707_at	AT5G05950	maternal effect embryo arrest 60;(source:Araport11)	18.85	44.84	0.42	5.92E-04	1.51E-03
5915	7	F2-3 vs. S2-3	250681_at	AT5G06590	hypothetical protein;(source:Araport11)	27.97	66.6	0.42	1.20E-04	4.46E-04
5916	7	F2-3 vs. S2-3	250269_at	AT5G12970	Calcium-dependent lipid-binding (CaLB domain) plant phosphoribosyltransferase family protein;(source:Araport11)	41.16	97.93	0.42	2.50E-05	1.43E-04
5917	7	F2-3 vs. S2-3	249795_at	AT5G23740	Encodes a putative ribosomal protein S11 (RPS11-beta).	73.33	174.66	0.42	1.08E-04	4.14E-04
5918	7	F2-3 vs. S2-3	246756_at	AT5G27930	EGR2 functions as a negative regulator of plant growth with prominent effect on plant growth during drought stress. EGR2 regulates microtubule organization and likely affects additional cytoskeleton and trafficking processes along the plasma membrane.	18.05	43.02	0.42	2.94E-04	8.81E-04

5919	7	F2-3 vs. S2-3	249115_at	AT5G43810	Encodes Argonaute10, a member of the EIF2C (elongation initiation factor 2c)/ Argonaute class of proteins. Required to establish the central-peripheral organization of the embryo apex. Along with WUS and CLV genes, controls the relative organization of central zone and peripheral zone cells in meristems. Acts in embryonic provascular tissue potentiating WUSCHEL function during meristem development in the embryo. AGO10 specifically sequesters miR166/165 to regulate shoot apical meristem development.	82.87	197	0.42	2.15E-04	6.94E-04
5920	7	F2-3 vs. S2-3	247175_at	AT5G65280	Encodes a protein with reported similarity to GCR2 a putative G protein coupled receptor thought to be an ABA receptor. Loss of function mutations in GCL1 show no ABA response defects based on assays of seed germination and seedling development.GCL1 also has similarity to LANCL1 and LANCL2, human homologs of bacterial lanthionine synthetase.	582.43	1397.34	0.42	6.53E-05	2.84E-04
5921	7	F2-3 vs. S2-3	244986_at	ATCG00820	Encodes a 6.8-kDa protein of the small ribosomal subunit.	548.16	1292.8	0.42	1.37E-04	4.93E-04
5922	7	F2-3 vs. S2-3	260714_at	AT1G14980	Encodes mitochondrial-localized chaperonin 10 that complements the E.coli groES mutant. Its mRNA is upregulated in response to heat shock treatment and is expressed uniformly in various organs.	28.66	69.79	0.41	1.89E-04	6.33E-04
5923	7	F2-3 vs. S2-3	260862_at	AT1G43850	Encodes a transcriptional co-regulator of AGAMOUS, that functions with LEUNIG to repress AG in the outer floral whorls.	30.19	72.99	0.41	7.54E-04	1.81E-03
5924	7	F2-3 vs. S2-3	263004_at	AT1G54510	Encodes AtNEK1, a member of the NIMA-related serine/threonine kinases (Neks) that have been linked to cell-cycle regulation in fungi and mammals. Plant Neks might be involved in plant development processes.	25.16	61.68	0.41	5.29E-05	2.47E-04
5925	7	F2-3 vs. S2-3	264159_at	AT1G65270	ER membrane protein complex subunit-like protein;(source:Araport11)	104.04	254.02	0.41	3.66E-05	1.89E-04
5926	7	F2-3 vs. S2-3	264233_at	AT1G67430	Ribosomal protein L22p/L17e family protein;(source:Araport11)	1787.59	4326.09	0.41	4.60E-06	4.33E-05
5927	7	F2-3 vs. S2-3	260006_at	AT1G68000	phosphatidylinositol synthase 1	23.6	57.09	0.41	2.80E-05	1.54E-04
5928	7	F2-3 vs. S2-3	259371_at	AT1G69080	Adenine nucleotide alpha hydrolases-like superfamily protein;(source:Araport11)	16.32	39.38	0.41	1.23E-05	8.61E-05
5929	7	F2-3 vs. S2-3	266348_at	AT2G01450	MPK17 Map kinase family member. Mutants have increased numbers of peroxisomes a phenotype that can be suppressed by mutations in PMD1. This and other treatments, suggests a function in control of peroxisome proliferation in salt stress.	112.9	274.18	0.41	1.16E-05	8.35E-05
5930	7	F2-3 vs. S2-3	263074_at	AT2G17560	Encodes a protein belonging to the subgroup of HMGB (high mobility group B) proteins that have a distinctive DNA-binding motif, the HMG-box domain. The motif confers non-sequence specific interaction with linear DNA and structure-specific binding to distorted DNA sites. The HMGB proteins are involved in the assembly of nucleoprotein complexes. Can be phosphorylated by CK2alpha.	127.53	313.3	0.41	1.06E-05	7.84E-05
5931	7	F2-3 vs. S2-3	267136_at	AT2G23460	encodes a novel G-alpha protein that shares similarity to plant, yeast, and animal G-alpha proteins at the C-terminus. It contains an N-terminus that is as large as the C-terminus, is a member of a small family, and is expressed in all tissues examined, including roots, leaves, stems, flowers, and fruits.	100.6	242.76	0.41	3.19E-04	9.39E-04
5932	7	F2-3 vs. S2-3	263640_at	AT2G25270	transmembrane protein;(source:Araport11)	34.39	83.53	0.41	4.44E-05	2.17E-04
5933	7	F2-3 vs. S2-3	265210_at	AT2G36620	RPL24A encodes ribosomal protein L24, homolog of cytosolic RPL24, found in archaea and higher eukaryotes. Arabidopsis has two RPL24 homologs, RPL24A (AT2G36620) and RPL24B (AT3G53020).	301.95	742.84	0.41	2.14E-05	1.27E-04
5934	7	F2-3 vs. S2-3	259062_at	AT3G07440	hypothetical protein;(source:Araport11)	14.45	34.92	0.41	4.87E-04	1.29E-03
5935	7	F2-3 vs. S2-3	257753_at	AT3G18740	The gene encodes a putative leucine-rich repeat (LRR)Receptor-like kinase (RLK).	2370.97	5806.46	0.41	5.30E-06	4.80E-05
5936	7	F2-3 vs. S2-3	256621_at	AT3G24450	Heavy metal transport/detoxification superfamily protein;(source:Araport11)	58.31	143.23	0.41	3.26E-05	1.73E-04
5937	7	F2-3 vs. S2-3	251738_at	AT3G56150	member of eIF3c - eukaryotic initiation factor 3c	60.34	146.45	0.41	1.63E-04	5.66E-04
5938	7	F2-3 vs. S2-3	251185_at	AT3G62870	Ribosomal protein L7Ae/L30e/S12e/Gadd45 family protein;(source:Araport11)	724.12	1770.47	0.41	4.77E-04	1.27E-03
5939	7	F2-3 vs. S2-3	255243_at	AT4G05590	Encodes NRGA1, a putative mitochondrial pyruvate carrier that mediates ABA regulation of guard cell ion channels and drought stress responses.	25.42	62.45	0.41	4.22E-04	1.16E-03
5940	7	F2-3 vs. S2-3	245316_at	AT4G14000	Putative methyltransferase family protein;(source:Araport11)	165.57	399.71	0.41	7.40E-06	6.07E-05
5941	7	F2-3 vs. S2-3	253243_at	AT4G34560	transmembrane protein;(source:Araport11)	12.5	30.46	0.41	4.36E-04	1.19E-03
5942	7	F2-3 vs. S2-3	246489_at	AT5G16050	Encodes GF14 upsilon chain, a 14-3-3 gene family member.	286.8	701.91	0.41	4.01E-04	1.11E-03
5943	7	F2-3 vs. S2-3	249788_at	AT5G24330	Encodes a SET-domain protein, a H3K27 monomethyltransferases required for chromatin structure and gene silencing. Regulates heterochromatic DNA replication. Contains a PCNA-binding domain. ATXR6 accumulates preferentially during the late G1 or S phase, suggesting that it plays a role in cell-cycle regulation or progression.	24.3	59.35	0.41	3.52E-04	1.01E-03

5944	7	F2-3 vs. S2-3	245861_at	AT5G28300	Encodes a Ca(2+)-dependent CaM-binding protein. AtGT2L specifically targets the nucleus and possesses both transcriptional activation and DNA-binding abilities, implicating its function as a nuclear transcription factor.	12.6	31.02	0.41	2.09E-05	1.25E-04
5945	7	F2-3 vs. S2-3	248512_at	AT5G50460	secE/sec61-gamma protein transport protein;(source:Araport11)	120.16	294.03	0.41	1.73E-05	1.09E-04
5946	7	F2-3 vs. S2-3	248471_at	AT5G50840	alpha-taxilin-like protein;(source:Araport11)	12.36	30.26	0.41	4.59E-04	1.24E-03
5947	7	F2-3 vs. S2-3	248413_at	AT5G51600	Mutant has defective roots. Essential for giant cell ontogenesis. Role in organizing the mitotic microtubule array during both early and late mitosis in all plant organs.	36.95	91.2	0.41	5.01E-04	1.33E-03
5948	7	F2-3 vs. S2-3	247968_at	AT5G56670	Ribosomal protein S30 family protein;(source:Araport11)	386.3	949.63	0.41	1.76E-04	5.99E-04
5949	7	F2-3 vs. S2-3	247667_at	AT5G60150	hypothetical protein;(source:Araport11)	88.21	212.97	0.41	1.33E-04	4.83E-04
5950	7	F2-3 vs. S2-3	264180_at	AT1G02190	Fatty acid hydroxylase superfamily;(source:Araport11)	69.88	173.3	0.4	3.37E-04	9.77E-04
5951	7	F2-3 vs. S2-3	261169_at	AT1G04920	Encodes a protein with putative sucrose-phosphate synthase activity.	40.74	101.5	0.4	5.42E-04	1.41E-03
5952	7	F2-3 vs. S2-3	256065_at	AT1G07070	Ribosomal protein L35Ae family protein;(source:Araport11)	60.55	151.58	0.4	8.88E-05	3.58E-04
5953	7	F2-3 vs. S2-3	245814_at	AT1G49910	Encodes a homolog of the yeast and human BUB3 (BUDDING UNINHIBITED BY BENZYMIDAZOL 3) protein. Yeast and human BUB3s function in spindle assembly checkpoint control.	19.73	49.77	0.4	2.36E-04	7.43E-04
5954	7	F2-3 vs. S2-3	264763_at	AT1G61450	CAP-gly domain linker;(source:Araport11)	12.21	30.59	0.4	7.10E-06	5.88E-05
5955	7	F2-3 vs. S2-3	262313_at	AT1G70900	hypothetical protein;(source:Araport11)	80.89	200.63	0.4	7.22E-04	1.75E-03
5956	7	F2-3 vs. S2-3	261911_at	AT1G80750	Cytosolic ribosomal 60S subunit protein.	169.76	421.24	0.4	2.23E-05	1.31E-04
5957	7	F2-3 vs. S2-3	265480_at	AT2G15970	encodes an alpha form of a protein similar to the cold acclimation protein WCOR413 in wheat. Expression is induced by short-term cold-treatment, water deprivation, and abscisic acid treatment. The mRNA is cell-to-cell mobile.	209.62	519.34	0.4	4.30E-06	4.17E-05
5958	7	F2-3 vs. S2-3	263526_at	AT2G24830	zinc finger (CCCH-type) family protein / D111/G-patch domain-containing protein;(source:Araport11)	46.58	116.04	0.4	1.47E-04	5.23E-04
5959	7	F2-3 vs. S2-3	264374_at	AT2G25180	Encodes an Arabidopsis response regulator (ARR) protein that acts in concert with other type-B ARRs in the cytokinin signaling pathway. Also involved in cytokinin-dependent inhibition of hypocotyl elongation and cytokinin-dependent greening and shooting in tissue culture. ARR1, ARR10, and ARR12 are redundant regulators of drought response, with ARR1 being the most critical.The retention of leaf water content, maintenance of cell membrane stability, and enhancement of anthocyanin biosynthesis were found to contribute to the enhanced drought tolerance of the arr1,10,12 triple mutant. ARR1, ARR10 and ARR12 redundantly bind to the promoter of WUSCHEL (WUS), directly activate its transcription. In parallel, ARR1, ARR10 and ARR12 repress the expression of YUCCAs (YUCs), which encode a key enzyme for auxin biosynthesis, indirectly promoting WUS induction. The regulation of ARR1, ARR10 and ARR12 on WUS and YUCs is required for regeneration and maintenance of shoot meristem.	20.8	51.45	0.4	4.93E-05	2.34E-04
5960	7	F2-3 vs. S2-3	267431_at	AT2G34870	hydroxyproline-rich glycoprotein family protein;(source:Araport11)	13.08	32.76	0.4	1.58E-05	1.03E-04
5961	7	F2-3 vs. S2-3	265798_at	AT2G35790	transmembrane protein;(source:Araport11)	84.23	210.05	0.4	2.69E-05	1.50E-04
5962	7	F2-3 vs. S2-3	257570_at	AT3G13662	Disease resistance-responsive (dirigent-like protein) family protein;(source:Araport11)	22.69	57.14	0.4	1.35E-05	9.22E-05
5963	7	F2-3 vs. S2-3	258206_at	AT3G14010	hydroxyproline-rich glycoprotein family protein, similar to Mrs16p (GI:2737884) (Saccharomyces cerevisiae); weak similarity to ataxin-2 related protein (GI:1679686) (Homo sapiens). Included in a family of CTC interacting domain proteins found to interact with PAB2.	25.25	62.6	0.4	5.33E-04	1.39E-03
5964	7	F2-3 vs. S2-3	258054_at	AT3G16240	Delta tonoplast intrinsic protein, functions as a water channel and ammonium (NH3) transporter. Highly expressed in flower, shoot, and stem. Expression shows diurnal regulation and is induced by ammonium (NH3). Protein localized to vacuolar membrane. The mRNA is cell-to-cell mobile.	236.55	595.31	0.4	7.77E-04	1.86E-03
5965	7	F2-3 vs. S2-3	252513_at	AT3G46220	E3 UFM1-protein ligase-like protein;(source:Araport11)	71.61	176.97	0.4	6.79E-04	1.68E-03
5966	7	F2-3 vs. S2-3	251707_at	AT3G56490	Encodes a protein that has adenylylsulfate sulfohydrolase activity (E.C. 3.6.2.1) in vitro.	420.44	1063.56	0.4	2.77E-04	8.40E-04
5967	7	F2-3 vs. S2-3	246289_at	AT3G56880	VQ motif-containing protein;(source:Araport11)	97.96	246.12	0.4	9.40E-06	7.23E-05
5968	7	F2-3 vs. S2-3	251656_at	AT3G57170	N-acetylglucosaminyl transferase component family protein / Gpi1 family protein;(source:Araport11)	19.78	50.01	0.4	2.10E-04	6.85E-04
5969	7	F2-3 vs. S2-3	255000_at	AT4G09800	encodes a ribosomal protein S18C, a constituent of the small subunit of the ribosomal complex	820.46	2042.56	0.4	5.30E-06	4.80E-05

5970	7	F2-3 vs. S2-3	250974_at	AT5G02820	Involved in the patterning and shape of leaf trichomes. Encodes the DNA topoisomerase VI SPO11-3, involved in endoreduplication	62.44	156.18	0.4	3.75E-04	1.06E-03
5971	7	F2-3 vs. S2-3	249984_at	AT5G18400	Cytokine-induced anti-apoptosis inhibitor 1, Fe-S biogenesi;(source:Araport11)	205.42	508.06	0.4	1.43E-04	5.11E-04
5972	7	F2-3 vs. S2-3	249832_at	AT5G23400	Leucine-rich repeat (LRR) family protein;(source:Araport11)	19.43	48.44	0.4	2.32E-04	7.34E-04
5973	7	F2-3 vs. S2-3	249755_at	AT5G24580	Heavy metal transport/detoxification superfamily protein;(source:Araport11)	29.81	75.35	0.4	4.30E-06	4.17E-05
5974	7	F2-3 vs. S2-3	249456_at	AT5G39410	Saccharopine dehydrogenase;(source:Araport11)	61.46	153.52	0.4	4.45E-04	1.21E-03
5975	7	F2-3 vs. S2-3	248387_at	AT5G51960	complex 1 protein, LYR family protein;(source:Araport11)	50.32	124.75	0.4	6.00E-06	5.18E-05
5976	7	F2-3 vs. S2-3	248349_at	AT5G52240	Encodes a protein with similarity to progesterone-binding proteins in animals. Has been shown to bind steroids in vitro. Expressed in aerial portions of the plant excluding mature flowers and siliques. Antisense experiments suggest a role in inhibition of hypocotyl cell elongation. Expression is suppressed light grown seedlings transferred to the dark. The mRNA is cell-to-cell mobile.	244.4	612.95	0.4	6.50E-06	5.49E-05
5977	7	F2-3 vs. S2-3	247935_at	AT5G56940	Ribosomal protein S16 family protein;(source:Araport11)	102.55	258.69	0.4	9.30E-06	7.18E-05
5978	7	F2-3 vs. S2-3	247787_at	AT5G58640	Selenoprotein, Rdx type;(source:Araport11)	85.59	215.14	0.4	3.32E-05	1.76E-04
5979	7	F2-3 vs. S2-3	247652_at	AT5G59910	Histone superfamily protein;(source:Araport11)	381.8	959.44	0.4	9.44E-05	3.75E-04
5980	7	F2-3 vs. S2-3	247148_at	AT5G65670	auxin (indole-3-acetic acid) induced gene The mRNA is cell-to-cell mobile.	497.08	1258.07	0.4	9.46E-05	3.75E-04
5981	7	F2-3 vs. S2-3	244960_at	ATCG01020	encodes a chloroplast ribosomal protein L32, a constituent of the large subunit of the ribosomal complex	822.99	2077.38	0.4	5.70E-06	5.00E-05
5982	7	F2-3 vs. S2-3	247864_s_a	AT1G25155	Glutamine amidotransferase type 1 family protein;(source:Araport11)	116.79	302.87	0.39	1.31E-05	9.05E-05
5983	7	F2-3 vs. S2-3	264066_at	AT2G27880	AGO5.Required for antiviral RNA silencing.Confers resistance to Potato virus X.	28.66	72.85	0.39	9.30E-06	7.18E-05
5984	7	F2-3 vs. S2-3	266471_at	AT2G31060	elongation factor family protein;(source:Araport11)	61.52	158.9	0.39	1.16E-04	4.37E-04
5985	7	F2-3 vs. S2-3	267062_at	AT2G32600	hydroxyproline-rich glycoprotein family protein;(source:Araport11)	40.33	102.2	0.39	4.62E-04	1.24E-03
5986	7	F2-3 vs. S2-3	267007_at	AT2G34480	Encodes a nuclear localized member of the ribosomal L18ae/LX protein family. Loss of function mutations show reduced transmission through the gametophytes and embryo lethality.	557.32	1429.67	0.39	8.89E-05	3.59E-04
5987	7	F2-3 vs. S2-3	260498_at	AT2G41710	Integrase-type DNA-binding superfamily protein;(source:Araport11)	25.59	65	0.39	7.95E-05	3.30E-04
5988	7	F2-3 vs. S2-3	266511_at	AT2G47680	zinc finger (CCCH type) helicase family protein;(source:Araport11)	33.84	86.52	0.39	2.50E-06	2.85E-05
5989	7	F2-3 vs. S2-3	259307_at	AT3G05230	Signal peptidase subunit;(source:Araport11)	25.74	66.14	0.39	6.96E-05	2.98E-04
5990	7	F2-3 vs. S2-3	256278_at	AT3G12130	KHZ1 is a CCCH zinc-finger and KH domain protein belonging to the VII subfamily. It is expressed throughout the plant. Highly similar to KHZ2. khz1 mutants are late flowering and double mutants with khz2 are even more late flowering. Overexpression leads to increased rates of leaf senescence.	27.25	69.09	0.39	2.32E-05	1.35E-04
5991	7	F2-3 vs. S2-3	257134_at	AT3G12870	transmembrane protein;(source:Araport11)	23.13	58.61	0.39	7.70E-06	6.20E-05
5992	7	F2-3 vs. S2-3	246328_at	AT3G43590	zinc knuckle (CCHC-type) family protein;(source:Araport11)	43.36	112.35	0.39	8.09E-04	1.92E-03
5993	7	F2-3 vs. S2-3	252055_at	AT3G52580	Ribosomal protein S11 family protein;(source:Araport11)	798.22	2065.51	0.39	1.95E-05	1.19E-04
5994	7	F2-3 vs. S2-3	251796_at	AT3G55360	Enoyl-CoA reductase is involved in all very long chain fatty acids (VLCFA) elongation reactions that are required for cuticular wax, storage lipid and sphingolipid metabolism. The protein is located in the ER, but in contrast to its yeast homolog TSC13 is not particularly enriched in the nuclear envelope-vacuole junction. Mutants in this gene show abnormal organ morphology and stem glossiness. Cells in all tissues are only about 1/3 of the size of wild type cells. The morphological changes are most likely to result from the reduction in the VLCFA content of sphingolipids. Mutants also show abnormalities in the endocytic membrane organization and transport as well as reduced trichome papillae.	99.17	254.5	0.39	2.59E-05	1.47E-04
5995	7	F2-3 vs. S2-3	261362_s_a	AT3G55750	Ribosomal protein L35Ae family protein;(source:Araport11)	26.59	68.49	0.39	5.20E-06	4.73E-05
5996	7	F2-3 vs. S2-3	245235_at	AT4G25550	Cleavage/polyadenylation specificity factor, 25kDa subunit;(source:Araport11)	126.74	326.22	0.39	3.20E-06	3.37E-05
5997	7	F2-3 vs. S2-3	253981_at	AT4G26670	Mitochondrial import inner membrane translocase subunit Tim17/Tim22/Tim23 family protein;(source:Araport11)	39.91	102.41	0.39	2.14E-05	1.27E-04
5998	7	F2-3 vs. S2-3	253264_at	AT4G33950	Encodes calcium-independent ABA-activated protein kinase, a member of SNF1-related protein kinases (SnRK2) whose activity is activated by ionic (salt) and non-ionic (mannitol) osmotic stress. Mutations disrupted ABA induction of stomatal closure as well as ABA inhibition of light-induced stomatal opening. However, regulation of stomatal opening/closing by light or CO(2) is not affected in these mutants. May act in the interval between ABA perception and reactive oxygen species production in the ABA signalling network.	58.76	151.79	0.39	8.08E-05	3.34E-04
5999	7	F2-3 vs. S2-3	245893_at	AT5G09270	transmembrane protein;(source:Araport11)	14.93	38.64	0.39	3.32E-05	1.76E-04
6000	7	F2-3 vs. S2-3	250397_at	AT5G10980	Histone superfamily protein;(source:Araport11)	522.17	1339.73	0.39	1.65E-04	5.69E-04

6001	7	F2-3 vs. S2-3	250109_at	AT5G15230	Encodes gibberellin-regulated protein GASA4. Promotes GA responses and exhibits redox activity.	431.46	1115.44	0.39	1.13E-04	4.28E-04
6002	7	F2-3 vs. S2-3	246978_at	AT5G24910	Member of CYP714A. Encodes one of the two tandemly duplicated gene pair ELA1 (CYP714A1) and ELA2 (CYP714A2), homologs of the rice cytochrome P450 monooxygenase gene EUI1. Double mutation of ELA1 and ELA2 results in increased biomass and enlarged organs.	56.53	145.69	0.39	6.44E-05	2.82E-04
6003	7	F2-3 vs. S2-3	248990_at	AT5G45210	Disease resistance protein (TIR-NBS-LRR class) family;(source:Araport11)	10.17	25.76	0.39	5.35E-05	2.49E-04
6004	7	F2-3 vs. S2-3	248958_at	AT5G45590	Ribosomal protein L35;(source:Araport11)	24.94	64.34	0.39	2.75E-05	1.53E-04
6005	7	F2-3 vs. S2-3	248521_s_a	AT5G50520	Major facilitator superfamily protein;(source:Araport11)	42.84	110.31	0.39	1.10E-04	4.20E-04
6006	7	F2-3 vs. S2-3	248453_at	AT5G51340	SCC4 is a tetratricopeptide repeat containing protein and a likely component of a plant cohesion loading complex along with its partner SSC2 It is expressed primarily in dividing cells. Loss of function mutants are embryo lethal, arresting by globular stage.	39.9	101.6	0.39	2.13E-04	6.89E-04
6007	7	F2-3 vs. S2-3	255776_at	AT1G18540	Ribosomal protein L6 family protein;(source:Araport11)	136.09	358.39	0.38	9.70E-06	7.40E-05
6008	7	F2-3 vs. S2-3	262488_at	AT1G21830	hypothetical protein;(source:Araport11)	11.44	30	0.38	8.81E-05	3.56E-04
6009	7	F2-3 vs. S2-3	260588_at	AT1G53320	Member of plant TLP family. TLP7 is tethered to the PM but detaches upon stimulus and translocates to the nucleus. Has DNA binding activity but lacks conservation of the transcription activation domain.	238.37	628.94	0.38	3.80E-06	3.79E-05
6010	7	F2-3 vs. S2-3	262331_at	AT1G64050	hypothetical protein;(source:Araport11)	11.04	28.84	0.38	3.00E-06	3.23E-05
6011	7	F2-3 vs. S2-3	260418_s_a	AT1G66590	Mitochondrial copper chaperone which influences copper and iron homeostasis.	12.75	33.92	0.38	4.75E-04	1.27E-03
6012	7	F2-3 vs. S2-3	260350_at	AT1G69410	Encodes eIF5A-2, a putative eukaryotic translation initiation factor. There are three eIF5A coding genes in Arabidopsis: eIF5A-1/At1g13950, eIF5A-2/At1g26630 and eIF5A-3/At1g69410.	105.49	277.72	0.38	1.73E-04	5.91E-04
6013	7	F2-3 vs. S2-3	245721_s_a	AT1G73450	Protein kinase superfamily protein;(source:Araport11)	182.79	484.29	0.38	7.00E-05	2.99E-04
6014	7	F2-3 vs. S2-3	266119_at	AT2G02100	Predicted to encode a PR (pathogenesis-related) protein. Belongs to the plant defensin (PDF) family with the following members: At1g75830/PDF1.1, At5g44420/PDF1.2a, At2g26020/PDF1.2b, At5g44430/PDF1.2c, At2g26010/PDF1.3, At1g19610/PDF1.4, At1g55010/PDF1.5, At2g02120/PDF2.1, At2g02100/PDF2.2, At2g02130/PDF2.3, At1g61070/PDF2.4, At5g63660/PDF2.5, At2g02140/PDF2.6, At5g38330/PDF3.1 and At4g30070/PDF3.2. The mRNA is cell-to-cell mobile.	594.61	1567.69	0.38	5.90E-06	5.12E-05
6015	7	F2-3 vs. S2-3	266693_at	AT2G19800	Encodes a myo-inositol oxygenase family gene.	36.87	97.47	0.38	1.36E-05	9.28E-05
6016	7	F2-3 vs. S2-3	257702_at	AT3G12670	Cytidine triphosphate synthase.	107.89	282.06	0.38	1.60E-05	1.04E-04
6017	7	F2-3 vs. S2-3	257126_at	AT3G20060	Encodes one of two ubiquitin-conjugating enzymes belonging to the E2-C gene family (the other being UBC19). Transcript is always found in dividing cells, but also in other non-dividing cells. Protein is localized to the cytoplasm as well as to the nucleus.	48.77	128.94	0.38	3.22E-05	1.71E-04
6018	7	F2-3 vs. S2-3	257225_s_a	AT3G27830	50S ribosomal protein L12-A The mRNA is cell-to-cell mobile.	69.21	183.84	0.38	2.62E-05	1.48E-04
6019	7	F2-3 vs. S2-3	252552_at	AT3G45900	Ribonuclease P protein subunit P38-like protein;(source:Araport11)	70.21	185.55	0.38	2.31E-05	1.35E-04
6020	7	F2-3 vs. S2-3	252359_at	AT3G48440	Zinc finger C-x8-C-x5-C-x3-H type family protein;(source:Araport11)	18.54	48.62	0.38	5.90E-04	1.51E-03
6021	7	F2-3 vs. S2-3	251184_at	AT3G62840	Small nuclear ribonucleoprotein family protein;(source:Araport11)	113.3	297.88	0.38	1.00E-04	3.93E-04
6022	7	F2-3 vs. S2-3	255128_at	AT4G08310	DNA ligase;(source:Araport11)	14.5	38.54	0.38	1.51E-04	5.33E-04
6023	7	F2-3 vs. S2-3	250511_at	AT5G09960	sorbin/SH3 domain protein;(source:Araport11)	66.11	175.93	0.38	7.37E-05	3.10E-04
6024	7	F2-3 vs. S2-3	250386_at	AT5G11510	Arabidopsis thaliana putative c-myb-like transcription factor MYB3R-4. Functions in powdery mildew induced host endoreduplication at the site of infection.	30.27	80.44	0.38	5.93E-05	2.67E-04
6025	7	F2-3 vs. S2-3	246920_at	AT5G25090	early nodulin-like protein 13;(source:Araport11)	15.15	40.24	0.38	7.80E-06	6.27E-05
6026	7	F2-3 vs. S2-3	246849_at	AT5G26850	Uncharacterized protein;(source:Araport11)	278.12	734.16	0.38	1.24E-05	8.66E-05
6027	7	F2-3 vs. S2-3	249528_at	AT5G38720	RRP7 shares limited sequence similarity to human and yeast RRP7. In Arabidopsis RRP7 functions in 18S ribosomal RNA maturation.	99.58	260.03	0.38	2.87E-05	1.57E-04
6028	7	F2-3 vs. S2-3	248691_at	AT5G48310	Protein of unknown function that may be involved in stress response. Strongly expressed in vascular tissues.Mutants are ABA- insensitive.	55.39	143.9	0.38	8.44E-05	3.44E-04
6029	7	F2-3 vs. S2-3	248511_at	AT5G50375	Converts pentacyclic cyclopropyl sterols to conventional tetracyclic sterols. CPI1 function during and just after division and support gravitropism by establishing polar PIN2 localization. Required for endocytosis of PIN2	48.03	126.11	0.38	1.03E-04	4.01E-04
6030	7	F2-3 vs. S2-3	247457_at	AT5G62170	LOW protein: M-phase inducer phosphatase-like protein;(source:Araport11)	12.95	33.87	0.38	1.35E-05	9.22E-05
6031	7	F2-3 vs. S2-3	264331_at	AT1G04130	Encodes one of the 36 carboxylate clamp (CC)-tetratricopeptide repeat (TPR) proteins (Prasad 2010, Pubmed ID: 20856808). Interacts with Hsp90/Hsp70 as co-chaperone.	24.67	66.07	0.37	6.20E-04	1.56E-03

6032	7	F2-3 vs. S2-3	261603_at	AT1G49600	RNA-binding protein 47A;(source:Araport11)	22.04	59.27	0.37	7.17E-05	3.04E-04
6033	7	F2-3 vs. S2-3	260511_at	AT1G51570	Required for maintenance of inflorescence and shoot SAMs and normal development of the derived vascular cambium, functions in the SAM to promote continuous organogenesis, affects SAM development through STM, where it affects intracellular localization of STM in SAM cells in the peripheral region and prevents STM localization toward the cell wall of SAM cells in the peripheral region.	16.66	44.7	0.37	2.36E-05	1.37E-04
6034	7	F2-3 vs. S2-3	263160_at	AT1G54080	oligouridylate-binding protein 1A;(source:Araport11)	60.11	164.05	0.37	9.40E-06	7.23E-05
6035	7	F2-3 vs. S2-3	263712_at	AT2G20585	nuclear fusion defective 6;(source:Araport11)	57.08	152.42	0.37	1.96E-05	1.19E-04
6036	7	F2-3 vs. S2-3	264377_at	AT2G25060	early nodulin-like protein 14;(source:Araport11)	46.88	125.23	0.37	1.15E-05	8.34E-05
6037	7	F2-3 vs. S2-3	265826_at	AT2G35795	Chaperone DnaJ-domain superfamily protein;(source:Araport11)	109.84	296.49	0.37	3.60E-06	3.67E-05
6038	7	F2-3 vs. S2-3	259308_at	AT3G05180	GDSL-motif esterase/acyltransferase/lipase. Enzyme group with broad substrate specificity that may catalyze acyltransfer or hydrolase reactions with lipid and non-lipid substrates.	14.5	38.79	0.37	9.23E-05	3.68E-04
6039	7	F2-3 vs. S2-3	258699_at	AT3G09455	dolichyl-diphosphooligosaccharide-protein glycosyltransferase subunit;(source:Araport11)	205.21	548.13	0.37	1.36E-04	4.90E-04
6040	7	F2-3 vs. S2-3	257654_at	AT3G13310	Chaperone DnaJ-domain superfamily protein;(source:Araport11)	23.23	63.17	0.37	1.77E-04	6.01E-04
6041	7	F2-3 vs. S2-3	256979_at	AT3G21055	Encodes photosystem II 5 kD protein subunit PSII-T. This is a nuclear-encoded gene (PsbTn) which also has a plastid-encoded paralog (PsbTc).	174.73	472.39	0.37	5.70E-06	5.00E-05
6042	7	F2-3 vs. S2-3	257813_at	AT3G25100	Required for normal meiosis, may act in the last round of DNA replication prior to meiosis, sequence similar to yeast CDC45	87.02	237.6	0.37	5.67E-05	2.59E-04
6043	7	F2-3 vs. S2-3	254595_at	AT4G18960	Floral homeotic gene encoding a MADS domain transcription factor. Specifies floral meristem and carpel and stamen identity. Binds CArG box sequences. It is the only C function gene. It interacts genetically with the other homeotic genes to specify the floral organs.	92.44	246.51	0.37	1.50E-04	5.32E-04
6044	7	F2-3 vs. S2-3	253558_at	AT4G31120	Involved in vernalization. Required for epigenetic silencing of FLC, and for vernalization-mediated histone modification.	167.03	456.35	0.37	8.92E-05	3.59E-04
6045	7	F2-3 vs. S2-3	253480_at	AT4G31840	early nodulin-like protein 15;(source:Araport11)	53.29	145.72	0.37	6.31E-05	2.77E-04
6046	7	F2-3 vs. S2-3	253270_at	AT4G34160	encodes a cyclin D-type protein involved in the switch from cell proliferation to the final stages of differentiation. The gene is transcriptionally regulated by cytokinin and brassinosteroid. Protein interacts with cyclin-dependent kinase inhibitor ICK1.	27.45	75.09	0.37	9.70E-06	7.40E-05
6047	7	F2-3 vs. S2-3	246506_at	AT5G16110	hypothetical protein;(source:Araport11)	62.38	166.49	0.37	1.21E-05	8.58E-05
6048	7	F2-3 vs. S2-3	249427_at	AT5G39850	Ribosomal protein S4;(source:Araport11)	202.46	553.33	0.37	2.60E-06	2.94E-05
6049	7	F2-3 vs. S2-3	248496_at	AT5G50790	Encodes a member of the SWEET sucrose efflux transporter family proteins. Transcriptionally activated by long photoperiods; activation depends on FT and SOC1. The ectopic expression of SWEET10 causes early flowering and leads to higher levels of transcription of flowering-time related genes in the shoot apex.	30.21	81.61	0.37	6.10E-06	5.23E-05
6050	7	F2-3 vs. S2-3	262603_at	AT1G15380	Glyoxalase which affects ABA?JA crosstalk.	173.92	478.59	0.36	2.52E-05	1.43E-04
6051	7	F2-3 vs. S2-3	259515_at	AT1G20430	hypothetical protein;(source:Araport11)	25.04	68.77	0.36	1.10E-05	8.08E-05
6052	7	F2-3 vs. S2-3	262245_at	AT1G48240	member of NPSN Gene Family	12.34	34.03	0.36	9.38E-05	3.73E-04
6053	7	F2-3 vs. S2-3	262467_at	AT1G50240	The FUSED (FU) gene belongs to Ser/Thr protein kinase family and has a key role in the hedgehog signaling pathway known to control cell proliferation and patterning in fruit flies and humans . Arabidopsis thaliana genome has a single Fu gene that is involved in male meiosis cytokinesis. Cytokinesis-defective mutants, named two-in-one (tio), result from mutations in Arabidopsis Fu. Phenotypic analysis of tio mutants reveals an essential role for TIO in conventional modes of cytokinesis in plant meristems and during male gametogenesis. TIO is tightly localized to the midline of the nascent phragmoplast and remains associated with the expanding phragmoplast ring. This gene was previously annotated as two gene models, AT1G50230.1 and AT1G50240.1, however the experimental evidence exists (Oh et al, Current Biology, 2005) showing that these two models are in fact single gene, named FUSED.	83.56	233.16	0.36	3.53E-05	1.84E-04
6054	7	F2-3 vs. S2-3	265930_at	AT2G18510	RNA-binding (RRM/RBD/RNP motifs) family protein;(source:Araport11)	16.11	45.3	0.36	4.45E-05	2.18E-04
6055	7	F2-3 vs. S2-3	266812_at	AT2G44830	AGCVIII kinase involved in the pulse-induced first positive phototropism. Plasma-membrane-associated element of a molecular rheostat that modulates auxin flux through developing protophloem sieve elements (PPSEs) while interacting with BRX, thereby timing PPSE differentiation. Activates PIN-mediated auxin efflux.	40.53	113.12	0.36	1.77E-05	1.11E-04
6056	7	F2-3 vs. S2-3	257701_at	AT3G12710	DNA glycosylase superfamily protein;(source:Araport11)	44.8	125.8	0.36	9.90E-06	7.47E-05

6057	7	F2-3 vs. S2-3	251194_at	AT3G62920	zinc metalloproteinase aureolysin;(source:Araport11)	9.8	27.49	0.36	1.11E-04	4.23E-04
6058	7	F2-3 vs. S2-3	253668_at	AT4G30220	small nuclear ribonucleoprotein F;(source:Araport11)	274.7	772.6	0.36	4.10E-06	4.02E-05
6059	7	F2-3 vs. S2-3	253304_at	AT4G33640	costars family protein;(source:Araport11)	96.35	269.97	0.36	6.10E-06	5.23E-05
6060	7	F2-3 vs. S2-3	250718_at	AT5G06240	embryo defective 2735;(source:Araport11)	13.08	35.85	0.36	5.70E-06	5.00E-05
6061	7	F2-3 vs. S2-3	249424_s_a	AT5G39800	Mitochondrial ribosomal protein L27;(source:Araport11)	56.74	158.28	0.36	1.30E-06	1.81E-05
6062	7	F2-3 vs. S2-3	247692_s_a	AT5G59970	Histone superfamily protein;(source:Araport11)	456.56	1283.75	0.36	2.98E-05	1.61E-04
6063	7	F2-3 vs. S2-3	247506_at	AT5G61960	A member of mei2-like gene family, predominantly plant-based family of genes encoding RNA binding proteins with characteristic presence of a highly conserved RNA binding motif first described in the mei2 gene of the fission yeast S. pombe. In silico analyses reveal nine mei2 -like genes in A. thaliana. They were grouped into four distinct clades, based on overall sequence similarity and subfamily-specific sequence elements. AML1 is a member of two sister clades of mei2-like gene family, AML1 through AML5 and belongs to the clade named ALM14. AML1 is expressed during early embryo development, particularly along embryonic axis at torpedo stage, in shoot apex (weaker expression) and in the organogenic regions of floral apices.	55.32	152.25	0.36	1.57E-05	1.02E-04
6064	7	F2-3 vs. S2-3	244981_at	ATCG00770	chloroplast 30S ribosomal protein S8	339.15	953.05	0.36	3.70E-06	3.73E-05
6065	7	F2-3 vs. S2-3	256062_at	AT1G07090	LIGHT-DEPENDENT SHORT HYPOCOTYLS-like protein (DUF640);(source:Araport11)	11.58	33.23	0.35	7.20E-06	5.94E-05
6066	7	F2-3 vs. S2-3	256460_at	AT1G36240	Ribosomal protein L7Ae/L30e/S12e/Gadd45 family protein;(source:Araport11)	940.82	2650.9	0.35	1.68E-05	1.07E-04
6067	7	F2-3 vs. S2-3	266700_at	AT2G19740	Ribosomal protein L31e family protein;(source:Araport11)	442.3	1246.67	0.35	6.80E-06	5.69E-05
6068	7	F2-3 vs. S2-3	265390_at	AT2G20820	hypothetical protein;(source:Araport11)	582.09	1652.12	0.35	7.30E-06	6.00E-05
6069	7	F2-3 vs. S2-3	266845_at	AT2G26110	bromodomain protein (DUF761);(source:Araport11)	17.66	50.86	0.35	1.00E-06	1.54E-05
6070	7	F2-3 vs. S2-3	267453_at	AT2G33880	Encodes a protein with similarity to WUS type homeodomain protein. Required for meristem growth and development and acts through positive regulation of WUS. Loss of function phenotypes include embryo lethality, hyponastic cotyledons, reduced root development and smaller meristems. Phenotypes can be rescued by addition of sucrose in the growth media. Overexpression can partially rescue the triple mutant cytokinin receptor phenotype suggesting HB-3 is a downstream effector of cytokinin signaling.	29.68	85.92	0.35	3.63E-04	1.04E-03
6071	7	F2-3 vs. S2-3	267507_at	AT2G45710	Zinc-binding ribosomal protein family protein;(source:Araport11)	431.41	1217.44	0.35	3.40E-06	3.52E-05
6072	7	F2-3 vs. S2-3	266460_at	AT2G47930	arabinogalactan protein 26;(source:Araport11)	23.29	67.03	0.35	4.82E-05	2.31E-04
6073	7	F2-3 vs. S2-3	257610_at	AT3G13810	indeterminate(ID)-domain 11;(source:Araport11)	17.78	50.15	0.35	1.77E-04	6.00E-04
6074	7	F2-3 vs. S2-3	258284_at	AT3G16080	Zinc-binding ribosomal protein family protein;(source:Araport11)	1592.86	4613.55	0.35	1.58E-05	1.03E-04
6075	7	F2-3 vs. S2-3	252089_at	AT3G52110	interferon-activable protein;(source:Araport11)	15.75	45.25	0.35	2.38E-04	7.47E-04
6076	7	F2-3 vs. S2-3	251921_at	AT3G53890	Cytosolic ribosomal protein. Mutants enhance the varigation effect of var2 mutations suggesting a link between cytosolic translation and chloroplast development.	18.15	51.37	0.35	2.68E-05	1.50E-04
6077	7	F2-3 vs. S2-3	255443_at	AT4G02700	sulfate transporter 3;(source:Araport11)	16.95	48.89	0.35	2.50E-06	2.85E-05
6078	7	F2-3 vs. S2-3	253703_at	AT4G29910	Origin Recognition Complex subunit 5. Involved in the initiation of DNA replication. Interacts strongly with all ORC subunits.	47.3	135.14	0.35	1.74E-05	1.10E-04
6079	7	F2-3 vs. S2-3	250777_at	AT5G05440	Encodes a member of the PYR (pyrabactin resistance)/PYL(PYR1-like)/RCAR (regulatory components of ABA receptor) family proteins with 14 members. PYR/PYL/RCAR family proteins function as abscisic acid sensors. Mediate ABA-dependent regulation of protein phosphatase 2Cs ABI1 and ABI2.	30.49	86.21	0.35	1.10E-05	8.08E-05
6080	7	F2-3 vs. S2-3	250756_at	AT5G05940	Encodes a member of KPP-like gene family, homolog of KPP (kinase partner protein) gene in tomato. Also a member of the RopGEF (guanine nucleotide exchange factor) family, containing the novel PRONE domain (plant-specific Rop nucleotide exchanger), which is exclusively active towards members of the Rop subfamily.	13.93	39.84	0.35	1.48E-04	5.26E-04
6081	7	F2-3 vs. S2-3	246125_at	AT5G19875	transmembrane protein;(source:Araport11)	18.83	53.53	0.35	8.80E-06	6.88E-05
6082	7	F2-3 vs. S2-3	249945_at	AT5G22440	Ribosomal protein L1p/L10e family;(source:Araport11)	115.05	333.45	0.35	5.20E-06	4.73E-05
6083	7	F2-3 vs. S2-3	249268_at	AT5G41685	Mitochondrial outer membrane translocase complex, subunit Tom7;(source:Araport11)	10.88	30.91	0.35	1.29E-05	8.96E-05
6084	7	F2-3 vs. S2-3	248613_at	AT5G49555	FAD/NAD(P)-binding oxidoreductase family protein;(source:Araport11)	108.95	311.02	0.35	1.52E-05	1.00E-04
6085	7	F2-3 vs. S2-3	248102_at	AT5G55140	ribosomal protein L30 family protein;(source:Araport11)	39.29	111.41	0.35	4.75E-05	2.28E-04
6086	7	F2-3 vs. S2-3	248041_at	AT5G55940	Uncharacterized protein family (UPF0172);(source:Araport11)	106.29	307.58	0.35	3.50E-06	3.60E-05

6087	7	F2-3 vs. S2-3	247858_at	AT5G58220	Encodes a transthyretin-like S-allantoin synthase protein that catalyzes two steps in the allantoin biosynthesis pathway by acting as a hydroxyisourate hydrolase and a 2-oxo-4-hydroxy-4-carboxy-5-ureidoimidazoline (OHCU) decarboxylase. Two alternatively spliced versions of the transcript give rise to a longer peroxisomally-targeted protein (AT5G58220.1 (called TTL1-)) and a slightly shorter cytoplasmic protein (AT5G58220.3 (called TTL2-)). Both have roughly equivalent enzymatic activity in vitro, but, allantoin biosynthesis is believed to occur in the peroxisome suggesting that the cytosolic form may participate in a different process.	26.29	75.77	0.35	1.53E-05	1.00E-04
6088	7	F2-3 vs. S2-3	247758_at	AT5G59120	SBT4.13 subtilase. Activity is inhibited by SPI-1.	21.31	61.64	0.35	3.74E-04	1.06E-03
6089	7	F2-3 vs. S2-3	247566_at	AT5G61170	Ribosomal protein S19e family protein;(source:Araport11)	846.08	2396.68	0.35	1.50E-06	2.00E-05
6090	7	F2-3 vs. S2-3	247375_at	AT5G63135	transcription termination factor;(source:Araport11)	22.17	62.78	0.35	5.13E-05	2.42E-04
6091	7	F2-3 vs. S2-3	247328_at	AT5G64130	cAMP-regulated phosphoprotein 19-related protein;(source:Araport11)	345.96	990.95	0.35	2.22E-04	7.11E-04
6092	7	F2-3 vs. S2-3	247208_at	AT5G64870	Belongs to the group of plant flotillins, which are plasma membrane proteins. Flot3 is found in membrane nanodomains.	74.09	214.23	0.35	3.40E-06	3.52E-05
6093	7	F2-3 vs. S2-3	264173_at	AT1G02160	Cox19 family protein (CHCH motif);(source:Araport11)	179.56	532.06	0.34	1.38E-05	9.39E-05
6094	7	F2-3 vs. S2-3	262490_at	AT1G21840	Encodes a urease accessory protein which is essential for the activation of plant urease.	23.85	71.05	0.34	2.30E-06	2.69E-05
6095	7	F2-3 vs. S2-3	260941_at	AT1G44970	Encodes a class III peroxidase that is genetically redundant with PRX40, expressed in the tapetum, and essential for proper anther and pollen development.	13.26	38.53	0.34	1.73E-04	5.91E-04
6096	7	F2-3 vs. S2-3	263727_at	AT2G13540	Encodes a nuclear cap-binding protein that forms a heterodimeric complex with CBP20 and is involved in ABA signaling and flowering. Mutants are early flowering and exhibit hypersensitive response to ABA in germination inhibition.Loss of ABH1 function results in abnormal processing of mRNAs for several important floral regulators (FLC, CO, FLM). Analysis of loss of function mutations suggests a role in pri-miRNA processing and mRNA splicing. Note that two different mutant alleles were given the same name abh1-7 (Kuhn et al 2007; Kim et al 2008). To avoid confusion, abh1-7 described in Kim et al (2008) has been renamed abh1-107 (other names: ensalada-1, ens-1).	77.1	226.44	0.34	1.21E-04	4.51E-04
6097	7	F2-3 vs. S2-3	245027_at	AT2G26550	Encodes a heme oxygenase-like protein lacking the conserved histidine residue at the active site that is usually involved in heme-iron coordination. It is unable to bind and degrade heme. Mutant analyses suggest a role in photomorphogenesis. The protein can bind the heme precursor, proto IX, which could be biologically significant and point to a role in the regulation of the two tetrapyrrole biosynthetic pathways.	19.83	58.53	0.34	4.83E-05	2.31E-04
6098	7	F2-3 vs. S2-3	265730_at	AT2G32220	Ribosomal L27e protein family;(source:Araport11)	21	61.46	0.34	1.60E-06	2.10E-05
6099	7	F2-3 vs. S2-3	258900_at	AT3G05590	Encodes cytoplasmic ribosomal protein L18.	1189.48	3464.47	0.34	7.50E-06	6.08E-05
6100	7	F2-3 vs. S2-3	258324_at	AT3G22780	CHC protein involved in cell cycle progression (positive regulator).	73.51	215.6	0.34	3.57E-05	1.86E-04
6101	7	F2-3 vs. S2-3	257311_at	AT3G26570	low affinity phosphate transporter	74.68	219.2	0.34	1.20E-06	1.75E-05
6102	7	F2-3 vs. S2-3	251843_x_a	AT3G54590	Encodes a hydroxyproline-rich glycoprotein.The mRNA is cell-to-cell mobile.	17.89	52.44	0.34	1.09E-04	4.19E-04
6103	7	F2-3 vs. S2-3	251581_at	AT3G58560	Encodes a protein that is involved in mRNA processing and localized to cytoplasmic p-bodies. Double mutants with CCR4b show decreased sensitivity to high concentrations of sucrose. Involved in starch and sucrose metabolism.	37.97	111	0.34	2.06E-05	1.24E-04
6104	7	F2-3 vs. S2-3	245401_at	AT4G17670	senescence-associated family protein (DUF581);(source:Araport11)	19.92	59.02	0.34	6.34E-05	2.78E-04
6105	7	F2-3 vs. S2-3	254049_at	AT4G25740	RNA binding Plectin/S10 domain-containing protein;(source:Araport11)	830.53	2473.96	0.34	5.00E-06	4.58E-05
6106	7	F2-3 vs. S2-3	253802_at	AT4G28180	hypothetical protein;(source:Araport11)	14.1	41.79	0.34	2.61E-05	1.47E-04
6107	7	F2-3 vs. S2-3	253715_at	AT4G29390	Ribosomal protein S30 family protein;(source:Araport11)	634.69	1889.05	0.34	2.01E-05	1.22E-04
6108	7	F2-3 vs. S2-3	250797_at	AT5G05310	TLC ATP/ADP transporter;(source:Araport11)	41.11	121.61	0.34	2.87E-05	1.57E-04
6109	7	F2-3 vs. S2-3	250703_at	AT5G06360	Ribosomal protein S8e family protein;(source:Araport11)	86.23	252.28	0.34	7.00E-07	1.20E-05
6110	7	F2-3 vs. S2-3	250433_at	AT5G10400	Histone superfamily protein;(source:Araport11)	71.93	212.8	0.34	2.50E-06	2.85E-05
6111	7	F2-3 vs. S2-3	250284_at	AT5G13290	Encodes a protein with predicted Ser/Thr kinase activity and membrane localization that is involved in the CLV3 signaling pathway that represses WUS expression in the meristem. Loss of function of CRN can suppress the phenotype caused by overexpression of CLV3. SOL2 isolated as a suppressor of root- specific overexpression of CLE19, a clavata3 like gene. sol2 partially suppresses the short root phenotype caused by CLE19 overexpression. Mutant flowers have extra carpels.	78.8	234.43	0.34	1.58E-04	5.54E-04
6112	7	F2-3 vs. S2-3	246863_at	AT5G25940	early nodulin-like protein;(source:Araport11)	28.47	83.56	0.34	1.37E-04	4.93E-04

6113	7	F2-3 vs. S2-3	247904_at	AT5G57390	Encodes a member of the AP2 family of transcriptional regulators. May be involved in germination and seedling growth. Mutants are resistant to ABA analogs and are resistant to high nitrogen concentrations.essential for the developmental transition between the embryonic and vegetative phases in plants. Overexpression results in the formation of somatic embryos on cotyledons. It is also required to maintain high levels of PIN1 expression at the periphery of the meristem and modulate local auxin production in the central region of the SAM which underlies phyllotactic transitions. Acts redundantly with PLT3 and 7 in lateral root pattern formation.	26.06	77.7	0.34	6.60E-06	5.55E-05
6114	7	F2-3 vs. S2-3	247482_at	AT5G62410	SMC2-1 (SMC2)	48.39	143.85	0.34	1.14E-04	4.32E-04
6115	7	F2-3 vs. S2-3	262109_at	AT1G02730	Encodes a gene similar to cellulose synthase. Knock-out mutant has reduced growth, reduced xylan level and reduced xylan synthase activity in stems.It's expression is cell cycle dependent and it appears to function in cell plate formation.	80.76	241.75	0.33	2.90E-06	3.14E-05
6116	7	F2-3 vs. S2-3	260787_at	AT1G06230	This gene is predicted to encode a bromodomain-containing protein. Plant lines expressing RNAi constructs targeted against GTE4 show some resistance to agrobacterium-mediated root transformation.	16.18	48.58	0.33	3.24E-04	9.47E-04
6117	7	F2-3 vs. S2-3	262593_at	AT1G15120	Ubiquinol-cytochrome C reductase hinge protein;(source:Araport11)	93.71	280.81	0.33	3.11E-05	1.67E-04
6118	7	F2-3 vs. S2-3	256091_at	AT1G20693	Encodes a protein belonging to the subgroup of HMGB (high mobility group B) proteins that have a distinctive DNA-binding motif, the HMG-box domain. The motif confers non-sequence specific interaction with linear DNA and structure-specific binding to distorted DNA sites. The HMGB proteins are involved in the assembly of nucleoprotein complexes. Can be phosphorylated by CK2alpha. The mRNA is cell-to-cell mobile.	91.57	275.53	0.33	2.30E-06	2.69E-05
6119	7	F2-3 vs. S2-3	261518_at	AT1G71695	Peroxidase superfamily protein;(source:Araport11)	73.74	221.22	0.33	2.80E-06	3.09E-05
6120	7	F2-3 vs. S2-3	260095_at	AT1G73230	Nascent polypeptide-associated complex NAC;(source:Araport11)	122.28	375.21	0.33	1.49E-05	9.94E-05
6121	7	F2-3 vs. S2-3	259845_at	AT1G73590	Encodes an auxin efflux carrier involved in shoot and root development. It is involved in the maintenance of embryonic auxin gradients. Loss of function severely affects organ initiation, pin1 mutants are characterised by an inflorescence meristem that does not initiate any flowers, resulting in the formation of a naked inflorescence stem. PIN1 is involved in the determination of leaf shape by actively promoting development of leaf margin serrations. In roots, the protein mainly resides at the basal end of the vascular cells, but weak signals can be detected in the epidermis and the cortex. Expression levels and polarity of this auxin efflux carrier change during primordium development suggesting that cycles of auxin build-up and depletion accompany, and may direct, different stages of primordium development. PIN1 action on plant development does not strictly require function of PGP1 and PGP19 proteins.	31.35	96.42	0.33	4.26E-05	2.12E-04
6122	7	F2-3 vs. S2-3	259971_at	AT1G76580	Squamosa promoter-binding protein-like (SBP domain) transcription factor family protein;(source:Araport11)	60.95	184.47	0.33	3.50E-06	3.60E-05
6123	7	F2-3 vs. S2-3	262043_at	AT1G80190	Similar to the PSF1 component of GINS complex, which in other organism was shown to be involved in the initiation of DNA replication.	56.21	170.34	0.33	7.00E-07	1.20E-05
6124	7	F2-3 vs. S2-3	263552_x_a	AT2G24980	Proline-rich extensin-like family protein;(source:Araport11)	15.31	46.23	0.33	5.40E-06	4.85E-05
6125	7	F2-3 vs. S2-3	265678_at	AT2G31970	Encodes the Arabidopsis RAD50 homologue. It is involved in double strand break repair. Component of the meiotic recombination complex that processes meiotic double-strand-breaks to produce single-stranded DNA ends, which act in the homology search and recombination. Accumulates in the nucleus during meiotic prophase, a process regulated by PHS1.	48.71	145.74	0.33	8.75E-05	3.54E-04
6126	7	F2-3 vs. S2-3	267316_at	AT2G34710	Dominant PHB mutations cause transformation of abaxial leaf fates into adaxial leaf fates. Encodes a member of HD-Zip family which contains homeodomain-leucine zipper domains and domain similar to a mammalian sterol binding domain. Has overlapping functions with PHAVOLUTA, REVOLUTA and CORONA.	69.55	208.5	0.33	1.60E-06	2.10E-05
6127	7	F2-3 vs. S2-3	265800_at	AT2G35630	Member of the MAP215 family of microtubule-associated proteins required to establish interphase arrays of cortical microtubules.Mutants have defects in cytokinesis during pollen development. Vegetative phenotypes observed in temperature sensitive mutants include left-handed organ twisting, isotropic cell expansion and impairment of root hair polarity. The mRNA is cell-to-cell mobile.	29.73	90.97	0.33	8.10E-06	6.48E-05

6128	7	F2-3 vs. S2-3	266984_at	AT2G39570	Encodes a ACT domain-containing protein. The ACT domain, named after bacterial aspartate kinase, chorismate mutase and TyrA (prephenate dehydrogenase), is a regulatory domain that serves as an amino acid-binding site in feedback-regulated amino acid metabolic enzymes.	51.12	155.74	0.33	2.96E-04	8.84E-04
6129	7	F2-3 vs. S2-3	263836_at	AT2G40330	Encodes a member of the PYR (pyrabactin resistance)/PYL(PYR1-like)/RCAR (regulatory components of ABA receptor) family proteins with 14 members. PYR/PYL/RCAR family proteins function as abscisic acid sensors. Mediate ABA-dependent regulation of protein phosphatase 2Cs ABI1 and ABI2.	36.72	112.41	0.33	3.60E-06	3.67E-05
6130	7	F2-3 vs. S2-3	259130_at	AT3G02190	Ribosomal protein L39 family protein;(source:Araport11)	134.46	411.65	0.33	4.67E-05	2.26E-04
6131	7	F2-3 vs. S2-3	258163_at	AT3G17940	Galactose mutarotase-like superfamily protein;(source:Araport11)	45.54	140.06	0.33	6.53E-05	2.84E-04
6132	7	F2-3 vs. S2-3	252676_at	AT3G44280	peptidyl-prolyl cis-trans isomerase G;(source:Araport11)	27.27	83.38	0.33	1.50E-05	9.94E-05
6133	7	F2-3 vs. S2-3	254609_at	AT4G18970	GDSL-motif esterase/acyltransferase/lipase. Enzyme group with broad substrate specificity that may catalyze acyltransfer or hydrolase reactions with lipid and non-lipid substrates.	79.44	242.65	0.33	3.82E-05	1.95E-04
6134	7	F2-3 vs. S2-3	253133_at	AT4G35800	Encodes the unique largest subunit of nuclear DNA-dependent RNA polymerase II; the ortholog of budding yeast RPB1 and a homolog of the E. coli RNA polymerase beta prime subunit.	13.73	41.25	0.33	9.30E-06	7.18E-05
6135	7	F2-3 vs. S2-3	252912_at	AT4G39200	Ribosomal protein S25 family protein;(source:Araport11)	165.41	505.68	0.33	2.20E-06	2.63E-05
6136	7	F2-3 vs. S2-3	250305_at	AT5G12150	Encodes a protein with similarity to REN1, a Rho GTPase activating protein.It is cytoplasmic and plasma membrane associated in interphase, but during mitosis localizes to the CDZ/CDS in a POK-dependent manner.	36.9	111.72	0.33	2.60E-06	2.94E-05
6137	7	F2-3 vs. S2-3	246479_at	AT5G16060	Cytochrome c oxidase biogenesis protein Cmc1-like protein;(source:Araport11)	22.06	66.96	0.33	1.40E-06	1.89E-05
6138	7	F2-3 vs. S2-3	249901_at	AT5G22650	Encodes a member of a plant-specific class of histone deacetylases. Controls the development of adaxial/abaxial leaf polarity. Its mRNA is widely expressed in stems, leaves, flowers and young siliques. Plant lines expressing RNAi constructs directed against this gene showed a marked reduction in agrobacterium-mediated root transformation.	168.63	511.01	0.33	3.90E-06	3.86E-05
6139	7	F2-3 vs. S2-3	246882_at	AT5G26180	S-adenosyl-L-methionine-dependent methyltransferases superfamily protein;(source:Araport11)	36.74	112.82	0.33	3.12E-05	1.67E-04
6140	7	F2-3 vs. S2-3	246730_at	AT5G28060	Ribosomal protein S24e family protein;(source:Araport11)	20.02	61.31	0.33	8.20E-06	6.55E-05
6141	7	F2-3 vs. S2-3	248767_at	AT5G47710	Calcium-dependent lipid-binding (CaLB domain) family protein;(source:Araport11)	124.43	375.35	0.33	6.00E-07	1.10E-05
6142	7	F2-3 vs. S2-3	247267_at	AT5G64140	Encodes a putative ribosomal protein S28.	49.02	150.21	0.33	4.50E-06	4.27E-05
6143	7	F2-3 vs. S2-3	260660_at	AT1G19485	Transducin/WD40 repeat-like superfamily protein;(source:Araport11)	20.59	64.59	0.32	1.20E-04	4.46E-04
6144	7	F2-3 vs. S2-3	262497_at	AT1G21720	20S proteasome beta subunit PBC1 truncated protein (PBC1)	142.87	442.46	0.32	7.10E-05	3.01E-04
6145	7	F2-3 vs. S2-3	264446_at	AT1G27310	Encodes an ortholog of yeast NTF2, a nuclear envelop transport protein that functions as the nuclear import receptor for RanGDP, an essential player in nucleocytoplasmic transport.	137.64	433.52	0.32	1.20E-06	1.75E-05
6146	7	F2-3 vs. S2-3	261598_at	AT1G49750	Leucine-rich repeat (LRR) family protein;(source:Araport11)	28.1	86.92	0.32	1.75E-05	1.10E-04
6147	7	F2-3 vs. S2-3	266001_at	AT2G24150	heptahelical transmembrane protein HHP3	38.03	120.64	0.32	8.40E-06	6.65E-05
6148	7	F2-3 vs. S2-3	256438_s_a	AT3G08520	Ribosomal protein L41 family;(source:Araport11)	1837.53	5685.77	0.32	4.13E-05	2.08E-04
6149	7	F2-3 vs. S2-3	252601_s_a	AT3G45030	Ribosomal protein S10p/S20e family protein;(source:Araport11)	327.21	1037.19	0.32	1.40E-06	1.89E-05
6150	7	F2-3 vs. S2-3	254054_at	AT4G25320	AT hook motif DNA-binding family protein;(source:Araport11)	34.31	107.2	0.32	7.35E-04	1.78E-03
6151	7	F2-3 vs. S2-3	253340_s_at	AT4G33260	Encodes a CDC20 protein that interacts with APC subunits, components of the mitochondrial checkpoint complex and mitotic cyclin substrates and is indispensable for normal plant development and fertility.	227.93	712.72	0.32	1.90E-04	6.33E-04
6152	7	F2-3 vs. S2-3	252883_at	AT4G39520	Encodes a member of the DRG (developmentally regulated G-protein) family. Has GTPase activity.	28.68	89.42	0.32	2.41E-04	7.52E-04
6153	7	F2-3 vs. S2-3	245886_at	AT5G09510	Ribosomal protein S19 family protein;(source:Araport11)	52.43	166.11	0.32	1.00E-06	1.54E-05
6154	7	F2-3 vs. S2-3	250384_at	AT5G11500	coiled-coil protein;(source:Araport11)	188.77	589.52	0.32	4.40E-06	4.21E-05
6155	7	F2-3 vs. S2-3	250228_at	AT5G13840	FIZZY-related 3;(source:Araport11)	58.45	183.5	0.32	2.80E-05	1.54E-04
6156	7	F2-3 vs. S2-3	250015_at	AT5G18070	encodes a novel protein involved in DNA repair from UV damage. Isolated by functional complementation of E. coli UV-sensitive mutants (UVR genes).	33.11	104.37	0.32	6.72E-05	2.89E-04
6157	7	F2-3 vs. S2-3	246758_at	AT5G27850	Ribosomal protein L18e/L15 superfamily protein;(source:Araport11)	306.17	952.65	0.32	1.04E-05	7.72E-05
6158	7	F2-3 vs. S2-3	249613_at	AT5G37380	Chaperone DnaJ-domain superfamily protein;(source:Araport11)	22.03	68.81	0.32	3.70E-06	3.73E-05
6159	7	F2-3 vs. S2-3	249332_at	AT5G40980	AT.I.24-6 protein, putative (DUF 3339);(source:Araport11)	24.95	78.89	0.32	3.20E-06	3.37E-05
6160	7	F2-3 vs. S2-3	248478_at	AT5G50870	ubiquitin-conjugating enzyme 27;(source:Araport11)	193.93	604.47	0.32	2.20E-06	2.63E-05
6161	7	F2-3 vs. S2-3	247216_at	AT5G64860	Encodes a maltotriose-metabolizing enzyme with chloroplastic α-1,4-glucanotransferase activity. Mutant has altered starch degradation.	56.64	174.37	0.32	2.35E-04	7.40E-04

6162	7	F2-3 vs. S2-3	262112_at	AT1G02870	nucleolar-like protein;(source:Araport11)	16.24	52.35	0.31	6.60E-06	5.55E-05
6163	7	F2-3 vs. S2-3	265037_at	AT1G03860	prohibitin 2	41.73	136.82	0.31	3.58E-05	1.86E-04
6164	7	F2-3 vs. S2-3	261418_at	AT1G07830	ribosomal protein L29 family protein;(source:Araport11)	169.02	543.9	0.31	2.00E-06	2.46E-05
6165	7	F2-3 vs. S2-3	261150_at	AT1G19640	Encodes a S-adenosyl-L-methionine:jasmonic acid carboxyl methyltransferase that catalyzes the formation of methyljasmonate from jasmonic acid. Its expression is induced in response to wounding or methyljasmonate treatment.	205.55	653.08	0.31	1.39E-04	4.97E-04
6166	7	F2-3 vs. S2-3	262417_at	AT1G50170	encodes sirohydrochlorin ferrochelatase catalyzing the last step of the siroheme biosynthesis	62.79	204.77	0.31	1.55E-05	1.01E-04
6167	7	F2-3 vs. S2-3	259612_at	AT1G52300	Zinc-binding ribosomal protein family protein;(source:Araport11)	687.09	2196.12	0.31	1.42E-05	9.57E-05
6168	7	F2-3 vs. S2-3	259662_at	AT1G55250	Gene encodes one of two orthologous E3 ubiquitin ligases in Arabidopsis that are involved in monoubiquitination of histone H2B.rRegulates expression of disease resistance genes.	28.03	91.33	0.31	5.02E-05	2.38E-04
6169	7	F2-3 vs. S2-3	260297_at	AT1G80280	alpha/beta-Hydrolases superfamily protein;(source:Araport11)	60.93	196.05	0.31	2.90E-06	3.14E-05
6170	7	F2-3 vs. S2-3	265873_at	AT2G01630	O-Glycosyl hydrolases family 17 protein;(source:Araport11)	35.73	114.8	0.31	2.90E-06	3.14E-05
6171	7	F2-3 vs. S2-3	266150_s_a	AT2G12290	hypothetical protein;(source:Araport11)	27.16	88.43	0.31	2.06E-05	1.24E-04
6172	7	F2-3 vs. S2-3	264056_at	AT2G28510	DOF transcription factor with a conserved zinc finger (ZF) DNA-binding domain.	19.83	64.3	0.31	5.30E-06	4.80E-05
6173	7	F2-3 vs. S2-3	267641_at	AT2G32940	Encodes a nuclear localized 879-amino-acid protein that contains conserved PAZ and PIWI domains that is important for the accumulation of specific heterochromatin-related siRNAs, and for DNA methylation and transcriptional gene silencing.	49.81	163.3	0.31	4.10E-06	4.02E-05
6174	7	F2-3 vs. S2-3	263868_at	AT2G36840	Encodes a ACT domain-containing protein. The ACT domain, named after bacterial aspartate kinase, chorismate mutase and TyrA (prephenate dehydrogenase), is a regulatory domain that serves as an amino acid-binding site in feedback-regulated amino acid metabolic enzymes.	13.38	42.66	0.31	6.03E-05	2.70E-04
6175	7	F2-3 vs. S2-3	258995_at	AT3G01790	Ribosomal protein L13 family protein;(source:Araport11)	102.22	325.93	0.31	9.60E-06	7.35E-05
6176	7	F2-3 vs. S2-3	258858_at	AT3G02080	Ribosomal protein S19e family protein;(source:Araport11)	202.08	658.7	0.31	1.80E-06	2.29E-05
6177	7	F2-3 vs. S2-3	258480_at	AT3G02640	transmembrane protein;(source:Araport11)	66.3	216.89	0.31	3.10E-04	9.14E-04
6178	7	F2-3 vs. S2-3	258846_at	AT3G03070	NADH-ubiquinone oxidoreductase-like protein;(source:Araport11)	14.21	46.55	0.31	6.30E-06	5.36E-05
6179	7	F2-3 vs. S2-3	258709_at	AT3G09500	Ribosomal L29 family protein;(source:Araport11)	270.18	867.28	0.31	1.13E-04	4.28E-04
6180	7	F2-3 vs. S2-3	258728_at	AT3G11800	Expp1 protein;(source:Araport11)	71.81	230.52	0.31	4.00E-07	8.26E-06
6181	7	F2-3 vs. S2-3	258166_at	AT3G21540	transducin family protein / WD-40 repeat family protein;(source:Araport11)	18.35	58.85	0.31	1.88E-05	1.16E-04
6182	7	F2-3 vs. S2-3	258067_at	AT3G25980	Encodes MAD2 (MITOTIC ARREST-DEFICIENT 2). May have the spindle assembly checkpoint protein functions conserved from yeast to humans.	25.48	81.76	0.31	6.60E-06	5.55E-05
6183	7	F2-3 vs. S2-3	252524_at	AT3G46430	Mitochondrial F1F0-ATP synthase.	200.08	642.92	0.31	3.30E-06	3.44E-05
6184	7	F2-3 vs. S2-3	245372_at	AT4G15000	Ribosomal L27e protein family;(source:Araport11)	499.86	1610.82	0.31	5.00E-07	9.67E-06
6185	7	F2-3 vs. S2-3	254363_at	AT4G22010	SKU5 similar 4;(source:Araport11)	121.74	394.7	0.31	1.60E-06	2.10E-05
6186	7	F2-3 vs. S2-3	250862_s_a	AT5G04800	Ribosomal S17 family protein;(source:Araport11)	645.55	2058.99	0.31	9.00E-07	1.44E-05
6187	7	F2-3 vs. S2-3	250752_at	AT5G05690	Encodes a member of the CP90A family, a cytochrome P450 monooxygenase which converts 6-deoxocathasterone to 6-deoxoteasterone in the late C6 oxidation pathway and cathasterone to teasterone in the early C6 oxidation pathway of brassinolide biosynthesis. Expressed in cotyledons and leaves. Mutants display de-etiolation and derepression of light-induced genes in the dark, dwarfism, male sterility and activation of stress-regulated genes in the light. The expression of the gene using a CPD promoter:LUC fusion construct was shown to be under circadian and light control. Additionally, the circadian regulation was shown to be independent of BR levels as it remains unchanged in <i>bri1</i> mutant lines. CPD appears to be involved in the autonomous pathway that regulates the transition to flowering, primarily through a BRI1-mediated signaling pathway that affects FLC expression levels, as uncovered by double mutant analyses.	108	343.06	0.31	2.03E-04	6.70E-04
6188	7	F2-3 vs. S2-3	250150_at	AT5G14710	proteasome assembly chaperone-like protein;(source:Araport11)	65.7	209.55	0.31	3.80E-06	3.79E-05
6189	7	F2-3 vs. S2-3	250080_at	AT5G16620	chloroplast protein import (Tic40)	185.65	606.04	0.31	2.53E-05	1.44E-04
6190	7	F2-3 vs. S2-3	246885_at	AT5G26230	Encodes a member of the MAKR (MEMBRANE-ASSOCIATED KINASE REGULATOR) gene family. MAKRs have putative kinase interacting motifs and membrane localization signals. Known members include: AT5G26230 (MAKR1), AT1G64080 (MAKR2), AT2G37380 (MAKR3), AT2G39370 (MAKR4), AT5G52870 (MAKR5) and AT5G52900 (MAKR6).	16.15	51.41	0.31	4.75E-05	2.28E-04
6191	7	F2-3 vs. S2-3	248465_at	AT5G51200	nuclear pore complex protein (DUF3414);(source:Araport11)	51.89	169.57	0.31	1.78E-04	6.02E-04

6192	7	F2-3 vs. S2-3	247213_at	AT5G64900	Encodes a putative 92-aa protein that is the precursor of AtPep1, a 23-aa peptide which activates transcription of the defensive gene defensin (PDF1.2) and activates the synthesis of H2O2, both being components of the innate immune response.	27.61	90.23	0.31	6.00E-07	1.10E-05
6193	7	F2-3 vs. S2-3	244993_s_a	ATCG01130	Ycf1 protein;(source:Araport11)	718.87	2349.95	0.31	7.70E-06	6.20E-05
6194	7	F2-3 vs. S2-3	264679_s_a	AT1G09690	Translation protein SH3-like family protein;(source:Araport11)	2238.87	7573	0.3	2.00E-06	2.46E-05
6195	7	F2-3 vs. S2-3	255767_at	AT1G16740	Ribosomal protein L20;(source:Araport11)	282.59	943.58	0.3	3.60E-06	3.67E-05
6196	7	F2-3 vs. S2-3	256112_at	AT1G16920	small GTP-binding protein (Rab11)similar to YPT3/RAB11 proteins in yeast and mammals, respectively. YPT3/RAB11 is involved in intracellular protein trafficking.	454.99	1517.04	0.3	2.00E-06	2.46E-05
6197	7	F2-3 vs. S2-3	262802_at	AT1G20930	Cyclin-dependent kinase, expressed in flowers and suspension cell culture, expression peaks during M phase in synchronized cultures. Required for proper organization of the shoot apical meristem and for hormone signaling. Expressed in the shoot apical meristem. Involved in regulation of the G2/M transition of the mitotic cell cycle.	39.45	133.66	0.3	7.08E-05	3.01E-04
6198	7	F2-3 vs. S2-3	262985_s_a t	AT1G23290	Encodes a ribosomal protein L27A, a constituent of the large subunit of the ribosomal complex. Regulated by TCP20. The mRNA is cell-to-cell mobile.	1541.87	5099.37	0.3	2.00E-06	2.46E-05
6199	7	F2-3 vs. S2-3	266118_at	AT2G02130	Predicted to encode a PR (pathogenesis-related) protein. Belongs to the plant defensin (PDF) family with the following members: At1g75830/PDF1.1, At5g44420/PDF1.2a, At2g26020/PDF1.2b, At5g44430/PDF1.2c, At2g26010/PDF1.3, At1g19610/PDF1.4, At1g55010/PDF1.5, At2g02120/PDF2.1, At2g02100/PDF2.2, At2g02130/PDF2.3, At1g61070/PDF2.4, At5g63660/PDF2.5, At2g02140/PDF2.6, At5g38330/PDF3.1 and At4g30070/PDF3.2. The mRNA is cell-to-cell mobile.	50.18	166.58	0.3	1.90E-06	2.37E-05
6200	7	F2-3 vs. S2-3	255789_at	AT2G33370	Ribosomal protein L14p/L23e family protein;(source:Araport11)	321.93	1067.58	0.3	1.40E-06	1.89E-05
6201	7	F2-3 vs. S2-3	258402_at	AT3G15450	aluminum induced protein with YGL and LRDR motifs;(source:Araport11)	14.54	48.62	0.3	6.13E-05	2.72E-04
6202	7	F2-3 vs. S2-3	258225_at	AT3G15630	plant/protein;(source:Araport11)	13.44	45.03	0.3	1.17E-05	8.37E-05
6203	7	F2-3 vs. S2-3	255642_at	AT4G00820	Member of IQ67 (CaM binding) domain containing family.	12.19	41.03	0.3	5.00E-06	4.58E-05
6204	7	F2-3 vs. S2-3	255569_at	AT4G01320	CAAX protease with broad substrate specificity. Localized exclusively to the endoplasmic reticulum.	270	898.8	0.3	8.00E-07	1.32E-05
6205	7	F2-3 vs. S2-3	254083_at	AT4G24920	secE/sec61-gamma protein transport protein;(source:Araport11)	236.68	783.15	0.3	7.00E-07	1.20E-05
6206	7	F2-3 vs. S2-3	253627_at	AT4G30650	Low temperature and salt responsive protein family;(source:Araport11)	64.05	216.57	0.3	2.90E-06	3.14E-05
6207	7	F2-3 vs. S2-3	253049_at	AT4G37300	maternal effect embryo arrest 59;(source:Araport11)	19.55	64.79	0.3	1.72E-05	1.09E-04
6208	7	F2-3 vs. S2-3	246526_at	AT5G15720	Contains lipase signature motif and GDSL domain.	45.61	151.65	0.3	2.44E-05	1.40E-04
6209	7	F2-3 vs. S2-3	250105_at	AT5G16630	DNA repair protein Rad4 family;(source:Araport11)	74	243.81	0.3	3.10E-06	3.28E-05
6210	7	F2-3 vs. S2-3	249829_at	AT5G23290	prefoldin 5;(source:Araport11)	96.85	326.43	0.3	1.90E-06	2.37E-05
6211	7	F2-3 vs. S2-3	254617_s_a	AT5G45775	Ribosomal L5P family protein;(source:Araport11)	764.05	2553.43	0.3	6.62E-05	2.87E-04
6212	7	F2-3 vs. S2-3	248647_at	AT5G49190	Encodes a sucrose synthase (SUS2). The activity of the enzyme could not be assayed as proved to be insoluble (PMID 17257168). However, analyses of an sus2 mutant revealed a deficiency in sucrose synthase activity 12 and 15 days after flowering. There are some reports that SUS2 transcript levels are increased in leaves specifically by O(2) deficiency whereas other reports indicate that SUS2 is expressed only in seeds. Immulocalization shows that SUS2 is present in the cytosol of developing seeds, but, it also associated with plastids, though not located within them.	28.85	95.78	0.3	2.80E-05	1.54E-04
6213	7	F2-3 vs. S2-3	248551_at	AT5G50200	Wound-responsive gene 3 (WR3). Encodes a high-affinity nitrate transporter. Up-regulated by nitrate. Involved in jasmonic acid-independent wound signal transduction.	16.27	54.89	0.3	8.19E-04	1.94E-03
6214	7	F2-3 vs. S2-3	245017_at	ATCG00510	Encodes subunit I of photosystem I.	396.76	1340.74	0.3	3.68E-04	1.05E-03
6215	7	F2-3 vs. S2-3	261001_at	AT1G26530	PIN domain-like family protein;(source:Araport11)	17.31	60.03	0.29	1.26E-04	4.63E-04
6216	7	F2-3 vs. S2-3	265386_at	AT2G20930	Part of multi-protein complex, acting as guanine nucleotide exchange factors (GEFs) and possibly as tethers, regulating intracellular trafficking.	28.93	100.83	0.29	1.70E-06	2.21E-05
6217	7	F2-3 vs. S2-3	251997_at	AT3G53020	RPL24B encodes ribosomal protein L24, homolog of cytosolic RPL24, found in archaea and higher eukaryotes. Arabidopsis has two RPL24 homologs, RPL24A (AT2G36620) and RPL24B. Mutants showed defects in apical-basal gynoecium patterning similar to previously described ett and mp mutants. Transformation of stv1-1 mutant with a uORF-eliminated ETT construct partially suppressed the stv1 gynoecium phenotype, implying that STV1 could influence ETT translation through its uORFs. Regulated by TCP20.	594.43	2018.22	0.29	1.20E-06	1.75E-05

6218	7	F2-3 vs. S2-3	251735_at	AT3G56090	Encodes FERRITIN 3, AtFER3. Ferritins are a class of 24-mer multi-meric proteins found in all kingdoms of life. Function as the main iron store in mammals. Evidence suggests that Arabidopsis ferritins are essential to protect cells against oxidative damage, but they do not constitute the major iron pool.	80.8	276.12	0.29	9.70E-06	7.40E-05
6219	7	F2-3 vs. S2-3	251737_at	AT3G56340	Small ribosomal subunit protein.	479.41	1679.67	0.29	5.60E-06	4.97E-05
6220	7	F2-3 vs. S2-3	251662_at	AT3G57080	Non-catalytic subunit unique to Nuclear DNA-dependent RNA polymerase V; homologous to budding yeast RPB5.	14.03	48.61	0.29	4.00E-07	8.26E-06
6221	7	F2-3 vs. S2-3	245362_at	AT4G17460	Encodes a class II HD-ZIP protein that regulates meristematic activity in different tissues, and that it is necessary for the correct formation of the gynoecium.	66.44	227.57	0.29	4.90E-06	4.55E-05
6222	7	F2-3 vs. S2-3	253225_at	AT4G35020	A member of ROP GTPase gene family; Encodes a Rho-like GTP binding protein.	90.24	313.15	0.29	1.30E-06	1.81E-05
6223	7	F2-3 vs. S2-3	249894_at	AT5G22580	Stress responsive A/B Barrel Domain-containing protein;(source:Araport11)	806.24	2792.81	0.29	5.25E-04	1.38E-03
6224	7	F2-3 vs. S2-3	264327_at	AT1G04170	protein synthesis initiation factor eIF2 gamma The mRNA is cell-to-cell mobile.	14.33	51.84	0.28	2.78E-05	1.53E-04
6225	7	F2-3 vs. S2-3	261254_at	AT1G05805	basic helix-loop-helix (bHLH) DNA-binding superfamily protein;(source:Araport11)	14.25	51.77	0.28	1.98E-05	1.20E-04
6226	7	F2-3 vs. S2-3	261738_s_a	AT1G47813	hypothetical protein;(source:Araport11)	57.67	202.83	0.28	3.00E-07	6.68E-06
6227	7	F2-3 vs. S2-3	263488_at	AT2G31840	Thioredoxin superfamily protein;(source:Araport11)	30.03	107.67	0.28	5.00E-06	4.58E-05
6228	7	F2-3 vs. S2-3	265960_at	AT2G37470	Histone superfamily protein;(source:Araport11)	18.56	67.29	0.28	1.23E-05	8.61E-05
6229	7	F2-3 vs. S2-3	255819_s_a	AT2G40510	Ribosomal protein S26e family protein;(source:Araport11)	14.73	51.86	0.28	5.40E-06	4.85E-05
6230	7	F2-3 vs. S2-3	259055_at	AT3G03340	LUC7 related protein;(source:Araport11)	170.9	609.82	0.28	1.40E-06	1.89E-05
6231	7	F2-3 vs. S2-3	252561_at	AT3G45980	Encodes a histone 2B (H2B) protein. This protein can be ubiquitinated in planta, and this modification depends on the HUB1 and HUB2 E3 ubiquitin ligases as well as the UBC1 and UBC2 E2 ubiquitin conjugating enzymes. Lysine 146 appears to be the site of the ubiquitin addition.	29.74	106.19	0.28	1.22E-05	8.58E-05
6232	7	F2-3 vs. S2-3	254464_at	AT4G20400	Encodes a histone H3K4 demethylase repressing floral transition.Coordinates and structure features have been deposited in the RCSB Protein Data Bank with the accession codes 5YKN (JMJ14CD) and 5YKO(JMJ14CD-NOG-H3K4em3 complex). Glu285 and Glu516 contribute to the recognition of H3R2. Asp312 contributes to the recognition of H3Q5. Phe171 and Val363 contribute to the recognition of H3A7. Ser290, Trp296, and Tyr298 contribute to the recognition of H3K4me3 (PMID:29233856).	27.84	99.37	0.28	1.90E-06	2.37E-05
6233	7	F2-3 vs. S2-3	253051_at	AT4G37490	Cyclin-dependent protein kinase CYCB1;1. Functions as an effector of growth control at G2/M. Regulated by TCP20.	34.2	121.06	0.28	2.70E-06	3.02E-05
6234	7	F2-3 vs. S2-3	250510_at	AT5G09920	Non-catalytic subunit specific to DNA-dependent RNA polymerase II; the ortholog of budding yeast RPB4)	79.22	285.71	0.28	1.08E-05	7.97E-05
6235	7	F2-3 vs. S2-3	250434_at	AT5G10390	Histone superfamily protein;(source:Araport11)	57.98	208.59	0.28	1.48E-05	9.91E-05
6236	7	F2-3 vs. S2-3	246140_at	AT5G19910	SOH1 family protein;(source:Araport11)	19.33	69.03	0.28	8.30E-06	6.60E-05
6237	7	F2-3 vs. S2-3	249313_at	AT5G41560	Encodes a substrate receptor for CRL4-CDD complexes that provides substrate specificity for CRL4 by interacting with ubiquitination targets. By its interaction and regulation of levels of PYL8 through proteasomal degradation, it negatively regulates ABA-mediated developmental responses, including inhibition of seed germination, seedling establishment, and root growth	13.63	49.33	0.28	1.30E-06	1.81E-05
6238	7	F2-3 vs. S2-3	249093_at	AT5G43880	methyl-coenzyme M reductase II subunit gamma, putative (DUF3741);(source:Araport11)	18.66	65.8	0.28	4.27E-05	2.12E-04
6239	7	F2-3 vs. S2-3	260946_at	AT1G06010	basic leucine zipper/W2 domain protein;(source:Araport11)	17.38	64.86	0.27	1.00E-06	1.54E-05
6240	7	F2-3 vs. S2-3	262000_at	AT1G33810	zinc finger/BTB domain protein;(source:Araport11)	54.59	205.75	0.27	5.00E-07	9.67E-06
6241	7	F2-3 vs. S2-3	261864_s_at	AT1G50480	10-formyltetrahydrofolate synthetase (THFS) mRNA, complete The mRNA is cell-to-cell mobile.	373.22	1361.47	0.27	2.25E-05	1.32E-04
6242	7	F2-3 vs. S2-3	262367_at	AT1G73030	Encodes an ESCRT-related protein: CHMP1A/AT1G73030; CHMP1B/AT1G17730. CHMP1A and B mediate multivesicular body sorting of auxin carriers and are required for plant development. ESCRT: Endosomal Sorting Complexes Required For Transport machinery; CHMP: Charged Multivesicular Body Protein/Chromatin Modifying Protein.	86.95	322.14	0.27	1.61E-05	1.04E-04
6243	7	F2-3 vs. S2-3	267174_at	AT2G37600	Ribosomal protein L36e family protein;(source:Araport11)	67.3	249.65	0.27	9.24E-04	2.14E-03
6244	7	F2-3 vs. S2-3	267360_at	AT2G40060	Encodes a clathrin that is localized to the cortical division zone and the cell plate and colocalizes with TPLATE during cell plate anchoring. The mRNA is cell-to-cell mobile.	180.1	665.83	0.27	2.00E-07	4.97E-06
6245	7	F2-3 vs. S2-3	254831_at	AT4G12600	Ribosomal protein L7Ae/L30e/S12e/Gadd45 family protein;(source:Araport11)	46.95	175.17	0.27	2.47E-05	1.42E-04

6246	7	F2-3 vs. S2-3	246090_at	AT5G20520	Encodes a Bem46-like protein. WAV2 negatively regulates root bending when roots alter their growth direction. It's not involved in sensing environmental stimuli (e.g. gravity, light, water, touch).	71.37	263.31	0.27	5.70E-06	5.00E-05
6247	7	F2-3 vs. S2-3	249492_at	AT5G39160	RmlC-like cupins superfamily protein;(source:Araport11)	9.17	34.02	0.27	1.20E-06	1.75E-05
6248	7	F2-3 vs. S2-3	249060_at	AT5G44560	SNF7 family protein;(source:Araport11)	45.27	166.2	0.27	8.00E-06	6.40E-05
6249	7	F2-3 vs. S2-3	244987_s_at	ATCG00830	encodes a chloroplast ribosomal protein L2, a constituent of the large subunit of the ribosomal complex	1697.38	6288.43	0.27	6.40E-06	5.42E-05
6250	7	F2-3 vs. S2-3	255902_at	AT1G17880	basic transcription factor 3;(source:Araport11)	145.98	560.55	0.26	4.90E-06	4.55E-05
6251	7	F2-3 vs. S2-3	261377_at	AT1G18850	PCP2 encodes a novel plant specific protein that is co-expressed with components of pre-rRNA processing complex. Co-localizes with NuGWD1 and SWA1.	16.95	66.33	0.26	3.05E-04	9.05E-04
6252	7	F2-3 vs. S2-3	245668_at	AT1G28330	dormancy-associated protein (DRM1)	20.7	80.29	0.26	1.00E-06	1.54E-05
6253	7	F2-3 vs. S2-3	262203_at	AT2G01060	myb-like HTH transcriptional regulator family protein;(source:Araport11)	36.72	139.64	0.26	1.54E-05	1.01E-04
6254	7	F2-3 vs. S2-3	265238_s_at	AT2G07696	Ribosomal protein S7p/S5e family protein;(source:Araport11)	17.95	68.89	0.26	2.89E-05	1.58E-04
6255	7	F2-3 vs. S2-3	266656_at	AT2G25900	Encodes a protein with two tandem-arrayed CCCH-type zinc fingers that binds RNA and is involved in RNA turnover. The mRNA is cell-to-cell mobile.	69.56	269.86	0.26	6.40E-06	5.42E-05
6256	7	F2-3 vs. S2-3	267619_at	AT2G26730	Leucine-rich repeat protein kinase family protein;(source:Araport11)	186.18	714.09	0.26	4.00E-07	8.26E-06
6257	7	F2-3 vs. S2-3	267636_at	AT2G42110	hypothetical protein;(source:Araport11)	12.86	48.91	0.26	6.75E-05	2.90E-04
6258	7	F2-3 vs. S2-3	259175_at	AT3G01560	proline-rich receptor-like kinase, putative (DUF1421);(source:Araport11)	17.43	65.9	0.26	3.48E-04	1.00E-03
6259	7	F2-3 vs. S2-3	259225_at	AT3G07590	SmD1a is one of two Yeast SmD1 orthologs, lower levels than SmD1b.It is localized to the nucleus and may play a minor role in RNA splicing and indirectly facilitating PTGS.	37.25	143.2	0.26	4.00E-07	8.26E-06
6260	7	F2-3 vs. S2-3	251783_at	AT3G55280	60S ribosomal protein L23A (RPL23aB). Paralog of RPL23aA.	178.02	680.48	0.26	1.22E-05	8.58E-05
6261	7	F2-3 vs. S2-3	255458_at	AT4G02840	SmD1b is one of two Yeast SmD1 orthologs, the other being SmD1a.SmD1b accumulates to higher levels than SmD1a.It is localized to the nucleolus and nuclear speckles and appears to have a role in RNA splicing and indirectly facilitating PTGS.	20.2	79.17	0.26	3.08E-05	1.66E-04
6262	7	F2-3 vs. S2-3	254964_at	AT4G11080	Encodes a protein containing three copies of the HMG (high mobility group)-box domain. The two Arabidopsis 3xHMG-box proteins are: AT4G11080 (3xHMG-box1) and AT4G23800 (3xHMG-box2). Interacts with mitotic and meiotic chromosomes.	10.54	40.15	0.26	1.75E-04	5.95E-04
6263	7	F2-3 vs. S2-3	253684_at	AT4G29690	Alkaline-phosphatase-like family protein;(source:Araport11)	23.92	91.18	0.26	7.00E-07	1.20E-05
6264	7	F2-3 vs. S2-3	253597_at	AT4G30690	SVR9-LIKE1 (SVR9L1)	48.09	183.26	0.26	8.00E-07	1.32E-05
6265	7	F2-3 vs. S2-3	250683_x_at	AT5G06640	Proline-rich extensin-like family protein;(source:Araport11)	17.54	66.64	0.26	1.62E-05	1.04E-04
6266	7	F2-3 vs. S2-3	250565_at	AT5G08000	Encodes a member of the X8-GPI family of proteins. It localizes to the plasmodesmata and binds callose.	151.24	586.64	0.26	2.00E-07	4.97E-06
6267	7	F2-3 vs. S2-3	246114_at	AT5G20250	encodes a member of glycosyl hydrolase family 36. Expression is induced within 3 hours of dark treatment, in senescing leaves and treatment with exogenous photosynthesis inhibitor. Induction of gene expression was suppressed in excised leaves supplied with sugar. The authors suggest that the gene's expression pattern is responding to the level of sugar in the cell. The mRNA is cell-to-cell mobile.	67.95	259.59	0.26	3.41E-05	1.79E-04
6268	7	F2-3 vs. S2-3	249235_at	AT5G42100	encodes a plasmodesmal (Pd)-associated membrane protein involved in plasmodesmal callose degradation, i.e. beta-1,3-glucanase (EC 3.2.1.39), and functions in the gating of Pd	105.74	403.46	0.26	3.02E-05	1.63E-04
6269	7	F2-3 vs. S2-3	248186_at	AT5G53880	hypothetical protein;(source:Araport11)	13.06	50.95	0.26	3.80E-05	1.94E-04
6270	7	F2-3 vs. S2-3	247563_at	AT5G61130	Encodes a plasmodesmatal localized protein with callose binding activity. The function of PDCB1 is unknown but overexpression leads to the hyperaccumulation of callose.	55.14	210.64	0.26	3.98E-05	2.01E-04
6271	7	F2-3 vs. S2-3	265085_at	AT1G03780	Homolog of vertebrate TPX2. Protein has three domains involved in nuclear targeting, one in nuclear export and two in microtubule binding. Involved in mitotic spindle assembly during late prophase and early prometaphase.	17.36	69.07	0.25	1.88E-04	6.31E-04
6272	7	F2-3 vs. S2-3	262504_at	AT1G21750	Encodes a protein disulfide isomerase-like (PDIL) protein, a member of a multigene family within the thioredoxin (TRX) superfamily; isoform contains non-consensus GA donor splice site at intron 9. Transcript levels for this gene are up-regulated in response to three different chemical inducers of ER stress (dithiothreitol, beta-mercaptoethanol, and tunicamycin). Neither AtIRE1-2 nor AtbZIP60 appear to be required for this response. The mRNA is cell-to-cell mobile.	43.89	178.57	0.25	2.10E-06	2.53E-05
6273	7	F2-3 vs. S2-3	263691_at	AT1G26880	Ribosomal protein L34e superfamily protein;(source:Araport11)	294.63	1160.03	0.25	8.80E-06	6.88E-05
6274	7	F2-3 vs. S2-3	255985_at	AT1G34150	Pseudouridine synthase family protein;(source:Araport11)	35.39	144.41	0.25	6.10E-06	5.23E-05
6275	7	F2-3 vs. S2-3	263585_at	AT2G25210	Ribosomal protein L39 family protein;(source:Araport11)	1050.52	4205.35	0.25	4.50E-06	4.27E-05

6276	7	F2-3 vs. S2-3	263785_at	AT2G46390	predicted to encode subunit 8 of mitochondrial complex II and to participate in the respiratory chain	79.81	315.51	0.25	3.90E-06	3.86E-05
6277	7	F2-3 vs. S2-3	258471_at	AT3G06030	NPK1-related protein kinase 3	12.9	50.73	0.25	8.00E-07	1.32E-05
6278	7	F2-3 vs. S2-3	251592_at	AT3G57670	Encodes a a C2H2/C2HC zinc finger transcription factor specifically expressed in the transmitting tract and involved in transmitting tract development and pollen tube growth.Acts redundantly with WIP4 and WIP5 to determine distal cell fate in the root. MP binds to regulatory elements within the NTT locus and likely regulates its expression.	13.36	53.27	0.25	3.06E-04	9.08E-04
6279	7	F2-3 vs. S2-3	253382_at	AT4G33040	Encodes a member of the CC-type glutaredoxin (ROXY) family that has been shown to interact with the transcription factor TGA2.	159.34	625.42	0.25	2.00E-07	4.97E-06
6280	7	F2-3 vs. S2-3	248103_at	AT5G55160	Encodes a small ubiquitin-like modifier (SUMO) polypeptide that becomes covalently attached to various intracellular protein targets, much like ubiquitination, leading to post-translational modification of those targets. SUMO2 can form SUMO chains through lysine residue 10 during in vitro assays.	364.86	1457.71	0.25	4.00E-07	8.26E-06
6281	7	F2-3 vs. S2-3	261259_at	AT1G26660	Prefoldin chaperone subunit family protein;(source:Araport11)	49.46	203.78	0.24	4.00E-07	8.26E-06
6282	7	F2-3 vs. S2-3	259697_at	AT1G68920	basic helix-loop-helix (bHLH) DNA-binding superfamily protein;(source:Araport11)	32.32	136.41	0.24	6.20E-05	2.75E-04
6283	7	F2-3 vs. S2-3	260180_at	AT1G70660	MMZ2/UEV1B encodes a protein that may play a role in DNA damage responses and error-free post-replicative DNA repair by participating in lysine-63-based polyubiquitination reactions. UEV1A can form diubiquitin and triubiquitin chains in combination with UBC13A/UBC35 in vitro. It can also functionally complement an mms2 mutation in budding yeast, both by increasing mms2 mutant viability in the presence of the DNA damaging agent MMS, and by reducing the rate of spontaneous DNA mutation. However, a combination of MMZ2/UEV1B and UBC13A do not do a good job of rescuing an mms2 ubc13 double mutant in yeast. MMZ2/UEV1B transcripts are found in most plant organs, but not in the pollen or in seedlings 6 hours or 2 days post-germination. The transcript levels do not appear to be stress-inducible. The mRNA is cell-to-cell mobile.	50.51	212.57	0.24	6.89E-05	2.95E-04
6284	7	F2-3 vs. S2-3	263631_at	AT2G04900	hypothetical protein;(source:Araport11)	44.26	182.73	0.24	3.00E-07	6.68E-06
6285	7	F2-3 vs. S2-3	266044_s_a	AT2G07725	Ribosomal L5P family protein;(source:Araport11)	15.94	65.68	0.24	2.00E-07	4.97E-06
6286	7	F2-3 vs. S2-3	263420_at	AT2G17240	Participates in the late stages of the biogenesis of 50S ribosomal subunits in plastids.	86.75	363.8	0.24	4.00E-07	8.26E-06
6287	7	F2-3 vs. S2-3	263371_at	AT2G20490	nucleolar RNA-binding Nop10p family protein;(source:Araport11)	202.58	852.71	0.24	9.14E-05	3.66E-04
6288	7	F2-3 vs. S2-3	266981_at	AT2G39460	Encodes a 60S ribosomal protein L23aA (AtrpL23aA). Paralog of RLPL23aB.	151.07	623.55	0.24	2.90E-06	3.14E-05
6289	7	F2-3 vs. S2-3	251555_at	AT3G58780	One of two genes (SHP1 and SHP2) that are required for fruit dehiscence. The two genes control dehiscence zone differentiation and promote the lignification of adjacent cells.	99.84	416.46	0.24	3.70E-06	3.73E-05
6290	7	F2-3 vs. S2-3	251365_at	AT3G61310	AT hook motif DNA-binding family protein;(source:Araport11)	84.17	352.15	0.24	7.00E-07	1.20E-05
6291	7	F2-3 vs. S2-3	254694_at	AT4G17900	PLATZ transcription factor family protein;(source:Araport11)	60.52	252.31	0.24	1.30E-06	1.81E-05
6292	7	F2-3 vs. S2-3	254262_at	AT4G23470	PLAC8 family protein;(source:Araport11)	17.71	73.57	0.24	1.00E-07	2.96E-06
6293	7	F2-3 vs. S2-3	252957_at	AT4G38680	Encodes a glycine-rich protein that binds nucleic acids and promotes DNA melting. Its transcript and protein levels are up-regulated in response to cold treatment with protein levels peaking earlier in shoots (~10-14 days) than in roots (~21 days). It is normally expressed in meristematic regions and developing tissues where cell division occurs. RNAi and antisense lines with lower levels of CSP2/GRP2 transcripts flower earlier than wild type plants and have some defects in anther and seed development.	87.79	362.07	0.24	1.16E-05	8.35E-05
6294	7	F2-3 vs. S2-3	248658_at	AT5G48600	member of SMC subfamily	64.81	273.19	0.24	1.00E-07	2.96E-06
6295	7	F2-3 vs. S2-3	259538_at	AT1G12310	Calcium-binding EF-hand family protein;(source:Araport11)	40.38	174.78	0.23	4.00E-07	8.26E-06
6296	7	F2-3 vs. S2-3	266074_at	AT2G18740	Putative temperature-specific splice regulator of development. Only the first splice form (PCP-alpha) has this function as result of C-terminal addition.	51.14	219.48	0.23	6.00E-07	1.10E-05
6297	7	F2-3 vs. S2-3	259331_at	AT3G03840	SAUR-like auxin-responsive protein family;(source:Araport11)	10.11	44.62	0.23	2.90E-06	3.14E-05
6298	7	F2-3 vs. S2-3	258090_at	AT3G14600	Ribosomal protein L18ae/LX family protein;(source:Araport11)	105.39	458.32	0.23	3.00E-07	6.68E-06
6299	7	F2-3 vs. S2-3	256794_at	AT3G22230	Ribosomal L27e protein family;(source:Araport11)	20.94	90.53	0.23	1.70E-05	1.08E-04
6300	7	F2-3 vs. S2-3	251926_at	AT3G53740	Ribosomal protein L36e family protein;(source:Araport11)	281.96	1243.06	0.23	4.40E-06	4.21E-05
6301	7	F2-3 vs. S2-3	255035_at	AT4G09550	Encodes a gamma-tubulin complex protein that plays a role in gamma-tubulin complex localization, spindle stability and chromosomal segregation.	43.46	186.96	0.23	2.00E-07	4.97E-06
6302	7	F2-3 vs. S2-3	250007_at	AT5G18670	putative beta-amylase BMY3 (BMY3)	805.84	3474.2	0.23	1.01E-05	7.57E-05

6303	7	F2-3 vs. S2-3	246028_at	AT5G21170	Encodes AKINbeta1, a subunit of the SnRK1 kinase (Sucrose non-fermenting-1-related protein kinase). Involved in regulation of nitrogen and sugar metabolism. As part of the regulatory subunit, it binds maltose which promotes kinase activity. Acts as a global regulator of genes involved in carbon, lipid and nitrogen metabolism.	43.13	189.08	0.23	2.80E-06	3.09E-05
6304	7	F2-3 vs. S2-3	265117_at	AT1G62500	Bifunctional inhibitor/lipid-transfer protein/seed storage 2S albumin superfamily protein;(source:Araport11)	112.89	502.23	0.22	1.53E-05	1.00E-04
6305	7	F2-3 vs. S2-3	257474_at	AT1G80850	DNA glycosylase superfamily protein;(source:Araport11)	42.14	191.72	0.22	2.24E-04	7.16E-04
6306	7	F2-3 vs. S2-3	266579_at	AT2G23930	Putative small nuclear ribonucleoprotein G;(source:Araport11)	179.82	813.38	0.22	1.20E-06	1.75E-05
6307	7	F2-3 vs. S2-3	263286_at	AT2G36160	Ribosomal protein S11 family protein;(source:Araport11)	39.83	184.86	0.22	4.00E-07	8.26E-06
6308	7	F2-3 vs. S2-3	263914_at	AT2G36400	Growth regulating factor encoding transcription activator. One of the nine members of a GRF gene family, containing nuclear targeting domain. Mutants result in smaller leaves indicating the role of the gene in leaf development. Expressed in root, shoot and flower.	19.39	87.43	0.22	9.00E-07	1.44E-05
6309	7	F2-3 vs. S2-3	263988_at	AT2G42830	AGAMOUS [AG]-like MADS box protein (AGL5) involved in fruit development (valve margin and dehiscence zone differentiation). A putative direct target of AG. SHP2 has been shown to be a downstream gene of the complex formed by AG and SEP proteins (SEP4 alone does not form a functional complex with AG).	119.47	549.38	0.22	2.00E-07	4.97E-06
6310	7	F2-3 vs. S2-3	259171_at	AT3G03590	SWIB/MDM2 domain superfamily protein;(source:Araport11)	69.67	318.82	0.22	2.10E-06	2.53E-05
6311	7	F2-3 vs. S2-3	252357_at	AT3G48410	alpha/beta-Hydrolases superfamily protein;(source:Araport11)	24.99	113.97	0.22	2.00E-07	4.97E-06
6312	7	F2-3 vs. S2-3	251446_at	AT3G59840	allyl alcohol dehydrogenase-like protein;(source:Araport11)	32.14	143.9	0.22	8.00E-07	1.32E-05
6313	7	F2-3 vs. S2-3	255513_at	AT4G02060	Member of the minichromosome maintenance complex, involved in DNA replication initiation. Abundant in proliferating and endocycling tissues. Localized in the nucleus during G1, S and G2 phases of the cell cycle, and are released into the cytoplasmic compartment during mitosis. Binds chromatin.	11.2	50.69	0.22	1.62E-04	5.63E-04
6314	7	F2-3 vs. S2-3	255433_at	AT4G03210	encodes a member of xyloglucan endotransglucosylase/hydrolases (XTHs) that catalyze the cleavage and molecular grafting of xyloglucan chains function in loosening and rearrangement of the cell wall. Gene is expressed in shoot apex region, flower buds, flower stalks and internodes bearing flowers.	48.47	216.44	0.22	2.30E-06	2.69E-05
6315	7	F2-3 vs. S2-3	254012_at	AT4G26230	Ribosomal protein L31e family protein;(source:Araport11)	346.66	1598.95	0.22	1.00E-07	2.96E-06
6316	7	F2-3 vs. S2-3	250682_x_a	AT5G06630	proline-rich extensin-like family protein;(source:Araport11)	13.95	64.2	0.22	1.90E-06	2.37E-05
6317	7	F2-3 vs. S2-3	244912_at	ATMG00830	cytochrome c biogenesis orf382	350.13	1573.85	0.22	2.40E-06	2.77E-05
6318	7	F2-3 vs. S2-3	261886_s_a	AT1G80700	stress response NST1-like protein;(source:Araport11)	54.25	256.65	0.21	6.00E-07	1.10E-05
6319	7	F2-3 vs. S2-3	256916_at	AT3G24050	Encodes a member of the GATA factor family of zinc finger transcription factors.	13.24	62.94	0.21	9.20E-06	7.13E-05
6320	7	F2-3 vs. S2-3	252235_at	AT3G49910	Translation protein SH3-like family protein;(source:Araport11)	830.52	4021.99	0.21	9.00E-07	1.44E-05
6321	7	F2-3 vs. S2-3	251778_at	AT3G55660	Encodes a member of KPP-like gene family, homolog of KPP (kinase partner protein) gene in tomato. Also a member of the RopGEF (guanine nucleotide exchange factor) family, containing the novel PRONE domain (plant-specific Rop nucleotide exchanger), which is exclusively active towards members of the Rop subfamily.	54.31	253.31	0.21	< 1e-07	2.96E-06
6322	7	F2-3 vs. S2-3	255140_x_a	AT4G08410	Proline-rich extensin-like family protein;(source:Araport11)	19.14	89.21	0.21	4.00E-07	8.26E-06
6323	7	F2-3 vs. S2-3	246212_at	AT4G36930	Encodes a transcription factor of the bHLH protein family. Mutants have abnormal, unfused carpels and reduced seed dormancy.	20.54	97.44	0.21	4.00E-07	8.26E-06
6324	7	F2-3 vs. S2-3	250102_at	AT5G16590	Leucine-rich repeat protein kinase family protein;(source:Araport11)	31.56	147.2	0.21	1.80E-06	2.29E-05
6325	7	F2-3 vs. S2-3	246422_at	AT5G17060	A member of ARF GTPase family. A thaliana has 21 members of this family, known to be essential for vesicle coating and uncoating and functions in GTP-binding. Gene encoding ADP-ribosylation factor and similar to ADP-ribosylation factor 1; ARF 1 (GP:385340) {Drosophila melanogaster), other ARFs and ARF-like proteins.	79.4	372.73	0.21	2.30E-06	2.69E-05
6326	7	F2-3 vs. S2-3	247540_at	AT5G61590	Encodes an AP2/ERF-type transcription factor that is preferentially expressed in the epidermis and induced by darkness and negatively regulates cuticular wax biosynthesis.	20.54	99.63	0.21	< 1e-07	2.96E-06
6327	7	F2-3 vs. S2-3	247355_at	AT5G63670	SPT4 homolog 2;(source:Araport11)	66.26	316.57	0.21	3.10E-06	3.28E-05
6328	7	F2-3 vs. S2-3	262594_at	AT1G15250	Zinc-binding ribosomal protein family protein;(source:Araport11)	39.07	198.11	0.2	2.60E-06	2.94E-05
6329	7	F2-3 vs. S2-3	259516_at	AT1G20450	Encodes a gene induced by low temperature and dehydration. Inhibits e.coli growth while overexpressed. Belongs to the dehydrin protein family, which contains highly conserved stretches of 7-17 residues that are repetitively scattered in their sequences, the K-, S-, Y- and lysine rich segments. LTI29 and LTI30 double overexpressors confer cold tolerance. Localized to membranes and cytoplasm.	122.15	598.26	0.2	7.35E-04	1.78E-03

6330	7	F2-3 vs. S2-3	264872_at	AT1G24260	Member of the MADs box transcription factor family. SEP3 is redundant with SEP1 and 2. Flowers of SEP1/2/3 triple mutants show a conversion of petals and stamens to sepals.SEP3 forms heterotetrameric complexes with other MADS box family members and binds to the CArG box motif.	128.49	656.26	0.2	2.00E-07	4.97E-06
6331	7	F2-3 vs. S2-3	245311_at	AT4G14320	Zinc-binding ribosomal protein family protein;(source:Araport11)	55.02	271.42	0.2	7.50E-06	6.08E-05
6332	7	F2-3 vs. S2-3	253482_at	AT4G31985	Expressed protein	509.54	2564.96	0.2	1.21E-05	8.58E-05
6333	7	F2-3 vs. S2-3	248085_at	AT5G55480	Glycerophosphoryl diester phosphodiesterase-like protein involved in cell wall cellulose accumulation and pectin linking. Impacts root hair, trichome and epidermal cell development. The mRNA is cell-to-cell mobile.	29.72	150.08	0.2	4.50E-05	2.19E-04
6334	7	F2-3 vs. S2-3	261019_at	AT1G26470	chromatin modification-like protein;(source:Araport11)	25.15	130.87	0.19	2.00E-07	4.97E-06
6335	7	F2-3 vs. S2-3	265844_at	AT2G35620	Encodes a plasma membrane localized leucine-rich repeat receptor kinase that is involved in cell wall elongation. Loss of function mutations of FEI1 and FEI2 exhibit defects in root and hypocotyl cell elongation. Double mutants are defective in cell wall biosynthesis and have thick hypocotyls, and short, thick roots.Mucilage is easily detached from fei2 mutants seeds, and forms a capsule that is >50% smaller relative to wild-type.	12.13	62.6	0.19	7.00E-07	1.20E-05
6336	7	F2-3 vs. S2-3	263979_at	AT2G42840	Encodes a putative extracellular proline-rich protein is exclusively expressed in the L1 layer of vegetative, inflorescence and floral meristems and the protoderm of organ primordia.	334.26	1752.42	0.19	2.06E-05	1.24E-04
6337	7	F2-3 vs. S2-3	258552_at	AT3G07010	Pectin lyase-like superfamily protein;(source:Araport11)	73.14	389.83	0.19	1.90E-06	2.37E-05
6338	7	F2-3 vs. S2-3	258296_at	AT3G23390	Zinc-binding ribosomal protein family protein;(source:Araport11)	38.9	209.77	0.19	2.80E-06	3.09E-05
6339	7	F2-3 vs. S2-3	255441_at	AT4G02570	Encodes a cullin that is a component of SCF ubiquitin ligase complexes involved in mediating responses to auxin and jasmonic acid. Homozygous auxin-resistant mutants arrest growth soon after germination, lacking a root and hypocotyl. Heterozygotes display a variety of phenotypes consistent with impaired auxin response.	88.28	469.99	0.19	7.00E-07	1.20E-05
6340	7	F2-3 vs. S2-3	255298_at	AT4G04840	methionine sulfoxide reductase B6;(source:Araport11)	24.16	124.41	0.19	7.00E-07	1.20E-05
6341	7	F2-3 vs. S2-3	253989_at	AT4G26130	cotton fiber protein;(source:Araport11)	22.3	116.33	0.19	1.10E-06	1.64E-05
6342	7	F2-3 vs. S2-3	253728_at	AT4G29410	Ribosomal L28e protein family;(source:Araport11)	42.55	222.84	0.19	5.00E-06	4.58E-05
6343	7	F2-3 vs. S2-3	253404_at	AT4G32840	phosphofructokinase 6;(source:Araport11)	63.35	339.45	0.19	8.60E-06	6.77E-05
6344	7	F2-3 vs. S2-3	250199_at	AT5G14180	Myzus persicae-induced lipase 1;(source:Araport11)	18.4	95.8	0.19	3.13E-05	1.67E-04
6345	7	F2-3 vs. S2-3	246923_at	AT5G25100	Endomembrane protein 70 protein family;(source:Araport11)	21.88	112.73	0.19	5.80E-06	5.07E-05
6346	7	F2-3 vs. S2-3	244985_at	ATCG00800	encodes a chloroplast ribosomal protein S3, a constituent of the small subunit of the ribosomal complex	41.37	221.59	0.19	1.42E-05	9.57E-05
6347	7	F2-3 vs. S2-3	259541_at	AT1G20650	Protein kinase superfamily protein;(source:Araport11)	24.64	138.98	0.18	6.14E-05	2.73E-04
6348	7	F2-3 vs. S2-3	259553_x_a	AT1G21310	Encodes extensin 3.	14.72	81.34	0.18	5.03E-05	2.38E-04
6349	7	F2-3 vs. S2-3	260181_at	AT1G70710	endo-1,4-beta-glucanase. Involved in cell elongation.	145.64	797.75	0.18	1.30E-06	1.81E-05
6350	7	F2-3 vs. S2-3	260077_at	AT1G73620	Pathogenesis-related thaumatin superfamily protein;(source:Araport11)	81.84	458.99	0.18	2.40E-06	2.77E-05
6351	7	F2-3 vs. S2-3	266366_at	AT2G41420	proline-rich family protein;(source:Araport11)	186.59	1057.85	0.18	4.90E-06	4.55E-05
6352	7	F2-3 vs. S2-3	251340_at	AT3G60830	Encodes an actin-related protein required for normal embryogenesis, plant architecture and floral organ abscission.	64.23	350.04	0.18	8.00E-07	1.32E-05
6353	7	F2-3 vs. S2-3	254915_s_a	AT4G11310	cysteine proteinase precursor-like protein	15.82	88.99	0.18	< 1e-07	< 1e-07
6354	7	F2-3 vs. S2-3	253202_at	AT4G34555	Ribosomal protein S25 family protein;(source:Araport11)	193.25	1103.02	0.18	6.10E-06	5.23E-05
6355	7	F2-3 vs. S2-3	251007_at	AT5G02610	Ribosomal L29 family protein;(source:Araport11)	81.43	462.04	0.18	< 1e-07	< 1e-07
6356	7	F2-3 vs. S2-3	257220_at	AT3G27810	Encodes a member of the R2R3-MYB transcription factor gene family. Induced by jasmonate. Involved in jasmonate response during stamen development. MYB21 interacts with JAZ proteins, and functions redundantly with MYB24 and MYB57 to regulate stamen development.	36.66	212.6	0.17	1.19E-05	8.49E-05
6357	7	F2-3 vs. S2-3	262558_at	AT1G31335	transmembrane protein;(source:Araport11)	48.11	277.11	0.17	2.00E-06	2.46E-05
6358	7	F2-3 vs. S2-3	264061_at	AT2G27970	CDK-subunit 2;(source:Araport11)	88.67	534.84	0.17	< 1e-07	< 1e-07
6359	7	F2-3 vs. S2-3	267639_at	AT2G42200	Encodes a putative transcriptional regulator that is involved in the vegetative to reproductive phase transition. Expression is regulated by MIR156b. SPL activity nonautonomously inhibits initiation of new leaves at the shoot apical meristem.	100.14	575.57	0.17	1.00E-07	2.96E-06
6360	7	F2-3 vs. S2-3	259090_at	AT3G04920	Ribosomal protein S24e family protein;(source:Araport11)	67.39	396.14	0.17	3.00E-07	6.68E-06
6361	7	F2-3 vs. S2-3	259156_at	AT3G10380	Subunit of the Putative Arabidopsis Exocyst Complex	40.23	243.56	0.17	7.00E-07	1.20E-05
6362	7	F2-3 vs. S2-3	253424_at	AT4G32330	WDL5 is an target of EIN3 that co-localizes with cortical microtubles. It its thought to function to stabilize microtubles during ethylene induced hypocotyl elongation.	29.46	168.6	0.17	5.00E-07	9.67E-06

6363	7	F2-3 vs. S2-3	247962_at	AT5G56580	Encodes a member of the MAP Kinase Kinase family of proteins. It can phosphorylate MPK12 in vitro and it can be dephosphorylated by MKP2 in vitro.	56.49	328.77	0.17	2.00E-07	4.97E-06
6364	7	F2-3 vs. S2-3	245640_at	AT1G25330	Encodes CESTA, a positive regulator of brassinosteroid biosynthesis.	22.84	144.65	0.16	< 1e-07	< 1e-07
6365	7	F2-3 vs. S2-3	260538_at	AT2G43460	Ribosomal L38e protein family;(source:Araport11)	548.31	3393.19	0.16	1.90E-06	2.37E-05
6366	7	F2-3 vs. S2-3	255014_at	AT4G09960	Encodes a MADS box transcription factor expressed in the carpel and ovules. Plays a maternal role in fertilization and seed development. Controls the structure and mechanical properties of the seed coat. Controls fruit size by regulating cytokinin levels and FRUITFULL.	32	206.38	0.16	1.00E-07	2.96E-06
6367	7	F2-3 vs. S2-3	248106_at	AT5G55100	SWAP (Suppressor-of-White-APricot)/surp domain-containing protein;(source:Araport11)	14.38	88.17	0.16	1.47E-04	5.21E-04
6368	7	F2-3 vs. S2-3	247468_at	AT5G62000	Encodes an auxin response factor. Mutants have many defects including enlarged rosette leaves, reduced fertility, later senescence, hypocotyl elongation defects, enlarged seeds and enlarged cotyledons. May not mediate auxin effects. Increase in seed size due to increased cell proliferation. The mRNA is cell-to-cell mobile.	193.98	1235.81	0.16	9.00E-07	1.44E-05
6369	7	F2-3 vs. S2-3	247192_at	AT5G65360	Histone superfamily protein;(source:Araport11)	13.66	84.99	0.16	< 1e-07	2.96E-06
6370	7	F2-3 vs. S2-3	262602_at	AT1G15270	Translation machinery associated TMA7;(source:Araport11)	61.45	406.35	0.15	6.00E-07	1.10E-05
6371	7	F2-3 vs. S2-3	256125_at	AT1G18250	encodes a thaumatin-like protein	46.85	318.47	0.15	2.00E-07	4.97E-06
6372	7	F2-3 vs. S2-3	252789_s_a	AT1G21930	transmembrane protein;(source:Araport11)	145.79	968.98	0.15	2.00E-07	4.97E-06
6373	7	F2-3 vs. S2-3	266229_at	AT2G28840	hypothetical protein;(source:Araport11)	30.27	205.02	0.15	1.30E-06	1.81E-05
6374	7	F2-3 vs. S2-3	266882_at	AT2G44670	senescence-associated family protein (DUF581);(source:Araport11)	52.84	355.08	0.15	3.30E-06	3.44E-05
6375	7	F2-3 vs. S2-3	250377_at	AT5G11560	catalytics;(source:Araport11)	103.79	705.25	0.15	1.10E-06	1.64E-05
6376	7	F2-3 vs. S2-3	248747_at	AT5G47930	Zinc-binding ribosomal protein family protein;(source:Araport11)	145.46	976.3	0.15	3.00E-07	6.68E-06
6377	7	F2-3 vs. S2-3	247478_at	AT5G62360	Pectin methylesterase inhibitor expressed throughout the plant.	28.45	186.62	0.15	7.00E-07	1.20E-05
6378	7	F2-3 vs. S2-3	262121_at	AT1G02800	Encodes a protein with similarity to endo-1,4-b-glucanases and is a member of Glycoside Hydrolase Family 9. CEL2 is induced by nemotodes and is expressed in syncitia induced by Heterodera schachtii.May be involved in the development and function of syncitia.	61.32	435.61	0.14	< 1e-07	< 1e-07
6379	7	F2-3 vs. S2-3	261489_at	AT1G14450	NADH dehydrogenase (ubiquinone)s;(source:Araport11)	43.88	315.96	0.14	1.00E-07	2.96E-06
6380	7	F2-3 vs. S2-3	255779_at	AT1G18650	Encodes a member of the X8-GPI family of proteins. It localizes to the plasmodesmata and is predicted to bind callose.	65.49	458.54	0.14	< 1e-07	< 1e-07
6381	7	F2-3 vs. S2-3	260369_at	AT1G69620	putative 60S ribosomal protein L34 The mRNA is cell-to-cell mobile.	383.49	2661.47	0.14	< 1e-07	2.96E-06
6382	7	F2-3 vs. S2-3	246557_at	AT5G15510	TPX2 (targeting protein for Xklp2) protein family;(source:Araport11)	27.98	199.17	0.14	9.00E-07	1.44E-05
6383	7	F2-3 vs. S2-3	244971_at	ATCG00670	Encodes the only ClpP (caseinolytic protease) encoded within the plastid genome. Contains a highly conserved catalytic triad of Ser-type proteases (Ser-His-Asp). Part of the 350 kDa chloroplast Clp complex. The name reflects nomenclature described in Adam et. al (2001).	487.62	3420.12	0.14	3.00E-07	6.68E-06
6384	7	F2-3 vs. S2-3	244933_at	ATCG01070	NADH dehydrogenase ND4L	138.7	1004.57	0.14	2.00E-07	4.97E-06
6385	7	F2-3 vs. S2-3	244982_at	ATCG00790	chloroplast gene encoding a ribosomal protein L16, which is a constituent of 50S large ribosomal subunit	434.59	3301.03	0.13	< 1e-07	2.96E-06
6386	7	F2-3 vs. S2-3	266768_s_a	AT2G47110	polyubiquitin gene The mRNA is cell-to-cell mobile.	623.56	4720.05	0.13	< 1e-07	< 1e-07
6387	7	F2-3 vs. S2-3	247900_at	AT5G57290	60S acidic ribosomal protein family;(source:Araport11)	134.51	1070.45	0.13	2.80E-06	3.09E-05
6388	7	F2-3 vs. S2-3	257334_at	ATMG01370	transmembrane protein;(source:Araport11)	31.58	235.64	0.13	1.20E-06	1.75E-05
6389	7	F2-3 vs. S2-3	260257_at	AT1G74340	Encodes a subunit of the dolichol phosphate mannase synthase (DPMS) complex that may serve as membrane anchors for the catalytic core, DPMS1, or provide catalytic assistance. It is localized in the ER and mediates isoprenyl-linked glycan biogenesis.	41.46	360.13	0.12	1.10E-06	1.64E-05
6390	7	F2-3 vs. S2-3	263064_at	AT2G18160	Encodes a b-ZIP transcription factor.	45.69	387.18	0.12	5.00E-07	9.67E-06
6391	7	F2-3 vs. S2-3	258632_s_at	AT3G07980	MAP3K epsilon protein kinase 2 is functionally redundant with MAP3Ke1. Required for pollen development but not essential.	34.79	300.78	0.12	1.16E-05	8.35E-05
6392	7	F2-3 vs. S2-3	262609_at	AT1G13930	Involved in response to salt stress. Knockout mutants are hypersensitive to salt stress. The mRNA is cell-to-cell mobile.	51.92	452.08	0.11	< 1e-07	< 1e-07
6393	7	F2-3 vs. S2-3	267239_at	AT2G02510	NADH dehydrogenase (ubiquinone)s;(source:Araport11)	109.53	984.95	0.11	1.00E-07	2.96E-06
6394	7	F2-3 vs. S2-3	265481_at	AT2G15960	Unknown protein. Expression decreased in response to proline.	49.85	454.4	0.11	< 1e-07	< 1e-07

6395	7	F2-3 vs. S2-3	252102_at	AT3G50970	Belongs to the dehydrin protein family, which contains highly conserved stretches of 7-17 residues that are repetitively scattered in their sequences, the K-, S-, Y- and lysine rich segments. LTI29 and LTI30 double overexpressors confer freeze tolerance. Located in membranes. mRNA upregulated by water deprivation and abscisic acid. The mRNA is cell-to-cell mobile.	33.91	329.24	0.1	1.72E-04	5.88E-04
6396	7	F2-3 vs. S2-3	255807_at	AT4G10270	Member of the wound-induced polypeptide (WIP) family.	14.74	143.92	0.1	1.10E-06	1.64E-05
6397	7	F2-3 vs. S2-3	246747_at	AT5G27700	Cytosolic ribosomal protein. Similar to EVR1 and redundant with EVR1. Also enhances VAR2 mutant varigation, but to a lesser extent than evr1.	193.15	1984.55	0.097	< 1e-07	< 1e-07
6398	7	F2-3 vs. S2-3	267256_s_a	AT2G23010	serine carboxypeptidase-like 9;(source:Araport11)	22.98	248.77	0.092	4.70E-06	4.42E-05
6399	7	F2-3 vs. S2-3	245138_at	AT2G45190	Encodes a member of the YABBY family of transcriptional regulators that is involved in abaxial cell type specification in leaves and fruits. YAB1 acts in a non-cell autonomous fashion within the meristem to affect phyllotactic patterning. The non-autonomous effect on the central region of the meristem is mediated through the activity if Lateral Suppressor (LAS).	41.65	455.16	0.092	< 1e-07	2.96E-06
6400	7	F2-3 vs. S2-3	260368_at	AT1G69700	Part of the AtHVA22 family. Protein expression is ABA- and stress-inducible. The mRNA is cell-to-cell mobile.	26.63	406.99	0.065	1.00E-07	2.96E-06
6401	7	F2-3 vs. S2-3	246550_at	AT5G14920	Encodes a GASA domain containing protein that regulates increases in plant growth through GA-induced and DELLA-dependent signal transduction and that can increase abiotic stress resistance by reducing ROS accumulation.	38.64	600.18	0.064	5.00E-07	9.67E-06
6402	7	F2-3 vs. S2-3	253874_at	AT4G27450	aluminum induced protein with YGL and LRDR motifs;(source:Araport11)	55.42	880.13	0.063	< 1e-07	< 1e-07
6403	7	F2-3 vs. S2-3	245009_at	ATCG00380	Chloroplast encoded ribosomal protein S4	70.96	1159.86	0.061	< 1e-07	< 1e-07
6404	7	F2-3 vs. S2-3	247494_at	AT5G61790	calnexin 1;(source:Araport11)	65.09	1105.77	0.059	2.00E-07	4.97E-06
6405	7	F2-3 vs. S2-3	254163_s_a	AT4G24350	Phosphorylase superfamily protein;(source:Araport11)	9.16	165.3	0.055	< 1e-07	< 1e-07
6406	7	F2-3 vs. S2-3	247553_at	AT5G60910	MADS box gene negatively regulated by APETALA1	21.7	398.67	0.054	< 1e-07	< 1e-07
6407	7	F2-3 vs. S2-3	249732_at	AT5G24420	Encodes a cytosolic 6-phosphogluconolactonase (PGL) thought to be involved in the oxidative pentose-phosphate pathway (OPPP).	10.1	195.99	0.052	< 1e-07	< 1e-07
6408	7	F2-3 vs. S2-3	265058_s_at	AT1G52030	Similar to myrosinase binding proteins which may be involved in metabolizing glucosinolates and forming defense compounds to protect against herbivory. Also similar to lectins and other agglutinating factors. Expressed only in flowers.	127.46	2710.8	0.047	< 1e-07	< 1e-07
6409	7	F2-3 vs. S2-3	252282_at	AT3G49360	Encodes a cytosolic 6-phosphogluconolactonase (PGL) thought to be involved in the oxidative pentose-phosphate pathway (OPPP).	101.6	2524.14	0.04	< 1e-07	< 1e-07
6410	7	F2-3 vs. S2-3	266123_at	AT2G45180	Bifunctional inhibitor/lipid-transfer protein/seed storage 2S albumin superfamily protein;(source:Araport11)	21.95	665.55	0.033	< 1e-07	< 1e-07
6411	7	F2-3 vs. S2-3	259381_s_at	AT3G16410	Encodes a nitrile-specifier protein NSP4. NSP4 is one out of five (At3g16400/NSP1, At2g33070/NSP2, At3g16390/NSP3, At3g16410/NSP4 and At5g48180/NSP5) A. thaliana epithiospecifier protein (ESP) homologues that promote simple nitrile, but not epithionitrile or thiocyanate formation. The mRNA is cell-to-cell mobile.	18.81	764.71	0.025	< 1e-07	< 1e-07
6412	7	F2-3 vs. S2-3	257629_at	AT3G26140	Cellulase (glycosyl hydrolase family 5) protein;(source:Araport11)	36.12	1562.86	0.023	< 1e-07	< 1e-07
