# Supplementary material for: Whether Gametophytes Are Reduced or Unreduced in Angiosperms Might Be Determined Metabolically
Source: Genes (Basel). 2020 Dec 2;11(12):1449. doi: 10.3390/genes11121449 (PMC7761559; doi:10.3390/genes11121449)
Supplement: Supplementary file 1 [file genes-11-01449-s001.zip › Table S7 OGOs.pdf]

Table S7. Overrepresented (+) and underrepresented (-) gene ontology (GO) terms by comparison using DEGs that were up-regulated in each treatment.

Order	Line no.	Comparison	DEGs used		GO term	GO no.	Group	Subgroup	Cat.	Ref. list	Obs.	Exp.	Fold	Raw P value	FDR
1	1	L1-3 vs. M1-3	L1-3 up	+	cytoplasm	GO:0005737	Development	Development	C	14837	118	77.93	1.51	3.19E-12	3.32E-09
1	2	L1-3 vs. M1-3	L1-3 up	+	cytosol	GO:0005829	Development	Development	C	3241	40	17.02	2.35	1.95E-07	1.01E-04
1	3	L1-3 vs. M1-3	M1-3 up	+	inner mitochondrial membrane protein complex	GO:0098800	Metabolism	Bioenergetics	C	177	6	0.9	6.64	3.55E-04	5.28E-02
1	4	L1-3 vs. M1-3	M1-3 up	+	mitochondrial envelope	GO:0005740	Metabolism	Bioenergetics	C	317	8	1.62	4.94	2.67E-04	4.63E-02
1	5	L1-3 vs. M1-3	M1-3 up	+	mitochondrial membrane	GO:0031966	Metabolism	Bioenergetics	C	297	8	1.52	5.27	1.74E-04	3.61E-02
1	6	L1-3 vs. M1-3	M1-3 up	+	cellular process	GO:0009987	Development	Development	P	12008	92	61.32	1.5	2.13E-07	1.28E-03
1	7	L1-3 vs. M1-3	M1-3 up	+	cytoplasm	GO:0005737	Development	Development	C	14837	104	75.77	1.37	1.39E-06	7.25E-04
1	8	L1-3 vs. M1-3	M1-3 up	+	cytosol	GO:0005829	Development	Development	C	3241	41	16.55	2.48	2.60E-08	2.71E-05
1	9	L1-3 vs. M1-3	M1-3 up	+	membrane	GO:0016020	Development	Development	C	5493	49	28.05	1.75	4.56E-05	1.19E-02
1	10	L1-3 vs. M1-3	M1-3 up	+	nuclear condensin complex	GO:0000799	Development	Development	C	4	2	0.02	97.91	3.79E-04	4.94E-02
1	11	L1-3 vs. M1-3	M1-3 up	+	organelle membrane	GO:0031090	Development	Development	C	1458	22	7.45	2.95	5.68E-06	1.97E-03
2	12	L4 vs. M4	L4 up	+	oxidoreductase activity	GO:0016491	Metabolism	Bioenergetics	F	1458	34	13.35	2.55	7.59E-07	2.44E-03
2	13	L4 vs. M4	L4 up	+	oxidoreductase activity, oxidizing metal ions	GO:0016722	Metabolism	Bioenergetics	F	15	4	0.14	29.13	2.30E-05	3.70E-02
2	14	L4 vs. M4	L4 up	+	oxidoreductase activity, oxidizing metal ions, oxygen as acceptor	GO:0016724	Metabolism	Bioenergetics	F	6	3	0.05	54.61	5.95E-05	4.78E-02
2	15	L4 vs. M4	L4 up	-	nucleus	GO:0005634	Development	Development	C	10540	70	96.5	0.73	5.32E-04	3.69E-02
2	16	L4 vs. M4	L4 up	+	cell wall	GO:0005618	Development	Development	C	565	16	5.17	3.09	9.08E-05	9.45E-03
2	17	L4 vs. M4	L4 up	+	cytosol	GO:0005829	Development	Development	C	3241	54	29.67	1.82	1.34E-05	6.99E-03
2	18	L4 vs. M4	L4 up	+	external encapsulating structure	GO:0030312	Development	Development	C	567	16	5.19	3.08	9.45E-05	8.94E-03
2	19	L4 vs. M4	L4 up	+	extracellular region	GO:0005576	Development	Development	C	3128	56	28.64	1.96	1.19E-06	1.24E-03
2	20	L4 vs. M4	L4 up	+	membrane	GO:0016020	Development	Development	C	5493	74	50.29	1.47	3.56E-04	3.08E-02
2	21	L4 vs. M4	L4 up	+	organelle subcompartment	GO:0031984	Development	Development	C	1156	23	10.58	2.17	7.07E-04	4.60E-02
2	22	L4 vs. M4	L4 up	+	ferroxidase activity	GO:0004322	Metabolism	Metabolism	F	6	3	0.05	54.61	5.95E-05	6.38E-02
2	23	L4 vs. M4	L4 up	+	polygalacturonase activity	GO:0004650	Metabolism	Metabolism	F	69	6	0.63	9.5	6.27E-05	4.03E-02
2	24	L4 vs. M4	L4 up	+	chloroplast stroma	GO:0009570	Metabolism	Photosynthesis	C	719	17	6.58	2.58	4.36E-04	3.49E-02
2	25	L4 vs. M4	L4 up	+	chloroplast thylakoid	GO:0009534	Metabolism	Photosynthesis	C	443	14	4.06	3.45	8.28E-05	1.23E-02
2	26	L4 vs. M4	L4 up	+	chloroplast thylakoid membrane	GO:0009535	Metabolism	Photosynthesis	C	358	13	3.28	3.97	3.85E-05	8.01E-03
2	27	L4 vs. M4	L4 up	+	photosynthesis	GO:0015979	Metabolism	Photosynthesis	P	189	11	1.73	6.36	2.37E-06	3.54E-03
2	28	L4 vs. M4	L4 up	+	photosynthetic membrane	GO:0034357	Metabolism	Photosynthesis	C	395	14	3.62	3.87	2.51E-05	6.53E-03
2	29	L4 vs. M4	L4 up	+	plastid stroma	GO:0009532	Metabolism	Photosynthesis	C	731	17	6.69	2.54	5.23E-04	3.89E-02
2	30	L4 vs. M4	L4 up	+	plastid thylakoid	GO:0031976	Metabolism	Photosynthesis	C	446	14	4.08	3.43	8.87E-05	1.15E-02
2	31	L4 vs. M4	L4 up	+	plastid thylakoid membrane	GO:0055035	Metabolism	Photosynthesis	C	363	13	3.32	3.91	4.41E-05	7.65E-03
2	32	L4 vs. M4	L4 up	+	thylakoid	GO:0009579	Metabolism	Photosynthesis	C	537	17	4.92	3.46	1.41E-05	4.90E-03
2	33	L4 vs. M4	L4 up	+	thylakoid membrane	GO:0042651	Metabolism	Photosynthesis	C	390	13	3.57	3.64	8.89E-05	1.03E-02
2	34	L4 vs. M4	L4 up	+	response to inorganic substance	GO:0010035	Signaling and response	Signaling	P	914	23	8.37	2.75	1.68E-05	2.00E-02
2	35	L4 vs. M4	L4 up	+	response to temperature stimulus	GO:0009266	Signaling and response	Signaling	P	617	21	5.65	3.72	4.40E-07	2.63E-03
2	36	L4 vs. M4	L4 up	+	response to cold	GO:0009409	Signaling and response	Stress	P	415	16	3.8	4.21	2.36E-06	4.71E-03
2	37	L4 vs. M4	L4 up	+	response to oxidative stress	GO:0006979	Signaling and response	Stress	P	460	17	4.21	4.04	1.98E-06	5.91E-03
2	38	L4 vs. M4	M4 up	+	ATPase activity	GO:0016887	Metabolism	Bioenergetics	F	512	18	6.54	2.75	1.56E-04	3.33E-02
2	39	L4 vs. M4	M4 up	+	carbohydrate metabolic process	GO:0005975	Metabolism	Bioenergetics	P	950	29	12.13	2.39	2.44E-05	3.40E-03
2	40	L4 vs. M4	M4 up	+	cellular carbohydrate metabolic process	GO:0044262	Metabolism	Bioenergetics	P	384	14	4.9	2.86	5.85E-04	4.22E-02
2	41	L4 vs. M4	M4 up	+	generation of precursor metabolites and energy	GO:0006091	Metabolism	Bioenergetics	P	378	14	4.83	2.9	5.04E-04	3.77E-02
2	42	L4 vs. M4	M4 up	+	oxidoreductase activity	GO:0016491	Metabolism	Bioenergetics	F	1458	36	18.61	1.93	1.89E-04	3.80E-02
2	43	L4 vs. M4	M4 up	+	primary metabolic process	GO:0044238	Metabolism	Bioenergetics	P	7219	155	92.16	1.68	7.60E-13	7.58E-10
2	44	L4 vs. M4	M4 up	+	alpha-amino acid catabolic process	GO:1901606	Metabolism	Catabolism	P	88	7	1.12	6.23	2.04E-04	1.79E-02
2	45	L4 vs. M4	M4 up	+	carboxylic acid catabolic process	GO:0046395	Metabolism	Catabolism	P	162	10	2.07	4.84	7.02E-05	7.63E-03
2	46	L4 vs. M4	M4 up	+	catabolic process	GO:0009056	Metabolism	Catabolism	P	1469	50	18.75	2.67	5.36E-10	2.91E-07
2	47	L4 vs. M4	M4 up	+	cellular amino acid catabolic process	GO:0009063	Metabolism	Catabolism	P	97	7	1.24	5.65	3.55E-04	2.91E-02
2	48	L4 vs. M4	M4 up	+	cellular catabolic process	GO:0044248	Metabolism	Catabolism	P	1223	43	15.61	2.75	3.97E-09	1.32E-06
2	49	L4 vs. M4	M4 up	+	cellular macromolecule catabolic process	GO:0044265	Metabolism	Catabolism	P	736	24	9.4	2.55	4.11E-05	5.01E-03
2	50	L4 vs. M4	M4 up	+	cysteine-type endopeptidase activity	GO:0004197	Metabolism	Catabolism	F	72	10	0.92	10.88	8.53E-08	1.37E-04
2	51	L4 vs. M4	M4 up	+	endopeptidase activity	GO:0004175	Metabolism	Catabolism	F	337	15	4.3	3.49	4.65E-05	1.36E-02
2	52	L4 vs. M4	M4 up	+	lysosome	GO:0005764	Metabolism	Catabolism	C	46	8	0.59	13.62	3.70E-07	5.50E-05
2	53	L4 vs. M4	M4 up	+	macromolecule catabolic process	GO:0009057	Metabolism	Catabolism	P	889	29	11.35	2.56	6.40E-06	1.13E-03
2	54	L4 vs. M4	M4 up	+	organic acid catabolic process	GO:0016054	Metabolism	Catabolism	P	162	10	2.07	4.84	7.02E-05	7.77E-03
2	55	L4 vs. M4	M4 up	+	organic cyclic compound catabolic process	GO:1901361	Metabolism	Catabolism	P	210	10	2.68	3.73	5.14E-04	3.79E-02
2	56	L4 vs. M4	M4 up	+	organic substance catabolic process	GO:1901575	Metabolism	Catabolism	P	1345	50	17.17	2.91	2.80E-11	2.09E-08

2	57	L4 vs. M4	M4 up	+	organonitrogen compound catabolic process	GO:1901565	Metabolism	Catabolism	P	836	33	10.67	3.09	2.20E-08	5.97E-06
2	58	L4 vs. M4	M4 up	+	peptidase activity	GO:0008233	Metabolism	Catabolism	F	504	20	6.43	3.11	1.32E-05	6.05E-03
2	59	L4 vs. M4	M4 up	+	protein catabolic process	GO:0030163	Metabolism	Catabolism	P	612	19	7.81	2.43	4.65E-04	3.52E-02
2	60	L4 vs. M4	M4 up	+	proteolysis	GO:0006508	Metabolism	Catabolism	P	812	26	10.37	2.51	2.64E-05	3.44E-03
2	61	L4 vs. M4	M4 up	+	small molecule catabolic process	GO:0044282	Metabolism	Catabolism	P	239	13	3.05	4.26	2.11E-05	3.01E-03
2	62	L4 vs. M4	M4 up	-	nucleus	GO:0005634	Development	Development	C	10540	105	134.56	0.78	1.30E-03	4.51E-02
2	63	L4 vs. M4	M4 up	+	bounding membrane of organelle	GO:0098588	Development	Development	C	923	24	11.78	2.04	1.48E-03	4.81E-02
2	64	L4 vs. M4	M4 up	+	cell junction	GO:0030054	Development	Development	C	944	29	12.05	2.41	2.24E-05	1.30E-03
2	65	L4 vs. M4	M4 up	+	cell periphery	GO:0071944	Development	Development	C	4012	87	51.22	1.7	6.36E-07	8.28E-05
2	66	L4 vs. M4	M4 up	+	cell-cell junction	GO:0005911	Development	Development	C	944	29	12.05	2.41	2.24E-05	1.56E-03
2	67	L4 vs. M4	M4 up	+	cellular anatomical entity	GO:0110165	Development	Development	C	24430	333	311.88	1.07	1.21E-04	5.74E-03
2	68	L4 vs. M4	M4 up	+	cellular process	GO:0009987	Development	Development	P	12008	230	153.3	1.5	3.77E-16	1.13E-12
2	69	L4 vs. M4	M4 up	+	cytoplasm	GO:0005737	Development	Development	C	14837	267	189.41	1.41	1.76E-17	1.83E-14
2	70	L4 vs. M4	M4 up	+	cytosol	GO:0005829	Development	Development	C	3241	85	41.38	2.05	1.32E-10	4.58E-08
2	71	L4 vs. M4	M4 up	+	extracellular space	GO:0005615	Development	Development	C	130	9	1.66	5.42	7.06E-05	3.68E-03
2	72	L4 vs. M4	M4 up	+	intracellular anatomical structure	GO:0005622	Development	Development	C	21464	308	274.02	1.12	4.11E-06	4.28E-04
2	73	L4 vs. M4	M4 up	+	lytic vacuole	GO:0000323	Development	Development	C	52	9	0.66	13.56	7.06E-08	1.47E-05
2	74	L4 vs. M4	M4 up	+	membrane	GO:0016020	Development	Development	C	5493	109	70.13	1.55	1.15E-06	1.33E-04
2	75	L4 vs. M4	M4 up	+	plasma membrane	GO:0005886	Development	Development	C	3534	73	45.12	1.62	3.90E-05	2.14E-03
2	76	L4 vs. M4	M4 up	+	symplast	GO:0055044	Development	Development	C	944	29	12.05	2.41	2.24E-05	1.46E-03
2	77	L4 vs. M4	M4 up	+	vacuolar membrane	GO:0005774	Development	Development	C	570	19	7.28	2.61	1.98E-04	8.97E-03
2	78	L4 vs. M4	M4 up	+	vacuole	GO:0005773	Development	Development	C	1083	44	13.83	3.18	3.31E-11	1.72E-08
2	79	L4 vs. M4	M4 up	+	alpha-amino acid biosynthetic process	GO:1901607	Metabolism	Metabolism	P	181	11	2.31	4.76	3.51E-05	4.38E-03
2	80	L4 vs. M4	M4 up	+	alpha-amino acid metabolic process	GO:1901605	Metabolism	Metabolism	P	278	15	3.55	4.23	5.44E-06	9.85E-04
2	81	L4 vs. M4	M4 up	+	anchoring junction	GO:0070161	Metabolism	Metabolism	C	944	29	12.05	2.41	2.24E-05	1.37E-03
2	82	L4 vs. M4	M4 up	+	aromatic amino acid family metabolic process	GO:0009072	Metabolism	Metabolism	P	87	9	1.11	8.1	3.59E-06	6.92E-04
2	83	L4 vs. M4	M4 up	+	biological_process	GO:0008150	Metabolism	Metabolism	P	21988	311	280.7	1.11	2.45E-05	3.33E-03
2	84	L4 vs. M4	M4 up	+	biosynthetic process	GO:0009058	Metabolism	Metabolism	P	2811	74	35.89	2.06	2.84E-09	1.00E-06
2	85	L4 vs. M4	M4 up	+	carboxylic acid biosynthetic process	GO:0046394	Metabolism	Metabolism	P	468	21	5.97	3.51	1.29E-06	2.75E-04
2	86	L4 vs. M4	M4 up	+	carboxylic acid metabolic process	GO:0019752	Metabolism	Metabolism	P	912	36	11.64	3.09	4.83E-09	1.52E-06
2	87	L4 vs. M4	M4 up	+	catalytic activity	GO:0003824	Metabolism	Metabolism	F	8380	182	106.98	1.7	1.64E-16	5.28E-13
2	88	L4 vs. M4	M4 up	+	cellular amino acid biosynthetic process	GO:0008652	Metabolism	Metabolism	P	200	13	2.55	5.09	3.48E-06	6.93E-04
2	89	L4 vs. M4	M4 up	+	cellular amino acid metabolic process	GO:0006520	Metabolism	Metabolism	P	391	20	4.99	4.01	3.35E-07	8.00E-05
2	90	L4 vs. M4	M4 up	+	cellular biosynthetic process	GO:0044249	Metabolism	Metabolism	P	2583	63	32.98	1.91	8.30E-07	1.91E-04
2	91	L4 vs. M4	M4 up	+	cellular metabolic process	GO:0044237	Metabolism	Metabolism	P	7649	163	97.65	1.67	2.03E-13	3.03E-10
2	92	L4 vs. M4	M4 up	+	cellular nitrogen compound metabolic process	GO:0034641	Metabolism	Metabolism	P	2872	61	36.66	1.66	1.02E-04	9.99E-03
2	93	L4 vs. M4	M4 up	+	cysteine-type peptidase activity	GO:0008234	Metabolism	Metabolism	F	101	10	1.29	7.76	1.49E-06	1.59E-03
2	94	L4 vs. M4	M4 up	+	hydrolase activity	GO:0016787	Metabolism	Metabolism	F	2995	69	38.23	1.8	1.75E-06	1.40E-03
2	95	L4 vs. M4	M4 up	+	macromolecule metabolic process	GO:0043170	Metabolism	Metabolism	P	5496	100	70.16	1.43	1.62E-04	1.44E-02
2	96	L4 vs. M4	M4 up	+	metabolic process	GO:0008152	Metabolism	Metabolism	P	8681	197	110.82	1.78	5.96E-21	3.56E-17
2	97	L4 vs. M4	M4 up	+	molecular_function	GO:0003674	Metabolism	Metabolism	F	21945	313	280.16	1.12	3.88E-06	2.08E-03
2	98	L4 vs. M4	M4 up	+	monocarboxylic acid metabolic process	GO:0032787	Metabolism	Metabolism	P	439	17	5.6	3.03	7.79E-05	8.03E-03
2	99	L4 vs. M4	M4 up	+	nitrogen compound metabolic process	GO:0006807	Metabolism	Metabolism	P	6150	129	78.51	1.64	1.47E-09	6.77E-07
2	100	L4 vs. M4	M4 up	+	organic acid biosynthetic process	GO:0016053	Metabolism	Metabolism	P	468	21	5.97	3.51	1.29E-06	2.85E-04
2	101	L4 vs. M4	M4 up	+	organic acid metabolic process	GO:0006082	Metabolism	Metabolism	P	1019	39	13.01	3	2.41E-09	9.00E-07
2	102	L4 vs. M4	M4 up	+	organic cyclic compound metabolic process	GO:1901360	Metabolism	Metabolism	P	2607	56	33.28	1.68	1.56E-04	1.42E-02
2	103	L4 vs. M4	M4 up	+	organic substance biosynthetic process	GO:1901576	Metabolism	Metabolism	P	2674	70	34.14	2.05	1.17E-08	3.34E-06
2	104	L4 vs. M4	M4 up	+	organic substance metabolic process	GO:0071704	Metabolism	Metabolism	P	7851	171	100.23	1.71	2.68E-15	5.34E-12
2	105	L4 vs. M4	M4 up	+	organonitrogen compound biosynthetic process	GO:1901566	Metabolism	Metabolism	P	1343	37	17.15	2.16	1.70E-05	2.61E-03
2	106	L4 vs. M4	M4 up	+	organonitrogen compound metabolic process	GO:1901564	Metabolism	Metabolism	P	4611	105	58.87	1.78	1.64E-09	7.00E-07
2	107	L4 vs. M4	M4 up	+	oxidation-reduction process	GO:0055114	Metabolism	Metabolism	P	768	26	9.8	2.65	1.05E-05	1.65E-03
2	108	L4 vs. M4	M4 up	+	oxoacid metabolic process	GO:0043436	Metabolism	Metabolism	P	1016	39	12.97	3.01	2.23E-09	8.88E-07
2	109	L4 vs. M4	M4 up	+	small molecule biosynthetic process	GO:0044283	Metabolism	Metabolism	P	645	24	8.23	2.91	5.17E-06	9.65E-04
2	110	L4 vs. M4	M4 up	+	small molecule metabolic process	GO:0044281	Metabolism	Metabolism	P	1520	51	19.4	2.63	5.67E-10	2.82E-07
2	111	L4 vs. M4	M4 up	+	chloroplast	GO:0009507	Metabolism	Photosynthesis	C	5013	105	64	1.64	1.48E-07	2.57E-05
2	112	L4 vs. M4	M4 up	+	chloroplast envelope	GO:0009941	Metabolism	Photosynthesis	C	613	19	7.83	2.43	4.74E-04	1.97E-02
2	113	L4 vs. M4	M4 up	+	chloroplast stroma	GO:0009570	Metabolism	Photosynthesis	C	719	25	9.18	2.72	1.01E-05	8.76E-04
2	114	L4 vs. M4	M4 up	+	photosynthesis	GO:0015979	Metabolism	Photosynthesis	P	189	10	2.41	4.14	2.32E-04	2.01E-02
2	115	L4 vs. M4	M4 up	+	photosynthetic electron transport in photosystem	GO:0009772	Metabolism	Photosynthesis	P	11	3	0.14	21.36	6.52E-04	4.59E-02
2	116	L4 vs. M4	M4 up	+	photosystem	GO:0009521	Metabolism	Photosynthesis	C	63	6	0.8	7.46	2.36E-04	1.02E-02
2	117	L4 vs. M4	M4 up	+	photosystem II reaction center	GO:0009539	Metabolism	Photosynthesis	C	11	3	0.14	21.36	6.52E-04	2.42E-02
2	118	L4 vs. M4	M4 up	+	plastid	GO:0009536	Metabolism	Photosynthesis	C	5432	119	69.35	1.72	6.10E-10	1.59E-07

2	119	L4 vs. M4	M4 up	+	plastid envelope	GO:0009526	Metabolism	Photosynthesis	C	637	21	8.13	2.58	1.07E-04	5.31E-03
2	120	L4 vs. M4	M4 up	+	plastid large ribosomal subunit	GO:0000311	Metabolism	Photosynthesis	C	15	3	0.19	15.67	1.41E-03	4.73E-02
2	121	L4 vs. M4	M4 up	+	plastid ribosome	GO:0009547	Metabolism	Photosynthesis	C	29	4	0.37	10.8	7.61E-04	2.73E-02
2	122	L4 vs. M4	M4 up	+	plastid stroma	GO:0009532	Metabolism	Photosynthesis	C	731	26	9.33	2.79	4.55E-06	4.30E-04
2	123	L4 vs. M4	M4 up	+	cellular response to chemical stimulus	GO:0070887	Signaling and response	Signaling	P	1085	29	13.85	2.09	2.70E-04	2.28E-02
2	124	L4 vs. M4	M4 up	+	cellular response to oxygen levels	GO:0071453	Signaling and response	Signaling	P	238	12	3.04	3.95	8.73E-05	8.70E-03
2	125	L4 vs. M4	M4 up	+	cellular response to stimulus	GO:0051716	Signaling and response	Signaling	P	2511	56	32.06	1.75	5.43E-05	6.37E-03
2	126	L4 vs. M4	M4 up	+	response to abiotic stimulus	GO:0009628	Signaling and response	Signaling	P	2131	60	27.2	2.21	1.08E-08	3.24E-06
2	127	L4 vs. M4	M4 up	+	response to biotic stimulus	GO:0009607	Signaling and response	Signaling	P	1098	33	14.02	2.35	8.12E-06	1.31E-03
2	128	L4 vs. M4	M4 up	+	response to chemical	GO:0042221	Signaling and response	Signaling	P	2681	79	34.23	2.31	3.45E-12	2.94E-09
2	129	L4 vs. M4	M4 up	+	response to external biotic stimulus	GO:0043207	Signaling and response	Signaling	P	1097	33	14	2.36	7.99E-06	1.36E-03
2	130	L4 vs. M4	M4 up	+	response to external stimulus	GO:0009605	Signaling and response	Signaling	P	1513	45	19.32	2.33	2.31E-07	5.75E-05
2	131	L4 vs. M4	M4 up	+	response to inorganic substance	GO:0010035	Signaling and response	Signaling	P	914	28	11.67	2.4	3.30E-05	4.20E-03
2	132	L4 vs. M4	M4 up	+	response to jasmonic acid	GO:0009753	Signaling and response	Signaling	P	175	10	2.23	4.48	1.28E-04	1.20E-02
2	133	L4 vs. M4	M4 up	+	response to lipid	GO:0033993	Signaling and response	Signaling	P	672	21	8.58	2.45	3.53E-04	2.93E-02
2	134	L4 vs. M4	M4 up	+	response to organic cyclic compound	GO:0014070	Signaling and response	Signaling	P	280	12	3.57	3.36	3.66E-04	2.92E-02
2	135	L4 vs. M4	M4 up	+	response to organic substance	GO:0010033	Signaling and response	Signaling	P	1705	49	21.77	2.25	1.78E-07	4.63E-05
2	136	L4 vs. M4	M4 up	+	response to oxygen levels	GO:0070482	Signaling and response	Signaling	P	267	13	3.41	3.81	6.27E-05	7.08E-03
2	137	L4 vs. M4	M4 up	+	response to oxygen-containing compound	GO:1901700	Signaling and response	Signaling	P	1514	53	19.33	2.74	5.62E-11	3.73E-08
2	138	L4 vs. M4	M4 up	+	response to salicylic acid	GO:0009751	Signaling and response	Signaling	P	155	9	1.98	4.55	2.48E-04	2.12E-02
2	139	L4 vs. M4	M4 up	+	response to stimulus	GO:0050896	Signaling and response	Signaling	P	5578	131	71.21	1.84	2.70E-13	3.23E-10
2	140	L4 vs. M4	M4 up	+	aging	GO:0007568	Signaling and response	Stress	P	132	8	1.69	4.75	4.15E-04	3.23E-02
2	141	L4 vs. M4	M4 up	+	cellular response to decreased oxygen levels	GO:0036294	Signaling and response	Stress	P	237	12	3.03	3.97	8.40E-05	8.51E-03
2	142	L4 vs. M4	M4 up	+	cellular response to hypoxia	GO:0071456	Signaling and response	Stress	P	235	12	3	4	7.78E-05	8.16E-03
2	143	L4 vs. M4	M4 up	+	cellular response to stress	GO:0033554	Signaling and response	Stress	P	1120	31	14.3	2.17	7.15E-05	7.63E-03
2	144	L4 vs. M4	M4 up	+	defense response	GO:0006952	Signaling and response	Stress	P	953	26	12.17	2.14	3.64E-04	2.94E-02
2	145	L4 vs. M4	M4 up	+	defense response to fungus	GO:0050832	Signaling and response	Stress	P	252	12	3.22	3.73	1.46E-04	1.34E-02
2	146	L4 vs. M4	M4 up	+	defense response to other organism	GO:0098542	Signaling and response	Stress	P	807	23	10.3	2.23	4.16E-04	3.19E-02
2	147	L4 vs. M4	M4 up	+	interspecies interaction between organisms	GO:0044419	Signaling and response	Stress	P	1125	33	14.36	2.3	1.74E-05	2.61E-03
2	148	L4 vs. M4	M4 up	+	response to abscisic acid	GO:0009737	Signaling and response	Stress	P	497	18	6.34	2.84	1.09E-04	1.05E-02
2	149	L4 vs. M4	M4 up	+	response to alcohol	GO:0097305	Signaling and response	Stress	P	501	18	6.4	2.81	1.20E-04	1.14E-02
2	150	L4 vs. M4	M4 up	+	response to decreased oxygen levels	GO:0036293	Signaling and response	Stress	P	265	13	3.38	3.84	5.83E-05	6.71E-03
2	151	L4 vs. M4	M4 up	+	response to fungus	GO:0009620	Signaling and response	Stress	P	350	16	4.47	3.58	1.92E-05	2.79E-03
2	152	L4 vs. M4	M4 up	+	response to hypoxia	GO:0001666	Signaling and response	Stress	P	261	13	3.33	3.9	5.03E-05	6.02E-03
2	153	L4 vs. M4	M4 up	+	response to other organism	GO:0051707	Signaling and response	Stress	P	1097	33	14	2.36	7.99E-06	1.33E-03
2	154	L4 vs. M4	M4 up	+	response to stress	GO:0006950	Signaling and response	Stress	P	3106	82	39.65	2.07	3.08E-10	1.84E-07
2	155	L4 vs. M4	M4 up	+	response to wounding	GO:0009611	Signaling and response	Stress	P	227	14	2.9	4.83	2.62E-06	5.40E-04
2	156	L4 vs. M4	M4 up	+	RNA binding	GO:0003723	Regulation	Transcription	F	1569	40	20.03	2	4.27E-05	1.37E-02
2	157	L4 vs. M4	M4 up	+	box C/D snoRNP complex	GO:0031428	Regulation	Translation	C	11	3	0.14	21.36	6.52E-04	2.51E-02
2	158	L4 vs. M4	M4 up	+	mRNA binding	GO:0003729	Regulation	Translation	F	1005	29	12.83	2.26	7.95E-05	1.96E-02
2	159	L4 vs. M4	M4 up	+	organellar ribosome	GO:0000313	Regulation	Translation	C	75	6	0.96	6.27	5.64E-04	2.26E-02
2	160	L4 vs. M4	M4 up	+	anion transport	GO:0006820	Metabolism	Transporters	P	283	12	3.61	3.32	4.01E-04	3.16E-02
2	161	L4 vs. M4	M4 up	+	ATPase-coupled transmembrane transporter activity	GO:0042626	Metabolism	Transporters	F	190	11	2.43	4.53	5.33E-05	1.43E-02
2	162	L4 vs. M4	M4 up	+	plasmodesma	GO:0009506	Metabolism	Transporters	C	944	29	12.05	2.41	2.24E-05	1.67E-03
2	163	L4 vs. M4	M4 up	+	primary active transmembrane transporter	GO:0015399	Metabolism	Transporters	F	213	11	2.72	4.05	1.40E-04	3.21E-02
2	164	L4 vs. M4	M4 up	+	secretory vesicle	GO:0099503	Metabolism	Transporters	C	170	11	2.17	5.07	2.04E-05	1.63E-03
2	165	L4 vs. M4	M4 up	+	transmembrane transport	GO:0055085	Metabolism	Transporters	P	859	24	10.97	2.19	5.33E-04	3.89E-02
2	166	L4 vs. M4	M4 up	+	transmembrane transporter activity	GO:0022857	Metabolism	Transporters	F	1242	35	15.86	2.21	1.64E-05	6.61E-03
2	167	L4 vs. M4	M4 up	+	transport	GO:0006810	Metabolism	Transporters	P	2261	48	28.86	1.66	5.93E-04	4.22E-02
2	168	L4 vs. M4	M4 up	+	transporter activity	GO:0005215	Metabolism	Transporters	F	1287	35	16.43	2.13	3.73E-05	1.33E-02
3	169	F2-3 vs. L2-3	F2-3 up	+	acetyl-CoA biosynthetic process	GO:0006085	Metabolism	Bioenergetics	P	15	5	0.6	8.29	8.15E-04	1.87E-02
3	170	F2-3 vs. L2-3	F2-3 up	+	acetyl-CoA metabolic process	GO:0006084	Metabolism	Bioenergetics	P	25	6	1	5.97	1.05E-03	2.29E-02
3	171	F2-3 vs. L2-3	F2-3 up	+	acyl-CoA biosynthetic process	GO:0071616	Metabolism	Bioenergetics	P	21	5	0.84	5.92	2.85E-03	4.90E-02
3	172	F2-3 vs. L2-3	F2-3 up	+	aerobic respiration	GO:0009060	Metabolism	Bioenergetics	P	89	15	3.58	4.19	1.11E-05	4.52E-04
3	173	F2-3 vs. L2-3	F2-3 up	+	aldehyde dehydrogenase (NAD+) activity	GO:0004029	Metabolism	Bioenergetics	F	12	6	0.48	12.44	4.09E-05	3.13E-03
3	174	F2-3 vs. L2-3	F2-3 up	+	aldehyde dehydrogenase [NAD(P)+] activity	GO:0004030	Metabolism	Bioenergetics	F	12	6	0.48	12.44	4.09E-05	3.20E-03
3	175	F2-3 vs. L2-3	F2-3 up	+	ATP metabolic process	GO:0046034	Metabolism	Bioenergetics	P	144	15	5.79	2.59	1.32E-03	2.66E-02
3	176	F2-3 vs. L2-3	F2-3 up	+	ATPase activity, coupled to transmembrane movement of ions, rotational mechanism	GO:0044769	Metabolism	Bioenergetics	F	25	8	1	7.96	2.84E-05	2.34E-03
3	177	F2-3 vs. L2-3	F2-3 up	+	carbohydrate biosynthetic process	GO:0016051	Metabolism	Bioenergetics	P	303	44	12.18	3.61	5.03E-12	9.12E-10
3	178	F2-3 vs. L2-3	F2-3 up	+	carbohydrate catabolic process	GO:0016052	Metabolism	Bioenergetics	P	234	27	9.41	2.87	3.71E-06	1.77E-04

3	179	F2-3 vs. L2-3	F2-3 up	+	carbohydrate derivative biosynthetic process	GO:1901137	Metabolism	Bioenergetics	P	458	42	18.41	2.28	2.98E-06	1.48E-04
3	180	F2-3 vs. L2-3	F2-3 up	+	carbohydrate derivative metabolic process	GO:1901135	Metabolism	Bioenergetics	P	746	69	29.99	2.3	1.50E-09	1.52E-07
3	181	F2-3 vs. L2-3	F2-3 up	+	carbohydrate metabolic process	GO:0005975	Metabolism	Bioenergetics	P	950	102	38.19	2.67	1.23E-17	4.60E-15
3	182	F2-3 vs. L2-3	F2-3 up	+	cellular carbohydrate biosynthetic process	GO:0034637	Metabolism	Bioenergetics	P	213	30	8.56	3.5	2.25E-08	1.82E-06
3	183	F2-3 vs. L2-3	F2-3 up	+	cellular carbohydrate metabolic process	GO:0044262	Metabolism	Bioenergetics	P	384	46	15.44	2.98	5.49E-10	6.32E-08
3	184	F2-3 vs. L2-3	F2-3 up	+	cellular polysaccharide biosynthetic process	GO:0033692	Metabolism	Bioenergetics	P	177	24	7.11	3.37	1.03E-06	6.16E-05
3	185	F2-3 vs. L2-3	F2-3 up	+	cellular polysaccharide metabolic process	GO:0044264	Metabolism	Bioenergetics	P	270	33	10.85	3.04	9.72E-08	7.35E-06
3	186	F2-3 vs. L2-3	F2-3 up	+	cellular respiration	GO:0045333	Metabolism	Bioenergetics	P	139	16	5.59	2.86	3.45E-04	9.18E-03
3	187	F2-3 vs. L2-3	F2-3 up	+	electron transport chain	GO:0022900	Metabolism	Bioenergetics	P	114	14	4.58	3.06	4.42E-04	1.12E-02
3	188	F2-3 vs. L2-3	F2-3 up	+	energy derivation by oxidation of organic compounds	GO:0015980	Metabolism	Bioenergetics	P	154	19	6.19	3.07	4.34E-05	1.54E-03
3	189	F2-3 vs. L2-3	F2-3 up	+	fatty acid biosynthetic process	GO:0006633	Metabolism	Bioenergetics	P	134	16	5.39	2.97	2.38E-04	6.65E-03
3	190	F2-3 vs. L2-3	F2-3 up	+	fatty acid derivative metabolic process	GO:1901568	Metabolism	Bioenergetics	P	43	7	1.73	4.05	2.95E-03	4.93E-02
3	191	F2-3 vs. L2-3	F2-3 up	+	fatty acid metabolic process	GO:0006631	Metabolism	Bioenergetics	P	221	25	8.88	2.81	1.14E-05	4.61E-04
3	192	F2-3 vs. L2-3	F2-3 up	+	generation of precursor metabolites and energy	GO:0006091	Metabolism	Bioenergetics	P	378	51	15.19	3.36	1.24E-12	2.48E-10
3	193	F2-3 vs. L2-3	F2-3 up	+	glucose 6-phosphate metabolic process	GO:0051156	Metabolism	Bioenergetics	P	42	7	1.69	4.15	2.63E-03	4.60E-02
3	194	F2-3 vs. L2-3	F2-3 up	+	glucose metabolic process	GO:0006006	Metabolism	Bioenergetics	P	42	8	1.69	4.74	6.15E-04	1.47E-02
3	195	F2-3 vs. L2-3	F2-3 up	+	glyceraldehyde-3-phosphate dehydrogenase (NAD+) (non-phosphorylating) activity	GO:0043878	Metabolism	Bioenergetics	F	8	4	0.32	12.44	8.56E-04	3.87E-02
3	196	F2-3 vs. L2-3	F2-3 up	+	hexose metabolic process	GO:0019318	Metabolism	Bioenergetics	P	88	11	3.54	3.11	1.54E-03	2.99E-02
3	197	F2-3 vs. L2-3	F2-3 up	+	monosaccharide biosynthetic process	GO:0046364	Metabolism	Bioenergetics	P	48	8	1.93	4.15	1.33E-03	2.66E-02
3	198	F2-3 vs. L2-3	F2-3 up	+	NAD binding	GO:0051287	Metabolism	Bioenergetics	F	77	13	3.1	4.2	4.15E-05	3.10E-03
3	199	F2-3 vs. L2-3	F2-3 up	+	NAD(P)H dehydrogenase complex	GO:0010598	Metabolism	Bioenergetics	C	12	4	0.48	8.29	2.78E-03	4.26E-02
3	200	F2-3 vs. L2-3	F2-3 up	+	NADH dehydrogenase activity	GO:0003954	Metabolism	Bioenergetics	F	49	9	1.97	4.57	3.60E-04	2.00E-02
3	201	F2-3 vs. L2-3	F2-3 up	+	NADP binding	GO:0050661	Metabolism	Bioenergetics	F	68	10	2.73	3.66	8.27E-04	3.80E-02
3	202	F2-3 vs. L2-3	F2-3 up	+	NADPH regeneration	GO:0006740	Metabolism	Bioenergetics	P	35	7	1.41	4.98	1.04E-03	2.28E-02
3	203	F2-3 vs. L2-3	F2-3 up	+	oxidoreductase activity	GO:0016491	Metabolism	Bioenergetics	F	1458	167	58.61	2.85	1.10E-31	1.76E-28
3	204	F2-3 vs. L2-3	F2-3 up	+	oxidoreductase activity, acting on CH-OH group of donors	GO:0016614	Metabolism	Bioenergetics	F	201	23	8.08	2.85	2.14E-05	1.91E-03
3	205	F2-3 vs. L2-3	F2-3 up	+	oxidoreductase activity, acting on NAD(P)H	GO:0016651	Metabolism	Bioenergetics	F	121	21	4.86	4.32	1.35E-07	2.07E-05
3	206	F2-3 vs. L2-3	F2-3 up	+	oxidoreductase activity, acting on paired donors, with incorporation or reduction of molecular	GO:0016705	Metabolism	Bioenergetics	F	392	36	15.76	2.28	1.93E-05	1.77E-03
3	207	F2-3 vs. L2-3	F2-3 up	+	oxidoreductase activity, acting on paired donors, with incorporation or reduction of molecular oxygen, NAD(P)H as one donor, and incorporation of one atom of oxygen	GO:0016709	Metabolism	Bioenergetics	F	147	16	5.91	2.71	6.05E-04	2.99E-02
3	208	F2-3 vs. L2-3	F2-3 up	+	oxidoreductase activity, acting on single donors with incorporation of molecular oxygen	GO:0016701	Metabolism	Bioenergetics	F	38	9	1.53	5.89	6.74E-05	4.81E-03
3	209	F2-3 vs. L2-3	F2-3 up	+	oxidoreductase activity, acting on single donors with incorporation of molecular oxygen, incorporation of two atoms of oxygen	GO:0016702	Metabolism	Bioenergetics	F	28	7	1.13	6.22	3.29E-04	1.89E-02
3	210	F2-3 vs. L2-3	F2-3 up	+	oxidoreductase activity, acting on the aldehyde or oxo group of donors	GO:0016903	Metabolism	Bioenergetics	F	62	14	2.49	5.62	1.12E-06	1.33E-04
3	211	F2-3 vs. L2-3	F2-3 up	+	oxidoreductase activity, acting on the aldehyde or oxo group of donors, NAD or NADP as	GO:0016620	Metabolism	Bioenergetics	F	43	12	1.73	6.94	9.86E-07	1.27E-04
3	212	F2-3 vs. L2-3	F2-3 up	+	oxidoreductase activity, acting on the CH-NH2 group of donors	GO:0016638	Metabolism	Bioenergetics	F	38	9	1.53	5.89	6.74E-05	4.71E-03
3	213	F2-3 vs. L2-3	F2-3 up	+	oxidoreductase activity, acting on the CH-OH group of donors, NAD or NADP as acceptor	GO:0016616	Metabolism	Bioenergetics	F	167	22	6.71	3.28	4.37E-06	4.39E-04
3	214	F2-3 vs. L2-3	F2-3 up	+	pentose-phosphate shunt	GO:0006098	Metabolism	Bioenergetics	P	33	7	1.33	5.28	7.70E-04	1.81E-02
3	215	F2-3 vs. L2-3	F2-3 up	+	polysaccharide biosynthetic process	GO:0000271	Metabolism	Bioenergetics	P	213	26	8.56	3.04	2.21E-06	1.17E-04
3	216	F2-3 vs. L2-3	F2-3 up	+	polysaccharide metabolic process	GO:0005976	Metabolism	Bioenergetics	P	419	51	16.84	3.03	3.78E-11	5.65E-09
3	217	F2-3 vs. L2-3	F2-3 up	+	primary metabolic process	GO:0044238	Metabolism	Bioenergetics	P	7219	361	290.17	1.24	3.61E-06	1.74E-04
3	218	F2-3 vs. L2-3	F2-3 up	+	proton transmembrane transport	GO:1902600	Metabolism	Bioenergetics	P	95	17	3.82	4.45	1.43E-06	7.98E-05
3	219	F2-3 vs. L2-3	F2-3 up	+	proton-exporting ATPase activity, phosphorylative mechanism	GO:0008553	Metabolism	Bioenergetics	F	20	6	0.8	7.46	3.89E-04	2.12E-02
3	220	F2-3 vs. L2-3	F2-3 up	+	proton-transporting ATPase activity, rotational mechanism	GO:0046961	Metabolism	Bioenergetics	F	25	8	1	7.96	2.84E-05	2.40E-03
3	221	F2-3 vs. L2-3	F2-3 up	+	proton-transporting two-sector ATPase complex	GO:0016469	Metabolism	Bioenergetics	C	65	11	2.61	4.21	1.57E-04	3.40E-03
3	222	F2-3 vs. L2-3	F2-3 up	+	proton-transporting two-sector ATPase complex, proton-transporting domain	GO:0033177	Metabolism	Bioenergetics	C	28	6	1.13	5.33	1.75E-03	2.84E-02
3	223	F2-3 vs. L2-3	F2-3 up	+	proton-transporting V-type ATPase complex	GO:0033176	Metabolism	Bioenergetics	C	23	6	0.92	6.49	7.27E-04	1.35E-02

3	224	F2-3 vs. L2-3	F2-3 up	+	pyrophosphate hydrolysis-driven proton transmembrane transporter activity	GO:0009678	Metabolism	Bioenergetics	F	44	13	1.77	7.35	2.01E-07	2.94E-05
3	225	F2-3 vs. L2-3	F2-3 up	+	pyruvate metabolic process	GO:0006090	Metabolism	Bioenergetics	P	79	10	3.18	3.15	2.29E-03	4.07E-02
3	226	F2-3 vs. L2-3	F2-3 up	+	reductive pentose-phosphate cycle	GO:0019253	Metabolism	Bioenergetics	P	14	8	0.56	14.22	9.56E-07	5.83E-05
3	227	F2-3 vs. L2-3	F2-3 up	+	tricarboxylic acid cycle	GO:0006099	Metabolism	Bioenergetics	P	46	8	1.85	4.33	1.04E-03	2.30E-02
3	228	F2-3 vs. L2-3	F2-3 up	+	unsaturated fatty acid biosynthetic process	GO:0006636	Metabolism	Bioenergetics	P	24	6	0.96	6.22	8.79E-04	1.99E-02
3	229	F2-3 vs. L2-3	F2-3 up	+	unsaturated fatty acid metabolic process	GO:0033559	Metabolism	Bioenergetics	P	25	6	1	5.97	1.05E-03	2.30E-02
3	230	F2-3 vs. L2-3	F2-3 up	-	modification-dependent macromolecule catabolic process	GO:0043632	Metabolism	Catabolism	P	536	7	21.54	0.32	6.47E-04	1.53E-02
3	231	F2-3 vs. L2-3	F2-3 up	-	modification-dependent protein catabolic	GO:0019941	Metabolism	Catabolism	P	524	7	21.06	0.33	8.65E-04	1.97E-02
3	232	F2-3 vs. L2-3	F2-3 up	-	protein-containing complex	GO:0032991	Metabolism	Catabolism	C	2958	79	118.9	0.66	7.97E-05	1.80E-03
3	233	F2-3 vs. L2-3	F2-3 up	-	ubiquitin-dependent protein catabolic process	GO:0006511	Metabolism	Catabolism	P	512	6	20.58	0.29	3.24E-04	8.68E-03
3	234	F2-3 vs. L2-3	F2-3 up	+	alpha-amino acid catabolic process	GO:1901606	Metabolism	Catabolism	P	88	15	3.54	4.24	9.85E-06	4.12E-04
3	235	F2-3 vs. L2-3	F2-3 up	+	aromatic amino acid family catabolic process	GO:0009074	Metabolism	Catabolism	P	21	5	0.84	5.92	2.85E-03	4.87E-02
3	236	F2-3 vs. L2-3	F2-3 up	+	carboxylic acid catabolic process	GO:0046395	Metabolism	Catabolism	P	162	23	6.51	3.53	8.46E-07	5.27E-05
3	237	F2-3 vs. L2-3	F2-3 up	+	catabolic process	GO:0009056	Metabolism	Catabolism	P	1469	95	59.05	1.61	1.39E-05	5.53E-04
3	238	F2-3 vs. L2-3	F2-3 up	+	cellular amino acid catabolic process	GO:0009063	Metabolism	Catabolism	P	97	15	3.9	3.85	2.74E-05	1.02E-03
3	239	F2-3 vs. L2-3	F2-3 up	+	cellular catabolic process	GO:0044248	Metabolism	Catabolism	P	1223	72	49.16	1.46	1.90E-03	3.45E-02
3	240	F2-3 vs. L2-3	F2-3 up	+	dicarboxylic acid catabolic process	GO:0043649	Metabolism	Catabolism	P	14	5	0.56	8.89	6.31E-04	1.50E-02
3	241	F2-3 vs. L2-3	F2-3 up	+	erythrose 4-phosphate/phosphoenolpyruvate family amino acid catabolic process	GO:1902222	Metabolism	Catabolism	P	12	4	0.48	8.29	2.78E-03	4.83E-02
3	242	F2-3 vs. L2-3	F2-3 up	+	leaf senescence	GO:0010150	Metabolism	Catabolism	P	106	12	4.26	2.82	2.07E-03	3.72E-02
3	243	F2-3 vs. L2-3	F2-3 up	+	L-phenylalanine catabolic process	GO:0006559	Metabolism	Catabolism	P	12	4	0.48	8.29	2.78E-03	4.85E-02
3	244	F2-3 vs. L2-3	F2-3 up	+	monocarboxylic acid catabolic process	GO:0072329	Metabolism	Catabolism	P	62	10	2.49	4.01	4.34E-04	1.12E-02
3	245	F2-3 vs. L2-3	F2-3 up	+	organic acid catabolic process	GO:0016054	Metabolism	Catabolism	P	162	23	6.51	3.53	8.46E-07	5.33E-05
3	246	F2-3 vs. L2-3	F2-3 up	+	organic hydroxy compound catabolic process	GO:1901616	Metabolism	Catabolism	P	44	8	1.77	4.52	8.05E-04	1.87E-02
3	247	F2-3 vs. L2-3	F2-3 up	+	organic substance catabolic process	GO:1901575	Metabolism	Catabolism	P	1345	86	54.06	1.59	5.46E-05	1.86E-03
3	248	F2-3 vs. L2-3	F2-3 up	+	pectin catabolic process	GO:0045490	Metabolism	Catabolism	P	99	13	3.98	3.27	3.93E-04	1.02E-02
3	249	F2-3 vs. L2-3	F2-3 up	+	peroxidase activity	GO:0004601	Metabolism	Catabolism	F	111	13	4.46	2.91	1.04E-03	4.64E-02
3	250	F2-3 vs. L2-3	F2-3 up	+	peroxisome	GO:0005777	Metabolism	Catabolism	C	320	31	12.86	2.41	1.97E-05	5.00E-04
3	251	F2-3 vs. L2-3	F2-3 up	+	phenol-containing compound catabolic process	GO:0019336	Metabolism	Catabolism	P	7	4	0.28	14.22	5.88E-04	1.42E-02
3	252	F2-3 vs. L2-3	F2-3 up	+	plant organ senescence	GO:0090693	Metabolism	Catabolism	P	112	13	4.5	2.89	1.12E-03	2.41E-02
3	253	F2-3 vs. L2-3	F2-3 up	+	polysaccharide catabolic process	GO:0000272	Metabolism	Catabolism	P	144	17	5.79	2.94	1.74E-04	5.16E-03
3	254	F2-3 vs. L2-3	F2-3 up	+	small molecule catabolic process	GO:0044282	Metabolism	Catabolism	P	239	29	9.61	3.02	6.54E-07	4.44E-05
3	255	F2-3 vs. L2-3	F2-3 up	-	nucleic acid metabolic process	GO:0090304	Development	Cell division	P	1705	26	68.53	0.38	5.10E-09	4.56E-07
3	256	F2-3 vs. L2-3	F2-3 up	-	RNA metabolic process	GO:0016070	Development	Cell division	P	1294	21	52.01	0.4	1.60E-06	8.69E-05
3	257	F2-3 vs. L2-3	F2-3 up	+	cytoskeleton-dependent cytokinesis	GO:0061640	Development	Cell division	P	55	10	2.21	4.52	1.85E-04	5.42E-03
3	258	F2-3 vs. L2-3	F2-3 up	+	gametophyte development	GO:0048229	Development	Cell division	P	461	34	18.53	1.83	1.32E-03	2.66E-02
3	259	F2-3 vs. L2-3	F2-3 up	+	mitotic cytokinesis	GO:0000281	Development	Cell division	P	40	8	1.61	4.98	4.62E-04	1.17E-02
3	260	F2-3 vs. L2-3	F2-3 up	+	nucleobase-containing small molecule metabolic process	GO:0055086	Development	Cell division	P	392	40	15.76	2.54	4.04E-07	2.95E-05
3	261	F2-3 vs. L2-3	F2-3 up	+	nucleoside phosphate binding	GO:1901265	Development	Cell division	F	1112	73	44.7	1.63	1.06E-04	6.69E-03
3	262	F2-3 vs. L2-3	F2-3 up	+	nucleotide binding	GO:0000166	Development	Cell division	F	1112	73	44.7	1.63	1.06E-04	6.83E-03
3	263	F2-3 vs. L2-3	F2-3 up	+	nucleotide-sugar biosynthetic process	GO:0009226	Development	Cell division	P	42	11	1.69	6.52	4.69E-06	2.21E-04
3	264	F2-3 vs. L2-3	F2-3 up	+	nucleotide-sugar metabolic process	GO:0009225	Development	Cell division	P	52	12	2.09	5.74	5.36E-06	2.43E-04
3	265	F2-3 vs. L2-3	F2-3 up	+	pollen germination	GO:0009846	Development	Cell division	P	63	10	2.53	3.95	4.86E-04	1.20E-02
3	266	F2-3 vs. L2-3	F2-3 up	+	pollen tube development	GO:0048868	Development	Cell division	P	192	26	7.72	3.37	3.79E-07	2.80E-05
3	267	F2-3 vs. L2-3	F2-3 up	+	pollen tube growth	GO:0009860	Development	Cell division	P	135	21	5.43	3.87	6.75E-07	4.49E-05
3	268	F2-3 vs. L2-3	F2-3 up	+	pollination	GO:0009856	Development	Cell division	P	287	30	11.54	2.6	1.02E-05	4.17E-04
3	269	F2-3 vs. L2-3	F2-3 up	+	reproduction	GO:0000003	Development	Cell division	P	1742	111	70.02	1.59	4.71E-06	2.18E-04
3	270	F2-3 vs. L2-3	F2-3 up	+	reproductive process	GO:0022414	Development	Cell division	P	1732	111	69.62	1.59	3.31E-06	1.62E-04
3	271	F2-3 vs. L2-3	F2-3 up	+	syncytium formation	GO:0006949	Development	Cell division	P	16	5	0.64	7.77	1.04E-03	2.30E-02
3	272	F2-3 vs. L2-3	F2-3 up	-	chromosome organization	GO:0051276	Development	Development	P	501	6	20.14	0.3	4.41E-04	1.13E-02
3	273	F2-3 vs. L2-3	F2-3 up	-	nuclear lumen	GO:0031981	Development	Development	C	1222	28	49.12	0.57	1.55E-03	2.60E-02
3	274	F2-3 vs. L2-3	F2-3 up	-	nucleoplasm	GO:0005654	Development	Development	C	466	5	18.73	0.27	4.04E-04	8.58E-03
3	275	F2-3 vs. L2-3	F2-3 up	-	nucleus	GO:0005634	Development	Development	C	10540	321	423.66	0.76	2.25E-10	9.78E-09
3	276	F2-3 vs. L2-3	F2-3 up	+	actin cytoskeleton	GO:0015629	Development	Development	C	63	9	2.53	3.55	1.78E-03	2.85E-02
3	277	F2-3 vs. L2-3	F2-3 up	+	actin cytoskeleton organization	GO:0030036	Development	Development	P	104	12	4.18	2.87	1.79E-03	3.35E-02
3	278	F2-3 vs. L2-3	F2-3 up	+	anatomical structure development	GO:0048856	Development	Development	P	2842	183	114.24	1.6	6.56E-10	7.14E-08
3	279	F2-3 vs. L2-3	F2-3 up	+	anatomical structure formation involved in morphogenesis	GO:0048646	Development	Development	P	204	19	8.2	2.32	1.27E-03	2.60E-02
3	280	F2-3 vs. L2-3	F2-3 up	+	anatomical structure morphogenesis	GO:0009653	Development	Development	P	896	70	36.02	1.94	5.95E-07	4.13E-05

3	281	F2-3 vs. L2-3	F2-3 up	+	anther dehiscence	GO:0009901	Development	Development	P	21	5	0.84	5.92	2.85E-03	4.86E-02
3	282	F2-3 vs. L2-3	F2-3 up	+	apoplast	GO:0048046	Development	Development	C	297	61	11.94	5.11	1.21E-22	4.21E-20
3	283	F2-3 vs. L2-3	F2-3 up	+	bounding membrane of organelle	GO:0098588	Development	Development	C	923	64	37.1	1.73	6.74E-05	1.56E-03
3	284	F2-3 vs. L2-3	F2-3 up	+	cell development	GO:0048468	Development	Development	P	319	30	12.82	2.34	5.71E-05	1.92E-03
3	285	F2-3 vs. L2-3	F2-3 up	+	cell differentiation	GO:0030154	Development	Development	P	613	46	24.64	1.87	1.29E-04	4.05E-03
3	286	F2-3 vs. L2-3	F2-3 up	+	cell growth	GO:0016049	Development	Development	P	386	44	15.52	2.84	5.12E-09	4.50E-07
3	287	F2-3 vs. L2-3	F2-3 up	+	cell junction	GO:0030054	Development	Development	C	944	61	37.94	1.61	5.85E-04	1.11E-02
3	288	F2-3 vs. L2-3	F2-3 up	+	cell morphogenesis	GO:0000902	Development	Development	P	369	37	14.83	2.49	1.56E-06	8.57E-05
3	289	F2-3 vs. L2-3	F2-3 up	+	cell morphogenesis involved in differentiation	GO:0000904	Development	Development	P	229	24	9.2	2.61	5.20E-05	1.78E-03
3	290	F2-3 vs. L2-3	F2-3 up	+	cell periphery	GO:0071944	Development	Development	C	4012	271	161.26	1.68	2.59E-17	2.45E-15
3	291	F2-3 vs. L2-3	F2-3 up	+	cell projection	GO:0042995	Development	Development	C	115	18	4.62	3.89	3.85E-06	1.25E-04
3	292	F2-3 vs. L2-3	F2-3 up	+	cell tip growth	GO:0009932	Development	Development	P	160	22	6.43	3.42	2.34E-06	1.23E-04
3	293	F2-3 vs. L2-3	F2-3 up	+	cell wall	GO:0005618	Development	Development	C	565	52	22.71	2.29	2.08E-07	7.72E-06
3	294	F2-3 vs. L2-3	F2-3 up	+	cell wall biogenesis	GO:0042546	Development	Development	P	238	22	9.57	2.3	5.58E-04	1.36E-02
3	295	F2-3 vs. L2-3	F2-3 up	+	cell wall modification	GO:0042545	Development	Development	P	173	24	6.95	3.45	7.15E-07	4.70E-05
3	296	F2-3 vs. L2-3	F2-3 up	+	cell wall organization	GO:0071555	Development	Development	P	274	39	11.01	3.54	1.38E-10	1.72E-08
3	297	F2-3 vs. L2-3	F2-3 up	+	cell wall organization or biogenesis	GO:0071554	Development	Development	P	507	57	20.38	2.8	4.52E-11	6.60E-09
3	298	F2-3 vs. L2-3	F2-3 up	+	cell wall pectin metabolic process	GO:0052546	Development	Development	P	27	6	1.09	5.53	1.49E-03	2.90E-02
3	299	F2-3 vs. L2-3	F2-3 up	+	cell-cell junction	GO:0005911	Development	Development	C	944	61	37.94	1.61	5.85E-04	1.17E-02
3	300	F2-3 vs. L2-3	F2-3 up	+	cellular anatomical entity	GO:0110165	Development	Development	C	24430	1024	981.98	1.04	2.94E-05	7.13E-04
3	301	F2-3 vs. L2-3	F2-3 up	+	cellular developmental process	GO:0048869	Development	Development	P	695	49	27.94	1.75	3.20E-04	8.62E-03
3	302	F2-3 vs. L2-3	F2-3 up	+	cellular process	GO:0009987	Development	Development	P	12008	683	482.67	1.42	1.84E-32	1.10E-28
3	303	F2-3 vs. L2-3	F2-3 up	+	circulatory system process	GO:0003013	Development	Development	P	21	6	0.84	7.11	4.84E-04	1.21E-02
3	304	F2-3 vs. L2-3	F2-3 up	+	cytoplasm	GO:0005737	Development	Development	C	14837	773	596.38	1.3	1.94E-26	2.02E-23
3	305	F2-3 vs. L2-3	F2-3 up	+	cytosol	GO:0005829	Development	Development	C	3241	235	130.27	1.8	3.48E-18	4.03E-16
3	306	F2-3 vs. L2-3	F2-3 up	+	developmental cell growth	GO:0048588	Development	Development	P	197	26	7.92	3.28	5.90E-07	4.20E-05
3	307	F2-3 vs. L2-3	F2-3 up	+	developmental growth	GO:0048589	Development	Development	P	385	46	15.48	2.97	5.93E-10	6.69E-08
3	308	F2-3 vs. L2-3	F2-3 up	+	developmental growth involved in	GO:0060560	Development	Development	P	335	40	13.47	2.97	7.92E-09	6.67E-07
3	309	F2-3 vs. L2-3	F2-3 up	+	developmental process	GO:0032502	Development	Development	P	2997	194	120.47	1.61	9.81E-11	1.30E-08
3	310	F2-3 vs. L2-3	F2-3 up	+	developmental process involved in reproduction	GO:0003006	Development	Development	P	1437	98	57.76	1.7	1.11E-06	6.52E-05
3	311	F2-3 vs. L2-3	F2-3 up	+	endomembrane system	GO:0012505	Development	Development	C	2286	138	91.89	1.5	4.36E-06	1.38E-04
3	312	F2-3 vs. L2-3	F2-3 up	+	envelope	GO:0031975	Development	Development	C	1025	97	41.2	2.35	1.59E-13	7.89E-12
3	313	F2-3 vs. L2-3	F2-3 up	+	external encapsulating structure	GO:0030312	Development	Development	C	567	54	22.79	2.37	3.58E-08	1.38E-06
3	314	F2-3 vs. L2-3	F2-3 up	+	external encapsulating structure organization	GO:0045229	Development	Development	P	320	43	12.86	3.34	8.18E-11	1.11E-08
3	315	F2-3 vs. L2-3	F2-3 up	+	extracellular region	GO:0005576	Development	Development	C	3128	190	125.73	1.51	2.33E-08	9.69E-07
3	316	F2-3 vs. L2-3	F2-3 up	+	floral organ development	GO:0048437	Development	Development	P	245	24	9.85	2.44	1.59E-04	4.80E-03
3	317	F2-3 vs. L2-3	F2-3 up	+	flower development	GO:0009908	Development	Development	P	356	32	14.31	2.24	8.22E-05	2.64E-03
3	318	F2-3 vs. L2-3	F2-3 up	+	Golgi apparatus subcompartment	GO:0098791	Development	Development	C	437	37	17.57	2.11	5.60E-05	1.33E-03
3	319	F2-3 vs. L2-3	F2-3 up	+	Golgi cisterna	GO:0031985	Development	Development	C	185	17	7.44	2.29	2.62E-03	4.06E-02
3	320	F2-3 vs. L2-3	F2-3 up	+	Golgi trans cisterna	GO:0000138	Development	Development	C	85	11	3.42	3.22	1.20E-03	2.11E-02
3	321	F2-3 vs. L2-3	F2-3 up	+	growth	GO:0040007	Development	Development	P	443	51	17.81	2.86	2.26E-10	2.71E-08
3	322	F2-3 vs. L2-3	F2-3 up	+	intracellular vesicle	GO:0097708	Development	Development	C	722	56	29.02	1.93	1.10E-05	3.09E-04
3	323	F2-3 vs. L2-3	F2-3 up	+	membrane	GO:0016020	Development	Development	C	5493	363	220.79	1.64	8.49E-23	4.42E-20
3	324	F2-3 vs. L2-3	F2-3 up	+	microbody	GO:0042579	Development	Development	C	320	31	12.86	2.41	1.97E-05	5.13E-04
3	325	F2-3 vs. L2-3	F2-3 up	+	multicellular organism development	GO:0007275	Development	Development	P	2457	147	98.76	1.49	3.15E-06	1.56E-04
3	326	F2-3 vs. L2-3	F2-3 up	+	multi-multicellular organism process	GO:0044706	Development	Development	P	287	30	11.54	2.6	1.02E-05	4.20E-04
3	327	F2-3 vs. L2-3	F2-3 up	+	organelle envelope	GO:0031967	Development	Development	C	1025	97	41.2	2.35	1.59E-13	7.53E-12
3	328	F2-3 vs. L2-3	F2-3 up	+	organelle envelope lumen	GO:0031970	Development	Development	C	16	5	0.64	7.77	1.04E-03	1.86E-02
3	329	F2-3 vs. L2-3	F2-3 up	+	organelle membrane	GO:0031090	Development	Development	C	1458	93	58.61	1.59	2.48E-05	6.14E-04
3	330	F2-3 vs. L2-3	F2-3 up	+	organelle subcompartment	GO:0031984	Development	Development	C	1156	114	46.47	2.45	6.08E-17	4.87E-15
3	331	F2-3 vs. L2-3	F2-3 up	+	phloem loading	GO:0110126	Development	Development	P	7	4	0.28	14.22	5.88E-04	1.42E-02
3	332	F2-3 vs. L2-3	F2-3 up	+	phyllome development	GO:0048827	Development	Development	P	495	40	19.9	2.01	9.09E-05	2.91E-03
3	333	F2-3 vs. L2-3	F2-3 up	+	plant organ development	GO:0099402	Development	Development	P	1009	69	40.56	1.7	4.89E-05	1.70E-03
3	334	F2-3 vs. L2-3	F2-3 up	+	plant-type cell wall	GO:0009505	Development	Development	C	233	21	9.37	2.24	1.42E-03	2.42E-02
3	335	F2-3 vs. L2-3	F2-3 up	+	plant-type cell wall biogenesis	GO:0009832	Development	Development	P	157	17	6.31	2.69	4.37E-04	1.12E-02
3	336	F2-3 vs. L2-3	F2-3 up	+	plant-type cell wall loosening	GO:0009828	Development	Development	P	37	7	1.49	4.71	1.38E-03	2.74E-02
3	337	F2-3 vs. L2-3	F2-3 up	+	plant-type cell wall modification	GO:0009827	Development	Development	P	64	11	2.57	4.28	1.39E-04	4.28E-03
3	338	F2-3 vs. L2-3	F2-3 up	+	plant-type cell wall organization	GO:0009664	Development	Development	P	150	22	6.03	3.65	8.97E-07	5.53E-05
3	339	F2-3 vs. L2-3	F2-3 up	+	plant-type cell wall organization or biogenesis	GO:0071669	Development	Development	P	265	31	10.65	2.91	5.58E-07	4.02E-05
3	340	F2-3 vs. L2-3	F2-3 up	+	plant-type primary cell wall biogenesis	GO:0009833	Development	Development	P	28	8	1.13	7.11	5.59E-05	1.89E-03
3	341	F2-3 vs. L2-3	F2-3 up	+	plasma membrane	GO:0005886	Development	Development	C	3534	226	142.05	1.59	5.23E-12	2.37E-10
3	342	F2-3 vs. L2-3	F2-3 up	+	plasma membrane bounded cell projection	GO:0120025	Development	Development	C	101	18	4.06	4.43	7.44E-07	2.58E-05

3	343	F2-3 vs. L2-3	F2-3 up	+	pollen development	GO:0009555	Development	Development	P	346	30	13.91	2.16	2.05E-04	5.82E-03
3	344	F2-3 vs. L2-3	F2-3 up	+	pollen tube	GO:0090406	Development	Development	C	83	17	3.34	5.1	2.73E-07	9.81E-06
3	345	F2-3 vs. L2-3	F2-3 up	+	post-embryonic development	GO:0009791	Development	Development	P	1482	86	59.57	1.44	1.17E-03	2.47E-02
3	346	F2-3 vs. L2-3	F2-3 up	+	reproductive shoot system development	GO:0090567	Development	Development	P	371	33	14.91	2.21	6.59E-05	2.17E-03
3	347	F2-3 vs. L2-3	F2-3 up	+	reproductive structure development	GO:0048608	Development	Development	P	1206	73	48.48	1.51	1.03E-03	2.30E-02
3	348	F2-3 vs. L2-3	F2-3 up	+	reproductive system development	GO:0061458	Development	Development	P	1208	73	48.56	1.5	1.04E-03	2.29E-02
3	349	F2-3 vs. L2-3	F2-3 up	+	shoot system development	GO:0048367	Development	Development	P	728	57	29.26	1.95	5.31E-06	2.42E-04
3	350	F2-3 vs. L2-3	F2-3 up	+	stromule	GO:0010319	Development	Development	C	36	13	1.45	8.98	2.87E-08	1.15E-06
3	351	F2-3 vs. L2-3	F2-3 up	+	symplast	GO:0055044	Development	Development	C	944	61	37.94	1.61	5.85E-04	1.15E-02
3	352	F2-3 vs. L2-3	F2-3 up	+	system development	GO:0048731	Development	Development	P	1867	114	75.05	1.52	2.13E-05	8.28E-04
3	353	F2-3 vs. L2-3	F2-3 up	+	system process	GO:0003008	Development	Development	P	21	6	0.84	7.11	4.84E-04	1.20E-02
3	354	F2-3 vs. L2-3	F2-3 up	+	unidimensional cell growth	GO:0009826	Development	Development	P	288	35	11.58	3.02	4.55E-08	3.63E-06
3	355	F2-3 vs. L2-3	F2-3 up	+	vacuolar membrane	GO:0005774	Development	Development	C	570	48	22.91	2.1	6.39E-06	1.90E-04
3	356	F2-3 vs. L2-3	F2-3 up	+	vacuole	GO:0005773	Development	Development	C	1083	102	43.53	2.34	3.61E-14	1.88E-12
3	357	F2-3 vs. L2-3	F2-3 up	+	vascular process in circulatory system	GO:0003018	Development	Development	P	21	6	0.84	7.11	4.84E-04	1.21E-02
3	358	F2-3 vs. L2-3	F2-3 up	-	DNA metabolic process	GO:0006259	Metabolism	Metabolism	P	448	5	18.01	0.28	7.73E-04	1.81E-02
3	359	F2-3 vs. L2-3	F2-3 up	+	actin binding	GO:0003779	Metabolism	Metabolism	F	114	14	4.58	3.06	4.42E-04	2.37E-02
3	360	F2-3 vs. L2-3	F2-3 up	+	actin filament-based process	GO:0030029	Metabolism	Metabolism	P	113	13	4.54	2.86	1.21E-03	2.51E-02
3	361	F2-3 vs. L2-3	F2-3 up	+	actin monomer binding	GO:0003785	Metabolism	Metabolism	F	7	4	0.28	14.22	5.88E-04	3.00E-02
3	362	F2-3 vs. L2-3	F2-3 up	+	alpha-amino acid biosynthetic process	GO:1901607	Metabolism	Metabolism	P	181	18	7.28	2.47	1.14E-03	2.42E-02
3	363	F2-3 vs. L2-3	F2-3 up	+	alpha-amino acid metabolic process	GO:1901605	Metabolism	Metabolism	P	278	36	11.17	3.22	6.71E-09	5.73E-07
3	364	F2-3 vs. L2-3	F2-3 up	+	aminoglycan biosynthetic process	GO:0006023	Metabolism	Metabolism	P	4	3	0.16	18.66	1.79E-03	3.29E-02
3	365	F2-3 vs. L2-3	F2-3 up	+	anchoring junction	GO:0070161	Metabolism	Metabolism	C	944	61	37.94	1.61	5.85E-04	1.13E-02
3	366	F2-3 vs. L2-3	F2-3 up	+	anion binding	GO:0043168	Metabolism	Metabolism	F	1369	85	55.03	1.54	1.51E-04	8.99E-03
3	367	F2-3 vs. L2-3	F2-3 up	+	aromatic compound biosynthetic process	GO:0019438	Metabolism	Metabolism	P	808	60	32.48	1.85	1.47E-05	5.82E-04
3	368	F2-3 vs. L2-3	F2-3 up	+	beta-glucan biosynthetic process	GO:0051274	Metabolism	Metabolism	P	62	11	2.49	4.41	1.08E-04	3.42E-03
3	369	F2-3 vs. L2-3	F2-3 up	+	beta-glucan metabolic process	GO:0051273	Metabolism	Metabolism	P	67	11	2.69	4.08	1.98E-04	5.65E-03
3	370	F2-3 vs. L2-3	F2-3 up	+	binding	GO:0005488	Metabolism	Metabolism	F	9843	468	395.64	1.18	1.15E-05	1.12E-03
3	371	F2-3 vs. L2-3	F2-3 up	+	biological_process	GO:0008150	Metabolism	Metabolism	P	21988	948	883.82	1.07	8.28E-07	5.27E-05
3	372	F2-3 vs. L2-3	F2-3 up	+	biosynthetic process	GO:0009058	Metabolism	Metabolism	P	2811	218	112.99	1.93	5.23E-20	2.61E-17
3	373	F2-3 vs. L2-3	F2-3 up	+	carbon utilization	GO:0015976	Metabolism	Metabolism	P	8	4	0.32	12.44	8.56E-04	1.95E-02
3	374	F2-3 vs. L2-3	F2-3 up	+	carbon-carbon lyase activity	GO:0016830	Metabolism	Metabolism	F	111	18	4.46	4.03	2.47E-06	2.74E-04
3	375	F2-3 vs. L2-3	F2-3 up	+	carboxylic acid biosynthetic process	GO:0046394	Metabolism	Metabolism	P	468	50	18.81	2.66	4.48E-09	4.06E-07
3	376	F2-3 vs. L2-3	F2-3 up	+	carboxylic acid metabolic process	GO:0019752	Metabolism	Metabolism	P	912	103	36.66	2.81	3.26E-19	1.30E-16
3	377	F2-3 vs. L2-3	F2-3 up	+	carboxy-lyase activity	GO:0016831	Metabolism	Metabolism	F	68	10	2.73	3.66	8.27E-04	3.85E-02
3	378	F2-3 vs. L2-3	F2-3 up	+	catalytic activity	GO:0003824	Metabolism	Metabolism	F	8380	569	336.84	1.69	8.73E-46	2.81E-42
3	379	F2-3 vs. L2-3	F2-3 up	+	cation binding	GO:0043169	Metabolism	Metabolism	F	1637	116	65.8	1.76	1.35E-08	2.90E-06
3	380	F2-3 vs. L2-3	F2-3 up	+	cellular amino acid biosynthetic process	GO:0008652	Metabolism	Metabolism	P	200	20	8.04	2.49	5.37E-04	1.31E-02
3	381	F2-3 vs. L2-3	F2-3 up	+	cellular amino acid metabolic process	GO:0006520	Metabolism	Metabolism	P	391	42	15.72	2.67	5.24E-08	4.07E-06
3	382	F2-3 vs. L2-3	F2-3 up	+	cellular biosynthetic process	GO:0044249	Metabolism	Metabolism	P	2583	186	103.82	1.79	4.41E-14	1.01E-11
3	383	F2-3 vs. L2-3	F2-3 up	+	cellular glucan metabolic process	GO:0006073	Metabolism	Metabolism	P	195	24	7.84	3.06	4.72E-06	2.17E-04
3	384	F2-3 vs. L2-3	F2-3 up	+	cellular lipid metabolic process	GO:0044255	Metabolism	Metabolism	P	689	57	27.69	2.06	1.08E-06	6.40E-05
3	385	F2-3 vs. L2-3	F2-3 up	+	cellular metabolic process	GO:0044237	Metabolism	Metabolism	P	7649	437	307.46	1.42	1.96E-16	6.50E-14
3	386	F2-3 vs. L2-3	F2-3 up	+	cellular modified amino acid metabolic process	GO:0006575	Metabolism	Metabolism	P	96	11	3.86	2.85	2.87E-03	4.86E-02
3	387	F2-3 vs. L2-3	F2-3 up	+	cellulose biosynthetic process	GO:0030244	Metabolism	Metabolism	P	50	10	2.01	4.98	9.27E-05	2.95E-03
3	388	F2-3 vs. L2-3	F2-3 up	+	cellulose metabolic process	GO:0030243	Metabolism	Metabolism	P	55	10	2.21	4.52	1.85E-04	5.36E-03
3	389	F2-3 vs. L2-3	F2-3 up	+	copper ion binding	GO:0005507	Metabolism	Metabolism	F	193	29	7.76	3.74	1.08E-08	2.48E-06
3	390	F2-3 vs. L2-3	F2-3 up	+	coumarin metabolic process	GO:0009804	Metabolism	Metabolism	P	9	4	0.36	11.06	1.20E-03	2.51E-02
3	391	F2-3 vs. L2-3	F2-3 up	+	dicarboxylic acid metabolic process	GO:0043648	Metabolism	Metabolism	P	89	16	3.58	4.47	2.74E-06	1.38E-04
3	392	F2-3 vs. L2-3	F2-3 up	+	dioxygenase activity	GO:0051213	Metabolism	Metabolism	F	116	17	4.66	3.65	1.53E-05	1.45E-03
3	393	F2-3 vs. L2-3	F2-3 up	+	erythrose 4-phosphate/phosphoenolpyruvate family amino acid metabolic process	GO:1902221	Metabolism	Metabolism	P	26	6	1.05	5.74	1.26E-03	2.60E-02
3	394	F2-3 vs. L2-3	F2-3 up	+	establishment of localization	GO:0051234	Metabolism	Metabolism	P	2306	148	92.69	1.6	4.89E-08	3.85E-06
3	395	F2-3 vs. L2-3	F2-3 up	+	galacturonan metabolic process	GO:0010393	Metabolism	Metabolism	P	163	21	6.55	3.21	9.73E-06	4.10E-04
3	396	F2-3 vs. L2-3	F2-3 up	+	glucan biosynthetic process	GO:0009250	Metabolism	Metabolism	P	118	16	4.74	3.37	6.27E-05	2.09E-03
3	397	F2-3 vs. L2-3	F2-3 up	+	glucan metabolic process	GO:0044042	Metabolism	Metabolism	P	203	24	8.16	2.94	8.73E-06	3.75E-04
3	398	F2-3 vs. L2-3	F2-3 up	+	glucosinolate biosynthetic process	GO:0019761	Metabolism	Metabolism	P	39	11	1.57	7.02	2.55E-06	1.32E-04
3	399	F2-3 vs. L2-3	F2-3 up	+	glucosinolate metabolic process	GO:0019760	Metabolism	Metabolism	P	112	16	4.5	3.55	3.57E-05	1.30E-03
3	400	F2-3 vs. L2-3	F2-3 up	+	glucosyltransferase activity	GO:0046527	Metabolism	Metabolism	F	190	19	7.64	2.49	7.71E-04	3.76E-02
3	401	F2-3 vs. L2-3	F2-3 up	+	glutamate metabolic process	GO:0006536	Metabolism	Metabolism	P	27	8	1.09	7.37	4.50E-05	1.57E-03
3	402	F2-3 vs. L2-3	F2-3 up	+	glutamine family amino acid metabolic process	GO:0009064	Metabolism	Metabolism	P	58	10	2.33	4.29	2.70E-04	7.35E-03
3	403	F2-3 vs. L2-3	F2-3 up	+	glycine metabolic process	GO:0006544	Metabolism	Metabolism	P	18	5	0.72	6.91	1.61E-03	3.08E-02

3	404	F2-3 vs. L2-3	F2-3 up	+	glycosaminoglycan biosynthetic process	GO:0006024	Metabolism	Metabolism	P	4	3	0.16	18.66	1.79E-03	3.30E-02
3	405	F2-3 vs. L2-3	F2-3 up	+	glycosaminoglycan metabolic process	GO:0030203	Metabolism	Metabolism	P	4	3	0.16	18.66	1.79E-03	3.32E-02
3	406	F2-3 vs. L2-3	F2-3 up	+	glycosinolate biosynthetic process	GO:0019758	Metabolism	Metabolism	P	39	11	1.57	7.02	2.55E-06	1.30E-04
3	407	F2-3 vs. L2-3	F2-3 up	+	glycosinolate metabolic process	GO:0019757	Metabolism	Metabolism	P	112	16	4.5	3.55	3.57E-05	1.29E-03
3	408	F2-3 vs. L2-3	F2-3 up	+	glycosyl compound biosynthetic process	GO:1901659	Metabolism	Metabolism	P	62	13	2.49	5.22	5.46E-06	2.45E-04
3	409	F2-3 vs. L2-3	F2-3 up	+	glycosyl compound metabolic process	GO:1901657	Metabolism	Metabolism	P	187	20	7.52	2.66	1.66E-04	4.94E-03
3	410	F2-3 vs. L2-3	F2-3 up	+	heme binding	GO:0020037	Metabolism	Metabolism	F	374	31	15.03	2.06	3.56E-04	2.01E-02
3	411	F2-3 vs. L2-3	F2-3 up	+	homogalacturonan metabolic process	GO:0010394	Metabolism	Metabolism	P	4	3	0.16	18.66	1.79E-03	3.28E-02
3	412	F2-3 vs. L2-3	F2-3 up	+	hydrolase activity	GO:0016787	Metabolism	Metabolism	F	2995	185	120.39	1.54	9.73E-09	2.61E-06
3	413	F2-3 vs. L2-3	F2-3 up	+	hydrolase activity, hydrolyzing O-glycosyl compounds	GO:0004553	Metabolism	Metabolism	F	396	33	15.92	2.07	1.97E-04	1.15E-02
3	414	F2-3 vs. L2-3	F2-3 up	+	ion binding	GO:0043167	Metabolism	Metabolism	F	2847	188	114.44	1.64	4.90E-11	1.97E-08
3	415	F2-3 vs. L2-3	F2-3 up	+	iron ion binding	GO:0005506	Metabolism	Metabolism	F	325	30	13.06	2.3	6.96E-05	4.66E-03
3	416	F2-3 vs. L2-3	F2-3 up	+	lignin biosynthetic process	GO:0009809	Metabolism	Metabolism	P	42	9	1.69	5.33	1.31E-04	4.06E-03
3	417	F2-3 vs. L2-3	F2-3 up	+	lignin metabolic process	GO:0009808	Metabolism	Metabolism	P	53	11	2.13	5.16	3.10E-05	1.14E-03
3	418	F2-3 vs. L2-3	F2-3 up	+	lipid biosynthetic process	GO:0008610	Metabolism	Metabolism	P	512	41	20.58	1.99	7.68E-05	2.48E-03
3	419	F2-3 vs. L2-3	F2-3 up	+	lipid metabolic process	GO:0006629	Metabolism	Metabolism	P	828	67	33.28	2.01	3.39E-07	2.54E-05
3	420	F2-3 vs. L2-3	F2-3 up	+	localization	GO:0051179	Metabolism	Metabolism	P	2471	159	99.32	1.6	1.11E-08	9.08E-07
3	421	F2-3 vs. L2-3	F2-3 up	+	L-phenylalanine metabolic process	GO:0006558	Metabolism	Metabolism	P	26	6	1.05	5.74	1.26E-03	2.61E-02
3	422	F2-3 vs. L2-3	F2-3 up	+	lyase activity	GO:0016829	Metabolism	Metabolism	F	339	40	13.63	2.94	1.07E-08	2.64E-06
3	423	F2-3 vs. L2-3	F2-3 up	+	metabolic process	GO:0008152	Metabolism	Metabolism	P	8681	525	348.94	1.5	4.42E-27	6.61E-24
3	424	F2-3 vs. L2-3	F2-3 up	+	metal ion binding	GO:0046872	Metabolism	Metabolism	F	1610	114	64.71	1.76	2.20E-08	4.41E-06
3	425	F2-3 vs. L2-3	F2-3 up	+	molecular_function	GO:0003674	Metabolism	Metabolism	F	21945	969	882.09	1.1	1.08E-11	5.78E-09
3	426	F2-3 vs. L2-3	F2-3 up	+	monocarboxylic acid biosynthetic process	GO:0072330	Metabolism	Metabolism	P	211	25	8.48	2.95	5.52E-06	2.46E-04
3	427	F2-3 vs. L2-3	F2-3 up	+	monocarboxylic acid metabolic process	GO:0032787	Metabolism	Metabolism	P	439	54	17.65	3.06	6.95E-12	1.22E-09
3	428	F2-3 vs. L2-3	F2-3 up	+	monooxygenase activity	GO:0004497	Metabolism	Metabolism	F	222	24	8.92	2.69	3.32E-05	2.66E-03
3	429	F2-3 vs. L2-3	F2-3 up	+	monosaccharide metabolic process	GO:0005996	Metabolism	Metabolism	P	125	17	5.02	3.38	3.62E-05	1.30E-03
3	430	F2-3 vs. L2-3	F2-3 up	+	mucilage biosynthetic process	GO:0010192	Metabolism	Metabolism	P	30	6	1.21	4.98	2.37E-03	4.19E-02
3	431	F2-3 vs. L2-3	F2-3 up	+	mucilage metabolic process	GO:0010191	Metabolism	Metabolism	P	37	7	1.49	4.71	1.38E-03	2.73E-02
3	432	F2-3 vs. L2-3	F2-3 up	+	organic acid biosynthetic process	GO:0016053	Metabolism	Metabolism	P	468	50	18.81	2.66	4.48E-09	4.12E-07
3	433	F2-3 vs. L2-3	F2-3 up	+	organic acid metabolic process	GO:0006082	Metabolism	Metabolism	P	1019	115	40.96	2.81	2.38E-21	1.42E-18
3	434	F2-3 vs. L2-3	F2-3 up	+	organic cyclic compound biosynthetic process	GO:1901362	Metabolism	Metabolism	P	899	70	36.14	1.94	6.24E-07	4.29E-05
3	435	F2-3 vs. L2-3	F2-3 up	+	organic hydroxy compound biosynthetic process	GO:1901617	Metabolism	Metabolism	P	172	19	6.91	2.75	1.64E-04	4.90E-03
3	436	F2-3 vs. L2-3	F2-3 up	+	organic hydroxy compound metabolic process	GO:1901615	Metabolism	Metabolism	P	292	32	11.74	2.73	1.34E-06	7.64E-05
3	437	F2-3 vs. L2-3	F2-3 up	+	organic substance biosynthetic process	GO:1901576	Metabolism	Metabolism	P	2674	201	107.48	1.87	5.61E-17	1.97E-14
3	438	F2-3 vs. L2-3	F2-3 up	+	organic substance metabolic process	GO:0071704	Metabolism	Metabolism	P	7851	425	315.57	1.35	4.17E-12	7.79E-10
3	439	F2-3 vs. L2-3	F2-3 up	+	organonitrogen compound biosynthetic process	GO:1901566	Metabolism	Metabolism	P	1343	79	53.98	1.46	1.43E-03	2.79E-02
3	440	F2-3 vs. L2-3	F2-3 up	+	organophosphate metabolic process	GO:0019637	Metabolism	Metabolism	P	583	48	23.43	2.05	8.82E-06	3.77E-04
3	441	F2-3 vs. L2-3	F2-3 up	+	oxidation-reduction process	GO:0055114	Metabolism	Metabolism	P	768	101	30.87	3.27	3.73E-23	2.79E-20
3	442	F2-3 vs. L2-3	F2-3 up	+	oxoacid metabolic process	GO:0043436	Metabolism	Metabolism	P	1016	115	40.84	2.82	1.92E-21	1.28E-18
3	443	F2-3 vs. L2-3	F2-3 up	+	oxylipin biosynthetic process	GO:0031408	Metabolism	Metabolism	P	17	5	0.68	7.32	1.30E-03	2.64E-02
3	444	F2-3 vs. L2-3	F2-3 up	+	oxylipin metabolic process	GO:0031407	Metabolism	Metabolism	P	19	5	0.76	6.55	1.97E-03	3.55E-02
3	445	F2-3 vs. L2-3	F2-3 up	+	pectin metabolic process	GO:0045488	Metabolism	Metabolism	P	162	21	6.51	3.22	8.94E-06	3.79E-04
3	446	F2-3 vs. L2-3	F2-3 up	+	phenylpropanoid biosynthetic process	GO:0009699	Metabolism	Metabolism	P	88	15	3.54	4.24	9.85E-06	4.09E-04
3	447	F2-3 vs. L2-3	F2-3 up	+	phenylpropanoid metabolic process	GO:0009698	Metabolism	Metabolism	P	109	17	4.38	3.88	7.40E-06	3.23E-04
3	448	F2-3 vs. L2-3	F2-3 up	+	phosphate-containing compound metabolic process	GO:0006796	Metabolism	Metabolism	P	1858	113	74.68	1.51	2.68E-05	1.01E-03
3	449	F2-3 vs. L2-3	F2-3 up	+	phosphorus metabolic process	GO:0006793	Metabolism	Metabolism	P	1903	123	76.49	1.61	5.92E-07	4.16E-05
3	450	F2-3 vs. L2-3	F2-3 up	+	proton export across plasma membrane	GO:0120029	Metabolism	Metabolism	P	10	5	0.4	12.44	1.85E-04	5.36E-03
3	451	F2-3 vs. L2-3	F2-3 up	+	purine ribonucleotide metabolic process	GO:0009150	Metabolism	Metabolism	P	200	19	8.04	2.36	1.08E-03	2.33E-02
3	452	F2-3 vs. L2-3	F2-3 up	+	purine-containing compound biosynthetic	GO:0072522	Metabolism	Metabolism	P	126	13	5.06	2.57	2.91E-03	4.92E-02
3	453	F2-3 vs. L2-3	F2-3 up	+	purine-containing compound metabolic process	GO:0072521	Metabolism	Metabolism	P	243	21	9.77	2.15	1.85E-03	3.37E-02
3	454	F2-3 vs. L2-3	F2-3 up	+	pyridoxal phosphate binding	GO:0030170	Metabolism	Metabolism	F	98	14	3.94	3.55	1.08E-04	6.54E-03
3	455	F2-3 vs. L2-3	F2-3 up	+	S-adenosylmethionine biosynthetic process	GO:0006556	Metabolism	Metabolism	P	4	3	0.16	18.66	1.79E-03	3.33E-02
3	456	F2-3 vs. L2-3	F2-3 up	+	S-adenosylmethionine metabolic process	GO:0046500	Metabolism	Metabolism	P	9	4	0.36	11.06	1.20E-03	2.51E-02
3	457	F2-3 vs. L2-3	F2-3 up	+	secondary metabolic process	GO:0019748	Metabolism	Metabolism	P	303	39	12.18	3.2	1.85E-09	1.79E-07
3	458	F2-3 vs. L2-3	F2-3 up	+	secondary metabolite biosynthetic process	GO:0044550	Metabolism	Metabolism	P	144	28	5.79	4.84	1.20E-10	1.52E-08
3	459	F2-3 vs. L2-3	F2-3 up	+	sequestering of actin monomers	GO:0042989	Metabolism	Metabolism	P	5	3	0.2	14.93	2.78E-03	4.81E-02
3	460	F2-3 vs. L2-3	F2-3 up	+	serine family amino acid metabolic process	GO:0009069	Metabolism	Metabolism	P	58	9	2.33	3.86	1.06E-03	2.30E-02
3	461	F2-3 vs. L2-3	F2-3 up	+	S-glycoside biosynthetic process	GO:0016144	Metabolism	Metabolism	P	39	11	1.57	7.02	2.55E-06	1.33E-04
3	462	F2-3 vs. L2-3	F2-3 up	+	S-glycoside metabolic process	GO:0016143	Metabolism	Metabolism	P	112	16	4.5	3.55	3.57E-05	1.31E-03
3	463	F2-3 vs. L2-3	F2-3 up	+	small molecule binding	GO:0036094	Metabolism	Metabolism	F	1340	93	53.86	1.73	1.01E-06	1.25E-04

3	464	F2-3 vs. L2-3	F2-3 up	+	small molecule biosynthetic process	GO:0044283	Metabolism	Metabolism	P	645	67	25.93	2.58	3.53E-11	5.41E-09
3	465	F2-3 vs. L2-3	F2-3 up	+	small molecule metabolic process	GO:0044281	Metabolism	Metabolism	P	1520	158	61.1	2.59	9.47E-26	1.13E-22
3	466	F2-3 vs. L2-3	F2-3 up	+	sulfur compound biosynthetic process	GO:0044272	Metabolism	Metabolism	P	142	31	5.71	5.43	9.04E-13	1.86E-10
3	467	F2-3 vs. L2-3	F2-3 up	+	sulfur compound metabolic process	GO:0006790	Metabolism	Metabolism	P	363	47	14.59	3.22	3.40E-11	5.35E-09
3	468	F2-3 vs. L2-3	F2-3 up	+	tetrapyrrole binding	GO:0046906	Metabolism	Metabolism	F	404	32	16.24	1.97	5.98E-04	3.00E-02
3	469	F2-3 vs. L2-3	F2-3 up	+	thioester biosynthetic process	GO:0035384	Metabolism	Metabolism	P	21	5	0.84	5.92	2.85E-03	4.89E-02
3	470	F2-3 vs. L2-3	F2-3 up	+	transferase activity, transferring nitrogenous groups	GO:0016769	Metabolism	Metabolism	F	52	9	2.09	4.31	5.29E-04	2.79E-02
3	471	F2-3 vs. L2-3	F2-3 up	+	transition metal ion binding	GO:0046914	Metabolism	Metabolism	F	1178	82	47.35	1.73	4.29E-06	4.45E-04
3	472	F2-3 vs. L2-3	F2-3 up	+	UDP-glucuronate biosynthetic process	GO:0006065	Metabolism	Metabolism	P	4	3	0.16	18.66	1.79E-03	3.31E-02
3	473	F2-3 vs. L2-3	F2-3 up	+	UDP-glucuronate metabolic process	GO:0046398	Metabolism	Metabolism	P	5	3	0.2	14.93	2.78E-03	4.83E-02
3	474	F2-3 vs. L2-3	F2-3 up	+	UDP-rhamnose biosynthetic process	GO:0010253	Metabolism	Metabolism	P	4	3	0.16	18.66	1.79E-03	3.27E-02
3	475	F2-3 vs. L2-3	F2-3 up	+	UDP-rhamnose metabolic process	GO:0033478	Metabolism	Metabolism	P	4	3	0.16	18.66	1.79E-03	3.34E-02
3	476	F2-3 vs. L2-3	F2-3 up	+	vitamin B6 binding	GO:0070279	Metabolism	Metabolism	F	98	14	3.94	3.55	1.08E-04	6.67E-03
3	477	F2-3 vs. L2-3	F2-3 up	+	carbon fixation	GO:0015977	Metabolism	Photosynthesis	P	16	8	0.64	12.44	2.05E-06	1.10E-04
3	478	F2-3 vs. L2-3	F2-3 up	+	chloroplast	GO:0009507	Metabolism	Photosynthesis	C	5013	329	201.5	1.63	9.22E-20	1.20E-17
3	479	F2-3 vs. L2-3	F2-3 up	+	chloroplast envelope	GO:0009941	Metabolism	Photosynthesis	C	613	76	24.64	3.08	2.19E-16	1.63E-14
3	480	F2-3 vs. L2-3	F2-3 up	+	chloroplast stroma	GO:0009570	Metabolism	Photosynthesis	C	719	92	28.9	3.18	1.93E-20	3.35E-18
3	481	F2-3 vs. L2-3	F2-3 up	+	chloroplast thylakoid	GO:0009534	Metabolism	Photosynthesis	C	443	65	17.81	3.65	2.45E-17	2.55E-15
3	482	F2-3 vs. L2-3	F2-3 up	+	chloroplast thylakoid membrane	GO:0009535	Metabolism	Photosynthesis	C	358	55	14.39	3.82	1.29E-15	7.49E-14
3	483	F2-3 vs. L2-3	F2-3 up	+	chloroplast thylakoid membrane protein complex	GO:0098807	Metabolism	Photosynthesis	C	24	6	0.96	6.22	8.79E-04	1.61E-02
3	484	F2-3 vs. L2-3	F2-3 up	+	photosynthesis	GO:0015979	Metabolism	Photosynthesis	P	189	38	7.6	5	2.39E-14	5.71E-12
3	485	F2-3 vs. L2-3	F2-3 up	+	photosynthesis, dark reaction	GO:0019685	Metabolism	Photosynthesis	P	15	8	0.6	13.27	1.42E-06	7.98E-05
3	486	F2-3 vs. L2-3	F2-3 up	+	photosynthesis, light reaction	GO:0019684	Metabolism	Photosynthesis	P	128	20	5.15	3.89	1.16E-06	6.68E-05
3	487	F2-3 vs. L2-3	F2-3 up	+	photosynthetic electron transport chain	GO:0009767	Metabolism	Photosynthesis	P	46	8	1.85	4.33	1.04E-03	2.29E-02
3	488	F2-3 vs. L2-3	F2-3 up	+	photosynthetic electron transport in photosystem	GO:0009773	Metabolism	Photosynthesis	P	17	5	0.68	7.32	1.30E-03	2.65E-02
3	489	F2-3 vs. L2-3	F2-3 up	+	photosynthetic membrane	GO:0034357	Metabolism	Photosynthesis	C	395	59	15.88	3.72	3.67E-16	2.38E-14
3	490	F2-3 vs. L2-3	F2-3 up	+	photosystem II assembly	GO:0010207	Metabolism	Photosynthesis	P	26	6	1.05	5.74	1.26E-03	2.59E-02
3	491	F2-3 vs. L2-3	F2-3 up	+	plastid	GO:0009536	Metabolism	Photosynthesis	C	5432	352	218.34	1.61	1.56E-20	3.24E-18
3	492	F2-3 vs. L2-3	F2-3 up	+	plastid envelope	GO:0009526	Metabolism	Photosynthesis	C	637	77	25.6	3.01	4.82E-16	2.95E-14
3	493	F2-3 vs. L2-3	F2-3 up	+	plastid stroma	GO:0009532	Metabolism	Photosynthesis	C	731	92	29.38	3.13	5.21E-20	7.75E-18
3	494	F2-3 vs. L2-3	F2-3 up	+	plastid thylakoid	GO:0031976	Metabolism	Photosynthesis	C	446	65	17.93	3.63	3.30E-17	2.87E-15
3	495	F2-3 vs. L2-3	F2-3 up	+	plastid thylakoid membrane	GO:0055035	Metabolism	Photosynthesis	C	363	55	14.59	3.77	2.19E-15	1.20E-13
3	496	F2-3 vs. L2-3	F2-3 up	+	plastoglobule	GO:0010287	Metabolism	Photosynthesis	C	76	11	3.05	3.6	5.21E-04	1.08E-02
3	497	F2-3 vs. L2-3	F2-3 up	+	thylakoid	GO:0009579	Metabolism	Photosynthesis	C	537	81	21.58	3.75	5.37E-22	1.40E-19
3	498	F2-3 vs. L2-3	F2-3 up	+	thylakoid lumen	GO:0031977	Metabolism	Photosynthesis	C	63	9	2.53	3.55	1.78E-03	2.81E-02
3	499	F2-3 vs. L2-3	F2-3 up	+	thylakoid membrane	GO:0042651	Metabolism	Photosynthesis	C	390	59	15.68	3.76	2.19E-16	1.52E-14
3	500	F2-3 vs. L2-3	F2-3 up	+	endoplasmic reticulum	GO:0005783	Regulation	Protein modification	C	1044	64	41.96	1.53	1.41E-03	2.45E-02
3	501	F2-3 vs. L2-3	F2-3 up	+	Golgi apparatus	GO:0005794	Regulation	Protein modification	C	1161	75	46.67	1.61	1.12E-04	2.47E-03
3	502	F2-3 vs. L2-3	F2-3 up	-	regulation of cellular macromolecule biosynthetic process	GO:2000112	Regulation	Regulation	P	2365	65	95.06	0.68	1.14E-03	2.44E-02
3	503	F2-3 vs. L2-3	F2-3 up	-	regulation of macromolecule biosynthetic	GO:0010556	Regulation	Regulation	P	2378	66	95.58	0.69	1.42E-03	2.79E-02
3	504	F2-3 vs. L2-3	F2-3 up	-	regulation of macromolecule metabolic process	GO:0060255	Regulation	Regulation	P	3058	81	122.92	0.66	4.39E-05	1.54E-03
3	505	F2-3 vs. L2-3	F2-3 up	-	regulation of nitrogen compound metabolic process	GO:0051171	Regulation	Regulation	P	2741	77	110.18	0.7	7.97E-04	1.86E-02
3	506	F2-3 vs. L2-3	F2-3 up	-	regulation of nucleobase-containing compound metabolic process	GO:0019219	Regulation	Regulation	P	2344	66	94.22	0.7	2.33E-03	4.13E-02
3	507	F2-3 vs. L2-3	F2-3 up	+	regulation of biological quality	GO:0065008	Regulation	Regulation	P	972	68	39.07	1.74	2.53E-05	9.57E-04
3	508	F2-3 vs. L2-3	F2-3 up	+	regulation of cell morphogenesis involved in differentiation	GO:0010769	Regulation	Regulation	P	40	10	1.61	6.22	1.79E-05	7.00E-04
3	509	F2-3 vs. L2-3	F2-3 up	+	regulation of pollen tube growth	GO:0080092	Regulation	Regulation	P	35	10	1.41	7.11	6.63E-06	2.91E-04
3	510	F2-3 vs. L2-3	F2-3 up	+	regulation of unidimensional cell growth	GO:0051510	Regulation	Regulation	P	74	11	2.97	3.7	4.26E-04	1.10E-02
3	511	F2-3 vs. L2-3	F2-3 up	+	auxin efflux	GO:0010315	Signaling and response	Signaling	P	15	5	0.6	8.29	8.15E-04	1.87E-02
3	512	F2-3 vs. L2-3	F2-3 up	+	cellular response to abiotic stimulus	GO:0071214	Signaling and response	Signaling	P	189	21	7.6	2.76	7.05E-05	2.29E-03
3	513	F2-3 vs. L2-3	F2-3 up	+	cellular response to blue light	GO:0071483	Signaling and response	Signaling	P	27	6	1.09	5.53	1.49E-03	2.89E-02
3	514	F2-3 vs. L2-3	F2-3 up	+	cellular response to chemical stimulus	GO:0070887	Signaling and response	Signaling	P	1085	71	43.61	1.63	1.24E-04	3.90E-03
3	515	F2-3 vs. L2-3	F2-3 up	+	cellular response to environmental stimulus	GO:0104004	Signaling and response	Signaling	P	189	21	7.6	2.76	7.05E-05	2.30E-03
3	516	F2-3 vs. L2-3	F2-3 up	+	cellular response to extracellular stimulus	GO:0031668	Signaling and response	Signaling	P	213	19	8.56	2.22	2.85E-03	4.85E-02
3	517	F2-3 vs. L2-3	F2-3 up	+	cellular response to light stimulus	GO:0071482	Signaling and response	Signaling	P	104	14	4.18	3.35	1.89E-04	5.44E-03
3	518	F2-3 vs. L2-3	F2-3 up	+	cellular response to oxygen levels	GO:0071453	Signaling and response	Signaling	P	238	21	9.57	2.2	1.60E-03	3.08E-02
3	519	F2-3 vs. L2-3	F2-3 up	+	cellular response to oxygen-containing	GO:1901701	Signaling and response	Signaling	P	509	36	20.46	1.76	2.20E-03	3.93E-02
3	520	F2-3 vs. L2-3	F2-3 up	+	cellular response to radiation	GO:0071478	Signaling and response	Signaling	P	110	14	4.42	3.17	3.19E-04	8.62E-03

3	521	F2-3 vs. L2-3	F2-3 up	+	jasmonic acid biosynthetic process	GO:0009695	Signaling and response	Signaling	P	26	7	1.05	6.7	2.23E-04	6.27E-03
3	522	F2-3 vs. L2-3	F2-3 up	+	jasmonic acid metabolic process	GO:0009694	Signaling and response	Signaling	P	50	8	2.01	3.98	1.67E-03	3.16E-02
3	523	F2-3 vs. L2-3	F2-3 up	+	multicellular organismal process	GO:0032501	Signaling and response	Signaling	P	2741	177	110.18	1.61	1.26E-09	1.30E-07
3	524	F2-3 vs. L2-3	F2-3 up	+	multi-organism process	GO:0051704	Signaling and response	Signaling	P	527	42	21.18	1.98	6.43E-05	2.12E-03
3	525	F2-3 vs. L2-3	F2-3 up	+	regulation of auxin mediated signaling pathway	GO:0010928	Signaling and response	Signaling	P	42	8	1.69	4.74	6.15E-04	1.48E-02
3	526	F2-3 vs. L2-3	F2-3 up	+	regulation of jasmonic acid mediated signaling pathway	GO:2000022	Signaling and response	Signaling	P	41	8	1.65	4.85	5.34E-04	1.31E-02
3	527	F2-3 vs. L2-3	F2-3 up	+	response to abiotic stimulus	GO:0009628	Signaling and response	Signaling	P	2131	189	85.66	2.21	3.01E-23	2.57E-20
3	528	F2-3 vs. L2-3	F2-3 up	+	response to acid chemical	GO:0001101	Signaling and response	Signaling	P	397	44	15.96	2.76	1.10E-08	9.11E-07
3	529	F2-3 vs. L2-3	F2-3 up	+	response to auxin	GO:0009733	Signaling and response	Signaling	P	300	25	12.06	2.07	1.18E-03	2.49E-02
3	530	F2-3 vs. L2-3	F2-3 up	+	response to bacterium	GO:0009617	Signaling and response	Signaling	P	508	62	20.42	3.04	2.56E-13	5.68E-11
3	531	F2-3 vs. L2-3	F2-3 up	+	response to biotic stimulus	GO:0009607	Signaling and response	Signaling	P	1098	106	44.13	2.4	2.33E-15	6.63E-13
3	532	F2-3 vs. L2-3	F2-3 up	+	response to cadmium ion	GO:0046686	Signaling and response	Signaling	P	315	43	12.66	3.4	5.22E-11	7.43E-09
3	533	F2-3 vs. L2-3	F2-3 up	+	response to carbohydrate	GO:0009743	Signaling and response	Signaling	P	153	17	6.15	2.76	3.33E-04	8.90E-03
3	534	F2-3 vs. L2-3	F2-3 up	+	response to chemical	GO:0042221	Signaling and response	Signaling	P	2681	237	107.76	2.2	2.30E-29	4.58E-26
3	535	F2-3 vs. L2-3	F2-3 up	+	response to chitin	GO:0010200	Signaling and response	Signaling	P	139	16	5.59	2.86	3.45E-04	9.14E-03
3	536	F2-3 vs. L2-3	F2-3 up	+	response to disaccharide	GO:0034285	Signaling and response	Signaling	P	72	13	2.89	4.49	2.23E-05	8.61E-04
3	537	F2-3 vs. L2-3	F2-3 up	+	response to endogenous stimulus	GO:0009719	Signaling and response	Signaling	P	1271	101	51.09	1.98	6.00E-10	6.65E-08
3	538	F2-3 vs. L2-3	F2-3 up	+	response to external biotic stimulus	GO:0043207	Signaling and response	Signaling	P	1097	106	44.09	2.4	2.23E-15	7.00E-13
3	539	F2-3 vs. L2-3	F2-3 up	+	response to external stimulus	GO:0009605	Signaling and response	Signaling	P	1513	143	60.82	2.35	9.13E-20	4.20E-17
3	540	F2-3 vs. L2-3	F2-3 up	+	response to extracellular stimulus	GO:0009991	Signaling and response	Signaling	P	262	25	10.53	2.37	1.56E-04	4.74E-03
3	541	F2-3 vs. L2-3	F2-3 up	+	response to far red light	GO:0010218	Signaling and response	Signaling	P	43	7	1.73	4.05	2.95E-03	4.92E-02
3	542	F2-3 vs. L2-3	F2-3 up	+	response to hormone	GO:0009725	Signaling and response	Signaling	P	1241	101	49.88	2.02	1.50E-10	1.83E-08
3	543	F2-3 vs. L2-3	F2-3 up	+	response to inorganic substance	GO:0010035	Signaling and response	Signaling	P	914	104	36.74	2.83	1.33E-19	5.68E-17
3	544	F2-3 vs. L2-3	F2-3 up	+	response to jasmonic acid	GO:0009753	Signaling and response	Signaling	P	175	30	7.03	4.26	3.88E-10	4.55E-08
3	545	F2-3 vs. L2-3	F2-3 up	+	response to karrikin	GO:0080167	Signaling and response	Signaling	P	128	25	5.15	4.86	1.08E-09	1.13E-07
3	546	F2-3 vs. L2-3	F2-3 up	+	response to light stimulus	GO:0009416	Signaling and response	Signaling	P	713	72	28.66	2.51	1.33E-11	2.21E-09
3	547	F2-3 vs. L2-3	F2-3 up	+	response to lipid	GO:0033993	Signaling and response	Signaling	P	672	53	27.01	1.96	1.33E-05	5.33E-04
3	548	F2-3 vs. L2-3	F2-3 up	+	response to metal ion	GO:0010038	Signaling and response	Signaling	P	433	51	17.4	2.93	1.09E-10	1.42E-08
3	549	F2-3 vs. L2-3	F2-3 up	+	response to nitrate	GO:0010167	Signaling and response	Signaling	P	26	6	1.05	5.74	1.26E-03	2.58E-02
3	550	F2-3 vs. L2-3	F2-3 up	+	response to nitrogen compound	GO:1901698	Signaling and response	Signaling	P	301	29	12.1	2.4	3.99E-05	1.42E-03
3	551	F2-3 vs. L2-3	F2-3 up	+	response to organic cyclic compound	GO:0014070	Signaling and response	Signaling	P	280	24	11.25	2.13	1.33E-03	2.67E-02
3	552	F2-3 vs. L2-3	F2-3 up	+	response to organic substance	GO:0010033	Signaling and response	Signaling	P	1705	142	68.53	2.07	3.37E-15	8.77E-13
3	553	F2-3 vs. L2-3	F2-3 up	+	response to oxygen levels	GO:0070482	Signaling and response	Signaling	P	267	22	10.73	2.05	2.93E-03	4.94E-02
3	554	F2-3 vs. L2-3	F2-3 up	+	response to oxygen-containing compound	GO:1901700	Signaling and response	Signaling	P	1514	144	60.86	2.37	4.52E-20	2.46E-17
3	555	F2-3 vs. L2-3	F2-3 up	+	response to stimulus	GO:0050896	Signaling and response	Signaling	P	5578	396	224.21	1.77	1.66E-31	4.98E-28
3	556	F2-3 vs. L2-3	F2-3 up	+	response to sucrose	GO:0009744	Signaling and response	Signaling	P	70	13	2.81	4.62	1.72E-05	6.75E-04
3	557	F2-3 vs. L2-3	F2-3 up	+	response to temperature stimulus	GO:0009266	Signaling and response	Signaling	P	617	60	24.8	2.42	3.24E-09	3.03E-07
3	558	F2-3 vs. L2-3	F2-3 up	+	response to UV	GO:0009411	Signaling and response	Signaling	P	125	14	5.02	2.79	1.01E-03	2.26E-02
3	559	F2-3 vs. L2-3	F2-3 up	+	response to water	GO:0009415	Signaling and response	Signaling	P	365	43	14.67	2.93	3.15E-09	2.99E-07
3	560	F2-3 vs. L2-3	F2-3 up	+	sterol biosynthetic process	GO:0016126	Signaling and response	Signaling	P	33	8	1.33	6.03	1.49E-04	4.56E-03
3	561	F2-3 vs. L2-3	F2-3 up	+	sterol metabolic process	GO:0016125	Signaling and response	Signaling	P	73	10	2.93	3.41	1.34E-03	2.68E-02
3	562	F2-3 vs. L2-3	F2-3 up	+	aging	GO:0007568	Signaling and response	Stress	P	132	19	5.31	3.58	6.23E-06	2.76E-04
3	563	F2-3 vs. L2-3	F2-3 up	+	anion homeostasis	GO:0055081	Signaling and response	Stress	P	43	7	1.73	4.05	2.95E-03	4.96E-02
3	564	F2-3 vs. L2-3	F2-3 up	+	antioxidant activity	GO:0016209	Signaling and response	Stress	F	140	18	5.63	3.2	4.21E-05	3.08E-03
3	565	F2-3 vs. L2-3	F2-3 up	+	cation homeostasis	GO:0055080	Signaling and response	Stress	P	236	22	9.49	2.32	5.09E-04	1.25E-02
3	566	F2-3 vs. L2-3	F2-3 up	+	cellular homeostasis	GO:0019725	Signaling and response	Stress	P	275	25	11.05	2.26	3.77E-04	9.88E-03
3	567	F2-3 vs. L2-3	F2-3 up	+	cellular response to decreased oxygen levels	GO:0036294	Signaling and response	Stress	P	237	21	9.53	2.2	1.56E-03	3.01E-02
3	568	F2-3 vs. L2-3	F2-3 up	+	cellular response to hypoxia	GO:0071456	Signaling and response	Stress	P	235	20	9.45	2.12	2.75E-03	4.81E-02
3	569	F2-3 vs. L2-3	F2-3 up	+	cellular response to light intensity	GO:0071484	Signaling and response	Stress	P	12	5	0.48	10.37	3.58E-04	9.43E-03
3	570	F2-3 vs. L2-3	F2-3 up	+	cellular response to toxic substance	GO:0097237	Signaling and response	Stress	P	40	7	1.61	4.35	2.06E-03	3.70E-02
3	571	F2-3 vs. L2-3	F2-3 up	+	chemical homeostasis	GO:0048878	Signaling and response	Stress	P	415	39	16.68	2.34	3.54E-06	1.72E-04
3	572	F2-3 vs. L2-3	F2-3 up	+	defense response	GO:0006952	Signaling and response	Stress	P	953	82	38.31	2.14	9.91E-10	1.06E-07
3	573	F2-3 vs. L2-3	F2-3 up	+	defense response to bacterium	GO:0042742	Signaling and response	Stress	P	414	51	16.64	3.06	2.55E-11	4.13E-09
3	574	F2-3 vs. L2-3	F2-3 up	+	defense response to fungus	GO:0050832	Signaling and response	Stress	P	252	24	10.13	2.37	2.18E-04	6.14E-03
3	575	F2-3 vs. L2-3	F2-3 up	+	defense response to Gram-negative bacterium	GO:0050829	Signaling and response	Stress	P	20	5	0.8	6.22	2.38E-03	4.20E-02
3	576	F2-3 vs. L2-3	F2-3 up	+	defense response to other organism	GO:0098542	Signaling and response	Stress	P	807	71	32.44	2.19	6.28E-09	5.45E-07
3	577	F2-3 vs. L2-3	F2-3 up	+	defense response, incompatible interaction	GO:0009814	Signaling and response	Stress	P	181	18	7.28	2.47	1.14E-03	2.43E-02
3	578	F2-3 vs. L2-3	F2-3 up	+	detoxification	GO:0098754	Signaling and response	Stress	P	100	14	4.02	3.48	1.31E-04	4.07E-03
3	579	F2-3 vs. L2-3	F2-3 up	+	flavonoid biosynthetic process	GO:0009813	Signaling and response	Stress	P	54	12	2.17	5.53	7.48E-06	3.24E-04
3	580	F2-3 vs. L2-3	F2-3 up	+	flavonoid metabolic process	GO:0009812	Signaling and response	Stress	P	63	12	2.53	4.74	2.88E-05	1.07E-03
3	581	F2-3 vs. L2-3	F2-3 up	+	gibberellic acid homeostasis	GO:0010336	Signaling and response	Stress	P	6	4	0.24	16.59	3.86E-04	1.01E-02

3	582	F2-3 vs. L2-3	F2-3 up	+	glutathione metabolic process	GO:0006749	Signaling and response	Stress	P	44	8	1.77	4.52	8.05E-04	1.87E-02
3	583	F2-3 vs. L2-3	F2-3 up	+	homeostatic process	GO:0042592	Signaling and response	Stress	P	537	48	21.58	2.22	1.13E-06	6.56E-05
3	584	F2-3 vs. L2-3	F2-3 up	+	inorganic ion homeostasis	GO:0098771	Signaling and response	Stress	P	250	23	10.05	2.29	6.65E-04	1.57E-02
3	585	F2-3 vs. L2-3	F2-3 up	+	interspecies interaction between organisms	GO:0044419	Signaling and response	Stress	P	1125	107	45.22	2.37	4.70E-15	1.17E-12
3	586	F2-3 vs. L2-3	F2-3 up	+	ion homeostasis	GO:0050801	Signaling and response	Stress	P	290	29	11.66	2.49	2.35E-05	8.94E-04
3	587	F2-3 vs. L2-3	F2-3 up	+	monovalent inorganic cation homeostasis	GO:0055067	Signaling and response	Stress	P	79	10	3.18	3.15	2.29E-03	4.08E-02
3	588	F2-3 vs. L2-3	F2-3 up	+	photoprotection	GO:0010117	Signaling and response	Stress	P	10	4	0.4	9.95	1.63E-03	3.11E-02
3	589	F2-3 vs. L2-3	F2-3 up	+	reactive oxygen species metabolic process	GO:0072593	Signaling and response	Stress	P	69	12	2.77	4.33	6.31E-05	2.10E-03
3	590	F2-3 vs. L2-3	F2-3 up	+	response to abscisic acid	GO:0009737	Signaling and response	Stress	P	497	39	19.98	1.95	1.62E-04	4.86E-03
3	591	F2-3 vs. L2-3	F2-3 up	+	response to alcohol	GO:0097305	Signaling and response	Stress	P	501	39	20.14	1.94	2.53E-04	6.97E-03
3	592	F2-3 vs. L2-3	F2-3 up	+	response to cold	GO:0009409	Signaling and response	Stress	P	415	52	16.68	3.12	9.22E-12	1.57E-09
3	593	F2-3 vs. L2-3	F2-3 up	+	response to decreased oxygen levels	GO:0036293	Signaling and response	Stress	P	265	22	10.65	2.07	2.82E-03	4.86E-02
3	594	F2-3 vs. L2-3	F2-3 up	+	response to desiccation	GO:0009269	Signaling and response	Stress	P	24	6	0.96	6.22	8.79E-04	1.98E-02
3	595	F2-3 vs. L2-3	F2-3 up	+	response to fungus	GO:0009620	Signaling and response	Stress	P	350	37	14.07	2.63	6.74E-07	4.53E-05
3	596	F2-3 vs. L2-3	F2-3 up	+	response to high light intensity	GO:0009644	Signaling and response	Stress	P	60	9	2.41	3.73	1.31E-03	2.66E-02
3	597	F2-3 vs. L2-3	F2-3 up	+	response to nematode	GO:0009624	Signaling and response	Stress	P	82	11	3.3	3.34	9.20E-04	2.07E-02
3	598	F2-3 vs. L2-3	F2-3 up	+	response to osmotic stress	GO:0006970	Signaling and response	Stress	P	528	43	21.22	2.03	3.74E-05	1.34E-03
3	599	F2-3 vs. L2-3	F2-3 up	+	response to other organism	GO:0051707	Signaling and response	Stress	P	1097	106	44.09	2.4	2.23E-15	6.65E-13
3	600	F2-3 vs. L2-3	F2-3 up	+	response to oxidative stress	GO:0006979	Signaling and response	Stress	P	460	43	18.49	2.33	1.66E-06	8.92E-05
3	601	F2-3 vs. L2-3	F2-3 up	+	response to ozone	GO:0010193	Signaling and response	Stress	P	33	8	1.33	6.03	1.49E-04	4.54E-03
3	602	F2-3 vs. L2-3	F2-3 up	+	response to radiation	GO:0009314	Signaling and response	Stress	P	738	72	29.66	2.43	5.78E-11	8.04E-09
3	603	F2-3 vs. L2-3	F2-3 up	+	response to reactive oxygen species	GO:0000302	Signaling and response	Stress	P	160	18	6.43	2.8	1.96E-04	5.62E-03
3	604	F2-3 vs. L2-3	F2-3 up	+	response to salt stress	GO:0009651	Signaling and response	Stress	P	447	36	17.97	2	1.84E-04	5.41E-03
3	605	F2-3 vs. L2-3	F2-3 up	+	response to stress	GO:0006950	Signaling and response	Stress	P	3106	246	124.85	1.97	6.29E-24	6.27E-21
3	606	F2-3 vs. L2-3	F2-3 up	+	response to toxic substance	GO:0009636	Signaling and response	Stress	P	120	17	4.82	3.52	2.27E-05	8.69E-04
3	607	F2-3 vs. L2-3	F2-3 up	+	response to UV-B	GO:0010224	Signaling and response	Stress	P	73	10	2.93	3.41	1.34E-03	2.67E-02
3	608	F2-3 vs. L2-3	F2-3 up	+	response to water deprivation	GO:0009414	Signaling and response	Stress	P	356	43	14.31	3	1.60E-09	1.59E-07
3	609	F2-3 vs. L2-3	F2-3 up	+	response to wounding	GO:0009611	Signaling and response	Stress	P	227	43	9.12	4.71	2.98E-15	8.10E-13
3	610	F2-3 vs. L2-3	F2-3 up	+	toxin metabolic process	GO:0009404	Signaling and response	Stress	P	59	9	2.37	3.8	1.18E-03	2.49E-02
3	611	F2-3 vs. L2-3	F2-3 up	-	gene expression	GO:0010467	Regulation	Transcription	P	1608	31	64.63	0.48	4.56E-06	2.17E-04
3	612	F2-3 vs. L2-3	F2-3 up	-	regulation of gene expression	GO:0010468	Regulation	Transcription	P	2679	77	107.68	0.72	1.77E-03	3.32E-02
3	613	F2-3 vs. L2-3	F2-3 up	-	regulation of nucleic acid-templated transcription	GO:1903506	Regulation	Transcription	P	2184	60	87.79	0.68	1.67E-03	3.17E-02
3	614	F2-3 vs. L2-3	F2-3 up	-	regulation of RNA biosynthetic process	GO:2001141	Regulation	Transcription	P	2186	60	87.87	0.68	1.67E-03	3.16E-02
3	615	F2-3 vs. L2-3	F2-3 up	-	regulation of transcription, DNA-templated	GO:0006355	Regulation	Transcription	P	2184	60	87.79	0.68	1.67E-03	3.18E-02
3	616	F2-3 vs. L2-3	F2-3 up	-	RNA modification	GO:0009451	Regulation	Transcription	P	283	2	11.38	0.18	1.76E-03	3.30E-02
3	617	F2-3 vs. L2-3	F2-3 up	-	ribonucleoprotein complex	GO:1990904	Regulation	Translation	C	677	7	27.21	0.26	1.17E-05	3.21E-04
3	618	F2-3 vs. L2-3	F2-3 up	-	RNA processing	GO:0006396	Regulation	Translation	P	787	11	31.63	0.35	5.15E-05	1.77E-03
3	619	F2-3 vs. L2-3	F2-3 up	-	RNA splicing	GO:0008380	Regulation	Translation	P	276	2	11.09	0.18	2.52E-03	4.43E-02
3	620	F2-3 vs. L2-3	F2-3 up	-	spliceosomal complex	GO:0005681	Regulation	Translation	C	160	0	6.43	<	3.20E-03	4.83E-02
3	621	F2-3 vs. L2-3	F2-3 up	+	mRNA binding	GO:0003729	Regulation	Translation	F	1005	68	40.4	1.68	6.81E-05	4.66E-03
3	622	F2-3 vs. L2-3	F2-3 up	+	active ion transmembrane transporter activity	GO:0022853	Metabolism	Transporters	F	215	30	8.64	3.47	2.72E-08	5.14E-06
3	623	F2-3 vs. L2-3	F2-3 up	+	active transmembrane transporter activity	GO:0022804	Metabolism	Transporters	F	482	53	19.37	2.74	4.49E-10	1.44E-07
3	624	F2-3 vs. L2-3	F2-3 up	+	amino acid transmembrane transport	GO:0003333	Metabolism	Transporters	P	27	6	1.09	5.53	1.49E-03	2.88E-02
3	625	F2-3 vs. L2-3	F2-3 up	+	anion transmembrane transport	GO:0098656	Metabolism	Transporters	P	107	14	4.3	3.26	2.47E-04	6.83E-03
3	626	F2-3 vs. L2-3	F2-3 up	+	anion transmembrane transporter activity	GO:0008509	Metabolism	Transporters	F	336	32	13.51	2.37	2.63E-05	2.28E-03
3	627	F2-3 vs. L2-3	F2-3 up	+	anion transport	GO:0006820	Metabolism	Transporters	P	283	32	11.38	2.81	7.25E-07	4.71E-05
3	628	F2-3 vs. L2-3	F2-3 up	+	ATPase-coupled cation transmembrane transporter activity	GO:0019829	Metabolism	Transporters	F	61	16	2.45	6.53	3.30E-08	5.90E-06
3	629	F2-3 vs. L2-3	F2-3 up	+	ATPase-coupled ion transmembrane transporter activity	GO:0042625	Metabolism	Transporters	F	76	17	3.05	5.56	9.14E-08	1.47E-05
3	630	F2-3 vs. L2-3	F2-3 up	+	ATPase-coupled transmembrane transporter activity	GO:0042626	Metabolism	Transporters	F	190	29	7.64	3.8	7.90E-09	2.31E-06
3	631	F2-3 vs. L2-3	F2-3 up	+	carboxylic acid transmembrane transport	GO:1905039	Metabolism	Transporters	P	55	10	2.21	4.52	1.85E-04	5.39E-03
3	632	F2-3 vs. L2-3	F2-3 up	+	carboxylic acid transmembrane transporter	GO:0046943	Metabolism	Transporters	F	122	14	4.9	2.85	8.13E-04	3.90E-02
3	633	F2-3 vs. L2-3	F2-3 up	+	cation transmembrane transport	GO:0098655	Metabolism	Transporters	P	229	27	9.2	2.93	2.57E-06	1.30E-04
3	634	F2-3 vs. L2-3	F2-3 up	+	cation transmembrane transporter activity	GO:0008324	Metabolism	Transporters	F	414	42	16.64	2.52	2.32E-07	3.24E-05
3	635	F2-3 vs. L2-3	F2-3 up	+	cation transport	GO:0006812	Metabolism	Transporters	P	412	40	16.56	2.42	1.48E-06	8.18E-05
3	636	F2-3 vs. L2-3	F2-3 up	+	cytoplasmic vesicle	GO:0031410	Metabolism	Transporters	C	720	55	28.94	1.9	1.69E-05	4.51E-04
3	637	F2-3 vs. L2-3	F2-3 up	+	endosome	GO:0005768	Metabolism	Transporters	C	411	31	16.52	1.88	1.62E-03	2.68E-02
3	638	F2-3 vs. L2-3	F2-3 up	+	export across plasma membrane	GO:0140115	Metabolism	Transporters	P	15	5	0.6	8.29	8.15E-04	1.88E-02
3	639	F2-3 vs. L2-3	F2-3 up	+	inorganic anion transport	GO:0015698	Metabolism	Transporters	P	108	14	4.34	3.22	2.69E-04	7.34E-03
3	640	F2-3 vs. L2-3	F2-3 up	+	inorganic cation transmembrane transport	GO:0098662	Metabolism	Transporters	P	223	26	8.96	2.9	4.71E-06	2.20E-04

3	641	F2-3 vs. L2-3	F2-3 up	+	inorganic cation transmembrane transporter activity	GO:0022890	Metabolism	Transporters	F	382	38	15.35	2.47	1.32E-06	1.51E-04
3	642	F2-3 vs. L2-3	F2-3 up	+	inorganic ion transmembrane transport	GO:0098660	Metabolism	Transporters	P	244	29	9.81	2.96	9.57E-07	5.78E-05
3	643	F2-3 vs. L2-3	F2-3 up	+	inorganic molecular entity transmembrane transporter activity	GO:0015318	Metabolism	Transporters	F	664	70	26.69	2.62	4.27E-12	2.74E-09
3	644	F2-3 vs. L2-3	F2-3 up	+	ion transmembrane transport	GO:0034220	Metabolism	Transporters	P	330	41	13.26	3.09	1.83E-09	1.79E-07
3	645	F2-3 vs. L2-3	F2-3 up	+	ion transmembrane transporter activity	GO:0015075	Metabolism	Transporters	F	718	69	28.86	2.39	3.80E-10	1.36E-07
3	646	F2-3 vs. L2-3	F2-3 up	+	ion transmembrane transporter activity, phosphorylative mechanism	GO:0015662	Metabolism	Transporters	F	50	10	2.01	4.98	9.27E-05	6.08E-03
3	647	F2-3 vs. L2-3	F2-3 up	+	ion transport	GO:0006811	Metabolism	Transporters	P	673	71	27.05	2.62	2.89E-12	5.58E-10
3	648	F2-3 vs. L2-3	F2-3 up	+	monovalent inorganic cation transmembrane transporter activity	GO:0015077	Metabolism	Transporters	F	220	26	8.84	2.94	3.77E-06	4.04E-04
3	649	F2-3 vs. L2-3	F2-3 up	+	monovalent inorganic cation transport	GO:0015672	Metabolism	Transporters	P	170	20	6.83	2.93	5.07E-05	1.75E-03
3	650	F2-3 vs. L2-3	F2-3 up	+	nitrate transport	GO:0015706	Metabolism	Transporters	P	43	7	1.73	4.05	2.95E-03	4.95E-02
3	651	F2-3 vs. L2-3	F2-3 up	+	organic acid transmembrane transport	GO:1903825	Metabolism	Transporters	P	57	10	2.29	4.36	2.39E-04	6.64E-03
3	652	F2-3 vs. L2-3	F2-3 up	+	organic acid transmembrane transporter activity	GO:0005342	Metabolism	Transporters	F	122	14	4.9	2.85	8.13E-04	3.84E-02
3	653	F2-3 vs. L2-3	F2-3 up	+	organic anion transmembrane transporter	GO:0008514	Metabolism	Transporters	F	200	20	8.04	2.49	5.37E-04	2.79E-02
3	654	F2-3 vs. L2-3	F2-3 up	+	organic anion transport	GO:0015711	Metabolism	Transporters	P	164	18	6.59	2.73	2.59E-04	7.10E-03
3	655	F2-3 vs. L2-3	F2-3 up	+	phloem transport	GO:0010233	Metabolism	Transporters	P	21	6	0.84	7.11	4.84E-04	1.22E-02
3	656	F2-3 vs. L2-3	F2-3 up	+	plasmodesma	GO:0009506	Metabolism	Transporters	C	944	61	37.94	1.61	5.85E-04	1.19E-02
3	657	F2-3 vs. L2-3	F2-3 up	+	primary active transmembrane transporter	GO:0015399	Metabolism	Transporters	F	213	29	8.56	3.39	7.42E-08	1.25E-05
3	658	F2-3 vs. L2-3	F2-3 up	+	proton transmembrane transporter activity	GO:0015078	Metabolism	Transporters	F	166	24	6.67	3.6	3.67E-07	4.91E-05
3	659	F2-3 vs. L2-3	F2-3 up	+	secondary active transmembrane transporter activity	GO:0015291	Metabolism	Transporters	F	266	23	10.69	2.15	1.06E-03	4.66E-02
3	660	F2-3 vs. L2-3	F2-3 up	+	secretory vesicle	GO:0099503	Metabolism	Transporters	C	170	22	6.83	3.22	5.65E-06	1.73E-04
3	661	F2-3 vs. L2-3	F2-3 up	+	trans-Golgi network	GO:0005802	Metabolism	Transporters	C	302	33	12.14	2.72	9.78E-07	3.28E-05
3	662	F2-3 vs. L2-3	F2-3 up	+	transmembrane transport	GO:0055085	Metabolism	Transporters	P	859	85	34.53	2.46	4.72E-13	1.01E-10
3	663	F2-3 vs. L2-3	F2-3 up	+	transmembrane transporter activity	GO:0022857	Metabolism	Transporters	F	1242	115	49.92	2.3	2.53E-15	2.03E-12
3	664	F2-3 vs. L2-3	F2-3 up	+	transport	GO:0006810	Metabolism	Transporters	P	2261	145	90.88	1.6	6.98E-08	5.35E-06
3	665	F2-3 vs. L2-3	F2-3 up	+	transporter activity	GO:0005215	Metabolism	Transporters	F	1287	119	51.73	2.3	7.37E-16	7.90E-13
3	666	F2-3 vs. L2-3	F2-3 up	+	vascular transport	GO:0010232	Metabolism	Transporters	P	21	6	0.84	7.11	4.84E-04	1.22E-02
3	667	F2-3 vs. L2-3	F2-3 up	+	vesicle	GO:0031982	Metabolism	Transporters	C	817	61	32.84	1.86	1.08E-05	3.11E-04
3	668	F2-3 vs. L2-3	L2-3 up	+	primary metabolic process	GO:0044238	Metabolism	Bioenergetics	P	7219	202	139.03	1.45	3.47E-09	2.31E-06
3	669	F2-3 vs. L2-3	L2-3 up	+	protein-containing complex	GO:0032991	Metabolism	Catabolism	C	2958	114	56.97	2	1.42E-12	1.48E-09
3	670	F2-3 vs. L2-3	L2-3 up	+	cell cycle	GO:0007049	Development	Cell division	P	458	24	8.82	2.72	1.90E-05	2.58E-03
3	671	F2-3 vs. L2-3	L2-3 up	+	cell cycle phase transition	GO:0044770	Development	Cell division	P	49	7	0.94	7.42	8.60E-05	8.43E-03
3	672	F2-3 vs. L2-3	L2-3 up	+	cell cycle process	GO:0022402	Development	Cell division	P	421	23	8.11	2.84	1.51E-05	2.32E-03
3	673	F2-3 vs. L2-3	L2-3 up	+	chromosome	GO:0005694	Development	Cell division	C	545	25	10.5	2.38	1.17E-04	6.42E-03
3	674	F2-3 vs. L2-3	L2-3 up	+	cyclin-dependent protein kinase holoenzyme complex	GO:0000307	Development	Cell division	C	62	7	1.19	5.86	3.24E-04	1.12E-02
3	675	F2-3 vs. L2-3	L2-3 up	+	DNA repair	GO:0006281	Development	Cell division	P	315	17	6.07	2.8	2.20E-04	1.75E-02
3	676	F2-3 vs. L2-3	L2-3 up	+	DNA replication	GO:0006260	Development	Cell division	P	125	11	2.41	4.57	5.75E-05	6.25E-03
3	677	F2-3 vs. L2-3	L2-3 up	+	DNA-dependent DNA replication	GO:0006261	Development	Cell division	P	113	10	2.18	4.6	1.19E-04	1.04E-02
3	678	F2-3 vs. L2-3	L2-3 up	+	mitotic cell cycle	GO:0000278	Development	Cell division	P	244	15	4.7	3.19	1.36E-04	1.18E-02
3	679	F2-3 vs. L2-3	L2-3 up	+	mitotic cell cycle phase transition	GO:0044772	Development	Cell division	P	48	7	0.92	7.57	7.65E-05	7.62E-03
3	680	F2-3 vs. L2-3	L2-3 up	+	mitotic cell cycle process	GO:1903047	Development	Cell division	P	203	14	3.91	3.58	7.25E-05	7.34E-03
3	681	F2-3 vs. L2-3	L2-3 up	+	nuclear origin of replication recognition complex	GO:0005664	Development	Cell division	C	7	3	0.13	22.25	7.29E-04	2.11E-02
3	682	F2-3 vs. L2-3	L2-3 up	+	nucleic acid metabolic process	GO:0090304	Development	Cell division	P	1705	66	32.84	2.01	1.71E-07	7.31E-05
3	683	F2-3 vs. L2-3	L2-3 up	+	nucleobase-containing compound metabolic process	GO:0006139	Development	Cell division	P	2093	76	40.31	1.89	1.65E-07	7.58E-05
3	684	F2-3 vs. L2-3	L2-3 up	+	origin recognition complex	GO:0000808	Development	Cell division	C	7	3	0.13	22.25	7.29E-04	2.00E-02
3	685	F2-3 vs. L2-3	L2-3 up	+	regulation of cell cycle	GO:0051726	Development	Cell division	P	241	17	4.64	3.66	9.58E-06	1.85E-03
3	686	F2-3 vs. L2-3	L2-3 up	+	regulation of cell cycle phase transition	GO:1901987	Development	Cell division	P	53	8	1.02	7.84	1.88E-05	2.68E-03
3	687	F2-3 vs. L2-3	L2-3 up	+	regulation of cell cycle process	GO:0010564	Development	Cell division	P	128	11	2.47	4.46	7.01E-05	7.23E-03
3	688	F2-3 vs. L2-3	L2-3 up	+	regulation of mitotic cell cycle	GO:0007346	Development	Cell division	P	89	9	1.71	5.25	1.02E-04	9.22E-03
3	689	F2-3 vs. L2-3	L2-3 up	+	regulation of mitotic cell cycle phase transition	GO:1901990	Development	Cell division	P	50	8	0.96	8.31	1.29E-05	2.08E-03
3	690	F2-3 vs. L2-3	L2-3 up	+	RNA metabolic process	GO:0016070	Development	Cell division	P	1294	47	24.92	1.89	5.01E-05	5.54E-03
3	691	F2-3 vs. L2-3	L2-3 up	+	anatomical structure development	GO:0048856	Development	Development	P	2842	83	54.73	1.52	1.76E-04	1.46E-02
3	692	F2-3 vs. L2-3	L2-3 up	+	cellular anatomical entity	GO:0110165	Development	Development	C	24430	499	470.49	1.06	2.61E-05	1.81E-03
3	693	F2-3 vs. L2-3	L2-3 up	+	cellular component assembly	GO:0022607	Development	Development	P	838	37	16.14	2.29	6.00E-06	1.38E-03
3	694	F2-3 vs. L2-3	L2-3 up	+	cellular component biogenesis	GO:0044085	Development	Development	P	1325	52	25.52	2.04	2.17E-06	6.19E-04
3	695	F2-3 vs. L2-3	L2-3 up	+	cellular component organization	GO:0016043	Development	Development	P	2405	74	46.32	1.6	9.99E-05	9.19E-03

3	696	F2-3 vs. L2-3	L2-3 up	+	cellular component organization or biogenesis	GO:0071840	Development	Development	P	2813	87	54.18	1.61	1.37E-05	2.15E-03
3	697	F2-3 vs. L2-3	L2-3 up	+	cellular process	GO:0009987	Development	Development	P	12008	310	231.26	1.34	1.26E-11	3.78E-08
3	698	F2-3 vs. L2-3	L2-3 up	+	chromosomal region	GO:0098687	Development	Development	C	64	6	1.23	4.87	2.09E-03	4.94E-02
3	699	F2-3 vs. L2-3	L2-3 up	+	cytoplasm	GO:0005737	Development	Development	C	14837	328	285.74	1.15	2.48E-04	9.20E-03
3	700	F2-3 vs. L2-3	L2-3 up	+	cytosol	GO:0005829	Development	Development	C	3241	101	62.42	1.62	1.79E-06	2.07E-04
3	701	F2-3 vs. L2-3	L2-3 up	+	developmental process	GO:0032502	Development	Development	P	2997	84	57.72	1.46	5.62E-04	3.87E-02
3	702	F2-3 vs. L2-3	L2-3 up	+	developmental process involved in reproduction	GO:0003006	Development	Development	P	1437	48	27.67	1.73	3.68E-04	2.72E-02
3	703	F2-3 vs. L2-3	L2-3 up	+	embryo development	GO:0009790	Development	Development	P	566	24	10.9	2.2	5.57E-04	3.87E-02
3	704	F2-3 vs. L2-3	L2-3 up	+	embryo development ending in seed dormancy	GO:0009793	Development	Development	P	541	24	10.42	2.3	2.39E-04	1.88E-02
3	705	F2-3 vs. L2-3	L2-3 up	+	fruit development	GO:0010154	Development	Development	P	749	30	14.42	2.08	2.50E-04	1.94E-02
3	706	F2-3 vs. L2-3	L2-3 up	+	intracellular anatomical structure	GO:0005622	Development	Development	C	21464	462	413.37	1.12	7.79E-08	1.35E-05
3	707	F2-3 vs. L2-3	L2-3 up	+	intracellular membrane-bounded organelle	GO:0043231	Development	Development	C	19384	413	373.31	1.11	1.30E-04	6.75E-03
3	708	F2-3 vs. L2-3	L2-3 up	+	intracellular non-membrane-bounded organelle	GO:0043232	Development	Development	C	1755	75	33.8	2.22	2.24E-10	1.17E-07
3	709	F2-3 vs. L2-3	L2-3 up	+	intracellular organelle	GO:0043229	Development	Development	C	19690	425	379.21	1.12	6.35E-06	5.51E-04
3	710	F2-3 vs. L2-3	L2-3 up	+	intracellular organelle lumen	GO:0070013	Development	Development	C	1386	48	26.69	1.8	1.33E-04	6.57E-03
3	711	F2-3 vs. L2-3	L2-3 up	+	membrane-bounded organelle	GO:0043227	Development	Development	C	19546	415	376.43	1.1	1.76E-04	7.64E-03
3	712	F2-3 vs. L2-3	L2-3 up	+	membrane-enclosed lumen	GO:0031974	Development	Development	C	1386	48	26.69	1.8	1.33E-04	6.27E-03
3	713	F2-3 vs. L2-3	L2-3 up	+	multicellular organism development	GO:0007275	Development	Development	P	2457	79	47.32	1.67	1.10E-05	1.93E-03
3	714	F2-3 vs. L2-3	L2-3 up	+	non-membrane-bounded organelle	GO:0043228	Development	Development	C	1755	75	33.8	2.22	2.24E-10	7.79E-08
3	715	F2-3 vs. L2-3	L2-3 up	+	nuclear chromosome	GO:0000228	Development	Development	C	369	17	7.11	2.39	1.80E-03	4.80E-02
3	716	F2-3 vs. L2-3	L2-3 up	+	nuclear lumen	GO:0031981	Development	Development	C	1222	42	23.53	1.78	4.26E-04	1.34E-02
3	717	F2-3 vs. L2-3	L2-3 up	+	nucleus	GO:0005634	Development	Development	C	10540	266	202.99	1.31	4.18E-08	8.70E-06
3	718	F2-3 vs. L2-3	L2-3 up	+	organelle	GO:0043226	Development	Development	C	19739	425	380.15	1.12	9.66E-06	7.74E-04
3	719	F2-3 vs. L2-3	L2-3 up	+	organelle lumen	GO:0043233	Development	Development	C	1386	48	26.69	1.8	1.33E-04	6.00E-03
3	720	F2-3 vs. L2-3	L2-3 up	+	plant organ development	GO:0099402	Development	Development	P	1009	40	19.43	2.06	3.75E-05	4.67E-03
3	721	F2-3 vs. L2-3	L2-3 up	+	plant organ morphogenesis	GO:1905392	Development	Development	P	413	20	7.95	2.51	2.50E-04	1.92E-02
3	722	F2-3 vs. L2-3	L2-3 up	+	plasma membrane	GO:0005886	Development	Development	C	3534	93	68.06	1.37	2.07E-03	5.13E-02
3	723	F2-3 vs. L2-3	L2-3 up	+	post-embryonic development	GO:0009791	Development	Development	P	1482	51	28.54	1.79	9.64E-05	9.15E-03
3	724	F2-3 vs. L2-3	L2-3 up	+	reproductive structure development	GO:0048608	Development	Development	P	1206	45	23.23	1.94	4.20E-05	4.83E-03
3	725	F2-3 vs. L2-3	L2-3 up	+	reproductive system development	GO:0061458	Development	Development	P	1208	45	23.26	1.93	4.29E-05	4.84E-03
3	726	F2-3 vs. L2-3	L2-3 up	+	seed development	GO:0048316	Development	Development	P	725	30	13.96	2.15	1.81E-04	1.47E-02
3	727	F2-3 vs. L2-3	L2-3 up	+	structural molecule activity	GO:0005198	Development	Development	F	443	27	8.53	3.16	3.88E-07	1.38E-04
3	728	F2-3 vs. L2-3	L2-3 up	+	system development	GO:0048731	Development	Development	P	1867	66	35.96	1.84	3.40E-06	8.85E-04
3	729	F2-3 vs. L2-3	L2-3 up	+	amide biosynthetic process	GO:0043604	Metabolism	Metabolism	P	591	29	11.38	2.55	8.67E-06	1.73E-03
3	730	F2-3 vs. L2-3	L2-3 up	+	binding	GO:0005488	Metabolism	Metabolism	F	9843	271	189.56	1.43	9.17E-13	2.95E-09
3	731	F2-3 vs. L2-3	L2-3 up	+	biological_process	GO:0008150	Metabolism	Metabolism	P	21988	460	423.46	1.09	4.07E-05	4.87E-03
3	732	F2-3 vs. L2-3	L2-3 up	+	biosynthetic process	GO:0009058	Metabolism	Metabolism	P	2811	88	54.14	1.63	7.09E-06	1.51E-03
3	733	F2-3 vs. L2-3	L2-3 up	+	catalytic complex	GO:1902494	Metabolism	Metabolism	C	1168	42	22.49	1.87	1.99E-04	8.30E-03
3	734	F2-3 vs. L2-3	L2-3 up	+	cellular amide metabolic process	GO:0043603	Metabolism	Metabolism	P	722	29	13.9	2.09	3.19E-04	2.38E-02
3	735	F2-3 vs. L2-3	L2-3 up	+	cellular aromatic compound metabolic process	GO:0006725	Metabolism	Metabolism	P	2519	83	48.51	1.71	2.25E-06	6.11E-04
3	736	F2-3 vs. L2-3	L2-3 up	+	cellular biosynthetic process	GO:0044249	Metabolism	Metabolism	P	2583	81	49.75	1.63	1.78E-05	2.59E-03
3	737	F2-3 vs. L2-3	L2-3 up	+	cellular macromolecule biosynthetic process	GO:0034645	Metabolism	Metabolism	P	1279	59	24.63	2.4	1.65E-09	1.41E-06
3	738	F2-3 vs. L2-3	L2-3 up	+	cellular macromolecule metabolic process	GO:0044260	Metabolism	Metabolism	P	4426	147	85.24	1.72	2.58E-11	5.15E-08
3	739	F2-3 vs. L2-3	L2-3 up	+	cellular metabolic process	GO:0044237	Metabolism	Metabolism	P	7649	207	147.31	1.41	2.88E-08	1.44E-05
3	740	F2-3 vs. L2-3	L2-3 up	+	cellular nitrogen compound biosynthetic process	GO:0044271	Metabolism	Metabolism	P	1241	51	23.9	2.13	8.91E-07	2.96E-04
3	741	F2-3 vs. L2-3	L2-3 up	+	cellular nitrogen compound metabolic process	GO:0034641	Metabolism	Metabolism	P	2872	106	55.31	1.92	1.26E-10	1.50E-07
3	742	F2-3 vs. L2-3	L2-3 up	+	cellular protein metabolic process	GO:0044267	Metabolism	Metabolism	P	3388	100	65.25	1.53	1.89E-05	2.63E-03
3	743	F2-3 vs. L2-3	L2-3 up	+	cellular protein-containing complex assembly	GO:0034622	Metabolism	Metabolism	P	474	25	9.13	2.74	1.15E-05	1.96E-03
3	744	F2-3 vs. L2-3	L2-3 up	+	DNA metabolic process	GO:0006259	Metabolism	Metabolism	P	448	21	8.63	2.43	3.89E-04	2.84E-02
3	745	F2-3 vs. L2-3	L2-3 up	+	heterocycle metabolic process	GO:0046483	Metabolism	Metabolism	P	2393	80	46.09	1.74	1.94E-06	5.81E-04
3	746	F2-3 vs. L2-3	L2-3 up	+	heterocyclic compound binding	GO:1901363	Metabolism	Metabolism	F	4765	147	91.77	1.6	4.14E-09	2.22E-06
3	747	F2-3 vs. L2-3	L2-3 up	+	macromolecule biosynthetic process	GO:0009059	Metabolism	Metabolism	P	1334	61	25.69	2.37	1.68E-09	1.25E-06
3	748	F2-3 vs. L2-3	L2-3 up	+	macromolecule metabolic process	GO:0043170	Metabolism	Metabolism	P	5496	179	105.85	1.69	1.98E-13	1.19E-09
3	749	F2-3 vs. L2-3	L2-3 up	+	metabolic process	GO:0008152	Metabolism	Metabolism	P	8681	232	167.19	1.39	5.55E-09	3.32E-06
3	750	F2-3 vs. L2-3	L2-3 up	+	nitrogen compound metabolic process	GO:0006807	Metabolism	Metabolism	P	6150	177	118.44	1.49	8.45E-09	4.59E-06
3	751	F2-3 vs. L2-3	L2-3 up	+	nucleic acid binding	GO:0003676	Metabolism	Metabolism	F	3299	118	63.53	1.86	6.59E-11	7.06E-08
3	752	F2-3 vs. L2-3	L2-3 up	+	organic cyclic compound binding	GO:0097159	Metabolism	Metabolism	F	4794	149	92.33	1.61	1.59E-09	1.28E-06
3	753	F2-3 vs. L2-3	L2-3 up	+	organic cyclic compound metabolic process	GO:1901360	Metabolism	Metabolism	P	2607	88	50.21	1.75	3.34E-07	1.33E-04
3	754	F2-3 vs. L2-3	L2-3 up	+	organic substance biosynthetic process	GO:1901576	Metabolism	Metabolism	P	2674	85	51.5	1.65	6.13E-06	1.36E-03
3	755	F2-3 vs. L2-3	L2-3 up	+	organic substance metabolic process	GO:0071704	Metabolism	Metabolism	P	7851	217	151.2	1.44	1.46E-09	1.45E-06
3	756	F2-3 vs. L2-3	L2-3 up	+	organonitrogen compound biosynthetic process	GO:1901566	Metabolism	Metabolism	P	1343	48	25.86	1.86	6.64E-05	6.96E-03
3	757	F2-3 vs. L2-3	L2-3 up	+	peptide metabolic process	GO:0006518	Metabolism	Metabolism	P	585	29	11.27	2.57	7.21E-06	1.49E-03

3	758	F2-3 vs. L2-3	L2-3 up	+	protein binding	GO:0005515	Metabolism	Metabolism	F	5446	172	104.88	1.64	9.98E-12	1.60E-08
3	759	F2-3 vs. L2-3	L2-3 up	+	protein metabolic process	GO:0019538	Metabolism	Metabolism	P	3544	101	68.25	1.48	6.51E-05	6.95E-03
3	760	F2-3 vs. L2-3	L2-3 up	+	Sm-like protein family complex	GO:0120114	Metabolism	Metabolism	C	86	10	1.66	6.04	1.40E-05	1.04E-03
3	761	F2-3 vs. L2-3	L2-3 up	+	protein-containing complex assembly	GO:0065003	Regulation	Protein modification	P	534	27	10.28	2.63	1.07E-05	1.93E-03
3	762	F2-3 vs. L2-3	L2-3 up	+	protein-containing complex subunit organization	GO:0043933	Regulation	Protein modification	P	609	27	11.73	2.3	9.65E-05	9.02E-03
3	763	F2-3 vs. L2-3	L2-3 up	+	negative regulation of biological process	GO:0048519	Regulation	Regulation	P	1102	45	21.22	2.12	4.02E-06	1.00E-03
3	764	F2-3 vs. L2-3	L2-3 up	+	negative regulation of macromolecule metabolic process	GO:0010605	Regulation	Regulation	P	603	29	11.61	2.5	1.75E-05	2.61E-03
3	765	F2-3 vs. L2-3	L2-3 up	+	negative regulation of metabolic process	GO:0009892	Regulation	Regulation	P	653	30	12.58	2.39	2.20E-05	2.87E-03
3	766	F2-3 vs. L2-3	L2-3 up	+	regulation of biological process	GO:0050789	Regulation	Regulation	P	5407	140	104.13	1.34	1.74E-04	1.47E-02
3	767	F2-3 vs. L2-3	L2-3 up	+	regulation of cellular amide metabolic process	GO:0034248	Regulation	Regulation	P	119	9	2.29	3.93	7.50E-04	4.87E-02
3	768	F2-3 vs. L2-3	L2-3 up	+	regulation of macromolecule metabolic process	GO:0060255	Regulation	Regulation	P	3058	89	58.89	1.51	9.45E-05	9.11E-03
3	769	F2-3 vs. L2-3	L2-3 up	+	regulation of meristem structural organization	GO:0009934	Regulation	Regulation	P	22	6	0.42	14.16	1.17E-05	1.94E-03
3	770	F2-3 vs. L2-3	L2-3 up	+	regulation of metabolic process	GO:0019222	Regulation	Regulation	P	3325	92	64.04	1.44	4.63E-04	3.30E-02
3	771	F2-3 vs. L2-3	L2-3 up	+	multicellular organismal process	GO:0032501	Signaling and response	Signaling	P	2741	81	52.79	1.53	1.39E-04	1.19E-02
3	772	F2-3 vs. L2-3	L2-3 up	+	protein kinase complex	GO:1902911	Signaling and response	Signaling	C	87	8	1.68	4.77	4.44E-04	1.36E-02
3	773	F2-3 vs. L2-3	L2-3 up	+	serine/threonine protein kinase complex	GO:1902554	Signaling and response	Signaling	C	79	8	1.52	5.26	2.44E-04	9.75E-03
3	774	F2-3 vs. L2-3	L2-3 up	+	cellular response to DNA damage stimulus	GO:0006974	Signaling and response	Stress	P	350	17	6.74	2.52	6.89E-04	4.63E-02
3	775	F2-3 vs. L2-3	L2-3 up	+	gene expression	GO:0010467	Regulation	Transcription	P	1608	73	30.97	2.36	3.01E-11	4.50E-08
3	776	F2-3 vs. L2-3	L2-3 up	+	negative regulation of gene expression	GO:0010629	Regulation	Transcription	P	493	25	9.49	2.63	2.15E-05	2.85E-03
3	777	F2-3 vs. L2-3	L2-3 up	+	regulation of gene expression	GO:0010468	Regulation	Transcription	P	2679	80	51.59	1.55	1.17E-04	1.04E-02
3	778	F2-3 vs. L2-3	L2-3 up	+	RNA binding	GO:0003723	Regulation	Transcription	F	1569	67	30.22	2.22	2.84E-09	1.83E-06
3	779	F2-3 vs. L2-3	L2-3 up	+	catalytic step 2 spliceosome	GO:0071013	Regulation	Translation	C	64	6	1.23	4.87	2.09E-03	5.05E-02
3	780	F2-3 vs. L2-3	L2-3 up	+	cytoplasmic ribonucleoprotein granule	GO:0036464	Regulation	Translation	C	84	8	1.62	4.95	3.57E-04	1.16E-02
3	781	F2-3 vs. L2-3	L2-3 up	+	cytoplasmic translation	GO:0002181	Regulation	Translation	P	72	7	1.39	5.05	7.41E-04	4.92E-02
3	782	F2-3 vs. L2-3	L2-3 up	+	cytosolic large ribosomal subunit	GO:0022625	Regulation	Translation	C	131	11	2.52	4.36	8.50E-05	4.92E-03
3	783	F2-3 vs. L2-3	L2-3 up	+	cytosolic ribosome	GO:0022626	Regulation	Translation	C	303	20	5.84	3.43	4.11E-06	4.28E-04
3	784	F2-3 vs. L2-3	L2-3 up	+	cytosolic small ribosomal subunit	GO:0022627	Regulation	Translation	C	102	9	1.96	4.58	2.63E-04	9.45E-03
3	785	F2-3 vs. L2-3	L2-3 up	+	large ribosomal subunit	GO:0015934	Regulation	Translation	C	180	15	3.47	4.33	5.03E-06	4.76E-04
3	786	F2-3 vs. L2-3	L2-3 up	+	Lsm1-7-Pat1 complex	GO:1990726	Regulation	Translation	C	7	3	0.13	22.25	7.29E-04	2.05E-02
3	787	F2-3 vs. L2-3	L2-3 up	+	mRNA binding	GO:0003729	Regulation	Translation	F	1005	46	19.36	2.38	1.65E-07	7.58E-05
3	788	F2-3 vs. L2-3	L2-3 up	+	ncRNA metabolic process	GO:0034660	Regulation	Translation	P	455	21	8.76	2.4	4.32E-04	3.11E-02
3	789	F2-3 vs. L2-3	L2-3 up	+	ncRNA processing	GO:0034470	Regulation	Translation	P	381	19	7.34	2.59	2.50E-04	1.89E-02
3	790	F2-3 vs. L2-3	L2-3 up	+	nuclear-transcribed mRNA catabolic process	GO:0000956	Regulation	Translation	P	72	7	1.39	5.05	7.41E-04	4.87E-02
3	791	F2-3 vs. L2-3	L2-3 up	+	peptide biosynthetic process	GO:0043043	Regulation	Translation	P	518	29	9.98	2.91	7.51E-07	2.64E-04
3	792	F2-3 vs. L2-3	L2-3 up	+	posttranscriptional regulation of gene expression	GO:0010608	Regulation	Translation	P	230	17	4.43	3.84	5.39E-06	1.29E-03
3	793	F2-3 vs. L2-3	L2-3 up	+	regulation of translation	GO:0006417	Regulation	Translation	P	116	9	2.23	4.03	6.32E-04	4.29E-02
3	794	F2-3 vs. L2-3	L2-3 up	+	ribonucleoprotein complex	GO:1990904	Regulation	Translation	C	677	36	13.04	2.76	1.15E-07	1.70E-05
3	795	F2-3 vs. L2-3	L2-3 up	+	ribonucleoprotein complex assembly	GO:0022618	Regulation	Translation	P	163	13	3.14	4.14	3.26E-05	4.15E-03
3	796	F2-3 vs. L2-3	L2-3 up	+	ribonucleoprotein complex biogenesis	GO:0022613	Regulation	Translation	P	447	26	8.61	3.02	1.45E-06	4.56E-04
3	797	F2-3 vs. L2-3	L2-3 up	+	ribonucleoprotein complex subunit organization	GO:0071826	Regulation	Translation	P	166	13	3.2	4.07	3.89E-05	4.75E-03
3	798	F2-3 vs. L2-3	L2-3 up	+	ribonucleoprotein granule	GO:0035770	Regulation	Translation	C	84	8	1.62	4.95	3.57E-04	1.20E-02
3	799	F2-3 vs. L2-3	L2-3 up	+	ribosomal subunit	GO:0044391	Regulation	Translation	C	316	25	6.09	4.11	9.99E-09	2.60E-06
3	800	F2-3 vs. L2-3	L2-3 up	+	ribosome	GO:0005840	Regulation	Translation	C	399	25	7.68	3.25	6.50E-07	8.46E-05
3	801	F2-3 vs. L2-3	L2-3 up	+	ribosome assembly	GO:0042255	Regulation	translation	P	90	8	1.73	4.62	5.48E-04	3.85E-02
3	802	F2-3 vs. L2-3	L2-3 up	+	ribosome biogenesis	GO:0042254	Regulation	Translation	P	370	19	7.13	2.67	1.76E-04	1.44E-02
3	803	F2-3 vs. L2-3	L2-3 up	+	RNA processing	GO:0006396	Regulation	Translation	P	787	35	15.16	2.31	1.02E-05	1.91E-03
3	804	F2-3 vs. L2-3	L2-3 up	+	small nuclear ribonucleoprotein complex	GO:0030532	Regulation	Translation	C	78	9	1.5	5.99	3.98E-05	2.44E-03
3	805	F2-3 vs. L2-3	L2-3 up	+	small ribosomal subunit	GO:0015935	Regulation	Translation	C	136	10	2.62	3.82	4.77E-04	1.42E-02
3	806	F2-3 vs. L2-3	L2-3 up	+	spliceosomal snRNP complex	GO:0097525	Regulation	Translation	C	78	9	1.5	5.99	3.98E-05	2.59E-03
3	807	F2-3 vs. L2-3	L2-3 up	+	spliceosomal tri-snRNP complex	GO:0097526	Regulation	Translation	C	41	6	0.79	7.6	2.46E-04	9.49E-03
3	808	F2-3 vs. L2-3	L2-3 up	+	structural constituent of ribosome	GO:0003735	Regulation	Translation	F	325	23	6.26	3.67	2.53E-07	1.02E-04
3	809	F2-3 vs. L2-3	L2-3 up	+	translation	GO:0006412	Regulation	Translation	P	513	29	9.88	2.94	6.23E-07	2.33E-04
3	810	F2-3 vs. L2-3	L2-3 up	+	U1 snRNP	GO:0005685	Regulation	Translation	C	24	4	0.46	8.65	1.80E-03	4.57E-02
3	811	F2-3 vs. L2-3	L2-3 up	+	U12-type spliceosomal complex	GO:0005689	Regulation	Translation	C	24	4	0.46	8.65	1.80E-03	4.68E-02
4	812	F2-3 vs. M2-3	F2-3 up	+	aldehyde dehydrogenase (NAD+) activity	GO:0004029	Metabolism	Bioenergetics	F	12	4	0.27	14.86	3.38E-04	2.31E-02
4	813	F2-3 vs. M2-3	F2-3 up	+	aldehyde dehydrogenase [NAD(P)+] activity	GO:0004030	Metabolism	Bioenergetics	F	12	4	0.27	14.86	3.38E-04	2.36E-02
4	814	F2-3 vs. M2-3	F2-3 up	+	carbohydrate biosynthetic process	GO:0016051	Metabolism	Bioenergetics	P	303	23	6.8	3.38	1.07E-06	2.00E-04
4	815	F2-3 vs. M2-3	F2-3 up	+	carbohydrate catabolic process	GO:0016052	Metabolism	Bioenergetics	P	234	21	5.25	4	2.55E-07	7.62E-05
4	816	F2-3 vs. M2-3	F2-3 up	+	carbohydrate metabolic process	GO:0005975	Metabolism	Bioenergetics	P	950	65	21.31	3.05	1.25E-14	3.75E-11
4	817	F2-3 vs. M2-3	F2-3 up	+	cellular carbohydrate biosynthetic process	GO:0034637	Metabolism	Bioenergetics	P	213	14	4.78	2.93	5.30E-04	2.33E-02
4	818	F2-3 vs. M2-3	F2-3 up	+	cellular carbohydrate metabolic process	GO:0044262	Metabolism	Bioenergetics	P	384	22	8.61	2.55	1.07E-04	6.88E-03

4	819	F2-3 vs. M2-3	F2-3 up	+	disaccharide biosynthetic process	GO:0046351	Metabolism	Bioenergetics	P	31	5	0.7	7.19	1.07E-03	3.55E-02
4	820	F2-3 vs. M2-3	F2-3 up	+	fatty acid derivative biosynthetic process	GO:1901570	Metabolism	Bioenergetics	P	30	5	0.67	7.43	9.42E-04	3.29E-02
4	821	F2-3 vs. M2-3	F2-3 up	+	fatty acid derivative metabolic process	GO:1901568	Metabolism	Bioenergetics	P	43	7	0.96	7.26	1.04E-04	6.77E-03
4	822	F2-3 vs. M2-3	F2-3 up	+	generation of precursor metabolites and energy	GO:0006091	Metabolism	Bioenergetics	P	378	23	8.48	2.71	3.16E-05	2.95E-03
4	823	F2-3 vs. M2-3	F2-3 up	+	gluconeogenesis	GO:0006094	Metabolism	Bioenergetics	P	23	5	0.52	9.69	3.24E-04	1.63E-02
4	824	F2-3 vs. M2-3	F2-3 up	+	glyceraldehyde-3-phosphate dehydrogenase (NAD+) (non-phosphorylating) activity	GO:0043878	Metabolism	Bioenergetics	F	8	4	0.18	22.29	9.87E-05	8.14E-03
4	825	F2-3 vs. M2-3	F2-3 up	+	hexose biosynthetic process	GO:0019319	Metabolism	Bioenergetics	P	26	5	0.58	8.57	5.30E-04	2.35E-02
4	826	F2-3 vs. M2-3	F2-3 up	+	monosaccharide biosynthetic process	GO:0046364	Metabolism	Bioenergetics	P	48	8	1.08	7.43	2.88E-05	2.74E-03
4	827	F2-3 vs. M2-3	F2-3 up	+	oxidoreductase activity	GO:0016491	Metabolism	Bioenergetics	F	1458	84	32.71	2.57	1.38E-14	2.22E-11
4	828	F2-3 vs. M2-3	F2-3 up	+	oxidoreductase activity, acting on CH-OH group of donors	GO:0016614	Metabolism	Bioenergetics	F	201	14	4.51	3.1	3.07E-04	2.20E-02
4	829	F2-3 vs. M2-3	F2-3 up	+	oxidoreductase activity, acting on paired donors, with incorporation or reduction of molecular oxygen, reduced flavin or flavoprotein as one donor, and incorporation of one atom of oxygen	GO:0016712	Metabolism	Bioenergetics	F	5	3	0.11	26.75	5.42E-04	3.42E-02
4	830	F2-3 vs. M2-3	F2-3 up	+	oxidoreductase activity, acting on the aldehyde or oxo group of donors	GO:0016903	Metabolism	Bioenergetics	F	62	7	1.39	5.03	7.87E-04	4.44E-02
4	831	F2-3 vs. M2-3	F2-3 up	+	oxidoreductase activity, acting on the aldehyde or oxo group of donors, NAD or NADP as	GO:0016620	Metabolism	Bioenergetics	F	43	7	0.96	7.26	1.04E-04	8.17E-03
4	832	F2-3 vs. M2-3	F2-3 up	+	oxidoreductase activity, acting on the CH-OH group of donors, NAD or NADP as acceptor	GO:0016616	Metabolism	Bioenergetics	F	167	12	3.75	3.2	6.27E-04	3.80E-02
4	833	F2-3 vs. M2-3	F2-3 up	+	photorespiration	GO:0009853	Metabolism	Bioenergetics	P	42	6	0.94	6.37	6.11E-04	2.50E-02
4	834	F2-3 vs. M2-3	F2-3 up	+	polysaccharide metabolic process	GO:0005976	Metabolism	Bioenergetics	P	419	31	9.4	3.3	2.54E-08	1.01E-05
4	835	F2-3 vs. M2-3	F2-3 up	+	proton transmembrane transport	GO:1902600	Metabolism	Bioenergetics	P	95	11	2.13	5.16	2.19E-05	2.18E-03
4	836	F2-3 vs. M2-3	F2-3 up	+	proton-exporting ATPase activity, phosphorylative mechanism	GO:0008553	Metabolism	Bioenergetics	F	20	6	0.45	13.37	1.72E-05	1.98E-03
4	837	F2-3 vs. M2-3	F2-3 up	+	pyrophosphate hydrolysis-driven proton transmembrane transporter activity	GO:0009678	Metabolism	Bioenergetics	F	44	8	0.99	8.11	1.65E-05	1.96E-03
4	838	F2-3 vs. M2-3	F2-3 up	+	reductive pentose-phosphate cycle	GO:0019253	Metabolism	Bioenergetics	P	14	4	0.31	12.74	5.50E-04	2.31E-02
4	839	F2-3 vs. M2-3	F2-3 up	+	sucrose biosynthetic process	GO:0005986	Metabolism	Bioenergetics	P	14	5	0.31	15.92	4.51E-05	3.64E-03
4	840	F2-3 vs. M2-3	F2-3 up	+	sucrose metabolic process	GO:0005985	Metabolism	Bioenergetics	P	46	6	1.03	5.81	9.41E-04	3.37E-02
4	841	F2-3 vs. M2-3	F2-3 up	+	sugar-phosphatase activity	GO:0050308	Metabolism	Bioenergetics	C	15	4	0.34	11.89	6.84E-04	4.00E-02
4	842	F2-3 vs. M2-3	F2-3 up	-	modification-dependent macromolecule catabolic process	GO:0043632	Metabolism	Catabolism	P	536	2	12.02	0.17	9.19E-04	3.31E-02
4	843	F2-3 vs. M2-3	F2-3 up	-	modification-dependent protein catabolic	GO:0019941	Metabolism	Catabolism	P	524	2	11.75	0.17	1.32E-03	4.07E-02
4	844	F2-3 vs. M2-3	F2-3 up	-	protein-containing complex	GO:0032991	Metabolism	Catabolism	C	2958	40	66.35	0.6	3.57E-04	9.52E-03
4	845	F2-3 vs. M2-3	F2-3 up	+	alpha-amino acid catabolic process	GO:1901606	Metabolism	Catabolism	P	88	10	1.97	5.07	5.95E-05	4.34E-03
4	846	F2-3 vs. M2-3	F2-3 up	+	carboxylic acid catabolic process	GO:0046395	Metabolism	Catabolism	P	162	15	3.63	4.13	9.19E-06	1.22E-03
4	847	F2-3 vs. M2-3	F2-3 up	+	catabolic process	GO:0009056	Metabolism	Catabolism	P	1469	60	32.95	1.82	1.35E-05	1.58E-03
4	848	F2-3 vs. M2-3	F2-3 up	+	cellular amino acid catabolic process	GO:0009063	Metabolism	Catabolism	P	97	10	2.18	4.6	1.25E-04	7.80E-03
4	849	F2-3 vs. M2-3	F2-3 up	+	glycine catabolic process	GO:0006546	Metabolism	Catabolism	P	8	3	0.18	16.72	1.52E-03	4.61E-02
4	850	F2-3 vs. M2-3	F2-3 up	+	organic acid catabolic process	GO:0016054	Metabolism	Catabolism	P	162	15	3.63	4.13	9.19E-06	1.25E-03
4	851	F2-3 vs. M2-3	F2-3 up	+	organic hydroxy compound catabolic process	GO:1901616	Metabolism	Catabolism	P	44	6	0.99	6.08	7.62E-04	2.86E-02
4	852	F2-3 vs. M2-3	F2-3 up	+	organic substance catabolic process	GO:1901575	Metabolism	Catabolism	P	1345	55	30.17	1.82	3.17E-05	2.92E-03
4	853	F2-3 vs. M2-3	F2-3 up	+	pectin catabolic process	GO:0045490	Metabolism	Catabolism	P	99	12	2.22	5.4	6.11E-06	8.70E-04
4	854	F2-3 vs. M2-3	F2-3 up	+	polysaccharide catabolic process	GO:0000272	Metabolism	Catabolism	P	144	15	3.23	4.64	2.46E-06	4.08E-04
4	855	F2-3 vs. M2-3	F2-3 up	+	small molecule catabolic process	GO:0044282	Metabolism	Catabolism	P	239	20	5.36	3.73	1.35E-06	2.37E-04
4	856	F2-3 vs. M2-3	F2-3 up	-	nucleic acid metabolic process	GO:0090304	Development	Cell division	P	1705	16	38.25	0.42	5.82E-05	4.35E-03
4	857	F2-3 vs. M2-3	F2-3 up	-	RNA metabolic process	GO:0016070	Development	Cell division	P	1294	13	29.03	0.45	1.31E-03	4.06E-02
4	858	F2-3 vs. M2-3	F2-3 up	+	nucleoside phosphate binding	GO:1901265	Development	Cell division	F	1112	43	24.94	1.72	9.15E-04	4.82E-02
4	859	F2-3 vs. M2-3	F2-3 up	+	nucleotide binding	GO:0000166	Development	Cell division	F	1112	43	24.94	1.72	9.15E-04	4.90E-02
4	860	F2-3 vs. M2-3	F2-3 up	+	nucleotide-sugar biosynthetic process	GO:0009226	Development	Cell division	P	42	6	0.94	6.37	6.11E-04	2.52E-02
4	861	F2-3 vs. M2-3	F2-3 up	+	nucleotide-sugar metabolic process	GO:0009225	Development	Cell division	P	52	6	1.17	5.14	1.67E-03	4.98E-02
4	862	F2-3 vs. M2-3	F2-3 up	+	pollen tube development	GO:0048868	Development	Cell division	P	192	15	4.31	3.48	5.77E-05	4.37E-03
4	863	F2-3 vs. M2-3	F2-3 up	+	pollen tube growth	GO:0009860	Development	Cell division	P	135	13	3.03	4.29	2.46E-05	2.37E-03
4	864	F2-3 vs. M2-3	F2-3 up	+	pollination	GO:0009856	Development	Cell division	P	287	17	6.44	2.64	4.41E-04	2.00E-02
4	865	F2-3 vs. M2-3	F2-3 up	+	syncytium formation	GO:0006949	Development	Cell division	P	16	4	0.36	11.14	8.40E-04	3.05E-02
4	866	F2-3 vs. M2-3	F2-3 up	-	chromatin organization	GO:0006325	Development	Development	P	333	0	7.47	<	1.05E-03	3.53E-02
4	867	F2-3 vs. M2-3	F2-3 up	-	chromosome organization	GO:0051276	Development	Development	P	501	0	11.24	<	3.37E-05	3.00E-03
4	868	F2-3 vs. M2-3	F2-3 up	-	nucleus	GO:0005634	Development	Development	C	10540	178	236.43	0.75	1.30E-06	4.38E-05
4	869	F2-3 vs. M2-3	F2-3 up	+	actin cytoskeleton organization	GO:0030036	Development	Development	P	104	11	2.33	4.72	4.70E-05	3.70E-03

4	870	F2-3 vs. M2-3	F2-3 up	+	anatomical structure development	GO:0048856	Development	Development	P	2842	90	63.75	1.41	1.06E-03	3.54E-02
4	871	F2-3 vs. M2-3	F2-3 up	+	apoplast	GO:0048046	Development	Development	C	297	32	6.66	4.8	2.44E-12	3.18E-10
4	872	F2-3 vs. M2-3	F2-3 up	+	bounding membrane of organelle	GO:0098588	Development	Development	C	923	42	20.7	2.03	2.74E-05	8.64E-04
4	873	F2-3 vs. M2-3	F2-3 up	+	cell development	GO:0048468	Development	Development	P	319	19	7.16	2.66	1.95E-04	1.12E-02
4	874	F2-3 vs. M2-3	F2-3 up	+	cell growth	GO:0016049	Development	Development	P	386	24	8.66	2.77	1.54E-05	1.73E-03
4	875	F2-3 vs. M2-3	F2-3 up	+	cell junction	GO:0030054	Development	Development	C	944	48	21.18	2.27	3.73E-07	1.39E-05
4	876	F2-3 vs. M2-3	F2-3 up	+	cell morphogenesis	GO:0000902	Development	Development	P	369	22	8.28	2.66	6.20E-05	4.41E-03
4	877	F2-3 vs. M2-3	F2-3 up	+	cell morphogenesis involved in differentiation	GO:0000904	Development	Development	P	229	15	5.14	2.92	3.52E-04	1.68E-02
4	878	F2-3 vs. M2-3	F2-3 up	+	cell periphery	GO:0071944	Development	Development	C	4012	166	90	1.84	4.61E-15	2.40E-12
4	879	F2-3 vs. M2-3	F2-3 up	+	cell tip growth	GO:0009932	Development	Development	P	160	15	3.59	4.18	8.01E-06	1.11E-03
4	880	F2-3 vs. M2-3	F2-3 up	+	cell wall	GO:0005618	Development	Development	C	565	30	12.67	2.37	2.67E-05	8.70E-04
4	881	F2-3 vs. M2-3	F2-3 up	+	cell wall modification	GO:0042545	Development	Development	P	173	12	3.88	3.09	8.38E-04	3.07E-02
4	882	F2-3 vs. M2-3	F2-3 up	+	cell wall organization	GO:0071555	Development	Development	P	274	16	6.15	2.6	7.42E-04	2.82E-02
4	883	F2-3 vs. M2-3	F2-3 up	+	cell-cell junction	GO:0005911	Development	Development	C	944	48	21.18	2.27	3.73E-07	1.55E-05
4	884	F2-3 vs. M2-3	F2-3 up	+	cellular anatomical entity	GO:0110165	Development	Development	C	24430	575	548.02	1.05	2.88E-04	7.89E-03
4	885	F2-3 vs. M2-3	F2-3 up	+	cellular process	GO:0009987	Development	Development	P	12008	355	269.37	1.32	9.37E-12	9.34E-09
4	886	F2-3 vs. M2-3	F2-3 up	+	cytoplasm	GO:0005737	Development	Development	C	14837	418	332.83	1.26	6.08E-12	7.03E-10
4	887	F2-3 vs. M2-3	F2-3 up	+	cytoskeleton organization	GO:0007010	Development	Development	P	283	18	6.35	2.84	1.33E-04	8.21E-03
4	888	F2-3 vs. M2-3	F2-3 up	+	cytosol	GO:0005829	Development	Development	C	3241	138	72.7	1.9	2.80E-13	7.28E-11
4	889	F2-3 vs. M2-3	F2-3 up	+	developmental cell growth	GO:0048588	Development	Development	P	197	16	4.42	3.62	2.13E-05	2.19E-03
4	890	F2-3 vs. M2-3	F2-3 up	+	developmental growth	GO:0048589	Development	Development	P	385	24	8.64	2.78	1.48E-05	1.70E-03
4	891	F2-3 vs. M2-3	F2-3 up	+	developmental growth involved in	GO:0060560	Development	Development	P	335	23	7.51	3.06	5.16E-06	7.91E-04
4	892	F2-3 vs. M2-3	F2-3 up	+	developmental process	GO:0032502	Development	Development	P	2997	94	67.23	1.4	1.10E-03	3.57E-02
4	893	F2-3 vs. M2-3	F2-3 up	+	endomembrane system	GO:0012505	Development	Development	C	2286	79	51.28	1.54	1.72E-04	4.96E-03
4	894	F2-3 vs. M2-3	F2-3 up	+	envelope	GO:0031975	Development	Development	C	1025	60	22.99	2.61	8.33E-11	8.67E-09
4	895	F2-3 vs. M2-3	F2-3 up	+	external encapsulating structure	GO:0030312	Development	Development	C	567	30	12.72	2.36	2.83E-05	8.66E-04
4	896	F2-3 vs. M2-3	F2-3 up	+	external encapsulating structure organization	GO:0045229	Development	Development	P	320	18	7.18	2.51	5.40E-04	2.34E-02
4	897	F2-3 vs. M2-3	F2-3 up	+	extracellular region	GO:0005576	Development	Development	C	3128	121	70.17	1.72	4.81E-09	2.64E-07
4	898	F2-3 vs. M2-3	F2-3 up	+	Golgi apparatus subcompartment	GO:0098791	Development	Development	C	437	23	9.8	2.35	2.78E-04	7.81E-03
4	899	F2-3 vs. M2-3	F2-3 up	+	growth	GO:0040007	Development	Development	P	443	25	9.94	2.52	7.52E-05	5.23E-03
4	900	F2-3 vs. M2-3	F2-3 up	+	intracellular vesicle	GO:0097708	Development	Development	C	722	31	16.2	1.91	8.90E-04	2.11E-02
4	901	F2-3 vs. M2-3	F2-3 up	+	membrane	GO:0016020	Development	Development	C	5493	221	123.22	1.79	1.61E-19	1.67E-16
4	902	F2-3 vs. M2-3	F2-3 up	+	multi-multicellular organism process	GO:0044706	Development	Development	P	287	17	6.44	2.64	4.41E-04	2.01E-02
4	903	F2-3 vs. M2-3	F2-3 up	+	organelle envelope	GO:0031967	Development	Development	C	1025	60	22.99	2.61	8.33E-11	7.88E-09
4	904	F2-3 vs. M2-3	F2-3 up	+	organelle subcompartment	GO:0031984	Development	Development	C	1156	62	25.93	2.39	1.23E-09	8.52E-08
4	905	F2-3 vs. M2-3	F2-3 up	+	phloem loading	GO:0110126	Development	Development	P	7	3	0.16	19.11	1.12E-03	3.59E-02
4	906	F2-3 vs. M2-3	F2-3 up	+	plasma membrane	GO:0005886	Development	Development	C	3534	144	79.28	1.82	2.03E-12	3.01E-10
4	907	F2-3 vs. M2-3	F2-3 up	+	stromule	GO:0010319	Development	Development	C	36	10	0.81	12.38	4.81E-08	2.50E-06
4	908	F2-3 vs. M2-3	F2-3 up	+	structural constituent of cytoskeleton	GO:0005200	Development	Development	F	28	6	0.63	9.55	8.67E-05	7.34E-03
4	909	F2-3 vs. M2-3	F2-3 up	+	symplast	GO:0055044	Development	Development	C	944	48	21.18	2.27	3.73E-07	1.49E-05
4	910	F2-3 vs. M2-3	F2-3 up	+	unidimensional cell growth	GO:0009826	Development	Development	P	288	21	6.46	3.25	5.58E-06	8.14E-04
4	911	F2-3 vs. M2-3	F2-3 up	+	vacuolar membrane	GO:0005774	Development	Development	C	570	27	12.79	2.11	5.30E-04	1.31E-02
4	912	F2-3 vs. M2-3	F2-3 up	+	vacuole	GO:0005773	Development	Development	C	1083	55	24.29	2.26	6.63E-08	3.29E-06
4	913	F2-3 vs. M2-3	F2-3 up	-	cellular macromolecule metabolic process	GO:0044260	Metabolism	Metabolism	P	4426	68	99.28	0.68	4.52E-04	2.03E-02
4	914	F2-3 vs. M2-3	F2-3 up	-	cellular protein metabolic process	GO:0044267	Metabolism	Metabolism	P	3388	47	76	0.62	2.34E-04	1.28E-02
4	915	F2-3 vs. M2-3	F2-3 up	-	protein metabolic process	GO:0019538	Metabolism	Metabolism	P	3544	49	79.5	0.62	1.48E-04	8.86E-03
4	916	F2-3 vs. M2-3	F2-3 up	+	actin binding	GO:0003779	Metabolism	Metabolism	F	114	11	2.56	4.3	1.01E-04	8.08E-03
4	917	F2-3 vs. M2-3	F2-3 up	+	actin filament binding	GO:0051015	Metabolism	Metabolism	F	75	8	1.68	4.76	4.77E-04	3.07E-02
4	918	F2-3 vs. M2-3	F2-3 up	+	actin filament bundle assembly	GO:0051017	Metabolism	Metabolism	P	20	4	0.45	8.92	1.72E-03	4.99E-02
4	919	F2-3 vs. M2-3	F2-3 up	+	actin filament bundle organization	GO:0061572	Metabolism	Metabolism	P	20	4	0.45	8.92	1.72E-03	5.02E-02
4	920	F2-3 vs. M2-3	F2-3 up	+	actin filament organization	GO:0007015	Metabolism	Metabolism	P	92	8	2.06	3.88	1.63E-03	4.89E-02
4	921	F2-3 vs. M2-3	F2-3 up	+	actin filament-based process	GO:0030029	Metabolism	Metabolism	P	113	11	2.53	4.34	9.35E-05	6.21E-03
4	922	F2-3 vs. M2-3	F2-3 up	+	actin monomer binding	GO:0003785	Metabolism	Metabolism	F	7	4	0.16	25.47	6.70E-05	5.98E-03
4	923	F2-3 vs. M2-3	F2-3 up	+	alpha-amino acid metabolic process	GO:1901605	Metabolism	Metabolism	P	278	20	6.24	3.21	1.12E-05	1.39E-03
4	924	F2-3 vs. M2-3	F2-3 up	+	anchoring junction	GO:0070161	Metabolism	Metabolism	C	944	48	21.18	2.27	3.73E-07	1.44E-05
4	925	F2-3 vs. M2-3	F2-3 up	+	anion binding	GO:0043168	Metabolism	Metabolism	F	1369	58	30.71	1.89	6.68E-06	9.33E-04
4	926	F2-3 vs. M2-3	F2-3 up	+	aromatase activity	GO:0070330	Metabolism	Metabolism	F	4	3	0.09	33.43	3.44E-04	2.31E-02
4	927	F2-3 vs. M2-3	F2-3 up	+	binding	GO:0005488	Metabolism	Metabolism	F	9843	291	220.8	1.32	1.12E-08	4.51E-06
4	928	F2-3 vs. M2-3	F2-3 up	+	biological_process	GO:0008150	Metabolism	Metabolism	P	21988	529	493.24	1.07	2.17E-04	1.23E-02
4	929	F2-3 vs. M2-3	F2-3 up	+	biosynthetic process	GO:0009058	Metabolism	Metabolism	P	2811	103	63.06	1.63	1.25E-06	2.27E-04
4	930	F2-3 vs. M2-3	F2-3 up	+	carbon-carbon lyase activity	GO:0016830	Metabolism	Metabolism	F	111	12	2.49	4.82	1.76E-05	1.95E-03
4	931	F2-3 vs. M2-3	F2-3 up	+	carboxylic acid biosynthetic process	GO:0046394	Metabolism	Metabolism	P	468	24	10.5	2.29	2.76E-04	1.45E-02

4	932	F2-3 vs. M2-3	F2-3 up	+	carboxylic acid metabolic process	GO:0019752	Metabolism	Metabolism	P	912	57	20.46	2.79	1.79E-11	1.53E-08
4	933	F2-3 vs. M2-3	F2-3 up	+	catalytic activity	GO:0003824	Metabolism	Metabolism	F	8380	310	187.98	1.65	4.83E-24	1.55E-20
4	934	F2-3 vs. M2-3	F2-3 up	+	cation binding	GO:0043169	Metabolism	Metabolism	F	1637	68	36.72	1.85	2.05E-06	3.67E-04
4	935	F2-3 vs. M2-3	F2-3 up	+	cellular aldehyde metabolic process	GO:0006081	Metabolism	Metabolism	P	48	6	1.08	5.57	1.15E-03	3.66E-02
4	936	F2-3 vs. M2-3	F2-3 up	+	cellular amino acid metabolic process	GO:0006520	Metabolism	Metabolism	P	391	24	8.77	2.74	1.87E-05	2.07E-03
4	937	F2-3 vs. M2-3	F2-3 up	+	cellular biosynthetic process	GO:0044249	Metabolism	Metabolism	P	2583	88	57.94	1.52	1.23E-04	7.76E-03
4	938	F2-3 vs. M2-3	F2-3 up	+	cellular lipid metabolic process	GO:0044255	Metabolism	Metabolism	P	689	30	15.46	1.94	1.05E-03	3.52E-02
4	939	F2-3 vs. M2-3	F2-3 up	+	cellular metabolic process	GO:0044237	Metabolism	Metabolism	P	7649	219	171.58	1.28	4.24E-05	3.52E-03
4	940	F2-3 vs. M2-3	F2-3 up	+	copper ion binding	GO:0005507	Metabolism	Metabolism	F	193	20	4.33	4.62	5.81E-08	1.87E-05
4	941	F2-3 vs. M2-3	F2-3 up	+	establishment of localization	GO:0051234	Metabolism	Metabolism	P	2306	91	51.73	1.76	2.56E-07	7.28E-05
4	942	F2-3 vs. M2-3	F2-3 up	+	ferrous iron binding	GO:0008198	Metabolism	Metabolism	F	16	4	0.36	11.14	8.40E-04	4.66E-02
4	943	F2-3 vs. M2-3	F2-3 up	+	galacturonan metabolic process	GO:0010393	Metabolism	Metabolism	P	163	17	3.66	4.65	5.23E-07	1.12E-04
4	944	F2-3 vs. M2-3	F2-3 up	+	glucosyltransferase activity	GO:0046527	Metabolism	Metabolism	F	190	13	4.26	3.05	5.83E-04	3.61E-02
4	945	F2-3 vs. M2-3	F2-3 up	+	glutamine family amino acid metabolic process	GO:0009064	Metabolism	Metabolism	P	58	7	1.3	5.38	5.47E-04	2.32E-02
4	946	F2-3 vs. M2-3	F2-3 up	+	glycine decarboxylation via glycine cleavage system	GO:0019464	Metabolism	Metabolism	P	7	3	0.16	19.11	1.12E-03	3.61E-02
4	947	F2-3 vs. M2-3	F2-3 up	+	glycine metabolic process	GO:0006544	Metabolism	Metabolism	P	18	4	0.4	9.91	1.23E-03	3.86E-02
4	948	F2-3 vs. M2-3	F2-3 up	+	hydrolase activity	GO:0016787	Metabolism	Metabolism	F	2995	108	67.18	1.61	1.28E-06	2.42E-04
4	949	F2-3 vs. M2-3	F2-3 up	+	hydrolase activity, acting on glycosyl bonds	GO:0016798	Metabolism	Metabolism	F	536	27	12.02	2.25	1.90E-04	1.42E-02
4	950	F2-3 vs. M2-3	F2-3 up	+	hydrolase activity, hydrolyzing O-glycosyl compounds	GO:0004553	Metabolism	Metabolism	F	396	25	8.88	2.81	8.03E-06	1.08E-03
4	951	F2-3 vs. M2-3	F2-3 up	+	ion binding	GO:0043167	Metabolism	Metabolism	F	2847	122	63.86	1.91	7.13E-12	7.64E-09
4	952	F2-3 vs. M2-3	F2-3 up	+	iron ion binding	GO:0005506	Metabolism	Metabolism	F	325	18	7.29	2.47	6.41E-04	3.82E-02
4	953	F2-3 vs. M2-3	F2-3 up	+	lignin biosynthetic process	GO:0009809	Metabolism	Metabolism	P	42	6	0.94	6.37	6.11E-04	2.48E-02
4	954	F2-3 vs. M2-3	F2-3 up	+	lignin metabolic process	GO:0009808	Metabolism	Metabolism	P	53	8	1.19	6.73	5.43E-05	4.22E-03
4	955	F2-3 vs. M2-3	F2-3 up	+	lipid metabolic process	GO:0006629	Metabolism	Metabolism	P	828	35	18.57	1.88	5.34E-04	2.33E-02
4	956	F2-3 vs. M2-3	F2-3 up	+	localization	GO:0051179	Metabolism	Metabolism	P	2471	97	55.43	1.75	1.27E-07	4.22E-05
4	957	F2-3 vs. M2-3	F2-3 up	+	lyase activity	GO:0016829	Metabolism	Metabolism	F	339	26	7.6	3.42	1.78E-07	4.76E-05
4	958	F2-3 vs. M2-3	F2-3 up	+	metabolic process	GO:0008152	Metabolism	Metabolism	P	8681	275	194.73	1.41	2.32E-11	1.73E-08
4	959	F2-3 vs. M2-3	F2-3 up	+	metal ion binding	GO:0046872	Metabolism	Metabolism	F	1610	67	36.12	1.86	2.59E-06	4.39E-04
4	960	F2-3 vs. M2-3	F2-3 up	+	methionine adenosyltransferase activity	GO:0004478	Metabolism	Metabolism	F	4	3	0.09	33.43	3.44E-04	2.26E-02
4	961	F2-3 vs. M2-3	F2-3 up	+	molecular_function	GO:0003674	Metabolism	Metabolism	F	21945	551	492.27	1.12	3.22E-10	1.73E-07
4	962	F2-3 vs. M2-3	F2-3 up	+	monocarboxylic acid metabolic process	GO:0032787	Metabolism	Metabolism	P	439	27	9.85	2.74	5.53E-06	8.26E-04
4	963	F2-3 vs. M2-3	F2-3 up	+	monooxygenase activity	GO:0004497	Metabolism	Metabolism	F	222	15	4.98	3.01	2.58E-04	1.89E-02
4	964	F2-3 vs. M2-3	F2-3 up	+	monosaccharide metabolic process	GO:0005996	Metabolism	Metabolism	P	125	11	2.8	3.92	2.13E-04	1.21E-02
4	965	F2-3 vs. M2-3	F2-3 up	+	organic acid biosynthetic process	GO:0016053	Metabolism	Metabolism	P	468	24	10.5	2.29	2.76E-04	1.46E-02
4	966	F2-3 vs. M2-3	F2-3 up	+	organic acid metabolic process	GO:0006082	Metabolism	Metabolism	P	1019	62	22.86	2.71	6.34E-12	9.47E-09
4	967	F2-3 vs. M2-3	F2-3 up	+	organic hydroxy compound metabolic process	GO:1901615	Metabolism	Metabolism	P	292	20	6.55	3.05	2.17E-05	2.20E-03
4	968	F2-3 vs. M2-3	F2-3 up	+	organic substance biosynthetic process	GO:1901576	Metabolism	Metabolism	P	2674	95	59.98	1.58	1.10E-05	1.39E-03
4	969	F2-3 vs. M2-3	F2-3 up	+	organic substance metabolic process	GO:0071704	Metabolism	Metabolism	P	7851	224	176.11	1.27	4.01E-05	3.43E-03
4	970	F2-3 vs. M2-3	F2-3 up	+	oxidation-reduction process	GO:0055114	Metabolism	Metabolism	P	768	48	17.23	2.79	7.65E-10	3.81E-07
4	971	F2-3 vs. M2-3	F2-3 up	+	oxoacid metabolic process	GO:0043436	Metabolism	Metabolism	P	1016	62	22.79	2.72	5.64E-12	1.12E-08
4	972	F2-3 vs. M2-3	F2-3 up	+	pectin metabolic process	GO:0045488	Metabolism	Metabolism	P	162	17	3.63	4.68	4.84E-07	1.11E-04
4	973	F2-3 vs. M2-3	F2-3 up	+	pectinesterase activity	GO:0030599	Metabolism	Metabolism	F	92	10	2.06	4.85	8.37E-05	7.27E-03
4	974	F2-3 vs. M2-3	F2-3 up	+	phenylpropanoid biosynthetic process	GO:0009699	Metabolism	Metabolism	P	88	9	1.97	4.56	2.86E-04	1.46E-02
4	975	F2-3 vs. M2-3	F2-3 up	+	phenylpropanoid metabolic process	GO:0009698	Metabolism	Metabolism	P	109	9	2.45	3.68	1.19E-03	3.78E-02
4	976	F2-3 vs. M2-3	F2-3 up	+	polygalacturonase activity	GO:0004650	Metabolism	Metabolism	F	69	9	1.55	5.81	5.27E-05	4.84E-03
4	977	F2-3 vs. M2-3	F2-3 up	+	proton export across plasma membrane	GO:0120029	Metabolism	Metabolism	P	10	5	0.22	22.29	1.25E-05	1.53E-03
4	978	F2-3 vs. M2-3	F2-3 up	+	S-adenosylmethionine biosynthetic process	GO:0006556	Metabolism	Metabolism	P	4	3	0.09	33.43	3.44E-04	1.70E-02
4	979	F2-3 vs. M2-3	F2-3 up	+	S-adenosylmethionine metabolic process	GO:0046500	Metabolism	Metabolism	P	9	4	0.2	19.81	1.40E-04	8.46E-03
4	980	F2-3 vs. M2-3	F2-3 up	+	secondary metabolic process	GO:0019748	Metabolism	Metabolism	P	303	18	6.8	2.65	2.93E-04	1.48E-02
4	981	F2-3 vs. M2-3	F2-3 up	+	secondary metabolite biosynthetic process	GO:0044550	Metabolism	Metabolism	P	144	14	3.23	4.33	1.09E-05	1.42E-03
4	982	F2-3 vs. M2-3	F2-3 up	+	sequestering of actin monomers	GO:0042989	Metabolism	Metabolism	P	5	3	0.11	26.75	5.42E-04	2.31E-02
4	983	F2-3 vs. M2-3	F2-3 up	+	small molecule binding	GO:0036094	Metabolism	Metabolism	F	1340	55	30.06	1.83	3.02E-05	3.23E-03
4	984	F2-3 vs. M2-3	F2-3 up	+	small molecule biosynthetic process	GO:0044283	Metabolism	Metabolism	P	645	36	14.47	2.49	1.58E-06	2.70E-04
4	985	F2-3 vs. M2-3	F2-3 up	+	small molecule metabolic process	GO:0044281	Metabolism	Metabolism	P	1520	80	34.1	2.35	6.39E-12	7.64E-09
4	986	F2-3 vs. M2-3	F2-3 up	+	sulfur compound biosynthetic process	GO:0044272	Metabolism	Metabolism	P	142	12	3.19	3.77	1.58E-04	9.28E-03
4	987	F2-3 vs. M2-3	F2-3 up	+	sulfur compound metabolic process	GO:0006790	Metabolism	Metabolism	P	363	19	8.14	2.33	1.07E-03	3.55E-02
4	988	F2-3 vs. M2-3	F2-3 up	+	transition metal ion binding	GO:0046914	Metabolism	Metabolism	F	1178	45	26.43	1.7	8.84E-04	4.82E-02
4	989	F2-3 vs. M2-3	F2-3 up	+	wax biosynthetic process	GO:0010025	Metabolism	Metabolism	P	27	5	0.61	8.26	6.17E-04	2.49E-02
4	990	F2-3 vs. M2-3	F2-3 up	+	wax metabolic process	GO:0010166	Metabolism	Metabolism	P	28	5	0.63	7.96	7.14E-04	2.75E-02
4	991	F2-3 vs. M2-3	F2-3 up	+	carbon fixation	GO:0015977	Metabolism	Photosynthesis	P	16	4	0.36	11.14	8.40E-04	3.06E-02

4	992	F2-3 vs. M2-3	F2-3 up	+	chloroplast	GO:0009507	Metabolism	Photosynthesis	C	5013	175	112.45	1.56	1.20E-09	8.94E-08
4	993	F2-3 vs. M2-3	F2-3 up	+	chloroplast envelope	GO:0009941	Metabolism	Photosynthesis	C	613	48	13.75	3.49	5.21E-13	9.03E-11
4	994	F2-3 vs. M2-3	F2-3 up	+	chloroplast stroma	GO:0009570	Metabolism	Photosynthesis	C	719	46	16.13	2.85	8.74E-10	7.00E-08
4	995	F2-3 vs. M2-3	F2-3 up	+	chloroplast thylakoid	GO:0009534	Metabolism	Photosynthesis	C	443	34	9.94	3.42	2.25E-09	1.38E-07
4	996	F2-3 vs. M2-3	F2-3 up	+	chloroplast thylakoid membrane	GO:0009535	Metabolism	Photosynthesis	C	358	26	8.03	3.24	4.72E-07	1.70E-05
4	997	F2-3 vs. M2-3	F2-3 up	+	fructose 1,6-bisphosphate metabolic process	GO:0030388	Metabolism	Photosynthesis	P	12	4	0.27	14.86	3.38E-04	1.69E-02
4	998	F2-3 vs. M2-3	F2-3 up	+	photosynthesis	GO:0015979	Metabolism	Photosynthesis	P	189	20	4.24	4.72	4.23E-08	1.58E-05
4	999	F2-3 vs. M2-3	F2-3 up	+	photosynthesis, dark reaction	GO:0019685	Metabolism	Photosynthesis	P	15	4	0.34	11.89	6.84E-04	2.69E-02
4	1000	F2-3 vs. M2-3	F2-3 up	+	photosynthesis, light reaction	GO:0019684	Metabolism	Photosynthesis	P	128	14	2.87	4.88	3.15E-06	5.09E-04
4	1001	F2-3 vs. M2-3	F2-3 up	+	photosynthetic electron transport chain	GO:0009767	Metabolism	Photosynthesis	P	46	7	1.03	6.78	1.52E-04	8.99E-03
4	1002	F2-3 vs. M2-3	F2-3 up	+	photosynthetic electron transport in photosystem	GO:0009773	Metabolism	Photosynthesis	P	17	4	0.38	10.49	1.02E-03	3.49E-02
4	1003	F2-3 vs. M2-3	F2-3 up	+	photosynthetic membrane	GO:0034357	Metabolism	Photosynthesis	C	395	28	8.86	3.16	2.73E-07	1.23E-05
4	1004	F2-3 vs. M2-3	F2-3 up	+	plastid	GO:0009536	Metabolism	Photosynthesis	C	5432	190	121.85	1.56	1.41E-10	1.22E-08
4	1005	F2-3 vs. M2-3	F2-3 up	+	plastid envelope	GO:0009526	Metabolism	Photosynthesis	C	637	50	14.29	3.5	1.53E-13	5.32E-11
4	1006	F2-3 vs. M2-3	F2-3 up	+	plastid stroma	GO:0009532	Metabolism	Photosynthesis	C	731	46	16.4	2.81	1.43E-09	9.31E-08
4	1007	F2-3 vs. M2-3	F2-3 up	+	plastid thylakoid	GO:0031976	Metabolism	Photosynthesis	C	446	34	10	3.4	2.64E-09	1.53E-07
4	1008	F2-3 vs. M2-3	F2-3 up	+	plastid thylakoid membrane	GO:0055035	Metabolism	Photosynthesis	C	363	26	8.14	3.19	6.04E-07	2.10E-05
4	1009	F2-3 vs. M2-3	F2-3 up	+	plastoglobule	GO:0010287	Metabolism	Photosynthesis	C	76	8	1.7	4.69	5.17E-04	1.31E-02
4	1010	F2-3 vs. M2-3	F2-3 up	+	thylakoid	GO:0009579	Metabolism	Photosynthesis	C	537	45	12.05	3.74	3.28E-13	6.83E-11
4	1011	F2-3 vs. M2-3	F2-3 up	+	thylakoid membrane	GO:0042651	Metabolism	Photosynthesis	C	390	28	8.75	3.2	2.14E-07	1.01E-05
4	1012	F2-3 vs. M2-3	F2-3 up	+	Golgi apparatus	GO:0005794	Regulation	Protein modification	C	1161	46	26.04	1.77	3.82E-04	9.93E-03
4	1013	F2-3 vs. M2-3	F2-3 up	+	maintenance of location in cell	GO:0051651	Regulation	Protein modification	P	46	6	1.03	5.81	9.41E-04	3.35E-02
4	1014	F2-3 vs. M2-3	F2-3 up	-	protein modification by small protein conjugation or removal	GO:0070647	Regulation	Protein modification	P	815	5	18.28	0.27	5.88E-04	2.44E-02
4	1015	F2-3 vs. M2-3	F2-3 up	+	negative regulation of protein polymerization	GO:0032272	Regulation	Protein modification	P	15	4	0.34	11.89	6.84E-04	2.71E-02
4	1016	F2-3 vs. M2-3	F2-3 up	+	negative regulation of protein-containing complex assembly	GO:0031333	Regulation	Protein modification	P	15	4	0.34	11.89	6.84E-04	2.73E-02
4	1017	F2-3 vs. M2-3	F2-3 up	-	regulation of cellular biosynthetic process	GO:0031326	Regulation	Regulation	P	2477	33	55.56	0.59	9.88E-04	3.43E-02
4	1018	F2-3 vs. M2-3	F2-3 up	-	regulation of cellular macromolecule biosynthetic process	GO:2000112	Regulation	Regulation	P	2365	28	53.05	0.53	1.40E-04	8.54E-03
4	1019	F2-3 vs. M2-3	F2-3 up	-	regulation of cellular metabolic process	GO:0031323	Regulation	Regulation	P	2975	43	66.74	0.64	1.56E-03	4.70E-02
4	1020	F2-3 vs. M2-3	F2-3 up	-	regulation of macromolecule biosynthetic	GO:0010556	Regulation	Regulation	P	2378	29	53.34	0.54	2.57E-04	1.39E-02
4	1021	F2-3 vs. M2-3	F2-3 up	-	regulation of macromolecule metabolic process	GO:0060255	Regulation	Regulation	P	3058	38	68.6	0.55	3.85E-05	3.34E-03
4	1022	F2-3 vs. M2-3	F2-3 up	-	regulation of metabolic process	GO:0019222	Regulation	Regulation	P	3325	48	74.59	0.64	6.99E-04	2.71E-02
4	1023	F2-3 vs. M2-3	F2-3 up	-	regulation of nitrogen compound metabolic process	GO:0051171	Regulation	Regulation	P	2741	36	61.49	0.59	3.69E-04	1.74E-02
4	1024	F2-3 vs. M2-3	F2-3 up	-	regulation of nucleobase-containing compound metabolic process	GO:0019219	Regulation	Regulation	P	2344	31	52.58	0.59	1.24E-03	3.87E-02
4	1025	F2-3 vs. M2-3	F2-3 up	-	regulation of primary metabolic process	GO:0080090	Regulation	Regulation	P	2825	37	63.37	0.58	2.64E-04	1.41E-02
4	1026	F2-3 vs. M2-3	F2-3 up	+	negative regulation of actin filament	GO:0030837	Regulation	Regulation	P	13	4	0.29	13.72	4.35E-04	2.02E-02
4	1027	F2-3 vs. M2-3	F2-3 up	+	negative regulation of cytoskeleton organization	GO:0051494	Regulation	Regulation	P	17	4	0.38	10.49	1.02E-03	3.51E-02
4	1028	F2-3 vs. M2-3	F2-3 up	+	negative regulation of supramolecular fiber organization	GO:1902904	Regulation	Regulation	P	17	4	0.38	10.49	1.02E-03	3.53E-02
4	1029	F2-3 vs. M2-3	F2-3 up	+	regulation of cell morphogenesis involved in differentiation	GO:0010769	Regulation	Regulation	P	40	7	0.9	7.8	6.94E-05	4.88E-03
4	1030	F2-3 vs. M2-3	F2-3 up	+	regulation of pollen tube growth	GO:0080092	Regulation	Regulation	P	35	7	0.79	8.92	3.28E-05	2.97E-03
4	1031	F2-3 vs. M2-3	F2-3 up	+	regulation of unidimensional cell growth	GO:0051510	Regulation	Regulation	P	74	8	1.66	4.82	4.40E-04	2.02E-02
4	1032	F2-3 vs. M2-3	F2-3 up	+	cellular response to abiotic stimulus	GO:0071214	Signaling and response	Signaling	P	189	12	4.24	2.83	1.71E-03	5.02E-02
4	1033	F2-3 vs. M2-3	F2-3 up	+	cellular response to blue light	GO:0071483	Signaling and response	Signaling	P	27	5	0.61	8.26	6.17E-04	2.47E-02
4	1034	F2-3 vs. M2-3	F2-3 up	+	cellular response to environmental stimulus	GO:0104004	Signaling and response	Signaling	P	189	12	4.24	2.83	1.71E-03	5.05E-02
4	1035	F2-3 vs. M2-3	F2-3 up	+	response to abiotic stimulus	GO:0009628	Signaling and response	Signaling	P	2131	96	47.8	2.01	1.95E-10	1.16E-07
4	1036	F2-3 vs. M2-3	F2-3 up	+	response to acid chemical	GO:0001101	Signaling and response	Signaling	P	397	20	8.91	2.25	1.10E-03	3.59E-02
4	1037	F2-3 vs. M2-3	F2-3 up	+	response to bacterium	GO:0009617	Signaling and response	Signaling	P	508	30	11.4	2.63	3.63E-06	5.71E-04
4	1038	F2-3 vs. M2-3	F2-3 up	+	response to biotic stimulus	GO:0009607	Signaling and response	Signaling	P	1098	48	24.63	1.95	1.96E-05	2.06E-03
4	1039	F2-3 vs. M2-3	F2-3 up	+	response to cadmium ion	GO:0046686	Signaling and response	Signaling	P	315	24	7.07	3.4	5.88E-07	1.21E-04
4	1040	F2-3 vs. M2-3	F2-3 up	+	response to chemical	GO:0042221	Signaling and response	Signaling	P	2681	108	60.14	1.8	4.81E-09	2.21E-06
4	1041	F2-3 vs. M2-3	F2-3 up	+	response to external biotic stimulus	GO:0043207	Signaling and response	Signaling	P	1097	48	24.61	1.95	1.93E-05	2.10E-03
4	1042	F2-3 vs. M2-3	F2-3 up	+	response to external stimulus	GO:0009605	Signaling and response	Signaling	P	1513	67	33.94	1.97	3.08E-07	8.01E-05
4	1043	F2-3 vs. M2-3	F2-3 up	+	response to extracellular stimulus	GO:0009991	Signaling and response	Signaling	P	262	15	5.88	2.55	1.29E-03	4.02E-02
4	1044	F2-3 vs. M2-3	F2-3 up	+	response to inorganic substance	GO:0010035	Signaling and response	Signaling	P	914	51	20.5	2.49	8.55E-09	3.65E-06
4	1045	F2-3 vs. M2-3	F2-3 up	+	response to karrikin	GO:0080167	Signaling and response	Signaling	P	128	15	2.87	5.22	6.39E-07	1.27E-04
4	1046	F2-3 vs. M2-3	F2-3 up	+	response to light stimulus	GO:0009416	Signaling and response	Signaling	P	713	41	15.99	2.56	1.61E-07	5.07E-05

4	1047	F2-3 vs. M2-3	F2-3 up	+	response to metal ion	GO:0010038	Signaling and response	Signaling	P	433	29	9.71	2.99	5.02E-07	1.11E-04
4	1048	F2-3 vs. M2-3	F2-3 up	+	response to oxygen-containing compound	GO:1901700	Signaling and response	Signaling	P	1514	68	33.96	2	1.22E-07	4.31E-05
4	1049	F2-3 vs. M2-3	F2-3 up	+	response to stimulus	GO:0050896	Signaling and response	Signaling	P	5578	209	125.13	1.67	6.88E-15	4.11E-11
4	1050	F2-3 vs. M2-3	F2-3 up	+	response to temperature stimulus	GO:0009266	Signaling and response	Signaling	P	617	31	13.84	2.24	5.74E-05	4.40E-03
4	1051	F2-3 vs. M2-3	F2-3 up	+	response to UV	GO:0009411	Signaling and response	Signaling	P	125	10	2.8	3.57	8.14E-04	3.02E-02
4	1052	F2-3 vs. M2-3	F2-3 up	+	response to water	GO:0009415	Signaling and response	Signaling	P	365	20	8.19	2.44	5.58E-04	2.33E-02
4	1053	F2-3 vs. M2-3	F2-3 up	+	small GTPase mediated signal transduction	GO:0007264	Signaling and response	Signaling	P	20	4	0.45	8.92	1.72E-03	5.04E-02
4	1054	F2-3 vs. M2-3	F2-3 up	+	cellular cation homeostasis	GO:0030003	Signaling and response	Stress	P	159	11	3.57	3.08	1.39E-03	4.25E-02
4	1055	F2-3 vs. M2-3	F2-3 up	+	cellular chemical homeostasis	GO:0055082	Signaling and response	Stress	P	195	13	4.37	2.97	7.32E-04	2.81E-02
4	1056	F2-3 vs. M2-3	F2-3 up	+	cellular homeostasis	GO:0019725	Signaling and response	Stress	P	275	16	6.17	2.59	7.69E-04	2.87E-02
4	1057	F2-3 vs. M2-3	F2-3 up	+	cellular ion homeostasis	GO:0006873	Signaling and response	Stress	P	169	12	3.79	3.17	6.91E-04	2.70E-02
4	1058	F2-3 vs. M2-3	F2-3 up	+	cellular monovalent inorganic cation	GO:0030004	Signaling and response	Stress	P	39	7	0.87	8	6.02E-05	4.34E-03
4	1059	F2-3 vs. M2-3	F2-3 up	+	chemical homeostasis	GO:0048878	Signaling and response	Stress	P	415	22	9.31	2.36	3.58E-04	1.70E-02
4	1060	F2-3 vs. M2-3	F2-3 up	+	defense response	GO:0006952	Signaling and response	Stress	P	953	43	21.38	2.01	3.39E-05	2.98E-03
4	1061	F2-3 vs. M2-3	F2-3 up	+	defense response to bacterium	GO:0042742	Signaling and response	Stress	P	414	24	9.29	2.58	4.44E-05	3.63E-03
4	1062	F2-3 vs. M2-3	F2-3 up	+	defense response to other organism	GO:0098542	Signaling and response	Stress	P	807	33	18.1	1.82	1.63E-03	4.89E-02
4	1063	F2-3 vs. M2-3	F2-3 up	+	homeostatic process	GO:0042592	Signaling and response	Stress	P	537	25	12.05	2.08	1.08E-03	3.54E-02
4	1064	F2-3 vs. M2-3	F2-3 up	+	inorganic ion homeostasis	GO:0098771	Signaling and response	Stress	P	250	15	5.61	2.67	8.28E-04	3.06E-02
4	1065	F2-3 vs. M2-3	F2-3 up	+	interspecies interaction between organisms	GO:0044419	Signaling and response	Stress	P	1125	49	25.24	1.94	2.28E-05	2.23E-03
4	1066	F2-3 vs. M2-3	F2-3 up	+	ion homeostasis	GO:0050801	Signaling and response	Stress	P	290	17	6.51	2.61	4.93E-04	2.20E-02
4	1067	F2-3 vs. M2-3	F2-3 up	+	reactive oxygen species metabolic process	GO:0072593	Signaling and response	Stress	P	69	8	1.55	5.17	2.86E-04	1.47E-02
4	1068	F2-3 vs. M2-3	F2-3 up	+	regulation of cellular pH	GO:0030641	Signaling and response	Stress	P	28	6	0.63	9.55	8.67E-05	5.89E-03
4	1069	F2-3 vs. M2-3	F2-3 up	+	regulation of intracellular pH	GO:0051453	Signaling and response	Stress	P	28	6	0.63	9.55	8.67E-05	5.83E-03
4	1070	F2-3 vs. M2-3	F2-3 up	+	response to cold	GO:0009409	Signaling and response	Stress	P	415	24	9.31	2.58	4.60E-05	3.67E-03
4	1071	F2-3 vs. M2-3	F2-3 up	+	response to desiccation	GO:0009269	Signaling and response	Stress	P	24	6	0.54	11.14	4.13E-05	3.47E-03
4	1072	F2-3 vs. M2-3	F2-3 up	+	response to other organism	GO:0051707	Signaling and response	Stress	P	1097	48	24.61	1.95	1.93E-05	2.06E-03
4	1073	F2-3 vs. M2-3	F2-3 up	+	response to oxidative stress	GO:0006979	Signaling and response	Stress	P	460	25	10.32	2.42	1.02E-04	6.70E-03
4	1074	F2-3 vs. M2-3	F2-3 up	+	response to radiation	GO:0009314	Signaling and response	Stress	P	738	41	16.55	2.48	3.04E-07	8.25E-05
4	1075	F2-3 vs. M2-3	F2-3 up	+	response to reactive oxygen species	GO:0000302	Signaling and response	Stress	P	160	11	3.59	3.06	1.45E-03	4.43E-02
4	1076	F2-3 vs. M2-3	F2-3 up	+	response to stress	GO:0006950	Signaling and response	Stress	P	3106	126	69.67	1.81	1.05E-10	6.96E-08
4	1077	F2-3 vs. M2-3	F2-3 up	+	response to UV-B	GO:0010224	Signaling and response	Stress	P	73	8	1.64	4.89	4.04E-04	1.89E-02
4	1078	F2-3 vs. M2-3	F2-3 up	+	response to water deprivation	GO:0009414	Signaling and response	Stress	P	356	20	7.99	2.5	2.76E-04	1.44E-02
4	1079	F2-3 vs. M2-3	F2-3 up	+	response to wounding	GO:0009611	Signaling and response	Stress	P	227	20	5.09	3.93	6.40E-07	1.24E-04
4	1080	F2-3 vs. M2-3	F2-3 up	-	macromolecule modification	GO:0043412	Regulation	Transcription	P	2832	39	63.53	0.61	7.52E-04	2.85E-02
4	1081	F2-3 vs. M2-3	F2-3 up	-	regulation of gene expression	GO:0010468	Regulation	Transcription	P	2679	34	60.1	0.57	2.40E-04	1.31E-02
4	1082	F2-3 vs. M2-3	F2-3 up	-	regulation of nucleic acid-templated transcription	GO:1903506	Regulation	Transcription	P	2184	26	48.99	0.53	3.50E-04	1.70E-02
4	1083	F2-3 vs. M2-3	F2-3 up	-	regulation of RNA biosynthetic process	GO:2001141	Regulation	Transcription	P	2186	26	49.04	0.53	3.51E-04	1.69E-02
4	1084	F2-3 vs. M2-3	F2-3 up	-	regulation of transcription, DNA-templated	GO:0006355	Regulation	Transcription	P	2184	26	48.99	0.53	3.50E-04	1.72E-02
4	1085	F2-3 vs. M2-3	F2-3 up	+	mRNA binding	GO:0003729	Regulation	Translation	F	1005	46	22.54	2.04	1.24E-05	1.60E-03
4	1086	F2-3 vs. M2-3	F2-3 up	+	active ion transmembrane transporter activity	GO:0022853	Metabolism	Transporters	F	215	19	4.82	3.94	1.17E-06	2.34E-04
4	1087	F2-3 vs. M2-3	F2-3 up	+	active transmembrane transporter activity	GO:0022804	Metabolism	Transporters	F	482	32	10.81	2.96	1.56E-07	4.55E-05
4	1088	F2-3 vs. M2-3	F2-3 up	+	anion transport	GO:0006820	Metabolism	Transporters	P	283	16	6.35	2.52	1.02E-03	3.48E-02
4	1089	F2-3 vs. M2-3	F2-3 up	+	ATPase-coupled cation transmembrane transporter activity	GO:0019829	Metabolism	Transporters	F	61	11	1.37	8.04	4.67E-07	1.07E-04
4	1090	F2-3 vs. M2-3	F2-3 up	+	ATPase-coupled ion transmembrane transporter activity	GO:0042625	Metabolism	Transporters	F	76	11	1.7	6.45	3.21E-06	5.16E-04
4	1091	F2-3 vs. M2-3	F2-3 up	+	ATPase-coupled transmembrane transporter activity	GO:0042626	Metabolism	Transporters	F	190	18	4.26	4.22	8.86E-07	1.90E-04
4	1092	F2-3 vs. M2-3	F2-3 up	+	cation transmembrane transport	GO:0098655	Metabolism	Transporters	P	229	16	5.14	3.11	1.14E-04	7.22E-03
4	1093	F2-3 vs. M2-3	F2-3 up	+	cation transmembrane transporter activity	GO:0008324	Metabolism	Transporters	F	414	25	9.29	2.69	1.63E-05	2.02E-03
4	1094	F2-3 vs. M2-3	F2-3 up	+	cation transport	GO:0006812	Metabolism	Transporters	P	412	23	9.24	2.49	1.68E-04	9.75E-03
4	1095	F2-3 vs. M2-3	F2-3 up	+	cytoplasmic vesicle	GO:0031410	Metabolism	Transporters	C	720	30	16.15	1.86	2.10E-03	4.87E-02
4	1096	F2-3 vs. M2-3	F2-3 up	+	export across plasma membrane	GO:0140115	Metabolism	Transporters	P	15	5	0.34	14.86	5.90E-05	4.36E-03
4	1097	F2-3 vs. M2-3	F2-3 up	+	fluid transport	GO:0042044	Metabolism	Transporters	P	30	5	0.67	7.43	9.42E-04	3.31E-02
4	1098	F2-3 vs. M2-3	F2-3 up	+	inorganic cation transmembrane transport	GO:0098662	Metabolism	Transporters	P	223	16	5	3.2	8.50E-05	5.84E-03
4	1099	F2-3 vs. M2-3	F2-3 up	+	inorganic cation transmembrane transporter activity	GO:0022890	Metabolism	Transporters	F	382	23	8.57	2.68	3.68E-05	3.82E-03
4	1100	F2-3 vs. M2-3	F2-3 up	+	inorganic ion transmembrane transport	GO:0098660	Metabolism	Transporters	P	244	16	5.47	2.92	2.24E-04	1.24E-02
4	1101	F2-3 vs. M2-3	F2-3 up	+	inorganic molecular entity transmembrane transporter activity	GO:0015318	Metabolism	Transporters	F	664	41	14.89	2.75	1.89E-08	6.75E-06
4	1102	F2-3 vs. M2-3	F2-3 up	+	ion transmembrane transport	GO:0034220	Metabolism	Transporters	P	330	22	7.4	2.97	1.27E-05	1.51E-03
4	1103	F2-3 vs. M2-3	F2-3 up	+	ion transmembrane transporter activity	GO:0015075	Metabolism	Transporters	F	718	41	16.11	2.55	1.79E-07	4.43E-05

4	1104	F2-3 vs. M2-3	F2-3 up	+	ion transmembrane transporter activity, phosphorylative mechanism	GO:0015662	Metabolism	Transporters	F	50	9	1.12	8.02	5.25E-06	7.67E-04
4	1105	F2-3 vs. M2-3	F2-3 up	+	ion transport	GO:0006811	Metabolism	Transporters	P	673	39	15.1	2.58	3.13E-07	7.79E-05
4	1106	F2-3 vs. M2-3	F2-3 up	+	monovalent inorganic cation transmembrane transporter activity	GO:0015077	Metabolism	Transporters	F	220	14	4.94	2.84	7.15E-04	4.10E-02
4	1107	F2-3 vs. M2-3	F2-3 up	+	one-carbon compound transport	GO:0019755	Metabolism	Transporters	P	7	3	0.16	19.11	1.12E-03	3.63E-02
4	1108	F2-3 vs. M2-3	F2-3 up	+	plasmodesma	GO:0009506	Metabolism	Transporters	C	944	48	21.18	2.27	3.73E-07	1.62E-05
4	1109	F2-3 vs. M2-3	F2-3 up	+	primary active transmembrane transporter	GO:0015399	Metabolism	Transporters	F	213	18	4.78	3.77	3.97E-06	6.08E-04
4	1110	F2-3 vs. M2-3	F2-3 up	+	proton transmembrane transporter activity	GO:0015078	Metabolism	Transporters	F	166	13	3.72	3.49	1.73E-04	1.33E-02
4	1111	F2-3 vs. M2-3	F2-3 up	+	trans-Golgi network	GO:0005802	Metabolism	Transporters	C	302	19	6.77	2.8	1.00E-04	2.98E-03
4	1112	F2-3 vs. M2-3	F2-3 up	+	transmembrane transport	GO:0055085	Metabolism	Transporters	P	859	52	19.27	2.7	4.19E-10	2.28E-07
4	1113	F2-3 vs. M2-3	F2-3 up	+	transmembrane transporter activity	GO:0022857	Metabolism	Transporters	F	1242	66	27.86	2.37	3.70E-10	1.70E-07
4	1114	F2-3 vs. M2-3	F2-3 up	+	transport	GO:0006810	Metabolism	Transporters	P	2261	89	50.72	1.75	4.33E-07	1.04E-04
4	1115	F2-3 vs. M2-3	F2-3 up	+	transporter activity	GO:0005215	Metabolism	Transporters	F	1287	70	28.87	2.42	3.32E-11	2.67E-08
4	1116	F2-3 vs. M2-3	F2-3 up	+	urea transmembrane transporter activity	GO:0015204	Metabolism	Transporters	F	6	4	0.13	29.72	4.34E-05	4.10E-03
4	1117	F2-3 vs. M2-3	F2-3 up	+	urea transport	GO:0015840	Metabolism	Transporters	P	5	3	0.11	26.75	5.42E-04	2.33E-02
4	1118	F2-3 vs. M2-3	F2-3 up	+	vesicle	GO:0031982	Metabolism	Transporters	C	817	34	18.33	1.86	7.94E-04	1.92E-02
4	1119	F2-3 vs. M2-3	F2-3 up	+	water channel activity	GO:0015250	Metabolism	Transporters	F	36	7	0.81	8.67	3.84E-05	3.74E-03
4	1120	F2-3 vs. M2-3	F2-3 up	+	water transmembrane transporter activity	GO:0005372	Metabolism	Transporters	F	36	7	0.81	8.67	3.84E-05	3.86E-03
4	1121	F2-3 vs. M2-3	F2-3 up	+	water transport	GO:0006833	Metabolism	Transporters	P	30	5	0.67	7.43	9.42E-04	3.33E-02
4	1122	F2-3 vs. M2-3	M2-3 up	+	primary metabolic process	GO:0044238	Metabolism	Bioenergetics	P	7219	81	55.03	1.47	1.02E-04	3.83E-02
4	1123	F2-3 vs. M2-3	M2-3 up	+	DNA biosynthetic process	GO:0071897	Development	Cell division	P	25	5	0.19	26.24	2.89E-06	8.63E-03
4	1124	F2-3 vs. M2-3	M2-3 up	+	DNA repair	GO:0006281	Development	Cell division	P	315	11	2.4	4.58	4.19E-05	2.79E-02
4	1125	F2-3 vs. M2-3	M2-3 up	+	double-strand break repair	GO:0006302	Development	Cell division	P	126	7	0.96	7.29	7.13E-05	3.28E-02
4	1126	F2-3 vs. M2-3	M2-3 up	+	carpel development	GO:0048440	Development	Development	P	78	6	0.59	10.09	4.34E-05	2.60E-02
4	1127	F2-3 vs. M2-3	M2-3 up	+	cellular process	GO:0009987	Development	Development	P	12008	127	91.54	1.39	1.12E-06	6.71E-03
4	1128	F2-3 vs. M2-3	M2-3 up	+	cytosol	GO:0005829	Development	Development	C	3241	47	24.71	1.9	1.39E-05	1.45E-02
4	1129	F2-3 vs. M2-3	M2-3 up	+	floral whorl development	GO:0048438	Development	Development	P	195	8	1.49	5.38	1.66E-04	4.95E-02
4	1130	F2-3 vs. M2-3	M2-3 up	+	gynoecium development	GO:0048467	Development	Development	P	91	7	0.69	10.09	9.91E-06	1.19E-02
4	1131	F2-3 vs. M2-3	M2-3 up	+	intracellular non-membrane-bounded organelle	GO:0043232	Development	Development	C	1755	28	13.38	2.09	2.99E-04	4.44E-02
4	1132	F2-3 vs. M2-3	M2-3 up	+	intracellular organelle lumen	GO:0070013	Development	Development	C	1386	24	10.57	2.27	1.99E-04	5.18E-02
4	1133	F2-3 vs. M2-3	M2-3 up	+	membrane-enclosed lumen	GO:0031974	Development	Development	C	1386	24	10.57	2.27	1.99E-04	4.14E-02
4	1134	F2-3 vs. M2-3	M2-3 up	+	non-membrane-bounded organelle	GO:0043228	Development	Development	C	1755	28	13.38	2.09	2.99E-04	3.89E-02
4	1135	F2-3 vs. M2-3	M2-3 up	+	nuclear lumen	GO:0031981	Development	Development	C	1222	23	9.32	2.47	7.55E-05	2.62E-02
4	1136	F2-3 vs. M2-3	M2-3 up	+	nucleus	GO:0005634	Development	Development	C	10540	111	80.35	1.38	2.26E-05	1.18E-02
4	1137	F2-3 vs. M2-3	M2-3 up	+	organelle lumen	GO:0043233	Development	Development	C	1386	24	10.57	2.27	1.99E-04	3.45E-02
4	1138	F2-3 vs. M2-3	M2-3 up	+	phyllome development	GO:0048827	Development	Development	P	495	14	3.77	3.71	3.68E-05	3.14E-02
4	1139	F2-3 vs. M2-3	M2-3 up	+	plant organ development	GO:0099402	Development	Development	P	1009	22	7.69	2.86	1.28E-05	1.27E-02
4	1140	F2-3 vs. M2-3	M2-3 up	+	plant ovule development	GO:0048481	Development	Development	P	55	6	0.42	14.31	6.85E-06	1.37E-02
4	1141	F2-3 vs. M2-3	M2-3 up	+	plant-type ovary development	GO:0035670	Development	Development	P	57	6	0.43	13.81	8.28E-06	1.24E-02
4	1142	F2-3 vs. M2-3	M2-3 up	+	shoot system development	GO:0048367	Development	Development	P	728	17	5.55	3.06	5.72E-05	2.85E-02
4	1143	F2-3 vs. M2-3	M2-3 up	+	system development	GO:0048731	Development	Development	P	1867	30	14.23	2.11	1.47E-04	4.61E-02
4	1144	F2-3 vs. M2-3	M2-3 up	+	binding	GO:0005488	Metabolism	Metabolism	F	9843	108	75.04	1.44	4.33E-06	6.95E-03
4	1145	F2-3 vs. M2-3	M2-3 up	+	cellular macromolecule metabolic process	GO:0044260	Metabolism	Metabolism	P	4426	56	33.74	1.66	9.74E-05	3.88E-02
4	1146	F2-3 vs. M2-3	M2-3 up	+	macromolecule metabolic process	GO:0043170	Metabolism	Metabolism	P	5496	66	41.9	1.58	8.65E-05	3.69E-02
4	1147	F2-3 vs. M2-3	M2-3 up	+	metabolic process	GO:0008152	Metabolism	Metabolism	P	8681	93	66.18	1.41	1.30E-04	4.31E-02
4	1148	F2-3 vs. M2-3	M2-3 up	+	organic cyclic compound metabolic process	GO:1901360	Metabolism	Metabolism	P	2607	39	19.87	1.96	4.68E-05	2.54E-02
4	1149	F2-3 vs. M2-3	M2-3 up	+	organic substance metabolic process	GO:0071704	Metabolism	Metabolism	P	7851	88	59.85	1.47	4.18E-05	3.12E-02
4	1150	F2-3 vs. M2-3	M2-3 up	+	protein binding	GO:0005515	Metabolism	Metabolism	F	5446	72	41.52	1.73	8.59E-07	2.76E-03
4	1151	F2-3 vs. M2-3	M2-3 up	+	cellular response to DNA damage stimulus	GO:0006974	Signaling and response	Stress	P	350	11	2.67	4.12	1.04E-04	3.66E-02
5	1152	L2-3 vs. S2-3	L2-3 up	+	organelle subcompartment	GO:0031984	Development	Development	C	1156	14	3.88	3.61	3.34E-05	3.47E-02
5	1153	L2-3 vs. S2-3	L2-3 up	+	metabolic process	GO:0008152	Metabolism	Metabolism	P	8681	50	29.13	1.72	8.28E-06	4.95E-02
5	1154	L2-3 vs. S2-3	S2-3 up	+	cytochrome complex	GO:0070069	Metabolism	Bioenergetics	C	30	5	0.24	21.15	7.47E-06	5.19E-04
5	1155	L2-3 vs. S2-3	S2-3 up	+	inner mitochondrial membrane protein complex	GO:0098800	Metabolism	Bioenergetics	C	177	8	1.39	5.74	1.10E-04	4.56E-03
5	1156	L2-3 vs. S2-3	S2-3 up	+	mitochondrial envelope	GO:0005740	Metabolism	Bioenergetics	C	317	12	2.5	4.8	1.20E-05	7.37E-04
5	1157	L2-3 vs. S2-3	S2-3 up	+	mitochondrial inner membrane	GO:0005743	Metabolism	Bioenergetics	C	245	10	1.93	5.18	3.52E-05	1.83E-03
5	1158	L2-3 vs. S2-3	S2-3 up	+	mitochondrial membrane	GO:0031966	Metabolism	Bioenergetics	C	297	11	2.34	4.7	3.38E-05	1.85E-03
5	1159	L2-3 vs. S2-3	S2-3 up	+	mitochondrial protein complex	GO:0098798	Metabolism	Bioenergetics	C	259	12	2.04	5.88	1.67E-06	1.34E-04
5	1160	L2-3 vs. S2-3	S2-3 up	+	mitochondrial respirasome	GO:0005746	Metabolism	Bioenergetics	C	111	7	0.87	8	4.09E-05	1.94E-03
5	1161	L2-3 vs. S2-3	S2-3 up	+	mitochondrial respiratory chain complex III	GO:0005750	Metabolism	Bioenergetics	C	19	5	0.15	33.4	1.05E-06	9.93E-05
5	1162	L2-3 vs. S2-3	S2-3 up	+	oxidoreductase activity, acting on diphenols and	GO:0016681	Metabolism	Bioenergetics	F	7	3	0.06	54.4	5.43E-05	4.36E-02
5	1163	L2-3 vs. S2-3	S2-3 up	+	oxidoreductase complex	GO:1990204	Metabolism	Bioenergetics	C	161	9	1.27	7.1	8.20E-06	5.33E-04

5	1164	L2-3 vs. S2-3	S2-3 up	+	respirasome	GO:0070469	Metabolism	Bioenergetics	C	116	7	0.91	7.66	5.34E-05	2.31E-03
5	1165	L2-3 vs. S2-3	S2-3 up	+	respiratory chain complex	GO:0098803	Metabolism	Bioenergetics	C	109	7	0.86	8.15	3.67E-05	1.82E-03
5	1166	L2-3 vs. S2-3	S2-3 up	+	respiratory chain complex III	GO:0045275	Metabolism	Bioenergetics	C	19	5	0.15	33.4	1.05E-06	9.10E-05
5	1167	L2-3 vs. S2-3	S2-3 up	+	ubiquinol-cytochrome-c reductase activity	GO:0008121	Metabolism	Bioenergetics	F	7	3	0.06	54.4	5.43E-05	3.49E-02
5	1168	L2-3 vs. S2-3	S2-3 up	+	protein-containing complex	GO:0032991	Metabolism	Catabolism	C	2958	53	23.3	2.27	1.53E-08	1.76E-06
5	1169	L2-3 vs. S2-3	S2-3 up	+	cellular process	GO:0009987	Development	Development	P	12008	124	94.61	1.31	7.98E-05	2.17E-02
5	1170	L2-3 vs. S2-3	S2-3 up	+	cytoplasm	GO:0005737	Development	Development	C	14837	142	116.89	1.21	5.93E-04	2.20E-02
5	1171	L2-3 vs. S2-3	S2-3 up	+	cytosol	GO:0005829	Development	Development	C	3241	55	25.53	2.15	3.62E-08	3.77E-06
5	1172	L2-3 vs. S2-3	S2-3 up	+	intracellular non-membrane-bounded organelle	GO:0043232	Development	Development	C	1755	42	13.83	3.04	1.44E-10	2.14E-08
5	1173	L2-3 vs. S2-3	S2-3 up	+	non-membrane-bounded organelle	GO:0043228	Development	Development	C	1755	42	13.83	3.04	1.44E-10	1.87E-08
5	1174	L2-3 vs. S2-3	S2-3 up	+	organelle inner membrane	GO:0019866	Development	Development	C	311	10	2.45	4.08	2.34E-04	9.36E-03
5	1175	L2-3 vs. S2-3	S2-3 up	+	organelle membrane	GO:0031090	Development	Development	C	1458	27	11.49	2.35	4.69E-05	2.12E-03
5	1176	L2-3 vs. S2-3	S2-3 up	+	plant-type cell wall organization	GO:0009664	Development	Development	P	150	8	1.18	6.77	3.61E-05	1.20E-02
5	1177	L2-3 vs. S2-3	S2-3 up	+	structural molecule activity	GO:0005198	Development	Development	F	443	25	3.49	7.16	4.95E-14	1.59E-10
5	1178	L2-3 vs. S2-3	S2-3 up	+	vacuole	GO:0005773	Development	Development	C	1083	20	8.53	2.34	5.87E-04	2.26E-02
5	1179	L2-3 vs. S2-3	S2-3 up	+	amide biosynthetic process	GO:0043604	Metabolism	Metabolism	P	591	27	4.66	5.8	5.46E-13	8.17E-10
5	1180	L2-3 vs. S2-3	S2-3 up	+	biosynthetic process	GO:0009058	Metabolism	Metabolism	P	2811	48	22.15	2.17	4.06E-07	1.87E-04
5	1181	L2-3 vs. S2-3	S2-3 up	+	cellular amide metabolic process	GO:0043603	Metabolism	Metabolism	P	722	28	5.69	4.92	8.40E-12	1.00E-08
5	1182	L2-3 vs. S2-3	S2-3 up	+	cellular biosynthetic process	GO:0044249	Metabolism	Metabolism	P	2583	46	20.35	2.26	1.56E-07	9.36E-05
5	1183	L2-3 vs. S2-3	S2-3 up	+	cellular macromolecule biosynthetic process	GO:0034645	Metabolism	Metabolism	P	1279	33	10.08	3.27	3.04E-09	3.03E-06
5	1184	L2-3 vs. S2-3	S2-3 up	+	cellular nitrogen compound biosynthetic process	GO:0044271	Metabolism	Metabolism	P	1241	32	9.78	3.27	5.52E-09	4.71E-06
5	1185	L2-3 vs. S2-3	S2-3 up	+	cellular nitrogen compound metabolic process	GO:0034641	Metabolism	Metabolism	P	2872	43	22.63	1.9	4.70E-05	1.48E-02
5	1186	L2-3 vs. S2-3	S2-3 up	+	macromolecule biosynthetic process	GO:0009059	Metabolism	Metabolism	P	1334	33	10.51	3.14	8.30E-09	6.20E-06
5	1187	L2-3 vs. S2-3	S2-3 up	+	organic substance biosynthetic process	GO:1901576	Metabolism	Metabolism	P	2674	45	21.07	2.14	1.42E-06	6.08E-04
5	1188	L2-3 vs. S2-3	S2-3 up	+	organonitrogen compound biosynthetic process	GO:1901566	Metabolism	Metabolism	P	1343	33	10.58	3.12	9.73E-09	6.46E-06
5	1189	L2-3 vs. S2-3	S2-3 up	+	peptide metabolic process	GO:0006518	Metabolism	Metabolism	P	585	28	4.61	6.08	6.61E-14	1.32E-10
5	1190	L2-3 vs. S2-3	S2-3 up	+	gene expression	GO:0010467	Regulation	Transcription	P	1608	34	12.67	2.68	1.95E-07	1.06E-04
5	1191	L2-3 vs. S2-3	S2-3 up	+	cytosolic large ribosomal subunit	GO:0022625	Regulation	Translation	C	131	14	1.03	13.56	9.22E-12	1.92E-09
5	1192	L2-3 vs. S2-3	S2-3 up	+	cytosolic ribosome	GO:0022626	Regulation	Translation	C	303	23	2.39	9.63	1.50E-15	5.20E-13
5	1193	L2-3 vs. S2-3	S2-3 up	+	cytosolic small ribosomal subunit	GO:0022627	Regulation	Translation	C	102	7	0.8	8.71	2.46E-05	1.42E-03
5	1194	L2-3 vs. S2-3	S2-3 up	+	large ribosomal subunit	GO:0015934	Regulation	Translation	C	180	16	1.42	11.28	3.89E-12	1.01E-09
5	1195	L2-3 vs. S2-3	S2-3 up	+	mRNA binding	GO:0003729	Regulation	Translation	F	1005	22	7.92	2.78	2.01E-05	2.16E-02
5	1196	L2-3 vs. S2-3	S2-3 up	+	peptide biosynthetic process	GO:0043043	Regulation	Translation	P	518	27	4.08	6.62	2.77E-14	8.28E-11
5	1197	L2-3 vs. S2-3	S2-3 up	+	ribonucleoprotein complex	GO:1990904	Regulation	Translation	C	677	27	5.33	5.06	1.11E-11	1.92E-09
5	1198	L2-3 vs. S2-3	S2-3 up	+	ribonucleoprotein complex assembly	GO:0022618	Regulation	Translation	P	163	8	1.28	6.23	6.32E-05	1.89E-02
5	1199	L2-3 vs. S2-3	S2-3 up	+	ribonucleoprotein complex biogenesis	GO:0022613	Regulation	Translation	P	447	15	3.52	4.26	4.02E-06	1.60E-03
5	1200	L2-3 vs. S2-3	S2-3 up	+	ribonucleoprotein complex subunit organization	GO:0071826	Regulation	Translation	P	166	8	1.31	6.12	7.14E-05	2.03E-02
5	1201	L2-3 vs. S2-3	S2-3 up	+	ribosomal large subunit biogenesis	GO:0042273	Regulation	Translation	P	109	9	0.86	10.48	3.95E-07	1.97E-04
5	1202	L2-3 vs. S2-3	S2-3 up	+	ribosomal subunit	GO:0044391	Regulation	Translation	C	316	25	2.49	10.04	3.31E-17	3.45E-14
5	1203	L2-3 vs. S2-3	S2-3 up	+	ribosome	GO:0005840	Regulation	Translation	C	399	27	3.14	8.59	6.59E-17	3.43E-14
5	1204	L2-3 vs. S2-3	S2-3 up	+	ribosome assembly	GO:0042255	Regulation	translation	P	90	7	0.71	9.87	1.15E-05	4.03E-03
5	1205	L2-3 vs. S2-3	S2-3 up	+	ribosome biogenesis	GO:0042254	Regulation	Translation	P	370	13	2.92	4.46	1.12E-05	4.19E-03
5	1206	L2-3 vs. S2-3	S2-3 up	+	small ribosomal subunit	GO:0015935	Regulation	Translation	C	136	9	1.07	8.4	2.23E-06	1.66E-04
5	1207	L2-3 vs. S2-3	S2-3 up	+	structural constituent of ribosome	GO:0003735	Regulation	Translation	F	325	22	2.56	8.59	5.70E-14	9.16E-11
5	1208	L2-3 vs. S2-3	S2-3 up	+	translation	GO:0006412	Regulation	Translation	P	513	27	4.04	6.68	2.22E-14	1.33E-10
6	1209	M2-3 vs. S2-3	M2-3 up	+	cytosol	GO:0005829	Development	Development	C	3241	26	10.28	2.53	5.53E-06	5.75E-03
6	1210	M2-3 vs. S2-3	M2-3 up	+	biosynthetic process	GO:0009058	Metabolism	Metabolism	P	2811	23	8.92	2.58	1.67E-05	3.33E-02
6	1211	M2-3 vs. S2-3	M2-3 up	+	cellular metabolic process	GO:0044237	Metabolism	Metabolism	P	7649	46	24.27	1.9	9.90E-07	2.96E-03
6	1212	M2-3 vs. S2-3	M2-3 up	+	metabolic process	GO:0008152	Metabolism	Metabolism	P	8681	50	27.55	1.82	7.58E-07	4.53E-03
6	1213	M2-3 vs. S2-3	S2-3 up	+	oxidoreductase complex	GO:1990204	Metabolism	Bioenergetics	C	161	7	1.04	6.73	1.10E-04	1.27E-02
6	1214	M2-3 vs. S2-3	S2-3 up	+	cytosol	GO:0005829	Development	Development	C	3241	50	20.92	2.39	3.45E-09	3.59E-06
6	1215	M2-3 vs. S2-3	S2-3 up	+	intracellular non-membrane-bounded organelle	GO:0043232	Development	Development	C	1755	26	11.33	2.29	7.99E-05	1.19E-02
6	1216	M2-3 vs. S2-3	S2-3 up	+	microtubule	GO:0005874	Development	Development	C	145	6	0.94	6.41	4.42E-04	4.60E-02
6	1217	M2-3 vs. S2-3	S2-3 up	+	non-membrane-bounded organelle	GO:0043228	Development	Development	C	1755	26	11.33	2.29	7.99E-05	1.04E-02
6	1218	M2-3 vs. S2-3	S2-3 up	+	polymeric cytoskeletal fiber	GO:0099513	Development	Development	C	155	6	1	6	6.20E-04	5.38E-02
6	1219	M2-3 vs. S2-3	S2-3 up	+	structural molecule activity	GO:0005198	Development	Development	F	443	14	2.86	4.9	1.65E-06	5.31E-03
6	1220	M2-3 vs. S2-3	S2-3 up	+	amide biosynthetic process	GO:0043604	Metabolism	Metabolism	P	591	14	3.82	3.67	3.88E-05	5.80E-02
6	1221	M2-3 vs. S2-3	S2-3 up	+	cellular amide metabolic process	GO:0043603	Metabolism	Metabolism	P	722	16	4.66	3.43	2.37E-05	7.10E-02
6	1222	M2-3 vs. S2-3	S2-3 up	+	peptide metabolic process	GO:0006518	Metabolism	Metabolism	P	585	15	3.78	3.97	8.25E-06	4.94E-02
6	1223	M2-3 vs. S2-3	S2-3 up	+	supramolecular fiber	GO:0099512	Metabolism	Metabolism	C	155	6	1	6	6.20E-04	4.97E-02
6	1224	M2-3 vs. S2-3	S2-3 up	+	supramolecular polymer	GO:0099081	Metabolism	Metabolism	C	155	6	1	6	6.20E-04	5.87E-02
6	1225	M2-3 vs. S2-3	S2-3 up	+	cytosolic ribosome	GO:0022626	Regulation	Translation	C	303	13	1.96	6.65	1.45E-07	7.57E-05

6	1226	M2-3 vs. S2-3	S2-3 up	+	cytosolic small ribosomal subunit	GO:0022627	Regulation	Translation	C	102	7	0.66	10.63	6.82E-06	1.42E-03
6	1227	M2-3 vs. S2-3	S2-3 up	+	peptide biosynthetic process	GO:0043043	Regulation	Translation	P	518	13	3.34	3.89	4.17E-05	4.99E-02
6	1228	M2-3 vs. S2-3	S2-3 up	+	ribosomal subunit	GO:0044391	Regulation	Translation	C	316	12	2.04	5.88	1.53E-06	5.30E-04
6	1229	M2-3 vs. S2-3	S2-3 up	+	ribosome	GO:0005840	Regulation	Translation	C	399	13	2.58	5.05	2.85E-06	7.42E-04
6	1230	M2-3 vs. S2-3	S2-3 up	+	small ribosomal subunit	GO:0015935	Regulation	Translation	C	136	7	0.88	7.97	3.99E-05	6.91E-03
6	1231	M2-3 vs. S2-3	S2-3 up	+	translation	GO:0006412	Regulation	Translation	P	513	13	3.31	3.93	3.79E-05	7.54E-02
7	1232	F2-3 vs. S2-3	F2-3 up	+	ADP metabolic process	GO:0046031	Metabolism	Bioenergetics	P	56	9	2.66	3.38	2.56E-03	4.42E-02
7	1233	F2-3 vs. S2-3	F2-3 up	+	aerobic respiration	GO:0009060	Metabolism	Bioenergetics	P	89	22	4.23	5.21	4.62E-09	3.95E-07
7	1234	F2-3 vs. S2-3	F2-3 up	+	alcohol dehydrogenase (NADP+) activity	GO:0008106	Metabolism	Bioenergetics	F	14	5	0.66	7.52	1.30E-03	3.56E-02
7	1235	F2-3 vs. S2-3	F2-3 up	+	aldehyde dehydrogenase (NAD+) activity	GO:0004029	Metabolism	Bioenergetics	F	12	7	0.57	12.28	1.21E-05	6.06E-04
7	1236	F2-3 vs. S2-3	F2-3 up	+	aldehyde dehydrogenase [NAD(P)+] activity	GO:0004030	Metabolism	Bioenergetics	F	12	7	0.57	12.28	1.21E-05	6.16E-04
7	1237	F2-3 vs. S2-3	F2-3 up	+	aldo-keto reductase (NADP) activity	GO:0004033	Metabolism	Bioenergetics	F	15	5	0.71	7.02	1.67E-03	4.42E-02
7	1238	F2-3 vs. S2-3	F2-3 up	+	ATP metabolic process	GO:0046034	Metabolism	Bioenergetics	P	144	19	6.84	2.78	1.57E-04	4.06E-03
7	1239	F2-3 vs. S2-3	F2-3 up	+	ATPase activity	GO:0016887	Metabolism	Bioenergetics	F	512	44	24.32	1.81	4.03E-04	1.28E-02
7	1240	F2-3 vs. S2-3	F2-3 up	+	ATPase activity, coupled to transmembrane movement of ions, rotational mechanism	GO:0044769	Metabolism	Bioenergetics	F	25	8	1.19	6.74	8.79E-05	3.53E-03
7	1241	F2-3 vs. S2-3	F2-3 up	+	carbohydrate biosynthetic process	GO:0016051	Metabolism	Bioenergetics	P	303	50	14.39	3.47	9.55E-13	1.36E-10
7	1242	F2-3 vs. S2-3	F2-3 up	+	carbohydrate catabolic process	GO:0016052	Metabolism	Bioenergetics	P	234	44	11.11	3.96	4.69E-13	7.38E-11
7	1243	F2-3 vs. S2-3	F2-3 up	+	carbohydrate derivative binding	GO:0097367	Metabolism	Bioenergetics	F	858	65	40.75	1.6	5.03E-04	1.57E-02
7	1244	F2-3 vs. S2-3	F2-3 up	+	carbohydrate derivative biosynthetic process	GO:1901137	Metabolism	Bioenergetics	P	458	45	21.75	2.07	1.79E-05	6.37E-04
7	1245	F2-3 vs. S2-3	F2-3 up	+	carbohydrate derivative metabolic process	GO:1901135	Metabolism	Bioenergetics	P	746	84	35.43	2.37	6.89E-12	9.37E-10
7	1246	F2-3 vs. S2-3	F2-3 up	+	carbohydrate metabolic process	GO:0005975	Metabolism	Bioenergetics	P	950	128	45.12	2.84	1.24E-23	6.18E-21
7	1247	F2-3 vs. S2-3	F2-3 up	+	carbohydrate phosphatase activity	GO:0019203	Metabolism	Bioenergetics	F	29	7	1.38	5.08	1.02E-03	2.82E-02
7	1248	F2-3 vs. S2-3	F2-3 up	+	cellular carbohydrate biosynthetic process	GO:0034637	Metabolism	Bioenergetics	P	213	34	10.12	3.36	8.53E-09	6.99E-07
7	1249	F2-3 vs. S2-3	F2-3 up	+	cellular carbohydrate catabolic process	GO:0044275	Metabolism	Bioenergetics	P	46	10	2.18	4.58	1.88E-04	4.66E-03
7	1250	F2-3 vs. S2-3	F2-3 up	+	cellular carbohydrate metabolic process	GO:0044262	Metabolism	Bioenergetics	P	384	58	18.24	3.18	4.03E-13	6.51E-11
7	1251	F2-3 vs. S2-3	F2-3 up	+	cellular polysaccharide biosynthetic process	GO:0033692	Metabolism	Bioenergetics	P	177	25	8.41	2.97	5.47E-06	2.15E-04
7	1252	F2-3 vs. S2-3	F2-3 up	+	cellular polysaccharide metabolic process	GO:0044264	Metabolism	Bioenergetics	P	270	37	12.82	2.89	6.84E-08	4.30E-06
7	1253	F2-3 vs. S2-3	F2-3 up	+	cellular respiration	GO:0045333	Metabolism	Bioenergetics	P	139	24	6.6	3.64	3.67E-07	1.89E-05
7	1254	F2-3 vs. S2-3	F2-3 up	+	disaccharide biosynthetic process	GO:0046351	Metabolism	Bioenergetics	P	31	8	1.47	5.43	3.06E-04	7.26E-03
7	1255	F2-3 vs. S2-3	F2-3 up	+	disaccharide metabolic process	GO:0005984	Metabolism	Bioenergetics	P	70	12	3.32	3.61	3.15E-04	7.42E-03
7	1256	F2-3 vs. S2-3	F2-3 up	+	electron transfer activity	GO:0009055	Metabolism	Bioenergetics	F	171	20	8.12	2.46	5.88E-04	1.77E-02
7	1257	F2-3 vs. S2-3	F2-3 up	+	electron transport chain	GO:0022900	Metabolism	Bioenergetics	P	114	18	5.41	3.32	2.98E-05	9.57E-04
7	1258	F2-3 vs. S2-3	F2-3 up	+	energy derivation by oxidation of organic compounds	GO:0015980	Metabolism	Bioenergetics	P	154	26	7.31	3.56	1.79E-07	9.82E-06
7	1259	F2-3 vs. S2-3	F2-3 up	+	fatty acid biosynthetic process	GO:0006633	Metabolism	Bioenergetics	P	134	19	6.36	2.99	6.72E-05	1.94E-03
7	1260	F2-3 vs. S2-3	F2-3 up	+	fatty acid derivative biosynthetic process	GO:1901570	Metabolism	Bioenergetics	P	30	7	1.42	4.91	1.21E-03	2.30E-02
7	1261	F2-3 vs. S2-3	F2-3 up	+	fatty acid derivative metabolic process	GO:1901568	Metabolism	Bioenergetics	P	43	10	2.04	4.9	1.16E-04	3.04E-03
7	1262	F2-3 vs. S2-3	F2-3 up	+	fatty acid metabolic process	GO:0006631	Metabolism	Bioenergetics	P	221	33	10.5	3.14	5.82E-08	3.74E-06
7	1263	F2-3 vs. S2-3	F2-3 up	+	generation of precursor metabolites and energy	GO:0006091	Metabolism	Bioenergetics	P	378	77	17.95	4.29	1.05E-23	5.68E-21
7	1264	F2-3 vs. S2-3	F2-3 up	+	gluconeogenesis	GO:0006094	Metabolism	Bioenergetics	P	23	8	1.09	7.32	5.42E-05	1.60E-03
7	1265	F2-3 vs. S2-3	F2-3 up	+	glucose 6-phosphate metabolic process	GO:0051156	Metabolism	Bioenergetics	P	42	11	1.99	5.51	2.10E-05	7.06E-04
7	1266	F2-3 vs. S2-3	F2-3 up	+	glucose metabolic process	GO:0006006	Metabolism	Bioenergetics	P	42	11	1.99	5.51	2.10E-05	7.10E-04
7	1267	F2-3 vs. S2-3	F2-3 up	+	glyceraldehyde-3-phosphate dehydrogenase (NAD+) (non-phosphorylating) activity	GO:0043878	Metabolism	Bioenergetics	F	8	5	0.38	13.16	1.80E-04	6.44E-03
7	1268	F2-3 vs. S2-3	F2-3 up	+	hexose biosynthetic process	GO:0019319	Metabolism	Bioenergetics	P	26	9	1.23	7.29	1.91E-05	6.63E-04
7	1269	F2-3 vs. S2-3	F2-3 up	+	hexose metabolic process	GO:0019318	Metabolism	Bioenergetics	P	88	18	4.18	4.31	1.27E-06	5.81E-05
7	1270	F2-3 vs. S2-3	F2-3 up	+	isocitrate metabolic process	GO:0006102	Metabolism	Bioenergetics	P	12	6	0.57	10.53	9.95E-05	2.72E-03
7	1271	F2-3 vs. S2-3	F2-3 up	+	monosaccharide biosynthetic process	GO:0046364	Metabolism	Bioenergetics	P	48	12	2.28	5.26	1.33E-05	4.86E-04
7	1272	F2-3 vs. S2-3	F2-3 up	+	NAD binding	GO:0051287	Metabolism	Bioenergetics	F	77	18	3.66	4.92	2.35E-07	1.89E-05
7	1273	F2-3 vs. S2-3	F2-3 up	+	NADP binding	GO:0050661	Metabolism	Bioenergetics	F	68	12	3.23	3.72	2.49E-04	8.25E-03
7	1274	F2-3 vs. S2-3	F2-3 up	+	NADP metabolic process	GO:0006739	Metabolism	Bioenergetics	P	44	13	2.09	6.22	1.22E-06	5.63E-05
7	1275	F2-3 vs. S2-3	F2-3 up	+	NADPH regeneration	GO:0006740	Metabolism	Bioenergetics	P	35	11	1.66	6.62	4.92E-06	2.00E-04
7	1276	F2-3 vs. S2-3	F2-3 up	+	oligosaccharide biosynthetic process	GO:0009312	Metabolism	Bioenergetics	P	37	8	1.76	4.55	8.43E-04	1.73E-02
7	1277	F2-3 vs. S2-3	F2-3 up	+	oligosaccharide metabolic process	GO:0009311	Metabolism	Bioenergetics	P	80	12	3.8	3.16	9.12E-04	1.82E-02
7	1278	F2-3 vs. S2-3	F2-3 up	+	oxidoreductase activity	GO:0016491	Metabolism	Bioenergetics	F	1458	181	69.24	2.61	2.81E-29	4.52E-26
7	1279	F2-3 vs. S2-3	F2-3 up	+	oxidoreductase activity, acting on CH-OH group of donors	GO:0016614	Metabolism	Bioenergetics	F	201	39	9.55	4.09	4.28E-12	1.15E-09
7	1280	F2-3 vs. S2-3	F2-3 up	+	oxidoreductase activity, acting on NAD(P)H	GO:0016651	Metabolism	Bioenergetics	F	121	23	5.75	4	1.43E-07	1.31E-05
7	1281	F2-3 vs. S2-3	F2-3 up	+	oxidoreductase activity, acting on paired donors, with incorporation or reduction of molecular	GO:0016705	Metabolism	Bioenergetics	F	392	37	18.62	1.99	2.31E-04	7.82E-03

7	1282	F2-3 vs. S2-3	F2-3 up	+	oxidoreductase activity, acting on single donors with incorporation of molecular oxygen	GO:0016701	Metabolism	Bioenergetics	F	38	9	1.8	4.99	2.26E-04	7.89E-03
7	1283	F2-3 vs. S2-3	F2-3 up	+	oxidoreductase activity, acting on the aldehyde or oxo group of donors	GO:0016903	Metabolism	Bioenergetics	F	62	15	2.94	5.09	1.62E-06	1.06E-04
7	1284	F2-3 vs. S2-3	F2-3 up	+	oxidoreductase activity, acting on the aldehyde or oxo group of donors, NAD or NADP as	GO:0016620	Metabolism	Bioenergetics	F	43	13	2.04	6.37	9.85E-07	6.88E-05
7	1285	F2-3 vs. S2-3	F2-3 up	+	oxidoreductase activity, acting on the CH-OH group of donors, NAD or NADP as acceptor	GO:0016616	Metabolism	Bioenergetics	F	167	35	7.93	4.41	8.26E-12	1.90E-09
7	1286	F2-3 vs. S2-3	F2-3 up	+	pentose-phosphate shunt	GO:0006098	Metabolism	Bioenergetics	P	33	11	1.57	7.02	3.07E-06	1.29E-04
7	1287	F2-3 vs. S2-3	F2-3 up	+	pentose-phosphate shunt, non-oxidative branch	GO:0009052	Metabolism	Bioenergetics	P	8	4	0.38	10.53	1.55E-03	2.87E-02
7	1288	F2-3 vs. S2-3	F2-3 up	+	photorespiration	GO:0009853	Metabolism	Bioenergetics	P	42	9	1.99	4.51	4.28E-04	9.85E-03
7	1289	F2-3 vs. S2-3	F2-3 up	+	polysaccharide biosynthetic process	GO:0000271	Metabolism	Bioenergetics	P	213	28	10.12	2.77	5.30E-06	2.10E-04
7	1290	F2-3 vs. S2-3	F2-3 up	+	polysaccharide metabolic process	GO:0005976	Metabolism	Bioenergetics	P	419	63	19.9	3.17	4.65E-14	9.27E-12
7	1291	F2-3 vs. S2-3	F2-3 up	+	primary metabolic process	GO:0044238	Metabolism	Bioenergetics	P	7219	453	342.83	1.32	5.43E-11	6.76E-09
7	1292	F2-3 vs. S2-3	F2-3 up	+	proton transmembrane transport	GO:1902600	Metabolism	Bioenergetics	P	95	22	4.51	4.88	1.28E-08	9.96E-07
7	1293	F2-3 vs. S2-3	F2-3 up	+	proton-exporting ATPase activity, phosphorylative mechanism	GO:0008553	Metabolism	Bioenergetics	F	20	7	0.95	7.37	1.55E-04	5.71E-03
7	1294	F2-3 vs. S2-3	F2-3 up	+	proton-transporting ATPase activity, rotational mechanism	GO:0046961	Metabolism	Bioenergetics	F	25	8	1.19	6.74	8.79E-05	3.58E-03
7	1295	F2-3 vs. S2-3	F2-3 up	+	proton-transporting two-sector ATPase complex	GO:0016469	Metabolism	Bioenergetics	C	65	12	3.09	3.89	1.72E-04	2.80E-03
7	1296	F2-3 vs. S2-3	F2-3 up	+	proton-transporting V-type ATPase complex	GO:0033176	Metabolism	Bioenergetics	C	23	7	1.09	6.41	3.15E-04	5.04E-03
7	1297	F2-3 vs. S2-3	F2-3 up	+	pyrophosphate hydrolysis-driven proton transmembrane transporter activity	GO:0009678	Metabolism	Bioenergetics	F	44	15	2.09	7.18	3.95E-08	4.37E-06
7	1298	F2-3 vs. S2-3	F2-3 up	+	pyruvate family amino acid metabolic process	GO:0009078	Metabolism	Bioenergetics	P	4	3	0.19	15.79	2.84E-03	4.71E-02
7	1299	F2-3 vs. S2-3	F2-3 up	+	pyruvate metabolic process	GO:0006090	Metabolism	Bioenergetics	P	79	12	3.75	3.2	8.27E-04	1.72E-02
7	1300	F2-3 vs. S2-3	F2-3 up	+	reductive pentose-phosphate cycle	GO:0019253	Metabolism	Bioenergetics	P	14	9	0.66	13.54	3.61E-07	1.88E-05
7	1301	F2-3 vs. S2-3	F2-3 up	+	sucrose biosynthetic process	GO:0005986	Metabolism	Bioenergetics	P	14	6	0.66	9.02	1.92E-04	4.75E-03
7	1302	F2-3 vs. S2-3	F2-3 up	+	sucrose metabolic process	GO:0005985	Metabolism	Bioenergetics	P	46	9	2.18	4.12	7.61E-04	1.59E-02
7	1303	F2-3 vs. S2-3	F2-3 up	+	tricarboxylic acid cycle	GO:0006099	Metabolism	Bioenergetics	P	46	16	2.18	7.32	1.11E-08	8.81E-07
7	1304	F2-3 vs. S2-3	F2-3 up	+	tricarboxylic acid metabolic process	GO:0072350	Metabolism	Bioenergetics	P	21	7	1	7.02	1.98E-04	4.82E-03
7	1305	F2-3 vs. S2-3	F2-3 up	+	alanine catabolic process	GO:0006524	Metabolism	Catabolism	P	4	3	0.19	15.79	2.84E-03	4.75E-02
7	1306	F2-3 vs. S2-3	F2-3 up	+	alpha-amino acid catabolic process	GO:1901606	Metabolism	Catabolism	P	88	24	4.18	5.74	1.72E-10	1.83E-08
7	1307	F2-3 vs. S2-3	F2-3 up	+	aminopeptidase activity	GO:0004177	Metabolism	Catabolism	F	23	6	1.09	5.49	1.66E-03	4.45E-02
7	1308	F2-3 vs. S2-3	F2-3 up	+	aromatic amino acid family catabolic process	GO:0009074	Metabolism	Catabolism	P	21	7	1	7.02	1.98E-04	4.84E-03
7	1309	F2-3 vs. S2-3	F2-3 up	+	aromatic compound catabolic process	GO:0019439	Metabolism	Catabolism	P	199	21	9.45	2.22	1.51E-03	2.81E-02
7	1310	F2-3 vs. S2-3	F2-3 up	+	carboxylic acid catabolic process	GO:0046395	Metabolism	Catabolism	P	162	33	7.69	4.29	6.22E-11	7.44E-09
7	1311	F2-3 vs. S2-3	F2-3 up	+	catabolic process	GO:0009056	Metabolism	Catabolism	P	1469	150	69.76	2.15	4.65E-17	1.21E-14
7	1312	F2-3 vs. S2-3	F2-3 up	+	cellular amino acid catabolic process	GO:0009063	Metabolism	Catabolism	P	97	24	4.61	5.21	9.17E-10	8.43E-08
7	1313	F2-3 vs. S2-3	F2-3 up	+	cellular catabolic process	GO:0044248	Metabolism	Catabolism	P	1223	113	58.08	1.95	1.51E-10	1.70E-08
7	1314	F2-3 vs. S2-3	F2-3 up	+	cysteine-type endopeptidase activity	GO:0004197	Metabolism	Catabolism	F	72	16	3.42	4.68	1.93E-06	1.17E-04
7	1315	F2-3 vs. S2-3	F2-3 up	+	endopeptidase activity	GO:0004175	Metabolism	Catabolism	F	337	39	16	2.44	1.96E-06	1.17E-04
7	1316	F2-3 vs. S2-3	F2-3 up	+	glycine catabolic process	GO:0006546	Metabolism	Catabolism	P	8	5	0.38	13.16	1.80E-04	4.49E-03
7	1317	F2-3 vs. S2-3	F2-3 up	+	L-alanine catabolic process	GO:0042853	Metabolism	Catabolism	P	4	3	0.19	15.79	2.84E-03	4.79E-02
7	1318	F2-3 vs. S2-3	F2-3 up	+	leaf senescence	GO:0010150	Metabolism	Catabolism	P	106	17	5.03	3.38	4.12E-05	1.26E-03
7	1319	F2-3 vs. S2-3	F2-3 up	+	lysosome	GO:0005764	Metabolism	Catabolism	C	46	13	2.18	5.95	1.86E-06	4.96E-05
7	1320	F2-3 vs. S2-3	F2-3 up	+	macromolecule catabolic process	GO:0009057	Metabolism	Catabolism	P	889	71	42.22	1.68	5.21E-05	1.56E-03
7	1321	F2-3 vs. S2-3	F2-3 up	+	monocarboxylic acid catabolic process	GO:0072329	Metabolism	Catabolism	P	62	11	2.94	3.74	4.29E-04	9.78E-03
7	1322	F2-3 vs. S2-3	F2-3 up	+	organic acid catabolic process	GO:0016054	Metabolism	Catabolism	P	162	33	7.69	4.29	6.22E-11	7.59E-09
7	1323	F2-3 vs. S2-3	F2-3 up	+	organic cyclic compound catabolic process	GO:1901361	Metabolism	Catabolism	P	210	22	9.97	2.21	1.17E-03	2.24E-02
7	1324	F2-3 vs. S2-3	F2-3 up	+	organic substance catabolic process	GO:1901575	Metabolism	Catabolism	P	1345	138	63.87	2.16	6.19E-16	1.42E-13
7	1325	F2-3 vs. S2-3	F2-3 up	+	organonitrogen compound catabolic process	GO:1901565	Metabolism	Catabolism	P	836	71	39.7	1.79	9.75E-06	3.67E-04
7	1326	F2-3 vs. S2-3	F2-3 up	+	pectin catabolic process	GO:0045490	Metabolism	Catabolism	P	99	21	4.7	4.47	1.00E-07	6.10E-06
7	1327	F2-3 vs. S2-3	F2-3 up	+	peptidase activity	GO:0008233	Metabolism	Catabolism	F	504	51	23.94	2.13	2.31E-06	1.33E-04
7	1328	F2-3 vs. S2-3	F2-3 up	+	peroxisome	GO:0005777	Metabolism	Catabolism	C	320	37	15.2	2.43	3.88E-06	9.40E-05
7	1329	F2-3 vs. S2-3	F2-3 up	+	plant organ senescence	GO:0090693	Metabolism	Catabolism	P	112	17	5.32	3.2	7.59E-05	2.11E-03
7	1330	F2-3 vs. S2-3	F2-3 up	+	polysaccharide catabolic process	GO:0000272	Metabolism	Catabolism	P	144	28	6.84	4.09	4.22E-09	3.66E-07
7	1331	F2-3 vs. S2-3	F2-3 up	+	pyruvate family amino acid catabolic process	GO:0009080	Metabolism	Catabolism	P	4	3	0.19	15.79	2.84E-03	4.72E-02
7	1332	F2-3 vs. S2-3	F2-3 up	+	serine family amino acid catabolic process	GO:0009071	Metabolism	Catabolism	P	17	6	0.81	7.43	4.45E-04	1.01E-02
7	1333	F2-3 vs. S2-3	F2-3 up	+	small molecule catabolic process	GO:0044282	Metabolism	Catabolism	P	239	44	11.35	3.88	8.82E-13	1.29E-10
7	1334	F2-3 vs. S2-3	F2-3 up	-	chromosome	GO:0005694	Development	Cell division	C	545	12	25.88	0.46	3.85E-03	4.95E-02
7	1335	F2-3 vs. S2-3	F2-3 up	-	DNA recombination	GO:0006310	Development	Cell division	P	155	0	7.36	<	1.43E-03	2.68E-02
7	1336	F2-3 vs. S2-3	F2-3 up	-	DNA repair	GO:0006281	Development	Cell division	P	315	2	14.96	0.13	1.04E-04	2.78E-03

7	1337	F2-3 vs. S2-3	F2-3 up	-	nucleic acid metabolic process	GO:0090304	Development	Cell division	P	1705	35	80.97	0.43	1.25E-08	9.83E-07
7	1338	F2-3 vs. S2-3	F2-3 up	-	RNA metabolic process	GO:0016070	Development	Cell division	P	1294	33	61.45	0.54	1.09E-04	2.89E-03
7	1339	F2-3 vs. S2-3	F2-3 up	+	gametophyte development	GO:0048229	Development	Cell division	P	461	39	21.89	1.78	1.04E-03	2.05E-02
7	1340	F2-3 vs. S2-3	F2-3 up	+	nucleobase-containing small molecule metabolic process	GO:0055086	Development	Cell division	P	392	46	18.62	2.47	1.53E-07	8.55E-06
7	1341	F2-3 vs. S2-3	F2-3 up	+	nucleoside phosphate binding	GO:1901265	Development	Cell division	F	1112	95	52.81	1.8	1.88E-07	1.59E-05
7	1342	F2-3 vs. S2-3	F2-3 up	+	nucleoside-triphosphatase activity	GO:0017111	Development	Cell division	F	734	60	34.86	1.72	1.24E-04	4.76E-03
7	1343	F2-3 vs. S2-3	F2-3 up	+	nucleotide binding	GO:0000166	Development	Cell division	F	1112	95	52.81	1.8	1.88E-07	1.64E-05
7	1344	F2-3 vs. S2-3	F2-3 up	+	nucleotide-sugar biosynthetic process	GO:0009226	Development	Cell division	P	42	13	1.99	6.52	7.89E-07	3.75E-05
7	1345	F2-3 vs. S2-3	F2-3 up	+	nucleotide-sugar metabolic process	GO:0009225	Development	Cell division	P	52	16	2.47	6.48	4.60E-08	3.12E-06
7	1346	F2-3 vs. S2-3	F2-3 up	+	pollen tube development	GO:0048868	Development	Cell division	P	192	24	9.12	2.63	5.02E-05	1.51E-03
7	1347	F2-3 vs. S2-3	F2-3 up	+	pollen tube growth	GO:0009860	Development	Cell division	P	135	22	6.41	3.43	2.58E-06	1.10E-04
7	1348	F2-3 vs. S2-3	F2-3 up	+	pollination	GO:0009856	Development	Cell division	P	287	30	13.63	2.2	1.77E-04	4.42E-03
7	1349	F2-3 vs. S2-3	F2-3 up	+	ribonucleotide metabolic process	GO:0009259	Development	Cell division	P	226	23	10.73	2.14	1.15E-03	2.20E-02
7	1350	F2-3 vs. S2-3	F2-3 up	+	ribose phosphate metabolic process	GO:0019693	Development	Cell division	P	235	23	11.16	2.06	2.18E-03	3.83E-02
7	1351	F2-3 vs. S2-3	F2-3 up	+	syncytium formation	GO:0006949	Development	Cell division	P	16	5	0.76	6.58	2.11E-03	3.71E-02
7	1352	F2-3 vs. S2-3	F2-3 up	-	chromatin organization	GO:0006325	Development	Development	P	333	4	15.81	0.25	9.05E-04	1.81E-02
7	1353	F2-3 vs. S2-3	F2-3 up	-	chromosome organization	GO:0051276	Development	Development	P	501	4	23.79	0.17	2.19E-06	9.65E-05
7	1354	F2-3 vs. S2-3	F2-3 up	-	intracellular organelle lumen	GO:0070013	Development	Development	C	1386	40	65.82	0.61	8.35E-04	1.24E-02
7	1355	F2-3 vs. S2-3	F2-3 up	-	membrane-enclosed lumen	GO:0031974	Development	Development	C	1386	40	65.82	0.61	8.35E-04	1.22E-02
7	1356	F2-3 vs. S2-3	F2-3 up	-	nuclear lumen	GO:0031981	Development	Development	C	1222	29	58.03	0.5	3.78E-05	6.55E-04
7	1357	F2-3 vs. S2-3	F2-3 up	-	nucleoplasm	GO:0005654	Development	Development	C	466	8	22.13	0.36	1.14E-03	1.63E-02
7	1358	F2-3 vs. S2-3	F2-3 up	-	nucleus	GO:0005634	Development	Development	C	10540	359	500.55	0.72	9.03E-16	4.09E-14
7	1359	F2-3 vs. S2-3	F2-3 up	-	organelle assembly	GO:0070925	Development	Development	P	193	1	9.17	0.11	2.61E-03	4.47E-02
7	1360	F2-3 vs. S2-3	F2-3 up	-	organelle lumen	GO:0043233	Development	Development	C	1386	40	65.82	0.61	8.35E-04	1.21E-02
7	1361	F2-3 vs. S2-3	F2-3 up	+	actin cytoskeleton	GO:0015629	Development	Development	C	63	10	2.99	3.34	1.64E-03	2.24E-02
7	1362	F2-3 vs. S2-3	F2-3 up	+	actin cytoskeleton organization	GO:0030036	Development	Development	P	104	14	4.94	2.83	9.27E-04	1.85E-02
7	1363	F2-3 vs. S2-3	F2-3 up	+	actin filament	GO:0005884	Development	Development	C	10	5	0.47	10.53	3.90E-04	6.15E-03
7	1364	F2-3 vs. S2-3	F2-3 up	+	anatomical structure development	GO:0048856	Development	Development	P	2842	189	134.97	1.4	5.68E-06	2.22E-04
7	1365	F2-3 vs. S2-3	F2-3 up	+	apoplast	GO:0048046	Development	Development	C	297	59	14.1	4.18	5.25E-18	2.60E-16
7	1366	F2-3 vs. S2-3	F2-3 up	+	bounding membrane of organelle	GO:0098588	Development	Development	C	923	84	43.83	1.92	7.53E-08	2.37E-06
7	1367	F2-3 vs. S2-3	F2-3 up	+	cell development	GO:0048468	Development	Development	P	319	34	15.15	2.24	4.40E-05	1.34E-03
7	1368	F2-3 vs. S2-3	F2-3 up	+	cell growth	GO:0016049	Development	Development	P	386	46	18.33	2.51	1.20E-07	7.18E-06
7	1369	F2-3 vs. S2-3	F2-3 up	+	cell junction	GO:0030054	Development	Development	C	944	78	44.83	1.74	7.60E-06	1.58E-04
7	1370	F2-3 vs. S2-3	F2-3 up	+	cell morphogenesis	GO:0000902	Development	Development	P	369	40	17.52	2.28	5.85E-06	2.27E-04
7	1371	F2-3 vs. S2-3	F2-3 up	+	cell morphogenesis involved in differentiation	GO:0000904	Development	Development	P	229	26	10.88	2.39	1.13E-04	2.99E-03
7	1372	F2-3 vs. S2-3	F2-3 up	+	cell periphery	GO:0071944	Development	Development	C	4012	324	190.53	1.7	3.84E-21	2.00E-19
7	1373	F2-3 vs. S2-3	F2-3 up	+	cell projection	GO:0042995	Development	Development	C	115	17	5.46	3.11	1.01E-04	1.73E-03
7	1374	F2-3 vs. S2-3	F2-3 up	+	cell tip growth	GO:0009932	Development	Development	P	160	24	7.6	3.16	3.35E-06	1.38E-04
7	1375	F2-3 vs. S2-3	F2-3 up	+	cell wall	GO:0005618	Development	Development	C	565	63	26.83	2.35	4.53E-09	1.52E-07
7	1376	F2-3 vs. S2-3	F2-3 up	+	cell wall biogenesis	GO:0042546	Development	Development	P	238	26	11.3	2.3	2.48E-04	5.97E-03
7	1377	F2-3 vs. S2-3	F2-3 up	+	cell wall modification	GO:0042545	Development	Development	P	173	28	8.22	3.41	1.33E-07	7.81E-06
7	1378	F2-3 vs. S2-3	F2-3 up	+	cell wall organization	GO:0071555	Development	Development	P	274	40	13.01	3.07	4.20E-09	3.69E-07
7	1379	F2-3 vs. S2-3	F2-3 up	+	cell wall organization or biogenesis	GO:0071554	Development	Development	P	507	63	24.08	2.62	7.74E-11	9.08E-09
7	1380	F2-3 vs. S2-3	F2-3 up	+	cell-cell junction	GO:0005911	Development	Development	C	944	78	44.83	1.74	7.60E-06	1.68E-04
7	1381	F2-3 vs. S2-3	F2-3 up	+	cellular anatomical entity	GO:0110165	Development	Development	C	24430	1230	1160.19	1.06	3.80E-11	1.41E-09
7	1382	F2-3 vs. S2-3	F2-3 up	+	cellular component organization or biogenesis	GO:0071840	Development	Development	P	2813	168	133.59	1.26	2.89E-03	4.78E-02
7	1383	F2-3 vs. S2-3	F2-3 up	+	cellular process	GO:0009987	Development	Development	P	12008	804	570.27	1.41	6.02E-37	1.20E-33
7	1384	F2-3 vs. S2-3	F2-3 up	+	cytoplasm	GO:0005737	Development	Development	C	14837	951	704.62	1.35	7.91E-43	8.24E-40
7	1385	F2-3 vs. S2-3	F2-3 up	+	cytosol	GO:0005829	Development	Development	C	3241	310	153.92	2.01	1.84E-31	2.74E-29
7	1386	F2-3 vs. S2-3	F2-3 up	+	developmental cell growth	GO:0048588	Development	Development	P	197	29	9.36	3.1	4.72E-07	2.35E-05
7	1387	F2-3 vs. S2-3	F2-3 up	+	developmental growth	GO:0048589	Development	Development	P	385	47	18.28	2.57	3.30E-08	2.35E-06
7	1388	F2-3 vs. S2-3	F2-3 up	+	developmental growth involved in	GO:0060560	Development	Development	P	335	43	15.91	2.7	5.49E-08	3.61E-06
7	1389	F2-3 vs. S2-3	F2-3 up	+	developmental process	GO:0032502	Development	Development	P	2997	197	142.33	1.38	6.13E-06	2.35E-04
7	1390	F2-3 vs. S2-3	F2-3 up	+	endomembrane system	GO:0012505	Development	Development	C	2286	185	108.56	1.7	8.28E-12	3.19E-10
7	1391	F2-3 vs. S2-3	F2-3 up	+	envelope	GO:0031975	Development	Development	C	1025	131	48.68	2.69	3.42E-22	1.98E-20
7	1392	F2-3 vs. S2-3	F2-3 up	+	external encapsulating structure	GO:0030312	Development	Development	C	567	63	26.93	2.34	4.94E-09	1.61E-07
7	1393	F2-3 vs. S2-3	F2-3 up	+	external encapsulating structure organization	GO:0045229	Development	Development	P	320	47	15.2	3.09	1.57E-10	1.74E-08
7	1394	F2-3 vs. S2-3	F2-3 up	+	extracellular region	GO:0005576	Development	Development	C	3128	228	148.55	1.53	2.92E-10	1.05E-08
7	1395	F2-3 vs. S2-3	F2-3 up	+	extracellular space	GO:0005615	Development	Development	C	130	22	6.17	3.56	1.49E-06	4.08E-05
7	1396	F2-3 vs. S2-3	F2-3 up	+	Golgi apparatus subcompartment	GO:0098791	Development	Development	C	437	44	20.75	2.12	1.16E-05	2.19E-04
7	1397	F2-3 vs. S2-3	F2-3 up	+	Golgi cisterna	GO:0031985	Development	Development	C	185	19	8.79	2.16	3.26E-03	4.30E-02

7	1398	F2-3 vs. S2-3	F2-3 up	+	Golgi trans cisterna	GO:0000138	Development	Development	C	85	12	4.04	2.97	1.46E-03	2.02E-02
7	1399	F2-3 vs. S2-3	F2-3 up	+	growth	GO:0040007	Development	Development	P	443	50	21.04	2.38	1.36E-07	7.92E-06
7	1400	F2-3 vs. S2-3	F2-3 up	+	integral component of plasma membrane	GO:0005887	Development	Development	C	107	13	5.08	2.56	3.15E-03	4.20E-02
7	1401	F2-3 vs. S2-3	F2-3 up	+	intracellular anatomical structure	GO:0005622	Development	Development	C	21464	1084	1019.34	1.06	1.32E-05	2.46E-04
7	1402	F2-3 vs. S2-3	F2-3 up	+	intracellular vesicle	GO:0097708	Development	Development	C	722	65	34.29	1.9	3.32E-06	8.43E-05
7	1403	F2-3 vs. S2-3	F2-3 up	+	lytic vacuole	GO:0000323	Development	Development	C	52	15	2.47	6.07	2.43E-07	7.45E-06
7	1404	F2-3 vs. S2-3	F2-3 up	+	membrane	GO:0016020	Development	Development	C	5493	448	260.87	1.72	5.90E-32	1.02E-29
7	1405	F2-3 vs. S2-3	F2-3 up	+	membrane protein complex	GO:0098796	Development	Development	C	597	51	28.35	1.8	1.67E-04	2.76E-03
7	1406	F2-3 vs. S2-3	F2-3 up	+	microbody	GO:0042579	Development	Development	C	320	37	15.2	2.43	3.88E-06	9.62E-05
7	1407	F2-3 vs. S2-3	F2-3 up	+	multicellular organism development	GO:0007275	Development	Development	P	2457	150	116.68	1.29	2.19E-03	3.84E-02
7	1408	F2-3 vs. S2-3	F2-3 up	+	multi-multicellular organism process	GO:0044706	Development	Development	P	287	30	13.63	2.2	1.77E-04	4.44E-03
7	1409	F2-3 vs. S2-3	F2-3 up	+	organelle envelope	GO:0031967	Development	Development	C	1025	131	48.68	2.69	3.42E-22	1.87E-20
7	1410	F2-3 vs. S2-3	F2-3 up	+	organelle membrane	GO:0031090	Development	Development	C	1458	114	69.24	1.65	7.12E-07	2.06E-05
7	1411	F2-3 vs. S2-3	F2-3 up	+	organelle subcompartment	GO:0031984	Development	Development	C	1156	143	54.9	2.6	4.36E-23	2.67E-21
7	1412	F2-3 vs. S2-3	F2-3 up	+	plant-type cell wall loosening	GO:0009828	Development	Development	P	37	8	1.76	4.55	8.43E-04	1.73E-02
7	1413	F2-3 vs. S2-3	F2-3 up	+	plant-type cell wall modification	GO:0009827	Development	Development	P	64	10	3.04	3.29	1.82E-03	3.26E-02
7	1414	F2-3 vs. S2-3	F2-3 up	+	plant-type cell wall organization	GO:0009664	Development	Development	P	150	20	7.12	2.81	9.41E-05	2.59E-03
7	1415	F2-3 vs. S2-3	F2-3 up	+	plant-type cell wall organization or biogenesis	GO:0071669	Development	Development	P	265	31	12.58	2.46	1.53E-05	5.53E-04
7	1416	F2-3 vs. S2-3	F2-3 up	+	plant-type vacuole	GO:0000325	Development	Development	C	173	23	8.22	2.8	3.03E-05	5.44E-04
7	1417	F2-3 vs. S2-3	F2-3 up	+	plasma membrane	GO:0005886	Development	Development	C	3534	273	167.83	1.63	3.32E-15	1.44E-13
7	1418	F2-3 vs. S2-3	F2-3 up	+	plasma membrane bounded cell projection	GO:0120025	Development	Development	C	101	17	4.8	3.54	2.39E-05	4.37E-04
7	1419	F2-3 vs. S2-3	F2-3 up	+	pollen development	GO:0009555	Development	Development	P	346	34	16.43	2.07	1.63E-04	4.19E-03
7	1420	F2-3 vs. S2-3	F2-3 up	+	pollen tube	GO:0090406	Development	Development	C	83	16	3.94	4.06	9.43E-06	1.85E-04
7	1421	F2-3 vs. S2-3	F2-3 up	+	stromule	GO:0010319	Development	Development	C	36	21	1.71	12.28	2.33E-14	9.70E-13
7	1422	F2-3 vs. S2-3	F2-3 up	+	structural constituent of cytoskeleton	GO:0005200	Development	Development	F	28	8	1.33	6.02	1.70E-04	6.13E-03
7	1423	F2-3 vs. S2-3	F2-3 up	+	symplast	GO:0055044	Development	Development	C	944	78	44.83	1.74	7.60E-06	1.65E-04
7	1424	F2-3 vs. S2-3	F2-3 up	+	unidimensional cell growth	GO:0009826	Development	Development	P	288	37	13.68	2.71	2.92E-07	1.56E-05
7	1425	F2-3 vs. S2-3	F2-3 up	+	vacuolar membrane	GO:0005774	Development	Development	C	570	58	27.07	2.14	3.07E-07	9.13E-06
7	1426	F2-3 vs. S2-3	F2-3 up	+	vacuole	GO:0005773	Development	Development	C	1083	124	51.43	2.41	1.17E-17	5.53E-16
7	1427	F2-3 vs. S2-3	F2-3 up	-	DNA binding	GO:0003677	Metabolism	Metabolism	F	1606	37	76.27	0.49	8.80E-07	6.28E-05
7	1428	F2-3 vs. S2-3	F2-3 up	-	DNA metabolic process	GO:0006259	Metabolism	Metabolism	P	448	3	21.28	0.14	3.10E-06	1.29E-04
7	1429	F2-3 vs. S2-3	F2-3 up	-	transferase complex	GO:1990234	Metabolism	Metabolism	C	698	17	33.15	0.51	3.41E-03	4.44E-02
7	1430	F2-3 vs. S2-3	F2-3 up	+	actin filament bundle assembly	GO:0051017	Metabolism	Metabolism	P	20	6	0.95	6.32	9.04E-04	1.82E-02
7	1431	F2-3 vs. S2-3	F2-3 up	+	actin filament bundle organization	GO:0061572	Metabolism	Metabolism	P	20	6	0.95	6.32	9.04E-04	1.83E-02
7	1432	F2-3 vs. S2-3	F2-3 up	+	actin filament-based process	GO:0030029	Metabolism	Metabolism	P	113	14	5.37	2.61	1.89E-03	3.36E-02
7	1433	F2-3 vs. S2-3	F2-3 up	+	alanine metabolic process	GO:0006522	Metabolism	Metabolism	P	4	3	0.19	15.79	2.84E-03	4.74E-02
7	1434	F2-3 vs. S2-3	F2-3 up	+	alcohol metabolic process	GO:0006066	Metabolism	Metabolism	P	119	16	5.65	2.83	4.19E-04	9.66E-03
7	1435	F2-3 vs. S2-3	F2-3 up	+	alpha-amino acid biosynthetic process	GO:1901607	Metabolism	Metabolism	P	181	24	8.6	2.79	2.14E-05	7.14E-04
7	1436	F2-3 vs. S2-3	F2-3 up	+	alpha-amino acid metabolic process	GO:1901605	Metabolism	Metabolism	P	278	48	13.2	3.64	6.44E-13	9.88E-11
7	1437	F2-3 vs. S2-3	F2-3 up	+	amine biosynthetic process	GO:0009309	Metabolism	Metabolism	P	44	11	2.09	5.26	3.03E-05	9.70E-04
7	1438	F2-3 vs. S2-3	F2-3 up	+	amine metabolic process	GO:0009308	Metabolism	Metabolism	P	115	17	5.46	3.11	1.01E-04	2.74E-03
7	1439	F2-3 vs. S2-3	F2-3 up	+	anchoring junction	GO:0070161	Metabolism	Metabolism	C	944	78	44.83	1.74	7.60E-06	1.62E-04
7	1440	F2-3 vs. S2-3	F2-3 up	+	anion binding	GO:0043168	Metabolism	Metabolism	F	1369	128	65.01	1.97	4.44E-12	1.10E-09
7	1441	F2-3 vs. S2-3	F2-3 up	+	aromatic amino acid family metabolic process	GO:0009072	Metabolism	Metabolism	P	87	13	4.13	3.15	5.86E-04	1.28E-02
7	1442	F2-3 vs. S2-3	F2-3 up	+	aromatic compound biosynthetic process	GO:0019438	Metabolism	Metabolism	P	808	67	38.37	1.75	3.17E-05	9.98E-04
7	1443	F2-3 vs. S2-3	F2-3 up	+	binding	GO:0005488	Metabolism	Metabolism	F	9843	572	467.45	1.22	5.85E-09	7.83E-07
7	1444	F2-3 vs. S2-3	F2-3 up	+	biological_process	GO:0008150	Metabolism	Metabolism	P	21988	1119	1044.22	1.07	1.37E-07	7.85E-06
7	1445	F2-3 vs. S2-3	F2-3 up	+	biosynthetic process	GO:0009058	Metabolism	Metabolism	P	2811	258	133.5	1.93	2.21E-23	1.02E-20
7	1446	F2-3 vs. S2-3	F2-3 up	+	capsule organization	GO:0045230	Metabolism	Metabolism	P	6	5	0.28	17.55	6.98E-05	1.99E-03
7	1447	F2-3 vs. S2-3	F2-3 up	+	capsule polysaccharide biosynthetic process	GO:0045227	Metabolism	Metabolism	P	6	5	0.28	17.55	6.98E-05	1.98E-03
7	1448	F2-3 vs. S2-3	F2-3 up	+	carbon utilization	GO:0015976	Metabolism	Metabolism	P	8	4	0.38	10.53	1.55E-03	2.88E-02
7	1449	F2-3 vs. S2-3	F2-3 up	+	carbon-carbon lyase activity	GO:0016830	Metabolism	Metabolism	F	111	23	5.27	4.36	3.63E-08	4.17E-06
7	1450	F2-3 vs. S2-3	F2-3 up	+	carboxylic acid biosynthetic process	GO:0046394	Metabolism	Metabolism	P	468	62	22.23	2.79	9.88E-12	1.28E-09
7	1451	F2-3 vs. S2-3	F2-3 up	+	carboxylic acid metabolic process	GO:0019752	Metabolism	Metabolism	P	912	132	43.31	3.05	6.13E-27	5.23E-24
7	1452	F2-3 vs. S2-3	F2-3 up	+	carboxylic ester hydrolase activity	GO:0052689	Metabolism	Metabolism	F	229	25	10.88	2.3	3.42E-04	1.11E-02
7	1453	F2-3 vs. S2-3	F2-3 up	+	carboxy-lyase activity	GO:0016831	Metabolism	Metabolism	F	68	15	3.23	4.64	4.34E-06	2.32E-04
7	1454	F2-3 vs. S2-3	F2-3 up	+	catalytic activity	GO:0003824	Metabolism	Metabolism	F	8380	716	397.97	1.8	8.81E-71	2.83E-67
7	1455	F2-3 vs. S2-3	F2-3 up	+	cation binding	GO:0043169	Metabolism	Metabolism	F	1637	157	77.74	2.02	1.51E-15	5.40E-13
7	1456	F2-3 vs. S2-3	F2-3 up	+	cellular aldehyde metabolic process	GO:0006081	Metabolism	Metabolism	P	48	13	2.28	5.7	2.77E-06	1.18E-04
7	1457	F2-3 vs. S2-3	F2-3 up	+	cellular amine metabolic process	GO:0044106	Metabolism	Metabolism	P	64	15	3.04	4.94	2.28E-06	9.87E-05
7	1458	F2-3 vs. S2-3	F2-3 up	+	cellular amino acid biosynthetic process	GO:0008652	Metabolism	Metabolism	P	200	27	9.5	2.84	5.00E-06	2.02E-04
7	1459	F2-3 vs. S2-3	F2-3 up	+	cellular amino acid metabolic process	GO:0006520	Metabolism	Metabolism	P	391	57	18.57	3.07	2.32E-12	3.23E-10

7	1460	F2-3 vs. S2-3	F2-3 up	+	cellular biogenic amine biosynthetic process	GO:0042401	Metabolism	Metabolism	P	44	11	2.09	5.26	3.03E-05	9.65E-04
7	1461	F2-3 vs. S2-3	F2-3 up	+	cellular biogenic amine metabolic process	GO:0006576	Metabolism	Metabolism	P	64	15	3.04	4.94	2.28E-06	9.94E-05
7	1462	F2-3 vs. S2-3	F2-3 up	+	cellular biosynthetic process	GO:0044249	Metabolism	Metabolism	P	2583	216	122.67	1.76	3.54E-15	7.84E-13
7	1463	F2-3 vs. S2-3	F2-3 up	+	cellular glucan metabolic process	GO:0006073	Metabolism	Metabolism	P	195	25	9.26	2.7	2.42E-05	7.96E-04
7	1464	F2-3 vs. S2-3	F2-3 up	+	cellular lipid metabolic process	GO:0044255	Metabolism	Metabolism	P	689	68	32.72	2.08	1.08E-07	6.55E-06
7	1465	F2-3 vs. S2-3	F2-3 up	+	cellular metabolic process	GO:0044237	Metabolism	Metabolism	P	7649	542	363.25	1.49	4.28E-25	2.84E-22
7	1466	F2-3 vs. S2-3	F2-3 up	+	cellulose synthase (UDP-forming) activity	GO:0016760	Metabolism	Metabolism	F	26	7	1.23	5.67	5.87E-04	1.78E-02
7	1467	F2-3 vs. S2-3	F2-3 up	+	cellulose synthase activity	GO:0016759	Metabolism	Metabolism	F	37	8	1.76	4.55	8.43E-04	2.46E-02
7	1468	F2-3 vs. S2-3	F2-3 up	+	copper ion binding	GO:0005507	Metabolism	Metabolism	F	193	35	9.17	3.82	2.67E-10	4.29E-08
7	1469	F2-3 vs. S2-3	F2-3 up	+	cysteine-type peptidase activity	GO:0008234	Metabolism	Metabolism	F	101	16	4.8	3.34	7.82E-05	3.26E-03
7	1470	F2-3 vs. S2-3	F2-3 up	+	dicarboxylic acid metabolic process	GO:0043648	Metabolism	Metabolism	P	89	16	4.23	3.79	2.02E-05	6.91E-04
7	1471	F2-3 vs. S2-3	F2-3 up	+	dioxygenase activity	GO:0051213	Metabolism	Metabolism	F	116	15	5.51	2.72	8.98E-04	2.55E-02
7	1472	F2-3 vs. S2-3	F2-3 up	+	establishment of localization	GO:0051234	Metabolism	Metabolism	P	2306	180	109.51	1.64	2.24E-10	2.27E-08
7	1473	F2-3 vs. S2-3	F2-3 up	+	extracellular polysaccharide biosynthetic	GO:0045226	Metabolism	Metabolism	P	6	5	0.28	17.55	6.98E-05	1.97E-03
7	1474	F2-3 vs. S2-3	F2-3 up	+	extracellular polysaccharide metabolic process	GO:0046379	Metabolism	Metabolism	P	6	5	0.28	17.55	6.98E-05	1.96E-03
7	1475	F2-3 vs. S2-3	F2-3 up	+	ferric iron binding	GO:0008199	Metabolism	Metabolism	F	11	5	0.52	9.57	5.46E-04	1.67E-02
7	1476	F2-3 vs. S2-3	F2-3 up	+	ferrous iron binding	GO:0008198	Metabolism	Metabolism	F	16	6	0.76	7.9	3.42E-04	1.10E-02
7	1477	F2-3 vs. S2-3	F2-3 up	+	galactose metabolic process	GO:0006012	Metabolism	Metabolism	P	26	6	1.23	4.86	2.82E-03	4.78E-02
7	1478	F2-3 vs. S2-3	F2-3 up	+	galacturonan metabolic process	GO:0010393	Metabolism	Metabolism	P	163	28	7.74	3.62	4.42E-08	3.04E-06
7	1479	F2-3 vs. S2-3	F2-3 up	+	glucan biosynthetic process	GO:0009250	Metabolism	Metabolism	P	118	16	5.6	2.86	3.85E-04	8.95E-03
7	1480	F2-3 vs. S2-3	F2-3 up	+	glucan metabolic process	GO:0044042	Metabolism	Metabolism	P	203	27	9.64	2.8	6.39E-06	2.42E-04
7	1481	F2-3 vs. S2-3	F2-3 up	+	glucosinolate biosynthetic process	GO:0019761	Metabolism	Metabolism	P	39	8	1.85	4.32	1.13E-03	2.20E-02
7	1482	F2-3 vs. S2-3	F2-3 up	+	glucosinolate metabolic process	GO:0019760	Metabolism	Metabolism	P	112	15	5.32	2.82	6.49E-04	1.41E-02
7	1483	F2-3 vs. S2-3	F2-3 up	+	glucosyltransferase activity	GO:0046527	Metabolism	Metabolism	F	190	28	9.02	3.1	7.25E-07	5.42E-05
7	1484	F2-3 vs. S2-3	F2-3 up	+	glutamine family amino acid metabolic process	GO:0009064	Metabolism	Metabolism	P	58	13	2.75	4.72	1.61E-05	5.77E-04
7	1485	F2-3 vs. S2-3	F2-3 up	+	glutamine metabolic process	GO:0006541	Metabolism	Metabolism	P	15	5	0.71	7.02	1.67E-03	3.03E-02
7	1486	F2-3 vs. S2-3	F2-3 up	+	glycine decarboxylation via glycine cleavage system	GO:0019464	Metabolism	Metabolism	P	7	4	0.33	12.03	1.07E-03	2.10E-02
7	1487	F2-3 vs. S2-3	F2-3 up	+	glycine metabolic process	GO:0006544	Metabolism	Metabolism	P	18	8	0.85	9.36	1.31E-05	4.82E-04
7	1488	F2-3 vs. S2-3	F2-3 up	+	glycosinolate biosynthetic process	GO:0019758	Metabolism	Metabolism	P	39	8	1.85	4.32	1.13E-03	2.19E-02
7	1489	F2-3 vs. S2-3	F2-3 up	+	glycosinolate metabolic process	GO:0019757	Metabolism	Metabolism	P	112	15	5.32	2.82	6.49E-04	1.40E-02
7	1490	F2-3 vs. S2-3	F2-3 up	+	glycosyl compound biosynthetic process	GO:1901659	Metabolism	Metabolism	P	62	10	2.94	3.4	1.47E-03	2.75E-02
7	1491	F2-3 vs. S2-3	F2-3 up	+	glycosyl compound metabolic process	GO:1901657	Metabolism	Metabolism	P	187	20	8.88	2.25	1.95E-03	3.45E-02
7	1492	F2-3 vs. S2-3	F2-3 up	+	heme binding	GO:0020037	Metabolism	Metabolism	F	374	33	17.76	1.86	1.57E-03	4.25E-02
7	1493	F2-3 vs. S2-3	F2-3 up	+	heterocycle biosynthetic process	GO:0018130	Metabolism	Metabolism	P	726	57	34.48	1.65	4.59E-04	1.04E-02
7	1494	F2-3 vs. S2-3	F2-3 up	+	homogalacturonan metabolic process	GO:0010394	Metabolism	Metabolism	P	4	3	0.19	15.79	2.84E-03	4.70E-02
7	1495	F2-3 vs. S2-3	F2-3 up	+	hydrolase activity	GO:0016787	Metabolism	Metabolism	F	2995	245	142.23	1.72	3.82E-16	1.53E-13
7	1496	F2-3 vs. S2-3	F2-3 up	+	hydrolase activity, acting on acid anhydrides	GO:0016817	Metabolism	Metabolism	F	802	67	38.09	1.76	2.93E-05	1.37E-03
7	1497	F2-3 vs. S2-3	F2-3 up	+	hydrolase activity, acting on acid anhydrides, in phosphorus-containing anhydrides	GO:0016818	Metabolism	Metabolism	F	796	67	37.8	1.77	1.94E-05	9.32E-04
7	1498	F2-3 vs. S2-3	F2-3 up	+	hydrolase activity, acting on ester bonds	GO:0016788	Metabolism	Metabolism	F	1044	80	49.58	1.61	7.12E-05	3.01E-03
7	1499	F2-3 vs. S2-3	F2-3 up	+	hydrolase activity, hydrolyzing O-glycosyl compounds	GO:0004553	Metabolism	Metabolism	F	396	38	18.81	2.02	1.07E-04	4.25E-03
7	1500	F2-3 vs. S2-3	F2-3 up	+	hydro-lyase activity	GO:0016836	Metabolism	Metabolism	F	88	12	4.18	2.87	1.90E-03	5.00E-02
7	1501	F2-3 vs. S2-3	F2-3 up	+	intramolecular oxidoreductase activity	GO:0016860	Metabolism	Metabolism	F	58	10	2.75	3.63	9.39E-04	2.62E-02
7	1502	F2-3 vs. S2-3	F2-3 up	+	intramolecular oxidoreductase activity, interconverting aldoses and ketoses	GO:0016861	Metabolism	Metabolism	F	19	6	0.9	6.65	7.23E-04	2.13E-02
7	1503	F2-3 vs. S2-3	F2-3 up	+	ion binding	GO:0043167	Metabolism	Metabolism	F	2847	261	135.21	1.93	1.45E-23	1.55E-20
7	1504	F2-3 vs. S2-3	F2-3 up	+	iron ion binding	GO:0005506	Metabolism	Metabolism	F	325	31	15.43	2.01	7.02E-04	2.09E-02
7	1505	F2-3 vs. S2-3	F2-3 up	+	isomerase activity	GO:0016853	Metabolism	Metabolism	F	258	30	12.25	2.45	2.32E-05	1.09E-03
7	1506	F2-3 vs. S2-3	F2-3 up	+	L-alanine metabolic process	GO:0042851	Metabolism	Metabolism	P	4	3	0.19	15.79	2.84E-03	4.78E-02
7	1507	F2-3 vs. S2-3	F2-3 up	+	lignin biosynthetic process	GO:0009809	Metabolism	Metabolism	P	42	9	1.99	4.51	4.28E-04	9.82E-03
7	1508	F2-3 vs. S2-3	F2-3 up	+	lignin metabolic process	GO:0009808	Metabolism	Metabolism	P	53	10	2.52	3.97	5.06E-04	1.13E-02
7	1509	F2-3 vs. S2-3	F2-3 up	+	lipid biosynthetic process	GO:0008610	Metabolism	Metabolism	P	512	51	24.32	2.1	2.95E-06	1.24E-04
7	1510	F2-3 vs. S2-3	F2-3 up	+	lipid metabolic process	GO:0006629	Metabolism	Metabolism	P	828	79	39.32	2.01	3.50E-08	2.46E-06
7	1511	F2-3 vs. S2-3	F2-3 up	+	localization	GO:0051179	Metabolism	Metabolism	P	2471	189	117.35	1.61	4.01E-10	3.75E-08
7	1512	F2-3 vs. S2-3	F2-3 up	+	L-serine biosynthetic process	GO:0006564	Metabolism	Metabolism	P	7	4	0.33	12.03	1.07E-03	2.11E-02
7	1513	F2-3 vs. S2-3	F2-3 up	+	L-serine metabolic process	GO:0006563	Metabolism	Metabolism	P	30	8	1.42	5.62	2.53E-04	6.08E-03
7	1514	F2-3 vs. S2-3	F2-3 up	+	lyase activity	GO:0016829	Metabolism	Metabolism	F	339	49	16.1	3.04	1.05E-10	1.78E-08
7	1515	F2-3 vs. S2-3	F2-3 up	+	magnesium ion binding	GO:0000287	Metabolism	Metabolism	F	126	17	5.98	2.84	2.70E-04	8.87E-03
7	1516	F2-3 vs. S2-3	F2-3 up	+	metabolic process	GO:0008152	Metabolism	Metabolism	P	8681	655	412.27	1.59	5.32E-42	3.18E-38
7	1517	F2-3 vs. S2-3	F2-3 up	+	metal ion binding	GO:0046872	Metabolism	Metabolism	F	1610	152	76.46	1.99	1.64E-14	5.26E-12

7	1518	F2-3 vs. S2-3	F2-3 up	+	molecular_function	GO:0003674	Metabolism	Metabolism	F	21945	1158	1042.18	1.11	5.12E-17	2.35E-14
7	1519	F2-3 vs. S2-3	F2-3 up	+	monocarboxylic acid biosynthetic process	GO:0072330	Metabolism	Metabolism	P	211	30	10.02	2.99	5.81E-07	2.78E-05
7	1520	F2-3 vs. S2-3	F2-3 up	+	monocarboxylic acid metabolic process	GO:0032787	Metabolism	Metabolism	P	439	65	20.85	3.12	3.46E-14	7.14E-12
7	1521	F2-3 vs. S2-3	F2-3 up	+	monosaccharide metabolic process	GO:0005996	Metabolism	Metabolism	P	125	25	5.94	4.21	1.73E-08	1.28E-06
7	1522	F2-3 vs. S2-3	F2-3 up	+	organic acid biosynthetic process	GO:0016053	Metabolism	Metabolism	P	468	62	22.23	2.79	9.88E-12	1.31E-09
7	1523	F2-3 vs. S2-3	F2-3 up	+	organic acid metabolic process	GO:0006082	Metabolism	Metabolism	P	1019	146	48.39	3.02	2.31E-29	2.30E-26
7	1524	F2-3 vs. S2-3	F2-3 up	+	organic cyclic compound biosynthetic process	GO:1901362	Metabolism	Metabolism	P	899	78	42.69	1.83	1.43E-06	6.47E-05
7	1525	F2-3 vs. S2-3	F2-3 up	+	organic hydroxy compound biosynthetic process	GO:1901617	Metabolism	Metabolism	P	172	21	8.17	2.57	1.94E-04	4.77E-03
7	1526	F2-3 vs. S2-3	F2-3 up	+	organic hydroxy compound metabolic process	GO:1901615	Metabolism	Metabolism	P	292	39	13.87	2.81	5.63E-08	3.66E-06
7	1527	F2-3 vs. S2-3	F2-3 up	+	organic substance biosynthetic process	GO:1901576	Metabolism	Metabolism	P	2674	237	126.99	1.87	1.56E-19	4.90E-17
7	1528	F2-3 vs. S2-3	F2-3 up	+	organic substance metabolic process	GO:0071704	Metabolism	Metabolism	P	7851	531	372.85	1.42	6.84E-20	2.41E-17
7	1529	F2-3 vs. S2-3	F2-3 up	+	organonitrogen compound biosynthetic process	GO:1901566	Metabolism	Metabolism	P	1343	95	63.78	1.49	2.55E-04	6.09E-03
7	1530	F2-3 vs. S2-3	F2-3 up	+	organonitrogen compound metabolic process	GO:1901564	Metabolism	Metabolism	P	4611	276	218.98	1.26	6.19E-05	1.81E-03
7	1531	F2-3 vs. S2-3	F2-3 up	+	organophosphate metabolic process	GO:0019637	Metabolism	Metabolism	P	583	59	27.69	2.13	3.74E-07	1.91E-05
7	1532	F2-3 vs. S2-3	F2-3 up	+	oxidation-reduction process	GO:0055114	Metabolism	Metabolism	P	768	118	36.47	3.24	4.18E-26	3.12E-23
7	1533	F2-3 vs. S2-3	F2-3 up	+	oxoacid metabolic process	GO:0043436	Metabolism	Metabolism	P	1016	146	48.25	3.03	1.75E-29	2.10E-26
7	1534	F2-3 vs. S2-3	F2-3 up	+	pectin metabolic process	GO:0045488	Metabolism	Metabolism	P	162	28	7.69	3.64	3.94E-08	2.74E-06
7	1535	F2-3 vs. S2-3	F2-3 up	+	pectinesterase activity	GO:0030599	Metabolism	Metabolism	F	92	17	4.37	3.89	8.20E-06	4.25E-04
7	1536	F2-3 vs. S2-3	F2-3 up	+	pectinesterase inhibitor activity	GO:0046910	Metabolism	Metabolism	F	92	13	4.37	2.98	9.34E-04	2.63E-02
7	1537	F2-3 vs. S2-3	F2-3 up	+	phenylpropanoid biosynthetic process	GO:0009699	Metabolism	Metabolism	P	88	14	4.18	3.35	2.06E-04	4.99E-03
7	1538	F2-3 vs. S2-3	F2-3 up	+	phenylpropanoid metabolic process	GO:0009698	Metabolism	Metabolism	P	109	16	5.18	3.09	1.73E-04	4.35E-03
7	1539	F2-3 vs. S2-3	F2-3 up	+	phosphate-containing compound metabolic process	GO:0006796	Metabolism	Metabolism	P	1858	128	88.24	1.45	5.39E-05	1.60E-03
7	1540	F2-3 vs. S2-3	F2-3 up	+	phosphatidylcholine biosynthetic process	GO:0006656	Metabolism	Metabolism	P	9	4	0.43	9.36	2.16E-03	3.80E-02
7	1541	F2-3 vs. S2-3	F2-3 up	+	phosphoric ester hydrolase activity	GO:0042578	Metabolism	Metabolism	F	392	37	18.62	1.99	2.31E-04	7.90E-03
7	1542	F2-3 vs. S2-3	F2-3 up	+	phosphorus metabolic process	GO:0006793	Metabolism	Metabolism	P	1903	142	90.37	1.57	3.38E-07	1.77E-05
7	1543	F2-3 vs. S2-3	F2-3 up	+	polyamine biosynthetic process	GO:0006596	Metabolism	Metabolism	P	15	7	0.71	9.83	3.62E-05	1.13E-03
7	1544	F2-3 vs. S2-3	F2-3 up	+	polyamine metabolic process	GO:0006595	Metabolism	Metabolism	P	25	9	1.19	7.58	1.48E-05	5.35E-04
7	1545	F2-3 vs. S2-3	F2-3 up	+	proton export across plasma membrane	GO:0120029	Metabolism	Metabolism	P	10	7	0.47	14.74	5.05E-06	2.03E-04
7	1546	F2-3 vs. S2-3	F2-3 up	+	purine nucleoside diphosphate metabolic	GO:0009135	Metabolism	Metabolism	P	56	9	2.66	3.38	2.56E-03	4.39E-02
7	1547	F2-3 vs. S2-3	F2-3 up	+	purine nucleotide metabolic process	GO:0006163	Metabolism	Metabolism	P	218	22	10.35	2.13	1.62E-03	2.96E-02
7	1548	F2-3 vs. S2-3	F2-3 up	+	purine ribonucleoside diphosphate metabolic process	GO:0009179	Metabolism	Metabolism	P	56	9	2.66	3.38	2.56E-03	4.41E-02
7	1549	F2-3 vs. S2-3	F2-3 up	+	purine ribonucleoside triphosphate binding	GO:0035639	Metabolism	Metabolism	F	624	52	29.63	1.75	2.32E-04	7.76E-03
7	1550	F2-3 vs. S2-3	F2-3 up	+	purine ribonucleotide metabolic process	GO:0009150	Metabolism	Metabolism	P	200	22	9.5	2.32	5.48E-04	1.21E-02
7	1551	F2-3 vs. S2-3	F2-3 up	+	purine-containing compound metabolic process	GO:0072521	Metabolism	Metabolism	P	243	24	11.54	2.08	1.56E-03	2.85E-02
7	1552	F2-3 vs. S2-3	F2-3 up	+	pyridoxal phosphate binding	GO:0030170	Metabolism	Metabolism	F	98	24	4.65	5.16	1.09E-09	1.53E-07
7	1553	F2-3 vs. S2-3	F2-3 up	+	pyrophosphatase activity	GO:0016462	Metabolism	Metabolism	F	793	67	37.66	1.78	1.84E-05	8.98E-04
7	1554	F2-3 vs. S2-3	F2-3 up	+	racemase and epimerase activity	GO:0016854	Metabolism	Metabolism	F	43	10	2.04	4.9	1.16E-04	4.51E-03
7	1555	F2-3 vs. S2-3	F2-3 up	+	racemase and epimerase activity, acting on carbohydrates and derivatives	GO:0016857	Metabolism	Metabolism	F	34	9	1.61	5.57	1.10E-04	4.31E-03
7	1556	F2-3 vs. S2-3	F2-3 up	+	S-adenosylmethionine biosynthetic process	GO:0006556	Metabolism	Metabolism	P	4	3	0.19	15.79	2.84E-03	4.76E-02
7	1557	F2-3 vs. S2-3	F2-3 up	+	S-adenosylmethionine metabolic process	GO:0046500	Metabolism	Metabolism	P	9	5	0.43	11.7	2.70E-04	6.43E-03
7	1558	F2-3 vs. S2-3	F2-3 up	+	secondary alcohol metabolic process	GO:1902652	Metabolism	Metabolism	P	12	6	0.57	10.53	9.95E-05	2.73E-03
7	1559	F2-3 vs. S2-3	F2-3 up	+	secondary metabolic process	GO:0019748	Metabolism	Metabolism	P	303	38	14.39	2.64	4.72E-07	2.37E-05
7	1560	F2-3 vs. S2-3	F2-3 up	+	secondary metabolite biosynthetic process	GO:0044550	Metabolism	Metabolism	P	144	23	6.84	3.36	2.09E-06	9.24E-05
7	1561	F2-3 vs. S2-3	F2-3 up	+	serine family amino acid biosynthetic process	GO:0009070	Metabolism	Metabolism	P	43	11	2.04	5.39	2.53E-05	8.27E-04
7	1562	F2-3 vs. S2-3	F2-3 up	+	serine family amino acid metabolic process	GO:0009069	Metabolism	Metabolism	P	58	15	2.75	5.45	7.93E-07	3.70E-05
7	1563	F2-3 vs. S2-3	F2-3 up	+	S-glycoside biosynthetic process	GO:0016144	Metabolism	Metabolism	P	39	8	1.85	4.32	1.13E-03	2.20E-02
7	1564	F2-3 vs. S2-3	F2-3 up	+	S-glycoside metabolic process	GO:0016143	Metabolism	Metabolism	P	112	15	5.32	2.82	6.49E-04	1.41E-02
7	1565	F2-3 vs. S2-3	F2-3 up	+	small molecule binding	GO:0036094	Metabolism	Metabolism	F	1340	123	63.64	1.93	4.12E-11	7.36E-09
7	1566	F2-3 vs. S2-3	F2-3 up	+	small molecule biosynthetic process	GO:0044283	Metabolism	Metabolism	P	645	92	30.63	3	1.22E-18	3.65E-16
7	1567	F2-3 vs. S2-3	F2-3 up	+	small molecule metabolic process	GO:0044281	Metabolism	Metabolism	P	1520	204	72.19	2.83	1.38E-37	4.13E-34
7	1568	F2-3 vs. S2-3	F2-3 up	+	spermidine metabolic process	GO:0008216	Metabolism	Metabolism	P	13	5	0.62	8.1	9.93E-04	1.97E-02
7	1569	F2-3 vs. S2-3	F2-3 up	+	spermine metabolic process	GO:0008215	Metabolism	Metabolism	P	11	5	0.52	9.57	5.46E-04	1.21E-02
7	1570	F2-3 vs. S2-3	F2-3 up	+	sulfur amino acid metabolic process	GO:0000096	Metabolism	Metabolism	P	53	9	2.52	3.58	1.83E-03	3.28E-02
7	1571	F2-3 vs. S2-3	F2-3 up	+	sulfur compound biosynthetic process	GO:0044272	Metabolism	Metabolism	P	142	30	6.74	4.45	2.13E-10	2.20E-08
7	1572	F2-3 vs. S2-3	F2-3 up	+	sulfur compound metabolic process	GO:0006790	Metabolism	Metabolism	P	363	53	17.24	3.07	1.30E-11	1.66E-09
7	1573	F2-3 vs. S2-3	F2-3 up	+	tetrapyrrole binding	GO:0046906	Metabolism	Metabolism	F	404	45	19.19	2.35	8.33E-07	6.08E-05
7	1574	F2-3 vs. S2-3	F2-3 up	+	transaminase activity	GO:0008483	Metabolism	Metabolism	F	51	13	2.42	5.37	4.89E-06	2.58E-04
7	1575	F2-3 vs. S2-3	F2-3 up	+	transferase activity	GO:0016740	Metabolism	Metabolism	F	3411	221	161.99	1.36	3.84E-06	2.09E-04

7	1576	F2-3 vs. S2-3	F2-3 up	+	transferase activity, transferring alkyl or aryl (other than methyl) groups	GO:0016765	Metabolism	Metabolism	F	155	20	7.36	2.72	1.40E-04	5.31E-03
7	1577	F2-3 vs. S2-3	F2-3 up	+	transferase activity, transferring glycosyl groups	GO:0016757	Metabolism	Metabolism	F	612	49	29.06	1.69	8.47E-04	2.45E-02
7	1578	F2-3 vs. S2-3	F2-3 up	+	transferase activity, transferring hexosyl groups	GO:0016758	Metabolism	Metabolism	F	353	40	16.76	2.39	2.01E-06	1.17E-04
7	1579	F2-3 vs. S2-3	F2-3 up	+	transferase activity, transferring nitrogenous groups	GO:0016769	Metabolism	Metabolism	F	52	14	2.47	5.67	1.23E-06	8.38E-05
7	1580	F2-3 vs. S2-3	F2-3 up	+	transition metal ion binding	GO:0046914	Metabolism	Metabolism	F	1178	113	55.94	2.02	2.56E-11	5.14E-09
7	1581	F2-3 vs. S2-3	F2-3 up	+	UDP-arabinose 4-epimerase activity	GO:0050373	Metabolism	Metabolism	F	6	5	0.28	17.55	6.98E-05	2.99E-03
7	1582	F2-3 vs. S2-3	F2-3 up	+	UDP-glucose 4-epimerase activity	GO:0003978	Metabolism	Metabolism	F	9	6	0.43	14.04	3.02E-05	1.39E-03
7	1583	F2-3 vs. S2-3	F2-3 up	+	UDP-glucosyltransferase activity	GO:0035251	Metabolism	Metabolism	F	143	23	6.79	3.39	1.88E-06	1.18E-04
7	1584	F2-3 vs. S2-3	F2-3 up	+	UDP-glycosyltransferase activity	GO:0008194	Metabolism	Metabolism	F	280	35	13.3	2.63	1.49E-06	9.95E-05
7	1585	F2-3 vs. S2-3	F2-3 up	+	UDP-L-arabinose biosynthetic process	GO:0033358	Metabolism	Metabolism	P	6	5	0.28	17.55	6.98E-05	2.00E-03
7	1586	F2-3 vs. S2-3	F2-3 up	+	UDP-L-arabinose metabolic process	GO:0033356	Metabolism	Metabolism	P	12	7	0.57	12.28	1.21E-05	4.48E-04
7	1587	F2-3 vs. S2-3	F2-3 up	+	vitamin B6 binding	GO:0070279	Metabolism	Metabolism	F	98	24	4.65	5.16	1.09E-09	1.60E-07
7	1588	F2-3 vs. S2-3	F2-3 up	+	vitamin binding	GO:0019842	Metabolism	Metabolism	F	134	25	6.36	3.93	5.71E-08	5.74E-06
7	1589	F2-3 vs. S2-3	F2-3 up	+	wax biosynthetic process	GO:0010025	Metabolism	Metabolism	P	27	7	1.28	5.46	7.10E-04	1.51E-02
7	1590	F2-3 vs. S2-3	F2-3 up	+	wax metabolic process	GO:0010166	Metabolism	Metabolism	P	28	7	1.33	5.26	8.53E-04	1.74E-02
7	1591	F2-3 vs. S2-3	F2-3 up	+	carbon fixation	GO:0015977	Metabolism	Photosynthesis	P	16	10	0.76	13.16	9.68E-08	6.03E-06
7	1592	F2-3 vs. S2-3	F2-3 up	+	chlorophyll binding	GO:0016168	Metabolism	Photosynthesis	F	28	12	1.33	9.02	1.23E-07	1.16E-05
7	1593	F2-3 vs. S2-3	F2-3 up	+	chloroplast	GO:0009507	Metabolism	Photosynthesis	C	5013	424	238.07	1.78	3.64E-33	7.59E-31
7	1594	F2-3 vs. S2-3	F2-3 up	+	chloroplast envelope	GO:0009941	Metabolism	Photosynthesis	C	613	104	29.11	3.57	4.83E-26	5.02E-24
7	1595	F2-3 vs. S2-3	F2-3 up	+	chloroplast stroma	GO:0009570	Metabolism	Photosynthesis	C	719	131	34.15	3.84	2.02E-35	7.02E-33
7	1596	F2-3 vs. S2-3	F2-3 up	+	chloroplast thylakoid	GO:0009534	Metabolism	Photosynthesis	C	443	86	21.04	4.09	4.14E-25	3.91E-23
7	1597	F2-3 vs. S2-3	F2-3 up	+	chloroplast thylakoid membrane	GO:0009535	Metabolism	Photosynthesis	C	358	76	17	4.47	2.30E-24	1.60E-22
7	1598	F2-3 vs. S2-3	F2-3 up	+	chloroplast thylakoid membrane protein complex	GO:0098807	Metabolism	Photosynthesis	C	24	7	1.14	6.14	3.91E-04	6.07E-03
7	1599	F2-3 vs. S2-3	F2-3 up	+	fructose 1,6-bisphosphate metabolic process	GO:0030388	Metabolism	Photosynthesis	P	12	5	0.57	8.77	7.45E-04	1.56E-02
7	1600	F2-3 vs. S2-3	F2-3 up	+	oxidative photosynthetic carbon pathway	GO:0009854	Metabolism	Photosynthesis	P	7	5	0.33	15.04	1.15E-04	3.04E-03
7	1601	F2-3 vs. S2-3	F2-3 up	+	photosynthesis	GO:0015979	Metabolism	Photosynthesis	P	189	50	8.98	5.57	7.40E-20	2.46E-17
7	1602	F2-3 vs. S2-3	F2-3 up	+	photosynthesis, dark reaction	GO:0019685	Metabolism	Photosynthesis	P	15	9	0.71	12.63	5.55E-07	2.70E-05
7	1603	F2-3 vs. S2-3	F2-3 up	+	photosynthesis, light harvesting	GO:0009765	Metabolism	Photosynthesis	P	42	13	1.99	6.52	7.89E-07	3.72E-05
7	1604	F2-3 vs. S2-3	F2-3 up	+	photosynthesis, light harvesting in photosystem I	GO:0009768	Metabolism	Photosynthesis	P	24	10	1.14	8.77	1.71E-06	7.71E-05
7	1605	F2-3 vs. S2-3	F2-3 up	+	photosynthesis, light reaction	GO:0019684	Metabolism	Photosynthesis	P	128	33	6.08	5.43	2.57E-13	4.81E-11
7	1606	F2-3 vs. S2-3	F2-3 up	+	photosynthetic electron transport chain	GO:0009767	Metabolism	Photosynthesis	P	46	11	2.18	5.04	4.31E-05	1.31E-03
7	1607	F2-3 vs. S2-3	F2-3 up	+	photosynthetic electron transport in photosystem	GO:0009773	Metabolism	Photosynthesis	P	17	5	0.81	6.19	2.62E-03	4.46E-02
7	1608	F2-3 vs. S2-3	F2-3 up	+	photosynthetic membrane	GO:0034357	Metabolism	Photosynthesis	C	395	81	18.76	4.32	4.80E-25	4.16E-23
7	1609	F2-3 vs. S2-3	F2-3 up	+	photosystem	GO:0009521	Metabolism	Photosynthesis	C	63	15	2.99	5.01	1.92E-06	5.01E-05
7	1610	F2-3 vs. S2-3	F2-3 up	+	photosystem I	GO:0009522	Metabolism	Photosynthesis	C	22	6	1.04	5.74	1.37E-03	1.93E-02
7	1611	F2-3 vs. S2-3	F2-3 up	+	photosystem II	GO:0009523	Metabolism	Photosynthesis	C	44	10	2.09	4.79	1.37E-04	2.30E-03
7	1612	F2-3 vs. S2-3	F2-3 up	+	photosystem II assembly	GO:0010207	Metabolism	Photosynthesis	P	26	9	1.23	7.29	1.91E-05	6.67E-04
7	1613	F2-3 vs. S2-3	F2-3 up	+	plastid	GO:0009536	Metabolism	Photosynthesis	C	5432	461	257.97	1.79	3.04E-37	1.58E-34
7	1614	F2-3 vs. S2-3	F2-3 up	+	plastid envelope	GO:0009526	Metabolism	Photosynthesis	C	637	111	30.25	3.67	1.30E-28	1.69E-26
7	1615	F2-3 vs. S2-3	F2-3 up	+	plastid stroma	GO:0009532	Metabolism	Photosynthesis	C	731	131	34.72	3.77	8.90E-35	2.32E-32
7	1616	F2-3 vs. S2-3	F2-3 up	+	plastid thylakoid	GO:0031976	Metabolism	Photosynthesis	C	446	86	21.18	4.06	6.17E-25	4.94E-23
7	1617	F2-3 vs. S2-3	F2-3 up	+	plastid thylakoid membrane	GO:0055035	Metabolism	Photosynthesis	C	363	76	17.24	4.41	4.81E-24	3.13E-22
7	1618	F2-3 vs. S2-3	F2-3 up	+	plastoglobule	GO:0010287	Metabolism	Photosynthesis	C	76	26	3.61	7.2	4.19E-13	1.68E-11
7	1619	F2-3 vs. S2-3	F2-3 up	+	regulation of photosynthesis	GO:0010109	Metabolism	Photosynthesis	P	40	9	1.9	4.74	3.14E-04	7.42E-03
7	1620	F2-3 vs. S2-3	F2-3 up	+	thylakoid	GO:0009579	Metabolism	Photosynthesis	C	537	101	25.5	3.96	2.23E-28	2.58E-26
7	1621	F2-3 vs. S2-3	F2-3 up	+	thylakoid lumen	GO:0031977	Metabolism	Photosynthesis	C	63	13	2.99	4.35	3.43E-05	6.06E-04
7	1622	F2-3 vs. S2-3	F2-3 up	+	thylakoid membrane	GO:0042651	Metabolism	Photosynthesis	C	390	80	18.52	4.32	9.32E-25	6.93E-23
7	1623	F2-3 vs. S2-3	F2-3 up	+	endoplasmic reticulum	GO:0005783	Regulation	Protein modification	C	1044	85	49.58	1.71	5.11E-06	1.21E-04
7	1624	F2-3 vs. S2-3	F2-3 up	+	Golgi apparatus	GO:0005794	Regulation	Protein modification	C	1161	105	55.14	1.9	2.77E-09	9.61E-08
7	1625	F2-3 vs. S2-3	F2-3 up	+	negative regulation of protein polymerization	GO:0032272	Regulation	Protein modification	P	15	5	0.71	7.02	1.67E-03	3.02E-02
7	1626	F2-3 vs. S2-3	F2-3 up	+	negative regulation of protein-containing complex assembly	GO:0031333	Regulation	Protein modification	P	15	5	0.71	7.02	1.67E-03	3.04E-02
7	1627	F2-3 vs. S2-3	F2-3 up	-	molecular function regulator	GO:0098772	Regulation	Regulation	F	2397	76	113.83	0.67	1.46E-04	5.44E-03
7	1628	F2-3 vs. S2-3	F2-3 up	-	regulation of biosynthetic process	GO:0009889	Regulation	Regulation	P	2514	76	119.39	0.64	1.94E-05	6.72E-04
7	1629	F2-3 vs. S2-3	F2-3 up	-	regulation of cellular biosynthetic process	GO:0031326	Regulation	Regulation	P	2477	72	117.63	0.61	5.26E-06	2.10E-04
7	1630	F2-3 vs. S2-3	F2-3 up	-	regulation of cellular macromolecule biosynthetic process	GO:2000112	Regulation	Regulation	P	2365	62	112.32	0.55	1.76E-07	9.75E-06
7	1631	F2-3 vs. S2-3	F2-3 up	-	regulation of cellular metabolic process	GO:0031323	Regulation	Regulation	P	2975	97	141.28	0.69	6.29E-05	1.83E-03
7	1632	F2-3 vs. S2-3	F2-3 up	-	regulation of macromolecule biosynthetic	GO:0010556	Regulation	Regulation	P	2378	62	112.93	0.55	1.41E-07	7.94E-06
7	1633	F2-3 vs. S2-3	F2-3 up	-	regulation of macromolecule metabolic process	GO:0060255	Regulation	Regulation	P	3058	77	145.23	0.53	2.09E-10	2.20E-08

7	1634	F2-3 vs. S2-3	F2-3 up	-	regulation of metabolic process	GO:0019222	Regulation	Regulation	P	3325	106	157.91	0.67	6.39E-06	2.43E-04
7	1635	F2-3 vs. S2-3	F2-3 up	-	regulation of nitrogen compound metabolic process	GO:0051171	Regulation	Regulation	P	2741	74	130.17	0.57	6.14E-08	3.91E-06
7	1636	F2-3 vs. S2-3	F2-3 up	-	regulation of nucleobase-containing compound metabolic process	GO:0019219	Regulation	Regulation	P	2344	59	111.32	0.53	4.80E-08	3.19E-06
7	1637	F2-3 vs. S2-3	F2-3 up	-	regulation of primary metabolic process	GO:0080090	Regulation	Regulation	P	2825	82	134.16	0.61	9.19E-07	4.26E-05
7	1638	F2-3 vs. S2-3	F2-3 up	+	negative regulation of cytoskeleton organization	GO:0051494	Regulation	Regulation	P	17	5	0.81	6.19	2.62E-03	4.47E-02
7	1639	F2-3 vs. S2-3	F2-3 up	+	negative regulation of supramolecular fiber organization	GO:1902904	Regulation	Regulation	P	17	5	0.81	6.19	2.62E-03	4.48E-02
7	1640	F2-3 vs. S2-3	F2-3 up	+	regulation of biological quality	GO:0065008	Regulation	Regulation	P	972	69	46.16	1.49	1.78E-03	3.20E-02
7	1641	F2-3 vs. S2-3	F2-3 up	+	regulation of cell morphogenesis involved in differentiation	GO:0010769	Regulation	Regulation	P	40	10	1.9	5.26	6.94E-05	1.99E-03
7	1642	F2-3 vs. S2-3	F2-3 up	+	regulation of pollen tube growth	GO:0080092	Regulation	Regulation	P	35	10	1.66	6.02	2.65E-05	8.56E-04
7	1643	F2-3 vs. S2-3	F2-3 up	+	regulation of unidimensional cell growth	GO:0051510	Regulation	Regulation	P	74	12	3.51	3.41	4.93E-04	1.11E-02
7	1644	F2-3 vs. S2-3	F2-3 up	+	acid phosphatase activity	GO:0003993	Signaling and response	Signaling	F	45	10	2.14	4.68	1.61E-04	5.88E-03
7	1645	F2-3 vs. S2-3	F2-3 up	+	cellular response to external stimulus	GO:0071496	Signaling and response	Signaling	P	219	23	10.4	2.21	8.58E-04	1.75E-02
7	1646	F2-3 vs. S2-3	F2-3 up	+	cellular response to extracellular stimulus	GO:0031668	Signaling and response	Signaling	P	213	23	10.12	2.27	6.99E-04	1.49E-02
7	1647	F2-3 vs. S2-3	F2-3 up	+	jasmonic acid biosynthetic process	GO:0009695	Signaling and response	Signaling	P	26	7	1.23	5.67	5.87E-04	1.28E-02
7	1648	F2-3 vs. S2-3	F2-3 up	+	multicellular organismal process	GO:0032501	Signaling and response	Signaling	P	2741	180	130.17	1.38	1.86E-05	6.59E-04
7	1649	F2-3 vs. S2-3	F2-3 up	+	multi-organism process	GO:0051704	Signaling and response	Signaling	P	527	45	25.03	1.8	3.43E-04	8.04E-03
7	1650	F2-3 vs. S2-3	F2-3 up	+	phosphatase activity	GO:0016791	Signaling and response	Signaling	F	328	34	15.58	2.18	6.21E-05	2.77E-03
7	1651	F2-3 vs. S2-3	F2-3 up	+	response to abiotic stimulus	GO:0009628	Signaling and response	Signaling	P	2131	213	101.2	2.1	3.37E-23	1.44E-20
7	1652	F2-3 vs. S2-3	F2-3 up	+	response to acid chemical	GO:0001101	Signaling and response	Signaling	P	397	43	18.85	2.28	2.46E-06	1.06E-04
7	1653	F2-3 vs. S2-3	F2-3 up	+	response to bacterium	GO:0009617	Signaling and response	Signaling	P	508	60	24.13	2.49	1.32E-09	1.18E-07
7	1654	F2-3 vs. S2-3	F2-3 up	+	response to biotic stimulus	GO:0009607	Signaling and response	Signaling	P	1098	114	52.14	2.19	1.35E-13	2.60E-11
7	1655	F2-3 vs. S2-3	F2-3 up	+	response to cadmium ion	GO:0046686	Signaling and response	Signaling	P	315	61	14.96	4.08	4.16E-18	1.18E-15
7	1656	F2-3 vs. S2-3	F2-3 up	+	response to carbohydrate	GO:0009743	Signaling and response	Signaling	P	153	17	7.27	2.34	2.29E-03	3.98E-02
7	1657	F2-3 vs. S2-3	F2-3 up	+	response to chemical	GO:0042221	Signaling and response	Signaling	P	2681	254	127.32	1.99	8.93E-25	5.34E-22
7	1658	F2-3 vs. S2-3	F2-3 up	+	response to disaccharide	GO:0034285	Signaling and response	Signaling	P	72	11	3.42	3.22	1.29E-03	2.44E-02
7	1659	F2-3 vs. S2-3	F2-3 up	+	response to endogenous stimulus	GO:0009719	Signaling and response	Signaling	P	1271	101	60.36	1.67	1.98E-06	8.82E-05
7	1660	F2-3 vs. S2-3	F2-3 up	+	response to external biotic stimulus	GO:0043207	Signaling and response	Signaling	P	1097	113	52.1	2.17	3.61E-13	6.16E-11
7	1661	F2-3 vs. S2-3	F2-3 up	+	response to external stimulus	GO:0009605	Signaling and response	Signaling	P	1513	151	71.85	2.1	2.18E-16	5.20E-14
7	1662	F2-3 vs. S2-3	F2-3 up	+	response to extracellular stimulus	GO:0009991	Signaling and response	Signaling	P	262	30	12.44	2.41	4.65E-05	1.40E-03
7	1663	F2-3 vs. S2-3	F2-3 up	+	response to far red light	GO:0010218	Signaling and response	Signaling	P	43	9	2.04	4.41	4.98E-04	1.11E-02
7	1664	F2-3 vs. S2-3	F2-3 up	+	response to hormone	GO:0009725	Signaling and response	Signaling	P	1241	101	58.94	1.71	5.56E-07	2.68E-05
7	1665	F2-3 vs. S2-3	F2-3 up	+	response to inorganic substance	GO:0010035	Signaling and response	Signaling	P	914	120	43.41	2.76	2.90E-21	1.16E-18
7	1666	F2-3 vs. S2-3	F2-3 up	+	response to jasmonic acid	GO:0009753	Signaling and response	Signaling	P	175	25	8.31	3.01	4.58E-06	1.87E-04
7	1667	F2-3 vs. S2-3	F2-3 up	+	response to karrikin	GO:0080167	Signaling and response	Signaling	P	128	26	6.08	4.28	6.83E-09	5.67E-07
7	1668	F2-3 vs. S2-3	F2-3 up	+	response to light stimulus	GO:0009416	Signaling and response	Signaling	P	713	77	33.86	2.27	3.15E-10	3.03E-08
7	1669	F2-3 vs. S2-3	F2-3 up	+	response to lipid	GO:0033993	Signaling and response	Signaling	P	672	59	31.91	1.85	1.94E-05	6.68E-04
7	1670	F2-3 vs. S2-3	F2-3 up	+	response to metal ion	GO:0010038	Signaling and response	Signaling	P	433	71	20.56	3.45	2.04E-17	5.55E-15
7	1671	F2-3 vs. S2-3	F2-3 up	+	response to nutrient levels	GO:0031667	Signaling and response	Signaling	P	222	25	10.54	2.37	1.70E-04	4.31E-03
7	1672	F2-3 vs. S2-3	F2-3 up	+	response to organic substance	GO:0010033	Signaling and response	Signaling	P	1705	143	80.97	1.77	2.99E-10	2.93E-08
7	1673	F2-3 vs. S2-3	F2-3 up	+	response to oxygen-containing compound	GO:1901700	Signaling and response	Signaling	P	1514	152	71.9	2.11	1.25E-16	3.11E-14
7	1674	F2-3 vs. S2-3	F2-3 up	+	response to stimulus	GO:0050896	Signaling and response	Signaling	P	5578	445	264.9	1.68	1.24E-29	1.86E-26
7	1675	F2-3 vs. S2-3	F2-3 up	+	response to sucrose	GO:0009744	Signaling and response	Signaling	P	70	11	3.32	3.31	1.05E-03	2.07E-02
7	1676	F2-3 vs. S2-3	F2-3 up	+	response to temperature stimulus	GO:0009266	Signaling and response	Signaling	P	617	77	29.3	2.63	7.04E-13	1.05E-10
7	1677	F2-3 vs. S2-3	F2-3 up	+	response to water	GO:0009415	Signaling and response	Signaling	P	365	43	17.33	2.48	3.98E-07	2.01E-05
7	1678	F2-3 vs. S2-3	F2-3 up	-	cellular response to DNA damage stimulus	GO:0006974	Signaling and response	Stress	P	350	4	16.62	0.24	6.83E-04	1.46E-02
7	1679	F2-3 vs. S2-3	F2-3 up	+	aging	GO:0007568	Signaling and response	Stress	P	132	23	6.27	3.67	5.54E-07	2.72E-05
7	1680	F2-3 vs. S2-3	F2-3 up	+	anion homeostasis	GO:0055081	Signaling and response	Stress	P	43	10	2.04	4.9	1.16E-04	3.05E-03
7	1681	F2-3 vs. S2-3	F2-3 up	+	cellular homeostasis	GO:0019725	Signaling and response	Stress	P	275	26	13.06	1.99	1.84E-03	3.28E-02
7	1682	F2-3 vs. S2-3	F2-3 up	+	cellular monovalent inorganic cation	GO:0030004	Signaling and response	Stress	P	39	8	1.85	4.32	1.13E-03	2.18E-02
7	1683	F2-3 vs. S2-3	F2-3 up	+	cellular response to phosphate starvation	GO:0016036	Signaling and response	Stress	P	75	12	3.56	3.37	5.48E-04	1.21E-02
7	1684	F2-3 vs. S2-3	F2-3 up	+	chemical homeostasis	GO:0048878	Signaling and response	Stress	P	415	42	19.71	2.13	1.91E-05	6.71E-04
7	1685	F2-3 vs. S2-3	F2-3 up	+	defense response	GO:0006952	Signaling and response	Stress	P	953	87	45.26	1.92	4.79E-08	3.22E-06
7	1686	F2-3 vs. S2-3	F2-3 up	+	defense response to bacterium	GO:0042742	Signaling and response	Stress	P	414	51	19.66	2.59	6.60E-09	5.56E-07
7	1687	F2-3 vs. S2-3	F2-3 up	+	defense response to fungus	GO:0050832	Signaling and response	Stress	P	252	26	11.97	2.17	6.58E-04	1.41E-02
7	1688	F2-3 vs. S2-3	F2-3 up	+	defense response to other organism	GO:0098542	Signaling and response	Stress	P	807	76	38.32	1.98	9.82E-08	6.05E-06
7	1689	F2-3 vs. S2-3	F2-3 up	+	flavonoid biosynthetic process	GO:0009813	Signaling and response	Stress	P	54	11	2.56	4.29	1.50E-04	3.88E-03
7	1690	F2-3 vs. S2-3	F2-3 up	+	flavonoid metabolic process	GO:0009812	Signaling and response	Stress	P	63	11	2.99	3.68	4.83E-04	1.09E-02
7	1691	F2-3 vs. S2-3	F2-3 up	+	glutathione metabolic process	GO:0006749	Signaling and response	Stress	P	44	8	2.09	3.83	2.22E-03	3.88E-02

7	1692	F2-3 vs. S2-3	F2-3 up	+	homeostatic process	GO:0042592	Signaling and response	Stress	P	537	51	25.5	2	1.23E-05	4.53E-04
7	1693	F2-3 vs. S2-3	F2-3 up	+	hydrogen peroxide biosynthetic process	GO:0050665	Signaling and response	Stress	P	8	4	0.38	10.53	1.55E-03	2.86E-02
7	1694	F2-3 vs. S2-3	F2-3 up	+	hydrogen peroxide metabolic process	GO:0042743	Signaling and response	Stress	P	36	8	1.71	4.68	7.21E-04	1.52E-02
7	1695	F2-3 vs. S2-3	F2-3 up	+	inorganic ion homeostasis	GO:0098771	Signaling and response	Stress	P	250	24	11.87	2.02	1.96E-03	3.47E-02
7	1696	F2-3 vs. S2-3	F2-3 up	+	interspecies interaction between organisms	GO:0044419	Signaling and response	Stress	P	1125	115	53.43	2.15	2.66E-13	4.82E-11
7	1697	F2-3 vs. S2-3	F2-3 up	+	ion homeostasis	GO:0050801	Signaling and response	Stress	P	290	30	13.77	2.18	1.94E-04	4.76E-03
7	1698	F2-3 vs. S2-3	F2-3 up	+	monovalent inorganic cation homeostasis	GO:0055067	Signaling and response	Stress	P	79	11	3.75	2.93	2.49E-03	4.32E-02
7	1699	F2-3 vs. S2-3	F2-3 up	+	reactive oxygen species metabolic process	GO:0072593	Signaling and response	Stress	P	69	14	3.28	4.27	2.04E-05	6.94E-04
7	1700	F2-3 vs. S2-3	F2-3 up	+	regulation of cellular pH	GO:0030641	Signaling and response	Stress	P	28	8	1.33	6.02	1.70E-04	4.34E-03
7	1701	F2-3 vs. S2-3	F2-3 up	+	regulation of intracellular pH	GO:0051453	Signaling and response	Stress	P	28	8	1.33	6.02	1.70E-04	4.32E-03
7	1702	F2-3 vs. S2-3	F2-3 up	+	regulation of pH	GO:0006885	Signaling and response	Stress	P	57	10	2.71	3.69	8.34E-04	1.73E-02
7	1703	F2-3 vs. S2-3	F2-3 up	+	regulation of stomatal movement	GO:0010119	Signaling and response	Stress	P	95	16	4.51	3.55	4.09E-05	1.26E-03
7	1704	F2-3 vs. S2-3	F2-3 up	+	response to abscisic acid	GO:0009737	Signaling and response	Stress	P	497	49	23.6	2.08	5.92E-06	2.28E-04
7	1705	F2-3 vs. S2-3	F2-3 up	+	response to alcohol	GO:0097305	Signaling and response	Stress	P	501	49	23.79	2.06	9.93E-06	3.71E-04
7	1706	F2-3 vs. S2-3	F2-3 up	+	response to cold	GO:0009409	Signaling and response	Stress	P	415	64	19.71	3.25	1.03E-14	2.20E-12
7	1707	F2-3 vs. S2-3	F2-3 up	+	response to desiccation	GO:0009269	Signaling and response	Stress	P	24	7	1.14	6.14	3.91E-04	9.06E-03
7	1708	F2-3 vs. S2-3	F2-3 up	+	response to fungus	GO:0009620	Signaling and response	Stress	P	350	36	16.62	2.17	5.70E-05	1.68E-03
7	1709	F2-3 vs. S2-3	F2-3 up	+	response to high light intensity	GO:0009644	Signaling and response	Stress	P	60	13	2.85	4.56	2.20E-05	7.26E-04
7	1710	F2-3 vs. S2-3	F2-3 up	+	response to nematode	GO:0009624	Signaling and response	Stress	P	82	15	3.89	3.85	3.05E-05	9.64E-04
7	1711	F2-3 vs. S2-3	F2-3 up	+	response to osmotic stress	GO:0006970	Signaling and response	Stress	P	528	45	25.07	1.79	3.50E-04	8.18E-03
7	1712	F2-3 vs. S2-3	F2-3 up	+	response to other organism	GO:0051707	Signaling and response	Stress	P	1097	113	52.1	2.17	3.61E-13	5.99E-11
7	1713	F2-3 vs. S2-3	F2-3 up	+	response to oxidative stress	GO:0006979	Signaling and response	Stress	P	460	44	21.85	2.01	3.37E-05	1.05E-03
7	1714	F2-3 vs. S2-3	F2-3 up	+	response to radiation	GO:0009314	Signaling and response	Stress	P	738	77	35.05	2.2	1.21E-09	1.09E-07
7	1715	F2-3 vs. S2-3	F2-3 up	+	response to reactive oxygen species	GO:0000302	Signaling and response	Stress	P	160	21	7.6	2.76	7.79E-05	2.16E-03
7	1716	F2-3 vs. S2-3	F2-3 up	+	response to salt stress	GO:0009651	Signaling and response	Stress	P	447	38	21.23	1.79	1.25E-03	2.37E-02
7	1717	F2-3 vs. S2-3	F2-3 up	+	response to starvation	GO:0042594	Signaling and response	Stress	P	195	21	9.26	2.27	1.36E-03	2.56E-02
7	1718	F2-3 vs. S2-3	F2-3 up	+	response to stress	GO:0006950	Signaling and response	Stress	P	3106	268	147.51	1.82	8.59E-21	3.21E-18
7	1719	F2-3 vs. S2-3	F2-3 up	+	response to water deprivation	GO:0009414	Signaling and response	Stress	P	356	42	16.91	2.48	5.52E-07	2.73E-05
7	1720	F2-3 vs. S2-3	F2-3 up	+	response to wounding	GO:0009611	Signaling and response	Stress	P	227	38	10.78	3.52	3.52E-10	3.35E-08
7	1721	F2-3 vs. S2-3	F2-3 up	+	vitamin biosynthetic process	GO:0009110	Signaling and response	Stress	P	59	10	2.8	3.57	1.05E-03	2.07E-02
7	1722	F2-3 vs. S2-3	F2-3 up	+	vitamin metabolic process	GO:0006766	Signaling and response	Stress	P	71	13	3.37	3.86	1.01E-04	2.73E-03
7	1723	F2-3 vs. S2-3	F2-3 up	+	water-soluble vitamin biosynthetic process	GO:0042364	Signaling and response	Stress	P	49	9	2.33	3.87	1.13E-03	2.20E-02
7	1724	F2-3 vs. S2-3	F2-3 up	+	water-soluble vitamin metabolic process	GO:0006767	Signaling and response	Stress	P	61	12	2.9	4.14	1.02E-04	2.74E-03
7	1725	F2-3 vs. S2-3	F2-3 up	-	DNA-binding transcription factor activity	GO:0003700	Regulation	Transcription	F	1650	43	78.36	0.55	1.74E-05	8.61E-04
7	1726	F2-3 vs. S2-3	F2-3 up	-	gene expression	GO:0010467	Regulation	Transcription	P	1608	44	76.36	0.58	7.11E-05	1.99E-03
7	1727	F2-3 vs. S2-3	F2-3 up	-	macromolecule modification	GO:0043412	Regulation	Transcription	P	2832	100	134.49	0.74	1.67E-03	3.02E-02
7	1728	F2-3 vs. S2-3	F2-3 up	-	regulation of gene expression	GO:0010468	Regulation	Transcription	P	2679	69	127.23	0.54	1.08E-08	8.71E-07
7	1729	F2-3 vs. S2-3	F2-3 up	-	regulation of nucleic acid-templated transcription	GO:1903506	Regulation	Transcription	P	2184	52	103.72	0.5	1.69E-08	1.28E-06
7	1730	F2-3 vs. S2-3	F2-3 up	-	regulation of RNA biosynthetic process	GO:2001141	Regulation	Transcription	P	2186	52	103.81	0.5	1.70E-08	1.27E-06
7	1731	F2-3 vs. S2-3	F2-3 up	-	regulation of transcription, DNA-templated	GO:0006355	Regulation	Transcription	P	2184	52	103.72	0.5	1.69E-08	1.30E-06
7	1732	F2-3 vs. S2-3	F2-3 up	-	RNA modification	GO:0009451	Regulation	Transcription	P	283	3	13.44	0.22	1.50E-03	2.79E-02
7	1733	F2-3 vs. S2-3	F2-3 up	-	sequence-specific DNA binding	GO:0043565	Regulation	Transcription	F	1029	27	48.87	0.55	8.87E-04	2.55E-02
7	1734	F2-3 vs. S2-3	F2-3 up	-	transcription regulator activity	GO:0140110	Regulation	Transcription	F	1818	43	86.34	0.5	2.58E-07	2.03E-05
7	1735	F2-3 vs. S2-3	F2-3 up	+	RNA binding	GO:0003723	Regulation	Transcription	F	1569	106	74.51	1.42	5.38E-04	1.66E-02
7	1736	F2-3 vs. S2-3	F2-3 up	-	large ribosomal subunit	GO:0015934	Regulation	Translation	C	180	0	8.55	<	4.36E-04	6.67E-03
7	1737	F2-3 vs. S2-3	F2-3 up	-	regulation of RNA metabolic process	GO:0051252	Regulation	Translation	P	2285	59	108.52	0.54	1.93E-07	1.05E-05
7	1738	F2-3 vs. S2-3	F2-3 up	-	ribonucleoprotein complex	GO:1990904	Regulation	Translation	C	677	8	32.15	0.25	9.54E-07	2.68E-05
7	1739	F2-3 vs. S2-3	F2-3 up	-	ribosomal subunit	GO:0044391	Regulation	Translation	C	316	3	15.01	0.2	5.71E-04	8.61E-03
7	1740	F2-3 vs. S2-3	F2-3 up	-	RNA processing	GO:0006396	Regulation	Translation	P	787	19	37.38	0.51	1.43E-03	2.68E-02
7	1741	F2-3 vs. S2-3	F2-3 up	-	structural constituent of ribosome	GO:0003735	Regulation	Translation	F	325	3	15.43	0.19	4.08E-04	1.28E-02
7	1742	F2-3 vs. S2-3	F2-3 up	+	mRNA binding	GO:0003729	Regulation	Translation	F	1005	88	47.73	1.84	2.34E-07	1.93E-05
7	1743	F2-3 vs. S2-3	F2-3 up	+	ribonucleotide binding	GO:0032553	Regulation	Translation	F	826	61	39.23	1.56	1.31E-03	3.58E-02
7	1744	F2-3 vs. S2-3	F2-3 up	+	active ion transmembrane transporter activity	GO:0022853	Metabolism	Transporters	F	215	37	10.21	3.62	3.04E-10	4.66E-08
7	1745	F2-3 vs. S2-3	F2-3 up	+	active transmembrane transporter activity	GO:0022804	Metabolism	Transporters	F	482	66	22.89	2.88	5.54E-13	1.62E-10
7	1746	F2-3 vs. S2-3	F2-3 up	+	amino acid transmembrane transport	GO:0003333	Metabolism	Transporters	P	27	7	1.28	5.46	7.10E-04	1.50E-02
7	1747	F2-3 vs. S2-3	F2-3 up	+	amino acid transmembrane transporter activity	GO:0015171	Metabolism	Transporters	F	78	13	3.7	3.51	2.30E-04	7.95E-03
7	1748	F2-3 vs. S2-3	F2-3 up	+	amino acid transport	GO:0006865	Metabolism	Transporters	P	48	11	2.28	4.83	6.01E-05	1.76E-03
7	1749	F2-3 vs. S2-3	F2-3 up	+	anion transmembrane transport	GO:0098656	Metabolism	Transporters	P	107	16	5.08	3.15	1.43E-04	3.71E-03
7	1750	F2-3 vs. S2-3	F2-3 up	+	anion transmembrane transporter activity	GO:0008509	Metabolism	Transporters	F	336	39	15.96	2.44	1.89E-06	1.17E-04
7	1751	F2-3 vs. S2-3	F2-3 up	+	anion transport	GO:0006820	Metabolism	Transporters	P	283	32	13.44	2.38	2.61E-05	8.48E-04
7	1752	F2-3 vs. S2-3	F2-3 up	+	antiporter activity	GO:0015297	Metabolism	Transporters	F	197	23	9.36	2.46	2.15E-04	7.58E-03

7	1753	F2-3 vs. S2-3	F2-3 up	+	ATPase-coupled cation transmembrane transporter activity	GO:0019829	Metabolism	Transporters	F	61	18	2.9	6.21	1.16E-08	1.49E-06
7	1754	F2-3 vs. S2-3	F2-3 up	+	ATPase-coupled ion transmembrane transporter activity	GO:0042625	Metabolism	Transporters	F	76	19	3.61	5.26	4.41E-08	4.72E-06
7	1755	F2-3 vs. S2-3	F2-3 up	+	ATPase-coupled transmembrane transporter activity	GO:0042626	Metabolism	Transporters	F	190	31	9.02	3.44	2.45E-08	2.92E-06
7	1756	F2-3 vs. S2-3	F2-3 up	+	carbohydrate transmembrane transporter	GO:0015144	Metabolism	Transporters	F	95	18	4.51	3.99	3.29E-06	1.86E-04
7	1757	F2-3 vs. S2-3	F2-3 up	+	carbohydrate transport	GO:0008643	Metabolism	Transporters	P	60	13	2.85	4.56	2.20E-05	7.30E-04
7	1758	F2-3 vs. S2-3	F2-3 up	+	carboxylic acid transmembrane transport	GO:1905039	Metabolism	Transporters	P	55	10	2.61	3.83	6.54E-04	1.41E-02
7	1759	F2-3 vs. S2-3	F2-3 up	+	carboxylic acid transmembrane transporter	GO:0046943	Metabolism	Transporters	F	122	18	5.79	3.11	6.56E-05	2.89E-03
7	1760	F2-3 vs. S2-3	F2-3 up	+	carboxylic acid transport	GO:0046942	Metabolism	Transporters	P	114	15	5.41	2.77	7.65E-04	1.59E-02
7	1761	F2-3 vs. S2-3	F2-3 up	+	cation transmembrane transport	GO:0098655	Metabolism	Transporters	P	229	33	10.88	3.03	1.22E-07	7.24E-06
7	1762	F2-3 vs. S2-3	F2-3 up	+	cation transmembrane transporter activity	GO:0008324	Metabolism	Transporters	F	414	49	19.66	2.49	4.76E-08	4.93E-06
7	1763	F2-3 vs. S2-3	F2-3 up	+	cation transport	GO:0006812	Metabolism	Transporters	P	412	47	19.57	2.4	3.20E-07	1.70E-05
7	1764	F2-3 vs. S2-3	F2-3 up	+	cytoplasmic vesicle	GO:0031410	Metabolism	Transporters	C	720	63	34.19	1.84	1.11E-05	2.15E-04
7	1765	F2-3 vs. S2-3	F2-3 up	+	endosome	GO:0005768	Metabolism	Transporters	C	411	35	19.52	1.79	1.76E-03	2.38E-02
7	1766	F2-3 vs. S2-3	F2-3 up	+	export across plasma membrane	GO:0140115	Metabolism	Transporters	P	15	7	0.71	9.83	3.62E-05	1.12E-03
7	1767	F2-3 vs. S2-3	F2-3 up	+	export from cell	GO:0140352	Metabolism	Transporters	P	98	14	4.65	3.01	5.48E-04	1.21E-02
7	1768	F2-3 vs. S2-3	F2-3 up	+	inorganic cation transmembrane transport	GO:0098662	Metabolism	Transporters	P	223	32	10.59	3.02	2.06E-07	1.11E-05
7	1769	F2-3 vs. S2-3	F2-3 up	+	inorganic cation transmembrane transporter activity	GO:0022890	Metabolism	Transporters	F	382	44	18.14	2.43	4.20E-07	3.21E-05
7	1770	F2-3 vs. S2-3	F2-3 up	+	inorganic ion transmembrane transport	GO:0098660	Metabolism	Transporters	P	244	36	11.59	3.11	1.94E-08	1.42E-06
7	1771	F2-3 vs. S2-3	F2-3 up	+	inorganic molecular entity transmembrane transporter activity	GO:0015318	Metabolism	Transporters	F	664	77	31.53	2.44	1.85E-11	3.97E-09
7	1772	F2-3 vs. S2-3	F2-3 up	+	ion transmembrane transport	GO:0034220	Metabolism	Transporters	P	330	48	15.67	3.06	1.35E-10	1.55E-08
7	1773	F2-3 vs. S2-3	F2-3 up	+	ion transmembrane transporter activity	GO:0015075	Metabolism	Transporters	F	718	80	34.1	2.35	3.41E-11	6.46E-09
7	1774	F2-3 vs. S2-3	F2-3 up	+	ion transmembrane transporter activity, phosphorylative mechanism	GO:0015662	Metabolism	Transporters	F	50	11	2.37	4.63	8.27E-05	3.41E-03
7	1775	F2-3 vs. S2-3	F2-3 up	+	ion transport	GO:0006811	Metabolism	Transporters	P	673	75	31.96	2.35	1.65E-10	1.79E-08
7	1776	F2-3 vs. S2-3	F2-3 up	+	monovalent inorganic cation transmembrane transporter activity	GO:0015077	Metabolism	Transporters	F	220	32	10.45	3.06	1.57E-07	1.40E-05
7	1777	F2-3 vs. S2-3	F2-3 up	+	monovalent inorganic cation transport	GO:0015672	Metabolism	Transporters	P	170	29	8.07	3.59	2.92E-08	2.11E-06
7	1778	F2-3 vs. S2-3	F2-3 up	+	organic acid transmembrane transport	GO:1903825	Metabolism	Transporters	P	57	10	2.71	3.69	8.34E-04	1.72E-02
7	1779	F2-3 vs. S2-3	F2-3 up	+	organic acid transmembrane transporter activity	GO:0005342	Metabolism	Transporters	F	122	18	5.79	3.11	6.56E-05	2.85E-03
7	1780	F2-3 vs. S2-3	F2-3 up	+	organic acid transport	GO:0015849	Metabolism	Transporters	P	116	15	5.51	2.72	8.98E-04	1.82E-02
7	1781	F2-3 vs. S2-3	F2-3 up	+	organic anion transmembrane transporter	GO:0008514	Metabolism	Transporters	F	200	28	9.5	2.95	1.79E-06	1.15E-04
7	1782	F2-3 vs. S2-3	F2-3 up	+	organic anion transport	GO:0015711	Metabolism	Transporters	P	164	21	7.79	2.7	1.07E-04	2.85E-03
7	1783	F2-3 vs. S2-3	F2-3 up	+	plasmodesma	GO:0009506	Metabolism	Transporters	C	944	78	44.83	1.74	7.60E-06	1.72E-04
7	1784	F2-3 vs. S2-3	F2-3 up	+	primary active transmembrane transporter	GO:0015399	Metabolism	Transporters	F	213	32	10.12	3.16	8.11E-08	7.90E-06
7	1785	F2-3 vs. S2-3	F2-3 up	+	proton transmembrane transporter activity	GO:0015078	Metabolism	Transporters	F	166	29	7.88	3.68	1.84E-08	2.28E-06
7	1786	F2-3 vs. S2-3	F2-3 up	+	secondary active transmembrane transporter activity	GO:0015291	Metabolism	Transporters	F	266	33	12.63	2.61	3.37E-06	1.87E-04
7	1787	F2-3 vs. S2-3	F2-3 up	+	secretory vesicle	GO:0099503	Metabolism	Transporters	C	170	24	8.07	2.97	8.42E-06	1.69E-04
7	1788	F2-3 vs. S2-3	F2-3 up	+	sugar transmembrane transporter activity	GO:0051119	Metabolism	Transporters	F	39	10	1.85	5.4	5.78E-05	2.62E-03
7	1789	F2-3 vs. S2-3	F2-3 up	+	trans-Golgi network	GO:0005802	Metabolism	Transporters	C	302	35	14.34	2.44	7.51E-06	1.74E-04
7	1790	F2-3 vs. S2-3	F2-3 up	+	transmembrane transport	GO:0055085	Metabolism	Transporters	P	859	96	40.79	2.35	2.93E-13	5.16E-11
7	1791	F2-3 vs. S2-3	F2-3 up	+	transmembrane transporter activity	GO:0022857	Metabolism	Transporters	F	1242	139	58.98	2.36	6.09E-19	4.89E-16
7	1792	F2-3 vs. S2-3	F2-3 up	+	transport	GO:0006810	Metabolism	Transporters	P	2261	177	107.38	1.65	2.93E-10	2.92E-08
7	1793	F2-3 vs. S2-3	F2-3 up	+	transporter activity	GO:0005215	Metabolism	Transporters	F	1287	141	61.12	2.31	2.07E-18	1.33E-15
7	1794	F2-3 vs. S2-3	F2-3 up	+	vesicle	GO:0031982	Metabolism	Transporters	C	817	70	38.8	1.8	7.79E-06	1.59E-04
7	1795	F2-3 vs. S2-3	S2-3 up	+	cytochrome complex	GO:0070069	Metabolism	Bioenergetics	C	30	6	1.23	4.87	2.61E-03	2.62E-02
7	1796	F2-3 vs. S2-3	S2-3 up	+	inner mitochondrial membrane protein complex	GO:0098800	Metabolism	Bioenergetics	C	177	20	7.26	2.75	1.10E-04	1.69E-03
7	1797	F2-3 vs. S2-3	S2-3 up	+	mitochondrial electron transport, ubiquinol to cytochrome c	GO:0006122	Metabolism	Bioenergetics	P	14	5	0.57	8.7	6.91E-04	3.30E-02
7	1798	F2-3 vs. S2-3	S2-3 up	+	mitochondrial envelope	GO:0005740	Metabolism	Bioenergetics	C	317	27	13.01	2.08	6.90E-04	8.16E-03
7	1799	F2-3 vs. S2-3	S2-3 up	+	mitochondrial inner membrane	GO:0005743	Metabolism	Bioenergetics	C	245	21	10.05	2.09	2.37E-03	2.41E-02
7	1800	F2-3 vs. S2-3	S2-3 up	+	mitochondrial matrix	GO:0005759	Metabolism	Bioenergetics	C	135	16	5.54	2.89	3.20E-04	4.32E-03
7	1801	F2-3 vs. S2-3	S2-3 up	+	mitochondrial membrane	GO:0031966	Metabolism	Bioenergetics	C	297	26	12.19	2.13	7.23E-04	8.45E-03
7	1802	F2-3 vs. S2-3	S2-3 up	+	mitochondrial protein complex	GO:0098798	Metabolism	Bioenergetics	C	259	33	10.63	3.11	6.38E-08	1.90E-06
7	1803	F2-3 vs. S2-3	S2-3 up	+	mitochondrial respirasome	GO:0005746	Metabolism	Bioenergetics	C	111	16	4.55	3.51	4.11E-05	7.01E-04
7	1804	F2-3 vs. S2-3	S2-3 up	+	mitochondrial respiratory chain complex I	GO:0005747	Metabolism	Bioenergetics	C	70	9	2.87	3.13	3.86E-03	3.56E-02
7	1805	F2-3 vs. S2-3	S2-3 up	+	mitochondrial respiratory chain complex III	GO:0005750	Metabolism	Bioenergetics	C	19	6	0.78	7.7	3.44E-04	4.53E-03

7	1806	F2-3 vs. S2-3	S2-3 up	+	NADH dehydrogenase complex	GO:0030964	Metabolism	Bioenergetics	C	77	10	3.16	3.16	2.23E-03	2.34E-02
7	1807	F2-3 vs. S2-3	S2-3 up	+	oxidoreductase complex	GO:1990204	Metabolism	Bioenergetics	C	161	19	6.61	2.88	9.67E-05	1.50E-03
7	1808	F2-3 vs. S2-3	S2-3 up	+	primary metabolic process	GO:0044238	Metabolism	Bioenergetics	P	7219	429	296.23	1.45	2.63E-17	4.92E-15
7	1809	F2-3 vs. S2-3	S2-3 up	+	respirasome	GO:0070469	Metabolism	Bioenergetics	C	116	16	4.76	3.36	6.59E-05	1.07E-03
7	1810	F2-3 vs. S2-3	S2-3 up	+	respiratory chain complex	GO:0098803	Metabolism	Bioenergetics	C	109	16	4.47	3.58	3.37E-05	5.85E-04
7	1811	F2-3 vs. S2-3	S2-3 up	+	respiratory chain complex I	GO:0045271	Metabolism	Bioenergetics	C	73	9	3	3	4.95E-03	4.37E-02
7	1812	F2-3 vs. S2-3	S2-3 up	+	respiratory chain complex III	GO:0045275	Metabolism	Bioenergetics	C	19	6	0.78	7.7	3.44E-04	4.42E-03
7	1813	F2-3 vs. S2-3	S2-3 up	+	protein-containing complex	GO:0032991	Metabolism	Catabolism	C	2958	315	121.38	2.6	2.92E-54	5.07E-52
7	1814	F2-3 vs. S2-3	S2-3 up	+	cell cycle	GO:0007049	Development	Cell division	P	458	49	18.79	2.61	9.87E-09	1.09E-06
7	1815	F2-3 vs. S2-3	S2-3 up	+	cell cycle process	GO:0022402	Development	Cell division	P	421	49	17.28	2.84	7.10E-10	9.23E-08
7	1816	F2-3 vs. S2-3	S2-3 up	+	cell division	GO:0051301	Development	Cell division	P	211	22	8.66	2.54	2.31E-04	1.29E-02
7	1817	F2-3 vs. S2-3	S2-3 up	+	chromosome	GO:0005694	Development	Cell division	C	545	40	22.36	1.79	7.84E-04	9.07E-03
7	1818	F2-3 vs. S2-3	S2-3 up	+	cyclin-dependent protein kinase holoenzyme complex	GO:0000307	Development	Cell division	C	62	9	2.54	3.54	1.85E-03	1.98E-02
7	1819	F2-3 vs. S2-3	S2-3 up	+	cytokinesis	GO:0000910	Development	Cell division	P	102	15	4.19	3.58	5.77E-05	3.56E-03
7	1820	F2-3 vs. S2-3	S2-3 up	+	cytokinesis by cell plate formation	GO:0000911	Development	Cell division	P	68	14	2.79	5.02	3.63E-06	2.78E-04
7	1821	F2-3 vs. S2-3	S2-3 up	+	G1/S transition of mitotic cell cycle	GO:0000082	Development	Cell division	P	8	4	0.33	12.18	9.22E-04	4.18E-02
7	1822	F2-3 vs. S2-3	S2-3 up	+	kinetochore	GO:0000776	Development	Cell division	C	24	5	0.98	5.08	5.09E-03	4.45E-02
7	1823	F2-3 vs. S2-3	S2-3 up	+	mitotic cell cycle	GO:0000278	Development	Cell division	P	244	26	10.01	2.6	2.79E-05	1.77E-03
7	1824	F2-3 vs. S2-3	S2-3 up	+	mitotic cell cycle process	GO:1903047	Development	Cell division	P	203	25	8.33	3	4.22E-06	3.19E-04
7	1825	F2-3 vs. S2-3	S2-3 up	+	mitotic spindle	GO:0072686	Development	Cell division	C	20	6	0.82	7.31	4.32E-04	5.49E-03
7	1826	F2-3 vs. S2-3	S2-3 up	+	nuclear division	GO:0000280	Development	Cell division	P	163	19	6.69	2.84	1.12E-04	6.50E-03
7	1827	F2-3 vs. S2-3	S2-3 up	+	nucleic acid metabolic process	GO:0090304	Development	Cell division	P	1705	131	69.96	1.87	3.15E-11	4.28E-09
7	1828	F2-3 vs. S2-3	S2-3 up	+	nucleobase-containing compound metabolic process	GO:0006139	Development	Cell division	P	2093	146	85.89	1.7	1.49E-09	1.82E-07
7	1829	F2-3 vs. S2-3	S2-3 up	+	preprophase band	GO:0009574	Development	Cell division	C	19	6	0.78	7.7	3.44E-04	4.59E-03
7	1830	F2-3 vs. S2-3	S2-3 up	+	regulation of cell cycle	GO:0051726	Development	Cell division	P	241	26	9.89	2.63	2.30E-05	1.49E-03
7	1831	F2-3 vs. S2-3	S2-3 up	+	regulation of cell division	GO:0051302	Development	Cell division	P	63	10	2.59	3.87	5.67E-04	2.75E-02
7	1832	F2-3 vs. S2-3	S2-3 up	+	regulation of mitotic cell cycle	GO:0007346	Development	Cell division	P	89	13	3.65	3.56	1.87E-04	1.07E-02
7	1833	F2-3 vs. S2-3	S2-3 up	+	reproduction	GO:0000003	Development	Cell division	P	1742	111	71.48	1.55	1.06E-05	7.53E-04
7	1834	F2-3 vs. S2-3	S2-3 up	+	reproductive process	GO:0022414	Development	Cell division	P	1732	110	71.07	1.55	1.33E-05	9.27E-04
7	1835	F2-3 vs. S2-3	S2-3 up	+	RNA metabolic process	GO:0016070	Development	Cell division	P	1294	101	53.1	1.9	4.73E-09	5.65E-07
7	1836	F2-3 vs. S2-3	S2-3 up	+	spindle	GO:0005819	Development	Cell division	C	75	17	3.08	5.52	1.03E-07	2.89E-06
7	1837	F2-3 vs. S2-3	S2-3 up	+	spindle microtubule	GO:0005876	Development	Cell division	C	19	6	0.78	7.7	3.44E-04	4.48E-03
7	1838	F2-3 vs. S2-3	S2-3 up	+	adaxial/abaxial pattern specification	GO:0009955	Development	Development	P	38	10	1.56	6.41	1.45E-05	9.98E-04
7	1839	F2-3 vs. S2-3	S2-3 up	+	anatomical structure development	GO:0048856	Development	Development	P	2842	175	116.62	1.5	1.41E-07	1.40E-05
7	1840	F2-3 vs. S2-3	S2-3 up	+	anatomical structure morphogenesis	GO:0009653	Development	Development	P	896	61	36.77	1.66	2.53E-04	1.37E-02
7	1841	F2-3 vs. S2-3	S2-3 up	+	bounding membrane of organelle	GO:0098588	Development	Development	C	923	65	37.87	1.72	5.77E-05	9.54E-04
7	1842	F2-3 vs. S2-3	S2-3 up	+	carpel development	GO:0048440	Development	Development	P	78	11	3.2	3.44	7.46E-04	3.54E-02
7	1843	F2-3 vs. S2-3	S2-3 up	+	cell cortex	GO:0005938	Development	Development	C	115	14	4.72	2.97	5.82E-04	7.29E-03
7	1844	F2-3 vs. S2-3	S2-3 up	+	cell junction	GO:0030054	Development	Development	C	944	100	38.74	2.58	2.26E-16	1.07E-14
7	1845	F2-3 vs. S2-3	S2-3 up	+	cell periphery	GO:0071944	Development	Development	C	4012	242	164.63	1.47	1.47E-09	5.66E-08
7	1846	F2-3 vs. S2-3	S2-3 up	+	cell wall	GO:0005618	Development	Development	C	565	48	23.18	2.07	7.49E-06	1.50E-04
7	1847	F2-3 vs. S2-3	S2-3 up	+	cell-cell junction	GO:0005911	Development	Development	C	944	100	38.74	2.58	2.26E-16	1.24E-14
7	1848	F2-3 vs. S2-3	S2-3 up	+	cellular anatomical entity	GO:0110165	Development	Development	C	24430	1061	1002.47	1.06	2.84E-09	1.06E-07
7	1849	F2-3 vs. S2-3	S2-3 up	+	cellular component assembly	GO:0022607	Development	Development	P	838	102	34.39	2.97	1.65E-20	4.12E-18
7	1850	F2-3 vs. S2-3	S2-3 up	+	cellular component biogenesis	GO:0044085	Development	Development	P	1325	144	54.37	2.65	2.76E-24	8.70E-22
7	1851	F2-3 vs. S2-3	S2-3 up	+	cellular component organization	GO:0016043	Development	Development	P	2405	190	98.69	1.93	3.63E-17	6.58E-15
7	1852	F2-3 vs. S2-3	S2-3 up	+	cellular component organization or biogenesis	GO:0071840	Development	Development	P	2813	228	115.43	1.98	4.05E-22	1.15E-19
7	1853	F2-3 vs. S2-3	S2-3 up	+	cellular localization	GO:0051641	Development	Development	P	934	61	38.33	1.59	6.45E-04	3.11E-02
7	1854	F2-3 vs. S2-3	S2-3 up	+	cellular process	GO:0009987	Development	Development	P	12008	667	492.74	1.35	1.91E-24	6.36E-22
7	1855	F2-3 vs. S2-3	S2-3 up	+	cellular_component	GO:0005575	Development	Development	C	25545	1079	1048.22	1.03	1.58E-04	2.17E-03
7	1856	F2-3 vs. S2-3	S2-3 up	+	cortical cytoskeleton	GO:0030863	Development	Development	C	47	8	1.93	4.15	1.33E-03	1.48E-02
7	1857	F2-3 vs. S2-3	S2-3 up	+	cortical microtubule	GO:0055028	Development	Development	C	41	7	1.68	4.16	2.60E-03	2.63E-02
7	1858	F2-3 vs. S2-3	S2-3 up	+	cortical microtubule cytoskeleton	GO:0030981	Development	Development	C	44	8	1.81	4.43	9.15E-04	1.05E-02
7	1859	F2-3 vs. S2-3	S2-3 up	+	cytoplasm	GO:0005737	Development	Development	C	14837	737	608.83	1.21	3.60E-14	1.56E-12
7	1860	F2-3 vs. S2-3	S2-3 up	+	cytoplasmic microtubule	GO:0005881	Development	Development	C	48	8	1.97	4.06	1.50E-03	1.65E-02
7	1861	F2-3 vs. S2-3	S2-3 up	+	cytoskeleton	GO:0005856	Development	Development	C	298	32	12.23	2.62	4.61E-06	9.41E-05
7	1862	F2-3 vs. S2-3	S2-3 up	+	cytoskeleton organization	GO:0007010	Development	Development	P	283	26	11.61	2.24	3.07E-04	1.59E-02
7	1863	F2-3 vs. S2-3	S2-3 up	+	cytosol	GO:0005829	Development	Development	C	3241	304	132.99	2.29	1.95E-41	2.54E-39
7	1864	F2-3 vs. S2-3	S2-3 up	+	developmental process	GO:0032502	Development	Development	P	2997	179	122.98	1.46	7.35E-07	6.19E-05
7	1865	F2-3 vs. S2-3	S2-3 up	+	developmental process involved in reproduction	GO:0003006	Development	Development	P	1437	94	58.97	1.59	1.94E-05	1.31E-03

7	1866	F2-3 vs. S2-3	S2-3 up	+	embryo development	GO:0009790	Development	Development	P	566	43	23.23	1.85	2.91E-04	1.55E-02
7	1867	F2-3 vs. S2-3	S2-3 up	+	embryo development ending in seed dormancy	GO:0009793	Development	Development	P	541	41	22.2	1.85	3.42E-04	1.73E-02
7	1868	F2-3 vs. S2-3	S2-3 up	+	endoplasmic reticulum subcompartment	GO:0098827	Development	Development	C	302	30	12.39	2.42	2.54E-05	4.63E-04
7	1869	F2-3 vs. S2-3	S2-3 up	+	envelope	GO:0031975	Development	Development	C	1025	69	42.06	1.64	1.30E-04	1.91E-03
7	1870	F2-3 vs. S2-3	S2-3 up	+	ESCRT III complex	GO:0000815	Development	Development	C	12	4	0.49	8.12	2.99E-03	2.91E-02
7	1871	F2-3 vs. S2-3	S2-3 up	+	external encapsulating structure	GO:0030312	Development	Development	C	567	48	23.27	2.06	7.90E-06	1.55E-04
7	1872	F2-3 vs. S2-3	S2-3 up	+	floral organ development	GO:0048437	Development	Development	P	245	27	10.05	2.69	1.14E-05	8.03E-04
7	1873	F2-3 vs. S2-3	S2-3 up	+	floral whorl development	GO:0048438	Development	Development	P	195	25	8	3.12	2.21E-06	1.74E-04
7	1874	F2-3 vs. S2-3	S2-3 up	+	flower development	GO:0009908	Development	Development	P	356	39	14.61	2.67	1.63E-07	1.57E-05
7	1875	F2-3 vs. S2-3	S2-3 up	+	fruit development	GO:0010154	Development	Development	P	749	57	30.73	1.85	2.92E-05	1.84E-03
7	1876	F2-3 vs. S2-3	S2-3 up	+	gynoecium development	GO:0048467	Development	Development	P	91	14	3.73	3.75	6.54E-05	3.87E-03
7	1877	F2-3 vs. S2-3	S2-3 up	+	inflorescence development	GO:0010229	Development	Development	P	25	7	1.03	6.82	2.06E-04	1.16E-02
7	1878	F2-3 vs. S2-3	S2-3 up	+	intracellular anatomical structure	GO:0005622	Development	Development	C	21464	990	880.76	1.12	1.46E-16	8.94E-15
7	1879	F2-3 vs. S2-3	S2-3 up	+	intracellular membrane-bounded organelle	GO:0043231	Development	Development	C	19384	873	795.41	1.1	3.37E-07	8.35E-06
7	1880	F2-3 vs. S2-3	S2-3 up	+	intracellular non-membrane-bounded organelle	GO:0043232	Development	Development	C	1755	235	72.02	3.26	1.58E-54	4.10E-52
7	1881	F2-3 vs. S2-3	S2-3 up	+	intracellular organelle	GO:0043229	Development	Development	C	19690	915	807.97	1.13	4.96E-13	2.06E-11
7	1882	F2-3 vs. S2-3	S2-3 up	+	intracellular organelle lumen	GO:0070013	Development	Development	C	1386	135	56.87	2.37	5.86E-19	4.70E-17
7	1883	F2-3 vs. S2-3	S2-3 up	+	kinetochore microtubule	GO:0005828	Development	Development	C	6	3	0.25	12.18	4.29E-03	3.92E-02
7	1884	F2-3 vs. S2-3	S2-3 up	+	leaf development	GO:0048366	Development	Development	P	333	29	13.66	2.12	3.22E-04	1.66E-02
7	1885	F2-3 vs. S2-3	S2-3 up	+	leaf morphogenesis	GO:0009965	Development	Development	P	94	13	3.86	3.37	3.03E-04	1.59E-02
7	1886	F2-3 vs. S2-3	S2-3 up	+	membrane	GO:0016020	Development	Development	C	5493	301	225.4	1.34	1.11E-07	3.03E-06
7	1887	F2-3 vs. S2-3	S2-3 up	+	membrane protein complex	GO:0098796	Development	Development	C	597	48	24.5	1.96	3.27E-05	5.77E-04
7	1888	F2-3 vs. S2-3	S2-3 up	+	membrane-bounded organelle	GO:0043227	Development	Development	C	19546	878	802.06	1.09	5.74E-07	1.36E-05
7	1889	F2-3 vs. S2-3	S2-3 up	+	membrane-enclosed lumen	GO:0031974	Development	Development	C	1386	135	56.87	2.37	5.86E-19	4.36E-17
7	1890	F2-3 vs. S2-3	S2-3 up	+	microtubule	GO:0005874	Development	Development	C	145	22	5.95	3.7	7.49E-07	1.73E-05
7	1891	F2-3 vs. S2-3	S2-3 up	+	microtubule associated complex	GO:0005875	Development	Development	C	67	9	2.75	3.27	2.97E-03	2.91E-02
7	1892	F2-3 vs. S2-3	S2-3 up	+	microtubule cytoskeleton	GO:0015630	Development	Development	C	216	30	8.86	3.38	4.61E-08	1.46E-06
7	1893	F2-3 vs. S2-3	S2-3 up	+	microtubule cytoskeleton organization	GO:0000226	Development	Development	P	164	22	6.73	3.27	4.60E-06	3.40E-04
7	1894	F2-3 vs. S2-3	S2-3 up	+	microtubule-based process	GO:0007017	Development	Development	P	228	28	9.36	2.99	1.20E-06	9.99E-05
7	1895	F2-3 vs. S2-3	S2-3 up	+	multicellular organism development	GO:0007275	Development	Development	P	2457	153	100.82	1.52	6.21E-07	5.38E-05
7	1896	F2-3 vs. S2-3	S2-3 up	+	non-membrane-bounded organelle	GO:0043228	Development	Development	C	1755	235	72.02	3.26	1.58E-54	3.28E-52
7	1897	F2-3 vs. S2-3	S2-3 up	+	nuclear body	GO:0016604	Development	Development	C	102	13	4.19	3.11	6.12E-04	7.59E-03
7	1898	F2-3 vs. S2-3	S2-3 up	+	nuclear chromosome	GO:0000228	Development	Development	C	369	27	15.14	1.78	5.84E-03	4.95E-02
7	1899	F2-3 vs. S2-3	S2-3 up	+	nuclear lumen	GO:0031981	Development	Development	C	1222	118	50.14	2.35	2.57E-16	1.16E-14
7	1900	F2-3 vs. S2-3	S2-3 up	+	nuclear outer membrane-endoplasmic reticulum membrane network	GO:0042175	Development	Development	C	301	31	12.35	2.51	1.07E-05	2.02E-04
7	1901	F2-3 vs. S2-3	S2-3 up	+	nucleoplasm	GO:0005654	Development	Development	C	466	36	19.12	1.88	6.71E-04	8.03E-03
7	1902	F2-3 vs. S2-3	S2-3 up	+	nucleus	GO:0005634	Development	Development	C	10540	531	432.5	1.23	4.96E-09	1.78E-07
7	1903	F2-3 vs. S2-3	S2-3 up	+	organelle	GO:0043226	Development	Development	C	19739	915	809.98	1.13	1.23E-12	4.91E-11
7	1904	F2-3 vs. S2-3	S2-3 up	+	organelle assembly	GO:0070925	Development	Development	P	193	43	7.92	5.43	3.84E-17	6.76E-15
7	1905	F2-3 vs. S2-3	S2-3 up	+	organelle envelope	GO:0031967	Development	Development	C	1025	69	42.06	1.64	1.30E-04	1.88E-03
7	1906	F2-3 vs. S2-3	S2-3 up	+	organelle fission	GO:0048285	Development	Development	P	206	21	8.45	2.48	3.80E-04	1.91E-02
7	1907	F2-3 vs. S2-3	S2-3 up	+	organelle inner membrane	GO:0019866	Development	Development	C	311	24	12.76	1.88	4.50E-03	4.04E-02
7	1908	F2-3 vs. S2-3	S2-3 up	+	organelle lumen	GO:0043233	Development	Development	C	1386	135	56.87	2.37	5.86E-19	4.07E-17
7	1909	F2-3 vs. S2-3	S2-3 up	+	organelle membrane	GO:0031090	Development	Development	C	1458	109	59.83	1.82	9.70E-09	3.37E-07
7	1910	F2-3 vs. S2-3	S2-3 up	+	organelle organization	GO:0006996	Development	Development	P	1691	133	69.39	1.92	6.55E-12	9.55E-10
7	1911	F2-3 vs. S2-3	S2-3 up	+	pattern specification process	GO:0007389	Development	Development	P	190	27	7.8	3.46	1.42E-07	1.39E-05
7	1912	F2-3 vs. S2-3	S2-3 up	+	phragmoplast	GO:0009524	Development	Development	C	61	16	2.5	6.39	4.34E-08	1.41E-06
7	1913	F2-3 vs. S2-3	S2-3 up	+	phyllome development	GO:0048827	Development	Development	P	495	47	20.31	2.31	5.19E-07	4.70E-05
7	1914	F2-3 vs. S2-3	S2-3 up	+	plant organ development	GO:0099402	Development	Development	P	1009	77	41.4	1.86	6.57E-07	5.61E-05
7	1915	F2-3 vs. S2-3	S2-3 up	+	plant ovule development	GO:0048481	Development	Development	P	55	9	2.26	3.99	8.72E-04	4.01E-02
7	1916	F2-3 vs. S2-3	S2-3 up	+	plant-type ovary development	GO:0035670	Development	Development	P	57	9	2.34	3.85	1.09E-03	4.91E-02
7	1917	F2-3 vs. S2-3	S2-3 up	+	plasma membrane	GO:0005886	Development	Development	C	3534	209	145.02	1.44	1.22E-07	3.26E-06
7	1918	F2-3 vs. S2-3	S2-3 up	+	polarity specification of adaxial/abaxial axis	GO:0009944	Development	Development	P	23	6	0.94	6.36	8.06E-04	3.77E-02
7	1919	F2-3 vs. S2-3	S2-3 up	+	polymeric cytoskeletal fiber	GO:0099513	Development	Development	C	155	22	6.36	3.46	2.02E-06	4.47E-05
7	1920	F2-3 vs. S2-3	S2-3 up	+	post-embryonic development	GO:0009791	Development	Development	P	1482	101	60.81	1.66	2.01E-06	1.60E-04
7	1921	F2-3 vs. S2-3	S2-3 up	+	protein storage vacuole	GO:0000326	Development	Development	C	13	4	0.53	7.5	3.79E-03	3.58E-02
7	1922	F2-3 vs. S2-3	S2-3 up	+	regionalization	GO:0003002	Development	Development	P	155	23	6.36	3.62	5.99E-07	5.34E-05
7	1923	F2-3 vs. S2-3	S2-3 up	+	reproductive shoot system development	GO:0090567	Development	Development	P	371	43	15.22	2.82	8.73E-09	1.00E-06
7	1924	F2-3 vs. S2-3	S2-3 up	+	reproductive structure development	GO:0048608	Development	Development	P	1206	89	49.49	1.8	3.59E-07	3.40E-05
7	1925	F2-3 vs. S2-3	S2-3 up	+	reproductive system development	GO:0061458	Development	Development	P	1208	89	49.57	1.8	3.71E-07	3.47E-05
7	1926	F2-3 vs. S2-3	S2-3 up	+	seed development	GO:0048316	Development	Development	P	725	52	29.75	1.75	2.34E-04	1.30E-02

7	1927	F2-3 vs. S2-3	S2-3 up	+	shoot system development	GO:0048367	Development	Development	P	728	71	29.87	2.38	2.24E-10	2.97E-08
7	1928	F2-3 vs. S2-3	S2-3 up	+	shoot system morphogenesis	GO:0010016	Development	Development	P	172	21	7.06	2.98	2.71E-05	1.74E-03
7	1929	F2-3 vs. S2-3	S2-3 up	+	stomatal complex development	GO:0010374	Development	Development	P	55	9	2.26	3.99	8.72E-04	4.04E-02
7	1930	F2-3 vs. S2-3	S2-3 up	+	structural molecule activity	GO:0005198	Development	Development	F	443	116	18.18	6.38	1.51E-50	2.42E-47
7	1931	F2-3 vs. S2-3	S2-3 up	+	symplast	GO:0055044	Development	Development	C	944	100	38.74	2.58	2.26E-16	1.18E-14
7	1932	F2-3 vs. S2-3	S2-3 up	+	system development	GO:0048731	Development	Development	P	1867	128	76.61	1.67	4.55E-08	4.61E-06
7	1933	F2-3 vs. S2-3	S2-3 up	+	vacuolar membrane	GO:0005774	Development	Development	C	570	44	23.39	1.88	1.40E-04	2.00E-03
7	1934	F2-3 vs. S2-3	S2-3 up	+	vacuole	GO:0005773	Development	Development	C	1083	79	44.44	1.78	3.12E-06	6.62E-05
7	1935	F2-3 vs. S2-3	S2-3 up	+	amide biosynthetic process	GO:0043604	Metabolism	Metabolism	P	591	126	24.25	5.2	1.44E-46	2.15E-43
7	1936	F2-3 vs. S2-3	S2-3 up	+	anchoring junction	GO:0070161	Metabolism	Metabolism	C	944	100	38.74	2.58	2.26E-16	1.12E-14
7	1937	F2-3 vs. S2-3	S2-3 up	+	binding	GO:0005488	Metabolism	Metabolism	F	9843	576	403.9	1.43	1.22E-24	6.54E-22
7	1938	F2-3 vs. S2-3	S2-3 up	+	biological_process	GO:0008150	Metabolism	Metabolism	P	21988	981	902.27	1.09	1.48E-09	1.84E-07
7	1939	F2-3 vs. S2-3	S2-3 up	+	biosynthetic process	GO:0009058	Metabolism	Metabolism	P	2811	221	115.35	1.92	7.97E-20	1.76E-17
7	1940	F2-3 vs. S2-3	S2-3 up	+	catalytic complex	GO:1902494	Metabolism	Metabolism	C	1168	90	47.93	1.88	4.67E-08	1.43E-06
7	1941	F2-3 vs. S2-3	S2-3 up	+	cellular amide metabolic process	GO:0043603	Metabolism	Metabolism	P	722	129	29.63	4.35	1.73E-40	1.48E-37
7	1942	F2-3 vs. S2-3	S2-3 up	+	cellular aromatic compound metabolic process	GO:0006725	Metabolism	Metabolism	P	2519	151	103.37	1.46	5.18E-06	3.78E-04
7	1943	F2-3 vs. S2-3	S2-3 up	+	cellular biosynthetic process	GO:0044249	Metabolism	Metabolism	P	2583	215	105.99	2.03	3.75E-22	1.12E-19
7	1944	F2-3 vs. S2-3	S2-3 up	+	cellular macromolecule biosynthetic process	GO:0034645	Metabolism	Metabolism	P	1279	178	52.48	3.39	1.16E-42	1.16E-39
7	1945	F2-3 vs. S2-3	S2-3 up	+	cellular macromolecule metabolic process	GO:0044260	Metabolism	Metabolism	P	4426	323	181.62	1.78	7.16E-25	2.68E-22
7	1946	F2-3 vs. S2-3	S2-3 up	+	cellular metabolic process	GO:0044237	Metabolism	Metabolism	P	7649	443	313.87	1.41	5.12E-16	8.75E-14
7	1947	F2-3 vs. S2-3	S2-3 up	+	cellular nitrogen compound biosynthetic process	GO:0044271	Metabolism	Metabolism	P	1241	161	50.92	3.16	3.63E-35	2.41E-32
7	1948	F2-3 vs. S2-3	S2-3 up	+	cellular nitrogen compound metabolic process	GO:0034641	Metabolism	Metabolism	P	2872	264	117.85	2.24	4.19E-34	2.50E-31
7	1949	F2-3 vs. S2-3	S2-3 up	+	cellular protein metabolic process	GO:0044267	Metabolism	Metabolism	P	3388	256	139.02	1.84	4.55E-21	1.18E-18
7	1950	F2-3 vs. S2-3	S2-3 up	+	cellular protein-containing complex assembly	GO:0034622	Metabolism	Metabolism	P	474	74	19.45	3.8	1.88E-20	4.50E-18
7	1951	F2-3 vs. S2-3	S2-3 up	+	DNA metabolic process	GO:0006259	Metabolism	Metabolism	P	448	35	18.38	1.9	5.53E-04	2.71E-02
7	1952	F2-3 vs. S2-3	S2-3 up	+	heterocycle metabolic process	GO:0046483	Metabolism	Metabolism	P	2393	150	98.2	1.53	6.01E-07	5.29E-05
7	1953	F2-3 vs. S2-3	S2-3 up	+	heterocyclic compound binding	GO:1901363	Metabolism	Metabolism	F	4765	311	195.53	1.59	7.64E-17	3.07E-14
7	1954	F2-3 vs. S2-3	S2-3 up	+	macromolecule biosynthetic process	GO:0009059	Metabolism	Metabolism	P	1334	178	54.74	3.25	1.87E-40	1.40E-37
7	1955	F2-3 vs. S2-3	S2-3 up	+	macromolecule metabolic process	GO:0043170	Metabolism	Metabolism	P	5496	382	225.53	1.69	1.75E-26	8.03E-24
7	1956	F2-3 vs. S2-3	S2-3 up	+	metabolic process	GO:0008152	Metabolism	Metabolism	P	8681	474	356.22	1.33	6.14E-13	9.18E-11
7	1957	F2-3 vs. S2-3	S2-3 up	+	microtubule binding	GO:0008017	Metabolism	Metabolism	F	162	19	6.65	2.86	1.04E-04	2.23E-02
7	1958	F2-3 vs. S2-3	S2-3 up	+	molecular_function	GO:0003674	Metabolism	Metabolism	F	21945	967	900.5	1.07	5.15E-07	1.38E-04
7	1959	F2-3 vs. S2-3	S2-3 up	+	nitrogen compound metabolic process	GO:0006807	Metabolism	Metabolism	P	6150	396	252.36	1.57	1.75E-21	4.75E-19
7	1960	F2-3 vs. S2-3	S2-3 up	+	nucleic acid binding	GO:0003676	Metabolism	Metabolism	F	3299	270	135.37	1.99	3.03E-27	1.95E-24
7	1961	F2-3 vs. S2-3	S2-3 up	+	organic cyclic compound binding	GO:0097159	Metabolism	Metabolism	F	4794	313	196.72	1.59	6.36E-17	2.92E-14
7	1962	F2-3 vs. S2-3	S2-3 up	+	organic cyclic compound metabolic process	GO:1901360	Metabolism	Metabolism	P	2607	156	106.98	1.46	4.31E-06	3.22E-04
7	1963	F2-3 vs. S2-3	S2-3 up	+	organic substance biosynthetic process	GO:1901576	Metabolism	Metabolism	P	2674	214	109.73	1.95	4.89E-20	1.12E-17
7	1964	F2-3 vs. S2-3	S2-3 up	+	organic substance metabolic process	GO:0071704	Metabolism	Metabolism	P	7851	445	322.16	1.38	1.67E-14	2.63E-12
7	1965	F2-3 vs. S2-3	S2-3 up	+	organonitrogen compound biosynthetic process	GO:1901566	Metabolism	Metabolism	P	1343	157	55.11	2.85	1.17E-29	5.85E-27
7	1966	F2-3 vs. S2-3	S2-3 up	+	organonitrogen compound metabolic process	GO:1901564	Metabolism	Metabolism	P	4611	289	189.21	1.53	2.09E-13	3.20E-11
7	1967	F2-3 vs. S2-3	S2-3 up	+	peptide metabolic process	GO:0006518	Metabolism	Metabolism	P	585	128	24.01	5.33	2.24E-48	4.46E-45
7	1968	F2-3 vs. S2-3	S2-3 up	+	protein binding	GO:0005515	Metabolism	Metabolism	F	5446	328	223.47	1.47	3.60E-13	1.28E-10
7	1969	F2-3 vs. S2-3	S2-3 up	+	protein metabolic process	GO:0019538	Metabolism	Metabolism	P	3544	260	145.43	1.79	8.95E-20	1.91E-17
7	1970	F2-3 vs. S2-3	S2-3 up	+	Sm-like protein family complex	GO:0120114	Metabolism	Metabolism	C	86	19	3.53	5.38	2.64E-08	8.86E-07
7	1971	F2-3 vs. S2-3	S2-3 up	+	supramolecular complex	GO:0099080	Metabolism	Metabolism	C	255	31	10.46	2.96	4.01E-07	9.72E-06
7	1972	F2-3 vs. S2-3	S2-3 up	+	supramolecular fiber	GO:0099512	Metabolism	Metabolism	C	155	22	6.36	3.46	2.02E-06	4.38E-05
7	1973	F2-3 vs. S2-3	S2-3 up	+	supramolecular polymer	GO:0099081	Metabolism	Metabolism	C	155	22	6.36	3.46	2.02E-06	4.57E-05
7	1974	F2-3 vs. S2-3	S2-3 up	+	transferase complex	GO:1990234	Metabolism	Metabolism	C	698	48	28.64	1.68	1.08E-03	1.21E-02
7	1975	F2-3 vs. S2-3	S2-3 up	+	transferase complex, transferring phosphorus-containing groups	GO:0061695	Metabolism	Metabolism	C	232	21	9.52	2.21	1.56E-03	1.69E-02
7	1976	F2-3 vs. S2-3	S2-3 up	+	tubulin binding	GO:0015631	Metabolism	Metabolism	F	172	20	7.06	2.83	7.71E-05	1.91E-02
7	1977	F2-3 vs. S2-3	S2-3 up	+	plastid large ribosomal subunit	GO:0000311	Metabolism	Photosynthesis	C	15	4	0.62	6.5	5.79E-03	4.94E-02
7	1978	F2-3 vs. S2-3	S2-3 up	+	plastid ribosome	GO:0009547	Metabolism	Photosynthesis	C	29	8	1.19	6.72	7.92E-05	1.25E-03
7	1979	F2-3 vs. S2-3	S2-3 up	+	plastid small ribosomal subunit	GO:0000312	Metabolism	Photosynthesis	C	13	4	0.53	7.5	3.79E-03	3.52E-02
7	1980	F2-3 vs. S2-3	S2-3 up	+	endoplasmic reticulum	GO:0005783	Regulation	Protein modification	C	1044	71	42.84	1.66	7.76E-05	1.24E-03
7	1981	F2-3 vs. S2-3	S2-3 up	+	endoplasmic reticulum membrane	GO:0005789	Regulation	Protein modification	C	297	30	12.19	2.46	1.97E-05	3.66E-04
7	1982	F2-3 vs. S2-3	S2-3 up	+	protein-containing complex assembly	GO:0065003	Regulation	Protein modification	P	534	77	21.91	3.51	1.98E-19	3.95E-17
7	1983	F2-3 vs. S2-3	S2-3 up	+	protein-containing complex subunit organization	GO:0043933	Regulation	Protein modification	P	609	79	24.99	3.16	1.67E-17	3.21E-15
7	1984	F2-3 vs. S2-3	S2-3 up	+	negative regulation of biological process	GO:0048519	Regulation	Regulation	P	1102	83	45.22	1.84	3.88E-07	3.57E-05
7	1985	F2-3 vs. S2-3	S2-3 up	+	negative regulation of cellular process	GO:0048523	Regulation	Regulation	P	743	55	30.49	1.8	6.31E-05	3.81E-03
7	1986	F2-3 vs. S2-3	S2-3 up	+	negative regulation of macromolecule metabolic process	GO:0010605	Regulation	Regulation	P	603	45	24.74	1.82	3.00E-04	1.59E-02

7	1987	F2-3 vs. S2-3	S2-3 up	+	negative regulation of metabolic process	GO:0009892	Regulation	Regulation	P	653	50	26.8	1.87	7.09E-05	4.16E-03
7	1988	F2-3 vs. S2-3	S2-3 up	+	regulation of biological process	GO:0050789	Regulation	Regulation	P	5407	272	221.87	1.23	3.37E-04	1.72E-02
7	1989	F2-3 vs. S2-3	S2-3 up	+	regulation of cellular process	GO:0050794	Regulation	Regulation	P	4751	240	194.95	1.23	7.57E-04	3.56E-02
7	1990	F2-3 vs. S2-3	S2-3 up	+	regulation of macromolecule metabolic process	GO:0060255	Regulation	Regulation	P	3058	168	125.48	1.34	1.73E-04	9.92E-03
7	1991	F2-3 vs. S2-3	S2-3 up	+	regulation of metabolic process	GO:0019222	Regulation	Regulation	P	3325	179	136.44	1.31	2.45E-04	1.34E-02
7	1992	F2-3 vs. S2-3	S2-3 up	+	multicellular organismal process	GO:0032501	Signaling and response	Signaling	P	2741	159	112.48	1.41	1.76E-05	1.20E-03
7	1993	F2-3 vs. S2-3	S2-3 up	+	protein kinase complex	GO:1902911	Signaling and response	Signaling	C	87	10	3.57	2.8	4.93E-03	4.38E-02
7	1994	F2-3 vs. S2-3	S2-3 up	+	serine/threonine protein kinase complex	GO:1902554	Signaling and response	Signaling	C	79	10	3.24	3.08	2.64E-03	2.61E-02
7	1995	F2-3 vs. S2-3	S2-3 up	+	cellular response to DNA damage stimulus	GO:0006974	Signaling and response	Stress	P	350	30	14.36	2.09	4.36E-04	2.17E-02
7	1996	F2-3 vs. S2-3	S2-3 up	+	endopeptidase Clp complex	GO:0009368	Signaling and response	Stress	C	11	4	0.45	8.86	2.31E-03	2.38E-02
7	1997	F2-3 vs. S2-3	S2-3 up	+	gene expression	GO:0010467	Regulation	Transcription	P	1608	208	65.98	3.15	9.70E-46	1.16E-42
7	1998	F2-3 vs. S2-3	S2-3 up	+	gene silencing	GO:0016458	Regulation	Transcription	P	153	16	6.28	2.55	1.10E-03	4.91E-02
7	1999	F2-3 vs. S2-3	S2-3 up	+	mRNA processing	GO:0006397	Regulation	Transcription	P	286	32	11.74	2.73	1.36E-06	1.12E-04
7	2000	F2-3 vs. S2-3	S2-3 up	+	negative regulation of gene expression	GO:0010629	Regulation	Transcription	P	493	41	20.23	2.03	6.46E-05	3.86E-03
7	2001	F2-3 vs. S2-3	S2-3 up	+	nuclear speck	GO:0016607	Regulation	Transcription	C	61	8	2.5	3.2	5.66E-03	4.87E-02
7	2002	F2-3 vs. S2-3	S2-3 up	+	nucleolus	GO:0005730	Regulation	Transcription	C	483	70	19.82	3.53	7.20E-18	4.69E-16
7	2003	F2-3 vs. S2-3	S2-3 up	+	regulation of gene expression	GO:0010468	Regulation	Transcription	P	2679	147	109.93	1.34	5.18E-04	2.56E-02
7	2004	F2-3 vs. S2-3	S2-3 up	+	RNA binding	GO:0003723	Regulation	Transcription	F	1569	178	64.38	2.76	2.96E-32	3.18E-29
7	2005	F2-3 vs. S2-3	S2-3 up	+	catalytic step 2 spliceosome	GO:0071013	Regulation	Translation	C	64	10	2.63	3.81	6.33E-04	7.75E-03
7	2006	F2-3 vs. S2-3	S2-3 up	+	cytoplasmic translation	GO:0002181	Regulation	Translation	P	72	18	2.95	6.09	1.19E-08	1.29E-06
7	2007	F2-3 vs. S2-3	S2-3 up	+	cytosolic large ribosomal subunit	GO:0022625	Regulation	Translation	C	131	58	5.38	10.79	1.57E-35	1.63E-33
7	2008	F2-3 vs. S2-3	S2-3 up	+	cytosolic ribosome	GO:0022626	Regulation	Translation	C	303	101	12.43	8.12	5.70E-52	8.48E-50
7	2009	F2-3 vs. S2-3	S2-3 up	+	cytosolic small ribosomal subunit	GO:0022627	Regulation	Translation	C	102	40	4.19	9.56	2.47E-23	2.14E-21
7	2010	F2-3 vs. S2-3	S2-3 up	+	large ribosomal subunit	GO:0015934	Regulation	Translation	C	180	70	7.39	9.48	9.87E-40	1.14E-37
7	2011	F2-3 vs. S2-3	S2-3 up	+	large ribosomal subunit rRNA binding	GO:0070180	Regulation	Translation	F	14	6	0.57	10.44	8.91E-05	2.05E-02
7	2012	F2-3 vs. S2-3	S2-3 up	+	Lsm1-7-Pat1 complex	GO:1990726	Regulation	Translation	C	7	3	0.29	10.44	5.95E-03	5.00E-02
7	2013	F2-3 vs. S2-3	S2-3 up	+	maturation of LSU-rRNA	GO:0000470	Regulation	Translation	P	46	10	1.89	5.3	5.94E-05	3.63E-03
7	2014	F2-3 vs. S2-3	S2-3 up	+	maturation of SSU-rRNA	GO:0030490	Regulation	Translation	P	54	13	2.22	5.87	1.81E-06	1.47E-04
7	2015	F2-3 vs. S2-3	S2-3 up	+	mitochondrial large ribosomal subunit	GO:0005762	Regulation	Translation	C	32	8	1.31	6.09	1.42E-04	2.00E-03
7	2016	F2-3 vs. S2-3	S2-3 up	+	mitochondrial ribosome	GO:0005761	Regulation	Translation	C	51	10	2.09	4.78	1.26E-04	1.87E-03
7	2017	F2-3 vs. S2-3	S2-3 up	+	mRNA binding	GO:0003729	Regulation	Translation	F	1005	135	41.24	3.27	8.90E-31	7.15E-28
7	2018	F2-3 vs. S2-3	S2-3 up	+	mRNA metabolic process	GO:0016071	Regulation	Translation	P	423	39	17.36	2.25	9.30E-06	6.70E-04
7	2019	F2-3 vs. S2-3	S2-3 up	+	mRNA splicing, via spliceosome	GO:0000398	Regulation	Translation	P	201	25	8.25	3.03	3.60E-06	2.80E-04
7	2020	F2-3 vs. S2-3	S2-3 up	+	ncRNA metabolic process	GO:0034660	Regulation	Translation	P	455	48	18.67	2.57	1.98E-08	2.04E-06
7	2021	F2-3 vs. S2-3	S2-3 up	+	ncRNA processing	GO:0034470	Regulation	Translation	P	381	43	15.63	2.75	1.76E-08	1.85E-06
7	2022	F2-3 vs. S2-3	S2-3 up	+	organellar large ribosomal subunit	GO:0000315	Regulation	Translation	C	45	11	1.85	5.96	9.92E-06	1.91E-04
7	2023	F2-3 vs. S2-3	S2-3 up	+	organellar ribosome	GO:0000313	Regulation	Translation	C	75	17	3.08	5.52	1.03E-07	2.97E-06
7	2024	F2-3 vs. S2-3	S2-3 up	+	organellar small ribosomal subunit	GO:0000314	Regulation	Translation	C	29	6	1.19	5.04	2.25E-03	2.35E-02
7	2025	F2-3 vs. S2-3	S2-3 up	+	peptide biosynthetic process	GO:0043043	Regulation	Translation	P	518	125	21.26	5.88	3.54E-51	1.06E-47
7	2026	F2-3 vs. S2-3	S2-3 up	+	pICln-Sm protein complex	GO:0034715	Regulation	Translation	C	6	3	0.25	12.18	4.29E-03	3.88E-02
7	2027	F2-3 vs. S2-3	S2-3 up	+	precatalytic spliceosome	GO:0071011	Regulation	Translation	C	31	8	1.27	6.29	1.18E-04	1.77E-03
7	2028	F2-3 vs. S2-3	S2-3 up	+	preribosome	GO:0030684	Regulation	Translation	C	90	11	3.69	2.98	2.12E-03	2.25E-02
7	2029	F2-3 vs. S2-3	S2-3 up	+	ribonucleoprotein complex	GO:1990904	Regulation	Translation	C	677	149	27.78	5.36	1.01E-56	3.50E-54
7	2030	F2-3 vs. S2-3	S2-3 up	+	ribonucleoprotein complex assembly	GO:0022618	Regulation	Translation	P	163	51	6.69	7.62	1.17E-25	5.02E-23
7	2031	F2-3 vs. S2-3	S2-3 up	+	ribonucleoprotein complex biogenesis	GO:0022613	Regulation	Translation	P	447	90	18.34	4.91	1.03E-31	5.59E-29
7	2032	F2-3 vs. S2-3	S2-3 up	+	ribonucleoprotein complex subunit organization	GO:0071826	Regulation	Translation	P	166	51	6.81	7.49	2.35E-25	9.38E-23
7	2033	F2-3 vs. S2-3	S2-3 up	+	ribosomal large subunit assembly	GO:0000027	Regulation	Translation	P	43	15	1.76	8.5	4.76E-09	5.58E-07
7	2034	F2-3 vs. S2-3	S2-3 up	+	ribosomal large subunit biogenesis	GO:0042273	Regulation	Translation	P	109	32	4.47	7.15	6.05E-16	1.00E-13
7	2035	F2-3 vs. S2-3	S2-3 up	+	ribosomal small subunit assembly	GO:0000028	Regulation	Translation	P	38	17	1.56	10.9	1.97E-11	2.80E-09
7	2036	F2-3 vs. S2-3	S2-3 up	+	ribosomal small subunit biogenesis	GO:0042274	Regulation	Translation	P	91	28	3.73	7.5	1.49E-14	2.41E-12
7	2037	F2-3 vs. S2-3	S2-3 up	+	ribosomal subunit	GO:0044391	Regulation	Translation	C	316	116	12.97	8.95	2.39E-63	2.48E-60
7	2038	F2-3 vs. S2-3	S2-3 up	+	ribosome	GO:0005840	Regulation	Translation	C	399	120	16.37	7.33	1.01E-57	5.26E-55
7	2039	F2-3 vs. S2-3	S2-3 up	+	ribosome assembly	GO:0042255	Regulation	translation	P	90	34	3.69	9.21	1.25E-19	2.58E-17
7	2040	F2-3 vs. S2-3	S2-3 up	+	ribosome biogenesis	GO:0042254	Regulation	Translation	P	370	72	15.18	4.74	8.76E-25	3.08E-22
7	2041	F2-3 vs. S2-3	S2-3 up	+	RNA processing	GO:0006396	Regulation	Translation	P	787	77	32.29	2.38	2.93E-11	4.08E-09
7	2042	F2-3 vs. S2-3	S2-3 up	+	RNA splicing	GO:0008380	Regulation	Translation	P	276	26	11.33	2.3	2.45E-04	1.33E-02
7	2043	F2-3 vs. S2-3	S2-3 up	+	RNA splicing, via transesterification reactions	GO:0000375	Regulation	Translation	P	225	25	9.23	2.71	2.10E-05	1.38E-03
7	2044	F2-3 vs. S2-3	S2-3 up	+	RNA splicing, via transesterification reactions with bulged adenosine as nucleophile	GO:0000377	Regulation	Translation	P	225	25	9.23	2.71	2.10E-05	1.39E-03
7	2045	F2-3 vs. S2-3	S2-3 up	+	rRNA binding	GO:0019843	Regulation	Translation	F	72	18	2.95	6.09	1.19E-08	3.83E-06
7	2046	F2-3 vs. S2-3	S2-3 up	+	rRNA metabolic process	GO:0016072	Regulation	Translation	P	263	35	10.79	3.24	9.57E-09	1.08E-06
7	2047	F2-3 vs. S2-3	S2-3 up	+	rRNA processing	GO:0006364	Regulation	Translation	P	254	34	10.42	3.26	1.36E-08	1.46E-06

7	2048	F2-3 vs. S2-3	S2-3 up	+	small nuclear ribonucleoprotein complex	GO:0030532	Regulation	Translation	C	78	17	3.2	5.31	1.67E-07	4.25E-06
7	2049	F2-3 vs. S2-3	S2-3 up	+	small ribosomal subunit	GO:0015935	Regulation	Translation	C	136	46	5.58	8.24	1.98E-24	1.87E-22
7	2050	F2-3 vs. S2-3	S2-3 up	+	spliceosomal complex	GO:0005681	Regulation	Translation	C	160	17	6.57	2.59	6.65E-04	8.04E-03
7	2051	F2-3 vs. S2-3	S2-3 up	+	spliceosomal snRNP assembly	GO:0000387	Regulation	Translation	P	25	8	1.03	7.8	3.27E-05	2.04E-03
7	2052	F2-3 vs. S2-3	S2-3 up	+	spliceosomal snRNP complex	GO:0097525	Regulation	Translation	C	78	17	3.2	5.31	1.67E-07	4.36E-06
7	2053	F2-3 vs. S2-3	S2-3 up	+	spliceosomal tri-snRNP complex	GO:0097526	Regulation	Translation	C	41	10	1.68	5.94	2.55E-05	4.57E-04
7	2054	F2-3 vs. S2-3	S2-3 up	+	structural constituent of ribosome	GO:0003735	Regulation	Translation	F	325	106	13.34	7.95	8.80E-54	2.83E-50
7	2055	F2-3 vs. S2-3	S2-3 up	+	translation	GO:0006412	Regulation	Translation	P	513	125	21.05	5.94	1.43E-51	8.57E-48
7	2056	F2-3 vs. S2-3	S2-3 up	+	U1 snRNP	GO:0005685	Regulation	Translation	C	24	9	0.98	9.14	3.65E-06	7.59E-05
7	2057	F2-3 vs. S2-3	S2-3 up	+	U12-type spliceosomal complex	GO:0005689	Regulation	Translation	C	24	5	0.98	5.08	5.09E-03	4.42E-02
7	2058	F2-3 vs. S2-3	S2-3 up	+	U2 snRNP	GO:0005686	Regulation	Translation	C	34	7	1.4	5.02	1.01E-03	1.14E-02
7	2059	F2-3 vs. S2-3	S2-3 up	+	U4 snRNP	GO:0005687	Regulation	Translation	C	12	4	0.49	8.12	2.99E-03	2.88E-02
7	2060	F2-3 vs. S2-3	S2-3 up	+	U4/U6 x U5 tri-snRNP complex	GO:0046540	Regulation	Translation	C	32	6	1.31	4.57	3.47E-03	3.31E-02
7	2061	F2-3 vs. S2-3	S2-3 up	+	U5 snRNP	GO:0005682	Regulation	translation	C	19	7	0.78	8.98	4.94E-05	8.29E-04
7	2062	F2-3 vs. S2-3	S2-3 up	+	macromolecule localization	GO:0033036	Metabolism	Transporters	P	941	61	38.61	1.58	8.82E-04	4.03E-02
7	2063	F2-3 vs. S2-3	S2-3 up	+	plasmodesma	GO:0009506	Metabolism	Transporters	C	944	100	38.74	2.58	2.26E-16	1.31E-14
7	2064	F2-3 vs. S2-3	S2-3 up	+	storage vacuole	GO:0000322	Metabolism	Transporters	C	13	4	0.53	7.5	3.79E-03	3.55E-02
