# Supplementary material for: Whether Gametophytes Are Reduced or Unreduced in Angiosperms Might Be Determined Metabolically
Source: Genes (Basel). 2020 Dec 2;11(12):1449. doi: 10.3390/genes11121449 (PMC7761559; doi:10.3390/genes11121449)
Supplement: Supplementary file 1 [file genes-11-01449-s001.zip › Table S8 EGOs.pdf]

Table S8. Enriched gene ontology terms (EGOs): +, genes upregulated in first taxon of each comparison; -, genes upregulated in second taxon of each comparison. GO terms are partitioned by groups and subgroups. L, *B. lignifera* ; M, *B. microphylla* ; F, *B. x formosa* ; S, *B. stricta* ; Stages: 1, megasporocyte (MMC); 2, meiocyte; 3, early gametophyte; 4, mature gametophyte.

Test	Line no.	Comparison	up or down	GO group	Go subgroup	EGO term	GO no.	GO type	Number of genes	p value	FDR
1	1	L1 vs. M1	+	Development	Cell division	nucleobase metabolic process	GO:0009112	P	29	1.69E-03	4.92E-02
1	2	L1 vs. M1	+	Development	Cell division	SCF ubiquitin ligase complex	GO:0019005	C	49	3.72E-04	9.65E-03
1	3	L1 vs. M1	+	Development	Development	cytosol	GO:0005829	C	2304	6.20E-05	2.35E-03
1	4	L1 vs. M1	+	Development	Development	extracellular space	GO:0005615	C	63	1.19E-03	2.55E-02
1	5	L1 vs. M1	+	Development	Development	lytic vacuole	GO:0000323	C	37	1.36E-05	7.06E-04
1	6	L1 vs. M1	+	Development	Development	membrane	GO:0016020	C	3472	1.13E-03	2.47E-02
1	7	L1 vs. M1	+	Development	Development	microbody	GO:0042579	C	201	3.64E-08	3.99E-06
1	8	L1 vs. M1	+	Development	Development	plant-type vacuole	GO:0000325	C	107	1.04E-04	3.65E-03
1	9	L1 vs. M1	+	Development	Development	plasma membrane	GO:0005886	C	2248	2.62E-03	4.45E-02
1	10	L1 vs. M1	+	Development	Development	vacuole	GO:0005773	C	736	9.05E-05	3.30E-03
1	11	L1 vs. M1	+	Metabolism	Bioenergetics	carbohydrate metabolic process	GO:0005975	P	593	1.16E-03	3.60E-02
1	12	L1 vs. M1	+	Metabolism	Bioenergetics	cellular carbohydrate catabolic process	GO:0044275	P	34	4.39E-06	4.12E-04
1	13	L1 vs. M1	+	Metabolism	Bioenergetics	cellular carbohydrate metabolic process	GO:0044262	P	262	2.69E-04	1.14E-02
1	14	L1 vs. M1	+	Metabolism	Bioenergetics	disaccharide metabolic process	GO:0005984	P	50	6.28E-04	2.34E-02
1	15	L1 vs. M1	+	Metabolism	Bioenergetics	fatty acid beta-oxidation	GO:0006635	P	26	4.04E-05	2.52E-03
1	16	L1 vs. M1	+	Metabolism	Bioenergetics	fatty acid catabolic process	GO:0009062	P	27	1.52E-05	1.10E-03
1	17	L1 vs. M1	+	Metabolism	Bioenergetics	fatty acid oxidation	GO:0019395	P	28	3.88E-04	1.57E-02
1	18	L1 vs. M1	+	Metabolism	Bioenergetics	lipid oxidation	GO:0034440	P	36	1.73E-03	4.97E-02
1	19	L1 vs. M1	+	Metabolism	Bioenergetics	oligosaccharide metabolic process	GO:0009311	P	57	7.96E-04	2.74E-02
1	20	L1 vs. M1	+	Metabolism	Bioenergetics	oxidoreductase activity, acting on CH-OH group of donors	GO:0016614	F	141	2.04E-04	2.97E-02
1	21	L1 vs. M1	+	Metabolism	Bioenergetics	oxidoreductase activity, acting on the CH-OH group of donors, NAD or NADP as acceptor	GO:0016616	F	124	2.98E-05	6.66E-03
1	22	L1 vs. M1	+	Metabolism	Bioenergetics	proton-transporting V-type ATPase complex	GO:0033176	C	16	2.90E-03	4.85E-02
1	23	L1 vs. M1	+	Metabolism	Catabolism	alpha-amino acid catabolic process	GO:1901606	P	64	5.67E-08	1.43E-05
1	24	L1 vs. M1	+	Metabolism	Catabolism	branched-chain amino acid catabolic process	GO:0009083	P	20	7.33E-04	2.57E-02
1	25	L1 vs. M1	+	Metabolism	Catabolism	carboxylic acid catabolic process	GO:0046395	P	117	7.44E-13	5.89E-10
1	26	L1 vs. M1	+	Metabolism	Catabolism	catabolic process	GO:0009056	P	935	2.22E-16	6.15E-13
1	27	L1 vs. M1	+	Metabolism	Catabolism	cellular amino acid catabolic process	GO:0009063	P	72	2.14E-08	6.59E-06
1	28	L1 vs. M1	+	Metabolism	Catabolism	cellular catabolic process	GO:0044248	P	785	0.00E+00	0.00E+00
1	29	L1 vs. M1	+	Metabolism	Catabolism	cellular lipid catabolic process	GO:0044242	P	64	3.57E-06	3.54E-04
1	30	L1 vs. M1	+	Metabolism	Catabolism	cellular macromolecule catabolic process	GO:0044265	P	467	4.23E-06	4.11E-04
1	31	L1 vs. M1	+	Metabolism	Catabolism	cellular protein catabolic process	GO:0044257	P	369	1.74E-06	2.05E-04
1	32	L1 vs. M1	+	Metabolism	Catabolism	extrinsic component of membrane	GO:0019898	C	50	2.59E-03	4.48E-02
1	33	L1 vs. M1	+	Metabolism	Catabolism	lipid catabolic process	GO:0016042	P	70	2.35E-05	1.52E-03
1	34	L1 vs. M1	+	Metabolism	Catabolism	lysosome	GO:0005764	C	34	1.36E-04	4.46E-03
1	35	L1 vs. M1	+	Metabolism	Catabolism	macromolecule catabolic process	GO:0009057	P	556	1.97E-06	2.23E-04
1	36	L1 vs. M1	+	Metabolism	Catabolism	modification-dependent protein catabolic process	GO:0019941	P	312	1.23E-03	3.79E-02
1	37	L1 vs. M1	+	Metabolism	Catabolism	monocarboxylic acid catabolic process	GO:0072329	P	44	4.40E-06	4.07E-04
1	38	L1 vs. M1	+	Metabolism	Catabolism	organic acid catabolic process	GO:0016054	P	117	7.44E-13	6.87E-10
1	39	L1 vs. M1	+	Metabolism	Catabolism	organic substance catabolic process	GO:1901575	P	861	6.44E-15	1.19E-11
1	40	L1 vs. M1	+	Metabolism	Catabolism	organonitrogen compound catabolic process	GO:1901565	P	524	5.46E-11	3.03E-08
1	41	L1 vs. M1	+	Metabolism	Catabolism	peroxisome	GO:0005777	C	201	3.64E-08	3.59E-06
1	42	L1 vs. M1	+	Metabolism	Catabolism	peroxisome organization	GO:0007031	P	25	5.04E-04	1.98E-02
1	43	L1 vs. M1	+	Metabolism	Catabolism	proteasomal protein catabolic process	GO:0010498	P	171	1.75E-03	5.00E-02
1	44	L1 vs. M1	+	Metabolism	Catabolism	protein catabolic process	GO:0030163	P	380	1.73E-06	2.08E-04
1	45	L1 vs. M1	+	Metabolism	Catabolism	protein ubiquitination	GO:0016567	P	362	1.15E-05	8.58E-04
1	46	L1 vs. M1	+	Metabolism	Catabolism	proteolysis	GO:0006508	P	500	9.80E-06	7.55E-04
1	47	L1 vs. M1	+	Metabolism	Catabolism	proteolysis involved in cellular protein catabolic process	GO:0051603	P	361	4.58E-06	4.16E-04
1	48	L1 vs. M1	+	Metabolism	Catabolism	SCF-dependent proteasomal ubiquitin-dependent protein catabolic process	GO:0031146	P	46	9.88E-05	5.26E-03
1	49	L1 vs. M1	+	Metabolism	Catabolism	small molecule catabolic process	GO:0044282	P	168	2.54E-14	3.52E-11
1	50	L1 vs. M1	+	Metabolism	Catabolism	ubiquitin-dependent protein catabolic process	GO:0006511	P	307	6.38E-04	2.36E-02
1	51	L1 vs. M1	+	Metabolism	Catabolism	ubiquitin-like protein transferase activity	GO:0019787	F	253	2.81E-05	7.43E-03
1	52	L1 vs. M1	+	Metabolism	Catabolism	ubiquitin-protein transferase activity	GO:0004842	F	249	1.42E-05	4.58E-03
1	53	L1 vs. M1	+	Metabolism	Metabolism	alcohol metabolic process	GO:0006066	P	77	1.76E-03	4.98E-02
1	54	L1 vs. M1	+	Metabolism	Metabolism	alpha-amino acid metabolic process	GO:1901605	P	206	1.03E-06	1.32E-04
1	55	L1 vs. M1	+	Metabolism	Metabolism	amine metabolic process	GO:0009308	P	67	1.54E-03	4.56E-02
1	56	L1 vs. M1	+	Metabolism	Metabolism	carboxylic acid metabolic process	GO:0019752	P	642	6.03E-08	1.45E-05
1	57	L1 vs. M1	+	Metabolism	Metabolism	catalytic activity	GO:0003824	F	4992	1.79E-05	5.21E-03

1	58	L1 vs. M1	+	Metabolism	Metabolism	catalytic activity, acting on a protein	GO:0140096	F	1414	1.33E-05	4.84E-03
1	59	L1 vs. M1	+	Metabolism	Metabolism	cellular amino acid metabolic process	GO:0006520	P	278	3.21E-07	5.56E-05
1	60	L1 vs. M1	+	Metabolism	Metabolism	cellular ketone metabolic process	GO:0042180	P	41	1.46E-04	7.10E-03
1	61	L1 vs. M1	+	Metabolism	Metabolism	cellular protein modification process	GO:0006464	P	1414	2.00E-04	8.85E-03
1	62	L1 vs. M1	+	Metabolism	Metabolism	dicarboxylic acid metabolic process	GO:0043648	P	66	1.10E-03	3.58E-02
1	63	L1 vs. M1	+	Metabolism	Metabolism	galactose metabolic process	GO:0006012	P	18	1.50E-03	4.48E-02
1	64	L1 vs. M1	+	Metabolism	Metabolism	glutamate metabolic process	GO:0006536	P	21	1.61E-03	4.73E-02
1	65	L1 vs. M1	+	Metabolism	Metabolism	glutamine family amino acid metabolic process	GO:0009064	P	45	1.39E-03	4.25E-02
1	66	L1 vs. M1	+	Metabolism	Metabolism	lipid modification	GO:0030258	P	69	9.49E-06	7.63E-04
1	67	L1 vs. M1	+	Metabolism	Metabolism	organic acid metabolic process	GO:0006082	P	706	9.67E-08	2.06E-05
1	68	L1 vs. M1	+	Metabolism	Metabolism	organic hydroxy compound metabolic process	GO:1901615	P	190	5.66E-05	3.34E-03
1	69	L1 vs. M1	+	Metabolism	Metabolism	organonitrogen compound metabolic process	GO:1901564	P	2816	3.62E-05	2.28E-03
1	70	L1 vs. M1	+	Metabolism	Metabolism	oxoacid metabolic process	GO:0043436	P	703	1.97E-07	3.89E-05
1	71	L1 vs. M1	+	Metabolism	Metabolism	phosphate-containing compound metabolic process	GO:0006796	P	1147	1.76E-03	5.01E-02
1	72	L1 vs. M1	+	Metabolism	Metabolism	phosphatidylinositol metabolic process	GO:0046488	P	54	5.57E-04	2.11E-02
1	73	L1 vs. M1	+	Metabolism	Metabolism	phosphoric ester hydrolase activity	GO:0042578	F	238	7.65E-07	4.45E-04
1	74	L1 vs. M1	+	Metabolism	Metabolism	phosphorus metabolic process	GO:0006793	P	1182	1.15E-03	3.60E-02
1	75	L1 vs. M1	+	Metabolism	Metabolism	pigment biosynthetic process	GO:0046148	P	78	5.20E-06	4.44E-04
1	76	L1 vs. M1	+	Metabolism	Metabolism	pigment metabolic process	GO:0042440	P	93	9.23E-06	7.53E-04
1	77	L1 vs. M1	+	Metabolism	Metabolism	protein binding	GO:0005515	F	3306	2.89E-05	7.00E-03
1	78	L1 vs. M1	+	Metabolism	Metabolism	small molecule biosynthetic process	GO:0044283	P	449	2.60E-04	1.12E-02
1	79	L1 vs. M1	+	Metabolism	Metabolism	small molecule metabolic process	GO:0044281	P	1035	3.26E-08	9.52E-06
1	80	L1 vs. M1	+	Metabolism	Photosynthesis	chloroplast envelope	GO:0009941	C	474	1.84E-04	5.50E-03
1	81	L1 vs. M1	+	Metabolism	Photosynthesis	chloroplast stroma	GO:0009570	C	548	3.65E-05	1.72E-03
1	82	L1 vs. M1	+	Metabolism	Photosynthesis	plastid	GO:0009536	C	3328	2.43E-03	4.36E-02
1	83	L1 vs. M1	+	Metabolism	Photosynthesis	plastid envelope	GO:0009526	C	490	2.95E-04	8.56E-03
1	84	L1 vs. M1	+	Metabolism	Photosynthesis	plastid stroma	GO:0009532	C	557	1.74E-04	5.37E-03
1	85	L1 vs. M1	+	Metabolism	Transporters	active ion transmembrane transporter activity	GO:0022853	F	146	2.49E-04	3.45E-02
1	86	L1 vs. M1	+	Metabolism	Transporters	establishment of protein localization to peroxisome	GO:0072663	P	10	8.41E-04	2.86E-02
1	87	L1 vs. M1	+	Metabolism	Transporters	protein localization to peroxisome	GO:0072662	P	10	8.41E-04	2.84E-02
1	88	L1 vs. M1	+	Metabolism	Transporters	protein targeting to peroxisome	GO:0006625	P	10	8.41E-04	2.88E-02
1	89	L1 vs. M1	+	Metabolism	Transporters	transporter activity	GO:0005215	F	816	1.95E-04	2.99E-02
1	90	L1 vs. M1	+	Metabolism	Transporters	water channel activity	GO:0015250	F	23	1.68E-04	2.88E-02
1	91	L1 vs. M1	+	Metabolism	Transporters	water transmembrane transporter activity	GO:0005372	F	23	1.68E-04	3.06E-02
1	92	L1 vs. M1	+	Regulation	Protein modification	protein modification by small protein conjugation	GO:0032446	P	380	4.31E-06	4.11E-04
1	93	L1 vs. M1	+	Regulation	Protein modification	protein modification by small protein conjugation or removal	GO:0070647	P	432	5.28E-06	4.43E-04
1	94	L1 vs. M1	+	Regulation	Protein modification	protein modification process	GO:0036211	P	1414	2.00E-04	8.92E-03
1	95	L1 vs. M1	+	Signaling and response	Signaling	cellular response to abiotic stimulus	GO:0071214	P	127	6.45E-04	2.34E-02
1	96	L1 vs. M1	+	Signaling and response	Signaling	cellular response to environmental stimulus	GO:0104004	P	127	6.45E-04	2.35E-02
1	97	L1 vs. M1	+	Signaling and response	Signaling	cellular response to external stimulus	GO:0071496	P	151	1.08E-04	5.72E-03
1	98	L1 vs. M1	+	Signaling and response	Signaling	cellular response to extracellular stimulus	GO:0031668	P	146	1.20E-04	6.16E-03
1	99	L1 vs. M1	+	Signaling and response	Signaling	cellular response to lipid	GO:0071396	P	156	9.87E-04	3.27E-02
1	100	L1 vs. M1	+	Signaling and response	Signaling	cellular response to nitrogen levels	GO:0043562	P	27	4.80E-04	1.91E-02
1	101	L1 vs. M1	+	Signaling and response	Signaling	circadian rhythm	GO:0007623	P	79	4.88E-06	4.36E-04
1	102	L1 vs. M1	+	Signaling and response	Signaling	dephosphorylation	GO:0016311	P	121	3.52E-06	3.54E-04
1	103	L1 vs. M1	+	Signaling and response	Signaling	phosphatase activity	GO:0016791	F	202	1.13E-08	1.10E-05
1	104	L1 vs. M1	+	Signaling and response	Signaling	phosphoprotein phosphatase activity	GO:0004721	F	110	3.12E-08	2.27E-05
1	105	L1 vs. M1	+	Signaling and response	Signaling	protein dephosphorylation	GO:0006470	P	81	1.57E-05	1.13E-03
1	106	L1 vs. M1	+	Signaling and response	Signaling	protein serine/threonine phosphatase activity	GO:0004722	F	74	9.67E-06	4.01E-03
1	107	L1 vs. M1	+	Signaling and response	Signaling	regulation of circadian rhythm	GO:0042752	P	37	1.42E-04	6.99E-03
1	108	L1 vs. M1	+	Signaling and response	Signaling	response to abiotic stimulus	GO:0009628	P	1362	3.30E-06	3.39E-04
1	109	L1 vs. M1	+	Signaling and response	Signaling	response to acid chemical	GO:0001101	P	267	5.39E-04	2.09E-02
1	110	L1 vs. M1	+	Signaling and response	Signaling	response to chemical	GO:0042221	P	1717	6.56E-06	5.43E-04
1	111	L1 vs. M1	+	Signaling and response	Signaling	response to endogenous stimulus	GO:0009719	P	804	1.31E-04	6.61E-03
1	112	L1 vs. M1	+	Signaling and response	Signaling	response to extracellular stimulus	GO:0009991	P	177	1.04E-05	7.87E-04
1	113	L1 vs. M1	+	Signaling and response	Signaling	response to hormone	GO:0009725	P	787	7.30E-05	4.09E-03
1	114	L1 vs. M1	+	Signaling and response	Signaling	response to inorganic substance	GO:0010035	P	631	1.31E-05	9.71E-04
1	115	L1 vs. M1	+	Signaling and response	Signaling	response to light intensity	GO:0009642	P	102	1.87E-04	8.50E-03
1	116	L1 vs. M1	+	Signaling and response	Signaling	response to light stimulus	GO:0009416	P	451	2.35E-05	1.53E-03
1	117	L1 vs. M1	+	Signaling and response	Signaling	response to lipid	GO:0033993	P	433	2.34E-07	4.19E-05
1	118	L1 vs. M1	+	Signaling and response	Signaling	response to nutrient levels	GO:0031667	P	147	1.84E-04	8.44E-03
1	119	L1 vs. M1	+	Signaling and response	Signaling	response to organic substance	GO:0010033	P	1069	2.05E-04	9.01E-03
1	120	L1 vs. M1	+	Signaling and response	Signaling	response to oxygen-containing compound	GO:1901700	P	956	3.60E-07	6.04E-05
1	121	L1 vs. M1	+	Signaling and response	Signaling	response to stimulus	GO:0050896	P	3374	1.42E-03	4.30E-02

1	122	L1 vs. M1	+	Signaling and response	Signaling	response to water	GO:0009415	P	246	1.35E-04	6.75E-03
1	123	L1 vs. M1	+	Signaling and response	Signaling	rhythmic process	GO:0048511	P	79	4.88E-06	4.29E-04
1	124	L1 vs. M1	+	Signaling and response	Stress	cellular monovalent inorganic cation homeostasis	GO:0030004	P	30	1.55E-04	7.35E-03
1	125	L1 vs. M1	+	Signaling and response	Stress	hyperosmotic response	GO:0006972	P	47	6.86E-04	2.44E-02
1	126	L1 vs. M1	+	Signaling and response	Stress	monovalent inorganic cation homeostasis	GO:0055067	P	50	1.83E-04	8.43E-03
1	127	L1 vs. M1	+	Signaling and response	Stress	potassium ion homeostasis	GO:0055075	P	15	6.53E-04	2.34E-02
1	128	L1 vs. M1	+	Signaling and response	Stress	regulation of abscisic acid-activated signaling pathway	GO:0009787	P	80	2.70E-04	1.13E-02
1	129	L1 vs. M1	+	Signaling and response	Stress	regulation of cellular pH	GO:0030641	P	21	5.47E-04	2.10E-02
1	130	L1 vs. M1	+	Signaling and response	Stress	regulation of cellular response to alcohol	GO:1905957	P	80	2.70E-04	1.13E-02
1	131	L1 vs. M1	+	Signaling and response	Stress	regulation of intracellular pH	GO:0051453	P	21	5.47E-04	2.09E-02
1	132	L1 vs. M1	+	Signaling and response	Stress	regulation of reactive oxygen species metabolic process	GO:2000377	P	39	9.78E-04	3.29E-02
1	133	L1 vs. M1	+	Signaling and response	Stress	regulation of response to alcohol	GO:1901419	P	80	2.70E-04	1.12E-02
1	134	L1 vs. M1	+	Signaling and response	Stress	regulation of stomatal closure	GO:0090333	P	17	1.72E-03	4.96E-02
1	135	L1 vs. M1	+	Signaling and response	Stress	regulation of stomatal movement	GO:0010119	P	57	6.10E-05	3.49E-03
1	136	L1 vs. M1	+	Signaling and response	Stress	response to abscisic acid	GO:0009737	P	328	4.73E-07	7.09E-05
1	137	L1 vs. M1	+	Signaling and response	Stress	response to absence of light	GO:0009646	P	26	2.12E-05	1.45E-03
1	138	L1 vs. M1	+	Signaling and response	Stress	response to alcohol	GO:0097305	P	331	6.99E-07	9.68E-05
1	139	L1 vs. M1	+	Signaling and response	Stress	response to osmotic stress	GO:0006970	P	356	1.80E-04	8.40E-03
1	140	L1 vs. M1	+	Signaling and response	Stress	response to radiation	GO:0009314	P	471	2.85E-04	1.17E-02
1	141	L1 vs. M1	+	Signaling and response	Stress	response to salt stress	GO:0009651	P	298	9.91E-04	3.27E-02
1	142	L1 vs. M1	+	Signaling and response	Stress	response to water deprivation	GO:0009414	P	242	1.38E-04	6.81E-03
1	143	L1 vs. M1	+	Signaling and response	Stress	vitamin biosynthetic process	GO:0009110	P	43	2.65E-04	1.13E-02
1	144	L1 vs. M1	+	Signaling and response	Stress	vitamin metabolic process	GO:0006766	P	52	1.15E-03	3.62E-02
1	145	L1 vs. M1	-	Development	Cell division	cell cycle	GO:0007049	P	292	2.77E-11	1.71E-08
1	146	L1 vs. M1	-	Development	Cell division	cell cycle G1/S phase transition	GO:0044843	P	7	5.85E-05	3.38E-03
1	147	L1 vs. M1	-	Development	Cell division	cell cycle phase transition	GO:0044770	P	28	4.45E-05	2.68E-03
1	148	L1 vs. M1	-	Development	Cell division	cell cycle process	GO:0022402	P	268	8.02E-10	3.70E-07
1	149	L1 vs. M1	-	Development	Cell division	chromosome	GO:0005694	C	289	7.42E-04	1.88E-02
1	150	L1 vs. M1	-	Development	Cell division	chromosome organization involved in meiotic cell cycle	GO:0070192	P	30	4.38E-04	1.76E-02
1	151	L1 vs. M1	-	Development	Cell division	chromosome segregation	GO:0007059	P	67	9.71E-06	7.58E-04
1	152	L1 vs. M1	-	Development	Cell division	condensed chromosome	GO:0000793	C	39	3.32E-05	1.64E-03
1	153	L1 vs. M1	-	Development	Cell division	condensed nuclear chromosome	GO:0000794	C	26	9.00E-04	2.11E-02
1	154	L1 vs. M1	-	Development	Cell division	condensin complex	GO:0000796	C	7	2.05E-03	3.82E-02
1	155	L1 vs. M1	-	Development	Cell division	cyclin-dependent protein kinase holoenzyme complex	GO:0000307	C	36	1.91E-03	3.63E-02
1	156	L1 vs. M1	-	Development	Cell division	DNA biosynthetic process	GO:0071897	P	17	6.08E-04	2.29E-02
1	157	L1 vs. M1	-	Development	Cell division	DNA packaging complex	GO:0044815	C	19	1.02E-03	2.30E-02
1	158	L1 vs. M1	-	Development	Cell division	DNA polymerase complex	GO:0042575	C	15	1.01E-03	2.32E-02
1	159	L1 vs. M1	-	Development	Cell division	DNA recombination	GO:0006310	P	84	8.26E-07	1.09E-04
1	160	L1 vs. M1	-	Development	Cell division	DNA replication	GO:0006260	P	82	7.19E-08	1.66E-05
1	161	L1 vs. M1	-	Development	Cell division	DNA-dependent DNA replication	GO:0006261	P	73	2.24E-07	4.14E-05
1	162	L1 vs. M1	-	Development	Cell division	double-strand break repair	GO:0006302	P	79	1.57E-06	1.98E-04
1	163	L1 vs. M1	-	Development	Cell division	G1/S transition of mitotic cell cycle	GO:0000082	P	6	1.53E-04	7.33E-03
1	164	L1 vs. M1	-	Development	Cell division	homologous chromosome pairing at meiosis	GO:0007129	P	17	1.33E-03	4.07E-02
1	165	L1 vs. M1	-	Development	Cell division	homologous chromosome segregation	GO:0045143	P	20	1.04E-03	3.40E-02
1	166	L1 vs. M1	-	Development	Cell division	homologous recombination	GO:0035825	P	27	1.12E-03	3.54E-02
1	167	L1 vs. M1	-	Development	Cell division	meiosis I	GO:0007127	P	36	1.98E-04	8.94E-03
1	168	L1 vs. M1	-	Development	Cell division	meiosis I cell cycle process	GO:0061982	P	40	6.10E-04	2.28E-02
1	169	L1 vs. M1	-	Development	Cell division	meiotic cell cycle	GO:0051321	P	99	2.07E-06	2.29E-04
1	170	L1 vs. M1	-	Development	Cell division	meiotic cell cycle process	GO:1903046	P	88	1.73E-05	1.21E-03
1	171	L1 vs. M1	-	Development	Cell division	meiotic chromosome segregation	GO:0045132	P	33	7.90E-04	2.75E-02
1	172	L1 vs. M1	-	Development	Cell division	meiotic nuclear division	GO:0140013	P	54	4.94E-05	2.94E-03
1	173	L1 vs. M1	-	Development	Cell division	microtubule motor activity	GO:0003777	F	38	7.26E-06	3.52E-03
1	174	L1 vs. M1	-	Development	Cell division	mitotic cell cycle	GO:0000278	P	160	8.08E-10	3.44E-07
1	175	L1 vs. M1	-	Development	Cell division	mitotic cell cycle phase transition	GO:0044772	P	27	9.76E-05	5.25E-03
1	176	L1 vs. M1	-	Development	Cell division	mitotic cell cycle process	GO:1903047	P	130	1.58E-09	5.84E-07
1	177	L1 vs. M1	-	Development	Cell division	nuclear chromosome segregation	GO:0098813	P	60	9.56E-06	7.57E-04
1	178	L1 vs. M1	-	Development	Cell division	nuclear division	GO:0000280	P	89	4.32E-07	6.65E-05
1	179	L1 vs. M1	-	Development	Cell division	nuclear DNA replication	GO:0033260	P	13	6.53E-04	2.35E-02
1	180	L1 vs. M1	-	Development	Cell division	nuclear replication fork	GO:0043596	C	18	4.39E-05	1.80E-03
1	181	L1 vs. M1	-	Development	Cell division	nucleic acid metabolic process	GO:0090304	P	1011	5.66E-07	8.04E-05
1	182	L1 vs. M1	-	Development	Cell division	nucleobase-containing compound metabolic process	GO:0006139	P	1292	9.50E-05	5.16E-03
1	183	L1 vs. M1	-	Development	Cell division	reciprocal homologous recombination	GO:0140527	P	27	1.12E-03	3.58E-02
1	184	L1 vs. M1	-	Development	Cell division	reciprocal meiotic recombination	GO:0007131	P	27	1.12E-03	3.56E-02
1	185	L1 vs. M1	-	Development	Cell division	recombinational repair	GO:0000725	P	55	1.58E-03	4.67E-02

1	186	L1 vs. M1	-	Development	Cell division	regulation of cell cycle	GO:0051726	P	142	1.17E-03	3.63E-02
1	187	L1 vs. M1	-	Development	Cell division	replication fork	GO:0005657	C	31	3.27E-04	8.95E-03
1	188	L1 vs. M1	-	Development	Cell division	replisome	GO:0030894	C	16	4.37E-05	1.87E-03
1	189	L1 vs. M1	-	Development	Cell division	RNA metabolic process	GO:0016070	P	764	2.33E-04	1.01E-02
1	190	L1 vs. M1	-	Development	Development	anchored component of membrane	GO:0031225	C	171	3.04E-03	4.99E-02
1	191	L1 vs. M1	-	Development	Development	anchored component of plasma membrane	GO:0046658	C	96	8.93E-04	2.15E-02
1	192	L1 vs. M1	-	Development	Development	cellular component biogenesis	GO:0044085	P	829	2.14E-05	1.44E-03
1	193	L1 vs. M1	-	Development	Development	cellular component organization or biogenesis	GO:0071840	P	1745	1.15E-04	5.94E-03
1	194	L1 vs. M1	-	Development	Development	chromosome organization	GO:0051276	P	301	1.61E-04	7.58E-03
1	195	L1 vs. M1	-	Development	Development	intracellular non-membrane-bounded organelle	GO:0043232	C	1113	1.43E-14	7.06E-12
1	196	L1 vs. M1	-	Development	Development	intracellular organelle lumen	GO:0070013	C	830	1.35E-06	1.02E-04
1	197	L1 vs. M1	-	Development	Development	membrane-enclosed lumen	GO:0031974	C	830	1.35E-06	9.49E-05
1	198	L1 vs. M1	-	Development	Development	microtubule	GO:0005874	C	88	1.61E-03	3.18E-02
1	199	L1 vs. M1	-	Development	Development	microtubule-based movement	GO:0007018	P	39	2.08E-05	1.44E-03
1	200	L1 vs. M1	-	Development	Development	microtubule-based process	GO:0007017	P	149	7.91E-04	2.74E-02
1	201	L1 vs. M1	-	Development	Development	movement of cell or subcellular component	GO:0006928	P	52	4.34E-05	2.64E-03
1	202	L1 vs. M1	-	Development	Development	non-membrane-bounded organelle	GO:0043228	C	1113	1.43E-14	4.71E-12
1	203	L1 vs. M1	-	Development	Development	nuclear lumen	GO:0031981	C	726	1.81E-06	1.11E-04
1	204	L1 vs. M1	-	Development	Development	nuclear replisome	GO:0043601	C	16	4.37E-05	1.96E-03
1	205	L1 vs. M1	-	Development	Development	nucleus organization	GO:0006997	P	29	6.94E-04	2.45E-02
1	206	L1 vs. M1	-	Development	Development	organelle assembly	GO:0070925	P	132	2.27E-05	1.50E-03
1	207	L1 vs. M1	-	Development	Development	organelle fission	GO:0048285	P	117	1.79E-06	2.07E-04
1	208	L1 vs. M1	-	Development	Development	organelle lumen	GO:0043233	C	830	1.35E-06	8.86E-05
1	209	L1 vs. M1	-	Development	Development	organelle organization	GO:0006996	P	1043	5.26E-04	2.05E-02
1	210	L1 vs. M1	-	Development	Development	structural molecule activity	GO:0005198	F	308	4.85E-10	7.05E-07
1	211	L1 vs. M1	-	Metabolism	Catabolism	protein-containing complex	GO:0032991	C	1910	1.71E-04	5.43E-03
1	212	L1 vs. M1	-	Metabolism	Catabolism	RNA phosphodiester bond hydrolysis, endonucleolytic	GO:0090502	P	13	1.66E-03	4.83E-02
1	213	L1 vs. M1	-	Metabolism	Metabolism	amide biosynthetic process	GO:0043604	P	408	7.41E-08	1.64E-05
1	214	L1 vs. M1	-	Metabolism	Metabolism	cellular amide metabolic process	GO:0043603	P	493	3.21E-06	3.36E-04
1	215	L1 vs. M1	-	Metabolism	Metabolism	cellular macromolecule biosynthetic process	GO:0034645	P	819	4.18E-08	1.10E-05
1	216	L1 vs. M1	-	Metabolism	Metabolism	cellular nitrogen compound biosynthetic process	GO:0044271	P	813	1.45E-03	4.37E-02
1	217	L1 vs. M1	-	Metabolism	Metabolism	cellular nitrogen compound metabolic process	GO:0034641	P	1813	2.97E-06	3.17E-04
1	218	L1 vs. M1	-	Metabolism	Metabolism	cellular protein-containing complex assembly	GO:0034622	P	306	4.25E-05	2.61E-03
1	219	L1 vs. M1	-	Metabolism	Metabolism	DNA metabolic process	GO:0006259	P	270	1.25E-04	6.33E-03
1	220	L1 vs. M1	-	Metabolism	Metabolism	macromolecule biosynthetic process	GO:0009059	P	858	1.25E-07	2.57E-05
1	221	L1 vs. M1	-	Metabolism	Metabolism	microtubule binding	GO:0008017	F	96	3.72E-05	7.71E-03
1	222	L1 vs. M1	-	Metabolism	Metabolism	motor activity	GO:0003774	F	49	4.07E-05	7.89E-03
1	223	L1 vs. M1	-	Metabolism	Metabolism	peptide metabolic process	GO:0006518	P	408	9.44E-09	3.27E-06
1	224	L1 vs. M1	-	Metabolism	Metabolism	transferase complex, transferring phosphorus-containing groups	GO:0061695	C	136	1.74E-03	3.37E-02
1	225	L1 vs. M1	-	Metabolism	Metabolism	tubulin binding	GO:0015631	F	103	1.74E-04	2.81E-02
1	226	L1 vs. M1	-	Metabolism	Photosynthesis	photosystem II reaction center	GO:0009539	C	8	3.05E-04	8.59E-03
1	227	L1 vs. M1	-	Metabolism	Photosynthesis	plastid large ribosomal subunit	GO:0000311	C	12	1.24E-03	2.60E-02
1	228	L1 vs. M1	-	Metabolism	Photosynthesis	plastid ribosome	GO:0009547	C	22	1.06E-05	6.17E-04
1	229	L1 vs. M1	-	Metabolism	Transporters	sulfate assimilation	GO:0000103	P	16	1.46E-04	7.04E-03
1	230	L1 vs. M1	-	Regulation	Protein modification	protein-containing complex assembly	GO:0065003	P	347	9.83E-04	3.28E-02
1	231	L1 vs. M1	-	Regulation	Protein modification	protein-containing complex subunit organization	GO:0043933	P	395	2.31E-04	1.01E-02
1	232	L1 vs. M1	-	Regulation	Transcription	DNA conformation change	GO:0071103	P	86	7.46E-05	4.14E-03
1	233	L1 vs. M1	-	Regulation	Transcription	gene expression	GO:0010467	P	1013	3.65E-08	1.01E-05
1	234	L1 vs. M1	-	Regulation	Transcription	nucleolus	GO:0005730	C	344	1.23E-09	2.02E-07
1	235	L1 vs. M1	-	Regulation	Transcription	nucleosome organization	GO:0034728	P	36	1.11E-03	3.58E-02
1	236	L1 vs. M1	-	Regulation	Transcription	protein-DNA complex	GO:0032993	C	41	1.55E-03	3.12E-02
1	237	L1 vs. M1	-	Regulation	Transcription	protein-DNA complex assembly	GO:0065004	P	49	4.86E-04	1.92E-02
1	238	L1 vs. M1	-	Regulation	Transcription	protein-DNA complex subunit organization	GO:0071824	P	59	5.83E-05	3.40E-03
1	239	L1 vs. M1	-	Regulation	Transcription	RNA modification	GO:0009451	P	151	2.81E-06	3.05E-04
1	240	L1 vs. M1	-	Regulation	Translation	90S preribosome	GO:0030686	C	17	1.34E-05	7.35E-04
1	241	L1 vs. M1	-	Regulation	Translation	cleavage involved in rRNA processing	GO:0000469	P	14	8.92E-05	4.89E-03
1	242	L1 vs. M1	-	Regulation	Translation	cytosolic large ribosomal subunit	GO:0022625	C	107	1.98E-09	2.44E-07
1	243	L1 vs. M1	-	Regulation	Translation	cytosolic ribosome	GO:0022626	C	241	2.51E-07	2.06E-05
1	244	L1 vs. M1	-	Regulation	Translation	cytosolic small ribosomal subunit	GO:0022627	C	78	2.46E-03	4.33E-02
1	245	L1 vs. M1	-	Regulation	Translation	endonucleolytic cleavage involved in rRNA processing	GO:0000478	P	7	1.48E-03	4.43E-02
1	246	L1 vs. M1	-	Regulation	Translation	large ribosomal subunit	GO:0015934	C	135	2.09E-11	4.13E-09
1	247	L1 vs. M1	-	Regulation	Translation	maturation of SSU-rRNA	GO:0030490	P	37	1.72E-05	1.22E-03

1	248	L1 vs. M1	-	Regulation	Translation	maturation of SSU-rRNA from tricistronic rRNA transcript (SSU-rRNA, 5.8S rRNA, LSU-rRNA)	GO:0000462	P	24	3.38E-04	1.38E-02
1	249	L1 vs. M1	-	Regulation	Translation	ncRNA metabolic process	GO:0034660	P	281	3.33E-05	2.12E-03
1	250	L1 vs. M1	-	Regulation	Translation	ncRNA processing	GO:0034470	P	235	5.03E-06	4.35E-04
1	251	L1 vs. M1	-	Regulation	Translation	organellar ribosome	GO:0000313	C	47	1.22E-04	4.16E-03
1	252	L1 vs. M1	-	Regulation	Translation	peptide biosynthetic process	GO:0043043	P	364	1.24E-09	4.90E-07
1	253	L1 vs. M1	-	Regulation	Translation	preribosome	GO:0030684	C	60	1.42E-07	1.28E-05
1	254	L1 vs. M1	-	Regulation	Translation	ribonucleoprotein complex	GO:1990904	C	459	6.33E-15	6.24E-12
1	255	L1 vs. M1	-	Regulation	Translation	ribonucleoprotein complex assembly	GO:0022618	P	124	3.82E-07	6.23E-05
1	256	L1 vs. M1	-	Regulation	Translation	ribonucleoprotein complex biogenesis	GO:0022613	P	307	6.69E-12	4.64E-09
1	257	L1 vs. M1	-	Regulation	Translation	ribonucleoprotein complex subunit organization	GO:0071826	P	127	4.13E-07	6.53E-05
1	258	L1 vs. M1	-	Regulation	Translation	ribosomal large subunit assembly	GO:0000027	P	36	2.21E-05	1.48E-03
1	259	L1 vs. M1	-	Regulation	Translation	ribosomal large subunit biogenesis	GO:0042273	P	79	8.13E-07	1.10E-04
1	260	L1 vs. M1	-	Regulation	Translation	ribosomal small subunit assembly	GO:0000028	P	30	1.09E-04	5.70E-03
1	261	L1 vs. M1	-	Regulation	Translation	ribosomal small subunit biogenesis	GO:0042274	P	67	5.44E-07	7.94E-05
1	262	L1 vs. M1	-	Regulation	Translation	ribosomal subunit	GO:0044391	C	238	1.65E-13	4.06E-11
1	263	L1 vs. M1	-	Regulation	Translation	ribosome	GO:0005840	C	303	1.48E-09	2.09E-07
1	264	L1 vs. M1	-	Regulation	Translation	ribosome assembly	GO:0042255	P	71	1.48E-08	4.84E-06
1	265	L1 vs. M1	-	Regulation	Translation	ribosome biogenesis	GO:0042254	P	253	6.36E-13	7.05E-10
1	266	L1 vs. M1	-	Regulation	Translation	RNA phosphodiester bond hydrolysis	GO:0090501	P	26	6.60E-05	3.73E-03
1	267	L1 vs. M1	-	Regulation	Translation	RNA processing	GO:0006396	P	495	6.44E-04	2.36E-02
1	268	L1 vs. M1	-	Regulation	Translation	rRNA metabolic process	GO:0016072	P	170	1.68E-06	2.07E-04
1	269	L1 vs. M1	-	Regulation	Translation	rRNA processing	GO:0006364	P	162	2.15E-07	4.11E-05
1	270	L1 vs. M1	-	Regulation	Translation	small nucleolar ribonucleoprotein complex	GO:0005732	C	17	1.49E-03	3.06E-02
1	271	L1 vs. M1	-	Regulation	Translation	small ribosomal subunit	GO:0015935	C	103	3.34E-04	8.89E-03
1	272	L1 vs. M1	-	Regulation	Translation	small-subunit processome	GO:0032040	C	37	4.85E-05	1.91E-03
1	273	L1 vs. M1	-	Regulation	Translation	snoRNA binding	GO:0030515	F	18	3.02E-04	3.99E-02
1	274	L1 vs. M1	-	Regulation	Translation	structural constituent of ribosome	GO:0003735	F	224	3.19E-12	9.26E-09
1	275	L1 vs. M1	-	Regulation	Translation	translation	GO:0006412	P	360	2.53E-10	1.28E-07
1	276	L1 vs. M1	-	Signaling and response	Signaling	protein kinase complex	GO:1902911	C	50	2.31E-03	4.21E-02
1	277	L1 vs. M1	-	Signaling and response	Signaling	serine/threonine protein kinase complex	GO:1902554	C	46	8.20E-04	2.02E-02
1	278	L1 vs. M1	-	Signaling and response	Stress	cellular response to DNA damage stimulus	GO:0006974	P	221	1.01E-03	3.30E-02
2	279	L2 vs. M2	+	Development	Cell division	nucleic acid metabolic process	GO:0090304	P	1011	1.85E-05	1.03E-02
2	280	L2 vs. M2	+	Development	Cell division	nucleobase-containing compound metabolic process	GO:0006139	P	1292	6.54E-05	2.01E-02
2	281	L2 vs. M2	+	Development	Cell division	nucleoside-triphosphatase activity	GO:0017111	F	496	1.01E-08	2.95E-05
2	282	L2 vs. M2	+	Development	Cell division	preprophase band	GO:0009574	C	13	7.70E-04	3.16E-02
2	283	L2 vs. M2	+	Development	Cell division	spindle	GO:0005819	C	50	5.91E-04	2.53E-02
2	284	L2 vs. M2	+	Development	Development	cellular component organization	GO:0016043	P	1494	3.69E-06	1.02E-02
2	285	L2 vs. M2	+	Development	Development	cellular component organization or biogenesis	GO:0071840	P	1745	4.66E-06	6.46E-03
2	286	L2 vs. M2	+	Development	Development	cytoskeletal protein binding	GO:0008092	F	180	2.29E-08	1.66E-05
2	287	L2 vs. M2	+	Development	Development	cytoskeleton	GO:0005856	C	192	4.69E-08	7.71E-06
2	288	L2 vs. M2	+	Development	Development	cytoskeleton organization	GO:0007010	P	189	4.29E-05	1.49E-02
2	289	L2 vs. M2	+	Development	Development	cytosol	GO:0005829	C	2304	2.13E-09	6.99E-07
2	290	L2 vs. M2	+	Development	Development	envelope	GO:0031975	C	740	1.06E-03	4.17E-02
2	291	L2 vs. M2	+	Development	Development	intracellular non-membrane-bounded organelle	GO:0043232	C	1113	2.49E-08	6.13E-06
2	292	L2 vs. M2	+	Development	Development	intracellular organelle lumen	GO:0070013	C	830	1.43E-04	8.29E-03
2	293	L2 vs. M2	+	Development	Development	membrane-enclosed lumen	GO:0031974	C	830	1.43E-04	7.83E-03
2	294	L2 vs. M2	+	Development	Development	microtubule	GO:0005874	C	88	3.19E-05	2.10E-03
2	295	L2 vs. M2	+	Development	Development	microtubule cytoskeleton	GO:0015630	C	139	7.37E-07	7.27E-05
2	296	L2 vs. M2	+	Development	Development	microtubule-based movement	GO:0007018	P	39	1.51E-04	2.61E-02
2	297	L2 vs. M2	+	Development	Development	microtubule-based process	GO:0007017	P	149	2.70E-05	1.15E-02
2	298	L2 vs. M2	+	Development	Development	movement of cell or subcellular component	GO:0006928	P	52	3.06E-04	4.47E-02
2	299	L2 vs. M2	+	Development	Development	non-membrane-bounded organelle	GO:0043228	C	1113	2.49E-08	4.91E-06
2	300	L2 vs. M2	+	Development	Development	nuclear lumen	GO:0031981	C	726	1.08E-04	6.65E-03
2	301	L2 vs. M2	+	Development	Development	nucleus	GO:0005634	C	5373	3.03E-04	1.42E-02
2	302	L2 vs. M2	+	Development	Development	organelle envelope	GO:0031967	C	740	1.06E-03	4.01E-02
2	303	L2 vs. M2	+	Development	Development	organelle lumen	GO:0043233	C	830	1.43E-04	7.42E-03
2	304	L2 vs. M2	+	Development	Development	organelle organization	GO:0006996	P	1043	8.39E-06	6.64E-03
2	305	L2 vs. M2	+	Development	Development	polymeric cytoskeletal fiber	GO:0099513	C	94	1.37E-05	1.12E-03
2	306	L2 vs. M2	+	Metabolism	Bioenergetics	ATPase activity	GO:0016887	F	346	5.01E-07	2.08E-04
2	307	L2 vs. M2	+	Metabolism	Bioenergetics	carbohydrate catabolic process	GO:0016052	P	154	9.66E-05	2.55E-02
2	308	L2 vs. M2	+	Metabolism	Bioenergetics	carbohydrate metabolic process	GO:0005975	P	593	6.45E-06	7.15E-03
2	309	L2 vs. M2	+	Metabolism	Bioenergetics	cellular carbohydrate metabolic process	GO:0044262	P	262	1.08E-04	2.60E-02
2	310	L2 vs. M2	+	Metabolism	Bioenergetics	polysaccharide metabolic process	GO:0005976	P	275	2.19E-04	3.46E-02

2	311	L2 vs. M2	+	Metabolism	Bioenergetics	primary metabolic process	GO:0044238	P	4375	4.30E-06	7.93E-03
2	312	L2 vs. M2	+	Metabolism	Catabolism	catabolic process	GO:0009056	P	935	8.28E-06	7.64E-03
2	313	L2 vs. M2	+	Metabolism	Catabolism	cellular catabolic process	GO:0044248	P	785	3.29E-04	4.67E-02
2	314	L2 vs. M2	+	Metabolism	Catabolism	organic substance catabolic process	GO:1901575	P	861	3.54E-05	1.31E-02
2	315	L2 vs. M2	+	Metabolism	Catabolism	protein-containing complex	GO:0032991	C	1910	1.86E-04	9.15E-03
2	316	L2 vs. M2	+	Metabolism	Metabolism	binding	GO:0005488	F	5829	1.27E-04	3.36E-02
2	317	L2 vs. M2	+	Metabolism	Metabolism	cellular aromatic compound metabolic process	GO:0006725	P	1558	2.50E-05	1.16E-02
2	318	L2 vs. M2	+	Metabolism	Metabolism	cellular macromolecule metabolic process	GO:0044260	P	2636	2.77E-04	4.15E-02
2	319	L2 vs. M2	+	Metabolism	Metabolism	cellular nitrogen compound metabolic process	GO:0034641	P	1813	1.51E-04	2.69E-02
2	320	L2 vs. M2	+	Metabolism	Metabolism	heterocycle metabolic process	GO:0046483	P	1486	1.66E-05	1.15E-02
2	321	L2 vs. M2	+	Metabolism	Metabolism	hydrolase activity	GO:0016787	F	1781	1.58E-05	4.58E-03
2	322	L2 vs. M2	+	Metabolism	Metabolism	hydrolase activity, acting on acid anhydrides	GO:0016817	F	535	3.00E-08	1.75E-05
2	323	L2 vs. M2	+	Metabolism	Metabolism	hydrolase activity, acting on acid anhydrides, in phosphorus-containing anhydrides	GO:0016818	F	530	1.70E-08	2.48E-05
2	324	L2 vs. M2	+	Metabolism	Metabolism	macromolecule metabolic process	GO:0043170	P	3269	2.30E-06	1.28E-02
2	325	L2 vs. M2	+	Metabolism	Metabolism	metabolic process	GO:0008152	P	5274	9.31E-05	2.58E-02
2	326	L2 vs. M2	+	Metabolism	Metabolism	microtubule binding	GO:0008017	F	96	2.33E-06	7.53E-04
2	327	L2 vs. M2	+	Metabolism	Metabolism	nitrogen compound metabolic process	GO:0006807	P	3724	2.23E-04	3.43E-02
2	328	L2 vs. M2	+	Metabolism	Metabolism	organic cyclic compound metabolic process	GO:1901360	P	1623	6.33E-05	2.06E-02
2	329	L2 vs. M2	+	Metabolism	Metabolism	organic substance metabolic process	GO:0071704	P	4781	1.76E-05	1.08E-02
2	330	L2 vs. M2	+	Metabolism	Metabolism	protein binding	GO:0005515	F	3306	4.56E-08	2.21E-05
2	331	L2 vs. M2	+	Metabolism	Metabolism	pyrophosphatase activity	GO:0016462	F	527	1.96E-08	1.90E-05
2	332	L2 vs. M2	+	Metabolism	Metabolism	supramolecular complex	GO:0099080	C	157	2.47E-05	1.74E-03
2	333	L2 vs. M2	+	Metabolism	Metabolism	supramolecular fiber	GO:0099512	C	94	1.37E-05	1.04E-03
2	334	L2 vs. M2	+	Metabolism	Metabolism	supramolecular polymer	GO:0099081	C	94	1.37E-05	1.23E-03
2	335	L2 vs. M2	+	Metabolism	Metabolism	tubulin binding	GO:0015631	F	103	2.12E-06	7.70E-04
2	336	L2 vs. M2	+	Metabolism	Photosynthesis	chlorophyll metabolic process	GO:0015994	P	45	3.40E-04	4.71E-02
2	337	L2 vs. M2	+	Metabolism	Photosynthesis	chloroplast envelope	GO:0009941	C	474	3.08E-07	3.80E-05
2	338	L2 vs. M2	+	Metabolism	Photosynthesis	chloroplast stroma	GO:0009570	C	548	2.29E-11	2.25E-08
2	339	L2 vs. M2	+	Metabolism	Photosynthesis	plastid	GO:0009536	C	3328	1.07E-03	3.75E-02
2	340	L2 vs. M2	+	Metabolism	Photosynthesis	plastid envelope	GO:0009526	C	490	1.80E-07	2.54E-05
2	341	L2 vs. M2	+	Metabolism	Photosynthesis	plastid stroma	GO:0009532	C	557	7.76E-11	3.83E-08
2	342	L2 vs. M2	+	Metabolism	Transporters	kinesin complex	GO:0005871	C	19	4.02E-04	1.80E-02
2	343	L2 vs. M2	+	Regulation	Transcription	gene expression	GO:0010467	P	1013	1.26E-04	2.58E-02
2	344	L2 vs. M2	+	Regulation	Transcription	RNA binding	GO:0003723	F	1050	1.69E-04	4.10E-02
2	345	L2 vs. M2	+	Regulation	Translation	mRNA metabolic process	GO:0016071	P	266	2.11E-04	3.45E-02
2	346	L2 vs. M2	+	Regulation	Translation	RNA splicing	GO:0008380	P	180	1.06E-04	2.68E-02
2	347	L2 vs. M2	+	Regulation	Translation	spliceosomal complex	GO:0005681	C	100	1.06E-03	3.87E-02
2	348	L2 vs. M2	-	Metabolism	Metabolism	glucosinolate biosynthetic process	GO:0019761	P	23	1.41E-04	2.69E-02
2	349	L2 vs. M2	-	Metabolism	Metabolism	glucosinolate metabolic process	GO:0019760	P	67	1.16E-04	2.58E-02
2	350	L2 vs. M2	-	Metabolism	Metabolism	glycosinolate biosynthetic process	GO:0019758	P	23	1.41E-04	2.60E-02
2	351	L2 vs. M2	-	Metabolism	Metabolism	glycosinolate metabolic process	GO:0019757	P	67	1.16E-04	2.48E-02
2	352	L2 vs. M2	-	Metabolism	Metabolism	glycosyl compound biosynthetic process	GO:1901659	P	40	8.76E-05	2.56E-02
2	353	L2 vs. M2	-	Metabolism	Metabolism	glycosyl compound metabolic process	GO:1901657	P	115	1.91E-04	3.20E-02
2	354	L2 vs. M2	-	Metabolism	Metabolism	S-glycoside biosynthetic process	GO:0016144	P	23	1.41E-04	2.78E-02
2	355	L2 vs. M2	-	Metabolism	Metabolism	S-glycoside metabolic process	GO:0016143	P	67	1.16E-04	2.69E-02
2	356	L2 vs. M2	-	Metabolism	Metabolism	sulfur compound metabolic process	GO:0006790	P	246	2.47E-05	1.25E-02
2	357	L2 vs. M2	-	Metabolism	Transporters	sulfate assimilation	GO:0000103	P	16	3.05E-05	1.21E-02
3	358	L3 vs. M3	+	Regulation	Translation	ribosomal subunit	GO:0044391	C	238	4.18E-05	4.12E-02
4	359	L4 vs. M4	+	Development	Cell division	syncytium formation	GO:0006949	P	9	4.31E-04	4.16E-02
4	360	L4 vs. M4	+	Development	Development	actin cytoskeleton organization	GO:0030036	P	52	3.73E-04	3.81E-02
4	361	L4 vs. M4	+	Development	Development	anatomical structure formation involved in morphogenesis	GO:0048646	P	100	1.81E-04	2.15E-02
4	362	L4 vs. M4	+	Development	Development	anchored component of membrane	GO:0031225	C	137	7.63E-05	1.74E-02
4	363	L4 vs. M4	+	Development	Development	cellular component assembly involved in morphogenesis	GO:0010927	P	31	3.69E-04	3.85E-02
4	364	L4 vs. M4	+	Development	Development	cellular component morphogenesis	GO:0032989	P	31	3.69E-04	3.93E-02
4	365	L4 vs. M4	+	Development	Development	external encapsulating structure organization	GO:0045229	P	176	1.09E-04	1.40E-02
4	366	L4 vs. M4	+	Development	Development	structural constituent of cell wall	GO:0005199	F	20	1.43E-05	2.87E-03
4	367	L4 vs. M4	+	Development	Development	structural molecule activity	GO:0005198	F	245	4.96E-06	1.08E-03
4	368	L4 vs. M4	+	Metabolism	Bioenergetics	oxidoreductase activity, oxidizing metal ions, oxygen as acceptor	GO:0016724	F	6	9.79E-05	1.50E-02
4	369	L4 vs. M4	+	Metabolism	Metabolism	ferroxidase activity	GO:0004322	F	6	9.79E-05	1.60E-02
4	370	L4 vs. M4	+	Metabolism	Metabolism	galactosyltransferase activity	GO:0008378	F	22	3.30E-04	3.59E-02
4	371	L4 vs. M4	+	Metabolism	Metabolism	sporopollenin biosynthetic process	GO:0080110	P	5	2.62E-04	3.04E-02
4	372	L4 vs. M4	+	Metabolism	Photosynthesis	chloroplast thylakoid	GO:0009534	C	256	4.06E-05	1.24E-02

4	373	L4 vs. M4	+	Metabolism	Photosynthesis	chloroplast thylakoid membrane	GO:0009535	C	207	2.15E-04	2.18E-02
4	374	L4 vs. M4	+	Metabolism	Photosynthesis	photosynthetic membrane	GO:0034357	C	225	1.78E-04	2.33E-02
4	375	L4 vs. M4	+	Metabolism	Photosynthesis	plastid thylakoid	GO:0031976	C	258	2.96E-05	1.35E-02
4	376	L4 vs. M4	+	Metabolism	Photosynthesis	plastid thylakoid membrane	GO:0055035	C	210	1.13E-04	1.73E-02
4	377	L4 vs. M4	+	Metabolism	Photosynthesis	thylakoid	GO:0009579	C	306	2.49E-04	2.28E-02
4	378	L4 vs. M4	+	Metabolism	Photosynthesis	thylakoid membrane	GO:0042651	C	221	2.01E-04	2.30E-02
4	379	L4 vs. M4	+	Metabolism	Transporters	Golgi vesicle transport	GO:0048193	P	77	2.61E-05	6.35E-03
4	380	L4 vs. M4	-	Development	Cell division	nucleobase-containing compound metabolic process	GO:0006139	P	939	5.58E-06	1.59E-03
4	381	L4 vs. M4	-	Development	Cell division	nucleoside phosphate binding	GO:1901265	F	475	3.49E-07	1.30E-04
4	382	L4 vs. M4	-	Development	Cell division	nucleoside-triphosphatase activity	GO:0017111	F	340	3.16E-08	2.75E-05
4	383	L4 vs. M4	-	Development	Cell division	nucleotide binding	GO:0000166	F	475	3.49E-07	1.52E-04
4	384	L4 vs. M4	-	Development	Development	nucleic acid phosphodiester bond hydrolysis	GO:0090305	P	39	4.28E-04	4.21E-02
4	385	L4 vs. M4	-	Metabolism	Bioenergetics	ATPase activity	GO:0016887	F	243	1.03E-08	1.35E-05
4	386	L4 vs. M4	-	Metabolism	Bioenergetics	carbohydrate derivative binding	GO:0097367	F	363	8.95E-05	1.56E-02
4	387	L4 vs. M4	-	Metabolism	Bioenergetics	primary metabolic process	GO:0044238	P	3260	8.07E-05	1.15E-02
4	388	L4 vs. M4	-	Metabolism	Bioenergetics	starch biosynthetic process	GO:0019252	P	22	5.17E-04	4.48E-02
4	389	L4 vs. M4	-	Metabolism	Catabolism	monocarboxylic acid catabolic process	GO:0072329	P	35	7.69E-05	1.16E-02
4	390	L4 vs. M4	-	Metabolism	Metabolism	anion binding	GO:0043168	F	587	3.14E-06	7.45E-04
4	391	L4 vs. M4	-	Metabolism	Metabolism	ATP binding	GO:0005524	F	211	3.32E-04	3.47E-02
4	392	L4 vs. M4	-	Metabolism	Metabolism	carboxylic acid metabolic process	GO:0019752	P	506	1.04E-06	6.64E-04
4	393	L4 vs. M4	-	Metabolism	Metabolism	catalytic activity	GO:0003824	F	3746	2.45E-06	6.40E-04
4	394	L4 vs. M4	-	Metabolism	Metabolism	cellular amino acid metabolic process	GO:0006520	P	215	4.95E-05	9.37E-03
4	395	L4 vs. M4	-	Metabolism	Metabolism	cellular aromatic compound metabolic process	GO:0006725	P	1153	8.18E-06	2.09E-03
4	396	L4 vs. M4	-	Metabolism	Metabolism	cellular metabolic process	GO:0044237	P	3499	5.14E-04	4.53E-02
4	397	L4 vs. M4	-	Metabolism	Metabolism	cellular nitrogen compound metabolic process	GO:0034641	P	1340	4.99E-04	4.55E-02
4	398	L4 vs. M4	-	Metabolism	Metabolism	glucosyltransferase activity	GO:0046527	F	98	1.01E-04	1.47E-02
4	399	L4 vs. M4	-	Metabolism	Metabolism	heterocycle metabolic process	GO:0046483	P	1083	2.44E-06	1.04E-03
4	400	L4 vs. M4	-	Metabolism	Metabolism	hydrolase activity, acting on acid anhydrides	GO:0016817	F	365	4.81E-07	1.57E-04
4	401	L4 vs. M4	-	Metabolism	Metabolism	hydrolase activity, acting on acid anhydrides, in phosphorus-containing anhydrides	GO:0016818	F	361	3.47E-07	2.26E-04
4	402	L4 vs. M4	-	Metabolism	Metabolism	ion binding	GO:0043167	F	1284	4.82E-04	4.66E-02
4	403	L4 vs. M4	-	Metabolism	Metabolism	lipid modification	GO:0030258	P	51	3.55E-04	3.95E-02
4	404	L4 vs. M4	-	Metabolism	Metabolism	metabolic process	GO:0008152	P	3955	3.43E-04	3.90E-02
4	405	L4 vs. M4	-	Metabolism	Metabolism	monocarboxylic acid metabolic process	GO:0032787	P	257	5.40E-05	9.86E-03
4	406	L4 vs. M4	-	Metabolism	Metabolism	nitrogen compound metabolic process	GO:0006807	P	2721	3.00E-05	6.97E-03
4	407	L4 vs. M4	-	Metabolism	Metabolism	organic acid metabolic process	GO:0006082	P	555	8.42E-07	8.61E-04
4	408	L4 vs. M4	-	Metabolism	Metabolism	organic cyclic compound metabolic process	GO:1901360	P	1194	4.42E-06	1.51E-03
4	409	L4 vs. M4	-	Metabolism	Metabolism	organic substance metabolic process	GO:0071704	P	3566	6.75E-05	1.11E-02
4	410	L4 vs. M4	-	Metabolism	Metabolism	oxoacid metabolic process	GO:0043436	P	554	1.09E-06	6.18E-04
4	411	L4 vs. M4	-	Metabolism	Metabolism	phosphorus metabolic process	GO:0006793	P	857	4.63E-04	4.38E-02
4	412	L4 vs. M4	-	Metabolism	Metabolism	purine nucleotide binding	GO:0017076	F	333	2.79E-04	3.16E-02
4	413	L4 vs. M4	-	Metabolism	Metabolism	purine ribonucleoside triphosphate binding	GO:0035639	F	275	1.06E-04	1.46E-02
4	414	L4 vs. M4	-	Metabolism	Metabolism	purine ribonucleotide binding	GO:0032555	F	326	2.12E-04	2.51E-02
4	415	L4 vs. M4	-	Metabolism	Metabolism	pyrophosphatase activity	GO:0016462	F	361	3.47E-07	1.81E-04
4	416	L4 vs. M4	-	Metabolism	Metabolism	small molecule binding	GO:0036094	F	579	9.71E-09	2.53E-05
4	417	L4 vs. M4	-	Metabolism	Metabolism	small molecule metabolic process	GO:0044281	P	806	3.48E-06	1.27E-03
4	418	L4 vs. M4	-	Metabolism	Metabolism	transferase activity	GO:0016740	F	1478	5.01E-07	1.45E-04
4	419	L4 vs. M4	-	Metabolism	Metabolism	UDP-glucosyltransferase activity	GO:0035251	F	75	2.28E-05	4.24E-03
4	420	L4 vs. M4	-	Metabolism	Metabolism	vitamin binding	GO:0019842	F	70	4.69E-04	4.71E-02
4	421	L4 vs. M4	-	Metabolism	Transporters	ATPase-coupled xenobiotic transmembrane transporter activity	GO:0008559	F	10	5.29E-04	4.93E-02
4	422	L4 vs. M4	-	Metabolism	Transporters	transmembrane transport	GO:0055085	P	408	7.55E-05	1.17E-02
4	423	L4 vs. M4	-	Metabolism	Transporters	zinc ion transmembrane transport	GO:0071577	P	12	3.82E-04	3.84E-02
4	424	L4 vs. M4	-	Regulation	Translation	90S preribosome	GO:0030686	C	12	1.08E-04	1.97E-02
4	425	L4 vs. M4	-	Regulation	Translation	ncRNA metabolic process	GO:0034660	P	221	7.86E-05	1.15E-02
4	426	L4 vs. M4	-	Regulation	Translation	ncRNA processing	GO:0034470	P	185	4.93E-04	4.58E-02
4	427	L4 vs. M4	-	Regulation	Translation	preribosome	GO:0030684	C	47	1.28E-06	1.17E-03
4	428	L4 vs. M4	-	Regulation	Translation	ribonucleotide binding	GO:0032553	F	346	1.19E-04	1.56E-02
4	429	L4 vs. M4	-	Regulation	Translation	small-subunit processome	GO:0032040	C	26	4.05E-04	3.36E-02
4	430	L4 vs. M4	-	Regulation	Translation	snoRNA binding	GO:0030515	F	16	1.93E-04	2.40E-02
4	431	L4 vs. M4	-	Regulation	Translation	tRNA metabolic process	GO:0006399	P	81	1.75E-04	2.13E-02
4	432	L4 vs. M4	-	Signaling and response	Signaling	auxin polar transport	GO:0009926	P	35	5.54E-04	4.72E-02
4	433	L4 vs. M4	-	Signaling and response	Signaling	cellular response to chemical stimulus	GO:0070887	P	486	1.17E-04	1.47E-02
4	434	L4 vs. M4	-	Signaling and response	Signaling	cellular response to oxygen levels	GO:0071453	P	105	6.70E-05	1.14E-02

4	435	L4 vs. M4	-	Signaling and response	Signaling	cellular response to stimulus	GO:0051716	P	1048	8.46E-07	7.21E-04
4	436	L4 vs. M4	-	Signaling and response	Signaling	protein autophosphorylation	GO:0046777	P	87	5.34E-06	1.61E-03
4	437	L4 vs. M4	-	Signaling and response	Signaling	response to cadmium ion	GO:0046686	P	183	3.99E-05	8.51E-03
4	438	L4 vs. M4	-	Signaling and response	Signaling	response to chemical	GO:0042221	P	1279	2.19E-09	1.12E-05
4	439	L4 vs. M4	-	Signaling and response	Signaling	response to chitin	GO:0010200	P	58	3.26E-06	1.28E-03
4	440	L4 vs. M4	-	Signaling and response	Signaling	response to inorganic substance	GO:0010035	P	501	3.26E-07	4.17E-04
4	441	L4 vs. M4	-	Signaling and response	Signaling	response to metal ion	GO:0010038	P	247	5.01E-04	4.50E-02
4	442	L4 vs. M4	-	Signaling and response	Signaling	response to nitrate	GO:0010167	P	11	3.64E-04	3.96E-02
4	443	L4 vs. M4	-	Signaling and response	Signaling	response to nitrogen compound	GO:1901698	P	138	8.88E-05	1.23E-02
4	444	L4 vs. M4	-	Signaling and response	Signaling	response to organic cyclic compound	GO:0014070	P	140	3.69E-05	8.19E-03
4	445	L4 vs. M4	-	Signaling and response	Signaling	response to organic substance	GO:0010033	P	779	2.23E-06	1.04E-03
4	446	L4 vs. M4	-	Signaling and response	Signaling	response to organonitrogen compound	GO:0010243	P	113	7.00E-05	1.12E-02
4	447	L4 vs. M4	-	Signaling and response	Signaling	response to oxygen levels	GO:0070482	P	123	1.07E-04	1.40E-02
4	448	L4 vs. M4	-	Signaling and response	Signaling	response to oxygen-containing compound	GO:1901700	P	737	1.63E-07	4.17E-04
4	449	L4 vs. M4	-	Signaling and response	Signaling	response to salicylic acid	GO:0009751	P	64	7.87E-06	2.12E-03
4	450	L4 vs. M4	-	Signaling and response	Signaling	response to stimulus	GO:0050896	P	2496	8.73E-07	6.38E-04
4	451	L4 vs. M4	-	Signaling and response	Stress	cellular response to decreased oxygen levels	GO:0036294	P	104	4.14E-05	8.14E-03
4	452	L4 vs. M4	-	Signaling and response	Stress	cellular response to hypoxia	GO:0071456	P	104	4.14E-05	8.47E-03
4	453	L4 vs. M4	-	Signaling and response	Stress	cellular response to stress	GO:0033554	P	498	4.60E-06	1.47E-03
4	454	L4 vs. M4	-	Signaling and response	Stress	response to decreased oxygen levels	GO:0036293	P	121	6.66E-05	1.17E-02
4	455	L4 vs. M4	-	Signaling and response	Stress	response to hypoxia	GO:0001666	P	119	9.93E-05	1.34E-02
4	456	L4 vs. M4	-	Signaling and response	Stress	response to stress	GO:0006950	P	1454	1.68E-07	2.87E-04
4	457	L4 vs. M4	-	Signaling and response	Stress	response to wounding	GO:0009611	P	121	1.84E-06	9.42E-04
5	458	F2 vs. L2	+	Development	Cell division	nucleobase-containing small molecule metabolic process	GO:0055086	P	246	4.15E-04	5.44E-03
5	459	F2 vs. L2	+	Development	Cell division	nucleotide-sugar biosynthetic process	GO:0009226	P	29	6.10E-05	1.13E-03
5	460	F2 vs. L2	+	Development	Cell division	nucleotide-sugar metabolic process	GO:0009225	P	33	9.62E-05	1.69E-03
5	461	F2 vs. L2	+	Development	Cell division	pollen germination	GO:0009846	P	37	3.22E-04	4.53E-03
5	462	F2 vs. L2	+	Development	Cell division	pollen tube development	GO:0048868	P	97	3.35E-09	2.08E-07
5	463	F2 vs. L2	+	Development	Cell division	pollen tube growth	GO:0009860	P	74	1.09E-08	5.80E-07
5	464	F2 vs. L2	+	Development	Cell division	pollination	GO:0009856	P	148	1.88E-07	7.97E-06
5	465	F2 vs. L2	+	Development	Cell division	syncytium formation	GO:0006949	P	10	3.88E-05	7.66E-04
5	466	F2 vs. L2	+	Development	Development	actin cytoskeleton	GO:0015629	C	36	1.26E-03	8.98E-03
5	467	F2 vs. L2	+	Development	Development	actin filament	GO:0005884	C	8	7.84E-04	5.82E-03
5	468	F2 vs. L2	+	Development	Development	apical part of cell	GO:0045177	C	15	1.43E-03	1.01E-02
5	469	F2 vs. L2	+	Development	Development	apical plasma membrane	GO:0016324	C	13	3.19E-04	2.61E-03
5	470	F2 vs. L2	+	Development	Development	apoplast	GO:0048046	C	166	0.00E+00	0.00E+00
5	471	F2 vs. L2	+	Development	Development	bounding membrane of organelle	GO:0098588	C	577	4.26E-04	3.34E-03
5	472	F2 vs. L2	+	Development	Development	cell development	GO:0048468	P	173	3.22E-04	4.53E-03
5	473	F2 vs. L2	+	Development	Development	cell growth	GO:0016049	P	230	1.88E-05	4.31E-04
5	474	F2 vs. L2	+	Development	Development	cell morphogenesis	GO:0000902	P	215	3.02E-03	2.86E-02
5	475	F2 vs. L2	+	Development	Development	cell morphogenesis involved in differentiation	GO:0000904	P	127	4.47E-04	5.80E-03
5	476	F2 vs. L2	+	Development	Development	cell periphery	GO:0071944	C	2265	1.14E-07	1.90E-06
5	477	F2 vs. L2	+	Development	Development	cell pole	GO:0060187	C	11	2.72E-03	1.64E-02
5	478	F2 vs. L2	+	Development	Development	cell projection	GO:0042995	C	63	1.33E-08	2.62E-07
5	479	F2 vs. L2	+	Development	Development	cell tip	GO:0051286	C	11	2.72E-03	1.63E-02
5	480	F2 vs. L2	+	Development	Development	cell tip growth	GO:0009932	P	85	2.85E-08	1.43E-06
5	481	F2 vs. L2	+	Development	Development	cell wall	GO:0005618	C	347	1.71E-03	1.16E-02
5	482	F2 vs. L2	+	Development	Development	cell wall biogenesis	GO:0042546	P	128	3.93E-04	5.23E-03
5	483	F2 vs. L2	+	Development	Development	cell wall modification	GO:0042545	P	97	2.45E-05	5.31E-04
5	484	F2 vs. L2	+	Development	Development	cell wall organization	GO:0071555	P	152	4.30E-08	2.05E-06
5	485	F2 vs. L2	+	Development	Development	cell wall organization or biogenesis	GO:0071554	P	285	2.36E-09	1.54E-07
5	486	F2 vs. L2	+	Development	Development	cell wall pectin metabolic process	GO:0052546	P	15	2.83E-03	2.77E-02
5	487	F2 vs. L2	+	Development	Development	cytosol	GO:0005829	C	2040	6.39E-03	3.30E-02
5	488	F2 vs. L2	+	Development	Development	developmental cell growth	GO:0048588	P	108	5.86E-09	3.49E-07
5	489	F2 vs. L2	+	Development	Development	developmental growth	GO:0048589	P	228	2.95E-06	8.59E-05
5	490	F2 vs. L2	+	Development	Development	developmental growth involved in morphogenesis	GO:0060560	P	195	1.24E-06	4.01E-05
5	491	F2 vs. L2	+	Development	Development	endomembrane system	GO:0012505	C	1266	4.04E-06	5.41E-05
5	492	F2 vs. L2	+	Development	Development	envelope	GO:0031975	C	655	4.31E-08	8.16E-07
5	493	F2 vs. L2	+	Development	Development	external encapsulating structure	GO:0030312	C	349	9.89E-04	7.12E-03
5	494	F2 vs. L2	+	Development	Development	external encapsulating structure organization	GO:0045229	P	178	2.69E-08	1.36E-06
5	495	F2 vs. L2	+	Development	Development	extracellular region	GO:0005576	C	1230	1.11E-16	5.36E-15
5	496	F2 vs. L2	+	Development	Development	Golgi apparatus subcompartment	GO:0098791	C	268	9.20E-06	1.17E-04
5	497	F2 vs. L2	+	Development	Development	Golgi trans cisterna	GO:0000138	C	55	1.54E-04	1.35E-03
5	498	F2 vs. L2	+	Development	Development	growing cell tip	GO:0035838	C	9	4.13E-03	2.30E-02

5	499	F2 vs. L2	+	Development	Development	growth	GO:0040007	P	266	2.11E-05	4.73E-04
5	500	F2 vs. L2	+	Development	Development	integral component of plasma membrane	GO:0005887	C	67	1.93E-03	1.28E-02
5	501	F2 vs. L2	+	Development	Development	intracellular vesicle	GO:0097708	C	411	8.82E-05	8.51E-04
5	502	F2 vs. L2	+	Development	Development	membrane	GO:0016020	C	3042	8.95E-13	2.98E-11
5	503	F2 vs. L2	+	Development	Development	membrane protein complex	GO:0098796	C	352	8.75E-03	4.24E-02
5	504	F2 vs. L2	+	Development	Development	microbody	GO:0042579	C	182	7.53E-05	7.65E-04
5	505	F2 vs. L2	+	Development	Development	multi-multicellular organism process	GO:0044706	P	148	1.88E-07	8.03E-06
5	506	F2 vs. L2	+	Development	Development	organelle envelope	GO:0031967	C	655	4.31E-08	8.00E-07
5	507	F2 vs. L2	+	Development	Development	organelle membrane	GO:0031090	C	880	5.64E-04	4.32E-03
5	508	F2 vs. L2	+	Development	Development	organelle subcompartment	GO:0031984	C	701	1.27E-14	4.52E-13
5	509	F2 vs. L2	+	Development	Development	plant-type cell wall biogenesis	GO:0009832	P	81	4.40E-04	5.73E-03
5	510	F2 vs. L2	+	Development	Development	plant-type cell wall loosening	GO:0009828	P	23	6.12E-04	7.69E-03
5	511	F2 vs. L2	+	Development	Development	plant-type cell wall modification	GO:0009827	P	41	2.51E-04	3.74E-03
5	512	F2 vs. L2	+	Development	Development	plant-type cell wall organization	GO:0009664	P	87	8.36E-06	2.09E-04
5	513	F2 vs. L2	+	Development	Development	plant-type cell wall organization or biogenesis	GO:0071669	P	145	1.56E-07	6.78E-06
5	514	F2 vs. L2	+	Development	Development	plant-type primary cell wall biogenesis	GO:0009833	P	19	2.28E-05	5.03E-04
5	515	F2 vs. L2	+	Development	Development	plant-type vacuole	GO:0000325	C	100	6.60E-04	4.94E-03
5	516	F2 vs. L2	+	Development	Development	plant-type vacuole membrane	GO:0009705	C	66	3.91E-03	2.20E-02
5	517	F2 vs. L2	+	Development	Development	plasma membrane	GO:0005886	C	1987	2.37E-06	3.31E-05
5	518	F2 vs. L2	+	Development	Development	plasma membrane bounded cell projection	GO:0120025	C	56	2.08E-11	6.29E-10
5	519	F2 vs. L2	+	Development	Development	pollen tube	GO:0090406	C	48	4.91E-11	1.39E-09
5	520	F2 vs. L2	+	Development	Development	pollen tube tip	GO:0090404	C	9	4.13E-03	2.31E-02
5	521	F2 vs. L2	+	Development	Development	stromule	GO:0010319	C	28	1.62E-09	4.23E-08
5	522	F2 vs. L2	+	Development	Development	unidimensional cell growth	GO:0009826	P	167	9.35E-06	2.33E-04
5	523	F2 vs. L2	+	Development	Development	vacuolar membrane	GO:0005774	C	372	7.34E-06	9.58E-05
5	524	F2 vs. L2	+	Development	Development	vacuole	GO:0005773	C	669	8.51E-13	2.93E-11
5	525	F2 vs. L2	+	Metabolism	Bioenergetics	(1->3)-beta-D-glucan binding (NAD+) activity	GO:0004029	F	9	9.97E-04	2.38E-02
5	526	F2 vs. L2	+	Metabolism	Bioenergetics	aerobic respiration	GO:0009060	P	54	7.15E-06	1.81E-04
5	527	F2 vs. L2	+	Metabolism	Bioenergetics	alcohol dehydrogenase (NADP+) activity	GO:0008106	F	7	6.41E-04	1.78E-02
5	528	F2 vs. L2	+	Metabolism	Bioenergetics	aldehyde dehydrogenase [NAD(P)+] activity	GO:0004030	F	9	9.97E-04	2.40E-02
5	529	F2 vs. L2	+	Metabolism	Bioenergetics	aldo-keto reductase (NADP) activity	GO:0004033	F	8	9.87E-04	2.40E-02
5	530	F2 vs. L2	+	Metabolism	Bioenergetics	ATP metabolic process	GO:0046034	P	90	2.49E-04	3.71E-03
5	531	F2 vs. L2	+	Metabolism	Bioenergetics	ATPase activity, coupled to transmembrane movement of ions, rotational mechanism	GO:0044769	F	19	3.26E-04	9.84E-03
5	532	F2 vs. L2	+	Metabolism	Bioenergetics	carbohydrate biosynthetic process	GO:0016051	P	177	9.63E-11	8.58E-09
5	533	F2 vs. L2	+	Metabolism	Bioenergetics	carbohydrate catabolic process	GO:0016052	P	146	1.73E-04	2.68E-03
5	534	F2 vs. L2	+	Metabolism	Bioenergetics	carbohydrate derivative metabolic process	GO:1901135	P	438	1.16E-05	2.79E-04
5	535	F2 vs. L2	+	Metabolism	Bioenergetics	carbohydrate metabolic process	GO:0005975	P	524	5.11E-13	6.07E-11
5	536	F2 vs. L2	+	Metabolism	Bioenergetics	cellular carbohydrate biosynthetic process	GO:0034637	P	121	5.54E-06	1.48E-04
5	537	F2 vs. L2	+	Metabolism	Bioenergetics	cellular carbohydrate metabolic process	GO:0044262	P	229	7.85E-06	1.97E-04
5	538	F2 vs. L2	+	Metabolism	Bioenergetics	cellular polysaccharide biosynthetic process	GO:0033692	P	101	1.14E-04	1.94E-03
5	539	F2 vs. L2	+	Metabolism	Bioenergetics	cellular polysaccharide metabolic process	GO:0044264	P	160	1.06E-03	1.21E-02
5	540	F2 vs. L2	+	Metabolism	Bioenergetics	cellular respiration	GO:0045333	P	82	1.27E-05	3.00E-04
5	541	F2 vs. L2	+	Metabolism	Bioenergetics	electron transfer activity	GO:0009055	F	84	4.47E-05	1.99E-03
5	542	F2 vs. L2	+	Metabolism	Bioenergetics	electron transport chain	GO:0022900	P	73	9.58E-06	2.37E-04
5	543	F2 vs. L2	+	Metabolism	Bioenergetics	energy derivation by oxidation of organic compounds	GO:0015980	P	95	3.64E-05	7.38E-04
5	544	F2 vs. L2	+	Metabolism	Bioenergetics	fatty acid biosynthetic process	GO:0006633	P	88	1.52E-05	3.52E-04
5	545	F2 vs. L2	+	Metabolism	Bioenergetics	fatty acid metabolic process	GO:0006631	P	145	1.03E-06	3.63E-05
5	546	F2 vs. L2	+	Metabolism	Bioenergetics	generation of precursor metabolites and energy	GO:0006091	P	249	1.11E-14	1.92E-12
5	547	F2 vs. L2	+	Metabolism	Bioenergetics	gluconeogenesis	GO:0006094	P	19	1.69E-04	2.64E-03
5	548	F2 vs. L2	+	Metabolism	Bioenergetics	glucose 6-phosphate metabolic process	GO:0051156	P	30	3.08E-04	4.37E-03
5	549	F2 vs. L2	+	Metabolism	Bioenergetics	glucose metabolic process	GO:0006006	P	30	3.16E-03	2.96E-02
5	550	F2 vs. L2	+	Metabolism	Bioenergetics	hexose biosynthetic process	GO:0019319	P	22	8.29E-05	1.48E-03
5	551	F2 vs. L2	+	Metabolism	Bioenergetics	mitochondrial envelope	GO:0005740	C	184	7.64E-03	3.82E-02
5	552	F2 vs. L2	+	Metabolism	Bioenergetics	mitochondrial respirasome	GO:0005746	C	69	7.87E-03	3.91E-02
5	553	F2 vs. L2	+	Metabolism	Bioenergetics	monosaccharide biosynthetic process	GO:0046364	P	33	5.65E-05	1.07E-03
5	554	F2 vs. L2	+	Metabolism	Bioenergetics	NAD binding	GO:0051287	F	58	8.39E-04	2.17E-02
5	555	F2 vs. L2	+	Metabolism	Bioenergetics	NAD(P)H dehydrogenase complex (plastoquinone)	GO:0010598	C	7	1.92E-03	1.27E-02
5	556	F2 vs. L2	+	Metabolism	Bioenergetics	NAD(P)H dehydrogenase complex assembly	GO:0010275	P	7	3.18E-03	2.98E-02
5	557	F2 vs. L2	+	Metabolism	Bioenergetics	NADH dehydrogenase complex (plastoquinone) assembly	GO:0010258	P	4	4.35E-03	3.88E-02
5	558	F2 vs. L2	+	Metabolism	Bioenergetics	NADP metabolic process	GO:0006739	P	34	2.37E-05	5.16E-04
5	559	F2 vs. L2	+	Metabolism	Bioenergetics	NADPH regeneration	GO:0006740	P	29	1.12E-04	1.90E-03
5	560	F2 vs. L2	+	Metabolism	Bioenergetics	oxidoreductase activity	GO:0016491	F	758	0.00E+00	0.00E+00
5	561	F2 vs. L2	+	Metabolism	Bioenergetics	oxidoreductase activity, acting on a sulfur group of donors	GO:0016667	F	58	1.49E-04	5.13E-03

5	562	F2 vs. L2	+	Metabolism	Bioenergetics	oxidoreductase activity, acting on CH-OH group of donors	GO:0016614	F	120	4.55E-05	2.00E-03
5	563	F2 vs. L2	+	Metabolism	Bioenergetics	oxidoreductase activity, acting on NAD(P)H	GO:0016651	F	70	1.24E-06	1.05E-04
5	564	F2 vs. L2	+	Metabolism	Bioenergetics	oxidoreductase activity, acting on NAD(P)H, quinone or similar compound as acceptor	GO:0016655	F	28	7.12E-04	1.96E-02
5	565	F2 vs. L2	+	Metabolism	Bioenergetics	oxidoreductase activity, acting on other nitrogenous compounds as donors	GO:0016661	F	4	2.28E-03	4.39E-02
5	566	F2 vs. L2	+	Metabolism	Bioenergetics	oxidoreductase activity, acting on paired donors, with incorporation or reduction of molecular oxygen	GO:0016705	F	182	9.25E-04	2.31E-02
5	567	F2 vs. L2	+	Metabolism	Bioenergetics	oxidoreductase activity, acting on single donors with incorporation of molecular oxygen	GO:0016701	F	21	3.55E-05	1.64E-03
5	568	F2 vs. L2	+	Metabolism	Bioenergetics	oxidoreductase activity, acting on the aldehyde or oxo group of donors	GO:0016903	F	39	5.76E-05	2.45E-03
5	569	F2 vs. L2	+	Metabolism	Bioenergetics	oxidoreductase activity, acting on the aldehyde or oxo group of donors, NAD or NADP as acceptor	GO:0016620	F	30	1.17E-04	4.32E-03
5	570	F2 vs. L2	+	Metabolism	Bioenergetics	oxidoreductase activity, acting on the CH-OH group of donors, NAD or NADP as acceptor	GO:0016616	F	106	1.75E-05	9.72E-04
5	571	F2 vs. L2	+	Metabolism	Bioenergetics	oxidoreductase complex	GO:1990204	C	100	2.65E-06	3.60E-05
5	572	F2 vs. L2	+	Metabolism	Bioenergetics	pentose-phosphate shunt	GO:0006098	P	27	5.91E-05	1.10E-03
5	573	F2 vs. L2	+	Metabolism	Bioenergetics	photorespiration	GO:0009853	P	29	2.28E-03	2.31E-02
5	574	F2 vs. L2	+	Metabolism	Bioenergetics	polysaccharide biosynthetic process	GO:0000271	P	120	3.32E-05	6.84E-04
5	575	F2 vs. L2	+	Metabolism	Bioenergetics	polysaccharide metabolic process	GO:0005976	P	243	3.80E-07	1.49E-05
5	576	F2 vs. L2	+	Metabolism	Bioenergetics	proton transmembrane transport	GO:1902600	P	59	4.60E-07	1.78E-05
5	577	F2 vs. L2	+	Metabolism	Bioenergetics	proton-transporting ATPase activity, rotational mechanism	GO:0046961	F	19	3.26E-04	9.95E-03
5	578	F2 vs. L2	+	Metabolism	Bioenergetics	proton-transporting two-sector ATPase complex	GO:0016469	C	41	3.91E-05	4.20E-04
5	579	F2 vs. L2	+	Metabolism	Bioenergetics	proton-transporting two-sector ATPase complex, catalytic domain	GO:0033178	C	17	3.79E-03	2.15E-02
5	580	F2 vs. L2	+	Metabolism	Bioenergetics	proton-transporting two-sector ATPase complex, proton-transporting domain	GO:0033177	C	17	4.28E-03	2.37E-02
5	581	F2 vs. L2	+	Metabolism	Bioenergetics	proton-transporting V-type ATPase complex	GO:0033176	C	18	3.45E-04	2.80E-03
5	582	F2 vs. L2	+	Metabolism	Bioenergetics	proton-transporting V-type ATPase, V1 domain	GO:0033180	C	5	9.31E-03	4.43E-02
5	583	F2 vs. L2	+	Metabolism	Bioenergetics	pyrophosphate hydrolysis-driven proton transmembrane transporter activity	GO:0009678	F	34	3.43E-05	1.64E-03
5	584	F2 vs. L2	+	Metabolism	Bioenergetics	pyruvate metabolic process	GO:0006090	P	58	1.43E-03	1.58E-02
5	585	F2 vs. L2	+	Metabolism	Bioenergetics	reductive pentose-phosphate cycle	GO:0019253	P	14	6.92E-06	1.77E-04
5	586	F2 vs. L2	+	Metabolism	Bioenergetics	regulation of carbohydrate metabolic process	GO:0006109	P	46	4.80E-03	4.21E-02
5	587	F2 vs. L2	+	Metabolism	Bioenergetics	respirasome	GO:0070469	C	72	1.91E-03	1.28E-02
5	588	F2 vs. L2	+	Metabolism	Bioenergetics	respiratory chain complex	GO:0098803	C	68	6.27E-03	3.26E-02
5	589	F2 vs. L2	+	Metabolism	Bioenergetics	tricarboxylic acid cycle	GO:0006099	P	31	5.85E-05	1.09E-03
5	590	F2 vs. L2	+	Metabolism	Bioenergetics	unsaturated fatty acid biosynthetic process	GO:0006636	P	18	1.46E-04	2.37E-03
5	591	F2 vs. L2	+	Metabolism	Bioenergetics	unsaturated fatty acid metabolic process	GO:0033559	P	19	6.53E-05	1.20E-03
5	592	F2 vs. L2	+	Metabolism	Catabolism	alpha-amino acid catabolic process	GO:1901606	P	62	3.14E-06	9.07E-05
5	593	F2 vs. L2	+	Metabolism	Catabolism	aromatic amino acid family catabolic process	GO:0009074	P	12	3.63E-04	4.89E-03
5	594	F2 vs. L2	+	Metabolism	Catabolism	carboxylic acid catabolic process	GO:0046395	P	107	1.05E-06	3.65E-05
5	595	F2 vs. L2	+	Metabolism	Catabolism	catabolic process	GO:0009056	P	803	2.65E-03	2.62E-02
5	596	F2 vs. L2	+	Metabolism	Catabolism	cellular amino acid catabolic process	GO:0009063	P	65	1.36E-05	3.20E-04
5	597	F2 vs. L2	+	Metabolism	Catabolism	cellular metabolic compound salvage	GO:0043094	P	48	9.43E-04	1.09E-02
5	598	F2 vs. L2	+	Metabolism	Catabolism	erythrose 4-phosphate/phosphoenolpyruvate family amino acid catabolic process	GO:1902222	P	9	4.15E-04	5.42E-03
5	599	F2 vs. L2	+	Metabolism	Catabolism	glycine catabolic process	GO:0006546	P	8	1.16E-04	1.95E-03
5	600	F2 vs. L2	+	Metabolism	Catabolism	L-phenylalanine catabolic process	GO:0006559	P	9	4.15E-04	5.43E-03
5	601	F2 vs. L2	+	Metabolism	Catabolism	monocarboxylic acid catabolic process	GO:0072329	P	43	3.62E-03	3.33E-02
5	602	F2 vs. L2	+	Metabolism	Catabolism	organic acid catabolic process	GO:0016054	P	107	1.05E-06	3.68E-05
5	603	F2 vs. L2	+	Metabolism	Catabolism	organic hydroxy compound catabolic process	GO:1901616	P	31	2.36E-03	2.38E-02
5	604	F2 vs. L2	+	Metabolism	Catabolism	pectin catabolic process	GO:0045490	P	55	6.53E-04	8.16E-03
5	605	F2 vs. L2	+	Metabolism	Catabolism	peroxisome	GO:0005777	C	182	7.53E-05	7.57E-04
5	606	F2 vs. L2	+	Metabolism	Catabolism	phenol-containing compound catabolic process	GO:0019336	P	6	4.66E-03	4.11E-02
5	607	F2 vs. L2	+	Metabolism	Catabolism	serine family amino acid catabolic process	GO:0009071	P	15	8.56E-04	1.02E-02
5	608	F2 vs. L2	+	Metabolism	Catabolism	small molecule catabolic process	GO:0044282	P	157	9.59E-07	3.47E-05
5	609	F2 vs. L2	+	Metabolism	Metabolism	actin filament bundle assembly	GO:0051017	P	15	1.77E-03	1.86E-02
5	610	F2 vs. L2	+	Metabolism	Metabolism	actin filament bundle organization	GO:0061572	P	15	1.77E-03	1.86E-02
5	611	F2 vs. L2	+	Metabolism	Metabolism	alcohol metabolic process	GO:0006066	P	63	4.65E-04	5.99E-03
5	612	F2 vs. L2	+	Metabolism	Metabolism	alpha-amino acid biosynthetic process	GO:1901607	P	117	1.49E-04	2.40E-03
5	613	F2 vs. L2	+	Metabolism	Metabolism	alpha-amino acid metabolic process	GO:1901605	P	184	1.53E-10	1.34E-08
5	614	F2 vs. L2	+	Metabolism	Metabolism	aminoglycan metabolic process	GO:0006022	P	10	5.32E-03	4.58E-02

5	615	F2 vs. L2	+	Metabolism	Metabolism	ammonia assimilation cycle	GO:0019676	P	4	3.97E-03	3.61E-02
5	616	F2 vs. L2	+	Metabolism	Metabolism	anion binding	GO:0043168	F	701	1.91E-03	3.90E-02
5	617	F2 vs. L2	+	Metabolism	Metabolism	anthocyanin-containing compound biosynthetic process	GO:0009718	P	10	5.38E-03	4.62E-02
5	618	F2 vs. L2	+	Metabolism	Metabolism	beta-glucan biosynthetic process	GO:0051274	P	41	5.98E-03	4.99E-02
5	619	F2 vs. L2	+	Metabolism	Metabolism	biosynthetic process	GO:0009058	P	1567	3.57E-03	3.30E-02
5	620	F2 vs. L2	+	Metabolism	Metabolism	carbon utilization	GO:0015976	P	7	3.95E-03	3.60E-02
5	621	F2 vs. L2	+	Metabolism	Metabolism	carbon-carbon lyase activity	GO:0016830	F	71	3.46E-05	1.62E-03
5	622	F2 vs. L2	+	Metabolism	Metabolism	carbon-oxygen lyase activity	GO:0016835	F	89	1.94E-03	3.91E-02
5	623	F2 vs. L2	+	Metabolism	Metabolism	carboxylic acid biosynthetic process	GO:0046394	P	283	1.82E-11	1.65E-09
5	624	F2 vs. L2	+	Metabolism	Metabolism	carboxylic acid metabolic process	GO:0019752	P	568	0.00E+00	0.00E+00
5	625	F2 vs. L2	+	Metabolism	Metabolism	carboxylic ester hydrolase activity	GO:0052689	F	106	1.38E-03	3.02E-02
5	626	F2 vs. L2	+	Metabolism	Metabolism	catalytic activity	GO:0003824	F	4291	2.01E-10	4.56E-08
5	627	F2 vs. L2	+	Metabolism	Metabolism	cation binding	GO:0043169	F	877	1.19E-06	1.05E-04
5	628	F2 vs. L2	+	Metabolism	Metabolism	cellular aldehyde metabolic process	GO:0006081	P	36	5.02E-03	4.37E-02
5	629	F2 vs. L2	+	Metabolism	Metabolism	cellular amino acid biosynthetic process	GO:0008652	P	129	3.29E-05	6.83E-04
5	630	F2 vs. L2	+	Metabolism	Metabolism	cellular amino acid metabolic process	GO:0006520	P	241	1.50E-07	6.58E-06
5	631	F2 vs. L2	+	Metabolism	Metabolism	cellular lipid metabolic process	GO:0044255	P	396	2.34E-08	1.19E-06
5	632	F2 vs. L2	+	Metabolism	Metabolism	cellular modified amino acid metabolic process	GO:0006575	P	51	5.39E-03	4.62E-02
5	633	F2 vs. L2	+	Metabolism	Metabolism	cellulose biosynthetic process	GO:0030244	P	32	1.57E-03	1.70E-02
5	634	F2 vs. L2	+	Metabolism	Metabolism	cellulose metabolic process	GO:0030243	P	33	4.64E-03	4.11E-02
5	635	F2 vs. L2	+	Metabolism	Metabolism	cellulose synthase (UDP-forming) activity	GO:0016760	F	17	9.68E-05	3.76E-03
5	636	F2 vs. L2	+	Metabolism	Metabolism	cellulose synthase activity	GO:0016759	F	20	4.07E-06	2.84E-04
5	637	F2 vs. L2	+	Metabolism	Metabolism	ceramide biosynthetic process	GO:0046513	P	6	5.23E-03	4.54E-02
5	638	F2 vs. L2	+	Metabolism	Metabolism	copper ion binding	GO:0005507	F	123	7.49E-08	1.02E-05
5	639	F2 vs. L2	+	Metabolism	Metabolism	coumarin metabolic process	GO:0009804	P	6	3.56E-03	3.30E-02
5	640	F2 vs. L2	+	Metabolism	Metabolism	dicarboxylic acid metabolic process	GO:0043648	P	62	2.21E-04	3.34E-03
5	641	F2 vs. L2	+	Metabolism	Metabolism	dioxygenase activity	GO:0051213	F	61	2.56E-03	4.80E-02
5	642	F2 vs. L2	+	Metabolism	Metabolism	enzyme inhibitor activity	GO:0004857	F	77	8.89E-04	2.24E-02
5	643	F2 vs. L2	+	Metabolism	Metabolism	erythrose 4-phosphate/phosphoenolpyruvate family amino acid metabolic process	GO:1902221	P	14	6.76E-04	8.38E-03
5	644	F2 vs. L2	+	Metabolism	Metabolism	establishment of localization	GO:0051234	P	1241	6.05E-06	1.61E-04
5	645	F2 vs. L2	+	Metabolism	Metabolism	FMN binding	GO:0010181	F	20	1.09E-03	2.58E-02
5	646	F2 vs. L2	+	Metabolism	Metabolism	galacturonan metabolic process	GO:0010393	P	89	2.66E-06	7.87E-05
5	647	F2 vs. L2	+	Metabolism	Metabolism	glucan biosynthetic process	GO:0009250	P	74	2.56E-03	2.55E-02
5	648	F2 vs. L2	+	Metabolism	Metabolism	glucan metabolic process	GO:0044042	P	123	4.98E-03	4.34E-02
5	649	F2 vs. L2	+	Metabolism	Metabolism	glucosinolate biosynthetic process	GO:0019761	P	23	1.01E-06	3.59E-05
5	650	F2 vs. L2	+	Metabolism	Metabolism	glucosinolate metabolic process	GO:0019760	P	60	1.50E-06	4.73E-05
5	651	F2 vs. L2	+	Metabolism	Metabolism	glucosyltransferase activity	GO:0046527	F	108	1.82E-05	9.88E-04
5	652	F2 vs. L2	+	Metabolism	Metabolism	glutamine metabolic process	GO:0006541	P	9	9.57E-04	1.10E-02
5	653	F2 vs. L2	+	Metabolism	Metabolism	glycine cleavage complex	GO:0005960	C	5	1.89E-03	1.27E-02
5	654	F2 vs. L2	+	Metabolism	Metabolism	glycine decarboxylation via glycine cleavage system	GO:0019464	P	7	3.45E-04	4.71E-03
5	655	F2 vs. L2	+	Metabolism	Metabolism	glycine metabolic process	GO:0006544	P	15	5.73E-05	1.08E-03
5	656	F2 vs. L2	+	Metabolism	Metabolism	glycosinolate biosynthetic process	GO:0019758	P	23	1.01E-06	3.57E-05
5	657	F2 vs. L2	+	Metabolism	Metabolism	glycosinolate metabolic process	GO:0019757	P	60	1.50E-06	4.70E-05
5	658	F2 vs. L2	+	Metabolism	Metabolism	glycosyl compound biosynthetic process	GO:1901659	P	37	4.94E-06	1.33E-04
5	659	F2 vs. L2	+	Metabolism	Metabolism	glycosyl compound metabolic process	GO:1901657	P	104	2.52E-04	3.72E-03
5	660	F2 vs. L2	+	Metabolism	Metabolism	heme binding	GO:0020037	F	168	1.89E-04	6.33E-03
5	661	F2 vs. L2	+	Metabolism	Metabolism	hydrolase activity, hydrolyzing O-glycosyl compounds	GO:0004553	F	182	3.11E-04	9.62E-03
5	662	F2 vs. L2	+	Metabolism	Metabolism	hydro-lyase activity	GO:0016836	F	50	7.35E-04	1.96E-02
5	663	F2 vs. L2	+	Metabolism	Metabolism	indole-containing compound metabolic process	GO:0042430	P	35	5.59E-04	7.09E-03
5	664	F2 vs. L2	+	Metabolism	Metabolism	ion binding	GO:0043167	F	1483	2.50E-06	1.94E-04
5	665	F2 vs. L2	+	Metabolism	Metabolism	iron ion binding	GO:0005506	F	148	1.31E-03	2.93E-02
5	666	F2 vs. L2	+	Metabolism	Metabolism	lignin biosynthetic process	GO:0009809	P	26	5.86E-03	4.92E-02
5	667	F2 vs. L2	+	Metabolism	Metabolism	lignin metabolic process	GO:0009808	P	35	3.11E-03	2.92E-02
5	668	F2 vs. L2	+	Metabolism	Metabolism	lipid biosynthetic process	GO:0008610	P	291	1.95E-05	4.44E-04
5	669	F2 vs. L2	+	Metabolism	Metabolism	lipid metabolic process	GO:0006629	P	464	3.35E-07	1.33E-05
5	670	F2 vs. L2	+	Metabolism	Metabolism	localization	GO:0051179	P	1320	6.31E-06	1.66E-04
5	671	F2 vs. L2	+	Metabolism	Metabolism	L-phenylalanine metabolic process	GO:0006558	P	14	6.76E-04	8.40E-03
5	672	F2 vs. L2	+	Metabolism	Metabolism	L-serine metabolic process	GO:0006563	P	21	2.50E-03	2.50E-02
5	673	F2 vs. L2	+	Metabolism	Metabolism	lyase activity	GO:0016829	F	194	2.42E-07	2.63E-05
5	674	F2 vs. L2	+	Metabolism	Metabolism	metal ion binding	GO:0046872	F	862	3.54E-06	2.60E-04
5	675	F2 vs. L2	+	Metabolism	Metabolism	methionine adenosyltransferase activity	GO:0004478	F	4	2.54E-03	4.80E-02
5	676	F2 vs. L2	+	Metabolism	Metabolism	monocarboxylic acid biosynthetic process	GO:0072330	P	130	3.81E-07	1.49E-05
5	677	F2 vs. L2	+	Metabolism	Metabolism	monocarboxylic acid metabolic process	GO:0032787	P	290	4.18E-13	5.32E-11

5	678	F2 vs. L2	+	Metabolism	Metabolism	monosaccharide metabolic process	GO:0005996	P	84	2.64E-04	3.86E-03
5	679	F2 vs. L2	+	Metabolism	Metabolism	nitrate assimilation	GO:0042128	P	9	2.96E-04	4.26E-03
5	680	F2 vs. L2	+	Metabolism	Metabolism	nitrate metabolic process	GO:0042126	P	9	2.96E-04	4.25E-03
5	681	F2 vs. L2	+	Metabolism	Metabolism	nitrogen cycle metabolic process	GO:0071941	P	11	6.19E-04	7.75E-03
5	682	F2 vs. L2	+	Metabolism	Metabolism	oligopeptide binding	GO:1900750	F	13	1.21E-04	4.27E-03
5	683	F2 vs. L2	+	Metabolism	Metabolism	organic acid biosynthetic process	GO:0016053	P	283	1.82E-11	1.68E-09
5	684	F2 vs. L2	+	Metabolism	Metabolism	organic acid metabolic process	GO:0006082	P	624	0.00E+00	0.00E+00
5	685	F2 vs. L2	+	Metabolism	Metabolism	organic hydroxy compound biosynthetic process	GO:1901617	P	89	8.87E-04	1.04E-02
5	686	F2 vs. L2	+	Metabolism	Metabolism	organic hydroxy compound metabolic process	GO:1901615	P	166	2.00E-05	4.52E-04
5	687	F2 vs. L2	+	Metabolism	Metabolism	organophosphate metabolic process	GO:0019637	P	364	5.73E-05	1.07E-03
5	688	F2 vs. L2	+	Metabolism	Metabolism	oxidation-reduction process	GO:0055114	P	442	0.00E+00	0.00E+00
5	689	F2 vs. L2	+	Metabolism	Metabolism	oxoacid metabolic process	GO:0043436	P	623	0.00E+00	0.00E+00
5	690	F2 vs. L2	+	Metabolism	Metabolism	oxylipin biosynthetic process	GO:0031408	P	11	2.52E-05	5.39E-04
5	691	F2 vs. L2	+	Metabolism	Metabolism	oxylipin metabolic process	GO:0031407	P	13	1.05E-04	1.82E-03
5	692	F2 vs. L2	+	Metabolism	Metabolism	pectin metabolic process	GO:0045488	P	88	1.10E-06	3.76E-05
5	693	F2 vs. L2	+	Metabolism	Metabolism	pectinesterase activity	GO:0030599	F	40	2.62E-03	4.89E-02
5	694	F2 vs. L2	+	Metabolism	Metabolism	pectinesterase inhibitor activity	GO:0046910	F	33	5.44E-04	1.57E-02
5	695	F2 vs. L2	+	Metabolism	Metabolism	phenylpropanoid biosynthetic process	GO:0009699	P	54	6.62E-04	8.25E-03
5	696	F2 vs. L2	+	Metabolism	Metabolism	phenylpropanoid metabolic process	GO:0009698	P	67	1.67E-04	2.62E-03
5	697	F2 vs. L2	+	Metabolism	Metabolism	phosphorus metabolic process	GO:0006793	P	1017	2.90E-03	2.84E-02
5	698	F2 vs. L2	+	Metabolism	Metabolism	pigment metabolic process	GO:0042440	P	77	4.02E-03	3.65E-02
5	699	F2 vs. L2	+	Metabolism	Metabolism	polyol metabolic process	GO:0019751	P	32	1.82E-03	1.90E-02
5	700	F2 vs. L2	+	Metabolism	Metabolism	purine nucleoside triphosphate biosynthetic process	GO:0009145	P	29	2.13E-03	2.18E-02
5	701	F2 vs. L2	+	Metabolism	Metabolism	purine nucleoside triphosphate metabolic process	GO:0009144	P	32	3.92E-03	3.58E-02
5	702	F2 vs. L2	+	Metabolism	Metabolism	purine nucleotide metabolic process	GO:0006163	P	139	2.04E-03	2.11E-02
5	703	F2 vs. L2	+	Metabolism	Metabolism	purine ribonucleoside triphosphate biosynthetic process	GO:0009206	P	28	4.64E-03	4.10E-02
5	704	F2 vs. L2	+	Metabolism	Metabolism	purine ribonucleoside triphosphate metabolic process	GO:0009205	P	28	4.64E-03	4.10E-02
5	705	F2 vs. L2	+	Metabolism	Metabolism	purine ribonucleotide metabolic process	GO:0009150	P	129	7.43E-04	9.04E-03
5	706	F2 vs. L2	+	Metabolism	Metabolism	pyridoxal phosphate binding	GO:0030170	F	65	1.99E-04	6.54E-03
5	707	F2 vs. L2	+	Metabolism	Metabolism	S-adenosylmethionine biosynthetic process	GO:0006556	P	4	2.54E-03	2.54E-02
5	708	F2 vs. L2	+	Metabolism	Metabolism	S-adenosylmethionine metabolic process	GO:0046500	P	8	2.22E-04	3.35E-03
5	709	F2 vs. L2	+	Metabolism	Metabolism	secondary metabolic process	GO:0019748	P	163	1.82E-10	1.54E-08
5	710	F2 vs. L2	+	Metabolism	Metabolism	secondary metabolite biosynthetic process	GO:0044550	P	87	1.60E-08	8.21E-07
5	711	F2 vs. L2	+	Metabolism	Metabolism	serine family amino acid biosynthetic process	GO:0009070	P	29	3.54E-04	4.78E-03
5	712	F2 vs. L2	+	Metabolism	Metabolism	serine family amino acid metabolic process	GO:0009069	P	42	6.02E-07	2.29E-05
5	713	F2 vs. L2	+	Metabolism	Metabolism	S-glycoside biosynthetic process	GO:0016144	P	23	1.01E-06	3.62E-05
5	714	F2 vs. L2	+	Metabolism	Metabolism	S-glycoside metabolic process	GO:0016143	P	60	1.50E-06	4.76E-05
5	715	F2 vs. L2	+	Metabolism	Metabolism	small molecule binding	GO:0036094	F	689	2.06E-03	4.02E-02
5	716	F2 vs. L2	+	Metabolism	Metabolism	small molecule biosynthetic process	GO:0044283	P	391	4.44E-16	1.13E-13
5	717	F2 vs. L2	+	Metabolism	Metabolism	small molecule metabolic process	GO:0044281	P	918	0.00E+00	0.00E+00
5	718	F2 vs. L2	+	Metabolism	Metabolism	sphingolipid biosynthetic process	GO:0030148	P	17	4.14E-04	5.46E-03
5	719	F2 vs. L2	+	Metabolism	Metabolism	sphingolipid metabolic process	GO:0006665	P	24	1.39E-04	2.29E-03
5	720	F2 vs. L2	+	Metabolism	Metabolism	sulfur amino acid metabolic process	GO:0000096	P	36	1.89E-04	2.91E-03
5	721	F2 vs. L2	+	Metabolism	Metabolism	sulfur compound binding	GO:1901681	F	22	3.77E-05	1.71E-03
5	722	F2 vs. L2	+	Metabolism	Metabolism	sulfur compound biosynthetic process	GO:0044272	P	94	3.68E-13	4.81E-11
5	723	F2 vs. L2	+	Metabolism	Metabolism	sulfur compound metabolic process	GO:0006790	P	212	1.67E-15	3.56E-13
5	724	F2 vs. L2	+	Metabolism	Metabolism	tetrapyrrole binding	GO:0046906	F	188	9.75E-05	3.73E-03
5	725	F2 vs. L2	+	Metabolism	Metabolism	transferase activity, transferring alkyl or aryl (other than methyl) groups	GO:0016765	F	73	7.66E-06	5.08E-04
5	726	F2 vs. L2	+	Metabolism	Metabolism	UDP-glucosyltransferase activity	GO:0035251	F	84	2.99E-05	1.48E-03
5	727	F2 vs. L2	+	Metabolism	Metabolism	UDP-glycosyltransferase activity	GO:0008194	F	153	1.31E-06	1.08E-04
5	728	F2 vs. L2	+	Metabolism	Metabolism	UDP-rhamnose biosynthetic process	GO:0010253	P	4	2.20E-03	2.23E-02
5	729	F2 vs. L2	+	Metabolism	Metabolism	UDP-rhamnose metabolic process	GO:0033478	P	4	2.20E-03	2.24E-02
5	730	F2 vs. L2	+	Metabolism	Metabolism	vitamin B6 binding	GO:0070279	F	65	1.99E-04	6.62E-03
5	731	F2 vs. L2	+	Metabolism	Metabolism	vitamin binding	GO:0019842	F	81	3.80E-04	1.14E-02
5	732	F2 vs. L2	+	Metabolism	Photosynthesis	carbon fixation	GO:0015977	P	15	6.38E-06	1.66E-04
5	733	F2 vs. L2	+	Metabolism	Photosynthesis	chloroplast	GO:0009507	C	2575	5.08E-10	1.36E-08
5	734	F2 vs. L2	+	Metabolism	Photosynthesis	chloroplast envelope	GO:0009941	C	418	5.62E-11	1.55E-09
5	735	F2 vs. L2	+	Metabolism	Photosynthesis	chloroplast stroma	GO:0009570	C	474	0.00E+00	0.00E+00
5	736	F2 vs. L2	+	Metabolism	Photosynthesis	chloroplast thylakoid	GO:0009534	C	282	0.00E+00	0.00E+00
5	737	F2 vs. L2	+	Metabolism	Photosynthesis	chloroplast thylakoid lumen	GO:0009543	C	32	6.16E-03	3.23E-02
5	738	F2 vs. L2	+	Metabolism	Photosynthesis	chloroplast thylakoid membrane	GO:0009535	C	227	4.44E-16	1.95E-14
5	739	F2 vs. L2	+	Metabolism	Photosynthesis	fructose 1,6-bisphosphate metabolic process	GO:0030388	P	11	3.41E-04	4.67E-03
5	740	F2 vs. L2	+	Metabolism	Photosynthesis	fructose-bisphosphate aldolase activity	GO:0004332	F	8	1.57E-03	3.36E-02

5	741	F2 vs. L2	+	Metabolism	Photosynthesis	oxidative photosynthetic carbon pathway	GO:0009854	P	5	2.09E-03	2.15E-02
5	742	F2 vs. L2	+	Metabolism	Photosynthesis	photosynthesis	GO:0015979	P	130	4.70E-13	5.84E-11
5	743	F2 vs. L2	+	Metabolism	Photosynthesis	photosynthesis, dark reaction	GO:0019685	P	14	6.92E-06	1.76E-04
5	744	F2 vs. L2	+	Metabolism	Photosynthesis	photosynthesis, light harvesting	GO:0009765	P	29	6.71E-04	8.35E-03
5	745	F2 vs. L2	+	Metabolism	Photosynthesis	photosynthesis, light reaction	GO:0019684	P	87	3.83E-09	2.35E-07
5	746	F2 vs. L2	+	Metabolism	Photosynthesis	photosynthetic electron transport chain	GO:0009767	P	30	2.49E-05	5.36E-04
5	747	F2 vs. L2	+	Metabolism	Photosynthesis	photosynthetic electron transport in photosystem I	GO:0009773	P	12	3.60E-04	4.85E-03
5	748	F2 vs. L2	+	Metabolism	Photosynthesis	photosynthetic membrane	GO:0034357	C	244	1.11E-16	6.30E-15
5	749	F2 vs. L2	+	Metabolism	Photosynthesis	photosystem II repair	GO:0010206	P	12	1.84E-03	1.92E-02
5	750	F2 vs. L2	+	Metabolism	Photosynthesis	plastid	GO:0009536	C	2853	1.13E-08	2.42E-07
5	751	F2 vs. L2	+	Metabolism	Photosynthesis	plastid envelope	GO:0009526	C	436	3.53E-11	1.03E-09
5	752	F2 vs. L2	+	Metabolism	Photosynthesis	plastid stroma	GO:0009532	C	480	1.11E-16	6.70E-15
5	753	F2 vs. L2	+	Metabolism	Photosynthesis	plastid thylakoid	GO:0031976	C	282	0.00E+00	0.00E+00
5	754	F2 vs. L2	+	Metabolism	Photosynthesis	plastid thylakoid lumen	GO:0031978	C	32	6.16E-03	3.21E-02
5	755	F2 vs. L2	+	Metabolism	Photosynthesis	plastid thylakoid membrane	GO:0055035	C	228	4.44E-16	1.86E-14
5	756	F2 vs. L2	+	Metabolism	Photosynthesis	plastid translation	GO:0032544	P	10	3.45E-03	3.21E-02
5	757	F2 vs. L2	+	Metabolism	Photosynthesis	plastoglobule	GO:0010287	C	48	1.12E-04	1.03E-03
5	758	F2 vs. L2	+	Metabolism	Photosynthesis	regulation of photosynthesis	GO:0010109	P	24	1.07E-03	1.21E-02
5	759	F2 vs. L2	+	Metabolism	Photosynthesis	thylakoid	GO:0009579	C	337	0.00E+00	0.00E+00
5	760	F2 vs. L2	+	Metabolism	Photosynthesis	thylakoid lumen	GO:0031977	C	43	8.14E-05	8.01E-04
5	761	F2 vs. L2	+	Metabolism	Photosynthesis	thylakoid membrane	GO:0042651	C	241	1.11E-16	5.10E-15
5	762	F2 vs. L2	+	Metabolism	Transporters	active ion transmembrane transporter activity	GO:0022853	F	128	5.35E-09	9.70E-07
5	763	F2 vs. L2	+	Metabolism	Transporters	active transmembrane transporter activity	GO:0022804	F	281	1.80E-13	8.16E-11
5	764	F2 vs. L2	+	Metabolism	Transporters	amino acid transmembrane transporter activity	GO:0015171	F	49	1.99E-03	3.96E-02
5	765	F2 vs. L2	+	Metabolism	Transporters	anion transmembrane transport	GO:0098656	P	68	1.47E-03	1.62E-02
5	766	F2 vs. L2	+	Metabolism	Transporters	anion transmembrane transporter activity	GO:0008509	F	193	3.63E-06	2.60E-04
5	767	F2 vs. L2	+	Metabolism	Transporters	anion transport	GO:0006820	P	155	9.79E-06	2.41E-04
5	768	F2 vs. L2	+	Metabolism	Transporters	antiporter activity	GO:0015297	F	115	2.42E-09	5.06E-07
5	769	F2 vs. L2	+	Metabolism	Transporters	ATPase-coupled cation transmembrane transporter activity	GO:0019829	F	43	1.73E-07	2.14E-05
5	770	F2 vs. L2	+	Metabolism	Transporters	ATPase-coupled ion transmembrane transporter activity	GO:0042625	F	52	1.85E-06	1.48E-04
5	771	F2 vs. L2	+	Metabolism	Transporters	ATPase-coupled transmembrane transporter activity	GO:0042626	F	122	2.44E-04	7.71E-03
5	772	F2 vs. L2	+	Metabolism	Transporters	calcium ion transmembrane transport	GO:0070588	P	25	4.98E-03	4.35E-02
5	773	F2 vs. L2	+	Metabolism	Transporters	carbohydrate transmembrane transport	GO:0034219	P	15	5.57E-03	4.71E-02
5	774	F2 vs. L2	+	Metabolism	Transporters	carbohydrate transmembrane transporter activity	GO:0015144	F	57	8.91E-06	5.63E-04
5	775	F2 vs. L2	+	Metabolism	Transporters	carbohydrate transport	GO:0008643	P	39	1.03E-04	1.78E-03
5	776	F2 vs. L2	+	Metabolism	Transporters	carboxylic acid transmembrane transporter activity	GO:0046943	F	80	9.38E-04	2.32E-02
5	777	F2 vs. L2	+	Metabolism	Transporters	cation transmembrane transport	GO:0098655	P	133	5.70E-08	2.65E-06
5	778	F2 vs. L2	+	Metabolism	Transporters	cation transmembrane transporter activity	GO:0008324	F	228	4.12E-09	8.01E-07
5	779	F2 vs. L2	+	Metabolism	Transporters	cation transport	GO:0006812	P	214	8.38E-08	3.83E-06
5	780	F2 vs. L2	+	Metabolism	Transporters	cytoplasmic vesicle	GO:0031410	C	409	1.27E-04	1.16E-03
5	781	F2 vs. L2	+	Metabolism	Transporters	endosome	GO:0005768	C	243	2.69E-03	1.64E-02
5	782	F2 vs. L2	+	Metabolism	Transporters	inorganic anion transmembrane transport	GO:0098661	P	13	2.08E-03	2.14E-02
5	783	F2 vs. L2	+	Metabolism	Transporters	inorganic anion transmembrane transporter activity	GO:0015103	F	59	7.90E-06	5.12E-04
5	784	F2 vs. L2	+	Metabolism	Transporters	inorganic anion transport	GO:0015698	P	51	2.80E-06	8.18E-05
5	785	F2 vs. L2	+	Metabolism	Transporters	inorganic cation transmembrane transport	GO:0098662	P	128	4.25E-08	2.05E-06
5	786	F2 vs. L2	+	Metabolism	Transporters	inorganic cation transmembrane transporter activity	GO:0022890	F	206	6.65E-09	1.13E-06
5	787	F2 vs. L2	+	Metabolism	Transporters	inorganic ion transmembrane transport	GO:0098660	P	140	9.72E-10	7.13E-08
5	788	F2 vs. L2	+	Metabolism	Transporters	inorganic molecular entity transmembrane transporter activity	GO:0015318	F	364	5.44E-15	3.70E-12
5	789	F2 vs. L2	+	Metabolism	Transporters	ion transmembrane transport	GO:0034220	P	197	4.72E-10	3.89E-08
5	790	F2 vs. L2	+	Metabolism	Transporters	ion transmembrane transporter activity	GO:0015075	F	398	1.25E-11	4.27E-09
5	791	F2 vs. L2	+	Metabolism	Transporters	ion transmembrane transporter activity, phosphorylative mechanism	GO:0015662	F	32	7.98E-04	2.09E-02
5	792	F2 vs. L2	+	Metabolism	Transporters	ion transport	GO:0006811	P	355	9.22E-12	9.14E-10
5	793	F2 vs. L2	+	Metabolism	Transporters	metal ion transmembrane transporter activity	GO:0046873	F	118	1.38E-03	3.00E-02
5	794	F2 vs. L2	+	Metabolism	Transporters	monovalent inorganic cation transmembrane transporter activity	GO:0015077	F	124	3.69E-07	3.72E-05
5	795	F2 vs. L2	+	Metabolism	Transporters	monovalent inorganic cation transport	GO:0015672	P	93	2.52E-07	1.03E-05
5	796	F2 vs. L2	+	Metabolism	Transporters	neutral amino acid transmembrane transporter activity	GO:0015175	F	12	6.40E-04	1.80E-02
5	797	F2 vs. L2	+	Metabolism	Transporters	organic acid transmembrane transporter activity	GO:0005342	F	80	9.38E-04	2.30E-02
5	798	F2 vs. L2	+	Metabolism	Transporters	organic anion transmembrane transporter activity	GO:0008514	F	130	1.53E-03	3.31E-02
5	799	F2 vs. L2	+	Metabolism	Transporters	phosphate ion transmembrane transport	GO:0035435	P	10	4.33E-03	3.87E-02
5	800	F2 vs. L2	+	Metabolism	Transporters	phosphate ion transport	GO:0006817	P	20	8.72E-04	1.03E-02
5	801	F2 vs. L2	+	Metabolism	Transporters	primary active transmembrane transporter activity	GO:0015399	F	136	1.75E-04	5.94E-03
5	802	F2 vs. L2	+	Metabolism	Transporters	proton transmembrane transporter activity	GO:0015078	F	95	3.04E-08	4.60E-06

5	803	F2 vs. L2	+	Metabolism	Transporters	secondary active transmembrane transporter activity	GO:0015291	F	143	5.48E-11	1.35E-08
5	804	F2 vs. L2	+	Metabolism	Transporters	secretory vesicle	GO:0099503	C	100	9.31E-04	6.75E-03
5	805	F2 vs. L2	+	Metabolism	Transporters	sugar transmembrane transporter activity	GO:0051119	F	24	6.46E-05	2.58E-03
5	806	F2 vs. L2	+	Metabolism	Transporters	trans-Golgi network	GO:0005802	C	185	8.82E-08	1.52E-06
5	807	F2 vs. L2	+	Metabolism	Transporters	transition metal ion transmembrane transporter activity	GO:0046915	F	38	2.00E-03	3.94E-02
5	808	F2 vs. L2	+	Metabolism	Transporters	transmembrane transport	GO:0055085	P	487	4.23E-14	6.47E-12
5	809	F2 vs. L2	+	Metabolism	Transporters	transmembrane transporter activity	GO:0022857	F	689	0.00E+00	0.00E+00
5	810	F2 vs. L2	+	Metabolism	Transporters	transport	GO:0006810	P	1219	6.91E-06	1.78E-04
5	811	F2 vs. L2	+	Metabolism	Transporters	transporter activity	GO:0005215	F	712	0.00E+00	0.00E+00
5	812	F2 vs. L2	+	Metabolism	Transporters	vesicle	GO:0031982	C	442	2.26E-05	2.56E-04
5	813	F2 vs. L2	+	Metabolism	Transporters	water channel activity	GO:0015250	F	22	1.27E-03	2.86E-02
5	814	F2 vs. L2	+	Metabolism	Transporters	water transmembrane transporter activity	GO:0005372	F	22	1.27E-03	2.88E-02
5	815	F2 vs. L2	+	Metabolism	Transporters	xenobiotic transmembrane transporter activity	GO:0042910	F	41	2.84E-04	8.88E-03
5	816	F2 vs. L2	+	Regulation	Protein modification	Golgi apparatus	GO:0005794	C	668	4.41E-09	1.04E-07
5	817	F2 vs. L2	+	Regulation	Protein modification	modified amino acid binding	GO:0072341	F	13	1.21E-04	4.39E-03
5	818	F2 vs. L2	+	Regulation	Regulation	regulation of cell morphogenesis involved in differentiation	GO:0010769	P	28	5.18E-07	1.99E-05
5	819	F2 vs. L2	+	Regulation	Regulation	regulation of pollen tube growth	GO:0080092	P	25	1.84E-07	7.94E-06
5	820	F2 vs. L2	+	Regulation	Regulation	regulation of secondary metabolite biosynthetic process	GO:1900376	P	18	2.17E-03	2.22E-02
5	821	F2 vs. L2	+	Regulation	Regulation	regulation of small molecule metabolic process	GO:0062012	P	61	1.46E-03	1.61E-02
5	822	F2 vs. L2	+	Regulation	Regulation	regulation of unidimensional cell growth	GO:0051510	P	46	1.61E-03	1.72E-02
5	823	F2 vs. L2	+	Regulation	Translation	poly(U) RNA binding	GO:0008266	F	19	2.10E-03	4.07E-02
5	824	F2 vs. L2	+	Signaling and response	Signaling	1-phosphatidylinositol-4-phosphate 5-kinase activity	GO:0016308	F	5	1.67E-03	3.52E-02
5	825	F2 vs. L2	+	Signaling and response	Signaling	calcium ion binding	GO:0005509	F	136	1.30E-05	7.55E-04
5	826	F2 vs. L2	+	Signaling and response	Signaling	cellular response to blue light	GO:0071483	P	19	3.53E-03	3.28E-02
5	827	F2 vs. L2	+	Signaling and response	Signaling	cellular response to chemical stimulus	GO:0070887	P	601	8.91E-04	1.04E-02
5	828	F2 vs. L2	+	Signaling and response	Signaling	cellular response to extracellular stimulus	GO:0031668	P	113	1.68E-03	1.79E-02
5	829	F2 vs. L2	+	Signaling and response	Signaling	cellular response to oxygen levels	GO:0071453	P	129	1.73E-03	1.83E-02
5	830	F2 vs. L2	+	Signaling and response	Signaling	disulfide oxidoreductase activity	GO:0015036	F	36	2.11E-04	6.74E-03
5	831	F2 vs. L2	+	Signaling and response	Signaling	jasmonic acid biosynthetic process	GO:0009695	P	17	9.54E-05	1.68E-03
5	832	F2 vs. L2	+	Signaling and response	Signaling	jasmonic acid metabolic process	GO:0009694	P	32	1.21E-04	2.02E-03
5	833	F2 vs. L2	+	Signaling and response	Signaling	multi-organism process	GO:0051704	P	254	5.65E-04	7.15E-03
5	834	F2 vs. L2	+	Signaling and response	Signaling	regulation of jasmonic acid mediated signaling pathway	GO:2000022	P	27	2.97E-04	4.24E-03
5	835	F2 vs. L2	+	Signaling and response	Signaling	response to abiotic stimulus	GO:0009628	P	1213	4.93E-08	2.31E-06
5	836	F2 vs. L2	+	Signaling and response	Signaling	response to acid chemical	GO:0001101	P	230	3.52E-08	1.74E-06
5	837	F2 vs. L2	+	Signaling and response	Signaling	response to bacterium	GO:0009617	P	303	1.08E-09	7.60E-08
5	838	F2 vs. L2	+	Signaling and response	Signaling	response to biotic stimulus	GO:0009607	P	619	8.44E-09	4.61E-07
5	839	F2 vs. L2	+	Signaling and response	Signaling	response to cadmium ion	GO:0046686	P	224	1.13E-06	3.81E-05
5	840	F2 vs. L2	+	Signaling and response	Signaling	response to chemical	GO:0042221	P	1528	0.00E+00	0.00E+00
5	841	F2 vs. L2	+	Signaling and response	Signaling	response to chitin	GO:0010200	P	73	3.25E-06	9.21E-05
5	842	F2 vs. L2	+	Signaling and response	Signaling	response to endogenous stimulus	GO:0009719	P	701	7.49E-06	1.89E-04
5	843	F2 vs. L2	+	Signaling and response	Signaling	response to external biotic stimulus	GO:0043207	P	618	8.02E-09	4.56E-07
5	844	F2 vs. L2	+	Signaling and response	Signaling	response to external stimulus	GO:0009605	P	845	4.98E-10	4.04E-08
5	845	F2 vs. L2	+	Signaling and response	Signaling	response to extracellular stimulus	GO:0009991	P	141	2.11E-04	3.22E-03
5	846	F2 vs. L2	+	Signaling and response	Signaling	response to hormone	GO:0009725	P	684	3.24E-06	9.23E-05
5	847	F2 vs. L2	+	Signaling and response	Signaling	response to inorganic substance	GO:0010035	P	564	1.02E-13	1.40E-11
5	848	F2 vs. L2	+	Signaling and response	Signaling	response to jasmonic acid	GO:0009753	P	104	6.09E-10	4.79E-08
5	849	F2 vs. L2	+	Signaling and response	Signaling	response to karrikin	GO:0080167	P	89	3.70E-05	7.42E-04
5	850	F2 vs. L2	+	Signaling and response	Signaling	response to light intensity	GO:0009642	P	80	4.07E-03	3.68E-02
5	851	F2 vs. L2	+	Signaling and response	Signaling	response to light stimulus	GO:0009416	P	403	1.49E-04	2.40E-03
5	852	F2 vs. L2	+	Signaling and response	Signaling	response to lipid	GO:0033993	P	395	1.51E-03	1.64E-02
5	853	F2 vs. L2	+	Signaling and response	Signaling	response to metal ion	GO:0010038	P	290	3.23E-07	1.29E-05
5	854	F2 vs. L2	+	Signaling and response	Signaling	response to nitrogen compound	GO:1901698	P	163	4.03E-03	3.66E-02
5	855	F2 vs. L2	+	Signaling and response	Signaling	response to nutrient levels	GO:0031667	P	119	3.60E-03	3.32E-02
5	856	F2 vs. L2	+	Signaling and response	Signaling	response to organic substance	GO:0010033	P	953	1.01E-08	5.41E-07
5	857	F2 vs. L2	+	Signaling and response	Signaling	response to oxygen levels	GO:0070482	P	149	2.42E-03	2.43E-02
5	858	F2 vs. L2	+	Signaling and response	Signaling	response to oxygen-containing compound	GO:1901700	P	874	1.60E-14	2.67E-12
5	859	F2 vs. L2	+	Signaling and response	Signaling	response to salicylic acid	GO:0009751	P	78	1.40E-05	3.26E-04
5	860	F2 vs. L2	+	Signaling and response	Signaling	response to stimulus	GO:0050896	P	2983	9.11E-10	6.77E-08
5	861	F2 vs. L2	+	Signaling and response	Signaling	response to temperature stimulus	GO:0009266	P	369	1.92E-04	2.94E-03
5	862	F2 vs. L2	+	Signaling and response	Signaling	response to water	GO:0009415	P	213	2.72E-09	1.71E-07
5	863	F2 vs. L2	+	Signaling and response	Stress	anion homeostasis	GO:0055081	P	29	1.25E-04	2.09E-03
5	864	F2 vs. L2	+	Signaling and response	Stress	antioxidant activity	GO:0016209	F	70	6.23E-05	2.53E-03
5	865	F2 vs. L2	+	Signaling and response	Stress	cation homeostasis	GO:0055080	P	127	9.77E-05	1.71E-03
5	866	F2 vs. L2	+	Signaling and response	Stress	cellular detoxification	GO:1990748	P	23	2.34E-04	3.50E-03

5	867	F2 vs. L2	+	Signaling and response	Stress	cellular homeostasis	GO:0019725	P	159	8.00E-04	9.64E-03
5	868	F2 vs. L2	+	Signaling and response	Stress	cellular oxidant detoxification	GO:0098869	P	15	6.37E-06	1.67E-04
5	869	F2 vs. L2	+	Signaling and response	Stress	cellular response to decreased oxygen levels	GO:0036294	P	128	1.38E-03	1.54E-02
5	870	F2 vs. L2	+	Signaling and response	Stress	cellular response to hypoxia	GO:0071456	P	126	2.16E-03	2.21E-02
5	871	F2 vs. L2	+	Signaling and response	Stress	cellular response to light intensity	GO:0071484	P	8	2.56E-03	2.55E-02
5	872	F2 vs. L2	+	Signaling and response	Stress	cellular response to oxidative stress	GO:0034599	P	41	3.02E-03	2.86E-02
5	873	F2 vs. L2	+	Signaling and response	Stress	cellular response to oxygen radical	GO:0071450	P	7	5.52E-03	4.69E-02
5	874	F2 vs. L2	+	Signaling and response	Stress	cellular response to phosphate starvation	GO:0016036	P	49	4.27E-03	3.83E-02
5	875	F2 vs. L2	+	Signaling and response	Stress	cellular response to superoxide	GO:0071451	P	7	5.52E-03	4.70E-02
5	876	F2 vs. L2	+	Signaling and response	Stress	cellular response to toxic substance	GO:0097237	P	24	7.94E-05	1.43E-03
5	877	F2 vs. L2	+	Signaling and response	Stress	chemical homeostasis	GO:0048878	P	219	2.07E-06	6.32E-05
5	878	F2 vs. L2	+	Signaling and response	Stress	defense response	GO:0006952	P	529	6.38E-06	1.66E-04
5	879	F2 vs. L2	+	Signaling and response	Stress	defense response to bacterium	GO:0042742	P	249	6.33E-09	3.72E-07
5	880	F2 vs. L2	+	Signaling and response	Stress	defense response to Gram-negative bacterium	GO:0050829	P	15	3.02E-03	2.86E-02
5	881	F2 vs. L2	+	Signaling and response	Stress	defense response to other organism	GO:0098542	P	451	3.84E-05	7.60E-04
5	882	F2 vs. L2	+	Signaling and response	Stress	detoxification	GO:0098754	P	49	1.40E-08	7.32E-07
5	883	F2 vs. L2	+	Signaling and response	Stress	flavonoid biosynthetic process	GO:0009813	P	32	1.10E-04	1.88E-03
5	884	F2 vs. L2	+	Signaling and response	Stress	flavonoid metabolic process	GO:0009812	P	38	3.26E-04	4.56E-03
5	885	F2 vs. L2	+	Signaling and response	Stress	glutathione binding	GO:0043295	F	13	1.21E-04	4.33E-03
5	886	F2 vs. L2	+	Signaling and response	Stress	glutathione metabolic process	GO:0006749	P	21	7.81E-04	9.46E-03
5	887	F2 vs. L2	+	Signaling and response	Stress	glutathione transferase activity	GO:0004364	F	25	2.42E-05	1.24E-03
5	888	F2 vs. L2	+	Signaling and response	Stress	homeostatic process	GO:0042592	P	287	4.21E-05	8.26E-04
5	889	F2 vs. L2	+	Signaling and response	Stress	hydrogen peroxide catabolic process	GO:0042744	P	15	2.92E-03	2.79E-02
5	890	F2 vs. L2	+	Signaling and response	Stress	hydrogen peroxide metabolic process	GO:0042743	P	21	1.16E-04	1.96E-03
5	891	F2 vs. L2	+	Signaling and response	Stress	hydrogen peroxide transmembrane transport	GO:0080170	P	5	1.37E-03	1.53E-02
5	892	F2 vs. L2	+	Signaling and response	Stress	inorganic ion homeostasis	GO:0098771	P	137	2.35E-05	5.14E-04
5	893	F2 vs. L2	+	Signaling and response	Stress	interspecies interaction between organisms	GO:0044419	P	630	8.16E-09	4.55E-07
5	894	F2 vs. L2	+	Signaling and response	Stress	ion homeostasis	GO:0050801	P	157	9.01E-07	3.30E-05
5	895	F2 vs. L2	+	Signaling and response	Stress	monovalent inorganic cation homeostasis	GO:0055067	P	41	6.97E-05	1.26E-03
5	896	F2 vs. L2	+	Signaling and response	Stress	protein repair	GO:0030091	P	19	1.07E-04	1.84E-03
5	897	F2 vs. L2	+	Signaling and response	Stress	reactive nitrogen species metabolic process	GO:2001057	P	11	4.76E-03	4.18E-02
5	898	F2 vs. L2	+	Signaling and response	Stress	reactive oxygen species metabolic process	GO:0072593	P	44	2.71E-06	7.97E-05
5	899	F2 vs. L2	+	Signaling and response	Stress	regulation of pH	GO:0006885	P	30	5.57E-04	7.07E-03
5	900	F2 vs. L2	+	Signaling and response	Stress	regulation of stomatal movement	GO:0010119	P	52	4.86E-03	4.25E-02
5	901	F2 vs. L2	+	Signaling and response	Stress	removal of superoxide radicals	GO:0019430	P	7	5.52E-03	4.68E-02
5	902	F2 vs. L2	+	Signaling and response	Stress	response to abscisic acid	GO:0009737	P	289	7.86E-05	1.42E-03
5	903	F2 vs. L2	+	Signaling and response	Stress	response to alcohol	GO:0097305	P	292	1.88E-04	2.90E-03
5	904	F2 vs. L2	+	Signaling and response	Stress	response to cold	GO:0009409	P	254	9.55E-09	5.16E-07
5	905	F2 vs. L2	+	Signaling and response	Stress	response to decreased oxygen levels	GO:0036293	P	148	1.99E-03	2.06E-02
5	906	F2 vs. L2	+	Signaling and response	Stress	response to desiccation	GO:0009269	P	16	1.63E-03	1.74E-02
5	907	F2 vs. L2	+	Signaling and response	Stress	response to fungus	GO:0009620	P	192	2.60E-03	2.58E-02
5	908	F2 vs. L2	+	Signaling and response	Stress	response to high light intensity	GO:0009644	P	39	3.96E-04	5.24E-03
5	909	F2 vs. L2	+	Signaling and response	Stress	response to hypoxia	GO:0001666	P	146	2.99E-03	2.85E-02
5	910	F2 vs. L2	+	Signaling and response	Stress	response to insect	GO:0009625	P	20	1.49E-03	1.63E-02
5	911	F2 vs. L2	+	Signaling and response	Stress	response to osmotic stress	GO:0006970	P	307	4.25E-03	3.81E-02
5	912	F2 vs. L2	+	Signaling and response	Stress	response to other organism	GO:0051707	P	618	8.02E-09	4.52E-07
5	913	F2 vs. L2	+	Signaling and response	Stress	response to oxidative stress	GO:0006979	P	255	4.82E-06	1.30E-04
5	914	F2 vs. L2	+	Signaling and response	Stress	response to oxygen radical	GO:0000305	P	9	5.46E-03	4.66E-02
5	915	F2 vs. L2	+	Signaling and response	Stress	response to ozone	GO:0010193	P	23	9.43E-04	1.09E-02
5	916	F2 vs. L2	+	Signaling and response	Stress	response to radiation	GO:0009314	P	419	1.30E-03	1.46E-02
5	917	F2 vs. L2	+	Signaling and response	Stress	response to reactive oxygen species	GO:0000302	P	95	3.91E-04	5.22E-03
5	918	F2 vs. L2	+	Signaling and response	Stress	response to stress	GO:0006950	P	1724	2.11E-06	6.34E-05
5	919	F2 vs. L2	+	Signaling and response	Stress	response to superoxide	GO:0000303	P	9	5.46E-03	4.66E-02
5	920	F2 vs. L2	+	Signaling and response	Stress	response to toxic substance	GO:0009636	P	60	6.78E-09	3.90E-07
5	921	F2 vs. L2	+	Signaling and response	Stress	response to UV-B	GO:0010224	P	48	1.59E-03	1.71E-02
5	922	F2 vs. L2	+	Signaling and response	Stress	response to water deprivation	GO:0009414	P	209	2.57E-09	1.66E-07
5	923	F2 vs. L2	+	Signaling and response	Stress	response to wounding	GO:0009611	P	137	2.12E-14	3.34E-12
5	924	F2 vs. L2	+	Signaling and response	Stress	superoxide metabolic process	GO:0006801	P	7	5.52E-03	4.71E-02
5	925	F2 vs. L2	+	Signaling and response	Stress	toxin catabolic process	GO:0009407	P	20	1.38E-04	2.28E-03
5	926	F2 vs. L2	+	Signaling and response	Stress	toxin metabolic process	GO:0009404	P	30	3.22E-06	9.23E-05
5	927	F2 vs. L2	-	Development	Cell division	alpha DNA polymerase:primase complex	GO:0005658	C	4	2.57E-03	1.59E-02
5	928	F2 vs. L2	-	Development	Cell division	ATP-dependent microtubule motor activity	GO:1990939	F	10	1.71E-03	3.57E-02
5	929	F2 vs. L2	-	Development	Cell division	base-excision repair	GO:0006284	P	17	3.71E-04	4.96E-03
5	930	F2 vs. L2	-	Development	Cell division	bub1-bub3 complex	GO:1990298	C	3	6.91E-03	3.53E-02

5	931	F2 vs. L2	-	Development	Cell division	Cajal body	GO:0015030	C	12	7.43E-03	3.76E-02
5	932	F2 vs. L2	-	Development	Cell division	cell cycle	GO:0007049	P	250	1.05E-14	1.88E-12
5	933	F2 vs. L2	-	Development	Cell division	cell cycle checkpoint	GO:0000075	P	24	4.39E-05	8.48E-04
5	934	F2 vs. L2	-	Development	Cell division	cell cycle DNA replication	GO:0044786	P	19	1.50E-03	1.64E-02
5	935	F2 vs. L2	-	Development	Cell division	cell cycle DNA replication initiation	GO:1902292	P	4	1.11E-03	1.25E-02
5	936	F2 vs. L2	-	Development	Cell division	cell cycle phase transition	GO:0044770	P	30	2.10E-06	6.36E-05
5	937	F2 vs. L2	-	Development	Cell division	cell cycle process	GO:0022402	P	227	1.11E-15	2.58E-13
5	938	F2 vs. L2	-	Development	Cell division	chromatin assembly	GO:0031497	P	42	1.10E-04	1.88E-03
5	939	F2 vs. L2	-	Development	Cell division	chromosome	GO:0005694	C	265	2.00E-15	7.42E-14
5	940	F2 vs. L2	-	Development	Cell division	chromosome passenger complex	GO:0032133	C	3	9.27E-03	4.43E-02
5	941	F2 vs. L2	-	Development	Cell division	chromosome segregation	GO:0007059	P	57	1.64E-04	2.58E-03
5	942	F2 vs. L2	-	Development	Cell division	chromosome, centromeric region	GO:0000775	C	27	7.64E-06	9.83E-05
5	943	F2 vs. L2	-	Development	Cell division	condensed chromosome	GO:0000793	C	35	1.37E-04	1.23E-03
5	944	F2 vs. L2	-	Development	Cell division	condensed chromosome kinetochore	GO:0000777	C	6	8.54E-03	4.20E-02
5	945	F2 vs. L2	-	Development	Cell division	condensed chromosome, centromeric region	GO:0000779	C	14	4.44E-03	2.42E-02
5	946	F2 vs. L2	-	Development	Cell division	condensed nuclear chromosome	GO:0000794	C	22	2.88E-03	1.71E-02
5	947	F2 vs. L2	-	Development	Cell division	condensed nuclear chromosome, centromeric region	GO:0000780	C	10	7.50E-03	3.77E-02
5	948	F2 vs. L2	-	Development	Cell division	cyclin-dependent protein kinase holoenzyme complex	GO:0000307	C	33	5.25E-05	5.50E-04
5	949	F2 vs. L2	-	Development	Cell division	cyclin-dependent protein serine/threonine kinase regulator activity	GO:0016538	F	23	9.81E-06	5.93E-04
5	950	F2 vs. L2	-	Development	Cell division	cytokinesis by cell plate formation	GO:0000911	P	42	2.94E-07	1.18E-05
5	951	F2 vs. L2	-	Development	Cell division	delta DNA polymerase complex	GO:0043625	C	4	9.15E-03	4.39E-02
5	952	F2 vs. L2	-	Development	Cell division	DNA biosynthetic process	GO:0071897	P	17	1.11E-05	2.69E-04
5	953	F2 vs. L2	-	Development	Cell division	DNA duplex unwinding	GO:0032508	P	22	7.78E-04	9.44E-03
5	954	F2 vs. L2	-	Development	Cell division	DNA helicase activity	GO:0003678	F	35	1.24E-05	7.31E-04
5	955	F2 vs. L2	-	Development	Cell division	DNA helicase complex	GO:0033202	C	9	1.46E-03	1.02E-02
5	956	F2 vs. L2	-	Development	Cell division	DNA integrity checkpoint	GO:0031570	P	11	4.59E-04	5.95E-03
5	957	F2 vs. L2	-	Development	Cell division	DNA packaging	GO:0006323	P	53	2.39E-03	2.40E-02
5	958	F2 vs. L2	-	Development	Cell division	DNA polymerase complex	GO:0042575	C	13	6.38E-05	6.62E-04
5	959	F2 vs. L2	-	Development	Cell division	DNA recombination	GO:0006310	P	73	2.63E-06	7.82E-05
5	960	F2 vs. L2	-	Development	Cell division	DNA repair	GO:0006281	P	172	6.22E-15	1.19E-12
5	961	F2 vs. L2	-	Development	Cell division	DNA replication	GO:0006260	P	69	5.70E-14	8.24E-12
5	962	F2 vs. L2	-	Development	Cell division	DNA replication checkpoint	GO:0000076	P	8	2.00E-03	2.07E-02
5	963	F2 vs. L2	-	Development	Cell division	DNA replication factor A complex	GO:0005662	C	8	6.45E-04	4.90E-03
5	964	F2 vs. L2	-	Development	Cell division	DNA replication factor C complex	GO:0005663	C	7	8.27E-03	4.09E-02
5	965	F2 vs. L2	-	Development	Cell division	DNA replication initiation	GO:0006270	P	14	5.97E-07	2.28E-05
5	966	F2 vs. L2	-	Development	Cell division	DNA replication origin binding	GO:0003688	F	12	7.61E-06	5.17E-04
5	967	F2 vs. L2	-	Development	Cell division	DNA strand elongation	GO:0022616	P	11	1.21E-06	3.99E-05
5	968	F2 vs. L2	-	Development	Cell division	DNA strand elongation involved in DNA replication	GO:0006271	P	11	1.21E-06	4.01E-05
5	969	F2 vs. L2	-	Development	Cell division	DNA unwinding involved in DNA replication	GO:0006268	P	13	2.58E-04	3.80E-03
5	970	F2 vs. L2	-	Development	Cell division	DNA-(apurinic or apyrimidinic site) endonuclease activity	GO:0003906	F	7	5.74E-04	1.64E-02
5	971	F2 vs. L2	-	Development	Cell division	DNA-dependent DNA replication	GO:0006261	P	60	4.71E-13	5.72E-11
5	972	F2 vs. L2	-	Development	Cell division	DNA-directed DNA polymerase activity	GO:0003887	F	12	2.49E-03	4.73E-02
5	973	F2 vs. L2	-	Development	Cell division	double-strand break repair	GO:0006302	P	70	1.12E-06	3.82E-05
5	974	F2 vs. L2	-	Development	Cell division	double-strand break repair via break-induced replication	GO:0000727	P	7	1.60E-03	1.71E-02
5	975	F2 vs. L2	-	Development	Cell division	double-strand break repair via homologous recombination	GO:0000724	P	48	4.30E-05	8.33E-04
5	976	F2 vs. L2	-	Development	Cell division	endonuclease activity	GO:0004519	F	49	1.13E-03	2.65E-02
5	977	F2 vs. L2	-	Development	Cell division	G1/S transition of mitotic cell cycle	GO:0000082	P	7	4.71E-03	4.15E-02
5	978	F2 vs. L2	-	Development	Cell division	kinetochore	GO:0000776	C	15	6.72E-05	6.90E-04
5	979	F2 vs. L2	-	Development	Cell division	MCM complex	GO:0042555	C	6	4.04E-04	3.25E-03
5	980	F2 vs. L2	-	Development	Cell division	meiotic cell cycle	GO:0051321	P	83	2.28E-04	3.42E-03
5	981	F2 vs. L2	-	Development	Cell division	meiotic cell cycle process	GO:1903046	P	74	1.22E-03	1.36E-02
5	982	F2 vs. L2	-	Development	Cell division	meiotic nuclear division	GO:0140013	P	49	3.79E-03	3.47E-02
5	983	F2 vs. L2	-	Development	Cell division	microtubule cytoskeleton organization involved in mitosis	GO:1902850	P	14	3.93E-05	7.72E-04
5	984	F2 vs. L2	-	Development	Cell division	microtubule motor activity	GO:0003777	F	34	2.47E-05	1.24E-03
5	985	F2 vs. L2	-	Development	Cell division	mismatch repair	GO:0006298	P	12	2.33E-03	2.36E-02
5	986	F2 vs. L2	-	Development	Cell division	mitotic cell cycle	GO:0000278	P	146	1.03E-07	4.60E-06
5	987	F2 vs. L2	-	Development	Cell division	mitotic cell cycle checkpoint	GO:0007093	P	19	3.98E-04	5.26E-03
5	988	F2 vs. L2	-	Development	Cell division	mitotic cell cycle phase transition	GO:0044772	P	29	1.08E-06	3.73E-05
5	989	F2 vs. L2	-	Development	Cell division	mitotic cell cycle process	GO:1903047	P	121	5.41E-09	3.25E-07
5	990	F2 vs. L2	-	Development	Cell division	mitotic checkpoint complex	GO:0033597	C	4	1.57E-03	1.08E-02
5	991	F2 vs. L2	-	Development	Cell division	mitotic DNA integrity checkpoint	GO:0044774	P	6	2.94E-03	2.81E-02
5	992	F2 vs. L2	-	Development	Cell division	mitotic DNA replication	GO:1902969	P	6	1.81E-03	1.89E-02
5	993	F2 vs. L2	-	Development	Cell division	mitotic DNA replication initiation	GO:1902975	P	4	1.11E-03	1.26E-02

5	994	F2 vs. L2	-	Development	Cell division	mitotic G2/M transition checkpoint	GO:0044818	P	5	5.24E-03	4.54E-02
5	995	F2 vs. L2	-	Development	Cell division	mitotic nuclear division	GO:0140014	P	35	2.59E-04	3.81E-03
5	996	F2 vs. L2	-	Development	Cell division	mitotic spindle	GO:0072686	C	7	2.67E-03	1.64E-02
5	997	F2 vs. L2	-	Development	Cell division	mitotic spindle assembly checkpoint	GO:0007094	P	9	2.92E-03	2.85E-02
5	998	F2 vs. L2	-	Development	Cell division	mitotic spindle checkpoint	GO:0071174	P	9	2.92E-03	2.82E-02
5	999	F2 vs. L2	-	Development	Cell division	mitotic spindle organization	GO:0007052	P	12	3.27E-04	4.56E-03
5	1000	F2 vs. L2	-	Development	Cell division	negative regulation of cell cycle	GO:0045786	P	38	2.11E-05	4.74E-04
5	1001	F2 vs. L2	-	Development	Cell division	negative regulation of cell cycle G2/M phase transition	GO:1902750	P	5	5.24E-03	4.53E-02
5	1002	F2 vs. L2	-	Development	Cell division	negative regulation of cell cycle phase transition	GO:1901988	P	15	2.28E-05	4.99E-04
5	1003	F2 vs. L2	-	Development	Cell division	negative regulation of cell cycle process	GO:0010948	P	26	6.58E-05	1.20E-03
5	1004	F2 vs. L2	-	Development	Cell division	negative regulation of chromosome organization	GO:2001251	P	11	3.02E-04	4.31E-03
5	1005	F2 vs. L2	-	Development	Cell division	negative regulation of chromosome segregation	GO:0051985	P	9	2.92E-03	2.84E-02
5	1006	F2 vs. L2	-	Development	Cell division	negative regulation of chromosome separation	GO:1905819	P	9	2.92E-03	2.79E-02
5	1007	F2 vs. L2	-	Development	Cell division	negative regulation of G2/M transition of mitotic cell cycle	GO:0010972	P	5	5.24E-03	4.53E-02
5	1008	F2 vs. L2	-	Development	Cell division	negative regulation of metaphase/anaphase transition of cell cycle	GO:1902100	P	9	2.92E-03	2.81E-02
5	1009	F2 vs. L2	-	Development	Cell division	negative regulation of mitotic cell cycle	GO:0045930	P	25	8.02E-04	9.64E-03
5	1010	F2 vs. L2	-	Development	Cell division	negative regulation of mitotic cell cycle phase transition	GO:1901991	P	15	2.28E-05	5.01E-04
5	1011	F2 vs. L2	-	Development	Cell division	negative regulation of mitotic metaphase/anaphase transition	GO:0045841	P	9	2.92E-03	2.80E-02
5	1012	F2 vs. L2	-	Development	Cell division	negative regulation of mitotic sister chromatid segregation	GO:0033048	P	9	2.92E-03	2.83E-02
5	1013	F2 vs. L2	-	Development	Cell division	negative regulation of mitotic sister chromatid separation	GO:2000816	P	9	2.92E-03	2.84E-02
5	1014	F2 vs. L2	-	Development	Cell division	negative regulation of nuclear division	GO:0051784	P	16	2.05E-03	2.12E-02
5	1015	F2 vs. L2	-	Development	Cell division	negative regulation of sister chromatid segregation	GO:0033046	P	9	2.92E-03	2.82E-02
5	1016	F2 vs. L2	-	Development	Cell division	nuclear cell cycle DNA replication initiation	GO:1902315	P	4	1.11E-03	1.25E-02
5	1017	F2 vs. L2	-	Development	Cell division	nuclear chromosome segregation	GO:0098813	P	50	3.53E-04	4.78E-03
5	1018	F2 vs. L2	-	Development	Cell division	nuclear division	GO:0000280	P	78	1.34E-06	4.29E-05
5	1019	F2 vs. L2	-	Development	Cell division	nuclear DNA replication	GO:0033260	P	9	1.62E-04	2.57E-03
5	1020	F2 vs. L2	-	Development	Cell division	nuclear origin of replication recognition complex	GO:0005664	C	4	4.35E-03	2.40E-02
5	1021	F2 vs. L2	-	Development	Cell division	nuclear replication fork	GO:0043596	C	18	1.99E-07	3.15E-06
5	1022	F2 vs. L2	-	Development	Cell division	nuclease activity	GO:0004518	F	90	2.06E-04	6.68E-03
5	1023	F2 vs. L2	-	Development	Cell division	nucleic acid metabolic process	GO:0090304	P	866	0.00E+00	0.00E+00
5	1024	F2 vs. L2	-	Development	Cell division	nucleobase-containing compound metabolic process	GO:0006139	P	1111	0.00E+00	0.00E+00
5	1025	F2 vs. L2	-	Development	Cell division	nucleosome assembly	GO:0006334	P	26	1.21E-03	1.36E-02
5	1026	F2 vs. L2	-	Development	Cell division	nucleotide-excision repair	GO:0006289	P	29	2.98E-05	6.29E-04
5	1027	F2 vs. L2	-	Development	Cell division	origin recognition complex	GO:0000808	C	4	4.35E-03	2.38E-02
5	1028	F2 vs. L2	-	Development	Cell division	preprophase band	GO:0009574	C	10	2.06E-03	1.33E-02
5	1029	F2 vs. L2	-	Development	Cell division	preprophase band assembly	GO:0000913	P	6	4.76E-03	4.17E-02
5	1030	F2 vs. L2	-	Development	Cell division	pre-replicative complex assembly	GO:0036388	P	5	1.40E-03	1.55E-02
5	1031	F2 vs. L2	-	Development	Cell division	pre-replicative complex assembly involved in cell cycle DNA replication	GO:1902299	P	5	1.40E-03	1.56E-02
5	1032	F2 vs. L2	-	Development	Cell division	pre-replicative complex assembly involved in nuclear cell cycle DNA replication	GO:0006267	P	5	1.40E-03	1.56E-02
5	1033	F2 vs. L2	-	Development	Cell division	recombinational repair	GO:0000725	P	51	3.39E-05	6.91E-04
5	1034	F2 vs. L2	-	Development	Cell division	regulation of cell cycle	GO:0051726	P	134	2.51E-13	3.36E-11
5	1035	F2 vs. L2	-	Development	Cell division	regulation of cell cycle G2/M phase transition	GO:1902749	P	18	3.46E-04	4.70E-03
5	1036	F2 vs. L2	-	Development	Cell division	regulation of cell cycle phase transition	GO:1901987	P	35	1.01E-07	4.55E-06
5	1037	F2 vs. L2	-	Development	Cell division	regulation of cell cycle process	GO:0010564	P	75	1.67E-10	1.44E-08
5	1038	F2 vs. L2	-	Development	Cell division	regulation of cell division	GO:0051302	P	35	1.04E-05	2.56E-04
5	1039	F2 vs. L2	-	Development	Cell division	regulation of cell population proliferation	GO:0042127	P	42	1.57E-03	1.70E-02
5	1040	F2 vs. L2	-	Development	Cell division	regulation of chromatin organization	GO:1902275	P	35	9.33E-04	1.09E-02
5	1041	F2 vs. L2	-	Development	Cell division	regulation of chromosome organization	GO:0033044	P	39	1.61E-06	5.00E-05
5	1042	F2 vs. L2	-	Development	Cell division	regulation of chromosome segregation	GO:0051983	P	16	4.84E-04	6.21E-03
5	1043	F2 vs. L2	-	Development	Cell division	regulation of chromosome separation	GO:1905818	P	15	3.34E-04	4.59E-03
5	1044	F2 vs. L2	-	Development	Cell division	regulation of cyclin-dependent protein kinase activity	GO:1904029	P	22	6.81E-04	8.39E-03
5	1045	F2 vs. L2	-	Development	Cell division	regulation of DNA metabolic process	GO:0051052	P	44	3.95E-04	5.24E-03
5	1046	F2 vs. L2	-	Development	Cell division	regulation of DNA replication	GO:0006275	P	31	2.72E-04	3.97E-03
5	1047	F2 vs. L2	-	Development	Cell division	regulation of DNA-dependent DNA replication	GO:0090329	P	25	3.30E-03	3.07E-02
5	1048	F2 vs. L2	-	Development	Cell division	regulation of G2/M transition of mitotic cell cycle	GO:0010389	P	18	3.46E-04	4.69E-03
5	1049	F2 vs. L2	-	Development	Cell division	regulation of metaphase/anaphase transition of cell cycle	GO:1902099	P	15	3.34E-04	4.61E-03
5	1050	F2 vs. L2	-	Development	Cell division	regulation of mitotic cell cycle	GO:0007346	P	50	4.40E-06	1.22E-04
5	1051	F2 vs. L2	-	Development	Cell division	regulation of mitotic cell cycle phase transition	GO:1901990	P	34	2.05E-07	8.48E-06
5	1052	F2 vs. L2	-	Development	Cell division	regulation of mitotic metaphase/anaphase transition	GO:0030071	P	15	3.34E-04	4.62E-03
5	1053	F2 vs. L2	-	Development	Cell division	regulation of mitotic nuclear division	GO:0007088	P	18	5.65E-03	4.75E-02
5	1054	F2 vs. L2	-	Development	Cell division	regulation of mitotic sister chromatid segregation	GO:0033047	P	9	2.92E-03	2.83E-02

5	1055	F2 vs. L2	-	Development	Cell division	regulation of mitotic sister chromatid separation	GO:0010965	P	15	3.34E-04	4.63E-03
5	1056	F2 vs. L2	-	Development	Cell division	regulation of nuclear division	GO:0051783	P	29	3.24E-04	4.54E-03
5	1057	F2 vs. L2	-	Development	Cell division	regulation of sister chromatid segregation	GO:0033045	P	16	4.84E-04	6.20E-03
5	1058	F2 vs. L2	-	Development	Cell division	replication fork	GO:0005657	C	28	3.93E-09	9.48E-08
5	1059	F2 vs. L2	-	Development	Cell division	replisome	GO:0030894	C	17	5.50E-08	9.83E-07
5	1060	F2 vs. L2	-	Development	Cell division	RNA metabolic process	GO:0016070	P	666	0.00E+00	0.00E+00
5	1061	F2 vs. L2	-	Development	Cell division	single-stranded DNA binding	GO:0003697	F	41	3.45E-06	2.60E-04
5	1062	F2 vs. L2	-	Development	Cell division	sister chromatid segregation	GO:0000819	P	35	8.64E-04	1.03E-02
5	1063	F2 vs. L2	-	Development	Cell division	site of double-strand break	GO:0035861	C	6	1.08E-02	4.96E-02
5	1064	F2 vs. L2	-	Development	Cell division	spindle	GO:0005819	C	43	3.52E-09	8.70E-08
5	1065	F2 vs. L2	-	Development	Cell division	spindle assembly	GO:0051225	P	21	3.68E-05	7.40E-04
5	1066	F2 vs. L2	-	Development	Cell division	spindle assembly checkpoint	GO:0071173	P	9	2.92E-03	2.81E-02
5	1067	F2 vs. L2	-	Development	Cell division	spindle checkpoint	GO:0031577	P	9	2.92E-03	2.80E-02
5	1068	F2 vs. L2	-	Development	Cell division	spindle microtubule	GO:0005876	C	13	1.76E-05	2.07E-04
5	1069	F2 vs. L2	-	Development	Cell division	spindle midzone	GO:0051233	C	4	2.10E-03	1.35E-02
5	1070	F2 vs. L2	-	Development	Cell division	spindle organization	GO:0007051	P	31	1.24E-06	3.99E-05
5	1071	F2 vs. L2	-	Development	Cell division	telomere maintenance	GO:0000723	P	16	1.46E-04	2.38E-03
5	1072	F2 vs. L2	-	Development	Cell division	telomere organization	GO:0032200	P	16	1.46E-04	2.37E-03
5	1073	F2 vs. L2	-	Development	Cell division	telomeric DNA binding	GO:0042162	F	14	1.92E-03	3.89E-02
5	1074	F2 vs. L2	-	Development	Development	anatomical structure homeostasis	GO:0060249	P	18	1.40E-04	2.31E-03
5	1075	F2 vs. L2	-	Development	Development	cellular component assembly	GO:0022607	P	475	1.15E-06	3.86E-05
5	1076	F2 vs. L2	-	Development	Development	cellular component biogenesis	GO:0044085	P	733	2.06E-06	6.34E-05
5	1077	F2 vs. L2	-	Development	Development	cellular component organization	GO:0016043	P	1327	7.43E-05	1.34E-03
5	1078	F2 vs. L2	-	Development	Development	cellular component organization or biogenesis	GO:0071840	P	1540	4.58E-06	1.26E-04
5	1079	F2 vs. L2	-	Development	Development	chromatin	GO:0000785	C	167	8.29E-05	8.08E-04
5	1080	F2 vs. L2	-	Development	Development	chromatin assembly or disassembly	GO:0006333	P	48	1.08E-05	2.63E-04
5	1081	F2 vs. L2	-	Development	Development	chromatin organization	GO:0006325	P	183	1.61E-14	2.61E-12
5	1082	F2 vs. L2	-	Development	Development	chromocenter	GO:0010369	C	6	9.38E-03	4.44E-02
5	1083	F2 vs. L2	-	Development	Development	chromosomal region	GO:0098687	C	35	9.86E-09	2.16E-07
5	1084	F2 vs. L2	-	Development	Development	chromosome organization	GO:0051276	P	275	0.00E+00	0.00E+00
5	1085	F2 vs. L2	-	Development	Development	chromosome, telomeric region	GO:0000781	C	7	6.48E-04	4.88E-03
5	1086	F2 vs. L2	-	Development	Development	cytoskeleton	GO:0005856	C	177	1.97E-03	1.29E-02
5	1087	F2 vs. L2	-	Development	Development	embryo development	GO:0009790	P	311	4.76E-06	1.29E-04
5	1088	F2 vs. L2	-	Development	Development	embryo development ending in seed dormancy	GO:0009793	P	304	6.65E-06	1.72E-04
5	1089	F2 vs. L2	-	Development	Development	fruit development	GO:0010154	P	428	3.66E-04	4.91E-03
5	1090	F2 vs. L2	-	Development	Development	Golgi cis cisterna	GO:0000137	C	26	9.62E-03	4.51E-02
5	1091	F2 vs. L2	-	Development	Development	heterochromatin	GO:0000792	C	8	5.54E-03	2.94E-02
5	1092	F2 vs. L2	-	Development	Development	heterochromatin organization	GO:0070828	P	22	9.58E-04	1.10E-02
5	1093	F2 vs. L2	-	Development	Development	intracellular non-membrane-bounded organelle	GO:0043232	C	974	0.00E+00	0.00E+00
5	1094	F2 vs. L2	-	Development	Development	intracellular organelle lumen	GO:0070013	C	749	0.00E+00	0.00E+00
5	1095	F2 vs. L2	-	Development	Development	kinetochore microtubule	GO:0005828	C	4	4.51E-03	2.43E-02
5	1096	F2 vs. L2	-	Development	Development	leaf vascular tissue pattern formation	GO:0010305	P	20	3.03E-03	2.86E-02
5	1097	F2 vs. L2	-	Development	Development	membrane-enclosed lumen	GO:0031974	C	749	0.00E+00	0.00E+00
5	1098	F2 vs. L2	-	Development	Development	meristem development	GO:0048507	P	113	1.28E-04	2.13E-03
5	1099	F2 vs. L2	-	Development	Development	microtubule	GO:0005874	C	87	3.21E-07	5.00E-06
5	1100	F2 vs. L2	-	Development	Development	microtubule associated complex	GO:0005875	C	44	1.59E-07	2.56E-06
5	1101	F2 vs. L2	-	Development	Development	microtubule cytoskeleton	GO:0015630	C	130	5.79E-09	1.33E-07
5	1102	F2 vs. L2	-	Development	Development	microtubule cytoskeleton organization	GO:0000226	P	97	1.54E-04	2.46E-03
5	1103	F2 vs. L2	-	Development	Development	microtubule-based movement	GO:0007018	P	35	2.18E-05	4.87E-04
5	1104	F2 vs. L2	-	Development	Development	microtubule-based process	GO:0007017	P	128	7.87E-08	3.63E-06
5	1105	F2 vs. L2	-	Development	Development	movement of cell or subcellular component	GO:0006928	P	48	5.44E-04	6.93E-03
5	1106	F2 vs. L2	-	Development	Development	non-membrane-bounded organelle	GO:0043228	C	974	0.00E+00	0.00E+00
5	1107	F2 vs. L2	-	Development	Development	nuclear body	GO:0016604	C	73	3.86E-05	4.19E-04
5	1108	F2 vs. L2	-	Development	Development	nuclear chromatin	GO:0000790	C	135	4.51E-03	2.44E-02
5	1109	F2 vs. L2	-	Development	Development	nuclear chromosome	GO:0000228	C	180	7.67E-09	1.72E-07
5	1110	F2 vs. L2	-	Development	Development	nuclear chromosome, telomeric region	GO:0000784	C	5	9.57E-03	4.51E-02
5	1111	F2 vs. L2	-	Development	Development	nuclear envelope	GO:0005635	C	57	3.04E-04	2.51E-03
5	1112	F2 vs. L2	-	Development	Development	nuclear lumen	GO:0031981	C	659	0.00E+00	0.00E+00
5	1113	F2 vs. L2	-	Development	Development	nuclear pore	GO:0005643	C	27	3.49E-03	2.01E-02
5	1114	F2 vs. L2	-	Development	Development	nuclear pore outer ring	GO:0031080	C	6	1.05E-02	4.86E-02
5	1115	F2 vs. L2	-	Development	Development	nuclear replisome	GO:0043601	C	17	5.50E-08	1.00E-06
5	1116	F2 vs. L2	-	Development	Development	nucleic acid phosphodiester bond hydrolysis	GO:0090305	P	41	4.74E-06	1.29E-04
5	1117	F2 vs. L2	-	Development	Development	nucleoplasm	GO:0005654	C	247	1.11E-16	7.14E-15
5	1118	F2 vs. L2	-	Development	Development	nucleus	GO:0005634	C	4575	0.00E+00	0.00E+00

5	1119	F2 vs. L2	-	Development	Development	organelle assembly	GO:0070925	P	105	6.62E-10	5.13E-08
5	1120	F2 vs. L2	-	Development	Development	organelle fission	GO:0048285	P	105	1.23E-06	4.02E-05
5	1121	F2 vs. L2	-	Development	Development	organelle lumen	GO:0043233	C	749	0.00E+00	0.00E+00
5	1122	F2 vs. L2	-	Development	Development	organelle organization	GO:0006996	P	920	9.63E-12	9.37E-10
5	1123	F2 vs. L2	-	Development	Development	pattern specification process	GO:0007389	P	108	1.75E-03	1.86E-02
5	1124	F2 vs. L2	-	Development	Development	phragmoplast	GO:0009524	C	40	2.43E-06	3.35E-05
5	1125	F2 vs. L2	-	Development	Development	plant epidermis morphogenesis	GO:0090626	P	53	3.09E-03	2.91E-02
5	1126	F2 vs. L2	-	Development	Development	polymeric cytoskeletal fiber	GO:0099513	C	95	8.93E-05	8.45E-04
5	1127	F2 vs. L2	-	Development	Development	post-embryonic development	GO:0009791	P	835	3.01E-03	2.86E-02
5	1128	F2 vs. L2	-	Development	Development	post-embryonic plant morphogenesis	GO:0090698	P	98	5.33E-03	4.58E-02
5	1129	F2 vs. L2	-	Development	Development	process utilizing autophagic mechanism	GO:0061919	P	28	3.63E-03	3.34E-02
5	1130	F2 vs. L2	-	Development	Development	regionalization	GO:0003002	P	92	9.71E-04	1.11E-02
5	1131	F2 vs. L2	-	Development	Development	reproductive structure development	GO:0048608	P	692	8.73E-04	1.03E-02
5	1132	F2 vs. L2	-	Development	Development	reproductive system development	GO:0061458	P	692	8.73E-04	1.03E-02
5	1133	F2 vs. L2	-	Development	Development	seed development	GO:0048316	P	413	2.64E-04	3.87E-03
5	1134	F2 vs. L2	-	Development	Development	structural molecule activity	GO:0005198	F	267	1.95E-08	3.12E-06
5	1135	F2 vs. L2	-	Development	Development	system development	GO:0048731	P	1021	4.57E-03	4.05E-02
5	1136	F2 vs. L2	-	Development	Development	tissue development	GO:0009888	P	321	1.77E-03	1.86E-02
5	1137	F2 vs. L2	-	Metabolism	Bioenergetics	ATPase activity	GO:0016887	F	318	1.78E-03	3.71E-02
5	1138	F2 vs. L2	-	Metabolism	Bioenergetics	ATPase complex	GO:1904949	C	22	4.33E-05	4.59E-04
5	1139	F2 vs. L2	-	Metabolism	Bioenergetics	DNA-dependent ATPase activity	GO:0008094	F	66	1.15E-07	1.49E-05
5	1140	F2 vs. L2	-	Metabolism	Bioenergetics	primary metabolic process	GO:0044238	P	3754	1.06E-04	1.83E-03
5	1141	F2 vs. L2	-	Metabolism	Catabolism	autophagy	GO:0006914	P	28	3.63E-03	3.34E-02
5	1142	F2 vs. L2	-	Metabolism	Catabolism	cellular macromolecule catabolic process	GO:0044265	P	377	1.76E-03	1.86E-02
5	1143	F2 vs. L2	-	Metabolism	Catabolism	cellular protein catabolic process	GO:0044257	P	295	1.50E-03	1.64E-02
5	1144	F2 vs. L2	-	Metabolism	Catabolism	Cul4-RING E3 ubiquitin ligase complex	GO:0080008	C	87	1.87E-04	1.60E-03
5	1145	F2 vs. L2	-	Metabolism	Catabolism	cullin-RING ubiquitin ligase complex	GO:0031461	C	142	7.26E-07	1.11E-05
5	1146	F2 vs. L2	-	Metabolism	Catabolism	endopeptidase complex	GO:1905369	C	49	4.36E-04	3.39E-03
5	1147	F2 vs. L2	-	Metabolism	Catabolism	exodeoxyribonuclease activity	GO:0004529	F	10	7.14E-04	1.92E-02
5	1148	F2 vs. L2	-	Metabolism	Catabolism	exodeoxyribonuclease activity, producing 5'-phosphomonoesters	GO:0016895	F	10	7.14E-04	1.94E-02
5	1149	F2 vs. L2	-	Metabolism	Catabolism	exonuclease activity	GO:0004527	F	42	4.27E-04	1.25E-02
5	1150	F2 vs. L2	-	Metabolism	Catabolism	exoribonuclease complex	GO:1905354	C	9	8.61E-03	4.20E-02
5	1151	F2 vs. L2	-	Metabolism	Catabolism	exosome (RNase complex)	GO:0000178	C	9	8.61E-03	4.22E-02
5	1152	F2 vs. L2	-	Metabolism	Catabolism	modification-dependent macromolecule catabolic process	GO:0043632	P	252	2.49E-05	5.36E-04
5	1153	F2 vs. L2	-	Metabolism	Catabolism	modification-dependent protein catabolic process	GO:0019941	P	248	4.45E-05	8.57E-04
5	1154	F2 vs. L2	-	Metabolism	Catabolism	peptidase complex	GO:1905368	C	58	2.55E-05	2.83E-04
5	1155	F2 vs. L2	-	Metabolism	Catabolism	phagophore assembly site	GO:0000407	C	7	6.05E-03	3.19E-02
5	1156	F2 vs. L2	-	Metabolism	Catabolism	proteasomal protein catabolic process	GO:0010498	P	139	5.72E-05	1.08E-03
5	1157	F2 vs. L2	-	Metabolism	Catabolism	proteasome complex	GO:0000502	C	45	2.79E-03	1.66E-02
5	1158	F2 vs. L2	-	Metabolism	Catabolism	proteasome core complex	GO:0005839	C	20	1.00E-02	4.66E-02
5	1159	F2 vs. L2	-	Metabolism	Catabolism	proteasome-mediated ubiquitin-dependent protein catabolic process	GO:0043161	P	132	1.97E-04	3.02E-03
5	1160	F2 vs. L2	-	Metabolism	Catabolism	protein catabolic process	GO:0030163	P	307	4.31E-03	3.86E-02
5	1161	F2 vs. L2	-	Metabolism	Catabolism	protein-containing complex	GO:0032991	C	1661	0.00E+00	0.00E+00
5	1162	F2 vs. L2	-	Metabolism	Catabolism	proteolysis	GO:0006508	P	414	3.82E-03	3.49E-02
5	1163	F2 vs. L2	-	Metabolism	Catabolism	proteolysis involved in cellular protein catabolic process	GO:0051603	P	286	7.87E-04	9.50E-03
5	1164	F2 vs. L2	-	Metabolism	Catabolism	RNA phosphodiester bond hydrolysis, endonucleolytic	GO:0090502	P	13	1.88E-03	1.96E-02
5	1165	F2 vs. L2	-	Metabolism	Catabolism	ubiquitin ligase complex	GO:0000151	C	174	1.14E-06	1.65E-05
5	1166	F2 vs. L2	-	Metabolism	Catabolism	ubiquitin-dependent protein catabolic process	GO:0006511	P	244	2.03E-05	4.58E-04
5	1167	F2 vs. L2	-	Metabolism	Metabolism	acetyltransferase complex	GO:1902493	C	24	3.10E-03	1.81E-02
5	1168	F2 vs. L2	-	Metabolism	Metabolism	amide biosynthetic process	GO:0043604	P	344	7.52E-07	2.81E-05
5	1169	F2 vs. L2	-	Metabolism	Metabolism	binding	GO:0005488	F	5021	6.00E-04	1.70E-02
5	1170	F2 vs. L2	-	Metabolism	Metabolism	catalytic activity, acting on DNA	GO:0140097	F	102	4.88E-11	1.33E-08
5	1171	F2 vs. L2	-	Metabolism	Metabolism	catalytic complex	GO:1902494	C	638	8.88E-16	3.43E-14
5	1172	F2 vs. L2	-	Metabolism	Metabolism	cellular amide metabolic process	GO:0043603	P	419	4.60E-04	5.95E-03
5	1173	F2 vs. L2	-	Metabolism	Metabolism	cellular aromatic compound metabolic process	GO:0006725	P	1339	1.33E-15	2.97E-13
5	1174	F2 vs. L2	-	Metabolism	Metabolism	cellular macromolecule biosynthetic process	GO:0034645	P	688	7.37E-14	1.04E-11
5	1175	F2 vs. L2	-	Metabolism	Metabolism	cellular macromolecule metabolic process	GO:0044260	P	2207	0.00E+00	0.00E+00
5	1176	F2 vs. L2	-	Metabolism	Metabolism	cellular nitrogen compound biosynthetic process	GO:0044271	P	691	3.29E-04	4.57E-03
5	1177	F2 vs. L2	-	Metabolism	Metabolism	cellular nitrogen compound metabolic process	GO:0034641	P	1546	0.00E+00	0.00E+00
5	1178	F2 vs. L2	-	Metabolism	Metabolism	cellular protein metabolic process	GO:0044267	P	1675	2.06E-10	1.72E-08
5	1179	F2 vs. L2	-	Metabolism	Metabolism	cellular protein modification process	GO:0006464	P	1182	4.33E-06	1.21E-04
5	1180	F2 vs. L2	-	Metabolism	Metabolism	cellular protein-containing complex assembly	GO:0034622	P	279	4.29E-05	8.37E-04

5	1181	F2 vs. L2	-	Metabolism	Metabolism	chromatin binding	GO:0003682	F	81	1.10E-04	4.15E-03
5	1182	F2 vs. L2	-	Metabolism	Metabolism	deoxyribonuclease activity	GO:0004536	F	19	3.12E-05	1.52E-03
5	1183	F2 vs. L2	-	Metabolism	Metabolism	DNA binding	GO:0003677	F	712	2.72E-07	2.85E-05
5	1184	F2 vs. L2	-	Metabolism	Metabolism	DNA metabolic process	GO:0006259	P	220	0.00E+00	0.00E+00
5	1185	F2 vs. L2	-	Metabolism	Metabolism	DNA secondary structure binding	GO:0000217	F	22	7.81E-05	3.08E-03
5	1186	F2 vs. L2	-	Metabolism	Metabolism	heterocycle metabolic process	GO:0046483	P	1270	0.00E+00	0.00E+00
5	1187	F2 vs. L2	-	Metabolism	Metabolism	heterocyclic compound binding	GO:1901363	F	2372	2.26E-05	1.18E-03
5	1188	F2 vs. L2	-	Metabolism	Metabolism	macromolecule biosynthetic process	GO:0009059	P	722	1.19E-11	1.14E-09
5	1189	F2 vs. L2	-	Metabolism	Metabolism	macromolecule metabolic process	GO:0043170	P	2776	0.00E+00	0.00E+00
5	1190	F2 vs. L2	-	Metabolism	Metabolism	microtubule binding	GO:0008017	F	83	4.16E-07	3.90E-05
5	1191	F2 vs. L2	-	Metabolism	Metabolism	motor activity	GO:0003774	F	44	1.18E-03	2.72E-02
5	1192	F2 vs. L2	-	Metabolism	Metabolism	nitrogen compound metabolic process	GO:0006807	P	3160	3.56E-12	3.97E-10
5	1193	F2 vs. L2	-	Metabolism	Metabolism	nucleic acid binding	GO:0003676	F	1637	4.95E-14	2.69E-11
5	1194	F2 vs. L2	-	Metabolism	Metabolism	organic cyclic compound binding	GO:0097159	F	2384	1.40E-05	7.93E-04
5	1195	F2 vs. L2	-	Metabolism	Metabolism	organic cyclic compound metabolic process	GO:1901360	P	1398	8.55E-15	1.58E-12
5	1196	F2 vs. L2	-	Metabolism	Metabolism	peptide metabolic process	GO:0006518	P	340	6.33E-07	2.39E-05
5	1197	F2 vs. L2	-	Metabolism	Metabolism	protein binding	GO:0005515	F	2895	1.10E-04	4.11E-03
5	1198	F2 vs. L2	-	Metabolism	Metabolism	protein metabolic process	GO:0019538	P	1763	1.40E-09	9.58E-08
5	1199	F2 vs. L2	-	Metabolism	Metabolism	Sm-like protein family complex	GO:0120114	C	44	1.28E-08	2.57E-07
5	1200	F2 vs. L2	-	Metabolism	Metabolism	supramolecular complex	GO:0099080	C	146	7.88E-07	1.19E-05
5	1201	F2 vs. L2	-	Metabolism	Metabolism	supramolecular fiber	GO:0099512	C	95	8.93E-05	8.37E-04
5	1202	F2 vs. L2	-	Metabolism	Metabolism	supramolecular polymer	GO:0099081	C	95	8.93E-05	8.53E-04
5	1203	F2 vs. L2	-	Metabolism	Metabolism	transferase complex	GO:1990234	C	369	1.11E-16	5.64E-15
5	1204	F2 vs. L2	-	Metabolism	Metabolism	transferase complex, transferring phosphorus-containing groups	GO:0061695	C	115	7.51E-12	2.42E-10
5	1205	F2 vs. L2	-	Metabolism	Metabolism	tubulin binding	GO:0015631	F	87	1.80E-07	2.13E-05
5	1206	F2 vs. L2	-	Metabolism	Transporters	kinesin complex	GO:0005871	C	22	8.02E-05	7.98E-04
5	1207	F2 vs. L2	-	Metabolism	Transporters	nuclear export	GO:0051168	P	39	7.32E-04	8.93E-03
5	1208	F2 vs. L2	-	Metabolism	Transporters	nuclear microtubule	GO:0005880	C	5	6.93E-03	3.52E-02
5	1209	F2 vs. L2	-	Metabolism	Transporters	nuclear transport	GO:0051169	P	64	8.66E-05	1.54E-03
5	1210	F2 vs. L2	-	Metabolism	Transporters	nucleic acid transport	GO:0050657	P	41	7.24E-04	8.84E-03
5	1211	F2 vs. L2	-	Metabolism	Transporters	nucleocytoplasmic transport	GO:0006913	P	64	8.66E-05	1.53E-03
5	1212	F2 vs. L2	-	Metabolism	Transporters	protein export from nucleus	GO:0006611	P	33	9.46E-04	1.09E-02
5	1213	F2 vs. L2	-	Metabolism	Transporters	RNA transport	GO:0050658	P	41	7.24E-04	8.86E-03
5	1214	F2 vs. L2	-	Regulation	Protein modification	lipoprotein metabolic process	GO:0042157	P	36	4.04E-03	3.66E-02
5	1215	F2 vs. L2	-	Regulation	Protein modification	methylation	GO:0032259	P	110	3.31E-05	6.85E-04
5	1216	F2 vs. L2	-	Regulation	Protein modification	protein alkylation	GO:0008213	P	45	2.82E-04	4.07E-03
5	1217	F2 vs. L2	-	Regulation	Protein modification	protein dealkylation	GO:0008214	P	8	1.70E-03	1.80E-02
5	1218	F2 vs. L2	-	Regulation	Protein modification	protein heterodimerization activity	GO:0046982	F	68	5.84E-05	2.45E-03
5	1219	F2 vs. L2	-	Regulation	Protein modification	protein methylation	GO:0006479	P	45	2.82E-04	4.09E-03
5	1220	F2 vs. L2	-	Regulation	Protein modification	regulation of protein metabolic process	GO:0051246	P	186	1.22E-04	2.04E-03
5	1221	F2 vs. L2	-	Regulation	Protein modification	peptidyl-amino acid modification	GO:0018193	P	190	4.15E-03	3.73E-02
5	1222	F2 vs. L2	-	Regulation	Protein modification	peptidyl-lysine modification	GO:0018205	P	68	8.17E-04	9.81E-03
5	1223	F2 vs. L2	-	Regulation	Protein modification	positive regulation of protein modification process	GO:0031401	P	31	1.35E-04	2.24E-03
5	1224	F2 vs. L2	-	Regulation	Protein modification	protein modification by small protein conjugation or removal	GO:0070647	P	351	8.82E-04	1.04E-02
5	1225	F2 vs. L2	-	Regulation	Protein modification	protein modification process	GO:0036211	P	1182	4.33E-06	1.21E-04
5	1226	F2 vs. L2	-	Regulation	Protein modification	protein-containing complex assembly	GO:0065003	P	317	2.17E-04	3.30E-03
5	1227	F2 vs. L2	-	Regulation	Protein modification	protein-containing complex localization	GO:0031503	P	39	9.77E-04	1.12E-02
5	1228	F2 vs. L2	-	Regulation	Protein modification	protein-containing complex subunit organization	GO:0043933	P	356	9.25E-04	1.08E-02
5	1229	F2 vs. L2	-	Regulation	Protein modification	regulation of protein modification process	GO:0031399	P	94	3.66E-05	7.39E-04
5	1230	F2 vs. L2	-	Regulation	Regulation	negative regulation of biological process	GO:0048519	P	600	2.05E-06	6.35E-05
5	1231	F2 vs. L2	-	Regulation	Regulation	negative regulation of biosynthetic process	GO:0009890	P	178	1.49E-05	3.46E-04
5	1232	F2 vs. L2	-	Regulation	Regulation	negative regulation of cellular biosynthetic process	GO:0031327	P	174	1.23E-05	2.93E-04
5	1233	F2 vs. L2	-	Regulation	Regulation	negative regulation of cellular macromolecule biosynthetic process	GO:2000113	P	164	4.69E-06	1.29E-04
5	1234	F2 vs. L2	-	Regulation	Regulation	negative regulation of cellular metabolic process	GO:0031324	P	233	3.40E-05	6.92E-04
5	1235	F2 vs. L2	-	Regulation	Regulation	negative regulation of cellular process	GO:0048523	P	395	2.62E-05	5.57E-04
5	1236	F2 vs. L2	-	Regulation	Regulation	negative regulation of macromolecule biosynthetic process	GO:0010558	P	165	3.42E-06	9.63E-05
5	1237	F2 vs. L2	-	Regulation	Regulation	negative regulation of macromolecule metabolic process	GO:0010605	P	305	3.67E-12	4.01E-10
5	1238	F2 vs. L2	-	Regulation	Regulation	negative regulation of metabolic process	GO:0009892	P	336	5.81E-10	4.64E-08
5	1239	F2 vs. L2	-	Regulation	Regulation	negative regulation of nitrogen compound metabolic process	GO:0051172	P	200	2.63E-07	1.06E-05
5	1240	F2 vs. L2	-	Regulation	Regulation	negative regulation of nucleobase-containing compound metabolic process	GO:0045934	P	155	7.87E-07	2.93E-05
5	1241	F2 vs. L2	-	Regulation	Regulation	positive regulation of macromolecule metabolic process	GO:0010604	P	351	3.31E-03	3.08E-02

5	1242	F2 vs. L2	-	Regulation	Regulation	regulation of biological process	GO:0050789	P	2539	3.12E-04	4.40E-03
5	1243	F2 vs. L2	-	Regulation	Regulation	regulation of biosynthetic process	GO:0009889	P	1100	3.39E-04	4.65E-03
5	1244	F2 vs. L2	-	Regulation	Regulation	regulation of cell shape	GO:0008360	P	12	5.73E-03	4.82E-02
5	1245	F2 vs. L2	-	Regulation	Regulation	regulation of cellular biosynthetic process	GO:0031326	P	1079	1.07E-04	1.84E-03
5	1246	F2 vs. L2	-	Regulation	Regulation	regulation of cellular macromolecule biosynthetic process	GO:2000112	P	1021	8.21E-07	3.03E-05
5	1247	F2 vs. L2	-	Regulation	Regulation	regulation of cellular metabolic process	GO:0031323	P	1344	2.20E-05	4.89E-04
5	1248	F2 vs. L2	-	Regulation	Regulation	regulation of cellular process	GO:0050794	P	2218	1.38E-03	1.54E-02
5	1249	F2 vs. L2	-	Regulation	Regulation	regulation of cellular protein metabolic process	GO:0032268	P	175	1.46E-04	2.38E-03
5	1250	F2 vs. L2	-	Regulation	Regulation	regulation of macromolecule biosynthetic process	GO:0010556	P	1029	9.28E-07	3.38E-05
5	1251	F2 vs. L2	-	Regulation	Regulation	regulation of macromolecule metabolic process	GO:0060255	P	1366	6.17E-12	6.60E-10
5	1252	F2 vs. L2	-	Regulation	Regulation	regulation of meristem structural organization	GO:0009934	P	17	2.77E-04	4.02E-03
5	1253	F2 vs. L2	-	Regulation	Regulation	regulation of metabolic process	GO:0019222	P	1523	2.10E-07	8.64E-06
5	1254	F2 vs. L2	-	Regulation	Regulation	regulation of nitrogen compound metabolic process	GO:0051171	P	1202	1.41E-07	6.24E-06
5	1255	F2 vs. L2	-	Regulation	Regulation	regulation of nucleobase-containing compound metabolic process	GO:0019219	P	1021	2.08E-06	6.34E-05
5	1256	F2 vs. L2	-	Regulation	Regulation	regulation of organelle organization	GO:0033043	P	109	1.20E-05	2.87E-04
5	1257	F2 vs. L2	-	Regulation	Regulation	regulation of primary metabolic process	GO:0080090	P	1251	2.59E-06	7.73E-05
5	1258	F2 vs. L2	-	Regulation	Regulation	regulation of transferase activity	GO:0051338	P	51	6.51E-05	1.20E-03
5	1259	F2 vs. L2	-	Regulation	Transcription	ATP-dependent chromatin remodeling	GO:0043044	P	14	1.92E-03	1.99E-02
5	1260	F2 vs. L2	-	Regulation	Transcription	chromatin organization involved in negative regulation of transcription	GO:0097549	P	38	3.32E-05	6.81E-04
5	1261	F2 vs. L2	-	Regulation	Transcription	chromatin organization involved in regulation of transcription	GO:0034401	P	39	6.04E-05	1.12E-03
5	1262	F2 vs. L2	-	Regulation	Transcription	chromatin remodeling	GO:0006338	P	49	2.57E-05	5.49E-04
5	1263	F2 vs. L2	-	Regulation	Transcription	chromatin silencing	GO:0006342	P	23	2.18E-04	3.31E-03
5	1264	F2 vs. L2	-	Regulation	Transcription	covalent chromatin modification	GO:0016569	P	88	4.52E-09	2.75E-07
5	1265	F2 vs. L2	-	Regulation	Transcription	demethylation	GO:0070988	P	16	1.59E-03	1.71E-02
5	1266	F2 vs. L2	-	Regulation	Transcription	DNA conformation change	GO:0071103	P	82	6.28E-06	1.66E-04
5	1267	F2 vs. L2	-	Regulation	Transcription	DNA geometric change	GO:0032392	P	23	4.14E-04	5.45E-03
5	1268	F2 vs. L2	-	Regulation	Transcription	DNA-binding transcription activator activity, RNA polymerase II-specific	GO:0001228	F	13	1.99E-03	3.97E-02
5	1269	F2 vs. L2	-	Regulation	Transcription	DNA-directed RNA polymerase complex	GO:0000428	C	60	1.46E-05	1.78E-04
5	1270	F2 vs. L2	-	Regulation	Transcription	dsRNA processing	GO:0031050	P	38	2.03E-07	8.57E-06
5	1271	F2 vs. L2	-	Regulation	Transcription	gene expression	GO:0010467	P	870	0.00E+00	0.00E+00
5	1272	F2 vs. L2	-	Regulation	Transcription	gene silencing	GO:0016458	P	91	1.00E-09	7.25E-08
5	1273	F2 vs. L2	-	Regulation	Transcription	gene silencing by miRNA	GO:0035195	P	23	4.75E-04	6.11E-03
5	1274	F2 vs. L2	-	Regulation	Transcription	gene silencing by RNA	GO:0031047	P	62	4.81E-08	2.28E-06
5	1275	F2 vs. L2	-	Regulation	Transcription	helicase activity	GO:0004386	F	83	5.82E-07	5.27E-05
5	1276	F2 vs. L2	-	Regulation	Transcription	histone binding	GO:0042393	F	49	3.83E-07	3.72E-05
5	1277	F2 vs. L2	-	Regulation	Transcription	histone deacetylase complex	GO:0000118	C	15	8.90E-03	4.29E-02
5	1278	F2 vs. L2	-	Regulation	Transcription	histone demethylation	GO:0016577	P	7	3.27E-03	3.06E-02
5	1279	F2 vs. L2	-	Regulation	Transcription	histone lysine demethylation	GO:0070076	P	7	3.27E-03	3.06E-02
5	1280	F2 vs. L2	-	Regulation	Transcription	histone lysine methylation	GO:0034968	P	27	9.84E-04	1.13E-02
5	1281	F2 vs. L2	-	Regulation	Transcription	histone methylation	GO:0016571	P	34	9.97E-05	1.74E-03
5	1282	F2 vs. L2	-	Regulation	Transcription	histone methyltransferase activity	GO:0042054	F	24	7.53E-04	1.99E-02
5	1283	F2 vs. L2	-	Regulation	Transcription	histone modification	GO:0016570	P	85	8.17E-09	4.51E-07
5	1284	F2 vs. L2	-	Regulation	Transcription	Ino80 complex	GO:0031011	C	9	1.46E-03	1.02E-02
5	1285	F2 vs. L2	-	Regulation	Transcription	INO80-type complex	GO:0097346	C	10	2.29E-03	1.46E-02
5	1286	F2 vs. L2	-	Regulation	Transcription	macromolecule deacylation	GO:0098732	P	12	5.58E-03	4.71E-02
5	1287	F2 vs. L2	-	Regulation	Transcription	macromolecule methylation	GO:0043414	P	86	6.54E-09	3.81E-07
5	1288	F2 vs. L2	-	Regulation	Transcription	macromolecule modification	GO:0043412	P	1339	6.87E-12	6.93E-10
5	1289	F2 vs. L2	-	Regulation	Transcription	mediator complex	GO:0016592	C	30	7.91E-04	5.83E-03
5	1290	F2 vs. L2	-	Regulation	Transcription	methylated histone binding	GO:0035064	F	10	8.82E-04	2.24E-02
5	1291	F2 vs. L2	-	Regulation	Transcription	methylation-dependent protein binding	GO:0140034	F	10	8.82E-04	2.26E-02
5	1292	F2 vs. L2	-	Regulation	Transcription	methyltransferase complex	GO:0034708	C	17	6.84E-03	3.51E-02
5	1293	F2 vs. L2	-	Regulation	Transcription	minor groove of adenine-thymine-rich DNA binding	GO:0003680	F	17	1.46E-04	5.10E-03
5	1294	F2 vs. L2	-	Regulation	Transcription	mRNA processing	GO:0006397	P	163	4.55E-15	9.02E-13
5	1295	F2 vs. L2	-	Regulation	Transcription	negative regulation of gene expression	GO:0010629	P	252	7.63E-10	5.83E-08
5	1296	F2 vs. L2	-	Regulation	Transcription	negative regulation of gene expression, epigenetic	GO:0045814	P	26	4.97E-05	9.54E-04
5	1297	F2 vs. L2	-	Regulation	Transcription	negative regulation of nucleic acid-templated transcription	GO:1903507	P	132	3.78E-05	7.51E-04
5	1298	F2 vs. L2	-	Regulation	Transcription	negative regulation of RNA biosynthetic process	GO:1902679	P	132	3.78E-05	7.54E-04
5	1299	F2 vs. L2	-	Regulation	Transcription	negative regulation of transcription, DNA-templated	GO:0045892	P	131	5.19E-05	9.89E-04
5	1300	F2 vs. L2	-	Regulation	Transcription	nuclear DNA-directed RNA polymerase complex	GO:0055029	C	52	1.08E-06	1.58E-05
5	1301	F2 vs. L2	-	Regulation	Transcription	nuclear speck	GO:0016607	C	46	1.49E-04	1.32E-03
5	1302	F2 vs. L2	-	Regulation	Transcription	nucleic acid-templated transcription	GO:0097659	P	114	1.63E-04	2.58E-03

5	1303	F2 vs. L2	-	Regulation	Transcription	nucleolus	GO:0005730	C	298	1.11E-16	5.95E-15
5	1304	F2 vs. L2	-	Regulation	Transcription	nucleosome organization	GO:0034728	P	33	7.09E-04	8.72E-03
5	1305	F2 vs. L2	-	Regulation	Transcription	peptidyl-lysine methylation	GO:0018022	P	32	2.75E-03	2.71E-02
5	1306	F2 vs. L2	-	Regulation	Transcription	positive regulation of chromatin organization	GO:1905269	P	10	4.56E-03	4.05E-02
5	1307	F2 vs. L2	-	Regulation	Transcription	positive regulation of chromosome organization	GO:2001252	P	12	2.61E-03	2.58E-02
5	1308	F2 vs. L2	-	Regulation	Transcription	positive regulation of RNA interference	GO:1900370	P	4	5.87E-03	4.91E-02
5	1309	F2 vs. L2	-	Regulation	Transcription	post-transcriptional gene silencing by RNA	GO:0035194	P	45	1.18E-06	3.94E-05
5	1310	F2 vs. L2	-	Regulation	Transcription	production of siRNA involved in RNA interference	GO:0030422	P	23	9.81E-05	1.72E-03
5	1311	F2 vs. L2	-	Regulation	Transcription	production of small RNA involved in gene silencing by RNA	GO:0070918	P	38	2.03E-07	8.51E-06
5	1312	F2 vs. L2	-	Regulation	Transcription	protein acetylation	GO:0006473	P	29	3.05E-04	4.35E-03
5	1313	F2 vs. L2	-	Regulation	Transcription	protein acetyltransferase complex	GO:0031248	C	24	3.10E-03	1.80E-02
5	1314	F2 vs. L2	-	Regulation	Transcription	protein acylation	GO:0043543	P	42	1.02E-03	1.16E-02
5	1315	F2 vs. L2	-	Regulation	Transcription	protein deacylation	GO:0035601	P	12	5.58E-03	4.71E-02
5	1316	F2 vs. L2	-	Regulation	Transcription	protein demethylation	GO:0006482	P	8	1.70E-03	1.80E-02
5	1317	F2 vs. L2	-	Regulation	Transcription	protein-DNA complex	GO:0032993	C	37	1.42E-06	2.01E-05
5	1318	F2 vs. L2	-	Regulation	Transcription	protein-DNA complex assembly	GO:0065004	P	43	5.06E-05	9.66E-04
5	1319	F2 vs. L2	-	Regulation	Transcription	protein-DNA complex subunit organization	GO:0071824	P	51	2.99E-05	6.27E-04
5	1320	F2 vs. L2	-	Regulation	Transcription	regulation of gene expression	GO:0010468	P	1194	1.07E-09	7.66E-08
5	1321	F2 vs. L2	-	Regulation	Transcription	regulation of gene expression, epigenetic	GO:0040029	P	51	1.26E-05	2.98E-04
5	1322	F2 vs. L2	-	Regulation	Transcription	regulation of gene silencing	GO:0060968	P	33	1.82E-04	2.82E-03
5	1323	F2 vs. L2	-	Regulation	Transcription	regulation of gene silencing by RNA	GO:0060966	P	11	5.66E-04	7.14E-03
5	1324	F2 vs. L2	-	Regulation	Transcription	regulation of nucleic acid-templated transcription	GO:1903506	P	930	3.11E-05	6.48E-04
5	1325	F2 vs. L2	-	Regulation	Transcription	regulation of RNA biosynthetic process	GO:2001141	P	932	4.29E-05	8.34E-04
5	1326	F2 vs. L2	-	Regulation	Transcription	regulation of RNA interference	GO:1900368	P	5	2.59E-03	2.57E-02
5	1327	F2 vs. L2	-	Regulation	Transcription	regulation of transcription by RNA polymerase II	GO:0006357	P	220	5.42E-04	6.92E-03
5	1328	F2 vs. L2	-	Regulation	Transcription	regulation of transcription, DNA-templated	GO:0006355	P	930	3.11E-05	6.51E-04
5	1329	F2 vs. L2	-	Regulation	Transcription	RNA 3'-end processing	GO:0031123	P	34	5.39E-05	1.02E-03
5	1330	F2 vs. L2	-	Regulation	Transcription	RNA binding	GO:0003723	F	895	1.91E-07	2.16E-05
5	1331	F2 vs. L2	-	Regulation	Transcription	RNA biosynthetic process	GO:0032774	P	116	6.90E-05	1.26E-03
5	1332	F2 vs. L2	-	Regulation	Transcription	RNA methylation	GO:0001510	P	35	1.95E-05	4.46E-04
5	1333	F2 vs. L2	-	Regulation	Transcription	RNA modification	GO:0009451	P	144	4.91E-14	7.29E-12
5	1334	F2 vs. L2	-	Regulation	Transcription	RNA polymerase complex	GO:0030880	C	63	2.13E-05	2.45E-04
5	1335	F2 vs. L2	-	Regulation	Transcription	RNA polymerase II CTD heptapeptide repeat phosphatase activity	GO:0008420	F	5	2.33E-03	4.46E-02
5	1336	F2 vs. L2	-	Regulation	Transcription	RNA polymerase II, core complex	GO:0005665	C	11	3.60E-03	2.07E-02
5	1337	F2 vs. L2	-	Regulation	Transcription	RNA polymerase II, holoenzyme	GO:0016591	C	35	2.61E-04	2.17E-03
5	1338	F2 vs. L2	-	Regulation	Transcription	RNA polymerase IV complex	GO:0000418	C	6	2.93E-03	1.72E-02
5	1339	F2 vs. L2	-	Regulation	Transcription	RNA polymerase V complex	GO:0000419	C	10	1.09E-04	1.01E-03
5	1340	F2 vs. L2	-	Regulation	Transcription	SWI/SNF superfamily-type complex	GO:0070603	C	18	4.45E-04	3.43E-03
5	1341	F2 vs. L2	-	Regulation	Transcription	THO complex	GO:0000347	C	10	1.28E-03	9.10E-03
5	1342	F2 vs. L2	-	Regulation	Transcription	transcription coregulator activity	GO:0003712	F	79	5.08E-08	7.27E-06
5	1343	F2 vs. L2	-	Regulation	Transcription	transcription corepressor activity	GO:0003714	F	12	1.34E-03	2.97E-02
5	1344	F2 vs. L2	-	Regulation	Transcription	transcription regulator complex	GO:0005667	C	59	2.00E-03	1.31E-02
5	1345	F2 vs. L2	-	Regulation	Transcription	transcription, DNA-templated	GO:0006351	P	114	1.63E-04	2.57E-03
5	1346	F2 vs. L2	-	Regulation	Translation	90S preribosome	GO:0030686	C	15	2.69E-03	1.64E-02
5	1347	F2 vs. L2	-	Regulation	Translation	box C/D snoRNP complex	GO:0031428	C	8	2.54E-03	1.60E-02
5	1348	F2 vs. L2	-	Regulation	Translation	catalytic activity, acting on a tRNA	GO:0140101	F	70	9.24E-06	5.71E-04
5	1349	F2 vs. L2	-	Regulation	Translation	catalytic activity, acting on RNA	GO:0140098	F	227	2.95E-12	1.15E-09
5	1350	F2 vs. L2	-	Regulation	Translation	catalytic step 2 spliceosome	GO:0071013	C	32	1.95E-05	2.26E-04
5	1351	F2 vs. L2	-	Regulation	Translation	cytoplasmic exosome (RNase complex)	GO:0000177	C	6	1.00E-02	4.67E-02
5	1352	F2 vs. L2	-	Regulation	Translation	cytosolic large ribosomal subunit	GO:0022625	C	92	1.28E-07	2.10E-06
5	1353	F2 vs. L2	-	Regulation	Translation	cytosolic ribosome	GO:0022626	C	212	2.28E-05	2.56E-04
5	1354	F2 vs. L2	-	Regulation	Translation	cytosolic small ribosomal subunit	GO:0022627	C	73	5.52E-06	7.30E-05
5	1355	F2 vs. L2	-	Regulation	Translation	establishment of RNA localization	GO:0051236	P	41	7.24E-04	8.88E-03
5	1356	F2 vs. L2	-	Regulation	Translation	large ribosomal subunit	GO:0015934	C	111	2.96E-09	7.52E-08
5	1357	F2 vs. L2	-	Regulation	Translation	maturation of 5.8S rRNA	GO:0000460	P	16	2.79E-03	2.74E-02
5	1358	F2 vs. L2	-	Regulation	Translation	maturation of LSU-rRNA	GO:0000470	P	30	2.52E-04	3.73E-03
5	1359	F2 vs. L2	-	Regulation	Translation	maturation of SSU-rRNA	GO:0030490	P	34	2.89E-05	6.12E-04
5	1360	F2 vs. L2	-	Regulation	Translation	maturation of SSU-rRNA from tricistronic rRNA transcript (SSU-rRNA, 5.8S rRNA, LSU-rRNA)	GO:0000462	P	23	1.72E-04	2.68E-03
5	1361	F2 vs. L2	-	Regulation	Translation	mitochondrial large ribosomal subunit	GO:0005762	C	13	4.09E-04	3.26E-03
5	1362	F2 vs. L2	-	Regulation	Translation	mitochondrial ribosome	GO:0005761	C	22	1.39E-04	1.24E-03
5	1363	F2 vs. L2	-	Regulation	Translation	mRNA cis splicing, via spliceosome	GO:0045292	P	25	8.34E-04	9.99E-03
5	1364	F2 vs. L2	-	Regulation	Translation	mRNA cleavage factor complex	GO:0005849	C	8	1.59E-03	1.09E-02

5	1365	F2 vs. L2	-	Regulation	Translation	mRNA export from nucleus	GO:0006406	P	20	8.67E-04	1.03E-02
5	1366	F2 vs. L2	-	Regulation	Translation	mRNA metabolic process	GO:0016071	P	224	2.22E-15	4.57E-13
5	1367	F2 vs. L2	-	Regulation	Translation	mRNA splicing, via spliceosome	GO:0000398	P	117	2.88E-12	3.28E-10
5	1368	F2 vs. L2	-	Regulation	Translation	mRNA transport	GO:0051028	P	21	3.08E-04	4.36E-03
5	1369	F2 vs. L2	-	Regulation	Translation	mRNA-containing ribonucleoprotein complex export from nucleus	GO:0071427	P	20	8.67E-04	1.03E-02
5	1370	F2 vs. L2	-	Regulation	Translation	ncRNA 3'-end processing	GO:0043628	P	18	5.73E-04	7.22E-03
5	1371	F2 vs. L2	-	Regulation	Translation	ncRNA metabolic process	GO:0034660	P	244	0.00E+00	0.00E+00
5	1372	F2 vs. L2	-	Regulation	Translation	ncRNA processing	GO:0034470	P	202	1.11E-16	2.97E-14
5	1373	F2 vs. L2	-	Regulation	Translation	negative regulation of RNA metabolic process	GO:0051253	P	139	1.13E-05	2.72E-04
5	1374	F2 vs. L2	-	Regulation	Translation	nuclear mRNA surveillance	GO:0071028	P	6	5.56E-03	4.71E-02
5	1375	F2 vs. L2	-	Regulation	Translation	organellar large ribosomal subunit	GO:0000315	C	19	4.85E-03	2.60E-02
5	1376	F2 vs. L2	-	Regulation	Translation	organellar ribosome	GO:0000313	C	34	2.48E-04	2.10E-03
5	1377	F2 vs. L2	-	Regulation	Translation	peptide biosynthetic process	GO:0043043	P	306	2.61E-09	1.66E-07
5	1378	F2 vs. L2	-	Regulation	Translation	posttranscriptional gene silencing	GO:0016441	P	50	1.13E-06	3.82E-05
5	1379	F2 vs. L2	-	Regulation	Translation	posttranscriptional regulation of gene expression	GO:0010608	P	121	3.14E-06	9.03E-05
5	1380	F2 vs. L2	-	Regulation	Translation	precatalytic spliceosome	GO:0071011	C	18	1.78E-04	1.53E-03
5	1381	F2 vs. L2	-	Regulation	Translation	preribosome	GO:0030684	C	51	1.08E-07	1.84E-06
5	1382	F2 vs. L2	-	Regulation	Translation	preribosome, large subunit precursor	GO:0030687	C	16	2.52E-04	2.11E-03
5	1383	F2 vs. L2	-	Regulation	Translation	prespliceosome	GO:0071010	C	14	2.56E-03	1.60E-02
5	1384	F2 vs. L2	-	Regulation	Translation	primary miRNA processing	GO:0031053	P	9	4.40E-03	3.92E-02
5	1385	F2 vs. L2	-	Regulation	Translation	production of miRNAs involved in gene silencing by miRNA	GO:0035196	P	17	8.57E-05	1.53E-03
5	1386	F2 vs. L2	-	Regulation	Translation	regulation of RNA metabolic process	GO:0051252	P	995	1.41E-05	3.28E-04
5	1387	F2 vs. L2	-	Regulation	Translation	regulation of RNA splicing	GO:0043484	P	40	2.53E-03	2.53E-02
5	1388	F2 vs. L2	-	Regulation	Translation	ribonucleoprotein complex	GO:1990904	C	392	0.00E+00	0.00E+00
5	1389	F2 vs. L2	-	Regulation	Translation	ribonucleoprotein complex assembly	GO:0022618	P	104	1.79E-09	1.20E-07
5	1390	F2 vs. L2	-	Regulation	Translation	ribonucleoprotein complex biogenesis	GO:0022613	P	256	6.66E-16	1.62E-13
5	1391	F2 vs. L2	-	Regulation	Translation	ribonucleoprotein complex export from nucleus	GO:0071426	P	32	1.55E-03	1.68E-02
5	1392	F2 vs. L2	-	Regulation	Translation	ribonucleoprotein complex localization	GO:0071166	P	32	1.55E-03	1.68E-02
5	1393	F2 vs. L2	-	Regulation	Translation	ribonucleoprotein complex subunit organization	GO:0071826	P	106	1.96E-09	1.29E-07
5	1394	F2 vs. L2	-	Regulation	Translation	ribosomal large subunit assembly	GO:0000027	P	30	1.54E-03	1.67E-02
5	1395	F2 vs. L2	-	Regulation	Translation	ribosomal large subunit biogenesis	GO:0042273	P	73	1.49E-08	7.72E-07
5	1396	F2 vs. L2	-	Regulation	Translation	ribosomal small subunit biogenesis	GO:0042274	P	58	1.47E-06	4.67E-05
5	1397	F2 vs. L2	-	Regulation	Translation	ribosomal subunit	GO:0044391	C	204	5.55E-16	2.23E-14
5	1398	F2 vs. L2	-	Regulation	Translation	ribosome	GO:0005840	C	259	8.42E-08	1.48E-06
5	1399	F2 vs. L2	-	Regulation	translation	ribosome assembly	GO:0042255	P	58	2.28E-04	3.43E-03
5	1400	F2 vs. L2	-	Regulation	Translation	ribosome biogenesis	GO:0042254	P	208	1.70E-11	1.59E-09
5	1401	F2 vs. L2	-	Regulation	Translation	RNA cap binding	GO:0000339	F	8	3.91E-04	1.16E-02
5	1402	F2 vs. L2	-	Regulation	Translation	RNA export from nucleus	GO:0006405	P	38	1.17E-03	1.32E-02
5	1403	F2 vs. L2	-	Regulation	Translation	RNA helicase activity	GO:0003724	F	51	1.25E-03	2.85E-02
5	1404	F2 vs. L2	-	Regulation	Translation	RNA interference	GO:0016246	P	24	1.62E-04	2.58E-03
5	1405	F2 vs. L2	-	Regulation	Translation	RNA localization	GO:0006403	P	43	2.92E-04	4.22E-03
5	1406	F2 vs. L2	-	Regulation	Translation	RNA methyltransferase activity	GO:0008173	F	36	2.12E-05	1.13E-03
5	1407	F2 vs. L2	-	Regulation	Translation	RNA phosphodiester bond hydrolysis	GO:0090501	P	22	1.11E-05	2.70E-04
5	1408	F2 vs. L2	-	Regulation	Translation	RNA processing	GO:0006396	P	428	0.00E+00	0.00E+00
5	1409	F2 vs. L2	-	Regulation	Translation	RNA splicing	GO:0008380	P	154	6.82E-13	7.93E-11
5	1410	F2 vs. L2	-	Regulation	Translation	RNA splicing, via transesterification reactions	GO:0000375	P	128	6.19E-12	6.37E-10
5	1411	F2 vs. L2	-	Regulation	Translation	RNA splicing, via transesterification reactions with bulged adenosine as nucleophile	GO:0000377	P	128	6.19E-12	6.50E-10
5	1412	F2 vs. L2	-	Regulation	Translation	rRNA binding	GO:0019843	F	37	1.16E-03	2.69E-02
5	1413	F2 vs. L2	-	Regulation	Translation	rRNA metabolic process	GO:0016072	P	139	3.71E-08	1.82E-06
5	1414	F2 vs. L2	-	Regulation	Translation	rRNA methylation	GO:0031167	P	12	5.92E-03	4.95E-02
5	1415	F2 vs. L2	-	Regulation	Translation	rRNA modification	GO:0000154	P	17	2.78E-03	2.73E-02
5	1416	F2 vs. L2	-	Regulation	Translation	rRNA processing	GO:0006364	P	134	9.55E-08	4.33E-06
5	1417	F2 vs. L2	-	Regulation	Translation	small nuclear ribonucleoprotein complex	GO:0030532	C	39	1.19E-08	2.44E-07
5	1418	F2 vs. L2	-	Regulation	Translation	small nucleolar ribonucleoprotein complex	GO:0005732	C	17	4.17E-04	3.30E-03
5	1419	F2 vs. L2	-	Regulation	Translation	small ribosomal subunit	GO:0015935	C	93	2.45E-08	4.73E-07
5	1420	F2 vs. L2	-	Regulation	Translation	small-subunit processome	GO:0032040	C	27	3.83E-05	4.20E-04
5	1421	F2 vs. L2	-	Regulation	Translation	snRNA metabolic process	GO:0016073	P	11	2.36E-03	2.38E-02
5	1422	F2 vs. L2	-	Regulation	Translation	snRNA processing	GO:0016180	P	9	5.08E-03	4.41E-02
5	1423	F2 vs. L2	-	Regulation	Translation	spliceosomal complex	GO:0005681	C	88	1.01E-11	3.15E-10
5	1424	F2 vs. L2	-	Regulation	Translation	spliceosomal snRNP assembly	GO:0000387	P	15	1.74E-04	2.70E-03
5	1425	F2 vs. L2	-	Regulation	Translation	spliceosomal snRNP complex	GO:0097525	C	39	1.19E-08	2.50E-07
5	1426	F2 vs. L2	-	Regulation	Translation	spliceosomal tri-snRNP complex	GO:0097526	C	20	8.75E-07	1.30E-05

5	1427	F2 vs. L2	-	Regulation	Translation	structural constituent of ribosome	GO:0003735	F	195	1.41E-11	4.27E-09
5	1428	F2 vs. L2	-	Regulation	Translation	translation	GO:0006412	P	304	1.18E-09	8.23E-08
5	1429	F2 vs. L2	-	Regulation	Translation	tRNA metabolic process	GO:0006399	P	91	8.52E-10	6.42E-08
5	1430	F2 vs. L2	-	Regulation	Translation	tRNA modification	GO:0006400	P	47	3.91E-08	1.90E-06
5	1431	F2 vs. L2	-	Regulation	Translation	tRNA processing	GO:0008033	P	56	1.62E-09	1.10E-07
5	1432	F2 vs. L2	-	Regulation	Translation	U1 snRNP	GO:0005685	C	16	8.50E-04	6.22E-03
5	1433	F2 vs. L2	-	Regulation	Translation	U12-type spliceosomal complex	GO:0005689	C	14	3.72E-03	2.12E-02
5	1434	F2 vs. L2	-	Regulation	Translation	U2 snRNP	GO:0005686	C	17	1.77E-04	1.54E-03
5	1435	F2 vs. L2	-	Regulation	Translation	U2-type prespliceosome	GO:0071004	C	14	2.56E-03	1.59E-02
5	1436	F2 vs. L2	-	Regulation	Translation	U2-type spliceosomal complex	GO:0005684	C	35	1.53E-05	1.83E-04
5	1437	F2 vs. L2	-	Regulation	Translation	U4 snRNP	GO:0005687	C	8	4.92E-03	2.62E-02
5	1438	F2 vs. L2	-	Regulation	Translation	U4/U6 x U5 tri-snRNP complex	GO:0046540	C	16	9.97E-06	1.25E-04
5	1439	F2 vs. L2	-	Regulation	translation	U5 snRNP	GO:0005682	C	11	2.18E-03	1.39E-02
5	1440	F2 vs. L2	-	Signaling and response	Signaling	cellular response to brassinosteroid stimulus	GO:0071367	P	35	5.45E-03	4.66E-02
5	1441	F2 vs. L2	-	Signaling and response	Signaling	kinase regulator activity	GO:0019207	F	40	6.04E-05	2.49E-03
5	1442	F2 vs. L2	-	Signaling and response	Signaling	negative regulation of long-day photoperiodism, flowering	GO:0048579	P	5	4.09E-03	3.69E-02
5	1443	F2 vs. L2	-	Signaling and response	Signaling	protein kinase complex	GO:1902911	C	42	1.04E-05	1.29E-04
5	1444	F2 vs. L2	-	Signaling and response	Signaling	protein kinase regulator activity	GO:0019887	F	39	4.74E-05	2.05E-03
5	1445	F2 vs. L2	-	Signaling and response	Signaling	regulation of cyclin-dependent protein serine/threonine kinase activity	GO:0000079	P	22	6.81E-04	8.41E-03
5	1446	F2 vs. L2	-	Signaling and response	Signaling	regulation of kinase activity	GO:0043549	P	39	1.44E-04	2.35E-03
5	1447	F2 vs. L2	-	Signaling and response	Signaling	regulation of protein kinase activity	GO:0045859	P	37	1.52E-04	2.43E-03
5	1448	F2 vs. L2	-	Signaling and response	Signaling	regulation of protein phosphorylation	GO:0001932	P	45	2.38E-03	2.39E-02
5	1449	F2 vs. L2	-	Signaling and response	Signaling	regulation of protein serine/threonine kinase activity	GO:0071900	P	31	8.72E-04	1.03E-02
5	1450	F2 vs. L2	-	Signaling and response	Signaling	response to brassinosteroid	GO:0009741	P	53	4.10E-03	3.69E-02
5	1451	F2 vs. L2	-	Signaling and response	Signaling	serine/threonine protein kinase complex	GO:1902554	C	40	1.53E-05	1.84E-04
5	1452	F2 vs. L2	-	Signaling and response	Stress	cellular response to DNA damage stimulus	GO:0006974	P	190	0.00E+00	0.00E+00
5	1453	F2 vs. L2	-	Signaling and response	Stress	damaged DNA binding	GO:0003684	F	26	1.89E-03	3.89E-02
5	1454	F2 vs. L2	-	Signaling and response	Stress	site of DNA damage	GO:0090734	C	6	1.08E-02	4.98E-02
6	1455	F3 vs. L3	+	Development	Cell division	nucleobase-containing small molecule metabolic process	GO:0055086	P	264	2.09E-06	5.34E-05
6	1456	F3 vs. L3	+	Development	Cell division	nucleoside diphosphate metabolic process	GO:0009132	P	55	5.30E-03	4.14E-02
6	1457	F3 vs. L3	+	Development	Cell division	nucleoside phosphate binding	GO:1901265	F	636	4.54E-05	1.70E-03
6	1458	F3 vs. L3	+	Development	Cell division	nucleoside phosphate metabolic process	GO:0006753	P	197	5.39E-03	4.20E-02
6	1459	F3 vs. L3	+	Development	Cell division	nucleotide binding	GO:0000166	F	636	4.54E-05	1.72E-03
6	1460	F3 vs. L3	+	Development	Cell division	nucleotide-sugar biosynthetic process	GO:0009226	P	32	5.55E-07	1.65E-05
6	1461	F3 vs. L3	+	Development	Cell division	nucleotide-sugar metabolic process	GO:0009225	P	36	8.25E-08	3.07E-06
6	1462	F3 vs. L3	+	Development	Cell division	pollen germination	GO:0009846	P	40	3.03E-05	5.40E-04
6	1463	F3 vs. L3	+	Development	Cell division	pollen tube development	GO:0048868	P	111	3.79E-13	4.37E-11
6	1464	F3 vs. L3	+	Development	Cell division	pollen tube growth	GO:0009860	P	81	6.83E-13	7.42E-11
6	1465	F3 vs. L3	+	Development	Cell division	pollen tube guidance	GO:0010183	P	23	6.43E-04	7.49E-03
6	1466	F3 vs. L3	+	Development	Cell division	pollination	GO:0009856	P	166	5.68E-10	3.35E-08
6	1467	F3 vs. L3	+	Development	Cell division	ribonucleotide metabolic process	GO:0009259	P	156	4.21E-03	3.44E-02
6	1468	F3 vs. L3	+	Development	Cell division	ribose phosphate metabolic process	GO:0019693	P	163	5.42E-03	4.22E-02
6	1469	F3 vs. L3	+	Development	Cell division	syncytium formation	GO:0006949	P	11	7.34E-05	1.18E-03
6	1470	F3 vs. L3	+	Development	Development	actin cytoskeleton	GO:0015629	C	38	8.43E-04	5.82E-03
6	1471	F3 vs. L3	+	Development	Development	actin cytoskeleton organization	GO:0030036	P	63	9.83E-04	1.06E-02
6	1472	F3 vs. L3	+	Development	Development	apical part of cell	GO:0045177	C	16	4.25E-04	3.39E-03
6	1473	F3 vs. L3	+	Development	Development	apical plasma membrane	GO:0016324	C	15	6.81E-04	5.02E-03
6	1474	F3 vs. L3	+	Development	Development	apoplast	GO:0048046	C	190	0.00E+00	0.00E+00
6	1475	F3 vs. L3	+	Development	Development	bounding membrane of organelle	GO:0098588	C	618	9.22E-08	1.40E-06
6	1476	F3 vs. L3	+	Development	Development	cell development	GO:0048468	P	192	7.68E-07	2.13E-05
6	1477	F3 vs. L3	+	Development	Development	cell differentiation	GO:0030154	P	337	8.47E-04	9.31E-03
6	1478	F3 vs. L3	+	Development	Development	cell growth	GO:0016049	P	246	1.15E-06	3.05E-05
6	1479	F3 vs. L3	+	Development	Development	cell junction	GO:0030054	C	610	3.07E-03	1.79E-02
6	1480	F3 vs. L3	+	Development	Development	cell morphogenesis	GO:0000902	P	227	1.88E-04	2.68E-03
6	1481	F3 vs. L3	+	Development	Development	cell morphogenesis involved in differentiation	GO:0000904	P	136	7.90E-07	2.16E-05
6	1482	F3 vs. L3	+	Development	Development	cell periphery	GO:0071944	C	2511	4.70E-14	1.69E-12
6	1483	F3 vs. L3	+	Development	Development	cell pole	GO:0060187	C	14	7.27E-04	5.28E-03
6	1484	F3 vs. L3	+	Development	Development	cell projection	GO:0042995	C	73	1.67E-10	4.17E-09
6	1485	F3 vs. L3	+	Development	Development	cell tip	GO:0051286	C	14	7.27E-04	5.24E-03
6	1486	F3 vs. L3	+	Development	Development	cell tip growth	GO:0009932	P	94	8.65E-12	7.99E-10
6	1487	F3 vs. L3	+	Development	Development	cell wall	GO:0005618	C	368	2.26E-06	2.90E-05
6	1488	F3 vs. L3	+	Development	Development	cell wall biogenesis	GO:0042546	P	156	3.31E-05	5.81E-04
6	1489	F3 vs. L3	+	Development	Development	cell wall modification	GO:0042545	P	121	2.75E-05	4.95E-04

6	1490	F3 vs. L3	+	Development	Development	cell wall organization	GO:0071555	P	184	7.87E-07	2.16E-05
6	1491	F3 vs. L3	+	Development	Development	cell wall organization or biogenesis	GO:0071554	P	333	3.93E-10	2.39E-08
6	1492	F3 vs. L3	+	Development	Development	cell wall pectin metabolic process	GO:0052546	P	16	2.04E-04	2.87E-03
6	1493	F3 vs. L3	+	Development	Development	cell-cell junction	GO:0005911	C	610	3.07E-03	1.82E-02
6	1494	F3 vs. L3	+	Development	Development	cellular developmental process	GO:0048869	P	390	2.83E-03	2.53E-02
6	1495	F3 vs. L3	+	Development	Development	cytoplasm	GO:0005737	C	8209	5.27E-06	6.18E-05
6	1496	F3 vs. L3	+	Development	Development	cytosol	GO:0005829	C	2250	9.96E-10	2.02E-08
6	1497	F3 vs. L3	+	Development	Development	developmental cell growth	GO:0048588	P	120	1.30E-12	1.36E-10
6	1498	F3 vs. L3	+	Development	Development	developmental growth	GO:0048589	P	247	8.62E-09	4.16E-07
6	1499	F3 vs. L3	+	Development	Development	developmental growth involved in morphogenesis	GO:0060560	P	211	9.10E-09	4.35E-07
6	1500	F3 vs. L3	+	Development	Development	endomembrane system	GO:0012505	C	1470	2.12E-09	4.05E-08
6	1501	F3 vs. L3	+	Development	Development	envelope	GO:0031975	C	697	1.11E-10	2.99E-09
6	1502	F3 vs. L3	+	Development	Development	external encapsulating structure	GO:0030312	C	370	1.03E-06	1.41E-05
6	1503	F3 vs. L3	+	Development	Development	external encapsulating structure organization	GO:0045229	P	213	1.44E-07	5.08E-06
6	1504	F3 vs. L3	+	Development	Development	extracellular region	GO:0005576	C	1453	0.00E+00	0.00E+00
6	1505	F3 vs. L3	+	Development	Development	extracellular space	GO:0005615	C	69	5.03E-04	3.83E-03
6	1506	F3 vs. L3	+	Development	Development	Golgi apparatus subcompartment	GO:0098791	C	308	7.13E-08	1.10E-06
6	1507	F3 vs. L3	+	Development	Development	Golgi cisterna	GO:0031985	C	136	4.80E-04	3.68E-03
6	1508	F3 vs. L3	+	Development	Development	Golgi stack	GO:0005795	C	143	5.45E-04	4.11E-03
6	1509	F3 vs. L3	+	Development	Development	Golgi trans cisterna	GO:0000138	C	62	1.41E-04	1.26E-03
6	1510	F3 vs. L3	+	Development	Development	growing cell tip	GO:0035838	C	11	4.32E-04	3.39E-03
6	1511	F3 vs. L3	+	Development	Development	growth	GO:0040007	P	285	2.89E-07	9.36E-06
6	1512	F3 vs. L3	+	Development	Development	hemicellulose metabolic process	GO:0010410	P	66	4.88E-03	3.87E-02
6	1513	F3 vs. L3	+	Development	Development	intracellular vesicle	GO:0097708	C	490	1.07E-08	1.85E-07
6	1514	F3 vs. L3	+	Development	Development	lytic vacuole	GO:0000323	C	39	8.72E-04	5.89E-03
6	1515	F3 vs. L3	+	Development	Development	membrane	GO:0016020	C	3430	0.00E+00	0.00E+00
6	1516	F3 vs. L3	+	Development	Development	microbody	GO:0042579	C	203	3.01E-05	3.02E-04
6	1517	F3 vs. L3	+	Development	Development	multi-multicellular organism process	GO:0044706	P	166	5.68E-10	3.38E-08
6	1518	F3 vs. L3	+	Development	Development	myosin complex	GO:0016459	C	4	9.13E-03	4.68E-02
6	1519	F3 vs. L3	+	Development	Development	organelle envelope	GO:0031967	C	697	1.11E-10	2.91E-09
6	1520	F3 vs. L3	+	Development	Development	organelle membrane	GO:0031090	C	959	2.73E-06	3.41E-05
6	1521	F3 vs. L3	+	Development	Development	organelle subcompartment	GO:0031984	C	809	5.70E-13	1.98E-11
6	1522	F3 vs. L3	+	Development	Development	plant-type cell wall biogenesis	GO:0009832	P	104	5.67E-05	9.32E-04
6	1523	F3 vs. L3	+	Development	Development	plant-type cell wall loosening	GO:0009828	P	26	2.07E-03	1.98E-02
6	1524	F3 vs. L3	+	Development	Development	plant-type cell wall modification	GO:0009827	P	47	1.64E-04	2.39E-03
6	1525	F3 vs. L3	+	Development	Development	plant-type cell wall organization	GO:0009664	P	101	5.12E-06	1.18E-04
6	1526	F3 vs. L3	+	Development	Development	plant-type cell wall organization or biogenesis	GO:0071669	P	176	1.66E-08	7.26E-07
6	1527	F3 vs. L3	+	Development	Development	plant-type primary cell wall biogenesis	GO:0009833	P	24	1.12E-04	1.71E-03
6	1528	F3 vs. L3	+	Development	Development	plant-type secondary cell wall biogenesis	GO:0009834	P	38	3.28E-03	2.82E-02
6	1529	F3 vs. L3	+	Development	Development	plant-type vacuole	GO:0000325	C	104	2.07E-06	2.68E-05
6	1530	F3 vs. L3	+	Development	Development	plant-type vacuole membrane	GO:0009705	C	71	2.04E-03	1.26E-02
6	1531	F3 vs. L3	+	Development	Development	plasma membrane	GO:0005886	C	2215	3.56E-11	1.02E-09
6	1532	F3 vs. L3	+	Development	Development	plasma membrane bounded cell projection	GO:0120025	C	63	4.30E-14	1.61E-12
6	1533	F3 vs. L3	+	Development	Development	plasma membrane region	GO:0098590	C	31	8.56E-03	4.50E-02
6	1534	F3 vs. L3	+	Development	Development	pollen tube	GO:0090406	C	55	6.37E-13	2.14E-11
6	1535	F3 vs. L3	+	Development	Development	pollen tube tip	GO:0090404	C	11	4.32E-04	3.41E-03
6	1536	F3 vs. L3	+	Development	Development	secretion	GO:0046903	P	52	1.50E-04	2.19E-03
6	1537	F3 vs. L3	+	Development	Development	secretion by cell	GO:0032940	P	47	1.17E-03	1.23E-02
6	1538	F3 vs. L3	+	Development	Development	site of polarized growth	GO:0030427	C	13	8.75E-03	4.57E-02
6	1539	F3 vs. L3	+	Development	Development	stamen filament development	GO:0080086	P	7	2.46E-04	3.31E-03
6	1540	F3 vs. L3	+	Development	Development	stromule	GO:0010319	C	32	2.79E-09	5.21E-08
6	1541	F3 vs. L3	+	Development	Development	symplast	GO:0055044	C	610	3.07E-03	1.81E-02
6	1542	F3 vs. L3	+	Development	Development	unidimensional cell growth	GO:0009826	P	176	3.07E-07	9.89E-06
6	1543	F3 vs. L3	+	Development	Development	vacuolar membrane	GO:0005774	C	389	1.31E-09	2.60E-08
6	1544	F3 vs. L3	+	Development	Development	vacuole	GO:0005773	C	737	0.00E+00	0.00E+00
6	1545	F3 vs. L3	+	Metabolism	Bioenergetics	(1->3)-beta-D-glucan binding (NAD+) activity	GO:0004029	F	10	1.01E-03	2.22E-02
6	1546	F3 vs. L3	+	Metabolism	Bioenergetics	ADP metabolic process	GO:0046031	P	43	3.14E-03	2.75E-02
6	1547	F3 vs. L3	+	Metabolism	Bioenergetics	aerobic respiration	GO:0009060	P	64	1.73E-05	3.23E-04
6	1548	F3 vs. L3	+	Metabolism	Bioenergetics	aldehyde dehydrogenase [NAD(P)+] activity	GO:0004030	F	10	1.01E-03	2.23E-02
6	1549	F3 vs. L3	+	Metabolism	Bioenergetics	aldo-keto reductase (NADP) activity	GO:0004033	F	9	2.85E-03	4.88E-02
6	1550	F3 vs. L3	+	Metabolism	Bioenergetics	ATP metabolic process	GO:0046034	P	95	5.61E-04	6.72E-03
6	1551	F3 vs. L3	+	Metabolism	Bioenergetics	ATPase activity, coupled to transmembrane movement of ions, rotational mechanism	GO:0044769	F	19	1.01E-03	2.19E-02
6	1552	F3 vs. L3	+	Metabolism	Bioenergetics	carbohydrate biosynthetic process	GO:0016051	P	219	1.17E-13	1.55E-11

6	1553	F3 vs. L3	+	Metabolism	Bioenergetics	carbohydrate catabolic process	GO:0016052	P	167	1.29E-08	6.13E-07
6	1554	F3 vs. L3	+	Metabolism	Bioenergetics	carbohydrate derivative biosynthetic process	GO:1901137	P	305	1.32E-03	1.36E-02
6	1555	F3 vs. L3	+	Metabolism	Bioenergetics	carbohydrate derivative metabolic process	GO:1901135	P	493	1.57E-07	5.47E-06
6	1556	F3 vs. L3	+	Metabolism	Bioenergetics	carbohydrate metabolic process	GO:0005975	P	626	0.00E+00	0.00E+00
6	1557	F3 vs. L3	+	Metabolism	Bioenergetics	cellular carbohydrate biosynthetic process	GO:0034637	P	152	9.91E-07	2.64E-05
6	1558	F3 vs. L3	+	Metabolism	Bioenergetics	cellular carbohydrate catabolic process	GO:0044275	P	37	4.26E-03	3.47E-02
6	1559	F3 vs. L3	+	Metabolism	Bioenergetics	cellular carbohydrate metabolic process	GO:0044262	P	273	4.45E-11	3.33E-09
6	1560	F3 vs. L3	+	Metabolism	Bioenergetics	cellular polysaccharide biosynthetic process	GO:0033692	P	124	3.74E-06	8.82E-05
6	1561	F3 vs. L3	+	Metabolism	Bioenergetics	cellular polysaccharide metabolic process	GO:0044264	P	184	1.21E-07	4.36E-06
6	1562	F3 vs. L3	+	Metabolism	Bioenergetics	cellular respiration	GO:0045333	P	95	8.36E-05	1.33E-03
6	1563	F3 vs. L3	+	Metabolism	Bioenergetics	CoA-ligase activity	GO:0016405	F	24	6.07E-05	2.13E-03
6	1564	F3 vs. L3	+	Metabolism	Bioenergetics	decanoate-CoA ligase activity	GO:0102391	F	8	1.46E-03	2.90E-02
6	1565	F3 vs. L3	+	Metabolism	Bioenergetics	disaccharide metabolic process	GO:0005984	P	57	4.52E-03	3.62E-02
6	1566	F3 vs. L3	+	Metabolism	Bioenergetics	electron transport chain	GO:0022900	P	81	3.21E-03	2.78E-02
6	1567	F3 vs. L3	+	Metabolism	Bioenergetics	energy derivation by oxidation of organic compounds	GO:0015980	P	106	1.04E-04	1.61E-03
6	1568	F3 vs. L3	+	Metabolism	Bioenergetics	fatty acid biosynthetic process	GO:0006633	P	93	9.40E-05	1.48E-03
6	1569	F3 vs. L3	+	Metabolism	Bioenergetics	fatty acid derivative metabolic process	GO:1901568	P	30	3.42E-03	2.93E-02
6	1570	F3 vs. L3	+	Metabolism	Bioenergetics	fatty acid ligase activity	GO:0015645	F	13	5.18E-04	1.30E-02
6	1571	F3 vs. L3	+	Metabolism	Bioenergetics	fatty acid metabolic process	GO:0006631	P	158	6.83E-08	2.59E-06
6	1572	F3 vs. L3	+	Metabolism	Bioenergetics	generation of precursor metabolites and energy	GO:0006091	P	267	1.59E-12	1.63E-10
6	1573	F3 vs. L3	+	Metabolism	Bioenergetics	gluconeogenesis	GO:0006094	P	21	1.02E-04	1.58E-03
6	1574	F3 vs. L3	+	Metabolism	Bioenergetics	glucose 6-phosphate metabolic process	GO:0051156	P	34	2.01E-05	3.69E-04
6	1575	F3 vs. L3	+	Metabolism	Bioenergetics	glucose metabolic process	GO:0006006	P	35	6.18E-04	7.29E-03
6	1576	F3 vs. L3	+	Metabolism	Bioenergetics	hexose biosynthetic process	GO:0019319	P	23	4.39E-05	7.46E-04
6	1577	F3 vs. L3	+	Metabolism	Bioenergetics	hexose metabolic process	GO:0019318	P	71	4.76E-03	3.78E-02
6	1578	F3 vs. L3	+	Metabolism	Bioenergetics	isocitrate metabolic process	GO:0006102	P	11	3.40E-03	2.91E-02
6	1579	F3 vs. L3	+	Metabolism	Bioenergetics	lipid oxidation	GO:0034440	P	35	2.45E-03	2.26E-02
6	1580	F3 vs. L3	+	Metabolism	Bioenergetics	long-chain fatty acid metabolic process	GO:0001676	P	10	1.92E-03	1.85E-02
6	1581	F3 vs. L3	+	Metabolism	Bioenergetics	long-chain fatty acid-CoA ligase activity	GO:0004467	F	9	1.80E-03	3.39E-02
6	1582	F3 vs. L3	+	Metabolism	Bioenergetics	malate metabolic process	GO:0006108	P	13	1.89E-03	1.83E-02
6	1583	F3 vs. L3	+	Metabolism	Bioenergetics	medium-chain fatty acid-CoA ligase activity	GO:0031956	F	12	1.51E-03	2.95E-02
6	1584	F3 vs. L3	+	Metabolism	Bioenergetics	monosaccharide biosynthetic process	GO:0046364	P	34	5.20E-07	1.58E-05
6	1585	F3 vs. L3	+	Metabolism	Bioenergetics	NAD binding	GO:0051287	F	57	1.69E-05	7.38E-04
6	1586	F3 vs. L3	+	Metabolism	Bioenergetics	NAD(P)H dehydrogenase complex (plastoquinone)	GO:0010598	C	8	8.55E-04	5.82E-03
6	1587	F3 vs. L3	+	Metabolism	Bioenergetics	NAD(P)H dehydrogenase complex assembly	GO:0010275	P	6	5.68E-03	4.40E-02
6	1588	F3 vs. L3	+	Metabolism	Bioenergetics	NADP metabolic process	GO:0006739	P	38	1.17E-05	2.25E-04
6	1589	F3 vs. L3	+	Metabolism	Bioenergetics	NADPH regeneration	GO:0006740	P	30	3.69E-05	6.39E-04
6	1590	F3 vs. L3	+	Metabolism	Bioenergetics	oligosaccharide metabolic process	GO:0009311	P	65	4.19E-03	3.43E-02
6	1591	F3 vs. L3	+	Metabolism	Bioenergetics	oxidoreductase activity	GO:0016491	F	891	0.00E+00	0.00E+00
6	1592	F3 vs. L3	+	Metabolism	Bioenergetics	oxidoreductase activity, acting on a sulfur group of donors	GO:0016667	F	83	5.02E-05	1.80E-03
6	1593	F3 vs. L3	+	Metabolism	Bioenergetics	oxidoreductase activity, acting on CH-OH group of donors	GO:0016614	F	132	2.47E-07	2.22E-05
6	1594	F3 vs. L3	+	Metabolism	Bioenergetics	oxidoreductase activity, acting on NAD(P)H	GO:0016651	F	77	8.27E-06	4.17E-04
6	1595	F3 vs. L3	+	Metabolism	Bioenergetics	oxidoreductase activity, acting on NAD(P)H, quinone or similar compound as acceptor	GO:0016655	F	30	1.96E-03	3.61E-02
6	1596	F3 vs. L3	+	Metabolism	Bioenergetics	oxidoreductase activity, acting on other nitrogenous compounds as donors	GO:0016661	F	4	2.52E-03	4.45E-02
6	1597	F3 vs. L3	+	Metabolism	Bioenergetics	oxidoreductase activity, acting on paired donors, with incorporation or reduction of molecular oxygen	GO:0016705	F	211	2.10E-04	5.93E-03
6	1598	F3 vs. L3	+	Metabolism	Bioenergetics	oxidoreductase activity, acting on single donors with incorporation of molecular oxygen	GO:0016701	F	32	6.45E-04	1.52E-02
6	1599	F3 vs. L3	+	Metabolism	Bioenergetics	oxidoreductase activity, acting on the aldehyde or oxo group of donors	GO:0016903	F	45	1.07E-04	3.31E-03
6	1600	F3 vs. L3	+	Metabolism	Bioenergetics	oxidoreductase activity, acting on the aldehyde or oxo group of donors, NAD or NADP as acceptor	GO:0016620	F	32	9.56E-05	3.06E-03
6	1601	F3 vs. L3	+	Metabolism	Bioenergetics	oxidoreductase activity, acting on the CH-NH2 group of donors	GO:0016638	F	23	2.56E-03	4.50E-02
6	1602	F3 vs. L3	+	Metabolism	Bioenergetics	oxidoreductase activity, acting on the CH-OH group of donors, NAD or NADP as acceptor	GO:0016616	F	116	1.15E-06	7.02E-05
6	1603	F3 vs. L3	+	Metabolism	Bioenergetics	oxidoreductase complex	GO:1990204	C	107	1.35E-04	1.22E-03
6	1604	F3 vs. L3	+	Metabolism	Bioenergetics	pentose-phosphate shunt	GO:0006098	P	28	4.91E-06	1.13E-04
6	1605	F3 vs. L3	+	Metabolism	Bioenergetics	photorespiration	GO:0009853	P	30	5.20E-03	4.08E-02
6	1606	F3 vs. L3	+	Metabolism	Bioenergetics	polysaccharide biosynthetic process	GO:0000271	P	150	1.52E-07	5.32E-06
6	1607	F3 vs. L3	+	Metabolism	Bioenergetics	polysaccharide metabolic process	GO:0005976	P	287	1.96E-13	2.52E-11
6	1608	F3 vs. L3	+	Metabolism	Bioenergetics	proton transmembrane transport	GO:1902600	P	62	1.11E-05	2.17E-04

6	1609	F3 vs. L3	+	Metabolism	Bioenergetics	proton-exporting ATPase activity, phosphorylative mechanism	GO:0008553	F	15	2.28E-04	6.31E-03
6	1610	F3 vs. L3	+	Metabolism	Bioenergetics	proton-transporting ATPase activity, rotational mechanism	GO:0046961	F	19	1.01E-03	2.20E-02
6	1611	F3 vs. L3	+	Metabolism	Bioenergetics	proton-transporting two-sector ATPase complex	GO:0016469	C	41	8.51E-04	5.83E-03
6	1612	F3 vs. L3	+	Metabolism	Bioenergetics	proton-transporting V-type ATPase complex	GO:0033176	C	18	2.58E-03	1.57E-02
6	1613	F3 vs. L3	+	Metabolism	Bioenergetics	pyrophosphate hydrolysis-driven proton transmembrane transporter activity	GO:0009678	F	34	2.41E-06	1.44E-04
6	1614	F3 vs. L3	+	Metabolism	Bioenergetics	pyruvate metabolic process	GO:0006090	P	63	3.05E-04	3.98E-03
6	1615	F3 vs. L3	+	Metabolism	Bioenergetics	reductive pentose-phosphate cycle	GO:0019253	P	14	8.82E-06	1.82E-04
6	1616	F3 vs. L3	+	Metabolism	Bioenergetics	sucrose metabolic process	GO:0005985	P	40	2.42E-03	2.25E-02
6	1617	F3 vs. L3	+	Metabolism	Bioenergetics	tricarboxylic acid cycle	GO:0006099	P	34	2.29E-06	5.83E-05
6	1618	F3 vs. L3	+	Metabolism	Bioenergetics	tricarboxylic acid metabolic process	GO:0072350	P	16	8.35E-04	9.22E-03
6	1619	F3 vs. L3	+	Metabolism	Bioenergetics	unsaturated fatty acid biosynthetic process	GO:0006636	P	16	1.20E-03	1.26E-02
6	1620	F3 vs. L3	+	Metabolism	Bioenergetics	unsaturated fatty acid metabolic process	GO:0033559	P	17	6.42E-04	7.54E-03
6	1621	F3 vs. L3	+	Metabolism	Catabolism	alcohol catabolic process	GO:0046164	P	23	2.52E-04	3.35E-03
6	1622	F3 vs. L3	+	Metabolism	Catabolism	alpha-amino acid catabolic process	GO:1901606	P	66	1.77E-11	1.56E-09
6	1623	F3 vs. L3	+	Metabolism	Catabolism	aminopeptidase activity	GO:0004177	F	16	1.02E-03	2.19E-02
6	1624	F3 vs. L3	+	Metabolism	Catabolism	aromatic amino acid family catabolic process	GO:0009074	P	16	6.21E-07	1.80E-05
6	1625	F3 vs. L3	+	Metabolism	Catabolism	aromatic compound catabolic process	GO:0019439	P	123	5.28E-03	4.14E-02
6	1626	F3 vs. L3	+	Metabolism	Catabolism	carboxylic acid catabolic process	GO:0046395	P	115	2.07E-13	2.55E-11
6	1627	F3 vs. L3	+	Metabolism	Catabolism	catabolic process	GO:0009056	P	905	2.30E-10	1.43E-08
6	1628	F3 vs. L3	+	Metabolism	Catabolism	cell wall macromolecule catabolic process	GO:0016998	P	18	9.30E-04	1.01E-02
6	1629	F3 vs. L3	+	Metabolism	Catabolism	cellular amino acid catabolic process	GO:0009063	P	72	5.26E-10	3.17E-08
6	1630	F3 vs. L3	+	Metabolism	Catabolism	cellular catabolic process	GO:0044248	P	738	3.34E-06	7.94E-05
6	1631	F3 vs. L3	+	Metabolism	Catabolism	cellular lipid catabolic process	GO:0044242	P	65	7.36E-04	8.33E-03
6	1632	F3 vs. L3	+	Metabolism	Catabolism	cellular metabolic compound salvage	GO:0043094	P	54	9.92E-05	1.55E-03
6	1633	F3 vs. L3	+	Metabolism	Catabolism	dicarboxylic acid catabolic process	GO:0043649	P	7	3.43E-03	2.93E-02
6	1634	F3 vs. L3	+	Metabolism	Catabolism	erythrose 4-phosphate/phosphoenolpyruvate family amino acid catabolic process	GO:1902222	P	10	9.06E-06	1.85E-04
6	1635	F3 vs. L3	+	Metabolism	Catabolism	glutamine family amino acid catabolic process	GO:0009065	P	10	6.21E-03	4.70E-02
6	1636	F3 vs. L3	+	Metabolism	Catabolism	glycine catabolic process	GO:0006546	P	8	5.65E-05	9.32E-04
6	1637	F3 vs. L3	+	Metabolism	Catabolism	lipid catabolic process	GO:0016042	P	69	1.41E-03	1.44E-02
6	1638	F3 vs. L3	+	Metabolism	Catabolism	L-phenylalanine catabolic process	GO:0006559	P	10	9.06E-06	1.86E-04
6	1639	F3 vs. L3	+	Metabolism	Catabolism	lysosome	GO:0005764	C	35	3.91E-04	3.22E-03
6	1640	F3 vs. L3	+	Metabolism	Catabolism	monocarboxylic acid catabolic process	GO:0072329	P	45	9.35E-07	2.52E-05
6	1641	F3 vs. L3	+	Metabolism	Catabolism	organic acid catabolic process	GO:0016054	P	115	2.07E-13	2.61E-11
6	1642	F3 vs. L3	+	Metabolism	Catabolism	organic cyclic compound catabolic process	GO:1901361	P	131	3.59E-03	3.01E-02
6	1643	F3 vs. L3	+	Metabolism	Catabolism	organic hydroxy compound catabolic process	GO:1901616	P	30	3.50E-07	1.10E-05
6	1644	F3 vs. L3	+	Metabolism	Catabolism	organic substance catabolic process	GO:1901575	P	839	6.03E-09	2.96E-07
6	1645	F3 vs. L3	+	Metabolism	Catabolism	pectin catabolic process	GO:0045490	P	66	9.57E-05	1.50E-03
6	1646	F3 vs. L3	+	Metabolism	Catabolism	peroxidase activity	GO:0004601	F	62	9.99E-05	3.16E-03
6	1647	F3 vs. L3	+	Metabolism	Catabolism	peroxisome	GO:0005777	C	203	3.01E-05	2.99E-04
6	1648	F3 vs. L3	+	Metabolism	Catabolism	phenol-containing compound catabolic process	GO:0019336	P	6	2.06E-04	2.89E-03
6	1649	F3 vs. L3	+	Metabolism	Catabolism	polyol catabolic process	GO:0046174	P	20	2.76E-03	2.48E-02
6	1650	F3 vs. L3	+	Metabolism	Catabolism	polysaccharide catabolic process	GO:0000272	P	96	1.13E-05	2.18E-04
6	1651	F3 vs. L3	+	Metabolism	Catabolism	serine family amino acid catabolic process	GO:0009071	P	14	8.33E-04	9.21E-03
6	1652	F3 vs. L3	+	Metabolism	Catabolism	small molecule catabolic process	GO:0044282	P	168	2.55E-15	5.06E-13
6	1653	F3 vs. L3	+	Metabolism	Catabolism	tyrosine catabolic process	GO:0006572	P	5	1.01E-03	1.08E-02
6	1654	F3 vs. L3	+	Metabolism	Metabolism	acid-thiol ligase activity	GO:0016878	F	28	1.45E-05	6.72E-04
6	1655	F3 vs. L3	+	Metabolism	Metabolism	actin binding	GO:0003779	F	68	7.04E-05	2.44E-03
6	1656	F3 vs. L3	+	Metabolism	Metabolism	actin filament binding	GO:0051015	F	45	1.88E-05	7.85E-04
6	1657	F3 vs. L3	+	Metabolism	Metabolism	actin filament-based process	GO:0030029	P	68	2.32E-04	3.16E-03
6	1658	F3 vs. L3	+	Metabolism	Metabolism	alcohol metabolic process	GO:0006066	P	82	5.52E-05	9.14E-04
6	1659	F3 vs. L3	+	Metabolism	Metabolism	alpha-amino acid biosynthetic process	GO:1901607	P	122	2.94E-08	1.20E-06
6	1660	F3 vs. L3	+	Metabolism	Metabolism	alpha-amino acid metabolic process	GO:1901605	P	195	0.00E+00	0.00E+00
6	1661	F3 vs. L3	+	Metabolism	Metabolism	amine biosynthetic process	GO:0009309	P	24	2.46E-03	2.26E-02
6	1662	F3 vs. L3	+	Metabolism	Metabolism	amine metabolic process	GO:0009308	P	60	2.43E-03	2.25E-02
6	1663	F3 vs. L3	+	Metabolism	Metabolism	aminoglycan biosynthetic process	GO:0006023	P	4	5.91E-03	4.52E-02
6	1664	F3 vs. L3	+	Metabolism	Metabolism	ammonia assimilation cycle	GO:0019676	P	6	2.32E-03	2.17E-02
6	1665	F3 vs. L3	+	Metabolism	Metabolism	anchoring junction	GO:0070161	C	610	3.07E-03	1.80E-02
6	1666	F3 vs. L3	+	Metabolism	Metabolism	anion binding	GO:0043168	F	788	1.10E-07	1.05E-05
6	1667	F3 vs. L3	+	Metabolism	Metabolism	anthocyanin-containing compound metabolic process	GO:0046283	P	12	1.85E-03	1.81E-02
6	1668	F3 vs. L3	+	Metabolism	Metabolism	aromatic amino acid family metabolic process	GO:0009072	P	51	7.92E-06	1.69E-04
6	1669	F3 vs. L3	+	Metabolism	Metabolism	aspartate family amino acid metabolic process	GO:0009066	P	46	1.91E-04	2.70E-03
6	1670	F3 vs. L3	+	Metabolism	Metabolism	benzene-containing compound metabolic process	GO:0042537	P	38	2.17E-04	2.97E-03

6	1671	F3 vs. L3	+	Metabolism	Metabolism	beta-glucan biosynthetic process	GO:0051274	P	51	3.28E-04	4.25E-03
6	1672	F3 vs. L3	+	Metabolism	Metabolism	beta-glucan metabolic process	GO:0051273	P	54	1.13E-03	1.20E-02
6	1673	F3 vs. L3	+	Metabolism	Metabolism	biosynthetic process	GO:0009058	P	1799	5.31E-07	1.60E-05
6	1674	F3 vs. L3	+	Metabolism	Metabolism	camalexin biosynthetic process	GO:0010120	P	7	2.31E-03	2.15E-02
6	1675	F3 vs. L3	+	Metabolism	Metabolism	camalexin metabolic process	GO:0052317	P	7	2.31E-03	2.16E-02
6	1676	F3 vs. L3	+	Metabolism	Metabolism	carbon utilization	GO:0015976	P	8	1.86E-04	2.65E-03
6	1677	F3 vs. L3	+	Metabolism	Metabolism	carbon-carbon lyase activity	GO:0016830	F	74	5.53E-08	6.36E-06
6	1678	F3 vs. L3	+	Metabolism	Metabolism	carbon-oxygen lyase activity	GO:0016835	F	97	8.79E-07	6.16E-05
6	1679	F3 vs. L3	+	Metabolism	Metabolism	carboxylic acid biosynthetic process	GO:0046394	P	313	3.66E-15	6.77E-13
6	1680	F3 vs. L3	+	Metabolism	Metabolism	carboxylic acid metabolic process	GO:0019752	P	636	0.00E+00	0.00E+00
6	1681	F3 vs. L3	+	Metabolism	Metabolism	carboxy-lyase activity	GO:0016831	F	42	1.45E-04	4.29E-03
6	1682	F3 vs. L3	+	Metabolism	Metabolism	catalytic activity	GO:0003824	F	5038	0.00E+00	0.00E+00
6	1683	F3 vs. L3	+	Metabolism	Metabolism	cation binding	GO:0043169	F	973	9.55E-15	3.43E-12
6	1684	F3 vs. L3	+	Metabolism	Metabolism	cell wall macromolecule biosynthetic process	GO:0044038	P	53	4.61E-03	3.67E-02
6	1685	F3 vs. L3	+	Metabolism	Metabolism	cell wall macromolecule metabolic process	GO:0044036	P	107	6.79E-04	7.82E-03
6	1686	F3 vs. L3	+	Metabolism	Metabolism	cell wall polysaccharide biosynthetic process	GO:0070592	P	50	5.01E-03	3.96E-02
6	1687	F3 vs. L3	+	Metabolism	Metabolism	cell wall polysaccharide metabolic process	GO:0010383	P	87	5.71E-04	6.81E-03
6	1688	F3 vs. L3	+	Metabolism	Metabolism	cellular amine metabolic process	GO:0044106	P	37	1.40E-04	2.06E-03
6	1689	F3 vs. L3	+	Metabolism	Metabolism	cellular amino acid biosynthetic process	GO:0008652	P	135	2.52E-09	1.30E-07
6	1690	F3 vs. L3	+	Metabolism	Metabolism	cellular amino acid metabolic process	GO:0006520	P	265	4.44E-16	1.12E-13
6	1691	F3 vs. L3	+	Metabolism	Metabolism	cellular biogenic amine biosynthetic process	GO:0042401	P	24	2.46E-03	2.26E-02
6	1692	F3 vs. L3	+	Metabolism	Metabolism	cellular biogenic amine metabolic process	GO:0006576	P	37	1.40E-04	2.07E-03
6	1693	F3 vs. L3	+	Metabolism	Metabolism	cellular biosynthetic process	GO:0044249	P	1644	4.39E-04	5.42E-03
6	1694	F3 vs. L3	+	Metabolism	Metabolism	cellular component macromolecule biosynthetic process	GO:0070589	P	53	4.61E-03	3.68E-02
6	1695	F3 vs. L3	+	Metabolism	Metabolism	cellular glucan metabolic process	GO:0006073	P	136	4.44E-05	7.52E-04
6	1696	F3 vs. L3	+	Metabolism	Metabolism	cellular lipid metabolic process	GO:0044255	P	446	4.77E-13	5.29E-11
6	1697	F3 vs. L3	+	Metabolism	Metabolism	cellular modified amino acid metabolic process	GO:0006575	P	60	1.09E-04	1.67E-03
6	1698	F3 vs. L3	+	Metabolism	Metabolism	cellulose biosynthetic process	GO:0030244	P	39	1.46E-04	2.15E-03
6	1699	F3 vs. L3	+	Metabolism	Metabolism	cellulose metabolic process	GO:0030243	P	42	6.95E-04	7.98E-03
6	1700	F3 vs. L3	+	Metabolism	Metabolism	cellulose synthase (UDP-forming) activity	GO:0016760	F	22	1.01E-04	3.16E-03
6	1701	F3 vs. L3	+	Metabolism	Metabolism	cellulose synthase activity	GO:0016759	F	28	3.24E-05	1.28E-03
6	1702	F3 vs. L3	+	Metabolism	Metabolism	cellulose synthase complex	GO:0010330	C	4	4.62E-03	2.65E-02
6	1703	F3 vs. L3	+	Metabolism	Metabolism	copper ion binding	GO:0005507	F	133	8.05E-10	1.45E-07
6	1704	F3 vs. L3	+	Metabolism	Metabolism	coumarin biosynthetic process	GO:0009805	P	4	2.22E-03	2.08E-02
6	1705	F3 vs. L3	+	Metabolism	Metabolism	coumarin metabolic process	GO:0009804	P	5	5.39E-04	6.48E-03
6	1706	F3 vs. L3	+	Metabolism	Metabolism	cysteine metabolic process	GO:0006534	P	18	4.89E-03	3.87E-02
6	1707	F3 vs. L3	+	Metabolism	Metabolism	dicarboxylic acid metabolic process	GO:0043648	P	63	5.47E-07	1.63E-05
6	1708	F3 vs. L3	+	Metabolism	Metabolism	endocytosis	GO:0006897	P	51	4.29E-03	3.49E-02
6	1709	F3 vs. L3	+	Metabolism	Metabolism	erythrose 4-phosphate/phosphoenolpyruvate family amino acid metabolic process	GO:1902221	P	19	1.90E-05	3.50E-04
6	1710	F3 vs. L3	+	Metabolism	Metabolism	establishment of localization	GO:0051234	P	1403	1.84E-11	1.52E-09
6	1711	F3 vs. L3	+	Metabolism	Metabolism	exocytosis	GO:0006887	P	37	5.16E-03	4.07E-02
6	1712	F3 vs. L3	+	Metabolism	Metabolism	flavin adenine dinucleotide binding	GO:0050660	F	96	2.35E-04	6.37E-03
6	1713	F3 vs. L3	+	Metabolism	Metabolism	FMN binding	GO:0010181	F	23	6.68E-04	1.56E-02
6	1714	F3 vs. L3	+	Metabolism	Metabolism	galactolipid metabolic process	GO:0019374	P	12	6.51E-03	4.91E-02
6	1715	F3 vs. L3	+	Metabolism	Metabolism	galacturonan metabolic process	GO:0010393	P	110	1.69E-09	9.10E-08
6	1716	F3 vs. L3	+	Metabolism	Metabolism	galacturonate metabolic process	GO:0019586	P	3	5.84E-03	4.49E-02
6	1717	F3 vs. L3	+	Metabolism	Metabolism	glucan biosynthetic process	GO:0009250	P	86	6.95E-04	7.99E-03
6	1718	F3 vs. L3	+	Metabolism	Metabolism	glucan metabolic process	GO:0044042	P	143	2.83E-05	5.08E-04
6	1719	F3 vs. L3	+	Metabolism	Metabolism	glucosinolate biosynthetic process	GO:0019761	P	29	7.95E-06	1.68E-04
6	1720	F3 vs. L3	+	Metabolism	Metabolism	glucosinolate metabolic process	GO:0019760	P	68	6.27E-05	1.02E-03
6	1721	F3 vs. L3	+	Metabolism	Metabolism	glucosyltransferase activity	GO:0046527	F	131	7.63E-07	5.49E-05
6	1722	F3 vs. L3	+	Metabolism	Metabolism	glutamate metabolic process	GO:0006536	P	19	3.34E-04	4.32E-03
6	1723	F3 vs. L3	+	Metabolism	Metabolism	glutamine family amino acid metabolic process	GO:0009064	P	42	6.56E-05	1.06E-03
6	1724	F3 vs. L3	+	Metabolism	Metabolism	glutamine metabolic process	GO:0006541	P	14	1.03E-03	1.11E-02
6	1725	F3 vs. L3	+	Metabolism	Metabolism	glycerolipid metabolic process	GO:0046486	P	121	1.79E-03	1.77E-02
6	1726	F3 vs. L3	+	Metabolism	Metabolism	glycerophospholipid metabolic process	GO:0006650	P	104	3.55E-03	2.99E-02
6	1727	F3 vs. L3	+	Metabolism	Metabolism	glycine biosynthetic process	GO:0006545	P	9	6.01E-03	4.58E-02
6	1728	F3 vs. L3	+	Metabolism	Metabolism	glycine cleavage complex	GO:0005960	C	5	1.58E-03	1.02E-02
6	1729	F3 vs. L3	+	Metabolism	Metabolism	glycine decarboxylation via glycine cleavage system	GO:0019464	P	7	1.79E-04	2.57E-03
6	1730	F3 vs. L3	+	Metabolism	Metabolism	glycine metabolic process	GO:0006544	P	16	4.57E-06	1.06E-04
6	1731	F3 vs. L3	+	Metabolism	Metabolism	glycosaminoglycan biosynthetic process	GO:0006024	P	4	5.91E-03	4.52E-02
6	1732	F3 vs. L3	+	Metabolism	Metabolism	glycosaminoglycan metabolic process	GO:0030203	P	4	5.91E-03	4.53E-02
6	1733	F3 vs. L3	+	Metabolism	Metabolism	glycosinolate biosynthetic process	GO:0019758	P	29	7.95E-06	1.68E-04

6	1734	F3 vs. L3	+	Metabolism	Metabolism	glycosinolate metabolic process	GO:0019757	P	68	6.27E-05	1.02E-03
6	1735	F3 vs. L3	+	Metabolism	Metabolism	glycosyl compound biosynthetic process	GO:1901659	P	47	6.39E-05	1.04E-03
6	1736	F3 vs. L3	+	Metabolism	Metabolism	glycosyl compound metabolic process	GO:1901657	P	118	3.44E-04	4.41E-03
6	1737	F3 vs. L3	+	Metabolism	Metabolism	heme binding	GO:0020037	F	196	8.48E-06	4.21E-04
6	1738	F3 vs. L3	+	Metabolism	Metabolism	hydrocarbon biosynthetic process	GO:0120251	P	35	2.90E-03	2.58E-02
6	1739	F3 vs. L3	+	Metabolism	Metabolism	hydrolase activity	GO:0016787	F	1794	1.05E-05	5.06E-04
6	1740	F3 vs. L3	+	Metabolism	Metabolism	hydrolase activity, acting on glycosyl bonds	GO:0016798	F	297	1.77E-04	5.10E-03
6	1741	F3 vs. L3	+	Metabolism	Metabolism	hydrolase activity, hydrolyzing O-glycosyl compounds	GO:0004553	F	232	4.03E-07	3.22E-05
6	1742	F3 vs. L3	+	Metabolism	Metabolism	hydro-lyase activity	GO:0016836	F	64	2.45E-05	9.77E-04
6	1743	F3 vs. L3	+	Metabolism	Metabolism	indole phytoalexin biosynthetic process	GO:0009700	P	9	1.78E-03	1.77E-02
6	1744	F3 vs. L3	+	Metabolism	Metabolism	indole-containing compound biosynthetic process	GO:0042435	P	31	3.78E-03	3.15E-02
6	1745	F3 vs. L3	+	Metabolism	Metabolism	indole-containing compound metabolic process	GO:0042430	P	49	1.84E-04	2.63E-03
6	1746	F3 vs. L3	+	Metabolism	Metabolism	inorganic diphosphatase activity	GO:0004427	F	10	9.28E-04	2.10E-02
6	1747	F3 vs. L3	+	Metabolism	Metabolism	inositol phosphate metabolic process	GO:0043647	P	23	3.50E-03	2.97E-02
6	1748	F3 vs. L3	+	Metabolism	Metabolism	ion binding	GO:0043167	F	1653	7.77E-16	3.73E-13
6	1749	F3 vs. L3	+	Metabolism	Metabolism	iron ion binding	GO:0005506	F	178	1.87E-04	5.33E-03
6	1750	F3 vs. L3	+	Metabolism	Metabolism	leucine biosynthetic process	GO:0009098	P	12	1.93E-03	1.86E-02
6	1751	F3 vs. L3	+	Metabolism	Metabolism	ligase activity, forming carbon-sulfur bonds	GO:0016877	F	38	1.13E-03	2.32E-02
6	1752	F3 vs. L3	+	Metabolism	Metabolism	lignin biosynthetic process	GO:0009809	P	29	1.91E-05	3.51E-04
6	1753	F3 vs. L3	+	Metabolism	Metabolism	lignin metabolic process	GO:0009808	P	39	5.83E-06	1.31E-04
6	1754	F3 vs. L3	+	Metabolism	Metabolism	lipid biosynthetic process	GO:0008610	P	328	5.45E-07	1.63E-05
6	1755	F3 vs. L3	+	Metabolism	Metabolism	lipid localization	GO:0010876	P	80	1.86E-03	1.82E-02
6	1756	F3 vs. L3	+	Metabolism	Metabolism	lipid metabolic process	GO:0006629	P	536	1.03E-11	9.38E-10
6	1757	F3 vs. L3	+	Metabolism	Metabolism	lipid modification	GO:0030258	P	74	1.29E-05	2.47E-04
6	1758	F3 vs. L3	+	Metabolism	Metabolism	localization	GO:0051179	P	1509	9.51E-11	6.43E-09
6	1759	F3 vs. L3	+	Metabolism	Metabolism	L-phenylalanine metabolic process	GO:0006558	P	19	1.90E-05	3.52E-04
6	1760	F3 vs. L3	+	Metabolism	Metabolism	L-serine metabolic process	GO:0006563	P	19	4.89E-04	6.01E-03
6	1761	F3 vs. L3	+	Metabolism	Metabolism	lyase activity	GO:0016829	F	209	2.72E-14	7.83E-12
6	1762	F3 vs. L3	+	Metabolism	Metabolism	magnesium ion binding	GO:0000287	F	69	1.71E-04	4.96E-03
6	1763	F3 vs. L3	+	Metabolism	Metabolism	membrane lipid metabolic process	GO:0006643	P	67	4.23E-03	3.46E-02
6	1764	F3 vs. L3	+	Metabolism	Metabolism	metabolic process	GO:0008152	P	5278	1.73E-03	1.74E-02
6	1765	F3 vs. L3	+	Metabolism	Metabolism	metal ion binding	GO:0046872	F	956	3.35E-14	8.77E-12
6	1766	F3 vs. L3	+	Metabolism	Metabolism	methionine adenosyltransferase activity	GO:0004478	F	4	1.50E-03	2.96E-02
6	1767	F3 vs. L3	+	Metabolism	Metabolism	methionine metabolic process	GO:0006555	P	19	1.48E-03	1.51E-02
6	1768	F3 vs. L3	+	Metabolism	Metabolism	monocarboxylic acid biosynthetic process	GO:0072330	P	148	2.35E-06	5.95E-05
6	1769	F3 vs. L3	+	Metabolism	Metabolism	monocarboxylic acid metabolic process	GO:0032787	P	325	9.77E-15	1.55E-12
6	1770	F3 vs. L3	+	Metabolism	Metabolism	monooxygenase activity	GO:0004497	F	135	1.69E-03	3.27E-02
6	1771	F3 vs. L3	+	Metabolism	Metabolism	monosaccharide metabolic process	GO:0005996	P	94	1.38E-05	2.64E-04
6	1772	F3 vs. L3	+	Metabolism	Metabolism	nitrate assimilation	GO:0042128	P	11	2.08E-04	2.91E-03
6	1773	F3 vs. L3	+	Metabolism	Metabolism	nitrate metabolic process	GO:0042126	P	11	2.08E-04	2.90E-03
6	1774	F3 vs. L3	+	Metabolism	Metabolism	nitrogen cycle metabolic process	GO:0071941	P	14	8.05E-05	1.29E-03
6	1775	F3 vs. L3	+	Metabolism	Metabolism	olefinic compound metabolic process	GO:0120254	P	36	2.46E-03	2.26E-02
6	1776	F3 vs. L3	+	Metabolism	Metabolism	oligopeptide binding	GO:1900750	F	11	4.76E-05	1.73E-03
6	1777	F3 vs. L3	+	Metabolism	Metabolism	organic acid biosynthetic process	GO:0016053	P	313	3.66E-15	7.00E-13
6	1778	F3 vs. L3	+	Metabolism	Metabolism	organic acid metabolic process	GO:0006082	P	700	0.00E+00	0.00E+00
6	1779	F3 vs. L3	+	Metabolism	Metabolism	organic cyclic compound biosynthetic process	GO:1901362	P	573	5.29E-03	4.14E-02
6	1780	F3 vs. L3	+	Metabolism	Metabolism	organic hydroxy compound biosynthetic process	GO:1901617	P	112	1.69E-04	2.45E-03
6	1781	F3 vs. L3	+	Metabolism	Metabolism	organic hydroxy compound metabolic process	GO:1901615	P	195	3.84E-08	1.53E-06
6	1782	F3 vs. L3	+	Metabolism	Metabolism	organic substance biosynthetic process	GO:1901576	P	1716	2.46E-05	4.47E-04
6	1783	F3 vs. L3	+	Metabolism	Metabolism	organophosphate metabolic process	GO:0019637	P	398	2.85E-08	1.18E-06
6	1784	F3 vs. L3	+	Metabolism	Metabolism	oxidation-reduction process	GO:0055114	P	492	0.00E+00	0.00E+00
6	1785	F3 vs. L3	+	Metabolism	Metabolism	oxoacid metabolic process	GO:0043436	P	698	0.00E+00	0.00E+00
6	1786	F3 vs. L3	+	Metabolism	Metabolism	oxylipin biosynthetic process	GO:0031408	P	12	8.46E-05	1.34E-03
6	1787	F3 vs. L3	+	Metabolism	Metabolism	oxylipin metabolic process	GO:0031407	P	14	1.28E-04	1.93E-03
6	1788	F3 vs. L3	+	Metabolism	Metabolism	pectate lyase activity	GO:0030570	F	16	8.75E-04	2.00E-02
6	1789	F3 vs. L3	+	Metabolism	Metabolism	pectin biosynthetic process	GO:0045489	P	37	2.53E-04	3.36E-03
6	1790	F3 vs. L3	+	Metabolism	Metabolism	pectin metabolic process	GO:0045488	P	109	7.09E-10	4.05E-08
6	1791	F3 vs. L3	+	Metabolism	Metabolism	pectinesterase activity	GO:0030599	F	57	2.26E-03	4.06E-02
6	1792	F3 vs. L3	+	Metabolism	Metabolism	phenylpropanoid biosynthetic process	GO:0009699	P	56	6.99E-06	1.54E-04
6	1793	F3 vs. L3	+	Metabolism	Metabolism	phenylpropanoid metabolic process	GO:0009698	P	73	6.52E-07	1.86E-05
6	1794	F3 vs. L3	+	Metabolism	Metabolism	phosphate-containing compound metabolic process	GO:0006796	P	1144	9.12E-06	1.86E-04
6	1795	F3 vs. L3	+	Metabolism	Metabolism	phospholipid metabolic process	GO:0006644	P	144	1.59E-03	1.61E-02
6	1796	F3 vs. L3	+	Metabolism	Metabolism	phosphorus metabolic process	GO:0006793	P	1176	2.09E-07	6.98E-06
6	1797	F3 vs. L3	+	Metabolism	Metabolism	pigment metabolic process	GO:0042440	P	89	2.04E-03	1.96E-02

6	1798	F3 vs. L3	+	Metabolism	Metabolism	polygalacturonase activity	GO:0004650	F	35	2.69E-03	4.66E-02
6	1799	F3 vs. L3	+	Metabolism	Metabolism	polyol metabolic process	GO:0019751	P	41	1.09E-04	1.67E-03
6	1800	F3 vs. L3	+	Metabolism	Metabolism	proton export across plasma membrane	GO:0120029	P	8	2.75E-03	2.47E-02
6	1801	F3 vs. L3	+	Metabolism	Metabolism	purine nucleoside diphosphate metabolic process	GO:0009135	P	43	3.14E-03	2.74E-02
6	1802	F3 vs. L3	+	Metabolism	Metabolism	purine nucleotide metabolic process	GO:0006163	P	144	1.76E-03	1.76E-02
6	1803	F3 vs. L3	+	Metabolism	Metabolism	purine ribonucleoside diphosphate metabolic process	GO:0009179	P	43	3.14E-03	2.75E-02
6	1804	F3 vs. L3	+	Metabolism	Metabolism	purine ribonucleotide metabolic process	GO:0009150	P	136	6.18E-04	7.28E-03
6	1805	F3 vs. L3	+	Metabolism	Metabolism	purine-containing compound metabolic process	GO:0072521	P	163	2.86E-03	2.55E-02
6	1806	F3 vs. L3	+	Metabolism	Metabolism	pyridoxal phosphate binding	GO:0030170	F	71	6.90E-08	7.35E-06
6	1807	F3 vs. L3	+	Metabolism	Metabolism	quercetin 7-O-glucosyltransferase activity	GO:0080044	F	33	5.39E-04	1.32E-02
6	1808	F3 vs. L3	+	Metabolism	Metabolism	racemase and epimerase activity	GO:0016854	F	33	1.17E-03	2.38E-02
6	1809	F3 vs. L3	+	Metabolism	Metabolism	S-adenosylmethionine biosynthetic process	GO:0006556	P	4	1.50E-03	1.53E-02
6	1810	F3 vs. L3	+	Metabolism	Metabolism	S-adenosylmethionine metabolic process	GO:0046500	P	8	1.35E-04	2.02E-03
6	1811	F3 vs. L3	+	Metabolism	Metabolism	secondary alcohol metabolic process	GO:1902652	P	11	3.40E-03	2.91E-02
6	1812	F3 vs. L3	+	Metabolism	Metabolism	secondary metabolic process	GO:0019748	P	185	4.38E-11	3.33E-09
6	1813	F3 vs. L3	+	Metabolism	Metabolism	secondary metabolite biosynthetic process	GO:0044550	P	97	1.31E-10	8.64E-09
6	1814	F3 vs. L3	+	Metabolism	Metabolism	serine family amino acid biosynthetic process	GO:0009070	P	31	8.80E-06	1.82E-04
6	1815	F3 vs. L3	+	Metabolism	Metabolism	serine family amino acid metabolic process	GO:0009069	P	44	1.52E-08	6.78E-07
6	1816	F3 vs. L3	+	Metabolism	Metabolism	S-glycoside biosynthetic process	GO:0016144	P	29	7.95E-06	1.69E-04
6	1817	F3 vs. L3	+	Metabolism	Metabolism	S-glycoside metabolic process	GO:0016143	P	68	6.27E-05	1.03E-03
6	1818	F3 vs. L3	+	Metabolism	Metabolism	small molecule binding	GO:0036094	F	785	1.56E-07	1.45E-05
6	1819	F3 vs. L3	+	Metabolism	Metabolism	small molecule biosynthetic process	GO:0044283	P	436	0.00E+00	0.00E+00
6	1820	F3 vs. L3	+	Metabolism	Metabolism	small molecule metabolic process	GO:0044281	P	1034	0.00E+00	0.00E+00
6	1821	F3 vs. L3	+	Metabolism	Metabolism	sphingolipid metabolic process	GO:0006665	P	28	7.37E-04	8.32E-03
6	1822	F3 vs. L3	+	Metabolism	Metabolism	sulfur amino acid biosynthetic process	GO:0000097	P	26	3.90E-04	4.90E-03
6	1823	F3 vs. L3	+	Metabolism	Metabolism	sulfur amino acid metabolic process	GO:0000096	P	39	9.94E-06	1.99E-04
6	1824	F3 vs. L3	+	Metabolism	Metabolism	sulfur compound binding	GO:1901681	F	26	7.94E-06	4.08E-04
6	1825	F3 vs. L3	+	Metabolism	Metabolism	sulfur compound biosynthetic process	GO:0044272	P	104	1.67E-15	3.42E-13
6	1826	F3 vs. L3	+	Metabolism	Metabolism	sulfur compound metabolic process	GO:0006790	P	238	0.00E+00	0.00E+00
6	1827	F3 vs. L3	+	Metabolism	Metabolism	terpenoid metabolic process	GO:0006721	P	90	6.48E-03	4.89E-02
6	1828	F3 vs. L3	+	Metabolism	Metabolism	tetrapyrrole binding	GO:0046906	F	221	1.47E-05	6.59E-04
6	1829	F3 vs. L3	+	Metabolism	Metabolism	transaminase activity	GO:0008483	F	39	2.22E-03	4.02E-02
6	1830	F3 vs. L3	+	Metabolism	Metabolism	transferase activity	GO:0016740	F	1999	5.64E-05	2.00E-03
6	1831	F3 vs. L3	+	Metabolism	Metabolism	transferase activity, transferring alkyl or aryl (other than methyl) groups	GO:0016765	F	85	1.50E-05	6.63E-04
6	1832	F3 vs. L3	+	Metabolism	Metabolism	transferase activity, transferring glycosyl groups	GO:0016757	F	367	1.85E-05	7.96E-04
6	1833	F3 vs. L3	+	Metabolism	Metabolism	transferase activity, transferring hexosyl groups	GO:0016758	F	235	1.46E-05	6.69E-04
6	1834	F3 vs. L3	+	Metabolism	Metabolism	transferase activity, transferring nitrogenous groups	GO:0016769	F	40	1.10E-03	2.32E-02
6	1835	F3 vs. L3	+	Metabolism	Metabolism	transition metal ion binding	GO:0046914	F	701	8.95E-07	6.13E-05
6	1836	F3 vs. L3	+	Metabolism	Metabolism	UDP-glucosyltransferase activity	GO:0035251	F	102	1.11E-06	7.07E-05
6	1837	F3 vs. L3	+	Metabolism	Metabolism	UDP-glucuronate biosynthetic process	GO:0006065	P	4	5.91E-03	4.53E-02
6	1838	F3 vs. L3	+	Metabolism	Metabolism	UDP-glucuronate metabolic process	GO:0046398	P	5	3.16E-03	2.75E-02
6	1839	F3 vs. L3	+	Metabolism	Metabolism	UDP-glycosyltransferase activity	GO:0008194	F	188	9.72E-09	1.33E-06
6	1840	F3 vs. L3	+	Metabolism	Metabolism	UDP-L-arabinose metabolic process	GO:0033356	P	9	1.29E-03	1.33E-02
6	1841	F3 vs. L3	+	Metabolism	Metabolism	UDP-rhamnose biosynthetic process	GO:0010253	P	4	3.92E-03	3.24E-02
6	1842	F3 vs. L3	+	Metabolism	Metabolism	UDP-rhamnose metabolic process	GO:0033478	P	4	3.92E-03	3.25E-02
6	1843	F3 vs. L3	+	Metabolism	Metabolism	uronic acid metabolic process	GO:0006063	P	3	5.84E-03	4.49E-02
6	1844	F3 vs. L3	+	Metabolism	Metabolism	vitamin B6 binding	GO:0070279	F	71	6.90E-08	7.63E-06
6	1845	F3 vs. L3	+	Metabolism	Metabolism	vitamin binding	GO:0019842	F	94	4.01E-07	3.30E-05
6	1846	F3 vs. L3	+	Metabolism	Photosynthesis	carbon fixation	GO:0015977	P	15	9.51E-06	1.92E-04
6	1847	F3 vs. L3	+	Metabolism	Photosynthesis	chloroplast	GO:0009507	C	3000	3.33E-16	1.62E-14
6	1848	F3 vs. L3	+	Metabolism	Photosynthesis	chloroplast avoidance movement	GO:0009903	P	17	6.56E-03	4.94E-02
6	1849	F3 vs. L3	+	Metabolism	Photosynthesis	chloroplast envelope	GO:0009941	C	442	1.89E-15	7.98E-14
6	1850	F3 vs. L3	+	Metabolism	Photosynthesis	chloroplast localization	GO:0019750	P	21	4.42E-03	3.55E-02
6	1851	F3 vs. L3	+	Metabolism	Photosynthesis	chloroplast relocation	GO:0009902	P	20	1.15E-03	1.21E-02
6	1852	F3 vs. L3	+	Metabolism	Photosynthesis	chloroplast stroma	GO:0009570	C	538	0.00E+00	0.00E+00
6	1853	F3 vs. L3	+	Metabolism	Photosynthesis	chloroplast thylakoid	GO:0009534	C	318	8.33E-12	2.46E-10
6	1854	F3 vs. L3	+	Metabolism	Photosynthesis	chloroplast thylakoid membrane	GO:0009535	C	259	8.47E-10	1.75E-08
6	1855	F3 vs. L3	+	Metabolism	Photosynthesis	establishment of plastid localization	GO:0051667	P	20	1.15E-03	1.22E-02
6	1856	F3 vs. L3	+	Metabolism	Photosynthesis	fructose 1,6-bisphosphate metabolic process	GO:0030388	P	11	1.22E-03	1.27E-02
6	1857	F3 vs. L3	+	Metabolism	Photosynthesis	fructose-bisphosphate aldolase activity	GO:0004332	F	7	6.19E-04	1.47E-02
6	1858	F3 vs. L3	+	Metabolism	Photosynthesis	photosynthesis	GO:0015979	P	136	8.52E-10	4.77E-08
6	1859	F3 vs. L3	+	Metabolism	Photosynthesis	photosynthesis, dark reaction	GO:0019685	P	15	3.39E-06	8.03E-05
6	1860	F3 vs. L3	+	Metabolism	Photosynthesis	photosynthesis, light reaction	GO:0019684	P	89	1.44E-05	2.75E-04

6	1861	F3 vs. L3	+	Metabolism	Photosynthesis	photosynthetic membrane	GO:0034357	C	283	2.38E-10	5.65E-09
6	1862	F3 vs. L3	+	Metabolism	Photosynthesis	plastid	GO:0009536	C	3297	2.22E-16	1.27E-14
6	1863	F3 vs. L3	+	Metabolism	Photosynthesis	plastid envelope	GO:0009526	C	460	8.88E-16	3.93E-14
6	1864	F3 vs. L3	+	Metabolism	Photosynthesis	plastid localization	GO:0051644	P	21	4.42E-03	3.55E-02
6	1865	F3 vs. L3	+	Metabolism	Photosynthesis	plastid stroma	GO:0009532	C	545	0.00E+00	0.00E+00
6	1866	F3 vs. L3	+	Metabolism	Photosynthesis	plastid thylakoid	GO:0031976	C	320	4.18E-12	1.31E-10
6	1867	F3 vs. L3	+	Metabolism	Photosynthesis	plastid thylakoid membrane	GO:0055035	C	262	4.55E-10	9.84E-09
6	1868	F3 vs. L3	+	Metabolism	Photosynthesis	plastoglobule	GO:0010287	C	60	1.17E-03	7.72E-03
6	1869	F3 vs. L3	+	Metabolism	Photosynthesis	thylakoid	GO:0009579	C	389	6.00E-15	2.43E-13
6	1870	F3 vs. L3	+	Metabolism	Photosynthesis	thylakoid lumen	GO:0031977	C	47	7.63E-04	5.38E-03
6	1871	F3 vs. L3	+	Metabolism	Photosynthesis	thylakoid membrane	GO:0042651	C	280	1.22E-10	3.12E-09
6	1872	F3 vs. L3	+	Metabolism	Transporters	active ion transmembrane transporter activity	GO:0022853	F	149	1.45E-10	2.78E-08
6	1873	F3 vs. L3	+	Metabolism	Transporters	active transmembrane transporter activity	GO:0022804	F	316	1.67E-14	5.32E-12
6	1874	F3 vs. L3	+	Metabolism	Transporters	amino acid transmembrane transport	GO:0003333	P	21	3.87E-03	3.22E-02
6	1875	F3 vs. L3	+	Metabolism	Transporters	amino acid transmembrane transporter activity	GO:0015171	F	59	2.59E-04	6.97E-03
6	1876	F3 vs. L3	+	Metabolism	Transporters	amino acid transport	GO:0006865	P	37	5.77E-03	4.46E-02
6	1877	F3 vs. L3	+	Metabolism	Transporters	anion transmembrane transport	GO:0098656	P	73	5.30E-04	6.40E-03
6	1878	F3 vs. L3	+	Metabolism	Transporters	anion transmembrane transporter activity	GO:0008509	F	235	4.04E-07	3.14E-05
6	1879	F3 vs. L3	+	Metabolism	Transporters	anion transport	GO:0006820	P	171	2.92E-08	1.20E-06
6	1880	F3 vs. L3	+	Metabolism	Transporters	antiporter activity	GO:0015297	F	135	5.07E-08	6.08E-06
6	1881	F3 vs. L3	+	Metabolism	Transporters	ATPase-coupled cation transmembrane transporter activity	GO:0019829	F	47	3.17E-07	2.76E-05
6	1882	F3 vs. L3	+	Metabolism	Transporters	ATPase-coupled ion transmembrane transporter activity	GO:0042625	F	53	3.17E-07	2.69E-05
6	1883	F3 vs. L3	+	Metabolism	Transporters	ATPase-coupled transmembrane transporter activity	GO:0042626	F	124	5.46E-07	4.03E-05
6	1884	F3 vs. L3	+	Metabolism	Transporters	calcium ion transmembrane transport	GO:0070588	P	26	1.89E-03	1.83E-02
6	1885	F3 vs. L3	+	Metabolism	Transporters	carbohydrate transmembrane transporter activity	GO:0015144	F	65	6.06E-04	1.48E-02
6	1886	F3 vs. L3	+	Metabolism	Transporters	carbohydrate transport	GO:0008643	P	40	3.02E-03	2.66E-02
6	1887	F3 vs. L3	+	Metabolism	Transporters	carboxylic acid transmembrane transport	GO:1905039	P	40	4.13E-03	3.39E-02
6	1888	F3 vs. L3	+	Metabolism	Transporters	carboxylic acid transmembrane transporter activity	GO:0046943	F	93	7.32E-05	2.51E-03
6	1889	F3 vs. L3	+	Metabolism	Transporters	carboxylic acid transport	GO:0046942	P	78	4.32E-03	3.50E-02
6	1890	F3 vs. L3	+	Metabolism	Transporters	cation transmembrane transport	GO:0098655	P	148	6.87E-07	1.93E-05
6	1891	F3 vs. L3	+	Metabolism	Transporters	cation transmembrane transporter activity	GO:0008324	F	270	2.17E-08	2.72E-06
6	1892	F3 vs. L3	+	Metabolism	Transporters	cation transport	GO:0006812	P	252	4.37E-07	1.35E-05
6	1893	F3 vs. L3	+	Metabolism	Transporters	chemotaxis	GO:0006935	P	23	6.43E-04	7.47E-03
6	1894	F3 vs. L3	+	Metabolism	Transporters	cytoplasmic vesicle	GO:0031410	C	488	1.75E-08	2.99E-07
6	1895	F3 vs. L3	+	Metabolism	Transporters	dicarboxylic acid transport	GO:0006835	P	17	5.78E-03	4.46E-02
6	1896	F3 vs. L3	+	Metabolism	Transporters	endosome	GO:0005768	C	293	1.85E-05	2.03E-04
6	1897	F3 vs. L3	+	Metabolism	Transporters	export across plasma membrane	GO:0140115	P	11	1.76E-03	1.76E-02
6	1898	F3 vs. L3	+	Metabolism	Transporters	export from cell	GO:0140352	P	61	1.11E-05	2.16E-04
6	1899	F3 vs. L3	+	Metabolism	Transporters	fluid transport	GO:0042044	P	22	6.06E-03	4.60E-02
6	1900	F3 vs. L3	+	Metabolism	Transporters	inorganic anion transmembrane transporter activity	GO:0015103	F	76	2.21E-05	8.96E-04
6	1901	F3 vs. L3	+	Metabolism	Transporters	inorganic anion transport	GO:0015698	P	57	1.23E-06	3.22E-05
6	1902	F3 vs. L3	+	Metabolism	Transporters	inorganic cation transmembrane transport	GO:0098662	P	144	6.47E-07	1.87E-05
6	1903	F3 vs. L3	+	Metabolism	Transporters	inorganic cation transmembrane transporter activity	GO:0022890	F	247	2.15E-08	2.81E-06
6	1904	F3 vs. L3	+	Metabolism	Transporters	inorganic ion transmembrane transport	GO:0098660	P	159	3.13E-08	1.26E-06
6	1905	F3 vs. L3	+	Metabolism	Transporters	inorganic molecular entity transmembrane transporter activity	GO:0015318	F	434	3.33E-16	1.92E-13
6	1906	F3 vs. L3	+	Metabolism	Transporters	ion transmembrane transport	GO:0034220	P	219	6.66E-10	3.85E-08
6	1907	F3 vs. L3	+	Metabolism	Transporters	ion transmembrane transporter activity	GO:0015075	F	472	1.84E-13	4.42E-11
6	1908	F3 vs. L3	+	Metabolism	Transporters	ion transmembrane transporter activity, phosphorylative mechanism	GO:0015662	F	34	7.56E-06	3.95E-04
6	1909	F3 vs. L3	+	Metabolism	Transporters	ion transport	GO:0006811	P	410	2.16E-13	2.60E-11
6	1910	F3 vs. L3	+	Metabolism	Transporters	locomotion	GO:0040011	P	25	1.27E-03	1.32E-02
6	1911	F3 vs. L3	+	Metabolism	Transporters	metal ion transmembrane transporter activity	GO:0046873	F	147	6.99E-04	1.62E-02
6	1912	F3 vs. L3	+	Metabolism	Transporters	metal ion transport	GO:0030001	P	154	2.92E-03	2.60E-02
6	1913	F3 vs. L3	+	Metabolism	Transporters	monovalent inorganic cation transmembrane transporter activity	GO:0015077	F	144	7.29E-08	7.49E-06
6	1914	F3 vs. L3	+	Metabolism	Transporters	monovalent inorganic cation transport	GO:0015672	P	107	1.70E-06	4.37E-05
6	1915	F3 vs. L3	+	Metabolism	Transporters	negative regulation of transport	GO:0051051	P	4	3.70E-03	3.09E-02
6	1916	F3 vs. L3	+	Metabolism	Transporters	neutral amino acid transmembrane transporter activity	GO:0015175	F	12	6.10E-04	1.47E-02
6	1917	F3 vs. L3	+	Metabolism	Transporters	neutral amino acid transport	GO:0015804	P	7	5.10E-03	4.02E-02
6	1918	F3 vs. L3	+	Metabolism	Transporters	nitrate transmembrane transporter activity	GO:0015112	F	16	2.11E-04	5.88E-03
6	1919	F3 vs. L3	+	Metabolism	Transporters	nitrate transport	GO:0015706	P	20	1.81E-04	2.59E-03
6	1920	F3 vs. L3	+	Metabolism	Transporters	organic acid transmembrane transport	GO:1903825	P	41	3.21E-03	2.78E-02
6	1921	F3 vs. L3	+	Metabolism	Transporters	organic acid transmembrane transporter activity	GO:0005342	F	93	7.32E-05	2.48E-03
6	1922	F3 vs. L3	+	Metabolism	Transporters	organic acid transport	GO:0015849	P	79	3.53E-03	2.98E-02

6	1923	F3 vs. L3	+	Metabolism	Transporters	organic anion transmembrane transporter activity	GO:0008514	F	149	4.32E-04	1.11E-02
6	1924	F3 vs. L3	+	Metabolism	Transporters	organic anion transport	GO:0015711	P	109	3.37E-04	4.34E-03
6	1925	F3 vs. L3	+	Metabolism	Transporters	organic substance transport	GO:0071702	P	635	5.93E-03	4.53E-02
6	1926	F3 vs. L3	+	Metabolism	Transporters	plasmodesma	GO:0009506	C	610	3.07E-03	1.83E-02
6	1927	F3 vs. L3	+	Metabolism	Transporters	positive chemotaxis	GO:0050918	P	23	6.43E-04	7.51E-03
6	1928	F3 vs. L3	+	Metabolism	Transporters	primary active transmembrane transporter activity	GO:0015399	F	139	9.92E-07	6.49E-05
6	1929	F3 vs. L3	+	Metabolism	Transporters	proton transmembrane transporter activity	GO:0015078	F	113	3.41E-09	5.78E-07
6	1930	F3 vs. L3	+	Metabolism	Transporters	regulation of ion transmembrane transporter activity	GO:0032412	P	7	3.58E-03	3.01E-02
6	1931	F3 vs. L3	+	Metabolism	Transporters	regulation of transmembrane transporter activity	GO:0022898	P	7	3.58E-03	3.02E-02
6	1932	F3 vs. L3	+	Metabolism	Transporters	regulation of transporter activity	GO:0032409	P	7	3.58E-03	3.01E-02
6	1933	F3 vs. L3	+	Metabolism	Transporters	secondary active transmembrane transporter activity	GO:0015291	F	174	6.30E-09	9.53E-07
6	1934	F3 vs. L3	+	Metabolism	Transporters	secretory vesicle	GO:0099503	C	114	2.12E-05	2.27E-04
6	1935	F3 vs. L3	+	Metabolism	Transporters	sulfate assimilation	GO:0000103	P	18	2.91E-03	2.59E-02
6	1936	F3 vs. L3	+	Metabolism	Transporters	trans-Golgi network	GO:0005802	C	221	9.04E-09	1.63E-07
6	1937	F3 vs. L3	+	Metabolism	Transporters	transmembrane transport	GO:0055085	P	546	1.17E-14	1.79E-12
6	1938	F3 vs. L3	+	Metabolism	Transporters	transmembrane transporter activity	GO:0022857	F	794	0.00E+00	0.00E+00
6	1939	F3 vs. L3	+	Metabolism	Transporters	transport	GO:0006810	P	1380	6.36E-11	4.52E-09
6	1940	F3 vs. L3	+	Metabolism	Transporters	transporter activity	GO:0005215	F	818	0.00E+00	0.00E+00
6	1941	F3 vs. L3	+	Metabolism	Transporters	vesicle	GO:0031982	C	529	1.70E-09	3.32E-08
6	1942	F3 vs. L3	+	Metabolism	Transporters	water channel activity	GO:0015250	F	22	4.92E-04	1.24E-02
6	1943	F3 vs. L3	+	Metabolism	Transporters	water transmembrane transporter activity	GO:0005372	F	22	4.92E-04	1.25E-02
6	1944	F3 vs. L3	+	Metabolism	Transporters	water transport	GO:0006833	P	22	6.06E-03	4.61E-02
6	1945	F3 vs. L3	+	Metabolism	Transporters	xenobiotic transmembrane transporter activity	GO:0042910	F	45	2.32E-04	6.35E-03
6	1946	F3 vs. L3	+	Regulation	Protein modification	endoplasmic reticulum	GO:0005783	C	668	9.02E-03	4.65E-02
6	1947	F3 vs. L3	+	Regulation	Protein modification	Golgi apparatus	GO:0005794	C	769	7.46E-12	2.27E-10
6	1948	F3 vs. L3	+	Regulation	Protein modification	modified amino acid binding	GO:0072341	F	15	9.88E-04	2.20E-02
6	1949	F3 vs. L3	+	Regulation	Regulation	regulation of biological quality	GO:0065008	P	609	2.33E-04	3.15E-03
6	1950	F3 vs. L3	+	Regulation	Regulation	regulation of cell morphogenesis involved in differentiation	GO:0010769	P	32	7.19E-07	2.01E-05
6	1951	F3 vs. L3	+	Regulation	Regulation	regulation of cellular ketone metabolic process	GO:0010565	P	60	4.19E-03	3.43E-02
6	1952	F3 vs. L3	+	Regulation	Regulation	regulation of pollen tube growth	GO:0080092	P	29	9.63E-08	3.51E-06
6	1953	F3 vs. L3	+	Regulation	Regulation	regulation of small molecule metabolic process	GO:0062012	P	76	6.43E-04	7.53E-03
6	1954	F3 vs. L3	+	Regulation	Regulation	regulation of unidimensional cell growth	GO:0051510	P	50	4.31E-04	5.33E-03
6	1955	F3 vs. L3	+	Regulation	Translation	poly(U) RNA binding	GO:0008266	F	19	1.28E-03	2.57E-02
6	1956	F3 vs. L3	+	Signaling and response	Signaling	auxin efflux	GO:0010315	P	14	9.12E-04	9.97E-03
6	1957	F3 vs. L3	+	Signaling and response	Signaling	calcium ion binding	GO:0005509	F	158	9.44E-09	1.36E-06
6	1958	F3 vs. L3	+	Signaling and response	Signaling	cellular response to abiotic stimulus	GO:0071214	P	121	3.21E-03	2.78E-02
6	1959	F3 vs. L3	+	Signaling and response	Signaling	cellular response to blue light	GO:0071483	P	18	1.82E-03	1.79E-02
6	1960	F3 vs. L3	+	Signaling and response	Signaling	cellular response to chemical stimulus	GO:0070887	P	659	2.18E-03	2.06E-02
6	1961	F3 vs. L3	+	Signaling and response	Signaling	cellular response to environmental stimulus	GO:0104004	P	121	3.21E-03	2.78E-02
6	1962	F3 vs. L3	+	Signaling and response	Signaling	cellular response to external stimulus	GO:0071496	P	136	1.15E-04	1.75E-03
6	1963	F3 vs. L3	+	Signaling and response	Signaling	cellular response to extracellular stimulus	GO:0031668	P	130	1.15E-05	2.21E-04
6	1964	F3 vs. L3	+	Signaling and response	Signaling	cellular response to jasmonic acid stimulus	GO:0071395	P	52	1.60E-03	1.62E-02
6	1965	F3 vs. L3	+	Signaling and response	Signaling	cellular response to light stimulus	GO:0071482	P	68	2.33E-03	2.17E-02
6	1966	F3 vs. L3	+	Signaling and response	Signaling	cellular response to nutrient levels	GO:0031669	P	103	4.23E-04	5.25E-03
6	1967	F3 vs. L3	+	Signaling and response	Signaling	cellular response to oxygen levels	GO:0071453	P	134	3.03E-03	2.66E-02
6	1968	F3 vs. L3	+	Signaling and response	Signaling	cellular response to oxygen-containing compound	GO:1901701	P	326	9.51E-04	1.03E-02
6	1969	F3 vs. L3	+	Signaling and response	Signaling	cellular response to salicylic acid stimulus	GO:0071446	P	36	3.92E-04	4.90E-03
6	1970	F3 vs. L3	+	Signaling and response	Signaling	cellular response to water stimulus	GO:0071462	P	27	3.05E-03	2.68E-02
6	1971	F3 vs. L3	+	Signaling and response	Signaling	cobalt ion binding	GO:0050897	F	44	2.05E-03	3.76E-02
6	1972	F3 vs. L3	+	Signaling and response	Signaling	dephosphorylation	GO:0016311	P	133	2.41E-03	2.24E-02
6	1973	F3 vs. L3	+	Signaling and response	Signaling	disulfide oxidoreductase activity	GO:0015036	F	53	2.77E-04	7.32E-03
6	1974	F3 vs. L3	+	Signaling and response	Signaling	jasmonic acid biosynthetic process	GO:0009695	P	17	2.95E-04	3.87E-03
6	1975	F3 vs. L3	+	Signaling and response	Signaling	jasmonic acid mediated signaling pathway	GO:0009867	P	49	4.12E-03	3.39E-02
6	1976	F3 vs. L3	+	Signaling and response	Signaling	jasmonic acid metabolic process	GO:0009694	P	34	5.63E-03	4.37E-02
6	1977	F3 vs. L3	+	Signaling and response	Signaling	multi-organism process	GO:0051704	P	298	9.13E-06	1.85E-04
6	1978	F3 vs. L3	+	Signaling and response	Signaling	phospholipase activity	GO:0004620	F	34	1.80E-03	3.41E-02
6	1979	F3 vs. L3	+	Signaling and response	Signaling	regulation of auxin metabolic process	GO:0090354	P	14	7.15E-04	8.13E-03
6	1980	F3 vs. L3	+	Signaling and response	Signaling	regulation of jasmonic acid mediated signaling pathway	GO:2000022	P	27	1.99E-04	2.81E-03
6	1981	F3 vs. L3	+	Signaling and response	Signaling	regulation of root development	GO:2000280	P	29	1.86E-03	1.82E-02
6	1982	F3 vs. L3	+	Signaling and response	Signaling	response to abiotic stimulus	GO:0009628	P	1375	8.92E-13	9.51E-11
6	1983	F3 vs. L3	+	Signaling and response	Signaling	response to acid chemical	GO:0001101	P	278	6.43E-10	3.75E-08
6	1984	F3 vs. L3	+	Signaling and response	Signaling	response to bacterium	GO:0009617	P	319	2.33E-09	1.22E-07
6	1985	F3 vs. L3	+	Signaling and response	Signaling	response to biotic stimulus	GO:0009607	P	683	1.79E-11	1.55E-09
6	1986	F3 vs. L3	+	Signaling and response	Signaling	response to cadmium ion	GO:0046686	P	237	8.89E-14	1.26E-11

6	1987	F3 vs. L3	+	Signaling and response	Signaling	response to chemical	GO:0042221	P	1685	0.00E+00	0.00E+00
6	1988	F3 vs. L3	+	Signaling and response	Signaling	response to chitin	GO:0010200	P	81	5.82E-06	1.32E-04
6	1989	F3 vs. L3	+	Signaling and response	Signaling	response to endogenous stimulus	GO:0009719	P	802	2.66E-07	8.66E-06
6	1990	F3 vs. L3	+	Signaling and response	Signaling	response to external biotic stimulus	GO:0043207	P	682	1.82E-11	1.55E-09
6	1991	F3 vs. L3	+	Signaling and response	Signaling	response to external stimulus	GO:0009605	P	940	3.33E-16	8.79E-14
6	1992	F3 vs. L3	+	Signaling and response	Signaling	response to extracellular stimulus	GO:0009991	P	165	3.28E-06	7.87E-05
6	1993	F3 vs. L3	+	Signaling and response	Signaling	response to hormone	GO:0009725	P	785	1.63E-07	5.65E-06
6	1994	F3 vs. L3	+	Signaling and response	Signaling	response to inorganic substance	GO:0010035	P	633	0.00E+00	0.00E+00
6	1995	F3 vs. L3	+	Signaling and response	Signaling	response to jasmonic acid	GO:0009753	P	123	6.34E-11	4.57E-09
6	1996	F3 vs. L3	+	Signaling and response	Signaling	response to karrikin	GO:0080167	P	96	1.32E-08	6.21E-07
6	1997	F3 vs. L3	+	Signaling and response	Signaling	response to light intensity	GO:0009642	P	103	4.18E-04	5.21E-03
6	1998	F3 vs. L3	+	Signaling and response	Signaling	response to light stimulus	GO:0009416	P	473	4.36E-06	1.02E-04
6	1999	F3 vs. L3	+	Signaling and response	Signaling	response to lipid	GO:0033993	P	438	1.09E-05	2.15E-04
6	2000	F3 vs. L3	+	Signaling and response	Signaling	response to metal ion	GO:0010038	P	306	3.93E-13	4.45E-11
6	2001	F3 vs. L3	+	Signaling and response	Signaling	response to nitrate	GO:0010167	P	18	1.62E-03	1.64E-02
6	2002	F3 vs. L3	+	Signaling and response	Signaling	response to nitrogen compound	GO:1901698	P	184	6.11E-04	7.22E-03
6	2003	F3 vs. L3	+	Signaling and response	Signaling	response to nutrient levels	GO:0031667	P	137	1.15E-04	1.76E-03
6	2004	F3 vs. L3	+	Signaling and response	Signaling	response to organic substance	GO:0010033	P	1068	1.18E-10	7.89E-09
6	2005	F3 vs. L3	+	Signaling and response	Signaling	response to oxygen levels	GO:0070482	P	156	5.80E-03	4.47E-02
6	2006	F3 vs. L3	+	Signaling and response	Signaling	response to oxygen-containing compound	GO:1901700	P	990	0.00E+00	0.00E+00
6	2007	F3 vs. L3	+	Signaling and response	Signaling	response to salicylic acid	GO:0009751	P	91	3.30E-06	7.89E-05
6	2008	F3 vs. L3	+	Signaling and response	Signaling	response to stimulus	GO:0050896	P	3365	1.33E-15	2.84E-13
6	2009	F3 vs. L3	+	Signaling and response	Signaling	response to temperature stimulus	GO:0009266	P	410	4.04E-06	9.48E-05
6	2010	F3 vs. L3	+	Signaling and response	Signaling	response to UV	GO:0009411	P	84	1.05E-03	1.12E-02
6	2011	F3 vs. L3	+	Signaling and response	Signaling	response to water	GO:0009415	P	259	1.55E-10	1.01E-08
6	2012	F3 vs. L3	+	Signaling and response	Signaling	salicylic acid mediated signaling pathway	GO:0009863	P	29	5.69E-04	6.79E-03
6	2013	F3 vs. L3	+	Signaling and response	Signaling	taxis	GO:0042330	P	23	6.43E-04	7.52E-03
6	2014	F3 vs. L3	+	Signaling and response	Stress	aging	GO:0007568	P	84	8.57E-04	9.41E-03
6	2015	F3 vs. L3	+	Signaling and response	Stress	anion homeostasis	GO:0055081	P	31	7.61E-04	8.51E-03
6	2016	F3 vs. L3	+	Signaling and response	Stress	antioxidant activity	GO:0016209	F	82	9.66E-07	6.46E-05
6	2017	F3 vs. L3	+	Signaling and response	Stress	auxin homeostasis	GO:0010252	P	26	3.18E-03	2.77E-02
6	2018	F3 vs. L3	+	Signaling and response	Stress	cation homeostasis	GO:0055080	P	147	4.20E-05	7.20E-04
6	2019	F3 vs. L3	+	Signaling and response	Stress	cell redox homeostasis	GO:0045454	P	54	1.07E-03	1.14E-02
6	2020	F3 vs. L3	+	Signaling and response	Stress	cellular detoxification	GO:1990748	P	27	2.87E-04	3.78E-03
6	2021	F3 vs. L3	+	Signaling and response	Stress	cellular homeostasis	GO:0019725	P	181	3.80E-04	4.79E-03
6	2022	F3 vs. L3	+	Signaling and response	Stress	cellular oxidant detoxification	GO:0098869	P	17	2.41E-06	6.08E-05
6	2023	F3 vs. L3	+	Signaling and response	Stress	cellular response to chemical stress	GO:0062197	P	76	4.62E-03	3.68E-02
6	2024	F3 vs. L3	+	Signaling and response	Stress	cellular response to decreased oxygen levels	GO:0036294	P	133	2.87E-03	2.56E-02
6	2025	F3 vs. L3	+	Signaling and response	Stress	cellular response to hypoxia	GO:0071456	P	132	4.32E-03	3.50E-02
6	2026	F3 vs. L3	+	Signaling and response	Stress	cellular response to oxidative stress	GO:0034599	P	47	3.70E-04	4.69E-03
6	2027	F3 vs. L3	+	Signaling and response	Stress	cellular response to oxygen radical	GO:0071450	P	8	2.13E-03	2.02E-02
6	2028	F3 vs. L3	+	Signaling and response	Stress	cellular response to phosphate starvation	GO:0016036	P	50	9.57E-04	1.03E-02
6	2029	F3 vs. L3	+	Signaling and response	Stress	cellular response to reactive oxygen species	GO:0034614	P	23	3.70E-03	3.10E-02
6	2030	F3 vs. L3	+	Signaling and response	Stress	cellular response to starvation	GO:0009267	P	94	8.98E-04	9.84E-03
6	2031	F3 vs. L3	+	Signaling and response	Stress	cellular response to superoxide	GO:0071451	P	8	2.13E-03	2.03E-02
6	2032	F3 vs. L3	+	Signaling and response	Stress	cellular response to toxic substance	GO:0097237	P	28	1.03E-04	1.61E-03
6	2033	F3 vs. L3	+	Signaling and response	Stress	cellular response to water deprivation	GO:0042631	P	26	3.30E-03	2.83E-02
6	2034	F3 vs. L3	+	Signaling and response	Stress	chemical homeostasis	GO:0048878	P	260	8.18E-07	2.22E-05
6	2035	F3 vs. L3	+	Signaling and response	Stress	defense response	GO:0006952	P	565	7.95E-10	4.50E-08
6	2036	F3 vs. L3	+	Signaling and response	Stress	defense response to bacterium	GO:0042742	P	259	6.85E-08	2.58E-06
6	2037	F3 vs. L3	+	Signaling and response	Stress	defense response to fungus	GO:0050832	P	149	2.66E-03	2.41E-02
6	2038	F3 vs. L3	+	Signaling and response	Stress	defense response to insect	GO:0002213	P	16	1.54E-03	1.57E-02
6	2039	F3 vs. L3	+	Signaling and response	Stress	defense response to other organism	GO:0098542	P	489	5.17E-08	2.00E-06
6	2040	F3 vs. L3	+	Signaling and response	Stress	defense response, incompatible interaction	GO:0009814	P	115	2.14E-04	2.96E-03
6	2041	F3 vs. L3	+	Signaling and response	Stress	detoxification	GO:0098754	P	60	1.48E-08	6.66E-07
6	2042	F3 vs. L3	+	Signaling and response	Stress	flavonoid biosynthetic process	GO:0009813	P	33	1.65E-05	3.10E-04
6	2043	F3 vs. L3	+	Signaling and response	Stress	flavonoid metabolic process	GO:0009812	P	37	4.62E-06	1.07E-04
6	2044	F3 vs. L3	+	Signaling and response	Stress	glutathione binding	GO:0043295	F	11	4.76E-05	1.75E-03
6	2045	F3 vs. L3	+	Signaling and response	Stress	glutathione metabolic process	GO:0006749	P	27	7.97E-05	1.28E-03
6	2046	F3 vs. L3	+	Signaling and response	Stress	glutathione transferase activity	GO:0004364	F	31	1.14E-04	3.42E-03
6	2047	F3 vs. L3	+	Signaling and response	Stress	homeostatic process	GO:0042592	P	342	2.68E-06	6.67E-05
6	2048	F3 vs. L3	+	Signaling and response	Stress	hydrogen peroxide catabolic process	GO:0042744	P	17	1.30E-04	1.96E-03
6	2049	F3 vs. L3	+	Signaling and response	Stress	hydrogen peroxide metabolic process	GO:0042743	P	24	1.17E-04	1.78E-03
6	2050	F3 vs. L3	+	Signaling and response	Stress	hydrogen peroxide transmembrane transport	GO:0080170	P	5	1.87E-03	1.83E-02

6	2051	F3 vs. L3	+	Signaling and response	Stress	immune system process	GO:0002376	P	214	4.44E-03	3.55E-02
6	2052	F3 vs. L3	+	Signaling and response	Stress	indole phytoalexin metabolic process	GO:0046217	P	9	1.78E-03	1.77E-02
6	2053	F3 vs. L3	+	Signaling and response	Stress	inorganic ion homeostasis	GO:0098771	P	157	1.65E-05	3.10E-04
6	2054	F3 vs. L3	+	Signaling and response	Stress	interspecies interaction between organisms	GO:0044419	P	696	4.87E-11	3.60E-09
6	2055	F3 vs. L3	+	Signaling and response	Stress	ion homeostasis	GO:0050801	P	184	1.06E-06	2.80E-05
6	2056	F3 vs. L3	+	Signaling and response	Stress	monovalent inorganic cation homeostasis	GO:0055067	P	50	2.54E-05	4.60E-04
6	2057	F3 vs. L3	+	Signaling and response	Stress	oxidoreductase activity, acting on peroxide as acceptor	GO:0016684	F	64	8.48E-05	2.77E-03
6	2058	F3 vs. L3	+	Signaling and response	Stress	phytoalexin biosynthetic process	GO:0052315	P	9	1.78E-03	1.78E-02
6	2059	F3 vs. L3	+	Signaling and response	Stress	phytoalexin metabolic process	GO:0052314	P	9	1.78E-03	1.77E-02
6	2060	F3 vs. L3	+	Signaling and response	Stress	reactive nitrogen species metabolic process	GO:2001057	P	12	2.26E-03	2.12E-02
6	2061	F3 vs. L3	+	Signaling and response	Stress	reactive oxygen species metabolic process	GO:0072593	P	49	4.43E-07	1.35E-05
6	2062	F3 vs. L3	+	Signaling and response	Stress	regulation of pH	GO:0006885	P	35	9.07E-05	1.43E-03
6	2063	F3 vs. L3	+	Signaling and response	Stress	regulation of stomatal movement	GO:0010119	P	68	1.83E-03	1.80E-02
6	2064	F3 vs. L3	+	Signaling and response	Stress	removal of superoxide radicals	GO:0019430	P	8	2.13E-03	2.02E-02
6	2065	F3 vs. L3	+	Signaling and response	Stress	response to abscisic acid	GO:0009737	P	317	1.86E-08	7.98E-07
6	2066	F3 vs. L3	+	Signaling and response	Stress	response to alcohol	GO:0097305	P	321	9.62E-08	3.53E-06
6	2067	F3 vs. L3	+	Signaling and response	Stress	response to cold	GO:0009409	P	294	3.70E-09	1.86E-07
6	2068	F3 vs. L3	+	Signaling and response	Stress	response to decreased oxygen levels	GO:0036293	P	155	5.55E-03	4.31E-02
6	2069	F3 vs. L3	+	Signaling and response	Stress	response to desiccation	GO:0009269	P	19	1.17E-03	1.23E-02
6	2070	F3 vs. L3	+	Signaling and response	Stress	response to fungus	GO:0009620	P	210	3.50E-05	6.09E-04
6	2071	F3 vs. L3	+	Signaling and response	Stress	response to herbivore	GO:0080027	P	8	1.22E-03	1.27E-02
6	2072	F3 vs. L3	+	Signaling and response	Stress	response to high light intensity	GO:0009644	P	48	2.41E-04	3.24E-03
6	2073	F3 vs. L3	+	Signaling and response	Stress	response to osmotic stress	GO:0006970	P	351	4.02E-05	6.95E-04
6	2074	F3 vs. L3	+	Signaling and response	Stress	response to other organism	GO:0051707	P	682	1.82E-11	1.52E-09
6	2075	F3 vs. L3	+	Signaling and response	Stress	response to oxidative stress	GO:0006979	P	294	2.58E-09	1.32E-07
6	2076	F3 vs. L3	+	Signaling and response	Stress	response to oxygen radical	GO:0000305	P	9	2.44E-03	2.26E-02
6	2077	F3 vs. L3	+	Signaling and response	Stress	response to ozone	GO:0010193	P	24	2.32E-04	3.16E-03
6	2078	F3 vs. L3	+	Signaling and response	Stress	response to radiation	GO:0009314	P	486	4.66E-05	7.85E-04
6	2079	F3 vs. L3	+	Signaling and response	Stress	response to reactive oxygen species	GO:0000302	P	105	2.13E-04	2.96E-03
6	2080	F3 vs. L3	+	Signaling and response	Stress	response to salt stress	GO:0009651	P	294	2.09E-04	2.91E-03
6	2081	F3 vs. L3	+	Signaling and response	Stress	response to starvation	GO:0042594	P	117	2.90E-04	3.80E-03
6	2082	F3 vs. L3	+	Signaling and response	Stress	response to stress	GO:0006950	P	1928	4.30E-12	4.11E-10
6	2083	F3 vs. L3	+	Signaling and response	Stress	response to superoxide	GO:0000303	P	9	2.44E-03	2.25E-02
6	2084	F3 vs. L3	+	Signaling and response	Stress	response to toxic substance	GO:0009636	P	73	1.86E-09	9.92E-08
6	2085	F3 vs. L3	+	Signaling and response	Stress	response to UV-B	GO:0010224	P	51	5.40E-04	6.48E-03
6	2086	F3 vs. L3	+	Signaling and response	Stress	response to water deprivation	GO:0009414	P	253	2.25E-10	1.42E-08
6	2087	F3 vs. L3	+	Signaling and response	Stress	response to wounding	GO:0009611	P	158	0.00E+00	0.00E+00
6	2088	F3 vs. L3	+	Signaling and response	Stress	superoxide metabolic process	GO:0006801	P	8	2.13E-03	2.03E-02
6	2089	F3 vs. L3	+	Signaling and response	Stress	toxin biosynthetic process	GO:0009403	P	9	1.78E-03	1.78E-02
6	2090	F3 vs. L3	+	Signaling and response	Stress	toxin catabolic process	GO:0009407	P	25	3.24E-04	4.21E-03
6	2091	F3 vs. L3	+	Signaling and response	Stress	toxin metabolic process	GO:0009404	P	34	2.76E-06	6.83E-05
6	2092	F3 vs. L3	-	Development	Cell division	anaphase-promoting complex	GO:0005680	C	14	5.52E-03	3.07E-02
6	2093	F3 vs. L3	-	Development	Cell division	anaphase-promoting complex-dependent catabolic process	GO:0031145	P	11	5.39E-03	4.20E-02
6	2094	F3 vs. L3	-	Development	Cell division	Cajal body	GO:0015030	C	13	1.60E-03	1.02E-02
6	2095	F3 vs. L3	-	Development	Cell division	cell cycle	GO:0007049	P	294	6.77E-15	1.10E-12
6	2096	F3 vs. L3	-	Development	Cell division	cell cycle checkpoint	GO:0000075	P	26	5.59E-06	1.28E-04
6	2097	F3 vs. L3	-	Development	Cell division	cell cycle phase transition	GO:0044770	P	31	1.18E-06	3.11E-05
6	2098	F3 vs. L3	-	Development	Cell division	cell cycle process	GO:0022402	P	270	1.22E-15	2.71E-13
6	2099	F3 vs. L3	-	Development	Cell division	chromatin assembly	GO:0031497	P	53	1.87E-05	3.48E-04
6	2100	F3 vs. L3	-	Development	Cell division	chromosome	GO:0005694	C	293	1.06E-10	2.94E-09
6	2101	F3 vs. L3	-	Development	Cell division	chromosome organization involved in meiotic cell cycle	GO:0070192	P	30	2.65E-03	2.41E-02
6	2102	F3 vs. L3	-	Development	Cell division	chromosome segregation	GO:0007059	P	63	5.02E-05	8.40E-04
6	2103	F3 vs. L3	-	Development	Cell division	chromosome, centromeric region	GO:0000775	C	28	4.12E-05	3.97E-04
6	2104	F3 vs. L3	-	Development	Cell division	condensed chromosome	GO:0000793	C	41	1.65E-03	1.04E-02
6	2105	F3 vs. L3	-	Development	Cell division	condensed nuclear chromosome, centromeric region	GO:0000780	C	10	7.64E-03	4.08E-02
6	2106	F3 vs. L3	-	Development	Cell division	cyclin-dependent protein kinase holoenzyme complex	GO:0000307	C	37	1.35E-04	1.23E-03
6	2107	F3 vs. L3	-	Development	Cell division	cyclin-dependent protein serine/threonine kinase regulator activity	GO:0016538	F	32	1.12E-05	5.28E-04
6	2108	F3 vs. L3	-	Development	Cell division	cytokinesis by cell plate formation	GO:0000911	P	51	6.76E-07	1.91E-05
6	2109	F3 vs. L3	-	Development	Cell division	DNA biosynthetic process	GO:0071897	P	18	4.20E-05	7.19E-04
6	2110	F3 vs. L3	-	Development	Cell division	DNA damage checkpoint	GO:0000077	P	8	5.86E-04	6.96E-03
6	2111	F3 vs. L3	-	Development	Cell division	DNA integrity checkpoint	GO:0031570	P	15	6.92E-05	1.11E-03
6	2112	F3 vs. L3	-	Development	Cell division	DNA packaging	GO:0006323	P	63	5.09E-04	6.18E-03
6	2113	F3 vs. L3	-	Development	Cell division	DNA polymerase complex	GO:0042575	C	13	3.57E-03	2.05E-02

6	2114	F3 vs. L3	-	Development	Cell division	DNA recombination	GO:0006310	P	85	2.33E-07	7.66E-06
6	2115	F3 vs. L3	-	Development	Cell division	DNA repair	GO:0006281	P	189	7.06E-14	1.03E-11
6	2116	F3 vs. L3	-	Development	Cell division	DNA replication	GO:0006260	P	78	7.34E-11	5.02E-09
6	2117	F3 vs. L3	-	Development	Cell division	DNA replication factor A complex	GO:0005662	C	8	6.33E-03	3.46E-02
6	2118	F3 vs. L3	-	Development	Cell division	DNA replication factor C complex	GO:0005663	C	5	9.64E-03	4.89E-02
6	2119	F3 vs. L3	-	Development	Cell division	DNA replication initiation	GO:0006270	P	16	9.53E-05	1.50E-03
6	2120	F3 vs. L3	-	Development	Cell division	DNA replication origin binding	GO:0003688	F	13	2.67E-04	7.11E-03
6	2121	F3 vs. L3	-	Development	Cell division	DNA strand elongation	GO:0022616	P	13	5.46E-05	9.06E-04
6	2122	F3 vs. L3	-	Development	Cell division	DNA strand elongation involved in DNA replication	GO:0006271	P	13	5.46E-05	9.09E-04
6	2123	F3 vs. L3	-	Development	Cell division	DNA unwinding involved in DNA replication	GO:0006268	P	12	1.36E-03	1.40E-02
6	2124	F3 vs. L3	-	Development	Cell division	DNA-dependent DNA replication	GO:0006261	P	68	1.69E-10	1.09E-08
6	2125	F3 vs. L3	-	Development	Cell division	double-strand break repair	GO:0006302	P	76	1.27E-07	4.53E-06
6	2126	F3 vs. L3	-	Development	Cell division	double-strand break repair via homologous recombination	GO:0000724	P	53	7.01E-06	1.54E-04
6	2127	F3 vs. L3	-	Development	Cell division	homologous chromosome pairing at meiosis	GO:0007129	P	20	2.71E-03	2.44E-02
6	2128	F3 vs. L3	-	Development	Cell division	homologous chromosome segregation	GO:0045143	P	21	2.81E-03	2.52E-02
6	2129	F3 vs. L3	-	Development	Cell division	homologous recombination	GO:0035825	P	25	7.61E-04	8.52E-03
6	2130	F3 vs. L3	-	Development	Cell division	kinetochore	GO:0000776	C	13	3.42E-04	2.89E-03
6	2131	F3 vs. L3	-	Development	Cell division	meiosis I	GO:0007127	P	33	1.04E-04	1.61E-03
6	2132	F3 vs. L3	-	Development	Cell division	meiosis I cell cycle process	GO:0061982	P	36	1.09E-05	2.14E-04
6	2133	F3 vs. L3	-	Development	Cell division	meiotic cell cycle	GO:0051321	P	92	6.43E-06	1.43E-04
6	2134	F3 vs. L3	-	Development	Cell division	meiotic cell cycle process	GO:1903046	P	83	4.15E-05	7.14E-04
6	2135	F3 vs. L3	-	Development	Cell division	meiotic chromosome segregation	GO:0045132	P	31	1.40E-04	2.06E-03
6	2136	F3 vs. L3	-	Development	Cell division	meiotic nuclear division	GO:0140013	P	53	1.54E-05	2.92E-04
6	2137	F3 vs. L3	-	Development	Cell division	microtubule cytoskeleton organization involved in mitosis	GO:1902850	P	22	1.39E-04	2.07E-03
6	2138	F3 vs. L3	-	Development	Cell division	mismatch repair	GO:0006298	P	13	7.02E-04	8.03E-03
6	2139	F3 vs. L3	-	Development	Cell division	mitotic cell cycle	GO:0000278	P	163	3.38E-07	1.07E-05
6	2140	F3 vs. L3	-	Development	Cell division	mitotic cell cycle checkpoint	GO:0007093	P	19	1.75E-04	2.52E-03
6	2141	F3 vs. L3	-	Development	Cell division	mitotic cell cycle phase transition	GO:0044772	P	30	5.89E-07	1.73E-05
6	2142	F3 vs. L3	-	Development	Cell division	mitotic cell cycle process	GO:1903047	P	139	2.60E-08	1.09E-06
6	2143	F3 vs. L3	-	Development	Cell division	mitotic DNA integrity checkpoint	GO:0044774	P	8	1.78E-03	1.78E-02
6	2144	F3 vs. L3	-	Development	Cell division	mitotic nuclear division	GO:0140014	P	40	7.09E-04	8.10E-03
6	2145	F3 vs. L3	-	Development	Cell division	mitotic spindle	GO:0072686	C	14	4.03E-04	3.30E-03
6	2146	F3 vs. L3	-	Development	Cell division	mitotic spindle assembly	GO:0090307	P	7	7.73E-04	8.62E-03
6	2147	F3 vs. L3	-	Development	Cell division	mitotic spindle organization	GO:0007052	P	19	1.81E-03	1.78E-02
6	2148	F3 vs. L3	-	Development	Cell division	negative regulation of cell cycle	GO:0045786	P	45	1.59E-05	3.01E-04
6	2149	F3 vs. L3	-	Development	Cell division	negative regulation of cell cycle phase transition	GO:1901988	P	13	3.55E-04	4.52E-03
6	2150	F3 vs. L3	-	Development	Cell division	negative regulation of cell cycle process	GO:0010948	P	25	1.39E-03	1.42E-02
6	2151	F3 vs. L3	-	Development	Cell division	negative regulation of chromosome organization	GO:2001251	P	9	1.87E-03	1.82E-02
6	2152	F3 vs. L3	-	Development	Cell division	negative regulation of mitotic cell cycle	GO:0045930	P	25	1.62E-04	2.35E-03
6	2153	F3 vs. L3	-	Development	Cell division	negative regulation of mitotic cell cycle phase transition	GO:1901991	P	13	3.55E-04	4.53E-03
6	2154	F3 vs. L3	-	Development	Cell division	nuclear chromosome segregation	GO:0098813	P	56	1.57E-04	2.30E-03
6	2155	F3 vs. L3	-	Development	Cell division	nuclear division	GO:0000280	P	90	1.45E-08	6.61E-07
6	2156	F3 vs. L3	-	Development	Cell division	nuclear DNA replication	GO:0033260	P	10	8.04E-04	8.95E-03
6	2157	F3 vs. L3	-	Development	Cell division	nuclear origin of replication recognition complex	GO:0005664	C	4	4.82E-03	2.73E-02
6	2158	F3 vs. L3	-	Development	Cell division	nuclear replication fork	GO:0043596	C	20	6.50E-05	5.96E-04
6	2159	F3 vs. L3	-	Development	Cell division	nuclease activity	GO:0004518	F	122	1.76E-03	3.38E-02
6	2160	F3 vs. L3	-	Development	Cell division	nucleic acid metabolic process	GO:0090304	P	1012	0.00E+00	0.00E+00
6	2161	F3 vs. L3	-	Development	Cell division	nucleobase-containing compound metabolic process	GO:0006139	P	1273	0.00E+00	0.00E+00
6	2162	F3 vs. L3	-	Development	Cell division	nucleosome assembly	GO:0006334	P	33	9.54E-04	1.03E-02
6	2163	F3 vs. L3	-	Development	Cell division	origin recognition complex	GO:0000808	C	4	4.82E-03	2.71E-02
6	2164	F3 vs. L3	-	Development	Cell division	positive regulation of cell cycle	GO:0045787	P	29	4.73E-03	3.76E-02
6	2165	F3 vs. L3	-	Development	Cell division	preprophase band	GO:0009574	C	14	1.19E-03	7.74E-03
6	2166	F3 vs. L3	-	Development	Cell division	preprophase band assembly	GO:0000913	P	6	4.08E-03	3.36E-02
6	2167	F3 vs. L3	-	Development	Cell division	reciprocal homologous recombination	GO:0140527	P	25	7.61E-04	8.55E-03
6	2168	F3 vs. L3	-	Development	Cell division	reciprocal meiotic recombination	GO:0007131	P	25	7.61E-04	8.54E-03
6	2169	F3 vs. L3	-	Development	Cell division	recombinational repair	GO:0000725	P	56	1.02E-05	2.03E-04
6	2170	F3 vs. L3	-	Development	Cell division	regulation of cell cycle	GO:0051726	P	146	9.48E-14	1.31E-11
6	2171	F3 vs. L3	-	Development	Cell division	regulation of cell cycle G2/M phase transition	GO:1902749	P	16	1.11E-03	1.18E-02
6	2172	F3 vs. L3	-	Development	Cell division	regulation of cell cycle phase transition	GO:1901987	P	33	6.52E-07	1.85E-05
6	2173	F3 vs. L3	-	Development	Cell division	regulation of cell cycle process	GO:0010564	P	73	1.41E-09	7.68E-08
6	2174	F3 vs. L3	-	Development	Cell division	regulation of cell division	GO:0051302	P	42	1.46E-04	2.15E-03
6	2175	F3 vs. L3	-	Development	Cell division	regulation of chromatin organization	GO:1902275	P	43	4.32E-03	3.51E-02
6	2176	F3 vs. L3	-	Development	Cell division	regulation of chromosome organization	GO:0033044	P	40	6.83E-06	1.51E-04
6	2177	F3 vs. L3	-	Development	Cell division	regulation of chromosome segregation	GO:0051983	P	16	2.15E-04	2.96E-03

6	2178	F3 vs. L3	-	Development	Cell division	regulation of chromosome separation	GO:1905818	P	15	5.08E-04	6.18E-03
6	2179	F3 vs. L3	-	Development	Cell division	regulation of cyclin-dependent protein kinase activity	GO:1904029	P	32	4.07E-03	3.36E-02
6	2180	F3 vs. L3	-	Development	Cell division	regulation of DNA metabolic process	GO:0051052	P	53	3.02E-06	7.42E-05
6	2181	F3 vs. L3	-	Development	Cell division	regulation of DNA replication	GO:0006275	P	34	5.30E-04	6.39E-03
6	2182	F3 vs. L3	-	Development	Cell division	regulation of DNA-dependent DNA replication	GO:0090329	P	28	3.84E-03	3.19E-02
6	2183	F3 vs. L3	-	Development	Cell division	regulation of G2/M transition of mitotic cell cycle	GO:0010389	P	15	1.26E-03	1.31E-02
6	2184	F3 vs. L3	-	Development	Cell division	regulation of helicase activity	GO:0051095	P	6	3.00E-03	2.65E-02
6	2185	F3 vs. L3	-	Development	Cell division	regulation of metaphase/anaphase transition of cell cycle	GO:1902099	P	15	5.08E-04	6.19E-03
6	2186	F3 vs. L3	-	Development	Cell division	regulation of mitotic cell cycle	GO:0007346	P	52	6.19E-07	1.81E-05
6	2187	F3 vs. L3	-	Development	Cell division	regulation of mitotic cell cycle phase transition	GO:1901990	P	31	1.50E-06	3.87E-05
6	2188	F3 vs. L3	-	Development	Cell division	regulation of mitotic metaphase/anaphase transition	GO:0030071	P	15	5.08E-04	6.21E-03
6	2189	F3 vs. L3	-	Development	Cell division	regulation of mitotic nuclear division	GO:0007088	P	18	3.24E-03	2.80E-02
6	2190	F3 vs. L3	-	Development	Cell division	regulation of mitotic sister chromatid separation	GO:0010965	P	15	5.08E-04	6.22E-03
6	2191	F3 vs. L3	-	Development	Cell division	regulation of mitotic spindle organization	GO:0060236	P	5	4.36E-03	3.52E-02
6	2192	F3 vs. L3	-	Development	Cell division	regulation of nuclear division	GO:0051783	P	28	5.95E-04	7.05E-03
6	2193	F3 vs. L3	-	Development	Cell division	regulation of sister chromatid segregation	GO:0033045	P	16	2.15E-04	2.95E-03
6	2194	F3 vs. L3	-	Development	Cell division	regulation of spindle organization	GO:0090224	P	5	4.36E-03	3.52E-02
6	2195	F3 vs. L3	-	Development	Cell division	replication fork	GO:0005657	C	32	4.67E-08	7.58E-07
6	2196	F3 vs. L3	-	Development	Cell division	replisome	GO:0030894	C	17	5.33E-05	4.94E-04
6	2197	F3 vs. L3	-	Development	Cell division	RNA metabolic process	GO:0016070	P	774	0.00E+00	0.00E+00
6	2198	F3 vs. L3	-	Development	Cell division	single-stranded DNA binding	GO:0003697	F	52	1.12E-06	7.00E-05
6	2199	F3 vs. L3	-	Development	Cell division	site of double-strand break	GO:0035861	C	6	6.66E-03	3.60E-02
6	2200	F3 vs. L3	-	Development	Cell division	spindle	GO:0005819	C	51	5.14E-10	1.09E-08
6	2201	F3 vs. L3	-	Development	Cell division	spindle assembly	GO:0051225	P	24	8.63E-06	1.79E-04
6	2202	F3 vs. L3	-	Development	Cell division	spindle microtubule	GO:0005876	C	13	5.57E-04	4.17E-03
6	2203	F3 vs. L3	-	Development	Cell division	spindle organization	GO:0007051	P	37	6.84E-06	1.51E-04
6	2204	F3 vs. L3	-	Development	Cell division	telomere maintenance	GO:0000723	P	20	2.49E-03	2.28E-02
6	2205	F3 vs. L3	-	Development	Cell division	telomere organization	GO:0032200	P	20	2.49E-03	2.28E-02
6	2206	F3 vs. L3	-	Development	Development	anatomical structure homeostasis	GO:0060249	P	23	1.36E-03	1.40E-02
6	2207	F3 vs. L3	-	Development	Development	cellular component assembly	GO:0022607	P	536	2.25E-08	9.52E-07
6	2208	F3 vs. L3	-	Development	Development	cellular component biogenesis	GO:0044085	P	840	9.43E-07	2.53E-05
6	2209	F3 vs. L3	-	Development	Development	cellular component organization	GO:0016043	P	1497	1.67E-03	1.68E-02
6	2210	F3 vs. L3	-	Development	Development	cellular component organization or biogenesis	GO:0071840	P	1752	3.21E-04	4.17E-03
6	2211	F3 vs. L3	-	Development	Development	chromatin	GO:0000785	C	182	6.16E-03	3.41E-02
6	2212	F3 vs. L3	-	Development	Development	chromatin assembly or disassembly	GO:0006333	P	61	1.08E-05	2.14E-04
6	2213	F3 vs. L3	-	Development	Development	chromatin organization	GO:0006325	P	210	3.29E-12	3.20E-10
6	2214	F3 vs. L3	-	Development	Development	chromosomal region	GO:0098687	C	37	1.72E-07	2.58E-06
6	2215	F3 vs. L3	-	Development	Development	chromosome organization	GO:0051276	P	307	0.00E+00	0.00E+00
6	2216	F3 vs. L3	-	Development	Development	chromosome, telomeric region	GO:0000781	C	8	3.02E-03	1.81E-02
6	2217	F3 vs. L3	-	Development	Development	cortical cytoskeleton organization	GO:0030865	P	43	5.25E-04	6.36E-03
6	2218	F3 vs. L3	-	Development	Development	cytoplasmic microtubule	GO:0005881	C	30	9.45E-03	4.81E-02
6	2219	F3 vs. L3	-	Development	Development	cytoskeleton	GO:0005856	C	195	2.18E-03	1.33E-02
6	2220	F3 vs. L3	-	Development	Development	embryo development	GO:0009790	P	348	4.51E-04	5.55E-03
6	2221	F3 vs. L3	-	Development	Development	embryo development ending in seed dormancy	GO:0009793	P	332	3.50E-04	4.48E-03
6	2222	F3 vs. L3	-	Development	Development	heterochromatin	GO:0000792	C	9	7.71E-03	4.10E-02
6	2223	F3 vs. L3	-	Development	Development	heterochromatin assembly	GO:0031507	P	19	1.02E-03	1.10E-02
6	2224	F3 vs. L3	-	Development	Development	heterochromatin organization	GO:0070828	P	23	2.41E-04	3.25E-03
6	2225	F3 vs. L3	-	Development	Development	intracellular non-membrane-bounded organelle	GO:0043232	C	1100	0.00E+00	0.00E+00
6	2226	F3 vs. L3	-	Development	Development	intracellular organelle lumen	GO:0070013	C	849	0.00E+00	0.00E+00
6	2227	F3 vs. L3	-	Development	Development	kinetochore microtubule	GO:0005828	C	4	3.30E-03	1.91E-02
6	2228	F3 vs. L3	-	Development	Development	maintenance of floral organ identity	GO:0048497	P	8	2.93E-03	2.59E-02
6	2229	F3 vs. L3	-	Development	Development	maintenance of plant organ identity	GO:0090700	P	8	2.93E-03	2.60E-02
6	2230	F3 vs. L3	-	Development	Development	membrane-enclosed lumen	GO:0031974	C	849	0.00E+00	0.00E+00
6	2231	F3 vs. L3	-	Development	Development	meristem development	GO:0048507	P	132	2.16E-03	2.04E-02
6	2232	F3 vs. L3	-	Development	Development	microtubule	GO:0005874	C	96	5.02E-06	6.03E-05
6	2233	F3 vs. L3	-	Development	Development	microtubule associated complex	GO:0005875	C	47	1.89E-05	2.04E-04
6	2234	F3 vs. L3	-	Development	Development	microtubule cytoskeleton	GO:0015630	C	143	1.78E-08	2.99E-07
6	2235	F3 vs. L3	-	Development	Development	microtubule cytoskeleton organization	GO:0000226	P	110	8.52E-06	1.78E-04
6	2236	F3 vs. L3	-	Development	Development	microtubule-based process	GO:0007017	P	148	1.98E-07	6.64E-06
6	2237	F3 vs. L3	-	Development	Development	non-membrane-bounded organelle	GO:0043228	C	1100	0.00E+00	0.00E+00
6	2238	F3 vs. L3	-	Development	Development	nuclear body	GO:0016604	C	71	2.83E-05	2.90E-04
6	2239	F3 vs. L3	-	Development	Development	nuclear chromosome	GO:0000228	C	192	2.43E-06	3.07E-05
6	2240	F3 vs. L3	-	Development	Development	nuclear envelope	GO:0005635	C	64	2.02E-03	1.25E-02
6	2241	F3 vs. L3	-	Development	Development	nuclear lumen	GO:0031981	C	741	0.00E+00	0.00E+00

6	2242	F3 vs. L3	-	Development	Development	nuclear pore	GO:0005643	C	28	1.60E-03	1.02E-02
6	2243	F3 vs. L3	-	Development	Development	nuclear pore outer ring	GO:0031080	C	5	8.88E-03	4.60E-02
6	2244	F3 vs. L3	-	Development	Development	nuclear replisome	GO:0043601	C	17	5.33E-05	4.99E-04
6	2245	F3 vs. L3	-	Development	Development	nucleoplasm	GO:0005654	C	277	1.79E-14	6.96E-13
6	2246	F3 vs. L3	-	Development	Development	nucleus	GO:0005634	C	5394	0.00E+00	0.00E+00
6	2247	F3 vs. L3	-	Development	Development	organelle assembly	GO:0070925	P	119	3.13E-08	1.26E-06
6	2248	F3 vs. L3	-	Development	Development	organelle fission	GO:0048285	P	118	3.19E-07	1.02E-05
6	2249	F3 vs. L3	-	Development	Development	organelle lumen	GO:0043233	C	849	0.00E+00	0.00E+00
6	2250	F3 vs. L3	-	Development	Development	organelle organization	GO:0006996	P	1028	1.33E-08	6.18E-07
6	2251	F3 vs. L3	-	Development	Development	phragmoplast	GO:0009524	C	45	2.17E-05	2.27E-04
6	2252	F3 vs. L3	-	Development	Development	phyllotactic patterning	GO:0060771	P	4	5.07E-03	4.00E-02
6	2253	F3 vs. L3	-	Development	Development	polymeric cytoskeletal fiber	GO:0099513	C	104	1.93E-04	1.67E-03
6	2254	F3 vs. L3	-	Development	Development	radial pattern formation	GO:0009956	P	16	2.68E-03	2.43E-02
6	2255	F3 vs. L3	-	Development	Development	structural constituent of nuclear pore	GO:0017056	F	14	8.06E-05	2.70E-03
6	2256	F3 vs. L3	-	Development	Development	structural molecule activity	GO:0005198	F	273	4.61E-09	7.37E-07
6	2257	F3 vs. L3	-	Metabolism	Bioenergetics	DNA-dependent ATPase activity	GO:0008094	F	63	3.16E-06	1.85E-04
6	2258	F3 vs. L3	-	Metabolism	Bioenergetics	positive regulation of ATPase activity	GO:0032781	P	10	5.17E-03	4.07E-02
6	2259	F3 vs. L3	-	Metabolism	Bioenergetics	regulation of ATPase activity	GO:0043462	P	13	1.17E-03	1.23E-02
6	2260	F3 vs. L3	-	Metabolism	Catabolism	Cul4-RING E3 ubiquitin ligase complex	GO:0080008	C	95	2.83E-05	2.93E-04
6	2261	F3 vs. L3	-	Metabolism	Catabolism	cullin-RING ubiquitin ligase complex	GO:0031461	C	163	6.52E-08	1.02E-06
6	2262	F3 vs. L3	-	Metabolism	Catabolism	endopeptidase complex	GO:1905369	C	44	9.77E-03	4.93E-02
6	2263	F3 vs. L3	-	Metabolism	Catabolism	modification-dependent macromolecule catabolic process	GO:0043632	P	292	2.12E-04	2.94E-03
6	2264	F3 vs. L3	-	Metabolism	Catabolism	modification-dependent protein catabolic process	GO:0019941	P	286	2.36E-04	3.19E-03
6	2265	F3 vs. L3	-	Metabolism	Catabolism	peptidase complex	GO:1905368	C	55	4.36E-04	3.39E-03
6	2266	F3 vs. L3	-	Metabolism	Catabolism	proteasomal protein catabolic process	GO:0010498	P	160	2.54E-03	2.32E-02
6	2267	F3 vs. L3	-	Metabolism	Catabolism	proteasome-mediated ubiquitin-dependent protein catabolic process	GO:0043161	P	153	3.73E-03	3.11E-02
6	2268	F3 vs. L3	-	Metabolism	Catabolism	protein-containing complex	GO:0032991	C	1865	0.00E+00	0.00E+00
6	2269	F3 vs. L3	-	Metabolism	Catabolism	ubiquitin ligase complex	GO:0000151	C	200	5.96E-08	9.51E-07
6	2270	F3 vs. L3	-	Metabolism	Catabolism	ubiquitin-dependent protein catabolic process	GO:0006511	P	283	2.15E-04	2.96E-03
6	2271	F3 vs. L3	-	Metabolism	Metabolism	acetyltransferase complex	GO:1902493	C	30	4.16E-04	3.37E-03
6	2272	F3 vs. L3	-	Metabolism	Metabolism	amide biosynthetic process	GO:0043604	P	370	7.02E-06	1.53E-04
6	2273	F3 vs. L3	-	Metabolism	Metabolism	catalytic activity, acting on DNA	GO:0140097	F	116	1.01E-07	9.98E-06
6	2274	F3 vs. L3	-	Metabolism	Metabolism	catalytic complex	GO:1902494	C	732	3.33E-16	1.71E-14
6	2275	F3 vs. L3	-	Metabolism	Metabolism	cellular aromatic compound metabolic process	GO:0006725	P	1545	1.38E-08	6.37E-07
6	2276	F3 vs. L3	-	Metabolism	Metabolism	cellular macromolecule biosynthetic process	GO:0034645	P	784	1.91E-10	1.21E-08
6	2277	F3 vs. L3	-	Metabolism	Metabolism	cellular macromolecule metabolic process	GO:0044260	P	2574	5.12E-11	3.73E-09
6	2278	F3 vs. L3	-	Metabolism	Metabolism	cellular nitrogen compound biosynthetic process	GO:0044271	P	777	5.45E-03	4.24E-02
6	2279	F3 vs. L3	-	Metabolism	Metabolism	cellular nitrogen compound metabolic process	GO:0034641	P	1758	7.77E-16	1.79E-13
6	2280	F3 vs. L3	-	Metabolism	Metabolism	cellular protein metabolic process	GO:0044267	P	1954	6.48E-07	1.86E-05
6	2281	F3 vs. L3	-	Metabolism	Metabolism	cellular protein modification process	GO:0006464	P	1413	7.19E-04	8.15E-03
6	2282	F3 vs. L3	-	Metabolism	Metabolism	cellular protein-containing complex assembly	GO:0034622	P	307	7.23E-07	2.01E-05
6	2283	F3 vs. L3	-	Metabolism	Metabolism	chromatin binding	GO:0003682	F	108	1.10E-03	2.31E-02
6	2284	F3 vs. L3	-	Metabolism	Metabolism	DNA binding	GO:0003677	F	848	1.18E-10	2.41E-08
6	2285	F3 vs. L3	-	Metabolism	Metabolism	DNA metabolic process	GO:0006259	P	260	0.00E+00	0.00E+00
6	2286	F3 vs. L3	-	Metabolism	Metabolism	DNA secondary structure binding	GO:0000217	F	25	1.89E-05	7.78E-04
6	2287	F3 vs. L3	-	Metabolism	Metabolism	double-stranded DNA binding	GO:0003690	F	420	5.35E-04	1.33E-02
6	2288	F3 vs. L3	-	Metabolism	Metabolism	heterocycle metabolic process	GO:0046483	P	1462	1.68E-12	1.69E-10
6	2289	F3 vs. L3	-	Metabolism	Metabolism	heterocyclic compound binding	GO:1901363	F	2711	1.83E-03	3.39E-02
6	2290	F3 vs. L3	-	Metabolism	Metabolism	lysine N-methyltransferase activity	GO:0016278	F	26	2.41E-03	4.29E-02
6	2291	F3 vs. L3	-	Metabolism	Metabolism	macromolecule biosynthetic process	GO:0009059	P	824	3.90E-08	1.54E-06
6	2292	F3 vs. L3	-	Metabolism	Metabolism	macromolecule metabolic process	GO:0043170	P	3227	6.66E-16	1.61E-13
6	2293	F3 vs. L3	-	Metabolism	Metabolism	methyltransferase activity	GO:0008168	F	149	1.63E-03	3.18E-02
6	2294	F3 vs. L3	-	Metabolism	Metabolism	microtubule binding	GO:0008017	F	95	1.10E-04	3.32E-03
6	2295	F3 vs. L3	-	Metabolism	Metabolism	microtubule polymerization or depolymerization	GO:0031109	P	12	3.25E-03	2.80E-02
6	2296	F3 vs. L3	-	Metabolism	Metabolism	nitrogen compound metabolic process	GO:0006807	P	3646	5.41E-06	1.24E-04
6	2297	F3 vs. L3	-	Metabolism	Metabolism	N-methyltransferase activity	GO:0008170	F	54	1.57E-04	4.61E-03
6	2298	F3 vs. L3	-	Metabolism	Metabolism	nucleic acid binding	GO:0003676	F	1881	8.10E-15	3.33E-12
6	2299	F3 vs. L3	-	Metabolism	Metabolism	organic cyclic compound binding	GO:0097159	F	2724	1.45E-03	2.89E-02
6	2300	F3 vs. L3	-	Metabolism	Metabolism	organic cyclic compound metabolic process	GO:1901360	P	1608	3.92E-07	1.21E-05
6	2301	F3 vs. L3	-	Metabolism	Metabolism	peptide metabolic process	GO:0006518	P	365	7.26E-06	1.57E-04
6	2302	F3 vs. L3	-	Metabolism	Metabolism	protein metabolic process	GO:0019538	P	2049	6.16E-06	1.38E-04
6	2303	F3 vs. L3	-	Metabolism	Metabolism	pseudouridine synthesis	GO:0001522	P	15	4.96E-04	6.08E-03
6	2304	F3 vs. L3	-	Metabolism	Metabolism	S-adenosylmethionine-dependent methyltransferase activity	GO:0008757	F	118	2.61E-03	4.56E-02

6	2305	F3 vs. L3	-	Metabolism	Metabolism	Sm-like protein family complex	GO:0120114	C	59	3.25E-10	7.19E-09
6	2306	F3 vs. L3	-	Metabolism	Metabolism	supramolecular complex	GO:0099080	C	168	4.60E-06	5.60E-05
6	2307	F3 vs. L3	-	Metabolism	Metabolism	supramolecular fiber	GO:0099512	C	104	1.93E-04	1.66E-03
6	2308	F3 vs. L3	-	Metabolism	Metabolism	supramolecular polymer	GO:0099081	C	104	1.93E-04	1.69E-03
6	2309	F3 vs. L3	-	Metabolism	Metabolism	transferase activity, transferring one-carbon groups	GO:0016741	F	161	1.81E-03	3.38E-02
6	2310	F3 vs. L3	-	Metabolism	Metabolism	transferase complex	GO:1990234	C	437	0.00E+00	0.00E+00
6	2311	F3 vs. L3	-	Metabolism	Metabolism	transferase complex, transferring phosphorus-containing groups	GO:0061695	C	140	1.46E-12	4.75E-11
6	2312	F3 vs. L3	-	Metabolism	Metabolism	tubulin binding	GO:0015631	F	101	3.64E-05	1.41E-03
6	2313	F3 vs. L3	-	Metabolism	Transporters	kinesin complex	GO:0005871	C	22	8.18E-03	4.32E-02
6	2314	F3 vs. L3	-	Metabolism	Transporters	nuclear export	GO:0051168	P	39	2.74E-03	2.47E-02
6	2315	F3 vs. L3	-	Metabolism	Transporters	nuclear microtubule	GO:0005880	C	9	6.03E-04	4.48E-03
6	2316	F3 vs. L3	-	Metabolism	Transporters	nuclear transport	GO:0051169	P	66	2.02E-03	1.94E-02
6	2317	F3 vs. L3	-	Metabolism	Transporters	nucleic acid transport	GO:0050657	P	41	1.11E-03	1.18E-02
6	2318	F3 vs. L3	-	Metabolism	Transporters	nucleocytoplasmic transport	GO:0006913	P	66	2.02E-03	1.94E-02
6	2319	F3 vs. L3	-	Metabolism	Transporters	RNA transport	GO:0050658	P	41	1.11E-03	1.18E-02
6	2320	F3 vs. L3	-	Regulation	Protein modification	internal peptidyl-lysine acetylation	GO:0018393	P	25	3.47E-03	2.94E-02
6	2321	F3 vs. L3	-	Regulation	Protein modification	internal protein amino acid acetylation	GO:0006475	P	25	3.47E-03	2.95E-02
6	2322	F3 vs. L3	-	Regulation	Protein modification	methylation	GO:0032259	P	136	2.11E-07	7.00E-06
6	2323	F3 vs. L3	-	Regulation	Protein modification	oligosaccharyltransferase complex	GO:0008250	C	9	4.95E-03	2.77E-02
6	2324	F3 vs. L3	-	Regulation	Protein modification	peptidyl-lysine acetylation	GO:0018394	P	25	3.47E-03	2.95E-02
6	2325	F3 vs. L3	-	Regulation	Protein modification	protein alkylation	GO:0008213	P	52	7.37E-06	1.58E-04
6	2326	F3 vs. L3	-	Regulation	Protein modification	protein heterodimerization activity	GO:0046982	F	79	3.23E-06	1.82E-04
6	2327	F3 vs. L3	-	Regulation	Protein modification	protein methylation	GO:0006479	P	52	7.37E-06	1.59E-04
6	2328	F3 vs. L3	-	Regulation	Protein modification	protein methyltransferase activity	GO:0008276	F	45	8.81E-05	2.85E-03
6	2329	F3 vs. L3	-	Regulation	Protein modification	protein-lysine N-methyltransferase activity	GO:0016279	F	26	2.41E-03	4.31E-02
6	2330	F3 vs. L3	-	Regulation	Protein modification	regulation of protein metabolic process	GO:0051246	P	237	8.27E-05	1.32E-03
6	2331	F3 vs. L3	-	Regulation	Protein modification	peptidyl-amino acid modification	GO:0018193	P	231	2.19E-03	2.06E-02
6	2332	F3 vs. L3	-	Regulation	Protein modification	peptidyl-lysine modification	GO:0018205	P	80	7.84E-07	2.16E-05
6	2333	F3 vs. L3	-	Regulation	Protein modification	positive regulation of protein modification process	GO:0031401	P	38	8.36E-04	9.22E-03
6	2334	F3 vs. L3	-	Regulation	Protein modification	protein modification by small protein conjugation or removal	GO:0070647	P	420	1.38E-03	1.42E-02
6	2335	F3 vs. L3	-	Regulation	Protein modification	protein modification process	GO:0036211	P	1413	7.19E-04	8.16E-03
6	2336	F3 vs. L3	-	Regulation	Protein modification	protein-containing complex assembly	GO:0065003	P	344	5.97E-06	1.34E-04
6	2337	F3 vs. L3	-	Regulation	Protein modification	protein-containing complex localization	GO:0031503	P	37	4.38E-03	3.53E-02
6	2338	F3 vs. L3	-	Regulation	Protein modification	protein-containing complex subunit organization	GO:0043933	P	393	3.48E-05	6.07E-04
6	2339	F3 vs. L3	-	Regulation	Protein modification	regulation of protein modification process	GO:0031399	P	117	1.72E-04	2.49E-03
6	2340	F3 vs. L3	-	Regulation	Regulation	negative regulation of biological process	GO:0048519	P	701	7.16E-08	2.68E-06
6	2341	F3 vs. L3	-	Regulation	Regulation	negative regulation of biosynthetic process	GO:0009890	P	218	4.99E-05	8.39E-04
6	2342	F3 vs. L3	-	Regulation	Regulation	negative regulation of cellular biosynthetic process	GO:0031327	P	214	3.47E-05	6.06E-04
6	2343	F3 vs. L3	-	Regulation	Regulation	negative regulation of cellular macromolecule biosynthetic process	GO:2000113	P	200	7.54E-06	1.61E-04
6	2344	F3 vs. L3	-	Regulation	Regulation	negative regulation of cellular metabolic process	GO:0031324	P	287	9.84E-06	1.98E-04
6	2345	F3 vs. L3	-	Regulation	Regulation	negative regulation of cellular process	GO:0048523	P	463	3.16E-07	1.01E-05
6	2346	F3 vs. L3	-	Regulation	Regulation	negative regulation of DNA metabolic process	GO:0051053	P	17	7.38E-04	8.31E-03
6	2347	F3 vs. L3	-	Regulation	Regulation	negative regulation of macromolecule biosynthetic process	GO:0010558	P	202	1.04E-05	2.07E-04
6	2348	F3 vs. L3	-	Regulation	Regulation	negative regulation of macromolecule metabolic process	GO:0010605	P	372	8.64E-12	8.12E-10
6	2349	F3 vs. L3	-	Regulation	Regulation	negative regulation of metabolic process	GO:0009892	P	406	1.23E-09	6.81E-08
6	2350	F3 vs. L3	-	Regulation	Regulation	negative regulation of nitrogen compound metabolic process	GO:0051172	P	253	2.15E-07	7.08E-06
6	2351	F3 vs. L3	-	Regulation	Regulation	negative regulation of nucleobase-containing compound metabolic process	GO:0045934	P	178	1.85E-07	6.34E-06
6	2352	F3 vs. L3	-	Regulation	Regulation	positive regulation of macromolecule metabolic process	GO:0010604	P	396	3.72E-04	4.70E-03
6	2353	F3 vs. L3	-	Regulation	Regulation	regulation of biological process	GO:0050789	P	2947	1.94E-04	2.74E-03
6	2354	F3 vs. L3	-	Regulation	Regulation	regulation of biosynthetic process	GO:0009889	P	1270	8.61E-06	1.79E-04
6	2355	F3 vs. L3	-	Regulation	Regulation	regulation of cellular biosynthetic process	GO:0031326	P	1244	2.96E-06	7.29E-05
6	2356	F3 vs. L3	-	Regulation	Regulation	regulation of cellular macromolecule biosynthetic process	GO:2000112	P	1173	1.85E-08	8.00E-07
6	2357	F3 vs. L3	-	Regulation	Regulation	regulation of cellular metabolic process	GO:0031323	P	1546	8.35E-07	2.26E-05
6	2358	F3 vs. L3	-	Regulation	Regulation	regulation of cellular process	GO:0050794	P	2562	8.30E-04	9.21E-03
6	2359	F3 vs. L3	-	Regulation	Regulation	regulation of cellular protein metabolic process	GO:0032268	P	225	2.98E-04	3.89E-03
6	2360	F3 vs. L3	-	Regulation	Regulation	regulation of macromolecule biosynthetic process	GO:0010556	P	1181	1.57E-08	6.98E-07
6	2361	F3 vs. L3	-	Regulation	Regulation	regulation of macromolecule metabolic process	GO:0060255	P	1560	6.66E-15	1.12E-12
6	2362	F3 vs. L3	-	Regulation	Regulation	regulation of meristem structural organization	GO:0009934	P	17	3.53E-03	2.98E-02
6	2363	F3 vs. L3	-	Regulation	Regulation	regulation of metabolic process	GO:0019222	P	1745	1.30E-09	7.14E-08
6	2364	F3 vs. L3	-	Regulation	Regulation	regulation of nitrogen compound metabolic process	GO:0051171	P	1393	2.18E-09	1.15E-07

6	2365	F3 vs. L3	-	Regulation	Regulation	regulation of nucleobase-containing compound metabolic process	GO:0019219	P	1160	2.35E-08	9.86E-07
6	2366	F3 vs. L3	-	Regulation	Regulation	regulation of organelle organization	GO:0033043	P	116	5.31E-05	8.86E-04
6	2367	F3 vs. L3	-	Regulation	Regulation	regulation of primary metabolic process	GO:0080090	P	1444	3.94E-08	1.54E-06
6	2368	F3 vs. L3	-	Regulation	Regulation	regulation of transferase activity	GO:0051338	P	61	3.07E-05	5.46E-04
6	2369	F3 vs. L3	-	Regulation	Transcription	chromatin organization involved in negative regulation of transcription	GO:0097549	P	44	1.93E-07	6.57E-06
6	2370	F3 vs. L3	-	Regulation	Transcription	chromatin organization involved in regulation of transcription	GO:0034401	P	44	1.93E-07	6.53E-06
6	2371	F3 vs. L3	-	Regulation	Transcription	chromatin remodeling	GO:0006338	P	51	4.23E-04	5.24E-03
6	2372	F3 vs. L3	-	Regulation	Transcription	chromatin silencing	GO:0006342	P	27	7.10E-06	1.54E-04
6	2373	F3 vs. L3	-	Regulation	Transcription	core mediator complex	GO:0070847	C	7	4.67E-03	2.66E-02
6	2374	F3 vs. L3	-	Regulation	Transcription	covalent chromatin modification	GO:0016569	P	101	9.52E-08	3.52E-06
6	2375	F3 vs. L3	-	Regulation	Transcription	DNA conformation change	GO:0071103	P	90	3.12E-05	5.52E-04
6	2376	F3 vs. L3	-	Regulation	Transcription	DNA modification	GO:0006304	P	30	2.69E-03	2.43E-02
6	2377	F3 vs. L3	-	Regulation	Transcription	DNA-binding transcription activator activity	GO:0001216	F	23	7.36E-04	1.69E-02
6	2378	F3 vs. L3	-	Regulation	Transcription	DNA-binding transcription activator activity, RNA polymerase II-specific	GO:0001228	F	19	3.77E-04	9.76E-03
6	2379	F3 vs. L3	-	Regulation	Transcription	DNA-directed RNA polymerase complex	GO:0000428	C	77	1.24E-06	1.68E-05
6	2380	F3 vs. L3	-	Regulation	Transcription	dsRNA processing	GO:0031050	P	43	6.25E-08	2.40E-06
6	2381	F3 vs. L3	-	Regulation	Transcription	gene expression	GO:0010467	P	987	0.00E+00	0.00E+00
6	2382	F3 vs. L3	-	Regulation	Transcription	gene silencing	GO:0016458	P	112	3.25E-11	2.53E-09
6	2383	F3 vs. L3	-	Regulation	Transcription	gene silencing by miRNA	GO:0035195	P	26	3.08E-03	2.70E-02
6	2384	F3 vs. L3	-	Regulation	Transcription	gene silencing by RNA	GO:0031047	P	75	3.92E-08	1.54E-06
6	2385	F3 vs. L3	-	Regulation	Transcription	gene silencing by RNA-directed DNA methylation	GO:0080188	P	13	1.88E-03	1.83E-02
6	2386	F3 vs. L3	-	Regulation	Transcription	heterochromatin assembly by small RNA	GO:0031048	P	9	1.32E-03	1.36E-02
6	2387	F3 vs. L3	-	Regulation	Transcription	histone acetylation	GO:0016573	P	25	3.47E-03	2.96E-02
6	2388	F3 vs. L3	-	Regulation	Transcription	histone acetyltransferase complex	GO:0000123	C	23	2.70E-03	1.63E-02
6	2389	F3 vs. L3	-	Regulation	Transcription	histone binding	GO:0042393	F	62	4.11E-05	1.58E-03
6	2390	F3 vs. L3	-	Regulation	Transcription	histone lysine methylation	GO:0034968	P	29	1.31E-04	1.96E-03
6	2391	F3 vs. L3	-	Regulation	Transcription	histone methylation	GO:0016571	P	36	2.72E-05	4.91E-04
6	2392	F3 vs. L3	-	Regulation	Transcription	histone methyltransferase activity	GO:0042054	F	25	1.09E-04	3.33E-03
6	2393	F3 vs. L3	-	Regulation	Transcription	histone modification	GO:0016570	P	97	1.16E-07	4.20E-06
6	2394	F3 vs. L3	-	Regulation	Transcription	histone-lysine N-methyltransferase activity	GO:0018024	F	18	2.10E-03	3.83E-02
6	2395	F3 vs. L3	-	Regulation	Transcription	macromolecule methylation	GO:0043414	P	107	3.81E-10	2.35E-08
6	2396	F3 vs. L3	-	Regulation	Transcription	macromolecule modification	GO:0043412	P	1596	1.44E-08	6.58E-07
6	2397	F3 vs. L3	-	Regulation	Transcription	maintenance of DNA repeat elements	GO:0043570	P	6	2.10E-03	2.00E-02
6	2398	F3 vs. L3	-	Regulation	Transcription	mediator complex	GO:0016592	C	33	1.15E-03	7.67E-03
6	2399	F3 vs. L3	-	Regulation	Transcription	methylated histone binding	GO:0035064	F	15	1.11E-03	2.29E-02
6	2400	F3 vs. L3	-	Regulation	Transcription	methylation-dependent protein binding	GO:0140034	F	15	1.11E-03	2.31E-02
6	2401	F3 vs. L3	-	Regulation	Transcription	methyltransferase complex	GO:0034708	C	22	1.27E-03	8.23E-03
6	2402	F3 vs. L3	-	Regulation	Transcription	minor groove of adenine-thymine-rich DNA binding	GO:0003680	F	18	8.46E-05	2.80E-03
6	2403	F3 vs. L3	-	Regulation	Transcription	mRNA polyadenylation	GO:0006378	P	16	4.38E-03	3.53E-02
6	2404	F3 vs. L3	-	Regulation	Transcription	mRNA processing	GO:0006397	P	191	2.32E-13	2.74E-11
6	2405	F3 vs. L3	-	Regulation	Transcription	negative regulation of gene expression	GO:0010629	P	302	4.88E-09	2.41E-07
6	2406	F3 vs. L3	-	Regulation	Transcription	negative regulation of gene expression, epigenetic	GO:0045814	P	28	3.26E-06	7.86E-05
6	2407	F3 vs. L3	-	Regulation	Transcription	negative regulation of nucleic acid-templated transcription	GO:1903507	P	154	3.21E-05	5.64E-04
6	2408	F3 vs. L3	-	Regulation	Transcription	negative regulation of RNA biosynthetic process	GO:1902679	P	154	3.21E-05	5.66E-04
6	2409	F3 vs. L3	-	Regulation	Transcription	negative regulation of transcription, DNA-templated	GO:0045892	P	152	2.24E-05	4.08E-04
6	2410	F3 vs. L3	-	Regulation	Transcription	nuclear DNA-directed RNA polymerase complex	GO:0055029	C	70	3.02E-07	4.33E-06
6	2411	F3 vs. L3	-	Regulation	Transcription	nuclear speck	GO:0016607	C	44	3.85E-04	3.20E-03
6	2412	F3 vs. L3	-	Regulation	Transcription	nucleic acid-templated transcription	GO:0097659	P	143	2.50E-04	3.34E-03
6	2413	F3 vs. L3	-	Regulation	Transcription	nucleolus	GO:0005730	C	339	2.22E-16	1.20E-14
6	2414	F3 vs. L3	-	Regulation	Transcription	nucleosome organization	GO:0034728	P	44	1.94E-03	1.86E-02
6	2415	F3 vs. L3	-	Regulation	Transcription	peptidyl-lysine methylation	GO:0018022	P	36	4.59E-05	7.75E-04
6	2416	F3 vs. L3	-	Regulation	Transcription	post-transcriptional gene silencing by RNA	GO:0035194	P	55	3.11E-06	7.60E-05
6	2417	F3 vs. L3	-	Regulation	Transcription	pre-transcriptional gene silencing by RNA	GO:0140458	P	13	1.88E-03	1.84E-02
6	2418	F3 vs. L3	-	Regulation	Transcription	production of siRNA involved in RNA interference	GO:0030422	P	29	4.24E-05	7.24E-04
6	2419	F3 vs. L3	-	Regulation	Transcription	production of small RNA involved in gene silencing by RNA	GO:0070918	P	43	6.25E-08	2.39E-06
6	2420	F3 vs. L3	-	Regulation	Transcription	production of ta-siRNAs involved in RNA interference	GO:0010267	P	14	6.04E-03	4.60E-02
6	2421	F3 vs. L3	-	Regulation	Transcription	protein acetylation	GO:0006473	P	33	2.87E-04	3.78E-03
6	2422	F3 vs. L3	-	Regulation	Transcription	protein acetyltransferase complex	GO:0031248	C	30	4.16E-04	3.35E-03
6	2423	F3 vs. L3	-	Regulation	Transcription	protein acylation	GO:0043543	P	50	2.82E-04	3.72E-03
6	2424	F3 vs. L3	-	Regulation	Transcription	protein-DNA complex	GO:0032993	C	40	3.99E-05	3.88E-04
6	2425	F3 vs. L3	-	Regulation	Transcription	protein-DNA complex assembly	GO:0065004	P	50	8.24E-06	1.73E-04

6	2426	F3 vs. L3	-	Regulation	Transcription	protein-DNA complex subunit organization	GO:0071824	P	62	1.79E-05	3.35E-04
6	2427	F3 vs. L3	-	Regulation	Transcription	regulation of gene expression	GO:0010468	P	1356	3.23E-11	2.56E-09
6	2428	F3 vs. L3	-	Regulation	Transcription	regulation of gene expression, epigenetic	GO:0040029	P	52	2.63E-06	6.55E-05
6	2429	F3 vs. L3	-	Regulation	Transcription	regulation of gene silencing	GO:0060968	P	34	2.53E-03	2.31E-02
6	2430	F3 vs. L3	-	Regulation	Transcription	regulation of nucleic acid-templated transcription	GO:1903506	P	1060	3.76E-07	1.17E-05
6	2431	F3 vs. L3	-	Regulation	Transcription	regulation of RNA biosynthetic process	GO:2001141	P	1061	4.43E-07	1.36E-05
6	2432	F3 vs. L3	-	Regulation	Transcription	regulation of transcription by RNA polymerase II	GO:0006357	P	237	2.10E-03	2.00E-02
6	2433	F3 vs. L3	-	Regulation	Transcription	regulation of transcription, DNA-templated	GO:0006355	P	1060	3.76E-07	1.18E-05
6	2434	F3 vs. L3	-	Regulation	Transcription	RNA 3'-end processing	GO:0031123	P	44	2.46E-06	6.18E-05
6	2435	F3 vs. L3	-	Regulation	Transcription	RNA binding	GO:0003723	F	1000	1.88E-05	7.95E-04
6	2436	F3 vs. L3	-	Regulation	Transcription	RNA biosynthetic process	GO:0032774	P	145	1.31E-04	1.96E-03
6	2437	F3 vs. L3	-	Regulation	Transcription	RNA methylation	GO:0001510	P	41	3.19E-06	7.72E-05
6	2438	F3 vs. L3	-	Regulation	Transcription	RNA modification	GO:0009451	P	163	6.33E-15	1.10E-12
6	2439	F3 vs. L3	-	Regulation	Transcription	RNA polymerase complex	GO:0030880	C	80	4.52E-07	6.37E-06
6	2440	F3 vs. L3	-	Regulation	Transcription	RNA polymerase II, core complex	GO:0005665	C	13	6.28E-03	3.45E-02
6	2441	F3 vs. L3	-	Regulation	Transcription	RNA polymerase II, holoenzyme	GO:0016591	C	42	8.26E-04	5.74E-03
6	2442	F3 vs. L3	-	Regulation	Transcription	RNA polymerase III complex	GO:0005666	C	18	7.41E-03	3.98E-02
6	2443	F3 vs. L3	-	Regulation	Transcription	RNA polymerase IV complex	GO:0000418	C	10	8.78E-03	4.57E-02
6	2444	F3 vs. L3	-	Regulation	Transcription	RNA polymerase V complex	GO:0000419	C	16	1.28E-05	1.42E-04
6	2445	F3 vs. L3	-	Regulation	Transcription	siRNA binding	GO:0035197	F	4	1.04E-03	2.22E-02
6	2446	F3 vs. L3	-	Regulation	Transcription	THO complex	GO:0000347	C	11	3.75E-04	3.14E-03
6	2447	F3 vs. L3	-	Regulation	Transcription	transcription coregulator activity	GO:0003712	F	81	7.53E-06	4.01E-04
6	2448	F3 vs. L3	-	Regulation	Transcription	transcription regulator complex	GO:0005667	C	73	1.07E-03	7.18E-03
6	2449	F3 vs. L3	-	Regulation	Transcription	transcription, DNA-templated	GO:0006351	P	143	2.50E-04	3.34E-03
6	2450	F3 vs. L3	-	Regulation	Translation	box C/D snoRNP complex	GO:0031428	C	10	1.90E-03	1.18E-02
6	2451	F3 vs. L3	-	Regulation	Translation	catalytic activity, acting on RNA	GO:0140098	F	278	4.90E-07	3.71E-05
6	2452	F3 vs. L3	-	Regulation	Translation	catalytic step 2 spliceosome	GO:0071013	C	39	6.22E-06	7.12E-05
6	2453	F3 vs. L3	-	Regulation	Translation	cytosolic large ribosomal subunit	GO:0022625	C	94	2.58E-08	4.25E-07
6	2454	F3 vs. L3	-	Regulation	Translation	cytosolic ribosome	GO:0022626	C	214	2.84E-05	2.88E-04
6	2455	F3 vs. L3	-	Regulation	Translation	cytosolic small ribosomal subunit	GO:0022627	C	68	2.94E-06	3.62E-05
6	2456	F3 vs. L3	-	Regulation	Translation	double-stranded RNA binding	GO:0003725	F	15	2.83E-03	4.88E-02
6	2457	F3 vs. L3	-	Regulation	Translation	establishment of RNA localization	GO:0051236	P	41	1.11E-03	1.18E-02
6	2458	F3 vs. L3	-	Regulation	Translation	large ribosomal subunit	GO:0015934	C	128	2.08E-10	5.07E-09
6	2459	F3 vs. L3	-	Regulation	Translation	maturation of LSU-rRNA	GO:0000470	P	32	9.36E-04	1.02E-02
6	2460	F3 vs. L3	-	Regulation	Translation	maturation of SSU-rRNA	GO:0030490	P	38	2.99E-05	5.35E-04
6	2461	F3 vs. L3	-	Regulation	Translation	maturation of SSU-rRNA from tricistronic rRNA transcript (SSU-rRNA, 5.8S rRNA, LSU-rRNA)	GO:0000462	P	26	3.77E-04	4.76E-03
6	2462	F3 vs. L3	-	Regulation	Translation	mitochondrial large ribosomal subunit	GO:0005762	C	19	1.18E-03	7.78E-03
6	2463	F3 vs. L3	-	Regulation	Translation	mitochondrial ribosome	GO:0005761	C	28	7.99E-04	5.59E-03
6	2464	F3 vs. L3	-	Regulation	Translation	mRNA 3'-end processing	GO:0031124	P	20	2.59E-03	2.36E-02
6	2465	F3 vs. L3	-	Regulation	Translation	mRNA cleavage factor complex	GO:0005849	C	11	4.69E-04	3.62E-03
6	2466	F3 vs. L3	-	Regulation	Translation	mRNA export from nucleus	GO:0006406	P	20	6.46E-04	7.48E-03
6	2467	F3 vs. L3	-	Regulation	Translation	mRNA metabolic process	GO:0016071	P	263	1.51E-11	1.35E-09
6	2468	F3 vs. L3	-	Regulation	Translation	mRNA splicing, via spliceosome	GO:0000398	P	138	3.96E-11	3.05E-09
6	2469	F3 vs. L3	-	Regulation	Translation	mRNA transport	GO:0051028	P	21	2.27E-04	3.11E-03
6	2470	F3 vs. L3	-	Regulation	Translation	mRNA-containing ribonucleoprotein complex export from nucleus	GO:0071427	P	20	6.46E-04	7.49E-03
6	2471	F3 vs. L3	-	Regulation	Translation	ncRNA 3'-end processing	GO:0043628	P	21	6.56E-04	7.58E-03
6	2472	F3 vs. L3	-	Regulation	Translation	ncRNA metabolic process	GO:0034660	P	290	2.53E-12	2.50E-10
6	2473	F3 vs. L3	-	Regulation	Translation	ncRNA processing	GO:0034470	P	245	1.17E-13	1.58E-11
6	2474	F3 vs. L3	-	Regulation	Translation	negative regulation of RNA metabolic process	GO:0051253	P	161	1.11E-05	2.16E-04
6	2475	F3 vs. L3	-	Regulation	Translation	organellar large ribosomal subunit	GO:0000315	C	30	1.67E-03	1.05E-02
6	2476	F3 vs. L3	-	Regulation	Translation	organellar ribosome	GO:0000313	C	46	7.07E-04	5.18E-03
6	2477	F3 vs. L3	-	Regulation	Translation	peptide biosynthetic process	GO:0043043	P	322	7.47E-09	3.63E-07
6	2478	F3 vs. L3	-	Regulation	Translation	posttranscriptional gene silencing	GO:0016441	P	60	3.15E-06	7.66E-05
6	2479	F3 vs. L3	-	Regulation	Translation	posttranscriptional regulation of gene expression	GO:0010608	P	152	2.17E-05	3.98E-04
6	2480	F3 vs. L3	-	Regulation	Translation	precatalytic spliceosome	GO:0071011	C	24	1.27E-06	1.69E-05
6	2481	F3 vs. L3	-	Regulation	Translation	preribosome	GO:0030684	C	62	6.06E-06	7.02E-05
6	2482	F3 vs. L3	-	Regulation	Translation	prespliceosome	GO:0071010	C	17	7.39E-04	5.29E-03
6	2483	F3 vs. L3	-	Regulation	Translation	production of miRNAs involved in gene silencing by miRNA	GO:0035196	P	19	9.23E-04	1.01E-02
6	2484	F3 vs. L3	-	Regulation	Translation	regulation of RNA metabolic process	GO:0051252	P	1129	5.77E-07	1.70E-05
6	2485	F3 vs. L3	-	Regulation	Translation	regulatory RNA binding	GO:0061980	F	6	2.91E-04	7.60E-03
6	2486	F3 vs. L3	-	Regulation	Translation	ribonucleoprotein complex	GO:1990904	C	443	0.00E+00	0.00E+00
6	2487	F3 vs. L3	-	Regulation	Translation	ribonucleoprotein complex assembly	GO:0022618	P	121	1.57E-08	6.93E-07

6	2488	F3 vs. L3	-	Regulation	Translation	ribonucleoprotein complex biogenesis	GO:0022613	P	295	1.69E-14	2.53E-12
6	2489	F3 vs. L3	-	Regulation	Translation	ribonucleoprotein complex subunit organization	GO:0071826	P	124	2.18E-08	9.30E-07
6	2490	F3 vs. L3	-	Regulation	Translation	ribosomal large subunit assembly	GO:0000027	P	34	7.13E-04	8.14E-03
6	2491	F3 vs. L3	-	Regulation	Translation	ribosomal large subunit biogenesis	GO:0042273	P	76	1.78E-07	6.12E-06
6	2492	F3 vs. L3	-	Regulation	Translation	ribosomal small subunit biogenesis	GO:0042274	P	62	5.71E-06	1.30E-04
6	2493	F3 vs. L3	-	Regulation	Translation	ribosomal subunit	GO:0044391	C	214	3.33E-16	1.54E-14
6	2494	F3 vs. L3	-	Regulation	Translation	ribosome	GO:0005840	C	274	2.00E-07	2.95E-06
6	2495	F3 vs. L3	-	Regulation	translation	ribosome assembly	GO:0042255	P	65	3.91E-04	4.90E-03
6	2496	F3 vs. L3	-	Regulation	Translation	ribosome biogenesis	GO:0042254	P	239	6.79E-11	4.70E-09
6	2497	F3 vs. L3	-	Regulation	Translation	RNA cap binding	GO:0000339	F	7	1.26E-03	2.55E-02
6	2498	F3 vs. L3	-	Regulation	Translation	RNA export from nucleus	GO:0006405	P	37	1.92E-03	1.85E-02
6	2499	F3 vs. L3	-	Regulation	Translation	RNA interference	GO:0016246	P	31	2.55E-04	3.38E-03
6	2500	F3 vs. L3	-	Regulation	Translation	RNA localization	GO:0006403	P	47	3.59E-04	4.57E-03
6	2501	F3 vs. L3	-	Regulation	Translation	RNA methyltransferase activity	GO:0008173	F	41	5.14E-06	2.85E-04
6	2502	F3 vs. L3	-	Regulation	Translation	RNA polyadenylation	GO:0043631	P	16	4.38E-03	3.52E-02
6	2503	F3 vs. L3	-	Regulation	Translation	RNA processing	GO:0006396	P	494	0.00E+00	0.00E+00
6	2504	F3 vs. L3	-	Regulation	Translation	RNA splicing	GO:0008380	P	174	6.60E-11	4.63E-09
6	2505	F3 vs. L3	-	Regulation	Translation	RNA splicing, via transesterification reactions	GO:0000375	P	150	2.10E-11	1.69E-09
6	2506	F3 vs. L3	-	Regulation	Translation	RNA splicing, via transesterification reactions with bulged adenosine as nucleophile	GO:0000377	P	150	2.10E-11	1.71E-09
6	2507	F3 vs. L3	-	Regulation	Translation	rRNA base methylation	GO:0070475	P	11	8.07E-04	8.96E-03
6	2508	F3 vs. L3	-	Regulation	Translation	rRNA metabolic process	GO:0016072	P	176	1.27E-06	3.29E-05
6	2509	F3 vs. L3	-	Regulation	Translation	rRNA methylation	GO:0031167	P	15	4.20E-04	5.23E-03
6	2510	F3 vs. L3	-	Regulation	Translation	rRNA modification	GO:0000154	P	24	5.74E-05	9.41E-04
6	2511	F3 vs. L3	-	Regulation	Translation	rRNA processing	GO:0006364	P	168	1.24E-06	3.23E-05
6	2512	F3 vs. L3	-	Regulation	Translation	small nuclear ribonucleoprotein complex	GO:0030532	C	54	3.23E-10	7.30E-09
6	2513	F3 vs. L3	-	Regulation	Translation	small nucleolar ribonucleoprotein complex	GO:0005732	C	21	1.31E-06	1.72E-05
6	2514	F3 vs. L3	-	Regulation	Translation	small ribosomal subunit	GO:0015935	C	86	2.48E-07	3.60E-06
6	2515	F3 vs. L3	-	Regulation	Translation	small-subunit processome	GO:0032040	C	37	4.65E-05	4.40E-04
6	2516	F3 vs. L3	-	Regulation	Translation	snoRNA binding	GO:0030515	F	20	6.18E-04	1.48E-02
6	2517	F3 vs. L3	-	Regulation	Translation	snRNA metabolic process	GO:0016073	P	17	6.16E-03	4.67E-02
6	2518	F3 vs. L3	-	Regulation	Translation	spliceosomal complex	GO:0005681	C	100	3.38E-09	6.20E-08
6	2519	F3 vs. L3	-	Regulation	Translation	spliceosomal snRNP assembly	GO:0000387	P	18	3.37E-04	4.34E-03
6	2520	F3 vs. L3	-	Regulation	Translation	spliceosomal snRNP complex	GO:0097525	C	54	3.23E-10	7.47E-09
6	2521	F3 vs. L3	-	Regulation	Translation	spliceosomal tri-snRNP complex	GO:0097526	C	32	9.33E-09	1.65E-07
6	2522	F3 vs. L3	-	Regulation	Translation	structural constituent of ribosome	GO:0003735	F	200	1.82E-12	4.02E-10
6	2523	F3 vs. L3	-	Regulation	Translation	translation	GO:0006412	P	317	2.72E-09	1.38E-07
6	2524	F3 vs. L3	-	Regulation	Translation	tRNA metabolic process	GO:0006399	P	104	9.67E-06	1.95E-04
6	2525	F3 vs. L3	-	Regulation	Translation	tRNA methylation	GO:0030488	P	21	5.81E-04	6.92E-03
6	2526	F3 vs. L3	-	Regulation	Translation	tRNA methyltransferase activity	GO:0008175	F	19	1.79E-03	3.40E-02
6	2527	F3 vs. L3	-	Regulation	Translation	tRNA modification	GO:0006400	P	53	1.32E-07	4.70E-06
6	2528	F3 vs. L3	-	Regulation	Translation	tRNA processing	GO:0008033	P	62	4.44E-09	2.22E-07
6	2529	F3 vs. L3	-	Regulation	Translation	U1 snRNP	GO:0005685	C	21	9.18E-06	1.04E-04
6	2530	F3 vs. L3	-	Regulation	Translation	U12-type spliceosomal complex	GO:0005689	C	17	3.39E-04	2.90E-03
6	2531	F3 vs. L3	-	Regulation	Translation	U2 snRNP	GO:0005686	C	21	5.10E-06	6.05E-05
6	2532	F3 vs. L3	-	Regulation	Translation	U2-type prespliceosome	GO:0071004	C	17	7.39E-04	5.25E-03
6	2533	F3 vs. L3	-	Regulation	Translation	U2-type spliceosomal complex	GO:0005684	C	38	1.42E-04	1.26E-03
6	2534	F3 vs. L3	-	Regulation	Translation	U4 snRNP	GO:0005687	C	11	4.59E-05	4.38E-04
6	2535	F3 vs. L3	-	Regulation	Translation	U4/U6 x U5 tri-snRNP complex	GO:0046540	C	24	9.48E-07	1.32E-05
6	2536	F3 vs. L3	-	Regulation	Translation	U5 snRNP	GO:0005682	C	15	2.14E-05	2.27E-04
6	2537	F3 vs. L3	-	Signaling and response	Signaling	kinase regulator activity	GO:0019207	F	55	3.20E-06	1.84E-04
6	2538	F3 vs. L3	-	Signaling and response	Signaling	protein kinase complex	GO:1902911	C	51	1.28E-05	1.43E-04
6	2539	F3 vs. L3	-	Signaling and response	Signaling	protein kinase regulator activity	GO:0019887	F	53	7.42E-06	4.03E-04
6	2540	F3 vs. L3	-	Signaling and response	Signaling	regulation of cyclin-dependent protein serine/threonine kinase activity	GO:0000079	P	32	4.07E-03	3.36E-02
6	2541	F3 vs. L3	-	Signaling and response	Signaling	regulation of kinase activity	GO:0043549	P	52	1.18E-04	1.79E-03
6	2542	F3 vs. L3	-	Signaling and response	Signaling	regulation of long-day photoperiodism, flowering	GO:0048586	P	17	6.35E-03	4.80E-02
6	2543	F3 vs. L3	-	Signaling and response	Signaling	regulation of phosphorylation	GO:0042325	P	68	3.97E-03	3.29E-02
6	2544	F3 vs. L3	-	Signaling and response	Signaling	regulation of protein kinase activity	GO:0045859	P	51	1.39E-04	2.07E-03
6	2545	F3 vs. L3	-	Signaling and response	Signaling	regulation of protein phosphorylation	GO:0001932	P	61	1.22E-03	1.27E-02
6	2546	F3 vs. L3	-	Signaling and response	Signaling	response to ionizing radiation	GO:0010212	P	15	3.01E-03	2.65E-02
6	2547	F3 vs. L3	-	Signaling and response	Signaling	serine/threonine protein kinase complex	GO:1902554	C	47	3.86E-05	3.79E-04
6	2548	F3 vs. L3	-	Signaling and response	Stress	cellular response to DNA damage stimulus	GO:0006974	P	209	4.88E-15	8.73E-13
6	2549	F3 vs. L3	-	Signaling and response	Stress	damaged DNA binding	GO:0003684	F	29	9.43E-04	2.12E-02

6	2550	F3 vs. L3	-	Signaling and response	Stress	site of DNA damage	GO:0090734	C	6	6.66E-03	3.62E-02
7	2551	F2 vs. M2	+	Development	Cell division	nucleobase-containing small molecule metabolic process	GO:0055086	P	237	3.93E-04	6.47E-03
7	2552	F2 vs. M2	+	Development	Cell division	nucleotide-sugar biosynthetic process	GO:0009226	P	30	3.53E-05	8.56E-04
7	2553	F2 vs. M2	+	Development	Cell division	nucleotide-sugar metabolic process	GO:0009225	P	34	5.85E-05	1.29E-03
7	2554	F2 vs. M2	+	Development	Cell division	pollen tube development	GO:0048868	P	96	9.07E-07	4.00E-05
7	2555	F2 vs. M2	+	Development	Cell division	pollen tube growth	GO:0009860	P	74	2.33E-06	8.83E-05
7	2556	F2 vs. M2	+	Development	Cell division	pollen tube guidance	GO:0010183	P	15	5.29E-03	4.54E-02
7	2557	F2 vs. M2	+	Development	Cell division	pollination	GO:0009856	P	142	1.49E-04	2.75E-03
7	2558	F2 vs. M2	+	Development	Cell division	syncytium formation	GO:0006949	P	12	5.63E-04	8.46E-03
7	2559	F2 vs. M2	+	Development	Development	actin cytoskeleton	GO:0015629	C	33	1.80E-04	1.58E-03
7	2560	F2 vs. M2	+	Development	Development	actin cytoskeleton organization	GO:0030036	P	58	1.06E-03	1.33E-02
7	2561	F2 vs. M2	+	Development	Development	actin filament	GO:0005884	C	5	1.89E-03	1.32E-02
7	2562	F2 vs. M2	+	Development	Development	apoplast	GO:0048046	C	169	0.00E+00	0.00E+00
7	2563	F2 vs. M2	+	Development	Development	bounding membrane of organelle	GO:0098588	C	553	6.45E-06	7.38E-05
7	2564	F2 vs. M2	+	Development	Development	cell development	GO:0048468	P	164	2.70E-04	4.60E-03
7	2565	F2 vs. M2	+	Development	Development	cell growth	GO:0016049	P	216	4.15E-06	1.40E-04
7	2566	F2 vs. M2	+	Development	Development	cell morphogenesis	GO:0000902	P	204	5.14E-03	4.45E-02
7	2567	F2 vs. M2	+	Development	Development	cell morphogenesis involved in differentiation	GO:0000904	P	118	1.32E-03	1.62E-02
7	2568	F2 vs. M2	+	Development	Development	cell periphery	GO:0071944	C	2123	2.51E-11	6.82E-10
7	2569	F2 vs. M2	+	Development	Development	cell pole	GO:0060187	C	9	5.74E-03	3.32E-02
7	2570	F2 vs. M2	+	Development	Development	cell projection	GO:0042995	C	55	4.18E-07	6.10E-06
7	2571	F2 vs. M2	+	Development	Development	cell tip	GO:0051286	C	9	5.74E-03	3.30E-02
7	2572	F2 vs. M2	+	Development	Development	cell tip growth	GO:0009932	P	86	1.28E-06	5.38E-05
7	2573	F2 vs. M2	+	Development	Development	cell wall	GO:0005618	C	325	6.82E-05	6.61E-04
7	2574	F2 vs. M2	+	Development	Development	cell wall biogenesis	GO:0042546	P	124	3.35E-04	5.60E-03
7	2575	F2 vs. M2	+	Development	Development	cell wall modification	GO:0042545	P	98	5.12E-03	4.45E-02
7	2576	F2 vs. M2	+	Development	Development	cell wall organization	GO:0071555	P	149	9.13E-06	2.74E-04
7	2577	F2 vs. M2	+	Development	Development	cell wall organization or biogenesis	GO:0071554	P	270	3.11E-08	2.21E-06
7	2578	F2 vs. M2	+	Development	Development	cell wall pectin metabolic process	GO:0052546	P	17	3.13E-04	5.28E-03
7	2579	F2 vs. M2	+	Development	Development	cytoplasm	GO:0005737	C	6876	5.41E-04	4.32E-03
7	2580	F2 vs. M2	+	Development	Development	cytosol	GO:0005829	C	1972	1.30E-06	1.65E-05
7	2581	F2 vs. M2	+	Development	Development	developmental cell growth	GO:0048588	P	101	7.33E-08	4.40E-06
7	2582	F2 vs. M2	+	Development	Development	developmental growth	GO:0048589	P	220	1.25E-05	3.54E-04
7	2583	F2 vs. M2	+	Development	Development	developmental growth involved in morphogenesis	GO:0060560	P	189	5.98E-06	1.94E-04
7	2584	F2 vs. M2	+	Development	Development	endomembrane system	GO:0012505	C	1212	5.41E-06	6.43E-05
7	2585	F2 vs. M2	+	Development	Development	envelope	GO:0031975	C	639	9.63E-13	2.86E-11
7	2586	F2 vs. M2	+	Development	Development	external encapsulating structure	GO:0030312	C	327	3.85E-05	3.93E-04
7	2587	F2 vs. M2	+	Development	Development	external encapsulating structure organization	GO:0045229	P	173	3.49E-06	1.23E-04
7	2588	F2 vs. M2	+	Development	Development	extracellular region	GO:0005576	C	1209	2.55E-15	8.98E-14
7	2589	F2 vs. M2	+	Development	Development	extracellular space	GO:0005615	C	48	7.46E-04	5.81E-03
7	2590	F2 vs. M2	+	Development	Development	Golgi apparatus subcompartment	GO:0098791	C	252	3.41E-09	7.03E-08
7	2591	F2 vs. M2	+	Development	Development	Golgi cisterna	GO:0031985	C	111	1.85E-03	1.30E-02
7	2592	F2 vs. M2	+	Development	Development	Golgi stack	GO:0005795	C	117	1.92E-03	1.33E-02
7	2593	F2 vs. M2	+	Development	Development	Golgi trans cisterna	GO:0000138	C	55	4.37E-04	3.61E-03
7	2594	F2 vs. M2	+	Development	Development	growing cell tip	GO:0035838	C	6	5.51E-03	3.25E-02
7	2595	F2 vs. M2	+	Development	Development	growth	GO:0040007	P	251	8.99E-06	2.71E-04
7	2596	F2 vs. M2	+	Development	Development	intracellular vesicle	GO:0097708	C	397	1.70E-07	2.70E-06
7	2597	F2 vs. M2	+	Development	Development	membrane	GO:0016020	C	2909	1.11E-16	4.39E-15
7	2598	F2 vs. M2	+	Development	Development	microbody	GO:0042579	C	162	1.12E-06	1.48E-05
7	2599	F2 vs. M2	+	Development	Development	multi-multicellular organism process	GO:0044706	P	142	1.49E-04	2.76E-03
7	2600	F2 vs. M2	+	Development	Development	organelle envelope	GO:0031967	C	639	9.63E-13	2.77E-11
7	2601	F2 vs. M2	+	Development	Development	organelle membrane	GO:0031090	C	843	1.64E-04	1.48E-03
7	2602	F2 vs. M2	+	Development	Development	organelle subcompartment	GO:0031984	C	693	0.00E+00	0.00E+00
7	2603	F2 vs. M2	+	Development	Development	plant-type cell wall biogenesis	GO:0009832	P	78	2.69E-04	4.61E-03
7	2604	F2 vs. M2	+	Development	Development	plant-type cell wall modification	GO:0009827	P	37	3.00E-03	3.02E-02
7	2605	F2 vs. M2	+	Development	Development	plant-type cell wall organization	GO:0009664	P	80	1.10E-05	3.15E-04
7	2606	F2 vs. M2	+	Development	Development	plant-type cell wall organization or biogenesis	GO:0071669	P	137	2.59E-07	1.30E-05
7	2607	F2 vs. M2	+	Development	Development	plant-type primary cell wall biogenesis	GO:0009833	P	18	8.10E-04	1.13E-02
7	2608	F2 vs. M2	+	Development	Development	plant-type vacuole	GO:0000325	C	96	9.70E-08	1.64E-06
7	2609	F2 vs. M2	+	Development	Development	plant-type vacuole membrane	GO:0009705	C	66	2.75E-05	2.87E-04
7	2610	F2 vs. M2	+	Development	Development	plasma membrane	GO:0005886	C	1864	2.93E-09	6.32E-08
7	2611	F2 vs. M2	+	Development	Development	plasma membrane bounded cell projection	GO:0120025	C	49	4.19E-09	8.47E-08
7	2612	F2 vs. M2	+	Development	Development	pollen tube	GO:0090406	C	41	3.14E-08	6.09E-07
7	2613	F2 vs. M2	+	Development	Development	pollen tube tip	GO:0090404	C	6	5.51E-03	3.27E-02

7	2614	F2 vs. M2	+	Development	Development	root epidermal cell differentiation	GO:0010053	P	68	4.35E-03	3.95E-02
7	2615	F2 vs. M2	+	Development	Development	root hair elongation	GO:0048767	P	32	3.16E-03	3.14E-02
7	2616	F2 vs. M2	+	Development	Development	stromule	GO:0010319	C	32	1.46E-10	3.76E-09
7	2617	F2 vs. M2	+	Development	Development	unidimensional cell growth	GO:0009826	P	165	1.18E-04	2.32E-03
7	2618	F2 vs. M2	+	Development	Development	vacuolar membrane	GO:0005774	C	355	4.18E-07	6.02E-06
7	2619	F2 vs. M2	+	Development	Development	vacuole	GO:0005773	C	657	4.54E-13	1.44E-11
7	2620	F2 vs. M2	+	Metabolism	Bioenergetics	(1->3)-beta-D-glucan binding (NAD+) activity	GO:0004029	F	10	1.26E-03	3.13E-02
7	2621	F2 vs. M2	+	Metabolism	Bioenergetics	aerobic respiration	GO:0009060	P	54	3.97E-05	9.55E-04
7	2622	F2 vs. M2	+	Metabolism	Bioenergetics	aldehyde dehydrogenase [NAD(P)+] activity	GO:0004030	F	10	1.26E-03	3.16E-02
7	2623	F2 vs. M2	+	Metabolism	Bioenergetics	ATPase activity, coupled to transmembrane movement of ions, rotational mechanism	GO:0044769	F	19	1.65E-03	3.74E-02
7	2624	F2 vs. M2	+	Metabolism	Bioenergetics	carbohydrate biosynthetic process	GO:0016051	P	177	1.25E-12	2.79E-10
7	2625	F2 vs. M2	+	Metabolism	Bioenergetics	carbohydrate catabolic process	GO:0016052	P	142	1.03E-06	4.43E-05
7	2626	F2 vs. M2	+	Metabolism	Bioenergetics	carbohydrate derivative metabolic process	GO:1901135	P	425	8.60E-05	1.80E-03
7	2627	F2 vs. M2	+	Metabolism	Bioenergetics	carbohydrate metabolic process	GO:0005975	P	514	0.00E+00	0.00E+00
7	2628	F2 vs. M2	+	Metabolism	Bioenergetics	cellular carbohydrate biosynthetic process	GO:0034637	P	123	2.60E-07	1.30E-05
7	2629	F2 vs. M2	+	Metabolism	Bioenergetics	cellular carbohydrate catabolic process	GO:0044275	P	33	3.15E-03	3.14E-02
7	2630	F2 vs. M2	+	Metabolism	Bioenergetics	cellular carbohydrate metabolic process	GO:0044262	P	226	1.35E-10	2.06E-08
7	2631	F2 vs. M2	+	Metabolism	Bioenergetics	cellular polysaccharide biosynthetic process	GO:0033692	P	103	2.56E-06	9.21E-05
7	2632	F2 vs. M2	+	Metabolism	Bioenergetics	cellular polysaccharide metabolic process	GO:0044264	P	160	3.87E-07	1.88E-05
7	2633	F2 vs. M2	+	Metabolism	Bioenergetics	cellular respiration	GO:0045333	P	80	1.24E-04	2.41E-03
7	2634	F2 vs. M2	+	Metabolism	Bioenergetics	CoA-ligase activity	GO:0016405	F	16	1.82E-04	7.58E-03
7	2635	F2 vs. M2	+	Metabolism	Bioenergetics	electron transport chain	GO:0022900	P	77	1.36E-04	2.60E-03
7	2636	F2 vs. M2	+	Metabolism	Bioenergetics	energy derivation by oxidation of organic compounds	GO:0015980	P	89	1.70E-04	3.12E-03
7	2637	F2 vs. M2	+	Metabolism	Bioenergetics	fatty acid metabolic process	GO:0006631	P	130	7.79E-05	1.66E-03
7	2638	F2 vs. M2	+	Metabolism	Bioenergetics	generation of precursor metabolites and energy	GO:0006091	P	235	2.22E-16	9.88E-14
7	2639	F2 vs. M2	+	Metabolism	Bioenergetics	gluconeogenesis	GO:0006094	P	19	6.50E-06	2.06E-04
7	2640	F2 vs. M2	+	Metabolism	Bioenergetics	glucose 6-phosphate metabolic process	GO:0051156	P	28	2.62E-04	4.53E-03
7	2641	F2 vs. M2	+	Metabolism	Bioenergetics	glucose metabolic process	GO:0006006	P	31	9.59E-05	1.90E-03
7	2642	F2 vs. M2	+	Metabolism	Bioenergetics	glyceraldehyde-3-phosphate dehydrogenase (NAD+) (non-phosphorylating) activity	GO:0043878	F	7	2.35E-03	4.88E-02
7	2643	F2 vs. M2	+	Metabolism	Bioenergetics	hexose biosynthetic process	GO:0019319	P	20	2.55E-06	9.33E-05
7	2644	F2 vs. M2	+	Metabolism	Bioenergetics	hexose metabolic process	GO:0019318	P	57	1.81E-04	3.28E-03
7	2645	F2 vs. M2	+	Metabolism	Bioenergetics	isocitrate metabolic process	GO:0006102	P	10	1.04E-03	1.32E-02
7	2646	F2 vs. M2	+	Metabolism	Bioenergetics	malate metabolic process	GO:0006108	P	9	4.77E-03	4.27E-02
7	2647	F2 vs. M2	+	Metabolism	Bioenergetics	monosaccharide biosynthetic process	GO:0046364	P	33	3.16E-06	1.13E-04
7	2648	F2 vs. M2	+	Metabolism	Bioenergetics	NAD binding	GO:0051287	F	52	2.65E-04	9.66E-03
7	2649	F2 vs. M2	+	Metabolism	Bioenergetics	NAD(P)H dehydrogenase complex (plastoquinone)	GO:0010598	C	8	1.17E-03	8.61E-03
7	2650	F2 vs. M2	+	Metabolism	Bioenergetics	NAD(P)H dehydrogenase complex assembly	GO:0010275	P	6	3.80E-03	3.55E-02
7	2651	F2 vs. M2	+	Metabolism	Bioenergetics	NADP metabolic process	GO:0006739	P	32	4.03E-06	1.40E-04
7	2652	F2 vs. M2	+	Metabolism	Bioenergetics	NADPH regeneration	GO:0006740	P	24	5.39E-05	1.21E-03
7	2653	F2 vs. M2	+	Metabolism	Bioenergetics	oxidoreductase activity	GO:0016491	F	739	0.00E+00	0.00E+00
7	2654	F2 vs. M2	+	Metabolism	Bioenergetics	oxidoreductase activity, acting on a sulfur group of donors	GO:0016667	F	56	1.30E-04	5.48E-03
7	2655	F2 vs. M2	+	Metabolism	Bioenergetics	oxidoreductase activity, acting on CH-OH group of donors	GO:0016614	F	114	2.34E-04	9.02E-03
7	2656	F2 vs. M2	+	Metabolism	Bioenergetics	oxidoreductase activity, acting on NAD(P)H	GO:0016651	F	66	1.14E-05	8.34E-04
7	2657	F2 vs. M2	+	Metabolism	Bioenergetics	oxidoreductase activity, acting on single donors with incorporation of molecular oxygen	GO:0016701	F	25	1.06E-05	8.18E-04
7	2658	F2 vs. M2	+	Metabolism	Bioenergetics	oxidoreductase activity, acting on single donors with incorporation of molecular oxygen, incorporation of two atoms of oxygen	GO:0016702	F	17	1.16E-03	2.99E-02
7	2659	F2 vs. M2	+	Metabolism	Bioenergetics	oxidoreductase activity, acting on the aldehyde or oxo group of donors	GO:0016903	F	38	1.65E-06	1.65E-04
7	2660	F2 vs. M2	+	Metabolism	Bioenergetics	oxidoreductase activity, acting on the aldehyde or oxo group of donors, NAD or NADP as acceptor	GO:0016620	F	28	1.46E-06	1.52E-04
7	2661	F2 vs. M2	+	Metabolism	Bioenergetics	oxidoreductase activity, acting on the CH-OH group of donors, NAD or NADP as acceptor	GO:0016616	F	102	1.02E-04	4.68E-03
7	2662	F2 vs. M2	+	Metabolism	Bioenergetics	oxidoreductase complex	GO:1990204	C	94	2.55E-05	2.69E-04
7	2663	F2 vs. M2	+	Metabolism	Bioenergetics	pentose-phosphate shunt	GO:0006098	P	23	5.20E-05	1.18E-03
7	2664	F2 vs. M2	+	Metabolism	Bioenergetics	photorespiration	GO:0009853	P	25	5.45E-04	8.25E-03
7	2665	F2 vs. M2	+	Metabolism	Bioenergetics	polysaccharide biosynthetic process	GO:0000271	P	119	1.51E-06	6.04E-05
7	2666	F2 vs. M2	+	Metabolism	Bioenergetics	polysaccharide metabolic process	GO:0005976	P	240	1.77E-10	2.62E-08
7	2667	F2 vs. M2	+	Metabolism	Bioenergetics	proton transmembrane transport	GO:1902600	P	62	3.92E-04	6.48E-03
7	2668	F2 vs. M2	+	Metabolism	Bioenergetics	proton-transporting ATPase activity, rotational mechanism	GO:0046961	F	19	1.65E-03	3.77E-02
7	2669	F2 vs. M2	+	Metabolism	Bioenergetics	proton-transporting two-sector ATPase complex	GO:0016469	C	43	3.39E-03	2.18E-02

7	2670	F2 vs. M2	+	Metabolism	Bioenergetics	proton-transporting two-sector ATPase complex, catalytic domain	GO:0033178	C	16	6.14E-03	3.45E-02
7	2671	F2 vs. M2	+	Metabolism	Bioenergetics	proton-transporting V-type ATPase complex	GO:0033176	C	17	2.58E-04	2.21E-03
7	2672	F2 vs. M2	+	Metabolism	Bioenergetics	pyrophosphate hydrolysis-driven proton transmembrane transporter activity	GO:0009678	F	33	7.28E-05	3.78E-03
7	2673	F2 vs. M2	+	Metabolism	Bioenergetics	pyruvate metabolic process	GO:0006090	P	53	3.29E-03	3.23E-02
7	2674	F2 vs. M2	+	Metabolism	Bioenergetics	reductive pentose-phosphate cycle	GO:0019253	P	14	7.15E-07	3.26E-05
7	2675	F2 vs. M2	+	Metabolism	Bioenergetics	sucrose metabolic process	GO:0005985	P	29	1.99E-03	2.23E-02
7	2676	F2 vs. M2	+	Metabolism	Bioenergetics	tricarboxylic acid cycle	GO:0006099	P	29	2.02E-05	5.36E-04
7	2677	F2 vs. M2	+	Metabolism	Bioenergetics	tricarboxylic acid metabolic process	GO:0072350	P	15	2.02E-04	3.61E-03
7	2678	F2 vs. M2	+	Metabolism	Bioenergetics	unsaturated fatty acid metabolic process	GO:0033559	P	18	4.99E-03	4.39E-02
7	2679	F2 vs. M2	+	Metabolism	Bioenergetics	vacuolar proton-transporting V-type ATPase complex	GO:0016471	C	7	4.26E-03	2.64E-02
7	2680	F2 vs. M2	+	Metabolism	Catabolism	alcohol catabolic process	GO:0046164	P	26	2.62E-04	4.54E-03
7	2681	F2 vs. M2	+	Metabolism	Catabolism	alpha-amino acid catabolic process	GO:1901606	P	56	1.58E-07	8.43E-06
7	2682	F2 vs. M2	+	Metabolism	Catabolism	aromatic amino acid family catabolic process	GO:0009074	P	13	1.48E-03	1.78E-02
7	2683	F2 vs. M2	+	Metabolism	Catabolism	carboxylic acid catabolic process	GO:0046395	P	98	2.90E-08	2.15E-06
7	2684	F2 vs. M2	+	Metabolism	Catabolism	catabolic process	GO:0009056	P	781	8.23E-06	2.52E-04
7	2685	F2 vs. M2	+	Metabolism	Catabolism	cell wall macromolecule catabolic process	GO:0016998	P	17	3.48E-03	3.35E-02
7	2686	F2 vs. M2	+	Metabolism	Catabolism	cellular amino acid catabolic process	GO:0009063	P	61	6.56E-06	2.07E-04
7	2687	F2 vs. M2	+	Metabolism	Catabolism	cellular catabolic process	GO:0044248	P	636	4.18E-03	3.83E-02
7	2688	F2 vs. M2	+	Metabolism	Catabolism	cellular lipid catabolic process	GO:0044242	P	52	2.74E-03	2.81E-02
7	2689	F2 vs. M2	+	Metabolism	Catabolism	cellular metabolic compound salvage	GO:0043094	P	47	7.68E-04	1.08E-02
7	2690	F2 vs. M2	+	Metabolism	Catabolism	erythrose 4-phosphate/phosphoenolpyruvate family amino acid catabolic process	GO:1902222	P	6	6.81E-04	9.90E-03
7	2691	F2 vs. M2	+	Metabolism	Catabolism	glycine catabolic process	GO:0006546	P	6	1.01E-03	1.30E-02
7	2692	F2 vs. M2	+	Metabolism	Catabolism	L-phenylalanine catabolic process	GO:0006559	P	6	6.81E-04	9.93E-03
7	2693	F2 vs. M2	+	Metabolism	Catabolism	monocarboxylic acid catabolic process	GO:0072329	P	36	8.47E-04	1.15E-02
7	2694	F2 vs. M2	+	Metabolism	Catabolism	organic acid catabolic process	GO:0016054	P	98	2.90E-08	2.18E-06
7	2695	F2 vs. M2	+	Metabolism	Catabolism	organic hydroxy compound catabolic process	GO:1901616	P	31	7.96E-06	2.47E-04
7	2696	F2 vs. M2	+	Metabolism	Catabolism	organic substance catabolic process	GO:1901575	P	719	1.46E-04	2.74E-03
7	2697	F2 vs. M2	+	Metabolism	Catabolism	organophosphate catabolic process	GO:0046434	P	33	5.57E-03	4.73E-02
7	2698	F2 vs. M2	+	Metabolism	Catabolism	pectin catabolic process	GO:0045490	P	56	7.14E-04	1.02E-02
7	2699	F2 vs. M2	+	Metabolism	Catabolism	peroxidase activity	GO:0004601	F	56	6.39E-04	1.94E-02
7	2700	F2 vs. M2	+	Metabolism	Catabolism	peroxisome	GO:0005777	C	162	1.12E-06	1.46E-05
7	2701	F2 vs. M2	+	Metabolism	Catabolism	phenol-containing compound catabolic process	GO:0019336	P	5	5.06E-03	4.41E-02
7	2702	F2 vs. M2	+	Metabolism	Catabolism	polyol catabolic process	GO:0046174	P	21	1.27E-03	1.57E-02
7	2703	F2 vs. M2	+	Metabolism	Catabolism	polysaccharide catabolic process	GO:0000272	P	81	1.24E-04	2.41E-03
7	2704	F2 vs. M2	+	Metabolism	Catabolism	serine family amino acid catabolic process	GO:0009071	P	10	2.81E-04	4.78E-03
7	2705	F2 vs. M2	+	Metabolism	Catabolism	small molecule catabolic process	GO:0044282	P	151	7.22E-09	6.76E-07
7	2706	F2 vs. M2	+	Metabolism	Metabolism	acid-ammonia (or amide) ligase activity	GO:0016880	F	8	2.01E-03	4.37E-02
7	2707	F2 vs. M2	+	Metabolism	Metabolism	acid-thiol ligase activity	GO:0016878	F	19	4.51E-04	1.56E-02
7	2708	F2 vs. M2	+	Metabolism	Metabolism	actin binding	GO:0003779	F	62	8.42E-05	4.14E-03
7	2709	F2 vs. M2	+	Metabolism	Metabolism	actin filament binding	GO:0051015	F	41	5.68E-06	4.80E-04
7	2710	F2 vs. M2	+	Metabolism	Metabolism	actin filament bundle assembly	GO:0051017	P	12	4.20E-03	3.82E-02
7	2711	F2 vs. M2	+	Metabolism	Metabolism	actin filament bundle organization	GO:0061572	P	12	4.20E-03	3.83E-02
7	2712	F2 vs. M2	+	Metabolism	Metabolism	actin filament depolymerization	GO:0030042	P	8	5.82E-03	4.89E-02
7	2713	F2 vs. M2	+	Metabolism	Metabolism	actin filament organization	GO:0007015	P	50	3.99E-03	3.68E-02
7	2714	F2 vs. M2	+	Metabolism	Metabolism	actin filament-based process	GO:0030029	P	62	2.07E-03	2.29E-02
7	2715	F2 vs. M2	+	Metabolism	Metabolism	alcohol metabolic process	GO:0006066	P	78	4.47E-05	1.05E-03
7	2716	F2 vs. M2	+	Metabolism	Metabolism	alpha-amino acid biosynthetic process	GO:1901607	P	114	4.23E-04	6.86E-03
7	2717	F2 vs. M2	+	Metabolism	Metabolism	alpha-amino acid metabolic process	GO:1901605	P	175	3.03E-10	4.26E-08
7	2718	F2 vs. M2	+	Metabolism	Metabolism	aminoglycan biosynthetic process	GO:0006023	P	4	3.68E-03	3.46E-02
7	2719	F2 vs. M2	+	Metabolism	Metabolism	ammonia assimilation cycle	GO:0019676	P	6	1.41E-03	1.71E-02
7	2720	F2 vs. M2	+	Metabolism	Metabolism	anion binding	GO:0043168	F	677	5.84E-05	3.22E-03
7	2721	F2 vs. M2	+	Metabolism	Metabolism	aromatic amino acid family metabolic process	GO:0009072	P	47	3.57E-03	3.42E-02
7	2722	F2 vs. M2	+	Metabolism	Metabolism	benzene-containing compound metabolic process	GO:0042537	P	25	2.36E-03	2.52E-02
7	2723	F2 vs. M2	+	Metabolism	Metabolism	beta-glucan biosynthetic process	GO:0051274	P	39	2.28E-04	4.03E-03
7	2724	F2 vs. M2	+	Metabolism	Metabolism	beta-glucan metabolic process	GO:0051273	P	42	8.97E-04	1.21E-02
7	2725	F2 vs. M2	+	Metabolism	Metabolism	biosynthetic process	GO:0009058	P	1591	1.15E-03	1.43E-02
7	2726	F2 vs. M2	+	Metabolism	Metabolism	carbon utilization	GO:0015976	P	8	5.09E-04	7.76E-03
7	2727	F2 vs. M2	+	Metabolism	Metabolism	carbon-carbon lyase activity	GO:0016830	F	65	8.46E-05	4.08E-03
7	2728	F2 vs. M2	+	Metabolism	Metabolism	carbon-oxygen lyase activity	GO:0016835	F	90	1.79E-03	4.00E-02
7	2729	F2 vs. M2	+	Metabolism	Metabolism	carboxylic acid biosynthetic process	GO:0046394	P	278	6.36E-08	3.90E-06
7	2730	F2 vs. M2	+	Metabolism	Metabolism	carboxylic acid metabolic process	GO:0019752	P	526	0.00E+00	0.00E+00

7	2731	F2 vs. M2	+	Metabolism	Metabolism	carboxy-lyase activity	GO:0016831	F	35	1.68E-03	3.78E-02
7	2732	F2 vs. M2	+	Metabolism	Metabolism	catalytic activity	GO:0003824	F	4128	6.32E-14	2.84E-11
7	2733	F2 vs. M2	+	Metabolism	Metabolism	cation binding	GO:0043169	F	826	2.61E-07	3.36E-05
7	2734	F2 vs. M2	+	Metabolism	Metabolism	cell wall macromolecule biosynthetic process	GO:0044038	P	43	1.70E-03	1.98E-02
7	2735	F2 vs. M2	+	Metabolism	Metabolism	cell wall macromolecule metabolic process	GO:0044036	P	93	1.28E-03	1.58E-02
7	2736	F2 vs. M2	+	Metabolism	Metabolism	cell wall polysaccharide biosynthetic process	GO:0070592	P	40	4.79E-03	4.28E-02
7	2737	F2 vs. M2	+	Metabolism	Metabolism	cell wall polysaccharide metabolic process	GO:0010383	P	76	3.33E-03	3.26E-02
7	2738	F2 vs. M2	+	Metabolism	Metabolism	cellular amino acid biosynthetic process	GO:0008652	P	126	5.72E-05	1.28E-03
7	2739	F2 vs. M2	+	Metabolism	Metabolism	cellular amino acid metabolic process	GO:0006520	P	230	8.87E-09	8.02E-07
7	2740	F2 vs. M2	+	Metabolism	Metabolism	cellular component macromolecule biosynthetic process	GO:0070589	P	43	1.70E-03	1.98E-02
7	2741	F2 vs. M2	+	Metabolism	Metabolism	cellular glucan metabolic process	GO:0006073	P	122	1.52E-04	2.79E-03
7	2742	F2 vs. M2	+	Metabolism	Metabolism	cellular lipid metabolic process	GO:0044255	P	368	2.19E-07	1.11E-05
7	2743	F2 vs. M2	+	Metabolism	Metabolism	cellulose biosynthetic process	GO:0030244	P	30	2.05E-04	3.64E-03
7	2744	F2 vs. M2	+	Metabolism	Metabolism	cellulose metabolic process	GO:0030243	P	33	1.04E-03	1.32E-02
7	2745	F2 vs. M2	+	Metabolism	Metabolism	cellulose synthase (UDP-forming) activity	GO:0016760	F	18	6.69E-04	1.98E-02
7	2746	F2 vs. M2	+	Metabolism	Metabolism	cellulose synthase activity	GO:0016759	F	23	5.55E-04	1.81E-02
7	2747	F2 vs. M2	+	Metabolism	Metabolism	copper ion binding	GO:0005507	F	116	9.47E-10	2.33E-07
7	2748	F2 vs. M2	+	Metabolism	Metabolism	dicarboxylic acid metabolic process	GO:0043648	P	53	1.44E-05	3.93E-04
7	2749	F2 vs. M2	+	Metabolism	Metabolism	dioxygenase activity	GO:0051213	F	58	2.03E-03	4.39E-02
7	2750	F2 vs. M2	+	Metabolism	Metabolism	enzyme inhibitor activity	GO:0004857	F	86	8.85E-05	4.20E-03
7	2751	F2 vs. M2	+	Metabolism	Metabolism	erythrose 4-phosphate/phosphoenolpyruvate family amino acid metabolic process	GO:1902221	P	15	3.69E-03	3.46E-02
7	2752	F2 vs. M2	+	Metabolism	Metabolism	establishment of localization	GO:0051234	P	1189	3.21E-08	2.26E-06
7	2753	F2 vs. M2	+	Metabolism	Metabolism	galacturonan metabolic process	GO:0010393	P	89	1.00E-06	4.39E-05
7	2754	F2 vs. M2	+	Metabolism	Metabolism	glucan biosynthetic process	GO:0009250	P	74	2.55E-04	4.46E-03
7	2755	F2 vs. M2	+	Metabolism	Metabolism	glucan metabolic process	GO:0044042	P	126	1.36E-04	2.59E-03
7	2756	F2 vs. M2	+	Metabolism	Metabolism	glucosinolate biosynthetic process	GO:0019761	P	20	8.24E-04	1.13E-02
7	2757	F2 vs. M2	+	Metabolism	Metabolism	glucosyltransferase activity	GO:0046527	F	110	8.40E-04	2.34E-02
7	2758	F2 vs. M2	+	Metabolism	Metabolism	glutamine biosynthetic process	GO:0006542	P	5	3.24E-03	3.19E-02
7	2759	F2 vs. M2	+	Metabolism	Metabolism	glutamine family amino acid metabolic process	GO:0009064	P	40	2.96E-03	2.99E-02
7	2760	F2 vs. M2	+	Metabolism	Metabolism	glutamine metabolic process	GO:0006541	P	13	9.49E-05	1.89E-03
7	2761	F2 vs. M2	+	Metabolism	Metabolism	glycerolipid metabolic process	GO:0046486	P	94	4.70E-03	4.22E-02
7	2762	F2 vs. M2	+	Metabolism	Metabolism	glycine decarboxylation via glycine cleavage system	GO:0019464	P	5	2.28E-03	2.47E-02
7	2763	F2 vs. M2	+	Metabolism	Metabolism	glycine metabolic process	GO:0006544	P	11	5.87E-05	1.29E-03
7	2764	F2 vs. M2	+	Metabolism	Metabolism	glycosaminoglycan biosynthetic process	GO:0006024	P	4	3.68E-03	3.47E-02
7	2765	F2 vs. M2	+	Metabolism	Metabolism	glycosaminoglycan metabolic process	GO:0030203	P	4	3.68E-03	3.48E-02
7	2766	F2 vs. M2	+	Metabolism	Metabolism	glycosinolate biosynthetic process	GO:0019758	P	20	8.24E-04	1.12E-02
7	2767	F2 vs. M2	+	Metabolism	Metabolism	glycosyl compound biosynthetic process	GO:1901659	P	33	2.18E-03	2.37E-02
7	2768	F2 vs. M2	+	Metabolism	Metabolism	hydrocarbon metabolic process	GO:0120252	P	37	1.81E-03	2.07E-02
7	2769	F2 vs. M2	+	Metabolism	Metabolism	hydrolase activity	GO:0016787	F	1476	2.40E-03	4.86E-02
7	2770	F2 vs. M2	+	Metabolism	Metabolism	hydrolase activity, hydrolyzing O-glycosyl compounds	GO:0004553	F	184	1.26E-04	5.39E-03
7	2771	F2 vs. M2	+	Metabolism	Metabolism	hydro-lyase activity	GO:0016836	F	54	9.92E-04	2.63E-02
7	2772	F2 vs. M2	+	Metabolism	Metabolism	indole-containing compound metabolic process	GO:0042430	P	39	2.83E-03	2.89E-02
7	2773	F2 vs. M2	+	Metabolism	Metabolism	inositol biosynthetic process	GO:0006021	P	4	4.93E-03	4.37E-02
7	2774	F2 vs. M2	+	Metabolism	Metabolism	inositol metabolic process	GO:0006020	P	11	2.85E-03	2.91E-02
7	2775	F2 vs. M2	+	Metabolism	Metabolism	intramolecular oxidoreductase activity, interconverting aldoses and ketoses	GO:0016861	F	14	1.61E-03	3.74E-02
7	2776	F2 vs. M2	+	Metabolism	Metabolism	ion binding	GO:0043167	F	1401	1.61E-08	2.72E-06
7	2777	F2 vs. M2	+	Metabolism	Metabolism	leucine metabolic process	GO:0006551	P	17	8.91E-04	1.20E-02
7	2778	F2 vs. M2	+	Metabolism	Metabolism	lignin biosynthetic process	GO:0009809	P	27	5.88E-06	1.91E-04
7	2779	F2 vs. M2	+	Metabolism	Metabolism	lignin metabolic process	GO:0009808	P	33	1.31E-06	5.44E-05
7	2780	F2 vs. M2	+	Metabolism	Metabolism	lipid biosynthetic process	GO:0008610	P	275	2.23E-03	2.42E-02
7	2781	F2 vs. M2	+	Metabolism	Metabolism	lipid metabolic process	GO:0006629	P	433	1.00E-06	4.35E-05
7	2782	F2 vs. M2	+	Metabolism	Metabolism	lipid modification	GO:0030258	P	59	4.89E-04	7.63E-03
7	2783	F2 vs. M2	+	Metabolism	Metabolism	localization	GO:0051179	P	1277	2.17E-07	1.12E-05
7	2784	F2 vs. M2	+	Metabolism	Metabolism	L-phenylalanine metabolic process	GO:0006558	P	15	3.69E-03	3.47E-02
7	2785	F2 vs. M2	+	Metabolism	Metabolism	lyase activity	GO:0016829	F	191	2.54E-07	3.43E-05
7	2786	F2 vs. M2	+	Metabolism	Metabolism	metal ion binding	GO:0046872	F	811	1.16E-06	1.25E-04
7	2787	F2 vs. M2	+	Metabolism	Metabolism	methionine adenosyltransferase activity	GO:0004478	F	4	2.16E-03	4.52E-02
7	2788	F2 vs. M2	+	Metabolism	Metabolism	monocarboxylic acid biosynthetic process	GO:0072330	P	129	2.67E-03	2.78E-02
7	2789	F2 vs. M2	+	Metabolism	Metabolism	monocarboxylic acid metabolic process	GO:0032787	P	260	3.07E-09	3.34E-07
7	2790	F2 vs. M2	+	Metabolism	Metabolism	monosaccharide metabolic process	GO:0005996	P	79	1.80E-06	6.95E-05
7	2791	F2 vs. M2	+	Metabolism	Metabolism	nitrate assimilation	GO:0042128	P	10	9.22E-05	1.88E-03
7	2792	F2 vs. M2	+	Metabolism	Metabolism	nitrate metabolic process	GO:0042126	P	10	9.22E-05	1.88E-03

7	2793	F2 vs. M2	+	Metabolism	Metabolism	nitrogen cycle metabolic process	GO:0071941	P	11	1.38E-04	2.61E-03
7	2794	F2 vs. M2	+	Metabolism	Metabolism	nitrogen utilization	GO:0019740	P	6	1.41E-03	1.71E-02
7	2795	F2 vs. M2	+	Metabolism	Metabolism	olefinic compound metabolic process	GO:0120254	P	31	2.03E-03	2.26E-02
7	2796	F2 vs. M2	+	Metabolism	Metabolism	organic acid biosynthetic process	GO:0016053	P	278	6.36E-08	3.95E-06
7	2797	F2 vs. M2	+	Metabolism	Metabolism	organic acid metabolic process	GO:0006082	P	574	0.00E+00	0.00E+00
7	2798	F2 vs. M2	+	Metabolism	Metabolism	organic hydroxy compound biosynthetic process	GO:1901617	P	97	1.04E-03	1.32E-02
7	2799	F2 vs. M2	+	Metabolism	Metabolism	organic hydroxy compound metabolic process	GO:1901615	P	164	5.08E-07	2.38E-05
7	2800	F2 vs. M2	+	Metabolism	Metabolism	organophosphate metabolic process	GO:0019637	P	335	1.39E-05	3.88E-04
7	2801	F2 vs. M2	+	Metabolism	Metabolism	oxidation-reduction process	GO:0055114	P	420	0.00E+00	0.00E+00
7	2802	F2 vs. M2	+	Metabolism	Metabolism	oxoacid metabolic process	GO:0043436	P	573	0.00E+00	0.00E+00
7	2803	F2 vs. M2	+	Metabolism	Metabolism	pectin metabolic process	GO:0045488	P	88	4.43E-07	2.13E-05
7	2804	F2 vs. M2	+	Metabolism	Metabolism	pectinesterase activity	GO:0030599	F	44	2.54E-03	4.97E-02
7	2805	F2 vs. M2	+	Metabolism	Metabolism	pectinesterase inhibitor activity	GO:0046910	F	36	5.05E-04	1.71E-02
7	2806	F2 vs. M2	+	Metabolism	Metabolism	phenylpropanoid biosynthetic process	GO:0009699	P	52	4.41E-05	1.04E-03
7	2807	F2 vs. M2	+	Metabolism	Metabolism	phenylpropanoid metabolic process	GO:0009698	P	65	9.44E-06	2.80E-04
7	2808	F2 vs. M2	+	Metabolism	Metabolism	phospholipid metabolic process	GO:0006644	P	112	4.86E-03	4.33E-02
7	2809	F2 vs. M2	+	Metabolism	Metabolism	phosphoric ester hydrolase activity	GO:0042578	F	191	7.84E-05	4.00E-03
7	2810	F2 vs. M2	+	Metabolism	Metabolism	phosphorus metabolic process	GO:0006793	P	969	7.68E-04	1.08E-02
7	2811	F2 vs. M2	+	Metabolism	Metabolism	pigment biosynthetic process	GO:0046148	P	74	2.06E-03	2.29E-02
7	2812	F2 vs. M2	+	Metabolism	Metabolism	pigment metabolic process	GO:0042440	P	89	1.13E-03	1.41E-02
7	2813	F2 vs. M2	+	Metabolism	Metabolism	polyol metabolic process	GO:0019751	P	36	1.35E-04	2.60E-03
7	2814	F2 vs. M2	+	Metabolism	Metabolism	poly-pyrimidine tract binding	GO:0008187	F	22	2.04E-03	4.31E-02
7	2815	F2 vs. M2	+	Metabolism	Metabolism	purine nucleotide metabolic process	GO:0006163	P	134	3.87E-03	3.60E-02
7	2816	F2 vs. M2	+	Metabolism	Metabolism	purine ribonucleotide metabolic process	GO:0009150	P	126	3.16E-03	3.14E-02
7	2817	F2 vs. M2	+	Metabolism	Metabolism	purine-containing compound metabolic process	GO:0072521	P	146	5.97E-03	4.98E-02
7	2818	F2 vs. M2	+	Metabolism	Metabolism	pyridoxal phosphate binding	GO:0030170	F	62	5.50E-04	1.81E-02
7	2819	F2 vs. M2	+	Metabolism	Metabolism	S-adenosylmethionine biosynthetic process	GO:0006556	P	4	2.16E-03	2.35E-02
7	2820	F2 vs. M2	+	Metabolism	Metabolism	S-adenosylmethionine metabolic process	GO:0046500	P	7	9.28E-05	1.86E-03
7	2821	F2 vs. M2	+	Metabolism	Metabolism	secondary alcohol metabolic process	GO:1902652	P	10	1.04E-03	1.32E-02
7	2822	F2 vs. M2	+	Metabolism	Metabolism	secondary metabolic process	GO:0019748	P	152	9.12E-08	5.18E-06
7	2823	F2 vs. M2	+	Metabolism	Metabolism	secondary metabolite biosynthetic process	GO:0044550	P	83	4.75E-08	3.13E-06
7	2824	F2 vs. M2	+	Metabolism	Metabolism	serine family amino acid biosynthetic process	GO:0009070	P	25	4.58E-03	4.12E-02
7	2825	F2 vs. M2	+	Metabolism	Metabolism	serine family amino acid metabolic process	GO:0009069	P	36	3.39E-05	8.34E-04
7	2826	F2 vs. M2	+	Metabolism	Metabolism	S-glycoside biosynthetic process	GO:0016144	P	20	8.24E-04	1.13E-02
7	2827	F2 vs. M2	+	Metabolism	Metabolism	sigma factor activity	GO:0016987	F	6	2.49E-03	4.99E-02
7	2828	F2 vs. M2	+	Metabolism	Metabolism	small molecule binding	GO:0036094	F	670	3.09E-04	1.08E-02
7	2829	F2 vs. M2	+	Metabolism	Metabolism	small molecule biosynthetic process	GO:0044283	P	387	5.35E-12	1.02E-09
7	2830	F2 vs. M2	+	Metabolism	Metabolism	small molecule metabolic process	GO:0044281	P	866	0.00E+00	0.00E+00
7	2831	F2 vs. M2	+	Metabolism	Metabolism	spermidine biosynthetic process	GO:0008295	P	3	5.01E-03	4.40E-02
7	2832	F2 vs. M2	+	Metabolism	Metabolism	sulfur amino acid metabolic process	GO:0000096	P	34	7.85E-04	1.10E-02
7	2833	F2 vs. M2	+	Metabolism	Metabolism	sulfur compound biosynthetic process	GO:0044272	P	86	6.84E-10	8.49E-08
7	2834	F2 vs. M2	+	Metabolism	Metabolism	sulfur compound metabolic process	GO:0006790	P	191	5.05E-10	6.74E-08
7	2835	F2 vs. M2	+	Metabolism	Metabolism	tetrapyrrole binding	GO:0046906	F	186	1.22E-03	3.07E-02
7	2836	F2 vs. M2	+	Metabolism	Metabolism	transferase activity, transferring hexosyl groups	GO:0016758	F	187	2.43E-03	4.90E-02
7	2837	F2 vs. M2	+	Metabolism	Metabolism	UDP-glucosyltransferase activity	GO:0035251	F	79	2.94E-04	1.06E-02
7	2838	F2 vs. M2	+	Metabolism	Metabolism	UDP-glucuronate biosynthetic process	GO:0006065	P	4	3.68E-03	3.48E-02
7	2839	F2 vs. M2	+	Metabolism	Metabolism	UDP-glucuronate metabolic process	GO:0046398	P	4	3.68E-03	3.46E-02
7	2840	F2 vs. M2	+	Metabolism	Metabolism	UDP-glycosyltransferase activity	GO:0008194	F	141	1.05E-04	4.74E-03
7	2841	F2 vs. M2	+	Metabolism	Metabolism	UDP-rhamnose biosynthetic process	GO:0010253	P	4	2.72E-03	2.82E-02
7	2842	F2 vs. M2	+	Metabolism	Metabolism	UDP-rhamnose metabolic process	GO:0033478	P	4	2.72E-03	2.82E-02
7	2843	F2 vs. M2	+	Metabolism	Metabolism	vitamin B6 binding	GO:0070279	F	62	5.50E-04	1.83E-02
7	2844	F2 vs. M2	+	Metabolism	Photosynthesis	carbon fixation	GO:0015977	P	15	3.42E-07	1.69E-05
7	2845	F2 vs. M2	+	Metabolism	Photosynthesis	chlorophyll metabolic process	GO:0015994	P	42	2.66E-03	2.78E-02
7	2846	F2 vs. M2	+	Metabolism	Photosynthesis	chloroplast	GO:0009507	C	2503	0.00E+00	0.00E+00
7	2847	F2 vs. M2	+	Metabolism	Photosynthesis	chloroplast envelope	GO:0009941	C	415	0.00E+00	0.00E+00
7	2848	F2 vs. M2	+	Metabolism	Photosynthesis	chloroplast stroma	GO:0009570	C	469	0.00E+00	0.00E+00
7	2849	F2 vs. M2	+	Metabolism	Photosynthesis	chloroplast thylakoid	GO:0009534	C	288	0.00E+00	0.00E+00
7	2850	F2 vs. M2	+	Metabolism	Photosynthesis	chloroplast thylakoid lumen	GO:0009543	C	29	1.07E-03	8.08E-03
7	2851	F2 vs. M2	+	Metabolism	Photosynthesis	chloroplast thylakoid membrane	GO:0009535	C	238	0.00E+00	0.00E+00
7	2852	F2 vs. M2	+	Metabolism	Photosynthesis	chloroplast thylakoid membrane protein complex	GO:0098807	C	20	3.29E-03	2.14E-02
7	2853	F2 vs. M2	+	Metabolism	Photosynthesis	fructose 1,6-bisphosphate metabolic process	GO:0030388	P	9	3.47E-04	5.76E-03
7	2854	F2 vs. M2	+	Metabolism	Photosynthesis	fructose-bisphosphate aldolase activity	GO:0004332	F	6	2.03E-03	4.36E-02
7	2855	F2 vs. M2	+	Metabolism	Photosynthesis	negative regulation of photosynthesis	GO:1905156	P	12	3.34E-03	3.25E-02
7	2856	F2 vs. M2	+	Metabolism	Photosynthesis	oxidative photosynthetic carbon pathway	GO:0009854	P	4	2.86E-03	2.91E-02

7	2857	F2 vs. M2	+	Metabolism	Photosynthesis	photosynthesis	GO:0015979	P	132	1.11E-16	5.93E-14
7	2858	F2 vs. M2	+	Metabolism	Photosynthesis	photosynthesis, dark reaction	GO:0019685	P	15	5.91E-07	2.72E-05
7	2859	F2 vs. M2	+	Metabolism	Photosynthesis	photosynthesis, light harvesting	GO:0009765	P	28	4.50E-05	1.04E-03
7	2860	F2 vs. M2	+	Metabolism	Photosynthesis	photosynthesis, light harvesting in photosystem I	GO:0009768	P	16	3.57E-03	3.42E-02
7	2861	F2 vs. M2	+	Metabolism	Photosynthesis	photosynthesis, light reaction	GO:0019684	P	87	1.31E-12	2.79E-10
7	2862	F2 vs. M2	+	Metabolism	Photosynthesis	photosynthetic electron transport chain	GO:0009767	P	33	1.49E-04	2.75E-03
7	2863	F2 vs. M2	+	Metabolism	Photosynthesis	photosynthetic electron transport in photosystem I	GO:0009773	P	13	1.73E-03	2.00E-02
7	2864	F2 vs. M2	+	Metabolism	Photosynthesis	photosynthetic membrane	GO:0034357	C	257	0.00E+00	0.00E+00
7	2865	F2 vs. M2	+	Metabolism	Photosynthesis	photosystem	GO:0009521	C	41	5.62E-03	3.30E-02
7	2866	F2 vs. M2	+	Metabolism	Photosynthesis	photosystem II	GO:0009523	C	27	5.68E-03	3.31E-02
7	2867	F2 vs. M2	+	Metabolism	Photosynthesis	photosystem II assembly	GO:0010207	P	20	1.64E-03	1.93E-02
7	2868	F2 vs. M2	+	Metabolism	Photosynthesis	photosystem II oxygen evolving complex	GO:0009654	C	12	3.63E-03	2.31E-02
7	2869	F2 vs. M2	+	Metabolism	Photosynthesis	photosystem II repair	GO:0010206	P	10	1.82E-03	2.07E-02
7	2870	F2 vs. M2	+	Metabolism	Photosynthesis	plastid	GO:0009536	C	2778	9.99E-16	3.65E-14
7	2871	F2 vs. M2	+	Metabolism	Photosynthesis	plastid envelope	GO:0009526	C	433	0.00E+00	0.00E+00
7	2872	F2 vs. M2	+	Metabolism	Photosynthesis	plastid stroma	GO:0009532	C	473	0.00E+00	0.00E+00
7	2873	F2 vs. M2	+	Metabolism	Photosynthesis	plastid thylakoid	GO:0031976	C	290	0.00E+00	0.00E+00
7	2874	F2 vs. M2	+	Metabolism	Photosynthesis	plastid thylakoid lumen	GO:0031978	C	29	1.07E-03	8.01E-03
7	2875	F2 vs. M2	+	Metabolism	Photosynthesis	plastid thylakoid membrane	GO:0055035	C	241	0.00E+00	0.00E+00
7	2876	F2 vs. M2	+	Metabolism	Photosynthesis	plastid translation	GO:0032544	P	17	3.57E-03	3.41E-02
7	2877	F2 vs. M2	+	Metabolism	Photosynthesis	plastoglobule	GO:0010287	C	51	5.27E-07	7.37E-06
7	2878	F2 vs. M2	+	Metabolism	Photosynthesis	regulation of generation of precursor metabolites and energy	GO:0043467	P	22	3.88E-03	3.60E-02
7	2879	F2 vs. M2	+	Metabolism	Photosynthesis	regulation of photosynthesis	GO:0010109	P	27	1.75E-05	4.69E-04
7	2880	F2 vs. M2	+	Metabolism	Photosynthesis	regulation of photosynthesis, light reaction	GO:0042548	P	17	1.10E-03	1.38E-02
7	2881	F2 vs. M2	+	Metabolism	Photosynthesis	thylakoid	GO:0009579	C	349	0.00E+00	0.00E+00
7	2882	F2 vs. M2	+	Metabolism	Photosynthesis	thylakoid lumen	GO:0031977	C	39	5.56E-06	6.52E-05
7	2883	F2 vs. M2	+	Metabolism	Photosynthesis	thylakoid membrane	GO:0042651	C	255	0.00E+00	0.00E+00
7	2884	F2 vs. M2	+	Metabolism	Photosynthesis	thylakoid membrane organization	GO:0010027	P	26	4.93E-03	4.37E-02
7	2885	F2 vs. M2	+	Metabolism	Transporters	active ion transmembrane transporter activity	GO:0022853	F	114	1.01E-12	3.91E-10
7	2886	F2 vs. M2	+	Metabolism	Transporters	active transmembrane transporter activity	GO:0022804	F	267	3.96E-14	2.68E-11
7	2887	F2 vs. M2	+	Metabolism	Transporters	amino acid transmembrane transport	GO:0003333	P	18	8.97E-04	1.21E-02
7	2888	F2 vs. M2	+	Metabolism	Transporters	anion transmembrane transport	GO:0098656	P	65	1.61E-03	1.91E-02
7	2889	F2 vs. M2	+	Metabolism	Transporters	anion transmembrane transporter activity	GO:0008509	F	197	1.13E-05	8.51E-04
7	2890	F2 vs. M2	+	Metabolism	Transporters	anion transport	GO:0006820	P	158	1.84E-04	3.32E-03
7	2891	F2 vs. M2	+	Metabolism	Transporters	anion:anion antiporter activity	GO:0015301	F	19	2.64E-05	1.66E-03
7	2892	F2 vs. M2	+	Metabolism	Transporters	antiporter activity	GO:0015297	F	102	9.81E-10	2.21E-07
7	2893	F2 vs. M2	+	Metabolism	Transporters	ATPase-coupled cation transmembrane transporter activity	GO:0019829	F	40	4.00E-05	2.35E-03
7	2894	F2 vs. M2	+	Metabolism	Transporters	ATPase-coupled ion transmembrane transporter activity	GO:0042625	F	48	2.85E-05	1.75E-03
7	2895	F2 vs. M2	+	Metabolism	Transporters	ATPase-coupled transmembrane transporter activity	GO:0042626	F	111	4.28E-05	2.46E-03
7	2896	F2 vs. M2	+	Metabolism	Transporters	C4-dicarboxylate transmembrane transporter activity	GO:0015556	F	6	6.36E-04	1.95E-02
7	2897	F2 vs. M2	+	Metabolism	Transporters	calcium ion transmembrane transport	GO:0070588	P	20	2.10E-03	2.31E-02
7	2898	F2 vs. M2	+	Metabolism	Transporters	carbohydrate transmembrane transporter activity	GO:0015144	F	66	3.45E-05	2.07E-03
7	2899	F2 vs. M2	+	Metabolism	Transporters	carbohydrate transport	GO:0008643	P	43	3.29E-03	3.23E-02
7	2900	F2 vs. M2	+	Metabolism	Transporters	carboxylic acid transmembrane transport	GO:1905039	P	36	5.40E-03	4.60E-02
7	2901	F2 vs. M2	+	Metabolism	Transporters	carboxylic acid transmembrane transporter activity	GO:0046943	F	80	2.35E-03	4.84E-02
7	2902	F2 vs. M2	+	Metabolism	Transporters	cation transmembrane transport	GO:0098655	P	132	1.42E-05	3.92E-04
7	2903	F2 vs. M2	+	Metabolism	Transporters	cation transmembrane transporter activity	GO:0008324	F	221	2.81E-09	5.83E-07
7	2904	F2 vs. M2	+	Metabolism	Transporters	cation transport	GO:0006812	P	205	1.09E-05	3.15E-04
7	2905	F2 vs. M2	+	Metabolism	Transporters	chemotaxis	GO:0006935	P	15	5.29E-03	4.53E-02
7	2906	F2 vs. M2	+	Metabolism	Transporters	cytoplasmic vesicle	GO:0031410	C	395	2.73E-07	4.19E-06
7	2907	F2 vs. M2	+	Metabolism	Transporters	dicarboxylic acid transport	GO:0006835	P	13	6.82E-04	9.89E-03
7	2908	F2 vs. M2	+	Metabolism	Transporters	endosome	GO:0005768	C	234	4.45E-05	4.50E-04
7	2909	F2 vs. M2	+	Metabolism	Transporters	inorganic anion transmembrane transporter activity	GO:0015103	F	62	6.05E-05	3.27E-03
7	2910	F2 vs. M2	+	Metabolism	Transporters	inorganic cation transmembrane transport	GO:0098662	P	126	1.28E-05	3.63E-04
7	2911	F2 vs. M2	+	Metabolism	Transporters	inorganic cation transmembrane transporter activity	GO:0022890	F	197	1.08E-08	1.94E-06
7	2912	F2 vs. M2	+	Metabolism	Transporters	inorganic ion transmembrane transport	GO:0098660	P	139	2.11E-06	8.09E-05
7	2913	F2 vs. M2	+	Metabolism	Transporters	inorganic molecular entity transmembrane transporter activity	GO:0015318	F	356	5.07E-14	2.74E-11
7	2914	F2 vs. M2	+	Metabolism	Transporters	ion transmembrane transport	GO:0034220	P	190	5.03E-08	3.27E-06
7	2915	F2 vs. M2	+	Metabolism	Transporters	ion transmembrane transporter activity	GO:0015075	F	396	1.70E-11	5.09E-09
7	2916	F2 vs. M2	+	Metabolism	Transporters	ion transport	GO:0006811	P	348	1.28E-08	1.07E-06
7	2917	F2 vs. M2	+	Metabolism	Transporters	malate transmembrane transporter activity	GO:0015140	F	6	6.36E-04	1.97E-02
7	2918	F2 vs. M2	+	Metabolism	Transporters	monovalent inorganic cation transmembrane transporter activity	GO:0015077	F	118	5.77E-08	8.65E-06
7	2919	F2 vs. M2	+	Metabolism	Transporters	monovalent inorganic cation transport	GO:0015672	P	91	3.27E-05	8.08E-04

7	2920	F2 vs. M2	+	Metabolism	Transporters	neutral amino acid transmembrane transporter activity	GO:0015175	F	11	6.17E-04	1.96E-02
7	2921	F2 vs. M2	+	Metabolism	Transporters	one-carbon compound transport	GO:0019755	P	6	2.11E-03	2.31E-02
7	2922	F2 vs. M2	+	Metabolism	Transporters	organic acid transmembrane transporter activity	GO:0005342	F	80	2.35E-03	4.80E-02
7	2923	F2 vs. M2	+	Metabolism	Transporters	organic anion transport	GO:0015711	P	102	1.81E-03	2.07E-02
7	2924	F2 vs. M2	+	Metabolism	Transporters	positive chemotaxis	GO:0050918	P	15	5.29E-03	4.55E-02
7	2925	F2 vs. M2	+	Metabolism	Transporters	primary active transmembrane transporter activity	GO:0015399	F	128	2.09E-04	8.43E-03
7	2926	F2 vs. M2	+	Metabolism	Transporters	proton transmembrane transporter activity	GO:0015078	F	92	2.65E-08	4.21E-06
7	2927	F2 vs. M2	+	Metabolism	Transporters	secondary active transmembrane transporter activity	GO:0015291	F	137	8.22E-12	2.78E-09
7	2928	F2 vs. M2	+	Metabolism	Transporters	secretory vesicle	GO:0099503	C	98	4.88E-04	3.96E-03
7	2929	F2 vs. M2	+	Metabolism	Transporters	solute:anion antiporter activity	GO:0140323	F	19	2.64E-05	1.70E-03
7	2930	F2 vs. M2	+	Metabolism	Transporters	solute:proton symporter activity	GO:0015295	F	16	1.18E-03	3.00E-02
7	2931	F2 vs. M2	+	Metabolism	Transporters	trans-Golgi network	GO:0005802	C	178	1.18E-09	2.66E-08
7	2932	F2 vs. M2	+	Metabolism	Transporters	transmembrane transport	GO:0055085	P	473	5.13E-14	1.61E-11
7	2933	F2 vs. M2	+	Metabolism	Transporters	transmembrane transporter activity	GO:0022857	F	665	0.00E+00	0.00E+00
7	2934	F2 vs. M2	+	Metabolism	Transporters	transport	GO:0006810	P	1165	4.35E-08	2.90E-06
7	2935	F2 vs. M2	+	Metabolism	Transporters	transporter activity	GO:0005215	F	688	0.00E+00	0.00E+00
7	2936	F2 vs. M2	+	Metabolism	Transporters	urea transmembrane transporter activity	GO:0015204	F	5	1.31E-03	3.15E-02
7	2937	F2 vs. M2	+	Metabolism	Transporters	urea transport	GO:0015840	P	4	5.93E-03	4.95E-02
7	2938	F2 vs. M2	+	Metabolism	Transporters	vesicle	GO:0031982	C	421	5.59E-08	1.00E-06
7	2939	F2 vs. M2	+	Metabolism	Transporters	water channel activity	GO:0015250	F	20	1.12E-04	4.89E-03
7	2940	F2 vs. M2	+	Metabolism	Transporters	water transmembrane transporter activity	GO:0005372	F	20	1.12E-04	4.97E-03
7	2941	F2 vs. M2	+	Metabolism	Transporters	xenobiotic transmembrane transporter activity	GO:0042910	F	40	6.63E-04	1.99E-02
7	2942	F2 vs. M2	+	Regulation	Protein modification	Golgi apparatus	GO:0005794	C	634	1.55E-10	3.86E-09
7	2943	F2 vs. M2	+	Regulation	Protein modification	Golgi membrane	GO:0000139	C	59	4.49E-03	2.75E-02
7	2944	F2 vs. M2	+	Regulation	Protein modification	protein disulfide oxidoreductase activity	GO:0015035	F	30	7.24E-04	2.10E-02
7	2945	F2 vs. M2	+	Regulation	Regulation	regulation of biological quality	GO:0065008	P	499	2.59E-03	2.73E-02
7	2946	F2 vs. M2	+	Regulation	Regulation	regulation of cell morphogenesis involved in differentiation	GO:0010769	P	26	8.31E-05	1.76E-03
7	2947	F2 vs. M2	+	Regulation	Regulation	regulation of pollen tube growth	GO:0080092	P	23	4.11E-06	1.40E-04
7	2948	F2 vs. M2	+	Regulation	Translation	poly(U) RNA binding	GO:0008266	F	20	4.58E-04	1.57E-02
7	2949	F2 vs. M2	+	Signaling and response	Signaling	acid phosphatase activity	GO:0003993	F	23	7.55E-04	2.15E-02
7	2950	F2 vs. M2	+	Signaling and response	Signaling	calcium ion binding	GO:0005509	F	134	1.02E-06	1.15E-04
7	2951	F2 vs. M2	+	Signaling and response	Signaling	cellular response to abiotic stimulus	GO:0071214	P	98	1.34E-03	1.63E-02
7	2952	F2 vs. M2	+	Signaling and response	Signaling	cellular response to acid chemical	GO:0071229	P	24	4.57E-03	4.12E-02
7	2953	F2 vs. M2	+	Signaling and response	Signaling	cellular response to chemical stimulus	GO:0070887	P	537	2.73E-03	2.81E-02
7	2954	F2 vs. M2	+	Signaling and response	Signaling	cellular response to cold	GO:0070417	P	17	2.61E-03	2.74E-02
7	2955	F2 vs. M2	+	Signaling and response	Signaling	cellular response to environmental stimulus	GO:0104004	P	98	1.34E-03	1.63E-02
7	2956	F2 vs. M2	+	Signaling and response	Signaling	cellular response to external stimulus	GO:0071496	P	125	2.70E-04	4.62E-03
7	2957	F2 vs. M2	+	Signaling and response	Signaling	cellular response to extracellular stimulus	GO:0031668	P	120	5.95E-05	1.30E-03
7	2958	F2 vs. M2	+	Signaling and response	Signaling	cellular response to light stimulus	GO:0071482	P	53	4.18E-03	3.82E-02
7	2959	F2 vs. M2	+	Signaling and response	Signaling	cellular response to oxygen levels	GO:0071453	P	116	3.41E-03	3.32E-02
7	2960	F2 vs. M2	+	Signaling and response	Signaling	cellular response to water stimulus	GO:0071462	P	21	4.93E-04	7.63E-03
7	2961	F2 vs. M2	+	Signaling and response	Signaling	dephosphorylation	GO:0016311	P	104	7.71E-05	1.65E-03
7	2962	F2 vs. M2	+	Signaling and response	Signaling	disulfide oxidoreductase activity	GO:0015036	F	39	8.09E-06	6.43E-04
7	2963	F2 vs. M2	+	Signaling and response	Signaling	phosphatase activity	GO:0016791	F	165	2.54E-04	9.41E-03
7	2964	F2 vs. M2	+	Signaling and response	Signaling	phosphatidylinositol dephosphorylation	GO:0046856	P	12	4.93E-03	4.38E-02
7	2965	F2 vs. M2	+	Signaling and response	Signaling	phospholipase C activity	GO:0004629	F	4	1.41E-03	3.38E-02
7	2966	F2 vs. M2	+	Signaling and response	Signaling	phospholipid dephosphorylation	GO:0046839	P	12	4.93E-03	4.38E-02
7	2967	F2 vs. M2	+	Signaling and response	Signaling	regulation of jasmonic acid mediated signaling pathway	GO:2000022	P	24	2.31E-03	2.48E-02
7	2968	F2 vs. M2	+	Signaling and response	Signaling	response to abiotic stimulus	GO:0009628	P	1185	3.31E-13	8.41E-11
7	2969	F2 vs. M2	+	Signaling and response	Signaling	response to acid chemical	GO:0001101	P	237	7.64E-08	4.53E-06
7	2970	F2 vs. M2	+	Signaling and response	Signaling	response to bacterium	GO:0009617	P	278	2.04E-07	1.07E-05
7	2971	F2 vs. M2	+	Signaling and response	Signaling	response to biotic stimulus	GO:0009607	P	578	6.67E-09	6.59E-07
7	2972	F2 vs. M2	+	Signaling and response	Signaling	response to cadmium ion	GO:0046686	P	221	1.22E-09	1.42E-07
7	2973	F2 vs. M2	+	Signaling and response	Signaling	response to chemical	GO:0042221	P	1442	6.99E-15	2.67E-12
7	2974	F2 vs. M2	+	Signaling and response	Signaling	response to chitin	GO:0010200	P	67	1.08E-05	3.12E-04
7	2975	F2 vs. M2	+	Signaling and response	Signaling	response to endogenous stimulus	GO:0009719	P	656	4.91E-04	7.63E-03
7	2976	F2 vs. M2	+	Signaling and response	Signaling	response to external biotic stimulus	GO:0043207	P	577	6.75E-09	6.55E-07
7	2977	F2 vs. M2	+	Signaling and response	Signaling	response to external stimulus	GO:0009605	P	801	4.42E-12	8.74E-10
7	2978	F2 vs. M2	+	Signaling and response	Signaling	response to extracellular stimulus	GO:0009991	P	144	1.29E-06	5.37E-05
7	2979	F2 vs. M2	+	Signaling and response	Signaling	response to hormone	GO:0009725	P	651	4.41E-04	7.07E-03
7	2980	F2 vs. M2	+	Signaling and response	Signaling	response to inorganic substance	GO:0010035	P	569	1.55E-15	6.38E-13
7	2981	F2 vs. M2	+	Signaling and response	Signaling	response to jasmonic acid	GO:0009753	P	109	1.40E-05	3.90E-04
7	2982	F2 vs. M2	+	Signaling and response	Signaling	response to karrikin	GO:0080167	P	78	3.06E-05	7.74E-04
7	2983	F2 vs. M2	+	Signaling and response	Signaling	response to light intensity	GO:0009642	P	87	8.88E-05	1.84E-03

7	2984	F2 vs. M2	+	Signaling and response	Signaling	response to light stimulus	GO:0009416	P	399	6.94E-08	4.21E-06
7	2985	F2 vs. M2	+	Signaling and response	Signaling	response to lipid	GO:0033993	P	381	9.02E-04	1.21E-02
7	2986	F2 vs. M2	+	Signaling and response	Signaling	response to metal ion	GO:0010038	P	283	1.05E-08	9.07E-07
7	2987	F2 vs. M2	+	Signaling and response	Signaling	response to nitrogen compound	GO:1901698	P	148	7.25E-04	1.04E-02
7	2988	F2 vs. M2	+	Signaling and response	Signaling	response to nutrient levels	GO:0031667	P	123	1.86E-04	3.34E-03
7	2989	F2 vs. M2	+	Signaling and response	Signaling	response to organic substance	GO:0010033	P	897	2.55E-06	9.27E-05
7	2990	F2 vs. M2	+	Signaling and response	Signaling	response to oxygen levels	GO:0070482	P	133	3.87E-03	3.60E-02
7	2991	F2 vs. M2	+	Signaling and response	Signaling	response to oxygen-containing compound	GO:1901700	P	839	5.11E-14	1.70E-11
7	2992	F2 vs. M2	+	Signaling and response	Signaling	response to stimulus	GO:0050896	P	2826	8.32E-12	1.43E-09
7	2993	F2 vs. M2	+	Signaling and response	Signaling	response to temperature stimulus	GO:0009266	P	374	1.32E-07	7.28E-06
7	2994	F2 vs. M2	+	Signaling and response	Signaling	response to water	GO:0009415	P	225	5.12E-08	3.25E-06
7	2995	F2 vs. M2	+	Signaling and response	Signaling	stomatal movement	GO:0010118	P	33	4.38E-03	3.97E-02
7	2996	F2 vs. M2	+	Signaling and response	Signaling	taxis	GO:0042330	P	15	5.29E-03	4.56E-02
7	2997	F2 vs. M2	+	Signaling and response	Stress	anion homeostasis	GO:0055081	P	30	6.38E-04	9.35E-03
7	2998	F2 vs. M2	+	Signaling and response	Stress	antioxidant activity	GO:0016209	F	70	3.03E-06	2.82E-04
7	2999	F2 vs. M2	+	Signaling and response	Stress	catalase activity	GO:0004096	F	5	2.50E-03	4.96E-02
7	3000	F2 vs. M2	+	Signaling and response	Stress	cation homeostasis	GO:0055080	P	112	2.26E-05	5.91E-04
7	3001	F2 vs. M2	+	Signaling and response	Stress	cell redox homeostasis	GO:0045454	P	44	2.82E-03	2.88E-02
7	3002	F2 vs. M2	+	Signaling and response	Stress	cellular cation homeostasis	GO:0030003	P	76	5.29E-04	8.04E-03
7	3003	F2 vs. M2	+	Signaling and response	Stress	cellular chemical homeostasis	GO:0055082	P	95	1.58E-03	1.88E-02
7	3004	F2 vs. M2	+	Signaling and response	Stress	cellular homeostasis	GO:0019725	P	143	9.26E-05	1.87E-03
7	3005	F2 vs. M2	+	Signaling and response	Stress	cellular ion homeostasis	GO:0006873	P	80	3.09E-04	5.23E-03
7	3006	F2 vs. M2	+	Signaling and response	Stress	cellular monovalent inorganic cation homeostasis	GO:0030004	P	22	5.31E-03	4.54E-02
7	3007	F2 vs. M2	+	Signaling and response	Stress	cellular oxidant detoxification	GO:0098869	P	16	2.52E-04	4.43E-03
7	3008	F2 vs. M2	+	Signaling and response	Stress	cellular response to decreased oxygen levels	GO:0036294	P	115	2.73E-03	2.81E-02
7	3009	F2 vs. M2	+	Signaling and response	Stress	cellular response to high light intensity	GO:0071486	P	5	3.23E-03	3.18E-02
7	3010	F2 vs. M2	+	Signaling and response	Stress	cellular response to hypoxia	GO:0071456	P	113	4.30E-03	3.91E-02
7	3011	F2 vs. M2	+	Signaling and response	Stress	cellular response to light intensity	GO:0071484	P	7	2.05E-03	2.27E-02
7	3012	F2 vs. M2	+	Signaling and response	Stress	cellular response to oxidative stress	GO:0034599	P	39	1.62E-03	1.92E-02
7	3013	F2 vs. M2	+	Signaling and response	Stress	cellular response to oxygen radical	GO:0071450	P	7	5.06E-03	4.42E-02
7	3014	F2 vs. M2	+	Signaling and response	Stress	cellular response to phosphate starvation	GO:0016036	P	43	2.65E-04	4.57E-03
7	3015	F2 vs. M2	+	Signaling and response	Stress	cellular response to superoxide	GO:0071451	P	7	5.06E-03	4.43E-02
7	3016	F2 vs. M2	+	Signaling and response	Stress	cellular response to toxic substance	GO:0097237	P	24	3.68E-03	3.49E-02
7	3017	F2 vs. M2	+	Signaling and response	Stress	cellular response to water deprivation	GO:0042631	P	21	4.93E-04	7.66E-03
7	3018	F2 vs. M2	+	Signaling and response	Stress	chemical homeostasis	GO:0048878	P	206	5.81E-07	2.70E-05
7	3019	F2 vs. M2	+	Signaling and response	Stress	defense response	GO:0006952	P	485	1.49E-06	6.03E-05
7	3020	F2 vs. M2	+	Signaling and response	Stress	defense response to bacterium	GO:0042742	P	227	7.94E-07	3.56E-05
7	3021	F2 vs. M2	+	Signaling and response	Stress	defense response to fungus, incompatible interaction	GO:0009817	P	19	2.29E-03	2.47E-02
7	3022	F2 vs. M2	+	Signaling and response	Stress	defense response to Gram-negative bacterium	GO:0050829	P	13	2.31E-03	2.48E-02
7	3023	F2 vs. M2	+	Signaling and response	Stress	defense response to other organism	GO:0098542	P	415	2.39E-06	8.87E-05
7	3024	F2 vs. M2	+	Signaling and response	Stress	defense response, incompatible interaction	GO:0009814	P	93	2.51E-03	2.66E-02
7	3025	F2 vs. M2	+	Signaling and response	Stress	detoxification	GO:0098754	P	47	5.12E-05	1.17E-03
7	3026	F2 vs. M2	+	Signaling and response	Stress	divalent inorganic anion homeostasis	GO:0072505	P	13	2.04E-03	2.26E-02
7	3027	F2 vs. M2	+	Signaling and response	Stress	flavonoid biosynthetic process	GO:0009813	P	33	3.01E-03	3.03E-02
7	3028	F2 vs. M2	+	Signaling and response	Stress	flavonoid metabolic process	GO:0009812	P	37	4.88E-03	4.35E-02
7	3029	F2 vs. M2	+	Signaling and response	Stress	glutathione metabolic process	GO:0006749	P	21	1.28E-03	1.58E-02
7	3030	F2 vs. M2	+	Signaling and response	Stress	homeostatic process	GO:0042592	P	269	3.44E-06	1.22E-04
7	3031	F2 vs. M2	+	Signaling and response	Stress	hydrogen peroxide catabolic process	GO:0042744	P	16	3.20E-05	7.94E-04
7	3032	F2 vs. M2	+	Signaling and response	Stress	hydrogen peroxide metabolic process	GO:0042743	P	21	4.42E-06	1.48E-04
7	3033	F2 vs. M2	+	Signaling and response	Stress	hydrogen peroxide transmembrane transport	GO:0080170	P	4	4.02E-03	3.70E-02
7	3034	F2 vs. M2	+	Signaling and response	Stress	inorganic ion homeostasis	GO:0098771	P	122	1.49E-06	6.06E-05
7	3035	F2 vs. M2	+	Signaling and response	Stress	interspecies interaction between organisms	GO:0044419	P	591	5.38E-09	5.63E-07
7	3036	F2 vs. M2	+	Signaling and response	Stress	ion homeostasis	GO:0050801	P	145	2.02E-07	1.07E-05
7	3037	F2 vs. M2	+	Signaling and response	Stress	metal ion homeostasis	GO:0055065	P	88	3.80E-03	3.55E-02
7	3038	F2 vs. M2	+	Signaling and response	Stress	monovalent inorganic anion homeostasis	GO:0055083	P	13	3.47E-03	3.35E-02
7	3039	F2 vs. M2	+	Signaling and response	Stress	monovalent inorganic cation homeostasis	GO:0055067	P	38	8.16E-05	1.74E-03
7	3040	F2 vs. M2	+	Signaling and response	Stress	oxidoreductase activity, acting on peroxide as acceptor	GO:0016684	F	60	2.41E-04	9.15E-03
7	3041	F2 vs. M2	+	Signaling and response	Stress	phosphate ion homeostasis	GO:0055062	P	12	2.72E-03	2.81E-02
7	3042	F2 vs. M2	+	Signaling and response	Stress	protein repair	GO:0030091	P	17	3.62E-03	3.45E-02
7	3043	F2 vs. M2	+	Signaling and response	Stress	reactive nitrogen species metabolic process	GO:2001057	P	13	1.93E-03	2.17E-02
7	3044	F2 vs. M2	+	Signaling and response	Stress	reactive oxygen species metabolic process	GO:0072593	P	44	2.93E-08	2.11E-06
7	3045	F2 vs. M2	+	Signaling and response	Stress	regulation of pH	GO:0006885	P	26	8.17E-04	1.13E-02
7	3046	F2 vs. M2	+	Signaling and response	Stress	regulation of stomatal movement	GO:0010119	P	59	5.57E-04	8.40E-03
7	3047	F2 vs. M2	+	Signaling and response	Stress	removal of superoxide radicals	GO:0019430	P	7	5.06E-03	4.42E-02

7	3048	F2 vs. M2	+	Signaling and response	Stress	response to abscisic acid	GO:0009737	P	282	5.75E-05	1.27E-03
7	3049	F2 vs. M2	+	Signaling and response	Stress	response to alcohol	GO:0097305	P	286	8.38E-05	1.77E-03
7	3050	F2 vs. M2	+	Signaling and response	Stress	response to cold	GO:0009409	P	269	6.20E-10	8.06E-08
7	3051	F2 vs. M2	+	Signaling and response	Stress	response to decreased oxygen levels	GO:0036293	P	132	3.16E-03	3.14E-02
7	3052	F2 vs. M2	+	Signaling and response	Stress	response to desiccation	GO:0009269	P	16	3.47E-03	3.35E-02
7	3053	F2 vs. M2	+	Signaling and response	Stress	response to fungus	GO:0009620	P	178	9.01E-05	1.86E-03
7	3054	F2 vs. M2	+	Signaling and response	Stress	response to high light intensity	GO:0009644	P	44	5.61E-06	1.84E-04
7	3055	F2 vs. M2	+	Signaling and response	Stress	response to osmotic stress	GO:0006970	P	293	8.07E-04	1.13E-02
7	3056	F2 vs. M2	+	Signaling and response	Stress	response to other organism	GO:0051707	P	577	6.75E-09	6.44E-07
7	3057	F2 vs. M2	+	Signaling and response	Stress	response to oxidative stress	GO:0006979	P	253	2.91E-08	2.13E-06
7	3058	F2 vs. M2	+	Signaling and response	Stress	response to oxygen radical	GO:0000305	P	9	3.62E-03	3.44E-02
7	3059	F2 vs. M2	+	Signaling and response	Stress	response to ozone	GO:0010193	P	23	2.55E-03	2.70E-02
7	3060	F2 vs. M2	+	Signaling and response	Stress	response to radiation	GO:0009314	P	418	1.60E-06	6.37E-05
7	3061	F2 vs. M2	+	Signaling and response	Stress	response to reactive oxygen species	GO:0000302	P	95	8.52E-05	1.79E-03
7	3062	F2 vs. M2	+	Signaling and response	Stress	response to salt stress	GO:0009651	P	249	3.46E-03	3.35E-02
7	3063	F2 vs. M2	+	Signaling and response	Stress	response to starvation	GO:0042594	P	104	1.36E-03	1.66E-02
7	3064	F2 vs. M2	+	Signaling and response	Stress	response to stress	GO:0006950	P	1638	3.46E-09	3.69E-07
7	3065	F2 vs. M2	+	Signaling and response	Stress	response to superoxide	GO:0000303	P	9	3.62E-03	3.44E-02
7	3066	F2 vs. M2	+	Signaling and response	Stress	response to toxic substance	GO:0009636	P	58	8.16E-06	2.52E-04
7	3067	F2 vs. M2	+	Signaling and response	Stress	response to water deprivation	GO:0009414	P	220	5.52E-08	3.47E-06
7	3068	F2 vs. M2	+	Signaling and response	Stress	response to wounding	GO:0009611	P	137	2.46E-08	1.90E-06
7	3069	F2 vs. M2	+	Signaling and response	Stress	superoxide metabolic process	GO:0006801	P	7	5.06E-03	4.44E-02
7	3070	F2 vs. M2	+	Signaling and response	Stress	toxin metabolic process	GO:0009404	P	30	8.65E-04	1.17E-02
7	3071	F2 vs. M2	+	Signaling and response	Stress	trivalent inorganic anion homeostasis	GO:0072506	P	12	2.72E-03	2.81E-02
7	3072	F2 vs. M2	-	Development	Cell division	alpha DNA polymerase:primase complex	GO:0005658	C	4	8.35E-03	4.56E-02
7	3073	F2 vs. M2	-	Development	Cell division	ATP-dependent microtubule motor activity	GO:1990939	F	8	5.66E-04	1.82E-02
7	3074	F2 vs. M2	-	Development	Cell division	base-excision repair	GO:0006284	P	17	1.41E-03	1.71E-02
7	3075	F2 vs. M2	-	Development	Cell division	Cajal body	GO:0015030	C	10	6.90E-03	3.85E-02
7	3076	F2 vs. M2	-	Development	Cell division	cell cycle	GO:0007049	P	250	6.29E-12	1.16E-09
7	3077	F2 vs. M2	-	Development	Cell division	cell cycle checkpoint	GO:0000075	P	25	1.43E-04	2.70E-03
7	3078	F2 vs. M2	-	Development	Cell division	cell cycle G1/S phase transition	GO:0044843	P	7	1.62E-03	1.92E-02
7	3079	F2 vs. M2	-	Development	Cell division	cell cycle phase transition	GO:0044770	P	32	7.63E-09	7.02E-07
7	3080	F2 vs. M2	-	Development	Cell division	cell cycle process	GO:0022402	P	229	1.40E-12	2.87E-10
7	3081	F2 vs. M2	-	Development	Cell division	chromatin assembly	GO:0031497	P	34	4.71E-03	4.22E-02
7	3082	F2 vs. M2	-	Development	Cell division	chromosome	GO:0005694	C	237	8.93E-12	2.49E-10
7	3083	F2 vs. M2	-	Development	Cell division	chromosome organization involved in meiotic cell cycle	GO:0070192	P	25	8.19E-04	1.13E-02
7	3084	F2 vs. M2	-	Development	Cell division	chromosome segregation	GO:0007059	P	53	2.74E-05	6.99E-04
7	3085	F2 vs. M2	-	Development	Cell division	chromosome, centromeric region	GO:0000775	C	21	1.47E-04	1.34E-03
7	3086	F2 vs. M2	-	Development	Cell division	condensed chromosome	GO:0000793	C	32	5.62E-07	7.63E-06
7	3087	F2 vs. M2	-	Development	Cell division	condensed chromosome kinetochore	GO:0000777	C	5	3.03E-03	2.00E-02
7	3088	F2 vs. M2	-	Development	Cell division	condensed chromosome, centromeric region	GO:0000779	C	11	6.54E-04	5.14E-03
7	3089	F2 vs. M2	-	Development	Cell division	condensed nuclear chromosome	GO:0000794	C	21	8.27E-07	1.11E-05
7	3090	F2 vs. M2	-	Development	Cell division	condensed nuclear chromosome kinetochore	GO:0000778	C	3	8.56E-03	4.65E-02
7	3091	F2 vs. M2	-	Development	Cell division	condensed nuclear chromosome, centromeric region	GO:0000780	C	8	3.32E-03	2.14E-02
7	3092	F2 vs. M2	-	Development	Cell division	cyclin-dependent protein kinase holoenzyme complex	GO:0000307	C	34	5.89E-08	1.04E-06
7	3093	F2 vs. M2	-	Development	Cell division	cyclin-dependent protein serine/threonine kinase regulator activity	GO:0016538	F	30	7.68E-06	6.29E-04
7	3094	F2 vs. M2	-	Development	Cell division	cytokinesis by cell plate formation	GO:0000911	P	38	3.10E-03	3.10E-02
7	3095	F2 vs. M2	-	Development	Cell division	DNA biosynthetic process	GO:0071897	P	13	4.62E-06	1.52E-04
7	3096	F2 vs. M2	-	Development	Cell division	DNA polymerase complex	GO:0042575	C	11	4.18E-04	3.51E-03
7	3097	F2 vs. M2	-	Development	Cell division	DNA recombination	GO:0006310	P	69	1.43E-05	3.90E-04
7	3098	F2 vs. M2	-	Development	Cell division	DNA repair	GO:0006281	P	159	1.40E-11	2.34E-09
7	3099	F2 vs. M2	-	Development	Cell division	DNA repair complex	GO:1990391	C	12	8.28E-03	4.55E-02
7	3100	F2 vs. M2	-	Development	Cell division	DNA replication	GO:0006260	P	71	3.05E-10	4.17E-08
7	3101	F2 vs. M2	-	Development	Cell division	DNA replication factor A complex	GO:0005662	C	5	4.65E-04	3.81E-03
7	3102	F2 vs. M2	-	Development	Cell division	DNA replication initiation	GO:0006270	P	14	8.75E-06	2.65E-04
7	3103	F2 vs. M2	-	Development	Cell division	DNA replication origin binding	GO:0003688	F	12	2.62E-05	1.72E-03
7	3104	F2 vs. M2	-	Development	Cell division	DNA strand elongation	GO:0022616	P	11	3.18E-05	7.93E-04
7	3105	F2 vs. M2	-	Development	Cell division	DNA strand elongation involved in DNA replication	GO:0006271	P	11	3.18E-05	7.96E-04
7	3106	F2 vs. M2	-	Development	Cell division	DNA unwinding involved in DNA replication	GO:0006268	P	8	9.57E-04	1.27E-02
7	3107	F2 vs. M2	-	Development	Cell division	DNA-dependent DNA replication	GO:0006261	P	62	1.35E-10	2.12E-08
7	3108	F2 vs. M2	-	Development	Cell division	double-strand break repair	GO:0006302	P	60	1.62E-06	6.35E-05
7	3109	F2 vs. M2	-	Development	Cell division	double-strand break repair via break-induced replication	GO:0000727	P	7	3.88E-03	3.60E-02
7	3110	F2 vs. M2	-	Development	Cell division	double-strand break repair via homologous recombination	GO:0000724	P	40	4.21E-04	6.87E-03

7	3111	F2 vs. M2	-	Development	Cell division	endonuclease activity	GO:0004519	F	52	5.19E-06	4.52E-04
7	3112	F2 vs. M2	-	Development	Cell division	G1/S transition of mitotic cell cycle	GO:0000082	P	6	1.71E-03	1.99E-02
7	3113	F2 vs. M2	-	Development	Cell division	kinetochore	GO:0000776	C	12	4.91E-04	3.95E-03
7	3114	F2 vs. M2	-	Development	Cell division	MCM complex	GO:0042555	C	6	9.02E-03	4.84E-02
7	3115	F2 vs. M2	-	Development	Cell division	meiotic cell cycle	GO:0051321	P	82	6.36E-05	1.38E-03
7	3116	F2 vs. M2	-	Development	Cell division	meiotic cell cycle process	GO:1903046	P	74	1.39E-03	1.69E-02
7	3117	F2 vs. M2	-	Development	Cell division	meiotic chromosome segregation	GO:0045132	P	26	5.71E-03	4.81E-02
7	3118	F2 vs. M2	-	Development	Cell division	microtubule cytoskeleton organization involved in mitosis	GO:1902850	P	14	2.63E-03	2.76E-02
7	3119	F2 vs. M2	-	Development	Cell division	mismatch repair	GO:0006298	P	11	4.49E-04	7.13E-03
7	3120	F2 vs. M2	-	Development	Cell division	mitotic cell cycle	GO:0000278	P	141	2.35E-08	1.87E-06
7	3121	F2 vs. M2	-	Development	Cell division	mitotic cell cycle checkpoint	GO:0007093	P	18	2.23E-03	2.42E-02
7	3122	F2 vs. M2	-	Development	Cell division	mitotic cell cycle phase transition	GO:0044772	P	31	8.87E-09	7.89E-07
7	3123	F2 vs. M2	-	Development	Cell division	mitotic cell cycle process	GO:1903047	P	116	6.42E-10	8.16E-08
7	3124	F2 vs. M2	-	Development	Cell division	mitotic checkpoint complex	GO:0033597	C	4	4.84E-03	2.93E-02
7	3125	F2 vs. M2	-	Development	Cell division	mitotic DNA replication	GO:1902969	P	6	3.44E-04	5.74E-03
7	3126	F2 vs. M2	-	Development	Cell division	mitotic nuclear division	GO:0140014	P	34	1.78E-03	2.04E-02
7	3127	F2 vs. M2	-	Development	Cell division	mitotic spindle assembly checkpoint	GO:0007094	P	8	9.85E-04	1.31E-02
7	3128	F2 vs. M2	-	Development	Cell division	mitotic spindle checkpoint	GO:0071174	P	8	9.85E-04	1.29E-02
7	3129	F2 vs. M2	-	Development	Cell division	negative regulation of cell cycle	GO:0045786	P	44	3.42E-05	8.38E-04
7	3130	F2 vs. M2	-	Development	Cell division	negative regulation of cell cycle phase transition	GO:1901988	P	14	8.19E-04	1.13E-02
7	3131	F2 vs. M2	-	Development	Cell division	negative regulation of cell cycle process	GO:0010948	P	26	4.23E-04	6.85E-03
7	3132	F2 vs. M2	-	Development	Cell division	negative regulation of chromosome organization	GO:2001251	P	10	1.82E-04	3.29E-03
7	3133	F2 vs. M2	-	Development	Cell division	negative regulation of chromosome segregation	GO:0051985	P	8	9.85E-04	1.30E-02
7	3134	F2 vs. M2	-	Development	Cell division	negative regulation of chromosome separation	GO:1905819	P	8	9.85E-04	1.27E-02
7	3135	F2 vs. M2	-	Development	Cell division	negative regulation of metaphase/anaphase transition of cell cycle	GO:1902100	P	8	9.85E-04	1.28E-02
7	3136	F2 vs. M2	-	Development	Cell division	negative regulation of mitotic cell cycle	GO:0045930	P	24	1.04E-03	1.32E-02
7	3137	F2 vs. M2	-	Development	Cell division	negative regulation of mitotic cell cycle phase transition	GO:1901991	P	14	8.19E-04	1.13E-02
7	3138	F2 vs. M2	-	Development	Cell division	negative regulation of mitotic metaphase/anaphase transition	GO:0045841	P	8	9.85E-04	1.28E-02
7	3139	F2 vs. M2	-	Development	Cell division	negative regulation of mitotic nuclear division	GO:0045839	P	12	5.78E-03	4.86E-02
7	3140	F2 vs. M2	-	Development	Cell division	negative regulation of mitotic sister chromatid segregation	GO:0033048	P	8	9.85E-04	1.30E-02
7	3141	F2 vs. M2	-	Development	Cell division	negative regulation of mitotic sister chromatid separation	GO:2000816	P	8	9.85E-04	1.30E-02
7	3142	F2 vs. M2	-	Development	Cell division	negative regulation of nuclear division	GO:0051784	P	16	5.78E-04	8.64E-03
7	3143	F2 vs. M2	-	Development	Cell division	negative regulation of reproductive process	GO:2000242	P	33	6.28E-04	9.23E-03
7	3144	F2 vs. M2	-	Development	Cell division	negative regulation of sister chromatid segregation	GO:0033046	P	8	9.85E-04	1.29E-02
7	3145	F2 vs. M2	-	Development	Cell division	nuclear chromosome segregation	GO:0098813	P	47	1.60E-04	2.94E-03
7	3146	F2 vs. M2	-	Development	Cell division	nuclear division	GO:0000280	P	77	1.01E-05	2.95E-04
7	3147	F2 vs. M2	-	Development	Cell division	nuclear DNA replication	GO:0033260	P	11	8.57E-04	1.16E-02
7	3148	F2 vs. M2	-	Development	Cell division	nuclear origin of replication recognition complex	GO:0005664	C	4	6.03E-03	3.43E-02
7	3149	F2 vs. M2	-	Development	Cell division	nuclear replication fork	GO:0043596	C	18	5.38E-07	7.41E-06
7	3150	F2 vs. M2	-	Development	Cell division	nuclease activity	GO:0004518	F	98	8.79E-07	1.03E-04
7	3151	F2 vs. M2	-	Development	Cell division	nucleic acid metabolic process	GO:0090304	P	854	0.00E+00	0.00E+00
7	3152	F2 vs. M2	-	Development	Cell division	nucleobase-containing compound metabolic process	GO:0006139	P	1089	7.33E-15	2.61E-12
7	3153	F2 vs. M2	-	Development	Cell division	nucleotide-excision repair	GO:0006289	P	29	1.95E-04	3.49E-03
7	3154	F2 vs. M2	-	Development	Cell division	origin recognition complex	GO:0000808	C	4	6.03E-03	3.41E-02
7	3155	F2 vs. M2	-	Development	Cell division	recombinational repair	GO:0000725	P	44	4.12E-04	6.74E-03
7	3156	F2 vs. M2	-	Development	Cell division	regulation of cell cycle	GO:0051726	P	139	2.08E-11	3.36E-09
7	3157	F2 vs. M2	-	Development	Cell division	regulation of cell cycle G2/M phase transition	GO:1902749	P	16	6.60E-04	9.65E-03
7	3158	F2 vs. M2	-	Development	Cell division	regulation of cell cycle phase transition	GO:1901987	P	31	5.10E-08	3.28E-06
7	3159	F2 vs. M2	-	Development	Cell division	regulation of cell cycle process	GO:0010564	P	71	1.11E-08	9.37E-07
7	3160	F2 vs. M2	-	Development	Cell division	regulation of cell division	GO:0051302	P	44	3.87E-06	1.36E-04
7	3161	F2 vs. M2	-	Development	Cell division	regulation of chromosome organization	GO:0033044	P	36	9.76E-06	2.88E-04
7	3162	F2 vs. M2	-	Development	Cell division	regulation of chromosome segregation	GO:0051983	P	15	8.86E-05	1.85E-03
7	3163	F2 vs. M2	-	Development	Cell division	regulation of chromosome separation	GO:1905818	P	14	4.47E-05	1.03E-03
7	3164	F2 vs. M2	-	Development	Cell division	regulation of cyclin-dependent protein kinase activity	GO:1904029	P	35	4.42E-04	7.04E-03
7	3165	F2 vs. M2	-	Development	Cell division	regulation of DNA metabolic process	GO:0051052	P	41	1.05E-03	1.33E-02
7	3166	F2 vs. M2	-	Development	Cell division	regulation of DNA replication	GO:0006275	P	32	2.52E-03	2.67E-02
7	3167	F2 vs. M2	-	Development	Cell division	regulation of G2/M transition of mitotic cell cycle	GO:0010389	P	15	5.00E-04	7.71E-03
7	3168	F2 vs. M2	-	Development	Cell division	regulation of metaphase/anaphase transition of cell cycle	GO:1902099	P	14	4.47E-05	1.04E-03
7	3169	F2 vs. M2	-	Development	Cell division	regulation of mitotic cell cycle	GO:0007346	P	46	9.38E-08	5.27E-06
7	3170	F2 vs. M2	-	Development	Cell division	regulation of mitotic cell cycle phase transition	GO:1901990	P	30	3.54E-08	2.39E-06
7	3171	F2 vs. M2	-	Development	Cell division	regulation of mitotic metaphase/anaphase transition	GO:0030071	P	14	4.47E-05	1.04E-03
7	3172	F2 vs. M2	-	Development	Cell division	regulation of mitotic nuclear division	GO:0007088	P	16	7.41E-04	1.05E-02
7	3173	F2 vs. M2	-	Development	Cell division	regulation of mitotic sister chromatid segregation	GO:0033047	P	8	9.85E-04	1.30E-02

7	3174	F2 vs. M2	-	Development	Cell division	regulation of mitotic sister chromatid separation	GO:0010965	P	14	4.47E-05	1.05E-03
7	3175	F2 vs. M2	-	Development	Cell division	regulation of nuclear division	GO:0051783	P	27	4.18E-05	9.95E-04
7	3176	F2 vs. M2	-	Development	Cell division	regulation of sister chromatid segregation	GO:0033045	P	15	8.86E-05	1.84E-03
7	3177	F2 vs. M2	-	Development	Cell division	replication fork	GO:0005657	C	26	1.16E-07	1.94E-06
7	3178	F2 vs. M2	-	Development	Cell division	replisome	GO:0030894	C	14	1.36E-07	2.19E-06
7	3179	F2 vs. M2	-	Development	Cell division	RNA metabolic process	GO:0016070	P	657	0.00E+00	0.00E+00
7	3180	F2 vs. M2	-	Development	Cell division	SCF ubiquitin ligase complex	GO:0019005	C	39	6.35E-04	5.03E-03
7	3181	F2 vs. M2	-	Development	Cell division	single-stranded DNA binding	GO:0003697	F	40	7.71E-07	9.46E-05
7	3182	F2 vs. M2	-	Development	Cell division	sister chromatid cohesion	GO:0007062	P	20	5.47E-03	4.65E-02
7	3183	F2 vs. M2	-	Development	Cell division	sister chromatid segregation	GO:0000819	P	30	1.64E-03	1.93E-02
7	3184	F2 vs. M2	-	Development	Cell division	spindle	GO:0005819	C	44	5.10E-07	7.23E-06
7	3185	F2 vs. M2	-	Development	Cell division	spindle assembly	GO:0051225	P	23	5.40E-04	8.18E-03
7	3186	F2 vs. M2	-	Development	Cell division	spindle assembly checkpoint	GO:0071173	P	8	9.85E-04	1.29E-02
7	3187	F2 vs. M2	-	Development	Cell division	spindle checkpoint	GO:0031577	P	8	9.85E-04	1.28E-02
7	3188	F2 vs. M2	-	Development	Cell division	spindle microtubule	GO:0005876	C	12	7.62E-05	7.24E-04
7	3189	F2 vs. M2	-	Development	Cell division	spindle organization	GO:0007051	P	31	4.36E-04	7.01E-03
7	3190	F2 vs. M2	-	Development	Cell division	telomere maintenance	GO:0000723	P	13	4.44E-03	4.02E-02
7	3191	F2 vs. M2	-	Development	Cell division	telomere maintenance via telomerase	GO:0007004	P	4	1.89E-03	2.14E-02
7	3192	F2 vs. M2	-	Development	Cell division	telomere maintenance via telomere lengthening	GO:0010833	P	4	1.89E-03	2.14E-02
7	3193	F2 vs. M2	-	Development	Cell division	telomere organization	GO:0032200	P	13	4.44E-03	4.01E-02
7	3194	F2 vs. M2	-	Development	Development	anatomical structure homeostasis	GO:0060249	P	14	2.75E-03	2.82E-02
7	3195	F2 vs. M2	-	Development	Development	anther wall tapetum development	GO:0048658	P	6	1.38E-04	2.61E-03
7	3196	F2 vs. M2	-	Development	Development	cellular component assembly	GO:0022607	P	450	3.33E-03	3.26E-02
7	3197	F2 vs. M2	-	Development	Development	chromatin	GO:0000785	C	146	7.47E-04	5.77E-03
7	3198	F2 vs. M2	-	Development	Development	chromatin assembly or disassembly	GO:0006333	P	40	3.53E-03	3.38E-02
7	3199	F2 vs. M2	-	Development	Development	chromatin organization	GO:0006325	P	168	1.33E-07	7.23E-06
7	3200	F2 vs. M2	-	Development	Development	chromosomal region	GO:0098687	C	31	9.53E-06	1.05E-04
7	3201	F2 vs. M2	-	Development	Development	chromosome organization	GO:0051276	P	249	1.12E-13	3.31E-11
7	3202	F2 vs. M2	-	Development	Development	cortical cytoskeleton organization	GO:0030865	P	40	1.75E-03	2.01E-02
7	3203	F2 vs. M2	-	Development	Development	intracellular non-membrane-bounded organelle	GO:0043232	C	948	0.00E+00	0.00E+00
7	3204	F2 vs. M2	-	Development	Development	intracellular organelle lumen	GO:0070013	C	719	0.00E+00	0.00E+00
7	3205	F2 vs. M2	-	Development	Development	kinetochore microtubule	GO:0005828	C	3	4.49E-03	2.73E-02
7	3206	F2 vs. M2	-	Development	Development	membrane-enclosed lumen	GO:0031974	C	719	0.00E+00	0.00E+00
7	3207	F2 vs. M2	-	Development	Development	microtubule	GO:0005874	C	75	9.43E-04	7.23E-03
7	3208	F2 vs. M2	-	Development	Development	microtubule associated complex	GO:0005875	C	37	6.66E-05	6.53E-04
7	3209	F2 vs. M2	-	Development	Development	microtubule cytoskeleton	GO:0015630	C	117	1.34E-05	1.47E-04
7	3210	F2 vs. M2	-	Development	Development	microtubule cytoskeleton organization	GO:0000226	P	99	1.74E-03	2.01E-02
7	3211	F2 vs. M2	-	Development	Development	microtubule-based process	GO:0007017	P	127	9.23E-05	1.87E-03
7	3212	F2 vs. M2	-	Development	Development	non-membrane-bounded organelle	GO:0043228	C	948	0.00E+00	0.00E+00
7	3213	F2 vs. M2	-	Development	Development	nuclear body	GO:0016604	C	67	1.28E-03	9.08E-03
7	3214	F2 vs. M2	-	Development	Development	nuclear chromatin	GO:0000790	C	115	3.80E-03	2.39E-02
7	3215	F2 vs. M2	-	Development	Development	nuclear chromosome	GO:0000228	C	162	5.68E-10	1.35E-08
7	3216	F2 vs. M2	-	Development	Development	nuclear lumen	GO:0031981	C	626	0.00E+00	0.00E+00
7	3217	F2 vs. M2	-	Development	Development	nuclear pore	GO:0005643	C	22	2.41E-03	1.63E-02
7	3218	F2 vs. M2	-	Development	Development	nuclear replisome	GO:0043601	C	14	1.36E-07	2.22E-06
7	3219	F2 vs. M2	-	Development	Development	nucleic acid phosphodiester bond hydrolysis	GO:0090305	P	42	1.07E-03	1.35E-02
7	3220	F2 vs. M2	-	Development	Development	nucleoplasm	GO:0005654	C	230	9.27E-10	2.15E-08
7	3221	F2 vs. M2	-	Development	Development	nucleus	GO:0005634	C	4325	5.03E-13	1.54E-11
7	3222	F2 vs. M2	-	Development	Development	organelle assembly	GO:0070925	P	112	2.65E-05	6.87E-04
7	3223	F2 vs. M2	-	Development	Development	organelle fission	GO:0048285	P	102	4.28E-06	1.44E-04
7	3224	F2 vs. M2	-	Development	Development	organelle lumen	GO:0043233	C	719	0.00E+00	0.00E+00
7	3225	F2 vs. M2	-	Development	Development	organelle organization	GO:0006996	P	895	5.74E-05	1.28E-03
7	3226	F2 vs. M2	-	Development	Development	phragmoplast	GO:0009524	C	36	1.24E-03	8.94E-03
7	3227	F2 vs. M2	-	Development	Development	reproductive shoot system development	GO:0090567	P	198	5.69E-03	4.80E-02
7	3228	F2 vs. M2	-	Development	Development	shoot system development	GO:0048367	P	376	3.45E-03	3.35E-02
7	3229	F2 vs. M2	-	Development	Development	stomatal complex development	GO:0010374	P	27	5.91E-03	4.94E-02
7	3230	F2 vs. M2	-	Development	Development	structural constituent of nuclear pore	GO:0017056	F	11	7.44E-04	2.14E-02
7	3231	F2 vs. M2	-	Development	Development	structural molecule activity	GO:0005198	F	267	2.14E-05	1.48E-03
7	3232	F2 vs. M2	-	Metabolism	Bioenergetics	ATP-dependent microtubule motor activity, plus-end-directed	GO:0008574	F	7	2.04E-03	4.33E-02
7	3233	F2 vs. M2	-	Metabolism	Bioenergetics	DNA-dependent ATPase activity	GO:0008094	F	58	3.06E-04	1.09E-02
7	3234	F2 vs. M2	-	Metabolism	Bioenergetics	positive regulation of ATPase activity	GO:0032781	P	10	3.20E-03	3.17E-02
7	3235	F2 vs. M2	-	Metabolism	Bioenergetics	regulation of ATPase activity	GO:0043462	P	12	3.18E-03	3.15E-02
7	3236	F2 vs. M2	-	Metabolism	Catabolism	cellular protein catabolic process	GO:0044257	P	288	5.48E-03	4.66E-02
7	3237	F2 vs. M2	-	Metabolism	Catabolism	Cul4-RING E3 ubiquitin ligase complex	GO:0080008	C	80	2.53E-05	2.70E-04

7	3238	F2 vs. M2	-	Metabolism	Catabolism	cullin-RING ubiquitin ligase complex	GO:0031461	C	133	3.36E-09	7.10E-08
7	3239	F2 vs. M2	-	Metabolism	Catabolism	endoribonuclease activity	GO:0004521	F	40	2.23E-04	8.72E-03
7	3240	F2 vs. M2	-	Metabolism	Catabolism	exonuclease activity	GO:0004527	F	45	1.94E-03	4.30E-02
7	3241	F2 vs. M2	-	Metabolism	Catabolism	modification-dependent macromolecule catabolic process	GO:0043632	P	255	7.88E-06	2.46E-04
7	3242	F2 vs. M2	-	Metabolism	Catabolism	modification-dependent protein catabolic process	GO:0019941	P	249	1.64E-05	4.43E-04
7	3243	F2 vs. M2	-	Metabolism	Catabolism	peptidase complex	GO:1905368	C	61	1.14E-03	8.49E-03
7	3244	F2 vs. M2	-	Metabolism	Catabolism	positive regulation of catabolic process	GO:0009896	P	38	1.77E-03	2.04E-02
7	3245	F2 vs. M2	-	Metabolism	Catabolism	positive regulation of cellular catabolic process	GO:0031331	P	37	1.13E-03	1.41E-02
7	3246	F2 vs. M2	-	Metabolism	Catabolism	positive regulation of cellular protein catabolic process	GO:1903364	P	21	2.87E-03	2.91E-02
7	3247	F2 vs. M2	-	Metabolism	Catabolism	positive regulation of proteasomal protein catabolic process	GO:1901800	P	19	2.10E-03	2.30E-02
7	3248	F2 vs. M2	-	Metabolism	Catabolism	positive regulation of proteasomal ubiquitin-dependent protein catabolic process	GO:0032436	P	19	2.10E-03	2.31E-02
7	3249	F2 vs. M2	-	Metabolism	Catabolism	positive regulation of protein catabolic process	GO:0045732	P	22	5.13E-03	4.45E-02
7	3250	F2 vs. M2	-	Metabolism	Catabolism	positive regulation of proteolysis	GO:0045862	P	22	2.08E-03	2.29E-02
7	3251	F2 vs. M2	-	Metabolism	Catabolism	positive regulation of proteolysis involved in cellular protein catabolic process	GO:1903052	P	21	2.87E-03	2.91E-02
7	3252	F2 vs. M2	-	Metabolism	Catabolism	positive regulation of ubiquitin-dependent protein catabolic process	GO:2000060	P	20	1.64E-03	1.93E-02
7	3253	F2 vs. M2	-	Metabolism	Catabolism	proteasomal protein catabolic process	GO:0010498	P	132	8.02E-04	1.12E-02
7	3254	F2 vs. M2	-	Metabolism	Catabolism	proteasome-mediated ubiquitin-dependent protein catabolic process	GO:0043161	P	124	4.34E-04	6.99E-03
7	3255	F2 vs. M2	-	Metabolism	Catabolism	protein-containing complex	GO:0032991	C	1615	0.00E+00	0.00E+00
7	3256	F2 vs. M2	-	Metabolism	Catabolism	proteolysis involved in cellular protein catabolic process	GO:0051603	P	282	1.71E-03	1.98E-02
7	3257	F2 vs. M2	-	Metabolism	Catabolism	regulation of catabolic process	GO:0009894	P	66	3.90E-03	3.61E-02
7	3258	F2 vs. M2	-	Metabolism	Catabolism	regulation of cellular protein catabolic process	GO:1903362	P	24	6.91E-04	9.97E-03
7	3259	F2 vs. M2	-	Metabolism	Catabolism	regulation of proteasomal protein catabolic process	GO:0061136	P	22	4.84E-04	7.57E-03
7	3260	F2 vs. M2	-	Metabolism	Catabolism	regulation of proteasomal ubiquitin-dependent protein catabolic process	GO:0032434	P	21	9.47E-04	1.27E-02
7	3261	F2 vs. M2	-	Metabolism	Catabolism	regulation of protein catabolic process	GO:0042176	P	32	3.63E-04	6.01E-03
7	3262	F2 vs. M2	-	Metabolism	Catabolism	regulation of proteolysis	GO:0030162	P	30	2.97E-03	2.99E-02
7	3263	F2 vs. M2	-	Metabolism	Catabolism	regulation of proteolysis involved in cellular protein catabolic process	GO:1903050	P	24	6.91E-04	9.94E-03
7	3264	F2 vs. M2	-	Metabolism	Catabolism	regulation of ubiquitin-dependent protein catabolic process	GO:2000058	P	22	7.39E-04	1.05E-02
7	3265	F2 vs. M2	-	Metabolism	Catabolism	ubiquitin ligase complex	GO:0000151	C	167	3.16E-10	7.70E-09
7	3266	F2 vs. M2	-	Metabolism	Catabolism	ubiquitin-dependent protein catabolic process	GO:0006511	P	244	2.09E-05	5.51E-04
7	3267	F2 vs. M2	-	Metabolism	Metabolism	amide biosynthetic process	GO:0043604	P	351	4.82E-04	7.57E-03
7	3268	F2 vs. M2	-	Metabolism	Metabolism	carboxy-terminal domain protein kinase complex	GO:0032806	C	8	5.82E-03	3.33E-02
7	3269	F2 vs. M2	-	Metabolism	Metabolism	catalytic activity, acting on DNA	GO:0140097	F	93	4.78E-06	4.30E-04
7	3270	F2 vs. M2	-	Metabolism	Metabolism	catalytic complex	GO:1902494	C	638	5.55E-16	2.11E-14
7	3271	F2 vs. M2	-	Metabolism	Metabolism	cellular aromatic compound metabolic process	GO:0006725	P	1319	2.26E-06	8.61E-05
7	3272	F2 vs. M2	-	Metabolism	Metabolism	cellular macromolecule biosynthetic process	GO:0034645	P	715	2.36E-08	1.85E-06
7	3273	F2 vs. M2	-	Metabolism	Metabolism	cellular macromolecule metabolic process	GO:0044260	P	2173	1.90E-10	2.74E-08
7	3274	F2 vs. M2	-	Metabolism	Metabolism	cellular nitrogen compound metabolic process	GO:0034641	P	1541	7.83E-12	1.39E-09
7	3275	F2 vs. M2	-	Metabolism	Metabolism	cellular protein metabolic process	GO:0044267	P	1636	8.25E-08	4.79E-06
7	3276	F2 vs. M2	-	Metabolism	Metabolism	cellular protein modification process	GO:0006464	P	1146	2.68E-05	6.88E-04
7	3277	F2 vs. M2	-	Metabolism	Metabolism	deoxyribonuclease activity	GO:0004536	F	16	7.07E-04	2.08E-02
7	3278	F2 vs. M2	-	Metabolism	Metabolism	DNA binding	GO:0003677	F	698	1.48E-07	2.11E-05
7	3279	F2 vs. M2	-	Metabolism	Metabolism	DNA metabolic process	GO:0006259	P	216	2.07E-13	5.53E-11
7	3280	F2 vs. M2	-	Metabolism	Metabolism	DNA secondary structure binding	GO:0000217	F	23	2.52E-04	9.47E-03
7	3281	F2 vs. M2	-	Metabolism	Metabolism	endoribonuclease activity, producing 5'-phosphomonoesters	GO:0016891	F	25	1.96E-03	4.31E-02
7	3282	F2 vs. M2	-	Metabolism	Metabolism	heterocycle metabolic process	GO:0046483	P	1256	2.51E-09	2.85E-07
7	3283	F2 vs. M2	-	Metabolism	Metabolism	heterocyclic compound binding	GO:1901363	F	2317	1.30E-03	3.17E-02
7	3284	F2 vs. M2	-	Metabolism	Metabolism	macromolecule biosynthetic process	GO:0009059	P	745	7.73E-07	3.50E-05
7	3285	F2 vs. M2	-	Metabolism	Metabolism	macromolecule metabolic process	GO:0043170	P	2713	1.75E-13	4.90E-11
7	3286	F2 vs. M2	-	Metabolism	Metabolism	microtubule binding	GO:0008017	F	80	1.00E-04	4.66E-03
7	3287	F2 vs. M2	-	Metabolism	Metabolism	nitrogen compound metabolic process	GO:0006807	P	3081	1.21E-06	5.12E-05
7	3288	F2 vs. M2	-	Metabolism	Metabolism	N-methyltransferase activity	GO:0008170	F	40	7.96E-05	3.98E-03
7	3289	F2 vs. M2	-	Metabolism	Metabolism	nucleic acid binding	GO:0003676	F	1619	3.41E-10	9.22E-08
7	3290	F2 vs. M2	-	Metabolism	Metabolism	organic cyclic compound binding	GO:0097159	F	2331	1.02E-03	2.67E-02
7	3291	F2 vs. M2	-	Metabolism	Metabolism	organic cyclic compound metabolic process	GO:1901360	P	1368	6.56E-06	2.06E-04
7	3292	F2 vs. M2	-	Metabolism	Metabolism	peptide metabolic process	GO:0006518	P	348	2.59E-04	4.51E-03
7	3293	F2 vs. M2	-	Metabolism	Metabolism	protein metabolic process	GO:0019538	P	1715	1.60E-06	6.32E-05
7	3294	F2 vs. M2	-	Metabolism	Metabolism	pseudouridine synthesis	GO:0001522	P	14	3.44E-03	3.34E-02
7	3295	F2 vs. M2	-	Metabolism	Metabolism	Sm-like protein family complex	GO:0120114	C	51	3.61E-08	6.86E-07

7	3296	F2 vs. M2	-	Metabolism	Metabolism	supramolecular complex	GO:0099080	C	135	1.13E-04	1.06E-03
7	3297	F2 vs. M2	-	Metabolism	Metabolism	transferase complex	GO:1990234	C	376	0.00E+00	0.00E+00
7	3298	F2 vs. M2	-	Metabolism	Metabolism	transferase complex, transferring phosphorus-containing groups	GO:0061695	C	126	1.28E-13	4.33E-12
7	3299	F2 vs. M2	-	Metabolism	Metabolism	tubulin binding	GO:0015631	F	83	1.85E-04	7.57E-03
7	3300	F2 vs. M2	-	Metabolism	Transporters	nuclear export	GO:0051168	P	31	2.36E-03	2.52E-02
7	3301	F2 vs. M2	-	Metabolism	Transporters	nuclear microtubule	GO:0005880	C	6	4.28E-03	2.64E-02
7	3302	F2 vs. M2	-	Metabolism	Transporters	nuclear transport	GO:0051169	P	56	4.18E-03	3.84E-02
7	3303	F2 vs. M2	-	Metabolism	Transporters	nucleic acid transport	GO:0050657	P	33	5.03E-04	7.69E-03
7	3304	F2 vs. M2	-	Metabolism	Transporters	nucleocytoplasmic transport	GO:0006913	P	56	4.18E-03	3.83E-02
7	3305	F2 vs. M2	-	Metabolism	Transporters	protein export from nucleus	GO:0006611	P	27	5.59E-03	4.74E-02
7	3306	F2 vs. M2	-	Metabolism	Transporters	RNA transport	GO:0050658	P	33	5.03E-04	7.71E-03
7	3307	F2 vs. M2	-	Regulation	Protein modification	methylation	GO:0032259	P	108	7.68E-05	1.65E-03
7	3308	F2 vs. M2	-	Regulation	Protein modification	protein alkylation	GO:0008213	P	41	5.33E-05	1.20E-03
7	3309	F2 vs. M2	-	Regulation	Protein modification	protein heterodimerization activity	GO:0046982	F	69	5.04E-05	2.84E-03
7	3310	F2 vs. M2	-	Regulation	Protein modification	protein methylation	GO:0006479	P	41	5.33E-05	1.21E-03
7	3311	F2 vs. M2	-	Regulation	Protein modification	protein methyltransferase activity	GO:0008276	F	35	7.70E-04	2.17E-02
7	3312	F2 vs. M2	-	Regulation	Protein modification	regulation of protein metabolic process	GO:0051246	P	208	3.53E-05	8.54E-04
7	3313	F2 vs. M2	-	Regulation	Protein modification	peptidyl-lysine modification	GO:0018205	P	67	2.08E-04	3.69E-03
7	3314	F2 vs. M2	-	Regulation	Protein modification	protein modification by small protein conjugation or removal	GO:0070647	P	330	8.10E-04	1.13E-02
7	3315	F2 vs. M2	-	Regulation	Protein modification	protein modification by small protein removal	GO:0070646	P	41	2.03E-03	2.27E-02
7	3316	F2 vs. M2	-	Regulation	Protein modification	protein modification process	GO:0036211	P	1146	2.68E-05	6.92E-04
7	3317	F2 vs. M2	-	Regulation	Protein modification	protein-containing complex localization	GO:0031503	P	32	2.71E-03	2.82E-02
7	3318	F2 vs. M2	-	Regulation	Protein modification	regulation of protein modification process	GO:0031399	P	105	2.18E-03	2.37E-02
7	3319	F2 vs. M2	-	Regulation	Regulation	biological regulation	GO:0065007	P	2712	5.32E-03	4.54E-02
7	3320	F2 vs. M2	-	Regulation	Regulation	negative regulation of biological process	GO:0048519	P	588	1.13E-05	3.21E-04
7	3321	F2 vs. M2	-	Regulation	Regulation	negative regulation of cellular process	GO:0048523	P	379	9.57E-04	1.28E-02
7	3322	F2 vs. M2	-	Regulation	Regulation	negative regulation of macromolecule metabolic process	GO:0010605	P	312	8.68E-07	3.86E-05
7	3323	F2 vs. M2	-	Regulation	Regulation	negative regulation of metabolic process	GO:0009892	P	347	1.01E-04	1.98E-03
7	3324	F2 vs. M2	-	Regulation	Regulation	positive regulation of biological process	GO:0048518	P	594	2.35E-04	4.14E-03
7	3325	F2 vs. M2	-	Regulation	Regulation	positive regulation of cellular metabolic process	GO:0031325	P	343	1.57E-03	1.87E-02
7	3326	F2 vs. M2	-	Regulation	Regulation	positive regulation of cellular process	GO:0048522	P	477	5.70E-04	8.54E-03
7	3327	F2 vs. M2	-	Regulation	Regulation	positive regulation of cellular protein metabolic process	GO:0032270	P	71	6.12E-04	9.05E-03
7	3328	F2 vs. M2	-	Regulation	Regulation	positive regulation of macromolecule metabolic process	GO:0010604	P	328	7.59E-05	1.64E-03
7	3329	F2 vs. M2	-	Regulation	Regulation	positive regulation of metabolic process	GO:0009893	P	366	1.28E-03	1.58E-02
7	3330	F2 vs. M2	-	Regulation	Regulation	positive regulation of nitrogen compound metabolic process	GO:0051173	P	314	9.68E-05	1.91E-03
7	3331	F2 vs. M2	-	Regulation	Regulation	positive regulation of protein metabolic process	GO:0051247	P	73	5.98E-04	8.88E-03
7	3332	F2 vs. M2	-	Regulation	Regulation	regulation of biological process	GO:0050789	P	2437	3.33E-04	5.60E-03
7	3333	F2 vs. M2	-	Regulation	Regulation	regulation of biosynthetic process	GO:0009889	P	1032	9.36E-05	1.87E-03
7	3334	F2 vs. M2	-	Regulation	Regulation	regulation of cellular biosynthetic process	GO:0031326	P	1011	3.47E-05	8.44E-04
7	3335	F2 vs. M2	-	Regulation	Regulation	regulation of cellular macromolecule biosynthetic process	GO:2000112	P	953	4.72E-07	2.25E-05
7	3336	F2 vs. M2	-	Regulation	Regulation	regulation of cellular metabolic process	GO:0031323	P	1272	4.53E-06	1.50E-04
7	3337	F2 vs. M2	-	Regulation	Regulation	regulation of cellular process	GO:0050794	P	2110	1.67E-03	1.95E-02
7	3338	F2 vs. M2	-	Regulation	Regulation	regulation of cellular protein metabolic process	GO:0032268	P	198	9.06E-05	1.86E-03
7	3339	F2 vs. M2	-	Regulation	Regulation	regulation of macromolecule biosynthetic process	GO:0010556	P	961	4.97E-07	2.35E-05
7	3340	F2 vs. M2	-	Regulation	Regulation	regulation of macromolecule metabolic process	GO:0060255	P	1299	5.89E-13	1.37E-10
7	3341	F2 vs. M2	-	Regulation	Regulation	regulation of meristem structural organization	GO:0009934	P	17	1.26E-03	1.57E-02
7	3342	F2 vs. M2	-	Regulation	Regulation	regulation of metabolic process	GO:0019222	P	1449	9.58E-08	5.32E-06
7	3343	F2 vs. M2	-	Regulation	Regulation	regulation of nitrogen compound metabolic process	GO:0051171	P	1144	1.92E-08	1.56E-06
7	3344	F2 vs. M2	-	Regulation	Regulation	regulation of nucleobase-containing compound metabolic process	GO:0019219	P	943	3.52E-07	1.72E-05
7	3345	F2 vs. M2	-	Regulation	Regulation	regulation of organelle organization	GO:0033043	P	115	4.36E-05	1.03E-03
7	3346	F2 vs. M2	-	Regulation	Regulation	regulation of primary metabolic process	GO:0080090	P	1190	2.18E-07	1.12E-05
7	3347	F2 vs. M2	-	Regulation	Regulation	regulation of transferase activity	GO:0051338	P	52	1.47E-04	2.75E-03
7	3348	F2 vs. M2	-	Regulation	Transcription	chromatin organization involved in negative regulation of transcription	GO:0097549	P	33	1.82E-03	2.07E-02
7	3349	F2 vs. M2	-	Regulation	Transcription	chromatin organization involved in regulation of transcription	GO:0034401	P	34	2.54E-03	2.69E-02
7	3350	F2 vs. M2	-	Regulation	Transcription	chromatin silencing	GO:0006342	P	22	1.00E-03	1.29E-02
7	3351	F2 vs. M2	-	Regulation	Transcription	covalent chromatin modification	GO:0016569	P	84	9.85E-06	2.89E-04
7	3352	F2 vs. M2	-	Regulation	Transcription	DNA conformation change	GO:0071103	P	67	1.02E-03	1.31E-02
7	3353	F2 vs. M2	-	Regulation	Transcription	DNA-binding transcription activator activity	GO:0001216	F	15	1.16E-03	3.02E-02
7	3354	F2 vs. M2	-	Regulation	Transcription	DNA-binding transcription activator activity, RNA polymerase II-specific	GO:0001228	F	11	8.62E-04	2.38E-02
7	3355	F2 vs. M2	-	Regulation	Transcription	DNA-directed RNA polymerase complex	GO:0000428	C	65	1.41E-06	1.76E-05

7	3356	F2 vs. M2	-	Regulation	Transcription	dsRNA processing	GO:0031050	P	34	2.36E-06	8.87E-05
7	3357	F2 vs. M2	-	Regulation	Transcription	gene expression	GO:0010467	P	870	0.00E+00	0.00E+00
7	3358	F2 vs. M2	-	Regulation	Transcription	gene silencing	GO:0016458	P	93	1.71E-08	1.40E-06
7	3359	F2 vs. M2	-	Regulation	Transcription	gene silencing by miRNA	GO:0035195	P	21	3.10E-03	3.10E-02
7	3360	F2 vs. M2	-	Regulation	Transcription	gene silencing by RNA	GO:0031047	P	62	2.54E-06	9.34E-05
7	3361	F2 vs. M2	-	Regulation	Transcription	histone binding	GO:0042393	F	44	9.89E-04	2.64E-02
7	3362	F2 vs. M2	-	Regulation	Transcription	histone lysine methylation	GO:0034968	P	24	4.10E-04	6.74E-03
7	3363	F2 vs. M2	-	Regulation	Transcription	histone methylation	GO:0016571	P	31	2.74E-05	6.97E-04
7	3364	F2 vs. M2	-	Regulation	Transcription	histone methyltransferase activity	GO:0042054	F	22	2.11E-04	8.38E-03
7	3365	F2 vs. M2	-	Regulation	Transcription	histone modification	GO:0016570	P	81	1.41E-05	3.90E-04
7	3366	F2 vs. M2	-	Regulation	Transcription	macromolecule methylation	GO:0043414	P	85	1.45E-06	5.96E-05
7	3367	F2 vs. M2	-	Regulation	Transcription	macromolecule modification	GO:0043412	P	1302	2.78E-09	3.09E-07
7	3368	F2 vs. M2	-	Regulation	Transcription	minor groove of adenine-thymine-rich DNA binding	GO:0003680	F	18	9.22E-04	2.49E-02
7	3369	F2 vs. M2	-	Regulation	Transcription	mRNA processing	GO:0006397	P	154	6.24E-09	6.28E-07
7	3370	F2 vs. M2	-	Regulation	Transcription	negative regulation of gene expression	GO:0010629	P	255	8.74E-06	2.67E-04
7	3371	F2 vs. M2	-	Regulation	Transcription	negative regulation of gene expression, epigenetic	GO:0045814	P	25	1.79E-04	3.26E-03
7	3372	F2 vs. M2	-	Regulation	Transcription	nuclear DNA-directed RNA polymerase complex	GO:0055029	C	60	1.80E-07	2.81E-06
7	3373	F2 vs. M2	-	Regulation	Transcription	nucleic acid-templated transcription	GO:0097659	P	127	7.31E-04	1.04E-02
7	3374	F2 vs. M2	-	Regulation	Transcription	nucleolus	GO:0005730	C	296	9.63E-11	2.54E-09
7	3375	F2 vs. M2	-	Regulation	Transcription	peptidyl-lysine methylation	GO:0018022	P	29	1.54E-03	1.84E-02
7	3376	F2 vs. M2	-	Regulation	Transcription	post-transcriptional gene silencing by RNA	GO:0035194	P	43	9.33E-06	2.78E-04
7	3377	F2 vs. M2	-	Regulation	Transcription	production of siRNA involved in RNA interference	GO:0030422	P	21	5.86E-04	8.73E-03
7	3378	F2 vs. M2	-	Regulation	Transcription	production of small RNA involved in gene silencing by RNA	GO:0070918	P	34	2.36E-06	8.81E-05
7	3379	F2 vs. M2	-	Regulation	Transcription	protein acylation	GO:0043543	P	39	1.69E-03	1.98E-02
7	3380	F2 vs. M2	-	Regulation	Transcription	protein-DNA complex	GO:0032993	C	32	2.73E-06	3.33E-05
7	3381	F2 vs. M2	-	Regulation	Transcription	protein-DNA complex assembly	GO:0065004	P	37	3.22E-04	5.42E-03
7	3382	F2 vs. M2	-	Regulation	Transcription	protein-DNA complex subunit organization	GO:0071824	P	46	4.80E-04	7.58E-03
7	3383	F2 vs. M2	-	Regulation	Transcription	regulation of DNA-templated transcription, elongation	GO:0032784	P	18	3.47E-03	3.35E-02
7	3384	F2 vs. M2	-	Regulation	Transcription	regulation of gene expression	GO:0010468	P	1122	8.12E-10	9.63E-08
7	3385	F2 vs. M2	-	Regulation	Transcription	regulation of gene expression, epigenetic	GO:0040029	P	43	5.89E-05	1.29E-03
7	3386	F2 vs. M2	-	Regulation	Transcription	regulation of nucleic acid-templated transcription	GO:1903506	P	853	4.05E-06	1.39E-04
7	3387	F2 vs. M2	-	Regulation	Transcription	regulation of RNA biosynthetic process	GO:2001141	P	855	5.99E-06	1.93E-04
7	3388	F2 vs. M2	-	Regulation	Transcription	regulation of transcription, DNA-templated	GO:0006355	P	853	4.05E-06	1.39E-04
7	3389	F2 vs. M2	-	Regulation	Transcription	RNA 3'-end processing	GO:0031123	P	40	6.84E-04	9.89E-03
7	3390	F2 vs. M2	-	Regulation	Transcription	RNA biosynthetic process	GO:0032774	P	130	6.22E-04	9.17E-03
7	3391	F2 vs. M2	-	Regulation	Transcription	RNA methylation	GO:0001510	P	33	6.00E-04	8.90E-03
7	3392	F2 vs. M2	-	Regulation	Transcription	RNA modification	GO:0009451	P	140	7.32E-10	8.88E-08
7	3393	F2 vs. M2	-	Regulation	Transcription	RNA polymerase complex	GO:0030880	C	68	2.36E-06	2.91E-05
7	3394	F2 vs. M2	-	Regulation	Transcription	RNA polymerase II, core complex	GO:0005665	C	13	1.95E-03	1.34E-02
7	3395	F2 vs. M2	-	Regulation	Transcription	RNA polymerase II, holoenzyme	GO:0016591	C	37	6.88E-05	6.60E-04
7	3396	F2 vs. M2	-	Regulation	Transcription	RNA polymerase III complex	GO:0005666	C	16	3.80E-03	2.38E-02
7	3397	F2 vs. M2	-	Regulation	Transcription	RNA polymerase IV complex	GO:0000418	C	9	1.21E-03	8.84E-03
7	3398	F2 vs. M2	-	Regulation	Transcription	RNA polymerase V complex	GO:0000419	C	14	4.35E-04	3.62E-03
7	3399	F2 vs. M2	-	Regulation	Transcription	siRNA binding	GO:0035197	F	4	8.75E-04	2.39E-02
7	3400	F2 vs. M2	-	Regulation	Transcription	THO complex	GO:0000347	C	10	2.60E-03	1.75E-02
7	3401	F2 vs. M2	-	Regulation	Transcription	transcription coregulator activity	GO:0003712	F	64	1.44E-03	3.38E-02
7	3402	F2 vs. M2	-	Regulation	Transcription	transcription factor TFIIH holo complex	GO:0005675	C	7	8.83E-03	4.76E-02
7	3403	F2 vs. M2	-	Regulation	Transcription	transcription, DNA-templated	GO:0006351	P	127	7.31E-04	1.04E-02
7	3404	F2 vs. M2	-	Regulation	Translation	90S preribosome	GO:0030686	C	18	1.97E-03	1.35E-02
7	3405	F2 vs. M2	-	Regulation	Translation	catalytic activity, acting on RNA	GO:0140098	F	223	1.92E-06	1.85E-04
7	3406	F2 vs. M2	-	Regulation	Translation	catalytic step 2 spliceosome	GO:0071013	C	34	5.00E-05	4.94E-04
7	3407	F2 vs. M2	-	Regulation	Translation	cleavage involved in rRNA processing	GO:0000469	P	13	2.37E-03	2.52E-02
7	3408	F2 vs. M2	-	Regulation	Translation	cytosolic large ribosomal subunit	GO:0022625	C	93	1.22E-06	1.57E-05
7	3409	F2 vs. M2	-	Regulation	Translation	cytosolic ribosome	GO:0022626	C	214	2.46E-04	2.12E-03
7	3410	F2 vs. M2	-	Regulation	Translation	cytosolic small ribosomal subunit	GO:0022627	C	68	6.35E-06	7.36E-05
7	3411	F2 vs. M2	-	Regulation	Translation	establishment of RNA localization	GO:0051236	P	33	5.03E-04	7.74E-03
7	3412	F2 vs. M2	-	Regulation	Translation	large ribosomal subunit	GO:0015934	C	118	8.02E-08	1.38E-06
7	3413	F2 vs. M2	-	Regulation	Translation	Lsm1-7-Pat1 complex	GO:1990726	C	7	8.04E-03	4.47E-02
7	3414	F2 vs. M2	-	Regulation	Translation	maturation of 5.8S rRNA from tricistronic rRNA transcript (SSU-rRNA, 5.8S rRNA, LSU-rRNA)	GO:0000466	P	10	2.62E-03	2.75E-02
7	3415	F2 vs. M2	-	Regulation	Translation	maturation of SSU-rRNA	GO:0030490	P	32	4.13E-05	9.88E-04
7	3416	F2 vs. M2	-	Regulation	Translation	maturation of SSU-rRNA from tricistronic rRNA transcript (SSU-rRNA, 5.8S rRNA, LSU-rRNA)	GO:0000462	P	21	1.83E-03	2.07E-02
7	3417	F2 vs. M2	-	Regulation	Translation	mitochondrial large ribosomal subunit	GO:0005762	C	16	1.02E-03	7.76E-03

7	3418	F2 vs. M2	-	Regulation	Translation	mitochondrial ribosome	GO:0005761	C	25	2.28E-04	1.99E-03
7	3419	F2 vs. M2	-	Regulation	Translation	mRNA export from nucleus	GO:0006406	P	18	4.96E-03	4.37E-02
7	3420	F2 vs. M2	-	Regulation	Translation	mRNA metabolic process	GO:0016071	P	224	1.03E-08	9.03E-07
7	3421	F2 vs. M2	-	Regulation	Translation	mRNA modification	GO:0016556	P	23	5.90E-03	4.94E-02
7	3422	F2 vs. M2	-	Regulation	Translation	mRNA splicing, via spliceosome	GO:0000398	P	114	2.81E-08	2.14E-06
7	3423	F2 vs. M2	-	Regulation	Translation	mRNA transport	GO:0051028	P	19	1.91E-03	2.15E-02
7	3424	F2 vs. M2	-	Regulation	Translation	mRNA-containing ribonucleoprotein complex export from nucleus	GO:0071427	P	18	4.96E-03	4.38E-02
7	3425	F2 vs. M2	-	Regulation	Translation	ncRNA 3'-end processing	GO:0043628	P	21	4.22E-04	6.87E-03
7	3426	F2 vs. M2	-	Regulation	Translation	ncRNA metabolic process	GO:0034660	P	247	8.39E-08	4.82E-06
7	3427	F2 vs. M2	-	Regulation	Translation	ncRNA processing	GO:0034470	P	211	7.72E-08	4.53E-06
7	3428	F2 vs. M2	-	Regulation	Translation	organellar large ribosomal subunit	GO:0000315	C	23	2.78E-03	1.86E-02
7	3429	F2 vs. M2	-	Regulation	Translation	organellar ribosome	GO:0000313	C	39	1.27E-03	9.07E-03
7	3430	F2 vs. M2	-	Regulation	Translation	peptide biosynthetic process	GO:0043043	P	311	6.09E-06	1.95E-04
7	3431	F2 vs. M2	-	Regulation	Translation	pICln-Sm protein complex	GO:0034715	C	5	3.64E-03	2.30E-02
7	3432	F2 vs. M2	-	Regulation	Translation	posttranscriptional gene silencing	GO:0016441	P	48	1.30E-05	3.66E-04
7	3433	F2 vs. M2	-	Regulation	Translation	posttranscriptional regulation of gene expression	GO:0010608	P	131	2.56E-04	4.46E-03
7	3434	F2 vs. M2	-	Regulation	Translation	precatalytic spliceosome	GO:0071011	C	22	1.25E-04	1.16E-03
7	3435	F2 vs. M2	-	Regulation	Translation	preribosome	GO:0030684	C	54	9.19E-06	1.03E-04
7	3436	F2 vs. M2	-	Regulation	Translation	preribosome, large subunit precursor	GO:0030687	C	15	2.87E-03	1.90E-02
7	3437	F2 vs. M2	-	Regulation	Translation	prespliceosome	GO:0071010	C	12	5.17E-03	3.11E-02
7	3438	F2 vs. M2	-	Regulation	Translation	production of miRNAs involved in gene silencing by miRNA	GO:0035196	P	14	1.75E-03	2.02E-02
7	3439	F2 vs. M2	-	Regulation	Translation	regulation of RNA metabolic process	GO:0051252	P	919	1.66E-06	6.48E-05
7	3440	F2 vs. M2	-	Regulation	Translation	regulation of RNA splicing	GO:0043484	P	38	5.09E-03	4.43E-02
7	3441	F2 vs. M2	-	Regulation	Translation	regulatory RNA binding	GO:0061980	F	7	1.61E-03	3.72E-02
7	3442	F2 vs. M2	-	Regulation	Translation	ribonuclease activity	GO:0004540	F	65	6.25E-04	1.96E-02
7	3443	F2 vs. M2	-	Regulation	Translation	ribonucleoprotein complex	GO:1990904	C	399	0.00E+00	0.00E+00
7	3444	F2 vs. M2	-	Regulation	Translation	ribonucleoprotein complex assembly	GO:0022618	P	104	2.65E-05	6.89E-04
7	3445	F2 vs. M2	-	Regulation	Translation	ribonucleoprotein complex biogenesis	GO:0022613	P	265	5.68E-09	5.83E-07
7	3446	F2 vs. M2	-	Regulation	Translation	ribonucleoprotein complex export from nucleus	GO:0071426	P	27	5.59E-03	4.73E-02
7	3447	F2 vs. M2	-	Regulation	Translation	ribonucleoprotein complex localization	GO:0071166	P	27	5.59E-03	4.72E-02
7	3448	F2 vs. M2	-	Regulation	Translation	ribonucleoprotein complex subunit organization	GO:0071826	P	107	1.57E-05	4.26E-04
7	3449	F2 vs. M2	-	Regulation	Translation	ribosomal large subunit biogenesis	GO:0042273	P	74	1.54E-03	1.84E-02
7	3450	F2 vs. M2	-	Regulation	Translation	ribosomal small subunit biogenesis	GO:0042274	P	59	1.45E-04	2.73E-03
7	3451	F2 vs. M2	-	Regulation	Translation	ribosomal subunit	GO:0044391	C	207	1.78E-13	5.83E-12
7	3452	F2 vs. M2	-	Regulation	Translation	ribosome	GO:0005840	C	268	7.26E-06	8.21E-05
7	3453	F2 vs. M2	-	Regulation	Translation	ribosome biogenesis	GO:0042254	P	221	1.08E-06	4.59E-05
7	3454	F2 vs. M2	-	Regulation	Translation	RNA cap binding	GO:0000339	F	6	1.43E-03	3.39E-02
7	3455	F2 vs. M2	-	Regulation	Translation	RNA export from nucleus	GO:0006405	P	31	2.36E-03	2.52E-02
7	3456	F2 vs. M2	-	Regulation	Translation	RNA interference	GO:0016246	P	23	4.01E-03	3.69E-02
7	3457	F2 vs. M2	-	Regulation	Translation	RNA localization	GO:0006403	P	37	1.23E-04	2.40E-03
7	3458	F2 vs. M2	-	Regulation	Translation	RNA methyltransferase activity	GO:0008173	F	31	2.54E-03	5.00E-02
7	3459	F2 vs. M2	-	Regulation	Translation	RNA phosphodiester bond hydrolysis	GO:0090501	P	25	4.57E-05	1.05E-03
7	3460	F2 vs. M2	-	Regulation	Translation	RNA processing	GO:0006396	P	418	1.11E-16	5.39E-14
7	3461	F2 vs. M2	-	Regulation	Translation	RNA splicing	GO:0008380	P	147	1.35E-07	7.29E-06
7	3462	F2 vs. M2	-	Regulation	Translation	RNA splicing, via transesterification reactions	GO:0000375	P	122	3.50E-08	2.39E-06
7	3463	F2 vs. M2	-	Regulation	Translation	RNA splicing, via transesterification reactions with bulged adenosine as nucleophile	GO:0000377	P	122	3.50E-08	2.42E-06
7	3464	F2 vs. M2	-	Regulation	Translation	rRNA metabolic process	GO:0016072	P	148	1.25E-04	2.41E-03
7	3465	F2 vs. M2	-	Regulation	Translation	rRNA processing	GO:0006364	P	142	1.30E-04	2.50E-03
7	3466	F2 vs. M2	-	Regulation	Translation	small nuclear ribonucleoprotein complex	GO:0030532	C	45	4.83E-08	8.83E-07
7	3467	F2 vs. M2	-	Regulation	Translation	small nucleolar ribonucleoprotein complex	GO:0005732	C	19	1.40E-04	1.29E-03
7	3468	F2 vs. M2	-	Regulation	Translation	small ribosomal subunit	GO:0015935	C	89	3.33E-07	4.94E-06
7	3469	F2 vs. M2	-	Regulation	Translation	small-subunit processome	GO:0032040	C	30	1.23E-03	8.94E-03
7	3470	F2 vs. M2	-	Regulation	Translation	snoRNA binding	GO:0030515	F	16	1.27E-03	3.11E-02
7	3471	F2 vs. M2	-	Regulation	Translation	spliceosomal complex	GO:0005681	C	80	3.42E-06	4.12E-05
7	3472	F2 vs. M2	-	Regulation	Translation	spliceosomal snRNP assembly	GO:0000387	P	17	1.43E-03	1.72E-02
7	3473	F2 vs. M2	-	Regulation	Translation	spliceosomal snRNP complex	GO:0097525	C	45	4.83E-08	9.00E-07
7	3474	F2 vs. M2	-	Regulation	Translation	spliceosomal tri-snRNP complex	GO:0097526	C	28	2.97E-07	4.47E-06
7	3475	F2 vs. M2	-	Regulation	Translation	structural constituent of ribosome	GO:0003735	F	200	4.56E-09	8.80E-07
7	3476	F2 vs. M2	-	Regulation	Translation	translation	GO:0006412	P	310	3.89E-06	1.36E-04
7	3477	F2 vs. M2	-	Regulation	Translation	tRNA metabolic process	GO:0006399	P	89	9.23E-05	1.87E-03
7	3478	F2 vs. M2	-	Regulation	Translation	tRNA modification	GO:0006400	P	46	1.00E-03	1.29E-02
7	3479	F2 vs. M2	-	Regulation	Translation	tRNA processing	GO:0008033	P	56	3.09E-05	7.78E-04

7	3480	F2 vs. M2	-	Regulation	Translation	U1 snRNP	GO:0005685	C	17	4.94E-05	4.94E-04
7	3481	F2 vs. M2	-	Regulation	Translation	U12-type spliceosomal complex	GO:0005689	C	16	2.77E-04	2.35E-03
7	3482	F2 vs. M2	-	Regulation	Translation	U2 snRNP	GO:0005686	C	21	1.69E-04	1.50E-03
7	3483	F2 vs. M2	-	Regulation	Translation	U2-type prespliceosome	GO:0071004	C	12	5.17E-03	3.09E-02
7	3484	F2 vs. M2	-	Regulation	Translation	U2-type spliceosomal complex	GO:0005684	C	30	3.08E-03	2.02E-02
7	3485	F2 vs. M2	-	Regulation	Translation	U4 snRNP	GO:0005687	C	10	1.68E-04	1.51E-03
7	3486	F2 vs. M2	-	Regulation	Translation	U4/U6 x U5 tri-snRNP complex	GO:0046540	C	20	2.46E-05	2.66E-04
7	3487	F2 vs. M2	-	Regulation	translation	U5 snRNP	GO:0005682	C	13	3.26E-05	3.37E-04
7	3488	F2 vs. M2	-	Regulation	Translation	U6 snRNP	GO:0005688	C	9	8.05E-03	4.45E-02
7	3489	F2 vs. M2	-	Signaling and response	Signaling	kinase regulator activity	GO:0019207	F	50	2.35E-05	1.59E-03
7	3490	F2 vs. M2	-	Signaling and response	Signaling	protein kinase complex	GO:1902911	C	49	1.03E-08	2.04E-07
7	3491	F2 vs. M2	-	Signaling and response	Signaling	protein kinase regulator activity	GO:0019887	F	49	1.35E-05	9.63E-04
7	3492	F2 vs. M2	-	Signaling and response	Signaling	regulation of cyclin-dependent protein serine/threonine kinase activity	GO:0000079	P	35	4.42E-04	7.06E-03
7	3493	F2 vs. M2	-	Signaling and response	Signaling	regulation of kinase activity	GO:0043549	P	45	2.23E-05	5.86E-04
7	3494	F2 vs. M2	-	Signaling and response	Signaling	regulation of phosphorylation	GO:0042325	P	64	2.57E-03	2.71E-02
7	3495	F2 vs. M2	-	Signaling and response	Signaling	regulation of protein kinase activity	GO:0045859	P	44	1.98E-05	5.28E-04
7	3496	F2 vs. M2	-	Signaling and response	Signaling	regulation of protein phosphorylation	GO:0001932	P	57	4.80E-04	7.56E-03
7	3497	F2 vs. M2	-	Signaling and response	Signaling	regulation of protein serine/threonine kinase activity	GO:0071900	P	39	4.63E-04	7.34E-03
7	3498	F2 vs. M2	-	Signaling and response	Signaling	serine/threonine protein kinase complex	GO:1902554	C	44	1.94E-09	4.29E-08
7	3499	F2 vs. M2	-	Signaling and response	Stress	cellular response to DNA damage stimulus	GO:0006974	P	180	4.63E-13	1.12E-10
7	3500	F2 vs. M2	-	Signaling and response	Stress	interaction with host	GO:0051701	P	18	3.06E-03	3.07E-02
7	3501	F2 vs. M2	-	Signaling and response	Stress	modulation by symbiont of host process	GO:0044003	P	9	5.19E-03	4.48E-02
7	3502	F2 vs. M2	-	Signaling and response	Stress	modulation of process of other organism	GO:0035821	P	9	5.19E-03	4.48E-02
7	3503	F2 vs. M2	-	Signaling and response	Stress	modulation of process of other organism involved in symbiotic interaction	GO:0051817	P	9	5.19E-03	4.49E-02
7	3504	F2 vs. M2	-	Signaling and response	Stress	RNA-dependent DNA biosynthetic process	GO:0006278	P	4	1.89E-03	2.13E-02
8	3505	F3 vs. M3	+	Development	Cell division	nucleobase-containing small molecule metabolic process	GO:0055086	P	279	9.21E-05	1.56E-03
8	3506	F3 vs. M3	+	Development	Cell division	nucleotide-sugar biosynthetic process	GO:0009226	P	39	3.50E-04	4.83E-03
8	3507	F3 vs. M3	+	Development	Cell division	nucleotide-sugar metabolic process	GO:0009225	P	45	3.65E-04	4.99E-03
8	3508	F3 vs. M3	+	Development	Cell division	pollen germination	GO:0009846	P	41	3.02E-03	2.75E-02
8	3509	F3 vs. M3	+	Development	Cell division	pollen tube development	GO:0048868	P	118	3.55E-10	2.41E-08
8	3510	F3 vs. M3	+	Development	Cell division	pollen tube growth	GO:0009860	P	93	5.30E-11	4.27E-09
8	3511	F3 vs. M3	+	Development	Cell division	pollination	GO:0009856	P	163	3.62E-08	1.58E-06
8	3512	F3 vs. M3	+	Development	Cell division	ribonucleotide metabolic process	GO:0009259	P	160	5.06E-03	4.21E-02
8	3513	F3 vs. M3	+	Development	Cell division	ribose phosphate metabolic process	GO:0019693	P	165	5.48E-03	4.44E-02
8	3514	F3 vs. M3	+	Development	Cell division	syncytium formation	GO:0006949	P	12	2.50E-06	6.74E-05
8	3515	F3 vs. M3	+	Development	Development	actin cytoskeleton	GO:0015629	C	40	4.42E-04	3.88E-03
8	3516	F3 vs. M3	+	Development	Development	actin cytoskeleton organization	GO:0030036	P	68	2.23E-04	3.31E-03
8	3517	F3 vs. M3	+	Development	Development	anatomical structure morphogenesis	GO:0009653	P	557	1.63E-04	2.52E-03
8	3518	F3 vs. M3	+	Development	Development	apical part of cell	GO:0045177	C	17	1.72E-04	1.63E-03
8	3519	F3 vs. M3	+	Development	Development	apical plasma membrane	GO:0016324	C	15	4.47E-05	4.67E-04
8	3520	F3 vs. M3	+	Development	Development	apoplast	GO:0048046	C	184	0.00E+00	0.00E+00
8	3521	F3 vs. M3	+	Development	Development	bounding membrane of organelle	GO:0098588	C	625	8.00E-09	1.59E-07
8	3522	F3 vs. M3	+	Development	Development	cell development	GO:0048468	P	207	5.73E-06	1.38E-04
8	3523	F3 vs. M3	+	Development	Development	cell growth	GO:0016049	P	255	5.31E-11	4.22E-09
8	3524	F3 vs. M3	+	Development	Development	cell junction	GO:0030054	C	654	7.73E-03	4.36E-02
8	3525	F3 vs. M3	+	Development	Development	cell morphogenesis	GO:0000902	P	241	9.52E-08	3.78E-06
8	3526	F3 vs. M3	+	Development	Development	cell morphogenesis involved in differentiation	GO:0000904	P	154	7.53E-07	2.30E-05
8	3527	F3 vs. M3	+	Development	Development	cell periphery	GO:0071944	C	2517	3.33E-16	1.14E-14
8	3528	F3 vs. M3	+	Development	Development	cell pole	GO:0060187	C	15	7.55E-04	6.19E-03
8	3529	F3 vs. M3	+	Development	Development	cell projection	GO:0042995	C	70	8.99E-10	1.98E-08
8	3530	F3 vs. M3	+	Development	Development	cell tip	GO:0051286	C	15	7.55E-04	6.14E-03
8	3531	F3 vs. M3	+	Development	Development	cell tip growth	GO:0009932	P	110	3.95E-12	4.22E-10
8	3532	F3 vs. M3	+	Development	Development	cell wall	GO:0005618	C	384	1.52E-05	1.71E-04
8	3533	F3 vs. M3	+	Development	Development	cell wall biogenesis	GO:0042546	P	155	5.08E-04	6.61E-03
8	3534	F3 vs. M3	+	Development	Development	cell wall modification	GO:0042545	P	110	1.59E-05	3.36E-04
8	3535	F3 vs. M3	+	Development	Development	cell wall organization	GO:0071555	P	176	2.42E-07	8.38E-06
8	3536	F3 vs. M3	+	Development	Development	cell wall organization or biogenesis	GO:0071554	P	323	1.16E-08	5.51E-07
8	3537	F3 vs. M3	+	Development	Development	cell wall pectin metabolic process	GO:0052546	P	19	3.73E-04	5.07E-03
8	3538	F3 vs. M3	+	Development	Development	cell-cell junction	GO:0005911	C	654	7.73E-03	4.43E-02
8	3539	F3 vs. M3	+	Development	Development	cellular process	GO:0009987	P	7297	2.51E-03	2.43E-02
8	3540	F3 vs. M3	+	Development	Development	cytoplasm	GO:0005737	C	8318	5.13E-07	7.60E-06
8	3541	F3 vs. M3	+	Development	Development	cytosol	GO:0005829	C	2260	2.43E-13	7.10E-12

8	3542	F3 vs. M3	+	Development	Development	developmental cell growth	GO:0048588	P	130	8.98E-12	8.32E-10
8	3543	F3 vs. M3	+	Development	Development	developmental growth	GO:0048589	P	250	8.80E-13	1.29E-10
8	3544	F3 vs. M3	+	Development	Development	developmental growth involved in morphogenesis	GO:0060560	P	218	1.04E-12	1.38E-10
8	3545	F3 vs. M3	+	Development	Development	endomembrane system	GO:0012505	C	1484	1.22E-08	2.28E-07
8	3546	F3 vs. M3	+	Development	Development	envelope	GO:0031975	C	727	0.00E+00	0.00E+00
8	3547	F3 vs. M3	+	Development	Development	external encapsulating structure	GO:0030312	C	386	8.19E-06	9.45E-05
8	3548	F3 vs. M3	+	Development	Development	external encapsulating structure organization	GO:0045229	P	210	3.12E-07	1.05E-05
8	3549	F3 vs. M3	+	Development	Development	extracellular region	GO:0005576	C	1456	9.22E-11	2.29E-09
8	3550	F3 vs. M3	+	Development	Development	Golgi apparatus subcompartment	GO:0098791	C	299	9.12E-09	1.74E-07
8	3551	F3 vs. M3	+	Development	Development	Golgi cisterna	GO:0031985	C	127	2.14E-03	1.48E-02
8	3552	F3 vs. M3	+	Development	Development	Golgi stack	GO:0005795	C	132	1.42E-03	1.04E-02
8	3553	F3 vs. M3	+	Development	Development	Golgi trans cisterna	GO:0000138	C	59	3.51E-04	3.14E-03
8	3554	F3 vs. M3	+	Development	Development	growing cell tip	GO:0035838	C	11	1.55E-03	1.13E-02
8	3555	F3 vs. M3	+	Development	Development	growth	GO:0040007	P	290	1.17E-11	1.03E-09
8	3556	F3 vs. M3	+	Development	Development	intracellular vesicle	GO:0097708	C	487	1.09E-07	1.80E-06
8	3557	F3 vs. M3	+	Development	Development	membrane	GO:0016020	C	3440	0.00E+00	0.00E+00
8	3558	F3 vs. M3	+	Development	Development	membrane protein complex	GO:0098796	C	402	7.14E-04	5.90E-03
8	3559	F3 vs. M3	+	Development	Development	microbody	GO:0042579	C	204	1.73E-06	2.23E-05
8	3560	F3 vs. M3	+	Development	Development	multi-multicellular organism process	GO:0044706	P	163	3.62E-08	1.59E-06
8	3561	F3 vs. M3	+	Development	Development	organelle envelope	GO:0031967	C	727	0.00E+00	0.00E+00
8	3562	F3 vs. M3	+	Development	Development	organelle inner membrane	GO:0019866	C	204	3.36E-03	2.12E-02
8	3563	F3 vs. M3	+	Development	Development	organelle membrane	GO:0031090	C	967	1.44E-07	2.30E-06
8	3564	F3 vs. M3	+	Development	Development	organelle subcompartment	GO:0031984	C	797	0.00E+00	0.00E+00
8	3565	F3 vs. M3	+	Development	Development	plant-type cell wall biogenesis	GO:0009832	P	100	2.84E-04	4.07E-03
8	3566	F3 vs. M3	+	Development	Development	plant-type cell wall loosening	GO:0009828	P	24	4.24E-05	7.98E-04
8	3567	F3 vs. M3	+	Development	Development	plant-type cell wall modification	GO:0009827	P	45	5.37E-06	1.31E-04
8	3568	F3 vs. M3	+	Development	Development	plant-type cell wall organization	GO:0009664	P	100	1.17E-06	3.46E-05
8	3569	F3 vs. M3	+	Development	Development	plant-type cell wall organization or biogenesis	GO:0071669	P	174	9.18E-08	3.67E-06
8	3570	F3 vs. M3	+	Development	Development	plant-type primary cell wall biogenesis	GO:0009833	P	21	3.11E-04	4.35E-03
8	3571	F3 vs. M3	+	Development	Development	plant-type vacuole	GO:0000325	C	108	1.67E-05	1.84E-04
8	3572	F3 vs. M3	+	Development	Development	plant-type vacuole membrane	GO:0009705	C	75	7.90E-04	6.37E-03
8	3573	F3 vs. M3	+	Development	Development	plasma membrane	GO:0005886	C	2208	4.39E-14	1.40E-12
8	3574	F3 vs. M3	+	Development	Development	plasma membrane bounded cell projection	GO:0120025	C	62	7.35E-13	2.08E-11
8	3575	F3 vs. M3	+	Development	Development	plasma membrane region	GO:0098590	C	33	3.95E-03	2.46E-02
8	3576	F3 vs. M3	+	Development	Development	pollen tube	GO:0090406	C	52	4.90E-11	1.28E-09
8	3577	F3 vs. M3	+	Development	Development	pollen tube tip	GO:0090404	C	11	1.55E-03	1.13E-02
8	3578	F3 vs. M3	+	Development	Development	root hair	GO:0035618	C	11	2.00E-03	1.41E-02
8	3579	F3 vs. M3	+	Development	Development	root hair elongation	GO:0048767	P	44	5.17E-03	4.28E-02
8	3580	F3 vs. M3	+	Development	Development	stromule	GO:0010319	C	30	1.01E-10	2.45E-09
8	3581	F3 vs. M3	+	Development	Development	structural constituent of cytoskeleton	GO:0005200	F	23	1.39E-03	2.98E-02
8	3582	F3 vs. M3	+	Development	Development	symplast	GO:0055044	C	654	7.73E-03	4.41E-02
8	3583	F3 vs. M3	+	Development	Development	unidimensional cell growth	GO:0009826	P	190	2.37E-11	2.02E-09
8	3584	F3 vs. M3	+	Development	Development	vacuolar membrane	GO:0005774	C	394	7.11E-10	1.60E-08
8	3585	F3 vs. M3	+	Development	Development	vacuole	GO:0005773	C	726	2.22E-16	7.87E-15
8	3586	F3 vs. M3	+	Metabolism	Bioenergetics	acetyl-CoA biosynthetic process	GO:0006085	P	14	5.76E-03	4.61E-02
8	3587	F3 vs. M3	+	Metabolism	Bioenergetics	acyl-CoA biosynthetic process	GO:0071616	P	18	3.37E-03	3.05E-02
8	3588	F3 vs. M3	+	Metabolism	Bioenergetics	aerobic respiration	GO:0009060	P	62	4.68E-06	1.17E-04
8	3589	F3 vs. M3	+	Metabolism	Bioenergetics	ATP metabolic process	GO:0046034	P	96	3.78E-04	5.10E-03
8	3590	F3 vs. M3	+	Metabolism	Bioenergetics	ATP synthesis coupled electron transport	GO:0042773	P	30	1.68E-03	1.77E-02
8	3591	F3 vs. M3	+	Metabolism	Bioenergetics	ATPase activity, coupled to transmembrane movement of ions, rotational mechanism	GO:0044769	F	20	9.93E-04	2.27E-02
8	3592	F3 vs. M3	+	Metabolism	Bioenergetics	carbohydrate biosynthetic process	GO:0016051	P	212	4.46E-12	4.42E-10
8	3593	F3 vs. M3	+	Metabolism	Bioenergetics	carbohydrate catabolic process	GO:0016052	P	158	6.97E-08	2.89E-06
8	3594	F3 vs. M3	+	Metabolism	Bioenergetics	carbohydrate derivative biosynthetic process	GO:1901137	P	319	1.16E-03	1.31E-02
8	3595	F3 vs. M3	+	Metabolism	Bioenergetics	carbohydrate derivative metabolic process	GO:1901135	P	524	6.01E-06	1.42E-04
8	3596	F3 vs. M3	+	Metabolism	Bioenergetics	carbohydrate metabolic process	GO:0005975	P	623	0.00E+00	0.00E+00
8	3597	F3 vs. M3	+	Metabolism	Bioenergetics	cellular carbohydrate biosynthetic process	GO:0034637	P	150	1.19E-05	2.58E-04
8	3598	F3 vs. M3	+	Metabolism	Bioenergetics	cellular carbohydrate catabolic process	GO:0044275	P	36	3.62E-03	3.24E-02
8	3599	F3 vs. M3	+	Metabolism	Bioenergetics	cellular carbohydrate metabolic process	GO:0044262	P	263	5.89E-09	3.06E-07
8	3600	F3 vs. M3	+	Metabolism	Bioenergetics	cellular polysaccharide biosynthetic process	GO:0033692	P	124	7.64E-05	1.33E-03
8	3601	F3 vs. M3	+	Metabolism	Bioenergetics	cellular polysaccharide metabolic process	GO:0044264	P	181	1.54E-05	3.27E-04
8	3602	F3 vs. M3	+	Metabolism	Bioenergetics	cellular respiration	GO:0045333	P	90	3.20E-07	1.07E-05
8	3603	F3 vs. M3	+	Metabolism	Bioenergetics	CoA-ligase activity	GO:0016405	F	23	7.26E-05	2.90E-03
8	3604	F3 vs. M3	+	Metabolism	Bioenergetics	electron transfer activity	GO:0009055	F	100	1.56E-03	3.30E-02

8	3605	F3 vs. M3	+	Metabolism	Bioenergetics	electron transport chain	GO:0022900	P	84	1.83E-06	5.15E-05
8	3606	F3 vs. M3	+	Metabolism	Bioenergetics	energy derivation by oxidation of organic compounds	GO:0015980	P	101	5.99E-06	1.43E-04
8	3607	F3 vs. M3	+	Metabolism	Bioenergetics	fatty acid biosynthetic process	GO:0006633	P	98	1.08E-04	1.81E-03
8	3608	F3 vs. M3	+	Metabolism	Bioenergetics	fatty acid derivative biosynthetic process	GO:1901570	P	22	5.65E-03	4.53E-02
8	3609	F3 vs. M3	+	Metabolism	Bioenergetics	fatty acid derivative metabolic process	GO:1901568	P	30	2.44E-03	2.39E-02
8	3610	F3 vs. M3	+	Metabolism	Bioenergetics	fatty acid ligase activity	GO:0015645	F	13	8.52E-04	2.06E-02
8	3611	F3 vs. M3	+	Metabolism	Bioenergetics	fatty acid metabolic process	GO:0006631	P	168	1.06E-07	4.19E-06
8	3612	F3 vs. M3	+	Metabolism	Bioenergetics	generation of precursor metabolites and energy	GO:0006091	P	275	5.00E-15	1.16E-12
8	3613	F3 vs. M3	+	Metabolism	Bioenergetics	gluconeogenesis	GO:0006094	P	21	7.02E-06	1.63E-04
8	3614	F3 vs. M3	+	Metabolism	Bioenergetics	glucose 6-phosphate metabolic process	GO:0051156	P	38	8.51E-04	1.01E-02
8	3615	F3 vs. M3	+	Metabolism	Bioenergetics	glucose metabolic process	GO:0006006	P	35	2.24E-04	3.32E-03
8	3616	F3 vs. M3	+	Metabolism	Bioenergetics	hexose biosynthetic process	GO:0019319	P	23	5.37E-06	1.31E-04
8	3617	F3 vs. M3	+	Metabolism	Bioenergetics	hexose metabolic process	GO:0019318	P	73	2.60E-04	3.81E-03
8	3618	F3 vs. M3	+	Metabolism	Bioenergetics	isocitrate metabolic process	GO:0006102	P	11	2.47E-03	2.41E-02
8	3619	F3 vs. M3	+	Metabolism	Bioenergetics	medium-chain fatty acid-CoA ligase activity	GO:0031956	F	12	2.44E-03	4.71E-02
8	3620	F3 vs. M3	+	Metabolism	Bioenergetics	mitochondrial envelope	GO:0005740	C	205	8.52E-04	6.76E-03
8	3621	F3 vs. M3	+	Metabolism	Bioenergetics	mitochondrial inner membrane	GO:0005743	C	163	2.48E-03	1.67E-02
8	3622	F3 vs. M3	+	Metabolism	Bioenergetics	mitochondrial membrane	GO:0031966	C	192	7.82E-03	4.36E-02
8	3623	F3 vs. M3	+	Metabolism	Bioenergetics	mitochondrial respirasome	GO:0005746	C	75	1.38E-04	1.32E-03
8	3624	F3 vs. M3	+	Metabolism	Bioenergetics	monosaccharide biosynthetic process	GO:0046364	P	37	7.94E-08	3.22E-06
8	3625	F3 vs. M3	+	Metabolism	Bioenergetics	NAD binding	GO:0051287	F	61	4.01E-05	1.83E-03
8	3626	F3 vs. M3	+	Metabolism	Bioenergetics	NAD(P)H dehydrogenase complex assembly	GO:0010275	P	6	1.87E-03	1.92E-02
8	3627	F3 vs. M3	+	Metabolism	Bioenergetics	NADH dehydrogenase complex	GO:0030964	C	47	2.44E-03	1.67E-02
8	3628	F3 vs. M3	+	Metabolism	Bioenergetics	NADP metabolic process	GO:0006739	P	39	5.63E-05	1.01E-03
8	3629	F3 vs. M3	+	Metabolism	Bioenergetics	NADPH regeneration	GO:0006740	P	32	2.93E-04	4.17E-03
8	3630	F3 vs. M3	+	Metabolism	Bioenergetics	oxidoreductase activity	GO:0016491	F	910	0.00E+00	0.00E+00
8	3631	F3 vs. M3	+	Metabolism	Bioenergetics	oxidoreductase activity, acting on a sulfur group of donors	GO:0016667	F	70	4.81E-05	2.10E-03
8	3632	F3 vs. M3	+	Metabolism	Bioenergetics	oxidoreductase activity, acting on CH-OH group of donors	GO:0016614	F	142	4.26E-07	3.96E-05
8	3633	F3 vs. M3	+	Metabolism	Bioenergetics	oxidoreductase activity, acting on NAD(P)H	GO:0016651	F	78	1.06E-05	6.36E-04
8	3634	F3 vs. M3	+	Metabolism	Bioenergetics	oxidoreductase activity, acting on single donors with incorporation of molecular oxygen	GO:0016701	F	28	1.78E-04	6.04E-03
8	3635	F3 vs. M3	+	Metabolism	Bioenergetics	oxidoreductase activity, acting on the aldehyde or oxo group of donors	GO:0016903	F	49	3.71E-05	1.78E-03
8	3636	F3 vs. M3	+	Metabolism	Bioenergetics	oxidoreductase activity, acting on the aldehyde or oxo group of donors, NAD or NADP as acceptor	GO:0016620	F	32	3.97E-05	1.84E-03
8	3637	F3 vs. M3	+	Metabolism	Bioenergetics	oxidoreductase activity, acting on the CH-OH group of donors, NAD or NADP as acceptor	GO:0016616	F	122	1.45E-06	1.13E-04
8	3638	F3 vs. M3	+	Metabolism	Bioenergetics	oxidoreductase complex	GO:1990204	C	115	4.62E-09	9.56E-08
8	3639	F3 vs. M3	+	Metabolism	Bioenergetics	pentose-phosphate shunt	GO:0006098	P	30	1.05E-04	1.75E-03
8	3640	F3 vs. M3	+	Metabolism	Bioenergetics	photorespiration	GO:0009853	P	33	3.64E-05	6.96E-04
8	3641	F3 vs. M3	+	Metabolism	Bioenergetics	polysaccharide biosynthetic process	GO:0000271	P	145	1.42E-05	3.06E-04
8	3642	F3 vs. M3	+	Metabolism	Bioenergetics	polysaccharide metabolic process	GO:0005976	P	274	1.41E-10	1.03E-08
8	3643	F3 vs. M3	+	Metabolism	Bioenergetics	proton transmembrane transport	GO:1902600	P	67	1.31E-04	2.09E-03
8	3644	F3 vs. M3	+	Metabolism	Bioenergetics	proton-exporting ATPase activity, phosphorylative mechanism	GO:0008553	F	17	2.58E-03	4.88E-02
8	3645	F3 vs. M3	+	Metabolism	Bioenergetics	proton-transporting ATPase activity, rotational mechanism	GO:0046961	F	20	9.93E-04	2.28E-02
8	3646	F3 vs. M3	+	Metabolism	Bioenergetics	proton-transporting two-sector ATPase complex	GO:0016469	C	47	7.97E-04	6.38E-03
8	3647	F3 vs. M3	+	Metabolism	Bioenergetics	proton-transporting V-type ATPase complex	GO:0033176	C	19	1.19E-04	1.16E-03
8	3648	F3 vs. M3	+	Metabolism	Bioenergetics	pyrophosphate hydrolysis-driven proton transmembrane transporter activity	GO:0009678	F	36	1.20E-05	6.93E-04
8	3649	F3 vs. M3	+	Metabolism	Bioenergetics	pyruvate metabolic process	GO:0006090	P	65	2.25E-03	2.25E-02
8	3650	F3 vs. M3	+	Metabolism	Bioenergetics	reductive pentose-phosphate cycle	GO:0019253	P	14	4.54E-06	1.14E-04
8	3651	F3 vs. M3	+	Metabolism	Bioenergetics	respirasome	GO:0070469	C	78	3.09E-05	3.27E-04
8	3652	F3 vs. M3	+	Metabolism	Bioenergetics	respiratory chain complex	GO:0098803	C	74	8.82E-05	8.93E-04
8	3653	F3 vs. M3	+	Metabolism	Bioenergetics	respiratory chain complex I	GO:0045271	C	46	2.75E-03	1.80E-02
8	3654	F3 vs. M3	+	Metabolism	Bioenergetics	respiratory electron transport chain	GO:0022904	P	37	2.20E-04	3.27E-03
8	3655	F3 vs. M3	+	Metabolism	Bioenergetics	sucrose metabolic process	GO:0005985	P	37	3.04E-04	4.28E-03
8	3656	F3 vs. M3	+	Metabolism	Bioenergetics	sugar-phosphatase activity	GO:0050308	F	12	2.35E-03	4.59E-02
8	3657	F3 vs. M3	+	Metabolism	Bioenergetics	tricarboxylic acid cycle	GO:0006099	P	34	8.04E-05	1.39E-03
8	3658	F3 vs. M3	+	Metabolism	Bioenergetics	tricarboxylic acid metabolic process	GO:0072350	P	17	5.99E-04	7.70E-03
8	3659	F3 vs. M3	+	Metabolism	Bioenergetics	vacuolar proton-transporting V-type ATPase complex	GO:0016471	C	8	9.32E-04	7.17E-03
8	3660	F3 vs. M3	+	Metabolism	Catabolism	alcohol catabolic process	GO:0046164	P	27	1.19E-04	1.93E-03
8	3661	F3 vs. M3	+	Metabolism	Catabolism	alpha-amino acid catabolic process	GO:1901606	P	71	1.92E-10	1.38E-08
8	3662	F3 vs. M3	+	Metabolism	Catabolism	aromatic amino acid family catabolic process	GO:0009074	P	18	5.31E-05	9.65E-04
8	3663	F3 vs. M3	+	Metabolism	Catabolism	carbonate dehydratase activity	GO:0004089	F	13	8.15E-04	1.99E-02

8	3664	F3 vs. M3	+	Metabolism	Catabolism	carboxylic acid catabolic process	GO:0046395	P	129	1.04E-11	9.32E-10
8	3665	F3 vs. M3	+	Metabolism	Catabolism	catabolic process	GO:0009056	P	942	3.70E-09	2.03E-07
8	3666	F3 vs. M3	+	Metabolism	Catabolism	cell wall macromolecule catabolic process	GO:0016998	P	18	1.54E-03	1.65E-02
8	3667	F3 vs. M3	+	Metabolism	Catabolism	cellular amino acid catabolic process	GO:0009063	P	78	2.44E-08	1.10E-06
8	3668	F3 vs. M3	+	Metabolism	Catabolism	cellular catabolic process	GO:0044248	P	775	2.70E-05	5.43E-04
8	3669	F3 vs. M3	+	Metabolism	Catabolism	cellular lipid catabolic process	GO:0044242	P	69	9.37E-04	1.09E-02
8	3670	F3 vs. M3	+	Metabolism	Catabolism	cellular metabolic compound salvage	GO:0043094	P	57	2.45E-06	6.70E-05
8	3671	F3 vs. M3	+	Metabolism	Catabolism	erythrose 4-phosphate/phosphoenolpyruvate family amino acid catabolic process	GO:1902222	P	11	1.53E-04	2.39E-03
8	3672	F3 vs. M3	+	Metabolism	Catabolism	glycine catabolic process	GO:0006546	P	7	6.40E-04	8.04E-03
8	3673	F3 vs. M3	+	Metabolism	Catabolism	lipid catabolic process	GO:0016042	P	74	1.23E-03	1.35E-02
8	3674	F3 vs. M3	+	Metabolism	Catabolism	L-phenylalanine catabolic process	GO:0006559	P	11	1.53E-04	2.39E-03
8	3675	F3 vs. M3	+	Metabolism	Catabolism	monocarboxylic acid catabolic process	GO:0072329	P	49	2.75E-05	5.49E-04
8	3676	F3 vs. M3	+	Metabolism	Catabolism	organic acid catabolic process	GO:0016054	P	129	1.04E-11	9.47E-10
8	3677	F3 vs. M3	+	Metabolism	Catabolism	organic hydroxy compound catabolic process	GO:1901616	P	35	1.37E-06	3.96E-05
8	3678	F3 vs. M3	+	Metabolism	Catabolism	organic substance catabolic process	GO:1901575	P	867	1.55E-07	5.82E-06
8	3679	F3 vs. M3	+	Metabolism	Catabolism	pectin catabolic process	GO:0045490	P	61	6.90E-05	1.22E-03
8	3680	F3 vs. M3	+	Metabolism	Catabolism	peroxisome	GO:0005777	C	204	1.73E-06	2.20E-05
8	3681	F3 vs. M3	+	Metabolism	Catabolism	phenol-containing compound catabolic process	GO:0019336	P	6	4.46E-03	3.78E-02
8	3682	F3 vs. M3	+	Metabolism	Catabolism	polyol catabolic process	GO:0046174	P	20	6.05E-04	7.74E-03
8	3683	F3 vs. M3	+	Metabolism	Catabolism	polysaccharide catabolic process	GO:0000272	P	90	1.76E-05	3.64E-04
8	3684	F3 vs. M3	+	Metabolism	Catabolism	serine family amino acid catabolic process	GO:0009071	P	13	9.42E-05	1.59E-03
8	3685	F3 vs. M3	+	Metabolism	Catabolism	small molecule catabolic process	GO:0044282	P	188	3.62E-12	3.94E-10
8	3686	F3 vs. M3	+	Metabolism	Metabolism	acid-ammonia (or amide) ligase activity	GO:0016880	F	8	6.21E-04	1.62E-02
8	3687	F3 vs. M3	+	Metabolism	Metabolism	acid-thiol ligase activity	GO:0016878	F	27	9.51E-05	3.70E-03
8	3688	F3 vs. M3	+	Metabolism	Metabolism	actin binding	GO:0003779	F	76	3.76E-05	1.77E-03
8	3689	F3 vs. M3	+	Metabolism	Metabolism	actin filament binding	GO:0051015	F	50	2.30E-05	1.22E-03
8	3690	F3 vs. M3	+	Metabolism	Metabolism	actin filament bundle assembly	GO:0051017	P	12	2.42E-03	2.38E-02
8	3691	F3 vs. M3	+	Metabolism	Metabolism	actin filament bundle organization	GO:0061572	P	12	2.42E-03	2.38E-02
8	3692	F3 vs. M3	+	Metabolism	Metabolism	actin filament depolymerization	GO:0030042	P	9	5.45E-03	4.44E-02
8	3693	F3 vs. M3	+	Metabolism	Metabolism	actin filament organization	GO:0007015	P	60	9.05E-04	1.06E-02
8	3694	F3 vs. M3	+	Metabolism	Metabolism	actin filament-based process	GO:0030029	P	74	1.07E-03	1.23E-02
8	3695	F3 vs. M3	+	Metabolism	Metabolism	alcohol metabolic process	GO:0006066	P	86	1.03E-05	2.26E-04
8	3696	F3 vs. M3	+	Metabolism	Metabolism	alpha-amino acid biosynthetic process	GO:1901607	P	119	4.92E-06	1.21E-04
8	3697	F3 vs. M3	+	Metabolism	Metabolism	alpha-amino acid metabolic process	GO:1901605	P	197	4.76E-14	9.45E-12
8	3698	F3 vs. M3	+	Metabolism	Metabolism	amine biosynthetic process	GO:0009309	P	26	1.47E-03	1.59E-02
8	3699	F3 vs. M3	+	Metabolism	Metabolism	amine metabolic process	GO:0009308	P	67	6.64E-04	8.32E-03
8	3700	F3 vs. M3	+	Metabolism	Metabolism	aminoglycan biosynthetic process	GO:0006023	P	4	5.31E-03	4.35E-02
8	3701	F3 vs. M3	+	Metabolism	Metabolism	ammonia assimilation cycle	GO:0019676	P	6	7.38E-04	9.03E-03
8	3702	F3 vs. M3	+	Metabolism	Metabolism	ammonia ligase activity	GO:0016211	F	8	6.21E-04	1.64E-02
8	3703	F3 vs. M3	+	Metabolism	Metabolism	anchoring junction	GO:0070161	C	654	7.73E-03	4.38E-02
8	3704	F3 vs. M3	+	Metabolism	Metabolism	anion binding	GO:0043168	F	820	1.09E-05	6.41E-04
8	3705	F3 vs. M3	+	Metabolism	Metabolism	aromatic amino acid family metabolic process	GO:0009072	P	56	1.18E-04	1.92E-03
8	3706	F3 vs. M3	+	Metabolism	Metabolism	aspartate family amino acid metabolic process	GO:0009066	P	41	1.98E-03	2.00E-02
8	3707	F3 vs. M3	+	Metabolism	Metabolism	benzene-containing compound metabolic process	GO:0042537	P	43	9.76E-04	1.13E-02
8	3708	F3 vs. M3	+	Metabolism	Metabolism	beta-galactosidase activity	GO:0004565	F	13	9.88E-04	2.29E-02
8	3709	F3 vs. M3	+	Metabolism	Metabolism	beta-glucan biosynthetic process	GO:0051274	P	46	1.16E-03	1.31E-02
8	3710	F3 vs. M3	+	Metabolism	Metabolism	beta-glucan metabolic process	GO:0051273	P	50	2.64E-03	2.51E-02
8	3711	F3 vs. M3	+	Metabolism	Metabolism	biosynthetic process	GO:0009058	P	1855	1.92E-07	6.88E-06
8	3712	F3 vs. M3	+	Metabolism	Metabolism	branched-chain amino acid biosynthetic process	GO:0009082	P	18	1.80E-03	1.85E-02
8	3713	F3 vs. M3	+	Metabolism	Metabolism	carbon utilization	GO:0015976	P	8	4.37E-05	8.19E-04
8	3714	F3 vs. M3	+	Metabolism	Metabolism	carbon-carbon lyase activity	GO:0016830	F	76	1.62E-06	1.20E-04
8	3715	F3 vs. M3	+	Metabolism	Metabolism	carbon-oxygen lyase activity	GO:0016835	F	99	1.65E-04	5.64E-03
8	3716	F3 vs. M3	+	Metabolism	Metabolism	carboxylic acid biosynthetic process	GO:0046394	P	323	4.08E-13	7.09E-11
8	3717	F3 vs. M3	+	Metabolism	Metabolism	carboxylic acid metabolic process	GO:0019752	P	652	0.00E+00	0.00E+00
8	3718	F3 vs. M3	+	Metabolism	Metabolism	carboxy-lyase activity	GO:0016831	F	43	1.55E-04	5.49E-03
8	3719	F3 vs. M3	+	Metabolism	Metabolism	catalytic activity	GO:0003824	F	5104	0.00E+00	0.00E+00
8	3720	F3 vs. M3	+	Metabolism	Metabolism	cation binding	GO:0043169	F	1013	2.69E-09	4.29E-07
8	3721	F3 vs. M3	+	Metabolism	Metabolism	cell wall macromolecule metabolic process	GO:0044036	P	109	1.95E-03	1.98E-02
8	3722	F3 vs. M3	+	Metabolism	Metabolism	cell wall polysaccharide metabolic process	GO:0010383	P	89	2.77E-03	2.62E-02
8	3723	F3 vs. M3	+	Metabolism	Metabolism	cellular amine metabolic process	GO:0044106	P	42	1.10E-04	1.83E-03
8	3724	F3 vs. M3	+	Metabolism	Metabolism	cellular amino acid biosynthetic process	GO:0008652	P	134	3.58E-07	1.17E-05
8	3725	F3 vs. M3	+	Metabolism	Metabolism	cellular amino acid metabolic process	GO:0006520	P	266	1.30E-12	1.64E-10
8	3726	F3 vs. M3	+	Metabolism	Metabolism	cellular biogenic amine biosynthetic process	GO:0042401	P	26	1.47E-03	1.58E-02

8	3727	F3 vs. M3	+	Metabolism	Metabolism	cellular biogenic amine metabolic process	GO:0006576	P	42	1.10E-04	1.83E-03
8	3728	F3 vs. M3	+	Metabolism	Metabolism	cellular biosynthetic process	GO:0044249	P	1701	6.90E-05	1.22E-03
8	3729	F3 vs. M3	+	Metabolism	Metabolism	cellular glucan metabolic process	GO:0006073	P	137	8.78E-04	1.03E-02
8	3730	F3 vs. M3	+	Metabolism	Metabolism	cellular ketone metabolic process	GO:0042180	P	47	3.73E-03	3.28E-02
8	3731	F3 vs. M3	+	Metabolism	Metabolism	cellular lipid metabolic process	GO:0044255	P	461	7.85E-13	1.21E-10
8	3732	F3 vs. M3	+	Metabolism	Metabolism	cellulose biosynthetic process	GO:0030244	P	36	3.57E-04	4.90E-03
8	3733	F3 vs. M3	+	Metabolism	Metabolism	cellulose metabolic process	GO:0030243	P	40	1.10E-03	1.25E-02
8	3734	F3 vs. M3	+	Metabolism	Metabolism	cellulose synthase (UDP-forming) activity	GO:0016760	F	20	3.36E-04	1.02E-02
8	3735	F3 vs. M3	+	Metabolism	Metabolism	cellulose synthase activity	GO:0016759	F	29	3.83E-04	1.14E-02
8	3736	F3 vs. M3	+	Metabolism	Metabolism	cinnamic acid metabolic process	GO:0009803	P	4	4.75E-03	3.99E-02
8	3737	F3 vs. M3	+	Metabolism	Metabolism	copper ion binding	GO:0005507	F	138	7.88E-11	1.89E-08
8	3738	F3 vs. M3	+	Metabolism	Metabolism	coumarin biosynthetic process	GO:0009805	P	4	2.47E-03	2.41E-02
8	3739	F3 vs. M3	+	Metabolism	Metabolism	coumarin metabolic process	GO:0009804	P	6	4.58E-04	6.07E-03
8	3740	F3 vs. M3	+	Metabolism	Metabolism	dicarboxylic acid metabolic process	GO:0043648	P	68	1.98E-06	5.49E-05
8	3741	F3 vs. M3	+	Metabolism	Metabolism	erythrose 4-phosphate/phosphoenolpyruvate family amino acid metabolic process	GO:1902221	P	20	1.78E-04	2.72E-03
8	3742	F3 vs. M3	+	Metabolism	Metabolism	establishment of localization	GO:0051234	P	1410	1.92E-09	1.12E-07
8	3743	F3 vs. M3	+	Metabolism	Metabolism	galactosidase activity	GO:0015925	F	18	2.09E-03	4.21E-02
8	3744	F3 vs. M3	+	Metabolism	Metabolism	galacturonan metabolic process	GO:0010393	P	103	9.37E-09	4.57E-07
8	3745	F3 vs. M3	+	Metabolism	Metabolism	glucan biosynthetic process	GO:0009250	P	85	2.18E-03	2.18E-02
8	3746	F3 vs. M3	+	Metabolism	Metabolism	glucan metabolic process	GO:0044042	P	143	5.76E-04	7.43E-03
8	3747	F3 vs. M3	+	Metabolism	Metabolism	glucosinolate biosynthetic process	GO:0019761	P	31	4.67E-04	6.17E-03
8	3748	F3 vs. M3	+	Metabolism	Metabolism	glucosyltransferase activity	GO:0046527	F	134	1.42E-04	5.24E-03
8	3749	F3 vs. M3	+	Metabolism	Metabolism	glutamate metabolic process	GO:0006536	P	23	1.57E-03	1.67E-02
8	3750	F3 vs. M3	+	Metabolism	Metabolism	glutamine family amino acid metabolic process	GO:0009064	P	46	2.04E-04	3.06E-03
8	3751	F3 vs. M3	+	Metabolism	Metabolism	glutamine metabolic process	GO:0006541	P	15	4.53E-04	6.02E-03
8	3752	F3 vs. M3	+	Metabolism	Metabolism	glycerolipid metabolic process	GO:0046486	P	120	2.47E-04	3.65E-03
8	3753	F3 vs. M3	+	Metabolism	Metabolism	glycerophospholipid metabolic process	GO:0006650	P	100	1.26E-04	2.02E-03
8	3754	F3 vs. M3	+	Metabolism	Metabolism	glycine cleavage complex	GO:0005960	C	5	7.07E-03	4.15E-02
8	3755	F3 vs. M3	+	Metabolism	Metabolism	glycine decarboxylation via glycine cleavage system	GO:0019464	P	6	2.33E-03	2.31E-02
8	3756	F3 vs. M3	+	Metabolism	Metabolism	glycine metabolic process	GO:0006544	P	15	1.93E-05	3.98E-04
8	3757	F3 vs. M3	+	Metabolism	Metabolism	glycosaminoglycan biosynthetic process	GO:0006024	P	4	5.31E-03	4.35E-02
8	3758	F3 vs. M3	+	Metabolism	Metabolism	glycosaminoglycan metabolic process	GO:0030203	P	4	5.31E-03	4.37E-02
8	3759	F3 vs. M3	+	Metabolism	Metabolism	glycosinolate biosynthetic process	GO:0019758	P	31	4.67E-04	6.15E-03
8	3760	F3 vs. M3	+	Metabolism	Metabolism	glycosyl compound biosynthetic process	GO:1901659	P	48	7.39E-04	9.03E-03
8	3761	F3 vs. M3	+	Metabolism	Metabolism	hydrolase activity	GO:0016787	F	1809	1.64E-04	5.69E-03
8	3762	F3 vs. M3	+	Metabolism	Metabolism	hydrolase activity, hydrolyzing O-glycosyl compounds	GO:0004553	F	233	1.31E-04	4.90E-03
8	3763	F3 vs. M3	+	Metabolism	Metabolism	hydro-lyase activity	GO:0016836	F	61	9.40E-04	2.22E-02
8	3764	F3 vs. M3	+	Metabolism	Metabolism	indole-containing compound metabolic process	GO:0042430	P	47	1.34E-03	1.46E-02
8	3765	F3 vs. M3	+	Metabolism	Metabolism	inorganic diphosphatase activity	GO:0004427	F	10	2.12E-04	6.93E-03
8	3766	F3 vs. M3	+	Metabolism	Metabolism	ion binding	GO:0043167	F	1724	7.58E-11	1.98E-08
8	3767	F3 vs. M3	+	Metabolism	Metabolism	leucine biosynthetic process	GO:0009098	P	12	3.72E-03	3.28E-02
8	3768	F3 vs. M3	+	Metabolism	Metabolism	leucine metabolic process	GO:0006551	P	19	7.93E-04	9.52E-03
8	3769	F3 vs. M3	+	Metabolism	Metabolism	lignin biosynthetic process	GO:0009809	P	30	4.54E-06	1.14E-04
8	3770	F3 vs. M3	+	Metabolism	Metabolism	lignin metabolic process	GO:0009808	P	38	5.19E-07	1.66E-05
8	3771	F3 vs. M3	+	Metabolism	Metabolism	lipid biosynthetic process	GO:0008610	P	339	6.79E-06	1.58E-04
8	3772	F3 vs. M3	+	Metabolism	Metabolism	lipid metabolic process	GO:0006629	P	548	4.11E-12	4.15E-10
8	3773	F3 vs. M3	+	Metabolism	Metabolism	lipid modification	GO:0030258	P	77	6.57E-06	1.55E-04
8	3774	F3 vs. M3	+	Metabolism	Metabolism	localization	GO:0051179	P	1507	3.20E-08	1.42E-06
8	3775	F3 vs. M3	+	Metabolism	Metabolism	L-phenylalanine metabolic process	GO:0006558	P	20	1.78E-04	2.73E-03
8	3776	F3 vs. M3	+	Metabolism	Metabolism	lyase activity	GO:0016829	F	214	2.00E-10	3.83E-08
8	3777	F3 vs. M3	+	Metabolism	Metabolism	membrane lipid biosynthetic process	GO:0046467	P	57	5.49E-03	4.43E-02
8	3778	F3 vs. M3	+	Metabolism	Metabolism	membrane lipid metabolic process	GO:0006643	P	67	6.68E-04	8.34E-03
8	3779	F3 vs. M3	+	Metabolism	Metabolism	metabolic process	GO:0008152	P	5391	1.93E-04	2.94E-03
8	3780	F3 vs. M3	+	Metabolism	Metabolism	metal ion binding	GO:0046872	F	994	6.56E-09	8.57E-07
8	3781	F3 vs. M3	+	Metabolism	Metabolism	methionine adenosyltransferase activity	GO:0004478	F	4	1.66E-03	3.49E-02
8	3782	F3 vs. M3	+	Metabolism	Metabolism	methionine metabolic process	GO:0006555	P	16	4.25E-03	3.61E-02
8	3783	F3 vs. M3	+	Metabolism	Metabolism	monocarboxylic acid biosynthetic process	GO:0072330	P	156	8.51E-06	1.93E-04
8	3784	F3 vs. M3	+	Metabolism	Metabolism	monocarboxylic acid metabolic process	GO:0032787	P	335	4.19E-13	7.05E-11
8	3785	F3 vs. M3	+	Metabolism	Metabolism	monosaccharide metabolic process	GO:0005996	P	102	5.45E-07	1.72E-05
8	3786	F3 vs. M3	+	Metabolism	Metabolism	nitrate assimilation	GO:0042128	P	10	1.22E-03	1.36E-02
8	3787	F3 vs. M3	+	Metabolism	Metabolism	nitrate metabolic process	GO:0042126	P	10	1.22E-03	1.36E-02
8	3788	F3 vs. M3	+	Metabolism	Metabolism	nitrogen cycle metabolic process	GO:0071941	P	13	4.03E-04	5.42E-03
8	3789	F3 vs. M3	+	Metabolism	Metabolism	nitrogen utilization	GO:0019740	P	6	7.38E-04	9.05E-03

8	3790	F3 vs. M3	+	Metabolism	Metabolism	olefinic compound metabolic process	GO:0120254	P	40	4.98E-04	6.50E-03
8	3791	F3 vs. M3	+	Metabolism	Metabolism	organic acid biosynthetic process	GO:0016053	P	323	4.08E-13	7.32E-11
8	3792	F3 vs. M3	+	Metabolism	Metabolism	organic acid metabolic process	GO:0006082	P	724	0.00E+00	0.00E+00
8	3793	F3 vs. M3	+	Metabolism	Metabolism	organic cyclic compound biosynthetic process	GO:1901362	P	591	4.17E-03	3.56E-02
8	3794	F3 vs. M3	+	Metabolism	Metabolism	organic hydroxy compound biosynthetic process	GO:1901617	P	118	4.64E-05	8.63E-04
8	3795	F3 vs. M3	+	Metabolism	Metabolism	organic hydroxy compound metabolic process	GO:1901615	P	207	2.31E-08	1.06E-06
8	3796	F3 vs. M3	+	Metabolism	Metabolism	organic substance biosynthetic process	GO:1901576	P	1771	4.35E-06	1.10E-04
8	3797	F3 vs. M3	+	Metabolism	Metabolism	organophosphate metabolic process	GO:0019637	P	404	1.21E-07	4.72E-06
8	3798	F3 vs. M3	+	Metabolism	Metabolism	oxaloacetate decarboxylase activity	GO:0008948	F	7	2.65E-03	4.97E-02
8	3799	F3 vs. M3	+	Metabolism	Metabolism	oxidation-reduction process	GO:0055114	P	500	0.00E+00	0.00E+00
8	3800	F3 vs. M3	+	Metabolism	Metabolism	oxoacid metabolic process	GO:0043436	P	721	0.00E+00	0.00E+00
8	3801	F3 vs. M3	+	Metabolism	Metabolism	oxylipin biosynthetic process	GO:0031408	P	13	1.95E-03	1.98E-02
8	3802	F3 vs. M3	+	Metabolism	Metabolism	oxylipin metabolic process	GO:0031407	P	15	2.49E-03	2.42E-02
8	3803	F3 vs. M3	+	Metabolism	Metabolism	pectin biosynthetic process	GO:0045489	P	34	3.89E-03	3.36E-02
8	3804	F3 vs. M3	+	Metabolism	Metabolism	pectin metabolic process	GO:0045488	P	102	4.18E-09	2.25E-07
8	3805	F3 vs. M3	+	Metabolism	Metabolism	pectinesterase activity	GO:0030599	F	50	6.16E-04	1.64E-02
8	3806	F3 vs. M3	+	Metabolism	Metabolism	pectinesterase inhibitor activity	GO:0046910	F	39	1.83E-03	3.81E-02
8	3807	F3 vs. M3	+	Metabolism	Metabolism	phenylpropanoid biosynthetic process	GO:0009699	P	55	1.68E-07	6.22E-06
8	3808	F3 vs. M3	+	Metabolism	Metabolism	phenylpropanoid metabolic process	GO:0009698	P	70	4.53E-09	2.42E-07
8	3809	F3 vs. M3	+	Metabolism	Metabolism	phosphate-containing compound metabolic process	GO:0006796	P	1149	5.09E-05	9.36E-04
8	3810	F3 vs. M3	+	Metabolism	Metabolism	phosphatidylinositol bisphosphate phosphatase activity	GO:0034593	F	17	2.36E-03	4.59E-02
8	3811	F3 vs. M3	+	Metabolism	Metabolism	phosphatidylinositol metabolic process	GO:0046488	P	58	1.68E-03	1.76E-02
8	3812	F3 vs. M3	+	Metabolism	Metabolism	phospholipid metabolic process	GO:0006644	P	144	1.95E-04	2.95E-03
8	3813	F3 vs. M3	+	Metabolism	Metabolism	phosphoric ester hydrolase activity	GO:0042578	F	251	1.28E-04	4.83E-03
8	3814	F3 vs. M3	+	Metabolism	Metabolism	phosphorus metabolic process	GO:0006793	P	1188	3.08E-06	8.06E-05
8	3815	F3 vs. M3	+	Metabolism	Metabolism	pigment biosynthetic process	GO:0046148	P	83	1.94E-04	2.95E-03
8	3816	F3 vs. M3	+	Metabolism	Metabolism	pigment metabolic process	GO:0042440	P	97	1.75E-05	3.65E-04
8	3817	F3 vs. M3	+	Metabolism	Metabolism	polyamine biosynthetic process	GO:0006596	P	12	2.04E-03	2.06E-02
8	3818	F3 vs. M3	+	Metabolism	Metabolism	polyamine metabolic process	GO:0006595	P	20	1.86E-03	1.91E-02
8	3819	F3 vs. M3	+	Metabolism	Metabolism	polyol metabolic process	GO:0019751	P	41	1.81E-04	2.77E-03
8	3820	F3 vs. M3	+	Metabolism	Metabolism	porphyrin-containing compound metabolic process	GO:0006778	P	56	2.39E-03	2.35E-02
8	3821	F3 vs. M3	+	Metabolism	Metabolism	purine nucleotide metabolic process	GO:0006163	P	154	8.84E-04	1.04E-02
8	3822	F3 vs. M3	+	Metabolism	Metabolism	purine ribonucleotide metabolic process	GO:0009150	P	143	4.45E-04	5.92E-03
8	3823	F3 vs. M3	+	Metabolism	Metabolism	purine-containing compound biosynthetic process	GO:0072522	P	87	5.63E-03	4.53E-02
8	3824	F3 vs. M3	+	Metabolism	Metabolism	purine-containing compound metabolic process	GO:0072521	P	170	1.05E-03	1.20E-02
8	3825	F3 vs. M3	+	Metabolism	Metabolism	pyridoxal phosphate binding	GO:0030170	F	68	6.43E-04	1.62E-02
8	3826	F3 vs. M3	+	Metabolism	Metabolism	quercetin 7-O-glucosyltransferase activity	GO:0080044	F	35	2.57E-03	4.90E-02
8	3827	F3 vs. M3	+	Metabolism	Metabolism	racemase and epimerase activity	GO:0016854	F	39	2.03E-03	4.14E-02
8	3828	F3 vs. M3	+	Metabolism	Metabolism	S-adenosylmethionine biosynthetic process	GO:0006556	P	4	1.66E-03	1.75E-02
8	3829	F3 vs. M3	+	Metabolism	Metabolism	S-adenosylmethionine metabolic process	GO:0046500	P	9	1.23E-04	1.99E-03
8	3830	F3 vs. M3	+	Metabolism	Metabolism	secondary alcohol metabolic process	GO:1902652	P	11	2.47E-03	2.41E-02
8	3831	F3 vs. M3	+	Metabolism	Metabolism	secondary metabolic process	GO:0019748	P	194	4.04E-10	2.71E-08
8	3832	F3 vs. M3	+	Metabolism	Metabolism	secondary metabolite biosynthetic process	GO:0044550	P	98	6.99E-12	6.58E-10
8	3833	F3 vs. M3	+	Metabolism	Metabolism	serine family amino acid biosynthetic process	GO:0009070	P	29	1.47E-03	1.58E-02
8	3834	F3 vs. M3	+	Metabolism	Metabolism	serine family amino acid metabolic process	GO:0009069	P	41	5.71E-06	1.38E-04
8	3835	F3 vs. M3	+	Metabolism	Metabolism	S-glycoside biosynthetic process	GO:0016144	P	31	4.67E-04	6.18E-03
8	3836	F3 vs. M3	+	Metabolism	Metabolism	sigma factor activity	GO:0016987	F	6	1.01E-03	2.28E-02
8	3837	F3 vs. M3	+	Metabolism	Metabolism	small molecule binding	GO:0036094	F	819	4.36E-04	1.23E-02
8	3838	F3 vs. M3	+	Metabolism	Metabolism	small molecule biosynthetic process	GO:0044283	P	448	0.00E+00	0.00E+00
8	3839	F3 vs. M3	+	Metabolism	Metabolism	small molecule metabolic process	GO:0044281	P	1071	0.00E+00	0.00E+00
8	3840	F3 vs. M3	+	Metabolism	Metabolism	spermidine biosynthetic process	GO:0008295	P	4	5.63E-03	4.52E-02
8	3841	F3 vs. M3	+	Metabolism	Metabolism	sphingolipid metabolic process	GO:0006665	P	30	2.90E-03	2.68E-02
8	3842	F3 vs. M3	+	Metabolism	Metabolism	sulfur amino acid biosynthetic process	GO:0000097	P	25	3.61E-03	3.24E-02
8	3843	F3 vs. M3	+	Metabolism	Metabolism	sulfur amino acid metabolic process	GO:0000096	P	37	3.78E-04	5.10E-03
8	3844	F3 vs. M3	+	Metabolism	Metabolism	sulfur compound biosynthetic process	GO:0044272	P	105	8.45E-13	1.27E-10
8	3845	F3 vs. M3	+	Metabolism	Metabolism	sulfur compound metabolic process	GO:0006790	P	248	3.16E-12	3.51E-10
8	3846	F3 vs. M3	+	Metabolism	Metabolism	tetrapyrrole metabolic process	GO:0033013	P	58	2.76E-03	2.61E-02
8	3847	F3 vs. M3	+	Metabolism	Metabolism	thioester biosynthetic process	GO:0035384	P	18	3.37E-03	3.05E-02
8	3848	F3 vs. M3	+	Metabolism	Metabolism	transferase activity	GO:0016740	F	2040	4.26E-04	1.24E-02
8	3849	F3 vs. M3	+	Metabolism	Metabolism	transferase activity, transferring hexosyl groups	GO:0016758	F	244	2.56E-04	8.28E-03
8	3850	F3 vs. M3	+	Metabolism	Metabolism	UDP-glucosyltransferase activity	GO:0035251	F	101	8.78E-05	3.46E-03
8	3851	F3 vs. M3	+	Metabolism	Metabolism	UDP-glucuronate biosynthetic process	GO:0006065	P	4	5.31E-03	4.36E-02
8	3852	F3 vs. M3	+	Metabolism	Metabolism	UDP-glucuronate metabolic process	GO:0046398	P	5	3.87E-03	3.35E-02
8	3853	F3 vs. M3	+	Metabolism	Metabolism	UDP-glycosyltransferase activity	GO:0008194	F	189	2.18E-06	1.57E-04

8	3854	F3 vs. M3	+	Metabolism	Metabolism	UDP-rhamnose biosynthetic process	GO:0010253	P	4	3.40E-03	3.07E-02
8	3855	F3 vs. M3	+	Metabolism	Metabolism	UDP-rhamnose metabolic process	GO:0033478	P	4	3.40E-03	3.07E-02
8	3856	F3 vs. M3	+	Metabolism	Metabolism	vitamin B6 binding	GO:0070279	F	68	6.43E-04	1.64E-02
8	3857	F3 vs. M3	+	Metabolism	Metabolism	vitamin binding	GO:0019842	F	84	1.19E-03	2.61E-02
8	3858	F3 vs. M3	+	Metabolism	Metabolism	wax biosynthetic process	GO:0010025	P	20	3.75E-03	3.28E-02
8	3859	F3 vs. M3	+	Metabolism	Metabolism	wax metabolic process	GO:0010166	P	20	3.75E-03	3.27E-02
8	3860	F3 vs. M3	+	Metabolism	Photosynthesis	carbon fixation	GO:0015977	P	15	3.14E-06	8.19E-05
8	3861	F3 vs. M3	+	Metabolism	Photosynthesis	chlorophyll metabolic process	GO:0015994	P	47	1.17E-03	1.32E-02
8	3862	F3 vs. M3	+	Metabolism	Photosynthesis	chloroplast	GO:0009507	C	3011	0.00E+00	0.00E+00
8	3863	F3 vs. M3	+	Metabolism	Photosynthesis	chloroplast envelope	GO:0009941	C	463	0.00E+00	0.00E+00
8	3864	F3 vs. M3	+	Metabolism	Photosynthesis	chloroplast stroma	GO:0009570	C	532	0.00E+00	0.00E+00
8	3865	F3 vs. M3	+	Metabolism	Photosynthesis	chloroplast thylakoid	GO:0009534	C	319	0.00E+00	0.00E+00
8	3866	F3 vs. M3	+	Metabolism	Photosynthesis	chloroplast thylakoid lumen	GO:0009543	C	37	1.61E-03	1.15E-02
8	3867	F3 vs. M3	+	Metabolism	Photosynthesis	chloroplast thylakoid membrane	GO:0009535	C	256	0.00E+00	0.00E+00
8	3868	F3 vs. M3	+	Metabolism	Photosynthesis	fructose 1,6-bisphosphate metabolic process	GO:0030388	P	11	1.12E-04	1.85E-03
8	3869	F3 vs. M3	+	Metabolism	Photosynthesis	fructose-bisphosphate aldolase activity	GO:0004332	F	7	5.70E-04	1.54E-02
8	3870	F3 vs. M3	+	Metabolism	Photosynthesis	photosynthesis	GO:0015979	P	146	3.28E-13	6.08E-11
8	3871	F3 vs. M3	+	Metabolism	Photosynthesis	photosynthesis, dark reaction	GO:0019685	P	15	1.60E-06	4.57E-05
8	3872	F3 vs. M3	+	Metabolism	Photosynthesis	photosynthesis, light harvesting	GO:0009765	P	32	1.45E-04	2.28E-03
8	3873	F3 vs. M3	+	Metabolism	Photosynthesis	photosynthesis, light reaction	GO:0019684	P	97	6.71E-09	3.36E-07
8	3874	F3 vs. M3	+	Metabolism	Photosynthesis	photosynthetic electron transport chain	GO:0009767	P	38	7.96E-04	9.53E-03
8	3875	F3 vs. M3	+	Metabolism	Photosynthesis	photosynthetic membrane	GO:0034357	C	280	0.00E+00	0.00E+00
8	3876	F3 vs. M3	+	Metabolism	Photosynthesis	photosystem II	GO:0009523	C	31	8.74E-03	4.79E-02
8	3877	F3 vs. M3	+	Metabolism	Photosynthesis	plastid	GO:0009536	C	3312	0.00E+00	0.00E+00
8	3878	F3 vs. M3	+	Metabolism	Photosynthesis	plastid envelope	GO:0009526	C	479	0.00E+00	0.00E+00
8	3879	F3 vs. M3	+	Metabolism	Photosynthesis	plastid stroma	GO:0009532	C	540	0.00E+00	0.00E+00
8	3880	F3 vs. M3	+	Metabolism	Photosynthesis	plastid thylakoid	GO:0031976	C	321	0.00E+00	0.00E+00
8	3881	F3 vs. M3	+	Metabolism	Photosynthesis	plastid thylakoid lumen	GO:0031978	C	37	1.61E-03	1.14E-02
8	3882	F3 vs. M3	+	Metabolism	Photosynthesis	plastid thylakoid membrane	GO:0055035	C	259	0.00E+00	0.00E+00
8	3883	F3 vs. M3	+	Metabolism	Photosynthesis	plastoglobule	GO:0010287	C	56	2.49E-07	3.74E-06
8	3884	F3 vs. M3	+	Metabolism	Photosynthesis	protein import into chloroplast thylakoid membrane	GO:0045038	P	5	2.86E-03	2.67E-02
8	3885	F3 vs. M3	+	Metabolism	Photosynthesis	regulation of photosynthesis	GO:0010109	P	32	2.68E-04	3.90E-03
8	3886	F3 vs. M3	+	Metabolism	Photosynthesis	thylakoid	GO:0009579	C	392	0.00E+00	0.00E+00
8	3887	F3 vs. M3	+	Metabolism	Photosynthesis	thylakoid lumen	GO:0031977	C	50	6.47E-06	7.64E-05
8	3888	F3 vs. M3	+	Metabolism	Photosynthesis	thylakoid membrane	GO:0042651	C	277	0.00E+00	0.00E+00
8	3889	F3 vs. M3	+	Metabolism	Transporters	acidic amino acid transport	GO:0015800	P	7	5.36E-03	4.37E-02
8	3890	F3 vs. M3	+	Metabolism	Transporters	active ion transmembrane transporter activity	GO:0022853	F	151	4.56E-14	1.64E-11
8	3891	F3 vs. M3	+	Metabolism	Transporters	active transmembrane transporter activity	GO:0022804	F	324	0.00E+00	0.00E+00
8	3892	F3 vs. M3	+	Metabolism	Transporters	anion transmembrane transport	GO:0098656	P	74	1.13E-04	1.85E-03
8	3893	F3 vs. M3	+	Metabolism	Transporters	anion transmembrane transporter activity	GO:0008509	F	232	1.89E-07	2.09E-05
8	3894	F3 vs. M3	+	Metabolism	Transporters	anion transport	GO:0006820	P	181	3.07E-05	5.99E-04
8	3895	F3 vs. M3	+	Metabolism	Transporters	anion:anion antiporter activity	GO:0015301	F	23	7.88E-07	6.66E-05
8	3896	F3 vs. M3	+	Metabolism	Transporters	antiporter activity	GO:0015297	F	127	1.01E-10	2.08E-08
8	3897	F3 vs. M3	+	Metabolism	Transporters	ATPase-coupled cation transmembrane transporter activity	GO:0019829	F	50	1.12E-06	8.98E-05
8	3898	F3 vs. M3	+	Metabolism	Transporters	ATPase-coupled ion transmembrane transporter activity	GO:0042625	F	62	3.11E-06	2.18E-04
8	3899	F3 vs. M3	+	Metabolism	Transporters	ATPase-coupled transmembrane transporter activity	GO:0042626	F	141	6.17E-06	4.13E-04
8	3900	F3 vs. M3	+	Metabolism	Transporters	C4-dicarboxylate transmembrane transporter activity	GO:0015556	F	8	9.76E-04	2.28E-02
8	3901	F3 vs. M3	+	Metabolism	Transporters	C4-dicarboxylate transport	GO:0015740	P	10	5.47E-03	4.44E-02
8	3902	F3 vs. M3	+	Metabolism	Transporters	calcium ion transmembrane transport	GO:0070588	P	26	2.89E-03	2.69E-02
8	3903	F3 vs. M3	+	Metabolism	Transporters	carbohydrate derivative transport	GO:1901264	P	39	2.00E-03	2.02E-02
8	3904	F3 vs. M3	+	Metabolism	Transporters	carbohydrate transmembrane transporter activity	GO:0015144	F	65	1.25E-04	4.80E-03
8	3905	F3 vs. M3	+	Metabolism	Transporters	carboxylic acid transmembrane transport	GO:1905039	P	39	1.63E-03	1.73E-02
8	3906	F3 vs. M3	+	Metabolism	Transporters	carboxylic acid transmembrane transporter activity	GO:0046943	F	91	2.66E-04	8.50E-03
8	3907	F3 vs. M3	+	Metabolism	Transporters	cation transmembrane transport	GO:0098655	P	144	4.96E-07	1.59E-05
8	3908	F3 vs. M3	+	Metabolism	Transporters	cation transmembrane transporter activity	GO:0008324	F	270	9.93E-11	2.20E-08
8	3909	F3 vs. M3	+	Metabolism	Transporters	cation transport	GO:0006812	P	249	8.24E-06	1.87E-04
8	3910	F3 vs. M3	+	Metabolism	Transporters	cytoplasmic vesicle	GO:0031410	C	486	1.60E-07	2.52E-06
8	3911	F3 vs. M3	+	Metabolism	Transporters	dicarboxylic acid transport	GO:0006835	P	16	1.42E-03	1.55E-02
8	3912	F3 vs. M3	+	Metabolism	Transporters	endosome	GO:0005768	C	289	2.81E-05	3.00E-04
8	3913	F3 vs. M3	+	Metabolism	Transporters	export from cell	GO:0140352	P	62	1.96E-03	1.98E-02
8	3914	F3 vs. M3	+	Metabolism	Transporters	fluid transport	GO:0042044	P	19	9.88E-06	2.19E-04
8	3915	F3 vs. M3	+	Metabolism	Transporters	inorganic anion transmembrane transporter activity	GO:0015103	F	77	2.69E-04	8.39E-03
8	3916	F3 vs. M3	+	Metabolism	Transporters	inorganic anion transport	GO:0015698	P	64	2.36E-03	2.33E-02
8	3917	F3 vs. M3	+	Metabolism	Transporters	inorganic cation transmembrane transport	GO:0098662	P	140	7.40E-07	2.28E-05

8	3918	F3 vs. M3	+	Metabolism	Transporters	inorganic cation transmembrane transporter activity	GO:0022890	F	243	2.76E-10	4.96E-08
8	3919	F3 vs. M3	+	Metabolism	Transporters	inorganic ion transmembrane transport	GO:0098660	P	157	4.23E-08	1.84E-06
8	3920	F3 vs. M3	+	Metabolism	Transporters	inorganic molecular entity transmembrane transporter activity	GO:0015318	F	430	2.22E-16	1.06E-13
8	3921	F3 vs. M3	+	Metabolism	Transporters	ion transmembrane transport	GO:0034220	P	216	2.93E-10	2.01E-08
8	3922	F3 vs. M3	+	Metabolism	Transporters	ion transmembrane transporter activity	GO:0015075	F	475	4.30E-14	1.76E-11
8	3923	F3 vs. M3	+	Metabolism	Transporters	ion transmembrane transporter activity, phosphorylative mechanism	GO:0015662	F	42	3.94E-04	1.15E-02
8	3924	F3 vs. M3	+	Metabolism	Transporters	ion transport	GO:0006811	P	416	3.99E-09	2.17E-07
8	3925	F3 vs. M3	+	Metabolism	Transporters	malate transmembrane transport	GO:0071423	P	4	4.49E-03	3.79E-02
8	3926	F3 vs. M3	+	Metabolism	Transporters	malate transmembrane transporter activity	GO:0015140	F	7	2.92E-04	8.92E-03
8	3927	F3 vs. M3	+	Metabolism	Transporters	metal ion transmembrane transporter activity	GO:0046873	F	142	1.47E-04	5.34E-03
8	3928	F3 vs. M3	+	Metabolism	Transporters	monovalent inorganic cation transmembrane transporter activity	GO:0015077	F	138	4.25E-09	6.11E-07
8	3929	F3 vs. M3	+	Metabolism	Transporters	monovalent inorganic cation transport	GO:0015672	P	101	1.55E-06	4.46E-05
8	3930	F3 vs. M3	+	Metabolism	Transporters	neutral amino acid transmembrane transporter activity	GO:0015175	F	13	1.19E-03	2.62E-02
8	3931	F3 vs. M3	+	Metabolism	Transporters	one-carbon compound transport	GO:0019755	P	7	4.10E-04	5.50E-03
8	3932	F3 vs. M3	+	Metabolism	Transporters	organic acid transmembrane transport	GO:1903825	P	41	2.91E-03	2.68E-02
8	3933	F3 vs. M3	+	Metabolism	Transporters	organic acid transmembrane transporter activity	GO:0005342	F	91	2.66E-04	8.40E-03
8	3934	F3 vs. M3	+	Metabolism	Transporters	organic anion transmembrane transporter activity	GO:0008514	F	146	5.26E-05	2.26E-03
8	3935	F3 vs. M3	+	Metabolism	Transporters	organic anion transport	GO:0015711	P	110	2.99E-04	4.21E-03
8	3936	F3 vs. M3	+	Metabolism	Transporters	organic substance transport	GO:0071702	P	653	2.01E-03	2.03E-02
8	3937	F3 vs. M3	+	Metabolism	Transporters	oxaloacetate transport	GO:0015729	P	4	4.49E-03	3.79E-02
8	3938	F3 vs. M3	+	Metabolism	Transporters	plasmodesma	GO:0009506	C	654	7.73E-03	4.46E-02
8	3939	F3 vs. M3	+	Metabolism	Transporters	primary active transmembrane transporter activity	GO:0015399	F	155	1.61E-06	1.22E-04
8	3940	F3 vs. M3	+	Metabolism	Transporters	proton transmembrane transporter activity	GO:0015078	F	108	4.99E-09	6.82E-07
8	3941	F3 vs. M3	+	Metabolism	Transporters	regulation of ion transmembrane transporter activity	GO:0032412	P	8	3.70E-03	3.27E-02
8	3942	F3 vs. M3	+	Metabolism	Transporters	regulation of transmembrane transporter activity	GO:0022898	P	8	3.70E-03	3.28E-02
8	3943	F3 vs. M3	+	Metabolism	Transporters	secondary active transmembrane transporter activity	GO:0015291	F	166	6.32E-13	2.02E-10
8	3944	F3 vs. M3	+	Metabolism	Transporters	secretory vesicle	GO:0099503	C	118	1.00E-03	7.67E-03
8	3945	F3 vs. M3	+	Metabolism	Transporters	solute:anion antiporter activity	GO:0140323	F	23	7.88E-07	6.86E-05
8	3946	F3 vs. M3	+	Metabolism	Transporters	solute:cation symporter activity	GO:0015294	F	21	4.31E-04	1.24E-02
8	3947	F3 vs. M3	+	Metabolism	Transporters	solute:proton symporter activity	GO:0015295	F	18	4.32E-04	1.23E-02
8	3948	F3 vs. M3	+	Metabolism	Transporters	sulfate transmembrane transporter activity	GO:0015116	F	13	1.15E-03	2.57E-02
8	3949	F3 vs. M3	+	Metabolism	Transporters	symporter activity	GO:0015293	F	22	1.11E-03	2.50E-02
8	3950	F3 vs. M3	+	Metabolism	Transporters	trans-Golgi network	GO:0005802	C	217	3.56E-10	8.21E-09
8	3951	F3 vs. M3	+	Metabolism	Transporters	transmembrane transport	GO:0055085	P	541	1.11E-16	3.25E-14
8	3952	F3 vs. M3	+	Metabolism	Transporters	transmembrane transporter activity	GO:0022857	F	799	0.00E+00	0.00E+00
8	3953	F3 vs. M3	+	Metabolism	Transporters	transport	GO:0006810	P	1386	1.29E-09	7.77E-08
8	3954	F3 vs. M3	+	Metabolism	Transporters	transporter activity	GO:0005215	F	827	0.00E+00	0.00E+00
8	3955	F3 vs. M3	+	Metabolism	Transporters	urea transmembrane transporter activity	GO:0015204	F	6	1.64E-04	5.75E-03
8	3956	F3 vs. M3	+	Metabolism	Transporters	urea transport	GO:0015840	P	5	7.43E-04	9.05E-03
8	3957	F3 vs. M3	+	Metabolism	Transporters	vesicle	GO:0031982	C	527	2.19E-08	3.88E-07
8	3958	F3 vs. M3	+	Metabolism	Transporters	water channel activity	GO:0015250	F	22	6.58E-06	4.21E-04
8	3959	F3 vs. M3	+	Metabolism	Transporters	water transmembrane transporter activity	GO:0005372	F	22	6.58E-06	4.30E-04
8	3960	F3 vs. M3	+	Metabolism	Transporters	water transport	GO:0006833	P	19	9.88E-06	2.20E-04
8	3961	F3 vs. M3	+	Metabolism	Transporters	xenobiotic transmembrane transporter activity	GO:0042910	F	47	1.48E-04	5.31E-03
8	3962	F3 vs. M3	+	Regulation	Protein modification	Golgi apparatus	GO:0005794	C	771	1.91E-11	5.12E-10
8	3963	F3 vs. M3	+	Regulation	Protein modification	peptidyl-serine modification	GO:0018209	P	71	3.28E-03	2.98E-02
8	3964	F3 vs. M3	+	Regulation	Protein modification	protein disulfide oxidoreductase activity	GO:0015035	F	33	1.48E-03	3.15E-02
8	3965	F3 vs. M3	+	Regulation	Regulation	regulation of biological quality	GO:0065008	P	607	1.19E-03	1.34E-02
8	3966	F3 vs. M3	+	Regulation	Regulation	regulation of cell growth	GO:0001558	P	81	2.56E-03	2.45E-02
8	3967	F3 vs. M3	+	Regulation	Regulation	regulation of cell morphogenesis involved in differentiation	GO:0010769	P	33	2.91E-07	9.91E-06
8	3968	F3 vs. M3	+	Regulation	Regulation	regulation of cellular ketone metabolic process	GO:0010565	P	57	4.05E-03	3.49E-02
8	3969	F3 vs. M3	+	Regulation	Regulation	regulation of pollen tube growth	GO:0080092	P	30	8.10E-08	3.26E-06
8	3970	F3 vs. M3	+	Regulation	Regulation	regulation of small molecule metabolic process	GO:0062012	P	71	5.18E-03	4.28E-02
8	3971	F3 vs. M3	+	Regulation	Regulation	regulation of unidimensional cell growth	GO:0051510	P	55	8.42E-05	1.44E-03
8	3972	F3 vs. M3	+	Regulation	Translation	poly(U) RNA binding	GO:0008266	F	18	8.78E-04	2.10E-02
8	3973	F3 vs. M3	+	Signaling and response	Signaling	1-phosphatidylinositol-4-phosphate 5-kinase activity	GO:0016308	F	6	2.07E-04	6.83E-03
8	3974	F3 vs. M3	+	Signaling and response	Signaling	calcium ion binding	GO:0005509	F	158	3.31E-11	9.51E-09
8	3975	F3 vs. M3	+	Signaling and response	Signaling	cellular response to abiotic stimulus	GO:0071214	P	128	1.35E-04	2.13E-03
8	3976	F3 vs. M3	+	Signaling and response	Signaling	cellular response to blue light	GO:0071483	P	20	3.00E-03	2.75E-02
8	3977	F3 vs. M3	+	Signaling and response	Signaling	cellular response to chemical stimulus	GO:0070887	P	655	3.58E-04	4.91E-03
8	3978	F3 vs. M3	+	Signaling and response	Signaling	cellular response to environmental stimulus	GO:0104004	P	128	1.35E-04	2.14E-03
8	3979	F3 vs. M3	+	Signaling and response	Signaling	cellular response to external stimulus	GO:0071496	P	134	2.51E-03	2.43E-02

8	3980	F3 vs. M3	+	Signaling and response	Signaling	cellular response to extracellular stimulus	GO:0031668	P	129	3.74E-04	5.07E-03
8	3981	F3 vs. M3	+	Signaling and response	Signaling	cellular response to light stimulus	GO:0071482	P	74	7.89E-04	9.49E-03
8	3982	F3 vs. M3	+	Signaling and response	Signaling	cellular response to radiation	GO:0071478	P	79	1.53E-03	1.64E-02
8	3983	F3 vs. M3	+	Signaling and response	Signaling	cellular response to water stimulus	GO:0071462	P	23	7.22E-04	8.89E-03
8	3984	F3 vs. M3	+	Signaling and response	Signaling	dephosphorylation	GO:0016311	P	134	1.71E-03	1.79E-02
8	3985	F3 vs. M3	+	Signaling and response	Signaling	disulfide oxidoreductase activity	GO:0015036	F	44	4.52E-05	2.00E-03
8	3986	F3 vs. M3	+	Signaling and response	Signaling	intracellular signal transduction	GO:0035556	P	229	7.71E-04	9.29E-03
8	3987	F3 vs. M3	+	Signaling and response	Signaling	jasmonic acid biosynthetic process	GO:0009695	P	21	5.34E-03	4.36E-02
8	3988	F3 vs. M3	+	Signaling and response	Signaling	multi-organism process	GO:0051704	P	286	3.50E-04	4.83E-03
8	3989	F3 vs. M3	+	Signaling and response	Signaling	oxidative phosphorylation	GO:0006119	P	33	6.15E-04	7.80E-03
8	3990	F3 vs. M3	+	Signaling and response	Signaling	peptidyl-serine phosphorylation	GO:0018105	P	70	3.50E-03	3.15E-02
8	3991	F3 vs. M3	+	Signaling and response	Signaling	phosphatase activity	GO:0016791	F	217	5.10E-04	1.41E-02
8	3992	F3 vs. M3	+	Signaling and response	Signaling	phosphatidylinositol dephosphorylation	GO:0046856	P	19	6.08E-04	7.76E-03
8	3993	F3 vs. M3	+	Signaling and response	Signaling	phosphatidylinositol phosphate kinase activity	GO:0016307	F	11	1.23E-03	2.67E-02
8	3994	F3 vs. M3	+	Signaling and response	Signaling	phosphatidylinositol phosphate phosphatase activity	GO:0052866	F	21	1.89E-03	3.88E-02
8	3995	F3 vs. M3	+	Signaling and response	Signaling	phosphatidylinositol phospholipase C activity	GO:0004435	F	4	2.27E-03	4.49E-02
8	3996	F3 vs. M3	+	Signaling and response	Signaling	phospholipase activity	GO:0004620	F	26	8.03E-04	1.97E-02
8	3997	F3 vs. M3	+	Signaling and response	Signaling	phospholipase C activity	GO:0004629	F	5	8.82E-04	2.10E-02
8	3998	F3 vs. M3	+	Signaling and response	Signaling	phospholipid dephosphorylation	GO:0046839	P	19	6.08E-04	7.75E-03
8	3999	F3 vs. M3	+	Signaling and response	Signaling	regulation of auxin metabolic process	GO:0090354	P	12	1.68E-03	1.77E-02
8	4000	F3 vs. M3	+	Signaling and response	Signaling	regulation of gibberellin biosynthetic process	GO:0010371	P	7	2.81E-03	2.64E-02
8	4001	F3 vs. M3	+	Signaling and response	Signaling	regulation of jasmonic acid mediated signaling pathway	GO:2000022	P	27	2.86E-03	2.67E-02
8	4002	F3 vs. M3	+	Signaling and response	Signaling	response to abiotic stimulus	GO:0009628	P	1391	1.37E-14	2.81E-12
8	4003	F3 vs. M3	+	Signaling and response	Signaling	response to acid chemical	GO:0001101	P	263	1.46E-09	8.70E-08
8	4004	F3 vs. M3	+	Signaling and response	Signaling	response to bacterium	GO:0009617	P	332	4.73E-09	2.50E-07
8	4005	F3 vs. M3	+	Signaling and response	Signaling	response to biotic stimulus	GO:0009607	P	693	1.51E-12	1.86E-10
8	4006	F3 vs. M3	+	Signaling and response	Signaling	response to cadmium ion	GO:0046686	P	234	9.12E-11	6.94E-09
8	4007	F3 vs. M3	+	Signaling and response	Signaling	response to chemical	GO:0042221	P	1678	0.00E+00	0.00E+00
8	4008	F3 vs. M3	+	Signaling and response	Signaling	response to chitin	GO:0010200	P	89	2.29E-05	4.67E-04
8	4009	F3 vs. M3	+	Signaling and response	Signaling	response to endogenous stimulus	GO:0009719	P	767	7.71E-07	2.34E-05
8	4010	F3 vs. M3	+	Signaling and response	Signaling	response to external biotic stimulus	GO:0043207	P	692	1.61E-12	1.95E-10
8	4011	F3 vs. M3	+	Signaling and response	Signaling	response to external stimulus	GO:0009605	P	949	1.11E-15	2.80E-13
8	4012	F3 vs. M3	+	Signaling and response	Signaling	response to extracellular stimulus	GO:0009991	P	164	9.50E-06	2.13E-04
8	4013	F3 vs. M3	+	Signaling and response	Signaling	response to hormone	GO:0009725	P	746	3.83E-07	1.25E-05
8	4014	F3 vs. M3	+	Signaling and response	Signaling	response to inorganic substance	GO:0010035	P	615	0.00E+00	0.00E+00
8	4015	F3 vs. M3	+	Signaling and response	Signaling	response to jasmonic acid	GO:0009753	P	121	1.19E-05	2.58E-04
8	4016	F3 vs. M3	+	Signaling and response	Signaling	response to karrikin	GO:0080167	P	97	1.81E-07	6.61E-06
8	4017	F3 vs. M3	+	Signaling and response	Signaling	response to light intensity	GO:0009642	P	102	6.88E-07	2.13E-05
8	4018	F3 vs. M3	+	Signaling and response	Signaling	response to light stimulus	GO:0009416	P	479	6.34E-09	3.23E-07
8	4019	F3 vs. M3	+	Signaling and response	Signaling	response to lipid	GO:0033993	P	418	4.43E-05	8.26E-04
8	4020	F3 vs. M3	+	Signaling and response	Signaling	response to metal ion	GO:0010038	P	304	8.97E-10	5.54E-08
8	4021	F3 vs. M3	+	Signaling and response	Signaling	response to nitrogen compound	GO:1901698	P	188	1.09E-03	1.25E-02
8	4022	F3 vs. M3	+	Signaling and response	Signaling	response to nutrient levels	GO:0031667	P	139	7.00E-04	8.70E-03
8	4023	F3 vs. M3	+	Signaling and response	Signaling	response to organic substance	GO:0010033	P	1042	7.51E-10	4.69E-08
8	4024	F3 vs. M3	+	Signaling and response	Signaling	response to organonitrogen compound	GO:0010243	P	157	4.15E-03	3.55E-02
8	4025	F3 vs. M3	+	Signaling and response	Signaling	response to oxygen-containing compound	GO:1901700	P	969	3.33E-16	8.81E-14
8	4026	F3 vs. M3	+	Signaling and response	Signaling	response to salicylic acid	GO:0009751	P	88	7.62E-04	9.20E-03
8	4027	F3 vs. M3	+	Signaling and response	Signaling	response to stimulus	GO:0050896	P	3388	3.44E-15	8.31E-13
8	4028	F3 vs. M3	+	Signaling and response	Signaling	response to temperature stimulus	GO:0009266	P	403	9.73E-09	4.70E-07
8	4029	F3 vs. M3	+	Signaling and response	Signaling	response to UV	GO:0009411	P	87	3.98E-03	3.44E-02
8	4030	F3 vs. M3	+	Signaling and response	Signaling	response to UV-A	GO:0070141	P	4	4.84E-03	4.03E-02
8	4031	F3 vs. M3	+	Signaling and response	Signaling	response to water	GO:0009415	P	244	9.82E-10	5.99E-08
8	4032	F3 vs. M3	+	Signaling and response	Signaling	stomatal movement	GO:0010118	P	37	4.14E-05	7.88E-04
8	4033	F3 vs. M3	+	Signaling and response	Stress	anion homeostasis	GO:0055081	P	31	2.61E-03	2.50E-02
8	4034	F3 vs. M3	+	Signaling and response	Stress	antioxidant activity	GO:0016209	F	82	7.93E-04	1.97E-02
8	4035	F3 vs. M3	+	Signaling and response	Stress	auxin homeostasis	GO:0010252	P	25	4.76E-03	3.99E-02
8	4036	F3 vs. M3	+	Signaling and response	Stress	cation homeostasis	GO:0055080	P	144	5.82E-06	1.39E-04
8	4037	F3 vs. M3	+	Signaling and response	Stress	cell redox homeostasis	GO:0045454	P	51	1.90E-03	1.94E-02
8	4038	F3 vs. M3	+	Signaling and response	Stress	cellular cation homeostasis	GO:0030003	P	96	3.27E-04	4.57E-03
8	4039	F3 vs. M3	+	Signaling and response	Stress	cellular chemical homeostasis	GO:0055082	P	119	3.29E-03	2.99E-02
8	4040	F3 vs. M3	+	Signaling and response	Stress	cellular homeostasis	GO:0019725	P	174	1.10E-04	1.83E-03
8	4041	F3 vs. M3	+	Signaling and response	Stress	cellular ion homeostasis	GO:0006873	P	101	3.33E-04	4.64E-03
8	4042	F3 vs. M3	+	Signaling and response	Stress	cellular monovalent inorganic cation homeostasis	GO:0030004	P	27	1.44E-03	1.56E-02
8	4043	F3 vs. M3	+	Signaling and response	Stress	cellular oxidant detoxification	GO:0098869	P	16	3.71E-03	3.28E-02

8	4044	F3 vs. M3	+	Signaling and response	Stress	cellular response to chemical stress	GO:0062197	P	75	1.09E-03	1.24E-02
8	4045	F3 vs. M3	+	Signaling and response	Stress	cellular response to high light intensity	GO:0071486	P	6	2.90E-03	2.68E-02
8	4046	F3 vs. M3	+	Signaling and response	Stress	cellular response to light intensity	GO:0071484	P	11	1.28E-03	1.40E-02
8	4047	F3 vs. M3	+	Signaling and response	Stress	cellular response to oxidative stress	GO:0034599	P	45	3.48E-04	4.82E-03
8	4048	F3 vs. M3	+	Signaling and response	Stress	cellular response to phosphate starvation	GO:0016036	P	47	2.59E-03	2.47E-02
8	4049	F3 vs. M3	+	Signaling and response	Stress	cellular response to UV-A	GO:0071492	P	4	4.84E-03	4.04E-02
8	4050	F3 vs. M3	+	Signaling and response	Stress	cellular response to water deprivation	GO:0042631	P	23	7.22E-04	8.91E-03
8	4051	F3 vs. M3	+	Signaling and response	Stress	chemical homeostasis	GO:0048878	P	255	1.35E-07	5.20E-06
8	4052	F3 vs. M3	+	Signaling and response	Stress	defense response	GO:0006952	P	584	9.17E-09	4.51E-07
8	4053	F3 vs. M3	+	Signaling and response	Stress	defense response to bacterium	GO:0042742	P	268	2.35E-08	1.07E-06
8	4054	F3 vs. M3	+	Signaling and response	Stress	defense response to Gram-negative bacterium	GO:0050829	P	14	3.10E-04	4.34E-03
8	4055	F3 vs. M3	+	Signaling and response	Stress	defense response to other organism	GO:0098542	P	508	6.70E-08	2.80E-06
8	4056	F3 vs. M3	+	Signaling and response	Stress	defense response, incompatible interaction	GO:0009814	P	127	1.46E-03	1.58E-02
8	4057	F3 vs. M3	+	Signaling and response	Stress	detoxification	GO:0098754	P	62	2.71E-04	3.94E-03
8	4058	F3 vs. M3	+	Signaling and response	Stress	flavonoid biosynthetic process	GO:0009813	P	36	1.64E-04	2.54E-03
8	4059	F3 vs. M3	+	Signaling and response	Stress	flavonoid metabolic process	GO:0009812	P	40	7.44E-05	1.30E-03
8	4060	F3 vs. M3	+	Signaling and response	Stress	glutathione metabolic process	GO:0006749	P	27	5.56E-03	4.48E-02
8	4061	F3 vs. M3	+	Signaling and response	Stress	homeostatic process	GO:0042592	P	333	2.54E-06	6.81E-05
8	4062	F3 vs. M3	+	Signaling and response	Stress	hydrogen peroxide catabolic process	GO:0042744	P	17	1.11E-05	2.41E-04
8	4063	F3 vs. M3	+	Signaling and response	Stress	hydrogen peroxide metabolic process	GO:0042743	P	24	2.59E-07	8.92E-06
8	4064	F3 vs. M3	+	Signaling and response	Stress	hydrogen peroxide transmembrane transport	GO:0080170	P	5	2.53E-03	2.44E-02
8	4065	F3 vs. M3	+	Signaling and response	Stress	inorganic ion homeostasis	GO:0098771	P	153	6.18E-07	1.93E-05
8	4066	F3 vs. M3	+	Signaling and response	Stress	interspecies interaction between organisms	GO:0044419	P	706	9.44E-13	1.35E-10
8	4067	F3 vs. M3	+	Signaling and response	Stress	ion homeostasis	GO:0050801	P	179	1.74E-07	6.39E-06
8	4068	F3 vs. M3	+	Signaling and response	Stress	metal ion homeostasis	GO:0055065	P	113	2.27E-03	2.25E-02
8	4069	F3 vs. M3	+	Signaling and response	Stress	monovalent inorganic cation homeostasis	GO:0055067	P	46	1.46E-05	3.13E-04
8	4070	F3 vs. M3	+	Signaling and response	Stress	reactive oxygen species metabolic process	GO:0072593	P	49	6.63E-09	3.35E-07
8	4071	F3 vs. M3	+	Signaling and response	Stress	regulation of anion channel activity	GO:0010359	P	5	3.84E-03	3.34E-02
8	4072	F3 vs. M3	+	Signaling and response	Stress	regulation of cellular pH	GO:0030641	P	23	3.74E-03	3.29E-02
8	4073	F3 vs. M3	+	Signaling and response	Stress	regulation of intracellular pH	GO:0051453	P	23	3.74E-03	3.28E-02
8	4074	F3 vs. M3	+	Signaling and response	Stress	regulation of pH	GO:0006885	P	35	5.14E-05	9.43E-04
8	4075	F3 vs. M3	+	Signaling and response	Stress	regulation of stomatal movement	GO:0010119	P	64	7.47E-06	1.73E-04
8	4076	F3 vs. M3	+	Signaling and response	Stress	response to abscisic acid	GO:0009737	P	319	2.54E-06	6.79E-05
8	4077	F3 vs. M3	+	Signaling and response	Stress	response to alcohol	GO:0097305	P	322	8.04E-06	1.84E-04
8	4078	F3 vs. M3	+	Signaling and response	Stress	response to cold	GO:0009409	P	282	4.46E-10	2.95E-08
8	4079	F3 vs. M3	+	Signaling and response	Stress	response to fungus	GO:0009620	P	209	4.22E-05	8.00E-04
8	4080	F3 vs. M3	+	Signaling and response	Stress	response to heat	GO:0009408	P	143	2.90E-03	2.68E-02
8	4081	F3 vs. M3	+	Signaling and response	Stress	response to high light intensity	GO:0009644	P	47	5.60E-06	1.36E-04
8	4082	F3 vs. M3	+	Signaling and response	Stress	response to nematode	GO:0009624	P	58	9.51E-04	1.11E-02
8	4083	F3 vs. M3	+	Signaling and response	Stress	response to osmotic stress	GO:0006970	P	350	8.39E-05	1.45E-03
8	4084	F3 vs. M3	+	Signaling and response	Stress	response to other organism	GO:0051707	P	692	1.61E-12	1.91E-10
8	4085	F3 vs. M3	+	Signaling and response	Stress	response to oxidative stress	GO:0006979	P	290	7.79E-08	3.18E-06
8	4086	F3 vs. M3	+	Signaling and response	Stress	response to ozone	GO:0010193	P	27	9.86E-04	1.14E-02
8	4087	F3 vs. M3	+	Signaling and response	Stress	response to radiation	GO:0009314	P	497	1.82E-07	6.62E-06
8	4088	F3 vs. M3	+	Signaling and response	Stress	response to reactive oxygen species	GO:0000302	P	102	2.38E-06	6.55E-05
8	4089	F3 vs. M3	+	Signaling and response	Stress	response to salt stress	GO:0009651	P	298	2.92E-04	4.16E-03
8	4090	F3 vs. M3	+	Signaling and response	Stress	response to starvation	GO:0042594	P	120	5.95E-03	4.74E-02
8	4091	F3 vs. M3	+	Signaling and response	Stress	response to stress	GO:0006950	P	1950	9.45E-11	7.00E-09
8	4092	F3 vs. M3	+	Signaling and response	Stress	response to toxic substance	GO:0009636	P	77	1.16E-04	1.90E-03
8	4093	F3 vs. M3	+	Signaling and response	Stress	response to water deprivation	GO:0009414	P	239	1.63E-09	9.62E-08
8	4094	F3 vs. M3	+	Signaling and response	Stress	response to wounding	GO:0009611	P	154	5.18E-14	9.93E-12
8	4095	F3 vs. M3	+	Signaling and response	Stress	toxin metabolic process	GO:0009404	P	37	6.09E-04	7.75E-03
8	4096	F3 vs. M3	+	Signaling and response	Stress	vitamin metabolic process	GO:0006766	P	46	3.61E-03	3.24E-02
8	4097	F3 vs. M3	+	Signaling and response	Stress	water-soluble vitamin metabolic process	GO:0006767	P	38	3.67E-03	3.27E-02
8	4098	F3 vs. M3	-	Development	Cell division	alpha DNA polymerase:primase complex	GO:0005658	C	4	7.75E-03	4.34E-02
8	4099	F3 vs. M3	-	Development	Cell division	ATP-dependent microtubule motor activity	GO:1990939	F	12	5.69E-04	1.56E-02
8	4100	F3 vs. M3	-	Development	Cell division	Cajal body	GO:0015030	C	10	9.01E-03	4.81E-02
8	4101	F3 vs. M3	-	Development	Cell division	cell cycle	GO:0007049	P	303	0.00E+00	0.00E+00
8	4102	F3 vs. M3	-	Development	Cell division	cell cycle arrest	GO:0007050	P	5	5.49E-03	4.43E-02
8	4103	F3 vs. M3	-	Development	Cell division	cell cycle checkpoint	GO:0000075	P	29	3.75E-05	7.15E-04
8	4104	F3 vs. M3	-	Development	Cell division	cell cycle DNA replication	GO:0044786	P	25	5.21E-03	4.30E-02
8	4105	F3 vs. M3	-	Development	Cell division	cell cycle G1/S phase transition	GO:0044843	P	8	1.08E-03	1.24E-02
8	4106	F3 vs. M3	-	Development	Cell division	cell cycle phase transition	GO:0044770	P	36	5.83E-10	3.72E-08
8	4107	F3 vs. M3	-	Development	Cell division	cell cycle process	GO:0022402	P	278	0.00E+00	0.00E+00

8	4108	F3 vs. M3	-	Development	Cell division	chiasma assembly	GO:0051026	P	9	1.89E-03	1.93E-02
8	4109	F3 vs. M3	-	Development	Cell division	chromatin assembly	GO:0031497	P	54	5.23E-05	9.55E-04
8	4110	F3 vs. M3	-	Development	Cell division	chromosome	GO:0005694	C	319	8.73E-14	2.71E-12
8	4111	F3 vs. M3	-	Development	Cell division	chromosome organization involved in meiotic cell cycle	GO:0070192	P	31	2.82E-05	5.60E-04
8	4112	F3 vs. M3	-	Development	Cell division	chromosome segregation	GO:0007059	P	67	4.52E-08	1.93E-06
8	4113	F3 vs. M3	-	Development	Cell division	chromosome, centromeric region	GO:0000775	C	26	1.03E-04	1.03E-03
8	4114	F3 vs. M3	-	Development	Cell division	condensed chromosome	GO:0000793	C	37	4.67E-09	9.46E-08
8	4115	F3 vs. M3	-	Development	Cell division	condensed chromosome, centromeric region	GO:0000779	C	12	1.23E-03	9.25E-03
8	4116	F3 vs. M3	-	Development	Cell division	condensed nuclear chromosome	GO:0000794	C	25	1.14E-07	1.86E-06
8	4117	F3 vs. M3	-	Development	Cell division	condensed nuclear chromosome, centromeric region	GO:0000780	C	9	4.86E-03	2.97E-02
8	4118	F3 vs. M3	-	Development	Cell division	condensin complex	GO:0000796	C	8	2.03E-03	1.41E-02
8	4119	F3 vs. M3	-	Development	Cell division	cyclin binding	GO:0030332	F	8	6.61E-05	2.68E-03
8	4120	F3 vs. M3	-	Development	Cell division	cyclin-dependent protein kinase holoenzyme complex	GO:0000307	C	39	1.39E-08	2.55E-07
8	4121	F3 vs. M3	-	Development	Cell division	cyclin-dependent protein serine/threonine kinase regulator activity	GO:0016538	F	35	2.09E-07	2.22E-05
8	4122	F3 vs. M3	-	Development	Cell division	cytokinesis by cell plate formation	GO:0000911	P	46	1.15E-06	3.42E-05
8	4123	F3 vs. M3	-	Development	Cell division	DNA biosynthetic process	GO:0071897	P	15	3.38E-06	8.76E-05
8	4124	F3 vs. M3	-	Development	Cell division	DNA damage checkpoint	GO:0000077	P	10	3.85E-03	3.34E-02
8	4125	F3 vs. M3	-	Development	Cell division	DNA duplex unwinding	GO:0032508	P	20	4.80E-03	4.02E-02
8	4126	F3 vs. M3	-	Development	Cell division	DNA integrity checkpoint	GO:0031570	P	17	2.64E-04	3.86E-03
8	4127	F3 vs. M3	-	Development	Cell division	DNA packaging	GO:0006323	P	67	1.72E-05	3.59E-04
8	4128	F3 vs. M3	-	Development	Cell division	DNA polymerase complex	GO:0042575	C	13	2.59E-04	2.38E-03
8	4129	F3 vs. M3	-	Development	Cell division	DNA recombination	GO:0006310	P	94	2.19E-10	1.56E-08
8	4130	F3 vs. M3	-	Development	Cell division	DNA repair	GO:0006281	P	202	1.14E-12	1.47E-10
8	4131	F3 vs. M3	-	Development	Cell division	DNA repair complex	GO:1990391	C	14	2.40E-03	1.65E-02
8	4132	F3 vs. M3	-	Development	Cell division	DNA replication	GO:0006260	P	76	6.31E-13	1.00E-10
8	4133	F3 vs. M3	-	Development	Cell division	DNA replication factor A complex	GO:0005662	C	7	1.24E-03	9.22E-03
8	4134	F3 vs. M3	-	Development	Cell division	DNA replication initiation	GO:0006270	P	20	3.12E-05	6.06E-04
8	4135	F3 vs. M3	-	Development	Cell division	DNA replication origin binding	GO:0003688	F	17	2.00E-04	6.69E-03
8	4136	F3 vs. M3	-	Development	Cell division	DNA strand elongation	GO:0022616	P	13	5.24E-04	6.78E-03
8	4137	F3 vs. M3	-	Development	Cell division	DNA strand elongation involved in DNA replication	GO:0006271	P	13	5.24E-04	6.80E-03
8	4138	F3 vs. M3	-	Development	Cell division	DNA unwinding involved in DNA replication	GO:0006268	P	10	2.82E-03	2.65E-02
8	4139	F3 vs. M3	-	Development	Cell division	DNA-dependent DNA replication	GO:0006261	P	69	6.64E-12	6.36E-10
8	4140	F3 vs. M3	-	Development	Cell division	double-strand break repair	GO:0006302	P	82	1.34E-11	1.16E-09
8	4141	F3 vs. M3	-	Development	Cell division	double-strand break repair via homologous recombination	GO:0000724	P	58	1.28E-06	3.73E-05
8	4142	F3 vs. M3	-	Development	Cell division	endonuclease activity	GO:0004519	F	74	2.47E-05	1.25E-03
8	4143	F3 vs. M3	-	Development	Cell division	G1/S transition of mitotic cell cycle	GO:0000082	P	7	7.04E-04	8.73E-03
8	4144	F3 vs. M3	-	Development	Cell division	homologous chromosome pairing at meiosis	GO:0007129	P	20	7.57E-04	9.16E-03
8	4145	F3 vs. M3	-	Development	Cell division	homologous chromosome segregation	GO:0045143	P	22	3.70E-04	5.04E-03
8	4146	F3 vs. M3	-	Development	Cell division	homologous recombination	GO:0035825	P	31	2.96E-05	5.83E-04
8	4147	F3 vs. M3	-	Development	Cell division	kinetochore	GO:0000776	C	13	6.55E-04	5.46E-03
8	4148	F3 vs. M3	-	Development	Cell division	male meiotic nuclear division	GO:0007140	P	16	1.37E-03	1.50E-02
8	4149	F3 vs. M3	-	Development	Cell division	meiosis I	GO:0007127	P	40	3.99E-06	1.02E-04
8	4150	F3 vs. M3	-	Development	Cell division	meiosis I cell cycle process	GO:0061982	P	43	3.52E-07	1.16E-05
8	4151	F3 vs. M3	-	Development	Cell division	meiotic cell cycle	GO:0051321	P	102	2.33E-10	1.64E-08
8	4152	F3 vs. M3	-	Development	Cell division	meiotic cell cycle process	GO:1903046	P	93	1.13E-08	5.39E-07
8	4153	F3 vs. M3	-	Development	Cell division	meiotic chromosome segregation	GO:0045132	P	33	3.60E-05	6.93E-04
8	4154	F3 vs. M3	-	Development	Cell division	meiotic DNA double-strand break formation	GO:0042138	P	6	1.78E-03	1.84E-02
8	4155	F3 vs. M3	-	Development	Cell division	meiotic mismatch repair	GO:0000710	P	4	5.33E-03	4.36E-02
8	4156	F3 vs. M3	-	Development	Cell division	meiotic nuclear division	GO:0140013	P	59	2.35E-07	8.20E-06
8	4157	F3 vs. M3	-	Development	Cell division	microtubule cytoskeleton organization involved in mitosis	GO:1902850	P	20	2.64E-03	2.51E-02
8	4158	F3 vs. M3	-	Development	Cell division	microtubule motor activity	GO:0003777	F	39	5.97E-05	2.45E-03
8	4159	F3 vs. M3	-	Development	Cell division	mismatch repair	GO:0006298	P	14	1.43E-04	2.24E-03
8	4160	F3 vs. M3	-	Development	Cell division	mismatch repair complex	GO:0032300	C	7	5.98E-03	3.58E-02
8	4161	F3 vs. M3	-	Development	Cell division	mitotic cell cycle	GO:0000278	P	171	7.31E-10	4.61E-08
8	4162	F3 vs. M3	-	Development	Cell division	mitotic cell cycle checkpoint	GO:0007093	P	23	9.60E-04	1.11E-02
8	4163	F3 vs. M3	-	Development	Cell division	mitotic cell cycle phase transition	GO:0044772	P	35	4.51E-10	2.95E-08
8	4164	F3 vs. M3	-	Development	Cell division	mitotic cell cycle process	GO:1903047	P	146	1.02E-12	1.39E-10
8	4165	F3 vs. M3	-	Development	Cell division	mitotic DNA replication	GO:1902969	P	7	2.81E-03	2.65E-02
8	4166	F3 vs. M3	-	Development	Cell division	mitotic nuclear division	GO:0140014	P	44	6.64E-06	1.56E-04
8	4167	F3 vs. M3	-	Development	Cell division	mitotic sister chromatid segregation	GO:0000070	P	30	7.57E-04	9.18E-03
8	4168	F3 vs. M3	-	Development	Cell division	mitotic spindle	GO:0072686	C	13	4.76E-04	4.10E-03
8	4169	F3 vs. M3	-	Development	Cell division	mitotic spindle assembly	GO:0090307	P	7	4.02E-03	3.47E-02
8	4170	F3 vs. M3	-	Development	Cell division	negative regulation of cell cycle	GO:0045786	P	52	2.82E-07	9.66E-06

8	4171	F3 vs. M3	-	Development	Cell division	negative regulation of cell cycle phase transition	GO:1901988	P	16	2.96E-04	4.18E-03
8	4172	F3 vs. M3	-	Development	Cell division	negative regulation of cell cycle process	GO:0010948	P	34	4.40E-05	8.22E-04
8	4173	F3 vs. M3	-	Development	Cell division	negative regulation of chromosome organization	GO:2001251	P	10	4.65E-03	3.91E-02
8	4174	F3 vs. M3	-	Development	Cell division	negative regulation of mitotic cell cycle	GO:0045930	P	33	2.54E-04	3.74E-03
8	4175	F3 vs. M3	-	Development	Cell division	negative regulation of mitotic cell cycle phase transition	GO:1901991	P	16	2.96E-04	4.19E-03
8	4176	F3 vs. M3	-	Development	Cell division	negative regulation of nuclear division	GO:0051784	P	19	1.20E-03	1.35E-02
8	4177	F3 vs. M3	-	Development	Cell division	negative regulation of reproductive process	GO:2000242	P	47	8.53E-04	1.01E-02
8	4178	F3 vs. M3	-	Development	Cell division	nuclear chromosome segregation	GO:0098813	P	61	1.60E-07	5.95E-06
8	4179	F3 vs. M3	-	Development	Cell division	nuclear division	GO:0000280	P	100	6.05E-12	5.89E-10
8	4180	F3 vs. M3	-	Development	Cell division	nuclear DNA replication	GO:0033260	P	12	6.28E-04	7.91E-03
8	4181	F3 vs. M3	-	Development	Cell division	nuclear origin of replication recognition complex	GO:0005664	C	5	8.95E-03	4.85E-02
8	4182	F3 vs. M3	-	Development	Cell division	nuclear replication fork	GO:0043596	C	17	2.67E-06	3.36E-05
8	4183	F3 vs. M3	-	Development	Cell division	nuclease activity	GO:0004518	F	125	4.08E-05	1.83E-03
8	4184	F3 vs. M3	-	Development	Cell division	nucleic acid metabolic process	GO:0090304	P	1036	0.00E+00	0.00E+00
8	4185	F3 vs. M3	-	Development	Cell division	nucleobase-containing compound metabolic process	GO:0006139	P	1315	0.00E+00	0.00E+00
8	4186	F3 vs. M3	-	Development	Cell division	nucleosome assembly	GO:0006334	P	32	2.34E-03	2.31E-02
8	4187	F3 vs. M3	-	Development	Cell division	nucleotide-excision repair	GO:0006289	P	33	5.46E-03	4.44E-02
8	4188	F3 vs. M3	-	Development	Cell division	nucleotidyltransferase activity	GO:0016779	F	93	7.45E-04	1.86E-02
8	4189	F3 vs. M3	-	Development	Cell division	origin recognition complex	GO:0000808	C	5	8.95E-03	4.83E-02
8	4190	F3 vs. M3	-	Development	Cell division	positive regulation of cell cycle	GO:0045787	P	26	2.80E-03	2.65E-02
8	4191	F3 vs. M3	-	Development	Cell division	preprophase band	GO:0009574	C	14	2.85E-03	1.85E-02
8	4192	F3 vs. M3	-	Development	Cell division	preprophase band assembly	GO:0000913	P	5	5.11E-03	4.24E-02
8	4193	F3 vs. M3	-	Development	Cell division	reciprocal homologous recombination	GO:0140527	P	31	2.96E-05	5.87E-04
8	4194	F3 vs. M3	-	Development	Cell division	reciprocal meiotic recombination	GO:0007131	P	31	2.96E-05	5.85E-04
8	4195	F3 vs. M3	-	Development	Cell division	recombinational repair	GO:0000725	P	64	1.99E-06	5.50E-05
8	4196	F3 vs. M3	-	Development	Cell division	regulation of cell cycle	GO:0051726	P	160	1.11E-16	3.08E-14
8	4197	F3 vs. M3	-	Development	Cell division	regulation of cell cycle G2/M phase transition	GO:1902749	P	20	3.44E-05	6.63E-04
8	4198	F3 vs. M3	-	Development	Cell division	regulation of cell cycle phase transition	GO:1901987	P	35	5.52E-08	2.32E-06
8	4199	F3 vs. M3	-	Development	Cell division	regulation of cell cycle process	GO:0010564	P	85	9.68E-13	1.34E-10
8	4200	F3 vs. M3	-	Development	Cell division	regulation of cell division	GO:0051302	P	39	2.76E-04	4.01E-03
8	4201	F3 vs. M3	-	Development	Cell division	regulation of chromatin organization	GO:1902275	P	52	3.78E-03	3.29E-02
8	4202	F3 vs. M3	-	Development	Cell division	regulation of chromosome organization	GO:0033044	P	51	3.45E-06	8.92E-05
8	4203	F3 vs. M3	-	Development	Cell division	regulation of chromosome segregation	GO:0051983	P	14	4.71E-04	6.18E-03
8	4204	F3 vs. M3	-	Development	Cell division	regulation of chromosome separation	GO:1905818	P	13	1.22E-03	1.35E-02
8	4205	F3 vs. M3	-	Development	Cell division	regulation of cyclin-dependent protein kinase activity	GO:1904029	P	36	2.45E-05	4.97E-04
8	4206	F3 vs. M3	-	Development	Cell division	regulation of DNA endoreduplication	GO:0032875	P	26	1.02E-03	1.18E-02
8	4207	F3 vs. M3	-	Development	Cell division	regulation of DNA metabolic process	GO:0051052	P	50	3.02E-05	5.93E-04
8	4208	F3 vs. M3	-	Development	Cell division	regulation of DNA replication	GO:0006275	P	38	1.90E-06	5.32E-05
8	4209	F3 vs. M3	-	Development	Cell division	regulation of DNA-dependent DNA replication	GO:0090329	P	32	8.40E-05	1.44E-03
8	4210	F3 vs. M3	-	Development	Cell division	regulation of G2/M transition of mitotic cell cycle	GO:0010389	P	19	5.96E-05	1.07E-03
8	4211	F3 vs. M3	-	Development	Cell division	regulation of meiotic cell cycle	GO:0051445	P	12	1.68E-04	2.59E-03
8	4212	F3 vs. M3	-	Development	Cell division	regulation of meiotic nuclear division	GO:0040020	P	9	1.27E-03	1.39E-02
8	4213	F3 vs. M3	-	Development	Cell division	regulation of metaphase/anaphase transition of cell cycle	GO:1902099	P	13	1.22E-03	1.35E-02
8	4214	F3 vs. M3	-	Development	Cell division	regulation of mitotic cell cycle	GO:0007346	P	58	1.89E-07	6.82E-06
8	4215	F3 vs. M3	-	Development	Cell division	regulation of mitotic cell cycle phase transition	GO:1901990	P	33	2.06E-07	7.33E-06
8	4216	F3 vs. M3	-	Development	Cell division	regulation of mitotic metaphase/anaphase transition	GO:0030071	P	13	1.22E-03	1.36E-02
8	4217	F3 vs. M3	-	Development	Cell division	regulation of mitotic nuclear division	GO:0007088	P	19	3.75E-03	3.28E-02
8	4218	F3 vs. M3	-	Development	Cell division	regulation of mitotic sister chromatid separation	GO:0010965	P	13	1.22E-03	1.36E-02
8	4219	F3 vs. M3	-	Development	Cell division	regulation of mitotic spindle organization	GO:0060236	P	5	2.53E-03	2.44E-02
8	4220	F3 vs. M3	-	Development	Cell division	regulation of nuclear division	GO:0051783	P	29	1.96E-05	4.03E-04
8	4221	F3 vs. M3	-	Development	Cell division	regulation of sister chromatid segregation	GO:0033045	P	14	4.71E-04	6.17E-03
8	4222	F3 vs. M3	-	Development	Cell division	regulation of spindle organization	GO:0090224	P	5	2.53E-03	2.44E-02
8	4223	F3 vs. M3	-	Development	Cell division	replication fork	GO:0005657	C	29	1.63E-07	2.49E-06
8	4224	F3 vs. M3	-	Development	Cell division	replisome	GO:0030894	C	14	6.04E-07	8.44E-06
8	4225	F3 vs. M3	-	Development	Cell division	RNA metabolic process	GO:0016070	P	781	0.00E+00	0.00E+00
8	4226	F3 vs. M3	-	Development	Cell division	SCF ubiquitin ligase complex	GO:0019005	C	49	6.39E-03	3.77E-02
8	4227	F3 vs. M3	-	Development	Cell division	single-stranded DNA binding	GO:0003697	F	50	1.70E-07	1.95E-05
8	4228	F3 vs. M3	-	Development	Cell division	sister chromatid cohesion	GO:0007062	P	25	2.87E-03	2.68E-02
8	4229	F3 vs. M3	-	Development	Cell division	sister chromatid segregation	GO:0000819	P	38	8.87E-05	1.51E-03
8	4230	F3 vs. M3	-	Development	Cell division	spindle	GO:0005819	C	47	5.14E-08	8.64E-07
8	4231	F3 vs. M3	-	Development	Cell division	spindle assembly	GO:0051225	P	27	7.27E-05	1.28E-03
8	4232	F3 vs. M3	-	Development	Cell division	spindle microtubule	GO:0005876	C	13	5.15E-04	4.37E-03
8	4233	F3 vs. M3	-	Development	Cell division	spindle organization	GO:0007051	P	40	2.52E-05	5.09E-04
8	4234	F3 vs. M3	-	Development	Cell division	telomere maintenance	GO:0000723	P	20	2.00E-04	3.02E-03

8	4235	F3 vs. M3	-	Development	Cell division	telomere maintenance via telomerase	GO:0007004	P	6	8.54E-04	1.01E-02
8	4236	F3 vs. M3	-	Development	Cell division	telomere maintenance via telomere lengthening	GO:0010833	P	6	8.54E-04	1.01E-02
8	4237	F3 vs. M3	-	Development	Cell division	telomere organization	GO:0032200	P	20	2.00E-04	3.01E-03
8	4238	F3 vs. M3	-	Development	Development	anatomical structure homeostasis	GO:0060249	P	22	1.23E-04	1.99E-03
8	4239	F3 vs. M3	-	Development	Development	anther wall tapetum development	GO:0048658	P	10	1.21E-03	1.35E-02
8	4240	F3 vs. M3	-	Development	Development	cellular component assembly	GO:0022607	P	535	3.25E-05	6.29E-04
8	4241	F3 vs. M3	-	Development	Development	cellular component biogenesis	GO:0044085	P	852	6.48E-05	1.15E-03
8	4242	F3 vs. M3	-	Development	Development	cellular component organization	GO:0016043	P	1531	4.08E-03	3.51E-02
8	4243	F3 vs. M3	-	Development	Development	cellular component organization or biogenesis	GO:0071840	P	1796	2.95E-04	4.19E-03
8	4244	F3 vs. M3	-	Development	Development	chromatin	GO:0000785	C	203	2.40E-04	2.23E-03
8	4245	F3 vs. M3	-	Development	Development	chromatin assembly or disassembly	GO:0006333	P	61	1.10E-04	1.83E-03
8	4246	F3 vs. M3	-	Development	Development	chromatin organization	GO:0006325	P	215	2.86E-10	1.99E-08
8	4247	F3 vs. M3	-	Development	Development	chromocenter	GO:0010369	C	9	8.83E-03	4.81E-02
8	4248	F3 vs. M3	-	Development	Development	chromosomal region	GO:0098687	C	39	5.77E-07	8.29E-06
8	4249	F3 vs. M3	-	Development	Development	chromosome organization	GO:0051276	P	315	0.00E+00	0.00E+00
8	4250	F3 vs. M3	-	Development	Development	chromosome, telomeric region	GO:0000781	C	12	4.65E-03	2.87E-02
8	4251	F3 vs. M3	-	Development	Development	intracellular non-membrane-bounded organelle	GO:0043232	C	1125	0.00E+00	0.00E+00
8	4252	F3 vs. M3	-	Development	Development	intracellular organelle lumen	GO:0070013	C	871	0.00E+00	0.00E+00
8	4253	F3 vs. M3	-	Development	Development	kinetochore microtubule	GO:0005828	C	5	7.93E-03	4.40E-02
8	4254	F3 vs. M3	-	Development	Development	membrane-enclosed lumen	GO:0031974	C	871	0.00E+00	0.00E+00
8	4255	F3 vs. M3	-	Development	Development	microtubule	GO:0005874	C	94	1.14E-04	1.12E-03
8	4256	F3 vs. M3	-	Development	Development	microtubule associated complex	GO:0005875	C	47	1.63E-07	2.53E-06
8	4257	F3 vs. M3	-	Development	Development	microtubule cytoskeleton	GO:0015630	C	136	6.89E-07	9.50E-06
8	4258	F3 vs. M3	-	Development	Development	microtubule cytoskeleton organization	GO:0000226	P	110	6.00E-04	7.69E-03
8	4259	F3 vs. M3	-	Development	Development	microtubule-based movement	GO:0007018	P	39	5.97E-05	1.07E-03
8	4260	F3 vs. M3	-	Development	Development	microtubule-based process	GO:0007017	P	148	1.41E-07	5.39E-06
8	4261	F3 vs. M3	-	Development	Development	non-membrane-bounded organelle	GO:0043228	C	1125	0.00E+00	0.00E+00
8	4262	F3 vs. M3	-	Development	Development	nuclear body	GO:0016604	C	68	1.05E-03	7.97E-03
8	4263	F3 vs. M3	-	Development	Development	nuclear chromatin	GO:0000790	C	154	8.86E-04	6.87E-03
8	4264	F3 vs. M3	-	Development	Development	nuclear chromosome	GO:0000228	C	210	5.47E-11	1.39E-09
8	4265	F3 vs. M3	-	Development	Development	nuclear lumen	GO:0031981	C	771	0.00E+00	0.00E+00
8	4266	F3 vs. M3	-	Development	Development	nuclear replisome	GO:0043601	C	14	6.04E-07	8.56E-06
8	4267	F3 vs. M3	-	Development	Development	nucleic acid phosphodiester bond hydrolysis	GO:0090305	P	55	4.24E-05	8.00E-04
8	4268	F3 vs. M3	-	Development	Development	nucleoplasm	GO:0005654	C	275	2.61E-09	5.62E-08
8	4269	F3 vs. M3	-	Development	Development	nucleus	GO:0005634	C	5493	0.00E+00	0.00E+00
8	4270	F3 vs. M3	-	Development	Development	organelle assembly	GO:0070925	P	124	5.26E-05	9.58E-04
8	4271	F3 vs. M3	-	Development	Development	organelle fission	GO:0048285	P	130	7.12E-11	5.49E-09
8	4272	F3 vs. M3	-	Development	Development	organelle lumen	GO:0043233	C	871	0.00E+00	0.00E+00
8	4273	F3 vs. M3	-	Development	Development	organelle organization	GO:0006996	P	1061	2.08E-07	7.35E-06
8	4274	F3 vs. M3	-	Development	Development	phragmoplast	GO:0009524	C	44	3.24E-06	4.01E-05
8	4275	F3 vs. M3	-	Development	Development	polymeric cytoskeletal fiber	GO:0099513	C	102	2.55E-03	1.69E-02
8	4276	F3 vs. M3	-	Metabolism	Bioenergetics	ADP binding	GO:0043531	F	73	5.70E-04	1.53E-02
8	4277	F3 vs. M3	-	Metabolism	Bioenergetics	ATP-dependent microtubule motor activity, plus-end-directed	GO:0008574	F	11	1.88E-03	3.88E-02
8	4278	F3 vs. M3	-	Metabolism	Bioenergetics	DNA-dependent ATPase activity	GO:0008094	F	71	1.91E-05	1.06E-03
8	4279	F3 vs. M3	-	Metabolism	Bioenergetics	positive regulation of ATPase activity	GO:0032781	P	9	2.33E-03	2.31E-02
8	4280	F3 vs. M3	-	Metabolism	Catabolism	Cul4-RING E3 ubiquitin ligase complex	GO:0080008	C	87	6.16E-03	3.66E-02
8	4281	F3 vs. M3	-	Metabolism	Catabolism	cullin-RING ubiquitin ligase complex	GO:0031461	C	155	6.13E-06	7.33E-05
8	4282	F3 vs. M3	-	Metabolism	Catabolism	endoribonuclease activity	GO:0004521	F	47	1.26E-03	2.73E-02
8	4283	F3 vs. M3	-	Metabolism	Catabolism	modification-dependent macromolecule catabolic process	GO:0043632	P	307	9.67E-04	1.12E-02
8	4284	F3 vs. M3	-	Metabolism	Catabolism	modification-dependent protein catabolic process	GO:0019941	P	300	1.75E-03	1.82E-02
8	4285	F3 vs. M3	-	Metabolism	Catabolism	peptidase complex	GO:1905368	C	66	6.01E-04	5.05E-03
8	4286	F3 vs. M3	-	Metabolism	Catabolism	protein-containing complex	GO:0032991	C	1909	0.00E+00	0.00E+00
8	4287	F3 vs. M3	-	Metabolism	Catabolism	RNA phosphodiester bond hydrolysis, endonucleolytic	GO:0090502	P	17	5.28E-03	4.35E-02
8	4288	F3 vs. M3	-	Metabolism	Catabolism	ubiquitin ligase complex	GO:0000151	C	203	3.63E-06	4.45E-05
8	4289	F3 vs. M3	-	Metabolism	Catabolism	ubiquitin-dependent protein catabolic process	GO:0006511	P	296	2.17E-03	2.18E-02
8	4290	F3 vs. M3	-	Metabolism	Metabolism	catalytic activity, acting on DNA	GO:0140097	F	113	5.41E-07	4.86E-05
8	4291	F3 vs. M3	-	Metabolism	Metabolism	catalytic complex	GO:1902494	C	755	1.53E-10	3.61E-09
8	4292	F3 vs. M3	-	Metabolism	Metabolism	cellular aromatic compound metabolic process	GO:0006725	P	1592	4.98E-08	2.11E-06
8	4293	F3 vs. M3	-	Metabolism	Metabolism	cellular macromolecule biosynthetic process	GO:0034645	P	813	4.78E-07	1.54E-05
8	4294	F3 vs. M3	-	Metabolism	Metabolism	cellular macromolecule metabolic process	GO:0044260	P	2641	7.45E-08	3.06E-06
8	4295	F3 vs. M3	-	Metabolism	Metabolism	cellular nitrogen compound metabolic process	GO:0034641	P	1834	4.09E-12	4.28E-10
8	4296	F3 vs. M3	-	Metabolism	Metabolism	cellular protein metabolic process	GO:0044267	P	1999	2.09E-04	3.13E-03
8	4297	F3 vs. M3	-	Metabolism	Metabolism	cellular protein modification process	GO:0006464	P	1439	1.59E-03	1.68E-02
8	4298	F3 vs. M3	-	Metabolism	Metabolism	cellular protein-containing complex assembly	GO:0034622	P	308	4.27E-04	5.70E-03

8	4299	F3 vs. M3	-	Metabolism	Metabolism	chromatin binding	GO:0003682	F	116	4.62E-04	1.29E-02
8	4300	F3 vs. M3	-	Metabolism	Metabolism	deoxyribonuclease activity	GO:0004536	F	25	2.88E-04	8.91E-03
8	4301	F3 vs. M3	-	Metabolism	Metabolism	DNA binding	GO:0003677	F	893	4.20E-09	6.35E-07
8	4302	F3 vs. M3	-	Metabolism	Metabolism	DNA metabolic process	GO:0006259	P	276	0.00E+00	0.00E+00
8	4303	F3 vs. M3	-	Metabolism	Metabolism	DNA secondary structure binding	GO:0000217	F	27	8.97E-06	5.60E-04
8	4304	F3 vs. M3	-	Metabolism	Metabolism	double-stranded DNA binding	GO:0003690	F	452	2.04E-03	4.14E-02
8	4305	F3 vs. M3	-	Metabolism	Metabolism	heterocycle metabolic process	GO:0046483	P	1511	2.68E-12	3.04E-10
8	4306	F3 vs. M3	-	Metabolism	Metabolism	lysine N-methyltransferase activity	GO:0016278	F	29	2.38E-05	1.22E-03
8	4307	F3 vs. M3	-	Metabolism	Metabolism	macromolecule biosynthetic process	GO:0009059	P	851	3.03E-05	5.93E-04
8	4308	F3 vs. M3	-	Metabolism	Metabolism	macromolecule metabolic process	GO:0043170	P	3292	1.78E-12	2.06E-10
8	4309	F3 vs. M3	-	Metabolism	Metabolism	microtubule binding	GO:0008017	F	97	7.68E-08	9.60E-06
8	4310	F3 vs. M3	-	Metabolism	Metabolism	microtubule depolymerization	GO:0007019	P	4	2.25E-03	2.25E-02
8	4311	F3 vs. M3	-	Metabolism	Metabolism	nitrogen compound metabolic process	GO:0006807	P	3759	1.33E-04	2.12E-03
8	4312	F3 vs. M3	-	Metabolism	Metabolism	N-methyltransferase activity	GO:0008170	F	50	2.89E-05	1.41E-03
8	4313	F3 vs. M3	-	Metabolism	Metabolism	nucleic acid binding	GO:0003676	F	1952	9.37E-10	1.58E-07
8	4314	F3 vs. M3	-	Metabolism	Metabolism	organic cyclic compound binding	GO:0097159	F	2827	2.33E-03	4.59E-02
8	4315	F3 vs. M3	-	Metabolism	Metabolism	organic cyclic compound metabolic process	GO:1901360	P	1659	3.30E-07	1.10E-05
8	4316	F3 vs. M3	-	Metabolism	Metabolism	protein metabolic process	GO:0019538	P	2100	1.29E-03	1.42E-02
8	4317	F3 vs. M3	-	Metabolism	Metabolism	pseudouridine synthesis	GO:0001522	P	16	1.73E-03	1.80E-02
8	4318	F3 vs. M3	-	Metabolism	Metabolism	Sm-like protein family complex	GO:0120114	C	52	1.76E-08	3.18E-07
8	4319	F3 vs. M3	-	Metabolism	Metabolism	supramolecular complex	GO:0099080	C	165	4.06E-04	3.60E-03
8	4320	F3 vs. M3	-	Metabolism	Metabolism	supramolecular fiber	GO:0099512	C	102	2.55E-03	1.68E-02
8	4321	F3 vs. M3	-	Metabolism	Metabolism	supramolecular polymer	GO:0099081	C	102	2.55E-03	1.70E-02
8	4322	F3 vs. M3	-	Metabolism	Metabolism	transferase complex	GO:1990234	C	439	1.11E-16	4.08E-15
8	4323	F3 vs. M3	-	Metabolism	Metabolism	transferase complex, transferring phosphorus-containing groups	GO:0061695	C	143	3.55E-15	1.17E-13
8	4324	F3 vs. M3	-	Metabolism	Metabolism	tubulin binding	GO:0015631	F	102	4.10E-07	3.93E-05
8	4325	F3 vs. M3	-	Metabolism	Transporters	kinesin complex	GO:0005871	C	21	5.01E-04	4.28E-03
8	4326	F3 vs. M3	-	Metabolism	Transporters	nuclear export	GO:0051168	P	45	1.52E-03	1.63E-02
8	4327	F3 vs. M3	-	Metabolism	Transporters	nuclear microtubule	GO:0005880	C	9	3.15E-03	2.01E-02
8	4328	F3 vs. M3	-	Metabolism	Transporters	nucleic acid transport	GO:0050657	P	47	2.82E-04	4.06E-03
8	4329	F3 vs. M3	-	Metabolism	Transporters	protein export from nucleus	GO:0006611	P	39	2.57E-03	2.46E-02
8	4330	F3 vs. M3	-	Metabolism	Transporters	RNA transport	GO:0050658	P	47	2.82E-04	4.07E-03
8	4331	F3 vs. M3	-	Regulation	Protein modification	methylation	GO:0032259	P	139	7.40E-05	1.30E-03
8	4332	F3 vs. M3	-	Regulation	Protein modification	protein alkylation	GO:0008213	P	54	2.49E-06	6.75E-05
8	4333	F3 vs. M3	-	Regulation	Protein modification	protein deubiquitination	GO:0016579	P	39	2.54E-03	2.44E-02
8	4334	F3 vs. M3	-	Regulation	Protein modification	protein heterodimerization activity	GO:0046982	F	86	3.89E-07	3.86E-05
8	4335	F3 vs. M3	-	Regulation	Protein modification	protein methylation	GO:0006479	P	54	2.49E-06	6.78E-05
8	4336	F3 vs. M3	-	Regulation	Protein modification	protein methyltransferase activity	GO:0008276	F	46	2.87E-05	1.42E-03
8	4337	F3 vs. M3	-	Regulation	Protein modification	protein-lysine N-methyltransferase activity	GO:0016279	F	29	2.38E-05	1.25E-03
8	4338	F3 vs. M3	-	Regulation	Protein modification	regulation of protein metabolic process	GO:0051246	P	256	1.12E-04	1.84E-03
8	4339	F3 vs. M3	-	Regulation	Protein modification	peptidyl-amino acid modification	GO:0018193	P	239	5.49E-03	4.44E-02
8	4340	F3 vs. M3	-	Regulation	Protein modification	peptidyl-lysine modification	GO:0018205	P	86	2.99E-07	1.01E-05
8	4341	F3 vs. M3	-	Regulation	Protein modification	positive regulation of protein modification process	GO:0031401	P	45	3.83E-03	3.34E-02
8	4342	F3 vs. M3	-	Regulation	Protein modification	protein modification by small protein conjugation or removal	GO:0070647	P	434	4.12E-03	3.53E-02
8	4343	F3 vs. M3	-	Regulation	Protein modification	protein modification by small protein removal	GO:0070646	P	53	5.02E-04	6.54E-03
8	4344	F3 vs. M3	-	Regulation	Protein modification	protein modification process	GO:0036211	P	1439	1.59E-03	1.69E-02
8	4345	F3 vs. M3	-	Regulation	Protein modification	protein-containing complex assembly	GO:0065003	P	346	3.54E-03	3.18E-02
8	4346	F3 vs. M3	-	Regulation	Protein modification	protein-containing complex localization	GO:0031503	P	43	6.92E-04	8.62E-03
8	4347	F3 vs. M3	-	Regulation	Protein modification	protein-containing complex subunit organization	GO:0043933	P	399	6.18E-03	4.92E-02
8	4348	F3 vs. M3	-	Regulation	Protein modification	regulation of protein modification process	GO:0031399	P	128	1.30E-04	2.09E-03
8	4349	F3 vs. M3	-	Regulation	Regulation	negative regulation of biological process	GO:0048519	P	692	1.33E-05	2.86E-04
8	4350	F3 vs. M3	-	Regulation	Regulation	negative regulation of cellular macromolecule biosynthetic process	GO:2000113	P	203	2.93E-03	2.69E-02
8	4351	F3 vs. M3	-	Regulation	Regulation	negative regulation of cellular process	GO:0048523	P	457	2.86E-03	2.67E-02
8	4352	F3 vs. M3	-	Regulation	Regulation	negative regulation of macromolecule biosynthetic process	GO:0010558	P	205	2.99E-03	2.74E-02
8	4353	F3 vs. M3	-	Regulation	Regulation	negative regulation of macromolecule metabolic process	GO:0010605	P	372	2.96E-08	1.33E-06
8	4354	F3 vs. M3	-	Regulation	Regulation	negative regulation of metabolic process	GO:0009892	P	408	4.79E-06	1.18E-04
8	4355	F3 vs. M3	-	Regulation	Regulation	negative regulation of nitrogen compound metabolic process	GO:0051172	P	249	6.24E-04	7.88E-03
8	4356	F3 vs. M3	-	Regulation	Regulation	negative regulation of nucleobase-containing compound metabolic process	GO:0045934	P	187	2.48E-04	3.66E-03
8	4357	F3 vs. M3	-	Regulation	Regulation	positive regulation of biological process	GO:0048518	P	744	3.45E-04	4.80E-03
8	4358	F3 vs. M3	-	Regulation	Regulation	positive regulation of cellular metabolic process	GO:0031325	P	447	2.89E-03	2.68E-02
8	4359	F3 vs. M3	-	Regulation	Regulation	positive regulation of cellular process	GO:0048522	P	602	1.47E-03	1.59E-02

8	4360	F3 vs. M3	-	Regulation	Regulation	positive regulation of macromolecule biosynthetic process	GO:0010557	P	311	2.94E-03	2.70E-02
8	4361	F3 vs. M3	-	Regulation	Regulation	positive regulation of macromolecule metabolic process	GO:0010604	P	432	5.35E-05	9.67E-04
8	4362	F3 vs. M3	-	Regulation	Regulation	positive regulation of metabolic process	GO:0009893	P	480	1.68E-03	1.76E-02
8	4363	F3 vs. M3	-	Regulation	Regulation	positive regulation of nitrogen compound metabolic process	GO:0051173	P	420	8.30E-04	9.89E-03
8	4364	F3 vs. M3	-	Regulation	Regulation	positive regulation of nucleobase-containing compound metabolic process	GO:0045935	P	306	3.68E-03	3.27E-02
8	4365	F3 vs. M3	-	Regulation	Regulation	regulation of biological process	GO:0050789	P	3004	2.63E-03	2.50E-02
8	4366	F3 vs. M3	-	Regulation	Regulation	regulation of biosynthetic process	GO:0009889	P	1332	2.66E-06	7.07E-05
8	4367	F3 vs. M3	-	Regulation	Regulation	regulation of cellular biosynthetic process	GO:0031326	P	1303	1.26E-06	3.70E-05
8	4368	F3 vs. M3	-	Regulation	Regulation	regulation of cellular macromolecule biosynthetic process	GO:2000112	P	1228	5.95E-09	3.06E-07
8	4369	F3 vs. M3	-	Regulation	Regulation	regulation of cellular metabolic process	GO:0031323	P	1626	3.89E-07	1.26E-05
8	4370	F3 vs. M3	-	Regulation	Regulation	regulation of cellular protein metabolic process	GO:0032268	P	243	2.84E-04	4.07E-03
8	4371	F3 vs. M3	-	Regulation	Regulation	regulation of cutin biosynthetic process	GO:1901957	P	5	1.69E-03	1.76E-02
8	4372	F3 vs. M3	-	Regulation	Regulation	regulation of flower development	GO:0009909	P	103	5.83E-03	4.65E-02
8	4373	F3 vs. M3	-	Regulation	Regulation	regulation of macromolecule biosynthetic process	GO:0010556	P	1238	5.40E-09	2.83E-07
8	4374	F3 vs. M3	-	Regulation	Regulation	regulation of macromolecule metabolic process	GO:0060255	P	1650	6.77E-15	1.50E-12
8	4375	F3 vs. M3	-	Regulation	Regulation	regulation of meristem structural organization	GO:0009934	P	14	2.26E-03	2.25E-02
8	4376	F3 vs. M3	-	Regulation	Regulation	regulation of metabolic process	GO:0019222	P	1836	3.04E-09	1.69E-07
8	4377	F3 vs. M3	-	Regulation	Regulation	regulation of multicellular organismal development	GO:2000026	P	234	4.56E-03	3.84E-02
8	4378	F3 vs. M3	-	Regulation	Regulation	regulation of nitrogen compound metabolic process	GO:0051171	P	1470	2.03E-09	1.18E-07
8	4379	F3 vs. M3	-	Regulation	Regulation	regulation of nucleobase-containing compound metabolic process	GO:0019219	P	1217	9.03E-09	4.48E-07
8	4380	F3 vs. M3	-	Regulation	Regulation	regulation of organelle organization	GO:0033043	P	136	2.68E-06	7.04E-05
8	4381	F3 vs. M3	-	Regulation	Regulation	regulation of phosphate metabolic process	GO:0019220	P	98	3.72E-03	3.28E-02
8	4382	F3 vs. M3	-	Regulation	Regulation	regulation of phosphorus metabolic process	GO:0051174	P	99	5.11E-03	4.24E-02
8	4383	F3 vs. M3	-	Regulation	Regulation	regulation of post-embryonic development	GO:0048580	P	223	2.45E-03	2.39E-02
8	4384	F3 vs. M3	-	Regulation	Regulation	regulation of primary metabolic process	GO:0080090	P	1524	1.22E-08	5.74E-07
8	4385	F3 vs. M3	-	Regulation	Regulation	regulation of transferase activity	GO:0051338	P	69	3.81E-06	9.80E-05
8	4386	F3 vs. M3	-	Regulation	Transcription	5'-3' RNA polymerase activity	GO:0034062	F	33	6.42E-04	1.66E-02
8	4387	F3 vs. M3	-	Regulation	Transcription	chromatin organization involved in negative regulation of transcription	GO:0097549	P	49	9.97E-06	2.21E-04
8	4388	F3 vs. M3	-	Regulation	Transcription	chromatin organization involved in regulation of transcription	GO:0034401	P	49	9.97E-06	2.20E-04
8	4389	F3 vs. M3	-	Regulation	Transcription	chromatin remodeling	GO:0006338	P	54	1.22E-03	1.36E-02
8	4390	F3 vs. M3	-	Regulation	Transcription	chromatin silencing	GO:0006342	P	30	5.83E-05	1.05E-03
8	4391	F3 vs. M3	-	Regulation	Transcription	covalent chromatin modification	GO:0016569	P	100	5.99E-06	1.42E-04
8	4392	F3 vs. M3	-	Regulation	Transcription	DNA conformation change	GO:0071103	P	95	8.77E-07	2.62E-05
8	4393	F3 vs. M3	-	Regulation	Transcription	DNA geometric change	GO:0032392	P	21	5.48E-03	4.44E-02
8	4394	F3 vs. M3	-	Regulation	Transcription	DNA-directed RNA polymerase complex	GO:0000428	C	78	6.96E-06	8.13E-05
8	4395	F3 vs. M3	-	Regulation	Transcription	dsRNA processing	GO:0031050	P	37	8.34E-07	2.52E-05
8	4396	F3 vs. M3	-	Regulation	Transcription	gene expression	GO:0010467	P	1013	0.00E+00	0.00E+00
8	4397	F3 vs. M3	-	Regulation	Transcription	gene silencing	GO:0016458	P	112	6.91E-11	5.40E-09
8	4398	F3 vs. M3	-	Regulation	Transcription	gene silencing by miRNA	GO:0035195	P	22	1.81E-03	1.86E-02
8	4399	F3 vs. M3	-	Regulation	Transcription	gene silencing by RNA	GO:0031047	P	72	4.28E-08	1.84E-06
8	4400	F3 vs. M3	-	Regulation	Transcription	gene silencing by RNA-directed DNA methylation	GO:0080188	P	18	1.73E-03	1.80E-02
8	4401	F3 vs. M3	-	Regulation	Transcription	H4 histone acetyltransferase complex	GO:1902562	C	11	7.54E-03	4.38E-02
8	4402	F3 vs. M3	-	Regulation	Transcription	histone binding	GO:0042393	F	70	5.45E-05	2.30E-03
8	4403	F3 vs. M3	-	Regulation	Transcription	histone H3-K27 methylation	GO:0070734	P	7	3.05E-03	2.79E-02
8	4404	F3 vs. M3	-	Regulation	Transcription	histone lysine methylation	GO:0034968	P	32	7.82E-05	1.36E-03
8	4405	F3 vs. M3	-	Regulation	Transcription	histone methylation	GO:0016571	P	40	7.54E-06	1.74E-04
8	4406	F3 vs. M3	-	Regulation	Transcription	histone methyltransferase activity	GO:0042054	F	27	5.59E-06	3.83E-04
8	4407	F3 vs. M3	-	Regulation	Transcription	histone methyltransferase activity (H3-K36 specific)	GO:0046975	F	4	2.56E-03	4.91E-02
8	4408	F3 vs. M3	-	Regulation	Transcription	histone modification	GO:0016570	P	97	1.05E-05	2.29E-04
8	4409	F3 vs. M3	-	Regulation	Transcription	histone-lysine N-methyltransferase activity	GO:0018024	F	20	2.25E-05	1.22E-03
8	4410	F3 vs. M3	-	Regulation	Transcription	macromolecule methylation	GO:0043414	P	108	5.46E-07	1.71E-05
8	4411	F3 vs. M3	-	Regulation	Transcription	macromolecule modification	GO:0043412	P	1617	5.29E-07	1.68E-05
8	4412	F3 vs. M3	-	Regulation	Transcription	maintenance of DNA repeat elements	GO:0043570	P	6	3.07E-03	2.80E-02
8	4413	F3 vs. M3	-	Regulation	Transcription	minor groove of adenine-thymine-rich DNA binding	GO:0003680	F	20	1.60E-05	9.03E-04
8	4414	F3 vs. M3	-	Regulation	Transcription	mRNA processing	GO:0006397	P	185	6.00E-13	9.79E-11
8	4415	F3 vs. M3	-	Regulation	Transcription	negative regulation of gene expression	GO:0010629	P	308	4.01E-06	1.02E-04
8	4416	F3 vs. M3	-	Regulation	Transcription	negative regulation of gene expression, epigenetic	GO:0045814	P	34	1.49E-05	3.16E-04
8	4417	F3 vs. M3	-	Regulation	Transcription	negative regulation of nucleic acid-templated transcription	GO:1903507	P	164	4.23E-03	3.60E-02
8	4418	F3 vs. M3	-	Regulation	Transcription	negative regulation of RNA biosynthetic process	GO:1902679	P	164	4.23E-03	3.60E-02
8	4419	F3 vs. M3	-	Regulation	Transcription	negative regulation of transcription, DNA-templated	GO:0045892	P	162	4.13E-03	3.53E-02
8	4420	F3 vs. M3	-	Regulation	Transcription	nuclear DNA-directed RNA polymerase complex	GO:0055029	C	69	5.43E-07	7.93E-06

8	4421	F3 vs. M3	-	Regulation	Transcription	nuclear speck	GO:0016607	C	41	4.96E-03	3.02E-02
8	4422	F3 vs. M3	-	Regulation	Transcription	nucleic acid-templated transcription	GO:0097659	P	148	9.23E-05	1.56E-03
8	4423	F3 vs. M3	-	Regulation	Transcription	nucleolus	GO:0005730	C	354	1.31E-11	3.60E-10
8	4424	F3 vs. M3	-	Regulation	Transcription	nucleosome organization	GO:0034728	P	43	8.21E-04	9.80E-03
8	4425	F3 vs. M3	-	Regulation	Transcription	peptidyl-lysine methylation	GO:0018022	P	40	1.62E-05	3.42E-04
8	4426	F3 vs. M3	-	Regulation	Transcription	positive regulation of chromosome organization	GO:2001252	P	21	1.78E-03	1.84E-02
8	4427	F3 vs. M3	-	Regulation	Transcription	post-transcriptional gene silencing by RNA	GO:0035194	P	48	8.79E-06	1.98E-04
8	4428	F3 vs. M3	-	Regulation	Transcription	pre-transcriptional gene silencing by RNA	GO:0140458	P	18	1.73E-03	1.80E-02
8	4429	F3 vs. M3	-	Regulation	Transcription	production of siRNA involved in RNA interference	GO:0030422	P	26	2.14E-04	3.20E-03
8	4430	F3 vs. M3	-	Regulation	Transcription	production of small RNA involved in gene silencing by RNA	GO:0070918	P	37	8.34E-07	2.50E-05
8	4431	F3 vs. M3	-	Regulation	Transcription	production of ta-siRNAs involved in RNA interference	GO:0010267	P	12	4.12E-03	3.53E-02
8	4432	F3 vs. M3	-	Regulation	Transcription	protein acetylation	GO:0006473	P	32	5.82E-03	4.65E-02
8	4433	F3 vs. M3	-	Regulation	Transcription	protein acylation	GO:0043543	P	52	4.17E-04	5.58E-03
8	4434	F3 vs. M3	-	Regulation	Transcription	protein-DNA complex	GO:0032993	C	40	1.58E-05	1.76E-04
8	4435	F3 vs. M3	-	Regulation	Transcription	protein-DNA complex assembly	GO:0065004	P	57	1.92E-06	5.36E-05
8	4436	F3 vs. M3	-	Regulation	Transcription	protein-DNA complex subunit organization	GO:0071824	P	69	7.42E-07	2.28E-05
8	4437	F3 vs. M3	-	Regulation	Transcription	regulation of gene expression	GO:0010468	P	1417	9.45E-11	7.09E-09
8	4438	F3 vs. M3	-	Regulation	Transcription	regulation of gene expression, epigenetic	GO:0040029	P	62	2.14E-05	4.39E-04
8	4439	F3 vs. M3	-	Regulation	Transcription	regulation of gene silencing	GO:0060968	P	38	5.00E-03	4.17E-02
8	4440	F3 vs. M3	-	Regulation	Transcription	regulation of nucleic acid-templated transcription	GO:1903506	P	1111	1.50E-07	5.65E-06
8	4441	F3 vs. M3	-	Regulation	Transcription	regulation of RNA biosynthetic process	GO:2001141	P	1113	2.33E-07	8.19E-06
8	4442	F3 vs. M3	-	Regulation	Transcription	regulation of transcription, DNA-templated	GO:0006355	P	1111	1.50E-07	5.69E-06
8	4443	F3 vs. M3	-	Regulation	Transcription	RNA 3'-end processing	GO:0031123	P	43	8.24E-06	1.88E-04
8	4444	F3 vs. M3	-	Regulation	Transcription	RNA biosynthetic process	GO:0032774	P	151	5.02E-05	9.26E-04
8	4445	F3 vs. M3	-	Regulation	Transcription	RNA modification	GO:0009451	P	156	2.58E-11	2.17E-09
8	4446	F3 vs. M3	-	Regulation	Transcription	RNA polymerase activity	GO:0097747	F	33	6.42E-04	1.65E-02
8	4447	F3 vs. M3	-	Regulation	Transcription	RNA polymerase complex	GO:0030880	C	81	1.40E-06	1.85E-05
8	4448	F3 vs. M3	-	Regulation	Transcription	RNA polymerase I complex	GO:0005736	C	10	9.03E-03	4.79E-02
8	4449	F3 vs. M3	-	Regulation	Transcription	RNA polymerase II, core complex	GO:0005665	C	14	3.06E-03	1.97E-02
8	4450	F3 vs. M3	-	Regulation	Transcription	RNA polymerase II, holoenzyme	GO:0016591	C	46	2.23E-04	2.09E-03
8	4451	F3 vs. M3	-	Regulation	Transcription	RNA polymerase III complex	GO:0005666	C	16	1.35E-03	9.98E-03
8	4452	F3 vs. M3	-	Regulation	Transcription	RNA polymerase V complex	GO:0000419	C	15	5.18E-03	3.11E-02
8	4453	F3 vs. M3	-	Regulation	Transcription	THO complex	GO:0000347	C	11	7.10E-03	4.14E-02
8	4454	F3 vs. M3	-	Regulation	Transcription	transcription coregulator activity	GO:0003712	F	85	3.49E-04	1.05E-02
8	4455	F3 vs. M3	-	Regulation	Transcription	transcription, DNA-templated	GO:0006351	P	148	9.23E-05	1.56E-03
8	4456	F3 vs. M3	-	Regulation	Translation	90S preribosome	GO:0030686	C	19	3.41E-03	2.14E-02
8	4457	F3 vs. M3	-	Regulation	Translation	catalytic activity, acting on RNA	GO:0140098	F	259	9.60E-06	5.87E-04
8	4458	F3 vs. M3	-	Regulation	Translation	catalytic step 2 spliceosome	GO:0071013	C	38	5.35E-05	5.52E-04
8	4459	F3 vs. M3	-	Regulation	Translation	cytosolic large ribosomal subunit	GO:0022625	C	96	5.08E-03	3.08E-02
8	4460	F3 vs. M3	-	Regulation	Translation	cytosolic small ribosomal subunit	GO:0022627	C	71	4.13E-03	2.56E-02
8	4461	F3 vs. M3	-	Regulation	Translation	establishment of RNA localization	GO:0051236	P	47	2.82E-04	4.08E-03
8	4462	F3 vs. M3	-	Regulation	Translation	large ribosomal subunit	GO:0015934	C	119	8.65E-04	6.81E-03
8	4463	F3 vs. M3	-	Regulation	Translation	maturation of 5.8S rRNA	GO:0000460	P	21	2.84E-03	2.66E-02
8	4464	F3 vs. M3	-	Regulation	Translation	maturation of SSU-rRNA	GO:0030490	P	41	8.59E-05	1.47E-03
8	4465	F3 vs. M3	-	Regulation	Translation	maturation of SSU-rRNA from tricistronic rRNA transcript (SSU-rRNA, 5.8S rRNA, LSU-rRNA)	GO:0000462	P	26	7.27E-04	8.93E-03
8	4466	F3 vs. M3	-	Regulation	Translation	mRNA 3'-end processing	GO:0031124	P	18	2.89E-03	2.69E-02
8	4467	F3 vs. M3	-	Regulation	Translation	mRNA cis splicing, via spliceosome	GO:0045292	P	28	4.75E-03	3.99E-02
8	4468	F3 vs. M3	-	Regulation	Translation	mRNA export from nucleus	GO:0006406	P	23	3.63E-03	3.23E-02
8	4469	F3 vs. M3	-	Regulation	Translation	mRNA metabolic process	GO:0016071	P	270	2.75E-11	2.28E-09
8	4470	F3 vs. M3	-	Regulation	Translation	mRNA splicing, via spliceosome	GO:0000398	P	133	2.69E-09	1.54E-07
8	4471	F3 vs. M3	-	Regulation	Translation	mRNA transport	GO:0051028	P	24	1.57E-03	1.67E-02
8	4472	F3 vs. M3	-	Regulation	Translation	mRNA-containing ribonucleoprotein complex export from nucleus	GO:0071427	P	23	3.63E-03	3.24E-02
8	4473	F3 vs. M3	-	Regulation	Translation	ncRNA 3'-end processing	GO:0043628	P	21	8.38E-04	9.97E-03
8	4474	F3 vs. M3	-	Regulation	Translation	ncRNA metabolic process	GO:0034660	P	290	4.85E-10	3.13E-08
8	4475	F3 vs. M3	-	Regulation	Translation	ncRNA processing	GO:0034470	P	244	4.95E-11	4.04E-09
8	4476	F3 vs. M3	-	Regulation	Translation	negative regulation of RNA metabolic process	GO:0051253	P	172	1.93E-03	1.96E-02
8	4477	F3 vs. M3	-	Regulation	Translation	organellar ribosome	GO:0000313	C	40	1.56E-03	1.12E-02
8	4478	F3 vs. M3	-	Regulation	Translation	pICln-Sm protein complex	GO:0034715	C	5	8.06E-03	4.44E-02
8	4479	F3 vs. M3	-	Regulation	Translation	posttranscriptional gene silencing	GO:0016441	P	55	2.74E-05	5.50E-04
8	4480	F3 vs. M3	-	Regulation	Translation	posttranscriptional regulation of gene expression	GO:0010608	P	150	1.55E-04	2.41E-03
8	4481	F3 vs. M3	-	Regulation	Translation	precatalytic spliceosome	GO:0071011	C	21	3.11E-04	2.80E-03
8	4482	F3 vs. M3	-	Regulation	Translation	preribosome	GO:0030684	C	65	7.32E-07	9.95E-06

8	4483	F3 vs. M3	-	Regulation	Translation	preribosome, large subunit precursor	GO:0030687	C	15	3.25E-03	2.07E-02
8	4484	F3 vs. M3	-	Regulation	Translation	prespliceosome	GO:0071010	C	14	2.48E-03	1.69E-02
8	4485	F3 vs. M3	-	Regulation	Translation	production of miRNAs involved in gene silencing by miRNA	GO:0035196	P	14	1.30E-04	2.09E-03
8	4486	F3 vs. M3	-	Regulation	Translation	regulation of RNA metabolic process	GO:0051252	P	1185	1.31E-07	5.08E-06
8	4487	F3 vs. M3	-	Regulation	Translation	ribonucleoprotein complex	GO:1990904	C	440	1.43E-13	4.29E-12
8	4488	F3 vs. M3	-	Regulation	Translation	ribonucleoprotein complex assembly	GO:0022618	P	116	1.81E-06	5.14E-05
8	4489	F3 vs. M3	-	Regulation	Translation	ribonucleoprotein complex biogenesis	GO:0022613	P	300	4.10E-12	4.22E-10
8	4490	F3 vs. M3	-	Regulation	Translation	ribonucleoprotein complex export from nucleus	GO:0071426	P	37	1.15E-03	1.31E-02
8	4491	F3 vs. M3	-	Regulation	Translation	ribonucleoprotein complex localization	GO:0071166	P	37	1.15E-03	1.31E-02
8	4492	F3 vs. M3	-	Regulation	Translation	ribonucleoprotein complex subunit organization	GO:0071826	P	119	1.27E-06	3.72E-05
8	4493	F3 vs. M3	-	Regulation	Translation	ribosomal large subunit biogenesis	GO:0042273	P	76	1.31E-03	1.44E-02
8	4494	F3 vs. M3	-	Regulation	Translation	ribosomal small subunit biogenesis	GO:0042274	P	66	1.52E-04	2.38E-03
8	4495	F3 vs. M3	-	Regulation	Translation	ribosomal subunit	GO:0044391	C	214	1.61E-06	2.11E-05
8	4496	F3 vs. M3	-	Regulation	Translation	ribosome biogenesis	GO:0042254	P	245	1.88E-08	8.69E-07
8	4497	F3 vs. M3	-	Regulation	Translation	RNA export from nucleus	GO:0006405	P	43	7.09E-04	8.77E-03
8	4498	F3 vs. M3	-	Regulation	Translation	RNA interference	GO:0016246	P	27	6.67E-04	8.34E-03
8	4499	F3 vs. M3	-	Regulation	Translation	RNA localization	GO:0006403	P	53	4.97E-05	9.20E-04
8	4500	F3 vs. M3	-	Regulation	Translation	RNA phosphodiester bond hydrolysis	GO:0090501	P	32	1.63E-05	3.41E-04
8	4501	F3 vs. M3	-	Regulation	Translation	RNA processing	GO:0006396	P	497	0.00E+00	0.00E+00
8	4502	F3 vs. M3	-	Regulation	Translation	RNA splicing	GO:0008380	P	177	1.72E-08	8.03E-07
8	4503	F3 vs. M3	-	Regulation	Translation	RNA splicing, via transesterification reactions	GO:0000375	P	145	2.78E-09	1.56E-07
8	4504	F3 vs. M3	-	Regulation	Translation	RNA splicing, via transesterification reactions with bulged adenosine as nucleophile	GO:0000377	P	145	2.78E-09	1.57E-07
8	4505	F3 vs. M3	-	Regulation	Translation	rRNA metabolic process	GO:0016072	P	177	3.84E-06	9.82E-05
8	4506	F3 vs. M3	-	Regulation	Translation	rRNA modification	GO:0000154	P	21	2.16E-03	2.17E-02
8	4507	F3 vs. M3	-	Regulation	Translation	rRNA processing	GO:0006364	P	170	1.69E-06	4.80E-05
8	4508	F3 vs. M3	-	Regulation	Translation	small nuclear ribonucleoprotein complex	GO:0030532	C	46	2.81E-08	4.80E-07
8	4509	F3 vs. M3	-	Regulation	Translation	small nucleolar ribonucleoprotein complex	GO:0005732	C	20	2.32E-05	2.50E-04
8	4510	F3 vs. M3	-	Regulation	Translation	small ribosomal subunit	GO:0015935	C	95	4.67E-04	4.07E-03
8	4511	F3 vs. M3	-	Regulation	Translation	small-subunit processome	GO:0032040	C	39	2.67E-04	2.43E-03
8	4512	F3 vs. M3	-	Regulation	Translation	snoRNA binding	GO:0030515	F	20	2.17E-03	4.33E-02
8	4513	F3 vs. M3	-	Regulation	Translation	spliceosomal complex	GO:0005681	C	93	3.86E-06	4.67E-05
8	4514	F3 vs. M3	-	Regulation	Translation	spliceosomal snRNP assembly	GO:0000387	P	18	7.56E-04	9.18E-03
8	4515	F3 vs. M3	-	Regulation	Translation	spliceosomal snRNP complex	GO:0097525	C	46	2.81E-08	4.88E-07
8	4516	F3 vs. M3	-	Regulation	Translation	spliceosomal tri-snRNP complex	GO:0097526	C	27	8.50E-07	1.14E-05
8	4517	F3 vs. M3	-	Regulation	Translation	structural constituent of ribosome	GO:0003735	F	212	5.87E-05	2.44E-03
8	4518	F3 vs. M3	-	Regulation	Translation	translation	GO:0006412	P	339	4.36E-03	3.70E-02
8	4519	F3 vs. M3	-	Regulation	Translation	tRNA metabolic process	GO:0006399	P	103	1.37E-04	2.16E-03
8	4520	F3 vs. M3	-	Regulation	Translation	tRNA modification	GO:0006400	P	47	6.24E-04	7.89E-03
8	4521	F3 vs. M3	-	Regulation	Translation	tRNA processing	GO:0008033	P	61	2.66E-06	7.04E-05
8	4522	F3 vs. M3	-	Regulation	Translation	U1 snRNP	GO:0005685	C	18	7.04E-05	7.20E-04
8	4523	F3 vs. M3	-	Regulation	Translation	U2 snRNP	GO:0005686	C	21	1.35E-05	1.54E-04
8	4524	F3 vs. M3	-	Regulation	Translation	U2-type prespliceosome	GO:0071004	C	14	2.48E-03	1.67E-02
8	4525	F3 vs. M3	-	Regulation	Translation	U2-type spliceosomal complex	GO:0005684	C	39	8.86E-04	6.92E-03
8	4526	F3 vs. M3	-	Regulation	Translation	U4 snRNP	GO:0005687	C	10	1.69E-04	1.61E-03
8	4527	F3 vs. M3	-	Regulation	Translation	U4/U6 x U5 tri-snRNP complex	GO:0046540	C	19	2.04E-05	2.22E-04
8	4528	F3 vs. M3	-	Regulation	translation	U5 snRNP	GO:0005682	C	14	9.94E-05	9.96E-04
8	4529	F3 vs. M3	-	Regulation	Translation	U6 snRNP	GO:0005688	C	7	8.95E-03	4.80E-02
8	4530	F3 vs. M3	-	Signaling and response	Signaling	kinase regulator activity	GO:0019207	F	58	3.12E-07	3.21E-05
8	4531	F3 vs. M3	-	Signaling and response	Signaling	protein kinase complex	GO:1902911	C	54	8.85E-09	1.72E-07
8	4532	F3 vs. M3	-	Signaling and response	Signaling	protein kinase regulator activity	GO:0019887	F	57	1.29E-07	1.55E-05
8	4533	F3 vs. M3	-	Signaling and response	Signaling	regulation of cyclin-dependent protein serine/threonine kinase activity	GO:0000079	P	36	2.45E-05	4.99E-04
8	4534	F3 vs. M3	-	Signaling and response	Signaling	regulation of kinase activity	GO:0043549	P	58	8.93E-06	2.01E-04
8	4535	F3 vs. M3	-	Signaling and response	Signaling	regulation of phosphorylation	GO:0042325	P	73	3.66E-04	5.00E-03
8	4536	F3 vs. M3	-	Signaling and response	Signaling	regulation of protein kinase activity	GO:0045859	P	56	4.72E-06	1.17E-04
8	4537	F3 vs. M3	-	Signaling and response	Signaling	regulation of protein phosphorylation	GO:0001932	P	65	7.71E-05	1.34E-03
8	4538	F3 vs. M3	-	Signaling and response	Signaling	regulation of protein serine/threonine kinase activity	GO:0071900	P	47	5.60E-04	7.23E-03
8	4539	F3 vs. M3	-	Signaling and response	Signaling	serine/threonine protein kinase complex	GO:1902554	C	50	3.73E-09	7.88E-08
8	4540	F3 vs. M3	-	Signaling and response	Stress	cellular response to DNA damage stimulus	GO:0006974	P	219	8.99E-15	1.92E-12
8	4541	F3 vs. M3	-	Signaling and response	Stress	RNA-dependent DNA biosynthetic process	GO:0006278	P	6	8.54E-04	1.01E-02
9	4542	L3 vs. S3	+	Metabolism	Bioenergetics	oxidoreductase activity, acting on CH-OH group of donors	GO:0016614	F	142	2.21E-04	2.58E-02
9	4543	L3 vs. S3	+	Metabolism	Bioenergetics	oxidoreductase activity, acting on the CH-OH group of donors, NAD or NADP as acceptor	GO:0016616	F	123	2.81E-04	2.93E-02

9	4544	L3 vs. S3	+	Metabolism	Catabolism	ubiquitin ligase complex	GO:0000151	C	205	1.98E-04	5.62E-03
9	4545	L3 vs. S3	+	Metabolism	Metabolism	catalytic activity	GO:0003824	F	5316	4.13E-04	4.14E-02
9	4546	L3 vs. S3	+	Metabolism	Metabolism	glycoprotein metabolic process	GO:0009100	P	99	3.58E-04	3.88E-02
9	4547	L3 vs. S3	+	Metabolism	Metabolism	transferase activity	GO:0016740	F	2129	2.71E-04	2.92E-02
9	4548	L3 vs. S3	+	Metabolism	Metabolism	transferase activity, transferring glycosyl groups	GO:0016757	F	397	1.34E-04	1.63E-02
9	4549	L3 vs. S3	+	Metabolism	Metabolism	UDP-glycosyltransferase activity	GO:0008194	F	193	8.22E-05	1.20E-02
9	4550	L3 vs. S3	+	Metabolism	Photosynthesis	chlorophyll binding	GO:0016168	F	21	6.38E-06	1.16E-03
9	4551	L3 vs. S3	+	Metabolism	Photosynthesis	plastoglobule	GO:0010287	C	54	7.95E-05	2.64E-03
9	4552	L3 vs. S3	-	Development	Cell division	cell cycle	GO:0007049	P	292	3.70E-04	3.93E-02
9	4553	L3 vs. S3	-	Development	Cell division	cell cycle process	GO:0022402	P	266	1.22E-04	1.43E-02
9	4554	L3 vs. S3	-	Development	Cell division	microtubule motor activity	GO:0003777	F	38	1.02E-06	2.98E-04
9	4555	L3 vs. S3	-	Development	Cell division	spindle	GO:0005819	C	48	4.97E-04	1.24E-02
9	4556	L3 vs. S3	-	Development	Development	cell junction	GO:0030054	C	652	3.88E-10	2.97E-08
9	4557	L3 vs. S3	-	Development	Development	cell periphery	GO:0071944	C	2649	1.37E-03	2.79E-02
9	4558	L3 vs. S3	-	Development	Development	cell wall	GO:0005618	C	390	2.09E-05	8.32E-04
9	4559	L3 vs. S3	-	Development	Development	cell-cell junction	GO:0005911	C	652	3.88E-10	3.86E-08
9	4560	L3 vs. S3	-	Development	Development	cellular component biogenesis	GO:0044085	P	878	1.87E-04	2.15E-02
9	4561	L3 vs. S3	-	Development	Development	cellular component organization	GO:0016043	P	1571	5.35E-06	8.69E-04
9	4562	L3 vs. S3	-	Development	Development	cellular component organization or biogenesis	GO:0071840	P	1843	6.41E-07	1.86E-04
9	4563	L3 vs. S3	-	Development	Development	cellular localization	GO:0051641	P	647	1.10E-04	1.35E-02
9	4564	L3 vs. S3	-	Development	Development	cortical cytoskeleton	GO:0030863	C	27	6.03E-04	1.37E-02
9	4565	L3 vs. S3	-	Development	Development	cortical microtubule	GO:0055028	C	22	6.18E-04	1.37E-02
9	4566	L3 vs. S3	-	Development	Development	cortical microtubule cytoskeleton	GO:0030981	C	25	1.07E-03	2.31E-02
9	4567	L3 vs. S3	-	Development	Development	cytoskeletal protein binding	GO:0008092	F	174	1.14E-06	3.01E-04
9	4568	L3 vs. S3	-	Development	Development	cytoskeleton	GO:0005856	C	192	1.06E-04	3.40E-03
9	4569	L3 vs. S3	-	Development	Development	cytosol	GO:0005829	C	2312	1.29E-07	7.55E-06
9	4570	L3 vs. S3	-	Development	Development	external encapsulating structure	GO:0030312	C	392	1.02E-05	4.25E-04
9	4571	L3 vs. S3	-	Development	Development	intracellular non-membrane-bounded organelle	GO:0043232	C	1125	0.00E+00	0.00E+00
9	4572	L3 vs. S3	-	Development	Development	intracellular organelle lumen	GO:0070013	C	862	1.40E-07	7.73E-06
9	4573	L3 vs. S3	-	Development	Development	membrane-enclosed lumen	GO:0031974	C	862	1.40E-07	7.32E-06
9	4574	L3 vs. S3	-	Development	Development	microtubule	GO:0005874	C	89	6.74E-05	2.31E-03
9	4575	L3 vs. S3	-	Development	Development	microtubule cytoskeleton	GO:0015630	C	137	4.89E-06	2.12E-04
9	4576	L3 vs. S3	-	Development	Development	microtubule-based movement	GO:0007018	P	38	1.02E-06	2.26E-04
9	4577	L3 vs. S3	-	Development	Development	microtubule-based process	GO:0007017	P	140	6.43E-08	2.73E-05
9	4578	L3 vs. S3	-	Development	Development	movement of cell or subcellular component	GO:0006928	P	55	5.17E-06	8.65E-04
9	4579	L3 vs. S3	-	Development	Development	non-membrane-bounded organelle	GO:0043228	C	1125	0.00E+00	0.00E+00
9	4580	L3 vs. S3	-	Development	Development	nuclear lumen	GO:0031981	C	755	9.32E-07	4.22E-05
9	4581	L3 vs. S3	-	Development	Development	nucleus	GO:0005634	C	5886	3.44E-04	9.02E-03
9	4582	L3 vs. S3	-	Development	Development	organelle assembly	GO:0070925	P	131	4.29E-09	3.39E-06
9	4583	L3 vs. S3	-	Development	Development	organelle lumen	GO:0043233	C	862	1.40E-07	6.95E-06
9	4584	L3 vs. S3	-	Development	Development	organelle membrane	GO:0031090	C	1001	4.23E-04	1.08E-02
9	4585	L3 vs. S3	-	Development	Development	organelle organization	GO:0006996	P	1093	1.59E-06	3.14E-04
9	4586	L3 vs. S3	-	Development	Development	plant-type cell wall organization	GO:0009664	P	113	3.10E-04	3.43E-02
9	4587	L3 vs. S3	-	Development	Development	polymeric cytoskeletal fiber	GO:0099513	C	97	5.30E-04	1.26E-02
9	4588	L3 vs. S3	-	Development	Development	structural constituent of cell wall	GO:0005199	F	23	1.85E-06	3.85E-04
9	4589	L3 vs. S3	-	Development	Development	structural molecule activity	GO:0005198	F	327	0.00E+00	0.00E+00
9	4590	L3 vs. S3	-	Development	Development	symplast	GO:0055044	C	652	3.88E-10	3.51E-08
9	4591	L3 vs. S3	-	Development	Development	vacuole	GO:0005773	C	747	4.99E-05	1.91E-03
9	4592	L3 vs. S3	-	Metabolism	Bioenergetics	inner mitochondrial membrane protein complex	GO:0098800	C	116	2.59E-03	4.69E-02
9	4593	L3 vs. S3	-	Metabolism	Bioenergetics	mitochondrial protein complex	GO:0098798	C	171	2.54E-04	7.03E-03
9	4594	L3 vs. S3	-	Metabolism	Bioenergetics	mitochondrial respirasome	GO:0005746	C	79	2.80E-04	7.55E-03
9	4595	L3 vs. S3	-	Metabolism	Bioenergetics	mitochondrial respiratory chain complex III	GO:0005750	C	17	2.42E-03	4.55E-02
9	4596	L3 vs. S3	-	Metabolism	Bioenergetics	oxidoreductase complex	GO:1990204	C	115	5.93E-05	2.11E-03
9	4597	L3 vs. S3	-	Metabolism	Bioenergetics	respirasome	GO:0070469	C	82	1.08E-03	2.29E-02
9	4598	L3 vs. S3	-	Metabolism	Bioenergetics	respiratory chain complex	GO:0098803	C	77	1.21E-03	2.52E-02
9	4599	L3 vs. S3	-	Metabolism	Bioenergetics	respiratory chain complex III	GO:0045275	C	17	2.42E-03	4.47E-02
9	4600	L3 vs. S3	-	Metabolism	Catabolism	protein-containing complex	GO:0032991	C	1958	3.04E-07	1.44E-05
9	4601	L3 vs. S3	-	Metabolism	Catabolism	ubiquitin conjugating enzyme activity	GO:0061631	F	28	9.92E-05	1.37E-02
9	4602	L3 vs. S3	-	Metabolism	Catabolism	ubiquitin-like protein conjugating enzyme activity	GO:0061650	F	29	7.80E-05	1.20E-02
9	4603	L3 vs. S3	-	Metabolism	Metabolism	amide biosynthetic process	GO:0043604	P	409	0.00E+00	0.00E+00
9	4604	L3 vs. S3	-	Metabolism	Metabolism	anchoring junction	GO:0070161	C	652	3.88E-10	3.22E-08
9	4605	L3 vs. S3	-	Metabolism	Metabolism	binding	GO:0005488	F	6149	6.96E-05	1.13E-02
9	4606	L3 vs. S3	-	Metabolism	Metabolism	cellular amide metabolic process	GO:0043603	P	498	6.44E-15	7.12E-12
9	4607	L3 vs. S3	-	Metabolism	Metabolism	cellular macromolecule biosynthetic process	GO:0034645	P	848	1.79E-08	1.10E-05

9	4608	L3 vs. S3	-	Metabolism	Metabolism	cellular nitrogen compound biosynthetic process	GO:0044271	P	830	5.88E-08	2.71E-05
9	4609	L3 vs. S3	-	Metabolism	Metabolism	cellular nitrogen compound metabolic process	GO:0034641	P	1858	2.10E-04	2.37E-02
9	4610	L3 vs. S3	-	Metabolism	Metabolism	cellular protein-containing complex assembly	GO:0034622	P	322	2.12E-05	2.86E-03
9	4611	L3 vs. S3	-	Metabolism	Metabolism	heterocyclic compound binding	GO:1901363	F	2939	1.23E-04	1.55E-02
9	4612	L3 vs. S3	-	Metabolism	Metabolism	macromolecule biosynthetic process	GO:0009059	P	891	5.71E-08	2.87E-05
9	4613	L3 vs. S3	-	Metabolism	Metabolism	microtubule binding	GO:0008017	F	90	3.90E-10	2.84E-07
9	4614	L3 vs. S3	-	Metabolism	Metabolism	motor activity	GO:0003774	F	52	1.79E-06	4.02E-04
9	4615	L3 vs. S3	-	Metabolism	Metabolism	nucleic acid binding	GO:0003676	F	2034	1.94E-07	6.28E-05
9	4616	L3 vs. S3	-	Metabolism	Metabolism	organic cyclic compound binding	GO:0097159	F	2956	1.06E-04	1.40E-02
9	4617	L3 vs. S3	-	Metabolism	Metabolism	organonitrogen compound biosynthetic process	GO:1901566	P	930	7.26E-06	1.11E-03
9	4618	L3 vs. S3	-	Metabolism	Metabolism	peptide metabolic process	GO:0006518	P	411	0.00E+00	0.00E+00
9	4619	L3 vs. S3	-	Metabolism	Metabolism	protein binding	GO:0005515	F	3492	2.55E-05	4.36E-03
9	4620	L3 vs. S3	-	Metabolism	Metabolism	supramolecular fiber	GO:0099512	C	97	5.30E-04	1.23E-02
9	4621	L3 vs. S3	-	Metabolism	Metabolism	supramolecular polymer	GO:0099081	C	97	5.30E-04	1.29E-02
9	4622	L3 vs. S3	-	Metabolism	Metabolism	tubulin binding	GO:0015631	F	94	1.16E-09	6.75E-07
9	4623	L3 vs. S3	-	Metabolism	Photosynthesis	plastid ribosome	GO:0009547	C	20	2.27E-03	4.35E-02
9	4624	L3 vs. S3	-	Metabolism	Transporters	plasmodesma	GO:0009506	C	652	3.88E-10	4.29E-08
9	4625	L3 vs. S3	-	Metabolism	Transporters	vesicle	GO:0031982	C	535	2.09E-03	4.08E-02
9	4626	L3 vs. S3	-	Regulation	Protein modification	protein dimerization activity	GO:0046983	F	341	2.61E-06	5.07E-04
9	4627	L3 vs. S3	-	Regulation	Regulation	biological regulation	GO:0065007	P	3580	4.62E-04	4.73E-02
9	4628	L3 vs. S3	-	Regulation	Regulation	molecular function regulator	GO:0098772	F	1336	1.47E-10	1.42E-07
9	4629	L3 vs. S3	-	Regulation	Regulation	regulation of biological process	GO:0050789	P	3217	5.95E-05	7.47E-03
9	4630	L3 vs. S3	-	Regulation	Regulation	regulation of biosynthetic process	GO:0009889	P	1426	1.25E-05	1.77E-03
9	4631	L3 vs. S3	-	Regulation	Regulation	regulation of cellular biosynthetic process	GO:0031326	P	1398	4.44E-06	7.66E-04
9	4632	L3 vs. S3	-	Regulation	Regulation	regulation of cellular macromolecule biosynthetic process	GO:2000112	P	1318	6.74E-06	1.06E-03
9	4633	L3 vs. S3	-	Regulation	Regulation	regulation of cellular metabolic process	GO:0031323	P	1726	3.53E-06	6.50E-04
9	4634	L3 vs. S3	-	Regulation	Regulation	regulation of cellular process	GO:0050794	P	2791	9.57E-06	1.39E-03
9	4635	L3 vs. S3	-	Regulation	Regulation	regulation of macromolecule biosynthetic process	GO:0010556	P	1328	8.64E-06	1.29E-03
9	4636	L3 vs. S3	-	Regulation	Regulation	regulation of macromolecule metabolic process	GO:0060255	P	1773	4.26E-06	7.59E-04
9	4637	L3 vs. S3	-	Regulation	Regulation	regulation of metabolic process	GO:0019222	P	1954	3.16E-05	4.15E-03
9	4638	L3 vs. S3	-	Regulation	Regulation	regulation of nitrogen compound metabolic process	GO:0051171	P	1572	9.50E-07	2.39E-04
9	4639	L3 vs. S3	-	Regulation	Regulation	regulation of nucleobase-containing compound metabolic process	GO:0019219	P	1315	1.20E-06	2.55E-04
9	4640	L3 vs. S3	-	Regulation	Regulation	regulation of primary metabolic process	GO:0080090	P	1622	1.26E-06	2.57E-04
9	4641	L3 vs. S3	-	Regulation	Transcription	DNA-binding transcription factor activity	GO:0003700	F	934	1.22E-07	4.44E-05
9	4642	L3 vs. S3	-	Regulation	Transcription	gene expression	GO:0010467	P	1063	7.97E-08	3.15E-05
9	4643	L3 vs. S3	-	Regulation	Transcription	nucleolus	GO:0005730	C	348	1.15E-07	7.14E-06
9	4644	L3 vs. S3	-	Regulation	Transcription	regulation of gene expression	GO:0010468	P	1537	1.67E-05	2.31E-03
9	4645	L3 vs. S3	-	Regulation	Transcription	regulation of nucleic acid-templated transcription	GO:1903506	P	1209	9.56E-07	2.20E-04
9	4646	L3 vs. S3	-	Regulation	Transcription	regulation of RNA biosynthetic process	GO:2001141	P	1210	7.91E-07	2.19E-04
9	4647	L3 vs. S3	-	Regulation	Transcription	regulation of transcription, DNA-templated	GO:0006355	P	1209	9.56E-07	2.30E-04
9	4648	L3 vs. S3	-	Regulation	Transcription	RNA binding	GO:0003723	F	1062	1.16E-06	2.82E-04
9	4649	L3 vs. S3	-	Regulation	Transcription	transcription regulator activity	GO:0140110	F	1012	2.01E-08	8.38E-06
9	4650	L3 vs. S3	-	Regulation	Translation	90S preribosome	GO:0030686	C	20	1.21E-04	3.76E-03
9	4651	L3 vs. S3	-	Regulation	Translation	cytosolic large ribosomal subunit	GO:0022625	C	103	0.00E+00	0.00E+00
9	4652	L3 vs. S3	-	Regulation	Translation	cytosolic ribosome	GO:0022626	C	230	0.00E+00	0.00E+00
9	4653	L3 vs. S3	-	Regulation	Translation	cytosolic small ribosomal subunit	GO:0022627	C	72	1.56E-09	1.11E-07
9	4654	L3 vs. S3	-	Regulation	Translation	large ribosomal subunit	GO:0015934	C	135	0.00E+00	0.00E+00
9	4655	L3 vs. S3	-	Regulation	Translation	maturation of SSU-rRNA	GO:0030490	P	36	1.10E-04	1.32E-02
9	4656	L3 vs. S3	-	Regulation	Translation	mRNA binding	GO:0003729	F	714	1.58E-08	7.68E-06
9	4657	L3 vs. S3	-	Regulation	Translation	organellar ribosome	GO:0000313	C	47	1.91E-04	5.60E-03
9	4658	L3 vs. S3	-	Regulation	Translation	peptide biosynthetic process	GO:0043043	P	366	0.00E+00	0.00E+00
9	4659	L3 vs. S3	-	Regulation	Translation	preribosome	GO:0030684	C	63	1.25E-04	3.78E-03
9	4660	L3 vs. S3	-	Regulation	Translation	regulation of RNA metabolic process	GO:0051252	P	1285	2.34E-06	4.45E-04
9	4661	L3 vs. S3	-	Regulation	Translation	ribonucleoprotein complex	GO:1990904	C	467	0.00E+00	0.00E+00
9	4662	L3 vs. S3	-	Regulation	Translation	ribonucleoprotein complex assembly	GO:0022618	P	124	2.00E-07	6.91E-05
9	4663	L3 vs. S3	-	Regulation	Translation	ribonucleoprotein complex biogenesis	GO:0022613	P	325	8.05E-08	2.96E-05
9	4664	L3 vs. S3	-	Regulation	Translation	ribonucleoprotein complex subunit organization	GO:0071826	P	127	2.78E-07	9.02E-05
9	4665	L3 vs. S3	-	Regulation	Translation	ribosomal large subunit assembly	GO:0000027	P	35	4.24E-05	5.45E-03
9	4666	L3 vs. S3	-	Regulation	Translation	ribosomal large subunit biogenesis	GO:0042273	P	86	8.27E-07	2.18E-04
9	4667	L3 vs. S3	-	Regulation	Translation	ribosomal small subunit assembly	GO:0000028	P	27	4.56E-07	1.40E-04
9	4668	L3 vs. S3	-	Regulation	Translation	ribosomal small subunit biogenesis	GO:0042274	P	66	5.75E-09	3.97E-06
9	4669	L3 vs. S3	-	Regulation	Translation	ribosomal subunit	GO:0044391	C	230	0.00E+00	0.00E+00
9	4670	L3 vs. S3	-	Regulation	Translation	ribosome	GO:0005840	C	293	0.00E+00	0.00E+00

9	4671	L3 vs. S3	-	Regulation	translation	ribosome assembly	GO:0042255	P	68	5.03E-10	4.64E-07
9	4672	L3 vs. S3	-	Regulation	Translation	ribosome biogenesis	GO:0042254	P	268	2.13E-08	1.18E-05
9	4673	L3 vs. S3	-	Regulation	Translation	rRNA binding	GO:0019843	F	48	2.53E-04	2.83E-02
9	4674	L3 vs. S3	-	Regulation	Translation	small ribosomal subunit	GO:0015935	C	95	1.07E-08	7.09E-07
9	4675	L3 vs. S3	-	Regulation	Translation	small-subunit processome	GO:0032040	C	33	1.61E-03	3.20E-02
9	4676	L3 vs. S3	-	Regulation	Translation	structural constituent of ribosome	GO:0003735	F	233	0.00E+00	0.00E+00
9	4677	L3 vs. S3	-	Regulation	Translation	translation	GO:0006412	P	361	0.00E+00	0.00E+00
9	4678	L3 vs. S3	-	Signaling and response	Stress	regulation of anion channel activity	GO:0010359	P	6	4.59E-04	4.78E-02
10	4679	M3 vs. S3	+	Development	Development	extracellular space	GO:0005615	C	76	2.26E-03	3.82E-02
10	4680	M3 vs. S3	+	Development	Development	lytic vacuole	GO:0000323	C	38	2.70E-03	4.40E-02
10	4681	M3 vs. S3	+	Metabolism	Bioenergetics	oxidoreductase activity, acting on CH-OH group of donors	GO:0016614	F	144	1.93E-05	2.96E-03
10	4682	M3 vs. S3	+	Metabolism	Bioenergetics	oxidoreductase activity, acting on the CH-OH group of donors, NAD or NADP as acceptor	GO:0016616	F	123	8.13E-06	1.39E-03
10	4683	M3 vs. S3	+	Metabolism	Catabolism	peptidase activity	GO:0008233	F	326	3.05E-04	2.78E-02
10	4684	M3 vs. S3	+	Metabolism	Catabolism	ubiquitin ligase complex	GO:0000151	C	208	4.31E-04	9.76E-03
10	4685	M3 vs. S3	+	Metabolism	Metabolism	capsule organization	GO:0045230	P	6	2.42E-04	2.25E-02
10	4686	M3 vs. S3	+	Metabolism	Metabolism	capsule polysaccharide biosynthetic process	GO:0045227	P	6	2.42E-04	2.22E-02
10	4687	M3 vs. S3	+	Metabolism	Metabolism	catalytic activity	GO:0003824	F	5364	2.94E-09	2.14E-06
10	4688	M3 vs. S3	+	Metabolism	Metabolism	cellular protein modification process	GO:0006464	P	1604	3.96E-04	3.08E-02
10	4689	M3 vs. S3	+	Metabolism	Metabolism	extracellular polysaccharide biosynthetic process	GO:0045226	P	6	2.42E-04	2.18E-02
10	4690	M3 vs. S3	+	Metabolism	Metabolism	extracellular polysaccharide metabolic process	GO:0046379	P	6	2.42E-04	2.15E-02
10	4691	M3 vs. S3	+	Metabolism	Metabolism	glucosyltransferase activity	GO:0046527	F	136	5.09E-05	6.45E-03
10	4692	M3 vs. S3	+	Metabolism	Metabolism	methyl-CpG binding	GO:0008327	F	12	4.71E-04	4.15E-02
10	4693	M3 vs. S3	+	Metabolism	Metabolism	quercetin 3-O-glucosyltransferase activity	GO:0080043	F	47	1.91E-07	6.19E-05
10	4694	M3 vs. S3	+	Metabolism	Metabolism	quercetin 7-O-glucosyltransferase activity	GO:0080044	F	40	3.32E-06	6.45E-04
10	4695	M3 vs. S3	+	Metabolism	Metabolism	transferase activity	GO:0016740	F	2203	7.56E-06	1.38E-03
10	4696	M3 vs. S3	+	Metabolism	Metabolism	transferase activity, transferring glycosyl groups	GO:0016757	F	407	5.21E-05	6.32E-03
10	4697	M3 vs. S3	+	Metabolism	Metabolism	transferase activity, transferring hexosyl groups	GO:0016758	F	252	2.39E-04	2.32E-02
10	4698	M3 vs. S3	+	Metabolism	Metabolism	UDP-arabinose 4-epimerase activity	GO:0050373	F	6	2.42E-04	2.27E-02
10	4699	M3 vs. S3	+	Metabolism	Metabolism	UDP-glucose 4-epimerase activity	GO:0003978	F	9	2.37E-04	2.38E-02
10	4700	M3 vs. S3	+	Metabolism	Metabolism	UDP-glucosyltransferase activity	GO:0035251	F	108	1.74E-05	2.82E-03
10	4701	M3 vs. S3	+	Metabolism	Metabolism	UDP-glycosyltransferase activity	GO:0008194	F	207	9.21E-07	2.06E-04
10	4702	M3 vs. S3	+	Metabolism	Metabolism	UDP-L-arabinose biosynthetic process	GO:0033358	P	6	2.42E-04	2.29E-02
10	4703	M3 vs. S3	+	Metabolism	Metabolism	UDP-L-arabinose metabolic process	GO:0033356	P	11	2.61E-05	3.95E-03
10	4704	M3 vs. S3	+	Metabolism	Transporters	amino acid transmembrane transport	GO:0003333	P	17	5.47E-04	4.14E-02
10	4705	M3 vs. S3	+	Metabolism	Transporters	amino acid transmembrane transporter activity	GO:0015171	F	54	1.15E-04	1.28E-02
10	4706	M3 vs. S3	+	Metabolism	Transporters	amino acid transport	GO:0006865	P	35	9.66E-07	2.35E-04
10	4707	M3 vs. S3	+	Regulation	Protein modification	protein modification process	GO:0036211	P	1604	3.96E-04	3.12E-02
10	4708	M3 vs. S3	+	Regulation	Transcription	macromolecule modification	GO:0043412	P	1810	7.80E-05	8.73E-03
10	4709	M3 vs. S3	-	Development	Cell division	syncytium formation	GO:0006949	P	15	6.32E-04	4.66E-02
10	4710	M3 vs. S3	-	Development	Development	cell junction	GO:0030054	C	671	5.49E-06	1.89E-04
10	4711	M3 vs. S3	-	Development	Development	cell-cell junction	GO:0005911	C	671	5.49E-06	2.10E-04
10	4712	M3 vs. S3	-	Development	Development	cellular component assembly	GO:0022607	P	547	2.65E-05	3.90E-03
10	4713	M3 vs. S3	-	Development	Development	cellular component biogenesis	GO:0044085	P	875	3.63E-05	4.96E-03
10	4714	M3 vs. S3	-	Development	Development	cellular component organization	GO:0016043	P	1568	1.57E-06	3.52E-04
10	4715	M3 vs. S3	-	Development	Development	cellular component organization or biogenesis	GO:0071840	P	1842	8.29E-07	2.11E-04
10	4716	M3 vs. S3	-	Development	Development	cortical cytoskeleton	GO:0030863	C	29	5.50E-04	1.19E-02
10	4717	M3 vs. S3	-	Development	Development	cortical microtubule	GO:0055028	C	26	1.79E-03	3.23E-02
10	4718	M3 vs. S3	-	Development	Development	cortical microtubule cytoskeleton	GO:0030981	C	27	7.65E-04	1.52E-02
10	4719	M3 vs. S3	-	Development	Development	cytoplasmic microtubule	GO:0005881	C	30	1.67E-03	3.09E-02
10	4720	M3 vs. S3	-	Development	Development	cytoskeletal protein binding	GO:0008092	F	180	5.57E-05	6.49E-03
10	4721	M3 vs. S3	-	Development	Development	cytoskeleton	GO:0005856	C	193	5.08E-06	2.11E-04
10	4722	M3 vs. S3	-	Development	Development	cytoskeleton organization	GO:0007010	P	185	1.49E-04	1.52E-02
10	4723	M3 vs. S3	-	Development	Development	cytosol	GO:0005829	C	2340	1.24E-09	1.03E-07
10	4724	M3 vs. S3	-	Development	Development	envelope	GO:0031975	C	754	3.37E-04	8.38E-03
10	4725	M3 vs. S3	-	Development	Development	floral organ development	GO:0048437	P	179	3.89E-04	3.11E-02
10	4726	M3 vs. S3	-	Development	Development	floral whorl development	GO:0048438	P	143	7.78E-05	8.88E-03
10	4727	M3 vs. S3	-	Development	Development	gynoecium development	GO:0048467	P	67	7.01E-04	4.96E-02
10	4728	M3 vs. S3	-	Development	Development	inflorescence development	GO:0010229	P	24	4.31E-06	8.94E-04
10	4729	M3 vs. S3	-	Development	Development	intracellular non-membrane-bounded organelle	GO:0043232	C	1137	0.00E+00	0.00E+00
10	4730	M3 vs. S3	-	Development	Development	intracellular organelle lumen	GO:0070013	C	870	1.36E-07	8.48E-06
10	4731	M3 vs. S3	-	Development	Development	leaf morphogenesis	GO:0009965	P	73	1.55E-05	2.55E-03
10	4732	M3 vs. S3	-	Development	Development	membrane protein complex	GO:0098796	C	410	2.18E-03	3.74E-02
10	4733	M3 vs. S3	-	Development	Development	membrane-enclosed lumen	GO:0031974	C	870	1.36E-07	7.99E-06

10	4734	M3 vs. S3	-	Development	Development	microbody membrane	GO:0031903	C	19	2.11E-03	3.69E-02
10	4735	M3 vs. S3	-	Development	Development	microtubule	GO:0005874	C	88	4.75E-06	2.06E-04
10	4736	M3 vs. S3	-	Development	Development	microtubule cytoskeleton	GO:0015630	C	135	3.30E-06	1.49E-04
10	4737	M3 vs. S3	-	Development	Development	microtubule cytoskeleton organization	GO:0000226	P	103	5.01E-05	6.22E-03
10	4738	M3 vs. S3	-	Development	Development	microtubule-based process	GO:0007017	P	139	1.20E-06	2.79E-04
10	4739	M3 vs. S3	-	Development	Development	non-membrane-bounded organelle	GO:0043228	C	1137	0.00E+00	0.00E+00
10	4740	M3 vs. S3	-	Development	Development	nuclear lumen	GO:0031981	C	756	6.50E-07	3.24E-05
10	4741	M3 vs. S3	-	Development	Development	nucleus	GO:0005634	C	5861	6.31E-06	2.10E-04
10	4742	M3 vs. S3	-	Development	Development	organelle assembly	GO:0070925	P	132	1.67E-08	6.67E-06
10	4743	M3 vs. S3	-	Development	Development	organelle envelope	GO:0031967	C	754	3.37E-04	8.17E-03
10	4744	M3 vs. S3	-	Development	Development	organelle lumen	GO:0043233	C	870	1.36E-07	7.54E-06
10	4745	M3 vs. S3	-	Development	Development	organelle organization	GO:0006996	P	1092	1.75E-07	5.16E-05
10	4746	M3 vs. S3	-	Development	Development	plant-type cell wall organization	GO:0009664	P	119	3.54E-04	2.92E-02
10	4747	M3 vs. S3	-	Development	Development	polymeric cytoskeletal fiber	GO:0099513	C	97	1.61E-04	4.44E-03
10	4748	M3 vs. S3	-	Development	Development	regionalization	GO:0003002	P	123	4.31E-04	3.31E-02
10	4749	M3 vs. S3	-	Development	Development	reproductive shoot system development	GO:0090567	P	276	2.42E-04	2.12E-02
10	4750	M3 vs. S3	-	Development	Development	shoot system morphogenesis	GO:0010016	P	122	3.13E-05	4.38E-03
10	4751	M3 vs. S3	-	Development	Development	specification of symmetry	GO:0009799	P	7	3.40E-04	2.84E-02
10	4752	M3 vs. S3	-	Development	Development	structural constituent of cell wall	GO:0005199	F	21	2.74E-05	3.62E-03
10	4753	M3 vs. S3	-	Development	Development	structural molecule activity	GO:0005198	F	326	0.00E+00	0.00E+00
10	4754	M3 vs. S3	-	Development	Development	symplast	GO:0055044	C	671	5.49E-06	2.03E-04
10	4755	M3 vs. S3	-	Metabolism	Bioenergetics	ATP synthesis coupled electron transport	GO:0042773	P	36	6.95E-04	4.98E-02
10	4756	M3 vs. S3	-	Metabolism	Bioenergetics	inner mitochondrial membrane protein complex	GO:0098800	C	128	3.10E-04	7.92E-03
10	4757	M3 vs. S3	-	Metabolism	Bioenergetics	mitochondrial ATP synthesis coupled electron transport	GO:0042775	P	31	3.59E-04	2.91E-02
10	4758	M3 vs. S3	-	Metabolism	Bioenergetics	mitochondrial electron transport, ubiquinol to cytochrome c	GO:0006122	P	13	2.19E-04	2.15E-02
10	4759	M3 vs. S3	-	Metabolism	Bioenergetics	mitochondrial envelope	GO:0005740	C	230	6.34E-04	1.32E-02
10	4760	M3 vs. S3	-	Metabolism	Bioenergetics	mitochondrial protein complex	GO:0098798	C	186	1.96E-05	6.30E-04
10	4761	M3 vs. S3	-	Metabolism	Bioenergetics	mitochondrial respirasome	GO:0005746	C	84	3.39E-08	2.25E-06
10	4762	M3 vs. S3	-	Metabolism	Bioenergetics	mitochondrial respiratory chain complex I	GO:0005747	C	51	1.63E-04	4.27E-03
10	4763	M3 vs. S3	-	Metabolism	Bioenergetics	mitochondrial respiratory chain complex III	GO:0005750	C	18	1.40E-04	4.23E-03
10	4764	M3 vs. S3	-	Metabolism	Bioenergetics	NADH dehydrogenase complex	GO:0030964	C	54	4.71E-04	1.04E-02
10	4765	M3 vs. S3	-	Metabolism	Bioenergetics	oxidoreductase complex	GO:1990204	C	117	7.43E-09	5.69E-07
10	4766	M3 vs. S3	-	Metabolism	Bioenergetics	respirasome	GO:0070469	C	87	3.59E-07	1.88E-05
10	4767	M3 vs. S3	-	Metabolism	Bioenergetics	respiratory chain complex	GO:0098803	C	84	1.08E-06	5.11E-05
10	4768	M3 vs. S3	-	Metabolism	Bioenergetics	respiratory chain complex I	GO:0045271	C	53	9.80E-04	1.88E-02
10	4769	M3 vs. S3	-	Metabolism	Bioenergetics	respiratory chain complex III	GO:0045275	C	18	1.40E-04	4.11E-03
10	4770	M3 vs. S3	-	Metabolism	Bioenergetics	respiratory electron transport chain	GO:0022904	P	43	2.31E-04	2.22E-02
10	4771	M3 vs. S3	-	Metabolism	Catabolism	peroxisomal membrane	GO:0005778	C	19	2.11E-03	3.76E-02
10	4772	M3 vs. S3	-	Metabolism	Catabolism	protein-containing complex	GO:0032991	C	1943	1.13E-08	8.04E-07
10	4773	M3 vs. S3	-	Metabolism	Catabolism	ubiquitin conjugating enzyme activity	GO:0061631	F	32	4.15E-07	1.10E-04
10	4774	M3 vs. S3	-	Metabolism	Catabolism	ubiquitin-like protein conjugating enzyme activity	GO:0061650	F	33	3.41E-07	9.92E-05
10	4775	M3 vs. S3	-	Metabolism	Metabolism	amide biosynthetic process	GO:0043604	P	411	0.00E+00	0.00E+00
10	4776	M3 vs. S3	-	Metabolism	Metabolism	anchoring junction	GO:0070161	C	671	5.49E-06	1.95E-04
10	4777	M3 vs. S3	-	Metabolism	Metabolism	cellular amide metabolic process	GO:0043603	P	497	1.32E-14	1.48E-11
10	4778	M3 vs. S3	-	Metabolism	Metabolism	cellular macromolecule biosynthetic process	GO:0034645	P	860	8.87E-10	6.20E-07
10	4779	M3 vs. S3	-	Metabolism	Metabolism	cellular nitrogen compound biosynthetic process	GO:0044271	P	838	5.03E-09	2.56E-06
10	4780	M3 vs. S3	-	Metabolism	Metabolism	cellular protein-containing complex assembly	GO:0034622	P	317	8.65E-08	3.02E-05
10	4781	M3 vs. S3	-	Metabolism	Metabolism	macromolecule biosynthetic process	GO:0009059	P	903	2.48E-09	1.54E-06
10	4782	M3 vs. S3	-	Metabolism	Metabolism	microtubule binding	GO:0008017	F	95	1.61E-04	1.74E-02
10	4783	M3 vs. S3	-	Metabolism	Metabolism	motor activity	GO:0003774	F	51	2.04E-04	2.12E-02
10	4784	M3 vs. S3	-	Metabolism	Metabolism	nucleic acid binding	GO:0003676	F	2066	1.55E-08	9.03E-06
10	4785	M3 vs. S3	-	Metabolism	Metabolism	organonitrogen compound biosynthetic process	GO:1901566	P	940	7.35E-07	1.96E-04
10	4786	M3 vs. S3	-	Metabolism	Metabolism	peptide metabolic process	GO:0006518	P	405	0.00E+00	0.00E+00
10	4787	M3 vs. S3	-	Metabolism	Metabolism	phosphatidylinositol metabolic process	GO:0046488	P	61	6.75E-04	4.91E-02
10	4788	M3 vs. S3	-	Metabolism	Metabolism	protein binding	GO:0005515	F	3536	2.15E-05	3.14E-03
10	4789	M3 vs. S3	-	Metabolism	Metabolism	Sm-like protein family complex	GO:0120114	C	52	2.44E-03	4.05E-02
10	4790	M3 vs. S3	-	Metabolism	Metabolism	supramolecular complex	GO:0099080	C	163	7.45E-04	1.51E-02
10	4791	M3 vs. S3	-	Metabolism	Metabolism	supramolecular fiber	GO:0099512	C	97	1.61E-04	4.32E-03
10	4792	M3 vs. S3	-	Metabolism	Metabolism	supramolecular polymer	GO:0099081	C	97	1.61E-04	4.57E-03
10	4793	M3 vs. S3	-	Metabolism	Metabolism	tubulin binding	GO:0015631	F	102	2.46E-05	3.41E-03
10	4794	M3 vs. S3	-	Metabolism	Transporters	plasmodesma	GO:0009506	C	671	5.49E-06	2.19E-04
10	4795	M3 vs. S3	-	Metabolism	Transporters	vesicle	GO:0031982	C	533	9.26E-04	1.81E-02
10	4796	M3 vs. S3	-	Metabolism	Transporters	vesicle-mediated transport	GO:0016192	P	316	1.15E-04	1.23E-02
10	4797	M3 vs. S3	-	Regulation	Protein modification	protein-containing complex assembly	GO:0065003	P	357	1.74E-05	2.78E-03

10	4798	M3 vs. S3	-	Regulation	Protein modification	protein-containing complex subunit organization	GO:0043933	P	409	1.23E-04	1.30E-02
10	4799	M3 vs. S3	-	Regulation	Regulation	biological regulation	GO:0065007	P	3590	5.89E-04	4.39E-02
10	4800	M3 vs. S3	-	Regulation	Regulation	molecular function regulator	GO:0098772	F	1343	8.94E-08	3.72E-05
10	4801	M3 vs. S3	-	Regulation	Regulation	regulation of biological process	GO:0050789	P	3230	7.31E-05	8.52E-03
10	4802	M3 vs. S3	-	Regulation	Regulation	regulation of biosynthetic process	GO:0009889	P	1436	1.44E-04	1.50E-02
10	4803	M3 vs. S3	-	Regulation	Regulation	regulation of cellular biosynthetic process	GO:0031326	P	1408	4.74E-05	6.17E-03
10	4804	M3 vs. S3	-	Regulation	Regulation	regulation of cellular macromolecule biosynthetic process	GO:2000112	P	1329	5.98E-05	7.12E-03
10	4805	M3 vs. S3	-	Regulation	Regulation	regulation of cellular metabolic process	GO:0031323	P	1750	5.05E-05	6.14E-03
10	4806	M3 vs. S3	-	Regulation	Regulation	regulation of cellular process	GO:0050794	P	2800	8.49E-06	1.48E-03
10	4807	M3 vs. S3	-	Regulation	Regulation	regulation of macromolecule biosynthetic process	GO:0010556	P	1338	8.23E-05	9.03E-03
10	4808	M3 vs. S3	-	Regulation	Regulation	regulation of macromolecule metabolic process	GO:0060255	P	1781	3.89E-05	5.18E-03
10	4809	M3 vs. S3	-	Regulation	Regulation	regulation of metabolic process	GO:0019222	P	1975	2.45E-04	2.11E-02
10	4810	M3 vs. S3	-	Regulation	Regulation	regulation of nitrogen compound metabolic process	GO:0051171	P	1586	2.53E-06	5.45E-04
10	4811	M3 vs. S3	-	Regulation	Regulation	regulation of nucleobase-containing compound metabolic process	GO:0019219	P	1332	7.69E-06	1.39E-03
10	4812	M3 vs. S3	-	Regulation	Regulation	regulation of primary metabolic process	GO:0080090	P	1640	1.75E-05	2.72E-03
10	4813	M3 vs. S3	-	Regulation	Transcription	DNA-binding transcription factor activity	GO:0003700	F	933	1.43E-06	2.98E-04
10	4814	M3 vs. S3	-	Regulation	Transcription	gene expression	GO:0010467	P	1063	4.89E-10	3.91E-07
10	4815	M3 vs. S3	-	Regulation	Transcription	nucleolus	GO:0005730	C	336	9.80E-10	8.88E-08
10	4816	M3 vs. S3	-	Regulation	Transcription	regulation of gene expression	GO:0010468	P	1551	2.96E-05	4.24E-03
10	4817	M3 vs. S3	-	Regulation	Transcription	regulation of nucleic acid-templated transcription	GO:1903506	P	1211	4.76E-06	9.18E-04
10	4818	M3 vs. S3	-	Regulation	Transcription	regulation of RNA biosynthetic process	GO:2001141	P	1213	4.94E-06	9.22E-04
10	4819	M3 vs. S3	-	Regulation	Transcription	regulation of transcription, DNA-templated	GO:0006355	P	1211	4.76E-06	9.51E-04
10	4820	M3 vs. S3	-	Regulation	Transcription	RNA binding	GO:0003723	F	1094	1.93E-08	9.37E-06
10	4821	M3 vs. S3	-	Regulation	Transcription	transcription regulator activity	GO:0140110	F	1016	9.50E-08	3.46E-05
10	4822	M3 vs. S3	-	Regulation	Translation	cytoplasmic translation	GO:0002181	P	55	1.69E-04	1.69E-02
10	4823	M3 vs. S3	-	Regulation	Translation	cytosolic large ribosomal subunit	GO:0022625	C	103	0.00E+00	0.00E+00
10	4824	M3 vs. S3	-	Regulation	Translation	cytosolic ribosome	GO:0022626	C	233	0.00E+00	0.00E+00
10	4825	M3 vs. S3	-	Regulation	Translation	cytosolic small ribosomal subunit	GO:0022627	C	76	5.91E-12	6.54E-10
10	4826	M3 vs. S3	-	Regulation	Translation	large ribosomal subunit	GO:0015934	C	134	0.00E+00	0.00E+00
10	4827	M3 vs. S3	-	Regulation	Translation	maturation of SSU-rRNA	GO:0030490	P	40	3.39E-04	2.87E-02
10	4828	M3 vs. S3	-	Regulation	Translation	mRNA binding	GO:0003729	F	732	3.07E-12	2.98E-09
10	4829	M3 vs. S3	-	Regulation	Translation	peptide biosynthetic process	GO:0043043	P	363	0.00E+00	0.00E+00
10	4830	M3 vs. S3	-	Regulation	Translation	precatalytic spliceosome	GO:0071011	C	21	1.00E-03	1.89E-02
10	4831	M3 vs. S3	-	Regulation	Translation	regulation of RNA metabolic process	GO:0051252	P	1293	1.04E-05	1.76E-03
10	4832	M3 vs. S3	-	Regulation	Translation	ribonucleoprotein complex	GO:1990904	C	465	0.00E+00	0.00E+00
10	4833	M3 vs. S3	-	Regulation	Translation	ribonucleoprotein complex assembly	GO:0022618	P	122	9.96E-08	3.28E-05
10	4834	M3 vs. S3	-	Regulation	Translation	ribonucleoprotein complex biogenesis	GO:0022613	P	314	7.71E-08	2.88E-05
10	4835	M3 vs. S3	-	Regulation	Translation	ribonucleoprotein complex subunit organization	GO:0071826	P	125	2.41E-07	6.74E-05
10	4836	M3 vs. S3	-	Regulation	Translation	ribosomal large subunit assembly	GO:0000027	P	35	4.95E-05	6.30E-03
10	4837	M3 vs. S3	-	Regulation	Translation	ribosomal large subunit biogenesis	GO:0042273	P	84	6.33E-09	2.95E-06
10	4838	M3 vs. S3	-	Regulation	Translation	ribosomal small subunit assembly	GO:0000028	P	28	1.74E-07	5.42E-05
10	4839	M3 vs. S3	-	Regulation	Translation	ribosomal small subunit biogenesis	GO:0042274	P	65	2.75E-09	1.54E-06
10	4840	M3 vs. S3	-	Regulation	Translation	ribosomal subunit	GO:0044391	C	235	0.00E+00	0.00E+00
10	4841	M3 vs. S3	-	Regulation	Translation	ribosome	GO:0005840	C	297	0.00E+00	0.00E+00
10	4842	M3 vs. S3	-	Regulation	Translation	ribosome assembly	GO:0042255	P	68	8.16E-11	7.61E-08
10	4843	M3 vs. S3	-	Regulation	Translation	ribosome biogenesis	GO:0042254	P	260	1.53E-08	6.57E-06
10	4844	M3 vs. S3	-	Regulation	Translation	rRNA binding	GO:0019843	F	51	5.40E-07	1.31E-04
10	4845	M3 vs. S3	-	Regulation	Translation	small nuclear ribonucleoprotein complex	GO:0030532	C	47	3.98E-04	9.23E-03
10	4846	M3 vs. S3	-	Regulation	Translation	small ribosomal subunit	GO:0015935	C	101	1.05E-11	1.05E-09
10	4847	M3 vs. S3	-	Regulation	Translation	spliceosomal snRNP complex	GO:0097525	C	47	3.98E-04	9.45E-03
10	4848	M3 vs. S3	-	Regulation	Translation	structural constituent of ribosome	GO:0003735	F	237	0.00E+00	0.00E+00
10	4849	M3 vs. S3	-	Regulation	Translation	translation	GO:0006412	P	359	0.00E+00	0.00E+00
10	4850	M3 vs. S3	-	Regulation	Translation	U1 snRNP	GO:0005685	C	17	5.63E-04	1.19E-02
11	4851	F3 vs. S3	+	Development	Cell division	nucleobase-containing small molecule metabolic process	GO:0055086	P	268	1.42E-07	5.12E-06
11	4852	F3 vs. S3	+	Development	Cell division	nucleoside bisphosphate metabolic process	GO:0033865	P	50	2.43E-03	2.39E-02
11	4853	F3 vs. S3	+	Development	Cell division	nucleoside phosphate binding	GO:1901265	F	699	2.82E-04	6.24E-03
11	4854	F3 vs. S3	+	Development	Cell division	nucleoside phosphate metabolic process	GO:0006753	P	201	7.66E-04	9.36E-03
11	4855	F3 vs. S3	+	Development	Cell division	nucleotide binding	GO:0000166	F	699	2.82E-04	6.29E-03
11	4856	F3 vs. S3	+	Development	Cell division	nucleotide metabolic process	GO:0009117	P	194	1.24E-03	1.41E-02
11	4857	F3 vs. S3	+	Development	Cell division	nucleotide-sugar biosynthetic process	GO:0009226	P	34	1.69E-06	4.84E-05
11	4858	F3 vs. S3	+	Development	Cell division	nucleotide-sugar metabolic process	GO:0009225	P	42	2.24E-06	6.20E-05
11	4859	F3 vs. S3	+	Development	Cell division	pollen germination	GO:0009846	P	40	1.49E-05	3.14E-04
11	4860	F3 vs. S3	+	Development	Cell division	pollen tube development	GO:0048868	P	121	4.67E-09	2.30E-07

11	4861	F3 vs. S3	+	Development	Cell division	pollen tube growth	GO:0009860	P	94	4.79E-09	2.32E-07
11	4862	F3 vs. S3	+	Development	Cell division	pollination	GO:0009856	P	171	6.09E-10	3.52E-08
11	4863	F3 vs. S3	+	Development	Cell division	ribonucleoside bisphosphate metabolic process	GO:0033875	P	50	2.43E-03	2.39E-02
11	4864	F3 vs. S3	+	Development	Cell division	ribonucleotide metabolic process	GO:0009259	P	156	5.13E-04	6.67E-03
11	4865	F3 vs. S3	+	Development	Cell division	ribose phosphate metabolic process	GO:0019693	P	162	4.50E-04	6.02E-03
11	4866	F3 vs. S3	+	Development	Development	actin cytoskeleton	GO:0015629	C	42	5.37E-03	3.01E-02
11	4867	F3 vs. S3	+	Development	Development	actin filament	GO:0005884	C	9	6.86E-03	3.58E-02
11	4868	F3 vs. S3	+	Development	Development	apical part of cell	GO:0045177	C	19	5.73E-04	4.27E-03
11	4869	F3 vs. S3	+	Development	Development	apical plasma membrane	GO:0016324	C	17	1.54E-04	1.34E-03
11	4870	F3 vs. S3	+	Development	Development	apoplast	GO:0048046	C	196	4.44E-16	1.63E-14
11	4871	F3 vs. S3	+	Development	Development	bounding membrane of organelle	GO:0098588	C	645	5.76E-03	3.19E-02
11	4872	F3 vs. S3	+	Development	Development	cell development	GO:0048468	P	217	8.66E-04	1.05E-02
11	4873	F3 vs. S3	+	Development	Development	cell growth	GO:0016049	P	272	1.97E-05	3.92E-04
11	4874	F3 vs. S3	+	Development	Development	cell morphogenesis	GO:0000902	P	252	4.42E-03	3.82E-02
11	4875	F3 vs. S3	+	Development	Development	cell morphogenesis involved in differentiation	GO:0000904	P	151	1.51E-03	1.63E-02
11	4876	F3 vs. S3	+	Development	Development	cell periphery	GO:0071944	C	2636	5.11E-08	9.21E-07
11	4877	F3 vs. S3	+	Development	Development	cell pole	GO:0060187	C	16	3.68E-03	2.23E-02
11	4878	F3 vs. S3	+	Development	Development	cell projection	GO:0042995	C	77	2.77E-08	5.49E-07
11	4879	F3 vs. S3	+	Development	Development	cell surface	GO:0009986	C	14	5.87E-03	3.23E-02
11	4880	F3 vs. S3	+	Development	Development	cell tip	GO:0051286	C	16	3.68E-03	2.21E-02
11	4881	F3 vs. S3	+	Development	Development	cell tip growth	GO:0009932	P	110	1.43E-07	5.11E-06
11	4882	F3 vs. S3	+	Development	Development	cell wall	GO:0005618	C	387	6.65E-03	3.53E-02
11	4883	F3 vs. S3	+	Development	Development	cell wall biogenesis	GO:0042546	P	165	1.52E-06	4.42E-05
11	4884	F3 vs. S3	+	Development	Development	cell wall modification	GO:0042545	P	125	1.73E-05	3.54E-04
11	4885	F3 vs. S3	+	Development	Development	cell wall organization	GO:0071555	P	202	2.04E-04	3.04E-03
11	4886	F3 vs. S3	+	Development	Development	cell wall organization or biogenesis	GO:0071554	P	361	4.70E-09	2.29E-07
11	4887	F3 vs. S3	+	Development	Development	cell wall pectin metabolic process	GO:0052546	P	17	5.96E-03	4.85E-02
11	4888	F3 vs. S3	+	Development	Development	cytoplasm	GO:0005737	C	8718	3.75E-05	4.09E-04
11	4889	F3 vs. S3	+	Development	Development	developmental cell growth	GO:0048588	P	135	3.57E-08	1.41E-06
11	4890	F3 vs. S3	+	Development	Development	developmental growth	GO:0048589	P	273	1.88E-05	3.81E-04
11	4891	F3 vs. S3	+	Development	Development	developmental growth involved in morphogenesis	GO:0060560	P	238	3.18E-06	8.35E-05
11	4892	F3 vs. S3	+	Development	Development	endomembrane system	GO:0012505	C	1530	1.50E-05	1.71E-04
11	4893	F3 vs. S3	+	Development	Development	envelope	GO:0031975	C	743	8.09E-05	7.71E-04
11	4894	F3 vs. S3	+	Development	Development	external encapsulating structure	GO:0030312	C	389	6.37E-03	3.43E-02
11	4895	F3 vs. S3	+	Development	Development	external encapsulating structure organization	GO:0045229	P	235	9.17E-06	2.12E-04
11	4896	F3 vs. S3	+	Development	Development	extracellular matrix	GO:0031012	C	14	6.08E-03	3.33E-02
11	4897	F3 vs. S3	+	Development	Development	extracellular region	GO:0005576	C	1547	7.77E-16	2.66E-14
11	4898	F3 vs. S3	+	Development	Development	extracellular space	GO:0005615	C	74	1.36E-04	1.23E-03
11	4899	F3 vs. S3	+	Development	Development	Golgi apparatus subcompartment	GO:0098791	C	312	1.41E-05	1.65E-04
11	4900	F3 vs. S3	+	Development	Development	Golgi cisterna	GO:0031985	C	132	8.71E-03	4.36E-02
11	4901	F3 vs. S3	+	Development	Development	Golgi medial cisterna	GO:0005797	C	45	8.99E-03	4.46E-02
11	4902	F3 vs. S3	+	Development	Development	Golgi trans cisterna	GO:0000138	C	58	2.16E-03	1.36E-02
11	4903	F3 vs. S3	+	Development	Development	growing cell tip	GO:0035838	C	12	4.21E-03	2.45E-02
11	4904	F3 vs. S3	+	Development	Development	growth	GO:0040007	P	310	1.04E-04	1.67E-03
11	4905	F3 vs. S3	+	Development	Development	hemicellulose metabolic process	GO:0010410	P	74	7.74E-06	1.83E-04
11	4906	F3 vs. S3	+	Development	Development	integral component of membrane	GO:0016021	C	294	3.06E-04	2.37E-03
11	4907	F3 vs. S3	+	Development	Development	integral component of plasma membrane	GO:0005887	C	76	3.67E-05	4.05E-04
11	4908	F3 vs. S3	+	Development	Development	intracellular vesicle	GO:0097708	C	503	1.15E-03	8.08E-03
11	4909	F3 vs. S3	+	Development	Development	lytic vacuole	GO:0000323	C	35	2.58E-05	2.91E-04
11	4910	F3 vs. S3	+	Development	Development	membrane	GO:0016020	C	3581	2.56E-11	6.19E-10
11	4911	F3 vs. S3	+	Development	Development	microbody	GO:0042579	C	215	2.93E-06	3.92E-05
11	4912	F3 vs. S3	+	Development	Development	monolayer-surrounded lipid storage body	GO:0012511	C	10	9.54E-03	4.68E-02
11	4913	F3 vs. S3	+	Development	Development	multi-multicellular organism process	GO:0044706	P	171	6.09E-10	3.56E-08
11	4914	F3 vs. S3	+	Development	Development	organelle envelope	GO:0031967	C	743	8.09E-05	7.64E-04
11	4915	F3 vs. S3	+	Development	Development	organelle subcompartment	GO:0031984	C	811	1.29E-11	3.21E-10
11	4916	F3 vs. S3	+	Development	Development	plant-type cell wall biogenesis	GO:0009832	P	107	2.71E-03	2.60E-02
11	4917	F3 vs. S3	+	Development	Development	plant-type cell wall organization or biogenesis	GO:0071669	P	190	3.30E-03	3.00E-02
11	4918	F3 vs. S3	+	Development	Development	plant-type primary cell wall biogenesis	GO:0009833	P	18	6.06E-03	4.92E-02
11	4919	F3 vs. S3	+	Development	Development	plant-type vacuole	GO:0000325	C	112	5.65E-06	6.93E-05
11	4920	F3 vs. S3	+	Development	Development	plant-type vacuole membrane	GO:0009705	C	80	4.50E-03	2.58E-02
11	4921	F3 vs. S3	+	Development	Development	plasma membrane	GO:0005886	C	2318	1.34E-06	1.95E-05
11	4922	F3 vs. S3	+	Development	Development	plasma membrane bounded cell projection	GO:0120025	C	66	8.68E-12	2.33E-10
11	4923	F3 vs. S3	+	Development	Development	plasma membrane region	GO:0098590	C	38	4.08E-03	2.41E-02
11	4924	F3 vs. S3	+	Development	Development	pollen tube	GO:0090406	C	55	1.54E-10	3.40E-09

11	4925	F3 vs. S3	+	Development	Development	pollen tube tip	GO:0090404	C	12	4.21E-03	2.46E-02
11	4926	F3 vs. S3	+	Development	Development	secretion	GO:0046903	P	53	3.15E-03	2.88E-02
11	4927	F3 vs. S3	+	Development	Development	stromule	GO:0010319	C	34	3.93E-09	8.12E-08
11	4928	F3 vs. S3	+	Development	Development	unidimensional cell growth	GO:0009826	P	203	1.40E-05	2.97E-04
11	4929	F3 vs. S3	+	Development	Development	vacuolar membrane	GO:0005774	C	405	4.76E-04	3.64E-03
11	4930	F3 vs. S3	+	Development	Development	vacuole	GO:0005773	C	757	4.19E-08	7.99E-07
11	4931	F3 vs. S3	+	Metabolism	Bioenergetics	(1->3)-beta-D-glucan binding (NAD+) activity	GO:0004029	F	9	7.44E-05	2.03E-03
11	4932	F3 vs. S3	+	Metabolism	Bioenergetics	acyl-CoA metabolic process	GO:0006637	P	36	1.62E-03	1.73E-02
11	4933	F3 vs. S3	+	Metabolism	Bioenergetics	aerobic respiration	GO:0009060	P	66	1.50E-03	1.63E-02
11	4934	F3 vs. S3	+	Metabolism	Bioenergetics	alcohol dehydrogenase (NADP+) activity	GO:0008106	F	10	4.40E-04	9.11E-03
11	4935	F3 vs. S3	+	Metabolism	Bioenergetics	aldehyde dehydrogenase [NAD(P)+] activity	GO:0004030	F	9	7.44E-05	2.05E-03
11	4936	F3 vs. S3	+	Metabolism	Bioenergetics	alditol:NADP+ 1-oxidoreductase activity	GO:0004032	F	6	1.73E-03	2.86E-02
11	4937	F3 vs. S3	+	Metabolism	Bioenergetics	aldo-keto reductase (NADP) activity	GO:0004033	F	11	2.29E-04	5.27E-03
11	4938	F3 vs. S3	+	Metabolism	Bioenergetics	ATPase activity, coupled to transmembrane movement of ions, rotational mechanism	GO:0044769	F	21	1.77E-04	4.42E-03
11	4939	F3 vs. S3	+	Metabolism	Bioenergetics	carbohydrate biosynthetic process	GO:0016051	P	214	5.36E-12	4.49E-10
11	4940	F3 vs. S3	+	Metabolism	Bioenergetics	carbohydrate catabolic process	GO:0016052	P	166	1.56E-10	1.03E-08
11	4941	F3 vs. S3	+	Metabolism	Bioenergetics	carbohydrate derivative biosynthetic process	GO:1901137	P	311	1.60E-05	3.28E-04
11	4942	F3 vs. S3	+	Metabolism	Bioenergetics	carbohydrate derivative metabolic process	GO:1901135	P	506	1.61E-10	1.05E-08
11	4943	F3 vs. S3	+	Metabolism	Bioenergetics	carbohydrate metabolic process	GO:0005975	P	640	0.00E+00	0.00E+00
11	4944	F3 vs. S3	+	Metabolism	Bioenergetics	carbohydrate phosphatase activity	GO:0019203	F	20	1.13E-03	2.04E-02
11	4945	F3 vs. S3	+	Metabolism	Bioenergetics	cellular carbohydrate biosynthetic process	GO:0034637	P	147	8.61E-07	2.71E-05
11	4946	F3 vs. S3	+	Metabolism	Bioenergetics	cellular carbohydrate catabolic process	GO:0044275	P	34	4.53E-03	3.88E-02
11	4947	F3 vs. S3	+	Metabolism	Bioenergetics	cellular carbohydrate metabolic process	GO:0044262	P	270	1.06E-11	8.60E-10
11	4948	F3 vs. S3	+	Metabolism	Bioenergetics	cellular polysaccharide biosynthetic process	GO:0033692	P	122	4.07E-05	7.38E-04
11	4949	F3 vs. S3	+	Metabolism	Bioenergetics	cellular polysaccharide metabolic process	GO:0044264	P	188	8.98E-07	2.81E-05
11	4950	F3 vs. S3	+	Metabolism	Bioenergetics	CoA-ligase activity	GO:0016405	F	21	3.81E-05	1.15E-03
11	4951	F3 vs. S3	+	Metabolism	Bioenergetics	disaccharide metabolic process	GO:0005984	P	54	2.15E-04	3.20E-03
11	4952	F3 vs. S3	+	Metabolism	Bioenergetics	fatty acid biosynthetic process	GO:0006633	P	103	5.75E-03	4.73E-02
11	4953	F3 vs. S3	+	Metabolism	Bioenergetics	fatty acid derivative metabolic process	GO:1901568	P	32	2.49E-03	2.43E-02
11	4954	F3 vs. S3	+	Metabolism	Bioenergetics	fatty acid ligase activity	GO:0015645	F	10	1.13E-03	2.05E-02
11	4955	F3 vs. S3	+	Metabolism	Bioenergetics	fatty acid metabolic process	GO:0006631	P	166	6.58E-07	2.11E-05
11	4956	F3 vs. S3	+	Metabolism	Bioenergetics	generation of precursor metabolites and energy	GO:0006091	P	266	2.70E-09	1.39E-07
11	4957	F3 vs. S3	+	Metabolism	Bioenergetics	gluconeogenesis	GO:0006094	P	20	4.39E-04	5.89E-03
11	4958	F3 vs. S3	+	Metabolism	Bioenergetics	glucose 6-phosphate metabolic process	GO:0051156	P	35	1.20E-03	1.37E-02
11	4959	F3 vs. S3	+	Metabolism	Bioenergetics	glucose metabolic process	GO:0006006	P	34	5.16E-03	4.33E-02
11	4960	F3 vs. S3	+	Metabolism	Bioenergetics	glyceraldehyde-3-phosphate dehydrogenase (NAD+) (non-phosphorylating) activity	GO:0043878	F	6	6.17E-04	1.20E-02
11	4961	F3 vs. S3	+	Metabolism	Bioenergetics	hexose biosynthetic process	GO:0019319	P	22	2.07E-04	3.08E-03
11	4962	F3 vs. S3	+	Metabolism	Bioenergetics	hexose metabolic process	GO:0019318	P	68	2.19E-05	4.28E-04
11	4963	F3 vs. S3	+	Metabolism	Bioenergetics	isocitrate dehydrogenase (NAD+) activity	GO:0004449	F	5	1.32E-03	2.31E-02
11	4964	F3 vs. S3	+	Metabolism	Bioenergetics	isocitrate dehydrogenase activity	GO:0004448	F	7	1.81E-04	4.44E-03
11	4965	F3 vs. S3	+	Metabolism	Bioenergetics	isocitrate metabolic process	GO:0006102	P	10	8.70E-05	1.43E-03
11	4966	F3 vs. S3	+	Metabolism	Bioenergetics	lipid oxidation	GO:0034440	P	38	2.18E-03	2.21E-02
11	4967	F3 vs. S3	+	Metabolism	Bioenergetics	medium-chain fatty acid-CoA ligase activity	GO:0031956	F	9	2.18E-03	3.46E-02
11	4968	F3 vs. S3	+	Metabolism	Bioenergetics	monosaccharide biosynthetic process	GO:0046364	P	37	2.51E-04	3.64E-03
11	4969	F3 vs. S3	+	Metabolism	Bioenergetics	NAD binding	GO:0051287	F	60	1.72E-05	5.81E-04
11	4970	F3 vs. S3	+	Metabolism	Bioenergetics	NAD(P)H dehydrogenase complex (plastoquinone)	GO:0010598	C	9	1.33E-03	8.88E-03
11	4971	F3 vs. S3	+	Metabolism	Bioenergetics	NADP binding	GO:0050661	F	50	2.15E-04	5.07E-03
11	4972	F3 vs. S3	+	Metabolism	Bioenergetics	NADP metabolic process	GO:0006739	P	38	2.65E-05	5.04E-04
11	4973	F3 vs. S3	+	Metabolism	Bioenergetics	NADPH regeneration	GO:0006740	P	31	3.28E-04	4.68E-03
11	4974	F3 vs. S3	+	Metabolism	Bioenergetics	oligosaccharide metabolic process	GO:0009311	P	60	3.46E-04	4.82E-03
11	4975	F3 vs. S3	+	Metabolism	Bioenergetics	oxidoreductase activity	GO:0016491	F	925	0.00E+00	0.00E+00
11	4976	F3 vs. S3	+	Metabolism	Bioenergetics	oxidoreductase activity, acting on CH-OH group of donors	GO:0016614	F	144	4.33E-15	8.97E-13
11	4977	F3 vs. S3	+	Metabolism	Bioenergetics	oxidoreductase activity, acting on NAD(P)H	GO:0016651	F	80	1.19E-04	3.07E-03
11	4978	F3 vs. S3	+	Metabolism	Bioenergetics	oxidoreductase activity, acting on paired donors, with incorporation or reduction of molecular oxygen	GO:0016705	F	217	6.06E-05	1.72E-03
11	4979	F3 vs. S3	+	Metabolism	Bioenergetics	oxidoreductase activity, acting on paired donors, with incorporation or reduction of molecular oxygen, reduced flavin or flavoprotein as one donor, and incorporation of one atom of oxygen	GO:0016712	F	4	1.19E-03	2.11E-02

11	4980	F3 vs. S3	+	Metabolism	Bioenergetics	oxidoreductase activity, acting on paired donors, with incorporation or reduction of molecular oxygen, reduced iron-sulfur protein as one donor, and incorporation of one atom of oxygen	GO:0016713	F	7	6.35E-04	1.22E-02
11	4981	F3 vs. S3	+	Metabolism	Bioenergetics	oxidoreductase activity, acting on single donors with incorporation of molecular oxygen	GO:0016701	F	30	1.88E-04	4.58E-03
11	4982	F3 vs. S3	+	Metabolism	Bioenergetics	oxidoreductase activity, acting on the aldehyde or oxo group of donors	GO:0016903	F	46	8.26E-07	3.68E-05
11	4983	F3 vs. S3	+	Metabolism	Bioenergetics	oxidoreductase activity, acting on the aldehyde or oxo group of donors, NAD or NADP as acceptor	GO:0016620	F	32	2.03E-07	1.13E-05
11	4984	F3 vs. S3	+	Metabolism	Bioenergetics	oxidoreductase activity, acting on the CH-CH group of donors	GO:0016627	F	57	3.17E-03	4.84E-02
11	4985	F3 vs. S3	+	Metabolism	Bioenergetics	oxidoreductase activity, acting on the CH-OH group of donors, NAD or NADP as acceptor	GO:0016616	F	124	4.22E-15	9.41E-13
11	4986	F3 vs. S3	+	Metabolism	Bioenergetics	pentose-phosphate shunt	GO:0006098	P	30	1.51E-04	2.31E-03
11	4987	F3 vs. S3	+	Metabolism	Bioenergetics	pentose-phosphate shunt, non-oxidative branch	GO:0009052	P	5	3.01E-03	2.79E-02
11	4988	F3 vs. S3	+	Metabolism	Bioenergetics	polysaccharide biosynthetic process	GO:0000271	P	148	1.52E-06	4.43E-05
11	4989	F3 vs. S3	+	Metabolism	Bioenergetics	polysaccharide metabolic process	GO:0005976	P	296	1.78E-13	1.88E-11
11	4990	F3 vs. S3	+	Metabolism	Bioenergetics	proton transmembrane transport	GO:1902600	P	60	5.23E-07	1.72E-05
11	4991	F3 vs. S3	+	Metabolism	Bioenergetics	proton-exporting ATPase activity, phosphorylative mechanism	GO:0008553	F	13	3.77E-05	1.15E-03
11	4992	F3 vs. S3	+	Metabolism	Bioenergetics	proton-transporting ATPase activity, rotational mechanism	GO:0046961	F	21	1.77E-04	4.46E-03
11	4993	F3 vs. S3	+	Metabolism	Bioenergetics	proton-transporting two-sector ATPase complex	GO:0016469	C	42	1.87E-03	1.20E-02
11	4994	F3 vs. S3	+	Metabolism	Bioenergetics	proton-transporting V-type ATPase complex	GO:0033176	C	19	8.98E-03	4.48E-02
11	4995	F3 vs. S3	+	Metabolism	Bioenergetics	pyrophosphate hydrolysis-driven proton transmembrane transporter activity	GO:0009678	F	34	1.12E-07	6.52E-06
11	4996	F3 vs. S3	+	Metabolism	Bioenergetics	pyruvate metabolic process	GO:0006090	P	57	1.35E-03	1.50E-02
11	4997	F3 vs. S3	+	Metabolism	Bioenergetics	reductive pentose-phosphate cycle	GO:0019253	P	14	3.08E-06	8.14E-05
11	4998	F3 vs. S3	+	Metabolism	Bioenergetics	sucrose metabolic process	GO:0005985	P	41	6.12E-05	1.07E-03
11	4999	F3 vs. S3	+	Metabolism	Bioenergetics	tricarboxylic acid cycle	GO:0006099	P	36	3.08E-05	5.68E-04
11	5000	F3 vs. S3	+	Metabolism	Bioenergetics	tricarboxylic acid metabolic process	GO:0072350	P	14	2.12E-05	4.19E-04
11	5001	F3 vs. S3	+	Metabolism	Bioenergetics	very long-chain fatty acid metabolic process	GO:0000038	P	32	1.41E-03	1.55E-02
11	5002	F3 vs. S3	+	Metabolism	Catabolism	alpha-amino acid catabolic process	GO:1901606	P	69	2.61E-09	1.36E-07
11	5003	F3 vs. S3	+	Metabolism	Catabolism	aminopeptidase activity	GO:0004177	F	18	3.27E-03	4.97E-02
11	5004	F3 vs. S3	+	Metabolism	Catabolism	aromatic amino acid family catabolic process	GO:0009074	P	18	3.64E-04	4.99E-03
11	5005	F3 vs. S3	+	Metabolism	Catabolism	carboxylic acid catabolic process	GO:0046395	P	124	3.29E-10	2.03E-08
11	5006	F3 vs. S3	+	Metabolism	Catabolism	catabolic process	GO:0009056	P	956	3.55E-10	2.16E-08
11	5007	F3 vs. S3	+	Metabolism	Catabolism	cellular amino acid catabolic process	GO:0009063	P	76	9.30E-08	3.48E-06
11	5008	F3 vs. S3	+	Metabolism	Catabolism	cellular catabolic process	GO:0044248	P	791	1.35E-05	2.94E-04
11	5009	F3 vs. S3	+	Metabolism	Catabolism	cysteine-type endopeptidase activity	GO:0004197	F	55	1.77E-03	2.91E-02
11	5010	F3 vs. S3	+	Metabolism	Catabolism	dicarboxylic acid catabolic process	GO:0043649	P	11	1.16E-03	1.34E-02
11	5011	F3 vs. S3	+	Metabolism	Catabolism	lipid catabolic process	GO:0016042	P	76	4.46E-03	3.84E-02
11	5012	F3 vs. S3	+	Metabolism	Catabolism	lysosome	GO:0005764	C	31	9.63E-06	1.15E-04
11	5013	F3 vs. S3	+	Metabolism	Catabolism	monocarboxylic acid catabolic process	GO:0072329	P	48	1.70E-04	2.59E-03
11	5014	F3 vs. S3	+	Metabolism	Catabolism	organic acid catabolic process	GO:0016054	P	124	3.29E-10	2.05E-08
11	5015	F3 vs. S3	+	Metabolism	Catabolism	organic cyclic compound catabolic process	GO:1901361	P	135	4.92E-03	4.16E-02
11	5016	F3 vs. S3	+	Metabolism	Catabolism	organic hydroxy compound catabolic process	GO:1901616	P	29	5.44E-03	4.52E-02
11	5017	F3 vs. S3	+	Metabolism	Catabolism	organic substance catabolic process	GO:1901575	P	874	2.42E-08	1.02E-06
11	5018	F3 vs. S3	+	Metabolism	Catabolism	pectin catabolic process	GO:0045490	P	70	1.76E-06	4.96E-05
11	5019	F3 vs. S3	+	Metabolism	Catabolism	peroxisome	GO:0005777	C	215	2.93E-06	3.87E-05
11	5020	F3 vs. S3	+	Metabolism	Catabolism	polysaccharide catabolic process	GO:0000272	P	101	2.57E-08	1.05E-06
11	5021	F3 vs. S3	+	Metabolism	Catabolism	PSII associated light-harvesting complex II catabolic process	GO:0010304	P	5	1.70E-03	1.80E-02
11	5022	F3 vs. S3	+	Metabolism	Catabolism	small molecule catabolic process	GO:0044282	P	176	1.67E-11	1.28E-09
11	5023	F3 vs. S3	+	Metabolism	Catabolism	tyrosine catabolic process	GO:0006572	P	5	4.53E-03	3.87E-02
11	5024	F3 vs. S3	+	Metabolism	Metabolism	acid-thiol ligase activity	GO:0016878	F	25	4.61E-06	1.61E-04
11	5025	F3 vs. S3	+	Metabolism	Metabolism	acylglycerol lipase activity	GO:0047372	F	11	1.13E-03	2.06E-02
11	5026	F3 vs. S3	+	Metabolism	Metabolism	alcohol biosynthetic process	GO:0046165	P	37	2.41E-03	2.38E-02
11	5027	F3 vs. S3	+	Metabolism	Metabolism	alcohol metabolic process	GO:0006066	P	79	3.03E-05	5.61E-04
11	5028	F3 vs. S3	+	Metabolism	Metabolism	aldehyde-lyase activity	GO:0016832	F	17	5.42E-04	1.08E-02
11	5029	F3 vs. S3	+	Metabolism	Metabolism	alkane 1-monooxygenase activity	GO:0018685	F	6	8.00E-04	1.48E-02
11	5030	F3 vs. S3	+	Metabolism	Metabolism	alpha-amino acid biosynthetic process	GO:1901607	P	124	1.42E-07	5.13E-06
11	5031	F3 vs. S3	+	Metabolism	Metabolism	alpha-amino acid metabolic process	GO:1901605	P	197	2.33E-15	2.97E-13
11	5032	F3 vs. S3	+	Metabolism	Metabolism	amine biosynthetic process	GO:0009309	P	27	1.03E-03	1.21E-02
11	5033	F3 vs. S3	+	Metabolism	Metabolism	amine metabolic process	GO:0009308	P	69	3.23E-04	4.64E-03
11	5034	F3 vs. S3	+	Metabolism	Metabolism	anion binding	GO:0043168	F	867	2.62E-06	1.03E-04
11	5035	F3 vs. S3	+	Metabolism	Metabolism	anthocyanin-containing compound biosynthetic process	GO:0009718	P	11	2.15E-03	2.18E-02

11	5036	F3 vs. S3	+	Metabolism	Metabolism	aromatase activity	GO:0070330	F	4	1.19E-03	2.12E-02
11	5037	F3 vs. S3	+	Metabolism	Metabolism	aromatic amino acid family metabolic process	GO:0009072	P	63	4.62E-04	6.13E-03
11	5038	F3 vs. S3	+	Metabolism	Metabolism	aromatic compound biosynthetic process	GO:0019438	P	533	3.91E-03	3.47E-02
11	5039	F3 vs. S3	+	Metabolism	Metabolism	aspartate family amino acid metabolic process	GO:0009066	P	41	1.53E-03	1.65E-02
11	5040	F3 vs. S3	+	Metabolism	Metabolism	capsule organization	GO:0045230	P	6	3.32E-04	4.72E-03
11	5041	F3 vs. S3	+	Metabolism	Metabolism	capsule polysaccharide biosynthetic process	GO:0045227	P	6	3.32E-04	4.71E-03
11	5042	F3 vs. S3	+	Metabolism	Metabolism	carbon utilization	GO:0015976	P	8	1.10E-03	1.29E-02
11	5043	F3 vs. S3	+	Metabolism	Metabolism	carbon-carbon lyase activity	GO:0016830	F	75	7.57E-10	6.10E-08
11	5044	F3 vs. S3	+	Metabolism	Metabolism	carbon-oxygen lyase activity	GO:0016835	F	108	1.80E-04	4.46E-03
11	5045	F3 vs. S3	+	Metabolism	Metabolism	carboxylic acid biosynthetic process	GO:0046394	P	336	1.40E-11	1.09E-09
11	5046	F3 vs. S3	+	Metabolism	Metabolism	carboxylic acid metabolic process	GO:0019752	P	651	0.00E+00	0.00E+00
11	5047	F3 vs. S3	+	Metabolism	Metabolism	carboxylic ester hydrolase activity	GO:0052689	F	136	4.88E-07	2.40E-05
11	5048	F3 vs. S3	+	Metabolism	Metabolism	carboxy-lyase activity	GO:0016831	F	40	4.27E-06	1.53E-04
11	5049	F3 vs. S3	+	Metabolism	Metabolism	catalytic activity	GO:0003824	F	5213	0.00E+00	0.00E+00
11	5050	F3 vs. S3	+	Metabolism	Metabolism	cation binding	GO:0043169	F	1019	1.32E-12	1.74E-10
11	5051	F3 vs. S3	+	Metabolism	Metabolism	cell wall macromolecule biosynthetic process	GO:0044038	P	56	2.24E-03	2.25E-02
11	5052	F3 vs. S3	+	Metabolism	Metabolism	cell wall macromolecule metabolic process	GO:0044036	P	118	2.17E-06	6.02E-05
11	5053	F3 vs. S3	+	Metabolism	Metabolism	cell wall polysaccharide biosynthetic process	GO:0070592	P	53	3.69E-03	3.29E-02
11	5054	F3 vs. S3	+	Metabolism	Metabolism	cell wall polysaccharide metabolic process	GO:0010383	P	97	1.12E-05	2.47E-04
11	5055	F3 vs. S3	+	Metabolism	Metabolism	cellular aldehyde metabolic process	GO:0006081	P	37	3.96E-04	5.39E-03
11	5056	F3 vs. S3	+	Metabolism	Metabolism	cellular amine metabolic process	GO:0044106	P	42	8.72E-05	1.43E-03
11	5057	F3 vs. S3	+	Metabolism	Metabolism	cellular amino acid biosynthetic process	GO:0008652	P	139	3.71E-08	1.44E-06
11	5058	F3 vs. S3	+	Metabolism	Metabolism	cellular amino acid metabolic process	GO:0006520	P	269	5.14E-13	4.89E-11
11	5059	F3 vs. S3	+	Metabolism	Metabolism	cellular biogenic amine biosynthetic process	GO:0042401	P	27	1.03E-03	1.21E-02
11	5060	F3 vs. S3	+	Metabolism	Metabolism	cellular biogenic amine metabolic process	GO:0006576	P	42	8.72E-05	1.43E-03
11	5061	F3 vs. S3	+	Metabolism	Metabolism	cellular component macromolecule biosynthetic process	GO:0070589	P	56	2.24E-03	2.25E-02
11	5062	F3 vs. S3	+	Metabolism	Metabolism	cellular glucan metabolic process	GO:0006073	P	142	4.35E-03	3.76E-02
11	5063	F3 vs. S3	+	Metabolism	Metabolism	cellular lipid metabolic process	GO:0044255	P	463	2.41E-09	1.27E-07
11	5064	F3 vs. S3	+	Metabolism	Metabolism	cellular modified amino acid metabolic process	GO:0006575	P	61	2.16E-04	3.20E-03
11	5065	F3 vs. S3	+	Metabolism	Metabolism	cellulose synthase (UDP-forming) activity	GO:0016760	F	18	2.14E-03	3.42E-02
11	5066	F3 vs. S3	+	Metabolism	Metabolism	copper ion binding	GO:0005507	F	146	4.21E-05	1.26E-03
11	5067	F3 vs. S3	+	Metabolism	Metabolism	dicarboxylic acid metabolic process	GO:0043648	P	63	1.59E-06	4.57E-05
11	5068	F3 vs. S3	+	Metabolism	Metabolism	erythrose 4-phosphate/phosphoenolpyruvate family amino acid metabolic process	GO:1902221	P	21	2.51E-03	2.43E-02
11	5069	F3 vs. S3	+	Metabolism	Metabolism	establishment of localization	GO:0051234	P	1508	1.79E-06	5.02E-05
11	5070	F3 vs. S3	+	Metabolism	Metabolism	extracellular polysaccharide biosynthetic process	GO:0045226	P	6	3.32E-04	4.70E-03
11	5071	F3 vs. S3	+	Metabolism	Metabolism	extracellular polysaccharide metabolic process	GO:0046379	P	6	3.32E-04	4.68E-03
11	5072	F3 vs. S3	+	Metabolism	Metabolism	flavin adenine dinucleotide binding	GO:0050660	F	106	1.64E-03	2.77E-02
11	5073	F3 vs. S3	+	Metabolism	Metabolism	flavonol 3-O-glucosyltransferase activity	GO:0047893	F	8	1.53E-03	2.59E-02
11	5074	F3 vs. S3	+	Metabolism	Metabolism	galactose metabolic process	GO:0006012	P	22	1.49E-03	1.62E-02
11	5075	F3 vs. S3	+	Metabolism	Metabolism	galacturonan metabolic process	GO:0010393	P	115	8.85E-09	4.07E-07
11	5076	F3 vs. S3	+	Metabolism	Metabolism	glucan metabolic process	GO:0044042	P	149	9.73E-04	1.16E-02
11	5077	F3 vs. S3	+	Metabolism	Metabolism	glucosinolate biosynthetic process	GO:0019761	P	29	5.67E-06	1.40E-04
11	5078	F3 vs. S3	+	Metabolism	Metabolism	glucosyltransferase activity	GO:0046527	F	143	4.01E-14	7.75E-12
11	5079	F3 vs. S3	+	Metabolism	Metabolism	glutamate metabolic process	GO:0006536	P	21	4.09E-04	5.54E-03
11	5080	F3 vs. S3	+	Metabolism	Metabolism	glutamine family amino acid biosynthetic process	GO:0009084	P	24	2.05E-03	2.09E-02
11	5081	F3 vs. S3	+	Metabolism	Metabolism	glutamine family amino acid metabolic process	GO:0009064	P	45	1.12E-06	3.40E-05
11	5082	F3 vs. S3	+	Metabolism	Metabolism	glutamine metabolic process	GO:0006541	P	13	6.74E-04	8.46E-03
11	5083	F3 vs. S3	+	Metabolism	Metabolism	glycine biosynthetic process	GO:0006545	P	9	4.32E-03	3.75E-02
11	5084	F3 vs. S3	+	Metabolism	Metabolism	glycine metabolic process	GO:0006544	P	15	6.75E-04	8.45E-03
11	5085	F3 vs. S3	+	Metabolism	Metabolism	glycosinolate biosynthetic process	GO:0019758	P	29	5.67E-06	1.39E-04
11	5086	F3 vs. S3	+	Metabolism	Metabolism	glycosyl compound biosynthetic process	GO:1901659	P	47	9.64E-05	1.56E-03
11	5087	F3 vs. S3	+	Metabolism	Metabolism	glycosyl compound metabolic process	GO:1901657	P	126	1.38E-03	1.53E-02
11	5088	F3 vs. S3	+	Metabolism	Metabolism	heme binding	GO:0020037	F	196	1.80E-05	5.99E-04
11	5089	F3 vs. S3	+	Metabolism	Metabolism	hydrogen sulfide metabolic process	GO:0070813	P	7	4.90E-03	4.15E-02
11	5090	F3 vs. S3	+	Metabolism	Metabolism	hydrolase activity	GO:0016787	F	1856	2.38E-09	1.77E-07
11	5091	F3 vs. S3	+	Metabolism	Metabolism	hydrolase activity, acting on ester bonds	GO:0016788	F	617	2.34E-06	9.57E-05
11	5092	F3 vs. S3	+	Metabolism	Metabolism	hydrolase activity, acting on glycosyl bonds	GO:0016798	F	307	8.29E-05	2.23E-03
11	5093	F3 vs. S3	+	Metabolism	Metabolism	hydrolase activity, hydrolyzing O-glycosyl compounds	GO:0004553	F	250	2.24E-07	1.20E-05
11	5094	F3 vs. S3	+	Metabolism	Metabolism	hydro-lyase activity	GO:0016836	F	61	2.00E-04	4.84E-03
11	5095	F3 vs. S3	+	Metabolism	Metabolism	indole-containing compound metabolic process	GO:0042430	P	48	1.54E-03	1.65E-02
11	5096	F3 vs. S3	+	Metabolism	Metabolism	intramolecular oxidoreductase activity	GO:0016860	F	43	6.62E-04	1.26E-02
11	5097	F3 vs. S3	+	Metabolism	Metabolism	intramolecular oxidoreductase activity, interconverting aldoses and ketoses	GO:0016861	F	15	4.47E-04	9.20E-03

11	5098	F3 vs. S3	+	Metabolism	Metabolism	ion binding	GO:0043167	F	1775	1.44E-13	2.46E-11
11	5099	F3 vs. S3	+	Metabolism	Metabolism	iron ion binding	GO:0005506	F	174	2.11E-04	5.02E-03
11	5100	F3 vs. S3	+	Metabolism	Metabolism	isoprenoid biosynthetic process	GO:0008299	P	101	1.67E-03	1.77E-02
11	5101	F3 vs. S3	+	Metabolism	Metabolism	isoprenoid metabolic process	GO:0006720	P	122	2.36E-03	2.35E-02
11	5102	F3 vs. S3	+	Metabolism	Metabolism	leucine biosynthetic process	GO:0009098	P	10	2.03E-03	2.09E-02
11	5103	F3 vs. S3	+	Metabolism	Metabolism	leucine metabolic process	GO:0006551	P	17	5.23E-04	6.77E-03
11	5104	F3 vs. S3	+	Metabolism	Metabolism	ligase activity	GO:0016874	F	141	3.12E-05	9.64E-04
11	5105	F3 vs. S3	+	Metabolism	Metabolism	ligase activity, forming carbon-sulfur bonds	GO:0016877	F	33	8.27E-05	2.24E-03
11	5106	F3 vs. S3	+	Metabolism	Metabolism	lignin biosynthetic process	GO:0009809	P	31	7.26E-05	1.24E-03
11	5107	F3 vs. S3	+	Metabolism	Metabolism	lignin metabolic process	GO:0009808	P	40	3.18E-06	8.36E-05
11	5108	F3 vs. S3	+	Metabolism	Metabolism	lipase activity	GO:0016298	F	67	1.90E-03	3.08E-02
11	5109	F3 vs. S3	+	Metabolism	Metabolism	lipid biosynthetic process	GO:0008610	P	349	7.37E-05	1.26E-03
11	5110	F3 vs. S3	+	Metabolism	Metabolism	lipid metabolic process	GO:0006629	P	551	6.82E-09	3.16E-07
11	5111	F3 vs. S3	+	Metabolism	Metabolism	lipid modification	GO:0030258	P	68	4.70E-04	6.23E-03
11	5112	F3 vs. S3	+	Metabolism	Metabolism	lipid storage	GO:0019915	P	15	5.76E-03	4.74E-02
11	5113	F3 vs. S3	+	Metabolism	Metabolism	localization	GO:0051179	P	1616	1.91E-05	3.86E-04
11	5114	F3 vs. S3	+	Metabolism	Metabolism	L-phenylalanine metabolic process	GO:0006558	P	21	2.51E-03	2.43E-02
11	5115	F3 vs. S3	+	Metabolism	Metabolism	L-serine metabolic process	GO:0006563	P	20	2.37E-03	2.35E-02
11	5116	F3 vs. S3	+	Metabolism	Metabolism	lyase activity	GO:0016829	F	229	2.12E-12	2.67E-10
11	5117	F3 vs. S3	+	Metabolism	Metabolism	magnesium ion binding	GO:0000287	F	82	2.69E-04	6.05E-03
11	5118	F3 vs. S3	+	Metabolism	Metabolism	manganese ion binding	GO:0030145	F	35	4.14E-04	8.69E-03
11	5119	F3 vs. S3	+	Metabolism	Metabolism	metabolic process	GO:0008152	P	5491	1.29E-05	2.82E-04
11	5120	F3 vs. S3	+	Metabolism	Metabolism	metal ion binding	GO:0046872	F	1001	6.97E-12	6.74E-10
11	5121	F3 vs. S3	+	Metabolism	Metabolism	monocarboxylic acid biosynthetic process	GO:0072330	P	164	2.39E-05	4.60E-04
11	5122	F3 vs. S3	+	Metabolism	Metabolism	monocarboxylic acid metabolic process	GO:0032787	P	334	1.13E-12	1.04E-10
11	5123	F3 vs. S3	+	Metabolism	Metabolism	monooxygenase activity	GO:0004497	F	130	1.65E-03	2.76E-02
11	5124	F3 vs. S3	+	Metabolism	Metabolism	monosaccharide metabolic process	GO:0005996	P	94	2.85E-07	9.82E-06
11	5125	F3 vs. S3	+	Metabolism	Metabolism	monovalent cation:proton antiporter activity	GO:0005451	F	35	1.50E-04	3.83E-03
11	5126	F3 vs. S3	+	Metabolism	Metabolism	oligopeptide binding	GO:1900750	F	14	2.00E-03	3.20E-02
11	5127	F3 vs. S3	+	Metabolism	Metabolism	organic acid biosynthetic process	GO:0016053	P	336	1.40E-11	1.11E-09
11	5128	F3 vs. S3	+	Metabolism	Metabolism	organic acid metabolic process	GO:0006082	P	725	0.00E+00	0.00E+00
11	5129	F3 vs. S3	+	Metabolism	Metabolism	organic cyclic compound biosynthetic process	GO:1901362	P	598	1.72E-03	1.80E-02
11	5130	F3 vs. S3	+	Metabolism	Metabolism	organic hydroxy compound biosynthetic process	GO:1901617	P	112	4.09E-04	5.55E-03
11	5131	F3 vs. S3	+	Metabolism	Metabolism	organic hydroxy compound metabolic process	GO:1901615	P	195	5.17E-06	1.30E-04
11	5132	F3 vs. S3	+	Metabolism	Metabolism	organophosphate biosynthetic process	GO:0090407	P	233	5.25E-03	4.37E-02
11	5133	F3 vs. S3	+	Metabolism	Metabolism	organophosphate metabolic process	GO:0019637	P	400	2.08E-07	7.26E-06
11	5134	F3 vs. S3	+	Metabolism	Metabolism	oxidation-reduction process	GO:0055114	P	526	1.24E-14	1.52E-12
11	5135	F3 vs. S3	+	Metabolism	Metabolism	oxoacid metabolic process	GO:0043436	P	723	0.00E+00	0.00E+00
11	5136	F3 vs. S3	+	Metabolism	Metabolism	pectin biosynthetic process	GO:0045489	P	36	6.49E-04	8.26E-03
11	5137	F3 vs. S3	+	Metabolism	Metabolism	pectin metabolic process	GO:0045488	P	114	3.26E-09	1.63E-07
11	5138	F3 vs. S3	+	Metabolism	Metabolism	pectinesterase activity	GO:0030599	F	53	2.46E-07	1.30E-05
11	5139	F3 vs. S3	+	Metabolism	Metabolism	pectinesterase inhibitor activity	GO:0046910	F	41	1.70E-06	7.15E-05
11	5140	F3 vs. S3	+	Metabolism	Metabolism	pentose metabolic process	GO:0019321	P	13	1.31E-03	1.47E-02
11	5141	F3 vs. S3	+	Metabolism	Metabolism	phenylpropanoid biosynthetic process	GO:0009699	P	63	4.19E-05	7.56E-04
11	5142	F3 vs. S3	+	Metabolism	Metabolism	phenylpropanoid metabolic process	GO:0009698	P	76	1.36E-06	4.05E-05
11	5143	F3 vs. S3	+	Metabolism	Metabolism	phosphate-containing compound metabolic process	GO:0006796	P	1169	3.40E-08	1.35E-06
11	5144	F3 vs. S3	+	Metabolism	Metabolism	phosphoric ester hydrolase activity	GO:0042578	F	252	2.28E-05	7.42E-04
11	5145	F3 vs. S3	+	Metabolism	Metabolism	phosphorus metabolic process	GO:0006793	P	1205	3.55E-10	2.14E-08
11	5146	F3 vs. S3	+	Metabolism	Metabolism	pigment biosynthetic process	GO:0046148	P	82	1.45E-03	1.59E-02
11	5147	F3 vs. S3	+	Metabolism	Metabolism	pigment metabolic process	GO:0042440	P	99	2.32E-04	3.39E-03
11	5148	F3 vs. S3	+	Metabolism	Metabolism	polyamine biosynthetic process	GO:0006596	P	11	3.87E-03	3.43E-02
11	5149	F3 vs. S3	+	Metabolism	Metabolism	polygalacturonate 4-alpha-galacturonosyltransferase activity	GO:0047262	F	17	1.44E-03	2.48E-02
11	5150	F3 vs. S3	+	Metabolism	Metabolism	poly-pyrimidine tract binding	GO:0008187	F	25	7.05E-05	1.98E-03
11	5151	F3 vs. S3	+	Metabolism	Metabolism	primary alcohol metabolic process	GO:0034308	P	7	4.77E-03	4.06E-02
11	5152	F3 vs. S3	+	Metabolism	Metabolism	proton export across plasma membrane	GO:0120029	P	8	2.68E-05	5.08E-04
11	5153	F3 vs. S3	+	Metabolism	Metabolism	purine nucleoside bisphosphate metabolic process	GO:0034032	P	50	2.43E-03	2.40E-02
11	5154	F3 vs. S3	+	Metabolism	Metabolism	purine nucleotide metabolic process	GO:0006163	P	149	1.21E-04	1.92E-03
11	5155	F3 vs. S3	+	Metabolism	Metabolism	purine ribonucleotide metabolic process	GO:0009150	P	139	1.92E-04	2.90E-03
11	5156	F3 vs. S3	+	Metabolism	Metabolism	purine-containing compound metabolic process	GO:0072521	P	160	7.73E-05	1.30E-03
11	5157	F3 vs. S3	+	Metabolism	Metabolism	pyridoxal phosphate binding	GO:0030170	F	70	1.03E-06	4.39E-05
11	5158	F3 vs. S3	+	Metabolism	Metabolism	quercetin 3-O-glucosyltransferase activity	GO:0080043	F	46	7.82E-10	6.13E-08
11	5159	F3 vs. S3	+	Metabolism	Metabolism	quercetin 7-O-glucosyltransferase activity	GO:0080044	F	41	1.98E-09	1.51E-07
11	5160	F3 vs. S3	+	Metabolism	Metabolism	secondary alcohol metabolic process	GO:1902652	P	10	8.70E-05	1.43E-03
11	5161	F3 vs. S3	+	Metabolism	Metabolism	secondary metabolic process	GO:0019748	P	197	1.43E-09	7.79E-08

11	5162	F3 vs. S3	+	Metabolism	Metabolism	secondary metabolite biosynthetic process	GO:0044550	P	105	1.12E-09	6.16E-08
11	5163	F3 vs. S3	+	Metabolism	Metabolism	serine family amino acid biosynthetic process	GO:0009070	P	30	1.75E-05	3.57E-04
11	5164	F3 vs. S3	+	Metabolism	Metabolism	serine family amino acid metabolic process	GO:0009069	P	40	1.04E-06	3.20E-05
11	5165	F3 vs. S3	+	Metabolism	Metabolism	S-glycoside biosynthetic process	GO:0016144	P	29	5.67E-06	1.41E-04
11	5166	F3 vs. S3	+	Metabolism	Metabolism	small molecule binding	GO:0036094	F	850	4.24E-06	1.54E-04
11	5167	F3 vs. S3	+	Metabolism	Metabolism	small molecule biosynthetic process	GO:0044283	P	458	0.00E+00	0.00E+00
11	5168	F3 vs. S3	+	Metabolism	Metabolism	small molecule metabolic process	GO:0044281	P	1055	0.00E+00	0.00E+00
11	5169	F3 vs. S3	+	Metabolism	Metabolism	sulfur amino acid metabolic process	GO:0000096	P	35	2.39E-04	3.48E-03
11	5170	F3 vs. S3	+	Metabolism	Metabolism	sulfur compound biosynthetic process	GO:0044272	P	104	2.92E-13	2.98E-11
11	5171	F3 vs. S3	+	Metabolism	Metabolism	sulfur compound metabolic process	GO:0006790	P	244	1.61E-12	1.41E-10
11	5172	F3 vs. S3	+	Metabolism	Metabolism	terpenoid metabolic process	GO:0006721	P	101	3.99E-03	3.53E-02
11	5173	F3 vs. S3	+	Metabolism	Metabolism	tetrapyrrole binding	GO:0046906	F	219	1.50E-07	8.55E-06
11	5174	F3 vs. S3	+	Metabolism	Metabolism	tetrapyrrole metabolic process	GO:0033013	P	59	4.11E-03	3.60E-02
11	5175	F3 vs. S3	+	Metabolism	Metabolism	thioester metabolic process	GO:0035383	P	36	1.62E-03	1.73E-02
11	5176	F3 vs. S3	+	Metabolism	Metabolism	transaminase activity	GO:0008483	F	39	1.26E-03	2.22E-02
11	5177	F3 vs. S3	+	Metabolism	Metabolism	transferase activity	GO:0016740	F	2083	3.41E-12	3.80E-10
11	5178	F3 vs. S3	+	Metabolism	Metabolism	transferase activity, transferring acyl groups	GO:0016746	F	248	8.77E-05	2.33E-03
11	5179	F3 vs. S3	+	Metabolism	Metabolism	transferase activity, transferring acyl groups other than amino-acyl groups	GO:0016747	F	221	1.65E-03	2.77E-02
11	5180	F3 vs. S3	+	Metabolism	Metabolism	transferase activity, transferring acyl groups, acyl groups converted into alkyl on transfer	GO:0046912	F	15	1.75E-03	2.89E-02
11	5181	F3 vs. S3	+	Metabolism	Metabolism	transferase activity, transferring alkyl or aryl (other than methyl) groups	GO:0016765	F	88	1.74E-06	7.23E-05
11	5182	F3 vs. S3	+	Metabolism	Metabolism	transferase activity, transferring glycosyl groups	GO:0016757	F	389	1.27E-11	1.18E-09
11	5183	F3 vs. S3	+	Metabolism	Metabolism	transferase activity, transferring hexosyl groups	GO:0016758	F	247	3.37E-12	3.91E-10
11	5184	F3 vs. S3	+	Metabolism	Metabolism	transferase activity, transferring nitrogenous groups	GO:0016769	F	40	7.17E-04	1.35E-02
11	5185	F3 vs. S3	+	Metabolism	Metabolism	transition metal ion binding	GO:0046914	F	740	4.92E-07	2.38E-05
11	5186	F3 vs. S3	+	Metabolism	Metabolism	tyrosine metabolic process	GO:0006570	P	11	1.71E-03	1.80E-02
11	5187	F3 vs. S3	+	Metabolism	Metabolism	UDP-arabinose 4-epimerase activity	GO:0050373	F	6	3.32E-04	7.14E-03
11	5188	F3 vs. S3	+	Metabolism	Metabolism	UDP-glucose 4-epimerase activity	GO:0003978	F	9	1.12E-04	2.94E-03
11	5189	F3 vs. S3	+	Metabolism	Metabolism	UDP-glucosyltransferase activity	GO:0035251	F	105	4.13E-13	6.66E-11
11	5190	F3 vs. S3	+	Metabolism	Metabolism	UDP-glycosyltransferase activity	GO:0008194	F	190	3.33E-16	9.66E-14
11	5191	F3 vs. S3	+	Metabolism	Metabolism	UDP-L-arabinose biosynthetic process	GO:0033358	P	6	3.32E-04	4.73E-03
11	5192	F3 vs. S3	+	Metabolism	Metabolism	UDP-L-arabinose metabolic process	GO:0033356	P	11	9.62E-06	2.18E-04
11	5193	F3 vs. S3	+	Metabolism	Metabolism	vitamin B6 binding	GO:0070279	F	70	1.03E-06	4.45E-05
11	5194	F3 vs. S3	+	Metabolism	Metabolism	vitamin binding	GO:0019842	F	90	3.06E-05	9.64E-04
11	5195	F3 vs. S3	+	Metabolism	Metabolism	xyloglucan metabolic process	GO:0010411	P	43	1.11E-03	1.29E-02
11	5196	F3 vs. S3	+	Metabolism	Metabolism	xyloglucan:xyloglucosyl transferase activity	GO:0016762	F	25	3.09E-03	4.77E-02
11	5197	F3 vs. S3	+	Metabolism	Photosynthesis	carbon fixation	GO:0015977	P	15	1.56E-06	4.52E-05
11	5198	F3 vs. S3	+	Metabolism	Photosynthesis	chlorophyll binding	GO:0016168	F	21	1.54E-05	5.27E-04
11	5199	F3 vs. S3	+	Metabolism	Photosynthesis	chlorophyll metabolic process	GO:0015994	P	48	2.37E-03	2.35E-02
11	5200	F3 vs. S3	+	Metabolism	Photosynthesis	chloroplast	GO:0009507	C	3158	0.00E+00	0.00E+00
11	5201	F3 vs. S3	+	Metabolism	Photosynthesis	chloroplast envelope	GO:0009941	C	470	4.38E-13	1.24E-11
11	5202	F3 vs. S3	+	Metabolism	Photosynthesis	chloroplast stroma	GO:0009570	C	537	0.00E+00	0.00E+00
11	5203	F3 vs. S3	+	Metabolism	Photosynthesis	chloroplast thylakoid	GO:0009534	C	313	0.00E+00	0.00E+00
11	5204	F3 vs. S3	+	Metabolism	Photosynthesis	chloroplast thylakoid membrane	GO:0009535	C	252	0.00E+00	0.00E+00
11	5205	F3 vs. S3	+	Metabolism	Photosynthesis	electron transporter, transferring electrons within the cyclic electron transport pathway of photosynthesis activity	GO:0045156	F	8	5.33E-04	1.07E-02
11	5206	F3 vs. S3	+	Metabolism	Photosynthesis	energy quenching	GO:1990066	P	11	5.78E-03	4.74E-02
11	5207	F3 vs. S3	+	Metabolism	Photosynthesis	fructose 1,6-bisphosphate metabolic process	GO:0030388	P	12	1.46E-04	2.27E-03
11	5208	F3 vs. S3	+	Metabolism	Photosynthesis	fructose-bisphosphate aldolase activity	GO:0004332	F	8	7.44E-04	1.39E-02
11	5209	F3 vs. S3	+	Metabolism	Photosynthesis	negative regulation of photosynthesis	GO:1905156	P	12	5.92E-03	4.83E-02
11	5210	F3 vs. S3	+	Metabolism	Photosynthesis	oxidative photosynthetic carbon pathway	GO:0009854	P	5	3.56E-04	4.92E-03
11	5211	F3 vs. S3	+	Metabolism	Photosynthesis	photosynthesis	GO:0015979	P	141	1.57E-12	1.40E-10
11	5212	F3 vs. S3	+	Metabolism	Photosynthesis	photosynthesis, dark reaction	GO:0019685	P	15	1.30E-06	3.89E-05
11	5213	F3 vs. S3	+	Metabolism	Photosynthesis	photosynthesis, light harvesting	GO:0009765	P	32	4.89E-04	6.45E-03
11	5214	F3 vs. S3	+	Metabolism	Photosynthesis	photosynthesis, light harvesting in photosystem I	GO:0009768	P	18	1.29E-03	1.47E-02
11	5215	F3 vs. S3	+	Metabolism	Photosynthesis	photosynthesis, light reaction	GO:0019684	P	92	8.91E-09	4.06E-07
11	5216	F3 vs. S3	+	Metabolism	Photosynthesis	photosynthetic electron transport chain	GO:0009767	P	33	3.56E-04	4.91E-03
11	5217	F3 vs. S3	+	Metabolism	Photosynthesis	photosynthetic membrane	GO:0034357	C	278	1.11E-16	4.24E-15
11	5218	F3 vs. S3	+	Metabolism	Photosynthesis	photosystem	GO:0009521	C	42	4.31E-04	3.31E-03
11	5219	F3 vs. S3	+	Metabolism	Photosynthesis	photosystem II	GO:0009523	C	29	6.25E-04	4.63E-03
11	5220	F3 vs. S3	+	Metabolism	Photosynthesis	plastid	GO:0009536	C	3456	3.22E-14	1.03E-12
11	5221	F3 vs. S3	+	Metabolism	Photosynthesis	plastid envelope	GO:0009526	C	490	5.47E-14	1.70E-12

11	5222	F3 vs. S3	+	Metabolism	Photosynthesis	plastid membrane organization	GO:0009668	P	36	3.92E-03	3.47E-02
11	5223	F3 vs. S3	+	Metabolism	Photosynthesis	plastid stroma	GO:0009532	C	545	0.00E+00	0.00E+00
11	5224	F3 vs. S3	+	Metabolism	Photosynthesis	plastid thylakoid	GO:0031976	C	315	0.00E+00	0.00E+00
11	5225	F3 vs. S3	+	Metabolism	Photosynthesis	plastid thylakoid membrane	GO:0055035	C	255	0.00E+00	0.00E+00
11	5226	F3 vs. S3	+	Metabolism	Photosynthesis	plastoglobule	GO:0010287	C	55	1.05E-10	2.41E-09
11	5227	F3 vs. S3	+	Metabolism	Photosynthesis	regulation of photosynthesis	GO:0010109	P	32	7.46E-05	1.26E-03
11	5228	F3 vs. S3	+	Metabolism	Photosynthesis	regulation of photosynthesis, light reaction	GO:0042548	P	17	1.33E-03	1.49E-02
11	5229	F3 vs. S3	+	Metabolism	Photosynthesis	terpenoid biosynthetic process	GO:0016114	P	82	3.40E-03	3.07E-02
11	5230	F3 vs. S3	+	Metabolism	Photosynthesis	thylakoid	GO:0009579	C	383	0.00E+00	0.00E+00
11	5231	F3 vs. S3	+	Metabolism	Photosynthesis	thylakoid lumen	GO:0031977	C	44	5.07E-05	5.35E-04
11	5232	F3 vs. S3	+	Metabolism	Photosynthesis	thylakoid membrane	GO:0042651	C	275	0.00E+00	0.00E+00
11	5233	F3 vs. S3	+	Metabolism	Photosynthesis	thylakoid membrane organization	GO:0010027	P	34	4.32E-03	3.75E-02
11	5234	F3 vs. S3	+	Metabolism	Transporters	active ion transmembrane transporter activity	GO:0022853	F	160	5.49E-13	7.58E-11
11	5235	F3 vs. S3	+	Metabolism	Transporters	active transmembrane transporter activity	GO:0022804	F	350	0.00E+00	0.00E+00
11	5236	F3 vs. S3	+	Metabolism	Transporters	amino acid transmembrane transport	GO:0003333	P	19	4.43E-05	7.97E-04
11	5237	F3 vs. S3	+	Metabolism	Transporters	amino acid transmembrane transporter activity	GO:0015171	F	62	3.57E-07	1.79E-05
11	5238	F3 vs. S3	+	Metabolism	Transporters	amino acid transport	GO:0006865	P	37	5.04E-06	1.27E-04
11	5239	F3 vs. S3	+	Metabolism	Transporters	anion transmembrane transporter activity	GO:0008509	F	255	5.56E-08	3.43E-06
11	5240	F3 vs. S3	+	Metabolism	Transporters	anion transport	GO:0006820	P	193	1.15E-05	2.53E-04
11	5241	F3 vs. S3	+	Metabolism	Transporters	antiporter activity	GO:0015297	F	144	1.07E-10	9.36E-09
11	5242	F3 vs. S3	+	Metabolism	Transporters	ATPase-coupled cation transmembrane transporter activity	GO:0019829	F	46	6.70E-07	3.09E-05
11	5243	F3 vs. S3	+	Metabolism	Transporters	ATPase-coupled ion transmembrane transporter activity	GO:0042625	F	58	3.69E-06	1.39E-04
11	5244	F3 vs. S3	+	Metabolism	Transporters	ATPase-coupled transmembrane transporter activity	GO:0042626	F	140	2.86E-07	1.46E-05
11	5245	F3 vs. S3	+	Metabolism	Transporters	calcium ion transmembrane transport	GO:0070588	P	24	2.04E-03	2.09E-02
11	5246	F3 vs. S3	+	Metabolism	Transporters	calcium ion transport	GO:0006816	P	41	4.44E-03	3.83E-02
11	5247	F3 vs. S3	+	Metabolism	Transporters	carbohydrate transmembrane transport	GO:0034219	P	25	2.93E-03	2.73E-02
11	5248	F3 vs. S3	+	Metabolism	Transporters	carbohydrate transmembrane transporter activity	GO:0015144	F	79	7.88E-08	4.66E-06
11	5249	F3 vs. S3	+	Metabolism	Transporters	carbohydrate transport	GO:0008643	P	51	1.50E-04	2.31E-03
11	5250	F3 vs. S3	+	Metabolism	Transporters	carboxylic acid transmembrane transport	GO:1905039	P	40	4.24E-03	3.69E-02
11	5251	F3 vs. S3	+	Metabolism	Transporters	carboxylic acid transmembrane transporter activity	GO:0046943	F	99	1.20E-08	8.48E-07
11	5252	F3 vs. S3	+	Metabolism	Transporters	carboxylic acid transport	GO:0046942	P	82	3.00E-05	5.57E-04
11	5253	F3 vs. S3	+	Metabolism	Transporters	cation transmembrane transport	GO:0098655	P	149	5.05E-10	2.98E-08
11	5254	F3 vs. S3	+	Metabolism	Transporters	cation transmembrane transporter activity	GO:0008324	F	289	4.60E-13	7.01E-11
11	5255	F3 vs. S3	+	Metabolism	Transporters	cation transport	GO:0006812	P	254	1.22E-11	9.78E-10
11	5256	F3 vs. S3	+	Metabolism	Transporters	cation:cation antiporter activity	GO:0015491	F	41	5.72E-05	1.66E-03
11	5257	F3 vs. S3	+	Metabolism	Transporters	cytoplasmic vesicle	GO:0031410	C	501	1.73E-03	1.12E-02
11	5258	F3 vs. S3	+	Metabolism	Transporters	disaccharide transport	GO:0015766	P	19	2.50E-03	2.43E-02
11	5259	F3 vs. S3	+	Metabolism	Transporters	divalent inorganic cation transmembrane transporter activity	GO:0072509	F	61	1.81E-03	2.95E-02
11	5260	F3 vs. S3	+	Metabolism	Transporters	divalent inorganic cation transport	GO:0072511	P	78	6.74E-04	8.48E-03
11	5261	F3 vs. S3	+	Metabolism	Transporters	divalent metal ion transport	GO:0070838	P	76	7.85E-04	9.57E-03
11	5262	F3 vs. S3	+	Metabolism	Transporters	endosome	GO:0005768	C	287	1.63E-03	1.07E-02
11	5263	F3 vs. S3	+	Metabolism	Transporters	export across plasma membrane	GO:0140115	P	12	2.21E-05	4.30E-04
11	5264	F3 vs. S3	+	Metabolism	Transporters	export from cell	GO:0140352	P	64	8.46E-06	1.98E-04
11	5265	F3 vs. S3	+	Metabolism	Transporters	inorganic anion transport	GO:0015698	P	70	3.34E-03	3.03E-02
11	5266	F3 vs. S3	+	Metabolism	Transporters	inorganic cation transmembrane transport	GO:0098662	P	143	1.96E-10	1.27E-08
11	5267	F3 vs. S3	+	Metabolism	Transporters	inorganic cation transmembrane transporter activity	GO:0022890	F	261	4.46E-12	4.62E-10
11	5268	F3 vs. S3	+	Metabolism	Transporters	inorganic ion transmembrane transport	GO:0098660	P	160	4.09E-10	2.44E-08
11	5269	F3 vs. S3	+	Metabolism	Transporters	inorganic molecular entity transmembrane transporter activity	GO:0015318	F	472	0.00E+00	0.00E+00
11	5270	F3 vs. S3	+	Metabolism	Transporters	ion transmembrane transport	GO:0034220	P	221	1.23E-10	8.33E-09
11	5271	F3 vs. S3	+	Metabolism	Transporters	ion transmembrane transporter activity	GO:0015075	F	512	8.88E-16	2.34E-13
11	5272	F3 vs. S3	+	Metabolism	Transporters	ion transmembrane transporter activity, phosphorylative mechanism	GO:0015662	F	35	1.39E-03	2.41E-02
11	5273	F3 vs. S3	+	Metabolism	Transporters	ion transport	GO:0006811	P	430	2.95E-14	3.31E-12
11	5274	F3 vs. S3	+	Metabolism	Transporters	L-alpha-amino acid transmembrane transport	GO:1902475	P	5	5.24E-03	4.38E-02
11	5275	F3 vs. S3	+	Metabolism	Transporters	L-amino acid transmembrane transporter activity	GO:0015179	F	17	3.06E-03	4.74E-02
11	5276	F3 vs. S3	+	Metabolism	Transporters	metal ion transmembrane transporter activity	GO:0046873	F	157	4.30E-06	1.52E-04
11	5277	F3 vs. S3	+	Metabolism	Transporters	metal ion transport	GO:0030001	P	158	5.12E-05	9.08E-04
11	5278	F3 vs. S3	+	Metabolism	Transporters	monosaccharide transmembrane transport	GO:0015749	P	14	5.88E-03	4.80E-02
11	5279	F3 vs. S3	+	Metabolism	Transporters	monovalent inorganic cation transmembrane transporter activity	GO:0015077	F	148	3.09E-12	3.74E-10
11	5280	F3 vs. S3	+	Metabolism	Transporters	monovalent inorganic cation transport	GO:0015672	P	104	2.52E-11	1.81E-09
11	5281	F3 vs. S3	+	Metabolism	Transporters	neutral amino acid transmembrane transporter activity	GO:0015175	F	15	1.48E-04	3.81E-03
11	5282	F3 vs. S3	+	Metabolism	Transporters	neutral amino acid transport	GO:0015804	P	9	2.64E-03	2.54E-02
11	5283	F3 vs. S3	+	Metabolism	Transporters	organic acid transmembrane transporter activity	GO:0005342	F	99	1.20E-08	8.27E-07

11	5284	F3 vs. S3	+	Metabolism	Transporters	organic acid transport	GO:0015849	P	84	5.88E-05	1.03E-03
11	5285	F3 vs. S3	+	Metabolism	Transporters	organic anion transmembrane transporter activity	GO:0008514	F	160	2.74E-07	1.42E-05
11	5286	F3 vs. S3	+	Metabolism	Transporters	organic anion transport	GO:0015711	P	119	1.00E-04	1.62E-03
11	5287	F3 vs. S3	+	Metabolism	Transporters	primary active transmembrane transporter activity	GO:0015399	F	159	2.85E-06	1.10E-04
11	5288	F3 vs. S3	+	Metabolism	Transporters	proton transmembrane transporter activity	GO:0015078	F	116	5.78E-12	5.78E-10
11	5289	F3 vs. S3	+	Metabolism	Transporters	secondary active transmembrane transporter activity	GO:0015291	F	189	1.11E-13	2.02E-11
11	5290	F3 vs. S3	+	Metabolism	Transporters	solute:cation antiporter activity	GO:0015298	F	43	2.21E-04	5.12E-03
11	5291	F3 vs. S3	+	Metabolism	Transporters	solute:cation symporter activity	GO:0015294	F	25	4.00E-04	8.53E-03
11	5292	F3 vs. S3	+	Metabolism	Transporters	solute:proton antiporter activity	GO:0015299	F	36	2.55E-04	5.77E-03
11	5293	F3 vs. S3	+	Metabolism	Transporters	solute:proton symporter activity	GO:0015295	F	22	2.16E-04	5.05E-03
11	5294	F3 vs. S3	+	Metabolism	Transporters	sucrose transmembrane transporter activity	GO:0008515	F	17	2.68E-03	4.18E-02
11	5295	F3 vs. S3	+	Metabolism	Transporters	sucrose transport	GO:0015770	P	19	2.50E-03	2.43E-02
11	5296	F3 vs. S3	+	Metabolism	Transporters	sugar transmembrane transporter activity	GO:0051119	F	34	6.92E-04	1.31E-02
11	5297	F3 vs. S3	+	Metabolism	Transporters	symporter activity	GO:0015293	F	25	4.00E-04	8.47E-03
11	5298	F3 vs. S3	+	Metabolism	Transporters	trans-Golgi network	GO:0005802	C	219	5.55E-06	6.88E-05
11	5299	F3 vs. S3	+	Metabolism	Transporters	transition metal ion transport	GO:0000041	P	51	4.15E-03	3.63E-02
11	5300	F3 vs. S3	+	Metabolism	Transporters	transmembrane transport	GO:0055085	P	574	0.00E+00	0.00E+00
11	5301	F3 vs. S3	+	Metabolism	Transporters	transmembrane transporter activity	GO:0022857	F	852	0.00E+00	0.00E+00
11	5302	F3 vs. S3	+	Metabolism	Transporters	transport	GO:0006810	P	1482	2.53E-06	6.82E-05
11	5303	F3 vs. S3	+	Metabolism	Transporters	transporter activity	GO:0005215	F	880	0.00E+00	0.00E+00
11	5304	F3 vs. S3	+	Metabolism	Transporters	vesicle	GO:0031982	C	544	7.87E-04	5.74E-03
11	5305	F3 vs. S3	+	Metabolism	Transporters	xenobiotic transmembrane transporter activity	GO:0042910	F	47	2.51E-06	1.01E-04
11	5306	F3 vs. S3	+	Regulation	Protein modification	Golgi apparatus	GO:0005794	C	812	1.15E-08	2.32E-07
11	5307	F3 vs. S3	+	Regulation	Regulation	regulation of biological quality	GO:0065008	P	632	2.50E-03	2.44E-02
11	5308	F3 vs. S3	+	Regulation	Regulation	regulation of cell morphogenesis involved in differentiation	GO:0010769	P	32	1.19E-07	4.37E-06
11	5309	F3 vs. S3	+	Regulation	Regulation	regulation of pollen tube growth	GO:0080092	P	29	4.86E-08	1.85E-06
11	5310	F3 vs. S3	+	Regulation	Regulation	regulation of unidimensional cell growth	GO:0051510	P	57	1.52E-03	1.64E-02
11	5311	F3 vs. S3	+	Regulation	Translation	poly(U) RNA binding	GO:0008266	F	22	7.14E-05	1.99E-03
11	5312	F3 vs. S3	+	Signaling and response	Signaling	calcium ion binding	GO:0005509	F	155	6.60E-06	2.28E-04
11	5313	F3 vs. S3	+	Signaling and response	Signaling	cellular response to external stimulus	GO:0071496	P	144	3.24E-04	4.63E-03
11	5314	F3 vs. S3	+	Signaling and response	Signaling	cellular response to extracellular stimulus	GO:0031668	P	139	8.16E-05	1.36E-03
11	5315	F3 vs. S3	+	Signaling and response	Signaling	cellular response to nutrient levels	GO:0031669	P	111	1.27E-04	2.00E-03
11	5316	F3 vs. S3	+	Signaling and response	Signaling	dephosphorylation	GO:0016311	P	128	1.13E-04	1.80E-03
11	5317	F3 vs. S3	+	Signaling and response	Signaling	jasmonic acid biosynthetic process	GO:0009695	P	22	1.49E-03	1.62E-02
11	5318	F3 vs. S3	+	Signaling and response	Signaling	multi-organism process	GO:0051704	P	294	1.10E-04	1.75E-03
11	5319	F3 vs. S3	+	Signaling and response	Signaling	phosphatase activity	GO:0016791	F	207	2.61E-06	1.04E-04
11	5320	F3 vs. S3	+	Signaling and response	Signaling	protein dephosphorylation	GO:0006470	P	81	9.38E-04	1.12E-02
11	5321	F3 vs. S3	+	Signaling and response	Signaling	protein serine/threonine phosphatase activity	GO:0004722	F	73	2.25E-03	3.54E-02
11	5322	F3 vs. S3	+	Signaling and response	Signaling	response to abiotic stimulus	GO:0009628	P	1424	1.53E-10	1.02E-08
11	5323	F3 vs. S3	+	Signaling and response	Signaling	response to acid chemical	GO:0001101	P	276	1.50E-04	2.31E-03
11	5324	F3 vs. S3	+	Signaling and response	Signaling	response to bacterium	GO:0009617	P	335	4.21E-07	1.40E-05
11	5325	F3 vs. S3	+	Signaling and response	Signaling	response to biotic stimulus	GO:0009607	P	733	9.67E-09	4.31E-07
11	5326	F3 vs. S3	+	Signaling and response	Signaling	response to cadmium ion	GO:0046686	P	241	5.86E-09	2.74E-07
11	5327	F3 vs. S3	+	Signaling and response	Signaling	response to chemical	GO:0042221	P	1750	4.61E-13	4.45E-11
11	5328	F3 vs. S3	+	Signaling and response	Signaling	response to chitin	GO:0010200	P	93	1.09E-06	3.34E-05
11	5329	F3 vs. S3	+	Signaling and response	Signaling	response to endogenous stimulus	GO:0009719	P	803	1.84E-03	1.93E-02
11	5330	F3 vs. S3	+	Signaling and response	Signaling	response to external biotic stimulus	GO:0043207	P	732	1.14E-08	4.99E-07
11	5331	F3 vs. S3	+	Signaling and response	Signaling	response to external stimulus	GO:0009605	P	1004	3.26E-11	2.31E-09
11	5332	F3 vs. S3	+	Signaling and response	Signaling	response to extracellular stimulus	GO:0009991	P	178	2.81E-05	5.27E-04
11	5333	F3 vs. S3	+	Signaling and response	Signaling	response to hormone	GO:0009725	P	782	1.87E-03	1.94E-02
11	5334	F3 vs. S3	+	Signaling and response	Signaling	response to inorganic substance	GO:0010035	P	644	1.00E-11	8.27E-10
11	5335	F3 vs. S3	+	Signaling and response	Signaling	response to jasmonic acid	GO:0009753	P	115	1.24E-05	2.72E-04
11	5336	F3 vs. S3	+	Signaling and response	Signaling	response to karrikin	GO:0080167	P	99	2.09E-06	5.85E-05
11	5337	F3 vs. S3	+	Signaling and response	Signaling	response to light intensity	GO:0009642	P	101	1.35E-03	1.50E-02
11	5338	F3 vs. S3	+	Signaling and response	Signaling	response to light stimulus	GO:0009416	P	491	1.29E-06	3.88E-05
11	5339	F3 vs. S3	+	Signaling and response	Signaling	response to lipid	GO:0033993	P	440	5.61E-03	4.65E-02
11	5340	F3 vs. S3	+	Signaling and response	Signaling	response to metal ion	GO:0010038	P	318	2.48E-08	1.03E-06
11	5341	F3 vs. S3	+	Signaling and response	Signaling	response to nitrogen compound	GO:1901698	P	193	2.20E-04	3.25E-03
11	5342	F3 vs. S3	+	Signaling and response	Signaling	response to nutrient levels	GO:0031667	P	149	6.09E-05	1.06E-03
11	5343	F3 vs. S3	+	Signaling and response	Signaling	response to organic substance	GO:0010033	P	1087	1.82E-05	3.69E-04
11	5344	F3 vs. S3	+	Signaling and response	Signaling	response to organonitrogen compound	GO:0010243	P	162	5.09E-04	6.65E-03
11	5345	F3 vs. S3	+	Signaling and response	Signaling	response to oxygen-containing compound	GO:1901700	P	1021	2.00E-10	1.27E-08
11	5346	F3 vs. S3	+	Signaling and response	Signaling	response to stimulus	GO:0050896	P	3525	4.14E-09	2.06E-07
11	5347	F3 vs. S3	+	Signaling and response	Signaling	response to temperature stimulus	GO:0009266	P	411	3.35E-08	1.34E-06

11	5348	F3 vs. S3	+	Signaling and response	Signaling	response to water	GO:0009415	P	259	7.17E-05	1.23E-03
11	5349	F3 vs. S3	+	Signaling and response	Stress	aging	GO:0007568	P	92	7.02E-04	8.69E-03
11	5350	F3 vs. S3	+	Signaling and response	Stress	cation homeostasis	GO:0055080	P	151	4.68E-05	8.34E-04
11	5351	F3 vs. S3	+	Signaling and response	Stress	cellular cation homeostasis	GO:0030003	P	107	5.00E-03	4.20E-02
11	5352	F3 vs. S3	+	Signaling and response	Stress	cellular detoxification	GO:1990748	P	30	3.12E-03	2.86E-02
11	5353	F3 vs. S3	+	Signaling and response	Stress	cellular homeostasis	GO:0019725	P	186	6.02E-03	4.90E-02
11	5354	F3 vs. S3	+	Signaling and response	Stress	cellular ion homeostasis	GO:0006873	P	112	3.37E-03	3.04E-02
11	5355	F3 vs. S3	+	Signaling and response	Stress	cellular oxidant detoxification	GO:0098869	P	19	3.47E-03	3.12E-02
11	5356	F3 vs. S3	+	Signaling and response	Stress	cellular response to phosphate starvation	GO:0016036	P	55	1.19E-03	1.36E-02
11	5357	F3 vs. S3	+	Signaling and response	Stress	cellular response to starvation	GO:0009267	P	101	6.89E-04	8.57E-03
11	5358	F3 vs. S3	+	Signaling and response	Stress	cellular response to toxic substance	GO:0097237	P	31	2.81E-03	2.64E-02
11	5359	F3 vs. S3	+	Signaling and response	Stress	chemical homeostasis	GO:0048878	P	265	3.90E-06	1.00E-04
11	5360	F3 vs. S3	+	Signaling and response	Stress	defense response	GO:0006952	P	611	5.63E-06	1.40E-04
11	5361	F3 vs. S3	+	Signaling and response	Stress	defense response to bacterium	GO:0042742	P	276	9.72E-07	3.01E-05
11	5362	F3 vs. S3	+	Signaling and response	Stress	defense response to Gram-negative bacterium	GO:0050829	P	18	3.08E-03	2.84E-02
11	5363	F3 vs. S3	+	Signaling and response	Stress	defense response to other organism	GO:0098542	P	527	9.47E-06	2.16E-04
11	5364	F3 vs. S3	+	Signaling and response	Stress	detoxification	GO:0098754	P	65	1.20E-07	4.38E-06
11	5365	F3 vs. S3	+	Signaling and response	Stress	flavonoid biosynthetic process	GO:0009813	P	32	7.91E-05	1.32E-03
11	5366	F3 vs. S3	+	Signaling and response	Stress	flavonoid metabolic process	GO:0009812	P	36	1.61E-04	2.46E-03
11	5367	F3 vs. S3	+	Signaling and response	Stress	glutathione binding	GO:0043295	F	14	2.00E-03	3.22E-02
11	5368	F3 vs. S3	+	Signaling and response	Stress	glutathione metabolic process	GO:0006749	P	28	1.22E-03	1.39E-02
11	5369	F3 vs. S3	+	Signaling and response	Stress	glutathione transferase activity	GO:0004364	F	35	3.31E-04	7.17E-03
11	5370	F3 vs. S3	+	Signaling and response	Stress	homeostatic process	GO:0042592	P	344	5.21E-05	9.22E-04
11	5371	F3 vs. S3	+	Signaling and response	Stress	inorganic ion homeostasis	GO:0098771	P	165	1.93E-05	3.88E-04
11	5372	F3 vs. S3	+	Signaling and response	Stress	interspecies interaction between organisms	GO:0044419	P	745	2.06E-08	8.77E-07
11	5373	F3 vs. S3	+	Signaling and response	Stress	ion homeostasis	GO:0050801	P	189	9.37E-06	2.15E-04
11	5374	F3 vs. S3	+	Signaling and response	Stress	monovalent inorganic cation homeostasis	GO:0055067	P	51	4.80E-04	6.35E-03
11	5375	F3 vs. S3	+	Signaling and response	Stress	nonphotochemical quenching	GO:0010196	P	11	5.78E-03	4.73E-02
11	5376	F3 vs. S3	+	Signaling and response	Stress	reactive oxygen species metabolic process	GO:0072593	P	50	1.04E-04	1.67E-03
11	5377	F3 vs. S3	+	Signaling and response	Stress	regulation of pH	GO:0006885	P	35	6.01E-06	1.46E-04
11	5378	F3 vs. S3	+	Signaling and response	Stress	regulation of stomatal movement	GO:0010119	P	63	2.97E-03	2.76E-02
11	5379	F3 vs. S3	+	Signaling and response	Stress	response to abscisic acid	GO:0009737	P	324	2.41E-05	4.61E-04
11	5380	F3 vs. S3	+	Signaling and response	Stress	response to alcohol	GO:0097305	P	328	7.71E-05	1.29E-03
11	5381	F3 vs. S3	+	Signaling and response	Stress	response to cold	GO:0009409	P	298	2.49E-08	1.03E-06
11	5382	F3 vs. S3	+	Signaling and response	Stress	response to desiccation	GO:0009269	P	16	1.33E-03	1.49E-02
11	5383	F3 vs. S3	+	Signaling and response	Stress	response to heat	GO:0009408	P	131	2.74E-05	5.18E-04
11	5384	F3 vs. S3	+	Signaling and response	Stress	response to high light intensity	GO:0009644	P	46	6.14E-04	7.84E-03
11	5385	F3 vs. S3	+	Signaling and response	Stress	response to nematode	GO:0009624	P	65	5.76E-03	4.73E-02
11	5386	F3 vs. S3	+	Signaling and response	Stress	response to osmotic stress	GO:0006970	P	365	2.61E-03	2.52E-02
11	5387	F3 vs. S3	+	Signaling and response	Stress	response to other organism	GO:0051707	P	732	1.14E-08	4.95E-07
11	5388	F3 vs. S3	+	Signaling and response	Stress	response to oxidative stress	GO:0006979	P	319	2.29E-05	4.41E-04
11	5389	F3 vs. S3	+	Signaling and response	Stress	response to radiation	GO:0009314	P	506	6.49E-06	1.57E-04
11	5390	F3 vs. S3	+	Signaling and response	Stress	response to reactive oxygen species	GO:0000302	P	112	3.77E-03	3.36E-02
11	5391	F3 vs. S3	+	Signaling and response	Stress	response to starvation	GO:0042594	P	129	6.52E-04	8.27E-03
11	5392	F3 vs. S3	+	Signaling and response	Stress	response to stress	GO:0006950	P	2009	1.32E-06	3.94E-05
11	5393	F3 vs. S3	+	Signaling and response	Stress	response to toxic substance	GO:0009636	P	81	5.51E-09	2.62E-07
11	5394	F3 vs. S3	+	Signaling and response	Stress	response to water deprivation	GO:0009414	P	252	1.00E-04	1.62E-03
11	5395	F3 vs. S3	+	Signaling and response	Stress	response to wounding	GO:0009611	P	156	1.36E-08	5.81E-07
11	5396	F3 vs. S3	+	Signaling and response	Stress	toxin catabolic process	GO:0009407	P	26	1.50E-04	2.31E-03
11	5397	F3 vs. S3	+	Signaling and response	Stress	toxin metabolic process	GO:0009404	P	36	1.36E-05	2.92E-04
11	5398	F3 vs. S3	+	Signaling and response	Stress	transition metal ion homeostasis	GO:0055076	P	65	4.64E-03	3.96E-02
11	5399	F3 vs. S3	+	Signaling and response	Stress	vitamin biosynthetic process	GO:0009110	P	41	4.11E-03	3.60E-02
11	5400	F3 vs. S3	+	Signaling and response	Stress	vitamin metabolic process	GO:0006766	P	48	1.15E-03	1.33E-02
11	5401	F3 vs. S3	+	Signaling and response	Stress	water-soluble vitamin metabolic process	GO:0006767	P	40	2.03E-03	2.09E-02
11	5402	F3 vs. S3	-	Development	Cell division	asymmetric cell division	GO:0008356	P	16	1.29E-03	1.46E-02
11	5403	F3 vs. S3	-	Development	Cell division	ATP-dependent microtubule motor activity	GO:1990939	F	15	3.08E-05	9.60E-04
11	5404	F3 vs. S3	-	Development	Cell division	base-excision repair	GO:0006284	P	15	4.15E-04	5.60E-03
11	5405	F3 vs. S3	-	Development	Cell division	cell cycle	GO:0007049	P	287	0.00E+00	0.00E+00
11	5406	F3 vs. S3	-	Development	Cell division	cell cycle checkpoint	GO:0000075	P	24	6.21E-05	1.08E-03
11	5407	F3 vs. S3	-	Development	Cell division	cell cycle DNA replication	GO:0044786	P	24	8.49E-04	1.03E-02
11	5408	F3 vs. S3	-	Development	Cell division	cell cycle phase transition	GO:0044770	P	32	2.43E-06	6.59E-05
11	5409	F3 vs. S3	-	Development	Cell division	cell cycle process	GO:0022402	P	264	0.00E+00	0.00E+00
11	5410	F3 vs. S3	-	Development	Cell division	cell division	GO:0051301	P	148	7.19E-04	8.85E-03
11	5411	F3 vs. S3	-	Development	Cell division	chromatin assembly	GO:0031497	P	49	5.38E-06	1.35E-04

11	5412	F3 vs. S3	-	Development	Cell division	chromosome	GO:0005694	C	312	1.05E-13	3.05E-12
11	5413	F3 vs. S3	-	Development	Cell division	chromosome organization involved in meiotic cell cycle	GO:0070192	P	26	1.30E-03	1.47E-02
11	5414	F3 vs. S3	-	Development	Cell division	chromosome segregation	GO:0007059	P	61	1.27E-06	3.84E-05
11	5415	F3 vs. S3	-	Development	Cell division	chromosome separation	GO:0051304	P	10	5.17E-04	6.71E-03
11	5416	F3 vs. S3	-	Development	Cell division	chromosome, centromeric region	GO:0000775	C	24	3.78E-05	4.08E-04
11	5417	F3 vs. S3	-	Development	Cell division	condensed chromosome	GO:0000793	C	38	7.55E-05	7.34E-04
11	5418	F3 vs. S3	-	Development	Cell division	condensed nuclear chromosome	GO:0000794	C	24	2.40E-04	2.00E-03
11	5419	F3 vs. S3	-	Development	Cell division	condensin complex	GO:0000796	C	7	2.71E-03	1.66E-02
11	5420	F3 vs. S3	-	Development	Cell division	cyclin-dependent protein kinase holoenzyme complex	GO:0000307	C	40	8.69E-06	1.05E-04
11	5421	F3 vs. S3	-	Development	Cell division	cyclin-dependent protein serine/threonine kinase regulator activity	GO:0016538	F	34	2.11E-07	1.16E-05
11	5422	F3 vs. S3	-	Development	Cell division	cytokinesis by cell plate formation	GO:0000911	P	48	3.31E-06	8.63E-05
11	5423	F3 vs. S3	-	Development	Cell division	DNA helicase activity	GO:0003678	F	33	4.32E-04	9.02E-03
11	5424	F3 vs. S3	-	Development	Cell division	DNA integrity checkpoint	GO:0031570	P	15	4.94E-04	6.49E-03
11	5425	F3 vs. S3	-	Development	Cell division	DNA packaging	GO:0006323	P	59	4.21E-06	1.08E-04
11	5426	F3 vs. S3	-	Development	Cell division	DNA packaging complex	GO:0044815	C	18	2.02E-04	1.70E-03
11	5427	F3 vs. S3	-	Development	Cell division	DNA recombination	GO:0006310	P	84	4.74E-07	1.56E-05
11	5428	F3 vs. S3	-	Development	Cell division	DNA repair	GO:0006281	P	191	2.41E-12	2.05E-10
11	5429	F3 vs. S3	-	Development	Cell division	DNA replication	GO:0006260	P	76	2.05E-07	7.20E-06
11	5430	F3 vs. S3	-	Development	Cell division	DNA replication checkpoint	GO:0000076	P	11	4.31E-03	3.75E-02
11	5431	F3 vs. S3	-	Development	Cell division	DNA replication initiation	GO:0006270	P	16	5.60E-05	9.88E-04
11	5432	F3 vs. S3	-	Development	Cell division	DNA replication origin binding	GO:0003688	F	13	2.31E-04	5.28E-03
11	5433	F3 vs. S3	-	Development	Cell division	DNA-dependent DNA replication	GO:0006261	P	67	1.83E-07	6.47E-06
11	5434	F3 vs. S3	-	Development	Cell division	double-strand break repair	GO:0006302	P	76	1.75E-06	4.95E-05
11	5435	F3 vs. S3	-	Development	Cell division	double-strand break repair via homologous recombination	GO:0000724	P	52	7.09E-05	1.22E-03
11	5436	F3 vs. S3	-	Development	Cell division	homologous recombination	GO:0035825	P	29	2.71E-04	3.90E-03
11	5437	F3 vs. S3	-	Development	Cell division	kinetochore	GO:0000776	C	14	5.29E-04	4.01E-03
11	5438	F3 vs. S3	-	Development	Cell division	male meiotic nuclear division	GO:0007140	P	13	6.53E-04	8.27E-03
11	5439	F3 vs. S3	-	Development	Cell division	meiosis I	GO:0007127	P	38	7.38E-05	1.25E-03
11	5440	F3 vs. S3	-	Development	Cell division	meiosis I cell cycle process	GO:0061982	P	41	1.09E-05	2.42E-04
11	5441	F3 vs. S3	-	Development	Cell division	meiotic cell cycle	GO:0051321	P	90	1.05E-09	5.83E-08
11	5442	F3 vs. S3	-	Development	Cell division	meiotic cell cycle process	GO:1903046	P	80	3.05E-08	1.23E-06
11	5443	F3 vs. S3	-	Development	Cell division	meiotic chromosome segregation	GO:0045132	P	29	5.91E-04	7.62E-03
11	5444	F3 vs. S3	-	Development	Cell division	meiotic chromosome separation	GO:0051307	P	8	1.86E-03	1.94E-02
11	5445	F3 vs. S3	-	Development	Cell division	meiotic nuclear division	GO:0140013	P	52	3.60E-07	1.22E-05
11	5446	F3 vs. S3	-	Development	Cell division	microtubule cytoskeleton organization involved in mitosis	GO:1902850	P	20	5.93E-05	1.04E-03
11	5447	F3 vs. S3	-	Development	Cell division	microtubule motor activity	GO:0003777	F	45	4.06E-06	1.49E-04
11	5448	F3 vs. S3	-	Development	Cell division	mitotic cell cycle	GO:0000278	P	160	1.75E-11	1.33E-09
11	5449	F3 vs. S3	-	Development	Cell division	mitotic cell cycle checkpoint	GO:0007093	P	17	1.11E-03	1.29E-02
11	5450	F3 vs. S3	-	Development	Cell division	mitotic cell cycle phase transition	GO:0044772	P	32	2.43E-06	6.62E-05
11	5451	F3 vs. S3	-	Development	Cell division	mitotic cell cycle process	GO:1903047	P	132	1.20E-10	8.18E-09
11	5452	F3 vs. S3	-	Development	Cell division	mitotic nuclear division	GO:0140014	P	39	7.92E-06	1.87E-04
11	5453	F3 vs. S3	-	Development	Cell division	mitotic spindle	GO:0072686	C	13	5.08E-06	6.38E-05
11	5454	F3 vs. S3	-	Development	Cell division	mitotic spindle assembly	GO:0090307	P	7	4.11E-04	5.55E-03
11	5455	F3 vs. S3	-	Development	Cell division	mitotic spindle assembly checkpoint	GO:0007094	P	6	2.79E-03	2.67E-02
11	5456	F3 vs. S3	-	Development	Cell division	mitotic spindle checkpoint	GO:0071174	P	6	2.79E-03	2.65E-02
11	5457	F3 vs. S3	-	Development	Cell division	mitotic spindle organization	GO:0007052	P	17	7.48E-05	1.26E-03
11	5458	F3 vs. S3	-	Development	Cell division	negative regulation of cell cycle	GO:0045786	P	49	1.53E-05	3.20E-04
11	5459	F3 vs. S3	-	Development	Cell division	negative regulation of cell cycle phase transition	GO:1901988	P	13	1.40E-03	1.54E-02
11	5460	F3 vs. S3	-	Development	Cell division	negative regulation of cell cycle process	GO:0010948	P	30	1.32E-03	1.49E-02
11	5461	F3 vs. S3	-	Development	Cell division	negative regulation of chromosome organization	GO:2001251	P	7	2.77E-03	2.65E-02
11	5462	F3 vs. S3	-	Development	Cell division	negative regulation of chromosome segregation	GO:0051985	P	6	2.79E-03	2.66E-02
11	5463	F3 vs. S3	-	Development	Cell division	negative regulation of chromosome separation	GO:1905819	P	6	2.79E-03	2.62E-02
11	5464	F3 vs. S3	-	Development	Cell division	negative regulation of metaphase/anaphase transition of cell cycle	GO:1902100	P	6	2.79E-03	2.64E-02
11	5465	F3 vs. S3	-	Development	Cell division	negative regulation of mitotic cell cycle	GO:0045930	P	27	3.59E-03	3.21E-02
11	5466	F3 vs. S3	-	Development	Cell division	negative regulation of mitotic cell cycle phase transition	GO:1901991	P	13	1.40E-03	1.54E-02
11	5467	F3 vs. S3	-	Development	Cell division	negative regulation of mitotic metaphase/anaphase transition	GO:0045841	P	6	2.79E-03	2.63E-02
11	5468	F3 vs. S3	-	Development	Cell division	negative regulation of mitotic sister chromatid segregation	GO:0033048	P	6	2.79E-03	2.66E-02
11	5469	F3 vs. S3	-	Development	Cell division	negative regulation of mitotic sister chromatid separation	GO:2000816	P	6	2.79E-03	2.66E-02
11	5470	F3 vs. S3	-	Development	Cell division	negative regulation of sister chromatid segregation	GO:0033046	P	6	2.79E-03	2.65E-02
11	5471	F3 vs. S3	-	Development	Cell division	nuclear chromosome segregation	GO:0098813	P	55	5.81E-06	1.42E-04
11	5472	F3 vs. S3	-	Development	Cell division	nuclear division	GO:0000280	P	88	2.25E-12	1.95E-10
11	5473	F3 vs. S3	-	Development	Cell division	nuclear DNA replication	GO:0033260	P	13	3.33E-04	4.67E-03

11	5474	F3 vs. S3	-	Development	Cell division	nuclear replication fork	GO:0043596	C	17	1.64E-04	1.42E-03
11	5475	F3 vs. S3	-	Development	Cell division	nucleic acid metabolic process	GO:0090304	P	1040	0.00E+00	0.00E+00
11	5476	F3 vs. S3	-	Development	Cell division	nucleobase-containing compound metabolic process	GO:0006139	P	1305	7.85E-14	8.47E-12
11	5477	F3 vs. S3	-	Development	Cell division	nucleosome assembly	GO:0006334	P	31	9.82E-06	2.21E-04
11	5478	F3 vs. S3	-	Development	Cell division	preprophase band	GO:0009574	C	14	1.09E-03	7.83E-03
11	5479	F3 vs. S3	-	Development	Cell division	reciprocal homologous recombination	GO:0140527	P	29	2.71E-04	3.92E-03
11	5480	F3 vs. S3	-	Development	Cell division	reciprocal meiotic recombination	GO:0007131	P	29	2.71E-04	3.91E-03
11	5481	F3 vs. S3	-	Development	Cell division	recombinational repair	GO:0000725	P	56	2.45E-04	3.56E-03
11	5482	F3 vs. S3	-	Development	Cell division	regulation of cell cycle	GO:0051726	P	160	5.00E-15	6.23E-13
11	5483	F3 vs. S3	-	Development	Cell division	regulation of cell cycle phase transition	GO:1901987	P	36	4.17E-05	7.54E-04
11	5484	F3 vs. S3	-	Development	Cell division	regulation of cell cycle process	GO:0010564	P	82	3.03E-08	1.23E-06
11	5485	F3 vs. S3	-	Development	Cell division	regulation of cell division	GO:0051302	P	41	1.37E-04	2.14E-03
11	5486	F3 vs. S3	-	Development	Cell division	regulation of cell population proliferation	GO:0042127	P	42	1.42E-03	1.55E-02
11	5487	F3 vs. S3	-	Development	Cell division	regulation of chromatin organization	GO:1902275	P	41	1.56E-03	1.67E-02
11	5488	F3 vs. S3	-	Development	Cell division	regulation of chromosome organization	GO:0033044	P	46	7.60E-06	1.81E-04
11	5489	F3 vs. S3	-	Development	Cell division	regulation of chromosome segregation	GO:0051983	P	17	7.06E-04	8.72E-03
11	5490	F3 vs. S3	-	Development	Cell division	regulation of chromosome separation	GO:1905818	P	16	1.38E-03	1.53E-02
11	5491	F3 vs. S3	-	Development	Cell division	regulation of cyclin-dependent protein kinase activity	GO:1904029	P	38	1.56E-05	3.24E-04
11	5492	F3 vs. S3	-	Development	Cell division	regulation of DNA metabolic process	GO:0051052	P	56	3.03E-07	1.04E-05
11	5493	F3 vs. S3	-	Development	Cell division	regulation of DNA recombination	GO:0000018	P	18	1.88E-03	1.95E-02
11	5494	F3 vs. S3	-	Development	Cell division	regulation of double-strand break repair	GO:2000779	P	6	3.10E-03	2.85E-02
11	5495	F3 vs. S3	-	Development	Cell division	regulation of meiotic cell cycle	GO:0051445	P	12	8.26E-04	1.00E-02
11	5496	F3 vs. S3	-	Development	Cell division	regulation of metaphase/anaphase transition of cell cycle	GO:1902099	P	16	1.38E-03	1.53E-02
11	5497	F3 vs. S3	-	Development	Cell division	regulation of mitotic cell cycle	GO:0007346	P	56	9.66E-06	2.18E-04
11	5498	F3 vs. S3	-	Development	Cell division	regulation of mitotic cell cycle phase transition	GO:1901990	P	34	1.58E-04	2.42E-03
11	5499	F3 vs. S3	-	Development	Cell division	regulation of mitotic metaphase/anaphase transition	GO:0030071	P	16	1.38E-03	1.53E-02
11	5500	F3 vs. S3	-	Development	Cell division	regulation of mitotic sister chromatid segregation	GO:0033047	P	7	6.54E-04	8.26E-03
11	5501	F3 vs. S3	-	Development	Cell division	regulation of mitotic sister chromatid separation	GO:0010965	P	16	1.38E-03	1.54E-02
11	5502	F3 vs. S3	-	Development	Cell division	regulation of mitotic spindle organization	GO:0060236	P	5	3.33E-03	3.03E-02
11	5503	F3 vs. S3	-	Development	Cell division	regulation of nuclear division	GO:0051783	P	33	1.01E-03	1.20E-02
11	5504	F3 vs. S3	-	Development	Cell division	regulation of sister chromatid segregation	GO:0033045	P	17	7.06E-04	8.70E-03
11	5505	F3 vs. S3	-	Development	Cell division	regulation of spindle organization	GO:0090224	P	5	3.33E-03	3.02E-02
11	5506	F3 vs. S3	-	Development	Cell division	replication fork	GO:0005657	C	28	3.25E-06	4.25E-05
11	5507	F3 vs. S3	-	Development	Cell division	replisome	GO:0030894	C	14	1.28E-03	8.68E-03
11	5508	F3 vs. S3	-	Development	Cell division	resolution of meiotic recombination intermediates	GO:0000712	P	7	3.44E-03	3.10E-02
11	5509	F3 vs. S3	-	Development	Cell division	RNA metabolic process	GO:0016070	P	803	0.00E+00	0.00E+00
11	5510	F3 vs. S3	-	Development	Cell division	single-stranded DNA binding	GO:0003697	F	57	2.07E-05	6.83E-04
11	5511	F3 vs. S3	-	Development	Cell division	sister chromatid segregation	GO:0000819	P	36	5.00E-04	6.55E-03
11	5512	F3 vs. S3	-	Development	Cell division	spindle	GO:0005819	C	54	2.21E-12	6.09E-11
11	5513	F3 vs. S3	-	Development	Cell division	spindle assembly	GO:0051225	P	28	7.91E-07	2.52E-05
11	5514	F3 vs. S3	-	Development	Cell division	spindle assembly checkpoint	GO:0071173	P	6	2.79E-03	2.64E-02
11	5515	F3 vs. S3	-	Development	Cell division	spindle checkpoint	GO:0031577	P	6	2.79E-03	2.63E-02
11	5516	F3 vs. S3	-	Development	Cell division	spindle microtubule	GO:0005876	C	15	1.39E-04	1.24E-03
11	5517	F3 vs. S3	-	Development	Cell division	spindle organization	GO:0007051	P	39	3.77E-08	1.46E-06
11	5518	F3 vs. S3	-	Development	Cell division	spindle pole	GO:0000922	C	5	2.32E-03	1.44E-02
11	5519	F3 vs. S3	-	Development	Development	adaxial/abaxial pattern specification	GO:0009955	P	32	7.25E-04	8.89E-03
11	5520	F3 vs. S3	-	Development	Development	carpel development	GO:0048440	P	59	1.21E-04	1.92E-03
11	5521	F3 vs. S3	-	Development	Development	cell fate commitment	GO:0045165	P	40	2.44E-03	2.39E-02
11	5522	F3 vs. S3	-	Development	Development	cell plate assembly	GO:0000919	P	9	4.74E-03	4.03E-02
11	5523	F3 vs. S3	-	Development	Development	cellular component assembly	GO:0022607	P	566	1.53E-12	1.39E-10
11	5524	F3 vs. S3	-	Development	Development	cellular component biogenesis	GO:0044085	P	893	2.14E-09	1.14E-07
11	5525	F3 vs. S3	-	Development	Development	cellular component organization	GO:0016043	P	1586	1.11E-08	4.89E-07
11	5526	F3 vs. S3	-	Development	Development	cellular component organization or biogenesis	GO:0071840	P	1856	2.13E-09	1.15E-07
11	5527	F3 vs. S3	-	Development	Development	cellular localization	GO:0051641	P	641	2.26E-03	2.27E-02
11	5528	F3 vs. S3	-	Development	Development	chromatin	GO:0000785	C	202	6.31E-05	6.38E-04
11	5529	F3 vs. S3	-	Development	Development	chromatin assembly or disassembly	GO:0006333	P	56	3.47E-07	1.18E-05
11	5530	F3 vs. S3	-	Development	Development	chromatin organization	GO:0006325	P	211	3.26E-13	3.26E-11
11	5531	F3 vs. S3	-	Development	Development	chromosomal region	GO:0098687	C	33	2.56E-06	3.53E-05
11	5532	F3 vs. S3	-	Development	Development	chromosome organization	GO:0051276	P	303	0.00E+00	0.00E+00
11	5533	F3 vs. S3	-	Development	Development	cortical cytoskeleton	GO:0030863	C	32	1.90E-04	1.63E-03
11	5534	F3 vs. S3	-	Development	Development	cortical cytoskeleton organization	GO:0030865	P	44	6.83E-05	1.18E-03
11	5535	F3 vs. S3	-	Development	Development	cortical microtubule	GO:0055028	C	30	1.53E-04	1.34E-03
11	5536	F3 vs. S3	-	Development	Development	cortical microtubule cytoskeleton	GO:0030981	C	31	6.23E-05	6.37E-04
11	5537	F3 vs. S3	-	Development	Development	cortical microtubule organization	GO:0043622	P	32	2.35E-03	2.35E-02

11	5538	F3 vs. S3	-	Development	Development	cytoplasmic microtubule	GO:0005881	C	36	7.13E-05	7.07E-04
11	5539	F3 vs. S3	-	Development	Development	cytoplasmic microtubule organization	GO:0031122	P	40	8.50E-04	1.03E-02
11	5540	F3 vs. S3	-	Development	Development	cytoskeletal protein binding	GO:0008092	F	206	6.11E-04	1.20E-02
11	5541	F3 vs. S3	-	Development	Development	cytoskeleton	GO:0005856	C	206	3.89E-07	6.04E-06
11	5542	F3 vs. S3	-	Development	Development	determination of bilateral symmetry	GO:0009855	P	6	3.64E-03	3.25E-02
11	5543	F3 vs. S3	-	Development	Development	embryo development	GO:0009790	P	349	7.49E-04	9.18E-03
11	5544	F3 vs. S3	-	Development	Development	embryo development ending in seed dormancy	GO:0009793	P	335	6.10E-04	7.83E-03
11	5545	F3 vs. S3	-	Development	Development	equatorial microtubule organizing center	GO:0000923	C	4	8.56E-03	4.33E-02
11	5546	F3 vs. S3	-	Development	Development	floral organ development	GO:0048437	P	178	1.28E-04	2.01E-03
11	5547	F3 vs. S3	-	Development	Development	floral whorl development	GO:0048438	P	142	3.83E-05	6.99E-04
11	5548	F3 vs. S3	-	Development	Development	flower development	GO:0009908	P	262	6.60E-05	1.14E-03
11	5549	F3 vs. S3	-	Development	Development	gamma-tubulin complex	GO:0000930	C	7	2.22E-03	1.39E-02
11	5550	F3 vs. S3	-	Development	Development	GPI anchor metabolic process	GO:0006505	P	19	5.22E-03	4.37E-02
11	5551	F3 vs. S3	-	Development	Development	guard cell differentiation	GO:0010052	P	11	4.96E-03	4.19E-02
11	5552	F3 vs. S3	-	Development	Development	gynoecium development	GO:0048467	P	68	2.77E-05	5.22E-04
11	5553	F3 vs. S3	-	Development	Development	host cell	GO:0043657	C	88	2.84E-04	2.33E-03
11	5554	F3 vs. S3	-	Development	Development	host cell nucleus	GO:0042025	C	88	2.84E-04	2.29E-03
11	5555	F3 vs. S3	-	Development	Development	host cell part	GO:0033643	C	88	2.84E-04	2.22E-03
11	5556	F3 vs. S3	-	Development	Development	host cellular component	GO:0018995	C	88	2.84E-04	2.35E-03
11	5557	F3 vs. S3	-	Development	Development	host intracellular membrane-bounded organelle	GO:0033648	C	88	2.84E-04	2.27E-03
11	5558	F3 vs. S3	-	Development	Development	host intracellular organelle	GO:0033647	C	88	2.84E-04	2.25E-03
11	5559	F3 vs. S3	-	Development	Development	host intracellular part	GO:0033646	C	88	2.84E-04	2.24E-03
11	5560	F3 vs. S3	-	Development	Development	host intracellular region	GO:0043656	C	88	2.84E-04	2.31E-03
11	5561	F3 vs. S3	-	Development	Development	intracellular membrane-bounded organelle	GO:0043231	C	10668	3.75E-03	2.24E-02
11	5562	F3 vs. S3	-	Development	Development	intracellular non-membrane-bounded organelle	GO:0043232	C	1145	0.00E+00	0.00E+00
11	5563	F3 vs. S3	-	Development	Development	intracellular organelle	GO:0043229	C	10881	1.12E-03	7.94E-03
11	5564	F3 vs. S3	-	Development	Development	intracellular organelle lumen	GO:0070013	C	868	0.00E+00	0.00E+00
11	5565	F3 vs. S3	-	Development	Development	kinetochore microtubule	GO:0005828	C	5	2.33E-03	1.44E-02
11	5566	F3 vs. S3	-	Development	Development	leaf morphogenesis	GO:0009965	P	69	4.98E-03	4.20E-02
11	5567	F3 vs. S3	-	Development	Development	membrane-bounded organelle	GO:0043227	C	10757	6.48E-03	3.47E-02
11	5568	F3 vs. S3	-	Development	Development	membrane-enclosed lumen	GO:0031974	C	868	0.00E+00	0.00E+00
11	5569	F3 vs. S3	-	Development	Development	meristem development	GO:0048507	P	137	4.92E-04	6.48E-03
11	5570	F3 vs. S3	-	Development	Development	microtubule	GO:0005874	C	102	1.31E-10	2.95E-09
11	5571	F3 vs. S3	-	Development	Development	microtubule associated complex	GO:0005875	C	52	4.80E-08	8.98E-07
11	5572	F3 vs. S3	-	Development	Development	microtubule cytoskeleton	GO:0015630	C	148	5.77E-15	1.91E-13
11	5573	F3 vs. S3	-	Development	Development	microtubule cytoskeleton organization	GO:0000226	P	113	6.22E-08	2.36E-06
11	5574	F3 vs. S3	-	Development	Development	microtubule nucleation	GO:0007020	P	9	2.03E-03	2.09E-02
11	5575	F3 vs. S3	-	Development	Development	microtubule nucleation by interphase microtubule organizing center	GO:0051415	P	6	1.03E-03	1.21E-02
11	5576	F3 vs. S3	-	Development	Development	microtubule nucleation by microtubule organizing center	GO:0051418	P	6	1.03E-03	1.21E-02
11	5577	F3 vs. S3	-	Development	Development	microtubule organizing center	GO:0005815	C	15	4.08E-03	2.40E-02
11	5578	F3 vs. S3	-	Development	Development	microtubule polymerization	GO:0046785	P	12	7.66E-05	1.29E-03
11	5579	F3 vs. S3	-	Development	Development	microtubule-based movement	GO:0007018	P	46	4.90E-06	1.25E-04
11	5580	F3 vs. S3	-	Development	Development	microtubule-based process	GO:0007017	P	158	2.54E-13	2.64E-11
11	5581	F3 vs. S3	-	Development	Development	movement of cell or subcellular component	GO:0006928	P	65	2.09E-03	2.12E-02
11	5582	F3 vs. S3	-	Development	Development	non-membrane-bounded organelle	GO:0043228	C	1145	0.00E+00	0.00E+00
11	5583	F3 vs. S3	-	Development	Development	nuclear body	GO:0016604	C	81	7.37E-05	7.23E-04
11	5584	F3 vs. S3	-	Development	Development	nuclear chromatin	GO:0000790	C	157	6.27E-03	3.40E-02
11	5585	F3 vs. S3	-	Development	Development	nuclear chromosome	GO:0000228	C	209	1.82E-07	2.96E-06
11	5586	F3 vs. S3	-	Development	Development	nuclear envelope	GO:0005635	C	67	2.15E-03	1.36E-02
11	5587	F3 vs. S3	-	Development	Development	nuclear lumen	GO:0031981	C	761	0.00E+00	0.00E+00
11	5588	F3 vs. S3	-	Development	Development	nuclear outer membrane-endoplasmic reticulum membrane network	GO:0042175	C	200	9.74E-03	4.76E-02
11	5589	F3 vs. S3	-	Development	Development	nuclear pore	GO:0005643	C	31	3.26E-03	1.99E-02
11	5590	F3 vs. S3	-	Development	Development	nuclear replisome	GO:0043601	C	14	1.28E-03	8.74E-03
11	5591	F3 vs. S3	-	Development	Development	nucleic acid phosphodiester bond hydrolysis	GO:0090305	P	52	6.29E-04	8.02E-03
11	5592	F3 vs. S3	-	Development	Development	nucleoplasm	GO:0005654	C	286	6.57E-14	1.98E-12
11	5593	F3 vs. S3	-	Development	Development	nucleus	GO:0005634	C	5749	0.00E+00	0.00E+00
11	5594	F3 vs. S3	-	Development	Development	organelle	GO:0043226	C	10889	1.24E-03	8.53E-03
11	5595	F3 vs. S3	-	Development	Development	organelle assembly	GO:0070925	P	136	0.00E+00	0.00E+00
11	5596	F3 vs. S3	-	Development	Development	organelle fission	GO:0048285	P	118	1.87E-11	1.38E-09
11	5597	F3 vs. S3	-	Development	Development	organelle lumen	GO:0043233	C	868	0.00E+00	0.00E+00
11	5598	F3 vs. S3	-	Development	Development	organelle organization	GO:0006996	P	1094	0.00E+00	0.00E+00
11	5599	F3 vs. S3	-	Development	Development	pattern specification process	GO:0007389	P	132	9.84E-06	2.21E-04

11	5600	F3 vs. S3	-	Development	Development	phragmoplast	GO:0009524	C	47	2.73E-06	3.72E-05
11	5601	F3 vs. S3	-	Development	Development	phyllome development	GO:0048827	P	355	4.52E-03	3.88E-02
11	5602	F3 vs. S3	-	Development	Development	plant epidermis morphogenesis	GO:0090626	P	55	1.80E-03	1.89E-02
11	5603	F3 vs. S3	-	Development	Development	plant ovule development	GO:0048481	P	46	1.98E-04	2.97E-03
11	5604	F3 vs. S3	-	Development	Development	plant-type ovary development	GO:0035670	P	46	1.98E-04	2.98E-03
11	5605	F3 vs. S3	-	Development	Development	polymeric cytoskeletal fiber	GO:0099513	C	111	7.00E-08	1.22E-06
11	5606	F3 vs. S3	-	Development	Development	post-embryonic development	GO:0009791	P	974	4.62E-03	3.94E-02
11	5607	F3 vs. S3	-	Development	Development	radial pattern formation	GO:0009956	P	16	1.18E-03	1.35E-02
11	5608	F3 vs. S3	-	Development	Development	regionalization	GO:0003002	P	109	9.55E-07	2.97E-05
11	5609	F3 vs. S3	-	Development	Development	reproductive shoot system development	GO:0090567	P	275	2.58E-05	4.93E-04
11	5610	F3 vs. S3	-	Development	Development	reproductive structure development	GO:0048608	P	790	2.25E-03	2.26E-02
11	5611	F3 vs. S3	-	Development	Development	reproductive system development	GO:0061458	P	791	2.41E-03	2.38E-02
11	5612	F3 vs. S3	-	Development	Development	shoot system development	GO:0048367	P	498	1.33E-04	2.08E-03
11	5613	F3 vs. S3	-	Development	Development	shoot system morphogenesis	GO:0010016	P	120	1.46E-03	1.59E-02
11	5614	F3 vs. S3	-	Development	Development	specification of symmetry	GO:0009799	P	7	3.17E-03	2.90E-02
11	5615	F3 vs. S3	-	Development	Development	stomatal complex development	GO:0010374	P	37	1.52E-03	1.64E-02
11	5616	F3 vs. S3	-	Development	Development	stomatal complex morphogenesis	GO:0010103	P	19	1.65E-03	1.75E-02
11	5617	F3 vs. S3	-	Development	Development	structural constituent of cell wall	GO:0005199	F	21	2.44E-05	7.85E-04
11	5618	F3 vs. S3	-	Development	Development	structural molecule activity	GO:0005198	F	319	0.00E+00	0.00E+00
11	5619	F3 vs. S3	-	Development	Development	tissue development	GO:0009888	P	387	5.06E-03	4.25E-02
11	5620	F3 vs. S3	-	Metabolism	Bioenergetics	ATP-dependent microtubule motor activity, plus-end-directed	GO:0008574	F	14	9.78E-05	2.58E-03
11	5621	F3 vs. S3	-	Metabolism	Bioenergetics	DNA-dependent ATPase activity	GO:0008094	F	67	4.54E-05	1.34E-03
11	5622	F3 vs. S3	-	Metabolism	Bioenergetics	inner mitochondrial membrane protein complex	GO:0098800	C	108	1.44E-03	9.47E-03
11	5623	F3 vs. S3	-	Metabolism	Bioenergetics	mitochondrial protein complex	GO:0098798	C	158	2.21E-06	3.09E-05
11	5624	F3 vs. S3	-	Metabolism	Bioenergetics	mitochondrial respirasome	GO:0005746	C	73	1.36E-03	8.97E-03
11	5625	F3 vs. S3	-	Metabolism	Bioenergetics	mitochondrial respiratory chain complex I	GO:0005747	C	44	6.57E-03	3.50E-02
11	5626	F3 vs. S3	-	Metabolism	Bioenergetics	mitochondrion organization	GO:0007005	P	123	1.30E-03	1.47E-02
11	5627	F3 vs. S3	-	Metabolism	Bioenergetics	respirasome	GO:0070469	C	76	8.61E-03	4.34E-02
11	5628	F3 vs. S3	-	Metabolism	Bioenergetics	respiratory chain complex	GO:0098803	C	73	4.29E-03	2.48E-02
11	5629	F3 vs. S3	-	Metabolism	Catabolism	modification-dependent macromolecule catabolic process	GO:0043632	P	314	4.88E-03	4.14E-02
11	5630	F3 vs. S3	-	Metabolism	Catabolism	peptidase complex	GO:1905368	C	60	1.02E-02	4.95E-02
11	5631	F3 vs. S3	-	Metabolism	Catabolism	protein-containing complex	GO:0032991	C	1905	0.00E+00	0.00E+00
11	5632	F3 vs. S3	-	Metabolism	Catabolism	ubiquitin conjugating enzyme activity	GO:0061631	F	30	3.09E-04	6.74E-03
11	5633	F3 vs. S3	-	Metabolism	Catabolism	ubiquitin-like protein conjugating enzyme activity	GO:0061650	F	31	3.07E-04	6.74E-03
11	5634	F3 vs. S3	-	Metabolism	Metabolism	acetyltransferase complex	GO:1902493	C	32	7.11E-03	3.69E-02
11	5635	F3 vs. S3	-	Metabolism	Metabolism	amide biosynthetic process	GO:0043604	P	415	0.00E+00	0.00E+00
11	5636	F3 vs. S3	-	Metabolism	Metabolism	binding	GO:0005488	F	6104	7.55E-04	1.40E-02
11	5637	F3 vs. S3	-	Metabolism	Metabolism	catalytic activity, acting on DNA	GO:0140097	F	110	3.19E-06	1.22E-04
11	5638	F3 vs. S3	-	Metabolism	Metabolism	catalytic complex	GO:1902494	C	721	6.40E-11	1.51E-09
11	5639	F3 vs. S3	-	Metabolism	Metabolism	cellular amide metabolic process	GO:0043603	P	504	5.97E-14	6.57E-12
11	5640	F3 vs. S3	-	Metabolism	Metabolism	cellular aromatic compound metabolic process	GO:0006725	P	1594	2.22E-04	3.28E-03
11	5641	F3 vs. S3	-	Metabolism	Metabolism	cellular macromolecule biosynthetic process	GO:0034645	P	846	0.00E+00	0.00E+00
11	5642	F3 vs. S3	-	Metabolism	Metabolism	cellular macromolecule metabolic process	GO:0044260	P	2704	9.54E-09	4.32E-07
11	5643	F3 vs. S3	-	Metabolism	Metabolism	cellular nitrogen compound biosynthetic process	GO:0044271	P	836	9.51E-10	5.39E-08
11	5644	F3 vs. S3	-	Metabolism	Metabolism	cellular nitrogen compound metabolic process	GO:0034641	P	1844	0.00E+00	0.00E+00
11	5645	F3 vs. S3	-	Metabolism	Metabolism	cellular protein localization	GO:0034613	P	450	3.19E-03	2.92E-02
11	5646	F3 vs. S3	-	Metabolism	Metabolism	cellular protein metabolic process	GO:0044267	P	2073	1.99E-05	3.94E-04
11	5647	F3 vs. S3	-	Metabolism	Metabolism	cellular protein-containing complex assembly	GO:0034622	P	333	1.59E-14	1.89E-12
11	5648	F3 vs. S3	-	Metabolism	Metabolism	chromatin binding	GO:0003682	F	107	2.07E-04	4.96E-03
11	5649	F3 vs. S3	-	Metabolism	Metabolism	DNA binding	GO:0003677	F	905	1.22E-15	2.95E-13
11	5650	F3 vs. S3	-	Metabolism	Metabolism	DNA metabolic process	GO:0006259	P	256	1.11E-15	1.48E-13
11	5651	F3 vs. S3	-	Metabolism	Metabolism	DNA N-glycosylase activity	GO:0019104	F	10	3.31E-03	4.99E-02
11	5652	F3 vs. S3	-	Metabolism	Metabolism	DNA secondary structure binding	GO:0000217	F	24	3.11E-03	4.78E-02
11	5653	F3 vs. S3	-	Metabolism	Metabolism	double-stranded DNA binding	GO:0003690	F	465	1.01E-06	4.43E-05
11	5654	F3 vs. S3	-	Metabolism	Metabolism	GPI anchor biosynthetic process	GO:0006506	P	19	5.22E-03	4.37E-02
11	5655	F3 vs. S3	-	Metabolism	Metabolism	heterocycle metabolic process	GO:0046483	P	1498	2.16E-07	7.48E-06
11	5656	F3 vs. S3	-	Metabolism	Metabolism	heterocyclic compound binding	GO:1901363	F	2891	2.41E-08	1.59E-06
11	5657	F3 vs. S3	-	Metabolism	Metabolism	macromolecule biosynthetic process	GO:0009059	P	888	0.00E+00	0.00E+00
11	5658	F3 vs. S3	-	Metabolism	Metabolism	macromolecule metabolic process	GO:0043170	P	3386	3.20E-10	2.01E-08
11	5659	F3 vs. S3	-	Metabolism	Metabolism	microtubule binding	GO:0008017	F	109	2.19E-10	1.87E-08
11	5660	F3 vs. S3	-	Metabolism	Metabolism	microtubule minus-end binding	GO:0051011	F	8	2.52E-03	3.94E-02
11	5661	F3 vs. S3	-	Metabolism	Metabolism	microtubule polymerization or depolymerization	GO:0031109	P	15	2.13E-05	4.20E-04
11	5662	F3 vs. S3	-	Metabolism	Metabolism	motor activity	GO:0003774	F	60	8.13E-04	1.49E-02
11	5663	F3 vs. S3	-	Metabolism	Metabolism	nitrogen compound metabolic process	GO:0006807	P	3812	2.45E-03	2.39E-02

11	5664	F3 vs. S3	-	Metabolism	Metabolism	nucleic acid binding	GO:0003676	F	2011	0.00E+00	0.00E+00
11	5665	F3 vs. S3	-	Metabolism	Metabolism	organic cyclic compound binding	GO:0097159	F	2911	1.59E-08	1.07E-06
11	5666	F3 vs. S3	-	Metabolism	Metabolism	organic cyclic compound metabolic process	GO:1901360	P	1653	3.51E-04	4.87E-03
11	5667	F3 vs. S3	-	Metabolism	Metabolism	peptide metabolic process	GO:0006518	P	410	0.00E+00	0.00E+00
11	5668	F3 vs. S3	-	Metabolism	Metabolism	protein binding	GO:0005515	F	3507	4.95E-05	1.45E-03
11	5669	F3 vs. S3	-	Metabolism	Metabolism	protein metabolic process	GO:0019538	P	2159	2.27E-04	3.33E-03
11	5670	F3 vs. S3	-	Metabolism	Metabolism	pseudouridine synthesis	GO:0001522	P	18	1.85E-03	1.94E-02
11	5671	F3 vs. S3	-	Metabolism	Metabolism	Sm-like protein family complex	GO:0120114	C	59	2.89E-10	6.24E-09
11	5672	F3 vs. S3	-	Metabolism	Metabolism	supramolecular complex	GO:0099080	C	169	1.65E-07	2.72E-06
11	5673	F3 vs. S3	-	Metabolism	Metabolism	supramolecular fiber	GO:0099512	C	111	7.00E-08	1.20E-06
11	5674	F3 vs. S3	-	Metabolism	Metabolism	supramolecular polymer	GO:0099081	C	111	7.00E-08	1.24E-06
11	5675	F3 vs. S3	-	Metabolism	Metabolism	transferase complex	GO:1990234	C	423	1.78E-06	2.53E-05
11	5676	F3 vs. S3	-	Metabolism	Metabolism	transferase complex, transferring phosphorus-containing groups	GO:0061695	C	136	4.18E-08	8.14E-07
11	5677	F3 vs. S3	-	Metabolism	Metabolism	tubulin binding	GO:0015631	F	117	8.55E-11	7.75E-09
11	5678	F3 vs. S3	-	Metabolism	Photosynthesis	plastid ribosome	GO:0009547	C	18	7.13E-05	7.14E-04
11	5679	F3 vs. S3	-	Metabolism	Photosynthesis	plastid small ribosomal subunit	GO:0000312	C	10	1.86E-03	1.20E-02
11	5680	F3 vs. S3	-	Metabolism	Transporters	cellular macromolecule localization	GO:0070727	P	475	2.21E-03	2.23E-02
11	5681	F3 vs. S3	-	Metabolism	Transporters	kinesin complex	GO:0005871	C	25	5.87E-05	6.07E-04
11	5682	F3 vs. S3	-	Metabolism	Transporters	nuclear export	GO:0051168	P	46	8.94E-04	1.07E-02
11	5683	F3 vs. S3	-	Metabolism	Transporters	nuclear microtubule	GO:0005880	C	8	8.35E-03	4.27E-02
11	5684	F3 vs. S3	-	Metabolism	Transporters	nuclear transport	GO:0051169	P	78	8.87E-04	1.07E-02
11	5685	F3 vs. S3	-	Metabolism	Transporters	nucleic acid transport	GO:0050657	P	48	3.37E-04	4.71E-03
11	5686	F3 vs. S3	-	Metabolism	Transporters	nucleocytoplasmic transport	GO:0006913	P	78	8.87E-04	1.07E-02
11	5687	F3 vs. S3	-	Metabolism	Transporters	protein export from nucleus	GO:0006611	P	42	2.50E-03	2.43E-02
11	5688	F3 vs. S3	-	Metabolism	Transporters	protein localization	GO:0008104	P	501	2.04E-03	2.09E-02
11	5689	F3 vs. S3	-	Metabolism	Transporters	protein localization to organelle	GO:0033365	P	217	1.13E-03	1.31E-02
11	5690	F3 vs. S3	-	Metabolism	Transporters	RNA transport	GO:0050658	P	48	3.37E-04	4.72E-03
11	5691	F3 vs. S3	-	Regulation	Protein modification	methylation	GO:0032259	P	141	1.21E-04	1.92E-03
11	5692	F3 vs. S3	-	Regulation	Protein modification	positive regulation of histone H3-K27 methylation	GO:0061087	P	6	5.56E-03	4.61E-02
11	5693	F3 vs. S3	-	Regulation	Protein modification	positive regulation of kinase activity	GO:0033674	P	13	1.14E-03	1.31E-02
11	5694	F3 vs. S3	-	Regulation	Protein modification	prefoldin complex	GO:0016272	C	7	8.29E-03	4.26E-02
11	5695	F3 vs. S3	-	Regulation	Protein modification	protein alkylation	GO:0008213	P	58	3.72E-04	5.08E-03
11	5696	F3 vs. S3	-	Regulation	Protein modification	protein dimerization activity	GO:0046983	F	357	4.72E-04	9.50E-03
11	5697	F3 vs. S3	-	Regulation	Protein modification	protein heterodimerization activity	GO:0046982	F	79	3.70E-06	1.37E-04
11	5698	F3 vs. S3	-	Regulation	Protein modification	protein methylation	GO:0006479	P	58	3.72E-04	5.09E-03
11	5699	F3 vs. S3	-	Regulation	Protein modification	regulation of catalytic activity	GO:0050790	P	181	1.84E-04	2.80E-03
11	5700	F3 vs. S3	-	Regulation	Protein modification	regulation of histone methylation	GO:0031060	P	21	5.75E-03	4.74E-02
11	5701	F3 vs. S3	-	Regulation	Protein modification	regulation of protein metabolic process	GO:0051246	P	252	7.52E-06	1.80E-04
11	5702	F3 vs. S3	-	Regulation	Protein modification	peptidyl-lysine modification	GO:0018205	P	84	6.81E-04	8.50E-03
11	5703	F3 vs. S3	-	Regulation	Protein modification	positive regulation of protein modification process	GO:0031401	P	38	4.60E-04	6.13E-03
11	5704	F3 vs. S3	-	Regulation	Protein modification	protein-containing complex assembly	GO:0065003	P	372	1.76E-11	1.31E-09
11	5705	F3 vs. S3	-	Regulation	Protein modification	protein-containing complex localization	GO:0031503	P	48	6.11E-04	7.83E-03
11	5706	F3 vs. S3	-	Regulation	Protein modification	protein-containing complex subunit organization	GO:0043933	P	419	3.86E-11	2.67E-09
11	5707	F3 vs. S3	-	Regulation	Protein modification	regulation of protein modification process	GO:0031399	P	123	5.96E-07	1.93E-05
11	5708	F3 vs. S3	-	Regulation	Regulation	biological regulation	GO:0065007	P	3498	1.05E-05	2.36E-04
11	5709	F3 vs. S3	-	Regulation	Regulation	molecular function regulator	GO:0098772	F	1291	5.50E-10	4.56E-08
11	5710	F3 vs. S3	-	Regulation	Regulation	negative regulation of biological process	GO:0048519	P	712	3.90E-07	1.31E-05
11	5711	F3 vs. S3	-	Regulation	Regulation	negative regulation of biosynthetic process	GO:0009890	P	222	3.16E-05	5.81E-04
11	5712	F3 vs. S3	-	Regulation	Regulation	negative regulation of cellular biosynthetic process	GO:0031327	P	216	1.47E-05	3.10E-04
11	5713	F3 vs. S3	-	Regulation	Regulation	negative regulation of cellular macromolecule biosynthetic process	GO:2000113	P	203	7.93E-06	1.86E-04
11	5714	F3 vs. S3	-	Regulation	Regulation	negative regulation of cellular metabolic process	GO:0031324	P	290	8.77E-06	2.04E-04
11	5715	F3 vs. S3	-	Regulation	Regulation	negative regulation of cellular process	GO:0048523	P	473	6.57E-07	2.12E-05
11	5716	F3 vs. S3	-	Regulation	Regulation	negative regulation of DNA metabolic process	GO:0051053	P	14	3.79E-03	3.36E-02
11	5717	F3 vs. S3	-	Regulation	Regulation	negative regulation of macromolecule biosynthetic process	GO:0010558	P	205	6.89E-06	1.66E-04
11	5718	F3 vs. S3	-	Regulation	Regulation	negative regulation of macromolecule metabolic process	GO:0010605	P	370	6.87E-10	3.93E-08
11	5719	F3 vs. S3	-	Regulation	Regulation	negative regulation of metabolic process	GO:0009892	P	406	1.16E-08	4.99E-07
11	5720	F3 vs. S3	-	Regulation	Regulation	negative regulation of nitrogen compound metabolic process	GO:0051172	P	254	1.71E-06	4.86E-05
11	5721	F3 vs. S3	-	Regulation	Regulation	negative regulation of nucleobase-containing compound metabolic process	GO:0045934	P	185	1.49E-06	4.38E-05
11	5722	F3 vs. S3	-	Regulation	Regulation	positive regulation of biological process	GO:0048518	P	787	6.66E-04	8.39E-03
11	5723	F3 vs. S3	-	Regulation	Regulation	positive regulation of biosynthetic process	GO:0009891	P	363	2.01E-03	2.07E-02
11	5724	F3 vs. S3	-	Regulation	Regulation	positive regulation of cellular biosynthetic process	GO:0031328	P	354	2.05E-03	2.09E-02

11	5725	F3 vs. S3	-	Regulation	Regulation	positive regulation of cellular metabolic process	GO:0031325	P	472	1.23E-04	1.94E-03
11	5726	F3 vs. S3	-	Regulation	Regulation	positive regulation of cellular process	GO:0048522	P	636	4.40E-04	5.89E-03
11	5727	F3 vs. S3	-	Regulation	Regulation	positive regulation of macromolecule biosynthetic process	GO:0010557	P	328	2.24E-04	3.30E-03
11	5728	F3 vs. S3	-	Regulation	Regulation	positive regulation of macromolecule metabolic process	GO:0010604	P	449	5.87E-06	1.43E-04
11	5729	F3 vs. S3	-	Regulation	Regulation	positive regulation of metabolic process	GO:0009893	P	508	8.29E-05	1.38E-03
11	5730	F3 vs. S3	-	Regulation	Regulation	positive regulation of nitrogen compound metabolic process	GO:0051173	P	430	9.13E-05	1.48E-03
11	5731	F3 vs. S3	-	Regulation	Regulation	positive regulation of nucleobase-containing compound metabolic process	GO:0045935	P	323	4.34E-04	5.84E-03
11	5732	F3 vs. S3	-	Regulation	Regulation	regulation of biological process	GO:0050789	P	3145	5.62E-09	2.65E-07
11	5733	F3 vs. S3	-	Regulation	Regulation	regulation of biosynthetic process	GO:0009889	P	1395	4.21E-13	4.14E-11
11	5734	F3 vs. S3	-	Regulation	Regulation	regulation of cellular biosynthetic process	GO:0031326	P	1375	2.09E-14	2.39E-12
11	5735	F3 vs. S3	-	Regulation	Regulation	regulation of cellular component organization	GO:0051128	P	255	2.72E-03	2.61E-02
11	5736	F3 vs. S3	-	Regulation	Regulation	regulation of cellular macromolecule biosynthetic process	GO:2000112	P	1297	1.11E-16	1.68E-14
11	5737	F3 vs. S3	-	Regulation	Regulation	regulation of cellular metabolic process	GO:0031323	P	1709	1.33E-15	1.74E-13
11	5738	F3 vs. S3	-	Regulation	Regulation	regulation of cellular process	GO:0050794	P	2736	2.14E-09	1.13E-07
11	5739	F3 vs. S3	-	Regulation	Regulation	regulation of cellular protein metabolic process	GO:0032268	P	239	2.16E-05	4.24E-04
11	5740	F3 vs. S3	-	Regulation	Regulation	regulation of flower development	GO:0009909	P	118	1.46E-04	2.27E-03
11	5741	F3 vs. S3	-	Regulation	Regulation	regulation of macromolecule biosynthetic process	GO:0010556	P	1301	0.00E+00	0.00E+00
11	5742	F3 vs. S3	-	Regulation	Regulation	regulation of macromolecule metabolic process	GO:0060255	P	1731	0.00E+00	0.00E+00
11	5743	F3 vs. S3	-	Regulation	Regulation	regulation of metabolic process	GO:0019222	P	1925	1.11E-16	1.89E-14
11	5744	F3 vs. S3	-	Regulation	Regulation	regulation of microtubule cytoskeleton organization	GO:0070507	P	15	3.51E-03	3.14E-02
11	5745	F3 vs. S3	-	Regulation	Regulation	regulation of microtubule-based process	GO:0032886	P	15	3.51E-03	3.15E-02
11	5746	F3 vs. S3	-	Regulation	Regulation	regulation of molecular function	GO:0065009	P	214	6.98E-04	8.66E-03
11	5747	F3 vs. S3	-	Regulation	Regulation	regulation of multicellular organismal development	GO:2000026	P	248	3.20E-03	2.92E-02
11	5748	F3 vs. S3	-	Regulation	Regulation	regulation of nitrogen compound metabolic process	GO:0051171	P	1542	0.00E+00	0.00E+00
11	5749	F3 vs. S3	-	Regulation	Regulation	regulation of nucleobase-containing compound metabolic process	GO:0019219	P	1289	0.00E+00	0.00E+00
11	5750	F3 vs. S3	-	Regulation	Regulation	regulation of organelle organization	GO:0033043	P	136	1.41E-06	4.15E-05
11	5751	F3 vs. S3	-	Regulation	Regulation	regulation of phosphate metabolic process	GO:0019220	P	101	7.68E-06	1.82E-04
11	5752	F3 vs. S3	-	Regulation	Regulation	regulation of phosphorus metabolic process	GO:0051174	P	102	1.36E-05	2.96E-04
11	5753	F3 vs. S3	-	Regulation	Regulation	regulation of primary metabolic process	GO:0080090	P	1595	0.00E+00	0.00E+00
11	5754	F3 vs. S3	-	Regulation	Regulation	regulation of shoot system development	GO:0048831	P	125	5.97E-04	7.67E-03
11	5755	F3 vs. S3	-	Regulation	Regulation	regulation of transferase activity	GO:0051338	P	64	4.62E-07	1.53E-05
11	5756	F3 vs. S3	-	Regulation	Transcription	ATP-dependent chromatin remodeling	GO:0043044	P	18	2.23E-03	2.25E-02
11	5757	F3 vs. S3	-	Regulation	Transcription	chromatin organization involved in negative regulation of transcription	GO:0097549	P	46	4.93E-06	1.25E-04
11	5758	F3 vs. S3	-	Regulation	Transcription	chromatin organization involved in regulation of transcription	GO:0034401	P	47	2.29E-06	6.31E-05
11	5759	F3 vs. S3	-	Regulation	Transcription	chromatin remodeling	GO:0006338	P	54	8.56E-05	1.42E-03
11	5760	F3 vs. S3	-	Regulation	Transcription	chromatin silencing	GO:0006342	P	29	2.32E-06	6.36E-05
11	5761	F3 vs. S3	-	Regulation	Transcription	covalent chromatin modification	GO:0016569	P	98	8.74E-05	1.43E-03
11	5762	F3 vs. S3	-	Regulation	Transcription	DNA conformation change	GO:0071103	P	86	8.01E-07	2.54E-05
11	5763	F3 vs. S3	-	Regulation	Transcription	DNA-binding transcription activator activity, RNA polymerase II-specific	GO:0001228	F	21	1.43E-03	2.47E-02
11	5764	F3 vs. S3	-	Regulation	Transcription	DNA-binding transcription factor activity	GO:0003700	F	888	3.30E-08	2.12E-06
11	5765	F3 vs. S3	-	Regulation	Transcription	DNA-binding transcription factor activity, RNA polymerase II-specific	GO:0000981	F	176	6.28E-04	1.22E-02
11	5766	F3 vs. S3	-	Regulation	Transcription	DNA-directed RNA polymerase complex	GO:0000428	C	74	7.81E-04	5.74E-03
11	5767	F3 vs. S3	-	Regulation	Transcription	dsRNA processing	GO:0031050	P	42	3.05E-03	2.82E-02
11	5768	F3 vs. S3	-	Regulation	Transcription	gene expression	GO:0010467	P	1051	0.00E+00	0.00E+00
11	5769	F3 vs. S3	-	Regulation	Transcription	gene silencing	GO:0016458	P	113	3.63E-08	1.43E-06
11	5770	F3 vs. S3	-	Regulation	Transcription	gene silencing by RNA	GO:0031047	P	78	1.42E-05	3.01E-04
11	5771	F3 vs. S3	-	Regulation	Transcription	gene silencing by RNA-directed DNA methylation	GO:0080188	P	18	2.95E-03	2.74E-02
11	5772	F3 vs. S3	-	Regulation	Transcription	H4 histone acetyltransferase complex	GO:1902562	C	11	6.23E-03	3.40E-02
11	5773	F3 vs. S3	-	Regulation	Transcription	H4/H2A histone acetyltransferase complex	GO:0043189	C	10	6.68E-03	3.52E-02
11	5774	F3 vs. S3	-	Regulation	Transcription	histone binding	GO:0042393	F	68	2.98E-05	9.50E-04
11	5775	F3 vs. S3	-	Regulation	Transcription	histone methylation	GO:0016571	P	41	2.31E-03	2.31E-02
11	5776	F3 vs. S3	-	Regulation	Transcription	histone modification	GO:0016570	P	94	1.85E-04	2.80E-03
11	5777	F3 vs. S3	-	Regulation	Transcription	macromolecule methylation	GO:0043414	P	110	2.97E-06	7.90E-05
11	5778	F3 vs. S3	-	Regulation	Transcription	mediator complex	GO:0016592	C	26	1.19E-03	8.24E-03
11	5779	F3 vs. S3	-	Regulation	Transcription	mRNA processing	GO:0006397	P	193	1.54E-07	5.47E-06
11	5780	F3 vs. S3	-	Regulation	Transcription	negative regulation of gene expression	GO:0010629	P	304	4.21E-08	1.62E-06
11	5781	F3 vs. S3	-	Regulation	Transcription	negative regulation of gene expression, epigenetic	GO:0045814	P	33	8.99E-06	2.08E-04
11	5782	F3 vs. S3	-	Regulation	Transcription	negative regulation of nucleic acid-templated transcription	GO:1903507	P	163	1.97E-05	3.93E-04
11	5783	F3 vs. S3	-	Regulation	Transcription	negative regulation of RNA biosynthetic process	GO:1902679	P	163	1.97E-05	3.95E-04

11	5784	F3 vs. S3	-	Regulation	Transcription	negative regulation of transcription, DNA-templated	GO:0045892	P	161	2.27E-05	4.40E-04
11	5785	F3 vs. S3	-	Regulation	Transcription	NuA4 histone acetyltransferase complex	GO:0035267	C	10	6.68E-03	3.51E-02
11	5786	F3 vs. S3	-	Regulation	Transcription	nuclear DNA-directed RNA polymerase complex	GO:0055029	C	66	8.12E-05	7.60E-04
11	5787	F3 vs. S3	-	Regulation	Transcription	nuclear speck	GO:0016607	C	46	1.09E-03	7.79E-03
11	5788	F3 vs. S3	-	Regulation	Transcription	nucleic acid-templated transcription	GO:0097659	P	151	1.36E-05	2.95E-04
11	5789	F3 vs. S3	-	Regulation	Transcription	nucleolus	GO:0005730	C	338	0.00E+00	0.00E+00
11	5790	F3 vs. S3	-	Regulation	Transcription	nucleosome organization	GO:0034728	P	41	3.46E-06	8.98E-05
11	5791	F3 vs. S3	-	Regulation	Transcription	positive regulation of chromatin organization	GO:1905269	P	16	3.06E-03	2.82E-02
11	5792	F3 vs. S3	-	Regulation	Transcription	positive regulation of chromosome organization	GO:2001252	P	18	5.71E-04	7.38E-03
11	5793	F3 vs. S3	-	Regulation	Transcription	positive regulation of gene expression	GO:0010628	P	346	3.53E-04	4.88E-03
11	5794	F3 vs. S3	-	Regulation	Transcription	positive regulation of histone methylation	GO:0031062	P	13	1.93E-03	2.00E-02
11	5795	F3 vs. S3	-	Regulation	Transcription	positive regulation of histone modification	GO:0031058	P	15	2.01E-03	2.07E-02
11	5796	F3 vs. S3	-	Regulation	Transcription	positive regulation of nucleic acid-templated transcription	GO:1903508	P	292	1.11E-03	1.29E-02
11	5797	F3 vs. S3	-	Regulation	Transcription	positive regulation of RNA biosynthetic process	GO:1902680	P	292	1.11E-03	1.29E-02
11	5798	F3 vs. S3	-	Regulation	Transcription	positive regulation of transcription by RNA polymerase II	GO:0045944	P	108	1.02E-03	1.22E-02
11	5799	F3 vs. S3	-	Regulation	Transcription	positive regulation of transcription, DNA-templated	GO:0045893	P	290	9.08E-04	1.09E-02
11	5800	F3 vs. S3	-	Regulation	Transcription	post-transcriptional gene silencing by RNA	GO:0035194	P	54	6.82E-04	8.50E-03
11	5801	F3 vs. S3	-	Regulation	Transcription	pre-transcriptional gene silencing by RNA	GO:0140458	P	18	2.95E-03	2.75E-02
11	5802	F3 vs. S3	-	Regulation	Transcription	production of small RNA involved in gene silencing by RNA	GO:0070918	P	42	3.05E-03	2.82E-02
11	5803	F3 vs. S3	-	Regulation	Transcription	protein acetyltransferase complex	GO:0031248	C	32	7.11E-03	3.67E-02
11	5804	F3 vs. S3	-	Regulation	Transcription	protein-DNA complex	GO:0032993	C	35	4.83E-05	5.15E-04
11	5805	F3 vs. S3	-	Regulation	Transcription	protein-DNA complex assembly	GO:0065004	P	54	7.62E-08	2.87E-06
11	5806	F3 vs. S3	-	Regulation	Transcription	protein-DNA complex subunit organization	GO:0071824	P	64	2.48E-08	1.04E-06
11	5807	F3 vs. S3	-	Regulation	Transcription	regulation of DNA methylation	GO:0044030	P	12	1.64E-03	1.74E-02
11	5808	F3 vs. S3	-	Regulation	Transcription	regulation of gene expression	GO:0010468	P	1499	0.00E+00	0.00E+00
11	5809	F3 vs. S3	-	Regulation	Transcription	regulation of gene expression, epigenetic	GO:0040029	P	57	2.95E-06	7.91E-05
11	5810	F3 vs. S3	-	Regulation	Transcription	regulation of gene silencing	GO:0060968	P	36	4.10E-03	3.60E-02
11	5811	F3 vs. S3	-	Regulation	Transcription	regulation of nucleic acid-templated transcription	GO:1903506	P	1176	1.11E-16	1.60E-14
11	5812	F3 vs. S3	-	Regulation	Transcription	regulation of RNA biosynthetic process	GO:2001141	P	1177	1.11E-16	1.83E-14
11	5813	F3 vs. S3	-	Regulation	Transcription	regulation of transcription by RNA polymerase II	GO:0006357	P	272	3.49E-06	9.02E-05
11	5814	F3 vs. S3	-	Regulation	Transcription	regulation of transcription, DNA-templated	GO:0006355	P	1176	1.11E-16	1.78E-14
11	5815	F3 vs. S3	-	Regulation	Transcription	regulatory region nucleic acid binding	GO:0001067	F	385	4.54E-04	9.27E-03
11	5816	F3 vs. S3	-	Regulation	Transcription	RNA 3'-end processing	GO:0031123	P	48	2.31E-04	3.39E-03
11	5817	F3 vs. S3	-	Regulation	Transcription	RNA binding	GO:0003723	F	1073	4.81E-13	6.98E-11
11	5818	F3 vs. S3	-	Regulation	Transcription	RNA biosynthetic process	GO:0032774	P	151	1.36E-05	2.93E-04
11	5819	F3 vs. S3	-	Regulation	Transcription	RNA modification	GO:0009451	P	166	3.60E-05	6.59E-04
11	5820	F3 vs. S3	-	Regulation	Transcription	RNA polymerase complex	GO:0030880	C	78	1.16E-03	8.11E-03
11	5821	F3 vs. S3	-	Regulation	Transcription	RNA polymerase II, core complex	GO:0005665	C	14	5.67E-03	3.16E-02
11	5822	F3 vs. S3	-	Regulation	Transcription	RNA polymerase II, holoenzyme	GO:0016591	C	44	1.92E-03	1.22E-02
11	5823	F3 vs. S3	-	Regulation	Transcription	RNA polymerase V complex	GO:0000419	C	14	4.64E-03	2.64E-02
11	5824	F3 vs. S3	-	Regulation	Transcription	sequence-specific DNA binding	GO:0043565	F	573	5.49E-07	2.61E-05
11	5825	F3 vs. S3	-	Regulation	Transcription	sequence-specific double-stranded DNA binding	GO:1990837	F	403	5.97E-05	1.71E-03
11	5826	F3 vs. S3	-	Regulation	Transcription	transcription by RNA polymerase II	GO:0006366	P	36	4.04E-03	3.55E-02
11	5827	F3 vs. S3	-	Regulation	Transcription	transcription coregulator activity	GO:0003712	F	82	6.77E-08	4.09E-06
11	5828	F3 vs. S3	-	Regulation	Transcription	transcription regulator activity	GO:0140110	F	973	3.58E-12	3.85E-10
11	5829	F3 vs. S3	-	Regulation	Transcription	transcription regulator complex	GO:0005667	C	68	5.45E-04	4.10E-03
11	5830	F3 vs. S3	-	Regulation	Transcription	transcription regulatory region sequence-specific DNA binding	GO:0000976	F	385	4.54E-04	9.21E-03
11	5831	F3 vs. S3	-	Regulation	Transcription	transcription, DNA-templated	GO:0006351	P	151	1.36E-05	2.92E-04
11	5832	F3 vs. S3	-	Regulation	Translation	90S preribosome	GO:0030686	C	20	1.71E-06	2.46E-05
11	5833	F3 vs. S3	-	Regulation	Translation	catalytic step 2 spliceosome	GO:0071013	C	46	5.52E-05	5.77E-04
11	5834	F3 vs. S3	-	Regulation	Translation	cleavage involved in rRNA processing	GO:0000469	P	16	1.67E-03	1.77E-02
11	5835	F3 vs. S3	-	Regulation	Translation	cytoplasmic translation	GO:0002181	P	59	2.27E-05	4.40E-04
11	5836	F3 vs. S3	-	Regulation	Translation	cytosolic large ribosomal subunit	GO:0022625	C	108	0.00E+00	0.00E+00
11	5837	F3 vs. S3	-	Regulation	Translation	cytosolic ribosome	GO:0022626	C	240	0.00E+00	0.00E+00
11	5838	F3 vs. S3	-	Regulation	Translation	cytosolic small ribosomal subunit	GO:0022627	C	80	4.44E-16	1.57E-14
11	5839	F3 vs. S3	-	Regulation	Translation	endonucleolytic cleavage in ITS1 to separate SSU-rRNA from 5.8S rRNA and LSU-rRNA from tricistronic rRNA transcript (SSU-rRNA, 5.8S rRNA, LSU-rRNA)	GO:0000447	P	4	1.89E-03	1.96E-02
11	5840	F3 vs. S3	-	Regulation	Translation	establishment of RNA localization	GO:0051236	P	48	3.37E-04	4.73E-03
11	5841	F3 vs. S3	-	Regulation	Translation	large ribosomal subunit	GO:0015934	C	137	0.00E+00	0.00E+00
11	5842	F3 vs. S3	-	Regulation	Translation	large ribosomal subunit rRNA binding	GO:0070180	F	11	6.06E-04	1.20E-02
11	5843	F3 vs. S3	-	Regulation	Translation	maturation of LSU-rRNA	GO:0000470	P	32	5.10E-04	6.66E-03
11	5844	F3 vs. S3	-	Regulation	Translation	maturation of SSU-rRNA	GO:0030490	P	38	1.00E-09	5.63E-08

11	5845	F3 vs. S3	-	Regulation	Translation	maturation of SSU-rRNA from tricistronic rRNA transcript (SSU-rRNA, 5.8S rRNA, LSU-rRNA)	GO:0000462	P	26	1.39E-05	2.96E-04
11	5846	F3 vs. S3	-	Regulation	Translation	mitochondrial large ribosomal subunit	GO:0005762	C	17	1.00E-03	7.26E-03
11	5847	F3 vs. S3	-	Regulation	Translation	mitochondrial ribosome	GO:0005761	C	27	1.27E-05	1.50E-04
11	5848	F3 vs. S3	-	Regulation	Translation	mitochondrial small ribosomal subunit	GO:0005763	C	10	3.91E-03	2.32E-02
11	5849	F3 vs. S3	-	Regulation	Translation	mRNA 3'-end processing	GO:0031124	P	21	4.03E-03	3.55E-02
11	5850	F3 vs. S3	-	Regulation	Translation	mRNA binding	GO:0003729	F	719	6.82E-07	3.09E-05
11	5851	F3 vs. S3	-	Regulation	Translation	mRNA cis splicing, via spliceosome	GO:0045292	P	28	2.40E-03	2.38E-02
11	5852	F3 vs. S3	-	Regulation	Translation	mRNA cleavage factor complex	GO:0005849	C	11	4.96E-03	2.80E-02
11	5853	F3 vs. S3	-	Regulation	Translation	mRNA export from nucleus	GO:0006406	P	25	5.68E-03	4.69E-02
11	5854	F3 vs. S3	-	Regulation	Translation	mRNA metabolic process	GO:0016071	P	280	1.15E-07	4.26E-06
11	5855	F3 vs. S3	-	Regulation	Translation	mRNA modification	GO:0016556	P	31	6.04E-03	4.91E-02
11	5856	F3 vs. S3	-	Regulation	Translation	mRNA splicing, via spliceosome	GO:0000398	P	141	9.22E-06	2.12E-04
11	5857	F3 vs. S3	-	Regulation	Translation	mRNA transport	GO:0051028	P	26	2.59E-03	2.50E-02
11	5858	F3 vs. S3	-	Regulation	Translation	mRNA-containing ribonucleoprotein complex export from nucleus	GO:0071427	P	25	5.68E-03	4.70E-02
11	5859	F3 vs. S3	-	Regulation	Translation	ncRNA metabolic process	GO:0034660	P	293	3.67E-11	2.58E-09
11	5860	F3 vs. S3	-	Regulation	Translation	ncRNA processing	GO:0034470	P	251	2.45E-11	1.79E-09
11	5861	F3 vs. S3	-	Regulation	Translation	negative regulation of RNA metabolic process	GO:0051253	P	171	2.87E-05	5.36E-04
11	5862	F3 vs. S3	-	Regulation	Translation	organellar large ribosomal subunit	GO:0000315	C	24	1.15E-04	1.05E-03
11	5863	F3 vs. S3	-	Regulation	Translation	organellar ribosome	GO:0000313	C	42	4.97E-08	9.13E-07
11	5864	F3 vs. S3	-	Regulation	Translation	organellar small ribosomal subunit	GO:0000314	C	18	1.04E-04	9.61E-04
11	5865	F3 vs. S3	-	Regulation	Translation	peptide biosynthetic process	GO:0043043	P	365	0.00E+00	0.00E+00
11	5866	F3 vs. S3	-	Regulation	Translation	polysomal ribosome	GO:0042788	C	3	8.37E-03	4.26E-02
11	5867	F3 vs. S3	-	Regulation	Translation	positive regulation of RNA metabolic process	GO:0051254	P	310	1.10E-03	1.29E-02
11	5868	F3 vs. S3	-	Regulation	Translation	posttranscriptional gene silencing	GO:0016441	P	59	1.45E-03	1.59E-02
11	5869	F3 vs. S3	-	Regulation	Translation	posttranscriptional regulation of gene expression	GO:0010608	P	157	4.43E-03	3.82E-02
11	5870	F3 vs. S3	-	Regulation	Translation	precatalytic spliceosome	GO:0071011	C	26	9.50E-07	1.43E-05
11	5871	F3 vs. S3	-	Regulation	Translation	preribosome	GO:0030684	C	65	1.50E-09	3.17E-08
11	5872	F3 vs. S3	-	Regulation	Translation	preribosome, large subunit precursor	GO:0030687	C	17	4.74E-03	2.69E-02
11	5873	F3 vs. S3	-	Regulation	Translation	prespliceosome	GO:0071010	C	19	1.31E-03	8.82E-03
11	5874	F3 vs. S3	-	Regulation	Translation	regulation of RNA metabolic process	GO:0051252	P	1248	1.11E-16	1.64E-14
11	5875	F3 vs. S3	-	Regulation	Translation	regulation of RNA splicing	GO:0043484	P	43	9.45E-04	1.13E-02
11	5876	F3 vs. S3	-	Regulation	Translation	ribonucleoprotein complex	GO:1990904	C	469	0.00E+00	0.00E+00
11	5877	F3 vs. S3	-	Regulation	Translation	ribonucleoprotein complex assembly	GO:0022618	P	125	0.00E+00	0.00E+00
11	5878	F3 vs. S3	-	Regulation	Translation	ribonucleoprotein complex biogenesis	GO:0022613	P	315	0.00E+00	0.00E+00
11	5879	F3 vs. S3	-	Regulation	Translation	ribonucleoprotein complex export from nucleus	GO:0071426	P	41	2.91E-03	2.72E-02
11	5880	F3 vs. S3	-	Regulation	Translation	ribonucleoprotein complex localization	GO:0071166	P	41	2.91E-03	2.72E-02
11	5881	F3 vs. S3	-	Regulation	Translation	ribonucleoprotein complex subunit organization	GO:0071826	P	127	0.00E+00	0.00E+00
11	5882	F3 vs. S3	-	Regulation	Translation	ribosomal large subunit assembly	GO:0000027	P	35	9.56E-09	4.29E-07
11	5883	F3 vs. S3	-	Regulation	Translation	ribosomal large subunit biogenesis	GO:0042273	P	85	1.98E-14	2.31E-12
11	5884	F3 vs. S3	-	Regulation	Translation	ribosomal small subunit assembly	GO:0000028	P	30	3.16E-09	1.61E-07
11	5885	F3 vs. S3	-	Regulation	Translation	ribosomal small subunit biogenesis	GO:0042274	P	69	3.33E-16	4.56E-14
11	5886	F3 vs. S3	-	Regulation	Translation	ribosomal subunit	GO:0044391	C	239	0.00E+00	0.00E+00
11	5887	F3 vs. S3	-	Regulation	Translation	ribosome	GO:0005840	C	300	0.00E+00	0.00E+00
11	5888	F3 vs. S3	-	Regulation	translation	ribosome assembly	GO:0042255	P	70	1.11E-16	1.73E-14
11	5889	F3 vs. S3	-	Regulation	Translation	ribosome biogenesis	GO:0042254	P	260	0.00E+00	0.00E+00
11	5890	F3 vs. S3	-	Regulation	Translation	RNA export from nucleus	GO:0006405	P	45	1.04E-03	1.22E-02
11	5891	F3 vs. S3	-	Regulation	Translation	RNA localization	GO:0006403	P	53	4.60E-05	8.22E-04
11	5892	F3 vs. S3	-	Regulation	Translation	RNA phosphodiester bond hydrolysis	GO:0090501	P	30	3.57E-04	4.91E-03
11	5893	F3 vs. S3	-	Regulation	Translation	RNA processing	GO:0006396	P	510	2.22E-16	3.11E-14
11	5894	F3 vs. S3	-	Regulation	Translation	RNA splicing	GO:0008380	P	179	2.96E-06	7.90E-05
11	5895	F3 vs. S3	-	Regulation	Translation	RNA splicing, via transesterification reactions	GO:0000375	P	151	1.60E-05	3.29E-04
11	5896	F3 vs. S3	-	Regulation	Translation	RNA splicing, via transesterification reactions with bulged adenosine as nucleophile	GO:0000377	P	151	1.60E-05	3.31E-04
11	5897	F3 vs. S3	-	Regulation	Translation	rRNA binding	GO:0019843	F	51	6.31E-07	2.95E-05
11	5898	F3 vs. S3	-	Regulation	Translation	rRNA metabolic process	GO:0016072	P	174	4.86E-09	2.33E-07
11	5899	F3 vs. S3	-	Regulation	Translation	rRNA modification	GO:0000154	P	21	4.47E-03	3.84E-02
11	5900	F3 vs. S3	-	Regulation	Translation	rRNA processing	GO:0006364	P	168	3.22E-09	1.63E-07
11	5901	F3 vs. S3	-	Regulation	Translation	small nuclear ribonucleoprotein complex	GO:0030532	C	54	1.25E-11	3.18E-10
11	5902	F3 vs. S3	-	Regulation	Translation	small nucleolar ribonucleoprotein complex	GO:0005732	C	20	1.91E-04	1.62E-03
11	5903	F3 vs. S3	-	Regulation	Translation	small ribosomal subunit	GO:0015935	C	102	0.00E+00	0.00E+00
11	5904	F3 vs. S3	-	Regulation	Translation	small-subunit processome	GO:0032040	C	39	1.27E-06	1.87E-05
11	5905	F3 vs. S3	-	Regulation	Translation	snoRNA binding	GO:0030515	F	19	1.16E-03	2.07E-02

11	5906	F3 vs. S3	-	Regulation	Translation	spliceosomal complex	GO:0005681	C	106	7.64E-08	1.28E-06
11	5907	F3 vs. S3	-	Regulation	Translation	spliceosomal snRNP assembly	GO:0000387	P	18	5.38E-03	4.48E-02
11	5908	F3 vs. S3	-	Regulation	Translation	spliceosomal snRNP complex	GO:0097525	C	54	1.25E-11	3.27E-10
11	5909	F3 vs. S3	-	Regulation	Translation	spliceosomal tri-snRNP complex	GO:0097526	C	31	3.10E-07	4.87E-06
11	5910	F3 vs. S3	-	Regulation	Translation	structural constituent of ribosome	GO:0003735	F	234	0.00E+00	0.00E+00
11	5911	F3 vs. S3	-	Regulation	Translation	translation	GO:0006412	P	361	0.00E+00	0.00E+00
11	5912	F3 vs. S3	-	Regulation	Translation	tRNA metabolic process	GO:0006399	P	104	3.36E-03	3.04E-02
11	5913	F3 vs. S3	-	Regulation	Translation	tRNA modification	GO:0006400	P	53	4.96E-03	4.19E-02
11	5914	F3 vs. S3	-	Regulation	Translation	tRNA processing	GO:0008033	P	66	1.59E-03	1.70E-02
11	5915	F3 vs. S3	-	Regulation	Translation	U1 snRNP	GO:0005685	C	20	5.32E-07	8.12E-06
11	5916	F3 vs. S3	-	Regulation	Translation	U12-type spliceosomal complex	GO:0005689	C	16	9.10E-03	4.49E-02
11	5917	F3 vs. S3	-	Regulation	Translation	U2 snRNP	GO:0005686	C	25	2.66E-07	4.26E-06
11	5918	F3 vs. S3	-	Regulation	Translation	U2-type prespliceosome	GO:0071004	C	19	1.31E-03	8.76E-03
11	5919	F3 vs. S3	-	Regulation	Translation	U2-type spliceosomal complex	GO:0005684	C	41	7.97E-05	7.68E-04
11	5920	F3 vs. S3	-	Regulation	Translation	U4 snRNP	GO:0005687	C	12	1.05E-04	9.68E-04
11	5921	F3 vs. S3	-	Regulation	Translation	U4/U6 x U5 tri-snRNP complex	GO:0046540	C	23	2.91E-05	3.24E-04
11	5922	F3 vs. S3	-	Regulation	Translation	U5 snRNP	GO:0005682	C	16	1.47E-05	1.70E-04
11	5923	F3 vs. S3	-	Signaling and response	Signaling	kinase regulator activity	GO:0019207	F	58	1.09E-08	7.93E-07
11	5924	F3 vs. S3	-	Signaling and response	Signaling	protein kinase complex	GO:1902911	C	55	4.72E-06	6.01E-05
11	5925	F3 vs. S3	-	Signaling and response	Signaling	protein kinase regulator activity	GO:0019887	F	55	4.04E-08	2.55E-06
11	5926	F3 vs. S3	-	Signaling and response	Signaling	regulation of cyclin-dependent protein serine/threonine kinase activity	GO:0000079	P	38	1.56E-05	3.26E-04
11	5927	F3 vs. S3	-	Signaling and response	Signaling	regulation of kinase activity	GO:0043549	P	54	1.13E-07	4.20E-06
11	5928	F3 vs. S3	-	Signaling and response	Signaling	regulation of phosphatase activity	GO:0010921	P	18	4.03E-03	3.55E-02
11	5929	F3 vs. S3	-	Signaling and response	Signaling	regulation of phosphorylation	GO:0042325	P	68	4.00E-05	7.28E-04
11	5930	F3 vs. S3	-	Signaling and response	Signaling	regulation of protein kinase activity	GO:0045859	P	52	5.62E-07	1.83E-05
11	5931	F3 vs. S3	-	Signaling and response	Signaling	regulation of protein phosphorylation	GO:0001932	P	61	4.51E-05	8.09E-04
11	5932	F3 vs. S3	-	Signaling and response	Signaling	regulation of protein serine/threonine kinase activity	GO:0071900	P	45	2.98E-05	5.55E-04
11	5933	F3 vs. S3	-	Signaling and response	Signaling	serine/threonine protein kinase complex	GO:1902554	C	51	4.14E-06	5.33E-05
11	5934	F3 vs. S3	-	Signaling and response	Stress	(1->3)-beta-D-glucan binding (NAD+) activity	GO:0001872	F	4	1.48E-03	2.52E-02
11	5935	F3 vs. S3	-	Signaling and response	Stress	cellular response to DNA damage stimulus	GO:0006974	P	211	6.03E-13	5.64E-11
11	5936	F3 vs. S3	-	Signaling and response	Stress	somatic cell DNA recombination	GO:0016444	P	7	4.22E-03	3.68E-02
