# Supplementary material for: Whether Gametophytes Are Reduced or Unreduced in Angiosperms Might Be Determined Metabolically
Source: Genes (Basel). 2020 Dec 2;11(12):1449. doi: 10.3390/genes11121449 (PMC7761559; doi:10.3390/genes11121449)
Supplement: Supplementary file 1 [file genes-11-01449-s001.zip › Table S9 key DEG literature.pdf]

Table S9. Genes by comparison identified by key word searches of DEG descriptions. L, B. lignifera; M, B. microphylla; F, B. x formosa; S, B. stricta; Stages: 1, megasporocyte (MMC); 2, meiocyte; 3, early gametophyte; 4, mature gametophyte.

Comparison	Gene	Name	Notes with additional references	Pathway	List	Fold	Gene description	Use	Key genes	FDR
F vs. M (23)	AT5G07410	**PME48**	Pectin methylesterase important to pollen function (TAIR)			621.32	Encodes a pectin methylesterase implicated in the remodeling of pectins in pollen. Its expression is restricted to the male gametophyte (dry pollen, imbibed pollen and pollen tube). Mutants do not display any phenotype in the vegetative tissues. Pollen germination of KO mutant is strongly delayed in vivo and in vitro. Extra calcium restores the wild type phenotype in vitro.	No	Ca, gametophyte	< 1e-07
F vs. S (23)	AT5G53820	**LEA AT5G53820**	Expression correlated with ABA and salt stress (doi.org/10.1186/s12870-018-1255-z)		ABA upregulated, LEA, Salt	130.05	Late embryogenesis abundant protein (LEA) family protein;(source:Araport11)	Yes	LEA	< 1e-07
F vs. M (23)	AT5G53820	**LEA AT5G53820**	Expression correlated with ABA and salt stress (doi.org/10.1186/s12870-018-1255-z)		ABA upregulated, LEA, Salt	103.63	Late embryogenesis abundant protein (LEA) family protein;(source:Araport11)	Use	LEA	4.88E-05
F vs. L (23)	AT5G53820	**LEA AT5G53820**	Expression correlated with ABA and salt stress (doi.org/10.1186/s12870-018-1255-z)		ABA upregulated, LEA, Salt	91.34	Late embryogenesis abundant protein (LEA) family protein;(source:Araport11)	Use	LEA	< 1e-07
M vs. S (23)	AT3G13580	**RPL7D (RP)**			ribosome	71.92	Ribosomal protein L30/L7 family protein;(source:Araport11)			7.54E-03
L vs. S (23)	AT3G13580	**RPL7D (RP)**			ribosome	66.1	Ribosomal protein L30/L7 family protein;(source:Araport11)			5.27E-03
F vs. M (23)	AT3G56240	**CCH**	Intracellular copper homeostasis (TAIR)		Homeostasis	46.79	CCH protein belongs to a family of eukaryotic proteins that participate in intracellular copper homeostasis by delivering this metal to the secretory pathway; mainly located along the vascular bundles of senescing leaves and petioles as well as in stem sieve elements; hypothesized to have a role in copper mobilization from decaying organs towards reproductive structures, as a result of metalloprotein breakdown. The plant-specific C-terminal domain of the CCH protein forms amyloid-like fibrils in vitro.	Use	Homeostasis	5.48E-05
F vs. L (23)	AT4G13770	**REF2**	Involved in auxin homeostasis (TAIR)		Homeostasis	37.10	Encodes a cytochrome p450 enzyme that catalyzes the initial conversion of aldoximes to thiohydroximates in the synthesis of glucosinolates not derived from tryptophan. Also has a role in auxin homeostasis.	Use	Homeostasis	< 1e-07
F vs. S (23)	AT4G13560	**LEA UNE15**	Involved in double fertilization (TAIR)	Flowering	LEA	36.51	Late embryogenesis abundant protein (LEA) family protein;(source:Araport11)	Yes	LEA	< 1e-07
L vs. S (23)	AT3G48510	**AITR2 (DIG1)**	AITR2 (DIG1) belongs to a group of six transcriptional repressors that are upregulated by ABA (DOI: 10.1126/science.aag1550). They strengthen ABA signaling (suppression of growth) by i) suppressing ABA induced downregulation of ABA receptor genes and ii) suppressing ABA induced upregulation of PP2C genes (DOI: 10.1111/pce.13058). Consistent with these functions, we found that PP2Cs were down-regulated in B. microphylla vs. B. lignifera ( ), and this may have enhanced ABA signaling in B. microphylla ovules.		ABA	29.48	ABA‐induced transcription repressor that acts as feedback regulator in ABA signalling.			9.20E-03
F vs. M (23)	AT4G13560	**LEA UNE15**	Involved in double fertilization (TAIR)	Flowering	LEA	22.70	Late embryogenesis abundant protein (LEA) family protein;(source:Araport11)	Use	LEA	6.51E-05
F vs. M (23)	AT2G44120	**RPL7C (RP)**			ribosome	22.44	Ribosomal protein L30/L7 family protein;(source:Araport11)		ribosome	2.06E-04
F vs. L (23)	AT3G03190	**GSTF11**	involved in responses to oxidative stress and toxin catabolic processes (TAIR)	ROS attenuation		20.87	Encodes glutathione transferase belonging to the phi class of GSTs. Naming convention according to Wagner et al. (2002).	Use	glutathione	< 1e-07
F vs. S (23)	AT3G56240	**CCH**	Intracellular copper homeostasis (TAIR)		Homeostasis	20.79	CCH protein belongs to a family of eukaryotic proteins that participate in intracellular copper homeostasis by delivering this metal to the secretory pathway; mainly located along the vascular bundles of senescing leaves and petioles as well as in stem sieve elements; hypothesized to have a role in copper mobilization from decaying organs towards reproductive structures, as a result of metalloprotein breakdown. The plant-specific C-terminal domain of the CCH protein forms amyloid-like fibrils in vitro.	Yes	Homeostasis	2.96E-06
F vs. M (23)	AT3G55610	**P5CS2**	Induced by dehydration, salt and ABA (TAIR)		ABA upregulated, Salt	17.11	encodes delta 1-pyrroline-5-carboxylate synthetase B. Gene expression is induced by dehydration, high salt and ABA. Knock-out mutations in P5CS2 are embryo-lethal. P5CS2 appears to be present in different cells and/or different subcellular locations from P5CS1 in a tissue-dependent manner. Mutants are defective in pollen development.	Use	ABA	6.79E-04
F vs. S (23)	AT2G26740	**SHE**	Induced by water stress (TAIR)			16.87	Encodes a soluble epoxide hydrolase whose expression is induced by auxin and water stress.	Yes	Stress	< 1e-07
F vs. L (23)	AT4G13560	**LEA UNE15**	Involved in double fertilization (TAIR)	Flowering	LEA	16.44	Late embryogenesis abundant protein (LEA) family protein;(source:Araport11)	Use	LEA	< 1e-07
F vs. S (23)	AT1G22760	**PAB3**	PAB3 is a poly(A) binding protein that is upregulated in floral organs and is probably involved in posttranslational regulation and translational initiation (TAIR)	Flowering, SnRK1 TOR		14.17	Putative poly(A) binding protein May there fore function in posttranscriptional regulation, including mRNA turnover and translational initiation. Expression detected only in floral organs.	Yes	Translation	< 1e-07
F vs. M (23)	AT3G51240		Involved in flavonoid biosynthesis (TAIR)		ET	13.98	Encodes flavanone 3-hydroxylase that is coordinately expressed with chalcone synthase and chalcone isomerases. Regulates flavonoid biosynthesis. Not responsive to auxin or ethylene stimulus (qRT-PCR).	Use	ethylene	7.94E-04
F vs. L (23)	AT2G44120	**RPL7C (RP)**	TAIR		ribosome	13.86	Ribosomal protein L30/L7 family protein;(source:Araport11)	Use	ribosome	< 1e-07
F vs. M (23)	AT4G36250	**AT4G36250**	Not involved in plant stress (TAIR)			13.62	Encodes a putative aldehyde dehydrogenase. The gene is not responsive to osmotic stress and is expressed constitutively at a low level in plantlets and root cultures.	No	Stress	1.30E-04
F vs. S (23)	AT1G27730	**ZAT10**	Expression levels of the transcriptional repressor ZAT10 are correlated with increases in salt and drought stresses (https://doi.org/10.1016/j.plaphy.2020.02.029)	Stress signaling	Salt	13.49	Related to Cys2/His2-type zinc-finger proteins found in higher plants. Compensated for a subset of calcineurin deficiency in yeast. Salt tolerance produced by ZAT10 appeared to be partially dependent on ENA1/PMR2, a P-type ATPase required for Li+ and Na+ efflux in yeast. The protein is localized to the nucleus, acts as a transcriptional repressor and is responsive to chitin oligomers. Also involved in response to photooxidative stress.	Yes	Stress	4.97E-06
F vs. M (23)	AT3G05890	**LT16b**	Salt responsive protein (TAIR)	Stress attenuation	Salt	13.02	Low temperature and salt responsive protein family;(source:Araport11)	Use	salt responsive	1.14E-03
F vs. S (23)	AT4G01610	**ATCATHB3**	ATCATHB3 is a cathepsin B capase that is upregulated by ER stress and positively regulates ER stress induced cell death (doi: 10.1111/nph.14676)	Stress attenuation		13	Encodes a capase involved in stress induced cell death. Activity detected in leaf and cell culture.	Yes	Stress	< 1e-07

F vs. S (23)	AT1G69120	AP1	Floral homeotic Class-A gene involved in sepal specification (TAIR)		Flowering	12.62	Floral homeotic gene encoding a MADS domain protein homologous to SRF transcription factors. Specifies floral meristem and sepal identity. Required for the transcriptional activation of AGAMOUS. Interacts with LEAFY.Binds to promoter and regulates the expression of flowering time genes SVP, SOC1 and AGL24.	Yes	LEA, AGL	< 1e-07
F vs. L (23)	AT1G22760	PAB3	PAB3 is a poly(A) binding protein that is upregulated in floral organs and is probably involved in posttranslational regulation and translational initiation (TAIR)	Flowering, SnRK1 TOR		12.45	Putative poly(A) binding protein May therefore function in posttranscriptional regulation, including mRNA turnover and translational initiation. Expression detected only in floral organs.	Use	translation	< 1e-07
F vs. M (23)	AT1G27730	ZAT10	Expression levels of the transcriptional repressor ZAT10 are correlated with increases in salt and drought stresses (https://doi.org/10.1016/j.plaphy.2020.02.029)	Stress signaling	Salt	12.33	Related to Cys2/His2-type zinc-finger proteins found in higher plants. Compensated for a subset of calcineurin deficiency in yeast. Salt tolerance produced by ZAT10 appeared to be partially dependent on ENA1/PMR2, a P-type ATPase required for Li+ and Na+ efflux in yeast. The protein is localized to the nucleus, acts as a transcriptional repressor and is responsive to chitin oligomers. Also involved in response to photooxidative stress.	Use	Stress	1.82E-03
F vs. M (23)	AT3G03190	GSTF11	Phi calls GST involved in responses to oxidative stress and toxin catabolic processes (TAIR)	ROS attenuation		12.28	Encodes glutathione transferase belonging to the phi class of GSTs. Naming convention according to Wagner et al. (2002).	Use	glutathione	3.69E-04
F vs. S (23)	AT4G37530	AT4G37530	AT4G37530 is a peroxidase that is upregulated in response to oxidative stress (TAIR)	ROS attenuation		12.27	Peroxidase superfamily protein;(source:Araport11)	Yes	peroxidase	< 1e-07
F vs. M (23)	AT4G25100	FSD1	chloroplastic SOD that removes superoxide radicals and is induced by salt stress; also functions as an osmoprotectant, DOI: 10.1111/pce.13773	ROS attenuation	Salt	12.23	Fe-superoxide dismutase	Use	H2O2, superoxide dismutase	4.10E-03
F vs. L (23)	AT1G69120	AP1	Floral homeotic Class-A gene involved in sepal specification (TAIR)		Flowering	12.10	Floral homeotic gene encoding a MADS domain protein homologous to SRF transcription factors. Specifies floral meristem and sepal identity. Required for the transcriptional activation of AGAMOUS. Interacts with LEAFY.Binds to promoter and regulates the expression of flowering time genes SVP, SOC1 and AGL24.	Use	LEA, AGL	< 1e-07
F vs. S (23)	AT2G30020	AP2C1	AP2C1 is activated by ABA and pathogens. It appears to modulate K+ homeostasis by dephosphorylation inactivating CIPK9, which normally enhances K+ ion uptake through K+ channels, particularly under low K+ conditions (doi:10.1093/jxb/ery182). It also moderates responses to pathogens by dephosphorylation inactivating MPK4,6 in the cytoplasm and nucleus. These MAPKs affect the expression of hundreds of genes by modifying the activity of 88 TFs from 21 families (doi:10.1093/jxb/erw485).	ABA signaling	Homeostasis	12.09	Encodes AP2C1. Belongs to the clade B of the PP2C-superfamily. Acts as a MAPK phosphatase that negatively regulates MPK4 and MPK6.	Yes	PP2C, MAPK	1.13E-04
F vs. S (23)	AT5G25610	RD22	RD22 transcriptions are upregulated by ABA, salt and desiccation. RD22 is also upregulated by C3H17, which does not belong to the zinc finger proteins normally involved in the ABA dependent salt stress-response pathway (https://doi.org/10.1016/j.bbrc.2018.03.088)		ABA upregulated, Salt	11.9	responsive to dehydration 22 (RD22) mediated by ABA	Yes	ABA	< 1e-07
F vs. M (23)	AT1G22760	PAB3		Flowering, SnRK1 TOR		11.82	Putative poly(A) binding protein May therefore function in posttranscriptional regulation, including mRNA turnover and translational initiation. Expression detected only in floral organs.		#N/A	< 1e-07
F vs. M (23)	AT1G69120	AP1	Floral homeotic Class-A gene involved in sepal specification (TAIR)		Flowering	11.72	Floral homeotic gene encoding a MADS domain protein homologous to SRF transcription factors. Specifies floral meristem and sepal identity. Required for the transcriptional activation of AGAMOUS. Interacts with LEAFY.Binds to promoter and regulates the expression of flowering time genes SVP, SOC1 and AGL24.	Use	AGL	4.88E-05
F vs. L (23)	AT5G05600	JOX2	JOX2 is induced by JA. It downregulates plant immunity signaling and responses by oxidizing JA (doi.org/10.1073/pnas.1701101114)			11.45	Encodes a protein with similarity to flavonol synthases that is involved in the detoxifcation polycyclic aromatic hydrocarbons.One of 4 paralogs encoding a 2-oxoglutarate/Fe(II)-dependent oxygenases that hydroxylates JA to 12-OH-JA.	No	flavonol	2.42E-05
F vs. S (23)	AT3G49110	PRXCA	Cell wall peroxidase that generates ROS. These ROS induce respiratory bursts, through RBOHD (DOI: 10.1111/mpp.12769), that exponentially increase H2O2 levels	ROS attenuation		11.41	Class III peroxidase Perx33. Expressed in roots. Located in the cell wall. Involved in cell elongation. Expression activated by light. May play a role in generating H2O2 during defense response. The mRNA is cell-to-cell mobile.	Yes	H2O2, Ca, peroxidase	1.81E-05
F vs. L (23)	AT3G56240	CCH	Intracellular copper homeostasis (TAIR)		Homeostasis	11.21	CCH protein belongs to a family of eukaryotic proteins that participate in intracellular copper homeostasis by delivering this metal to the secretory pathway; mainly located along the vascular bundles of senescing leaves and petioles as well as in stem sieve elements; hypothesized to have a role in copper mobilization from decaying organs towards reproductive structures, as a result of metalloprotein breakdown. The plant-specific C-terminal domain of the CCH protein forms amyloid-like fibrils in vitro.	Use	Homeostasis	1.91E-03
F vs. L (23)	AT1G27730	ZAT10	Expression levels of the transcriptional repressor ZAT10 are correlated with increases in salt and drought stresses (https://doi.org/10.1016/j.plaphy.2020.02.029)	Stress signaling	Salt	11.07	Related to Cys2/His2-type zinc-finger proteins found in higher plants. Compensated for a subset of calcineurin deficiency in yeast. Salt tolerance produced by ZAT10 appeared to be partially dependent on ENA1/PMR2, a P-type ATPase required for Li+ and Na+ efflux in yeast. The protein is localized to the nucleus, acts as a transcriptional repressor and is responsive to chitin oligomers. Also involved in response to photooxidative stress.	Use	Stress	2.42E-05
F vs. L (23)	AT1G51090	HMAD1	Upregulated by reactive nitrogen species (DOI: 10.3389/fpls.2016.01712)	Stress signaling		10.79	Heavy metal associated domain containing protein involved in plant immunity.Mutants show an increase in root length under NO stress and reduction in root length under H2O2 stress conditions. Mutants show increases in defense responses to pathogens including hypersensitive lesions, increased resistance and induction of SAR genes.	Use	Stress, H2O2, Ca	< 1e-07
F vs. L (23)	AT5G51720	NEET	Fe-S clusters mediate ETC reactions, but they also react with oxygen to produce ROS. NEET proteins participate in regulating the biogenesis of Fe-S clusters and maintaining Fe and ROS homeostasis (10.1111/tpj.14581)	ROS attenuation	Homeostasis	10.68	Encodes a protein with biochemical, structural, and biophysical characteristics of a NEET protein. It plays a key role in plant development, senescence, reactive oxygen homeostasis, and Fe metabolism.	Use	Homeostasis	< 1e-07
F vs. S (23)	AT1G78370	GSTU20	GSTU20 encodes a Tau class glutathione transferase. It is upregulated by stress and interacts with the phytochrome signaling protein far-red insensitive 219. GSTU20 is required for plant responses to far red light (doi.org/10.1199/tab.0131)	ROS attenuation		10.62	Encodes glutathione transferase belonging to the tau class of GSTs. Naming convention according to Wagner et al. (2002).	Yes	glutathione	6.68E-06

F vs. M (23)	AT1G51090	HMAD1	Upregulated by reactive nitrogen species (DOI: 10.3389/fpls.2016.01712)	Stress signaling		10.49	Heavy metal associated domain containing protein involved in plant immunity.Mutants show an increase in root length under NO stress and reduction in root length under H2O2 stress conditions. Mutants show increases in defense responses to pathogens including hypersensitive lesions, increased resistance and induction of SAR genes.	Use	Stress, H2O2, Ca	2.17E-05
L vs M (123)	AT3G42570	AT3G42570	response to oxidative stress (TAIR)	ROS attenuation		10.29	peroxidase family protein;(source:Araport11)	Use	peroxidase	1.17E-03
F vs. M (23)	AT3G49110	PRXCA	Cell wall peroxidase that generates ROS. These ROS induce respiratory bursts, through RBOHD (DOI: 10.1111/mpp.12769), that exponentially increase H2O2 levels	ROS attenuation		10.04	Class III peroxidase Perx33. Expressed in roots. Located in the cell wall. Involved in cell elongation. Expression activated by light. May play a role in generating H2O2 during defense response. The mRNA is cell-to-cell mobile.	Use	H2O2, Ca, peroxidase	1.65E-03
F vs. S (23)	AT3G55610	P5CS2	Induced by dehydration, salt and ABA (TAIR)		ABA upregulated, Salt	9.97	encodes delta 1-pyrroline-5-carboxylate synthetase B. Gene expression is induced by dehydration, high salt and ABA. Knock-out mutations in P5CS2 are embryo-lethal. P5CS2 appears to be present in different cells and/or different subcellular locations from P5CS1 in a tissue-dependent manner. Mutants are defective in pollen development.	Yes	ABA	1.06E-04
F vs. M (23)	AT1G56580	SVB		Stress attenuation		9.92	Encodes SMALLER WITH VARIABLE BRANCHES (SVB), a protein with a conserved domain of unknown function (DUF538). The trichomes of the SVB mutants are smaller and exhibit branches of variable length and number.		#N/A	1.36E-03
F vs. M (23)	AT3G05960	STP6	sugar transporter known to function during pollen development and function (TAIR)	SnRK1 TOR		9.57	Encodes a hexose sugar transporter that is expressed in pollen. STP6 may play a role in providing sugars during late pollen maturation or pollen tube germination.	Use	sugar transporter	1.13E-03
F vs. L (23)	AT3G09440	HSP70 AT3G09440	HSP70 (TAIR)	Stress attenuation	HSP	9.56	Heat shock protein 70 (Hsp 70) family protein;(source:Araport11)	Use	HSP	1.25E-05
F vs. M (23)	AT5G25610	RD22	RD22 transcriptions are upregulated by ABA, salt and desiccation. RD22 is also upregulated by C3H17, which does not belong to the zinc finger proteins normally involved in the ABA dependent salt stress-response pathway (https://doi.org/10.1016/j.bbrc.2018.03.088)		ABA upregulated, Salt	9.42	responsive to dehydration 22 (RD22) mediated by ABA	Use	ABA	1.46E-03
F vs. S (23)	AT2G37760	AKR4C8	NADPH dependent reductases that are upregulated by cold, salt and drought stress (TAIR)	Stress signaling	Salt	8.95	Encodes an NADPH-dependent aldo-keto reductase that can act on a wide variety of substrates in vitro including aliphatic and aromatic aldehydes and steroids. Transcript levels for this gene are up-regulated in response to cold, salt, and drought stress.	Yes	Stress	2.96E-06
F vs. S (23)	AT4G38970	FBA2	Upregulated in response to drought stress (http://dx.doi.org/10.1016%2Fj.gene.2012.04.042)		Stress upregulated	8.81	Protein is tyrosine-phosphorylated and its phosphorylation state is modulated in response to ABA in Arabidopsis thaliana seeds.	Yes	ABA	< 1e-07
F vs. M (23)	AT4G38970	FBA2	Upregulated in response to drought stress (http://dx.doi.org/10.1016%2Fj.gene.2012.04.042)		Stress upregulated	8.78	Protein is tyrosine-phosphorylated and its phosphorylation state is modulated in response to ABA in Arabidopsis thaliana seeds.	Use	ABA	6.85E-04
F vs. L (23)	AT3G49110	PRXCA	Cell wall peroxidase that generates ROS. These ROS induce respiratory bursts, through RBOHD (DOI: 10.1111/mpp.12769), that exponentially increase H2O2 levels	ROS attenuation		8.47	Class III peroxidase Perx33. Expressed in roots. Located in the cell wall. Involved in cell elongation. Expression activated by light. May play a role in generating H2O2 during defense response. The mRNA is cell-to-cell mobile.	Use	H2O2, Ca, peroxidase	1.65E-05
F vs. L (23)	AT5G47910	RBOHD	Responsible for superoxide production during the HR and in response to heat and wounding (TAIR)	ABA signaling		8.17	NADPH/respiratory burst oxidase protein D (RbohD).Interacts with AtrbohF gene to fine tune the spatial control of ROI production and hypersensitive response to cell in and around infection site. The mRNA is cell-to-cell mobile.	Use	Rboh	< 1e-07
M vs. S (23)	AT2G26740	SHE			Stress	8.16	Encodes a soluble epoxide hydrolase whose expression is induced by auxin and water stress.			1.42E-02
F vs. M (23)	AT4G31700	RPS6A (RP)	Ribosomal protein required for gametogenesis; activated by TOR in the light (doi.org/10.1073/pnas.1809526115; 10.3389/fpls.2016.01611)	SnRK1 TOR	ribosome	8.07	Encodes a putative ribosomal protein S6 (rps6a). RPS6A and RPS6B are fully redundant and essential during gametogenesis.	Use	ribosome	1.19E-03
F vs. M (23)	AT2G37760	AKR4C8	NADPH dependent reductases that are upregulated by cold, salt and drought stress (TAIR)	Stress signaling	Salt	8.01	Encodes an NADPH-dependent aldo-keto reductase that can act on a wide variety of substrates in vitro including aliphatic and aromatic aldehydes and steroids. Transcript levels for this gene are up-regulated in response to cold, salt, and drought stress.	Use	Stress	1.73E-03
F vs. S (23)	AT1G51090	HMAD1	Upregulated by reactive nitrogen species (DOI: 10.3389/fpls.2016.01712)	Stress signaling		7.9	Heavy metal associated domain containing protein involved in plant immunity.Mutants show an increase in root length under NO stress and reduction in root length under H2O2 stress conditions. Mutants show increases in defense responses to pathogens including hypersensitive lesions, increased resistance and induction of SAR genes.	Yes	Stress, H2O2, Ca	2.96E-06
F vs. S (23)	AT5G51720	NEET	Fe-S clusters mediate ETC reactions, but they also react with oxygen to produce ROS. NEET proteins participate in regulating the biogenesis of Fe-S clusters and maintaining Fe and ROS homeostasis (10.1111/tpj.14581)	ROS attenuation	Homeostasis	7.84	Encodes a protein with biochemical, structural, and biophysical characteristics of a NEET protein. It plays a key role in plant development, senescence, reactive oxygen homeostasis, and Fe metabolism.	Yes	Homeostasis	2.29E-05
L vs. S (23)	AT2G26740	SHE			Stress	7.83	Encodes a soluble epoxide hydrolase whose expression is induced by auxin and water stress.			9.27E-03
F vs. S (23)	AT1G32640	MYC2	Transcriptional activator induced by dehydration stress, oxidative stress, JA, ABA and blue light. It positively regulates flavonoid biosynthesis and responses to oxidative stress (TAIR)	ABA signaling	ABA upregulated	7.8	Encodes a MYC-related transcriptional activator with a typical DNA binding domain of a basic helix-loop-helix leucine zipper motif. Binds to an extended G-Box promoter motif and interacts with Jasmonate ZIM-domain proteins. MYC2 interacts with EIN3 and EIL1 to repress hook curvature and resistance to Botrytis cinera.Its transcription is induced by dehydration stress, ABA treatment and blue light via CRY1. Negative regulator of blue light-mediated photomorphogenic growth and blue and far-red-light-regulated gene expression. Positive regulator of lateral root formation. Regulates diverse JA-dependent functions. Negatively regulates Trp metabolism and biosynthesis of Trp-derived secondary metabolites. Positively regulates flavonoid biosynthesis, resistance to insects, and response to oxidative stress. Regulates other transcription factors, and negatively regulates its own expression. For example it binds to and regulates the expression of NST1. Its stability is modulated by PUB10 through polyubiquitination.	Yes	Stress, ABA	2.96E-06
F vs. M (23)	AT3G54660	GR	A plastid glutathione reductase that maintains a highly reduced glutathione pool that balances ROS and enables the redox buffering required to maintain efficient photosynthesis (doi.org/10.1111/tpj.14791)	ROS attenuation	glutathione	7.70	Encodes glutathione reductase that is most likely localized in the chloroplast.	Use	glutathione	1.08E-04
F vs. L (23)	AT4G29780	AT4G29780	Responsive to hypoxia (TAIR)	Stress signaling		7.52	Expression of the gene is affected by multiple stresses. Knockout and overexpression lines show no obvious phenotypes.	Use	Stress	4.91E-05

F vs. M (23)	AT4G13770	REF2	NA		Homeostasis	7.51	Encodes a cytochrome p450 enzyme that catalyzes the initial conversion of aldoximes to thiohydroximates in the synthesis of glucosinolates not derived from tryptophan. Also has a role in auxin homeostasis.	Use	Homeostasis	1.92E-03
F vs. S (23)	AT3G03190	GSTF11	involved in responses to oxidative stress and toxin catabolic processes (TAIR)	ROS attenuation		7.5	Encodes glutathione transferase belonging to the phi class of GSTs. Naming convention according to Wagner et al. (2002).	Yes	glutathione	< 1e-07
F vs. L (23)	AT4G38970	FBA2	Upregulated in response to drought stress (http://dx.doi.org/10.1016%2Fj.gene.2012.04.042)		Stress upregulated	7.28	Protein is tyrosine-phosphorylated and its phosphorylation state is modulated in response to ABA in Arabidopsis thaliana seeds.	Use	ABA	3.93E-05
F vs. L (23)	AT3G54660	GR	A plastic glutathione reductase that maintains a highly reduced glutathione pool that balances ROS and enables the redox buffering required to maintain efficient photosynthesis (doi.org/10.1111/tpj.14791)	ROS attenuation	glutathione	7.26	Encodes glutathione reductase that is most likely localized in the chloroplast.	Use	glutathione	< 1e-07
L vs M (123)	AT4G31700	RPS6A (RP)	Ribosomal protein required for gametogenesis; activated by TOR in the light (doi.org/10.1073/pnas.1809526115; 10.3389/fpls.2016.01611)	SnRK1 TOR	ribosome	7.15	Encodes a putative ribosomal protein S6 (rps6a). RPS6A and RPS6B are fully redundant and essential during gametogenesis.	Use	ribosome	< 1e-07
F vs. M (23)	AT4G18920		NA		Histones	7.11	histone acetyltransferase (DUF1264);(source:Araport11)	Use	Histone	1.08E-04
F vs. M (23)	AT2G36830	GAMMA-TIP	NA			7.09	Encodes a tonoplast intrinsic protein, which functions as water channel. It has also been shown to be able to facilitate the transport of urea and hydrogen peroxide. Highly expressed in vascular tissues of the root, stem, cauline leaves and flowers but not in the apical meristems. The mRNA is cell-to-cell mobile.	No	H2O2	2.99E-03
L vs M (123)	AT3G12500	PR3	ethylene mediated signaling pathway		ET	6.78	encodes a basic chitinase involved in ethylene/jasmonic acid mediated signalling pathway during systemic acquired resistance based on expression	Use	ethylene	8.73E-05
F vs. L (23)	AT5G22580	AT5G22580	Stress responsive (TAIR)	Stress signaling		6.65	Stress responsive A/B Barrel Domain-containing protein;(source:Araport11)	Use	Stress	1.70E-03
F vs. S (23)	AT2G18470	PERK4	PERK4 enhances ROS accumulation by enabling ABA induced expression of RBOHC (doi.org/10.1071/FP18219)	ROS attenuation	Homeostasis	6.65	Proline-rich extensin-like receptor kinase 4. Functions at an early stage of ABA signalling inhibiting primary root cell elongation by perturbing Ca2+	Yes	ABA, Homeostasis	1.49E-04
F vs. L (23)	AT3G55610	P5CS2	Induced by dehydration, salt and ABA (TAIR)		ABA upregulated, Salt	6.63	encodes delta 1-pyrroline-5-carboxylate synthetase B. Gene expression is induced by dehydration, high salt and ABA. Knock-out mutations in P5CS2 are embryo-lethal. P5CS2 appears to be present in different cells and/or different subcellular locations from P5CS1 in a tissue-dependent manner. Mutants are defective in pollen development.	Use	ABA	2.52E-03
F vs. S (23)	AT4G29780	AT4G29780	Responsive to hypoxia (TAIR)	Stress signaling		6.6	Expression of the gene is affected by multiple stresses. Knockout and overexpression lines show no obvious phenotypes.	Yes	Stress	< 1e-07
F vs. L (23)	AT2G18470	PERK4	PERK4 enhances ROS accumulation by enabling ABA induced expression of RBOHC (doi.org/10.1071/FP18219)	ROS attenuation	Homeostasis	6.52	Proline-rich extensin-like receptor kinase 4. Functions at an early stage of ABA signalling inhibiting primary root cell elongation by perturbing Ca2+	Use	ABA, Homeostasis	1.75E-04
F vs. L (23)	AT2G35940	BLH1	BLH overexpression leads to multiple eggs within the female gametophyte (TAIR)		Ovule gametophyte	6.51	Encodes a member of the BEL-like homeodomain protein family. Ecotopic expression in the embryo sac leads to defects in nuclear migration and cellularization and embryo sacs with multiple egg cells. Loss of function alleles have no female gametophyte defects. The ecotopic expression phenotype requires KNAT3 because it can be suppressed by loss of KNAT3 function alleles. Localized to the nucleus but interaction with OFP1 relocates it to the cytoplasm.	Use	gametophyte	4.57E-06
F vs. L (23)	AT3G48610	NPC6	NPC6 and NPT2 are required for gametophyte formation (10.1111/nph.15147)		Ovule gametophyte	6.42	Non-specific phospholipase C6 involved in gametophyte development.	Use	gametophyte	< 1e-07
F vs. L (23)	AT3G05890	LT16b	Salt responsive protein (TAIR)	Stress attenuation	Salt	6.35	Low temperature and salt responsive protein family;(source:Araport11)	Use	salt responsive	1.31E-03
F vs. L (23)	AT5G25610	RD22	RD22 transcriptions are upregulated by ABA, salt and desiccation. RD22 is also upregulated by C3H17, which does not belong to the zinc finger proteins normally involved in the ABA dependent salt stress-response pathway (https://doi.org/10.1016/j.bbrc.2018.03.088)		ABA upregulated, Salt	6.34	responsive to dehydration 22 (RD22) mediated by ABA	Use	ABA	4.57E-06
F vs. S (23)	AT3G12580	HSP70 AT3G12580	HSP70 is a HSP chaperone that is upregulated by unfolded proteins and is involved in protein folding and refolding (TAIR)	Stress attenuation	HSP	6.25	heat shock protein 70;(source:Araport11)	Yes	Hsp	2.96E-06
F vs. S (23)	AT3G52880	MDAR1	MDAR1 is a peroxisomal monodehydroascorbate reductase that functions in the ascorbate-glutathione cycle to remove toxic H2O2 (TAIR)	ROS attenuation		6.19	Encodes a peroxisomal monodehydroascorbate reductase, involved in the ascorbate-glutathione cycle which removes toxic H2O2	Yes	glutathione, H2O2, Ca, Ascorbate	8.26E-06
F vs. M (23)	AT3G09440	HSP70 AT3G09440	HSP70 (TAIR)	Stress attenuation	HSP	6.11	Heat shock protein 70 (Hsp 70) family protein;(source:Araport11)	Use	HSP	8.40E-04
F vs. L (23)	AT2G30020	AP2C1	AP2C1 is activated by ABA and pathogens. It appears to modulate K+ homeostasis by dephosphorylation inactivating CIPK9, which normally enhances K+ ion uptake through K+ channels, particularly under low K+ conditions (doi:10.1093/jxb/ery182). It also moderates responses to pathogens by dephosphorylation inactivating MPK4,6 in the cytoplasm and nucleus. These MAPKs affect the expression of hundreds of genes by modifying the activity of 88 TFs from 21 families (doi:10.1093/jxb/erw485).	ABA signaling	Homeostasis	6.08	Encodes AP2C1. Belongs to the clade B of the PP2C-superfamily. Acts as a MAPK phosphatase that negatively regulates MPK4 and MPK6.	Use	PP2C, MAPK	9.99E-04
F vs. M (23)	AT1G32640	MYC2	Transcriptional activator induced by dehydration stress, oxidative stress, JA, ABA and blue light. It positively regulates flavonoid biosynthesis and responses to oxidative stress (TAIR)	ABA signaling	ABA upregulated	6.03	Encodes a MYC-related transcriptional activator with a typical DNA binding domain of a basic helix-loop-helix leucine zipper motif. Binds to an extended G-Box promoter motif and interacts with Jasmonate ZIM-domain proteins. MYC2 interacts with EIN3 and EIL1 to repress hook curvature and resistance to Botrytis cinera.Its transcription is induced by dehydration stress, ABA treatment and blue light via CRY1. Negative regulator of blue light-mediated photomorphogenic growth and blue and far-red-light-regulated gene expression. Positive regulator of lateral root formation. Regulates diverse JA-dependent functions. Negatively regulates Trp metabolism and biosynthesis of Trp-derived secondary metabolites. Positively regulates flavonoid biosynthesis, resistance to insects, and response to oxidative stress. Regulates other transcription factors, and negatively regulates its own expression. For example it binds to and regulates the expression of NST1. Its stability is modulated by PUB10 through polyubiquitination.	Use	Stress, ABA	2.02E-03
M vs. S (23)	AT3G12580	HSP70 AT3G12580	HSP70 is a HSP chaperone that is upregulated by unfolded proteins and is involved in protein folding and refolding (TAIR)		HSP	5.93	heat shock protein 70;(source:Araport11)			1.98E-02
M vs. S (23)	AT1G78370	GSTU20	GSTU20 encodes a Tau class glutathione transferase. It is upregulated by stress and interacts with the phytochrome signaling protein far-red insensitive 219. GSTU20 is required for plant responses to far red light (doi.org/10.1199/tab.0131)		glutathione	5.91	Encodes glutathione transferase belonging to the tau class of GSTs. Naming convention according to Wagner et al. (2002).			1.94E-02

F vs. S (23)	AT4G36250	AT4G36250	Not involved in plant stress (TAIR)			5.88	Encodes a putative aldehyde dehydrogenase. The gene is not responsive to osmotic stress and is expressed constitutively at a low level in plantlets and root cultures.	Yes	Stress	6.68E-06
L vs M (123)	AT4G25100	FSD1	chloroplastic SOD that removes superoxide radicals and is induced by salt stress; also functions as an osmoprotectant, DOI: 10.1111/pce.13773	ROS attenuation	Salt	5.84	Fe-superoxide dismutase	Use	H2O2, superoxide dismutase	5.18E-03
F vs. S (23)	AT5G38760	LEA AT5G38760	LEA (TAIR)		LEA	5.82	Late embryogenesis abundant protein (LEA) family protein;(source:Araport11)	Yes	LEA	< 1e-07
F vs. M (23)	AT1G70790	CAR9	CAR proteins postively regulate ABA signaling by interacting with PYR/PYL and ABA at the plasma membrane (doi/10.1105/tpc.114.129973). This complex inactivates PP2Cs, thus activating SnRKs.	ABA signaling		5.80	C2-domain ABA-related (CAR) protein, involved in the recruitment of ABA receptors to the plasma membrane to facilitate ABA signaling. Its stability and dynamic localization is regulated by LOT1.	Use	ABA	3.50E-04
F vs. S (23)	AT3G46620	RDUF1	RDUF1 is upregulated by salt and positively regulates plant tolerances to osmotic and dehydration stress (doi:10.1371/journal.pone.0071078)	Stress signaling	Salt	5.79	Encodes an ABA- and drought-induced RING-DUF1117 gene whose mutation results in hyposensitive phenotypes toward ABA in terms of germination rate and stomatal closure and markedly reduced tolerance to drought stress relative to wild-type plants.	Yes	Stress, ABA	< 1e-07
F vs. S (23)	AT4G13770	REF2	Involved in auxin homeostasis (TAIR)		Homeostasis	5.77	Encodes a cytochrome p450 enzyme that catalyzes the initial conversion of aldoximes to thiohydroximates in the synthesis of glucosinolates not derived from tryptophan. Also has a role in auxin homeostasis.	Yes	Homeostasis	< 1e-07
F vs. M (23)	AT1G19550	DHAR	A major dehydroascorbate reductase with glutathione dehydrogenase (ascorbate) activity. Functions with APX6 in cellular ROS detoxification by coupling H2O2 to reduced glutathione. Small increases in DHAR and APX6 significantly enhance tolerance to abiotic stresses, reviewed in (doi.org/10.1093/jxb/eraa107)	ROS attenuation		5.72	Glutathione S-transferase family protein;(source:Araport11)	Use	glutathione	< 1e-07
F vs. M (23)	AT5G05600	JOX2	JOX2 is induced by JA. It downregulates plant immunity signaling and responses by oxidizing JA (doi.org/10.1073/pnas.1701101114)			5.71	Encodes a protein with similarity to flavonol synthases that is involved in the detoxifcation polycyclic aromatic hydrocarbons.One of 4 paralogs encoding a 2-oxoglutarate/Fe(II)-dependent oxygenases that hydroxylates JA to 12-OH-JA.	No	flavonol	4.10E-03
F vs. L (23)	AT3G26520	TIP2	TIP2 is a tonoplast protein that is upregulated by ABA or salt stress (TAIR)	ABA signaling	ABA upregulated, Salt	5.69	gamma tonoplast intrinsic protein 2 (TIP2). expressed throughout the plant and transcript level is increased upon NaCl or ABA treatments. NaCl stress-sensitive yeast mutant strains exhibit more resistance to salt when expressing this protein.	Use	Stress, ABA	1.05E-05
F vs. M (23)	AT3G07700	SIA1	SIA1 is induced by salt stress and mediates salt-induces changes in lipid membranes (TAIR)	Stress signaling	Salt	5.65	ABC1K7 is a member of an atypical protein kinase family that is induced by salt stress. Loss of function mutations affect the metabolic profile of chloroplast lipids. It appears to function along with ABC1K8 in mediating lipid membrane changes in response to stress.	Use	Stress	3.31E-03
F vs. M (23)	AT5G42050	NRP	ABA signaling is negatively regulated by FyPP3, which deactivates ABI5 by dephosphorylating it. NRP facilitates the degradation of FyPP3, thus positively regulating ABA signaling. FyPP3 can also dephosphorylate NRP (10.1016/j.molp.2017.11.006)	ABA signaling	ABA upregulated	5.62	Stress responsive asparagine-rich protein. Binds to PevD (Verticillium dahliae) fungal effector protein. NRP interacts with CRY2, leading to increased cytoplasmic accumulation of CRY2 in a blue light-independent manner (PMID:28633330).NRP also binds FyPP3 and recruits it to endosomes and thus targets it for degradation.	Use	Stress	8.23E-04
F vs. S (23)	AT4G25100	FSD1	chloroplastic SOD that removes superoxide radicals and is induced by salt stress; also functions as an osmoprotectant, DOI: 10.1111/pce.13773	ROS attenuation	Salt	5.59	Fe-superoxide dismutase	Yes	H2O2, superoxide dismutase	2.46E-05
F vs. M (23)	AT2G18470	PERK4	PERK4 enhances ROS accumulation by enabling ABA induced expression of RBOHC (doi.org/10.1071/FP18219)	ROS attenuation	Homeostasis	5.55	Proline-rich extensin-like receptor kinase 4. Functions at an early stage of ABA signalling inhibiting primary root cell elongation by perturbing Ca2+	Use	ABA, Homeostasis	6.16E-04
F vs. S (23)	AT2G13680	CALS5	CALS5 is responsible for callose deposits in the walls of meiocytes (TAIR)	Flowering, Stress attenuation	Meiosis	5.54	Responsible for the synthesis of callose deposited at the primary cell wall of meiocytes, tetrads and microspores. Required for exine formation during microgametogenesis and for pollen viability. Highest expression in meiocytes, tetrads, microspores and mature pollen.	Yes	meiosis	4.48E-04
F vs. L (23)	AT2G37760	AKR4C8	NADPH dependent reductases that are upregulated by cold, salt and drought stress (TAIR)	Stress signaling	Salt	5.49	Encodes an NADPH-dependent aldo-keto reductase that can act on a wide variety of substrates in vitro including aliphatic and aromatic aldehydes and steroids. Transcript levels for this gene are up-regulated in response to cold, salt, and drought stress.	Use	Stress	1.23E-04
F vs. M (23)	AT2G13680	CALS5	CALS5 is responsible for callose deposits in the walls of meiocytes (TAIR)	Flowering, Stress attenuation	Meiosis	5.45	Responsible for the synthesis of callose deposited at the primary cell wall of meiocytes, tetrads and microspores. Required for exine formation during microgametogenesis and for pollen viability. Highest expression in meiocytes, tetrads, microspores and mature pollen.	Use	Meiocyte callose deposition	5.05E-03
F vs. M (23)	AT4G34710	ADC2	Is osmotic stress induced; upregulated by exogenous sucrose - osmotic effect? (doi.org/10.1016/j.jplph.2019.04.012)	Stress signaling	Salt	5.43	Encodes a arginine decarboxylase (ADC), a rate-limiting enzyme that catalyzes the first step of polyamine (PA) biosynthesis via ADC pathway in Arabidopsis thaliana. Arabidopsis genome has two ADC paralogs, ADC1 and ADC2. ADC2 is stress-inducible (osmotic stress). Double mutant analysis showed that ADC genes are essential for the production of PA, and are required for normal seed development. Overexpression causes phenotypes similar to GA-deficient plants and these plants show reduced levels of GA due to lower expression levels of AtGA20ox1, AtGA3ox3 and AtGA3ox1.	Use	Stress	2.52E-03
F vs. L (23)	AT3G53420	PIP2A	PIP2A is a water and H2O2 transporting transmembrane aquaporin that is downregulated by salt stress. RBOHF generated ROS in the apoplast causes PIP2A clustering and subsequent endocytosis of the PIP2A cluster thus potentially regulating levels of cytoplasmic ROS (doi/10.1104/pp.18.01065)	ROS attenuation	downregulated by salt stress	5.29	a member of the plasma membrane intrinsic protein subfamily PIP2. localizes to the plasma membrane and exhibits water transport activity in Xenopus oocyte. expressed specifically in the vascular bundles and protein level increases slightly during leaf dev. When expressed in yeast cells can conduct hydrogen peroxide into those cells.	Use	H2O2	8.91E-05
F vs. M (23)	AT4G35060	HIPP25	NA			5.27	Heavy metal transport/detoxification superfamily protein;(source:Araport11)		detoxification	4.37E-03
F vs. L (23)	AT5G38760	LEA AT5G38760	LEA (TAIR)		LEA	5.25	Late embryogenesis abundant protein (LEA) family protein;(source:Araport11)	Use	LEA	< 1e-07
F vs. L (23)	AT2G36830	GAMMA-TIP	NA			5.19	Encodes a tonoplast intrinsic protein, which functions as water channel. It has also been shown to be able to facilitate the transport of urea and hydrogen peroxide. Highly expressed in vascular tissues of the root, stem, cauline leaves and flowers but not in the apical meristems. The mRNA is cell-to-cell mobile.	No	H2O2	2.22E-05
F vs. L (23)	AT3G45640	MPK3	MPK3 is upregulated by ABA and phospholipase D alpha 1 (PLDα1) signaling in response to salinity stress. MPK3 and PLDα1 function together to suppress growth during salinity stress (doi.org/10.3389/fpls.2019.00275)	ABA signaling	ABA upregulated	5.16	Encodes a mitogen-activated kinase whose mRNA levels increase in response to touch, cold, salinity stress and chitin oligomers.Also functions in ovule development. Heterozygous MPK3 mutants in a homozygous MPK6 background are female sterile due to defects in integument development. MPK3 can be dephosphorylated by MKP2 in vitro. The mRNA is cell-to-cell	Use	Stress	1.22E-04

F vs. M (23)	AT5G47910	RBOHD	Responsible for superoxide production during the HR and in response to heat and wounding (TAIR)	ABA signaling		5.10	NADPH/respiratory burst oxidase protein D (RbohD).Interacts with AtrbohF gene to fine tune the spatial control of ROI production and hypersensitive response to cell in and around infection site. The mRNA is cell-to-cell mobile.	Use	Rboh	3.68E-04
F vs. L (23)	AT2G18390	ARL2	NA		ARF	5.04	Encodes a member of ARF-like GTPase family. A thaliana has 21 members, in two subfamilies, ARF and ARF-like (ARL) GTPases. Mutant has abnormal mitosis and cell cycle control during seed development.	Use	ARF	7.75E-06
F vs. S (23)	AT1G59730	TH7	TH7 functions as a redox sensor/transmitter in the ER where it is involved in protein folding and the transport of NADPH to peroxisomes	ROS attenuation	Thioredoxin	5.04	Thioredoxin H-type 7 , oxidoreductase located in cytosol and ER. Interacts with GPT1.	Yes	Thioredoxin	7.07E-05
F vs. L (23)	AT4G26970	ACON2	May participate in the TCA cycle. May play a role in the response to ROS by upregulating CSD2 (superoxide dismutase)	ROS attenuation		4.99	Encodes an aconitase that can catalyze the conversion of citrate to isocitrate through a cis-aconitate intermediate, indicating that it may participate in the TCA cycle and other primary metabolic pathways. The protein is believed to accumulate in the mitochondria and the cytosol. It affects CSD2 (At2g28190 - a superoxide dismutase) transcript levels and may play a role in the response to oxidative stress. One member of the family (ACO1 - At35830) was shown to specifically bind to the 5' UTR of CSD2 in vitro. The mRNA is cell-to-cell	Use	Stress, H2O2, superoxide dismutase	5.35E-05
F vs. M (23)	AT1G18150	MPK8	MPK8 promotes seed germination possibly by enhancing TCP14 activity, a TF regulating germination (10.1111/tpj.14461). It is also involved in regulating Ca2+ and ROS in the wound signaling pathway (TAIR)	ROS attenuation		4.95	Encodes mitogen-activated protein kinase 8 (MPK8). MPK8 connects protein phosphorylation, Ca2+, and ROS in the wound-signaling pathway.	Use	ROS	1.24E-03
F vs. L (23)	AT3G07700	SIA1	SIA1 is induced by salt stress and mediates salt-induces changes in lipid membranes (TAIR)	Stress signaling	Salt	4.90	ABC1K7 is a member of an atypical protein kinase family that is induced by salt stress. Loss of function mutations affect the metabolic profile of chloroplast lipids. It appears to function along with ABC1K8 in mediating lipid membrane changes in response to stress.	Use	Stress	6.09E-04
F vs. M (23)	AT5G51720	NEET	Fe-S clusters mediate ETC reactions, but they also react with oxygen to produce ROS. NEET proteins participate in regulating the biogenesis of Fe-S clusters and maintaining Fe and ROS homeostasis (10.1111/tpj.14581)	ROS attenuation	Homeostasis	4.86	Encodes a protein with biochemical, structural, and biophysical characteristics of a NEET protein. It plays a key role in plant development, senescence, reactive oxygen homeostasis, and Fe metabolism.	Use	Homeostasis	1.21E-03
F vs. L (23)	AT1G59730	TH7	TH7 functions as a redox sensor/transmitter in the ER where it is involved in protein folding and the transport of NADPH to peroxisomes	ROS attenuation	Thioredoxin	4.86	Thioredoxin H-type 7 , oxidoreductase located in cytosol and ER. Interacts with GPT1.	Use	Thioredoxin	5.02E-05
F vs. M (23)	AT5G23060	CAS	Calcium sensing protein localized to choloroplasts that is involved in regulating stomatal closure as well as other photoacclimation processes following phosphorylation by Ca2+ dependent kinases (doi.org/10.3389/fpls.2019.00974)			4.71	Encodes a chloroplast-localized protein that modulates cytoplasmic Ca2+ concentration and is crucial for proper stomatal regulation in response to elevations of external Ca2+. Phosphorylation of this protein is dependent on calcium.	No	Ca	8.70E-04
F vs. S (23)	AT5G66400	RAB18	RAB18 is an ABA upregulated dehydrin that may interact with aquaporins in drought stress tolerance responses (doi.org/10.1016/j.bbrc.2019.01.095)		ABA, upregulated, stress attenuation	4.71	Belongs to the dehydrin protein family, which contains highly conserved stretches of 7-17 residues that are repetitively scattered in their sequences, the K-, S-, Y- and lysine rich segments. ABA- and drought-induced glycine-rice dehydrin protein. The ABA-induced expression of RAB18 was reduced following ACC application, indicating that ethylene inhibits the ABA signaling pathway. RAB18 is also expressed in response to the formation of the phospholipid diacylglycerol pyrophosphate. COR47 and RAB18 double overexpressor plants are cold tolerant. Expressed in guard cells.	Yes	ABA, ethylene	6.68E-06
F vs. S (23)	AT4G26260	MIOX	Important enzyme in the production of ascorbate, upregulation leads to increased growth, photosynthesis, intracellular glucose levels, defense response genes, and stress response genes (doi.org/10.1002/pld3.165)	ROS attenuation		4.68	Encodes a myo-inositol oxygenase, which is the first enzyme in the inositol route to ascorbate (L‐ascorbic acid, AsA, vitamin C). Overexpression results in enhanced biomass and abiotic stress tolerance.	Yes	Stress, Ascorbate	8.21E-05
F vs. M (23)	AT3G23920	BAM1	BAM1 is Involved in starch degradation to yield cytoplasmic glucose pools for bioenergetic and metabolic functions (doi.org/10.1105/tpc.18.00802)	Stress attenuation		4.67	Encodes a chloroplast beta-amylase. Is necessary for leaf starch breakdown in the absence of BAM3. Activity of BAM1 increases 4 days after osmotic stress. BAM1 has a higher temperature optimum than BAM3 (PMID:25293962).	Use	Stress	4.88E-03
L vs. S (23)	AT2G25940	AT2G25940	Vacuolar-processing enzyme alpha-isozyme		Stress	4.62	Encodes a vacuolar processing enzyme belonging to a novel group of cysteine proteinases that is expressed in vegetative organs and is upregulated in association with various types of cell death and under stressed conditions.			1.07E-02
F vs. L (23)	AT3G15353	MT3	MT3 limits oxidative damage by detoxifying excess copper and other metals (TAIR)			4.60	metallothionein, binds to and detoxifies excess copper and other metals, limiting oxidative damage	No	oxidative damage	1.05E-04
F vs. M (23)	AT3G21180	ACA9	NA			4.58	one of the type IIB calcium pump isoforms. encodes an autoinhibited Ca(2+)-ATPase that contains an N-terminal calmodulin binding autoinhibitory domain.	No	Ca, Calmodulin	1.68E-03
F vs. M (23)	AT4G25670	DOB1	Upregulated early by onset of salt stress (doi.org/10.1016/j.isci.2019.10.043)	Stress signaling	Salt	4.58	stress response NST1-like protein;(source:Araport11)	Use	Stress	1.88E-03
F vs. L (23)	AT4G13230	LEA AT4G13230	LEA (TAIR)		LEA	4.56	Late embryogenesis abundant protein (LEA) family protein;(source:Araport11)	Use	LEA	4.54E-05
F vs. M (23)	AT1G59730	TH7	TH7 functions as a redox sensor/transmitter in the ER where it is involved in protein folding and the transport of NADPH to peroxisomes	ROS attenuation	Thioredoxin	4.54	Thioredoxin H-type 7 , oxidoreductase located in cytosol and ER. Interacts with GPT1.	Use	Thioredoxin	2.49E-03
F vs. L (23)	AT2G37770	ChlAKR	Upregulated by cold, salt and drought, involved in detoxifying sugar derived reactive carbonyl compounds (TAIR)	Stress signaling	Salt	4.53	Encodes an NADPH-dependent aldo-keto reductase that can act on a wide variety of substrates in vitro including saturated and unsaturated aldehydes, steroids, and sugars. GFP-tagged AKR4C9 localizes to the chloroplast where it may play a role in detoxifying reactive carbonyl compounds that threaten to impair the photosynthetic process. Transcript levels for this gene are up-regulated in response to cold, salt, and drought stress.	Use	Stress	7.75E-06
F vs. S (23)	AT1G68440	AT1G68440	Transmembrane protein upregulated by ABA and multiple stresses including heat, salt and drought (TAIR)	ABA signaling	ABA upregulated, Salt	4.51	Transmembrane protein;(source:Araport11). Expression induced by abiotic stressors such as ABA, drought, heat, light, NaCl, osmotic stress and wounding.	Yes	Stress, ABA	9.67E-06
F vs. M (23)	AT2G37770	ChlAKR	Upregulated by cold, salt and drought, involved in detoxifying sugar derived reactive carbonyl compounds (TAIR)	Stress signaling	Salt	4.51	Encodes an NADPH-dependent aldo-keto reductase that can act on a wide variety of substrates in vitro including saturated and unsaturated aldehydes, steroids, and sugars. GFP-tagged AKR4C9 localizes to the chloroplast where it may play a role in detoxifying reactive carbonyl compounds that threaten to impair the photosynthetic process. Transcript levels for this gene are up-regulated in response to cold, salt, and drought stress.	Use	Stress	4.55E-04
F vs. M (23)	AT5G01600	FER1	NA			4.51	Encodes a ferretin protein that is targeted to the chloroplast. Member of a Ferritin gene family. Gene expression is induced in response to iron overload and by nitric oxide. Expression of the gene is downregulated in the presence of paraquat, an inducer of photoxidative stress.	No	Stress	4.13E-03
F vs. M (23)	AT2G39770	VTC1	Essential enzyme in ascorbic acid biosynthesis. High levels of VTC1 are correlated with high levels of ascorbic acid (doi.org/10.1093/jxb/erw223)	ROS attenuation		4.51	Encodes a GDP-mannose pyrophosphorylase/ mannose-1-pyrophosphatase. This enzyme provides GDP-mannose, which is used for cell wall carbohydrate biosynthesis and protein glycosylation as well as for ascorbate (vitamin C) biosynthesis. Mutations in this gene confer hypersensitivity to NH4+.	Use	Ascorbate	5.21E-03

F vs. S (23)	AT3G23920	BAM1	BAM1 is Involved in starch degradation to yield cytoplasmic glucose pools for bioenergetic and metabolic functions (doi.org/10.1105/tpc.18.00802)	Stress attenuation		4.46	Encodes a chloroplast beta-amylase. Is necessary for leaf starch breakdown in the absence of BAM3.Activity of BAM1 increases 4 days after osmotic stress. BAM1 has a higher temperature optimum than BAM3 (PMID:25293962).	Yes	Stress	6.03E-04
F vs. L (23)	AT1G20620	CAT3	CAT3 catalyzes the breakdown of H2O2, but it also transnitrosylates GSNOR1 to activate redox signaling in the presence of NO (doi.org/10.1016/j.devcel.2020.03.020)	ROS attenuation, REDOX signaling		4.39	Catalase, catalyzes the breakdown of hydrogen peroxide (H2O2) into water and oxygen. The mRNA is cell-to-cell mobile.	Use	H2O2, Ca, catalase	8.86E-04
F vs. L (23)	AT5G60450	ARF4	auxin response gene (TAIR)			4.34	Encodes a member of the ARF family of transcription factors which mediate auxin responses. ARF4 appears to have redundant function with ETT(ARF3) in specifying abaxial cell identity.	No	ABA, ARF	1.88E-04
F vs. S (23)	AT4G33150	LKR	LKR is a reductase and dehydrogenase involved in lysine degradation. It is upregulated by ABA, JA and sucrose starvation (TAIR)		ABA upregulated	4.34	This is a splice variant of the LKR/SDH locus. It encodes a bifunctional polypeptide lysine-ketoglutarate reductase and saccharopine dehydrogenase involved in lysine degradation. There is another splice variant that encodes a mono saccharopine dehydrogenase protein. Gene expression is induced by abscisic acid, jasmonate, and under sucrose starvation.	Yes	ABA	6.65E-05
F vs. M (23)	AT3G04120	GADPH subunit	Potentially involved in ROS signaling (TAIR)	ROS attenuation		4.31	encodes cytosolic GADPH (C subunit) involved in the glycolytic pathway but also interacts with H2O2 potentially placing it in a signalling cascade induced by ROS. The mRNA is cell-to-cell mobile.	Use	H2O2, Ca, ROS	1.95E-03
F vs. S (23)	AT1G70790	CAR9	CAR proteins postively regulate ABA signaling by interacting with PYR/PYL and ABA at the plasma membrane (doi/10.1105/tpc.114.129973). This complex inactivates PP2Cs, thus activating SnRKs.	ABA signaling		4.3	C2-domain ABA-related (CAR) protein, involved in the recruitment of ABA receptors to the plasma membrane to facilitate ABA signaling. Its stability and dynamic localization is regulated by LOT1.	Yes	ABA	3.79E-05
F vs. L (23)	AT4G23100	GSH1	GSH1 catalyzes the rate-limiting step in glutathione biosynthesis (TAIR)	ROS attenuation		4.29	Encodes the enzyme glutamate-cysteine ligase catalyzing the first, and rate-limiting, step of glutathione biosynthesis. Required for cell proliferation at the root tip. Involved in susceptibility to the bacterial pathogen Pseudomonas syringae. Mutants are phytoalexin defective.	Use	glutathione	1.05E-05
F vs. M (23)	AT4G15440	CYP74B2	NA	Stress signaling		4.24	Encodes a hydroperoxide lyase. Also a member of the CYP74B cytochrome p450 family. In the ecotype Columbia (Col) the gene contains a 10-nucleotide deletion in its first exon that causes it to code for a truncated protein that results in a non-functional hydroperoxide lyase.	Use	H2O2	3.28E-03
F vs. M (23)	AT4G13230	LEA AT4G13230	LEA (TAIR)		LEA	4.23	Late embryogenesis abundant protein (LEA) family protein;(source:Araport11)	Use	LEA	2.54E-03
F vs. S (23)	AT4G13230	LEA AT4G13230	LEA (TAIR)		LEA	4.23	Late embryogenesis abundant protein (LEA) family protein;(source:Araport11)	Yes	LEA	2.12E-04
F vs. S (23)	AT4G27410	RD26	RD26 is a NAC TF that upregulates drought response genes. It is upregulated by BIN2, which is upregulated when ABA accumulates in response to drought stress and relieves PP2C inhibition of BIN2 (doi: 10.1111/tpj.14484)	ABA BR signaling	ABA upregulated	4.22	Encodes a NAC transcription factor induced in response to desiccation. It is localized to the nucleus and acts as a transcriptional activator in ABA-mediated dehydration response.	Yes	ABA	4.48E-05
F vs. M (23)	AT1G64060	RBOHF	RBOHF and D produce apoplastic superoxide in response to ABA. Their expression (superoxide signaling) is critical to cell survival under ER stress (doi: 10.1111/tpj.14091)	ABA signaling	ABA upregulated	4.20	Interacts with AtrbohD gene to fine tune the spatial control of ROI production and hypersensitive response to cell in and around infection site.	Use	Rboh	5.79E-03
F vs. L (23)	AT1G18150	MPK8	MPK8 promotes seed germination possibly by enhancing TCP14 activity, a TF regulating germination (10.1111/tpj.14461). It is also involved in regulating Ca2+ and ROS in the wound signaling pathway (TAIR)	ROS attenuation		4.14	Encodes mitogen-activated protein kinase 8 (MPK8). MPK8 connects protein phosphorylation, Ca2+, and ROS in the wound-signaling pathway.	Use	ROS	5.24E-04
F vs. L (23)	AT5G42050	NRP	ABA signaling is negatively regulated by FyPP3, which deactivates ABI5 by dephosphorylating it. NRP facilitates the degradation of FyPP3, thus positively regulating ABA signaling. FyPP3 can also dephosphorylate NRP (10.1016/j.molp.2017.11.006)	ABA signaling	ABA upregulated	4.12	Stress responsive asparagine-rich protein. Binds to PevD (Verticillium dahliae) fungal effector protein. NRP interacts with CRY2, leading to increased cytoplasmic accumulation of CRY2 in a blue light-independent manner (PMID:28633330).NRP also binds FyPP3 and recruits it to endosomes and thus targets it for degradation.	Use	Stress	4.51E-04
F vs. L (23)	AT4G25670	DOB1	Upregulated early by onset of salt stress (doi.org/10.1016/j.isci.2019.10.043)	Stress signaling	Salt	4.11	stress response NST1-like protein;(source:Araport11)	Use	Stress	1.65E-05
F vs. L (23)	AT1G32640	MYC2	Transcriptional activator induced by dehydration stress, oxidative stress, JA, ABA and blue light. It positively regulates flavonoid biosynthesis and responses to oxidative stress (TAIR)	ABA signaling	ABA upregulated	4.11	Encodes a MYC-related transcriptional activator with a typical DNA binding domain of a basic helix-loop-helix leucine zipper motif. Binds to an extended G-Box promoter motif and interacts with Jasmonate ZIM-domain proteins. MYC2 interacts with EIN3 and EIL1 to repress hook curvature and resistance to Botrytis cinera.Its transcription is induced by dehydration stress, ABA treatment and blue light via CRY1. Negative regulator of blue light-mediated photomorphogenic growth and blue and far-red-light-regulated gene expression. Positive regulator of lateral root formation. Regulates diverse JA-dependent functions. Negatively regulates Trp metabolism and biosynthesis of Trp-derived secondary metabolites. Positively regulates flavonoid biosynthesis, resistance to insects, and response to oxidative stress. Regulates other transcription factors, and negatively regulates its own expression. For example it binds to and regulates the expression of NST1. Its stability is modulated by PUB10 through polyubiquitination.	Use	Stress, ABA	3.64E-05
F vs. S (23)	AT2G38750	ANN4	ANN4 is an annexin that binds to plasma membranes where it enhances Ca2+ uptake during stress. It is upregulated by ABA, heat, drought, salt and dehydration and is downregulated by the TF MYB30 (doi: 10.1111/nph.14679)	ABA	ABA upregulated, Ca, Salt	4.09	Annexins are a family of calcium dependent membrane binding proteins though to be involved in Golgi mediated secretion. This is one of four annexins identified in Arabidopsis.	Yes	Ca	2.71E-04
F vs. M (23)	AT5G38760	LEA AT5G38760	LEA (TAIR)		LEA	4.07	Late embryogenesis abundant protein (LEA) family protein;(source:Araport11)	Use	LEA	1.07E-03
F vs. M (23)	AT4G26970	ACON2	May participate in the TCA cycle. May play a role in the response to ROS by upregulating CSD2 (superoxide dismutase)	ROS attenuation		4.06	Encodes an aconitase that can catalyze the conversion of citrate to isocitrate through a cis-aconitate intermediate, indicating that it may participate in the TCA cycle and other primary metabolic pathways. The protein is believed to accumulate in the mitochondria and the cytosol. It affects CSD2 (At2g28190 - a superoxide dismutase) transcript levels and may play a role in the response to oxidative stress. One member of the family (ACO1 - At35830) was shown to specifically bind to the 5' UTR of CSD2 in vitro. The mRNA is cell-to-cell	Use	Stress, H2O2, superoxide dismutase	4.98E-03
F vs. M (23)	AT2G18390	ARL2	NA		ARF	4.04	Encodes a member of ARF-like GTPase family. A thaliana has 21 members, in two subfamilies, ARF and ARF-like (ARL) GTPases. Mutant has abnormal mitosis and cell cycle control during seed development.	Use	ARF	1.68E-03
F vs. M (23)	AT1G78320	GSTU23	Especially adapted for detoxifying ROS under high oxidative stress conditions (10.1016/j.bbagen.2017.10.007)	ROS attenuation		4.04	Encodes glutathione transferase belonging to the tau class of GSTs. Naming convention according to Wagner et al. (2002).	Use	glutathione	3.22E-03
F vs. M (23)	AT2G18400	AT2G18400 (RP)			ribosome	3.98	ribosomal protein L6 family protein;(source:Araport11)		#N/A	3.95E-04

F vs. M (23)	AT1G59640	BPE	NA		Flowering	3.97	A basic helix-loop-helix encoding gene (BIGPETAL, BPE) involved in the control of petal size. BPE is expressed via two mRNAs derived from an alternative splicing event. The BPEub (AT1G59640.1)transcript is expressed ubiquitously, whereas the BPEp (AT1G59640.2) transcript is preferentially expressed in petals. Plants that lack the petal-expressed variant BPEp have larger petals as a result of increased cell size. BPEp is positively regulated downstream of APETALA3, PISTILLATA, APETALA1 and PISTILLATA3 and is negatively regulated downstream of AGAMOUS.	Use	AGL	3.99E-04
F vs. L (23)	AT4G17090	BAM3	The primary function of BAM3 is nocturnal degradation of starch to maltose in chloroplasts. Its transcription is regulated by numerous TFs, which suggests that *BAM3* transcription is complex (doi.org/10.1016/j.plantsci.2018.08.016)	SnRK1 TOR		3.95	Encodes a beta-amylase targeted to the chloroplast. Transgenic BMY8 RNAi lines fail to accumulate maltose during cold shock suggesting that maltose accumulation coincides with BMY8 expression. Apart from maltose, the sugar content of the RNAi lines were similar to wildtype (glucose and sucrose unaffected).BAM3 activity declines 2 and 4 days after start of cold stress despite an increase in transcript levels. BAM3 activity has a lower temperature optimum than BAM1 (PMID:25293962).	Use	Stress, RNAi	2.08E-04
F vs. S (23)	AT2G37770	ChlAKR	Upregulated by cold, salt and drought, involved in detoxifying sugar derived reactive carbonyl compounds (TAIR)	Stress signaling	Salt	3.95	Encodes an NADPH-dependent aldo-keto reductase that can act on a wide variety of substrates in vitro including saturated and unsaturated aldehydes, steroids, and sugars. GFP-tagged AKR4C9 localizes to the chloroplast where it may play a role in detoxifying reactive carbonyl compounds that threaten to impair the photosynthetic process. Transcript levels for this gene are up-regulated in response to cold, salt, and drought stress.	Yes	Stress	3.14E-05
F vs. L (23)	AT3G08510	PLC2	Involved in the ER stress response (www.plantphysiol.org/cgi/doi/10.1104/pp.19.01488)	ROS attenuation		3.94	Phosphoinositide-specific phospholipase C (PI-PLC), catalyzes hydrolysis of phosphatidylinositol 4,5-bisphosphate into inositol 1,4,5-trisphosphate and diacylglycerol. It is involved in auxin biosynthesis and signaling, modulating development of both male and female gametophytes. It also regulates MAMP-triggered immunity by modulating ROS production.	Use	ROS, gametophyte	9.97E-05
F vs. S (23)	AT3G09440	HSP70 AT3G09440	HSP70 (TAIR)	Stress attenuation	HSP	3.91	Heat shock protein 70 (Hsp 70) family protein;(source:Araport11)	Yes	Hsp	3.28E-05
F vs. S (23)	AT4G32320	APX6	A major cytosolic ascorbate peroxidase that catalyzes the reduction of H2O2 thus reducing the effect of ROS on ABA and auxin signaling (10.1104/pp.114.245324)	ROS attenuation		3.9	Encodes a cytosolic ascorbate peroxidase APX6. Ascorbate peroxidases are enzymes that scavenge hydrogen peroxide in plant cells. Eight types of APX have been described for Arabidopsis: three cytosolic (APX1, APX2, APX6), two chloroplastic types (stromal sAPX, thylakoid tAPX), and three microsomal (APX3, APX4, APX5) isoforms.	Yes	H2O2, peroxidase, Ascorbate	4.97E-06
F vs. S (23)	AT3G04720	PR4	TAIR			3.87	Encodes a protein similar to the antifungal chitin-binding protein hevein from rubber tree latex. mRNA levels increase in response to ethylene and turnip crinkle virus infection. The mRNA is cell-to-cell mobile.	Yes	ethylene	9.67E-06
F vs. L (23)	AT1G78320	GSTU23	Especially adapted for detoxifying ROS under high oxidative stress conditions (10.1016/j.bbagen.2017.10.007)	ROS attenuation		3.85	Encodes glutathione transferase belonging to the tau class of GSTs. Naming convention according to Wagner et al. (2002).	Use	glutathione	1.08E-03
F vs. S (23)	AT5G15450	APG6	APG6 is an Hsp101 that functions as a molecular chaperone during plastid differentiation. It is upregulated by heat shock (TAIR)	Stress attenuation		3.83	Encodes a chloroplast-targeted Hsp101 homologue. Functions as a molecular chaperone involved in plastid differentiation mediating internal thylakoid membrane formation and conferring thermotolerance to chloroplasts during heat stress. APG6 is constitutively expressed in the root tips, the organ boundary region, the reproductive tissues of mature plants where plastids exist as proplastids, and slightly in the stems and leaves. APG6 expression is upregulated in response to heat shock in various organs, but not in response to other abiotic stresses. Apg6 mutants have a pale-green phenotype.	Yes	Stress	4.30E-04
F vs. S (23)	AT1G78320	GSTU23	Especially adapted for detoxifying ROS under high oxidative stress conditions (10.1016/j.bbagen.2017.10.007)	ROS attenuation		3.8	Encodes glutathione transferase belonging to the tau class of GSTs. Naming convention according to Wagner et al. (2002).	Yes	glutathione	4.97E-06
F vs. M (23)	AT3G08510	PLC2	Involved in the ER stress response (www.plantphysiol.org/cgi/doi/10.1104/pp.19.01488)	ROS attenuation		3.77	Phosphoinositide-specific phospholipase C (PI-PLC), catalyzes hydrolysis of phosphatidylinositol 4,5-bisphosphate into inositol 1,4,5-trisphosphate and diacylglycerol. It is involved in auxin biosynthesis and signaling, modulating development of both male and female gametophytes. It also regulates MAMP-triggered immunity by modulating ROS production.	Use	ROS, gametophyte	2.82E-04
F vs. L (23)	AT4G20360	SVR11	Nucleus transcribed chloropast translation elongation factor involved in leaf variegation (10.3389/fpls.2019.00295)	SnRK1 TOR		3.77	Nuclear transcribed, plastid localized EF-Tu translation elongation factor. Referred to as AtRabE1b in DOI:10.1104/pp.013052. However, wider community usage and more publications assign the symbol RabE1b to At5g59840.	Use	translation	< 1e-07
F vs. L (23)	AT3G23920	BAM1	BAM1 is Involved in starch degradation to yield cytoplasmic glucose pools for bioenergetic and metabolic functions (doi.org/10.1105/tpc.18.00802)	Stress attenuation		3.72	Encodes a chloroplast beta-amylase. Is necessary for leaf starch breakdown in the absence of BAM3.Activity of BAM1 increases 4 days after osmotic stress. BAM1 has a higher temperature optimum than BAM3 (PMID:25293962).	Use	Stress	3.16E-05
F vs. S (23)	AT5G06760	LEA4-5	LEA4-5, like many other LEA proteins, is highly hydrophilic and is involved in protecting cells from damage during freeze-thaw cycles (TAIR)		LEA	3.72	Encodes LEA4-5, a member of the Late Embryogenesis Abundant (LEA) proteins which typically accumulate in response to low water availability conditions imposed during development or by the environment. Most of the diverse set of LEA proteins can be grouped according to properties such as high hydrophilicity and high content of glycine or other small amino acids in what has been termed hydrophilins. LEA4-5 protects enzyme activities from the adverse effects induced by freeze-thaw cycles in vitro.	Yes	LEA	1.44E-05
F vs. M (23)	AT3G19130	RBP47B	Major stress granule marker protein. Stress granules medate stress by harboring enzymes that moderate stress as well as major metabolites that may moderate stress responses, e.g., CDKA (10.1111/nph.15690)	Stress attenuation		3.72	RBP47B, is a component of the stress granule proteome and interacts with 2',3'-cAMP.	Use	Stress	4.51E-03
F vs. S (23)	AT2G18960	HA1	HA1 is a plasma membrane proton ATPase that acidifies the apoplast while increasing cytosolic pH, which decreases ROS levels and enhances plant growth (doi.org/10.3390/ijms21031173)	ROS attenuation		3.69	Encodes a plasma membrane proton ATPase. Mutants have a reduced ability to close their stomata in response to drought and blue light, and are affected in stomatal but not seed responsiveness to ABA.It is the major isoform involved in the blue light mediated stomatal respsonse.The mRNA is cell-to-cell mobile.	Yes	ROS	1.89E-05
F vs. M (23)	AT4G26260	MIOX	Important enzyme in the production of ascorbate, upregulation leads to increased growth, photosynthesis, intracellular glucose levels, defense response genes, and stress response genes (doi.org/10.1002/pld3.165)	ROS attenuation		3.67	Encodes a myo-inositol oxygenase, which is the first enzyme in the inositol route to ascorbate (L‐ascorbic acid, AsA, vitamin C). Overexpression results in enhanced biomass and abiotic stress tolerance.	Use	Stress, Ascorbate	3.72E-03
F vs. M (23)	AT3G12620	PP2C.D3	Involved in auxin regulated cell expansion by modulating plasma membrane ATPases (10.1104/pp.19.00496)	ABA signaling		3.63	Protein phosphatase 2C family protein;(source:Araport11)	Use	PP2C	2.17E-05

L vs. S (23)	AT1G78370	GSTU20	GSTU20 encodes a Tau class glutathione transferase. It is upregulated by stress and interacts with the phytochrome signaling protein far-red insensitive 219. GSTU20 is required for plant responses to far red light (doi.org/10.1199/tab.0131)		glutathione	3.62	Encodes glutathione transferase belonging to the tau class of GSTs. Naming convention according to Wagner et al. (2002).			1.24E-02
F vs. L (23)	AT3G46620	RDUF1	RDUF1 is upregulated by salt and positively regulates plant tolerances to osmotic and dehydration stress (doi:10.1371/journal.pone.0071078)	Stress signaling	Salt	3.62	Encodes an ABA- and drought-induced RING-DUF1117 gene whose mutation results in hyposensitive phenotypes toward ABA in terms of germination rate and stomatal closure and markedly reduced tolerance to drought stress relative to wild-type plants.	Use	Stress, ABA	1.31E-03
F vs. S (23)	AT1G47128	RD21	RD21 is a dehydration response protein (TAIR)	Stress signaling		3.61	cysteine proteinase precursor-like protein/ dehydration stress-responsive gene (RD21). Has been shown to have peptide ligase activity and protease activity in vitro. RD21 is involved in immunity to the necrotrophic fungal pathogen Botrytis cinerea.Activity detected in root, leaf, flower and cell culture.	Yes	Stress	8.26E-06
F vs. M (23)	AT1G16310		NA			3.56	Cation efflux family protein which affects ABA-JA crosstalk and susceptibility to Mamestra brassicae herbivory.		ABA	2.60E-04
F vs. L (23)	AT1G64060	RBOHF	RBOHF and D produce apoplastic superoxide in response to ABA. Their expression (superoxide signaling) is critical to cell survival under ER stress (doi: 10.1111/tpj.14091)	ABA signaling	ABA upregulated	3.54	Interacts with AtrbohD gene to fine tune the spatial control of ROI production and hypersensitive response to cell in and around infection site.	Use	Rboh	3.89E-05
F vs. S (23)	AT3G11410	PP2CA	PP2CA is a protein phosphatase that is upregulated by water deprivation and ABA and functions as a negative regulator of ABA signaling by inactivating SnRK2 (doi: 10.1111/tpj.14274)	ABA signaling	ABA upregulated	3.53	Encodes protein phosphatase 2C. Negative regulator of ABA signalling. Expressed in seeds during germination. mRNA up-regulated by drought and ABA.	Yes	PP2C, ABA	4.90E-05
F vs. L (23)	AT2G13680	CALS5	CALS5 is responsible for callose deposits in the walls of meiocytes (TAIR)	Flowering, Stress attenuation	Meiosis	3.51	Responsible for the synthesis of callose deposited at the primary cell wall of meiocytes, tetrads and microspores. Required for exine formation during microgametogenesis and for pollen viability. Highest expression in meiocytes, tetrads, microspores and mature pollen.	Use	meiosis	2.23E-03
F vs. S (23)	AT1G18150	MPK8	MPK8 promotes seed germination possibly by enhancing TCP14 activity, a TF regulating germination (10.1111/tpj.14461). It is also involved in regulating Ca2+ and ROS in the wound signaling pathway (TAIR)	ROS attenuation		3.51	Encodes mitogen-activated protein kinase 8 (MPK8). MPK8 connects protein phosphorylation, Ca2+, and ROS in the wound-signaling pathway.	Yes	ROS	5.42E-05
F vs. S (23)	AT1G56580	SVB	SVB, a phophoinositide binding protein involved in ER stress tolerance (doi/10.1104/pp.19.01488)	Stress attenuation		3.49	Encodes SMALLER WITH VARIABLE BRANCHES (SVB), a protein with a conserved domain of unknown function (DUF538). The trichomes of the SVB mutants are smaller and exhibit branches of variable length and number.	Yes	Stress, ER	1.64E-05
F vs. S (23)	AT1G15470	XIW1	Positively regulates ABA signaling by stabilizing ABI5 (doi.org/10.1016%2Fj.molp.2019.07.001	ABA signaling	ABA upregulated	3.49	WD40 nucleoplasmic shuttling protein that positively regulates the Abscisic acid (ABA) response by interacting with and maintaining the stability of ABI5 in the nucleus. Nuclear export of XIW1 is XPO1-dependent. Involved in regulating seed germination, primary root growth, and drought stress	Yes	Stress, ABA	4.33E-05
F vs. L (23)	AT4G15440	CYP74B2	Produces galactolipids that accumulate in response to biotic and abiotic stress (doi:10.1093/jxb/erw278)	Stress signaling		3.41	Encodes a hydroperoxide lyase. Also a member of the CYP74B cytochrome p450 family. In the ecotype Columbia (Col) the gene contains a 10-nucleotide deletion in its first exon that causes it to code for a truncated protein that results in a non-functional hydroperoxide lyase.	Use	H2O2	3.89E-05
L vs. S (23)	AT3G50660	DWF4			BR	3.4	Encodes a 22α hydroxylase whose reaction is a rate-limiting step in brassinosteroid biosynthetic pathway. The protein is a member of CYP90B gene family. CLM is an epi-allele with small, compressed rosette, reduced internode length, and reduced fertility, appears in selfed ddm mutant plants possibly due to loss of cytosine methylation. Transcripts accumulate in actively growing tissues, and GUS expression is negatively regulated by brassinosteroids. Localized in the endoplasmic reticulum. The in vitro expressed protein can perform the C-22 hydroxylation of a variety of C27-, C28- and C29-sterols. Cholesterol was the best substrate, followed by campesterol.			6.15E-03
F vs. L (23)	AT1G32080	PLGG1	Upregulated by ABA or drought. It participates in the detoxification of 2-phosphoglycolate in the chloroplast during photorespiration by facilitating the export of glycolate from chloroplasts and the import of glycerate (10.1105/tpc.16.00775)		ABA upregulated	3.38	Encodes a plant LrgAB/CidAB protein localized to the chloroplast envelope that is involved in chloroplast development, carbon partitioning, ABA/drought response, and leaf senescence. The gene may have evolved from gene fusion of bacterial lrgA and lrgB.	Use	ABA	5.09E-05
F vs. M (23)	AT1G30330	ARF6	NA		ARF	3.37	Encodes a member of the auxin response factor family. Mediates auxin response via expression of auxin regulated genes. Acts redundantly with ARF8 to control stamen elongation and flower maturation. Expression of ARF6 is controlled by miR167.	Use	ARF	6.35E-04
F vs. M (23)	AT1G32080	PLGG1	Upregulated by ABA or drought. It participates in the detoxification of 2-phosphoglycolate in the chloroplast during photorespiration by facilitating the export of glycolate from chloroplasts and the import of glycerate (10.1105/tpc.16.00775)		ABA upregulated	3.36	Encodes a plant LrgAB/CidAB protein localized to the chloroplast envelope that is involved in chloroplast development, carbon partitioning, ABA/drought response, and leaf senescence. The gene may have evolved from gene fusion of bacterial lrgA and lrgB.	Use	ABA	1.19E-03
F vs. L (23)	AT2G17290	CPK6	CPK6 is transiently upregulated by ABA, oxidative stress and dehydration and is activated by Ca2+. CPK6 functions similarly to OST1 (SnRK2.6) by enhancing ABA sensitivity and confering drought tolerance through phosphorylating core ABA responsive TFs including ABF3 and ABI5. This leads to the expression of downstream stress response genes including LEA-like chaperone genes, S-type anion channels, and methyl jasmonate (doi:10.1093/jxb/erz432)	ABA signaling	ABA upregulated	3.34	Encodes calcium-dependent protein kinase 6 (CPK6), a member of the Arabidopsis CDPK gene family. CDPKs contain an intrinsic Ca2+-activation domain with four EF hand Ca2+-binding sites. CDPKs protein kinases have been proposed to function in multiple plant signal transduction pathways downstream of [Ca2+]cyt elevations, thus transducing various physiological responses. CPK6 is expressed in both guard cells and mesophyll cells. Functions in guard cell ion channel regulation. ABA and Ca(2+) activation of slow-type anion channels and, interestingly, ABA activation of plasma membrane Ca(2+)-permeable channels were impaired in independent alleles of single and double cpk3cpk6 mutant guard cells. Furthermore, ABA- and Ca(2+)-induced stomatal closing were partially impaired in these cpk3cpk6 mutant alleles. The protein kinase CPK6 is shown in biochemical assays to be directly activated by elevations in calcium concentrations in the physiological range (Laanements et al., 2013 PlantPhys.; PMID: 23766366). These data correlate with the in vivo function of CPK6 in Ca2+ and ABA activation of S-type anion channels (Mori et al., 2006 PLoS Biol.; PMID: 17032064) and the ability of CPK6 to mediate ABA activation of SLAC1 (Brandt et al., 2012 PNAS; PMID: 22689970).The mRNA is cell-to-cell mobile.	Use	ABA, Ca	1.56E-03
F vs. L (23)	AT2G30860	GSTF9	Upregulated by osmotic stress, mitigates oxidative damage caused by osmotic stress, protected from deactivation by LEA dehydrin proteins (doi.org/10.1016/j.bbagen.2019.129506)	ROS attenuation		3.32	Encodes glutathione transferase belonging to the phi class of GSTs. Naming convention according to Wagner et al. (2002).	Use	glutathione	2.55E-04

F vs. L (23)	AT4G26260	MIOX	Important enzyme in the production of ascorbate, upregulation leads to increased growth, photosynthesis, intracellular glucose levels, defense response genes, and stress response genes (doi.org/10.1002/pld3.165)	ROS attenuation		3.28	Encodes a myo-inositol oxygenase, which is the first enzyme in the inositol route to ascorbate (L‐ascorbic acid, AsA, vitamin C). Overexpression results in enhanced biomass and abiotic stress tolerance.	Use	Stress, Ascorbate	2.34E-04
F vs. M (23)	AT5G17480	PC1	Pollen Ca binding protein			3.28	pollen calcium-binding protein 1;(source:Araport11)	No	Ca	1.08E-04
F vs. L (23)	AT1G59640	BPE	NA		Flowering	3.27	A basic helix-loop-helix encoding gene (BIGPETAL, BPE) involved in the control of petal size. BPE is expressed via two mRNAs derived from an alternative splicing event. The BPEub (AT1G59640.1)transcript is expressed ubiquitously, whereas the BPEp (AT1G59640.2) transcript is preferentially expressed in petals. Plants that lack the petal-expressed variant BPEp have larger petals as a result of increased cell size. BPEp is positively regulated downstream of APETALA3, PISTILLATA, APETALA1 and PISTILLATA3 and is negatively regulated downstream of AGAMOUS.	Use	AGL	6.01E-05
F vs. M (23)	AT1G30360	ERD4	Early response to dehydration.	Stress signaling		3.27	Early-responsive to dehydration stress protein (ERD4);(source:Araport11)	Use	Stress	4.27E-03
F vs. L (23)	AT1G30330	ARF6	NA		ARF	3.25	Encodes a member of the auxin response factor family. Mediates auxin response via expression of auxin regulated genes. Acts redundantly with ARF8 to control stamen elongation and flower maturation. Expression of ARF6 is controlled by miR167.	Use	ARF	1.83E-05
F vs. S (23)	AT1G49670	AT1G49670	Function not know, upregulated in response to oxidative stress (TAIR)	Stress signaling		3.22	molecular function has not been defined. Was shown involved in oxidative stress tolerance.	Yes	Stress	1.75E-05
F vs. S (23)	AT1G20620	CAT3	CAT3 catalyzes the breakdown of H_2O_2, but it also transnitrosylates GSNOR1 to activate redox signaling in the presence of NO (doi.org/10.1016/j.devcel.2020.03.020)	ROS attenuation, REDOX signaling		3.22	Catalase, catalyzes the breakdown of hydrogen peroxide (H_2O_2) into water and oxygen. The mRNA is cell-to-cell mobile.	Yes	H_2O_2, Ca, catalase	4.58E-05
F vs. L (23)	AT1G08830	CSD1	CSD1 is a cytosolic copper/zinc superoxide dismutase that converts superoxide free radicals to O_2 and H_2O_2. Under nonstress conditions, its transcripts are cleaved by miR398, which decreases CSD1 levels. Under stress conditions, miR398 expression is suppressed, and CSD1 levels increase (DOI 10.1007/s10646-017-1843-y)	ROS attenuation		3.22	Encodes a cytosolic copper/zinc superoxide dismutase CSD1 that can detoxify superoxide radicals. Its expression is affected by miR398-directed mRNA cleavage. Regulated by biotic and abiotic stress. Activation of CSD1 in the cytoplasm involves both a CCS-dependent and -independent pathway.	Use	Stress, H_2O_2, superoxide dismutase	2.23E-03
F vs. L (23)	AT2G30360	SnRK3.22	SnRK3.22 phophorylation activates ABI5 (doi/10.1104/pp.114.255455). In the absence of salt stress, SnRK3.22 inactivates the H^+ ATPase driven SOS2 Na^+/H^+ antiporter by complexing it with 14-3-3 proteins by phosphorylation. During salt stress, SOS2 is activated by Ca^{2+} signaling, which upregulates SOS3 and SCaBP8. These proteins then bind to SOS2 causing the latter to release the 14-3-3 proteins. SOS2 is then activated (doi.org/10.1038/s41467-019-09181-2)	ABA signaling	Salt	3.22	Encodes a SOS2-like protein kinase that is a member of the CBL-interacting protein kinase family.Loss of function mutants show a decrease in sensitivity to high pH.Phosphorylates AHA2, a plasma membrane H^+ ATPase.This phosphorylation appears to regulate the activity of the proton transporter.	Use	SnRK	7.05E-04
F vs. L (23)	AT1G78370	GSTU20	GSTU20 encodes a Tau class glutathione transferase. It is upregulated by stress and interacts with the phytochrome signaling protein far-red insensitive 219. GSTU20 is required for plant responses to far red light (doi.org/10.1199/tab.0131)	ROS attenuation		3.19	Encodes glutathione transferase belonging to the tau class of GSTs. Naming convention according to Wagner et al. (2002).	Use	glutathione	1.44E-05
F vs. L (23)	AT4G16660	HSP70 AT4G16660	Coats the surfaces of stress induced unfolded protein aggregates (stress granules) and targets them for proteosome complex breakdown (doi.org/10.1104/pp.19.00263)	Stress attenuation	HSP	3.19	heat shock protein 70 (Hsp 70) family protein;(source:Araport11)	Use	HSP	9.51E-05
F vs. S (23)	AT4G25670	DOB1	Upregulated early by onset of salt stress (doi.org/10.1016/j.isci.2019.10.043)	Stress signaling	Salt	3.17	stress response NST1-like protein;(source:Araport11)	Yes	Stress	2.49E-04
F vs. S (23)	AT1G20630	CAT1	CAT1 is upregulated by ROS and catalyzes the reduction of H_2O_2 (TAIR)	ROS attenuation		3.14	Catalyzes the reduction of hydrogen peroxide using heme group as cofactor. Protects cells from toxicity by H_2O_2.	Yes	H_2O_2, Ca	1.81E-05
F vs. S (23)	AT4G26970	ACON2	May participate in the TCA cycle. May play a role in the response to ROS by upregulating CSD2 (superoxide dismutase)	ROS attenuation		3.11	Encodes an aconitase that can catalyze the conversion of citrate to isocitrate through a cis-aconitate intermediate, indicating that it may participate in the TCA cycle and other primary metabolic pathways. The protein is believed to accumulate in the mitochondria and the cytosol. It affects CSD2 (At2g28190 - a superoxide dismutase) transcript levels and may play a role in the response to oxidative stress. One member of the family (ACO1 - At35830) was shown to specifically bind to the 5' UTR of CSD2 in vitro. The mRNA is cell-to-cell	Yes	Stress, H_2O_2, superoxide dismutase	4.85E-04
F vs. S (23)	AT1G01720	ATAF1	ATAF1 is a SNAC-A TF that is upregulated by carbon starvation. It upregulates carbon starvation responses, similar to those that occur when SnRK1 is upregulated. It specifically upregulates TREHALASE1 (TRE1), which catabolizes sugars and induces global transcriptome changes associated with carbon starvation. It also attenuates ABA signaling and synthesis (doi/10.1104/pp.15.00917; doi:10.1093/pcp/pcz015)	SnRK1 TOR		3.11	Belongs to a large family of putative transcriptional activators with NAC domain. Transcript level increases in response to wounding and abscisic acid. ATAF1 attentuates ABA signaling and sythesis. Mutants are hyposensitive to ABA. The mRNA is cell-to-cell mobile.	Yes	ABA	4.59E-04
F vs. S (23)	AT3G08510	PLC2	Involved in the ER stress response (www.plantphysiol.org/cgi/doi/10.1104/pp.19.01488)	ROS attenuation		3.1	Phosphoinositide-specific phospholipase C (PI-PLC), catalyzes hydrolysis of phosphatidylinositol 4,5-bisphosphate into inositol 1,4,5-trisphosphate and diacylglycerol. It is involved in auxin biosynthesis and signaling, modulating development of both male and female gametophytes. It also regulates MAMP-triggered immunity by modulating ROS production.	Yes	ROS, gametophyte	2.29E-05
F vs. L (23)	AT3G57600	DREB A-2 gene	Dehydration response ERF/AP2 gene (TAIR)		ET	3.08	encodes a member of the DREB subfamily A-2 of ERF/AP2 transcription factor family. The protein contains one AP2 domain. There are eight members in this subfamily including DREB2A AND DREB2B that are involved in response to drought.	Use	ERF	1.83E-05
F vs. S (23)	AT1G63940	MDAR6	MDAR6 is a monohydroascorbate reductase that participates during oxidative stress in transfering electrons from NADPH through glutathione and ascorbate to reduce H_2O_2 to H_2O (TAIR)	ROS attenuation		3.05	monodehydroascorbate reductase 6;(source:Araport11)	Yes	Ascorbate	6.40E-05
F vs. S (23)	AT2G27050	EIL1	EIL1 is an ethylene response TF that is upregulated by ET. ET signaling is suppressed by TOR (doi.org/10.3390/ijms21082680)	Stress signaling, SnRK1	ET	3	ethylene-insensitive3-like1 (EIL1) The mRNA is cell-to-cell mobile.	Yes	ethylene, TOR	5.83E-04
F vs. S (23)	AT2G30360	SnRK3.22	SnRK3.22 phophorylation activates ABI5 (doi/10.1104/pp.114.255455). In the absence of salt stress, SnRK3.22 inactivates the H^+ ATPase driven SOS2 Na^+/H^+ antiporter by complexing it with 14-3-3 proteins by phosphorylation. During salt stress, SOS2 is activated by Ca^{2+} signaling, which upregulates SOS3 and SCaBP8. These proteins then bind to SOS2 causing the latter to release the 14-3-3 proteins. SOS2 is then activated (doi.org/10.1038/s41467-019-09181-2)	ABA signaling	Salt	3	Encodes a SOS2-like protein kinase that is a member of the CBL-interacting protein kinase family.Loss of function mutants show a decrease in sensitivity to high pH.Phosphorylates AHA2, a plasma membrane H^+ ATPase.This phosphorylation appears to regulate the activity of the proton transporter.	Yes	SnRK	1.74E-03

F vs. S (23)	AT5G42050	NRP	ABA signaling is negatively regulated by FyPP3, which deactivates ABI5 by dephosphorylating it. NRP facilitates the degradation of FyPP3, thus positively regulating ABA signaling. FyPP3 can also dephosphorylate NRP (10.1016/j.molp.2017.11.006)	ABA signaling	ABA upregulated	2.99	Stress responsive asparagine-rich protein. Binds to PevD (Verticillium dahliae) fungal effector protein. NRP interacts with CRY2, leading to increased cytoplasmic accumulation of CRY2 in a blue light-independent manner (PMID:28633330).NRP also binds FyPP3 and recruits it to endosomes and thus targets it for degradation.	Yes	Stress	1.79E-04
F vs. L (23)	AT5G51890	PRX66	PRX66 is a peroxidase that is induced by oxidative stress and detoxifies ROS (TAIR)	ROS attenuation		2.99	encodes peroxidase involved in the lignification of tracheary elements (TE) in roots	Use	peroxidase	3.21E-04
F vs. S (23)	AT5G05730	ASA1	TAIR			2.98	ASA1 encodes the alpha subunit of anthranilate synthase, which catalyzes the rate-limiting step of tryptophan synthesis. ASA1 is induced by ethylene, and forms a link between ethylene signalling and auxin synthesis in roots.	Yes	ethylene	2.31E-04
F vs. S (23)	AT5G47000	AT5G47000	Peroxidase that is upregulated in response to oxidative stress (TAIR)	ROS attenuation		2.98	Peroxidase superfamily protein;(source:Araport11)	Yes	peroxidase	3.52E-05
F vs. M (23)	AT5G19450	CPK8	Functions with CAT3 to establish ROS homeostasis in response to ABA signaling (10.1105/tpc.15.00144)	ROS attenuation	ABA upregulated, Ca, homeostasis	2.94	calcium-dependent protein kinase (CDPK19) mRNA, complete	Use	Ca, ROS homeostasis	5.07E-03
F vs. S (23)	AT1G64060	RBOHF	RBOHF and D produce apoplastic superoxide in response to ABA. Their expression (superoxide signaling) is critical to cell survival under ER stress (doi: 10.1111/tpj.14091)	ABA signaling	ABA upregulated	2.94	Interacts with AtrbohD gene to fine tune the spatial control of ROI production and hypersensitive response to cell in and around infection site.	Yes	Rboh	6.88E-04
F vs. S (23)	AT3G54660	GR	GR is a plastid glutathione reductase that maintains a highly reduced glutathione pool that balances ROS and enables the redox buffering required to maintain efficient photosynthesis (doi.org/10.1111/tpj.14791)	ROS attenuation	glutathione	2.92	Encodes glutathione reductase that is most likely localized in the chloroplast.	Yes	glutathione	1.29E-04
F vs. S (23)	AT5G47910	RBOHD	Responsible for superoxide production during the HR and in response to heat and wounding (TAIR)	ABA signaling		2.92	NADPH/respiratory burst oxidase protein D (RbohD).Interacts with AtrbohF gene to fine tune the spatial control of ROI production and hypersensitive response to cell in and around infection site. The mRNA is cell-to-cell mobile.	Yes	Rboh	1.51E-04
F vs. S (23)	AT4G20260	PCaP1	PCaP1 is a Ca2+ binding protein that is upregulated by osmotic stress and may participate in transducing water potential signals (doi:10.1093/pcp/pcz042)		Ca	2.91	Encodes a Ca2+ and Cu2+ binding protein. N-terminal myristylation on glycine 2 appears to enable it to associate tightly with the plasma membrane. Recombinant PCaP1 interacts strongly with phosphatidylinositol 3,5-bisphosphate (PtdIns(3,5)P2) and PtdIns (3,4,5)P3, and weakly with PtdIns(3,5)P2 and PtdIns(4,5). It also interacts with calmodulin (CaM) in a calcium-dependent manner. CaM does not interfere with PCaP1 membrane localization but does weaken interactions between it and the PtdInsPs. PCaP1 has an apparent Kd of 10 uM for Cu2+ and can bind six ions per protein. Transcript levels for PCaP1 first fall and then rise following exposure to CuCl2. Mannitol, sorbitol, and the flg22 oligopeptide also increase expression levels.The mRNA is cell-to-cell mobile.	Yes	Ca, Calmodulin	9.71E-04
F vs. S (23)	AT2G18400	RP AT2G18400	TAIR		ribosome	2.91	ribosomal protein L6 family protein;(source:Araport11)	Yes	Ribosome	2.69E-05
F vs. L (23)	AT2G38170	CAX1	Involved in maintaining Ca and ROS homeostasis in response to heavy metal stress (doi.org/10.1080/15592324.2016.1183861)		Ca, homeostasis	2.90	Encodes a high affinity vacuolar calcium antiporter. The residue His 338 is critical to Ca2+ transport activity. Disruption of CAX1 reduces manganese and zinc of shoot tissue and results in a decrease in the activity of vacuolar V-type proton ATPase.	Use	Ca	2.61E-05
F vs. S (23)	AT3G16800	EGR3	EGR3 is a PP2C that restrains growth during drought stress and enhances recovery. As a growth suppressor, it dephosphorylation inactivates MASP1, which is also upregulated by drought. MASP1 activates growth by stabilizing microtubule bundles that are responsible for cell expansion (doi/10.1105/tpc.16.00847)	Stress attenuation		2.9	EGR3 functions as a negative regulator of plant growth with prominent effect on plant growth during drought stress, EGR3 regulates microtubule organization and likely affects additional cytoskeleton and trafficking processes along the plasma membrane.	Yes	Stress	3.60E-05
L vs M (123)	AT1G67600					2.90	Acid phosphatase/vanadium-dependent haloperoxidase-related protein;(source:Araport11)	No	peroxidase	4.26E-05
F vs. M (23)	AT2G38170	CAX1	Involved in maintaining Ca and ROS homeostasis in response to heavy metal stress (doi.org/10.1080/15592324.2016.1183861)		Ca, homeostasis	2.89	Encodes a high affinity vacuolar calcium antiporter. The residue His 338 is critical to Ca2+ transport activity. Disruption of CAX1 reduces manganese and zinc of shoot tissue and results in a decrease in the activity of vacuolar V-type proton ATPase.	Use	Ca	3.05E-03
F vs. L (23)	AT4G10040	CYTC-2	CYTC-2 encodes the mitochondrial electron carrier chytochrome c. Increased levels of CYTC enhance growth probably by increasing mitochondrial energy (ATP) and gibberellin signaling and homeostasis (doi: 10.1111/tpj.13845)		Homeostasis	2.88	Encodes cytochrome c. Promoter directs preferential expression in vascular tissues of cotyledons, leaves, roots, and hypocotyls, and in anthers. Double mutants with CYTC-1 accumulate starch during the day, have delayed growth and development and reduced GA and DELLA proteins linking cellular metabolism and GA homeostasis.	Use	Homeostasis	3.48E-04
F vs. S (23)	AT5G04760	DIV2	DIV2 is a TF that is upregulated by ABA, GA, SA and salt. It negatively regulates ABA biosynthesis and signaling probably by downregulating the ABA synthesis genes ABA1,2 (doi.org/10.1007/s00299-018-2321-6)	ABA signaling	ABA upregulated, Salt	2.87	R-R-type MYB protein which plays negative roles in salt stress and is required for ABA signaling in Arabidopsis.	Yes	Stress, ABA	1.46E-04
F vs. S (23)	AT1G23190	PGM3	PGM3 together with PGM2 are essential for male and female gametophyte formation (TAIR)		Ovule gametophyte	2.87	Encodes a cytosolic phosphoglucomutase (PGM). Two Arabidopsis PGM proteins (AT1G70730/PGM2 and AT1G23190/PGM3) have high sequence similarities and redundant functions. Mature plants possessing a single cPGM allele had a major reduction in cPGM activity. Whereas pgm2 and pgm3 single mutants are undistinguishable from the wild type, loss of both PGM2 and PGM3 severely impairs male and female gametophyte development. The mRNA is cell-to-cell mobile.	Yes	gametophyte	6.81E-04
M vs. S (23)	AT1G23740	AOR			Atten	2.85	AOR is an alkenal/one oxidoreductase that acts on compounds with unsaturated alpha,beta-carbonyls. The activity of this enzyme with a number of substrates, including acrolein and 3-buten-2-one, was demonstrated in vitro using a truncated form of the protein that lacked approximately 80 of the first amino acids. This protein appears to localize to the chloroplast where it likely helps to maintain the photosynthetic process by detoxifying reactive carbonyls formed during lipid peroxidation.			1.97E-02
F vs. S (23)	AT4G23100	GSH1	GSH1 catalyzes the rate-limiting step in glutathione biosynthesis (TAIR)	ROS attenuation		2.85	Encodes the enzyme glutamate-cysteine ligase catalyzing the first, and rate-limiting, step of glutathione biosynthesis. Required for cell proliferation at the root tip. Involved in susceptibility to the bacterial pathogen Pseudomonas syringae. Mutants are phytoalexin defective.	Yes	glutathione	3.34E-04

F vs. M (23)	AT1G70730	PGM2	PGM2 and PGM3 together are required for male and female gametophyte formation (TAIR)		Ovule gametophyte	2.84	Encodes a cytosolic phosphoglucomutase (PGM). Two Arabidopsis PGM proteins (AT1G70730/PGM2 and AT1G23190/PGM3) have high sequence similarities and redundant functions. Mature plants possessing a single cPGM allele had a major reduction in cPGM activity. Whereas pgm2 and pgm3 single mutants are undistinguishable from the wild type, loss of both PGM2 and PGM3 severely impairs male and female gametophyte development.	Use	gametophyte	1.06E-03
F vs. S (23)	AT3G48170	ALDA10A9	ALDA10A9 is a peroxisome localized NAD+-dependent aminoaldehyde dehydrogenase that is upregulated by salt stress. It performs the final step in catabolizing polyamines to produce 4-aminobutyrate (GABA), which ameliorates the effects of salt stress (DOI: 10.1038/srep35115)	Stress attenuation	ABA upregulated, Salt	2.81	ALDH10A9 encodes a protein that can function as a betaine aldehyde dehydrogenase in vitro. The C-terminal amino acids of this protein direct GFP to the peroxisome suggesting that ALDH10A9 accumulates in this organelle. ALDH10A9 transcript levels rise in response to ABA, NaCl, chilling, methyl viologen, and dehydration stress. The enzyme can catalyze the formation of glycine betaine in vitro, but there are still questions about whether Arabidopsis makes this protective compound under natural conditions. This enzyme may be involved in oxidizing aminoaldehydes formed through polyamine metabolism.	Yes	Stress, ABA	1.89E-04
F vs. L (23)	AT2G28190	CSD2 (SOD2)	SOD2 is a chloroplastic copper/zinc superoxide dismutase that converts superoxide free radicals to O2 and H2O2. Under nonstress conditions, its transcripts are cleaved by miR398, which decreases SOD2 levels. Under stress conditions, miR398 expression is suppressed, and SOD2 levels increase (DOI 10.1007/s10646-017-1843-y)	ROS attenuation		2.81	Encodes a chloroplastic copper/zinc superoxide dismutase CSD2 that can detoxify superoxide radicals. Its expression is affected by miR398-directed mRNA cleavage. Activation depends totally on CCS. Overexpression of a miR398-resistant form of CSD2 leads to more dramatic improvements in stress (hight light, Cu2+ and methyl viologen) tolerance than overexpression of wild-type CSD2. The mRNA is cell-to-cell mobile.	Use	Stress, H2O2, superoxide dismutase	1.20E-03
F vs. M (23)	AT5G08640	FLS1	Enzyme that catalyzes the formation of flavonols			2.81	Encodes a flavonol synthase that catalyzes formation of flavonols from dihydroflavonols. Co-expressed with CHI and CHS (qRT-PCR).	No	flavonol	1.34E-03
F vs. L (23)	AT5G05730	ASA1	TAIR			2.80	ASA1 encodes the alpha subunit of anthranilate synthase, which catalyzes the rate-limiting step of tryptophan synthesis. ASA1 is induced by ethylene, and forms a link between ethylene signalling and auxin synthesis in roots.	No	ethylene	1.28E-03
M vs. S (23)	AT5G40820	ATR	ATR responds to DNA damage and was identified as a meiosis important gene by Walker et al. (doi.org/10.1038/s41588-017-0008-5)		meiosis	2.8	Encodes a Arabidopsis ortholog of the ATR protein kinase that is involved in a wide range of responses to DNA damage and plays a central role in cell-cycle regulation. Dominant loss of function alleles identified as suppressors of ALS also exhibit increased tolerance to aluminum. This may be due to the inhibition of terminal differentiation of the root apex upon exposure to Al.			3.85E-03
F vs. S (23)	AT4G02380	LEA38	LEA38 is upregulated by ABA, ROS, water deprivation, ethylene (TAIR)		ABA, upregulated, LEA	2.8	Encodes AtLEA5 (late embryogenesis abundant like protein). Also known as SENESCENCE-ASSOCIATED GENE 21 (SAG21). Has a role on oxidative stress tolerance. mRNA levels are elevated in response to various stresses.	Yes	LEA Stress	3.09E-05
F vs. S (23)	AT3G53420	PIP2A	PIP2A is a water and H2O2 transporting transmembrane aquaporin that is downregulated by salt stress. RBOHF generated ROS in the apoplast causes PIP2A clustering and subsequent endocytosis of the PIP2A cluster thus potentially regulating levels of cytoplasmic ROS (doi/10.1104/pp.18.01065)	ROS attenuation		2.78	a member of the plasma membrane intrinsic protein subfamily PIP2. localizes to the plasma membrane and exhibits water transport activity in Xenopus oocyte. expressed specifically in the vascular bundles and protein level increases slightly during leaf dev. When expressed in yeast cells can conduct hydrogen peroxide into those cells.	Yes	H2O2	4.58E-05
L vs. S (23)	AT1G23740	AOR	NADPH-dependent alkenal/one oxidoreductase, chloroplastic		Atten	2.76	AOR is an alkenal/one oxidoreductase that acts on compounds with unsaturated alpha,beta-carbonyls. The activity of this enzyme with a number of substrates, including acrolein and 3-buten-2-one, was demonstrated in vitro using a truncated form of the protein that lacked approximately 80 of the first amino acids. This protein appears to localize to the chloroplast where it likely helps to maintain the photosynthetic process by detoxifying reactive carbonyls formed during lipid peroxidation.			1.09E-02
F vs. L (23)	AT1G19550	DHAR	A major dehydroascorbate reductase with glutathione dehydrogenase (ascorbate) activity. Functions with APX6 in cellular ROS detoxification by coupling H2O2 to reduced glutathione. Small increases in DHAR and APX6 significantly enhance tolerance to abiotic stresses, reviewed in (doi.org/10.1093/jxb/eraa107)	ROS attenuation		2.76	Glutathione S-transferase family protein;(source:Araport11)	Use	glutathione	6.26E-05
F vs. M (23)	AT1G15470	XIW1	Positively regulates ABA signaling by stabilizing ABI5 (doi.org/10.1016%2Fj.molp.2019.07.001	ABA signaling	ABA upregulated	2.76	WD40 nucleoplasmic shuttling protein that positively regulates the Abscisic acid (ABA) response by interacting with and maintaining the stability of ABI5 in the nucleus. Nuclear export of XIW1 is XPO1-dependent. Involved in regulating seed germination, primary root growth, and drought stress	Use	Stress, ABA	1.62E-04
F vs. S (23)	AT5G66650	cMCU	cMCU is a mitochondrial and chloroplast Ca2+ uniporter involved in maintaining cytosolic Ca2+ homeostasis during stress (doi.org/10.1038/s41477-019-0434-8) Nature Plants		Ca, Stress attenuation, homeostasis	2.75	Chloroplast localized mitochondrial calcium uniporter.	Yes	Ca	1.14E-03
F vs. M (23)	AT3G57600	DREB A-2 gene	Dehydration response ERF/AP2 gene (TAIR)		ET	2.75	encodes a member of the DREB subfamily A-2 of ERF/AP2 transcription factor family. The protein contains one AP2 domain. There are eight members in this subfamily including DREB2A AND DREB2B that are involved in response to drought.	Use	ERF	2.00E-03
F vs. L (23)	AT2G41480	PRX25	PRX66 is a cell wall bound peroxidase that detoxifies ROS and is induced by oxidative stress (TAIR)	ROS attenuation		2.75	Encodes a cationic cell-wall-bound peroxidase homolog that is involved in the lignification of cell walls. Regulated by COG1, involved in seed longevity.	Use	peroxidase	1.78E-04
F vs. L (23)	AT2G46300	LEA AT2G46300	TAIR		LEA	2.74	Late embryogenesis abundant (LEA) hydroxyproline-rich glycoprotein family;(source:Araport11)	Use	LEA	9.42E-04
L vs. S (23)	AT3G66658	no	Aldehyde dehydrogenase 22A1		Stress	2.74	Encodes a putative aldehyde dehydrogenase. The gene is not responsive to osmotic stress and is expressed constitutively at a low level in plantlets and root cultures.			1.13E-02
F vs. L (23)	ATCG00820	RPS19 (RP)	TAIR		ribosome	2.74	Encodes a 6.8-kDa protein of the small ribosomal subunit.	Use	ribosome	1.92E-04
L vs. S (23)	AT1G49670	AT1G49670	Function not know, upregulated in response to oxidative stress (TAIR)	Stress signaling		2.72	molecular function has not been defined. Was shown involved in oxidative stress tolerance.			1.30E-02
F vs. S (23)	AT3G57600	DREB A-2 gene	Dehydration response ERF/AP2 gene (TAIR)		ET	2.71	encodes a member of the DREB subfamily A-2 of ERF/AP2 transcription factor family. The protein contains one AP2 domain. There are eight members in this subfamily including DREB2A AND DREB2B that are involved in response to drought.	Yes	ERF	5.18E-05
F vs. L (23)	AT2G17840	ERD7	ERD7 is upregulated by drought and salt stress proobably by the nuclear C2H2 zinc finger protein ZAT18, which is upregulated by drought	Stress signaling	Salt	2.71	Identified as drought-inducible gene by differential hybridization. Upregulated by high light, drought, cold and salt stress determined by microarray analysis.	Use	Stress	2.33E-04

F vs. S (23)	AT2G39770	VTC1	Essential enzyme in ascorbic acid biosynthesis. High levels of VTC1 are correlated with high levels of ascorbic acid (doi.org/10.1093/jxb/erw223)	ROS attenuation		2.71	Encodes a GDP-mannose pyrophosphorylase/ mannose-1-pyrophosphatase. This enzyme provides GDP-mannose, which is used for cell wall carbohydrate biosynthesis and protein glycosylation as well as for ascorbate (vitamin C) biosynthesis. Mutations in this gene confer hypersensitivity to NH_4^+.	Yes	Ascorbate	1.09E-04
F vs. S (23)	AT2G35940	BLH1	BLH overexpression leads to multiple eggs within the female gametophyte (TAIR)		Ovule gametophyte	2.68	Encodes a member of the BEL-like homeodomain protein family. Ecotopic expression in the embryo sac leads to defects in nuclear migration and cellularization and embryo sacs with multiple egg cells. Loss of function alleles have no female gametophyte defects. The ecotopic expression phenotype requires KNAT3 because it can be suppressed by loss of KNAT3 function alleles. Localized to the nucleus but interaction with OFP1 relocates it to the cytoplasm.	Yes	gametophyte	9.22E-05
F vs. M (23)	AT1G49670	AT1G49670	Function not know, upregulated in response to oxidative stress (TAIR)	Stress signaling		2.67	molecular function has not been defined. Was shown involved in oxidative stress tolerance.	Use	Stress	3.05E-03
F vs. L (23)	AT2G18660	PNP-A	Induced by stress, functions with PNP-R2 to suppress SA signaling and cell death (10.1105/tpc.20.00018)	Stress attenuation		2.67	Encodes PNP-A (Plant Natriuretic Peptide A). PNPs are a class of systemically mobile molecules distantly related to expansins; their biological role has remained elusive. PNP-A contains a signal peptide domain and is secreted into the extracellular space. Co-expression analyses using microarray data suggest that PNP-A may function as a component of plant defence response and SAR in particular, and could be classified as a newly identified PR protein. It is stress responsive and can enhance its own expression.	Use	Stress	3.29E-04
F vs. S (23)	AT2G46300	LEA AT2G46300	TAIR		LEA	2.65	Late embryogenesis abundant (LEA) hydroxyproline-rich glycoprotein family;(source:Araport11)	Yes	LEA	1.82E-03
F vs. L (23)	AT1G07180	NDA1	NDA1 is an alternative NAD(P)H dehydrogenase (ND) that may function in the plant mitochondrial alternative pathway in response to oxidative stress (doi/10.1104/pp.19.00877)	ROS attenuation		2.65	Internal NAD(P)H dehydrogenase in mitochondria. The predicted protein sequence has high homology with other designated NAD(P)H DHs from microorganisms; the capacity for matrix NAD(P)H oxidation via the rotenone-insensitive pathway is significantly reduced in the Atndi1 mutant plant line; the in vitro translation product of AtNDI1 is imported into isolated mitochondria and located on the inside of the inner membrane.	Use	translation	5.94E-05
F vs. L (23)	AT3G04840	S3Ae (RP)	TAIR		ribosome	2.62	Ribosomal protein S3Ae;(source:Araport11)	Use	ribosome	6.27E-04
F vs. L (23)	AT5G50800	SWEET13	TAIR	SnRK1 TOR		2.61	Encodes a member of the SWEET sucrose efflux transporter family proteins, together with RPG1, it is involved in pollen development. Together with SWEET14, it is likely involved in modulating the GA response and is required for proper development of anthers, seeds and seedlings.	Use	SWEET transporter	4.85E-04
F vs. M (23)	AT5G47000	AT5G47000	Upregulated in response to oxidative stress (TAIR)	ROS attenuation		2.60	Peroxidase superfamily protein;(source:Araport11)	Use	peroxidase	3.44E-03
F vs. M (23)	AT1G77980	AGL66	NA		Flowering	2.59	Encodes a member of the MIKC (MADS box, Keratin binding domain, and C terminal domain containing) family of transcriptional regulators. AGL66 is expressed in pollen. It forms heterodimers with other MICK family members (AGL104). Involved in late stages of pollen development and pollen tube	Use	AGL	3.36E-03
F vs. M (23)	AT1G01250	DREB A-4 gene	NA		ET	2.58	encodes a member of the DREB subfamily A-4 of ERF/AP2 transcription factor family. The protein contains one AP2 domain. There are 17 members in this subfamily including TINY.	Use	ERF	2.49E-03
F vs. L (23)	AT3G52880	MDAR1	MDAR1 is a peroxisomal monodehydroascorbate reductase that functions in the ascorbate-glutathione cycle to remove toxic H_2O_2 (TAIR)	ROS attenuation		2.58	Encodes a peroxisomal monodehydroascorbate reductase, involved in the ascorbate-glutathione cycle which removes toxic H_2O_2	Use	glutathione, H_2O_2, Ca, Ascorbate	2.06E-04
F vs. S (23)	AT4G10040	CYTC-2	CYTC-2 encodes the mitochondrial electron carrier chytochrome c. Increased levels of CYTC enhance growth probably by increasing mitochondrial energy (ATP) and gibberellin signaling and homeostasis (doi: 10.1111/tpj.13845)		Homeostasis	2.57	Encodes cytochrome c. Promoter directs preferential expression in vascular tissues of cotyledons, leaves, roots, and hypocotyls, and in anthers. Double mutants with CYTC-1 accumulate starch during the day, have delayed growth and development and reduced GA and DELLA proteins linking cellular metabolism and GA homeostasis.	Yes	Homeostasis	7.47E-05
F vs. S (23)	AT1G59640	BPE	NA		Flowering	2.56	A basic helix-loop-helix encoding gene (BIGPETAL, BPE) involved in the control of petal size. BPE is expressed via two mRNAs derived from an alternative splicing event. The BPEub (AT1G59640.1)transcript is expressed ubiquitously, whereas the BPEp (AT1G59640.2) transcript is preferentially expressed in petals. Plants that lack the petal-expressed variant BPEp have larger petals as a result of increased cell size. BPEp is positively regulated downstream of APETALA3, PISTILLATA, APETALA1 and PISTILLATA3 and is negatively regulated downstream of AGAMOUS.	Yes	AGL	6.93E-04
F vs. L (23)	AT5G47000	AT5G47000	Upregulated in response to oxidative stress (TAIR)	ROS attenuation		2.55	Peroxidase superfamily protein;(source:Araport11)	Use	peroxidase	8.66E-05
F vs. S (23)	AT3G47940	HSP AT3G47940	TAIR	Stress attenuation	HSP	2.53	DNAJ heat shock family protein;(source:Araport11)	Yes	Hsp	5.88E-05
F vs. L (23)	AT1G60030	NAT7	Transmembrane ascorbate transport (TAIR)	ROS attenuation		2.53	nucleobase-ascorbate transporter 7;(source:Araport11)	Use	Ascorbate	1.90E-04
F vs. L (23)	AT3G19980	FyPP3	FyPP3 dephosphorylates the TF ABI5, thus negatively regulating ABA signaling. FyPP3 degradation is facilitated by NRP, but FyPP3 can also deactivate NRP by dephosphorylating it (doi.org/10.1016/j.molp.2017.11.006). Photomorphogenesis is repressed in the dark by kinase activated PHYTOCHROME INTERACTING FACTORS (PIFs). By dephosphorylating PIFs, PP6 (FyPP3, catalytic subunit) regulates photomorphogenesis (doi.org/10.1073/pnas.1907540116)	ABA signaling		2.52	Encodes catalytic subunit of serine/threonine protein phosphatase 2A. It can associate with phytochromes A and B in vitro. Mutant plants display an accelerated flowering phenotype.Acts antagonistically to SnRK2 to regulate ABI5 phosphorylation. It inteacts with NRP which results in tethering to endosomes leading to its degradation.	Use	SnRK	1.33E-03
F vs. L (23)	AT3G54960	PDI1	PDI1 is upregulated by ABA, ER stress, and abiotic stress. It is a thiol-disulfide oxidoreductase (thioredoxin) that participates in protein folding and unfolding and in establishing cell redox homeostasis (doi.org/10.3389/fpls.2018.00913)	ABA signaling, ROS attenuation	ABA upregulated, thioredoxin	2.52	Encodes a protein disulfide isomerase-like (PDIL) protein, a member of a multigene family within the thioredoxin (TRX) superfamily. Transcript levels for this gene are up-regulated in response to three different chemical inducers of ER stress (dithiothreitol, beta-mercaptoethanol, and tunicamycin). Neither AtIRE1-2 nor AtbZIP60 appear to be required for this response.	Use	Stress, Thioredoxin	2.91E-03
F vs. L (23)	AT3G52470	LEA AT3G52470	LEA (TAIR)		LEA	2.51	Late embryogenesis abundant (LEA) hydroxyproline-rich glycoprotein family;(source:Araport11)	Use	LEA	9.66E-04
F vs. M (23)	AT4G30996	NKS1	Ion homeostasis regulation pathway upregulated in response to hyperosmotic salinity stress (TAIR)	Stress signaling	Homeostasis	2.51	NKS1, a plant-specific gene, encodes a 19 kDa endomembrane-localized protein. It is part of SOS independent ion homeostasis regulation pathway.	Use	Homeostasis	1.05E-03

L vs M (123)	AT5G54160	**OMT1**	OMT1 expression is correlated with ROS levels (DOI: 10.1111/jpi.12640)	Stress signaling		2.51	A caffeic acid/5-hydroxyferulic acid O-methyltransferase. Interacts with 14-4-3 proteins in yeast 2 hybrid assay. AtOMT1 (At5g54160) encodes a flavonol 3?-O-methyltransferase that is highly active towards quercetin and myricetin. The substrate specificity identifies the enzyme as flavonol 3?-methyltransferase which replaces the former annotation of the gene to encode a caffeic acid/5-hydroxyferulic acid O-methyltransferase The mRNA is cell-to-cell mobile.	No	flavonol	9.79E-05
F vs. S (23)	AT4G40010	**SnRK2.7**	SnRK2.7 is upregulated and activated by salt and non-ionic osmotic stress (TAIR)	ABA signaling, Stress signaling	ABA upregulated, Salt	2.49	encodes a member of SNF1-related protein kinases (SnRK2.7) whose activity is activated by ionic (salt) and non-ionic (mannitol) osmotic stress.	Yes	SnRK, Stress	7.45E-04
F vs. S (23)	AT2G05710	**ACO3**	ACO3 is an aconitase the catalyzes citrate to isocitrate in the TCA cycle. It may play a role in the response to ROS by upregulating CSD2 (superoxide dismutase) (TAIR)	ROS attenuation		2.48	Encodes an aconitase that can catalyze the conversion of citrate to isocitrate through a cis-aconitate intermediate, indicating that it may participate in the TCA cycle and other primary metabolic pathways. The protein is believed to accumulate in the mitochondria and the cytosol. It affects CSD2 (At2g28190 - a superoxide dismutase) transcript levels and may play a role in the response to oxidative stress. One member of the family (ACO1 - At35830) was shown to specifically bind to the 5' UTR of CSD2 in vitro. ACO3 is tyrosine-phosphorylated and its phosphorylation state is modulated in response to ABA in Arabidopsis thaliana seeds. The mRNA is cell-to-cell mobile.	Yes	Stress, H_2O_2, ABA, superoxide dismutase	1.29E-04
F vs. S (23)	AT1G77980	**AGL66**	NA		Flowering	2.48	Encodes a member of the MIKC (MADS box, Keratin binding domain, and C terminal domain containing)family of transcriptional regulators. AGL66 is expressed in pollen.It forms heterodimers with other MICK family members (AGL104). Involved in late stages of pollen development and pollen tube	Yes	AGL	1.10E-04
F vs. L (23)	AT2G18400	**AT2G18400 (RP)**	TAIR		ribosome	2.48	ribosomal protein L6 family protein;(source:Araport11)	Use	ribosome	2.80E-05
F vs. L (23)	AT4G01610	**ATCATHB3**	ATCATHB3 is a cathepsin B capase that is upregulated by ER stress and positively regulates ER stress induced cell death (doi: 10.1111/nph.14676)	Stress attenuation		2.48	Encodes a capase involved in stress induced cell death. Activity detected in leaf and cell culture.	Use	Stress	1.24E-03
F vs. L (23)	AT1G01250	**DREB A-4 gene**	NA		ET	2.47	encodes a member of the DREB subfamily A-4 of ERF/AP2 transcription factor family. The protein contains one AP2 domain. There are 17 members in this subfamily including TINY.	Use	ERF	1.05E-04
F vs. M (23)	AT5G54160	**OMT1**	OMT1 expression is correlated with ROS levels (DOI: 10.1111/jpi.12640)	Stress signaling		2.46	A caffeic acid/5-hydroxyferulic acid O-methyltransferase. Interacts with 14-4-3 proteins in yeast 2 hybrid assay. AtOMT1 (At5g54160) encodes a flavonol 3?-O-methyltransferase that is highly active towards quercetin and myricetin. The substrate specificity identifies the enzyme as flavonol 3?-methyltransferase which replaces the former annotation of the gene to encode a caffeic acid/5-hydroxyferulic acid O-methyltransferase The mRNA is cell-to-cell mobile.	No	flavonol	1.21E-03
F vs. M (23)	AT2G47730	**GSTF8**	Reduces oxidative damage caused by heavy metals by binding to them.	ROS attenuation		2.45	Encodes glutathione transferase belonging to the phi class of GSTs. Naming convention according to Wagner et al. (2002).	Use	glutathione	3.28E-03
L vs. S (23)	AT5G64610	**no**			Histone	2.44	Encodes an enzyme with histone acetyltransferase activity. HAM1 primarily acetylate histone H4, but also display some ability to acetylate H3. Prior acetylation of lysine 5 on histone H4 reduces radioactive acetylation by either HAM1. HAM1 acetylates histone H4 lysine 5.			1.77E-02
F vs. S (23)	AT1G14920	**GAI**	GAI is a DELLA transcriptional regulator that interacts with TFs to regulate downstream genes. It is upregulated by ABA, ethylene, and salt stress. It increases drought tolerance by restraining cell proliferation and expansion (GA inhibition), and it likely reduces ROS accumulation by upregulating superoxide dismutases (TAIR)	ABA signaling	ABA upregulated, Salt	2.43	Similar to a putative transcription factor and transcriptional coactivators. Repressor of GA responses and involved in gibberellic acid mediated signaling. Member of the DELLA proteins that restrain the cell proliferation and expansion that drives plant growth. The protein undergoes degradation in response to GA via the 26S proteasome. GAI may be involved in reducing ROS accumulation in response to stress by up-regulating the transcription of superoxide dismutases. Represses GA-induced vegetative growth and floral initiation. Rapidly degraded in response to GA.	Yes	Stress, H_2O_2, ROS, superoxide dismutase	1.28E-03
F vs. L (23)	AT4G30996	**NKS1**	Ion homeostasis regulation pathway upregulated in response to hyperosmotic salinity stress (TAIR)	Stress signaling	Homeostasis	2.43	NKS1, a plant-specific gene, encodes a 19 kDa endomembrane-localized protein. It is part of SOS independent ion homeostasis regulation pathway.	Use	Homeostasis	6.65E-05
F vs. S (23)	ATCG00490	**RBCL**	RBCL is the large subunit of RUBISCO.	ABA response		2.43	large subunit of RUBISCO. Protein is tyrosine-phosphorylated and its phosphorylation state is modulated in response to ABA in Arabidopsis thaliana seeds.	Yes	ABA	2.19E-04
F vs. S (23)	AT1G04530	**TPR4 (HSP)**	TRP4 interacts with HSP90/70 as co-chaperones (TAIR)	Stress attenuation	HSP	2.43	Encodes one of the 36 carboxylate clamp (CC)-tetratricopeptide repeat (TPR) proteins (Prasad 2010, Pubmed ID: 20856808) with potential to interact with Hsp90/Hsp70 as co-chaperones.	Yes	Hsp	1.58E-04
F vs. M (23)	AT5G02790		Response to cadmium ions	ROS attenuation		2.43	Glutathione S-transferase family protein;(source:Araport11)	Use	glutathione	2.10E-03
F vs. S (23)	AT5G50800	**SWEET13**	TAIR	SnRK1 TOR		2.42	Encodes a member of the SWEET sucrose efflux transporter family proteins, together with RPG1, it is involved in pollen development. Together with SWEET14, it is likely involved in modulating the GA response and is required for proper development of anthers, seeds and seedlings.	Yes	SWEET transporter	1.50E-04
F vs. S (23)	AT2G42750	**DJC77 (HSP)**	TAIR	Stress attenuation	HSP	2.41	DNAJ heat shock N-terminal domain-containing protein;(source:Araport11)	Yes	Hsp	1.23E-04
F vs. L (23)	AT1G30810	**JMJ18**	The jumonji domain containing histone H3K4 demethylase JMJ18 suppresses the expression of genes that suppress flowering by reversing H3K4 methylation in their promoter regions (doi:10.1242/dev.183905)	Gene silencing, flowering	Histones	2.41	JMJ18 encodes a novel JmjC domain- containing histone H3K4 demethylase.	Use	Histone, H3K4	2.30E-04
F vs. S (23)	AT4G28680	**TYDC**	TAIR			2.41	Encodes a stress-induced tyrosine decarboxylase (TyrDC). Recombinant (His)6-TyrDC expressed in E. coli catalyzes the conversion of L-tyrosine to tyramine. Recombinant TyrDC forms tetramers.	Yes	Stress	1.11E-04
F vs. L (23)	AT3G17310	**DRM3**	DRM3, maintains non-CG DNA methylation, RDDM triggered by repeat sequences, and promotes Pol V transcription or assists in stabilizing Pol V transcripts (TAIR)	Gene silencing		2.40	Encodes DRM3 (Domains Rearranged Methyltransferase3), a catalytically mutated paralog of the cytosine methyltransferase DRM2. Despite being catalytically mutated, DRM3 is required for normal maintenance of non-CG DNA methylation, establishment of RNA-directed DNA methylation triggered by repeat sequences and accumulation of repeat-associated small RNAs.	Use	RDDM	4.47E-04
F vs. S (23)	AT3G47950	**HA4**	HA4 is a plasma membrane proton ATPase that acidifies the apoplast while increasing cytosolic pH (TAIR)	Stress attenuation		2.4	mutant has Slight reduction in root and shoot growth; Exaggerated defects in salt stress; Plasma Membrane H^+ ATPase	Yes	Stress	2.81E-04

F vs. S (23)	AT4G14880	CYS-3A	CYS-3A encodes a key enzyme in cysteine biosynthesis that is upregulated by salt and heavy metals (TAIR)	Stress signaling	Salt	2.39	Encodes a cytosolic isoform of cytosolic O-acetylserine(thiol)lyase, a key enzyme in cysteine biosynthesis and for the fixation of inorganic sulfide. It catalyzes the formation of cysteine from O-acetylserine and inorganic sulfide. Gene expression is predominant in the root cortex and the xylem parenchyma. Gene expression is induced in leave, stems and roots by high salt and heavy metal stresses, mediated by ABA. Lines carrying semi-dominant mutations exhibit early senescence. Required for pollen tube growth and/or fertilization.	Yes	Stress, ABA	1.03E-04
F vs. L (23)	AT4G14880	CYS-3A	CYS-3A encodes a key enzyme in cysteine biosynthesis that is upregulated by salt and heavy metals (TAIR)	Stress signaling	Salt	2.36	Encodes a cytosolic isoform of cytosolic O-acetylserine(thiol)lyase, a key enzyme in cysteine biosynthesis and for the fixation of inorganic sulfide. It catalyzes the formation of cysteine from O-acetylserine and inorganic sulfide. Gene expression is predominant in the root cortex and the xylem parenchyma. Gene expression is induced in leave, stems and roots by high salt and heavy metal stresses, mediated by ABA. Lines carrying semi-dominant mutations exhibit early senescence. Required for pollen tube growth and/or fertilization.	Use	Stress, ABA	8.13E-04
F vs. L (23)	AT3G08730	S6K1	Interacts with RAPTOR in the TOR signaling pathway, upregulated in metabolically active cells, activates ribosomal proteins through phosphorylation (doi.org/10.1016/j.bbrc.2018.05.027)	SnRK1 TOR	ribosome	2.36	Encodes a protein-serine kinase that phosphorylates ribosomal protein in vitro. Activation of AtS6k is regulated by 1-naphthylacetic acid and kinetin, at least in part, via a lipid kinase-dependent pathway. Involved in translational up-regulation of ribosomal proteins. Phosphorylated by PDK1. Interacts with RAPTOR1, which in turn interacts with TOR. SPK6 activity is affected by osmotic stress, and plants overexpressing S6k1 are hypersensitive to osmotic stress. The gene is expressed in all tissues examined, with highest expression level detected in metabolically active tissues.	Use	Stress, translation, ribosome	5.59E-04
F vs. M (23)	AT3G08730	S6K1	Interacts with RAPTOR in the TOR signaling pathway, upregulated in metabolically active cells, activates ribosomal proteins through phosphorylation (doi.org/10.1016/j.bbrc.2018.05.027)	SnRK1 TOR	ribosome	2.36	Encodes a protein-serine kinase that phosphorylates ribosomal protein in vitro. Activation of AtS6k is regulated by 1-naphthylacetic acid and kinetin, at least in part, via a lipid kinase-dependent pathway. Involved in translational up-regulation of ribosomal proteins. Phosphorylated by PDK1. Interacts with RAPTOR1, which in turn interacts with TOR. SPK6 activity is affected by osmotic stress, and plants overexpressing S6k1 are hypersensitive to osmotic stress. The gene is expressed in all tissues examined, with highest expression level detected in metabolically active tissues.	Use	Stress, translation, ribosome	3.16E-03
F vs. L (23)	AT1G77980	AGL66	NA		Flowering	2.35	Encodes a member of the MIKC (MADS box, Keratin binding domain, and C terminal domain containing) family of transcriptional regulators. AGL66 is expressed in pollen.It forms heterodimers with other MICK family members (AGL104). Involved in late stages of pollen development and pollen tube	Use	AGL	1.00E-03
F vs. L (23)	AT4G39730	PLAT	PLAT1 is upregulated by abiotic stress and localizes in the ER where it positively regulates growth (DOI 10.1007/s11248-015-9868-6)	Stress attenuation		2.35	PLAT1 domain stress protein family member. Involved in mediating response to stresses such as pathogen infection. It is found in endoplasmic reticulum bodies. PLAT1 is induced by pathogenic fungi and induces the production of	Use	Stress	1.36E-03
M vs. S (23)	AT1G52340	ABA2	Involved in ABA synthesis (TAIR)		ABA	2.34	Encodes a cytosolic short-chain dehydrogenase/reductase involved in the conversion of xanthoxin to ABA-aldehyde during ABA biosynthesis. Mutants are insensitive to sucrose and glucose.			1.37E-02
F vs. M (23)	AT3G17310	DRM3	DRM3, maintains non-CG DNA methylation, RdDM triggered by repeat sequences, and promotes Pol V transcription or assists in stabilizing Pol V transcripts (TAIR)	Gene silencing		2.34	Encodes DRM3 (Domains Rearranged Methyltransferase3), a catalytically mutated paralog of the cytosine methyltransferase DRM2. Despite being catalytically mutated, DRM3 is required for normal maintenance of non-CG DNA methylation, establishment of RNA-directed DNA methylation triggered by repeat sequences and accumulation of repeat-associated small RNAs.	Use	RDDM	4.13E-03
F vs. M (23)	AT4G39640	GGT1	Upregulated by ER and oxidative stress, degrades glutathione in the apoplast but also mitigates oxidative stress by metabolizing oxidized GSH (GSSH) (doi.org/10.1071/FP17151)	ROS attenuation		2.32	The gene encodes a gamma-glutamyltransferase (AKA gamma-glutamyl transpeptidase, EC 2.3.2.2) that is located in vascular tissues (predominantly phloem) of leaves and is involved in the degradation of glutathione. The encoded enzyme also mitigates oxidative stress by metabolizing GSSG (oxidized form of GSH - glutathione) in the apoplast.	Use	glutathione, Stress, ER stress	1.19E-03
F vs. S (23)	AT4G26080	ABI1	ABI1 is a PP2C that is upregulated by drought, salt, ABA and ethylene. It negatively regulates ABA and BR signaling (doi/10.1104/pp.105.070128; doi:10.1242/dev.151894)	ABA signaling	ABA upregulated, Salt	2.3	Involved in abscisic acid (ABA) signal transduction. Negative regulator of ABA promotion of stomatal closure.	Yes	ABA	8.58E-05
F vs. L (23)	AT3G07940	ARF AT3G07940	Ca dependent ARF (TAIR)		ARF	2.27	Calcium-dependent ARF-type GTPase activating protein family;(source:Araport11)	Use	ARF	1.99E-03
F vs. L (23)	AT3G01420	DOX1	DOX1 is an alpha dioxygenase that is upregulated by and provides protection from oxidative stress (TAIR)	ROS attenuation		2.27	Encodes an alpha-dioxygenase involved in protection against oxidative stress and cell death. Induced in response to Salicylic acid and oxidative stress. Independent of NPR1 in induction by salicylic acid. The mRNA is cell-to-cell mobile.	Use	Stress	1.07E-03
F vs. M (23)	AT1G44170	ALDH3H1	Upregulated by ABA, salt and desiccation; involved in detoxifying reactive aldehydes that form due to ROS-induced peroxidation of polyunsaturated fatty acids; subject to nitric oxide degradation (doi.org/10.1016/j.plantsci.2019.110389)	ROS attenuation	Salt, ABA upregulated	2.26	Encodes an aldehyde dehydrogenase induced by ABA and dehydration that can oxidize saturated aliphatic aldehydes. It is also able to oxidize beta-unsaturated aldehydes, but not aromatic aldehydes. Activity of ALDH3H1 is NAD +-dependent.	Use	ABA	2.13E-03
F vs. S (23)	AT2G15310	ARFB1a	ARF (TAIR)		ARF	2.26	A member of ARF GTPase family. A thaliana has 21 members of this family, known to be essential for vesicle coating and uncoating and functions in GTP-binding. Gene encoding ADP-ribosylation factor and similar to ADP-ribosylation factor (GI:861205) (Chlamydomonas reinhardtii), other ARFs and ARF-like proteins.	Yes	ARF	4.01E-04
F vs. S (23)	AT1G27760	SAT32	SAT32 is upregulated in response to salt stress and is required for salt tolerance and fertility (TAIR)	Stress signaling	Salt	2.26	Encodes a protein with similarity to human interferon-related developmental regulator (IFRD)that is involved in salt tolerance. Loss of function mutations are hypersensitive to salt stress and have reduced fertility. SAT32 is found in the cytoplasm but appears to translocate to the nucleus when plants are subject to salt stress.	Yes	Stress	1.80E-03
F vs. L (23)	AT4G02380	LEA38	LEA38 is upregulated by ABA, ROS, water deprivation, ethylene (TAIR)		ABA, upregulated, LEA	2.25	Encodes AtLEA5 (late embryogenesis abundant like protein). Also known as SENESCENCE-ASSOCIATED GENE 21 (SAG21). Has a role on oxidative stress tolerance. mRNA levels are elevated in response to various stresses.	Use	LEA, Stress	2.91E-03

F vs. S (23)	AT3G05500	SRP3	TAIR			2.25	Encodes a protein that associates with lipid droplet surfaces and shares sequence homology with family of small rubber particle proteins. Plays dual roles as positive factors for tissue growth and development and in drought stress responses. The mRNA is cell-to-cell mobile.	Yes	Stress	1.70E-03
F vs. L (23)	AT1G76080	CDSP32	CDSP32 is a thioredoxin that is upregulated by oxidative stress and drought. It participates in maintaining cell redox homeostasis (TAIR)	ROS attenuation	Homeostasis, thioredoxin	2.24	Encodes a thioredoxin localized in chloroplast stroma. Known as CDSP32 (CHLOROPLASTIC DROUGHT-INDUCED STRESS PROTEIN OF 32 KD).	Use	Thioredoxin	2.81E-03
F vs. S (23)	AT1G73500	MKK9	MPK9 is upregulated by salt stress through the lipid second messenger phosphatidic acid (PA). MPK9 then phosphorylation activates MPK3,6 that upregulates several ERFs and Na+ H+ antiporter SOS1 (doi.org/10.1016/j.plantsci.2019.110190)	Stress signaling	ET, Salt	2.24	member of MAP Kinase Kinase family. Autophosphorylates and also phosphorylates MPK3 and MPK6. Independently involved in ethylene and calmalexin biosynthesis. Induces transcription of ACS2, ACS6, ERF1, ERF2, ERF5, ERF6, CYP79B2, CYP79B3, CYP71A13 and PAD3.	Yes	ethylene, ERF, MAPK	2.18E-04
F vs. L (23)	AT1G17370	UBP1B	UBP1B is an RNA binding protein that protects mRNAs in stress granules during abiotic stress. Upregulation of UBP1B increased ABA signaling (hypersensitivity) and upregulated the ABA receptor genes *PYL5,6* (doi.org/10.1080/15592324.2017.1282591)	ABA signaling, Stress signaling	ABA upregulated	2.24	Encodes an RNA?binding protein involved in stress granule formation. Regulated by a transposable element small RNA.	Use	Stress	2.64E-03
F vs. S (23)	AT3G22950	ARFC1	TAIR		ARF	2.23	A member of ARF GTPase family. A thaliana has 21 members of this family, known to be essential for vesicle coating and uncoating and functions in GTP-binding. Gene encoding ADP-ribosylation factor and similar to ADP-ribosylation factor GB:P91924 (Dugesia japonica), other ARFs and ARF-like proteins.	Yes	ARF	3.75E-04
M vs. S (23)	AT4G27890	BOB2 (HSP)			HSP	2.23	HSP20-like chaperones superfamily protein;(source:Araport11)			1.42E-02
F vs. L (23)	AT4G34710	ADC2	Is osmotic stress induced; upregulated by exogenous sucrose - osmotic effect? (doi.org/10.1016/j.jplph.2019.04.012)	Stress signaling	Salt	2.22	Encodes a arginine decarboxylase (ADC), a rate-limiting enzyme that catalyzes the first step of polyamine (PA) biosynthesis via ADC pathway in Arabidopsis thaliana. Arabidopsis genome has two ADC paralogs, ADC1 and ADC2. ADC2 is stress-inducible (osmotic stress). Double mutant analysis showed that ADC genes are essential for the production of PA, and are required for normal seed development. Overexpression causes phenotypes similar to GA-deficient plants and these plants show reduced levels of GA due to lower expression levels of AtGA20ox1, AtGA3ox3 and AtGA3ox1.	Use	Stress	4.55E-04
F vs. L (23)	AT4G17230	SCL13 (HSP)	Upregulated by hypoxia (TAIR)	Stress attenuation	HSP	2.22	Encodes a scarecrow-like protein (SCL13). Member of GRAS gene family. Regulated by heat shock.	Use	HSP	9.84E-04
F vs. M (23)	AT3G26060	PRXQ	Upregulated by oxidative stress, decomposes peroxides, necessary for the synthesis of certain thylacoid membrane lipids (doi.org/10.1111%2Ftpj.14657)	ROS attenuation		2.20	encodes periredoxin Q which decomposes peroxides and plays a role in the protection of the photosynthetic apparatus	Use	H2O2	2.70E-03
F vs. S (23)	AT5G01520	AIRP2	AIRP2 is upregulated by ABA and salt stress and is a cytosolic ubiquitin ligase necessary for ABA and salinity responses. It targets for ubiquitination ATP1/SDIRIP1, which in the absence of stress negatively regulates ABA signaling by silencing ABI5 (ABA and high salinity responses in seeds) (doi.org/10.1104%2Fpp.17.00467)	ABA signaling	ABA upregulated, Salt	2.19	Encodes a cytosolic RING-type E3 ubiquitin (Ub) ligase that is critical for ABA and high salinity responses during germination. AtAIRP2 and SDIR1 likely play a combinatory role in ABA signaling and the response to high salt in Arabidopsis.	Yes	ABA	7.13E-05
F vs. S (23)	AT3G30180	CYP85A2	CYP85A2 catalyzes the last step in BR synthesis. Upregulation of CYP85A2 increases BR signaling and reduces ABA signaling (doi.org/10.1038/nplants.2016.114) Nature Plants	BR		2.19	Encodes a cytochrome p450 enzyme that catalyzes the last reaction in the production of brassinolide. It is capable of converting 6-deoxocastasterone into castasterone, a C-6 oxidation, as well as the further conversion of castasterone into brassinolide by a Baeyer-Villinger oxidation reaction at C-6, resulting in the formation of an unusual seven-membered lactone ring. The enzyme possesses high affinity for both C28- and C27-Brassinosteroids. The expression of the gene using a CYP85A2 promoter:LUC fusion construct was shown to be under circadian and light control.	Yes	BR	1.01E-03
F vs. L (23)	AT1G14920	GAI	GAI is a DELLA transcriptional regulator that interacts with TFs to regulate downstream genes. It is upregulated by ABA, ethylene, and salt stress. It increases drought tolerance by restraining cell proliferation and expansion (GA inhibition), and it likely reduces ROS accumulation by upregulating superoxide dismutases (TAIR)	ABA signaling	ABA upregulated, Salt	2.18	Similar to a putative transcription factor and transcriptional coactivators. Repressor of GA responses and involved in gibberellic acid mediated signaling. Member of the DELLA proteins that restrain the cell proliferation and expansion that drives plant growth. The protein undergoes degradation in response to GA via the 26S proteasome. GAI may be involved in reducing ROS accumulation in response to stress by up-regulating the transcription of superoxide dismutases. Represses GA-induced vegetative growth and floral initiation. Rapidly degraded in response to GA.	Use	Stress, H2O2, ROS, superoxide dismutase	2.83E-04
F vs. M (23)	AT5G30510	RPS1 (RP)	Plastid ribosomal protein (TAIR)		ribosome	2.16	ribosomal protein S1;(source:Araport11)	Use	ribosome	1.21E-03
F vs. S (23)	AT4G34710	ADC2	Is osmotic stress induced; upregulated by exogenous sucrose - osmotic effect? (doi.org/10.1016/j.jplph.2019.04.012)	Stress signaling	Salt	2.15	Encodes a arginine decarboxylase (ADC), a rate-limiting enzyme that catalyzes the first step of polyamine (PA) biosynthesis via ADC pathway in Arabidopsis thaliana. Arabidopsis genome has two ADC paralogs, ADC1 and ADC2. ADC2 is stress-inducible (osmotic stress). Double mutant analysis showed that ADC genes are essential for the production of PA, and are required for normal seed development. Overexpression causes phenotypes similar to GA-deficient plants and these plants show reduced levels of GA due to lower expression levels of AtGA20ox1, AtGA3ox3 and AtGA3ox1.	Yes	Stress	9.45E-04
F vs. L (23)	AT5G41790	CIP1	CIP1 locates to the plasma membrane, is upregulated by ABA and osmotic stress, and is a positive regulator of ABA responses (http://dx.doi.org/10.1016/j.bbrc.2016.06.147)	ABA signaling	ABA upregulated	2.15	CIP1 physically interacts specifically with the putative coiled-coil region of COP1 in vitro. In hypocotyl and cotyledon protoplasts, it is associated to the cytoskeleton, but not in the root. IN the root the protein is found around epidermal cells. Expression is induced by ABA and osmotic stress but is not regulated by light. Loss of function mutants are also sensitive to osmotic stress and are somewhat ABA insensitive. The mRNA is cell-to-cell mobile.	Use	Stress, ABA	2.45E-03
F vs. L (23)	AT2G46140	LEA27	TAIR		LEA	2.15	Late embryogenesis abundant protein;(source:Araport11)	Use	LEA	1.85E-03
F vs. L (23)	AT5G30510	RPS1 (RP)	Plastid ribosomal protein (TAIR)		ribosome	2.14	ribosomal protein S1;(source:Araport11)	Use	ribosome	3.93E-05
F vs. L (23)	AT5G64400	AT12CYS-1	TAIR			2.13	CHCH domain protein;(source:Araport11) involved in mechanotransduction. Loss of both At12cys-1 and At12cys-2 lead to enhanced tolerance to drought and light stress and increased anti-oxidant capacity.	No	Stress	2.02E-03
F vs. S (23)	AT2G24800	AT2G24800	AT2G24800 is a peroxidase that is upregulated in response to oxidative stress (TAIR)	ROS attenuation		2.13	Peroxidase superfamily protein;(source:Araport11)	Yes	peroxidase	3.36E-04
F vs. L (23)	AT5G17860	CAX7	Ca transport (TAIR)			2.13	calcium exchanger 7;(source:Araport11)	No	Ca	6.65E-04

F vs. S (23)	AT3G08730	**S6K1**	Interacts with RAPTOR in the TOR signaling pathway, upregulated in metabolically active cells, activates ribosomal proteins through phosphorylation (doi.org/10.1016/j.bbrc.2018.05.027)	SnRK1 TOR	ribosome	2.13	Encodes a protein-serine kinase that phosphorylates ribosomal protein in vitro. Activation of AtS6k is regulated by 1-naphthylacetic acid and kinetin, at least in part, via a lipid kinase-dependent pathway. Involved in translational up-regulation of ribosomal proteins. Phosphorylated by PDK1. Interacts with RAPTOR1, which in turn interacts with TOR. SPK6 activity is affected by osmotic stress, and plants overexpressing S6k1 are hypersensitive to osmotic stress. The gene is expressed in all tissues examined, with highest expression level detected in metabolically active tissues.	Yes	Stress	8.40E-04
F vs. L (23)	AT4G19230	**CYP707A1**	CYP707A1 is an hydroxylase that inactivates ABA. It is downregulated by drought through the expression of HISTONE DEACETYLASE 9 (HDA9) and ABA INSENSITIVE 4 (ABI4). Hence, during drought, ABA accumulates. It's upregulation in B. x formosa ovules suggests that ABA signaling is weaker in B. microphylla (stronger in B. x. formosa) (doi.org/10.3389/fpls.2020.00143)	ABA signaling		2.12	Encodes a protein with ABA 8'-hydroxylase activity, involved in ABA catabolism. Member of the CYP707A gene family. CYP707A1 appears to play an important role in determining the ABA levels in dry seeds. Gene involved in postgermination growth. Overexpression of CYP707A1 leads to a decrease in ABA levels and a reduction in after-ripening period to break dormancy.	Use	ABA	2.29E-04
F vs. S (23)	AT4G27630	**GTG2**	GTG2 is a G-protein with nine transmembrane domains. It binds ABA and may function as an ABA receptor (TAIR)	ABA signaling		2.12	Encodes a GPCR-type G protein receptor with nine predicted transmembrane domains. The protein binds abscisic acid (ABA) and is predicted to function as an ABA receptor. It has GTP-binding and GTPase activity and binds to ABA more effectively in the presence of GDP. GTG2 binds to GPA1, the alpha subunit of the heterotrimeric G protein. GPA1 (in its GTP-bound state) affects the GTP binding and GTPase activity of GTG2 and may act to down-regulate GTG2 binding to ABA. GTG2 is widely expressed throughout the plant and appears to be involved in the regulation of several ABA-dependent responses including seed germination, plant development, and promotion of stomatal closure. GTG2 transcript levels do not appear to change in response to ABA or	Yes	Stress, ABA	2.28E-03
M vs. S (23)	AT1G07500	**SMR5**			Stress	2.12	SMR5 is a member of the SIAMESE-RELATED Cyclin-Dependent Kinase Inhibitor family. It is induced by ROS/oxidative stress.			7.94E-03
F vs. S (23)	AT1G63010	**VPT1**	VPT1 is a Pi transporter that regulates phosphate homeostasis by regulating Pi levels in vacuoles (TAIR)		Homeostasis	2.12	Encodes an SPX domain protein that transports Pi into the vacuole and is essential for phosphate homeostasis.	Yes	Homeostasis	4.32E-04
L vs M (123)	AT1G68820	**PPRT1**	Negative regulator of ABA: induced by salt stress and causes increased sensitivity to salt stress (https://doi.org/10.1080/15592324.2020.1732103); downregulates stess inducible genes by upregulating the ABA catabolism genes AtCYP707A1 and AtCYP707A3 (doi.org/10.3390/ijms20020394)	ABA signaling	ABA upregulated, Salt	2.11	Putative C3HC4 zinc-finger ubiquitin E3 ligase, negative regulator in ABA and drought stress response.	Use	Stress, ABA	4.63E-03
L vs M (123)	AT2G36530	**ENO2 (MBP-1 like)**	Increases tolerance to salt and drought stress (doi.org/10.1111/ppl.13013; 10.1007/s11033-018-4292-7	Stress attenuation	Salt	2.10	Involved in light-dependent cold tolerance and encodes an enolase. Protein is tyrosine-phosphorylated and its phosphorylation state is modulated in response to ABA in Arabidopsis thaliana seeds.	Use	ABA	1.10E-03
F vs. S (23)	AT3G52180	**SEX4**	SEX4 is a glucan phosphatase that is involved in starch catabolism. It also interacts with SnRK1.2 possibly phosphorylation inactating it (TAIR)	SnRK1-TOR		2.1	Encodes a plant-specific glucan phosphatase that contains a noncatalytic carbohydrate-binding module as well as a dual specificity protein phosphatase domain. SEX4 can dephosphorylate C6- and C3-glucosyl residues on native starch grains and related maltodextrin compounds in vitro. This protein interacts with the plant SnRK AKIN11 (SnRK1.2), binds starch, and is localized in the chloroplast. sex4 mutants have elevated levels of starch.	Yes	SnRK	1.65E-03
F vs. L (23)	AT3G05500	**SRP3**	TAIR			2.10	Encodes a protein that associates with lipid droplet surfaces and shares sequence homology with family of small rubber particle proteins. Plays dual roles as positive factors for tissue growth and development and in drought stress responses. The mRNA is cell-to-cell mobile.	No	Stress	8.16E-04
F vs. L (23)	AT1G04530	**TPR4 (HSP)**	TRP4 interacts with HSP90/70 as co-chaperones (TAIR)	Stress attenuation	HSP	2.10	Encodes one of the 36 carboxylate clamp (CC)-tetratricopeptide repeat (TPR) proteins (Prasad 2010, Pubmed ID: 20856808) with potential to interact with Hsp90/Hsp70 as co-chaperones.	Use	HSP	3.89E-05
F vs. S (23)	AT1G21760	**FBP7**	FPB7 is an F-box protein that is upregulated by temperature stress and regulates translation (TAIR)	SnRK1 TOR, Stress attenuation		2.09	This gene is predicted to encode an F-box protein that is evolutionarily conserved between Arabidopsis and other eukaryotes including S.cerevisiae and humans. It may play a role in regulating translation under conditions of temperature stress. FBP7 transcript levels are increased at high and low temperatures. The mRNA is cell-to-cell mobile.	Yes	Stress	1.50E-04
M vs. S (23)	AT4G32320	**APX6**	A major cytosolic ascorbate peroxidase that catalyzes the reduction of H2O2 thus reducing the effect of ROS on ABA and auxin signaling (10.1104/pp.114.245324)		H2O2, peroxidase, Ascorbate	2.08	Encodes a cytosolic ascorbate peroxidase APX6. Ascorbate peroxidases are enzymes that scavenge hydrogen peroxide in plant cells. Eight types of APX have been described for Arabidopsis: three cytosolic (APX1, APX2, APX6), two chloroplastic types (stromal sAPX, thylakoid tAPX), and three microsomal (APX3, APX4, APX5) isoforms.			1.16E-02
F vs. L (23)	AT2G42750	**DJC77 (HSP)**	TAIR	Stress attenuation	HSP	2.08	DNAJ heat shock N-terminal domain-containing protein;(source:Araport11)	Use	HSP	1.26E-03
F vs. S (23)	AT4G01370	**MPK4**	MPK4 and the TF MYB42 are upregulated by salt stress. MPK4 phosphorylation activates MYB42, which upregulates SOS2. SOS2 is a protein kinase that activates the Na+/H+ antiporter SOS1 (doi: 10.1111/nph.16538)	Flowering, Stress attenuation	Meiosis, Salt	2.08	Encodes a nuclear and cytoplasmically localized MAP kinase involved in mediating responses to pathogens. Its substrates include MKS1 and probably MAP65-1.The MAP65-1 interaction is involved in mediating cortical microtuble organization. Required for male-specific meiotic cytokinesis. The mRNA is cell-to-cell mobile.	Yes	meiosis, MAPK	1.52E-04
F vs. M (23)	AT4G05590	**NRGA1**	Mitochondrial pyruvate carrier that mediates ABA signaling, drought stress and Cd toxicity by sustaining the TCA cycle and glutathione synthesis (doi.org/10.1104%2Fpp.18.01610)	ABA signaling	ABA upregulated	2.08	Encodes NRGA1, a putative mitochondrial pyruvate carrier that mediates ABA regulation of guard cell ion channels and drought stress responses.	Use	Stress, ABA	2.23E-03
L vs M (123)	AT1G17280	**UBC34**	Negative regulator of ABA: prevents abscisic acid-mediated stomatal closure thus reducing tolerance to drought stress (10.1016/j.jplph.2018.08.010); reduces sucrose transport across membranes by ubiquitinating SUC2 (doi.org/10.1073/pnas.1912754117)	ABA signaling, Stress signaling	ABA upregulated	2.08	Group XIV ubiquitin-conjugating enzyme that functions negative regulation of drought stress.	Use	Stress	5.68E-03
F vs. L (23)	AT2G39770	**VTC1**	Essential enzyme in ascorbic acid biosynthesis. High levels of VTC1 are correlated with high levels of ascorbic acid (doi.org/10.1093/jxb/erw223)	ROS attenuation		2.07	Encodes a GDP-mannose pyrophosphorylase/ mannose-1-pyrophosphatase. This enzyme provides GDP-mannose, which is used for cell wall carbohydrate biosynthesis and protein glycosylation as well as for ascorbate (vitamin C) biosynthesis. Mutations in this gene confer hypersensitivity to NH4+.	Use	Ascorbate	1.22E-04

F vs. S (23)	AT3G48510	**AITR2 (DIG1)**	AITR2 (DIG1) belongs to a group of six transcriptional repressors that are upregulated by ABA (DOI: 10.1126/science.aag1550). They strengthen ABA signaling (suppression of growth) by i) suppressing ABA induced downregulation of ABA receptor genes and ii) suppressing ABA induced upregulation of PP2C genes (DOI: 10.1111/pce.13058). Consistent with these functions, we found that PP2Cs were down-regulated in B. microphylla vs. B. lignifera ( ), and this may have enhanced ABA signaling in B. microphylla ovules.	ABA signaling		2.06	ABA‐induced transcription repressor that acts as feedback regulator in ABA signalling.	Yes	ABA	3.64E-04
F vs. S (23)	AT4G19810	**ChiC**	TAIR	ABA signaling		2.06	ChiC encodes a Class V chitinase that is a part of glycoside hydrolase family 18 based on CAZy groupings. It appears to primarily act as an exochitinase in vitro where it predominantly cleaves a chitobiose (GlcNAc)2 residue from the non-reducing end of a chitin oligosaccharide. However, it shows some minor endochitinase activity in vitro, as well. A putative 24 amino-acid signal peptide may direct this protein to the secretory system and it has been detected in cell wall apoplastic fluid. RT-PCR experiments demonstrate that ChiC transcript levels are increased in response to abscisisc acid, jasmonic acid, and NaCl stress. Microarray results also suggest that transcript levels rise in response to osmotic stress, two fungal pathogens, a bacterial pathogen, and the elicitor flagellin. The mRNA is cell-to-cell mobile.	Yes	Stress	4.00E-04
F vs. L (23)	AT3G08720	**S6K2**	S6K2 is a ribosomal protein that is upregulated under favorable growth conditions by TOR. Unlike S6K1, which primarily functions in the cytoplasm to upregulate translation, S6K2 is primarily localized in the nucleus where it activates the BR transcription factors BZR1 and BES1 by phosphorylation inactivation of BIN2 (doi: 10.1111/nph.14118)	SnRK1 TOR, BR	ribosome	2.06	Encodes a ribosomal-protein S6 kinase. Gene expression is induced by cold and salt (NaCl). Activation of AtS6k is regulated by 1-naphthylacetic acid and kinetin, at least in part, via a lipid kinase-dependent pathway. Phosphorylates specifically mammalian and plant S6 at 25 degrees C but not at 37 degrees C. Involved in translational up-regulation of ribosomal proteins.	Use	NaCl, translation, ribosome	1.61E-03
F vs. L (23)	AT3G22370	**AOX1a**	AOX1a is highly suppressed at the transcriptional and post transcriptional levels under favorable growing conditions. During stress, AOX1a is upregulated by multiple stress related TFs including SnRK1 (https://doi.org/10.1016/j.tplants.2018.03.012)	SnRK1 TOR		2.05	Encodes AOX1a, an isoform of alternative oxidase that is expressed in rosettes, flowers, and root. The alternative oxidase of plant mitochondria transfers electrons from the ubiquinone pool to oxygen without energy conservations. It is regulated through transcriptional control and by pyruvate. Plays a role in shoot acclimation to low temperature. Also is capable of ameliorating reactive oxygen species production when the cytochrome pathway is inhibited. AOX1a also functions as a marker for mitochondrial retrograde response. The mRNA is cell-to-cell mobile.	Use	AOX	4.38E-04
F vs. S (23)	AT1G63060	**NEP1-like**	Ribosome biogenesis (TAIR)		ribosome	2.05	ribosome biogenesis NEP1-like protein;(source:Araport11)	Yes	Ribosome	1.03E-04
F vs. L (23)	AT1G15470	**XIW1**	Positively regulates ABA signaling by stabilizing ABI5 (doi.org/10.1016%2Fj.molp.2019.07.001	ABA signaling	ABA upregulated	2.05	WD40 nucleoplasmic shuttling protein that positively regulates the Abscisic acid (ABA) response by interacting with and maintaining the stability of ABI5 in the nucleus. Nuclear export of XIW1 is XPO1-dependent. Involved in regulating seed germination, primary root growth, and drought stress	Use	Stress, ABA	4.38E-04
F vs. S (23)	AT5G27150	**NHX1**	NHX1 is a Na+/H+ antiporter that is induced by salt stress and regulates Na+ homeostasis (TAIR)	Stress signaling	Homeostasis, Salt	2.04	Encodes a vacuolar sodium/proton antiporter involved in salt tolerance, ion homeostasis, and leaf development. The mRNA is cell-to-cell mobile.	Yes	Homeostasis	2.89E-04
F vs. M (23)	AT5G42270	**VAR1**	NA			2.04	VAR1 contains a conserved motif for ATPase and a metalloprotease characteristic to FtsH proteins, and is targeted into chloroplasts. A VAR1-fusion protein synthesized in vitro exhibited ATPase activity and partial metalloprotease activity. This protein is located to the thylakoid membrane and forms a complex with VAR2. FtsH1 (VAR1) and FtsH5 are interchangeable in thylakoid membranes. Phosphorylation of this protein is dependent on calcium. The mRNA is cell-to-cell mobile.	No	Ca	2.20E-03
F vs. M (23)	AT1G78160	**APUM7**		SnRK1 TOR		2.02	Encodes a member of the Arabidopsis Pumilio (APUM) proteins containing PUF domain (eight repeats of approximately 36 amino acids each). PUF proteins regulate both mRNA stability and translation through sequence-specific binding to the 3' UTR of target mRNA transcripts.		#N/A	1.54E-03
F vs. S (23)	AT4G24160	**AT4G24160**	AT4G24160 maintains lipid homeostasis by regulating phopholipid and neutral lipid levels (TAIR)		Homeostasis	2.02	Encodes a soluble lysophosphatidic acid acyltransferase with additional triacylglycerol lipase and phosphatidylcholine hydrolyzing enzymatic activities. Plays a pivotal role in maintaining the lipid homeostasis by regulating both phospholipid and neutral lipid levels.	Yes	Homeostasis	2.84E-04
F vs. S (23)	AT1G75220	**ERDL6**	ERDL6 is a vacuolar glucose exporter that is upregulated under low energy conditions to facilitate glucose homeostasis during stress (doi/10.1104/pp.111.186825)	Stress signaling	Homeostasis	2.02	Encodes a vacuolar glucose exporter that is induced in response to factors that activate vacuolar glucose pools like darkness, heat stress and wounding and repressed during conditions that trigger glucose accumulation in the vacuole like cold stress and external sugar supply.	Yes	Stress	4.38E-04
F vs. L (23)	AT2G15310	**ARFB1a**	ARF (TAIR)		ARF	2.01	A member of ARF GTPase family. A thaliana has 21 members of this family, known to be essential for vesicle coating and uncoating and functions in GTP-binding. Gene encoding ADP-ribosylation factor and similar to ADP-ribosylation factor (GI:861205) (Chlamydomonas reinhardtii), other ARFs and ARF-like proteins.	Use	ARF	1.92E-04
F vs. L (23)	AT1G68440	**AT1G68440**	Transmembrane protein upregulated by ABA and multiple stresses including heat, salt and drought (TAIR)	ABA signaling	ABA upregulated, Salt	2.01	Transmembrane protein;(source:Araport11). Expression induced by abiotic stressors such as ABA, drought, heat, light, NaCl, osmotic stress and wounding.	Use	Stress, ABA	4.24E-04
F vs. M (23)	AT3G52470	**LEA AT3G52470**	LEA (TAIR)		LEA	2.01	Late embryogenesis abundant (LEA) hydroxyproline-rich glycoprotein family;(source:Araport11)	Use	LEA	3.64E-03
F vs. L (23)	AT3G09940	**MDAR3**	TAIR			2.01	Encodes a member of the monodehydroascorbate reductase gene family. Critical for a mutualistic symbiosis between the host Arabidopsis and the root colonizing fungus Piriformospora indica.	No	Ascorbate	9.97E-05
L vs. S (23)	AT1G23190	**PGM3**			gametophyte	2	Encodes a cytosolic phosphoglucomutase (PGM). Two Arabidopsis PGM proteins (AT1G70730/PGM2 and AT1G23190/PGM3) have high sequence similarities and redundant functions. Mature plants possessing a single cPGM allele had a major reduction in cPGM activity. Whereas pgm2 and pgm3 single mutants are undistinguishable from the wild type, loss of both PGM2 and PGM3 severely impairs male and female gametophyte development. The mRNA is cell-to-cell mobile.			1.07E-02

F vs. M (23)	AT1G21980	PIP5K1	NA	ABA signaling	ABA upregulated	2.00	Type I phosphatidylinositol-4-phosphate 5-kinase. Preferentially phosphorylates PtdIns4P. Induced by water stress and abscisic acid in Arabidopsis thaliana. Expressed in procambial cells of leaves, flowers and roots. A N-terminal Membrane Occupation and Recognition Nexus (MORN)affects enzyme activity and distribution.	Use	Stress, ABA	4.80E-03
L vs M (123)	AT4G34710	ADC2	Is osmotic stress induced; upregulated by exogenous sucrose - osmotic effect? (doi.org/10.1016/j.jplph.2019.04.012)	Stress signaling	Salt	1.99	Encodes a arginine decarboxylase (ADC), a rate-limiting enzyme that catalyzes the first step of polyamine (PA) biosynthesis via ADC pathway in Arabidopsis thaliana. Arabidopsis genome has two ADC paralogs, ADC1 and ADC2. ADC2 is stress-inducible (osmotic stress). Double mutant analysis showed that ADC genes are essential for the production of PA, and are required for normal seed development. Overexpression causes phenotypes similar to GA-deficient plants and these plants show reduced levels of GA due to lower expression levels of AtGA20ox1, AtGA3ox3 and AtGA3ox1.	Use	Stress	7.14E-03
F vs. S (23)	AT1G80030	DJA7 (HSP)	DAJ7 is a HSP chaperone involved in protein folding and refolding (TAIR)	Stress attenuation	HSP	1.99	Molecular chaperone Hsp40/DnaJ family protein;(source:Araport11)	Yes	Hsp	4.05E-04
F vs. S (23)	AT2G47730	GSTF8	Reduces oxidative damage caused by heavy metals by binding to them.	ROS attenuation		1.99	Encodes glutathione transferase belonging to the phi class of GSTs. Naming convention according to Wagner et al. (2002).	Yes	glutathione	3.07E-04
F vs. L (23)	AT3G04720	PR4	TAIR			1.99	Encodes a protein similar to the antifungal chitin-binding protein hevein from rubber tree latex. mRNA levels increase in response to ethylene and turnip crinkle virus infection. The mRNA is cell-to-cell mobile.	No	ethylene	1.16E-03
F vs. S (23)	AT5G64940	ABC1K8	ABC1K8 is a chloroplast localized protein kinase that is upregulated by heavy metals and mediates iron distribution and lipid membrane changes during oxidative stress (doi: 10.1111/nph.12533)	ROS attenuation		1.98	ABC1K8 is a member of an atypical protein kinase family that is induced by heavy metals. Loss of function mutations affect the metabolic profile of chloroplast lipids. It appears to function along with ABC1K7 in mediating lipid membrane changes in response to stress. The mRNA is cell-to-cell mobile.	Yes	Stress	9.53E-04
L vs M (123)	AT1G23490	ARF1	involved in vesicle trafficking (10.1105/tpc.20.00240); invovled in ethylene sensing (10.1105/tpc.16.00743)		ARF	1.98	Gene encoding ADP-ribosylation factor and similar to other ARFs and ARF-like proteins. A member of ARF GTPase family. Arabidopsis has 21 known members, known to be essential for vesicle coating and uncoating and functions in GTP-binding. The gene is shown to play a role in cell division, cell expansion and cellulose production using antisense construct.	Use	ARF	2.35E-03
F vs. L (23)	AT1G61660	BHLH112	BHLH112 is a TF that is upregulated by salt, drought and ABA. It reduces ROS levels by upregulating POD and SOD scavenging genes and proline biosynthesis genes (doi: 10.1111/nph.13387)	ABA signaling	ABA upregulated, Salt	1.98	Encodes a transcriptional activator that regulates the expression of genes by binding to their GCG- or E-boxes to mediate physiological responses, including proline biosynthesis and ROS scavenging pathways, to enhance stress tolerance.	Use	Stress, ROS	1.17E-03
L vs M (123)	AT4G32320	APX6	A major cytosolic ascorbate peroxidase that catalyzes the reduction of H2O2 thus reducing the effect of ROS on ABA and auxin signaling (10.1104/pp.114.245324)	ROS attenuation		1.97	Encodes a cytosolic ascorbate peroxidase APX6. Ascorbate peroxidases are enzymes that scavenge hydrogen peroxide in plant cells. Eight types of APX have been described for Arabidopsis: three cytosolic (APX1, APX2, APX6), two chloroplastic types (stromal sAPX, thylakoid tAPX), and three microsomal (APX3, APX4, APX5) isoforms.	Use	H2O2, peroxidase, Ascorbate	2.53E-03
F vs. L (23)	AT5G23060	CAS	Calcium sensing protein localized to choloroplasts that is involved in regulating stomatal closure as well as other photoacclimation processes following phosphorylation by Ca2+ dependent kinases (doi.org/10.3389/fpls.2019.00974)			1.97	Encodes a chloroplast-localized protein that modulates cytoplasmic Ca2+ concentration and is crucial for proper stomatal regulation in response to elevations of external Ca2+. Phosphorylation of this protein is dependent on calcium.	No	Ca	2.89E-04
F vs. L (23)	AT5G62520	SRO5	SRO5 is upregulated by salt stress and generates 24 and 21 nt siRNAs that function together to regulate ROS production and responses (TAIR)	Stress signaling	Salt	1.97	Encodes a protein with similarity to RCD1 but without the WWE domain. The protein does have a PARP signature upstream of the C-terminal protein interaction domain. The PARP signature may bind NAD+ and attach the ADP-ribose-moiety from NAD+ to the target molecule. Its presence suggests a role for the protein in ADP ribosylation. Up-regulated by NaCl. SRO5 and P5CDH (an overlapping gene in the antisense orientation) generate 24-nt and 21-nt siRNAs, which together are components of a regulatory loop controlling reactive oxygen species (ROS) production and stress response.	Use	Stress, ROS, siRNA, NaCl	1.95E-04
F vs. L (23)	AT3G11630	2CPA	2CPA is a chloroplast peroxiredoxin that, together with a thylakoid and a stromal ascorbate peroxidase, is upregulated by the redox sensitive TF RAP2.4a (DOI 10.1186/s12870-017-1092-5)	ROS attenuation		1.96	Encodes a 2-Cys peroxiredoxin (2-Cys PrxA) that contains two catalytic Cys residues. Functions in redox cascade with TrxL2 via the ferredoxin-thioredoxin reductase (FTR)/thioredoxin (Trx) pathway to mediate the light-responsive reductive control of target proteins. Continuously transfers reducing power from TrxL2 to H2O2.	Use	H2O2, Ca, Thioredoxin	4.26E-04
F vs. M (23)	AT1G20823	ATL80	NA			1.96	Encodes a RING E3 ubiquitin ligase ATL80. Involved in phosphate mobilization and cold stress response in sufficient phosphate growth conditions. The mRNA is cell-to-cell mobile.	No	Stress	3.07E-03
F vs. L (23)	AT5G12170	CLT3	CLT3 is a CRT-like glutethione transporter involved in maintaining glutethione homeostasis (TAIR)	ROS attenuation	Homeostasis	1.96	Encodes one of the CRT-Like transporters (CLT1/AT5G19380, CLT2/AT4G24460, CLT3/AT5G12170). Required for glutathione homeostasis and stress responses. Mutants lacking these transporters are heavy metal-sensitive, glutathione(GSH)-deficient, and hypersensitive to Phytophthora infection. The mRNA is cell-to-cell mobile.	Use	glutathione, Stress, Homeostasis	9.75E-04
F vs. L (23)	AT1G78290	SnRK2.8	SnRK2.8 is an ABA responsive kinase that is activated by dehydration and osmotic stress. It is upregulated by stress through the removal of repressive H3K27me3 marks by jumonji-C domain containing demethylases (doi.org/10.1080/15592324.2019.1604019)	ABA signaling	ABA upregulated	1.96	encodes a member of SNF1-related protein kinase (SnRK2) family whose activity is activated by ionic (salt) and non-ionic (mannitol) osmotic stress and dehydration.	Use	SnRK, Stress	2.72E-04
F vs. S (23)	AT4G25490	DREB1B	TAIR	ABA response		1.95	Transcriptional activator that binds to the DRE/CRT regulatory element and induces COR (cold-regulated) gene expression increasing plant freezing tolerance. It encodes a member of the DREB subfamily A-1 of ERF/AP2 transcription factor family (CBF1). The protein contains one AP2 domain. There are six members in this subfamily, including CBF1, CBF2, and CBF3. This gene is involved in response to low temperature and abscisic acid.	Yes	ABA, ERF	1.58E-03
F vs. L (23)	AT2G26740	SHE	Induced by water stress (TAIR)			1.95	Encodes a soluble epoxide hydrolase whose expression is induced by auxin and water stress.	No	Stress	1.06E-03
F vs. S (23)	AT2G37240	AT2G37240	A thioredoxin that participates in maintaining cell redox homeostasis (TAIR)	ROS attenuation	Homeostasis, thioredoxin	1.94	Thioredoxin superfamily protein;(source:Araport11)	Yes	Thioredoxin	1.10E-03

F vs. S (23)	AT5G23060	CAS	Calcium sensing protein localized to choloroplasts that is involved in regulating stomatal closure as well as other photoacclimation processes following phosphorylation by Ca2+ dependent kinases (doi.org/10.3389/fpls.2019.00974)			1.94	Encodes a chloroplast-localized protein that modulates cytoplasmic Ca2+ concentration and is crucial for proper stomatal regulation in response to elevations of external Ca2+. Phosphorylation of this protein is dependent on calcium.	Yes	Ca	1.15E-03
F vs. S (23)	AT3G44680	HDA9	HDA9 is a histone deacetylase that is upregulated by drought and forms a complex with ABI4 that downregulates the ABA catabolism genes *CYP707A1,2* (https://doi.org/10.3389/fpls.2020.00143)	ABA signaling	Histones	1.94	Encodes HDA9 (a RPD3-like histone deacetylase). Functions in promoting the onset of leaf senescence.The hda9 mutant shows enhanced H3K9 acetylation levels,based on immunodetection using H3K9ac antibodies.	Yes	Histone, ABA	5.75E-04
L vs M (123)	AT3G55170	L35-3 (RP)			ribosome	1.94	Ribosomal L29 family protein;(source:Araport11)	Use	ribosome	8.34E-03
F vs. L (23)	AT1G70730	PGM2	PGM2 and PGM3 together are required for male and female gametophyte formation (TAIR)		Ovule gametophyte	1.94	Encodes a cytosolic phosphoglucomutase (PGM). Two Arabidopsis PGM proteins (AT1G70730/PGM2 and AT1G23190/PGM3) have high sequence similarities and redundant functions. Mature plants possessing a single cPGM allele had a major reduction in cPGM activity. Whereas pgm2 and pgm3 single mutants are undistinguishable from the wild type, loss of both PGM2 and PGM3 severely impairs male and female gametophyte development.	Use	gametophyte	7.07E-04
F vs. L (23)	AT1G05850	CTL1	CTL1 is an endo chitinase-like protein that is required for tolerance to heat, salt and drought stresses (TAIR)	Stress signaling	Salt	1.93	Encodes an endo chitinase-like protein AtCTL1. Essential for tolerance to heat, salt and drought stresses. Also involved in root hair development, cell expansion and response to cytokinin. Allelic to erh2. 11 alleles described in Hauser (1995). Mutant is defective in acquired thermotolerance, appears semidwarf throughout its life cycle and has extra lateral branches. There are two EMS alleles. Expression of AtHSP101 is not affected in the mutants.	Use	Stress, HSP	3.68E-04
F vs. L (23)	AT4G05590	NRGA1	Mitochondrial pyruvate carrier that mediates ABA signaling, drought stress and Cd toxicity by sustaining the TCA cycle and glutathione synthesis (doi.org/10.1104%2Fpp.18.01610)	ABA signaling	ABA upregulated	1.93	Encodes NRGA1, a putative mitochondrial pyruvate carrier that mediates ABA regulation of guard cell ion channels and drought stress responses.	Use	Stress, ABA	2.31E-03
F vs. L (23)	AT1G14000	VIK	VIK is a MAP kinase kinase kinase like protein that modifies brassinosteroid signaling by interacting with the BRI1 related BR transmembrane receptor kinase BRL2 (TAIR)	BR		1.93	Encodes a protein with similarity to members of the C1 subgroup of MAP kinase kinase kinases. Interacts physically with the receptor kinase BRL2/VH1 and appears to be involved in auxin and brassinosteroid signaling. The mRNA is cell-to-cell mobile.	Use	MAPK	6.55E-05
F vs. M (23)	AT4G32320	APX6	A major cytosolic ascorbate peroxidase that catalyzes the reduction of H2O2 thus reducing the effect of ROS on ABA and auxin signaling (10.1104/pp.114.245324)	ROS attenuation		1.92	Encodes a cytosolic ascorbate peroxidase APX6. Ascorbate peroxidases are enzymes that scavenge hydrogen peroxide in plant cells. Eight types of APX have been described for Arabidopsis: three cytosolic (APX1, APX2, APX6), two chloroplastic types (stromal sAPX, thylakoid tAPX), and three microsomal (APX3, APX4, APX5) isoforms.	Use	H2O2, peroxidase, Ascorbate	2.98E-04
F vs. S (23)	AT4G16160	OEP16L	TAIR	ABA response		1.92	Homologous to pea OEP16 and barley pPORA (OEP16), a member of Arabidopsis OEP16 family. Two OEP16 genes are closely related to each other and are conserved in all land plants, OEP16-2, also named OEP16-S, and OEP16-1 (renamed OEP16-L) are result of the gene duplication event that occurred prior to divergence of bryophytes and seed plants. Predominantly expressed in seed and is not inducible by cold treatment. atOEP16-S gained an additional exon. The promoter region of atOEP16-S (but not atOEP16-L) contains multiple G-box ABA-responsive elements. The atOEP16-S promoter conferred developmentally regulated seed- and pollen-specific GUS expression in tobacco.	Yes	ABA	1.19E-03
F vs. M (23)	AT1G70490	ARF AT1G70490	NA		ARF	1.91	A member of ARF GTPase family. A thaliana has 21 members of this family, known to be essential for vesicle coating and uncoating and functions in GTP-binding. Gene encoding ADP-ribosylation factor and similar to other ARFs and ARF-like proteins. The gene is shown to play a role in cell division, cell expansion and cellulose production using antisense construct.	Use	ARF	4.60E-03
F vs. S (23)	AT3G24170	GR1	GR1 is upregulated by drought, dehydration and ABA and is the major peroxisomal glutathione reductase that functions in the ascorbate glutathione cycle to detoxify H2O2 by converting it to H2O (doi:10.1093/jxb/ery266)	ROS attenuation	ABA upregulated	1.91	Encodes a cytosolic glutathione reductase.	Yes	glutathione	1.06E-03
F vs. S (23)	AT2G44060	LEA26	upregulated by dessication and cadmium (TAIR)		LEA	1.91	Late embryogenesis abundant protein, group 2;(source:Araport11)	Yes	LEA	5.41E-04
L vs M (123)	AT3G23000	SnRK3.10	upregulated in response to cold, increases cold tolererance (10.1007/s11033-012-1638-4); phosphorylates proteins in the C/N response pathway (10.1016/j.molp.2017.01.005)	ABA signaling		1.91	Encodes a serine/threonine protein kinase with similarities to CBL-interacting protein kinases, SNF1 and SOS2. The mRNA is cell-to-cell mobile.	Use	SnRK	5.30E-04
F vs. S (23)	AT2G37130	AT2G37130	AT2G37130 is a peroxidase that is upregulated in response to oxidative stress (TAIR)	ROS attenuation		1.9	Peroxidase superfamily protein;(source:Araport11)	Yes	peroxidase	1.33E-03
F vs. S (23)	AT1G42990	bZIP60	bZIP60 is a TF that is upregulated by ER stress. As part of the unfolded protein response, ERI1 splices bZIP60 into an active TF that localizes in the nucleus and upregulates SVB, a phophoinositide binding protein involved in ER stress tolerance (doi/10.1104/pp.19.01488)	Stress attenuation		1.9	bZIP60 consists of a bZIP DNA binding domain followed by a putative transmembrane domain. bZIP60 mRNA is upregulated by the addition of ER stress inducers, tunicamycin (inhibitor of N-linked glycosylation), DTT (inhibitor of disulfide bond formation) and azetin-2-carboxylate (proline analog perturbing protein structure). Upon ER stress, bZIP60 mRNA is spliced by IRE1A and IRE1B to produce bZIP60-S, an active transcription factor without the transmembrane domain. bZIP60-U, a product of unspliced form of bZIP60 mRNA, is localized at the ER membrane and bZIP60-S is localized in the nucleus.	Yes	Stress, ER	1.98E-03
L vs M (123)	AT1G19550	DHAR	A major dehydroascorbate reductase with glutathione dehydrogenase (ascorbate) activity. Functions with APX6 in cellular ROS detoxification by coupling H2O2 to reduced glutathione. Small increases in DHAR and APX6 significantly enhance tolerance to abiotic stresses, reviewed in (doi.org/10.1093/jxb/eraa107)	ROS attenuation		1.90	Glutathione S-transferase family protein;(source:Araport11)	Use	glutathione	9.38E-03
F vs. S (23)	AT5G09230	SRT2	SRT2 is a histone deacetylase that interacts with ENAP1 in the nucleus to repress certain ethyene responsive genes by binding to their promoter regions where they reducing H3K9Ac levels (doi/10.1105/tpc.17.00671)	Gene silencing	ET, Histones	1.9	Encodes SRT2, a member of the SIR2 (sirtuin) family HDAC (histone deacetylase) (SRT1/AT5g55760, SRT2/AT5G09230).	Yes	Histone, HDAC	1.75E-03

F vs. L (23)	AT3G48170	ALDA10A9	ALDA10A9 is a peroxisome localized NAD+-dependent aminoaldehyde dehydrogenase that is upregulated by salt stress. It performs the final step in catabolizing polyamines to produce 4-aminobutyrate (GABA), which ameliorates the effects of salt stress (DOI: 10.1038/srep35115)	Stress attenuation	ABA upregulated, Salt	1.89	ALDH10A9 encodes a protein that can function as a betaine aldehyde dehydrogenase in vitro. The C-terminal amino acids of this protein direct GFP to the peroxisome suggesting that ALDH10A9 accumulates in this organelle. ALDH10A9 transcript levels rise in response to ABA, NaCl, chilling, methyl viologen, and dehydration stress. The enzyme can catalyze the formation of glycine betaine in vitro, but there are still questions about whether Arabidopsis makes this protective compound under natural conditions. This enzyme may be involved in oxidizing aminoaldehydes formed through polyamine metabolism.	Use	Stress, ABA	3.18E-03
F vs. M (23)	AT1G79340	MC4	Positive regulation of programmed cell death by Ca2+ dependent activation (doi.org/10.1038/s41467-020-15830-8)	Stress attenuation		1.89	Encodes MCP2d, the predominant and constitutively expressed member of type II metacaspases (MCPs). MCP2d plays a positive regulatory role in biotic and abiotic stress-induced programmed cell death (PCD). Arabidopsis contains three type I MCP genes (MCP1a-c) and six type II MCP genes (MCP2a?f): AtMCP1a/At5g64240, AtMCP1b/At1g02170, AtMCP1c/At4g25110, AtMCP2a/At1g79310, AtMCP2b/At1g79330, AtMCP2c/At1g79320, AtMCP2d/At1g79340, AtMCP2e/At1g16420, AtMCP2f/At5g04200. The mRNA is cell-to-cell mobile.	Use	Stress	5.86E-03
M vs. S (23)	AT4G04020	PAP1			ABA	1.88	Fibrillin precursor protein. The fibrillin preprotein, but not the mature protein interacts with ABI2. Regulated by abscisic acid response regulators. Involved in abscisic acid-mediated photoprotection. The mRNA is cell-to-cell mobile.			1.47E-02
F vs. L (23)	AT1G54570	PES1	NA		Homeostasis (photosynthetic membrane)	1.88	Encodes a protein with phytyl ester synthesis and diacylglycerol acyltransferase activities that is involved in the deposition of free phytol and free fatty acids in the form of phytyl esters in chloroplasts, a process involved in maintaining the integrity of the photosynthetic membrane during abiotic	No	Stress	1.50E-04
F vs. L (23)	AT1G22840	CYTC-1	CYTC-1 encodes the mitochondrial electron carrier chytochrome c. Increased levels of CYTC enhance growth probably by increasing mitochondrial energy (ATP) and gibberellin signaling and homeostasis (doi: 10.1111/tpj.13845)		Homeostasis	1.87	Encodes cytochrome c. Contains two site II (TGGGCC/T) elements, which interact with a TCP-domain transcription factor, and a downstream internal telomeric repeat, and are required for expression of the Cytc-1 gene. Promoter directs preferential expression in root and shoot meristems and in anthers. Double mutants with CYTC-2 accumulate starch during the day, have delayed growth and development and reduced GA and DELLA proteins linking cellular metabolism and GA homeostasis.	Use	Homeostasis	2.01E-03
L vs M (123)	AT1G71130	ERF070	an AP2/ERF family member TF that is Induced by Pi starvation, which appears to function in maintaining Pi homeostasis (doi/10.1104/pp.113.231183)		ET, homeostasis	1.87	encodes a member of the ERF (ethylene response factor) subfamily B-5 of ERF/AP2 transcription factor family. The protein contains one AP2 domain. There are 7 members in this subfamily.	Use	ethylene, ERF	1.53E-03
L vs M (123)	AT5G19940	FBN6	Protein involved in maintaining expression levels of genes involved in removing superoxide radicals, maintaining ROS homeostasis, and regulating glutathione levels in chloroplasts (doi: 10.1111/nph.16246)	ROS attenuation	Homeostasis	1.87	Enables plants to cope with moderate light stress and affects cadmium tolerance.	Use	Stress	3.84E-03
F vs. S (23)	AT3G62290	ARFA1e	ARF (TAIR)		ARF	1.86	A member of ARF GTPase family. A thaliana has 21 members of this family, known to be essential for vesicle coating and uncoating and functions in GTP-binding. The gene is shown to play a role in cell division, cell expansion and cellulose production using antisense construct. The mRNA is cell-to-cell	Yes	ARF	1.97E-03
F vs. M (23)	AT2G44060	LEA26	upregulated by dessication and cadmium (TAIR)		LEA	1.86	Late embryogenesis abundant protein, group 2;(source:Araport11)	Use	LEA	4.26E-03
F vs. S (23)	AT1G32080	PLGG1	Upregulated by ABA or drought. It participates in the detoxification of 2-phosphoglycolate in the chloroplast during photorespiration by facilitating the export of glycolate from chloroplasts and the import of glycerate (10.1105/tpc.16.00775)		ABA upregulated	1.86	Encodes a plant LrgAB/CidAB protein localized to the chloroplast envelope that is involved in chloroplast development, carbon partitioning, ABA/drought response, and leaf senescence. The gene may have evolved from gene fusion of bacterial lrgA and lrgB.	Yes	ABA	2.11E-03
F vs. S (23)	AT2G37550	AGD7	TAIR		ARF	1.85	A member of ARF GAP domain (AGD), A thaliana has 15 members, grouped into four classes.	Yes	ARF	9.08E-04
F vs. L (23)	AT4G11600	GPX6	Functions in the ascorbate-glutathione cycle to remove toxic H2O2 in mitochondria (doi.org/10.1111/pce.12919)	ROS attenuation		1.85	Encodes glutathione peroxidase.	Use	glutathione, peroxidase	1.77E-03
F vs. S (23)	AT2G17420	NTRA	NTRA is a major cytosolic isoform of NADPH-dependent thioredoxin reductases, which redundantly regulate many enzymes involved in multiple metabolic processes including photorespiration, ATP synthesis, stress related reactions, redox homeostasis processes, and TCA cycle enzymes (doi:10.1093/pcp/pcy194)	ROS attenuation	Homeostasis, thioredoxin	1.85	NADPH-dependent thioredoxin reductase, major cytosolic isoformThe mRNA is cell-to-cell mobile.	Yes	Thioredoxin	8.40E-04
L vs M (123)	AT2G41900	OXS2	OXS2 is a salinity induced zinc finger protein that enters the nucleus in response to salt stress and enhances the expression of salt stress attenuation genes, e.g., *CA1* and *Araport11* (doi.org/10.1038/s41598-019-56456-1)	Stress signaling	Salt	1.84	AtOXS2 specifcally entered the nucleus under salt stress. Te specific nuclear localization of AtOXS2 could play a role in salt tolerance at the molecular level. Tese results implied that AtOXS2 might target some downstream cis-elements which are required for salt stress responses	Use	Stress	3.60E-04
F vs. M (23)	AT5G60660	PIP2;4	H2O2 transfer into cells (TAIR)	ROS attenuation		1.84	A member of the plasma membrane intrinsic protein subfamily PIP2.When expressed in yeast cells can conduct hydrogen peroxide into those cells. Mutants exhibit longer root hairs.	Use	H2O2	2.95E-03
F vs. L (23)	AT5G50720	HVA22e	HVA22e is upregulated by dehydration, salt stress or ABA treatments (TAIR)	ABA signaling	ABA upregulated, Salt	1.83	Encodes one of five HVA22 homologs in Arabidopsis. HVA22 is an ABA- and stress-inducible gene first isolated from barley. Members of this gene family have only been found in eukaryotes. AtHVA22e mRNA is upregulated to varying degrees in response to cold stress, salt stress, ABA treatment or	Use	Stress, ABA	6.62E-04
F vs. M (23)	AT2G28190	SOD2 (CSD2)	SOD2 is a chloroplastic copper/zinc superoxide dismutase that converts superoxide free radicals to O2 and H2O2. Under nonstress conditions, its transcripts are cleaved by miR398, which decreases SOD2 levels. Under stress conditions, miR398 expression is suppressed, and SOD2 levels increase (DOI 10.1007/s10646-017-1843-y)	ROS attenuation		1.83	Encodes a chloroplastic copper/zinc superoxide dismutase CSD2 that can detoxify superoxide radicals. Its expression is affected by miR398-directed mRNA cleavage. Activation depends totally on CCS. Overexpression of a miR398-resistant form of CSD2 leads to more dramatic improvements in stress (hight light, Cu2+ and methyl viologen) tolerance than overexpression of wild-type CSD2. The mRNA is cell-to-cell mobile.	Use	Stress, H2O2, superoxide dismutase	3.66E-03
F vs. L (23)	AT3G62290	ARFA1e	ARF (TAIR)		ARF	1.82	A member of ARF GTPase family. A thaliana has 21 members of this family, known to be essential for vesicle coating and uncoating and functions in GTP-binding. The gene is shown to play a role in cell division, cell expansion and cellulose production using antisense construct. The mRNA is cell-to-cell	Use	ARF	3.59E-04
L vs M (123)	AT1G49670	AT1G49670	Function not know, upregulated in response to oxidative stress (TAIR)	Stress signaling		1.82	molecular function has not been defined. Was shown involved in oxidative stress tolerance.	Use	Stress	4.26E-05
F vs. S (23)	AT1G76080	CDSP32	CDSP32 is a thioredoxin that is upregulated by oxidative stress and drought. It participates in maintaining cell redox homeostasis (TAIR)	ROS attenuation	Homeostasis, thioredoxin	1.82	Encodes a thioredoxin localized in chloroplast stroma. Known as CDSP32 (CHLOROPLASTIC DROUGHT-INDUCED STRESS PROTEIN OF 32 KD).	Yes	Thioredoxin	9.42E-04

F vs. M (23)	AT5G63860	**UVR8**	NA		Histones	1.82	UV-B-specific signaling component that orchestrates expression of a range of genes with vital UV-protective functions. Located in the nucleus and the cytosol. Associates with chromatin via histones. UV-B light promotes URV8 protein accumulation in the nucleus. UVR8 interaction with COP1 is negatively regulated by RUP1 and RUP2.	Use	Histone	4.10E-03
F vs. L (23)	AT1G78160	**APUM7**	PUM7 is a Pumilio protein involved in mRNA stabilizaion and translation (TAIR)	SnRK1 TOR		1.81	Encodes a member of the Arabidopsis Pumilio (APUM) proteins containing PUF domain (eight repeats of approximately 36 amino acids each). PUF proteins regulate both mRNA stability and translation through sequence-specific binding to the 3' UTR of target mRNA transcripts.	Use	translation	6.08E-04
L vs M (123)	AT5G23060	**CAS**	Calcium sensing protein localized to choloroplasts that is involved in regulating stomatal closure as well as other photoacclimation processes following phosphorylation by Ca2+ dependent kinases (doi.org/10.3389/fpls.2019.00974)			1.81	Encodes a chloroplast-localized protein that modulates cytoplasmic Ca2+ concentration and is crucial for proper stomatal regulation in response to elevations of external Ca2+. Phosphorylation of this protein is dependent on calcium.	No	Ca	2.12E-03
F vs. L (23)	AT1G21760	**FBP7**	FPB7 is an F-box protein that is upregulated by temperature stress and regulates translation (TAIR)	SnRK1 TOR, Stress attenuation		1.81	This gene is predicted to encode an F-box protein that is evolutionarily conserved between Arabidopsis and other eukaryotes including S.cerevisiae and humans. It may play a role in regulating translation under conditions of temperature stress. FBP7 transcript levels are increased at high and low temperatures. The mRNA is cell-to-cell mobile.	Use	Stress, translation	4.33E-04
F vs. L (23)	AT4G39640	**GGT1**	Upregulated by ER and oxidative stress, degrades glutathione in the apoplast but also mitigates oxidative stress my metabolizing oxidized GSH (GSSH) (doi.org/10.1071/FP17151)	ROS attenuation		1.81	The gene encodes a gamma-glutamyltransferase (AKA gamma-glutamyl transpeptidase, EC 2.3.2.2) that is located in vascular tissues (predominantly phloem) of leaves and is involved in the degradation of glutathione. The encoded enzyme also mitigates oxidative stress by metabolizing GSSG (oxidized form of GSH - glutathione) in the apoplast.	Use	glutathione, Stress	4.19E-04
F vs. L (23)	AT5G11790	**GINT1**	TAIR			1.80	Plays a role in dehydration stress response.	No	Stress	7.28E-04
F vs. L (23)	AT3G43210	**NACK2**	NACK2 is a kinesin involved in cell plate formation of meiocytes and gametophytes (TAIR)	Flowering	Meiosis	1.80	Encodes a kinesin TETRASPORE. Required for cytokinesis in pollen. In mutants, all four microspore nuclei remain within the same cytoplasm after meiosis.	Use	meiosis	1.02E-04
F vs. M (23)	AT4G19230	**CYP707A1**	CYP707A1 is an hydroxylase that inactivates ABA. It is downregulated by drought through the expression of HISTONE DEACETYLASE 9 (HDA9) and ABA INSENSITIVE 4 (ABI4). Hence, during drought, ABA accumulates. It's upregulation in *B. x formosa* ovules suggests that ABA signaling is weaker in *B. microphylla* (stronger in *B. x. formosa*) (doi.org/10.3389/fpls.2020.00143)	ABA signaling		1.79	Encodes a protein with ABA 8'-hydroxylase activity, involved in ABA catabolism. Member of the CYP707A gene family. CYP707A1 appears to play an important role in determining the ABA levels in dry seeds. Gene involved in postgermination growth. Overexpression of CYP707A1 leads to a decrease in ABA levels and a reduction in after-ripening period to break dormancy.	Use	ABA	1.82E-03
F vs. S (23)	AT2G01270	**QSOX2**	QSOX2 is a thioredoxin involved in protein folding and in maintaining cell redox homeostasis (TAIR)	ROS attenuation	Homeostasis, thioredoxin	1.78	Encodes a protein disulfide isomerase-like (PDIL) protein, a member of a multigene family within the thioredoxin (TRX) superfamily. This protein also belongs to the quiescin-sulfhydryl oxidase (QSOX) family, which possess an Erv1-like domain at the COOH terminus in addition to a TRX domain.	Yes	Thioredoxin	5.79E-04
F vs. S (23)	AT5G30510	**RPS1 (RP)**	Plastid ribosomal protein (TAIR)		ribosome	1.78	ribosomal protein S1;(source:Araport11)	Yes	Ribosome	4.33E-04
F vs. M (23)	AT1G06890	**UXT3**	TAIR	SnRK1 TOR		1.78	UXT3 is a member of the NST-KT subfamily of nucleotide/sugar transporters. It is localized to the golgi and functions as a UDP-Xyl transporter.	Use	sugar transporter	6.17E-03
F vs. S (23)	AT1G07500	**SMR5**	SMR5 is a cyclin dependent kinase inhibitor that is upregulated by DNA damage and oxidative stress (TAIR)	ROS attenuation		1.77	SMR5 is a member of the SIAMESE-RELATED Cyclin-Dependent Kinase Inhibitor family. It is induced by ROS/oxidative stress.	Yes	Stress, ROS	3.41E-04
F vs. M (23)	AT1G56500	**SOQ1**	TAIR		Thioredoxin	1.77	Encodes a thylakoid membrane protein with thioredoxin-like and beta-propeller domains located in the lumen and a haloacid-dehalogenase domain exposed to the chloroplast stroma. The protein Primes role may be to prevent formation of a slowly reversible form of antenna quenching, thereby maintaining the efficiency of light harvesting.The mRNA is cell-to-cell mobile.	Use	Thioredoxin	4.72E-03
F vs. L (23)	AT5G09230	**SRT2**	SRT2 is a histone deacetylase that interacts with ENAP1 in the nucleus to repress certain ethyene responsive genes by binding to their promoter regions where they reduce H3K9Ac levels (doi/10.1105/tpc.17.00671)	Gene silencing	ET, Histones	1.76	Encodes SRT2, a member of the SIR2 (sirtuin) family HDAC (histone deacetylase) (SRT1/AT5g55760, SRT2/AT5G09230).	Use	Histone, HDAC	1.31E-03
F vs. L (23)	AT1G44170	**ALDH3H1**	Upregulated by ABA, salt and desiccation; involved in detoxifying highly reactive aldehydes that form due to ROS-induced peroxidation of polyunsaturated fatty acids; subject to nitric oxide degradation (doi.org/10.1016/j.plantsci.2019.110389)	ROS attenuation	Salt, ABA upregulated	1.75	Encodes an aldehyde dehydrogenase induced by ABA and dehydration that can oxidize saturated aliphatic aldehydes. It is also able to oxidize beta-unsaturated aldehydes, but not aromatic aldehydes. Activity of ALDH3H1 is NAD +-dependent.	Use	ABA	2.08E-04
F vs. M (23)	AT5G66070	**ARRE**	ARRE is an ubiquitination E3 ligase that is upregulated by ABA and NaCl. It is a negative regulator of ABA possibly by inducing the catabolism of ABI5 (doi.org/10.1007/s00299-018-2311-8)	ABA signaling	ABA upregulated	1.75	E3 ubiquitin ligase that functions in negative regulation of ABA signaling.	Use	ABA	3.88E-03
F vs. L (23)	AT1G10370	**GSTU17**	GSTU17 encodes a glutathione S-transferase that reduces resistance to drought and salt stress by reducing glutethione and ABA levels (10.1104/pp.111.181875)	ROS attenuation	Salt	1.75	Encodes GSTU17 (Glutathione S-Transferase U17). Functions as a negative component of stress-mediated signal transduction pathways in drought and salt stress responses.	Use	glutathione, Stress	1.14E-04
F vs. S (23)	AT3G22370	**AOX1a**	AOX1a is highly suppressed at the transcriptional and post transcriptional levels under favorable growing conditions. During stress, AOX1a is upregulated by multiple stress related TFs including SnRK1 (https://doi.org/10.1016/j.tplants.2018.03.012)	SnRK1 TOR		1.74	Encodes AOX1a, an isoform of alternative oxidase that is expressed in rosettes, flowers, and root. The alternative oxidase of plant mitochondria transfers electrons from the ubiquinone pool to oxygen without energy conservations. It is regulated through transcriptional control and by pyruvate. Plays a role in shoot acclimation to low temperature. Also is capable of ameliorating reactive oxygen species production when the cytochrome pathway is inhibited. AOX1a also functions as a marker for mitochondrial retrograde response. The mRNA is cell-to-cell mobile.	Yes	AOX	8.81E-04
F vs. L (23)	AT5G66070	**ARRE**	ARRE is an ubiquitination E3 ligase that is upregulated by ABA and NaCl. It is a negative regulator of ABA possibly by inducing the catabolism of ABI5 (doi.org/10.1007/s00299-018-2311-8)	ABA signaling	ABA upregulated	1.74	E3 ubiquitin ligase that functions in negative regulation of ABA signaling.	Use	ABA	6.80E-04
F vs. S (23)	AT3G63310	**BIL4**	BIL4 encodes a transmembrane protein that interacts with BRI1 to positively regulate BR signaling and cell elongation by suppressing BRI1 degradation and maintaining its intracellular location near the plasma membrane (DOI:10.1038/s41598-017-06016-2)	BR		1.74	Mediates cell elongation in brassinosteroid signaling.	Yes	BR	1.73E-03
F vs. L (23)	AT1G63060	**NEP1-like**	Ribosome biogenesis (TAIR)		ribosome	1.74	ribosome biogenesis NEP1-like protein;(source:Araport11)	Use	ribosome	5.03E-04
F vs. M (23)	AT5G38710	**PDH2**	upregulated by osmotic stress and water deprivation (TAIR)	Stress signaling	ET	1.74	Methylenetetrahydrofolate reductase family protein;(source:Araport11)	Use	ethylene	4.20E-03

F vs. L (23)	AT4G04770	ABC1	Involved in Fe homeostasis (TAIR)			1.73	Encodes an iron-stimulated ATPase. A member of the NAP subfamily of ABC transporters. Involved in Fe-S cluster assembly. Similar to SufB. Involved in the regulation of iron homeostasis. Able to form homodimers. Interacts with AtNAP7 inside the chloroplast.	No	Homeostasis	1.93E-03
M vs. S (23)	AT3G54250	MVD2			Steroid biosynthesis	1.73	Encodes mevalonate diphosphate decarboxylase, the enzyme that catalyzes the synthesis of isopentenyl diphosphate, used in sterol and isoprenoid			5.76E-04
L vs M (123)	AT5G65360	H3.1 AT5G65360	Histone 3 variant H3.1s are expressed and incorporated into DNA during S phase where they are methylated to retain specific epigenetic states (doi.org/10.1016/j.cub.2010.11.012; 10.1126/science.1248357)	Gene silencing	Histones	1.72	Histone superfamily protein;(source:Araport11)	Use	Histone	8.34E-03
F vs. L (23)	AT3G11410	PP2CA	PP2CA is a protein phosphatase that is upregulated by water deprivation and ABA and functions as a negative regulator of ABA signaling by inactivating SnRK2 (doi: 10.1111/tpj.14274)	ABA signaling	ABA upregulated	1.72	Encodes protein phosphatase 2C. Negative regulator of ABA signalling. Expressed in seeds during germination. mRNA up-regulated by drought and ABA.	Use	PP2C, ABA	9.51E-05
L vs M (123)	AT2G15760		Calcium binding protein (TAIR)			1.71	calmodulin-binding protein (DUF1645);(source:Araport11)		Calmodulin	1.10E-03
F vs. L (23)	AT3G51920	CML9	Upregulated by ABA, salt stress and water deprivation	ABA signaling	ABA upregulated, Salt	1.70	encodes a divergent member of calmodulin, which is an EF-hand family of Ca^{2+}-binding proteins. This gene is expressed in leaves, flowers and siliques. The gene functionally complements yeast calmodulin 1 (CAM1) but only when selected against the plasmid harboring wild-type yeast sequences. Also the protein does not form formed a complex with a basic amphiphilic helical peptide in the presence of Ca^{2+} in vitro. Authors suggest that this gene may represent a Ca^{2+}-binding sensor protein that interacts with a more limited set of target proteins than do more conventional CaM isoforms. Mutations in this gene alter plant responses to abiotic stress and abscisic acid.	Use	Stress, ABA, Calmodulin	4.05E-04
L vs M (123)	AT5G48760	L13a-4 (RP)			ribosome	1.70	Ribosomal protein L13 family protein;(source:Araport11)	Use	ribosome	3.69E-03
F vs. M (23)	AT5G64040	PSAN	NA			1.70	Encodes the only subunit of photosystem I located entirely in the thylakoid lumen. May be involved in the interaction between plastocyanin and the photosystem I complex. Phosphorylation of this protein is dependent on	No	Ca	6.09E-03
F vs. S (23)	AT4G04770	ABC1	Involved in Fe homeostasis (TAIR)			1.69	Encodes an iron-stimulated ATPase. A member of the NAP subfamily of ABC transporters. Involved in Fe-S cluster assembly. Similar to SufB. Involved in the regulation of iron homeostasis. Able to form homodimers. Interacts with AtNAP7 inside the chloroplast.	Yes	Homeostasis	1.76E-03
F vs. M (23)	AT3G51920	CML9	Upregulated by ABA, salt stress and water deprivation	ABA signaling	ABA upregulated, Salt	1.69	encodes a divergent member of calmodulin, which is an EF-hand family of Ca^{2+}-binding proteins. This gene is expressed in leaves, flowers and siliques. The gene functionally complements yeast calmodulin 1 (CAM1) but only when selected against the plasmid harboring wild-type yeast sequences. Also the protein does not form formed a complex with a basic amphiphilic helical peptide in the presence of Ca^{2+} in vitro. Authors suggest that this gene may represent a Ca^{2+}-binding sensor protein that interacts with a more limited set of target proteins than do more conventional CaM isoforms. Mutations in this gene alter plant responses to abiotic stress and abscisic acid.	Use	Stress, ABA, Calmodulin	1.74E-03
F vs. M (23)	AT1G54570	PES1	TAIR			1.69	Encodes a protein with phytyl ester synthesis and diacylglycerol acyltransferase activities that is involved in the deposition of free phytol and free fatty acids in the form of phytyl esters in chloroplasts, a process involved in maintaining the integrity of the photosynthetic membrane during abiotic	Use	Stress	5.12E-03
L vs M (123)	AT1G10130		Ca ion transmembrane transport and Mn and Ca ion homeostasis (TAIR)		Homeostasis, Ca	1.69	Encodes a golgi localized P2A-type Ca^{2+} ATPase involved in Mn nutrition and homeostasis.	Use	Homeostasis	6.28E-03
F vs. L (23)	AT3G24506	CGL20B (RP)	Plastic ribosome biogenesis (TAIR)		ribosome	1.68	Participates in the late stages of the biogenesis of 50S ribosomal subunits in plastids.	Use	ribosome	1.33E-03
F vs. S (23)	AT1G78380	GSTU19	GSTU19 is a plant specific glutathione transferase that is upregulated by salt, drought and oxidative stress and functions redundanctly with other GSTs to maintain redox homeostasis (doi.org/10.3390/ijms21072349)	ROS attenuation	Homeostasis, Salt	1.68	Encodes a glutathione transferase that is a member of Tau GST gene family. Expression is induced by drought stress, oxidative stress, and high doses of auxin and cytokinin. naming convention according to Wagner et al. (2002) The expression of this gene is upregulated by herbicide safeners such as benoxacor and fenclorim.	Yes	glutathione, Stress	2.08E-03
M vs. S (23)	AT4G25890	RPP3A (RP)			ribosome	1.68	60S acidic ribosomal protein family;(source:Araport11)			2.10E-02
F vs. S (23)	AT1G07260	UGT71C3	TAIR			1.68	Encodes a uridine diphosphate-glycosyltransferase that acts on methyl salicylate (MeSA) to form MeSA glucosides in vitro and in vivo and facilitates negative regulation of the SAR response by modulating homeostasis of MeSA	Yes	Homeostasis	1.11E-03
F vs. L (23)	AT4G32800	ERF043	TAIR		ET	1.67	encodes a member of the DREB subfamily A-4 of ERF/AP2 transcription factor family. The protein contains one AP2 domain. There are 17 members in this subfamily including TINY.	Use	ERF	8.35E-04
F vs. L (23)	AT1G21980	PIP5K1	Induced by water stress and ABA	ABA signaling	ABA upregulated	1.67	Type I phosphatidylinositol-4-phosphate 5-kinase. Preferentially phosphorylates PtdIns4P. Induced by water stress and abscisic acid in Arabidopsis thaliana. Expressed in procambial cells of leaves, flowers and roots. A N-terminal Membrane Occupation and Recognition Nexus (MORN)affects enzyme activity and distribution.	Use	Stress, ABA	1.42E-03
F vs. L (23)	AT1G64510	PRPS6	Translation elongation factor (TAIR)	SnRK1 TOR		1.67	Translation elongation factor EF1B/ribosomal protein S6 family protein;(source:Araport11)	Use	Translation	6.78E-04
F vs. S (23)	AT3G51920	CML9	Upregulated by ABA, salt stress and water deprivation	ABA signaling	ABA upregulated, Salt	1.66	encodes a divergent member of calmodulin, which is an EF-hand family of Ca^{2+}-binding proteins. This gene is expressed in leaves, flowers and siliques. The gene functionally complements yeast calmodulin 1 (CAM1) but only when selected against the plasmid harboring wild-type yeast sequences. Also the protein does not form formed a complex with a basic amphiphilic helical peptide in the presence of Ca^{2+} in vitro. Authors suggest that this gene may represent a Ca^{2+}-binding sensor protein that interacts with a more limited set of target proteins than do more conventional CaM isoforms. Mutations in this gene alter plant responses to abiotic stress and abscisic acid.	Yes	Stress, ABA, Calmodulin	1.31E-03
F vs. L (23)	AT5G62020	HSFB2A	HSFB2A is a heat stress transcription factor the expression of which is essential to female gametophyte formation (DOI 10.1007/s11103-014-0202-0)	Stress attenuation, Ovule		1.66	member of Heat Stress Transcription Factor (Hsf) family The mRNA is cell-to-cell mobile.	Use	Stress, gametophyte	2.62E-03

F vs. S (23)	AT4G02900		ERD is upregulated by dehyration and has Ca2+ activated cation channel activity (TAIR)	Stress attenuation		1.66	ERD (early-responsive to dehydration stress) family protein;(source:Araport11)	Yes	Stress	7.69E-04
F vs. M (23)	AT4G17500	ERF1A	NA		ET	1.65	Encodes a member of the ERF (ethylene response factor) subfamily B-3 of ERF/AP2 transcription factor family (ATERF-1). The protein contains one AP2 domain. There are 18 members in this subfamily including ATERF-1, ATERF-2, AND ATERF-5. The mRNA is cell-to-cell mobile.	Use	ethylene, ERF	4.16E-03
F vs. L (23)	AT2G17950	WUS	Involved with AG in regulating stem cell activity. It's expression is absent in MMCs (doi:10.1093/jxb/erz212)		Ovule gametophyte	1.65	Homeobox gene controlling the stem cell pool. Expressed in the stem cell organizing center of meristems. Required to keep the stem cells in an undifferentiated state. Regulation of WUS transcription is a central checkpoint in stem cell control. The size of the WUS expression domain controls the size of the stem cell population through WUS indirectly activating the expression of CLAVATA3 (CLV3) in the stem cells and CLV3 repressing WUS transcription through the CLV1 receptor kinase signaling pathway. Repression of WUS transcription through AGAMOUS (AG) activity controls stem cell activity in the determinate floral meristem. Binds to TAAT element core motif. WUS is also involved in cell differentiation during anther development.	Use	AGL	2.26E-04
F vs. S (23)	AT2G20560	DNAJ (HSP)	DNAJ is a chaperone protein involved in Protein refolding	Stress attenuation	HSP	1.64	DNAJ heat shock family protein;(source:Araport11)	Yes	Hsp	1.44E-03
F vs. S (23)	AT4G17500	ERF1A	NA		ET	1.64	Encodes a member of the ERF (ethylene response factor) subfamily B-3 of ERF/AP2 transcription factor family (ATERF-1). The protein contains one AP2 domain. There are 18 members in this subfamily including ATERF-1, ATERF-2, AND ATERF-5. The mRNA is cell-to-cell mobile.	Yes	ethylene, ERF	2.18E-03
M vs. S (23)	AT3G47940	HSP AT3G47940	TAIR		HSP	1.64	DNAJ heat shock family protein;(source:Araport11)			1.36E-02
F vs. L (23)	AT1G62380	ACO2	Involved in ethylene synthesis from ACC (TAIR)		ET	1.63	Encodes a protein similar to 1-aminocyclopropane-1-carboxylic oxidase (ACC oxidase). Expression of the AtACO2 transcripts is affected by ethylene.	Use	ethylene	2.48E-03
F vs. L (23)	AT3G54510	ERD4	ERD4 is upregulated by drought stress (TAIR)	Stress signaling		1.62	Early-responsive to dehydration stress protein (ERD4);(source:Araport11)	Use	Stress	1.72E-03
F vs. M (23)	AT3G55170	RPL35C (RP)			ribosome	1.61	Ribosomal L29 family protein;(source:Araport11)	Use	ribosome	4.45E-03
L vs M (123)	AT5G23720	PHS1	PHS1 is a MAPK phosphatase that functions as a positive regulator of flowering by modulating *CO* and *FLC* mRNA levels (DOI 10.1007/s00425-015-2447-5). It may also suppress ABA signaling (TAIR)	Flowering		1.60	Encodes a protein tyrosine phosphatase Propyzamide-Hypersensitive 1 (PHS1). One of the mutant alleles, phs1-1, is hypersensitive to the microtubule-destabilizing drug propyzamide, suggesting that PHS1 may be involved in phosphorylation cascades that control the dynamics of cortical microtubules in plant cells. A second allele, phs1-3, is hypersensitive to abscisic acid, indicating a possible involvement of PHS1 in ABA signalling.	Use	ABA	5.72E-03
L vs M (123)	AT4G34190	SEP1	Upregulated by high light intensity stress. Repressed prior to the vegetative to floral transition (doi: 10.1111/j.1365-313X.2012.05074.x)	Stress signaling		1.60	Encodes a stress enhanced protein that localizes to the thylakoid membrane and whose mRNA is upregulated in response to high light intensity. It may be involved in chlorophyll binding.	Use	Stress	2.13E-03
F vs. L (23)	AT1G50320	THX	THX is a thioredoxin that participates in maintaining cell redox homeostasis (TAIR)	ROS attenuation	Homeostasis, thioredoxin	1.60	encodes a prokaryotic thioredoxin	Use	Thioredoxin	4.33E-04
F vs. L (23)	AT2G37240	AT2G37240	A thioredoxin that participates in maintaining cell redox homeostasis (TAIR)	ROS attenuation	Homeostasis, thioredoxin	1.59	Thioredoxin superfamily protein;(source:Araport11)	Use	Thioredoxin	2.88E-03
F vs. L (23)	AT1G01720	ATAF1	ATAF1 is a SNAC-A TF that is upregulated by carbon starvation. It upregulates carbon starvation responses, similar to those that occur when SnRK1 is upregulated. It specifically upregulates *TREHALASE1* (*TRE1*), which catabolizes sugars and induces global transcriptome changes associated with carbon starvation. It also attenuates ABA signaling and synthesis (doi/10.1104/pp.15.00917; doi:10.1093/pcp/pcz015)	SnRK1 TOR		1.56	Belongs to a large family of putative transcriptional activators with NAC domain. Transcript level increases in response to wounding and abscisic acid. ATAF1 attentuates ABA signaling and sythesis. Mutants are hyposensitive to ABA. The mRNA is cell-to-cell mobile.	Use	ABA	1.00E-03
F vs. S (23)	AT3G50060	MYB77 (ARF)	TAIR		ARF	1.56	Encodes a member of the R2R3 transcription factor gene family. Expressed in response to potassium deprivation and auxin. Involved in lateral root development. Interacts with ARF7 and regulates the expression of some auxin responsive genes.	Yes	ARF	1.58E-03
F vs. L (23)	AT2G31570	GPX2	Functions in the ascorbate-glutathione cycle to remove toxic H2O2 in the cytosol and nucleus (doi.org/10.1111/pce.12919)	ROS attenuation		1.55	glutathione peroxidase GPx	Use	glutathione, peroxidase	1.35E-03
F vs. L (23)	AT3G47940	HSP AT3G47940	TAIR	Stress attenuation	HSP	1.55	DNAJ heat shock family protein;(source:Araport11)	Use	HSP	1.27E-03
L vs M (123)	AT1G79340	MC4	Positive regulation of programmed cell death by Ca2+ dependent activation (doi.org/10.1038/s41467-020-15830-8)	Stress attenuation		1.54	Encodes MCP2d, the predominant and constitutively expressed member of type II metacaspases (MCPs). MCP2d plays a positive regulatory role in biotic and abiotic stress-induced programmed cell death (PCD). Arabidopsis contains three type I MCP genes (MCP1a-c) and six type II MCP genes (MCP2a?f): AtMCP1a/At5g64240, AtMCP1b/At1g02170, AtMCP1c/At4g25110, AtMCP2a/At1g79310, AtMCP2b/At1g79330, AtMCP2c/At1g79320, AtMCP2d/At1g79340, AtMCP2e/At1g16420, AtMCP2f/At5g04200. The mRNA is cell-to-cell mobile.	Use	Stress	3.28E-03
F vs. L (23)	AT3G56950	SIP2;1	SIP2;1 is an aquaporin involved in ER stress (TAIR)			1.53	One of the Major Intrinsic Proteins(MIPs) which facilitate the passive transport of small molecules across membranes.Belongs to a family of plant aquaporins.Similar to yeast and radish aquaporins. Located on ER. Probably involved in the alleviation of ER stress; the lack of SIP2;1 reduces both pollen germination and pollen tube elongation.	No	Stress	1.97E-03
F vs. L (23)	AT1G68590	PSRP3/1 (RP)	TAIR		ribosome	1.52	Ribosomal protein PSRP-3/Ycf65;(source:Araport11)	Use	ribosome	2.46E-03
L vs M (123)	AT1G56310	ATRM2	In RNA-induced silencing complex assembly, ATRM2 appears to interact with AGO1 in trimming unmethylated (unprotected) regions of small RNAs prior to DNA silencing (doi.org/10.1073/pnas.1721917115)	Gene silencing		1.51	DEDDy-type 3′ -> 5′ exoribonuclease involved in miRNA degradation.	Use	miRNA	7.41E-03
F vs. L (23)	AT1G44160	HSP AT1G44160	TAIR	Stress attenuation	HSP	1.49	HSP40/DnaJ peptide-binding protein;(source:Araport11)	Use	HSP	4.30E-04
F vs. L (23)	AT3G26060	PRXQ	Upregulated by oxidative stress, decomposes peroxides, necessary for the synthesis of certain thylacoid membrane lipids (doi.org/10.1111%2Ftpj.14657)	ROS attenuation		1.49	encodes periredoxin Q which decomposes peroxides and plays a role in the protection of the photosynthetic apparatus	Use	H2O2	3.15E-03
F vs. L (23)	AT5G47650	NUDT2	Responsive to oxidative stress (TAIR)			1.48	Encodes an ADP-ribose pyrophosphatase that confers enhanced tolerance to oxidative stress.	No	Stress	2.33E-03

M vs. S (23)	AT3G49810	PUB30	PUB30 is an E3 ubiquitin ligase that facilitates BKI1 degradation through the ubiquitin/26S proteasome-mediated protein degradation pathway. BKI1 is an inhibitor of BRI1. Hence, the upregulated PUB30 in B. microphylla ovules should have enhanced BR signaling by facilitating BRI1 silencing of BIN2. With BIN2 silenced, the BR growth response TFs BZR1 and BES1 should have produced downstream BR growth responses (doi.org/10.1111/pce.13064), possibly including the observed aposporous gametophyte formation		Stress	1.41	Encodes a protein with E3 ubiquitin ligase activity that is involved in negative regulation of salt stress tolerance during germination.			1.33E-02
L vs. S (23)	AT3G21180	No			Ca, Calmodulir	0.72	one of the type IIB calcium pump isoforms. encodes an autoinhibited Ca(2+)-ATPase that contains an N-terminal calmodulin binding autoinhibitory domain.			1.70E-02
F vs. L (23)	AT3G59380	FTA	TAIR			0.68	Encodes the alpha-subunit shared between protein farnesyltransferase and protein geranylgeranyltransferase-I. Involved in protein prenylation: covalent attachment of the C-15 isoprene farnesyl or the C-20 isoprene geranylgeranyl groups to the C-terminal end of some proteins. Involved in shoot and flower meristem homeostasis, and response to ABA and drought. Also regulates leaf cell shape. Mutant is epistatic to era1.	No	ABA, Homeostasis	2.00E-03
F vs. S (23)	AT1G07400	HSP17.8	HSP17.8 is upregulated by heat and ROS (TAIR)	Stress attenuation	HSP	0.66	HSP20-like chaperones superfamily protein;(source:Araport11)	Yes	HSP	2.11E-03
F vs. L (23)	AT5G48760	L13a-4 (RP)	TAIR		ribosome	0.66	Ribosomal protein L13 family protein;(source:Araport11)	Use	ribosome	2.91E-03
F vs. L (23)	AT4G39090	RD19	RD19 is a cysteine proteinase that is upregulated by desiccation, osmotic stess and salt stress independently of ABA (TAIR)	Stress signaling	Salt	0.66	Similar to cysteine proteinases, induced by desiccation but not abscisic acid. Required for RRS1-R mediated resistance against Ralstonia solanacearum. Interacts with the R. solanacearum type III effector PopP2. RD19 associates with PopP2 to form a nuclear complex that is required for activation of the RRS1-R?mediated resistance response.	Use	ABA	8.86E-04
L vs M (123)	AT5G65020	ANNAT2	Required for post phloem sugar transport in sink cells (10.1104/pp.18.00713)		Ca	0.65	Annexins are calcium binding proteins that are localized in the cytoplasm. When cytosolic Ca2+ increases, they relocate to the plasma membrane. They may be involved in the Golgi-mediated secretion of polysaccharides.	Use	Ca	9.22E-04
F vs. L (23)	AT5G64670	AT5G64670 (RP)	Ribosome protein (TAIR)		ribosome	0.65	Ribosomal protein L18e/L15 superfamily protein;(source:Araport11)	Use	ribosome	7.22E-04
L vs M (123)	AT2G05250	HSP AT2G05250	DNAJ heat shock protein (TAIR)	Stress attenuation	HSP	0.65	DNAJ heat shock N-terminal domain-containing protein;(source:Araport11)	Use	Hsp	6.79E-03
F vs. S (23)	AT5G59310	LTP4	LTP4 is a lipid transfer protein that is upregulated by ABA (TAIR)		ABA upregulated	0.65	Encodes a member of the lipid transfer protein family. Proteins of this family are generally small (~9 kD), basic, expressed abundantly and contain eight Cys residues. The proteins can bind fatty acids and acylCoA esters and can transfer several different phospholipids. They are localized to the cell wall. The mRNA is present in flowers and siliques, and is strongly up-regulated by abscisic acid. Predicted to be a member of PR-14 pathogenesis-related protein family with the following members: At2g38540/LTP1, At2g38530/LTP2, At5g59320/LTP3, At5g59310/LTP4, At3g51600/LTP5, At3g08770/LTP6, At2g15050/LTP7, At2g18370/LTP8, At2g15325/LTP9, At5g01870/LTP10, At4g33355/LTP11, At3g51590/LTP12, At5g44265/LTP13, At5g62065/LTP14,	Yes	ABA	1.50E-03
L vs M (123)	AT1G06790	NRPC7	Subunit of RNA polymerase III, which transcribes genes required for translaction, including 5S rRNA and tRNAs. It also transcribes many small RNAs (doi:10.1093/jxb/erw020)		Homeostasis	0.65	Encodes a subunit of RNA polymerase III involved in maintaining global RNA homeostasis, not just that of genes transcribed by RNA pol III.	Use	Homeostasis	3.51E-04
L vs M (123)	AT3G13170	SPO11-1	SPO11-1 is a DNA topoisomerase subunit that accumulates and degrades rapidly during a 1-5 h period shortly after S phase where it generates double stand breaks along the DNA as a first step in meiotic recombination. It is also responsible for the expression of 1660 meiosis specific sRNAs, which associate with cross over hotspots during recombination (doi/10.1105/tpc.18.00511)	Flowering	Meiosis	0.65	Encodes AtSPO11-1, one of the three Arabidopsis homologues of the archaeal DNA topoisomerase VIA subunit (topo VIA). Required for meiotic recombination. AtSPO11-1 and AtSPO11-2 have overlapping functions (i.e. both required for meiotic recombination) whereas AtSPO11-3 functions in DNA replication. AtSPO11-1 accumulates in foci in early G2. At 1 h post-S phase, no foci are observed, but by 3 h a majority (80%) of meiocytes at this time point contain >50 foci. However, by 5 h, AtSPO11-1 foci are no longer detectable. This suggests that the protein undergoes a rapid cycle of accumulation and disappearance in meiocytes over a period of between 1 and 5 h post-S phase.	Use	meiosis, SPO	3.29E-03
F vs. L (23)	AT4G29830	VIP3	Involved in histone methylation (TAIR)	Gene silencing		0.65	VIP3 protein is composed of repeats of WD motif which is involved in protein complex formation. The gene is involved in flower timing and flower development. This gene is predicted to encode a protein with a DWD motif. It can bind to DDB1a in Y2H assays, and DDB1b in co-IP assays, and may be involved in the formation of a CUL4-based E3 ubiquitin ligase. Loss of gene function leads to a redistribution of H3K4me3 and K3K36me2 modifications within genes but not a change in the overall abundance of these modifications within chromatin. Also known as SKI8, a component of the SKI complex involved in exosome mediated RNA degradation. Member of PAF-C complex.	Use	H3K4	1.38E-03
L vs. S (23)	AT3G54090	No	Fructokinase-like 1, chloroplastic		Thioredoxin	0.64	Encodes a fructokinase-like protein (AT3G54090/FLN1, AT1G69200/FLN2), a member of the pfkB-carbohydrate kinase family. FLN1 and FLN2 are potential plastidial thioredoxin z (TRX z) targets. Mutants display mutant chloroplast development, general plant growth and development defects and defects in PEP-dependent transcription.			6.15E-03
L vs. S (23)	AT1G07180	No			#N/A	0.64	Internal NAD(P)H dehydrogenase in mitochondria. The predicted protein sequence has high homology with other designated NAD(P)H DHs from microorganisms; the capacity for matrix NAD(P)H oxidation via the rotenone-insensitive pathway is significantly reduced in the Atndi1 mutant plant line; the in vitro translation product of AtNDI1 is imported into isolated mitochondria and located on the inside of the inner membrane.			1.45E-02
F vs. L (23)	AT1G07070	AT1G07070 (RP)	Ribosome protein (TAIR)		ribosome	0.63	Ribosomal protein L35Ae family protein;(source:Araport11)	Use	ribosome	2.90E-03
M vs. S (23)	AT4G19230	CYP707A1	CYP707A1 is an hydroxylase that inactivates ABA. It is downregulated by drought through the expression of HISTONE DEACETYLASE 9 (HDA9) and ABA INSENSITIVE 4 (ABI4). Hence, during drought, ABA accumulates. It's upregulation in B. x formosa ovules suggests that ABA signaling is weaker in B. microphylla (stronger in B. x. formosa) (doi.org/10.3389/fpls.2020.00143)		ABA	0.63	Encodes a protein with ABA 8'-hydroxylase activity, involved in ABA catabolism. Member of the CYP707A gene family. CYP707A1 appears to play an important role in determining the ABA levels in dry seeds. Gene involved in postgermination growth. Overexpression of CYP707A1 leads to a decrease in ABA levels and a reduction in after-ripening period to break dormancy.			1.94E-02

F vs. S (23)	AT3G13170	**SPO11-1**	SPO11-1 is a DNA topoisomerase subunit that accumulates and degrades rapidly during a 1-5 h period shortly after S phase where it generates double stand breaks along the DNA as a first step in meiotic recombination. It is also responsible for the expression of 1660 meiosis specific sRNAs, which associate with cross over hotspots during recombination (doi/10.1105/tpc.18.00511)	Flowering	Meiosis	0.63	Encodes AtSPO11-1, one of the three Arabidopsis homologues of the archaeal DNA topoisomerase VIA subunit (topo VIA). Required for meiotic recombination. AtSPO11-1 and AtSPO11-2 have overlapping functions (i.e. both required for meiotic recombination) whereas AtSPO11-3 functions in DNA replication. AtSPO11-1 accumulates in foci in early G2. At 1 h post-S phase, no foci are observed, but by 3 h a majority (80%) of meiocytes at this time point contain >50 foci. However, by 5 h, AtSPO11-1 foci are no longer detectable. This suggests that the protein undergoes a rapid cycle of accumulation and disappearance in meiocytes over a period of between 1 and 5 h post-S phase.	Yes	meiosis, SPO	1.43E-03
F vs. L (23)	AT5G23580	**CPK12**	CPK12 is a Ca2+ dependent protein kinase responsible for reducing salt and H2O2 levels in cells during salt stress (doi.org/10.3390/ijms19124062)		Ca, Salt	0.62	Member of a unique family of enzymes containing a single polypeptide chain with a kinase domain at the amino terminus and a putative calcium-binding EF hands structure at the carboxyl terminus; recombinant protein is fully active and induced by Ca2+	Use	Ca	1.47E-03
M vs. S (23)	AT4G14730	**GAAP1**	The Arabidopsis anti apoptotic protein 1 (GAAP1) is a stress induced protein that participates in ER stress mitigation (doi:10.1093/jxb/erz402)		Stress	0.62	Stress induced membrane protein. Mutants show enhanced cell death under stress.			1.61E-02
F vs. S (23)	AT3G46210	**AT3G46210 (RP)**	Ribosome protein (TAIR)		ribosome	0.61	Ribosomal protein S5 domain 2-like superfamily protein;(source:Araport11)	Yes	Ribosome	2.00E-03
F vs. L (23)	AT3G53430	**AT3G53430 (RP)**	Ribosome protein (TAIR)		ribosome	0.61	Ribosomal protein L11 family protein;(source:Araport11)	Use	ribosome	8.86E-04
L vs M (123)	AT4G15890	**CAP-3D**	CAP-3D is a component of the meiotic chromosome condensin complex and is upregulated during meiotic prophase by the meiotic H3K4me2 histone-reading PHD-finger MMD1/DUET. This process is responsible for achieving the thin thread-like chromosomes of the leptotene stage (10.1105/tpc.16.00040).	Flowering	Meiosis	0.61	CAP-3D is a subunit of condensin. It a target of MMD1 regulation and also involved in meiotic chromosome condensation. Mutants have reduced fertility. Required for the correct spatial relationship between centromeres and rDNA arrays.	Use	meiosis	8.69E-04
F vs. L (23)	AT3G62800	**DRB4**	DRB4 is a dsRNA-binding protein that participates with Pol IV and DCL4 in producing siRNAs and miRNAs for gene silencing (TAIR)	Gene silencing		0.61	Encodes a nuclear dsRNA-binding protein DRB4 that interacts specifically with DCL4. May regulate DCL4 function and thereby affect miRNA biogenesis. Also has an impact on polymerase IV-dependent siRNA levels. DRB4 interacts with the P6 viral protein from Cauliflower mosaic virus and may be a target of viral silencing suppression.	Use	siRNA, silencing	6.94E-04
F vs. S (23)	AT2G05250	**HSP AT2G05250**	DNAJ heat shock protein (TAIR)	Stress attenuation	HSP	0.61	DNAJ heat shock N-terminal domain-containing protein;(source:Araport11)	Yes	Hsp	8.88E-04
L vs. S (23)	AT5G23900	**RPL13D (RP)**	60S ribosomal protein L13-3		ribosome	0.61	Ribosomal protein L13e family protein;(source:Araport11)			1.30E-02
L vs M (123)	AT1G12500		organic anion transmembrane transporter (TAIR)	SnRK1 TOR		0.61	Nucleotide-sugar transporter family protein;(source:Araport11)	Use	sugar transporter	5.88E-03
F vs. S (23)	AT3G10500	**ANACO53**	ANACO53 is a NAC domain TF that is upregulated by stress and by ANAC017. It may be responsible for drought induced senescence through ROS accumulation and for the activation of the UPR via increased expression of numerous genes that prevent proteotoxic stress during episodes of UPRs (doi/10.1105/tpc.15.01022)	ROS attenuation		0.6	Encodes a transcriptional activator that is associated with the plasma membrane in a dormant form and is proteolytically cleaved to create a form that can enter the nucleus. It is thought to promote ROS production by binding directly to the promoters of genes encoding ROS biosynthetic enzymes during drought-induced leaf senescence.The mRNA is cell-to-cell mobile.	Yes	ROS	6.33E-04
F vs. S (23)	AT3G01740	**AT3G01740 (RP)**	Ribosome protein (TAIR)		ribosome	0.6	Mitochondrial ribosomal protein L37;(source:Araport11)	Yes	Ribosome	1.79E-03
F vs. L (23)	AT5G46160	**AT5G46160 (RP)**	Ribosome protein (TAIR)		ribosome	0.60	Ribosomal protein L14p/L23e family protein;(source:Araport11)	Use	ribosome	6.58E-04
L vs. S (23)	AT5G04560	**DME**			Imprinting	0.6	Encodes a DNA glycosylase DEMETER (DME). Responsible for endosperm maternal-allele-specific hypomethylation at the MEDEA (MEA) gene. DME can excise 5-methylcytosine in vitro and when expressed in E. coli. DME establishes MEA imprinting by removing 5-methylcytosine to activate the			1.68E-02
F vs. L (23)	AT3G53890	**EVR1 (RP)**	TAIR	SnRK1 TOR	ribosome	0.60	Cytosolic ribosomal protein. Mutants enhance the varigation effect of var2 mutations suggesting a link between cytosolic translation and chloroplast development.	Use	translation, ribosome	4.05E-04
L vs M (123)	AT1G67230	**LINC1**	Negatively regulates ABA during seed germination by regulating rapid degradation of ABI5 following seed stratification (doi: 10.1111/jipb.12448); also involved in triggering the immunity response during pathogenic attack by activating MAPKs and suppressing growth by positively regulating JA signaling and the biosynthesis of secondary metabolites including pungent glucosinolates (doi.org/10.3389/fpls.2019.01639)	ABA signaling		0.60	Encodes a nuclear coiled-coil protein related to the carrot peripheral nuclear protein NMCP1 that is involved in the determination of plant nuclear structure. Member of a small gene family in Arabidopsis containing 4 proteins (LNC1-4 or CRWN 1-4) with redundant functions in protection from oxidative damage, control of nuclear morphology and degradation of ABI5.	Use	oxidative damage	5.72E-03
F vs. S (23)	AT2G40030	**NRPE1**	NRPE1 is the largest subunit of DNA-dependent RNA polymerase V, which is required for RdDM (TAIR)	Gene silencing		0.6	Encodes the unique largest subunit of nuclear DNA-dependent RNA polymerase V; homologous to budding yeast RPB1 and the E. coli RNA polymerase beta prime subunit. Required for normal RNA-directed DNA methylation at non-CG methylation sites and transgene silencing. The nrpe1 mutant is more resistant to biotrophic pathogens and is primed to activate salicylic acid-dependent defence genes.	Yes	silencing, RDDM	6.51E-04
F vs. S (23)	AT5G26210	**AL4**	AL4 is an Alfin1-like nuclear-localized plant homeodomain (PHD) protein that binds to di or trimethylated histone H3 (H3K4me3/2) and regulates transcription (TAIR)	Gene silencing	Histones	0.59	Encodes a member of the Alfin1-like family of nuclear-localized PHD (plant homeodomain) domain containing proteins. All AL proteins except AL3 bind to di- or trimethylated histone H3 (H3K4me3/2). Members of this family include: AT5G05610 (AL1), AT3G11200 (AL2), AT3G42790 (AL3), AT5G26210 (AL4), AT5G20510 (AL5), AT2G02470 (AL6), AT1G14510 (AL7).	Yes	Histone, H3K4	1.21E-03
F vs. S (23)	AT5G09740	**HAM2**	HAM2 is an H4 specific acetyltransferase involved in achieving high transcription levels, through acetylation, of the floral initiation gene *FT* and the floral repressor *FLC* , which is epigenetically regulated through multiple pathways (doi.org/10.1016/j.tplants.2012.05.001). For FT, this process is initiated by MRG2, which trimethylates histone H3 (H3K36me3) in the 5' coding region. MRG1/2 then targets HAM1/2 to this region for acetylation. Research suggests that these processes also occur at the *FLC* locus, but that *FLC* is also being suppressed by H3K27me3 by PRC2 (doi: 10.1093/nar/gku781; doi: 10.1126/sciadv.aau7246).	Flowering, gene silencing	Histones	0.59	Encodes an enzyme with histone acetyltransferase activity. HAM2 primarily acetylate histone H4, but also display some ability to acetylate H3. Prior acetylation of lysine 5 on histone H4 reduces radioactive acetylation by either HAM2.	Yes	Histone	1.86E-03
F vs. L (23)	AT1G69440	**AGO7**	AGO7 belongs to the ARGONAUTE family of PAZ and PIWI domain containing proteins. It participates in regulating development and uses vsiRNAs to silence viral RNA (doi/10.1104/pp.19.00121)	Gene silencing	AGO	0.58	Encodes ARGONAUTE7, a member of the ARGONAUTE family, characterised by the presence of PAZ and PIWI domains. Involved in the regulation of developmental timing. Required for the accumulation of TAS3 ta-siRNAs but not for accumulation of miR171, miR173, miR390 or mi391. Localized in mature rosette leaves and floral buds.	Use	siRNA	6.29E-04
F vs. M (23)	AT5G02410	**ALG10**	ALG10 is an ER localized glucosyltransferase required for lipid-linked oligosaccharide synthesis. It is upregulated by salt stress and its absence induces UPRs (doi.org/10.1111%2Fj.1365-313X.2011.04688.x)	Stress signaling	Salt	0.58	Encodes ALG10, an ER-resident alpha1,2-glucosyltransferase that is required for lipid-linked oligosaccharide biosynthesis and subsequently for normal leaf development and abiotic stress response.	Use	Stress	4.87E-03
F vs. L (23)	AT1G52300	**AT1G52300 (RP)**	Ribosome protein (TAIR)		ribosome	0.58	Zinc-binding ribosomal protein family protein;(source:Araport11)	Use	ribosome	1.80E-03

F vs. S (23)	AT2G33560	BUBR1	BUBR1 was identified as a meiosis important gene by Walker et al. (doi.org/10.1038/s41588-017-0008-5)	Flowering	Meiosis	0.58	Encodes BUBR1. May have the spindle assembly checkpoint protein functions conserved from yeast to humans.	Yes	meiosis	1.81E-03
F vs. L (23)	AT4G39150	HSP AT4G39150	Heat shock protein (TAIR)	Stress attenuation	HSP	0.58	DNAJ heat shock N-terminal domain-containing protein;(source:Araport11)	Use	HSP	5.30E-04
L vs. S (23)	AT3G44380	LEA AT3G44380			LEA	0.58	Late embryogenesis abundant (LEA) hydroxyproline-rich glycoprotein family;(source:Araport11)			1.73E-02
M vs. S (23)	AT3G61460	BRH1			BR downregulates it	0.57	Encodes a novel ring finger protein and forms an N-terminal hydrophobic domain and a C-terminal RING-H2 signature. Expression is down regulated by brassinolide.			1.35E-02
F vs. L (23)	AT3G12550	FDM3	FDM3 participates in gene silencing by RNA-directed DNA methylation (TAIR)	Gene silencing		0.57	Belongs to a subgroup of SGS3-like proteins that act redundantly in RNA-directed DNA methylation: AT1G15910 (FDM1), AT4G00380 (FDM2), AT3G12550 (FDM3), AT1G13790 (FDM4), AT1G80790 (FDM5).	Use	RDDM	3.03E-04
L vs M (123)	AT3G03190	GSTF11	involved in responses to oxidative stress and toxin catabolic processes (TAIR)	ROS attenuation		0.57	Encodes glutathione transferase belonging to the phi class of GSTs. Naming convention according to Wagner et al. (2002).	Use	glutathione	1.77E-03
F vs. L (23)	AT3G44530	HIRA	A histone chaperone that functions complemenary with ATRX to modify H3.1/H3.3 occupancy thus fine tuning genome expression during meristem and embryo development (doi.org/10.1105/tpc.16.00877)	Gene silencing, ovule and gametophyte	Histones	0.57	Encodes a nuclear localized WD-repeat containing protein involved in negative regulation of knox gene expression via epigenetic mechanism of chromatin re-organization. It is a part of the HISTONE REGULATOR complex that deposits histones in a DNA synthesis-independent manner and affects both nucleosome occupancy and the maintenance of transcriptional silencing. Interacts physically and genetically with AS1. Expressed in meristem and leaf primordia. Homozygous mutants are embryo lethal. Phenotype of cosuppressed lines is variable but show effects on leaf development similar to as1/as2.	Use	Histone, silencing, epigenetic	5.21E-04
F vs. M (23)	AT4G39150	HSP AT4G39150	Heat shock protein (TAIR)	Stress attenuation	HSP	0.57	DNAJ heat shock N-terminal domain-containing protein;(source:Araport11)	Use	HSP	3.03E-03
F vs. S (23)	AT5G01390	HSP AT5G01390	Heat shock protein associated with protein folding and refolding (TAIR)	Stress attenuation	HSP	0.57	DNAJ heat shock family protein;(source:Araport11)	Yes	Hsp	6.49E-04
F vs. S (23)	AT5G48760	L13a-4 (RP)	TAIR		ribosome	0.57	Ribosomal protein L13 family protein;(source:Araport11)	Yes	Ribosome	4.41E-04
F vs. S (23)	AT2G18170	MPK7	MPK7 is upregulated by ABA and ROS (TAIR)	ABA signaling	ABA upregulated	0.57	MAP kinase 7;(source:Araport11)	Yes	MAPK	9.04E-04
F vs. L (23)	AT3G13120	PRPS10 (RP)	Ribosome protein (TAIR)		ribosome	0.57	Ribosomal protein S10p/S20e family protein;(source:Araport11)	Use	ribosome	2.30E-03
L vs. S (23)	AT4G22380	RP AT4G22380	At4g22380		ribosome	0.57	Ribosomal protein L7Ae/L30e/S12e/Gadd45 family protein;(source:Araport11)			1.79E-02
F vs. S (23)	AT2G24490	RPA2	TAIR			0.57	Encodes a component of Replication Protein A. Component of transcriptional gene silencing which does not affect endogenous small RNA accumulation nor DNA methylation. Localized in the nucleus. Involved in DNA repair. Interacts physically with ROS1.	Yes	silencing	2.15E-03
F vs. S (23)	AT1G63130	RPF6	TAIR			0.57	Transacting siRNA generating locus. Its derived siR9as targets AT1G62930 for cleavage. Itself is targeted by TAS2-derived ta-siR2140 for cleavage.	Yes	siRNA	4.33E-04
L vs. S (23)	AT5G28060	RPS24B (RP)	40S ribosomal protein S24-2		ribosome	0.57	Ribosomal protein S24e family protein;(source:Araport11)			1.07E-02
F vs. L (23)	AT3G04770	RPSAB (RP)	Ribosome protein (TAIR)		ribosome	0.57	40s ribosomal protein SA B;(source:Araport11)	Use	ribosome	5.77E-04
F vs. M (23)	AT2G47900	TLP3	TLP3 is tethered to the PM at PIP2 sites. Under abiotic stess, TLP3 detaches from the PM and enters the nucleus where it facilitates ABA signaling (TAIR)	ABA signaling	ABA upregulated	0.57	Member of plant TLP family which differs in having an F box domain. Plasma membrane tethering is mediated by PIP2 binding domain. Under abiotic stress TLP3 detaches from the PM and translocates to the nucleus. Mutants are insensitive to ABA.	Use	Stress, ABA	3.05E-03
F vs. M (23)	AT5G65950	TRAPPC11	TRAPPC11 is involved in regulating endosomal function and salt stress responses (10.1105/tpc.20.00044)	Stress signaling	Salt	0.57	TRAPPIII complex protein which regulates TGN integrity, by altered TGN/EE association of several residents, including SYNTAXIN OF PLANTS 61 (SYP61), and altered vesicle morphology. Involved in regulation of endosomal function and salt stress response.	Use	Stress	2.67E-03
F vs. L (23)	AT4G39400	BRI1	BRI1 is a membrane spanning protein that binds BR extracellularly and triggers a phosphorelay that traverses the membrane and cytoplasm to upregulate 1000s of BR-responsive genes. BIN2 is the centrally located major regulator of the BR TFs BZR1 and BES1. Active BIN2 inactivates these BR TFs by phosphorylating them. However, In the presence of BR, BIN2 is inactivated, and BZRI1 and BES1 are then activated by PP2A dephosphorylation. BR inactivates BIN2 by inducing BRI1 and BAK1 heterodimerization. The resulting complex phosphorylates BSU1 intracellularly. BSU1 then inactivates BIN2 by phosphorylation. In the absence of ABA, BIN2 is also inactivated by PP2Cs. This also upregulates BR induced growth. BZRI1 controls many genes including suppression of the major ABA stress response TF ABI5. In the absence of BR and the presence of ABA, BIN2 activates growth supressing SnRK2 kinases (and the ABI5 TF) while suppressing the growth promoting TFs BZRI1 and BES1 (doi:10.1242/dev.151894)	BR		0.56	Encodes a plasma membrane localized leucine-rich repeat receptor kinase involved in brassinosteroid signal transduction. BRI1 ligand is brassinolide which binds at the extracellular domain. Binding results in phosphorylation of the kinase domain which activates the BRI1 protein leading to BR responses. Residue T-1049 and either S-1044 or T-1045 were essential for kinase function in vitro and normal BRI1 signaling in planta. The structure of BRI1 ligand-binding domain has been determined at 2.5A resolution. Although BAK1 and BRI1 alone localize in the plasma membrane, when BAK1 and BRI1 are coexpressed, the heterodimer BAK1/BRI1 they form is localized in the endosome. BRI1 appears to be involved in the autonomous pathway that regulates the transition to flowering, primarily through its effects on FLC expression levels, as uncovered by double mutant analyses. This most likely occurs as a result of BRI1-dependent effects on histone acetylation, but not histone triMeH3K4 methylation, at the FLC locus. The mRNA is cell-to-cell	Use	BR, Histone	1.61E-03
F vs. M (23)	AT1G35140	EXL1	EXL1 is upregulated by BR and sugar starvation conditions. It suppresses BR induced growth during low energy conditions (doi/10.1104/pp.111.177204)	BR		0.56	EXL1 is involved in the C-starvation response. Phenotypic changes of an exl1 loss of function mutant became evident only under corresponding experimental conditions. For example, the mutant showed diminished biomass production in a short-day/low light growth regime, impaired survival during extended night, and impaired survival of anoxia stress.	Use	BR, Stress	4.20E-03
F vs. S (23)	AT4G14730	GAAP1	The *Arabidopsis* anti apoptotic protein 1 (GAAP1) is a stress induced protein that participates in ER stress mitigation (doi:10.1093/jxb/erz402)	Stress attenuation		0.56	Stress induced membrane protein. Mutants show enhanced cell death under stress.	Yes	Stress	6.42E-04
F vs. M (23)	AT2G43350	GPX3	GPX3 is a peroxidase of the secratory pathway where it is bound to the membrane by an n-terminal transmembrane domain. It is upregulated by ABA and drought stress and functions as a redox transducer and scavenger (	ROS attenuation	ABA upregulated	0.56	Glutathione peroxidase. Functions as both a redox transducer and a scavenger in abscisic acid and drought stress responses. Interacts with ABI2 and ABI1.	Use	glutathione, Stress, ABA, peroxidase	1.01E-03
M vs. S (23)	AT1G07400	HSP17.8			HSP	0.56	HSP20-like chaperones superfamily protein;(source:Araport11)			1.37E-02
F vs. L (23)	AT3G16780	RPL19B (RP)	TAIR		ribosome	0.56	Ribosomal protein L19e family protein;(source:Araport11)	Use	ribosome	7.62E-04

F vs. L (23)	AT5G42920	**THO5**	THO5 has been implicated in siRNA biosynthesis and in facilitating the export from the nucleus of mRNA precursers (TAIR)	Gene silencing		0.56	Encodes a component of the putative Arabidopsis THO/TREX complex: THO1 or HPR1 (At5g09860), THO2 (At1g24706), THO3 or TEX1 (At5g56130), THO5 (At5g42920, At1g45233), THO6 (At2g19430), and THO7 (At5g16790, At3g02950). THO/TREX complexes in animals have been implicated in the transport of mRNA precursors. Mutants of THO3/TEX1, THO1, THO6 accumulate reduced amount of small interfering (si)RNA, suggesting a role of the putative Arabidopsis THO/TREX in siRNA biosynthesis.	Use	siRNA	1.61E-04
L vs M (123)	AT5G16970	**AER**	involved in responses to oxidative stress (TAIR)	ROS attenuation		0.55	encodes a 2-alkenal reductase (EC 1.3.1.74), plays a key role in the detoxification of reactive carbonyls	Use	detoxification	3.87E-03
F vs. L (23)	AT3G57390	**AGL18**	MADS-box gene expressed in gametophytes (TAIR)			0.55	encodes a MADS-box containing protein likely to be a transcription factor that is expressed in endosperm and developing gametophytes. The protein sequence is most similar to that of AGL15, which is expressed in developing	No	AGL, gametophyte	8.09E-04
F vs. S (23)	AT2G41430	**ERD15**	DRD15 is an early response to dehydration gene (TAIR)	Stress signaling		0.55	Encodes hydrophilic protein lacking Cys residues that is expressed in response to drought stress, light stress and treatment with plant-growth-promoting rhizobacteria (Paenibacillus polymyxa), possibly revealing a connection between responses to biotic and abiotic stress. Also identified as a CTC Interacting Domain (CID) protein in a yeast two hybrid screen using the PAB2 protein as bait. Contains PAM2 like domain which mediates interaction with PABC domain in PAB2.	Yes	Stress	1.99E-03
F vs. S (23)	AT3G30775	**ERD5**	PRODH1 is an early response to dehydration gene and functions as a proline dehydrogenase that catalyzes the first and rate limiting step of proline catabolism. Here, proline may serve as an alternative substrate in respiration with PRODH1 functioning in energy recovery by participating in the conversion of proline to glutamate (doi:10.1093/jxb/erz351)	Stress attenuation		0.55	Encodes a proline oxidase that is predicted to localize to the inner mitochondrial membrane, its mRNA expression induced by high levels of Al and by osmotic stress. The promoter contains an L-proline-inducible element.	Yes	Stress	2.88E-04
M vs. S (23)	AT5G10450	**GRF6**			Stress, BR, peroxidase, Ascorbate	0.55	Encodes a member of the 14-3-3 gene family that is a lambda isoform (14-3-3λ). Interacts with APX3 (ascorbate peroxidase) and AKR2 , suggesting a role in mediating oxidative metabolism in stress response. This protein was shown to colocalize and interact with SERK1 by which it is phosphorylated. This protein is also reported to interact with the phosphorylated form of the BZR1 transcription factor involved in brassinosteroid signaling and may affect the nucleocytoplasmic shuttling of BZR1. Interacts with JAZ10.4 which lacks the Jas motif. It is also phosphorylated by CRPK1 as part of the response to cold and translocates to the nucleus after phosphorylation.			1.98E-02
F vs. S (23)	AT1G68820	**PPRT1**	Negative regulator of ABA: induced by salt stress and causes increased sensitivity to salt stress (https://doi.org/10.1080/15592324.2020.1732103); downregulates stress inducible genes by upregulating the ABA catabolism genes AtCYP707A1 and AtCYP707A3 (doi.org/10.3390/ijms20020394)	ABA signaling	ABA upregulated, Salt	0.55	Putative C3HC4 zinc-finger ubiquitin E3 ligase, negative regulator in ABA and drought stress response.	Yes	Stress, ABA	7.37E-04
F vs. L (23)	AT4G30480	**TPR1 (HSP)**	TPR1 is a tetratricopeptide repeat containing protein that functions with Hsp90/Hsp70 as a co-chaperone. It also functions as a transcriptional co-repressor with SNC1 to repress the accumulation of miRNAs and phased siRNA that suppress the expression of pathogen resistance genes under pathogen free conditions (DOI: 10.1038/s41467-018-07516-z)	Stress attenuation	HSP	0.55	Encodes one of the 36 carboxylate clamp (CC)-tetratricopeptide repeat (TPR) proteins (Prasad 2010, Pubmed ID: 20856808). Interacts with Hsp90/Hsp70 as co-chaperone.	Use	HSP	1.07E-03
F vs. S (23)	AT1G08680	**AGD14**	AGD14 is an ARF that is upregulated in carpels. Hence, its upregulation in *B. stricta* pistils compared to *B. x. formosa* ovules is uninformative with regard to reprodutive mode regulation.		ARF	0.54	A member of ARF GAP domain (AGD), A thaliana has 15 members, grouped into four classes. AGD14 belongs to the class 4, together with AGD15.	Yes	ARF	1.94E-03
F vs. S (23)	AT1G14510	**AL7**	AL7 is one of two Alfin1-like nuclear-localized H3K4me3 binding PHD (plant homeodomain) containing proteins recently identified as contributing to the functional structure of polycomb repressive complex 1 (PCR1s). AL7 and AL2 target PRC1s to transcriptionally active chromatin where the expressed H3K4me3 state is switched to H2Aub1 repressive marks. PRC2 is then recruited to these sites to stabilize gene silencing through H3K27me3 (doi.org/10.1016/j.jmb.2018.08.021)	Gene silencing	Histones	0.54	Encodes a member of the Alfin1-like family of nuclear-localized PHD (plant homeodomain) domain containing proteins. All AL proteins except AL3 bind to di- or trimethylated histone H3 (H3K4me3/2). Members of this family include: AT5G05610 (AL1), AT3G11200 (AL2), AT3G42790 (AL3), AT5G26210 (AL4), AT5G20510 (AL5), AT2G02470 (AL6), AT1G14510 (AL7).	Yes	Histone, H3K4	4.35E-04
L vs. S (23)	AT5G42190	**ASK2**			meiosis	0.54	Similar to SKP1 in yeast and humans which are involved in mitotic cell cycle control and ubiquitin mediated proteolysis.			1.02E-02
L vs. S (23)	AT1G52300	**AT1G52300**			#N/A	0.54	Zinc-binding ribosomal protein family protein;(source:Araport11)			1.11E-02
L vs M (123)	AT1G08860	**BON3**	BON3 overlaps with BON1 in maintaining cellular homeopstasis and inhibiting cell death (TAIR)		Ca, homeostasis	0.54	Encodes a copine-like protein, which is a member of a newly identified class of calcium-dependent, phospholipid binding proteins that are present in a wide range of organisms. Overexpression of this gene suppresses bon1-1 phenotypes. Double mutant analyses with bon1-1 suggest that BON1 and BON3 have overlapping functions in maintaining cellular homeostasis and inhibiting cell death.	Use	Ca, Homeostasis	3.74E-03
F vs. M (23)	AT5G17800	**BRAVO**	BRAVO is an MYB TF that is specifically expressed during tissue development in cells that normally display a reduced level of cell division, e.g., the quiescent centers of root meristems and organ boundary cells. In these cells, the BRAVO TF functions as a cell-specific repressor of cell division possibly by repressing BES1 and BZR1 activity. Cell division resumes in quiescent cells and organ boundary cells when BRAVO is repressed due to complexes that form in these cells and consist of BES1 and the coprepressor TOPLESS (TPL)	BR		0.54	Member of the R2R3 factor gene family that acts as a cell-specific repressor of quiescent center (QC) divisions in the primary root, acting through the BR signaling pathway. Works with BES1 to regulate QC division in the root.	Use	BR	5.17E-03
F vs. S (23)	AT1G62810	**CuAO1**	CuAO1 is a copper amine oxidase involved in polyamine catabolism that is upregulated by ABA and SA and upon recovery from dehydration stress. It may be involved in maintaining water balance homeostasis during drought stress (https://doi.org/10.1016/j.plaphy.2019.11.037)		ABA upregulated, homeostasis	0.54	Encodes COPPER AMINE OXIDASE1 (CuAO1). Contributes to abscisic acid- and polyamine-induced nitric oxide biosynthesis and abscisic acid signal transduction.	Yes	ABA	2.08E-03
F vs. S (23)	AT1G54410	**HIRD11**	HIRD11 is a dehydrin that reduces the formation of Cu involved ROS formation (TAIR)	ROS attenuation		0.54	Encodes a KS-type dehydrin can reduce the formation of reactive oxygen species (ROS) from Cu.	Yes	ROS	1.11E-03
F vs. S (23)	AT1G60200	**RMB25**	RMB25 is upregulated by ABA and abiotic stresses. It is an alternative splicing factor that regulates the concentration of ABA signaling pathway participants such as the PP2C HAB1.1 and HAB1.2 isoforms (doi.org/10.3389/fpls.2017.00292)	ABA signaling	ABA upregulated	0.54	RMB25 is an alternative splicing factor involved in mediation of abiotic stress response and ABA response. Its expression is modulated by a variety of stressors and it in turn appears to affect the ratio of splice variants of stress responsive genes such as HAB1.2/HAB1.1.	Yes	Stress, ABA	4.00E-04
F vs. M (23)	AT1G48830	**RPS7A (RP)**			ribosome	0.54	Ribosomal protein S7e family protein;(source:Araport11)	Use	ribosome	1.97E-03

F vs. S (23)	AT1G71830	SERK1	SERK1 is a receptor-like kinase that is expressed during floral development. It phosphorylation activates EMS1 to regulate whether anther cells develop as meiocytes (pollen mother cells) or develop as anther tapetum cells. THE SERK1/EMS1 pathway also activates the BES1 TF in a BR independent manner (doi/10.1104/pp.16.01219)	Flowering	Ovule gametophyte	0.54	Plasma membrane LRR receptor-like serine threonine kinase expressed during embryogenesis in locules until stage 6 anthers, with higher expression in the tapetal cell layer. SERK1 and SERK2 receptor kinases function redundantly as an important control point for sporophytic development controlling male gametophyte production. SERK1 interacts with and transphosphorylates EMS1	Yes	gametophyte	2.13E-03
F vs. L (23)	AT4G29520	SES1	SES is an ER localized chaperone protein that is upregulated by heat and salt stress and facilitates normal protein folding durng stress (doi.org/10.1016/j.bbrc.2019.04.015)	Stress signaling	Salt	0.54	SES1 is an ER localized chaperone involved in salt and heat stress response.	Use	Stress	2.48E-03
F vs. S (23)	AT5G65950	TRAPPC11	TRAPPC11 is involved in regulating endosomal function and salt stress responses (10.1105/tpc.20.00044)	Stress signaling	Salt	0.54	TRAPPIII complex protein which regulates TGN integrity, by altered TGN/EE association of several residents, including SYNTAXIN OF PLANTS 61 (SYP61), and altered vesicle morphology. Involved in regulation of endosomal function and salt stress response.	Yes	Stress	3.33E-04
F vs. L (23)	AT1G36240	AT1G36240 (RP)	Ribosome protein (TAIR)		ribosome	0.53	Ribosomal protein L7Ae/L30e/S12e/Gadd45 family protein;(source:Araport11)	Use	ribosome	7.65E-04
F vs. L (23)	AT5G24490	AT5G24490 (RP)	Ribosome protein (TAIR)		ribosome	0.53	30S ribosomal protein;(source:Araport11)	Use	ribosome	2.99E-03
F vs. L (23)	AT5G17800	BRAVO	BRAVO is an MYB TF that is specifically expressed during tissue development in cells that normally display a reduced level of cell division, e.g., the quiescent centers of root meristems and organ boundary cells. In these cells, the BRAVO TF functions as a cell-specific repressor of cell division possibly by repressing BES1 and BZR1 activity. Cell division resumes in quiescent cells and organ boundary cells when BRAVO is repressed due to complexes that form in these cells and consist of BES1 and the coprepressor TOPLESS (TPL)	BR		0.53	Member of the R2R3 factor gene family that acts as a cell-specific repressor of quiescent center (QC) divisions in the primary root, acting through the BR signaling pathway. Works with BES1 to regulate QC division in the root.	Use	BR	1.50E-03
F vs. S (23)	AT1G17760	CSTF77	CSTF77 is a subunit of a polyadenylation factor that processes *FLC* antisense transcripts, which induces flowering by reducing *FLC* sense transcription (10.1126/science.1180278)	Flowering, Gene silencing		0.53	Encodes a homolog of the mammalian protein CstF77, a polyadenylation factor subunit. RNA 3′-end?processing factor of antisense FLC transcript. Mediates silencing of the floral repressor gene FLC. Member of CstF complex.	Yes	silencing	3.40E-04
F vs. S (23)	AT5G04560	DME	DME is a DNA glycosylase that demethylates small transposons that flank coding genes in the female gametophyte central cell (maternal genome) prior to fertilization. The affected (imprinted) genes are then preferentially expressed during endosperm formation (doi/10.1073/pnas.1907290116)	Flowering	Ovule gametophyte	0.53	Encodes a DNA glycosylase DEMETER (DME). Responsible for endosperm maternal-allele-specific hypomethylation at the MEDEA (MEA) gene. DME can excise 5-methylcytosine in vitro and when expressed in E. coli. DME establishes MEA imprinting by removing 5-methylcytosine to activate the	Yes	Imprinting	1.80E-03
F vs. M (23)	AT3G24010	ING1	ING and alfin1-like proteins bind to H3K4 di or trimethylated DNA where they are involved in chromatin regulation (TAIR)	Gene silencing		0.53	ING1 encodes a member of the Inhibitor of Growth family of nuclear-localized PhD domain containing homeodomain proteins. Binds to H3K4 di or trimethylated DNA.	Use	H3K4	4.84E-03
F vs. S (23)	AT3G44380	LEA AT3G44380	TAIR		LEA	0.53	Late embryogenesis abundant (LEA) hydroxyproline-rich glycoprotein family;(source:Araport11)	Yes	LEA	4.05E-04
L vs. S (23)	AT3G50770	No	Probable calcium-binding protein CML41		Calmodulin	0.53	calmodulin-like 41;(source:Araport11)			1.79E-02
L vs. S (23)	AT5G15760	RP AT5G15760	30S ribosomal protein 3-2, chloroplastic		ribosome	0.53	Ribosomal protein PSRP-3/Ycf65;(source:Araport11)			1.41E-02
F vs. M (23)	AT4G30480	TPR1 (HSP)	TPR1 is a tetratricopeptide repeat containing protein that functions with Hsp90/Hsp70 as a co-chaperone. It also functions as a transcriptional co-repressor with SNC1 to repress the accumulation of miRNAs and phased siRNA that suppress the expression of pathogen resistance genes under pathogen free conditions (DOI: 10.1038/s41467-018-07516-z)	Stress attenuation	HSP	0.53	Encodes one of the 36 carboxylate clamp (CC)-tetratricopeptide repeat (TPR) proteins (Prasad 2010, Pubmed ID: 20856808). Interacts with Hsp90/Hsp70 as co-chaperone.	Use	HSP	3.77E-03
F vs. M (23)	AT1G52340	ABA2	Involved in ABA synthesis (TAIR)	ABA signaling		0.52	Encodes a cytosolic short-chain dehydrogenase/reductase involved in the conversion of xanthoxin to ABA-aldehyde during ABA biosynthesis. Mutants are insensitive to sucrose and glucose.	Use	ABA	2.99E-03
L vs M (123)	AT2G07680	ABCC13	Expression during seedling development is upregulated by nitrate, phosphate and sucrose (DOI: 10.1007/s10535-013-0387-0)	No		0.52	Encodes ABCC13/MRP11, a member of the multidrug resistance associated protein MRP/ABCC subfamily. Its expression is induced by gibberellic acid and downregulated by naphthalene acetic acid, abscisic acid, and zeatin.	Use	ABA	3.60E-04
F vs. M (23)	AT3G10500	ANACO53	ANACO53 is a NAC domain TF that is upregulated by stress and by ANAC017. It may be responsible for drought induced senescence through ROS accumulation and for the activation of the UPR via increased expression of numerous genes that prevent proteotoxic stress during episodes of UPRs (doi/10.1105/tpc.15.01022)	ROS attenuation		0.52	Encodes a transcriptional activator that is associated with the plasma membrane in a dormant form and is proteolytically cleaved to create a form that can enter the nucleus. It is thought to promote ROS production by binding directly to the promoters of genes encoding ROS biosynthetic enzymes during drought-induced leaf senescence.The mRNA is cell-to-cell mobile.	Use	ROS	3.36E-03
F vs. M (23)	AT3G02580	DWF7	Involved in BR synthesis (TAIR)	BR		0.52	Brassinosteroid biosynthetic enzyme, catalyzes delta7 sterol C-5 desaturation step. Mutant has dwarf phenotype.	Use	BR	4.10E-03
F vs. S (23)	AT1G80790	FDM5	FDM5 belongs to a group of SGS3-like proteins that function redundantly in RdDM (TAIR)	Gene silencing		0.52	Belongs to a subgroup of SGS3-like proteins that act redundantly in RNA-directed DNA methylation: AT1G15910 (FDM1), AT4G00380 (FDM2), AT3G12550 (FDM3), AT1G13790 (FDM4), AT1G80790 (FDM5).The mRNA is cell-to-cell mobile.	Yes	RDDM	7.80E-04
F vs. L (23)	AT3G24010	ING1	ING and alfin1-like proteins bind to H3K4 di or trimethylated DNA where they are involved in chromatin regulation (TAIR)	Gene silencing		0.52	ING1 encodes a member of the Inhibitor of Growth family of nuclear-localized PhD domain containing homeodomain proteins. Binds to H3K4 di or trimethylated DNA.	Use	H3K4	2.20E-03
F vs. M (23)	AT2G13540	ABH1	ABH1 is an ABA induced cap-binding protein involved in proper splicing of ABA signaling and flowering related mRNAs (TAIR)		ABA upregulated	0.51	Encodes a nuclear cap-binding protein that forms a heterodimeric complex with CBP20 and is involved in ABA signaling and flowering. Mutants are early flowering and exhibit hypersensitive response to ABA in germination inhibition.Loss of ABH1 function results in abnormal processing of mRNAs for several important floral regulators (FLC, CO, FLM). Analysis of loss of function mutations suggests a role in pri-miRNA processing and mRNA splicing. Note that two different mutant alleles were given the same name abh1-7 (Kuhn et al 2007; Kim et al 2008). To avoid confusion, abh1-7 described in Kim et al (2008) has been renamed abh1-107 (other names: ensalada-1, ens-1).	Use	ABA, miRNA	6.15E-03
F vs. L (23)	AT1G34190	ANAC017	ANAC017 is a NAC domain TF that responds to mitochondrial retrograde signaling by upregulating genes that attenuate mitochondrial stress, induce autophagy, senescence, and suppression of chloroplast and auxin function, and reprogram metabolism associated genes that attenuate low energy conditions possibly through the activation of SnRK1 (doi/10.1104/pp.18.01603)	SnRK1 TOR signaling, Stress signaling		0.51	Encodes a NAC domain transcription factor that regulates the mitochondrial retrograde response and coordinates organellar functions and stress responses.	Use	Stress	3.16E-05
F vs. L (23)	AT3G10500	ANACO53	ANACO53 is a NAC domain TF that is upregulated by stress and by ANAC017. It may be responsible for drought induced senescence through ROS accumulation and for the activation of the UPR via increased expression of numerous genes that prevent proteotoxic stress during episodes of UPRs (doi/10.1105/tpc.15.01022)	ROS attenuation		0.51	Encodes a transcriptional activator that is associated with the plasma membrane in a dormant form and is proteolytically cleaved to create a form that can enter the nucleus. It is thought to promote ROS production by binding directly to the promoters of genes encoding ROS biosynthetic enzymes during drought-induced leaf senescence.The mRNA is cell-to-cell mobile.	Use	ROS	1.48E-04

F vs. L (23)	AT3G01740	**AT3G01740 (RP)**	Ribosome protein (TAIR)		ribosome	0.51	Mitochondrial ribosomal protein L37;(source:Araport11)	Use	ribosome	1.03E-04
F vs. S (23)	AT1G18400	**BEE1**	BEE1 is directly upregulated by BES1. In blue light, BEE1 interacts with the blue light photoreceptor CRY2 and binds directly to *FT* to initiate flowering (doi: 10.1111/nph.15866)	Flowering		0.51	Encodes the brassinosteroid signaling component BEE1 (BR-ENHANCED EXPRESSION 1). Positively modulates the shade avoidance syndrome in Arabidopsis seedlings.	Yes	BR	2.00E-03
F vs. S (23)	AT4G16280	**FCA**	FCA is an RNA binding protein that binds nascent transcripts of the antisense gene *COOLAIR* . This complex interacts with the polycomb repressive complex 2 (PRC2) subunit CURLY LEAF (CLF) to deposit repressive H3K27me3 histone marks at FLOWERING LOCUS C (FLC), the central floral transcription repressor (10.1126/sciadv.aau7246). Up and down regulation of FCA in sexual *B. stricta* and diplosporous *B. x. formosa* is correlated with meiosis and apomeiosis, respectively, making this pathway of interest in studying sex apomixis regulation.	Flowering, Gene silencing		0.51	Involved in the promotion of the transition of the vegetative meristem to reproductive development. Four forms of the protein (alpha, beta, delta and gamma) are produced by alternative splicing. Involved in RNA-mediated chromatin silencing. At one point it was believed to act as an abscisic acid receptor but the paper describing that function was retracted.	Yes	silencing	4.05E-04
L vs M (123)	AT1G42970	**GAPB**	Chloroplast localized GLYCERALDEHYDE-3-PHOSPHATE DEHYDROGENASE B SUBUNIT; Involved in responses to sucrose (TAIR), active in the light under reducing conditions		Thioredoxin	0.51	Encodes chloroplast localized glyceraldehyde-3-phosphate dehydrogenase that can use both NADH and NADPH to reduce 1,3-diphosphate glycerate. It forms A2B2 heterotetramers with GapA forms of the GADPH enzyme. These complexes are active in the light under reducing conditions, but show reduced NADPH-dependent activity in response to oxidized thioredoxins and increased NAD(H)/NADP(H) ratios due to the formation of inactive A8B8 hexadecamers. The mRNA is cell-to-cell mobile.	Use	Thioredoxin	5.44E-03
F vs. S (23)	AT2G43350	**GPX3**	GPX3 is a peroxidase of the secratory pathway where it is bound to the membrane by an n-terminal transmembrane domain. It is upregulated by ABA and drought stress and functions as a redox transducer and scavenger (	ROS attenuation	ABA upregulated	0.51	Glutathione peroxidase. Functions as both a redox transducer and a scavenger in abscisic acid and drought stress responses. Interacts with ABI2 and ABI1.	Yes	glutathione, Stress, ABA, peroxidase	4.83E-04
L vs. S (23)	AT3G04840	**S3Ae (RP)**			ribosome	0.51	Ribosomal protein S3Ae;(source:Araport11)			1.09E-02
F vs. L (23)	AT5G02410	**ALG10**	ALG10 is an ER localized glucosyltransferase required for lipid-linked oligosaccharide synthesis. It is upregulated by salt stress and its absence induces UPRs (doi.org/10.1111%2Fj.1365-313X.2011.04688.x)	Stress signaling	Salt	0.50	Encodes ALG10, an ER-resident alpha1,2-glucosyltransferase that is required for lipid-linked oligosaccharide biosynthesis and subsequently for normal leaf development and abiotic stress response.	Use	Stress	6.29E-04
F vs. S (23)	AT2G36830	**GAMMA-TIP**	NA			0.5	Encodes a tonoplast intrinsic protein, which functions as water channel. It has also been shown to be able to facilitate the transport of urea and hydrogen peroxide. Highly expressed in vascular tissues of the root, stem, cauline leaves and flowers but not in the apical meristems. The mRNA is cell-to-cell mobile.	Yes	H2O2	1.92E-03
L vs. S (23)	AT1G51940	**LYK3**			#N/A	0.5	Encodes a LysM containing receptor-like kinase. Induction of chitin responsive genes by chitin treatment is not blocked in the mutant. Based on protein sequence alignment analysis, it has a typical RD signaling domain in its catalytic loop and possesses autophosphorylation activity.It is required for the suppression of defense responses in absence of pathogen infection or upon abscisic acid treatment. Loss-of-function mutants display enhanced resistance to Botrytis cinerea and Pectobacterium carotovorum. Its expression is repressed by pathogen infection and biological elicitors and is induced abscisic acid.Expression is strongly repressed by elicitors and fungal infection, and is induced by the hormone abscisic acid (ABA). Insertional mutants show increased expression of PHYTOALEXIN-DEFICIENT 3 (PAD3), enhanced resistance to Botrytis cinerea and Pectobacterium carotovorum infection and reduced physiological responses to ABA, suggesting that LYK3 is important for the cross-talk between signaling pathways activated by ABA and pathogens (PMID:24639336).			9.20E-03
L vs. S (23)	AT4G35490	**MRPL11 (RP)**	At4g35490		ribosome	0.5	mitochondrial ribosomal protein L11;(source:Araport11)			1.51E-02
F vs. S (23)	AT4G36380	**ROT3**	ROT3 is a P-450 protein that is upregulated by ET, induces leaf length expansion and catalyzes hydroxylation reactions in the BR biosynthesis pathway (TAIR)	BR		0.5	Encodes a cytochrome P-450 gene that is involved in leaf blade expansion by controlling polar cell expansion in the leaf length direction. Member of the CYP90C CYP450 family. ROT3 was shown to be involved in brassinosteroid biosynthesis, most likely in the conversion step of typhasterol (TY) to castasterone (CS). As 6-deoxo-CS was unable to restore the phenotype of rot3-1, it has been postulated that ROT3 might be specifically involved in the conversion of TY to CS in the C6-oxidation pathway of brassinolide. Recently, CYP90C1 was shown to catalyse the C-23 hydroxylation of several brassinosteroids (the enzyme has a broad specificity for 22-hydroxylated substrates).	Yes	BR	1.49E-03
M vs. S (23)	AT2G19720	**RPS15AB (RP)**			ribosome	0.5	ribosomal protein S15A B;(source:Araport11)			1.48E-02
F vs. S (23)	AT4G28190	**ULT1**	ULT1 is a trithorax group (trxG) factor that regulates over 700 genes, most of which are downregulated, including genes associated with reproductive development in floral apices (doi.org/10.1534/g3.119.400559)	Gene silencing, ovule gametophyte		0.5	Encodes a novel Cys-rich protein with a B-box like domain that acts as a negative regulator of meristem cell accumulation in inflorescence and floral meristems as loss-of-function ult1 mutations cause inflorescence meristem enlargement, the production of extra flowers and floral organs, and a decrease in floral meristem determinacy. Acts opposite to CLF which represses AG, but preventing deposition of CLF repressive methylation marks.ULT1 acts as an anti-repressor that counteracts EMF1 action through modulation of histone marks on target genes. Regulates developmental as well as biotic and abiotic stress response genes.	Yes	Stress, Histone	4.34E-04
F vs. L (23)	AT1G18540	**AT1G18540 (RP)**	Ribosome protein (TAIR)		ribosome	0.49	Ribosomal protein L6 family protein;(source:Araport11)	Use	ribosome	1.47E-03
M vs. S (23)	AT1G61660	**BHLH112**	#N/A		Stress, ROS	0.49	Encodes a transcriptional activator that regulates the expression of genes by binding to their GCG- or E-boxes to mediate physiological responses, including proline biosynthesis and ROS scavenging pathways, to enhance stress tolerance.			2.04E-02
F vs. L (23)	AT3G13320	**CAX2**	TAIR			0.49	low affinity calcium antiporter CAX2 The mRNA is cell-to-cell mobile.	No	Ca	5.49E-04
F vs. S (23)	AT1G66750	**CDKD;2**	CDK;2	Flowering		0.49	Encodes a CDK-activating kinase that interacts with SPT5, a regulator of transcription and histone methylation.	Yes	Histone	1.88E-04
L vs M (123)	AT2G28190	**CSD2**	SOD2 is a chloroplastic copper/zinc superoxide dismutase that converts superoxide free radicals to O2 and H2O2. Under nonstress conditions, its transcripts are cleaved by miR398, which decreases SOD2 levels. Under stress conditions, miR398 expression is suppressed, and SOD2 levels increase (DOI 10.1007/s10646-017-1843-y)	ROS attenuation		0.49	Encodes a chloroplastic copper/zinc superoxide dismutase CSD2 that can detoxify superoxide radicals. Its expression is affected by miR398-directed mRNA cleavage. Activation depends totally on CCS. Overexpression of a miR398-resistant form of CSD2 leads to more dramatic improvements in stress (hight light, Cu2+ and methyl viologen) tolerance than overexpression of wild-type CSD2. The mRNA is cell-to-cell mobile.	Use	Stress, H2O2, superoxide dismutase	7.29E-03

F vs. S (23)	AT5G51230	**EMF2**	EMBRYONIC FLOWER 2 (EMF2) is part of a polycomb group complex involved in negative regulation of reproductive development (TAIR)	Flowering		0.49	Polycomb group protein with zinc finger domain involved in negative regulation of reproductive development. Forms a complex with FIE, CLF, and MSI1. This complex modulates the expression of target genes including AG, PI and AP3.	Yes	MSI1	1.52E-03
L vs M (123)	AT3G19980	**FyPP3**	FyPP3 dephosphorylates the TF ABI5, thus negatively regulating ABA signaling. FyPP3 degradation is facilitated by NRP, but FyPP3 can also deactivate NRP by dephosphorylating it (doi.org/10.1016/j.molp.2017.11.006). Photomorphogenesis is repressed in the dark by kinase activated PHYTOCHROME INTERACTING FACTORS (PIFs). By dephosphorylating PIFs, PP6 (FyPP3, catalytic subunit) regulates photomorphogenesis (doi.org/10.1073/pnas.1907540116)	ABA signaling		0.49	Encodes catalytic subunit of serine/threonine protein phosphatase 2A. It can associate with phytochromes A and B in vitro. Mutant plants display an accelerated flowering phenotype.Acts antagonistically to SnRK2 to regulate ABI5 phosphorylation. It inteacts with NRP which results in tethering to endosomes leading to its degradation.	Use	PP2A, SnRK	1.57E-03
L vs M (123)	AT3G28900	**L34-3 (RP)**			ribosome	0.49	Ribosomal protein L34e superfamily protein;(source:Araport11)	Use	ribosome	6.39E-03
L vs M (123)	AT3G02190	**L39-2 (RP)**			ribosome	0.49	Ribosomal protein L39 family protein;(source:Araport11)	Use	ribosome	5.02E-04
L vs M (123)	AT5G51720	**NEET**	Fe-S clusters mediate ETC reactions, but they also react with oxygen to produce ROS. NEET proteins participate in regulating the biogenesis of Fe-S clusters and maintaining Fe and ROS homeostasis (10.1111/tpj.14581)	ROS attenuation	Homeostasis	0.49	Encodes a protein with biochemical, structural, and biophysical characteristics of a NEET protein. It plays a key role in plant development, senescence, reactive oxygen homeostasis, and Fe metabolism.	Use	Homeostasis	1.60E-03
F vs. L (23)	AT1G09180	**SAR1A**	TAIR			0.49	A member of ARF-like GTPase family. A thaliana has 21 members, in two subfamilies, ARF and ARF-like (ARL) GTPases.	No	ARF	1.21E-03
F vs. L (23)	AT4G32330	**WDL5**	WDL5 is upregulated by ethylene during salt stress and is involved in microtubule bundle formation. It is a target of the ethylene induced TF EIN3 (doi/10.1104/pp.17.01124)	Stress signaling	ET, Salt	0.49	WDL5 is an target of EIN3 that co-localizes with cortical microtubles. It its thought to function to stabilize microtubles during ethylene induced hypocotyl elongation.	Use	ethylene	6.46E-04
F vs. M (23)	AT1G14510	**AL7**	AL7 is one of two Alfin1-like nuclear-localized H3K4me3 binding PHD (plant homeodomain) containing proteins recently identified as contributing to the functional structure of polycomb repressive complex 1 (PCR1s). AL7 and AL2 target PRC1s to transcriptionally active chromatin where the expressed H3K4me3 state is switched to H2Aub1 repressive marks. PRC2 is then recruited to these sites to stabilize gene silencing through H3K27me3 (doi.org/10.1016/j.jmb.2018.08.021)	Gene silencing	Histones	0.48	Encodes a member of the Alfin1-like family of nuclear-localized PHD (plant homeodomain) domain containing proteins. All AL proteins except AL3 bind to di- or trimethylated histone H3 (H3K4me3/2). Members of this family include: AT5G05610 (AL1), AT3G11200 (AL2), AT3G42790 (AL3), AT5G26210 (AL4), AT5G20510 (AL5), AT2G02470 (AL6), AT1G14510 (AL7).	Use	Histone, H3K4	2.99E-04
F vs. M (23)	AT1G34190	**ANAC017**	ANAC017 is a NAC domain TF that responds to mitochondrial retrograde signaling by upregulating genes that attenuate mitochondrial stress, induce autophagy, senescence, and suppression of chloroplast and auxin function, and reprogram metabolism associated genes that attenuate low energy conditions possibly through the activation of SnRK1 (doi/10.1104/pp.18.01603)	SnRK1 TOR signaling, Stress signaling		0.48	Encodes a NAC domain transcription factor that regulates the mitochondrial retrograde response and coordinates organellar functions and stress responses.	Use	Stress	9.26E-04
F vs. L (23)	AT1G80700	**AT1G80700**	TAIR			0.48	stress response NST1-like protein;(source:Araport11)	No	Stress	3.84E-04
F vs. L (23)	AT5G15220	**AT5G15220 (RP)**	Ribosome protein (TAIR)		ribosome	0.48	Ribosomal protein L27 family protein;(source:Araport11)	Use	ribosome	3.95E-04
F vs. L (23)	AT1G56310	**ATRM2**	In RNA-induced silencing complex assembly, ATRM2 appears to interact with AGO1 in trimming unmethylated (unprotected) regions of small RNAs prior to DNA silencing (doi.org/10.1073/pnas.1721917115)	Gene silencing		0.48	DEDDy-type 3′ -> 5′ exoribonuclease involved in miRNA degradation.	Use	miRNA	3.35E-04
L vs M (123)	AT1G09200	**H3.1 AT1G09200**	Histone 3 variant H3.1s are expressed and incorporated into DNA during S phase where they are methylated to retain specific epigenetic states (doi.org/10.1016/j.cub.2010.11.012; 10.1126/science.1248357)	Gene silencing	Histones	0.48	Histone superfamily protein;(source:Araport11)	Use	Histone	2.45E-03
F vs. S (23)	AT3G13120	**PRPS10 (RP)**	Ribosome protein (TAIR)		ribosome	0.48	Ribosomal protein S10p/S20e family protein;(source:Araport11)	Yes	Ribosome	3.17E-04
F vs. L (23)	ATCG00830	**RPL2.1 (RP)**	Ribosome protein (TAIR)		ribosome	0.48	encodes a chloroplast ribosomal protein L2, a constituent of the large subunit of the ribosomal complex	Use	ribosome	7.65E-04
F vs. M (23)	AT2G07680	**ABCC13**	Expression during seedling development is upregulated by nitrate, phosphate and sucrose (DOI: 10.1007/s10535-013-0387-0)	No		0.47	Encodes ABCC13/MRP11, a member of the multidrug resistance associated protein MRP/ABCC subfamily. Its expression is induced by gibberellic acid and downregulated by naphthalene acetic acid, abscisic acid, and zeatin.	Use	ABA	5.50E-03
F vs. S (23)	AT1G34190	**ANAC017**	ANAC017 is a NAC domain TF that responds to mitochondrial retrograde signaling by upregulating genes that attenuate mitochondrial stress, induce autophagy, senescence, and suppression of chloroplast and auxin function, and reprogram metabolism associated genes that attenuate low energy conditions possibly through the activation of SnRK1 (doi/10.1104/pp.18.01603)	SnRK1 TOR signaling, Stress signaling		0.47	Encodes a NAC domain transcription factor that regulates the mitochondrial retrograde response and coordinates organellar functions and stress responses.	Yes	Stress	6.05E-04
F vs. M (23)	AT3G03860	**APRL5**	NA		Thioredoxin	0.47	Encodes a protein disulfide isomerase-like (PDIL) protein, a member of a multigene family within the thioredoxin (TRX) superfamily. This protein also belongs to the adenosine 5'-phosphosulfate reductase-like (APRL) group. The mRNA is cell-to-cell mobile.	Use	Thioredoxin	4.37E-03
F vs. L (23)	AT1G15250	**AT1G15250 (RP)**	Ribosome protein (TAIR)		ribosome	0.47	Zinc-binding ribosomal protein family protein;(source:Araport11)	Use	ribosome	2.04E-04
F vs. L (23)	AT1G16740	**AT1G16740 (RP)**	Ribosome protein (TAIR)		ribosome	0.47	Ribosomal protein L20;(source:Araport11)	Use	ribosome	1.17E-04
F vs. S (23)	AT3G53430	**AT3G53430 (RP)**	Ribosome protein (TAIR)		ribosome	0.47	Ribosomal protein L11 family protein;(source:Araport11)	Yes	Ribosome	1.76E-03
F vs. M (23)	AT3G48530	**KING1**	SnRK1 subunit gamma (TAIR)	SnRK1-TOR		0.47	SNF1-related protein kinase regulatory subunit gamma 1;(source:Araport11)	Use	SnRK	2.30E-03
L vs. S (23)	AT5G04080	**No**	At5g04080		Stress	0.47	cysteine-rich TM module stress tolerance protein;(source:Araport11)			1.02E-02
F vs. M (23)	AT3G49810	**PUB30**	PUB30 is an E3 ubiquitin ligase that facilitates BKI1 degradation through the ubiquitin/26S proteasome-mediated protein degradation pathway. BKI1 is an inhibitor of BRI1. Hence, the upregulated PUB30 in *B. microphylla* ovules should have enhanced BR signaling by facilitating BRI1 silencing of BIN2. With BIN2 silenced, the BR growth response TFs BZR1 and BES1 should have produced downstream BR growth responses (doi.org/10.1111/pce.13064), possibly including the observed aposporous gametophyte formation	BR signaling		0.47	Encodes a protein with E3 ubiquitin ligase activity that is involved in negative regulation of salt stress tolerance during germination.	Use	Stress, BR	5.31E-03
M vs. S (23)	AT1G79650	**RAD23B**			Gametophyte	0.47	Encodes a member of the RADIATION SENSITIVE23 (RAD23) family: AT1G16190(RAD23A), AT1G79650(RAD23B), AT3G02540(RAD23C), AT5G38470(RAD23D). RAD23 proteins play an essential role in the cell cycle, morphology, and fertility of plants through their delivery of UPS (ubiquitin/26S proteasome system) substrates to the 26S proteasome.			1.35E-02
F vs. S (23)	AT3G22680	**RDM1**	RDM1 with DMS3 and DRD1 form the DDR complex that is required for Pol V transcription of sRNAs and RdDM silencing of TEs. RDM1 may also participate in reconstituting histone modifications after DNA replication (doi/10.1073/pnas.1816652116)	Gene silencing		0.47	Encodes RNA-DIRECTED DNA METHYLATION 1 (RDM1), forming a complex with DMS3 (AT3G49250) and DRD1 (AT2G16390). This complex is termed DDR. The DDR complex is required for polymerase V transcripts and RNA-directed DNA methylation.	Yes	RDDM	1.68E-03
L vs. S (23)	AT2G40010	**RPP0A (RP)**	60S acidic ribosomal protein P0-1		ribosome	0.47	Ribosomal protein L10 family protein;(source:Araport11)			1.63E-02
M vs. S (23)	AT5G28060	**RPS24B (RP)**			ribosome	0.47	Ribosomal protein S24e family protein;(source:Araport11)			1.42E-02
L vs M (123)	AT5G64140	**RPS28 (RP)**	TAIR		ribosome	0.47	Encodes a putative ribosomal protein S28.	Use	ribosome	2.09E-03
L vs M (123)	AT5G50740		Heavy metal transporter (TAIR)			0.47	Heavy metal transport/detoxification superfamily protein;(source:Araport11)	No	detoxification	5.44E-03

F vs. S (23)	AT2G27040	AGO4	AGO4,6,9 participate in programming one of the sporogenous cells in the ovule (nucellar cells) for meiosis and gametophyte formation and the remaining nucellar cells for death (doi: 10.1534/genetics.116.188151). Downregulation of AGO4 and 6 in *B. x. formosa* may contribute to its apomixis phenotype. AGO4 also participates in spatialtemporally regulated maternal gene imprinting associated RdDM in endosperm (doi/10.1073/pnas.1807621116).	Gene silencing, ovule and gametophyte		0.46	AGO4 is a member of a class of PAZ/PIWI domain containing proteins involved in siRNA mediated gene silencing.Loss of function mutations have reduced site specific CpNpG and CpHpH methylation and increased susceptibility to bacterial pathogens including Tobacco rattle virus.	Yes	siRNA, silencing, AGO	6.60E-05
F vs. S (23)	AT5G42190	ASK2	ASK2 was identified as a meiosis important gene by Walker et al. (doi.org/10.1038/s41588-017-0008-5)	Flowering	Meiosis	0.46	Similar to SKP1 in yeast and humans which are involved in mitotic cell cycle control and ubiquitin mediated proteolysis.	Yes	meiosis	9.98E-05
F vs. M (23)	AT4G33430	BAK1	BAK1 is a cell surface receptor kinase that forms a complex with BRI1 that perceives BL and its precursor CS (doi.org/10.3390/ijms21082737)	BR		0.46	Leu-rich receptor Serine/threonine protein kinase. Component of BR signaling that interacts with BRI1 in vitro and in vivo to form a heterodimer. Brassinolide-dependent association of BRI1 and BAK1 in vivo. Phosphorylation of both BRI1 and BAK1 on Thr residues was BR dependent. Although BAK1 and BRI1 alone localize in the plasma membrane, when BAK1 and BRI1 are coexpressed, the heterodimer BAK1/BRI1 they form is localized in the	Use	BR	5.58E-03
F vs. S (23)	AT4G26630	DEK3	DEK3 binds H3 and H4 and participates in chromatin remodeling (TAIR)	Gene silencing	Histones	0.46	Encodes a chromatin-associated protein that specifically binds histones H3 and H4 and contributes to modulation of Arabidopsis chromatin structure and function.	Yes	Histone	2.55E-04
F vs. S (23)	AT4G15910	DI21	DI21 is upregulated by drought stress and ABA (TAIR)	ABA signaling	ABA upregulated	0.46	encodes a gene whose transcript level in root and leaves increases to progressive drought stress. The transcript level is also affected by changes of endogenous or exogenous abscisic acid level. It appears to be a member of plant-specific gene family that includes late embryo-abundant and zinc- IAA-induced proteins in other plants. The mRNA is cell-to-cell mobile.	Yes	Stress, ABA	9.23E-04
F vs. M (23)	AT5G64610	HAM1	NA		Histones	0.46	Encodes an enzyme with histone acetyltransferase activity. HAM1 primarily acetylate histone H4, but also display some ability to acetylate H3. Prior acetylation of lysine 5 on histone H4 reduces radioactive acetylation by either HAM1. HAM1 acetylates histone H4 lysine 5.	Use	Histone	4.45E-03
F vs. M (23)	AT3G44530	HIRA	A histone chaperone that functions complemenary with ATRX to modify H3.1/H3.3 occupancy thus fine tuning genome expression during meristem and embryo development (doi.org/10.1105/tpc.16.00877)	Gene silencing, ovule and gametophyte	Histones	0.46	Encodes a nuclear localized WD-repeat containing protein involved in negative regulation of knox gene expression via epigenetic mechanism of chromatin re-organization. It is a part of the HISTONE REGULATOR complex that deposits histones in a DNA synthesis-independent manner and affects both nucleosome occupancy and the maintenance of transcriptional silencing. Interacts physically and genetically with AS1. Expressed in meristem and leaf primordia. Homozygous mutants are embryo lethal. Phenotype of cosuppressed lines is variable but show effects on leaf development similar to as1/as2.	Use	Histone, silencing, epigenetic	3.02E-04
F vs. L (23)	ATCG00790	RPL16 (RP)	Ribosome protein (TAIR)		ribosome	0.46	chloroplast gene encoding a ribosomal protein L16, which is a constituent of 50S large ribosomal subunit	Use	ribosome	8.35E-05
F vs. L (23)	ATCG00770	RPS8 (RP)	Ribosome protein (TAIR)		ribosome	0.46	chloroplast 30S ribosomal protein S8	Use	ribosome	2.61E-04
F vs. S (23)	AT2G45640	SAP18	SAP18 is upregulated by salt stress, drought, cold, ABA and ET (TAIR)	ABA signaling	ABA upregulated, Salt	0.46	Involved in the regulation of salt stress. Expression of AtSAP18 is induced by NaCl, cold, drought, ABA, and ethylene treatment. AtSAP18 and HDA19 associate with ERF3 and ERF4 both in vitro and in vivo.	Yes	Stress, ABA, ethylene, ERF	6.77E-05
F vs. S (23)	AT2G44080	ARL	ARL is an ARGOS gene that enhances growth by cell expansion. It is upregulated by stress and by multiple hormones, including BR and ET, and is a negative regulator of ET signaling (https://doi.org/10.1016/j.jplph.2019.153033)	BR		0.45	Encodes ARL, a gene similar to ARGOS involved in cell expansion-dependent organ growth. Upregulated by brassinosteroid. Acts downstream of BRI1. The mRNA is cell-to-cell mobile.	Yes	BR	1.66E-04
F vs. S (23)	AT3G23150	ETR2	ETR1 is an ET sensor that also suppresses ABA signaling (doi/10.1104/pp.114.241695)		ET	0.45	Involved in ethylene perception in ArabidopsisThe mRNA is cell-to-cell mobile.	Yes	ethylene	5.36E-04
F vs. L (23)	AT5G64610	HAM1	NA		Histones	0.45	Encodes an enzyme with histone acetyltransferase activity. HAM1 primarily acetylate histone H4, but also display some ability to acetylate H3. Prior acetylation of lysine 5 on histone H4 reduces radioactive acetylation by either HAM1. HAM1 acetylates histone H4 lysine 5.	Use	Histone	1.96E-04
L vs M (123)	AT5G01390	HSP AT5G01390	Heat shock protein associated with protein folding and refolding (TAIR)	Stress attenuation	HSP	0.45	DNAJ heat shock family protein;(source:Araport11)	Use	Hsp	3.43E-03
F vs. S (23)	AT5G05100	MUG13.4	MUG13.4 is an RNA binding protein that binds with and enhances the slicing activity of AGO2 to enhance salinity tolerance	Gene silencing		0.45	R3H RNA binding protein that interacts with AGO2 and miRNA.	Yes	miRNA, AGO	2.24E-03
F vs. S (23)	AT5G42920	THO5	THO5 has been implicated in siRNA biosynthesis and in facilitating the export from the nucleus of mRNA precursers (TAIR)	Gene silencing		0.45	Encodes a component of the putative Arabidopsis THO/TREX complex: THO1 or HPR1 (At5g09860), THO2 (At1g24706), THO3 or TEX1 (At5g56130), THO5 (At5g42920, At1g45233), THO6 (At2g19430), and THO7 (At5g16790, At3g02950). THO/TREX complexes in animals have been implicated in the transport of mRNA precursers. Mutants of THO3/TEX1, THO1, THO6 accumulate reduced amount of small interfering (si)RNA, suggesting a role of the putative Arabidopsis THO/TREX in siRNA biosynthesis.	Yes	siRNA	5.32E-04
F vs. L (23)	AT3G46210	AT3G46210 (RP)	Ribosome protein (TAIR)		ribosome	0.44	Ribosomal protein S5 domain 2-like superfamily protein;(source:Araport11)	Use	ribosome	3.93E-05
F vs. S (23)	AT4G33430	BAK1	BAK1 is a cell surface receptor kinase that forms a complex with BRI1 that perceives BL and its precursor CS (doi.org/10.3390/ijms21082737)	BR		0.44	Leu-rich receptor Serine/threonine protein kinase. Component of BR signaling that interacts with BRI1 in vitro and in vivo to form a heterodimer. Brassinolide-dependent association of BRI1 and BAK1 in vivo. Phosphorylation of both BRI1 and BAK1 on Thr residues was BR dependent. Although BAK1 and BRI1 alone localize in the plasma membrane, when BAK1 and BRI1 are coexpressed, the heterodimer BAK1/BRI1 they form is localized in the	Yes	BR	1.59E-04

F vs. S (23)	AT3G49670	**BAM2**	Required for meristem formation and possibly required for the completion of meiosis (TAIR)	Flowering	Meiosis	0.44	Encodes a CLAVATA1-related receptor kinase-like protein required for both shoot and flower meristem function. Very similar to BAM1,with more than 85% a.a. identity. It has a broad expression pattern and is involved in vascular strand development in the leaf, control of leaf shape, size and symmetry, male gametophyte development and ovule specification and function. Anthers of double mutants (bam1bam2) appeared abnormal at a very early stage and lack the endothecium, middle, and tapetum layers. Further analyses revealed that cells interior to the epidermis (in anther tissue) acquire some characteristics of pollen mother cells (PMCs), suggesting defects in cell fate specification. The pollen mother-like cells degenerate before the completion of meiosis, suggesting that these cells are defective. In addition, the BAM2 expression pattern supports both an early role in promoting somatic cell fates and a subsequent function in the PMCs. The mRNA is cell-to-cell mobile.	Yes	meiosis, gametophyte	4.63E-04
L vs M (123)	AT3G48750	**CDKA;1**	CDKA;1 phosphorylates ASY1 for synaptonemal complex assemby (DOI 10.15252/embj.2019101625)	Flowering	Meiosis	0.44	A-type cyclin-dependent kinase. Together with its specific inhibitor, the Kip-related protein, KRP2 they regulate the mitosis-to-endocycle transition during leaf development. Dominant negative mutations abolish cell division. Loss of function phenotype has reduced fertility with failure to transmit via pollen. Pollen development is arrested at the second mitotic division. Expression is regulated by environmental and chemical signals. Part of the promoter is responsible for expression in trichomes. Functions as a positive regulator of cell proliferation during development of the male gametophyte, embryo and endosperm. Phosphorylation of threonine 161 is required for activation of its associated kinase.	Use	meiosis, gametophyte	2.96E-03
F vs. S (23)	AT4G32551	**LEUNIG**	LEUNIG is a transcriptional co-regulator of AG that is expressed preferentially in carpel tissues (doi:10.1093/molbev/msw229). Hence, its upregulation in *B. stricta* pistils compared to *B. x. formosa* ovules is uninformative with regard to reproductive mode regulation.		Flowering	0.44	LEUNIG regulates floral organ identity,gynoecium and ovule development. Negatively regulates AGAMOUS . Encodes a glutamine-rich protein with seven WD repeats similar to transcriptional corepressors.	Yes	AGL	7.82E-04
F vs. S (23)	AT1G51940	**LYK3**	LYK3 is a receptor-like kinase that suppresses defense responses in the absence of infection and is upregulated by ABA (TAIR)		ABA upregulated	0.44	Encodes a LysM-containing receptor-like kinase. Induction of chitin-responsive genes by chitin treatment is not blocked in the mutant. Based on protein sequence alignment analysis, it has a typical RD signaling domain in its catalytic loop and possesses autophosphorylation activity.It is required for the suppression of defense responses in absence of pathogen infection or upon abscisic acid treatment. Loss-of-function mutants display enhanced resistance to Botrytis cinerea and Pectobacterium carotovorum. Its expression is repressed by pathogen infection and biological elicitors and is induced abscisic acid.Expression is strongly repressed by elicitors and fungal infection, and is induced by the hormone abscisic acid (ABA). Insertional mutants show increased expression of PHYTOALEXIN-DEFICIENT 3 (PAD3), enhanced resistance to Botrytis cinerea and Pectobacterium carotovorum infection and reduced physiological responses to ABA, suggesting that LYK3 is important for the cross-talk between signaling pathways activated by ABA and pathogens (PMID:24639336).	Yes	ABA	5.18E-05
L vs. S (23)	AT3G02080	**RPS19A (RP)**	40S ribosomal protein S19-1		ribosome	0.44	Ribosomal protein S19e family protein;(source:Araport11)			1.68E-02
L vs. S (23)	AT3G45030	**RPS20C (RP)**	40S ribosomal protein S20-1		ribosome	0.44	Ribosomal protein S10p/S20e family protein;(source:Araport11)			1.67E-02
F vs. S (23)	AT1G19330	**AFR2**	AFR2 is an evening expressed key component of the Sin3-HDAC complex that suppresses circadian clock associated gene expression by binding to their promotors and catalyzing histone 3 deacetylation (TAIR)		Histones	0.43	Evening-expressed key component of Sin3-HDAC complex, which bind directly to the CIRCADIAN CLOCK ASSOCIATED 1 (CCA1) and PSEUDO-RESPONSE REGULATOR 9 (PRR9) promoters and catalyze histone 3 (H3) deacetylation at the cognate regions to repress expression, allowing the declining phase of their expression at dusk.	Yes	Histone, SPO, HDAC	2.10E-04
F vs. M (23)	AT5G65020	**ANNAT2**	Required for post phloem sugar transport in sink cells (10.1104/pp.18.00713)		Ca	0.43	Annexins are calcium binding proteins that are localized in the cytoplasm. When cytosolic Ca2+ increases, they relocate to the plasma membrane. They may be involved in the Golgi-mediated secretion of polysaccharides.	Use	Ca	1.03E-03
L vs. S (23)	AT1G80700	**AT1G80700**			#N/A	0.43	stress response NST1-like protein;(source:Araport11)			1.02E-02
F vs. S (23)	AT5G19450	**CPK8**	Functions with CAT3 to establish ROS homeostasis in response to ABA signaling (10.1105/tpc.15.00144)	ROS attenuation	ABA upregulated, Ca, homeostasis	0.43	calcium-dependent protein kinase (CDPK19) mRNA, complete	Yes	Ca	5.59E-04
M vs. S (23)	AT4G26630	**DEK3**	DEK3 binds H3 and H4 and participates in chromatin remodeling (TAIR)		Histone	0.43	Encodes a chromatin-associated protein that specifically binds histones H3 and H4 and contributes to modulation of Arabidopsis chromatin structure and function.			1.42E-02
F vs. L (23)	AT3G50660	**DWF4**	DWF4 is the rate limiting enzyme in BR synthesis. DWF4 is a negative feedback repression target of BES1/BZR1 (doi.org/10.1038%2Fs41477-019-0436-6)	BR		0.43	Encodes a 22α hydroxylase whose reaction is a rate-limiting step in brassinosteroid biosynthetic pathway. The protein is a member of CYP90B gene family. CLM is an epi-allele with small, compressed rosette, reduced internode length, and reduced fertility, appears in selfed ddm mutant plants possibly due to loss of cytosine methylation. Transcripts accumulate in actively growing tissues, and GUS expression is negatively regulated by brassinosteroids. Localized in the endoplasmic reticulum. The in vitro expressed protein can perform the C-22 hydroxylation of a variety of C27-, C28- and C29-sterols. Cholesterol was the best substrate, followed by campesterol.	Use	BR	5.69E-05
F vs. L (23)	AT5G51230	**EMF2**	EMBRYONIC FLOWER 2 (EMF2) is part of a polycomb group complex involved in negative regulation of reproductive development (TAIR)	Flowering		0.43	Polycomb group protein with zinc finger domain involved in negative regulation of reproductive development. Forms a complex with FIE, CLF, and MSI1. This complex modulates the expression of target genes including AG, PI and AP3.	Use	MSI1	1.43E-03
M vs. S (23)	AT5G37380	**MNJ8.20**			Chaperone	0.43	Chaperone DnaJ-domain superfamily protein;(source:Araport11)			1.75E-02
F vs. L (23)	AT1G69620	**RPL34 (RP)**	Ribosome protein (TAIR)		ribosome	0.43	putative 60S ribosomal protein L34 The mRNA is cell-to-cell mobile.	Use	ribosome	2.11E-04
F vs. L (23)	AT5G65950	**TRAPPC11**	TRAPPC11 is involved in regulating endosomal function and salt stress responses (10.1105/tpc.20.00044)	Stress signaling	Salt	0.43	TRAPPIII complex protein which regulates TGN integrity, by altered TGN/EE association of several residents, including SYNTAXIN OF PLANTS 61 (SYP61), and altered vesicle morphology. Involved in regulation of endosomal function and salt stress response.	Use	Stress	4.63E-04

F vs. M (23)	AT1G45145	**TRX5**	Upregulated by oxidative stress (TAIR)		Thioredoxin	0.43	encodes a cytosolic thioredoxin that reduces disulfide bridges of target proteins by the reversible formation of a disulfide bridge between two neighboring Cys residues present in the active site. Thioredoxins have been found to regulate a variety of biological reactions in prokaryotic and eukaryotic cells.	Use	Thioredoxin	5.97E-03
L vs M (123)	AT1G45145	**TRX5**	Upregulated by oxidative stress (TAIR)	ROS attenuation	Thioredoxin	0.43	encodes a cytosolic thioredoxin that reduces disulfide bridges of target proteins by the reversible formation of a disulfide bridge between two neighboring Cys residues present in the active site. Thioredoxins have been found to regulate a variety of biological reactions in prokaryotic and eukaryotic cells.	Use	Thioredoxin	2.27E-03
F vs. S (23)	AT5G43810	**AGO10**	AGO10 functions with AGO1 to establish and localize stem cells within meristems by regulating miR165/6 function and stability. AGO1 and AGO10 suppress and enhance miR165/6 degradation, respectively (doi:10.1371/journal)		AGO	0.42	Encodes Argonaute10, a member of the EIF2C (elongation initiation factor 2c)/ Argonaute class of proteins. Required to establish the central-peripheral organization of the embryo apex. Along with WUS and CLV genes, controls the relative organization of central zone and peripheral zone cells in meristems. Acts in embryonic provascular tissue potentiating WUSCHEL function during meristem development in the embryo. AGO10 specifically sequesters miR166/165 to regulate shoot apical meristem development.	Yes	AGO	6.94E-04
F vs. S (23)	AT4G21270	**ATK1**	TAIR	Flowering	Meiosis	0.42	Encodes a kinesin-like motor protein heavy chain. Loss of function mutations have reduced fertility and are defective in spindle formation in male meiosis.	Yes	meiosis	8.01E-04
F vs. S (23)	AT5G27930	**EGR2**	EGR2 is a myristoylation activated phosphatase that inactivates SnRK2.6 (OST1) in warm weather. In cold weather, myristoylation of EGR2 fails to occur, which allows SnRK2.6 to become activated leading to the expression of stress response genes, including freezing tolerance genes (DOI 10.15252/embj.201899819)	ABA signaling		0.42	EGR2 functions as a negative regulator of plant growth with prominent effect on plant growth during drought stress. EGR2 regulates microtubule organization and likely affects additional cytoskeleton and trafficking processes along the plasma membrane.	Yes	Stress	8.81E-04
L vs. S (23)	AT4G39640	**GGT1**			glutathione, Str	0.42	The gene encodes a gamma-glutamyltransferase (AKA gamma-glutamyl transpeptidase, EC 2.3.2.2) that is located in vascular tissues (predominantly phloem) of leaves and is involved in the degradation of glutathione. The encoded enzyme also mitigates oxidative stress by metabolizing GSSG (oxidized form of GSH - glutathione) in the apoplast.			1.29E-02
F vs. L (23)	AT3G20810	**JMJD5**	JMJD5 is a jumonji-C containing histone demethylase involved in regulating circadian rhythm (TAIR)	Gene silencing	Histones	0.42	JMJD5 encodes a protein which contains a jumonji-C (jmjC) domain. jmjd5 mutant plants have a short-period circadian phenotype. JMJD5 has histone demethylase activity and interacts with EFM to repress FT.	Use	Histone	1.78E-03
L vs. S (23)	AT2G32220	**RPL27A (RP)**	60S ribosomal protein L27-1		ribosome	0.42	Ribosomal L27e protein family;(source:Araport11)			1.05E-02
F vs. S (23)	ATCG00820	**RPS19 (RP)**	TAIR		ribosome	0.42	Encodes a 6.8-kDa protein of the small ribosomal subunit.	Yes	Ribosome	4.93E-04
M vs. S (23)	AT1G24260	**SEP3**			flowering	0.42	Member of the MADs box transcription factor family. SEP3 is redundant with SEP1 and 2. Flowers of SEP1/2/3 triple mutants show a conversion of petals and stamens to sepals.SEP3 forms heterotetrameric complexes with other MADS box family members and binds to the CArG box motif.			1.29E-02
F vs. L (23)	AT2G13540	**ABH1**	ABH1 is an ABA induced cap-binding protein involved in proper splicing of ABA signaling and flowering related mRNAs (TAIR)		ABA upregulated	0.41	Encodes a nuclear cap-binding protein that forms a heterodimeric complex with CBP20 and is involved in ABA signaling and flowering. Mutants are early flowering and exhibit hypersensitive response to ABA in germination inhibition.Loss of ABH1 function results in abnormal processing of mRNAs for several important floral regulators (FLC, CO, FLM). Analysis of loss of function mutations suggests a role in pri-miRNA processing and mRNA splicing. Note that two different mutant alleles were given the same name abh1-7 (Kuhn et al 2007; Kim et al 2008). To avoid confusion, abh1-7 described in Kim et al (2008) has been renamed abh1-107 (other names: ensalada-1, ens-1).	Use	ABA, miRNA	1.64E-04
F vs. S (23)	AT5G24330	**ATXR6**	ATXR6 is a monomethyltransferase that binds to the proliferating cell nuclear antigen PCNA. It is indirectly involved in the reestablishment of histone methylation following DNA replication (doi.org/10.1016/j.jmb.2019.02.020)	Gene silencing		0.41	Encodes a SET-domain protein, a H3K27 monomethyltransferases required for chromatin structure and gene silencing. Regulates heterochromatic DNA replication. Contains a PCNA-binding domain. ATXR6 accumulates preferentially during the late G1 or S phase, suggesting that it plays a role in cell-cycle regulation or progression.	Yes	silencing	1.01E-03
F vs. S (23)	AT1G14980	**CPN10 (HSP)**		Stress attenuation	HSP	0.41	Encodes mitochondrial-localized chaperonin 10 that complements the E.coli groES mutant. Its mRNA is upregulated in response to heat shock treatment and is expressed uniformly in various organs.	Yes	Hsp	6.33E-04
M vs. S (23)	AT3G23490	**CYN**			NaCl	0.41	Encodes a cyanase that catalyzes the bicarbonate-dependent breakdown of cyanate to ammonia and bicarbonate. CYN forms a hexadecamer and is believed to be a cytosolic protein. Long-term exposure to NaCl increases CYN transcript levels. It is also expressed at higher levels in flowers relative to stems, roots, and seedlings.			2.81E-03
M vs. S (23)	AT5G41560	**DDA1**	DDA1 negatively regulates ABA signaling by targeting ABA receptors (PYL8) for proteasomal degradation (TAIR)		ABA	0.41	Encodes a substrate receptor for CRL4-CDD complexes that provides substrate specificity for CRL4 by interacting with ubiquitination targets. By its interaction and regulation of levels of PYL8 through proteasomal degradation, it negatively regulates ABA-mediated developmental responses, including inhibition of seed germination, seedling establishment, and root growth			2.04E-02
F vs. S (23)	AT2G01450	**MPK17**	MPK17 is a MAP kinase regulates and participates with PEROXISOME AND MITOCHONDRIAL DIVISION FACTOR1 (PMD1) in proliferating peroxisomes in response to salt stress (doi.org/10.1080/15592324.2018.1428518)	Stress signaling	Salt	0.41	MPK17 Map kinase family member. Mutants have increased numbers of peroxisomes a phenotype that can be suppressed by mutations in PMD1. This and other treatments, suggests a function in control of peroxisome proliferation in salt stress.	Yes	Stress	8.35E-05
F vs. S (23)	AT4G05590	**NRGA1**	Mitochondrial pyruvate carrier that mediates ABA signaling, drought stress and Cd toxicity by sustaining the TCA cycle and glutathione synthesis (doi.org/10.1104%2Fpp.18.01610)	ABA signaling	ABA upregulated	0.41	Encodes NRGA1, a putative mitochondrial pyruvate carrier that mediates ABA regulation of guard cell ion channels and drought stress responses.	Yes	Stress, ABA	1.16E-03
F vs. S (23)	AT1G43850	**SEUSS**	SEUSS is a transcriptional co-regulator of AG that is expressed preferentially in carpel tissues (doi:10.1093/molbev/msw229). Hence, its upregulation in *B. stricta* pistils compared to *B. x. formosa* ovules is uninformative with regard to reprodutive mode regulation.		Flowering	0.41	Encodes a transcriptional co-regulator of AGAMOUS, that functions with LEUNIG to repress AG in the outer floral whorls.	Yes	AGL	1.81E-03

F vs. L (23)	AT1G14510	**AL7**	AL7 is one of two Alfin1-like nuclear-localized H3K4me3 binding PHD (plant homeodomain) containing proteins recently identified as contributing to the functional structure of polycomb repressive complex 1 (PCR1s). AL7 and AL2 target PRC1s to transcriptionally active chromatin where the expressed H3K4me3 state is switched to H2Aub1 repressive marks. PRC2 is then recruited to these sites to stabilize gene silencing through H3K27me3 (doi.org/10.1016/j.jmb.2018.08.021)	Gene silencing	Histones	0.40	Encodes a member of the Alfin1-like family of nuclear-localized PHD (plant homeodomain) containing proteins. All AL proteins except AL3 bind to di- or trimethylated histone H3 (H3K4me3/2). Members of this family include: AT5G05610 (AL1), AT3G11200 (AL2), AT3G42790 (AL3), AT5G26210 (AL4), AT5G20510 (AL5), AT2G02470 (AL6), AT1G14510 (AL7).	Use	Histone, H3K4	3.93E-05
L vs M (123)	AT1G67370	**ASY1**	required for synaptonemal complex assemby; phosphorylation activated by CDKA;1 (DOI 10.15252/embj.2019101625)	Flowering	Meiosis	0.40	meiotic asynaptic mutant 1 (ASY1). ASY1 protein is initially distributed as numerous foci throughout the chromatin. During early G2, the foci are juxtaposed to the nascent chromosome axes to form a continuous axis associated signal.	Use	meiosis	2.33E-03
F vs. S (23)	AT1G07070	**AT1G07070 (RP)**	Ribosome protein (TAIR)		ribosome	0.4	Ribosomal protein L35Ae family protein;(source:Araport11)	Yes	Ribosome	3.58E-04
L vs. S (23)	AT5G24890	**AT5G24890**	At5g24890		Stress	0.4	stress response NST1-like protein;(source:Araport11)			1.74E-02
F vs. S (23)	AT5G02820	**BIN5**	BIN5 encodes the DNA topoisomerase VI SPO11-3, which is involved in endoreduplication, meiotic double strand break processing and reciprical meitoic recombination (TAIR)	Flowering	Meiosis	0.4	Involved in the patterning and shape of leaf trichomes. Encodes the DNA topoisomerase VI SPO11-3, involved in endoreduplication	Yes	Meiosis	1.06E-03
F vs. L (23)	AT5G38970	**BR6OX**	BR6OX is upregulated by light and is necessary for BR biosynthesis (TAIR)	BR		0.40	Encodes a polypeptide involved in the C-6 oxidation of brassinosteroids. Heterologous expression of the protein in yeast conferred the ability to catalyze multiple reactions in which the C-6 position of 6-deoxocastasterone, 6-deoxytyphasterol, 3-dehydro-6-deoxoteasterone and 6-deoxoteasterone are oxidized.	Use	BR	1.67E-03
F vs. L (23)	AT2G36530	**ENO2 (MBP-1 like)**	Increases tolerance to salt and drought stress (doi.org/10.1111/ppl.13013; 10.1007/s11033-018-4292-7	Stress attenuation	Salt	0.40	Involved in light-dependent cold tolerance and encodes an enolase. Protein is tyrosine-phosphorylated and its phosphorylation state is modulated in response to ABA in Arabidopsis thaliana seeds.	Use	ABA	1.41E-03
F vs. L (23)	AT5G10400	**H3.1 AT5G10400**	NA		Histones	0.40	Histone superfamily protein;(source:Araport11)	Use	Histone	2.45E-04
F vs. M (23)	AT2G31840	**MRL7L**	NA		Thioredoxin	0.40	Thioredoxin superfamily protein;(source:Araport11)	Use	Thioredoxin	6.30E-04
L vs M (123)	AT2G18660	**PNP-A**	Induced by stress, functions with PNP-R2 to suppress SA signaling and cell death (10.1105/tpc.20.00018)	Stress attenuation		0.40	Encodes PNP-A (Plant Natriuretic Peptide A). PNPs are a class of systemically mobile molecules distantly related to expansins; their biological role has remained elusive. PNP-A contains a signal peptide domain and is secreted into the extracellular space. Co-expression analyses using microarray data suggest that PNP-A may function as a component of plant defence response and SAR in particular, and could be classified as a newly identified PR protein. It is stress responsive and can enhance its own expression.	Use	Stress	5.02E-04
L vs. S (23)	AT3G09500	**RPL35A (RP)**	60S ribosomal protein L35-1		ribosome	0.4	Ribosomal L29 family protein;(source:Araport11)			1.19E-02
M vs. S (23)	AT3G09500	**RPL35A (RP)**			ribosome	0.4	Ribosomal L29 family protein;(source:Araport11)			1.37E-02
L vs M (123)	AT4G20360	**SVR11**	Nucleus transcripbed chloropast translation elongation factor involved in leaf variegation (10.3389/fpls.2019.00295)	SnRK1 TOR		0.40	Nuclear transcribed, plastid localized EF-Tu translation elongation factor. Referred to as AtRabE1b in DOI:10.1104/pp.013052. However, wider community usage and more publications assign the symbol RabE1b to At5g59840.	Use	translation	3.78E-05
F vs. L (23)	AT4G28190	**ULT1**	ULT1 is a trithorax group (trxG) factor that regulates over 700 genes, most of which are downregulated, including genes associated with reproductive development in floral apices (doi.org/10.1534/g3.119.400559)	Gene silencing, ovule gametophyte		0.40	Encodes a novel Cys-rich protein with a B-box like domain that acts as a negative regulator of meristem cell accumulation in inflorescence and floral meristems as loss-of-function ult1 mutations cause inflorescence meristem enlargement, the production of extra flowers and floral organs, and a decrease in floral meristem determinacy. Acts opposite to CLF which represses AG, but preventing deposition of CLF repressive methylation marks.ULT1 acts as an anti-repressor that counteracts EMF1 action through modulation of histone marks on target genes. Regulates developmental as well as biotic and abiotic stress response genes.	Use	Stress, Histone	3.40E-04
F vs. S (23)	AT2G15970	**WCOR413**	WCOR413 is upregulated by cold, dehydration and ABA (TAIR)		ABA upregulated	0.4	encodes an alpha form of a protein similar to the cold acclimation protein WCOR413 in wheat. Expression is induced by short-term cold-treatment, water deprivation, and abscisic acid treatment. The mRNA is cell-to-cell mobile.	Yes	ABA	4.17E-05
F vs. S (23)	AT2G27880	**AGO5**	AGO5 delays flowering by interacting with miRNA156 to suppress transcription of the SQUAMOSA-PROMOTOR BINDING LIKE (SPL) TFs in shoot apical meristems (doi:10.1093/pcp/pcaa022)	Gene silencing	AGO	0.39	AGO5.Required for antiviral RNA silencing.Confers resistance to Potato virus X.	Yes	silencing, AGO	7.18E-05
F vs. S (23)	AT2G34480	**L18AB**	L18AB is a ribosomal protein involved in sexual reproduction. It is required for normal male gametophyte formation and early embryo development (DOI: 10.1038/srep31195)		Ovule gametophyte	0.39	Encodes a nuclear localized member of the ribosomal L18ae/LX protein family. Loss of function mutations show reduced transmission through the gametophytes and embryo lethality.	Yes	gametophyte	3.59E-04
F vs. L (23)	AT1G10540	**NAT8**	Ascorbate transporter (TAIR)	ROS attenuation		0.39	nucleobase-ascorbate transporter 8;(source:Araport11)	Use	Ascorbate	2.88E-04
F vs. S (23)	AT4G33950	**SnRK2.6 (OST1)**	SnRK2.6 is a major SnRK2 that remains phosphatase inactivated in the absence of stress due to the action of PP2Cs but are activated when ABA binds with ABA receptors and deactivates PP2Cs. Recent evidence indicates that SnRK2s can also be activated by osmostress responsive Raf-like kinases (ARKs) (doi: 10.1111/tpj.14756)	ABA signaling, Stress signaling	ABA upregulated	0.39	Encodes calcium-independent ABA-activated protein kinase, a member of SNF1-related protein kinases (SnRK2) whose activity is activated by ionic (salt) and non-ionic (mannitol) osmotic stress. Mutations disrupted ABA induction of stomatal closure as well as ABA inhibition of light-induced stomatal opening. However, regulation of stomatal opening/closing by light or CO(2) is not affected in these mutants. May act in the interval between ABA perception and reactive oxygen species production in the ABA signalling network.	Yes	SnRK, Stress, Ca, ABA	3.34E-04
F vs. L (23)	AT5G61960	**AML1**	AML1, a mei2-like gene, was identified as a meiosis important gene by Walker et al. (doi.org/10.1038/s41588-017-0008-5)	Flowering, Stress attenuation	Meiosis	0.38	A member of mei2-like gene family, predominantly plant-based family of genes encoding RNA binding proteins with characteristic presence of a highly conserved RNA binding motif first described in the mei2 gene of the fission yeast S. pombe. In silico analyses reveal nine mei2 -like genes in A. thaliana. They were grouped into four distinct clades, based on overall sequence similarity and subfamily-specific sequence elements. AML1 is a member of two sister clades of mei2-like gene family, AML1 through AML5 and belongs to the clade named ALM14. AML1 is expressed during early embryo development, particularly along embryonic axis at torpedo stage, in shoot apex (weaker expression) and in the organogenic regions of floral apices.	Use	meiosis	1.05E-05
F vs. S (23)	AT1G18540	**AT1G18540 (RP)**	Ribosome protein (TAIR)		ribosome	0.38	Ribosomal protein L6 family protein;(source:Araport11)	Yes	Ribosome	7.40E-05
F vs. S (23)	AT1G66590	**COX19-1**	TAIR		homeostasis	0.38	homeostasis.	Yes	Homeostasis	1.27E-03

M vs. S (23)	AT5G19940	**FBN6**	Protein involved in maintaining expression levels of genes involved in removing superoxide radicals, maintaining ROS homeostasis, and regulating glutathione levels in chloroplasts (doi: 10.1111/nph.16246)		Stress	0.38	Enables plants to cope with moderate light stress and affects cadmium tolerance.			1.94E-02
L vs. S (23)	AT3G19980	**FyPP3**	FyPP3 dephosphorylates the TF ABI5, thus negatively regulating ABA signaling. FyPP3 degradation is facilitated by NRP, but FyPP3 can also deactivate NRP by dephosphorylating it (doi.org/10.1016/j.molp.2017.11.006). Photomorphogenesis is repressed in the dark by kinase activated PHYTOCHROME INTERACTING FACTORS (PIFs). By dephosphorylating PIFs, PP6 (FyPP3, catalytic subunit) regulates photomorphogenesis (doi.org/10.1073/pnas.1907540116)		SnRK	0.38	Encodes catalytic subunit of serine/threonine protein phosphatase 2A. It can associate with phytochromes A and B in vitro. Mutant plants display an accelerated flowering phenotype.Acts antagonistically to SnRK2 to regulate ABI5 phosphorylation. It inteacts with NRP which results in tethering to endosomes leading to its degradation.			1.74E-02
L vs. S (23)	AT4G39730	**No**			Stress	0.38	PLAT1 domain stress protein family member. Involved in mediating response to stresses such as pathogen infection. It is found in endoplasmic reticulum bodies. PLAT1 is induced by pathogenic fungi and induces the production of			1.69E-02
F vs. L (23)	AT3G49810	**PUB30**	PUB30 is an E3 ubiquitin ligase that facilitates BKI1 degradation through the ubiquitin/26S proteasome-mediated protein degradation pathway. BKI1 is an inhibitor of BRI1. Hence, the upregulated PUB30 in B. microphylla ovules should have enhanced BR signaling by facilitating BRI1 silencing of BIN2. With BIN2 silenced, the BR growth response TFs BZR1 and BES1 should have produced downstream BR growth responses (doi.org/10.1111/pce.13064), possibly including the observed aposporous gametophyte formation	BR signaling		0.38	Encodes a protein with E3 ubiquitin ligase activity that is involved in negative regulation of salt stress tolerance during germination.	Use	Stress	3.89E-05
F vs. S (23)	AT5G48310	**RASD1**	RASD1 is upregulated by ABA, salt and drought stress (TAIR)	ABA signaling	ABA upregulated, Salt	0.38	Protein of unknown function that may be involved in stress response. Strongly expressed in vascular tissues.Mutants are ABA- insensitive.	Yes	Stress, ABA	3.44E-04
M vs. S (23)	AT3G06040	**RP AT3G06040**			ribosome	0.38	Ribosomal protein L12/ ATP-dependent Clp protease adaptor protein ClpS family protein;(source:Araport11)			1.97E-02
F vs. M (23)	AT5G39850	**RPS9C (RP)**			ribosome	0.38	Ribosomal protein S4;(source:Araport11)	Use	ribosome	1.59E-04
M vs. S (23)	AT1G80700	**AT1G80700**			Stress	0.37	stress response NST1-like protein;(source:Araport11)			1.75E-02
F vs. L (23)	AT4G12600	**AT4G12600 (RP)**	Ribosome protein (TAIR)		ribosome	0.37	Ribosomal protein L7Ae/L30e/S12e/Gadd45 family protein;(source:Araport11)	Use	ribosome	1.32E-03
F vs. M (23)	AT5G40820	**ATR**	ATR responds to DNA damage and was identified as a meiosis important gene by Walker et al. (doi.org/10.1038/s41588-017-0008-5)	Flowering, Stress attenuation	Meiosis	0.37	Encodes a Arabidopsis ortholog of the ATR protein kinase that is involved in a wide range of responses to DNA damage and plays a central role in cell-cycle regulation. Dominant loss of function alleles identified as suppressors of ALS also exhibit increased tolerance to aluminum. This may be due to the inhibition of terminal differentiation of the root apex upon exposure to Al.	Use	meiosis	1.13E-03
L vs M (123)	AT3G49670	**BAM2**	Required for meristem formation and possibly required for the completion of meiosis (TAIR)	Flowering	Meiosis	0.37	Encodes a CLAVATA1-related receptor kinase-like protein required for both shoot and flower meristem function. Very similar to BAM1,with more than 85% a.a. identity. It has a broad expression pattern and is involved in vascular strand development in the leaf, control of leaf shape, size and symmetry, male gametophyte development and ovule specification and function. Anthers of double mutants (bam1bam2) appeared abnormal at a very early stage and lack the endothecium, middle, and tapetum layers. Further analyses revealed that cells interior to the epidermis (in anther tissue) acquire some characteristics of pollen mother cells (PMCs), suggesting defects in cell fate specification. The pollen mother-like cells degenerate before the completion of meiosis, suggesting that these cells are defective. In addition, the BAM2 expression pattern supports both an early role in promoting somatic cell fates and a subsequent function in the PMCs. The mRNA is cell-to-cell mobile.	Use	meiosis, gametophyte	5.71E-04
F vs. S (23)	AT4G31120	**CAU1**	CAU1 is a methylase that suppresses transcription by H4R3sme2-type histone methylation at promotor regions including the *P5CS1* promotor. *P5CS1/2* encode redundant enzymes important to proline biosynthesis. Proline enhances drought resistance (doi:10.1093/jxb/erx419). The observed 3-fold increase in *CAU1* mRNA in *B. stricta* compared to *B. x. formosa* is consistent with the observed 10-fold increase in *P5CS2* mRNA in *B. x formosa*.	Gene silencing	Histones	0.37	Involved in vernalization. Required for epigenetic silencing of FLC, and for vernalization-mediated histone modification.	Yes	Histone, silencing, epigenetic	3.59E-04
F vs. S (23)	AT3G25100	**CDC45**	(TAIR)	Flowering	Meiosis	0.37	Required for normal meiosis, may act in the last round of DNA replication prior to meiosis, sequence similar to yeast CDC45	Yes	meiosis	2.59E-04
F vs. S (23)	AT4G34160	**CYCD3**	CYCD3 is a D-type cyclin that reinitiates mitosis when upregulated (doi: 10.1111/nph.16309). It is upregulated by cytokinin and BR	BR		0.37	encodes a cyclin D-type protein involved in the switch from cell proliferation to the final stages of differentiation. The gene is transcriptionally regulated by cytokinin and brassinosteroid. Protein interacts with cyclin-dependent kinase inhibitor ICK1.	Yes	BR	7.40E-05
F vs. M (23)	AT5G51230	**EMF2**	EMBRYONIC FLOWER 2 (EMF2) is part of a polycomb group complex involved in negative regulation of reproductive development (TAIR)	Flowering		0.37	Polycomb group protein with zinc finger domain involved in negative regulation of reproductive development. Forms a complex with FIE, CLF, and MSI1. This complex modulates the expression of target genes including AG, PI and AP3.	Use	EMF2	3.09E-03
L vs M (123)	AT1G69700	**HVA22C**	ABA and stress inducible (TAIR)	ABA signaling	ABA upregulated	0.37	Part of the AtHVA22 family. Protein expression is ABA- and stress-inducible. The mRNA is cell-to-cell mobile.	Use	Stress, ABA	6.16E-03
L vs. S (23)	AT3G57080	**NRPE5A**	DNA-directed RNA polymerase V subunit 5A		Pol V	0.37	Non-catalytic subunit unique to Nuclear DNA-dependent RNA polymerase V; homologous to budding yeast RPB5.			6.43E-03
F vs. L (23)	AT1G21750	**PDI5**	PDI5 is a disulfide isomerase-like thioredoxin that is upregulated by ER stress. It participates in cell redox homeostasis maintenance, protein folding and regulating programmed cell death (TAIR)	ROS attenuation	Homeostasis, thioredoxin	0.37	Encodes a protein disulfide isomerase-like (PDIL) protein, a member of a multigene family within the thioredoxin (TRX) superfamily; isoform contains non-consensus GA donor splice site at intron 9. Transcript levels for this gene are up-regulated in response to three different chemical inducers of ER stress (dithiothreitol, beta-mercaptoethanol, and tunicamycin). Neither AtIRE1-2 nor AtbZIP60 appear to be required for this response. The mRNA is cell-to-cell mobile.	Use	Stress, Thioredoxin	2.74E-04
F vs. M (23)	AT3G16780	**RPL19B (RP)**			ribosome	0.37	Ribosomal protein L19e family protein;(source:Araport11)	Use	ribosome	9.38E-04
F vs. S (23)	AT5G39850	**RPS9C (RP)**	TAIR		ribosome	0.37	Ribosomal protein S4;(source:Araport11)	Yes	Ribosome	2.94E-05
F vs. S (23)	AT5G50790	**SWEET10**	SWEET10 is a sucrose efflux transporter that is upregulated in source leaves by FT (doi.org/10.1186/s12870-020-2266-0)	Flowering		0.37	Encodes a member of the SWEET sucrose efflux transporter family proteins. Transcriptionally activated by long photoperiods; activation depends on FT and SOC1. The ectopic expression of SWEET10 causes early flowering and leads to higher levels of transcription of flowering-time related genes in the shoot	Yes	SWEET transporter, flowering	5.23E-05

F vs. S (23)	AT5G61960	**AML1**	AML1, a mei2-like gene, was identified as a meiosis important gene by Walker et al. (doi.org/10.1038/s41588-017-0008-5)	Flowering, Stress attenuation	Meiosis	0.36	A member of mei2-like gene family, predominantly plant-based family of genes encoding RNA binding proteins with characteristic presence of a highly conserved RNA binding motif first described in the mei2 gene of the fission yeast S. pombe. In silico analyses reveal nine mei2 -like genes in A. thaliana. They were grouped into four distinct clades, based on overall sequence similarity and subfamily-specific sequence elements. AML1 is a member of two sister clades of mei2-like gene family, AML1 through AML5 and belongs to the clade named ALM14. AML1 is expressed during early embryo development, particularly along embryonic axis at torpedo stage, in shoot apex (weaker expression) and in the organogenic regions of floral apices.	Yes	meiosis	1.02E-04
L vs. S (23)	AT5G37020	**ARF8**	Auxin response factor 8		ARF	0.36	Encodes a member of the auxin response factor family. Mediates auxin response via expression of auxin regulated genes. Acts redundantly with ARF6 to control stamen elongation and flower maturation. Expression of ARF8 is controlled by miR167.			1.07E-02
M vs. S (23)	AT5G37020	**ARF8**			ARF	0.36	Encodes a member of the auxin response factor family. Mediates auxin response via expression of auxin regulated genes. Acts redundantly with ARF6 to control stamen elongation and flower maturation. Expression of ARF8 is controlled by miR167.			1.94E-02
F vs. L (23)	AT2G36160	**AT2G36160 (RP)**	Ribosome protein (TAIR)		ribosome	0.36	Ribosomal protein S11 family protein;(source:Araport11)	Use	ribosome	5.93E-04
F vs. S (23)	AT1G50240	**FU**	TAIR	Flowering	Meiosis	0.36	The FUSED (FU) gene belongs to Ser/Thr protein kinase family and has a key role in the hedgehog signaling pathway known to control cell proliferation and patterning in fruit flies and humans . Arabidopsis thaliana genome has a single Fu gene that is involved in male meiosis cytokinesis. Cytokinesis-defective mutants, named two-in-one (tio), result from mutations in Arabidopsis Fu. Phenotypic analysis of tio mutants reveals an essential role for TIO in conventional modes of cytokinesis in plant meristems and during male gametogenesis. TIO is tightly localized to the midline of the nascent phragmoplast and remains associated with the expanding phragmoplast ring. This gene was previously annotated as two gene models, AT1G50230.1 and AT1G50240.1, however the experimental evidence exists (Oh et al, Current Biology, 2005) showing that these two models are in fact single gene, named FUSED.	Yes	meiosis	1.84E-04
M vs. S (23)	AT4G39640	**GGT1**	Upregulated by ER and oxidative stress, degrades glutathione in the apoplast but also mitigates oxidative stress my metabolizing oxidized GSH (GSSH) (doi.org/10.1071/FP17151)		glutathione, Stress	0.36	The gene encodes a gamma-glutamyltransferase (AKA gamma-glutamyl transpeptidase, EC 2.3.2.2) that is located in vascular tissues (predominantly phloem) of leaves and is involved in the degradation of glutathione. The encoded enzyme also mitigates oxidative stress by metabolizing GSSG (oxidized form of GSH - glutathione) in the apoplast.			1.09E-02
F vs. L (23)	AT2G29500	**HSP17.6B**	TAIR	Stress attenuation	HSP	0.36	HSP20-like chaperones superfamily protein;(source:Araport11)	Use	HSP	2.30E-04
L vs. S (23)	AT3G02190	**L39-2 (RP)**			ribosome	0.36	Ribosomal protein L39 family protein;(source:Araport11)			1.37E-02
L vs. S (23)	AT2G36620	**RPL24A (RP)**	60S ribosomal protein L24-1		ribosome	0.36	RPL24A encodes ribosomal protein L24, homolog of cytosolic RPL24, found in archaea and higher eukaryotes. Arabidopsis has two RPL24 homologs, RPL24A (AT2G36620) and RPL24B (AT3G53020).			1.13E-02
M vs. S (23)	AT3G45030	**RPS20C (RP)**			ribosome	0.36	Ribosomal protein S10p/S20e family protein;(source:Araport11)			6.32E-03
F vs. S (23)	ATCG00770	**RPS8 (RP)**	Ribosome protein (TAIR)		ribosome	0.36	chloroplast 30S ribosomal protein S8	Yes	Ribosome	3.73E-05
F vs. S (23)	AT1G36240	**AT1G36240 (RP)**	Ribosome protein (TAIR)		ribosome	0.35	Ribosomal protein L7Ae/L30e/S12e/Gadd45 family protein;(source:Araport11)	Yes	Ribosome	1.07E-04
F vs. M (23)	AT3G25100	**CDC45**	(TAIR)	Flowering	Meiosis	0.35	Required for normal meiosis, may act in the last round of DNA replication prior to meiosis, sequence similar to yeast CDC45	Use	meiosis	3.88E-03
L vs M (123)	AT1G08830	**CSD1**	CSD1 is a cytosolic copper/zinc superoxide dismutase that converts superoxide free radicals to O_2 and H_2O_2. Under nonstress conditions, its transcripts are cleaved by miR398, which decreases CSD1 levels. Under stress conditions, miR398 expression is suppressed, and CSD1 levels increase (DOI 10.1007/s10646-017-1843-y)	ROS attenuation		0.35	Encodes a cytosolic copper/zinc superoxide dismutase CSD1 that can detoxify superoxide radicals. Its expression is affected by miR398-directed mRNA cleavage. Regulated by biotic and abiotic stress. Activation of CSD1 in the cytoplasm involves both a CCS-dependent and -independent pathway.	Use	Stress, H_2O_2, superoxide dismutase	4.32E-03
F vs. S (23)	AT3G53890	**EVR1 (RP)**	TAIR	SnRK1 TOR	ribosome	0.35	Cytosolic ribosomal protein. Mutants enhance the varigation effect of var2 mutations suggesting a link between cytosolic translation and chloroplast development.	Yes	Ribosome	1.50E-04
L vs. S (23)	AT5G26030	**FC1**	Ferrochelatase-1, chloroplastic/mitochondrial		Stress	0.35	encodes ferrochelatase I located in plastids. Involved in heme biosynthesis in non-photosynthetic tissues and induced by oxidative stress in photosynthetic tissues to supply heme for defensive hemoproteins The mRNA is cell-to-cell mobile.			1.18E-02
M vs. S (23)	AT3G54660	**GR**	A plastic glutathione reductase that maintains a highly reduced glutathione pool that balances ROS and enables the redox buffering required to maintain efficient photosynthesis (doi.org/10.1111/tpj.14791)		glutathione	0.35	Encodes glutathione reductase that is most likely localized in the chloroplast.			1.75E-02
L vs. S (23)	AT3G04120	**No**	Glyceraldehyde-3-phosphate dehydrogenase GAPC1, cytosolic		H_2O_2, Ca, RO[illegible]	0.35	encodes cytosolic GADPH (C subunit) involved in the glycolytic pathway but also interacts with H_2O_2 potentially placing it in a signalling cascade induced by ROS. The mRNA is cell-to-cell mobile.			1.51E-02
F vs. S (23)	AT5G05440	**PYL5**	PYL5 belongs to a 14 member family of ABA sensors that interact with ABA to inhibit PP2Cs from inhibiting SnRKs (TAIR)	ABA signaling	PP2C, ABA	0.35	Encodes a member of the PYR (pyrabactin resistance)/PYL(PYR1-like)/RCAR (regulatory components of ABA receptor) family proteins with 14 members. PYR/PYL/RCAR family proteins function as abscisic acid sensors. Mediate ABA-dependent regulation of protein phosphatase 2Cs ABI1 and ABI2.	Yes	PP2C, ABA	8.08E-05
F vs. M (23)	AT3G24450		NA			0.35	Heavy metal transport/detoxification superfamily protein;(source:Araport11)		detoxification	4.37E-03

F vs. S (23)	AT2G13540	**ABH1**	ABH1 is an ABA induced cap-binding protein involved in proper splicing of ABA signaling and flowering related mRNAs (TAIR)		ABA upregulated	0.34	Encodes a nuclear cap-binding protein that forms a heterodimeric complex with CBP20 and is involved in ABA signaling and flowering. Mutants are early flowering and exhibit hypersensitive response to ABA in germination inhibition.Loss of ABH1 function results in abnormal processing of mRNAs for several important floral regulators (FLC, CO, FLM). Analysis of loss of function mutations suggests a role in pri-miRNA processing and mRNA splicing. Note that two different mutant alleles were given the same name abh1-7 (Kuhn et al 2007; Kim et al 2008). To avoid confusion, abh1-7 described in Kim et al (2008) has been renamed abh1-107 (other names: ensalada-1, ens-1).	Yes	ABA, miRNA	4.51E-04
F vs. M (23)	AT5G16970	**AER**	involved in responses to oxidative stress (TAIR)	ROS attenuation		0.34	encodes a 2-alkenal reductase (EC 1.3.1.74), plays a key role in the detoxification of reactive carbonyls	Use	detoxification	3.06E-03
F vs. S (23)	AT5G57390	**AIL5**	AIL5 is an aintegumenta-like AP2 family protein involved in organogenesis of floral organs. Overexpression leads to somatic embryo formation on cotyledons (TAIR)		Flowering	0.34	Encodes a member of the AP2 family of transcriptional regulators. May be involved in germination and seedling growth. Mutants are resistant to ABA analogs and are resistant to high nitrogen concentrations.essential for the developmental transition between the embryonic and vegetative phases in plants. Overexpression results in the formation of somatic embryos on cotyledons. It is also required to maintain high levels of PIN1 expression at the periphery of the meristem and modulate local auxin production in the central region of the SAM which underlies phyllotactic transitions. Acts redundantly with PLT3 and 7 in lateral root pattern formation.	Yes	ABA	5.55E-05
F vs. L (23)	AT3G03860	**APRL5**	NA		Thioredoxin	0.34	Encodes a protein disulfide isomerase-like (PDIL) protein, a member of a multigene family within the thioredoxin (TRX) superfamily. This protein also belongs to the adenosine 5'-phosphosulfate reductase-like (APRL) group. The mRNA is cell-to-cell mobile.	Use	Thioredoxin	2.41E-04
F vs. M (23)	AT1G08860	**BON3**	BON3 overlaps with BON1 in maintaining cellular homeostasis and inhibiting cell death (TAIR)		Ca, homeostasis	0.34	Encodes a copine-like protein, which is a member of a newly identified class of calcium-dependent, phospholipid binding proteins that are present in a wide range of organisms. Overexpression of this gene suppresses bon1-1 phenotypes. Double mutant analyses with bon1-1 suggest that BON1 and BON3 have overlapping functions in maintaining cellular homeostasis and inhibiting cell death.	Use	Ca, Homeostasis	4.07E-03
F vs. L (23)	AT3G24340	**CLSY4**	CLSY4 is a master regulator of locus specific RdDM through Pol-IV-dependent siRNA production (DOI 10.1038/s41421-018-0056-8)	Gene silencing		0.34	Involved in gene silencing. Locus-specific regulator of 24nt-siRNA expression, works together with CLSY1-3 as the master regulators of essentially all Pol-IV-dependent 24nt-siRNAs.	Use	siRNA, silencing	2.31E-03
F vs. S (23)	AT5G10400	**H3.1 AT5G10400**	NA		Histones	0.34	Histone superfamily protein;(source:Araport11)	Yes	Histone	2.85E-05
L vs. S (23)	AT4G08150	**KNAT1**	Homeobox protein knotted-1-like 1		flowering	0.34	A member of class I knotted1-like homeobox gene family (together with KNAT2). Similar to the knotted1 (kn1) homeobox gene of maize. Normally expressed in the peripheral and rib zone of shoot apical meristem but not in the leaf primordia. It is also expressed in the fourth floral whorl, in the region that would become style, particularly in the cell surrounding the transmitting tissue. No expression was detected in the first three floral whorls. Expression is repressed by auxin and AS1 which results in the promotion of leaf fate.			1.07E-02
F vs. S (23)	AT1G44970	**PRX9**	PRX9 is a peroxidase that is upregulated during oxidative stress (TAIR)	ROS attenuation		0.34	Encodes a class III peroxidase that is genetically redundant with PRX40, expressed in the tapetum, and essential for proper anther and pollen development.	Yes	peroxidase	5.91E-04
M vs. S (23)	AT5G05440	**PYL5**	PYL5 belongs to a 14 member family of ABA sensors that interact with ABA to inhibit PP2Cs from inhibiting SnRKs (TAIR)	ABA signaling	PP2C, ABA	0.34	Encodes a member of the PYR (pyrabactin resistance)/PYL(PYR1-like)/RCAR (regulatory components of ABA receptor) family proteins with 14 members. PYR/PYL/RCAR family proteins function as abscisic acid sensors. Mediate ABA-dependent regulation of protein phosphatase 2Cs ABI1 and ABI2.			1.42E-02
L vs M (123)	ATCG00660	**RPS4 (RP)**			ribosome	0.34	encodes a chloroplast ribosomal protein L20, a constituent of the large subunit of the ribosomal complex	Use	ribosome	3.10E-03
L vs. S (23)	AT1G24260	**SEP3**	Developmental protein SEPALLATA 3		flowering	0.34	Member of the MADs box transcription factor family. SEP3 is redundant with SEP1 and 2. Flowers of SEP1/2/3 triple mutants show a conversion of petals and stamens to sepals.SEP3 forms heterotetrameric complexes with other MADS box family members and binds to the CArG box motif.			7.69E-03
F vs. S (23)	AT5G62410	**SMC2**	SMC2 was identified as a meiosis important gene by Walker et al. (doi.org/10.1038/s41588-017-0008-5)	Flowering	Meiosis	0.34	SMC2-1 (SMC2)	Yes	meiosis	4.32E-04
F vs. L (23)	AT5G26210	**AL4**	AL4 is an Alfin1-like nuclear-localized plant homeodomain (PHD) protein that binds to di or trimethylated histone H3 (H3K4me3/2) and regulates transcription (TAIR)	Gene silencing		0.33	Encodes a member of the Alfin1-like family of nuclear-localized PHD (plant homeodomain) domain containing proteins. All AL proteins except AL3 bind to di- or trimethylated histone H3 (H3K4me3/2). Members of this family include: AT5G05610 (AL1), AT3G11200 (AL2), AT3G42790 (AL3), AT5G26210 (AL4), AT5G20510 (AL5), AT2G02470 (AL6), AT1G14510 (AL7).	Use	Histone, H3K4	1.89E-04
F vs. L (23)	AT1G13930	**AT1G13930**	AT1G13930 is upregulated by salt stress and is required for salt stress tolerance (TAIR)	Stress signaling	Salt	0.33	Involved in response to salt stress. Knockout mutants are hypersensitive to salt stress. The mRNA is cell-to-cell mobile.	Use	Stress	4.30E-04
F vs. S (23)	AT1G71695	**AT1G71695**	AT1G71695 is a peroxidase that is upregulated during oxidative stress (TAIR)	ROS attenuation		0.33	Peroxidase superfamily protein;(source:Araport11)	Yes	peroxidase	3.09E-05
L vs. S (23)	AT5G41560	**DDA1**			ABA	0.33	Encodes a substrate receptor for CRL4-CDD complexes that provides substrate specificity for CRL4 by interacting with ubiquitination targets. By its interaction and regulation of levels of PYL8 through proteasomal degradation, it negatively regulates ABA-mediated developmental responses, including inhibition of seed germination, seedling establishment, and root growth			7.98E-03
F vs. S (23)	AT5G22650	**HD2**	HD2 is a histone deacetylase involved in rDNA silencing and transcriptional repression of ribosome biogenesis (doi/10.1105/tpc.17.00626)	SnRK1 TOR	ribosome	0.33	Encodes a member of a plant-specific class of histone deacetylases. Controls the development of adaxial/abaxial leaf polarity. Its mRNA is widely expressed in stems, leaves, flowers and young siliques. Plant lines expressing RNAi constructs directed against this gene showed a marked reduction in agrobacterium-mediated root transformation.	Yes	ABA, Histone, RNAi	3.86E-05
F vs. S (23)	AT3G02190	**L39-2 (RP)**	TAIR		ribosome	0.33	Ribosomal protein L39 family protein;(source:Araport11)	Yes	Ribosome	2.26E-04
L vs. S (23)	AT5G05440	**PYL5**	PYL5 belongs to a 14 member family of ABA sensors that interact with ABA to inhibit PP2Cs from inhibiting SnRKs (TAIR)	ABA signaling	PP2C, ABA	0.33	Encodes a member of the PYR (pyrabactin resistance)/PYL(PYR1-like)/RCAR (regulatory components of ABA receptor) family proteins with 14 members. PYR/PYL/RCAR family proteins function as abscisic acid sensors. Mediate ABA-dependent regulation of protein phosphatase 2Cs ABI1 and ABI2.			1.15E-02

F vs. S (23)	AT2G40330	**PYL6**	PYL6 belongs to a 14 member family of ABA sensors that interact with ABA to inhibit PP2Cs from inhibiting SnRKs (TAIR)	ABA signaling	PP2C, ABA	0.33	Encodes a member of the PYR (pyrabactin resistance)/PYL(PYR1-like)/RCAR (regulatory components of ABA receptor) family proteins with 14 members. PYR/PYL/RCAR family proteins function as abscisic acid sensors. Mediate ABA-dependent regulation of protein phosphatase 2Cs ABI1 and ABI2.	Yes	PP2C, ABA	3.67E-05
F vs. S (23)	AT2G31970	**RAD50**	RAD50 is involved in double strand break repair and meiotic recombination (TAIR)	Flowering	Meiosis	0.33	Encodes the Arabidopsis RAD50 homologue. It is involved in double strand break repair. Component of the meiotic recombination complex that processes meiotic double-strand-breaks to produce single-stranded DNA ends, which act in the homology search and recombination. Accumulates in the nucleus during meiotic prophase, a process regulated by PHS1.	Yes	meiosis	3.54E-04
L vs. S (23)	AT2G07725	**RPL5 (RP)**	At2g07725		ribosome	0.33	Ribosomal L5P family protein;(source:Araport11)			1.30E-02
F vs. S (23)	AT5G64140	**RPS28 (RP)**	TAIR		ribosome	0.33	Encodes a putative ribosomal protein S28.	Yes	Ribosome	4.27E-05
F vs. L (23)	AT1G19850	**ARF5**	ARF5 is a TF that binds to auxin response elements that participate as important network components in regulating meristem and vascular tissue development (doi:10.1242/dev.186130). It also induces growth of early seedlings when upregulated by BR regulated BES1 and BEH4 TFs (DOI: 10.1002/pld3.166)	BR	ARF	0.32	Encodes a transcription factor (IAA24) mediating embryo axis formation and vascular development. Similar to AUXIN RESPONSIVE FACTOR 1 (ARF1) shown to bind to auxin responsive elements (AREs), and to the maize transcriptional activator VIVIPAROUS 1(VP1). In situ hybridization shows expression in provascular tissue of embryos, the emerging shoot primordia, then is restricted to provascular tissue, and in the root central vascular cylinder.	Use	ARF	2.18E-03
F vs. M (23)	AT5G10400	**H3.1 AT5G10400**	NA		Histones	0.32	Histone superfamily protein;(source:Araport11)	Use	Histone	4.01E-03
M vs. S (23)	AT3G57080	**NRPE5A**			Pol V	0.32	Non-catalytic subunit unique to Nuclear DNA-dependent RNA polymerase V; homologous to budding yeast RPB5.			6.43E-03
L vs. S (23)	AT3G15270	**SPL5**	Squamosa promoter-binding-like protein 5		flowering	0.32	Encodes a member of the SPL (squamosa-promoter binding protein-like)gene family, a novel gene family encoding DNA binding proteins and putative transcription factors. Contains the SBP-box, which encodes the SBP-domain, required and sufficient for interaction with DNA. It is involved in regulation of flowering and vegetative phase change. Its temporal expression is regulated by the microRNA miR156. The target site for the microRNA is in the 3'UTR.			1.77E-02
F vs. M (23)	AT2G42830	**AGL5**	NA		Flowering	0.31	AGAMOUS [AG]-like MADS box protein (AGL5) involved in fruit development (valve margin and dehiscence zone differentiation). A putative direct target of AG. SHP2 has been shown to be a downstream gene of the complex formed by AG and SEP proteins (SEP4 alone does not form a functional complex with	Use	AGL	1.70E-03
F vs. S (23)	AT2G32940	**AGO6**	AGO4,6,9 participate in programming one of the sporogenous cells in the ovule (nucellar cells) for meiosis and gametophyte formation and the remaining nucellar cells for death (doi: 10.1534/genetics.116.188151). Downregulation of AGO4 and 6 in *B. x. formosa* may contribute to its apomixis phenotype.	Gene silencing, ovule and gametophyte	AGO	0.31	Encodes a nuclear localized 879-amino-acid protein that contains conserved PAZ and PIWI domains that is important for the accumulation of specific heterochromatin-related siRNAs, and for DNA methylation and transcriptional gene silencing.	Yes	siRNA, silencing	4.02E-05
F vs. S (23)	AT1G52300	**AT1G52300 (RP)**	Ribosome protein (TAIR)		ribosome	0.31	Zinc-binding ribosomal protein family protein;(source:Araport11)	Yes	Ribosome	9.57E-05
F vs. S (23)	AT5G05690	**CPD**	CPD is a central enzyme of BR biosynthesis. It participates in the autonomous pathway of flowering through BRI1 induced suppression of FLC expression (TAIR)	BR signaling		0.31	Encodes a member of the CP90A family, a cytochrome P450 monooxygenase which converts 6-deoxocathasterone to 6-deoxoteasterone in the late C6 oxidation pathway and cathasterone to teasterone in the early C6 oxidation pathway of brassinolide biosynthesis. Expressed in cotyledons and leaves. Mutants display de-etiolation and derepression of light-induced genes in the dark, dwarfism, male sterility and activation of stress-regulated genes in the light. The expression of the gene using a CPD promoter:LUC fusion construct was shown to be under circadian and light control. Additionally, the circadian regulation was shown to be independent of BR levels as it remains unchanged in <i>bri1</i> mutant lines. CPD appears to be involved in the autonomous pathway that regulates the transition to flowering, primarily through a BRI1-mediated signaling pathway that affects FLC expression levels, as uncovered by double mutant analyses.	Yes	Stress, BR, flowering	6.70E-04
F vs. M (23)	AT3G50660	**DWF4**	DWF4 is the rate limiting enzyme in BR synthesis. DWF4 is a negative feedback repression target of BES1/BZR1 (doi.org/10.1038%2Fs41477-019-0436-6)	BR		0.31	Encodes a 22α hydroxylase whose reaction is a rate-limiting step in brassinosteroid biosynthetic pathway. The protein is a member of CYP90B gene family. CLM is an epi-allele with small, compressed rosette, reduced internode length, and reduced fertility, appears in selfed ddm mutant plants possibly due to loss of cytosine methylation. Transcripts accumulate in actively growing tissues, and GUS expression is negatively regulated by brassinosteroids. Localized in the endoplasmic reticulum. The in vitro expressed protein can perform the C-22 hydroxylation of a variety of C27-, C28- and C29-sterols. Cholesterol was the best substrate, followed by campesterol.	Use	BR	4.94E-03
L vs. S (23)	AT1G52030	**F-ATMBP**	Myrosinase-binding protein 2		flowering	0.31	Similar to myrosinase binding proteins which may be involved in metabolizing glucosinolates and forming defense compounds to protect against herbivory. Also similar to lectins and other agglutinating factors. Expressed only in			1.18E-02
F vs. M (23)	AT3G56090	**FER3**	NA	ROS attenuation		0.31	Encodes FERRITIN 3, AtFER3. Ferritins are a class of 24-mer multi-meric proteins found in all kingdoms of life. Function as the main iron store in mammals. Evidence suggests that Arabidopsis ferritins are essential to protect cells against oxidative damage, but they do not constitute the major iron pool.	Use	oxidative damage	5.68E-03
F vs. L (23)	AT5G65360	**H3.1 AT5G65360**	Histone 3 variant H3.1s are expressed and incorporated into DNA during S phase where they are methylated to retain specific epigenetic states (doi.org/10.1016/j.cub.2010.11.012; 10.1126/science.1248357)	Gene silencing	Histones	0.31	Histone superfamily protein;(source:Araport11)	Use	Histone	1.42E-03
F vs. S (23)	AT3G25980	**MAD2**	MAD2 was identified as a meiosis important gene by Walker et al. (doi.org/10.1038/s41588-017-0008-5)	Flowering	Meiosis	0.31	Encodes MAD2 (MITOTIC ARREST-DEFICIENT 2). May have the spindle assembly checkpoint protein functions conserved from yeast to humans.	Yes	meiosis	5.55E-05
L vs. S (23)	ATCG00790	**RPL16 (RP)**			ribosome	0.31	chloroplast gene encoding a ribosomal protein L16, which is a constituent of 50S large ribosomal subunit			1.07E-02
L vs. S (23)	AT3G22230	**RPL27B (RP)**	60S ribosomal protein L27-2		ribosome	0.31	Ribosomal L27e protein family;(source:Araport11)			1.21E-02
F vs. L (23)	AT1G26470	**SNS1**	SNRK2-SUBSTRATE 1 (SNS1) is upregulated by ABA and inhibits ABA responses (10.1126/scisignal.2003509)	ABA signaling	ABA upregulated	0.31	chromatin modification-like protein;(source:Araport11)	Use	chromatin modification	3.89E-05
F vs. L (23)	AT1G19330	**AFR2**	AFR2 is an evening expressed key component of the Sin3-HDAC complex that suppresses circadian clock associated gene expression by binding to their promotors and catalyzing histone 3 deacetylation (TAIR)		Histones	0.30	Evening-expressed key component of Sin3-HDAC complex, which bind directly to the CIRCADIAN CLOCK ASSOCIATED 1 (CCA1) and PSEUDO-RESPONSE REGULATOR 9 (PRR9) promoters and catalyze histone 3 (H3) deacetylation at the cognate regions to repress expression, allowing the declining phase of their expression at dusk.	Use	Histone, SPO, HDAC	3.54E-04

F vs. S (23)	AT1G16740	**AT1G16740 (RP)**	Ribosome protein (TAIR)		ribosome	0.3	Ribosomal protein L20;(source:Araport11)	Yes	Ribosome	3.67E-05
F vs. L (23)	AT3G57490	**AT3G57490 (RP)**	Ribosome protein (TAIR)		ribosome	0.30	Ribosomal protein S5 family protein;(source:Araport11)	Use	ribosome	1.60E-04
F vs. S (23)	AT4G30650	**AT4G30650**	TAIR	Stress response		0.3	Low temperature and salt responsive protein family;(source:Araport11)	Yes	responsive	3.14E-05
L vs. S (23)	AT3G03190	**GSTF11**			glutathione	0.3	Encodes glutathione transferase belonging to the phi class of GSTs. Naming convention according to Wagner et al. (2002).			1.02E-02
L vs. S (23)	AT4G13770	**REF2**			Homeostasis	0.3	Encodes a cytochrome p450 enzyme that catalyzes the initial conversion of aldoximes to thiohydroximates in the synthesis of glucosinolates not derived from tryptophan. Also has a role in auxin homeostasis.			8.72E-03
F vs. S (23)	AT5G22580	**AT5G22580**	Stress responsive (TAIR)	Stress signaling		0.29	Stress responsive A/B Barrel Domain-containing protein;(source:Araport11)	Yes	Stress	1.38E-03
F vs. S (23)	AT3G56090	**FER3**	Induced by Fe, protects cells against oxidative stress (doi.org/10.1016/j.molp.2014.11.014)	ROS attenuation		0.29	Encodes FERRITIN 3, AtFER3. Ferritins are a class of 24-mer multi-meric proteins found in all kingdoms of life. Function as the main iron store in mammals. Evidence suggests that Arabidopsis ferritins are essential to protect cells against oxidative damage, but they do not constitute the major iron pool.	Yes	oxidative damage	7.40E-05
L vs M (123)	AT2G30860	**GSTF9**	Upregulated by osmotic stress, mitigates oxidative damage caused by osmotic stress, protected from deactivation by LEA dehydrin proteins (doi.org/10.1016/j.bbagen.2019.129506)	ROS attenuation		0.29	Encodes glutathione transferase belonging to the phi class of GSTs. Naming convention according to Wagner et al. (2002).	Use	glutathione	5.41E-05
F vs. M (23)	AT1G26470	**SNS1**	SNRK2-SUBSTRATE 1 (SNS1) is upregulated by ABA and inhibits ABA responses (10.1126/scisignal.2003509)	ABA signaling	ABA upregulated	0.29	chromatin modification-like protein;(source:Araport11)	Use	chromatin modification	4.10E-03
F vs. S (23)	AT5G41560	**DDA1**	DDA1 negatively regulates ABA signaling by targeting ABA receptors (PYL8) for proteasomal degradation (TAIR)	ABA signaling		0.28	Encodes a substrate receptor for CRL4-CDD complexes that provides substrate specificity for CRL4 by interacting with ubiquitination targets. By its interaction and regulation of levels of PYL8 through proteasomal degradation, it negatively regulates ABA-mediated developmental responses, including inhibition of seed germination, seedling establishment, and root growth	Yes	ABA	1.81E-05
L vs M (123)	AT4G16660	**HSP70 AT4G16660**	Coats the surfaces of stress induced unfolded protein aggregates (stress granules) and targets them for proteosome complex breakdown (doi.org/10.1104/pp.19.00263)	Stress attenuation	HSP	0.28	heat shock protein 70 (Hsp 70) family protein;(source:Araport11)	Use	Hsp	1.53E-03
F vs. S (23)	AT4G20400	**JMJ14**	JMJ14 is a histone demethylase that catalyzes the demethylation of active H3K4 transcription marks in the promotor regions of hundreds of genes and is directed to these genes by the TFs NAC050 and NAC052. JMJ14 delays flowering by suppressing floral transition genes (doi: 10.1093/nar/gku1382)	Gene silencing, flowering	Histones	0.28	Encodes a histone H3K4 demethylase repressing floral transition.Coordinates and structure features have been deposited in the RCSB Protein Data Bank with the accession codes 5YKN (JMJ14CD) and 5YKO(JMJ14CD-NOG-H3K4em3 complex). Glu285 and Glu516 contribute to the recognition of H3R2. Asp312 contributes to the recognition of H3Q5. Phe171 and Val363 contribute to the recognition of H3A7. Ser290, Trp296, and Tyr298 contribute to the recognition of H3K4me3 (PMID:29233856).	Yes	Histone, H3K4	2.37E-05
F vs. S (23)	AT2G31840	**MRL7L**	NA		Thioredoxin	0.28	Thioredoxin superfamily protein;(source:Araport11)	Yes	Thioredoxin	4.58E-05
M vs. S (23)	AT5G61170	**RPS19C (RP)**			ribosome	0.28	Ribosomal protein S19e family protein;(source:Araport11)			1.34E-02
L vs. S (23)	AT4G34555	**RPS25D (RP)**	40S ribosomal protein S25-3		ribosome	0.28	Ribosomal protein S25 family protein;(source:Araport11)			1.15E-02
F vs. S (23)	AT4G12600	**AT4G12600 (RP)**	Ribosome protein (TAIR)		ribosome	0.27	Ribosomal protein L7Ae/L30e/S12e/Gadd45 family protein;(source:Araport11)	Yes	Ribosome	1.42E-04
F vs. M (23)	AT5G56580	**MKK6**	Involved in meiotic cytokinesis; interacts with MAPKKK5, which is phosphorylated by the receptor-like cytoplasmic kinase BRASSINOSTEROID-SIGNALING KINASE1 (BSK1), which is activated by FLAGENNIN SENSING2 (FLS2) as part of the immune response (doi.org/10.1104%2Fpp.17.01757)	Flowering, BR, Stress attenuation	Meiosis	0.27	Encodes a member of the MAP Kinase Kinase family of proteins. It can phosphorylate MPK12 in vitro and it can be dephosphorylated by MKP2 in vitro.	Use	MAPK	3.54E-03
F vs. S (23)	ATCG00830	**RPL2.1 (RP)**	Ribosome protein (TAIR)		ribosome	0.27	encodes a chloroplast ribosomal protein L2, a constituent of the large subunit of the ribosomal complex	Yes	Ribosome	5.42E-05
M vs. S (23)	AT3G53020	**RPL24B**			translation, ribosome	0.27	RPL24B encodes ribosomal protein L24, homolog of cytosolic RPL24, found in archaea and higher eukaryotes. Arabidopsis has two RPL24 homologs, RPL24A (AT2G36620) and RPL24B. Mutants showed defects in apical-basal gynoecium patterning similar to previously described ett and mp mutants. Transformation of stv1-1 mutant with a uORF-eliminated ETT construct partially suppressed the stv1 gynoecium phenotype, implying that STV1 could influence ETT translation through its uORFs. Regulated by TCP20.			1.49E-02
L vs. S (23)	AT2G43460	**RPL38B (RP)**	60S ribosomal protein L38		ribosome	0.27	Ribosomal L38e protein family;(source:Araport11)			9.20E-03
F vs. L (23)	AT5G39850	**RPS9C (RP)**	TAIR		ribosome	0.27	Ribosomal protein S4;(source:Araport11)	Use	ribosome	7.75E-06
M vs. S (23)	AT4G33950	**SnRK2.6 (OST1)**	SnRK2.6 is a major SnRK2 that remains phosphatase inactivated in the absence of stress due to the action of PP2Cs but are activated when ABA binds with ABA receptors and deactivates PP2Cs. Recent evidence indicates that SnRK2s can also be activated by osmostress responsive Raf-like kinases (ARKs) (doi: 10.1111/tpj.14756)		SnRK, Stress, Ca, ABA	0.27	Encodes calcium-independent ABA-activated protein kinase, a member of SNF1-related protein kinases (SnRK2) whose activity is activated by ionic (salt) and non-ionic (mannitol) osmotic stress. Mutations disrupted ABA induction of stomatal closure as well as ABA inhibition of light-induced stomatal opening. However, regulation of stomatal opening/closing by light or CO(2) is not affected in these mutants. May act in the interval between ABA perception and reactive oxygen species production in the ABA signalling network.			2.10E-02
F vs. S (23)	AT4G11080	**3xHMG-box2**	TAIR	Flowering	Meiosis	0.26	Encodes a protein containing three copies of the HMG (high mobility group)-box domain. The two Arabidopsis 3xHMG-box proteins are: AT4G11080 (3xHMG-box1) and AT4G23800 (3xHMG-box2). Interacts with mitotic and meiotic chromosomes.	Yes	meiosis	5.95E-04
M vs. S (23)	AT1G01170	**AT1G01170**			Stress	0.25	ozone-responsive stress-like protein (DUF1138);(source:Araport11)			1.37E-02
L vs. S (23)	AT4G30650	**AT4G30650**			salt responsive	0.25	Low temperature and salt responsive protein family;(source:Araport11)			1.75E-02
M vs. S (23)	AT5G65670	**IAA9 (ARF)**			ARF	0.25	auxin (indole-3-acetic acid) induced gene The mRNA is cell-to-cell mobile.			1.97E-02
F vs. S (23)	AT1G21750	**PDI5**	PDI5 is a disulfide isomerase-like thioredoxin that is upregulated by ER stress. It participates in cell redox homeostasis maintenance, protein folding and regulating programmed cell death (TAIR)	ROS attenuation	Homeostasis, thioredoxin	0.25	Encodes a protein disulfide isomerase-like (PDIL) protein, a member of a multigene family within the thioredoxin (TRX) superfamily; isoform contains non-consensus GA donor splice site at intron 9. Transcript levels for this gene are up-regulated in response to three different chemical inducers of ER stress (dithiothreitol, beta-mercaptoethanol, and tunicamycin). Neither AtIRE1-2 nor AtbZIP60 appear to be required for this response. The mRNA is cell-to-cell mobile.	Yes	Stress, Thioredoxin	2.53E-05
M vs. S (23)	AT1G30330	**ARF6**			ARF	0.24	Encodes a member of the auxin response factor family. Mediates auxin response via expression of auxin regulated genes. Acts redundantly with ARF8 to control stamen elongation and flower maturation. Expression of ARF6 is controlled by miR167.			1.30E-02

M vs. S (23)	AT5G65360	**H3.1 AT5G65360**	Histone 3 variant H3.1s are expressed and incorporated into DNA during S phase where they are methylated to retain specific epigenetic states (doi.org/10.1016/j.cub.2010.11.012; 10.1126/science.1248357)		Histone	0.24	Histone superfamily protein;(source:Araport11)			1.57E-02
F vs. L (23)	AT5G56580	**MKK6**	Involved in meiotic cytokinesis; interacts with MAPKKK5, which is phosphorylated by the receptor-like cytoplasmic kinase BRASSINOSTEROID-SIGNALINGKINASE1 (BSK1), which is activated by FLAGENNIN SENSING2 (FLS2) as part of the immune response (doi.org/10.1104%2Fpp.17.01757)	Flowering, BR, Stress attenuation	Meiosis	0.24	Encodes a member of the MAP Kinase Kinase family of proteins. It can phosphorylate MPK12 in vitro and it can be dephosphorylated by MKP2 in vitro.	Use	MAPK	1.83E-05
F vs. S (23)	AT1G70660	**MMZ2**	MMZ2 is a protein that responds to DNA damage and participates in post replicative DNA repair (TAIR)	Stress attenuation		0.24	MMZ2/UEV1B encodes a protein that may play a role in DNA damage responses and error-free post-replicative DNA repair by participating in lysine-63-based polyubiquitination reactions. UEV1A can form diubiquitin and triubiquitin chains in combination with UBC13A/UBC35 in vitro. It can also functionally complement an mms2 mutation in budding yeast, both by increasing mms2 mutant viability in the presence of the DNA damaging agent MMS, and by reducing the rate of spontaneous DNA mutation. However, a combination of MMZ2/UEV1B and UBC13A do not do a good job of rescuing an mms2 ubc13 double mutant in yeast. MMZ2/UEV1B transcripts are found in most plant organs, but not in the pollen or in seedlings 6 hours or 2 days post-germination. The transcript levels do not appear to be stress-inducible. The	Yes	Stress, DNA damage	2.95E-04
L vs. S (23)	AT3G04920	**RPS24A (RP)**	40S ribosomal protein S24-1		ribosome	0.24	Ribosomal protein S24e family protein;(source:Araport11)			1.02E-02
F vs. S (23)	AT5G21170	**AKINβ1**	KINβ1 is a major regulatory SnRK1 subunit that targets active SnRK1 to the nucleus for regulating gene expression and, upon *N* -myristoylation, suppresses SnRK1 activity by dissociating from SnRK1 and relocating individually to the Golgi (doi:10.1093/jxb/erz460)	SnRK1-TOR		0.23	Encodes AKINbeta1, a subunit of the SnRK1 kinase (Sucrose non-fermenting-1-related protein kinase). Involved in regulation of nitrogen and sugar metabolism. As part of the regulatory subunit, it binds maltose which promotes kinase activity. Acts as a global regulator of genes involved in carbon, lipid and nitrogen metabolism.	Yes	SnRK	3.09E-05
L vs. S (23)	AT1G30330	**ARF6**			#N/A	0.23	Encodes a member of the auxin response factor family. Mediates auxin response via expression of auxin regulated genes. Acts redundantly with ARF8 to control stamen elongation and flower maturation. Expression of ARF6 is controlled by miR167.			7.69E-03
M vs. S (23)	AT4G15440	**CYP74B2**	Produces galactolipids that accumulate in response to biotic and abiotic stress (doi:10.1093/jxb/erw278)		H2O2	0.23	Encodes a hydroperoxide lyase. Also a member of the CYP74B cytochrome p450 family. In the ecotype Columbia (Col) the gene contains a 10-nucleotide deletion in its first exon that causes it to code for a truncated protein that results in a non-functional hydroperoxide lyase.			3.17E-03
F vs. L (23)	AT3G25980	**MAD2**	MAD2 was identified as a meiosis important gene by Walker et al. (doi.org/10.1038/s41588-017-0008-5)	Flowering	Meiosis	0.23	Encodes MAD2 (MITOTIC ARREST-DEFICIENT 2). May have the spindle assembly checkpoint protein functions conserved from yeast to humans.	Use	meiosis	8.57E-05
F vs. L (23)	AT2G31840	**MRL7L**	NA		Thioredoxin	0.23	Thioredoxin superfamily protein;(source:Araport11)	Use	Thioredoxin	4.57E-06
L vs. S (23)	AT1G62360	**STM**	Homeobox protein SHOOT MERISTEMLESS		seed	0.23	Class I knotted-like homeodomain protein that is required for shoot apical meristem (SAM) formation during embryogenesis and for SAM function throughout the lifetime of the plant. Functions by preventing incorporation of cells in the meristem center into differentiating organ primordia. It has also been shown to have a role in the specification of flower meristem identity.			1.30E-02
F vs. L (23)	AT2G42830	**AGL5**	NA		Flowering	0.22	AGAMOUS [AG]-like MADS box protein (AGL5) involved in fruit development (valve margin and dehiscence zone differentiation). A putative direct target of AG. SHP2 has been shown to be a downstream gene of the complex formed by AG and SEP proteins (SEP4 alone does not form a functional complex with	Use	AGL	9.59E-05
F vs. S (23)	AT2G42830	**AGL5**	AGL		Flowering	0.22	AGAMOUS [AG]-like MADS box protein (AGL5) involved in fruit development (valve margin and dehiscence zone differentiation). A putative direct target of AG. SHP2 has been shown to be a downstream gene of the complex formed by AG and SEP proteins (SEP4 alone does not form a functional complex with	Yes	AGL	4.97E-06
F vs. S (23)	AT2G36160	**AT2G36160 (RP)**	Ribosome protein (TAIR)		ribosome	0.22	Ribosomal protein S11 family protein;(source:Araport11)	Yes	Ribosome	8.26E-06
L vs. S (23)	AT2G17240	**RP AT2G17240**	Arginine/serine-rich-like splicing factor		ribosome	0.22	Participates in the late stages of the biogenesis of 50S ribosomal subunits in plastids.			1.13E-02
L vs M (123)	ATCG00380	**RP ATCG00380**			ribosome	0.22	Chloroplast encoded ribosomal protein S4	Use	ribosome	2.73E-04
M vs. S (23)	AT5G47930	**RPS27D (RP)**			ribosome	0.22	Zinc-binding ribosomal protein family protein;(source:Araport11)			1.76E-02
F vs. S (23)	AT5G17060	**ARFB1B**	ARFB1B is an ARF that is upregulated in carpels. Hence, its upregulation in *B. stricta* pistils compared to *B. x. formosa* ovules is uninformative with regard to reproductive mode regulation.		ARF	0.21	A member of ARF GTPase family. A thaliana has 21 members of this family, known to be essential for vesicle coating and uncoating and functions in GTP-binding. Gene encoding ADP-ribosylation factor and similar to ADP-ribosylation factor 1; ARF 1 (GP:385340) {Drosophila melanogaster}, other ARFs and ARF-like proteins.	Yes	ARF	2.69E-05
F vs. S (23)	AT1G80700	**AT1G80700**	TAIR			0.21	stress response NST1-like protein;(source:Araport11)	Yes	Stress	1.10E-05
M vs. S (23)	AT3G49910	**RPL26A**			Translation	0.21	Translation protein SH3-like family protein;(source:Araport11)			2.04E-02
L vs. S (23)	AT5G02610	**RPL35D (RP)**	60S ribosomal protein L35-4		ribosome	0.21	Ribosomal L29 family protein;(source:Araport11)			5.44E-03
M vs. S (23)	AT5G21170	**AKINβ1**	KINβ1 is a major regulatory SnRK1 subunit that targets active SnRK1 to the nucleus for regulating gene expression and, upon N-myristoylation, suppresses SnRK1 activity by dissociating from SnRK1 and relocating individually to the Golgi (doi:10.1093/jxb/erz460)		SnRK	0.2	Encodes AKINbeta1, a subunit of the SnRK1 kinase (Sucrose non-fermenting-1-related protein kinase). Involved in regulation of nitrogen and sugar metabolism. As part of the regulatory subunit, it binds maltose which promotes kinase activity. Acts as a global regulator of genes involved in carbon, lipid and nitrogen metabolism.			1.37E-02
F vs. S (23)	AT1G15250	**AT1G15250 (RP)**	Ribosome protein (TAIR)		ribosome	0.2	Zinc-binding ribosomal protein family protein;(source:Araport11)	Yes	Ribosome	2.94E-05
F vs. L (23)	AT1G62810	**CuAO1**	CuAO1 is a copper amine oxidase involved in polyamine catabolism that is upregulated by ABA and SA and upon recovery from dehydration stress. It may be involved in maintaining water balance homeostasis during drought stress (https://doi.org/10.1016/j.plaphy.2019.11.037)		ABA upregulated, homeostasis	0.20	Encodes COPPER AMINE OXIDASE1 (CuAO1). Contributes to abscisic acid- and polyamine-induced nitric oxide biosynthesis and abscisic acid signal transduction.	Use	ABA	1.97E-03
M vs. S (23)	AT1G19550	**DHAR**	A major dehydroascorbate reductase with glutathione dehydrogenase (ascorbate) activity. Functions with APX6 in cellular ROS detoxification by coupling H2O2 to reduced glutathione. Small increases in DHAR and APX6 significantly enhance tolerance to abiotic stresses, reviewed in (doi.org/10.1093/jxb/eraa107)		glutathione	0.2	Glutathione S-transferase family protein;(source:Araport11)			1.67E-03
L vs M (123)	AT1G60030	**NAT7**	Transmembrane ascorbate transport (TAIR)	ROS attenuation		0.20	nucleobase-ascorbate transporter 7;(source:Araport11)	Use	Ascorbate	< 1e-07
M vs. S (23)	AT4G05590	**NRGA1**	Mitochondrial pyruvate carrier that mediates ABA signaling, drought stress and Cd toxicity by sustaining the TCA cycle and glutathione synthesis (doi.org/10.1104%2Fpp.18.01610)		Stress, ABA	0.2	Encodes NRGA1, a putative mitochondrial pyruvate carrier that mediates ABA regulation of guard cell ion channels and drought stress responses.			1.35E-02

L vs M (123)	AT1G05260	**RCI3**	As cationic peroxidase, RCI3 participates in the K deficiency signaling pathway possibly by producing sufficient H2O2 to induce a plasma membrane induced oxidative burst involving NADPH oxidases that generate superoxide free radicals. RCI3 expression is induced by ABA (doi: 10.1093/mp/ssp121)	ABA signaling	ABA upregulated	0.20	Encodes a cold-inducible cationic peroxidase that is involved in the stress response. In response to low temperature, RCI3 transcripts accumulate in the aerial part and in roots of etiolated seedlings but only in roots of light-grown seedlings. The mRNA is cell-to-cell mobile.	Use	Stress, peroxidase	1.64E-04
M vs. S (23)	AT5G02610	**RPL35D (RP)**			ribosome	0.2	Ribosomal L29 family protein;(source:Araport11)			1.26E-02
M vs. S (23)	AT3G03840	**SAUR27 (ARF)**			ARF	0.2	SAUR-like auxin-responsive protein family;(source:Araport11)			1.42E-02
F vs. L (23)	AT5G56300	**GAMT2**	GAMT2 is a methyltransferase that methylation inactivates GAs. It is highly expressed in the shoot apical meristem during floral transitioning and is also highly expressed during ovule and seed development. It is thought to suppress precocious flowering by inactivating GAs (doi:10.1111/ppl.13146)	Flowering	Ovule gametophyte	0.19	A member of the Arabidopsis SABATH methyltransferase gene family. Encodes GAMT2, a methyltransferase that uses S-adenosine-L-methionine (SAM) as a methyl donor to methylate the carboxyl group of GAs, resulting in the methyl esters of GAs (MeGAs). Expressed most highly in the siliques during seed development.	Use	Gametophyte	1.46E-03
L vs. S (23)	AT5G18670	**No**	Inactive beta-amylase 9		amalase	0.19	putative beta-amylase BMY3 (BMY3)			1.75E-02
L vs. S (23)	AT5G57290	**RPP3B (RP)**	60S acidic ribosomal protein P3-2		ribosome	0.19	60S acidic ribosomal protein family;(source:Araport11)			9.04E-03
F vs. S (23)	AT1G26470	**SNS1**	SNRK2-SUBSTRATE 1 (SNS1) is upregulated by ABA and inhibits ABA responses (10.1126/scisignal.2003509)	ABA signaling	ABA upregulated	0.19	chromatin modification-like protein;(source:Araport11)	Yes	chromatin modification	4.97E-06
L vs. S (23)	AT4G05590	**NRGA1**			Stress, ABA	0.18	Encodes NRGA1, a putative mitochondrial pyruvate carrier that mediates ABA regulation of guard cell ion channels and drought stress responses.			1.62E-02
F vs. S (23)	AT5G56580	**MKK6**	Involved in meiotic cytokinesis; interacts with MAPKKK5, which is phosphorylated by the receptor-like cytoplasmic kinase BRASSINOSTEROID-SIGNALINGKINASE1 (BSK1), which is activated by FLAGENNIN SENSING2 (FLS2) as part of the immune response (doi.org/10.1104%2Fpp.17.01757)	Flowering, BR, Stress attenuation	Meiosis	0.17	Encodes a member of the MAP Kinase Kinase family of proteins. It can phosphorylate MPK12 in vitro and it can be dephosphorylated by MKP2 in vitro.	Yes	MAPK	4.97E-06
M vs. S (23)	AT2G17240	**RP AT2G17240**			ribosome	0.17	Participates in the late stages of the biogenesis of 50S ribosomal subunits in plastids.			1.49E-02
F vs. S (23)	AT4G32330	**WDL5**	WDL5 is upregulated by ethylene during salt stress and is involved in microtubule bundle formation. It is a target of the ethylene induced TF EIN3 (doi/10.1104/pp.17.01124)	Stress signaling	ET, Salt	0.17	WDL5 is an target of EIN3 that co-localizes with cortical microtubules. It its thought to function to stabilize microtubles during ethylene induced hypocotyl elongation.	Yes	ethylene	9.67E-06
M vs. S (23)	AT5G62000	**ARF2**			ARF	0.16	Encodes an auxin response factor. Mutants have many defects including enlarged rosette leaves, reduced fertility, later senescence, hypocotyl elongation defects, enlarged seeds and enlarged cotyledons. May not mediate auxin effects. Increase in seed size due to increased cell proliferation. The mRNA is cell-to-cell mobile.			1.04E-02
F vs. S (23)	AT1G25330	**CESTA**	CESTA is a positive regulator of BR synthesis (TAIR)	BR		0.16	Encodes CESTA, a positive regulator of brassinosteroid biosynthesis.	Yes	BR	< 1e-07
M vs. S (23)	AT5G19450	**CPK8**	Functions with CAT3 to establish ROS homeostasis in response to ABA signaling (10.1105/tpc.15.00144)		Ca	0.16	calcium-dependent protein kinase (CDPK19) mRNA, complete			1.34E-02
F vs. S (23)	AT5G65360	**H3.1 AT5G65360**	Histone 3 variant H3.1s are expressed and incorporated into DNA during S phase where they are methylated to retain specific epigenetic states (doi.org/10.1016/j.cub.2010.11.012; 10.1126/science.1248357)	Gene silencing	Histones	0.16	Histone superfamily protein;(source:Araport11)	Yes	Histone	2.96E-06
F vs. L (23)	AT3G12500	**PR3**	ethylene mediated signaling pathway		ET	0.16	encodes a basic chitinase involved in ethylene/jasmonic acid mediated signalling pathway during systemic acquired resistance based on expression	Use	ethylene	< 1e-07
L vs. S (23)	AT5G17060	**ARFB1B**			ARF	0.15	A member of ARF GTPase family. A thaliana has 21 members of this family, known to be essential for vesicle coating and uncoating and functions in GTP-binding. Gene encoding ADP-ribosylation factor and similar to ADP-ribosylation factor 1; ARF 1 (GP:385340) {Drosophila melanogaster}, other ARFs and ARF-like proteins.			1.77E-02
F vs. L (23)	AT2G26710	**BAS1**	BAS1 is a circadian regulated BR catabolic enzyme (inactivating cytochrome P450) that reduces the availability of bioactive BRs in a tissue specific manner (doi:10.1093/jxb/erz468)	BR		0.15	Encodes a member of the cytochrome p450 family that serves as a control point between multiple photoreceptor systems and brassinosteroid signal transduction. Involved in brassinolide metabolism. Mediates response to a variety of light signals including hypocotyl elongation and cotyledon expansion.	Use	BR	7.75E-06
L vs. S (23)	AT5G27700	**RPS21C (RP)**	40S ribosomal protein S21-2		ribosome	0.15	Cytosolic ribosomal protein. Similar to EVR1 and redundant with EVR1. Also enhances VAR2 mutant varigation, but to a lesser extent than evr1.			9.58E-03
F vs. S (23)	AT1G69620	**RPL34 (RP)**	Ribosome protein (TAIR)		ribosome	0.14	putative 60S ribosomal protein L34 The mRNA is cell-to-cell mobile.	Yes	Ribosome	2.96E-06
L vs. S (23)	ATCG00820	**RPS19 (RP)**			ribosome	0.14	Encodes a 6.8-kDa protein of the small ribosomal subunit.			3.31E-03
M vs. S (23)	AT5G27700	**RPS21C (RP)**			ribosome	0.14	Cytosolic ribosomal protein. Similar to EVR1 and redundant with EVR1. Also enhances VAR2 mutant varigation, but to a lesser extent than evr1.			1.37E-02
F vs. M (23)	AT1G05260	**RCI3**	As cationic peroxidase, RCI3 participates in the K deficiency signaling pathway possibly by producing sufficient H2O2 to induce a plasma membrane induced oxidative burst involving NADPH oxidases that generate superoxide free radicals. RCI3 expression is induced by ABA (doi: 10.1093/mp/ssp121)	ABA signaling	ABA upregulated	0.13	Encodes a cold-inducible cationic peroxidase that is involved in the stress response. In response to low temperature, RCI3 transcripts accumulate in the aerial part and in roots of etiolated seedlings but only in roots of light-grown seedlings. The mRNA is cell-to-cell mobile.	Use	Stress, peroxidase	1.45E-03
F vs. S (23)	ATCG00790	**RPL16 (RP)**	Ribosome protein (TAIR)		ribosome	0.13	chloroplast gene encoding a ribosomal protein L16, which is a constituent of 50S large ribosomal subunit	Yes	Ribosome	2.96E-06
F vs. S (23)	AT1G13930	**AT1G13930**	AT1G13930 is upregulated by salt stress and is required for salt stress tolerance (TAIR)	Stress signaling	Salt	0.11	Involved in response to salt stress. Knockout mutants are hypersensitive to salt stress. The mRNA is cell-to-cell mobile.	Yes	Stress	< 1e-07
F vs. M (23)	AT2G26710	**BAS1**	BAS1 is a circadian regulated BR catabolic enzyme (inactivating cytochrome P450) that reduces the availability of bioactive BRs in a tissue specific manner (doi:10.1093/jxb/erz468)	BR		0.11	Encodes a member of the cytochrome p450 family that serves as a control point between multiple photoreceptor systems and brassinosteroid signal transduction. Involved in brassinolide metabolism. Mediates response to a variety of light signals including hypocotyl elongation and cotyledon expansion.	Use	BR	1.36E-03
L vs. S (23)	AT1G69700	**HVA22C**	ABA and stress inducible (TAIR)	ABA signaling	ABA upregulated	0.1	Part of the AtHVA22 family. Protein expression is ABA- and stress-inducible. The mRNA is cell-to-cell mobile.			1.02E-02
F vs. S (23)	AT3G50970	**LTI30**	LT130 is a dehydrin protein (protects membranes by crosslinks lipids during drought stress). It is upregulated by dehydration and ABA (TAIR)		ABA upregulated, Stress attenuation	0.1	Belongs to the dehydrin protein family, which contains highly conserved stretches of 7-17 residues that are repetitively scattered in their sequences, the K-, S-, Y- and lysine rich segments. LTI29 and LTI30 double overexpressors confer freeze tolerance. Located in membranes. mRNA upregulated by water deprivation and abscisic acid. The mRNA is cell-to-cell mobile.	Yes	ABA	5.88E-04
L vs. S (23)	AT2G36830	**GAMMA-TIP**			#N/A	0.09	Encodes a tonoplast intrinsic protein, which functions as water channel. It has also been shown to be able to facilitate the transport of urea and hydrogen peroxide. Highly expressed in vascular tissues of the root, stem, cauline leaves and flowers but not in the apical meristems. The mRNA is cell-to-cell mobile.			7.12E-03

F vs. L (23)	AT5G14920	**GASA14**	GASA14 is upregulated by salt stress and by GA and is required for GA-induced growth. It also increases abiotic stress resistances by reducing ROS accumulation. BR upregulates the biosynthesis of GAs (doi: 10.1105/tpc.114.132092; doi:10.1093/jxb/ert021)	Stress signaling	Salt	0.08	Encodes a GASA domain containing protein that regulates increases in plant growth through GA-induced and DELLA-dependent signal transduction and that can increase abiotic stress resistance by reducing ROS accumulation.	Use	Stress, ROS	5.94E-05
F vs. L (23)	AT3G48510	**AITR2 (DIG1)**	AITR2 (DIG1) belongs to a group of six transcriptional repressors that are upregulated by ABA (DOI: 10.1126/science.aag1550). They strengthen ABA signaling (suppression of growth) by i) suppressing ABA induced downregulation of ABA receptor genes and ii) suppressing ABA induced upregulation of PP2C genes (DOI: 10.1111/pce.13058). Consistent with these functions, we found that PP2Cs were down-regulated in B. microphylla vs. B. lignifera ( ), and this may have enhanced ABA signaling in B. microphylla ovules.	ABA signaling		0.07	ABA‐induced transcription repressor that acts as feedback regulator in ABA signalling.	Use	ABA	< 1e-07
M vs. S (23)	AT2G36830	**GAMMA-TIP**			H2O2	0.07	Encodes a tonoplast intrinsic protein, which functions as water channel. It has also been shown to be able to facilitate the transport of urea and hydrogen peroxide. Highly expressed in vascular tissues of the root, stem, cauline leaves and flowers but not in the apical meristems. The mRNA is cell-to-cell mobile.			1.34E-02
L vs. S (23)	ATCG00380	**RP ATCG00380**			ribosome	0.066	Chloroplast encoded ribosomal protein S4			1.51E-02
F vs. S (23)	AT1G69700	**HVA22C**	ABA and stress inducible (TAIR)	ABA signaling	ABA upregulated	0.065	Part of the AtHVA22 family. Protein expression is ABA- and stress-inducible. The mRNA is cell-to-cell mobile.	Yes	Stress, ABA	2.96E-06
F vs. S (23)	AT5G14920	**GASA14**	GASA14 is upregulated by salt stress and by GA and is required for GA-induced growth. It also increases abiotic stress resistances by reducing ROS accumulation. BR upregulates the biosynthesis of GAs (doi: 10.1105/tpc.114.132092). As one of the most highly upregulated genes in B. microphylla, it may contribute to the apospory phenotype (doi:10.1093/jxb/ert021)	Stress signaling	Salt	0.064	Encodes a GASA domain containing protein that regulates increases in plant growth through GA-induced and DELLA-dependent signal transduction and that can increase abiotic stress resistance by reducing ROS accumulation.	Yes	Stress, ROS	9.67E-06
F vs. S (23)	ATCG00380	**ATCG00380 (RP)**	TAIR		ribosome	0.061	Chloroplast encoded ribosomal protein S4	Yes	Ribosome	< 1e-07
L vs. S (23)	AT2G44120	**RPL7C (RP)**			ribosome	0.043	Ribosomal protein L30/L7 family protein;(source:Araport11)			5.44E-03
F vs. L (23)	AT3G42570	**AT3G42570**	response to oxidative stress (TAIR)			0.04	peroxidase family protein;(source:Araport11)	Use	peroxidase	1.83E-05
M vs. S (23)	AT5G60910	**AGL8**			flowering	0.04	MADS box gene negatively regulated by APETALA1			1.25E-03
M vs. S (23)	AT2G44120	**RPL7C (RP)**			ribosome	0.039	Ribosomal protein L30/L7 family protein;(source:Araport11)			2.31E-03
M vs. S (23)	AT2G42200	**SPL9**			flowering	0.039	Encodes a putative transcriptional regulator that is involved in the vegetative to reproductive phase transition. Expression is regulated by MIR156b. SPL activity nonautonomously inhibits initiation of new leaves at the shoot apical			3.85E-03
L vs. S (23)	AT5G60910	**AGL8**	Agamous-like MADS-box protein AGL8		flowering	0.035	MADS box gene negatively regulated by APETALA1			2.59E-03
F vs. M (23)	AT3G13580	**RPL7D (RP)**			ribosome	0.01	Ribosomal protein L30/L7 family protein;(source:Araport11)	Use	ribosome	2.16E-04
F vs. L (23)	AT3G13580	**RPL7D (RP)**	TAIR		ribosome	0.01	Ribosomal protein L30/L7 family protein;(source:Araport11)	Use	ribosome	< 1e-07
