# Supplementary figures and images for: Whether Gametophytes Are Reduced or Unreduced in Angiosperms Might Be Determined Metabolically
Source: Genes (Basel). 2020 Dec 2;11(12):1449. doi: 10.3390/genes11121449 (PMC7761559; doi:10.3390/genes11121449)

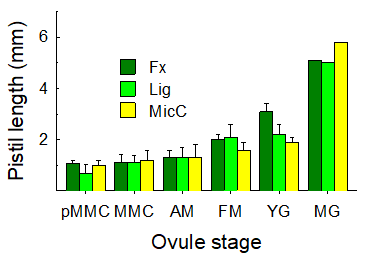

Supplement: Supplementary file 1 [file genes-11-01449-s001.zip › Fig S1.tif]

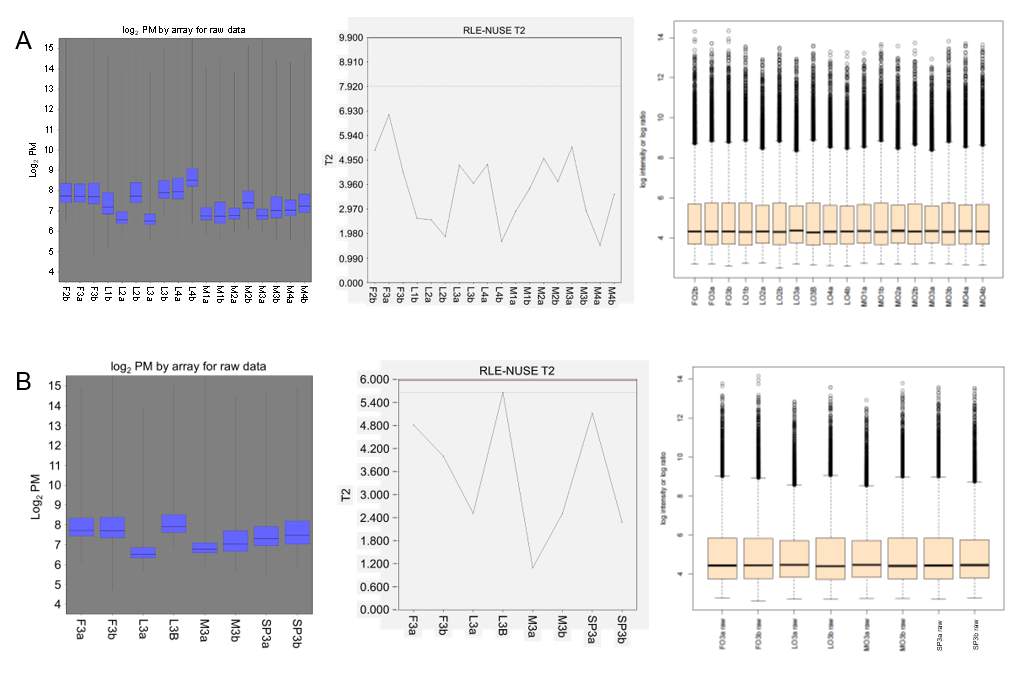

Supplement: Supplementary file 1 [file genes-11-01449-s001.zip › Fig S2.tif]

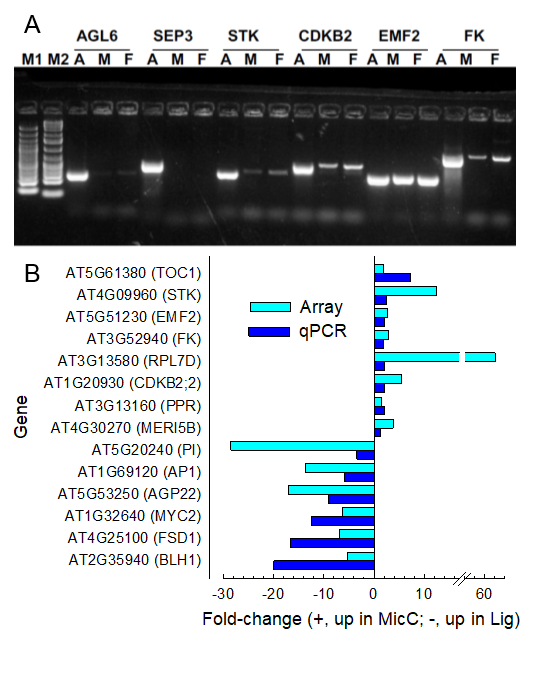

Supplement: Supplementary file 1 [file genes-11-01449-s001.zip › Fig S3.tif]
