# Supplementary material for: Whether Gametophytes Are Reduced or Unreduced in Angiosperms Might Be Determined Metabolically
Source: Genes (Basel). 2020 Dec 2;11(12):1449. doi: 10.3390/genes11121449 (PMC7761559; doi:10.3390/genes11121449)
Supplement: Supplementary file 1 [file Table S6 All GOs of DEGs.pdf › Table S6 All GOs of DEGs.pdf]

Table S6. All gene ontology categories (GOs) of all differentially expressed genes (DEGs) by comparison, GO group and GO subgroup. L, *B. lignifera* ; M, *B. microphylla* ; F, *B. x formosa* ; S, *B. stricta* ; 1, megasporocyte (MMC) stage; 2, meiocyte stage; 3, young gametophyte stage; 4, mature gametophyte stage; GO types: P, biological process; F, molecular function, C, cellular component.

Line no.		Comparison	DEGs used	GO no.	GO group	GO subgroup	GO description	Type	DEG
1	1	L123 vs. M123	L123 up	GO:0008283	Development	Cell division	cell population proliferation	P	AT4G02060
2	1	L123 vs. M123	L123 up	GO:0030261	Development	Cell division	chromosome condensation	P	AT2G30620
3	1	L123 vs. M123	L123 up	GO:0000910	Development	Cell division	cytokinesis	P	AT3G50960
4	1	L123 vs. M123	L123 up	GO:0000910	Development	Cell division	cytokinesis	P	AT3G60740
5	1	L123 vs. M123	L123 up	GO:0006260	Development	Cell division	DNA replication	P	AT4G02060
6	1	L123 vs. M123	L123 up	GO:0006260	Development	Cell division	DNA replication	P	AT4G12620
7	1	L123 vs. M123	L123 up	GO:0006270	Development	Cell division	DNA replication initiation	P	AT4G02060
8	1	L123 vs. M123	L123 up	GO:0006270	Development	Cell division	DNA replication initiation	P	AT4G12620
9	1	L123 vs. M123	L123 up	GO:0003688	Development	Cell division	DNA replication origin binding	F	AT4G02060
10	1	L123 vs. M123	L123 up	GO:0003688	Development	Cell division	DNA replication origin binding	F	AT4G12620
11	1	L123 vs. M123	L123 up	GO:0006271	Development	Cell division	DNA strand elongation involved in DNA replication	P	AT4G02060
12	1	L123 vs. M123	L123 up	GO:0006268	Development	Cell division	DNA unwinding involved in DNA replication	P	AT4G02060
13	1	L123 vs. M123	L123 up	GO:0006261	Development	Cell division	DNA-dependent DNA replication	P	AT1G34380
14	1	L123 vs. M123	L123 up	GO:0003887	Development	Cell division	DNA-directed DNA polymerase activity	F	AT1G34380
15	1	L123 vs. M123	L123 up	GO:0000727	Development	Cell division	double-strand break repair via break-induced replication	P	AT4G02060
16	1	L123 vs. M123	L123 up	GO:0010385	Development	Cell division	double-stranded methylated DNA binding	F	AT4G12620
17	1	L123 vs. M123	L123 up	GO:0042555	Development	Cell division	MCM complex	C	AT4G02060
18	1	L123 vs. M123	L123 up	GO:0000278	Development	Cell division	mitotic cell cycle	P	AT1G64740
19	1	L123 vs. M123	L123 up	GO:0000278	Development	Cell division	mitotic cell cycle	P	AT4G12620
20	1	L123 vs. M123	L123 up	GO:0033314	Development	Cell division	mitotic DNA replication checkpoint	P	AT4G12620
21	1	L123 vs. M123	L123 up	GO:0045910	Development	Cell division	negative regulation of DNA recombination	P	AT2G30620
22	1	L123 vs. M123	L123 up	GO:0000280	Development	Cell division	nuclear division	P	AT3G50960
23	1	L123 vs. M123	L123 up	GO:0005664	Development	Cell division	nuclear origin of replication recognition complex	C	AT4G12620
24	1	L123 vs. M123	L123 up	GO:0031492	Development	Cell division	nucleosomal DNA binding	F	AT2G30620
25	1	L123 vs. M123	L123 up	GO:0000786	Development	Cell division	nucleosome	C	AT2G30620
26	1	L123 vs. M123	L123 up	GO:0000786	Development	Cell division	nucleosome	C	AT5G65360
27	1	L123 vs. M123	L123 up	GO:0006334	Development	Cell division	nucleosome assembly	P	AT2G30620
28	1	L123 vs. M123	L123 up	GO:0016584	Development	Cell division	nucleosome positioning	P	AT2G30620
29	1	L123 vs. M123	L123 up	GO:0008252	Development	Cell division	nucleotidase activity	F	AT4G14930
30	1	L123 vs. M123	L123 up	GO:0000808	Development	Cell division	origin recognition complex	C	AT4G12620
31	1	L123 vs. M123	L123 up	GO:0006267	Development	Cell division	pre-replicative complex assembly involved in nuclear cell cycle DNA replication	P	AT4G02060
32	1	L123 vs. M123	L123 up	GO:0048544	Development	Cell division	recognition of pollen	P	AT1G11410
33	1	L123 vs. M123	L123 up	GO:1990518	Development	Cell division	single-stranded 3'-5' DNA helicase activity	F	AT4G02060
34	1	L123 vs. M123	L123 up	GO:0003697	Development	Cell division	single-stranded DNA binding	F	AT4G02060
35	1	L123 vs. M123	L123 up	GO:0005819	Development	Cell division	spindle	C	AT4G35020
36	1	L123 vs. M123	L123 up	GO:0030036	Development	Development	actin cytoskeleton organization	P	AT4G35020
37	1	L123 vs. M123	L123 up	GO:0031225	Development	Development	anchored component of membrane	C	AT1G72230
38	1	L123 vs. M123	L123 up	GO:0046658	Development	Development	anchored component of plasma membrane	C	AT1G72230
39	1	L123 vs. M123	L123 up	GO:0051211	Development	Development	anisotropic cell growth	P	AT3G50960
40	1	L123 vs. M123	L123 up	GO:0048046	Development	Development	apoplast	C	AT1G16080
41	1	L123 vs. M123	L123 up	GO:0048046	Development	Development	apoplast	C	AT2G36530
42	1	L123 vs. M123	L123 up	GO:0048046	Development	Development	apoplast	C	AT5G09440
43	1	L123 vs. M123	L123 up	GO:0005938	Development	Development	cell cortex	C	AT4G35020
44	1	L123 vs. M123	L123 up	GO:0042995	Development	Development	cell projection	C	AT4G35020
45	1	L123 vs. M123	L123 up	GO:0005618	Development	Development	cell wall	C	AT3G16240
46	1	L123 vs. M123	L123 up	GO:0005575	Development	Development	cellular_component	C	AT3G15430
47	1	L123 vs. M123	L123 up	GO:0042807	Development	Development	central vacuole	C	AT3G16240
48	1	L123 vs. M123	L123 up	GO:0000785	Development	Development	chromatin	C	AT1G20693
49	1	L123 vs. M123	L123 up	GO:0006333	Development	Development	chromatin assembly or disassembly	P	AT1G20693

50	1	L123 vs. M123	L123 up	GO:0010369	Development	Development	chromocenter	C	AT5G65360
51	1	L123 vs. M123	L123 up	GO:0005801	Development	Development	cis-Golgi network	C	AT1G15880
52	1	L123 vs. M123	L123 up	GO:0043622	Development	Development	cortical microtubule organization	P	AT3G50960
53	1	L123 vs. M123	L123 up	GO:0043622	Development	Development	cortical microtubule organization	P	AT5G23720
54	1	L123 vs. M123	L123 up	GO:0048825	Development	Development	cotyledon development	P	AT2G32280
55	1	L123 vs. M123	L123 up	GO:0010588	Development	Development	cotyledon vascular tissue pattern formation	P	AT2G32280
56	1	L123 vs. M123	L123 up	GO:0005737	Development	Development	cytoplasm	C	AT1G09270
57	1	L123 vs. M123	L123 up	GO:0005737	Development	Development	cytoplasm	C	AT1G16470
58	1	L123 vs. M123	L123 up	GO:0005737	Development	Development	cytoplasm	C	AT1G23490
59	1	L123 vs. M123	L123 up	GO:0005737	Development	Development	cytoplasm	C	AT1G34380
60	1	L123 vs. M123	L123 up	GO:0005737	Development	Development	cytoplasm	C	AT1G49670
61	1	L123 vs. M123	L123 up	GO:0005737	Development	Development	cytoplasm	C	AT1G50440
62	1	L123 vs. M123	L123 up	GO:0005737	Development	Development	cytoplasm	C	AT1G64740
63	1	L123 vs. M123	L123 up	GO:0005737	Development	Development	cytoplasm	C	AT1G79340
64	1	L123 vs. M123	L123 up	GO:0005737	Development	Development	cytoplasm	C	AT2G01350
65	1	L123 vs. M123	L123 up	GO:0005737	Development	Development	cytoplasm	C	AT2G01670
66	1	L123 vs. M123	L123 up	GO:0005737	Development	Development	cytoplasm	C	AT2G36530
67	1	L123 vs. M123	L123 up	GO:0005737	Development	Development	cytoplasm	C	AT2G41140
68	1	L123 vs. M123	L123 up	GO:0005737	Development	Development	cytoplasm	C	AT2G41900
69	1	L123 vs. M123	L123 up	GO:0005737	Development	Development	cytoplasm	C	AT2G42690
70	1	L123 vs. M123	L123 up	GO:0005737	Development	Development	cytoplasm	C	AT3G14130
71	1	L123 vs. M123	L123 up	GO:0005737	Development	Development	cytoplasm	C	AT3G17020
72	1	L123 vs. M123	L123 up	GO:0005737	Development	Development	cytoplasm	C	AT3G19240
73	1	L123 vs. M123	L123 up	GO:0005737	Development	Development	cytoplasm	C	AT3G23000
74	1	L123 vs. M123	L123 up	GO:0005737	Development	Development	cytoplasm	C	AT3G50960
75	1	L123 vs. M123	L123 up	GO:0005737	Development	Development	cytoplasm	C	AT3G53620
76	1	L123 vs. M123	L123 up	GO:0005737	Development	Development	cytoplasm	C	AT3G55170
77	1	L123 vs. M123	L123 up	GO:0005737	Development	Development	cytoplasm	C	AT3G60740
78	1	L123 vs. M123	L123 up	GO:0005737	Development	Development	cytoplasm	C	AT4G02060
79	1	L123 vs. M123	L123 up	GO:0005737	Development	Development	cytoplasm	C	AT4G14930
80	1	L123 vs. M123	L123 up	GO:0005737	Development	Development	cytoplasm	C	AT4G15093
81	1	L123 vs. M123	L123 up	GO:0005737	Development	Development	cytoplasm	C	AT4G25100
82	1	L123 vs. M123	L123 up	GO:0005737	Development	Development	cytoplasm	C	AT4G33640
83	1	L123 vs. M123	L123 up	GO:0005737	Development	Development	cytoplasm	C	AT4G35020
84	1	L123 vs. M123	L123 up	GO:0005737	Development	Development	cytoplasm	C	AT4G37870
85	1	L123 vs. M123	L123 up	GO:0005737	Development	Development	cytoplasm	C	AT4G39280
86	1	L123 vs. M123	L123 up	GO:0005737	Development	Development	cytoplasm	C	AT5G01660
87	1	L123 vs. M123	L123 up	GO:0005737	Development	Development	cytoplasm	C	AT5G17760
88	1	L123 vs. M123	L123 up	GO:0005737	Development	Development	cytoplasm	C	AT5G23720
89	1	L123 vs. M123	L123 up	GO:0005737	Development	Development	cytoplasm	C	AT5G47060
90	1	L123 vs. M123	L123 up	GO:0005737	Development	Development	cytoplasm	C	AT5G48760
91	1	L123 vs. M123	L123 up	GO:0005737	Development	Development	cytoplasm	C	AT5G54160
92	1	L123 vs. M123	L123 up	GO:0005737	Development	Development	cytoplasm	C	AT5G57610
93	1	L123 vs. M123	L123 up	GO:0005737	Development	Development	cytoplasm	C	AT5G60940
94	1	L123 vs. M123	L123 up	GO:0005737	Development	Development	cytoplasm	C	ATCG00670
95	1	L123 vs. M123	L123 up	GO:0005829	Development	Development	cytosol	C	AT1G09270
96	1	L123 vs. M123	L123 up	GO:0005829	Development	Development	cytosol	C	AT1G16470
97	1	L123 vs. M123	L123 up	GO:0005829	Development	Development	cytosol	C	AT1G19920
98	1	L123 vs. M123	L123 up	GO:0005829	Development	Development	cytosol	C	AT1G20693
99	1	L123 vs. M123	L123 up	GO:0005829	Development	Development	cytosol	C	AT1G31190
100	1	L123 vs. M123	L123 up	GO:0005829	Development	Development	cytosol	C	AT1G64740
101	1	L123 vs. M123	L123 up	GO:0005829	Development	Development	cytosol	C	AT1G72680
102	1	L123 vs. M123	L123 up	GO:0005829	Development	Development	cytosol	C	AT1G79340
103	1	L123 vs. M123	L123 up	GO:0005829	Development	Development	cytosol	C	AT2G29370
104	1	L123 vs. M123	L123 up	GO:0005829	Development	Development	cytosol	C	AT2G36530
105	1	L123 vs. M123	L123 up	GO:0005829	Development	Development	cytosol	C	AT2G42690

106	1	L123 vs. M123	L123 up	GO:0005829	Development	Development	cytosol	C	AT2G45740
107	1	L123 vs. M123	L123 up	GO:0005829	Development	Development	cytosol	C	AT2G47970
108	1	L123 vs. M123	L123 up	GO:0005829	Development	Development	cytosol	C	AT3G06690
109	1	L123 vs. M123	L123 up	GO:0005829	Development	Development	cytosol	C	AT3G09300
110	1	L123 vs. M123	L123 up	GO:0005829	Development	Development	cytosol	C	AT3G12500
111	1	L123 vs. M123	L123 up	GO:0005829	Development	Development	cytosol	C	AT3G16100
112	1	L123 vs. M123	L123 up	GO:0005829	Development	Development	cytosol	C	AT3G17020
113	1	L123 vs. M123	L123 up	GO:0005829	Development	Development	cytosol	C	AT3G48000
114	1	L123 vs. M123	L123 up	GO:0005829	Development	Development	cytosol	C	AT3G51000
115	1	L123 vs. M123	L123 up	GO:0005829	Development	Development	cytosol	C	AT3G53620
116	1	L123 vs. M123	L123 up	GO:0005829	Development	Development	cytosol	C	AT3G60740
117	1	L123 vs. M123	L123 up	GO:0005829	Development	Development	cytosol	C	AT3G61990
118	1	L123 vs. M123	L123 up	GO:0005829	Development	Development	cytosol	C	AT3G62310
119	1	L123 vs. M123	L123 up	GO:0005829	Development	Development	cytosol	C	AT4G14930
120	1	L123 vs. M123	L123 up	GO:0005829	Development	Development	cytosol	C	AT4G25100
121	1	L123 vs. M123	L123 up	GO:0005829	Development	Development	cytosol	C	AT4G31700
122	1	L123 vs. M123	L123 up	GO:0005829	Development	Development	cytosol	C	AT4G32320
123	1	L123 vs. M123	L123 up	GO:0005829	Development	Development	cytosol	C	AT4G33640
124	1	L123 vs. M123	L123 up	GO:0005829	Development	Development	cytosol	C	AT4G34710
125	1	L123 vs. M123	L123 up	GO:0005829	Development	Development	cytosol	C	AT4G37870
126	1	L123 vs. M123	L123 up	GO:0005829	Development	Development	cytosol	C	AT4G39280
127	1	L123 vs. M123	L123 up	GO:0005829	Development	Development	cytosol	C	AT5G10070
128	1	L123 vs. M123	L123 up	GO:0005829	Development	Development	cytosol	C	AT5G18110
129	1	L123 vs. M123	L123 up	GO:0005829	Development	Development	cytosol	C	AT5G21060
130	1	L123 vs. M123	L123 up	GO:0005829	Development	Development	cytosol	C	AT5G47240
131	1	L123 vs. M123	L123 up	GO:0005829	Development	Development	cytosol	C	AT5G50960
132	1	L123 vs. M123	L123 up	GO:0005829	Development	Development	cytosol	C	AT5G54160
133	1	L123 vs. M123	L123 up	GO:0009855	Development	Development	determination of bilateral symmetry	P	AT2G32280
134	1	L123 vs. M123	L123 up	GO:0009567	Development	Development	double fertilization forming a zygote and endosperm	P	AT4G12620
135	1	L123 vs. M123	L123 up	GO:0009790	Development	Development	embryo development	P	AT4G02060
136	1	L123 vs. M123	L123 up	GO:0009793	Development	Development	embryo development ending in seed dormancy	P	AT1G32930
137	1	L123 vs. M123	L123 up	GO:0009793	Development	Development	embryo development ending in seed dormancy	P	AT1G53580
138	1	L123 vs. M123	L123 up	GO:0009793	Development	Development	embryo development ending in seed dormancy	P	AT3G60740
139	1	L123 vs. M123	L123 up	GO:0012505	Development	Development	endomembrane system	C	AT1G10130
140	1	L123 vs. M123	L123 up	GO:0009960	Development	Development	endosperm development	P	AT1G53580
141	1	L123 vs. M123	L123 up	GO:0007163	Development	Development	establishment or maintenance of cell polarity	P	AT4G35020
142	1	L123 vs. M123	L123 up	GO:0005576	Development	Development	extracellular region	C	AT1G11410
143	1	L123 vs. M123	L123 up	GO:0005576	Development	Development	extracellular region	C	AT1G58440
144	1	L123 vs. M123	L123 up	GO:0005576	Development	Development	extracellular region	C	AT2G01610
145	1	L123 vs. M123	L123 up	GO:0005576	Development	Development	extracellular region	C	AT3G06500
146	1	L123 vs. M123	L123 up	GO:0005576	Development	Development	extracellular region	C	AT3G08770
147	1	L123 vs. M123	L123 up	GO:0005576	Development	Development	extracellular region	C	AT3G12500
148	1	L123 vs. M123	L123 up	GO:0005576	Development	Development	extracellular region	C	AT3G61990
149	1	L123 vs. M123	L123 up	GO:0005576	Development	Development	extracellular region	C	AT4G38690
150	1	L123 vs. M123	L123 up	GO:0005615	Development	Development	extracellular space	C	AT5G09440
151	1	L123 vs. M123	L123 up	GO:0042406	Development	Development	extrinsic component of endoplasmic reticulum membrane	C	AT4G16710
152	1	L123 vs. M123	L123 up	GO:0005797	Development	Development	Golgi medial cisterna	C	AT1G15880
153	1	L123 vs. M123	L123 up	GO:0030176	Development	Development	integral component of endoplasmic reticulum membrane	C	AT1G14270
154	1	L123 vs. M123	L123 up	GO:0016021	Development	Development	integral component of membrane	C	AT1G10130
155	1	L123 vs. M123	L123 up	GO:0016021	Development	Development	integral component of membrane	C	AT1G11410
156	1	L123 vs. M123	L123 up	GO:0016021	Development	Development	integral component of membrane	C	AT1G15880
157	1	L123 vs. M123	L123 up	GO:0016021	Development	Development	integral component of membrane	C	AT1G17280
158	1	L123 vs. M123	L123 up	GO:0016021	Development	Development	integral component of membrane	C	AT1G24470

159	1	L123 vs. M123	L123 up	GO:0016021	Development	Development	integral component of membrane	C	AT1G29930
160	1	L123 vs. M123	L123 up	GO:0016021	Development	Development	integral component of membrane	C	AT1G32930
161	1	L123 vs. M123	L123 up	GO:0016021	Development	Development	integral component of membrane	C	AT1G50440
162	1	L123 vs. M123	L123 up	GO:0016021	Development	Development	integral component of membrane	C	AT1G58440
163	1	L123 vs. M123	L123 up	GO:0016021	Development	Development	integral component of membrane	C	AT1G67600
164	1	L123 vs. M123	L123 up	GO:0016021	Development	Development	integral component of membrane	C	AT1G68820
165	1	L123 vs. M123	L123 up	GO:0016021	Development	Development	integral component of membrane	C	AT2G32280
166	1	L123 vs. M123	L123 up	GO:0016021	Development	Development	integral component of membrane	C	AT2G34585
167	1	L123 vs. M123	L123 up	GO:0016021	Development	Development	integral component of membrane	C	AT2G38330
168	1	L123 vs. M123	L123 up	GO:0016021	Development	Development	integral component of membrane	C	AT2G41700
169	1	L123 vs. M123	L123 up	GO:0016021	Development	Development	integral component of membrane	C	AT2G47380
170	1	L123 vs. M123	L123 up	GO:0016021	Development	Development	integral component of membrane	C	AT3G16240
171	1	L123 vs. M123	L123 up	GO:0016021	Development	Development	integral component of membrane	C	AT3G22290
172	1	L123 vs. M123	L123 up	GO:0016021	Development	Development	integral component of membrane	C	AT3G50170
173	1	L123 vs. M123	L123 up	GO:0016021	Development	Development	integral component of membrane	C	AT4G18230
174	1	L123 vs. M123	L123 up	GO:0016021	Development	Development	integral component of membrane	C	AT4G19450
175	1	L123 vs. M123	L123 up	GO:0016021	Development	Development	integral component of membrane	C	AT4G26550
176	1	L123 vs. M123	L123 up	GO:0016021	Development	Development	integral component of membrane	C	AT4G28390
177	1	L123 vs. M123	L123 up	GO:0016021	Development	Development	integral component of membrane	C	AT4G34190
178	1	L123 vs. M123	L123 up	GO:0016021	Development	Development	integral component of membrane	C	AT5G17760
179	1	L123 vs. M123	L123 up	GO:0016021	Development	Development	integral component of membrane	C	AT5G23060
180	1	L123 vs. M123	L123 up	GO:0016021	Development	Development	integral component of membrane	C	AT5G36290
181	1	L123 vs. M123	L123 up	GO:0016021	Development	Development	integral component of membrane	C	ATMG00830
182	1	L123 vs. M123	L123 up	GO:0005887	Development	Development	integral component of plasma membrane	C	AT3G47420
183	1	L123 vs. M123	L123 up	GO:0005887	Development	Development	integral component of plasma membrane	C	AT4G02700
184	1	L123 vs. M123	L123 up	GO:0005887	Development	Development	integral component of plasma membrane	C	AT5G16150
185	1	L123 vs. M123	L123 up	GO:0005622	Development	Development	intracellular	C	AT3G12500
186	1	L123 vs. M123	L123 up	GO:0005622	Development	Development	intracellular	C	AT5G49890
187	1	L123 vs. M123	L123 up	GO:0043231	Development	Development	intracellular membrane-bounded organelle	C	AT2G41700
188	1	L123 vs. M123	L123 up	GO:0043231	Development	Development	intracellular membrane-bounded organelle	C	AT3G09300
189	1	L123 vs. M123	L123 up	GO:0043231	Development	Development	intracellular membrane-bounded organelle	C	AT4G15490
190	1	L123 vs. M123	L123 up	GO:0043231	Development	Development	intracellular membrane-bounded organelle	C	AT5G49890
191	1	L123 vs. M123	L123 up	GO:0031902	Development	Development	late endosome membrane	C	AT1G10130
192	1	L123 vs. M123	L123 up	GO:0048366	Development	Development	leaf development	P	AT2G32280
193	1	L123 vs. M123	L123 up	GO:0010305	Development	Development	leaf vascular tissue pattern formation	P	AT2G32280
194	1	L123 vs. M123	L123 up	GO:0016020	Development	Development	membrane	C	AT3G09300
195	1	L123 vs. M123	L123 up	GO:0016020	Development	Development	membrane	C	AT4G28390
196	1	L123 vs. M123	L123 up	GO:0005874	Development	Development	microtubule	C	AT1G64740
197	1	L123 vs. M123	L123 up	GO:0005874	Development	Development	microtubule	C	AT3G50960
198	1	L123 vs. M123	L123 up	GO:0015630	Development	Development	microtubule cytoskeleton	C	AT1G64740
199	1	L123 vs. M123	L123 up	GO:0000226	Development	Development	microtubule cytoskeleton organization	P	AT1G64740
200	1	L123 vs. M123	L123 up	GO:0000226	Development	Development	microtubule cytoskeleton organization	P	AT3G60740
201	1	L123 vs. M123	L123 up	GO:0007017	Development	Development	microtubule-based process	P	AT1G64740
202	1	L123 vs. M123	L123 up	GO:0007017	Development	Development	microtubule-based process	P	AT3G60740
203	1	L123 vs. M123	L123 up	GO:0000790	Development	Development	nuclear chromatin	C	AT4G02060
204	1	L123 vs. M123	L123 up	GO:0000784	Development	Development	nuclear chromosome, telomeric region	C	AT4G12620
205	1	L123 vs. M123	L123 up	GO:0005643	Development	Development	nuclear pore	C	AT1G09270
206	1	L123 vs. M123	L123 up	GO:0090305	Development	Development	nucleic acid phosphodiester bond hydrolysis	P	AT1G56310
207	1	L123 vs. M123	L123 up	GO:0007000	Development	Development	nucleolus organization	P	AT3G50960
208	1	L123 vs. M123	L123 up	GO:0005654	Development	Development	nucleoplasm	C	AT1G09270
209	1	L123 vs. M123	L123 up	GO:0005634	Development	Development	nucleus	C	AT1G01350
210	1	L123 vs. M123	L123 up	GO:0005634	Development	Development	nucleus	C	AT1G10130
211	1	L123 vs. M123	L123 up	GO:0005634	Development	Development	nucleus	C	AT1G16080
212	1	L123 vs. M123	L123 up	GO:0005634	Development	Development	nucleus	C	AT1G16470
213	1	L123 vs. M123	L123 up	GO:0005634	Development	Development	nucleus	C	AT1G16920
214	1	L123 vs. M123	L123 up	GO:0005634	Development	Development	nucleus	C	AT1G19550

215	1	L123 vs. M123	L123 up	GO:0005634	Development	Development	nucleus	C	AT1G19920
216	1	L123 vs. M123	L123 up	GO:0005634	Development	Development	nucleus	C	AT1G20693
217	1	L123 vs. M123	L123 up	GO:0005634	Development	Development	nucleus	C	AT1G23490
218	1	L123 vs. M123	L123 up	GO:0005634	Development	Development	nucleus	C	AT1G29930
219	1	L123 vs. M123	L123 up	GO:0005634	Development	Development	nucleus	C	AT1G34380
220	1	L123 vs. M123	L123 up	GO:0005634	Development	Development	nucleus	C	AT1G49670
221	1	L123 vs. M123	L123 up	GO:0005634	Development	Development	nucleus	C	AT1G53580
222	1	L123 vs. M123	L123 up	GO:0005634	Development	Development	nucleus	C	AT1G56310
223	1	L123 vs. M123	L123 up	GO:0005634	Development	Development	nucleus	C	AT1G64740
224	1	L123 vs. M123	L123 up	GO:0005634	Development	Development	nucleus	C	AT1G67520
225	1	L123 vs. M123	L123 up	GO:0005634	Development	Development	nucleus	C	AT1G68820
226	1	L123 vs. M123	L123 up	GO:0005634	Development	Development	nucleus	C	AT1G71130
227	1	L123 vs. M123	L123 up	GO:0005634	Development	Development	nucleus	C	AT1G79340
228	1	L123 vs. M123	L123 up	GO:0005634	Development	Development	nucleus	C	AT2G01670
229	1	L123 vs. M123	L123 up	GO:0005634	Development	Development	nucleus	C	AT2G15760
230	1	L123 vs. M123	L123 up	GO:0005634	Development	Development	nucleus	C	AT2G20670
231	1	L123 vs. M123	L123 up	GO:0005634	Development	Development	nucleus	C	AT2G30620
232	1	L123 vs. M123	L123 up	GO:0005634	Development	Development	nucleus	C	AT2G36530
233	1	L123 vs. M123	L123 up	GO:0005634	Development	Development	nucleus	C	AT2G41140
234	1	L123 vs. M123	L123 up	GO:0005634	Development	Development	nucleus	C	AT2G41900
235	1	L123 vs. M123	L123 up	GO:0005634	Development	Development	nucleus	C	AT2G42690
236	1	L123 vs. M123	L123 up	GO:0005634	Development	Development	nucleus	C	AT2G47970
237	1	L123 vs. M123	L123 up	GO:0005634	Development	Development	nucleus	C	AT3G06690
238	1	L123 vs. M123	L123 up	GO:0005634	Development	Development	nucleus	C	AT3G09300
239	1	L123 vs. M123	L123 up	GO:0005634	Development	Development	nucleus	C	AT3G16100
240	1	L123 vs. M123	L123 up	GO:0005634	Development	Development	nucleus	C	AT3G19240
241	1	L123 vs. M123	L123 up	GO:0005634	Development	Development	nucleus	C	AT3G23000
242	1	L123 vs. M123	L123 up	GO:0005634	Development	Development	nucleus	C	AT3G49640
243	1	L123 vs. M123	L123 up	GO:0005634	Development	Development	nucleus	C	AT3G50960
244	1	L123 vs. M123	L123 up	GO:0005634	Development	Development	nucleus	C	AT3G51000
245	1	L123 vs. M123	L123 up	GO:0005634	Development	Development	nucleus	C	AT3G62310
246	1	L123 vs. M123	L123 up	GO:0005634	Development	Development	nucleus	C	AT3G63390
247	1	L123 vs. M123	L123 up	GO:0005634	Development	Development	nucleus	C	AT4G02060
248	1	L123 vs. M123	L123 up	GO:0005634	Development	Development	nucleus	C	AT4G12620
249	1	L123 vs. M123	L123 up	GO:0005634	Development	Development	nucleus	C	AT4G31700
250	1	L123 vs. M123	L123 up	GO:0005634	Development	Development	nucleus	C	AT4G34710
251	1	L123 vs. M123	L123 up	GO:0005634	Development	Development	nucleus	C	AT4G35020
252	1	L123 vs. M123	L123 up	GO:0005634	Development	Development	nucleus	C	AT4G37870
253	1	L123 vs. M123	L123 up	GO:0005634	Development	Development	nucleus	C	AT5G01660
254	1	L123 vs. M123	L123 up	GO:0005634	Development	Development	nucleus	C	AT5G10070
255	1	L123 vs. M123	L123 up	GO:0005634	Development	Development	nucleus	C	AT5G18110
256	1	L123 vs. M123	L123 up	GO:0005634	Development	Development	nucleus	C	AT5G47060
257	1	L123 vs. M123	L123 up	GO:0005634	Development	Development	nucleus	C	AT5G50960
258	1	L123 vs. M123	L123 up	GO:0005634	Development	Development	nucleus	C	AT5G54160
259	1	L123 vs. M123	L123 up	GO:0005634	Development	Development	nucleus	C	AT5G56880
260	1	L123 vs. M123	L123 up	GO:0005634	Development	Development	nucleus	C	AT5G57610
261	1	L123 vs. M123	L123 up	GO:0005634	Development	Development	nucleus	C	AT5G59960
262	1	L123 vs. M123	L123 up	GO:0005634	Development	Development	nucleus	C	AT5G65360
263	1	L123 vs. M123	L123 up	GO:0010087	Development	Development	phloem or xylem histogenesis	P	AT2G32280
264	1	L123 vs. M123	L123 up	GO:0009505	Development	Development	plant-type cell wall	C	AT3G16240
265	1	L123 vs. M123	L123 up	GO:0009505	Development	Development	plant-type cell wall	C	AT3G42570
266	1	L123 vs. M123	L123 up	GO:0009705	Development	Development	plant-type vacuole membrane	C	AT3G16240
267	1	L123 vs. M123	L123 up	GO:0009705	Development	Development	plant-type vacuole membrane	C	AT5G49890
268	1	L123 vs. M123	L123 up	GO:0005886	Development	Development	plasma membrane	C	AT1G11410
269	1	L123 vs. M123	L123 up	GO:0005886	Development	Development	plasma membrane	C	AT1G14270
270	1	L123 vs. M123	L123 up	GO:0005886	Development	Development	plasma membrane	C	AT1G16920

271	1	L123 vs. M123	L123 up	GO:0005886	Development	Development	plasma membrane	C	AT1G64740
272	1	L123 vs. M123	L123 up	GO:0005886	Development	Development	plasma membrane	C	AT1G67520
273	1	L123 vs. M123	L123 up	GO:0005886	Development	Development	plasma membrane	C	AT1G79340
274	1	L123 vs. M123	L123 up	GO:0005886	Development	Development	plasma membrane	C	AT2G15760
275	1	L123 vs. M123	L123 up	GO:0005886	Development	Development	plasma membrane	C	AT2G36530
276	1	L123 vs. M123	L123 up	GO:0005886	Development	Development	plasma membrane	C	AT2G41140
277	1	L123 vs. M123	L123 up	GO:0005886	Development	Development	plasma membrane	C	AT3G16100
278	1	L123 vs. M123	L123 up	GO:0005886	Development	Development	plasma membrane	C	AT3G16240
279	1	L123 vs. M123	L123 up	GO:0005886	Development	Development	plasma membrane	C	AT3G17020
280	1	L123 vs. M123	L123 up	GO:0005886	Development	Development	plasma membrane	C	AT3G26980
281	1	L123 vs. M123	L123 up	GO:0005886	Development	Development	plasma membrane	C	AT4G16620
282	1	L123 vs. M123	L123 up	GO:0005886	Development	Development	plasma membrane	C	AT4G25100
283	1	L123 vs. M123	L123 up	GO:0005886	Development	Development	plasma membrane	C	AT4G26550
284	1	L123 vs. M123	L123 up	GO:0005886	Development	Development	plasma membrane	C	AT4G28390
285	1	L123 vs. M123	L123 up	GO:0005886	Development	Development	plasma membrane	C	AT4G30010
286	1	L123 vs. M123	L123 up	GO:0005886	Development	Development	plasma membrane	C	AT4G31700
287	1	L123 vs. M123	L123 up	GO:0005886	Development	Development	plasma membrane	C	AT4G35020
288	1	L123 vs. M123	L123 up	GO:0005886	Development	Development	plasma membrane	C	AT4G38690
289	1	L123 vs. M123	L123 up	GO:0005886	Development	Development	plasma membrane	C	AT4G39280
290	1	L123 vs. M123	L123 up	GO:0005886	Development	Development	plasma membrane	C	AT5G17760
291	1	L123 vs. M123	L123 up	GO:0005886	Development	Development	plasma membrane	C	AT5G54160
292	1	L123 vs. M123	L123 up	GO:0009555	Development	Development	pollen development	P	AT4G02060
293	1	L123 vs. M123	L123 up	GO:0000326	Development	Development	protein storage vacuole	C	AT3G16240
294	1	L123 vs. M123	L123 up	GO:0048364	Development	Development	root development	P	AT1G10130
295	1	L123 vs. M123	L123 up	GO:0048316	Development	Development	seed development	P	AT1G53580
296	1	L123 vs. M123	L123 up	GO:0048316	Development	Development	seed development	P	AT4G34710
297	1	L123 vs. M123	L123 up	GO:0009845	Development	Development	seed germination	P	AT4G32320
298	1	L123 vs. M123	L123 up	GO:0010431	Development	Development	seed maturation	P	AT4G32320
299	1	L123 vs. M123	L123 up	GO:0030427	Development	Development	site of polarized growth	C	AT4G35020
300	1	L123 vs. M123	L123 up	GO:0010319	Development	Development	stromule	C	AT1G67280
301	1	L123 vs. M123	L123 up	GO:0030527	Development	Development	structural constituent of chromatin	F	AT1G20693
302	1	L123 vs. M123	L123 up	GO:0005200	Development	Development	structural constituent of cytoskeleton	F	AT1G64740
303	1	L123 vs. M123	L123 up	GO:0007021	Development	Development	tubulin complex assembly	P	AT3G60740
304	1	L123 vs. M123	L123 up	GO:0005774	Development	Development	vacuolar membrane	C	AT1G16920
305	1	L123 vs. M123	L123 up	GO:0005774	Development	Development	vacuolar membrane	C	AT2G34585
306	1	L123 vs. M123	L123 up	GO:0005774	Development	Development	vacuolar membrane	C	AT2G41700
307	1	L123 vs. M123	L123 up	GO:0005774	Development	Development	vacuolar membrane	C	AT3G12500
308	1	L123 vs. M123	L123 up	GO:0005774	Development	Development	vacuolar membrane	C	AT3G16100
309	1	L123 vs. M123	L123 up	GO:0005774	Development	Development	vacuolar membrane	C	AT3G16240
310	1	L123 vs. M123	L123 up	GO:0005774	Development	Development	vacuolar membrane	C	AT5G49890
311	1	L123 vs. M123	L123 up	GO:0005773	Development	Development	vacuole	C	AT1G16920
312	1	L123 vs. M123	L123 up	GO:0005773	Development	Development	vacuole	C	AT2G34585
313	1	L123 vs. M123	L123 up	GO:0005773	Development	Development	vacuole	C	AT3G12500
314	1	L123 vs. M123	L123 up	GO:0005773	Development	Development	vacuole	C	AT3G16240
315	1	L123 vs. M123	L123 up	GO:0005773	Development	Development	vacuole	C	AT4G29690
316	1	L123 vs. M123	L123 up	GO:0006906	Development	Development	vesicle fusion	P	AT1G15880
317	1	L123 vs. M123	L123 up	GO:0003997	Metabolism	Bioenergetics	acyl-CoA oxidase activity	F	AT3G06690
318	1	L123 vs. M123	L123 up	GO:0043531	Metabolism	Bioenergetics	ADP binding	F	AT4G12010
319	1	L123 vs. M123	L123 up	GO:0047631	Metabolism	Bioenergetics	ADP-ribose diphosphatase activity	F	AT5G47240
320	1	L123 vs. M123	L123 up	GO:0004029	Metabolism	Bioenergetics	aldehyde dehydrogenase (NAD+) activity	F	AT3G48000
321	1	L123 vs. M123	L123 up	GO:0016887	Metabolism	Bioenergetics	ATPase activity	F	AT2G41700
322	1	L123 vs. M123	L123 up	GO:0016887	Metabolism	Bioenergetics	ATPase activity	F	AT5G17760
323	1	L123 vs. M123	L123 up	GO:0051117	Metabolism	Bioenergetics	ATPase binding	F	AT1G11750
324	1	L123 vs. M123	L123 up	GO:0051117	Metabolism	Bioenergetics	ATPase binding	F	ATCG00670
325	1	L123 vs. M123	L123 up	GO:0030246	Metabolism	Bioenergetics	carbohydrate binding	F	AT1G11410
326	1	L123 vs. M123	L123 up	GO:0017004	Metabolism	Bioenergetics	cytochrome complex assembly	P	ATMG00830

327	1	L123 vs. M123	L123 up	GO:0009055	Metabolism	Bioenergetics	electron transfer activity	F	AT1G60950
328	1	L123 vs. M123	L123 up	GO:0009055	Metabolism	Bioenergetics	electron transfer activity	F	AT1G72230
329	1	L123 vs. M123	L123 up	GO:0033540	Metabolism	Bioenergetics	fatty acid beta-oxidation using acyl-CoA oxidase	P	AT3G06690
330	1	L123 vs. M123	L123 up	GO:0005504	Metabolism	Bioenergetics	fatty acid binding	F	AT3G06690
331	1	L123 vs. M123	L123 up	GO:0006094	Metabolism	Bioenergetics	gluconeogenesis	P	AT4G37870
332	1	L123 vs. M123	L123 up	GO:0043878	Metabolism	Bioenergetics	glyceraldehyde-3-phosphate dehydrogenase (NAD+) (non-phosphorylating) activity	F	AT3G48000
333	1	L123 vs. M123	L123 up	GO:0006096	Metabolism	Bioenergetics	glycolytic process	P	AT2G36530
334	1	L123 vs. M123	L123 up	GO:0052853	Metabolism	Bioenergetics	long-chain-(S)-2-hydroxy-long-chain-acid oxidase activity	F	AT3G14130
335	1	L123 vs. M123	L123 up	GO:0006120	Metabolism	Bioenergetics	mitochondrial electron transport, NADH to ubiquinone	P	AT3G03070
336	1	L123 vs. M123	L123 up	GO:0005740	Metabolism	Bioenergetics	mitochondrial envelope	C	AT2G36530
337	1	L123 vs. M123	L123 up	GO:0005743	Metabolism	Bioenergetics	mitochondrial inner membrane	C	AT4G28390
338	1	L123 vs. M123	L123 up	GO:0005743	Metabolism	Bioenergetics	mitochondrial inner membrane	C	ATMG00830
339	1	L123 vs. M123	L123 up	GO:0005759	Metabolism	Bioenergetics	mitochondrial matrix	C	AT3G48000
340	1	L123 vs. M123	L123 up	GO:0005759	Metabolism	Bioenergetics	mitochondrial matrix	C	AT5G49840
341	1	L123 vs. M123	L123 up	GO:0005741	Metabolism	Bioenergetics	mitochondrial outer membrane	C	AT2G36530
342	1	L123 vs. M123	L123 up	GO:0005753	Metabolism	Bioenergetics	mitochondrial proton-transporting ATP synthase complex	C	AT4G30010
343	1	L123 vs. M123	L123 up	GO:0005746	Metabolism	Bioenergetics	mitochondrial respirasome	C	AT2G47380
344	1	L123 vs. M123	L123 up	GO:0005747	Metabolism	Bioenergetics	mitochondrial respiratory chain complex I	C	AT3G03070
345	1	L123 vs. M123	L123 up	GO:0005739	Metabolism	Bioenergetics	mitochondrion	C	AT1G14890
346	1	L123 vs. M123	L123 up	GO:0005739	Metabolism	Bioenergetics	mitochondrion	C	AT1G49670
347	1	L123 vs. M123	L123 up	GO:0005739	Metabolism	Bioenergetics	mitochondrion	C	AT1G53580
348	1	L123 vs. M123	L123 up	GO:0005739	Metabolism	Bioenergetics	mitochondrion	C	AT1G67280
349	1	L123 vs. M123	L123 up	GO:0005739	Metabolism	Bioenergetics	mitochondrion	C	AT1G68820
350	1	L123 vs. M123	L123 up	GO:0005739	Metabolism	Bioenergetics	mitochondrion	C	AT1G79340
351	1	L123 vs. M123	L123 up	GO:0005739	Metabolism	Bioenergetics	mitochondrion	C	AT2G01670
352	1	L123 vs. M123	L123 up	GO:0005739	Metabolism	Bioenergetics	mitochondrion	C	AT2G29370
353	1	L123 vs. M123	L123 up	GO:0005739	Metabolism	Bioenergetics	mitochondrion	C	AT2G30620
354	1	L123 vs. M123	L123 up	GO:0005739	Metabolism	Bioenergetics	mitochondrion	C	AT2G36530
355	1	L123 vs. M123	L123 up	GO:0005739	Metabolism	Bioenergetics	mitochondrion	C	AT2G41700
356	1	L123 vs. M123	L123 up	GO:0005739	Metabolism	Bioenergetics	mitochondrion	C	AT2G43020
357	1	L123 vs. M123	L123 up	GO:0005739	Metabolism	Bioenergetics	mitochondrion	C	AT2G45740
358	1	L123 vs. M123	L123 up	GO:0005739	Metabolism	Bioenergetics	mitochondrion	C	AT2G47380
359	1	L123 vs. M123	L123 up	GO:0005739	Metabolism	Bioenergetics	mitochondrion	C	AT3G03070
360	1	L123 vs. M123	L123 up	GO:0005739	Metabolism	Bioenergetics	mitochondrion	C	AT3G06500
361	1	L123 vs. M123	L123 up	GO:0005739	Metabolism	Bioenergetics	mitochondrion	C	AT3G48000
362	1	L123 vs. M123	L123 up	GO:0005739	Metabolism	Bioenergetics	mitochondrion	C	AT4G16620
363	1	L123 vs. M123	L123 up	GO:0005739	Metabolism	Bioenergetics	mitochondrion	C	AT4G18230
364	1	L123 vs. M123	L123 up	GO:0005739	Metabolism	Bioenergetics	mitochondrion	C	AT4G19450
365	1	L123 vs. M123	L123 up	GO:0005739	Metabolism	Bioenergetics	mitochondrion	C	AT4G25100
366	1	L123 vs. M123	L123 up	GO:0005739	Metabolism	Bioenergetics	mitochondrion	C	AT4G28390
367	1	L123 vs. M123	L123 up	GO:0005739	Metabolism	Bioenergetics	mitochondrion	C	AT4G30010
368	1	L123 vs. M123	L123 up	GO:0005739	Metabolism	Bioenergetics	mitochondrion	C	AT4G31700
369	1	L123 vs. M123	L123 up	GO:0005739	Metabolism	Bioenergetics	mitochondrion	C	AT4G35020
370	1	L123 vs. M123	L123 up	GO:0005739	Metabolism	Bioenergetics	mitochondrion	C	AT5G09950
371	1	L123 vs. M123	L123 up	GO:0005739	Metabolism	Bioenergetics	mitochondrion	C	AT5G23060
372	1	L123 vs. M123	L123 up	GO:0005739	Metabolism	Bioenergetics	mitochondrion	C	AT5G59960
373	1	L123 vs. M123	L123 up	GO:0005739	Metabolism	Bioenergetics	mitochondrion	C	AT5G65685
374	1	L123 vs. M123	L123 up	GO:0005739	Metabolism	Bioenergetics	mitochondrion	C	ATMG00830
375	1	L123 vs. M123	L123 up	GO:0051287	Metabolism	Bioenergetics	NAD binding	F	AT5G47240
376	1	L123 vs. M123	L123 up	GO:0009435	Metabolism	Bioenergetics	NAD biosynthetic process	P	AT2G01350
377	1	L123 vs. M123	L123 up	GO:0010275	Metabolism	Bioenergetics	NAD(P)H dehydrogenase complex assembly	P	AT5G13120
378	1	L123 vs. M123	L123 up	GO:0035529	Metabolism	Bioenergetics	NADH pyrophosphatase activity	F	AT4G29690

379	1	L123 vs. M123	L123 up	GO:0035529	Metabolism	Bioenergetics	NADH pyrophosphatase activity	F	AT5G47240
380	1	L123 vs. M123	L123 up	GO:0016491	Metabolism	Bioenergetics	oxidoreductase activity	F	AT1G49670
381	1	L123 vs. M123	L123 up	GO:0016491	Metabolism	Bioenergetics	oxidoreductase activity	F	AT2G29370
382	1	L123 vs. M123	L123 up	GO:0016491	Metabolism	Bioenergetics	oxidoreductase activity	F	AT2G43020
383	1	L123 vs. M123	L123 up	GO:0016701	Metabolism	Bioenergetics	oxidoreductase activity, acting on single donors with incorporation of molecular oxygen	F	AT4G15093
384	1	L123 vs. M123	L123 up	GO:0016616	Metabolism	Bioenergetics	oxidoreductase activity, acting on the CH-OH group of donors, NAD or NADP as acceptor	F	AT1G72680
385	1	L123 vs. M123	L123 up	GO:0000015	Metabolism	Bioenergetics	phosphopyruvate hydratase complex	C	AT2G36530
386	1	L123 vs. M123	L123 up	GO:0008553	Metabolism	Bioenergetics	proton-exporting ATPase activity, phosphorylative mechanism	F	AT1G10130
387	1	L123 vs. M123	L123 up	GO:0009011	Metabolism	Bioenergetics	starch synthase activity	F	AT5G65685
388	1	L123 vs. M123	L123 up	GO:0005987	Metabolism	Bioenergetics	sucrose catabolic process	P	AT3G06500
389	1	L123 vs. M123	L123 up	GO:0052852	Metabolism	Bioenergetics	very-long-chain-(S)-2-hydroxy-acid oxidase activity	F	AT3G14130
390	1	L123 vs. M123	L123 up	GO:0006527	Metabolism	Catabolism	arginine catabolic process	P	AT4G34710
391	1	L123 vs. M123	L123 up	GO:0016998	Metabolism	Catabolism	cell wall macromolecule catabolic process	P	AT3G12500
392	1	L123 vs. M123	L123 up	GO:0006032	Metabolism	Catabolism	chitin catabolic process	P	AT3G12500
393	1	L123 vs. M123	L123 up	GO:0080008	Metabolism	Catabolism	Cul4-RING E3 ubiquitin ligase complex	C	AT5G60940
394	1	L123 vs. M123	L123 up	GO:0004197	Metabolism	Catabolism	cysteine-type endopeptidase activity	F	AT1G14270
395	1	L123 vs. M123	L123 up	GO:0004197	Metabolism	Catabolism	cysteine-type endopeptidase activity	F	AT1G79340
396	1	L123 vs. M123	L123 up	GO:0004175	Metabolism	Catabolism	endopeptidase activity	F	AT1G16470
397	1	L123 vs. M123	L123 up	GO:0004527	Metabolism	Catabolism	exonuclease activity	F	AT1G34380
398	1	L123 vs. M123	L123 up	GO:0005779	Metabolism	Catabolism	integral component of peroxisomal membrane	C	AT2G45740
399	1	L123 vs. M123	L123 up	GO:0016042	Metabolism	Catabolism	lipid catabolic process	P	AT2G42690
400	1	L123 vs. M123	L123 up	GO:0008233	Metabolism	Catabolism	peptidase activity	F	AT4G17486
401	1	L123 vs. M123	L123 up	GO:0004601	Metabolism	Catabolism	peroxidase activity	F	AT1G67600
402	1	L123 vs. M123	L123 up	GO:0004601	Metabolism	Catabolism	peroxidase activity	F	AT3G42570
403	1	L123 vs. M123	L123 up	GO:0004601	Metabolism	Catabolism	peroxidase activity	F	AT4G32320
404	1	L123 vs. M123	L123 up	GO:0005778	Metabolism	Catabolism	peroxisomal membrane	C	AT2G45740
405	1	L123 vs. M123	L123 up	GO:0005777	Metabolism	Catabolism	peroxisome	C	AT1G49670
406	1	L123 vs. M123	L123 up	GO:0005777	Metabolism	Catabolism	peroxisome	C	AT2G29370
407	1	L123 vs. M123	L123 up	GO:0005777	Metabolism	Catabolism	peroxisome	C	AT2G43020
408	1	L123 vs. M123	L123 up	GO:0005777	Metabolism	Catabolism	peroxisome	C	AT2G45740
409	1	L123 vs. M123	L123 up	GO:0005777	Metabolism	Catabolism	peroxisome	C	AT3G06690
410	1	L123 vs. M123	L123 up	GO:0005777	Metabolism	Catabolism	peroxisome	C	AT3G14130
411	1	L123 vs. M123	L123 up	GO:0005777	Metabolism	Catabolism	peroxisome	C	AT3G50170
412	1	L123 vs. M123	L123 up	GO:0016559	Metabolism	Catabolism	peroxisome fission	P	AT2G45740
413	1	L123 vs. M123	L123 up	GO:0007031	Metabolism	Catabolism	peroxisome organization	P	AT2G45740
414	1	L123 vs. M123	L123 up	GO:0006598	Metabolism	Catabolism	polyamine catabolic process	P	AT2G43020
415	1	L123 vs. M123	L123 up	GO:0000272	Metabolism	Catabolism	polysaccharide catabolic process	P	AT3G12500
416	1	L123 vs. M123	L123 up	GO:0010498	Metabolism	Catabolism	proteasomal protein catabolic process	P	AT1G16470
417	1	L123 vs. M123	L123 up	GO:0010499	Metabolism	Catabolism	proteasomal ubiquitin-independent protein catabolic process	P	AT1G16470
418	1	L123 vs. M123	L123 up	GO:0000502	Metabolism	Catabolism	proteasome complex	C	AT1G16470
419	1	L123 vs. M123	L123 up	GO:0005839	Metabolism	Catabolism	proteasome core complex	C	AT1G16470
420	1	L123 vs. M123	L123 up	GO:0019773	Metabolism	Catabolism	proteasome core complex, alpha-subunit complex	C	AT1G16470
421	1	L123 vs. M123	L123 up	GO:0043161	Metabolism	Catabolism	proteasome-mediated ubiquitin-dependent protein catabolic process	P	AT1G16470
422	1	L123 vs. M123	L123 up	GO:0043161	Metabolism	Catabolism	proteasome-mediated ubiquitin-dependent protein catabolic process	P	AT2G47970
423	1	L123 vs. M123	L123 up	GO:0030163	Metabolism	Catabolism	protein catabolic process	P	AT5G49840
424	1	L123 vs. M123	L123 up	GO:0000209	Metabolism	Catabolism	protein polyubiquitination	P	AT1G17280
425	1	L123 vs. M123	L123 up	GO:0006508	Metabolism	Catabolism	proteolysis	P	AT4G17486
426	1	L123 vs. M123	L123 up	GO:0006508	Metabolism	Catabolism	proteolysis	P	ATCG00670
427	1	L123 vs. M123	L123 up	GO:0034213	Metabolism	Catabolism	quinolinate catabolic process	P	AT2G01350
428	1	L123 vs. M123	L123 up	GO:0044375	Metabolism	Catabolism	regulation of peroxisome size	P	AT2G45740

429	1	L123 vs. M123	L123 up	GO:0004252	Metabolism	Catabolism	serine-type endopeptidase activity	F	AT1G11750
430	1	L123 vs. M123	L123 up	GO:0004252	Metabolism	Catabolism	serine-type endopeptidase activity	F	ATCG00670
431	1	L123 vs. M123	L123 up	GO:0046203	Metabolism	Catabolism	spermidine catabolic process	P	AT2G43020
432	1	L123 vs. M123	L123 up	GO:0046208	Metabolism	Catabolism	spermine catabolic process	P	AT2G43020
433	1	L123 vs. M123	L123 up	GO:0004298	Metabolism	Catabolism	threonine-type endopeptidase activity	F	AT1G16470
434	1	L123 vs. M123	L123 up	GO:0043130	Metabolism	Catabolism	ubiquitin binding	F	AT2G47970
435	1	L123 vs. M123	L123 up	GO:0061631	Metabolism	Catabolism	ubiquitin conjugating enzyme activity	F	AT1G17280
436	1	L123 vs. M123	L123 up	GO:0031625	Metabolism	Catabolism	ubiquitin protein ligase binding	F	AT2G47970
437	1	L123 vs. M123	L123 up	GO:0006511	Metabolism	Catabolism	ubiquitin-dependent protein catabolic process	P	AT1G16470
438	1	L123 vs. M123	L123 up	GO:0006511	Metabolism	Catabolism	ubiquitin-dependent protein catabolic process	P	AT1G17280
439	1	L123 vs. M123	L123 up	GO:0006511	Metabolism	Catabolism	ubiquitin-dependent protein catabolic process	P	AT2G47970
440	1	L123 vs. M123	L123 up	GO:0004842	Metabolism	Catabolism	ubiquitin-protein transferase activity	F	AT1G17280
441	1	L123 vs. M123	L123 up	GO:0051537	Metabolism	Metabolism	2 iron, 2 sulfur cluster binding	F	AT1G60950
442	1	L123 vs. M123	L123 up	GO:0008408	Metabolism	Metabolism	3'-5' exonuclease activity	F	AT1G56310
443	1	L123 vs. M123	L123 up	GO:0008409	Metabolism	Metabolism	5'-3' exonuclease activity	F	AT1G34380
444	1	L123 vs. M123	L123 up	GO:0017096	Metabolism	Metabolism	acetylserotonin O-methyltransferase activity	F	AT5G54160
445	1	L123 vs. M123	L123 up	GO:0007015	Metabolism	Metabolism	actin filament organization	P	AT4G35020
446	1	L123 vs. M123	L123 up	GO:0008792	Metabolism	Metabolism	arginine decarboxylase activity	F	AT4G34710
447	1	L123 vs. M123	L123 up	GO:0019438	Metabolism	Metabolism	aromatic compound biosynthetic process	P	AT5G54160
448	1	L123 vs. M123	L123 up	GO:0005524	Metabolism	Metabolism	ATP binding	F	AT1G10130
449	1	L123 vs. M123	L123 up	GO:0005524	Metabolism	Metabolism	ATP binding	F	AT1G11410
450	1	L123 vs. M123	L123 up	GO:0005524	Metabolism	Metabolism	ATP binding	F	AT1G17280
451	1	L123 vs. M123	L123 up	GO:0005524	Metabolism	Metabolism	ATP binding	F	AT1G19920
452	1	L123 vs. M123	L123 up	GO:0005524	Metabolism	Metabolism	ATP binding	F	AT2G41140
453	1	L123 vs. M123	L123 up	GO:0005524	Metabolism	Metabolism	ATP binding	F	AT2G41700
454	1	L123 vs. M123	L123 up	GO:0005524	Metabolism	Metabolism	ATP binding	F	AT3G23000
455	1	L123 vs. M123	L123 up	GO:0005524	Metabolism	Metabolism	ATP binding	F	AT3G48000
456	1	L123 vs. M123	L123 up	GO:0005524	Metabolism	Metabolism	ATP binding	F	AT3G62310
457	1	L123 vs. M123	L123 up	GO:0005524	Metabolism	Metabolism	ATP binding	F	AT4G02060
458	1	L123 vs. M123	L123 up	GO:0005524	Metabolism	Metabolism	ATP binding	F	AT4G12010
459	1	L123 vs. M123	L123 up	GO:0005524	Metabolism	Metabolism	ATP binding	F	AT4G12620
460	1	L123 vs. M123	L123 up	GO:0005524	Metabolism	Metabolism	ATP binding	F	AT4G37870
461	1	L123 vs. M123	L123 up	GO:0005524	Metabolism	Metabolism	ATP binding	F	AT4G39280
462	1	L123 vs. M123	L123 up	GO:0005524	Metabolism	Metabolism	ATP binding	F	AT5G17760
463	1	L123 vs. M123	L123 up	GO:0005524	Metabolism	Metabolism	ATP binding	F	AT5G49840
464	1	L123 vs. M123	L123 up	GO:0005524	Metabolism	Metabolism	ATP binding	F	AT5G50960
465	1	L123 vs. M123	L123 up	GO:0005524	Metabolism	Metabolism	ATP binding	F	AT5G57610
466	1	L123 vs. M123	L123 up	GO:0004176	Metabolism	Metabolism	ATP-dependent peptidase activity	F	AT1G11750
467	1	L123 vs. M123	L123 up	GO:0004176	Metabolism	Metabolism	ATP-dependent peptidase activity	F	AT5G49840
468	1	L123 vs. M123	L123 up	GO:0004176	Metabolism	Metabolism	ATP-dependent peptidase activity	F	ATCG00670
469	1	L123 vs. M123	L123 up	GO:0048487	Metabolism	Metabolism	beta-tubulin binding	F	AT3G50960
470	1	L123 vs. M123	L123 up	GO:0048487	Metabolism	Metabolism	beta-tubulin binding	F	AT3G60740
471	1	L123 vs. M123	L123 up	GO:0008150	Metabolism	Metabolism	biological_process	P	AT1G67600
472	1	L123 vs. M123	L123 up	GO:0008150	Metabolism	Metabolism	biological_process	P	AT2G15760
473	1	L123 vs. M123	L123 up	GO:0008150	Metabolism	Metabolism	biological_process	P	AT2G34585
474	1	L123 vs. M123	L123 up	GO:0008150	Metabolism	Metabolism	biological_process	P	AT2G41950
475	1	L123 vs. M123	L123 up	GO:0008150	Metabolism	Metabolism	biological_process	P	AT3G15430
476	1	L123 vs. M123	L123 up	GO:0008150	Metabolism	Metabolism	biological_process	P	AT3G22540
477	1	L123 vs. M123	L123 up	GO:0008150	Metabolism	Metabolism	biological_process	P	AT3G50170
478	1	L123 vs. M123	L123 up	GO:0008150	Metabolism	Metabolism	biological_process	P	AT3G63390
479	1	L123 vs. M123	L123 up	GO:0008150	Metabolism	Metabolism	biological_process	P	AT4G18975
480	1	L123 vs. M123	L123 up	GO:0008150	Metabolism	Metabolism	biological_process	P	AT4G19450
481	1	L123 vs. M123	L123 up	GO:0008150	Metabolism	Metabolism	biological_process	P	AT4G30010
482	1	L123 vs. M123	L123 up	GO:0008150	Metabolism	Metabolism	biological_process	P	AT5G19940
483	1	L123 vs. M123	L123 up	GO:0008150	Metabolism	Metabolism	biological_process	P	AT5G45350
484	1	L123 vs. M123	L123 up	GO:0008150	Metabolism	Metabolism	biological_process	P	AT5G48330

485	1	L123 vs. M123	L123 up	GO:0008150	Metabolism	Metabolism	biological_process	P	AT5G56880
486	1	L123 vs. M123	L123 up	GO:0008150	Metabolism	Metabolism	biological_process	P	AT5G59960
487	1	L123 vs. M123	L123 up	GO:0047763	Metabolism	Metabolism	caffeate O-methyltransferase activity	F	AT5G54160
488	1	L123 vs. M123	L123 up	GO:0006725	Metabolism	Metabolism	cellular aromatic compound metabolic process	P	AT4G15093
489	1	L123 vs. M123	L123 up	GO:0008061	Metabolism	Metabolism	chitin binding	F	AT3G12500
490	1	L123 vs. M123	L123 up	GO:0004568	Metabolism	Metabolism	chitinase activity	F	AT3G12500
491	1	L123 vs. M123	L123 up	GO:0003682	Metabolism	Metabolism	chromatin binding	F	AT1G20693
492	1	L123 vs. M123	L123 up	GO:0003682	Metabolism	Metabolism	chromatin binding	F	AT4G12620
493	1	L123 vs. M123	L123 up	GO:0050412	Metabolism	Metabolism	cinnamate beta-D-glucosyltransferase activity	F	AT4G15490
494	1	L123 vs. M123	L123 up	GO:0045551	Metabolism	Metabolism	cinnamyl-alcohol dehydrogenase activity	F	AT1G72680
495	1	L123 vs. M123	L123 up	GO:0005507	Metabolism	Metabolism	copper ion binding	F	AT2G36530
496	1	L123 vs. M123	L123 up	GO:0005507	Metabolism	Metabolism	copper ion binding	F	AT4G25100
497	1	L123 vs. M123	L123 up	GO:0016018	Metabolism	Metabolism	cyclosporin A binding	F	AT5G13120
498	1	L123 vs. M123	L123 up	GO:0008234	Metabolism	Metabolism	cysteine-type peptidase activity	F	AT1G79340
499	1	L123 vs. M123	L123 up	GO:0009704	Metabolism	Metabolism	de-etiolation	P	AT5G23060
500	1	L123 vs. M123	L123 up	GO:0051213	Metabolism	Metabolism	dioxygenase activity	F	AT4G15093
501	1	L123 vs. M123	L123 up	GO:0003677	Metabolism	Metabolism	DNA binding	F	AT1G01350
502	1	L123 vs. M123	L123 up	GO:0003677	Metabolism	Metabolism	DNA binding	F	AT1G20693
503	1	L123 vs. M123	L123 up	GO:0003677	Metabolism	Metabolism	DNA binding	F	AT1G34380
504	1	L123 vs. M123	L123 up	GO:0003677	Metabolism	Metabolism	DNA binding	F	AT2G36530
505	1	L123 vs. M123	L123 up	GO:0003677	Metabolism	Metabolism	DNA binding	F	AT2G41900
506	1	L123 vs. M123	L123 up	GO:0003677	Metabolism	Metabolism	DNA binding	F	AT5G65360
507	1	L123 vs. M123	L123 up	GO:0006488	Metabolism	Metabolism	dolichol-linked oligosaccharide biosynthetic process	P	AT4G16710
508	1	L123 vs. M123	L123 up	GO:0006488	Metabolism	Metabolism	dolichol-linked oligosaccharide biosynthetic process	P	AT4G18230
509	1	L123 vs. M123	L123 up	GO:0003690	Metabolism	Metabolism	double-stranded DNA binding	F	AT2G30620
510	1	L123 vs. M123	L123 up	GO:0004857	Metabolism	Metabolism	enzyme inhibitor activity	F	AT1G14890
511	1	L123 vs. M123	L123 up	GO:0004857	Metabolism	Metabolism	enzyme inhibitor activity	F	AT2G01610
512	1	L123 vs. M123	L123 up	GO:0008198	Metabolism	Metabolism	ferrous iron binding	F	AT4G15093
513	1	L123 vs. M123	L123 up	GO:0050660	Metabolism	Metabolism	flavin adenine dinucleotide binding	F	AT1G58440
514	1	L123 vs. M123	L123 up	GO:0050660	Metabolism	Metabolism	flavin adenine dinucleotide binding	F	AT3G06690
515	1	L123 vs. M123	L123 up	GO:0050660	Metabolism	Metabolism	flavin adenine dinucleotide binding	F	AT3G49640
516	1	L123 vs. M123	L123 up	GO:0010181	Metabolism	Metabolism	FMN binding	F	AT3G14130
517	1	L123 vs. M123	L123 up	GO:0008378	Metabolism	Metabolism	galactosyltransferase activity	F	AT1G32930
518	1	L123 vs. M123	L123 up	GO:0019003	Metabolism	Metabolism	GDP binding	F	AT4G35020
519	1	L123 vs. M123	L123 up	GO:0005525	Metabolism	Metabolism	GTP binding	F	AT1G16920
520	1	L123 vs. M123	L123 up	GO:0005525	Metabolism	Metabolism	GTP binding	F	AT1G23490
521	1	L123 vs. M123	L123 up	GO:0005525	Metabolism	Metabolism	GTP binding	F	AT1G64740
522	1	L123 vs. M123	L123 up	GO:0005525	Metabolism	Metabolism	GTP binding	F	AT3G16100
523	1	L123 vs. M123	L123 up	GO:0005525	Metabolism	Metabolism	GTP binding	F	AT4G35020
524	1	L123 vs. M123	L123 up	GO:0005096	Metabolism	Metabolism	GTPase activator activity	F	AT3G60740
525	1	L123 vs. M123	L123 up	GO:0003924	Metabolism	Metabolism	GTPase activity	F	AT1G16920
526	1	L123 vs. M123	L123 up	GO:0003924	Metabolism	Metabolism	GTPase activity	F	AT1G64740
527	1	L123 vs. M123	L123 up	GO:0003924	Metabolism	Metabolism	GTPase activity	F	AT3G16100
528	1	L123 vs. M123	L123 up	GO:0003924	Metabolism	Metabolism	GTPase activity	F	AT4G35020
529	1	L123 vs. M123	L123 up	GO:0020037	Metabolism	Metabolism	heme binding	F	AT4G32320
530	1	L123 vs. M123	L123 up	GO:0020037	Metabolism	Metabolism	heme binding	F	ATMG00830
531	1	L123 vs. M123	L123 up	GO:0004412	Metabolism	Metabolism	homoserine dehydrogenase activity	F	AT5G21060
532	1	L123 vs. M123	L123 up	GO:0070814	Metabolism	Metabolism	hydrogen sulfide biosynthetic process	P	AT1G19920
533	1	L123 vs. M123	L123 up	GO:0070813	Metabolism	Metabolism	hydrogen sulfide metabolic process	P	AT1G53580
534	1	L123 vs. M123	L123 up	GO:0016787	Metabolism	Metabolism	hydrolase activity	F	AT2G01670
535	1	L123 vs. M123	L123 up	GO:0016787	Metabolism	Metabolism	hydrolase activity	F	AT3G17020
536	1	L123 vs. M123	L123 up	GO:0016787	Metabolism	Metabolism	hydrolase activity	F	AT3G51000
537	1	L123 vs. M123	L123 up	GO:0016788	Metabolism	Metabolism	hydrolase activity, acting on ester bonds	F	AT1G53580
538	1	L123 vs. M123	L123 up	GO:0042802	Metabolism	Metabolism	identical protein binding	F	AT1G79340

539	1	L123 vs. M123	L123 up	GO:0042802	Metabolism	Metabolism	identical protein binding	F	AT2G45740
540	1	L123 vs. M123	L123 up	GO:0042802	Metabolism	Metabolism	identical protein binding	F	AT3G16240
541	1	L123 vs. M123	L123 up	GO:0004427	Metabolism	Metabolism	inorganic diphosphatase activity	F	AT3G53620
542	1	L123 vs. M123	L123 up	GO:0006021	Metabolism	Metabolism	inositol biosynthetic process	P	AT1G31190
543	1	L123 vs. M123	L123 up	GO:0008934	Metabolism	Metabolism	inositol monophosphate 1-phosphatase activity	F	AT1G31190
544	1	L123 vs. M123	L123 up	GO:0052832	Metabolism	Metabolism	inositol monophosphate 3-phosphatase activity	F	AT1G31190
545	1	L123 vs. M123	L123 up	GO:0052833	Metabolism	Metabolism	inositol monophosphate 4-phosphatase activity	F	AT1G31190
546	1	L123 vs. M123	L123 up	GO:0016226	Metabolism	Metabolism	iron-sulfur cluster assembly	P	AT5G50960
547	1	L123 vs. M123	L123 up	GO:0051536	Metabolism	Metabolism	iron-sulfur cluster binding	F	AT5G50960
548	1	L123 vs. M123	L123 up	GO:0009097	Metabolism	Metabolism	isoleucine biosynthetic process	P	AT5G21060
549	1	L123 vs. M123	L123 up	GO:0045703	Metabolism	Metabolism	ketoreductase activity	F	AT1G24470
550	1	L123 vs. M123	L123 up	GO:0019516	Metabolism	Metabolism	lactate oxidation	P	AT3G14130
551	1	L123 vs. M123	L123 up	GO:0009809	Metabolism	Metabolism	lignin biosynthetic process	P	AT1G72680
552	1	L123 vs. M123	L123 up	GO:0009809	Metabolism	Metabolism	lignin biosynthetic process	P	AT5G54160
553	1	L123 vs. M123	L123 up	GO:0008289	Metabolism	Metabolism	lipid binding	F	AT3G08770
554	1	L123 vs. M123	L123 up	GO:0008289	Metabolism	Metabolism	lipid binding	F	AT3G09300
555	1	L123 vs. M123	L123 up	GO:0006629	Metabolism	Metabolism	lipid metabolic process	P	AT4G38690
556	1	L123 vs. M123	L123 up	GO:0004459	Metabolism	Metabolism	L-lactate dehydrogenase activity	F	AT3G14130
557	1	L123 vs. M123	L123 up	GO:0030744	Metabolism	Metabolism	luteolin O-methyltransferase activity	F	AT5G54160
558	1	L123 vs. M123	L123 up	GO:0000287	Metabolism	Metabolism	magnesium ion binding	F	AT2G36530
559	1	L123 vs. M123	L123 up	GO:0000287	Metabolism	Metabolism	magnesium ion binding	F	AT3G53620
560	1	L123 vs. M123	L123 up	GO:0052854	Metabolism	Metabolism	medium-chain-(S)-2-hydroxy-acid oxidase activity	F	AT3G14130
561	1	L123 vs. M123	L123 up	GO:0030187	Metabolism	Metabolism	melatonin biosynthetic process	P	AT5G54160
562	1	L123 vs. M123	L123 up	GO:0046872	Metabolism	Metabolism	metal ion binding	F	AT1G01350
563	1	L123 vs. M123	L123 up	GO:0046872	Metabolism	Metabolism	metal ion binding	F	AT1G10130
564	1	L123 vs. M123	L123 up	GO:0046872	Metabolism	Metabolism	metal ion binding	F	AT1G29930
565	1	L123 vs. M123	L123 up	GO:0046872	Metabolism	Metabolism	metal ion binding	F	AT1G31190
566	1	L123 vs. M123	L123 up	GO:0046872	Metabolism	Metabolism	metal ion binding	F	AT1G53580
567	1	L123 vs. M123	L123 up	GO:0046872	Metabolism	Metabolism	metal ion binding	F	AT1G60950
568	1	L123 vs. M123	L123 up	GO:0046872	Metabolism	Metabolism	metal ion binding	F	AT1G67280
569	1	L123 vs. M123	L123 up	GO:0046872	Metabolism	Metabolism	metal ion binding	F	AT2G01670
570	1	L123 vs. M123	L123 up	GO:0046872	Metabolism	Metabolism	metal ion binding	F	AT2G41140
571	1	L123 vs. M123	L123 up	GO:0046872	Metabolism	Metabolism	metal ion binding	F	AT2G41900
572	1	L123 vs. M123	L123 up	GO:0046872	Metabolism	Metabolism	metal ion binding	F	AT4G02060
573	1	L123 vs. M123	L123 up	GO:0046872	Metabolism	Metabolism	metal ion binding	F	AT4G12620
574	1	L123 vs. M123	L123 up	GO:0046872	Metabolism	Metabolism	metal ion binding	F	AT4G32320
575	1	L123 vs. M123	L123 up	GO:0046872	Metabolism	Metabolism	metal ion binding	F	AT5G47060
576	1	L123 vs. M123	L123 up	GO:0046872	Metabolism	Metabolism	metal ion binding	F	AT5G47240
577	1	L123 vs. M123	L123 up	GO:0046872	Metabolism	Metabolism	metal ion binding	F	AT5G50960
578	1	L123 vs. M123	L123 up	GO:0009086	Metabolism	Metabolism	methionine biosynthetic process	P	AT5G21060
579	1	L123 vs. M123	L123 up	GO:0003674	Metabolism	Metabolism	molecular_function	F	AT2G34585
580	1	L123 vs. M123	L123 up	GO:0003674	Metabolism	Metabolism	molecular_function	F	AT3G19240
581	1	L123 vs. M123	L123 up	GO:0003674	Metabolism	Metabolism	molecular_function	F	AT3G22290
582	1	L123 vs. M123	L123 up	GO:0003674	Metabolism	Metabolism	molecular_function	F	AT3G22540
583	1	L123 vs. M123	L123 up	GO:0003674	Metabolism	Metabolism	molecular_function	F	AT3G26980
584	1	L123 vs. M123	L123 up	GO:0003674	Metabolism	Metabolism	molecular_function	F	AT3G50170
585	1	L123 vs. M123	L123 up	GO:0003674	Metabolism	Metabolism	molecular_function	F	AT4G18975
586	1	L123 vs. M123	L123 up	GO:0003674	Metabolism	Metabolism	molecular_function	F	AT4G19450
587	1	L123 vs. M123	L123 up	GO:0003674	Metabolism	Metabolism	molecular_function	F	AT4G26550
588	1	L123 vs. M123	L123 up	GO:0003674	Metabolism	Metabolism	molecular_function	F	AT5G09440
589	1	L123 vs. M123	L123 up	GO:0003674	Metabolism	Metabolism	molecular_function	F	AT5G10070
590	1	L123 vs. M123	L123 up	GO:0003674	Metabolism	Metabolism	molecular_function	F	AT5G36290
591	1	L123 vs. M123	L123 up	GO:0003674	Metabolism	Metabolism	molecular_function	F	AT5G45350
592	1	L123 vs. M123	L123 up	GO:0003674	Metabolism	Metabolism	molecular_function	F	AT5G56880
593	1	L123 vs. M123	L123 up	GO:0003674	Metabolism	Metabolism	molecular_function	F	AT5G59960
594	1	L123 vs. M123	L123 up	GO:0033799	Metabolism	Metabolism	myricetin 3'-O-methyltransferase activity	F	AT5G54160

595	1	L123 vs. M123	L123 up	GO:0004577	Metabolism	Metabolism	N-acetylglucosaminyldiphosphodolichol N-acetylglucosaminyltransferase activity	F	AT4G18230
596	1	L123 vs. M123	L123 up	GO:0004514	Metabolism	Metabolism	nicotinate-nucleotide diphosphorylase (carboxylating) activity	F	AT2G01350
597	1	L123 vs. M123	L123 up	GO:0006607	Metabolism	Metabolism	NLS-bearing protein import into nucleus	P	AT1G09270
598	1	L123 vs. M123	L123 up	GO:0017196	Metabolism	Metabolism	N-terminal peptidyl-methionine acetylation	P	AT1G03150
599	1	L123 vs. M123	L123 up	GO:0008171	Metabolism	Metabolism	O-methyltransferase activity	F	AT3G61990
600	1	L123 vs. M123	L123 up	GO:0008171	Metabolism	Metabolism	O-methyltransferase activity	F	AT5G54160
601	1	L123 vs. M123	L123 up	GO:0055114	Metabolism	Metabolism	oxidation-reduction process	P	AT1G19550
602	1	L123 vs. M123	L123 up	GO:0055114	Metabolism	Metabolism	oxidation-reduction process	P	AT1G24470
603	1	L123 vs. M123	L123 up	GO:0055114	Metabolism	Metabolism	oxidation-reduction process	P	AT1G49670
604	1	L123 vs. M123	L123 up	GO:0055114	Metabolism	Metabolism	oxidation-reduction process	P	AT1G53580
605	1	L123 vs. M123	L123 up	GO:0055114	Metabolism	Metabolism	oxidation-reduction process	P	AT1G58440
606	1	L123 vs. M123	L123 up	GO:0055114	Metabolism	Metabolism	oxidation-reduction process	P	AT1G72680
607	1	L123 vs. M123	L123 up	GO:0055114	Metabolism	Metabolism	oxidation-reduction process	P	AT2G29370
608	1	L123 vs. M123	L123 up	GO:0055114	Metabolism	Metabolism	oxidation-reduction process	P	AT2G43020
609	1	L123 vs. M123	L123 up	GO:0055114	Metabolism	Metabolism	oxidation-reduction process	P	AT3G48000
610	1	L123 vs. M123	L123 up	GO:0055114	Metabolism	Metabolism	oxidation-reduction process	P	AT3G49640
611	1	L123 vs. M123	L123 up	GO:0055114	Metabolism	Metabolism	oxidation-reduction process	P	AT4G15093
612	1	L123 vs. M123	L123 up	GO:0055114	Metabolism	Metabolism	oxidation-reduction process	P	AT4G25100
613	1	L123 vs. M123	L123 up	GO:0055114	Metabolism	Metabolism	oxidation-reduction process	P	AT4G32320
614	1	L123 vs. M123	L123 up	GO:0055114	Metabolism	Metabolism	oxidation-reduction process	P	AT5G21060
615	1	L123 vs. M123	L123 up	GO:0006796	Metabolism	Metabolism	phosphate-containing compound metabolic process	P	AT3G53620
616	1	L123 vs. M123	L123 up	GO:0004612	Metabolism	Metabolism	phosphoenolpyruvate carboxykinase (ATP) activity	F	AT4G37870
617	1	L123 vs. M123	L123 up	GO:0004634	Metabolism	Metabolism	phosphopyruvate hydratase activity	F	AT2G36530
618	1	L123 vs. M123	L123 up	GO:0008081	Metabolism	Metabolism	phosphoric diester hydrolase activity	F	AT4G38690
619	1	L123 vs. M123	L123 up	GO:0006596	Metabolism	Metabolism	polyamine biosynthetic process	P	AT4G34710
620	1	L123 vs. M123	L123 up	GO:0046592	Metabolism	Metabolism	polyamine oxidase activity	F	AT2G43020
621	1	L123 vs. M123	L123 up	GO:0005515	Metabolism	Metabolism	protein binding	F	AT1G09270
622	1	L123 vs. M123	L123 up	GO:0005515	Metabolism	Metabolism	protein binding	F	AT1G17280
623	1	L123 vs. M123	L123 up	GO:0005515	Metabolism	Metabolism	protein binding	F	AT1G23490
624	1	L123 vs. M123	L123 up	GO:0005515	Metabolism	Metabolism	protein binding	F	AT1G32930
625	1	L123 vs. M123	L123 up	GO:0005515	Metabolism	Metabolism	protein binding	F	AT1G56310
626	1	L123 vs. M123	L123 up	GO:0005515	Metabolism	Metabolism	protein binding	F	AT1G71130
627	1	L123 vs. M123	L123 up	GO:0005515	Metabolism	Metabolism	protein binding	F	AT2G32280
628	1	L123 vs. M123	L123 up	GO:0005515	Metabolism	Metabolism	protein binding	F	AT2G41140
629	1	L123 vs. M123	L123 up	GO:0005515	Metabolism	Metabolism	protein binding	F	AT2G45740
630	1	L123 vs. M123	L123 up	GO:0005515	Metabolism	Metabolism	protein binding	F	AT3G23000
631	1	L123 vs. M123	L123 up	GO:0005515	Metabolism	Metabolism	protein binding	F	AT4G02060
632	1	L123 vs. M123	L123 up	GO:0005515	Metabolism	Metabolism	protein binding	F	AT4G12010
633	1	L123 vs. M123	L123 up	GO:0005515	Metabolism	Metabolism	protein binding	F	AT4G12620
634	1	L123 vs. M123	L123 up	GO:0005515	Metabolism	Metabolism	protein binding	F	AT4G25100
635	1	L123 vs. M123	L123 up	GO:0005515	Metabolism	Metabolism	protein binding	F	AT4G34710
636	1	L123 vs. M123	L123 up	GO:0005515	Metabolism	Metabolism	protein binding	F	AT4G35020
637	1	L123 vs. M123	L123 up	GO:0005515	Metabolism	Metabolism	protein binding	F	AT5G13120
638	1	L123 vs. M123	L123 up	GO:0005515	Metabolism	Metabolism	protein binding	F	AT5G23720
639	1	L123 vs. M123	L123 up	GO:0005515	Metabolism	Metabolism	protein binding	F	AT5G49890
640	1	L123 vs. M123	L123 up	GO:0005515	Metabolism	Metabolism	protein binding	F	AT5G65360
641	1	L123 vs. M123	L123 up	GO:0005515	Metabolism	Metabolism	protein binding	F	ATCG00360
642	1	L123 vs. M123	L123 up	GO:0005515	Metabolism	Metabolism	protein binding	F	ATMG00830
643	1	L123 vs. M123	L123 up	GO:0043424	Metabolism	Metabolism	protein histidine kinase binding	F	AT5G13120
644	1	L123 vs. M123	L123 up	GO:0009446	Metabolism	Metabolism	putrescine biosynthetic process	P	AT4G34710
645	1	L123 vs. M123	L123 up	GO:0033388	Metabolism	Metabolism	putrescine biosynthetic process from arginine	P	AT4G34710
646	1	L123 vs. M123	L123 up	GO:0080043	Metabolism	Metabolism	quercetin 3-O-glucosyltransferase activity	F	AT4G15490
647	1	L123 vs. M123	L123 up	GO:0102822	Metabolism	Metabolism	quercetin 3'-O-methyltransferase activity	F	AT5G54160

648	1	L123 vs. M123	L123 up	GO:0030755	Metabolism	Metabolism	quercetin 3-O-methyltransferase activity	F	AT5G54160
649	1	L123 vs. M123	L123 up	GO:0080044	Metabolism	Metabolism	quercetin 7-O-glucosyltransferase activity	F	AT4G15490
650	1	L123 vs. M123	L123 up	GO:0008757	Metabolism	Metabolism	S-adenosylmethionine-dependent methyltransferase activity	F	AT5G54160
651	1	L123 vs. M123	L123 up	GO:0008236	Metabolism	Metabolism	serine-type peptidase activity	F	ATCG00670
652	1	L123 vs. M123	L123 up	GO:0050284	Metabolism	Metabolism	sinapate 1-glucosyltransferase activity	F	AT4G15490
653	1	L123 vs. M123	L123 up	GO:0052747	Metabolism	Metabolism	sinapyl alcohol dehydrogenase activity	F	AT1G72680
654	1	L123 vs. M123	L123 up	GO:0008295	Metabolism	Metabolism	spermidine biosynthetic process	P	AT4G34710
655	1	L123 vs. M123	L123 up	GO:0004506	Metabolism	Metabolism	squalene monooxygenase activity	F	AT1G58440
656	1	L123 vs. M123	L123 up	GO:0004575	Metabolism	Metabolism	sucrose alpha-glucosidase activity	F	AT3G06500
657	1	L123 vs. M123	L123 up	GO:0004781	Metabolism	Metabolism	sulfate adenylyltransferase (ATP) activity	F	AT1G19920
658	1	L123 vs. M123	L123 up	GO:0050313	Metabolism	Metabolism	sulfur dioxygenase activity	F	AT1G53580
659	1	L123 vs. M123	L123 up	GO:0009088	Metabolism	Metabolism	threonine biosynthetic process	P	AT5G21060
660	1	L123 vs. M123	L123 up	GO:0016740	Metabolism	Metabolism	transferase activity	F	AT1G19550
661	1	L123 vs. M123	L123 up	GO:0016757	Metabolism	Metabolism	transferase activity, transferring glycosyl groups	F	AT1G32930
662	1	L123 vs. M123	L123 up	GO:0016757	Metabolism	Metabolism	transferase activity, transferring glycosyl groups	F	AT4G15490
663	1	L123 vs. M123	L123 up	GO:0016758	Metabolism	Metabolism	transferase activity, transferring hexosyl groups	F	AT4G16710
664	1	L123 vs. M123	L123 up	GO:0015631	Metabolism	Metabolism	tubulin binding	F	AT3G60740
665	1	L123 vs. M123	L123 up	GO:0008194	Metabolism	Metabolism	UDP-glycosyltransferase activity	F	AT4G15490
666	1	L123 vs. M123	L123 up	GO:0008270	Metabolism	Metabolism	zinc ion binding	F	AT1G49670
667	1	L123 vs. M123	L123 up	GO:0008270	Metabolism	Metabolism	zinc ion binding	F	AT1G50440
668	1	L123 vs. M123	L123 up	GO:0008270	Metabolism	Metabolism	zinc ion binding	F	AT1G72680
669	1	L123 vs. M123	L123 up	GO:0008270	Metabolism	Metabolism	zinc ion binding	F	AT4G15093
670	1	L123 vs. M123	L123 up	GO:0008270	Metabolism	Metabolism	zinc ion binding	F	AT5G09950
671	1	L123 vs. M123	L123 up	GO:0016168	Metabolism	Photosynthesis	chlorophyll binding	F	AT1G29930
672	1	L123 vs. M123	L123 up	GO:0016168	Metabolism	Photosynthesis	chlorophyll binding	F	AT4G34190
673	1	L123 vs. M123	L123 up	GO:0009507	Metabolism	Photosynthesis	chloroplast	C	AT1G11750
674	1	L123 vs. M123	L123 up	GO:0009507	Metabolism	Photosynthesis	chloroplast	C	AT1G16080
675	1	L123 vs. M123	L123 up	GO:0009507	Metabolism	Photosynthesis	chloroplast	C	AT1G16470
676	1	L123 vs. M123	L123 up	GO:0009507	Metabolism	Photosynthesis	chloroplast	C	AT1G19550
677	1	L123 vs. M123	L123 up	GO:0009507	Metabolism	Photosynthesis	chloroplast	C	AT1G19920
678	1	L123 vs. M123	L123 up	GO:0009507	Metabolism	Photosynthesis	chloroplast	C	AT1G29930
679	1	L123 vs. M123	L123 up	GO:0009507	Metabolism	Photosynthesis	chloroplast	C	AT1G31190
680	1	L123 vs. M123	L123 up	GO:0009507	Metabolism	Photosynthesis	chloroplast	C	AT1G60950
681	1	L123 vs. M123	L123 up	GO:0009507	Metabolism	Photosynthesis	chloroplast	C	AT1G67280
682	1	L123 vs. M123	L123 up	GO:0009507	Metabolism	Photosynthesis	chloroplast	C	AT1G67600
683	1	L123 vs. M123	L123 up	GO:0009507	Metabolism	Photosynthesis	chloroplast	C	AT1G72680
684	1	L123 vs. M123	L123 up	GO:0009507	Metabolism	Photosynthesis	chloroplast	C	AT2G01350
685	1	L123 vs. M123	L123 up	GO:0009507	Metabolism	Photosynthesis	chloroplast	C	AT2G20670
686	1	L123 vs. M123	L123 up	GO:0009507	Metabolism	Photosynthesis	chloroplast	C	AT2G36530
687	1	L123 vs. M123	L123 up	GO:0009507	Metabolism	Photosynthesis	chloroplast	C	AT2G36730
688	1	L123 vs. M123	L123 up	GO:0009507	Metabolism	Photosynthesis	chloroplast	C	AT2G38330
689	1	L123 vs. M123	L123 up	GO:0009507	Metabolism	Photosynthesis	chloroplast	C	AT2G41950
690	1	L123 vs. M123	L123 up	GO:0009507	Metabolism	Photosynthesis	chloroplast	C	AT2G45740
691	1	L123 vs. M123	L123 up	GO:0009507	Metabolism	Photosynthesis	chloroplast	C	AT3G48000
692	1	L123 vs. M123	L123 up	GO:0009507	Metabolism	Photosynthesis	chloroplast	C	AT3G49640
693	1	L123 vs. M123	L123 up	GO:0009507	Metabolism	Photosynthesis	chloroplast	C	AT4G02700
694	1	L123 vs. M123	L123 up	GO:0009507	Metabolism	Photosynthesis	chloroplast	C	AT4G16620
695	1	L123 vs. M123	L123 up	GO:0009507	Metabolism	Photosynthesis	chloroplast	C	AT4G17486
696	1	L123 vs. M123	L123 up	GO:0009507	Metabolism	Photosynthesis	chloroplast	C	AT4G18975
697	1	L123 vs. M123	L123 up	GO:0009507	Metabolism	Photosynthesis	chloroplast	C	AT4G25100
698	1	L123 vs. M123	L123 up	GO:0009507	Metabolism	Photosynthesis	chloroplast	C	AT4G32320
699	1	L123 vs. M123	L123 up	GO:0009507	Metabolism	Photosynthesis	chloroplast	C	AT4G34710
700	1	L123 vs. M123	L123 up	GO:0009507	Metabolism	Photosynthesis	chloroplast	C	AT5G09440
701	1	L123 vs. M123	L123 up	GO:0009507	Metabolism	Photosynthesis	chloroplast	C	AT5G13120
702	1	L123 vs. M123	L123 up	GO:0009507	Metabolism	Photosynthesis	chloroplast	C	AT5G16150

703	1	L123 vs. M123	L123 up	GO:0009507	Metabolism	Photosynthesis	chloroplast	C	AT5G19940
704	1	L123 vs. M123	L123 up	GO:0009507	Metabolism	Photosynthesis	chloroplast	C	AT5G21060
705	1	L123 vs. M123	L123 up	GO:0009507	Metabolism	Photosynthesis	chloroplast	C	AT5G23060
706	1	L123 vs. M123	L123 up	GO:0009507	Metabolism	Photosynthesis	chloroplast	C	AT5G48330
707	1	L123 vs. M123	L123 up	GO:0009507	Metabolism	Photosynthesis	chloroplast	C	AT5G49840
708	1	L123 vs. M123	L123 up	GO:0009507	Metabolism	Photosynthesis	chloroplast	C	AT5G65685
709	1	L123 vs. M123	L123 up	GO:0009507	Metabolism	Photosynthesis	chloroplast	C	ATCG00360
710	1	L123 vs. M123	L123 up	GO:0009507	Metabolism	Photosynthesis	chloroplast	C	ATCG00670
711	1	L123 vs. M123	L123 up	GO:0009941	Metabolism	Photosynthesis	chloroplast envelope	C	AT1G11750
712	1	L123 vs. M123	L123 up	GO:0009941	Metabolism	Photosynthesis	chloroplast envelope	C	AT1G16080
713	1	L123 vs. M123	L123 up	GO:0009941	Metabolism	Photosynthesis	chloroplast envelope	C	AT1G29930
714	1	L123 vs. M123	L123 up	GO:0009941	Metabolism	Photosynthesis	chloroplast envelope	C	AT3G16240
715	1	L123 vs. M123	L123 up	GO:0009941	Metabolism	Photosynthesis	chloroplast envelope	C	AT4G25100
716	1	L123 vs. M123	L123 up	GO:0009941	Metabolism	Photosynthesis	chloroplast envelope	C	AT4G28390
717	1	L123 vs. M123	L123 up	GO:0009941	Metabolism	Photosynthesis	chloroplast envelope	C	AT5G16150
718	1	L123 vs. M123	L123 up	GO:0009941	Metabolism	Photosynthesis	chloroplast envelope	C	AT5G19940
719	1	L123 vs. M123	L123 up	GO:0009941	Metabolism	Photosynthesis	chloroplast envelope	C	ATCG00670
720	1	L123 vs. M123	L123 up	GO:0009706	Metabolism	Photosynthesis	chloroplast inner membrane	C	AT5G16150
721	1	L123 vs. M123	L123 up	GO:0031969	Metabolism	Photosynthesis	chloroplast membrane	C	AT2G38330
722	1	L123 vs. M123	L123 up	GO:0031969	Metabolism	Photosynthesis	chloroplast membrane	C	AT4G25100
723	1	L123 vs. M123	L123 up	GO:0042644	Metabolism	Photosynthesis	chloroplast nucleoid	C	AT4G25100
724	1	L123 vs. M123	L123 up	GO:0009658	Metabolism	Photosynthesis	chloroplast organization	P	AT1G11750
725	1	L123 vs. M123	L123 up	GO:0009570	Metabolism	Photosynthesis	chloroplast stroma	C	AT1G11750
726	1	L123 vs. M123	L123 up	GO:0009570	Metabolism	Photosynthesis	chloroplast stroma	C	AT1G16080
727	1	L123 vs. M123	L123 up	GO:0009570	Metabolism	Photosynthesis	chloroplast stroma	C	AT1G19920
728	1	L123 vs. M123	L123 up	GO:0009570	Metabolism	Photosynthesis	chloroplast stroma	C	AT1G31190
729	1	L123 vs. M123	L123 up	GO:0009570	Metabolism	Photosynthesis	chloroplast stroma	C	AT1G60950
730	1	L123 vs. M123	L123 up	GO:0009570	Metabolism	Photosynthesis	chloroplast stroma	C	AT1G67280
731	1	L123 vs. M123	L123 up	GO:0009570	Metabolism	Photosynthesis	chloroplast stroma	C	AT2G41950
732	1	L123 vs. M123	L123 up	GO:0009570	Metabolism	Photosynthesis	chloroplast stroma	C	AT4G25100
733	1	L123 vs. M123	L123 up	GO:0009570	Metabolism	Photosynthesis	chloroplast stroma	C	ATCG00670
734	1	L123 vs. M123	L123 up	GO:0009533	Metabolism	Photosynthesis	chloroplast stromal thylakoid	C	AT5G13120
735	1	L123 vs. M123	L123 up	GO:0009534	Metabolism	Photosynthesis	chloroplast thylakoid	C	AT1G11750
736	1	L123 vs. M123	L123 up	GO:0009534	Metabolism	Photosynthesis	chloroplast thylakoid	C	AT1G29930
737	1	L123 vs. M123	L123 up	GO:0009534	Metabolism	Photosynthesis	chloroplast thylakoid	C	AT5G13120
738	1	L123 vs. M123	L123 up	GO:0009534	Metabolism	Photosynthesis	chloroplast thylakoid	C	AT5G19940
739	1	L123 vs. M123	L123 up	GO:0009534	Metabolism	Photosynthesis	chloroplast thylakoid	C	AT5G23060
740	1	L123 vs. M123	L123 up	GO:0009543	Metabolism	Photosynthesis	chloroplast thylakoid lumen	C	AT5G13120
741	1	L123 vs. M123	L123 up	GO:0009535	Metabolism	Photosynthesis	chloroplast thylakoid membrane	C	AT1G29930
742	1	L123 vs. M123	L123 up	GO:0009535	Metabolism	Photosynthesis	chloroplast thylakoid membrane	C	AT4G34190
743	1	L123 vs. M123	L123 up	GO:0009535	Metabolism	Photosynthesis	chloroplast thylakoid membrane	C	AT5G13120
744	1	L123 vs. M123	L123 up	GO:0009535	Metabolism	Photosynthesis	chloroplast thylakoid membrane	C	AT5G23060
745	1	L123 vs. M123	L123 up	GO:0009535	Metabolism	Photosynthesis	chloroplast thylakoid membrane	C	ATCG00360
746	1	L123 vs. M123	L123 up	GO:0009535	Metabolism	Photosynthesis	chloroplast thylakoid membrane	C	ATCG00670
747	1	L123 vs. M123	L123 up	GO:0009840	Metabolism	Photosynthesis	chloroplastic endopeptidase Clp complex	C	AT1G11750
748	1	L123 vs. M123	L123 up	GO:0009840	Metabolism	Photosynthesis	chloroplastic endopeptidase Clp complex	C	ATCG00670
749	1	L123 vs. M123	L123 up	GO:0009854	Metabolism	Photosynthesis	oxidative photosynthetic carbon pathway	P	AT3G14130
750	1	L123 vs. M123	L123 up	GO:0015979	Metabolism	Photosynthesis	photosynthesis	P	AT1G11750
751	1	L123 vs. M123	L123 up	GO:0015979	Metabolism	Photosynthesis	photosynthesis	P	AT4G34190
752	1	L123 vs. M123	L123 up	GO:0009768	Metabolism	Photosynthesis	photosynthesis, light harvesting in photosystem I	P	AT1G29930
753	1	L123 vs. M123	L123 up	GO:0009643	Metabolism	Photosynthesis	photosynthetic acclimation	P	AT1G60950
754	1	L123 vs. M123	L123 up	GO:0009767	Metabolism	Photosynthesis	photosynthetic electron transport chain	P	AT1G60950
755	1	L123 vs. M123	L123 up	GO:0009522	Metabolism	Photosynthesis	photosystem I	C	AT1G29930
756	1	L123 vs. M123	L123 up	GO:0048564	Metabolism	Photosynthesis	photosystem I assembly	P	ATCG00360
757	1	L123 vs. M123	L123 up	GO:0009523	Metabolism	Photosynthesis	photosystem II	C	AT1G29930
758	1	L123 vs. M123	L123 up	GO:0009523	Metabolism	Photosynthesis	photosystem II	C	AT4G34190

759	1	L123 vs. M123	L123 up	GO:0009536	Metabolism	Photosynthesis	plastid	C	AT3G63390
760	1	L123 vs. M123	L123 up	GO:0009536	Metabolism	Photosynthesis	plastid	C	AT4G30010
761	1	L123 vs. M123	L123 up	GO:0009536	Metabolism	Photosynthesis	plastid	C	AT5G13120
762	1	L123 vs. M123	L123 up	GO:0009536	Metabolism	Photosynthesis	plastid	C	AT5G16150
763	1	L123 vs. M123	L123 up	GO:0009536	Metabolism	Photosynthesis	plastid	C	AT5G19940
764	1	L123 vs. M123	L123 up	GO:0009536	Metabolism	Photosynthesis	plastid	C	AT5G21060
765	1	L123 vs. M123	L123 up	GO:0009536	Metabolism	Photosynthesis	plastid	C	AT5G23060
766	1	L123 vs. M123	L123 up	GO:0009536	Metabolism	Photosynthesis	plastid	C	AT5G48760
767	1	L123 vs. M123	L123 up	GO:0009536	Metabolism	Photosynthesis	plastid	C	AT5G49890
768	1	L123 vs. M123	L123 up	GO:0009536	Metabolism	Photosynthesis	plastid	C	AT5G54160
769	1	L123 vs. M123	L123 up	GO:0009536	Metabolism	Photosynthesis	plastid	C	AT5G65360
770	1	L123 vs. M123	L123 up	GO:0009532	Metabolism	Photosynthesis	plastid stroma	C	AT1G11750
771	1	L123 vs. M123	L123 up	GO:0009532	Metabolism	Photosynthesis	plastid stroma	C	ATCG00670
772	1	L123 vs. M123	L123 up	GO:0010287	Metabolism	Photosynthesis	plastoglobule	C	AT1G29930
773	1	L123 vs. M123	L123 up	GO:0018298	Metabolism	Photosynthesis	protein-chromophore linkage	P	AT1G29930
774	1	L123 vs. M123	L123 up	GO:0009579	Metabolism	Photosynthesis	thylakoid	C	AT1G29930
775	1	L123 vs. M123	L123 up	GO:0009579	Metabolism	Photosynthesis	thylakoid	C	AT1G67280
776	1	L123 vs. M123	L123 up	GO:0009579	Metabolism	Photosynthesis	thylakoid	C	AT4G25100
777	1	L123 vs. M123	L123 up	GO:0009579	Metabolism	Photosynthesis	thylakoid	C	AT5G13120
778	1	L123 vs. M123	L123 up	GO:0009579	Metabolism	Photosynthesis	thylakoid	C	AT5G23060
779	1	L123 vs. M123	L123 up	GO:0031977	Metabolism	Photosynthesis	thylakoid lumen	C	AT1G67280
780	1	L123 vs. M123	L123 up	GO:0031977	Metabolism	Photosynthesis	thylakoid lumen	C	AT5G13120
781	1	L123 vs. M123	L123 up	GO:0015171	Metabolism	Transporters	amino acid transmembrane transporter activity	F	AT2G41700
782	1	L123 vs. M123	L123 up	GO:0008519	Metabolism	Transporters	ammonium transmembrane transporter activity	F	AT3G16240
783	1	L123 vs. M123	L123 up	GO:0005253	Metabolism	Transporters	anion channel activity	F	AT5G49890
784	1	L123 vs. M123	L123 up	GO:0015301	Metabolism	Transporters	anion:anion antiporter activity	F	AT4G02700
785	1	L123 vs. M123	L123 up	GO:0015297	Metabolism	Transporters	antiporter activity	F	AT2G38330
786	1	L123 vs. M123	L123 up	GO:0005471	Metabolism	Transporters	ATP:ADP antiporter activity	F	AT4G28390
787	1	L123 vs. M123	L123 up	GO:0042626	Metabolism	Transporters	ATPase-coupled transmembrane transporter activity	F	AT2G41700
788	1	L123 vs. M123	L123 up	GO:0006816	Metabolism	Transporters	calcium ion transport	P	AT1G10130
789	1	L123 vs. M123	L123 up	GO:0005388	Metabolism	Transporters	calcium transmembrane transporter activity, phosphorylative mechanism	F	AT1G10130
790	1	L123 vs. M123	L123 up	GO:0015144	Metabolism	Transporters	carbohydrate transmembrane transporter activity	F	AT5G16150
791	1	L123 vs. M123	L123 up	GO:0008643	Metabolism	Transporters	carbohydrate transport	P	AT3G47420
792	1	L123 vs. M123	L123 up	GO:0005351	Metabolism	Transporters	carbohydrate:proton symporter activity	F	AT5G16150
793	1	L123 vs. M123	L123 up	GO:0034707	Metabolism	Transporters	chloride channel complex	C	AT5G49890
794	1	L123 vs. M123	L123 up	GO:0015108	Metabolism	Transporters	chloride transmembrane transporter activity	F	AT5G49890
795	1	L123 vs. M123	L123 up	GO:0030134	Metabolism	Transporters	COPII-coated ER to Golgi transport vesicle	C	AT3G22290
796	1	L123 vs. M123	L123 up	GO:0006888	Metabolism	Transporters	endoplasmic reticulum to Golgi vesicle-mediated transport	P	AT1G15880
797	1	L123 vs. M123	L123 up	GO:0006888	Metabolism	Transporters	endoplasmic reticulum to Golgi vesicle-mediated transport	P	AT3G22290
798	1	L123 vs. M123	L123 up	GO:0005768	Metabolism	Transporters	endosome	C	AT1G10130
799	1	L123 vs. M123	L123 up	GO:0005768	Metabolism	Transporters	endosome	C	AT4G26550
800	1	L123 vs. M123	L123 up	GO:0015886	Metabolism	Transporters	heme transport	P	ATMG00830
801	1	L123 vs. M123	L123 up	GO:0015232	Metabolism	Transporters	heme transporter activity	F	ATMG00830
802	1	L123 vs. M123	L123 up	GO:0006886	Metabolism	Transporters	intracellular protein transport	P	AT1G16920
803	1	L123 vs. M123	L123 up	GO:0006886	Metabolism	Transporters	intracellular protein transport	P	AT1G23490
804	1	L123 vs. M123	L123 up	GO:0006886	Metabolism	Transporters	intracellular protein transport	P	AT3G16100
805	1	L123 vs. M123	L123 up	GO:0006891	Metabolism	Transporters	intra-Golgi vesicle-mediated transport	P	AT1G15880
806	1	L123 vs. M123	L123 up	GO:0006869	Metabolism	Transporters	lipid transport	P	AT2G41700
807	1	L123 vs. M123	L123 up	GO:0006869	Metabolism	Transporters	lipid transport	P	AT3G08770
808	1	L123 vs. M123	L123 up	GO:0006869	Metabolism	Transporters	lipid transport	P	AT3G09300
809	1	L123 vs. M123	L123 up	GO:0005319	Metabolism	Transporters	lipid transporter activity	F	AT2G41700
810	1	L123 vs. M123	L123 up	GO:0006828	Metabolism	Transporters	manganese ion transport	P	AT1G10130

811	1	L123 vs. M123	L123 up	GO:0015410	Metabolism	Transporters	manganese transmembrane transporter activity, phosphorylative mechanism	F	AT1G10130
812	1	L123 vs. M123	L123 up	GO:0015200	Metabolism	Transporters	methylammonium transmembrane transporter activity	F	AT3G16240
813	1	L123 vs. M123	L123 up	GO:0008139	Metabolism	Transporters	nuclear localization sequence binding	F	AT1G09270
814	1	L123 vs. M123	L123 up	GO:0009506	Metabolism	Transporters	plasmodesma	C	AT1G79340
815	1	L123 vs. M123	L123 up	GO:0009506	Metabolism	Transporters	plasmodesma	C	AT2G36530
816	1	L123 vs. M123	L123 up	GO:0009506	Metabolism	Transporters	plasmodesma	C	AT3G16240
817	1	L123 vs. M123	L123 up	GO:0009506	Metabolism	Transporters	plasmodesma	C	AT3G42570
818	1	L123 vs. M123	L123 up	GO:0009506	Metabolism	Transporters	plasmodesma	C	AT3G60740
819	1	L123 vs. M123	L123 up	GO:0009506	Metabolism	Transporters	plasmodesma	C	AT4G31700
820	1	L123 vs. M123	L123 up	GO:0009506	Metabolism	Transporters	plasmodesma	C	AT5G09440
821	1	L123 vs. M123	L123 up	GO:0009506	Metabolism	Transporters	plasmodesma	C	AT5G54160
822	1	L123 vs. M123	L123 up	GO:0015031	Metabolism	Transporters	protein transport	P	AT1G15880
823	1	L123 vs. M123	L123 up	GO:0015031	Metabolism	Transporters	protein transport	P	AT4G26550
824	1	L123 vs. M123	L123 up	GO:0015865	Metabolism	Transporters	purine nucleotide transport	P	AT4G28390
825	1	L123 vs. M123	L123 up	GO:0034765	Metabolism	Transporters	regulation of ion transmembrane transport	P	AT5G49890
826	1	L123 vs. M123	L123 up	GO:0048209	Metabolism	Transporters	regulation of vesicle targeting, to, from or within Golgi	P	AT1G15880
827	1	L123 vs. M123	L123 up	GO:0060627	Metabolism	Transporters	regulation of vesicle-mediated transport	P	AT1G16920
828	1	L123 vs. M123	L123 up	GO:0008271	Metabolism	Transporters	secondary active sulfate transmembrane transporter activity	F	AT4G02700
829	1	L123 vs. M123	L123 up	GO:0099503	Metabolism	Transporters	secretory vesicle	C	AT3G12500
830	1	L123 vs. M123	L123 up	GO:0005484	Metabolism	Transporters	SNAP receptor activity	F	AT1G15880
831	1	L123 vs. M123	L123 up	GO:0000149	Metabolism	Transporters	SNARE binding	F	AT1G15880
832	1	L123 vs. M123	L123 up	GO:0031201	Metabolism	Transporters	SNARE complex	C	AT1G15880
833	1	L123 vs. M123	L123 up	GO:0000103	Metabolism	Transporters	sulfate assimilation	P	AT1G19920
834	1	L123 vs. M123	L123 up	GO:0015116	Metabolism	Transporters	sulfate transmembrane transporter activity	F	AT4G02700
835	1	L123 vs. M123	L123 up	GO:0030581	Metabolism	Transporters	symbiont intracellular protein transport in host	P	AT1G09270
836	1	L123 vs. M123	L123 up	GO:0015293	Metabolism	Transporters	symporter activity	F	AT4G02700
837	1	L123 vs. M123	L123 up	GO:0005802	Metabolism	Transporters	trans-Golgi network	C	AT1G10130
838	1	L123 vs. M123	L123 up	GO:0005802	Metabolism	Transporters	trans-Golgi network	C	AT1G16920
839	1	L123 vs. M123	L123 up	GO:0005802	Metabolism	Transporters	trans-Golgi network	C	AT4G26550
840	1	L123 vs. M123	L123 up	GO:0055085	Metabolism	Transporters	transmembrane transport	P	AT2G38330
841	1	L123 vs. M123	L123 up	GO:0055085	Metabolism	Transporters	transmembrane transport	P	AT2G41700
842	1	L123 vs. M123	L123 up	GO:0055085	Metabolism	Transporters	transmembrane transport	P	AT3G16240
843	1	L123 vs. M123	L123 up	GO:0055085	Metabolism	Transporters	transmembrane transport	P	AT3G47420
844	1	L123 vs. M123	L123 up	GO:0055085	Metabolism	Transporters	transmembrane transport	P	AT4G28390
845	1	L123 vs. M123	L123 up	GO:0022857	Metabolism	Transporters	transmembrane transporter activity	F	AT3G47420
846	1	L123 vs. M123	L123 up	GO:0022857	Metabolism	Transporters	transmembrane transporter activity	F	AT5G49890
847	1	L123 vs. M123	L123 up	GO:0015204	Metabolism	Transporters	urea transmembrane transporter activity	F	AT3G16240
848	1	L123 vs. M123	L123 up	GO:0015840	Metabolism	Transporters	urea transport	P	AT3G16240
849	1	L123 vs. M123	L123 up	GO:0016192	Metabolism	Transporters	vesicle-mediated transport	P	AT1G23490
850	1	L123 vs. M123	L123 up	GO:0016192	Metabolism	Transporters	vesicle-mediated transport	P	AT4G26550
851	1	L123 vs. M123	L123 up	GO:0005247	Metabolism	Transporters	voltage-gated chloride channel activity	F	AT5G49890
852	1	L123 vs. M123	L123 up	GO:0015250	Metabolism	Transporters	water channel activity	F	AT3G16240
853	1	L123 vs. M123	L123 up	GO:0006833	Metabolism	Transporters	water transport	P	AT3G16240
854	1	L123 vs. M123	L123 up	GO:0042910	Metabolism	Transporters	xenobiotic transmembrane transporter activity	F	AT2G38330
855	1	L123 vs. M123	L123 up	GO:0005783	Regulation	Protein modification	endoplasmic reticulum	C	AT1G17280
856	1	L123 vs. M123	L123 up	GO:0005783	Regulation	Protein modification	endoplasmic reticulum	C	AT1G24470
857	1	L123 vs. M123	L123 up	GO:0005783	Regulation	Protein modification	endoplasmic reticulum	C	AT1G58440
858	1	L123 vs. M123	L123 up	GO:0005783	Regulation	Protein modification	endoplasmic reticulum	C	AT2G32280
859	1	L123 vs. M123	L123 up	GO:0005783	Regulation	Protein modification	endoplasmic reticulum	C	AT2G34585
860	1	L123 vs. M123	L123 up	GO:0005783	Regulation	Protein modification	endoplasmic reticulum	C	AT2G36530
861	1	L123 vs. M123	L123 up	GO:0005783	Regulation	Protein modification	endoplasmic reticulum	C	AT3G22290
862	1	L123 vs. M123	L123 up	GO:0005783	Regulation	Protein modification	endoplasmic reticulum	C	AT3G61990

863	1	L123 vs. M123	L123 up	GO:0005783	Regulation	Protein modification	endoplasmic reticulum	C	AT5G59960
864	1	L123 vs. M123	L123 up	GO:0005789	Regulation	Protein modification	endoplasmic reticulum membrane	C	AT1G24470
865	1	L123 vs. M123	L123 up	GO:0005794	Regulation	Protein modification	Golgi apparatus	C	AT1G10130
866	1	L123 vs. M123	L123 up	GO:0005794	Regulation	Protein modification	Golgi apparatus	C	AT1G15880
867	1	L123 vs. M123	L123 up	GO:0005794	Regulation	Protein modification	Golgi apparatus	C	AT1G23490
868	1	L123 vs. M123	L123 up	GO:0005794	Regulation	Protein modification	Golgi apparatus	C	AT1G32930
869	1	L123 vs. M123	L123 up	GO:0005794	Regulation	Protein modification	Golgi apparatus	C	AT3G16100
870	1	L123 vs. M123	L123 up	GO:0005794	Regulation	Protein modification	Golgi apparatus	C	AT3G16240
871	1	L123 vs. M123	L123 up	GO:0005794	Regulation	Protein modification	Golgi apparatus	C	AT4G26550
872	1	L123 vs. M123	L123 up	GO:0005794	Regulation	Protein modification	Golgi apparatus	C	AT5G36290
873	1	L123 vs. M123	L123 up	GO:0000139	Regulation	Protein modification	Golgi membrane	C	AT1G10130
874	1	L123 vs. M123	L123 up	GO:0000139	Regulation	Protein modification	Golgi membrane	C	AT1G15880
875	1	L123 vs. M123	L123 up	GO:0000139	Regulation	Protein modification	Golgi membrane	C	AT1G32930
876	1	L123 vs. M123	L123 up	GO:0032259	Regulation	Protein modification	methylation	P	AT5G54160
877	1	L123 vs. M123	L123 up	GO:0031416	Regulation	Protein modification	NatB complex	C	AT1G03150
878	1	L123 vs. M123	L123 up	GO:0006499	Regulation	Protein modification	N-terminal protein myristoylation	P	AT1G23490
879	1	L123 vs. M123	L123 up	GO:0006499	Regulation	Protein modification	N-terminal protein myristoylation	P	AT3G19240
880	1	L123 vs. M123	L123 up	GO:0003755	Regulation	Protein modification	peptidyl-prolyl cis-trans isomerase activity	F	AT5G13120
881	1	L123 vs. M123	L123 up	GO:0007023	Regulation	Protein modification	post-chaperonin tubulin folding pathway	P	AT3G60740
882	1	L123 vs. M123	L123 up	GO:0016540	Regulation	Protein modification	protein autoprocessing	P	AT1G79340
883	1	L123 vs. M123	L123 up	GO:0046983	Regulation	Protein modification	protein dimerization activity	F	AT5G54160
884	1	L123 vs. M123	L123 up	GO:0006457	Regulation	Protein modification	protein folding	P	AT3G60740
885	1	L123 vs. M123	L123 up	GO:0010731	Regulation	Protein modification	protein glutathionylation	P	AT1G19550
886	1	L123 vs. M123	L123 up	GO:0006486	Regulation	Protein modification	protein glycosylation	P	AT1G32930
887	1	L123 vs. M123	L123 up	GO:0046982	Regulation	Protein modification	protein heterodimerization activity	F	AT5G65360
888	1	L123 vs. M123	L123 up	GO:0042803	Regulation	Protein modification	protein homodimerization activity	F	AT4G34710
889	1	L123 vs. M123	L123 up	GO:0042803	Regulation	Protein modification	protein homodimerization activity	F	AT5G50960
890	1	L123 vs. M123	L123 up	GO:0008276	Regulation	Protein modification	protein methyltransferase activity	F	AT3G61990
891	1	L123 vs. M123	L123 up	GO:0000413	Regulation	Protein modification	protein peptidyl-prolyl isomerization	P	AT5G13120
892	1	L123 vs. M123	L123 up	GO:0043541	Regulation	Protein modification	UDP-N-acetylglucosamine transferase complex	C	AT4G16710
893	1	L123 vs. M123	L123 up	GO:0043541	Regulation	Protein modification	UDP-N-acetylglucosamine transferase complex	C	AT4G18230
894	1	L123 vs. M123	L123 up	GO:0004596	Regulation	Protein modification	peptide alpha-N-acetyltransferase activity	F	AT1G03150
895	1	L123 vs. M123	L123 up	GO:0043086	Regulation	Regulation	negative regulation of catalytic activity	P	AT1G14890
896	1	L123 vs. M123	L123 up	GO:0043086	Regulation	Regulation	negative regulation of catalytic activity	P	AT2G01610
897	1	L123 vs. M123	L123 up	GO:0032956	Regulation	Regulation	regulation of actin cytoskeleton organization	P	AT4G35020
898	1	L123 vs. M123	L123 up	GO:0032970	Regulation	Regulation	regulation of actin filament-based process	P	AT4G33640
899	1	L123 vs. M123	L123 up	GO:0008360	Regulation	Regulation	regulation of cell shape	P	AT4G35020
900	1	L123 vs. M123	L123 up	GO:0003700	Regulation	Transcription	DNA-binding transcription factor activity	F	AT1G20693
901	1	L123 vs. M123	L123 up	GO:0003700	Regulation	Transcription	DNA-binding transcription factor activity	F	AT1G71130
902	1	L123 vs. M123	L123 up	GO:0003700	Regulation	Transcription	DNA-binding transcription factor activity	F	AT2G41900
903	1	L123 vs. M123	L123 up	GO:0003899	Regulation	Transcription	DNA-directed 5'-3' RNA polymerase activity	F	AT2G41950
904	1	L123 vs. M123	L123 up	GO:0004386	Regulation	Transcription	helicase activity	F	AT4G30010
905	1	L123 vs. M123	L123 up	GO:0042393	Regulation	Transcription	histone binding	F	AT4G12620
906	1	L123 vs. M123	L123 up	GO:0006397	Regulation	Transcription	mRNA processing	P	AT3G62310
907	1	L123 vs. M123	L123 up	GO:0006397	Regulation	Transcription	mRNA processing	P	AT5G60940
908	1	L123 vs. M123	L123 up	GO:0005730	Regulation	Transcription	nucleolus	C	AT2G30620
909	1	L123 vs. M123	L123 up	GO:0005730	Regulation	Transcription	nucleolus	C	AT3G62310
910	1	L123 vs. M123	L123 up	GO:0005730	Regulation	Transcription	nucleolus	C	AT4G31700
911	1	L123 vs. M123	L123 up	GO:0005730	Regulation	Transcription	nucleolus	C	AT4G35020
912	1	L123 vs. M123	L123 up	GO:0005730	Regulation	Transcription	nucleolus	C	AT4G37870
913	1	L123 vs. M123	L123 up	GO:0030422	Regulation	Transcription	production of siRNA involved in RNA interference	P	AT1G56310
914	1	L123 vs. M123	L123 up	GO:0010468	Regulation	Transcription	regulation of gene expression	P	AT5G23720
915	1	L123 vs. M123	L123 up	GO:0006355	Regulation	Transcription	regulation of transcription, DNA-templated	P	AT1G71130
916	1	L123 vs. M123	L123 up	GO:0006355	Regulation	Transcription	regulation of transcription, DNA-templated	P	AT2G30620
917	1	L123 vs. M123	L123 up	GO:0006355	Regulation	Transcription	regulation of transcription, DNA-templated	P	AT2G41900
918	1	L123 vs. M123	L123 up	GO:0006355	Regulation	Transcription	regulation of transcription, DNA-templated	P	AT4G12620

919	1	L123 vs. M123	L123 up	GO:0003723	Regulation	Transcription	RNA binding	F	AT3G62310
920	1	L123 vs. M123	L123 up	GO:0009451	Regulation	Transcription	RNA modification	P	AT2G36730
921	1	L123 vs. M123	L123 up	GO:0009451	Regulation	Transcription	RNA modification	P	AT5G09950
922	1	L123 vs. M123	L123 up	GO:0043565	Regulation	Transcription	sequence-specific DNA binding	F	AT1G71130
923	1	L123 vs. M123	L123 up	GO:0000347	Regulation	Transcription	THO complex	C	AT4G02060
924	1	L123 vs. M123	L123 up	GO:0044212	Regulation	Transcription	transcription regulatory region DNA binding	F	AT1G71130
925	1	L123 vs. M123	L123 up	GO:0003743	Regulation	Transcription	translation initiation factor activity	F	AT5G18110
926	1	L123 vs. M123	L123 up	GO:0022625	Regulation	Translation	cytosolic large ribosomal subunit	C	AT3G55170
927	1	L123 vs. M123	L123 up	GO:0022625	Regulation	Translation	cytosolic large ribosomal subunit	C	AT5G48760
928	1	L123 vs. M123	L123 up	GO:0022626	Regulation	Translation	cytosolic ribosome	C	AT1G16470
929	1	L123 vs. M123	L123 up	GO:0022626	Regulation	Translation	cytosolic ribosome	C	AT4G31700
930	1	L123 vs. M123	L123 up	GO:0022626	Regulation	Translation	cytosolic ribosome	C	AT5G48760
931	1	L123 vs. M123	L123 up	GO:0022627	Regulation	Translation	cytosolic small ribosomal subunit	C	AT4G31700
932	1	L123 vs. M123	L123 up	GO:0016281	Regulation	Translation	eukaryotic translation initiation factor 4F complex	C	AT5G18110
933	1	L123 vs. M123	L123 up	GO:0000463	Regulation	Translation	maturation of LSU-rRNA from tricistronic rRNA transcript (SSU-rRNA, 5.8S rRNA, LSU-rRNA)	P	AT3G55170
934	1	L123 vs. M123	L123 up	GO:0003729	Regulation	Translation	mRNA binding	F	AT3G55170
935	1	L123 vs. M123	L123 up	GO:0003729	Regulation	Translation	mRNA binding	F	AT4G31700
936	1	L123 vs. M123	L123 up	GO:0003729	Regulation	Translation	mRNA binding	F	AT5G48760
937	1	L123 vs. M123	L123 up	GO:0005848	Regulation	Translation	mRNA cleavage stimulating factor complex	C	AT5G60940
938	1	L123 vs. M123	L123 up	GO:0004826	Regulation	Translation	phenylalanine-tRNA ligase activity	F	AT4G39280
939	1	L123 vs. M123	L123 up	GO:0009328	Regulation	Translation	phenylalanine-tRNA ligase complex	C	AT4G39280
940	1	L123 vs. M123	L123 up	GO:0006432	Regulation	Translation	phenylalanyl-tRNA aminoacylation	P	AT4G39280
941	1	L123 vs. M123	L123 up	GO:0006417	Regulation	Translation	regulation of translation	P	AT5G18110
942	1	L123 vs. M123	L123 up	GO:0005840	Regulation	Translation	ribosome	C	AT5G48760
943	1	L123 vs. M123	L123 up	GO:0000340	Regulation	Translation	RNA 7-methylguanosine cap binding	F	AT5G18110
944	1	L123 vs. M123	L123 up	GO:0003724	Regulation	Translation	RNA helicase activity	F	AT3G62310
945	1	L123 vs. M123	L123 up	GO:0008380	Regulation	Translation	RNA splicing	P	AT3G62310
946	1	L123 vs. M123	L123 up	GO:0034247	Regulation	Translation	snoRNA splicing	P	AT1G01350
947	1	L123 vs. M123	L123 up	GO:0005681	Regulation	Translation	spliceosomal complex	C	AT3G62310
948	1	L123 vs. M123	L123 up	GO:0003735	Regulation	Translation	structural constituent of ribosome	F	AT5G48760
949	1	L123 vs. M123	L123 up	GO:0006412	Regulation	Translation	translation	P	AT3G55170
950	1	L123 vs. M123	L123 up	GO:0006412	Regulation	Translation	translation	P	AT4G31700
951	1	L123 vs. M123	L123 up	GO:0006412	Regulation	Translation	translation	P	AT5G48760
952	1	L123 vs. M123	L123 up	GO:0006413	Regulation	Translation	translational initiation	P	AT5G18110
953	1	L123 vs. M123	L123 up	GO:0000049	Regulation	Translation	tRNA binding	F	AT4G39280
954	1	L123 vs. M123	L123 up	GO:0017150	Regulation	Translation	tRNA dihydrouridine synthase activity	F	AT3G49640
955	1	L123 vs. M123	L123 up	GO:0005684	Regulation	Translation	U2-type spliceosomal complex	C	AT1G01350
956	1	L123 vs. M123	L123 up	GO:0009734	Signaling and response	Signaling	auxin-activated signaling pathway	P	AT4G35020
957	1	L123 vs. M123	L123 up	GO:0009931	Signaling and response	Signaling	calcium-dependent protein serine/threonine kinase activity	F	AT2G41140
958	1	L123 vs. M123	L123 up	GO:0005516	Signaling and response	Signaling	calmodulin binding	F	AT2G15760
959	1	L123 vs. M123	L123 up	GO:0005516	Signaling and response	Signaling	calmodulin binding	F	AT2G41140
960	1	L123 vs. M123	L123 up	GO:0004683	Signaling and response	Signaling	calmodulin-dependent protein kinase activity	F	AT2G41140
961	1	L123 vs. M123	L123 up	GO:0071277	Signaling and response	Signaling	cellular response to calcium ion	P	AT5G23060
962	1	L123 vs. M123	L123 up	GO:0007623	Signaling and response	Signaling	circadian rhythm	P	AT3G06500
963	1	L123 vs. M123	L123 up	GO:0007623	Signaling and response	Signaling	circadian rhythm	P	AT4G25100
964	1	L123 vs. M123	L123 up	GO:0046855	Signaling and response	Signaling	inositol phosphate dephosphorylation	P	AT1G31190
965	1	L123 vs. M123	L123 up	GO:0035556	Signaling and response	Signaling	intracellular signal transduction	P	AT2G41140
966	1	L123 vs. M123	L123 up	GO:0035556	Signaling and response	Signaling	intracellular signal transduction	P	AT3G23000
967	1	L123 vs. M123	L123 up	GO:0016301	Signaling and response	Signaling	kinase activity	F	AT5G23720
968	1	L123 vs. M123	L123 up	GO:0019900	Signaling and response	Signaling	kinase binding	F	AT5G47060
969	1	L123 vs. M123	L123 up	GO:1902457	Signaling and response	Signaling	negative regulation of stomatal opening	P	AT1G17280
970	1	L123 vs. M123	L123 up	GO:0061608	Signaling and response	Signaling	nuclear import signal receptor activity	F	AT1G09270
971	1	L123 vs. M123	L123 up	GO:0018105	Signaling and response	Signaling	peptidyl-serine phosphorylation	P	AT2G41140
972	1	L123 vs. M123	L123 up	GO:0019902	Signaling and response	Signaling	phosphatase binding	F	AT5G47060

973	1	L123 vs. M123	L123 up	GO:0008970	Signaling and response	Signaling	phospholipase A1 activity	F	AT2G42690
974	1	L123 vs. M123	L123 up	GO:0016004	Signaling and response	Signaling	phospholipase activator activity	F	AT1G23490
975	1	L123 vs. M123	L123 up	GO:0004721	Signaling and response	Signaling	phosphoprotein phosphatase activity	F	AT5G23720
976	1	L123 vs. M123	L123 up	GO:0009958	Signaling and response	Signaling	positive gravitropism	P	AT4G35020
977	1	L123 vs. M123	L123 up	GO:0046777	Signaling and response	Signaling	protein autophosphorylation	P	AT2G41140
978	1	L123 vs. M123	L123 up	GO:0019901	Signaling and response	Signaling	protein kinase binding	F	AT4G35020
979	1	L123 vs. M123	L123 up	GO:0006468	Signaling and response	Signaling	protein phosphorylation	P	AT1G11410
980	1	L123 vs. M123	L123 up	GO:0006468	Signaling and response	Signaling	protein phosphorylation	P	AT1G67520
981	1	L123 vs. M123	L123 up	GO:0006468	Signaling and response	Signaling	protein phosphorylation	P	AT3G23000
982	1	L123 vs. M123	L123 up	GO:0006468	Signaling and response	Signaling	protein phosphorylation	P	AT5G57610
983	1	L123 vs. M123	L123 up	GO:0004674	Signaling and response	Signaling	protein serine/threonine kinase activity	F	AT1G11410
984	1	L123 vs. M123	L123 up	GO:0004674	Signaling and response	Signaling	protein serine/threonine kinase activity	F	AT1G67520
985	1	L123 vs. M123	L123 up	GO:0004674	Signaling and response	Signaling	protein serine/threonine kinase activity	F	AT3G23000
986	1	L123 vs. M123	L123 up	GO:0004712	Signaling and response	Signaling	protein serine/threonine/tyrosine kinase activity	F	AT5G57610
987	1	L123 vs. M123	L123 up	GO:0004725	Signaling and response	Signaling	protein tyrosine phosphatase activity	F	AT5G23720
988	1	L123 vs. M123	L123 up	GO:0008138	Signaling and response	Signaling	protein tyrosine/serine/threonine phosphatase activity	F	AT5G23720
989	1	L123 vs. M123	L123 up	GO:0032482	Signaling and response	Signaling	Rab protein signal transduction	P	AT1G16920
990	1	L123 vs. M123	L123 up	GO:0032482	Signaling and response	Signaling	Rab protein signal transduction	P	AT3G16100
991	1	L123 vs. M123	L123 up	GO:0010928	Signaling and response	Signaling	regulation of auxin mediated signaling pathway	P	AT4G35020
992	1	L123 vs. M123	L123 up	GO:0010029	Signaling and response	Signaling	regulation of seed germination	P	AT3G06500
993	1	L123 vs. M123	L123 up	GO:0048510	Signaling and response	Signaling	regulation of timing of transition from vegetative to reproductive phase	P	AT3G06500
994	1	L123 vs. M123	L123 up	GO:0046686	Signaling and response	Signaling	response to cadmium ion	P	AT1G23490
995	1	L123 vs. M123	L123 up	GO:0046686	Signaling and response	Signaling	response to cadmium ion	P	AT2G36530
996	1	L123 vs. M123	L123 up	GO:0046686	Signaling and response	Signaling	response to cadmium ion	P	AT3G12500
997	1	L123 vs. M123	L123 up	GO:0046686	Signaling and response	Signaling	response to cadmium ion	P	AT3G48000
998	1	L123 vs. M123	L123 up	GO:0046686	Signaling and response	Signaling	response to cadmium ion	P	AT3G53620
999	1	L123 vs. M123	L123 up	GO:0046686	Signaling and response	Signaling	response to cadmium ion	P	AT4G25100
1000	1	L123 vs. M123	L123 up	GO:0046686	Signaling and response	Signaling	response to cadmium ion	P	AT4G37870
1001	1	L123 vs. M123	L123 up	GO:0046688	Signaling and response	Signaling	response to copper ion	P	AT4G25100
1002	1	L123 vs. M123	L123 up	GO:0009749	Signaling and response	Signaling	response to glucose	P	AT5G47060
1003	1	L123 vs. M123	L123 up	GO:0009753	Signaling and response	Signaling	response to jasmonic acid	P	AT4G34710
1004	1	L123 vs. M123	L123 up	GO:0080167	Signaling and response	Signaling	response to karrikin	P	AT4G34710
1005	1	L123 vs. M123	L123 up	GO:0009642	Signaling and response	Signaling	response to light intensity	P	AT4G25100
1006	1	L123 vs. M123	L123 up	GO:0009416	Signaling and response	Signaling	response to light stimulus	P	AT1G29930
1007	1	L123 vs. M123	L123 up	GO:0009416	Signaling and response	Signaling	response to light stimulus	P	AT1G60950
1008	1	L123 vs. M123	L123 up	GO:0009416	Signaling and response	Signaling	response to light stimulus	P	AT2G36530
1009	1	L123 vs. M123	L123 up	GO:1905582	Signaling and response	Signaling	response to mannose	P	AT5G47060
1010	1	L123 vs. M123	L123 up	GO:0009744	Signaling and response	Signaling	response to sucrose	P	AT5G47060
1011	1	L123 vs. M123	L123 up	GO:0010043	Signaling and response	Signaling	response to zinc ion	P	AT1G16470
1012	1	L123 vs. M123	L123 up	GO:0006890	Signaling and response	Signaling	retrograde vesicle-mediated transport, Golgi to endoplasmic reticulum	P	AT3G22290
1013	1	L123 vs. M123	L123 up	GO:0007266	Signaling and response	Signaling	Rho protein signal transduction	P	AT4G35020
1014	1	L123 vs. M123	L123 up	GO:0007165	Signaling and response	Signaling	signal transduction	P	AT1G31190
1015	1	L123 vs. M123	L123 up	GO:0007165	Signaling and response	Signaling	signal transduction	P	AT4G12010
1016	1	L123 vs. M123	L123 up	GO:0032934	Signaling and response	Signaling	sterol binding	F	AT3G09300
1017	1	L123 vs. M123	L123 up	GO:0016126	Signaling and response	Signaling	sterol biosynthetic process	P	AT1G58440
1018	1	L123 vs. M123	L123 up	GO:0010182	Signaling and response	Signaling	sugar mediated signaling pathway	P	AT4G02060
1019	1	L123 vs. M123	L123 up	GO:0009738	Signaling and response	Stress	abscisic acid-activated signaling pathway	P	AT5G23720
1020	1	L123 vs. M123	L123 up	GO:0071586	Signaling and response	Stress	CAAX-box protein processing	P	AT1G14270
1021	1	L123 vs. M123	L123 up	GO:0045454	Signaling and response	Stress	cell redox homeostasis	P	AT3G50960
1022	1	L123 vs. M123	L123 up	GO:0098869	Signaling and response	Stress	cellular oxidant detoxification	P	AT1G19550
1023	1	L123 vs. M123	L123 up	GO:0071486	Signaling and response	Stress	cellular response to high light intensity	P	AT4G34190
1024	1	L123 vs. M123	L123 up	GO:0034599	Signaling and response	Stress	cellular response to oxidative stress	P	AT4G32320
1025	1	L123 vs. M123	L123 up	GO:0016036	Signaling and response	Stress	cellular response to phosphate starvation	P	AT4G37870

1026	1	L123 vs. M123	L123 up	GO:0009970	Signaling and response	Stress	cellular response to sulfate starvation	P	AT1G19920
1027	1	L123 vs. M123	L123 up	GO:0071492	Signaling and response	Stress	cellular response to UV-A	P	AT4G34190
1028	1	L123 vs. M123	L123 up	GO:0071493	Signaling and response	Stress	cellular response to UV-B	P	AT2G42690
1029	1	L123 vs. M123	L123 up	GO:0042631	Signaling and response	Stress	cellular response to water deprivation	P	AT1G17280
1030	1	L123 vs. M123	L123 up	GO:0010494	Signaling and response	Stress	cytoplasmic stress granule	C	AT1G56310
1031	1	L123 vs. M123	L123 up	GO:0006952	Signaling and response	Stress	defense response	P	AT1G79340
1032	1	L123 vs. M123	L123 up	GO:0042742	Signaling and response	Stress	defense response to bacterium	P	AT3G14130
1033	1	L123 vs. M123	L123 up	GO:0042742	Signaling and response	Stress	defense response to bacterium	P	AT4G12010
1034	1	L123 vs. M123	L123 up	GO:0050832	Signaling and response	Stress	defense response to fungus	P	AT3G12500
1035	1	L123 vs. M123	L123 up	GO:0050832	Signaling and response	Stress	defense response to fungus	P	AT5G09440
1036	1	L123 vs. M123	L123 up	GO:0009817	Signaling and response	Stress	defense response to fungus, incompatible interaction	P	AT4G37870
1037	1	L123 vs. M123	L123 up	GO:0009368	Signaling and response	Stress	endopeptidase Clp complex	C	AT1G11750
1038	1	L123 vs. M123	L123 up	GO:0009368	Signaling and response	Stress	endopeptidase Clp complex	C	ATCG00670
1039	1	L123 vs. M123	L123 up	GO:0009873	Signaling and response	Stress	ethylene-activated signaling pathway	P	AT1G71130
1040	1	L123 vs. M123	L123 up	GO:0051555	Signaling and response	Stress	flavonol biosynthetic process	P	AT5G54160
1041	1	L123 vs. M123	L123 up	GO:0045174	Signaling and response	Stress	glutathione dehydrogenase (ascorbate) activity	F	AT1G19550
1042	1	L123 vs. M123	L123 up	GO:0006749	Signaling and response	Stress	glutathione metabolic process	P	AT1G53580
1043	1	L123 vs. M123	L123 up	GO:0010286	Signaling and response	Stress	heat acclimation	P	AT2G41140
1044	1	L123 vs. M123	L123 up	GO:0080034	Signaling and response	Stress	host response to induction by symbiont of tumor, nodule or growth in host	P	AT1G09270
1045	1	L123 vs. M123	L123 up	GO:0050665	Signaling and response	Stress	hydrogen peroxide biosynthetic process	P	AT3G14130
1046	1	L123 vs. M123	L123 up	GO:0042744	Signaling and response	Stress	hydrogen peroxide catabolic process	P	AT4G32320
1047	1	L123 vs. M123	L123 up	GO:0042538	Signaling and response	Stress	hyperosmotic salinity response	P	AT1G16920
1048	1	L123 vs. M123	L123 up	GO:0009871	Signaling and response	Stress	jasmonic acid and ethylene-dependent systemic resistance, ethylene mediated signaling pathway	P	AT3G12500
1049	1	L123 vs. M123	L123 up	GO:0031640	Signaling and response	Stress	killing of cells of other organism	P	AT3G12500
1050	1	L123 vs. M123	L123 up	GO:0004462	Signaling and response	Stress	lactoylglutathione lyase activity	F	AT1G67280
1051	1	L123 vs. M123	L123 up	GO:0016688	Signaling and response	Stress	L-ascorbate peroxidase activity	F	AT4G32320
1052	1	L123 vs. M123	L123 up	GO:0055088	Signaling and response	Stress	lipid homeostasis	P	AT3G06690
1053	1	L123 vs. M123	L123 up	GO:0055071	Signaling and response	Stress	manganese ion homeostasis	P	AT1G10130
1054	1	L123 vs. M123	L123 up	GO:0019243	Signaling and response	Stress	methylglyoxal catabolic process to D-lactate via S-lactoyl-glutathione	P	AT1G67280
1055	1	L123 vs. M123	L123 up	GO:0010587	Signaling and response	Stress	miRNA catabolic process	P	AT1G56310
1056	1	L123 vs. M123	L123 up	GO:0031936	Signaling and response	Stress	negative regulation of chromatin silencing	P	AT2G30620
1057	1	L123 vs. M123	L123 up	GO:0017148	Signaling and response	Stress	negative regulation of translation	P	AT5G48760
1058	1	L123 vs. M123	L123 up	GO:0055062	Signaling and response	Stress	phosphate ion homeostasis	P	AT3G47420
1059	1	L123 vs. M123	L123 up	GO:0046854	Signaling and response	Stress	phosphatidylinositol phosphorylation	P	AT1G31190
1060	1	L123 vs. M123	L123 up	GO:0009626	Signaling and response	Stress	plant-type hypersensitive response	P	AT3G12500
1061	1	L123 vs. M123	L123 up	GO:0009626	Signaling and response	Stress	plant-type hypersensitive response	P	AT4G12010
1062	1	L123 vs. M123	L123 up	GO:0043068	Signaling and response	Stress	positive regulation of programmed cell death	P	AT1G79340
1063	1	L123 vs. M123	L123 up	GO:0006515	Signaling and response	Stress	protein quality control for misfolded or incompletely synthesized proteins	P	AT1G11750
1064	1	L123 vs. M123	L123 up	GO:0006515	Signaling and response	Stress	protein quality control for misfolded or incompletely synthesized proteins	P	ATCG00670
1065	1	L123 vs. M123	L123 up	GO:0042026	Signaling and response	Stress	protein refolding	P	AT5G13120
1066	1	L123 vs. M123	L123 up	GO:0090333	Signaling and response	Stress	regulation of stomatal closure	P	AT5G23060
1067	1	L123 vs. M123	L123 up	GO:0010119	Signaling and response	Stress	regulation of stomatal movement	P	AT5G23720
1068	1	L123 vs. M123	L123 up	GO:0019430	Signaling and response	Stress	removal of superoxide radicals	P	AT4G25100
1069	1	L123 vs. M123	L123 up	GO:0009737	Signaling and response	Stress	response to abscisic acid	P	AT2G36530
1070	1	L123 vs. M123	L123 up	GO:0009737	Signaling and response	Stress	response to abscisic acid	P	AT4G34710
1071	1	L123 vs. M123	L123 up	GO:0009737	Signaling and response	Stress	response to abscisic acid	P	AT5G23720
1072	1	L123 vs. M123	L123 up	GO:0009409	Signaling and response	Stress	response to cold	P	AT1G67280
1073	1	L123 vs. M123	L123 up	GO:0009409	Signaling and response	Stress	response to cold	P	AT2G36530
1074	1	L123 vs. M123	L123 up	GO:0009409	Signaling and response	Stress	response to cold	P	AT3G17020
1075	1	L123 vs. M123	L123 up	GO:0009409	Signaling and response	Stress	response to cold	P	AT3G23000

1076	1	L123 vs. M123	L123 up	GO:0009409	Signaling and response	Stress	response to cold	P	AT4G34710
1077	1	L123 vs. M123	L123 up	GO:0009644	Signaling and response	Stress	response to high light intensity	P	AT4G34190
1078	1	L123 vs. M123	L123 up	GO:0006970	Signaling and response	Stress	response to osmotic stress	P	AT4G34710
1079	1	L123 vs. M123	L123 up	GO:0006979	Signaling and response	Stress	response to oxidative stress	P	AT1G49670
1080	1	L123 vs. M123	L123 up	GO:0006979	Signaling and response	Stress	response to oxidative stress	P	AT4G25100
1081	1	L123 vs. M123	L123 up	GO:0006979	Signaling and response	Stress	response to oxidative stress	P	AT4G34710
1082	1	L123 vs. M123	L123 up	GO:0010193	Signaling and response	Stress	response to ozone	P	AT4G25100
1083	1	L123 vs. M123	L123 up	GO:0000302	Signaling and response	Stress	response to reactive oxygen species	P	AT4G32320
1084	1	L123 vs. M123	L123 up	GO:0009651	Signaling and response	Stress	response to salt stress	P	AT4G34710
1085	1	L123 vs. M123	L123 up	GO:0042594	Signaling and response	Stress	response to starvation	P	AT5G47060
1086	1	L123 vs. M123	L123 up	GO:0010353	Signaling and response	Stress	response to trehalose	P	AT5G16150
1087	1	L123 vs. M123	L123 up	GO:0009414	Signaling and response	Stress	response to water deprivation	P	AT1G58440
1088	1	L123 vs. M123	L123 up	GO:0009414	Signaling and response	Stress	response to water deprivation	P	AT1G68820
1089	1	L123 vs. M123	L123 up	GO:0009414	Signaling and response	Stress	response to water deprivation	P	AT3G08770
1090	1	L123 vs. M123	L123 up	GO:0009611	Signaling and response	Stress	response to wounding	P	AT4G34190
1091	1	L123 vs. M123	L123 up	GO:0009611	Signaling and response	Stress	response to wounding	P	AT4G34710
1092	1	L123 vs. M123	L123 up	GO:0009611	Signaling and response	Stress	response to wounding	P	AT5G47240
1093	1	L123 vs. M123	L123 up	GO:0004784	Signaling and response	Stress	superoxide dismutase activity	F	AT4G25100
1094	1	L123 vs. M123	L123 up	GO:0030433	Signaling and response	Stress	ubiquitin-dependent ERAD pathway	P	AT1G17280
1095	1	L123 vs. M123	L123 up	GO:0051082	Signaling and response	Stress	unfolded protein binding	F	AT5G13120
1096	1	L123 vs. M123	L123 up	GO:0051082	Signaling and response	Stress	unfolded protein binding	F	ATCG00360
1097	1	L123 vs. M123	L123 up	GO:0009650	Signaling and response	Stress	UV protection	P	AT2G42690
1098	1	L123 vs. M123	M123 up	GO:0008356	Development	Cell division	asymmetric cell division	P	AT3G48750
1099	1	L123 vs. M123	M123 up	GO:0051301	Development	Cell division	cell division	P	AT2G26760
1100	1	L123 vs. M123	M123 up	GO:0051301	Development	Cell division	cell division	P	AT5G17620
1101	1	L123 vs. M123	M123 up	GO:0051301	Development	Cell division	cell division	P	AT5G48600
1102	1	L123 vs. M123	M123 up	GO:0051026	Development	Cell division	chiasma assembly	P	AT1G67370
1103	1	L123 vs. M123	M123 up	GO:0051026	Development	Cell division	chiasma assembly	P	AT3G13170
1104	1	L123 vs. M123	M123 up	GO:0005694	Development	Cell division	chromosome	C	AT1G67370
1105	1	L123 vs. M123	M123 up	GO:0005694	Development	Cell division	chromosome	C	AT3G13170
1106	1	L123 vs. M123	M123 up	GO:0005694	Development	Cell division	chromosome	C	AT5G48600
1107	1	L123 vs. M123	M123 up	GO:0051304	Development	Cell division	chromosome separation	P	AT4G15890
1108	1	L123 vs. M123	M123 up	GO:0000779	Development	Cell division	condensed chromosome, centromeric region	C	AT4G15890
1109	1	L123 vs. M123	M123 up	GO:0000794	Development	Cell division	condensed nuclear chromosome	C	AT1G67370
1110	1	L123 vs. M123	M123 up	GO:0030332	Development	Cell division	cyclin binding	F	AT3G48750
1111	1	L123 vs. M123	M123 up	GO:0000307	Development	Cell division	cyclin-dependent protein kinase holoenzyme complex	C	AT2G26760
1112	1	L123 vs. M123	M123 up	GO:0000307	Development	Cell division	cyclin-dependent protein kinase holoenzyme complex	C	AT3G48750
1113	1	L123 vs. M123	M123 up	GO:0004693	Development	Cell division	cyclin-dependent protein serine/threonine kinase activity	F	AT3G48750
1114	1	L123 vs. M123	M123 up	GO:0016538	Development	Cell division	cyclin-dependent protein serine/threonine kinase regulator activity	F	AT2G26760
1115	1	L123 vs. M123	M123 up	GO:0000910	Development	Cell division	cytokinesis	P	AT3G48750
1116	1	L123 vs. M123	M123 up	GO:0042023	Development	Cell division	DNA endoreduplication	P	AT3G48750
1117	1	L123 vs. M123	M123 up	GO:0004519	Development	Cell division	endonuclease activity	F	AT1G11290
1118	1	L123 vs. M123	M123 up	GO:0000082	Development	Cell division	G1/S transition of mitotic cell cycle	P	AT3G48750
1119	1	L123 vs. M123	M123 up	GO:0048229	Development	Cell division	gametophyte development	P	AT3G48750
1120	1	L123 vs. M123	M123 up	GO:0048229	Development	Cell division	gametophyte development	P	AT3G49670
1121	1	L123 vs. M123	M123 up	GO:0070652	Development	Cell division	HAUS complex	C	AT5G17620
1122	1	L123 vs. M123	M123 up	GO:0034090	Development	Cell division	maintenance of meiotic sister chromatid cohesion	P	AT3G13170
1123	1	L123 vs. M123	M123 up	GO:0048232	Development	Cell division	male gamete generation	P	AT4G39940
1124	1	L123 vs. M123	M123 up	GO:0051321	Development	Cell division	meiotic cell cycle	P	AT5G48600
1125	1	L123 vs. M123	M123 up	GO:0010032	Development	Cell division	meiotic chromosome condensation	P	AT4G15890
1126	1	L123 vs. M123	M123 up	GO:0042138	Development	Cell division	meiotic DNA double-strand break formation	P	AT3G13170
1127	1	L123 vs. M123	M123 up	GO:0000706	Development	Cell division	meiotic DNA double-strand break processing	P	AT3G13170

1128	1	L123 vs. M123	M123 up	GO:0010480	Development	Cell division	microsporocyte differentiation	P	AT3G49670
1129	1	L123 vs. M123	M123 up	GO:0009556	Development	Cell division	microsporogenesis	P	AT4G15890
1130	1	L123 vs. M123	M123 up	GO:0003777	Development	Cell division	microtubule motor activity	F	AT5G65460
1131	1	L123 vs. M123	M123 up	GO:0044772	Development	Cell division	mitotic cell cycle phase transition	P	AT2G26760
1132	1	L123 vs. M123	M123 up	GO:0007076	Development	Cell division	mitotic chromosome condensation	P	AT4G15890
1133	1	L123 vs. M123	M123 up	GO:0007076	Development	Cell division	mitotic chromosome condensation	P	AT5G48600
1134	1	L123 vs. M123	M123 up	GO:0000070	Development	Cell division	mitotic sister chromatid segregation	P	AT5G48600
1135	1	L123 vs. M123	M123 up	GO:0031022	Development	Cell division	nuclear migration along microfilament	P	AT5G65460
1136	1	L123 vs. M123	M123 up	GO:0048868	Development	Cell division	pollen tube development	P	AT3G05000
1137	1	L123 vs. M123	M123 up	GO:0008284	Development	Cell division	positive regulation of cell population proliferation	P	AT3G48750
1138	1	L123 vs. M123	M123 up	GO:0009574	Development	Cell division	preprophase band	C	AT3G48750
1139	1	L123 vs. M123	M123 up	GO:0007131	Development	Cell division	reciprocal meiotic recombination	P	AT3G13170
1140	1	L123 vs. M123	M123 up	GO:1902806	Development	Cell division	regulation of cell cycle G1/S phase transition	P	AT3G48750
1141	1	L123 vs. M123	M123 up	GO:0010389	Development	Cell division	regulation of G2/M transition of mitotic cell cycle	P	AT3G48750
1142	1	L123 vs. M123	M123 up	GO:0040020	Development	Cell division	regulation of meiotic nuclear division	P	AT3G48750
1143	1	L123 vs. M123	M123 up	GO:0016070	Development	Cell division	RNA metabolic process	P	AT1G06790
1144	1	L123 vs. M123	M123 up	GO:0051225	Development	Cell division	spindle assembly	P	AT5G17620
1145	1	L123 vs. M123	M123 up	GO:0005876	Development	Cell division	spindle microtubule	C	AT5G17620
1146	1	L123 vs. M123	M123 up	GO:0098725	Development	Cell division	symmetric cell division	P	AT3G48750
1147	1	L123 vs. M123	M123 up	GO:0007129	Development	Cell division	synapsis	P	AT1G67370
1148	1	L123 vs. M123	M123 up	GO:0007129	Development	Cell division	synapsis	P	AT3G13170
1149	1	L123 vs. M123	M123 up	GO:0007130	Development	Cell division	synaptonemal complex assembly	P	AT1G67370
1150	1	L123 vs. M123	M123 up	GO:0048830	Development	Development	adventitious root development	P	AT4G31500
1151	1	L123 vs. M123	M123 up	GO:0031225	Development	Development	anchored component of membrane	C	AT1G65240
1152	1	L123 vs. M123	M123 up	GO:0046658	Development	Development	anchored component of plasma membrane	C	AT1G65240
1153	1	L123 vs. M123	M123 up	GO:0046658	Development	Development	anchored component of plasma membrane	C	AT4G12880
1154	1	L123 vs. M123	M123 up	GO:0048653	Development	Development	anther development	P	AT3G49670
1155	1	L123 vs. M123	M123 up	GO:0048046	Development	Development	apoplast	C	AT1G14720
1156	1	L123 vs. M123	M123 up	GO:0048046	Development	Development	apoplast	C	AT1G42970
1157	1	L123 vs. M123	M123 up	GO:0048046	Development	Development	apoplast	C	AT2G18660
1158	1	L123 vs. M123	M123 up	GO:0048046	Development	Development	apoplast	C	AT2G28190
1159	1	L123 vs. M123	M123 up	GO:0048046	Development	Development	apoplast	C	AT2G30860
1160	1	L123 vs. M123	M123 up	GO:0048046	Development	Development	apoplast	C	AT4G12880
1161	1	L123 vs. M123	M123 up	GO:0048046	Development	Development	apoplast	C	AT4G20360
1162	1	L123 vs. M123	M123 up	GO:0005623	Development	Development	cell	C	AT5G65460
1163	1	L123 vs. M123	M123 up	GO:0030154	Development	Development	cell differentiation	P	AT3G49670
1164	1	L123 vs. M123	M123 up	GO:0009986	Development	Development	cell surface	C	AT5G65020
1165	1	L123 vs. M123	M123 up	GO:0005618	Development	Development	cell wall	C	AT1G14720
1166	1	L123 vs. M123	M123 up	GO:0005618	Development	Development	cell wall	C	AT2G18660
1167	1	L123 vs. M123	M123 up	GO:0005618	Development	Development	cell wall	C	AT4G19410
1168	1	L123 vs. M123	M123 up	GO:0005618	Development	Development	cell wall	C	AT4G24890
1169	1	L123 vs. M123	M123 up	GO:0042546	Development	Development	cell wall biogenesis	P	AT1G14720
1170	1	L123 vs. M123	M123 up	GO:0071555	Development	Development	cell wall organization	P	AT1G14720
1171	1	L123 vs. M123	M123 up	GO:0071555	Development	Development	cell wall organization	P	AT4G19410
1172	1	L123 vs. M123	M123 up	GO:0098653	Development	Development	centromere clustering	P	AT4G15890
1173	1	L123 vs. M123	M123 up	GO:0005813	Development	Development	centrosome	C	AT2G26760
1174	1	L123 vs. M123	M123 up	GO:0007098	Development	Development	centrosome cycle	P	AT5G17620
1175	1	L123 vs. M123	M123 up	GO:0000785	Development	Development	chromatin	C	AT1G67370
1176	1	L123 vs. M123	M123 up	GO:0005801	Development	Development	cis-Golgi network	C	AT3G05000
1177	1	L123 vs. M123	M123 up	GO:0010005	Development	Development	cortical microtubule, transverse to long axis	C	AT3G48750
1178	1	L123 vs. M123	M123 up	GO:0042335	Development	Development	cuticle development	P	AT3G55360
1179	1	L123 vs. M123	M123 up	GO:0005737	Development	Development	cytoplasm	C	AT1G01800
1180	1	L123 vs. M123	M123 up	GO:0005737	Development	Development	cytoplasm	C	AT1G01940
1181	1	L123 vs. M123	M123 up	GO:0005737	Development	Development	cytoplasm	C	AT1G08830
1182	1	L123 vs. M123	M123 up	GO:0005737	Development	Development	cytoplasm	C	AT1G25350
1183	1	L123 vs. M123	M123 up	GO:0005737	Development	Development	cytoplasm	C	AT1G45145

1184	1	L123 vs. M123	M123 up	GO:0005737	Development	Development	cytoplasm	C	AT1G53650
1185	1	L123 vs. M123	M123 up	GO:0005737	Development	Development	cytoplasm	C	AT1G54650
1186	1	L123 vs. M123	M123 up	GO:0005737	Development	Development	cytoplasm	C	AT1G79690
1187	1	L123 vs. M123	M123 up	GO:0005737	Development	Development	cytoplasm	C	AT1G80280
1188	1	L123 vs. M123	M123 up	GO:0005737	Development	Development	cytoplasm	C	AT2G05250
1189	1	L123 vs. M123	M123 up	GO:0005737	Development	Development	cytoplasm	C	AT2G26760
1190	1	L123 vs. M123	M123 up	GO:0005737	Development	Development	cytoplasm	C	AT2G30860
1191	1	L123 vs. M123	M123 up	GO:0005737	Development	Development	cytoplasm	C	AT2G38860
1192	1	L123 vs. M123	M123 up	GO:0005737	Development	Development	cytoplasm	C	AT3G03190
1193	1	L123 vs. M123	M123 up	GO:0005737	Development	Development	cytoplasm	C	AT3G16410
1194	1	L123 vs. M123	M123 up	GO:0005737	Development	Development	cytoplasm	C	AT3G19980
1195	1	L123 vs. M123	M123 up	GO:0005737	Development	Development	cytoplasm	C	AT3G24503
1196	1	L123 vs. M123	M123 up	GO:0005737	Development	Development	cytoplasm	C	AT3G28900
1197	1	L123 vs. M123	M123 up	GO:0005737	Development	Development	cytoplasm	C	AT3G48750
1198	1	L123 vs. M123	M123 up	GO:0005737	Development	Development	cytoplasm	C	AT3G52190
1199	1	L123 vs. M123	M123 up	GO:0005737	Development	Development	cytoplasm	C	AT3G53520
1200	1	L123 vs. M123	M123 up	GO:0005737	Development	Development	cytoplasm	C	AT3G61580
1201	1	L123 vs. M123	M123 up	GO:0005737	Development	Development	cytoplasm	C	AT3G61760
1202	1	L123 vs. M123	M123 up	GO:0005737	Development	Development	cytoplasm	C	AT4G02890
1203	1	L123 vs. M123	M123 up	GO:0005737	Development	Development	cytoplasm	C	AT4G17640
1204	1	L123 vs. M123	M123 up	GO:0005737	Development	Development	cytoplasm	C	AT4G26270
1205	1	L123 vs. M123	M123 up	GO:0005737	Development	Development	cytoplasm	C	AT4G39520
1206	1	L123 vs. M123	M123 up	GO:0005737	Development	Development	cytoplasm	C	AT5G01390
1207	1	L123 vs. M123	M123 up	GO:0005737	Development	Development	cytoplasm	C	AT5G16970
1208	1	L123 vs. M123	M123 up	GO:0005737	Development	Development	cytoplasm	C	AT5G50740
1209	1	L123 vs. M123	M123 up	GO:0005737	Development	Development	cytoplasm	C	AT5G56630
1210	1	L123 vs. M123	M123 up	GO:0005737	Development	Development	cytoplasm	C	AT5G63680
1211	1	L123 vs. M123	M123 up	GO:0005737	Development	Development	cytoplasm	C	AT5G64140
1212	1	L123 vs. M123	M123 up	GO:0005737	Development	Development	cytoplasm	C	AT5G64350
1213	1	L123 vs. M123	M123 up	GO:0005829	Development	Development	cytosol	C	AT1G01800
1214	1	L123 vs. M123	M123 up	GO:0005829	Development	Development	cytosol	C	AT1G08830
1215	1	L123 vs. M123	M123 up	GO:0005829	Development	Development	cytosol	C	AT1G25350
1216	1	L123 vs. M123	M123 up	GO:0005829	Development	Development	cytosol	C	AT1G45145
1217	1	L123 vs. M123	M123 up	GO:0005829	Development	Development	cytosol	C	AT1G47970
1218	1	L123 vs. M123	M123 up	GO:0005829	Development	Development	cytosol	C	AT1G60070
1219	1	L123 vs. M123	M123 up	GO:0005829	Development	Development	cytosol	C	AT1G67230
1220	1	L123 vs. M123	M123 up	GO:0005829	Development	Development	cytosol	C	AT1G79690
1221	1	L123 vs. M123	M123 up	GO:0005829	Development	Development	cytosol	C	AT2G30860
1222	1	L123 vs. M123	M123 up	GO:0005829	Development	Development	cytosol	C	AT2G38280
1223	1	L123 vs. M123	M123 up	GO:0005829	Development	Development	cytosol	C	AT3G02190
1224	1	L123 vs. M123	M123 up	GO:0005829	Development	Development	cytosol	C	AT3G03190
1225	1	L123 vs. M123	M123 up	GO:0005829	Development	Development	cytosol	C	AT3G05000
1226	1	L123 vs. M123	M123 up	GO:0005829	Development	Development	cytosol	C	AT3G16410
1227	1	L123 vs. M123	M123 up	GO:0005829	Development	Development	cytosol	C	AT3G24503
1228	1	L123 vs. M123	M123 up	GO:0005829	Development	Development	cytosol	C	AT3G28900
1229	1	L123 vs. M123	M123 up	GO:0005829	Development	Development	cytosol	C	AT3G46430
1230	1	L123 vs. M123	M123 up	GO:0005829	Development	Development	cytosol	C	AT3G48750
1231	1	L123 vs. M123	M123 up	GO:0005829	Development	Development	cytosol	C	AT3G53520
1232	1	L123 vs. M123	M123 up	GO:0005829	Development	Development	cytosol	C	AT3G55360
1233	1	L123 vs. M123	M123 up	GO:0005829	Development	Development	cytosol	C	AT4G02890
1234	1	L123 vs. M123	M123 up	GO:0005829	Development	Development	cytosol	C	AT4G15210
1235	1	L123 vs. M123	M123 up	GO:0005829	Development	Development	cytosol	C	AT4G16660
1236	1	L123 vs. M123	M123 up	GO:0005829	Development	Development	cytosol	C	AT4G16800
1237	1	L123 vs. M123	M123 up	GO:0005829	Development	Development	cytosol	C	AT4G17640
1238	1	L123 vs. M123	M123 up	GO:0005829	Development	Development	cytosol	C	AT4G26270
1239	1	L123 vs. M123	M123 up	GO:0005829	Development	Development	cytosol	C	AT4G39520

1240	1	L123 vs. M123	M123 up	GO:0005829	Development	Development	cytosol	C	AT5G01390
1241	1	L123 vs. M123	M123 up	GO:0005829	Development	Development	cytosol	C	AT5G08160
1242	1	L123 vs. M123	M123 up	GO:0005829	Development	Development	cytosol	C	AT5G16970
1243	1	L123 vs. M123	M123 up	GO:0005829	Development	Development	cytosol	C	AT5G42250
1244	1	L123 vs. M123	M123 up	GO:0005829	Development	Development	cytosol	C	AT5G51720
1245	1	L123 vs. M123	M123 up	GO:0005829	Development	Development	cytosol	C	AT5G56630
1246	1	L123 vs. M123	M123 up	GO:0005829	Development	Development	cytosol	C	AT5G63680
1247	1	L123 vs. M123	M123 up	GO:0005829	Development	Development	cytosol	C	AT5G64350
1248	1	L123 vs. M123	M123 up	GO:0005829	Development	Development	cytosol	C	AT5G65020
1249	1	L123 vs. M123	M123 up	GO:0005829	Development	Development	cytosol	C	AT5G65460
1250	1	L123 vs. M123	M123 up	GO:0009793	Development	Development	embryo development ending in seed dormancy	P	AT2G38280
1251	1	L123 vs. M123	M123 up	GO:0009793	Development	Development	embryo development ending in seed dormancy	P	AT3G19980
1252	1	L123 vs. M123	M123 up	GO:0009793	Development	Development	embryo development ending in seed dormancy	P	AT3G48750
1253	1	L123 vs. M123	M123 up	GO:0009793	Development	Development	embryo development ending in seed dormancy	P	AT5G48600
1254	1	L123 vs. M123	M123 up	GO:0090158	Development	Development	endoplasmic reticulum membrane organization	P	AT4G00170
1255	1	L123 vs. M123	M123 up	GO:0061817	Development	Development	endoplasmic reticulum-plasma membrane tethering	P	AT4G00170
1256	1	L123 vs. M123	M123 up	GO:0005576	Development	Development	extracellular region	C	AT1G05260
1257	1	L123 vs. M123	M123 up	GO:0005576	Development	Development	extracellular region	C	AT1G08830
1258	1	L123 vs. M123	M123 up	GO:0005576	Development	Development	extracellular region	C	AT1G09200
1259	1	L123 vs. M123	M123 up	GO:0005576	Development	Development	extracellular region	C	AT1G10740
1260	1	L123 vs. M123	M123 up	GO:0005576	Development	Development	extracellular region	C	AT1G14720
1261	1	L123 vs. M123	M123 up	GO:0005576	Development	Development	extracellular region	C	AT1G31335
1262	1	L123 vs. M123	M123 up	GO:0005576	Development	Development	extracellular region	C	AT1G59990
1263	1	L123 vs. M123	M123 up	GO:0005576	Development	Development	extracellular region	C	AT1G67230
1264	1	L123 vs. M123	M123 up	GO:0005576	Development	Development	extracellular region	C	AT2G07680
1265	1	L123 vs. M123	M123 up	GO:0005576	Development	Development	extracellular region	C	AT2G14900
1266	1	L123 vs. M123	M123 up	GO:0005576	Development	Development	extracellular region	C	AT2G18660
1267	1	L123 vs. M123	M123 up	GO:0005576	Development	Development	extracellular region	C	AT2G28190
1268	1	L123 vs. M123	M123 up	GO:0005576	Development	Development	extracellular region	C	AT3G48460
1269	1	L123 vs. M123	M123 up	GO:0005576	Development	Development	extracellular region	C	AT3G50610
1270	1	L123 vs. M123	M123 up	GO:0005576	Development	Development	extracellular region	C	AT4G12390
1271	1	L123 vs. M123	M123 up	GO:0005576	Development	Development	extracellular region	C	AT4G15210
1272	1	L123 vs. M123	M123 up	GO:0005576	Development	Development	extracellular region	C	AT4G19410
1273	1	L123 vs. M123	M123 up	GO:0005576	Development	Development	extracellular region	C	AT4G24890
1274	1	L123 vs. M123	M123 up	GO:0005576	Development	Development	extracellular region	C	AT5G02140
1275	1	L123 vs. M123	M123 up	GO:0005615	Development	Development	extracellular space	C	AT1G08830
1276	1	L123 vs. M123	M123 up	GO:0005615	Development	Development	extracellular space	C	AT2G28190
1277	1	L123 vs. M123	M123 up	GO:0048437	Development	Development	floral organ development	P	AT3G49670
1278	1	L123 vs. M123	M123 up	GO:0010154	Development	Development	fruit development	P	AT1G14720
1279	1	L123 vs. M123	M123 up	GO:0005796	Development	Development	Golgi lumen	C	AT3G03210
1280	1	L123 vs. M123	M123 up	GO:0000138	Development	Development	Golgi trans cisterna	C	AT3G53520
1281	1	L123 vs. M123	M123 up	GO:0010444	Development	Development	guard mother cell differentiation	P	AT3G48750
1282	1	L123 vs. M123	M123 up	GO:0030176	Development	Development	integral component of endoplasmic reticulum membrane	C	AT1G43580
1283	1	L123 vs. M123	M123 up	GO:0030176	Development	Development	integral component of endoplasmic reticulum membrane	C	AT3G52190
1284	1	L123 vs. M123	M123 up	GO:0016021	Development	Development	integral component of membrane	C	AT1G04760
1285	1	L123 vs. M123	M123 up	GO:0016021	Development	Development	integral component of membrane	C	AT1G06350
1286	1	L123 vs. M123	M123 up	GO:0016021	Development	Development	integral component of membrane	C	AT1G10740
1287	1	L123 vs. M123	M123 up	GO:0016021	Development	Development	integral component of membrane	C	AT1G12500
1288	1	L123 vs. M123	M123 up	GO:0016021	Development	Development	integral component of membrane	C	AT1G27770
1289	1	L123 vs. M123	M123 up	GO:0016021	Development	Development	integral component of membrane	C	AT1G31335
1290	1	L123 vs. M123	M123 up	GO:0016021	Development	Development	integral component of membrane	C	AT1G54990
1291	1	L123 vs. M123	M123 up	GO:0016021	Development	Development	integral component of membrane	C	AT1G57680
1292	1	L123 vs. M123	M123 up	GO:0016021	Development	Development	integral component of membrane	C	AT1G60030

1293	1	L123 vs. M123	M123 up	GO:0016021	Development	Development	integral component of membrane	C	AT1G69700
1294	1	L123 vs. M123	M123 up	GO:0016021	Development	Development	integral component of membrane	C	AT1G80280
1295	1	L123 vs. M123	M123 up	GO:0016021	Development	Development	integral component of membrane	C	AT2G03240
1296	1	L123 vs. M123	M123 up	GO:0016021	Development	Development	integral component of membrane	C	AT2G07680
1297	1	L123 vs. M123	M123 up	GO:0016021	Development	Development	integral component of membrane	C	AT2G22330
1298	1	L123 vs. M123	M123 up	GO:0016021	Development	Development	integral component of membrane	C	AT2G34070
1299	1	L123 vs. M123	M123 up	GO:0016021	Development	Development	integral component of membrane	C	AT3G01980
1300	1	L123 vs. M123	M123 up	GO:0016021	Development	Development	integral component of membrane	C	AT3G03210
1301	1	L123 vs. M123	M123 up	GO:0016021	Development	Development	integral component of membrane	C	AT3G09700
1302	1	L123 vs. M123	M123 up	GO:0016021	Development	Development	integral component of membrane	C	AT3G24660
1303	1	L123 vs. M123	M123 up	GO:0016021	Development	Development	integral component of membrane	C	AT3G27870
1304	1	L123 vs. M123	M123 up	GO:0016021	Development	Development	integral component of membrane	C	AT3G49670
1305	1	L123 vs. M123	M123 up	GO:0016021	Development	Development	integral component of membrane	C	AT3G53520
1306	1	L123 vs. M123	M123 up	GO:0016021	Development	Development	integral component of membrane	C	AT3G55360
1307	1	L123 vs. M123	M123 up	GO:0016021	Development	Development	integral component of membrane	C	AT3G61580
1308	1	L123 vs. M123	M123 up	GO:0016021	Development	Development	integral component of membrane	C	AT4G00170
1309	1	L123 vs. M123	M123 up	GO:0016021	Development	Development	integral component of membrane	C	AT4G03320
1310	1	L123 vs. M123	M123 up	GO:0016021	Development	Development	integral component of membrane	C	AT4G31500
1311	1	L123 vs. M123	M123 up	GO:0016021	Development	Development	integral component of membrane	C	AT5G22540
1312	1	L123 vs. M123	M123 up	GO:0016021	Development	Development	integral component of membrane	C	AT5G24650
1313	1	L123 vs. M123	M123 up	GO:0016021	Development	Development	integral component of membrane	C	AT5G40670
1314	1	L123 vs. M123	M123 up	GO:0016021	Development	Development	integral component of membrane	C	AT5G41000
1315	1	L123 vs. M123	M123 up	GO:0016021	Development	Development	integral component of membrane	C	AT5G55370
1316	1	L123 vs. M123	M123 up	GO:0016021	Development	Development	integral component of membrane	C	AT5G62620
1317	1	L123 vs. M123	M123 up	GO:0016021	Development	Development	integral component of membrane	C	ATCG00560
1318	1	L123 vs. M123	M123 up	GO:0016021	Development	Development	integral component of membrane	C	ATCG00680
1319	1	L123 vs. M123	M123 up	GO:0016021	Development	Development	integral component of membrane	C	ATCG01040
1320	1	L123 vs. M123	M123 up	GO:0016021	Development	Development	integral component of membrane	C	ATMG00410
1321	1	L123 vs. M123	M123 up	GO:0016021	Development	Development	integral component of membrane	C	ATMG00480
1322	1	L123 vs. M123	M123 up	GO:0016021	Development	Development	integral component of membrane	C	ATMG01370
1323	1	L123 vs. M123	M123 up	GO:0005887	Development	Development	integral component of plasma membrane	C	AT1G43580
1324	1	L123 vs. M123	M123 up	GO:0005887	Development	Development	integral component of plasma membrane	C	AT3G03160
1325	1	L123 vs. M123	M123 up	GO:0005622	Development	Development	intracellular	C	AT5G65460
1326	1	L123 vs. M123	M123 up	GO:0043231	Development	Development	intracellular membrane-bounded organelle	C	AT2G38280
1327	1	L123 vs. M123	M123 up	GO:0048366	Development	Development	leaf development	P	AT4G20360
1328	1	L123 vs. M123	M123 up	GO:0005765	Development	Development	lysosomal membrane	C	AT5G40670
1329	1	L123 vs. M123	M123 up	GO:0016020	Development	Development	membrane	C	AT2G07680
1330	1	L123 vs. M123	M123 up	GO:0016020	Development	Development	membrane	C	AT3G53520
1331	1	L123 vs. M123	M123 up	GO:0016020	Development	Development	membrane	C	AT3G61760
1332	1	L123 vs. M123	M123 up	GO:0016020	Development	Development	membrane	C	AT5G65020
1333	1	L123 vs. M123	M123 up	GO:0005874	Development	Development	microtubule	C	AT3G61760
1334	1	L123 vs. M123	M123 up	GO:0005874	Development	Development	microtubule	C	AT5G65460
1335	1	L123 vs. M123	M123 up	GO:0007018	Development	Development	microtubule-based movement	P	AT5G65460
1336	1	L123 vs. M123	M123 up	GO:0000799	Development	Development	nuclear condensin complex	C	AT4G15890
1337	1	L123 vs. M123	M123 up	GO:0005652	Development	Development	nuclear lamina	C	AT1G67230
1338	1	L123 vs. M123	M123 up	GO:0034399	Development	Development	nuclear periphery	C	AT1G67230
1339	1	L123 vs. M123	M123 up	GO:0009295	Development	Development	nucleoid	C	AT4G20360
1340	1	L123 vs. M123	M123 up	GO:0005654	Development	Development	nucleoplasm	C	AT1G67370
1341	1	L123 vs. M123	M123 up	GO:0005654	Development	Development	nucleoplasm	C	AT5G16970
1342	1	L123 vs. M123	M123 up	GO:0005634	Development	Development	nucleus	C	AT1G01940
1343	1	L123 vs. M123	M123 up	GO:0005634	Development	Development	nucleus	C	AT1G06790
1344	1	L123 vs. M123	M123 up	GO:0005634	Development	Development	nucleus	C	AT1G08830
1345	1	L123 vs. M123	M123 up	GO:0005634	Development	Development	nucleus	C	AT1G09200
1346	1	L123 vs. M123	M123 up	GO:0005634	Development	Development	nucleus	C	AT1G12140
1347	1	L123 vs. M123	M123 up	GO:0005634	Development	Development	nucleus	C	AT1G12200
1348	1	L123 vs. M123	M123 up	GO:0005634	Development	Development	nucleus	C	AT1G17100

1349	1	L123 vs. M123	M123 up	GO:0005634	Development	Development	nucleus	C	AT1G25350
1350	1	L123 vs. M123	M123 up	GO:0005634	Development	Development	nucleus	C	AT1G27770
1351	1	L123 vs. M123	M123 up	GO:0005634	Development	Development	nucleus	C	AT1G42970
1352	1	L123 vs. M123	M123 up	GO:0005634	Development	Development	nucleus	C	AT1G45145
1353	1	L123 vs. M123	M123 up	GO:0005634	Development	Development	nucleus	C	AT1G47970
1354	1	L123 vs. M123	M123 up	GO:0005634	Development	Development	nucleus	C	AT1G53650
1355	1	L123 vs. M123	M123 up	GO:0005634	Development	Development	nucleus	C	AT1G54990
1356	1	L123 vs. M123	M123 up	GO:0005634	Development	Development	nucleus	C	AT1G67230
1357	1	L123 vs. M123	M123 up	GO:0005634	Development	Development	nucleus	C	AT1G69700
1358	1	L123 vs. M123	M123 up	GO:0005634	Development	Development	nucleus	C	AT2G26760
1359	1	L123 vs. M123	M123 up	GO:0005634	Development	Development	nucleus	C	AT2G27590
1360	1	L123 vs. M123	M123 up	GO:0005634	Development	Development	nucleus	C	AT2G27980
1361	1	L123 vs. M123	M123 up	GO:0005634	Development	Development	nucleus	C	AT2G38280
1362	1	L123 vs. M123	M123 up	GO:0005634	Development	Development	nucleus	C	AT3G13170
1363	1	L123 vs. M123	M123 up	GO:0005634	Development	Development	nucleus	C	AT3G16410
1364	1	L123 vs. M123	M123 up	GO:0005634	Development	Development	nucleus	C	AT3G19980
1365	1	L123 vs. M123	M123 up	GO:0005634	Development	Development	nucleus	C	AT3G24050
1366	1	L123 vs. M123	M123 up	GO:0005634	Development	Development	nucleus	C	AT3G48750
1367	1	L123 vs. M123	M123 up	GO:0005634	Development	Development	nucleus	C	AT4G02890
1368	1	L123 vs. M123	M123 up	GO:0005634	Development	Development	nucleus	C	AT4G10925
1369	1	L123 vs. M123	M123 up	GO:0005634	Development	Development	nucleus	C	AT4G15890
1370	1	L123 vs. M123	M123 up	GO:0005634	Development	Development	nucleus	C	AT4G17640
1371	1	L123 vs. M123	M123 up	GO:0005634	Development	Development	nucleus	C	AT4G20360
1372	1	L123 vs. M123	M123 up	GO:0005634	Development	Development	nucleus	C	AT4G39235
1373	1	L123 vs. M123	M123 up	GO:0005634	Development	Development	nucleus	C	AT5G08160
1374	1	L123 vs. M123	M123 up	GO:0005634	Development	Development	nucleus	C	AT5G16970
1375	1	L123 vs. M123	M123 up	GO:0005634	Development	Development	nucleus	C	AT5G41810
1376	1	L123 vs. M123	M123 up	GO:0005634	Development	Development	nucleus	C	AT5G48600
1377	1	L123 vs. M123	M123 up	GO:0005634	Development	Development	nucleus	C	AT5G60570
1378	1	L123 vs. M123	M123 up	GO:0005634	Development	Development	nucleus	C	AT5G65020
1379	1	L123 vs. M123	M123 up	GO:0005634	Development	Development	nucleus	C	AT5G65460
1380	1	L123 vs. M123	M123 up	GO:0006997	Development	Development	nucleus organization	P	AT1G67230
1381	1	L123 vs. M123	M123 up	GO:0009524	Development	Development	phragmoplast	C	AT5G17620
1382	1	L123 vs. M123	M123 up	GO:0048481	Development	Development	plant ovule development	P	AT1G25350
1383	1	L123 vs. M123	M123 up	GO:0009505	Development	Development	plant-type cell wall	C	AT1G01800
1384	1	L123 vs. M123	M123 up	GO:0009505	Development	Development	plant-type cell wall	C	AT1G05260
1385	1	L123 vs. M123	M123 up	GO:0009505	Development	Development	plant-type cell wall	C	AT4G19410
1386	1	L123 vs. M123	M123 up	GO:0005886	Development	Development	plasma membrane	C	AT1G04760
1387	1	L123 vs. M123	M123 up	GO:0005886	Development	Development	plasma membrane	C	AT1G08860
1388	1	L123 vs. M123	M123 up	GO:0005886	Development	Development	plasma membrane	C	AT1G12500
1389	1	L123 vs. M123	M123 up	GO:0005886	Development	Development	plasma membrane	C	AT1G27770
1390	1	L123 vs. M123	M123 up	GO:0005886	Development	Development	plasma membrane	C	AT1G31335
1391	1	L123 vs. M123	M123 up	GO:0005886	Development	Development	plasma membrane	C	AT1G45145
1392	1	L123 vs. M123	M123 up	GO:0005886	Development	Development	plasma membrane	C	AT1G60030
1393	1	L123 vs. M123	M123 up	GO:0005886	Development	Development	plasma membrane	C	AT1G65240
1394	1	L123 vs. M123	M123 up	GO:0005886	Development	Development	plasma membrane	C	AT2G03240
1395	1	L123 vs. M123	M123 up	GO:0005886	Development	Development	plasma membrane	C	AT2G30860
1396	1	L123 vs. M123	M123 up	GO:0005886	Development	Development	plasma membrane	C	AT3G24660
1397	1	L123 vs. M123	M123 up	GO:0005886	Development	Development	plasma membrane	C	AT3G27870
1398	1	L123 vs. M123	M123 up	GO:0005886	Development	Development	plasma membrane	C	AT3G48750
1399	1	L123 vs. M123	M123 up	GO:0005886	Development	Development	plasma membrane	C	AT3G49670
1400	1	L123 vs. M123	M123 up	GO:0005886	Development	Development	plasma membrane	C	AT3G52190
1401	1	L123 vs. M123	M123 up	GO:0005886	Development	Development	plasma membrane	C	AT3G53520
1402	1	L123 vs. M123	M123 up	GO:0005886	Development	Development	plasma membrane	C	AT3G55360
1403	1	L123 vs. M123	M123 up	GO:0005886	Development	Development	plasma membrane	C	AT4G00170
1404	1	L123 vs. M123	M123 up	GO:0005886	Development	Development	plasma membrane	C	AT4G31500

1405	1	L123 vs. M123	M123 up	GO:0005886	Development	Development	plasma membrane	C	AT5G24650
1406	1	L123 vs. M123	M123 up	GO:0005886	Development	Development	plasma membrane	C	AT5G50740
1407	1	L123 vs. M123	M123 up	GO:0005886	Development	Development	plasma membrane	C	AT5G63680
1408	1	L123 vs. M123	M123 up	GO:0005886	Development	Development	plasma membrane	C	AT5G65460
1409	1	L123 vs. M123	M123 up	GO:0009555	Development	Development	pollen development	P	AT3G48750
1410	1	L123 vs. M123	M123 up	GO:0080022	Development	Development	primary root development	P	AT5G65020
1411	1	L123 vs. M123	M123 up	GO:0010053	Development	Development	root epidermal cell differentiation	P	AT4G26270
1412	1	L123 vs. M123	M123 up	GO:0080086	Development	Development	stamen filament development	P	AT1G14720
1413	1	L123 vs. M123	M123 up	GO:0010319	Development	Development	stromule	C	AT1G42970
1414	1	L123 vs. M123	M123 up	GO:0010091	Development	Development	trichome branching	P	AT3G55360
1415	1	L123 vs. M123	M123 up	GO:1905499	Development	Development	trichome papilla formation	P	AT3G55360
1416	1	L123 vs. M123	M123 up	GO:0005774	Development	Development	vacuolar membrane	C	AT1G17100
1417	1	L123 vs. M123	M123 up	GO:0005774	Development	Development	vacuolar membrane	C	AT1G54990
1418	1	L123 vs. M123	M123 up	GO:0005774	Development	Development	vacuolar membrane	C	AT2G07680
1419	1	L123 vs. M123	M123 up	GO:0005774	Development	Development	vacuolar membrane	C	AT4G16660
1420	1	L123 vs. M123	M123 up	GO:0005774	Development	Development	vacuolar membrane	C	AT5G24650
1421	1	L123 vs. M123	M123 up	GO:0005774	Development	Development	vacuolar membrane	C	AT5G40670
1422	1	L123 vs. M123	M123 up	GO:0005774	Development	Development	vacuolar membrane	C	AT5G41000
1423	1	L123 vs. M123	M123 up	GO:0005773	Development	Development	vacuole	C	AT1G17100
1424	1	L123 vs. M123	M123 up	GO:0005773	Development	Development	vacuole	C	AT1G79690
1425	1	L123 vs. M123	M123 up	GO:0005773	Development	Development	vacuole	C	AT2G30860
1426	1	L123 vs. M123	M123 up	GO:0005773	Development	Development	vacuole	C	AT4G02890
1427	1	L123 vs. M123	M123 up	GO:0005773	Development	Development	vacuole	C	AT5G24650
1428	1	L123 vs. M123	M123 up	GO:0005773	Development	Development	vacuole	C	AT5G24780
1429	1	L123 vs. M123	M123 up	GO:0032440	Metabolism	Bioenergetics	2-alkenal reductase [NAD(P)+] activity	F	AT5G16970
1430	1	L123 vs. M123	M123 up	GO:0003872	Metabolism	Bioenergetics	6-phosphofructokinase activity	F	AT4G26270
1431	1	L123 vs. M123	M123 up	GO:0003872	Metabolism	Bioenergetics	6-phosphofructokinase activity	F	AT5G56630
1432	1	L123 vs. M123	M123 up	GO:0047617	Metabolism	Bioenergetics	acyl-CoA hydrolase activity	F	AT5G48370
1433	1	L123 vs. M123	M123 up	GO:0006637	Metabolism	Bioenergetics	acyl-CoA metabolic process	P	AT5G48370
1434	1	L123 vs. M123	M123 up	GO:0004029	Metabolism	Bioenergetics	aldehyde dehydrogenase (NAD+) activity	F	AT3G24503
1435	1	L123 vs. M123	M123 up	GO:0102966	Metabolism	Bioenergetics	arachidoyl-CoA:1-dodecanol O-acyltransferase activity	F	AT5G55370
1436	1	L123 vs. M123	M123 up	GO:0006754	Metabolism	Bioenergetics	ATP biosynthetic process	P	ATMG00480
1437	1	L123 vs. M123	M123 up	GO:0015986	Metabolism	Bioenergetics	ATP synthesis coupled proton transport	P	ATMG00410
1438	1	L123 vs. M123	M123 up	GO:0015986	Metabolism	Bioenergetics	ATP synthesis coupled proton transport	P	ATMG00480
1439	1	L123 vs. M123	M123 up	GO:0001671	Metabolism	Bioenergetics	ATPase activator activity	F	AT3G09700
1440	1	L123 vs. M123	M123 up	GO:0016887	Metabolism	Bioenergetics	ATPase activity	F	AT2G07680
1441	1	L123 vs. M123	M123 up	GO:0016887	Metabolism	Bioenergetics	ATPase activity	F	AT5G65460
1442	1	L123 vs. M123	M123 up	GO:0016161	Metabolism	Bioenergetics	beta-amylase activity	F	AT4G15210
1443	1	L123 vs. M123	M123 up	GO:0030246	Metabolism	Bioenergetics	carbohydrate binding	F	AT3G16410
1444	1	L123 vs. M123	M123 up	GO:0030246	Metabolism	Bioenergetics	carbohydrate binding	F	AT5G62620
1445	1	L123 vs. M123	M123 up	GO:0005975	Metabolism	Bioenergetics	carbohydrate metabolic process	P	AT3G03210
1446	1	L123 vs. M123	M123 up	GO:0045333	Metabolism	Bioenergetics	cellular respiration	P	ATMG00410
1447	1	L123 vs. M123	M123 up	GO:0009055	Metabolism	Bioenergetics	electron transfer activity	F	AT4G12880
1448	1	L123 vs. M123	M123 up	GO:0004300	Metabolism	Bioenergetics	enoyl-CoA hydratase activity	F	AT4G16800
1449	1	L123 vs. M123	M123 up	GO:0006635	Metabolism	Bioenergetics	fatty acid beta-oxidation	P	AT4G16800
1450	1	L123 vs. M123	M123 up	GO:0006633	Metabolism	Bioenergetics	fatty acid biosynthetic process	P	AT1G08510
1451	1	L123 vs. M123	M123 up	GO:0009922	Metabolism	Bioenergetics	fatty acid elongase activity	F	AT3G55360
1452	1	L123 vs. M123	M123 up	GO:0009923	Metabolism	Bioenergetics	fatty acid elongase complex	C	AT3G55360
1453	1	L123 vs. M123	M123 up	GO:0006631	Metabolism	Bioenergetics	fatty acid metabolic process	P	AT3G48460
1454	1	L123 vs. M123	M123 up	GO:0006006	Metabolism	Bioenergetics	glucose metabolic process	P	AT1G42970
1455	1	L123 vs. M123	M123 up	GO:0043878	Metabolism	Bioenergetics	glyceraldehyde-3-phosphate dehydrogenase (NAD+) (non-phosphorylating) activity	F	AT3G24503
1456	1	L123 vs. M123	M123 up	GO:0004365	Metabolism	Bioenergetics	glyceraldehyde-3-phosphate dehydrogenase (NAD+) (phosphorylating) activity	F	AT1G42970

1457	1	L123 vs. M123	M123 up	GO:0047100	Metabolism	Bioenergetics	glyceraldehyde-3-phosphate dehydrogenase (NADP+) (phosphorylating) activity	F	AT1G42970
1458	1	L123 vs. M123	M123 up	GO:0006096	Metabolism	Bioenergetics	glycolytic process	P	AT4G26270
1459	1	L123 vs. M123	M123 up	GO:0006096	Metabolism	Bioenergetics	glycolytic process	P	AT5G56630
1460	1	L123 vs. M123	M123 up	GO:0006096	Metabolism	Bioenergetics	glycolytic process	P	AT5G63680
1461	1	L123 vs. M123	M123 up	GO:0031307	Metabolism	Bioenergetics	integral component of mitochondrial outer membrane	C	AT2G38280
1462	1	L123 vs. M123	M123 up	GO:0047196	Metabolism	Bioenergetics	long-chain-alcohol O-fatty-acyltransferase activity	F	AT5G55370
1463	1	L123 vs. M123	M123 up	GO:0004490	Metabolism	Bioenergetics	methylglutaconyl-CoA hydratase activity	F	AT4G16800
1464	1	L123 vs. M123	M123 up	GO:0042776	Metabolism	Bioenergetics	mitochondrial ATP synthesis coupled proton transport	P	ATMG00410
1465	1	L123 vs. M123	M123 up	GO:0000266	Metabolism	Bioenergetics	mitochondrial fission	P	AT3G61760
1466	1	L123 vs. M123	M123 up	GO:0005743	Metabolism	Bioenergetics	mitochondrial inner membrane	C	ATMG00410
1467	1	L123 vs. M123	M123 up	GO:0031966	Metabolism	Bioenergetics	mitochondrial membrane	C	ATMG01370
1468	1	L123 vs. M123	M123 up	GO:0005753	Metabolism	Bioenergetics	mitochondrial proton-transporting ATP synthase complex	C	AT3G46430
1469	1	L123 vs. M123	M123 up	GO:0005753	Metabolism	Bioenergetics	mitochondrial proton-transporting ATP synthase complex	C	ATMG00410
1470	1	L123 vs. M123	M123 up	GO:0005753	Metabolism	Bioenergetics	mitochondrial proton-transporting ATP synthase complex	C	ATMG00480
1471	1	L123 vs. M123	M123 up	GO:0000276	Metabolism	Bioenergetics	mitochondrial proton-transporting ATP synthase complex, coupling factor F(o)	C	ATMG00480
1472	1	L123 vs. M123	M123 up	GO:0005747	Metabolism	Bioenergetics	mitochondrial respiratory chain complex I	C	AT5G47890
1473	1	L123 vs. M123	M123 up	GO:0005750	Metabolism	Bioenergetics	mitochondrial respiratory chain complex III	C	AT3G46430
1474	1	L123 vs. M123	M123 up	GO:0005739	Metabolism	Bioenergetics	mitochondrion	C	AT1G06350
1475	1	L123 vs. M123	M123 up	GO:0005739	Metabolism	Bioenergetics	mitochondrion	C	AT1G43580
1476	1	L123 vs. M123	M123 up	GO:0005739	Metabolism	Bioenergetics	mitochondrion	C	AT1G54120
1477	1	L123 vs. M123	M123 up	GO:0005739	Metabolism	Bioenergetics	mitochondrion	C	AT1G54990
1478	1	L123 vs. M123	M123 up	GO:0005739	Metabolism	Bioenergetics	mitochondrion	C	AT1G67230
1479	1	L123 vs. M123	M123 up	GO:0005739	Metabolism	Bioenergetics	mitochondrion	C	AT1G79690
1480	1	L123 vs. M123	M123 up	GO:0005739	Metabolism	Bioenergetics	mitochondrion	C	AT3G01980
1481	1	L123 vs. M123	M123 up	GO:0005739	Metabolism	Bioenergetics	mitochondrion	C	AT3G02010
1482	1	L123 vs. M123	M123 up	GO:0005739	Metabolism	Bioenergetics	mitochondrion	C	AT3G03160
1483	1	L123 vs. M123	M123 up	GO:0005739	Metabolism	Bioenergetics	mitochondrion	C	AT3G09700
1484	1	L123 vs. M123	M123 up	GO:0005739	Metabolism	Bioenergetics	mitochondrion	C	AT3G27870
1485	1	L123 vs. M123	M123 up	GO:0005739	Metabolism	Bioenergetics	mitochondrion	C	AT3G46430
1486	1	L123 vs. M123	M123 up	GO:0005739	Metabolism	Bioenergetics	mitochondrion	C	AT4G03320
1487	1	L123 vs. M123	M123 up	GO:0005739	Metabolism	Bioenergetics	mitochondrion	C	AT4G16800
1488	1	L123 vs. M123	M123 up	GO:0005739	Metabolism	Bioenergetics	mitochondrion	C	AT4G31500
1489	1	L123 vs. M123	M123 up	GO:0005739	Metabolism	Bioenergetics	mitochondrion	C	AT5G24650
1490	1	L123 vs. M123	M123 up	GO:0005739	Metabolism	Bioenergetics	mitochondrion	C	AT5G47890
1491	1	L123 vs. M123	M123 up	GO:0005739	Metabolism	Bioenergetics	mitochondrion	C	AT5G48370
1492	1	L123 vs. M123	M123 up	GO:0005739	Metabolism	Bioenergetics	mitochondrion	C	AT5G51720
1493	1	L123 vs. M123	M123 up	GO:0005739	Metabolism	Bioenergetics	mitochondrion	C	ATMG00410
1494	1	L123 vs. M123	M123 up	GO:0005739	Metabolism	Bioenergetics	mitochondrion	C	ATMG00480
1495	1	L123 vs. M123	M123 up	GO:0005739	Metabolism	Bioenergetics	mitochondrion	C	ATMG01370
1496	1	L123 vs. M123	M123 up	GO:0007005	Metabolism	Bioenergetics	mitochondrion organization	P	AT5G24650
1497	1	L123 vs. M123	M123 up	GO:0051287	Metabolism	Bioenergetics	NAD binding	F	AT1G42970
1498	1	L123 vs. M123	M123 up	GO:0070403	Metabolism	Bioenergetics	NAD+ binding	F	AT3G53520
1499	1	L123 vs. M123	M123 up	GO:0050661	Metabolism	Bioenergetics	NADP binding	F	AT1G12140
1500	1	L123 vs. M123	M123 up	GO:0050661	Metabolism	Bioenergetics	NADP binding	F	AT1G12200
1501	1	L123 vs. M123	M123 up	GO:0050661	Metabolism	Bioenergetics	NADP binding	F	AT1G42970
1502	1	L123 vs. M123	M123 up	GO:0016491	Metabolism	Bioenergetics	oxidoreductase activity	F	AT3G55360
1503	1	L123 vs. M123	M123 up	GO:0016671	Metabolism	Bioenergetics	oxidoreductase activity, acting on a sulfur group of donors, disulfide as acceptor	F	AT1G45145

1504	1	L123 vs. M123	M123 up	GO:0016709	Metabolism	Bioenergetics	oxidoreductase activity, acting on paired donors, with incorporation or reduction of molecular oxygen, NAD(P)H as one donor, and incorporation of one atom of oxygen	F	AT2G22330
1505	1	L123 vs. M123	M123 up	GO:0016709	Metabolism	Bioenergetics	oxidoreductase activity, acting on paired donors, with incorporation or reduction of molecular oxygen, NAD(P)H as one donor, and incorporation of one atom of oxygen	F	AT4G31500
1506	1	L123 vs. M123	M123 up	GO:0016717	Metabolism	Bioenergetics	oxidoreductase activity, acting on paired donors, with oxidation of a pair of donors resulting in the reduction of molecular oxygen to two molecules of water	F	AT1G06350
1507	1	L123 vs. M123	M123 up	GO:0001405	Metabolism	Bioenergetics	PAM complex, Tim23 associated import motor	C	AT3G09700
1508	1	L123 vs. M123	M123 up	GO:0046933	Metabolism	Bioenergetics	proton-transporting ATP synthase activity, rotational mechanism	F	ATMG00410
1509	1	L123 vs. M123	M123 up	GO:0046933	Metabolism	Bioenergetics	proton-transporting ATP synthase activity, rotational mechanism	F	ATMG00480
1510	1	L123 vs. M123	M123 up	GO:0045259	Metabolism	Bioenergetics	proton-transporting ATP synthase complex	C	ATMG00410
1511	1	L123 vs. M123	M123 up	GO:0045263	Metabolism	Bioenergetics	proton-transporting ATP synthase complex, coupling factor F(o)	C	ATMG00410
1512	1	L123 vs. M123	M123 up	GO:0004743	Metabolism	Bioenergetics	pyruvate kinase activity	F	AT5G63680
1513	1	L123 vs. M123	M123 up	GO:0019253	Metabolism	Bioenergetics	reductive pentose-phosphate cycle	P	AT1G42970
1514	1	L123 vs. M123	M123 up	GO:0005983	Metabolism	Bioenergetics	starch catabolic process	P	AT4G15210
1515	1	L123 vs. M123	M123 up	GO:0042721	Metabolism	Bioenergetics	TIM22 mitochondrial import inner membrane insertion complex	C	AT5G24650
1516	1	L123 vs. M123	M123 up	GO:0005744	Metabolism	Bioenergetics	TIM23 mitochondrial import inner membrane translocase complex	C	AT5G24650
1517	1	L123 vs. M123	M123 up	GO:0019166	Metabolism	Bioenergetics	trans-2-enoyl-CoA reductase (NADPH) activity	F	AT3G55360
1518	1	L123 vs. M123	M123 up	GO:0006636	Metabolism	Bioenergetics	unsaturated fatty acid biosynthetic process	P	AT1G06350
1519	1	L123 vs. M123	M123 up	GO:0042761	Metabolism	Bioenergetics	very long-chain fatty acid biosynthetic process	P	AT3G55360
1520	1	L123 vs. M123	M123 up	GO:0102758	Metabolism	Bioenergetics	very-long-chain enoyl-CoA reductase activity	F	AT3G55360
1521	1	L123 vs. M123	M123 up	GO:0009083	Metabolism	Catabolism	branched-chain amino acid catabolic process	P	AT4G16800
1522	1	L123 vs. M123	M123 up	GO:0000737	Metabolism	Catabolism	DNA catabolic process, endonucleolytic	P	AT3G13170
1523	1	L123 vs. M123	M123 up	GO:0046294	Metabolism	Catabolism	formaldehyde catabolic process	P	AT5G42250
1524	1	L123 vs. M123	M123 up	GO:0019762	Metabolism	Catabolism	glucosinolate catabolic process	P	AT3G16410
1525	1	L123 vs. M123	M123 up	GO:0032264	Metabolism	Catabolism	IMP salvage	P	AT2G38280
1526	1	L123 vs. M123	M123 up	GO:0010150	Metabolism	Catabolism	leaf senescence	P	AT5G51720
1527	1	L123 vs. M123	M123 up	GO:0016042	Metabolism	Catabolism	lipid catabolic process	P	AT3G48460
1528	1	L123 vs. M123	M123 up	GO:0019941	Metabolism	Catabolism	modification-dependent protein catabolic process	P	AT4G02890
1529	1	L123 vs. M123	M123 up	GO:1903298	Metabolism	Catabolism	negative regulation of hypoxia-induced intrinsic apoptotic signaling pathway	P	AT4G16660
1530	1	L123 vs. M123	M123 up	GO:1900056	Metabolism	Catabolism	negative regulation of leaf senescence	P	AT5G62620
1531	1	L123 vs. M123	M123 up	GO:0004601	Metabolism	Catabolism	peroxidase activity	F	AT1G05260
1532	1	L123 vs. M123	M123 up	GO:0005777	Metabolism	Catabolism	peroxisome	C	AT2G30860
1533	1	L123 vs. M123	M123 up	GO:0005777	Metabolism	Catabolism	peroxisome	C	AT3G01980
1534	1	L123 vs. M123	M123 up	GO:0005777	Metabolism	Catabolism	peroxisome	C	AT5G48370
1535	1	L123 vs. M123	M123 up	GO:0030163	Metabolism	Catabolism	protein catabolic process	P	AT1G65240
1536	1	L123 vs. M123	M123 up	GO:0016567	Metabolism	Catabolism	protein ubiquitination	P	AT4G02890
1537	1	L123 vs. M123	M123 up	GO:0016567	Metabolism	Catabolism	protein ubiquitination	P	AT4G10925
1538	1	L123 vs. M123	M123 up	GO:0006508	Metabolism	Catabolism	proteolysis	P	AT1G65240
1539	1	L123 vs. M123	M123 up	GO:0006569	Metabolism	Catabolism	tryptophan catabolic process	P	AT2G22330
1540	1	L123 vs. M123	M123 up	GO:0031625	Metabolism	Catabolism	ubiquitin protein ligase binding	F	AT4G02890
1541	1	L123 vs. M123	M123 up	GO:0006511	Metabolism	Catabolism	ubiquitin-dependent protein catabolic process	P	AT4G02890
1542	1	L123 vs. M123	M123 up	GO:0009979	Metabolism	Metabolism	16:0 monogalactosyldiacylglycerol desaturase activity	F	AT1G06350
1543	1	L123 vs. M123	M123 up	GO:0051537	Metabolism	Metabolism	2 iron, 2 sulfur cluster binding	F	AT5G51720

1544	1	L123 vs. M123	M123 up	GO:0080107	Metabolism	Metabolism	8-methylthiopropyl glucosinolate S-oxygenase activity	F	AT1G12140
1545	1	L123 vs. M123	M123 up	GO:0008126	Metabolism	Metabolism	acetylesterase activity	F	AT3G48460
1546	1	L123 vs. M123	M123 up	GO:0016407	Metabolism	Metabolism	acetyltransferase activity	F	AT3G03210
1547	1	L123 vs. M123	M123 up	GO:0000036	Metabolism	Metabolism	acyl carrier activity	F	AT1G08510
1548	1	L123 vs. M123	M123 up	GO:0016297	Metabolism	Metabolism	acyl-[acyl-carrier-protein] hydrolase activity	F	AT1G08510
1549	1	L123 vs. M123	M123 up	GO:0004020	Metabolism	Metabolism	adenylylsulfate kinase activity	F	AT4G39940
1550	1	L123 vs. M123	M123 up	GO:0004024	Metabolism	Metabolism	alcohol dehydrogenase activity, zinc-dependent	F	AT5G42250
1551	1	L123 vs. M123	M123 up	GO:0003876	Metabolism	Metabolism	AMP deaminase activity	F	AT2G38280
1552	1	L123 vs. M123	M123 up	GO:0046033	Metabolism	Metabolism	AMP metabolic process	P	AT2G38280
1553	1	L123 vs. M123	M123 up	GO:0102229	Metabolism	Metabolism	amylopectin maltohydrolase activity	F	AT4G15210
1554	1	L123 vs. M123	M123 up	GO:0010405	Metabolism	Metabolism	arabinogalactan protein metabolic process	P	AT5G62620
1555	1	L123 vs. M123	M123 up	GO:0004190	Metabolism	Metabolism	aspartic-type endopeptidase activity	F	AT1G65240
1556	1	L123 vs. M123	M123 up	GO:0005524	Metabolism	Metabolism	ATP binding	F	AT1G25350
1557	1	L123 vs. M123	M123 up	GO:0005524	Metabolism	Metabolism	ATP binding	F	AT1G27770
1558	1	L123 vs. M123	M123 up	GO:0005524	Metabolism	Metabolism	ATP binding	F	AT1G59990
1559	1	L123 vs. M123	M123 up	GO:0005524	Metabolism	Metabolism	ATP binding	F	AT2G07680
1560	1	L123 vs. M123	M123 up	GO:0005524	Metabolism	Metabolism	ATP binding	F	AT2G38280
1561	1	L123 vs. M123	M123 up	GO:0005524	Metabolism	Metabolism	ATP binding	F	AT3G13170
1562	1	L123 vs. M123	M123 up	GO:0005524	Metabolism	Metabolism	ATP binding	F	AT3G24660
1563	1	L123 vs. M123	M123 up	GO:0005524	Metabolism	Metabolism	ATP binding	F	AT3G27870
1564	1	L123 vs. M123	M123 up	GO:0005524	Metabolism	Metabolism	ATP binding	F	AT3G48750
1565	1	L123 vs. M123	M123 up	GO:0005524	Metabolism	Metabolism	ATP binding	F	AT3G49670
1566	1	L123 vs. M123	M123 up	GO:0005524	Metabolism	Metabolism	ATP binding	F	AT4G26270
1567	1	L123 vs. M123	M123 up	GO:0005524	Metabolism	Metabolism	ATP binding	F	AT4G39940
1568	1	L123 vs. M123	M123 up	GO:0005524	Metabolism	Metabolism	ATP binding	F	AT5G08160
1569	1	L123 vs. M123	M123 up	GO:0005524	Metabolism	Metabolism	ATP binding	F	AT5G48600
1570	1	L123 vs. M123	M123 up	GO:0005524	Metabolism	Metabolism	ATP binding	F	AT5G56630
1571	1	L123 vs. M123	M123 up	GO:0005524	Metabolism	Metabolism	ATP binding	F	AT5G65460
1572	1	L123 vs. M123	M123 up	GO:0005524	Metabolism	Metabolism	ATP binding	F	ATMG00480
1573	1	L123 vs. M123	M123 up	GO:0008150	Metabolism	Metabolism	biological_process	P	AT1G17100
1574	1	L123 vs. M123	M123 up	GO:0008150	Metabolism	Metabolism	biological_process	P	AT1G27385
1575	1	L123 vs. M123	M123 up	GO:0008150	Metabolism	Metabolism	biological_process	P	AT1G31335
1576	1	L123 vs. M123	M123 up	GO:0008150	Metabolism	Metabolism	biological_process	P	AT1G47970
1577	1	L123 vs. M123	M123 up	GO:0008150	Metabolism	Metabolism	biological_process	P	AT1G54120
1578	1	L123 vs. M123	M123 up	GO:0008150	Metabolism	Metabolism	biological_process	P	AT1G57680
1579	1	L123 vs. M123	M123 up	GO:0008150	Metabolism	Metabolism	biological_process	P	AT2G36300
1580	1	L123 vs. M123	M123 up	GO:0008150	Metabolism	Metabolism	biological_process	P	AT3G46430
1581	1	L123 vs. M123	M123 up	GO:0008150	Metabolism	Metabolism	biological_process	P	AT4G39235
1582	1	L123 vs. M123	M123 up	GO:0008150	Metabolism	Metabolism	biological_process	P	AT5G22540
1583	1	L123 vs. M123	M123 up	GO:0008150	Metabolism	Metabolism	biological_process	P	AT5G41810
1584	1	L123 vs. M123	M123 up	GO:0008150	Metabolism	Metabolism	biological_process	P	AT5G60570
1585	1	L123 vs. M123	M123 up	GO:0008150	Metabolism	Metabolism	biological_process	P	ATMG01370
1586	1	L123 vs. M123	M123 up	GO:0009058	Metabolism	Metabolism	biosynthetic process	P	AT1G03210
1587	1	L123 vs. M123	M123 up	GO:0010120	Metabolism	Metabolism	camalexin biosynthetic process	P	AT2G22330
1588	1	L123 vs. M123	M123 up	GO:0003824	Metabolism	Metabolism	catalytic activity	F	AT1G03210
1589	1	L123 vs. M123	M123 up	GO:0046513	Metabolism	Metabolism	ceramide biosynthetic process	P	AT1G43580
1590	1	L123 vs. M123	M123 up	GO:0047493	Metabolism	Metabolism	ceramide cholinephosphotransferase activity	F	AT1G43580
1591	1	L123 vs. M123	M123 up	GO:0003682	Metabolism	Metabolism	chromatin binding	F	AT2G27980
1592	1	L123 vs. M123	M123 up	GO:0003682	Metabolism	Metabolism	chromatin binding	F	AT4G15890
1593	1	L123 vs. M123	M123 up	GO:0050269	Metabolism	Metabolism	coniferyl-aldehyde dehydrogenase activity	F	AT3G24503
1594	1	L123 vs. M123	M123 up	GO:0005507	Metabolism	Metabolism	copper ion binding	F	AT1G08830
1595	1	L123 vs. M123	M123 up	GO:0005507	Metabolism	Metabolism	copper ion binding	F	AT2G28190
1596	1	L123 vs. M123	M123 up	GO:0005507	Metabolism	Metabolism	copper ion binding	F	AT2G30860
1597	1	L123 vs. M123	M123 up	GO:0019344	Metabolism	Metabolism	cysteine biosynthetic process	P	AT4G39940
1598	1	L123 vs. M123	M123 up	GO:0008239	Metabolism	Metabolism	dipeptidyl-peptidase activity	F	AT1G79690

1599	1	L123 vs. M123	M123 up	GO:0003677	Metabolism	Metabolism	DNA binding	F	AT3G13170
1600	1	L123 vs. M123	M123 up	GO:0042732	Metabolism	Metabolism	D-xylose metabolic process	P	AT3G53520
1601	1	L123 vs. M123	M123 up	GO:0016889	Metabolism	Metabolism	endodeoxyribonuclease activity, producing 3'-phosphomonoesters	F	AT3G13170
1602	1	L123 vs. M123	M123 up	GO:0004857	Metabolism	Metabolism	enzyme inhibitor activity	F	AT4G12390
1603	1	L123 vs. M123	M123 up	GO:0050660	Metabolism	Metabolism	flavin adenine dinucleotide binding	F	AT1G12140
1604	1	L123 vs. M123	M123 up	GO:0050660	Metabolism	Metabolism	flavin adenine dinucleotide binding	F	AT1G12200
1605	1	L123 vs. M123	M123 up	GO:0019003	Metabolism	Metabolism	GDP binding	F	AT4G39520
1606	1	L123 vs. M123	M123 up	GO:0019761	Metabolism	Metabolism	glucosinolate biosynthetic process	P	AT1G12140
1607	1	L123 vs. M123	M123 up	GO:0019761	Metabolism	Metabolism	glucosinolate biosynthetic process	P	AT2G22330
1608	1	L123 vs. M123	M123 up	GO:0006541	Metabolism	Metabolism	glutamine metabolic process	P	AT2G38860
1609	1	L123 vs. M123	M123 up	GO:0006662	Metabolism	Metabolism	glycerol ether metabolic process	P	AT1G45145
1610	1	L123 vs. M123	M123 up	GO:0032580	Metabolism	Metabolism	Golgi cisterna membrane	C	AT3G53520
1611	1	L123 vs. M123	M123 up	GO:0005525	Metabolism	Metabolism	GTP binding	F	AT3G61760
1612	1	L123 vs. M123	M123 up	GO:0005525	Metabolism	Metabolism	GTP binding	F	AT4G20360
1613	1	L123 vs. M123	M123 up	GO:0005525	Metabolism	Metabolism	GTP binding	F	AT4G39520
1614	1	L123 vs. M123	M123 up	GO:0005096	Metabolism	Metabolism	GTPase activator activity	F	AT3G52190
1615	1	L123 vs. M123	M123 up	GO:0003924	Metabolism	Metabolism	GTPase activity	F	AT3G61760
1616	1	L123 vs. M123	M123 up	GO:0003924	Metabolism	Metabolism	GTPase activity	F	AT4G20360
1617	1	L123 vs. M123	M123 up	GO:0003924	Metabolism	Metabolism	GTPase activity	F	AT4G39520
1618	1	L123 vs. M123	M123 up	GO:0020037	Metabolism	Metabolism	heme binding	F	AT1G05260
1619	1	L123 vs. M123	M123 up	GO:0020037	Metabolism	Metabolism	heme binding	F	AT1G17100
1620	1	L123 vs. M123	M123 up	GO:0020037	Metabolism	Metabolism	heme binding	F	AT2G22330
1621	1	L123 vs. M123	M123 up	GO:0020037	Metabolism	Metabolism	heme binding	F	AT4G31500
1622	1	L123 vs. M123	M123 up	GO:0020037	Metabolism	Metabolism	heme binding	F	ATCG01040
1623	1	L123 vs. M123	M123 up	GO:0070814	Metabolism	Metabolism	hydrogen sulfide biosynthetic process	P	AT4G39940
1624	1	L123 vs. M123	M123 up	GO:0016787	Metabolism	Metabolism	hydrolase activity	F	AT1G10740
1625	1	L123 vs. M123	M123 up	GO:0016787	Metabolism	Metabolism	hydrolase activity	F	AT1G80280
1626	1	L123 vs. M123	M123 up	GO:0004553	Metabolism	Metabolism	hydrolase activity, hydrolyzing O-glycosyl compounds	F	AT1G14720
1627	1	L123 vs. M123	M123 up	GO:1990714	Metabolism	Metabolism	hydroxyproline O-galactosyltransferase activity	F	AT5G62620
1628	1	L123 vs. M123	M123 up	GO:0006188	Metabolism	Metabolism	IMP biosynthetic process	P	AT2G38280
1629	1	L123 vs. M123	M123 up	GO:0009759	Metabolism	Metabolism	indole glucosinolate biosynthetic process	P	AT4G31500
1630	1	L123 vs. M123	M123 up	GO:0000822	Metabolism	Metabolism	inositol hexakisphosphate binding	F	AT2G03240
1631	1	L123 vs. M123	M123 up	GO:0045140	Metabolism	Metabolism	inositol phosphoceramide synthase activity	F	AT1G43580
1632	1	L123 vs. M123	M123 up	GO:0005506	Metabolism	Metabolism	iron ion binding	F	AT2G22330
1633	1	L123 vs. M123	M123 up	GO:0005506	Metabolism	Metabolism	iron ion binding	F	AT4G31500
1634	1	L123 vs. M123	M123 up	GO:0016853	Metabolism	Metabolism	isomerase activity	F	AT3G13170
1635	1	L123 vs. M123	M123 up	GO:0016853	Metabolism	Metabolism	isomerase activity	F	AT5G48370
1636	1	L123 vs. M123	M123 up	GO:0006629	Metabolism	Metabolism	lipid metabolic process	P	AT3G61580
1637	1	L123 vs. M123	M123 up	GO:0006629	Metabolism	Metabolism	lipid metabolic process	P	AT5G55370
1638	1	L123 vs. M123	M123 up	GO:0000287	Metabolism	Metabolism	magnesium ion binding	F	AT3G27870
1639	1	L123 vs. M123	M123 up	GO:0000287	Metabolism	Metabolism	magnesium ion binding	F	AT5G63680
1640	1	L123 vs. M123	M123 up	GO:0046872	Metabolism	Metabolism	metal ion binding	F	AT1G05260
1641	1	L123 vs. M123	M123 up	GO:0046872	Metabolism	Metabolism	metal ion binding	F	AT1G27770
1642	1	L123 vs. M123	M123 up	GO:0046872	Metabolism	Metabolism	metal ion binding	F	AT1G79690
1643	1	L123 vs. M123	M123 up	GO:0046872	Metabolism	Metabolism	metal ion binding	F	AT2G38280
1644	1	L123 vs. M123	M123 up	GO:0046872	Metabolism	Metabolism	metal ion binding	F	AT3G13170
1645	1	L123 vs. M123	M123 up	GO:0046872	Metabolism	Metabolism	metal ion binding	F	AT3G19980
1646	1	L123 vs. M123	M123 up	GO:0046872	Metabolism	Metabolism	metal ion binding	F	AT3G61580
1647	1	L123 vs. M123	M123 up	GO:0046872	Metabolism	Metabolism	metal ion binding	F	AT4G24890
1648	1	L123 vs. M123	M123 up	GO:0046872	Metabolism	Metabolism	metal ion binding	F	AT4G26270
1649	1	L123 vs. M123	M123 up	GO:0046872	Metabolism	Metabolism	metal ion binding	F	AT5G50740
1650	1	L123 vs. M123	M123 up	GO:0046872	Metabolism	Metabolism	metal ion binding	F	AT5G51720
1651	1	L123 vs. M123	M123 up	GO:0046872	Metabolism	Metabolism	metal ion binding	F	AT5G56630
1652	1	L123 vs. M123	M123 up	GO:0008168	Metabolism	Metabolism	methyltransferase activity	F	AT2G27590

1653	1	L123 vs. M123	M123 up	GO:0008168	Metabolism	Metabolism	methyltransferase activity	F	AT4G14000
1654	1	L123 vs. M123	M123 up	GO:0008017	Metabolism	Metabolism	microtubule binding	F	AT3G61760
1655	1	L123 vs. M123	M123 up	GO:0008017	Metabolism	Metabolism	microtubule binding	F	AT5G65460
1656	1	L123 vs. M123	M123 up	GO:0051011	Metabolism	Metabolism	microtubule minus-end binding	F	AT5G17620
1657	1	L123 vs. M123	M123 up	GO:0003674	Metabolism	Metabolism	molecular_function	F	AT1G27385
1658	1	L123 vs. M123	M123 up	GO:0003674	Metabolism	Metabolism	molecular_function	F	AT1G31335
1659	1	L123 vs. M123	M123 up	GO:0003674	Metabolism	Metabolism	molecular_function	F	AT1G47970
1660	1	L123 vs. M123	M123 up	GO:0003674	Metabolism	Metabolism	molecular_function	F	AT1G54120
1661	1	L123 vs. M123	M123 up	GO:0003674	Metabolism	Metabolism	molecular_function	F	AT1G57680
1662	1	L123 vs. M123	M123 up	GO:0003674	Metabolism	Metabolism	molecular_function	F	AT1G69700
1663	1	L123 vs. M123	M123 up	GO:0003674	Metabolism	Metabolism	molecular_function	F	AT2G14900
1664	1	L123 vs. M123	M123 up	GO:0003674	Metabolism	Metabolism	molecular_function	F	AT2G36300
1665	1	L123 vs. M123	M123 up	GO:0003674	Metabolism	Metabolism	molecular_function	F	AT3G03160
1666	1	L123 vs. M123	M123 up	GO:0003674	Metabolism	Metabolism	molecular_function	F	AT3G46430
1667	1	L123 vs. M123	M123 up	GO:0003674	Metabolism	Metabolism	molecular_function	F	AT3G50610
1668	1	L123 vs. M123	M123 up	GO:0003674	Metabolism	Metabolism	molecular_function	F	AT4G10925
1669	1	L123 vs. M123	M123 up	GO:0003674	Metabolism	Metabolism	molecular_function	F	AT4G39235
1670	1	L123 vs. M123	M123 up	GO:0003674	Metabolism	Metabolism	molecular_function	F	AT5G02140
1671	1	L123 vs. M123	M123 up	GO:0003674	Metabolism	Metabolism	molecular_function	F	AT5G22540
1672	1	L123 vs. M123	M123 up	GO:0003674	Metabolism	Metabolism	molecular_function	F	AT5G41810
1673	1	L123 vs. M123	M123 up	GO:0003674	Metabolism	Metabolism	molecular_function	F	AT5G60570
1674	1	L123 vs. M123	M123 up	GO:0003674	Metabolism	Metabolism	molecular_function	F	ATMG01370
1675	1	L123 vs. M123	M123 up	GO:0048354	Metabolism	Metabolism	mucilage biosynthetic process involved in seed coat development	P	AT5G62620
1676	1	L123 vs. M123	M123 up	GO:0004499	Metabolism	Metabolism	N,N-dimethylaniline monooxygenase activity	F	AT1G12140
1677	1	L123 vs. M123	M123 up	GO:0004499	Metabolism	Metabolism	N,N-dimethylaniline monooxygenase activity	F	AT1G12200
1678	1	L123 vs. M123	M123 up	GO:0080028	Metabolism	Metabolism	nitrile biosynthetic process	P	AT3G16410
1679	1	L123 vs. M123	M123 up	GO:0045735	Metabolism	Metabolism	nutrient reservoir activity	F	AT5G24780
1680	1	L123 vs. M123	M123 up	GO:0016413	Metabolism	Metabolism	O-acetyltransferase activity	F	AT2G34070
1681	1	L123 vs. M123	M123 up	GO:0055114	Metabolism	Metabolism	oxidation-reduction process	P	AT1G01800
1682	1	L123 vs. M123	M123 up	GO:0055114	Metabolism	Metabolism	oxidation-reduction process	P	AT1G05260
1683	1	L123 vs. M123	M123 up	GO:0055114	Metabolism	Metabolism	oxidation-reduction process	P	AT1G06350
1684	1	L123 vs. M123	M123 up	GO:0055114	Metabolism	Metabolism	oxidation-reduction process	P	AT1G08830
1685	1	L123 vs. M123	M123 up	GO:0055114	Metabolism	Metabolism	oxidation-reduction process	P	AT1G12140
1686	1	L123 vs. M123	M123 up	GO:0055114	Metabolism	Metabolism	oxidation-reduction process	P	AT1G12200
1687	1	L123 vs. M123	M123 up	GO:0055114	Metabolism	Metabolism	oxidation-reduction process	P	AT1G42970
1688	1	L123 vs. M123	M123 up	GO:0055114	Metabolism	Metabolism	oxidation-reduction process	P	AT1G45145
1689	1	L123 vs. M123	M123 up	GO:0055114	Metabolism	Metabolism	oxidation-reduction process	P	AT2G22330
1690	1	L123 vs. M123	M123 up	GO:0055114	Metabolism	Metabolism	oxidation-reduction process	P	AT2G28190
1691	1	L123 vs. M123	M123 up	GO:0055114	Metabolism	Metabolism	oxidation-reduction process	P	AT2G30860
1692	1	L123 vs. M123	M123 up	GO:0055114	Metabolism	Metabolism	oxidation-reduction process	P	AT3G24503
1693	1	L123 vs. M123	M123 up	GO:0055114	Metabolism	Metabolism	oxidation-reduction process	P	AT3G55360
1694	1	L123 vs. M123	M123 up	GO:0055114	Metabolism	Metabolism	oxidation-reduction process	P	AT3G61580
1695	1	L123 vs. M123	M123 up	GO:0055114	Metabolism	Metabolism	oxidation-reduction process	P	AT4G31500
1696	1	L123 vs. M123	M123 up	GO:0055114	Metabolism	Metabolism	oxidation-reduction process	P	AT5G16970
1697	1	L123 vs. M123	M123 up	GO:0055114	Metabolism	Metabolism	oxidation-reduction process	P	AT5G42250
1698	1	L123 vs. M123	M123 up	GO:0055114	Metabolism	Metabolism	oxidation-reduction process	P	AT5G47890
1699	1	L123 vs. M123	M123 up	GO:0052793	Metabolism	Metabolism	pectin acetylesterase activity	F	AT4G19410
1700	1	L123 vs. M123	M123 up	GO:0009699	Metabolism	Metabolism	phenylpropanoid biosynthetic process	P	AT3G24503
1701	1	L123 vs. M123	M123 up	GO:0016310	Metabolism	Metabolism	phosphorylation	P	AT4G39940
1702	1	L123 vs. M123	M123 up	GO:0030955	Metabolism	Metabolism	potassium ion binding	F	AT5G63680
1703	1	L123 vs. M123	M123 up	GO:0005515	Metabolism	Metabolism	protein binding	F	AT1G08830
1704	1	L123 vs. M123	M123 up	GO:0005515	Metabolism	Metabolism	protein binding	F	AT1G53650
1705	1	L123 vs. M123	M123 up	GO:0005515	Metabolism	Metabolism	protein binding	F	AT1G67230
1706	1	L123 vs. M123	M123 up	GO:0005515	Metabolism	Metabolism	protein binding	F	AT1G79690
1707	1	L123 vs. M123	M123 up	GO:0005515	Metabolism	Metabolism	protein binding	F	AT2G26760

1708	1	L123 vs. M123	M123 up	GO:0005515	Metabolism	Metabolism	protein binding	F	AT2G38280
1709	1	L123 vs. M123	M123 up	GO:0005515	Metabolism	Metabolism	protein binding	F	AT3G13170
1710	1	L123 vs. M123	M123 up	GO:0005515	Metabolism	Metabolism	protein binding	F	AT3G19980
1711	1	L123 vs. M123	M123 up	GO:0005515	Metabolism	Metabolism	protein binding	F	AT3G48750
1712	1	L123 vs. M123	M123 up	GO:0005515	Metabolism	Metabolism	protein binding	F	AT3G49670
1713	1	L123 vs. M123	M123 up	GO:0005515	Metabolism	Metabolism	protein binding	F	AT3G52190
1714	1	L123 vs. M123	M123 up	GO:0005515	Metabolism	Metabolism	protein binding	F	AT3G55360
1715	1	L123 vs. M123	M123 up	GO:0005515	Metabolism	Metabolism	protein binding	F	AT4G20360
1716	1	L123 vs. M123	M123 up	GO:0005515	Metabolism	Metabolism	protein binding	F	AT5G17620
1717	1	L123 vs. M123	M123 up	GO:0005515	Metabolism	Metabolism	protein binding	F	AT5G24650
1718	1	L123 vs. M123	M123 up	GO:0005515	Metabolism	Metabolism	protein binding	F	AT5G45680
1719	1	L123 vs. M123	M123 up	GO:0005515	Metabolism	Metabolism	protein binding	F	AT5G64350
1720	1	L123 vs. M123	M123 up	GO:0005515	Metabolism	Metabolism	protein binding	F	AT5G65460
1721	1	L123 vs. M123	M123 up	GO:0005515	Metabolism	Metabolism	protein binding	F	ATCG00680
1722	1	L123 vs. M123	M123 up	GO:0043424	Metabolism	Metabolism	protein histidine kinase binding	F	AT2G38280
1723	1	L123 vs. M123	M123 up	GO:0005090	Metabolism	Metabolism	Sar guanyl-nucleotide exchange factor activity	F	AT3G52190
1724	1	L123 vs. M123	M123 up	GO:0030148	Metabolism	Metabolism	sphingolipid biosynthetic process	P	AT1G43580
1725	1	L123 vs. M123	M123 up	GO:0030148	Metabolism	Metabolism	sphingolipid biosynthetic process	P	AT3G61580
1726	1	L123 vs. M123	M123 up	GO:0052631	Metabolism	Metabolism	sphingolipid delta-8 desaturase activity	F	AT3G61580
1727	1	L123 vs. M123	M123 up	GO:0006665	Metabolism	Metabolism	sphingolipid metabolic process	P	AT3G55360
1728	1	L123 vs. M123	M123 up	GO:0033188	Metabolism	Metabolism	sphingomyelin synthase activity	F	AT1G43580
1729	1	L123 vs. M123	M123 up	GO:0016740	Metabolism	Metabolism	transferase activity	F	AT2G38860
1730	1	L123 vs. M123	M123 up	GO:0000162	Metabolism	Metabolism	tryptophan biosynthetic process	P	AT4G31500
1731	1	L123 vs. M123	M123 up	GO:0033320	Metabolism	Metabolism	UDP-D-xylose biosynthetic process	P	AT3G53520
1732	1	L123 vs. M123	M123 up	GO:0048040	Metabolism	Metabolism	UDP-glucuronate decarboxylase activity	F	AT3G53520
1733	1	L123 vs. M123	M123 up	GO:0010025	Metabolism	Metabolism	wax biosynthetic process	P	AT3G55360
1734	1	L123 vs. M123	M123 up	GO:0045492	Metabolism	Metabolism	xylan biosynthetic process	P	AT3G03210
1735	1	L123 vs. M123	M123 up	GO:1990538	Metabolism	Metabolism	xylan O-acetyltransferase activity	F	AT3G03210
1736	1	L123 vs. M123	M123 up	GO:0010411	Metabolism	Metabolism	xyloglucan metabolic process	P	AT1G14720
1737	1	L123 vs. M123	M123 up	GO:0016762	Metabolism	Metabolism	xyloglucan:xyloglucosyl transferase activity	F	AT1G14720
1738	1	L123 vs. M123	M123 up	GO:0008270	Metabolism	Metabolism	zinc ion binding	F	AT1G11290
1739	1	L123 vs. M123	M123 up	GO:0008270	Metabolism	Metabolism	zinc ion binding	F	AT3G02010
1740	1	L123 vs. M123	M123 up	GO:0008270	Metabolism	Metabolism	zinc ion binding	F	AT5G42250
1741	1	L123 vs. M123	M123 up	GO:0016168	Metabolism	Photosynthesis	chlorophyll binding	F	ATCG00680
1742	1	L123 vs. M123	M123 up	GO:0009507	Metabolism	Photosynthesis	chloroplast	C	AT1G01800
1743	1	L123 vs. M123	M123 up	GO:0009507	Metabolism	Photosynthesis	chloroplast	C	AT1G08510
1744	1	L123 vs. M123	M123 up	GO:0009507	Metabolism	Photosynthesis	chloroplast	C	AT1G11290
1745	1	L123 vs. M123	M123 up	GO:0009507	Metabolism	Photosynthesis	chloroplast	C	AT1G17100
1746	1	L123 vs. M123	M123 up	GO:0009507	Metabolism	Photosynthesis	chloroplast	C	AT1G19170
1747	1	L123 vs. M123	M123 up	GO:0009507	Metabolism	Photosynthesis	chloroplast	C	AT1G27770
1748	1	L123 vs. M123	M123 up	GO:0009507	Metabolism	Photosynthesis	chloroplast	C	AT1G42970
1749	1	L123 vs. M123	M123 up	GO:0009507	Metabolism	Photosynthesis	chloroplast	C	AT1G59990
1750	1	L123 vs. M123	M123 up	GO:0009507	Metabolism	Photosynthesis	chloroplast	C	AT2G22330
1751	1	L123 vs. M123	M123 up	GO:0009507	Metabolism	Photosynthesis	chloroplast	C	AT2G28190
1752	1	L123 vs. M123	M123 up	GO:0009507	Metabolism	Photosynthesis	chloroplast	C	AT2G30860
1753	1	L123 vs. M123	M123 up	GO:0009507	Metabolism	Photosynthesis	chloroplast	C	AT2G38280
1754	1	L123 vs. M123	M123 up	GO:0009507	Metabolism	Photosynthesis	chloroplast	C	AT3G48460
1755	1	L123 vs. M123	M123 up	GO:0009507	Metabolism	Photosynthesis	chloroplast	C	AT4G03320
1756	1	L123 vs. M123	M123 up	GO:0009507	Metabolism	Photosynthesis	chloroplast	C	AT4G14000
1757	1	L123 vs. M123	M123 up	GO:0009507	Metabolism	Photosynthesis	chloroplast	C	AT4G20360
1758	1	L123 vs. M123	M123 up	GO:0009507	Metabolism	Photosynthesis	chloroplast	C	AT4G24890
1759	1	L123 vs. M123	M123 up	GO:0009507	Metabolism	Photosynthesis	chloroplast	C	AT4G31500
1760	1	L123 vs. M123	M123 up	GO:0009507	Metabolism	Photosynthesis	chloroplast	C	AT4G39940
1761	1	L123 vs. M123	M123 up	GO:0009507	Metabolism	Photosynthesis	chloroplast	C	AT5G24650
1762	1	L123 vs. M123	M123 up	GO:0009507	Metabolism	Photosynthesis	chloroplast	C	AT5G24780
1763	1	L123 vs. M123	M123 up	GO:0009507	Metabolism	Photosynthesis	chloroplast	C	AT5G40670

1764	1	L123 vs. M123	M123 up	GO:0009507	Metabolism	Photosynthesis	chloroplast	C	AT5G41000
1765	1	L123 vs. M123	M123 up	GO:0009507	Metabolism	Photosynthesis	chloroplast	C	AT5G42250
1766	1	L123 vs. M123	M123 up	GO:0009507	Metabolism	Photosynthesis	chloroplast	C	AT5G45680
1767	1	L123 vs. M123	M123 up	GO:0009507	Metabolism	Photosynthesis	chloroplast	C	AT5G47890
1768	1	L123 vs. M123	M123 up	GO:0009507	Metabolism	Photosynthesis	chloroplast	C	AT5G48370
1769	1	L123 vs. M123	M123 up	GO:0009507	Metabolism	Photosynthesis	chloroplast	C	AT5G51720
1770	1	L123 vs. M123	M123 up	GO:0009507	Metabolism	Photosynthesis	chloroplast	C	AT5G65020
1771	1	L123 vs. M123	M123 up	GO:0009507	Metabolism	Photosynthesis	chloroplast	C	AT5G65460
1772	1	L123 vs. M123	M123 up	GO:0009507	Metabolism	Photosynthesis	chloroplast	C	ATCG00380
1773	1	L123 vs. M123	M123 up	GO:0009507	Metabolism	Photosynthesis	chloroplast	C	ATCG00560
1774	1	L123 vs. M123	M123 up	GO:0009507	Metabolism	Photosynthesis	chloroplast	C	ATCG00660
1775	1	L123 vs. M123	M123 up	GO:0009507	Metabolism	Photosynthesis	chloroplast	C	ATCG00680
1776	1	L123 vs. M123	M123 up	GO:0009507	Metabolism	Photosynthesis	chloroplast	C	ATCG01040
1777	1	L123 vs. M123	M123 up	GO:0009507	Metabolism	Photosynthesis	chloroplast	C	ATMG00480
1778	1	L123 vs. M123	M123 up	GO:0009904	Metabolism	Photosynthesis	chloroplast accumulation movement	P	AT5G65460
1779	1	L123 vs. M123	M123 up	GO:0009903	Metabolism	Photosynthesis	chloroplast avoidance movement	P	AT5G65460
1780	1	L123 vs. M123	M123 up	GO:0009941	Metabolism	Photosynthesis	chloroplast envelope	C	AT1G42970
1781	1	L123 vs. M123	M123 up	GO:0009941	Metabolism	Photosynthesis	chloroplast envelope	C	AT4G20360
1782	1	L123 vs. M123	M123 up	GO:0009941	Metabolism	Photosynthesis	chloroplast envelope	C	AT5G24650
1783	1	L123 vs. M123	M123 up	GO:0009941	Metabolism	Photosynthesis	chloroplast envelope	C	ATCG00660
1784	1	L123 vs. M123	M123 up	GO:0009706	Metabolism	Photosynthesis	chloroplast inner membrane	C	AT1G27770
1785	1	L123 vs. M123	M123 up	GO:0009706	Metabolism	Photosynthesis	chloroplast inner membrane	C	AT4G03320
1786	1	L123 vs. M123	M123 up	GO:0009706	Metabolism	Photosynthesis	chloroplast inner membrane	C	AT5G24650
1787	1	L123 vs. M123	M123 up	GO:0031969	Metabolism	Photosynthesis	chloroplast membrane	C	AT1G42970
1788	1	L123 vs. M123	M123 up	GO:0009658	Metabolism	Photosynthesis	chloroplast organization	P	AT4G20360
1789	1	L123 vs. M123	M123 up	GO:0009570	Metabolism	Photosynthesis	chloroplast stroma	C	AT1G42970
1790	1	L123 vs. M123	M123 up	GO:0009570	Metabolism	Photosynthesis	chloroplast stroma	C	AT2G28190
1791	1	L123 vs. M123	M123 up	GO:0009570	Metabolism	Photosynthesis	chloroplast stroma	C	AT2G30860
1792	1	L123 vs. M123	M123 up	GO:0009570	Metabolism	Photosynthesis	chloroplast stroma	C	AT4G20360
1793	1	L123 vs. M123	M123 up	GO:0009570	Metabolism	Photosynthesis	chloroplast stroma	C	AT5G51720
1794	1	L123 vs. M123	M123 up	GO:0009570	Metabolism	Photosynthesis	chloroplast stroma	C	ATCG00380
1795	1	L123 vs. M123	M123 up	GO:0009570	Metabolism	Photosynthesis	chloroplast stroma	C	ATCG00660
1796	1	L123 vs. M123	M123 up	GO:0009534	Metabolism	Photosynthesis	chloroplast thylakoid	C	AT4G20360
1797	1	L123 vs. M123	M123 up	GO:0009534	Metabolism	Photosynthesis	chloroplast thylakoid	C	ATCG00560
1798	1	L123 vs. M123	M123 up	GO:0009534	Metabolism	Photosynthesis	chloroplast thylakoid	C	ATCG00680
1799	1	L123 vs. M123	M123 up	GO:0009543	Metabolism	Photosynthesis	chloroplast thylakoid lumen	C	AT5G45680
1800	1	L123 vs. M123	M123 up	GO:0009535	Metabolism	Photosynthesis	chloroplast thylakoid membrane	C	AT1G42970
1801	1	L123 vs. M123	M123 up	GO:0009535	Metabolism	Photosynthesis	chloroplast thylakoid membrane	C	AT4G20360
1802	1	L123 vs. M123	M123 up	GO:0009535	Metabolism	Photosynthesis	chloroplast thylakoid membrane	C	ATCG00560
1803	1	L123 vs. M123	M123 up	GO:0009535	Metabolism	Photosynthesis	chloroplast thylakoid membrane	C	ATCG00680
1804	1	L123 vs. M123	M123 up	GO:0009535	Metabolism	Photosynthesis	chloroplast thylakoid membrane	C	ATCG01040
1805	1	L123 vs. M123	M123 up	GO:0045156	Metabolism	Photosynthesis	electron transporter, transferring electrons within the cyclic electron transport pathway of photosynthesis activity	F	ATCG00680
1806	1	L123 vs. M123	M123 up	GO:0006002	Metabolism	Photosynthesis	fructose 6-phosphate metabolic process	P	AT4G26270
1807	1	L123 vs. M123	M123 up	GO:0006002	Metabolism	Photosynthesis	fructose 6-phosphate metabolic process	P	AT5G56630
1808	1	L123 vs. M123	M123 up	GO:0110128	Metabolism	Photosynthesis	phloem sucrose unloading	P	AT5G65020
1809	1	L123 vs. M123	M123 up	GO:0019684	Metabolism	Photosynthesis	photosynthesis, light reaction	P	ATCG00680
1810	1	L123 vs. M123	M123 up	GO:0009772	Metabolism	Photosynthesis	photosynthetic electron transport in photosystem II	P	ATCG00680
1811	1	L123 vs. M123	M123 up	GO:0009523	Metabolism	Photosynthesis	photosystem II	C	ATCG00680
1812	1	L123 vs. M123	M123 up	GO:0010207	Metabolism	Photosynthesis	photosystem II assembly	P	ATCG00680
1813	1	L123 vs. M123	M123 up	GO:0009539	Metabolism	Photosynthesis	photosystem II reaction center	C	ATCG00560
1814	1	L123 vs. M123	M123 up	GO:0009539	Metabolism	Photosynthesis	photosystem II reaction center	C	ATCG00680
1815	1	L123 vs. M123	M123 up	GO:0009536	Metabolism	Photosynthesis	plastid	C	AT1G08510
1816	1	L123 vs. M123	M123 up	GO:0009536	Metabolism	Photosynthesis	plastid	C	AT4G20360
1817	1	L123 vs. M123	M123 up	GO:0009536	Metabolism	Photosynthesis	plastid	C	AT4G39940

1818	1	L123 vs. M123	M123 up	GO:0009536	Metabolism	Photosynthesis	plastid	C	AT5G16970
1819	1	L123 vs. M123	M123 up	GO:0009536	Metabolism	Photosynthesis	plastid	C	AT5G24650
1820	1	L123 vs. M123	M123 up	GO:0009536	Metabolism	Photosynthesis	plastid	C	AT5G45680
1821	1	L123 vs. M123	M123 up	GO:0009536	Metabolism	Photosynthesis	plastid	C	AT5G47890
1822	1	L123 vs. M123	M123 up	GO:0009536	Metabolism	Photosynthesis	plastid	C	AT5G51720
1823	1	L123 vs. M123	M123 up	GO:0009536	Metabolism	Photosynthesis	plastid	C	AT5G63680
1824	1	L123 vs. M123	M123 up	GO:0009536	Metabolism	Photosynthesis	plastid	C	AT5G64140
1825	1	L123 vs. M123	M123 up	GO:0009536	Metabolism	Photosynthesis	plastid	C	AT5G64350
1826	1	L123 vs. M123	M123 up	GO:0009536	Metabolism	Photosynthesis	plastid	C	AT5G65020
1827	1	L123 vs. M123	M123 up	GO:0009536	Metabolism	Photosynthesis	plastid	C	ATCG00380
1828	1	L123 vs. M123	M123 up	GO:0009536	Metabolism	Photosynthesis	plastid	C	ATCG00660
1829	1	L123 vs. M123	M123 up	GO:0009536	Metabolism	Photosynthesis	plastid	C	ATCG00680
1830	1	L123 vs. M123	M123 up	GO:0000311	Metabolism	Photosynthesis	plastid large ribosomal subunit	C	ATCG00660
1831	1	L123 vs. M123	M123 up	GO:0000312	Metabolism	Photosynthesis	plastid small ribosomal subunit	C	ATCG00380
1832	1	L123 vs. M123	M123 up	GO:0010287	Metabolism	Photosynthesis	plastoglobule	C	ATCG00680
1833	1	L123 vs. M123	M123 up	GO:0045037	Metabolism	Photosynthesis	protein import into chloroplast stroma	P	AT4G03320
1834	1	L123 vs. M123	M123 up	GO:0045036	Metabolism	Photosynthesis	protein targeting to chloroplast	P	AT5G24650
1835	1	L123 vs. M123	M123 up	GO:0018298	Metabolism	Photosynthesis	protein-chromophore linkage	P	ATCG00680
1836	1	L123 vs. M123	M123 up	GO:0009579	Metabolism	Photosynthesis	thylakoid	C	AT2G28190
1837	1	L123 vs. M123	M123 up	GO:0009579	Metabolism	Photosynthesis	thylakoid	C	AT2G30860
1838	1	L123 vs. M123	M123 up	GO:0009579	Metabolism	Photosynthesis	thylakoid	C	AT5G65020
1839	1	L123 vs. M123	M123 up	GO:0009579	Metabolism	Photosynthesis	thylakoid	C	ATCG00560
1840	1	L123 vs. M123	M123 up	GO:0009579	Metabolism	Photosynthesis	thylakoid	C	ATCG00680
1841	1	L123 vs. M123	M123 up	GO:0031977	Metabolism	Photosynthesis	thylakoid lumen	C	AT5G45680
1842	1	L123 vs. M123	M123 up	GO:0015297	Metabolism	Transporters	antiporter activity	F	AT1G12500
1843	1	L123 vs. M123	M123 up	GO:0030121	Metabolism	Transporters	AP-1 adaptor complex	C	AT1G60070
1844	1	L123 vs. M123	M123 up	GO:0140326	Metabolism	Transporters	ATPase-coupled intramembrane lipid transporter activity	F	AT3G27870
1845	1	L123 vs. M123	M123 up	GO:0042626	Metabolism	Transporters	ATPase-coupled transmembrane transporter activity	F	AT2G07680
1846	1	L123 vs. M123	M123 up	GO:0008559	Metabolism	Transporters	ATPase-coupled xenobiotic transmembrane transporter activity	F	AT2G07680
1847	1	L123 vs. M123	M123 up	GO:0070588	Metabolism	Transporters	calcium ion transmembrane transport	P	AT1G27770
1848	1	L123 vs. M123	M123 up	GO:0005388	Metabolism	Transporters	calcium transmembrane transporter activity, phosphorylative mechanism	F	AT1G27770
1849	1	L123 vs. M123	M123 up	GO:0008643	Metabolism	Transporters	carbohydrate transport	P	AT1G12500
1850	1	L123 vs. M123	M123 up	GO:0031901	Metabolism	Transporters	early endosome membrane	C	AT1G04760
1851	1	L123 vs. M123	M123 up	GO:0006888	Metabolism	Transporters	endoplasmic reticulum to Golgi vesicle-mediated transport	P	AT3G03160
1852	1	L123 vs. M123	M123 up	GO:0006888	Metabolism	Transporters	endoplasmic reticulum to Golgi vesicle-mediated transport	P	AT3G05000
1853	1	L123 vs. M123	M123 up	GO:0006888	Metabolism	Transporters	endoplasmic reticulum to Golgi vesicle-mediated transport	P	AT3G52190
1854	1	L123 vs. M123	M123 up	GO:0005768	Metabolism	Transporters	endosome	C	AT1G04760
1855	1	L123 vs. M123	M123 up	GO:0005768	Metabolism	Transporters	endosome	C	AT2G03240
1856	1	L123 vs. M123	M123 up	GO:0005768	Metabolism	Transporters	endosome	C	AT3G53520
1857	1	L123 vs. M123	M123 up	GO:0015886	Metabolism	Transporters	heme transport	P	ATCG01040
1858	1	L123 vs. M123	M123 up	GO:0006886	Metabolism	Transporters	intracellular protein transport	P	AT1G60070
1859	1	L123 vs. M123	M123 up	GO:0006886	Metabolism	Transporters	intracellular protein transport	P	AT3G03160
1860	1	L123 vs. M123	M123 up	GO:0015662	Metabolism	Transporters	ion transmembrane transporter activity, phosphorylative mechanism	F	AT3G27870
1861	1	L123 vs. M123	M123 up	GO:0006811	Metabolism	Transporters	ion transport	P	ATMG00480
1862	1	L123 vs. M123	M123 up	GO:0005871	Metabolism	Transporters	kinesin complex	C	AT5G65460
1863	1	L123 vs. M123	M123 up	GO:0015184	Metabolism	Transporters	L-cystine transmembrane transporter activity	F	AT5G40670
1864	1	L123 vs. M123	M123 up	GO:0015811	Metabolism	Transporters	L-cystine transport	P	AT5G40670
1865	1	L123 vs. M123	M123 up	GO:0030001	Metabolism	Transporters	metal ion transport	P	AT5G50740

1866	1	L123 vs. M123	M123 up	GO:0008514	Metabolism	Transporters	organic anion transmembrane transporter activity	F	AT1G12500
1867	1	L123 vs. M123	M123 up	GO:0015114	Metabolism	Transporters	phosphate ion transmembrane transporter activity	F	AT2G03240
1868	1	L123 vs. M123	M123 up	GO:0006817	Metabolism	Transporters	phosphate ion transport	P	AT2G03240
1869	1	L123 vs. M123	M123 up	GO:0006817	Metabolism	Transporters	phosphate ion transport	P	AT3G52190
1870	1	L123 vs. M123	M123 up	GO:0045332	Metabolism	Transporters	phospholipid translocation	P	AT3G27870
1871	1	L123 vs. M123	M123 up	GO:0009506	Metabolism	Transporters	plasmodesma	C	AT1G05260
1872	1	L123 vs. M123	M123 up	GO:0009506	Metabolism	Transporters	plasmodesma	C	AT1G43580
1873	1	L123 vs. M123	M123 up	GO:0009506	Metabolism	Transporters	plasmodesma	C	AT1G45145
1874	1	L123 vs. M123	M123 up	GO:0009506	Metabolism	Transporters	plasmodesma	C	AT1G60030
1875	1	L123 vs. M123	M123 up	GO:0009506	Metabolism	Transporters	plasmodesma	C	AT1G69700
1876	1	L123 vs. M123	M123 up	GO:0009506	Metabolism	Transporters	plasmodesma	C	AT2G30860
1877	1	L123 vs. M123	M123 up	GO:0009506	Metabolism	Transporters	plasmodesma	C	AT3G16410
1878	1	L123 vs. M123	M123 up	GO:0009506	Metabolism	Transporters	plasmodesma	C	AT3G24660
1879	1	L123 vs. M123	M123 up	GO:0009506	Metabolism	Transporters	plasmodesma	C	AT4G12390
1880	1	L123 vs. M123	M123 up	GO:0009506	Metabolism	Transporters	plasmodesma	C	AT4G19410
1881	1	L123 vs. M123	M123 up	GO:0015774	Metabolism	Transporters	polysaccharide transport	P	AT5G65020
1882	1	L123 vs. M123	M123 up	GO:0030150	Metabolism	Transporters	protein import into mitochondrial matrix	P	AT3G09700
1883	1	L123 vs. M123	M123 up	GO:0045039	Metabolism	Transporters	protein insertion into mitochondrial inner membrane	P	AT5G24650
1884	1	L123 vs. M123	M123 up	GO:0070973	Metabolism	Transporters	protein localization to endoplasmic reticulum exit site	P	AT3G03160
1885	1	L123 vs. M123	M123 up	GO:0009306	Metabolism	Transporters	protein secretion	P	AT3G52190
1886	1	L123 vs. M123	M123 up	GO:0008320	Metabolism	Transporters	protein transmembrane transporter activity	F	AT5G24650
1887	1	L123 vs. M123	M123 up	GO:0015031	Metabolism	Transporters	protein transport	P	AT1G04760
1888	1	L123 vs. M123	M123 up	GO:0015078	Metabolism	Transporters	proton transmembrane transporter activity	F	ATMG00410
1889	1	L123 vs. M123	M123 up	GO:0015165	Metabolism	Transporters	pyrimidine nucleotide-sugar transmembrane transporter activity	F	AT1G12500
1890	1	L123 vs. M123	M123 up	GO:0003400	Metabolism	Transporters	regulation of COPII vesicle coating	P	AT3G52190
1891	1	L123 vs. M123	M123 up	GO:0099503	Metabolism	Transporters	secretory vesicle	C	AT1G17100
1892	1	L123 vs. M123	M123 up	GO:0099503	Metabolism	Transporters	secretory vesicle	C	AT4G15210
1893	1	L123 vs. M123	M123 up	GO:0099503	Metabolism	Transporters	secretory vesicle	C	AT4G16660
1894	1	L123 vs. M123	M123 up	GO:0000103	Metabolism	Transporters	sulfate assimilation	P	AT4G39940
1895	1	L123 vs. M123	M123 up	GO:0005802	Metabolism	Transporters	trans-Golgi network	C	AT1G43580
1896	1	L123 vs. M123	M123 up	GO:0005802	Metabolism	Transporters	trans-Golgi network	C	AT2G03240
1897	1	L123 vs. M123	M123 up	GO:0005802	Metabolism	Transporters	trans-Golgi network	C	AT3G05000
1898	1	L123 vs. M123	M123 up	GO:0005802	Metabolism	Transporters	trans-Golgi network	C	AT3G53520
1899	1	L123 vs. M123	M123 up	GO:0055085	Metabolism	Transporters	transmembrane transport	P	AT1G60030
1900	1	L123 vs. M123	M123 up	GO:0055085	Metabolism	Transporters	transmembrane transport	P	AT2G07680
1901	1	L123 vs. M123	M123 up	GO:0055085	Metabolism	Transporters	transmembrane transport	P	AT5G41000
1902	1	L123 vs. M123	M123 up	GO:0022857	Metabolism	Transporters	transmembrane transporter activity	F	AT1G12500
1903	1	L123 vs. M123	M123 up	GO:0022857	Metabolism	Transporters	transmembrane transporter activity	F	AT1G60030
1904	1	L123 vs. M123	M123 up	GO:0005215	Metabolism	Transporters	transporter activity	F	AT1G60030
1905	1	L123 vs. M123	M123 up	GO:0005215	Metabolism	Transporters	transporter activity	F	AT5G48600
1906	1	L123 vs. M123	M123 up	GO:0016192	Metabolism	Transporters	vesicle-mediated transport	P	AT1G04760
1907	1	L123 vs. M123	M123 up	GO:0016192	Metabolism	Transporters	vesicle-mediated transport	P	AT1G60070
1908	1	L123 vs. M123	M123 up	GO:0051087	Regulation	Protein modification	chaperone binding	F	AT5G01390
1909	1	L123 vs. M123	M123 up	GO:0061077	Regulation	Protein modification	chaperone-mediated protein folding	P	AT5G64350
1910	1	L123 vs. M123	M123 up	GO:0005783	Regulation	Protein modification	endoplasmic reticulum	C	AT1G05260
1911	1	L123 vs. M123	M123 up	GO:0005783	Regulation	Protein modification	endoplasmic reticulum	C	AT1G27770
1912	1	L123 vs. M123	M123 up	GO:0005783	Regulation	Protein modification	endoplasmic reticulum	C	AT1G54990
1913	1	L123 vs. M123	M123 up	GO:0005783	Regulation	Protein modification	endoplasmic reticulum	C	AT2G38280
1914	1	L123 vs. M123	M123 up	GO:0005783	Regulation	Protein modification	endoplasmic reticulum	C	AT3G52190
1915	1	L123 vs. M123	M123 up	GO:0005783	Regulation	Protein modification	endoplasmic reticulum	C	AT3G55360
1916	1	L123 vs. M123	M123 up	GO:0005783	Regulation	Protein modification	endoplasmic reticulum	C	AT3G61580
1917	1	L123 vs. M123	M123 up	GO:0005783	Regulation	Protein modification	endoplasmic reticulum	C	AT4G00170
1918	1	L123 vs. M123	M123 up	GO:0005783	Regulation	Protein modification	endoplasmic reticulum	C	AT4G16660

1919	1	L123 vs. M123	M123 up	GO:0005783	Regulation	Protein modification	endoplasmic reticulum	C	AT4G31500
1920	1	L123 vs. M123	M123 up	GO:0034663	Regulation	Protein modification	endoplasmic reticulum chaperone complex	C	AT4G16660
1921	1	L123 vs. M123	M123 up	GO:0005789	Regulation	Protein modification	endoplasmic reticulum membrane	C	AT1G06350
1922	1	L123 vs. M123	M123 up	GO:0005789	Regulation	Protein modification	endoplasmic reticulum membrane	C	AT1G54990
1923	1	L123 vs. M123	M123 up	GO:0005789	Regulation	Protein modification	endoplasmic reticulum membrane	C	AT3G03160
1924	1	L123 vs. M123	M123 up	GO:0005789	Regulation	Protein modification	endoplasmic reticulum membrane	C	AT3G55360
1925	1	L123 vs. M123	M123 up	GO:0005789	Regulation	Protein modification	endoplasmic reticulum membrane	C	AT3G61580
1926	1	L123 vs. M123	M123 up	GO:0005789	Regulation	Protein modification	endoplasmic reticulum membrane	C	AT4G00170
1927	1	L123 vs. M123	M123 up	GO:0030234	Regulation	Protein modification	enzyme regulator activity	F	AT3G16410
1928	1	L123 vs. M123	M123 up	GO:0005794	Regulation	Protein modification	Golgi apparatus	C	AT1G12500
1929	1	L123 vs. M123	M123 up	GO:0005794	Regulation	Protein modification	Golgi apparatus	C	AT2G03240
1930	1	L123 vs. M123	M123 up	GO:0005794	Regulation	Protein modification	Golgi apparatus	C	AT2G34070
1931	1	L123 vs. M123	M123 up	GO:0005794	Regulation	Protein modification	Golgi apparatus	C	AT2G38860
1932	1	L123 vs. M123	M123 up	GO:0005794	Regulation	Protein modification	Golgi apparatus	C	AT3G03210
1933	1	L123 vs. M123	M123 up	GO:0005794	Regulation	Protein modification	Golgi apparatus	C	AT3G05000
1934	1	L123 vs. M123	M123 up	GO:0005794	Regulation	Protein modification	Golgi apparatus	C	AT3G53520
1935	1	L123 vs. M123	M123 up	GO:0005794	Regulation	Protein modification	Golgi apparatus	C	AT4G16660
1936	1	L123 vs. M123	M123 up	GO:0005794	Regulation	Protein modification	Golgi apparatus	C	AT5G62620
1937	1	L123 vs. M123	M123 up	GO:0000139	Regulation	Protein modification	Golgi membrane	C	AT5G62620
1938	1	L123 vs. M123	M123 up	GO:0030173	Regulation	Protein modification	integral component of Golgi membrane	C	AT1G43580
1939	1	L123 vs. M123	M123 up	GO:0032259	Regulation	Protein modification	methylation	P	AT1G54650
1940	1	L123 vs. M123	M123 up	GO:0032259	Regulation	Protein modification	methylation	P	AT2G27590
1941	1	L123 vs. M123	M123 up	GO:0032259	Regulation	Protein modification	methylation	P	AT4G14000
1942	1	L123 vs. M123	M123 up	GO:0018119	Regulation	Protein modification	peptidyl-cysteine S-nitrosylation	P	AT4G20360
1943	1	L123 vs. M123	M123 up	GO:0003755	Regulation	Protein modification	peptidyl-prolyl cis-trans isomerase activity	F	AT1G01940
1944	1	L123 vs. M123	M123 up	GO:0003755	Regulation	Protein modification	peptidyl-prolyl cis-trans isomerase activity	F	AT5G45680
1945	1	L123 vs. M123	M123 up	GO:0003755	Regulation	Protein modification	peptidyl-prolyl cis-trans isomerase activity	F	AT5G64350
1946	1	L123 vs. M123	M123 up	GO:0015035	Regulation	Protein modification	protein disulfide oxidoreductase activity	F	AT1G45145
1947	1	L123 vs. M123	M123 up	GO:0006457	Regulation	Protein modification	protein folding	P	AT1G01940
1948	1	L123 vs. M123	M123 up	GO:0006457	Regulation	Protein modification	protein folding	P	AT5G01390
1949	1	L123 vs. M123	M123 up	GO:0005956	Regulation	Protein modification	protein kinase CK2 complex	C	AT4G17640
1950	1	L123 vs. M123	M123 up	GO:0018258	Regulation	Protein modification	protein O-linked glycosylation via hydroxyproline	P	AT5G62620
1951	1	L123 vs. M123	M123 up	GO:0000413	Regulation	Protein modification	protein peptidyl-prolyl isomerization	P	AT1G01940
1952	1	L123 vs. M123	M123 up	GO:0000413	Regulation	Protein modification	protein peptidyl-prolyl isomerization	P	AT5G64350
1953	1	L123 vs. M123	M123 up	GO:0043621	Regulation	Protein modification	protein self-association	F	AT5G24650
1954	1	L123 vs. M123	M123 up	GO:0031386	Regulation	Protein modification	protein tag	F	AT4G02890
1955	1	L123 vs. M123	M123 up	GO:0043086	Regulation	Regulation	negative regulation of catalytic activity	P	AT4G12390
1956	1	L123 vs. M123	M123 up	GO:0009910	Regulation	Regulation	negative regulation of flower development	P	AT3G19980
1957	1	L123 vs. M123	M123 up	GO:0010439	Regulation	Regulation	regulation of glucosinolate biosynthetic process	P	AT4G39940
1958	1	L123 vs. M123	M123 up	GO:0040008	Regulation	Regulation	regulation of growth	P	AT4G31500
1959	1	L123 vs. M123	M123 up	GO:0010075	Regulation	Regulation	regulation of meristem growth	P	AT3G49670
1960	1	L123 vs. M123	M123 up	GO:0009934	Regulation	Regulation	regulation of meristem structural organization	P	AT3G49670
1961	1	L123 vs. M123	M123 up	GO:0003700	Regulation	Transcription	DNA-binding transcription factor activity	F	AT3G24050
1962	1	L123 vs. M123	M123 up	GO:0003899	Regulation	Transcription	DNA-directed 5'-3' RNA polymerase activity	F	AT1G06790
1963	1	L123 vs. M123	M123 up	GO:0035195	Regulation	Transcription	gene silencing by miRNA	P	AT1G08830
1964	1	L123 vs. M123	M123 up	GO:0035195	Regulation	Transcription	gene silencing by miRNA	P	AT2G28190
1965	1	L123 vs. M123	M123 up	GO:0042393	Regulation	Transcription	histone binding	F	AT2G27980
1966	1	L123 vs. M123	M123 up	GO:0042393	Regulation	Transcription	histone binding	F	AT4G15890
1967	1	L123 vs. M123	M123 up	GO:0006397	Regulation	Transcription	mRNA processing	P	AT1G11290
1968	1	L123 vs. M123	M123 up	GO:0005730	Regulation	Transcription	nucleolus	C	AT4G20360
1969	1	L123 vs. M123	M123 up	GO:0045944	Regulation	Transcription	positive regulation of transcription by RNA polymerase II	P	AT2G27980
1970	1	L123 vs. M123	M123 up	GO:0006355	Regulation	Transcription	regulation of transcription, DNA-templated	P	AT3G24050
1971	1	L123 vs. M123	M123 up	GO:0003723	Regulation	Transcription	RNA binding	F	AT1G53650
1972	1	L123 vs. M123	M123 up	GO:0003723	Regulation	Transcription	RNA binding	F	AT1G59990
1973	1	L123 vs. M123	M123 up	GO:0009451	Regulation	Transcription	RNA modification	P	AT1G11290

1974	1	L123 vs. M123	M123 up	GO:0009451	Regulation	Transcription	RNA modification	P	AT3G02010
1975	1	L123 vs. M123	M123 up	GO:0008353	Regulation	Transcription	RNA polymerase II CTD heptapeptide repeat kinase activity	F	AT3G48750
1976	1	L123 vs. M123	M123 up	GO:0000977	Regulation	Transcription	RNA polymerase II regulatory region sequence-specific DNA binding	F	AT2G27980
1977	1	L123 vs. M123	M123 up	GO:0001056	Regulation	Transcription	RNA polymerase III activity	F	AT1G06790
1978	1	L123 vs. M123	M123 up	GO:0005666	Regulation	Transcription	RNA polymerase III complex	C	AT1G06790
1979	1	L123 vs. M123	M123 up	GO:0008134	Regulation	Transcription	transcription factor binding	F	AT5G24780
1980	1	L123 vs. M123	M123 up	GO:0006384	Regulation	Transcription	transcription initiation from RNA polymerase III promoter	P	AT1G06790
1981	1	L123 vs. M123	M123 up	GO:0044212	Regulation	Transcription	transcription regulatory region DNA binding	F	AT2G27980
1982	1	L123 vs. M123	M123 up	GO:0044212	Regulation	Transcription	transcription regulatory region DNA binding	F	AT3G24050
1983	1	L123 vs. M123	M123 up	GO:0003746	Regulation	Transcription	translation elongation factor activity	F	AT4G20360
1984	1	L123 vs. M123	M123 up	GO:0002181	Regulation	Translation	cytoplasmic translation	P	AT4G39520
1985	1	L123 vs. M123	M123 up	GO:0022625	Regulation	Translation	cytosolic large ribosomal subunit	C	AT3G02190
1986	1	L123 vs. M123	M123 up	GO:0022625	Regulation	Translation	cytosolic large ribosomal subunit	C	AT3G28900
1987	1	L123 vs. M123	M123 up	GO:0022626	Regulation	Translation	cytosolic ribosome	C	AT3G28900
1988	1	L123 vs. M123	M123 up	GO:0022626	Regulation	Translation	cytosolic ribosome	C	AT5G64140
1989	1	L123 vs. M123	M123 up	GO:0022627	Regulation	Translation	cytosolic small ribosomal subunit	C	AT5G64140
1990	1	L123 vs. M123	M123 up	GO:0004819	Regulation	Translation	glutamine-tRNA ligase activity	F	AT1G25350
1991	1	L123 vs. M123	M123 up	GO:0006425	Regulation	Translation	glutaminyl-tRNA aminoacylation	P	AT1G25350
1992	1	L123 vs. M123	M123 up	GO:0006424	Regulation	Translation	glutamyl-tRNA aminoacylation	P	AT1G25350
1993	1	L123 vs. M123	M123 up	GO:0030490	Regulation	Translation	maturation of SSU-rRNA	P	AT5G64140
1994	1	L123 vs. M123	M123 up	GO:0003729	Regulation	Translation	mRNA binding	F	AT5G64140
1995	1	L123 vs. M123	M123 up	GO:0016556	Regulation	Translation	mRNA modification	P	AT1G11290
1996	1	L123 vs. M123	M123 up	GO:0045903	Regulation	Translation	positive regulation of translational fidelity	P	ATCG00380
1997	1	L123 vs. M123	M123 up	GO:0000027	Regulation	Translation	ribosomal large subunit assembly	P	ATCG00660
1998	1	L123 vs. M123	M123 up	GO:0000028	Regulation	Translation	ribosomal small subunit assembly	P	AT5G64140
1999	1	L123 vs. M123	M123 up	GO:0005840	Regulation	Translation	ribosome	C	ATCG00660
2000	1	L123 vs. M123	M123 up	GO:0042254	Regulation	Translation	ribosome biogenesis	P	AT3G28900
2001	1	L123 vs. M123	M123 up	GO:0003724	Regulation	Translation	RNA helicase activity	F	AT1G59990
2002	1	L123 vs. M123	M123 up	GO:0008380	Regulation	Translation	RNA splicing	P	AT1G06790
2003	1	L123 vs. M123	M123 up	GO:0019843	Regulation	Translation	rRNA binding	F	ATCG00380
2004	1	L123 vs. M123	M123 up	GO:0015935	Regulation	Translation	small ribosomal subunit	C	ATCG00380
2005	1	L123 vs. M123	M123 up	GO:0003735	Regulation	Translation	structural constituent of ribosome	F	AT5G64140
2006	1	L123 vs. M123	M123 up	GO:0003735	Regulation	Translation	structural constituent of ribosome	F	ATCG00380
2007	1	L123 vs. M123	M123 up	GO:0003735	Regulation	Translation	structural constituent of ribosome	F	ATCG00660
2008	1	L123 vs. M123	M123 up	GO:0006412	Regulation	Translation	translation	P	AT1G25350
2009	1	L123 vs. M123	M123 up	GO:0006412	Regulation	Translation	translation	P	AT3G02190
2010	1	L123 vs. M123	M123 up	GO:0006412	Regulation	Translation	translation	P	AT3G28900
2011	1	L123 vs. M123	M123 up	GO:0006412	Regulation	Translation	translation	P	AT5G64140
2012	1	L123 vs. M123	M123 up	GO:0006412	Regulation	Translation	translation	P	ATCG00380
2013	1	L123 vs. M123	M123 up	GO:0006412	Regulation	Translation	translation	P	ATCG00660
2014	1	L123 vs. M123	M123 up	GO:0006414	Regulation	Translation	translational elongation	P	AT4G20360
2015	1	L123 vs. M123	M123 up	GO:0016031	Regulation	Translation	tRNA import into mitochondrion	P	AT5G24650
2016	1	L123 vs. M123	M123 up	GO:0003993	Signaling and response	Signaling	acid phosphatase activity	F	AT4G24890
2017	1	L123 vs. M123	M123 up	GO:0003993	Signaling and response	Signaling	acid phosphatase activity	F	AT5G24780
2018	1	L123 vs. M123	M123 up	GO:0009926	Signaling and response	Signaling	auxin polar transport	P	AT1G54990
2019	1	L123 vs. M123	M123 up	GO:0005509	Signaling and response	Signaling	calcium ion binding	F	AT5G65020
2020	1	L123 vs. M123	M123 up	GO:0005516	Signaling and response	Signaling	calmodulin binding	F	AT1G27770
2021	1	L123 vs. M123	M123 up	GO:0071277	Signaling and response	Signaling	cellular response to calcium ion	P	AT1G08860
2022	1	L123 vs. M123	M123 up	GO:0070417	Signaling and response	Signaling	cellular response to cold	P	AT3G61580
2023	1	L123 vs. M123	M123 up	GO:0071280	Signaling and response	Signaling	cellular response to copper ion	P	AT1G08830
2024	1	L123 vs. M123	M123 up	GO:0071329	Signaling and response	Signaling	cellular response to sucrose stimulus	P	AT1G08830
2025	1	L123 vs. M123	M123 up	GO:0071329	Signaling and response	Signaling	cellular response to sucrose stimulus	P	AT2G28190
2026	1	L123 vs. M123	M123 up	GO:0007623	Signaling and response	Signaling	circadian rhythm	P	AT3G24050

2027	1	L123 vs. M123	M123 up	GO:0016311	Signaling and response	Signaling	dephosphorylation	P	AT4G24890
2028	1	L123 vs. M123	M123 up	GO:0016311	Signaling and response	Signaling	dephosphorylation	P	AT5G24780
2029	1	L123 vs. M123	M123 up	GO:0009740	Signaling and response	Signaling	gibberellic acid mediated signaling pathway	P	AT2G14900
2030	1	L123 vs. M123	M123 up	GO:0051020	Signaling and response	Signaling	GTPase binding	F	AT3G52190
2031	1	L123 vs. M123	M123 up	GO:0009684	Signaling and response	Signaling	indoleacetic acid biosynthetic process	P	AT2G22330
2032	1	L123 vs. M123	M123 up	GO:0009684	Signaling and response	Signaling	indoleacetic acid biosynthetic process	P	AT4G31500
2033	1	L123 vs. M123	M123 up	GO:0016301	Signaling and response	Signaling	kinase activity	F	AT3G24660
2034	1	L123 vs. M123	M123 up	GO:0016301	Signaling and response	Signaling	kinase activity	F	AT3G48750
2035	1	L123 vs. M123	M123 up	GO:0016301	Signaling and response	Signaling	kinase activity	F	AT5G63680
2036	1	L123 vs. M123	M123 up	GO:0004721	Signaling and response	Signaling	phosphoprotein phosphatase activity	F	AT3G19980
2037	1	L123 vs. M123	M123 up	GO:0006470	Signaling and response	Signaling	protein dephosphorylation	P	AT3G19980
2038	1	L123 vs. M123	M123 up	GO:0004672	Signaling and response	Signaling	protein kinase activity	F	AT2G26760
2039	1	L123 vs. M123	M123 up	GO:0004672	Signaling and response	Signaling	protein kinase activity	F	AT3G24660
2040	1	L123 vs. M123	M123 up	GO:0004672	Signaling and response	Signaling	protein kinase activity	F	AT3G48750
2041	1	L123 vs. M123	M123 up	GO:0019901	Signaling and response	Signaling	protein kinase binding	F	AT2G26760
2042	1	L123 vs. M123	M123 up	GO:0019887	Signaling and response	Signaling	protein kinase regulator activity	F	AT4G17640
2043	1	L123 vs. M123	M123 up	GO:0000159	Signaling and response	Signaling	protein phosphatase type 2A complex	C	AT3G19980
2044	1	L123 vs. M123	M123 up	GO:0006468	Signaling and response	Signaling	protein phosphorylation	P	AT3G24660
2045	1	L123 vs. M123	M123 up	GO:0006468	Signaling and response	Signaling	protein phosphorylation	P	AT3G48750
2046	1	L123 vs. M123	M123 up	GO:0006468	Signaling and response	Signaling	protein phosphorylation	P	AT3G49670
2047	1	L123 vs. M123	M123 up	GO:0006468	Signaling and response	Signaling	protein phosphorylation	P	AT4G17640
2048	1	L123 vs. M123	M123 up	GO:0006468	Signaling and response	Signaling	protein phosphorylation	P	AT5G08160
2049	1	L123 vs. M123	M123 up	GO:0004674	Signaling and response	Signaling	protein serine/threonine kinase activity	F	AT3G19980
2050	1	L123 vs. M123	M123 up	GO:0004674	Signaling and response	Signaling	protein serine/threonine kinase activity	F	AT3G49670
2051	1	L123 vs. M123	M123 up	GO:0004674	Signaling and response	Signaling	protein serine/threonine kinase activity	F	AT4G17640
2052	1	L123 vs. M123	M123 up	GO:0004674	Signaling and response	Signaling	protein serine/threonine kinase activity	F	AT5G08160
2053	1	L123 vs. M123	M123 up	GO:0033612	Signaling and response	Signaling	receptor serine/threonine kinase binding	F	AT3G49670
2054	1	L123 vs. M123	M123 up	GO:0000079	Signaling and response	Signaling	regulation of cyclin-dependent protein serine/threonine kinase activity	P	AT2G26760
2055	1	L123 vs. M123	M123 up	GO:0043087	Signaling and response	Signaling	regulation of GTPase activity	P	AT3G05000
2056	1	L123 vs. M123	M123 up	GO:0009733	Signaling and response	Signaling	response to auxin	P	AT1G54990
2057	1	L123 vs. M123	M123 up	GO:0009617	Signaling and response	Signaling	response to bacterium	P	AT2G22330
2058	1	L123 vs. M123	M123 up	GO:0046686	Signaling and response	Signaling	response to cadmium ion	P	AT1G42970
2059	1	L123 vs. M123	M123 up	GO:0046686	Signaling and response	Signaling	response to cadmium ion	P	AT1G45145
2060	1	L123 vs. M123	M123 up	GO:0046686	Signaling and response	Signaling	response to cadmium ion	P	AT2G30860
2061	1	L123 vs. M123	M123 up	GO:0046686	Signaling and response	Signaling	response to cadmium ion	P	AT5G16970
2062	1	L123 vs. M123	M123 up	GO:0046686	Signaling and response	Signaling	response to cadmium ion	P	AT5G63680
2063	1	L123 vs. M123	M123 up	GO:0046688	Signaling and response	Signaling	response to copper ion	P	AT1G08830
2064	1	L123 vs. M123	M123 up	GO:0046688	Signaling and response	Signaling	response to copper ion	P	AT2G28190
2065	1	L123 vs. M123	M123 up	GO:0009725	Signaling and response	Signaling	response to hormone	P	AT2G40710
2066	1	L123 vs. M123	M123 up	GO:0010039	Signaling and response	Signaling	response to iron ion	P	AT1G08830
2067	1	L123 vs. M123	M123 up	GO:0010039	Signaling and response	Signaling	response to iron ion	P	AT2G28190
2068	1	L123 vs. M123	M123 up	GO:0009753	Signaling and response	Signaling	response to jasmonic acid	P	AT5G24780
2069	1	L123 vs. M123	M123 up	GO:0009416	Signaling and response	Signaling	response to light stimulus	P	AT1G42970
2070	1	L123 vs. M123	M123 up	GO:0009416	Signaling and response	Signaling	response to light stimulus	P	AT2G28190
2071	1	L123 vs. M123	M123 up	GO:0010114	Signaling and response	Signaling	response to red light	P	AT4G31500
2072	1	L123 vs. M123	M123 up	GO:0009744	Signaling and response	Signaling	response to sucrose	P	AT1G42970
2073	1	L123 vs. M123	M123 up	GO:0010043	Signaling and response	Signaling	response to zinc ion	P	AT2G30860
2074	1	L123 vs. M123	M123 up	GO:0009641	Signaling and response	Signaling	shade avoidance	P	AT4G31500
2075	1	L123 vs. M123	M123 up	GO:0038023	Signaling and response	Signaling	signaling receptor activity	F	AT2G40710
2076	1	L123 vs. M123	M123 up	GO:0010230	Signaling and response	Stress	alternative respiration	P	AT2G18660
2077	1	L123 vs. M123	M123 up	GO:0005262	Signaling and response	Stress	calcium channel activity	F	AT1G27770
2078	1	L123 vs. M123	M123 up	GO:0005544	Signaling and response	Stress	calcium-dependent phospholipid binding	F	AT1G08860
2079	1	L123 vs. M123	M123 up	GO:0005544	Signaling and response	Stress	calcium-dependent phospholipid binding	F	AT5G65020
2080	1	L123 vs. M123	M123 up	GO:0045454	Signaling and response	Stress	cell redox homeostasis	P	AT1G45145
2081	1	L123 vs. M123	M123 up	GO:0019725	Signaling and response	Stress	cellular homeostasis	P	AT1G08860

2082	1	L123 vs. M123	M123 up	GO:0071456	Signaling and response	Stress	cellular response to hypoxia	P	AT4G16660
2083	1	L123 vs. M123	M123 up	GO:0071484	Signaling and response	Stress	cellular response to light intensity	P	AT1G08830
2084	1	L123 vs. M123	M123 up	GO:0071484	Signaling and response	Stress	cellular response to light intensity	P	AT2G28190
2085	1	L123 vs. M123	M123 up	GO:0034599	Signaling and response	Stress	cellular response to oxidative stress	P	AT1G08830
2086	1	L123 vs. M123	M123 up	GO:0034599	Signaling and response	Stress	cellular response to oxidative stress	P	AT2G28190
2087	1	L123 vs. M123	M123 up	GO:0071457	Signaling and response	Stress	cellular response to ozone	P	AT1G08830
2088	1	L123 vs. M123	M123 up	GO:0071457	Signaling and response	Stress	cellular response to ozone	P	AT2G28190
2089	1	L123 vs. M123	M123 up	GO:0016036	Signaling and response	Stress	cellular response to phosphate starvation	P	AT2G03240
2090	1	L123 vs. M123	M123 up	GO:0016036	Signaling and response	Stress	cellular response to phosphate starvation	P	AT3G52190
2091	1	L123 vs. M123	M123 up	GO:0071472	Signaling and response	Stress	cellular response to salt stress	P	AT1G08830
2092	1	L123 vs. M123	M123 up	GO:0071472	Signaling and response	Stress	cellular response to salt stress	P	AT2G28190
2093	1	L123 vs. M123	M123 up	GO:0071493	Signaling and response	Stress	cellular response to UV-B	P	AT1G08830
2094	1	L123 vs. M123	M123 up	GO:0071493	Signaling and response	Stress	cellular response to UV-B	P	AT2G28190
2095	1	L123 vs. M123	M123 up	GO:0051085	Signaling and response	Stress	chaperone cofactor-dependent protein refolding	P	AT5G01390
2096	1	L123 vs. M123	M123 up	GO:0006952	Signaling and response	Stress	defense response	P	AT1G08860
2097	1	L123 vs. M123	M123 up	GO:0006952	Signaling and response	Stress	defense response	P	AT2G22330
2098	1	L123 vs. M123	M123 up	GO:0006952	Signaling and response	Stress	defense response	P	AT2G30860
2099	1	L123 vs. M123	M123 up	GO:0006952	Signaling and response	Stress	defense response	P	AT5G02140
2100	1	L123 vs. M123	M123 up	GO:0006952	Signaling and response	Stress	defense response	P	AT5G24780
2101	1	L123 vs. M123	M123 up	GO:0052544	Signaling and response	Stress	defense response by callose deposition in cell wall	P	AT2G22330
2102	1	L123 vs. M123	M123 up	GO:0052544	Signaling and response	Stress	defense response by callose deposition in cell wall	P	AT4G31500
2103	1	L123 vs. M123	M123 up	GO:0042742	Signaling and response	Stress	defense response to bacterium	P	AT1G08830
2104	1	L123 vs. M123	M123 up	GO:0042742	Signaling and response	Stress	defense response to bacterium	P	AT2G22330
2105	1	L123 vs. M123	M123 up	GO:0042742	Signaling and response	Stress	defense response to bacterium	P	AT2G30860
2106	1	L123 vs. M123	M123 up	GO:0042742	Signaling and response	Stress	defense response to bacterium	P	AT4G31500
2107	1	L123 vs. M123	M123 up	GO:0050832	Signaling and response	Stress	defense response to fungus	P	AT1G12200
2108	1	L123 vs. M123	M123 up	GO:0050832	Signaling and response	Stress	defense response to fungus	P	AT1G45145
2109	1	L123 vs. M123	M123 up	GO:0002229	Signaling and response	Stress	defense response to oomycetes	P	AT2G22330
2110	1	L123 vs. M123	M123 up	GO:0043295	Signaling and response	Stress	glutathione binding	F	AT2G30860
2111	1	L123 vs. M123	M123 up	GO:0043295	Signaling and response	Stress	glutathione binding	F	AT3G03190
2112	1	L123 vs. M123	M123 up	GO:0006749	Signaling and response	Stress	glutathione metabolic process	P	AT2G30860
2113	1	L123 vs. M123	M123 up	GO:0006749	Signaling and response	Stress	glutathione metabolic process	P	AT3G03190
2114	1	L123 vs. M123	M123 up	GO:0004602	Signaling and response	Stress	glutathione peroxidase activity	F	AT2G30860
2115	1	L123 vs. M123	M123 up	GO:0004364	Signaling and response	Stress	glutathione transferase activity	F	AT2G30860
2116	1	L123 vs. M123	M123 up	GO:0004364	Signaling and response	Stress	glutathione transferase activity	F	AT3G03190
2117	1	L123 vs. M123	M123 up	GO:0010235	Signaling and response	Stress	guard mother cell cytokinesis	P	AT3G48750
2118	1	L123 vs. M123	M123 up	GO:0042744	Signaling and response	Stress	hydrogen peroxide catabolic process	P	AT1G05260
2119	1	L123 vs. M123	M123 up	GO:0042538	Signaling and response	Stress	hyperosmotic salinity response	P	AT1G05260
2120	1	L123 vs. M123	M123 up	GO:0042538	Signaling and response	Stress	hyperosmotic salinity response	P	AT1G69700
2121	1	L123 vs. M123	M123 up	GO:0009682	Signaling and response	Stress	induced systemic resistance	P	AT2G22330
2122	1	L123 vs. M123	M123 up	GO:0009682	Signaling and response	Stress	induced systemic resistance	P	AT4G31500
2123	1	L123 vs. M123	M123 up	GO:0055072	Signaling and response	Stress	iron ion homeostasis	P	AT5G51720
2124	1	L123 vs. M123	M123 up	GO:0060548	Signaling and response	Stress	negative regulation of cell death	P	AT1G08860
2125	1	L123 vs. M123	M123 up	GO:0010186	Signaling and response	Stress	positive regulation of cellular defense response	P	AT1G08860
2126	1	L123 vs. M123	M123 up	GO:0072593	Signaling and response	Stress	reactive oxygen species metabolic process	P	AT5G51720
2127	1	L123 vs. M123	M123 up	GO:0010112	Signaling and response	Stress	regulation of systemic acquired resistance	P	AT2G22330
2128	1	L123 vs. M123	M123 up	GO:0019430	Signaling and response	Stress	removal of superoxide radicals	P	AT1G08830
2129	1	L123 vs. M123	M123 up	GO:0019430	Signaling and response	Stress	removal of superoxide radicals	P	AT2G28190
2130	1	L123 vs. M123	M123 up	GO:0009737	Signaling and response	Stress	response to abscisic acid	P	AT1G69700
2131	1	L123 vs. M123	M123 up	GO:0009737	Signaling and response	Stress	response to abscisic acid	P	AT2G38280
2132	1	L123 vs. M123	M123 up	GO:0009737	Signaling and response	Stress	response to abscisic acid	P	AT3G05000
2133	1	L123 vs. M123	M123 up	GO:0009409	Signaling and response	Stress	response to cold	P	AT1G05260
2134	1	L123 vs. M123	M123 up	GO:0009409	Signaling and response	Stress	response to cold	P	AT1G42970
2135	1	L123 vs. M123	M123 up	GO:0009409	Signaling and response	Stress	response to cold	P	AT3G48750
2136	1	L123 vs. M123	M123 up	GO:0009409	Signaling and response	Stress	response to cold	P	AT5G65020
2137	1	L123 vs. M123	M123 up	GO:0009269	Signaling and response	Stress	response to desiccation	P	AT1G05260

2138	1	L123 vs. M123	M123 up	GO:0009408	Signaling and response	Stress	response to heat	P	AT5G65020
2139	1	L123 vs. M123	M123 up	GO:0080027	Signaling and response	Stress	response to herbivore	P	AT4G15210
2140	1	L123 vs. M123	M123 up	GO:0009625	Signaling and response	Stress	response to insect	P	AT2G22330
2141	1	L123 vs. M123	M123 up	GO:0009625	Signaling and response	Stress	response to insect	P	AT4G31500
2142	1	L123 vs. M123	M123 up	GO:0009612	Signaling and response	Stress	response to mechanical stimulus	P	AT1G54990
2143	1	L123 vs. M123	M123 up	GO:0010188	Signaling and response	Stress	response to microbial phytotoxin	P	AT1G45145
2144	1	L123 vs. M123	M123 up	GO:0006979	Signaling and response	Stress	response to oxidative stress	P	AT1G05260
2145	1	L123 vs. M123	M123 up	GO:0006979	Signaling and response	Stress	response to oxidative stress	P	AT1G08830
2146	1	L123 vs. M123	M123 up	GO:0006979	Signaling and response	Stress	response to oxidative stress	P	AT1G45145
2147	1	L123 vs. M123	M123 up	GO:0006979	Signaling and response	Stress	response to oxidative stress	P	AT2G28190
2148	1	L123 vs. M123	M123 up	GO:0006979	Signaling and response	Stress	response to oxidative stress	P	AT3G03190
2149	1	L123 vs. M123	M123 up	GO:0006979	Signaling and response	Stress	response to oxidative stress	P	AT5G16970
2150	1	L123 vs. M123	M123 up	GO:0010193	Signaling and response	Stress	response to ozone	P	AT1G08830
2151	1	L123 vs. M123	M123 up	GO:0009651	Signaling and response	Stress	response to salt stress	P	AT1G08830
2152	1	L123 vs. M123	M123 up	GO:0009651	Signaling and response	Stress	response to salt stress	P	AT5G65020
2153	1	L123 vs. M123	M123 up	GO:0009414	Signaling and response	Stress	response to water deprivation	P	AT1G69700
2154	1	L123 vs. M123	M123 up	GO:0009414	Signaling and response	Stress	response to water deprivation	P	AT5G65020
2155	1	L123 vs. M123	M123 up	GO:0009611	Signaling and response	Stress	response to wounding	P	AT2G22330
2156	1	L123 vs. M123	M123 up	GO:0051903	Signaling and response	Stress	S-(hydroxymethyl)glutathione dehydrogenase activity	F	AT5G42250
2157	1	L123 vs. M123	M123 up	GO:0090332	Signaling and response	Stress	stomatal closure	P	AT1G08860
2158	1	L123 vs. M123	M123 up	GO:0004784	Signaling and response	Stress	superoxide dismutase activity	F	AT1G08830
2159	1	L123 vs. M123	M123 up	GO:0004784	Signaling and response	Stress	superoxide dismutase activity	F	AT2G28190
2160	1	L123 vs. M123	M123 up	GO:0009627	Signaling and response	Stress	systemic acquired resistance	P	AT2G18660
2161	1	L123 vs. M123	M123 up	GO:0009407	Signaling and response	Stress	toxin catabolic process	P	AT2G30860
2162	1	L123 vs. M123	M123 up	GO:0009407	Signaling and response	Stress	toxin catabolic process	P	AT3G03190
2163	1	L123 vs. M123	M123 up	GO:0051082	Signaling and response	Stress	unfolded protein binding	F	AT5G01390
2164	2	L4 vs. M4	L4 Up	GO:0006284	Development	Cell division	base-excision repair	P	AT3G12040
2165	2	L4 vs. M4	L4 Up	GO:0051301	Development	Cell division	cell division	P	AT2G20190
2166	2	L4 vs. M4	L4 Up	GO:0032508	Development	Cell division	DNA duplex unwinding	P	AT2G21660
2167	2	L4 vs. M4	L4 Up	GO:0006260	Development	Cell division	DNA replication	P	AT4G12620
2168	2	L4 vs. M4	L4 Up	GO:0006270	Development	Cell division	DNA replication initiation	P	AT4G12620
2169	2	L4 vs. M4	L4 Up	GO:0003688	Development	Cell division	DNA replication origin binding	F	AT4G12620
2170	2	L4 vs. M4	L4 Up	GO:0006261	Development	Cell division	DNA-dependent DNA replication	P	AT1G34380
2171	2	L4 vs. M4	L4 Up	GO:0003887	Development	Cell division	DNA-directed DNA polymerase activity	F	AT1G34380
2172	2	L4 vs. M4	L4 Up	GO:0010385	Development	Cell division	double-stranded methylated DNA binding	F	AT4G12620
2173	2	L4 vs. M4	L4 Up	GO:0000278	Development	Cell division	mitotic cell cycle	P	AT2G20190
2174	2	L4 vs. M4	L4 Up	GO:0000278	Development	Cell division	mitotic cell cycle	P	AT4G12620
2175	2	L4 vs. M4	L4 Up	GO:0033314	Development	Cell division	mitotic DNA replication checkpoint	P	AT4G12620
2176	2	L4 vs. M4	L4 Up	GO:0005664	Development	Cell division	nuclear origin of replication recognition complex	C	AT4G12620
2177	2	L4 vs. M4	L4 Up	GO:0008252	Development	Cell division	nucleotidase activity	F	AT4G14930
2178	2	L4 vs. M4	L4 Up	GO:0000808	Development	Cell division	origin recognition complex	C	AT4G12620
2179	2	L4 vs. M4	L4 Up	GO:0009846	Development	Cell division	pollen germination	P	AT5G07410
2180	2	L4 vs. M4	L4 Up	GO:0009860	Development	Cell division	pollen tube growth	P	AT1G04950
2181	2	L4 vs. M4	L4 Up	GO:0009860	Development	Cell division	pollen tube growth	P	AT2G41970
2182	2	L4 vs. M4	L4 Up	GO:0009860	Development	Cell division	pollen tube growth	P	AT2G47040
2183	2	L4 vs. M4	L4 Up	GO:0009860	Development	Cell division	pollen tube growth	P	AT5G52360
2184	2	L4 vs. M4	L4 Up	GO:0051781	Development	Cell division	positive regulation of cell division	P	AT2G20190
2185	2	L4 vs. M4	L4 Up	GO:0019005	Development	Cell division	SCF ubiquitin ligase complex	C	AT2G25490
2186	2	L4 vs. M4	L4 Up	GO:0019953	Development	Cell division	sexual reproduction	P	AT5G07550
2187	2	L4 vs. M4	L4 Up	GO:0007338	Development	Cell division	single fertilization	P	AT5G02100
2188	2	L4 vs. M4	L4 Up	GO:0003697	Development	Cell division	single-stranded DNA binding	F	AT2G21660
2189	2	L4 vs. M4	L4 Up	GO:0003697	Development	Cell division	single-stranded DNA binding	F	AT5G59180
2190	2	L4 vs. M4	L4 Up	GO:0005876	Development	Cell division	spindle microtubule	C	AT2G20190
2191	2	L4 vs. M4	L4 Up	GO:0006949	Development	Cell division	syncytium formation	P	AT4G26260
2192	2	L4 vs. M4	L4 Up	GO:0006949	Development	Cell division	syncytium formation	P	AT5G56640

2193	2	L4 vs. M4	L4 Up	GO:0015629	Development	Development	actin cytoskeleton	C	AT1G01780
2194	2	L4 vs. M4	L4 Up	GO:0005884	Development	Development	actin filament	C	AT4G25590
2195	2	L4 vs. M4	L4 Up	GO:0005884	Development	Development	actin filament	C	AT5G52360
2196	2	L4 vs. M4	L4 Up	GO:0031225	Development	Development	anchored component of membrane	C	AT1G69295
2197	2	L4 vs. M4	L4 Up	GO:0031225	Development	Development	anchored component of membrane	C	AT3G20580
2198	2	L4 vs. M4	L4 Up	GO:0031225	Development	Development	anchored component of membrane	C	AT3G20865
2199	2	L4 vs. M4	L4 Up	GO:0031225	Development	Development	anchored component of membrane	C	AT3G57690
2200	2	L4 vs. M4	L4 Up	GO:0031225	Development	Development	anchored component of membrane	C	AT5G08000
2201	2	L4 vs. M4	L4 Up	GO:0031225	Development	Development	anchored component of membrane	C	AT5G40730
2202	2	L4 vs. M4	L4 Up	GO:0046658	Development	Development	anchored component of plasma membrane	C	AT5G08000
2203	2	L4 vs. M4	L4 Up	GO:0009901	Development	Development	anther dehiscence	P	AT5G50800
2204	2	L4 vs. M4	L4 Up	GO:0048046	Development	Development	apoplast	C	AT1G28270
2205	2	L4 vs. M4	L4 Up	GO:0048046	Development	Development	apoplast	C	AT1G72610
2206	2	L4 vs. M4	L4 Up	GO:0048046	Development	Development	apoplast	C	AT3G01500
2207	2	L4 vs. M4	L4 Up	GO:0048046	Development	Development	apoplast	C	AT3G13750
2208	2	L4 vs. M4	L4 Up	GO:0048046	Development	Development	apoplast	C	AT3G52840
2209	2	L4 vs. M4	L4 Up	GO:0048046	Development	Development	apoplast	C	AT4G05180
2210	2	L4 vs. M4	L4 Up	GO:0048046	Development	Development	apoplast	C	AT4G35010
2211	2	L4 vs. M4	L4 Up	GO:0048046	Development	Development	apoplast	C	AT5G09550
2212	2	L4 vs. M4	L4 Up	GO:0048046	Development	Development	apoplast	C	AT5G20630
2213	2	L4 vs. M4	L4 Up	GO:0048046	Development	Development	apoplast	C	AT5G26700
2214	2	L4 vs. M4	L4 Up	GO:0048046	Development	Development	apoplast	C	AT5G52360
2215	2	L4 vs. M4	L4 Up	GO:0048046	Development	Development	apoplast	C	ATCG00490
2216	2	L4 vs. M4	L4 Up	GO:0005623	Development	Development	cell	C	AT1G54030
2217	2	L4 vs. M4	L4 Up	GO:0005623	Development	Development	cell	C	AT4G24920
2218	2	L4 vs. M4	L4 Up	GO:0005623	Development	Development	cell	C	AT5G54750
2219	2	L4 vs. M4	L4 Up	GO:0007155	Development	Development	cell adhesion	P	AT3G25140
2220	2	L4 vs. M4	L4 Up	GO:0005938	Development	Development	cell cortex	C	AT2G20190
2221	2	L4 vs. M4	L4 Up	GO:0005938	Development	Development	cell cortex	C	AT4G29340
2222	2	L4 vs. M4	L4 Up	GO:0016049	Development	Development	cell growth	P	AT1G78020
2223	2	L4 vs. M4	L4 Up	GO:0009932	Development	Development	cell tip growth	P	AT3G07880
2224	2	L4 vs. M4	L4 Up	GO:0005618	Development	Development	cell wall	C	AT1G07890
2225	2	L4 vs. M4	L4 Up	GO:0005618	Development	Development	cell wall	C	AT1G71695
2226	2	L4 vs. M4	L4 Up	GO:0005618	Development	Development	cell wall	C	AT1G72610
2227	2	L4 vs. M4	L4 Up	GO:0005618	Development	Development	cell wall	C	AT2G21140
2228	2	L4 vs. M4	L4 Up	GO:0005618	Development	Development	cell wall	C	AT2G39460
2229	2	L4 vs. M4	L4 Up	GO:0005618	Development	Development	cell wall	C	AT2G47040
2230	2	L4 vs. M4	L4 Up	GO:0005618	Development	Development	cell wall	C	AT3G07850
2231	2	L4 vs. M4	L4 Up	GO:0005618	Development	Development	cell wall	C	AT3G13750
2232	2	L4 vs. M4	L4 Up	GO:0005618	Development	Development	cell wall	C	AT3G16240
2233	2	L4 vs. M4	L4 Up	GO:0005618	Development	Development	cell wall	C	AT3G52840
2234	2	L4 vs. M4	L4 Up	GO:0005618	Development	Development	cell wall	C	AT3G62170
2235	2	L4 vs. M4	L4 Up	GO:0005618	Development	Development	cell wall	C	AT4G35010
2236	2	L4 vs. M4	L4 Up	GO:0005618	Development	Development	cell wall	C	AT5G07410
2237	2	L4 vs. M4	L4 Up	GO:0005618	Development	Development	cell wall	C	AT5G14040
2238	2	L4 vs. M4	L4 Up	GO:0005618	Development	Development	cell wall	C	AT5G15200
2239	2	L4 vs. M4	L4 Up	GO:0005618	Development	Development	cell wall	C	AT5G20630
2240	2	L4 vs. M4	L4 Up	GO:0005618	Development	Development	cell wall	C	AT5G26700
2241	2	L4 vs. M4	L4 Up	GO:0005618	Development	Development	cell wall	C	ATCG00490
2242	2	L4 vs. M4	L4 Up	GO:0042545	Development	Development	cell wall modification	P	AT2G47040
2243	2	L4 vs. M4	L4 Up	GO:0042545	Development	Development	cell wall modification	P	AT3G62170
2244	2	L4 vs. M4	L4 Up	GO:0042545	Development	Development	cell wall modification	P	AT5G07410
2245	2	L4 vs. M4	L4 Up	GO:0071555	Development	Development	cell wall organization	P	AT1G02460
2246	2	L4 vs. M4	L4 Up	GO:0071555	Development	Development	cell wall organization	P	AT1G02800
2247	2	L4 vs. M4	L4 Up	GO:0071555	Development	Development	cell wall organization	P	AT1G60590
2248	2	L4 vs. M4	L4 Up	GO:0071555	Development	Development	cell wall organization	P	AT3G07830

2249	2	L4 vs. M4	L4 Up	GO:0071555	Development	Development	cell wall organization	P	AT3G07840
2250	2	L4 vs. M4	L4 Up	GO:0071555	Development	Development	cell wall organization	P	AT3G07850
2251	2	L4 vs. M4	L4 Up	GO:0071555	Development	Development	cell wall organization	P	AT5G48140
2252	2	L4 vs. M4	L4 Up	GO:0005575	Development	Development	cellular_component	C	AT1G62510
2253	2	L4 vs. M4	L4 Up	GO:0005575	Development	Development	cellular_component	C	AT3G28550
2254	2	L4 vs. M4	L4 Up	GO:0005575	Development	Development	cellular_component	C	AT3G54580
2255	2	L4 vs. M4	L4 Up	GO:0005575	Development	Development	cellular_component	C	AT5G08250
2256	2	L4 vs. M4	L4 Up	GO:0005575	Development	Development	cellular_component	C	AT5G19810
2257	2	L4 vs. M4	L4 Up	GO:0005575	Development	Development	cellular_component	C	AT5G59170
2258	2	L4 vs. M4	L4 Up	GO:0042807	Development	Development	central vacuole	C	AT3G16240
2259	2	L4 vs. M4	L4 Up	GO:0006325	Development	Development	chromatin organization	P	AT1G04950
2260	2	L4 vs. M4	L4 Up	GO:0005801	Development	Development	cis-Golgi network	C	AT1G15880
2261	2	L4 vs. M4	L4 Up	GO:0043622	Development	Development	cortical microtubule organization	P	AT2G20190
2262	2	L4 vs. M4	L4 Up	GO:0048825	Development	Development	cotyledon development	P	AT1G64520
2263	2	L4 vs. M4	L4 Up	GO:0010588	Development	Development	cotyledon vascular tissue pattern formation	P	AT2G21050
2264	2	L4 vs. M4	L4 Up	GO:0042335	Development	Development	cuticle development	P	AT2G46720
2265	2	L4 vs. M4	L4 Up	GO:0042335	Development	Development	cuticle development	P	AT5G49070
2266	2	L4 vs. M4	L4 Up	GO:0005737	Development	Development	cytoplasm	C	AT1G01780
2267	2	L4 vs. M4	L4 Up	GO:0005737	Development	Development	cytoplasm	C	AT1G04170
2268	2	L4 vs. M4	L4 Up	GO:0005737	Development	Development	cytoplasm	C	AT1G07890
2269	2	L4 vs. M4	L4 Up	GO:0005737	Development	Development	cytoplasm	C	AT1G14880
2270	2	L4 vs. M4	L4 Up	GO:0005737	Development	Development	cytoplasm	C	AT1G16470
2271	2	L4 vs. M4	L4 Up	GO:0005737	Development	Development	cytoplasm	C	AT1G27450
2272	2	L4 vs. M4	L4 Up	GO:0005737	Development	Development	cytoplasm	C	AT1G27970
2273	2	L4 vs. M4	L4 Up	GO:0005737	Development	Development	cytoplasm	C	AT1G34380
2274	2	L4 vs. M4	L4 Up	GO:0005737	Development	Development	cytoplasm	C	AT1G60140
2275	2	L4 vs. M4	L4 Up	GO:0005737	Development	Development	cytoplasm	C	AT1G62940
2276	2	L4 vs. M4	L4 Up	GO:0005737	Development	Development	cytoplasm	C	AT1G68500
2277	2	L4 vs. M4	L4 Up	GO:0005737	Development	Development	cytoplasm	C	AT1G78020
2278	2	L4 vs. M4	L4 Up	GO:0005737	Development	Development	cytoplasm	C	AT1G78300
2279	2	L4 vs. M4	L4 Up	GO:0005737	Development	Development	cytoplasm	C	AT1G79210
2280	2	L4 vs. M4	L4 Up	GO:0005737	Development	Development	cytoplasm	C	AT2G20190
2281	2	L4 vs. M4	L4 Up	GO:0005737	Development	Development	cytoplasm	C	AT2G21660
2282	2	L4 vs. M4	L4 Up	GO:0005737	Development	Development	cytoplasm	C	AT2G25490
2283	2	L4 vs. M4	L4 Up	GO:0005737	Development	Development	cytoplasm	C	AT2G25900
2284	2	L4 vs. M4	L4 Up	GO:0005737	Development	Development	cytoplasm	C	AT2G34555
2285	2	L4 vs. M4	L4 Up	GO:0005737	Development	Development	cytoplasm	C	AT2G38310
2286	2	L4 vs. M4	L4 Up	GO:0005737	Development	Development	cytoplasm	C	AT2G39460
2287	2	L4 vs. M4	L4 Up	GO:0005737	Development	Development	cytoplasm	C	AT2G39770
2288	2	L4 vs. M4	L4 Up	GO:0005737	Development	Development	cytoplasm	C	AT2G41970
2289	2	L4 vs. M4	L4 Up	GO:0005737	Development	Development	cytoplasm	C	AT2G46225
2290	2	L4 vs. M4	L4 Up	GO:0005737	Development	Development	cytoplasm	C	AT2G46720
2291	2	L4 vs. M4	L4 Up	GO:0005737	Development	Development	cytoplasm	C	AT2G46880
2292	2	L4 vs. M4	L4 Up	GO:0005737	Development	Development	cytoplasm	C	AT3G01500
2293	2	L4 vs. M4	L4 Up	GO:0005737	Development	Development	cytoplasm	C	AT3G07880
2294	2	L4 vs. M4	L4 Up	GO:0005737	Development	Development	cytoplasm	C	AT3G07890
2295	2	L4 vs. M4	L4 Up	GO:0005737	Development	Development	cytoplasm	C	AT3G11050
2296	2	L4 vs. M4	L4 Up	GO:0005737	Development	Development	cytoplasm	C	AT3G22960
2297	2	L4 vs. M4	L4 Up	GO:0005737	Development	Development	cytoplasm	C	AT3G44680
2298	2	L4 vs. M4	L4 Up	GO:0005737	Development	Development	cytoplasm	C	AT3G52580
2299	2	L4 vs. M4	L4 Up	GO:0005737	Development	Development	cytoplasm	C	AT3G55970
2300	2	L4 vs. M4	L4 Up	GO:0005737	Development	Development	cytoplasm	C	AT3G56090
2301	2	L4 vs. M4	L4 Up	GO:0005737	Development	Development	cytoplasm	C	AT4G02250
2302	2	L4 vs. M4	L4 Up	GO:0005737	Development	Development	cytoplasm	C	AT4G05050
2303	2	L4 vs. M4	L4 Up	GO:0005737	Development	Development	cytoplasm	C	AT4G14930
2304	2	L4 vs. M4	L4 Up	GO:0005737	Development	Development	cytoplasm	C	AT4G25100

2305	2	L4 vs. M4	L4 Up	GO:0005737	Development	Development	cytoplasm	C	AT4G25590
2306	2	L4 vs. M4	L4 Up	GO:0005737	Development	Development	cytoplasm	C	AT4G26260
2307	2	L4 vs. M4	L4 Up	GO:0005737	Development	Development	cytoplasm	C	AT4G29340
2308	2	L4 vs. M4	L4 Up	GO:0005737	Development	Development	cytoplasm	C	AT4G37610
2309	2	L4 vs. M4	L4 Up	GO:0005737	Development	Development	cytoplasm	C	AT5G01600
2310	2	L4 vs. M4	L4 Up	GO:0005737	Development	Development	cytoplasm	C	AT5G02840
2311	2	L4 vs. M4	L4 Up	GO:0005737	Development	Development	cytoplasm	C	AT5G04180
2312	2	L4 vs. M4	L4 Up	GO:0005737	Development	Development	cytoplasm	C	AT5G04800
2313	2	L4 vs. M4	L4 Up	GO:0005737	Development	Development	cytoplasm	C	AT5G09550
2314	2	L4 vs. M4	L4 Up	GO:0005737	Development	Development	cytoplasm	C	AT5G13350
2315	2	L4 vs. M4	L4 Up	GO:0005737	Development	Development	cytoplasm	C	AT5G16650
2316	2	L4 vs. M4	L4 Up	GO:0005737	Development	Development	cytoplasm	C	AT5G16970
2317	2	L4 vs. M4	L4 Up	GO:0005737	Development	Development	cytoplasm	C	AT5G20250
2318	2	L4 vs. M4	L4 Up	GO:0005737	Development	Development	cytoplasm	C	AT5G21170
2319	2	L4 vs. M4	L4 Up	GO:0005737	Development	Development	cytoplasm	C	AT5G52360
2320	2	L4 vs. M4	L4 Up	GO:0005737	Development	Development	cytoplasm	C	AT5G54160
2321	2	L4 vs. M4	L4 Up	GO:0005737	Development	Development	cytoplasm	C	AT5G54750
2322	2	L4 vs. M4	L4 Up	GO:0005737	Development	Development	cytoplasm	C	AT5G56640
2323	2	L4 vs. M4	L4 Up	GO:0005856	Development	Development	cytoskeleton	C	AT2G46225
2324	2	L4 vs. M4	L4 Up	GO:0005856	Development	Development	cytoskeleton	C	AT4G29340
2325	2	L4 vs. M4	L4 Up	GO:0005829	Development	Development	cytosol	C	AT1G04170
2326	2	L4 vs. M4	L4 Up	GO:0005829	Development	Development	cytosol	C	AT1G07890
2327	2	L4 vs. M4	L4 Up	GO:0005829	Development	Development	cytosol	C	AT1G13930
2328	2	L4 vs. M4	L4 Up	GO:0005829	Development	Development	cytosol	C	AT1G16470
2329	2	L4 vs. M4	L4 Up	GO:0005829	Development	Development	cytosol	C	AT1G27450
2330	2	L4 vs. M4	L4 Up	GO:0005829	Development	Development	cytosol	C	AT1G27970
2331	2	L4 vs. M4	L4 Up	GO:0005829	Development	Development	cytosol	C	AT1G30820
2332	2	L4 vs. M4	L4 Up	GO:0005829	Development	Development	cytosol	C	AT1G35580
2333	2	L4 vs. M4	L4 Up	GO:0005829	Development	Development	cytosol	C	AT1G60140
2334	2	L4 vs. M4	L4 Up	GO:0005829	Development	Development	cytosol	C	AT1G64230
2335	2	L4 vs. M4	L4 Up	GO:0005829	Development	Development	cytosol	C	AT1G64520
2336	2	L4 vs. M4	L4 Up	GO:0005829	Development	Development	cytosol	C	AT1G78020
2337	2	L4 vs. M4	L4 Up	GO:0005829	Development	Development	cytosol	C	AT1G78300
2338	2	L4 vs. M4	L4 Up	GO:0005829	Development	Development	cytosol	C	AT1G79210
2339	2	L4 vs. M4	L4 Up	GO:0005829	Development	Development	cytosol	C	AT2G21660
2340	2	L4 vs. M4	L4 Up	GO:0005829	Development	Development	cytosol	C	AT2G35370
2341	2	L4 vs. M4	L4 Up	GO:0005829	Development	Development	cytosol	C	AT2G39460
2342	2	L4 vs. M4	L4 Up	GO:0005829	Development	Development	cytosol	C	AT2G39770
2343	2	L4 vs. M4	L4 Up	GO:0005829	Development	Development	cytosol	C	AT2G43400
2344	2	L4 vs. M4	L4 Up	GO:0005829	Development	Development	cytosol	C	AT2G46540
2345	2	L4 vs. M4	L4 Up	GO:0005829	Development	Development	cytosol	C	AT3G01500
2346	2	L4 vs. M4	L4 Up	GO:0005829	Development	Development	cytosol	C	AT3G01740
2347	2	L4 vs. M4	L4 Up	GO:0005829	Development	Development	cytosol	C	AT3G07880
2348	2	L4 vs. M4	L4 Up	GO:0005829	Development	Development	cytosol	C	AT3G12500
2349	2	L4 vs. M4	L4 Up	GO:0005829	Development	Development	cytosol	C	AT3G13750
2350	2	L4 vs. M4	L4 Up	GO:0005829	Development	Development	cytosol	C	AT3G26935
2351	2	L4 vs. M4	L4 Up	GO:0005829	Development	Development	cytosol	C	AT3G47470
2352	2	L4 vs. M4	L4 Up	GO:0005829	Development	Development	cytosol	C	AT3G52580
2353	2	L4 vs. M4	L4 Up	GO:0005829	Development	Development	cytosol	C	AT3G52840
2354	2	L4 vs. M4	L4 Up	GO:0005829	Development	Development	cytosol	C	AT3G56090
2355	2	L4 vs. M4	L4 Up	GO:0005829	Development	Development	cytosol	C	AT3G62580
2356	2	L4 vs. M4	L4 Up	GO:0005829	Development	Development	cytosol	C	AT4G05050
2357	2	L4 vs. M4	L4 Up	GO:0005829	Development	Development	cytosol	C	AT4G05180
2358	2	L4 vs. M4	L4 Up	GO:0005829	Development	Development	cytosol	C	AT4G13560
2359	2	L4 vs. M4	L4 Up	GO:0005829	Development	Development	cytosol	C	AT4G14930
2360	2	L4 vs. M4	L4 Up	GO:0005829	Development	Development	cytosol	C	AT4G22930

2361	2	L4 vs. M4	L4 Up	GO:0005829	Development	Development	cytosol	C	AT4G24920
2362	2	L4 vs. M4	L4 Up	GO:0005829	Development	Development	cytosol	C	AT4G25100
2363	2	L4 vs. M4	L4 Up	GO:0005829	Development	Development	cytosol	C	AT4G26910
2364	2	L4 vs. M4	L4 Up	GO:0005829	Development	Development	cytosol	C	AT4G37550
2365	2	L4 vs. M4	L4 Up	GO:0005829	Development	Development	cytosol	C	AT5G02100
2366	2	L4 vs. M4	L4 Up	GO:0005829	Development	Development	cytosol	C	AT5G08160
2367	2	L4 vs. M4	L4 Up	GO:0005829	Development	Development	cytosol	C	AT5G15200
2368	2	L4 vs. M4	L4 Up	GO:0005829	Development	Development	cytosol	C	AT5G16970
2369	2	L4 vs. M4	L4 Up	GO:0005829	Development	Development	cytosol	C	AT5G20250
2370	2	L4 vs. M4	L4 Up	GO:0005829	Development	Development	cytosol	C	AT5G39850
2371	2	L4 vs. M4	L4 Up	GO:0005829	Development	Development	cytosol	C	AT5G45620
2372	2	L4 vs. M4	L4 Up	GO:0005829	Development	Development	cytosol	C	AT5G54160
2373	2	L4 vs. M4	L4 Up	GO:0005829	Development	Development	cytosol	C	AT5G63190
2374	2	L4 vs. M4	L4 Up	GO:0009567	Development	Development	double fertilization forming a zygote and endosperm	P	AT2G01110
2375	2	L4 vs. M4	L4 Up	GO:0009567	Development	Development	double fertilization forming a zygote and endosperm	P	AT3G21180
2376	2	L4 vs. M4	L4 Up	GO:0009567	Development	Development	double fertilization forming a zygote and endosperm	P	AT4G12620
2377	2	L4 vs. M4	L4 Up	GO:0009567	Development	Development	double fertilization forming a zygote and endosperm	P	AT4G13560
2378	2	L4 vs. M4	L4 Up	GO:0009567	Development	Development	double fertilization forming a zygote and endosperm	P	AT5G02100
2379	2	L4 vs. M4	L4 Up	GO:0009793	Development	Development	embryo development ending in seed dormancy	P	AT1G07890
2380	2	L4 vs. M4	L4 Up	GO:0009793	Development	Development	embryo development ending in seed dormancy	P	AT1G61110
2381	2	L4 vs. M4	L4 Up	GO:0009793	Development	Development	embryo development ending in seed dormancy	P	AT5G23660
2382	2	L4 vs. M4	L4 Up	GO:0007029	Development	Development	endoplasmic reticulum organization	P	AT1G54030
2383	2	L4 vs. M4	L4 Up	GO:0044432	Development	Development	endoplasmic reticulum part	C	AT1G78020
2384	2	L4 vs. M4	L4 Up	GO:0005793	Development	Development	endoplasmic reticulum-Golgi intermediate compartment	C	AT1G11890
2385	2	L4 vs. M4	L4 Up	GO:0010168	Development	Development	ER body	C	AT1G54030
2386	2	L4 vs. M4	L4 Up	GO:0031012	Development	Development	extracellular matrix	C	AT1G72610
2387	2	L4 vs. M4	L4 Up	GO:0031012	Development	Development	extracellular matrix	C	AT5G20630
2388	2	L4 vs. M4	L4 Up	GO:0005576	Development	Development	extracellular region	C	AT1G02305
2389	2	L4 vs. M4	L4 Up	GO:0005576	Development	Development	extracellular region	C	AT1G02460
2390	2	L4 vs. M4	L4 Up	GO:0005576	Development	Development	extracellular region	C	AT1G02800
2391	2	L4 vs. M4	L4 Up	GO:0005576	Development	Development	extracellular region	C	AT1G04170
2392	2	L4 vs. M4	L4 Up	GO:0005576	Development	Development	extracellular region	C	AT1G07890
2393	2	L4 vs. M4	L4 Up	GO:0005576	Development	Development	extracellular region	C	AT1G11890
2394	2	L4 vs. M4	L4 Up	GO:0005576	Development	Development	extracellular region	C	AT1G13930
2395	2	L4 vs. M4	L4 Up	GO:0005576	Development	Development	extracellular region	C	AT1G14880
2396	2	L4 vs. M4	L4 Up	GO:0005576	Development	Development	extracellular region	C	AT1G28270
2397	2	L4 vs. M4	L4 Up	GO:0005576	Development	Development	extracellular region	C	AT1G32540
2398	2	L4 vs. M4	L4 Up	GO:0005576	Development	Development	extracellular region	C	AT1G50170
2399	2	L4 vs. M4	L4 Up	GO:0005576	Development	Development	extracellular region	C	AT1G55570
2400	2	L4 vs. M4	L4 Up	GO:0005576	Development	Development	extracellular region	C	AT1G56360
2401	2	L4 vs. M4	L4 Up	GO:0005576	Development	Development	extracellular region	C	AT1G60590
2402	2	L4 vs. M4	L4 Up	GO:0005576	Development	Development	extracellular region	C	AT1G66850
2403	2	L4 vs. M4	L4 Up	GO:0005576	Development	Development	extracellular region	C	AT1G67290
2404	2	L4 vs. M4	L4 Up	GO:0005576	Development	Development	extracellular region	C	AT1G69295
2405	2	L4 vs. M4	L4 Up	GO:0005576	Development	Development	extracellular region	C	AT1G71695
2406	2	L4 vs. M4	L4 Up	GO:0005576	Development	Development	extracellular region	C	AT1G72260
2407	2	L4 vs. M4	L4 Up	GO:0005576	Development	Development	extracellular region	C	AT1G72610
2408	2	L4 vs. M4	L4 Up	GO:0005576	Development	Development	extracellular region	C	AT2G19000
2409	2	L4 vs. M4	L4 Up	GO:0005576	Development	Development	extracellular region	C	AT2G19980
2410	2	L4 vs. M4	L4 Up	GO:0005576	Development	Development	extracellular region	C	AT2G21140

2411	2	L4 vs. M4	L4 Up	GO:0005576	Development	Development	extracellular region	C	AT2G23010
2412	2	L4 vs. M4	L4 Up	GO:0005576	Development	Development	extracellular region	C	AT2G45180
2413	2	L4 vs. M4	L4 Up	GO:0005576	Development	Development	extracellular region	C	AT2G46880
2414	2	L4 vs. M4	L4 Up	GO:0005576	Development	Development	extracellular region	C	AT2G47040
2415	2	L4 vs. M4	L4 Up	GO:0005576	Development	Development	extracellular region	C	AT3G06100
2416	2	L4 vs. M4	L4 Up	GO:0005576	Development	Development	extracellular region	C	AT3G07830
2417	2	L4 vs. M4	L4 Up	GO:0005576	Development	Development	extracellular region	C	AT3G07840
2418	2	L4 vs. M4	L4 Up	GO:0005576	Development	Development	extracellular region	C	AT3G07850
2419	2	L4 vs. M4	L4 Up	GO:0005576	Development	Development	extracellular region	C	AT3G12500
2420	2	L4 vs. M4	L4 Up	GO:0005576	Development	Development	extracellular region	C	AT3G13390
2421	2	L4 vs. M4	L4 Up	GO:0005576	Development	Development	extracellular region	C	AT3G13400
2422	2	L4 vs. M4	L4 Up	GO:0005576	Development	Development	extracellular region	C	AT3G13750
2423	2	L4 vs. M4	L4 Up	GO:0005576	Development	Development	extracellular region	C	AT3G48050
2424	2	L4 vs. M4	L4 Up	GO:0005576	Development	Development	extracellular region	C	AT3G51070
2425	2	L4 vs. M4	L4 Up	GO:0005576	Development	Development	extracellular region	C	AT3G51590
2426	2	L4 vs. M4	L4 Up	GO:0005576	Development	Development	extracellular region	C	AT3G52840
2427	2	L4 vs. M4	L4 Up	GO:0005576	Development	Development	extracellular region	C	AT3G62170
2428	2	L4 vs. M4	L4 Up	GO:0005576	Development	Development	extracellular region	C	AT4G25780
2429	2	L4 vs. M4	L4 Up	GO:0005576	Development	Development	extracellular region	C	AT4G35010
2430	2	L4 vs. M4	L4 Up	GO:0005576	Development	Development	extracellular region	C	AT5G07410
2431	2	L4 vs. M4	L4 Up	GO:0005576	Development	Development	extracellular region	C	AT5G07550
2432	2	L4 vs. M4	L4 Up	GO:0005576	Development	Development	extracellular region	C	AT5G15110
2433	2	L4 vs. M4	L4 Up	GO:0005576	Development	Development	extracellular region	C	AT5G19580
2434	2	L4 vs. M4	L4 Up	GO:0005576	Development	Development	extracellular region	C	AT5G20630
2435	2	L4 vs. M4	L4 Up	GO:0005576	Development	Development	extracellular region	C	AT5G23660
2436	2	L4 vs. M4	L4 Up	GO:0005576	Development	Development	extracellular region	C	AT5G26700
2437	2	L4 vs. M4	L4 Up	GO:0005576	Development	Development	extracellular region	C	AT5G36910
2438	2	L4 vs. M4	L4 Up	GO:0005576	Development	Development	extracellular region	C	AT5G45650
2439	2	L4 vs. M4	L4 Up	GO:0005576	Development	Development	extracellular region	C	AT5G45880
2440	2	L4 vs. M4	L4 Up	GO:0005576	Development	Development	extracellular region	C	AT5G48140
2441	2	L4 vs. M4	L4 Up	GO:0005576	Development	Development	extracellular region	C	AT5G50800
2442	2	L4 vs. M4	L4 Up	GO:0005615	Development	Development	extracellular space	C	AT1G02305
2443	2	L4 vs. M4	L4 Up	GO:0005615	Development	Development	extracellular space	C	AT2G19980
2444	2	L4 vs. M4	L4 Up	GO:0005615	Development	Development	extracellular space	C	AT4G25780
2445	2	L4 vs. M4	L4 Up	GO:0005615	Development	Development	extracellular space	C	AT5G45880
2446	2	L4 vs. M4	L4 Up	GO:0009908	Development	Development	flower development	P	AT1G64520
2447	2	L4 vs. M4	L4 Up	GO:0009908	Development	Development	flower development	P	AT3G11050
2448	2	L4 vs. M4	L4 Up	GO:0009908	Development	Development	flower development	P	AT3G56090
2449	2	L4 vs. M4	L4 Up	GO:0009908	Development	Development	flower development	P	AT4G24960
2450	2	L4 vs. M4	L4 Up	GO:0009908	Development	Development	flower development	P	AT5G01600
2451	2	L4 vs. M4	L4 Up	GO:0005797	Development	Development	Golgi medial cisterna	C	AT1G15880
2452	2	L4 vs. M4	L4 Up	GO:0000138	Development	Development	Golgi trans cisterna	C	AT3G25140
2453	2	L4 vs. M4	L4 Up	GO:0016021	Development	Development	integral component of membrane	C	AT1G11890
2454	2	L4 vs. M4	L4 Up	GO:0016021	Development	Development	integral component of membrane	C	AT1G13150
2455	2	L4 vs. M4	L4 Up	GO:0016021	Development	Development	integral component of membrane	C	AT1G14880
2456	2	L4 vs. M4	L4 Up	GO:0016021	Development	Development	integral component of membrane	C	AT1G15880
2457	2	L4 vs. M4	L4 Up	GO:0016021	Development	Development	integral component of membrane	C	AT1G24400
2458	2	L4 vs. M4	L4 Up	GO:0016021	Development	Development	integral component of membrane	C	AT1G29930
2459	2	L4 vs. M4	L4 Up	GO:0016021	Development	Development	integral component of membrane	C	AT1G48460
2460	2	L4 vs. M4	L4 Up	GO:0016021	Development	Development	integral component of membrane	C	AT1G52220
2461	2	L4 vs. M4	L4 Up	GO:0016021	Development	Development	integral component of membrane	C	AT1G59740
2462	2	L4 vs. M4	L4 Up	GO:0016021	Development	Development	integral component of membrane	C	AT1G67600
2463	2	L4 vs. M4	L4 Up	GO:0016021	Development	Development	integral component of membrane	C	AT1G68820
2464	2	L4 vs. M4	L4 Up	GO:0016021	Development	Development	integral component of membrane	C	AT1G74520
2465	2	L4 vs. M4	L4 Up	GO:0016021	Development	Development	integral component of membrane	C	AT1G74540
2466	2	L4 vs. M4	L4 Up	GO:0016021	Development	Development	integral component of membrane	C	AT2G02510

2467	2	L4 vs. M4	L4 Up	GO:0016021	Development	Development	integral component of membrane	C	AT2G07560
2468	2	L4 vs. M4	L4 Up	GO:0016021	Development	Development	integral component of membrane	C	AT2G19980
2469	2	L4 vs. M4	L4 Up	GO:0016021	Development	Development	integral component of membrane	C	AT2G21050
2470	2	L4 vs. M4	L4 Up	GO:0016021	Development	Development	integral component of membrane	C	AT2G34430
2471	2	L4 vs. M4	L4 Up	GO:0016021	Development	Development	integral component of membrane	C	AT2G38330
2472	2	L4 vs. M4	L4 Up	GO:0016021	Development	Development	integral component of membrane	C	AT2G46210
2473	2	L4 vs. M4	L4 Up	GO:0016021	Development	Development	integral component of membrane	C	AT2G46720
2474	2	L4 vs. M4	L4 Up	GO:0016021	Development	Development	integral component of membrane	C	AT3G03860
2475	2	L4 vs. M4	L4 Up	GO:0016021	Development	Development	integral component of membrane	C	AT3G05890
2476	2	L4 vs. M4	L4 Up	GO:0016021	Development	Development	integral component of membrane	C	AT3G06100
2477	2	L4 vs. M4	L4 Up	GO:0016021	Development	Development	integral component of membrane	C	AT3G08040
2478	2	L4 vs. M4	L4 Up	GO:0016021	Development	Development	integral component of membrane	C	AT3G16240
2479	2	L4 vs. M4	L4 Up	GO:0016021	Development	Development	integral component of membrane	C	AT3G21180
2480	2	L4 vs. M4	L4 Up	GO:0016021	Development	Development	integral component of membrane	C	AT3G25140
2481	2	L4 vs. M4	L4 Up	GO:0016021	Development	Development	integral component of membrane	C	AT3G26935
2482	2	L4 vs. M4	L4 Up	GO:0016021	Development	Development	integral component of membrane	C	AT3G47470
2483	2	L4 vs. M4	L4 Up	GO:0016021	Development	Development	integral component of membrane	C	AT3G50580
2484	2	L4 vs. M4	L4 Up	GO:0016021	Development	Development	integral component of membrane	C	AT3G51070
2485	2	L4 vs. M4	L4 Up	GO:0016021	Development	Development	integral component of membrane	C	AT4G18230
2486	2	L4 vs. M4	L4 Up	GO:0016021	Development	Development	integral component of membrane	C	AT4G20870
2487	2	L4 vs. M4	L4 Up	GO:0016021	Development	Development	integral component of membrane	C	AT4G24960
2488	2	L4 vs. M4	L4 Up	GO:0016021	Development	Development	integral component of membrane	C	AT4G25040
2489	2	L4 vs. M4	L4 Up	GO:0016021	Development	Development	integral component of membrane	C	AT4G30650
2490	2	L4 vs. M4	L4 Up	GO:0016021	Development	Development	integral component of membrane	C	AT5G01530
2491	2	L4 vs. M4	L4 Up	GO:0016021	Development	Development	integral component of membrane	C	AT5G05740
2492	2	L4 vs. M4	L4 Up	GO:0016021	Development	Development	integral component of membrane	C	AT5G07550
2493	2	L4 vs. M4	L4 Up	GO:0016021	Development	Development	integral component of membrane	C	AT5G17170
2494	2	L4 vs. M4	L4 Up	GO:0016021	Development	Development	integral component of membrane	C	AT5G23660
2495	2	L4 vs. M4	L4 Up	GO:0016021	Development	Development	integral component of membrane	C	AT5G42000
2496	2	L4 vs. M4	L4 Up	GO:0016021	Development	Development	integral component of membrane	C	AT5G49070
2497	2	L4 vs. M4	L4 Up	GO:0016021	Development	Development	integral component of membrane	C	AT5G50800
2498	2	L4 vs. M4	L4 Up	GO:0016021	Development	Development	integral component of membrane	C	AT5G53190
2499	2	L4 vs. M4	L4 Up	GO:0016021	Development	Development	integral component of membrane	C	ATCG00700
2500	2	L4 vs. M4	L4 Up	GO:0016021	Development	Development	integral component of membrane	C	ATMG01360
2501	2	L4 vs. M4	L4 Up	GO:0005887	Development	Development	integral component of plasma membrane	C	AT5G23660
2502	2	L4 vs. M4	L4 Up	GO:0005887	Development	Development	integral component of plasma membrane	C	AT5G50800
2503	2	L4 vs. M4	L4 Up	GO:0005887	Development	Development	integral component of plasma membrane	C	AT5G53190
2504	2	L4 vs. M4	L4 Up	GO:0005622	Development	Development	intracellular	C	AT1G54030
2505	2	L4 vs. M4	L4 Up	GO:0005622	Development	Development	intracellular	C	AT3G12500
2506	2	L4 vs. M4	L4 Up	GO:0005622	Development	Development	intracellular	C	AT4G05050
2507	2	L4 vs. M4	L4 Up	GO:0005622	Development	Development	intracellular	C	AT4G24920
2508	2	L4 vs. M4	L4 Up	GO:0005622	Development	Development	intracellular	C	AT5G54750
2509	2	L4 vs. M4	L4 Up	GO:0043231	Development	Development	intracellular membrane-bounded organelle	C	AT2G33380
2510	2	L4 vs. M4	L4 Up	GO:0043231	Development	Development	intracellular membrane-bounded organelle	C	AT5G02100
2511	2	L4 vs. M4	L4 Up	GO:0048366	Development	Development	leaf development	P	AT1G64520
2512	2	L4 vs. M4	L4 Up	GO:0048366	Development	Development	leaf development	P	AT3G11050
2513	2	L4 vs. M4	L4 Up	GO:0048366	Development	Development	leaf development	P	AT3G56090
2514	2	L4 vs. M4	L4 Up	GO:0048366	Development	Development	leaf development	P	AT5G01600
2515	2	L4 vs. M4	L4 Up	GO:0016020	Development	Development	membrane	C	AT1G35580
2516	2	L4 vs. M4	L4 Up	GO:0016020	Development	Development	membrane	C	AT3G07880
2517	2	L4 vs. M4	L4 Up	GO:0016020	Development	Development	membrane	C	AT3G21180
2518	2	L4 vs. M4	L4 Up	GO:0016020	Development	Development	membrane	C	AT5G02100
2519	2	L4 vs. M4	L4 Up	GO:0016020	Development	Development	membrane	C	AT5G55990
2520	2	L4 vs. M4	L4 Up	GO:0012511	Development	Development	monolayer-surrounded lipid storage body	C	AT2G33380
2521	2	L4 vs. M4	L4 Up	GO:0012511	Development	Development	monolayer-surrounded lipid storage body	C	AT5G07550
2522	2	L4 vs. M4	L4 Up	GO:0007275	Development	Development	multicellular organism development	P	AT1G67290

2523	2	L4 vs. M4	L4 Up	GO:0000784	Development	Development	nuclear chromosome, telomeric region	C	AT4G12620
2524	2	L4 vs. M4	L4 Up	GO:0005635	Development	Development	nuclear envelope	C	AT1G27970
2525	2	L4 vs. M4	L4 Up	GO:0005654	Development	Development	nucleoplasm	C	AT5G16970
2526	2	L4 vs. M4	L4 Up	GO:0005634	Development	Development	nucleus	C	AT1G04950
2527	2	L4 vs. M4	L4 Up	GO:0005634	Development	Development	nucleus	C	AT1G14330
2528	2	L4 vs. M4	L4 Up	GO:0005634	Development	Development	nucleus	C	AT1G16290
2529	2	L4 vs. M4	L4 Up	GO:0005634	Development	Development	nucleus	C	AT1G16470
2530	2	L4 vs. M4	L4 Up	GO:0005634	Development	Development	nucleus	C	AT1G26610
2531	2	L4 vs. M4	L4 Up	GO:0005634	Development	Development	nucleus	C	AT1G27450
2532	2	L4 vs. M4	L4 Up	GO:0005634	Development	Development	nucleus	C	AT1G27970
2533	2	L4 vs. M4	L4 Up	GO:0005634	Development	Development	nucleus	C	AT1G29930
2534	2	L4 vs. M4	L4 Up	GO:0005634	Development	Development	nucleus	C	AT1G32540
2535	2	L4 vs. M4	L4 Up	GO:0005634	Development	Development	nucleus	C	AT1G34310
2536	2	L4 vs. M4	L4 Up	GO:0005634	Development	Development	nucleus	C	AT1G34380
2537	2	L4 vs. M4	L4 Up	GO:0005634	Development	Development	nucleus	C	AT1G35580
2538	2	L4 vs. M4	L4 Up	GO:0005634	Development	Development	nucleus	C	AT1G49010
2539	2	L4 vs. M4	L4 Up	GO:0005634	Development	Development	nucleus	C	AT1G52220
2540	2	L4 vs. M4	L4 Up	GO:0005634	Development	Development	nucleus	C	AT1G54080
2541	2	L4 vs. M4	L4 Up	GO:0005634	Development	Development	nucleus	C	AT1G61110
2542	2	L4 vs. M4	L4 Up	GO:0005634	Development	Development	nucleus	C	AT1G64230
2543	2	L4 vs. M4	L4 Up	GO:0005634	Development	Development	nucleus	C	AT1G64520
2544	2	L4 vs. M4	L4 Up	GO:0005634	Development	Development	nucleus	C	AT1G67520
2545	2	L4 vs. M4	L4 Up	GO:0005634	Development	Development	nucleus	C	AT1G68200
2546	2	L4 vs. M4	L4 Up	GO:0005634	Development	Development	nucleus	C	AT1G68820
2547	2	L4 vs. M4	L4 Up	GO:0005634	Development	Development	nucleus	C	AT1G74520
2548	2	L4 vs. M4	L4 Up	GO:0005634	Development	Development	nucleus	C	AT1G78020
2549	2	L4 vs. M4	L4 Up	GO:0005634	Development	Development	nucleus	C	AT1G78300
2550	2	L4 vs. M4	L4 Up	GO:0005634	Development	Development	nucleus	C	AT1G79210
2551	2	L4 vs. M4	L4 Up	GO:0005634	Development	Development	nucleus	C	AT1G79700
2552	2	L4 vs. M4	L4 Up	GO:0005634	Development	Development	nucleus	C	AT1G80920
2553	2	L4 vs. M4	L4 Up	GO:0005634	Development	Development	nucleus	C	AT2G18160
2554	2	L4 vs. M4	L4 Up	GO:0005634	Development	Development	nucleus	C	AT2G20670
2555	2	L4 vs. M4	L4 Up	GO:0005634	Development	Development	nucleus	C	AT2G21660
2556	2	L4 vs. M4	L4 Up	GO:0005634	Development	Development	nucleus	C	AT2G21880
2557	2	L4 vs. M4	L4 Up	GO:0005634	Development	Development	nucleus	C	AT2G25490
2558	2	L4 vs. M4	L4 Up	GO:0005634	Development	Development	nucleus	C	AT2G25900
2559	2	L4 vs. M4	L4 Up	GO:0005634	Development	Development	nucleus	C	AT2G35370
2560	2	L4 vs. M4	L4 Up	GO:0005634	Development	Development	nucleus	C	AT2G38310
2561	2	L4 vs. M4	L4 Up	GO:0005634	Development	Development	nucleus	C	AT2G39770
2562	2	L4 vs. M4	L4 Up	GO:0005634	Development	Development	nucleus	C	AT2G41830
2563	2	L4 vs. M4	L4 Up	GO:0005634	Development	Development	nucleus	C	AT2G41970
2564	2	L4 vs. M4	L4 Up	GO:0005634	Development	Development	nucleus	C	AT2G42940
2565	2	L4 vs. M4	L4 Up	GO:0005634	Development	Development	nucleus	C	AT3G12040
2566	2	L4 vs. M4	L4 Up	GO:0005634	Development	Development	nucleus	C	AT3G44680
2567	2	L4 vs. M4	L4 Up	GO:0005634	Development	Development	nucleus	C	AT3G48050
2568	2	L4 vs. M4	L4 Up	GO:0005634	Development	Development	nucleus	C	AT3G49640
2569	2	L4 vs. M4	L4 Up	GO:0005634	Development	Development	nucleus	C	AT3G51070
2570	2	L4 vs. M4	L4 Up	GO:0005634	Development	Development	nucleus	C	AT4G05050
2571	2	L4 vs. M4	L4 Up	GO:0005634	Development	Development	nucleus	C	AT4G09830
2572	2	L4 vs. M4	L4 Up	GO:0005634	Development	Development	nucleus	C	AT4G12620
2573	2	L4 vs. M4	L4 Up	GO:0005634	Development	Development	nucleus	C	AT4G19160
2574	2	L4 vs. M4	L4 Up	GO:0005634	Development	Development	nucleus	C	AT4G25590
2575	2	L4 vs. M4	L4 Up	GO:0005634	Development	Development	nucleus	C	AT4G27590
2576	2	L4 vs. M4	L4 Up	GO:0005634	Development	Development	nucleus	C	AT4G28240
2577	2	L4 vs. M4	L4 Up	GO:0005634	Development	Development	nucleus	C	AT4G29340
2578	2	L4 vs. M4	L4 Up	GO:0005634	Development	Development	nucleus	C	AT4G30100

2579	2	L4 vs. M4	L4 Up	GO:0005634	Development	Development	nucleus	C	AT4G35420
2580	2	L4 vs. M4	L4 Up	GO:0005634	Development	Development	nucleus	C	AT4G35770
2581	2	L4 vs. M4	L4 Up	GO:0005634	Development	Development	nucleus	C	AT4G36040
2582	2	L4 vs. M4	L4 Up	GO:0005634	Development	Development	nucleus	C	AT4G37610
2583	2	L4 vs. M4	L4 Up	GO:0005634	Development	Development	nucleus	C	AT4G40000
2584	2	L4 vs. M4	L4 Up	GO:0005634	Development	Development	nucleus	C	AT5G02100
2585	2	L4 vs. M4	L4 Up	GO:0005634	Development	Development	nucleus	C	AT5G02840
2586	2	L4 vs. M4	L4 Up	GO:0005634	Development	Development	nucleus	C	AT5G08160
2587	2	L4 vs. M4	L4 Up	GO:0005634	Development	Development	nucleus	C	AT5G13350
2588	2	L4 vs. M4	L4 Up	GO:0005634	Development	Development	nucleus	C	AT5G13360
2589	2	L4 vs. M4	L4 Up	GO:0005634	Development	Development	nucleus	C	AT5G16970
2590	2	L4 vs. M4	L4 Up	GO:0005634	Development	Development	nucleus	C	AT5G20630
2591	2	L4 vs. M4	L4 Up	GO:0005634	Development	Development	nucleus	C	AT5G25280
2592	2	L4 vs. M4	L4 Up	GO:0005634	Development	Development	nucleus	C	AT5G45620
2593	2	L4 vs. M4	L4 Up	GO:0005634	Development	Development	nucleus	C	AT5G48590
2594	2	L4 vs. M4	L4 Up	GO:0005634	Development	Development	nucleus	C	AT5G52360
2595	2	L4 vs. M4	L4 Up	GO:0005634	Development	Development	nucleus	C	AT5G54160
2596	2	L4 vs. M4	L4 Up	GO:0005634	Development	Development	nucleus	C	AT5G58670
2597	2	L4 vs. M4	L4 Up	GO:0005634	Development	Development	nucleus	C	AT5G59180
2598	2	L4 vs. M4	L4 Up	GO:0005634	Development	Development	nucleus	C	AT5G63190
2599	2	L4 vs. M4	L4 Up	GO:0007389	Development	Development	pattern specification process	P	AT1G02800
2600	2	L4 vs. M4	L4 Up	GO:0009524	Development	Development	phragmoplast	C	AT2G20190
2601	2	L4 vs. M4	L4 Up	GO:0009505	Development	Development	plant-type cell wall	C	AT1G27450
2602	2	L4 vs. M4	L4 Up	GO:0009505	Development	Development	plant-type cell wall	C	AT1G71695
2603	2	L4 vs. M4	L4 Up	GO:0009505	Development	Development	plant-type cell wall	C	AT3G13750
2604	2	L4 vs. M4	L4 Up	GO:0009505	Development	Development	plant-type cell wall	C	AT3G15400
2605	2	L4 vs. M4	L4 Up	GO:0009505	Development	Development	plant-type cell wall	C	AT3G16240
2606	2	L4 vs. M4	L4 Up	GO:0009505	Development	Development	plant-type cell wall	C	AT3G51590
2607	2	L4 vs. M4	L4 Up	GO:0009505	Development	Development	plant-type cell wall	C	AT3G52840
2608	2	L4 vs. M4	L4 Up	GO:0009831	Development	Development	plant-type cell wall modification involved in multidimensional cell growth	P	AT1G61110
2609	2	L4 vs. M4	L4 Up	GO:0009664	Development	Development	plant-type cell wall organization	P	AT2G21140
2610	2	L4 vs. M4	L4 Up	GO:0009705	Development	Development	plant-type vacuole membrane	C	AT1G54030
2611	2	L4 vs. M4	L4 Up	GO:0009705	Development	Development	plant-type vacuole membrane	C	AT3G16240
2612	2	L4 vs. M4	L4 Up	GO:0009705	Development	Development	plant-type vacuole membrane	C	AT5G55990
2613	2	L4 vs. M4	L4 Up	GO:0005886	Development	Development	plasma membrane	C	AT1G01780
2614	2	L4 vs. M4	L4 Up	GO:0005886	Development	Development	plasma membrane	C	AT1G07890
2615	2	L4 vs. M4	L4 Up	GO:0005886	Development	Development	plasma membrane	C	AT1G11890
2616	2	L4 vs. M4	L4 Up	GO:0005886	Development	Development	plasma membrane	C	AT1G13930
2617	2	L4 vs. M4	L4 Up	GO:0005886	Development	Development	plasma membrane	C	AT1G14880
2618	2	L4 vs. M4	L4 Up	GO:0005886	Development	Development	plasma membrane	C	AT1G24400
2619	2	L4 vs. M4	L4 Up	GO:0005886	Development	Development	plasma membrane	C	AT1G27450
2620	2	L4 vs. M4	L4 Up	GO:0005886	Development	Development	plasma membrane	C	AT1G27970
2621	2	L4 vs. M4	L4 Up	GO:0005886	Development	Development	plasma membrane	C	AT1G35580
2622	2	L4 vs. M4	L4 Up	GO:0005886	Development	Development	plasma membrane	C	AT1G54080
2623	2	L4 vs. M4	L4 Up	GO:0005886	Development	Development	plasma membrane	C	AT1G55570
2624	2	L4 vs. M4	L4 Up	GO:0005886	Development	Development	plasma membrane	C	AT1G59740
2625	2	L4 vs. M4	L4 Up	GO:0005886	Development	Development	plasma membrane	C	AT1G64230
2626	2	L4 vs. M4	L4 Up	GO:0005886	Development	Development	plasma membrane	C	AT1G67520
2627	2	L4 vs. M4	L4 Up	GO:0005886	Development	Development	plasma membrane	C	AT1G69295
2628	2	L4 vs. M4	L4 Up	GO:0005886	Development	Development	plasma membrane	C	AT1G78300
2629	2	L4 vs. M4	L4 Up	GO:0005886	Development	Development	plasma membrane	C	AT2G07560
2630	2	L4 vs. M4	L4 Up	GO:0005886	Development	Development	plasma membrane	C	AT2G20190
2631	2	L4 vs. M4	L4 Up	GO:0005886	Development	Development	plasma membrane	C	AT2G21050
2632	2	L4 vs. M4	L4 Up	GO:0005886	Development	Development	plasma membrane	C	AT2G21880
2633	2	L4 vs. M4	L4 Up	GO:0005886	Development	Development	plasma membrane	C	AT2G38310

2634	2	L4 vs. M4	L4 Up	GO:0005886	Development	Development	plasma membrane	C	AT2G41830
2635	2	L4 vs. M4	L4 Up	GO:0005886	Development	Development	plasma membrane	C	AT2G41970
2636	2	L4 vs. M4	L4 Up	GO:0005886	Development	Development	plasma membrane	C	AT2G47040
2637	2	L4 vs. M4	L4 Up	GO:0005886	Development	Development	plasma membrane	C	AT3G01500
2638	2	L4 vs. M4	L4 Up	GO:0005886	Development	Development	plasma membrane	C	AT3G08040
2639	2	L4 vs. M4	L4 Up	GO:0005886	Development	Development	plasma membrane	C	AT3G13390
2640	2	L4 vs. M4	L4 Up	GO:0005886	Development	Development	plasma membrane	C	AT3G13400
2641	2	L4 vs. M4	L4 Up	GO:0005886	Development	Development	plasma membrane	C	AT3G15400
2642	2	L4 vs. M4	L4 Up	GO:0005886	Development	Development	plasma membrane	C	AT3G16240
2643	2	L4 vs. M4	L4 Up	GO:0005886	Development	Development	plasma membrane	C	AT3G20580
2644	2	L4 vs. M4	L4 Up	GO:0005886	Development	Development	plasma membrane	C	AT3G20865
2645	2	L4 vs. M4	L4 Up	GO:0005886	Development	Development	plasma membrane	C	AT3G21180
2646	2	L4 vs. M4	L4 Up	GO:0005886	Development	Development	plasma membrane	C	AT3G26935
2647	2	L4 vs. M4	L4 Up	GO:0005886	Development	Development	plasma membrane	C	AT3G57690
2648	2	L4 vs. M4	L4 Up	GO:0005886	Development	Development	plasma membrane	C	AT4G25040
2649	2	L4 vs. M4	L4 Up	GO:0005886	Development	Development	plasma membrane	C	AT4G25100
2650	2	L4 vs. M4	L4 Up	GO:0005886	Development	Development	plasma membrane	C	AT4G28240
2651	2	L4 vs. M4	L4 Up	GO:0005886	Development	Development	plasma membrane	C	AT4G28940
2652	2	L4 vs. M4	L4 Up	GO:0005886	Development	Development	plasma membrane	C	AT4G37550
2653	2	L4 vs. M4	L4 Up	GO:0005886	Development	Development	plasma membrane	C	AT5G01530
2654	2	L4 vs. M4	L4 Up	GO:0005886	Development	Development	plasma membrane	C	AT5G01600
2655	2	L4 vs. M4	L4 Up	GO:0005886	Development	Development	plasma membrane	C	AT5G02100
2656	2	L4 vs. M4	L4 Up	GO:0005886	Development	Development	plasma membrane	C	AT5G04800
2657	2	L4 vs. M4	L4 Up	GO:0005886	Development	Development	plasma membrane	C	AT5G08000
2658	2	L4 vs. M4	L4 Up	GO:0005886	Development	Development	plasma membrane	C	AT5G11770
2659	2	L4 vs. M4	L4 Up	GO:0005886	Development	Development	plasma membrane	C	AT5G21170
2660	2	L4 vs. M4	L4 Up	GO:0005886	Development	Development	plasma membrane	C	AT5G23660
2661	2	L4 vs. M4	L4 Up	GO:0005886	Development	Development	plasma membrane	C	AT5G40730
2662	2	L4 vs. M4	L4 Up	GO:0005886	Development	Development	plasma membrane	C	AT5G50800
2663	2	L4 vs. M4	L4 Up	GO:0005886	Development	Development	plasma membrane	C	AT5G54160
2664	2	L4 vs. M4	L4 Up	GO:0005886	Development	Development	plasma membrane	C	AT5G58670
2665	2	L4 vs. M4	L4 Up	GO:0009555	Development	Development	pollen development	P	AT3G21180
2666	2	L4 vs. M4	L4 Up	GO:0009555	Development	Development	pollen development	P	AT4G24960
2667	2	L4 vs. M4	L4 Up	GO:0009555	Development	Development	pollen development	P	AT4G35420
2668	2	L4 vs. M4	L4 Up	GO:0009555	Development	Development	pollen development	P	AT5G50800
2669	2	L4 vs. M4	L4 Up	GO:0010584	Development	Development	pollen exine formation	P	AT1G62940
2670	2	L4 vs. M4	L4 Up	GO:0010584	Development	Development	pollen exine formation	P	AT4G35420
2671	2	L4 vs. M4	L4 Up	GO:0090406	Development	Development	pollen tube	C	AT2G47040
2672	2	L4 vs. M4	L4 Up	GO:0090406	Development	Development	pollen tube	C	AT3G62170
2673	2	L4 vs. M4	L4 Up	GO:0090406	Development	Development	pollen tube	C	AT5G07410
2674	2	L4 vs. M4	L4 Up	GO:0010208	Development	Development	pollen wall assembly	P	AT2G42940
2675	2	L4 vs. M4	L4 Up	GO:0048528	Development	Development	post-embryonic root development	P	AT1G64520
2676	2	L4 vs. M4	L4 Up	GO:0000326	Development	Development	protein storage vacuole	C	AT3G16240
2677	2	L4 vs. M4	L4 Up	GO:0048829	Development	Development	root cap development	P	AT2G21050
2678	2	L4 vs. M4	L4 Up	GO:0048364	Development	Development	root development	P	AT1G35580
2679	2	L4 vs. M4	L4 Up	GO:0010053	Development	Development	root epidermal cell differentiation	P	AT3G07880
2680	2	L4 vs. M4	L4 Up	GO:0048768	Development	Development	root hair cell tip growth	P	AT2G41970
2681	2	L4 vs. M4	L4 Up	GO:0031209	Development	Development	SCAR complex	C	AT2G46225
2682	2	L4 vs. M4	L4 Up	GO:0048316	Development	Development	seed development	P	AT4G35420
2683	2	L4 vs. M4	L4 Up	GO:0080112	Development	Development	seed growth	P	AT5G50800
2684	2	L4 vs. M4	L4 Up	GO:0010431	Development	Development	seed maturation	P	AT3G22960
2685	2	L4 vs. M4	L4 Up	GO:0010431	Development	Development	seed maturation	P	AT5G23660
2686	2	L4 vs. M4	L4 Up	GO:0090351	Development	Development	seedling development	P	AT1G78020
2687	2	L4 vs. M4	L4 Up	GO:0009647	Development	Development	skotomorphogenesis	P	AT1G64520
2688	2	L4 vs. M4	L4 Up	GO:0010319	Development	Development	stromule	C	AT3G01500
2689	2	L4 vs. M4	L4 Up	GO:0005198	Development	Development	structural molecule activity	F	AT5G45620

2690	2	L4 vs. M4	L4 Up	GO:0010090	Development	Development	trichome morphogenesis	P	AT2G46225
2691	2	L4 vs. M4	L4 Up	GO:0009826	Development	Development	unidimensional cell growth	P	AT2G20190
2692	2	L4 vs. M4	L4 Up	GO:0005774	Development	Development	vacuolar membrane	C	AT1G78300
2693	2	L4 vs. M4	L4 Up	GO:0005774	Development	Development	vacuolar membrane	C	AT2G21880
2694	2	L4 vs. M4	L4 Up	GO:0005774	Development	Development	vacuolar membrane	C	AT3G12500
2695	2	L4 vs. M4	L4 Up	GO:0005774	Development	Development	vacuolar membrane	C	AT3G16240
2696	2	L4 vs. M4	L4 Up	GO:0005774	Development	Development	vacuolar membrane	C	AT5G14040
2697	2	L4 vs. M4	L4 Up	GO:0005774	Development	Development	vacuolar membrane	C	AT5G15200
2698	2	L4 vs. M4	L4 Up	GO:0005773	Development	Development	vacuole	C	AT1G02305
2699	2	L4 vs. M4	L4 Up	GO:0005773	Development	Development	vacuole	C	AT1G14880
2700	2	L4 vs. M4	L4 Up	GO:0005773	Development	Development	vacuole	C	AT1G16290
2701	2	L4 vs. M4	L4 Up	GO:0005773	Development	Development	vacuole	C	AT1G54030
2702	2	L4 vs. M4	L4 Up	GO:0005773	Development	Development	vacuole	C	AT1G71695
2703	2	L4 vs. M4	L4 Up	GO:0005773	Development	Development	vacuole	C	AT1G78300
2704	2	L4 vs. M4	L4 Up	GO:0005773	Development	Development	vacuole	C	AT2G21660
2705	2	L4 vs. M4	L4 Up	GO:0005773	Development	Development	vacuole	C	AT2G21880
2706	2	L4 vs. M4	L4 Up	GO:0005773	Development	Development	vacuole	C	AT2G25830
2707	2	L4 vs. M4	L4 Up	GO:0005773	Development	Development	vacuole	C	AT2G33380
2708	2	L4 vs. M4	L4 Up	GO:0005773	Development	Development	vacuole	C	AT3G12500
2709	2	L4 vs. M4	L4 Up	GO:0005773	Development	Development	vacuole	C	AT3G13750
2710	2	L4 vs. M4	L4 Up	GO:0005773	Development	Development	vacuole	C	AT3G16240
2711	2	L4 vs. M4	L4 Up	GO:0005773	Development	Development	vacuole	C	AT3G52840
2712	2	L4 vs. M4	L4 Up	GO:0005773	Development	Development	vacuole	C	AT4G35010
2713	2	L4 vs. M4	L4 Up	GO:0005773	Development	Development	vacuole	C	AT4G37550
2714	2	L4 vs. M4	L4 Up	GO:0005773	Development	Development	vacuole	C	AT5G55990
2715	2	L4 vs. M4	L4 Up	GO:0010228	Development	Development	vegetative to reproductive phase transition of meristem	P	AT2G21660
2716	2	L4 vs. M4	L4 Up	GO:0010228	Development	Development	vegetative to reproductive phase transition of meristem	P	AT2G42940
2717	2	L4 vs. M4	L4 Up	GO:0006906	Development	Development	vesicle fusion	P	AT1G15880
2718	2	L4 vs. M4	L4 Up	GO:0048280	Development	Development	vesicle fusion with Golgi apparatus	P	AT1G11890
2719	2	L4 vs. M4	L4 Up	GO:0032440	Metabolism	Bioenergetics	2-alkenal reductase [NAD(P)+] activity	F	AT5G16970
2720	2	L4 vs. M4	L4 Up	GO:0102336	Metabolism	Bioenergetics	3-oxo-arachidoyl-CoA synthase activity	F	AT2G46720
2721	2	L4 vs. M4	L4 Up	GO:0102336	Metabolism	Bioenergetics	3-oxo-arachidoyl-CoA synthase activity	F	AT5G49070
2722	2	L4 vs. M4	L4 Up	GO:0102337	Metabolism	Bioenergetics	3-oxo-cerotoyl-CoA synthase activity	F	AT2G46720
2723	2	L4 vs. M4	L4 Up	GO:0102337	Metabolism	Bioenergetics	3-oxo-cerotoyl-CoA synthase activity	F	AT5G49070
2724	2	L4 vs. M4	L4 Up	GO:0102338	Metabolism	Bioenergetics	3-oxo-lignoceronyl-CoA synthase activity	F	AT2G46720
2725	2	L4 vs. M4	L4 Up	GO:0102338	Metabolism	Bioenergetics	3-oxo-lignoceronyl-CoA synthase activity	F	AT5G49070
2726	2	L4 vs. M4	L4 Up	GO:0016207	Metabolism	Bioenergetics	4-coumarate-CoA ligase activity	F	AT1G62940
2727	2	L4 vs. M4	L4 Up	GO:0047617	Metabolism	Bioenergetics	acyl-CoA hydrolase activity	F	AT1G01710
2728	2	L4 vs. M4	L4 Up	GO:0006637	Metabolism	Bioenergetics	acyl-CoA metabolic process	P	AT1G01710
2729	2	L4 vs. M4	L4 Up	GO:0009060	Metabolism	Bioenergetics	aerobic respiration	P	AT5G11770
2730	2	L4 vs. M4	L4 Up	GO:0009060	Metabolism	Bioenergetics	aerobic respiration	P	ATMG01360
2731	2	L4 vs. M4	L4 Up	GO:0003825	Metabolism	Bioenergetics	alpha,alpha-trehalose-phosphate synthase (UDP-forming) activity	F	AT1G60140
2732	2	L4 vs. M4	L4 Up	GO:0016887	Metabolism	Bioenergetics	ATPase activity	F	AT2G07560
2733	2	L4 vs. M4	L4 Up	GO:0030246	Metabolism	Bioenergetics	carbohydrate binding	F	AT3G13750
2734	2	L4 vs. M4	L4 Up	GO:0030246	Metabolism	Bioenergetics	carbohydrate binding	F	AT4G35010
2735	2	L4 vs. M4	L4 Up	GO:0005975	Metabolism	Bioenergetics	carbohydrate metabolic process	P	AT1G02460
2736	2	L4 vs. M4	L4 Up	GO:0005975	Metabolism	Bioenergetics	carbohydrate metabolic process	P	AT1G35580
2737	2	L4 vs. M4	L4 Up	GO:0005975	Metabolism	Bioenergetics	carbohydrate metabolic process	P	AT1G60590
2738	2	L4 vs. M4	L4 Up	GO:0005975	Metabolism	Bioenergetics	carbohydrate metabolic process	P	AT3G07830
2739	2	L4 vs. M4	L4 Up	GO:0005975	Metabolism	Bioenergetics	carbohydrate metabolic process	P	AT3G07840
2740	2	L4 vs. M4	L4 Up	GO:0005975	Metabolism	Bioenergetics	carbohydrate metabolic process	P	AT3G07850
2741	2	L4 vs. M4	L4 Up	GO:0005975	Metabolism	Bioenergetics	carbohydrate metabolic process	P	AT3G13750
2742	2	L4 vs. M4	L4 Up	GO:0005975	Metabolism	Bioenergetics	carbohydrate metabolic process	P	AT3G52840

2743	2	L4 vs. M4	L4 Up	GO:0005975	Metabolism	Bioenergetics	carbohydrate metabolic process	P	AT4G35010
2744	2	L4 vs. M4	L4 Up	GO:0005975	Metabolism	Bioenergetics	carbohydrate metabolic process	P	AT5G20250
2745	2	L4 vs. M4	L4 Up	GO:0005975	Metabolism	Bioenergetics	carbohydrate metabolic process	P	AT5G21170
2746	2	L4 vs. M4	L4 Up	GO:0005975	Metabolism	Bioenergetics	carbohydrate metabolic process	P	AT5G48140
2747	2	L4 vs. M4	L4 Up	GO:0050662	Metabolism	Bioenergetics	coenzyme binding	F	AT4G35420
2748	2	L4 vs. M4	L4 Up	GO:0004129	Metabolism	Bioenergetics	cytochrome-c oxidase activity	F	ATMG01360
2749	2	L4 vs. M4	L4 Up	GO:0009055	Metabolism	Bioenergetics	electron transfer activity	F	AT1G60950
2750	2	L4 vs. M4	L4 Up	GO:0009055	Metabolism	Bioenergetics	electron transfer activity	F	AT2G43400
2751	2	L4 vs. M4	L4 Up	GO:0022900	Metabolism	Bioenergetics	electron transport chain	P	AT2G02510
2752	2	L4 vs. M4	L4 Up	GO:0015990	Metabolism	Bioenergetics	electron transport coupled proton transport	P	AT5G11770
2753	2	L4 vs. M4	L4 Up	GO:0015990	Metabolism	Bioenergetics	electron transport coupled proton transport	P	ATMG01360
2754	2	L4 vs. M4	L4 Up	GO:0004174	Metabolism	Bioenergetics	electron-transferring-flavoprotein dehydrogenase activity	F	AT2G43400
2755	2	L4 vs. M4	L4 Up	GO:0080132	Metabolism	Bioenergetics	fatty acid alpha-hydroxylase activity	F	AT4G20870
2756	2	L4 vs. M4	L4 Up	GO:0005504	Metabolism	Bioenergetics	fatty acid binding	F	AT3G07450
2757	2	L4 vs. M4	L4 Up	GO:0006633	Metabolism	Bioenergetics	fatty acid biosynthetic process	P	AT2G46720
2758	2	L4 vs. M4	L4 Up	GO:0006633	Metabolism	Bioenergetics	fatty acid biosynthetic process	P	AT4G20870
2759	2	L4 vs. M4	L4 Up	GO:0006633	Metabolism	Bioenergetics	fatty acid biosynthetic process	P	AT5G21170
2760	2	L4 vs. M4	L4 Up	GO:0006633	Metabolism	Bioenergetics	fatty acid biosynthetic process	P	AT5G49070
2761	2	L4 vs. M4	L4 Up	GO:0009062	Metabolism	Bioenergetics	fatty acid catabolic process	P	AT1G01710
2762	2	L4 vs. M4	L4 Up	GO:0030497	Metabolism	Bioenergetics	fatty acid elongation	P	AT2G46720
2763	2	L4 vs. M4	L4 Up	GO:0006631	Metabolism	Bioenergetics	fatty acid metabolic process	P	AT4G20870
2764	2	L4 vs. M4	L4 Up	GO:0046949	Metabolism	Bioenergetics	fatty-acyl-CoA biosynthetic process	P	AT1G62940
2765	2	L4 vs. M4	L4 Up	GO:0006096	Metabolism	Bioenergetics	glycolytic process	P	AT3G22960
2766	2	L4 vs. M4	L4 Up	GO:0031305	Metabolism	Bioenergetics	integral component of mitochondrial inner membrane	C	AT2G43400
2767	2	L4 vs. M4	L4 Up	GO:0031305	Metabolism	Bioenergetics	integral component of mitochondrial inner membrane	C	AT5G14040
2768	2	L4 vs. M4	L4 Up	GO:0004467	Metabolism	Bioenergetics	long-chain fatty acid-CoA ligase activity	F	AT1G62940
2769	2	L4 vs. M4	L4 Up	GO:0031956	Metabolism	Bioenergetics	medium-chain fatty acid-CoA ligase activity	F	AT1G62940
2770	2	L4 vs. M4	L4 Up	GO:0006123	Metabolism	Bioenergetics	mitochondrial electron transport, cytochrome c to oxygen	P	ATMG01360
2771	2	L4 vs. M4	L4 Up	GO:0005747	Metabolism	Bioenergetics	mitochondrial respiratory chain complex I	C	AT2G02510
2772	2	L4 vs. M4	L4 Up	GO:0005747	Metabolism	Bioenergetics	mitochondrial respiratory chain complex I	C	AT5G11770
2773	2	L4 vs. M4	L4 Up	GO:0032981	Metabolism	Bioenergetics	mitochondrial respiratory chain complex I assembly	P	AT2G02510
2774	2	L4 vs. M4	L4 Up	GO:0032981	Metabolism	Bioenergetics	mitochondrial respiratory chain complex I assembly	P	AT5G11770
2775	2	L4 vs. M4	L4 Up	GO:0005751	Metabolism	Bioenergetics	mitochondrial respiratory chain complex IV	C	ATMG01360
2776	2	L4 vs. M4	L4 Up	GO:0005739	Metabolism	Bioenergetics	mitochondrion	C	AT1G35580
2777	2	L4 vs. M4	L4 Up	GO:0005739	Metabolism	Bioenergetics	mitochondrion	C	AT1G48460
2778	2	L4 vs. M4	L4 Up	GO:0005739	Metabolism	Bioenergetics	mitochondrion	C	AT1G68820
2779	2	L4 vs. M4	L4 Up	GO:0005739	Metabolism	Bioenergetics	mitochondrion	C	AT1G69295
2780	2	L4 vs. M4	L4 Up	GO:0005739	Metabolism	Bioenergetics	mitochondrion	C	AT1G71695
2781	2	L4 vs. M4	L4 Up	GO:0005739	Metabolism	Bioenergetics	mitochondrion	C	AT1G72610
2782	2	L4 vs. M4	L4 Up	GO:0005739	Metabolism	Bioenergetics	mitochondrion	C	AT1G74540
2783	2	L4 vs. M4	L4 Up	GO:0005739	Metabolism	Bioenergetics	mitochondrion	C	AT1G78300
2784	2	L4 vs. M4	L4 Up	GO:0005739	Metabolism	Bioenergetics	mitochondrion	C	AT1G79210
2785	2	L4 vs. M4	L4 Up	GO:0005739	Metabolism	Bioenergetics	mitochondrion	C	AT1G79420
2786	2	L4 vs. M4	L4 Up	GO:0005739	Metabolism	Bioenergetics	mitochondrion	C	AT2G02510
2787	2	L4 vs. M4	L4 Up	GO:0005739	Metabolism	Bioenergetics	mitochondrion	C	AT2G12190
2788	2	L4 vs. M4	L4 Up	GO:0005739	Metabolism	Bioenergetics	mitochondrion	C	AT2G20190
2789	2	L4 vs. M4	L4 Up	GO:0005739	Metabolism	Bioenergetics	mitochondrion	C	AT2G35370
2790	2	L4 vs. M4	L4 Up	GO:0005739	Metabolism	Bioenergetics	mitochondrion	C	AT2G43400
2791	2	L4 vs. M4	L4 Up	GO:0005739	Metabolism	Bioenergetics	mitochondrion	C	AT2G46540
2792	2	L4 vs. M4	L4 Up	GO:0005739	Metabolism	Bioenergetics	mitochondrion	C	AT3G01740
2793	2	L4 vs. M4	L4 Up	GO:0005739	Metabolism	Bioenergetics	mitochondrion	C	AT3G07450
2794	2	L4 vs. M4	L4 Up	GO:0005739	Metabolism	Bioenergetics	mitochondrion	C	AT3G25140

2795	2	L4 vs. M4	L4 Up	GO:0005739	Metabolism	Bioenergetics	mitochondrion	C	AT3G30775
2796	2	L4 vs. M4	L4 Up	GO:0005739	Metabolism	Bioenergetics	mitochondrion	C	AT3G52130
2797	2	L4 vs. M4	L4 Up	GO:0005739	Metabolism	Bioenergetics	mitochondrion	C	AT4G02380
2798	2	L4 vs. M4	L4 Up	GO:0005739	Metabolism	Bioenergetics	mitochondrion	C	AT4G02920
2799	2	L4 vs. M4	L4 Up	GO:0005739	Metabolism	Bioenergetics	mitochondrion	C	AT4G12590
2800	2	L4 vs. M4	L4 Up	GO:0005739	Metabolism	Bioenergetics	mitochondrion	C	AT4G18230
2801	2	L4 vs. M4	L4 Up	GO:0005739	Metabolism	Bioenergetics	mitochondrion	C	AT4G20870
2802	2	L4 vs. M4	L4 Up	GO:0005739	Metabolism	Bioenergetics	mitochondrion	C	AT4G22930
2803	2	L4 vs. M4	L4 Up	GO:0005739	Metabolism	Bioenergetics	mitochondrion	C	AT4G24920
2804	2	L4 vs. M4	L4 Up	GO:0005739	Metabolism	Bioenergetics	mitochondrion	C	AT4G24960
2805	2	L4 vs. M4	L4 Up	GO:0005739	Metabolism	Bioenergetics	mitochondrion	C	AT4G25100
2806	2	L4 vs. M4	L4 Up	GO:0005739	Metabolism	Bioenergetics	mitochondrion	C	AT4G26910
2807	2	L4 vs. M4	L4 Up	GO:0005739	Metabolism	Bioenergetics	mitochondrion	C	AT5G01080
2808	2	L4 vs. M4	L4 Up	GO:0005739	Metabolism	Bioenergetics	mitochondrion	C	AT5G01600
2809	2	L4 vs. M4	L4 Up	GO:0005739	Metabolism	Bioenergetics	mitochondrion	C	AT5G11770
2810	2	L4 vs. M4	L4 Up	GO:0005739	Metabolism	Bioenergetics	mitochondrion	C	AT5G13630
2811	2	L4 vs. M4	L4 Up	GO:0005739	Metabolism	Bioenergetics	mitochondrion	C	AT5G14040
2812	2	L4 vs. M4	L4 Up	GO:0005739	Metabolism	Bioenergetics	mitochondrion	C	AT5G14550
2813	2	L4 vs. M4	L4 Up	GO:0005739	Metabolism	Bioenergetics	mitochondrion	C	AT5G15200
2814	2	L4 vs. M4	L4 Up	GO:0005739	Metabolism	Bioenergetics	mitochondrion	C	AT5G38710
2815	2	L4 vs. M4	L4 Up	GO:0005739	Metabolism	Bioenergetics	mitochondrion	C	AT5G42000
2816	2	L4 vs. M4	L4 Up	GO:0005739	Metabolism	Bioenergetics	mitochondrion	C	AT5G65685
2817	2	L4 vs. M4	L4 Up	GO:0005739	Metabolism	Bioenergetics	mitochondrion	C	ATMG01360
2818	2	L4 vs. M4	L4 Up	GO:0008137	Metabolism	Bioenergetics	NADH dehydrogenase (ubiquinone) activity	F	AT5G11770
2819	2	L4 vs. M4	L4 Up	GO:0003954	Metabolism	Bioenergetics	NADH dehydrogenase activity	F	AT5G11770
2820	2	L4 vs. M4	L4 Up	GO:0031588	Metabolism	Bioenergetics	nucleotide-activated protein kinase complex	C	AT5G21170
2821	2	L4 vs. M4	L4 Up	GO:0016491	Metabolism	Bioenergetics	oxidoreductase activity	F	AT1G55570
2822	2	L4 vs. M4	L4 Up	GO:0016491	Metabolism	Bioenergetics	oxidoreductase activity	F	AT3G13390
2823	2	L4 vs. M4	L4 Up	GO:0016491	Metabolism	Bioenergetics	oxidoreductase activity	F	AT3G13400
2824	2	L4 vs. M4	L4 Up	GO:0016491	Metabolism	Bioenergetics	oxidoreductase activity	F	AT4G35420
2825	2	L4 vs. M4	L4 Up	GO:0016705	Metabolism	Bioenergetics	oxidoreductase activity, acting on paired donors, with incorporation or reduction of molecular oxygen	F	AT1G13150
2826	2	L4 vs. M4	L4 Up	GO:0016705	Metabolism	Bioenergetics	oxidoreductase activity, acting on paired donors, with incorporation or reduction of molecular oxygen	F	AT5G08250
2827	2	L4 vs. M4	L4 Up	GO:0016709	Metabolism	Bioenergetics	oxidoreductase activity, acting on paired donors, with incorporation or reduction of molecular oxygen, NAD(P)H as one donor, and incorporation of one atom of oxygen	F	AT1G74540
2828	2	L4 vs. M4	L4 Up	GO:0016709	Metabolism	Bioenergetics	oxidoreductase activity, acting on paired donors, with incorporation or reduction of molecular oxygen, NAD(P)H as one donor, and incorporation of one atom of oxygen	F	AT2G12190
2829	2	L4 vs. M4	L4 Up	GO:0016616	Metabolism	Bioenergetics	oxidoreductase activity, acting on the CH-OH group of donors, NAD or NADP as acceptor	F	AT4G35420
2830	2	L4 vs. M4	L4 Up	GO:0045252	Metabolism	Bioenergetics	oxoglutarate dehydrogenase complex	C	AT4G26910
2831	2	L4 vs. M4	L4 Up	GO:0009853	Metabolism	Bioenergetics	photorespiration	P	AT2G35370
2832	2	L4 vs. M4	L4 Up	GO:0009853	Metabolism	Bioenergetics	photorespiration	P	ATCG00490
2833	2	L4 vs. M4	L4 Up	GO:0045723	Metabolism	Bioenergetics	positive regulation of fatty acid biosynthetic process	P	AT1G79700
2834	2	L4 vs. M4	L4 Up	GO:1902600	Metabolism	Bioenergetics	proton transmembrane transport	P	AT2G07560
2835	2	L4 vs. M4	L4 Up	GO:0008553	Metabolism	Bioenergetics	proton-exporting ATPase activity, phosphorylative mechanism	F	AT2G07560
2836	2	L4 vs. M4	L4 Up	GO:0004743	Metabolism	Bioenergetics	pyruvate kinase activity	F	AT3G22960
2837	2	L4 vs. M4	L4 Up	GO:0019253	Metabolism	Bioenergetics	reductive pentose-phosphate cycle	P	ATCG00490
2838	2	L4 vs. M4	L4 Up	GO:0022904	Metabolism	Bioenergetics	respiratory electron transport chain	P	AT2G43400
2839	2	L4 vs. M4	L4 Up	GO:0022904	Metabolism	Bioenergetics	respiratory electron transport chain	P	ATMG01360
2840	2	L4 vs. M4	L4 Up	GO:0009011	Metabolism	Bioenergetics	starch synthase activity	F	AT5G65685

2841	2	L4 vs. M4	L4 Up	GO:0005987	Metabolism	Bioenergetics	sucrose catabolic process	P	AT1G35580
2842	2	L4 vs. M4	L4 Up	GO:0006099	Metabolism	Bioenergetics	tricarboxylic acid cycle	P	AT4G26910
2843	2	L4 vs. M4	L4 Up	GO:0048039	Metabolism	Bioenergetics	ubiquinone binding	F	AT2G43400
2844	2	L4 vs. M4	L4 Up	GO:0000038	Metabolism	Bioenergetics	very long-chain fatty acid metabolic process	P	AT2G46720
2845	2	L4 vs. M4	L4 Up	GO:0000038	Metabolism	Bioenergetics	very long-chain fatty acid metabolic process	P	AT5G49070
2846	2	L4 vs. M4	L4 Up	GO:0102756	Metabolism	Bioenergetics	very-long-chain 3-ketoacyl-CoA synthase activity	F	AT2G46720
2847	2	L4 vs. M4	L4 Up	GO:0102756	Metabolism	Bioenergetics	very-long-chain 3-ketoacyl-CoA synthase activity	F	AT5G49070
2848	2	L4 vs. M4	L4 Up	GO:0006168	Metabolism	Catabolism	adenine salvage	P	AT1G27450
2849	2	L4 vs. M4	L4 Up	GO:0004089	Metabolism	Catabolism	carbonate dehydratase activity	F	AT3G01500
2850	2	L4 vs. M4	L4 Up	GO:0004089	Metabolism	Catabolism	carbonate dehydratase activity	F	AT5G04180
2851	2	L4 vs. M4	L4 Up	GO:0016998	Metabolism	Catabolism	cell wall macromolecule catabolic process	P	AT3G12500
2852	2	L4 vs. M4	L4 Up	GO:0030245	Metabolism	Catabolism	cellulose catabolic process	P	AT1G02800
2853	2	L4 vs. M4	L4 Up	GO:0006032	Metabolism	Catabolism	chitin catabolic process	P	AT3G12500
2854	2	L4 vs. M4	L4 Up	GO:0009440	Metabolism	Catabolism	cyanate catabolic process	P	AT3G01500
2855	2	L4 vs. M4	L4 Up	GO:0004197	Metabolism	Catabolism	cysteine-type endopeptidase activity	F	AT1G02305
2856	2	L4 vs. M4	L4 Up	GO:0004175	Metabolism	Catabolism	endopeptidase activity	F	AT1G16470
2857	2	L4 vs. M4	L4 Up	GO:0004175	Metabolism	Catabolism	endopeptidase activity	F	AT1G79210
2858	2	L4 vs. M4	L4 Up	GO:0004527	Metabolism	Catabolism	exonuclease activity	F	AT1G34380
2859	2	L4 vs. M4	L4 Up	GO:0019898	Metabolism	Catabolism	extrinsic component of membrane	C	AT4G05180
2860	2	L4 vs. M4	L4 Up	GO:0045487	Metabolism	Catabolism	gibberellin catabolic process	P	AT2G34555
2861	2	L4 vs. M4	L4 Up	GO:0019310	Metabolism	Catabolism	inositol catabolic process	P	AT4G26260
2862	2	L4 vs. M4	L4 Up	GO:0019310	Metabolism	Catabolism	inositol catabolic process	P	AT5G56640
2863	2	L4 vs. M4	L4 Up	GO:0010150	Metabolism	Catabolism	leaf senescence	P	AT4G02380
2864	2	L4 vs. M4	L4 Up	GO:0010150	Metabolism	Catabolism	leaf senescence	P	AT5G42000
2865	2	L4 vs. M4	L4 Up	GO:0006552	Metabolism	Catabolism	leucine catabolic process	P	AT2G43400
2866	2	L4 vs. M4	L4 Up	GO:0016042	Metabolism	Catabolism	lipid catabolic process	P	AT5G58670
2867	2	L4 vs. M4	L4 Up	GO:0033512	Metabolism	Catabolism	L-lysine catabolic process to acetyl-CoA via saccharopine	P	AT4G26910
2868	2	L4 vs. M4	L4 Up	GO:0005764	Metabolism	Catabolism	lysosome	C	AT1G02305
2869	2	L4 vs. M4	L4 Up	GO:0019941	Metabolism	Catabolism	modification-dependent protein catabolic process	P	AT4G05050
2870	2	L4 vs. M4	L4 Up	GO:0061157	Metabolism	Catabolism	mRNA destabilization	P	AT2G25900
2871	2	L4 vs. M4	L4 Up	GO:0043069	Metabolism	Catabolism	negative regulation of programmed cell death	P	AT4G20870
2872	2	L4 vs. M4	L4 Up	GO:0045490	Metabolism	Catabolism	pectin catabolic process	P	AT2G47040
2873	2	L4 vs. M4	L4 Up	GO:0045490	Metabolism	Catabolism	pectin catabolic process	P	AT3G62170
2874	2	L4 vs. M4	L4 Up	GO:0045490	Metabolism	Catabolism	pectin catabolic process	P	AT5G07410
2875	2	L4 vs. M4	L4 Up	GO:0045490	Metabolism	Catabolism	pectin catabolic process	P	AT5G15110
2876	2	L4 vs. M4	L4 Up	GO:0004601	Metabolism	Catabolism	peroxidase activity	F	AT1G07890
2877	2	L4 vs. M4	L4 Up	GO:0004601	Metabolism	Catabolism	peroxidase activity	F	AT1G67600
2878	2	L4 vs. M4	L4 Up	GO:0004601	Metabolism	Catabolism	peroxidase activity	F	AT1G71695
2879	2	L4 vs. M4	L4 Up	GO:0005777	Metabolism	Catabolism	peroxisome	C	AT1G01710
2880	2	L4 vs. M4	L4 Up	GO:0005777	Metabolism	Catabolism	peroxisome	C	AT2G21660
2881	2	L4 vs. M4	L4 Up	GO:1990137	Metabolism	Catabolism	plant seed peroxidase activity	F	AT2G33380
2882	2	L4 vs. M4	L4 Up	GO:0000272	Metabolism	Catabolism	polysaccharide catabolic process	P	AT3G12500
2883	2	L4 vs. M4	L4 Up	GO:0006562	Metabolism	Catabolism	proline catabolic process	P	AT3G30775
2884	2	L4 vs. M4	L4 Up	GO:0006562	Metabolism	Catabolism	proline catabolic process	P	AT5G38710
2885	2	L4 vs. M4	L4 Up	GO:0010133	Metabolism	Catabolism	proline catabolic process to glutamate	P	AT3G30775
2886	2	L4 vs. M4	L4 Up	GO:0010133	Metabolism	Catabolism	proline catabolic process to glutamate	P	AT5G38710
2887	2	L4 vs. M4	L4 Up	GO:0010498	Metabolism	Catabolism	proteasomal protein catabolic process	P	AT1G16470
2888	2	L4 vs. M4	L4 Up	GO:0010498	Metabolism	Catabolism	proteasomal protein catabolic process	P	AT1G79210
2889	2	L4 vs. M4	L4 Up	GO:0010499	Metabolism	Catabolism	proteasomal ubiquitin-independent protein catabolic process	P	AT1G16470
2890	2	L4 vs. M4	L4 Up	GO:0010499	Metabolism	Catabolism	proteasomal ubiquitin-independent protein catabolic process	P	AT1G79210
2891	2	L4 vs. M4	L4 Up	GO:0043248	Metabolism	Catabolism	proteasome assembly	P	AT1G64520
2892	2	L4 vs. M4	L4 Up	GO:0043248	Metabolism	Catabolism	proteasome assembly	P	AT5G45620
2893	2	L4 vs. M4	L4 Up	GO:0000502	Metabolism	Catabolism	proteasome complex	C	AT1G16470

2894	2	L4 vs. M4	L4 Up	GO:0000502	Metabolism	Catabolism	proteasome complex	C	AT1G64520
2895	2	L4 vs. M4	L4 Up	GO:0000502	Metabolism	Catabolism	proteasome complex	C	AT1G79210
2896	2	L4 vs. M4	L4 Up	GO:0000502	Metabolism	Catabolism	proteasome complex	C	AT5G45620
2897	2	L4 vs. M4	L4 Up	GO:0005839	Metabolism	Catabolism	proteasome core complex	C	AT1G16470
2898	2	L4 vs. M4	L4 Up	GO:0005839	Metabolism	Catabolism	proteasome core complex	C	AT1G79210
2899	2	L4 vs. M4	L4 Up	GO:0019773	Metabolism	Catabolism	proteasome core complex, alpha-subunit complex	C	AT1G16470
2900	2	L4 vs. M4	L4 Up	GO:0019773	Metabolism	Catabolism	proteasome core complex, alpha-subunit complex	C	AT1G79210
2901	2	L4 vs. M4	L4 Up	GO:0008541	Metabolism	Catabolism	proteasome regulatory particle, lid subcomplex	C	AT1G64520
2902	2	L4 vs. M4	L4 Up	GO:0008541	Metabolism	Catabolism	proteasome regulatory particle, lid subcomplex	C	AT5G45620
2903	2	L4 vs. M4	L4 Up	GO:0043161	Metabolism	Catabolism	proteasome-mediated ubiquitin-dependent protein catabolic process	P	AT1G16470
2904	2	L4 vs. M4	L4 Up	GO:0043161	Metabolism	Catabolism	proteasome-mediated ubiquitin-dependent protein catabolic process	P	AT1G64520
2905	2	L4 vs. M4	L4 Up	GO:0043161	Metabolism	Catabolism	proteasome-mediated ubiquitin-dependent protein catabolic process	P	AT1G79210
2906	2	L4 vs. M4	L4 Up	GO:0030163	Metabolism	Catabolism	protein catabolic process	P	AT1G64520
2907	2	L4 vs. M4	L4 Up	GO:0016567	Metabolism	Catabolism	protein ubiquitination	P	AT1G64230
2908	2	L4 vs. M4	L4 Up	GO:0016567	Metabolism	Catabolism	protein ubiquitination	P	AT2G25490
2909	2	L4 vs. M4	L4 Up	GO:0016567	Metabolism	Catabolism	protein ubiquitination	P	AT4G05050
2910	2	L4 vs. M4	L4 Up	GO:0016567	Metabolism	Catabolism	protein ubiquitination	P	AT4G37610
2911	2	L4 vs. M4	L4 Up	GO:0016567	Metabolism	Catabolism	protein ubiquitination	P	AT5G05080
2912	2	L4 vs. M4	L4 Up	GO:0006508	Metabolism	Catabolism	proteolysis	P	AT1G02305
2913	2	L4 vs. M4	L4 Up	GO:0006508	Metabolism	Catabolism	proteolysis	P	AT2G23010
2914	2	L4 vs. M4	L4 Up	GO:0006508	Metabolism	Catabolism	proteolysis	P	AT5G05740
2915	2	L4 vs. M4	L4 Up	GO:0006508	Metabolism	Catabolism	proteolysis	P	AT5G45650
2916	2	L4 vs. M4	L4 Up	GO:0051603	Metabolism	Catabolism	proteolysis involved in cellular protein catabolic process	P	AT1G02305
2917	2	L4 vs. M4	L4 Up	GO:0006166	Metabolism	Catabolism	purine ribonucleoside salvage	P	AT1G27450
2918	2	L4 vs. M4	L4 Up	GO:1900055	Metabolism	Catabolism	regulation of leaf senescence	P	AT3G44680
2919	2	L4 vs. M4	L4 Up	GO:0031146	Metabolism	Catabolism	SCF-dependent proteasomal ubiquitin-dependent protein catabolic process	P	AT2G25490
2920	2	L4 vs. M4	L4 Up	GO:0004185	Metabolism	Catabolism	serine-type carboxypeptidase activity	F	AT2G23010
2921	2	L4 vs. M4	L4 Up	GO:0004252	Metabolism	Catabolism	serine-type endopeptidase activity	F	AT5G45650
2922	2	L4 vs. M4	L4 Up	GO:0004298	Metabolism	Catabolism	threonine-type endopeptidase activity	F	AT1G16470
2923	2	L4 vs. M4	L4 Up	GO:0004298	Metabolism	Catabolism	threonine-type endopeptidase activity	F	AT1G79210
2924	2	L4 vs. M4	L4 Up	GO:0061631	Metabolism	Catabolism	ubiquitin conjugating enzyme activity	F	AT1G64230
2925	2	L4 vs. M4	L4 Up	GO:0061631	Metabolism	Catabolism	ubiquitin conjugating enzyme activity	F	AT5G05080
2926	2	L4 vs. M4	L4 Up	GO:0031625	Metabolism	Catabolism	ubiquitin protein ligase binding	F	AT4G05050
2927	2	L4 vs. M4	L4 Up	GO:0006511	Metabolism	Catabolism	ubiquitin-dependent protein catabolic process	P	AT1G16470
2928	2	L4 vs. M4	L4 Up	GO:0006511	Metabolism	Catabolism	ubiquitin-dependent protein catabolic process	P	AT1G64230
2929	2	L4 vs. M4	L4 Up	GO:0006511	Metabolism	Catabolism	ubiquitin-dependent protein catabolic process	P	AT1G64520
2930	2	L4 vs. M4	L4 Up	GO:0006511	Metabolism	Catabolism	ubiquitin-dependent protein catabolic process	P	AT2G25490
2931	2	L4 vs. M4	L4 Up	GO:0006511	Metabolism	Catabolism	ubiquitin-dependent protein catabolic process	P	AT4G05050
2932	2	L4 vs. M4	L4 Up	GO:0006511	Metabolism	Catabolism	ubiquitin-dependent protein catabolic process	P	AT5G05080
2933	2	L4 vs. M4	L4 Up	GO:0006511	Metabolism	Catabolism	ubiquitin-dependent protein catabolic process	P	AT5G45620
2934	2	L4 vs. M4	L4 Up	GO:0004842	Metabolism	Catabolism	ubiquitin-protein transferase activity	F	AT1G64230
2935	2	L4 vs. M4	L4 Up	GO:0004842	Metabolism	Catabolism	ubiquitin-protein transferase activity	F	AT2G25490
2936	2	L4 vs. M4	L4 Up	GO:0004842	Metabolism	Catabolism	ubiquitin-protein transferase activity	F	AT5G05080
2937	2	L4 vs. M4	L4 Up	GO:1902609	Metabolism	Metabolism	(R)-2-hydroxy-alpha-linolenic acid biosynthetic process	P	AT2G33380
2938	2	L4 vs. M4	L4 Up	GO:0051537	Metabolism	Metabolism	2 iron, 2 sulfur cluster binding	F	AT1G60950
2939	2	L4 vs. M4	L4 Up	GO:0051539	Metabolism	Metabolism	4 iron, 4 sulfur cluster binding	F	AT1G50170
2940	2	L4 vs. M4	L4 Up	GO:0051539	Metabolism	Metabolism	4 iron, 4 sulfur cluster binding	F	AT2G43400
2941	2	L4 vs. M4	L4 Up	GO:0051539	Metabolism	Metabolism	4 iron, 4 sulfur cluster binding	F	AT4G25040
2942	2	L4 vs. M4	L4 Up	GO:0051539	Metabolism	Metabolism	4 iron, 4 sulfur cluster binding	F	AT5G11770
2943	2	L4 vs. M4	L4 Up	GO:0008839	Metabolism	Metabolism	4-hydroxy-tetrahydrodipicolinate reductase	F	AT5G52100

2944	2	L4 vs. M4	L4 Up	GO:0008409	Metabolism	Metabolism	5'-3' exonuclease activity	F	AT1G34380
2945	2	L4 vs. M4	L4 Up	GO:0008375	Metabolism	Metabolism	acetylglucosaminyltransferase activity	F	AT5G14550
2946	2	L4 vs. M4	L4 Up	GO:0017096	Metabolism	Metabolism	acetylserotonin O-methyltransferase activity	F	AT5G54160
2947	2	L4 vs. M4	L4 Up	GO:0016881	Metabolism	Metabolism	acid-amino acid ligase activity	F	AT5G13350
2948	2	L4 vs. M4	L4 Up	GO:0003779	Metabolism	Metabolism	actin binding	F	AT4G25590
2949	2	L4 vs. M4	L4 Up	GO:0003779	Metabolism	Metabolism	actin binding	F	AT5G52360
2950	2	L4 vs. M4	L4 Up	GO:0051015	Metabolism	Metabolism	actin filament binding	F	AT1G01780
2951	2	L4 vs. M4	L4 Up	GO:0051017	Metabolism	Metabolism	actin filament bundle assembly	P	AT1G01780
2952	2	L4 vs. M4	L4 Up	GO:0030042	Metabolism	Metabolism	actin filament depolymerization	P	AT4G25590
2953	2	L4 vs. M4	L4 Up	GO:0030042	Metabolism	Metabolism	actin filament depolymerization	P	AT5G52360
2954	2	L4 vs. M4	L4 Up	GO:0003785	Metabolism	Metabolism	actin monomer binding	F	AT4G29340
2955	2	L4 vs. M4	L4 Up	GO:0045010	Metabolism	Metabolism	actin nucleation	P	AT2G46225
2956	2	L4 vs. M4	L4 Up	GO:0003999	Metabolism	Metabolism	adenine phosphoribosyltransferase activity	F	AT1G27450
2957	2	L4 vs. M4	L4 Up	GO:0004031	Metabolism	Metabolism	aldehyde oxidase activity	F	AT1G67290
2958	2	L4 vs. M4	L4 Up	GO:0003905	Metabolism	Metabolism	alkylbase DNA N-glycosylase activity	F	AT3G12040
2959	2	L4 vs. M4	L4 Up	GO:0016597	Metabolism	Metabolism	amino acid binding	F	AT2G03730
2960	2	L4 vs. M4	L4 Up	GO:0044209	Metabolism	Metabolism	AMP salvage	P	AT1G27450
2961	2	L4 vs. M4	L4 Up	GO:0019438	Metabolism	Metabolism	aromatic compound biosynthetic process	P	AT5G54160
2962	2	L4 vs. M4	L4 Up	GO:0045330	Metabolism	Metabolism	aspartyl esterase activity	F	AT2G47040
2963	2	L4 vs. M4	L4 Up	GO:0045330	Metabolism	Metabolism	aspartyl esterase activity	F	AT3G62170
2964	2	L4 vs. M4	L4 Up	GO:0045330	Metabolism	Metabolism	aspartyl esterase activity	F	AT5G07410
2965	2	L4 vs. M4	L4 Up	GO:0005524	Metabolism	Metabolism	ATP binding	F	AT1G62940
2966	2	L4 vs. M4	L4 Up	GO:0005524	Metabolism	Metabolism	ATP binding	F	AT1G64230
2967	2	L4 vs. M4	L4 Up	GO:0005524	Metabolism	Metabolism	ATP binding	F	AT2G07560
2968	2	L4 vs. M4	L4 Up	GO:0005524	Metabolism	Metabolism	ATP binding	F	AT2G41970
2969	2	L4 vs. M4	L4 Up	GO:0005524	Metabolism	Metabolism	ATP binding	F	AT3G21180
2970	2	L4 vs. M4	L4 Up	GO:0005524	Metabolism	Metabolism	ATP binding	F	AT3G22960
2971	2	L4 vs. M4	L4 Up	GO:0005524	Metabolism	Metabolism	ATP binding	F	AT4G12620
2972	2	L4 vs. M4	L4 Up	GO:0005524	Metabolism	Metabolism	ATP binding	F	AT5G05080
2973	2	L4 vs. M4	L4 Up	GO:0005524	Metabolism	Metabolism	ATP binding	F	AT5G08160
2974	2	L4 vs. M4	L4 Up	GO:0005524	Metabolism	Metabolism	ATP binding	F	AT5G13630
2975	2	L4 vs. M4	L4 Up	GO:0005524	Metabolism	Metabolism	ATP binding	F	AT5G21170
2976	2	L4 vs. M4	L4 Up	GO:0004564	Metabolism	Metabolism	beta-fructofuranosidase activity	F	AT1G35580
2977	2	L4 vs. M4	L4 Up	GO:0004565	Metabolism	Metabolism	beta-galactosidase activity	F	AT3G13750
2978	2	L4 vs. M4	L4 Up	GO:0004565	Metabolism	Metabolism	beta-galactosidase activity	F	AT3G52840
2979	2	L4 vs. M4	L4 Up	GO:0004565	Metabolism	Metabolism	beta-galactosidase activity	F	AT4G35010
2980	2	L4 vs. M4	L4 Up	GO:0008150	Metabolism	Metabolism	biological_process	P	AT1G14330
2981	2	L4 vs. M4	L4 Up	GO:0008150	Metabolism	Metabolism	biological_process	P	AT1G14880
2982	2	L4 vs. M4	L4 Up	GO:0008150	Metabolism	Metabolism	biological_process	P	AT1G16290
2983	2	L4 vs. M4	L4 Up	GO:0008150	Metabolism	Metabolism	biological_process	P	AT1G48460
2984	2	L4 vs. M4	L4 Up	GO:0008150	Metabolism	Metabolism	biological_process	P	AT1G52220
2985	2	L4 vs. M4	L4 Up	GO:0008150	Metabolism	Metabolism	biological_process	P	AT1G62510
2986	2	L4 vs. M4	L4 Up	GO:0008150	Metabolism	Metabolism	biological_process	P	AT1G67600
2987	2	L4 vs. M4	L4 Up	GO:0008150	Metabolism	Metabolism	biological_process	P	AT1G69295
2988	2	L4 vs. M4	L4 Up	GO:0008150	Metabolism	Metabolism	biological_process	P	AT1G79420
2989	2	L4 vs. M4	L4 Up	GO:0008150	Metabolism	Metabolism	biological_process	P	AT2G19000
2990	2	L4 vs. M4	L4 Up	GO:0008150	Metabolism	Metabolism	biological_process	P	AT2G19980
2991	2	L4 vs. M4	L4 Up	GO:0008150	Metabolism	Metabolism	biological_process	P	AT2G36020
2992	2	L4 vs. M4	L4 Up	GO:0008150	Metabolism	Metabolism	biological_process	P	AT2G44360
2993	2	L4 vs. M4	L4 Up	GO:0008150	Metabolism	Metabolism	biological_process	P	AT3G01740
2994	2	L4 vs. M4	L4 Up	GO:0008150	Metabolism	Metabolism	biological_process	P	AT3G15400
2995	2	L4 vs. M4	L4 Up	GO:0008150	Metabolism	Metabolism	biological_process	P	AT3G20865
2996	2	L4 vs. M4	L4 Up	GO:0008150	Metabolism	Metabolism	biological_process	P	AT3G28550
2997	2	L4 vs. M4	L4 Up	GO:0008150	Metabolism	Metabolism	biological_process	P	AT3G54580
2998	2	L4 vs. M4	L4 Up	GO:0008150	Metabolism	Metabolism	biological_process	P	AT3G57690
2999	2	L4 vs. M4	L4 Up	GO:0008150	Metabolism	Metabolism	biological_process	P	AT3G62580

3000	2	L4 vs. M4	L4 Up	GO:0008150	Metabolism	Metabolism	biological_process	P	AT4G02920
3001	2	L4 vs. M4	L4 Up	GO:0008150	Metabolism	Metabolism	biological_process	P	AT4G09830
3002	2	L4 vs. M4	L4 Up	GO:0008150	Metabolism	Metabolism	biological_process	P	AT4G19160
3003	2	L4 vs. M4	L4 Up	GO:0008150	Metabolism	Metabolism	biological_process	P	AT4G25780
3004	2	L4 vs. M4	L4 Up	GO:0008150	Metabolism	Metabolism	biological_process	P	AT4G28240
3005	2	L4 vs. M4	L4 Up	GO:0008150	Metabolism	Metabolism	biological_process	P	AT4G30100
3006	2	L4 vs. M4	L4 Up	GO:0008150	Metabolism	Metabolism	biological_process	P	AT4G33480
3007	2	L4 vs. M4	L4 Up	GO:0008150	Metabolism	Metabolism	biological_process	P	AT5G19580
3008	2	L4 vs. M4	L4 Up	GO:0008150	Metabolism	Metabolism	biological_process	P	AT5G19810
3009	2	L4 vs. M4	L4 Up	GO:0008150	Metabolism	Metabolism	biological_process	P	AT5G25280
3010	2	L4 vs. M4	L4 Up	GO:0008150	Metabolism	Metabolism	biological_process	P	AT5G38760
3011	2	L4 vs. M4	L4 Up	GO:0008150	Metabolism	Metabolism	biological_process	P	AT5G40730
3012	2	L4 vs. M4	L4 Up	GO:0008150	Metabolism	Metabolism	biological_process	P	AT5G45880
3013	2	L4 vs. M4	L4 Up	GO:0008150	Metabolism	Metabolism	biological_process	P	AT5G48590
3014	2	L4 vs. M4	L4 Up	GO:0008150	Metabolism	Metabolism	biological_process	P	AT5G53820
3015	2	L4 vs. M4	L4 Up	GO:0008150	Metabolism	Metabolism	biological_process	P	AT5G59170
3016	2	L4 vs. M4	L4 Up	GO:0047763	Metabolism	Metabolism	caffeate O-methyltransferase activity	F	AT5G54160
3017	2	L4 vs. M4	L4 Up	GO:0015976	Metabolism	Metabolism	carbon utilization	P	AT3G01500
3018	2	L4 vs. M4	L4 Up	GO:0006520	Metabolism	Metabolism	cellular amino acid metabolic process	P	AT1G35580
3019	2	L4 vs. M4	L4 Up	GO:0008810	Metabolism	Metabolism	cellulase activity	F	AT1G02800
3020	2	L4 vs. M4	L4 Up	GO:0030244	Metabolism	Metabolism	cellulose biosynthetic process	P	AT2G39770
3021	2	L4 vs. M4	L4 Up	GO:0010215	Metabolism	Metabolism	cellulose microfibril organization	P	AT3G20580
3022	2	L4 vs. M4	L4 Up	GO:0006672	Metabolism	Metabolism	ceramide metabolic process	P	AT5G42000
3023	2	L4 vs. M4	L4 Up	GO:0008061	Metabolism	Metabolism	chitin binding	F	AT3G12500
3024	2	L4 vs. M4	L4 Up	GO:0004568	Metabolism	Metabolism	chitinase activity	F	AT3G12500
3025	2	L4 vs. M4	L4 Up	GO:0003682	Metabolism	Metabolism	chromatin binding	F	AT4G12620
3026	2	L4 vs. M4	L4 Up	GO:0009236	Metabolism	Metabolism	cobalamin biosynthetic process	P	AT1G50170
3027	2	L4 vs. M4	L4 Up	GO:0005507	Metabolism	Metabolism	copper ion binding	F	AT1G55570
3028	2	L4 vs. M4	L4 Up	GO:0005507	Metabolism	Metabolism	copper ion binding	F	AT3G13390
3029	2	L4 vs. M4	L4 Up	GO:0005507	Metabolism	Metabolism	copper ion binding	F	AT3G13400
3030	2	L4 vs. M4	L4 Up	GO:0005507	Metabolism	Metabolism	copper ion binding	F	AT4G25100
3031	2	L4 vs. M4	L4 Up	GO:0006241	Metabolism	Metabolism	CTP biosynthetic process	P	AT1G30820
3032	2	L4 vs. M4	L4 Up	GO:0003883	Metabolism	Metabolism	CTP synthase activity	F	AT1G30820
3033	2	L4 vs. M4	L4 Up	GO:0006207	Metabolism	Metabolism	'de novo' pyrimidine nucleobase biosynthetic process	P	AT4G22930
3034	2	L4 vs. M4	L4 Up	GO:0044205	Metabolism	Metabolism	'de novo' UMP biosynthetic process	P	AT4G22930
3035	2	L4 vs. M4	L4 Up	GO:0019877	Metabolism	Metabolism	diaminopimelate biosynthetic process	P	AT5G52100
3036	2	L4 vs. M4	L4 Up	GO:0072548	Metabolism	Metabolism	dicoumaroyl monocaffeoyl spermidine meta-hydroxylase activity	F	AT1G74540
3037	2	L4 vs. M4	L4 Up	GO:0072551	Metabolism	Metabolism	diferuloyl mono-(hydroxyferuloyl) spermidine meta-hydroxylase activity	F	AT1G74540
3038	2	L4 vs. M4	L4 Up	GO:0004149	Metabolism	Metabolism	dihydrolipoyllysine-residue succinyltransferase activity	F	AT4G26910
3039	2	L4 vs. M4	L4 Up	GO:0004151	Metabolism	Metabolism	dihydroorotase activity	F	AT4G22930
3040	2	L4 vs. M4	L4 Up	GO:0051213	Metabolism	Metabolism	dioxygenase activity	F	AT2G34555
3041	2	L4 vs. M4	L4 Up	GO:0051213	Metabolism	Metabolism	dioxygenase activity	F	AT3G55970
3042	2	L4 vs. M4	L4 Up	GO:0003677	Metabolism	Metabolism	DNA binding	F	AT1G32540
3043	2	L4 vs. M4	L4 Up	GO:0003677	Metabolism	Metabolism	DNA binding	F	AT1G34380
3044	2	L4 vs. M4	L4 Up	GO:0003677	Metabolism	Metabolism	DNA binding	F	AT1G79700
3045	2	L4 vs. M4	L4 Up	GO:0003677	Metabolism	Metabolism	DNA binding	F	AT2G25900
3046	2	L4 vs. M4	L4 Up	GO:0003677	Metabolism	Metabolism	DNA binding	F	AT3G12040
3047	2	L4 vs. M4	L4 Up	GO:0008725	Metabolism	Metabolism	DNA-3-methyladenine glycosylase activity	F	AT3G12040
3048	2	L4 vs. M4	L4 Up	GO:0052822	Metabolism	Metabolism	DNA-3-methylguanine glycosylase activity	F	AT3G12040
3049	2	L4 vs. M4	L4 Up	GO:0052821	Metabolism	Metabolism	DNA-7-methyladenine glycosylase activity	F	AT3G12040
3050	2	L4 vs. M4	L4 Up	GO:0043916	Metabolism	Metabolism	DNA-7-methylguanine glycosylase activity	F	AT3G12040

3051	2	L4 vs. M4	L4 Up	GO:0006488	Metabolism	Metabolism	dolichol-linked oligosaccharide biosynthetic process	P	AT4G18230
3052	2	L4 vs. M4	L4 Up	GO:0003690	Metabolism	Metabolism	double-stranded DNA binding	F	AT2G21660
3053	2	L4 vs. M4	L4 Up	GO:0004857	Metabolism	Metabolism	enzyme inhibitor activity	F	AT2G47050
3054	2	L4 vs. M4	L4 Up	GO:0071949	Metabolism	Metabolism	FAD binding	F	AT3G30775
3055	2	L4 vs. M4	L4 Up	GO:0071949	Metabolism	Metabolism	FAD binding	F	AT5G38710
3056	2	L4 vs. M4	L4 Up	GO:0008199	Metabolism	Metabolism	ferric iron binding	F	AT3G11050
3057	2	L4 vs. M4	L4 Up	GO:0008199	Metabolism	Metabolism	ferric iron binding	F	AT3G56090
3058	2	L4 vs. M4	L4 Up	GO:0008199	Metabolism	Metabolism	ferric iron binding	F	AT5G01600
3059	2	L4 vs. M4	L4 Up	GO:0008198	Metabolism	Metabolism	ferrous iron binding	F	AT3G11050
3060	2	L4 vs. M4	L4 Up	GO:0008198	Metabolism	Metabolism	ferrous iron binding	F	AT3G56090
3061	2	L4 vs. M4	L4 Up	GO:0008198	Metabolism	Metabolism	ferrous iron binding	F	AT5G01600
3062	2	L4 vs. M4	L4 Up	GO:0004322	Metabolism	Metabolism	ferroxidase activity	F	AT1G55570
3063	2	L4 vs. M4	L4 Up	GO:0004322	Metabolism	Metabolism	ferroxidase activity	F	AT3G11050
3064	2	L4 vs. M4	L4 Up	GO:0004322	Metabolism	Metabolism	ferroxidase activity	F	AT3G13390
3065	2	L4 vs. M4	L4 Up	GO:0004322	Metabolism	Metabolism	ferroxidase activity	F	AT3G13400
3066	2	L4 vs. M4	L4 Up	GO:0004322	Metabolism	Metabolism	ferroxidase activity	F	AT3G56090
3067	2	L4 vs. M4	L4 Up	GO:0004322	Metabolism	Metabolism	ferroxidase activity	F	AT5G01600
3068	2	L4 vs. M4	L4 Up	GO:0050660	Metabolism	Metabolism	flavin adenine dinucleotide binding	F	AT3G49640
3069	2	L4 vs. M4	L4 Up	GO:0004328	Metabolism	Metabolism	formamidase activity	F	AT4G37550
3070	2	L4 vs. M4	L4 Up	GO:0047274	Metabolism	Metabolism	galactinol-sucrose galactosyltransferase activity	F	AT5G20250
3071	2	L4 vs. M4	L4 Up	GO:0047911	Metabolism	Metabolism	galacturan 1,4-alpha-galacturonidase activity	F	AT3G07850
3072	2	L4 vs. M4	L4 Up	GO:0009298	Metabolism	Metabolism	GDP-mannose biosynthetic process	P	AT2G39770
3073	2	L4 vs. M4	L4 Up	GO:0102797	Metabolism	Metabolism	geranial:oxygen oxidoreductase activity	F	AT1G67290
3074	2	L4 vs. M4	L4 Up	GO:0005960	Metabolism	Metabolism	glycine cleavage complex	C	AT2G35370
3075	2	L4 vs. M4	L4 Up	GO:0019464	Metabolism	Metabolism	glycine decarboxylation via glycine cleavage system	P	AT2G35370
3076	2	L4 vs. M4	L4 Up	GO:0004375	Metabolism	Metabolism	glycine dehydrogenase (decarboxylating) activity	F	AT2G35370
3077	2	L4 vs. M4	L4 Up	GO:0033926	Metabolism	Metabolism	glycopeptide alpha-N-acetylgalactosaminidase activity	F	AT1G35580
3078	2	L4 vs. M4	L4 Up	GO:0005525	Metabolism	Metabolism	GTP binding	F	AT1G04170
3079	2	L4 vs. M4	L4 Up	GO:0005525	Metabolism	Metabolism	GTP binding	F	AT2G21880
3080	2	L4 vs. M4	L4 Up	GO:0005525	Metabolism	Metabolism	GTP binding	F	AT2G39770
3081	2	L4 vs. M4	L4 Up	GO:0005096	Metabolism	Metabolism	GTPase activator activity	F	AT3G07880
3082	2	L4 vs. M4	L4 Up	GO:0005096	Metabolism	Metabolism	GTPase activator activity	F	AT3G07890
3083	2	L4 vs. M4	L4 Up	GO:0005096	Metabolism	Metabolism	GTPase activator activity	F	AT5G09550
3084	2	L4 vs. M4	L4 Up	GO:0003924	Metabolism	Metabolism	GTPase activity	F	AT1G04170
3085	2	L4 vs. M4	L4 Up	GO:0003924	Metabolism	Metabolism	GTPase activity	F	AT2G21880
3086	2	L4 vs. M4	L4 Up	GO:0020037	Metabolism	Metabolism	heme binding	F	AT1G07890
3087	2	L4 vs. M4	L4 Up	GO:0020037	Metabolism	Metabolism	heme binding	F	AT1G13150
3088	2	L4 vs. M4	L4 Up	GO:0020037	Metabolism	Metabolism	heme binding	F	AT1G71695
3089	2	L4 vs. M4	L4 Up	GO:0020037	Metabolism	Metabolism	heme binding	F	AT1G74540
3090	2	L4 vs. M4	L4 Up	GO:0020037	Metabolism	Metabolism	heme binding	F	AT2G12190
3091	2	L4 vs. M4	L4 Up	GO:0020037	Metabolism	Metabolism	heme binding	F	AT5G08250
3092	2	L4 vs. M4	L4 Up	GO:0020037	Metabolism	Metabolism	heme binding	F	ATMG01360
3093	2	L4 vs. M4	L4 Up	GO:0102798	Metabolism	Metabolism	heptaldehyde:oxygen oxidoreductase activity	F	AT1G67290
3094	2	L4 vs. M4	L4 Up	GO:0010289	Metabolism	Metabolism	homogalacturonan biosynthetic process	P	AT3G25140
3095	2	L4 vs. M4	L4 Up	GO:0016787	Metabolism	Metabolism	hydrolase activity	F	AT4G30100
3096	2	L4 vs. M4	L4 Up	GO:0016811	Metabolism	Metabolism	hydrolase activity, acting on carbon-nitrogen (but not peptide) bonds, in linear amides	F	AT4G37550
3097	2	L4 vs. M4	L4 Up	GO:0016788	Metabolism	Metabolism	hydrolase activity, acting on ester bonds	F	AT1G54030
3098	2	L4 vs. M4	L4 Up	GO:0016788	Metabolism	Metabolism	hydrolase activity, acting on ester bonds	F	AT2G46880
3099	2	L4 vs. M4	L4 Up	GO:0042802	Metabolism	Metabolism	identical protein binding	F	AT1G30820
3100	2	L4 vs. M4	L4 Up	GO:0042802	Metabolism	Metabolism	identical protein binding	F	AT1G50170
3101	2	L4 vs. M4	L4 Up	GO:0042802	Metabolism	Metabolism	identical protein binding	F	AT3G11050
3102	2	L4 vs. M4	L4 Up	GO:0042802	Metabolism	Metabolism	identical protein binding	F	AT3G16240
3103	2	L4 vs. M4	L4 Up	GO:0042802	Metabolism	Metabolism	identical protein binding	F	AT3G56090

3104	2	L4 vs. M4	L4 Up	GO:0042802	Metabolism	Metabolism	identical protein binding	F	AT5G01600
3105	2	L4 vs. M4	L4 Up	GO:0006880	Metabolism	Metabolism	intracellular sequestering of iron ion	P	AT3G11050
3106	2	L4 vs. M4	L4 Up	GO:0006880	Metabolism	Metabolism	intracellular sequestering of iron ion	P	AT3G56090
3107	2	L4 vs. M4	L4 Up	GO:0006880	Metabolism	Metabolism	intracellular sequestering of iron ion	P	AT5G01600
3108	2	L4 vs. M4	L4 Up	GO:0005506	Metabolism	Metabolism	iron ion binding	F	AT1G13150
3109	2	L4 vs. M4	L4 Up	GO:0005506	Metabolism	Metabolism	iron ion binding	F	AT1G74540
3110	2	L4 vs. M4	L4 Up	GO:0005506	Metabolism	Metabolism	iron ion binding	F	AT2G12190
3111	2	L4 vs. M4	L4 Up	GO:0005506	Metabolism	Metabolism	iron ion binding	F	AT3G11050
3112	2	L4 vs. M4	L4 Up	GO:0005506	Metabolism	Metabolism	iron ion binding	F	AT3G56090
3113	2	L4 vs. M4	L4 Up	GO:0005506	Metabolism	Metabolism	iron ion binding	F	AT4G20870
3114	2	L4 vs. M4	L4 Up	GO:0005506	Metabolism	Metabolism	iron ion binding	F	AT4G26260
3115	2	L4 vs. M4	L4 Up	GO:0005506	Metabolism	Metabolism	iron ion binding	F	AT5G01600
3116	2	L4 vs. M4	L4 Up	GO:0005506	Metabolism	Metabolism	iron ion binding	F	AT5G08250
3117	2	L4 vs. M4	L4 Up	GO:0005506	Metabolism	Metabolism	iron ion binding	F	AT5G17170
3118	2	L4 vs. M4	L4 Up	GO:0005506	Metabolism	Metabolism	iron ion binding	F	AT5G56640
3119	2	L4 vs. M4	L4 Up	GO:0051536	Metabolism	Metabolism	iron-sulfur cluster binding	F	AT1G50170
3120	2	L4 vs. M4	L4 Up	GO:0009809	Metabolism	Metabolism	lignin biosynthetic process	P	AT5G54160
3121	2	L4 vs. M4	L4 Up	GO:0008289	Metabolism	Metabolism	lipid binding	F	AT3G51590
3122	2	L4 vs. M4	L4 Up	GO:0008289	Metabolism	Metabolism	lipid binding	F	AT5G02100
3123	2	L4 vs. M4	L4 Up	GO:0008289	Metabolism	Metabolism	lipid binding	F	AT5G07550
3124	2	L4 vs. M4	L4 Up	GO:0006629	Metabolism	Metabolism	lipid metabolic process	P	AT3G22960
3125	2	L4 vs. M4	L4 Up	GO:0019915	Metabolism	Metabolism	lipid storage	P	AT5G07550
3126	2	L4 vs. M4	L4 Up	GO:0030744	Metabolism	Metabolism	luteolin O-methyltransferase activity	F	AT5G54160
3127	2	L4 vs. M4	L4 Up	GO:0016829	Metabolism	Metabolism	lyase activity	F	AT1G02460
3128	2	L4 vs. M4	L4 Up	GO:0016829	Metabolism	Metabolism	lyase activity	F	AT1G60590
3129	2	L4 vs. M4	L4 Up	GO:0016829	Metabolism	Metabolism	lyase activity	F	AT3G07830
3130	2	L4 vs. M4	L4 Up	GO:0016829	Metabolism	Metabolism	lyase activity	F	AT3G07840
3131	2	L4 vs. M4	L4 Up	GO:0016829	Metabolism	Metabolism	lyase activity	F	AT5G15110
3132	2	L4 vs. M4	L4 Up	GO:0016829	Metabolism	Metabolism	lyase activity	F	AT5G48140
3133	2	L4 vs. M4	L4 Up	GO:0009089	Metabolism	Metabolism	lysine biosynthetic process via diaminopimelate	P	AT5G52100
3134	2	L4 vs. M4	L4 Up	GO:0016851	Metabolism	Metabolism	magnesium chelatase activity	F	AT5G13630
3135	2	L4 vs. M4	L4 Up	GO:0000287	Metabolism	Metabolism	magnesium ion binding	F	AT3G22960
3136	2	L4 vs. M4	L4 Up	GO:1901982	Metabolism	Metabolism	maltose binding	F	AT5G21170
3137	2	L4 vs. M4	L4 Up	GO:0030145	Metabolism	Metabolism	manganese ion binding	F	AT1G72610
3138	2	L4 vs. M4	L4 Up	GO:0030145	Metabolism	Metabolism	manganese ion binding	F	AT5G20630
3139	2	L4 vs. M4	L4 Up	GO:0030145	Metabolism	Metabolism	manganese ion binding	F	AT5G26700
3140	2	L4 vs. M4	L4 Up	GO:0004475	Metabolism	Metabolism	mannose-1-phosphate guanylyltransferase activity	F	AT2G39770
3141	2	L4 vs. M4	L4 Up	GO:0030187	Metabolism	Metabolism	melatonin biosynthetic process	P	AT5G54160
3142	2	L4 vs. M4	L4 Up	GO:0046872	Metabolism	Metabolism	metal ion binding	F	AT1G07890
3143	2	L4 vs. M4	L4 Up	GO:0046872	Metabolism	Metabolism	metal ion binding	F	AT1G29930
3144	2	L4 vs. M4	L4 Up	GO:0046872	Metabolism	Metabolism	metal ion binding	F	AT1G50170
3145	2	L4 vs. M4	L4 Up	GO:0046872	Metabolism	Metabolism	metal ion binding	F	AT1G56360
3146	2	L4 vs. M4	L4 Up	GO:0046872	Metabolism	Metabolism	metal ion binding	F	AT1G60950
3147	2	L4 vs. M4	L4 Up	GO:0046872	Metabolism	Metabolism	metal ion binding	F	AT1G71695
3148	2	L4 vs. M4	L4 Up	GO:0046872	Metabolism	Metabolism	metal ion binding	F	AT1G78020
3149	2	L4 vs. M4	L4 Up	GO:0046872	Metabolism	Metabolism	metal ion binding	F	AT2G07560
3150	2	L4 vs. M4	L4 Up	GO:0046872	Metabolism	Metabolism	metal ion binding	F	AT2G25900
3151	2	L4 vs. M4	L4 Up	GO:0046872	Metabolism	Metabolism	metal ion binding	F	AT2G34555
3152	2	L4 vs. M4	L4 Up	GO:0046872	Metabolism	Metabolism	metal ion binding	F	AT2G43400
3153	2	L4 vs. M4	L4 Up	GO:0046872	Metabolism	Metabolism	metal ion binding	F	AT2G46210
3154	2	L4 vs. M4	L4 Up	GO:0046872	Metabolism	Metabolism	metal ion binding	F	AT2G46880
3155	2	L4 vs. M4	L4 Up	GO:0046872	Metabolism	Metabolism	metal ion binding	F	AT3G21180
3156	2	L4 vs. M4	L4 Up	GO:0046872	Metabolism	Metabolism	metal ion binding	F	AT3G47470
3157	2	L4 vs. M4	L4 Up	GO:0046872	Metabolism	Metabolism	metal ion binding	F	AT3G55970
3158	2	L4 vs. M4	L4 Up	GO:0046872	Metabolism	Metabolism	metal ion binding	F	AT4G12620
3159	2	L4 vs. M4	L4 Up	GO:0046872	Metabolism	Metabolism	metal ion binding	F	AT4G22930

3160	2	L4 vs. M4	L4 Up	GO:0046872	Metabolism	Metabolism	metal ion binding	F	AT5G01530
3161	2	L4 vs. M4	L4 Up	GO:0046872	Metabolism	Metabolism	metal ion binding	F	AT5G15110
3162	2	L4 vs. M4	L4 Up	GO:0046872	Metabolism	Metabolism	metal ion binding	F	ATMG01360
3163	2	L4 vs. M4	L4 Up	GO:0008237	Metabolism	Metabolism	metallopeptidase activity	F	AT5G05740
3164	2	L4 vs. M4	L4 Up	GO:0008168	Metabolism	Metabolism	methyltransferase activity	F	AT3G51070
3165	2	L4 vs. M4	L4 Up	GO:0008168	Metabolism	Metabolism	methyltransferase activity	F	AT4G40000
3166	2	L4 vs. M4	L4 Up	GO:0051010	Metabolism	Metabolism	microtubule plus-end binding	F	AT2G20190
3167	2	L4 vs. M4	L4 Up	GO:0003674	Metabolism	Metabolism	molecular_function	F	AT1G13930
3168	2	L4 vs. M4	L4 Up	GO:0003674	Metabolism	Metabolism	molecular_function	F	AT1G14330
3169	2	L4 vs. M4	L4 Up	GO:0003674	Metabolism	Metabolism	molecular_function	F	AT1G14880
3170	2	L4 vs. M4	L4 Up	GO:0003674	Metabolism	Metabolism	molecular_function	F	AT1G16290
3171	2	L4 vs. M4	L4 Up	GO:0003674	Metabolism	Metabolism	molecular_function	F	AT1G48460
3172	2	L4 vs. M4	L4 Up	GO:0003674	Metabolism	Metabolism	molecular_function	F	AT1G52220
3173	2	L4 vs. M4	L4 Up	GO:0003674	Metabolism	Metabolism	molecular_function	F	AT1G62510
3174	2	L4 vs. M4	L4 Up	GO:0003674	Metabolism	Metabolism	molecular_function	F	AT1G70900
3175	2	L4 vs. M4	L4 Up	GO:0003674	Metabolism	Metabolism	molecular_function	F	AT1G74520
3176	2	L4 vs. M4	L4 Up	GO:0003674	Metabolism	Metabolism	molecular_function	F	AT1G79420
3177	2	L4 vs. M4	L4 Up	GO:0003674	Metabolism	Metabolism	molecular_function	F	AT2G19000
3178	2	L4 vs. M4	L4 Up	GO:0003674	Metabolism	Metabolism	molecular_function	F	AT2G19980
3179	2	L4 vs. M4	L4 Up	GO:0003674	Metabolism	Metabolism	molecular_function	F	AT2G36020
3180	2	L4 vs. M4	L4 Up	GO:0003674	Metabolism	Metabolism	molecular_function	F	AT2G44360
3181	2	L4 vs. M4	L4 Up	GO:0003674	Metabolism	Metabolism	molecular_function	F	AT2G46540
3182	2	L4 vs. M4	L4 Up	GO:0003674	Metabolism	Metabolism	molecular_function	F	AT3G15400
3183	2	L4 vs. M4	L4 Up	GO:0003674	Metabolism	Metabolism	molecular_function	F	AT3G20865
3184	2	L4 vs. M4	L4 Up	GO:0003674	Metabolism	Metabolism	molecular_function	F	AT3G28550
3185	2	L4 vs. M4	L4 Up	GO:0003674	Metabolism	Metabolism	molecular_function	F	AT3G54580
3186	2	L4 vs. M4	L4 Up	GO:0003674	Metabolism	Metabolism	molecular_function	F	AT3G62580
3187	2	L4 vs. M4	L4 Up	GO:0003674	Metabolism	Metabolism	molecular_function	F	AT4G02380
3188	2	L4 vs. M4	L4 Up	GO:0003674	Metabolism	Metabolism	molecular_function	F	AT4G02920
3189	2	L4 vs. M4	L4 Up	GO:0003674	Metabolism	Metabolism	molecular_function	F	AT4G09830
3190	2	L4 vs. M4	L4 Up	GO:0003674	Metabolism	Metabolism	molecular_function	F	AT4G12590
3191	2	L4 vs. M4	L4 Up	GO:0003674	Metabolism	Metabolism	molecular_function	F	AT4G13560
3192	2	L4 vs. M4	L4 Up	GO:0003674	Metabolism	Metabolism	molecular_function	F	AT4G24960
3193	2	L4 vs. M4	L4 Up	GO:0003674	Metabolism	Metabolism	molecular_function	F	AT4G25780
3194	2	L4 vs. M4	L4 Up	GO:0003674	Metabolism	Metabolism	molecular_function	F	AT4G28240
3195	2	L4 vs. M4	L4 Up	GO:0003674	Metabolism	Metabolism	molecular_function	F	AT4G33480
3196	2	L4 vs. M4	L4 Up	GO:0003674	Metabolism	Metabolism	molecular_function	F	AT4G35770
3197	2	L4 vs. M4	L4 Up	GO:0003674	Metabolism	Metabolism	molecular_function	F	AT5G19580
3198	2	L4 vs. M4	L4 Up	GO:0003674	Metabolism	Metabolism	molecular_function	F	AT5G19810
3199	2	L4 vs. M4	L4 Up	GO:0003674	Metabolism	Metabolism	molecular_function	F	AT5G25280
3200	2	L4 vs. M4	L4 Up	GO:0003674	Metabolism	Metabolism	molecular_function	F	AT5G38760
3201	2	L4 vs. M4	L4 Up	GO:0003674	Metabolism	Metabolism	molecular_function	F	AT5G45880
3202	2	L4 vs. M4	L4 Up	GO:0003674	Metabolism	Metabolism	molecular_function	F	AT5G53820
3203	2	L4 vs. M4	L4 Up	GO:0003674	Metabolism	Metabolism	molecular_function	F	AT5G54750
3204	2	L4 vs. M4	L4 Up	GO:0003674	Metabolism	Metabolism	molecular_function	F	AT5G59170
3205	2	L4 vs. M4	L4 Up	GO:0072549	Metabolism	Metabolism	monocoumaroyl dicaffeoyl spermidine meta-hydroxylase activity	F	AT1G74540
3206	2	L4 vs. M4	L4 Up	GO:0072552	Metabolism	Metabolism	monoferuloyl di-(hydroxyferuloyl) spermidine meta-hydroxylase activity	F	AT1G74540
3207	2	L4 vs. M4	L4 Up	GO:0004497	Metabolism	Metabolism	monooxygenase activity	F	AT1G13150
3208	2	L4 vs. M4	L4 Up	GO:0004497	Metabolism	Metabolism	monooxygenase activity	F	AT2G33380
3209	2	L4 vs. M4	L4 Up	GO:0004497	Metabolism	Metabolism	monooxygenase activity	F	AT5G08250
3210	2	L4 vs. M4	L4 Up	GO:0004497	Metabolism	Metabolism	monooxygenase activity	F	ATCG00490
3211	2	L4 vs. M4	L4 Up	GO:0033799	Metabolism	Metabolism	myricetin 3'-O-methyltransferase activity	F	AT5G54160
3212	2	L4 vs. M4	L4 Up	GO:0004577	Metabolism	Metabolism	N-acetylglucosaminyldiphosphodolichol N-acetylglucosaminyltransferase activity	F	AT4G18230

3213	2	L4 vs. M4	L4 Up	GO:0042128	Metabolism	Metabolism	nitrate assimilation	P	AT5G21170
3214	2	L4 vs. M4	L4 Up	GO:0017196	Metabolism	Metabolism	N-terminal peptidyl-methionine acetylation	P	AT1G03150
3215	2	L4 vs. M4	L4 Up	GO:0045735	Metabolism	Metabolism	nutrient reservoir activity	F	AT1G72610
3216	2	L4 vs. M4	L4 Up	GO:0045735	Metabolism	Metabolism	nutrient reservoir activity	F	AT5G20630
3217	2	L4 vs. M4	L4 Up	GO:0045735	Metabolism	Metabolism	nutrient reservoir activity	F	AT5G26700
3218	2	L4 vs. M4	L4 Up	GO:0008171	Metabolism	Metabolism	O-methyltransferase activity	F	AT5G54160
3219	2	L4 vs. M4	L4 Up	GO:0055114	Metabolism	Metabolism	oxidation-reduction process	P	AT1G07890
3220	2	L4 vs. M4	L4 Up	GO:0055114	Metabolism	Metabolism	oxidation-reduction process	P	AT1G13150
3221	2	L4 vs. M4	L4 Up	GO:0055114	Metabolism	Metabolism	oxidation-reduction process	P	AT1G55570
3222	2	L4 vs. M4	L4 Up	GO:0055114	Metabolism	Metabolism	oxidation-reduction process	P	AT1G67290
3223	2	L4 vs. M4	L4 Up	GO:0055114	Metabolism	Metabolism	oxidation-reduction process	P	AT1G71695
3224	2	L4 vs. M4	L4 Up	GO:0055114	Metabolism	Metabolism	oxidation-reduction process	P	AT1G74540
3225	2	L4 vs. M4	L4 Up	GO:0055114	Metabolism	Metabolism	oxidation-reduction process	P	AT2G12190
3226	2	L4 vs. M4	L4 Up	GO:0055114	Metabolism	Metabolism	oxidation-reduction process	P	AT2G33380
3227	2	L4 vs. M4	L4 Up	GO:0055114	Metabolism	Metabolism	oxidation-reduction process	P	AT2G34555
3228	2	L4 vs. M4	L4 Up	GO:0055114	Metabolism	Metabolism	oxidation-reduction process	P	AT2G46210
3229	2	L4 vs. M4	L4 Up	GO:0055114	Metabolism	Metabolism	oxidation-reduction process	P	AT3G11050
3230	2	L4 vs. M4	L4 Up	GO:0055114	Metabolism	Metabolism	oxidation-reduction process	P	AT3G13390
3231	2	L4 vs. M4	L4 Up	GO:0055114	Metabolism	Metabolism	oxidation-reduction process	P	AT3G13400
3232	2	L4 vs. M4	L4 Up	GO:0055114	Metabolism	Metabolism	oxidation-reduction process	P	AT3G30775
3233	2	L4 vs. M4	L4 Up	GO:0055114	Metabolism	Metabolism	oxidation-reduction process	P	AT3G49640
3234	2	L4 vs. M4	L4 Up	GO:0055114	Metabolism	Metabolism	oxidation-reduction process	P	AT3G55970
3235	2	L4 vs. M4	L4 Up	GO:0055114	Metabolism	Metabolism	oxidation-reduction process	P	AT3G56090
3236	2	L4 vs. M4	L4 Up	GO:0055114	Metabolism	Metabolism	oxidation-reduction process	P	AT4G20870
3237	2	L4 vs. M4	L4 Up	GO:0055114	Metabolism	Metabolism	oxidation-reduction process	P	AT4G25100
3238	2	L4 vs. M4	L4 Up	GO:0055114	Metabolism	Metabolism	oxidation-reduction process	P	AT4G26260
3239	2	L4 vs. M4	L4 Up	GO:0055114	Metabolism	Metabolism	oxidation-reduction process	P	AT4G35420
3240	2	L4 vs. M4	L4 Up	GO:0055114	Metabolism	Metabolism	oxidation-reduction process	P	AT5G01600
3241	2	L4 vs. M4	L4 Up	GO:0055114	Metabolism	Metabolism	oxidation-reduction process	P	AT5G02540
3242	2	L4 vs. M4	L4 Up	GO:0055114	Metabolism	Metabolism	oxidation-reduction process	P	AT5G08250
3243	2	L4 vs. M4	L4 Up	GO:0055114	Metabolism	Metabolism	oxidation-reduction process	P	AT5G16970
3244	2	L4 vs. M4	L4 Up	GO:0055114	Metabolism	Metabolism	oxidation-reduction process	P	AT5G38710
3245	2	L4 vs. M4	L4 Up	GO:0055114	Metabolism	Metabolism	oxidation-reduction process	P	AT5G52100
3246	2	L4 vs. M4	L4 Up	GO:0055114	Metabolism	Metabolism	oxidation-reduction process	P	AT5G56640
3247	2	L4 vs. M4	L4 Up	GO:0055114	Metabolism	Metabolism	oxidation-reduction process	P	ATCG00490
3248	2	L4 vs. M4	L4 Up	GO:0031407	Metabolism	Metabolism	oxylipin metabolic process	P	AT2G33380
3249	2	L4 vs. M4	L4 Up	GO:0030570	Metabolism	Metabolism	pectate lyase activity	F	AT5G15110
3250	2	L4 vs. M4	L4 Up	GO:0045489	Metabolism	Metabolism	pectin biosynthetic process	P	AT3G25140
3251	2	L4 vs. M4	L4 Up	GO:0030599	Metabolism	Metabolism	pectinesterase activity	F	AT2G47040
3252	2	L4 vs. M4	L4 Up	GO:0030599	Metabolism	Metabolism	pectinesterase activity	F	AT3G62170
3253	2	L4 vs. M4	L4 Up	GO:0030599	Metabolism	Metabolism	pectinesterase activity	F	AT4G02250
3254	2	L4 vs. M4	L4 Up	GO:0030599	Metabolism	Metabolism	pectinesterase activity	F	AT5G07410
3255	2	L4 vs. M4	L4 Up	GO:0046910	Metabolism	Metabolism	pectinesterase inhibitor activity	F	AT2G47040
3256	2	L4 vs. M4	L4 Up	GO:0046910	Metabolism	Metabolism	pectinesterase inhibitor activity	F	AT3G62170
3257	2	L4 vs. M4	L4 Up	GO:0046910	Metabolism	Metabolism	pectinesterase inhibitor activity	F	AT4G02250
3258	2	L4 vs. M4	L4 Up	GO:0004650	Metabolism	Metabolism	polygalacturonase activity	F	AT1G02460
3259	2	L4 vs. M4	L4 Up	GO:0004650	Metabolism	Metabolism	polygalacturonase activity	F	AT1G60590
3260	2	L4 vs. M4	L4 Up	GO:0004650	Metabolism	Metabolism	polygalacturonase activity	F	AT3G07830
3261	2	L4 vs. M4	L4 Up	GO:0004650	Metabolism	Metabolism	polygalacturonase activity	F	AT3G07840
3262	2	L4 vs. M4	L4 Up	GO:0004650	Metabolism	Metabolism	polygalacturonase activity	F	AT3G07850
3263	2	L4 vs. M4	L4 Up	GO:0004650	Metabolism	Metabolism	polygalacturonase activity	F	AT5G48140
3264	2	L4 vs. M4	L4 Up	GO:0047262	Metabolism	Metabolism	polygalacturonate 4-alpha-galacturonosyltransferase activity	F	AT3G25140
3265	2	L4 vs. M4	L4 Up	GO:0030247	Metabolism	Metabolism	polysaccharide binding	F	AT1G69295
3266	2	L4 vs. M4	L4 Up	GO:0030247	Metabolism	Metabolism	polysaccharide binding	F	AT5G08000
3267	2	L4 vs. M4	L4 Up	GO:0030955	Metabolism	Metabolism	potassium ion binding	F	AT3G22960

3268	2	L4 vs. M4	L4 Up	GO:0004657	Metabolism	Metabolism	proline dehydrogenase activity	F	AT3G30775
3269	2	L4 vs. M4	L4 Up	GO:0004657	Metabolism	Metabolism	proline dehydrogenase activity	F	AT5G38710
3270	2	L4 vs. M4	L4 Up	GO:0005515	Metabolism	Metabolism	protein binding	F	AT1G07890
3271	2	L4 vs. M4	L4 Up	GO:0005515	Metabolism	Metabolism	protein binding	F	AT1G27970
3272	2	L4 vs. M4	L4 Up	GO:0005515	Metabolism	Metabolism	protein binding	F	AT1G28270
3273	2	L4 vs. M4	L4 Up	GO:0005515	Metabolism	Metabolism	protein binding	F	AT1G35580
3274	2	L4 vs. M4	L4 Up	GO:0005515	Metabolism	Metabolism	protein binding	F	AT1G49010
3275	2	L4 vs. M4	L4 Up	GO:0005515	Metabolism	Metabolism	protein binding	F	AT1G62940
3276	2	L4 vs. M4	L4 Up	GO:0005515	Metabolism	Metabolism	protein binding	F	AT1G64520
3277	2	L4 vs. M4	L4 Up	GO:0005515	Metabolism	Metabolism	protein binding	F	AT1G78020
3278	2	L4 vs. M4	L4 Up	GO:0005515	Metabolism	Metabolism	protein binding	F	AT2G07560
3279	2	L4 vs. M4	L4 Up	GO:0005515	Metabolism	Metabolism	protein binding	F	AT2G18160
3280	2	L4 vs. M4	L4 Up	GO:0005515	Metabolism	Metabolism	protein binding	F	AT2G21660
3281	2	L4 vs. M4	L4 Up	GO:0005515	Metabolism	Metabolism	protein binding	F	AT2G25490
3282	2	L4 vs. M4	L4 Up	GO:0005515	Metabolism	Metabolism	protein binding	F	AT2G38310
3283	2	L4 vs. M4	L4 Up	GO:0005515	Metabolism	Metabolism	protein binding	F	AT2G39770
3284	2	L4 vs. M4	L4 Up	GO:0005515	Metabolism	Metabolism	protein binding	F	AT2G42940
3285	2	L4 vs. M4	L4 Up	GO:0005515	Metabolism	Metabolism	protein binding	F	AT2G46225
3286	2	L4 vs. M4	L4 Up	GO:0005515	Metabolism	Metabolism	protein binding	F	AT2G47040
3287	2	L4 vs. M4	L4 Up	GO:0005515	Metabolism	Metabolism	protein binding	F	AT3G07880
3288	2	L4 vs. M4	L4 Up	GO:0005515	Metabolism	Metabolism	protein binding	F	AT3G44680
3289	2	L4 vs. M4	L4 Up	GO:0005515	Metabolism	Metabolism	protein binding	F	AT4G12620
3290	2	L4 vs. M4	L4 Up	GO:0005515	Metabolism	Metabolism	protein binding	F	AT4G20870
3291	2	L4 vs. M4	L4 Up	GO:0005515	Metabolism	Metabolism	protein binding	F	AT4G25100
3292	2	L4 vs. M4	L4 Up	GO:0005515	Metabolism	Metabolism	protein binding	F	AT4G35420
3293	2	L4 vs. M4	L4 Up	GO:0005515	Metabolism	Metabolism	protein binding	F	AT5G02100
3294	2	L4 vs. M4	L4 Up	GO:0005515	Metabolism	Metabolism	protein binding	F	AT5G13630
3295	2	L4 vs. M4	L4 Up	GO:0005515	Metabolism	Metabolism	protein binding	F	AT5G15200
3296	2	L4 vs. M4	L4 Up	GO:0005515	Metabolism	Metabolism	protein binding	F	AT5G21170
3297	2	L4 vs. M4	L4 Up	GO:0005515	Metabolism	Metabolism	protein binding	F	AT5G23660
3298	2	L4 vs. M4	L4 Up	GO:0005515	Metabolism	Metabolism	protein binding	F	AT5G39850
3299	2	L4 vs. M4	L4 Up	GO:0005515	Metabolism	Metabolism	protein binding	F	AT5G42000
3300	2	L4 vs. M4	L4 Up	GO:0005515	Metabolism	Metabolism	protein binding	F	AT5G50800
3301	2	L4 vs. M4	L4 Up	GO:0005515	Metabolism	Metabolism	protein binding	F	AT5G53190
3302	2	L4 vs. M4	L4 Up	GO:0005515	Metabolism	Metabolism	protein binding	F	AT5G55990
3303	2	L4 vs. M4	L4 Up	GO:0005515	Metabolism	Metabolism	protein binding	F	AT5G63190
3304	2	L4 vs. M4	L4 Up	GO:0005515	Metabolism	Metabolism	protein binding	F	ATCG00490
3305	2	L4 vs. M4	L4 Up	GO:0120029	Metabolism	Metabolism	proton export across plasma membrane	P	AT2G07560
3306	2	L4 vs. M4	L4 Up	GO:0019856	Metabolism	Metabolism	pyrimidine nucleobase biosynthetic process	P	AT1G30820
3307	2	L4 vs. M4	L4 Up	GO:0006221	Metabolism	Metabolism	pyrimidine nucleotide biosynthetic process	P	AT4G22930
3308	2	L4 vs. M4	L4 Up	GO:0102822	Metabolism	Metabolism	quercetin 3'-O-methyltransferase activity	F	AT5G54160
3309	2	L4 vs. M4	L4 Up	GO:0030755	Metabolism	Metabolism	quercetin 3-O-methyltransferase activity	F	AT5G54160
3310	2	L4 vs. M4	L4 Up	GO:0048038	Metabolism	Metabolism	quinone binding	F	AT5G11770
3311	2	L4 vs. M4	L4 Up	GO:0008757	Metabolism	Metabolism	S-adenosylmethionine-dependent methyltransferase activity	F	AT5G54160
3312	2	L4 vs. M4	L4 Up	GO:0019748	Metabolism	Metabolism	secondary metabolic process	P	AT2G23010
3313	2	L4 vs. M4	L4 Up	GO:0042989	Metabolism	Metabolism	sequestering of actin monomers	P	AT4G29340
3314	2	L4 vs. M4	L4 Up	GO:0047158	Metabolism	Metabolism	sinapoylglucose-sinapoylglucose O-sinapoyltransferase activity	F	AT2G23010
3315	2	L4 vs. M4	L4 Up	GO:0019354	Metabolism	Metabolism	siroheme biosynthetic process	P	AT1G50170
3316	2	L4 vs. M4	L4 Up	GO:0016852	Metabolism	Metabolism	sirohydrochlorin cobaltochelatase activity	F	AT1G50170
3317	2	L4 vs. M4	L4 Up	GO:0051266	Metabolism	Metabolism	sirohydrochlorin ferrochelatase activity	F	AT1G50170
3318	2	L4 vs. M4	L4 Up	GO:0080088	Metabolism	Metabolism	spermidine hydroxycinnamate conjugate biosynthetic process	P	AT1G74540
3319	2	L4 vs. M4	L4 Up	GO:0008216	Metabolism	Metabolism	spermidine metabolic process	P	AT1G74540
3320	2	L4 vs. M4	L4 Up	GO:0030148	Metabolism	Metabolism	sphingolipid biosynthetic process	P	AT2G46210

3321	2	L4 vs. M4	L4 Up	GO:0030148	Metabolism	Metabolism	sphingolipid biosynthetic process	P	AT5G42000
3322	2	L4 vs. M4	L4 Up	GO:0052631	Metabolism	Metabolism	sphingolipid delta-8 desaturase activity	F	AT2G46210
3323	2	L4 vs. M4	L4 Up	GO:0080110	Metabolism	Metabolism	sporopollenin biosynthetic process	P	AT1G62940
3324	2	L4 vs. M4	L4 Up	GO:0080110	Metabolism	Metabolism	sporopollenin biosynthetic process	P	AT4G35420
3325	2	L4 vs. M4	L4 Up	GO:0035339	Metabolism	Metabolism	SPOTS complex	C	AT5G42000
3326	2	L4 vs. M4	L4 Up	GO:0004575	Metabolism	Metabolism	sucrose alpha-glucosidase activity	F	AT1G35580
3327	2	L4 vs. M4	L4 Up	GO:0008483	Metabolism	Metabolism	transaminase activity	F	AT5G48590
3328	2	L4 vs. M4	L4 Up	GO:0016757	Metabolism	Metabolism	transferase activity, transferring glycosyl groups	F	AT1G60140
3329	2	L4 vs. M4	L4 Up	GO:0016757	Metabolism	Metabolism	transferase activity, transferring glycosyl groups	F	AT3G25140
3330	2	L4 vs. M4	L4 Up	GO:0016757	Metabolism	Metabolism	transferase activity, transferring glycosyl groups	F	AT5G14550
3331	2	L4 vs. M4	L4 Up	GO:0072532	Metabolism	Metabolism	tri-(feruloyl or hydroxyferuloyl) spermidine meta-hydroxylase activity	F	AT1G74540
3332	2	L4 vs. M4	L4 Up	GO:0072547	Metabolism	Metabolism	tricoumaroylspermidine meta-hydroxylase activity	F	AT1G74540
3333	2	L4 vs. M4	L4 Up	GO:0072550	Metabolism	Metabolism	triferuloylspermidine meta-hydroxylase activity	F	AT1G74540
3334	2	L4 vs. M4	L4 Up	GO:0008270	Metabolism	Metabolism	zinc ion binding	F	AT3G01500
3335	2	L4 vs. M4	L4 Up	GO:0008270	Metabolism	Metabolism	zinc ion binding	F	AT4G26910
3336	2	L4 vs. M4	L4 Up	GO:0008270	Metabolism	Metabolism	zinc ion binding	F	AT4G37610
3337	2	L4 vs. M4	L4 Up	GO:0008270	Metabolism	Metabolism	zinc ion binding	F	AT5G04180
3338	2	L4 vs. M4	L4 Up	GO:0008270	Metabolism	Metabolism	zinc ion binding	F	AT5G11770
3339	2	L4 vs. M4	L4 Up	GO:0015977	Metabolism	Photosynthesis	carbon fixation	P	ATCG00490
3340	2	L4 vs. M4	L4 Up	GO:0016168	Metabolism	Photosynthesis	chlorophyll binding	F	AT1G29930
3341	2	L4 vs. M4	L4 Up	GO:0016168	Metabolism	Photosynthesis	chlorophyll binding	F	AT2G34430
3342	2	L4 vs. M4	L4 Up	GO:0016168	Metabolism	Photosynthesis	chlorophyll binding	F	AT3G47470
3343	2	L4 vs. M4	L4 Up	GO:0016168	Metabolism	Photosynthesis	chlorophyll binding	F	AT5G01530
3344	2	L4 vs. M4	L4 Up	GO:0015995	Metabolism	Photosynthesis	chlorophyll biosynthetic process	P	AT5G13630
3345	2	L4 vs. M4	L4 Up	GO:0009507	Metabolism	Photosynthesis	chloroplast	C	AT1G07890
3346	2	L4 vs. M4	L4 Up	GO:0009507	Metabolism	Photosynthesis	chloroplast	C	AT1G13930
3347	2	L4 vs. M4	L4 Up	GO:0009507	Metabolism	Photosynthesis	chloroplast	C	AT1G16470
3348	2	L4 vs. M4	L4 Up	GO:0009507	Metabolism	Photosynthesis	chloroplast	C	AT1G27450
3349	2	L4 vs. M4	L4 Up	GO:0009507	Metabolism	Photosynthesis	chloroplast	C	AT1G29930
3350	2	L4 vs. M4	L4 Up	GO:0009507	Metabolism	Photosynthesis	chloroplast	C	AT1G35580
3351	2	L4 vs. M4	L4 Up	GO:0009507	Metabolism	Photosynthesis	chloroplast	C	AT1G50170
3352	2	L4 vs. M4	L4 Up	GO:0009507	Metabolism	Photosynthesis	chloroplast	C	AT1G52220
3353	2	L4 vs. M4	L4 Up	GO:0009507	Metabolism	Photosynthesis	chloroplast	C	AT1G54030
3354	2	L4 vs. M4	L4 Up	GO:0009507	Metabolism	Photosynthesis	chloroplast	C	AT1G60950
3355	2	L4 vs. M4	L4 Up	GO:0009507	Metabolism	Photosynthesis	chloroplast	C	AT1G62940
3356	2	L4 vs. M4	L4 Up	GO:0009507	Metabolism	Photosynthesis	chloroplast	C	AT1G67600
3357	2	L4 vs. M4	L4 Up	GO:0009507	Metabolism	Photosynthesis	chloroplast	C	AT1G79210
3358	2	L4 vs. M4	L4 Up	GO:0009507	Metabolism	Photosynthesis	chloroplast	C	AT1G80920
3359	2	L4 vs. M4	L4 Up	GO:0009507	Metabolism	Photosynthesis	chloroplast	C	AT2G01110
3360	2	L4 vs. M4	L4 Up	GO:0009507	Metabolism	Photosynthesis	chloroplast	C	AT2G03730
3361	2	L4 vs. M4	L4 Up	GO:0009507	Metabolism	Photosynthesis	chloroplast	C	AT2G20670
3362	2	L4 vs. M4	L4 Up	GO:0009507	Metabolism	Photosynthesis	chloroplast	C	AT2G21660
3363	2	L4 vs. M4	L4 Up	GO:0009507	Metabolism	Photosynthesis	chloroplast	C	AT2G25830
3364	2	L4 vs. M4	L4 Up	GO:0009507	Metabolism	Photosynthesis	chloroplast	C	AT2G34430
3365	2	L4 vs. M4	L4 Up	GO:0009507	Metabolism	Photosynthesis	chloroplast	C	AT2G38330
3366	2	L4 vs. M4	L4 Up	GO:0009507	Metabolism	Photosynthesis	chloroplast	C	AT2G43400
3367	2	L4 vs. M4	L4 Up	GO:0009507	Metabolism	Photosynthesis	chloroplast	C	AT2G46880
3368	2	L4 vs. M4	L4 Up	GO:0009507	Metabolism	Photosynthesis	chloroplast	C	AT2G47050
3369	2	L4 vs. M4	L4 Up	GO:0009507	Metabolism	Photosynthesis	chloroplast	C	AT3G01500
3370	2	L4 vs. M4	L4 Up	GO:0009507	Metabolism	Photosynthesis	chloroplast	C	AT3G11050
3371	2	L4 vs. M4	L4 Up	GO:0009507	Metabolism	Photosynthesis	chloroplast	C	AT3G15400
3372	2	L4 vs. M4	L4 Up	GO:0009507	Metabolism	Photosynthesis	chloroplast	C	AT3G15450
3373	2	L4 vs. M4	L4 Up	GO:0009507	Metabolism	Photosynthesis	chloroplast	C	AT3G22960
3374	2	L4 vs. M4	L4 Up	GO:0009507	Metabolism	Photosynthesis	chloroplast	C	AT3G47470
3375	2	L4 vs. M4	L4 Up	GO:0009507	Metabolism	Photosynthesis	chloroplast	C	AT3G49640

3376	2	L4 vs. M4	L4 Up	GO:0009507	Metabolism	Photosynthesis	chloroplast	C	AT3G56090
3377	2	L4 vs. M4	L4 Up	GO:0009507	Metabolism	Photosynthesis	chloroplast	C	AT3G62580
3378	2	L4 vs. M4	L4 Up	GO:0009507	Metabolism	Photosynthesis	chloroplast	C	AT4G02380
3379	2	L4 vs. M4	L4 Up	GO:0009507	Metabolism	Photosynthesis	chloroplast	C	AT4G05180
3380	2	L4 vs. M4	L4 Up	GO:0009507	Metabolism	Photosynthesis	chloroplast	C	AT4G10000
3381	2	L4 vs. M4	L4 Up	GO:0009507	Metabolism	Photosynthesis	chloroplast	C	AT4G19160
3382	2	L4 vs. M4	L4 Up	GO:0009507	Metabolism	Photosynthesis	chloroplast	C	AT4G22930
3383	2	L4 vs. M4	L4 Up	GO:0009507	Metabolism	Photosynthesis	chloroplast	C	AT4G25100
3384	2	L4 vs. M4	L4 Up	GO:0009507	Metabolism	Photosynthesis	chloroplast	C	AT4G33480
3385	2	L4 vs. M4	L4 Up	GO:0009507	Metabolism	Photosynthesis	chloroplast	C	AT4G35770
3386	2	L4 vs. M4	L4 Up	GO:0009507	Metabolism	Photosynthesis	chloroplast	C	AT4G36040
3387	2	L4 vs. M4	L4 Up	GO:0009507	Metabolism	Photosynthesis	chloroplast	C	AT4G37550
3388	2	L4 vs. M4	L4 Up	GO:0009507	Metabolism	Photosynthesis	chloroplast	C	AT5G01530
3389	2	L4 vs. M4	L4 Up	GO:0009507	Metabolism	Photosynthesis	chloroplast	C	AT5G01600
3390	2	L4 vs. M4	L4 Up	GO:0009507	Metabolism	Photosynthesis	chloroplast	C	AT5G02100
3391	2	L4 vs. M4	L4 Up	GO:0009507	Metabolism	Photosynthesis	chloroplast	C	AT5G05740
3392	2	L4 vs. M4	L4 Up	GO:0009507	Metabolism	Photosynthesis	chloroplast	C	AT5G07550
3393	2	L4 vs. M4	L4 Up	GO:0009507	Metabolism	Photosynthesis	chloroplast	C	AT5G13630
3394	2	L4 vs. M4	L4 Up	GO:0009507	Metabolism	Photosynthesis	chloroplast	C	AT5G14040
3395	2	L4 vs. M4	L4 Up	GO:0009507	Metabolism	Photosynthesis	chloroplast	C	AT5G15200
3396	2	L4 vs. M4	L4 Up	GO:0009507	Metabolism	Photosynthesis	chloroplast	C	AT5G17170
3397	2	L4 vs. M4	L4 Up	GO:0009507	Metabolism	Photosynthesis	chloroplast	C	AT5G20250
3398	2	L4 vs. M4	L4 Up	GO:0009507	Metabolism	Photosynthesis	chloroplast	C	AT5G21170
3399	2	L4 vs. M4	L4 Up	GO:0009507	Metabolism	Photosynthesis	chloroplast	C	AT5G39850
3400	2	L4 vs. M4	L4 Up	GO:0009507	Metabolism	Photosynthesis	chloroplast	C	AT5G49070
3401	2	L4 vs. M4	L4 Up	GO:0009507	Metabolism	Photosynthesis	chloroplast	C	AT5G52100
3402	2	L4 vs. M4	L4 Up	GO:0009507	Metabolism	Photosynthesis	chloroplast	C	AT5G53820
3403	2	L4 vs. M4	L4 Up	GO:0009507	Metabolism	Photosynthesis	chloroplast	C	AT5G65685
3404	2	L4 vs. M4	L4 Up	GO:0009507	Metabolism	Photosynthesis	chloroplast	C	ATCG00490
3405	2	L4 vs. M4	L4 Up	GO:0009941	Metabolism	Photosynthesis	chloroplast envelope	C	AT1G13930
3406	2	L4 vs. M4	L4 Up	GO:0009941	Metabolism	Photosynthesis	chloroplast envelope	C	AT1G29930
3407	2	L4 vs. M4	L4 Up	GO:0009941	Metabolism	Photosynthesis	chloroplast envelope	C	AT1G48460
3408	2	L4 vs. M4	L4 Up	GO:0009941	Metabolism	Photosynthesis	chloroplast envelope	C	AT1G64520
3409	2	L4 vs. M4	L4 Up	GO:0009941	Metabolism	Photosynthesis	chloroplast envelope	C	AT2G34430
3410	2	L4 vs. M4	L4 Up	GO:0009941	Metabolism	Photosynthesis	chloroplast envelope	C	AT3G01500
3411	2	L4 vs. M4	L4 Up	GO:0009941	Metabolism	Photosynthesis	chloroplast envelope	C	AT3G16240
3412	2	L4 vs. M4	L4 Up	GO:0009941	Metabolism	Photosynthesis	chloroplast envelope	C	AT3G47470
3413	2	L4 vs. M4	L4 Up	GO:0009941	Metabolism	Photosynthesis	chloroplast envelope	C	AT3G56090
3414	2	L4 vs. M4	L4 Up	GO:0009941	Metabolism	Photosynthesis	chloroplast envelope	C	AT4G25100
3415	2	L4 vs. M4	L4 Up	GO:0009941	Metabolism	Photosynthesis	chloroplast envelope	C	AT5G01530
3416	2	L4 vs. M4	L4 Up	GO:0009941	Metabolism	Photosynthesis	chloroplast envelope	C	AT5G17170
3417	2	L4 vs. M4	L4 Up	GO:0009941	Metabolism	Photosynthesis	chloroplast envelope	C	ATCG00490
3418	2	L4 vs. M4	L4 Up	GO:0009706	Metabolism	Photosynthesis	chloroplast inner membrane	C	AT5G13630
3419	2	L4 vs. M4	L4 Up	GO:0031969	Metabolism	Photosynthesis	chloroplast membrane	C	AT2G33380
3420	2	L4 vs. M4	L4 Up	GO:0031969	Metabolism	Photosynthesis	chloroplast membrane	C	AT2G38330
3421	2	L4 vs. M4	L4 Up	GO:0031969	Metabolism	Photosynthesis	chloroplast membrane	C	AT4G25100
3422	2	L4 vs. M4	L4 Up	GO:0031969	Metabolism	Photosynthesis	chloroplast membrane	C	AT5G05740
3423	2	L4 vs. M4	L4 Up	GO:0042644	Metabolism	Photosynthesis	chloroplast nucleoid	C	AT4G25100
3424	2	L4 vs. M4	L4 Up	GO:0009570	Metabolism	Photosynthesis	chloroplast stroma	C	AT1G07890
3425	2	L4 vs. M4	L4 Up	GO:0009570	Metabolism	Photosynthesis	chloroplast stroma	C	AT1G13930
3426	2	L4 vs. M4	L4 Up	GO:0009570	Metabolism	Photosynthesis	chloroplast stroma	C	AT1G27450
3427	2	L4 vs. M4	L4 Up	GO:0009570	Metabolism	Photosynthesis	chloroplast stroma	C	AT1G60950
3428	2	L4 vs. M4	L4 Up	GO:0009570	Metabolism	Photosynthesis	chloroplast stroma	C	AT1G80920
3429	2	L4 vs. M4	L4 Up	GO:0009570	Metabolism	Photosynthesis	chloroplast stroma	C	AT3G01500
3430	2	L4 vs. M4	L4 Up	GO:0009570	Metabolism	Photosynthesis	chloroplast stroma	C	AT3G22960
3431	2	L4 vs. M4	L4 Up	GO:0009570	Metabolism	Photosynthesis	chloroplast stroma	C	AT3G56090

3432	2	L4 vs. M4	L4 Up	GO:0009570	Metabolism	Photosynthesis	chloroplast stroma	C	AT4G05180
3433	2	L4 vs. M4	L4 Up	GO:0009570	Metabolism	Photosynthesis	chloroplast stroma	C	AT4G25100
3434	2	L4 vs. M4	L4 Up	GO:0009570	Metabolism	Photosynthesis	chloroplast stroma	C	AT4G36040
3435	2	L4 vs. M4	L4 Up	GO:0009570	Metabolism	Photosynthesis	chloroplast stroma	C	AT5G01600
3436	2	L4 vs. M4	L4 Up	GO:0009570	Metabolism	Photosynthesis	chloroplast stroma	C	AT5G04180
3437	2	L4 vs. M4	L4 Up	GO:0009570	Metabolism	Photosynthesis	chloroplast stroma	C	AT5G13630
3438	2	L4 vs. M4	L4 Up	GO:0009570	Metabolism	Photosynthesis	chloroplast stroma	C	AT5G52100
3439	2	L4 vs. M4	L4 Up	GO:0009570	Metabolism	Photosynthesis	chloroplast stroma	C	ATCG00490
3440	2	L4 vs. M4	L4 Up	GO:0009534	Metabolism	Photosynthesis	chloroplast thylakoid	C	AT1G29930
3441	2	L4 vs. M4	L4 Up	GO:0009534	Metabolism	Photosynthesis	chloroplast thylakoid	C	AT2G34430
3442	2	L4 vs. M4	L4 Up	GO:0009534	Metabolism	Photosynthesis	chloroplast thylakoid	C	AT3G47470
3443	2	L4 vs. M4	L4 Up	GO:0009534	Metabolism	Photosynthesis	chloroplast thylakoid	C	AT4G04330
3444	2	L4 vs. M4	L4 Up	GO:0009534	Metabolism	Photosynthesis	chloroplast thylakoid	C	AT4G05180
3445	2	L4 vs. M4	L4 Up	GO:0009534	Metabolism	Photosynthesis	chloroplast thylakoid	C	AT5G01530
3446	2	L4 vs. M4	L4 Up	GO:0009534	Metabolism	Photosynthesis	chloroplast thylakoid	C	AT5G17170
3447	2	L4 vs. M4	L4 Up	GO:0009543	Metabolism	Photosynthesis	chloroplast thylakoid lumen	C	AT4G05180
3448	2	L4 vs. M4	L4 Up	GO:0009535	Metabolism	Photosynthesis	chloroplast thylakoid membrane	C	AT1G29930
3449	2	L4 vs. M4	L4 Up	GO:0009535	Metabolism	Photosynthesis	chloroplast thylakoid membrane	C	AT1G52220
3450	2	L4 vs. M4	L4 Up	GO:0009535	Metabolism	Photosynthesis	chloroplast thylakoid membrane	C	AT2G01110
3451	2	L4 vs. M4	L4 Up	GO:0009535	Metabolism	Photosynthesis	chloroplast thylakoid membrane	C	AT2G34430
3452	2	L4 vs. M4	L4 Up	GO:0009535	Metabolism	Photosynthesis	chloroplast thylakoid membrane	C	AT2G45180
3453	2	L4 vs. M4	L4 Up	GO:0009535	Metabolism	Photosynthesis	chloroplast thylakoid membrane	C	AT3G01500
3454	2	L4 vs. M4	L4 Up	GO:0009535	Metabolism	Photosynthesis	chloroplast thylakoid membrane	C	AT3G47470
3455	2	L4 vs. M4	L4 Up	GO:0009535	Metabolism	Photosynthesis	chloroplast thylakoid membrane	C	AT4G05180
3456	2	L4 vs. M4	L4 Up	GO:0009535	Metabolism	Photosynthesis	chloroplast thylakoid membrane	C	AT5G01530
3457	2	L4 vs. M4	L4 Up	GO:0009535	Metabolism	Photosynthesis	chloroplast thylakoid membrane	C	AT5G01600
3458	2	L4 vs. M4	L4 Up	GO:0009535	Metabolism	Photosynthesis	chloroplast thylakoid membrane	C	AT5G05740
3459	2	L4 vs. M4	L4 Up	GO:0009535	Metabolism	Photosynthesis	chloroplast thylakoid membrane	C	AT5G17170
3460	2	L4 vs. M4	L4 Up	GO:0009535	Metabolism	Photosynthesis	chloroplast thylakoid membrane	C	ATCG00490
3461	2	L4 vs. M4	L4 Up	GO:0009535	Metabolism	Photosynthesis	chloroplast thylakoid membrane	C	ATCG00700
3462	2	L4 vs. M4	L4 Up	GO:0045156	Metabolism	Photosynthesis	electron transporter, transferring electrons within the cyclic electron transport pathway of photosynthesis activity	F	AT4G05180
3463	2	L4 vs. M4	L4 Up	GO:0031361	Metabolism	Photosynthesis	integral component of thylakoid membrane	C	AT2G01110
3464	2	L4 vs. M4	L4 Up	GO:0010007	Metabolism	Photosynthesis	magnesium chelatase complex	C	AT5G13630
3465	2	L4 vs. M4	L4 Up	GO:0015979	Metabolism	Photosynthesis	photosynthesis	P	AT2G34430
3466	2	L4 vs. M4	L4 Up	GO:0015979	Metabolism	Photosynthesis	photosynthesis	P	AT3G01500
3467	2	L4 vs. M4	L4 Up	GO:0015979	Metabolism	Photosynthesis	photosynthesis	P	AT3G11050
3468	2	L4 vs. M4	L4 Up	GO:0015979	Metabolism	Photosynthesis	photosynthesis	P	AT3G56090
3469	2	L4 vs. M4	L4 Up	GO:0015979	Metabolism	Photosynthesis	photosynthesis	P	AT5G01600
3470	2	L4 vs. M4	L4 Up	GO:0015979	Metabolism	Photosynthesis	photosynthesis	P	AT5G13630
3471	2	L4 vs. M4	L4 Up	GO:0015979	Metabolism	Photosynthesis	photosynthesis	P	ATCG00700
3472	2	L4 vs. M4	L4 Up	GO:0009768	Metabolism	Photosynthesis	photosynthesis, light harvesting in photosystem I	P	AT1G29930
3473	2	L4 vs. M4	L4 Up	GO:0009768	Metabolism	Photosynthesis	photosynthesis, light harvesting in photosystem I	P	AT2G34430
3474	2	L4 vs. M4	L4 Up	GO:0009768	Metabolism	Photosynthesis	photosynthesis, light harvesting in photosystem I	P	AT3G47470
3475	2	L4 vs. M4	L4 Up	GO:0009768	Metabolism	Photosynthesis	photosynthesis, light harvesting in photosystem I	P	AT5G01530
3476	2	L4 vs. M4	L4 Up	GO:0009769	Metabolism	Photosynthesis	photosynthesis, light harvesting in photosystem II	P	AT2G34430
3477	2	L4 vs. M4	L4 Up	GO:0019684	Metabolism	Photosynthesis	photosynthesis, light reaction	P	AT5G52100
3478	2	L4 vs. M4	L4 Up	GO:0009643	Metabolism	Photosynthesis	photosynthetic acclimation	P	AT1G60950
3479	2	L4 vs. M4	L4 Up	GO:0009767	Metabolism	Photosynthesis	photosynthetic electron transport chain	P	AT1G60950
3480	2	L4 vs. M4	L4 Up	GO:0009767	Metabolism	Photosynthesis	photosynthetic electron transport chain	P	AT4G05180
3481	2	L4 vs. M4	L4 Up	GO:0009522	Metabolism	Photosynthesis	photosystem I	C	AT1G29930
3482	2	L4 vs. M4	L4 Up	GO:0009522	Metabolism	Photosynthesis	photosystem I	C	AT2G34430
3483	2	L4 vs. M4	L4 Up	GO:0009522	Metabolism	Photosynthesis	photosystem I	C	AT3G47470
3484	2	L4 vs. M4	L4 Up	GO:0009522	Metabolism	Photosynthesis	photosystem I	C	AT5G01530
3485	2	L4 vs. M4	L4 Up	GO:0009523	Metabolism	Photosynthesis	photosystem II	C	AT1G29930

3486	2	L4 vs. M4	L4 Up	GO:0009523	Metabolism	Photosynthesis	photosystem II	C	AT2G34430
3487	2	L4 vs. M4	L4 Up	GO:0009523	Metabolism	Photosynthesis	photosystem II	C	AT5G01530
3488	2	L4 vs. M4	L4 Up	GO:0009654	Metabolism	Photosynthesis	photosystem II oxygen evolving complex	C	AT4G05180
3489	2	L4 vs. M4	L4 Up	GO:0009539	Metabolism	Photosynthesis	photosystem II reaction center	C	ATCG00700
3490	2	L4 vs. M4	L4 Up	GO:0009536	Metabolism	Photosynthesis	plastid	C	AT1G48460
3491	2	L4 vs. M4	L4 Up	GO:0009536	Metabolism	Photosynthesis	plastid	C	AT4G36040
3492	2	L4 vs. M4	L4 Up	GO:0009536	Metabolism	Photosynthesis	plastid	C	AT5G13630
3493	2	L4 vs. M4	L4 Up	GO:0009536	Metabolism	Photosynthesis	plastid	C	AT5G15200
3494	2	L4 vs. M4	L4 Up	GO:0009536	Metabolism	Photosynthesis	plastid	C	AT5G16970
3495	2	L4 vs. M4	L4 Up	GO:0009536	Metabolism	Photosynthesis	plastid	C	AT5G17170
3496	2	L4 vs. M4	L4 Up	GO:0009536	Metabolism	Photosynthesis	plastid	C	AT5G20630
3497	2	L4 vs. M4	L4 Up	GO:0009536	Metabolism	Photosynthesis	plastid	C	AT5G39850
3498	2	L4 vs. M4	L4 Up	GO:0009536	Metabolism	Photosynthesis	plastid	C	AT5G54160
3499	2	L4 vs. M4	L4 Up	GO:0009536	Metabolism	Photosynthesis	plastid	C	AT5G54750
3500	2	L4 vs. M4	L4 Up	GO:0009536	Metabolism	Photosynthesis	plastid	C	AT5G55990
3501	2	L4 vs. M4	L4 Up	GO:0009536	Metabolism	Photosynthesis	plastid	C	ATCG00490
3502	2	L4 vs. M4	L4 Up	GO:0010287	Metabolism	Photosynthesis	plastoglobule	C	AT1G29930
3503	2	L4 vs. M4	L4 Up	GO:0010287	Metabolism	Photosynthesis	plastoglobule	C	AT2G34430
3504	2	L4 vs. M4	L4 Up	GO:0010287	Metabolism	Photosynthesis	plastoglobule	C	AT3G47470
3505	2	L4 vs. M4	L4 Up	GO:0010287	Metabolism	Photosynthesis	plastoglobule	C	AT5G01530
3506	2	L4 vs. M4	L4 Up	GO:0010287	Metabolism	Photosynthesis	plastoglobule	C	ATCG00490
3507	2	L4 vs. M4	L4 Up	GO:0018298	Metabolism	Photosynthesis	protein-chromophore linkage	P	AT1G29930
3508	2	L4 vs. M4	L4 Up	GO:0018298	Metabolism	Photosynthesis	protein-chromophore linkage	P	AT2G34430
3509	2	L4 vs. M4	L4 Up	GO:0018298	Metabolism	Photosynthesis	protein-chromophore linkage	P	AT3G47470
3510	2	L4 vs. M4	L4 Up	GO:0018298	Metabolism	Photosynthesis	protein-chromophore linkage	P	AT5G01530
3511	2	L4 vs. M4	L4 Up	GO:0031540	Metabolism	Photosynthesis	regulation of anthocyanin biosynthetic process	P	AT1G64520
3512	2	L4 vs. M4	L4 Up	GO:0016984	Metabolism	Photosynthesis	ribulose-bisphosphate carboxylase activity	F	ATCG00490
3513	2	L4 vs. M4	L4 Up	GO:0009579	Metabolism	Photosynthesis	thylakoid	C	AT1G29930
3514	2	L4 vs. M4	L4 Up	GO:0009579	Metabolism	Photosynthesis	thylakoid	C	AT2G34430
3515	2	L4 vs. M4	L4 Up	GO:0009579	Metabolism	Photosynthesis	thylakoid	C	AT2G35370
3516	2	L4 vs. M4	L4 Up	GO:0009579	Metabolism	Photosynthesis	thylakoid	C	AT3G01500
3517	2	L4 vs. M4	L4 Up	GO:0009579	Metabolism	Photosynthesis	thylakoid	C	AT3G47470
3518	2	L4 vs. M4	L4 Up	GO:0009579	Metabolism	Photosynthesis	thylakoid	C	AT4G05180
3519	2	L4 vs. M4	L4 Up	GO:0009579	Metabolism	Photosynthesis	thylakoid	C	AT4G25100
3520	2	L4 vs. M4	L4 Up	GO:0009579	Metabolism	Photosynthesis	thylakoid	C	AT4G35770
3521	2	L4 vs. M4	L4 Up	GO:0009579	Metabolism	Photosynthesis	thylakoid	C	AT5G01530
3522	2	L4 vs. M4	L4 Up	GO:0009579	Metabolism	Photosynthesis	thylakoid	C	AT5G01600
3523	2	L4 vs. M4	L4 Up	GO:0009579	Metabolism	Photosynthesis	thylakoid	C	ATCG00490
3524	2	L4 vs. M4	L4 Up	GO:0031977	Metabolism	Photosynthesis	thylakoid lumen	C	AT4G05180
3525	2	L4 vs. M4	L4 Up	GO:0042651	Metabolism	Photosynthesis	thylakoid membrane	C	AT2G34430
3526	2	L4 vs. M4	L4 Up	GO:0010027	Metabolism	Photosynthesis	thylakoid membrane organization	P	AT2G01110
3527	2	L4 vs. M4	L4 Up	GO:0015172	Metabolism	Transporters	acidic amino acid transmembrane transporter activity	F	AT1G24400
3528	2	L4 vs. M4	L4 Up	GO:0046715	Metabolism	Transporters	active borate transmembrane transporter activity	F	AT3G06100
3529	2	L4 vs. M4	L4 Up	GO:0003333	Metabolism	Transporters	amino acid transmembrane transport	P	AT1G24400
3530	2	L4 vs. M4	L4 Up	GO:0003333	Metabolism	Transporters	amino acid transmembrane transport	P	AT2G21050
3531	2	L4 vs. M4	L4 Up	GO:0015171	Metabolism	Transporters	amino acid transmembrane transporter activity	F	AT1G24400
3532	2	L4 vs. M4	L4 Up	GO:0015171	Metabolism	Transporters	amino acid transmembrane transporter activity	F	AT2G21050
3533	2	L4 vs. M4	L4 Up	GO:0008519	Metabolism	Transporters	ammonium transmembrane transporter activity	F	AT3G16240
3534	2	L4 vs. M4	L4 Up	GO:0015297	Metabolism	Transporters	antiporter activity	F	AT2G38330
3535	2	L4 vs. M4	L4 Up	GO:0015297	Metabolism	Transporters	antiporter activity	F	AT3G08040
3536	2	L4 vs. M4	L4 Up	GO:0035445	Metabolism	Transporters	borate transmembrane transport	P	AT3G06100
3537	2	L4 vs. M4	L4 Up	GO:0070588	Metabolism	Transporters	calcium ion transmembrane transport	P	AT3G21180
3538	2	L4 vs. M4	L4 Up	GO:0005388	Metabolism	Transporters	calcium transmembrane transporter activity, phosphorylative mechanism	F	AT3G21180
3539	2	L4 vs. M4	L4 Up	GO:0008643	Metabolism	Transporters	carbohydrate transport	P	AT5G23660

3540	2	L4 vs. M4	L4 Up	GO:0008643	Metabolism	Transporters	carbohydrate transport	P	AT5G50800
3541	2	L4 vs. M4	L4 Up	GO:0008643	Metabolism	Transporters	carbohydrate transport	P	AT5G53190
3542	2	L4 vs. M4	L4 Up	GO:1905039	Metabolism	Transporters	carboxylic acid transmembrane transport	P	AT5G50800
3543	2	L4 vs. M4	L4 Up	GO:0015267	Metabolism	Transporters	channel activity	F	AT3G06100
3544	2	L4 vs. M4	L4 Up	GO:0015137	Metabolism	Transporters	citrate transmembrane transporter activity	F	AT3G08040
3545	2	L4 vs. M4	L4 Up	GO:0006888	Metabolism	Transporters	endoplasmic reticulum to Golgi vesicle-mediated transport	P	AT1G11890
3546	2	L4 vs. M4	L4 Up	GO:0006888	Metabolism	Transporters	endoplasmic reticulum to Golgi vesicle-mediated transport	P	AT1G15880
3547	2	L4 vs. M4	L4 Up	GO:0006888	Metabolism	Transporters	endoplasmic reticulum to Golgi vesicle-mediated transport	P	AT5G02280
3548	2	L4 vs. M4	L4 Up	GO:0005768	Metabolism	Transporters	endosome	C	AT3G25140
3549	2	L4 vs. M4	L4 Up	GO:0005768	Metabolism	Transporters	endosome	C	AT3G51070
3550	2	L4 vs. M4	L4 Up	GO:0012507	Metabolism	Transporters	ER to Golgi transport vesicle membrane	C	AT1G11890
3551	2	L4 vs. M4	L4 Up	GO:0048193	Metabolism	Transporters	Golgi vesicle transport	P	AT5G54750
3552	2	L4 vs. M4	L4 Up	GO:0005315	Metabolism	Transporters	inorganic phosphate transmembrane transporter activity	F	AT5G14040
3553	2	L4 vs. M4	L4 Up	GO:0065002	Metabolism	Transporters	intracellular protein transmembrane transport	P	AT2G01110
3554	2	L4 vs. M4	L4 Up	GO:0006886	Metabolism	Transporters	intracellular protein transport	P	AT1G54030
3555	2	L4 vs. M4	L4 Up	GO:0006886	Metabolism	Transporters	intracellular protein transport	P	AT2G21880
3556	2	L4 vs. M4	L4 Up	GO:0006886	Metabolism	Transporters	intracellular protein transport	P	AT3G07890
3557	2	L4 vs. M4	L4 Up	GO:0006891	Metabolism	Transporters	intra-Golgi vesicle-mediated transport	P	AT1G15880
3558	2	L4 vs. M4	L4 Up	GO:0005381	Metabolism	Transporters	iron ion transmembrane transporter activity	F	AT3G08040
3559	2	L4 vs. M4	L4 Up	GO:0006826	Metabolism	Transporters	iron ion transport	P	AT1G55570
3560	2	L4 vs. M4	L4 Up	GO:0006826	Metabolism	Transporters	iron ion transport	P	AT3G11050
3561	2	L4 vs. M4	L4 Up	GO:0006826	Metabolism	Transporters	iron ion transport	P	AT3G13390
3562	2	L4 vs. M4	L4 Up	GO:0006826	Metabolism	Transporters	iron ion transport	P	AT3G13400
3563	2	L4 vs. M4	L4 Up	GO:0006826	Metabolism	Transporters	iron ion transport	P	AT3G56090
3564	2	L4 vs. M4	L4 Up	GO:0006826	Metabolism	Transporters	iron ion transport	P	AT5G01600
3565	2	L4 vs. M4	L4 Up	GO:0006869	Metabolism	Transporters	lipid transport	P	AT1G66850
3566	2	L4 vs. M4	L4 Up	GO:0006869	Metabolism	Transporters	lipid transport	P	AT3G51590
3567	2	L4 vs. M4	L4 Up	GO:0006869	Metabolism	Transporters	lipid transport	P	AT5G02100
3568	2	L4 vs. M4	L4 Up	GO:0006869	Metabolism	Transporters	lipid transport	P	AT5G50800
3569	2	L4 vs. M4	L4 Up	GO:0046873	Metabolism	Transporters	metal ion transmembrane transporter activity	F	AT3G08040
3570	2	L4 vs. M4	L4 Up	GO:0030001	Metabolism	Transporters	metal ion transport	P	AT3G08040
3571	2	L4 vs. M4	L4 Up	GO:0015200	Metabolism	Transporters	methylammonium transmembrane transporter activity	F	AT3G16240
3572	2	L4 vs. M4	L4 Up	GO:0015175	Metabolism	Transporters	neutral amino acid transmembrane transporter activity	F	AT1G24400
3573	2	L4 vs. M4	L4 Up	GO:0015804	Metabolism	Transporters	neutral amino acid transport	P	AT1G24400
3574	2	L4 vs. M4	L4 Up	GO:0044613	Metabolism	Transporters	nuclear pore central transport channel	C	AT1G27970
3575	2	L4 vs. M4	L4 Up	GO:0006913	Metabolism	Transporters	nucleocytoplasmic transport	P	AT1G27970
3576	2	L4 vs. M4	L4 Up	GO:0035673	Metabolism	Transporters	oligopeptide transmembrane transporter activity	F	AT1G59740
3577	2	L4 vs. M4	L4 Up	GO:1904680	Metabolism	Transporters	peptide transmembrane transporter activity	F	AT1G59740
3578	2	L4 vs. M4	L4 Up	GO:0015333	Metabolism	Transporters	peptide:proton symporter activity	F	AT1G59740
3579	2	L4 vs. M4	L4 Up	GO:0035435	Metabolism	Transporters	phosphate ion transmembrane transport	P	AT5G14040
3580	2	L4 vs. M4	L4 Up	GO:0009506	Metabolism	Transporters	plasmodesma	C	AT1G07890
3581	2	L4 vs. M4	L4 Up	GO:0009506	Metabolism	Transporters	plasmodesma	C	AT1G28270
3582	2	L4 vs. M4	L4 Up	GO:0009506	Metabolism	Transporters	plasmodesma	C	AT1G54030
3583	2	L4 vs. M4	L4 Up	GO:0009506	Metabolism	Transporters	plasmodesma	C	AT1G69295
3584	2	L4 vs. M4	L4 Up	GO:0009506	Metabolism	Transporters	plasmodesma	C	AT1G71695
3585	2	L4 vs. M4	L4 Up	GO:0009506	Metabolism	Transporters	plasmodesma	C	AT2G20190
3586	2	L4 vs. M4	L4 Up	GO:0009506	Metabolism	Transporters	plasmodesma	C	AT2G21660
3587	2	L4 vs. M4	L4 Up	GO:0009506	Metabolism	Transporters	plasmodesma	C	AT2G39460
3588	2	L4 vs. M4	L4 Up	GO:0009506	Metabolism	Transporters	plasmodesma	C	AT2G46880
3589	2	L4 vs. M4	L4 Up	GO:0009506	Metabolism	Transporters	plasmodesma	C	AT3G13750

3590	2	L4 vs. M4	L4 Up	GO:0009506	Metabolism	Transporters	plasmodesma	C	AT3G16240
3591	2	L4 vs. M4	L4 Up	GO:0009506	Metabolism	Transporters	plasmodesma	C	AT4G30100
3592	2	L4 vs. M4	L4 Up	GO:0009506	Metabolism	Transporters	plasmodesma	C	AT5G08000
3593	2	L4 vs. M4	L4 Up	GO:0009506	Metabolism	Transporters	plasmodesma	C	AT5G15200
3594	2	L4 vs. M4	L4 Up	GO:0009506	Metabolism	Transporters	plasmodesma	C	AT5G20250
3595	2	L4 vs. M4	L4 Up	GO:0009506	Metabolism	Transporters	plasmodesma	C	AT5G26700
3596	2	L4 vs. M4	L4 Up	GO:0009506	Metabolism	Transporters	plasmodesma	C	AT5G39850
3597	2	L4 vs. M4	L4 Up	GO:0009506	Metabolism	Transporters	plasmodesma	C	AT5G54160
3598	2	L4 vs. M4	L4 Up	GO:0010497	Metabolism	Transporters	plasmodesmata-mediated intercellular transport	P	AT5G26700
3599	2	L4 vs. M4	L4 Up	GO:0032527	Metabolism	Transporters	protein exit from endoplasmic reticulum	P	AT1G54030
3600	2	L4 vs. M4	L4 Up	GO:0006606	Metabolism	Transporters	protein import into nucleus	P	AT1G27970
3601	2	L4 vs. M4	L4 Up	GO:0015031	Metabolism	Transporters	protein transport	P	AT1G11890
3602	2	L4 vs. M4	L4 Up	GO:0015031	Metabolism	Transporters	protein transport	P	AT1G15880
3603	2	L4 vs. M4	L4 Up	GO:0015031	Metabolism	Transporters	protein transport	P	AT5G09550
3604	2	L4 vs. M4	L4 Up	GO:0043953	Metabolism	Transporters	protein transport by the Tat complex	P	AT2G01110
3605	2	L4 vs. M4	L4 Up	GO:0009977	Metabolism	Transporters	proton motive force dependent protein transmembrane transporter activity	F	AT2G01110
3606	2	L4 vs. M4	L4 Up	GO:0048209	Metabolism	Transporters	regulation of vesicle targeting, to, from or within Golgi	P	AT1G15880
3607	2	L4 vs. M4	L4 Up	GO:0099503	Metabolism	Transporters	secretory vesicle	C	AT3G12500
3608	2	L4 vs. M4	L4 Up	GO:0099503	Metabolism	Transporters	secretory vesicle	C	AT3G52840
3609	2	L4 vs. M4	L4 Up	GO:0099503	Metabolism	Transporters	secretory vesicle	C	AT5G20630
3610	2	L4 vs. M4	L4 Up	GO:0005484	Metabolism	Transporters	SNAP receptor activity	F	AT1G11890
3611	2	L4 vs. M4	L4 Up	GO:0005484	Metabolism	Transporters	SNAP receptor activity	F	AT1G15880
3612	2	L4 vs. M4	L4 Up	GO:0000149	Metabolism	Transporters	SNARE binding	F	AT1G15880
3613	2	L4 vs. M4	L4 Up	GO:0031201	Metabolism	Transporters	SNARE complex	C	AT1G11890
3614	2	L4 vs. M4	L4 Up	GO:0031201	Metabolism	Transporters	SNARE complex	C	AT1G15880
3615	2	L4 vs. M4	L4 Up	GO:0008515	Metabolism	Transporters	sucrose transmembrane transporter activity	F	AT5G23660
3616	2	L4 vs. M4	L4 Up	GO:0008515	Metabolism	Transporters	sucrose transmembrane transporter activity	F	AT5G50800
3617	2	L4 vs. M4	L4 Up	GO:0015770	Metabolism	Transporters	sucrose transport	P	AT5G23660
3618	2	L4 vs. M4	L4 Up	GO:0015770	Metabolism	Transporters	sucrose transport	P	AT5G50800
3619	2	L4 vs. M4	L4 Up	GO:0051119	Metabolism	Transporters	sugar transmembrane transporter activity	F	AT5G23660
3620	2	L4 vs. M4	L4 Up	GO:0051119	Metabolism	Transporters	sugar transmembrane transporter activity	F	AT5G50800
3621	2	L4 vs. M4	L4 Up	GO:0051119	Metabolism	Transporters	sugar transmembrane transporter activity	F	AT5G53190
3622	2	L4 vs. M4	L4 Up	GO:0015293	Metabolism	Transporters	symporter activity	F	AT1G24400
3623	2	L4 vs. M4	L4 Up	GO:0015293	Metabolism	Transporters	symporter activity	F	AT2G21050
3624	2	L4 vs. M4	L4 Up	GO:0033281	Metabolism	Transporters	TAT protein transport complex	C	AT2G01110
3625	2	L4 vs. M4	L4 Up	GO:0005802	Metabolism	Transporters	trans-Golgi network	C	AT3G25140
3626	2	L4 vs. M4	L4 Up	GO:0005802	Metabolism	Transporters	trans-Golgi network	C	AT3G51070
3627	2	L4 vs. M4	L4 Up	GO:0055085	Metabolism	Transporters	transmembrane transport	P	AT2G38330
3628	2	L4 vs. M4	L4 Up	GO:0055085	Metabolism	Transporters	transmembrane transport	P	AT3G16240
3629	2	L4 vs. M4	L4 Up	GO:0030008	Metabolism	Transporters	TRAPP complex	C	AT5G02280
3630	2	L4 vs. M4	L4 Up	GO:0030008	Metabolism	Transporters	TRAPP complex	C	AT5G54750
3631	2	L4 vs. M4	L4 Up	GO:0015204	Metabolism	Transporters	urea transmembrane transporter activity	F	AT3G16240
3632	2	L4 vs. M4	L4 Up	GO:0015840	Metabolism	Transporters	urea transport	P	AT3G16240
3633	2	L4 vs. M4	L4 Up	GO:0016192	Metabolism	Transporters	vesicle-mediated transport	P	AT5G09550
3634	2	L4 vs. M4	L4 Up	GO:0015250	Metabolism	Transporters	water channel activity	F	AT3G06100
3635	2	L4 vs. M4	L4 Up	GO:0015250	Metabolism	Transporters	water channel activity	F	AT3G16240
3636	2	L4 vs. M4	L4 Up	GO:0006833	Metabolism	Transporters	water transport	P	AT3G16240
3637	2	L4 vs. M4	L4 Up	GO:0042910	Metabolism	Transporters	xenobiotic transmembrane transporter activity	F	AT2G38330
3638	2	L4 vs. M4	L4 Up	GO:0042910	Metabolism	Transporters	xenobiotic transmembrane transporter activity	F	AT3G08040
3639	2	L4 vs. M4	L4 Up	GO:0061077	Regulation	Protein modification	chaperone-mediated protein folding	P	AT4G04330
3640	2	L4 vs. M4	L4 Up	GO:0005783	Regulation	Protein modification	endoplasmic reticulum	C	AT1G11890
3641	2	L4 vs. M4	L4 Up	GO:0005783	Regulation	Protein modification	endoplasmic reticulum	C	AT1G54030
3642	2	L4 vs. M4	L4 Up	GO:0005783	Regulation	Protein modification	endoplasmic reticulum	C	AT1G62940
3643	2	L4 vs. M4	L4 Up	GO:0005783	Regulation	Protein modification	endoplasmic reticulum	C	AT1G78020

3644	2	L4 vs. M4	L4 Up	GO:0005783	Regulation	Protein modification	endoplasmic reticulum	C	AT2G33380
3645	2	L4 vs. M4	L4 Up	GO:0005783	Regulation	Protein modification	endoplasmic reticulum	C	AT2G34430
3646	2	L4 vs. M4	L4 Up	GO:0005783	Regulation	Protein modification	endoplasmic reticulum	C	AT2G35370
3647	2	L4 vs. M4	L4 Up	GO:0005783	Regulation	Protein modification	endoplasmic reticulum	C	AT2G46210
3648	2	L4 vs. M4	L4 Up	GO:0005783	Regulation	Protein modification	endoplasmic reticulum	C	AT3G26935
3649	2	L4 vs. M4	L4 Up	GO:0005783	Regulation	Protein modification	endoplasmic reticulum	C	AT3G62580
3650	2	L4 vs. M4	L4 Up	GO:0005783	Regulation	Protein modification	endoplasmic reticulum	C	AT4G12590
3651	2	L4 vs. M4	L4 Up	GO:0005783	Regulation	Protein modification	endoplasmic reticulum	C	AT4G20870
3652	2	L4 vs. M4	L4 Up	GO:0005783	Regulation	Protein modification	endoplasmic reticulum	C	AT4G35420
3653	2	L4 vs. M4	L4 Up	GO:0005783	Regulation	Protein modification	endoplasmic reticulum	C	AT5G02100
3654	2	L4 vs. M4	L4 Up	GO:0005783	Regulation	Protein modification	endoplasmic reticulum	C	AT5G42000
3655	2	L4 vs. M4	L4 Up	GO:0005789	Regulation	Protein modification	endoplasmic reticulum membrane	C	AT1G11890
3656	2	L4 vs. M4	L4 Up	GO:0005789	Regulation	Protein modification	endoplasmic reticulum membrane	C	AT2G46210
3657	2	L4 vs. M4	L4 Up	GO:0005789	Regulation	Protein modification	endoplasmic reticulum membrane	C	AT3G51070
3658	2	L4 vs. M4	L4 Up	GO:0005789	Regulation	Protein modification	endoplasmic reticulum membrane	C	AT4G20870
3659	2	L4 vs. M4	L4 Up	GO:0072546	Regulation	Protein modification	ER membrane protein complex	C	AT4G12590
3660	2	L4 vs. M4	L4 Up	GO:0005794	Regulation	Protein modification	Golgi apparatus	C	AT1G07890
3661	2	L4 vs. M4	L4 Up	GO:0005794	Regulation	Protein modification	Golgi apparatus	C	AT1G11890
3662	2	L4 vs. M4	L4 Up	GO:0005794	Regulation	Protein modification	Golgi apparatus	C	AT1G15880
3663	2	L4 vs. M4	L4 Up	GO:0005794	Regulation	Protein modification	Golgi apparatus	C	AT1G27450
3664	2	L4 vs. M4	L4 Up	GO:0005794	Regulation	Protein modification	Golgi apparatus	C	AT1G78300
3665	2	L4 vs. M4	L4 Up	GO:0005794	Regulation	Protein modification	Golgi apparatus	C	AT2G39460
3666	2	L4 vs. M4	L4 Up	GO:0005794	Regulation	Protein modification	Golgi apparatus	C	AT3G16240
3667	2	L4 vs. M4	L4 Up	GO:0005794	Regulation	Protein modification	Golgi apparatus	C	AT3G25140
3668	2	L4 vs. M4	L4 Up	GO:0005794	Regulation	Protein modification	Golgi apparatus	C	AT3G26935
3669	2	L4 vs. M4	L4 Up	GO:0005794	Regulation	Protein modification	Golgi apparatus	C	AT5G02100
3670	2	L4 vs. M4	L4 Up	GO:0005794	Regulation	Protein modification	Golgi apparatus	C	AT5G02280
3671	2	L4 vs. M4	L4 Up	GO:0005794	Regulation	Protein modification	Golgi apparatus	C	AT5G54750
3672	2	L4 vs. M4	L4 Up	GO:0000139	Regulation	Protein modification	Golgi membrane	C	AT1G11890
3673	2	L4 vs. M4	L4 Up	GO:0000139	Regulation	Protein modification	Golgi membrane	C	AT1G15880
3674	2	L4 vs. M4	L4 Up	GO:0000139	Regulation	Protein modification	Golgi membrane	C	AT2G47040
3675	2	L4 vs. M4	L4 Up	GO:0000139	Regulation	Protein modification	Golgi membrane	C	AT3G25140
3676	2	L4 vs. M4	L4 Up	GO:0032259	Regulation	Protein modification	methylation	P	AT3G51070
3677	2	L4 vs. M4	L4 Up	GO:0032259	Regulation	Protein modification	methylation	P	AT5G54160
3678	2	L4 vs. M4	L4 Up	GO:0031416	Regulation	Protein modification	NatB complex	C	AT1G03150
3679	2	L4 vs. M4	L4 Up	GO:0006499	Regulation	Protein modification	N-terminal protein myristoylation	P	AT5G21170
3680	2	L4 vs. M4	L4 Up	GO:0018119	Regulation	Protein modification	peptidyl-cysteine S-nitrosylation	P	AT4G05180
3681	2	L4 vs. M4	L4 Up	GO:0018119	Regulation	Protein modification	peptidyl-cysteine S-nitrosylation	P	AT5G20630
3682	2	L4 vs. M4	L4 Up	GO:0018119	Regulation	Protein modification	peptidyl-cysteine S-nitrosylation	P	ATCG00490
3683	2	L4 vs. M4	L4 Up	GO:0018230	Regulation	Protein modification	peptidyl-L-cysteine S-palmitoylation	P	AT3G26935
3684	2	L4 vs. M4	L4 Up	GO:0046983	Regulation	Protein modification	protein dimerization activity	F	AT5G54160
3685	2	L4 vs. M4	L4 Up	GO:0019904	Regulation	Protein modification	protein domain specific binding	F	AT1G78300
3686	2	L4 vs. M4	L4 Up	GO:0046982	Regulation	Protein modification	protein heterodimerization activity	F	AT1G04950
3687	2	L4 vs. M4	L4 Up	GO:0046982	Regulation	Protein modification	protein heterodimerization activity	F	AT2G18160
3688	2	L4 vs. M4	L4 Up	GO:0051260	Regulation	Protein modification	protein homooligomerization	P	AT5G23660
3689	2	L4 vs. M4	L4 Up	GO:0050821	Regulation	Protein modification	protein stabilization	P	AT2G20190
3690	2	L4 vs. M4	L4 Up	GO:0031386	Regulation	Protein modification	protein tag	F	AT4G05050
3691	2	L4 vs. M4	L4 Up	GO:0006612	Regulation	Protein modification	protein targeting to membrane	P	AT3G26935
3692	2	L4 vs. M4	L4 Up	GO:0019706	Regulation	Protein modification	protein-cysteine S-palmitoyltransferase activity	F	AT3G26935
3693	2	L4 vs. M4	L4 Up	GO:0050790	Regulation	Protein modification	regulation of catalytic activity	P	AT1G02305
3694	2	L4 vs. M4	L4 Up	GO:0043541	Regulation	Protein modification	UDP-N-acetylglucosamine transferase complex	C	AT4G18230
3695	2	L4 vs. M4	L4 Up	GO:0004596	Regulation	Protein modification	peptide alpha-N-acetyltransferase activity	F	AT1G03150
3696	2	L4 vs. M4	L4 Up	GO:0043086	Regulation	Regulation	negative regulation of catalytic activity	P	AT2G47050
3697	2	L4 vs. M4	L4 Up	GO:0043086	Regulation	Regulation	negative regulation of catalytic activity	P	AT4G02250
3698	2	L4 vs. M4	L4 Up	GO:1900060	Regulation	Regulation	negative regulation of ceramide biosynthetic process	P	AT5G42000

3699	2	L4 vs. M4	L4 Up	GO:0007026	Regulation	Regulation	negative regulation of microtubule depolymerization	P	AT2G20190
3700	2	L4 vs. M4	L4 Up	GO:2000122	Regulation	Regulation	negative regulation of stomatal complex development	P	AT3G01500
3701	2	L4 vs. M4	L4 Up	GO:1901959	Regulation	Regulation	positive regulation of cutin biosynthetic process	P	AT1G79700
3702	2	L4 vs. M4	L4 Up	GO:1904278	Regulation	Regulation	positive regulation of wax biosynthetic process	P	AT1G79700
3703	2	L4 vs. M4	L4 Up	GO:0080092	Regulation	Regulation	regulation of pollen tube growth	P	AT1G28270
3704	2	L4 vs. M4	L4 Up	GO:0003680	Regulation	Transcription	AT DNA binding	F	AT2G42940
3705	2	L4 vs. M4	L4 Up	GO:0031490	Regulation	Transcription	chromatin DNA binding	F	AT2G42940
3706	2	L4 vs. M4	L4 Up	GO:0003700	Regulation	Transcription	DNA-binding transcription factor activity	F	AT1G26610
3707	2	L4 vs. M4	L4 Up	GO:0003700	Regulation	Transcription	DNA-binding transcription factor activity	F	AT1G34310
3708	2	L4 vs. M4	L4 Up	GO:0003700	Regulation	Transcription	DNA-binding transcription factor activity	F	AT1G49010
3709	2	L4 vs. M4	L4 Up	GO:0003700	Regulation	Transcription	DNA-binding transcription factor activity	F	AT1G61110
3710	2	L4 vs. M4	L4 Up	GO:0003700	Regulation	Transcription	DNA-binding transcription factor activity	F	AT1G68200
3711	2	L4 vs. M4	L4 Up	GO:0003700	Regulation	Transcription	DNA-binding transcription factor activity	F	AT1G79700
3712	2	L4 vs. M4	L4 Up	GO:0003700	Regulation	Transcription	DNA-binding transcription factor activity	F	AT2G18160
3713	2	L4 vs. M4	L4 Up	GO:0003700	Regulation	Transcription	DNA-binding transcription factor activity	F	AT2G25900
3714	2	L4 vs. M4	L4 Up	GO:0003700	Regulation	Transcription	DNA-binding transcription factor activity	F	AT2G42940
3715	2	L4 vs. M4	L4 Up	GO:0003700	Regulation	Transcription	DNA-binding transcription factor activity	F	AT5G02840
3716	2	L4 vs. M4	L4 Up	GO:0000981	Regulation	Transcription	DNA-binding transcription factor activity, RNA polymerase II-specific	F	AT1G79700
3717	2	L4 vs. M4	L4 Up	GO:0003899	Regulation	Transcription	DNA-directed 5'-3' RNA polymerase activity	F	AT5G59180
3718	2	L4 vs. M4	L4 Up	GO:0004402	Regulation	Transcription	histone acetyltransferase activity	F	AT4G37610
3719	2	L4 vs. M4	L4 Up	GO:0042393	Regulation	Transcription	histone binding	F	AT4G12620
3720	2	L4 vs. M4	L4 Up	GO:0004407	Regulation	Transcription	histone deacetylase activity	F	AT3G44680
3721	2	L4 vs. M4	L4 Up	GO:0000118	Regulation	Transcription	histone deacetylase complex	C	AT3G44680
3722	2	L4 vs. M4	L4 Up	GO:0070932	Regulation	Transcription	histone H3 deacetylation	P	AT3G44680
3723	2	L4 vs. M4	L4 Up	GO:1990619	Regulation	Transcription	histone H3-K9 deacetylation	P	AT3G44680
3724	2	L4 vs. M4	L4 Up	GO:0070933	Regulation	Transcription	histone H4 deacetylation	P	AT3G44680
3725	2	L4 vs. M4	L4 Up	GO:0048027	Regulation	Transcription	mRNA 5'-UTR binding	F	AT3G52580
3726	2	L4 vs. M4	L4 Up	GO:0006397	Regulation	Transcription	mRNA processing	P	AT1G54080
3727	2	L4 vs. M4	L4 Up	GO:0006397	Regulation	Transcription	mRNA processing	P	AT2G21660
3728	2	L4 vs. M4	L4 Up	GO:0032041	Regulation	Transcription	NAD-dependent histone deacetylase activity (H3-K14 specific)	F	AT3G44680
3729	2	L4 vs. M4	L4 Up	GO:0035067	Regulation	Transcription	negative regulation of histone acetylation	P	AT2G42940
3730	2	L4 vs. M4	L4 Up	GO:0045892	Regulation	Transcription	negative regulation of transcription, DNA-templated	P	AT5G63190
3731	2	L4 vs. M4	L4 Up	GO:0010529	Regulation	Transcription	negative regulation of transposition	P	AT2G42940
3732	2	L4 vs. M4	L4 Up	GO:0005730	Regulation	Transcription	nucleolus	C	AT2G39460
3733	2	L4 vs. M4	L4 Up	GO:0005730	Regulation	Transcription	nucleolus	C	AT5G15200
3734	2	L4 vs. M4	L4 Up	GO:0000932	Regulation	Transcription	P-body	C	AT3G48050
3735	2	L4 vs. M4	L4 Up	GO:0000932	Regulation	Transcription	P-body	C	AT5G59180
3736	2	L4 vs. M4	L4 Up	GO:0010628	Regulation	Transcription	positive regulation of gene expression	P	AT1G61110
3737	2	L4 vs. M4	L4 Up	GO:1900111	Regulation	Transcription	positive regulation of histone H3-K9 dimethylation	P	AT2G42940
3738	2	L4 vs. M4	L4 Up	GO:0060213	Regulation	Transcription	positive regulation of nuclear-transcribed mRNA poly(A) tail shortening	P	AT5G59180
3739	2	L4 vs. M4	L4 Up	GO:0045944	Regulation	Transcription	positive regulation of transcription by RNA polymerase II	P	AT3G44680
3740	2	L4 vs. M4	L4 Up	GO:0045893	Regulation	Transcription	positive regulation of transcription, DNA-templated	P	AT2G18160
3741	2	L4 vs. M4	L4 Up	GO:0006357	Regulation	Transcription	regulation of transcription by RNA polymerase II	P	AT1G04950
3742	2	L4 vs. M4	L4 Up	GO:0006355	Regulation	Transcription	regulation of transcription, DNA-templated	P	AT1G26610
3743	2	L4 vs. M4	L4 Up	GO:0006355	Regulation	Transcription	regulation of transcription, DNA-templated	P	AT1G34310
3744	2	L4 vs. M4	L4 Up	GO:0006355	Regulation	Transcription	regulation of transcription, DNA-templated	P	AT1G61110
3745	2	L4 vs. M4	L4 Up	GO:0006355	Regulation	Transcription	regulation of transcription, DNA-templated	P	AT1G68200
3746	2	L4 vs. M4	L4 Up	GO:0006355	Regulation	Transcription	regulation of transcription, DNA-templated	P	AT1G79700
3747	2	L4 vs. M4	L4 Up	GO:0006355	Regulation	Transcription	regulation of transcription, DNA-templated	P	AT2G25900
3748	2	L4 vs. M4	L4 Up	GO:0006355	Regulation	Transcription	regulation of transcription, DNA-templated	P	AT4G12620
3749	2	L4 vs. M4	L4 Up	GO:0006355	Regulation	Transcription	regulation of transcription, DNA-templated	P	AT4G37610

3750	2	L4 vs. M4	L4 Up	GO:0003723	Regulation	Transcription	RNA binding	F	AT2G21660
3751	2	L4 vs. M4	L4 Up	GO:0003723	Regulation	Transcription	RNA binding	F	AT2G25900
3752	2	L4 vs. M4	L4 Up	GO:0003723	Regulation	Transcription	RNA binding	F	AT4G30100
3753	2	L4 vs. M4	L4 Up	GO:0051123	Regulation	Transcription	RNA polymerase II preinitiation complex assembly	P	AT1G04950
3754	2	L4 vs. M4	L4 Up	GO:0005665	Regulation	Transcription	RNA polymerase II, core complex	C	AT5G59180
3755	2	L4 vs. M4	L4 Up	GO:0000124	Regulation	Transcription	SAGA complex	C	AT1G04950
3756	2	L4 vs. M4	L4 Up	GO:0043565	Regulation	Transcription	sequence-specific DNA binding	F	AT1G61110
3757	2	L4 vs. M4	L4 Up	GO:0043565	Regulation	Transcription	sequence-specific DNA binding	F	AT1G79700
3758	2	L4 vs. M4	L4 Up	GO:0043565	Regulation	Transcription	sequence-specific DNA binding	F	AT2G18160
3759	2	L4 vs. M4	L4 Up	GO:0043565	Regulation	Transcription	sequence-specific DNA binding	F	AT3G44680
3760	2	L4 vs. M4	L4 Up	GO:0046695	Regulation	Transcription	SLIK (SAGA-like) complex	C	AT1G04950
3761	2	L4 vs. M4	L4 Up	GO:0006366	Regulation	Transcription	transcription by RNA polymerase II	P	AT1G04950
3762	2	L4 vs. M4	L4 Up	GO:0003712	Regulation	Transcription	transcription coregulator activity	F	AT4G37610
3763	2	L4 vs. M4	L4 Up	GO:0005669	Regulation	Transcription	transcription factor TFIID complex	C	AT1G04950
3764	2	L4 vs. M4	L4 Up	GO:0006367	Regulation	Transcription	transcription initiation from RNA polymerase II promoter	P	AT5G59180
3765	2	L4 vs. M4	L4 Up	GO:0044212	Regulation	Transcription	transcription regulatory region DNA binding	F	AT1G01780
3766	2	L4 vs. M4	L4 Up	GO:0044212	Regulation	Transcription	transcription regulatory region DNA binding	F	AT1G26610
3767	2	L4 vs. M4	L4 Up	GO:0044212	Regulation	Transcription	transcription regulatory region DNA binding	F	AT1G34310
3768	2	L4 vs. M4	L4 Up	GO:0044212	Regulation	Transcription	transcription regulatory region DNA binding	F	AT1G49010
3769	2	L4 vs. M4	L4 Up	GO:0044212	Regulation	Transcription	transcription regulatory region DNA binding	F	AT1G68200
3770	2	L4 vs. M4	L4 Up	GO:0044212	Regulation	Transcription	transcription regulatory region DNA binding	F	AT2G18160
3771	2	L4 vs. M4	L4 Up	GO:0044212	Regulation	Transcription	transcription regulatory region DNA binding	F	AT5G02840
3772	2	L4 vs. M4	L4 Up	GO:0000976	Regulation	Transcription	transcription regulatory region sequence-specific DNA binding	F	AT1G79700
3773	2	L4 vs. M4	L4 Up	GO:0003743	Regulation	Transcription	translation initiation factor activity	F	AT1G04170
3774	2	L4 vs. M4	L4 Up	GO:0003743	Regulation	Transcription	translation initiation factor activity	F	AT1G49500
3775	2	L4 vs. M4	L4 Up	GO:0000380	Regulation	Translation	alternative mRNA splicing, via spliceosome	P	AT2G21660
3776	2	L4 vs. M4	L4 Up	GO:0022625	Regulation	Translation	cytosolic large ribosomal subunit	C	AT2G39460
3777	2	L4 vs. M4	L4 Up	GO:0022625	Regulation	Translation	cytosolic large ribosomal subunit	C	AT5G63190
3778	2	L4 vs. M4	L4 Up	GO:0022626	Regulation	Translation	cytosolic ribosome	C	AT1G16470
3779	2	L4 vs. M4	L4 Up	GO:0022626	Regulation	Translation	cytosolic ribosome	C	AT2G39460
3780	2	L4 vs. M4	L4 Up	GO:0022626	Regulation	Translation	cytosolic ribosome	C	AT5G15200
3781	2	L4 vs. M4	L4 Up	GO:0022626	Regulation	Translation	cytosolic ribosome	C	AT5G55990
3782	2	L4 vs. M4	L4 Up	GO:0022626	Regulation	Translation	cytosolic ribosome	C	ATCG00490
3783	2	L4 vs. M4	L4 Up	GO:0022627	Regulation	Translation	cytosolic small ribosomal subunit	C	AT3G52580
3784	2	L4 vs. M4	L4 Up	GO:0022627	Regulation	Translation	cytosolic small ribosomal subunit	C	AT5G15200
3785	2	L4 vs. M4	L4 Up	GO:0022627	Regulation	Translation	cytosolic small ribosomal subunit	C	AT5G39850
3786	2	L4 vs. M4	L4 Up	GO:0005850	Regulation	Translation	eukaryotic translation initiation factor 2 complex	C	AT1G04170
3787	2	L4 vs. M4	L4 Up	GO:0001731	Regulation	Translation	formation of translation preinitiation complex	P	AT1G04170
3788	2	L4 vs. M4	L4 Up	GO:0015934	Regulation	Translation	large ribosomal subunit	C	AT2G39460
3789	2	L4 vs. M4	L4 Up	GO:0000462	Regulation	Translation	maturation of SSU-rRNA from tricistronic rRNA transcript (SSU-rRNA, 5.8S rRNA, LSU-rRNA)	P	AT3G52580
3790	2	L4 vs. M4	L4 Up	GO:0035278	Regulation	Translation	miRNA mediated inhibition of translation	P	AT3G48050
3791	2	L4 vs. M4	L4 Up	GO:0005762	Regulation	Translation	mitochondrial large ribosomal subunit	C	AT3G01740
3792	2	L4 vs. M4	L4 Up	GO:0003729	Regulation	Translation	mRNA binding	F	AT1G54080
3793	2	L4 vs. M4	L4 Up	GO:0003729	Regulation	Translation	mRNA binding	F	AT2G21660
3794	2	L4 vs. M4	L4 Up	GO:0003729	Regulation	Translation	mRNA binding	F	AT5G04800
3795	2	L4 vs. M4	L4 Up	GO:0003729	Regulation	Translation	mRNA binding	F	AT5G15200
3796	2	L4 vs. M4	L4 Up	GO:0003729	Regulation	Translation	mRNA binding	F	AT5G20250
3797	2	L4 vs. M4	L4 Up	GO:0006406	Regulation	Translation	mRNA export from nucleus	P	AT2G21660
3798	2	L4 vs. M4	L4 Up	GO:0000291	Regulation	Translation	nuclear-transcribed mRNA catabolic process, exonucleolytic	P	AT5G59180
3799	2	L4 vs. M4	L4 Up	GO:0045903	Regulation	Translation	positive regulation of translational fidelity	P	AT1G04170
3800	2	L4 vs. M4	L4 Up	GO:0045903	Regulation	Translation	positive regulation of translational fidelity	P	AT5G15200
3801	2	L4 vs. M4	L4 Up	GO:0045903	Regulation	Translation	positive regulation of translational fidelity	P	AT5G39850

3802	2	L4 vs. M4	L4 Up	GO:0045948	Regulation	Translation	positive regulation of translational initiation	P	AT5G59180
3803	2	L4 vs. M4	L4 Up	GO:0006417	Regulation	Translation	regulation of translation	P	AT5G63190
3804	2	L4 vs. M4	L4 Up	GO:1990904	Regulation	Translation	ribonucleoprotein complex	C	AT2G21660
3805	2	L4 vs. M4	L4 Up	GO:0000027	Regulation	Translation	ribosomal large subunit assembly	P	AT2G39460
3806	2	L4 vs. M4	L4 Up	GO:0000028	Regulation	Translation	ribosomal small subunit assembly	P	AT3G52580
3807	2	L4 vs. M4	L4 Up	GO:0005840	Regulation	Translation	ribosome	C	AT5G04800
3808	2	L4 vs. M4	L4 Up	GO:0043022	Regulation	Translation	ribosome binding	F	AT5G63190
3809	2	L4 vs. M4	L4 Up	GO:0010501	Regulation	Translation	RNA secondary structure unwinding	P	AT2G21660
3810	2	L4 vs. M4	L4 Up	GO:0019843	Regulation	Translation	rRNA binding	F	AT2G39460
3811	2	L4 vs. M4	L4 Up	GO:0019843	Regulation	Translation	rRNA binding	F	AT5G15200
3812	2	L4 vs. M4	L4 Up	GO:0019843	Regulation	Translation	rRNA binding	F	AT5G39850
3813	2	L4 vs. M4	L4 Up	GO:0003727	Regulation	Translation	single-stranded RNA binding	F	AT2G25900
3814	2	L4 vs. M4	L4 Up	GO:0003727	Regulation	Translation	single-stranded RNA binding	F	AT5G59180
3815	2	L4 vs. M4	L4 Up	GO:0070181	Regulation	Translation	small ribosomal subunit rRNA binding	F	AT3G52580
3816	2	L4 vs. M4	L4 Up	GO:0003735	Regulation	Translation	structural constituent of ribosome	F	AT2G39460
3817	2	L4 vs. M4	L4 Up	GO:0003735	Regulation	Translation	structural constituent of ribosome	F	AT3G01740
3818	2	L4 vs. M4	L4 Up	GO:0003735	Regulation	Translation	structural constituent of ribosome	F	AT3G52580
3819	2	L4 vs. M4	L4 Up	GO:0003735	Regulation	Translation	structural constituent of ribosome	F	AT5G04800
3820	2	L4 vs. M4	L4 Up	GO:0003735	Regulation	Translation	structural constituent of ribosome	F	AT5G15200
3821	2	L4 vs. M4	L4 Up	GO:0003735	Regulation	Translation	structural constituent of ribosome	F	AT5G39850
3822	2	L4 vs. M4	L4 Up	GO:0006412	Regulation	Translation	translation	P	AT2G39460
3823	2	L4 vs. M4	L4 Up	GO:0006412	Regulation	Translation	translation	P	AT3G52580
3824	2	L4 vs. M4	L4 Up	GO:0006412	Regulation	Translation	translation	P	AT5G04800
3825	2	L4 vs. M4	L4 Up	GO:0006412	Regulation	Translation	translation	P	AT5G15200
3826	2	L4 vs. M4	L4 Up	GO:0006412	Regulation	Translation	translation	P	AT5G39850
3827	2	L4 vs. M4	L4 Up	GO:0031369	Regulation	Translation	translation initiation factor binding	F	AT5G59180
3828	2	L4 vs. M4	L4 Up	GO:0006413	Regulation	Translation	translational initiation	P	AT1G49500
3829	2	L4 vs. M4	L4 Up	GO:0000049	Regulation	Translation	tRNA binding	F	AT1G04170
3830	2	L4 vs. M4	L4 Up	GO:0017150	Regulation	Translation	tRNA dihydrouridine synthase activity	F	AT3G49640
3831	2	L4 vs. M4	L4 Up	GO:0003993	Signaling and response	Signaling	acid phosphatase activity	F	AT1G56360
3832	2	L4 vs. M4	L4 Up	GO:0003993	Signaling and response	Signaling	acid phosphatase activity	F	AT2G46880
3833	2	L4 vs. M4	L4 Up	GO:0090630	Signaling and response	Signaling	activation of GTPase activity	P	AT3G07890
3834	2	L4 vs. M4	L4 Up	GO:0009734	Signaling and response	Signaling	auxin-activated signaling pathway	P	AT1G34310
3835	2	L4 vs. M4	L4 Up	GO:0009734	Signaling and response	Signaling	auxin-activated signaling pathway	P	AT2G21050
3836	2	L4 vs. M4	L4 Up	GO:0009742	Signaling and response	Signaling	brassinosteroid mediated signaling pathway	P	AT1G78300
3837	2	L4 vs. M4	L4 Up	GO:0052634	Signaling and response	Signaling	C-19 gibberellin 2-beta-dioxygenase activity	F	AT2G34555
3838	2	L4 vs. M4	L4 Up	GO:0005509	Signaling and response	Signaling	calcium ion binding	F	AT2G33380
3839	2	L4 vs. M4	L4 Up	GO:0005509	Signaling and response	Signaling	calcium ion binding	F	AT4G05180
3840	2	L4 vs. M4	L4 Up	GO:0005509	Signaling and response	Signaling	calcium ion binding	F	AT5G55990
3841	2	L4 vs. M4	L4 Up	GO:0019722	Signaling and response	Signaling	calcium-mediated signaling	P	AT1G28270
3842	2	L4 vs. M4	L4 Up	GO:0019722	Signaling and response	Signaling	calcium-mediated signaling	P	AT5G55990
3843	2	L4 vs. M4	L4 Up	GO:0005516	Signaling and response	Signaling	calmodulin binding	F	AT3G21180
3844	2	L4 vs. M4	L4 Up	GO:0005516	Signaling and response	Signaling	calmodulin binding	F	AT4G37610
3845	2	L4 vs. M4	L4 Up	GO:0007267	Signaling and response	Signaling	cell-cell signaling	P	AT1G28270
3846	2	L4 vs. M4	L4 Up	GO:0070417	Signaling and response	Signaling	cellular response to cold	P	AT2G46210
3847	2	L4 vs. M4	L4 Up	GO:0043562	Signaling and response	Signaling	cellular response to nitrogen levels	P	AT5G21170
3848	2	L4 vs. M4	L4 Up	GO:0007623	Signaling and response	Signaling	circadian rhythm	P	AT1G27450
3849	2	L4 vs. M4	L4 Up	GO:0007623	Signaling and response	Signaling	circadian rhythm	P	AT2G21660
3850	2	L4 vs. M4	L4 Up	GO:0007623	Signaling and response	Signaling	circadian rhythm	P	AT4G25100
3851	2	L4 vs. M4	L4 Up	GO:0007623	Signaling and response	Signaling	circadian rhythm	P	AT5G02840
3852	2	L4 vs. M4	L4 Up	GO:0009690	Signaling and response	Signaling	cytokinin metabolic process	P	AT1G27450
3853	2	L4 vs. M4	L4 Up	GO:0009736	Signaling and response	Signaling	cytokinin-activated signaling pathway	P	AT1G64520
3854	2	L4 vs. M4	L4 Up	GO:0016311	Signaling and response	Signaling	dephosphorylation	P	AT1G56360
3855	2	L4 vs. M4	L4 Up	GO:1905200	Signaling and response	Signaling	gibberellic acid transmembrane transport	P	AT5G50800
3856	2	L4 vs. M4	L4 Up	GO:0009686	Signaling and response	Signaling	gibberellin biosynthetic process	P	AT2G34555
3857	2	L4 vs. M4	L4 Up	GO:1905201	Signaling and response	Signaling	gibberellin transmembrane transporter activity	F	AT5G50800

3858	2	L4 vs. M4	L4 Up	GO:0120091	Signaling and response	Signaling	jasmonic acid hydrolase	F	AT3G55970
3859	2	L4 vs. M4	L4 Up	GO:0009867	Signaling and response	Signaling	jasmonic acid mediated signaling pathway	P	AT1G72260
3860	2	L4 vs. M4	L4 Up	GO:0016301	Signaling and response	Signaling	kinase activity	F	AT3G22960
3861	2	L4 vs. M4	L4 Up	GO:0019900	Signaling and response	Signaling	kinase binding	F	AT1G78020
3862	2	L4 vs. M4	L4 Up	GO:0019900	Signaling and response	Signaling	kinase binding	F	AT5G55990
3863	2	L4 vs. M4	L4 Up	GO:0004435	Signaling and response	Signaling	phosphatidylinositol phospholipase C activity	F	AT5G58670
3864	2	L4 vs. M4	L4 Up	GO:0004629	Signaling and response	Signaling	phospholipase C activity	F	AT5G58670
3865	2	L4 vs. M4	L4 Up	GO:0019887	Signaling and response	Signaling	protein kinase regulator activity	F	AT5G21170
3866	2	L4 vs. M4	L4 Up	GO:0004864	Signaling and response	Signaling	protein phosphatase inhibitor activity	F	AT2G38310
3867	2	L4 vs. M4	L4 Up	GO:0045309	Signaling and response	Signaling	protein phosphorylated amino acid binding	F	AT1G78300
3868	2	L4 vs. M4	L4 Up	GO:0006468	Signaling and response	Signaling	protein phosphorylation	P	AT1G67520
3869	2	L4 vs. M4	L4 Up	GO:0006468	Signaling and response	Signaling	protein phosphorylation	P	AT2G41970
3870	2	L4 vs. M4	L4 Up	GO:0006468	Signaling and response	Signaling	protein phosphorylation	P	AT5G08160
3871	2	L4 vs. M4	L4 Up	GO:0004674	Signaling and response	Signaling	protein serine/threonine kinase activity	F	AT1G67520
3872	2	L4 vs. M4	L4 Up	GO:0004674	Signaling and response	Signaling	protein serine/threonine kinase activity	F	AT5G08160
3873	2	L4 vs. M4	L4 Up	GO:0004713	Signaling and response	Signaling	protein tyrosine kinase activity	F	AT2G41970
3874	2	L4 vs. M4	L4 Up	GO:0005093	Signaling and response	Signaling	Rab GDP-dissociation inhibitor activity	F	AT5G09550
3875	2	L4 vs. M4	L4 Up	GO:0017137	Signaling and response	Signaling	Rab GTPase binding	F	AT3G07890
3876	2	L4 vs. M4	L4 Up	GO:0017137	Signaling and response	Signaling	Rab GTPase binding	F	AT5G09550
3877	2	L4 vs. M4	L4 Up	GO:0017112	Signaling and response	Signaling	Rab guanyl-nucleotide exchange factor activity	F	AT5G02280
3878	2	L4 vs. M4	L4 Up	GO:0032482	Signaling and response	Signaling	Rab protein signal transduction	P	AT2G21880
3879	2	L4 vs. M4	L4 Up	GO:0048365	Signaling and response	Signaling	Rac GTPase binding	F	AT3G07880
3880	2	L4 vs. M4	L4 Up	GO:0008536	Signaling and response	Signaling	Ran GTPase binding	F	AT1G27970
3881	2	L4 vs. M4	L4 Up	GO:0043235	Signaling and response	Signaling	receptor complex	C	AT2G01110
3882	2	L4 vs. M4	L4 Up	GO:0042752	Signaling and response	Signaling	regulation of circadian rhythm	P	AT5G02840
3883	2	L4 vs. M4	L4 Up	GO:2000022	Signaling and response	Signaling	regulation of jasmonic acid mediated signaling pathway	P	AT3G55970
3884	2	L4 vs. M4	L4 Up	GO:0080163	Signaling and response	Signaling	regulation of protein serine/threonine phosphatase activity	P	AT2G38310
3885	2	L4 vs. M4	L4 Up	GO:2000280	Signaling and response	Signaling	regulation of root development	P	AT5G26700
3886	2	L4 vs. M4	L4 Up	GO:0060359	Signaling and response	Signaling	response to ammonium ion	P	AT2G39770
3887	2	L4 vs. M4	L4 Up	GO:0009733	Signaling and response	Signaling	response to auxin	P	AT1G49010
3888	2	L4 vs. M4	L4 Up	GO:0009733	Signaling and response	Signaling	response to auxin	P	AT1G64520
3889	2	L4 vs. M4	L4 Up	GO:0009733	Signaling and response	Signaling	response to auxin	P	AT4G37610
3890	2	L4 vs. M4	L4 Up	GO:0009733	Signaling and response	Signaling	response to auxin	P	AT5G02840
3891	2	L4 vs. M4	L4 Up	GO:0009617	Signaling and response	Signaling	response to bacterium	P	AT5G01600
3892	2	L4 vs. M4	L4 Up	GO:0009637	Signaling and response	Signaling	response to blue light	P	AT5G01530
3893	2	L4 vs. M4	L4 Up	GO:0046686	Signaling and response	Signaling	response to cadmium ion	P	AT1G07890
3894	2	L4 vs. M4	L4 Up	GO:0046686	Signaling and response	Signaling	response to cadmium ion	P	AT1G13930
3895	2	L4 vs. M4	L4 Up	GO:0046686	Signaling and response	Signaling	response to cadmium ion	P	AT1G27450
3896	2	L4 vs. M4	L4 Up	GO:0046686	Signaling and response	Signaling	response to cadmium ion	P	AT1G49010
3897	2	L4 vs. M4	L4 Up	GO:0046686	Signaling and response	Signaling	response to cadmium ion	P	AT2G21660
3898	2	L4 vs. M4	L4 Up	GO:0046686	Signaling and response	Signaling	response to cadmium ion	P	AT3G12500
3899	2	L4 vs. M4	L4 Up	GO:0046686	Signaling and response	Signaling	response to cadmium ion	P	AT3G22960
3900	2	L4 vs. M4	L4 Up	GO:0046686	Signaling and response	Signaling	response to cadmium ion	P	AT4G25100
3901	2	L4 vs. M4	L4 Up	GO:0046686	Signaling and response	Signaling	response to cadmium ion	P	AT5G02840
3902	2	L4 vs. M4	L4 Up	GO:0046686	Signaling and response	Signaling	response to cadmium ion	P	AT5G16970
3903	2	L4 vs. M4	L4 Up	GO:0046686	Signaling and response	Signaling	response to cadmium ion	P	ATCG00490
3904	2	L4 vs. M4	L4 Up	GO:0010037	Signaling and response	Signaling	response to carbon dioxide	P	AT3G01500
3905	2	L4 vs. M4	L4 Up	GO:0010037	Signaling and response	Signaling	response to carbon dioxide	P	AT5G04180
3906	2	L4 vs. M4	L4 Up	GO:0010200	Signaling and response	Signaling	response to chitin	P	AT4G37610
3907	2	L4 vs. M4	L4 Up	GO:0046688	Signaling and response	Signaling	response to copper ion	P	AT4G25100
3908	2	L4 vs. M4	L4 Up	GO:0009735	Signaling and response	Signaling	response to cytokinin	P	AT1G64520
3909	2	L4 vs. M4	L4 Up	GO:0010218	Signaling and response	Signaling	response to far red light	P	AT5G01530
3910	2	L4 vs. M4	L4 Up	GO:0009739	Signaling and response	Signaling	response to gibberellin	P	AT1G49010
3911	2	L4 vs. M4	L4 Up	GO:0009739	Signaling and response	Signaling	response to gibberellin	P	AT5G02840

3912	2	L4 vs. M4	L4 Up	GO:0010039	Signaling and response	Signaling	response to iron ion	P	AT3G11050
3913	2	L4 vs. M4	L4 Up	GO:0010039	Signaling and response	Signaling	response to iron ion	P	AT3G56090
3914	2	L4 vs. M4	L4 Up	GO:0010039	Signaling and response	Signaling	response to iron ion	P	AT5G01600
3915	2	L4 vs. M4	L4 Up	GO:0009753	Signaling and response	Signaling	response to jasmonic acid	P	AT1G49010
3916	2	L4 vs. M4	L4 Up	GO:0009753	Signaling and response	Signaling	response to jasmonic acid	P	AT2G39770
3917	2	L4 vs. M4	L4 Up	GO:0009753	Signaling and response	Signaling	response to jasmonic acid	P	AT4G35770
3918	2	L4 vs. M4	L4 Up	GO:0009753	Signaling and response	Signaling	response to jasmonic acid	P	AT5G02840
3919	2	L4 vs. M4	L4 Up	GO:0080167	Signaling and response	Signaling	response to karrikin	P	AT3G47470
3920	2	L4 vs. M4	L4 Up	GO:0080167	Signaling and response	Signaling	response to karrikin	P	AT5G20250
3921	2	L4 vs. M4	L4 Up	GO:0009642	Signaling and response	Signaling	response to light intensity	P	AT4G25100
3922	2	L4 vs. M4	L4 Up	GO:0009416	Signaling and response	Signaling	response to light stimulus	P	AT1G29930
3923	2	L4 vs. M4	L4 Up	GO:0009416	Signaling and response	Signaling	response to light stimulus	P	AT1G60950
3924	2	L4 vs. M4	L4 Up	GO:0009416	Signaling and response	Signaling	response to light stimulus	P	AT1G80920
3925	2	L4 vs. M4	L4 Up	GO:0009416	Signaling and response	Signaling	response to light stimulus	P	AT2G34430
3926	2	L4 vs. M4	L4 Up	GO:0009416	Signaling and response	Signaling	response to light stimulus	P	AT3G47470
3927	2	L4 vs. M4	L4 Up	GO:0009416	Signaling and response	Signaling	response to light stimulus	P	AT4G02380
3928	2	L4 vs. M4	L4 Up	GO:0009416	Signaling and response	Signaling	response to light stimulus	P	AT4G35770
3929	2	L4 vs. M4	L4 Up	GO:0009416	Signaling and response	Signaling	response to light stimulus	P	AT5G01530
3930	2	L4 vs. M4	L4 Up	GO:0009416	Signaling and response	Signaling	response to light stimulus	P	AT5G20250
3931	2	L4 vs. M4	L4 Up	GO:0010114	Signaling and response	Signaling	response to red light	P	AT5G01530
3932	2	L4 vs. M4	L4 Up	GO:0009751	Signaling and response	Signaling	response to salicylic acid	P	AT1G49010
3933	2	L4 vs. M4	L4 Up	GO:0009751	Signaling and response	Signaling	response to salicylic acid	P	AT2G33380
3934	2	L4 vs. M4	L4 Up	GO:0009751	Signaling and response	Signaling	response to salicylic acid	P	AT4G37610
3935	2	L4 vs. M4	L4 Up	GO:0009751	Signaling and response	Signaling	response to salicylic acid	P	AT5G02840
3936	2	L4 vs. M4	L4 Up	GO:0009744	Signaling and response	Signaling	response to sucrose	P	AT4G35770
3937	2	L4 vs. M4	L4 Up	GO:0009744	Signaling and response	Signaling	response to sucrose	P	AT5G20250
3938	2	L4 vs. M4	L4 Up	GO:0010043	Signaling and response	Signaling	response to zinc ion	P	AT1G16470
3939	2	L4 vs. M4	L4 Up	GO:0010043	Signaling and response	Signaling	response to zinc ion	P	AT2G21660
3940	2	L4 vs. M4	L4 Up	GO:0010043	Signaling and response	Signaling	response to zinc ion	P	AT5G01600
3941	2	L4 vs. M4	L4 Up	GO:0006890	Signaling and response	Signaling	retrograde vesicle-mediated transport, Golgi to endoplasmic reticulum	P	AT1G11890
3942	2	L4 vs. M4	L4 Up	GO:0007266	Signaling and response	Signaling	Rho protein signal transduction	P	AT3G07880
3943	2	L4 vs. M4	L4 Up	GO:0038023	Signaling and response	Signaling	signaling receptor activity	F	AT2G38310
3944	2	L4 vs. M4	L4 Up	GO:0007264	Signaling and response	Signaling	small GTPase mediated signal transduction	P	AT5G09550
3945	2	L4 vs. M4	L4 Up	GO:0032934	Signaling and response	Signaling	sterol binding	F	AT5G02100
3946	2	L4 vs. M4	L4 Up	GO:0010118	Signaling and response	Signaling	stomatal movement	P	AT2G33380
3947	2	L4 vs. M4	L4 Up	GO:0001872	Signaling and response	Stress	(1->3)-beta-D-glucan binding	F	AT1G69295
3948	2	L4 vs. M4	L4 Up	GO:0001872	Signaling and response	Stress	(1->3)-beta-D-glucan binding	F	AT5G08000
3949	2	L4 vs. M4	L4 Up	GO:0010427	Signaling and response	Stress	abscisic acid binding	F	AT2G38310
3950	2	L4 vs. M4	L4 Up	GO:0009738	Signaling and response	Stress	abscisic acid-activated signaling pathway	P	AT2G38310
3951	2	L4 vs. M4	L4 Up	GO:0009738	Signaling and response	Stress	abscisic acid-activated signaling pathway	P	AT5G58670
3952	2	L4 vs. M4	L4 Up	GO:0007568	Signaling and response	Stress	aging	P	AT4G35770
3953	2	L4 vs. M4	L4 Up	GO:0052543	Signaling and response	Stress	callose deposition in cell wall	P	AT5G50800
3954	2	L4 vs. M4	L4 Up	GO:0045454	Signaling and response	Stress	cell redox homeostasis	P	AT3G03860
3955	2	L4 vs. M4	L4 Up	GO:0006879	Signaling and response	Stress	cellular iron ion homeostasis	P	AT3G08040
3956	2	L4 vs. M4	L4 Up	GO:0034599	Signaling and response	Stress	cellular response to oxidative stress	P	AT1G07890
3957	2	L4 vs. M4	L4 Up	GO:0016036	Signaling and response	Stress	cellular response to phosphate starvation	P	AT3G08040
3958	2	L4 vs. M4	L4 Up	GO:0042631	Signaling and response	Stress	cellular response to water deprivation	P	AT4G02380
3959	2	L4 vs. M4	L4 Up	GO:0090156	Signaling and response	Stress	cellular sphingolipid homeostasis	P	AT5G42000
3960	2	L4 vs. M4	L4 Up	GO:0006952	Signaling and response	Stress	defense response	P	AT1G02305
3961	2	L4 vs. M4	L4 Up	GO:0006952	Signaling and response	Stress	defense response	P	AT1G72260
3962	2	L4 vs. M4	L4 Up	GO:0006952	Signaling and response	Stress	defense response	P	AT4G02380
3963	2	L4 vs. M4	L4 Up	GO:0006952	Signaling and response	Stress	defense response	P	AT5G36910
3964	2	L4 vs. M4	L4 Up	GO:0042742	Signaling and response	Stress	defense response to bacterium	P	AT1G79210
3965	2	L4 vs. M4	L4 Up	GO:0042742	Signaling and response	Stress	defense response to bacterium	P	AT2G39770
3966	2	L4 vs. M4	L4 Up	GO:0042742	Signaling and response	Stress	defense response to bacterium	P	AT3G01500

3967	2	L4 vs. M4	L4 Up	GO:0042742	Signaling and response	Stress	defense response to bacterium	P	AT3G30775
3968	2	L4 vs. M4	L4 Up	GO:0042742	Signaling and response	Stress	defense response to bacterium	P	AT5G42000
3969	2	L4 vs. M4	L4 Up	GO:0050832	Signaling and response	Stress	defense response to fungus	P	AT2G33380
3970	2	L4 vs. M4	L4 Up	GO:0050832	Signaling and response	Stress	defense response to fungus	P	AT3G12500
3971	2	L4 vs. M4	L4 Up	GO:0050832	Signaling and response	Stress	defense response to fungus	P	AT4G30650
3972	2	L4 vs. M4	L4 Up	GO:0009817	Signaling and response	Stress	defense response to fungus, incompatible interaction	P	AT3G01500
3973	2	L4 vs. M4	L4 Up	GO:0009814	Signaling and response	Stress	defense response, incompatible interaction	P	AT1G32540
3974	2	L4 vs. M4	L4 Up	GO:0009873	Signaling and response	Stress	ethylene-activated signaling pathway	P	AT1G79700
3975	2	L4 vs. M4	L4 Up	GO:0009873	Signaling and response	Stress	ethylene-activated signaling pathway	P	AT2G25490
3976	2	L4 vs. M4	L4 Up	GO:0009813	Signaling and response	Stress	flavonoid biosynthetic process	P	AT3G55970
3977	2	L4 vs. M4	L4 Up	GO:0051555	Signaling and response	Stress	flavonol biosynthetic process	P	AT5G54160
3978	2	L4 vs. M4	L4 Up	GO:0042744	Signaling and response	Stress	hydrogen peroxide catabolic process	P	AT1G07890
3979	2	L4 vs. M4	L4 Up	GO:0042744	Signaling and response	Stress	hydrogen peroxide catabolic process	P	AT1G71695
3980	2	L4 vs. M4	L4 Up	GO:0042538	Signaling and response	Stress	hyperosmotic salinity response	P	AT1G74520
3981	2	L4 vs. M4	L4 Up	GO:0042538	Signaling and response	Stress	hyperosmotic salinity response	P	AT4G24960
3982	2	L4 vs. M4	L4 Up	GO:0045087	Signaling and response	Stress	innate immune response	P	AT2G21660
3983	2	L4 vs. M4	L4 Up	GO:0050113	Signaling and response	Stress	inositol oxygenase activity	F	AT4G26260
3984	2	L4 vs. M4	L4 Up	GO:0050113	Signaling and response	Stress	inositol oxygenase activity	F	AT5G56640
3985	2	L4 vs. M4	L4 Up	GO:0055072	Signaling and response	Stress	iron ion homeostasis	P	AT1G55570
3986	2	L4 vs. M4	L4 Up	GO:0055072	Signaling and response	Stress	iron ion homeostasis	P	AT3G08040
3987	2	L4 vs. M4	L4 Up	GO:0055072	Signaling and response	Stress	iron ion homeostasis	P	AT3G13390
3988	2	L4 vs. M4	L4 Up	GO:0055072	Signaling and response	Stress	iron ion homeostasis	P	AT3G13400
3989	2	L4 vs. M4	L4 Up	GO:0055072	Signaling and response	Stress	iron ion homeostasis	P	AT3G56090
3990	2	L4 vs. M4	L4 Up	GO:0055072	Signaling and response	Stress	iron ion homeostasis	P	AT5G01600
3991	2	L4 vs. M4	L4 Up	GO:0009871	Signaling and response	Stress	jasmonic acid and ethylene-dependent systemic resistance, ethylene mediated signaling pathway	P	AT3G12500
3992	2	L4 vs. M4	L4 Up	GO:0031640	Signaling and response	Stress	killing of cells of other organism	P	AT3G12500
3993	2	L4 vs. M4	L4 Up	GO:0016688	Signaling and response	Stress	L-ascorbate peroxidase activity	F	AT1G07890
3994	2	L4 vs. M4	L4 Up	GO:0019853	Signaling and response	Stress	L-ascorbic acid biosynthetic process	P	AT2G39770
3995	2	L4 vs. M4	L4 Up	GO:0019853	Signaling and response	Stress	L-ascorbic acid biosynthetic process	P	AT4G26260
3996	2	L4 vs. M4	L4 Up	GO:0019853	Signaling and response	Stress	L-ascorbic acid biosynthetic process	P	AT5G56640
3997	2	L4 vs. M4	L4 Up	GO:0010507	Signaling and response	Stress	negative regulation of autophagy	P	AT4G24960
3998	2	L4 vs. M4	L4 Up	GO:0010105	Signaling and response	Stress	negative regulation of ethylene-activated signaling pathway	P	AT2G25490
3999	2	L4 vs. M4	L4 Up	GO:0010187	Signaling and response	Stress	negative regulation of seed germination	P	AT3G44680
4000	2	L4 vs. M4	L4 Up	GO:0009405	Signaling and response	Stress	pathogenesis	P	AT5G36910
4001	2	L4 vs. M4	L4 Up	GO:0048015	Signaling and response	Stress	phosphatidylinositol-mediated signaling	P	AT5G58670
4002	2	L4 vs. M4	L4 Up	GO:0009626	Signaling and response	Stress	plant-type hypersensitive response	P	AT1G32540
4003	2	L4 vs. M4	L4 Up	GO:0009626	Signaling and response	Stress	plant-type hypersensitive response	P	AT3G12500
4004	2	L4 vs. M4	L4 Up	GO:0034052	Signaling and response	Stress	positive regulation of plant-type hypersensitive response	P	AT1G32540
4005	2	L4 vs. M4	L4 Up	GO:0043068	Signaling and response	Stress	positive regulation of programmed cell death	P	AT1G32540
4006	2	L4 vs. M4	L4 Up	GO:0055075	Signaling and response	Stress	potassium ion homeostasis	P	AT5G55990
4007	2	L4 vs. M4	L4 Up	GO:0044183	Signaling and response	Stress	protein folding chaperone	F	AT4G04330
4008	2	L4 vs. M4	L4 Up	GO:0010115	Signaling and response	Stress	regulation of abscisic acid biosynthetic process	P	AT1G13930
4009	2	L4 vs. M4	L4 Up	GO:0051453	Signaling and response	Stress	regulation of intracellular pH	P	AT2G07560
4010	2	L4 vs. M4	L4 Up	GO:2000377	Signaling and response	Stress	regulation of reactive oxygen species metabolic process	P	AT2G33380
4011	2	L4 vs. M4	L4 Up	GO:2000121	Signaling and response	Stress	regulation of removal of superoxide radicals	P	AT1G32540
4012	2	L4 vs. M4	L4 Up	GO:0010119	Signaling and response	Stress	regulation of stomatal movement	P	AT2G21660
4013	2	L4 vs. M4	L4 Up	GO:0010119	Signaling and response	Stress	regulation of stomatal movement	P	AT3G01500
4014	2	L4 vs. M4	L4 Up	GO:0051209	Signaling and response	Stress	release of sequestered calcium ion into cytosol	P	AT5G58670
4015	2	L4 vs. M4	L4 Up	GO:0019430	Signaling and response	Stress	removal of superoxide radicals	P	AT4G25100
4016	2	L4 vs. M4	L4 Up	GO:0009737	Signaling and response	Stress	response to abscisic acid	P	AT1G74520
4017	2	L4 vs. M4	L4 Up	GO:0009737	Signaling and response	Stress	response to abscisic acid	P	AT2G33380

4018	2	L4 vs. M4	L4 Up	GO:0009737	Signaling and response	Stress	response to abscisic acid	P	AT3G08040
4019	2	L4 vs. M4	L4 Up	GO:0009737	Signaling and response	Stress	response to abscisic acid	P	AT3G11050
4020	2	L4 vs. M4	L4 Up	GO:0009737	Signaling and response	Stress	response to abscisic acid	P	AT4G02380
4021	2	L4 vs. M4	L4 Up	GO:0009737	Signaling and response	Stress	response to abscisic acid	P	AT4G24960
4022	2	L4 vs. M4	L4 Up	GO:0009737	Signaling and response	Stress	response to abscisic acid	P	AT5G02840
4023	2	L4 vs. M4	L4 Up	GO:0009737	Signaling and response	Stress	response to abscisic acid	P	AT5G58670
4024	2	L4 vs. M4	L4 Up	GO:0009737	Signaling and response	Stress	response to abscisic acid	P	ATCG00490
4025	2	L4 vs. M4	L4 Up	GO:0009646	Signaling and response	Stress	response to absence of light	P	AT2G43400
4026	2	L4 vs. M4	L4 Up	GO:0009646	Signaling and response	Stress	response to absence of light	P	AT4G02380
4027	2	L4 vs. M4	L4 Up	GO:0009646	Signaling and response	Stress	response to absence of light	P	AT5G63190
4028	2	L4 vs. M4	L4 Up	GO:0090549	Signaling and response	Stress	response to carbon starvation	P	AT5G63190
4029	2	L4 vs. M4	L4 Up	GO:0009409	Signaling and response	Stress	response to cold	P	AT1G74520
4030	2	L4 vs. M4	L4 Up	GO:0009409	Signaling and response	Stress	response to cold	P	AT2G21660
4031	2	L4 vs. M4	L4 Up	GO:0009409	Signaling and response	Stress	response to cold	P	AT2G33380
4032	2	L4 vs. M4	L4 Up	GO:0009409	Signaling and response	Stress	response to cold	P	AT2G39460
4033	2	L4 vs. M4	L4 Up	GO:0009409	Signaling and response	Stress	response to cold	P	AT3G01500
4034	2	L4 vs. M4	L4 Up	GO:0009409	Signaling and response	Stress	response to cold	P	AT3G05890
4035	2	L4 vs. M4	L4 Up	GO:0009409	Signaling and response	Stress	response to cold	P	AT3G47470
4036	2	L4 vs. M4	L4 Up	GO:0009409	Signaling and response	Stress	response to cold	P	AT4G02380
4037	2	L4 vs. M4	L4 Up	GO:0009409	Signaling and response	Stress	response to cold	P	AT4G04330
4038	2	L4 vs. M4	L4 Up	GO:0009409	Signaling and response	Stress	response to cold	P	AT4G24960
4039	2	L4 vs. M4	L4 Up	GO:0009409	Signaling and response	Stress	response to cold	P	AT4G37610
4040	2	L4 vs. M4	L4 Up	GO:0009409	Signaling and response	Stress	response to cold	P	AT5G01600
4041	2	L4 vs. M4	L4 Up	GO:0009409	Signaling and response	Stress	response to cold	P	AT5G20250
4042	2	L4 vs. M4	L4 Up	GO:0009409	Signaling and response	Stress	response to cold	P	AT5G20630
4043	2	L4 vs. M4	L4 Up	GO:0009409	Signaling and response	Stress	response to cold	P	AT5G58670
4044	2	L4 vs. M4	L4 Up	GO:0009269	Signaling and response	Stress	response to desiccation	P	AT2G33380
4045	2	L4 vs. M4	L4 Up	GO:0034976	Signaling and response	Stress	response to endoplasmic reticulum stress	P	AT5G42000
4046	2	L4 vs. M4	L4 Up	GO:0009723	Signaling and response	Stress	response to ethylene	P	AT2G25490
4047	2	L4 vs. M4	L4 Up	GO:0009723	Signaling and response	Stress	response to ethylene	P	AT4G02380
4048	2	L4 vs. M4	L4 Up	GO:0009723	Signaling and response	Stress	response to ethylene	P	AT5G02840
4049	2	L4 vs. M4	L4 Up	GO:0009620	Signaling and response	Stress	response to fungus	P	AT2G33380
4050	2	L4 vs. M4	L4 Up	GO:0009408	Signaling and response	Stress	response to heat	P	AT1G07890
4051	2	L4 vs. M4	L4 Up	GO:0009408	Signaling and response	Stress	response to heat	P	AT1G13930
4052	2	L4 vs. M4	L4 Up	GO:0009408	Signaling and response	Stress	response to heat	P	AT1G64520
4053	2	L4 vs. M4	L4 Up	GO:0009408	Signaling and response	Stress	response to heat	P	AT2G39770
4054	2	L4 vs. M4	L4 Up	GO:0009408	Signaling and response	Stress	response to heat	P	AT5G08000
4055	2	L4 vs. M4	L4 Up	GO:0009644	Signaling and response	Stress	response to high light intensity	P	AT2G39460
4056	2	L4 vs. M4	L4 Up	GO:0009644	Signaling and response	Stress	response to high light intensity	P	AT3G47470
4057	2	L4 vs. M4	L4 Up	GO:0042542	Signaling and response	Stress	response to hydrogen peroxide	P	AT1G35580
4058	2	L4 vs. M4	L4 Up	GO:0042542	Signaling and response	Stress	response to hydrogen peroxide	P	AT4G37610
4059	2	L4 vs. M4	L4 Up	GO:0042542	Signaling and response	Stress	response to hydrogen peroxide	P	AT5G01600
4060	2	L4 vs. M4	L4 Up	GO:0009625	Signaling and response	Stress	response to insect	P	AT4G02380
4061	2	L4 vs. M4	L4 Up	GO:0009645	Signaling and response	Stress	response to low light intensity stimulus	P	AT3G47470
4062	2	L4 vs. M4	L4 Up	GO:0051788	Signaling and response	Stress	response to misfolded protein	P	AT1G64520
4063	2	L4 vs. M4	L4 Up	GO:0009624	Signaling and response	Stress	response to nematode	P	AT1G02800
4064	2	L4 vs. M4	L4 Up	GO:0009624	Signaling and response	Stress	response to nematode	P	AT1G59740
4065	2	L4 vs. M4	L4 Up	GO:0009624	Signaling and response	Stress	response to nematode	P	AT2G21050
4066	2	L4 vs. M4	L4 Up	GO:0006970	Signaling and response	Stress	response to osmotic stress	P	AT2G21660
4067	2	L4 vs. M4	L4 Up	GO:0006970	Signaling and response	Stress	response to osmotic stress	P	AT5G38710
4068	2	L4 vs. M4	L4 Up	GO:0006979	Signaling and response	Stress	response to oxidative stress	P	AT1G50170
4069	2	L4 vs. M4	L4 Up	GO:0006979	Signaling and response	Stress	response to oxidative stress	P	AT1G71695
4070	2	L4 vs. M4	L4 Up	GO:0006979	Signaling and response	Stress	response to oxidative stress	P	AT2G39460
4071	2	L4 vs. M4	L4 Up	GO:0006979	Signaling and response	Stress	response to oxidative stress	P	AT3G11050
4072	2	L4 vs. M4	L4 Up	GO:0006979	Signaling and response	Stress	response to oxidative stress	P	AT3G30775
4073	2	L4 vs. M4	L4 Up	GO:0006979	Signaling and response	Stress	response to oxidative stress	P	AT4G02380

4074	2	L4 vs. M4	L4 Up	GO:0006979	Signaling and response	Stress	response to oxidative stress	P	AT4G25100
4075	2	L4 vs. M4	L4 Up	GO:0006979	Signaling and response	Stress	response to oxidative stress	P	AT4G35770
4076	2	L4 vs. M4	L4 Up	GO:0006979	Signaling and response	Stress	response to oxidative stress	P	AT5G16970
4077	2	L4 vs. M4	L4 Up	GO:0006979	Signaling and response	Stress	response to oxidative stress	P	AT5G20250
4078	2	L4 vs. M4	L4 Up	GO:0006979	Signaling and response	Stress	response to oxidative stress	P	AT5G42000
4079	2	L4 vs. M4	L4 Up	GO:0010193	Signaling and response	Stress	response to ozone	P	AT2G39770
4080	2	L4 vs. M4	L4 Up	GO:0010193	Signaling and response	Stress	response to ozone	P	AT4G25100
4081	2	L4 vs. M4	L4 Up	GO:0000302	Signaling and response	Stress	response to reactive oxygen species	P	AT1G07890
4082	2	L4 vs. M4	L4 Up	GO:0000302	Signaling and response	Stress	response to reactive oxygen species	P	AT3G11050
4083	2	L4 vs. M4	L4 Up	GO:0000302	Signaling and response	Stress	response to reactive oxygen species	P	AT3G56090
4084	2	L4 vs. M4	L4 Up	GO:0000302	Signaling and response	Stress	response to reactive oxygen species	P	AT4G02380
4085	2	L4 vs. M4	L4 Up	GO:0000302	Signaling and response	Stress	response to reactive oxygen species	P	AT5G01600
4086	2	L4 vs. M4	L4 Up	GO:0009651	Signaling and response	Stress	response to salt stress	P	AT1G13930
4087	2	L4 vs. M4	L4 Up	GO:0009651	Signaling and response	Stress	response to salt stress	P	AT1G49010
4088	2	L4 vs. M4	L4 Up	GO:0009651	Signaling and response	Stress	response to salt stress	P	AT2G21660
4089	2	L4 vs. M4	L4 Up	GO:0009651	Signaling and response	Stress	response to salt stress	P	AT2G33380
4090	2	L4 vs. M4	L4 Up	GO:0009651	Signaling and response	Stress	response to salt stress	P	AT2G39770
4091	2	L4 vs. M4	L4 Up	GO:0009651	Signaling and response	Stress	response to salt stress	P	AT4G04330
4092	2	L4 vs. M4	L4 Up	GO:0009651	Signaling and response	Stress	response to salt stress	P	AT5G02840
4093	2	L4 vs. M4	L4 Up	GO:0009651	Signaling and response	Stress	response to salt stress	P	AT5G14040
4094	2	L4 vs. M4	L4 Up	GO:0009651	Signaling and response	Stress	response to salt stress	P	AT5G58670
4095	2	L4 vs. M4	L4 Up	GO:0042594	Signaling and response	Stress	response to starvation	P	AT1G78020
4096	2	L4 vs. M4	L4 Up	GO:0009414	Signaling and response	Stress	response to water deprivation	P	AT1G68820
4097	2	L4 vs. M4	L4 Up	GO:0009414	Signaling and response	Stress	response to water deprivation	P	AT1G74520
4098	2	L4 vs. M4	L4 Up	GO:0009414	Signaling and response	Stress	response to water deprivation	P	AT2G21660
4099	2	L4 vs. M4	L4 Up	GO:0009414	Signaling and response	Stress	response to water deprivation	P	AT3G30775
4100	2	L4 vs. M4	L4 Up	GO:0009414	Signaling and response	Stress	response to water deprivation	P	AT4G02380
4101	2	L4 vs. M4	L4 Up	GO:0009414	Signaling and response	Stress	response to water deprivation	P	AT4G04330
4102	2	L4 vs. M4	L4 Up	GO:0009414	Signaling and response	Stress	response to water deprivation	P	AT4G24960
4103	2	L4 vs. M4	L4 Up	GO:0009414	Signaling and response	Stress	response to water deprivation	P	AT5G38710
4104	2	L4 vs. M4	L4 Up	GO:0009414	Signaling and response	Stress	response to water deprivation	P	AT5G58670
4105	2	L4 vs. M4	L4 Up	GO:0009611	Signaling and response	Stress	response to wounding	P	AT4G35770
4106	2	L4 vs. M4	L4 Up	GO:0005094	Signaling and response	Stress	Rho GDP-dissociation inhibitor activity	F	AT3G07880
4107	2	L4 vs. M4	L4 Up	GO:0004784	Signaling and response	Stress	superoxide dismutase activity	F	AT4G25100
4108	2	L4 vs. M4	L4 Up	GO:0009627	Signaling and response	Stress	systemic acquired resistance	P	AT3G07450
4109	2	L4 vs. M4	L4 Up	GO:0090729	Signaling and response	Stress	toxin activity	F	AT1G72260
4110	2	L4 vs. M4	L4 Up	GO:0090729	Signaling and response	Stress	toxin activity	F	AT5G36910
4111	2	L4 vs. M4	L4 Up	GO:0004675	Signaling and response	Stress	transmembrane receptor protein serine/threonine kinase activity	F	AT2G41970
4112	2	L4 vs. M4	M4 up	GO:0006284	Development	Cell division	base-excision repair	P	AT3G12710
4113	2	L4 vs. M4	M4 up	GO:0015030	Development	Cell division	Cajal body	C	AT4G25630
4114	2	L4 vs. M4	M4 up	GO:0044843	Development	Cell division	cell cycle G1/S phase transition	P	AT3G51800
4115	2	L4 vs. M4	M4 up	GO:0051301	Development	Cell division	cell division	P	AT1G78580
4116	2	L4 vs. M4	M4 up	GO:0005694	Development	Cell division	chromosome	C	AT1G09450
4117	2	L4 vs. M4	M4 up	GO:0000243	Development	Cell division	commitment complex	C	AT4G36690
4118	2	L4 vs. M4	M4 up	GO:0004693	Development	Cell division	cyclin-dependent protein serine/threonine kinase activity	F	AT5G53450
4119	2	L4 vs. M4	M4 up	GO:0000910	Development	Cell division	cytokinesis	P	AT3G01780
4120	2	L4 vs. M4	M4 up	GO:0003678	Development	Cell division	DNA helicase activity	F	AT1G03190
4121	2	L4 vs. M4	M4 up	GO:0003678	Development	Cell division	DNA helicase activity	F	AT3G09660
4122	2	L4 vs. M4	M4 up	GO:0009294	Development	Cell division	DNA mediated transformation	P	AT2G19480
4123	2	L4 vs. M4	M4 up	GO:0006281	Development	Cell division	DNA repair	P	AT1G03190
4124	2	L4 vs. M4	M4 up	GO:0006281	Development	Cell division	DNA repair	P	AT2G19480
4125	2	L4 vs. M4	M4 up	GO:0006281	Development	Cell division	DNA repair	P	AT4G05420
4126	2	L4 vs. M4	M4 up	GO:0006270	Development	Cell division	DNA replication initiation	P	AT3G09660
4127	2	L4 vs. M4	M4 up	GO:0003688	Development	Cell division	DNA replication origin binding	F	AT3G09660

4128	2	L4 vs. M4	M4 up	GO:0000724	Development	Cell division	double-strand break repair via homologous recombination	P	AT2G19480
4129	2	L4 vs. M4	M4 up	GO:0000724	Development	Cell division	double-strand break repair via homologous recombination	P	AT3G09660
4130	2	L4 vs. M4	M4 up	GO:0008309	Development	Cell division	double-stranded DNA exodeoxyribonuclease activity	F	AT1G11190
4131	2	L4 vs. M4	M4 up	GO:0004519	Development	Cell division	endonuclease activity	F	AT1G11190
4132	2	L4 vs. M4	M4 up	GO:0004519	Development	Cell division	endonuclease activity	F	AT1G67660
4133	2	L4 vs. M4	M4 up	GO:0007292	Development	Cell division	female gamete generation	P	AT4G29040
4134	2	L4 vs. M4	M4 up	GO:0007143	Development	Cell division	female meiotic nuclear division	P	AT3G09660
4135	2	L4 vs. M4	M4 up	GO:0007276	Development	Cell division	gamete generation	P	AT2G40360
4136	2	L4 vs. M4	M4 up	GO:0048229	Development	Cell division	gametophyte development	P	AT3G49080
4137	2	L4 vs. M4	M4 up	GO:0048229	Development	Cell division	gametophyte development	P	AT3G49670
4138	2	L4 vs. M4	M4 up	GO:0048232	Development	Cell division	male gamete generation	P	AT4G29040
4139	2	L4 vs. M4	M4 up	GO:0007140	Development	Cell division	male meiotic nuclear division	P	AT3G09660
4140	2	L4 vs. M4	M4 up	GO:0042555	Development	Cell division	MCM complex	C	AT3G09660
4141	2	L4 vs. M4	M4 up	GO:0009554	Development	Cell division	megasporogenesis	P	AT2G41420
4142	2	L4 vs. M4	M4 up	GO:0010480	Development	Cell division	microsporocyte differentiation	P	AT3G49670
4143	2	L4 vs. M4	M4 up	GO:0008568	Development	Cell division	microtubule-severing ATPase activity	F	AT3G27120
4144	2	L4 vs. M4	M4 up	GO:0000278	Development	Cell division	mitotic cell cycle	P	AT1G09450
4145	2	L4 vs. M4	M4 up	GO:0045128	Development	Cell division	negative regulation of reciprocal meiotic recombination	P	AT3G27120
4146	2	L4 vs. M4	M4 up	GO:0006334	Development	Cell division	nucleosome assembly	P	AT2G19480
4147	2	L4 vs. M4	M4 up	GO:0000717	Development	Cell division	nucleotide-excision repair, DNA duplex unwinding	P	AT1G03190
4148	2	L4 vs. M4	M4 up	GO:0033683	Development	Cell division	nucleotide-excision repair, DNA incision	P	AT1G03190
4149	2	L4 vs. M4	M4 up	GO:0009860	Development	Cell division	pollen tube growth	P	AT1G56430
4150	2	L4 vs. M4	M4 up	GO:0010183	Development	Cell division	pollen tube guidance	P	AT3G45640
4151	2	L4 vs. M4	M4 up	GO:0045951	Development	Cell division	positive regulation of mitotic recombination	P	AT1G03190
4152	2	L4 vs. M4	M4 up	GO:0007131	Development	Cell division	reciprocal meiotic recombination	P	AT5G66030
4153	2	L4 vs. M4	M4 up	GO:0051302	Development	Cell division	regulation of cell division	P	AT2G40360
4154	2	L4 vs. M4	M4 up	GO:0051302	Development	Cell division	regulation of cell division	P	AT3G51800
4155	2	L4 vs. M4	M4 up	GO:0010569	Development	Cell division	regulation of double-strand break repair via homologous recombination	P	AT3G27120
4156	2	L4 vs. M4	M4 up	GO:0000019	Development	Cell division	regulation of mitotic recombination	P	AT1G03190
4157	2	L4 vs. M4	M4 up	GO:2000035	Development	Cell division	regulation of stem cell division	P	AT5G03730
4158	2	L4 vs. M4	M4 up	GO:0043021	Development	Cell division	ribonucleoprotein complex binding	F	AT2G40360
4159	2	L4 vs. M4	M4 up	GO:0003697	Development	Cell division	single-stranded DNA binding	F	AT3G09660
4160	2	L4 vs. M4	M4 up	GO:0003697	Development	Cell division	single-stranded DNA binding	F	AT5G61030
4161	2	L4 vs. M4	M4 up	GO:0000014	Development	Cell division	single-stranded DNA endodeoxyribonuclease activity	F	AT1G11190
4162	2	L4 vs. M4	M4 up	GO:0043765	Development	Cell division	T/G mismatch-specific endonuclease activity	F	AT1G11190
4163	2	L4 vs. M4	M4 up	GO:0030479	Development	Development	actin cortical patch	C	AT1G13180
4164	2	L4 vs. M4	M4 up	GO:0046658	Development	Development	anchored component of plasma membrane	C	AT5G06320
4165	2	L4 vs. M4	M4 up	GO:0048653	Development	Development	anther development	P	AT3G49670
4166	2	L4 vs. M4	M4 up	GO:0048658	Development	Development	anther wall tapetum development	P	AT5G50260
4167	2	L4 vs. M4	M4 up	GO:0048046	Development	Development	apoplast	C	AT1G09310
4168	2	L4 vs. M4	M4 up	GO:0048046	Development	Development	apoplast	C	AT1G35140
4169	2	L4 vs. M4	M4 up	GO:0048046	Development	Development	apoplast	C	AT1G45130
4170	2	L4 vs. M4	M4 up	GO:0048046	Development	Development	apoplast	C	AT1G47128
4171	2	L4 vs. M4	M4 up	GO:0048046	Development	Development	apoplast	C	AT1G50480
4172	2	L4 vs. M4	M4 up	GO:0048046	Development	Development	apoplast	C	AT2G44450
4173	2	L4 vs. M4	M4 up	GO:0048046	Development	Development	apoplast	C	AT3G04120
4174	2	L4 vs. M4	M4 up	GO:0048046	Development	Development	apoplast	C	AT3G08590
4175	2	L4 vs. M4	M4 up	GO:0048046	Development	Development	apoplast	C	AT3G09440
4176	2	L4 vs. M4	M4 up	GO:0048046	Development	Development	apoplast	C	AT3G10740
4177	2	L4 vs. M4	M4 up	GO:0048046	Development	Development	apoplast	C	AT4G25000

4178	2	L4 vs. M4	M4 up	GO:0048046	Development	Development	apoplast	C	AT5G04140
4179	2	L4 vs. M4	M4 up	GO:0048046	Development	Development	apoplast	C	AT5G08380
4180	2	L4 vs. M4	M4 up	GO:0048046	Development	Development	apoplast	C	AT5G20740
4181	2	L4 vs. M4	M4 up	GO:0048046	Development	Development	apoplast	C	AT5G49360
4182	2	L4 vs. M4	M4 up	GO:0005938	Development	Development	cell cortex	C	AT3G45640
4183	2	L4 vs. M4	M4 up	GO:0030154	Development	Development	cell differentiation	P	AT3G49670
4184	2	L4 vs. M4	M4 up	GO:0000902	Development	Development	cell morphogenesis	P	AT1G13180
4185	2	L4 vs. M4	M4 up	GO:0009504	Development	Development	cell plate	C	AT1G04750
4186	2	L4 vs. M4	M4 up	GO:0009504	Development	Development	cell plate	C	AT1G05570
4187	2	L4 vs. M4	M4 up	GO:0009504	Development	Development	cell plate	C	AT3G01780
4188	2	L4 vs. M4	M4 up	GO:0009920	Development	Development	cell plate formation involved in plant-type cell wall biogenesis	P	AT1G04750
4189	2	L4 vs. M4	M4 up	GO:0009920	Development	Development	cell plate formation involved in plant-type cell wall biogenesis	P	AT1G05560
4190	2	L4 vs. M4	M4 up	GO:0005618	Development	Development	cell wall	C	AT1G02810
4191	2	L4 vs. M4	M4 up	GO:0005618	Development	Development	cell wall	C	AT1G20190
4192	2	L4 vs. M4	M4 up	GO:0005618	Development	Development	cell wall	C	AT1G45130
4193	2	L4 vs. M4	M4 up	GO:0005618	Development	Development	cell wall	C	AT1G56110
4194	2	L4 vs. M4	M4 up	GO:0005618	Development	Development	cell wall	C	AT1G78580
4195	2	L4 vs. M4	M4 up	GO:0005618	Development	Development	cell wall	C	AT2G02990
4196	2	L4 vs. M4	M4 up	GO:0005618	Development	Development	cell wall	C	AT2G44350
4197	2	L4 vs. M4	M4 up	GO:0005618	Development	Development	cell wall	C	AT2G44450
4198	2	L4 vs. M4	M4 up	GO:0005618	Development	Development	cell wall	C	AT3G09440
4199	2	L4 vs. M4	M4 up	GO:0005618	Development	Development	cell wall	C	AT4G11310
4200	2	L4 vs. M4	M4 up	GO:0005618	Development	Development	cell wall	C	AT4G19810
4201	2	L4 vs. M4	M4 up	GO:0005618	Development	Development	cell wall	C	AT5G08380
4202	2	L4 vs. M4	M4 up	GO:0005618	Development	Development	cell wall	C	AT5G37600
4203	2	L4 vs. M4	M4 up	GO:0005618	Development	Development	cell wall	C	AT5G45280
4204	2	L4 vs. M4	M4 up	GO:0005618	Development	Development	cell wall	C	AT5G66760
4205	2	L4 vs. M4	M4 up	GO:0042545	Development	Development	cell wall modification	P	AT1G02810
4206	2	L4 vs. M4	M4 up	GO:0071555	Development	Development	cell wall organization	P	AT1G05570
4207	2	L4 vs. M4	M4 up	GO:0071555	Development	Development	cell wall organization	P	AT2G32530
4208	2	L4 vs. M4	M4 up	GO:0071555	Development	Development	cell wall organization	P	AT3G28340
4209	2	L4 vs. M4	M4 up	GO:0071555	Development	Development	cell wall organization	P	AT5G08380
4210	2	L4 vs. M4	M4 up	GO:0071555	Development	Development	cell wall organization	P	AT5G45280
4211	2	L4 vs. M4	M4 up	GO:0005575	Development	Development	cellular_component	C	AT4G29780
4212	2	L4 vs. M4	M4 up	GO:0051276	Development	Development	chromosome organization	P	AT5G58270
4213	2	L4 vs. M4	M4 up	GO:0010588	Development	Development	cotyledon vascular tissue pattern formation	P	AT5G16780
4214	2	L4 vs. M4	M4 up	GO:0042335	Development	Development	cuticle development	P	AT1G01120
4215	2	L4 vs. M4	M4 up	GO:0005737	Development	Development	cytoplasm	C	AT1G01120
4216	2	L4 vs. M4	M4 up	GO:0005737	Development	Development	cytoplasm	C	AT1G01800
4217	2	L4 vs. M4	M4 up	GO:0005737	Development	Development	cytoplasm	C	AT1G02590
4218	2	L4 vs. M4	M4 up	GO:0005737	Development	Development	cytoplasm	C	AT1G06410
4219	2	L4 vs. M4	M4 up	GO:0005737	Development	Development	cytoplasm	C	AT1G09450
4220	2	L4 vs. M4	M4 up	GO:0005737	Development	Development	cytoplasm	C	AT1G13180
4221	2	L4 vs. M4	M4 up	GO:0005737	Development	Development	cytoplasm	C	AT1G14320
4222	2	L4 vs. M4	M4 up	GO:0005737	Development	Development	cytoplasm	C	AT1G15500
4223	2	L4 vs. M4	M4 up	GO:0005737	Development	Development	cytoplasm	C	AT1G17430
4224	2	L4 vs. M4	M4 up	GO:0005737	Development	Development	cytoplasm	C	AT1G18740
4225	2	L4 vs. M4	M4 up	GO:0005737	Development	Development	cytoplasm	C	AT1G19640
4226	2	L4 vs. M4	M4 up	GO:0005737	Development	Development	cytoplasm	C	AT1G20380
4227	2	L4 vs. M4	M4 up	GO:0005737	Development	Development	cytoplasm	C	AT1G25155
4228	2	L4 vs. M4	M4 up	GO:0005737	Development	Development	cytoplasm	C	AT1G28680
4229	2	L4 vs. M4	M4 up	GO:0005737	Development	Development	cytoplasm	C	AT1G29340
4230	2	L4 vs. M4	M4 up	GO:0005737	Development	Development	cytoplasm	C	AT1G32340
4231	2	L4 vs. M4	M4 up	GO:0005737	Development	Development	cytoplasm	C	AT1G45145

4232	2	L4 vs. M4	M4 up	GO:0005737	Development	Development	cytoplasm	C	AT1G48430
4233	2	L4 vs. M4	M4 up	GO:0005737	Development	Development	cytoplasm	C	AT1G50480
4234	2	L4 vs. M4	M4 up	GO:0005737	Development	Development	cytoplasm	C	AT1G60810
4235	2	L4 vs. M4	M4 up	GO:0005737	Development	Development	cytoplasm	C	AT1G62740
4236	2	L4 vs. M4	M4 up	GO:0005737	Development	Development	cytoplasm	C	AT1G65450
4237	2	L4 vs. M4	M4 up	GO:0005737	Development	Development	cytoplasm	C	AT1G70410
4238	2	L4 vs. M4	M4 up	GO:0005737	Development	Development	cytoplasm	C	AT1G72660
4239	2	L4 vs. M4	M4 up	GO:0005737	Development	Development	cytoplasm	C	AT1G78580
4240	2	L4 vs. M4	M4 up	GO:0005737	Development	Development	cytoplasm	C	AT1G80280
4241	2	L4 vs. M4	M4 up	GO:0005737	Development	Development	cytoplasm	C	AT2G17290
4242	2	L4 vs. M4	M4 up	GO:0005737	Development	Development	cytoplasm	C	AT2G19480
4243	2	L4 vs. M4	M4 up	GO:0005737	Development	Development	cytoplasm	C	AT2G19620
4244	2	L4 vs. M4	M4 up	GO:0005737	Development	Development	cytoplasm	C	AT2G21820
4245	2	L4 vs. M4	M4 up	GO:0005737	Development	Development	cytoplasm	C	AT2G22360
4246	2	L4 vs. M4	M4 up	GO:0005737	Development	Development	cytoplasm	C	AT2G24360
4247	2	L4 vs. M4	M4 up	GO:0005737	Development	Development	cytoplasm	C	AT2G35840
4248	2	L4 vs. M4	M4 up	GO:0005737	Development	Development	cytoplasm	C	AT2G37040
4249	2	L4 vs. M4	M4 up	GO:0005737	Development	Development	cytoplasm	C	AT2G39330
4250	2	L4 vs. M4	M4 up	GO:0005737	Development	Development	cytoplasm	C	AT2G39890
4251	2	L4 vs. M4	M4 up	GO:0005737	Development	Development	cytoplasm	C	AT2G40000
4252	2	L4 vs. M4	M4 up	GO:0005737	Development	Development	cytoplasm	C	AT3G01780
4253	2	L4 vs. M4	M4 up	GO:0005737	Development	Development	cytoplasm	C	AT3G04120
4254	2	L4 vs. M4	M4 up	GO:0005737	Development	Development	cytoplasm	C	AT3G07750
4255	2	L4 vs. M4	M4 up	GO:0005737	Development	Development	cytoplasm	C	AT3G08590
4256	2	L4 vs. M4	M4 up	GO:0005737	Development	Development	cytoplasm	C	AT3G09440
4257	2	L4 vs. M4	M4 up	GO:0005737	Development	Development	cytoplasm	C	AT3G12640
4258	2	L4 vs. M4	M4 up	GO:0005737	Development	Development	cytoplasm	C	AT3G16410
4259	2	L4 vs. M4	M4 up	GO:0005737	Development	Development	cytoplasm	C	AT3G16470
4260	2	L4 vs. M4	M4 up	GO:0005737	Development	Development	cytoplasm	C	AT3G20250
4261	2	L4 vs. M4	M4 up	GO:0005737	Development	Development	cytoplasm	C	AT3G45640
4262	2	L4 vs. M4	M4 up	GO:0005737	Development	Development	cytoplasm	C	AT3G50530
4263	2	L4 vs. M4	M4 up	GO:0005737	Development	Development	cytoplasm	C	AT3G51240
4264	2	L4 vs. M4	M4 up	GO:0005737	Development	Development	cytoplasm	C	AT3G51800
4265	2	L4 vs. M4	M4 up	GO:0005737	Development	Development	cytoplasm	C	AT3G53520
4266	2	L4 vs. M4	M4 up	GO:0005737	Development	Development	cytoplasm	C	AT3G58670
4267	2	L4 vs. M4	M4 up	GO:0005737	Development	Development	cytoplasm	C	AT3G60240
4268	2	L4 vs. M4	M4 up	GO:0005737	Development	Development	cytoplasm	C	AT4G02890
4269	2	L4 vs. M4	M4 up	GO:0005737	Development	Development	cytoplasm	C	AT4G13930
4270	2	L4 vs. M4	M4 up	GO:0005737	Development	Development	cytoplasm	C	AT4G16370
4271	2	L4 vs. M4	M4 up	GO:0005737	Development	Development	cytoplasm	C	AT4G18220
4272	2	L4 vs. M4	M4 up	GO:0005737	Development	Development	cytoplasm	C	AT4G22870
4273	2	L4 vs. M4	M4 up	GO:0005737	Development	Development	cytoplasm	C	AT4G24110
4274	2	L4 vs. M4	M4 up	GO:0005737	Development	Development	cytoplasm	C	AT4G24130
4275	2	L4 vs. M4	M4 up	GO:0005737	Development	Development	cytoplasm	C	AT4G24400
4276	2	L4 vs. M4	M4 up	GO:0005737	Development	Development	cytoplasm	C	AT4G25310
4277	2	L4 vs. M4	M4 up	GO:0005737	Development	Development	cytoplasm	C	AT4G29160
4278	2	L4 vs. M4	M4 up	GO:0005737	Development	Development	cytoplasm	C	AT4G32840
4279	2	L4 vs. M4	M4 up	GO:0005737	Development	Development	cytoplasm	C	AT4G33090
4280	2	L4 vs. M4	M4 up	GO:0005737	Development	Development	cytoplasm	C	AT4G33120
4281	2	L4 vs. M4	M4 up	GO:0005737	Development	Development	cytoplasm	C	AT4G33150
4282	2	L4 vs. M4	M4 up	GO:0005737	Development	Development	cytoplasm	C	AT4G36250
4283	2	L4 vs. M4	M4 up	GO:0005737	Development	Development	cytoplasm	C	AT4G36750
4284	2	L4 vs. M4	M4 up	GO:0005737	Development	Development	cytoplasm	C	AT5G03430
4285	2	L4 vs. M4	M4 up	GO:0005737	Development	Development	cytoplasm	C	AT5G05570
4286	2	L4 vs. M4	M4 up	GO:0005737	Development	Development	cytoplasm	C	AT5G05600
4287	2	L4 vs. M4	M4 up	GO:0005737	Development	Development	cytoplasm	C	AT5G08590

4288	2	L4 vs. M4	M4 up	GO:0005737	Development	Development	cytoplasm	C	AT5G15740
4289	2	L4 vs. M4	M4 up	GO:0005737	Development	Development	cytoplasm	C	AT5G16570
4290	2	L4 vs. M4	M4 up	GO:0005737	Development	Development	cytoplasm	C	AT5G28900
4291	2	L4 vs. M4	M4 up	GO:0005737	Development	Development	cytoplasm	C	AT5G36160
4292	2	L4 vs. M4	M4 up	GO:0005737	Development	Development	cytoplasm	C	AT5G37490
4293	2	L4 vs. M4	M4 up	GO:0005737	Development	Development	cytoplasm	C	AT5G37600
4294	2	L4 vs. M4	M4 up	GO:0005737	Development	Development	cytoplasm	C	AT5G40780
4295	2	L4 vs. M4	M4 up	GO:0005737	Development	Development	cytoplasm	C	AT5G42050
4296	2	L4 vs. M4	M4 up	GO:0005737	Development	Development	cytoplasm	C	AT5G43380
4297	2	L4 vs. M4	M4 up	GO:0005737	Development	Development	cytoplasm	C	AT5G47540
4298	2	L4 vs. M4	M4 up	GO:0005737	Development	Development	cytoplasm	C	AT5G47930
4299	2	L4 vs. M4	M4 up	GO:0005737	Development	Development	cytoplasm	C	AT5G54810
4300	2	L4 vs. M4	M4 up	GO:0005737	Development	Development	cytoplasm	C	AT5G58430
4301	2	L4 vs. M4	M4 up	GO:0005737	Development	Development	cytoplasm	C	AT5G58590
4302	2	L4 vs. M4	M4 up	GO:0005737	Development	Development	cytoplasm	C	AT5G60160
4303	2	L4 vs. M4	M4 up	GO:0005737	Development	Development	cytoplasm	C	AT5G64140
4304	2	L4 vs. M4	M4 up	GO:0005737	Development	Development	cytoplasm	C	AT5G64350
4305	2	L4 vs. M4	M4 up	GO:0005737	Development	Development	cytoplasm	C	AT5G66030
4306	2	L4 vs. M4	M4 up	GO:0005737	Development	Development	cytoplasm	C	AT5G66420
4307	2	L4 vs. M4	M4 up	GO:0005737	Development	Development	cytoplasm	C	AT5G67480
4308	2	L4 vs. M4	M4 up	GO:0031122	Development	Development	cytoplasmic microtubule organization	P	AT3G27120
4309	2	L4 vs. M4	M4 up	GO:0008092	Development	Development	cytoskeletal protein binding	F	AT1G68370
4310	2	L4 vs. M4	M4 up	GO:0005856	Development	Development	cytoskeleton	C	AT1G05560
4311	2	L4 vs. M4	M4 up	GO:0005856	Development	Development	cytoskeleton	C	AT1G09450
4312	2	L4 vs. M4	M4 up	GO:0005856	Development	Development	cytoskeleton	C	AT1G68370
4313	2	L4 vs. M4	M4 up	GO:0005829	Development	Development	cytosol	C	AT1G01800
4314	2	L4 vs. M4	M4 up	GO:0005829	Development	Development	cytosol	C	AT1G06410
4315	2	L4 vs. M4	M4 up	GO:0005829	Development	Development	cytosol	C	AT1G09310
4316	2	L4 vs. M4	M4 up	GO:0005829	Development	Development	cytosol	C	AT1G13180
4317	2	L4 vs. M4	M4 up	GO:0005829	Development	Development	cytosol	C	AT1G14320
4318	2	L4 vs. M4	M4 up	GO:0005829	Development	Development	cytosol	C	AT1G14610
4319	2	L4 vs. M4	M4 up	GO:0005829	Development	Development	cytosol	C	AT1G20380
4320	2	L4 vs. M4	M4 up	GO:0005829	Development	Development	cytosol	C	AT1G45145
4321	2	L4 vs. M4	M4 up	GO:0005829	Development	Development	cytosol	C	AT1G48300
4322	2	L4 vs. M4	M4 up	GO:0005829	Development	Development	cytosol	C	AT1G48430
4323	2	L4 vs. M4	M4 up	GO:0005829	Development	Development	cytosol	C	AT1G50480
4324	2	L4 vs. M4	M4 up	GO:0005829	Development	Development	cytosol	C	AT1G54100
4325	2	L4 vs. M4	M4 up	GO:0005829	Development	Development	cytosol	C	AT1G60810
4326	2	L4 vs. M4	M4 up	GO:0005829	Development	Development	cytosol	C	AT1G62740
4327	2	L4 vs. M4	M4 up	GO:0005829	Development	Development	cytosol	C	AT1G70410
4328	2	L4 vs. M4	M4 up	GO:0005829	Development	Development	cytosol	C	AT1G76130
4329	2	L4 vs. M4	M4 up	GO:0005829	Development	Development	cytosol	C	AT1G78580
4330	2	L4 vs. M4	M4 up	GO:0005829	Development	Development	cytosol	C	AT2G04400
4331	2	L4 vs. M4	M4 up	GO:0005829	Development	Development	cytosol	C	AT2G17290
4332	2	L4 vs. M4	M4 up	GO:0005829	Development	Development	cytosol	C	AT2G19480
4333	2	L4 vs. M4	M4 up	GO:0005829	Development	Development	cytosol	C	AT2G25670
4334	2	L4 vs. M4	M4 up	GO:0005829	Development	Development	cytosol	C	AT2G31390
4335	2	L4 vs. M4	M4 up	GO:0005829	Development	Development	cytosol	C	AT2G33210
4336	2	L4 vs. M4	M4 up	GO:0005829	Development	Development	cytosol	C	AT2G35040
4337	2	L4 vs. M4	M4 up	GO:0005829	Development	Development	cytosol	C	AT2G35840
4338	2	L4 vs. M4	M4 up	GO:0005829	Development	Development	cytosol	C	AT2G42790
4339	2	L4 vs. M4	M4 up	GO:0005829	Development	Development	cytosol	C	AT2G43820
4340	2	L4 vs. M4	M4 up	GO:0005829	Development	Development	cytosol	C	AT2G44350
4341	2	L4 vs. M4	M4 up	GO:0005829	Development	Development	cytosol	C	AT2G47060
4342	2	L4 vs. M4	M4 up	GO:0005829	Development	Development	cytosol	C	AT2G47390
4343	2	L4 vs. M4	M4 up	GO:0005829	Development	Development	cytosol	C	AT3G01780

4344	2	L4 vs. M4	M4 up	GO:0005829	Development	Development	cytosol	C	AT3G04120
4345	2	L4 vs. M4	M4 up	GO:0005829	Development	Development	cytosol	C	AT3G04520
4346	2	L4 vs. M4	M4 up	GO:0005829	Development	Development	cytosol	C	AT3G08590
4347	2	L4 vs. M4	M4 up	GO:0005829	Development	Development	cytosol	C	AT3G09440
4348	2	L4 vs. M4	M4 up	GO:0005829	Development	Development	cytosol	C	AT3G12070
4349	2	L4 vs. M4	M4 up	GO:0005829	Development	Development	cytosol	C	AT3G14620
4350	2	L4 vs. M4	M4 up	GO:0005829	Development	Development	cytosol	C	AT3G16410
4351	2	L4 vs. M4	M4 up	GO:0005829	Development	Development	cytosol	C	AT3G16470
4352	2	L4 vs. M4	M4 up	GO:0005829	Development	Development	cytosol	C	AT3G16950
4353	2	L4 vs. M4	M4 up	GO:0005829	Development	Development	cytosol	C	AT3G20250
4354	2	L4 vs. M4	M4 up	GO:0005829	Development	Development	cytosol	C	AT3G21250
4355	2	L4 vs. M4	M4 up	GO:0005829	Development	Development	cytosol	C	AT3G25760
4356	2	L4 vs. M4	M4 up	GO:0005829	Development	Development	cytosol	C	AT3G45310
4357	2	L4 vs. M4	M4 up	GO:0005829	Development	Development	cytosol	C	AT3G49080
4358	2	L4 vs. M4	M4 up	GO:0005829	Development	Development	cytosol	C	AT3G49470
4359	2	L4 vs. M4	M4 up	GO:0005829	Development	Development	cytosol	C	AT3G51800
4360	2	L4 vs. M4	M4 up	GO:0005829	Development	Development	cytosol	C	AT3G53520
4361	2	L4 vs. M4	M4 up	GO:0005829	Development	Development	cytosol	C	AT3G53580
4362	2	L4 vs. M4	M4 up	GO:0005829	Development	Development	cytosol	C	AT3G53730
4363	2	L4 vs. M4	M4 up	GO:0005829	Development	Development	cytosol	C	AT3G54960
4364	2	L4 vs. M4	M4 up	GO:0005829	Development	Development	cytosol	C	AT3G55760
4365	2	L4 vs. M4	M4 up	GO:0005829	Development	Development	cytosol	C	AT3G56190
4366	2	L4 vs. M4	M4 up	GO:0005829	Development	Development	cytosol	C	AT3G58620
4367	2	L4 vs. M4	M4 up	GO:0005829	Development	Development	cytosol	C	AT3G59140
4368	2	L4 vs. M4	M4 up	GO:0005829	Development	Development	cytosol	C	AT3G62150
4369	2	L4 vs. M4	M4 up	GO:0005829	Development	Development	cytosol	C	AT4G01610
4370	2	L4 vs. M4	M4 up	GO:0005829	Development	Development	cytosol	C	AT4G02890
4371	2	L4 vs. M4	M4 up	GO:0005829	Development	Development	cytosol	C	AT4G05420
4372	2	L4 vs. M4	M4 up	GO:0005829	Development	Development	cytosol	C	AT4G10120
4373	2	L4 vs. M4	M4 up	GO:0005829	Development	Development	cytosol	C	AT4G13930
4374	2	L4 vs. M4	M4 up	GO:0005829	Development	Development	cytosol	C	AT4G16370
4375	2	L4 vs. M4	M4 up	GO:0005829	Development	Development	cytosol	C	AT4G16660
4376	2	L4 vs. M4	M4 up	GO:0005829	Development	Development	cytosol	C	AT4G25630
4377	2	L4 vs. M4	M4 up	GO:0005829	Development	Development	cytosol	C	AT4G32840
4378	2	L4 vs. M4	M4 up	GO:0005829	Development	Development	cytosol	C	AT4G33090
4379	2	L4 vs. M4	M4 up	GO:0005829	Development	Development	cytosol	C	AT4G33150
4380	2	L4 vs. M4	M4 up	GO:0005829	Development	Development	cytosol	C	AT5G05600
4381	2	L4 vs. M4	M4 up	GO:0005829	Development	Development	cytosol	C	AT5G16260
4382	2	L4 vs. M4	M4 up	GO:0005829	Development	Development	cytosol	C	AT5G16570
4383	2	L4 vs. M4	M4 up	GO:0005829	Development	Development	cytosol	C	AT5G17380
4384	2	L4 vs. M4	M4 up	GO:0005829	Development	Development	cytosol	C	AT5G27380
4385	2	L4 vs. M4	M4 up	GO:0005829	Development	Development	cytosol	C	AT5G36160
4386	2	L4 vs. M4	M4 up	GO:0005829	Development	Development	cytosol	C	AT5G36210
4387	2	L4 vs. M4	M4 up	GO:0005829	Development	Development	cytosol	C	AT5G37600
4388	2	L4 vs. M4	M4 up	GO:0005829	Development	Development	cytosol	C	AT5G42250
4389	2	L4 vs. M4	M4 up	GO:0005829	Development	Development	cytosol	C	AT5G45280
4390	2	L4 vs. M4	M4 up	GO:0005829	Development	Development	cytosol	C	AT5G53140
4391	2	L4 vs. M4	M4 up	GO:0005829	Development	Development	cytosol	C	AT5G57330
4392	2	L4 vs. M4	M4 up	GO:0005829	Development	Development	cytosol	C	AT5G58430
4393	2	L4 vs. M4	M4 up	GO:0005829	Development	Development	cytosol	C	AT5G58590
4394	2	L4 vs. M4	M4 up	GO:0005829	Development	Development	cytosol	C	AT5G60160
4395	2	L4 vs. M4	M4 up	GO:0005829	Development	Development	cytosol	C	AT5G62290
4396	2	L4 vs. M4	M4 up	GO:0005829	Development	Development	cytosol	C	AT5G64350
4397	2	L4 vs. M4	M4 up	GO:0005829	Development	Development	cytosol	C	AT5G64460
4398	2	L4 vs. M4	M4 up	GO:0005829	Development	Development	cytosol	C	AT5G66420
4399	2	L4 vs. M4	M4 up	GO:0032502	Development	Development	developmental process	P	AT1G14320

4400	2	L4 vs. M4	M4 up	GO:0009567	Development	Development	double fertilization forming a zygote and endosperm	P	AT1G65450
4401	2	L4 vs. M4	M4 up	GO:0009790	Development	Development	embryo development	P	AT1G78580
4402	2	L4 vs. M4	M4 up	GO:0009793	Development	Development	embryo development ending in seed dormancy	P	AT1G03910
4403	2	L4 vs. M4	M4 up	GO:0009793	Development	Development	embryo development ending in seed dormancy	P	AT1G14610
4404	2	L4 vs. M4	M4 up	GO:0009793	Development	Development	embryo development ending in seed dormancy	P	AT1G78580
4405	2	L4 vs. M4	M4 up	GO:0009793	Development	Development	embryo development ending in seed dormancy	P	AT2G31530
4406	2	L4 vs. M4	M4 up	GO:0009793	Development	Development	embryo development ending in seed dormancy	P	AT3G18290
4407	2	L4 vs. M4	M4 up	GO:0009793	Development	Development	embryo development ending in seed dormancy	P	AT5G13010
4408	2	L4 vs. M4	M4 up	GO:0009793	Development	Development	embryo development ending in seed dormancy	P	AT5G49930
4409	2	L4 vs. M4	M4 up	GO:0009559	Development	Development	embryo sac central cell differentiation	P	AT3G49080
4410	2	L4 vs. M4	M4 up	GO:0012505	Development	Development	endomembrane system	C	AT5G58430
4411	2	L4 vs. M4	M4 up	GO:0005788	Development	Development	endoplasmic reticulum lumen	C	AT3G54960
4412	2	L4 vs. M4	M4 up	GO:0000815	Development	Development	ESCRT III complex	C	AT2G06530
4413	2	L4 vs. M4	M4 up	GO:0000815	Development	Development	ESCRT III complex	C	AT4G29160
4414	2	L4 vs. M4	M4 up	GO:0000145	Development	Development	exocyst	C	AT5G58430
4415	2	L4 vs. M4	M4 up	GO:0070062	Development	Development	extracellular exosome	C	AT5G58430
4416	2	L4 vs. M4	M4 up	GO:0005576	Development	Development	extracellular region	C	AT1G02520
4417	2	L4 vs. M4	M4 up	GO:0005576	Development	Development	extracellular region	C	AT1G02810
4418	2	L4 vs. M4	M4 up	GO:0005576	Development	Development	extracellular region	C	AT1G05300
4419	2	L4 vs. M4	M4 up	GO:0005576	Development	Development	extracellular region	C	AT1G09310
4420	2	L4 vs. M4	M4 up	GO:0005576	Development	Development	extracellular region	C	AT1G11190
4421	2	L4 vs. M4	M4 up	GO:0005576	Development	Development	extracellular region	C	AT1G14000
4422	2	L4 vs. M4	M4 up	GO:0005576	Development	Development	extracellular region	C	AT1G14320
4423	2	L4 vs. M4	M4 up	GO:0005576	Development	Development	extracellular region	C	AT1G14610
4424	2	L4 vs. M4	M4 up	GO:0005576	Development	Development	extracellular region	C	AT1G20190
4425	2	L4 vs. M4	M4 up	GO:0005576	Development	Development	extracellular region	C	AT1G34040
4426	2	L4 vs. M4	M4 up	GO:0005576	Development	Development	extracellular region	C	AT1G35140
4427	2	L4 vs. M4	M4 up	GO:0005576	Development	Development	extracellular region	C	AT1G45130
4428	2	L4 vs. M4	M4 up	GO:0005576	Development	Development	extracellular region	C	AT1G47128
4429	2	L4 vs. M4	M4 up	GO:0005576	Development	Development	extracellular region	C	AT1G62290
4430	2	L4 vs. M4	M4 up	GO:0005576	Development	Development	extracellular region	C	AT1G73260
4431	2	L4 vs. M4	M4 up	GO:0005576	Development	Development	extracellular region	C	AT1G75880
4432	2	L4 vs. M4	M4 up	GO:0005576	Development	Development	extracellular region	C	AT1G76130
4433	2	L4 vs. M4	M4 up	GO:0005576	Development	Development	extracellular region	C	AT1G78580
4434	2	L4 vs. M4	M4 up	GO:0005576	Development	Development	extracellular region	C	AT2G02990
4435	2	L4 vs. M4	M4 up	GO:0005576	Development	Development	extracellular region	C	AT2G05830
4436	2	L4 vs. M4	M4 up	GO:0005576	Development	Development	extracellular region	C	AT2G07680
4437	2	L4 vs. M4	M4 up	GO:0005576	Development	Development	extracellular region	C	AT2G14900
4438	2	L4 vs. M4	M4 up	GO:0005576	Development	Development	extracellular region	C	AT2G23810
4439	2	L4 vs. M4	M4 up	GO:0005576	Development	Development	extracellular region	C	AT2G25940
4440	2	L4 vs. M4	M4 up	GO:0005576	Development	Development	extracellular region	C	AT2G37870
4441	2	L4 vs. M4	M4 up	GO:0005576	Development	Development	extracellular region	C	AT2G39850
4442	2	L4 vs. M4	M4 up	GO:0005576	Development	Development	extracellular region	C	AT2G44450
4443	2	L4 vs. M4	M4 up	GO:0005576	Development	Development	extracellular region	C	AT2G45580
4444	2	L4 vs. M4	M4 up	GO:0005576	Development	Development	extracellular region	C	AT3G10740
4445	2	L4 vs. M4	M4 up	GO:0005576	Development	Development	extracellular region	C	AT3G19390
4446	2	L4 vs. M4	M4 up	GO:0005576	Development	Development	extracellular region	C	AT3G23410
4447	2	L4 vs. M4	M4 up	GO:0005576	Development	Development	extracellular region	C	AT3G24670
4448	2	L4 vs. M4	M4 up	GO:0005576	Development	Development	extracellular region	C	AT3G26140
4449	2	L4 vs. M4	M4 up	GO:0005576	Development	Development	extracellular region	C	AT3G45010
4450	2	L4 vs. M4	M4 up	GO:0005576	Development	Development	extracellular region	C	AT3G45310
4451	2	L4 vs. M4	M4 up	GO:0005576	Development	Development	extracellular region	C	AT3G53400
4452	2	L4 vs. M4	M4 up	GO:0005576	Development	Development	extracellular region	C	AT3G57630
4453	2	L4 vs. M4	M4 up	GO:0005576	Development	Development	extracellular region	C	AT3G60240
4454	2	L4 vs. M4	M4 up	GO:0005576	Development	Development	extracellular region	C	AT4G01610

4455	2	L4 vs. M4	M4 up	GO:0005576	Development	Development	extracellular region	C	AT4G11310
4456	2	L4 vs. M4	M4 up	GO:0005576	Development	Development	extracellular region	C	AT4G19810
4457	2	L4 vs. M4	M4 up	GO:0005576	Development	Development	extracellular region	C	AT4G25000
4458	2	L4 vs. M4	M4 up	GO:0005576	Development	Development	extracellular region	C	AT4G32940
4459	2	L4 vs. M4	M4 up	GO:0005576	Development	Development	extracellular region	C	AT4G39480
4460	2	L4 vs. M4	M4 up	GO:0005576	Development	Development	extracellular region	C	AT5G10695
4461	2	L4 vs. M4	M4 up	GO:0005576	Development	Development	extracellular region	C	AT5G36210
4462	2	L4 vs. M4	M4 up	GO:0005576	Development	Development	extracellular region	C	AT5G45280
4463	2	L4 vs. M4	M4 up	GO:0005576	Development	Development	extracellular region	C	AT5G45950
4464	2	L4 vs. M4	M4 up	GO:0005576	Development	Development	extracellular region	C	AT5G49360
4465	2	L4 vs. M4	M4 up	GO:0005576	Development	Development	extracellular region	C	AT5G50260
4466	2	L4 vs. M4	M4 up	GO:0005576	Development	Development	extracellular region	C	AT5G50400
4467	2	L4 vs. M4	M4 up	GO:0005576	Development	Development	extracellular region	C	AT5G60360
4468	2	L4 vs. M4	M4 up	GO:0005576	Development	Development	extracellular region	C	AT5G67480
4469	2	L4 vs. M4	M4 up	GO:0005615	Development	Development	extracellular space	C	AT1G35140
4470	2	L4 vs. M4	M4 up	GO:0005615	Development	Development	extracellular space	C	AT1G47128
4471	2	L4 vs. M4	M4 up	GO:0005615	Development	Development	extracellular space	C	AT3G19390
4472	2	L4 vs. M4	M4 up	GO:0005615	Development	Development	extracellular space	C	AT3G45310
4473	2	L4 vs. M4	M4 up	GO:0005615	Development	Development	extracellular space	C	AT4G01610
4474	2	L4 vs. M4	M4 up	GO:0005615	Development	Development	extracellular space	C	AT4G11310
4475	2	L4 vs. M4	M4 up	GO:0005615	Development	Development	extracellular space	C	AT5G45890
4476	2	L4 vs. M4	M4 up	GO:0005615	Development	Development	extracellular space	C	AT5G50260
4477	2	L4 vs. M4	M4 up	GO:0005615	Development	Development	extracellular space	C	AT5G60360
4478	2	L4 vs. M4	M4 up	GO:0042406	Development	Development	extrinsic component of endoplasmic reticulum membrane	C	AT5G42800
4479	2	L4 vs. M4	M4 up	GO:0048437	Development	Development	floral organ development	P	AT3G49670
4480	2	L4 vs. M4	M4 up	GO:0009908	Development	Development	flower development	P	AT5G16780
4481	2	L4 vs. M4	M4 up	GO:0010154	Development	Development	fruit development	P	AT3G04120
4482	2	L4 vs. M4	M4 up	GO:0010154	Development	Development	fruit development	P	AT5G40010
4483	2	L4 vs. M4	M4 up	GO:0005797	Development	Development	Golgi medial cisterna	C	AT2G34300
4484	2	L4 vs. M4	M4 up	GO:0000138	Development	Development	Golgi trans cisterna	C	AT3G53520
4485	2	L4 vs. M4	M4 up	GO:0000138	Development	Development	Golgi trans cisterna	C	AT4G30996
4486	2	L4 vs. M4	M4 up	GO:0000138	Development	Development	Golgi trans cisterna	C	AT5G01710
4487	2	L4 vs. M4	M4 up	GO:0030660	Development	Development	Golgi-associated vesicle membrane	C	AT4G33410
4488	2	L4 vs. M4	M4 up	GO:0010229	Development	Development	inflorescence development	P	AT3G45640
4489	2	L4 vs. M4	M4 up	GO:0071458	Development	Development	integral component of cytoplasmic side of endoplasmic reticulum membrane	C	AT4G33410
4490	2	L4 vs. M4	M4 up	GO:0071556	Development	Development	integral component of lumenal side of endoplasmic reticulum membrane	C	AT4G33410
4491	2	L4 vs. M4	M4 up	GO:0016021	Development	Development	integral component of membrane	C	AT1G01120
4492	2	L4 vs. M4	M4 up	GO:0016021	Development	Development	integral component of membrane	C	AT1G02520
4493	2	L4 vs. M4	M4 up	GO:0016021	Development	Development	integral component of membrane	C	AT1G04750
4494	2	L4 vs. M4	M4 up	GO:0016021	Development	Development	integral component of membrane	C	AT1G05300
4495	2	L4 vs. M4	M4 up	GO:0016021	Development	Development	integral component of membrane	C	AT1G05570
4496	2	L4 vs. M4	M4 up	GO:0016021	Development	Development	integral component of membrane	C	AT1G06350
4497	2	L4 vs. M4	M4 up	GO:0016021	Development	Development	integral component of membrane	C	AT1G10970
4498	2	L4 vs. M4	M4 up	GO:0016021	Development	Development	integral component of membrane	C	AT1G15500
4499	2	L4 vs. M4	M4 up	GO:0016021	Development	Development	integral component of membrane	C	AT1G17430
4500	2	L4 vs. M4	M4 up	GO:0016021	Development	Development	integral component of membrane	C	AT1G18700
4501	2	L4 vs. M4	M4 up	GO:0016021	Development	Development	integral component of membrane	C	AT1G18740
4502	2	L4 vs. M4	M4 up	GO:0016021	Development	Development	integral component of membrane	C	AT1G27770
4503	2	L4 vs. M4	M4 up	GO:0016021	Development	Development	integral component of membrane	C	AT1G34040
4504	2	L4 vs. M4	M4 up	GO:0016021	Development	Development	integral component of membrane	C	AT1G34470
4505	2	L4 vs. M4	M4 up	GO:0016021	Development	Development	integral component of membrane	C	AT1G58360
4506	2	L4 vs. M4	M4 up	GO:0016021	Development	Development	integral component of membrane	C	AT1G61800
4507	2	L4 vs. M4	M4 up	GO:0016021	Development	Development	integral component of membrane	C	AT1G67300

4508	2	L4 vs. M4	M4 up	GO:0016021	Development	Development	integral component of membrane	C	AT1G69870
4509	2	L4 vs. M4	M4 up	GO:0016021	Development	Development	integral component of membrane	C	AT1G73080
4510	2	L4 vs. M4	M4 up	GO:0016021	Development	Development	integral component of membrane	C	AT1G73650
4511	2	L4 vs. M4	M4 up	GO:0016021	Development	Development	integral component of membrane	C	AT1G80280
4512	2	L4 vs. M4	M4 up	GO:0016021	Development	Development	integral component of membrane	C	AT1G80760
4513	2	L4 vs. M4	M4 up	GO:0016021	Development	Development	integral component of membrane	C	AT2G01320
4514	2	L4 vs. M4	M4 up	GO:0016021	Development	Development	integral component of membrane	C	AT2G03240
4515	2	L4 vs. M4	M4 up	GO:0016021	Development	Development	integral component of membrane	C	AT2G07680
4516	2	L4 vs. M4	M4 up	GO:0016021	Development	Development	integral component of membrane	C	AT2G19620
4517	2	L4 vs. M4	M4 up	GO:0016021	Development	Development	integral component of membrane	C	AT2G20890
4518	2	L4 vs. M4	M4 up	GO:0016021	Development	Development	integral component of membrane	C	AT2G22500
4519	2	L4 vs. M4	M4 up	GO:0016021	Development	Development	integral component of membrane	C	AT2G23810
4520	2	L4 vs. M4	M4 up	GO:0016021	Development	Development	integral component of membrane	C	AT2G32530
4521	2	L4 vs. M4	M4 up	GO:0016021	Development	Development	integral component of membrane	C	AT2G34300
4522	2	L4 vs. M4	M4 up	GO:0016021	Development	Development	integral component of membrane	C	AT2G34660
4523	2	L4 vs. M4	M4 up	GO:0016021	Development	Development	integral component of membrane	C	AT2G39890
4524	2	L4 vs. M4	M4 up	GO:0016021	Development	Development	integral component of membrane	C	AT2G41420
4525	2	L4 vs. M4	M4 up	GO:0016021	Development	Development	integral component of membrane	C	AT2G45580
4526	2	L4 vs. M4	M4 up	GO:0016021	Development	Development	integral component of membrane	C	AT2G46440
4527	2	L4 vs. M4	M4 up	GO:0016021	Development	Development	integral component of membrane	C	AT2G48010
4528	2	L4 vs. M4	M4 up	GO:0016021	Development	Development	integral component of membrane	C	AT3G01350
4529	2	L4 vs. M4	M4 up	GO:0016021	Development	Development	integral component of membrane	C	AT3G12750
4530	2	L4 vs. M4	M4 up	GO:0016021	Development	Development	integral component of membrane	C	AT3G14620
4531	2	L4 vs. M4	M4 up	GO:0016021	Development	Development	integral component of membrane	C	AT3G18290
4532	2	L4 vs. M4	M4 up	GO:0016021	Development	Development	integral component of membrane	C	AT3G21250
4533	2	L4 vs. M4	M4 up	GO:0016021	Development	Development	integral component of membrane	C	AT3G22240
4534	2	L4 vs. M4	M4 up	GO:0016021	Development	Development	integral component of membrane	C	AT3G22370
4535	2	L4 vs. M4	M4 up	GO:0016021	Development	Development	integral component of membrane	C	AT3G23410
4536	2	L4 vs. M4	M4 up	GO:0016021	Development	Development	integral component of membrane	C	AT3G28340
4537	2	L4 vs. M4	M4 up	GO:0016021	Development	Development	integral component of membrane	C	AT3G49310
4538	2	L4 vs. M4	M4 up	GO:0016021	Development	Development	integral component of membrane	C	AT3G49530
4539	2	L4 vs. M4	M4 up	GO:0016021	Development	Development	integral component of membrane	C	AT3G49670
4540	2	L4 vs. M4	M4 up	GO:0016021	Development	Development	integral component of membrane	C	AT3G50170
4541	2	L4 vs. M4	M4 up	GO:0016021	Development	Development	integral component of membrane	C	AT3G50660
4542	2	L4 vs. M4	M4 up	GO:0016021	Development	Development	integral component of membrane	C	AT3G53400
4543	2	L4 vs. M4	M4 up	GO:0016021	Development	Development	integral component of membrane	C	AT3G53420
4544	2	L4 vs. M4	M4 up	GO:0016021	Development	Development	integral component of membrane	C	AT3G53520
4545	2	L4 vs. M4	M4 up	GO:0016021	Development	Development	integral component of membrane	C	AT3G54140
4546	2	L4 vs. M4	M4 up	GO:0016021	Development	Development	integral component of membrane	C	AT3G57630
4547	2	L4 vs. M4	M4 up	GO:0016021	Development	Development	integral component of membrane	C	AT3G59140
4548	2	L4 vs. M4	M4 up	GO:0016021	Development	Development	integral component of membrane	C	AT3G62150
4549	2	L4 vs. M4	M4 up	GO:0016021	Development	Development	integral component of membrane	C	AT4G16370
4550	2	L4 vs. M4	M4 up	GO:0016021	Development	Development	integral component of membrane	C	AT4G18220
4551	2	L4 vs. M4	M4 up	GO:0016021	Development	Development	integral component of membrane	C	AT4G24110
4552	2	L4 vs. M4	M4 up	GO:0016021	Development	Development	integral component of membrane	C	AT4G25700
4553	2	L4 vs. M4	M4 up	GO:0016021	Development	Development	integral component of membrane	C	AT4G30996
4554	2	L4 vs. M4	M4 up	GO:0016021	Development	Development	integral component of membrane	C	AT4G36380
4555	2	L4 vs. M4	M4 up	GO:0016021	Development	Development	integral component of membrane	C	AT4G39480
4556	2	L4 vs. M4	M4 up	GO:0016021	Development	Development	integral component of membrane	C	AT4G39850
4557	2	L4 vs. M4	M4 up	GO:0016021	Development	Development	integral component of membrane	C	AT5G05570
4558	2	L4 vs. M4	M4 up	GO:0016021	Development	Development	integral component of membrane	C	AT5G06320
4559	2	L4 vs. M4	M4 up	GO:0016021	Development	Development	integral component of membrane	C	AT5G12080
4560	2	L4 vs. M4	M4 up	GO:0016021	Development	Development	integral component of membrane	C	AT5G14940
4561	2	L4 vs. M4	M4 up	GO:0016021	Development	Development	integral component of membrane	C	AT5G15410
4562	2	L4 vs. M4	M4 up	GO:0016021	Development	Development	integral component of membrane	C	AT5G15740
4563	2	L4 vs. M4	M4 up	GO:0016021	Development	Development	integral component of membrane	C	AT5G35735

4564	2	L4 vs. M4	M4 up	GO:0016021	Development	Development	integral component of membrane	C	AT5G38460
4565	2	L4 vs. M4	M4 up	GO:0016021	Development	Development	integral component of membrane	C	AT5G40010
4566	2	L4 vs. M4	M4 up	GO:0016021	Development	Development	integral component of membrane	C	AT5G40780
4567	2	L4 vs. M4	M4 up	GO:0016021	Development	Development	integral component of membrane	C	AT5G52540
4568	2	L4 vs. M4	M4 up	GO:0016021	Development	Development	integral component of membrane	C	AT5G57350
4569	2	L4 vs. M4	M4 up	GO:0016021	Development	Development	integral component of membrane	C	AT5G58270
4570	2	L4 vs. M4	M4 up	GO:0016021	Development	Development	integral component of membrane	C	AT5G67370
4571	2	L4 vs. M4	M4 up	GO:0016021	Development	Development	integral component of membrane	C	ATCG00270
4572	2	L4 vs. M4	M4 up	GO:0016021	Development	Development	integral component of membrane	C	ATCG00340
4573	2	L4 vs. M4	M4 up	GO:0016021	Development	Development	integral component of membrane	C	ATCG00350
4574	2	L4 vs. M4	M4 up	GO:0016021	Development	Development	integral component of membrane	C	ATCG00560
4575	2	L4 vs. M4	M4 up	GO:0016021	Development	Development	integral component of membrane	C	ATCG00680
4576	2	L4 vs. M4	M4 up	GO:0016021	Development	Development	integral component of membrane	C	ATCG00690
4577	2	L4 vs. M4	M4 up	GO:0016021	Development	Development	integral component of membrane	C	ATCG01100
4578	2	L4 vs. M4	M4 up	GO:0043231	Development	Development	intracellular membrane-bounded organelle	C	AT1G05560
4579	2	L4 vs. M4	M4 up	GO:0043231	Development	Development	intracellular membrane-bounded organelle	C	AT2G18570
4580	2	L4 vs. M4	M4 up	GO:0043231	Development	Development	intracellular membrane-bounded organelle	C	AT2G43820
4581	2	L4 vs. M4	M4 up	GO:0043231	Development	Development	intracellular membrane-bounded organelle	C	AT3G23410
4582	2	L4 vs. M4	M4 up	GO:0043231	Development	Development	intracellular membrane-bounded organelle	C	AT4G34135
4583	2	L4 vs. M4	M4 up	GO:0070676	Development	Development	intralumenal vesicle formation	P	AT2G06530
4584	2	L4 vs. M4	M4 up	GO:0070676	Development	Development	intralumenal vesicle formation	P	AT4G29160
4585	2	L4 vs. M4	M4 up	GO:0005770	Development	Development	late endosome	C	AT2G06530
4586	2	L4 vs. M4	M4 up	GO:0010311	Development	Development	lateral root formation	P	AT4G29040
4587	2	L4 vs. M4	M4 up	GO:0048366	Development	Development	leaf development	P	AT3G50660
4588	2	L4 vs. M4	M4 up	GO:0048366	Development	Development	leaf development	P	AT4G36380
4589	2	L4 vs. M4	M4 up	GO:0048366	Development	Development	leaf development	P	AT5G16780
4590	2	L4 vs. M4	M4 up	GO:0009965	Development	Development	leaf morphogenesis	P	AT4G29040
4591	2	L4 vs. M4	M4 up	GO:0009965	Development	Development	leaf morphogenesis	P	AT4G36380
4592	2	L4 vs. M4	M4 up	GO:0010358	Development	Development	leaf shaping	P	AT3G50660
4593	2	L4 vs. M4	M4 up	GO:0010305	Development	Development	leaf vascular tissue pattern formation	P	AT1G14000
4594	2	L4 vs. M4	M4 up	GO:0010305	Development	Development	leaf vascular tissue pattern formation	P	AT5G16780
4595	2	L4 vs. M4	M4 up	GO:0000323	Development	Development	lytic vacuole	C	AT4G32940
4596	2	L4 vs. M4	M4 up	GO:0010078	Development	Development	maintenance of root meristem identity	P	AT4G29040
4597	2	L4 vs. M4	M4 up	GO:0016020	Development	Development	membrane	C	AT1G01120
4598	2	L4 vs. M4	M4 up	GO:0016020	Development	Development	membrane	C	AT1G20190
4599	2	L4 vs. M4	M4 up	GO:0016020	Development	Development	membrane	C	AT2G07680
4600	2	L4 vs. M4	M4 up	GO:0016020	Development	Development	membrane	C	AT2G17290
4601	2	L4 vs. M4	M4 up	GO:0016020	Development	Development	membrane	C	AT2G34660
4602	2	L4 vs. M4	M4 up	GO:0016020	Development	Development	membrane	C	AT3G21250
4603	2	L4 vs. M4	M4 up	GO:0016020	Development	Development	membrane	C	AT3G53520
4604	2	L4 vs. M4	M4 up	GO:0016020	Development	Development	membrane	C	AT3G59140
4605	2	L4 vs. M4	M4 up	GO:0016020	Development	Development	membrane	C	AT4G16370
4606	2	L4 vs. M4	M4 up	GO:0035266	Development	Development	meristem growth	P	AT4G29040
4607	2	L4 vs. M4	M4 up	GO:0009933	Development	Development	meristem structural organization	P	AT4G29040
4608	2	L4 vs. M4	M4 up	GO:0009933	Development	Development	meristem structural organization	P	AT5G16780
4609	2	L4 vs. M4	M4 up	GO:0005874	Development	Development	microtubule	C	AT2G35880
4610	2	L4 vs. M4	M4 up	GO:0042814	Development	Development	monopolar cell growth	P	AT4G36380
4611	2	L4 vs. M4	M4 up	GO:0007275	Development	Development	multicellular organism development	P	AT1G13180
4612	2	L4 vs. M4	M4 up	GO:0007275	Development	Development	multicellular organism development	P	AT3G50660
4613	2	L4 vs. M4	M4 up	GO:0007275	Development	Development	multicellular organism development	P	AT3G51800
4614	2	L4 vs. M4	M4 up	GO:0007275	Development	Development	multicellular organism development	P	AT4G16370
4615	2	L4 vs. M4	M4 up	GO:0007275	Development	Development	multicellular organism development	P	AT4G36380
4616	2	L4 vs. M4	M4 up	GO:0009825	Development	Development	multidimensional cell growth	P	AT1G13180
4617	2	L4 vs. M4	M4 up	GO:0005771	Development	Development	multivesicular body	C	AT2G06530
4618	2	L4 vs. M4	M4 up	GO:0071836	Development	Development	nectar secretion	P	AT5G11110
4619	2	L4 vs. M4	M4 up	GO:0000176	Development	Development	nuclear exosome (RNase complex)	C	AT3G07750

4620	2	L4 vs. M4	M4 up	GO:0016363	Development	Development	nuclear matrix	C	AT3G09440
4621	2	L4 vs. M4	M4 up	GO:0005643	Development	Development	nuclear pore	C	AT5G58590
4622	2	L4 vs. M4	M4 up	GO:0009295	Development	Development	nucleoid	C	ATCG00180
4623	2	L4 vs. M4	M4 up	GO:0005634	Development	Development	nucleus	C	AT1G02520
4624	2	L4 vs. M4	M4 up	GO:0005634	Development	Development	nucleus	C	AT1G03190
4625	2	L4 vs. M4	M4 up	GO:0005634	Development	Development	nucleus	C	AT1G03910
4626	2	L4 vs. M4	M4 up	GO:0005634	Development	Development	nucleus	C	AT1G09310
4627	2	L4 vs. M4	M4 up	GO:0005634	Development	Development	nucleus	C	AT1G09450
4628	2	L4 vs. M4	M4 up	GO:0005634	Development	Development	nucleus	C	AT1G14000
4629	2	L4 vs. M4	M4 up	GO:0005634	Development	Development	nucleus	C	AT1G14320
4630	2	L4 vs. M4	M4 up	GO:0005634	Development	Development	nucleus	C	AT1G18700
4631	2	L4 vs. M4	M4 up	GO:0005634	Development	Development	nucleus	C	AT1G18740
4632	2	L4 vs. M4	M4 up	GO:0005634	Development	Development	nucleus	C	AT1G19640
4633	2	L4 vs. M4	M4 up	GO:0005634	Development	Development	nucleus	C	AT1G22190
4634	2	L4 vs. M4	M4 up	GO:0005634	Development	Development	nucleus	C	AT1G23710
4635	2	L4 vs. M4	M4 up	GO:0005634	Development	Development	nucleus	C	AT1G27770
4636	2	L4 vs. M4	M4 up	GO:0005634	Development	Development	nucleus	C	AT1G29340
4637	2	L4 vs. M4	M4 up	GO:0005634	Development	Development	nucleus	C	AT1G32340
4638	2	L4 vs. M4	M4 up	GO:0005634	Development	Development	nucleus	C	AT1G45130
4639	2	L4 vs. M4	M4 up	GO:0005634	Development	Development	nucleus	C	AT1G45145
4640	2	L4 vs. M4	M4 up	GO:0005634	Development	Development	nucleus	C	AT1G47128
4641	2	L4 vs. M4	M4 up	GO:0005634	Development	Development	nucleus	C	AT1G50480
4642	2	L4 vs. M4	M4 up	GO:0005634	Development	Development	nucleus	C	AT1G51980
4643	2	L4 vs. M4	M4 up	GO:0005634	Development	Development	nucleus	C	AT1G56110
4644	2	L4 vs. M4	M4 up	GO:0005634	Development	Development	nucleus	C	AT1G56430
4645	2	L4 vs. M4	M4 up	GO:0005634	Development	Development	nucleus	C	AT1G59640
4646	2	L4 vs. M4	M4 up	GO:0005634	Development	Development	nucleus	C	AT1G62290
4647	2	L4 vs. M4	M4 up	GO:0005634	Development	Development	nucleus	C	AT1G62740
4648	2	L4 vs. M4	M4 up	GO:0005634	Development	Development	nucleus	C	AT1G65450
4649	2	L4 vs. M4	M4 up	GO:0005634	Development	Development	nucleus	C	AT1G67660
4650	2	L4 vs. M4	M4 up	GO:0005634	Development	Development	nucleus	C	AT1G67920
4651	2	L4 vs. M4	M4 up	GO:0005634	Development	Development	nucleus	C	AT1G80840
4652	2	L4 vs. M4	M4 up	GO:0005634	Development	Development	nucleus	C	AT2G03470
4653	2	L4 vs. M4	M4 up	GO:0005634	Development	Development	nucleus	C	AT2G05830
4654	2	L4 vs. M4	M4 up	GO:0005634	Development	Development	nucleus	C	AT2G17290
4655	2	L4 vs. M4	M4 up	GO:0005634	Development	Development	nucleus	C	AT2G19480
4656	2	L4 vs. M4	M4 up	GO:0005634	Development	Development	nucleus	C	AT2G25670
4657	2	L4 vs. M4	M4 up	GO:0005634	Development	Development	nucleus	C	AT2G26190
4658	2	L4 vs. M4	M4 up	GO:0005634	Development	Development	nucleus	C	AT2G27590
4659	2	L4 vs. M4	M4 up	GO:0005634	Development	Development	nucleus	C	AT2G30020
4660	2	L4 vs. M4	M4 up	GO:0005634	Development	Development	nucleus	C	AT2G30360
4661	2	L4 vs. M4	M4 up	GO:0005634	Development	Development	nucleus	C	AT2G35840
4662	2	L4 vs. M4	M4 up	GO:0005634	Development	Development	nucleus	C	AT2G35880
4663	2	L4 vs. M4	M4 up	GO:0005634	Development	Development	nucleus	C	AT2G39650
4664	2	L4 vs. M4	M4 up	GO:0005634	Development	Development	nucleus	C	AT2G40360
4665	2	L4 vs. M4	M4 up	GO:0005634	Development	Development	nucleus	C	AT2G42760
4666	2	L4 vs. M4	M4 up	GO:0005634	Development	Development	nucleus	C	AT2G46600
4667	2	L4 vs. M4	M4 up	GO:0005634	Development	Development	nucleus	C	AT2G47060
4668	2	L4 vs. M4	M4 up	GO:0005634	Development	Development	nucleus	C	AT3G01780
4669	2	L4 vs. M4	M4 up	GO:0005634	Development	Development	nucleus	C	AT3G04120
4670	2	L4 vs. M4	M4 up	GO:0005634	Development	Development	nucleus	C	AT3G04350
4671	2	L4 vs. M4	M4 up	GO:0005634	Development	Development	nucleus	C	AT3G04520
4672	2	L4 vs. M4	M4 up	GO:0005634	Development	Development	nucleus	C	AT3G09660
4673	2	L4 vs. M4	M4 up	GO:0005634	Development	Development	nucleus	C	AT3G12640
4674	2	L4 vs. M4	M4 up	GO:0005634	Development	Development	nucleus	C	AT3G12860
4675	2	L4 vs. M4	M4 up	GO:0005634	Development	Development	nucleus	C	AT3G16410

4676	2	L4 vs. M4	M4 up	GO:0005634	Development	Development	nucleus	C	AT3G16470
4677	2	L4 vs. M4	M4 up	GO:0005634	Development	Development	nucleus	C	AT3G17040
4678	2	L4 vs. M4	M4 up	GO:0005634	Development	Development	nucleus	C	AT3G17310
4679	2	L4 vs. M4	M4 up	GO:0005634	Development	Development	nucleus	C	AT3G18290
4680	2	L4 vs. M4	M4 up	GO:0005634	Development	Development	nucleus	C	AT3G22240
4681	2	L4 vs. M4	M4 up	GO:0005634	Development	Development	nucleus	C	AT3G27120
4682	2	L4 vs. M4	M4 up	GO:0005634	Development	Development	nucleus	C	AT3G45640
4683	2	L4 vs. M4	M4 up	GO:0005634	Development	Development	nucleus	C	AT3G49470
4684	2	L4 vs. M4	M4 up	GO:0005634	Development	Development	nucleus	C	AT3G49530
4685	2	L4 vs. M4	M4 up	GO:0005634	Development	Development	nucleus	C	AT3G50530
4686	2	L4 vs. M4	M4 up	GO:0005634	Development	Development	nucleus	C	AT3G51800
4687	2	L4 vs. M4	M4 up	GO:0005634	Development	Development	nucleus	C	AT3G53730
4688	2	L4 vs. M4	M4 up	GO:0005634	Development	Development	nucleus	C	AT3G54140
4689	2	L4 vs. M4	M4 up	GO:0005634	Development	Development	nucleus	C	AT3G58620
4690	2	L4 vs. M4	M4 up	GO:0005634	Development	Development	nucleus	C	AT3G58670
4691	2	L4 vs. M4	M4 up	GO:0005634	Development	Development	nucleus	C	AT3G62150
4692	2	L4 vs. M4	M4 up	GO:0005634	Development	Development	nucleus	C	AT3G62550
4693	2	L4 vs. M4	M4 up	GO:0005634	Development	Development	nucleus	C	AT4G02890
4694	2	L4 vs. M4	M4 up	GO:0005634	Development	Development	nucleus	C	AT4G05420
4695	2	L4 vs. M4	M4 up	GO:0005634	Development	Development	nucleus	C	AT4G10120
4696	2	L4 vs. M4	M4 up	GO:0005634	Development	Development	nucleus	C	AT4G12280
4697	2	L4 vs. M4	M4 up	GO:0005634	Development	Development	nucleus	C	AT4G24130
4698	2	L4 vs. M4	M4 up	GO:0005634	Development	Development	nucleus	C	AT4G24400
4699	2	L4 vs. M4	M4 up	GO:0005634	Development	Development	nucleus	C	AT4G25630
4700	2	L4 vs. M4	M4 up	GO:0005634	Development	Development	nucleus	C	AT4G27410
4701	2	L4 vs. M4	M4 up	GO:0005634	Development	Development	nucleus	C	AT4G29040
4702	2	L4 vs. M4	M4 up	GO:0005634	Development	Development	nucleus	C	AT4G33090
4703	2	L4 vs. M4	M4 up	GO:0005634	Development	Development	nucleus	C	AT4G36690
4704	2	L4 vs. M4	M4 up	GO:0005634	Development	Development	nucleus	C	AT4G37260
4705	2	L4 vs. M4	M4 up	GO:0005634	Development	Development	nucleus	C	AT5G03730
4706	2	L4 vs. M4	M4 up	GO:0005634	Development	Development	nucleus	C	AT5G04150
4707	2	L4 vs. M4	M4 up	GO:0005634	Development	Development	nucleus	C	AT5G05570
4708	2	L4 vs. M4	M4 up	GO:0005634	Development	Development	nucleus	C	AT5G08590
4709	2	L4 vs. M4	M4 up	GO:0005634	Development	Development	nucleus	C	AT5G11110
4710	2	L4 vs. M4	M4 up	GO:0005634	Development	Development	nucleus	C	AT5G12080
4711	2	L4 vs. M4	M4 up	GO:0005634	Development	Development	nucleus	C	AT5G13010
4712	2	L4 vs. M4	M4 up	GO:0005634	Development	Development	nucleus	C	AT5G13180
4713	2	L4 vs. M4	M4 up	GO:0005634	Development	Development	nucleus	C	AT5G13800
4714	2	L4 vs. M4	M4 up	GO:0005634	Development	Development	nucleus	C	AT5G15410
4715	2	L4 vs. M4	M4 up	GO:0005634	Development	Development	nucleus	C	AT5G16260
4716	2	L4 vs. M4	M4 up	GO:0005634	Development	Development	nucleus	C	AT5G16780
4717	2	L4 vs. M4	M4 up	GO:0005634	Development	Development	nucleus	C	AT5G27660
4718	2	L4 vs. M4	M4 up	GO:0005634	Development	Development	nucleus	C	AT5G28900
4719	2	L4 vs. M4	M4 up	GO:0005634	Development	Development	nucleus	C	AT5G40010
4720	2	L4 vs. M4	M4 up	GO:0005634	Development	Development	nucleus	C	AT5G42050
4721	2	L4 vs. M4	M4 up	GO:0005634	Development	Development	nucleus	C	AT5G43380
4722	2	L4 vs. M4	M4 up	GO:0005634	Development	Development	nucleus	C	AT5G49930
4723	2	L4 vs. M4	M4 up	GO:0005634	Development	Development	nucleus	C	AT5G53450
4724	2	L4 vs. M4	M4 up	GO:0005634	Development	Development	nucleus	C	AT5G57340
4725	2	L4 vs. M4	M4 up	GO:0005634	Development	Development	nucleus	C	AT5G58590
4726	2	L4 vs. M4	M4 up	GO:0005634	Development	Development	nucleus	C	AT5G61030
4727	2	L4 vs. M4	M4 up	GO:0005634	Development	Development	nucleus	C	AT5G62290
4728	2	L4 vs. M4	M4 up	GO:0005634	Development	Development	nucleus	C	AT5G67300
4729	2	L4 vs. M4	M4 up	GO:0005634	Development	Development	nucleus	C	AT5G67480
4730	2	L4 vs. M4	M4 up	GO:0031090	Development	Development	organelle membrane	C	AT4G33090
4731	2	L4 vs. M4	M4 up	GO:0048471	Development	Development	perinuclear region of cytoplasm	C	AT1G05560

4732	2	L4 vs. M4	M4 up	GO:0048471	Development	Development	perinuclear region of cytoplasm	C	AT1G09450
4733	2	L4 vs. M4	M4 up	GO:0048441	Development	Development	petal development	P	AT4G36380
4734	2	L4 vs. M4	M4 up	GO:0048446	Development	Development	petal morphogenesis	P	AT1G59640
4735	2	L4 vs. M4	M4 up	GO:0045335	Development	Development	phagocytic vesicle	C	AT5G58430
4736	2	L4 vs. M4	M4 up	GO:0010087	Development	Development	phloem or xylem histogenesis	P	AT5G16780
4737	2	L4 vs. M4	M4 up	GO:0009524	Development	Development	phragmoplast	C	AT1G05560
4738	2	L4 vs. M4	M4 up	GO:0009524	Development	Development	phragmoplast	C	AT1G09450
4739	2	L4 vs. M4	M4 up	GO:0009524	Development	Development	phragmoplast	C	AT3G01780
4740	2	L4 vs. M4	M4 up	GO:0048827	Development	Development	phyllome development	P	AT4G29040
4741	2	L4 vs. M4	M4 up	GO:0048481	Development	Development	plant ovule development	P	AT3G45640
4742	2	L4 vs. M4	M4 up	GO:0009505	Development	Development	plant-type cell wall	C	AT1G01800
4743	2	L4 vs. M4	M4 up	GO:0009505	Development	Development	plant-type cell wall	C	AT1G35140
4744	2	L4 vs. M4	M4 up	GO:0009505	Development	Development	plant-type cell wall	C	AT1G45130
4745	2	L4 vs. M4	M4 up	GO:0009505	Development	Development	plant-type cell wall	C	AT2G44450
4746	2	L4 vs. M4	M4 up	GO:0009505	Development	Development	plant-type cell wall	C	AT5G08380
4747	2	L4 vs. M4	M4 up	GO:0009505	Development	Development	plant-type cell wall	C	AT5G45280
4748	2	L4 vs. M4	M4 up	GO:0009505	Development	Development	plant-type cell wall	C	AT5G49360
4749	2	L4 vs. M4	M4 up	GO:0009505	Development	Development	plant-type cell wall	C	AT5G50260
4750	2	L4 vs. M4	M4 up	GO:0009832	Development	Development	plant-type cell wall biogenesis	P	AT1G78580
4751	2	L4 vs. M4	M4 up	GO:0009828	Development	Development	plant-type cell wall loosening	P	AT1G20190
4752	2	L4 vs. M4	M4 up	GO:0009664	Development	Development	plant-type cell wall organization	P	AT1G20190
4753	2	L4 vs. M4	M4 up	GO:0009833	Development	Development	plant-type primary cell wall biogenesis	P	AT2G32530
4754	2	L4 vs. M4	M4 up	GO:0000325	Development	Development	plant-type vacuole	C	AT2G34660
4755	2	L4 vs. M4	M4 up	GO:0000325	Development	Development	plant-type vacuole	C	AT3G21250
4756	2	L4 vs. M4	M4 up	GO:0000325	Development	Development	plant-type vacuole	C	AT3G59140
4757	2	L4 vs. M4	M4 up	GO:0000325	Development	Development	plant-type vacuole	C	AT5G50260
4758	2	L4 vs. M4	M4 up	GO:0005886	Development	Development	plasma membrane	C	AT1G02520
4759	2	L4 vs. M4	M4 up	GO:0005886	Development	Development	plasma membrane	C	AT1G04750
4760	2	L4 vs. M4	M4 up	GO:0005886	Development	Development	plasma membrane	C	AT1G05300
4761	2	L4 vs. M4	M4 up	GO:0005886	Development	Development	plasma membrane	C	AT1G05570
4762	2	L4 vs. M4	M4 up	GO:0005886	Development	Development	plasma membrane	C	AT1G10970
4763	2	L4 vs. M4	M4 up	GO:0005886	Development	Development	plasma membrane	C	AT1G23020
4764	2	L4 vs. M4	M4 up	GO:0005886	Development	Development	plasma membrane	C	AT1G27770
4765	2	L4 vs. M4	M4 up	GO:0005886	Development	Development	plasma membrane	C	AT1G45145
4766	2	L4 vs. M4	M4 up	GO:0005886	Development	Development	plasma membrane	C	AT1G50480
4767	2	L4 vs. M4	M4 up	GO:0005886	Development	Development	plasma membrane	C	AT1G58360
4768	2	L4 vs. M4	M4 up	GO:0005886	Development	Development	plasma membrane	C	AT1G62740
4769	2	L4 vs. M4	M4 up	GO:0005886	Development	Development	plasma membrane	C	AT1G67300
4770	2	L4 vs. M4	M4 up	GO:0005886	Development	Development	plasma membrane	C	AT1G69870
4771	2	L4 vs. M4	M4 up	GO:0005886	Development	Development	plasma membrane	C	AT1G70410
4772	2	L4 vs. M4	M4 up	GO:0005886	Development	Development	plasma membrane	C	AT1G73080
4773	2	L4 vs. M4	M4 up	GO:0005886	Development	Development	plasma membrane	C	AT1G73650
4774	2	L4 vs. M4	M4 up	GO:0005886	Development	Development	plasma membrane	C	AT1G80760
4775	2	L4 vs. M4	M4 up	GO:0005886	Development	Development	plasma membrane	C	AT2G01320
4776	2	L4 vs. M4	M4 up	GO:0005886	Development	Development	plasma membrane	C	AT2G02990
4777	2	L4 vs. M4	M4 up	GO:0005886	Development	Development	plasma membrane	C	AT2G03240
4778	2	L4 vs. M4	M4 up	GO:0005886	Development	Development	plasma membrane	C	AT2G17290
4779	2	L4 vs. M4	M4 up	GO:0005886	Development	Development	plasma membrane	C	AT2G19480
4780	2	L4 vs. M4	M4 up	GO:0005886	Development	Development	plasma membrane	C	AT2G23810
4781	2	L4 vs. M4	M4 up	GO:0005886	Development	Development	plasma membrane	C	AT2G24360
4782	2	L4 vs. M4	M4 up	GO:0005886	Development	Development	plasma membrane	C	AT2G31390
4783	2	L4 vs. M4	M4 up	GO:0005886	Development	Development	plasma membrane	C	AT2G32530
4784	2	L4 vs. M4	M4 up	GO:0005886	Development	Development	plasma membrane	C	AT2G33210
4785	2	L4 vs. M4	M4 up	GO:0005886	Development	Development	plasma membrane	C	AT2G34300
4786	2	L4 vs. M4	M4 up	GO:0005886	Development	Development	plasma membrane	C	AT2G39890
4787	2	L4 vs. M4	M4 up	GO:0005886	Development	Development	plasma membrane	C	AT2G41420

4788	2	L4 vs. M4	M4 up	GO:0005886	Development	Development	plasma membrane	C	AT2G46440
4789	2	L4 vs. M4	M4 up	GO:0005886	Development	Development	plasma membrane	C	AT2G47060
4790	2	L4 vs. M4	M4 up	GO:0005886	Development	Development	plasma membrane	C	AT2G48010
4791	2	L4 vs. M4	M4 up	GO:0005886	Development	Development	plasma membrane	C	AT3G01780
4792	2	L4 vs. M4	M4 up	GO:0005886	Development	Development	plasma membrane	C	AT3G04120
4793	2	L4 vs. M4	M4 up	GO:0005886	Development	Development	plasma membrane	C	AT3G09440
4794	2	L4 vs. M4	M4 up	GO:0005886	Development	Development	plasma membrane	C	AT3G12750
4795	2	L4 vs. M4	M4 up	GO:0005886	Development	Development	plasma membrane	C	AT3G14050
4796	2	L4 vs. M4	M4 up	GO:0005886	Development	Development	plasma membrane	C	AT3G17810
4797	2	L4 vs. M4	M4 up	GO:0005886	Development	Development	plasma membrane	C	AT3G22240
4798	2	L4 vs. M4	M4 up	GO:0005886	Development	Development	plasma membrane	C	AT3G49530
4799	2	L4 vs. M4	M4 up	GO:0005886	Development	Development	plasma membrane	C	AT3G49670
4800	2	L4 vs. M4	M4 up	GO:0005886	Development	Development	plasma membrane	C	AT3G50530
4801	2	L4 vs. M4	M4 up	GO:0005886	Development	Development	plasma membrane	C	AT3G51800
4802	2	L4 vs. M4	M4 up	GO:0005886	Development	Development	plasma membrane	C	AT3G53420
4803	2	L4 vs. M4	M4 up	GO:0005886	Development	Development	plasma membrane	C	AT3G53520
4804	2	L4 vs. M4	M4 up	GO:0005886	Development	Development	plasma membrane	C	AT3G54140
4805	2	L4 vs. M4	M4 up	GO:0005886	Development	Development	plasma membrane	C	AT3G56190
4806	2	L4 vs. M4	M4 up	GO:0005886	Development	Development	plasma membrane	C	AT3G62150
4807	2	L4 vs. M4	M4 up	GO:0005886	Development	Development	plasma membrane	C	AT4G10120
4808	2	L4 vs. M4	M4 up	GO:0005886	Development	Development	plasma membrane	C	AT4G13930
4809	2	L4 vs. M4	M4 up	GO:0005886	Development	Development	plasma membrane	C	AT4G16370
4810	2	L4 vs. M4	M4 up	GO:0005886	Development	Development	plasma membrane	C	AT4G24400
4811	2	L4 vs. M4	M4 up	GO:0005886	Development	Development	plasma membrane	C	AT4G29040
4812	2	L4 vs. M4	M4 up	GO:0005886	Development	Development	plasma membrane	C	AT4G29160
4813	2	L4 vs. M4	M4 up	GO:0005886	Development	Development	plasma membrane	C	AT4G33090
4814	2	L4 vs. M4	M4 up	GO:0005886	Development	Development	plasma membrane	C	AT4G33120
4815	2	L4 vs. M4	M4 up	GO:0005886	Development	Development	plasma membrane	C	AT4G33410
4816	2	L4 vs. M4	M4 up	GO:0005886	Development	Development	plasma membrane	C	AT4G35230
4817	2	L4 vs. M4	M4 up	GO:0005886	Development	Development	plasma membrane	C	AT4G36250
4818	2	L4 vs. M4	M4 up	GO:0005886	Development	Development	plasma membrane	C	AT4G36400
4819	2	L4 vs. M4	M4 up	GO:0005886	Development	Development	plasma membrane	C	AT4G36750
4820	2	L4 vs. M4	M4 up	GO:0005886	Development	Development	plasma membrane	C	AT5G04140
4821	2	L4 vs. M4	M4 up	GO:0005886	Development	Development	plasma membrane	C	AT5G05570
4822	2	L4 vs. M4	M4 up	GO:0005886	Development	Development	plasma membrane	C	AT5G06320
4823	2	L4 vs. M4	M4 up	GO:0005886	Development	Development	plasma membrane	C	AT5G08590
4824	2	L4 vs. M4	M4 up	GO:0005886	Development	Development	plasma membrane	C	AT5G11000
4825	2	L4 vs. M4	M4 up	GO:0005886	Development	Development	plasma membrane	C	AT5G11110
4826	2	L4 vs. M4	M4 up	GO:0005886	Development	Development	plasma membrane	C	AT5G12080
4827	2	L4 vs. M4	M4 up	GO:0005886	Development	Development	plasma membrane	C	AT5G15410
4828	2	L4 vs. M4	M4 up	GO:0005886	Development	Development	plasma membrane	C	AT5G35735
4829	2	L4 vs. M4	M4 up	GO:0005886	Development	Development	plasma membrane	C	AT5G37600
4830	2	L4 vs. M4	M4 up	GO:0005886	Development	Development	plasma membrane	C	AT5G40780
4831	2	L4 vs. M4	M4 up	GO:0005886	Development	Development	plasma membrane	C	AT5G45280
4832	2	L4 vs. M4	M4 up	GO:0005886	Development	Development	plasma membrane	C	AT5G47930
4833	2	L4 vs. M4	M4 up	GO:0005886	Development	Development	plasma membrane	C	AT5G54810
4834	2	L4 vs. M4	M4 up	GO:0005886	Development	Development	plasma membrane	C	AT5G57350
4835	2	L4 vs. M4	M4 up	GO:0005886	Development	Development	plasma membrane	C	AT5G58430
4836	2	L4 vs. M4	M4 up	GO:0005886	Development	Development	plasma membrane	C	AT5G62290
4837	2	L4 vs. M4	M4 up	GO:0009555	Development	Development	pollen development	P	AT1G56430
4838	2	L4 vs. M4	M4 up	GO:0009555	Development	Development	pollen development	P	AT2G37040
4839	2	L4 vs. M4	M4 up	GO:0009555	Development	Development	pollen development	P	AT3G01780
4840	2	L4 vs. M4	M4 up	GO:0009555	Development	Development	pollen development	P	AT3G08590
4841	2	L4 vs. M4	M4 up	GO:0009555	Development	Development	pollen development	P	AT3G09660
4842	2	L4 vs. M4	M4 up	GO:0009555	Development	Development	pollen development	P	AT3G12070
4843	2	L4 vs. M4	M4 up	GO:0009555	Development	Development	pollen development	P	AT3G45640

4844	2	L4 vs. M4	M4 up	GO:0009555	Development	Development	pollen development	P	AT3G49080
4845	2	L4 vs. M4	M4 up	GO:0009555	Development	Development	pollen development	P	AT5G50260
4846	2	L4 vs. M4	M4 up	GO:0009555	Development	Development	pollen development	P	AT5G58270
4847	2	L4 vs. M4	M4 up	GO:0010208	Development	Development	pollen wall assembly	P	AT5G11110
4848	2	L4 vs. M4	M4 up	GO:0048528	Development	Development	post-embryonic root development	P	AT5G16780
4849	2	L4 vs. M4	M4 up	GO:0048829	Development	Development	root cap development	P	AT4G29040
4850	2	L4 vs. M4	M4 up	GO:0048364	Development	Development	root development	P	AT3G12070
4851	2	L4 vs. M4	M4 up	GO:0048364	Development	Development	root development	P	AT4G24400
4852	2	L4 vs. M4	M4 up	GO:0048364	Development	Development	root development	P	AT4G29040
4853	2	L4 vs. M4	M4 up	GO:0048364	Development	Development	root development	P	AT5G16780
4854	2	L4 vs. M4	M4 up	GO:0048364	Development	Development	root development	P	AT5G58270
4855	2	L4 vs. M4	M4 up	GO:0080147	Development	Development	root hair cell development	P	AT3G57630
4856	2	L4 vs. M4	M4 up	GO:0048767	Development	Development	root hair elongation	P	AT5G13010
4857	2	L4 vs. M4	M4 up	GO:0010015	Development	Development	root morphogenesis	P	AT4G29040
4858	2	L4 vs. M4	M4 up	GO:0010223	Development	Development	secondary shoot formation	P	AT2G26170
4859	2	L4 vs. M4	M4 up	GO:0010214	Development	Development	seed coat development	P	AT5G15740
4860	2	L4 vs. M4	M4 up	GO:0010214	Development	Development	seed coat development	P	AT5G49360
4861	2	L4 vs. M4	M4 up	GO:0048316	Development	Development	seed development	P	AT3G04120
4862	2	L4 vs. M4	M4 up	GO:0010431	Development	Development	seed maturation	P	AT5G40010
4863	2	L4 vs. M4	M4 up	GO:0010344	Development	Development	seed oilbody biogenesis	P	AT2G24360
4864	2	L4 vs. M4	M4 up	GO:0090351	Development	Development	seedling development	P	AT4G29040
4865	2	L4 vs. M4	M4 up	GO:0048367	Development	Development	shoot system development	P	AT4G29040
4866	2	L4 vs. M4	M4 up	GO:0048367	Development	Development	shoot system development	P	AT5G16780
4867	2	L4 vs. M4	M4 up	GO:0048443	Development	Development	stamen development	P	AT2G06050
4868	2	L4 vs. M4	M4 up	GO:0048443	Development	Development	stamen development	P	AT4G36380
4869	2	L4 vs. M4	M4 up	GO:0010319	Development	Development	stromule	C	AT2G20890
4870	2	L4 vs. M4	M4 up	GO:0010319	Development	Development	stromule	C	AT2G35040
4871	2	L4 vs. M4	M4 up	GO:0010091	Development	Development	trichome branching	P	AT2G46600
4872	2	L4 vs. M4	M4 up	GO:0010090	Development	Development	trichome morphogenesis	P	AT1G13180
4873	2	L4 vs. M4	M4 up	GO:0009826	Development	Development	unidimensional cell growth	P	AT1G20190
4874	2	L4 vs. M4	M4 up	GO:0009826	Development	Development	unidimensional cell growth	P	AT3G50660
4875	2	L4 vs. M4	M4 up	GO:0005774	Development	Development	vacuolar membrane	C	AT1G14320
4876	2	L4 vs. M4	M4 up	GO:0005774	Development	Development	vacuolar membrane	C	AT1G51980
4877	2	L4 vs. M4	M4 up	GO:0005774	Development	Development	vacuolar membrane	C	AT1G61800
4878	2	L4 vs. M4	M4 up	GO:0005774	Development	Development	vacuolar membrane	C	AT1G73650
4879	2	L4 vs. M4	M4 up	GO:0005774	Development	Development	vacuolar membrane	C	AT2G07680
4880	2	L4 vs. M4	M4 up	GO:0005774	Development	Development	vacuolar membrane	C	AT2G33210
4881	2	L4 vs. M4	M4 up	GO:0005774	Development	Development	vacuolar membrane	C	AT2G34660
4882	2	L4 vs. M4	M4 up	GO:0005774	Development	Development	vacuolar membrane	C	AT3G04120
4883	2	L4 vs. M4	M4 up	GO:0005774	Development	Development	vacuolar membrane	C	AT3G09440
4884	2	L4 vs. M4	M4 up	GO:0005774	Development	Development	vacuolar membrane	C	AT3G17040
4885	2	L4 vs. M4	M4 up	GO:0005774	Development	Development	vacuolar membrane	C	AT3G21250
4886	2	L4 vs. M4	M4 up	GO:0005774	Development	Development	vacuolar membrane	C	AT3G54960
4887	2	L4 vs. M4	M4 up	GO:0005774	Development	Development	vacuolar membrane	C	AT3G56190
4888	2	L4 vs. M4	M4 up	GO:0005774	Development	Development	vacuolar membrane	C	AT3G59140
4889	2	L4 vs. M4	M4 up	GO:0005774	Development	Development	vacuolar membrane	C	AT3G62150
4890	2	L4 vs. M4	M4 up	GO:0005774	Development	Development	vacuolar membrane	C	AT4G16660
4891	2	L4 vs. M4	M4 up	GO:0005774	Development	Development	vacuolar membrane	C	AT5G06320
4892	2	L4 vs. M4	M4 up	GO:0005774	Development	Development	vacuolar membrane	C	AT5G60160
4893	2	L4 vs. M4	M4 up	GO:0005774	Development	Development	vacuolar membrane	C	AT5G67480
4894	2	L4 vs. M4	M4 up	GO:0005773	Development	Development	vacuole	C	AT1G14320
4895	2	L4 vs. M4	M4 up	GO:0005773	Development	Development	vacuole	C	AT1G45130
4896	2	L4 vs. M4	M4 up	GO:0005773	Development	Development	vacuole	C	AT1G47128
4897	2	L4 vs. M4	M4 up	GO:0005773	Development	Development	vacuole	C	AT1G62290
4898	2	L4 vs. M4	M4 up	GO:0005773	Development	Development	vacuole	C	AT1G73650
4899	2	L4 vs. M4	M4 up	GO:0005773	Development	Development	vacuole	C	AT1G78580

4900	2	L4 vs. M4	M4 up	GO:0005773	Development	Development	vacuole	C	AT2G22360
4901	2	L4 vs. M4	M4 up	GO:0005773	Development	Development	vacuole	C	AT2G23810
4902	2	L4 vs. M4	M4 up	GO:0005773	Development	Development	vacuole	C	AT2G25670
4903	2	L4 vs. M4	M4 up	GO:0005773	Development	Development	vacuole	C	AT2G25940
4904	2	L4 vs. M4	M4 up	GO:0005773	Development	Development	vacuole	C	AT2G34660
4905	2	L4 vs. M4	M4 up	GO:0005773	Development	Development	vacuole	C	AT2G39330
4906	2	L4 vs. M4	M4 up	GO:0005773	Development	Development	vacuole	C	AT3G09440
4907	2	L4 vs. M4	M4 up	GO:0005773	Development	Development	vacuole	C	AT3G10740
4908	2	L4 vs. M4	M4 up	GO:0005773	Development	Development	vacuole	C	AT3G16470
4909	2	L4 vs. M4	M4 up	GO:0005773	Development	Development	vacuole	C	AT3G20250
4910	2	L4 vs. M4	M4 up	GO:0005773	Development	Development	vacuole	C	AT3G45010
4911	2	L4 vs. M4	M4 up	GO:0005773	Development	Development	vacuole	C	AT3G53420
4912	2	L4 vs. M4	M4 up	GO:0005773	Development	Development	vacuole	C	AT3G56190
4913	2	L4 vs. M4	M4 up	GO:0005773	Development	Development	vacuole	C	AT3G57010
4914	2	L4 vs. M4	M4 up	GO:0005773	Development	Development	vacuole	C	AT3G62550
4915	2	L4 vs. M4	M4 up	GO:0005773	Development	Development	vacuole	C	AT4G01610
4916	2	L4 vs. M4	M4 up	GO:0005773	Development	Development	vacuole	C	AT4G02890
4917	2	L4 vs. M4	M4 up	GO:0005773	Development	Development	vacuole	C	AT4G32940
4918	2	L4 vs. M4	M4 up	GO:0005773	Development	Development	vacuole	C	AT4G35230
4919	2	L4 vs. M4	M4 up	GO:0005773	Development	Development	vacuole	C	AT5G19860
4920	2	L4 vs. M4	M4 up	GO:0005773	Development	Development	vacuole	C	AT5G60160
4921	2	L4 vs. M4	M4 up	GO:0005773	Development	Development	vacuole	C	AT5G60360
4922	2	L4 vs. M4	M4 up	GO:0005773	Development	Development	vacuole	C	AT5G65760
4923	2	L4 vs. M4	M4 up	GO:0010228	Development	Development	vegetative to reproductive phase transition of meristem	P	AT3G16470
4924	2	L4 vs. M4	M4 up	GO:0010051	Development	Development	xylem and phloem pattern formation	P	AT5G13010
4925	2	L4 vs. M4	M4 up	GO:0102336	Metabolism	Bioenergetics	3-oxo-arachidoyl-CoA synthase activity	F	AT1G01120
4926	2	L4 vs. M4	M4 up	GO:0102337	Metabolism	Bioenergetics	3-oxo-cerotoyl-CoA synthase activity	F	AT1G01120
4927	2	L4 vs. M4	M4 up	GO:0102338	Metabolism	Bioenergetics	3-oxo-lignoceronyl-CoA synthase activity	F	AT1G01120
4928	2	L4 vs. M4	M4 up	GO:0003866	Metabolism	Bioenergetics	3-phosphoshikimate 1-carboxyvinyltransferase activity	F	AT1G48860
4929	2	L4 vs. M4	M4 up	GO:0003872	Metabolism	Bioenergetics	6-phosphofructokinase activity	F	AT4G32840
4930	2	L4 vs. M4	M4 up	GO:0006085	Metabolism	Bioenergetics	acetyl-CoA biosynthetic process	P	AT1G60810
4931	2	L4 vs. M4	M4 up	GO:0006086	Metabolism	Bioenergetics	acetyl-CoA biosynthetic process from pyruvate	P	AT3G16950
4932	2	L4 vs. M4	M4 up	GO:0009060	Metabolism	Bioenergetics	aerobic respiration	P	ATCG01100
4933	2	L4 vs. M4	M4 up	GO:0004029	Metabolism	Bioenergetics	aldehyde dehydrogenase (NAD+) activity	F	AT4G36250
4934	2	L4 vs. M4	M4 up	GO:0003825	Metabolism	Bioenergetics	alpha,alpha-trehalose-phosphate synthase (UDP-forming) activity	F	AT1G06410
4935	2	L4 vs. M4	M4 up	GO:0003825	Metabolism	Bioenergetics	alpha,alpha-trehalose-phosphate synthase (UDP-forming) activity	F	AT1G78580
4936	2	L4 vs. M4	M4 up	GO:0004556	Metabolism	Bioenergetics	alpha-amylase activity	F	AT1G76130
4937	2	L4 vs. M4	M4 up	GO:0004556	Metabolism	Bioenergetics	alpha-amylase activity	F	AT4G25000
4938	2	L4 vs. M4	M4 up	GO:0103025	Metabolism	Bioenergetics	alpha-amylase activity (releasing maltohexaose)	F	AT1G76130
4939	2	L4 vs. M4	M4 up	GO:0103025	Metabolism	Bioenergetics	alpha-amylase activity (releasing maltohexaose)	F	AT4G25000
4940	2	L4 vs. M4	M4 up	GO:0003878	Metabolism	Bioenergetics	ATP citrate synthase activity	F	AT1G60810
4941	2	L4 vs. M4	M4 up	GO:0016887	Metabolism	Bioenergetics	ATPase activity	F	AT1G02520
4942	2	L4 vs. M4	M4 up	GO:0016887	Metabolism	Bioenergetics	ATPase activity	F	AT2G01320
4943	2	L4 vs. M4	M4 up	GO:0016887	Metabolism	Bioenergetics	ATPase activity	F	AT2G07680
4944	2	L4 vs. M4	M4 up	GO:0016887	Metabolism	Bioenergetics	ATPase activity	F	AT2G34660
4945	2	L4 vs. M4	M4 up	GO:0016887	Metabolism	Bioenergetics	ATPase activity	F	AT3G09440
4946	2	L4 vs. M4	M4 up	GO:0016887	Metabolism	Bioenergetics	ATPase activity	F	AT3G21250
4947	2	L4 vs. M4	M4 up	GO:0016887	Metabolism	Bioenergetics	ATPase activity	F	AT3G27120
4948	2	L4 vs. M4	M4 up	GO:0016887	Metabolism	Bioenergetics	ATPase activity	F	AT3G59140
4949	2	L4 vs. M4	M4 up	GO:0016887	Metabolism	Bioenergetics	ATPase activity	F	AT3G62150
4950	2	L4 vs. M4	M4 up	GO:0016887	Metabolism	Bioenergetics	ATPase activity	F	AT4G29040
4951	2	L4 vs. M4	M4 up	GO:0016887	Metabolism	Bioenergetics	ATPase activity	F	AT4G39850

4952	2	L4 vs. M4	M4 up	GO:0016887	Metabolism	Bioenergetics	ATPase activity	F	AT5G40010
4953	2	L4 vs. M4	M4 up	GO:0016887	Metabolism	Bioenergetics	ATPase activity	F	AT5G57350
4954	2	L4 vs. M4	M4 up	GO:0016887	Metabolism	Bioenergetics	ATPase activity	F	AT5G58270
4955	2	L4 vs. M4	M4 up	GO:0042623	Metabolism	Bioenergetics	ATPase activity, coupled	F	AT3G09440
4956	2	L4 vs. M4	M4 up	GO:0030246	Metabolism	Bioenergetics	carbohydrate binding	F	AT2G39330
4957	2	L4 vs. M4	M4 up	GO:0030246	Metabolism	Bioenergetics	carbohydrate binding	F	AT3G16410
4958	2	L4 vs. M4	M4 up	GO:0030246	Metabolism	Bioenergetics	carbohydrate binding	F	AT3G16470
4959	2	L4 vs. M4	M4 up	GO:0019200	Metabolism	Bioenergetics	carbohydrate kinase activity	F	AT2G31390
4960	2	L4 vs. M4	M4 up	GO:0019200	Metabolism	Bioenergetics	carbohydrate kinase activity	F	AT5G26570
4961	2	L4 vs. M4	M4 up	GO:0005975	Metabolism	Bioenergetics	carbohydrate metabolic process	P	AT1G45130
4962	2	L4 vs. M4	M4 up	GO:0005975	Metabolism	Bioenergetics	carbohydrate metabolic process	P	AT1G76130
4963	2	L4 vs. M4	M4 up	GO:0005975	Metabolism	Bioenergetics	carbohydrate metabolic process	P	AT2G42790
4964	2	L4 vs. M4	M4 up	GO:0005975	Metabolism	Bioenergetics	carbohydrate metabolic process	P	AT2G44350
4965	2	L4 vs. M4	M4 up	GO:0005975	Metabolism	Bioenergetics	carbohydrate metabolic process	P	AT2G44450
4966	2	L4 vs. M4	M4 up	GO:0005975	Metabolism	Bioenergetics	carbohydrate metabolic process	P	AT3G26140
4967	2	L4 vs. M4	M4 up	GO:0005975	Metabolism	Bioenergetics	carbohydrate metabolic process	P	AT4G25000
4968	2	L4 vs. M4	M4 up	GO:0005975	Metabolism	Bioenergetics	carbohydrate metabolic process	P	AT5G08380
4969	2	L4 vs. M4	M4 up	GO:0005975	Metabolism	Bioenergetics	carbohydrate metabolic process	P	AT5G57330
4970	2	L4 vs. M4	M4 up	GO:0044262	Metabolism	Bioenergetics	cellular carbohydrate metabolic process	P	AT3G08590
4971	2	L4 vs. M4	M4 up	GO:0045333	Metabolism	Bioenergetics	cellular respiration	P	AT3G22370
4972	2	L4 vs. M4	M4 up	GO:0004108	Metabolism	Bioenergetics	citrate (Si)-synthase activity	F	AT2G42790
4973	2	L4 vs. M4	M4 up	GO:0004108	Metabolism	Bioenergetics	citrate (Si)-synthase activity	F	AT2G44350
4974	2	L4 vs. M4	M4 up	GO:0009346	Metabolism	Bioenergetics	citrate lyase complex	C	AT1G60810
4975	2	L4 vs. M4	M4 up	GO:0006101	Metabolism	Bioenergetics	citrate metabolic process	P	AT2G44350
4976	2	L4 vs. M4	M4 up	GO:0050662	Metabolism	Bioenergetics	coenzyme binding	F	AT5G42800
4977	2	L4 vs. M4	M4 up	GO:0017113	Metabolism	Bioenergetics	dihydropyrimidine dehydrogenase (NADP+) activity	F	AT3G17810
4978	2	L4 vs. M4	M4 up	GO:0009055	Metabolism	Bioenergetics	electron transfer activity	F	AT5G66760
4979	2	L4 vs. M4	M4 up	GO:0001561	Metabolism	Bioenergetics	fatty acid alpha-oxidation	P	AT5G17380
4980	2	L4 vs. M4	M4 up	GO:0006635	Metabolism	Bioenergetics	fatty acid beta-oxidation	P	AT2G42790
4981	2	L4 vs. M4	M4 up	GO:0006635	Metabolism	Bioenergetics	fatty acid beta-oxidation	P	AT4G39850
4982	2	L4 vs. M4	M4 up	GO:0005504	Metabolism	Bioenergetics	fatty acid binding	F	AT2G37870
4983	2	L4 vs. M4	M4 up	GO:0006633	Metabolism	Bioenergetics	fatty acid biosynthetic process	P	AT1G01120
4984	2	L4 vs. M4	M4 up	GO:0006633	Metabolism	Bioenergetics	fatty acid biosynthetic process	P	AT3G02630
4985	2	L4 vs. M4	M4 up	GO:0009922	Metabolism	Bioenergetics	fatty acid elongase activity	F	AT1G01120
4986	2	L4 vs. M4	M4 up	GO:0030497	Metabolism	Bioenergetics	fatty acid elongation	P	AT1G01120
4987	2	L4 vs. M4	M4 up	GO:0006631	Metabolism	Bioenergetics	fatty acid metabolic process	P	AT3G02630
4988	2	L4 vs. M4	M4 up	GO:0008865	Metabolism	Bioenergetics	fructokinase activity	F	AT2G31390
4989	2	L4 vs. M4	M4 up	GO:0006000	Metabolism	Bioenergetics	fructose metabolic process	P	AT2G31390
4990	2	L4 vs. M4	M4 up	GO:0006094	Metabolism	Bioenergetics	gluconeogenesis	P	AT3G04120
4991	2	L4 vs. M4	M4 up	GO:0006007	Metabolism	Bioenergetics	glucose catabolic process	P	AT3G08590
4992	2	L4 vs. M4	M4 up	GO:0047938	Metabolism	Bioenergetics	glucose-6-phosphate 1-epimerase activity	F	AT5G57330
4993	2	L4 vs. M4	M4 up	GO:0043878	Metabolism	Bioenergetics	glyceraldehyde-3-phosphate dehydrogenase (NAD+) (non-phosphorylating) activity	F	AT1G54100
4994	2	L4 vs. M4	M4 up	GO:0043878	Metabolism	Bioenergetics	glyceraldehyde-3-phosphate dehydrogenase (NAD+) (non-phosphorylating) activity	F	AT4G36250
4995	2	L4 vs. M4	M4 up	GO:0004365	Metabolism	Bioenergetics	glyceraldehyde-3-phosphate dehydrogenase (NAD+) (phosphorylating) activity	F	AT3G04120
4996	2	L4 vs. M4	M4 up	GO:0008886	Metabolism	Bioenergetics	glyceraldehyde-3-phosphate dehydrogenase (NADP+) (non-phosphorylating) activity	F	AT3G04120
4997	2	L4 vs. M4	M4 up	GO:0008891	Metabolism	Bioenergetics	glycolate oxidase activity	F	AT4G18360
4998	2	L4 vs. M4	M4 up	GO:0006096	Metabolism	Bioenergetics	glycolytic process	P	AT3G04120
4999	2	L4 vs. M4	M4 up	GO:0006096	Metabolism	Bioenergetics	glycolytic process	P	AT3G08590
5000	2	L4 vs. M4	M4 up	GO:0006096	Metabolism	Bioenergetics	glycolytic process	P	AT4G32840
5001	2	L4 vs. M4	M4 up	GO:0046861	Metabolism	Bioenergetics	glyoxysomal membrane	C	AT4G39850
5002	2	L4 vs. M4	M4 up	GO:0004419	Metabolism	Bioenergetics	hydroxymethylglutaryl-CoA lyase activity	F	AT2G26800

5003	2	L4 vs. M4	M4 up	GO:0052853	Metabolism	Bioenergetics	long-chain-(S)-2-hydroxy-long-chain-acid oxidase activity	F	AT4G18360
5004	2	L4 vs. M4	M4 up	GO:0004488	Metabolism	Bioenergetics	methylenetetrahydrofolate dehydrogenase (NADP+) activity	F	AT1G50480
5005	2	L4 vs. M4	M4 up	GO:0006121	Metabolism	Bioenergetics	mitochondrial electron transport, succinate to ubiquinone	P	AT5G66760
5006	2	L4 vs. M4	M4 up	GO:0005740	Metabolism	Bioenergetics	mitochondrial envelope	C	AT3G04120
5007	2	L4 vs. M4	M4 up	GO:0005743	Metabolism	Bioenergetics	mitochondrial inner membrane	C	AT1G51980
5008	2	L4 vs. M4	M4 up	GO:0005743	Metabolism	Bioenergetics	mitochondrial inner membrane	C	AT2G22500
5009	2	L4 vs. M4	M4 up	GO:0005743	Metabolism	Bioenergetics	mitochondrial inner membrane	C	AT3G22370
5010	2	L4 vs. M4	M4 up	GO:0005743	Metabolism	Bioenergetics	mitochondrial inner membrane	C	AT5G58270
5011	2	L4 vs. M4	M4 up	GO:0005759	Metabolism	Bioenergetics	mitochondrial matrix	C	AT2G26800
5012	2	L4 vs. M4	M4 up	GO:0005759	Metabolism	Bioenergetics	mitochondrial matrix	C	AT2G42790
5013	2	L4 vs. M4	M4 up	GO:0005759	Metabolism	Bioenergetics	mitochondrial matrix	C	AT2G44350
5014	2	L4 vs. M4	M4 up	GO:0005759	Metabolism	Bioenergetics	mitochondrial matrix	C	AT5G04140
5015	2	L4 vs. M4	M4 up	GO:0005759	Metabolism	Bioenergetics	mitochondrial matrix	C	AT5G53350
5016	2	L4 vs. M4	M4 up	GO:0031966	Metabolism	Bioenergetics	mitochondrial membrane	C	AT5G40010
5017	2	L4 vs. M4	M4 up	GO:0017087	Metabolism	Bioenergetics	mitochondrial processing peptidase complex	C	AT1G51980
5018	2	L4 vs. M4	M4 up	GO:0005749	Metabolism	Bioenergetics	mitochondrial respiratory chain complex II, succinate dehydrogenase complex (ubiquinone)	C	AT5G66760
5019	2	L4 vs. M4	M4 up	GO:0005750	Metabolism	Bioenergetics	mitochondrial respiratory chain complex III	C	AT1G51980
5020	2	L4 vs. M4	M4 up	GO:0005739	Metabolism	Bioenergetics	mitochondrion	C	AT1G06350
5021	2	L4 vs. M4	M4 up	GO:0005739	Metabolism	Bioenergetics	mitochondrion	C	AT1G14610
5022	2	L4 vs. M4	M4 up	GO:0005739	Metabolism	Bioenergetics	mitochondrion	C	AT1G15500
5023	2	L4 vs. M4	M4 up	GO:0005739	Metabolism	Bioenergetics	mitochondrion	C	AT1G48200
5024	2	L4 vs. M4	M4 up	GO:0005739	Metabolism	Bioenergetics	mitochondrion	C	AT1G51980
5025	2	L4 vs. M4	M4 up	GO:0005739	Metabolism	Bioenergetics	mitochondrion	C	AT1G70410
5026	2	L4 vs. M4	M4 up	GO:0005739	Metabolism	Bioenergetics	mitochondrion	C	AT1G72660
5027	2	L4 vs. M4	M4 up	GO:0005739	Metabolism	Bioenergetics	mitochondrion	C	AT1G73260
5028	2	L4 vs. M4	M4 up	GO:0005739	Metabolism	Bioenergetics	mitochondrion	C	AT1G73650
5029	2	L4 vs. M4	M4 up	GO:0005739	Metabolism	Bioenergetics	mitochondrion	C	AT1G80270
5030	2	L4 vs. M4	M4 up	GO:0005739	Metabolism	Bioenergetics	mitochondrion	C	AT2G01320
5031	2	L4 vs. M4	M4 up	GO:0005739	Metabolism	Bioenergetics	mitochondrion	C	AT2G05830
5032	2	L4 vs. M4	M4 up	GO:0005739	Metabolism	Bioenergetics	mitochondrion	C	AT2G06530
5033	2	L4 vs. M4	M4 up	GO:0005739	Metabolism	Bioenergetics	mitochondrion	C	AT2G17290
5034	2	L4 vs. M4	M4 up	GO:0005739	Metabolism	Bioenergetics	mitochondrion	C	AT2G17710
5035	2	L4 vs. M4	M4 up	GO:0005739	Metabolism	Bioenergetics	mitochondrion	C	AT2G19480
5036	2	L4 vs. M4	M4 up	GO:0005739	Metabolism	Bioenergetics	mitochondrion	C	AT2G20890
5037	2	L4 vs. M4	M4 up	GO:0005739	Metabolism	Bioenergetics	mitochondrion	C	AT2G22500
5038	2	L4 vs. M4	M4 up	GO:0005739	Metabolism	Bioenergetics	mitochondrion	C	AT2G26170
5039	2	L4 vs. M4	M4 up	GO:0005739	Metabolism	Bioenergetics	mitochondrion	C	AT2G26800
5040	2	L4 vs. M4	M4 up	GO:0005739	Metabolism	Bioenergetics	mitochondrion	C	AT2G33210
5041	2	L4 vs. M4	M4 up	GO:0005739	Metabolism	Bioenergetics	mitochondrion	C	AT2G40000
5042	2	L4 vs. M4	M4 up	GO:0005739	Metabolism	Bioenergetics	mitochondrion	C	AT2G42790
5043	2	L4 vs. M4	M4 up	GO:0005739	Metabolism	Bioenergetics	mitochondrion	C	AT2G44350
5044	2	L4 vs. M4	M4 up	GO:0005739	Metabolism	Bioenergetics	mitochondrion	C	AT2G46440
5045	2	L4 vs. M4	M4 up	GO:0005739	Metabolism	Bioenergetics	mitochondrion	C	AT3G04120
5046	2	L4 vs. M4	M4 up	GO:0005739	Metabolism	Bioenergetics	mitochondrion	C	AT3G17470
5047	2	L4 vs. M4	M4 up	GO:0005739	Metabolism	Bioenergetics	mitochondrion	C	AT3G22370
5048	2	L4 vs. M4	M4 up	GO:0005739	Metabolism	Bioenergetics	mitochondrion	C	AT3G49080
5049	2	L4 vs. M4	M4 up	GO:0005739	Metabolism	Bioenergetics	mitochondrion	C	AT3G62650
5050	2	L4 vs. M4	M4 up	GO:0005739	Metabolism	Bioenergetics	mitochondrion	C	AT4G33150
5051	2	L4 vs. M4	M4 up	GO:0005739	Metabolism	Bioenergetics	mitochondrion	C	AT4G36400
5052	2	L4 vs. M4	M4 up	GO:0005739	Metabolism	Bioenergetics	mitochondrion	C	AT5G04140
5053	2	L4 vs. M4	M4 up	GO:0005739	Metabolism	Bioenergetics	mitochondrion	C	AT5G06320
5054	2	L4 vs. M4	M4 up	GO:0005739	Metabolism	Bioenergetics	mitochondrion	C	AT5G10695

5055	2	L4 vs. M4	M4 up	GO:0005739	Metabolism	Bioenergetics	mitochondrion	C	AT5G27660
5056	2	L4 vs. M4	M4 up	GO:0005739	Metabolism	Bioenergetics	mitochondrion	C	AT5G40010
5057	2	L4 vs. M4	M4 up	GO:0005739	Metabolism	Bioenergetics	mitochondrion	C	AT5G43380
5058	2	L4 vs. M4	M4 up	GO:0005739	Metabolism	Bioenergetics	mitochondrion	C	AT5G53350
5059	2	L4 vs. M4	M4 up	GO:0005739	Metabolism	Bioenergetics	mitochondrion	C	AT5G53450
5060	2	L4 vs. M4	M4 up	GO:0005739	Metabolism	Bioenergetics	mitochondrion	C	AT5G58270
5061	2	L4 vs. M4	M4 up	GO:0005739	Metabolism	Bioenergetics	mitochondrion	C	AT5G61030
5062	2	L4 vs. M4	M4 up	GO:0005739	Metabolism	Bioenergetics	mitochondrion	C	AT5G66760
5063	2	L4 vs. M4	M4 up	GO:0005739	Metabolism	Bioenergetics	mitochondrion	C	ATCG00270
5064	2	L4 vs. M4	M4 up	GO:0051287	Metabolism	Bioenergetics	NAD binding	F	AT3G04120
5065	2	L4 vs. M4	M4 up	GO:0051287	Metabolism	Bioenergetics	NAD binding	F	ATCG01110
5066	2	L4 vs. M4	M4 up	GO:0003955	Metabolism	Bioenergetics	NAD(P)H dehydrogenase (quinone) activity	F	AT4G36750
5067	2	L4 vs. M4	M4 up	GO:0070403	Metabolism	Bioenergetics	NAD+ binding	F	AT3G53520
5068	2	L4 vs. M4	M4 up	GO:0003954	Metabolism	Bioenergetics	NADH dehydrogenase activity	F	ATCG01100
5069	2	L4 vs. M4	M4 up	GO:0050661	Metabolism	Bioenergetics	NADP binding	F	AT3G04120
5070	2	L4 vs. M4	M4 up	GO:0050661	Metabolism	Bioenergetics	NADP binding	F	AT3G17810
5071	2	L4 vs. M4	M4 up	GO:0003959	Metabolism	Bioenergetics	NADPH dehydrogenase activity	F	ATCG01110
5072	2	L4 vs. M4	M4 up	GO:0016491	Metabolism	Bioenergetics	oxidoreductase activity	F	AT1G51980
5073	2	L4 vs. M4	M4 up	GO:0016491	Metabolism	Bioenergetics	oxidoreductase activity	F	AT1G76470
5074	2	L4 vs. M4	M4 up	GO:0016491	Metabolism	Bioenergetics	oxidoreductase activity	F	AT2G06050
5075	2	L4 vs. M4	M4 up	GO:0016491	Metabolism	Bioenergetics	oxidoreductase activity	F	AT4G22870
5076	2	L4 vs. M4	M4 up	GO:0016491	Metabolism	Bioenergetics	oxidoreductase activity	F	AT4G25700
5077	2	L4 vs. M4	M4 up	GO:0016491	Metabolism	Bioenergetics	oxidoreductase activity	F	AT5G42800
5078	2	L4 vs. M4	M4 up	GO:0016671	Metabolism	Bioenergetics	oxidoreductase activity, acting on a sulfur group of donors, disulfide as acceptor	F	AT1G45145
5079	2	L4 vs. M4	M4 up	GO:0016651	Metabolism	Bioenergetics	oxidoreductase activity, acting on NAD(P)H	F	ATCG01100
5080	2	L4 vs. M4	M4 up	GO:0016705	Metabolism	Bioenergetics	oxidoreductase activity, acting on paired donors, with incorporation or reduction of molecular oxygen	F	AT3G14620
5081	2	L4 vs. M4	M4 up	GO:0016705	Metabolism	Bioenergetics	oxidoreductase activity, acting on paired donors, with incorporation or reduction of molecular oxygen	F	AT3G50660
5082	2	L4 vs. M4	M4 up	GO:0016705	Metabolism	Bioenergetics	oxidoreductase activity, acting on paired donors, with incorporation or reduction of molecular oxygen	F	AT4G39480
5083	2	L4 vs. M4	M4 up	GO:0016709	Metabolism	Bioenergetics	oxidoreductase activity, acting on paired donors, with incorporation or reduction of molecular oxygen, NAD(P)H as one donor, and incorporation of one atom of oxygen	F	AT2G45580
5084	2	L4 vs. M4	M4 up	GO:0016709	Metabolism	Bioenergetics	oxidoreductase activity, acting on paired donors, with incorporation or reduction of molecular oxygen, NAD(P)H as one donor, and incorporation of one atom of oxygen	F	AT4G36380
5085	2	L4 vs. M4	M4 up	GO:0016717	Metabolism	Bioenergetics	oxidoreductase activity, acting on paired donors, with oxidation of a pair of donors resulting in the reduction of molecular oxygen to two molecules of water	F	AT1G06350
5086	2	L4 vs. M4	M4 up	GO:0016627	Metabolism	Bioenergetics	oxidoreductase activity, acting on the CH-CH group of donors	F	AT1G73650
5087	2	L4 vs. M4	M4 up	GO:0016616	Metabolism	Bioenergetics	oxidoreductase activity, acting on the CH-OH group of donors, NAD or NADP as acceptor	F	AT1G76470
5088	2	L4 vs. M4	M4 up	GO:0016616	Metabolism	Bioenergetics	oxidoreductase activity, acting on the CH-OH group of donors, NAD or NADP as acceptor	F	AT5G42800
5089	2	L4 vs. M4	M4 up	GO:0009853	Metabolism	Bioenergetics	photorespiration	P	AT4G36400
5090	2	L4 vs. M4	M4 up	GO:0009853	Metabolism	Bioenergetics	photorespiration	P	AT5G04140
5091	2	L4 vs. M4	M4 up	GO:1902600	Metabolism	Bioenergetics	proton transmembrane transport	P	AT2G22500
5092	2	L4 vs. M4	M4 up	GO:1902600	Metabolism	Bioenergetics	proton transmembrane transport	P	AT5G57350
5093	2	L4 vs. M4	M4 up	GO:0008553	Metabolism	Bioenergetics	proton-exporting ATPase activity, phosphorylative mechanism	F	AT5G57350

5094	2	L4 vs. M4	M4 up	GO:0070469	Metabolism	Bioenergetics	respirasome	C	AT3G22370
5095	2	L4 vs. M4	M4 up	GO:0045273	Metabolism	Bioenergetics	respiratory chain complex II	C	AT5G66760
5096	2	L4 vs. M4	M4 up	GO:0047131	Metabolism	Bioenergetics	saccharopine dehydrogenase (NAD+, L-glutamate-forming) activity	F	AT4G33150
5097	2	L4 vs. M4	M4 up	GO:0047130	Metabolism	Bioenergetics	saccharopine dehydrogenase (NADP+, L-lysine-forming) activity	F	AT4G33150
5098	2	L4 vs. M4	M4 up	GO:2001070	Metabolism	Bioenergetics	starch binding	F	AT3G55760
5099	2	L4 vs. M4	M4 up	GO:2001070	Metabolism	Bioenergetics	starch binding	F	AT5G26570
5100	2	L4 vs. M4	M4 up	GO:0019252	Metabolism	Bioenergetics	starch biosynthetic process	P	AT2G31390
5101	2	L4 vs. M4	M4 up	GO:0005983	Metabolism	Bioenergetics	starch catabolic process	P	AT5G26570
5102	2	L4 vs. M4	M4 up	GO:0043036	Metabolism	Bioenergetics	starch grain	C	AT3G55760
5103	2	L4 vs. M4	M4 up	GO:0005982	Metabolism	Bioenergetics	starch metabolic process	P	AT3G55760
5104	2	L4 vs. M4	M4 up	GO:0005982	Metabolism	Bioenergetics	starch metabolic process	P	AT5G26570
5105	2	L4 vs. M4	M4 up	GO:0008177	Metabolism	Bioenergetics	succinate dehydrogenase (ubiquinone) activity	F	AT5G66760
5106	2	L4 vs. M4	M4 up	GO:0005986	Metabolism	Bioenergetics	sucrose biosynthetic process	P	AT2G35840
5107	2	L4 vs. M4	M4 up	GO:0005986	Metabolism	Bioenergetics	sucrose biosynthetic process	P	AT5G11110
5108	2	L4 vs. M4	M4 up	GO:0016157	Metabolism	Bioenergetics	sucrose synthase activity	F	AT5G11110
5109	2	L4 vs. M4	M4 up	GO:0046524	Metabolism	Bioenergetics	sucrose-phosphate synthase activity	F	AT4G10120
5110	2	L4 vs. M4	M4 up	GO:0046524	Metabolism	Bioenergetics	sucrose-phosphate synthase activity	F	AT5G11110
5111	2	L4 vs. M4	M4 up	GO:0005992	Metabolism	Bioenergetics	trehalose biosynthetic process	P	AT1G78580
5112	2	L4 vs. M4	M4 up	GO:0005991	Metabolism	Bioenergetics	trehalose metabolic process	P	AT1G78580
5113	2	L4 vs. M4	M4 up	GO:0004805	Metabolism	Bioenergetics	trehalose-phosphatase activity	F	AT1G78580
5114	2	L4 vs. M4	M4 up	GO:0006099	Metabolism	Bioenergetics	tricarboxylic acid cycle	P	AT2G42790
5115	2	L4 vs. M4	M4 up	GO:0006099	Metabolism	Bioenergetics	tricarboxylic acid cycle	P	AT2G44350
5116	2	L4 vs. M4	M4 up	GO:0006099	Metabolism	Bioenergetics	tricarboxylic acid cycle	P	AT5G66760
5117	2	L4 vs. M4	M4 up	GO:0035436	Metabolism	Bioenergetics	triose phosphate transmembrane transport	P	AT1G61800
5118	2	L4 vs. M4	M4 up	GO:0102721	Metabolism	Bioenergetics	ubiquinol:oxygen oxidoreductase activity	F	AT3G22370
5119	2	L4 vs. M4	M4 up	GO:0006744	Metabolism	Bioenergetics	ubiquinone biosynthetic process	P	AT4G39850
5120	2	L4 vs. M4	M4 up	GO:0006636	Metabolism	Bioenergetics	unsaturated fatty acid biosynthetic process	P	AT1G06350
5121	2	L4 vs. M4	M4 up	GO:0000038	Metabolism	Bioenergetics	very long-chain fatty acid metabolic process	P	AT1G01120
5122	2	L4 vs. M4	M4 up	GO:0102756	Metabolism	Bioenergetics	very-long-chain 3-ketoacyl-CoA synthase activity	F	AT1G01120
5123	2	L4 vs. M4	M4 up	GO:0052852	Metabolism	Bioenergetics	very-long-chain-(S)-2-hydroxy-acid oxidase activity	F	AT4G18360
5124	2	L4 vs. M4	M4 up	GO:0061158	Metabolism	Catabolism	3'-UTR-mediated mRNA destabilization	P	AT3G20250
5125	2	L4 vs. M4	M4 up	GO:0046556	Metabolism	Catabolism	alpha-L-arabinofuranosidase activity	F	AT3G10740
5126	2	L4 vs. M4	M4 up	GO:0046556	Metabolism	Catabolism	alpha-L-arabinofuranosidase activity	F	AT5G49360
5127	2	L4 vs. M4	M4 up	GO:0004177	Metabolism	Catabolism	aminopeptidase activity	F	AT4G33090
5128	2	L4 vs. M4	M4 up	GO:0031222	Metabolism	Catabolism	arabinan catabolic process	P	AT5G49360
5129	2	L4 vs. M4	M4 up	GO:0004089	Metabolism	Catabolism	carbonate dehydratase activity	F	AT1G70410
5130	2	L4 vs. M4	M4 up	GO:0006032	Metabolism	Catabolism	chitin catabolic process	P	AT4G19810
5131	2	L4 vs. M4	M4 up	GO:0015996	Metabolism	Catabolism	chlorophyll catabolic process	P	AT2G20890
5132	2	L4 vs. M4	M4 up	GO:0015996	Metabolism	Catabolism	chlorophyll catabolic process	P	AT5G13800
5133	2	L4 vs. M4	M4 up	GO:0080008	Metabolism	Catabolism	Cul4-RING E3 ubiquitin ligase complex	C	AT4G05420
5134	2	L4 vs. M4	M4 up	GO:0009440	Metabolism	Catabolism	cyanate catabolic process	P	AT1G70410
5135	2	L4 vs. M4	M4 up	GO:0004197	Metabolism	Catabolism	cysteine-type endopeptidase activity	F	AT1G47128
5136	2	L4 vs. M4	M4 up	GO:0004197	Metabolism	Catabolism	cysteine-type endopeptidase activity	F	AT2G25940
5137	2	L4 vs. M4	M4 up	GO:0004197	Metabolism	Catabolism	cysteine-type endopeptidase activity	F	AT3G19390
5138	2	L4 vs. M4	M4 up	GO:0004197	Metabolism	Catabolism	cysteine-type endopeptidase activity	F	AT3G45310
5139	2	L4 vs. M4	M4 up	GO:0004197	Metabolism	Catabolism	cysteine-type endopeptidase activity	F	AT4G01610
5140	2	L4 vs. M4	M4 up	GO:0004197	Metabolism	Catabolism	cysteine-type endopeptidase activity	F	AT4G11310
5141	2	L4 vs. M4	M4 up	GO:0004197	Metabolism	Catabolism	cysteine-type endopeptidase activity	F	AT4G32940
5142	2	L4 vs. M4	M4 up	GO:0004197	Metabolism	Catabolism	cysteine-type endopeptidase activity	F	AT5G45890
5143	2	L4 vs. M4	M4 up	GO:0004197	Metabolism	Catabolism	cysteine-type endopeptidase activity	F	AT5G50260
5144	2	L4 vs. M4	M4 up	GO:0004197	Metabolism	Catabolism	cysteine-type endopeptidase activity	F	AT5G60360
5145	2	L4 vs. M4	M4 up	GO:0006308	Metabolism	Catabolism	DNA catabolic process	P	AT1G11190
5146	2	L4 vs. M4	M4 up	GO:0004175	Metabolism	Catabolism	endopeptidase activity	F	AT1G20380
5147	2	L4 vs. M4	M4 up	GO:0004175	Metabolism	Catabolism	endopeptidase activity	F	AT1G51980

5148	2	L4 vs. M4	M4 up	GO:0004175	Metabolism	Catabolism	endopeptidase activity	F	AT4G32940
5149	2	L4 vs. M4	M4 up	GO:0004521	Metabolism	Catabolism	endoribonuclease activity	F	AT1G11190
5150	2	L4 vs. M4	M4 up	GO:0004521	Metabolism	Catabolism	endoribonuclease activity	F	AT2G02990
5151	2	L4 vs. M4	M4 up	GO:0043928	Metabolism	Catabolism	exonucleolytic catabolism of deadenylated mRNA	P	AT3G07750
5152	2	L4 vs. M4	M4 up	GO:0080187	Metabolism	Catabolism	floral organ senescence	P	AT1G11190
5153	2	L4 vs. M4	M4 up	GO:0080187	Metabolism	Catabolism	floral organ senescence	P	AT5G45890
5154	2	L4 vs. M4	M4 up	GO:0046294	Metabolism	Catabolism	formaldehyde catabolic process	P	AT5G42250
5155	2	L4 vs. M4	M4 up	GO:0019762	Metabolism	Catabolism	glucosinolate catabolic process	P	AT2G44450
5156	2	L4 vs. M4	M4 up	GO:0019762	Metabolism	Catabolism	glucosinolate catabolic process	P	AT3G16410
5157	2	L4 vs. M4	M4 up	GO:0019563	Metabolism	Catabolism	glycerol catabolic process	P	AT1G48430
5158	2	L4 vs. M4	M4 up	GO:0010150	Metabolism	Catabolism	leaf senescence	P	AT1G11190
5159	2	L4 vs. M4	M4 up	GO:0010150	Metabolism	Catabolism	leaf senescence	P	AT4G32940
5160	2	L4 vs. M4	M4 up	GO:0010150	Metabolism	Catabolism	leaf senescence	P	AT5G12080
5161	2	L4 vs. M4	M4 up	GO:0010150	Metabolism	Catabolism	leaf senescence	P	AT5G13180
5162	2	L4 vs. M4	M4 up	GO:0010150	Metabolism	Catabolism	leaf senescence	P	AT5G37600
5163	2	L4 vs. M4	M4 up	GO:0010150	Metabolism	Catabolism	leaf senescence	P	AT5G45890
5164	2	L4 vs. M4	M4 up	GO:0006552	Metabolism	Catabolism	leucine catabolic process	P	AT2G26800
5165	2	L4 vs. M4	M4 up	GO:0046274	Metabolism	Catabolism	lignin catabolic process	P	AT2G37040
5166	2	L4 vs. M4	M4 up	GO:0016042	Metabolism	Catabolism	lipid catabolic process	P	AT1G75880
5167	2	L4 vs. M4	M4 up	GO:0016042	Metabolism	Catabolism	lipid catabolic process	P	AT4G39850
5168	2	L4 vs. M4	M4 up	GO:0016042	Metabolism	Catabolism	lipid catabolic process	P	AT5G45950
5169	2	L4 vs. M4	M4 up	GO:0019477	Metabolism	Catabolism	L-lysine catabolic process	P	AT4G33150
5170	2	L4 vs. M4	M4 up	GO:0033512	Metabolism	Catabolism	L-lysine catabolic process to acetyl-CoA via saccharopine	P	AT4G33150
5171	2	L4 vs. M4	M4 up	GO:0019509	Metabolism	Catabolism	L-methionine salvage from methylthioadenosine	P	AT2G05830
5172	2	L4 vs. M4	M4 up	GO:0006559	Metabolism	Catabolism	L-phenylalanine catabolic process	P	AT2G37040
5173	2	L4 vs. M4	M4 up	GO:0006559	Metabolism	Catabolism	L-phenylalanine catabolic process	P	AT5G36160
5174	2	L4 vs. M4	M4 up	GO:0006565	Metabolism	Catabolism	L-serine catabolic process	P	AT4G13930
5175	2	L4 vs. M4	M4 up	GO:0005764	Metabolism	Catabolism	lysosome	C	AT1G47128
5176	2	L4 vs. M4	M4 up	GO:0005764	Metabolism	Catabolism	lysosome	C	AT3G19390
5177	2	L4 vs. M4	M4 up	GO:0005764	Metabolism	Catabolism	lysosome	C	AT3G45310
5178	2	L4 vs. M4	M4 up	GO:0005764	Metabolism	Catabolism	lysosome	C	AT4G01610
5179	2	L4 vs. M4	M4 up	GO:0005764	Metabolism	Catabolism	lysosome	C	AT4G11310
5180	2	L4 vs. M4	M4 up	GO:0005764	Metabolism	Catabolism	lysosome	C	AT5G45890
5181	2	L4 vs. M4	M4 up	GO:0005764	Metabolism	Catabolism	lysosome	C	AT5G50260
5182	2	L4 vs. M4	M4 up	GO:0005764	Metabolism	Catabolism	lysosome	C	AT5G60360
5183	2	L4 vs. M4	M4 up	GO:0033619	Metabolism	Catabolism	membrane protein proteolysis	P	AT4G33410
5184	2	L4 vs. M4	M4 up	GO:0004222	Metabolism	Catabolism	metalloendopeptidase activity	F	AT1G51980
5185	2	L4 vs. M4	M4 up	GO:0019941	Metabolism	Catabolism	modification-dependent protein catabolic process	P	AT4G02890
5186	2	L4 vs. M4	M4 up	GO:1903298	Metabolism	Catabolism	negative regulation of hypoxia-induced intrinsic apoptotic signaling pathway	P	AT4G16660
5187	2	L4 vs. M4	M4 up	GO:0045490	Metabolism	Catabolism	pectin catabolic process	P	AT1G02810
5188	2	L4 vs. M4	M4 up	GO:0045490	Metabolism	Catabolism	pectin catabolic process	P	AT3G24670
5189	2	L4 vs. M4	M4 up	GO:0008233	Metabolism	Catabolism	peptidase activity	F	AT1G47128
5190	2	L4 vs. M4	M4 up	GO:0008233	Metabolism	Catabolism	peptidase activity	F	AT5G27660
5191	2	L4 vs. M4	M4 up	GO:0043171	Metabolism	Catabolism	peptide catabolic process	P	AT4G33090
5192	2	L4 vs. M4	M4 up	GO:0005777	Metabolism	Catabolism	peroxisome	C	AT2G06050
5193	2	L4 vs. M4	M4 up	GO:0005777	Metabolism	Catabolism	peroxisome	C	AT2G31390
5194	2	L4 vs. M4	M4 up	GO:0005777	Metabolism	Catabolism	peroxisome	C	AT2G42790
5195	2	L4 vs. M4	M4 up	GO:0005777	Metabolism	Catabolism	peroxisome	C	AT3G50170
5196	2	L4 vs. M4	M4 up	GO:0005777	Metabolism	Catabolism	peroxisome	C	AT4G18360
5197	2	L4 vs. M4	M4 up	GO:0005777	Metabolism	Catabolism	peroxisome	C	AT4G39850
5198	2	L4 vs. M4	M4 up	GO:0005777	Metabolism	Catabolism	peroxisome	C	AT5G17380
5199	2	L4 vs. M4	M4 up	GO:0000272	Metabolism	Catabolism	polysaccharide catabolic process	P	AT4G19810
5200	2	L4 vs. M4	M4 up	GO:0032436	Metabolism	Catabolism	positive regulation of proteasomal ubiquitin-dependent protein catabolic process	P	AT1G32340

5201	2	L4 vs. M4	M4 up	GO:0012501	Metabolism	Catabolism	programmed cell death	P	AT1G73260
5202	2	L4 vs. M4	M4 up	GO:0010623	Metabolism	Catabolism	programmed cell death involved in cell development	P	AT5G50260
5203	2	L4 vs. M4	M4 up	GO:0043248	Metabolism	Catabolism	proteasome assembly	P	AT4G29040
5204	2	L4 vs. M4	M4 up	GO:0000502	Metabolism	Catabolism	proteasome complex	C	AT4G29040
5205	2	L4 vs. M4	M4 up	GO:0008540	Metabolism	Catabolism	proteasome regulatory particle, base subcomplex	C	AT4G29040
5206	2	L4 vs. M4	M4 up	GO:0036402	Metabolism	Catabolism	proteasome-activating ATPase activity	F	AT4G29040
5207	2	L4 vs. M4	M4 up	GO:0043161	Metabolism	Catabolism	proteasome-mediated ubiquitin-dependent protein catabolic process	P	AT4G05420
5208	2	L4 vs. M4	M4 up	GO:0043161	Metabolism	Catabolism	proteasome-mediated ubiquitin-dependent protein catabolic process	P	AT4G29040
5209	2	L4 vs. M4	M4 up	GO:0030163	Metabolism	Catabolism	protein catabolic process	P	AT1G62290
5210	2	L4 vs. M4	M4 up	GO:0030163	Metabolism	Catabolism	protein catabolic process	P	AT5G53350
5211	2	L4 vs. M4	M4 up	GO:0000209	Metabolism	Catabolism	protein polyubiquitination	P	AT1G32340
5212	2	L4 vs. M4	M4 up	GO:0016567	Metabolism	Catabolism	protein ubiquitination	P	AT1G29340
5213	2	L4 vs. M4	M4 up	GO:0016567	Metabolism	Catabolism	protein ubiquitination	P	AT3G18290
5214	2	L4 vs. M4	M4 up	GO:0016567	Metabolism	Catabolism	protein ubiquitination	P	AT4G02890
5215	2	L4 vs. M4	M4 up	GO:0016567	Metabolism	Catabolism	protein ubiquitination	P	AT4G05420
5216	2	L4 vs. M4	M4 up	GO:0016567	Metabolism	Catabolism	protein ubiquitination	P	AT5G37490
5217	2	L4 vs. M4	M4 up	GO:0016567	Metabolism	Catabolism	protein ubiquitination	P	AT5G67480
5218	2	L4 vs. M4	M4 up	GO:0006508	Metabolism	Catabolism	proteolysis	P	AT1G47128
5219	2	L4 vs. M4	M4 up	GO:0006508	Metabolism	Catabolism	proteolysis	P	AT1G62290
5220	2	L4 vs. M4	M4 up	GO:0006508	Metabolism	Catabolism	proteolysis	P	AT2G47390
5221	2	L4 vs. M4	M4 up	GO:0006508	Metabolism	Catabolism	proteolysis	P	AT3G45310
5222	2	L4 vs. M4	M4 up	GO:0006508	Metabolism	Catabolism	proteolysis	P	AT4G01610
5223	2	L4 vs. M4	M4 up	GO:0006508	Metabolism	Catabolism	proteolysis	P	AT4G33090
5224	2	L4 vs. M4	M4 up	GO:0006508	Metabolism	Catabolism	proteolysis	P	AT5G27660
5225	2	L4 vs. M4	M4 up	GO:0006508	Metabolism	Catabolism	proteolysis	P	AT5G53350
5226	2	L4 vs. M4	M4 up	GO:0006508	Metabolism	Catabolism	proteolysis	P	AT5G60160
5227	2	L4 vs. M4	M4 up	GO:0006508	Metabolism	Catabolism	proteolysis	P	AT5G60360
5228	2	L4 vs. M4	M4 up	GO:0006508	Metabolism	Catabolism	proteolysis	P	AT5G65760
5229	2	L4 vs. M4	M4 up	GO:0051603	Metabolism	Catabolism	proteolysis involved in cellular protein catabolic process	P	AT1G47128
5230	2	L4 vs. M4	M4 up	GO:0051603	Metabolism	Catabolism	proteolysis involved in cellular protein catabolic process	P	AT2G25940
5231	2	L4 vs. M4	M4 up	GO:0051603	Metabolism	Catabolism	proteolysis involved in cellular protein catabolic process	P	AT3G19390
5232	2	L4 vs. M4	M4 up	GO:0051603	Metabolism	Catabolism	proteolysis involved in cellular protein catabolic process	P	AT3G45010
5233	2	L4 vs. M4	M4 up	GO:0051603	Metabolism	Catabolism	proteolysis involved in cellular protein catabolic process	P	AT3G45310
5234	2	L4 vs. M4	M4 up	GO:0051603	Metabolism	Catabolism	proteolysis involved in cellular protein catabolic process	P	AT4G01610
5235	2	L4 vs. M4	M4 up	GO:0051603	Metabolism	Catabolism	proteolysis involved in cellular protein catabolic process	P	AT4G11310
5236	2	L4 vs. M4	M4 up	GO:0051603	Metabolism	Catabolism	proteolysis involved in cellular protein catabolic process	P	AT4G32940
5237	2	L4 vs. M4	M4 up	GO:0051603	Metabolism	Catabolism	proteolysis involved in cellular protein catabolic process	P	AT5G27660
5238	2	L4 vs. M4	M4 up	GO:0051603	Metabolism	Catabolism	proteolysis involved in cellular protein catabolic process	P	AT5G45890
5239	2	L4 vs. M4	M4 up	GO:0051603	Metabolism	Catabolism	proteolysis involved in cellular protein catabolic process	P	AT5G50260
5240	2	L4 vs. M4	M4 up	GO:0051603	Metabolism	Catabolism	proteolysis involved in cellular protein catabolic process	P	AT5G60360
5241	2	L4 vs. M4	M4 up	GO:0052692	Metabolism	Catabolism	raffinose alpha-galactosidase activity	F	AT5G08380

5242	2	L4 vs. M4	M4 up	GO:1990116	Metabolism	Catabolism	ribosome-associated ubiquitin-dependent protein catabolic process	P	AT5G49930
5243	2	L4 vs. M4	M4 up	GO:0006401	Metabolism	Catabolism	RNA catabolic process	P	AT2G02990
5244	2	L4 vs. M4	M4 up	GO:0090502	Metabolism	Catabolism	RNA phosphodiester bond hydrolysis, endonucleolytic	P	AT1G11190
5245	2	L4 vs. M4	M4 up	GO:0016075	Metabolism	Catabolism	rRNA catabolic process	P	AT3G07750
5246	2	L4 vs. M4	M4 up	GO:0046244	Metabolism	Catabolism	salicylic acid catabolic process	P	AT2G37040
5247	2	L4 vs. M4	M4 up	GO:0010282	Metabolism	Catabolism	senescence-associated vacuole	C	AT5G45890
5248	2	L4 vs. M4	M4 up	GO:0004185	Metabolism	Catabolism	serine-type carboxypeptidase activity	F	AT3G45010
5249	2	L4 vs. M4	M4 up	GO:0004252	Metabolism	Catabolism	serine-type endopeptidase activity	F	AT2G47390
5250	2	L4 vs. M4	M4 up	GO:0004252	Metabolism	Catabolism	serine-type endopeptidase activity	F	AT5G27660
5251	2	L4 vs. M4	M4 up	GO:0006567	Metabolism	Catabolism	threonine catabolic process	P	AT3G04520
5252	2	L4 vs. M4	M4 up	GO:0006210	Metabolism	Catabolism	thymine catabolic process	P	AT3G17810
5253	2	L4 vs. M4	M4 up	GO:0019441	Metabolism	Catabolism	tryptophan catabolic process to kynurenine	P	AT2G40000
5254	2	L4 vs. M4	M4 up	GO:0006572	Metabolism	Catabolism	tyrosine catabolic process	P	AT5G36160
5255	2	L4 vs. M4	M4 up	GO:0031624	Metabolism	Catabolism	ubiquitin conjugating enzyme binding	F	AT1G32340
5256	2	L4 vs. M4	M4 up	GO:0000151	Metabolism	Catabolism	ubiquitin ligase complex	C	AT1G32340
5257	2	L4 vs. M4	M4 up	GO:0061630	Metabolism	Catabolism	ubiquitin protein ligase activity	F	AT1G32340
5258	2	L4 vs. M4	M4 up	GO:0061630	Metabolism	Catabolism	ubiquitin protein ligase activity	F	AT3G18290
5259	2	L4 vs. M4	M4 up	GO:0031625	Metabolism	Catabolism	ubiquitin protein ligase binding	F	AT3G53420
5260	2	L4 vs. M4	M4 up	GO:0031625	Metabolism	Catabolism	ubiquitin protein ligase binding	F	AT4G02890
5261	2	L4 vs. M4	M4 up	GO:0006511	Metabolism	Catabolism	ubiquitin-dependent protein catabolic process	P	AT1G32340
5262	2	L4 vs. M4	M4 up	GO:0006511	Metabolism	Catabolism	ubiquitin-dependent protein catabolic process	P	AT4G02890
5263	2	L4 vs. M4	M4 up	GO:0006511	Metabolism	Catabolism	ubiquitin-dependent protein catabolic process	P	AT4G29040
5264	2	L4 vs. M4	M4 up	GO:0004842	Metabolism	Catabolism	ubiquitin-protein transferase activity	F	AT1G29340
5265	2	L4 vs. M4	M4 up	GO:0004842	Metabolism	Catabolism	ubiquitin-protein transferase activity	F	AT5G37490
5266	2	L4 vs. M4	M4 up	GO:0006212	Metabolism	Catabolism	uracil catabolic process	P	AT3G17810
5267	2	L4 vs. M4	M4 up	GO:0009044	Metabolism	Catabolism	xylan 1,4-beta-xylosidase activity	F	AT3G10740
5268	2	L4 vs. M4	M4 up	GO:0009044	Metabolism	Catabolism	xylan 1,4-beta-xylosidase activity	F	AT5G49360
5269	2	L4 vs. M4	M4 up	GO:0045493	Metabolism	Catabolism	xylan catabolic process	P	AT3G10740
5270	2	L4 vs. M4	M4 up	GO:0045493	Metabolism	Catabolism	xylan catabolic process	P	AT5G49360
5271	2	L4 vs. M4	M4 up	GO:0051990	Metabolism	Metabolism	(R)-2-hydroxyglutarate dehydrogenase activity	F	AT4G36400
5272	2	L4 vs. M4	M4 up	GO:0009257	Metabolism	Metabolism	10-formyltetrahydrofolate biosynthetic process	P	AT1G50480
5273	2	L4 vs. M4	M4 up	GO:0016629	Metabolism	Metabolism	12-oxophytodienoate reductase activity	F	AT2G06050
5274	2	L4 vs. M4	M4 up	GO:0009979	Metabolism	Metabolism	16:0 monogalactosyldiacylglycerol desaturase activity	F	AT1G06350
5275	2	L4 vs. M4	M4 up	GO:0046537	Metabolism	Metabolism	2,3-bisphosphoglycerate-independent phosphoglycerate mutase activity	F	AT3G08590
5276	2	L4 vs. M4	M4 up	GO:0047545	Metabolism	Metabolism	2-hydroxyglutarate dehydrogenase activity	F	AT4G36400
5277	2	L4 vs. M4	M4 up	GO:0051538	Metabolism	Metabolism	3 iron, 4 sulfur cluster binding	F	AT5G04140
5278	2	L4 vs. M4	M4 up	GO:0004028	Metabolism	Metabolism	3-chloroallyl aldehyde dehydrogenase activity	F	AT4G36250
5279	2	L4 vs. M4	M4 up	GO:0051539	Metabolism	Metabolism	4 iron, 4 sulfur cluster binding	F	AT1G03190
5280	2	L4 vs. M4	M4 up	GO:0051539	Metabolism	Metabolism	4 iron, 4 sulfur cluster binding	F	ATCG00340
5281	2	L4 vs. M4	M4 up	GO:0051539	Metabolism	Metabolism	4 iron, 4 sulfur cluster binding	F	ATCG00350
5282	2	L4 vs. M4	M4 up	GO:0102217	Metabolism	Metabolism	6-phosphoglucan, water dikinase activity	F	AT5G26570
5283	2	L4 vs. M4	M4 up	GO:0051015	Metabolism	Metabolism	actin filament binding	F	AT1G13180
5284	2	L4 vs. M4	M4 up	GO:0007015	Metabolism	Metabolism	actin filament organization	P	AT1G13180
5285	2	L4 vs. M4	M4 up	GO:0045300	Metabolism	Metabolism	acyl-[acyl-carrier-protein] desaturase activity	F	AT3G02630
5286	2	L4 vs. M4	M4 up	GO:0004024	Metabolism	Metabolism	alcohol dehydrogenase activity, zinc-dependent	F	AT5G42250
5287	2	L4 vs. M4	M4 up	GO:0006066	Metabolism	Metabolism	alcohol metabolic process	P	AT3G23410
5288	2	L4 vs. M4	M4 up	GO:0046423	Metabolism	Metabolism	allene-oxide cyclase activity	F	AT3G25760
5289	2	L4 vs. M4	M4 up	GO:0009308	Metabolism	Metabolism	amine metabolic process	P	AT4G12280
5290	2	L4 vs. M4	M4 up	GO:0016597	Metabolism	Metabolism	amino acid binding	F	AT4G13930
5291	2	L4 vs. M4	M4 up	GO:0019676	Metabolism	Metabolism	ammonia assimilation cycle	P	AT5G04140
5292	2	L4 vs. M4	M4 up	GO:0009718	Metabolism	Metabolism	anthocyanin-containing compound biosynthetic process	P	AT2G02990

5293	2	L4 vs. M4	M4 up	GO:0009718	Metabolism	Metabolism	anthocyanin-containing compound biosynthetic process	P	AT5G42800
5294	2	L4 vs. M4	M4 up	GO:0009073	Metabolism	Metabolism	aromatic amino acid family biosynthetic process	P	AT1G48860
5295	2	L4 vs. M4	M4 up	GO:0034314	Metabolism	Metabolism	Arp2/3 complex-mediated actin nucleation	P	AT1G13180
5296	2	L4 vs. M4	M4 up	GO:0005885	Metabolism	Metabolism	Arp2/3 protein complex	C	AT1G13180
5297	2	L4 vs. M4	M4 up	GO:0042500	Metabolism	Metabolism	aspartic endopeptidase activity, intramembrane cleaving	F	AT4G33410
5298	2	L4 vs. M4	M4 up	GO:0004190	Metabolism	Metabolism	aspartic-type endopeptidase activity	F	AT1G62290
5299	2	L4 vs. M4	M4 up	GO:0045330	Metabolism	Metabolism	aspartyl esterase activity	F	AT1G02810
5300	2	L4 vs. M4	M4 up	GO:0005524	Metabolism	Metabolism	ATP binding	F	AT1G02520
5301	2	L4 vs. M4	M4 up	GO:0005524	Metabolism	Metabolism	ATP binding	F	AT1G03190
5302	2	L4 vs. M4	M4 up	GO:0005524	Metabolism	Metabolism	ATP binding	F	AT1G09450
5303	2	L4 vs. M4	M4 up	GO:0005524	Metabolism	Metabolism	ATP binding	F	AT1G13180
5304	2	L4 vs. M4	M4 up	GO:0005524	Metabolism	Metabolism	ATP binding	F	AT1G14000
5305	2	L4 vs. M4	M4 up	GO:0005524	Metabolism	Metabolism	ATP binding	F	AT1G14610
5306	2	L4 vs. M4	M4 up	GO:0005524	Metabolism	Metabolism	ATP binding	F	AT1G15500
5307	2	L4 vs. M4	M4 up	GO:0005524	Metabolism	Metabolism	ATP binding	F	AT1G27770
5308	2	L4 vs. M4	M4 up	GO:0005524	Metabolism	Metabolism	ATP binding	F	AT1G50480
5309	2	L4 vs. M4	M4 up	GO:0005524	Metabolism	Metabolism	ATP binding	F	AT1G51980
5310	2	L4 vs. M4	M4 up	GO:0005524	Metabolism	Metabolism	ATP binding	F	AT1G60810
5311	2	L4 vs. M4	M4 up	GO:0005524	Metabolism	Metabolism	ATP binding	F	AT1G73080
5312	2	L4 vs. M4	M4 up	GO:0005524	Metabolism	Metabolism	ATP binding	F	AT2G01320
5313	2	L4 vs. M4	M4 up	GO:0005524	Metabolism	Metabolism	ATP binding	F	AT2G07680
5314	2	L4 vs. M4	M4 up	GO:0005524	Metabolism	Metabolism	ATP binding	F	AT2G17290
5315	2	L4 vs. M4	M4 up	GO:0005524	Metabolism	Metabolism	ATP binding	F	AT2G22360
5316	2	L4 vs. M4	M4 up	GO:0005524	Metabolism	Metabolism	ATP binding	F	AT2G24360
5317	2	L4 vs. M4	M4 up	GO:0005524	Metabolism	Metabolism	ATP binding	F	AT2G30360
5318	2	L4 vs. M4	M4 up	GO:0005524	Metabolism	Metabolism	ATP binding	F	AT2G31390
5319	2	L4 vs. M4	M4 up	GO:0005524	Metabolism	Metabolism	ATP binding	F	AT2G33210
5320	2	L4 vs. M4	M4 up	GO:0005524	Metabolism	Metabolism	ATP binding	F	AT2G34660
5321	2	L4 vs. M4	M4 up	GO:0005524	Metabolism	Metabolism	ATP binding	F	AT2G44350
5322	2	L4 vs. M4	M4 up	GO:0005524	Metabolism	Metabolism	ATP binding	F	AT2G47060
5323	2	L4 vs. M4	M4 up	GO:0005524	Metabolism	Metabolism	ATP binding	F	AT2G48010
5324	2	L4 vs. M4	M4 up	GO:0005524	Metabolism	Metabolism	ATP binding	F	AT3G09440
5325	2	L4 vs. M4	M4 up	GO:0005524	Metabolism	Metabolism	ATP binding	F	AT3G09660
5326	2	L4 vs. M4	M4 up	GO:0005524	Metabolism	Metabolism	ATP binding	F	AT3G14050
5327	2	L4 vs. M4	M4 up	GO:0005524	Metabolism	Metabolism	ATP binding	F	AT3G17470
5328	2	L4 vs. M4	M4 up	GO:0005524	Metabolism	Metabolism	ATP binding	F	AT3G21250
5329	2	L4 vs. M4	M4 up	GO:0005524	Metabolism	Metabolism	ATP binding	F	AT3G45640
5330	2	L4 vs. M4	M4 up	GO:0005524	Metabolism	Metabolism	ATP binding	F	AT3G49670
5331	2	L4 vs. M4	M4 up	GO:0005524	Metabolism	Metabolism	ATP binding	F	AT3G50530
5332	2	L4 vs. M4	M4 up	GO:0005524	Metabolism	Metabolism	ATP binding	F	AT3G51240
5333	2	L4 vs. M4	M4 up	GO:0005524	Metabolism	Metabolism	ATP binding	F	AT3G59140
5334	2	L4 vs. M4	M4 up	GO:0005524	Metabolism	Metabolism	ATP binding	F	AT3G62150
5335	2	L4 vs. M4	M4 up	GO:0005524	Metabolism	Metabolism	ATP binding	F	AT4G14680
5336	2	L4 vs. M4	M4 up	GO:0005524	Metabolism	Metabolism	ATP binding	F	AT4G24400
5337	2	L4 vs. M4	M4 up	GO:0005524	Metabolism	Metabolism	ATP binding	F	AT4G29040
5338	2	L4 vs. M4	M4 up	GO:0005524	Metabolism	Metabolism	ATP binding	F	AT4G32840
5339	2	L4 vs. M4	M4 up	GO:0005524	Metabolism	Metabolism	ATP binding	F	AT4G35230
5340	2	L4 vs. M4	M4 up	GO:0005524	Metabolism	Metabolism	ATP binding	F	AT4G39850
5341	2	L4 vs. M4	M4 up	GO:0005524	Metabolism	Metabolism	ATP binding	F	AT5G03730
5342	2	L4 vs. M4	M4 up	GO:0005524	Metabolism	Metabolism	ATP binding	F	AT5G08590
5343	2	L4 vs. M4	M4 up	GO:0005524	Metabolism	Metabolism	ATP binding	F	AT5G16570
5344	2	L4 vs. M4	M4 up	GO:0005524	Metabolism	Metabolism	ATP binding	F	AT5G26570
5345	2	L4 vs. M4	M4 up	GO:0005524	Metabolism	Metabolism	ATP binding	F	AT5G37600
5346	2	L4 vs. M4	M4 up	GO:0005524	Metabolism	Metabolism	ATP binding	F	AT5G40010

5347	2	L4 vs. M4	M4 up	GO:0005524	Metabolism	Metabolism	ATP binding	F	AT5G53350
5348	2	L4 vs. M4	M4 up	GO:0005524	Metabolism	Metabolism	ATP binding	F	AT5G53450
5349	2	L4 vs. M4	M4 up	GO:0005524	Metabolism	Metabolism	ATP binding	F	AT5G57350
5350	2	L4 vs. M4	M4 up	GO:0005524	Metabolism	Metabolism	ATP binding	F	AT5G58270
5351	2	L4 vs. M4	M4 up	GO:0005524	Metabolism	Metabolism	ATP binding	F	AT5G61030
5352	2	L4 vs. M4	M4 up	GO:0005524	Metabolism	Metabolism	ATP binding	F	AT5G66760
5353	2	L4 vs. M4	M4 up	GO:0004176	Metabolism	Metabolism	ATP-dependent peptidase activity	F	AT5G53350
5354	2	L4 vs. M4	M4 up	GO:0018874	Metabolism	Metabolism	benzoate metabolic process	P	AT2G43820
5355	2	L4 vs. M4	M4 up	GO:0052641	Metabolism	Metabolism	benzoic acid glucosyltransferase activity	F	AT2G43820
5356	2	L4 vs. M4	M4 up	GO:0019483	Metabolism	Metabolism	beta-alanine biosynthetic process	P	AT3G17810
5357	2	L4 vs. M4	M4 up	GO:0004565	Metabolism	Metabolism	beta-galactosidase activity	F	AT1G45130
5358	2	L4 vs. M4	M4 up	GO:0008422	Metabolism	Metabolism	beta-glucosidase activity	F	AT2G44450
5359	2	L4 vs. M4	M4 up	GO:0008150	Metabolism	Metabolism	biological_process	P	AT1G07040
5360	2	L4 vs. M4	M4 up	GO:0008150	Metabolism	Metabolism	biological_process	P	AT1G09310
5361	2	L4 vs. M4	M4 up	GO:0008150	Metabolism	Metabolism	biological_process	P	AT1G48200
5362	2	L4 vs. M4	M4 up	GO:0008150	Metabolism	Metabolism	biological_process	P	AT1G67920
5363	2	L4 vs. M4	M4 up	GO:0008150	Metabolism	Metabolism	biological_process	P	AT1G74640
5364	2	L4 vs. M4	M4 up	GO:0008150	Metabolism	Metabolism	biological_process	P	AT2G17710
5365	2	L4 vs. M4	M4 up	GO:0008150	Metabolism	Metabolism	biological_process	P	AT2G21820
5366	2	L4 vs. M4	M4 up	GO:0008150	Metabolism	Metabolism	biological_process	P	AT2G25670
5367	2	L4 vs. M4	M4 up	GO:0008150	Metabolism	Metabolism	biological_process	P	AT2G39650
5368	2	L4 vs. M4	M4 up	GO:0008150	Metabolism	Metabolism	biological_process	P	AT2G42760
5369	2	L4 vs. M4	M4 up	GO:0008150	Metabolism	Metabolism	biological_process	P	AT3G04350
5370	2	L4 vs. M4	M4 up	GO:0008150	Metabolism	Metabolism	biological_process	P	AT3G22240
5371	2	L4 vs. M4	M4 up	GO:0008150	Metabolism	Metabolism	biological_process	P	AT3G50170
5372	2	L4 vs. M4	M4 up	GO:0008150	Metabolism	Metabolism	biological_process	P	AT4G24130
5373	2	L4 vs. M4	M4 up	GO:0008150	Metabolism	Metabolism	biological_process	P	AT4G28260
5374	2	L4 vs. M4	M4 up	GO:0008150	Metabolism	Metabolism	biological_process	P	AT4G29780
5375	2	L4 vs. M4	M4 up	GO:0008150	Metabolism	Metabolism	biological_process	P	AT4G34080
5376	2	L4 vs. M4	M4 up	GO:0008150	Metabolism	Metabolism	biological_process	P	AT5G05250
5377	2	L4 vs. M4	M4 up	GO:0008150	Metabolism	Metabolism	biological_process	P	AT5G10695
5378	2	L4 vs. M4	M4 up	GO:0008150	Metabolism	Metabolism	biological_process	P	AT5G11000
5379	2	L4 vs. M4	M4 up	GO:0008150	Metabolism	Metabolism	biological_process	P	AT5G19860
5380	2	L4 vs. M4	M4 up	GO:0008150	Metabolism	Metabolism	biological_process	P	AT5G43390
5381	2	L4 vs. M4	M4 up	GO:0008150	Metabolism	Metabolism	biological_process	P	AT5G57340
5382	2	L4 vs. M4	M4 up	GO:0008150	Metabolism	Metabolism	biological_process	P	AT5G64460
5383	2	L4 vs. M4	M4 up	GO:0009058	Metabolism	Metabolism	biosynthetic process	P	AT3G57010
5384	2	L4 vs. M4	M4 up	GO:0009058	Metabolism	Metabolism	biosynthetic process	P	AT5G36160
5385	2	L4 vs. M4	M4 up	GO:0010120	Metabolism	Metabolism	camalexin biosynthetic process	P	AT3G45640
5386	2	L4 vs. M4	M4 up	GO:0030552	Metabolism	Metabolism	cAMP binding	F	AT2G46440
5387	2	L4 vs. M4	M4 up	GO:0030552	Metabolism	Metabolism	cAMP binding	F	AT5G15410
5388	2	L4 vs. M4	M4 up	GO:0015976	Metabolism	Metabolism	carbon utilization	P	AT1G70410
5389	2	L4 vs. M4	M4 up	GO:0016830	Metabolism	Metabolism	carbon-carbon lyase activity	F	AT5G17380
5390	2	L4 vs. M4	M4 up	GO:0016846	Metabolism	Metabolism	carbon-sulfur lyase activity	F	AT1G34040
5391	2	L4 vs. M4	M4 up	GO:0016831	Metabolism	Metabolism	carboxy-lyase activity	F	AT5G17380
5392	2	L4 vs. M4	M4 up	GO:0004180	Metabolism	Metabolism	carboxypeptidase activity	F	AT5G65760
5393	2	L4 vs. M4	M4 up	GO:0010291	Metabolism	Metabolism	carotene beta-ring hydroxylase activity	F	AT4G25700
5394	2	L4 vs. M4	M4 up	GO:0016119	Metabolism	Metabolism	carotene metabolic process	P	AT4G25700
5395	2	L4 vs. M4	M4 up	GO:0003824	Metabolism	Metabolism	catalytic activity	F	AT5G66420
5396	2	L4 vs. M4	M4 up	GO:0006081	Metabolism	Metabolism	cellular aldehyde metabolic process	P	AT4G36250
5397	2	L4 vs. M4	M4 up	GO:0006520	Metabolism	Metabolism	cellular amino acid metabolic process	P	AT5G36160
5398	2	L4 vs. M4	M4 up	GO:0044249	Metabolism	Metabolism	cellular biosynthetic process	P	AT2G05830
5399	2	L4 vs. M4	M4 up	GO:0030244	Metabolism	Metabolism	cellulose biosynthetic process	P	AT2G32530
5400	2	L4 vs. M4	M4 up	GO:0016760	Metabolism	Metabolism	cellulose synthase (UDP-forming) activity	F	AT2G32530
5401	2	L4 vs. M4	M4 up	GO:0016759	Metabolism	Metabolism	cellulose synthase activity	F	AT2G32530
5402	2	L4 vs. M4	M4 up	GO:0030553	Metabolism	Metabolism	cGMP binding	F	AT2G46440

5403	2	L4 vs. M4	M4 up	GO:0030553	Metabolism	Metabolism	cGMP binding	F	AT5G15410
5404	2	L4 vs. M4	M4 up	GO:0008061	Metabolism	Metabolism	chitin binding	F	AT4G19810
5405	2	L4 vs. M4	M4 up	GO:0004568	Metabolism	Metabolism	chitinase activity	F	AT4G19810
5406	2	L4 vs. M4	M4 up	GO:0009423	Metabolism	Metabolism	chorismate biosynthetic process	P	AT1G48860
5407	2	L4 vs. M4	M4 up	GO:0009800	Metabolism	Metabolism	cinnamic acid biosynthetic process	P	AT2G37040
5408	2	L4 vs. M4	M4 up	GO:0005507	Metabolism	Metabolism	copper ion binding	F	AT1G50480
5409	2	L4 vs. M4	M4 up	GO:0005507	Metabolism	Metabolism	copper ion binding	F	AT2G04400
5410	2	L4 vs. M4	M4 up	GO:0005507	Metabolism	Metabolism	copper ion binding	F	AT2G33210
5411	2	L4 vs. M4	M4 up	GO:0005507	Metabolism	Metabolism	copper ion binding	F	AT3G04120
5412	2	L4 vs. M4	M4 up	GO:0005507	Metabolism	Metabolism	copper ion binding	F	AT4G12280
5413	2	L4 vs. M4	M4 up	GO:0005507	Metabolism	Metabolism	copper ion binding	F	AT5G37600
5414	2	L4 vs. M4	M4 up	GO:0005507	Metabolism	Metabolism	copper ion binding	F	AT5G61030
5415	2	L4 vs. M4	M4 up	GO:0017172	Metabolism	Metabolism	cysteine dioxygenase activity	F	AT3G58670
5416	2	L4 vs. M4	M4 up	GO:0008234	Metabolism	Metabolism	cysteine-type peptidase activity	F	AT1G47128
5417	2	L4 vs. M4	M4 up	GO:0008234	Metabolism	Metabolism	cysteine-type peptidase activity	F	AT5G45890
5418	2	L4 vs. M4	M4 up	GO:0008234	Metabolism	Metabolism	cysteine-type peptidase activity	F	AT5G60360
5419	2	L4 vs. M4	M4 up	GO:0016554	Metabolism	Metabolism	cytidine to uridine editing	P	AT5G61030
5420	2	L4 vs. M4	M4 up	GO:0006189	Metabolism	Metabolism	'de novo' IMP biosynthetic process	P	AT2G35040
5421	2	L4 vs. M4	M4 up	GO:0004144	Metabolism	Metabolism	diacylglycerol O-acyltransferase activity	F	AT1G48300
5422	2	L4 vs. M4	M4 up	GO:0004144	Metabolism	Metabolism	diacylglycerol O-acyltransferase activity	F	AT1G54570
5423	2	L4 vs. M4	M4 up	GO:0008837	Metabolism	Metabolism	diaminopimelate epimerase activity	F	AT3G53580
5424	2	L4 vs. M4	M4 up	GO:0045552	Metabolism	Metabolism	dihydrokaempferol 4-reductase activity	F	AT5G42800
5425	2	L4 vs. M4	M4 up	GO:0004148	Metabolism	Metabolism	dihydrolipoyl dehydrogenase activity	F	AT3G16950
5426	2	L4 vs. M4	M4 up	GO:0051213	Metabolism	Metabolism	dioxygenase activity	F	AT3G51240
5427	2	L4 vs. M4	M4 up	GO:0051213	Metabolism	Metabolism	dioxygenase activity	F	AT4G25310
5428	2	L4 vs. M4	M4 up	GO:0051213	Metabolism	Metabolism	dioxygenase activity	F	AT5G05600
5429	2	L4 vs. M4	M4 up	GO:0008239	Metabolism	Metabolism	dipeptidyl-peptidase activity	F	AT5G65760
5430	2	L4 vs. M4	M4 up	GO:0003677	Metabolism	Metabolism	DNA binding	F	AT1G22190
5431	2	L4 vs. M4	M4 up	GO:0003677	Metabolism	Metabolism	DNA binding	F	AT3G04120
5432	2	L4 vs. M4	M4 up	GO:0003677	Metabolism	Metabolism	DNA binding	F	AT3G17310
5433	2	L4 vs. M4	M4 up	GO:0003677	Metabolism	Metabolism	DNA binding	F	AT5G13180
5434	2	L4 vs. M4	M4 up	GO:0008725	Metabolism	Metabolism	DNA-3-methyladenine glycosylase activity	F	AT3G12710
5435	2	L4 vs. M4	M4 up	GO:0006488	Metabolism	Metabolism	dolichol-linked oligosaccharide biosynthetic process	P	AT5G38460
5436	2	L4 vs. M4	M4 up	GO:0042281	Metabolism	Metabolism	dolichyl pyrophosphate Man9GlcNAc2 alpha-1,3-glucosyltransferase activity	F	AT5G38460
5437	2	L4 vs. M4	M4 up	GO:0042732	Metabolism	Metabolism	D-xylose metabolic process	P	AT3G53520
5438	2	L4 vs. M4	M4 up	GO:0008843	Metabolism	Metabolism	endochitinase activity	F	AT4G19810
5439	2	L4 vs. M4	M4 up	GO:0006897	Metabolism	Metabolism	endocytosis	P	AT3G01780
5440	2	L4 vs. M4	M4 up	GO:0004866	Metabolism	Metabolism	endopeptidase inhibitor activity	F	AT1G73260
5441	2	L4 vs. M4	M4 up	GO:0016891	Metabolism	Metabolism	endoribonuclease activity, producing 5'-phosphomonoesters	F	AT1G11190
5442	2	L4 vs. M4	M4 up	GO:0004857	Metabolism	Metabolism	enzyme inhibitor activity	F	AT5G20740
5443	2	L4 vs. M4	M4 up	GO:0035885	Metabolism	Metabolism	exochitinase activity	F	AT4G19810
5444	2	L4 vs. M4	M4 up	GO:0006887	Metabolism	Metabolism	exocytosis	P	AT5G58430
5445	2	L4 vs. M4	M4 up	GO:0010409	Metabolism	Metabolism	extensin metabolic process	P	AT3G57630
5446	2	L4 vs. M4	M4 up	GO:0071949	Metabolism	Metabolism	FAD binding	F	AT4G36400
5447	2	L4 vs. M4	M4 up	GO:0006747	Metabolism	Metabolism	FAD biosynthetic process	P	AT5G03430
5448	2	L4 vs. M4	M4 up	GO:0000293	Metabolism	Metabolism	ferric-chelate reductase activity	F	AT1G23020
5449	2	L4 vs. M4	M4 up	GO:0047890	Metabolism	Metabolism	flavanone 4-reductase activity	F	AT5G42800
5450	2	L4 vs. M4	M4 up	GO:0050660	Metabolism	Metabolism	flavin adenine dinucleotide binding	F	AT3G16950
5451	2	L4 vs. M4	M4 up	GO:0050660	Metabolism	Metabolism	flavin adenine dinucleotide binding	F	AT3G23410
5452	2	L4 vs. M4	M4 up	GO:0050660	Metabolism	Metabolism	flavin adenine dinucleotide binding	F	AT5G66760
5453	2	L4 vs. M4	M4 up	GO:0047893	Metabolism	Metabolism	flavonol 3-O-glucosyltransferase activity	F	AT4G34135
5454	2	L4 vs. M4	M4 up	GO:0033836	Metabolism	Metabolism	flavonol 7-O-beta-glucosyltransferase activity	F	AT4G34135
5455	2	L4 vs. M4	M4 up	GO:0003919	Metabolism	Metabolism	FMN adenylyltransferase activity	F	AT5G03430

5456	2	L4 vs. M4	M4 up	GO:0010181	Metabolism	Metabolism	FMN binding	F	AT2G06050
5457	2	L4 vs. M4	M4 up	GO:0010181	Metabolism	Metabolism	FMN binding	F	AT4G18360
5458	2	L4 vs. M4	M4 up	GO:0010181	Metabolism	Metabolism	FMN binding	F	AT4G36750
5459	2	L4 vs. M4	M4 up	GO:0046655	Metabolism	Metabolism	folic acid metabolic process	P	AT4G13930
5460	2	L4 vs. M4	M4 up	GO:0004329	Metabolism	Metabolism	formate-tetrahydrofolate ligase activity	F	AT1G50480
5461	2	L4 vs. M4	M4 up	GO:0006004	Metabolism	Metabolism	fucose metabolic process	P	AT5G15740
5462	2	L4 vs. M4	M4 up	GO:0019003	Metabolism	Metabolism	GDP binding	F	AT1G72660
5463	2	L4 vs. M4	M4 up	GO:0019760	Metabolism	Metabolism	glucosinolate metabolic process	P	AT4G37260
5464	2	L4 vs. M4	M4 up	GO:0006537	Metabolism	Metabolism	glutamate biosynthetic process	P	AT5G04140
5465	2	L4 vs. M4	M4 up	GO:0016041	Metabolism	Metabolism	glutamate synthase (ferredoxin) activity	F	AT5G04140
5466	2	L4 vs. M4	M4 up	GO:0004356	Metabolism	Metabolism	glutamate-ammonia ligase activity	F	AT5G16570
5467	2	L4 vs. M4	M4 up	GO:0004356	Metabolism	Metabolism	glutamate-ammonia ligase activity	F	AT5G37600
5468	2	L4 vs. M4	M4 up	GO:0006542	Metabolism	Metabolism	glutamine biosynthetic process	P	AT5G16570
5469	2	L4 vs. M4	M4 up	GO:0006542	Metabolism	Metabolism	glutamine biosynthetic process	P	AT5G37600
5470	2	L4 vs. M4	M4 up	GO:0006662	Metabolism	Metabolism	glycerol ether metabolic process	P	AT1G45145
5471	2	L4 vs. M4	M4 up	GO:0004371	Metabolism	Metabolism	glycerone kinase activity	F	AT1G48430
5472	2	L4 vs. M4	M4 up	GO:0006545	Metabolism	Metabolism	glycine biosynthetic process	P	AT3G04520
5473	2	L4 vs. M4	M4 up	GO:0019264	Metabolism	Metabolism	glycine biosynthetic process from serine	P	AT4G13930
5474	2	L4 vs. M4	M4 up	GO:0004372	Metabolism	Metabolism	glycine hydroxymethyltransferase activity	F	AT4G13930
5475	2	L4 vs. M4	M4 up	GO:0006544	Metabolism	Metabolism	glycine metabolic process	P	AT4G13930
5476	2	L4 vs. M4	M4 up	GO:0018920	Metabolism	Metabolism	glyphosate metabolic process	P	AT1G48860
5477	2	L4 vs. M4	M4 up	GO:0032580	Metabolism	Metabolism	Golgi cisterna membrane	C	AT3G53520
5478	2	L4 vs. M4	M4 up	GO:0005525	Metabolism	Metabolism	GTP binding	F	AT1G72660
5479	2	L4 vs. M4	M4 up	GO:0005525	Metabolism	Metabolism	GTP binding	F	AT3G14050
5480	2	L4 vs. M4	M4 up	GO:0005525	Metabolism	Metabolism	GTP binding	F	AT3G17470
5481	2	L4 vs. M4	M4 up	GO:0005525	Metabolism	Metabolism	GTP binding	F	AT4G02790
5482	2	L4 vs. M4	M4 up	GO:0005096	Metabolism	Metabolism	GTPase activator activity	F	AT5G05570
5483	2	L4 vs. M4	M4 up	GO:0005096	Metabolism	Metabolism	GTPase activator activity	F	AT5G58590
5484	2	L4 vs. M4	M4 up	GO:0003924	Metabolism	Metabolism	GTPase activity	F	AT1G72660
5485	2	L4 vs. M4	M4 up	GO:0003924	Metabolism	Metabolism	GTPase activity	F	AT4G02790
5486	2	L4 vs. M4	M4 up	GO:0015969	Metabolism	Metabolism	guanosine tetraphosphate metabolic process	P	AT3G14050
5487	2	L4 vs. M4	M4 up	GO:0015969	Metabolism	Metabolism	guanosine tetraphosphate metabolic process	P	AT3G17470
5488	2	L4 vs. M4	M4 up	GO:0004383	Metabolism	Metabolism	guanylate cyclase activity	F	AT1G73080
5489	2	L4 vs. M4	M4 up	GO:0020037	Metabolism	Metabolism	heme binding	F	AT2G40000
5490	2	L4 vs. M4	M4 up	GO:0020037	Metabolism	Metabolism	heme binding	F	AT2G45580
5491	2	L4 vs. M4	M4 up	GO:0020037	Metabolism	Metabolism	heme binding	F	AT3G14620
5492	2	L4 vs. M4	M4 up	GO:0020037	Metabolism	Metabolism	heme binding	F	AT3G50660
5493	2	L4 vs. M4	M4 up	GO:0020037	Metabolism	Metabolism	heme binding	F	AT4G36380
5494	2	L4 vs. M4	M4 up	GO:0020037	Metabolism	Metabolism	heme binding	F	AT4G39480
5495	2	L4 vs. M4	M4 up	GO:0070814	Metabolism	Metabolism	hydrogen sulfide biosynthetic process	P	AT4G14680
5496	2	L4 vs. M4	M4 up	GO:0016787	Metabolism	Metabolism	hydrolase activity	F	AT1G17430
5497	2	L4 vs. M4	M4 up	GO:0016787	Metabolism	Metabolism	hydrolase activity	F	AT1G74640
5498	2	L4 vs. M4	M4 up	GO:0016787	Metabolism	Metabolism	hydrolase activity	F	AT1G80280
5499	2	L4 vs. M4	M4 up	GO:0016787	Metabolism	Metabolism	hydrolase activity	F	AT3G02875
5500	2	L4 vs. M4	M4 up	GO:0016787	Metabolism	Metabolism	hydrolase activity	F	AT3G15650
5501	2	L4 vs. M4	M4 up	GO:0016787	Metabolism	Metabolism	hydrolase activity	F	AT3G62550
5502	2	L4 vs. M4	M4 up	GO:0016787	Metabolism	Metabolism	hydrolase activity	F	AT4G25700
5503	2	L4 vs. M4	M4 up	GO:0016788	Metabolism	Metabolism	hydrolase activity, acting on ester bonds	F	AT5G45950
5504	2	L4 vs. M4	M4 up	GO:0004553	Metabolism	Metabolism	hydrolase activity, hydrolyzing O-glycosyl compounds	F	AT3G26140
5505	2	L4 vs. M4	M4 up	GO:0042802	Metabolism	Metabolism	identical protein binding	F	AT2G19480
5506	2	L4 vs. M4	M4 up	GO:0003937	Metabolism	Metabolism	IMP cyclohydrolase activity	F	AT2G35040
5507	2	L4 vs. M4	M4 up	GO:0004425	Metabolism	Metabolism	indole-3-glycerol-phosphate synthase activity	F	AT2G04400
5508	2	L4 vs. M4	M4 up	GO:0000822	Metabolism	Metabolism	inositol hexakisphosphate binding	F	AT2G03240
5509	2	L4 vs. M4	M4 up	GO:0005242	Metabolism	Metabolism	inward rectifier potassium channel activity	F	AT5G15410
5510	2	L4 vs. M4	M4 up	GO:0005506	Metabolism	Metabolism	iron ion binding	F	AT2G45580

5511	2	L4 vs. M4	M4 up	GO:0005506	Metabolism	Metabolism	iron ion binding	F	AT3G14620
5512	2	L4 vs. M4	M4 up	GO:0005506	Metabolism	Metabolism	iron ion binding	F	AT3G18290
5513	2	L4 vs. M4	M4 up	GO:0005506	Metabolism	Metabolism	iron ion binding	F	AT3G50660
5514	2	L4 vs. M4	M4 up	GO:0005506	Metabolism	Metabolism	iron ion binding	F	AT4G25700
5515	2	L4 vs. M4	M4 up	GO:0005506	Metabolism	Metabolism	iron ion binding	F	AT4G36380
5516	2	L4 vs. M4	M4 up	GO:0005506	Metabolism	Metabolism	iron ion binding	F	AT4G39480
5517	2	L4 vs. M4	M4 up	GO:0051536	Metabolism	Metabolism	iron-sulfur cluster binding	F	AT3G17810
5518	2	L4 vs. M4	M4 up	GO:0046951	Metabolism	Metabolism	ketone body biosynthetic process	P	AT2G26800
5519	2	L4 vs. M4	M4 up	GO:0019516	Metabolism	Metabolism	lactate oxidation	P	AT4G18360
5520	2	L4 vs. M4	M4 up	GO:0008732	Metabolism	Metabolism	L-allo-threonine aldolase activity	F	AT3G04520
5521	2	L4 vs. M4	M4 up	GO:0046373	Metabolism	Metabolism	L-arabinose metabolic process	P	AT3G10740
5522	2	L4 vs. M4	M4 up	GO:0097054	Metabolism	Metabolism	L-glutamate biosynthetic process	P	AT5G04140
5523	2	L4 vs. M4	M4 up	GO:0016874	Metabolism	Metabolism	ligase activity	F	AT3G18290
5524	2	L4 vs. M4	M4 up	GO:0016298	Metabolism	Metabolism	lipase activity	F	AT1G75880
5525	2	L4 vs. M4	M4 up	GO:0006629	Metabolism	Metabolism	lipid metabolic process	P	AT1G60810
5526	2	L4 vs. M4	M4 up	GO:0006629	Metabolism	Metabolism	lipid metabolic process	P	AT1G62290
5527	2	L4 vs. M4	M4 up	GO:0006629	Metabolism	Metabolism	lipid metabolic process	P	AT1G73650
5528	2	L4 vs. M4	M4 up	GO:0006629	Metabolism	Metabolism	lipid metabolic process	P	AT2G26800
5529	2	L4 vs. M4	M4 up	GO:0004459	Metabolism	Metabolism	L-lactate dehydrogenase activity	F	AT4G18360
5530	2	L4 vs. M4	M4 up	GO:0046577	Metabolism	Metabolism	long-chain-alcohol oxidase activity	F	AT3G23410
5531	2	L4 vs. M4	M4 up	GO:0004838	Metabolism	Metabolism	L-tyrosine:2-oxoglutarate aminotransferase activity	F	AT5G36160
5532	2	L4 vs. M4	M4 up	GO:0016829	Metabolism	Metabolism	lyase activity	F	AT2G02990
5533	2	L4 vs. M4	M4 up	GO:0019878	Metabolism	Metabolism	lysine biosynthetic process via aminoadipic acid	P	AT4G33150
5534	2	L4 vs. M4	M4 up	GO:0009089	Metabolism	Metabolism	lysine biosynthetic process via diaminopimelate	P	AT3G53580
5535	2	L4 vs. M4	M4 up	GO:0000287	Metabolism	Metabolism	magnesium ion binding	F	AT2G35840
5536	2	L4 vs. M4	M4 up	GO:0000287	Metabolism	Metabolism	magnesium ion binding	F	AT5G17380
5537	2	L4 vs. M4	M4 up	GO:0000287	Metabolism	Metabolism	magnesium ion binding	F	AT5G57350
5538	2	L4 vs. M4	M4 up	GO:0030145	Metabolism	Metabolism	manganese ion binding	F	AT3G08590
5539	2	L4 vs. M4	M4 up	GO:0008381	Metabolism	Metabolism	mechanosensitive ion channel activity	F	AT5G12080
5540	2	L4 vs. M4	M4 up	GO:0052854	Metabolism	Metabolism	medium-chain-(S)-2-hydroxy-acid oxidase activity	F	AT4G18360
5541	2	L4 vs. M4	M4 up	GO:0046872	Metabolism	Metabolism	metal ion binding	F	AT1G03190
5542	2	L4 vs. M4	M4 up	GO:0046872	Metabolism	Metabolism	metal ion binding	F	AT1G11190
5543	2	L4 vs. M4	M4 up	GO:0046872	Metabolism	Metabolism	metal ion binding	F	AT1G27770
5544	2	L4 vs. M4	M4 up	GO:0046872	Metabolism	Metabolism	metal ion binding	F	AT1G32340
5545	2	L4 vs. M4	M4 up	GO:0046872	Metabolism	Metabolism	metal ion binding	F	AT1G51980
5546	2	L4 vs. M4	M4 up	GO:0046872	Metabolism	Metabolism	metal ion binding	F	AT2G22360
5547	2	L4 vs. M4	M4 up	GO:0046872	Metabolism	Metabolism	metal ion binding	F	AT2G30020
5548	2	L4 vs. M4	M4 up	GO:0046872	Metabolism	Metabolism	metal ion binding	F	AT2G40000
5549	2	L4 vs. M4	M4 up	GO:0046872	Metabolism	Metabolism	metal ion binding	F	AT3G02630
5550	2	L4 vs. M4	M4 up	GO:0046872	Metabolism	Metabolism	metal ion binding	F	AT3G02875
5551	2	L4 vs. M4	M4 up	GO:0046872	Metabolism	Metabolism	metal ion binding	F	AT3G09660
5552	2	L4 vs. M4	M4 up	GO:0046872	Metabolism	Metabolism	metal ion binding	F	AT3G12070
5553	2	L4 vs. M4	M4 up	GO:0046872	Metabolism	Metabolism	metal ion binding	F	AT3G22370
5554	2	L4 vs. M4	M4 up	GO:0046872	Metabolism	Metabolism	metal ion binding	F	AT3G24670
5555	2	L4 vs. M4	M4 up	GO:0046872	Metabolism	Metabolism	metal ion binding	F	AT3G50530
5556	2	L4 vs. M4	M4 up	GO:0046872	Metabolism	Metabolism	metal ion binding	F	AT3G51240
5557	2	L4 vs. M4	M4 up	GO:0046872	Metabolism	Metabolism	metal ion binding	F	AT3G58670
5558	2	L4 vs. M4	M4 up	GO:0046872	Metabolism	Metabolism	metal ion binding	F	AT4G25310
5559	2	L4 vs. M4	M4 up	GO:0046872	Metabolism	Metabolism	metal ion binding	F	AT4G32840
5560	2	L4 vs. M4	M4 up	GO:0046872	Metabolism	Metabolism	metal ion binding	F	AT5G04140
5561	2	L4 vs. M4	M4 up	GO:0046872	Metabolism	Metabolism	metal ion binding	F	AT5G05600
5562	2	L4 vs. M4	M4 up	GO:0046872	Metabolism	Metabolism	metal ion binding	F	AT5G26570
5563	2	L4 vs. M4	M4 up	GO:0046872	Metabolism	Metabolism	metal ion binding	F	AT5G35735
5564	2	L4 vs. M4	M4 up	GO:0046872	Metabolism	Metabolism	metal ion binding	F	AT5G43380
5565	2	L4 vs. M4	M4 up	GO:0046872	Metabolism	Metabolism	metal ion binding	F	AT5G47930
5566	2	L4 vs. M4	M4 up	GO:0046872	Metabolism	Metabolism	metal ion binding	F	AT5G50400

5567	2	L4 vs. M4	M4 up	GO:0046872	Metabolism	Metabolism	metal ion binding	F	AT5G53140
5568	2	L4 vs. M4	M4 up	GO:0070006	Metabolism	Metabolism	metalloaminopeptidase activity	F	AT4G33090
5569	2	L4 vs. M4	M4 up	GO:0070006	Metabolism	Metabolism	metalloaminopeptidase activity	F	AT5G60160
5570	2	L4 vs. M4	M4 up	GO:0004477	Metabolism	Metabolism	methenyltetrahydrofolate cyclohydrolase activity	F	AT1G50480
5571	2	L4 vs. M4	M4 up	GO:0102078	Metabolism	Metabolism	methyl jasmonate methylesterase activity	F	AT1G19640
5572	2	L4 vs. M4	M4 up	GO:0008168	Metabolism	Metabolism	methyltransferase activity	F	AT2G27590
5573	2	L4 vs. M4	M4 up	GO:0008168	Metabolism	Metabolism	methyltransferase activity	F	AT2G34300
5574	2	L4 vs. M4	M4 up	GO:0008168	Metabolism	Metabolism	methyltransferase activity	F	AT3G05390
5575	2	L4 vs. M4	M4 up	GO:0008168	Metabolism	Metabolism	methyltransferase activity	F	AT3G17310
5576	2	L4 vs. M4	M4 up	GO:0008168	Metabolism	Metabolism	methyltransferase activity	F	AT3G53400
5577	2	L4 vs. M4	M4 up	GO:0008168	Metabolism	Metabolism	methyltransferase activity	F	AT4G33120
5578	2	L4 vs. M4	M4 up	GO:0008168	Metabolism	Metabolism	methyltransferase activity	F	AT5G01710
5579	2	L4 vs. M4	M4 up	GO:0008168	Metabolism	Metabolism	methyltransferase activity	F	AT5G05570
5580	2	L4 vs. M4	M4 up	GO:0003674	Metabolism	Metabolism	molecular_function	F	AT1G23710
5581	2	L4 vs. M4	M4 up	GO:0003674	Metabolism	Metabolism	molecular_function	F	AT1G35140
5582	2	L4 vs. M4	M4 up	GO:0003674	Metabolism	Metabolism	molecular_function	F	AT1G48200
5583	2	L4 vs. M4	M4 up	GO:0003674	Metabolism	Metabolism	molecular_function	F	AT1G67920
5584	2	L4 vs. M4	M4 up	GO:0003674	Metabolism	Metabolism	molecular_function	F	AT2G14900
5585	2	L4 vs. M4	M4 up	GO:0003674	Metabolism	Metabolism	molecular_function	F	AT2G17710
5586	2	L4 vs. M4	M4 up	GO:0003674	Metabolism	Metabolism	molecular_function	F	AT2G21820
5587	2	L4 vs. M4	M4 up	GO:0003674	Metabolism	Metabolism	molecular_function	F	AT2G23810
5588	2	L4 vs. M4	M4 up	GO:0003674	Metabolism	Metabolism	molecular_function	F	AT2G25670
5589	2	L4 vs. M4	M4 up	GO:0003674	Metabolism	Metabolism	molecular_function	F	AT2G35880
5590	2	L4 vs. M4	M4 up	GO:0003674	Metabolism	Metabolism	molecular_function	F	AT2G39650
5591	2	L4 vs. M4	M4 up	GO:0003674	Metabolism	Metabolism	molecular_function	F	AT2G42760
5592	2	L4 vs. M4	M4 up	GO:0003674	Metabolism	Metabolism	molecular_function	F	AT3G04350
5593	2	L4 vs. M4	M4 up	GO:0003674	Metabolism	Metabolism	molecular_function	F	AT3G22240
5594	2	L4 vs. M4	M4 up	GO:0003674	Metabolism	Metabolism	molecular_function	F	AT3G49470
5595	2	L4 vs. M4	M4 up	GO:0003674	Metabolism	Metabolism	molecular_function	F	AT3G50170
5596	2	L4 vs. M4	M4 up	GO:0003674	Metabolism	Metabolism	molecular_function	F	AT4G24110
5597	2	L4 vs. M4	M4 up	GO:0003674	Metabolism	Metabolism	molecular_function	F	AT4G29780
5598	2	L4 vs. M4	M4 up	GO:0003674	Metabolism	Metabolism	molecular_function	F	AT4G30996
5599	2	L4 vs. M4	M4 up	GO:0003674	Metabolism	Metabolism	molecular_function	F	AT4G34080
5600	2	L4 vs. M4	M4 up	GO:0003674	Metabolism	Metabolism	molecular_function	F	AT5G05250
5601	2	L4 vs. M4	M4 up	GO:0003674	Metabolism	Metabolism	molecular_function	F	AT5G11000
5602	2	L4 vs. M4	M4 up	GO:0003674	Metabolism	Metabolism	molecular_function	F	AT5G16780
5603	2	L4 vs. M4	M4 up	GO:0003674	Metabolism	Metabolism	molecular_function	F	AT5G42680
5604	2	L4 vs. M4	M4 up	GO:0003674	Metabolism	Metabolism	molecular_function	F	AT5G43390
5605	2	L4 vs. M4	M4 up	GO:0003674	Metabolism	Metabolism	molecular_function	F	AT5G57340
5606	2	L4 vs. M4	M4 up	GO:0003674	Metabolism	Metabolism	molecular_function	F	AT5G67370
5607	2	L4 vs. M4	M4 up	GO:0006777	Metabolism	Metabolism	Mo-molybdopterin cofactor biosynthetic process	P	AT5G58270
5608	2	L4 vs. M4	M4 up	GO:0004497	Metabolism	Metabolism	monooxygenase activity	F	AT3G14620
5609	2	L4 vs. M4	M4 up	GO:0004497	Metabolism	Metabolism	monooxygenase activity	F	AT3G50660
5610	2	L4 vs. M4	M4 up	GO:0004497	Metabolism	Metabolism	monooxygenase activity	F	AT4G36380
5611	2	L4 vs. M4	M4 up	GO:0004497	Metabolism	Metabolism	monooxygenase activity	F	AT4G39480
5612	2	L4 vs. M4	M4 up	GO:0045159	Metabolism	Metabolism	myosin II binding	F	AT5G05570
5613	2	L4 vs. M4	M4 up	GO:0010013	Metabolism	Metabolism	N-1-naphthylphthalamic acid binding	F	AT4G33090
5614	2	L4 vs. M4	M4 up	GO:0045486	Metabolism	Metabolism	naringenin 3-dioxygenase activity	F	AT3G51240
5615	2	L4 vs. M4	M4 up	GO:0030418	Metabolism	Metabolism	nicotianamine biosynthetic process	P	AT1G56430
5616	2	L4 vs. M4	M4 up	GO:0030410	Metabolism	Metabolism	nicotianamine synthase activity	F	AT1G56430
5617	2	L4 vs. M4	M4 up	GO:0090704	Metabolism	Metabolism	nicotinate-O-glucosyltransferase activity	F	AT2G43820
5618	2	L4 vs. M4	M4 up	GO:0042128	Metabolism	Metabolism	nitrate assimilation	P	AT1G69870
5619	2	L4 vs. M4	M4 up	GO:0042128	Metabolism	Metabolism	nitrate assimilation	P	AT5G16570
5620	2	L4 vs. M4	M4 up	GO:0042128	Metabolism	Metabolism	nitrate assimilation	P	AT5G37600
5621	2	L4 vs. M4	M4 up	GO:0080028	Metabolism	Metabolism	nitrile biosynthetic process	P	AT3G16410
5622	2	L4 vs. M4	M4 up	GO:0006807	Metabolism	Metabolism	nitrogen compound metabolic process	P	AT3G54140

5623	2	L4 vs. M4	M4 up	GO:0006807	Metabolism	Metabolism	nitrogen compound metabolic process	P	AT4G36400
5624	2	L4 vs. M4	M4 up	GO:0003676	Metabolism	Metabolism	nucleic acid binding	F	AT1G11190
5625	2	L4 vs. M4	M4 up	GO:0003676	Metabolism	Metabolism	nucleic acid binding	F	AT3G51800
5626	2	L4 vs. M4	M4 up	GO:0003676	Metabolism	Metabolism	nucleic acid binding	F	AT5G05570
5627	2	L4 vs. M4	M4 up	GO:0070012	Metabolism	Metabolism	oligopeptidase activity	F	AT1G20380
5628	2	L4 vs. M4	M4 up	GO:0006490	Metabolism	Metabolism	oligosaccharide-lipid intermediate biosynthetic process	P	AT5G38460
5629	2	L4 vs. M4	M4 up	GO:0006730	Metabolism	Metabolism	one-carbon metabolic process	P	AT4G13930
5630	2	L4 vs. M4	M4 up	GO:0055114	Metabolism	Metabolism	oxidation-reduction process	P	AT1G01800
5631	2	L4 vs. M4	M4 up	GO:0055114	Metabolism	Metabolism	oxidation-reduction process	P	AT1G06350
5632	2	L4 vs. M4	M4 up	GO:0055114	Metabolism	Metabolism	oxidation-reduction process	P	AT1G23020
5633	2	L4 vs. M4	M4 up	GO:0055114	Metabolism	Metabolism	oxidation-reduction process	P	AT1G45145
5634	2	L4 vs. M4	M4 up	GO:0055114	Metabolism	Metabolism	oxidation-reduction process	P	AT1G51980
5635	2	L4 vs. M4	M4 up	GO:0055114	Metabolism	Metabolism	oxidation-reduction process	P	AT1G54100
5636	2	L4 vs. M4	M4 up	GO:0055114	Metabolism	Metabolism	oxidation-reduction process	P	AT1G76470
5637	2	L4 vs. M4	M4 up	GO:0055114	Metabolism	Metabolism	oxidation-reduction process	P	AT2G06050
5638	2	L4 vs. M4	M4 up	GO:0055114	Metabolism	Metabolism	oxidation-reduction process	P	AT2G45580
5639	2	L4 vs. M4	M4 up	GO:0055114	Metabolism	Metabolism	oxidation-reduction process	P	AT3G02630
5640	2	L4 vs. M4	M4 up	GO:0055114	Metabolism	Metabolism	oxidation-reduction process	P	AT3G04120
5641	2	L4 vs. M4	M4 up	GO:0055114	Metabolism	Metabolism	oxidation-reduction process	P	AT3G14620
5642	2	L4 vs. M4	M4 up	GO:0055114	Metabolism	Metabolism	oxidation-reduction process	P	AT3G16950
5643	2	L4 vs. M4	M4 up	GO:0055114	Metabolism	Metabolism	oxidation-reduction process	P	AT3G17810
5644	2	L4 vs. M4	M4 up	GO:0055114	Metabolism	Metabolism	oxidation-reduction process	P	AT3G22370
5645	2	L4 vs. M4	M4 up	GO:0055114	Metabolism	Metabolism	oxidation-reduction process	P	AT3G23410
5646	2	L4 vs. M4	M4 up	GO:0055114	Metabolism	Metabolism	oxidation-reduction process	P	AT3G50660
5647	2	L4 vs. M4	M4 up	GO:0055114	Metabolism	Metabolism	oxidation-reduction process	P	AT3G51240
5648	2	L4 vs. M4	M4 up	GO:0055114	Metabolism	Metabolism	oxidation-reduction process	P	AT3G58670
5649	2	L4 vs. M4	M4 up	GO:0055114	Metabolism	Metabolism	oxidation-reduction process	P	AT4G12280
5650	2	L4 vs. M4	M4 up	GO:0055114	Metabolism	Metabolism	oxidation-reduction process	P	AT4G22870
5651	2	L4 vs. M4	M4 up	GO:0055114	Metabolism	Metabolism	oxidation-reduction process	P	AT4G25310
5652	2	L4 vs. M4	M4 up	GO:0055114	Metabolism	Metabolism	oxidation-reduction process	P	AT4G25700
5653	2	L4 vs. M4	M4 up	GO:0055114	Metabolism	Metabolism	oxidation-reduction process	P	AT4G33150
5654	2	L4 vs. M4	M4 up	GO:0055114	Metabolism	Metabolism	oxidation-reduction process	P	AT4G36250
5655	2	L4 vs. M4	M4 up	GO:0055114	Metabolism	Metabolism	oxidation-reduction process	P	AT4G36380
5656	2	L4 vs. M4	M4 up	GO:0055114	Metabolism	Metabolism	oxidation-reduction process	P	AT4G36400
5657	2	L4 vs. M4	M4 up	GO:0055114	Metabolism	Metabolism	oxidation-reduction process	P	AT4G36750
5658	2	L4 vs. M4	M4 up	GO:0055114	Metabolism	Metabolism	oxidation-reduction process	P	AT4G39480
5659	2	L4 vs. M4	M4 up	GO:0055114	Metabolism	Metabolism	oxidation-reduction process	P	AT5G04140
5660	2	L4 vs. M4	M4 up	GO:0055114	Metabolism	Metabolism	oxidation-reduction process	P	AT5G05600
5661	2	L4 vs. M4	M4 up	GO:0055114	Metabolism	Metabolism	oxidation-reduction process	P	AT5G35735
5662	2	L4 vs. M4	M4 up	GO:0055114	Metabolism	Metabolism	oxidation-reduction process	P	AT5G42250
5663	2	L4 vs. M4	M4 up	GO:0055114	Metabolism	Metabolism	oxidation-reduction process	P	AT5G42800
5664	2	L4 vs. M4	M4 up	GO:0055114	Metabolism	Metabolism	oxidation-reduction process	P	ATCG01100
5665	2	L4 vs. M4	M4 up	GO:0017077	Metabolism	Metabolism	oxidative phosphorylation uncoupler activity	F	AT2G22500
5666	2	L4 vs. M4	M4 up	GO:0031408	Metabolism	Metabolism	oxylipin biosynthetic process	P	AT1G19640
5667	2	L4 vs. M4	M4 up	GO:0031408	Metabolism	Metabolism	oxylipin biosynthetic process	P	AT2G06050
5668	2	L4 vs. M4	M4 up	GO:0046482	Metabolism	Metabolism	para-aminobenzoic acid metabolic process	P	AT1G05560
5669	2	L4 vs. M4	M4 up	GO:0046482	Metabolism	Metabolism	para-aminobenzoic acid metabolic process	P	AT2G43820
5670	2	L4 vs. M4	M4 up	GO:0030570	Metabolism	Metabolism	pectate lyase activity	F	AT3G24670
5671	2	L4 vs. M4	M4 up	GO:0052793	Metabolism	Metabolism	pectin acetylesterase activity	F	AT5G45280
5672	2	L4 vs. M4	M4 up	GO:0045489	Metabolism	Metabolism	pectin biosynthetic process	P	AT3G28340
5673	2	L4 vs. M4	M4 up	GO:0045489	Metabolism	Metabolism	pectin biosynthetic process	P	AT5G15740
5674	2	L4 vs. M4	M4 up	GO:0030599	Metabolism	Metabolism	pectinesterase activity	F	AT1G02810
5675	2	L4 vs. M4	M4 up	GO:0046910	Metabolism	Metabolism	pectinesterase inhibitor activity	F	AT1G02810
5676	2	L4 vs. M4	M4 up	GO:0042277	Metabolism	Metabolism	peptide binding	F	AT4G33090
5677	2	L4 vs. M4	M4 up	GO:0045548	Metabolism	Metabolism	phenylalanine ammonia-lyase activity	F	AT2G37040

5678	2	L4 vs. M4	M4 up	GO:0009699	Metabolism	Metabolism	phenylpropanoid biosynthetic process	P	AT2G37040
5679	2	L4 vs. M4	M4 up	GO:0080124	Metabolism	Metabolism	pheophytinase activity	F	AT5G13800
5680	2	L4 vs. M4	M4 up	GO:0051752	Metabolism	Metabolism	phosphoglucan, water dikinase activity	F	AT5G26570
5681	2	L4 vs. M4	M4 up	GO:0102219	Metabolism	Metabolism	phosphogluco-amylopectin water dikinase activity	F	AT5G26570
5682	2	L4 vs. M4	M4 up	GO:0004643	Metabolism	Metabolism	phosphoribosylaminoimidazolecarboxamide formyltransferase activity	F	AT2G35040
5683	2	L4 vs. M4	M4 up	GO:0004640	Metabolism	Metabolism	phosphoribosylanthranilate isomerase activity	F	AT2G04400
5684	2	L4 vs. M4	M4 up	GO:0016310	Metabolism	Metabolism	phosphorylation	P	AT3G14050
5685	2	L4 vs. M4	M4 up	GO:0016310	Metabolism	Metabolism	phosphorylation	P	AT3G17470
5686	2	L4 vs. M4	M4 up	GO:0033306	Metabolism	Metabolism	phytol metabolic process	P	AT1G54570
5687	2	L4 vs. M4	M4 up	GO:0047262	Metabolism	Metabolism	polygalacturonate 4-alpha-galacturonosyltransferase activity	F	AT3G28340
5688	2	L4 vs. M4	M4 up	GO:0008187	Metabolism	Metabolism	poly-pyrimidine tract binding	F	AT4G36690
5689	2	L4 vs. M4	M4 up	GO:0008131	Metabolism	Metabolism	primary amine oxidase activity	F	AT4G12280
5690	2	L4 vs. M4	M4 up	GO:0005515	Metabolism	Metabolism	protein binding	F	AT1G03190
5691	2	L4 vs. M4	M4 up	GO:0005515	Metabolism	Metabolism	protein binding	F	AT1G04750
5692	2	L4 vs. M4	M4 up	GO:0005515	Metabolism	Metabolism	protein binding	F	AT1G05560
5693	2	L4 vs. M4	M4 up	GO:0005515	Metabolism	Metabolism	protein binding	F	AT1G10970
5694	2	L4 vs. M4	M4 up	GO:0005515	Metabolism	Metabolism	protein binding	F	AT1G14000
5695	2	L4 vs. M4	M4 up	GO:0005515	Metabolism	Metabolism	protein binding	F	AT1G14320
5696	2	L4 vs. M4	M4 up	GO:0005515	Metabolism	Metabolism	protein binding	F	AT1G18740
5697	2	L4 vs. M4	M4 up	GO:0005515	Metabolism	Metabolism	protein binding	F	AT1G22190
5698	2	L4 vs. M4	M4 up	GO:0005515	Metabolism	Metabolism	protein binding	F	AT1G47128
5699	2	L4 vs. M4	M4 up	GO:0005515	Metabolism	Metabolism	protein binding	F	AT1G70410
5700	2	L4 vs. M4	M4 up	GO:0005515	Metabolism	Metabolism	protein binding	F	AT1G73080
5701	2	L4 vs. M4	M4 up	GO:0005515	Metabolism	Metabolism	protein binding	F	AT1G80840
5702	2	L4 vs. M4	M4 up	GO:0005515	Metabolism	Metabolism	protein binding	F	AT2G06530
5703	2	L4 vs. M4	M4 up	GO:0005515	Metabolism	Metabolism	protein binding	F	AT2G17290
5704	2	L4 vs. M4	M4 up	GO:0005515	Metabolism	Metabolism	protein binding	F	AT2G19480
5705	2	L4 vs. M4	M4 up	GO:0005515	Metabolism	Metabolism	protein binding	F	AT2G19620
5706	2	L4 vs. M4	M4 up	GO:0005515	Metabolism	Metabolism	protein binding	F	AT2G20890
5707	2	L4 vs. M4	M4 up	GO:0005515	Metabolism	Metabolism	protein binding	F	AT2G30360
5708	2	L4 vs. M4	M4 up	GO:0005515	Metabolism	Metabolism	protein binding	F	AT2G37040
5709	2	L4 vs. M4	M4 up	GO:0005515	Metabolism	Metabolism	protein binding	F	AT2G40000
5710	2	L4 vs. M4	M4 up	GO:0005515	Metabolism	Metabolism	protein binding	F	AT2G46600
5711	2	L4 vs. M4	M4 up	GO:0005515	Metabolism	Metabolism	protein binding	F	AT2G47060
5712	2	L4 vs. M4	M4 up	GO:0005515	Metabolism	Metabolism	protein binding	F	AT3G04120
5713	2	L4 vs. M4	M4 up	GO:0005515	Metabolism	Metabolism	protein binding	F	AT3G09440
5714	2	L4 vs. M4	M4 up	GO:0005515	Metabolism	Metabolism	protein binding	F	AT3G16470
5715	2	L4 vs. M4	M4 up	GO:0005515	Metabolism	Metabolism	protein binding	F	AT3G18290
5716	2	L4 vs. M4	M4 up	GO:0005515	Metabolism	Metabolism	protein binding	F	AT3G19390
5717	2	L4 vs. M4	M4 up	GO:0005515	Metabolism	Metabolism	protein binding	F	AT3G27120
5718	2	L4 vs. M4	M4 up	GO:0005515	Metabolism	Metabolism	protein binding	F	AT3G45640
5719	2	L4 vs. M4	M4 up	GO:0005515	Metabolism	Metabolism	protein binding	F	AT3G49080
5720	2	L4 vs. M4	M4 up	GO:0005515	Metabolism	Metabolism	protein binding	F	AT3G49670
5721	2	L4 vs. M4	M4 up	GO:0005515	Metabolism	Metabolism	protein binding	F	AT3G51800
5722	2	L4 vs. M4	M4 up	GO:0005515	Metabolism	Metabolism	protein binding	F	AT4G05420
5723	2	L4 vs. M4	M4 up	GO:0005515	Metabolism	Metabolism	protein binding	F	AT4G13930
5724	2	L4 vs. M4	M4 up	GO:0005515	Metabolism	Metabolism	protein binding	F	AT4G29160
5725	2	L4 vs. M4	M4 up	GO:0005515	Metabolism	Metabolism	protein binding	F	AT4G35230
5726	2	L4 vs. M4	M4 up	GO:0005515	Metabolism	Metabolism	protein binding	F	AT4G36690
5727	2	L4 vs. M4	M4 up	GO:0005515	Metabolism	Metabolism	protein binding	F	AT4G37260
5728	2	L4 vs. M4	M4 up	GO:0005515	Metabolism	Metabolism	protein binding	F	AT4G39850
5729	2	L4 vs. M4	M4 up	GO:0005515	Metabolism	Metabolism	protein binding	F	AT5G03730
5730	2	L4 vs. M4	M4 up	GO:0005515	Metabolism	Metabolism	protein binding	F	AT5G04140
5731	2	L4 vs. M4	M4 up	GO:0005515	Metabolism	Metabolism	protein binding	F	AT5G04150

5732	2	L4 vs. M4	M4 up	GO:0005515	Metabolism	Metabolism	protein binding	F	AT5G08590
5733	2	L4 vs. M4	M4 up	GO:0005515	Metabolism	Metabolism	protein binding	F	AT5G13180
5734	2	L4 vs. M4	M4 up	GO:0005515	Metabolism	Metabolism	protein binding	F	AT5G13800
5735	2	L4 vs. M4	M4 up	GO:0005515	Metabolism	Metabolism	protein binding	F	AT5G16570
5736	2	L4 vs. M4	M4 up	GO:0005515	Metabolism	Metabolism	protein binding	F	AT5G37600
5737	2	L4 vs. M4	M4 up	GO:0005515	Metabolism	Metabolism	protein binding	F	AT5G42050
5738	2	L4 vs. M4	M4 up	GO:0005515	Metabolism	Metabolism	protein binding	F	AT5G42800
5739	2	L4 vs. M4	M4 up	GO:0005515	Metabolism	Metabolism	protein binding	F	AT5G58430
5740	2	L4 vs. M4	M4 up	GO:0005515	Metabolism	Metabolism	protein binding	F	AT5G58590
5741	2	L4 vs. M4	M4 up	GO:0005515	Metabolism	Metabolism	protein binding	F	AT5G61030
5742	2	L4 vs. M4	M4 up	GO:0005515	Metabolism	Metabolism	protein binding	F	AT5G64350
5743	2	L4 vs. M4	M4 up	GO:0005515	Metabolism	Metabolism	protein binding	F	AT5G66030
5744	2	L4 vs. M4	M4 up	GO:0005515	Metabolism	Metabolism	protein binding	F	AT5G67300
5745	2	L4 vs. M4	M4 up	GO:0005515	Metabolism	Metabolism	protein binding	F	AT5G67480
5746	2	L4 vs. M4	M4 up	GO:0005515	Metabolism	Metabolism	protein binding	F	ATCG00270
5747	2	L4 vs. M4	M4 up	GO:0005515	Metabolism	Metabolism	protein binding	F	ATCG00680
5748	2	L4 vs. M4	M4 up	GO:0120029	Metabolism	Metabolism	proton export across plasma membrane	P	AT5G57350
5749	2	L4 vs. M4	M4 up	GO:0009113	Metabolism	Metabolism	purine nucleobase biosynthetic process	P	AT1G50480
5750	2	L4 vs. M4	M4 up	GO:0030170	Metabolism	Metabolism	pyridoxal phosphate binding	F	AT4G13930
5751	2	L4 vs. M4	M4 up	GO:0030170	Metabolism	Metabolism	pyridoxal phosphate binding	F	AT5G36160
5752	2	L4 vs. M4	M4 up	GO:0080043	Metabolism	Metabolism	quercetin 3-O-glucosyltransferase activity	F	AT1G05560
5753	2	L4 vs. M4	M4 up	GO:0080043	Metabolism	Metabolism	quercetin 3-O-glucosyltransferase activity	F	AT2G43820
5754	2	L4 vs. M4	M4 up	GO:0080044	Metabolism	Metabolism	quercetin 7-O-glucosyltransferase activity	F	AT1G05560
5755	2	L4 vs. M4	M4 up	GO:0080044	Metabolism	Metabolism	quercetin 7-O-glucosyltransferase activity	F	AT2G43820
5756	2	L4 vs. M4	M4 up	GO:0080044	Metabolism	Metabolism	quercetin 7-O-glucosyltransferase activity	F	AT4G34135
5757	2	L4 vs. M4	M4 up	GO:0048038	Metabolism	Metabolism	quinone binding	F	AT4G12280
5758	2	L4 vs. M4	M4 up	GO:0048038	Metabolism	Metabolism	quinone binding	F	ATCG01100
5759	2	L4 vs. M4	M4 up	GO:0048038	Metabolism	Metabolism	quinone binding	F	ATCG01110
5760	2	L4 vs. M4	M4 up	GO:0010395	Metabolism	Metabolism	rhamnogalacturonan I metabolic process	P	AT5G15740
5761	2	L4 vs. M4	M4 up	GO:0004753	Metabolism	Metabolism	saccharopine dehydrogenase activity	F	AT4G33150
5762	2	L4 vs. M4	M4 up	GO:0008757	Metabolism	Metabolism	S-adenosylmethionine-dependent methyltransferase activity	F	AT1G19640
5763	2	L4 vs. M4	M4 up	GO:0102483	Metabolism	Metabolism	scopolin beta-glucosidase activity	F	AT2G44450
5764	2	L4 vs. M4	M4 up	GO:0070905	Metabolism	Metabolism	serine binding	F	AT4G13930
5765	2	L4 vs. M4	M4 up	GO:0008236	Metabolism	Metabolism	serine-type peptidase activity	F	AT5G65760
5766	2	L4 vs. M4	M4 up	GO:0016987	Metabolism	Metabolism	sigma factor activity	F	AT3G53920
5767	2	L4 vs. M4	M4 up	GO:0046523	Metabolism	Metabolism	S-methyl-5-thioribose-1-phosphate isomerase activity	F	AT2G05830
5768	2	L4 vs. M4	M4 up	GO:0005483	Metabolism	Metabolism	soluble NSF attachment protein activity	F	AT3G56190
5769	2	L4 vs. M4	M4 up	GO:0102786	Metabolism	Metabolism	stearoyl-[acp] desaturase activity	F	AT3G02630
5770	2	L4 vs. M4	M4 up	GO:0000104	Metabolism	Metabolism	succinate dehydrogenase activity	F	AT5G66760
5771	2	L4 vs. M4	M4 up	GO:0050307	Metabolism	Metabolism	sucrose-phosphate phosphatase activity	F	AT2G35840
5772	2	L4 vs. M4	M4 up	GO:0004781	Metabolism	Metabolism	sulfate adenylyltransferase (ATP) activity	F	AT4G14680
5773	2	L4 vs. M4	M4 up	GO:0019905	Metabolism	Metabolism	syntaxin binding	F	AT3G56190
5774	2	L4 vs. M4	M4 up	GO:0019905	Metabolism	Metabolism	syntaxin binding	F	AT5G05570
5775	2	L4 vs. M4	M4 up	GO:0017025	Metabolism	Metabolism	TBP-class protein binding	F	AT4G29040
5776	2	L4 vs. M4	M4 up	GO:0035999	Metabolism	Metabolism	tetrahydrofolate interconversion	P	AT1G50480
5777	2	L4 vs. M4	M4 up	GO:0035999	Metabolism	Metabolism	tetrahydrofolate interconversion	P	AT4G13930
5778	2	L4 vs. M4	M4 up	GO:0046653	Metabolism	Metabolism	tetrahydrofolate metabolic process	P	AT4G13930
5779	2	L4 vs. M4	M4 up	GO:0030976	Metabolism	Metabolism	thiamine pyrophosphate binding	F	AT5G17380
5780	2	L4 vs. M4	M4 up	GO:0004793	Metabolism	Metabolism	threonine aldolase activity	F	AT3G04520
5781	2	L4 vs. M4	M4 up	GO:0008483	Metabolism	Metabolism	transaminase activity	F	AT1G34040
5782	2	L4 vs. M4	M4 up	GO:0016740	Metabolism	Metabolism	transferase activity	F	AT1G25155
5783	2	L4 vs. M4	M4 up	GO:0016740	Metabolism	Metabolism	transferase activity	F	AT2G05830
5784	2	L4 vs. M4	M4 up	GO:0016746	Metabolism	Metabolism	transferase activity, transferring acyl groups	F	AT1G75880
5785	2	L4 vs. M4	M4 up	GO:0016746	Metabolism	Metabolism	transferase activity, transferring acyl groups	F	AT4G28260

5786	2	L4 vs. M4	M4 up	GO:0016747	Metabolism	Metabolism	transferase activity, transferring acyl groups other than amino-acyl groups	F	AT1G28680
5787	2	L4 vs. M4	M4 up	GO:0016747	Metabolism	Metabolism	transferase activity, transferring acyl groups other than amino-acyl groups	F	AT1G65450
5788	2	L4 vs. M4	M4 up	GO:0016757	Metabolism	Metabolism	transferase activity, transferring glycosyl groups	F	AT1G05560
5789	2	L4 vs. M4	M4 up	GO:0016757	Metabolism	Metabolism	transferase activity, transferring glycosyl groups	F	AT1G05570
5790	2	L4 vs. M4	M4 up	GO:0016757	Metabolism	Metabolism	transferase activity, transferring glycosyl groups	F	AT1G06410
5791	2	L4 vs. M4	M4 up	GO:0016757	Metabolism	Metabolism	transferase activity, transferring glycosyl groups	F	AT1G78580
5792	2	L4 vs. M4	M4 up	GO:0016757	Metabolism	Metabolism	transferase activity, transferring glycosyl groups	F	AT2G18570
5793	2	L4 vs. M4	M4 up	GO:0016757	Metabolism	Metabolism	transferase activity, transferring glycosyl groups	F	AT2G32530
5794	2	L4 vs. M4	M4 up	GO:0016757	Metabolism	Metabolism	transferase activity, transferring glycosyl groups	F	AT2G43820
5795	2	L4 vs. M4	M4 up	GO:0016757	Metabolism	Metabolism	transferase activity, transferring glycosyl groups	F	AT3G57630
5796	2	L4 vs. M4	M4 up	GO:0016757	Metabolism	Metabolism	transferase activity, transferring glycosyl groups	F	AT4G10120
5797	2	L4 vs. M4	M4 up	GO:0016757	Metabolism	Metabolism	transferase activity, transferring glycosyl groups	F	AT5G15740
5798	2	L4 vs. M4	M4 up	GO:0016758	Metabolism	Metabolism	transferase activity, transferring hexosyl groups	F	AT2G18570
5799	2	L4 vs. M4	M4 up	GO:0019432	Metabolism	Metabolism	triglyceride biosynthetic process	P	AT1G48300
5800	2	L4 vs. M4	M4 up	GO:0019432	Metabolism	Metabolism	triglyceride biosynthetic process	P	AT1G54570
5801	2	L4 vs. M4	M4 up	GO:0000162	Metabolism	Metabolism	tryptophan biosynthetic process	P	AT2G04400
5802	2	L4 vs. M4	M4 up	GO:0000162	Metabolism	Metabolism	tryptophan biosynthetic process	P	AT5G54810
5803	2	L4 vs. M4	M4 up	GO:0004834	Metabolism	Metabolism	tryptophan synthase activity	F	AT5G54810
5804	2	L4 vs. M4	M4 up	GO:0033320	Metabolism	Metabolism	UDP-D-xylose biosynthetic process	P	AT3G53520
5805	2	L4 vs. M4	M4 up	GO:0080002	Metabolism	Metabolism	UDP-glucose:4-aminobenzoate acylglucosyltransferase activity	F	AT1G05560
5806	2	L4 vs. M4	M4 up	GO:0080002	Metabolism	Metabolism	UDP-glucose:4-aminobenzoate acylglucosyltransferase activity	F	AT2G43820
5807	2	L4 vs. M4	M4 up	GO:0035251	Metabolism	Metabolism	UDP-glucosyltransferase activity	F	AT1G05560
5808	2	L4 vs. M4	M4 up	GO:0035251	Metabolism	Metabolism	UDP-glucosyltransferase activity	F	AT2G43820
5809	2	L4 vs. M4	M4 up	GO:0035251	Metabolism	Metabolism	UDP-glucosyltransferase activity	F	AT4G34135
5810	2	L4 vs. M4	M4 up	GO:0048040	Metabolism	Metabolism	UDP-glucuronate decarboxylase activity	F	AT3G53520
5811	2	L4 vs. M4	M4 up	GO:0008194	Metabolism	Metabolism	UDP-glycosyltransferase activity	F	AT1G05560
5812	2	L4 vs. M4	M4 up	GO:0008194	Metabolism	Metabolism	UDP-glycosyltransferase activity	F	AT2G18570
5813	2	L4 vs. M4	M4 up	GO:0008194	Metabolism	Metabolism	UDP-glycosyltransferase activity	F	AT2G43820
5814	2	L4 vs. M4	M4 up	GO:0008194	Metabolism	Metabolism	UDP-glycosyltransferase activity	F	AT4G34135
5815	2	L4 vs. M4	M4 up	GO:0008194	Metabolism	Metabolism	UDP-glycosyltransferase activity	F	AT5G15740
5816	2	L4 vs. M4	M4 up	GO:0002058	Metabolism	Metabolism	uracil binding	F	AT3G17810
5817	2	L4 vs. M4	M4 up	GO:0006624	Metabolism	Metabolism	vacuolar protein processing	P	AT2G25940
5818	2	L4 vs. M4	M4 up	GO:0006624	Metabolism	Metabolism	vacuolar protein processing	P	AT4G32940
5819	2	L4 vs. M4	M4 up	GO:0010025	Metabolism	Metabolism	wax biosynthetic process	P	AT1G01120
5820	2	L4 vs. M4	M4 up	GO:0016123	Metabolism	Metabolism	xanthophyll biosynthetic process	P	AT4G25700
5821	2	L4 vs. M4	M4 up	GO:0010089	Metabolism	Metabolism	xylem development	P	AT5G13180
5822	2	L4 vs. M4	M4 up	GO:0008270	Metabolism	Metabolism	zinc ion binding	F	AT1G70410
5823	2	L4 vs. M4	M4 up	GO:0008270	Metabolism	Metabolism	zinc ion binding	F	AT2G44350
5824	2	L4 vs. M4	M4 up	GO:0008270	Metabolism	Metabolism	zinc ion binding	F	AT3G18290
5825	2	L4 vs. M4	M4 up	GO:0008270	Metabolism	Metabolism	zinc ion binding	F	AT4G13930
5826	2	L4 vs. M4	M4 up	GO:0008270	Metabolism	Metabolism	zinc ion binding	F	AT4G33090
5827	2	L4 vs. M4	M4 up	GO:0008270	Metabolism	Metabolism	zinc ion binding	F	AT5G42250
5828	2	L4 vs. M4	M4 up	GO:0008270	Metabolism	Metabolism	zinc ion binding	F	AT5G49930
5829	2	L4 vs. M4	M4 up	GO:0008270	Metabolism	Metabolism	zinc ion binding	F	AT5G60160
5830	2	L4 vs. M4	M4 up	GO:0008270	Metabolism	Metabolism	zinc ion binding	F	AT5G67480
5831	2	L4 vs. M4	M4 up	GO:0016117	Metabolism	Photosynthesis	carotenoid biosynthetic process	P	AT2G26170
5832	2	L4 vs. M4	M4 up	GO:0016168	Metabolism	Photosynthesis	chlorophyll binding	F	ATCG00340
5833	2	L4 vs. M4	M4 up	GO:0016168	Metabolism	Photosynthesis	chlorophyll binding	F	ATCG00350
5834	2	L4 vs. M4	M4 up	GO:0016168	Metabolism	Photosynthesis	chlorophyll binding	F	ATCG00680
5835	2	L4 vs. M4	M4 up	GO:0016168	Metabolism	Photosynthesis	chlorophyll binding	F	ATCG00690
5836	2	L4 vs. M4	M4 up	GO:0009507	Metabolism	Photosynthesis	chloroplast	C	AT1G01800
5837	2	L4 vs. M4	M4 up	GO:0009507	Metabolism	Photosynthesis	chloroplast	C	AT1G02810

5838	2	L4 vs. M4	M4 up	GO:0009507	Metabolism	Photosynthesis	chloroplast	C	AT1G05300
5839	2	L4 vs. M4	M4 up	GO:0009507	Metabolism	Photosynthesis	chloroplast	C	AT1G05560
5840	2	L4 vs. M4	M4 up	GO:0009507	Metabolism	Photosynthesis	chloroplast	C	AT1G07040
5841	2	L4 vs. M4	M4 up	GO:0009507	Metabolism	Photosynthesis	chloroplast	C	AT1G10970
5842	2	L4 vs. M4	M4 up	GO:0009507	Metabolism	Photosynthesis	chloroplast	C	AT1G14610
5843	2	L4 vs. M4	M4 up	GO:0009507	Metabolism	Photosynthesis	chloroplast	C	AT1G15500
5844	2	L4 vs. M4	M4 up	GO:0009507	Metabolism	Photosynthesis	chloroplast	C	AT1G18700
5845	2	L4 vs. M4	M4 up	GO:0009507	Metabolism	Photosynthesis	chloroplast	C	AT1G19170
5846	2	L4 vs. M4	M4 up	GO:0009507	Metabolism	Photosynthesis	chloroplast	C	AT1G27770
5847	2	L4 vs. M4	M4 up	GO:0009507	Metabolism	Photosynthesis	chloroplast	C	AT1G29340
5848	2	L4 vs. M4	M4 up	GO:0009507	Metabolism	Photosynthesis	chloroplast	C	AT1G34040
5849	2	L4 vs. M4	M4 up	GO:0009507	Metabolism	Photosynthesis	chloroplast	C	AT1G48200
5850	2	L4 vs. M4	M4 up	GO:0009507	Metabolism	Photosynthesis	chloroplast	C	AT1G48860
5851	2	L4 vs. M4	M4 up	GO:0009507	Metabolism	Photosynthesis	chloroplast	C	AT1G50480
5852	2	L4 vs. M4	M4 up	GO:0009507	Metabolism	Photosynthesis	chloroplast	C	AT1G54100
5853	2	L4 vs. M4	M4 up	GO:0009507	Metabolism	Photosynthesis	chloroplast	C	AT1G54570
5854	2	L4 vs. M4	M4 up	GO:0009507	Metabolism	Photosynthesis	chloroplast	C	AT1G67660
5855	2	L4 vs. M4	M4 up	GO:0009507	Metabolism	Photosynthesis	chloroplast	C	AT1G69870
5856	2	L4 vs. M4	M4 up	GO:0009507	Metabolism	Photosynthesis	chloroplast	C	AT1G70410
5857	2	L4 vs. M4	M4 up	GO:0009507	Metabolism	Photosynthesis	chloroplast	C	AT1G73650
5858	2	L4 vs. M4	M4 up	GO:0009507	Metabolism	Photosynthesis	chloroplast	C	AT1G74640
5859	2	L4 vs. M4	M4 up	GO:0009507	Metabolism	Photosynthesis	chloroplast	C	AT1G78580
5860	2	L4 vs. M4	M4 up	GO:0009507	Metabolism	Photosynthesis	chloroplast	C	AT1G80270
5861	2	L4 vs. M4	M4 up	GO:0009507	Metabolism	Photosynthesis	chloroplast	C	AT2G01320
5862	2	L4 vs. M4	M4 up	GO:0009507	Metabolism	Photosynthesis	chloroplast	C	AT2G04400
5863	2	L4 vs. M4	M4 up	GO:0009507	Metabolism	Photosynthesis	chloroplast	C	AT2G18570
5864	2	L4 vs. M4	M4 up	GO:0009507	Metabolism	Photosynthesis	chloroplast	C	AT2G20890
5865	2	L4 vs. M4	M4 up	GO:0009507	Metabolism	Photosynthesis	chloroplast	C	AT2G22360
5866	2	L4 vs. M4	M4 up	GO:0009507	Metabolism	Photosynthesis	chloroplast	C	AT2G26170
5867	2	L4 vs. M4	M4 up	GO:0009507	Metabolism	Photosynthesis	chloroplast	C	AT2G26190
5868	2	L4 vs. M4	M4 up	GO:0009507	Metabolism	Photosynthesis	chloroplast	C	AT2G26800
5869	2	L4 vs. M4	M4 up	GO:0009507	Metabolism	Photosynthesis	chloroplast	C	AT2G30020
5870	2	L4 vs. M4	M4 up	GO:0009507	Metabolism	Photosynthesis	chloroplast	C	AT2G31390
5871	2	L4 vs. M4	M4 up	GO:0009507	Metabolism	Photosynthesis	chloroplast	C	AT2G31530
5872	2	L4 vs. M4	M4 up	GO:0009507	Metabolism	Photosynthesis	chloroplast	C	AT2G35040
5873	2	L4 vs. M4	M4 up	GO:0009507	Metabolism	Photosynthesis	chloroplast	C	AT2G44350
5874	2	L4 vs. M4	M4 up	GO:0009507	Metabolism	Photosynthesis	chloroplast	C	AT2G47390
5875	2	L4 vs. M4	M4 up	GO:0009507	Metabolism	Photosynthesis	chloroplast	C	AT3G02630
5876	2	L4 vs. M4	M4 up	GO:0009507	Metabolism	Photosynthesis	chloroplast	C	AT3G02875
5877	2	L4 vs. M4	M4 up	GO:0009507	Metabolism	Photosynthesis	chloroplast	C	AT3G04120
5878	2	L4 vs. M4	M4 up	GO:0009507	Metabolism	Photosynthesis	chloroplast	C	AT3G09440
5879	2	L4 vs. M4	M4 up	GO:0009507	Metabolism	Photosynthesis	chloroplast	C	AT3G14050
5880	2	L4 vs. M4	M4 up	GO:0009507	Metabolism	Photosynthesis	chloroplast	C	AT3G15650
5881	2	L4 vs. M4	M4 up	GO:0009507	Metabolism	Photosynthesis	chloroplast	C	AT3G16470
5882	2	L4 vs. M4	M4 up	GO:0009507	Metabolism	Photosynthesis	chloroplast	C	AT3G16950
5883	2	L4 vs. M4	M4 up	GO:0009507	Metabolism	Photosynthesis	chloroplast	C	AT3G17040
5884	2	L4 vs. M4	M4 up	GO:0009507	Metabolism	Photosynthesis	chloroplast	C	AT3G17470
5885	2	L4 vs. M4	M4 up	GO:0009507	Metabolism	Photosynthesis	chloroplast	C	AT3G17810
5886	2	L4 vs. M4	M4 up	GO:0009507	Metabolism	Photosynthesis	chloroplast	C	AT3G21250
5887	2	L4 vs. M4	M4 up	GO:0009507	Metabolism	Photosynthesis	chloroplast	C	AT3G25760
5888	2	L4 vs. M4	M4 up	GO:0009507	Metabolism	Photosynthesis	chloroplast	C	AT3G28340
5889	2	L4 vs. M4	M4 up	GO:0009507	Metabolism	Photosynthesis	chloroplast	C	AT3G53580
5890	2	L4 vs. M4	M4 up	GO:0009507	Metabolism	Photosynthesis	chloroplast	C	AT3G53920
5891	2	L4 vs. M4	M4 up	GO:0009507	Metabolism	Photosynthesis	chloroplast	C	AT3G54960
5892	2	L4 vs. M4	M4 up	GO:0009507	Metabolism	Photosynthesis	chloroplast	C	AT3G55760
5893	2	L4 vs. M4	M4 up	GO:0009507	Metabolism	Photosynthesis	chloroplast	C	AT3G56190

5894	2	L4 vs. M4	M4 up	GO:0009507	Metabolism	Photosynthesis	chloroplast	C	AT3G57010
5895	2	L4 vs. M4	M4 up	GO:0009507	Metabolism	Photosynthesis	chloroplast	C	AT3G59140
5896	2	L4 vs. M4	M4 up	GO:0009507	Metabolism	Photosynthesis	chloroplast	C	AT3G62910
5897	2	L4 vs. M4	M4 up	GO:0009507	Metabolism	Photosynthesis	chloroplast	C	AT4G02790
5898	2	L4 vs. M4	M4 up	GO:0009507	Metabolism	Photosynthesis	chloroplast	C	AT4G14680
5899	2	L4 vs. M4	M4 up	GO:0009507	Metabolism	Photosynthesis	chloroplast	C	AT4G25700
5900	2	L4 vs. M4	M4 up	GO:0009507	Metabolism	Photosynthesis	chloroplast	C	AT4G36380
5901	2	L4 vs. M4	M4 up	GO:0009507	Metabolism	Photosynthesis	chloroplast	C	AT5G04140
5902	2	L4 vs. M4	M4 up	GO:0009507	Metabolism	Photosynthesis	chloroplast	C	AT5G11000
5903	2	L4 vs. M4	M4 up	GO:0009507	Metabolism	Photosynthesis	chloroplast	C	AT5G13100
5904	2	L4 vs. M4	M4 up	GO:0009507	Metabolism	Photosynthesis	chloroplast	C	AT5G13800
5905	2	L4 vs. M4	M4 up	GO:0009507	Metabolism	Photosynthesis	chloroplast	C	AT5G17380
5906	2	L4 vs. M4	M4 up	GO:0009507	Metabolism	Photosynthesis	chloroplast	C	AT5G19860
5907	2	L4 vs. M4	M4 up	GO:0009507	Metabolism	Photosynthesis	chloroplast	C	AT5G20740
5908	2	L4 vs. M4	M4 up	GO:0009507	Metabolism	Photosynthesis	chloroplast	C	AT5G26570
5909	2	L4 vs. M4	M4 up	GO:0009507	Metabolism	Photosynthesis	chloroplast	C	AT5G27380
5910	2	L4 vs. M4	M4 up	GO:0009507	Metabolism	Photosynthesis	chloroplast	C	AT5G36170
5911	2	L4 vs. M4	M4 up	GO:0009507	Metabolism	Photosynthesis	chloroplast	C	AT5G36210
5912	2	L4 vs. M4	M4 up	GO:0009507	Metabolism	Photosynthesis	chloroplast	C	AT5G42250
5913	2	L4 vs. M4	M4 up	GO:0009507	Metabolism	Photosynthesis	chloroplast	C	AT5G42680
5914	2	L4 vs. M4	M4 up	GO:0009507	Metabolism	Photosynthesis	chloroplast	C	AT5G43390
5915	2	L4 vs. M4	M4 up	GO:0009507	Metabolism	Photosynthesis	chloroplast	C	AT5G45890
5916	2	L4 vs. M4	M4 up	GO:0009507	Metabolism	Photosynthesis	chloroplast	C	AT5G50400
5917	2	L4 vs. M4	M4 up	GO:0009507	Metabolism	Photosynthesis	chloroplast	C	AT5G52540
5918	2	L4 vs. M4	M4 up	GO:0009507	Metabolism	Photosynthesis	chloroplast	C	AT5G53140
5919	2	L4 vs. M4	M4 up	GO:0009507	Metabolism	Photosynthesis	chloroplast	C	AT5G53350
5920	2	L4 vs. M4	M4 up	GO:0009507	Metabolism	Photosynthesis	chloroplast	C	AT5G53450
5921	2	L4 vs. M4	M4 up	GO:0009507	Metabolism	Photosynthesis	chloroplast	C	AT5G54810
5922	2	L4 vs. M4	M4 up	GO:0009507	Metabolism	Photosynthesis	chloroplast	C	AT5G57350
5923	2	L4 vs. M4	M4 up	GO:0009507	Metabolism	Photosynthesis	chloroplast	C	AT5G58270
5924	2	L4 vs. M4	M4 up	GO:0009507	Metabolism	Photosynthesis	chloroplast	C	AT5G60160
5925	2	L4 vs. M4	M4 up	GO:0009507	Metabolism	Photosynthesis	chloroplast	C	AT5G65760
5926	2	L4 vs. M4	M4 up	GO:0009507	Metabolism	Photosynthesis	chloroplast	C	AT5G66420
5927	2	L4 vs. M4	M4 up	GO:0009507	Metabolism	Photosynthesis	chloroplast	C	AT5G67370
5928	2	L4 vs. M4	M4 up	GO:0009507	Metabolism	Photosynthesis	chloroplast	C	ATCG00180
5929	2	L4 vs. M4	M4 up	GO:0009507	Metabolism	Photosynthesis	chloroplast	C	ATCG00270
5930	2	L4 vs. M4	M4 up	GO:0009507	Metabolism	Photosynthesis	chloroplast	C	ATCG00340
5931	2	L4 vs. M4	M4 up	GO:0009507	Metabolism	Photosynthesis	chloroplast	C	ATCG00350
5932	2	L4 vs. M4	M4 up	GO:0009507	Metabolism	Photosynthesis	chloroplast	C	ATCG00380
5933	2	L4 vs. M4	M4 up	GO:0009507	Metabolism	Photosynthesis	chloroplast	C	ATCG00560
5934	2	L4 vs. M4	M4 up	GO:0009507	Metabolism	Photosynthesis	chloroplast	C	ATCG00640
5935	2	L4 vs. M4	M4 up	GO:0009507	Metabolism	Photosynthesis	chloroplast	C	ATCG00660
5936	2	L4 vs. M4	M4 up	GO:0009507	Metabolism	Photosynthesis	chloroplast	C	ATCG00680
5937	2	L4 vs. M4	M4 up	GO:0009507	Metabolism	Photosynthesis	chloroplast	C	ATCG00690
5938	2	L4 vs. M4	M4 up	GO:0009507	Metabolism	Photosynthesis	chloroplast	C	ATCG00790
5939	2	L4 vs. M4	M4 up	GO:0009507	Metabolism	Photosynthesis	chloroplast	C	ATCG00820
5940	2	L4 vs. M4	M4 up	GO:0009507	Metabolism	Photosynthesis	chloroplast	C	ATCG01100
5941	2	L4 vs. M4	M4 up	GO:0009507	Metabolism	Photosynthesis	chloroplast	C	ATCG01110
5942	2	L4 vs. M4	M4 up	GO:0009941	Metabolism	Photosynthesis	chloroplast envelope	C	AT1G15500
5943	2	L4 vs. M4	M4 up	GO:0009941	Metabolism	Photosynthesis	chloroplast envelope	C	AT1G70410
5944	2	L4 vs. M4	M4 up	GO:0009941	Metabolism	Photosynthesis	chloroplast envelope	C	AT1G74640
5945	2	L4 vs. M4	M4 up	GO:0009941	Metabolism	Photosynthesis	chloroplast envelope	C	AT1G80270
5946	2	L4 vs. M4	M4 up	GO:0009941	Metabolism	Photosynthesis	chloroplast envelope	C	AT2G01320
5947	2	L4 vs. M4	M4 up	GO:0009941	Metabolism	Photosynthesis	chloroplast envelope	C	AT2G20890
5948	2	L4 vs. M4	M4 up	GO:0009941	Metabolism	Photosynthesis	chloroplast envelope	C	AT2G22360
5949	2	L4 vs. M4	M4 up	GO:0009941	Metabolism	Photosynthesis	chloroplast envelope	C	AT2G33210

5950	2	L4 vs. M4	M4 up	GO:0009941	Metabolism	Photosynthesis	chloroplast envelope	C	AT3G16950
5951	2	L4 vs. M4	M4 up	GO:0009941	Metabolism	Photosynthesis	chloroplast envelope	C	AT3G17040
5952	2	L4 vs. M4	M4 up	GO:0009941	Metabolism	Photosynthesis	chloroplast envelope	C	AT3G25760
5953	2	L4 vs. M4	M4 up	GO:0009941	Metabolism	Photosynthesis	chloroplast envelope	C	AT5G04140
5954	2	L4 vs. M4	M4 up	GO:0009941	Metabolism	Photosynthesis	chloroplast envelope	C	AT5G52540
5955	2	L4 vs. M4	M4 up	GO:0009941	Metabolism	Photosynthesis	chloroplast envelope	C	AT5G58270
5956	2	L4 vs. M4	M4 up	GO:0009941	Metabolism	Photosynthesis	chloroplast envelope	C	ATCG00340
5957	2	L4 vs. M4	M4 up	GO:0009941	Metabolism	Photosynthesis	chloroplast envelope	C	ATCG00350
5958	2	L4 vs. M4	M4 up	GO:0009941	Metabolism	Photosynthesis	chloroplast envelope	C	ATCG00660
5959	2	L4 vs. M4	M4 up	GO:0009941	Metabolism	Photosynthesis	chloroplast envelope	C	ATCG00790
5960	2	L4 vs. M4	M4 up	GO:0009941	Metabolism	Photosynthesis	chloroplast envelope	C	ATCG00820
5961	2	L4 vs. M4	M4 up	GO:0009706	Metabolism	Photosynthesis	chloroplast inner membrane	C	AT1G27770
5962	2	L4 vs. M4	M4 up	GO:0031969	Metabolism	Photosynthesis	chloroplast membrane	C	AT1G15500
5963	2	L4 vs. M4	M4 up	GO:0031969	Metabolism	Photosynthesis	chloroplast membrane	C	AT1G61800
5964	2	L4 vs. M4	M4 up	GO:0031969	Metabolism	Photosynthesis	chloroplast membrane	C	AT1G67300
5965	2	L4 vs. M4	M4 up	GO:0031969	Metabolism	Photosynthesis	chloroplast membrane	C	AT3G17040
5966	2	L4 vs. M4	M4 up	GO:0031969	Metabolism	Photosynthesis	chloroplast membrane	C	AT4G25700
5967	2	L4 vs. M4	M4 up	GO:0009658	Metabolism	Photosynthesis	chloroplast organization	P	AT2G31530
5968	2	L4 vs. M4	M4 up	GO:0009658	Metabolism	Photosynthesis	chloroplast organization	P	AT3G62910
5969	2	L4 vs. M4	M4 up	GO:0009658	Metabolism	Photosynthesis	chloroplast organization	P	AT5G58270
5970	2	L4 vs. M4	M4 up	GO:0009707	Metabolism	Photosynthesis	chloroplast outer membrane	C	AT2G20890
5971	2	L4 vs. M4	M4 up	GO:0043253	Metabolism	Photosynthesis	chloroplast ribosome	C	AT4G02790
5972	2	L4 vs. M4	M4 up	GO:0009570	Metabolism	Photosynthesis	chloroplast stroma	C	AT2G04400
5973	2	L4 vs. M4	M4 up	GO:0009570	Metabolism	Photosynthesis	chloroplast stroma	C	AT2G20890
5974	2	L4 vs. M4	M4 up	GO:0009570	Metabolism	Photosynthesis	chloroplast stroma	C	AT2G33210
5975	2	L4 vs. M4	M4 up	GO:0009570	Metabolism	Photosynthesis	chloroplast stroma	C	AT2G35040
5976	2	L4 vs. M4	M4 up	GO:0009570	Metabolism	Photosynthesis	chloroplast stroma	C	AT2G47390
5977	2	L4 vs. M4	M4 up	GO:0009570	Metabolism	Photosynthesis	chloroplast stroma	C	AT3G02630
5978	2	L4 vs. M4	M4 up	GO:0009570	Metabolism	Photosynthesis	chloroplast stroma	C	AT3G16950
5979	2	L4 vs. M4	M4 up	GO:0009570	Metabolism	Photosynthesis	chloroplast stroma	C	AT3G17040
5980	2	L4 vs. M4	M4 up	GO:0009570	Metabolism	Photosynthesis	chloroplast stroma	C	AT3G17810
5981	2	L4 vs. M4	M4 up	GO:0009570	Metabolism	Photosynthesis	chloroplast stroma	C	AT3G53580
5982	2	L4 vs. M4	M4 up	GO:0009570	Metabolism	Photosynthesis	chloroplast stroma	C	AT3G55760
5983	2	L4 vs. M4	M4 up	GO:0009570	Metabolism	Photosynthesis	chloroplast stroma	C	AT4G14680
5984	2	L4 vs. M4	M4 up	GO:0009570	Metabolism	Photosynthesis	chloroplast stroma	C	AT5G04140
5985	2	L4 vs. M4	M4 up	GO:0009570	Metabolism	Photosynthesis	chloroplast stroma	C	AT5G13800
5986	2	L4 vs. M4	M4 up	GO:0009570	Metabolism	Photosynthesis	chloroplast stroma	C	AT5G26570
5987	2	L4 vs. M4	M4 up	GO:0009570	Metabolism	Photosynthesis	chloroplast stroma	C	AT5G36170
5988	2	L4 vs. M4	M4 up	GO:0009570	Metabolism	Photosynthesis	chloroplast stroma	C	AT5G54810
5989	2	L4 vs. M4	M4 up	GO:0009570	Metabolism	Photosynthesis	chloroplast stroma	C	ATCG00380
5990	2	L4 vs. M4	M4 up	GO:0009570	Metabolism	Photosynthesis	chloroplast stroma	C	ATCG00660
5991	2	L4 vs. M4	M4 up	GO:0009570	Metabolism	Photosynthesis	chloroplast stroma	C	ATCG00790
5992	2	L4 vs. M4	M4 up	GO:0009570	Metabolism	Photosynthesis	chloroplast stroma	C	ATCG00820
5993	2	L4 vs. M4	M4 up	GO:0009570	Metabolism	Photosynthesis	chloroplast stroma	C	ATCG01110
5994	2	L4 vs. M4	M4 up	GO:0009534	Metabolism	Photosynthesis	chloroplast thylakoid	C	AT2G20890
5995	2	L4 vs. M4	M4 up	GO:0009534	Metabolism	Photosynthesis	chloroplast thylakoid	C	ATCG00270
5996	2	L4 vs. M4	M4 up	GO:0009534	Metabolism	Photosynthesis	chloroplast thylakoid	C	ATCG00340
5997	2	L4 vs. M4	M4 up	GO:0009534	Metabolism	Photosynthesis	chloroplast thylakoid	C	ATCG00350
5998	2	L4 vs. M4	M4 up	GO:0009534	Metabolism	Photosynthesis	chloroplast thylakoid	C	ATCG00560
5999	2	L4 vs. M4	M4 up	GO:0009534	Metabolism	Photosynthesis	chloroplast thylakoid	C	ATCG00680
6000	2	L4 vs. M4	M4 up	GO:0009534	Metabolism	Photosynthesis	chloroplast thylakoid	C	ATCG01110
6001	2	L4 vs. M4	M4 up	GO:0009535	Metabolism	Photosynthesis	chloroplast thylakoid membrane	C	AT1G10970
6002	2	L4 vs. M4	M4 up	GO:0009535	Metabolism	Photosynthesis	chloroplast thylakoid membrane	C	AT1G74640
6003	2	L4 vs. M4	M4 up	GO:0009535	Metabolism	Photosynthesis	chloroplast thylakoid membrane	C	AT2G20890
6004	2	L4 vs. M4	M4 up	GO:0009535	Metabolism	Photosynthesis	chloroplast thylakoid membrane	C	AT2G22360
6005	2	L4 vs. M4	M4 up	GO:0009535	Metabolism	Photosynthesis	chloroplast thylakoid membrane	C	AT3G25760

6006	2	L4 vs. M4	M4 up	GO:0009535	Metabolism	Photosynthesis	chloroplast thylakoid membrane	C	AT5G13800
6007	2	L4 vs. M4	M4 up	GO:0009535	Metabolism	Photosynthesis	chloroplast thylakoid membrane	C	ATCG00270
6008	2	L4 vs. M4	M4 up	GO:0009535	Metabolism	Photosynthesis	chloroplast thylakoid membrane	C	ATCG00340
6009	2	L4 vs. M4	M4 up	GO:0009535	Metabolism	Photosynthesis	chloroplast thylakoid membrane	C	ATCG00350
6010	2	L4 vs. M4	M4 up	GO:0009535	Metabolism	Photosynthesis	chloroplast thylakoid membrane	C	ATCG00560
6011	2	L4 vs. M4	M4 up	GO:0009535	Metabolism	Photosynthesis	chloroplast thylakoid membrane	C	ATCG00680
6012	2	L4 vs. M4	M4 up	GO:0009535	Metabolism	Photosynthesis	chloroplast thylakoid membrane	C	ATCG00690
6013	2	L4 vs. M4	M4 up	GO:0009535	Metabolism	Photosynthesis	chloroplast thylakoid membrane	C	ATCG01100
6014	2	L4 vs. M4	M4 up	GO:0009535	Metabolism	Photosynthesis	chloroplast thylakoid membrane	C	ATCG01110
6015	2	L4 vs. M4	M4 up	GO:0045156	Metabolism	Photosynthesis	electron transporter, transferring electrons within the cyclic electron transport pathway of photosynthesis activity	F	ATCG00270
6016	2	L4 vs. M4	M4 up	GO:0045156	Metabolism	Photosynthesis	electron transporter, transferring electrons within the cyclic electron transport pathway of photosynthesis activity	F	ATCG00680
6017	2	L4 vs. M4	M4 up	GO:0045157	Metabolism	Photosynthesis	electron transporter, transferring electrons within the noncyclic electron transport pathway of photosynthesis activity	F	ATCG00270
6018	2	L4 vs. M4	M4 up	GO:0006002	Metabolism	Photosynthesis	fructose 6-phosphate metabolic process	P	AT4G32840
6019	2	L4 vs. M4	M4 up	GO:0009854	Metabolism	Photosynthesis	oxidative photosynthetic carbon pathway	P	AT4G18360
6020	2	L4 vs. M4	M4 up	GO:0015979	Metabolism	Photosynthesis	photosynthesis	P	AT1G61800
6021	2	L4 vs. M4	M4 up	GO:0015979	Metabolism	Photosynthesis	photosynthesis	P	ATCG00350
6022	2	L4 vs. M4	M4 up	GO:0015979	Metabolism	Photosynthesis	photosynthesis	P	ATCG00690
6023	2	L4 vs. M4	M4 up	GO:0015979	Metabolism	Photosynthesis	photosynthesis	P	ATCG01100
6024	2	L4 vs. M4	M4 up	GO:0019685	Metabolism	Photosynthesis	photosynthesis, dark reaction	P	AT3G53920
6025	2	L4 vs. M4	M4 up	GO:0009768	Metabolism	Photosynthesis	photosynthesis, light harvesting in photosystem I	P	ATCG00340
6026	2	L4 vs. M4	M4 up	GO:0009769	Metabolism	Photosynthesis	photosynthesis, light harvesting in photosystem II	P	ATCG00270
6027	2	L4 vs. M4	M4 up	GO:0019684	Metabolism	Photosynthesis	photosynthesis, light reaction	P	ATCG00680
6028	2	L4 vs. M4	M4 up	GO:0009643	Metabolism	Photosynthesis	photosynthetic acclimation	P	AT1G61800
6029	2	L4 vs. M4	M4 up	GO:0009772	Metabolism	Photosynthesis	photosynthetic electron transport in photosystem II	P	ATCG00270
6030	2	L4 vs. M4	M4 up	GO:0009772	Metabolism	Photosynthesis	photosynthetic electron transport in photosystem II	P	ATCG00680
6031	2	L4 vs. M4	M4 up	GO:0009772	Metabolism	Photosynthesis	photosynthetic electron transport in photosystem II	P	ATCG01110
6032	2	L4 vs. M4	M4 up	GO:0009522	Metabolism	Photosynthesis	photosystem I	C	ATCG00340
6033	2	L4 vs. M4	M4 up	GO:0009522	Metabolism	Photosynthesis	photosystem I	C	ATCG00350
6034	2	L4 vs. M4	M4 up	GO:0009538	Metabolism	Photosynthesis	photosystem I reaction center	C	ATCG00340
6035	2	L4 vs. M4	M4 up	GO:0009538	Metabolism	Photosynthesis	photosystem I reaction center	C	ATCG00350
6036	2	L4 vs. M4	M4 up	GO:0009523	Metabolism	Photosynthesis	photosystem II	C	ATCG00270
6037	2	L4 vs. M4	M4 up	GO:0009523	Metabolism	Photosynthesis	photosystem II	C	ATCG00680
6038	2	L4 vs. M4	M4 up	GO:0009523	Metabolism	Photosynthesis	photosystem II	C	ATCG00690
6039	2	L4 vs. M4	M4 up	GO:0010207	Metabolism	Photosynthesis	photosystem II assembly	P	AT2G20890
6040	2	L4 vs. M4	M4 up	GO:0010207	Metabolism	Photosynthesis	photosystem II assembly	P	ATCG00680
6041	2	L4 vs. M4	M4 up	GO:0009539	Metabolism	Photosynthesis	photosystem II reaction center	C	ATCG00560
6042	2	L4 vs. M4	M4 up	GO:0009539	Metabolism	Photosynthesis	photosystem II reaction center	C	ATCG00680
6043	2	L4 vs. M4	M4 up	GO:0009539	Metabolism	Photosynthesis	photosystem II reaction center	C	ATCG00690
6044	2	L4 vs. M4	M4 up	GO:0009536	Metabolism	Photosynthesis	plastid	C	AT1G15500
6045	2	L4 vs. M4	M4 up	GO:0009536	Metabolism	Photosynthesis	plastid	C	AT1G51980
6046	2	L4 vs. M4	M4 up	GO:0009536	Metabolism	Photosynthesis	plastid	C	AT2G30020
6047	2	L4 vs. M4	M4 up	GO:0009536	Metabolism	Photosynthesis	plastid	C	AT2G35040
6048	2	L4 vs. M4	M4 up	GO:0009536	Metabolism	Photosynthesis	plastid	C	AT3G17810
6049	2	L4 vs. M4	M4 up	GO:0009536	Metabolism	Photosynthesis	plastid	C	AT3G62910
6050	2	L4 vs. M4	M4 up	GO:0009536	Metabolism	Photosynthesis	plastid	C	AT5G16570
6051	2	L4 vs. M4	M4 up	GO:0009536	Metabolism	Photosynthesis	plastid	C	AT5G17380
6052	2	L4 vs. M4	M4 up	GO:0009536	Metabolism	Photosynthesis	plastid	C	AT5G19860
6053	2	L4 vs. M4	M4 up	GO:0009536	Metabolism	Photosynthesis	plastid	C	AT5G26570
6054	2	L4 vs. M4	M4 up	GO:0009536	Metabolism	Photosynthesis	plastid	C	AT5G27380
6055	2	L4 vs. M4	M4 up	GO:0009536	Metabolism	Photosynthesis	plastid	C	AT5G36160

6056	2	L4 vs. M4	M4 up	GO:0009536	Metabolism	Photosynthesis	plastid	C	AT5G36210
6057	2	L4 vs. M4	M4 up	GO:0009536	Metabolism	Photosynthesis	plastid	C	AT5G37600
6058	2	L4 vs. M4	M4 up	GO:0009536	Metabolism	Photosynthesis	plastid	C	AT5G40780
6059	2	L4 vs. M4	M4 up	GO:0009536	Metabolism	Photosynthesis	plastid	C	AT5G47540
6060	2	L4 vs. M4	M4 up	GO:0009536	Metabolism	Photosynthesis	plastid	C	AT5G47930
6061	2	L4 vs. M4	M4 up	GO:0009536	Metabolism	Photosynthesis	plastid	C	AT5G49360
6062	2	L4 vs. M4	M4 up	GO:0009536	Metabolism	Photosynthesis	plastid	C	AT5G49930
6063	2	L4 vs. M4	M4 up	GO:0009536	Metabolism	Photosynthesis	plastid	C	AT5G53140
6064	2	L4 vs. M4	M4 up	GO:0009536	Metabolism	Photosynthesis	plastid	C	AT5G57350
6065	2	L4 vs. M4	M4 up	GO:0009536	Metabolism	Photosynthesis	plastid	C	AT5G58270
6066	2	L4 vs. M4	M4 up	GO:0009536	Metabolism	Photosynthesis	plastid	C	AT5G58590
6067	2	L4 vs. M4	M4 up	GO:0009536	Metabolism	Photosynthesis	plastid	C	AT5G60160
6068	2	L4 vs. M4	M4 up	GO:0009536	Metabolism	Photosynthesis	plastid	C	AT5G60360
6069	2	L4 vs. M4	M4 up	GO:0009536	Metabolism	Photosynthesis	plastid	C	AT5G64140
6070	2	L4 vs. M4	M4 up	GO:0009536	Metabolism	Photosynthesis	plastid	C	AT5G64350
6071	2	L4 vs. M4	M4 up	GO:0009536	Metabolism	Photosynthesis	plastid	C	AT5G64460
6072	2	L4 vs. M4	M4 up	GO:0009536	Metabolism	Photosynthesis	plastid	C	AT5G66760
6073	2	L4 vs. M4	M4 up	GO:0009536	Metabolism	Photosynthesis	plastid	C	ATCG00270
6074	2	L4 vs. M4	M4 up	GO:0009536	Metabolism	Photosynthesis	plastid	C	ATCG00340
6075	2	L4 vs. M4	M4 up	GO:0009536	Metabolism	Photosynthesis	plastid	C	ATCG00350
6076	2	L4 vs. M4	M4 up	GO:0009536	Metabolism	Photosynthesis	plastid	C	ATCG00380
6077	2	L4 vs. M4	M4 up	GO:0009536	Metabolism	Photosynthesis	plastid	C	ATCG00640
6078	2	L4 vs. M4	M4 up	GO:0009536	Metabolism	Photosynthesis	plastid	C	ATCG00660
6079	2	L4 vs. M4	M4 up	GO:0009536	Metabolism	Photosynthesis	plastid	C	ATCG00680
6080	2	L4 vs. M4	M4 up	GO:0009536	Metabolism	Photosynthesis	plastid	C	ATCG00790
6081	2	L4 vs. M4	M4 up	GO:0009536	Metabolism	Photosynthesis	plastid	C	ATCG00820
6082	2	L4 vs. M4	M4 up	GO:0009526	Metabolism	Photosynthesis	plastid envelope	C	AT2G31530
6083	2	L4 vs. M4	M4 up	GO:0009528	Metabolism	Photosynthesis	plastid inner membrane	C	AT2G20890
6084	2	L4 vs. M4	M4 up	GO:0000311	Metabolism	Photosynthesis	plastid large ribosomal subunit	C	ATCG00640
6085	2	L4 vs. M4	M4 up	GO:0000311	Metabolism	Photosynthesis	plastid large ribosomal subunit	C	ATCG00660
6086	2	L4 vs. M4	M4 up	GO:0000311	Metabolism	Photosynthesis	plastid large ribosomal subunit	C	ATCG00790
6087	2	L4 vs. M4	M4 up	GO:0009657	Metabolism	Photosynthesis	plastid organization	P	AT3G17040
6088	2	L4 vs. M4	M4 up	GO:0009657	Metabolism	Photosynthesis	plastid organization	P	AT5G36170
6089	2	L4 vs. M4	M4 up	GO:0009527	Metabolism	Photosynthesis	plastid outer membrane	C	AT2G20890
6090	2	L4 vs. M4	M4 up	GO:0009547	Metabolism	Photosynthesis	plastid ribosome	C	ATCG00820
6091	2	L4 vs. M4	M4 up	GO:0000312	Metabolism	Photosynthesis	plastid small ribosomal subunit	C	ATCG00380
6092	2	L4 vs. M4	M4 up	GO:0000312	Metabolism	Photosynthesis	plastid small ribosomal subunit	C	ATCG00820
6093	2	L4 vs. M4	M4 up	GO:0009532	Metabolism	Photosynthesis	plastid stroma	C	AT2G20890
6094	2	L4 vs. M4	M4 up	GO:0032544	Metabolism	Photosynthesis	plastid translation	P	AT3G62910
6095	2	L4 vs. M4	M4 up	GO:0010287	Metabolism	Photosynthesis	plastoglobule	C	AT1G54570
6096	2	L4 vs. M4	M4 up	GO:0010287	Metabolism	Photosynthesis	plastoglobule	C	ATCG00270
6097	2	L4 vs. M4	M4 up	GO:0010287	Metabolism	Photosynthesis	plastoglobule	C	ATCG00340
6098	2	L4 vs. M4	M4 up	GO:0010287	Metabolism	Photosynthesis	plastoglobule	C	ATCG00350
6099	2	L4 vs. M4	M4 up	GO:0010287	Metabolism	Photosynthesis	plastoglobule	C	ATCG00680
6100	2	L4 vs. M4	M4 up	GO:0045037	Metabolism	Photosynthesis	protein import into chloroplast stroma	P	AT2G20890
6101	2	L4 vs. M4	M4 up	GO:0045038	Metabolism	Photosynthesis	protein import into chloroplast thylakoid membrane	P	AT2G20890
6102	2	L4 vs. M4	M4 up	GO:0072598	Metabolism	Photosynthesis	protein localization to chloroplast	P	AT2G31530
6103	2	L4 vs. M4	M4 up	GO:0018298	Metabolism	Photosynthesis	protein-chromophore linkage	P	ATCG00270
6104	2	L4 vs. M4	M4 up	GO:0018298	Metabolism	Photosynthesis	protein-chromophore linkage	P	ATCG00340
6105	2	L4 vs. M4	M4 up	GO:0018298	Metabolism	Photosynthesis	protein-chromophore linkage	P	ATCG00350
6106	2	L4 vs. M4	M4 up	GO:0018298	Metabolism	Photosynthesis	protein-chromophore linkage	P	ATCG00680
6107	2	L4 vs. M4	M4 up	GO:0010380	Metabolism	Photosynthesis	regulation of chlorophyll biosynthetic process	P	AT5G58270
6108	2	L4 vs. M4	M4 up	GO:0010109	Metabolism	Photosynthesis	regulation of photosynthesis	P	AT1G61800
6109	2	L4 vs. M4	M4 up	GO:0009579	Metabolism	Photosynthesis	thylakoid	C	ATCG00270
6110	2	L4 vs. M4	M4 up	GO:0009579	Metabolism	Photosynthesis	thylakoid	C	ATCG00340
6111	2	L4 vs. M4	M4 up	GO:0009579	Metabolism	Photosynthesis	thylakoid	C	ATCG00350

6112	2	L4 vs. M4	M4 up	GO:0009579	Metabolism	Photosynthesis	thylakoid	C	ATCG00560
6113	2	L4 vs. M4	M4 up	GO:0009579	Metabolism	Photosynthesis	thylakoid	C	ATCG00680
6114	2	L4 vs. M4	M4 up	GO:0009579	Metabolism	Photosynthesis	thylakoid	C	ATCG00690
6115	2	L4 vs. M4	M4 up	GO:0010027	Metabolism	Photosynthesis	thylakoid membrane organization	P	AT2G20890
6116	2	L4 vs. M4	M4 up	GO:0010027	Metabolism	Photosynthesis	thylakoid membrane organization	P	AT2G31530
6117	2	L4 vs. M4	M4 up	GO:0010027	Metabolism	Photosynthesis	thylakoid membrane organization	P	AT3G62910
6118	2	L4 vs. M4	M4 up	GO:0046715	Metabolism	Transporters	active borate transmembrane transporter activity	F	AT1G80760
6119	2	L4 vs. M4	M4 up	GO:0043090	Metabolism	Transporters	amino acid import	P	AT1G58360
6120	2	L4 vs. M4	M4 up	GO:0043090	Metabolism	Transporters	amino acid import	P	AT5G40780
6121	2	L4 vs. M4	M4 up	GO:0003333	Metabolism	Transporters	amino acid transmembrane transport	P	AT1G58360
6122	2	L4 vs. M4	M4 up	GO:0003333	Metabolism	Transporters	amino acid transmembrane transport	P	AT2G39890
6123	2	L4 vs. M4	M4 up	GO:0003333	Metabolism	Transporters	amino acid transmembrane transport	P	AT5G40780
6124	2	L4 vs. M4	M4 up	GO:0015171	Metabolism	Transporters	amino acid transmembrane transporter activity	F	AT1G58360
6125	2	L4 vs. M4	M4 up	GO:0015171	Metabolism	Transporters	amino acid transmembrane transporter activity	F	AT2G39890
6126	2	L4 vs. M4	M4 up	GO:0015171	Metabolism	Transporters	amino acid transmembrane transporter activity	F	AT5G14940
6127	2	L4 vs. M4	M4 up	GO:0015171	Metabolism	Transporters	amino acid transmembrane transporter activity	F	AT5G40780
6128	2	L4 vs. M4	M4 up	GO:0006820	Metabolism	Transporters	anion transport	P	AT5G12080
6129	2	L4 vs. M4	M4 up	GO:0015297	Metabolism	Transporters	antiporter activity	F	AT1G61800
6130	2	L4 vs. M4	M4 up	GO:0015297	Metabolism	Transporters	antiporter activity	F	AT2G22500
6131	2	L4 vs. M4	M4 up	GO:0005471	Metabolism	Transporters	ATP:ADP antiporter activity	F	AT1G15500
6132	2	L4 vs. M4	M4 up	GO:0042626	Metabolism	Transporters	ATPase-coupled transmembrane transporter activity	F	AT1G02520
6133	2	L4 vs. M4	M4 up	GO:0042626	Metabolism	Transporters	ATPase-coupled transmembrane transporter activity	F	AT2G01320
6134	2	L4 vs. M4	M4 up	GO:0042626	Metabolism	Transporters	ATPase-coupled transmembrane transporter activity	F	AT2G07680
6135	2	L4 vs. M4	M4 up	GO:0042626	Metabolism	Transporters	ATPase-coupled transmembrane transporter activity	F	AT2G34660
6136	2	L4 vs. M4	M4 up	GO:0042626	Metabolism	Transporters	ATPase-coupled transmembrane transporter activity	F	AT3G21250
6137	2	L4 vs. M4	M4 up	GO:0042626	Metabolism	Transporters	ATPase-coupled transmembrane transporter activity	F	AT3G59140
6138	2	L4 vs. M4	M4 up	GO:0042626	Metabolism	Transporters	ATPase-coupled transmembrane transporter activity	F	AT3G62150
6139	2	L4 vs. M4	M4 up	GO:0042626	Metabolism	Transporters	ATPase-coupled transmembrane transporter activity	F	AT4G39850
6140	2	L4 vs. M4	M4 up	GO:0042626	Metabolism	Transporters	ATPase-coupled transmembrane transporter activity	F	AT5G58270
6141	2	L4 vs. M4	M4 up	GO:0008559	Metabolism	Transporters	ATPase-coupled xenobiotic transmembrane transporter activity	F	AT2G07680
6142	2	L4 vs. M4	M4 up	GO:0008559	Metabolism	Transporters	ATPase-coupled xenobiotic transmembrane transporter activity	F	AT2G34660
6143	2	L4 vs. M4	M4 up	GO:0008559	Metabolism	Transporters	ATPase-coupled xenobiotic transmembrane transporter activity	F	AT3G21250
6144	2	L4 vs. M4	M4 up	GO:0008559	Metabolism	Transporters	ATPase-coupled xenobiotic transmembrane transporter activity	F	AT3G59140
6145	2	L4 vs. M4	M4 up	GO:0046713	Metabolism	Transporters	borate transport	P	AT1G80760
6146	2	L4 vs. M4	M4 up	GO:0070509	Metabolism	Transporters	calcium ion import	P	AT5G15410
6147	2	L4 vs. M4	M4 up	GO:0070588	Metabolism	Transporters	calcium ion transmembrane transport	P	AT1G27770
6148	2	L4 vs. M4	M4 up	GO:0005388	Metabolism	Transporters	calcium transmembrane transporter activity, phosphorylative mechanism	F	AT1G27770
6149	2	L4 vs. M4	M4 up	GO:0015144	Metabolism	Transporters	carbohydrate transmembrane transporter activity	F	AT1G67300
6150	2	L4 vs. M4	M4 up	GO:0008643	Metabolism	Transporters	carbohydrate transport	P	AT1G67300
6151	2	L4 vs. M4	M4 up	GO:0005261	Metabolism	Transporters	cation channel activity	F	AT2G46440
6152	2	L4 vs. M4	M4 up	GO:0098655	Metabolism	Transporters	cation transmembrane transport	P	AT1G05300
6153	2	L4 vs. M4	M4 up	GO:0015267	Metabolism	Transporters	channel activity	F	AT1G80760

6154	2	L4 vs. M4	M4 up	GO:0006821	Metabolism	Transporters	chloride transport	P	AT5G62290
6155	2	L4 vs. M4	M4 up	GO:0005375	Metabolism	Transporters	copper ion transmembrane transporter activity	F	AT1G10970
6156	2	L4 vs. M4	M4 up	GO:0005310	Metabolism	Transporters	dicarboxylic acid transmembrane transporter activity	F	AT2G22500
6157	2	L4 vs. M4	M4 up	GO:0042938	Metabolism	Transporters	dipeptide transport	P	AT3G54140
6158	2	L4 vs. M4	M4 up	GO:0031901	Metabolism	Transporters	early endosome membrane	C	AT1G04750
6159	2	L4 vs. M4	M4 up	GO:0005768	Metabolism	Transporters	endosome	C	AT1G04750
6160	2	L4 vs. M4	M4 up	GO:0005768	Metabolism	Transporters	endosome	C	AT2G03240
6161	2	L4 vs. M4	M4 up	GO:0005768	Metabolism	Transporters	endosome	C	AT2G34300
6162	2	L4 vs. M4	M4 up	GO:0005768	Metabolism	Transporters	endosome	C	AT3G53520
6163	2	L4 vs. M4	M4 up	GO:0005768	Metabolism	Transporters	endosome	C	AT4G30996
6164	2	L4 vs. M4	M4 up	GO:0005768	Metabolism	Transporters	endosome	C	AT4G33410
6165	2	L4 vs. M4	M4 up	GO:0005768	Metabolism	Transporters	endosome	C	AT5G42050
6166	2	L4 vs. M4	M4 up	GO:0010008	Metabolism	Transporters	endosome membrane	C	AT2G06530
6167	2	L4 vs. M4	M4 up	GO:0010008	Metabolism	Transporters	endosome membrane	C	AT4G29160
6168	2	L4 vs. M4	M4 up	GO:0010008	Metabolism	Transporters	endosome membrane	C	AT4G33410
6169	2	L4 vs. M4	M4 up	GO:0032509	Metabolism	Transporters	endosome transport via multivesicular body sorting pathway	P	AT2G06530
6170	2	L4 vs. M4	M4 up	GO:0015916	Metabolism	Transporters	fatty-acyl-CoA transport	P	AT4G39850
6171	2	L4 vs. M4	M4 up	GO:0015152	Metabolism	Transporters	glucose-6-phosphate transmembrane transporter activity	F	AT1G61800
6172	2	L4 vs. M4	M4 up	GO:0015760	Metabolism	Transporters	glucose-6-phosphate transport	P	AT1G61800
6173	2	L4 vs. M4	M4 up	GO:0015168	Metabolism	Transporters	glycerol transmembrane transporter activity	F	AT1G80760
6174	2	L4 vs. M4	M4 up	GO:0048193	Metabolism	Transporters	Golgi vesicle transport	P	AT5G66030
6175	2	L4 vs. M4	M4 up	GO:0005315	Metabolism	Transporters	inorganic phosphate transmembrane transporter activity	F	AT1G61800
6176	2	L4 vs. M4	M4 up	GO:0005222	Metabolism	Transporters	intracellular cAMP-activated cation channel activity	F	AT5G15410
6177	2	L4 vs. M4	M4 up	GO:0005221	Metabolism	Transporters	intracellular cyclic nucleotide activated cation channel activity	F	AT5G15410
6178	2	L4 vs. M4	M4 up	GO:0006886	Metabolism	Transporters	intracellular protein transport	P	AT3G56190
6179	2	L4 vs. M4	M4 up	GO:0005216	Metabolism	Transporters	ion channel activity	F	AT2G46440
6180	2	L4 vs. M4	M4 up	GO:0006811	Metabolism	Transporters	ion transport	P	AT5G58270
6181	2	L4 vs. M4	M4 up	GO:0015180	Metabolism	Transporters	L-alanine transmembrane transporter activity	F	AT1G58360
6182	2	L4 vs. M4	M4 up	GO:0015808	Metabolism	Transporters	L-alanine transport	P	AT1G58360
6183	2	L4 vs. M4	M4 up	GO:0045324	Metabolism	Transporters	late endosome to vacuole transport	P	AT2G06530
6184	2	L4 vs. M4	M4 up	GO:0098712	Metabolism	Transporters	L-glutamate import across plasma membrane	P	AT1G58360
6185	2	L4 vs. M4	M4 up	GO:0005313	Metabolism	Transporters	L-glutamate transmembrane transporter activity	F	AT1G58360
6186	2	L4 vs. M4	M4 up	GO:0015186	Metabolism	Transporters	L-glutamine transmembrane transporter activity	F	AT1G58360
6187	2	L4 vs. M4	M4 up	GO:0006869	Metabolism	Transporters	lipid transport	P	AT2G37870
6188	2	L4 vs. M4	M4 up	GO:0080054	Metabolism	Transporters	low-affinity nitrate transmembrane transporter activity	F	AT1G69870
6189	2	L4 vs. M4	M4 up	GO:0015193	Metabolism	Transporters	L-proline transmembrane transporter activity	F	AT1G58360
6190	2	L4 vs. M4	M4 up	GO:0015193	Metabolism	Transporters	L-proline transmembrane transporter activity	F	AT2G39890
6191	2	L4 vs. M4	M4 up	GO:0015194	Metabolism	Transporters	L-serine transmembrane transporter activity	F	AT1G58360
6192	2	L4 vs. M4	M4 up	GO:0015095	Metabolism	Transporters	magnesium ion transmembrane transporter activity	F	AT1G34470
6193	2	L4 vs. M4	M4 up	GO:0015693	Metabolism	Transporters	magnesium ion transport	P	AT1G34470
6194	2	L4 vs. M4	M4 up	GO:0071423	Metabolism	Transporters	malate transmembrane transport	P	AT2G22500
6195	2	L4 vs. M4	M4 up	GO:0015140	Metabolism	Transporters	malate transmembrane transporter activity	F	AT2G22500
6196	2	L4 vs. M4	M4 up	GO:0046873	Metabolism	Transporters	metal ion transmembrane transporter activity	F	AT1G05300
6197	2	L4 vs. M4	M4 up	GO:0006839	Metabolism	Transporters	mitochondrial transport	P	AT2G22500
6198	2	L4 vs. M4	M4 up	GO:0015098	Metabolism	Transporters	molybdate ion transmembrane transporter activity	F	AT3G49310
6199	2	L4 vs. M4	M4 up	GO:0015689	Metabolism	Transporters	molybdate ion transport	P	AT3G49310
6200	2	L4 vs. M4	M4 up	GO:0015175	Metabolism	Transporters	neutral amino acid transmembrane transporter activity	F	AT1G58360
6201	2	L4 vs. M4	M4 up	GO:0015804	Metabolism	Transporters	neutral amino acid transport	P	AT1G58360
6202	2	L4 vs. M4	M4 up	GO:0015706	Metabolism	Transporters	nitrate transport	P	AT1G69870

6203	2	L4 vs. M4	M4 up	GO:0035673	Metabolism	Transporters	oligopeptide transmembrane transporter activity	F	AT1G69870
6204	2	L4 vs. M4	M4 up	GO:0035673	Metabolism	Transporters	oligopeptide transmembrane transporter activity	F	AT3G01350
6205	2	L4 vs. M4	M4 up	GO:0035673	Metabolism	Transporters	oligopeptide transmembrane transporter activity	F	AT3G54140
6206	2	L4 vs. M4	M4 up	GO:0035673	Metabolism	Transporters	oligopeptide transmembrane transporter activity	F	AT5G14940
6207	2	L4 vs. M4	M4 up	GO:0015131	Metabolism	Transporters	oxaloacetate transmembrane transporter activity	F	AT2G22500
6208	2	L4 vs. M4	M4 up	GO:0015729	Metabolism	Transporters	oxaloacetate transport	P	AT2G22500
6209	2	L4 vs. M4	M4 up	GO:1904680	Metabolism	Transporters	peptide transmembrane transporter activity	F	AT1G69870
6210	2	L4 vs. M4	M4 up	GO:1904680	Metabolism	Transporters	peptide transmembrane transporter activity	F	AT3G01350
6211	2	L4 vs. M4	M4 up	GO:1904680	Metabolism	Transporters	peptide transmembrane transporter activity	F	AT3G54140
6212	2	L4 vs. M4	M4 up	GO:1904680	Metabolism	Transporters	peptide transmembrane transporter activity	F	AT5G14940
6213	2	L4 vs. M4	M4 up	GO:0015333	Metabolism	Transporters	peptide:proton symporter activity	F	AT1G69870
6214	2	L4 vs. M4	M4 up	GO:0015333	Metabolism	Transporters	peptide:proton symporter activity	F	AT3G01350
6215	2	L4 vs. M4	M4 up	GO:0015333	Metabolism	Transporters	peptide:proton symporter activity	F	AT3G54140
6216	2	L4 vs. M4	M4 up	GO:0015333	Metabolism	Transporters	peptide:proton symporter activity	F	AT5G14940
6217	2	L4 vs. M4	M4 up	GO:0010233	Metabolism	Transporters	phloem transport	P	AT1G56430
6218	2	L4 vs. M4	M4 up	GO:0035435	Metabolism	Transporters	phosphate ion transmembrane transport	P	AT2G22500
6219	2	L4 vs. M4	M4 up	GO:0015114	Metabolism	Transporters	phosphate ion transmembrane transporter activity	F	AT2G03240
6220	2	L4 vs. M4	M4 up	GO:0006817	Metabolism	Transporters	phosphate ion transport	P	AT2G03240
6221	2	L4 vs. M4	M4 up	GO:0015714	Metabolism	Transporters	phosphoenolpyruvate transport	P	AT1G61800
6222	2	L4 vs. M4	M4 up	GO:0015713	Metabolism	Transporters	phosphoglycerate transmembrane transport	P	AT1G61800
6223	2	L4 vs. M4	M4 up	GO:0015120	Metabolism	Transporters	phosphoglycerate transmembrane transporter activity	F	AT1G61800
6224	2	L4 vs. M4	M4 up	GO:0009506	Metabolism	Transporters	plasmodesma	C	AT1G02520
6225	2	L4 vs. M4	M4 up	GO:0009506	Metabolism	Transporters	plasmodesma	C	AT1G04750
6226	2	L4 vs. M4	M4 up	GO:0009506	Metabolism	Transporters	plasmodesma	C	AT1G05570
6227	2	L4 vs. M4	M4 up	GO:0009506	Metabolism	Transporters	plasmodesma	C	AT1G45145
6228	2	L4 vs. M4	M4 up	GO:0009506	Metabolism	Transporters	plasmodesma	C	AT1G47128
6229	2	L4 vs. M4	M4 up	GO:0009506	Metabolism	Transporters	plasmodesma	C	AT1G56110
6230	2	L4 vs. M4	M4 up	GO:0009506	Metabolism	Transporters	plasmodesma	C	AT1G73080
6231	2	L4 vs. M4	M4 up	GO:0009506	Metabolism	Transporters	plasmodesma	C	AT1G73650
6232	2	L4 vs. M4	M4 up	GO:0009506	Metabolism	Transporters	plasmodesma	C	AT2G05830
6233	2	L4 vs. M4	M4 up	GO:0009506	Metabolism	Transporters	plasmodesma	C	AT2G23810
6234	2	L4 vs. M4	M4 up	GO:0009506	Metabolism	Transporters	plasmodesma	C	AT2G34300
6235	2	L4 vs. M4	M4 up	GO:0009506	Metabolism	Transporters	plasmodesma	C	AT2G35840
6236	2	L4 vs. M4	M4 up	GO:0009506	Metabolism	Transporters	plasmodesma	C	AT2G37870
6237	2	L4 vs. M4	M4 up	GO:0009506	Metabolism	Transporters	plasmodesma	C	AT2G39330
6238	2	L4 vs. M4	M4 up	GO:0009506	Metabolism	Transporters	plasmodesma	C	AT2G44450
6239	2	L4 vs. M4	M4 up	GO:0009506	Metabolism	Transporters	plasmodesma	C	AT3G01780
6240	2	L4 vs. M4	M4 up	GO:0009506	Metabolism	Transporters	plasmodesma	C	AT3G08590
6241	2	L4 vs. M4	M4 up	GO:0009506	Metabolism	Transporters	plasmodesma	C	AT3G09440
6242	2	L4 vs. M4	M4 up	GO:0009506	Metabolism	Transporters	plasmodesma	C	AT3G16410
6243	2	L4 vs. M4	M4 up	GO:0009506	Metabolism	Transporters	plasmodesma	C	AT3G16470
6244	2	L4 vs. M4	M4 up	GO:0009506	Metabolism	Transporters	plasmodesma	C	AT3G53420
6245	2	L4 vs. M4	M4 up	GO:0009506	Metabolism	Transporters	plasmodesma	C	AT3G54140
6246	2	L4 vs. M4	M4 up	GO:0009506	Metabolism	Transporters	plasmodesma	C	AT3G56190
6247	2	L4 vs. M4	M4 up	GO:0009506	Metabolism	Transporters	plasmodesma	C	AT4G13930
6248	2	L4 vs. M4	M4 up	GO:0009506	Metabolism	Transporters	plasmodesma	C	AT4G16370
6249	2	L4 vs. M4	M4 up	GO:0009506	Metabolism	Transporters	plasmodesma	C	AT4G35230
6250	2	L4 vs. M4	M4 up	GO:0009506	Metabolism	Transporters	plasmodesma	C	AT5G06320
6251	2	L4 vs. M4	M4 up	GO:0009506	Metabolism	Transporters	plasmodesma	C	AT5G37600
6252	2	L4 vs. M4	M4 up	GO:0009506	Metabolism	Transporters	plasmodesma	C	AT5G45280
6253	2	L4 vs. M4	M4 up	GO:0009506	Metabolism	Transporters	plasmodesma	C	AT5G57350
6254	2	L4 vs. M4	M4 up	GO:0015824	Metabolism	Transporters	proline transport	P	AT2G39890
6255	2	L4 vs. M4	M4 up	GO:0045041	Metabolism	Transporters	protein import into mitochondrial intermembrane space	P	AT2G33210
6256	2	L4 vs. M4	M4 up	GO:0006606	Metabolism	Transporters	protein import into nucleus	P	AT5G58590

6257	2	L4 vs. M4	M4 up	GO:0006627	Metabolism	Transporters	protein processing involved in protein targeting to mitochondrion	P	AT1G51980
6258	2	L4 vs. M4	M4 up	GO:0008320	Metabolism	Transporters	protein transmembrane transporter activity	F	AT2G31530
6259	2	L4 vs. M4	M4 up	GO:0015031	Metabolism	Transporters	protein transport	P	AT1G04750
6260	2	L4 vs. M4	M4 up	GO:0015031	Metabolism	Transporters	protein transport	P	AT2G06530
6261	2	L4 vs. M4	M4 up	GO:0015031	Metabolism	Transporters	protein transport	P	AT3G49470
6262	2	L4 vs. M4	M4 up	GO:0015031	Metabolism	Transporters	protein transport	P	AT3G54140
6263	2	L4 vs. M4	M4 up	GO:0015031	Metabolism	Transporters	protein transport	P	AT4G16370
6264	2	L4 vs. M4	M4 up	GO:0015031	Metabolism	Transporters	protein transport	P	AT4G29160
6265	2	L4 vs. M4	M4 up	GO:0005345	Metabolism	Transporters	purine nucleobase transmembrane transporter activity	F	AT4G18220
6266	2	L4 vs. M4	M4 up	GO:0015211	Metabolism	Transporters	purine nucleoside transmembrane transporter activity	F	AT4G18220
6267	2	L4 vs. M4	M4 up	GO:0099503	Metabolism	Transporters	secretory vesicle	C	AT1G47128
6268	2	L4 vs. M4	M4 up	GO:0099503	Metabolism	Transporters	secretory vesicle	C	AT1G62290
6269	2	L4 vs. M4	M4 up	GO:0099503	Metabolism	Transporters	secretory vesicle	C	AT1G73260
6270	2	L4 vs. M4	M4 up	GO:0099503	Metabolism	Transporters	secretory vesicle	C	AT3G10740
6271	2	L4 vs. M4	M4 up	GO:0099503	Metabolism	Transporters	secretory vesicle	C	AT3G54960
6272	2	L4 vs. M4	M4 up	GO:0099503	Metabolism	Transporters	secretory vesicle	C	AT4G01610
6273	2	L4 vs. M4	M4 up	GO:0099503	Metabolism	Transporters	secretory vesicle	C	AT4G16660
6274	2	L4 vs. M4	M4 up	GO:0099503	Metabolism	Transporters	secretory vesicle	C	AT4G19810
6275	2	L4 vs. M4	M4 up	GO:0099503	Metabolism	Transporters	secretory vesicle	C	AT5G08380
6276	2	L4 vs. M4	M4 up	GO:0099503	Metabolism	Transporters	secretory vesicle	C	AT5G19860
6277	2	L4 vs. M4	M4 up	GO:0099503	Metabolism	Transporters	secretory vesicle	C	AT5G60360
6278	2	L4 vs. M4	M4 up	GO:0099503	Metabolism	Transporters	secretory vesicle	C	AT5G65760
6279	2	L4 vs. M4	M4 up	GO:0031201	Metabolism	Transporters	SNARE complex	C	AT3G56190
6280	2	L4 vs. M4	M4 up	GO:0035494	Metabolism	Transporters	SNARE complex disassembly	P	AT3G56190
6281	2	L4 vs. M4	M4 up	GO:0006616	Metabolism	Transporters	SRP-dependent cotranslational protein targeting to membrane, translocation	P	AT2G31530
6282	2	L4 vs. M4	M4 up	GO:0071422	Metabolism	Transporters	succinate transmembrane transport	P	AT2G22500
6283	2	L4 vs. M4	M4 up	GO:0015141	Metabolism	Transporters	succinate transmembrane transporter activity	F	AT2G22500
6284	2	L4 vs. M4	M4 up	GO:0000103	Metabolism	Transporters	sulfate assimilation	P	AT4G14680
6285	2	L4 vs. M4	M4 up	GO:0015116	Metabolism	Transporters	sulfate transmembrane transporter activity	F	AT2G22500
6286	2	L4 vs. M4	M4 up	GO:0008272	Metabolism	Transporters	sulfate transport	P	AT2G22500
6287	2	L4 vs. M4	M4 up	GO:0015293	Metabolism	Transporters	symporter activity	F	AT1G58360
6288	2	L4 vs. M4	M4 up	GO:0015293	Metabolism	Transporters	symporter activity	F	AT5G40780
6289	2	L4 vs. M4	M4 up	GO:0015117	Metabolism	Transporters	thiosulfate transmembrane transporter activity	F	AT2G22500
6290	2	L4 vs. M4	M4 up	GO:0015709	Metabolism	Transporters	thiosulfate transport	P	AT2G22500
6291	2	L4 vs. M4	M4 up	GO:0005802	Metabolism	Transporters	trans-Golgi network	C	AT2G03240
6292	2	L4 vs. M4	M4 up	GO:0005802	Metabolism	Transporters	trans-Golgi network	C	AT2G32530
6293	2	L4 vs. M4	M4 up	GO:0005802	Metabolism	Transporters	trans-Golgi network	C	AT2G34300
6294	2	L4 vs. M4	M4 up	GO:0005802	Metabolism	Transporters	trans-Golgi network	C	AT3G53520
6295	2	L4 vs. M4	M4 up	GO:0005802	Metabolism	Transporters	trans-Golgi network	C	AT4G30996
6296	2	L4 vs. M4	M4 up	GO:0005802	Metabolism	Transporters	trans-Golgi network	C	AT4G33410
6297	2	L4 vs. M4	M4 up	GO:0005802	Metabolism	Transporters	trans-Golgi network	C	AT5G66030
6298	2	L4 vs. M4	M4 up	GO:0046915	Metabolism	Transporters	transition metal ion transmembrane transporter activity	F	AT4G16370
6299	2	L4 vs. M4	M4 up	GO:0055085	Metabolism	Transporters	transmembrane transport	P	AT1G02520
6300	2	L4 vs. M4	M4 up	GO:0055085	Metabolism	Transporters	transmembrane transport	P	AT1G67300
6301	2	L4 vs. M4	M4 up	GO:0055085	Metabolism	Transporters	transmembrane transport	P	AT2G01320
6302	2	L4 vs. M4	M4 up	GO:0055085	Metabolism	Transporters	transmembrane transport	P	AT2G07680
6303	2	L4 vs. M4	M4 up	GO:0055085	Metabolism	Transporters	transmembrane transport	P	AT2G34660
6304	2	L4 vs. M4	M4 up	GO:0055085	Metabolism	Transporters	transmembrane transport	P	AT3G21250
6305	2	L4 vs. M4	M4 up	GO:0055085	Metabolism	Transporters	transmembrane transport	P	AT3G53420
6306	2	L4 vs. M4	M4 up	GO:0055085	Metabolism	Transporters	transmembrane transport	P	AT3G59140
6307	2	L4 vs. M4	M4 up	GO:0055085	Metabolism	Transporters	transmembrane transport	P	AT3G62150

6308	2	L4 vs. M4	M4 up	GO:0055085	Metabolism	Transporters	transmembrane transport	P	AT4G16370
6309	2	L4 vs. M4	M4 up	GO:0055085	Metabolism	Transporters	transmembrane transport	P	AT4G39850
6310	2	L4 vs. M4	M4 up	GO:0055085	Metabolism	Transporters	transmembrane transport	P	AT5G12080
6311	2	L4 vs. M4	M4 up	GO:0055085	Metabolism	Transporters	transmembrane transport	P	AT5G58270
6312	2	L4 vs. M4	M4 up	GO:0022857	Metabolism	Transporters	transmembrane transporter activity	F	AT1G61800
6313	2	L4 vs. M4	M4 up	GO:0022857	Metabolism	Transporters	transmembrane transporter activity	F	AT1G67300
6314	2	L4 vs. M4	M4 up	GO:0005215	Metabolism	Transporters	transporter activity	F	AT5G58270
6315	2	L4 vs. M4	M4 up	GO:0071917	Metabolism	Transporters	triose-phosphate transmembrane transporter activity	F	AT1G61800
6316	2	L4 vs. M4	M4 up	GO:0042937	Metabolism	Transporters	tripeptide transmembrane transporter activity	F	AT3G54140
6317	2	L4 vs. M4	M4 up	GO:0042939	Metabolism	Transporters	tripeptide transport	P	AT3G54140
6318	2	L4 vs. M4	M4 up	GO:0015204	Metabolism	Transporters	urea transmembrane transporter activity	F	AT1G80760
6319	2	L4 vs. M4	M4 up	GO:0043182	Metabolism	Transporters	vacuolar sequestering of sodium ion	P	AT4G30996
6320	2	L4 vs. M4	M4 up	GO:0007034	Metabolism	Transporters	vacuolar transport	P	AT4G29160
6321	2	L4 vs. M4	M4 up	GO:0016192	Metabolism	Transporters	vesicle-mediated transport	P	AT1G04750
6322	2	L4 vs. M4	M4 up	GO:0016192	Metabolism	Transporters	vesicle-mediated transport	P	AT2G06530
6323	2	L4 vs. M4	M4 up	GO:0016192	Metabolism	Transporters	vesicle-mediated transport	P	AT4G29160
6324	2	L4 vs. M4	M4 up	GO:0016192	Metabolism	Transporters	vesicle-mediated transport	P	AT5G05570
6325	2	L4 vs. M4	M4 up	GO:0015250	Metabolism	Transporters	water channel activity	F	AT1G80760
6326	2	L4 vs. M4	M4 up	GO:0015250	Metabolism	Transporters	water channel activity	F	AT3G53420
6327	2	L4 vs. M4	M4 up	GO:0006833	Metabolism	Transporters	water transport	P	AT3G53420
6328	2	L4 vs. M4	M4 up	GO:1990388	Metabolism	Transporters	xylem-to-phloem iron transport	P	AT4G16370
6329	2	L4 vs. M4	M4 up	GO:0071577	Metabolism	Transporters	zinc ion transmembrane transport	P	AT1G05300
6330	2	L4 vs. M4	M4 up	GO:0071577	Metabolism	Transporters	zinc ion transmembrane transport	P	AT1G10970
6331	2	L4 vs. M4	M4 up	GO:0071577	Metabolism	Transporters	zinc ion transmembrane transport	P	AT3G12750
6332	2	L4 vs. M4	M4 up	GO:0005385	Metabolism	Transporters	zinc ion transmembrane transporter activity	F	AT1G05300
6333	2	L4 vs. M4	M4 up	GO:0005385	Metabolism	Transporters	zinc ion transmembrane transporter activity	F	AT1G10970
6334	2	L4 vs. M4	M4 up	GO:0005385	Metabolism	Transporters	zinc ion transmembrane transporter activity	F	AT3G12750
6335	2	L4 vs. M4	M4 up	GO:0006829	Metabolism	Transporters	zinc ion transport	P	AT3G12750
6336	2	L4 vs. M4	M4 up	GO:0061077	Regulation	Protein modification	chaperone-mediated protein folding	P	AT5G60160
6337	2	L4 vs. M4	M4 up	GO:0061077	Regulation	Protein modification	chaperone-mediated protein folding	P	AT5G64350
6338	2	L4 vs. M4	M4 up	GO:0006458	Regulation	Protein modification	'de novo' protein folding	P	AT2G33210
6339	2	L4 vs. M4	M4 up	GO:0005783	Regulation	Protein modification	endoplasmic reticulum	C	AT1G01120
6340	2	L4 vs. M4	M4 up	GO:0005783	Regulation	Protein modification	endoplasmic reticulum	C	AT1G18700
6341	2	L4 vs. M4	M4 up	GO:0005783	Regulation	Protein modification	endoplasmic reticulum	C	AT1G27770
6342	2	L4 vs. M4	M4 up	GO:0005783	Regulation	Protein modification	endoplasmic reticulum	C	AT1G73260
6343	2	L4 vs. M4	M4 up	GO:0005783	Regulation	Protein modification	endoplasmic reticulum	C	AT1G73650
6344	2	L4 vs. M4	M4 up	GO:0005783	Regulation	Protein modification	endoplasmic reticulum	C	AT2G32530
6345	2	L4 vs. M4	M4 up	GO:0005783	Regulation	Protein modification	endoplasmic reticulum	C	AT2G33210
6346	2	L4 vs. M4	M4 up	GO:0005783	Regulation	Protein modification	endoplasmic reticulum	C	AT2G34300
6347	2	L4 vs. M4	M4 up	GO:0005783	Regulation	Protein modification	endoplasmic reticulum	C	AT2G34660
6348	2	L4 vs. M4	M4 up	GO:0005783	Regulation	Protein modification	endoplasmic reticulum	C	AT2G35840
6349	2	L4 vs. M4	M4 up	GO:0005783	Regulation	Protein modification	endoplasmic reticulum	C	AT2G35880
6350	2	L4 vs. M4	M4 up	GO:0005783	Regulation	Protein modification	endoplasmic reticulum	C	AT3G02875
6351	2	L4 vs. M4	M4 up	GO:0005783	Regulation	Protein modification	endoplasmic reticulum	C	AT3G50660
6352	2	L4 vs. M4	M4 up	GO:0005783	Regulation	Protein modification	endoplasmic reticulum	C	AT3G54960
6353	2	L4 vs. M4	M4 up	GO:0005783	Regulation	Protein modification	endoplasmic reticulum	C	AT3G57010
6354	2	L4 vs. M4	M4 up	GO:0005783	Regulation	Protein modification	endoplasmic reticulum	C	AT4G16660
6355	2	L4 vs. M4	M4 up	GO:0005783	Regulation	Protein modification	endoplasmic reticulum	C	AT4G30996
6356	2	L4 vs. M4	M4 up	GO:0005783	Regulation	Protein modification	endoplasmic reticulum	C	AT4G33090
6357	2	L4 vs. M4	M4 up	GO:0005783	Regulation	Protein modification	endoplasmic reticulum	C	AT4G36250
6358	2	L4 vs. M4	M4 up	GO:0005783	Regulation	Protein modification	endoplasmic reticulum	C	AT5G40010
6359	2	L4 vs. M4	M4 up	GO:0005783	Regulation	Protein modification	endoplasmic reticulum	C	AT5G50260
6360	2	L4 vs. M4	M4 up	GO:0034663	Regulation	Protein modification	endoplasmic reticulum chaperone complex	C	AT4G16660
6361	2	L4 vs. M4	M4 up	GO:0005789	Regulation	Protein modification	endoplasmic reticulum membrane	C	AT1G01120
6362	2	L4 vs. M4	M4 up	GO:0005789	Regulation	Protein modification	endoplasmic reticulum membrane	C	AT1G06350

6363	2	L4 vs. M4	M4 up	GO:0005789	Regulation	Protein modification	endoplasmic reticulum membrane	C	AT1G68370
6364	2	L4 vs. M4	M4 up	GO:0005789	Regulation	Protein modification	endoplasmic reticulum membrane	C	AT4G36380
6365	2	L4 vs. M4	M4 up	GO:0005789	Regulation	Protein modification	endoplasmic reticulum membrane	C	AT5G03730
6366	2	L4 vs. M4	M4 up	GO:0005789	Regulation	Protein modification	endoplasmic reticulum membrane	C	AT5G38460
6367	2	L4 vs. M4	M4 up	GO:0030234	Regulation	Protein modification	enzyme regulator activity	F	AT3G16410
6368	2	L4 vs. M4	M4 up	GO:0005794	Regulation	Protein modification	Golgi apparatus	C	AT1G05560
6369	2	L4 vs. M4	M4 up	GO:0005794	Regulation	Protein modification	Golgi apparatus	C	AT1G35140
6370	2	L4 vs. M4	M4 up	GO:0005794	Regulation	Protein modification	Golgi apparatus	C	AT1G47128
6371	2	L4 vs. M4	M4 up	GO:0005794	Regulation	Protein modification	Golgi apparatus	C	AT1G61800
6372	2	L4 vs. M4	M4 up	GO:0005794	Regulation	Protein modification	Golgi apparatus	C	AT1G73650
6373	2	L4 vs. M4	M4 up	GO:0005794	Regulation	Protein modification	Golgi apparatus	C	AT2G03240
6374	2	L4 vs. M4	M4 up	GO:0005794	Regulation	Protein modification	Golgi apparatus	C	AT2G23810
6375	2	L4 vs. M4	M4 up	GO:0005794	Regulation	Protein modification	Golgi apparatus	C	AT2G32530
6376	2	L4 vs. M4	M4 up	GO:0005794	Regulation	Protein modification	Golgi apparatus	C	AT2G34300
6377	2	L4 vs. M4	M4 up	GO:0005794	Regulation	Protein modification	Golgi apparatus	C	AT2G44450
6378	2	L4 vs. M4	M4 up	GO:0005794	Regulation	Protein modification	Golgi apparatus	C	AT3G09440
6379	2	L4 vs. M4	M4 up	GO:0005794	Regulation	Protein modification	Golgi apparatus	C	AT3G28340
6380	2	L4 vs. M4	M4 up	GO:0005794	Regulation	Protein modification	Golgi apparatus	C	AT3G49310
6381	2	L4 vs. M4	M4 up	GO:0005794	Regulation	Protein modification	Golgi apparatus	C	AT3G53520
6382	2	L4 vs. M4	M4 up	GO:0005794	Regulation	Protein modification	Golgi apparatus	C	AT3G57630
6383	2	L4 vs. M4	M4 up	GO:0005794	Regulation	Protein modification	Golgi apparatus	C	AT4G16660
6384	2	L4 vs. M4	M4 up	GO:0005794	Regulation	Protein modification	Golgi apparatus	C	AT4G30996
6385	2	L4 vs. M4	M4 up	GO:0005794	Regulation	Protein modification	Golgi apparatus	C	AT4G33410
6386	2	L4 vs. M4	M4 up	GO:0005794	Regulation	Protein modification	Golgi apparatus	C	AT5G06320
6387	2	L4 vs. M4	M4 up	GO:0005794	Regulation	Protein modification	Golgi apparatus	C	AT5G15740
6388	2	L4 vs. M4	M4 up	GO:0000139	Regulation	Protein modification	Golgi membrane	C	AT1G68370
6389	2	L4 vs. M4	M4 up	GO:0000139	Regulation	Protein modification	Golgi membrane	C	AT2G32530
6390	2	L4 vs. M4	M4 up	GO:0000139	Regulation	Protein modification	Golgi membrane	C	AT2G34300
6391	2	L4 vs. M4	M4 up	GO:0000139	Regulation	Protein modification	Golgi membrane	C	AT3G28340
6392	2	L4 vs. M4	M4 up	GO:0000139	Regulation	Protein modification	Golgi membrane	C	AT5G15740
6393	2	L4 vs. M4	M4 up	GO:0032259	Regulation	Protein modification	methylation	P	AT1G19640
6394	2	L4 vs. M4	M4 up	GO:0032259	Regulation	Protein modification	methylation	P	AT2G27590
6395	2	L4 vs. M4	M4 up	GO:0032259	Regulation	Protein modification	methylation	P	AT2G34300
6396	2	L4 vs. M4	M4 up	GO:0032259	Regulation	Protein modification	methylation	P	AT3G05390
6397	2	L4 vs. M4	M4 up	GO:0032259	Regulation	Protein modification	methylation	P	AT4G33120
6398	2	L4 vs. M4	M4 up	GO:0032259	Regulation	Protein modification	methylation	P	AT5G01710
6399	2	L4 vs. M4	M4 up	GO:0032259	Regulation	Protein modification	methylation	P	AT5G05570
6400	2	L4 vs. M4	M4 up	GO:0006499	Regulation	Protein modification	N-terminal protein myristoylation	P	AT4G29040
6401	2	L4 vs. M4	M4 up	GO:0018119	Regulation	Protein modification	peptidyl-cysteine S-nitrosylation	P	AT3G25760
6402	2	L4 vs. M4	M4 up	GO:0003755	Regulation	Protein modification	peptidyl-prolyl cis-trans isomerase activity	F	AT5G64350
6403	2	L4 vs. M4	M4 up	GO:0046983	Regulation	Protein modification	protein dimerization activity	F	AT1G59640
6404	2	L4 vs. M4	M4 up	GO:0046983	Regulation	Protein modification	protein dimerization activity	F	AT5G04150
6405	2	L4 vs. M4	M4 up	GO:0003756	Regulation	Protein modification	protein disulfide isomerase activity	F	AT3G54960
6406	2	L4 vs. M4	M4 up	GO:0015035	Regulation	Protein modification	protein disulfide oxidoreductase activity	F	AT1G45145
6407	2	L4 vs. M4	M4 up	GO:0006457	Regulation	Protein modification	protein folding	P	AT3G09440
6408	2	L4 vs. M4	M4 up	GO:0006457	Regulation	Protein modification	protein folding	P	AT3G54960
6409	2	L4 vs. M4	M4 up	GO:0006457	Regulation	Protein modification	protein folding	P	AT5G53350
6410	2	L4 vs. M4	M4 up	GO:0018344	Regulation	Protein modification	protein geranylgeranylation	P	AT3G12070
6411	2	L4 vs. M4	M4 up	GO:0006486	Regulation	Protein modification	protein glycosylation	P	AT3G57630
6412	2	L4 vs. M4	M4 up	GO:0006487	Regulation	Protein modification	protein N-linked glycosylation	P	AT5G38460
6413	2	L4 vs. M4	M4 up	GO:0000413	Regulation	Protein modification	protein peptidyl-prolyl isomerization	P	AT5G64350
6414	2	L4 vs. M4	M4 up	GO:0018342	Regulation	Protein modification	protein prenylation	P	AT3G12070
6415	2	L4 vs. M4	M4 up	GO:0043621	Regulation	Protein modification	protein self-association	F	AT3G17040
6416	2	L4 vs. M4	M4 up	GO:0043621	Regulation	Protein modification	protein self-association	F	AT3G49530
6417	2	L4 vs. M4	M4 up	GO:0031386	Regulation	Protein modification	protein tag	F	AT4G02890
6418	2	L4 vs. M4	M4 up	GO:0070206	Regulation	Protein modification	protein trimerization	P	AT4G14680

6419	2	L4 vs. M4	M4 up	GO:0050790	Regulation	Protein modification	regulation of catalytic activity	P	AT4G01610
6420	2	L4 vs. M4	M4 up	GO:0050790	Regulation	Protein modification	regulation of catalytic activity	P	AT5G58270
6421	2	L4 vs. M4	M4 up	GO:0043086	Regulation	Regulation	negative regulation of catalytic activity	P	AT5G20740
6422	2	L4 vs. M4	M4 up	GO:1901918	Regulation	Regulation	negative regulation of exoribonuclease activity	P	AT3G17040
6423	2	L4 vs. M4	M4 up	GO:0009910	Regulation	Regulation	negative regulation of flower development	P	AT5G16260
6424	2	L4 vs. M4	M4 up	GO:2000122	Regulation	Regulation	negative regulation of stomatal complex development	P	AT1G70410
6425	2	L4 vs. M4	M4 up	GO:0080114	Regulation	Regulation	positive regulation of glycine hydroxymethyltransferase activity	P	AT5G04140
6426	2	L4 vs. M4	M4 up	GO:0031334	Regulation	Regulation	positive regulation of protein complex assembly	P	AT5G13010
6427	2	L4 vs. M4	M4 up	GO:0010030	Regulation	Regulation	positive regulation of seed germination	P	AT2G43820
6428	2	L4 vs. M4	M4 up	GO:0010030	Regulation	Regulation	positive regulation of seed germination	P	AT4G39850
6429	2	L4 vs. M4	M4 up	GO:1902458	Regulation	Regulation	positive regulation of stomatal opening	P	AT2G20890
6430	2	L4 vs. M4	M4 up	GO:0001558	Regulation	Regulation	regulation of cell growth	P	AT3G51800
6431	2	L4 vs. M4	M4 up	GO:0008360	Regulation	Regulation	regulation of cell shape	P	AT1G05570
6432	2	L4 vs. M4	M4 up	GO:0045995	Regulation	Regulation	regulation of embryonic development	P	AT2G24360
6433	2	L4 vs. M4	M4 up	GO:0017157	Regulation	Regulation	regulation of exocytosis	P	AT5G05570
6434	2	L4 vs. M4	M4 up	GO:0040008	Regulation	Regulation	regulation of growth	P	AT2G19620
6435	2	L4 vs. M4	M4 up	GO:0040008	Regulation	Regulation	regulation of growth	P	AT3G62910
6436	2	L4 vs. M4	M4 up	GO:0010075	Regulation	Regulation	regulation of meristem growth	P	AT3G49670
6437	2	L4 vs. M4	M4 up	GO:0009934	Regulation	Regulation	regulation of meristem structural organization	P	AT2G26170
6438	2	L4 vs. M4	M4 up	GO:0009934	Regulation	Regulation	regulation of meristem structural organization	P	AT3G49670
6439	2	L4 vs. M4	M4 up	GO:0040014	Regulation	Regulation	regulation of multicellular organism growth	P	AT5G58270
6440	2	L4 vs. M4	M4 up	GO:2000069	Regulation	Regulation	regulation of post-embryonic root development	P	AT5G03730
6441	2	L4 vs. M4	M4 up	GO:1902183	Regulation	Regulation	regulation of shoot apical meristem development	P	AT5G20740
6442	2	L4 vs. M4	M4 up	GO:2000038	Regulation	Regulation	regulation of stomatal complex development	P	AT3G45640
6443	2	L4 vs. M4	M4 up	GO:2000037	Regulation	Regulation	regulation of stomatal complex patterning	P	AT3G45640
6444	2	L4 vs. M4	M4 up	GO:0006306	Regulation	Transcription	DNA methylation	P	AT3G17310
6445	2	L4 vs. M4	M4 up	GO:0003700	Regulation	Transcription	DNA-binding transcription factor activity	F	AT1G22190
6446	2	L4 vs. M4	M4 up	GO:0003700	Regulation	Transcription	DNA-binding transcription factor activity	F	AT1G59640
6447	2	L4 vs. M4	M4 up	GO:0003700	Regulation	Transcription	DNA-binding transcription factor activity	F	AT1G80840
6448	2	L4 vs. M4	M4 up	GO:0003700	Regulation	Transcription	DNA-binding transcription factor activity	F	AT3G49530
6449	2	L4 vs. M4	M4 up	GO:0003700	Regulation	Transcription	DNA-binding transcription factor activity	F	AT4G27410
6450	2	L4 vs. M4	M4 up	GO:0003700	Regulation	Transcription	DNA-binding transcription factor activity	F	AT4G37260
6451	2	L4 vs. M4	M4 up	GO:0003700	Regulation	Transcription	DNA-binding transcription factor activity	F	AT5G04150
6452	2	L4 vs. M4	M4 up	GO:0003700	Regulation	Transcription	DNA-binding transcription factor activity	F	AT5G13180
6453	2	L4 vs. M4	M4 up	GO:0003700	Regulation	Transcription	DNA-binding transcription factor activity	F	AT5G67300
6454	2	L4 vs. M4	M4 up	GO:0003899	Regulation	Transcription	DNA-directed 5'-3' RNA polymerase activity	F	AT3G53920
6455	2	L4 vs. M4	M4 up	GO:0003899	Regulation	Transcription	DNA-directed 5'-3' RNA polymerase activity	F	ATCG00180
6456	2	L4 vs. M4	M4 up	GO:0006354	Regulation	Transcription	DNA-templated transcription, elongation	P	ATCG00180
6457	2	L4 vs. M4	M4 up	GO:0006352	Regulation	Transcription	DNA-templated transcription, initiation	P	AT3G53920
6458	2	L4 vs. M4	M4 up	GO:0004402	Regulation	Transcription	histone acetyltransferase activity	F	AT5G67480
6459	2	L4 vs. M4	M4 up	GO:0042393	Regulation	Transcription	histone binding	F	AT2G19480
6460	2	L4 vs. M4	M4 up	GO:1990258	Regulation	Transcription	histone glutamine methylation	P	AT4G25630
6461	2	L4 vs. M4	M4 up	GO:0035407	Regulation	Transcription	histone H3-T11 phosphorylation	P	AT1G09450
6462	2	L4 vs. M4	M4 up	GO:0072355	Regulation	Transcription	histone H3-T3 phosphorylation	P	AT1G09450
6463	2	L4 vs. M4	M4 up	GO:0035402	Regulation	Transcription	histone kinase activity (H3-T11 specific)	F	AT1G09450
6464	2	L4 vs. M4	M4 up	GO:0072354	Regulation	Transcription	histone kinase activity (H3-T3 specific)	F	AT1G09450
6465	2	L4 vs. M4	M4 up	GO:1990259	Regulation	Transcription	histone-glutamine methyltransferase activity	F	AT4G25630
6466	2	L4 vs. M4	M4 up	GO:0016592	Regulation	Transcription	mediator complex	C	AT4G25630
6467	2	L4 vs. M4	M4 up	GO:0006397	Regulation	Transcription	mRNA processing	P	AT3G12640
6468	2	L4 vs. M4	M4 up	GO:0006397	Regulation	Transcription	mRNA processing	P	AT3G17040
6469	2	L4 vs. M4	M4 up	GO:0006397	Regulation	Transcription	mRNA processing	P	AT5G61030
6470	2	L4 vs. M4	M4 up	GO:1900364	Regulation	Transcription	negative regulation of mRNA polyadenylation	P	AT3G12640
6471	2	L4 vs. M4	M4 up	GO:0045892	Regulation	Transcription	negative regulation of transcription, DNA-templated	P	AT1G80840
6472	2	L4 vs. M4	M4 up	GO:0045892	Regulation	Transcription	negative regulation of transcription, DNA-templated	P	AT4G05420

6473	2	L4 vs. M4	M4 up	GO:0045892	Regulation	Transcription	negative regulation of transcription, DNA-templated	P	AT5G13180
6474	2	L4 vs. M4	M4 up	GO:0016607	Regulation	Transcription	nuclear speck	C	AT4G36690
6475	2	L4 vs. M4	M4 up	GO:0005730	Regulation	Transcription	nucleolus	C	AT1G14320
6476	2	L4 vs. M4	M4 up	GO:0005730	Regulation	Transcription	nucleolus	C	AT1G56110
6477	2	L4 vs. M4	M4 up	GO:0005730	Regulation	Transcription	nucleolus	C	AT3G16950
6478	2	L4 vs. M4	M4 up	GO:0005730	Regulation	Transcription	nucleolus	C	AT3G51800
6479	2	L4 vs. M4	M4 up	GO:0005730	Regulation	Transcription	nucleolus	C	AT4G25630
6480	2	L4 vs. M4	M4 up	GO:0005730	Regulation	Transcription	nucleolus	C	AT5G16780
6481	2	L4 vs. M4	M4 up	GO:0000932	Regulation	Transcription	P-body	C	AT1G47128
6482	2	L4 vs. M4	M4 up	GO:0008143	Regulation	Transcription	poly(A) binding	F	AT3G12640
6483	2	L4 vs. M4	M4 up	GO:0045893	Regulation	Transcription	positive regulation of transcription, DNA-templated	P	AT1G03190
6484	2	L4 vs. M4	M4 up	GO:0045893	Regulation	Transcription	positive regulation of transcription, DNA-templated	P	AT4G27410
6485	2	L4 vs. M4	M4 up	GO:0010468	Regulation	Transcription	regulation of gene expression	P	AT3G45640
6486	2	L4 vs. M4	M4 up	GO:0060968	Regulation	Transcription	regulation of gene silencing	P	AT4G29040
6487	2	L4 vs. M4	M4 up	GO:2001141	Regulation	Transcription	regulation of RNA biosynthetic process	P	AT3G53920
6488	2	L4 vs. M4	M4 up	GO:0006357	Regulation	Transcription	regulation of transcription by RNA polymerase II	P	AT5G04150
6489	2	L4 vs. M4	M4 up	GO:0006355	Regulation	Transcription	regulation of transcription, DNA-templated	P	AT1G22190
6490	2	L4 vs. M4	M4 up	GO:0006355	Regulation	Transcription	regulation of transcription, DNA-templated	P	AT1G59640
6491	2	L4 vs. M4	M4 up	GO:0006355	Regulation	Transcription	regulation of transcription, DNA-templated	P	AT3G49530
6492	2	L4 vs. M4	M4 up	GO:0006355	Regulation	Transcription	regulation of transcription, DNA-templated	P	AT4G27410
6493	2	L4 vs. M4	M4 up	GO:0006355	Regulation	Transcription	regulation of transcription, DNA-templated	P	AT4G37260
6494	2	L4 vs. M4	M4 up	GO:0006355	Regulation	Transcription	regulation of transcription, DNA-templated	P	AT5G04150
6495	2	L4 vs. M4	M4 up	GO:0006355	Regulation	Transcription	regulation of transcription, DNA-templated	P	AT5G67300
6496	2	L4 vs. M4	M4 up	GO:0006355	Regulation	Transcription	regulation of transcription, DNA-templated	P	AT5G67480
6497	2	L4 vs. M4	M4 up	GO:0003723	Regulation	Transcription	RNA binding	F	AT2G02990
6498	2	L4 vs. M4	M4 up	GO:0003723	Regulation	Transcription	RNA binding	F	AT2G33210
6499	2	L4 vs. M4	M4 up	GO:0003723	Regulation	Transcription	RNA binding	F	AT3G49080
6500	2	L4 vs. M4	M4 up	GO:0003723	Regulation	Transcription	RNA binding	F	AT3G51800
6501	2	L4 vs. M4	M4 up	GO:0003723	Regulation	Transcription	RNA binding	F	AT4G25630
6502	2	L4 vs. M4	M4 up	GO:0003723	Regulation	Transcription	RNA binding	F	AT5G13010
6503	2	L4 vs. M4	M4 up	GO:0003723	Regulation	Transcription	RNA binding	F	AT5G61030
6504	2	L4 vs. M4	M4 up	GO:0001510	Regulation	Transcription	RNA methylation	P	AT4G25630
6505	2	L4 vs. M4	M4 up	GO:0008353	Regulation	Transcription	RNA polymerase II CTD heptapeptide repeat kinase activity	F	AT1G03190
6506	2	L4 vs. M4	M4 up	GO:0000977	Regulation	Transcription	RNA polymerase II regulatory region sequence-specific DNA binding	F	AT5G04150
6507	2	L4 vs. M4	M4 up	GO:0090575	Regulation	Transcription	RNA polymerase II transcription factor complex	C	AT5G04150
6508	2	L4 vs. M4	M4 up	GO:0043565	Regulation	Transcription	sequence-specific DNA binding	F	AT1G22190
6509	2	L4 vs. M4	M4 up	GO:0043565	Regulation	Transcription	sequence-specific DNA binding	F	AT1G59640
6510	2	L4 vs. M4	M4 up	GO:0043565	Regulation	Transcription	sequence-specific DNA binding	F	AT4G27410
6511	2	L4 vs. M4	M4 up	GO:0043565	Regulation	Transcription	sequence-specific DNA binding	F	AT4G37260
6512	2	L4 vs. M4	M4 up	GO:0043565	Regulation	Transcription	sequence-specific DNA binding	F	AT5G67300
6513	2	L4 vs. M4	M4 up	GO:0006366	Regulation	Transcription	transcription by RNA polymerase II	P	AT1G03190
6514	2	L4 vs. M4	M4 up	GO:0003712	Regulation	Transcription	transcription coregulator activity	F	AT5G67480
6515	2	L4 vs. M4	M4 up	GO:0008134	Regulation	Transcription	transcription factor binding	F	AT5G04150
6516	2	L4 vs. M4	M4 up	GO:0044212	Regulation	Transcription	transcription regulatory region DNA binding	F	AT1G22190
6517	2	L4 vs. M4	M4 up	GO:0044212	Regulation	Transcription	transcription regulatory region DNA binding	F	AT1G80840
6518	2	L4 vs. M4	M4 up	GO:0044212	Regulation	Transcription	transcription regulatory region DNA binding	F	AT3G49530
6519	2	L4 vs. M4	M4 up	GO:0044212	Regulation	Transcription	transcription regulatory region DNA binding	F	AT3G53920
6520	2	L4 vs. M4	M4 up	GO:0044212	Regulation	Transcription	transcription regulatory region DNA binding	F	AT4G27410
6521	2	L4 vs. M4	M4 up	GO:0044212	Regulation	Transcription	transcription regulatory region DNA binding	F	AT4G37260
6522	2	L4 vs. M4	M4 up	GO:0044212	Regulation	Transcription	transcription regulatory region DNA binding	F	AT5G67300
6523	2	L4 vs. M4	M4 up	GO:0003743	Regulation	Transcription	translation initiation factor activity	F	AT3G60240
6524	2	L4 vs. M4	M4 up	GO:0002161	Regulation	Translation	aminoacyl-tRNA editing activity	F	AT1G14610
6525	2	L4 vs. M4	M4 up	GO:0004812	Regulation	Translation	aminoacyl-tRNA ligase activity	F	AT5G10695
6526	2	L4 vs. M4	M4 up	GO:0000494	Regulation	Translation	box C/D snoRNA 3'-end processing	P	AT4G25630

6527	2	L4 vs. M4	M4 up	GO:0031428	Regulation	Translation	box C/D snoRNP complex	C	AT1G56110
6528	2	L4 vs. M4	M4 up	GO:0031428	Regulation	Translation	box C/D snoRNP complex	C	AT3G12860
6529	2	L4 vs. M4	M4 up	GO:0031428	Regulation	Translation	box C/D snoRNP complex	C	AT4G25630
6530	2	L4 vs. M4	M4 up	GO:0000177	Regulation	Translation	cytoplasmic exosome (RNase complex)	C	AT3G07750
6531	2	L4 vs. M4	M4 up	GO:0002181	Regulation	Translation	cytoplasmic translation	P	AT1G72660
6532	2	L4 vs. M4	M4 up	GO:0022625	Regulation	Translation	cytosolic large ribosomal subunit	C	AT1G14320
6533	2	L4 vs. M4	M4 up	GO:0022626	Regulation	Translation	cytosolic ribosome	C	AT1G01120
6534	2	L4 vs. M4	M4 up	GO:0022626	Regulation	Translation	cytosolic ribosome	C	AT1G14320
6535	2	L4 vs. M4	M4 up	GO:0022626	Regulation	Translation	cytosolic ribosome	C	AT3G09440
6536	2	L4 vs. M4	M4 up	GO:0022626	Regulation	Translation	cytosolic ribosome	C	AT5G37600
6537	2	L4 vs. M4	M4 up	GO:0022626	Regulation	Translation	cytosolic ribosome	C	AT5G47930
6538	2	L4 vs. M4	M4 up	GO:0022626	Regulation	Translation	cytosolic ribosome	C	AT5G64140
6539	2	L4 vs. M4	M4 up	GO:0022627	Regulation	Translation	cytosolic small ribosomal subunit	C	AT5G47930
6540	2	L4 vs. M4	M4 up	GO:0022627	Regulation	Translation	cytosolic small ribosomal subunit	C	AT5G64140
6541	2	L4 vs. M4	M4 up	GO:0016281	Regulation	Translation	eukaryotic translation initiation factor 4F complex	C	AT3G60240
6542	2	L4 vs. M4	M4 up	GO:0000467	Regulation	Translation	exonucleolytic trimming to generate mature 3'-end of 5.8S rRNA from tricistronic rRNA transcript (SSU-rRNA, 5.8S rRNA, LSU-rRNA)	P	AT3G07750
6543	2	L4 vs. M4	M4 up	GO:0000481	Regulation	Translation	maturation of 5S rRNA	P	AT5G16780
6544	2	L4 vs. M4	M4 up	GO:0000463	Regulation	Translation	maturation of LSU-rRNA from tricistronic rRNA transcript (SSU-rRNA, 5.8S rRNA, LSU-rRNA)	P	AT2G40360
6545	2	L4 vs. M4	M4 up	GO:0030490	Regulation	Translation	maturation of SSU-rRNA	P	AT5G64140
6546	2	L4 vs. M4	M4 up	GO:0034709	Regulation	Translation	methylosome	C	AT5G62290
6547	2	L4 vs. M4	M4 up	GO:0005762	Regulation	Translation	mitochondrial large ribosomal subunit	C	ATCG00790
6548	2	L4 vs. M4	M4 up	GO:0080156	Regulation	Translation	mitochondrial mRNA modification	P	AT5G61030
6549	2	L4 vs. M4	M4 up	GO:0005763	Regulation	Translation	mitochondrial small ribosomal subunit	C	AT3G49080
6550	2	L4 vs. M4	M4 up	GO:0005763	Regulation	Translation	mitochondrial small ribosomal subunit	C	ATCG00820
6551	2	L4 vs. M4	M4 up	GO:0032543	Regulation	Translation	mitochondrial translation	P	ATCG00790
6552	2	L4 vs. M4	M4 up	GO:0003729	Regulation	Translation	mRNA binding	F	AT1G14320
6553	2	L4 vs. M4	M4 up	GO:0003729	Regulation	Translation	mRNA binding	F	AT3G20250
6554	2	L4 vs. M4	M4 up	GO:0003729	Regulation	Translation	mRNA binding	F	AT3G53420
6555	2	L4 vs. M4	M4 up	GO:0003729	Regulation	Translation	mRNA binding	F	AT3G60240
6556	2	L4 vs. M4	M4 up	GO:0003729	Regulation	Translation	mRNA binding	F	AT5G16260
6557	2	L4 vs. M4	M4 up	GO:0003729	Regulation	Translation	mRNA binding	F	AT5G64140
6558	2	L4 vs. M4	M4 up	GO:0045292	Regulation	Translation	mRNA cis splicing, via spliceosome	P	AT5G16780
6559	2	L4 vs. M4	M4 up	GO:0000398	Regulation	Translation	mRNA splicing, via spliceosome	P	AT4G36690
6560	2	L4 vs. M4	M4 up	GO:0000398	Regulation	Translation	mRNA splicing, via spliceosome	P	AT5G13010
6561	2	L4 vs. M4	M4 up	GO:0000398	Regulation	Translation	mRNA splicing, via spliceosome	P	AT5G16260
6562	2	L4 vs. M4	M4 up	GO:0051028	Regulation	Translation	mRNA transport	P	AT5G58590
6563	2	L4 vs. M4	M4 up	GO:0005854	Regulation	Translation	nascent polypeptide-associated complex	C	AT3G49470
6564	2	L4 vs. M4	M4 up	GO:0071028	Regulation	Translation	nuclear mRNA surveillance	P	AT3G07750
6565	2	L4 vs. M4	M4 up	GO:0071042	Regulation	Translation	nuclear polyadenylation-dependent mRNA catabolic process	P	AT3G07750
6566	2	L4 vs. M4	M4 up	GO:0071035	Regulation	Translation	nuclear polyadenylation-dependent rRNA catabolic process	P	AT3G07750
6567	2	L4 vs. M4	M4 up	GO:0071038	Regulation	Translation	nuclear polyadenylation-dependent tRNA catabolic process	P	AT3G07750
6568	2	L4 vs. M4	M4 up	GO:0034427	Regulation	Translation	nuclear-transcribed mRNA catabolic process, exonucleolytic, 3'-5'	P	AT3G07750
6569	2	L4 vs. M4	M4 up	GO:0000184	Regulation	Translation	nuclear-transcribed mRNA catabolic process, nonsense-mediated decay	P	AT5G16260
6570	2	L4 vs. M4	M4 up	GO:0070545	Regulation	Translation	PeBoW complex	C	AT2G40360
6571	2	L4 vs. M4	M4 up	GO:0034715	Regulation	Translation	pICln-Sm protein complex	C	AT5G62290
6572	2	L4 vs. M4	M4 up	GO:0045903	Regulation	Translation	positive regulation of translational fidelity	P	ATCG00380
6573	2	L4 vs. M4	M4 up	GO:0030628	Regulation	Translation	pre-mRNA 3'-splice site binding	F	AT4G36690
6574	2	L4 vs. M4	M4 up	GO:0030687	Regulation	Translation	preribosome, large subunit precursor	C	AT2G40360

6575	2	L4 vs. M4	M4 up	GO:0031053	Regulation	Translation	primary miRNA processing	P	AT5G13010
6576	2	L4 vs. M4	M4 up	GO:0043488	Regulation	Translation	regulation of mRNA stability	P	AT3G12640
6577	2	L4 vs. M4	M4 up	GO:0006417	Regulation	Translation	regulation of translation	P	AT3G17040
6578	2	L4 vs. M4	M4 up	GO:0006417	Regulation	Translation	regulation of translation	P	AT3G60240
6579	2	L4 vs. M4	M4 up	GO:0072344	Regulation	Translation	rescue of stalled ribosome	P	AT5G49930
6580	2	L4 vs. M4	M4 up	GO:0004540	Regulation	Translation	ribonuclease activity	F	AT2G02990
6581	2	L4 vs. M4	M4 up	GO:0004540	Regulation	Translation	ribonuclease activity	F	AT4G25630
6582	2	L4 vs. M4	M4 up	GO:0033897	Regulation	Translation	ribonuclease T2 activity	F	AT2G02990
6583	2	L4 vs. M4	M4 up	GO:1990904	Regulation	Translation	ribonucleoprotein complex	C	AT5G61030
6584	2	L4 vs. M4	M4 up	GO:0000027	Regulation	Translation	ribosomal large subunit assembly	P	AT1G14320
6585	2	L4 vs. M4	M4 up	GO:0000027	Regulation	Translation	ribosomal large subunit assembly	P	ATCG00660
6586	2	L4 vs. M4	M4 up	GO:0043023	Regulation	Translation	ribosomal large subunit binding	F	AT5G49930
6587	2	L4 vs. M4	M4 up	GO:0000028	Regulation	Translation	ribosomal small subunit assembly	P	AT5G47930
6588	2	L4 vs. M4	M4 up	GO:0000028	Regulation	Translation	ribosomal small subunit assembly	P	AT5G64140
6589	2	L4 vs. M4	M4 up	GO:0000028	Regulation	Translation	ribosomal small subunit assembly	P	ATCG00820
6590	2	L4 vs. M4	M4 up	GO:0005840	Regulation	Translation	ribosome	C	AT1G14320
6591	2	L4 vs. M4	M4 up	GO:0005840	Regulation	Translation	ribosome	C	AT3G49080
6592	2	L4 vs. M4	M4 up	GO:0005840	Regulation	Translation	ribosome	C	ATCG00640
6593	2	L4 vs. M4	M4 up	GO:0005840	Regulation	Translation	ribosome	C	ATCG00660
6594	2	L4 vs. M4	M4 up	GO:0043022	Regulation	Translation	ribosome binding	F	AT3G62910
6595	2	L4 vs. M4	M4 up	GO:0006396	Regulation	Translation	RNA processing	P	AT3G17040
6596	2	L4 vs. M4	M4 up	GO:0006396	Regulation	Translation	RNA processing	P	AT5G36170
6597	2	L4 vs. M4	M4 up	GO:0008380	Regulation	Translation	RNA splicing	P	AT5G13010
6598	2	L4 vs. M4	M4 up	GO:0019843	Regulation	Translation	rRNA binding	F	ATCG00380
6599	2	L4 vs. M4	M4 up	GO:0019843	Regulation	Translation	rRNA binding	F	ATCG00790
6600	2	L4 vs. M4	M4 up	GO:0031167	Regulation	Translation	rRNA methylation	P	AT4G25630
6601	2	L4 vs. M4	M4 up	GO:0008649	Regulation	Translation	rRNA methyltransferase activity	F	AT4G25630
6602	2	L4 vs. M4	M4 up	GO:0006364	Regulation	Translation	rRNA processing	P	AT3G51800
6603	2	L4 vs. M4	M4 up	GO:0006364	Regulation	Translation	rRNA processing	P	AT4G25630
6604	2	L4 vs. M4	M4 up	GO:0003727	Regulation	Translation	single-stranded RNA binding	F	AT3G17040
6605	2	L4 vs. M4	M4 up	GO:0003727	Regulation	Translation	single-stranded RNA binding	F	AT3G20250
6606	2	L4 vs. M4	M4 up	GO:0015935	Regulation	Translation	small ribosomal subunit	C	ATCG00380
6607	2	L4 vs. M4	M4 up	GO:0015935	Regulation	Translation	small ribosomal subunit	C	ATCG00820
6608	2	L4 vs. M4	M4 up	GO:0032040	Regulation	Translation	small-subunit processome	C	AT1G56110
6609	2	L4 vs. M4	M4 up	GO:0032040	Regulation	Translation	small-subunit processome	C	AT3G12860
6610	2	L4 vs. M4	M4 up	GO:0032040	Regulation	Translation	small-subunit processome	C	AT4G25630
6611	2	L4 vs. M4	M4 up	GO:0030515	Regulation	Translation	snoRNA binding	F	AT1G56110
6612	2	L4 vs. M4	M4 up	GO:0030515	Regulation	Translation	snoRNA binding	F	AT3G12860
6613	2	L4 vs. M4	M4 up	GO:0030515	Regulation	Translation	snoRNA binding	F	AT4G25630
6614	2	L4 vs. M4	M4 up	GO:0000387	Regulation	Translation	spliceosomal snRNP assembly	P	AT5G62290
6615	2	L4 vs. M4	M4 up	GO:0003735	Regulation	Translation	structural constituent of ribosome	F	AT1G14320
6616	2	L4 vs. M4	M4 up	GO:0003735	Regulation	Translation	structural constituent of ribosome	F	AT3G49080
6617	2	L4 vs. M4	M4 up	GO:0003735	Regulation	Translation	structural constituent of ribosome	F	AT5G47930
6618	2	L4 vs. M4	M4 up	GO:0003735	Regulation	Translation	structural constituent of ribosome	F	AT5G64140
6619	2	L4 vs. M4	M4 up	GO:0003735	Regulation	Translation	structural constituent of ribosome	F	ATCG00380
6620	2	L4 vs. M4	M4 up	GO:0003735	Regulation	Translation	structural constituent of ribosome	F	ATCG00640
6621	2	L4 vs. M4	M4 up	GO:0003735	Regulation	Translation	structural constituent of ribosome	F	ATCG00660
6622	2	L4 vs. M4	M4 up	GO:0003735	Regulation	Translation	structural constituent of ribosome	F	ATCG00790
6623	2	L4 vs. M4	M4 up	GO:0003735	Regulation	Translation	structural constituent of ribosome	F	ATCG00820
6624	2	L4 vs. M4	M4 up	GO:0006412	Regulation	Translation	translation	P	AT1G14320
6625	2	L4 vs. M4	M4 up	GO:0006412	Regulation	Translation	translation	P	AT3G49080
6626	2	L4 vs. M4	M4 up	GO:0006412	Regulation	Translation	translation	P	AT5G47930
6627	2	L4 vs. M4	M4 up	GO:0006412	Regulation	Translation	translation	P	AT5G64140
6628	2	L4 vs. M4	M4 up	GO:0006412	Regulation	Translation	translation	P	ATCG00380
6629	2	L4 vs. M4	M4 up	GO:0006412	Regulation	Translation	translation	P	ATCG00640
6630	2	L4 vs. M4	M4 up	GO:0006412	Regulation	Translation	translation	P	ATCG00660

6631	2	L4 vs. M4	M4 up	GO:0006412	Regulation	Translation	translation	P	ATCG00790
6632	2	L4 vs. M4	M4 up	GO:0006412	Regulation	Translation	translation	P	ATCG00820
6633	2	L4 vs. M4	M4 up	GO:0003747	Regulation	Translation	translation release factor activity	F	AT3G62910
6634	2	L4 vs. M4	M4 up	GO:0016149	Regulation	Translation	translation release factor activity, codon specific	F	AT3G62910
6635	2	L4 vs. M4	M4 up	GO:0016149	Regulation	Translation	translation release factor activity, codon specific	F	AT5G36170
6636	2	L4 vs. M4	M4 up	GO:0006413	Regulation	Translation	translational initiation	P	AT3G60240
6637	2	L4 vs. M4	M4 up	GO:0006415	Regulation	Translation	translational termination	P	AT3G62910
6638	2	L4 vs. M4	M4 up	GO:0006415	Regulation	Translation	translational termination	P	AT5G36170
6639	2	L4 vs. M4	M4 up	GO:0000049	Regulation	Translation	tRNA binding	F	AT5G49930
6640	2	L4 vs. M4	M4 up	GO:0034473	Regulation	Translation	U1 snRNA 3'-end processing	P	AT3G07750
6641	2	L4 vs. M4	M4 up	GO:0005686	Regulation	Translation	U2 snRNP	C	AT5G16260
6642	2	L4 vs. M4	M4 up	GO:0089701	Regulation	Translation	U2AF	C	AT4G36690
6643	2	L4 vs. M4	M4 up	GO:0071004	Regulation	Translation	U2-type prespliceosome	C	AT4G36690
6644	2	L4 vs. M4	M4 up	GO:0005684	Regulation	Translation	U2-type spliceosomal complex	C	AT5G16260
6645	2	L4 vs. M4	M4 up	GO:0034475	Regulation	Translation	U4 snRNA 3'-end processing	P	AT3G07750
6646	2	L4 vs. M4	M4 up	GO:0046540	Regulation	Translation	U4/U6 x U5 tri-snRNP complex	C	AT5G16780
6647	2	L4 vs. M4	M4 up	GO:0034476	Regulation	Translation	U5 snRNA 3'-end processing	P	AT3G07750
6648	2	L4 vs. M4	M4 up	GO:0004832	Regulation	Translation	valine-tRNA ligase activity	F	AT1G14610
6649	2	L4 vs. M4	M4 up	GO:0006438	Regulation	Translation	valyl-tRNA aminoacylation	P	AT1G14610
6650	2	L4 vs. M4	M4 up	GO:0003993	Signaling and response	Signaling	acid phosphatase activity	F	AT5G50400
6651	2	L4 vs. M4	M4 up	GO:0010315	Signaling and response	Signaling	auxin efflux	P	AT3G62150
6652	2	L4 vs. M4	M4 up	GO:0010329	Signaling and response	Signaling	auxin efflux transmembrane transporter activity	F	AT1G02520
6653	2	L4 vs. M4	M4 up	GO:0010329	Signaling and response	Signaling	auxin efflux transmembrane transporter activity	F	AT3G62150
6654	2	L4 vs. M4	M4 up	GO:0060919	Signaling and response	Signaling	auxin influx	P	AT3G62150
6655	2	L4 vs. M4	M4 up	GO:0010328	Signaling and response	Signaling	auxin influx transmembrane transporter activity	F	AT3G62150
6656	2	L4 vs. M4	M4 up	GO:0009850	Signaling and response	Signaling	auxin metabolic process	P	AT3G02875
6657	2	L4 vs. M4	M4 up	GO:0009926	Signaling and response	Signaling	auxin polar transport	P	AT2G26170
6658	2	L4 vs. M4	M4 up	GO:0009926	Signaling and response	Signaling	auxin polar transport	P	AT4G33090
6659	2	L4 vs. M4	M4 up	GO:0009734	Signaling and response	Signaling	auxin-activated signaling pathway	P	AT1G14000
6660	2	L4 vs. M4	M4 up	GO:0009734	Signaling and response	Signaling	auxin-activated signaling pathway	P	AT3G51800
6661	2	L4 vs. M4	M4 up	GO:0010540	Signaling and response	Signaling	basipetal auxin transport	P	AT1G02520
6662	2	L4 vs. M4	M4 up	GO:0010540	Signaling and response	Signaling	basipetal auxin transport	P	AT3G62150
6663	2	L4 vs. M4	M4 up	GO:0016132	Signaling and response	Signaling	brassinosteroid biosynthetic process	P	AT3G50660
6664	2	L4 vs. M4	M4 up	GO:0016132	Signaling and response	Signaling	brassinosteroid biosynthetic process	P	AT4G36380
6665	2	L4 vs. M4	M4 up	GO:0009742	Signaling and response	Signaling	brassinosteroid mediated signaling pathway	P	AT1G14000
6666	2	L4 vs. M4	M4 up	GO:0009742	Signaling and response	Signaling	brassinosteroid mediated signaling pathway	P	AT4G35230
6667	2	L4 vs. M4	M4 up	GO:0005509	Signaling and response	Signaling	calcium ion binding	F	AT1G76130
6668	2	L4 vs. M4	M4 up	GO:0005509	Signaling and response	Signaling	calcium ion binding	F	AT2G17290
6669	2	L4 vs. M4	M4 up	GO:0005509	Signaling and response	Signaling	calcium ion binding	F	AT2G46600
6670	2	L4 vs. M4	M4 up	GO:0005509	Signaling and response	Signaling	calcium ion binding	F	AT3G17470
6671	2	L4 vs. M4	M4 up	GO:0005509	Signaling and response	Signaling	calcium ion binding	F	AT4G25000
6672	2	L4 vs. M4	M4 up	GO:0005509	Signaling and response	Signaling	calcium ion binding	F	AT5G28900
6673	2	L4 vs. M4	M4 up	GO:0009931	Signaling and response	Signaling	calcium-dependent protein serine/threonine kinase activity	F	AT2G17290
6674	2	L4 vs. M4	M4 up	GO:0009931	Signaling and response	Signaling	calcium-dependent protein serine/threonine kinase activity	F	AT3G50530
6675	2	L4 vs. M4	M4 up	GO:0005516	Signaling and response	Signaling	calmodulin binding	F	AT1G27770
6676	2	L4 vs. M4	M4 up	GO:0005516	Signaling and response	Signaling	calmodulin binding	F	AT2G17290
6677	2	L4 vs. M4	M4 up	GO:0005516	Signaling and response	Signaling	calmodulin binding	F	AT2G26190
6678	2	L4 vs. M4	M4 up	GO:0005516	Signaling and response	Signaling	calmodulin binding	F	AT2G34660
6679	2	L4 vs. M4	M4 up	GO:0005516	Signaling and response	Signaling	calmodulin binding	F	AT2G46440
6680	2	L4 vs. M4	M4 up	GO:0005516	Signaling and response	Signaling	calmodulin binding	F	AT3G50530
6681	2	L4 vs. M4	M4 up	GO:0005516	Signaling and response	Signaling	calmodulin binding	F	AT5G15410
6682	2	L4 vs. M4	M4 up	GO:0005516	Signaling and response	Signaling	calmodulin binding	F	AT5G67480
6683	2	L4 vs. M4	M4 up	GO:0004683	Signaling and response	Signaling	calmodulin-dependent protein kinase activity	F	AT2G17290
6684	2	L4 vs. M4	M4 up	GO:0004683	Signaling and response	Signaling	calmodulin-dependent protein kinase activity	F	AT3G50530

6685	2	L4 vs. M4	M4 up	GO:0080029	Signaling and response	Signaling	cellular response to boron-containing substance levels	P	AT1G80760
6686	2	L4 vs. M4	M4 up	GO:0070417	Signaling and response	Signaling	cellular response to cold	P	AT3G49530
6687	2	L4 vs. M4	M4 up	GO:0010106	Signaling and response	Signaling	cellular response to iron ion starvation	P	AT3G18290
6688	2	L4 vs. M4	M4 up	GO:0010106	Signaling and response	Signaling	cellular response to iron ion starvation	P	AT5G04150
6689	2	L4 vs. M4	M4 up	GO:0071482	Signaling and response	Signaling	cellular response to light stimulus	P	AT3G53920
6690	2	L4 vs. M4	M4 up	GO:0043562	Signaling and response	Signaling	cellular response to nitrogen levels	P	AT3G17810
6691	2	L4 vs. M4	M4 up	GO:1904482	Signaling and response	Signaling	cellular response to tetrahydrofolate	P	AT4G13930
6692	2	L4 vs. M4	M4 up	GO:0007623	Signaling and response	Signaling	circadian rhythm	P	AT3G14620
6693	2	L4 vs. M4	M4 up	GO:0007623	Signaling and response	Signaling	circadian rhythm	P	AT4G13930
6694	2	L4 vs. M4	M4 up	GO:0050897	Signaling and response	Signaling	cobalt ion binding	F	AT4G13930
6695	2	L4 vs. M4	M4 up	GO:0050897	Signaling and response	Signaling	cobalt ion binding	F	AT5G66760
6696	2	L4 vs. M4	M4 up	GO:0016311	Signaling and response	Signaling	dephosphorylation	P	AT5G50400
6697	2	L4 vs. M4	M4 up	GO:0050982	Signaling and response	Signaling	detection of mechanical stimulus	P	AT5G12080
6698	2	L4 vs. M4	M4 up	GO:0007186	Signaling and response	Signaling	G protein-coupled receptor signaling pathway	P	AT2G20890
6699	2	L4 vs. M4	M4 up	GO:0009740	Signaling and response	Signaling	gibberellic acid mediated signaling pathway	P	AT2G14900
6700	2	L4 vs. M4	M4 up	GO:0009686	Signaling and response	Signaling	gibberellin biosynthetic process	P	AT5G03730
6701	2	L4 vs. M4	M4 up	GO:0008728	Signaling and response	Signaling	GTP diphosphokinase activity	F	AT3G14050
6702	2	L4 vs. M4	M4 up	GO:0008728	Signaling and response	Signaling	GTP diphosphokinase activity	F	AT3G17470
6703	2	L4 vs. M4	M4 up	GO:0010274	Signaling and response	Signaling	hydrotropism	P	AT5G42680
6704	2	L4 vs. M4	M4 up	GO:0010179	Signaling and response	Signaling	IAA-Ala conjugate hydrolase activity	F	AT3G02875
6705	2	L4 vs. M4	M4 up	GO:0010211	Signaling and response	Signaling	IAA-Leu conjugate hydrolase activity	F	AT3G02875
6706	2	L4 vs. M4	M4 up	GO:0010210	Signaling and response	Signaling	IAA-Phe conjugate hydrolase activity	F	AT3G02875
6707	2	L4 vs. M4	M4 up	GO:0047215	Signaling and response	Signaling	indole-3-acetate beta-glucosyltransferase activity	F	AT1G05560
6708	2	L4 vs. M4	M4 up	GO:0009684	Signaling and response	Signaling	indoleacetic acid biosynthetic process	P	AT5G54810
6709	2	L4 vs. M4	M4 up	GO:0035556	Signaling and response	Signaling	intracellular signal transduction	P	AT1G09450
6710	2	L4 vs. M4	M4 up	GO:0035556	Signaling and response	Signaling	intracellular signal transduction	P	AT2G17290
6711	2	L4 vs. M4	M4 up	GO:0035556	Signaling and response	Signaling	intracellular signal transduction	P	AT3G45640
6712	2	L4 vs. M4	M4 up	GO:0035556	Signaling and response	Signaling	intracellular signal transduction	P	AT3G50530
6713	2	L4 vs. M4	M4 up	GO:0035556	Signaling and response	Signaling	intracellular signal transduction	P	AT4G24400
6714	2	L4 vs. M4	M4 up	GO:0035556	Signaling and response	Signaling	intracellular signal transduction	P	AT5G08590
6715	2	L4 vs. M4	M4 up	GO:0030795	Signaling and response	Signaling	jasmonate O-methyltransferase activity	F	AT1G19640
6716	2	L4 vs. M4	M4 up	GO:0009695	Signaling and response	Signaling	jasmonic acid biosynthetic process	P	AT1G19640
6717	2	L4 vs. M4	M4 up	GO:0009695	Signaling and response	Signaling	jasmonic acid biosynthetic process	P	AT2G06050
6718	2	L4 vs. M4	M4 up	GO:0009695	Signaling and response	Signaling	jasmonic acid biosynthetic process	P	AT3G25760
6719	2	L4 vs. M4	M4 up	GO:0120091	Signaling and response	Signaling	jasmonic acid hydrolase	F	AT5G05600
6720	2	L4 vs. M4	M4 up	GO:0009867	Signaling and response	Signaling	jasmonic acid mediated signaling pathway	P	AT1G19640
6721	2	L4 vs. M4	M4 up	GO:0009867	Signaling and response	Signaling	jasmonic acid mediated signaling pathway	P	AT3G50660
6722	2	L4 vs. M4	M4 up	GO:0009694	Signaling and response	Signaling	jasmonic acid metabolic process	P	AT1G19640
6723	2	L4 vs. M4	M4 up	GO:0016301	Signaling and response	Signaling	kinase activity	F	AT1G73080
6724	2	L4 vs. M4	M4 up	GO:0016301	Signaling and response	Signaling	kinase activity	F	AT2G48010
6725	2	L4 vs. M4	M4 up	GO:0016301	Signaling and response	Signaling	kinase activity	F	AT3G14050
6726	2	L4 vs. M4	M4 up	GO:0016301	Signaling and response	Signaling	kinase activity	F	AT3G17470
6727	2	L4 vs. M4	M4 up	GO:0016301	Signaling and response	Signaling	kinase activity	F	AT5G08590
6728	2	L4 vs. M4	M4 up	GO:0004724	Signaling and response	Signaling	magnesium-dependent protein serine/threonine phosphatase activity	F	AT2G30020
6729	2	L4 vs. M4	M4 up	GO:0004724	Signaling and response	Signaling	magnesium-dependent protein serine/threonine phosphatase activity	F	AT5G53140
6730	2	L4 vs. M4	M4 up	GO:0004707	Signaling and response	Signaling	MAP kinase activity	F	AT3G45640
6731	2	L4 vs. M4	M4 up	GO:0031930	Signaling and response	Signaling	mitochondria-nucleus signaling pathway	P	AT3G22370
6732	2	L4 vs. M4	M4 up	GO:0010100	Signaling and response	Signaling	negative regulation of photomorphogenesis	P	AT4G05420
6733	2	L4 vs. M4	M4 up	GO:0001653	Signaling and response	Signaling	peptide receptor activity	F	AT1G73080
6734	2	L4 vs. M4	M4 up	GO:0018105	Signaling and response	Signaling	peptidyl-serine phosphorylation	P	AT2G17290
6735	2	L4 vs. M4	M4 up	GO:0018105	Signaling and response	Signaling	peptidyl-serine phosphorylation	P	AT3G50530
6736	2	L4 vs. M4	M4 up	GO:0009958	Signaling and response	Signaling	positive gravitropism	P	AT1G68370

6737	2	L4 vs. M4	M4 up	GO:0010929	Signaling and response	Signaling	positive regulation of auxin mediated signaling pathway	P	AT4G37260
6738	2	L4 vs. M4	M4 up	GO:0010929	Signaling and response	Signaling	positive regulation of auxin mediated signaling pathway	P	AT5G67300
6739	2	L4 vs. M4	M4 up	GO:0046777	Signaling and response	Signaling	protein autophosphorylation	P	AT2G17290
6740	2	L4 vs. M4	M4 up	GO:0046777	Signaling and response	Signaling	protein autophosphorylation	P	AT3G50530
6741	2	L4 vs. M4	M4 up	GO:0046777	Signaling and response	Signaling	protein autophosphorylation	P	AT4G24400
6742	2	L4 vs. M4	M4 up	GO:0046777	Signaling and response	Signaling	protein autophosphorylation	P	AT5G03730
6743	2	L4 vs. M4	M4 up	GO:0046777	Signaling and response	Signaling	protein autophosphorylation	P	AT5G26570
6744	2	L4 vs. M4	M4 up	GO:0006470	Signaling and response	Signaling	protein dephosphorylation	P	AT2G30020
6745	2	L4 vs. M4	M4 up	GO:0006470	Signaling and response	Signaling	protein dephosphorylation	P	AT5G28900
6746	2	L4 vs. M4	M4 up	GO:0006470	Signaling and response	Signaling	protein dephosphorylation	P	AT5G43380
6747	2	L4 vs. M4	M4 up	GO:0006470	Signaling and response	Signaling	protein dephosphorylation	P	AT5G53140
6748	2	L4 vs. M4	M4 up	GO:0004672	Signaling and response	Signaling	protein kinase activity	F	AT2G30360
6749	2	L4 vs. M4	M4 up	GO:0004672	Signaling and response	Signaling	protein kinase activity	F	AT3G45640
6750	2	L4 vs. M4	M4 up	GO:0004672	Signaling and response	Signaling	protein kinase activity	F	AT4G24400
6751	2	L4 vs. M4	M4 up	GO:0004672	Signaling and response	Signaling	protein kinase activity	F	AT4G35230
6752	2	L4 vs. M4	M4 up	GO:0004672	Signaling and response	Signaling	protein kinase activity	F	AT5G08590
6753	2	L4 vs. M4	M4 up	GO:0019888	Signaling and response	Signaling	protein phosphatase regulator activity	F	AT5G28900
6754	2	L4 vs. M4	M4 up	GO:0000159	Signaling and response	Signaling	protein phosphatase type 2A complex	C	AT5G28900
6755	2	L4 vs. M4	M4 up	GO:0006468	Signaling and response	Signaling	protein phosphorylation	P	AT1G14000
6756	2	L4 vs. M4	M4 up	GO:0006468	Signaling and response	Signaling	protein phosphorylation	P	AT1G73080
6757	2	L4 vs. M4	M4 up	GO:0006468	Signaling and response	Signaling	protein phosphorylation	P	AT2G17290
6758	2	L4 vs. M4	M4 up	GO:0006468	Signaling and response	Signaling	protein phosphorylation	P	AT2G24360
6759	2	L4 vs. M4	M4 up	GO:0006468	Signaling and response	Signaling	protein phosphorylation	P	AT2G30360
6760	2	L4 vs. M4	M4 up	GO:0006468	Signaling and response	Signaling	protein phosphorylation	P	AT2G47060
6761	2	L4 vs. M4	M4 up	GO:0006468	Signaling and response	Signaling	protein phosphorylation	P	AT2G48010
6762	2	L4 vs. M4	M4 up	GO:0006468	Signaling and response	Signaling	protein phosphorylation	P	AT3G45640
6763	2	L4 vs. M4	M4 up	GO:0006468	Signaling and response	Signaling	protein phosphorylation	P	AT3G49670
6764	2	L4 vs. M4	M4 up	GO:0006468	Signaling and response	Signaling	protein phosphorylation	P	AT4G24400
6765	2	L4 vs. M4	M4 up	GO:0006468	Signaling and response	Signaling	protein phosphorylation	P	AT4G35230
6766	2	L4 vs. M4	M4 up	GO:0006468	Signaling and response	Signaling	protein phosphorylation	P	AT5G08590
6767	2	L4 vs. M4	M4 up	GO:0006468	Signaling and response	Signaling	protein phosphorylation	P	AT5G53450
6768	2	L4 vs. M4	M4 up	GO:0004674	Signaling and response	Signaling	protein serine/threonine kinase activity	F	AT1G09450
6769	2	L4 vs. M4	M4 up	GO:0004674	Signaling and response	Signaling	protein serine/threonine kinase activity	F	AT1G14000
6770	2	L4 vs. M4	M4 up	GO:0004674	Signaling and response	Signaling	protein serine/threonine kinase activity	F	AT1G73080
6771	2	L4 vs. M4	M4 up	GO:0004674	Signaling and response	Signaling	protein serine/threonine kinase activity	F	AT2G17290
6772	2	L4 vs. M4	M4 up	GO:0004674	Signaling and response	Signaling	protein serine/threonine kinase activity	F	AT2G24360
6773	2	L4 vs. M4	M4 up	GO:0004674	Signaling and response	Signaling	protein serine/threonine kinase activity	F	AT2G30360
6774	2	L4 vs. M4	M4 up	GO:0004674	Signaling and response	Signaling	protein serine/threonine kinase activity	F	AT3G49670
6775	2	L4 vs. M4	M4 up	GO:0004674	Signaling and response	Signaling	protein serine/threonine kinase activity	F	AT4G24400
6776	2	L4 vs. M4	M4 up	GO:0004674	Signaling and response	Signaling	protein serine/threonine kinase activity	F	AT4G35230
6777	2	L4 vs. M4	M4 up	GO:0004674	Signaling and response	Signaling	protein serine/threonine kinase activity	F	AT5G03730
6778	2	L4 vs. M4	M4 up	GO:0004674	Signaling and response	Signaling	protein serine/threonine kinase activity	F	AT5G08590
6779	2	L4 vs. M4	M4 up	GO:0004674	Signaling and response	Signaling	protein serine/threonine kinase activity	F	AT5G47540
6780	2	L4 vs. M4	M4 up	GO:0004722	Signaling and response	Signaling	protein serine/threonine phosphatase activity	F	AT2G30020
6781	2	L4 vs. M4	M4 up	GO:0004722	Signaling and response	Signaling	protein serine/threonine phosphatase activity	F	AT5G43380
6782	2	L4 vs. M4	M4 up	GO:0004722	Signaling and response	Signaling	protein serine/threonine phosphatase activity	F	AT5G53140
6783	2	L4 vs. M4	M4 up	GO:0004712	Signaling and response	Signaling	protein serine/threonine/tyrosine kinase activity	F	AT1G14000
6784	2	L4 vs. M4	M4 up	GO:0004712	Signaling and response	Signaling	protein serine/threonine/tyrosine kinase activity	F	AT2G24360
6785	2	L4 vs. M4	M4 up	GO:0004712	Signaling and response	Signaling	protein serine/threonine/tyrosine kinase activity	F	AT5G03730
6786	2	L4 vs. M4	M4 up	GO:0004713	Signaling and response	Signaling	protein tyrosine kinase activity	F	AT2G24360
6787	2	L4 vs. M4	M4 up	GO:0004663	Signaling and response	Signaling	Rab geranylgeranyltransferase activity	F	AT3G12070
6788	2	L4 vs. M4	M4 up	GO:0017137	Signaling and response	Signaling	Rab GTPase binding	F	AT5G05570
6789	2	L4 vs. M4	M4 up	GO:0005968	Signaling and response	Signaling	Rab-protein geranylgeranyltransferase complex	C	AT3G12070
6790	2	L4 vs. M4	M4 up	GO:0008536	Signaling and response	Signaling	Ran GTPase binding	F	AT5G58590

6791	2	L4 vs. M4	M4 up	GO:0033612	Signaling and response	Signaling	receptor serine/threonine kinase binding	F	AT3G49670
6792	2	L4 vs. M4	M4 up	GO:0009585	Signaling and response	Signaling	red, far-red light phototransduction	P	AT4G05420
6793	2	L4 vs. M4	M4 up	GO:2000012	Signaling and response	Signaling	regulation of auxin polar transport	P	AT2G19620
6794	2	L4 vs. M4	M4 up	GO:2000022	Signaling and response	Signaling	regulation of jasmonic acid mediated signaling pathway	P	AT5G05600
6795	2	L4 vs. M4	M4 up	GO:2000022	Signaling and response	Signaling	regulation of jasmonic acid mediated signaling pathway	P	AT5G67300
6796	2	L4 vs. M4	M4 up	GO:2000031	Signaling and response	Signaling	regulation of salicylic acid mediated signaling pathway	P	AT5G67300
6797	2	L4 vs. M4	M4 up	GO:0048510	Signaling and response	Signaling	regulation of timing of transition from vegetative to reproductive phase	P	AT5G03730
6798	2	L4 vs. M4	M4 up	GO:0009733	Signaling and response	Signaling	response to auxin	P	AT5G45890
6799	2	L4 vs. M4	M4 up	GO:0009733	Signaling and response	Signaling	response to auxin	P	AT5G67300
6800	2	L4 vs. M4	M4 up	GO:0009733	Signaling and response	Signaling	response to auxin	P	AT5G67480
6801	2	L4 vs. M4	M4 up	GO:0009617	Signaling and response	Signaling	response to bacterium	P	AT2G46440
6802	2	L4 vs. M4	M4 up	GO:0009617	Signaling and response	Signaling	response to bacterium	P	AT3G45640
6803	2	L4 vs. M4	M4 up	GO:0009617	Signaling and response	Signaling	response to bacterium	P	AT5G06320
6804	2	L4 vs. M4	M4 up	GO:0009741	Signaling and response	Signaling	response to brassinosteroid	P	AT3G50660
6805	2	L4 vs. M4	M4 up	GO:0046686	Signaling and response	Signaling	response to cadmium ion	P	AT1G45145
6806	2	L4 vs. M4	M4 up	GO:0046686	Signaling and response	Signaling	response to cadmium ion	P	AT1G50480
6807	2	L4 vs. M4	M4 up	GO:0046686	Signaling and response	Signaling	response to cadmium ion	P	AT1G62740
6808	2	L4 vs. M4	M4 up	GO:0046686	Signaling and response	Signaling	response to cadmium ion	P	AT2G19480
6809	2	L4 vs. M4	M4 up	GO:0046686	Signaling and response	Signaling	response to cadmium ion	P	AT2G35840
6810	2	L4 vs. M4	M4 up	GO:0046686	Signaling and response	Signaling	response to cadmium ion	P	AT2G44350
6811	2	L4 vs. M4	M4 up	GO:0046686	Signaling and response	Signaling	response to cadmium ion	P	AT3G04120
6812	2	L4 vs. M4	M4 up	GO:0046686	Signaling and response	Signaling	response to cadmium ion	P	AT3G08590
6813	2	L4 vs. M4	M4 up	GO:0046686	Signaling and response	Signaling	response to cadmium ion	P	AT3G09440
6814	2	L4 vs. M4	M4 up	GO:0046686	Signaling and response	Signaling	response to cadmium ion	P	AT4G13930
6815	2	L4 vs. M4	M4 up	GO:0046686	Signaling and response	Signaling	response to cadmium ion	P	AT4G29040
6816	2	L4 vs. M4	M4 up	GO:0046686	Signaling and response	Signaling	response to cadmium ion	P	AT4G37260
6817	2	L4 vs. M4	M4 up	GO:0046686	Signaling and response	Signaling	response to cadmium ion	P	AT5G58270
6818	2	L4 vs. M4	M4 up	GO:0046686	Signaling and response	Signaling	response to cadmium ion	P	AT5G60160
6819	2	L4 vs. M4	M4 up	GO:0046686	Signaling and response	Signaling	response to cadmium ion	P	AT5G67300
6820	2	L4 vs. M4	M4 up	GO:0010037	Signaling and response	Signaling	response to carbon dioxide	P	AT1G70410
6821	2	L4 vs. M4	M4 up	GO:0010200	Signaling and response	Signaling	response to chitin	P	AT1G80840
6822	2	L4 vs. M4	M4 up	GO:0010200	Signaling and response	Signaling	response to chitin	P	AT3G45640
6823	2	L4 vs. M4	M4 up	GO:0010200	Signaling and response	Signaling	response to chitin	P	AT3G49530
6824	2	L4 vs. M4	M4 up	GO:0010200	Signaling and response	Signaling	response to chitin	P	AT4G37260
6825	2	L4 vs. M4	M4 up	GO:0010200	Signaling and response	Signaling	response to chitin	P	AT5G37490
6826	2	L4 vs. M4	M4 up	GO:0010200	Signaling and response	Signaling	response to chitin	P	AT5G67300
6827	2	L4 vs. M4	M4 up	GO:0009735	Signaling and response	Signaling	response to cytokinin	P	AT5G45890
6828	2	L4 vs. M4	M4 up	GO:0009750	Signaling and response	Signaling	response to fructose	P	AT5G03730
6829	2	L4 vs. M4	M4 up	GO:0009750	Signaling and response	Signaling	response to fructose	P	AT5G45890
6830	2	L4 vs. M4	M4 up	GO:0009739	Signaling and response	Signaling	response to gibberellin	P	AT4G25000
6831	2	L4 vs. M4	M4 up	GO:0009739	Signaling and response	Signaling	response to gibberellin	P	AT5G67300
6832	2	L4 vs. M4	M4 up	GO:0009739	Signaling and response	Signaling	response to gibberellin	P	AT5G67480
6833	2	L4 vs. M4	M4 up	GO:0009749	Signaling and response	Signaling	response to glucose	P	AT1G61800
6834	2	L4 vs. M4	M4 up	GO:0009749	Signaling and response	Signaling	response to glucose	P	AT4G24400
6835	2	L4 vs. M4	M4 up	GO:0009749	Signaling and response	Signaling	response to glucose	P	AT5G45890
6836	2	L4 vs. M4	M4 up	GO:0009753	Signaling and response	Signaling	response to jasmonic acid	P	AT1G73080
6837	2	L4 vs. M4	M4 up	GO:0009753	Signaling and response	Signaling	response to jasmonic acid	P	AT3G16470
6838	2	L4 vs. M4	M4 up	GO:0009753	Signaling and response	Signaling	response to jasmonic acid	P	AT3G50660
6839	2	L4 vs. M4	M4 up	GO:0009753	Signaling and response	Signaling	response to jasmonic acid	P	AT4G19810
6840	2	L4 vs. M4	M4 up	GO:0009753	Signaling and response	Signaling	response to jasmonic acid	P	AT4G32940
6841	2	L4 vs. M4	M4 up	GO:0009753	Signaling and response	Signaling	response to jasmonic acid	P	AT4G37260
6842	2	L4 vs. M4	M4 up	GO:0009753	Signaling and response	Signaling	response to jasmonic acid	P	AT5G27380

6843	2	L4 vs. M4	M4 up	GO:0009753	Signaling and response	Signaling	response to jasmonic acid	P	AT5G67300
6844	2	L4 vs. M4	M4 up	GO:0009753	Signaling and response	Signaling	response to jasmonic acid	P	AT5G67480
6845	2	L4 vs. M4	M4 up	GO:0080167	Signaling and response	Signaling	response to karrikin	P	AT1G61800
6846	2	L4 vs. M4	M4 up	GO:0080167	Signaling and response	Signaling	response to karrikin	P	AT2G37040
6847	2	L4 vs. M4	M4 up	GO:0080167	Signaling and response	Signaling	response to karrikin	P	AT3G09440
6848	2	L4 vs. M4	M4 up	GO:0080167	Signaling and response	Signaling	response to karrikin	P	AT4G24110
6849	2	L4 vs. M4	M4 up	GO:0080167	Signaling and response	Signaling	response to karrikin	P	AT4G33150
6850	2	L4 vs. M4	M4 up	GO:0080167	Signaling and response	Signaling	response to karrikin	P	AT5G05600
6851	2	L4 vs. M4	M4 up	GO:0080167	Signaling and response	Signaling	response to karrikin	P	AT5G40780
6852	2	L4 vs. M4	M4 up	GO:1902065	Signaling and response	Signaling	response to L-glutamate	P	AT3G45640
6853	2	L4 vs. M4	M4 up	GO:0009416	Signaling and response	Signaling	response to light stimulus	P	AT1G01120
6854	2	L4 vs. M4	M4 up	GO:0009416	Signaling and response	Signaling	response to light stimulus	P	AT5G04140
6855	2	L4 vs. M4	M4 up	GO:0010167	Signaling and response	Signaling	response to nitrate	P	AT4G24400
6856	2	L4 vs. M4	M4 up	GO:0010114	Signaling and response	Signaling	response to red light	P	AT3G17040
6857	2	L4 vs. M4	M4 up	GO:0009751	Signaling and response	Signaling	response to salicylic acid	P	AT1G05560
6858	2	L4 vs. M4	M4 up	GO:0009751	Signaling and response	Signaling	response to salicylic acid	P	AT1G73260
6859	2	L4 vs. M4	M4 up	GO:0009751	Signaling and response	Signaling	response to salicylic acid	P	AT1G80840
6860	2	L4 vs. M4	M4 up	GO:0009751	Signaling and response	Signaling	response to salicylic acid	P	AT2G40000
6861	2	L4 vs. M4	M4 up	GO:0009751	Signaling and response	Signaling	response to salicylic acid	P	AT4G32940
6862	2	L4 vs. M4	M4 up	GO:0009751	Signaling and response	Signaling	response to salicylic acid	P	AT4G37260
6863	2	L4 vs. M4	M4 up	GO:0009751	Signaling and response	Signaling	response to salicylic acid	P	AT5G06320
6864	2	L4 vs. M4	M4 up	GO:0009751	Signaling and response	Signaling	response to salicylic acid	P	AT5G67300
6865	2	L4 vs. M4	M4 up	GO:0009751	Signaling and response	Signaling	response to salicylic acid	P	AT5G67480
6866	2	L4 vs. M4	M4 up	GO:0009744	Signaling and response	Signaling	response to sucrose	P	AT1G61800
6867	2	L4 vs. M4	M4 up	GO:0009744	Signaling and response	Signaling	response to sucrose	P	AT3G04120
6868	2	L4 vs. M4	M4 up	GO:0009744	Signaling and response	Signaling	response to sucrose	P	AT5G03730
6869	2	L4 vs. M4	M4 up	GO:0009744	Signaling and response	Signaling	response to sucrose	P	AT5G04140
6870	2	L4 vs. M4	M4 up	GO:0009744	Signaling and response	Signaling	response to sucrose	P	AT5G45890
6871	2	L4 vs. M4	M4 up	GO:0009411	Signaling and response	Signaling	response to UV	P	AT1G03190
6872	2	L4 vs. M4	M4 up	GO:0010043	Signaling and response	Signaling	response to zinc ion	P	AT3G12750
6873	2	L4 vs. M4	M4 up	GO:0052639	Signaling and response	Signaling	salicylic acid glucosyltransferase (ester-forming) activity	F	AT2G43820
6874	2	L4 vs. M4	M4 up	GO:0052640	Signaling and response	Signaling	salicylic acid glucosyltransferase (glucoside-forming) activity	F	AT2G43820
6875	2	L4 vs. M4	M4 up	GO:0009696	Signaling and response	Signaling	salicylic acid metabolic process	P	AT2G43820
6876	2	L4 vs. M4	M4 up	GO:0006465	Signaling and response	Signaling	signal peptide processing	P	AT4G33410
6877	2	L4 vs. M4	M4 up	GO:0005048	Signaling and response	Signaling	signal sequence binding	F	AT2G31530
6878	2	L4 vs. M4	M4 up	GO:0007165	Signaling and response	Signaling	signal transduction	P	AT2G30360
6879	2	L4 vs. M4	M4 up	GO:0007165	Signaling and response	Signaling	signal transduction	P	AT3G45640
6880	2	L4 vs. M4	M4 up	GO:0023014	Signaling and response	Signaling	signal transduction by protein phosphorylation	P	AT5G47540
6881	2	L4 vs. M4	M4 up	GO:0010012	Signaling and response	Signaling	steroid 22-alpha hydroxylase activity	F	AT3G50660
6882	2	L4 vs. M4	M4 up	GO:0008395	Signaling and response	Signaling	steroid hydroxylase activity	F	AT4G36380
6883	2	L4 vs. M4	M4 up	GO:0016125	Signaling and response	Signaling	sterol metabolic process	P	AT3G50660
6884	2	L4 vs. M4	M4 up	GO:0016125	Signaling and response	Signaling	sterol metabolic process	P	AT4G36380
6885	2	L4 vs. M4	M4 up	GO:0010118	Signaling and response	Signaling	stomatal movement	P	AT3G08590
6886	2	L4 vs. M4	M4 up	GO:0010182	Signaling and response	Signaling	sugar mediated signaling pathway	P	AT1G78580
6887	2	L4 vs. M4	M4 up	GO:0010182	Signaling and response	Signaling	sugar mediated signaling pathway	P	AT2G20890
6888	2	L4 vs. M4	M4 up	GO:0010182	Signaling and response	Signaling	sugar mediated signaling pathway	P	AT5G03730
6889	2	L4 vs. M4	M4 up	GO:1902418	Signaling and response	Stress	(+)-abscisic acid D-glucopyranosyl ester transmembrane transport	P	AT2G34660
6890	2	L4 vs. M4	M4 up	GO:1902417	Signaling and response	Stress	(+)-abscisic acid D-glucopyranosyl ester transmembrane transporter activity	F	AT2G34660
6891	2	L4 vs. M4	M4 up	GO:0006075	Signaling and response	Stress	(1->3)-beta-D-glucan biosynthetic process	P	AT1G05570
6892	2	L4 vs. M4	M4 up	GO:0003843	Signaling and response	Stress	1,3-beta-D-glucan synthase activity	F	AT1G05570
6893	2	L4 vs. M4	M4 up	GO:0000148	Signaling and response	Stress	1,3-beta-D-glucan synthase complex	C	AT1G05570
6894	2	L4 vs. M4	M4 up	GO:0010294	Signaling and response	Stress	abscisic acid glucosyltransferase activity	F	AT1G05560

6895	2	L4 vs. M4	M4 up	GO:0009738	Signaling and response	Stress	abscisic acid-activated signaling pathway	P	AT2G17290
6896	2	L4 vs. M4	M4 up	GO:0009738	Signaling and response	Stress	abscisic acid-activated signaling pathway	P	AT2G30020
6897	2	L4 vs. M4	M4 up	GO:0009738	Signaling and response	Stress	abscisic acid-activated signaling pathway	P	AT3G45640
6898	2	L4 vs. M4	M4 up	GO:0007568	Signaling and response	Stress	aging	P	AT2G02990
6899	2	L4 vs. M4	M4 up	GO:0007568	Signaling and response	Stress	aging	P	AT5G45890
6900	2	L4 vs. M4	M4 up	GO:0007568	Signaling and response	Stress	aging	P	AT5G60360
6901	2	L4 vs. M4	M4 up	GO:0009916	Signaling and response	Stress	alternative oxidase activity	F	AT3G22370
6902	2	L4 vs. M4	M4 up	GO:0010230	Signaling and response	Stress	alternative respiration	P	AT3G22370
6903	2	L4 vs. M4	M4 up	GO:0010268	Signaling and response	Stress	brassinosteroid homeostasis	P	AT3G50660
6904	2	L4 vs. M4	M4 up	GO:0010268	Signaling and response	Stress	brassinosteroid homeostasis	P	AT4G36380
6905	2	L4 vs. M4	M4 up	GO:0005262	Signaling and response	Stress	calcium channel activity	F	AT1G27770
6906	2	L4 vs. M4	M4 up	GO:0005262	Signaling and response	Stress	calcium channel activity	F	AT5G15410
6907	2	L4 vs. M4	M4 up	GO:0045454	Signaling and response	Stress	cell redox homeostasis	P	AT1G45145
6908	2	L4 vs. M4	M4 up	GO:0045454	Signaling and response	Stress	cell redox homeostasis	P	AT3G16950
6909	2	L4 vs. M4	M4 up	GO:0045454	Signaling and response	Stress	cell redox homeostasis	P	AT3G54960
6910	2	L4 vs. M4	M4 up	GO:0045454	Signaling and response	Stress	cell redox homeostasis	P	AT3G58620
6911	2	L4 vs. M4	M4 up	GO:0006884	Signaling and response	Stress	cell volume homeostasis	P	AT5G62290
6912	2	L4 vs. M4	M4 up	GO:0006879	Signaling and response	Stress	cellular iron ion homeostasis	P	AT5G58270
6913	2	L4 vs. M4	M4 up	GO:0006875	Signaling and response	Stress	cellular metal ion homeostasis	P	AT4G16370
6914	2	L4 vs. M4	M4 up	GO:0034605	Signaling and response	Stress	cellular response to heat	P	AT3G09440
6915	2	L4 vs. M4	M4 up	GO:0034605	Signaling and response	Stress	cellular response to heat	P	AT5G27660
6916	2	L4 vs. M4	M4 up	GO:0071456	Signaling and response	Stress	cellular response to hypoxia	P	AT4G16660
6917	2	L4 vs. M4	M4 up	GO:0016036	Signaling and response	Stress	cellular response to phosphate starvation	P	AT2G02990
6918	2	L4 vs. M4	M4 up	GO:0016036	Signaling and response	Stress	cellular response to phosphate starvation	P	AT2G03240
6919	2	L4 vs. M4	M4 up	GO:0009970	Signaling and response	Stress	cellular response to sulfate starvation	P	AT4G14680
6920	2	L4 vs. M4	M4 up	GO:0097237	Signaling and response	Stress	cellular response to toxic substance	P	AT5G05600
6921	2	L4 vs. M4	M4 up	GO:0034620	Signaling and response	Stress	cellular response to unfolded protein	P	AT3G09440
6922	2	L4 vs. M4	M4 up	GO:0071493	Signaling and response	Stress	cellular response to UV-B	P	AT1G14320
6923	2	L4 vs. M4	M4 up	GO:0051085	Signaling and response	Stress	chaperone cofactor-dependent protein refolding	P	AT2G22360
6924	2	L4 vs. M4	M4 up	GO:0051085	Signaling and response	Stress	chaperone cofactor-dependent protein refolding	P	AT3G09440
6925	2	L4 vs. M4	M4 up	GO:0009631	Signaling and response	Stress	cold acclimation	P	AT1G18740
6926	2	L4 vs. M4	M4 up	GO:0004869	Signaling and response	Stress	cysteine-type endopeptidase inhibitor activity	F	AT1G73260
6927	2	L4 vs. M4	M4 up	GO:0010494	Signaling and response	Stress	cytoplasmic stress granule	C	AT1G47128
6928	2	L4 vs. M4	M4 up	GO:0003684	Signaling and response	Stress	damaged DNA binding	F	AT1G03190
6929	2	L4 vs. M4	M4 up	GO:0003684	Signaling and response	Stress	damaged DNA binding	F	AT4G05420
6930	2	L4 vs. M4	M4 up	GO:0006952	Signaling and response	Stress	defense response	P	AT2G37040
6931	2	L4 vs. M4	M4 up	GO:0006952	Signaling and response	Stress	defense response	P	AT2G46440
6932	2	L4 vs. M4	M4 up	GO:0006952	Signaling and response	Stress	defense response	P	AT4G01610
6933	2	L4 vs. M4	M4 up	GO:0006952	Signaling and response	Stress	defense response	P	AT5G15410
6934	2	L4 vs. M4	M4 up	GO:0010204	Signaling and response	Stress	defense response signaling pathway, resistance gene-independent	P	AT4G18360
6935	2	L4 vs. M4	M4 up	GO:0042742	Signaling and response	Stress	defense response to bacterium	P	AT1G73260
6936	2	L4 vs. M4	M4 up	GO:0042742	Signaling and response	Stress	defense response to bacterium	P	AT1G80840
6937	2	L4 vs. M4	M4 up	GO:0042742	Signaling and response	Stress	defense response to bacterium	P	AT4G18360
6938	2	L4 vs. M4	M4 up	GO:0042742	Signaling and response	Stress	defense response to bacterium	P	AT4G35230
6939	2	L4 vs. M4	M4 up	GO:0042742	Signaling and response	Stress	defense response to bacterium	P	AT4G36690
6940	2	L4 vs. M4	M4 up	GO:0042742	Signaling and response	Stress	defense response to bacterium	P	AT5G06320
6941	2	L4 vs. M4	M4 up	GO:0042742	Signaling and response	Stress	defense response to bacterium	P	AT5G58430
6942	2	L4 vs. M4	M4 up	GO:0042742	Signaling and response	Stress	defense response to bacterium	P	AT5G67300
6943	2	L4 vs. M4	M4 up	GO:0009816	Signaling and response	Stress	defense response to bacterium, incompatible interaction	P	AT2G40000
6944	2	L4 vs. M4	M4 up	GO:0009816	Signaling and response	Stress	defense response to bacterium, incompatible interaction	P	AT5G06320
6945	2	L4 vs. M4	M4 up	GO:0050832	Signaling and response	Stress	defense response to fungus	P	AT1G45145
6946	2	L4 vs. M4	M4 up	GO:0050832	Signaling and response	Stress	defense response to fungus	P	AT1G47128
6947	2	L4 vs. M4	M4 up	GO:0050832	Signaling and response	Stress	defense response to fungus	P	AT1G80840

6948	2	L4 vs. M4	M4 up	GO:0050832	Signaling and response	Stress	defense response to fungus	P	AT2G30020
6949	2	L4 vs. M4	M4 up	GO:0050832	Signaling and response	Stress	defense response to fungus	P	AT4G35230
6950	2	L4 vs. M4	M4 up	GO:0050832	Signaling and response	Stress	defense response to fungus	P	AT5G50260
6951	2	L4 vs. M4	M4 up	GO:0050832	Signaling and response	Stress	defense response to fungus	P	AT5G58430
6952	2	L4 vs. M4	M4 up	GO:0050832	Signaling and response	Stress	defense response to fungus	P	AT5G67300
6953	2	L4 vs. M4	M4 up	GO:0009817	Signaling and response	Stress	defense response to fungus, incompatible interaction	P	AT1G29340
6954	2	L4 vs. M4	M4 up	GO:0009817	Signaling and response	Stress	defense response to fungus, incompatible interaction	P	AT5G13010
6955	2	L4 vs. M4	M4 up	GO:0009817	Signaling and response	Stress	defense response to fungus, incompatible interaction	P	AT5G45890
6956	2	L4 vs. M4	M4 up	GO:0050829	Signaling and response	Stress	defense response to Gram-negative bacterium	P	AT2G23810
6957	2	L4 vs. M4	M4 up	GO:0051607	Signaling and response	Stress	defense response to virus	P	AT3G20250
6958	2	L4 vs. M4	M4 up	GO:0051607	Signaling and response	Stress	defense response to virus	P	AT5G06320
6959	2	L4 vs. M4	M4 up	GO:0009814	Signaling and response	Stress	defense response, incompatible interaction	P	AT1G29340
6960	2	L4 vs. M4	M4 up	GO:0009814	Signaling and response	Stress	defense response, incompatible interaction	P	AT3G49530
6961	2	L4 vs. M4	M4 up	GO:0009819	Signaling and response	Stress	drought recovery	P	AT2G37040
6962	2	L4 vs. M4	M4 up	GO:0009819	Signaling and response	Stress	drought recovery	P	AT3G20250
6963	2	L4 vs. M4	M4 up	GO:0009873	Signaling and response	Stress	ethylene-activated signaling pathway	P	AT1G22190
6964	2	L4 vs. M4	M4 up	GO:0009873	Signaling and response	Stress	ethylene-activated signaling pathway	P	AT5G03730
6965	2	L4 vs. M4	M4 up	GO:0009813	Signaling and response	Stress	flavonoid biosynthetic process	P	AT3G51240
6966	2	L4 vs. M4	M4 up	GO:0009813	Signaling and response	Stress	flavonoid biosynthetic process	P	AT5G05600
6967	2	L4 vs. M4	M4 up	GO:0051555	Signaling and response	Stress	flavonol biosynthetic process	P	AT4G34135
6968	2	L4 vs. M4	M4 up	GO:0043295	Signaling and response	Stress	glutathione binding	F	AT5G27380
6969	2	L4 vs. M4	M4 up	GO:0006750	Signaling and response	Stress	glutathione biosynthetic process	P	AT5G27380
6970	2	L4 vs. M4	M4 up	GO:0004363	Signaling and response	Stress	glutathione synthase activity	F	AT5G27380
6971	2	L4 vs. M4	M4 up	GO:0031072	Signaling and response	Stress	heat shock protein binding	F	AT2G22360
6972	2	L4 vs. M4	M4 up	GO:0031072	Signaling and response	Stress	heat shock protein binding	F	AT3G09440
6973	2	L4 vs. M4	M4 up	GO:0051879	Signaling and response	Stress	Hsp90 protein binding	F	AT1G62740
6974	2	L4 vs. M4	M4 up	GO:0050665	Signaling and response	Stress	hydrogen peroxide biosynthetic process	P	AT4G18360
6975	2	L4 vs. M4	M4 up	GO:0080170	Signaling and response	Stress	hydrogen peroxide transmembrane transport	P	AT3G53420
6976	2	L4 vs. M4	M4 up	GO:0042538	Signaling and response	Stress	hyperosmotic salinity response	P	AT4G30996
6977	2	L4 vs. M4	M4 up	GO:0006955	Signaling and response	Stress	immune response	P	AT1G73080
6978	2	L4 vs. M4	M4 up	GO:0045087	Signaling and response	Stress	innate immune response	P	AT1G73080
6979	2	L4 vs. M4	M4 up	GO:0045087	Signaling and response	Stress	innate immune response	P	AT4G29040
6980	2	L4 vs. M4	M4 up	GO:0055072	Signaling and response	Stress	iron ion homeostasis	P	AT4G16370
6981	2	L4 vs. M4	M4 up	GO:0055072	Signaling and response	Stress	iron ion homeostasis	P	AT5G04150
6982	2	L4 vs. M4	M4 up	GO:0031418	Signaling and response	Stress	L-ascorbic acid binding	F	AT3G51240
6983	2	L4 vs. M4	M4 up	GO:0051787	Signaling and response	Stress	misfolded protein binding	F	AT3G09440
6984	2	L4 vs. M4	M4 up	GO:0060586	Signaling and response	Stress	multicellular organismal iron ion homeostasis	P	AT3G18290
6985	2	L4 vs. M4	M4 up	GO:0010105	Signaling and response	Stress	negative regulation of ethylene-activated signaling pathway	P	AT5G03730
6986	2	L4 vs. M4	M4 up	GO:1901001	Signaling and response	Stress	negative regulation of response to salt stress	P	AT4G37260
6987	2	L4 vs. M4	M4 up	GO:0007263	Signaling and response	Stress	nitric oxide mediated signal transduction	P	AT5G15410
6988	2	L4 vs. M4	M4 up	GO:0009626	Signaling and response	Stress	plant-type hypersensitive response	P	AT1G29340
6989	2	L4 vs. M4	M4 up	GO:0009626	Signaling and response	Stress	plant-type hypersensitive response	P	AT3G45640
6990	2	L4 vs. M4	M4 up	GO:0009626	Signaling and response	Stress	plant-type hypersensitive response	P	AT5G15410
6991	2	L4 vs. M4	M4 up	GO:0009626	Signaling and response	Stress	plant-type hypersensitive response	P	AT5G50260
6992	2	L4 vs. M4	M4 up	GO:0009789	Signaling and response	Stress	positive regulation of abscisic acid-activated signaling pathway	P	AT5G58430
6993	2	L4 vs. M4	M4 up	GO:1900103	Signaling and response	Stress	positive regulation of endoplasmic reticulum unfolded protein response	P	AT3G49530
6994	2	L4 vs. M4	M4 up	GO:0009963	Signaling and response	Stress	positive regulation of flavonoid biosynthetic process	P	AT2G26170
6995	2	L4 vs. M4	M4 up	GO:0080136	Signaling and response	Stress	priming of cellular response to stress	P	AT3G45640
6996	2	L4 vs. M4	M4 up	GO:0097468	Signaling and response	Stress	programmed cell death in response to reactive oxygen species	P	AT5G12080

6997	2	L4 vs. M4	M4 up	GO:0002020	Signaling and response	Stress	protease binding	F	AT3G09440
6998	2	L4 vs. M4	M4 up	GO:0044183	Signaling and response	Stress	protein folding chaperone	F	AT3G09440
6999	2	L4 vs. M4	M4 up	GO:0042026	Signaling and response	Stress	protein refolding	P	AT2G22360
7000	2	L4 vs. M4	M4 up	GO:0042026	Signaling and response	Stress	protein refolding	P	AT2G33210
7001	2	L4 vs. M4	M4 up	GO:0042026	Signaling and response	Stress	protein refolding	P	AT3G09440
7002	2	L4 vs. M4	M4 up	GO:0010359	Signaling and response	Stress	regulation of anion channel activity	P	AT2G17290
7003	2	L4 vs. M4	M4 up	GO:0031347	Signaling and response	Stress	regulation of defense response	P	AT1G80840
7004	2	L4 vs. M4	M4 up	GO:1900150	Signaling and response	Stress	regulation of defense response to fungus	P	AT5G67300
7005	2	L4 vs. M4	M4 up	GO:0050691	Signaling and response	Stress	regulation of defense response to virus by host	P	AT1G80840
7006	2	L4 vs. M4	M4 up	GO:0051453	Signaling and response	Stress	regulation of intracellular pH	P	AT5G57350
7007	2	L4 vs. M4	M4 up	GO:1903426	Signaling and response	Stress	regulation of reactive oxygen species biosynthetic process	P	AT2G20890
7008	2	L4 vs. M4	M4 up	GO:2000070	Signaling and response	Stress	regulation of response to water deprivation	P	AT2G20890
7009	2	L4 vs. M4	M4 up	GO:0090333	Signaling and response	Stress	regulation of stomatal closure	P	AT5G58430
7010	2	L4 vs. M4	M4 up	GO:0010119	Signaling and response	Stress	regulation of stomatal movement	P	AT1G70410
7011	2	L4 vs. M4	M4 up	GO:0010119	Signaling and response	Stress	regulation of stomatal movement	P	AT2G17290
7012	2	L4 vs. M4	M4 up	GO:1902456	Signaling and response	Stress	regulation of stomatal opening	P	AT2G17290
7013	2	L4 vs. M4	M4 up	GO:0009737	Signaling and response	Stress	response to abscisic acid	P	AT1G54100
7014	2	L4 vs. M4	M4 up	GO:0009737	Signaling and response	Stress	response to abscisic acid	P	AT2G17290
7015	2	L4 vs. M4	M4 up	GO:0009737	Signaling and response	Stress	response to abscisic acid	P	AT2G26190
7016	2	L4 vs. M4	M4 up	GO:0009737	Signaling and response	Stress	response to abscisic acid	P	AT3G14050
7017	2	L4 vs. M4	M4 up	GO:0009737	Signaling and response	Stress	response to abscisic acid	P	AT3G53420
7018	2	L4 vs. M4	M4 up	GO:0009737	Signaling and response	Stress	response to abscisic acid	P	AT3G60240
7019	2	L4 vs. M4	M4 up	GO:0009737	Signaling and response	Stress	response to abscisic acid	P	AT4G19810
7020	2	L4 vs. M4	M4 up	GO:0009737	Signaling and response	Stress	response to abscisic acid	P	AT4G25000
7021	2	L4 vs. M4	M4 up	GO:0009737	Signaling and response	Stress	response to abscisic acid	P	AT4G27410
7022	2	L4 vs. M4	M4 up	GO:0009737	Signaling and response	Stress	response to abscisic acid	P	AT4G35230
7023	2	L4 vs. M4	M4 up	GO:0009737	Signaling and response	Stress	response to abscisic acid	P	AT4G37260
7024	2	L4 vs. M4	M4 up	GO:0009737	Signaling and response	Stress	response to abscisic acid	P	AT5G13180
7025	2	L4 vs. M4	M4 up	GO:0009737	Signaling and response	Stress	response to abscisic acid	P	AT5G40010
7026	2	L4 vs. M4	M4 up	GO:0009737	Signaling and response	Stress	response to abscisic acid	P	AT5G57330
7027	2	L4 vs. M4	M4 up	GO:0009737	Signaling and response	Stress	response to abscisic acid	P	AT5G67300
7028	2	L4 vs. M4	M4 up	GO:0046685	Signaling and response	Stress	response to arsenic-containing substance	P	AT3G16950
7029	2	L4 vs. M4	M4 up	GO:0046685	Signaling and response	Stress	response to arsenic-containing substance	P	AT4G33540
7030	2	L4 vs. M4	M4 up	GO:0009409	Signaling and response	Stress	response to cold	P	AT1G01120
7031	2	L4 vs. M4	M4 up	GO:0009409	Signaling and response	Stress	response to cold	P	AT1G22190
7032	2	L4 vs. M4	M4 up	GO:0009409	Signaling and response	Stress	response to cold	P	AT2G35040
7033	2	L4 vs. M4	M4 up	GO:0009409	Signaling and response	Stress	response to cold	P	AT3G16470
7034	2	L4 vs. M4	M4 up	GO:0009409	Signaling and response	Stress	response to cold	P	AT3G22370
7035	2	L4 vs. M4	M4 up	GO:0009409	Signaling and response	Stress	response to cold	P	AT3G45640
7036	2	L4 vs. M4	M4 up	GO:0009409	Signaling and response	Stress	response to cold	P	AT5G40010
7037	2	L4 vs. M4	M4 up	GO:0009409	Signaling and response	Stress	response to cold	P	AT5G61030
7038	2	L4 vs. M4	M4 up	GO:0009269	Signaling and response	Stress	response to desiccation	P	AT1G54100
7039	2	L4 vs. M4	M4 up	GO:0009269	Signaling and response	Stress	response to desiccation	P	AT3G25760
7040	2	L4 vs. M4	M4 up	GO:0034976	Signaling and response	Stress	response to endoplasmic reticulum stress	P	AT1G04750
7041	2	L4 vs. M4	M4 up	GO:0034976	Signaling and response	Stress	response to endoplasmic reticulum stress	P	AT3G54960
7042	2	L4 vs. M4	M4 up	GO:0009723	Signaling and response	Stress	response to ethylene	P	AT4G32940
7043	2	L4 vs. M4	M4 up	GO:0009723	Signaling and response	Stress	response to ethylene	P	AT4G37260
7044	2	L4 vs. M4	M4 up	GO:0009723	Signaling and response	Stress	response to ethylene	P	AT5G03730
7045	2	L4 vs. M4	M4 up	GO:0009723	Signaling and response	Stress	response to ethylene	P	AT5G45890
7046	2	L4 vs. M4	M4 up	GO:0009723	Signaling and response	Stress	response to ethylene	P	AT5G60360
7047	2	L4 vs. M4	M4 up	GO:0009723	Signaling and response	Stress	response to ethylene	P	AT5G67300
7048	2	L4 vs. M4	M4 up	GO:0050826	Signaling and response	Stress	response to freezing	P	AT3G45640
7049	2	L4 vs. M4	M4 up	GO:0009620	Signaling and response	Stress	response to fungus	P	AT2G06050
7050	2	L4 vs. M4	M4 up	GO:0009620	Signaling and response	Stress	response to fungus	P	AT2G30020
7051	2	L4 vs. M4	M4 up	GO:0009620	Signaling and response	Stress	response to fungus	P	AT2G46440

7052	2	L4 vs. M4	M4 up	GO:0009408	Signaling and response	Stress	response to heat	P	AT1G03190
7053	2	L4 vs. M4	M4 up	GO:0009408	Signaling and response	Stress	response to heat	P	AT2G22360
7054	2	L4 vs. M4	M4 up	GO:0009408	Signaling and response	Stress	response to heat	P	AT3G04120
7055	2	L4 vs. M4	M4 up	GO:0009408	Signaling and response	Stress	response to heat	P	AT3G09440
7056	2	L4 vs. M4	M4 up	GO:0009408	Signaling and response	Stress	response to heat	P	AT4G29040
7057	2	L4 vs. M4	M4 up	GO:0009408	Signaling and response	Stress	response to heat	P	AT5G67300
7058	2	L4 vs. M4	M4 up	GO:0042542	Signaling and response	Stress	response to hydrogen peroxide	P	AT1G73260
7059	2	L4 vs. M4	M4 up	GO:0042542	Signaling and response	Stress	response to hydrogen peroxide	P	AT3G04120
7060	2	L4 vs. M4	M4 up	GO:0042542	Signaling and response	Stress	response to hydrogen peroxide	P	AT5G67480
7061	2	L4 vs. M4	M4 up	GO:0001666	Signaling and response	Stress	response to hypoxia	P	AT1G35140
7062	2	L4 vs. M4	M4 up	GO:0001666	Signaling and response	Stress	response to hypoxia	P	AT5G03730
7063	2	L4 vs. M4	M4 up	GO:0009625	Signaling and response	Stress	response to insect	P	AT1G73260
7064	2	L4 vs. M4	M4 up	GO:1990641	Signaling and response	Stress	response to iron ion starvation	P	AT5G67370
7065	2	L4 vs. M4	M4 up	GO:0010288	Signaling and response	Stress	response to lead ion	P	AT5G58270
7066	2	L4 vs. M4	M4 up	GO:0010188	Signaling and response	Stress	response to microbial phytotoxin	P	AT1G45145
7067	2	L4 vs. M4	M4 up	GO:0051788	Signaling and response	Stress	response to misfolded protein	P	AT4G29040
7068	2	L4 vs. M4	M4 up	GO:0002237	Signaling and response	Stress	response to molecule of bacterial origin	P	AT1G73260
7069	2	L4 vs. M4	M4 up	GO:0002237	Signaling and response	Stress	response to molecule of bacterial origin	P	AT1G80840
7070	2	L4 vs. M4	M4 up	GO:0002238	Signaling and response	Stress	response to molecule of fungal origin	P	AT1G73260
7071	2	L4 vs. M4	M4 up	GO:0009624	Signaling and response	Stress	response to nematode	P	AT1G58360
7072	2	L4 vs. M4	M4 up	GO:0009624	Signaling and response	Stress	response to nematode	P	AT1G61800
7073	2	L4 vs. M4	M4 up	GO:0009624	Signaling and response	Stress	response to nematode	P	AT1G73260
7074	2	L4 vs. M4	M4 up	GO:0009624	Signaling and response	Stress	response to nematode	P	AT5G14940
7075	2	L4 vs. M4	M4 up	GO:0006970	Signaling and response	Stress	response to osmotic stress	P	AT1G22190
7076	2	L4 vs. M4	M4 up	GO:0006970	Signaling and response	Stress	response to osmotic stress	P	AT3G20250
7077	2	L4 vs. M4	M4 up	GO:0006970	Signaling and response	Stress	response to osmotic stress	P	AT3G45640
7078	2	L4 vs. M4	M4 up	GO:0006970	Signaling and response	Stress	response to osmotic stress	P	AT3G58620
7079	2	L4 vs. M4	M4 up	GO:0006970	Signaling and response	Stress	response to osmotic stress	P	AT5G08590
7080	2	L4 vs. M4	M4 up	GO:0051707	Signaling and response	Stress	response to other organism	P	AT4G34135
7081	2	L4 vs. M4	M4 up	GO:0006979	Signaling and response	Stress	response to oxidative stress	P	AT1G03190
7082	2	L4 vs. M4	M4 up	GO:0006979	Signaling and response	Stress	response to oxidative stress	P	AT1G45145
7083	2	L4 vs. M4	M4 up	GO:0006979	Signaling and response	Stress	response to oxidative stress	P	AT2G37040
7084	2	L4 vs. M4	M4 up	GO:0006979	Signaling and response	Stress	response to oxidative stress	P	AT2G40000
7085	2	L4 vs. M4	M4 up	GO:0006979	Signaling and response	Stress	response to oxidative stress	P	AT2G47060
7086	2	L4 vs. M4	M4 up	GO:0006979	Signaling and response	Stress	response to oxidative stress	P	AT3G04120
7087	2	L4 vs. M4	M4 up	GO:0006979	Signaling and response	Stress	response to oxidative stress	P	AT3G28340
7088	2	L4 vs. M4	M4 up	GO:0006979	Signaling and response	Stress	response to oxidative stress	P	AT3G45640
7089	2	L4 vs. M4	M4 up	GO:0006979	Signaling and response	Stress	response to oxidative stress	P	AT5G54810
7090	2	L4 vs. M4	M4 up	GO:0010193	Signaling and response	Stress	response to ozone	P	AT2G06050
7091	2	L4 vs. M4	M4 up	GO:0009268	Signaling and response	Stress	response to pH	P	AT2G30360
7092	2	L4 vs. M4	M4 up	GO:0051775	Signaling and response	Stress	response to redox state	P	AT3G04120
7093	2	L4 vs. M4	M4 up	GO:0009651	Signaling and response	Stress	response to salt stress	P	AT1G54100
7094	2	L4 vs. M4	M4 up	GO:0009651	Signaling and response	Stress	response to salt stress	P	AT2G26190
7095	2	L4 vs. M4	M4 up	GO:0009651	Signaling and response	Stress	response to salt stress	P	AT2G44450
7096	2	L4 vs. M4	M4 up	GO:0009651	Signaling and response	Stress	response to salt stress	P	AT3G20250
7097	2	L4 vs. M4	M4 up	GO:0009651	Signaling and response	Stress	response to salt stress	P	AT3G53420
7098	2	L4 vs. M4	M4 up	GO:0009651	Signaling and response	Stress	response to salt stress	P	AT4G19810
7099	2	L4 vs. M4	M4 up	GO:0009651	Signaling and response	Stress	response to salt stress	P	AT5G08590
7100	2	L4 vs. M4	M4 up	GO:0009651	Signaling and response	Stress	response to salt stress	P	AT5G13180
7101	2	L4 vs. M4	M4 up	GO:0009651	Signaling and response	Stress	response to salt stress	P	AT5G40010
7102	2	L4 vs. M4	M4 up	GO:0009651	Signaling and response	Stress	response to salt stress	P	AT5G67300
7103	2	L4 vs. M4	M4 up	GO:0009651	Signaling and response	Stress	response to salt stress	P	AT5G67480
7104	2	L4 vs. M4	M4 up	GO:0006986	Signaling and response	Stress	response to unfolded protein	P	AT3G09440
7105	2	L4 vs. M4	M4 up	GO:0010224	Signaling and response	Stress	response to UV-B	P	AT2G37040
7106	2	L4 vs. M4	M4 up	GO:0010224	Signaling and response	Stress	response to UV-B	P	AT3G45640
7107	2	L4 vs. M4	M4 up	GO:0010224	Signaling and response	Stress	response to UV-B	P	AT3G51240

7108	2	L4 vs. M4	M4 up	GO:0010224	Signaling and response	Stress	response to UV-B	P	AT5G45890
7109	2	L4 vs. M4	M4 up	GO:0009615	Signaling and response	Stress	response to virus	P	AT3G09440
7110	2	L4 vs. M4	M4 up	GO:0009615	Signaling and response	Stress	response to virus	P	AT3G60240
7111	2	L4 vs. M4	M4 up	GO:0009414	Signaling and response	Stress	response to water deprivation	P	AT1G22190
7112	2	L4 vs. M4	M4 up	GO:0009414	Signaling and response	Stress	response to water deprivation	P	AT1G47128
7113	2	L4 vs. M4	M4 up	GO:0009414	Signaling and response	Stress	response to water deprivation	P	AT3G53420
7114	2	L4 vs. M4	M4 up	GO:0009414	Signaling and response	Stress	response to water deprivation	P	AT4G27410
7115	2	L4 vs. M4	M4 up	GO:0009414	Signaling and response	Stress	response to water deprivation	P	AT5G40010
7116	2	L4 vs. M4	M4 up	GO:0009414	Signaling and response	Stress	response to water deprivation	P	AT5G58430
7117	2	L4 vs. M4	M4 up	GO:0009414	Signaling and response	Stress	response to water deprivation	P	AT5G67300
7118	2	L4 vs. M4	M4 up	GO:0009611	Signaling and response	Stress	response to wounding	P	AT1G19640
7119	2	L4 vs. M4	M4 up	GO:0009611	Signaling and response	Stress	response to wounding	P	AT1G73080
7120	2	L4 vs. M4	M4 up	GO:0009611	Signaling and response	Stress	response to wounding	P	AT1G73260
7121	2	L4 vs. M4	M4 up	GO:0009611	Signaling and response	Stress	response to wounding	P	AT1G80840
7122	2	L4 vs. M4	M4 up	GO:0009611	Signaling and response	Stress	response to wounding	P	AT2G02990
7123	2	L4 vs. M4	M4 up	GO:0009611	Signaling and response	Stress	response to wounding	P	AT2G06050
7124	2	L4 vs. M4	M4 up	GO:0009611	Signaling and response	Stress	response to wounding	P	AT2G30020
7125	2	L4 vs. M4	M4 up	GO:0009611	Signaling and response	Stress	response to wounding	P	AT2G37040
7126	2	L4 vs. M4	M4 up	GO:0009611	Signaling and response	Stress	response to wounding	P	AT3G14050
7127	2	L4 vs. M4	M4 up	GO:0009611	Signaling and response	Stress	response to wounding	P	AT3G16470
7128	2	L4 vs. M4	M4 up	GO:0009611	Signaling and response	Stress	response to wounding	P	AT3G45640
7129	2	L4 vs. M4	M4 up	GO:0009611	Signaling and response	Stress	response to wounding	P	AT4G32940
7130	2	L4 vs. M4	M4 up	GO:0009611	Signaling and response	Stress	response to wounding	P	AT5G06320
7131	2	L4 vs. M4	M4 up	GO:0009611	Signaling and response	Stress	response to wounding	P	AT5G67480
7132	2	L4 vs. M4	M4 up	GO:1990112	Signaling and response	Stress	RQC complex	C	AT5G49930
7133	2	L4 vs. M4	M4 up	GO:0051903	Signaling and response	Stress	S-(hydroxymethyl)glutathione dehydrogenase activity	F	AT5G42250
7134	2	L4 vs. M4	M4 up	GO:0004867	Signaling and response	Stress	serine-type endopeptidase inhibitor activity	F	AT1G73260
7135	2	L4 vs. M4	M4 up	GO:0016444	Signaling and response	Stress	somatic cell DNA recombination	P	AT2G19480
7136	2	L4 vs. M4	M4 up	GO:0009627	Signaling and response	Stress	systemic acquired resistance	P	AT2G37870
7137	2	L4 vs. M4	M4 up	GO:0004675	Signaling and response	Stress	transmembrane receptor protein serine/threonine kinase activity	F	AT2G47060
7138	2	L4 vs. M4	M4 up	GO:0004675	Signaling and response	Stress	transmembrane receptor protein serine/threonine kinase activity	F	AT2G48010
7139	2	L4 vs. M4	M4 up	GO:0070413	Signaling and response	Stress	trehalose metabolism in response to stress	P	AT1G78580
7140	2	L4 vs. M4	M4 up	GO:0051082	Signaling and response	Stress	unfolded protein binding	F	AT2G22360
7141	2	L4 vs. M4	M4 up	GO:0051082	Signaling and response	Stress	unfolded protein binding	F	AT2G33210
7142	2	L4 vs. M4	M4 up	GO:0051082	Signaling and response	Stress	unfolded protein binding	F	AT3G09440
7143	2	L4 vs. M4	M4 up	GO:0051082	Signaling and response	Stress	unfolded protein binding	F	AT5G53350
7144	2	L4 vs. M4	M4 up	GO:0016032	Signaling and response	Stress	viral process	P	AT5G13180
7145	3	F23 vs. L23	F2-3 up	GO:0008356	Development	Cell division	asymmetric cell division	P	AT2G37630
7146	3	F23 vs. L23	F2-3 up	GO:0006284	Development	Cell division	base-excision repair	P	AT3G12710
7147	3	F23 vs. L23	F2-3 up	GO:0006284	Development	Cell division	base-excision repair	P	AT5G44680
7148	3	F23 vs. L23	F2-3 up	GO:0051301	Development	Cell division	cell division	P	AT3G18780
7149	3	F23 vs. L23	F2-3 up	GO:0051301	Development	Cell division	cell division	P	AT4G11280
7150	3	F23 vs. L23	F2-3 up	GO:0051301	Development	Cell division	cell division	P	AT5G09810
7151	3	F23 vs. L23	F2-3 up	GO:0008283	Development	Cell division	cell population proliferation	P	AT5G65870
7152	3	F23 vs. L23	F2-3 up	GO:0000793	Development	Cell division	condensed chromosome	C	AT2G37630
7153	3	F23 vs. L23	F2-3 up	GO:0000307	Development	Cell division	cyclin-dependent protein kinase holoenzyme complex	C	AT1G09600
7154	3	F23 vs. L23	F2-3 up	GO:0004693	Development	Cell division	cyclin-dependent protein serine/threonine kinase activity	F	AT1G09600
7155	3	F23 vs. L23	F2-3 up	GO:0000910	Development	Cell division	cytokinesis	P	AT3G43210
7156	3	F23 vs. L23	F2-3 up	GO:0032508	Development	Cell division	DNA duplex unwinding	P	AT3G23700
7157	3	F23 vs. L23	F2-3 up	GO:0006281	Development	Cell division	DNA repair	P	AT3G12610
7158	3	F23 vs. L23	F2-3 up	GO:0006281	Development	Cell division	DNA repair	P	AT3G50000

7159	3	F23 vs. L23	F2-3 up	GO:0004519	Development	Cell division	endonuclease activity	F	AT5G54130
7160	3	F23 vs. L23	F2-3 up	GO:0048229	Development	Cell division	gametophyte development	P	AT1G70730
7161	3	F23 vs. L23	F2-3 up	GO:0048229	Development	Cell division	gametophyte development	P	AT3G43210
7162	3	F23 vs. L23	F2-3 up	GO:0048232	Development	Cell division	male gamete generation	P	AT2G14750
7163	3	F23 vs. L23	F2-3 up	GO:0048232	Development	Cell division	male gamete generation	P	AT4G39940
7164	3	F23 vs. L23	F2-3 up	GO:0007112	Development	Cell division	male meiosis cytokinesis	P	AT3G43210
7165	3	F23 vs. L23	F2-3 up	GO:0033206	Development	Cell division	meiotic cytokinesis	P	AT1G14920
7166	3	F23 vs. L23	F2-3 up	GO:0009556	Development	Cell division	microsporogenesis	P	AT2G13680
7167	3	F23 vs. L23	F2-3 up	GO:0009556	Development	Cell division	microsporogenesis	P	AT3G08510
7168	3	F23 vs. L23	F2-3 up	GO:0003777	Development	Cell division	microtubule motor activity	F	AT3G43210
7169	3	F23 vs. L23	F2-3 up	GO:0003777	Development	Cell division	microtubule motor activity	F	AT5G27000
7170	3	F23 vs. L23	F2-3 up	GO:0003777	Development	Cell division	microtubule motor activity	F	AT5G65460
7171	3	F23 vs. L23	F2-3 up	GO:0000278	Development	Cell division	mitotic cell cycle	P	AT1G64740
7172	3	F23 vs. L23	F2-3 up	GO:0008285	Development	Cell division	negative regulation of cell population proliferation	P	AT3G08730
7173	3	F23 vs. L23	F2-3 up	GO:0031022	Development	Cell division	nuclear migration along microfilament	P	AT5G65460
7174	3	F23 vs. L23	F2-3 up	GO:0043224	Development	Cell division	nuclear SCF ubiquitin ligase complex	C	AT4G03190
7175	3	F23 vs. L23	F2-3 up	GO:0017110	Development	Cell division	nucleoside-diphosphatase activity	F	AT5G18280
7176	3	F23 vs. L23	F2-3 up	GO:0000166	Development	Cell division	nucleotide binding	F	AT3G12610
7177	3	F23 vs. L23	F2-3 up	GO:0010197	Development	Cell division	polar nucleus fusion	P	AT2G35940
7178	3	F23 vs. L23	F2-3 up	GO:0009846	Development	Cell division	pollen germination	P	AT1G64980
7179	3	F23 vs. L23	F2-3 up	GO:0009846	Development	Cell division	pollen germination	P	AT2G13680
7180	3	F23 vs. L23	F2-3 up	GO:0009846	Development	Cell division	pollen germination	P	AT2G26490
7181	3	F23 vs. L23	F2-3 up	GO:0009846	Development	Cell division	pollen germination	P	AT2G33100
7182	3	F23 vs. L23	F2-3 up	GO:0009846	Development	Cell division	pollen germination	P	AT2G41210
7183	3	F23 vs. L23	F2-3 up	GO:0009846	Development	Cell division	pollen germination	P	AT3G45100
7184	3	F23 vs. L23	F2-3 up	GO:0009846	Development	Cell division	pollen germination	P	AT3G56950
7185	3	F23 vs. L23	F2-3 up	GO:0009846	Development	Cell division	pollen germination	P	AT3G56960
7186	3	F23 vs. L23	F2-3 up	GO:0009846	Development	Cell division	pollen germination	P	AT5G18280
7187	3	F23 vs. L23	F2-3 up	GO:0048235	Development	Cell division	pollen sperm cell differentiation	P	AT1G24520
7188	3	F23 vs. L23	F2-3 up	GO:0048235	Development	Cell division	pollen sperm cell differentiation	P	AT1G68610
7189	3	F23 vs. L23	F2-3 up	GO:0048235	Development	Cell division	pollen sperm cell differentiation	P	AT2G32460
7190	3	F23 vs. L23	F2-3 up	GO:0048235	Development	Cell division	pollen sperm cell differentiation	P	AT5G57800
7191	3	F23 vs. L23	F2-3 up	GO:0048868	Development	Cell division	pollen tube development	P	AT2G34850
7192	3	F23 vs. L23	F2-3 up	GO:0009860	Development	Cell division	pollen tube growth	P	AT1G03050
7193	3	F23 vs. L23	F2-3 up	GO:0009860	Development	Cell division	pollen tube growth	P	AT1G54280
7194	3	F23 vs. L23	F2-3 up	GO:0009860	Development	Cell division	pollen tube growth	P	AT1G54560
7195	3	F23 vs. L23	F2-3 up	GO:0009860	Development	Cell division	pollen tube growth	P	AT1G61290
7196	3	F23 vs. L23	F2-3 up	GO:0009860	Development	Cell division	pollen tube growth	P	AT1G64980
7197	3	F23 vs. L23	F2-3 up	GO:0009860	Development	Cell division	pollen tube growth	P	AT2G13680
7198	3	F23 vs. L23	F2-3 up	GO:0009860	Development	Cell division	pollen tube growth	P	AT2G36880
7199	3	F23 vs. L23	F2-3 up	GO:0009860	Development	Cell division	pollen tube growth	P	AT2G41210
7200	3	F23 vs. L23	F2-3 up	GO:0009860	Development	Cell division	pollen tube growth	P	AT3G05580
7201	3	F23 vs. L23	F2-3 up	GO:0009860	Development	Cell division	pollen tube growth	P	AT3G12690
7202	3	F23 vs. L23	F2-3 up	GO:0009860	Development	Cell division	pollen tube growth	P	AT3G45100
7203	3	F23 vs. L23	F2-3 up	GO:0009860	Development	Cell division	pollen tube growth	P	AT3G56950
7204	3	F23 vs. L23	F2-3 up	GO:0009860	Development	Cell division	pollen tube growth	P	AT3G56960
7205	3	F23 vs. L23	F2-3 up	GO:0009860	Development	Cell division	pollen tube growth	P	AT4G14880
7206	3	F23 vs. L23	F2-3 up	GO:0009860	Development	Cell division	pollen tube growth	P	AT4G17530
7207	3	F23 vs. L23	F2-3 up	GO:0009860	Development	Cell division	pollen tube growth	P	AT5G15470
7208	3	F23 vs. L23	F2-3 up	GO:0009860	Development	Cell division	pollen tube growth	P	AT5G52360
7209	3	F23 vs. L23	F2-3 up	GO:0010183	Development	Cell division	pollen tube guidance	P	AT2G41630
7210	3	F23 vs. L23	F2-3 up	GO:0010183	Development	Cell division	pollen tube guidance	P	AT3G45640
7211	3	F23 vs. L23	F2-3 up	GO:0010183	Development	Cell division	pollen tube guidance	P	AT5G45840
7212	3	F23 vs. L23	F2-3 up	GO:0008284	Development	Cell division	positive regulation of cell population proliferation	P	AT4G32880
7213	3	F23 vs. L23	F2-3 up	GO:0048544	Development	Cell division	recognition of pollen	P	AT5G24080
7214	3	F23 vs. L23	F2-3 up	GO:0051726	Development	Cell division	regulation of cell cycle	P	AT3G50000

7215	3	F23 vs. L23	F2-3 up	GO:0042127	Development	Cell division	regulation of cell population proliferation	P	AT1G78570
7216	3	F23 vs. L23	F2-3 up	GO:0004751	Development	Cell division	ribose-5-phosphate isomerase activity	F	AT2G01290
7217	3	F23 vs. L23	F2-3 up	GO:0004751	Development	Cell division	ribose-5-phosphate isomerase activity	F	AT3G04790
7218	3	F23 vs. L23	F2-3 up	GO:0004750	Development	Cell division	ribulose-phosphate 3-epimerase activity	F	AT5G61410
7219	3	F23 vs. L23	F2-3 up	GO:0019005	Development	Cell division	SCF ubiquitin ligase complex	C	AT1G21760
7220	3	F23 vs. L23	F2-3 up	GO:0019005	Development	Cell division	SCF ubiquitin ligase complex	C	AT3G23050
7221	3	F23 vs. L23	F2-3 up	GO:0019953	Development	Cell division	sexual reproduction	P	AT4G17030
7222	3	F23 vs. L23	F2-3 up	GO:0019953	Development	Cell division	sexual reproduction	P	AT4G38400
7223	3	F23 vs. L23	F2-3 up	GO:0019953	Development	Cell division	sexual reproduction	P	AT5G07550
7224	3	F23 vs. L23	F2-3 up	GO:0007338	Development	Cell division	single fertilization	P	AT1G64980
7225	3	F23 vs. L23	F2-3 up	GO:0005819	Development	Cell division	spindle	C	AT5G67470
7226	3	F23 vs. L23	F2-3 up	GO:0006949	Development	Cell division	syncytium formation	P	AT1G26770
7227	3	F23 vs. L23	F2-3 up	GO:0006949	Development	Cell division	syncytium formation	P	AT1G69530
7228	3	F23 vs. L23	F2-3 up	GO:0006949	Development	Cell division	syncytium formation	P	AT2G28950
7229	3	F23 vs. L23	F2-3 up	GO:0006949	Development	Cell division	syncytium formation	P	AT2G39700
7230	3	F23 vs. L23	F2-3 up	GO:0006949	Development	Cell division	syncytium formation	P	AT4G26260
7231	3	F23 vs. L23	F2-3 up	GO:0019079	Development	Cell division	viral genome replication	P	AT3G02470
7232	3	F23 vs. L23	F2-3 up	GO:0010158	Development	Development	abaxial cell fate specification	P	AT2G45190
7233	3	F23 vs. L23	F2-3 up	GO:0010158	Development	Development	abaxial cell fate specification	P	AT5G60450
7234	3	F23 vs. L23	F2-3 up	GO:0015629	Development	Development	actin cytoskeleton	C	AT1G01780
7235	3	F23 vs. L23	F2-3 up	GO:0015629	Development	Development	actin cytoskeleton	C	AT2G45800
7236	3	F23 vs. L23	F2-3 up	GO:0015629	Development	Development	actin cytoskeleton	C	AT3G61230
7237	3	F23 vs. L23	F2-3 up	GO:0030036	Development	Development	actin cytoskeleton organization	P	AT2G19770
7238	3	F23 vs. L23	F2-3 up	GO:0030036	Development	Development	actin cytoskeleton organization	P	AT5G59370
7239	3	F23 vs. L23	F2-3 up	GO:0005884	Development	Development	actin filament	C	AT1G64500
7240	3	F23 vs. L23	F2-3 up	GO:0005884	Development	Development	actin filament	C	AT4G25590
7241	3	F23 vs. L23	F2-3 up	GO:0005884	Development	Development	actin filament	C	AT5G52360
7242	3	F23 vs. L23	F2-3 up	GO:0042643	Development	Development	actomyosin, actin portion	C	AT2G01330
7243	3	F23 vs. L23	F2-3 up	GO:0009943	Development	Development	adaxial/abaxial axis specification	P	AT1G79440
7244	3	F23 vs. L23	F2-3 up	GO:0009501	Development	Development	amyloplast	C	AT3G01180
7245	3	F23 vs. L23	F2-3 up	GO:0031225	Development	Development	anchored component of membrane	C	AT1G24520
7246	3	F23 vs. L23	F2-3 up	GO:0031225	Development	Development	anchored component of membrane	C	AT1G65240
7247	3	F23 vs. L23	F2-3 up	GO:0031225	Development	Development	anchored component of membrane	C	AT2G24450
7248	3	F23 vs. L23	F2-3 up	GO:0031225	Development	Development	anchored component of membrane	C	AT3G04010
7249	3	F23 vs. L23	F2-3 up	GO:0031225	Development	Development	anchored component of membrane	C	AT3G06035
7250	3	F23 vs. L23	F2-3 up	GO:0031225	Development	Development	anchored component of membrane	C	AT3G20580
7251	3	F23 vs. L23	F2-3 up	GO:0031225	Development	Development	anchored component of membrane	C	AT3G20865
7252	3	F23 vs. L23	F2-3 up	GO:0031225	Development	Development	anchored component of membrane	C	AT5G22430
7253	3	F23 vs. L23	F2-3 up	GO:0031225	Development	Development	anchored component of membrane	C	AT5G36260
7254	3	F23 vs. L23	F2-3 up	GO:0031225	Development	Development	anchored component of membrane	C	AT5G58050
7255	3	F23 vs. L23	F2-3 up	GO:0046658	Development	Development	anchored component of plasma membrane	C	AT1G65240
7256	3	F23 vs. L23	F2-3 up	GO:0046658	Development	Development	anchored component of plasma membrane	C	AT2G24450
7257	3	F23 vs. L23	F2-3 up	GO:0046658	Development	Development	anchored component of plasma membrane	C	AT3G04010
7258	3	F23 vs. L23	F2-3 up	GO:0046658	Development	Development	anchored component of plasma membrane	C	AT3G52470
7259	3	F23 vs. L23	F2-3 up	GO:0046658	Development	Development	anchored component of plasma membrane	C	AT5G06320
7260	3	F23 vs. L23	F2-3 up	GO:0009887	Development	Development	animal organ morphogenesis	P	AT5G65870
7261	3	F23 vs. L23	F2-3 up	GO:0051211	Development	Development	anisotropic cell growth	P	AT2G22125
7262	3	F23 vs. L23	F2-3 up	GO:0009901	Development	Development	anther dehiscence	P	AT1G17420
7263	3	F23 vs. L23	F2-3 up	GO:0009901	Development	Development	anther dehiscence	P	AT1G72520
7264	3	F23 vs. L23	F2-3 up	GO:0009901	Development	Development	anther dehiscence	P	AT4G25640
7265	3	F23 vs. L23	F2-3 up	GO:0009901	Development	Development	anther dehiscence	P	AT5G50800
7266	3	F23 vs. L23	F2-3 up	GO:0048653	Development	Development	anther development	P	AT1G17420
7267	3	F23 vs. L23	F2-3 up	GO:0048653	Development	Development	anther development	P	AT1G72520
7268	3	F23 vs. L23	F2-3 up	GO:0048653	Development	Development	anther development	P	AT2G17950
7269	3	F23 vs. L23	F2-3 up	GO:0048653	Development	Development	anther development	P	AT2G42200
7270	3	F23 vs. L23	F2-3 up	GO:0048653	Development	Development	anther development	P	AT3G48520

7271	3	F23 vs. L23	F2-3 up	GO:0016324	Development	Development	apical plasma membrane	C	AT2G41210
7272	3	F23 vs. L23	F2-3 up	GO:0016324	Development	Development	apical plasma membrane	C	AT3G07960
7273	3	F23 vs. L23	F2-3 up	GO:0016324	Development	Development	apical plasma membrane	C	AT3G56960
7274	3	F23 vs. L23	F2-3 up	GO:0016324	Development	Development	apical plasma membrane	C	AT4G39110
7275	3	F23 vs. L23	F2-3 up	GO:0048046	Development	Development	apoplast	C	AT1G05850
7276	3	F23 vs. L23	F2-3 up	GO:0048046	Development	Development	apoplast	C	AT1G09310
7277	3	F23 vs. L23	F2-3 up	GO:0048046	Development	Development	apoplast	C	AT1G09340
7278	3	F23 vs. L23	F2-3 up	GO:0048046	Development	Development	apoplast	C	AT1G11860
7279	3	F23 vs. L23	F2-3 up	GO:0048046	Development	Development	apoplast	C	AT1G20020
7280	3	F23 vs. L23	F2-3 up	GO:0048046	Development	Development	apoplast	C	AT1G20620
7281	3	F23 vs. L23	F2-3 up	GO:0048046	Development	Development	apoplast	C	AT1G23310
7282	3	F23 vs. L23	F2-3 up	GO:0048046	Development	Development	apoplast	C	AT1G23740
7283	3	F23 vs. L23	F2-3 up	GO:0048046	Development	Development	apoplast	C	AT1G28270
7284	3	F23 vs. L23	F2-3 up	GO:0048046	Development	Development	apoplast	C	AT1G32060
7285	3	F23 vs. L23	F2-3 up	GO:0048046	Development	Development	apoplast	C	AT1G35720
7286	3	F23 vs. L23	F2-3 up	GO:0048046	Development	Development	apoplast	C	AT1G41830
7287	3	F23 vs. L23	F2-3 up	GO:0048046	Development	Development	apoplast	C	AT1G42970
7288	3	F23 vs. L23	F2-3 up	GO:0048046	Development	Development	apoplast	C	AT1G45130
7289	3	F23 vs. L23	F2-3 up	GO:0048046	Development	Development	apoplast	C	AT1G48030
7290	3	F23 vs. L23	F2-3 up	GO:0048046	Development	Development	apoplast	C	AT1G68010
7291	3	F23 vs. L23	F2-3 up	GO:0048046	Development	Development	apoplast	C	AT1G72610
7292	3	F23 vs. L23	F2-3 up	GO:0048046	Development	Development	apoplast	C	AT1G78370
7293	3	F23 vs. L23	F2-3 up	GO:0048046	Development	Development	apoplast	C	AT1G78820
7294	3	F23 vs. L23	F2-3 up	GO:0048046	Development	Development	apoplast	C	AT2G10940
7295	3	F23 vs. L23	F2-3 up	GO:0048046	Development	Development	apoplast	C	AT2G15620
7296	3	F23 vs. L23	F2-3 up	GO:0048046	Development	Development	apoplast	C	AT2G18660
7297	3	F23 vs. L23	F2-3 up	GO:0048046	Development	Development	apoplast	C	AT2G21330
7298	3	F23 vs. L23	F2-3 up	GO:0048046	Development	Development	apoplast	C	AT2G28190
7299	3	F23 vs. L23	F2-3 up	GO:0048046	Development	Development	apoplast	C	AT2G30860
7300	3	F23 vs. L23	F2-3 up	GO:0048046	Development	Development	apoplast	C	AT2G39730
7301	3	F23 vs. L23	F2-3 up	GO:0048046	Development	Development	apoplast	C	AT2G43570
7302	3	F23 vs. L23	F2-3 up	GO:0048046	Development	Development	apoplast	C	AT3G01500
7303	3	F23 vs. L23	F2-3 up	GO:0048046	Development	Development	apoplast	C	AT3G04120
7304	3	F23 vs. L23	F2-3 up	GO:0048046	Development	Development	apoplast	C	AT3G05930
7305	3	F23 vs. L23	F2-3 up	GO:0048046	Development	Development	apoplast	C	AT3G09440
7306	3	F23 vs. L23	F2-3 up	GO:0048046	Development	Development	apoplast	C	AT3G11630
7307	3	F23 vs. L23	F2-3 up	GO:0048046	Development	Development	apoplast	C	AT3G12780
7308	3	F23 vs. L23	F2-3 up	GO:0048046	Development	Development	apoplast	C	AT3G14310
7309	3	F23 vs. L23	F2-3 up	GO:0048046	Development	Development	apoplast	C	AT3G14420
7310	3	F23 vs. L23	F2-3 up	GO:0048046	Development	Development	apoplast	C	AT3G14940
7311	3	F23 vs. L23	F2-3 up	GO:0048046	Development	Development	apoplast	C	AT3G16370
7312	3	F23 vs. L23	F2-3 up	GO:0048046	Development	Development	apoplast	C	AT3G25170
7313	3	F23 vs. L23	F2-3 up	GO:0048046	Development	Development	apoplast	C	AT3G26650
7314	3	F23 vs. L23	F2-3 up	GO:0048046	Development	Development	apoplast	C	AT3G44990
7315	3	F23 vs. L23	F2-3 up	GO:0048046	Development	Development	apoplast	C	AT3G48990
7316	3	F23 vs. L23	F2-3 up	GO:0048046	Development	Development	apoplast	C	AT3G52600
7317	3	F23 vs. L23	F2-3 up	GO:0048046	Development	Development	apoplast	C	AT3G52880
7318	3	F23 vs. L23	F2-3 up	GO:0048046	Development	Development	apoplast	C	AT3G55800
7319	3	F23 vs. L23	F2-3 up	GO:0048046	Development	Development	apoplast	C	AT3G63140
7320	3	F23 vs. L23	F2-3 up	GO:0048046	Development	Development	apoplast	C	AT4G14880
7321	3	F23 vs. L23	F2-3 up	GO:0048046	Development	Development	apoplast	C	AT4G20360
7322	3	F23 vs. L23	F2-3 up	GO:0048046	Development	Development	apoplast	C	AT4G33010
7323	3	F23 vs. L23	F2-3 up	GO:0048046	Development	Development	apoplast	C	AT4G35010
7324	3	F23 vs. L23	F2-3 up	GO:0048046	Development	Development	apoplast	C	AT4G38740
7325	3	F23 vs. L23	F2-3 up	GO:0048046	Development	Development	apoplast	C	AT4G38970
7326	3	F23 vs. L23	F2-3 up	GO:0048046	Development	Development	apoplast	C	AT4G39640

7327	3	F23 vs. L23	F2-3 up	GO:0048046	Development	Development	apoplast	C	AT5G04140
7328	3	F23 vs. L23	F2-3 up	GO:0048046	Development	Development	apoplast	C	AT5G08370
7329	3	F23 vs. L23	F2-3 up	GO:0048046	Development	Development	apoplast	C	AT5G09550
7330	3	F23 vs. L23	F2-3 up	GO:0048046	Development	Development	apoplast	C	AT5G09660
7331	3	F23 vs. L23	F2-3 up	GO:0048046	Development	Development	apoplast	C	AT5G14740
7332	3	F23 vs. L23	F2-3 up	GO:0048046	Development	Development	apoplast	C	AT5G25980
7333	3	F23 vs. L23	F2-3 up	GO:0048046	Development	Development	apoplast	C	AT5G35630
7334	3	F23 vs. L23	F2-3 up	GO:0048046	Development	Development	apoplast	C	AT5G38420
7335	3	F23 vs. L23	F2-3 up	GO:0048046	Development	Development	apoplast	C	AT5G48300
7336	3	F23 vs. L23	F2-3 up	GO:0048046	Development	Development	apoplast	C	AT5G49360
7337	3	F23 vs. L23	F2-3 up	GO:0048046	Development	Development	apoplast	C	AT5G52360
7338	3	F23 vs. L23	F2-3 up	GO:0048046	Development	Development	apoplast	C	AT5G57560
7339	3	F23 vs. L23	F2-3 up	GO:0048046	Development	Development	apoplast	C	AT5G58330
7340	3	F23 vs. L23	F2-3 up	GO:0048046	Development	Development	apoplast	C	AT5G60020
7341	3	F23 vs. L23	F2-3 up	GO:0048046	Development	Development	apoplast	C	AT5G61410
7342	3	F23 vs. L23	F2-3 up	GO:0048046	Development	Development	apoplast	C	AT5G65730
7343	3	F23 vs. L23	F2-3 up	GO:0090506	Development	Development	axillary shoot meristem initiation	P	AT2G17950
7344	3	F23 vs. L23	F2-3 up	GO:0048440	Development	Development	carpel development	P	AT1G62360
7345	3	F23 vs. L23	F2-3 up	GO:0048226	Development	Development	Casparian strip	C	AT4G15620
7346	3	F23 vs. L23	F2-3 up	GO:0048226	Development	Development	Casparian strip	C	AT4G15630
7347	3	F23 vs. L23	F2-3 up	GO:0005623	Development	Development	cell	C	AT3G07560
7348	3	F23 vs. L23	F2-3 up	GO:0005623	Development	Development	cell	C	AT3G15353
7349	3	F23 vs. L23	F2-3 up	GO:0005623	Development	Development	cell	C	AT3G25690
7350	3	F23 vs. L23	F2-3 up	GO:0005623	Development	Development	cell	C	AT4G17170
7351	3	F23 vs. L23	F2-3 up	GO:0005623	Development	Development	cell	C	AT5G65460
7352	3	F23 vs. L23	F2-3 up	GO:0007155	Development	Development	cell adhesion	P	AT1G78240
7353	3	F23 vs. L23	F2-3 up	GO:0007155	Development	Development	cell adhesion	P	AT3G25140
7354	3	F23 vs. L23	F2-3 up	GO:0005938	Development	Development	cell cortex	C	AT2G19770
7355	3	F23 vs. L23	F2-3 up	GO:0005938	Development	Development	cell cortex	C	AT3G45640
7356	3	F23 vs. L23	F2-3 up	GO:0005938	Development	Development	cell cortex	C	AT4G29340
7357	3	F23 vs. L23	F2-3 up	GO:0005938	Development	Development	cell cortex	C	AT4G29350
7358	3	F23 vs. L23	F2-3 up	GO:0030154	Development	Development	cell differentiation	P	AT1G69120
7359	3	F23 vs. L23	F2-3 up	GO:0030154	Development	Development	cell differentiation	P	AT1G78570
7360	3	F23 vs. L23	F2-3 up	GO:0030154	Development	Development	cell differentiation	P	AT2G17950
7361	3	F23 vs. L23	F2-3 up	GO:0030154	Development	Development	cell differentiation	P	AT2G32460
7362	3	F23 vs. L23	F2-3 up	GO:0030154	Development	Development	cell differentiation	P	AT2G46410
7363	3	F23 vs. L23	F2-3 up	GO:0030154	Development	Development	cell differentiation	P	AT3G15030
7364	3	F23 vs. L23	F2-3 up	GO:0030154	Development	Development	cell differentiation	P	AT3G54340
7365	3	F23 vs. L23	F2-3 up	GO:0030154	Development	Development	cell differentiation	P	AT4G32880
7366	3	F23 vs. L23	F2-3 up	GO:0030154	Development	Development	cell differentiation	P	AT4G36710
7367	3	F23 vs. L23	F2-3 up	GO:0030154	Development	Development	cell differentiation	P	AT5G16600
7368	3	F23 vs. L23	F2-3 up	GO:0030154	Development	Development	cell differentiation	P	AT5G20240
7369	3	F23 vs. L23	F2-3 up	GO:0030154	Development	Development	cell differentiation	P	AT5G60910
7370	3	F23 vs. L23	F2-3 up	GO:0030154	Development	Development	cell differentiation	P	AT5G65870
7371	3	F23 vs. L23	F2-3 up	GO:0045165	Development	Development	cell fate commitment	P	AT2G45190
7372	3	F23 vs. L23	F2-3 up	GO:0045165	Development	Development	cell fate commitment	P	AT4G08150
7373	3	F23 vs. L23	F2-3 up	GO:0001708	Development	Development	cell fate specification	P	AT4G08150
7374	3	F23 vs. L23	F2-3 up	GO:0009504	Development	Development	cell plate	C	AT5G49720
7375	3	F23 vs. L23	F2-3 up	GO:0009986	Development	Development	cell surface	C	AT1G70940
7376	3	F23 vs. L23	F2-3 up	GO:0009986	Development	Development	cell surface	C	AT3G17790
7377	3	F23 vs. L23	F2-3 up	GO:0009986	Development	Development	cell surface	C	AT3G45780
7378	3	F23 vs. L23	F2-3 up	GO:0005618	Development	Development	cell wall	C	AT1G02790
7379	3	F23 vs. L23	F2-3 up	GO:0005618	Development	Development	cell wall	C	AT1G03230
7380	3	F23 vs. L23	F2-3 up	GO:0005618	Development	Development	cell wall	C	AT1G20190
7381	3	F23 vs. L23	F2-3 up	GO:0005618	Development	Development	cell wall	C	AT1G20620
7382	3	F23 vs. L23	F2-3 up	GO:0005618	Development	Development	cell wall	C	AT1G20850

7383	3	F23 vs. L23	F2-3 up	GO:0005618	Development	Development	cell wall	C	AT1G26770
7384	3	F23 vs. L23	F2-3 up	GO:0005618	Development	Development	cell wall	C	AT1G35720
7385	3	F23 vs. L23	F2-3 up	GO:0005618	Development	Development	cell wall	C	AT1G41830
7386	3	F23 vs. L23	F2-3 up	GO:0005618	Development	Development	cell wall	C	AT1G45130
7387	3	F23 vs. L23	F2-3 up	GO:0005618	Development	Development	cell wall	C	AT1G62380
7388	3	F23 vs. L23	F2-3 up	GO:0005618	Development	Development	cell wall	C	AT1G69530
7389	3	F23 vs. L23	F2-3 up	GO:0005618	Development	Development	cell wall	C	AT1G72610
7390	3	F23 vs. L23	F2-3 up	GO:0005618	Development	Development	cell wall	C	AT1G78820
7391	3	F23 vs. L23	F2-3 up	GO:0005618	Development	Development	cell wall	C	AT2G02990
7392	3	F23 vs. L23	F2-3 up	GO:0005618	Development	Development	cell wall	C	AT2G16430
7393	3	F23 vs. L23	F2-3 up	GO:0005618	Development	Development	cell wall	C	AT2G18660
7394	3	F23 vs. L23	F2-3 up	GO:0005618	Development	Development	cell wall	C	AT2G20870
7395	3	F23 vs. L23	F2-3 up	GO:0005618	Development	Development	cell wall	C	AT2G21140
7396	3	F23 vs. L23	F2-3 up	GO:0005618	Development	Development	cell wall	C	AT2G38870
7397	3	F23 vs. L23	F2-3 up	GO:0005618	Development	Development	cell wall	C	AT2G39730
7398	3	F23 vs. L23	F2-3 up	GO:0005618	Development	Development	cell wall	C	AT2G45220
7399	3	F23 vs. L23	F2-3 up	GO:0005618	Development	Development	cell wall	C	AT3G05620
7400	3	F23 vs. L23	F2-3 up	GO:0005618	Development	Development	cell wall	C	AT3G05930
7401	3	F23 vs. L23	F2-3 up	GO:0005618	Development	Development	cell wall	C	AT3G07850
7402	3	F23 vs. L23	F2-3 up	GO:0005618	Development	Development	cell wall	C	AT3G09440
7403	3	F23 vs. L23	F2-3 up	GO:0005618	Development	Development	cell wall	C	AT3G12780
7404	3	F23 vs. L23	F2-3 up	GO:0005618	Development	Development	cell wall	C	AT3G14310
7405	3	F23 vs. L23	F2-3 up	GO:0005618	Development	Development	cell wall	C	AT3G15360
7406	3	F23 vs. L23	F2-3 up	GO:0005618	Development	Development	cell wall	C	AT3G17060
7407	3	F23 vs. L23	F2-3 up	GO:0005618	Development	Development	cell wall	C	AT3G17390
7408	3	F23 vs. L23	F2-3 up	GO:0005618	Development	Development	cell wall	C	AT3G18080
7409	3	F23 vs. L23	F2-3 up	GO:0005618	Development	Development	cell wall	C	AT3G29030
7410	3	F23 vs. L23	F2-3 up	GO:0005618	Development	Development	cell wall	C	AT3G43270
7411	3	F23 vs. L23	F2-3 up	GO:0005618	Development	Development	cell wall	C	AT3G44990
7412	3	F23 vs. L23	F2-3 up	GO:0005618	Development	Development	cell wall	C	AT3G51600
7413	3	F23 vs. L23	F2-3 up	GO:0005618	Development	Development	cell wall	C	AT3G52600
7414	3	F23 vs. L23	F2-3 up	GO:0005618	Development	Development	cell wall	C	AT3G62170
7415	3	F23 vs. L23	F2-3 up	GO:0005618	Development	Development	cell wall	C	AT4G00230
7416	3	F23 vs. L23	F2-3 up	GO:0005618	Development	Development	cell wall	C	AT4G01850
7417	3	F23 vs. L23	F2-3 up	GO:0005618	Development	Development	cell wall	C	AT4G13990
7418	3	F23 vs. L23	F2-3 up	GO:0005618	Development	Development	cell wall	C	AT4G19410
7419	3	F23 vs. L23	F2-3 up	GO:0005618	Development	Development	cell wall	C	AT4G24890
7420	3	F23 vs. L23	F2-3 up	GO:0005618	Development	Development	cell wall	C	AT4G33220
7421	3	F23 vs. L23	F2-3 up	GO:0005618	Development	Development	cell wall	C	AT4G35010
7422	3	F23 vs. L23	F2-3 up	GO:0005618	Development	Development	cell wall	C	AT4G38770
7423	3	F23 vs. L23	F2-3 up	GO:0005618	Development	Development	cell wall	C	AT5G07430
7424	3	F23 vs. L23	F2-3 up	GO:0005618	Development	Development	cell wall	C	AT5G09810
7425	3	F23 vs. L23	F2-3 up	GO:0005618	Development	Development	cell wall	C	AT5G11420
7426	3	F23 vs. L23	F2-3 up	GO:0005618	Development	Development	cell wall	C	AT5G15490
7427	3	F23 vs. L23	F2-3 up	GO:0005618	Development	Development	cell wall	C	AT5G45280
7428	3	F23 vs. L23	F2-3 up	GO:0005618	Development	Development	cell wall	C	AT5G57560
7429	3	F23 vs. L23	F2-3 up	GO:0005618	Development	Development	cell wall	C	AT5G65730
7430	3	F23 vs. L23	F2-3 up	GO:0005618	Development	Development	cell wall	C	AT5G67470
7431	3	F23 vs. L23	F2-3 up	GO:0042546	Development	Development	cell wall biogenesis	P	AT3G44990
7432	3	F23 vs. L23	F2-3 up	GO:0042546	Development	Development	cell wall biogenesis	P	AT5G57560
7433	3	F23 vs. L23	F2-3 up	GO:0042546	Development	Development	cell wall biogenesis	P	AT5G65730
7434	3	F23 vs. L23	F2-3 up	GO:0042545	Development	Development	cell wall modification	P	AT2G45220
7435	3	F23 vs. L23	F2-3 up	GO:0042545	Development	Development	cell wall modification	P	AT3G05620
7436	3	F23 vs. L23	F2-3 up	GO:0042545	Development	Development	cell wall modification	P	AT3G14310
7437	3	F23 vs. L23	F2-3 up	GO:0042545	Development	Development	cell wall modification	P	AT3G17060
7438	3	F23 vs. L23	F2-3 up	GO:0042545	Development	Development	cell wall modification	P	AT3G43270

7439	3	F23 vs. L23	F2-3 up	GO:0042545	Development	Development	cell wall modification	P	AT3G62170
7440	3	F23 vs. L23	F2-3 up	GO:0042545	Development	Development	cell wall modification	P	AT4G15620
7441	3	F23 vs. L23	F2-3 up	GO:0042545	Development	Development	cell wall modification	P	AT4G15630
7442	3	F23 vs. L23	F2-3 up	GO:0042545	Development	Development	cell wall modification	P	AT4G33220
7443	3	F23 vs. L23	F2-3 up	GO:0042545	Development	Development	cell wall modification	P	AT5G07430
7444	3	F23 vs. L23	F2-3 up	GO:0042545	Development	Development	cell wall modification	P	AT5G53370
7445	3	F23 vs. L23	F2-3 up	GO:0071555	Development	Development	cell wall organization	P	AT1G02790
7446	3	F23 vs. L23	F2-3 up	GO:0071555	Development	Development	cell wall organization	P	AT1G63000
7447	3	F23 vs. L23	F2-3 up	GO:0071555	Development	Development	cell wall organization	P	AT1G64390
7448	3	F23 vs. L23	F2-3 up	GO:0071555	Development	Development	cell wall organization	P	AT1G75500
7449	3	F23 vs. L23	F2-3 up	GO:0071555	Development	Development	cell wall organization	P	AT1G78570
7450	3	F23 vs. L23	F2-3 up	GO:0071555	Development	Development	cell wall organization	P	AT2G13680
7451	3	F23 vs. L23	F2-3 up	GO:0071555	Development	Development	cell wall organization	P	AT2G32990
7452	3	F23 vs. L23	F2-3 up	GO:0071555	Development	Development	cell wall organization	P	AT2G33100
7453	3	F23 vs. L23	F2-3 up	GO:0071555	Development	Development	cell wall organization	P	AT3G05910
7454	3	F23 vs. L23	F2-3 up	GO:0071555	Development	Development	cell wall organization	P	AT3G07820
7455	3	F23 vs. L23	F2-3 up	GO:0071555	Development	Development	cell wall organization	P	AT3G07830
7456	3	F23 vs. L23	F2-3 up	GO:0071555	Development	Development	cell wall organization	P	AT3G07840
7457	3	F23 vs. L23	F2-3 up	GO:0071555	Development	Development	cell wall organization	P	AT3G07850
7458	3	F23 vs. L23	F2-3 up	GO:0071555	Development	Development	cell wall organization	P	AT3G44990
7459	3	F23 vs. L23	F2-3 up	GO:0071555	Development	Development	cell wall organization	P	AT4G16590
7460	3	F23 vs. L23	F2-3 up	GO:0071555	Development	Development	cell wall organization	P	AT4G19410
7461	3	F23 vs. L23	F2-3 up	GO:0071555	Development	Development	cell wall organization	P	AT4G19420
7462	3	F23 vs. L23	F2-3 up	GO:0071555	Development	Development	cell wall organization	P	AT4G24510
7463	3	F23 vs. L23	F2-3 up	GO:0071555	Development	Development	cell wall organization	P	AT4G31590
7464	3	F23 vs. L23	F2-3 up	GO:0071555	Development	Development	cell wall organization	P	AT4G32410
7465	3	F23 vs. L23	F2-3 up	GO:0071555	Development	Development	cell wall organization	P	AT4G39350
7466	3	F23 vs. L23	F2-3 up	GO:0071555	Development	Development	cell wall organization	P	AT5G08370
7467	3	F23 vs. L23	F2-3 up	GO:0071555	Development	Development	cell wall organization	P	AT5G09870
7468	3	F23 vs. L23	F2-3 up	GO:0071555	Development	Development	cell wall organization	P	AT5G16910
7469	3	F23 vs. L23	F2-3 up	GO:0071555	Development	Development	cell wall organization	P	AT5G45280
7470	3	F23 vs. L23	F2-3 up	GO:0071555	Development	Development	cell wall organization	P	AT5G48140
7471	3	F23 vs. L23	F2-3 up	GO:0071555	Development	Development	cell wall organization	P	AT5G49720
7472	3	F23 vs. L23	F2-3 up	GO:0071555	Development	Development	cell wall organization	P	AT5G64740
7473	3	F23 vs. L23	F2-3 up	GO:0071555	Development	Development	cell wall organization	P	AT5G65730
7474	3	F23 vs. L23	F2-3 up	GO:0052546	Development	Development	cell wall pectin metabolic process	P	AT3G29360
7475	3	F23 vs. L23	F2-3 up	GO:0052546	Development	Development	cell wall pectin metabolic process	P	AT5G15490
7476	3	F23 vs. L23	F2-3 up	GO:0007043	Development	Development	cell-cell junction assembly	P	AT4G15620
7477	3	F23 vs. L23	F2-3 up	GO:0007043	Development	Development	cell-cell junction assembly	P	AT4G15630
7478	3	F23 vs. L23	F2-3 up	GO:0005575	Development	Development	cellular_component	C	AT3G50560
7479	3	F23 vs. L23	F2-3 up	GO:0005575	Development	Development	cellular_component	C	AT4G29780
7480	3	F23 vs. L23	F2-3 up	GO:0005575	Development	Development	cellular_component	C	AT5G55620
7481	3	F23 vs. L23	F2-3 up	GO:0042807	Development	Development	central vacuole	C	AT2G36830
7482	3	F23 vs. L23	F2-3 up	GO:0000785	Development	Development	chromatin	C	AT1G11950
7483	3	F23 vs. L23	F2-3 up	GO:0006325	Development	Development	chromatin organization	P	AT1G04400
7484	3	F23 vs. L23	F2-3 up	GO:0006325	Development	Development	chromatin organization	P	AT3G50000
7485	3	F23 vs. L23	F2-3 up	GO:0009661	Development	Development	chromoplast organization	P	AT5G61670
7486	3	F23 vs. L23	F2-3 up	GO:0048268	Development	Development	clathrin coat assembly	P	AT1G03050
7487	3	F23 vs. L23	F2-3 up	GO:0032050	Development	Development	clathrin heavy chain binding	F	AT1G03050
7488	3	F23 vs. L23	F2-3 up	GO:0045334	Development	Development	clathrin-coated endocytic vesicle	C	AT1G03050
7489	3	F23 vs. L23	F2-3 up	GO:0005905	Development	Development	clathrin-coated pit	C	AT1G03050
7490	3	F23 vs. L23	F2-3 up	GO:0030136	Development	Development	clathrin-coated vesicle	C	AT1G03050
7491	3	F23 vs. L23	F2-3 up	GO:0072583	Development	Development	clathrin-dependent endocytosis	P	AT1G03050
7492	3	F23 vs. L23	F2-3 up	GO:0072583	Development	Development	clathrin-dependent endocytosis	P	AT3G07960
7493	3	F23 vs. L23	F2-3 up	GO:0043674	Development	Development	columella	C	AT2G47160
7494	3	F23 vs. L23	F2-3 up	GO:0043674	Development	Development	columella	C	AT3G26520

7495	3	F23 vs. L23	F2-3 up	GO:0030864	Development	Development	cortical actin cytoskeleton	C	AT2G01330
7496	3	F23 vs. L23	F2-3 up	GO:0043622	Development	Development	cortical microtubule organization	P	AT5G49720
7497	3	F23 vs. L23	F2-3 up	GO:0043622	Development	Development	cortical microtubule organization	P	AT5G64740
7498	3	F23 vs. L23	F2-3 up	GO:0010005	Development	Development	cortical microtubule, transverse to long axis	C	AT5G64740
7499	3	F23 vs. L23	F2-3 up	GO:0048825	Development	Development	cotyledon development	P	AT1G79440
7500	3	F23 vs. L23	F2-3 up	GO:0048825	Development	Development	cotyledon development	P	AT3G07050
7501	3	F23 vs. L23	F2-3 up	GO:0048825	Development	Development	cotyledon development	P	AT3G15030
7502	3	F23 vs. L23	F2-3 up	GO:0048826	Development	Development	cotyledon morphogenesis	P	AT3G15030
7503	3	F23 vs. L23	F2-3 up	GO:0042335	Development	Development	cuticle development	P	AT1G02205
7504	3	F23 vs. L23	F2-3 up	GO:0042335	Development	Development	cuticle development	P	AT1G68530
7505	3	F23 vs. L23	F2-3 up	GO:0042335	Development	Development	cuticle development	P	AT5G33370
7506	3	F23 vs. L23	F2-3 up	GO:0042335	Development	Development	cuticle development	P	AT5G57800
7507	3	F23 vs. L23	F2-3 up	GO:0005737	Development	Development	cytoplasm	C	AT1G01780
7508	3	F23 vs. L23	F2-3 up	GO:0005737	Development	Development	cytoplasm	C	AT1G03495
7509	3	F23 vs. L23	F2-3 up	GO:0005737	Development	Development	cytoplasm	C	AT1G03620
7510	3	F23 vs. L23	F2-3 up	GO:0005737	Development	Development	cytoplasm	C	AT1G03905
7511	3	F23 vs. L23	F2-3 up	GO:0005737	Development	Development	cytoplasm	C	AT1G04400
7512	3	F23 vs. L23	F2-3 up	GO:0005737	Development	Development	cytoplasm	C	AT1G05010
7513	3	F23 vs. L23	F2-3 up	GO:0005737	Development	Development	cytoplasm	C	AT1G06640
7514	3	F23 vs. L23	F2-3 up	GO:0005737	Development	Development	cytoplasm	C	AT1G08830
7515	3	F23 vs. L23	F2-3 up	GO:0005737	Development	Development	cytoplasm	C	AT1G09430
7516	3	F23 vs. L23	F2-3 up	GO:0005737	Development	Development	cytoplasm	C	AT1G10370
7517	3	F23 vs. L23	F2-3 up	GO:0005737	Development	Development	cytoplasm	C	AT1G12070
7518	3	F23 vs. L23	F2-3 up	GO:0005737	Development	Development	cytoplasm	C	AT1G13890
7519	3	F23 vs. L23	F2-3 up	GO:0005737	Development	Development	cytoplasm	C	AT1G15470
7520	3	F23 vs. L23	F2-3 up	GO:0005737	Development	Development	cytoplasm	C	AT1G18150
7521	3	F23 vs. L23	F2-3 up	GO:0005737	Development	Development	cytoplasm	C	AT1G18660
7522	3	F23 vs. L23	F2-3 up	GO:0005737	Development	Development	cytoplasm	C	AT1G18740
7523	3	F23 vs. L23	F2-3 up	GO:0005737	Development	Development	cytoplasm	C	AT1G21760
7524	3	F23 vs. L23	F2-3 up	GO:0005737	Development	Development	cytoplasm	C	AT1G21980
7525	3	F23 vs. L23	F2-3 up	GO:0005737	Development	Development	cytoplasm	C	AT1G22710
7526	3	F23 vs. L23	F2-3 up	GO:0005737	Development	Development	cytoplasm	C	AT1G22760
7527	3	F23 vs. L23	F2-3 up	GO:0005737	Development	Development	cytoplasm	C	AT1G23310
7528	3	F23 vs. L23	F2-3 up	GO:0005737	Development	Development	cytoplasm	C	AT1G23750
7529	3	F23 vs. L23	F2-3 up	GO:0005737	Development	Development	cytoplasm	C	AT1G25350
7530	3	F23 vs. L23	F2-3 up	GO:0005737	Development	Development	cytoplasm	C	AT1G26480
7531	3	F23 vs. L23	F2-3 up	GO:0005737	Development	Development	cytoplasm	C	AT1G28480
7532	3	F23 vs. L23	F2-3 up	GO:0005737	Development	Development	cytoplasm	C	AT1G28680
7533	3	F23 vs. L23	F2-3 up	GO:0005737	Development	Development	cytoplasm	C	AT1G29340
7534	3	F23 vs. L23	F2-3 up	GO:0005737	Development	Development	cytoplasm	C	AT1G31490
7535	3	F23 vs. L23	F2-3 up	GO:0005737	Development	Development	cytoplasm	C	AT1G35190
7536	3	F23 vs. L23	F2-3 up	GO:0005737	Development	Development	cytoplasm	C	AT1G42680
7537	3	F23 vs. L23	F2-3 up	GO:0005737	Development	Development	cytoplasm	C	AT1G44160
7538	3	F23 vs. L23	F2-3 up	GO:0005737	Development	Development	cytoplasm	C	AT1G44170
7539	3	F23 vs. L23	F2-3 up	GO:0005737	Development	Development	cytoplasm	C	AT1G47530
7540	3	F23 vs. L23	F2-3 up	GO:0005737	Development	Development	cytoplasm	C	AT1G48600
7541	3	F23 vs. L23	F2-3 up	GO:0005737	Development	Development	cytoplasm	C	AT1G50320
7542	3	F23 vs. L23	F2-3 up	GO:0005737	Development	Development	cytoplasm	C	AT1G54290
7543	3	F23 vs. L23	F2-3 up	GO:0005737	Development	Development	cytoplasm	C	AT1G56500
7544	3	F23 vs. L23	F2-3 up	GO:0005737	Development	Development	cytoplasm	C	AT1G56580
7545	3	F23 vs. L23	F2-3 up	GO:0005737	Development	Development	cytoplasm	C	AT1G58180
7546	3	F23 vs. L23	F2-3 up	GO:0005737	Development	Development	cytoplasm	C	AT1G59730
7547	3	F23 vs. L23	F2-3 up	GO:0005737	Development	Development	cytoplasm	C	AT1G60810
7548	3	F23 vs. L23	F2-3 up	GO:0005737	Development	Development	cytoplasm	C	AT1G62360
7549	3	F23 vs. L23	F2-3 up	GO:0005737	Development	Development	cytoplasm	C	AT1G62380
7550	3	F23 vs. L23	F2-3 up	GO:0005737	Development	Development	cytoplasm	C	AT1G62450

7551	3	F23 vs. L23	F2-3 up	GO:0005737	Development	Development	cytoplasm	C	AT1G64190
7552	3	F23 vs. L23	F2-3 up	GO:0005737	Development	Development	cytoplasm	C	AT1G64740
7553	3	F23 vs. L23	F2-3 up	GO:0005737	Development	Development	cytoplasm	C	AT1G68530
7554	3	F23 vs. L23	F2-3 up	GO:0005737	Development	Development	cytoplasm	C	AT1G68570
7555	3	F23 vs. L23	F2-3 up	GO:0005737	Development	Development	cytoplasm	C	AT1G70580
7556	3	F23 vs. L23	F2-3 up	GO:0005737	Development	Development	cytoplasm	C	AT1G70730
7557	3	F23 vs. L23	F2-3 up	GO:0005737	Development	Development	cytoplasm	C	AT1G71050
7558	3	F23 vs. L23	F2-3 up	GO:0005737	Development	Development	cytoplasm	C	AT1G71697
7559	3	F23 vs. L23	F2-3 up	GO:0005737	Development	Development	cytoplasm	C	AT1G71770
7560	3	F23 vs. L23	F2-3 up	GO:0005737	Development	Development	cytoplasm	C	AT1G72730
7561	3	F23 vs. L23	F2-3 up	GO:0005737	Development	Development	cytoplasm	C	AT1G74090
7562	3	F23 vs. L23	F2-3 up	GO:0005737	Development	Development	cytoplasm	C	AT1G78160
7563	3	F23 vs. L23	F2-3 up	GO:0005737	Development	Development	cytoplasm	C	AT1G78290
7564	3	F23 vs. L23	F2-3 up	GO:0005737	Development	Development	cytoplasm	C	AT1G78320
7565	3	F23 vs. L23	F2-3 up	GO:0005737	Development	Development	cytoplasm	C	AT1G78370
7566	3	F23 vs. L23	F2-3 up	GO:0005737	Development	Development	cytoplasm	C	AT1G78780
7567	3	F23 vs. L23	F2-3 up	GO:0005737	Development	Development	cytoplasm	C	AT1G80280
7568	3	F23 vs. L23	F2-3 up	GO:0005737	Development	Development	cytoplasm	C	AT2G01290
7569	3	F23 vs. L23	F2-3 up	GO:0005737	Development	Development	cytoplasm	C	AT2G01330
7570	3	F23 vs. L23	F2-3 up	GO:0005737	Development	Development	cytoplasm	C	AT2G02390
7571	3	F23 vs. L23	F2-3 up	GO:0005737	Development	Development	cytoplasm	C	AT2G02800
7572	3	F23 vs. L23	F2-3 up	GO:0005737	Development	Development	cytoplasm	C	AT2G03410
7573	3	F23 vs. L23	F2-3 up	GO:0005737	Development	Development	cytoplasm	C	AT2G15310
7574	3	F23 vs. L23	F2-3 up	GO:0005737	Development	Development	cytoplasm	C	AT2G17290
7575	3	F23 vs. L23	F2-3 up	GO:0005737	Development	Development	cytoplasm	C	AT2G18390
7576	3	F23 vs. L23	F2-3 up	GO:0005737	Development	Development	cytoplasm	C	AT2G19770
7577	3	F23 vs. L23	F2-3 up	GO:0005737	Development	Development	cytoplasm	C	AT2G22125
7578	3	F23 vs. L23	F2-3 up	GO:0005737	Development	Development	cytoplasm	C	AT2G22240
7579	3	F23 vs. L23	F2-3 up	GO:0005737	Development	Development	cytoplasm	C	AT2G23170
7580	3	F23 vs. L23	F2-3 up	GO:0005737	Development	Development	cytoplasm	C	AT2G24270
7581	3	F23 vs. L23	F2-3 up	GO:0005737	Development	Development	cytoplasm	C	AT2G24540
7582	3	F23 vs. L23	F2-3 up	GO:0005737	Development	Development	cytoplasm	C	AT2G25530
7583	3	F23 vs. L23	F2-3 up	GO:0005737	Development	Development	cytoplasm	C	AT2G26490
7584	3	F23 vs. L23	F2-3 up	GO:0005737	Development	Development	cytoplasm	C	AT2G29630
7585	3	F23 vs. L23	F2-3 up	GO:0005737	Development	Development	cytoplasm	C	AT2G30520
7586	3	F23 vs. L23	F2-3 up	GO:0005737	Development	Development	cytoplasm	C	AT2G30830
7587	3	F23 vs. L23	F2-3 up	GO:0005737	Development	Development	cytoplasm	C	AT2G30860
7588	3	F23 vs. L23	F2-3 up	GO:0005737	Development	Development	cytoplasm	C	AT2G31500
7589	3	F23 vs. L23	F2-3 up	GO:0005737	Development	Development	cytoplasm	C	AT2G31570
7590	3	F23 vs. L23	F2-3 up	GO:0005737	Development	Development	cytoplasm	C	AT2G31830
7591	3	F23 vs. L23	F2-3 up	GO:0005737	Development	Development	cytoplasm	C	AT2G34810
7592	3	F23 vs. L23	F2-3 up	GO:0005737	Development	Development	cytoplasm	C	AT2G35840
7593	3	F23 vs. L23	F2-3 up	GO:0005737	Development	Development	cytoplasm	C	AT2G35940
7594	3	F23 vs. L23	F2-3 up	GO:0005737	Development	Development	cytoplasm	C	AT2G36830
7595	3	F23 vs. L23	F2-3 up	GO:0005737	Development	Development	cytoplasm	C	AT2G36880
7596	3	F23 vs. L23	F2-3 up	GO:0005737	Development	Development	cytoplasm	C	AT2G37040
7597	3	F23 vs. L23	F2-3 up	GO:0005737	Development	Development	cytoplasm	C	AT2G37760
7598	3	F23 vs. L23	F2-3 up	GO:0005737	Development	Development	cytoplasm	C	AT2G37770
7599	3	F23 vs. L23	F2-3 up	GO:0005737	Development	Development	cytoplasm	C	AT2G37970
7600	3	F23 vs. L23	F2-3 up	GO:0005737	Development	Development	cytoplasm	C	AT2G38240
7601	3	F23 vs. L23	F2-3 up	GO:0005737	Development	Development	cytoplasm	C	AT2G39330
7602	3	F23 vs. L23	F2-3 up	GO:0005737	Development	Development	cytoplasm	C	AT2G39770
7603	3	F23 vs. L23	F2-3 up	GO:0005737	Development	Development	cytoplasm	C	AT2G40000
7604	3	F23 vs. L23	F2-3 up	GO:0005737	Development	Development	cytoplasm	C	AT2G40230
7605	3	F23 vs. L23	F2-3 up	GO:0005737	Development	Development	cytoplasm	C	AT2G40670
7606	3	F23 vs. L23	F2-3 up	GO:0005737	Development	Development	cytoplasm	C	AT2G42200

7607	3	F23 vs. L23	F2-3 up	GO:0005737	Development	Development	cytoplasm	C	AT2G44120
7608	3	F23 vs. L23	F2-3 up	GO:0005737	Development	Development	cytoplasm	C	AT2G45800
7609	3	F23 vs. L23	F2-3 up	GO:0005737	Development	Development	cytoplasm	C	AT2G46860
7610	3	F23 vs. L23	F2-3 up	GO:0005737	Development	Development	cytoplasm	C	AT2G47160
7611	3	F23 vs. L23	F2-3 up	GO:0005737	Development	Development	cytoplasm	C	AT2G47650
7612	3	F23 vs. L23	F2-3 up	GO:0005737	Development	Development	cytoplasm	C	AT3G01400
7613	3	F23 vs. L23	F2-3 up	GO:0005737	Development	Development	cytoplasm	C	AT3G01500
7614	3	F23 vs. L23	F2-3 up	GO:0005737	Development	Development	cytoplasm	C	AT3G02570
7615	3	F23 vs. L23	F2-3 up	GO:0005737	Development	Development	cytoplasm	C	AT3G02770
7616	3	F23 vs. L23	F2-3 up	GO:0005737	Development	Development	cytoplasm	C	AT3G03190
7617	3	F23 vs. L23	F2-3 up	GO:0005737	Development	Development	cytoplasm	C	AT3G03250
7618	3	F23 vs. L23	F2-3 up	GO:0005737	Development	Development	cytoplasm	C	AT3G03430
7619	3	F23 vs. L23	F2-3 up	GO:0005737	Development	Development	cytoplasm	C	AT3G04120
7620	3	F23 vs. L23	F2-3 up	GO:0005737	Development	Development	cytoplasm	C	AT3G04840
7621	3	F23 vs. L23	F2-3 up	GO:0005737	Development	Development	cytoplasm	C	AT3G05500
7622	3	F23 vs. L23	F2-3 up	GO:0005737	Development	Development	cytoplasm	C	AT3G05580
7623	3	F23 vs. L23	F2-3 up	GO:0005737	Development	Development	cytoplasm	C	AT3G07960
7624	3	F23 vs. L23	F2-3 up	GO:0005737	Development	Development	cytoplasm	C	AT3G08730
7625	3	F23 vs. L23	F2-3 up	GO:0005737	Development	Development	cytoplasm	C	AT3G09440
7626	3	F23 vs. L23	F2-3 up	GO:0005737	Development	Development	cytoplasm	C	AT3G09940
7627	3	F23 vs. L23	F2-3 up	GO:0005737	Development	Development	cytoplasm	C	AT3G10700
7628	3	F23 vs. L23	F2-3 up	GO:0005737	Development	Development	cytoplasm	C	AT3G11050
7629	3	F23 vs. L23	F2-3 up	GO:0005737	Development	Development	cytoplasm	C	AT3G11630
7630	3	F23 vs. L23	F2-3 up	GO:0005737	Development	Development	cytoplasm	C	AT3G12690
7631	3	F23 vs. L23	F2-3 up	GO:0005737	Development	Development	cytoplasm	C	AT3G12780
7632	3	F23 vs. L23	F2-3 up	GO:0005737	Development	Development	cytoplasm	C	AT3G13930
7633	3	F23 vs. L23	F2-3 up	GO:0005737	Development	Development	cytoplasm	C	AT3G14310
7634	3	F23 vs. L23	F2-3 up	GO:0005737	Development	Development	cytoplasm	C	AT3G14420
7635	3	F23 vs. L23	F2-3 up	GO:0005737	Development	Development	cytoplasm	C	AT3G14940
7636	3	F23 vs. L23	F2-3 up	GO:0005737	Development	Development	cytoplasm	C	AT3G15360
7637	3	F23 vs. L23	F2-3 up	GO:0005737	Development	Development	cytoplasm	C	AT3G16800
7638	3	F23 vs. L23	F2-3 up	GO:0005737	Development	Development	cytoplasm	C	AT3G17020
7639	3	F23 vs. L23	F2-3 up	GO:0005737	Development	Development	cytoplasm	C	AT3G17390
7640	3	F23 vs. L23	F2-3 up	GO:0005737	Development	Development	cytoplasm	C	AT3G18780
7641	3	F23 vs. L23	F2-3 up	GO:0005737	Development	Development	cytoplasm	C	AT3G19370
7642	3	F23 vs. L23	F2-3 up	GO:0005737	Development	Development	cytoplasm	C	AT3G19710
7643	3	F23 vs. L23	F2-3 up	GO:0005737	Development	Development	cytoplasm	C	AT3G19980
7644	3	F23 vs. L23	F2-3 up	GO:0005737	Development	Development	cytoplasm	C	AT3G21260
7645	3	F23 vs. L23	F2-3 up	GO:0005737	Development	Development	cytoplasm	C	AT3G21560
7646	3	F23 vs. L23	F2-3 up	GO:0005737	Development	Development	cytoplasm	C	AT3G22400
7647	3	F23 vs. L23	F2-3 up	GO:0005737	Development	Development	cytoplasm	C	AT3G23810
7648	3	F23 vs. L23	F2-3 up	GO:0005737	Development	Development	cytoplasm	C	AT3G24503
7649	3	F23 vs. L23	F2-3 up	GO:0005737	Development	Development	cytoplasm	C	AT3G26060
7650	3	F23 vs. L23	F2-3 up	GO:0005737	Development	Development	cytoplasm	C	AT3G27300
7651	3	F23 vs. L23	F2-3 up	GO:0005737	Development	Development	cytoplasm	C	AT3G29360
7652	3	F23 vs. L23	F2-3 up	GO:0005737	Development	Development	cytoplasm	C	AT3G44190
7653	3	F23 vs. L23	F2-3 up	GO:0005737	Development	Development	cytoplasm	C	AT3G45140
7654	3	F23 vs. L23	F2-3 up	GO:0005737	Development	Development	cytoplasm	C	AT3G45640
7655	3	F23 vs. L23	F2-3 up	GO:0005737	Development	Development	cytoplasm	C	AT3G45780
7656	3	F23 vs. L23	F2-3 up	GO:0005737	Development	Development	cytoplasm	C	AT3G46520
7657	3	F23 vs. L23	F2-3 up	GO:0005737	Development	Development	cytoplasm	C	AT3G46620
7658	3	F23 vs. L23	F2-3 up	GO:0005737	Development	Development	cytoplasm	C	AT3G47940
7659	3	F23 vs. L23	F2-3 up	GO:0005737	Development	Development	cytoplasm	C	AT3G48170
7660	3	F23 vs. L23	F2-3 up	GO:0005737	Development	Development	cytoplasm	C	AT3G48990
7661	3	F23 vs. L23	F2-3 up	GO:0005737	Development	Development	cytoplasm	C	AT3G50210
7662	3	F23 vs. L23	F2-3 up	GO:0005737	Development	Development	cytoplasm	C	AT3G51240

7663	3	F23 vs. L23	F2-3 up	GO:0005737	Development	Development	cytoplasm	C	AT3G52720
7664	3	F23 vs. L23	F2-3 up	GO:0005737	Development	Development	cytoplasm	C	AT3G52880
7665	3	F23 vs. L23	F2-3 up	GO:0005737	Development	Development	cytoplasm	C	AT3G53960
7666	3	F23 vs. L23	F2-3 up	GO:0005737	Development	Development	cytoplasm	C	AT3G54660
7667	3	F23 vs. L23	F2-3 up	GO:0005737	Development	Development	cytoplasm	C	AT3G54700
7668	3	F23 vs. L23	F2-3 up	GO:0005737	Development	Development	cytoplasm	C	AT3G55610
7669	3	F23 vs. L23	F2-3 up	GO:0005737	Development	Development	cytoplasm	C	AT3G55800
7670	3	F23 vs. L23	F2-3 up	GO:0005737	Development	Development	cytoplasm	C	AT3G56150
7671	3	F23 vs. L23	F2-3 up	GO:0005737	Development	Development	cytoplasm	C	AT3G56240
7672	3	F23 vs. L23	F2-3 up	GO:0005737	Development	Development	cytoplasm	C	AT3G57050
7673	3	F23 vs. L23	F2-3 up	GO:0005737	Development	Development	cytoplasm	C	AT3G61220
7674	3	F23 vs. L23	F2-3 up	GO:0005737	Development	Development	cytoplasm	C	AT3G61230
7675	3	F23 vs. L23	F2-3 up	GO:0005737	Development	Development	cytoplasm	C	AT3G61760
7676	3	F23 vs. L23	F2-3 up	GO:0005737	Development	Development	cytoplasm	C	AT3G62290
7677	3	F23 vs. L23	F2-3 up	GO:0005737	Development	Development	cytoplasm	C	AT3G62960
7678	3	F23 vs. L23	F2-3 up	GO:0005737	Development	Development	cytoplasm	C	AT4G01480
7679	3	F23 vs. L23	F2-3 up	GO:0005737	Development	Development	cytoplasm	C	AT4G01850
7680	3	F23 vs. L23	F2-3 up	GO:0005737	Development	Development	cytoplasm	C	AT4G02890
7681	3	F23 vs. L23	F2-3 up	GO:0005737	Development	Development	cytoplasm	C	AT4G03190
7682	3	F23 vs. L23	F2-3 up	GO:0005737	Development	Development	cytoplasm	C	AT4G04040
7683	3	F23 vs. L23	F2-3 up	GO:0005737	Development	Development	cytoplasm	C	AT4G11280
7684	3	F23 vs. L23	F2-3 up	GO:0005737	Development	Development	cytoplasm	C	AT4G13930
7685	3	F23 vs. L23	F2-3 up	GO:0005737	Development	Development	cytoplasm	C	AT4G14710
7686	3	F23 vs. L23	F2-3 up	GO:0005737	Development	Development	cytoplasm	C	AT4G14880
7687	3	F23 vs. L23	F2-3 up	GO:0005737	Development	Development	cytoplasm	C	AT4G15700
7688	3	F23 vs. L23	F2-3 up	GO:0005737	Development	Development	cytoplasm	C	AT4G16143
7689	3	F23 vs. L23	F2-3 up	GO:0005737	Development	Development	cytoplasm	C	AT4G17080
7690	3	F23 vs. L23	F2-3 up	GO:0005737	Development	Development	cytoplasm	C	AT4G17170
7691	3	F23 vs. L23	F2-3 up	GO:0005737	Development	Development	cytoplasm	C	AT4G17230
7692	3	F23 vs. L23	F2-3 up	GO:0005737	Development	Development	cytoplasm	C	AT4G18220
7693	3	F23 vs. L23	F2-3 up	GO:0005737	Development	Development	cytoplasm	C	AT4G18950
7694	3	F23 vs. L23	F2-3 up	GO:0005737	Development	Development	cytoplasm	C	AT4G21990
7695	3	F23 vs. L23	F2-3 up	GO:0005737	Development	Development	cytoplasm	C	AT4G22870
7696	3	F23 vs. L23	F2-3 up	GO:0005737	Development	Development	cytoplasm	C	AT4G24380
7697	3	F23 vs. L23	F2-3 up	GO:0005737	Development	Development	cytoplasm	C	AT4G25590
7698	3	F23 vs. L23	F2-3 up	GO:0005737	Development	Development	cytoplasm	C	AT4G25640
7699	3	F23 vs. L23	F2-3 up	GO:0005737	Development	Development	cytoplasm	C	AT4G26100
7700	3	F23 vs. L23	F2-3 up	GO:0005737	Development	Development	cytoplasm	C	AT4G26260
7701	3	F23 vs. L23	F2-3 up	GO:0005737	Development	Development	cytoplasm	C	AT4G26470
7702	3	F23 vs. L23	F2-3 up	GO:0005737	Development	Development	cytoplasm	C	AT4G28000
7703	3	F23 vs. L23	F2-3 up	GO:0005737	Development	Development	cytoplasm	C	AT4G29340
7704	3	F23 vs. L23	F2-3 up	GO:0005737	Development	Development	cytoplasm	C	AT4G29350
7705	3	F23 vs. L23	F2-3 up	GO:0005737	Development	Development	cytoplasm	C	AT4G30530
7706	3	F23 vs. L23	F2-3 up	GO:0005737	Development	Development	cytoplasm	C	AT4G31290
7707	3	F23 vs. L23	F2-3 up	GO:0005737	Development	Development	cytoplasm	C	AT4G31800
7708	3	F23 vs. L23	F2-3 up	GO:0005737	Development	Development	cytoplasm	C	AT4G36250
7709	3	F23 vs. L23	F2-3 up	GO:0005737	Development	Development	cytoplasm	C	AT4G37990
7710	3	F23 vs. L23	F2-3 up	GO:0005737	Development	Development	cytoplasm	C	AT4G38740
7711	3	F23 vs. L23	F2-3 up	GO:0005737	Development	Development	cytoplasm	C	AT4G39800
7712	3	F23 vs. L23	F2-3 up	GO:0005737	Development	Development	cytoplasm	C	AT5G01410
7713	3	F23 vs. L23	F2-3 up	GO:0005737	Development	Development	cytoplasm	C	AT5G01600
7714	3	F23 vs. L23	F2-3 up	GO:0005737	Development	Development	cytoplasm	C	AT5G02270
7715	3	F23 vs. L23	F2-3 up	GO:0005737	Development	Development	cytoplasm	C	AT5G02790
7716	3	F23 vs. L23	F2-3 up	GO:0005737	Development	Development	cytoplasm	C	AT5G04710
7717	3	F23 vs. L23	F2-3 up	GO:0005737	Development	Development	cytoplasm	C	AT5G04980
7718	3	F23 vs. L23	F2-3 up	GO:0005737	Development	Development	cytoplasm	C	AT5G05600

7719	3	F23 vs. L23	F2-3 up	GO:0005737	Development	Development	cytoplasm	C	AT5G07440
7720	3	F23 vs. L23	F2-3 up	GO:0005737	Development	Development	cytoplasm	C	AT5G07460
7721	3	F23 vs. L23	F2-3 up	GO:0005737	Development	Development	cytoplasm	C	AT5G08100
7722	3	F23 vs. L23	F2-3 up	GO:0005737	Development	Development	cytoplasm	C	AT5G08640
7723	3	F23 vs. L23	F2-3 up	GO:0005737	Development	Development	cytoplasm	C	AT5G09230
7724	3	F23 vs. L23	F2-3 up	GO:0005737	Development	Development	cytoplasm	C	AT5G09550
7725	3	F23 vs. L23	F2-3 up	GO:0005737	Development	Development	cytoplasm	C	AT5G09660
7726	3	F23 vs. L23	F2-3 up	GO:0005737	Development	Development	cytoplasm	C	AT5G09810
7727	3	F23 vs. L23	F2-3 up	GO:0005737	Development	Development	cytoplasm	C	AT5G11790
7728	3	F23 vs. L23	F2-3 up	GO:0005737	Development	Development	cytoplasm	C	AT5G13930
7729	3	F23 vs. L23	F2-3 up	GO:0005737	Development	Development	cytoplasm	C	AT5G14740
7730	3	F23 vs. L23	F2-3 up	GO:0005737	Development	Development	cytoplasm	C	AT5G15490
7731	3	F23 vs. L23	F2-3 up	GO:0005737	Development	Development	cytoplasm	C	AT5G16450
7732	3	F23 vs. L23	F2-3 up	GO:0005737	Development	Development	cytoplasm	C	AT5G17480
7733	3	F23 vs. L23	F2-3 up	GO:0005737	Development	Development	cytoplasm	C	AT5G18280
7734	3	F23 vs. L23	F2-3 up	GO:0005737	Development	Development	cytoplasm	C	AT5G18600
7735	3	F23 vs. L23	F2-3 up	GO:0005737	Development	Development	cytoplasm	C	AT5G20240
7736	3	F23 vs. L23	F2-3 up	GO:0005737	Development	Development	cytoplasm	C	AT5G20700
7737	3	F23 vs. L23	F2-3 up	GO:0005737	Development	Development	cytoplasm	C	AT5G22030
7738	3	F23 vs. L23	F2-3 up	GO:0005737	Development	Development	cytoplasm	C	AT5G24530
7739	3	F23 vs. L23	F2-3 up	GO:0005737	Development	Development	cytoplasm	C	AT5G27000
7740	3	F23 vs. L23	F2-3 up	GO:0005737	Development	Development	cytoplasm	C	AT5G36160
7741	3	F23 vs. L23	F2-3 up	GO:0005737	Development	Development	cytoplasm	C	AT5G39320
7742	3	F23 vs. L23	F2-3 up	GO:0005737	Development	Development	cytoplasm	C	AT5G41790
7743	3	F23 vs. L23	F2-3 up	GO:0005737	Development	Development	cytoplasm	C	AT5G42050
7744	3	F23 vs. L23	F2-3 up	GO:0005737	Development	Development	cytoplasm	C	AT5G43380
7745	3	F23 vs. L23	F2-3 up	GO:0005737	Development	Development	cytoplasm	C	AT5G44110
7746	3	F23 vs. L23	F2-3 up	GO:0005737	Development	Development	cytoplasm	C	AT5G47650
7747	3	F23 vs. L23	F2-3 up	GO:0005737	Development	Development	cytoplasm	C	AT5G52360
7748	3	F23 vs. L23	F2-3 up	GO:0005737	Development	Development	cytoplasm	C	AT5G54080
7749	3	F23 vs. L23	F2-3 up	GO:0005737	Development	Development	cytoplasm	C	AT5G54510
7750	3	F23 vs. L23	F2-3 up	GO:0005737	Development	Development	cytoplasm	C	AT5G56350
7751	3	F23 vs. L23	F2-3 up	GO:0005737	Development	Development	cytoplasm	C	AT5G58330
7752	3	F23 vs. L23	F2-3 up	GO:0005737	Development	Development	cytoplasm	C	AT5G59370
7753	3	F23 vs. L23	F2-3 up	GO:0005737	Development	Development	cytoplasm	C	AT5G62360
7754	3	F23 vs. L23	F2-3 up	GO:0005737	Development	Development	cytoplasm	C	AT5G63680
7755	3	F23 vs. L23	F2-3 up	GO:0005737	Development	Development	cytoplasm	C	AT5G64350
7756	3	F23 vs. L23	F2-3 up	GO:0005737	Development	Development	cytoplasm	C	AT5G64370
7757	3	F23 vs. L23	F2-3 up	GO:0005737	Development	Development	cytoplasm	C	AT5G67160
7758	3	F23 vs. L23	F2-3 up	GO:0005737	Development	Development	cytoplasm	C	AT5G67470
7759	3	F23 vs. L23	F2-3 up	GO:0005737	Development	Development	cytoplasm	C	AT5G67480
7760	3	F23 vs. L23	F2-3 up	GO:0009898	Development	Development	cytoplasmic side of plasma membrane	C	AT3G45780
7761	3	F23 vs. L23	F2-3 up	GO:0005856	Development	Development	cytoskeleton	C	AT2G19770
7762	3	F23 vs. L23	F2-3 up	GO:0005856	Development	Development	cytoskeleton	C	AT3G18780
7763	3	F23 vs. L23	F2-3 up	GO:0005856	Development	Development	cytoskeleton	C	AT3G46520
7764	3	F23 vs. L23	F2-3 up	GO:0005856	Development	Development	cytoskeleton	C	AT4G29340
7765	3	F23 vs. L23	F2-3 up	GO:0005856	Development	Development	cytoskeleton	C	AT4G29350
7766	3	F23 vs. L23	F2-3 up	GO:0005856	Development	Development	cytoskeleton	C	AT5G09810
7767	3	F23 vs. L23	F2-3 up	GO:0005856	Development	Development	cytoskeleton	C	AT5G41790
7768	3	F23 vs. L23	F2-3 up	GO:0005856	Development	Development	cytoskeleton	C	AT5G59370
7769	3	F23 vs. L23	F2-3 up	GO:0007010	Development	Development	cytoskeleton organization	P	AT3G46520
7770	3	F23 vs. L23	F2-3 up	GO:0007010	Development	Development	cytoskeleton organization	P	AT5G09810
7771	3	F23 vs. L23	F2-3 up	GO:0007010	Development	Development	cytoskeleton organization	P	AT5G59370
7772	3	F23 vs. L23	F2-3 up	GO:0005829	Development	Development	cytosol	C	AT1G01320
7773	3	F23 vs. L23	F2-3 up	GO:0005829	Development	Development	cytosol	C	AT1G03905
7774	3	F23 vs. L23	F2-3 up	GO:0005829	Development	Development	cytosol	C	AT1G05010

7775	3	F23 vs. L23	F2-3 up	GO:0005829	Development	Development	cytosol	C	AT1G08830
7776	3	F23 vs. L23	F2-3 up	GO:0005829	Development	Development	cytosol	C	AT1G09310
7777	3	F23 vs. L23	F2-3 up	GO:0005829	Development	Development	cytosol	C	AT1G09340
7778	3	F23 vs. L23	F2-3 up	GO:0005829	Development	Development	cytosol	C	AT1G09430
7779	3	F23 vs. L23	F2-3 up	GO:0005829	Development	Development	cytosol	C	AT1G10370
7780	3	F23 vs. L23	F2-3 up	GO:0005829	Development	Development	cytosol	C	AT1G11840
7781	3	F23 vs. L23	F2-3 up	GO:0005829	Development	Development	cytosol	C	AT1G12070
7782	3	F23 vs. L23	F2-3 up	GO:0005829	Development	Development	cytosol	C	AT1G20620
7783	3	F23 vs. L23	F2-3 up	GO:0005829	Development	Development	cytosol	C	AT1G22760
7784	3	F23 vs. L23	F2-3 up	GO:0005829	Development	Development	cytosol	C	AT1G22840
7785	3	F23 vs. L23	F2-3 up	GO:0005829	Development	Development	cytosol	C	AT1G23310
7786	3	F23 vs. L23	F2-3 up	GO:0005829	Development	Development	cytosol	C	AT1G25350
7787	3	F23 vs. L23	F2-3 up	GO:0005829	Development	Development	cytosol	C	AT1G35720
7788	3	F23 vs. L23	F2-3 up	GO:0005829	Development	Development	cytosol	C	AT1G44160
7789	3	F23 vs. L23	F2-3 up	GO:0005829	Development	Development	cytosol	C	AT1G53280
7790	3	F23 vs. L23	F2-3 up	GO:0005829	Development	Development	cytosol	C	AT1G53500
7791	3	F23 vs. L23	F2-3 up	GO:0005829	Development	Development	cytosol	C	AT1G54290
7792	3	F23 vs. L23	F2-3 up	GO:0005829	Development	Development	cytosol	C	AT1G60810
7793	3	F23 vs. L23	F2-3 up	GO:0005829	Development	Development	cytosol	C	AT1G62380
7794	3	F23 vs. L23	F2-3 up	GO:0005829	Development	Development	cytosol	C	AT1G62450
7795	3	F23 vs. L23	F2-3 up	GO:0005829	Development	Development	cytosol	C	AT1G63000
7796	3	F23 vs. L23	F2-3 up	GO:0005829	Development	Development	cytosol	C	AT1G64190
7797	3	F23 vs. L23	F2-3 up	GO:0005829	Development	Development	cytosol	C	AT1G64740
7798	3	F23 vs. L23	F2-3 up	GO:0005829	Development	Development	cytosol	C	AT1G64980
7799	3	F23 vs. L23	F2-3 up	GO:0005829	Development	Development	cytosol	C	AT1G65960
7800	3	F23 vs. L23	F2-3 up	GO:0005829	Development	Development	cytosol	C	AT1G68010
7801	3	F23 vs. L23	F2-3 up	GO:0005829	Development	Development	cytosol	C	AT1G70310
7802	3	F23 vs. L23	F2-3 up	GO:0005829	Development	Development	cytosol	C	AT1G70580
7803	3	F23 vs. L23	F2-3 up	GO:0005829	Development	Development	cytosol	C	AT1G70730
7804	3	F23 vs. L23	F2-3 up	GO:0005829	Development	Development	cytosol	C	AT1G71770
7805	3	F23 vs. L23	F2-3 up	GO:0005829	Development	Development	cytosol	C	AT1G72730
7806	3	F23 vs. L23	F2-3 up	GO:0005829	Development	Development	cytosol	C	AT1G74450
7807	3	F23 vs. L23	F2-3 up	GO:0005829	Development	Development	cytosol	C	AT1G76130
7808	3	F23 vs. L23	F2-3 up	GO:0005829	Development	Development	cytosol	C	AT1G76990
7809	3	F23 vs. L23	F2-3 up	GO:0005829	Development	Development	cytosol	C	AT1G77090
7810	3	F23 vs. L23	F2-3 up	GO:0005829	Development	Development	cytosol	C	AT1G78320
7811	3	F23 vs. L23	F2-3 up	GO:0005829	Development	Development	cytosol	C	AT1G78370
7812	3	F23 vs. L23	F2-3 up	GO:0005829	Development	Development	cytosol	C	AT1G78570
7813	3	F23 vs. L23	F2-3 up	GO:0005829	Development	Development	cytosol	C	AT1G79440
7814	3	F23 vs. L23	F2-3 up	GO:0005829	Development	Development	cytosol	C	AT1G80460
7815	3	F23 vs. L23	F2-3 up	GO:0005829	Development	Development	cytosol	C	AT2G01520
7816	3	F23 vs. L23	F2-3 up	GO:0005829	Development	Development	cytosol	C	AT2G02390
7817	3	F23 vs. L23	F2-3 up	GO:0005829	Development	Development	cytosol	C	AT2G16430
7818	3	F23 vs. L23	F2-3 up	GO:0005829	Development	Development	cytosol	C	AT2G17290
7819	3	F23 vs. L23	F2-3 up	GO:0005829	Development	Development	cytosol	C	AT2G19570
7820	3	F23 vs. L23	F2-3 up	GO:0005829	Development	Development	cytosol	C	AT2G21330
7821	3	F23 vs. L23	F2-3 up	GO:0005829	Development	Development	cytosol	C	AT2G22125
7822	3	F23 vs. L23	F2-3 up	GO:0005829	Development	Development	cytosol	C	AT2G24270
7823	3	F23 vs. L23	F2-3 up	GO:0005829	Development	Development	cytosol	C	AT2G26740
7824	3	F23 vs. L23	F2-3 up	GO:0005829	Development	Development	cytosol	C	AT2G28305
7825	3	F23 vs. L23	F2-3 up	GO:0005829	Development	Development	cytosol	C	AT2G30860
7826	3	F23 vs. L23	F2-3 up	GO:0005829	Development	Development	cytosol	C	AT2G30970
7827	3	F23 vs. L23	F2-3 up	GO:0005829	Development	Development	cytosol	C	AT2G31570
7828	3	F23 vs. L23	F2-3 up	GO:0005829	Development	Development	cytosol	C	AT2G31750
7829	3	F23 vs. L23	F2-3 up	GO:0005829	Development	Development	cytosol	C	AT2G35370
7830	3	F23 vs. L23	F2-3 up	GO:0005829	Development	Development	cytosol	C	AT2G35840

7831	3	F23 vs. L23	F2-3 up	GO:0005829	Development	Development	cytosol	C	AT2G36880
7832	3	F23 vs. L23	F2-3 up	GO:0005829	Development	Development	cytosol	C	AT2G37760
7833	3	F23 vs. L23	F2-3 up	GO:0005829	Development	Development	cytosol	C	AT2G37770
7834	3	F23 vs. L23	F2-3 up	GO:0005829	Development	Development	cytosol	C	AT2G38240
7835	3	F23 vs. L23	F2-3 up	GO:0005829	Development	Development	cytosol	C	AT2G38280
7836	3	F23 vs. L23	F2-3 up	GO:0005829	Development	Development	cytosol	C	AT2G38870
7837	3	F23 vs. L23	F2-3 up	GO:0005829	Development	Development	cytosol	C	AT2G39700
7838	3	F23 vs. L23	F2-3 up	GO:0005829	Development	Development	cytosol	C	AT2G39770
7839	3	F23 vs. L23	F2-3 up	GO:0005829	Development	Development	cytosol	C	AT2G40890
7840	3	F23 vs. L23	F2-3 up	GO:0005829	Development	Development	cytosol	C	AT2G41210
7841	3	F23 vs. L23	F2-3 up	GO:0005829	Development	Development	cytosol	C	AT2G42750
7842	3	F23 vs. L23	F2-3 up	GO:0005829	Development	Development	cytosol	C	AT2G43760
7843	3	F23 vs. L23	F2-3 up	GO:0005829	Development	Development	cytosol	C	AT2G44120
7844	3	F23 vs. L23	F2-3 up	GO:0005829	Development	Development	cytosol	C	AT2G45770
7845	3	F23 vs. L23	F2-3 up	GO:0005829	Development	Development	cytosol	C	AT2G46140
7846	3	F23 vs. L23	F2-3 up	GO:0005829	Development	Development	cytosol	C	AT2G46820
7847	3	F23 vs. L23	F2-3 up	GO:0005829	Development	Development	cytosol	C	AT2G46860
7848	3	F23 vs. L23	F2-3 up	GO:0005829	Development	Development	cytosol	C	AT2G48020
7849	3	F23 vs. L23	F2-3 up	GO:0005829	Development	Development	cytosol	C	AT3G01500
7850	3	F23 vs. L23	F2-3 up	GO:0005829	Development	Development	cytosol	C	AT3G02470
7851	3	F23 vs. L23	F2-3 up	GO:0005829	Development	Development	cytosol	C	AT3G02570
7852	3	F23 vs. L23	F2-3 up	GO:0005829	Development	Development	cytosol	C	AT3G02770
7853	3	F23 vs. L23	F2-3 up	GO:0005829	Development	Development	cytosol	C	AT3G02780
7854	3	F23 vs. L23	F2-3 up	GO:0005829	Development	Development	cytosol	C	AT3G03190
7855	3	F23 vs. L23	F2-3 up	GO:0005829	Development	Development	cytosol	C	AT3G03250
7856	3	F23 vs. L23	F2-3 up	GO:0005829	Development	Development	cytosol	C	AT3G04120
7857	3	F23 vs. L23	F2-3 up	GO:0005829	Development	Development	cytosol	C	AT3G04790
7858	3	F23 vs. L23	F2-3 up	GO:0005829	Development	Development	cytosol	C	AT3G04840
7859	3	F23 vs. L23	F2-3 up	GO:0005829	Development	Development	cytosol	C	AT3G05580
7860	3	F23 vs. L23	F2-3 up	GO:0005829	Development	Development	cytosol	C	AT3G06035
7861	3	F23 vs. L23	F2-3 up	GO:0005829	Development	Development	cytosol	C	AT3G06510
7862	3	F23 vs. L23	F2-3 up	GO:0005829	Development	Development	cytosol	C	AT3G08560
7863	3	F23 vs. L23	F2-3 up	GO:0005829	Development	Development	cytosol	C	AT3G09440
7864	3	F23 vs. L23	F2-3 up	GO:0005829	Development	Development	cytosol	C	AT3G09810
7865	3	F23 vs. L23	F2-3 up	GO:0005829	Development	Development	cytosol	C	AT3G09940
7866	3	F23 vs. L23	F2-3 up	GO:0005829	Development	Development	cytosol	C	AT3G10700
7867	3	F23 vs. L23	F2-3 up	GO:0005829	Development	Development	cytosol	C	AT3G11170
7868	3	F23 vs. L23	F2-3 up	GO:0005829	Development	Development	cytosol	C	AT3G11410
7869	3	F23 vs. L23	F2-3 up	GO:0005829	Development	Development	cytosol	C	AT3G11630
7870	3	F23 vs. L23	F2-3 up	GO:0005829	Development	Development	cytosol	C	AT3G12780
7871	3	F23 vs. L23	F2-3 up	GO:0005829	Development	Development	cytosol	C	AT3G13110
7872	3	F23 vs. L23	F2-3 up	GO:0005829	Development	Development	cytosol	C	AT3G13930
7873	3	F23 vs. L23	F2-3 up	GO:0005829	Development	Development	cytosol	C	AT3G14310
7874	3	F23 vs. L23	F2-3 up	GO:0005829	Development	Development	cytosol	C	AT3G14420
7875	3	F23 vs. L23	F2-3 up	GO:0005829	Development	Development	cytosol	C	AT3G14620
7876	3	F23 vs. L23	F2-3 up	GO:0005829	Development	Development	cytosol	C	AT3G14940
7877	3	F23 vs. L23	F2-3 up	GO:0005829	Development	Development	cytosol	C	AT3G15360
7878	3	F23 vs. L23	F2-3 up	GO:0005829	Development	Development	cytosol	C	AT3G16170
7879	3	F23 vs. L23	F2-3 up	GO:0005829	Development	Development	cytosol	C	AT3G17020
7880	3	F23 vs. L23	F2-3 up	GO:0005829	Development	Development	cytosol	C	AT3G17390
7881	3	F23 vs. L23	F2-3 up	GO:0005829	Development	Development	cytosol	C	AT3G18780
7882	3	F23 vs. L23	F2-3 up	GO:0005829	Development	Development	cytosol	C	AT3G19710
7883	3	F23 vs. L23	F2-3 up	GO:0005829	Development	Development	cytosol	C	AT3G21260
7884	3	F23 vs. L23	F2-3 up	GO:0005829	Development	Development	cytosol	C	AT3G21670
7885	3	F23 vs. L23	F2-3 up	GO:0005829	Development	Development	cytosol	C	AT3G23810
7886	3	F23 vs. L23	F2-3 up	GO:0005829	Development	Development	cytosol	C	AT3G23920

7887	3	F23 vs. L23	F2-3 up	GO:0005829	Development	Development	cytosol	C	AT3G24503
7888	3	F23 vs. L23	F2-3 up	GO:0005829	Development	Development	cytosol	C	AT3G25690
7889	3	F23 vs. L23	F2-3 up	GO:0005829	Development	Development	cytosol	C	AT3G25760
7890	3	F23 vs. L23	F2-3 up	GO:0005829	Development	Development	cytosol	C	AT3G26060
7891	3	F23 vs. L23	F2-3 up	GO:0005829	Development	Development	cytosol	C	AT3G26520
7892	3	F23 vs. L23	F2-3 up	GO:0005829	Development	Development	cytosol	C	AT3G26650
7893	3	F23 vs. L23	F2-3 up	GO:0005829	Development	Development	cytosol	C	AT3G27300
7894	3	F23 vs. L23	F2-3 up	GO:0005829	Development	Development	cytosol	C	AT3G27380
7895	3	F23 vs. L23	F2-3 up	GO:0005829	Development	Development	cytosol	C	AT3G29360
7896	3	F23 vs. L23	F2-3 up	GO:0005829	Development	Development	cytosol	C	AT3G45140
7897	3	F23 vs. L23	F2-3 up	GO:0005829	Development	Development	cytosol	C	AT3G46520
7898	3	F23 vs. L23	F2-3 up	GO:0005829	Development	Development	cytosol	C	AT3G46620
7899	3	F23 vs. L23	F2-3 up	GO:0005829	Development	Development	cytosol	C	AT3G47940
7900	3	F23 vs. L23	F2-3 up	GO:0005829	Development	Development	cytosol	C	AT3G47960
7901	3	F23 vs. L23	F2-3 up	GO:0005829	Development	Development	cytosol	C	AT3G48170
7902	3	F23 vs. L23	F2-3 up	GO:0005829	Development	Development	cytosol	C	AT3G48420
7903	3	F23 vs. L23	F2-3 up	GO:0005829	Development	Development	cytosol	C	AT3G48990
7904	3	F23 vs. L23	F2-3 up	GO:0005829	Development	Development	cytosol	C	AT3G49110
7905	3	F23 vs. L23	F2-3 up	GO:0005829	Development	Development	cytosol	C	AT3G50000
7906	3	F23 vs. L23	F2-3 up	GO:0005829	Development	Development	cytosol	C	AT3G51600
7907	3	F23 vs. L23	F2-3 up	GO:0005829	Development	Development	cytosol	C	AT3G51820
7908	3	F23 vs. L23	F2-3 up	GO:0005829	Development	Development	cytosol	C	AT3G51920
7909	3	F23 vs. L23	F2-3 up	GO:0005829	Development	Development	cytosol	C	AT3G52880
7910	3	F23 vs. L23	F2-3 up	GO:0005829	Development	Development	cytosol	C	AT3G54660
7911	3	F23 vs. L23	F2-3 up	GO:0005829	Development	Development	cytosol	C	AT3G54960
7912	3	F23 vs. L23	F2-3 up	GO:0005829	Development	Development	cytosol	C	AT3G55610
7913	3	F23 vs. L23	F2-3 up	GO:0005829	Development	Development	cytosol	C	AT3G55800
7914	3	F23 vs. L23	F2-3 up	GO:0005829	Development	Development	cytosol	C	AT3G56150
7915	3	F23 vs. L23	F2-3 up	GO:0005829	Development	Development	cytosol	C	AT3G56240
7916	3	F23 vs. L23	F2-3 up	GO:0005829	Development	Development	cytosol	C	AT3G56960
7917	3	F23 vs. L23	F2-3 up	GO:0005829	Development	Development	cytosol	C	AT3G57020
7918	3	F23 vs. L23	F2-3 up	GO:0005829	Development	Development	cytosol	C	AT3G58730
7919	3	F23 vs. L23	F2-3 up	GO:0005829	Development	Development	cytosol	C	AT3G59970
7920	3	F23 vs. L23	F2-3 up	GO:0005829	Development	Development	cytosol	C	AT3G60100
7921	3	F23 vs. L23	F2-3 up	GO:0005829	Development	Development	cytosol	C	AT3G61160
7922	3	F23 vs. L23	F2-3 up	GO:0005829	Development	Development	cytosol	C	AT3G61220
7923	3	F23 vs. L23	F2-3 up	GO:0005829	Development	Development	cytosol	C	AT3G61990
7924	3	F23 vs. L23	F2-3 up	GO:0005829	Development	Development	cytosol	C	AT3G62290
7925	3	F23 vs. L23	F2-3 up	GO:0005829	Development	Development	cytosol	C	AT3G62410
7926	3	F23 vs. L23	F2-3 up	GO:0005829	Development	Development	cytosol	C	AT3G63140
7927	3	F23 vs. L23	F2-3 up	GO:0005829	Development	Development	cytosol	C	AT4G01480
7928	3	F23 vs. L23	F2-3 up	GO:0005829	Development	Development	cytosol	C	AT4G01610
7929	3	F23 vs. L23	F2-3 up	GO:0005829	Development	Development	cytosol	C	AT4G01850
7930	3	F23 vs. L23	F2-3 up	GO:0005829	Development	Development	cytosol	C	AT4G02890
7931	3	F23 vs. L23	F2-3 up	GO:0005829	Development	Development	cytosol	C	AT4G03280
7932	3	F23 vs. L23	F2-3 up	GO:0005829	Development	Development	cytosol	C	AT4G04040
7933	3	F23 vs. L23	F2-3 up	GO:0005829	Development	Development	cytosol	C	AT4G10040
7934	3	F23 vs. L23	F2-3 up	GO:0005829	Development	Development	cytosol	C	AT4G10120
7935	3	F23 vs. L23	F2-3 up	GO:0005829	Development	Development	cytosol	C	AT4G10440
7936	3	F23 vs. L23	F2-3 up	GO:0005829	Development	Development	cytosol	C	AT4G11600
7937	3	F23 vs. L23	F2-3 up	GO:0005829	Development	Development	cytosol	C	AT4G11960
7938	3	F23 vs. L23	F2-3 up	GO:0005829	Development	Development	cytosol	C	AT4G13230
7939	3	F23 vs. L23	F2-3 up	GO:0005829	Development	Development	cytosol	C	AT4G13560
7940	3	F23 vs. L23	F2-3 up	GO:0005829	Development	Development	cytosol	C	AT4G13770
7941	3	F23 vs. L23	F2-3 up	GO:0005829	Development	Development	cytosol	C	AT4G13930
7942	3	F23 vs. L23	F2-3 up	GO:0005829	Development	Development	cytosol	C	AT4G14710

7943	3	F23 vs. L23	F2-3 up	GO:0005829	Development	Development	cytosol	C	AT4G14880
7944	3	F23 vs. L23	F2-3 up	GO:0005829	Development	Development	cytosol	C	AT4G15210
7945	3	F23 vs. L23	F2-3 up	GO:0005829	Development	Development	cytosol	C	AT4G15530
7946	3	F23 vs. L23	F2-3 up	GO:0005829	Development	Development	cytosol	C	AT4G16143
7947	3	F23 vs. L23	F2-3 up	GO:0005829	Development	Development	cytosol	C	AT4G16660
7948	3	F23 vs. L23	F2-3 up	GO:0005829	Development	Development	cytosol	C	AT4G17090
7949	3	F23 vs. L23	F2-3 up	GO:0005829	Development	Development	cytosol	C	AT4G17530
7950	3	F23 vs. L23	F2-3 up	GO:0005829	Development	Development	cytosol	C	AT4G18050
7951	3	F23 vs. L23	F2-3 up	GO:0005829	Development	Development	cytosol	C	AT4G18950
7952	3	F23 vs. L23	F2-3 up	GO:0005829	Development	Development	cytosol	C	AT4G20760
7953	3	F23 vs. L23	F2-3 up	GO:0005829	Development	Development	cytosol	C	AT4G23100
7954	3	F23 vs. L23	F2-3 up	GO:0005829	Development	Development	cytosol	C	AT4G23890
7955	3	F23 vs. L23	F2-3 up	GO:0005829	Development	Development	cytosol	C	AT4G24930
7956	3	F23 vs. L23	F2-3 up	GO:0005829	Development	Development	cytosol	C	AT4G26970
7957	3	F23 vs. L23	F2-3 up	GO:0005829	Development	Development	cytosol	C	AT4G29350
7958	3	F23 vs. L23	F2-3 up	GO:0005829	Development	Development	cytosol	C	AT4G30530
7959	3	F23 vs. L23	F2-3 up	GO:0005829	Development	Development	cytosol	C	AT4G33010
7960	3	F23 vs. L23	F2-3 up	GO:0005829	Development	Development	cytosol	C	AT4G34050
7961	3	F23 vs. L23	F2-3 up	GO:0005829	Development	Development	cytosol	C	AT4G34710
7962	3	F23 vs. L23	F2-3 up	GO:0005829	Development	Development	cytosol	C	AT4G35150
7963	3	F23 vs. L23	F2-3 up	GO:0005829	Development	Development	cytosol	C	AT4G37180
7964	3	F23 vs. L23	F2-3 up	GO:0005829	Development	Development	cytosol	C	AT4G37550
7965	3	F23 vs. L23	F2-3 up	GO:0005829	Development	Development	cytosol	C	AT4G37560
7966	3	F23 vs. L23	F2-3 up	GO:0005829	Development	Development	cytosol	C	AT4G38740
7967	3	F23 vs. L23	F2-3 up	GO:0005829	Development	Development	cytosol	C	AT4G38970
7968	3	F23 vs. L23	F2-3 up	GO:0005829	Development	Development	cytosol	C	AT5G01410
7969	3	F23 vs. L23	F2-3 up	GO:0005829	Development	Development	cytosol	C	AT5G02270
7970	3	F23 vs. L23	F2-3 up	GO:0005829	Development	Development	cytosol	C	AT5G02790
7971	3	F23 vs. L23	F2-3 up	GO:0005829	Development	Development	cytosol	C	AT5G05600
7972	3	F23 vs. L23	F2-3 up	GO:0005829	Development	Development	cytosol	C	AT5G07460
7973	3	F23 vs. L23	F2-3 up	GO:0005829	Development	Development	cytosol	C	AT5G08100
7974	3	F23 vs. L23	F2-3 up	GO:0005829	Development	Development	cytosol	C	AT5G08160
7975	3	F23 vs. L23	F2-3 up	GO:0005829	Development	Development	cytosol	C	AT5G09810
7976	3	F23 vs. L23	F2-3 up	GO:0005829	Development	Development	cytosol	C	AT5G11520
7977	3	F23 vs. L23	F2-3 up	GO:0005829	Development	Development	cytosol	C	AT5G13990
7978	3	F23 vs. L23	F2-3 up	GO:0005829	Development	Development	cytosol	C	AT5G14200
7979	3	F23 vs. L23	F2-3 up	GO:0005829	Development	Development	cytosol	C	AT5G14740
7980	3	F23 vs. L23	F2-3 up	GO:0005829	Development	Development	cytosol	C	AT5G15490
7981	3	F23 vs. L23	F2-3 up	GO:0005829	Development	Development	cytosol	C	AT5G15950
7982	3	F23 vs. L23	F2-3 up	GO:0005829	Development	Development	cytosol	C	AT5G22580
7983	3	F23 vs. L23	F2-3 up	GO:0005829	Development	Development	cytosol	C	AT5G36160
7984	3	F23 vs. L23	F2-3 up	GO:0005829	Development	Development	cytosol	C	AT5G36880
7985	3	F23 vs. L23	F2-3 up	GO:0005829	Development	Development	cytosol	C	AT5G38900
7986	3	F23 vs. L23	F2-3 up	GO:0005829	Development	Development	cytosol	C	AT5G39320
7987	3	F23 vs. L23	F2-3 up	GO:0005829	Development	Development	cytosol	C	AT5G41790
7988	3	F23 vs. L23	F2-3 up	GO:0005829	Development	Development	cytosol	C	AT5G44110
7989	3	F23 vs. L23	F2-3 up	GO:0005829	Development	Development	cytosol	C	AT5G45280
7990	3	F23 vs. L23	F2-3 up	GO:0005829	Development	Development	cytosol	C	AT5G47650
7991	3	F23 vs. L23	F2-3 up	GO:0005829	Development	Development	cytosol	C	AT5G51720
7992	3	F23 vs. L23	F2-3 up	GO:0005829	Development	Development	cytosol	C	AT5G54080
7993	3	F23 vs. L23	F2-3 up	GO:0005829	Development	Development	cytosol	C	AT5G56350
7994	3	F23 vs. L23	F2-3 up	GO:0005829	Development	Development	cytosol	C	AT5G58690
7995	3	F23 vs. L23	F2-3 up	GO:0005829	Development	Development	cytosol	C	AT5G58700
7996	3	F23 vs. L23	F2-3 up	GO:0005829	Development	Development	cytosol	C	AT5G59730
7997	3	F23 vs. L23	F2-3 up	GO:0005829	Development	Development	cytosol	C	AT5G61410
7998	3	F23 vs. L23	F2-3 up	GO:0005829	Development	Development	cytosol	C	AT5G63680

7999	3	F23 vs. L23	F2-3 up	GO:0005829	Development	Development	cytosol	C	AT5G64350
8000	3	F23 vs. L23	F2-3 up	GO:0005829	Development	Development	cytosol	C	AT5G64370
8001	3	F23 vs. L23	F2-3 up	GO:0005829	Development	Development	cytosol	C	AT5G65460
8002	3	F23 vs. L23	F2-3 up	GO:0005829	Development	Development	cytosol	C	ATCG00120
8003	3	F23 vs. L23	F2-3 up	GO:0048589	Development	Development	developmental growth	P	AT4G03190
8004	3	F23 vs. L23	F2-3 up	GO:0048589	Development	Development	developmental growth	P	AT5G53460
8005	3	F23 vs. L23	F2-3 up	GO:0060560	Development	Development	developmental growth involved in morphogenesis	P	AT5G60910
8006	3	F23 vs. L23	F2-3 up	GO:0032502	Development	Development	developmental process	P	AT2G30490
8007	3	F23 vs. L23	F2-3 up	GO:0080186	Development	Development	developmental vegetative growth	P	AT3G05500
8008	3	F23 vs. L23	F2-3 up	GO:0009567	Development	Development	double fertilization forming a zygote and endosperm	P	AT3G21180
8009	3	F23 vs. L23	F2-3 up	GO:0009567	Development	Development	double fertilization forming a zygote and endosperm	P	AT4G13560
8010	3	F23 vs. L23	F2-3 up	GO:0009567	Development	Development	double fertilization forming a zygote and endosperm	P	AT4G14880
8011	3	F23 vs. L23	F2-3 up	GO:0009793	Development	Development	embryo development ending in seed dormancy	P	AT2G18390
8012	3	F23 vs. L23	F2-3 up	GO:0009793	Development	Development	embryo development ending in seed dormancy	P	AT2G22240
8013	3	F23 vs. L23	F2-3 up	GO:0009793	Development	Development	embryo development ending in seed dormancy	P	AT2G34850
8014	3	F23 vs. L23	F2-3 up	GO:0009793	Development	Development	embryo development ending in seed dormancy	P	AT2G38280
8015	3	F23 vs. L23	F2-3 up	GO:0009793	Development	Development	embryo development ending in seed dormancy	P	AT3G02570
8016	3	F23 vs. L23	F2-3 up	GO:0009793	Development	Development	embryo development ending in seed dormancy	P	AT3G15030
8017	3	F23 vs. L23	F2-3 up	GO:0009793	Development	Development	embryo development ending in seed dormancy	P	AT3G19980
8018	3	F23 vs. L23	F2-3 up	GO:0009793	Development	Development	embryo development ending in seed dormancy	P	AT3G48740
8019	3	F23 vs. L23	F2-3 up	GO:0009793	Development	Development	embryo development ending in seed dormancy	P	AT3G55610
8020	3	F23 vs. L23	F2-3 up	GO:0009793	Development	Development	embryo development ending in seed dormancy	P	AT4G04040
8021	3	F23 vs. L23	F2-3 up	GO:0009793	Development	Development	embryo development ending in seed dormancy	P	AT4G39800
8022	3	F23 vs. L23	F2-3 up	GO:0009793	Development	Development	embryo development ending in seed dormancy	P	AT5G13710
8023	3	F23 vs. L23	F2-3 up	GO:0009793	Development	Development	embryo development ending in seed dormancy	P	AT5G61410
8024	3	F23 vs. L23	F2-3 up	GO:0009558	Development	Development	embryo sac cellularization	P	AT2G18390
8025	3	F23 vs. L23	F2-3 up	GO:0009558	Development	Development	embryo sac cellularization	P	AT3G43210
8026	3	F23 vs. L23	F2-3 up	GO:0009553	Development	Development	embryo sac development	P	AT3G08510
8027	3	F23 vs. L23	F2-3 up	GO:0009553	Development	Development	embryo sac development	P	AT5G14200
8028	3	F23 vs. L23	F2-3 up	GO:0009553	Development	Development	embryo sac development	P	AT5G24120
8029	3	F23 vs. L23	F2-3 up	GO:0012505	Development	Development	endomembrane system	C	AT1G54280
8030	3	F23 vs. L23	F2-3 up	GO:0012505	Development	Development	endomembrane system	C	AT1G61290
8031	3	F23 vs. L23	F2-3 up	GO:0012505	Development	Development	endomembrane system	C	AT3G03800
8032	3	F23 vs. L23	F2-3 up	GO:0012505	Development	Development	endomembrane system	C	AT3G17630
8033	3	F23 vs. L23	F2-3 up	GO:0012505	Development	Development	endomembrane system	C	AT4G25030
8034	3	F23 vs. L23	F2-3 up	GO:0012505	Development	Development	endomembrane system	C	AT5G01410
8035	3	F23 vs. L23	F2-3 up	GO:0012505	Development	Development	endomembrane system	C	AT5G01690
8036	3	F23 vs. L23	F2-3 up	GO:0012505	Development	Development	endomembrane system	C	AT5G45840
8037	3	F23 vs. L23	F2-3 up	GO:0005788	Development	Development	endoplasmic reticulum lumen	C	AT3G54960
8038	3	F23 vs. L23	F2-3 up	GO:0009960	Development	Development	endosperm development	P	AT2G18390
8039	3	F23 vs. L23	F2-3 up	GO:0009960	Development	Development	endosperm development	P	AT2G41630
8040	3	F23 vs. L23	F2-3 up	GO:0009913	Development	Development	epidermal cell differentiation	P	AT2G46410
8041	3	F23 vs. L23	F2-3 up	GO:0009957	Development	Development	epidermal cell fate specification	P	AT1G63650
8042	3	F23 vs. L23	F2-3 up	GO:0007164	Development	Development	establishment of tissue polarity	P	AT3G56960
8043	3	F23 vs. L23	F2-3 up	GO:0000145	Development	Development	exocyst	C	AT5G13990
8044	3	F23 vs. L23	F2-3 up	GO:0000145	Development	Development	exocyst	C	AT5G59730
8045	3	F23 vs. L23	F2-3 up	GO:0070062	Development	Development	extracellular exosome	C	AT4G01850
8046	3	F23 vs. L23	F2-3 up	GO:0031012	Development	Development	extracellular matrix	C	AT1G72610
8047	3	F23 vs. L23	F2-3 up	GO:0031012	Development	Development	extracellular matrix	C	AT5G65870
8048	3	F23 vs. L23	F2-3 up	GO:0005576	Development	Development	extracellular region	C	AT1G01320
8049	3	F23 vs. L23	F2-3 up	GO:0005576	Development	Development	extracellular region	C	AT1G02790
8050	3	F23 vs. L23	F2-3 up	GO:0005576	Development	Development	extracellular region	C	AT1G03230
8051	3	F23 vs. L23	F2-3 up	GO:0005576	Development	Development	extracellular region	C	AT1G04530

8052	3	F23 vs. L23	F2-3 up	GO:0005576	Development	Development	extracellular region	C	AT1G05010
8053	3	F23 vs. L23	F2-3 up	GO:0005576	Development	Development	extracellular region	C	AT1G05850
8054	3	F23 vs. L23	F2-3 up	GO:0005576	Development	Development	extracellular region	C	AT1G06690
8055	3	F23 vs. L23	F2-3 up	GO:0005576	Development	Development	extracellular region	C	AT1G07440
8056	3	F23 vs. L23	F2-3 up	GO:0005576	Development	Development	extracellular region	C	AT1G08830
8057	3	F23 vs. L23	F2-3 up	GO:0005576	Development	Development	extracellular region	C	AT1G09310
8058	3	F23 vs. L23	F2-3 up	GO:0005576	Development	Development	extracellular region	C	AT1G09340
8059	3	F23 vs. L23	F2-3 up	GO:0005576	Development	Development	extracellular region	C	AT1G09430
8060	3	F23 vs. L23	F2-3 up	GO:0005576	Development	Development	extracellular region	C	AT1G11840
8061	3	F23 vs. L23	F2-3 up	GO:0005576	Development	Development	extracellular region	C	AT1G11860
8062	3	F23 vs. L23	F2-3 up	GO:0005576	Development	Development	extracellular region	C	AT1G12250
8063	3	F23 vs. L23	F2-3 up	GO:0005576	Development	Development	extracellular region	C	AT1G12840
8064	3	F23 vs. L23	F2-3 up	GO:0005576	Development	Development	extracellular region	C	AT1G13270
8065	3	F23 vs. L23	F2-3 up	GO:0005576	Development	Development	extracellular region	C	AT1G14000
8066	3	F23 vs. L23	F2-3 up	GO:0005576	Development	Development	extracellular region	C	AT1G15470
8067	3	F23 vs. L23	F2-3 up	GO:0005576	Development	Development	extracellular region	C	AT1G17360
8068	3	F23 vs. L23	F2-3 up	GO:0005576	Development	Development	extracellular region	C	AT1G19610
8069	3	F23 vs. L23	F2-3 up	GO:0005576	Development	Development	extracellular region	C	AT1G20190
8070	3	F23 vs. L23	F2-3 up	GO:0005576	Development	Development	extracellular region	C	AT1G22750
8071	3	F23 vs. L23	F2-3 up	GO:0005576	Development	Development	extracellular region	C	AT1G26770
8072	3	F23 vs. L23	F2-3 up	GO:0005576	Development	Development	extracellular region	C	AT1G28270
8073	3	F23 vs. L23	F2-3 up	GO:0005576	Development	Development	extracellular region	C	AT1G29140
8074	3	F23 vs. L23	F2-3 up	GO:0005576	Development	Development	extracellular region	C	AT1G34040
8075	3	F23 vs. L23	F2-3 up	GO:0005576	Development	Development	extracellular region	C	AT1G41830
8076	3	F23 vs. L23	F2-3 up	GO:0005576	Development	Development	extracellular region	C	AT1G45130
8077	3	F23 vs. L23	F2-3 up	GO:0005576	Development	Development	extracellular region	C	AT1G47980
8078	3	F23 vs. L23	F2-3 up	GO:0005576	Development	Development	extracellular region	C	AT1G49800
8079	3	F23 vs. L23	F2-3 up	GO:0005576	Development	Development	extracellular region	C	AT1G55570
8080	3	F23 vs. L23	F2-3 up	GO:0005576	Development	Development	extracellular region	C	AT1G58430
8081	3	F23 vs. L23	F2-3 up	GO:0005576	Development	Development	extracellular region	C	AT1G61820
8082	3	F23 vs. L23	F2-3 up	GO:0005576	Development	Development	extracellular region	C	AT1G62290
8083	3	F23 vs. L23	F2-3 up	GO:0005576	Development	Development	extracellular region	C	AT1G64140
8084	3	F23 vs. L23	F2-3 up	GO:0005576	Development	Development	extracellular region	C	AT1G64390
8085	3	F23 vs. L23	F2-3 up	GO:0005576	Development	Development	extracellular region	C	AT1G67870
8086	3	F23 vs. L23	F2-3 up	GO:0005576	Development	Development	extracellular region	C	AT1G69530
8087	3	F23 vs. L23	F2-3 up	GO:0005576	Development	Development	extracellular region	C	AT1G72610
8088	3	F23 vs. L23	F2-3 up	GO:0005576	Development	Development	extracellular region	C	AT1G74670
8089	3	F23 vs. L23	F2-3 up	GO:0005576	Development	Development	extracellular region	C	AT1G75800
8090	3	F23 vs. L23	F2-3 up	GO:0005576	Development	Development	extracellular region	C	AT1G75880
8091	3	F23 vs. L23	F2-3 up	GO:0005576	Development	Development	extracellular region	C	AT1G76130
8092	3	F23 vs. L23	F2-3 up	GO:0005576	Development	Development	extracellular region	C	AT1G78820
8093	3	F23 vs. L23	F2-3 up	GO:0005576	Development	Development	extracellular region	C	AT2G02140
8094	3	F23 vs. L23	F2-3 up	GO:0005576	Development	Development	extracellular region	C	AT2G02990
8095	3	F23 vs. L23	F2-3 up	GO:0005576	Development	Development	extracellular region	C	AT2G03200
8096	3	F23 vs. L23	F2-3 up	GO:0005576	Development	Development	extracellular region	C	AT2G05530
8097	3	F23 vs. L23	F2-3 up	GO:0005576	Development	Development	extracellular region	C	AT2G14900
8098	3	F23 vs. L23	F2-3 up	GO:0005576	Development	Development	extracellular region	C	AT2G16430
8099	3	F23 vs. L23	F2-3 up	GO:0005576	Development	Development	extracellular region	C	AT2G18660
8100	3	F23 vs. L23	F2-3 up	GO:0005576	Development	Development	extracellular region	C	AT2G19980
8101	3	F23 vs. L23	F2-3 up	GO:0005576	Development	Development	extracellular region	C	AT2G20870
8102	3	F23 vs. L23	F2-3 up	GO:0005576	Development	Development	extracellular region	C	AT2G21140
8103	3	F23 vs. L23	F2-3 up	GO:0005576	Development	Development	extracellular region	C	AT2G28190
8104	3	F23 vs. L23	F2-3 up	GO:0005576	Development	Development	extracellular region	C	AT2G28950
8105	3	F23 vs. L23	F2-3 up	GO:0005576	Development	Development	extracellular region	C	AT2G30490
8106	3	F23 vs. L23	F2-3 up	GO:0005576	Development	Development	extracellular region	C	AT2G32990
8107	3	F23 vs. L23	F2-3 up	GO:0005576	Development	Development	extracellular region	C	AT2G34810

8108	3	F23 vs. L23	F2-3 up	GO:0005576	Development	Development	extracellular region	C	AT2G37240
8109	3	F23 vs. L23	F2-3 up	GO:0005576	Development	Development	extracellular region	C	AT2G38870
8110	3	F23 vs. L23	F2-3 up	GO:0005576	Development	Development	extracellular region	C	AT2G39700
8111	3	F23 vs. L23	F2-3 up	GO:0005576	Development	Development	extracellular region	C	AT2G39850
8112	3	F23 vs. L23	F2-3 up	GO:0005576	Development	Development	extracellular region	C	AT2G40090
8113	3	F23 vs. L23	F2-3 up	GO:0005576	Development	Development	extracellular region	C	AT2G41480
8114	3	F23 vs. L23	F2-3 up	GO:0005576	Development	Development	extracellular region	C	AT2G43570
8115	3	F23 vs. L23	F2-3 up	GO:0005576	Development	Development	extracellular region	C	AT2G43590
8116	3	F23 vs. L23	F2-3 up	GO:0005576	Development	Development	extracellular region	C	AT2G44210
8117	3	F23 vs. L23	F2-3 up	GO:0005576	Development	Development	extracellular region	C	AT2G44890
8118	3	F23 vs. L23	F2-3 up	GO:0005576	Development	Development	extracellular region	C	AT2G45220
8119	3	F23 vs. L23	F2-3 up	GO:0005576	Development	Development	extracellular region	C	AT3G01420
8120	3	F23 vs. L23	F2-3 up	GO:0005576	Development	Development	extracellular region	C	AT3G04010
8121	3	F23 vs. L23	F2-3 up	GO:0005576	Development	Development	extracellular region	C	AT3G04360
8122	3	F23 vs. L23	F2-3 up	GO:0005576	Development	Development	extracellular region	C	AT3G04720
8123	3	F23 vs. L23	F2-3 up	GO:0005576	Development	Development	extracellular region	C	AT3G05620
8124	3	F23 vs. L23	F2-3 up	GO:0005576	Development	Development	extracellular region	C	AT3G05910
8125	3	F23 vs. L23	F2-3 up	GO:0005576	Development	Development	extracellular region	C	AT3G05930
8126	3	F23 vs. L23	F2-3 up	GO:0005576	Development	Development	extracellular region	C	AT3G07820
8127	3	F23 vs. L23	F2-3 up	GO:0005576	Development	Development	extracellular region	C	AT3G07830
8128	3	F23 vs. L23	F2-3 up	GO:0005576	Development	Development	extracellular region	C	AT3G07840
8129	3	F23 vs. L23	F2-3 up	GO:0005576	Development	Development	extracellular region	C	AT3G07850
8130	3	F23 vs. L23	F2-3 up	GO:0005576	Development	Development	extracellular region	C	AT3G13065
8131	3	F23 vs. L23	F2-3 up	GO:0005576	Development	Development	extracellular region	C	AT3G13390
8132	3	F23 vs. L23	F2-3 up	GO:0005576	Development	Development	extracellular region	C	AT3G15353
8133	3	F23 vs. L23	F2-3 up	GO:0005576	Development	Development	extracellular region	C	AT3G17060
8134	3	F23 vs. L23	F2-3 up	GO:0005576	Development	Development	extracellular region	C	AT3G17180
8135	3	F23 vs. L23	F2-3 up	GO:0005576	Development	Development	extracellular region	C	AT3G17790
8136	3	F23 vs. L23	F2-3 up	GO:0005576	Development	Development	extracellular region	C	AT3G18080
8137	3	F23 vs. L23	F2-3 up	GO:0005576	Development	Development	extracellular region	C	AT3G18280
8138	3	F23 vs. L23	F2-3 up	GO:0005576	Development	Development	extracellular region	C	AT3G19390
8139	3	F23 vs. L23	F2-3 up	GO:0005576	Development	Development	extracellular region	C	AT3G23920
8140	3	F23 vs. L23	F2-3 up	GO:0005576	Development	Development	extracellular region	C	AT3G24670
8141	3	F23 vs. L23	F2-3 up	GO:0005576	Development	Development	extracellular region	C	AT3G25170
8142	3	F23 vs. L23	F2-3 up	GO:0005576	Development	Development	extracellular region	C	AT3G29030
8143	3	F23 vs. L23	F2-3 up	GO:0005576	Development	Development	extracellular region	C	AT3G43270
8144	3	F23 vs. L23	F2-3 up	GO:0005576	Development	Development	extracellular region	C	AT3G44990
8145	3	F23 vs. L23	F2-3 up	GO:0005576	Development	Development	extracellular region	C	AT3G45010
8146	3	F23 vs. L23	F2-3 up	GO:0005576	Development	Development	extracellular region	C	AT3G48460
8147	3	F23 vs. L23	F2-3 up	GO:0005576	Development	Development	extracellular region	C	AT3G48520
8148	3	F23 vs. L23	F2-3 up	GO:0005576	Development	Development	extracellular region	C	AT3G48610
8149	3	F23 vs. L23	F2-3 up	GO:0005576	Development	Development	extracellular region	C	AT3G49110
8150	3	F23 vs. L23	F2-3 up	GO:0005576	Development	Development	extracellular region	C	AT3G50620
8151	3	F23 vs. L23	F2-3 up	GO:0005576	Development	Development	extracellular region	C	AT3G51070
8152	3	F23 vs. L23	F2-3 up	GO:0005576	Development	Development	extracellular region	C	AT3G51600
8153	3	F23 vs. L23	F2-3 up	GO:0005576	Development	Development	extracellular region	C	AT3G52000
8154	3	F23 vs. L23	F2-3 up	GO:0005576	Development	Development	extracellular region	C	AT3G52370
8155	3	F23 vs. L23	F2-3 up	GO:0005576	Development	Development	extracellular region	C	AT3G53100
8156	3	F23 vs. L23	F2-3 up	GO:0005576	Development	Development	extracellular region	C	AT3G53980
8157	3	F23 vs. L23	F2-3 up	GO:0005576	Development	Development	extracellular region	C	AT3G56950
8158	3	F23 vs. L23	F2-3 up	GO:0005576	Development	Development	extracellular region	C	AT3G57680
8159	3	F23 vs. L23	F2-3 up	GO:0005576	Development	Development	extracellular region	C	AT3G61990
8160	3	F23 vs. L23	F2-3 up	GO:0005576	Development	Development	extracellular region	C	AT3G62170
8161	3	F23 vs. L23	F2-3 up	GO:0005576	Development	Development	extracellular region	C	AT4G00230
8162	3	F23 vs. L23	F2-3 up	GO:0005576	Development	Development	extracellular region	C	AT4G00360
8163	3	F23 vs. L23	F2-3 up	GO:0005576	Development	Development	extracellular region	C	AT4G01610

8164	3	F23 vs. L23	F2-3 up	GO:0005576	Development	Development	extracellular region	C	AT4G04460
8165	3	F23 vs. L23	F2-3 up	GO:0005576	Development	Development	extracellular region	C	AT4G11760
8166	3	F23 vs. L23	F2-3 up	GO:0005576	Development	Development	extracellular region	C	AT4G15210
8167	3	F23 vs. L23	F2-3 up	GO:0005576	Development	Development	extracellular region	C	AT4G17030
8168	3	F23 vs. L23	F2-3 up	GO:0005576	Development	Development	extracellular region	C	AT4G17090
8169	3	F23 vs. L23	F2-3 up	GO:0005576	Development	Development	extracellular region	C	AT4G19410
8170	3	F23 vs. L23	F2-3 up	GO:0005576	Development	Development	extracellular region	C	AT4G19420
8171	3	F23 vs. L23	F2-3 up	GO:0005576	Development	Development	extracellular region	C	AT4G24890
8172	3	F23 vs. L23	F2-3 up	GO:0005576	Development	Development	extracellular region	C	AT4G27435
8173	3	F23 vs. L23	F2-3 up	GO:0005576	Development	Development	extracellular region	C	AT4G28780
8174	3	F23 vs. L23	F2-3 up	GO:0005576	Development	Development	extracellular region	C	AT4G33220
8175	3	F23 vs. L23	F2-3 up	GO:0005576	Development	Development	extracellular region	C	AT4G33530
8176	3	F23 vs. L23	F2-3 up	GO:0005576	Development	Development	extracellular region	C	AT4G33550
8177	3	F23 vs. L23	F2-3 up	GO:0005576	Development	Development	extracellular region	C	AT4G35010
8178	3	F23 vs. L23	F2-3 up	GO:0005576	Development	Development	extracellular region	C	AT4G35350
8179	3	F23 vs. L23	F2-3 up	GO:0005576	Development	Development	extracellular region	C	AT4G37760
8180	3	F23 vs. L23	F2-3 up	GO:0005576	Development	Development	extracellular region	C	AT4G38400
8181	3	F23 vs. L23	F2-3 up	GO:0005576	Development	Development	extracellular region	C	AT4G38770
8182	3	F23 vs. L23	F2-3 up	GO:0005576	Development	Development	extracellular region	C	AT4G39480
8183	3	F23 vs. L23	F2-3 up	GO:0005576	Development	Development	extracellular region	C	AT4G39640
8184	3	F23 vs. L23	F2-3 up	GO:0005576	Development	Development	extracellular region	C	AT4G39730
8185	3	F23 vs. L23	F2-3 up	GO:0005576	Development	Development	extracellular region	C	AT5G07430
8186	3	F23 vs. L23	F2-3 up	GO:0005576	Development	Development	extracellular region	C	AT5G07550
8187	3	F23 vs. L23	F2-3 up	GO:0005576	Development	Development	extracellular region	C	AT5G08260
8188	3	F23 vs. L23	F2-3 up	GO:0005576	Development	Development	extracellular region	C	AT5G11420
8189	3	F23 vs. L23	F2-3 up	GO:0005576	Development	Development	extracellular region	C	AT5G24080
8190	3	F23 vs. L23	F2-3 up	GO:0005576	Development	Development	extracellular region	C	AT5G25610
8191	3	F23 vs. L23	F2-3 up	GO:0005576	Development	Development	extracellular region	C	AT5G25980
8192	3	F23 vs. L23	F2-3 up	GO:0005576	Development	Development	extracellular region	C	AT5G26060
8193	3	F23 vs. L23	F2-3 up	GO:0005576	Development	Development	extracellular region	C	AT5G33370
8194	3	F23 vs. L23	F2-3 up	GO:0005576	Development	Development	extracellular region	C	AT5G36260
8195	3	F23 vs. L23	F2-3 up	GO:0005576	Development	Development	extracellular region	C	AT5G45280
8196	3	F23 vs. L23	F2-3 up	GO:0005576	Development	Development	extracellular region	C	AT5G47000
8197	3	F23 vs. L23	F2-3 up	GO:0005576	Development	Development	extracellular region	C	AT5G48140
8198	3	F23 vs. L23	F2-3 up	GO:0005576	Development	Development	extracellular region	C	AT5G49360
8199	3	F23 vs. L23	F2-3 up	GO:0005576	Development	Development	extracellular region	C	AT5G50800
8200	3	F23 vs. L23	F2-3 up	GO:0005576	Development	Development	extracellular region	C	AT5G51890
8201	3	F23 vs. L23	F2-3 up	GO:0005576	Development	Development	extracellular region	C	AT5G53370
8202	3	F23 vs. L23	F2-3 up	GO:0005576	Development	Development	extracellular region	C	AT5G55720
8203	3	F23 vs. L23	F2-3 up	GO:0005576	Development	Development	extracellular region	C	AT5G60020
8204	3	F23 vs. L23	F2-3 up	GO:0005576	Development	Development	extracellular region	C	AT5G60360
8205	3	F23 vs. L23	F2-3 up	GO:0005576	Development	Development	extracellular region	C	AT5G63180
8206	3	F23 vs. L23	F2-3 up	GO:0005576	Development	Development	extracellular region	C	AT5G65730
8207	3	F23 vs. L23	F2-3 up	GO:0005576	Development	Development	extracellular region	C	AT5G65870
8208	3	F23 vs. L23	F2-3 up	GO:0005576	Development	Development	extracellular region	C	AT5G67480
8209	3	F23 vs. L23	F2-3 up	GO:0005615	Development	Development	extracellular space	C	AT1G08830
8210	3	F23 vs. L23	F2-3 up	GO:0005615	Development	Development	extracellular space	C	AT1G20850
8211	3	F23 vs. L23	F2-3 up	GO:0005615	Development	Development	extracellular space	C	AT2G19980
8212	3	F23 vs. L23	F2-3 up	GO:0005615	Development	Development	extracellular space	C	AT2G28190
8213	3	F23 vs. L23	F2-3 up	GO:0005615	Development	Development	extracellular space	C	AT3G19390
8214	3	F23 vs. L23	F2-3 up	GO:0005615	Development	Development	extracellular space	C	AT4G01610
8215	3	F23 vs. L23	F2-3 up	GO:0005615	Development	Development	extracellular space	C	AT4G35350
8216	3	F23 vs. L23	F2-3 up	GO:0005615	Development	Development	extracellular space	C	AT5G45890
8217	3	F23 vs. L23	F2-3 up	GO:0005615	Development	Development	extracellular space	C	AT5G60360
8218	3	F23 vs. L23	F2-3 up	GO:0042406	Development	Development	extrinsic component of endoplasmic reticulum membrane	C	AT5G42800

8219	3	F23 vs. L23	F2-3 up	GO:0010582	Development	Development	floral meristem determinacy	P	AT1G62360
8220	3	F23 vs. L23	F2-3 up	GO:0010582	Development	Development	floral meristem determinacy	P	AT1G69120
8221	3	F23 vs. L23	F2-3 up	GO:0048437	Development	Development	floral organ development	P	AT3G08510
8222	3	F23 vs. L23	F2-3 up	GO:0048449	Development	Development	floral organ formation	P	AT4G37180
8223	3	F23 vs. L23	F2-3 up	GO:0048444	Development	Development	floral organ morphogenesis	P	AT3G07050
8224	3	F23 vs. L23	F2-3 up	GO:0009908	Development	Development	flower development	P	AT1G19180
8225	3	F23 vs. L23	F2-3 up	GO:0009908	Development	Development	flower development	P	AT1G30330
8226	3	F23 vs. L23	F2-3 up	GO:0009908	Development	Development	flower development	P	AT1G69120
8227	3	F23 vs. L23	F2-3 up	GO:0009908	Development	Development	flower development	P	AT2G37630
8228	3	F23 vs. L23	F2-3 up	GO:0009908	Development	Development	flower development	P	AT2G38110
8229	3	F23 vs. L23	F2-3 up	GO:0009908	Development	Development	flower development	P	AT3G02780
8230	3	F23 vs. L23	F2-3 up	GO:0009908	Development	Development	flower development	P	AT3G10570
8231	3	F23 vs. L23	F2-3 up	GO:0009908	Development	Development	flower development	P	AT3G11050
8232	3	F23 vs. L23	F2-3 up	GO:0009908	Development	Development	flower development	P	AT4G23100
8233	3	F23 vs. L23	F2-3 up	GO:0009908	Development	Development	flower development	P	AT5G01600
8234	3	F23 vs. L23	F2-3 up	GO:0009908	Development	Development	flower development	P	AT5G06850
8235	3	F23 vs. L23	F2-3 up	GO:0009908	Development	Development	flower development	P	AT5G60910
8236	3	F23 vs. L23	F2-3 up	GO:0010154	Development	Development	fruit development	P	AT2G45190
8237	3	F23 vs. L23	F2-3 up	GO:0010154	Development	Development	fruit development	P	AT3G04120
8238	3	F23 vs. L23	F2-3 up	GO:0010154	Development	Development	fruit development	P	AT3G48520
8239	3	F23 vs. L23	F2-3 up	GO:0010154	Development	Development	fruit development	P	AT3G61880
8240	3	F23 vs. L23	F2-3 up	GO:0010154	Development	Development	fruit development	P	AT5G40010
8241	3	F23 vs. L23	F2-3 up	GO:0010154	Development	Development	fruit development	P	AT5G60910
8242	3	F23 vs. L23	F2-3 up	GO:0009835	Development	Development	fruit ripening	P	AT1G05010
8243	3	F23 vs. L23	F2-3 up	GO:0009835	Development	Development	fruit ripening	P	AT4G11280
8244	3	F23 vs. L23	F2-3 up	GO:0005797	Development	Development	Golgi medial cisterna	C	AT1G27930
8245	3	F23 vs. L23	F2-3 up	GO:0005797	Development	Development	Golgi medial cisterna	C	AT1G31850
8246	3	F23 vs. L23	F2-3 up	GO:0005797	Development	Development	Golgi medial cisterna	C	AT3G21190
8247	3	F23 vs. L23	F2-3 up	GO:0005797	Development	Development	Golgi medial cisterna	C	AT3G23300
8248	3	F23 vs. L23	F2-3 up	GO:0005797	Development	Development	Golgi medial cisterna	C	AT4G22750
8249	3	F23 vs. L23	F2-3 up	GO:0005797	Development	Development	Golgi medial cisterna	C	AT5G18280
8250	3	F23 vs. L23	F2-3 up	GO:0000138	Development	Development	Golgi trans cisterna	C	AT1G04430
8251	3	F23 vs. L23	F2-3 up	GO:0000138	Development	Development	Golgi trans cisterna	C	AT1G74380
8252	3	F23 vs. L23	F2-3 up	GO:0000138	Development	Development	Golgi trans cisterna	C	AT1G78240
8253	3	F23 vs. L23	F2-3 up	GO:0000138	Development	Development	Golgi trans cisterna	C	AT2G47650
8254	3	F23 vs. L23	F2-3 up	GO:0000138	Development	Development	Golgi trans cisterna	C	AT3G23820
8255	3	F23 vs. L23	F2-3 up	GO:0000138	Development	Development	Golgi trans cisterna	C	AT3G25140
8256	3	F23 vs. L23	F2-3 up	GO:0000138	Development	Development	Golgi trans cisterna	C	AT3G27230
8257	3	F23 vs. L23	F2-3 up	GO:0000138	Development	Development	Golgi trans cisterna	C	AT3G47960
8258	3	F23 vs. L23	F2-3 up	GO:0000138	Development	Development	Golgi trans cisterna	C	AT4G19120
8259	3	F23 vs. L23	F2-3 up	GO:0000138	Development	Development	Golgi trans cisterna	C	AT4G27720
8260	3	F23 vs. L23	F2-3 up	GO:0000138	Development	Development	Golgi trans cisterna	C	AT4G30996
8261	3	F23 vs. L23	F2-3 up	GO:0010229	Development	Development	inflorescence development	P	AT3G45640
8262	3	F23 vs. L23	F2-3 up	GO:0010229	Development	Development	inflorescence development	P	AT4G29350
8263	3	F23 vs. L23	F2-3 up	GO:0010450	Development	Development	inflorescence meristem growth	P	AT2G45190
8264	3	F23 vs. L23	F2-3 up	GO:0016021	Development	Development	integral component of membrane	C	AT1G01600
8265	3	F23 vs. L23	F2-3 up	GO:0016021	Development	Development	integral component of membrane	C	AT1G02390
8266	3	F23 vs. L23	F2-3 up	GO:0016021	Development	Development	integral component of membrane	C	AT1G03550
8267	3	F23 vs. L23	F2-3 up	GO:0016021	Development	Development	integral component of membrane	C	AT1G04430
8268	3	F23 vs. L23	F2-3 up	GO:0016021	Development	Development	integral component of membrane	C	AT1G04760
8269	3	F23 vs. L23	F2-3 up	GO:0016021	Development	Development	integral component of membrane	C	AT1G06090
8270	3	F23 vs. L23	F2-3 up	GO:0016021	Development	Development	integral component of membrane	C	AT1G12110
8271	3	F23 vs. L23	F2-3 up	GO:0016021	Development	Development	integral component of membrane	C	AT1G12240
8272	3	F23 vs. L23	F2-3 up	GO:0016021	Development	Development	integral component of membrane	C	AT1G13860
8273	3	F23 vs. L23	F2-3 up	GO:0016021	Development	Development	integral component of membrane	C	AT1G16310
8274	3	F23 vs. L23	F2-3 up	GO:0016021	Development	Development	integral component of membrane	C	AT1G16370

8275	3	F23 vs. L23	F2-3 up	GO:0016021	Development	Development	integral component of membrane	C	AT1G16410
8276	3	F23 vs. L23	F2-3 up	GO:0016021	Development	Development	integral component of membrane	C	AT1G18740
8277	3	F23 vs. L23	F2-3 up	GO:0016021	Development	Development	integral component of membrane	C	AT1G19150
8278	3	F23 vs. L23	F2-3 up	GO:0016021	Development	Development	integral component of membrane	C	AT1G19380
8279	3	F23 vs. L23	F2-3 up	GO:0016021	Development	Development	integral component of membrane	C	AT1G20823
8280	3	F23 vs. L23	F2-3 up	GO:0016021	Development	Development	integral component of membrane	C	AT1G21070
8281	3	F23 vs. L23	F2-3 up	GO:0016021	Development	Development	integral component of membrane	C	AT1G22550
8282	3	F23 vs. L23	F2-3 up	GO:0016021	Development	Development	integral component of membrane	C	AT1G22750
8283	3	F23 vs. L23	F2-3 up	GO:0016021	Development	Development	integral component of membrane	C	AT1G24400
8284	3	F23 vs. L23	F2-3 up	GO:0016021	Development	Development	integral component of membrane	C	AT1G24520
8285	3	F23 vs. L23	F2-3 up	GO:0016021	Development	Development	integral component of membrane	C	AT1G27930
8286	3	F23 vs. L23	F2-3 up	GO:0016021	Development	Development	integral component of membrane	C	AT1G27980
8287	3	F23 vs. L23	F2-3 up	GO:0016021	Development	Development	integral component of membrane	C	AT1G31260
8288	3	F23 vs. L23	F2-3 up	GO:0016021	Development	Development	integral component of membrane	C	AT1G31850
8289	3	F23 vs. L23	F2-3 up	GO:0016021	Development	Development	integral component of membrane	C	AT1G32080
8290	3	F23 vs. L23	F2-3 up	GO:0016021	Development	Development	integral component of membrane	C	AT1G32450
8291	3	F23 vs. L23	F2-3 up	GO:0016021	Development	Development	integral component of membrane	C	AT1G33170
8292	3	F23 vs. L23	F2-3 up	GO:0016021	Development	Development	integral component of membrane	C	AT1G34040
8293	3	F23 vs. L23	F2-3 up	GO:0016021	Development	Development	integral component of membrane	C	AT1G44575
8294	3	F23 vs. L23	F2-3 up	GO:0016021	Development	Development	integral component of membrane	C	AT1G45474
8295	3	F23 vs. L23	F2-3 up	GO:0016021	Development	Development	integral component of membrane	C	AT1G47530
8296	3	F23 vs. L23	F2-3 up	GO:0016021	Development	Development	integral component of membrane	C	AT1G50250
8297	3	F23 vs. L23	F2-3 up	GO:0016021	Development	Development	integral component of membrane	C	AT1G50610
8298	3	F23 vs. L23	F2-3 up	GO:0016021	Development	Development	integral component of membrane	C	AT1G52190
8299	3	F23 vs. L23	F2-3 up	GO:0016021	Development	Development	integral component of membrane	C	AT1G52580
8300	3	F23 vs. L23	F2-3 up	GO:0016021	Development	Development	integral component of membrane	C	AT1G54280
8301	3	F23 vs. L23	F2-3 up	GO:0016021	Development	Development	integral component of membrane	C	AT1G56500
8302	3	F23 vs. L23	F2-3 up	GO:0016021	Development	Development	integral component of membrane	C	AT1G59870
8303	3	F23 vs. L23	F2-3 up	GO:0016021	Development	Development	integral component of membrane	C	AT1G60030
8304	3	F23 vs. L23	F2-3 up	GO:0016021	Development	Development	integral component of membrane	C	AT1G61290
8305	3	F23 vs. L23	F2-3 up	GO:0016021	Development	Development	integral component of membrane	C	AT1G61740
8306	3	F23 vs. L23	F2-3 up	GO:0016021	Development	Development	integral component of membrane	C	AT1G61800
8307	3	F23 vs. L23	F2-3 up	GO:0016021	Development	Development	integral component of membrane	C	AT1G63060
8308	3	F23 vs. L23	F2-3 up	GO:0016021	Development	Development	integral component of membrane	C	AT1G63710
8309	3	F23 vs. L23	F2-3 up	GO:0016021	Development	Development	integral component of membrane	C	AT1G64060
8310	3	F23 vs. L23	F2-3 up	GO:0016021	Development	Development	integral component of membrane	C	AT1G64150
8311	3	F23 vs. L23	F2-3 up	GO:0016021	Development	Development	integral component of membrane	C	AT1G68440
8312	3	F23 vs. L23	F2-3 up	GO:0016021	Development	Development	integral component of membrane	C	AT1G68530
8313	3	F23 vs. L23	F2-3 up	GO:0016021	Development	Development	integral component of membrane	C	AT1G68570
8314	3	F23 vs. L23	F2-3 up	GO:0016021	Development	Development	integral component of membrane	C	AT1G68610
8315	3	F23 vs. L23	F2-3 up	GO:0016021	Development	Development	integral component of membrane	C	AT1G69640
8316	3	F23 vs. L23	F2-3 up	GO:0016021	Development	Development	integral component of membrane	C	AT1G70940
8317	3	F23 vs. L23	F2-3 up	GO:0016021	Development	Development	integral component of membrane	C	AT1G72180
8318	3	F23 vs. L23	F2-3 up	GO:0016021	Development	Development	integral component of membrane	C	AT1G74380
8319	3	F23 vs. L23	F2-3 up	GO:0016021	Development	Development	integral component of membrane	C	AT1G74450
8320	3	F23 vs. L23	F2-3 up	GO:0016021	Development	Development	integral component of membrane	C	AT1G75020
8321	3	F23 vs. L23	F2-3 up	GO:0016021	Development	Development	integral component of membrane	C	AT1G75500
8322	3	F23 vs. L23	F2-3 up	GO:0016021	Development	Development	integral component of membrane	C	AT1G75690
8323	3	F23 vs. L23	F2-3 up	GO:0016021	Development	Development	integral component of membrane	C	AT1G76090
8324	3	F23 vs. L23	F2-3 up	GO:0016021	Development	Development	integral component of membrane	C	AT1G76490
8325	3	F23 vs. L23	F2-3 up	GO:0016021	Development	Development	integral component of membrane	C	AT1G76670
8326	3	F23 vs. L23	F2-3 up	GO:0016021	Development	Development	integral component of membrane	C	AT1G78240
8327	3	F23 vs. L23	F2-3 up	GO:0016021	Development	Development	integral component of membrane	C	AT1G80280
8328	3	F23 vs. L23	F2-3 up	GO:0016021	Development	Development	integral component of membrane	C	AT1G80660
8329	3	F23 vs. L23	F2-3 up	GO:0016021	Development	Development	integral component of membrane	C	AT1G80760
8330	3	F23 vs. L23	F2-3 up	GO:0016021	Development	Development	integral component of membrane	C	AT2G01320

8331	3	F23 vs. L23	F2-3 up	GO:0016021	Development	Development	integral component of membrane	C	AT2G01420
8332	3	F23 vs. L23	F2-3 up	GO:0016021	Development	Development	integral component of membrane	C	AT2G03240
8333	3	F23 vs. L23	F2-3 up	GO:0016021	Development	Development	integral component of membrane	C	AT2G07560
8334	3	F23 vs. L23	F2-3 up	GO:0016021	Development	Development	integral component of membrane	C	AT2G13680
8335	3	F23 vs. L23	F2-3 up	GO:0016021	Development	Development	integral component of membrane	C	AT2G17370
8336	3	F23 vs. L23	F2-3 up	GO:0016021	Development	Development	integral component of membrane	C	AT2G18470
8337	3	F23 vs. L23	F2-3 up	GO:0016021	Development	Development	integral component of membrane	C	AT2G19980
8338	3	F23 vs. L23	F2-3 up	GO:0016021	Development	Development	integral component of membrane	C	AT2G20840
8339	3	F23 vs. L23	F2-3 up	GO:0016021	Development	Development	integral component of membrane	C	AT2G21960
8340	3	F23 vs. L23	F2-3 up	GO:0016021	Development	Development	integral component of membrane	C	AT2G22900
8341	3	F23 vs. L23	F2-3 up	GO:0016021	Development	Development	integral component of membrane	C	AT2G22950
8342	3	F23 vs. L23	F2-3 up	GO:0016021	Development	Development	integral component of membrane	C	AT2G24180
8343	3	F23 vs. L23	F2-3 up	GO:0016021	Development	Development	integral component of membrane	C	AT2G24820
8344	3	F23 vs. L23	F2-3 up	GO:0016021	Development	Development	integral component of membrane	C	AT2G26500
8345	3	F23 vs. L23	F2-3 up	GO:0016021	Development	Development	integral component of membrane	C	AT2G26690
8346	3	F23 vs. L23	F2-3 up	GO:0016021	Development	Development	integral component of membrane	C	AT2G29980
8347	3	F23 vs. L23	F2-3 up	GO:0016021	Development	Development	integral component of membrane	C	AT2G30010
8348	3	F23 vs. L23	F2-3 up	GO:0016021	Development	Development	integral component of membrane	C	AT2G30490
8349	3	F23 vs. L23	F2-3 up	GO:0016021	Development	Development	integral component of membrane	C	AT2G31360
8350	3	F23 vs. L23	F2-3 up	GO:0016021	Development	Development	integral component of membrane	C	AT2G33100
8351	3	F23 vs. L23	F2-3 up	GO:0016021	Development	Development	integral component of membrane	C	AT2G33670
8352	3	F23 vs. L23	F2-3 up	GO:0016021	Development	Development	integral component of membrane	C	AT2G34850
8353	3	F23 vs. L23	F2-3 up	GO:0016021	Development	Development	integral component of membrane	C	AT2G36630
8354	3	F23 vs. L23	F2-3 up	GO:0016021	Development	Development	integral component of membrane	C	AT2G36830
8355	3	F23 vs. L23	F2-3 up	GO:0016021	Development	Development	integral component of membrane	C	AT2G37180
8356	3	F23 vs. L23	F2-3 up	GO:0016021	Development	Development	integral component of membrane	C	AT2G38110
8357	3	F23 vs. L23	F2-3 up	GO:0016021	Development	Development	integral component of membrane	C	AT2G38170
8358	3	F23 vs. L23	F2-3 up	GO:0016021	Development	Development	integral component of membrane	C	AT2G38460
8359	3	F23 vs. L23	F2-3 up	GO:0016021	Development	Development	integral component of membrane	C	AT2G39510
8360	3	F23 vs. L23	F2-3 up	GO:0016021	Development	Development	integral component of membrane	C	AT2G40460
8361	3	F23 vs. L23	F2-3 up	GO:0016021	Development	Development	integral component of membrane	C	AT2G40890
8362	3	F23 vs. L23	F2-3 up	GO:0016021	Development	Development	integral component of membrane	C	AT2G46210
8363	3	F23 vs. L23	F2-3 up	GO:0016021	Development	Development	integral component of membrane	C	AT2G46300
8364	3	F23 vs. L23	F2-3 up	GO:0016021	Development	Development	integral component of membrane	C	AT2G46820
8365	3	F23 vs. L23	F2-3 up	GO:0016021	Development	Development	integral component of membrane	C	AT2G47160
8366	3	F23 vs. L23	F2-3 up	GO:0016021	Development	Development	integral component of membrane	C	AT2G47650
8367	3	F23 vs. L23	F2-3 up	GO:0016021	Development	Development	integral component of membrane	C	AT3G01980
8368	3	F23 vs. L23	F2-3 up	GO:0016021	Development	Development	integral component of membrane	C	AT3G03770
8369	3	F23 vs. L23	F2-3 up	GO:0016021	Development	Development	integral component of membrane	C	AT3G03800
8370	3	F23 vs. L23	F2-3 up	GO:0016021	Development	Development	integral component of membrane	C	AT3G05890
8371	3	F23 vs. L23	F2-3 up	GO:0016021	Development	Development	integral component of membrane	C	AT3G07560
8372	3	F23 vs. L23	F2-3 up	GO:0016021	Development	Development	integral component of membrane	C	AT3G07700
8373	3	F23 vs. L23	F2-3 up	GO:0016021	Development	Development	integral component of membrane	C	AT3G08610
8374	3	F23 vs. L23	F2-3 up	GO:0016021	Development	Development	integral component of membrane	C	AT3G10570
8375	3	F23 vs. L23	F2-3 up	GO:0016021	Development	Development	integral component of membrane	C	AT3G11170
8376	3	F23 vs. L23	F2-3 up	GO:0016021	Development	Development	integral component of membrane	C	AT3G11900
8377	3	F23 vs. L23	F2-3 up	GO:0016021	Development	Development	integral component of membrane	C	AT3G13065
8378	3	F23 vs. L23	F2-3 up	GO:0016021	Development	Development	integral component of membrane	C	AT3G14620
8379	3	F23 vs. L23	F2-3 up	GO:0016021	Development	Development	integral component of membrane	C	AT3G14770
8380	3	F23 vs. L23	F2-3 up	GO:0016021	Development	Development	integral component of membrane	C	AT3G16340
8381	3	F23 vs. L23	F2-3 up	GO:0016021	Development	Development	integral component of membrane	C	AT3G17611
8382	3	F23 vs. L23	F2-3 up	GO:0016021	Development	Development	integral component of membrane	C	AT3G17630
8383	3	F23 vs. L23	F2-3 up	GO:0016021	Development	Development	integral component of membrane	C	AT3G20190
8384	3	F23 vs. L23	F2-3 up	GO:0016021	Development	Development	integral component of membrane	C	AT3G21180
8385	3	F23 vs. L23	F2-3 up	GO:0016021	Development	Development	integral component of membrane	C	AT3G21190
8386	3	F23 vs. L23	F2-3 up	GO:0016021	Development	Development	integral component of membrane	C	AT3G21670

8387	3	F23 vs. L23	F2-3 up	GO:0016021	Development	Development	integral component of membrane	C	AT3G22370
8388	3	F23 vs. L23	F2-3 up	GO:0016021	Development	Development	integral component of membrane	C	AT3G22840
8389	3	F23 vs. L23	F2-3 up	GO:0016021	Development	Development	integral component of membrane	C	AT3G23300
8390	3	F23 vs. L23	F2-3 up	GO:0016021	Development	Development	integral component of membrane	C	AT3G23820
8391	3	F23 vs. L23	F2-3 up	GO:0016021	Development	Development	integral component of membrane	C	AT3G25140
8392	3	F23 vs. L23	F2-3 up	GO:0016021	Development	Development	integral component of membrane	C	AT3G25290
8393	3	F23 vs. L23	F2-3 up	GO:0016021	Development	Development	integral component of membrane	C	AT3G26520
8394	3	F23 vs. L23	F2-3 up	GO:0016021	Development	Development	integral component of membrane	C	AT3G27230
8395	3	F23 vs. L23	F2-3 up	GO:0016021	Development	Development	integral component of membrane	C	AT3G28150
8396	3	F23 vs. L23	F2-3 up	GO:0016021	Development	Development	integral component of membrane	C	AT3G29060
8397	3	F23 vs. L23	F2-3 up	GO:0016021	Development	Development	integral component of membrane	C	AT3G30390
8398	3	F23 vs. L23	F2-3 up	GO:0016021	Development	Development	integral component of membrane	C	AT3G42640
8399	3	F23 vs. L23	F2-3 up	GO:0016021	Development	Development	integral component of membrane	C	AT3G45100
8400	3	F23 vs. L23	F2-3 up	GO:0016021	Development	Development	integral component of membrane	C	AT3G47960
8401	3	F23 vs. L23	F2-3 up	GO:0016021	Development	Development	integral component of membrane	C	AT3G48520
8402	3	F23 vs. L23	F2-3 up	GO:0016021	Development	Development	integral component of membrane	C	AT3G48740
8403	3	F23 vs. L23	F2-3 up	GO:0016021	Development	Development	integral component of membrane	C	AT3G50620
8404	3	F23 vs. L23	F2-3 up	GO:0016021	Development	Development	integral component of membrane	C	AT3G50910
8405	3	F23 vs. L23	F2-3 up	GO:0016021	Development	Development	integral component of membrane	C	AT3G51070
8406	3	F23 vs. L23	F2-3 up	GO:0016021	Development	Development	integral component of membrane	C	AT3G51820
8407	3	F23 vs. L23	F2-3 up	GO:0016021	Development	Development	integral component of membrane	C	AT3G52470
8408	3	F23 vs. L23	F2-3 up	GO:0016021	Development	Development	integral component of membrane	C	AT3G52950
8409	3	F23 vs. L23	F2-3 up	GO:0016021	Development	Development	integral component of membrane	C	AT3G53420
8410	3	F23 vs. L23	F2-3 up	GO:0016021	Development	Development	integral component of membrane	C	AT3G53960
8411	3	F23 vs. L23	F2-3 up	GO:0016021	Development	Development	integral component of membrane	C	AT3G54510
8412	3	F23 vs. L23	F2-3 up	GO:0016021	Development	Development	integral component of membrane	C	AT3G54700
8413	3	F23 vs. L23	F2-3 up	GO:0016021	Development	Development	integral component of membrane	C	AT3G55100
8414	3	F23 vs. L23	F2-3 up	GO:0016021	Development	Development	integral component of membrane	C	AT3G56950
8415	3	F23 vs. L23	F2-3 up	GO:0016021	Development	Development	integral component of membrane	C	AT3G57330
8416	3	F23 vs. L23	F2-3 up	GO:0016021	Development	Development	integral component of membrane	C	AT3G60330
8417	3	F23 vs. L23	F2-3 up	GO:0016021	Development	Development	integral component of membrane	C	AT3G61880
8418	3	F23 vs. L23	F2-3 up	GO:0016021	Development	Development	integral component of membrane	C	AT4G00360
8419	3	F23 vs. L23	F2-3 up	GO:0016021	Development	Development	integral component of membrane	C	AT4G01470
8420	3	F23 vs. L23	F2-3 up	GO:0016021	Development	Development	integral component of membrane	C	AT4G03280
8421	3	F23 vs. L23	F2-3 up	GO:0016021	Development	Development	integral component of membrane	C	AT4G10440
8422	3	F23 vs. L23	F2-3 up	GO:0016021	Development	Development	integral component of membrane	C	AT4G11960
8423	3	F23 vs. L23	F2-3 up	GO:0016021	Development	Development	integral component of membrane	C	AT4G13770
8424	3	F23 vs. L23	F2-3 up	GO:0016021	Development	Development	integral component of membrane	C	AT4G13800
8425	3	F23 vs. L23	F2-3 up	GO:0016021	Development	Development	integral component of membrane	C	AT4G13990
8426	3	F23 vs. L23	F2-3 up	GO:0016021	Development	Development	integral component of membrane	C	AT4G14615
8427	3	F23 vs. L23	F2-3 up	GO:0016021	Development	Development	integral component of membrane	C	AT4G14690
8428	3	F23 vs. L23	F2-3 up	GO:0016021	Development	Development	integral component of membrane	C	AT4G15620
8429	3	F23 vs. L23	F2-3 up	GO:0016021	Development	Development	integral component of membrane	C	AT4G15630
8430	3	F23 vs. L23	F2-3 up	GO:0016021	Development	Development	integral component of membrane	C	AT4G16590
8431	3	F23 vs. L23	F2-3 up	GO:0016021	Development	Development	integral component of membrane	C	AT4G17080
8432	3	F23 vs. L23	F2-3 up	GO:0016021	Development	Development	integral component of membrane	C	AT4G18050
8433	3	F23 vs. L23	F2-3 up	GO:0016021	Development	Development	integral component of membrane	C	AT4G18220
8434	3	F23 vs. L23	F2-3 up	GO:0016021	Development	Development	integral component of membrane	C	AT4G19120
8435	3	F23 vs. L23	F2-3 up	GO:0016021	Development	Development	integral component of membrane	C	AT4G19230
8436	3	F23 vs. L23	F2-3 up	GO:0016021	Development	Development	integral component of membrane	C	AT4G20460
8437	3	F23 vs. L23	F2-3 up	GO:0016021	Development	Development	integral component of membrane	C	AT4G24570
8438	3	F23 vs. L23	F2-3 up	GO:0016021	Development	Development	integral component of membrane	C	AT4G25030
8439	3	F23 vs. L23	F2-3 up	GO:0016021	Development	Development	integral component of membrane	C	AT4G25040
8440	3	F23 vs. L23	F2-3 up	GO:0016021	Development	Development	integral component of membrane	C	AT4G27030
8441	3	F23 vs. L23	F2-3 up	GO:0016021	Development	Development	integral component of membrane	C	AT4G27435
8442	3	F23 vs. L23	F2-3 up	GO:0016021	Development	Development	integral component of membrane	C	AT4G27720

8443	3	F23 vs. L23	F2-3 up	GO:0016021	Development	Development	integral component of membrane	C	AT4G28490
8444	3	F23 vs. L23	F2-3 up	GO:0016021	Development	Development	integral component of membrane	C	AT4G30996
8445	3	F23 vs. L23	F2-3 up	GO:0016021	Development	Development	integral component of membrane	C	AT4G31330
8446	3	F23 vs. L23	F2-3 up	GO:0016021	Development	Development	integral component of membrane	C	AT4G31590
8447	3	F23 vs. L23	F2-3 up	GO:0016021	Development	Development	integral component of membrane	C	AT4G32260
8448	3	F23 vs. L23	F2-3 up	GO:0016021	Development	Development	integral component of membrane	C	AT4G32410
8449	3	F23 vs. L23	F2-3 up	GO:0016021	Development	Development	integral component of membrane	C	AT4G33530
8450	3	F23 vs. L23	F2-3 up	GO:0016021	Development	Development	integral component of membrane	C	AT4G34720
8451	3	F23 vs. L23	F2-3 up	GO:0016021	Development	Development	integral component of membrane	C	AT4G34950
8452	3	F23 vs. L23	F2-3 up	GO:0016021	Development	Development	integral component of membrane	C	AT4G37430
8453	3	F23 vs. L23	F2-3 up	GO:0016021	Development	Development	integral component of membrane	C	AT4G37760
8454	3	F23 vs. L23	F2-3 up	GO:0016021	Development	Development	integral component of membrane	C	AT4G38100
8455	3	F23 vs. L23	F2-3 up	GO:0016021	Development	Development	integral component of membrane	C	AT4G39030
8456	3	F23 vs. L23	F2-3 up	GO:0016021	Development	Development	integral component of membrane	C	AT4G39110
8457	3	F23 vs. L23	F2-3 up	GO:0016021	Development	Development	integral component of membrane	C	AT4G39350
8458	3	F23 vs. L23	F2-3 up	GO:0016021	Development	Development	integral component of membrane	C	AT4G39480
8459	3	F23 vs. L23	F2-3 up	GO:0016021	Development	Development	integral component of membrane	C	AT4G39510
8460	3	F23 vs. L23	F2-3 up	GO:0016021	Development	Development	integral component of membrane	C	AT5G01430
8461	3	F23 vs. L23	F2-3 up	GO:0016021	Development	Development	integral component of membrane	C	AT5G01690
8462	3	F23 vs. L23	F2-3 up	GO:0016021	Development	Development	integral component of membrane	C	AT5G04500
8463	3	F23 vs. L23	F2-3 up	GO:0016021	Development	Development	integral component of membrane	C	AT5G06320
8464	3	F23 vs. L23	F2-3 up	GO:0016021	Development	Development	integral component of membrane	C	AT5G06530
8465	3	F23 vs. L23	F2-3 up	GO:0016021	Development	Development	integral component of membrane	C	AT5G06850
8466	3	F23 vs. L23	F2-3 up	GO:0016021	Development	Development	integral component of membrane	C	AT5G07550
8467	3	F23 vs. L23	F2-3 up	GO:0016021	Development	Development	integral component of membrane	C	AT5G09870
8468	3	F23 vs. L23	F2-3 up	GO:0016021	Development	Development	integral component of membrane	C	AT5G11960
8469	3	F23 vs. L23	F2-3 up	GO:0016021	Development	Development	integral component of membrane	C	AT5G12170
8470	3	F23 vs. L23	F2-3 up	GO:0016021	Development	Development	integral component of membrane	C	AT5G13490
8471	3	F23 vs. L23	F2-3 up	GO:0016021	Development	Development	integral component of membrane	C	AT5G14120
8472	3	F23 vs. L23	F2-3 up	GO:0016021	Development	Development	integral component of membrane	C	AT5G14940
8473	3	F23 vs. L23	F2-3 up	GO:0016021	Development	Development	integral component of membrane	C	AT5G15470
8474	3	F23 vs. L23	F2-3 up	GO:0016021	Development	Development	integral component of membrane	C	AT5G16010
8475	3	F23 vs. L23	F2-3 up	GO:0016021	Development	Development	integral component of membrane	C	AT5G17860
8476	3	F23 vs. L23	F2-3 up	GO:0016021	Development	Development	integral component of membrane	C	AT5G18280
8477	3	F23 vs. L23	F2-3 up	GO:0016021	Development	Development	integral component of membrane	C	AT5G23060
8478	3	F23 vs. L23	F2-3 up	GO:0016021	Development	Development	integral component of membrane	C	AT5G24080
8479	3	F23 vs. L23	F2-3 up	GO:0016021	Development	Development	integral component of membrane	C	AT5G24140
8480	3	F23 vs. L23	F2-3 up	GO:0016021	Development	Development	integral component of membrane	C	AT5G24650
8481	3	F23 vs. L23	F2-3 up	GO:0016021	Development	Development	integral component of membrane	C	AT5G25120
8482	3	F23 vs. L23	F2-3 up	GO:0016021	Development	Development	integral component of membrane	C	AT5G35735
8483	3	F23 vs. L23	F2-3 up	GO:0016021	Development	Development	integral component of membrane	C	AT5G36220
8484	3	F23 vs. L23	F2-3 up	GO:0016021	Development	Development	integral component of membrane	C	AT5G36260
8485	3	F23 vs. L23	F2-3 up	GO:0016021	Development	Development	integral component of membrane	C	AT5G37300
8486	3	F23 vs. L23	F2-3 up	GO:0016021	Development	Development	integral component of membrane	C	AT5G37810
8487	3	F23 vs. L23	F2-3 up	GO:0016021	Development	Development	integral component of membrane	C	AT5G38660
8488	3	F23 vs. L23	F2-3 up	GO:0016021	Development	Development	integral component of membrane	C	AT5G40010
8489	3	F23 vs. L23	F2-3 up	GO:0016021	Development	Development	integral component of membrane	C	AT5G43745
8490	3	F23 vs. L23	F2-3 up	GO:0016021	Development	Development	integral component of membrane	C	AT5G45840
8491	3	F23 vs. L23	F2-3 up	GO:0016021	Development	Development	integral component of membrane	C	AT5G46800
8492	3	F23 vs. L23	F2-3 up	GO:0016021	Development	Development	integral component of membrane	C	AT5G47910
8493	3	F23 vs. L23	F2-3 up	GO:0016021	Development	Development	integral component of membrane	C	AT5G49130
8494	3	F23 vs. L23	F2-3 up	GO:0016021	Development	Development	integral component of membrane	C	AT5G49720
8495	3	F23 vs. L23	F2-3 up	GO:0016021	Development	Development	integral component of membrane	C	AT5G49950
8496	3	F23 vs. L23	F2-3 up	GO:0016021	Development	Development	integral component of membrane	C	AT5G50720
8497	3	F23 vs. L23	F2-3 up	GO:0016021	Development	Development	integral component of membrane	C	AT5G50800
8498	3	F23 vs. L23	F2-3 up	GO:0016021	Development	Development	integral component of membrane	C	AT5G52450

8499	3	F23 vs. L23	F2-3 up	GO:0016021	Development	Development	integral component of membrane	C	AT5G53370
8500	3	F23 vs. L23	F2-3 up	GO:0016021	Development	Development	integral component of membrane	C	AT5G53890
8501	3	F23 vs. L23	F2-3 up	GO:0016021	Development	Development	integral component of membrane	C	AT5G54250
8502	3	F23 vs. L23	F2-3 up	GO:0016021	Development	Development	integral component of membrane	C	AT5G54980
8503	3	F23 vs. L23	F2-3 up	GO:0016021	Development	Development	integral component of membrane	C	AT5G55340
8504	3	F23 vs. L23	F2-3 up	GO:0016021	Development	Development	integral component of membrane	C	AT5G55960
8505	3	F23 vs. L23	F2-3 up	GO:0016021	Development	Development	integral component of membrane	C	AT5G57350
8506	3	F23 vs. L23	F2-3 up	GO:0016021	Development	Development	integral component of membrane	C	AT5G57800
8507	3	F23 vs. L23	F2-3 up	GO:0016021	Development	Development	integral component of membrane	C	AT5G58050
8508	3	F23 vs. L23	F2-3 up	GO:0016021	Development	Development	integral component of membrane	C	AT5G60740
8509	3	F23 vs. L23	F2-3 up	GO:0016021	Development	Development	integral component of membrane	C	AT5G61670
8510	3	F23 vs. L23	F2-3 up	GO:0016021	Development	Development	integral component of membrane	C	AT5G61700
8511	3	F23 vs. L23	F2-3 up	GO:0016021	Development	Development	integral component of membrane	C	AT5G62680
8512	3	F23 vs. L23	F2-3 up	GO:0016021	Development	Development	integral component of membrane	C	AT5G64280
8513	3	F23 vs. L23	F2-3 up	GO:0016021	Development	Development	integral component of membrane	C	AT5G65650
8514	3	F23 vs. L23	F2-3 up	GO:0016021	Development	Development	integral component of membrane	C	AT5G65810
8515	3	F23 vs. L23	F2-3 up	GO:0016021	Development	Development	integral component of membrane	C	AT5G66070
8516	3	F23 vs. L23	F2-3 up	GO:0016021	Development	Development	integral component of membrane	C	AT5G67210
8517	3	F23 vs. L23	F2-3 up	GO:0016021	Development	Development	integral component of membrane	C	AT5G67370
8518	3	F23 vs. L23	F2-3 up	GO:0016021	Development	Development	integral component of membrane	C	AT5G67470
8519	3	F23 vs. L23	F2-3 up	GO:0016021	Development	Development	integral component of membrane	C	ATCG00150
8520	3	F23 vs. L23	F2-3 up	GO:0016021	Development	Development	integral component of membrane	C	ATCG00220
8521	3	F23 vs. L23	F2-3 up	GO:0016021	Development	Development	integral component of membrane	C	ATCG00560
8522	3	F23 vs. L23	F2-3 up	GO:0016021	Development	Development	integral component of membrane	C	ATCG01100
8523	3	F23 vs. L23	F2-3 up	GO:0016021	Development	Development	integral component of membrane	C	ATMG00480
8524	3	F23 vs. L23	F2-3 up	GO:0016021	Development	Development	integral component of membrane	C	ATMG00650
8525	3	F23 vs. L23	F2-3 up	GO:0005887	Development	Development	integral component of plasma membrane	C	AT1G22710
8526	3	F23 vs. L23	F2-3 up	GO:0005887	Development	Development	integral component of plasma membrane	C	AT1G50310
8527	3	F23 vs. L23	F2-3 up	GO:0005887	Development	Development	integral component of plasma membrane	C	AT1G64060
8528	3	F23 vs. L23	F2-3 up	GO:0005887	Development	Development	integral component of plasma membrane	C	AT2G30440
8529	3	F23 vs. L23	F2-3 up	GO:0005887	Development	Development	integral component of plasma membrane	C	AT2G39210
8530	3	F23 vs. L23	F2-3 up	GO:0005887	Development	Development	integral component of plasma membrane	C	AT2G48020
8531	3	F23 vs. L23	F2-3 up	GO:0005887	Development	Development	integral component of plasma membrane	C	AT3G03160
8532	3	F23 vs. L23	F2-3 up	GO:0005887	Development	Development	integral component of plasma membrane	C	AT3G05960
8533	3	F23 vs. L23	F2-3 up	GO:0005887	Development	Development	integral component of plasma membrane	C	AT3G14770
8534	3	F23 vs. L23	F2-3 up	GO:0005887	Development	Development	integral component of plasma membrane	C	AT3G48740
8535	3	F23 vs. L23	F2-3 up	GO:0005887	Development	Development	integral component of plasma membrane	C	AT3G51490
8536	3	F23 vs. L23	F2-3 up	GO:0005887	Development	Development	integral component of plasma membrane	C	AT5G09220
8537	3	F23 vs. L23	F2-3 up	GO:0005887	Development	Development	integral component of plasma membrane	C	AT5G50800
8538	3	F23 vs. L23	F2-3 up	GO:0005887	Development	Development	integral component of plasma membrane	C	AT5G64740
8539	3	F23 vs. L23	F2-3 up	GO:0005622	Development	Development	intracellular	C	AT3G07560
8540	3	F23 vs. L23	F2-3 up	GO:0005622	Development	Development	intracellular	C	AT4G17170
8541	3	F23 vs. L23	F2-3 up	GO:0005622	Development	Development	intracellular	C	AT5G40890
8542	3	F23 vs. L23	F2-3 up	GO:0005622	Development	Development	intracellular	C	AT5G65460
8543	3	F23 vs. L23	F2-3 up	GO:0043231	Development	Development	intracellular membrane-bounded organelle	C	AT2G17370
8544	3	F23 vs. L23	F2-3 up	GO:0043231	Development	Development	intracellular membrane-bounded organelle	C	AT2G18570
8545	3	F23 vs. L23	F2-3 up	GO:0043231	Development	Development	intracellular membrane-bounded organelle	C	AT2G31750
8546	3	F23 vs. L23	F2-3 up	GO:0043231	Development	Development	intracellular membrane-bounded organelle	C	AT2G31790
8547	3	F23 vs. L23	F2-3 up	GO:0043231	Development	Development	intracellular membrane-bounded organelle	C	AT2G36790
8548	3	F23 vs. L23	F2-3 up	GO:0043231	Development	Development	intracellular membrane-bounded organelle	C	AT2G38280
8549	3	F23 vs. L23	F2-3 up	GO:0043231	Development	Development	intracellular membrane-bounded organelle	C	AT2G40890
8550	3	F23 vs. L23	F2-3 up	GO:0043231	Development	Development	intracellular membrane-bounded organelle	C	AT3G21560
8551	3	F23 vs. L23	F2-3 up	GO:0043231	Development	Development	intracellular membrane-bounded organelle	C	AT3G21760
8552	3	F23 vs. L23	F2-3 up	GO:0043231	Development	Development	intracellular membrane-bounded organelle	C	AT3G46670
8553	3	F23 vs. L23	F2-3 up	GO:0043231	Development	Development	intracellular membrane-bounded organelle	C	AT4G01070
8554	3	F23 vs. L23	F2-3 up	GO:0043231	Development	Development	intracellular membrane-bounded organelle	C	AT5G40890

8555	3	F23 vs. L23	F2-3 up	GO:0043231	Development	Development	intracellular membrane-bounded organelle	C	AT5G61690
8556	3	F23 vs. L23	F2-3 up	GO:0043231	Development	Development	intracellular membrane-bounded organelle	C	AT5G61700
8557	3	F23 vs. L23	F2-3 up	GO:0097708	Development	Development	intracellular vesicle	C	AT1G68570
8558	3	F23 vs. L23	F2-3 up	GO:0005770	Development	Development	late endosome	C	AT3G17630
8559	3	F23 vs. L23	F2-3 up	GO:0031902	Development	Development	late endosome membrane	C	AT1G12240
8560	3	F23 vs. L23	F2-3 up	GO:0016328	Development	Development	lateral plasma membrane	C	AT1G70940
8561	3	F23 vs. L23	F2-3 up	GO:0016328	Development	Development	lateral plasma membrane	C	AT2G47160
8562	3	F23 vs. L23	F2-3 up	GO:0048527	Development	Development	lateral root development	P	AT1G10370
8563	3	F23 vs. L23	F2-3 up	GO:0048527	Development	Development	lateral root development	P	AT1G32080
8564	3	F23 vs. L23	F2-3 up	GO:0048527	Development	Development	lateral root development	P	AT4G29350
8565	3	F23 vs. L23	F2-3 up	GO:0010311	Development	Development	lateral root formation	P	AT3G22400
8566	3	F23 vs. L23	F2-3 up	GO:0010311	Development	Development	lateral root formation	P	AT5G05730
8567	3	F23 vs. L23	F2-3 up	GO:0010102	Development	Development	lateral root morphogenesis	P	AT4G28490
8568	3	F23 vs. L23	F2-3 up	GO:0048366	Development	Development	leaf development	P	AT1G79440
8569	3	F23 vs. L23	F2-3 up	GO:0048366	Development	Development	leaf development	P	AT2G42200
8570	3	F23 vs. L23	F2-3 up	GO:0048366	Development	Development	leaf development	P	AT3G11050
8571	3	F23 vs. L23	F2-3 up	GO:0048366	Development	Development	leaf development	P	AT3G14940
8572	3	F23 vs. L23	F2-3 up	GO:0048366	Development	Development	leaf development	P	AT3G15030
8573	3	F23 vs. L23	F2-3 up	GO:0048366	Development	Development	leaf development	P	AT4G20360
8574	3	F23 vs. L23	F2-3 up	GO:0048366	Development	Development	leaf development	P	AT4G29350
8575	3	F23 vs. L23	F2-3 up	GO:0048366	Development	Development	leaf development	P	AT5G01600
8576	3	F23 vs. L23	F2-3 up	GO:0010338	Development	Development	leaf formation	P	AT2G37630
8577	3	F23 vs. L23	F2-3 up	GO:0009965	Development	Development	leaf morphogenesis	P	AT2G23760
8578	3	F23 vs. L23	F2-3 up	GO:0009965	Development	Development	leaf morphogenesis	P	AT2G37630
8579	3	F23 vs. L23	F2-3 up	GO:0009965	Development	Development	leaf morphogenesis	P	AT3G15030
8580	3	F23 vs. L23	F2-3 up	GO:0009965	Development	Development	leaf morphogenesis	P	AT5G08370
8581	3	F23 vs. L23	F2-3 up	GO:0009965	Development	Development	leaf morphogenesis	P	AT5G65810
8582	3	F23 vs. L23	F2-3 up	GO:0010305	Development	Development	leaf vascular tissue pattern formation	P	AT1G14000
8583	3	F23 vs. L23	F2-3 up	GO:0010305	Development	Development	leaf vascular tissue pattern formation	P	AT1G27340
8584	3	F23 vs. L23	F2-3 up	GO:0005811	Development	Development	lipid droplet	C	AT3G05500
8585	3	F23 vs. L23	F2-3 up	GO:0010076	Development	Development	maintenance of floral meristem identity	P	AT1G69120
8586	3	F23 vs. L23	F2-3 up	GO:0010077	Development	Development	maintenance of inflorescence meristem identity	P	AT3G07050
8587	3	F23 vs. L23	F2-3 up	GO:0010077	Development	Development	maintenance of inflorescence meristem identity	P	AT5G60910
8588	3	F23 vs. L23	F2-3 up	GO:0010492	Development	Development	maintenance of shoot apical meristem identity	P	AT1G79440
8589	3	F23 vs. L23	F2-3 up	GO:0010492	Development	Development	maintenance of shoot apical meristem identity	P	AT2G17950
8590	3	F23 vs. L23	F2-3 up	GO:0016020	Development	Development	membrane	C	AT1G02390
8591	3	F23 vs. L23	F2-3 up	GO:0016020	Development	Development	membrane	C	AT1G12070
8592	3	F23 vs. L23	F2-3 up	GO:0016020	Development	Development	membrane	C	AT1G12240
8593	3	F23 vs. L23	F2-3 up	GO:0016020	Development	Development	membrane	C	AT1G16310
8594	3	F23 vs. L23	F2-3 up	GO:0016020	Development	Development	membrane	C	AT1G20190
8595	3	F23 vs. L23	F2-3 up	GO:0016020	Development	Development	membrane	C	AT1G22710
8596	3	F23 vs. L23	F2-3 up	GO:0016020	Development	Development	membrane	C	AT1G26770
8597	3	F23 vs. L23	F2-3 up	GO:0016020	Development	Development	membrane	C	AT1G35720
8598	3	F23 vs. L23	F2-3 up	GO:0016020	Development	Development	membrane	C	AT1G47530
8599	3	F23 vs. L23	F2-3 up	GO:0016020	Development	Development	membrane	C	AT1G62450
8600	3	F23 vs. L23	F2-3 up	GO:0016020	Development	Development	membrane	C	AT1G69530
8601	3	F23 vs. L23	F2-3 up	GO:0016020	Development	Development	membrane	C	AT1G71050
8602	3	F23 vs. L23	F2-3 up	GO:0016020	Development	Development	membrane	C	AT1G76490
8603	3	F23 vs. L23	F2-3 up	GO:0016020	Development	Development	membrane	C	AT2G17290
8604	3	F23 vs. L23	F2-3 up	GO:0016020	Development	Development	membrane	C	AT2G28950
8605	3	F23 vs. L23	F2-3 up	GO:0016020	Development	Development	membrane	C	AT2G38110
8606	3	F23 vs. L23	F2-3 up	GO:0016020	Development	Development	membrane	C	AT2G39420
8607	3	F23 vs. L23	F2-3 up	GO:0016020	Development	Development	membrane	C	AT2G39700
8608	3	F23 vs. L23	F2-3 up	GO:0016020	Development	Development	membrane	C	AT2G45770
8609	3	F23 vs. L23	F2-3 up	GO:0016020	Development	Development	membrane	C	AT2G47650
8610	3	F23 vs. L23	F2-3 up	GO:0016020	Development	Development	membrane	C	AT3G11900

8611	3	F23 vs. L23	F2-3 up	GO:0016020	Development	Development	membrane	C	AT3G21180
8612	3	F23 vs. L23	F2-3 up	GO:0016020	Development	Development	membrane	C	AT3G29030
8613	3	F23 vs. L23	F2-3 up	GO:0016020	Development	Development	membrane	C	AT3G61760
8614	3	F23 vs. L23	F2-3 up	GO:0016020	Development	Development	membrane	C	AT4G03280
8615	3	F23 vs. L23	F2-3 up	GO:0016020	Development	Development	membrane	C	AT4G13990
8616	3	F23 vs. L23	F2-3 up	GO:0016020	Development	Development	membrane	C	AT4G35060
8617	3	F23 vs. L23	F2-3 up	GO:0016020	Development	Development	membrane	C	AT5G17450
8618	3	F23 vs. L23	F2-3 up	GO:0016020	Development	Development	membrane	C	AT5G39210
8619	3	F23 vs. L23	F2-3 up	GO:0016020	Development	Development	membrane	C	AT5G49130
8620	3	F23 vs. L23	F2-3 up	GO:0016020	Development	Development	membrane	C	AT5G52450
8621	3	F23 vs. L23	F2-3 up	GO:0016020	Development	Development	membrane	C	AT5G54250
8622	3	F23 vs. L23	F2-3 up	GO:0016020	Development	Development	membrane	C	AT5G57800
8623	3	F23 vs. L23	F2-3 up	GO:0061025	Development	Development	membrane fusion	P	AT1G13890
8624	3	F23 vs. L23	F2-3 up	GO:0061025	Development	Development	membrane fusion	P	AT1G61290
8625	3	F23 vs. L23	F2-3 up	GO:0061025	Development	Development	membrane fusion	P	AT3G03800
8626	3	F23 vs. L23	F2-3 up	GO:0010014	Development	Development	meristem initiation	P	AT4G32880
8627	3	F23 vs. L23	F2-3 up	GO:0010073	Development	Development	meristem maintenance	P	AT1G23380
8628	3	F23 vs. L23	F2-3 up	GO:0009933	Development	Development	meristem structural organization	P	AT1G69120
8629	3	F23 vs. L23	F2-3 up	GO:0009933	Development	Development	meristem structural organization	P	AT2G45190
8630	3	F23 vs. L23	F2-3 up	GO:0042579	Development	Development	microbody	C	AT5G09660
8631	3	F23 vs. L23	F2-3 up	GO:0005874	Development	Development	microtubule	C	AT1G64740
8632	3	F23 vs. L23	F2-3 up	GO:0005874	Development	Development	microtubule	C	AT3G43210
8633	3	F23 vs. L23	F2-3 up	GO:0005874	Development	Development	microtubule	C	AT3G61760
8634	3	F23 vs. L23	F2-3 up	GO:0005874	Development	Development	microtubule	C	AT5G27000
8635	3	F23 vs. L23	F2-3 up	GO:0005874	Development	Development	microtubule	C	AT5G65460
8636	3	F23 vs. L23	F2-3 up	GO:0015630	Development	Development	microtubule cytoskeleton	C	AT1G62360
8637	3	F23 vs. L23	F2-3 up	GO:0015630	Development	Development	microtubule cytoskeleton	C	AT1G64740
8638	3	F23 vs. L23	F2-3 up	GO:0015630	Development	Development	microtubule cytoskeleton	C	AT2G18390
8639	3	F23 vs. L23	F2-3 up	GO:0000226	Development	Development	microtubule cytoskeleton organization	P	AT1G64740
8640	3	F23 vs. L23	F2-3 up	GO:0000226	Development	Development	microtubule cytoskeleton organization	P	AT2G18390
8641	3	F23 vs. L23	F2-3 up	GO:0000226	Development	Development	microtubule cytoskeleton organization	P	AT3G16800
8642	3	F23 vs. L23	F2-3 up	GO:0007018	Development	Development	microtubule-based movement	P	AT3G43210
8643	3	F23 vs. L23	F2-3 up	GO:0007018	Development	Development	microtubule-based movement	P	AT5G27000
8644	3	F23 vs. L23	F2-3 up	GO:0007018	Development	Development	microtubule-based movement	P	AT5G65460
8645	3	F23 vs. L23	F2-3 up	GO:0007017	Development	Development	microtubule-based process	P	AT1G64740
8646	3	F23 vs. L23	F2-3 up	GO:0012511	Development	Development	monolayer-surrounded lipid storage body	C	AT3G01420
8647	3	F23 vs. L23	F2-3 up	GO:0012511	Development	Development	monolayer-surrounded lipid storage body	C	AT5G07550
8648	3	F23 vs. L23	F2-3 up	GO:0007275	Development	Development	multicellular organism development	P	AT1G14920
8649	3	F23 vs. L23	F2-3 up	GO:0007275	Development	Development	multicellular organism development	P	AT1G49800
8650	3	F23 vs. L23	F2-3 up	GO:0007275	Development	Development	multicellular organism development	P	AT1G50250
8651	3	F23 vs. L23	F2-3 up	GO:0007275	Development	Development	multicellular organism development	P	AT1G63650
8652	3	F23 vs. L23	F2-3 up	GO:0007275	Development	Development	multicellular organism development	P	AT1G69120
8653	3	F23 vs. L23	F2-3 up	GO:0007275	Development	Development	multicellular organism development	P	AT1G77980
8654	3	F23 vs. L23	F2-3 up	GO:0007275	Development	Development	multicellular organism development	P	AT3G23290
8655	3	F23 vs. L23	F2-3 up	GO:0007275	Development	Development	multicellular organism development	P	AT3G54340
8656	3	F23 vs. L23	F2-3 up	GO:0007275	Development	Development	multicellular organism development	P	AT5G60910
8657	3	F23 vs. L23	F2-3 up	GO:0007275	Development	Development	multicellular organism development	P	AT5G62520
8658	3	F23 vs. L23	F2-3 up	GO:0007275	Development	Development	multicellular organism development	P	AT5G65870
8659	3	F23 vs. L23	F2-3 up	GO:0035264	Development	Development	multicellular organism growth	P	AT1G27730
8660	3	F23 vs. L23	F2-3 up	GO:0009825	Development	Development	multidimensional cell growth	P	AT1G05850
8661	3	F23 vs. L23	F2-3 up	GO:0009825	Development	Development	multidimensional cell growth	P	AT4G32410
8662	3	F23 vs. L23	F2-3 up	GO:0009825	Development	Development	multidimensional cell growth	P	AT5G64740
8663	3	F23 vs. L23	F2-3 up	GO:0016459	Development	Development	myosin complex	C	AT1G54560
8664	3	F23 vs. L23	F2-3 up	GO:0016604	Development	Development	nuclear body	C	AT1G04400
8665	3	F23 vs. L23	F2-3 up	GO:0000790	Development	Development	nuclear chromatin	C	AT1G09600
8666	3	F23 vs. L23	F2-3 up	GO:0016363	Development	Development	nuclear matrix	C	AT3G09440

8667	3	F23 vs. L23	F2-3 up	GO:0016363	Development	Development	nuclear matrix	C	AT5G62520
8668	3	F23 vs. L23	F2-3 up	GO:0005643	Development	Development	nuclear pore	C	AT4G16143
8669	3	F23 vs. L23	F2-3 up	GO:0009295	Development	Development	nucleoid	C	AT2G46820
8670	3	F23 vs. L23	F2-3 up	GO:0009295	Development	Development	nucleoid	C	AT4G20360
8671	3	F23 vs. L23	F2-3 up	GO:0005654	Development	Development	nucleoplasm	C	AT4G16143
8672	3	F23 vs. L23	F2-3 up	GO:0005634	Development	Development	nucleus	C	AT1G01250
8673	3	F23 vs. L23	F2-3 up	GO:0005634	Development	Development	nucleus	C	AT1G01320
8674	3	F23 vs. L23	F2-3 up	GO:0005634	Development	Development	nucleus	C	AT1G01630
8675	3	F23 vs. L23	F2-3 up	GO:0005634	Development	Development	nucleus	C	AT1G01720
8676	3	F23 vs. L23	F2-3 up	GO:0005634	Development	Development	nucleus	C	AT1G03050
8677	3	F23 vs. L23	F2-3 up	GO:0005634	Development	Development	nucleus	C	AT1G03620
8678	3	F23 vs. L23	F2-3 up	GO:0005634	Development	Development	nucleus	C	AT1G04400
8679	3	F23 vs. L23	F2-3 up	GO:0005634	Development	Development	nucleus	C	AT1G04530
8680	3	F23 vs. L23	F2-3 up	GO:0005634	Development	Development	nucleus	C	AT1G08830
8681	3	F23 vs. L23	F2-3 up	GO:0005634	Development	Development	nucleus	C	AT1G09250
8682	3	F23 vs. L23	F2-3 up	GO:0005634	Development	Development	nucleus	C	AT1G09310
8683	3	F23 vs. L23	F2-3 up	GO:0005634	Development	Development	nucleus	C	AT1G09600
8684	3	F23 vs. L23	F2-3 up	GO:0005634	Development	Development	nucleus	C	AT1G11950
8685	3	F23 vs. L23	F2-3 up	GO:0005634	Development	Development	nucleus	C	AT1G13860
8686	3	F23 vs. L23	F2-3 up	GO:0005634	Development	Development	nucleus	C	AT1G13890
8687	3	F23 vs. L23	F2-3 up	GO:0005634	Development	Development	nucleus	C	AT1G14000
8688	3	F23 vs. L23	F2-3 up	GO:0005634	Development	Development	nucleus	C	AT1G14920
8689	3	F23 vs. L23	F2-3 up	GO:0005634	Development	Development	nucleus	C	AT1G15470
8690	3	F23 vs. L23	F2-3 up	GO:0005634	Development	Development	nucleus	C	AT1G15980
8691	3	F23 vs. L23	F2-3 up	GO:0005634	Development	Development	nucleus	C	AT1G16290
8692	3	F23 vs. L23	F2-3 up	GO:0005634	Development	Development	nucleus	C	AT1G16410
8693	3	F23 vs. L23	F2-3 up	GO:0005634	Development	Development	nucleus	C	AT1G16880
8694	3	F23 vs. L23	F2-3 up	GO:0005634	Development	Development	nucleus	C	AT1G17370
8695	3	F23 vs. L23	F2-3 up	GO:0005634	Development	Development	nucleus	C	AT1G17380
8696	3	F23 vs. L23	F2-3 up	GO:0005634	Development	Development	nucleus	C	AT1G18150
8697	3	F23 vs. L23	F2-3 up	GO:0005634	Development	Development	nucleus	C	AT1G18660
8698	3	F23 vs. L23	F2-3 up	GO:0005634	Development	Development	nucleus	C	AT1G18740
8699	3	F23 vs. L23	F2-3 up	GO:0005634	Development	Development	nucleus	C	AT1G19180
8700	3	F23 vs. L23	F2-3 up	GO:0005634	Development	Development	nucleus	C	AT1G19550
8701	3	F23 vs. L23	F2-3 up	GO:0005634	Development	Development	nucleus	C	AT1G20020
8702	3	F23 vs. L23	F2-3 up	GO:0005634	Development	Development	nucleus	C	AT1G20620
8703	3	F23 vs. L23	F2-3 up	GO:0005634	Development	Development	nucleus	C	AT1G21010
8704	3	F23 vs. L23	F2-3 up	GO:0005634	Development	Development	nucleus	C	AT1G21060
8705	3	F23 vs. L23	F2-3 up	GO:0005634	Development	Development	nucleus	C	AT1G21760
8706	3	F23 vs. L23	F2-3 up	GO:0005634	Development	Development	nucleus	C	AT1G22710
8707	3	F23 vs. L23	F2-3 up	GO:0005634	Development	Development	nucleus	C	AT1G22760
8708	3	F23 vs. L23	F2-3 up	GO:0005634	Development	Development	nucleus	C	AT1G22840
8709	3	F23 vs. L23	F2-3 up	GO:0005634	Development	Development	nucleus	C	AT1G23310
8710	3	F23 vs. L23	F2-3 up	GO:0005634	Development	Development	nucleus	C	AT1G23380
8711	3	F23 vs. L23	F2-3 up	GO:0005634	Development	Development	nucleus	C	AT1G23460
8712	3	F23 vs. L23	F2-3 up	GO:0005634	Development	Development	nucleus	C	AT1G23710
8713	3	F23 vs. L23	F2-3 up	GO:0005634	Development	Development	nucleus	C	AT1G23740
8714	3	F23 vs. L23	F2-3 up	GO:0005634	Development	Development	nucleus	C	AT1G23750
8715	3	F23 vs. L23	F2-3 up	GO:0005634	Development	Development	nucleus	C	AT1G23800
8716	3	F23 vs. L23	F2-3 up	GO:0005634	Development	Development	nucleus	C	AT1G25350
8717	3	F23 vs. L23	F2-3 up	GO:0005634	Development	Development	nucleus	C	AT1G26480
8718	3	F23 vs. L23	F2-3 up	GO:0005634	Development	Development	nucleus	C	AT1G26610
8719	3	F23 vs. L23	F2-3 up	GO:0005634	Development	Development	nucleus	C	AT1G27730
8720	3	F23 vs. L23	F2-3 up	GO:0005634	Development	Development	nucleus	C	AT1G27930
8721	3	F23 vs. L23	F2-3 up	GO:0005634	Development	Development	nucleus	C	AT1G28480
8722	3	F23 vs. L23	F2-3 up	GO:0005634	Development	Development	nucleus	C	AT1G29340

8723	3	F23 vs. L23	F2-3 up	GO:0005634	Development	Development	nucleus	C	AT1G30330
8724	3	F23 vs. L23	F2-3 up	GO:0005634	Development	Development	nucleus	C	AT1G30810
8725	3	F23 vs. L23	F2-3 up	GO:0005634	Development	Development	nucleus	C	AT1G32060
8726	3	F23 vs. L23	F2-3 up	GO:0005634	Development	Development	nucleus	C	AT1G32450
8727	3	F23 vs. L23	F2-3 up	GO:0005634	Development	Development	nucleus	C	AT1G32640
8728	3	F23 vs. L23	F2-3 up	GO:0005634	Development	Development	nucleus	C	AT1G33170
8729	3	F23 vs. L23	F2-3 up	GO:0005634	Development	Development	nucleus	C	AT1G35490
8730	3	F23 vs. L23	F2-3 up	GO:0005634	Development	Development	nucleus	C	AT1G35720
8731	3	F23 vs. L23	F2-3 up	GO:0005634	Development	Development	nucleus	C	AT1G42970
8732	3	F23 vs. L23	F2-3 up	GO:0005634	Development	Development	nucleus	C	AT1G44170
8733	3	F23 vs. L23	F2-3 up	GO:0005634	Development	Development	nucleus	C	AT1G44575
8734	3	F23 vs. L23	F2-3 up	GO:0005634	Development	Development	nucleus	C	AT1G44820
8735	3	F23 vs. L23	F2-3 up	GO:0005634	Development	Development	nucleus	C	AT1G45130
8736	3	F23 vs. L23	F2-3 up	GO:0005634	Development	Development	nucleus	C	AT1G47530
8737	3	F23 vs. L23	F2-3 up	GO:0005634	Development	Development	nucleus	C	AT1G47570
8738	3	F23 vs. L23	F2-3 up	GO:0005634	Development	Development	nucleus	C	AT1G48030
8739	3	F23 vs. L23	F2-3 up	GO:0005634	Development	Development	nucleus	C	AT1G50250
8740	3	F23 vs. L23	F2-3 up	GO:0005634	Development	Development	nucleus	C	AT1G50610
8741	3	F23 vs. L23	F2-3 up	GO:0005634	Development	Development	nucleus	C	AT1G50940
8742	3	F23 vs. L23	F2-3 up	GO:0005634	Development	Development	nucleus	C	AT1G51090
8743	3	F23 vs. L23	F2-3 up	GO:0005634	Development	Development	nucleus	C	AT1G51110
8744	3	F23 vs. L23	F2-3 up	GO:0005634	Development	Development	nucleus	C	AT1G52190
8745	3	F23 vs. L23	F2-3 up	GO:0005634	Development	Development	nucleus	C	AT1G53280
8746	3	F23 vs. L23	F2-3 up	GO:0005634	Development	Development	nucleus	C	AT1G53500
8747	3	F23 vs. L23	F2-3 up	GO:0005634	Development	Development	nucleus	C	AT1G54290
8748	3	F23 vs. L23	F2-3 up	GO:0005634	Development	Development	nucleus	C	AT1G54560
8749	3	F23 vs. L23	F2-3 up	GO:0005634	Development	Development	nucleus	C	AT1G55480
8750	3	F23 vs. L23	F2-3 up	GO:0005634	Development	Development	nucleus	C	AT1G56500
8751	3	F23 vs. L23	F2-3 up	GO:0005634	Development	Development	nucleus	C	AT1G57770
8752	3	F23 vs. L23	F2-3 up	GO:0005634	Development	Development	nucleus	C	AT1G59640
8753	3	F23 vs. L23	F2-3 up	GO:0005634	Development	Development	nucleus	C	AT1G61660
8754	3	F23 vs. L23	F2-3 up	GO:0005634	Development	Development	nucleus	C	AT1G62290
8755	3	F23 vs. L23	F2-3 up	GO:0005634	Development	Development	nucleus	C	AT1G62360
8756	3	F23 vs. L23	F2-3 up	GO:0005634	Development	Development	nucleus	C	AT1G62380
8757	3	F23 vs. L23	F2-3 up	GO:0005634	Development	Development	nucleus	C	AT1G62540
8758	3	F23 vs. L23	F2-3 up	GO:0005634	Development	Development	nucleus	C	AT1G62570
8759	3	F23 vs. L23	F2-3 up	GO:0005634	Development	Development	nucleus	C	AT1G63650
8760	3	F23 vs. L23	F2-3 up	GO:0005634	Development	Development	nucleus	C	AT1G63930
8761	3	F23 vs. L23	F2-3 up	GO:0005634	Development	Development	nucleus	C	AT1G64500
8762	3	F23 vs. L23	F2-3 up	GO:0005634	Development	Development	nucleus	C	AT1G64510
8763	3	F23 vs. L23	F2-3 up	GO:0005634	Development	Development	nucleus	C	AT1G64740
8764	3	F23 vs. L23	F2-3 up	GO:0005634	Development	Development	nucleus	C	AT1G64800
8765	3	F23 vs. L23	F2-3 up	GO:0005634	Development	Development	nucleus	C	AT1G64980
8766	3	F23 vs. L23	F2-3 up	GO:0005634	Development	Development	nucleus	C	AT1G65960
8767	3	F23 vs. L23	F2-3 up	GO:0005634	Development	Development	nucleus	C	AT1G68440
8768	3	F23 vs. L23	F2-3 up	GO:0005634	Development	Development	nucleus	C	AT1G68610
8769	3	F23 vs. L23	F2-3 up	GO:0005634	Development	Development	nucleus	C	AT1G69120
8770	3	F23 vs. L23	F2-3 up	GO:0005634	Development	Development	nucleus	C	AT1G70310
8771	3	F23 vs. L23	F2-3 up	GO:0005634	Development	Development	nucleus	C	AT1G70480
8772	3	F23 vs. L23	F2-3 up	GO:0005634	Development	Development	nucleus	C	AT1G70700
8773	3	F23 vs. L23	F2-3 up	GO:0005634	Development	Development	nucleus	C	AT1G70730
8774	3	F23 vs. L23	F2-3 up	GO:0005634	Development	Development	nucleus	C	AT1G71770
8775	3	F23 vs. L23	F2-3 up	GO:0005634	Development	Development	nucleus	C	AT1G73390
8776	3	F23 vs. L23	F2-3 up	GO:0005634	Development	Development	nucleus	C	AT1G74450
8777	3	F23 vs. L23	F2-3 up	GO:0005634	Development	Development	nucleus	C	AT1G74510
8778	3	F23 vs. L23	F2-3 up	GO:0005634	Development	Development	nucleus	C	AT1G75160

8779	3	F23 vs. L23	F2-3 up	GO:0005634	Development	Development	nucleus	C	AT1G77980
8780	3	F23 vs. L23	F2-3 up	GO:0005634	Development	Development	nucleus	C	AT1G78240
8781	3	F23 vs. L23	F2-3 up	GO:0005634	Development	Development	nucleus	C	AT1G78290
8782	3	F23 vs. L23	F2-3 up	GO:0005634	Development	Development	nucleus	C	AT1G78370
8783	3	F23 vs. L23	F2-3 up	GO:0005634	Development	Development	nucleus	C	AT1G78940
8784	3	F23 vs. L23	F2-3 up	GO:0005634	Development	Development	nucleus	C	AT1G79270
8785	3	F23 vs. L23	F2-3 up	GO:0005634	Development	Development	nucleus	C	AT1G80840
8786	3	F23 vs. L23	F2-3 up	GO:0005634	Development	Development	nucleus	C	AT2G01520
8787	3	F23 vs. L23	F2-3 up	GO:0005634	Development	Development	nucleus	C	AT2G02800
8788	3	F23 vs. L23	F2-3 up	GO:0005634	Development	Development	nucleus	C	AT2G13570
8789	3	F23 vs. L23	F2-3 up	GO:0005634	Development	Development	nucleus	C	AT2G16750
8790	3	F23 vs. L23	F2-3 up	GO:0005634	Development	Development	nucleus	C	AT2G17220
8791	3	F23 vs. L23	F2-3 up	GO:0005634	Development	Development	nucleus	C	AT2G17290
8792	3	F23 vs. L23	F2-3 up	GO:0005634	Development	Development	nucleus	C	AT2G17370
8793	3	F23 vs. L23	F2-3 up	GO:0005634	Development	Development	nucleus	C	AT2G17840
8794	3	F23 vs. L23	F2-3 up	GO:0005634	Development	Development	nucleus	C	AT2G17950
8795	3	F23 vs. L23	F2-3 up	GO:0005634	Development	Development	nucleus	C	AT2G18470
8796	3	F23 vs. L23	F2-3 up	GO:0005634	Development	Development	nucleus	C	AT2G19570
8797	3	F23 vs. L23	F2-3 up	GO:0005634	Development	Development	nucleus	C	AT2G19770
8798	3	F23 vs. L23	F2-3 up	GO:0005634	Development	Development	nucleus	C	AT2G20320
8799	3	F23 vs. L23	F2-3 up	GO:0005634	Development	Development	nucleus	C	AT2G20470
8800	3	F23 vs. L23	F2-3 up	GO:0005634	Development	Development	nucleus	C	AT2G21880
8801	3	F23 vs. L23	F2-3 up	GO:0005634	Development	Development	nucleus	C	AT2G22125
8802	3	F23 vs. L23	F2-3 up	GO:0005634	Development	Development	nucleus	C	AT2G23170
8803	3	F23 vs. L23	F2-3 up	GO:0005634	Development	Development	nucleus	C	AT2G23760
8804	3	F23 vs. L23	F2-3 up	GO:0005634	Development	Development	nucleus	C	AT2G23910
8805	3	F23 vs. L23	F2-3 up	GO:0005634	Development	Development	nucleus	C	AT2G25740
8806	3	F23 vs. L23	F2-3 up	GO:0005634	Development	Development	nucleus	C	AT2G26490
8807	3	F23 vs. L23	F2-3 up	GO:0005634	Development	Development	nucleus	C	AT2G26740
8808	3	F23 vs. L23	F2-3 up	GO:0005634	Development	Development	nucleus	C	AT2G27590
8809	3	F23 vs. L23	F2-3 up	GO:0005634	Development	Development	nucleus	C	AT2G28305
8810	3	F23 vs. L23	F2-3 up	GO:0005634	Development	Development	nucleus	C	AT2G30020
8811	3	F23 vs. L23	F2-3 up	GO:0005634	Development	Development	nucleus	C	AT2G30360
8812	3	F23 vs. L23	F2-3 up	GO:0005634	Development	Development	nucleus	C	AT2G30520
8813	3	F23 vs. L23	F2-3 up	GO:0005634	Development	Development	nucleus	C	AT2G31380
8814	3	F23 vs. L23	F2-3 up	GO:0005634	Development	Development	nucleus	C	AT2G31500
8815	3	F23 vs. L23	F2-3 up	GO:0005634	Development	Development	nucleus	C	AT2G31570
8816	3	F23 vs. L23	F2-3 up	GO:0005634	Development	Development	nucleus	C	AT2G32460
8817	3	F23 vs. L23	F2-3 up	GO:0005634	Development	Development	nucleus	C	AT2G34600
8818	3	F23 vs. L23	F2-3 up	GO:0005634	Development	Development	nucleus	C	AT2G35370
8819	3	F23 vs. L23	F2-3 up	GO:0005634	Development	Development	nucleus	C	AT2G35840
8820	3	F23 vs. L23	F2-3 up	GO:0005634	Development	Development	nucleus	C	AT2G35940
8821	3	F23 vs. L23	F2-3 up	GO:0005634	Development	Development	nucleus	C	AT2G36880
8822	3	F23 vs. L23	F2-3 up	GO:0005634	Development	Development	nucleus	C	AT2G37630
8823	3	F23 vs. L23	F2-3 up	GO:0005634	Development	Development	nucleus	C	AT2G38280
8824	3	F23 vs. L23	F2-3 up	GO:0005634	Development	Development	nucleus	C	AT2G38640
8825	3	F23 vs. L23	F2-3 up	GO:0005634	Development	Development	nucleus	C	AT2G39130
8826	3	F23 vs. L23	F2-3 up	GO:0005634	Development	Development	nucleus	C	AT2G39650
8827	3	F23 vs. L23	F2-3 up	GO:0005634	Development	Development	nucleus	C	AT2G39730
8828	3	F23 vs. L23	F2-3 up	GO:0005634	Development	Development	nucleus	C	AT2G39770
8829	3	F23 vs. L23	F2-3 up	GO:0005634	Development	Development	nucleus	C	AT2G40140
8830	3	F23 vs. L23	F2-3 up	GO:0005634	Development	Development	nucleus	C	AT2G40670
8831	3	F23 vs. L23	F2-3 up	GO:0005634	Development	Development	nucleus	C	AT2G41630
8832	3	F23 vs. L23	F2-3 up	GO:0005634	Development	Development	nucleus	C	AT2G42200
8833	3	F23 vs. L23	F2-3 up	GO:0005634	Development	Development	nucleus	C	AT2G42320
8834	3	F23 vs. L23	F2-3 up	GO:0005634	Development	Development	nucleus	C	AT2G43760

8835	3	F23 vs. L23	F2-3 up	GO:0005634	Development	Development	nucleus	C	AT2G45190
8836	3	F23 vs. L23	F2-3 up	GO:0005634	Development	Development	nucleus	C	AT2G45610
8837	3	F23 vs. L23	F2-3 up	GO:0005634	Development	Development	nucleus	C	AT2G45800
8838	3	F23 vs. L23	F2-3 up	GO:0005634	Development	Development	nucleus	C	AT2G46410
8839	3	F23 vs. L23	F2-3 up	GO:0005634	Development	Development	nucleus	C	AT2G46830
8840	3	F23 vs. L23	F2-3 up	GO:0005634	Development	Development	nucleus	C	AT2G47590
8841	3	F23 vs. L23	F2-3 up	GO:0005634	Development	Development	nucleus	C	AT3G01400
8842	3	F23 vs. L23	F2-3 up	GO:0005634	Development	Development	nucleus	C	AT3G01680
8843	3	F23 vs. L23	F2-3 up	GO:0005634	Development	Development	nucleus	C	AT3G04120
8844	3	F23 vs. L23	F2-3 up	GO:0005634	Development	Development	nucleus	C	AT3G05580
8845	3	F23 vs. L23	F2-3 up	GO:0005634	Development	Development	nucleus	C	AT3G06490
8846	3	F23 vs. L23	F2-3 up	GO:0005634	Development	Development	nucleus	C	AT3G07050
8847	3	F23 vs. L23	F2-3 up	GO:0005634	Development	Development	nucleus	C	AT3G07960
8848	3	F23 vs. L23	F2-3 up	GO:0005634	Development	Development	nucleus	C	AT3G08560
8849	3	F23 vs. L23	F2-3 up	GO:0005634	Development	Development	nucleus	C	AT3G08720
8850	3	F23 vs. L23	F2-3 up	GO:0005634	Development	Development	nucleus	C	AT3G08730
8851	3	F23 vs. L23	F2-3 up	GO:0005634	Development	Development	nucleus	C	AT3G08990
8852	3	F23 vs. L23	F2-3 up	GO:0005634	Development	Development	nucleus	C	AT3G11410
8853	3	F23 vs. L23	F2-3 up	GO:0005634	Development	Development	nucleus	C	AT3G12690
8854	3	F23 vs. L23	F2-3 up	GO:0005634	Development	Development	nucleus	C	AT3G12780
8855	3	F23 vs. L23	F2-3 up	GO:0005634	Development	Development	nucleus	C	AT3G14420
8856	3	F23 vs. L23	F2-3 up	GO:0005634	Development	Development	nucleus	C	AT3G15030
8857	3	F23 vs. L23	F2-3 up	GO:0005634	Development	Development	nucleus	C	AT3G15540
8858	3	F23 vs. L23	F2-3 up	GO:0005634	Development	Development	nucleus	C	AT3G16170
8859	3	F23 vs. L23	F2-3 up	GO:0005634	Development	Development	nucleus	C	AT3G16800
8860	3	F23 vs. L23	F2-3 up	GO:0005634	Development	Development	nucleus	C	AT3G17310
8861	3	F23 vs. L23	F2-3 up	GO:0005634	Development	Development	nucleus	C	AT3G17800
8862	3	F23 vs. L23	F2-3 up	GO:0005634	Development	Development	nucleus	C	AT3G18780
8863	3	F23 vs. L23	F2-3 up	GO:0005634	Development	Development	nucleus	C	AT3G19090
8864	3	F23 vs. L23	F2-3 up	GO:0005634	Development	Development	nucleus	C	AT3G19800
8865	3	F23 vs. L23	F2-3 up	GO:0005634	Development	Development	nucleus	C	AT3G19980
8866	3	F23 vs. L23	F2-3 up	GO:0005634	Development	Development	nucleus	C	AT3G20220
8867	3	F23 vs. L23	F2-3 up	GO:0005634	Development	Development	nucleus	C	AT3G21890
8868	3	F23 vs. L23	F2-3 up	GO:0005634	Development	Development	nucleus	C	AT3G23050
8869	3	F23 vs. L23	F2-3 up	GO:0005634	Development	Development	nucleus	C	AT3G23290
8870	3	F23 vs. L23	F2-3 up	GO:0005634	Development	Development	nucleus	C	AT3G23300
8871	3	F23 vs. L23	F2-3 up	GO:0005634	Development	Development	nucleus	C	AT3G23700
8872	3	F23 vs. L23	F2-3 up	GO:0005634	Development	Development	nucleus	C	AT3G23920
8873	3	F23 vs. L23	F2-3 up	GO:0005634	Development	Development	nucleus	C	AT3G25690
8874	3	F23 vs. L23	F2-3 up	GO:0005634	Development	Development	nucleus	C	AT3G29360
8875	3	F23 vs. L23	F2-3 up	GO:0005634	Development	Development	nucleus	C	AT3G43120
8876	3	F23 vs. L23	F2-3 up	GO:0005634	Development	Development	nucleus	C	AT3G43210
8877	3	F23 vs. L23	F2-3 up	GO:0005634	Development	Development	nucleus	C	AT3G45100
8878	3	F23 vs. L23	F2-3 up	GO:0005634	Development	Development	nucleus	C	AT3G45640
8879	3	F23 vs. L23	F2-3 up	GO:0005634	Development	Development	nucleus	C	AT3G45780
8880	3	F23 vs. L23	F2-3 up	GO:0005634	Development	Development	nucleus	C	AT3G46600
8881	3	F23 vs. L23	F2-3 up	GO:0005634	Development	Development	nucleus	C	AT3G46620
8882	3	F23 vs. L23	F2-3 up	GO:0005634	Development	Development	nucleus	C	AT3G46670
8883	3	F23 vs. L23	F2-3 up	GO:0005634	Development	Development	nucleus	C	AT3G47960
8884	3	F23 vs. L23	F2-3 up	GO:0005634	Development	Development	nucleus	C	AT3G50000
8885	3	F23 vs. L23	F2-3 up	GO:0005634	Development	Development	nucleus	C	AT3G50910
8886	3	F23 vs. L23	F2-3 up	GO:0005634	Development	Development	nucleus	C	AT3G51070
8887	3	F23 vs. L23	F2-3 up	GO:0005634	Development	Development	nucleus	C	AT3G51290
8888	3	F23 vs. L23	F2-3 up	GO:0005634	Development	Development	nucleus	C	AT3G51920
8889	3	F23 vs. L23	F2-3 up	GO:0005634	Development	Development	nucleus	C	AT3G52800
8890	3	F23 vs. L23	F2-3 up	GO:0005634	Development	Development	nucleus	C	AT3G54340

8891	3	F23 vs. L23	F2-3 up	GO:0005634	Development	Development	nucleus	C	AT3G56150
8892	3	F23 vs. L23	F2-3 up	GO:0005634	Development	Development	nucleus	C	AT3G57600
8893	3	F23 vs. L23	F2-3 up	GO:0005634	Development	Development	nucleus	C	AT3G61160
8894	3	F23 vs. L23	F2-3 up	GO:0005634	Development	Development	nucleus	C	AT3G62960
8895	3	F23 vs. L23	F2-3 up	GO:0005634	Development	Development	nucleus	C	AT4G00480
8896	3	F23 vs. L23	F2-3 up	GO:0005634	Development	Development	nucleus	C	AT4G02890
8897	3	F23 vs. L23	F2-3 up	GO:0005634	Development	Development	nucleus	C	AT4G03190
8898	3	F23 vs. L23	F2-3 up	GO:0005634	Development	Development	nucleus	C	AT4G03200
8899	3	F23 vs. L23	F2-3 up	GO:0005634	Development	Development	nucleus	C	AT4G04980
8900	3	F23 vs. L23	F2-3 up	GO:0005634	Development	Development	nucleus	C	AT4G08150
8901	3	F23 vs. L23	F2-3 up	GO:0005634	Development	Development	nucleus	C	AT4G10120
8902	3	F23 vs. L23	F2-3 up	GO:0005634	Development	Development	nucleus	C	AT4G14710
8903	3	F23 vs. L23	F2-3 up	GO:0005634	Development	Development	nucleus	C	AT4G14880
8904	3	F23 vs. L23	F2-3 up	GO:0005634	Development	Development	nucleus	C	AT4G15530
8905	3	F23 vs. L23	F2-3 up	GO:0005634	Development	Development	nucleus	C	AT4G15700
8906	3	F23 vs. L23	F2-3 up	GO:0005634	Development	Development	nucleus	C	AT4G17230
8907	3	F23 vs. L23	F2-3 up	GO:0005634	Development	Development	nucleus	C	AT4G18050
8908	3	F23 vs. L23	F2-3 up	GO:0005634	Development	Development	nucleus	C	AT4G18920
8909	3	F23 vs. L23	F2-3 up	GO:0005634	Development	Development	nucleus	C	AT4G18950
8910	3	F23 vs. L23	F2-3 up	GO:0005634	Development	Development	nucleus	C	AT4G19120
8911	3	F23 vs. L23	F2-3 up	GO:0005634	Development	Development	nucleus	C	AT4G20300
8912	3	F23 vs. L23	F2-3 up	GO:0005634	Development	Development	nucleus	C	AT4G20360
8913	3	F23 vs. L23	F2-3 up	GO:0005634	Development	Development	nucleus	C	AT4G24510
8914	3	F23 vs. L23	F2-3 up	GO:0005634	Development	Development	nucleus	C	AT4G25030
8915	3	F23 vs. L23	F2-3 up	GO:0005634	Development	Development	nucleus	C	AT4G25590
8916	3	F23 vs. L23	F2-3 up	GO:0005634	Development	Development	nucleus	C	AT4G26100
8917	3	F23 vs. L23	F2-3 up	GO:0005634	Development	Development	nucleus	C	AT4G29340
8918	3	F23 vs. L23	F2-3 up	GO:0005634	Development	Development	nucleus	C	AT4G30470
8919	3	F23 vs. L23	F2-3 up	GO:0005634	Development	Development	nucleus	C	AT4G31080
8920	3	F23 vs. L23	F2-3 up	GO:0005634	Development	Development	nucleus	C	AT4G31230
8921	3	F23 vs. L23	F2-3 up	GO:0005634	Development	Development	nucleus	C	AT4G31290
8922	3	F23 vs. L23	F2-3 up	GO:0005634	Development	Development	nucleus	C	AT4G31590
8923	3	F23 vs. L23	F2-3 up	GO:0005634	Development	Development	nucleus	C	AT4G31800
8924	3	F23 vs. L23	F2-3 up	GO:0005634	Development	Development	nucleus	C	AT4G32020
8925	3	F23 vs. L23	F2-3 up	GO:0005634	Development	Development	nucleus	C	AT4G32800
8926	3	F23 vs. L23	F2-3 up	GO:0005634	Development	Development	nucleus	C	AT4G32880
8927	3	F23 vs. L23	F2-3 up	GO:0005634	Development	Development	nucleus	C	AT4G33530
8928	3	F23 vs. L23	F2-3 up	GO:0005634	Development	Development	nucleus	C	AT4G34050
8929	3	F23 vs. L23	F2-3 up	GO:0005634	Development	Development	nucleus	C	AT4G34710
8930	3	F23 vs. L23	F2-3 up	GO:0005634	Development	Development	nucleus	C	AT4G35060
8931	3	F23 vs. L23	F2-3 up	GO:0005634	Development	Development	nucleus	C	AT4G35350
8932	3	F23 vs. L23	F2-3 up	GO:0005634	Development	Development	nucleus	C	AT4G35500
8933	3	F23 vs. L23	F2-3 up	GO:0005634	Development	Development	nucleus	C	AT4G36710
8934	3	F23 vs. L23	F2-3 up	GO:0005634	Development	Development	nucleus	C	AT4G37180
8935	3	F23 vs. L23	F2-3 up	GO:0005634	Development	Development	nucleus	C	AT4G39800
8936	3	F23 vs. L23	F2-3 up	GO:0005634	Development	Development	nucleus	C	AT5G06850
8937	3	F23 vs. L23	F2-3 up	GO:0005634	Development	Development	nucleus	C	AT5G08160
8938	3	F23 vs. L23	F2-3 up	GO:0005634	Development	Development	nucleus	C	AT5G08640
8939	3	F23 vs. L23	F2-3 up	GO:0005634	Development	Development	nucleus	C	AT5G09230
8940	3	F23 vs. L23	F2-3 up	GO:0005634	Development	Development	nucleus	C	AT5G12000
8941	3	F23 vs. L23	F2-3 up	GO:0005634	Development	Development	nucleus	C	AT5G13160
8942	3	F23 vs. L23	F2-3 up	GO:0005634	Development	Development	nucleus	C	AT5G13930
8943	3	F23 vs. L23	F2-3 up	GO:0005634	Development	Development	nucleus	C	AT5G13990
8944	3	F23 vs. L23	F2-3 up	GO:0005634	Development	Development	nucleus	C	AT5G15490
8945	3	F23 vs. L23	F2-3 up	GO:0005634	Development	Development	nucleus	C	AT5G15950
8946	3	F23 vs. L23	F2-3 up	GO:0005634	Development	Development	nucleus	C	AT5G16600

8947	3	F23 vs. L23	F2-3 up	GO:0005634	Development	Development	nucleus	C	AT5G17450
8948	3	F23 vs. L23	F2-3 up	GO:0005634	Development	Development	nucleus	C	AT5G20240
8949	3	F23 vs. L23	F2-3 up	GO:0005634	Development	Development	nucleus	C	AT5G20700
8950	3	F23 vs. L23	F2-3 up	GO:0005634	Development	Development	nucleus	C	AT5G22030
8951	3	F23 vs. L23	F2-3 up	GO:0005634	Development	Development	nucleus	C	AT5G22250
8952	3	F23 vs. L23	F2-3 up	GO:0005634	Development	Development	nucleus	C	AT5G24080
8953	3	F23 vs. L23	F2-3 up	GO:0005634	Development	Development	nucleus	C	AT5G37300
8954	3	F23 vs. L23	F2-3 up	GO:0005634	Development	Development	nucleus	C	AT5G39210
8955	3	F23 vs. L23	F2-3 up	GO:0005634	Development	Development	nucleus	C	AT5G39320
8956	3	F23 vs. L23	F2-3 up	GO:0005634	Development	Development	nucleus	C	AT5G40010
8957	3	F23 vs. L23	F2-3 up	GO:0005634	Development	Development	nucleus	C	AT5G40890
8958	3	F23 vs. L23	F2-3 up	GO:0005634	Development	Development	nucleus	C	AT5G41810
8959	3	F23 vs. L23	F2-3 up	GO:0005634	Development	Development	nucleus	C	AT5G42050
8960	3	F23 vs. L23	F2-3 up	GO:0005634	Development	Development	nucleus	C	AT5G43380
8961	3	F23 vs. L23	F2-3 up	GO:0005634	Development	Development	nucleus	C	AT5G44300
8962	3	F23 vs. L23	F2-3 up	GO:0005634	Development	Development	nucleus	C	AT5G44680
8963	3	F23 vs. L23	F2-3 up	GO:0005634	Development	Development	nucleus	C	AT5G47670
8964	3	F23 vs. L23	F2-3 up	GO:0005634	Development	Development	nucleus	C	AT5G47910
8965	3	F23 vs. L23	F2-3 up	GO:0005634	Development	Development	nucleus	C	AT5G52360
8966	3	F23 vs. L23	F2-3 up	GO:0005634	Development	Development	nucleus	C	AT5G53370
8967	3	F23 vs. L23	F2-3 up	GO:0005634	Development	Development	nucleus	C	AT5G54490
8968	3	F23 vs. L23	F2-3 up	GO:0005634	Development	Development	nucleus	C	AT5G54510
8969	3	F23 vs. L23	F2-3 up	GO:0005634	Development	Development	nucleus	C	AT5G54980
8970	3	F23 vs. L23	F2-3 up	GO:0005634	Development	Development	nucleus	C	AT5G56140
8971	3	F23 vs. L23	F2-3 up	GO:0005634	Development	Development	nucleus	C	AT5G56860
8972	3	F23 vs. L23	F2-3 up	GO:0005634	Development	Development	nucleus	C	AT5G59480
8973	3	F23 vs. L23	F2-3 up	GO:0005634	Development	Development	nucleus	C	AT5G59730
8974	3	F23 vs. L23	F2-3 up	GO:0005634	Development	Development	nucleus	C	AT5G60450
8975	3	F23 vs. L23	F2-3 up	GO:0005634	Development	Development	nucleus	C	AT5G60600
8976	3	F23 vs. L23	F2-3 up	GO:0005634	Development	Development	nucleus	C	AT5G60910
8977	3	F23 vs. L23	F2-3 up	GO:0005634	Development	Development	nucleus	C	AT5G61670
8978	3	F23 vs. L23	F2-3 up	GO:0005634	Development	Development	nucleus	C	AT5G62020
8979	3	F23 vs. L23	F2-3 up	GO:0005634	Development	Development	nucleus	C	AT5G62520
8980	3	F23 vs. L23	F2-3 up	GO:0005634	Development	Development	nucleus	C	AT5G62570
8981	3	F23 vs. L23	F2-3 up	GO:0005634	Development	Development	nucleus	C	AT5G64660
8982	3	F23 vs. L23	F2-3 up	GO:0005634	Development	Development	nucleus	C	AT5G65410
8983	3	F23 vs. L23	F2-3 up	GO:0005634	Development	Development	nucleus	C	AT5G65460
8984	3	F23 vs. L23	F2-3 up	GO:0005634	Development	Development	nucleus	C	AT5G65810
8985	3	F23 vs. L23	F2-3 up	GO:0005634	Development	Development	nucleus	C	AT5G66070
8986	3	F23 vs. L23	F2-3 up	GO:0005634	Development	Development	nucleus	C	AT5G67300
8987	3	F23 vs. L23	F2-3 up	GO:0005634	Development	Development	nucleus	C	AT5G67470
8988	3	F23 vs. L23	F2-3 up	GO:0005634	Development	Development	nucleus	C	AT5G67480
8989	3	F23 vs. L23	F2-3 up	GO:0051640	Development	Development	organelle localization	P	AT1G54560
8990	3	F23 vs. L23	F2-3 up	GO:0007389	Development	Development	pattern specification process	P	AT1G70940
8991	3	F23 vs. L23	F2-3 up	GO:0007389	Development	Development	pattern specification process	P	AT2G01420
8992	3	F23 vs. L23	F2-3 up	GO:0048446	Development	Development	petal morphogenesis	P	AT1G59640
8993	3	F23 vs. L23	F2-3 up	GO:0010088	Development	Development	phloem development	P	AT3G01680
8994	3	F23 vs. L23	F2-3 up	GO:0090449	Development	Development	phloem glucosinolate loading	P	AT3G47960
8995	3	F23 vs. L23	F2-3 up	GO:0090449	Development	Development	phloem glucosinolate loading	P	AT5G62680
8996	3	F23 vs. L23	F2-3 up	GO:0110126	Development	Development	phloem loading	P	AT5G09220
8997	3	F23 vs. L23	F2-3 up	GO:0009524	Development	Development	phragmoplast	C	AT2G19770
8998	3	F23 vs. L23	F2-3 up	GO:0009524	Development	Development	phragmoplast	C	AT3G43210
8999	3	F23 vs. L23	F2-3 up	GO:0009524	Development	Development	phragmoplast	C	AT5G67470
9000	3	F23 vs. L23	F2-3 up	GO:0099402	Development	Development	plant organ development	P	AT3G02470
9001	3	F23 vs. L23	F2-3 up	GO:0048481	Development	Development	plant ovule development	P	AT1G25350
9002	3	F23 vs. L23	F2-3 up	GO:0048481	Development	Development	plant ovule development	P	AT3G45640

9003	3	F23 vs. L23	F2-3 up	GO:0009505	Development	Development	plant-type cell wall	C	AT1G26770
9004	3	F23 vs. L23	F2-3 up	GO:0009505	Development	Development	plant-type cell wall	C	AT1G45130
9005	3	F23 vs. L23	F2-3 up	GO:0009505	Development	Development	plant-type cell wall	C	AT1G69530
9006	3	F23 vs. L23	F2-3 up	GO:0009505	Development	Development	plant-type cell wall	C	AT1G72730
9007	3	F23 vs. L23	F2-3 up	GO:0009505	Development	Development	plant-type cell wall	C	AT2G16430
9008	3	F23 vs. L23	F2-3 up	GO:0009505	Development	Development	plant-type cell wall	C	AT2G28950
9009	3	F23 vs. L23	F2-3 up	GO:0009505	Development	Development	plant-type cell wall	C	AT2G30490
9010	3	F23 vs. L23	F2-3 up	GO:0009505	Development	Development	plant-type cell wall	C	AT2G39700
9011	3	F23 vs. L23	F2-3 up	GO:0009505	Development	Development	plant-type cell wall	C	AT2G41480
9012	3	F23 vs. L23	F2-3 up	GO:0009505	Development	Development	plant-type cell wall	C	AT3G05910
9013	3	F23 vs. L23	F2-3 up	GO:0009505	Development	Development	plant-type cell wall	C	AT3G15400
9014	3	F23 vs. L23	F2-3 up	GO:0009505	Development	Development	plant-type cell wall	C	AT3G18080
9015	3	F23 vs. L23	F2-3 up	GO:0009505	Development	Development	plant-type cell wall	C	AT3G49110
9016	3	F23 vs. L23	F2-3 up	GO:0009505	Development	Development	plant-type cell wall	C	AT4G19410
9017	3	F23 vs. L23	F2-3 up	GO:0009505	Development	Development	plant-type cell wall	C	AT4G19420
9018	3	F23 vs. L23	F2-3 up	GO:0009505	Development	Development	plant-type cell wall	C	AT4G38400
9019	3	F23 vs. L23	F2-3 up	GO:0009505	Development	Development	plant-type cell wall	C	AT5G08370
9020	3	F23 vs. L23	F2-3 up	GO:0009505	Development	Development	plant-type cell wall	C	AT5G11420
9021	3	F23 vs. L23	F2-3 up	GO:0009505	Development	Development	plant-type cell wall	C	AT5G45280
9022	3	F23 vs. L23	F2-3 up	GO:0009505	Development	Development	plant-type cell wall	C	AT5G49360
9023	3	F23 vs. L23	F2-3 up	GO:0009505	Development	Development	plant-type cell wall	C	AT5G51890
9024	3	F23 vs. L23	F2-3 up	GO:0009832	Development	Development	plant-type cell wall biogenesis	P	AT4G32410
9025	3	F23 vs. L23	F2-3 up	GO:0009832	Development	Development	plant-type cell wall biogenesis	P	AT5G09870
9026	3	F23 vs. L23	F2-3 up	GO:0009832	Development	Development	plant-type cell wall biogenesis	P	AT5G64740
9027	3	F23 vs. L23	F2-3 up	GO:0009832	Development	Development	plant-type cell wall biogenesis	P	AT5G65810
9028	3	F23 vs. L23	F2-3 up	GO:0009828	Development	Development	plant-type cell wall loosening	P	AT1G20190
9029	3	F23 vs. L23	F2-3 up	GO:0009828	Development	Development	plant-type cell wall loosening	P	AT1G26770
9030	3	F23 vs. L23	F2-3 up	GO:0009828	Development	Development	plant-type cell wall loosening	P	AT1G69530
9031	3	F23 vs. L23	F2-3 up	GO:0009828	Development	Development	plant-type cell wall loosening	P	AT2G28950
9032	3	F23 vs. L23	F2-3 up	GO:0009828	Development	Development	plant-type cell wall loosening	P	AT2G39700
9033	3	F23 vs. L23	F2-3 up	GO:0009828	Development	Development	plant-type cell wall loosening	P	AT3G29030
9034	3	F23 vs. L23	F2-3 up	GO:0009828	Development	Development	plant-type cell wall loosening	P	AT4G38400
9035	3	F23 vs. L23	F2-3 up	GO:0009827	Development	Development	plant-type cell wall modification	P	AT1G27930
9036	3	F23 vs. L23	F2-3 up	GO:0009827	Development	Development	plant-type cell wall modification	P	AT2G41210
9037	3	F23 vs. L23	F2-3 up	GO:0009827	Development	Development	plant-type cell wall modification	P	AT3G56960
9038	3	F23 vs. L23	F2-3 up	GO:0009827	Development	Development	plant-type cell wall modification	P	AT5G62360
9039	3	F23 vs. L23	F2-3 up	GO:0009664	Development	Development	plant-type cell wall organization	P	AT1G20190
9040	3	F23 vs. L23	F2-3 up	GO:0009664	Development	Development	plant-type cell wall organization	P	AT1G26770
9041	3	F23 vs. L23	F2-3 up	GO:0009664	Development	Development	plant-type cell wall organization	P	AT1G69530
9042	3	F23 vs. L23	F2-3 up	GO:0009664	Development	Development	plant-type cell wall organization	P	AT2G21140
9043	3	F23 vs. L23	F2-3 up	GO:0009664	Development	Development	plant-type cell wall organization	P	AT2G28950
9044	3	F23 vs. L23	F2-3 up	GO:0009664	Development	Development	plant-type cell wall organization	P	AT2G39700
9045	3	F23 vs. L23	F2-3 up	GO:0009664	Development	Development	plant-type cell wall organization	P	AT2G41480
9046	3	F23 vs. L23	F2-3 up	GO:0009664	Development	Development	plant-type cell wall organization	P	AT3G29030
9047	3	F23 vs. L23	F2-3 up	GO:0009664	Development	Development	plant-type cell wall organization	P	AT5G57560
9048	3	F23 vs. L23	F2-3 up	GO:0009664	Development	Development	plant-type cell wall organization	P	AT5G65810
9049	3	F23 vs. L23	F2-3 up	GO:0009833	Development	Development	plant-type primary cell wall biogenesis	P	AT1G76670
9050	3	F23 vs. L23	F2-3 up	GO:0009833	Development	Development	plant-type primary cell wall biogenesis	P	AT2G33100
9051	3	F23 vs. L23	F2-3 up	GO:0009833	Development	Development	plant-type primary cell wall biogenesis	P	AT4G32410
9052	3	F23 vs. L23	F2-3 up	GO:0009833	Development	Development	plant-type primary cell wall biogenesis	P	AT4G39350
9053	3	F23 vs. L23	F2-3 up	GO:0009833	Development	Development	plant-type primary cell wall biogenesis	P	AT5G09870
9054	3	F23 vs. L23	F2-3 up	GO:0009833	Development	Development	plant-type primary cell wall biogenesis	P	AT5G16910
9055	3	F23 vs. L23	F2-3 up	GO:0009833	Development	Development	plant-type primary cell wall biogenesis	P	AT5G64740
9056	3	F23 vs. L23	F2-3 up	GO:0009834	Development	Development	plant-type secondary cell wall biogenesis	P	AT1G27930
9057	3	F23 vs. L23	F2-3 up	GO:0009834	Development	Development	plant-type secondary cell wall biogenesis	P	AT1G75500
9058	3	F23 vs. L23	F2-3 up	GO:0009834	Development	Development	plant-type secondary cell wall biogenesis	P	AT5G67210

9059	3	F23 vs. L23	F2-3 up	GO:0000325	Development	Development	plant-type vacuole	C	AT1G12240
9060	3	F23 vs. L23	F2-3 up	GO:0000325	Development	Development	plant-type vacuole	C	AT1G12840
9061	3	F23 vs. L23	F2-3 up	GO:0000325	Development	Development	plant-type vacuole	C	AT3G58730
9062	3	F23 vs. L23	F2-3 up	GO:0000325	Development	Development	plant-type vacuole	C	AT4G33530
9063	3	F23 vs. L23	F2-3 up	GO:0000325	Development	Development	plant-type vacuole	C	AT4G35350
9064	3	F23 vs. L23	F2-3 up	GO:0009705	Development	Development	plant-type vacuole membrane	C	AT1G16370
9065	3	F23 vs. L23	F2-3 up	GO:0009705	Development	Development	plant-type vacuole membrane	C	AT1G22550
9066	3	F23 vs. L23	F2-3 up	GO:0009705	Development	Development	plant-type vacuole membrane	C	AT1G75500
9067	3	F23 vs. L23	F2-3 up	GO:0009705	Development	Development	plant-type vacuole membrane	C	AT2G36830
9068	3	F23 vs. L23	F2-3 up	GO:0009705	Development	Development	plant-type vacuole membrane	C	AT2G38170
9069	3	F23 vs. L23	F2-3 up	GO:0009705	Development	Development	plant-type vacuole membrane	C	AT2G48020
9070	3	F23 vs. L23	F2-3 up	GO:0009705	Development	Development	plant-type vacuole membrane	C	AT3G51490
9071	3	F23 vs. L23	F2-3 up	GO:0009705	Development	Development	plant-type vacuole membrane	C	AT3G57330
9072	3	F23 vs. L23	F2-3 up	GO:0009705	Development	Development	plant-type vacuole membrane	C	AT5G13930
9073	3	F23 vs. L23	F2-3 up	GO:0009705	Development	Development	plant-type vacuole membrane	C	AT5G40890
9074	3	F23 vs. L23	F2-3 up	GO:0005886	Development	Development	plasma membrane	C	AT1G01780
9075	3	F23 vs. L23	F2-3 up	GO:0005886	Development	Development	plasma membrane	C	AT1G03050
9076	3	F23 vs. L23	F2-3 up	GO:0005886	Development	Development	plasma membrane	C	AT1G03230
9077	3	F23 vs. L23	F2-3 up	GO:0005886	Development	Development	plasma membrane	C	AT1G03550
9078	3	F23 vs. L23	F2-3 up	GO:0005886	Development	Development	plasma membrane	C	AT1G04760
9079	3	F23 vs. L23	F2-3 up	GO:0005886	Development	Development	plasma membrane	C	AT1G11840
9080	3	F23 vs. L23	F2-3 up	GO:0005886	Development	Development	plasma membrane	C	AT1G12110
9081	3	F23 vs. L23	F2-3 up	GO:0005886	Development	Development	plasma membrane	C	AT1G12840
9082	3	F23 vs. L23	F2-3 up	GO:0005886	Development	Development	plasma membrane	C	AT1G13890
9083	3	F23 vs. L23	F2-3 up	GO:0005886	Development	Development	plasma membrane	C	AT1G16310
9084	3	F23 vs. L23	F2-3 up	GO:0005886	Development	Development	plasma membrane	C	AT1G16370
9085	3	F23 vs. L23	F2-3 up	GO:0005886	Development	Development	plasma membrane	C	AT1G18150
9086	3	F23 vs. L23	F2-3 up	GO:0005886	Development	Development	plasma membrane	C	AT1G18660
9087	3	F23 vs. L23	F2-3 up	GO:0005886	Development	Development	plasma membrane	C	AT1G20620
9088	3	F23 vs. L23	F2-3 up	GO:0005886	Development	Development	plasma membrane	C	AT1G20823
9089	3	F23 vs. L23	F2-3 up	GO:0005886	Development	Development	plasma membrane	C	AT1G20850
9090	3	F23 vs. L23	F2-3 up	GO:0005886	Development	Development	plasma membrane	C	AT1G21070
9091	3	F23 vs. L23	F2-3 up	GO:0005886	Development	Development	plasma membrane	C	AT1G21980
9092	3	F23 vs. L23	F2-3 up	GO:0005886	Development	Development	plasma membrane	C	AT1G22710
9093	3	F23 vs. L23	F2-3 up	GO:0005886	Development	Development	plasma membrane	C	AT1G24400
9094	3	F23 vs. L23	F2-3 up	GO:0005886	Development	Development	plasma membrane	C	AT1G24520
9095	3	F23 vs. L23	F2-3 up	GO:0005886	Development	Development	plasma membrane	C	AT1G27850
9096	3	F23 vs. L23	F2-3 up	GO:0005886	Development	Development	plasma membrane	C	AT1G31260
9097	3	F23 vs. L23	F2-3 up	GO:0005886	Development	Development	plasma membrane	C	AT1G32450
9098	3	F23 vs. L23	F2-3 up	GO:0005886	Development	Development	plasma membrane	C	AT1G35720
9099	3	F23 vs. L23	F2-3 up	GO:0005886	Development	Development	plasma membrane	C	AT1G41830
9100	3	F23 vs. L23	F2-3 up	GO:0005886	Development	Development	plasma membrane	C	AT1G42470
9101	3	F23 vs. L23	F2-3 up	GO:0005886	Development	Development	plasma membrane	C	AT1G47530
9102	3	F23 vs. L23	F2-3 up	GO:0005886	Development	Development	plasma membrane	C	AT1G50610
9103	3	F23 vs. L23	F2-3 up	GO:0005886	Development	Development	plasma membrane	C	AT1G52190
9104	3	F23 vs. L23	F2-3 up	GO:0005886	Development	Development	plasma membrane	C	AT1G54280
9105	3	F23 vs. L23	F2-3 up	GO:0005886	Development	Development	plasma membrane	C	AT1G55570
9106	3	F23 vs. L23	F2-3 up	GO:0005886	Development	Development	plasma membrane	C	AT1G59870
9107	3	F23 vs. L23	F2-3 up	GO:0005886	Development	Development	plasma membrane	C	AT1G60030
9108	3	F23 vs. L23	F2-3 up	GO:0005886	Development	Development	plasma membrane	C	AT1G61290
9109	3	F23 vs. L23	F2-3 up	GO:0005886	Development	Development	plasma membrane	C	AT1G62360
9110	3	F23 vs. L23	F2-3 up	GO:0005886	Development	Development	plasma membrane	C	AT1G62380
9111	3	F23 vs. L23	F2-3 up	GO:0005886	Development	Development	plasma membrane	C	AT1G63000
9112	3	F23 vs. L23	F2-3 up	GO:0005886	Development	Development	plasma membrane	C	AT1G64060
9113	3	F23 vs. L23	F2-3 up	GO:0005886	Development	Development	plasma membrane	C	AT1G64740
9114	3	F23 vs. L23	F2-3 up	GO:0005886	Development	Development	plasma membrane	C	AT1G65240

9115	3	F23 vs. L23	F2-3 up	GO:0005886	Development	Development	plasma membrane	C	AT1G67940
9116	3	F23 vs. L23	F2-3 up	GO:0005886	Development	Development	plasma membrane	C	AT1G68570
9117	3	F23 vs. L23	F2-3 up	GO:0005886	Development	Development	plasma membrane	C	AT1G70310
9118	3	F23 vs. L23	F2-3 up	GO:0005886	Development	Development	plasma membrane	C	AT1G70730
9119	3	F23 vs. L23	F2-3 up	GO:0005886	Development	Development	plasma membrane	C	AT1G70940
9120	3	F23 vs. L23	F2-3 up	GO:0005886	Development	Development	plasma membrane	C	AT1G72180
9121	3	F23 vs. L23	F2-3 up	GO:0005886	Development	Development	plasma membrane	C	AT1G75500
9122	3	F23 vs. L23	F2-3 up	GO:0005886	Development	Development	plasma membrane	C	AT1G76370
9123	3	F23 vs. L23	F2-3 up	GO:0005886	Development	Development	plasma membrane	C	AT1G76670
9124	3	F23 vs. L23	F2-3 up	GO:0005886	Development	Development	plasma membrane	C	AT1G80660
9125	3	F23 vs. L23	F2-3 up	GO:0005886	Development	Development	plasma membrane	C	AT1G80760
9126	3	F23 vs. L23	F2-3 up	GO:0005886	Development	Development	plasma membrane	C	AT2G01320
9127	3	F23 vs. L23	F2-3 up	GO:0005886	Development	Development	plasma membrane	C	AT2G01420
9128	3	F23 vs. L23	F2-3 up	GO:0005886	Development	Development	plasma membrane	C	AT2G02800
9129	3	F23 vs. L23	F2-3 up	GO:0005886	Development	Development	plasma membrane	C	AT2G02990
9130	3	F23 vs. L23	F2-3 up	GO:0005886	Development	Development	plasma membrane	C	AT2G03240
9131	3	F23 vs. L23	F2-3 up	GO:0005886	Development	Development	plasma membrane	C	AT2G07180
9132	3	F23 vs. L23	F2-3 up	GO:0005886	Development	Development	plasma membrane	C	AT2G07560
9133	3	F23 vs. L23	F2-3 up	GO:0005886	Development	Development	plasma membrane	C	AT2G10940
9134	3	F23 vs. L23	F2-3 up	GO:0005886	Development	Development	plasma membrane	C	AT2G16750
9135	3	F23 vs. L23	F2-3 up	GO:0005886	Development	Development	plasma membrane	C	AT2G17220
9136	3	F23 vs. L23	F2-3 up	GO:0005886	Development	Development	plasma membrane	C	AT2G17290
9137	3	F23 vs. L23	F2-3 up	GO:0005886	Development	Development	plasma membrane	C	AT2G17840
9138	3	F23 vs. L23	F2-3 up	GO:0005886	Development	Development	plasma membrane	C	AT2G18470
9139	3	F23 vs. L23	F2-3 up	GO:0005886	Development	Development	plasma membrane	C	AT2G20840
9140	3	F23 vs. L23	F2-3 up	GO:0005886	Development	Development	plasma membrane	C	AT2G20870
9141	3	F23 vs. L23	F2-3 up	GO:0005886	Development	Development	plasma membrane	C	AT2G21880
9142	3	F23 vs. L23	F2-3 up	GO:0005886	Development	Development	plasma membrane	C	AT2G22125
9143	3	F23 vs. L23	F2-3 up	GO:0005886	Development	Development	plasma membrane	C	AT2G22450
9144	3	F23 vs. L23	F2-3 up	GO:0005886	Development	Development	plasma membrane	C	AT2G22950
9145	3	F23 vs. L23	F2-3 up	GO:0005886	Development	Development	plasma membrane	C	AT2G24180
9146	3	F23 vs. L23	F2-3 up	GO:0005886	Development	Development	plasma membrane	C	AT2G30440
9147	3	F23 vs. L23	F2-3 up	GO:0005886	Development	Development	plasma membrane	C	AT2G30490
9148	3	F23 vs. L23	F2-3 up	GO:0005886	Development	Development	plasma membrane	C	AT2G30860
9149	3	F23 vs. L23	F2-3 up	GO:0005886	Development	Development	plasma membrane	C	AT2G31500
9150	3	F23 vs. L23	F2-3 up	GO:0005886	Development	Development	plasma membrane	C	AT2G31570
9151	3	F23 vs. L23	F2-3 up	GO:0005886	Development	Development	plasma membrane	C	AT2G33100
9152	3	F23 vs. L23	F2-3 up	GO:0005886	Development	Development	plasma membrane	C	AT2G36880
9153	3	F23 vs. L23	F2-3 up	GO:0005886	Development	Development	plasma membrane	C	AT2G37180
9154	3	F23 vs. L23	F2-3 up	GO:0005886	Development	Development	plasma membrane	C	AT2G37970
9155	3	F23 vs. L23	F2-3 up	GO:0005886	Development	Development	plasma membrane	C	AT2G38460
9156	3	F23 vs. L23	F2-3 up	GO:0005886	Development	Development	plasma membrane	C	AT2G39130
9157	3	F23 vs. L23	F2-3 up	GO:0005886	Development	Development	plasma membrane	C	AT2G39510
9158	3	F23 vs. L23	F2-3 up	GO:0005886	Development	Development	plasma membrane	C	AT2G40890
9159	3	F23 vs. L23	F2-3 up	GO:0005886	Development	Development	plasma membrane	C	AT2G45800
9160	3	F23 vs. L23	F2-3 up	GO:0005886	Development	Development	plasma membrane	C	AT2G46140
9161	3	F23 vs. L23	F2-3 up	GO:0005886	Development	Development	plasma membrane	C	AT2G46300
9162	3	F23 vs. L23	F2-3 up	GO:0005886	Development	Development	plasma membrane	C	AT2G47160
9163	3	F23 vs. L23	F2-3 up	GO:0005886	Development	Development	plasma membrane	C	AT2G47650
9164	3	F23 vs. L23	F2-3 up	GO:0005886	Development	Development	plasma membrane	C	AT3G01500
9165	3	F23 vs. L23	F2-3 up	GO:0005886	Development	Development	plasma membrane	C	AT3G03250
9166	3	F23 vs. L23	F2-3 up	GO:0005886	Development	Development	plasma membrane	C	AT3G03770
9167	3	F23 vs. L23	F2-3 up	GO:0005886	Development	Development	plasma membrane	C	AT3G03800
9168	3	F23 vs. L23	F2-3 up	GO:0005886	Development	Development	plasma membrane	C	AT3G04120
9169	3	F23 vs. L23	F2-3 up	GO:0005886	Development	Development	plasma membrane	C	AT3G04840
9170	3	F23 vs. L23	F2-3 up	GO:0005886	Development	Development	plasma membrane	C	AT3G06035

9171	3	F23 vs. L23	F2-3 up	GO:0005886	Development	Development	plasma membrane	C	AT3G06510
9172	3	F23 vs. L23	F2-3 up	GO:0005886	Development	Development	plasma membrane	C	AT3G08510
9173	3	F23 vs. L23	F2-3 up	GO:0005886	Development	Development	plasma membrane	C	AT3G09440
9174	3	F23 vs. L23	F2-3 up	GO:0005886	Development	Development	plasma membrane	C	AT3G12610
9175	3	F23 vs. L23	F2-3 up	GO:0005886	Development	Development	plasma membrane	C	AT3G12620
9176	3	F23 vs. L23	F2-3 up	GO:0005886	Development	Development	plasma membrane	C	AT3G12690
9177	3	F23 vs. L23	F2-3 up	GO:0005886	Development	Development	plasma membrane	C	AT3G13065
9178	3	F23 vs. L23	F2-3 up	GO:0005886	Development	Development	plasma membrane	C	AT3G13390
9179	3	F23 vs. L23	F2-3 up	GO:0005886	Development	Development	plasma membrane	C	AT3G14310
9180	3	F23 vs. L23	F2-3 up	GO:0005886	Development	Development	plasma membrane	C	AT3G15400
9181	3	F23 vs. L23	F2-3 up	GO:0005886	Development	Development	plasma membrane	C	AT3G16340
9182	3	F23 vs. L23	F2-3 up	GO:0005886	Development	Development	plasma membrane	C	AT3G16800
9183	3	F23 vs. L23	F2-3 up	GO:0005886	Development	Development	plasma membrane	C	AT3G17020
9184	3	F23 vs. L23	F2-3 up	GO:0005886	Development	Development	plasma membrane	C	AT3G17390
9185	3	F23 vs. L23	F2-3 up	GO:0005886	Development	Development	plasma membrane	C	AT3G17611
9186	3	F23 vs. L23	F2-3 up	GO:0005886	Development	Development	plasma membrane	C	AT3G18780
9187	3	F23 vs. L23	F2-3 up	GO:0005886	Development	Development	plasma membrane	C	AT3G19370
9188	3	F23 vs. L23	F2-3 up	GO:0005886	Development	Development	plasma membrane	C	AT3G20530
9189	3	F23 vs. L23	F2-3 up	GO:0005886	Development	Development	plasma membrane	C	AT3G20580
9190	3	F23 vs. L23	F2-3 up	GO:0005886	Development	Development	plasma membrane	C	AT3G20865
9191	3	F23 vs. L23	F2-3 up	GO:0005886	Development	Development	plasma membrane	C	AT3G21180
9192	3	F23 vs. L23	F2-3 up	GO:0005886	Development	Development	plasma membrane	C	AT3G22890
9193	3	F23 vs. L23	F2-3 up	GO:0005886	Development	Development	plasma membrane	C	AT3G23810
9194	3	F23 vs. L23	F2-3 up	GO:0005886	Development	Development	plasma membrane	C	AT3G25290
9195	3	F23 vs. L23	F2-3 up	GO:0005886	Development	Development	plasma membrane	C	AT3G26520
9196	3	F23 vs. L23	F2-3 up	GO:0005886	Development	Development	plasma membrane	C	AT3G29060
9197	3	F23 vs. L23	F2-3 up	GO:0005886	Development	Development	plasma membrane	C	AT3G30390
9198	3	F23 vs. L23	F2-3 up	GO:0005886	Development	Development	plasma membrane	C	AT3G42640
9199	3	F23 vs. L23	F2-3 up	GO:0005886	Development	Development	plasma membrane	C	AT3G44990
9200	3	F23 vs. L23	F2-3 up	GO:0005886	Development	Development	plasma membrane	C	AT3G45780
9201	3	F23 vs. L23	F2-3 up	GO:0005886	Development	Development	plasma membrane	C	AT3G47960
9202	3	F23 vs. L23	F2-3 up	GO:0005886	Development	Development	plasma membrane	C	AT3G48610
9203	3	F23 vs. L23	F2-3 up	GO:0005886	Development	Development	plasma membrane	C	AT3G48740
9204	3	F23 vs. L23	F2-3 up	GO:0005886	Development	Development	plasma membrane	C	AT3G50000
9205	3	F23 vs. L23	F2-3 up	GO:0005886	Development	Development	plasma membrane	C	AT3G52880
9206	3	F23 vs. L23	F2-3 up	GO:0005886	Development	Development	plasma membrane	C	AT3G53420
9207	3	F23 vs. L23	F2-3 up	GO:0005886	Development	Development	plasma membrane	C	AT3G53960
9208	3	F23 vs. L23	F2-3 up	GO:0005886	Development	Development	plasma membrane	C	AT3G54700
9209	3	F23 vs. L23	F2-3 up	GO:0005886	Development	Development	plasma membrane	C	AT3G54950
9210	3	F23 vs. L23	F2-3 up	GO:0005886	Development	Development	plasma membrane	C	AT3G55100
9211	3	F23 vs. L23	F2-3 up	GO:0005886	Development	Development	plasma membrane	C	AT3G55640
9212	3	F23 vs. L23	F2-3 up	GO:0005886	Development	Development	plasma membrane	C	AT3G56960
9213	3	F23 vs. L23	F2-3 up	GO:0005886	Development	Development	plasma membrane	C	AT3G57330
9214	3	F23 vs. L23	F2-3 up	GO:0005886	Development	Development	plasma membrane	C	AT3G57680
9215	3	F23 vs. L23	F2-3 up	GO:0005886	Development	Development	plasma membrane	C	AT3G58730
9216	3	F23 vs. L23	F2-3 up	GO:0005886	Development	Development	plasma membrane	C	AT3G60330
9217	3	F23 vs. L23	F2-3 up	GO:0005886	Development	Development	plasma membrane	C	AT3G61220
9218	3	F23 vs. L23	F2-3 up	GO:0005886	Development	Development	plasma membrane	C	AT3G61230
9219	3	F23 vs. L23	F2-3 up	GO:0005886	Development	Development	plasma membrane	C	AT4G03280
9220	3	F23 vs. L23	F2-3 up	GO:0005886	Development	Development	plasma membrane	C	AT4G10060
9221	3	F23 vs. L23	F2-3 up	GO:0005886	Development	Development	plasma membrane	C	AT4G10120
9222	3	F23 vs. L23	F2-3 up	GO:0005886	Development	Development	plasma membrane	C	AT4G11600
9223	3	F23 vs. L23	F2-3 up	GO:0005886	Development	Development	plasma membrane	C	AT4G13800
9224	3	F23 vs. L23	F2-3 up	GO:0005886	Development	Development	plasma membrane	C	AT4G13930
9225	3	F23 vs. L23	F2-3 up	GO:0005886	Development	Development	plasma membrane	C	AT4G14710
9226	3	F23 vs. L23	F2-3 up	GO:0005886	Development	Development	plasma membrane	C	AT4G14880

9227	3	F23 vs. L23	F2-3 up	GO:0005886	Development	Development	plasma membrane	C	AT4G15620
9228	3	F23 vs. L23	F2-3 up	GO:0005886	Development	Development	plasma membrane	C	AT4G15630
9229	3	F23 vs. L23	F2-3 up	GO:0005886	Development	Development	plasma membrane	C	AT4G17170
9230	3	F23 vs. L23	F2-3 up	GO:0005886	Development	Development	plasma membrane	C	AT4G17530
9231	3	F23 vs. L23	F2-3 up	GO:0005886	Development	Development	plasma membrane	C	AT4G25040
9232	3	F23 vs. L23	F2-3 up	GO:0005886	Development	Development	plasma membrane	C	AT4G25950
9233	3	F23 vs. L23	F2-3 up	GO:0005886	Development	Development	plasma membrane	C	AT4G26470
9234	3	F23 vs. L23	F2-3 up	GO:0005886	Development	Development	plasma membrane	C	AT4G27720
9235	3	F23 vs. L23	F2-3 up	GO:0005886	Development	Development	plasma membrane	C	AT4G28490
9236	3	F23 vs. L23	F2-3 up	GO:0005886	Development	Development	plasma membrane	C	AT4G28940
9237	3	F23 vs. L23	F2-3 up	GO:0005886	Development	Development	plasma membrane	C	AT4G29350
9238	3	F23 vs. L23	F2-3 up	GO:0005886	Development	Development	plasma membrane	C	AT4G29480
9239	3	F23 vs. L23	F2-3 up	GO:0005886	Development	Development	plasma membrane	C	AT4G30530
9240	3	F23 vs. L23	F2-3 up	GO:0005886	Development	Development	plasma membrane	C	AT4G31750
9241	3	F23 vs. L23	F2-3 up	GO:0005886	Development	Development	plasma membrane	C	AT4G32260
9242	3	F23 vs. L23	F2-3 up	GO:0005886	Development	Development	plasma membrane	C	AT4G32410
9243	3	F23 vs. L23	F2-3 up	GO:0005886	Development	Development	plasma membrane	C	AT4G33010
9244	3	F23 vs. L23	F2-3 up	GO:0005886	Development	Development	plasma membrane	C	AT4G33530
9245	3	F23 vs. L23	F2-3 up	GO:0005886	Development	Development	plasma membrane	C	AT4G34050
9246	3	F23 vs. L23	F2-3 up	GO:0005886	Development	Development	plasma membrane	C	AT4G34950
9247	3	F23 vs. L23	F2-3 up	GO:0005886	Development	Development	plasma membrane	C	AT4G35250
9248	3	F23 vs. L23	F2-3 up	GO:0005886	Development	Development	plasma membrane	C	AT4G35350
9249	3	F23 vs. L23	F2-3 up	GO:0005886	Development	Development	plasma membrane	C	AT4G36250
9250	3	F23 vs. L23	F2-3 up	GO:0005886	Development	Development	plasma membrane	C	AT4G36490
9251	3	F23 vs. L23	F2-3 up	GO:0005886	Development	Development	plasma membrane	C	AT4G37550
9252	3	F23 vs. L23	F2-3 up	GO:0005886	Development	Development	plasma membrane	C	AT4G37990
9253	3	F23 vs. L23	F2-3 up	GO:0005886	Development	Development	plasma membrane	C	AT4G38510
9254	3	F23 vs. L23	F2-3 up	GO:0005886	Development	Development	plasma membrane	C	AT4G38740
9255	3	F23 vs. L23	F2-3 up	GO:0005886	Development	Development	plasma membrane	C	AT4G38970
9256	3	F23 vs. L23	F2-3 up	GO:0005886	Development	Development	plasma membrane	C	AT4G39110
9257	3	F23 vs. L23	F2-3 up	GO:0005886	Development	Development	plasma membrane	C	AT4G39350
9258	3	F23 vs. L23	F2-3 up	GO:0005886	Development	Development	plasma membrane	C	AT4G39640
9259	3	F23 vs. L23	F2-3 up	GO:0005886	Development	Development	plasma membrane	C	AT4G39730
9260	3	F23 vs. L23	F2-3 up	GO:0005886	Development	Development	plasma membrane	C	AT4G39955
9261	3	F23 vs. L23	F2-3 up	GO:0005886	Development	Development	plasma membrane	C	AT5G01410
9262	3	F23 vs. L23	F2-3 up	GO:0005886	Development	Development	plasma membrane	C	AT5G01430
9263	3	F23 vs. L23	F2-3 up	GO:0005886	Development	Development	plasma membrane	C	AT5G01600
9264	3	F23 vs. L23	F2-3 up	GO:0005886	Development	Development	plasma membrane	C	AT5G02790
9265	3	F23 vs. L23	F2-3 up	GO:0005886	Development	Development	plasma membrane	C	AT5G04140
9266	3	F23 vs. L23	F2-3 up	GO:0005886	Development	Development	plasma membrane	C	AT5G04710
9267	3	F23 vs. L23	F2-3 up	GO:0005886	Development	Development	plasma membrane	C	AT5G05200
9268	3	F23 vs. L23	F2-3 up	GO:0005886	Development	Development	plasma membrane	C	AT5G05370
9269	3	F23 vs. L23	F2-3 up	GO:0005886	Development	Development	plasma membrane	C	AT5G06320
9270	3	F23 vs. L23	F2-3 up	GO:0005886	Development	Development	plasma membrane	C	AT5G06530
9271	3	F23 vs. L23	F2-3 up	GO:0005886	Development	Development	plasma membrane	C	AT5G07440
9272	3	F23 vs. L23	F2-3 up	GO:0005886	Development	Development	plasma membrane	C	AT5G07460
9273	3	F23 vs. L23	F2-3 up	GO:0005886	Development	Development	plasma membrane	C	AT5G08100
9274	3	F23 vs. L23	F2-3 up	GO:0005886	Development	Development	plasma membrane	C	AT5G09220
9275	3	F23 vs. L23	F2-3 up	GO:0005886	Development	Development	plasma membrane	C	AT5G09650
9276	3	F23 vs. L23	F2-3 up	GO:0005886	Development	Development	plasma membrane	C	AT5G09660
9277	3	F23 vs. L23	F2-3 up	GO:0005886	Development	Development	plasma membrane	C	AT5G09810
9278	3	F23 vs. L23	F2-3 up	GO:0005886	Development	Development	plasma membrane	C	AT5G09870
9279	3	F23 vs. L23	F2-3 up	GO:0005886	Development	Development	plasma membrane	C	AT5G11420
9280	3	F23 vs. L23	F2-3 up	GO:0005886	Development	Development	plasma membrane	C	AT5G11520
9281	3	F23 vs. L23	F2-3 up	GO:0005886	Development	Development	plasma membrane	C	AT5G11960
9282	3	F23 vs. L23	F2-3 up	GO:0005886	Development	Development	plasma membrane	C	AT5G13160

9283	3	F23 vs. L23	F2-3 up	GO:0005886	Development	Development	plasma membrane	C	AT5G15460
9284	3	F23 vs. L23	F2-3 up	GO:0005886	Development	Development	plasma membrane	C	AT5G16910
9285	3	F23 vs. L23	F2-3 up	GO:0005886	Development	Development	plasma membrane	C	AT5G17860
9286	3	F23 vs. L23	F2-3 up	GO:0005886	Development	Development	plasma membrane	C	AT5G24080
9287	3	F23 vs. L23	F2-3 up	GO:0005886	Development	Development	plasma membrane	C	AT5G24650
9288	3	F23 vs. L23	F2-3 up	GO:0005886	Development	Development	plasma membrane	C	AT5G25120
9289	3	F23 vs. L23	F2-3 up	GO:0005886	Development	Development	plasma membrane	C	AT5G35735
9290	3	F23 vs. L23	F2-3 up	GO:0005886	Development	Development	plasma membrane	C	AT5G37300
9291	3	F23 vs. L23	F2-3 up	GO:0005886	Development	Development	plasma membrane	C	AT5G37810
9292	3	F23 vs. L23	F2-3 up	GO:0005886	Development	Development	plasma membrane	C	AT5G40230
9293	3	F23 vs. L23	F2-3 up	GO:0005886	Development	Development	plasma membrane	C	AT5G41790
9294	3	F23 vs. L23	F2-3 up	GO:0005886	Development	Development	plasma membrane	C	AT5G44110
9295	3	F23 vs. L23	F2-3 up	GO:0005886	Development	Development	plasma membrane	C	AT5G45280
9296	3	F23 vs. L23	F2-3 up	GO:0005886	Development	Development	plasma membrane	C	AT5G45840
9297	3	F23 vs. L23	F2-3 up	GO:0005886	Development	Development	plasma membrane	C	AT5G47910
9298	3	F23 vs. L23	F2-3 up	GO:0005886	Development	Development	plasma membrane	C	AT5G49720
9299	3	F23 vs. L23	F2-3 up	GO:0005886	Development	Development	plasma membrane	C	AT5G50800
9300	3	F23 vs. L23	F2-3 up	GO:0005886	Development	Development	plasma membrane	C	AT5G53890
9301	3	F23 vs. L23	F2-3 up	GO:0005886	Development	Development	plasma membrane	C	AT5G54250
9302	3	F23 vs. L23	F2-3 up	GO:0005886	Development	Development	plasma membrane	C	AT5G54980
9303	3	F23 vs. L23	F2-3 up	GO:0005886	Development	Development	plasma membrane	C	AT5G55960
9304	3	F23 vs. L23	F2-3 up	GO:0005886	Development	Development	plasma membrane	C	AT5G57350
9305	3	F23 vs. L23	F2-3 up	GO:0005886	Development	Development	plasma membrane	C	AT5G57800
9306	3	F23 vs. L23	F2-3 up	GO:0005886	Development	Development	plasma membrane	C	AT5G58690
9307	3	F23 vs. L23	F2-3 up	GO:0005886	Development	Development	plasma membrane	C	AT5G58700
9308	3	F23 vs. L23	F2-3 up	GO:0005886	Development	Development	plasma membrane	C	AT5G60020
9309	3	F23 vs. L23	F2-3 up	GO:0005886	Development	Development	plasma membrane	C	AT5G60740
9310	3	F23 vs. L23	F2-3 up	GO:0005886	Development	Development	plasma membrane	C	AT5G60790
9311	3	F23 vs. L23	F2-3 up	GO:0005886	Development	Development	plasma membrane	C	AT5G62360
9312	3	F23 vs. L23	F2-3 up	GO:0005886	Development	Development	plasma membrane	C	AT5G62680
9313	3	F23 vs. L23	F2-3 up	GO:0005886	Development	Development	plasma membrane	C	AT5G62750
9314	3	F23 vs. L23	F2-3 up	GO:0005886	Development	Development	plasma membrane	C	AT5G63680
9315	3	F23 vs. L23	F2-3 up	GO:0005886	Development	Development	plasma membrane	C	AT5G64740
9316	3	F23 vs. L23	F2-3 up	GO:0005886	Development	Development	plasma membrane	C	AT5G65460
9317	3	F23 vs. L23	F2-3 up	GO:0005886	Development	Development	plasma membrane	C	AT5G66070
9318	3	F23 vs. L23	F2-3 up	GO:0005886	Development	Development	plasma membrane	C	AT5G67470
9319	3	F23 vs. L23	F2-3 up	GO:0009511	Development	Development	plasmodesmatal endoplasmic reticulum	C	AT5G06850
9320	3	F23 vs. L23	F2-3 up	GO:0016605	Development	Development	PML body	C	AT1G04400
9321	3	F23 vs. L23	F2-3 up	GO:0009944	Development	Development	polarity specification of adaxial/abaxial axis	P	AT2G37630
9322	3	F23 vs. L23	F2-3 up	GO:0009944	Development	Development	polarity specification of adaxial/abaxial axis	P	AT2G45190
9323	3	F23 vs. L23	F2-3 up	GO:0009555	Development	Development	pollen development	P	AT1G17420
9324	3	F23 vs. L23	F2-3 up	GO:0009555	Development	Development	pollen development	P	AT1G19180
9325	3	F23 vs. L23	F2-3 up	GO:0009555	Development	Development	pollen development	P	AT1G42470
9326	3	F23 vs. L23	F2-3 up	GO:0009555	Development	Development	pollen development	P	AT1G72520
9327	3	F23 vs. L23	F2-3 up	GO:0009555	Development	Development	pollen development	P	AT1G74450
9328	3	F23 vs. L23	F2-3 up	GO:0009555	Development	Development	pollen development	P	AT1G77980
9329	3	F23 vs. L23	F2-3 up	GO:0009555	Development	Development	pollen development	P	AT2G22950
9330	3	F23 vs. L23	F2-3 up	GO:0009555	Development	Development	pollen development	P	AT2G30490
9331	3	F23 vs. L23	F2-3 up	GO:0009555	Development	Development	pollen development	P	AT2G32460
9332	3	F23 vs. L23	F2-3 up	GO:0009555	Development	Development	pollen development	P	AT2G37040
9333	3	F23 vs. L23	F2-3 up	GO:0009555	Development	Development	pollen development	P	AT3G03250
9334	3	F23 vs. L23	F2-3 up	GO:0009555	Development	Development	pollen development	P	AT3G21180
9335	3	F23 vs. L23	F2-3 up	GO:0009555	Development	Development	pollen development	P	AT3G43210
9336	3	F23 vs. L23	F2-3 up	GO:0009555	Development	Development	pollen development	P	AT3G45640
9337	3	F23 vs. L23	F2-3 up	GO:0009555	Development	Development	pollen development	P	AT3G48520
9338	3	F23 vs. L23	F2-3 up	GO:0009555	Development	Development	pollen development	P	AT3G55610

9339	3	F23 vs. L23	F2-3 up	GO:0009555	Development	Development	pollen development	P	AT3G59530
9340	3	F23 vs. L23	F2-3 up	GO:0009555	Development	Development	pollen development	P	AT4G17530
9341	3	F23 vs. L23	F2-3 up	GO:0009555	Development	Development	pollen development	P	AT4G24510
9342	3	F23 vs. L23	F2-3 up	GO:0009555	Development	Development	pollen development	P	AT4G25640
9343	3	F23 vs. L23	F2-3 up	GO:0009555	Development	Development	pollen development	P	AT5G14200
9344	3	F23 vs. L23	F2-3 up	GO:0009555	Development	Development	pollen development	P	AT5G15470
9345	3	F23 vs. L23	F2-3 up	GO:0009555	Development	Development	pollen development	P	AT5G50800
9346	3	F23 vs. L23	F2-3 up	GO:0010584	Development	Development	pollen exine formation	P	AT3G59530
9347	3	F23 vs. L23	F2-3 up	GO:0010152	Development	Development	pollen maturation	P	AT1G77980
9348	3	F23 vs. L23	F2-3 up	GO:0010152	Development	Development	pollen maturation	P	AT4G03190
9349	3	F23 vs. L23	F2-3 up	GO:0090406	Development	Development	pollen tube	C	AT1G03050
9350	3	F23 vs. L23	F2-3 up	GO:0090406	Development	Development	pollen tube	C	AT1G21760
9351	3	F23 vs. L23	F2-3 up	GO:0090406	Development	Development	pollen tube	C	AT2G01330
9352	3	F23 vs. L23	F2-3 up	GO:0090406	Development	Development	pollen tube	C	AT2G32460
9353	3	F23 vs. L23	F2-3 up	GO:0090406	Development	Development	pollen tube	C	AT2G41210
9354	3	F23 vs. L23	F2-3 up	GO:0090406	Development	Development	pollen tube	C	AT3G03250
9355	3	F23 vs. L23	F2-3 up	GO:0090406	Development	Development	pollen tube	C	AT3G07960
9356	3	F23 vs. L23	F2-3 up	GO:0090406	Development	Development	pollen tube	C	AT3G56960
9357	3	F23 vs. L23	F2-3 up	GO:0090406	Development	Development	pollen tube	C	AT3G62170
9358	3	F23 vs. L23	F2-3 up	GO:0090406	Development	Development	pollen tube	C	AT5G13990
9359	3	F23 vs. L23	F2-3 up	GO:0090406	Development	Development	pollen tube	C	AT5G15470
9360	3	F23 vs. L23	F2-3 up	GO:0090406	Development	Development	pollen tube	C	AT5G45840
9361	3	F23 vs. L23	F2-3 up	GO:0090404	Development	Development	pollen tube tip	C	AT1G03050
9362	3	F23 vs. L23	F2-3 up	GO:0090404	Development	Development	pollen tube tip	C	AT3G21700
9363	3	F23 vs. L23	F2-3 up	GO:0010208	Development	Development	pollen wall assembly	P	AT2G13680
9364	3	F23 vs. L23	F2-3 up	GO:0009791	Development	Development	post-embryonic development	P	AT4G39800
9365	3	F23 vs. L23	F2-3 up	GO:0090698	Development	Development	post-embryonic plant morphogenesis	P	AT3G23290
9366	3	F23 vs. L23	F2-3 up	GO:0080022	Development	Development	primary root development	P	AT1G12240
9367	3	F23 vs. L23	F2-3 up	GO:0080022	Development	Development	primary root development	P	AT1G35720
9368	3	F23 vs. L23	F2-3 up	GO:0080022	Development	Development	primary root development	P	AT3G29030
9369	3	F23 vs. L23	F2-3 up	GO:0010072	Development	Development	primary shoot apical meristem specification	P	AT4G32880
9370	3	F23 vs. L23	F2-3 up	GO:0010067	Development	Development	procambium histogenesis	P	AT4G32880
9371	3	F23 vs. L23	F2-3 up	GO:0000326	Development	Development	protein storage vacuole	C	AT2G36830
9372	3	F23 vs. L23	F2-3 up	GO:1990019	Development	Development	protein storage vacuole organization	P	AT2G32460
9373	3	F23 vs. L23	F2-3 up	GO:0009946	Development	Development	proximal/distal axis specification	P	AT2G37630
9374	3	F23 vs. L23	F2-3 up	GO:0010245	Development	Development	radial microtubular system formation	P	AT3G43210
9375	3	F23 vs. L23	F2-3 up	GO:0055038	Development	Development	recycling endosome membrane	C	AT1G03550
9376	3	F23 vs. L23	F2-3 up	GO:0055038	Development	Development	recycling endosome membrane	C	AT2G20840
9377	3	F23 vs. L23	F2-3 up	GO:0048364	Development	Development	root development	P	AT1G32640
9378	3	F23 vs. L23	F2-3 up	GO:0048364	Development	Development	root development	P	AT1G70940
9379	3	F23 vs. L23	F2-3 up	GO:0048364	Development	Development	root development	P	AT1G78240
9380	3	F23 vs. L23	F2-3 up	GO:0048364	Development	Development	root development	P	AT2G01420
9381	3	F23 vs. L23	F2-3 up	GO:0048364	Development	Development	root development	P	AT2G18470
9382	3	F23 vs. L23	F2-3 up	GO:0048364	Development	Development	root development	P	AT3G22400
9383	3	F23 vs. L23	F2-3 up	GO:0048364	Development	Development	root development	P	AT5G09810
9384	3	F23 vs. L23	F2-3 up	GO:0010053	Development	Development	root epidermal cell differentiation	P	AT1G05850
9385	3	F23 vs. L23	F2-3 up	GO:0010053	Development	Development	root epidermal cell differentiation	P	AT3G18780
9386	3	F23 vs. L23	F2-3 up	GO:0010053	Development	Development	root epidermal cell differentiation	P	AT4G31230
9387	3	F23 vs. L23	F2-3 up	GO:0010053	Development	Development	root epidermal cell differentiation	P	AT5G09810
9388	3	F23 vs. L23	F2-3 up	GO:0048768	Development	Development	root hair cell tip growth	P	AT3G05580
9389	3	F23 vs. L23	F2-3 up	GO:0048768	Development	Development	root hair cell tip growth	P	AT3G18780
9390	3	F23 vs. L23	F2-3 up	GO:0048767	Development	Development	root hair elongation	P	AT1G70940
9391	3	F23 vs. L23	F2-3 up	GO:0048767	Development	Development	root hair elongation	P	AT1G74380
9392	3	F23 vs. L23	F2-3 up	GO:0048767	Development	Development	root hair elongation	P	AT3G18780
9393	3	F23 vs. L23	F2-3 up	GO:0048767	Development	Development	root hair elongation	P	AT5G09810
9394	3	F23 vs. L23	F2-3 up	GO:0048767	Development	Development	root hair elongation	P	AT5G16910

9395	3	F23 vs. L23	F2-3 up	GO:0048766	Development	Development	root hair initiation	P	AT1G70940
9396	3	F23 vs. L23	F2-3 up	GO:0010214	Development	Development	seed coat development	P	AT1G53500
9397	3	F23 vs. L23	F2-3 up	GO:0010214	Development	Development	seed coat development	P	AT1G63930
9398	3	F23 vs. L23	F2-3 up	GO:0010214	Development	Development	seed coat development	P	AT2G22900
9399	3	F23 vs. L23	F2-3 up	GO:0010214	Development	Development	seed coat development	P	AT3G48990
9400	3	F23 vs. L23	F2-3 up	GO:0010214	Development	Development	seed coat development	P	AT5G49360
9401	3	F23 vs. L23	F2-3 up	GO:0048316	Development	Development	seed development	P	AT2G39510
9402	3	F23 vs. L23	F2-3 up	GO:0048316	Development	Development	seed development	P	AT3G04120
9403	3	F23 vs. L23	F2-3 up	GO:0048316	Development	Development	seed development	P	AT3G61880
9404	3	F23 vs. L23	F2-3 up	GO:0048316	Development	Development	seed development	P	AT4G34710
9405	3	F23 vs. L23	F2-3 up	GO:0009845	Development	Development	seed germination	P	AT2G18470
9406	3	F23 vs. L23	F2-3 up	GO:0009845	Development	Development	seed germination	P	AT5G04500
9407	3	F23 vs. L23	F2-3 up	GO:0009845	Development	Development	seed germination	P	AT5G09810
9408	3	F23 vs. L23	F2-3 up	GO:0080112	Development	Development	seed growth	P	AT5G50800
9409	3	F23 vs. L23	F2-3 up	GO:0010431	Development	Development	seed maturation	P	AT3G48740
9410	3	F23 vs. L23	F2-3 up	GO:0010431	Development	Development	seed maturation	P	AT5G40010
9411	3	F23 vs. L23	F2-3 up	GO:0010431	Development	Development	seed maturation	P	AT5G65410
9412	3	F23 vs. L23	F2-3 up	GO:0048367	Development	Development	shoot system development	P	AT1G78240
9413	3	F23 vs. L23	F2-3 up	GO:0048367	Development	Development	shoot system development	P	AT5G49720
9414	3	F23 vs. L23	F2-3 up	GO:0060776	Development	Development	simple leaf morphogenesis	P	AT1G27340
9415	3	F23 vs. L23	F2-3 up	GO:0010093	Development	Development	specification of floral organ identity	P	AT2G45190
9416	3	F23 vs. L23	F2-3 up	GO:0010093	Development	Development	specification of floral organ identity	P	AT3G54340
9417	3	F23 vs. L23	F2-3 up	GO:0010093	Development	Development	specification of floral organ identity	P	AT5G20240
9418	3	F23 vs. L23	F2-3 up	GO:0090701	Development	Development	specification of plant organ identity	P	AT4G37180
9419	3	F23 vs. L23	F2-3 up	GO:0090706	Development	Development	specification of plant organ position	P	AT2G45190
9420	3	F23 vs. L23	F2-3 up	GO:0048443	Development	Development	stamen development	P	AT2G06050
9421	3	F23 vs. L23	F2-3 up	GO:0048443	Development	Development	stamen development	P	AT3G54340
9422	3	F23 vs. L23	F2-3 up	GO:0048443	Development	Development	stamen development	P	AT4G03190
9423	3	F23 vs. L23	F2-3 up	GO:0080086	Development	Development	stamen filament development	P	AT1G17420
9424	3	F23 vs. L23	F2-3 up	GO:0080086	Development	Development	stamen filament development	P	AT1G72520
9425	3	F23 vs. L23	F2-3 up	GO:0080086	Development	Development	stamen filament development	P	AT3G15540
9426	3	F23 vs. L23	F2-3 up	GO:0019827	Development	Development	stem cell population maintenance	P	AT1G62360
9427	3	F23 vs. L23	F2-3 up	GO:0019827	Development	Development	stem cell population maintenance	P	AT2G17950
9428	3	F23 vs. L23	F2-3 up	GO:0048480	Development	Development	stigma development	P	AT3G48520
9429	3	F23 vs. L23	F2-3 up	GO:0010374	Development	Development	stomatal complex development	P	AT1G32640
9430	3	F23 vs. L23	F2-3 up	GO:0010376	Development	Development	stomatal complex formation	P	AT2G46410
9431	3	F23 vs. L23	F2-3 up	GO:0080166	Development	Development	stomium development	P	AT2G17950
9432	3	F23 vs. L23	F2-3 up	GO:0010319	Development	Development	stromule	C	AT1G09340
9433	3	F23 vs. L23	F2-3 up	GO:0010319	Development	Development	stromule	C	AT1G16880
9434	3	F23 vs. L23	F2-3 up	GO:0010319	Development	Development	stromule	C	AT1G23740
9435	3	F23 vs. L23	F2-3 up	GO:0010319	Development	Development	stromule	C	AT1G32060
9436	3	F23 vs. L23	F2-3 up	GO:0010319	Development	Development	stromule	C	AT1G42970
9437	3	F23 vs. L23	F2-3 up	GO:0010319	Development	Development	stromule	C	AT2G39730
9438	3	F23 vs. L23	F2-3 up	GO:0010319	Development	Development	stromule	C	AT3G01500
9439	3	F23 vs. L23	F2-3 up	GO:0010319	Development	Development	stromule	C	AT3G11630
9440	3	F23 vs. L23	F2-3 up	GO:0010319	Development	Development	stromule	C	AT3G12780
9441	3	F23 vs. L23	F2-3 up	GO:0010319	Development	Development	stromule	C	AT3G26650
9442	3	F23 vs. L23	F2-3 up	GO:0010319	Development	Development	stromule	C	AT5G61410
9443	3	F23 vs. L23	F2-3 up	GO:0010319	Development	Development	stromule	C	ATCG00120
9444	3	F23 vs. L23	F2-3 up	GO:0005199	Development	Development	structural constituent of cell wall	F	AT1G26770
9445	3	F23 vs. L23	F2-3 up	GO:0005200	Development	Development	structural constituent of cytoskeleton	F	AT1G64740
9446	3	F23 vs. L23	F2-3 up	GO:0005200	Development	Development	structural constituent of cytoskeleton	F	AT3G18780
9447	3	F23 vs. L23	F2-3 up	GO:0005200	Development	Development	structural constituent of cytoskeleton	F	AT3G46520
9448	3	F23 vs. L23	F2-3 up	GO:0005200	Development	Development	structural constituent of cytoskeleton	F	AT5G09810
9449	3	F23 vs. L23	F2-3 up	GO:0005200	Development	Development	structural constituent of cytoskeleton	F	AT5G59370
9450	3	F23 vs. L23	F2-3 up	GO:0032588	Development	Development	trans-Golgi network membrane	C	AT1G03550

9451	3	F23 vs. L23	F2-3 up	GO:0032588	Development	Development	trans-Golgi network membrane	C	AT2G20840
9452	3	F23 vs. L23	F2-3 up	GO:0010148	Development	Development	transpiration	P	AT5G06530
9453	3	F23 vs. L23	F2-3 up	GO:0010026	Development	Development	trichome differentiation	P	AT1G63650
9454	3	F23 vs. L23	F2-3 up	GO:0010090	Development	Development	trichome morphogenesis	P	AT1G56580
9455	3	F23 vs. L23	F2-3 up	GO:0048629	Development	Development	trichome patterning	P	AT4G00480
9456	3	F23 vs. L23	F2-3 up	GO:0007021	Development	Development	tubulin complex assembly	P	AT2G18390
9457	3	F23 vs. L23	F2-3 up	GO:0009826	Development	Development	unidimensional cell growth	P	AT1G12840
9458	3	F23 vs. L23	F2-3 up	GO:0009826	Development	Development	unidimensional cell growth	P	AT1G20190
9459	3	F23 vs. L23	F2-3 up	GO:0009826	Development	Development	unidimensional cell growth	P	AT1G26770
9460	3	F23 vs. L23	F2-3 up	GO:0009826	Development	Development	unidimensional cell growth	P	AT1G68530
9461	3	F23 vs. L23	F2-3 up	GO:0009826	Development	Development	unidimensional cell growth	P	AT1G69530
9462	3	F23 vs. L23	F2-3 up	GO:0009826	Development	Development	unidimensional cell growth	P	AT1G75500
9463	3	F23 vs. L23	F2-3 up	GO:0009826	Development	Development	unidimensional cell growth	P	AT2G28950
9464	3	F23 vs. L23	F2-3 up	GO:0009826	Development	Development	unidimensional cell growth	P	AT2G39700
9465	3	F23 vs. L23	F2-3 up	GO:0009826	Development	Development	unidimensional cell growth	P	AT3G12690
9466	3	F23 vs. L23	F2-3 up	GO:0009826	Development	Development	unidimensional cell growth	P	AT3G29030
9467	3	F23 vs. L23	F2-3 up	GO:0009826	Development	Development	unidimensional cell growth	P	AT3G49110
9468	3	F23 vs. L23	F2-3 up	GO:0009826	Development	Development	unidimensional cell growth	P	AT4G38400
9469	3	F23 vs. L23	F2-3 up	GO:0009826	Development	Development	unidimensional cell growth	P	AT5G49720
9470	3	F23 vs. L23	F2-3 up	GO:0009826	Development	Development	unidimensional cell growth	P	AT5G54510
9471	3	F23 vs. L23	F2-3 up	GO:0005775	Development	Development	vacuolar lumen	C	AT1G12240
9472	3	F23 vs. L23	F2-3 up	GO:0005774	Development	Development	vacuolar membrane	C	AT1G04430
9473	3	F23 vs. L23	F2-3 up	GO:0005774	Development	Development	vacuolar membrane	C	AT1G12240
9474	3	F23 vs. L23	F2-3 up	GO:0005774	Development	Development	vacuolar membrane	C	AT1G12840
9475	3	F23 vs. L23	F2-3 up	GO:0005774	Development	Development	vacuolar membrane	C	AT1G13860
9476	3	F23 vs. L23	F2-3 up	GO:0005774	Development	Development	vacuolar membrane	C	AT1G16310
9477	3	F23 vs. L23	F2-3 up	GO:0005774	Development	Development	vacuolar membrane	C	AT1G22840
9478	3	F23 vs. L23	F2-3 up	GO:0005774	Development	Development	vacuolar membrane	C	AT1G35720
9479	3	F23 vs. L23	F2-3 up	GO:0005774	Development	Development	vacuolar membrane	C	AT1G47530
9480	3	F23 vs. L23	F2-3 up	GO:0005774	Development	Development	vacuolar membrane	C	AT1G51420
9481	3	F23 vs. L23	F2-3 up	GO:0005774	Development	Development	vacuolar membrane	C	AT1G59870
9482	3	F23 vs. L23	F2-3 up	GO:0005774	Development	Development	vacuolar membrane	C	AT1G61800
9483	3	F23 vs. L23	F2-3 up	GO:0005774	Development	Development	vacuolar membrane	C	AT1G67940
9484	3	F23 vs. L23	F2-3 up	GO:0005774	Development	Development	vacuolar membrane	C	AT1G72730
9485	3	F23 vs. L23	F2-3 up	GO:0005774	Development	Development	vacuolar membrane	C	AT1G75500
9486	3	F23 vs. L23	F2-3 up	GO:0005774	Development	Development	vacuolar membrane	C	AT1G80660
9487	3	F23 vs. L23	F2-3 up	GO:0005774	Development	Development	vacuolar membrane	C	AT2G21880
9488	3	F23 vs. L23	F2-3 up	GO:0005774	Development	Development	vacuolar membrane	C	AT2G30490
9489	3	F23 vs. L23	F2-3 up	GO:0005774	Development	Development	vacuolar membrane	C	AT2G36830
9490	3	F23 vs. L23	F2-3 up	GO:0005774	Development	Development	vacuolar membrane	C	AT2G38170
9491	3	F23 vs. L23	F2-3 up	GO:0005774	Development	Development	vacuolar membrane	C	AT2G39130
9492	3	F23 vs. L23	F2-3 up	GO:0005774	Development	Development	vacuolar membrane	C	AT2G47650
9493	3	F23 vs. L23	F2-3 up	GO:0005774	Development	Development	vacuolar membrane	C	AT2G48020
9494	3	F23 vs. L23	F2-3 up	GO:0005774	Development	Development	vacuolar membrane	C	AT3G04120
9495	3	F23 vs. L23	F2-3 up	GO:0005774	Development	Development	vacuolar membrane	C	AT3G08560
9496	3	F23 vs. L23	F2-3 up	GO:0005774	Development	Development	vacuolar membrane	C	AT3G09440
9497	3	F23 vs. L23	F2-3 up	GO:0005774	Development	Development	vacuolar membrane	C	AT3G23300
9498	3	F23 vs. L23	F2-3 up	GO:0005774	Development	Development	vacuolar membrane	C	AT3G26520
9499	3	F23 vs. L23	F2-3 up	GO:0005774	Development	Development	vacuolar membrane	C	AT3G30390
9500	3	F23 vs. L23	F2-3 up	GO:0005774	Development	Development	vacuolar membrane	C	AT3G54960
9501	3	F23 vs. L23	F2-3 up	GO:0005774	Development	Development	vacuolar membrane	C	AT3G57330
9502	3	F23 vs. L23	F2-3 up	GO:0005774	Development	Development	vacuolar membrane	C	AT3G58730
9503	3	F23 vs. L23	F2-3 up	GO:0005774	Development	Development	vacuolar membrane	C	AT4G01470
9504	3	F23 vs. L23	F2-3 up	GO:0005774	Development	Development	vacuolar membrane	C	AT4G14880
9505	3	F23 vs. L23	F2-3 up	GO:0005774	Development	Development	vacuolar membrane	C	AT4G16660
9506	3	F23 vs. L23	F2-3 up	GO:0005774	Development	Development	vacuolar membrane	C	AT4G25640

9507	3	F23 vs. L23	F2-3 up	GO:0005774	Development	Development	vacuolar membrane	C	AT4G33530
9508	3	F23 vs. L23	F2-3 up	GO:0005774	Development	Development	vacuolar membrane	C	AT4G38510
9509	3	F23 vs. L23	F2-3 up	GO:0005774	Development	Development	vacuolar membrane	C	AT4G39730
9510	3	F23 vs. L23	F2-3 up	GO:0005774	Development	Development	vacuolar membrane	C	AT5G06320
9511	3	F23 vs. L23	F2-3 up	GO:0005774	Development	Development	vacuolar membrane	C	AT5G07440
9512	3	F23 vs. L23	F2-3 up	GO:0005774	Development	Development	vacuolar membrane	C	AT5G13490
9513	3	F23 vs. L23	F2-3 up	GO:0005774	Development	Development	vacuolar membrane	C	AT5G14120
9514	3	F23 vs. L23	F2-3 up	GO:0005774	Development	Development	vacuolar membrane	C	AT5G17860
9515	3	F23 vs. L23	F2-3 up	GO:0005774	Development	Development	vacuolar membrane	C	AT5G24650
9516	3	F23 vs. L23	F2-3 up	GO:0005774	Development	Development	vacuolar membrane	C	AT5G41790
9517	3	F23 vs. L23	F2-3 up	GO:0005774	Development	Development	vacuolar membrane	C	AT5G50850
9518	3	F23 vs. L23	F2-3 up	GO:0005774	Development	Development	vacuolar membrane	C	AT5G52450
9519	3	F23 vs. L23	F2-3 up	GO:0005774	Development	Development	vacuolar membrane	C	AT5G67480
9520	3	F23 vs. L23	F2-3 up	GO:0005773	Development	Development	vacuole	C	AT1G04400
9521	3	F23 vs. L23	F2-3 up	GO:0005773	Development	Development	vacuole	C	AT1G09340
9522	3	F23 vs. L23	F2-3 up	GO:0005773	Development	Development	vacuole	C	AT1G11840
9523	3	F23 vs. L23	F2-3 up	GO:0005773	Development	Development	vacuole	C	AT1G12240
9524	3	F23 vs. L23	F2-3 up	GO:0005773	Development	Development	vacuole	C	AT1G12840
9525	3	F23 vs. L23	F2-3 up	GO:0005773	Development	Development	vacuole	C	AT1G16290
9526	3	F23 vs. L23	F2-3 up	GO:0005773	Development	Development	vacuole	C	AT1G20620
9527	3	F23 vs. L23	F2-3 up	GO:0005773	Development	Development	vacuole	C	AT1G22710
9528	3	F23 vs. L23	F2-3 up	GO:0005773	Development	Development	vacuole	C	AT1G22750
9529	3	F23 vs. L23	F2-3 up	GO:0005773	Development	Development	vacuole	C	AT1G23310
9530	3	F23 vs. L23	F2-3 up	GO:0005773	Development	Development	vacuole	C	AT1G35720
9531	3	F23 vs. L23	F2-3 up	GO:0005773	Development	Development	vacuole	C	AT1G44170
9532	3	F23 vs. L23	F2-3 up	GO:0005773	Development	Development	vacuole	C	AT1G45130
9533	3	F23 vs. L23	F2-3 up	GO:0005773	Development	Development	vacuole	C	AT1G62290
9534	3	F23 vs. L23	F2-3 up	GO:0005773	Development	Development	vacuole	C	AT2G21880
9535	3	F23 vs. L23	F2-3 up	GO:0005773	Development	Development	vacuole	C	AT2G21960
9536	3	F23 vs. L23	F2-3 up	GO:0005773	Development	Development	vacuole	C	AT2G24180
9537	3	F23 vs. L23	F2-3 up	GO:0005773	Development	Development	vacuole	C	AT2G24270
9538	3	F23 vs. L23	F2-3 up	GO:0005773	Development	Development	vacuole	C	AT2G24820
9539	3	F23 vs. L23	F2-3 up	GO:0005773	Development	Development	vacuole	C	AT2G30860
9540	3	F23 vs. L23	F2-3 up	GO:0005773	Development	Development	vacuole	C	AT2G36830
9541	3	F23 vs. L23	F2-3 up	GO:0005773	Development	Development	vacuole	C	AT2G37970
9542	3	F23 vs. L23	F2-3 up	GO:0005773	Development	Development	vacuole	C	AT2G38170
9543	3	F23 vs. L23	F2-3 up	GO:0005773	Development	Development	vacuole	C	AT2G39330
9544	3	F23 vs. L23	F2-3 up	GO:0005773	Development	Development	vacuole	C	AT2G44120
9545	3	F23 vs. L23	F2-3 up	GO:0005773	Development	Development	vacuole	C	AT2G47160
9546	3	F23 vs. L23	F2-3 up	GO:0005773	Development	Development	vacuole	C	AT2G47650
9547	3	F23 vs. L23	F2-3 up	GO:0005773	Development	Development	vacuole	C	AT2G48020
9548	3	F23 vs. L23	F2-3 up	GO:0005773	Development	Development	vacuole	C	AT3G05500
9549	3	F23 vs. L23	F2-3 up	GO:0005773	Development	Development	vacuole	C	AT3G08560
9550	3	F23 vs. L23	F2-3 up	GO:0005773	Development	Development	vacuole	C	AT3G09440
9551	3	F23 vs. L23	F2-3 up	GO:0005773	Development	Development	vacuole	C	AT3G17180
9552	3	F23 vs. L23	F2-3 up	GO:0005773	Development	Development	vacuole	C	AT3G23810
9553	3	F23 vs. L23	F2-3 up	GO:0005773	Development	Development	vacuole	C	AT3G26520
9554	3	F23 vs. L23	F2-3 up	GO:0005773	Development	Development	vacuole	C	AT3G30390
9555	3	F23 vs. L23	F2-3 up	GO:0005773	Development	Development	vacuole	C	AT3G45010
9556	3	F23 vs. L23	F2-3 up	GO:0005773	Development	Development	vacuole	C	AT3G45780
9557	3	F23 vs. L23	F2-3 up	GO:0005773	Development	Development	vacuole	C	AT3G49110
9558	3	F23 vs. L23	F2-3 up	GO:0005773	Development	Development	vacuole	C	AT3G52000
9559	3	F23 vs. L23	F2-3 up	GO:0005773	Development	Development	vacuole	C	AT3G53420
9560	3	F23 vs. L23	F2-3 up	GO:0005773	Development	Development	vacuole	C	AT3G57010
9561	3	F23 vs. L23	F2-3 up	GO:0005773	Development	Development	vacuole	C	AT3G57020
9562	3	F23 vs. L23	F2-3 up	GO:0005773	Development	Development	vacuole	C	AT3G57330

9563	3	F23 vs. L23	F2-3 up	GO:0005773	Development	Development	vacuole	C	AT3G58730
9564	3	F23 vs. L23	F2-3 up	GO:0005773	Development	Development	vacuole	C	AT3G59530
9565	3	F23 vs. L23	F2-3 up	GO:0005773	Development	Development	vacuole	C	AT3G62290
9566	3	F23 vs. L23	F2-3 up	GO:0005773	Development	Development	vacuole	C	AT4G01610
9567	3	F23 vs. L23	F2-3 up	GO:0005773	Development	Development	vacuole	C	AT4G02890
9568	3	F23 vs. L23	F2-3 up	GO:0005773	Development	Development	vacuole	C	AT4G10060
9569	3	F23 vs. L23	F2-3 up	GO:0005773	Development	Development	vacuole	C	AT4G17170
9570	3	F23 vs. L23	F2-3 up	GO:0005773	Development	Development	vacuole	C	AT4G17530
9571	3	F23 vs. L23	F2-3 up	GO:0005773	Development	Development	vacuole	C	AT4G24640
9572	3	F23 vs. L23	F2-3 up	GO:0005773	Development	Development	vacuole	C	AT4G25950
9573	3	F23 vs. L23	F2-3 up	GO:0005773	Development	Development	vacuole	C	AT4G34720
9574	3	F23 vs. L23	F2-3 up	GO:0005773	Development	Development	vacuole	C	AT4G35010
9575	3	F23 vs. L23	F2-3 up	GO:0005773	Development	Development	vacuole	C	AT4G37550
9576	3	F23 vs. L23	F2-3 up	GO:0005773	Development	Development	vacuole	C	AT4G38510
9577	3	F23 vs. L23	F2-3 up	GO:0005773	Development	Development	vacuole	C	AT4G39730
9578	3	F23 vs. L23	F2-3 up	GO:0005773	Development	Development	vacuole	C	AT5G08260
9579	3	F23 vs. L23	F2-3 up	GO:0005773	Development	Development	vacuole	C	AT5G09660
9580	3	F23 vs. L23	F2-3 up	GO:0005773	Development	Development	vacuole	C	AT5G13710
9581	3	F23 vs. L23	F2-3 up	GO:0005773	Development	Development	vacuole	C	AT5G14120
9582	3	F23 vs. L23	F2-3 up	GO:0005773	Development	Development	vacuole	C	AT5G24650
9583	3	F23 vs. L23	F2-3 up	GO:0005773	Development	Development	vacuole	C	AT5G24780
9584	3	F23 vs. L23	F2-3 up	GO:0005773	Development	Development	vacuole	C	AT5G25980
9585	3	F23 vs. L23	F2-3 up	GO:0005773	Development	Development	vacuole	C	AT5G41790
9586	3	F23 vs. L23	F2-3 up	GO:0005773	Development	Development	vacuole	C	AT5G60360
9587	3	F23 vs. L23	F2-3 up	GO:0005773	Development	Development	vacuole	C	AT5G65760
9588	3	F23 vs. L23	F2-3 up	GO:0001944	Development	Development	vasculature development	P	AT4G11280
9589	3	F23 vs. L23	F2-3 up	GO:0010228	Development	Development	vegetative to reproductive phase transition of meristem	P	AT2G01290
9590	3	F23 vs. L23	F2-3 up	GO:0010228	Development	Development	vegetative to reproductive phase transition of meristem	P	AT2G01520
9591	3	F23 vs. L23	F2-3 up	GO:0010228	Development	Development	vegetative to reproductive phase transition of meristem	P	AT2G42200
9592	3	F23 vs. L23	F2-3 up	GO:0010228	Development	Development	vegetative to reproductive phase transition of meristem	P	AT5G06850
9593	3	F23 vs. L23	F2-3 up	GO:0006900	Development	Development	vesicle budding from membrane	P	AT1G03050
9594	3	F23 vs. L23	F2-3 up	GO:0048278	Development	Development	vesicle docking	P	AT1G61290
9595	3	F23 vs. L23	F2-3 up	GO:0048278	Development	Development	vesicle docking	P	AT3G03800
9596	3	F23 vs. L23	F2-3 up	GO:0006906	Development	Development	vesicle fusion	P	AT1G61290
9597	3	F23 vs. L23	F2-3 up	GO:0006906	Development	Development	vesicle fusion	P	AT3G03800
9598	3	F23 vs. L23	F2-3 up	GO:0012506	Development	Development	vesicle membrane	C	AT1G70940
9599	3	F23 vs. L23	F2-3 up	GO:0010051	Development	Development	xylem and phloem pattern formation	P	AT4G08150
9600	3	F23 vs. L23	F2-3 up	GO:0035798	Metabolism	Bioenergetics	2-alkenal reductase (NADP+) activity	F	AT1G23740
9601	3	F23 vs. L23	F2-3 up	GO:0102336	Metabolism	Bioenergetics	3-oxo-arachidoyl-CoA synthase activity	F	AT1G68530
9602	3	F23 vs. L23	F2-3 up	GO:0102337	Metabolism	Bioenergetics	3-oxo-cerotoyl-CoA synthase activity	F	AT1G68530
9603	3	F23 vs. L23	F2-3 up	GO:0102338	Metabolism	Bioenergetics	3-oxo-lignoceronyl-CoA synthase activity	F	AT1G68530
9604	3	F23 vs. L23	F2-3 up	GO:0016207	Metabolism	Bioenergetics	4-coumarate-CoA ligase activity	F	AT3G21230
9605	3	F23 vs. L23	F2-3 up	GO:0016207	Metabolism	Bioenergetics	4-coumarate-CoA ligase activity	F	AT5G63380
9606	3	F23 vs. L23	F2-3 up	GO:0003987	Metabolism	Bioenergetics	acetate-CoA ligase activity	F	AT5G36880
9607	3	F23 vs. L23	F2-3 up	GO:0010327	Metabolism	Bioenergetics	acetyl CoA:(Z)-3-hexen-1-ol acetyltransferase activity	F	AT3G03480
9608	3	F23 vs. L23	F2-3 up	GO:0006085	Metabolism	Bioenergetics	acetyl-CoA biosynthetic process	P	AT1G09430
9609	3	F23 vs. L23	F2-3 up	GO:0006085	Metabolism	Bioenergetics	acetyl-CoA biosynthetic process	P	AT1G60810
9610	3	F23 vs. L23	F2-3 up	GO:0019427	Metabolism	Bioenergetics	acetyl-CoA biosynthetic process from acetate	P	AT5G36880
9611	3	F23 vs. L23	F2-3 up	GO:0006086	Metabolism	Bioenergetics	acetyl-CoA biosynthetic process from pyruvate	P	AT1G01090
9612	3	F23 vs. L23	F2-3 up	GO:0006086	Metabolism	Bioenergetics	acetyl-CoA biosynthetic process from pyruvate	P	AT5G50850
9613	3	F23 vs. L23	F2-3 up	GO:0047617	Metabolism	Bioenergetics	acyl-CoA hydrolase activity	F	AT3G54950

9614	3	F23 vs. L23	F2-3 up	GO:0003996	Metabolism	Bioenergetics	acyl-CoA ligase activity	F	AT4G11030
9615	3	F23 vs. L23	F2-3 up	GO:0043531	Metabolism	Bioenergetics	ADP binding	F	AT2G39730
9616	3	F23 vs. L23	F2-3 up	GO:0043531	Metabolism	Bioenergetics	ADP binding	F	AT3G12780
9617	3	F23 vs. L23	F2-3 up	GO:0043531	Metabolism	Bioenergetics	ADP binding	F	AT5G04720
9618	3	F23 vs. L23	F2-3 up	GO:0047631	Metabolism	Bioenergetics	ADP-ribose diphosphatase activity	F	AT5G47650
9619	3	F23 vs. L23	F2-3 up	GO:0080041	Metabolism	Bioenergetics	ADP-ribose pyrophosphohydrolase activity	F	AT2G29630
9620	3	F23 vs. L23	F2-3 up	GO:0009060	Metabolism	Bioenergetics	aerobic respiration	P	AT3G27380
9621	3	F23 vs. L23	F2-3 up	GO:0009060	Metabolism	Bioenergetics	aerobic respiration	P	AT5G40650
9622	3	F23 vs. L23	F2-3 up	GO:0009060	Metabolism	Bioenergetics	aerobic respiration	P	ATCG00430
9623	3	F23 vs. L23	F2-3 up	GO:0009060	Metabolism	Bioenergetics	aerobic respiration	P	ATCG01100
9624	3	F23 vs. L23	F2-3 up	GO:0004022	Metabolism	Bioenergetics	alcohol dehydrogenase (NAD+) activity	F	AT1G51410
9625	3	F23 vs. L23	F2-3 up	GO:0008106	Metabolism	Bioenergetics	alcohol dehydrogenase (NADP+) activity	F	AT2G37760
9626	3	F23 vs. L23	F2-3 up	GO:0008106	Metabolism	Bioenergetics	alcohol dehydrogenase (NADP+) activity	F	AT2G37770
9627	3	F23 vs. L23	F2-3 up	GO:0008106	Metabolism	Bioenergetics	alcohol dehydrogenase (NADP+) activity	F	AT3G61220
9628	3	F23 vs. L23	F2-3 up	GO:0004029	Metabolism	Bioenergetics	aldehyde dehydrogenase (NAD+) activity	F	AT1G23800
9629	3	F23 vs. L23	F2-3 up	GO:0004029	Metabolism	Bioenergetics	aldehyde dehydrogenase (NAD+) activity	F	AT1G44170
9630	3	F23 vs. L23	F2-3 up	GO:0004029	Metabolism	Bioenergetics	aldehyde dehydrogenase (NAD+) activity	F	AT3G24503
9631	3	F23 vs. L23	F2-3 up	GO:0004029	Metabolism	Bioenergetics	aldehyde dehydrogenase (NAD+) activity	F	AT3G48170
9632	3	F23 vs. L23	F2-3 up	GO:0004029	Metabolism	Bioenergetics	aldehyde dehydrogenase (NAD+) activity	F	AT4G36250
9633	3	F23 vs. L23	F2-3 up	GO:0004032	Metabolism	Bioenergetics	alditol:NADP+ 1-oxidoreductase activity	F	AT2G37760
9634	3	F23 vs. L23	F2-3 up	GO:0004032	Metabolism	Bioenergetics	alditol:NADP+ 1-oxidoreductase activity	F	AT2G37770
9635	3	F23 vs. L23	F2-3 up	GO:0004033	Metabolism	Bioenergetics	aldo-keto reductase (NADP) activity	F	AT2G37760
9636	3	F23 vs. L23	F2-3 up	GO:0004033	Metabolism	Bioenergetics	aldo-keto reductase (NADP) activity	F	AT2G37770
9637	3	F23 vs. L23	F2-3 up	GO:0004556	Metabolism	Bioenergetics	alpha-amylase activity	F	AT1G76130
9638	3	F23 vs. L23	F2-3 up	GO:0103025	Metabolism	Bioenergetics	alpha-amylase activity (releasing maltohexaose)	F	AT1G76130
9639	3	F23 vs. L23	F2-3 up	GO:0102966	Metabolism	Bioenergetics	arachidoyl-CoA:1-dodecanol O-acyltransferase activity	F	AT5G37300
9640	3	F23 vs. L23	F2-3 up	GO:0102966	Metabolism	Bioenergetics	arachidoyl-CoA:1-dodecanol O-acyltransferase activity	F	AT5G55340
9641	3	F23 vs. L23	F2-3 up	GO:0047681	Metabolism	Bioenergetics	aryl-alcohol dehydrogenase (NADP+) activity	F	AT4G37990
9642	3	F23 vs. L23	F2-3 up	GO:0006754	Metabolism	Bioenergetics	ATP biosynthetic process	P	ATMG00480
9643	3	F23 vs. L23	F2-3 up	GO:0003878	Metabolism	Bioenergetics	ATP citrate synthase activity	F	AT1G09430
9644	3	F23 vs. L23	F2-3 up	GO:0003878	Metabolism	Bioenergetics	ATP citrate synthase activity	F	AT1G60810
9645	3	F23 vs. L23	F2-3 up	GO:0046034	Metabolism	Bioenergetics	ATP metabolic process	P	AT4G38510
9646	3	F23 vs. L23	F2-3 up	GO:0042773	Metabolism	Bioenergetics	ATP synthesis coupled electron transport	P	ATMG00650
9647	3	F23 vs. L23	F2-3 up	GO:0015986	Metabolism	Bioenergetics	ATP synthesis coupled proton transport	P	AT4G29480
9648	3	F23 vs. L23	F2-3 up	GO:0015986	Metabolism	Bioenergetics	ATP synthesis coupled proton transport	P	AT4G32260
9649	3	F23 vs. L23	F2-3 up	GO:0015986	Metabolism	Bioenergetics	ATP synthesis coupled proton transport	P	ATMG00480
9650	3	F23 vs. L23	F2-3 up	GO:0016887	Metabolism	Bioenergetics	ATPase activity	F	AT1G03905
9651	3	F23 vs. L23	F2-3 up	GO:0016887	Metabolism	Bioenergetics	ATPase activity	F	AT1G59870
9652	3	F23 vs. L23	F2-3 up	GO:0016887	Metabolism	Bioenergetics	ATPase activity	F	AT1G67940
9653	3	F23 vs. L23	F2-3 up	GO:0016887	Metabolism	Bioenergetics	ATPase activity	F	AT1G70610
9654	3	F23 vs. L23	F2-3 up	GO:0016887	Metabolism	Bioenergetics	ATPase activity	F	AT2G01320
9655	3	F23 vs. L23	F2-3 up	GO:0016887	Metabolism	Bioenergetics	ATPase activity	F	AT2G07560
9656	3	F23 vs. L23	F2-3 up	GO:0016887	Metabolism	Bioenergetics	ATPase activity	F	AT2G25530
9657	3	F23 vs. L23	F2-3 up	GO:0016887	Metabolism	Bioenergetics	ATPase activity	F	AT3G09440
9658	3	F23 vs. L23	F2-3 up	GO:0016887	Metabolism	Bioenergetics	ATPase activity	F	AT3G16340
9659	3	F23 vs. L23	F2-3 up	GO:0016887	Metabolism	Bioenergetics	ATPase activity	F	AT3G42640
9660	3	F23 vs. L23	F2-3 up	GO:0016887	Metabolism	Bioenergetics	ATPase activity	F	AT3G43210
9661	3	F23 vs. L23	F2-3 up	GO:0016887	Metabolism	Bioenergetics	ATPase activity	F	AT3G55100
9662	3	F23 vs. L23	F2-3 up	GO:0016887	Metabolism	Bioenergetics	ATPase activity	F	AT4G18050
9663	3	F23 vs. L23	F2-3 up	GO:0016887	Metabolism	Bioenergetics	ATPase activity	F	AT4G34720
9664	3	F23 vs. L23	F2-3 up	GO:0016887	Metabolism	Bioenergetics	ATPase activity	F	AT5G02270
9665	3	F23 vs. L23	F2-3 up	GO:0016887	Metabolism	Bioenergetics	ATPase activity	F	AT5G06530
9666	3	F23 vs. L23	F2-3 up	GO:0016887	Metabolism	Bioenergetics	ATPase activity	F	AT5G18280
9667	3	F23 vs. L23	F2-3 up	GO:0016887	Metabolism	Bioenergetics	ATPase activity	F	AT5G27000

9668	3	F23 vs. L23	F2-3 up	GO:0016887	Metabolism	Bioenergetics	ATPase activity	F	AT5G40010
9669	3	F23 vs. L23	F2-3 up	GO:0016887	Metabolism	Bioenergetics	ATPase activity	F	AT5G44110
9670	3	F23 vs. L23	F2-3 up	GO:0016887	Metabolism	Bioenergetics	ATPase activity	F	AT5G57350
9671	3	F23 vs. L23	F2-3 up	GO:0016887	Metabolism	Bioenergetics	ATPase activity	F	AT5G60740
9672	3	F23 vs. L23	F2-3 up	GO:0016887	Metabolism	Bioenergetics	ATPase activity	F	AT5G60790
9673	3	F23 vs. L23	F2-3 up	GO:0016887	Metabolism	Bioenergetics	ATPase activity	F	AT5G61700
9674	3	F23 vs. L23	F2-3 up	GO:0016887	Metabolism	Bioenergetics	ATPase activity	F	AT5G64840
9675	3	F23 vs. L23	F2-3 up	GO:0016887	Metabolism	Bioenergetics	ATPase activity	F	AT5G65460
9676	3	F23 vs. L23	F2-3 up	GO:0042623	Metabolism	Bioenergetics	ATPase activity, coupled	F	AT3G09440
9677	3	F23 vs. L23	F2-3 up	GO:0016161	Metabolism	Bioenergetics	beta-amylase activity	F	AT3G23920
9678	3	F23 vs. L23	F2-3 up	GO:0016161	Metabolism	Bioenergetics	beta-amylase activity	F	AT4G15210
9679	3	F23 vs. L23	F2-3 up	GO:0016161	Metabolism	Bioenergetics	beta-amylase activity	F	AT4G17090
9680	3	F23 vs. L23	F2-3 up	GO:0042409	Metabolism	Bioenergetics	caffeoyl-CoA O-methyltransferase activity	F	AT4G34050
9681	3	F23 vs. L23	F2-3 up	GO:0030246	Metabolism	Bioenergetics	carbohydrate binding	F	AT1G64390
9682	3	F23 vs. L23	F2-3 up	GO:0030246	Metabolism	Bioenergetics	carbohydrate binding	F	AT1G73040
9683	3	F23 vs. L23	F2-3 up	GO:0030246	Metabolism	Bioenergetics	carbohydrate binding	F	AT1G78820
9684	3	F23 vs. L23	F2-3 up	GO:0030246	Metabolism	Bioenergetics	carbohydrate binding	F	AT2G39330
9685	3	F23 vs. L23	F2-3 up	GO:0030246	Metabolism	Bioenergetics	carbohydrate binding	F	AT4G19840
9686	3	F23 vs. L23	F2-3 up	GO:0030246	Metabolism	Bioenergetics	carbohydrate binding	F	AT4G35010
9687	3	F23 vs. L23	F2-3 up	GO:0030246	Metabolism	Bioenergetics	carbohydrate binding	F	AT5G24080
9688	3	F23 vs. L23	F2-3 up	GO:0016051	Metabolism	Bioenergetics	carbohydrate biosynthetic process	P	AT3G55800
9689	3	F23 vs. L23	F2-3 up	GO:1901135	Metabolism	Bioenergetics	carbohydrate derivative metabolic process	P	AT3G54690
9690	3	F23 vs. L23	F2-3 up	GO:0005975	Metabolism	Bioenergetics	carbohydrate metabolic process	P	AT1G02790
9691	3	F23 vs. L23	F2-3 up	GO:0005975	Metabolism	Bioenergetics	carbohydrate metabolic process	P	AT1G45130
9692	3	F23 vs. L23	F2-3 up	GO:0005975	Metabolism	Bioenergetics	carbohydrate metabolic process	P	AT1G61820
9693	3	F23 vs. L23	F2-3 up	GO:0005975	Metabolism	Bioenergetics	carbohydrate metabolic process	P	AT1G70730
9694	3	F23 vs. L23	F2-3 up	GO:0005975	Metabolism	Bioenergetics	carbohydrate metabolic process	P	AT1G76130
9695	3	F23 vs. L23	F2-3 up	GO:0005975	Metabolism	Bioenergetics	carbohydrate metabolic process	P	AT3G02570
9696	3	F23 vs. L23	F2-3 up	GO:0005975	Metabolism	Bioenergetics	carbohydrate metabolic process	P	AT3G03640
9697	3	F23 vs. L23	F2-3 up	GO:0005975	Metabolism	Bioenergetics	carbohydrate metabolic process	P	AT3G04010
9698	3	F23 vs. L23	F2-3 up	GO:0005975	Metabolism	Bioenergetics	carbohydrate metabolic process	P	AT3G06510
9699	3	F23 vs. L23	F2-3 up	GO:0005975	Metabolism	Bioenergetics	carbohydrate metabolic process	P	AT3G07820
9700	3	F23 vs. L23	F2-3 up	GO:0005975	Metabolism	Bioenergetics	carbohydrate metabolic process	P	AT3G07830
9701	3	F23 vs. L23	F2-3 up	GO:0005975	Metabolism	Bioenergetics	carbohydrate metabolic process	P	AT3G07840
9702	3	F23 vs. L23	F2-3 up	GO:0005975	Metabolism	Bioenergetics	carbohydrate metabolic process	P	AT3G07850
9703	3	F23 vs. L23	F2-3 up	GO:0005975	Metabolism	Bioenergetics	carbohydrate metabolic process	P	AT3G18080
9704	3	F23 vs. L23	F2-3 up	GO:0005975	Metabolism	Bioenergetics	carbohydrate metabolic process	P	AT3G29360
9705	3	F23 vs. L23	F2-3 up	GO:0005975	Metabolism	Bioenergetics	carbohydrate metabolic process	P	AT3G52600
9706	3	F23 vs. L23	F2-3 up	GO:0005975	Metabolism	Bioenergetics	carbohydrate metabolic process	P	AT3G54690
9707	3	F23 vs. L23	F2-3 up	GO:0005975	Metabolism	Bioenergetics	carbohydrate metabolic process	P	AT3G60100
9708	3	F23 vs. L23	F2-3 up	GO:0005975	Metabolism	Bioenergetics	carbohydrate metabolic process	P	AT4G24450
9709	3	F23 vs. L23	F2-3 up	GO:0005975	Metabolism	Bioenergetics	carbohydrate metabolic process	P	AT4G35010
9710	3	F23 vs. L23	F2-3 up	GO:0005975	Metabolism	Bioenergetics	carbohydrate metabolic process	P	AT5G08370
9711	3	F23 vs. L23	F2-3 up	GO:0005975	Metabolism	Bioenergetics	carbohydrate metabolic process	P	AT5G15490
9712	3	F23 vs. L23	F2-3 up	GO:0005975	Metabolism	Bioenergetics	carbohydrate metabolic process	P	AT5G25980
9713	3	F23 vs. L23	F2-3 up	GO:0005975	Metabolism	Bioenergetics	carbohydrate metabolic process	P	AT5G48140
9714	3	F23 vs. L23	F2-3 up	GO:0005975	Metabolism	Bioenergetics	carbohydrate metabolic process	P	AT5G58330
9715	3	F23 vs. L23	F2-3 up	GO:0046835	Metabolism	Bioenergetics	carbohydrate phosphorylation	P	AT3G10700
9716	3	F23 vs. L23	F2-3 up	GO:0044262	Metabolism	Bioenergetics	cellular carbohydrate metabolic process	P	AT5G61410
9717	3	F23 vs. L23	F2-3 up	GO:0045333	Metabolism	Bioenergetics	cellular respiration	P	AT3G22370
9718	3	F23 vs. L23	F2-3 up	GO:0045333	Metabolism	Bioenergetics	cellular respiration	P	ATMG00650
9719	3	F23 vs. L23	F2-3 up	GO:0004108	Metabolism	Bioenergetics	citrate (Si)-synthase activity	F	AT3G60100
9720	3	F23 vs. L23	F2-3 up	GO:0009346	Metabolism	Bioenergetics	citrate lyase complex	C	AT1G09430
9721	3	F23 vs. L23	F2-3 up	GO:0009346	Metabolism	Bioenergetics	citrate lyase complex	C	AT1G60810
9722	3	F23 vs. L23	F2-3 up	GO:0006101	Metabolism	Bioenergetics	citrate metabolic process	P	AT3G60100
9723	3	F23 vs. L23	F2-3 up	GO:0006101	Metabolism	Bioenergetics	citrate metabolic process	P	AT4G26970

9724	3	F23 vs. L23	F2-3 up	GO:0015936	Metabolism	Bioenergetics	coenzyme A metabolic process	P	AT1G76490
9725	3	F23 vs. L23	F2-3 up	GO:0015936	Metabolism	Bioenergetics	coenzyme A metabolic process	P	AT2G17370
9726	3	F23 vs. L23	F2-3 up	GO:0050662	Metabolism	Bioenergetics	coenzyme binding	F	AT1G09340
9727	3	F23 vs. L23	F2-3 up	GO:0050662	Metabolism	Bioenergetics	coenzyme binding	F	AT1G51410
9728	3	F23 vs. L23	F2-3 up	GO:0050662	Metabolism	Bioenergetics	coenzyme binding	F	AT1G76490
9729	3	F23 vs. L23	F2-3 up	GO:0050662	Metabolism	Bioenergetics	coenzyme binding	F	AT2G17370
9730	3	F23 vs. L23	F2-3 up	GO:0050662	Metabolism	Bioenergetics	coenzyme binding	F	AT3G63140
9731	3	F23 vs. L23	F2-3 up	GO:0050662	Metabolism	Bioenergetics	coenzyme binding	F	AT5G42800
9732	3	F23 vs. L23	F2-3 up	GO:0006176	Metabolism	Bioenergetics	dATP biosynthetic process from ADP	P	ATCG00120
9733	3	F23 vs. L23	F2-3 up	GO:0006176	Metabolism	Bioenergetics	dATP biosynthetic process from ADP	P	ATCG00150
9734	3	F23 vs. L23	F2-3 up	GO:0102391	Metabolism	Bioenergetics	decanoate-CoA ligase activity	F	AT4G11030
9735	3	F23 vs. L23	F2-3 up	GO:0009055	Metabolism	Bioenergetics	electron transfer activity	F	AT1G10960
9736	3	F23 vs. L23	F2-3 up	GO:0009055	Metabolism	Bioenergetics	electron transfer activity	F	AT1G22840
9737	3	F23 vs. L23	F2-3 up	GO:0009055	Metabolism	Bioenergetics	electron transfer activity	F	AT1G28480
9738	3	F23 vs. L23	F2-3 up	GO:0009055	Metabolism	Bioenergetics	electron transfer activity	F	AT1G48030
9739	3	F23 vs. L23	F2-3 up	GO:0009055	Metabolism	Bioenergetics	electron transfer activity	F	AT1G50940
9740	3	F23 vs. L23	F2-3 up	GO:0009055	Metabolism	Bioenergetics	electron transfer activity	F	AT1G64500
9741	3	F23 vs. L23	F2-3 up	GO:0009055	Metabolism	Bioenergetics	electron transfer activity	F	AT2G05620
9742	3	F23 vs. L23	F2-3 up	GO:0009055	Metabolism	Bioenergetics	electron transfer activity	F	AT2G42750
9743	3	F23 vs. L23	F2-3 up	GO:0009055	Metabolism	Bioenergetics	electron transfer activity	F	AT3G27380
9744	3	F23 vs. L23	F2-3 up	GO:0009055	Metabolism	Bioenergetics	electron transfer activity	F	AT3G54660
9745	3	F23 vs. L23	F2-3 up	GO:0009055	Metabolism	Bioenergetics	electron transfer activity	F	AT3G62960
9746	3	F23 vs. L23	F2-3 up	GO:0009055	Metabolism	Bioenergetics	electron transfer activity	F	AT4G10040
9747	3	F23 vs. L23	F2-3 up	GO:0009055	Metabolism	Bioenergetics	electron transfer activity	F	AT4G15700
9748	3	F23 vs. L23	F2-3 up	GO:0009055	Metabolism	Bioenergetics	electron transfer activity	F	AT5G18600
9749	3	F23 vs. L23	F2-3 up	GO:0009055	Metabolism	Bioenergetics	electron transfer activity	F	AT5G40650
9750	3	F23 vs. L23	F2-3 up	GO:0022900	Metabolism	Bioenergetics	electron transport chain	P	AT1G10960
9751	3	F23 vs. L23	F2-3 up	GO:0022900	Metabolism	Bioenergetics	electron transport chain	P	AT5G05370
9752	3	F23 vs. L23	F2-3 up	GO:0015990	Metabolism	Bioenergetics	electron transport coupled proton transport	P	ATCG00430
9753	3	F23 vs. L23	F2-3 up	GO:0004174	Metabolism	Bioenergetics	electron-transferring-flavoprotein dehydrogenase activity	F	AT3G44190
9754	3	F23 vs. L23	F2-3 up	GO:0031314	Metabolism	Bioenergetics	extrinsic component of mitochondrial inner membrane	C	AT3G03420
9755	3	F23 vs. L23	F2-3 up	GO:0031314	Metabolism	Bioenergetics	extrinsic component of mitochondrial inner membrane	C	AT4G05020
9756	3	F23 vs. L23	F2-3 up	GO:0001561	Metabolism	Bioenergetics	fatty acid alpha-oxidation	P	AT3G01420
9757	3	F23 vs. L23	F2-3 up	GO:0006635	Metabolism	Bioenergetics	fatty acid beta-oxidation	P	AT3G07560
9758	3	F23 vs. L23	F2-3 up	GO:0033539	Metabolism	Bioenergetics	fatty acid beta-oxidation using acyl-CoA dehydrogenase	P	AT1G50940
9759	3	F23 vs. L23	F2-3 up	GO:0005504	Metabolism	Bioenergetics	fatty acid binding	F	AT3G53980
9760	3	F23 vs. L23	F2-3 up	GO:0006633	Metabolism	Bioenergetics	fatty acid biosynthetic process	P	AT1G08510
9761	3	F23 vs. L23	F2-3 up	GO:0006633	Metabolism	Bioenergetics	fatty acid biosynthetic process	P	AT1G68530
9762	3	F23 vs. L23	F2-3 up	GO:0006633	Metabolism	Bioenergetics	fatty acid biosynthetic process	P	AT3G11170
9763	3	F23 vs. L23	F2-3 up	GO:0006633	Metabolism	Bioenergetics	fatty acid biosynthetic process	P	AT3G16170
9764	3	F23 vs. L23	F2-3 up	GO:0006633	Metabolism	Bioenergetics	fatty acid biosynthetic process	P	AT4G11030
9765	3	F23 vs. L23	F2-3 up	GO:0052722	Metabolism	Bioenergetics	fatty acid in-chain hydroxylase activity	F	AT3G10570
9766	3	F23 vs. L23	F2-3 up	GO:0006631	Metabolism	Bioenergetics	fatty acid metabolic process	P	AT1G01600
9767	3	F23 vs. L23	F2-3 up	GO:0006631	Metabolism	Bioenergetics	fatty acid metabolic process	P	AT1G63710
9768	3	F23 vs. L23	F2-3 up	GO:0006631	Metabolism	Bioenergetics	fatty acid metabolic process	P	AT3G48460
9769	3	F23 vs. L23	F2-3 up	GO:0006631	Metabolism	Bioenergetics	fatty acid metabolic process	P	AT4G00360
9770	3	F23 vs. L23	F2-3 up	GO:0006631	Metabolism	Bioenergetics	fatty acid metabolic process	P	AT4G11030
9771	3	F23 vs. L23	F2-3 up	GO:0006631	Metabolism	Bioenergetics	fatty acid metabolic process	P	AT4G15440
9772	3	F23 vs. L23	F2-3 up	GO:0006631	Metabolism	Bioenergetics	fatty acid metabolic process	P	AT5G36880
9773	3	F23 vs. L23	F2-3 up	GO:0004321	Metabolism	Bioenergetics	fatty-acyl-CoA synthase activity	F	AT5G63380
9774	3	F23 vs. L23	F2-3 up	GO:0006000	Metabolism	Bioenergetics	fructose metabolic process	P	AT3G55800
9775	3	F23 vs. L23	F2-3 up	GO:0006094	Metabolism	Bioenergetics	gluconeogenesis	P	AT2G21330

9776	3	F23 vs. L23	F2-3 up	GO:0006094	Metabolism	Bioenergetics	gluconeogenesis	P	AT3G04120
9777	3	F23 vs. L23	F2-3 up	GO:0006094	Metabolism	Bioenergetics	gluconeogenesis	P	AT3G12780
9778	3	F23 vs. L23	F2-3 up	GO:0006094	Metabolism	Bioenergetics	gluconeogenesis	P	AT3G55800
9779	3	F23 vs. L23	F2-3 up	GO:0006094	Metabolism	Bioenergetics	gluconeogenesis	P	AT4G38970
9780	3	F23 vs. L23	F2-3 up	GO:0006006	Metabolism	Bioenergetics	glucose metabolic process	P	AT1G42970
9781	3	F23 vs. L23	F2-3 up	GO:0006006	Metabolism	Bioenergetics	glucose metabolic process	P	AT1G70730
9782	3	F23 vs. L23	F2-3 up	GO:0006006	Metabolism	Bioenergetics	glucose metabolic process	P	AT3G26650
9783	3	F23 vs. L23	F2-3 up	GO:0006006	Metabolism	Bioenergetics	glucose metabolic process	P	AT3G27300
9784	3	F23 vs. L23	F2-3 up	GO:0008878	Metabolism	Bioenergetics	glucose-1-phosphate adenylyltransferase activity	F	AT5G19220
9785	3	F23 vs. L23	F2-3 up	GO:0008878	Metabolism	Bioenergetics	glucose-1-phosphate adenylyltransferase activity	F	AT5G48300
9786	3	F23 vs. L23	F2-3 up	GO:0004345	Metabolism	Bioenergetics	glucose-6-phosphate dehydrogenase activity	F	AT3G27300
9787	3	F23 vs. L23	F2-3 up	GO:0004352	Metabolism	Bioenergetics	glutamate dehydrogenase (NAD+) activity	F	AT5G07440
9788	3	F23 vs. L23	F2-3 up	GO:0004353	Metabolism	Bioenergetics	glutamate dehydrogenase [NAD(P)+] activity	F	AT5G07440
9789	3	F23 vs. L23	F2-3 up	GO:0016040	Metabolism	Bioenergetics	glutamate synthase (NADH) activity	F	AT5G53460
9790	3	F23 vs. L23	F2-3 up	GO:0043878	Metabolism	Bioenergetics	glyceraldehyde-3-phosphate dehydrogenase (NAD+) (non-phosphorylating) activity	F	AT1G23800
9791	3	F23 vs. L23	F2-3 up	GO:0043878	Metabolism	Bioenergetics	glyceraldehyde-3-phosphate dehydrogenase (NAD+) (non-phosphorylating) activity	F	AT1G44170
9792	3	F23 vs. L23	F2-3 up	GO:0043878	Metabolism	Bioenergetics	glyceraldehyde-3-phosphate dehydrogenase (NAD+) (non-phosphorylating) activity	F	AT3G24503
9793	3	F23 vs. L23	F2-3 up	GO:0043878	Metabolism	Bioenergetics	glyceraldehyde-3-phosphate dehydrogenase (NAD+) (non-phosphorylating) activity	F	AT4G36250
9794	3	F23 vs. L23	F2-3 up	GO:0004365	Metabolism	Bioenergetics	glyceraldehyde-3-phosphate dehydrogenase (NAD+) (phosphorylating) activity	F	AT1G42970
9795	3	F23 vs. L23	F2-3 up	GO:0004365	Metabolism	Bioenergetics	glyceraldehyde-3-phosphate dehydrogenase (NAD+) (phosphorylating) activity	F	AT3G04120
9796	3	F23 vs. L23	F2-3 up	GO:0004365	Metabolism	Bioenergetics	glyceraldehyde-3-phosphate dehydrogenase (NAD+) (phosphorylating) activity	F	AT3G26650
9797	3	F23 vs. L23	F2-3 up	GO:0008886	Metabolism	Bioenergetics	glyceraldehyde-3-phosphate dehydrogenase (NADP+) (non-phosphorylating) activity	F	AT2G24270
9798	3	F23 vs. L23	F2-3 up	GO:0008886	Metabolism	Bioenergetics	glyceraldehyde-3-phosphate dehydrogenase (NADP+) (non-phosphorylating) activity	F	AT3G04120
9799	3	F23 vs. L23	F2-3 up	GO:0047100	Metabolism	Bioenergetics	glyceraldehyde-3-phosphate dehydrogenase (NADP+) (phosphorylating) activity	F	AT1G42970
9800	3	F23 vs. L23	F2-3 up	GO:0047100	Metabolism	Bioenergetics	glyceraldehyde-3-phosphate dehydrogenase (NADP+) (phosphorylating) activity	F	AT3G26650
9801	3	F23 vs. L23	F2-3 up	GO:0004373	Metabolism	Bioenergetics	glycogen (starch) synthase activity	F	AT3G01180
9802	3	F23 vs. L23	F2-3 up	GO:0008891	Metabolism	Bioenergetics	glycolate oxidase activity	F	AT3G14420
9803	3	F23 vs. L23	F2-3 up	GO:0006096	Metabolism	Bioenergetics	glycolytic process	P	AT1G01090
9804	3	F23 vs. L23	F2-3 up	GO:0006096	Metabolism	Bioenergetics	glycolytic process	P	AT2G21330
9805	3	F23 vs. L23	F2-3 up	GO:0006096	Metabolism	Bioenergetics	glycolytic process	P	AT3G04120
9806	3	F23 vs. L23	F2-3 up	GO:0006096	Metabolism	Bioenergetics	glycolytic process	P	AT3G12780
9807	3	F23 vs. L23	F2-3 up	GO:0006096	Metabolism	Bioenergetics	glycolytic process	P	AT3G13930
9808	3	F23 vs. L23	F2-3 up	GO:0006096	Metabolism	Bioenergetics	glycolytic process	P	AT4G38970
9809	3	F23 vs. L23	F2-3 up	GO:0006096	Metabolism	Bioenergetics	glycolytic process	P	AT5G50850
9810	3	F23 vs. L23	F2-3 up	GO:0006096	Metabolism	Bioenergetics	glycolytic process	P	AT5G56350
9811	3	F23 vs. L23	F2-3 up	GO:0006096	Metabolism	Bioenergetics	glycolytic process	P	AT5G63680
9812	3	F23 vs. L23	F2-3 up	GO:0006097	Metabolism	Bioenergetics	glyoxylate cycle	P	AT4G26970
9813	3	F23 vs. L23	F2-3 up	GO:0006097	Metabolism	Bioenergetics	glyoxylate cycle	P	AT5G09660
9814	3	F23 vs. L23	F2-3 up	GO:0030267	Metabolism	Bioenergetics	glyoxylate reductase (NADP) activity	F	AT1G68010
9815	3	F23 vs. L23	F2-3 up	GO:0009514	Metabolism	Bioenergetics	glyoxysome	C	AT5G36880
9816	3	F23 vs. L23	F2-3 up	GO:0030931	Metabolism	Bioenergetics	heterotetrameric ADPG pyrophosphorylase complex	C	AT5G48300
9817	3	F23 vs. L23	F2-3 up	GO:0050505	Metabolism	Bioenergetics	hydroquinone glucosyltransferase activity	F	AT4G01070
9818	3	F23 vs. L23	F2-3 up	GO:0052716	Metabolism	Bioenergetics	hydroquinone:oxygen oxidoreductase activity	F	AT5G60020

9819	3	F23 vs. L23	F2-3 up	GO:0004420	Metabolism	Bioenergetics	hydroxymethylglutaryl-CoA reductase (NADPH) activity	F	AT1G76490
9820	3	F23 vs. L23	F2-3 up	GO:0004420	Metabolism	Bioenergetics	hydroxymethylglutaryl-CoA reductase (NADPH) activity	F	AT2G17370
9821	3	F23 vs. L23	F2-3 up	GO:0042282	Metabolism	Bioenergetics	hydroxymethylglutaryl-CoA reductase activity	F	AT1G76490
9822	3	F23 vs. L23	F2-3 up	GO:0042282	Metabolism	Bioenergetics	hydroxymethylglutaryl-CoA reductase activity	F	AT2G17370
9823	3	F23 vs. L23	F2-3 up	GO:0031305	Metabolism	Bioenergetics	integral component of mitochondrial inner membrane	C	AT4G05590
9824	3	F23 vs. L23	F2-3 up	GO:0031307	Metabolism	Bioenergetics	integral component of mitochondrial outer membrane	C	AT2G38280
9825	3	F23 vs. L23	F2-3 up	GO:0031304	Metabolism	Bioenergetics	intrinsic component of mitochondrial inner membrane	C	AT1G07180
9826	3	F23 vs. L23	F2-3 up	GO:0031304	Metabolism	Bioenergetics	intrinsic component of mitochondrial inner membrane	C	AT2G29990
9827	3	F23 vs. L23	F2-3 up	GO:0004449	Metabolism	Bioenergetics	isocitrate dehydrogenase (NAD+) activity	F	AT3G09810
9828	3	F23 vs. L23	F2-3 up	GO:0006102	Metabolism	Bioenergetics	isocitrate metabolic process	P	AT3G09810
9829	3	F23 vs. L23	F2-3 up	GO:0006102	Metabolism	Bioenergetics	isocitrate metabolic process	P	AT4G26970
9830	3	F23 vs. L23	F2-3 up	GO:0034440	Metabolism	Bioenergetics	lipid oxidation	P	AT1G17420
9831	3	F23 vs. L23	F2-3 up	GO:0034440	Metabolism	Bioenergetics	lipid oxidation	P	AT1G72520
9832	3	F23 vs. L23	F2-3 up	GO:0034440	Metabolism	Bioenergetics	lipid oxidation	P	AT3G22400
9833	3	F23 vs. L23	F2-3 up	GO:0034440	Metabolism	Bioenergetics	lipid oxidation	P	AT3G45140
9834	3	F23 vs. L23	F2-3 up	GO:0030060	Metabolism	Bioenergetics	L-malate dehydrogenase activity	F	AT5G09660
9835	3	F23 vs. L23	F2-3 up	GO:0030060	Metabolism	Bioenergetics	L-malate dehydrogenase activity	F	AT5G58330
9836	3	F23 vs. L23	F2-3 up	GO:0004467	Metabolism	Bioenergetics	long-chain fatty acid-CoA ligase activity	F	AT4G11030
9837	3	F23 vs. L23	F2-3 up	GO:0052853	Metabolism	Bioenergetics	long-chain-(S)-2-hydroxy-long-chain-acid oxidase activity	F	AT3G14420
9838	3	F23 vs. L23	F2-3 up	GO:0047196	Metabolism	Bioenergetics	long-chain-alcohol O-fatty-acyltransferase activity	F	AT5G37300
9839	3	F23 vs. L23	F2-3 up	GO:0047196	Metabolism	Bioenergetics	long-chain-alcohol O-fatty-acyltransferase activity	F	AT5G55340
9840	3	F23 vs. L23	F2-3 up	GO:0046554	Metabolism	Bioenergetics	malate dehydrogenase (NADP+) activity	F	AT5G58330
9841	3	F23 vs. L23	F2-3 up	GO:0016615	Metabolism	Bioenergetics	malate dehydrogenase activity	F	AT5G09660
9842	3	F23 vs. L23	F2-3 up	GO:0006108	Metabolism	Bioenergetics	malate metabolic process	P	AT5G09660
9843	3	F23 vs. L23	F2-3 up	GO:0006108	Metabolism	Bioenergetics	malate metabolic process	P	AT5G58330
9844	3	F23 vs. L23	F2-3 up	GO:0090409	Metabolism	Bioenergetics	malonyl-CoA synthetase activity	F	AT3G16170
9845	3	F23 vs. L23	F2-3 up	GO:0004489	Metabolism	Bioenergetics	methylenetetrahydrofolate reductase (NAD(P)H) activity	F	AT3G59970
9846	3	F23 vs. L23	F2-3 up	GO:0006123	Metabolism	Bioenergetics	mitochondrial electron transport, cytochrome c to oxygen	P	AT1G22840
9847	3	F23 vs. L23	F2-3 up	GO:0006123	Metabolism	Bioenergetics	mitochondrial electron transport, cytochrome c to oxygen	P	AT4G10040
9848	3	F23 vs. L23	F2-3 up	GO:0006121	Metabolism	Bioenergetics	mitochondrial electron transport, succinate to ubiquinone	P	AT3G27380
9849	3	F23 vs. L23	F2-3 up	GO:0006121	Metabolism	Bioenergetics	mitochondrial electron transport, succinate to ubiquinone	P	AT5G40650
9850	3	F23 vs. L23	F2-3 up	GO:0006122	Metabolism	Bioenergetics	mitochondrial electron transport, ubiquinol to cytochrome c	P	AT1G22840
9851	3	F23 vs. L23	F2-3 up	GO:0006122	Metabolism	Bioenergetics	mitochondrial electron transport, ubiquinol to cytochrome c	P	AT4G10040
9852	3	F23 vs. L23	F2-3 up	GO:0005740	Metabolism	Bioenergetics	mitochondrial envelope	C	AT3G04120
9853	3	F23 vs. L23	F2-3 up	GO:0005740	Metabolism	Bioenergetics	mitochondrial envelope	C	AT5G13490
9854	3	F23 vs. L23	F2-3 up	GO:0000266	Metabolism	Bioenergetics	mitochondrial fission	P	AT3G61760
9855	3	F23 vs. L23	F2-3 up	GO:0005743	Metabolism	Bioenergetics	mitochondrial inner membrane	C	AT3G22370
9856	3	F23 vs. L23	F2-3 up	GO:0005743	Metabolism	Bioenergetics	mitochondrial inner membrane	C	AT3G27380
9857	3	F23 vs. L23	F2-3 up	GO:0005743	Metabolism	Bioenergetics	mitochondrial inner membrane	C	AT4G05020
9858	3	F23 vs. L23	F2-3 up	GO:0005743	Metabolism	Bioenergetics	mitochondrial inner membrane	C	AT4G24570
9859	3	F23 vs. L23	F2-3 up	GO:0005743	Metabolism	Bioenergetics	mitochondrial inner membrane	C	AT5G13490
9860	3	F23 vs. L23	F2-3 up	GO:0005743	Metabolism	Bioenergetics	mitochondrial inner membrane	C	AT5G46800

9861	3	F23 vs. L23	F2-3 up	GO:0005758	Metabolism	Bioenergetics	mitochondrial intermembrane space	C	AT1G22840
9862	3	F23 vs. L23	F2-3 up	GO:0005758	Metabolism	Bioenergetics	mitochondrial intermembrane space	C	AT4G10040
9863	3	F23 vs. L23	F2-3 up	GO:0005759	Metabolism	Bioenergetics	mitochondrial matrix	C	AT1G23800
9864	3	F23 vs. L23	F2-3 up	GO:0005759	Metabolism	Bioenergetics	mitochondrial matrix	C	AT1G48030
9865	3	F23 vs. L23	F2-3 up	GO:0005759	Metabolism	Bioenergetics	mitochondrial matrix	C	AT1G50940
9866	3	F23 vs. L23	F2-3 up	GO:0005759	Metabolism	Bioenergetics	mitochondrial matrix	C	AT1G79440
9867	3	F23 vs. L23	F2-3 up	GO:0005759	Metabolism	Bioenergetics	mitochondrial matrix	C	AT2G30970
9868	3	F23 vs. L23	F2-3 up	GO:0005759	Metabolism	Bioenergetics	mitochondrial matrix	C	AT3G13930
9869	3	F23 vs. L23	F2-3 up	GO:0005759	Metabolism	Bioenergetics	mitochondrial matrix	C	AT3G60100
9870	3	F23 vs. L23	F2-3 up	GO:0005759	Metabolism	Bioenergetics	mitochondrial matrix	C	AT5G04140
9871	3	F23 vs. L23	F2-3 up	GO:0005759	Metabolism	Bioenergetics	mitochondrial matrix	C	AT5G09230
9872	3	F23 vs. L23	F2-3 up	GO:0005759	Metabolism	Bioenergetics	mitochondrial matrix	C	AT5G50850
9873	3	F23 vs. L23	F2-3 up	GO:0031966	Metabolism	Bioenergetics	mitochondrial membrane	C	AT3G17611
9874	3	F23 vs. L23	F2-3 up	GO:0031966	Metabolism	Bioenergetics	mitochondrial membrane	C	AT5G40010
9875	3	F23 vs. L23	F2-3 up	GO:0031966	Metabolism	Bioenergetics	mitochondrial membrane	C	ATMG00650
9876	3	F23 vs. L23	F2-3 up	GO:0034982	Metabolism	Bioenergetics	mitochondrial protein processing	P	AT3G03420
9877	3	F23 vs. L23	F2-3 up	GO:0005753	Metabolism	Bioenergetics	mitochondrial proton-transporting ATP synthase complex	C	ATMG00480
9878	3	F23 vs. L23	F2-3 up	GO:0033615	Metabolism	Bioenergetics	mitochondrial proton-transporting ATP synthase complex assembly	P	AT3G03420
9879	3	F23 vs. L23	F2-3 up	GO:0000276	Metabolism	Bioenergetics	mitochondrial proton-transporting ATP synthase complex, coupling factor F(o)	C	AT4G29480
9880	3	F23 vs. L23	F2-3 up	GO:0000276	Metabolism	Bioenergetics	mitochondrial proton-transporting ATP synthase complex, coupling factor F(o)	C	ATMG00480
9881	3	F23 vs. L23	F2-3 up	GO:0006850	Metabolism	Bioenergetics	mitochondrial pyruvate transmembrane transport	P	AT4G05590
9882	3	F23 vs. L23	F2-3 up	GO:0005747	Metabolism	Bioenergetics	mitochondrial respiratory chain complex I	C	AT1G48030
9883	3	F23 vs. L23	F2-3 up	GO:0005747	Metabolism	Bioenergetics	mitochondrial respiratory chain complex I	C	AT3G08610
9884	3	F23 vs. L23	F2-3 up	GO:0005747	Metabolism	Bioenergetics	mitochondrial respiratory chain complex I	C	AT5G47890
9885	3	F23 vs. L23	F2-3 up	GO:0005749	Metabolism	Bioenergetics	mitochondrial respiratory chain complex II, succinate dehydrogenase complex (ubiquinone)	C	AT5G40650
9886	3	F23 vs. L23	F2-3 up	GO:0005750	Metabolism	Bioenergetics	mitochondrial respiratory chain complex III	C	AT5G05370
9887	3	F23 vs. L23	F2-3 up	GO:0033617	Metabolism	Bioenergetics	mitochondrial respiratory chain complex IV assembly	P	AT4G14615
9888	3	F23 vs. L23	F2-3 up	GO:0005739	Metabolism	Bioenergetics	mitochondrion	C	AT1G01090
9889	3	F23 vs. L23	F2-3 up	GO:0005739	Metabolism	Bioenergetics	mitochondrion	C	AT1G01600
9890	3	F23 vs. L23	F2-3 up	GO:0005739	Metabolism	Bioenergetics	mitochondrion	C	AT1G02205
9891	3	F23 vs. L23	F2-3 up	GO:0005739	Metabolism	Bioenergetics	mitochondrion	C	AT1G02390
9892	3	F23 vs. L23	F2-3 up	GO:0005739	Metabolism	Bioenergetics	mitochondrion	C	AT1G06090
9893	3	F23 vs. L23	F2-3 up	GO:0005739	Metabolism	Bioenergetics	mitochondrion	C	AT1G07180
9894	3	F23 vs. L23	F2-3 up	GO:0005739	Metabolism	Bioenergetics	mitochondrion	C	AT1G11840
9895	3	F23 vs. L23	F2-3 up	GO:0005739	Metabolism	Bioenergetics	mitochondrion	C	AT1G11860
9896	3	F23 vs. L23	F2-3 up	GO:0005739	Metabolism	Bioenergetics	mitochondrion	C	AT1G13270
9897	3	F23 vs. L23	F2-3 up	GO:0005739	Metabolism	Bioenergetics	mitochondrion	C	AT1G13970
9898	3	F23 vs. L23	F2-3 up	GO:0005739	Metabolism	Bioenergetics	mitochondrion	C	AT1G14560
9899	3	F23 vs. L23	F2-3 up	GO:0005739	Metabolism	Bioenergetics	mitochondrion	C	AT1G20620
9900	3	F23 vs. L23	F2-3 up	GO:0005739	Metabolism	Bioenergetics	mitochondrion	C	AT1G21000
9901	3	F23 vs. L23	F2-3 up	GO:0005739	Metabolism	Bioenergetics	mitochondrion	C	AT1G21760
9902	3	F23 vs. L23	F2-3 up	GO:0005739	Metabolism	Bioenergetics	mitochondrion	C	AT1G22750
9903	3	F23 vs. L23	F2-3 up	GO:0005739	Metabolism	Bioenergetics	mitochondrion	C	AT1G22840
9904	3	F23 vs. L23	F2-3 up	GO:0005739	Metabolism	Bioenergetics	mitochondrion	C	AT1G27340
9905	3	F23 vs. L23	F2-3 up	GO:0005739	Metabolism	Bioenergetics	mitochondrion	C	AT1G35720
9906	3	F23 vs. L23	F2-3 up	GO:0005739	Metabolism	Bioenergetics	mitochondrion	C	AT1G44000
9907	3	F23 vs. L23	F2-3 up	GO:0005739	Metabolism	Bioenergetics	mitochondrion	C	AT1G48030
9908	3	F23 vs. L23	F2-3 up	GO:0005739	Metabolism	Bioenergetics	mitochondrion	C	AT1G50940
9909	3	F23 vs. L23	F2-3 up	GO:0005739	Metabolism	Bioenergetics	mitochondrion	C	AT1G54280
9910	3	F23 vs. L23	F2-3 up	GO:0005739	Metabolism	Bioenergetics	mitochondrion	C	AT1G58180

9911	3	F23 vs. L23	F2-3 up	GO:0005739	Metabolism	Bioenergetics	mitochondrion	C	AT1G58265
9912	3	F23 vs. L23	F2-3 up	GO:0005739	Metabolism	Bioenergetics	mitochondrion	C	AT1G59870
9913	3	F23 vs. L23	F2-3 up	GO:0005739	Metabolism	Bioenergetics	mitochondrion	C	AT1G63060
9914	3	F23 vs. L23	F2-3 up	GO:0005739	Metabolism	Bioenergetics	mitochondrion	C	AT1G68010
9915	3	F23 vs. L23	F2-3 up	GO:0005739	Metabolism	Bioenergetics	mitochondrion	C	AT1G68530
9916	3	F23 vs. L23	F2-3 up	GO:0005739	Metabolism	Bioenergetics	mitochondrion	C	AT1G68590
9917	3	F23 vs. L23	F2-3 up	GO:0005739	Metabolism	Bioenergetics	mitochondrion	C	AT1G69530
9918	3	F23 vs. L23	F2-3 up	GO:0005739	Metabolism	Bioenergetics	mitochondrion	C	AT1G70310
9919	3	F23 vs. L23	F2-3 up	GO:0005739	Metabolism	Bioenergetics	mitochondrion	C	AT1G70580
9920	3	F23 vs. L23	F2-3 up	GO:0005739	Metabolism	Bioenergetics	mitochondrion	C	AT1G70610
9921	3	F23 vs. L23	F2-3 up	GO:0005739	Metabolism	Bioenergetics	mitochondrion	C	AT1G70730
9922	3	F23 vs. L23	F2-3 up	GO:0005739	Metabolism	Bioenergetics	mitochondrion	C	AT1G70940
9923	3	F23 vs. L23	F2-3 up	GO:0005739	Metabolism	Bioenergetics	mitochondrion	C	AT1G71500
9924	3	F23 vs. L23	F2-3 up	GO:0005739	Metabolism	Bioenergetics	mitochondrion	C	AT1G71770
9925	3	F23 vs. L23	F2-3 up	GO:0005739	Metabolism	Bioenergetics	mitochondrion	C	AT1G72240
9926	3	F23 vs. L23	F2-3 up	GO:0005739	Metabolism	Bioenergetics	mitochondrion	C	AT1G72610
9927	3	F23 vs. L23	F2-3 up	GO:0005739	Metabolism	Bioenergetics	mitochondrion	C	AT1G72730
9928	3	F23 vs. L23	F2-3 up	GO:0005739	Metabolism	Bioenergetics	mitochondrion	C	AT1G73390
9929	3	F23 vs. L23	F2-3 up	GO:0005739	Metabolism	Bioenergetics	mitochondrion	C	AT1G75020
9930	3	F23 vs. L23	F2-3 up	GO:0005739	Metabolism	Bioenergetics	mitochondrion	C	AT1G75500
9931	3	F23 vs. L23	F2-3 up	GO:0005739	Metabolism	Bioenergetics	mitochondrion	C	AT1G75690
9932	3	F23 vs. L23	F2-3 up	GO:0005739	Metabolism	Bioenergetics	mitochondrion	C	AT1G76080
9933	3	F23 vs. L23	F2-3 up	GO:0005739	Metabolism	Bioenergetics	mitochondrion	C	AT1G77090
9934	3	F23 vs. L23	F2-3 up	GO:0005739	Metabolism	Bioenergetics	mitochondrion	C	AT1G78320
9935	3	F23 vs. L23	F2-3 up	GO:0005739	Metabolism	Bioenergetics	mitochondrion	C	AT1G78370
9936	3	F23 vs. L23	F2-3 up	GO:0005739	Metabolism	Bioenergetics	mitochondrion	C	AT1G78570
9937	3	F23 vs. L23	F2-3 up	GO:0005739	Metabolism	Bioenergetics	mitochondrion	C	AT1G79440
9938	3	F23 vs. L23	F2-3 up	GO:0005739	Metabolism	Bioenergetics	mitochondrion	C	AT1G80460
9939	3	F23 vs. L23	F2-3 up	GO:0005739	Metabolism	Bioenergetics	mitochondrion	C	AT1G80660
9940	3	F23 vs. L23	F2-3 up	GO:0005739	Metabolism	Bioenergetics	mitochondrion	C	AT2G01320
9941	3	F23 vs. L23	F2-3 up	GO:0005739	Metabolism	Bioenergetics	mitochondrion	C	AT2G01420
9942	3	F23 vs. L23	F2-3 up	GO:0005739	Metabolism	Bioenergetics	mitochondrion	C	AT2G01520
9943	3	F23 vs. L23	F2-3 up	GO:0005739	Metabolism	Bioenergetics	mitochondrion	C	AT2G04039
9944	3	F23 vs. L23	F2-3 up	GO:0005739	Metabolism	Bioenergetics	mitochondrion	C	AT2G05530
9945	3	F23 vs. L23	F2-3 up	GO:0005739	Metabolism	Bioenergetics	mitochondrion	C	AT2G07708
9946	3	F23 vs. L23	F2-3 up	GO:0005739	Metabolism	Bioenergetics	mitochondrion	C	AT2G10940
9947	3	F23 vs. L23	F2-3 up	GO:0005739	Metabolism	Bioenergetics	mitochondrion	C	AT2G10970
9948	3	F23 vs. L23	F2-3 up	GO:0005739	Metabolism	Bioenergetics	mitochondrion	C	AT2G15620
9949	3	F23 vs. L23	F2-3 up	GO:0005739	Metabolism	Bioenergetics	mitochondrion	C	AT2G15680
9950	3	F23 vs. L23	F2-3 up	GO:0005739	Metabolism	Bioenergetics	mitochondrion	C	AT2G17290
9951	3	F23 vs. L23	F2-3 up	GO:0005739	Metabolism	Bioenergetics	mitochondrion	C	AT2G19330
9952	3	F23 vs. L23	F2-3 up	GO:0005739	Metabolism	Bioenergetics	mitochondrion	C	AT2G19570
9953	3	F23 vs. L23	F2-3 up	GO:0005739	Metabolism	Bioenergetics	mitochondrion	C	AT2G19770
9954	3	F23 vs. L23	F2-3 up	GO:0005739	Metabolism	Bioenergetics	mitochondrion	C	AT2G20470
9955	3	F23 vs. L23	F2-3 up	GO:0005739	Metabolism	Bioenergetics	mitochondrion	C	AT2G21220
9956	3	F23 vs. L23	F2-3 up	GO:0005739	Metabolism	Bioenergetics	mitochondrion	C	AT2G21330
9957	3	F23 vs. L23	F2-3 up	GO:0005739	Metabolism	Bioenergetics	mitochondrion	C	AT2G24180
9958	3	F23 vs. L23	F2-3 up	GO:0005739	Metabolism	Bioenergetics	mitochondrion	C	AT2G25530
9959	3	F23 vs. L23	F2-3 up	GO:0005739	Metabolism	Bioenergetics	mitochondrion	C	AT2G29990
9960	3	F23 vs. L23	F2-3 up	GO:0005739	Metabolism	Bioenergetics	mitochondrion	C	AT2G30970
9961	3	F23 vs. L23	F2-3 up	GO:0005739	Metabolism	Bioenergetics	mitochondrion	C	AT2G35370
9962	3	F23 vs. L23	F2-3 up	GO:0005739	Metabolism	Bioenergetics	mitochondrion	C	AT2G38110
9963	3	F23 vs. L23	F2-3 up	GO:0005739	Metabolism	Bioenergetics	mitochondrion	C	AT2G38400
9964	3	F23 vs. L23	F2-3 up	GO:0005739	Metabolism	Bioenergetics	mitochondrion	C	AT2G40000
9965	3	F23 vs. L23	F2-3 up	GO:0005739	Metabolism	Bioenergetics	mitochondrion	C	AT2G40890
9966	3	F23 vs. L23	F2-3 up	GO:0005739	Metabolism	Bioenergetics	mitochondrion	C	AT2G41380

9967	3	F23 vs. L23	F2-3 up	GO:0005739	Metabolism	Bioenergetics	mitochondrion	C	AT3G01980
9968	3	F23 vs. L23	F2-3 up	GO:0005739	Metabolism	Bioenergetics	mitochondrion	C	AT3G02010
9969	3	F23 vs. L23	F2-3 up	GO:0005739	Metabolism	Bioenergetics	mitochondrion	C	AT3G02780
9970	3	F23 vs. L23	F2-3 up	GO:0005739	Metabolism	Bioenergetics	mitochondrion	C	AT3G03160
9971	3	F23 vs. L23	F2-3 up	GO:0005739	Metabolism	Bioenergetics	mitochondrion	C	AT3G03420
9972	3	F23 vs. L23	F2-3 up	GO:0005739	Metabolism	Bioenergetics	mitochondrion	C	AT3G03430
9973	3	F23 vs. L23	F2-3 up	GO:0005739	Metabolism	Bioenergetics	mitochondrion	C	AT3G04120
9974	3	F23 vs. L23	F2-3 up	GO:0005739	Metabolism	Bioenergetics	mitochondrion	C	AT3G08610
9975	3	F23 vs. L23	F2-3 up	GO:0005739	Metabolism	Bioenergetics	mitochondrion	C	AT3G08730
9976	3	F23 vs. L23	F2-3 up	GO:0005739	Metabolism	Bioenergetics	mitochondrion	C	AT3G09810
9977	3	F23 vs. L23	F2-3 up	GO:0005739	Metabolism	Bioenergetics	mitochondrion	C	AT3G10570
9978	3	F23 vs. L23	F2-3 up	GO:0005739	Metabolism	Bioenergetics	mitochondrion	C	AT3G12780
9979	3	F23 vs. L23	F2-3 up	GO:0005739	Metabolism	Bioenergetics	mitochondrion	C	AT3G13110
9980	3	F23 vs. L23	F2-3 up	GO:0005739	Metabolism	Bioenergetics	mitochondrion	C	AT3G13930
9981	3	F23 vs. L23	F2-3 up	GO:0005739	Metabolism	Bioenergetics	mitochondrion	C	AT3G16170
9982	3	F23 vs. L23	F2-3 up	GO:0005739	Metabolism	Bioenergetics	mitochondrion	C	AT3G16340
9983	3	F23 vs. L23	F2-3 up	GO:0005739	Metabolism	Bioenergetics	mitochondrion	C	AT3G18280
9984	3	F23 vs. L23	F2-3 up	GO:0005739	Metabolism	Bioenergetics	mitochondrion	C	AT3G20190
9985	3	F23 vs. L23	F2-3 up	GO:0005739	Metabolism	Bioenergetics	mitochondrion	C	AT3G20530
9986	3	F23 vs. L23	F2-3 up	GO:0005739	Metabolism	Bioenergetics	mitochondrion	C	AT3G21700
9987	3	F23 vs. L23	F2-3 up	GO:0005739	Metabolism	Bioenergetics	mitochondrion	C	AT3G22370
9988	3	F23 vs. L23	F2-3 up	GO:0005739	Metabolism	Bioenergetics	mitochondrion	C	AT3G23290
9989	3	F23 vs. L23	F2-3 up	GO:0005739	Metabolism	Bioenergetics	mitochondrion	C	AT3G25140
9990	3	F23 vs. L23	F2-3 up	GO:0005739	Metabolism	Bioenergetics	mitochondrion	C	AT3G27380
9991	3	F23 vs. L23	F2-3 up	GO:0005739	Metabolism	Bioenergetics	mitochondrion	C	AT3G46520
9992	3	F23 vs. L23	F2-3 up	GO:0005739	Metabolism	Bioenergetics	mitochondrion	C	AT3G48170
9993	3	F23 vs. L23	F2-3 up	GO:0005739	Metabolism	Bioenergetics	mitochondrion	C	AT3G48990
9994	3	F23 vs. L23	F2-3 up	GO:0005739	Metabolism	Bioenergetics	mitochondrion	C	AT3G52000
9995	3	F23 vs. L23	F2-3 up	GO:0005739	Metabolism	Bioenergetics	mitochondrion	C	AT3G54510
9996	3	F23 vs. L23	F2-3 up	GO:0005739	Metabolism	Bioenergetics	mitochondrion	C	AT3G54660
9997	3	F23 vs. L23	F2-3 up	GO:0005739	Metabolism	Bioenergetics	mitochondrion	C	AT3G55610
9998	3	F23 vs. L23	F2-3 up	GO:0005739	Metabolism	Bioenergetics	mitochondrion	C	AT3G55640
9999	3	F23 vs. L23	F2-3 up	GO:0005739	Metabolism	Bioenergetics	mitochondrion	C	AT3G59530
10000	3	F23 vs. L23	F2-3 up	GO:0005739	Metabolism	Bioenergetics	mitochondrion	C	AT3G60100
10001	3	F23 vs. L23	F2-3 up	GO:0005739	Metabolism	Bioenergetics	mitochondrion	C	AT3G61160
10002	3	F23 vs. L23	F2-3 up	GO:0005739	Metabolism	Bioenergetics	mitochondrion	C	AT4G00880
10003	3	F23 vs. L23	F2-3 up	GO:0005739	Metabolism	Bioenergetics	mitochondrion	C	AT4G02380
10004	3	F23 vs. L23	F2-3 up	GO:0005739	Metabolism	Bioenergetics	mitochondrion	C	AT4G05020
10005	3	F23 vs. L23	F2-3 up	GO:0005739	Metabolism	Bioenergetics	mitochondrion	C	AT4G05590
10006	3	F23 vs. L23	F2-3 up	GO:0005739	Metabolism	Bioenergetics	mitochondrion	C	AT4G10040
10007	3	F23 vs. L23	F2-3 up	GO:0005739	Metabolism	Bioenergetics	mitochondrion	C	AT4G11600
10008	3	F23 vs. L23	F2-3 up	GO:0005739	Metabolism	Bioenergetics	mitochondrion	C	AT4G12030
10009	3	F23 vs. L23	F2-3 up	GO:0005739	Metabolism	Bioenergetics	mitochondrion	C	AT4G12410
10010	3	F23 vs. L23	F2-3 up	GO:0005739	Metabolism	Bioenergetics	mitochondrion	C	AT4G14615
10011	3	F23 vs. L23	F2-3 up	GO:0005739	Metabolism	Bioenergetics	mitochondrion	C	AT4G15620
10012	3	F23 vs. L23	F2-3 up	GO:0005739	Metabolism	Bioenergetics	mitochondrion	C	AT4G20760
10013	3	F23 vs. L23	F2-3 up	GO:0005739	Metabolism	Bioenergetics	mitochondrion	C	AT4G21490
10014	3	F23 vs. L23	F2-3 up	GO:0005739	Metabolism	Bioenergetics	mitochondrion	C	AT4G24380
10015	3	F23 vs. L23	F2-3 up	GO:0005739	Metabolism	Bioenergetics	mitochondrion	C	AT4G24570
10016	3	F23 vs. L23	F2-3 up	GO:0005739	Metabolism	Bioenergetics	mitochondrion	C	AT4G26970
10017	3	F23 vs. L23	F2-3 up	GO:0005739	Metabolism	Bioenergetics	mitochondrion	C	AT4G29480
10018	3	F23 vs. L23	F2-3 up	GO:0005739	Metabolism	Bioenergetics	mitochondrion	C	AT4G31330
10019	3	F23 vs. L23	F2-3 up	GO:0005739	Metabolism	Bioenergetics	mitochondrion	C	AT4G33010
10020	3	F23 vs. L23	F2-3 up	GO:0005739	Metabolism	Bioenergetics	mitochondrion	C	AT4G37430
10021	3	F23 vs. L23	F2-3 up	GO:0005739	Metabolism	Bioenergetics	mitochondrion	C	AT4G39030
10022	3	F23 vs. L23	F2-3 up	GO:0005739	Metabolism	Bioenergetics	mitochondrion	C	AT5G01600

10023	3	F23 vs. L23	F2-3 up	GO:0005739	Metabolism	Bioenergetics	mitochondrion	C	AT5G04140
10024	3	F23 vs. L23	F2-3 up	GO:0005739	Metabolism	Bioenergetics	mitochondrion	C	AT5G05370
10025	3	F23 vs. L23	F2-3 up	GO:0005739	Metabolism	Bioenergetics	mitochondrion	C	AT5G06320
10026	3	F23 vs. L23	F2-3 up	GO:0005739	Metabolism	Bioenergetics	mitochondrion	C	AT5G06530
10027	3	F23 vs. L23	F2-3 up	GO:0005739	Metabolism	Bioenergetics	mitochondrion	C	AT5G07440
10028	3	F23 vs. L23	F2-3 up	GO:0005739	Metabolism	Bioenergetics	mitochondrion	C	AT5G09810
10029	3	F23 vs. L23	F2-3 up	GO:0005739	Metabolism	Bioenergetics	mitochondrion	C	AT5G11070
10030	3	F23 vs. L23	F2-3 up	GO:0005739	Metabolism	Bioenergetics	mitochondrion	C	AT5G13490
10031	3	F23 vs. L23	F2-3 up	GO:0005739	Metabolism	Bioenergetics	mitochondrion	C	AT5G14660
10032	3	F23 vs. L23	F2-3 up	GO:0005739	Metabolism	Bioenergetics	mitochondrion	C	AT5G15910
10033	3	F23 vs. L23	F2-3 up	GO:0005739	Metabolism	Bioenergetics	mitochondrion	C	AT5G17480
10034	3	F23 vs. L23	F2-3 up	GO:0005739	Metabolism	Bioenergetics	mitochondrion	C	AT5G23060
10035	3	F23 vs. L23	F2-3 up	GO:0005739	Metabolism	Bioenergetics	mitochondrion	C	AT5G24120
10036	3	F23 vs. L23	F2-3 up	GO:0005739	Metabolism	Bioenergetics	mitochondrion	C	AT5G24650
10037	3	F23 vs. L23	F2-3 up	GO:0005739	Metabolism	Bioenergetics	mitochondrion	C	AT5G35630
10038	3	F23 vs. L23	F2-3 up	GO:0005739	Metabolism	Bioenergetics	mitochondrion	C	AT5G37300
10039	3	F23 vs. L23	F2-3 up	GO:0005739	Metabolism	Bioenergetics	mitochondrion	C	AT5G38660
10040	3	F23 vs. L23	F2-3 up	GO:0005739	Metabolism	Bioenergetics	mitochondrion	C	AT5G40010
10041	3	F23 vs. L23	F2-3 up	GO:0005739	Metabolism	Bioenergetics	mitochondrion	C	AT5G40650
10042	3	F23 vs. L23	F2-3 up	GO:0005739	Metabolism	Bioenergetics	mitochondrion	C	AT5G43380
10043	3	F23 vs. L23	F2-3 up	GO:0005739	Metabolism	Bioenergetics	mitochondrion	C	AT5G43745
10044	3	F23 vs. L23	F2-3 up	GO:0005739	Metabolism	Bioenergetics	mitochondrion	C	AT5G46800
10045	3	F23 vs. L23	F2-3 up	GO:0005739	Metabolism	Bioenergetics	mitochondrion	C	AT5G47890
10046	3	F23 vs. L23	F2-3 up	GO:0005739	Metabolism	Bioenergetics	mitochondrion	C	AT5G50850
10047	3	F23 vs. L23	F2-3 up	GO:0005739	Metabolism	Bioenergetics	mitochondrion	C	AT5G51720
10048	3	F23 vs. L23	F2-3 up	GO:0005739	Metabolism	Bioenergetics	mitochondrion	C	AT5G54780
10049	3	F23 vs. L23	F2-3 up	GO:0005739	Metabolism	Bioenergetics	mitochondrion	C	AT5G57785
10050	3	F23 vs. L23	F2-3 up	GO:0005739	Metabolism	Bioenergetics	mitochondrion	C	AT5G58330
10051	3	F23 vs. L23	F2-3 up	GO:0005739	Metabolism	Bioenergetics	mitochondrion	C	AT5G58690
10052	3	F23 vs. L23	F2-3 up	GO:0005739	Metabolism	Bioenergetics	mitochondrion	C	AT5G58700
10053	3	F23 vs. L23	F2-3 up	GO:0005739	Metabolism	Bioenergetics	mitochondrion	C	AT5G58770
10054	3	F23 vs. L23	F2-3 up	GO:0005739	Metabolism	Bioenergetics	mitochondrion	C	AT5G59370
10055	3	F23 vs. L23	F2-3 up	GO:0005739	Metabolism	Bioenergetics	mitochondrion	C	AT5G61710
10056	3	F23 vs. L23	F2-3 up	GO:0005739	Metabolism	Bioenergetics	mitochondrion	C	AT5G62520
10057	3	F23 vs. L23	F2-3 up	GO:0005739	Metabolism	Bioenergetics	mitochondrion	C	AT5G62570
10058	3	F23 vs. L23	F2-3 up	GO:0005739	Metabolism	Bioenergetics	mitochondrion	C	AT5G63380
10059	3	F23 vs. L23	F2-3 up	GO:0005739	Metabolism	Bioenergetics	mitochondrion	C	AT5G64400
10060	3	F23 vs. L23	F2-3 up	GO:0005739	Metabolism	Bioenergetics	mitochondrion	C	ATCG00120
10061	3	F23 vs. L23	F2-3 up	GO:0005739	Metabolism	Bioenergetics	mitochondrion	C	ATMG00480
10062	3	F23 vs. L23	F2-3 up	GO:0005739	Metabolism	Bioenergetics	mitochondrion	C	ATMG00650
10063	3	F23 vs. L23	F2-3 up	GO:0007005	Metabolism	Bioenergetics	mitochondrion organization	P	AT5G24650
10064	3	F23 vs. L23	F2-3 up	GO:0051287	Metabolism	Bioenergetics	NAD binding	F	AT1G42970
10065	3	F23 vs. L23	F2-3 up	GO:0051287	Metabolism	Bioenergetics	NAD binding	F	AT1G68010
10066	3	F23 vs. L23	F2-3 up	GO:0051287	Metabolism	Bioenergetics	NAD binding	F	AT1G79440
10067	3	F23 vs. L23	F2-3 up	GO:0051287	Metabolism	Bioenergetics	NAD binding	F	AT3G04120
10068	3	F23 vs. L23	F2-3 up	GO:0051287	Metabolism	Bioenergetics	NAD binding	F	AT3G09810
10069	3	F23 vs. L23	F2-3 up	GO:0051287	Metabolism	Bioenergetics	NAD binding	F	AT3G26650
10070	3	F23 vs. L23	F2-3 up	GO:0051287	Metabolism	Bioenergetics	NAD binding	F	AT3G29360
10071	3	F23 vs. L23	F2-3 up	GO:0051287	Metabolism	Bioenergetics	NAD binding	F	AT5G14200
10072	3	F23 vs. L23	F2-3 up	GO:0051287	Metabolism	Bioenergetics	NAD binding	F	AT5G15490
10073	3	F23 vs. L23	F2-3 up	GO:0051287	Metabolism	Bioenergetics	NAD binding	F	AT5G39320
10074	3	F23 vs. L23	F2-3 up	GO:0051287	Metabolism	Bioenergetics	NAD binding	F	AT5G47650
10075	3	F23 vs. L23	F2-3 up	GO:0008746	Metabolism	Bioenergetics	NAD(P)+ transhydrogenase activity	F	AT5G58330
10076	3	F23 vs. L23	F2-3 up	GO:0010598	Metabolism	Bioenergetics	NAD(P)H dehydrogenase complex (plastoquinone)	C	AT1G15980
10077	3	F23 vs. L23	F2-3 up	GO:0010598	Metabolism	Bioenergetics	NAD(P)H dehydrogenase complex (plastoquinone)	C	AT4G23890
10078	3	F23 vs. L23	F2-3 up	GO:0010598	Metabolism	Bioenergetics	NAD(P)H dehydrogenase complex (plastoquinone)	C	AT5G39210

10079	3	F23 vs. L23	F2-3 up	GO:0010598	Metabolism	Bioenergetics	NAD(P)H dehydrogenase complex (plastoquinone)	C	AT5G58260
10080	3	F23 vs. L23	F2-3 up	GO:0010275	Metabolism	Bioenergetics	NAD(P)H dehydrogenase complex assembly	P	AT5G39210
10081	3	F23 vs. L23	F2-3 up	GO:0016174	Metabolism	Bioenergetics	NAD(P)H oxidase activity	F	AT1G64060
10082	3	F23 vs. L23	F2-3 up	GO:0016174	Metabolism	Bioenergetics	NAD(P)H oxidase activity	F	AT5G47910
10083	3	F23 vs. L23	F2-3 up	GO:0003950	Metabolism	Bioenergetics	NAD+ ADP-ribosyltransferase activity	F	AT5G62520
10084	3	F23 vs. L23	F2-3 up	GO:0070403	Metabolism	Bioenergetics	NAD+ binding	F	AT2G47650
10085	3	F23 vs. L23	F2-3 up	GO:0000210	Metabolism	Bioenergetics	NAD+ diphosphatase activity	F	AT5G47650
10086	3	F23 vs. L23	F2-3 up	GO:0008137	Metabolism	Bioenergetics	NADH dehydrogenase (ubiquinone) activity	F	ATCG00420
10087	3	F23 vs. L23	F2-3 up	GO:0008137	Metabolism	Bioenergetics	NADH dehydrogenase (ubiquinone) activity	F	ATCG00430
10088	3	F23 vs. L23	F2-3 up	GO:0008137	Metabolism	Bioenergetics	NADH dehydrogenase (ubiquinone) activity	F	ATCG01090
10089	3	F23 vs. L23	F2-3 up	GO:0008137	Metabolism	Bioenergetics	NADH dehydrogenase (ubiquinone) activity	F	ATMG00650
10090	3	F23 vs. L23	F2-3 up	GO:0003954	Metabolism	Bioenergetics	NADH dehydrogenase activity	F	AT1G07180
10091	3	F23 vs. L23	F2-3 up	GO:0003954	Metabolism	Bioenergetics	NADH dehydrogenase activity	F	AT4G21490
10092	3	F23 vs. L23	F2-3 up	GO:0003954	Metabolism	Bioenergetics	NADH dehydrogenase activity	F	AT5G15910
10093	3	F23 vs. L23	F2-3 up	GO:0003954	Metabolism	Bioenergetics	NADH dehydrogenase activity	F	ATCG00430
10094	3	F23 vs. L23	F2-3 up	GO:0003954	Metabolism	Bioenergetics	NADH dehydrogenase activity	F	ATCG01100
10095	3	F23 vs. L23	F2-3 up	GO:0003954	Metabolism	Bioenergetics	NADH dehydrogenase activity	F	ATMG00650
10096	3	F23 vs. L23	F2-3 up	GO:0030964	Metabolism	Bioenergetics	NADH dehydrogenase complex	C	ATMG00650
10097	3	F23 vs. L23	F2-3 up	GO:0010258	Metabolism	Bioenergetics	NADH dehydrogenase complex (plastoquinone) assembly	P	AT5G58260
10098	3	F23 vs. L23	F2-3 up	GO:0006734	Metabolism	Bioenergetics	NADH metabolic process	P	AT5G58330
10099	3	F23 vs. L23	F2-3 up	GO:0035529	Metabolism	Bioenergetics	NADH pyrophosphatase activity	F	AT5G47650
10100	3	F23 vs. L23	F2-3 up	GO:0050661	Metabolism	Bioenergetics	NADP binding	F	AT1G42970
10101	3	F23 vs. L23	F2-3 up	GO:0050661	Metabolism	Bioenergetics	NADP binding	F	AT1G58290
10102	3	F23 vs. L23	F2-3 up	GO:0050661	Metabolism	Bioenergetics	NADP binding	F	AT1G62540
10103	3	F23 vs. L23	F2-3 up	GO:0050661	Metabolism	Bioenergetics	NADP binding	F	AT1G62570
10104	3	F23 vs. L23	F2-3 up	GO:0050661	Metabolism	Bioenergetics	NADP binding	F	AT1G64190
10105	3	F23 vs. L23	F2-3 up	GO:0050661	Metabolism	Bioenergetics	NADP binding	F	AT3G04120
10106	3	F23 vs. L23	F2-3 up	GO:0050661	Metabolism	Bioenergetics	NADP binding	F	AT3G27300
10107	3	F23 vs. L23	F2-3 up	GO:0050661	Metabolism	Bioenergetics	NADP binding	F	AT3G54660
10108	3	F23 vs. L23	F2-3 up	GO:0070401	Metabolism	Bioenergetics	NADP+ binding	F	AT2G37760
10109	3	F23 vs. L23	F2-3 up	GO:0070401	Metabolism	Bioenergetics	NADP+ binding	F	AT2G37770
10110	3	F23 vs. L23	F2-3 up	GO:0003959	Metabolism	Bioenergetics	NADPH dehydrogenase activity	F	AT1G20020
10111	3	F23 vs. L23	F2-3 up	GO:0003959	Metabolism	Bioenergetics	NADPH dehydrogenase activity	F	ATCG01090
10112	3	F23 vs. L23	F2-3 up	GO:0008942	Metabolism	Bioenergetics	nitrite reductase [NAD(P)H] activity	F	AT1G71500
10113	3	F23 vs. L23	F2-3 up	GO:0042389	Metabolism	Bioenergetics	omega-3 fatty acid desaturase activity	F	AT2G29980
10114	3	F23 vs. L23	F2-3 up	GO:0042389	Metabolism	Bioenergetics	omega-3 fatty acid desaturase activity	F	AT3G11170
10115	3	F23 vs. L23	F2-3 up	GO:0050203	Metabolism	Bioenergetics	oxalate-CoA ligase activity	F	AT3G48990
10116	3	F23 vs. L23	F2-3 up	GO:0016491	Metabolism	Bioenergetics	oxidoreductase activity	F	AT1G06690
10117	3	F23 vs. L23	F2-3 up	GO:0016491	Metabolism	Bioenergetics	oxidoreductase activity	F	AT1G41830
10118	3	F23 vs. L23	F2-3 up	GO:0016491	Metabolism	Bioenergetics	oxidoreductase activity	F	AT1G50940
10119	3	F23 vs. L23	F2-3 up	GO:0016491	Metabolism	Bioenergetics	oxidoreductase activity	F	AT1G51410
10120	3	F23 vs. L23	F2-3 up	GO:0016491	Metabolism	Bioenergetics	oxidoreductase activity	F	AT1G55570
10121	3	F23 vs. L23	F2-3 up	GO:0016491	Metabolism	Bioenergetics	oxidoreductase activity	F	AT1G57770
10122	3	F23 vs. L23	F2-3 up	GO:0016491	Metabolism	Bioenergetics	oxidoreductase activity	F	AT1G63000
10123	3	F23 vs. L23	F2-3 up	GO:0016491	Metabolism	Bioenergetics	oxidoreductase activity	F	AT1G69640
10124	3	F23 vs. L23	F2-3 up	GO:0016491	Metabolism	Bioenergetics	oxidoreductase activity	F	AT1G78570
10125	3	F23 vs. L23	F2-3 up	GO:0016491	Metabolism	Bioenergetics	oxidoreductase activity	F	AT2G06050
10126	3	F23 vs. L23	F2-3 up	GO:0016491	Metabolism	Bioenergetics	oxidoreductase activity	F	AT2G23910
10127	3	F23 vs. L23	F2-3 up	GO:0016491	Metabolism	Bioenergetics	oxidoreductase activity	F	AT2G34810
10128	3	F23 vs. L23	F2-3 up	GO:0016491	Metabolism	Bioenergetics	oxidoreductase activity	F	AT2G37760
10129	3	F23 vs. L23	F2-3 up	GO:0016491	Metabolism	Bioenergetics	oxidoreductase activity	F	AT2G37770
10130	3	F23 vs. L23	F2-3 up	GO:0016491	Metabolism	Bioenergetics	oxidoreductase activity	F	AT3G13390
10131	3	F23 vs. L23	F2-3 up	GO:0016491	Metabolism	Bioenergetics	oxidoreductase activity	F	AT4G22870
10132	3	F23 vs. L23	F2-3 up	GO:0016491	Metabolism	Bioenergetics	oxidoreductase activity	F	AT4G30470
10133	3	F23 vs. L23	F2-3 up	GO:0016491	Metabolism	Bioenergetics	oxidoreductase activity	F	AT5G16010

10134	3	F23 vs. L23	F2-3 up	GO:0016491	Metabolism	Bioenergetics	oxidoreductase activity	F	AT5G42800
10135	3	F23 vs. L23	F2-3 up	GO:0016491	Metabolism	Bioenergetics	oxidoreductase activity	F	AT5G57800
10136	3	F23 vs. L23	F2-3 up	GO:0016491	Metabolism	Bioenergetics	oxidoreductase activity	F	AT5G60020
10137	3	F23 vs. L23	F2-3 up	GO:0016671	Metabolism	Bioenergetics	oxidoreductase activity, acting on a sulfur group of donors, disulfide as acceptor	F	AT1G50320
10138	3	F23 vs. L23	F2-3 up	GO:0016671	Metabolism	Bioenergetics	oxidoreductase activity, acting on a sulfur group of donors, disulfide as acceptor	F	AT3G15360
10139	3	F23 vs. L23	F2-3 up	GO:0016730	Metabolism	Bioenergetics	oxidoreductase activity, acting on iron-sulfur proteins as donors	F	AT4G11960
10140	3	F23 vs. L23	F2-3 up	GO:0016651	Metabolism	Bioenergetics	oxidoreductase activity, acting on NAD(P)H	F	ATCG01100
10141	3	F23 vs. L23	F2-3 up	GO:0016655	Metabolism	Bioenergetics	oxidoreductase activity, acting on NAD(P)H, quinone or similar compound as acceptor	F	AT5G58260
10142	3	F23 vs. L23	F2-3 up	GO:0016705	Metabolism	Bioenergetics	oxidoreductase activity, acting on paired donors, with incorporation or reduction of molecular oxygen	F	AT1G58265
10143	3	F23 vs. L23	F2-3 up	GO:0016705	Metabolism	Bioenergetics	oxidoreductase activity, acting on paired donors, with incorporation or reduction of molecular oxygen	F	AT3G14620
10144	3	F23 vs. L23	F2-3 up	GO:0016705	Metabolism	Bioenergetics	oxidoreductase activity, acting on paired donors, with incorporation or reduction of molecular oxygen	F	AT3G48520
10145	3	F23 vs. L23	F2-3 up	GO:0016705	Metabolism	Bioenergetics	oxidoreductase activity, acting on paired donors, with incorporation or reduction of molecular oxygen	F	AT4G15440
10146	3	F23 vs. L23	F2-3 up	GO:0016705	Metabolism	Bioenergetics	oxidoreductase activity, acting on paired donors, with incorporation or reduction of molecular oxygen	F	AT4G39480
10147	3	F23 vs. L23	F2-3 up	GO:0016705	Metabolism	Bioenergetics	oxidoreductase activity, acting on paired donors, with incorporation or reduction of molecular oxygen	F	AT4G39510
10148	3	F23 vs. L23	F2-3 up	GO:0016709	Metabolism	Bioenergetics	oxidoreductase activity, acting on paired donors, with incorporation or reduction of molecular oxygen, NAD(P)H as one donor, and incorporation of one atom of oxygen	F	AT1G16410
10149	3	F23 vs. L23	F2-3 up	GO:0016709	Metabolism	Bioenergetics	oxidoreductase activity, acting on paired donors, with incorporation or reduction of molecular oxygen, NAD(P)H as one donor, and incorporation of one atom of oxygen	F	AT2G24180
10150	3	F23 vs. L23	F2-3 up	GO:0016709	Metabolism	Bioenergetics	oxidoreductase activity, acting on paired donors, with incorporation or reduction of molecular oxygen, NAD(P)H as one donor, and incorporation of one atom of oxygen	F	AT2G30490
10151	3	F23 vs. L23	F2-3 up	GO:0016709	Metabolism	Bioenergetics	oxidoreductase activity, acting on paired donors, with incorporation or reduction of molecular oxygen, NAD(P)H as one donor, and incorporation of one atom of oxygen	F	AT2G40890
10152	3	F23 vs. L23	F2-3 up	GO:0016709	Metabolism	Bioenergetics	oxidoreductase activity, acting on paired donors, with incorporation or reduction of molecular oxygen, NAD(P)H as one donor, and incorporation of one atom of oxygen	F	AT3G10570
10153	3	F23 vs. L23	F2-3 up	GO:0016709	Metabolism	Bioenergetics	oxidoreductase activity, acting on paired donors, with incorporation or reduction of molecular oxygen, NAD(P)H as one donor, and incorporation of one atom of oxygen	F	AT3G61880
10154	3	F23 vs. L23	F2-3 up	GO:0016709	Metabolism	Bioenergetics	oxidoreductase activity, acting on paired donors, with incorporation or reduction of molecular oxygen, NAD(P)H as one donor, and incorporation of one atom of oxygen	F	AT4G13770
10155	3	F23 vs. L23	F2-3 up	GO:0016709	Metabolism	Bioenergetics	oxidoreductase activity, acting on paired donors, with incorporation or reduction of molecular oxygen, NAD(P)H as one donor, and incorporation of one atom of oxygen	F	AT4G37430

10156	3	F23 vs. L23	F2-3 up	GO:0016709	Metabolism	Bioenergetics	oxidoreductase activity, acting on paired donors, with incorporation or reduction of molecular oxygen, NAD(P)H as one donor, and incorporation of one atom of oxygen	F	AT5G25120
10157	3	F23 vs. L23	F2-3 up	GO:0016709	Metabolism	Bioenergetics	oxidoreductase activity, acting on paired donors, with incorporation or reduction of molecular oxygen, NAD(P)H as one donor, and incorporation of one atom of oxygen	F	AT5G36220
10158	3	F23 vs. L23	F2-3 up	GO:0016717	Metabolism	Bioenergetics	oxidoreductase activity, acting on paired donors, with oxidation of a pair of donors resulting in the reduction of molecular oxygen to two molecules of water	F	AT1G06090
10159	3	F23 vs. L23	F2-3 up	GO:0016717	Metabolism	Bioenergetics	oxidoreductase activity, acting on paired donors, with oxidation of a pair of donors resulting in the reduction of molecular oxygen to two molecules of water	F	AT2G29980
10160	3	F23 vs. L23	F2-3 up	GO:0016717	Metabolism	Bioenergetics	oxidoreductase activity, acting on paired donors, with oxidation of a pair of donors resulting in the reduction of molecular oxygen to two molecules of water	F	AT2G31360
10161	3	F23 vs. L23	F2-3 up	GO:0016717	Metabolism	Bioenergetics	oxidoreductase activity, acting on paired donors, with oxidation of a pair of donors resulting in the reduction of molecular oxygen to two molecules of water	F	AT3G11170
10162	3	F23 vs. L23	F2-3 up	GO:0016702	Metabolism	Bioenergetics	oxidoreductase activity, acting on single donors with incorporation of molecular oxygen, incorporation of two atoms of oxygen	F	AT3G01420
10163	3	F23 vs. L23	F2-3 up	GO:0016627	Metabolism	Bioenergetics	oxidoreductase activity, acting on the CH-CH group of donors	F	AT5G16010
10164	3	F23 vs. L23	F2-3 up	GO:0016616	Metabolism	Bioenergetics	oxidoreductase activity, acting on the CH-OH group of donors, NAD or NADP as acceptor	F	AT1G51410
10165	3	F23 vs. L23	F2-3 up	GO:0016616	Metabolism	Bioenergetics	oxidoreductase activity, acting on the CH-OH group of donors, NAD or NADP as acceptor	F	AT1G63000
10166	3	F23 vs. L23	F2-3 up	GO:0016616	Metabolism	Bioenergetics	oxidoreductase activity, acting on the CH-OH group of donors, NAD or NADP as acceptor	F	AT2G23910
10167	3	F23 vs. L23	F2-3 up	GO:0016616	Metabolism	Bioenergetics	oxidoreductase activity, acting on the CH-OH group of donors, NAD or NADP as acceptor	F	AT4G30470
10168	3	F23 vs. L23	F2-3 up	GO:0016616	Metabolism	Bioenergetics	oxidoreductase activity, acting on the CH-OH group of donors, NAD or NADP as acceptor	F	AT4G37990
10169	3	F23 vs. L23	F2-3 up	GO:0016616	Metabolism	Bioenergetics	oxidoreductase activity, acting on the CH-OH group of donors, NAD or NADP as acceptor	F	AT5G42800
10170	3	F23 vs. L23	F2-3 up	GO:0006098	Metabolism	Bioenergetics	pentose-phosphate shunt	P	AT2G21330
10171	3	F23 vs. L23	F2-3 up	GO:0006098	Metabolism	Bioenergetics	pentose-phosphate shunt	P	AT4G38970
10172	3	F23 vs. L23	F2-3 up	GO:0009052	Metabolism	Bioenergetics	pentose-phosphate shunt, non-oxidative branch	P	AT2G01290
10173	3	F23 vs. L23	F2-3 up	GO:0009052	Metabolism	Bioenergetics	pentose-phosphate shunt, non-oxidative branch	P	AT3G04790
10174	3	F23 vs. L23	F2-3 up	GO:0009052	Metabolism	Bioenergetics	pentose-phosphate shunt, non-oxidative branch	P	AT5G61410
10175	3	F23 vs. L23	F2-3 up	GO:0009051	Metabolism	Bioenergetics	pentose-phosphate shunt, oxidative branch	P	AT1G64190
10176	3	F23 vs. L23	F2-3 up	GO:0009051	Metabolism	Bioenergetics	pentose-phosphate shunt, oxidative branch	P	AT3G27300
10177	3	F23 vs. L23	F2-3 up	GO:0009853	Metabolism	Bioenergetics	photorespiration	P	AT1G23310
10178	3	F23 vs. L23	F2-3 up	GO:0009853	Metabolism	Bioenergetics	photorespiration	P	AT1G68010
10179	3	F23 vs. L23	F2-3 up	GO:0009853	Metabolism	Bioenergetics	photorespiration	P	AT1G70580
10180	3	F23 vs. L23	F2-3 up	GO:0009853	Metabolism	Bioenergetics	photorespiration	P	AT2G35370
10181	3	F23 vs. L23	F2-3 up	GO:0009853	Metabolism	Bioenergetics	photorespiration	P	AT2G38400
10182	3	F23 vs. L23	F2-3 up	GO:0009853	Metabolism	Bioenergetics	photorespiration	P	AT5G04140
10183	3	F23 vs. L23	F2-3 up	GO:0009853	Metabolism	Bioenergetics	photorespiration	P	AT5G38420
10184	3	F23 vs. L23	F2-3 up	GO:0045272	Metabolism	Bioenergetics	plasma membrane respiratory chain complex I	C	ATCG00430

10185	3	F23 vs. L23	F2-3 up	GO:1903862	Metabolism	Bioenergetics	positive regulation of oxidative phosphorylation	P	AT3G12780
10186	3	F23 vs. L23	F2-3 up	GO:1903942	Metabolism	Bioenergetics	positive regulation of respiratory gaseous exchange	P	AT5G65810
10187	3	F23 vs. L23	F2-3 up	GO:1902600	Metabolism	Bioenergetics	proton transmembrane transport	P	AT1G12840
10188	3	F23 vs. L23	F2-3 up	GO:1902600	Metabolism	Bioenergetics	proton transmembrane transport	P	AT1G32450
10189	3	F23 vs. L23	F2-3 up	GO:1902600	Metabolism	Bioenergetics	proton transmembrane transport	P	AT1G80660
10190	3	F23 vs. L23	F2-3 up	GO:1902600	Metabolism	Bioenergetics	proton transmembrane transport	P	AT2G07560
10191	3	F23 vs. L23	F2-3 up	GO:1902600	Metabolism	Bioenergetics	proton transmembrane transport	P	AT3G08560
10192	3	F23 vs. L23	F2-3 up	GO:1902600	Metabolism	Bioenergetics	proton transmembrane transport	P	AT3G42640
10193	3	F23 vs. L23	F2-3 up	GO:1902600	Metabolism	Bioenergetics	proton transmembrane transport	P	AT3G60330
10194	3	F23 vs. L23	F2-3 up	GO:1902600	Metabolism	Bioenergetics	proton transmembrane transport	P	AT4G25950
10195	3	F23 vs. L23	F2-3 up	GO:1902600	Metabolism	Bioenergetics	proton transmembrane transport	P	AT4G38510
10196	3	F23 vs. L23	F2-3 up	GO:1902600	Metabolism	Bioenergetics	proton transmembrane transport	P	AT5G57350
10197	3	F23 vs. L23	F2-3 up	GO:0008553	Metabolism	Bioenergetics	proton-exporting ATPase activity, phosphorylative mechanism	F	AT1G12840
10198	3	F23 vs. L23	F2-3 up	GO:0008553	Metabolism	Bioenergetics	proton-exporting ATPase activity, phosphorylative mechanism	F	AT1G80660
10199	3	F23 vs. L23	F2-3 up	GO:0008553	Metabolism	Bioenergetics	proton-exporting ATPase activity, phosphorylative mechanism	F	AT2G07560
10200	3	F23 vs. L23	F2-3 up	GO:0008553	Metabolism	Bioenergetics	proton-exporting ATPase activity, phosphorylative mechanism	F	AT3G42640
10201	3	F23 vs. L23	F2-3 up	GO:0008553	Metabolism	Bioenergetics	proton-exporting ATPase activity, phosphorylative mechanism	F	AT3G60330
10202	3	F23 vs. L23	F2-3 up	GO:0008553	Metabolism	Bioenergetics	proton-exporting ATPase activity, phosphorylative mechanism	F	AT4G25950
10203	3	F23 vs. L23	F2-3 up	GO:0008553	Metabolism	Bioenergetics	proton-exporting ATPase activity, phosphorylative mechanism	F	AT5G57350
10204	3	F23 vs. L23	F2-3 up	GO:0046933	Metabolism	Bioenergetics	proton-transporting ATP synthase activity, rotational mechanism	F	AT4G29480
10205	3	F23 vs. L23	F2-3 up	GO:0046933	Metabolism	Bioenergetics	proton-transporting ATP synthase activity, rotational mechanism	F	ATMG00480
10206	3	F23 vs. L23	F2-3 up	GO:0045261	Metabolism	Bioenergetics	proton-transporting ATP synthase complex, catalytic core F(1)	C	ATCG00120
10207	3	F23 vs. L23	F2-3 up	GO:0045263	Metabolism	Bioenergetics	proton-transporting ATP synthase complex, coupling factor F(o)	C	AT4G32260
10208	3	F23 vs. L23	F2-3 up	GO:0045263	Metabolism	Bioenergetics	proton-transporting ATP synthase complex, coupling factor F(o)	C	ATCG00150
10209	3	F23 vs. L23	F2-3 up	GO:0046961	Metabolism	Bioenergetics	proton-transporting ATPase activity, rotational mechanism	F	AT1G12840
10210	3	F23 vs. L23	F2-3 up	GO:0046961	Metabolism	Bioenergetics	proton-transporting ATPase activity, rotational mechanism	F	AT3G08560
10211	3	F23 vs. L23	F2-3 up	GO:0046961	Metabolism	Bioenergetics	proton-transporting ATPase activity, rotational mechanism	F	AT3G58730
10212	3	F23 vs. L23	F2-3 up	GO:0046961	Metabolism	Bioenergetics	proton-transporting ATPase activity, rotational mechanism	F	AT4G32260
10213	3	F23 vs. L23	F2-3 up	GO:0046961	Metabolism	Bioenergetics	proton-transporting ATPase activity, rotational mechanism	F	AT4G34720
10214	3	F23 vs. L23	F2-3 up	GO:0046961	Metabolism	Bioenergetics	proton-transporting ATPase activity, rotational mechanism	F	ATCG00120
10215	3	F23 vs. L23	F2-3 up	GO:0033178	Metabolism	Bioenergetics	proton-transporting two-sector ATPase complex, catalytic domain	C	AT3G08560
10216	3	F23 vs. L23	F2-3 up	GO:0033176	Metabolism	Bioenergetics	proton-transporting V-type ATPase complex	C	AT3G58730
10217	3	F23 vs. L23	F2-3 up	GO:0033180	Metabolism	Bioenergetics	proton-transporting V-type ATPase, V1 domain	C	AT4G38510
10218	3	F23 vs. L23	F2-3 up	GO:0010318	Metabolism	Bioenergetics	pyrophosphate-dependent phosphofructokinase complex, beta-subunit complex	C	AT4G04040
10219	3	F23 vs. L23	F2-3 up	GO:0004739	Metabolism	Bioenergetics	pyruvate dehydrogenase (acetyl-transferring)	F	AT1G01090

10220	3	F23 vs. L23	F2-3 up	GO:0004739	Metabolism	Bioenergetics	pyruvate dehydrogenase (acetyl-transferring)	F	AT5G50850
10221	3	F23 vs. L23	F2-3 up	GO:0045254	Metabolism	Bioenergetics	pyruvate dehydrogenase complex	C	AT3G13930
10222	3	F23 vs. L23	F2-3 up	GO:0004743	Metabolism	Bioenergetics	pyruvate kinase activity	F	AT5G56350
10223	3	F23 vs. L23	F2-3 up	GO:0004743	Metabolism	Bioenergetics	pyruvate kinase activity	F	AT5G63680
10224	3	F23 vs. L23	F2-3 up	GO:0006090	Metabolism	Bioenergetics	pyruvate metabolic process	P	AT4G15530
10225	3	F23 vs. L23	F2-3 up	GO:0019253	Metabolism	Bioenergetics	reductive pentose-phosphate cycle	P	AT1G32060
10226	3	F23 vs. L23	F2-3 up	GO:0019253	Metabolism	Bioenergetics	reductive pentose-phosphate cycle	P	AT1G42970
10227	3	F23 vs. L23	F2-3 up	GO:0019253	Metabolism	Bioenergetics	reductive pentose-phosphate cycle	P	AT3G04790
10228	3	F23 vs. L23	F2-3 up	GO:0019253	Metabolism	Bioenergetics	reductive pentose-phosphate cycle	P	AT3G12780
10229	3	F23 vs. L23	F2-3 up	GO:0019253	Metabolism	Bioenergetics	reductive pentose-phosphate cycle	P	AT3G26650
10230	3	F23 vs. L23	F2-3 up	GO:0019253	Metabolism	Bioenergetics	reductive pentose-phosphate cycle	P	AT3G55800
10231	3	F23 vs. L23	F2-3 up	GO:0019253	Metabolism	Bioenergetics	reductive pentose-phosphate cycle	P	AT3G62410
10232	3	F23 vs. L23	F2-3 up	GO:0019253	Metabolism	Bioenergetics	reductive pentose-phosphate cycle	P	AT5G38420
10233	3	F23 vs. L23	F2-3 up	GO:0043255	Metabolism	Bioenergetics	regulation of carbohydrate biosynthetic process	P	AT1G05850
10234	3	F23 vs. L23	F2-3 up	GO:0043255	Metabolism	Bioenergetics	regulation of carbohydrate biosynthetic process	P	AT1G12840
10235	3	F23 vs. L23	F2-3 up	GO:0006109	Metabolism	Bioenergetics	regulation of carbohydrate metabolic process	P	AT3G15360
10236	3	F23 vs. L23	F2-3 up	GO:0043610	Metabolism	Bioenergetics	regulation of carbohydrate utilization	P	AT5G56860
10237	3	F23 vs. L23	F2-3 up	GO:0031998	Metabolism	Bioenergetics	regulation of fatty acid beta-oxidation	P	AT5G09660
10238	3	F23 vs. L23	F2-3 up	GO:0080093	Metabolism	Bioenergetics	regulation of photorespiration	P	AT5G09660
10239	3	F23 vs. L23	F2-3 up	GO:0070469	Metabolism	Bioenergetics	respirasome	C	AT1G22840
10240	3	F23 vs. L23	F2-3 up	GO:0070469	Metabolism	Bioenergetics	respirasome	C	AT3G22370
10241	3	F23 vs. L23	F2-3 up	GO:0070469	Metabolism	Bioenergetics	respirasome	C	AT4G10040
10242	3	F23 vs. L23	F2-3 up	GO:0070469	Metabolism	Bioenergetics	respirasome	C	ATMG00650
10243	3	F23 vs. L23	F2-3 up	GO:0045271	Metabolism	Bioenergetics	respiratory chain complex I	C	ATCG00430
10244	3	F23 vs. L23	F2-3 up	GO:0045271	Metabolism	Bioenergetics	respiratory chain complex I	C	ATMG00650
10245	3	F23 vs. L23	F2-3 up	GO:0045273	Metabolism	Bioenergetics	respiratory chain complex II	C	AT3G27380
10246	3	F23 vs. L23	F2-3 up	GO:0045273	Metabolism	Bioenergetics	respiratory chain complex II	C	AT5G40650
10247	3	F23 vs. L23	F2-3 up	GO:0022904	Metabolism	Bioenergetics	respiratory electron transport chain	P	AT3G27380
10248	3	F23 vs. L23	F2-3 up	GO:0022904	Metabolism	Bioenergetics	respiratory electron transport chain	P	AT5G40650
10249	3	F23 vs. L23	F2-3 up	GO:0102420	Metabolism	Bioenergetics	sn-1-glycerol-3-phosphate C16:0-DCA-CoA acyl transferase activity	F	AT1G02390
10250	3	F23 vs. L23	F2-3 up	GO:0102419	Metabolism	Bioenergetics	sn-2-glycerol-3-phosphate omega-OH-C22:0-CoA acyl transferase activity	F	AT2G38110
10251	3	F23 vs. L23	F2-3 up	GO:0019252	Metabolism	Bioenergetics	starch biosynthetic process	P	AT3G01180
10252	3	F23 vs. L23	F2-3 up	GO:0019252	Metabolism	Bioenergetics	starch biosynthetic process	P	AT3G55800
10253	3	F23 vs. L23	F2-3 up	GO:0019252	Metabolism	Bioenergetics	starch biosynthetic process	P	AT5G19220
10254	3	F23 vs. L23	F2-3 up	GO:0019252	Metabolism	Bioenergetics	starch biosynthetic process	P	AT5G48300
10255	3	F23 vs. L23	F2-3 up	GO:0005983	Metabolism	Bioenergetics	starch catabolic process	P	AT3G23920
10256	3	F23 vs. L23	F2-3 up	GO:0005983	Metabolism	Bioenergetics	starch catabolic process	P	AT4G15210
10257	3	F23 vs. L23	F2-3 up	GO:0005983	Metabolism	Bioenergetics	starch catabolic process	P	AT4G17090
10258	3	F23 vs. L23	F2-3 up	GO:0009011	Metabolism	Bioenergetics	starch synthase activity	F	AT3G01180
10259	3	F23 vs. L23	F2-3 up	GO:0102218	Metabolism	Bioenergetics	starch, H2O dikinase activity	F	AT4G24450
10260	3	F23 vs. L23	F2-3 up	GO:0008177	Metabolism	Bioenergetics	succinate dehydrogenase (ubiquinone) activity	F	AT3G27380
10261	3	F23 vs. L23	F2-3 up	GO:0008177	Metabolism	Bioenergetics	succinate dehydrogenase (ubiquinone) activity	F	AT5G40650
10262	3	F23 vs. L23	F2-3 up	GO:0045281	Metabolism	Bioenergetics	succinate dehydrogenase complex	C	AT3G27380
10263	3	F23 vs. L23	F2-3 up	GO:0045281	Metabolism	Bioenergetics	succinate dehydrogenase complex	C	AT5G40650
10264	3	F23 vs. L23	F2-3 up	GO:0004777	Metabolism	Bioenergetics	succinate-semialdehyde dehydrogenase (NAD+) activity	F	AT1G79440
10265	3	F23 vs. L23	F2-3 up	GO:0009013	Metabolism	Bioenergetics	succinate-semialdehyde dehydrogenase [NAD(P)+] activity	F	AT1G79440
10266	3	F23 vs. L23	F2-3 up	GO:0005986	Metabolism	Bioenergetics	sucrose biosynthetic process	P	AT1G51420
10267	3	F23 vs. L23	F2-3 up	GO:0005986	Metabolism	Bioenergetics	sucrose biosynthetic process	P	AT2G35840
10268	3	F23 vs. L23	F2-3 up	GO:0005986	Metabolism	Bioenergetics	sucrose biosynthetic process	P	AT3G55800
10269	3	F23 vs. L23	F2-3 up	GO:0005985	Metabolism	Bioenergetics	sucrose metabolic process	P	AT1G12240
10270	3	F23 vs. L23	F2-3 up	GO:0005985	Metabolism	Bioenergetics	sucrose metabolic process	P	AT1G22710
10271	3	F23 vs. L23	F2-3 up	GO:0005985	Metabolism	Bioenergetics	sucrose metabolic process	P	AT3G03250

10272	3	F23 vs. L23	F2-3 up	GO:0046524	Metabolism	Bioenergetics	sucrose-phosphate synthase activity	F	AT4G10120
10273	3	F23 vs. L23	F2-3 up	GO:0042721	Metabolism	Bioenergetics	TIM22 mitochondrial import inner membrane insertion complex	C	AT5G24650
10274	3	F23 vs. L23	F2-3 up	GO:0005744	Metabolism	Bioenergetics	TIM23 mitochondrial import inner membrane translocase complex	C	AT5G24650
10275	3	F23 vs. L23	F2-3 up	GO:0006099	Metabolism	Bioenergetics	tricarboxylic acid cycle	P	AT3G09810
10276	3	F23 vs. L23	F2-3 up	GO:0006099	Metabolism	Bioenergetics	tricarboxylic acid cycle	P	AT3G14940
10277	3	F23 vs. L23	F2-3 up	GO:0006099	Metabolism	Bioenergetics	tricarboxylic acid cycle	P	AT3G27380
10278	3	F23 vs. L23	F2-3 up	GO:0006099	Metabolism	Bioenergetics	tricarboxylic acid cycle	P	AT3G60100
10279	3	F23 vs. L23	F2-3 up	GO:0006099	Metabolism	Bioenergetics	tricarboxylic acid cycle	P	AT4G26970
10280	3	F23 vs. L23	F2-3 up	GO:0006099	Metabolism	Bioenergetics	tricarboxylic acid cycle	P	AT5G09660
10281	3	F23 vs. L23	F2-3 up	GO:0006099	Metabolism	Bioenergetics	tricarboxylic acid cycle	P	AT5G40650
10282	3	F23 vs. L23	F2-3 up	GO:0006099	Metabolism	Bioenergetics	tricarboxylic acid cycle	P	AT5G58330
10283	3	F23 vs. L23	F2-3 up	GO:0035436	Metabolism	Bioenergetics	triose phosphate transmembrane transport	P	AT1G61800
10284	3	F23 vs. L23	F2-3 up	GO:0102721	Metabolism	Bioenergetics	ubiquinol:oxygen oxidoreductase activity	F	AT3G22370
10285	3	F23 vs. L23	F2-3 up	GO:0008121	Metabolism	Bioenergetics	ubiquinol-cytochrome-c reductase activity	F	AT4G03280
10286	3	F23 vs. L23	F2-3 up	GO:0008121	Metabolism	Bioenergetics	ubiquinol-cytochrome-c reductase activity	F	AT5G05370
10287	3	F23 vs. L23	F2-3 up	GO:0006744	Metabolism	Bioenergetics	ubiquinone biosynthetic process	P	AT2G30490
10288	3	F23 vs. L23	F2-3 up	GO:1901006	Metabolism	Bioenergetics	ubiquinone-6 biosynthetic process	P	AT5G15910
10289	3	F23 vs. L23	F2-3 up	GO:0006636	Metabolism	Bioenergetics	unsaturated fatty acid biosynthetic process	P	AT1G06090
10290	3	F23 vs. L23	F2-3 up	GO:0006636	Metabolism	Bioenergetics	unsaturated fatty acid biosynthetic process	P	AT2G29980
10291	3	F23 vs. L23	F2-3 up	GO:0006636	Metabolism	Bioenergetics	unsaturated fatty acid biosynthetic process	P	AT2G31360
10292	3	F23 vs. L23	F2-3 up	GO:0006636	Metabolism	Bioenergetics	unsaturated fatty acid biosynthetic process	P	AT3G11170
10293	3	F23 vs. L23	F2-3 up	GO:0006636	Metabolism	Bioenergetics	unsaturated fatty acid biosynthetic process	P	AT4G27030
10294	3	F23 vs. L23	F2-3 up	GO:0016471	Metabolism	Bioenergetics	vacuolar proton-transporting V-type ATPase complex	C	AT4G25950
10295	3	F23 vs. L23	F2-3 up	GO:0000221	Metabolism	Bioenergetics	vacuolar proton-transporting V-type ATPase, V1 domain	C	AT1G12840
10296	3	F23 vs. L23	F2-3 up	GO:0042761	Metabolism	Bioenergetics	very long-chain fatty acid biosynthetic process	P	AT2G31360
10297	3	F23 vs. L23	F2-3 up	GO:0042761	Metabolism	Bioenergetics	very long-chain fatty acid biosynthetic process	P	AT4G24510
10298	3	F23 vs. L23	F2-3 up	GO:0000038	Metabolism	Bioenergetics	very long-chain fatty acid metabolic process	P	AT1G68530
10299	3	F23 vs. L23	F2-3 up	GO:0102756	Metabolism	Bioenergetics	very-long-chain 3-ketoacyl-CoA synthase activity	F	AT1G68530
10300	3	F23 vs. L23	F2-3 up	GO:0052852	Metabolism	Bioenergetics	very-long-chain-(S)-2-hydroxy-acid oxidase activity	F	AT3G14420
10301	3	F23 vs. L23	F2-3 up	GO:0046185	Metabolism	Catabolism	aldehyde catabolic process	P	AT1G02205
10302	3	F23 vs. L23	F2-3 up	GO:0071771	Metabolism	Catabolism	aldehyde decarbonylase activity	F	AT1G02205
10303	3	F23 vs. L23	F2-3 up	GO:0043447	Metabolism	Catabolism	alkane biosynthetic process	P	AT1G02205
10304	3	F23 vs. L23	F2-3 up	GO:0043447	Metabolism	Catabolism	alkane biosynthetic process	P	AT5G57800
10305	3	F23 vs. L23	F2-3 up	GO:0046556	Metabolism	Catabolism	alpha-L-arabinofuranosidase activity	F	AT5G49360
10306	3	F23 vs. L23	F2-3 up	GO:0031222	Metabolism	Catabolism	arabinan catabolic process	P	AT5G49360
10307	3	F23 vs. L23	F2-3 up	GO:0006527	Metabolism	Catabolism	arginine catabolic process	P	AT4G34710
10308	3	F23 vs. L23	F2-3 up	GO:0004089	Metabolism	Catabolism	carbonate dehydratase activity	F	AT1G58180
10309	3	F23 vs. L23	F2-3 up	GO:0004089	Metabolism	Catabolism	carbonate dehydratase activity	F	AT3G01500
10310	3	F23 vs. L23	F2-3 up	GO:0004089	Metabolism	Catabolism	carbonate dehydratase activity	F	AT3G52720
10311	3	F23 vs. L23	F2-3 up	GO:0004089	Metabolism	Catabolism	carbonate dehydratase activity	F	AT5G14740
10312	3	F23 vs. L23	F2-3 up	GO:0009056	Metabolism	Catabolism	catabolic process	P	AT2G45610
10313	3	F23 vs. L23	F2-3 up	GO:0008219	Metabolism	Catabolism	cell death	P	AT2G01290
10314	3	F23 vs. L23	F2-3 up	GO:0008219	Metabolism	Catabolism	cell death	P	AT3G01420
10315	3	F23 vs. L23	F2-3 up	GO:0008219	Metabolism	Catabolism	cell death	P	AT3G06490
10316	3	F23 vs. L23	F2-3 up	GO:0016998	Metabolism	Catabolism	cell wall macromolecule catabolic process	P	AT1G05850
10317	3	F23 vs. L23	F2-3 up	GO:0016998	Metabolism	Catabolism	cell wall macromolecule catabolic process	P	AT2G43570
10318	3	F23 vs. L23	F2-3 up	GO:0016998	Metabolism	Catabolism	cell wall macromolecule catabolic process	P	AT2G43590
10319	3	F23 vs. L23	F2-3 up	GO:0016998	Metabolism	Catabolism	cell wall macromolecule catabolic process	P	AT3G44990
10320	3	F23 vs. L23	F2-3 up	GO:0030245	Metabolism	Catabolism	cellulose catabolic process	P	AT1G64390
10321	3	F23 vs. L23	F2-3 up	GO:0030245	Metabolism	Catabolism	cellulose catabolic process	P	AT2G32990
10322	3	F23 vs. L23	F2-3 up	GO:0030245	Metabolism	Catabolism	cellulose catabolic process	P	AT5G49720
10323	3	F23 vs. L23	F2-3 up	GO:0006032	Metabolism	Catabolism	chitin catabolic process	P	AT1G05850

10324	3	F23 vs. L23	F2-3 up	GO:0006032	Metabolism	Catabolism	chitin catabolic process	P	AT2G43570
10325	3	F23 vs. L23	F2-3 up	GO:0006032	Metabolism	Catabolism	chitin catabolic process	P	AT2G43590
10326	3	F23 vs. L23	F2-3 up	GO:0015996	Metabolism	Catabolism	chlorophyll catabolic process	P	AT1G44000
10327	3	F23 vs. L23	F2-3 up	GO:0031464	Metabolism	Catabolism	Cul4A-RING E3 ubiquitin ligase complex	C	AT1G61740
10328	3	F23 vs. L23	F2-3 up	GO:0031464	Metabolism	Catabolism	Cul4A-RING E3 ubiquitin ligase complex	C	AT2G25740
10329	3	F23 vs. L23	F2-3 up	GO:0031464	Metabolism	Catabolism	Cul4A-RING E3 ubiquitin ligase complex	C	AT2G36630
10330	3	F23 vs. L23	F2-3 up	GO:0080008	Metabolism	Catabolism	Cul4-RING E3 ubiquitin ligase complex	C	AT1G15470
10331	3	F23 vs. L23	F2-3 up	GO:0080008	Metabolism	Catabolism	Cul4-RING E3 ubiquitin ligase complex	C	AT2G01330
10332	3	F23 vs. L23	F2-3 up	GO:0009440	Metabolism	Catabolism	cyanate catabolic process	P	AT1G58180
10333	3	F23 vs. L23	F2-3 up	GO:0009440	Metabolism	Catabolism	cyanate catabolic process	P	AT3G01500
10334	3	F23 vs. L23	F2-3 up	GO:0009440	Metabolism	Catabolism	cyanate catabolic process	P	AT5G14740
10335	3	F23 vs. L23	F2-3 up	GO:0004197	Metabolism	Catabolism	cysteine-type endopeptidase activity	F	AT1G20850
10336	3	F23 vs. L23	F2-3 up	GO:0004197	Metabolism	Catabolism	cysteine-type endopeptidase activity	F	AT3G19390
10337	3	F23 vs. L23	F2-3 up	GO:0004197	Metabolism	Catabolism	cysteine-type endopeptidase activity	F	AT4G01610
10338	3	F23 vs. L23	F2-3 up	GO:0004197	Metabolism	Catabolism	cysteine-type endopeptidase activity	F	AT4G35350
10339	3	F23 vs. L23	F2-3 up	GO:0004197	Metabolism	Catabolism	cysteine-type endopeptidase activity	F	AT5G45890
10340	3	F23 vs. L23	F2-3 up	GO:0004197	Metabolism	Catabolism	cysteine-type endopeptidase activity	F	AT5G60360
10341	3	F23 vs. L23	F2-3 up	GO:0006216	Metabolism	Catabolism	cytidine catabolic process	P	AT2G19570
10342	3	F23 vs. L23	F2-3 up	GO:0046177	Metabolism	Catabolism	D-gluconate catabolic process	P	AT1G64190
10343	3	F23 vs. L23	F2-3 up	GO:0004175	Metabolism	Catabolism	endopeptidase activity	F	AT2G30440
10344	3	F23 vs. L23	F2-3 up	GO:0004175	Metabolism	Catabolism	endopeptidase activity	F	AT3G17611
10345	3	F23 vs. L23	F2-3 up	GO:0004175	Metabolism	Catabolism	endopeptidase activity	F	AT3G57680
10346	3	F23 vs. L23	F2-3 up	GO:0004521	Metabolism	Catabolism	endoribonuclease activity	F	AT2G02990
10347	3	F23 vs. L23	F2-3 up	GO:0004527	Metabolism	Catabolism	exonuclease activity	F	AT5G54130
10348	3	F23 vs. L23	F2-3 up	GO:0043928	Metabolism	Catabolism	exonucleolytic catabolism of deadenylated mRNA	P	AT5G22250
10349	3	F23 vs. L23	F2-3 up	GO:0019898	Metabolism	Catabolism	extrinsic component of membrane	C	AT1G77090
10350	3	F23 vs. L23	F2-3 up	GO:0019898	Metabolism	Catabolism	extrinsic component of membrane	C	AT2G39470
10351	3	F23 vs. L23	F2-3 up	GO:0010227	Metabolism	Catabolism	floral organ abscission	P	AT4G28490
10352	3	F23 vs. L23	F2-3 up	GO:0080187	Metabolism	Catabolism	floral organ senescence	P	AT5G45890
10353	3	F23 vs. L23	F2-3 up	GO:0019388	Metabolism	Catabolism	galactose catabolic process	P	AT1G09340
10354	3	F23 vs. L23	F2-3 up	GO:0019388	Metabolism	Catabolism	galactose catabolic process	P	AT1G70730
10355	3	F23 vs. L23	F2-3 up	GO:0019388	Metabolism	Catabolism	galactose catabolic process	P	AT3G63140
10356	3	F23 vs. L23	F2-3 up	GO:0033499	Metabolism	Catabolism	galactose catabolic process via UDP-galactose	P	AT1G09340
10357	3	F23 vs. L23	F2-3 up	GO:0033499	Metabolism	Catabolism	galactose catabolic process via UDP-galactose	P	AT3G10700
10358	3	F23 vs. L23	F2-3 up	GO:0033499	Metabolism	Catabolism	galactose catabolic process via UDP-galactose	P	AT3G63140
10359	3	F23 vs. L23	F2-3 up	GO:0009450	Metabolism	Catabolism	gamma-aminobutyric acid catabolic process	P	AT1G79440
10360	3	F23 vs. L23	F2-3 up	GO:0034722	Metabolism	Catabolism	gamma-glutamyl-peptidase activity	F	AT4G30530
10361	3	F23 vs. L23	F2-3 up	GO:0019762	Metabolism	Catabolism	glucosinolate catabolic process	P	AT3G03640
10362	3	F23 vs. L23	F2-3 up	GO:0019762	Metabolism	Catabolism	glucosinolate catabolic process	P	AT5G25980
10363	3	F23 vs. L23	F2-3 up	GO:0006538	Metabolism	Catabolism	glutamate catabolic process	P	AT1G65960
10364	3	F23 vs. L23	F2-3 up	GO:0006751	Metabolism	Catabolism	glutathione catabolic process	P	AT4G31290
10365	3	F23 vs. L23	F2-3 up	GO:0006751	Metabolism	Catabolism	glutathione catabolic process	P	AT4G39640
10366	3	F23 vs. L23	F2-3 up	GO:0019563	Metabolism	Catabolism	glycerol catabolic process	P	AT1G80460
10367	3	F23 vs. L23	F2-3 up	GO:0006546	Metabolism	Catabolism	glycine catabolic process	P	AT4G33010
10368	3	F23 vs. L23	F2-3 up	GO:1902000	Metabolism	Catabolism	homogentisate catabolic process	P	AT2G02390
10369	3	F23 vs. L23	F2-3 up	GO:1902000	Metabolism	Catabolism	homogentisate catabolic process	P	AT5G54080
10370	3	F23 vs. L23	F2-3 up	GO:0032264	Metabolism	Catabolism	IMP salvage	P	AT2G38280
10371	3	F23 vs. L23	F2-3 up	GO:0042344	Metabolism	Catabolism	indole glucosinolate catabolic process	P	AT1G59870
10372	3	F23 vs. L23	F2-3 up	GO:0019310	Metabolism	Catabolism	inositol catabolic process	P	AT4G26260
10373	3	F23 vs. L23	F2-3 up	GO:0042853	Metabolism	Catabolism	L-alanine catabolic process	P	AT1G23310
10374	3	F23 vs. L23	F2-3 up	GO:0042853	Metabolism	Catabolism	L-alanine catabolic process	P	AT1G70580
10375	3	F23 vs. L23	F2-3 up	GO:0060866	Metabolism	Catabolism	leaf abscission	P	AT4G28490
10376	3	F23 vs. L23	F2-3 up	GO:0010150	Metabolism	Catabolism	leaf senescence	P	AT1G32450
10377	3	F23 vs. L23	F2-3 up	GO:0010150	Metabolism	Catabolism	leaf senescence	P	AT2G13810
10378	3	F23 vs. L23	F2-3 up	GO:0010150	Metabolism	Catabolism	leaf senescence	P	AT2G39730
10379	3	F23 vs. L23	F2-3 up	GO:0010150	Metabolism	Catabolism	leaf senescence	P	AT2G43570

10380	3	F23 vs. L23	F2-3 up	GO:0010150	Metabolism	Catabolism	leaf senescence	P	AT3G10985
10381	3	F23 vs. L23	F2-3 up	GO:0010150	Metabolism	Catabolism	leaf senescence	P	AT3G15030
10382	3	F23 vs. L23	F2-3 up	GO:0010150	Metabolism	Catabolism	leaf senescence	P	AT4G02380
10383	3	F23 vs. L23	F2-3 up	GO:0010150	Metabolism	Catabolism	leaf senescence	P	AT4G22750
10384	3	F23 vs. L23	F2-3 up	GO:0010150	Metabolism	Catabolism	leaf senescence	P	AT5G11520
10385	3	F23 vs. L23	F2-3 up	GO:0010150	Metabolism	Catabolism	leaf senescence	P	AT5G24530
10386	3	F23 vs. L23	F2-3 up	GO:0010150	Metabolism	Catabolism	leaf senescence	P	AT5G45890
10387	3	F23 vs. L23	F2-3 up	GO:0010150	Metabolism	Catabolism	leaf senescence	P	AT5G51720
10388	3	F23 vs. L23	F2-3 up	GO:0046274	Metabolism	Catabolism	lignin catabolic process	P	AT2G37040
10389	3	F23 vs. L23	F2-3 up	GO:0046274	Metabolism	Catabolism	lignin catabolic process	P	AT5G60020
10390	3	F23 vs. L23	F2-3 up	GO:0016042	Metabolism	Catabolism	lipid catabolic process	P	AT1G58430
10391	3	F23 vs. L23	F2-3 up	GO:0016042	Metabolism	Catabolism	lipid catabolic process	P	AT1G75880
10392	3	F23 vs. L23	F2-3 up	GO:0016042	Metabolism	Catabolism	lipid catabolic process	P	AT3G08510
10393	3	F23 vs. L23	F2-3 up	GO:0016042	Metabolism	Catabolism	lipid catabolic process	P	AT3G16370
10394	3	F23 vs. L23	F2-3 up	GO:0016042	Metabolism	Catabolism	lipid catabolic process	P	AT3G48460
10395	3	F23 vs. L23	F2-3 up	GO:0016042	Metabolism	Catabolism	lipid catabolic process	P	AT3G53100
10396	3	F23 vs. L23	F2-3 up	GO:0016042	Metabolism	Catabolism	lipid catabolic process	P	AT3G54950
10397	3	F23 vs. L23	F2-3 up	GO:0016042	Metabolism	Catabolism	lipid catabolic process	P	AT4G28780
10398	3	F23 vs. L23	F2-3 up	GO:0016042	Metabolism	Catabolism	lipid catabolic process	P	AT5G33370
10399	3	F23 vs. L23	F2-3 up	GO:0016042	Metabolism	Catabolism	lipid catabolic process	P	AT5G58690
10400	3	F23 vs. L23	F2-3 up	GO:0016042	Metabolism	Catabolism	lipid catabolic process	P	AT5G58700
10401	3	F23 vs. L23	F2-3 up	GO:0019509	Metabolism	Catabolism	L-methionine salvage from methylthioadenosine	P	AT4G14710
10402	3	F23 vs. L23	F2-3 up	GO:0006559	Metabolism	Catabolism	L-phenylalanine catabolic process	P	AT2G37040
10403	3	F23 vs. L23	F2-3 up	GO:0006559	Metabolism	Catabolism	L-phenylalanine catabolic process	P	AT5G36160
10404	3	F23 vs. L23	F2-3 up	GO:0006559	Metabolism	Catabolism	L-phenylalanine catabolic process	P	AT5G54080
10405	3	F23 vs. L23	F2-3 up	GO:0006565	Metabolism	Catabolism	L-serine catabolic process	P	AT4G13930
10406	3	F23 vs. L23	F2-3 up	GO:0005764	Metabolism	Catabolism	lysosome	C	AT1G20850
10407	3	F23 vs. L23	F2-3 up	GO:0005764	Metabolism	Catabolism	lysosome	C	AT3G19390
10408	3	F23 vs. L23	F2-3 up	GO:0005764	Metabolism	Catabolism	lysosome	C	AT4G01610
10409	3	F23 vs. L23	F2-3 up	GO:0005764	Metabolism	Catabolism	lysosome	C	AT4G35350
10410	3	F23 vs. L23	F2-3 up	GO:0005764	Metabolism	Catabolism	lysosome	C	AT5G45890
10411	3	F23 vs. L23	F2-3 up	GO:0005764	Metabolism	Catabolism	lysosome	C	AT5G60360
10412	3	F23 vs. L23	F2-3 up	GO:0090410	Metabolism	Catabolism	malonate catabolic process	P	AT3G16170
10413	3	F23 vs. L23	F2-3 up	GO:0004222	Metabolism	Catabolism	metalloendopeptidase activity	F	AT1G50250
10414	3	F23 vs. L23	F2-3 up	GO:0004222	Metabolism	Catabolism	metalloendopeptidase activity	F	AT2G21960
10415	3	F23 vs. L23	F2-3 up	GO:0004222	Metabolism	Catabolism	metalloendopeptidase activity	F	AT3G03420
10416	3	F23 vs. L23	F2-3 up	GO:0019941	Metabolism	Catabolism	modification-dependent protein catabolic process	P	AT4G02890
10417	3	F23 vs. L23	F2-3 up	GO:1903298	Metabolism	Catabolism	negative regulation of hypoxia-induced intrinsic apoptotic signaling pathway	P	AT4G16660
10418	3	F23 vs. L23	F2-3 up	GO:0043069	Metabolism	Catabolism	negative regulation of programmed cell death	P	AT1G64060
10419	3	F23 vs. L23	F2-3 up	GO:0043069	Metabolism	Catabolism	negative regulation of programmed cell death	P	AT3G57330
10420	3	F23 vs. L23	F2-3 up	GO:0043069	Metabolism	Catabolism	negative regulation of programmed cell death	P	AT5G47910
10421	3	F23 vs. L23	F2-3 up	GO:0033611	Metabolism	Catabolism	oxalate catabolic process	P	AT3G48990
10422	3	F23 vs. L23	F2-3 up	GO:0045490	Metabolism	Catabolism	pectin catabolic process	P	AT2G45220
10423	3	F23 vs. L23	F2-3 up	GO:0045490	Metabolism	Catabolism	pectin catabolic process	P	AT3G05620
10424	3	F23 vs. L23	F2-3 up	GO:0045490	Metabolism	Catabolism	pectin catabolic process	P	AT3G14310
10425	3	F23 vs. L23	F2-3 up	GO:0045490	Metabolism	Catabolism	pectin catabolic process	P	AT3G17060
10426	3	F23 vs. L23	F2-3 up	GO:0045490	Metabolism	Catabolism	pectin catabolic process	P	AT3G24670
10427	3	F23 vs. L23	F2-3 up	GO:0045490	Metabolism	Catabolism	pectin catabolic process	P	AT3G43270
10428	3	F23 vs. L23	F2-3 up	GO:0045490	Metabolism	Catabolism	pectin catabolic process	P	AT3G62170
10429	3	F23 vs. L23	F2-3 up	GO:0045490	Metabolism	Catabolism	pectin catabolic process	P	AT4G28490
10430	3	F23 vs. L23	F2-3 up	GO:0045490	Metabolism	Catabolism	pectin catabolic process	P	AT4G33220
10431	3	F23 vs. L23	F2-3 up	GO:0045490	Metabolism	Catabolism	pectin catabolic process	P	AT5G07430
10432	3	F23 vs. L23	F2-3 up	GO:0045490	Metabolism	Catabolism	pectin catabolic process	P	AT5G53370
10433	3	F23 vs. L23	F2-3 up	GO:0045490	Metabolism	Catabolism	pectin catabolic process	P	AT5G55720
10434	3	F23 vs. L23	F2-3 up	GO:0045490	Metabolism	Catabolism	pectin catabolic process	P	AT5G63180

10435	3	F23 vs. L23	F2-3 up	GO:0019323	Metabolism	Catabolism	pentose catabolic process	P	AT5G61410
10436	3	F23 vs. L23	F2-3 up	GO:0008233	Metabolism	Catabolism	peptidase activity	F	AT2G03200
10437	3	F23 vs. L23	F2-3 up	GO:0008233	Metabolism	Catabolism	peptidase activity	F	AT2G25740
10438	3	F23 vs. L23	F2-3 up	GO:0008233	Metabolism	Catabolism	peptidase activity	F	AT2G38870
10439	3	F23 vs. L23	F2-3 up	GO:0008233	Metabolism	Catabolism	peptidase activity	F	AT4G30530
10440	3	F23 vs. L23	F2-3 up	GO:0004601	Metabolism	Catabolism	peroxidase activity	F	AT1G35720
10441	3	F23 vs. L23	F2-3 up	GO:0004601	Metabolism	Catabolism	peroxidase activity	F	AT1G64060
10442	3	F23 vs. L23	F2-3 up	GO:0004601	Metabolism	Catabolism	peroxidase activity	F	AT2G02390
10443	3	F23 vs. L23	F2-3 up	GO:0004601	Metabolism	Catabolism	peroxidase activity	F	AT2G31570
10444	3	F23 vs. L23	F2-3 up	GO:0004601	Metabolism	Catabolism	peroxidase activity	F	AT2G41480
10445	3	F23 vs. L23	F2-3 up	GO:0004601	Metabolism	Catabolism	peroxidase activity	F	AT3G01420
10446	3	F23 vs. L23	F2-3 up	GO:0004601	Metabolism	Catabolism	peroxidase activity	F	AT3G11630
10447	3	F23 vs. L23	F2-3 up	GO:0004601	Metabolism	Catabolism	peroxidase activity	F	AT3G17790
10448	3	F23 vs. L23	F2-3 up	GO:0004601	Metabolism	Catabolism	peroxidase activity	F	AT3G49110
10449	3	F23 vs. L23	F2-3 up	GO:0004601	Metabolism	Catabolism	peroxidase activity	F	AT4G11600
10450	3	F23 vs. L23	F2-3 up	GO:0004601	Metabolism	Catabolism	peroxidase activity	F	AT5G47000
10451	3	F23 vs. L23	F2-3 up	GO:0004601	Metabolism	Catabolism	peroxidase activity	F	AT5G47910
10452	3	F23 vs. L23	F2-3 up	GO:0004601	Metabolism	Catabolism	peroxidase activity	F	AT5G51890
10453	3	F23 vs. L23	F2-3 up	GO:0005782	Metabolism	Catabolism	peroxisomal matrix	C	AT3G52880
10454	3	F23 vs. L23	F2-3 up	GO:0005778	Metabolism	Catabolism	peroxisomal membrane	C	AT1G76490
10455	3	F23 vs. L23	F2-3 up	GO:0005778	Metabolism	Catabolism	peroxisomal membrane	C	AT2G17370
10456	3	F23 vs. L23	F2-3 up	GO:0005778	Metabolism	Catabolism	peroxisomal membrane	C	AT3G07560
10457	3	F23 vs. L23	F2-3 up	GO:0005777	Metabolism	Catabolism	peroxisome	C	AT1G07180
10458	3	F23 vs. L23	F2-3 up	GO:0005777	Metabolism	Catabolism	peroxisome	C	AT1G09340
10459	3	F23 vs. L23	F2-3 up	GO:0005777	Metabolism	Catabolism	peroxisome	C	AT1G11840
10460	3	F23 vs. L23	F2-3 up	GO:0005777	Metabolism	Catabolism	peroxisome	C	AT1G20620
10461	3	F23 vs. L23	F2-3 up	GO:0005777	Metabolism	Catabolism	peroxisome	C	AT1G23310
10462	3	F23 vs. L23	F2-3 up	GO:0005777	Metabolism	Catabolism	peroxisome	C	AT1G68010
10463	3	F23 vs. L23	F2-3 up	GO:0005777	Metabolism	Catabolism	peroxisome	C	AT1G70580
10464	3	F23 vs. L23	F2-3 up	GO:0005777	Metabolism	Catabolism	peroxisome	C	AT2G06050
10465	3	F23 vs. L23	F2-3 up	GO:0005777	Metabolism	Catabolism	peroxisome	C	AT2G26500
10466	3	F23 vs. L23	F2-3 up	GO:0005777	Metabolism	Catabolism	peroxisome	C	AT2G26740
10467	3	F23 vs. L23	F2-3 up	GO:0005777	Metabolism	Catabolism	peroxisome	C	AT2G29990
10468	3	F23 vs. L23	F2-3 up	GO:0005777	Metabolism	Catabolism	peroxisome	C	AT2G30490
10469	3	F23 vs. L23	F2-3 up	GO:0005777	Metabolism	Catabolism	peroxisome	C	AT2G30520
10470	3	F23 vs. L23	F2-3 up	GO:0005777	Metabolism	Catabolism	peroxisome	C	AT2G30860
10471	3	F23 vs. L23	F2-3 up	GO:0005777	Metabolism	Catabolism	peroxisome	C	AT2G30970
10472	3	F23 vs. L23	F2-3 up	GO:0005777	Metabolism	Catabolism	peroxisome	C	AT2G31570
10473	3	F23 vs. L23	F2-3 up	GO:0005777	Metabolism	Catabolism	peroxisome	C	AT2G31750
10474	3	F23 vs. L23	F2-3 up	GO:0005777	Metabolism	Catabolism	peroxisome	C	AT2G31790
10475	3	F23 vs. L23	F2-3 up	GO:0005777	Metabolism	Catabolism	peroxisome	C	AT3G01980
10476	3	F23 vs. L23	F2-3 up	GO:0005777	Metabolism	Catabolism	peroxisome	C	AT3G14420
10477	3	F23 vs. L23	F2-3 up	GO:0005777	Metabolism	Catabolism	peroxisome	C	AT3G20530
10478	3	F23 vs. L23	F2-3 up	GO:0005777	Metabolism	Catabolism	peroxisome	C	AT3G48170
10479	3	F23 vs. L23	F2-3 up	GO:0005777	Metabolism	Catabolism	peroxisome	C	AT3G52880
10480	3	F23 vs. L23	F2-3 up	GO:0005777	Metabolism	Catabolism	peroxisome	C	AT4G05020
10481	3	F23 vs. L23	F2-3 up	GO:0005777	Metabolism	Catabolism	peroxisome	C	AT4G14880
10482	3	F23 vs. L23	F2-3 up	GO:0005777	Metabolism	Catabolism	peroxisome	C	AT5G09660
10483	3	F23 vs. L23	F2-3 up	GO:0005777	Metabolism	Catabolism	peroxisome	C	AT5G11520
10484	3	F23 vs. L23	F2-3 up	GO:0005777	Metabolism	Catabolism	peroxisome	C	AT5G25980
10485	3	F23 vs. L23	F2-3 up	GO:0005777	Metabolism	Catabolism	peroxisome	C	AT5G63380
10486	3	F23 vs. L23	F2-3 up	GO:0009395	Metabolism	Catabolism	phospholipid catabolic process	P	AT3G48610
10487	3	F23 vs. L23	F2-3 up	GO:0090693	Metabolism	Catabolism	plant organ senescence	P	AT5G56860
10488	3	F23 vs. L23	F2-3 up	GO:0000272	Metabolism	Catabolism	polysaccharide catabolic process	P	AT1G09340
10489	3	F23 vs. L23	F2-3 up	GO:0000272	Metabolism	Catabolism	polysaccharide catabolic process	P	AT2G43570
10490	3	F23 vs. L23	F2-3 up	GO:0000272	Metabolism	Catabolism	polysaccharide catabolic process	P	AT2G43590

10491	3	F23 vs. L23	F2-3 up	GO:0043085	Metabolism	Catabolism	positive regulation of catalytic activity	P	AT1G50320
10492	3	F23 vs. L23	F2-3 up	GO:0043085	Metabolism	Catabolism	positive regulation of catalytic activity	P	AT3G15360
10493	3	F23 vs. L23	F2-3 up	GO:0010623	Metabolism	Catabolism	programmed cell death involved in cell development	P	AT1G20850
10494	3	F23 vs. L23	F2-3 up	GO:0010623	Metabolism	Catabolism	programmed cell death involved in cell development	P	AT4G35350
10495	3	F23 vs. L23	F2-3 up	GO:0000502	Metabolism	Catabolism	proteasome complex	C	AT3G23050
10496	3	F23 vs. L23	F2-3 up	GO:0043161	Metabolism	Catabolism	proteasome-mediated ubiquitin-dependent protein catabolic process	P	AT1G56500
10497	3	F23 vs. L23	F2-3 up	GO:0051865	Metabolism	Catabolism	protein autoubiquitination	P	AT3G46620
10498	3	F23 vs. L23	F2-3 up	GO:0030163	Metabolism	Catabolism	protein catabolic process	P	AT1G03230
10499	3	F23 vs. L23	F2-3 up	GO:0030163	Metabolism	Catabolism	protein catabolic process	P	AT1G62290
10500	3	F23 vs. L23	F2-3 up	GO:0030163	Metabolism	Catabolism	protein catabolic process	P	AT1G65240
10501	3	F23 vs. L23	F2-3 up	GO:0030163	Metabolism	Catabolism	protein catabolic process	P	AT2G03200
10502	3	F23 vs. L23	F2-3 up	GO:0030163	Metabolism	Catabolism	protein catabolic process	P	AT4G04460
10503	3	F23 vs. L23	F2-3 up	GO:0030163	Metabolism	Catabolism	protein catabolic process	P	AT5G36260
10504	3	F23 vs. L23	F2-3 up	GO:0016558	Metabolism	Catabolism	protein import into peroxisome matrix	P	AT3G07560
10505	3	F23 vs. L23	F2-3 up	GO:0000209	Metabolism	Catabolism	protein polyubiquitination	P	AT1G56500
10506	3	F23 vs. L23	F2-3 up	GO:0016567	Metabolism	Catabolism	protein ubiquitination	P	AT1G20823
10507	3	F23 vs. L23	F2-3 up	GO:0016567	Metabolism	Catabolism	protein ubiquitination	P	AT1G21760
10508	3	F23 vs. L23	F2-3 up	GO:0016567	Metabolism	Catabolism	protein ubiquitination	P	AT1G29340
10509	3	F23 vs. L23	F2-3 up	GO:0016567	Metabolism	Catabolism	protein ubiquitination	P	AT1G61740
10510	3	F23 vs. L23	F2-3 up	GO:0016567	Metabolism	Catabolism	protein ubiquitination	P	AT2G24540
10511	3	F23 vs. L23	F2-3 up	GO:0016567	Metabolism	Catabolism	protein ubiquitination	P	AT2G25740
10512	3	F23 vs. L23	F2-3 up	GO:0016567	Metabolism	Catabolism	protein ubiquitination	P	AT2G30520
10513	3	F23 vs. L23	F2-3 up	GO:0016567	Metabolism	Catabolism	protein ubiquitination	P	AT2G36630
10514	3	F23 vs. L23	F2-3 up	GO:0016567	Metabolism	Catabolism	protein ubiquitination	P	AT3G46620
10515	3	F23 vs. L23	F2-3 up	GO:0016567	Metabolism	Catabolism	protein ubiquitination	P	AT4G02890
10516	3	F23 vs. L23	F2-3 up	GO:0016567	Metabolism	Catabolism	protein ubiquitination	P	AT4G03190
10517	3	F23 vs. L23	F2-3 up	GO:0016567	Metabolism	Catabolism	protein ubiquitination	P	AT5G64660
10518	3	F23 vs. L23	F2-3 up	GO:0016567	Metabolism	Catabolism	protein ubiquitination	P	AT5G67480
10519	3	F23 vs. L23	F2-3 up	GO:0032991	Metabolism	Catabolism	protein-containing complex	C	AT3G62410
10520	3	F23 vs. L23	F2-3 up	GO:0006508	Metabolism	Catabolism	proteolysis	P	AT1G03230
10521	3	F23 vs. L23	F2-3 up	GO:0006508	Metabolism	Catabolism	proteolysis	P	AT1G13270
10522	3	F23 vs. L23	F2-3 up	GO:0006508	Metabolism	Catabolism	proteolysis	P	AT1G50250
10523	3	F23 vs. L23	F2-3 up	GO:0006508	Metabolism	Catabolism	proteolysis	P	AT1G52580
10524	3	F23 vs. L23	F2-3 up	GO:0006508	Metabolism	Catabolism	proteolysis	P	AT1G62290
10525	3	F23 vs. L23	F2-3 up	GO:0006508	Metabolism	Catabolism	proteolysis	P	AT1G65240
10526	3	F23 vs. L23	F2-3 up	GO:0006508	Metabolism	Catabolism	proteolysis	P	AT2G03200
10527	3	F23 vs. L23	F2-3 up	GO:0006508	Metabolism	Catabolism	proteolysis	P	AT2G21960
10528	3	F23 vs. L23	F2-3 up	GO:0006508	Metabolism	Catabolism	proteolysis	P	AT2G25740
10529	3	F23 vs. L23	F2-3 up	GO:0006508	Metabolism	Catabolism	proteolysis	P	AT2G38870
10530	3	F23 vs. L23	F2-3 up	GO:0006508	Metabolism	Catabolism	proteolysis	P	AT3G17611
10531	3	F23 vs. L23	F2-3 up	GO:0006508	Metabolism	Catabolism	proteolysis	P	AT4G00230
10532	3	F23 vs. L23	F2-3 up	GO:0006508	Metabolism	Catabolism	proteolysis	P	AT4G01610
10533	3	F23 vs. L23	F2-3 up	GO:0006508	Metabolism	Catabolism	proteolysis	P	AT4G04460
10534	3	F23 vs. L23	F2-3 up	GO:0006508	Metabolism	Catabolism	proteolysis	P	AT4G30530
10535	3	F23 vs. L23	F2-3 up	GO:0006508	Metabolism	Catabolism	proteolysis	P	AT5G04710
10536	3	F23 vs. L23	F2-3 up	GO:0006508	Metabolism	Catabolism	proteolysis	P	AT5G36260
10537	3	F23 vs. L23	F2-3 up	GO:0006508	Metabolism	Catabolism	proteolysis	P	AT5G60360
10538	3	F23 vs. L23	F2-3 up	GO:0006508	Metabolism	Catabolism	proteolysis	P	AT5G65760
10539	3	F23 vs. L23	F2-3 up	GO:0051603	Metabolism	Catabolism	proteolysis involved in cellular protein catabolic process	P	AT1G20850
10540	3	F23 vs. L23	F2-3 up	GO:0051603	Metabolism	Catabolism	proteolysis involved in cellular protein catabolic process	P	AT3G17180

10541	3	F23 vs. L23	F2-3 up	GO:0051603	Metabolism	Catabolism	proteolysis involved in cellular protein catabolic process	P	AT3G19390
10542	3	F23 vs. L23	F2-3 up	GO:0051603	Metabolism	Catabolism	proteolysis involved in cellular protein catabolic process	P	AT3G45010
10543	3	F23 vs. L23	F2-3 up	GO:0051603	Metabolism	Catabolism	proteolysis involved in cellular protein catabolic process	P	AT3G52000
10544	3	F23 vs. L23	F2-3 up	GO:0051603	Metabolism	Catabolism	proteolysis involved in cellular protein catabolic process	P	AT4G01610
10545	3	F23 vs. L23	F2-3 up	GO:0051603	Metabolism	Catabolism	proteolysis involved in cellular protein catabolic process	P	AT4G35350
10546	3	F23 vs. L23	F2-3 up	GO:0051603	Metabolism	Catabolism	proteolysis involved in cellular protein catabolic process	P	AT5G08260
10547	3	F23 vs. L23	F2-3 up	GO:0051603	Metabolism	Catabolism	proteolysis involved in cellular protein catabolic process	P	AT5G45890
10548	3	F23 vs. L23	F2-3 up	GO:0051603	Metabolism	Catabolism	proteolysis involved in cellular protein catabolic process	P	AT5G60360
10549	3	F23 vs. L23	F2-3 up	GO:0010304	Metabolism	Catabolism	PSII associated light-harvesting complex II catabolic process	P	AT1G50250
10550	3	F23 vs. L23	F2-3 up	GO:0052692	Metabolism	Catabolism	raffinose alpha-galactosidase activity	F	AT5G08370
10551	3	F23 vs. L23	F2-3 up	GO:0006401	Metabolism	Catabolism	RNA catabolic process	P	AT2G02990
10552	3	F23 vs. L23	F2-3 up	GO:0046244	Metabolism	Catabolism	salicylic acid catabolic process	P	AT2G37040
10553	3	F23 vs. L23	F2-3 up	GO:0031146	Metabolism	Catabolism	SCF-dependent proteasomal ubiquitin-dependent protein catabolic process	P	AT1G21760
10554	3	F23 vs. L23	F2-3 up	GO:0031146	Metabolism	Catabolism	SCF-dependent proteasomal ubiquitin-dependent protein catabolic process	P	AT1G27340
10555	3	F23 vs. L23	F2-3 up	GO:0010282	Metabolism	Catabolism	senescence-associated vacuole	C	AT5G45890
10556	3	F23 vs. L23	F2-3 up	GO:0004185	Metabolism	Catabolism	serine-type carboxypeptidase activity	F	AT3G17180
10557	3	F23 vs. L23	F2-3 up	GO:0004185	Metabolism	Catabolism	serine-type carboxypeptidase activity	F	AT3G45010
10558	3	F23 vs. L23	F2-3 up	GO:0004185	Metabolism	Catabolism	serine-type carboxypeptidase activity	F	AT3G52000
10559	3	F23 vs. L23	F2-3 up	GO:0004185	Metabolism	Catabolism	serine-type carboxypeptidase activity	F	AT5G08260
10560	3	F23 vs. L23	F2-3 up	GO:0004252	Metabolism	Catabolism	serine-type endopeptidase activity	F	AT1G52580
10561	3	F23 vs. L23	F2-3 up	GO:0004252	Metabolism	Catabolism	serine-type endopeptidase activity	F	AT2G30440
10562	3	F23 vs. L23	F2-3 up	GO:0004252	Metabolism	Catabolism	serine-type endopeptidase activity	F	AT3G17611
10563	3	F23 vs. L23	F2-3 up	GO:0004252	Metabolism	Catabolism	serine-type endopeptidase activity	F	AT4G00230
10564	3	F23 vs. L23	F2-3 up	GO:0030149	Metabolism	Catabolism	sphingolipid catabolic process	P	AT1G27980
10565	3	F23 vs. L23	F2-3 up	GO:0036459	Metabolism	Catabolism	thiol-dependent ubiquitinyl hydrolase activity	F	AT5G22030
10566	3	F23 vs. L23	F2-3 up	GO:0019441	Metabolism	Catabolism	tryptophan catabolic process to kynurenine	P	AT2G40000
10567	3	F23 vs. L23	F2-3 up	GO:0006572	Metabolism	Catabolism	tyrosine catabolic process	P	AT5G36160
10568	3	F23 vs. L23	F2-3 up	GO:0006572	Metabolism	Catabolism	tyrosine catabolic process	P	AT5G54080
10569	3	F23 vs. L23	F2-3 up	GO:0000151	Metabolism	Catabolism	ubiquitin ligase complex	C	AT3G08990
10570	3	F23 vs. L23	F2-3 up	GO:0061630	Metabolism	Catabolism	ubiquitin protein ligase activity	F	AT1G20823
10571	3	F23 vs. L23	F2-3 up	GO:0061630	Metabolism	Catabolism	ubiquitin protein ligase activity	F	AT1G56500
10572	3	F23 vs. L23	F2-3 up	GO:0061630	Metabolism	Catabolism	ubiquitin protein ligase activity	F	AT3G46620
10573	3	F23 vs. L23	F2-3 up	GO:0061630	Metabolism	Catabolism	ubiquitin protein ligase activity	F	AT5G66070
10574	3	F23 vs. L23	F2-3 up	GO:0031625	Metabolism	Catabolism	ubiquitin protein ligase binding	F	AT3G53420
10575	3	F23 vs. L23	F2-3 up	GO:0031625	Metabolism	Catabolism	ubiquitin protein ligase binding	F	AT4G02890
10576	3	F23 vs. L23	F2-3 up	GO:0006511	Metabolism	Catabolism	ubiquitin-dependent protein catabolic process	P	AT4G02890
10577	3	F23 vs. L23	F2-3 up	GO:0006511	Metabolism	Catabolism	ubiquitin-dependent protein catabolic process	P	AT4G03190
10578	3	F23 vs. L23	F2-3 up	GO:0006511	Metabolism	Catabolism	ubiquitin-dependent protein catabolic process	P	AT5G22030
10579	3	F23 vs. L23	F2-3 up	GO:0004842	Metabolism	Catabolism	ubiquitin-protein transferase activity	F	AT1G27340
10580	3	F23 vs. L23	F2-3 up	GO:0004842	Metabolism	Catabolism	ubiquitin-protein transferase activity	F	AT1G29340
10581	3	F23 vs. L23	F2-3 up	GO:0004842	Metabolism	Catabolism	ubiquitin-protein transferase activity	F	AT3G46620
10582	3	F23 vs. L23	F2-3 up	GO:0004842	Metabolism	Catabolism	ubiquitin-protein transferase activity	F	AT4G03190
10583	3	F23 vs. L23	F2-3 up	GO:0004842	Metabolism	Catabolism	ubiquitin-protein transferase activity	F	AT5G64660
10584	3	F23 vs. L23	F2-3 up	GO:0006212	Metabolism	Catabolism	uracil catabolic process	P	AT5G64370
10585	3	F23 vs. L23	F2-3 up	GO:0042178	Metabolism	Catabolism	xenobiotic catabolic process	P	AT4G01070

10586	3	F23 vs. L23	F2-3 up	GO:0009044	Metabolism	Catabolism	xylan 1,4-beta-xylosidase activity	F	AT5G47000
10587	3	F23 vs. L23	F2-3 up	GO:0009044	Metabolism	Catabolism	xylan 1,4-beta-xylosidase activity	F	AT5G49360
10588	3	F23 vs. L23	F2-3 up	GO:0045493	Metabolism	Catabolism	xylan catabolic process	P	AT5G49360
10589	3	F23 vs. L23	F2-3 up	GO:0047504	Metabolism	Metabolism	(-)-menthol dehydrogenase activity	F	AT3G61220
10590	3	F23 vs. L23	F2-3 up	GO:0047501	Metabolism	Metabolism	(+)-neomenthol dehydrogenase activity	F	AT3G61220
10591	3	F23 vs. L23	F2-3 up	GO:0034768	Metabolism	Metabolism	(E)-beta-ocimene synthase activity	F	AT2G24210
10592	3	F23 vs. L23	F2-3 up	GO:1902609	Metabolism	Metabolism	(R)-2-hydroxy-alpha-linolenic acid biosynthetic process	P	AT3G01420
10593	3	F23 vs. L23	F2-3 up	GO:0102165	Metabolism	Metabolism	(Z)-3-hexen-1-ol acetyltransferase activity	F	AT3G03480
10594	3	F23 vs. L23	F2-3 up	GO:0016629	Metabolism	Metabolism	12-oxophytodienoate reductase activity	F	AT2G06050
10595	3	F23 vs. L23	F2-3 up	GO:0009979	Metabolism	Metabolism	16:0 monogalactosyldiacylglycerol desaturase activity	F	AT1G06090
10596	3	F23 vs. L23	F2-3 up	GO:0009979	Metabolism	Metabolism	16:0 monogalactosyldiacylglycerol desaturase activity	F	AT2G31360
10597	3	F23 vs. L23	F2-3 up	GO:0003841	Metabolism	Metabolism	1-acylglycerol-3-phosphate O-acyltransferase activity	F	AT1G75020
10598	3	F23 vs. L23	F2-3 up	GO:0009815	Metabolism	Metabolism	1-aminocyclopropane-1-carboxylate oxidase activity	F	AT1G05010
10599	3	F23 vs. L23	F2-3 up	GO:0009815	Metabolism	Metabolism	1-aminocyclopropane-1-carboxylate oxidase activity	F	AT1G62380
10600	3	F23 vs. L23	F2-3 up	GO:0009815	Metabolism	Metabolism	1-aminocyclopropane-1-carboxylate oxidase activity	F	AT2G30830
10601	3	F23 vs. L23	F2-3 up	GO:0016847	Metabolism	Metabolism	1-aminocyclopropane-1-carboxylate synthase activity	F	AT4G11280
10602	3	F23 vs. L23	F2-3 up	GO:0005545	Metabolism	Metabolism	1-phosphatidylinositol binding	F	AT1G03050
10603	3	F23 vs. L23	F2-3 up	GO:0051537	Metabolism	Metabolism	2 iron, 2 sulfur cluster binding	F	AT1G10960
10604	3	F23 vs. L23	F2-3 up	GO:0051537	Metabolism	Metabolism	2 iron, 2 sulfur cluster binding	F	AT1G71500
10605	3	F23 vs. L23	F2-3 up	GO:0051537	Metabolism	Metabolism	2 iron, 2 sulfur cluster binding	F	AT2G24820
10606	3	F23 vs. L23	F2-3 up	GO:0051537	Metabolism	Metabolism	2 iron, 2 sulfur cluster binding	F	AT3G27380
10607	3	F23 vs. L23	F2-3 up	GO:0051537	Metabolism	Metabolism	2 iron, 2 sulfur cluster binding	F	AT4G03280
10608	3	F23 vs. L23	F2-3 up	GO:0051537	Metabolism	Metabolism	2 iron, 2 sulfur cluster binding	F	AT4G15700
10609	3	F23 vs. L23	F2-3 up	GO:0051537	Metabolism	Metabolism	2 iron, 2 sulfur cluster binding	F	AT5G18600
10610	3	F23 vs. L23	F2-3 up	GO:0051537	Metabolism	Metabolism	2 iron, 2 sulfur cluster binding	F	AT5G40650
10611	3	F23 vs. L23	F2-3 up	GO:0051537	Metabolism	Metabolism	2 iron, 2 sulfur cluster binding	F	AT5G51720
10612	3	F23 vs. L23	F2-3 up	GO:0016706	Metabolism	Metabolism	2-oxoglutarate-dependent dioxygenase activity	F	AT1G06640
10613	3	F23 vs. L23	F2-3 up	GO:0051538	Metabolism	Metabolism	3 iron, 4 sulfur cluster binding	F	AT3G27380
10614	3	F23 vs. L23	F2-3 up	GO:0051538	Metabolism	Metabolism	3 iron, 4 sulfur cluster binding	F	AT5G04140
10615	3	F23 vs. L23	F2-3 up	GO:0051538	Metabolism	Metabolism	3 iron, 4 sulfur cluster binding	F	AT5G40650
10616	3	F23 vs. L23	F2-3 up	GO:0051538	Metabolism	Metabolism	3 iron, 4 sulfur cluster binding	F	AT5G53460
10617	3	F23 vs. L23	F2-3 up	GO:0008686	Metabolism	Metabolism	3,4-dihydroxy-2-butanone-4-phosphate synthase activity	F	AT2G22450
10618	3	F23 vs. L23	F2-3 up	GO:0008408	Metabolism	Metabolism	3'-5' exonuclease activity	F	AT5G22250
10619	3	F23 vs. L23	F2-3 up	GO:0000175	Metabolism	Metabolism	3'-5'-exoribonuclease activity	F	AT5G54130
10620	3	F23 vs. L23	F2-3 up	GO:0004028	Metabolism	Metabolism	3-chloroallyl aldehyde dehydrogenase activity	F	AT1G44170
10621	3	F23 vs. L23	F2-3 up	GO:0004028	Metabolism	Metabolism	3-chloroallyl aldehyde dehydrogenase activity	F	AT4G36250
10622	3	F23 vs. L23	F2-3 up	GO:0003849	Metabolism	Metabolism	3-deoxy-7-phosphoheptulonate synthase activity	F	AT1G22410
10623	3	F23 vs. L23	F2-3 up	GO:0003861	Metabolism	Metabolism	3-isopropylmalate dehydratase activity	F	AT2G43100
10624	3	F23 vs. L23	F2-3 up	GO:0003861	Metabolism	Metabolism	3-isopropylmalate dehydratase activity	F	AT3G58990
10625	3	F23 vs. L23	F2-3 up	GO:0003862	Metabolism	Metabolism	3-isopropylmalate dehydrogenase activity	F	AT5G14200
10626	3	F23 vs. L23	F2-3 up	GO:0080102	Metabolism	Metabolism	3-methylthiopropyl glucosinolate S-oxygenase activity	F	AT1G62540
10627	3	F23 vs. L23	F2-3 up	GO:0080066	Metabolism	Metabolism	3-methylthiopropyl-desulfoglucosinolate sulfotransferase activity	F	AT1G74090
10628	3	F23 vs. L23	F2-3 up	GO:0051539	Metabolism	Metabolism	4 iron, 4 sulfur cluster binding	F	AT2G15620
10629	3	F23 vs. L23	F2-3 up	GO:0051539	Metabolism	Metabolism	4 iron, 4 sulfur cluster binding	F	AT2G29630
10630	3	F23 vs. L23	F2-3 up	GO:0051539	Metabolism	Metabolism	4 iron, 4 sulfur cluster binding	F	AT3G27380
10631	3	F23 vs. L23	F2-3 up	GO:0051539	Metabolism	Metabolism	4 iron, 4 sulfur cluster binding	F	AT4G15620
10632	3	F23 vs. L23	F2-3 up	GO:0051539	Metabolism	Metabolism	4 iron, 4 sulfur cluster binding	F	AT4G15630
10633	3	F23 vs. L23	F2-3 up	GO:0051539	Metabolism	Metabolism	4 iron, 4 sulfur cluster binding	F	AT4G21990

10634	3	F23 vs. L23	F2-3 up	GO:0051539	Metabolism	Metabolism	4 iron, 4 sulfur cluster binding	F	AT4G25040
10635	3	F23 vs. L23	F2-3 up	GO:0051539	Metabolism	Metabolism	4 iron, 4 sulfur cluster binding	F	AT4G26970
10636	3	F23 vs. L23	F2-3 up	GO:0051539	Metabolism	Metabolism	4 iron, 4 sulfur cluster binding	F	AT5G40650
10637	3	F23 vs. L23	F2-3 up	GO:0051539	Metabolism	Metabolism	4 iron, 4 sulfur cluster binding	F	AT5G54980
10638	3	F23 vs. L23	F2-3 up	GO:0051539	Metabolism	Metabolism	4 iron, 4 sulfur cluster binding	F	AT5G60600
10639	3	F23 vs. L23	F2-3 up	GO:0051539	Metabolism	Metabolism	4 iron, 4 sulfur cluster binding	F	ATCG00430
10640	3	F23 vs. L23	F2-3 up	GO:0051539	Metabolism	Metabolism	4 iron, 4 sulfur cluster binding	F	ATCG01090
10641	3	F23 vs. L23	F2-3 up	GO:0046429	Metabolism	Metabolism	4-hydroxy-3-methylbut-2-en-1-yl diphosphate synthase activity	F	AT5G60600
10642	3	F23 vs. L23	F2-3 up	GO:0047443	Metabolism	Metabolism	4-hydroxy-4-methyl-2-oxoglutarate aldolase activity	F	AT3G02770
10643	3	F23 vs. L23	F2-3 up	GO:0047443	Metabolism	Metabolism	4-hydroxy-4-methyl-2-oxoglutarate aldolase activity	F	AT5G16450
10644	3	F23 vs. L23	F2-3 up	GO:0008839	Metabolism	Metabolism	4-hydroxy-tetrahydrodipicolinate reductase	F	AT5G52100
10645	3	F23 vs. L23	F2-3 up	GO:0080067	Metabolism	Metabolism	4-methylthiobutyl-desulfoglucosinolate sulfotransferase activity	F	AT1G74090
10646	3	F23 vs. L23	F2-3 up	GO:0080103	Metabolism	Metabolism	4-methylthiopropyl glucosinolate S-oxygenase activity	F	AT1G62540
10647	3	F23 vs. L23	F2-3 up	GO:0080103	Metabolism	Metabolism	4-methylthiopropyl glucosinolate S-oxygenase activity	F	AT1G62570
10648	3	F23 vs. L23	F2-3 up	GO:0080081	Metabolism	Metabolism	4-methylumbelliferyl-beta-D-glucopyranoside beta-glucosidase activity	F	AT3G18080
10649	3	F23 vs. L23	F2-3 up	GO:0080068	Metabolism	Metabolism	5-methylthiopentyl-desulfoglucosinolate sulfotransferase activity	F	AT1G74090
10650	3	F23 vs. L23	F2-3 up	GO:0080104	Metabolism	Metabolism	5-methylthiopropyl glucosinolate S-oxygenase activity	F	AT1G62540
10651	3	F23 vs. L23	F2-3 up	GO:0080069	Metabolism	Metabolism	7-methylthioheptyl-desulfoglucosinolate sulfotransferase activity	F	AT1G74090
10652	3	F23 vs. L23	F2-3 up	GO:0080106	Metabolism	Metabolism	7-methylthiopropyl glucosinolate S-oxygenase activity	F	AT1G62540
10653	3	F23 vs. L23	F2-3 up	GO:0080070	Metabolism	Metabolism	8-methylthiooctyl-desulfoglucosinolate sulfotransferase activity	F	AT1G74090
10654	3	F23 vs. L23	F2-3 up	GO:0080107	Metabolism	Metabolism	8-methylthiopropyl glucosinolate S-oxygenase activity	F	AT1G62540
10655	3	F23 vs. L23	F2-3 up	GO:0080107	Metabolism	Metabolism	8-methylthiopropyl glucosinolate S-oxygenase activity	F	AT1G62570
10656	3	F23 vs. L23	F2-3 up	GO:0102490	Metabolism	Metabolism	8-oxo-dGTP phosphohydrolase activity	F	AT5G18280
10657	3	F23 vs. L23	F2-3 up	GO:0006083	Metabolism	Metabolism	acetate metabolic process	P	AT5G36880
10658	3	F23 vs. L23	F2-3 up	GO:0008126	Metabolism	Metabolism	acetylesterase activity	F	AT3G48460
10659	3	F23 vs. L23	F2-3 up	GO:0016407	Metabolism	Metabolism	acetyltransferase activity	F	AT3G13930
10660	3	F23 vs. L23	F2-3 up	GO:0016881	Metabolism	Metabolism	acid-amino acid ligase activity	F	AT2G23170
10661	3	F23 vs. L23	F2-3 up	GO:0016881	Metabolism	Metabolism	acid-amino acid ligase activity	F	AT5G54510
10662	3	F23 vs. L23	F2-3 up	GO:0010309	Metabolism	Metabolism	acireductone dioxygenase [iron(II)-requiring] activity	F	AT4G14710
10663	3	F23 vs. L23	F2-3 up	GO:0003994	Metabolism	Metabolism	aconitate hydratase activity	F	AT4G26970
10664	3	F23 vs. L23	F2-3 up	GO:0003779	Metabolism	Metabolism	actin binding	F	AT4G25590
10665	3	F23 vs. L23	F2-3 up	GO:0003779	Metabolism	Metabolism	actin binding	F	AT5G52360
10666	3	F23 vs. L23	F2-3 up	GO:0003779	Metabolism	Metabolism	actin binding	F	AT5G67470
10667	3	F23 vs. L23	F2-3 up	GO:0051015	Metabolism	Metabolism	actin filament binding	F	AT1G01780
10668	3	F23 vs. L23	F2-3 up	GO:0051015	Metabolism	Metabolism	actin filament binding	F	AT1G21980
10669	3	F23 vs. L23	F2-3 up	GO:0051015	Metabolism	Metabolism	actin filament binding	F	AT2G01330
10670	3	F23 vs. L23	F2-3 up	GO:0051015	Metabolism	Metabolism	actin filament binding	F	AT2G45800
10671	3	F23 vs. L23	F2-3 up	GO:0051015	Metabolism	Metabolism	actin filament binding	F	AT3G61230
10672	3	F23 vs. L23	F2-3 up	GO:0051015	Metabolism	Metabolism	actin filament binding	F	AT4G38510
10673	3	F23 vs. L23	F2-3 up	GO:0051017	Metabolism	Metabolism	actin filament bundle assembly	P	AT1G01780
10674	3	F23 vs. L23	F2-3 up	GO:0051017	Metabolism	Metabolism	actin filament bundle assembly	P	AT1G64500
10675	3	F23 vs. L23	F2-3 up	GO:0051017	Metabolism	Metabolism	actin filament bundle assembly	P	AT2G45800
10676	3	F23 vs. L23	F2-3 up	GO:0051017	Metabolism	Metabolism	actin filament bundle assembly	P	AT3G61230
10677	3	F23 vs. L23	F2-3 up	GO:0051017	Metabolism	Metabolism	actin filament bundle assembly	P	AT4G38510

10678	3	F23 vs. L23	F2-3 up	GO:0051693	Metabolism	Metabolism	actin filament capping	P	AT4G38510
10679	3	F23 vs. L23	F2-3 up	GO:0030042	Metabolism	Metabolism	actin filament depolymerization	P	AT2G01330
10680	3	F23 vs. L23	F2-3 up	GO:0030042	Metabolism	Metabolism	actin filament depolymerization	P	AT4G25590
10681	3	F23 vs. L23	F2-3 up	GO:0030042	Metabolism	Metabolism	actin filament depolymerization	P	AT5G52360
10682	3	F23 vs. L23	F2-3 up	GO:0007015	Metabolism	Metabolism	actin filament organization	P	AT1G54560
10683	3	F23 vs. L23	F2-3 up	GO:0030048	Metabolism	Metabolism	actin filament-based movement	P	AT1G54560
10684	3	F23 vs. L23	F2-3 up	GO:0003785	Metabolism	Metabolism	actin monomer binding	F	AT1G21980
10685	3	F23 vs. L23	F2-3 up	GO:0003785	Metabolism	Metabolism	actin monomer binding	F	AT2G19770
10686	3	F23 vs. L23	F2-3 up	GO:0003785	Metabolism	Metabolism	actin monomer binding	F	AT4G29340
10687	3	F23 vs. L23	F2-3 up	GO:0003785	Metabolism	Metabolism	actin monomer binding	F	AT4G29350
10688	3	F23 vs. L23	F2-3 up	GO:0008154	Metabolism	Metabolism	actin polymerization or depolymerization	P	AT4G29350
10689	3	F23 vs. L23	F2-3 up	GO:0000036	Metabolism	Metabolism	acyl carrier activity	F	AT1G08510
10690	3	F23 vs. L23	F2-3 up	GO:0016297	Metabolism	Metabolism	acyl-[acyl-carrier-protein] hydrolase activity	F	AT1G08510
10691	3	F23 vs. L23	F2-3 up	GO:0047372	Metabolism	Metabolism	acylglycerol lipase activity	F	AT2G39420
10692	3	F23 vs. L23	F2-3 up	GO:0004013	Metabolism	Metabolism	adenosylhomocysteinase activity	F	AT3G23810
10693	3	F23 vs. L23	F2-3 up	GO:0004014	Metabolism	Metabolism	adenosylmethionine decarboxylase activity	F	AT3G02470
10694	3	F23 vs. L23	F2-3 up	GO:0004014	Metabolism	Metabolism	adenosylmethionine decarboxylase activity	F	AT5G15950
10695	3	F23 vs. L23	F2-3 up	GO:0004020	Metabolism	Metabolism	adenylylsulfate kinase activity	F	AT2G14750
10696	3	F23 vs. L23	F2-3 up	GO:0004020	Metabolism	Metabolism	adenylylsulfate kinase activity	F	AT4G39940
10697	3	F23 vs. L23	F2-3 up	GO:0009973	Metabolism	Metabolism	adenylyl-sulfate reductase activity	F	AT4G21990
10698	3	F23 vs. L23	F2-3 up	GO:0008453	Metabolism	Metabolism	alanine-glyoxylate transaminase activity	F	AT1G23310
10699	3	F23 vs. L23	F2-3 up	GO:0008453	Metabolism	Metabolism	alanine-glyoxylate transaminase activity	F	AT1G70580
10700	3	F23 vs. L23	F2-3 up	GO:0008453	Metabolism	Metabolism	alanine-glyoxylate transaminase activity	F	AT2G38400
10701	3	F23 vs. L23	F2-3 up	GO:1990465	Metabolism	Metabolism	aldehyde oxygenase (deformylating) activity	F	AT5G57800
10702	3	F23 vs. L23	F2-3 up	GO:0018685	Metabolism	Metabolism	alkane 1-monooxygenase activity	F	AT1G01600
10703	3	F23 vs. L23	F2-3 up	GO:0018685	Metabolism	Metabolism	alkane 1-monooxygenase activity	F	AT1G63710
10704	3	F23 vs. L23	F2-3 up	GO:0018685	Metabolism	Metabolism	alkane 1-monooxygenase activity	F	AT4G00360
10705	3	F23 vs. L23	F2-3 up	GO:0046423	Metabolism	Metabolism	allene-oxide cyclase activity	F	AT3G25760
10706	3	F23 vs. L23	F2-3 up	GO:0033201	Metabolism	Metabolism	alpha-1,4-glucan synthase activity	F	AT3G01180
10707	3	F23 vs. L23	F2-3 up	GO:0004557	Metabolism	Metabolism	alpha-galactosidase activity	F	AT5G08370
10708	3	F23 vs. L23	F2-3 up	GO:0009308	Metabolism	Metabolism	amine metabolic process	P	AT1G31490
10709	3	F23 vs. L23	F2-3 up	GO:0016843	Metabolism	Metabolism	amine-lyase activity	F	AT5G01410
10710	3	F23 vs. L23	F2-3 up	GO:0016597	Metabolism	Metabolism	amino acid binding	F	AT1G76990
10711	3	F23 vs. L23	F2-3 up	GO:0016597	Metabolism	Metabolism	amino acid binding	F	AT4G13930
10712	3	F23 vs. L23	F2-3 up	GO:0004046	Metabolism	Metabolism	aminoacylase activity	F	AT1G44820
10713	3	F23 vs. L23	F2-3 up	GO:0004047	Metabolism	Metabolism	aminomethyltransferase activity	F	AT1G11860
10714	3	F23 vs. L23	F2-3 up	GO:0019676	Metabolism	Metabolism	ammonia assimilation cycle	P	AT5G04140
10715	3	F23 vs. L23	F2-3 up	GO:0019676	Metabolism	Metabolism	ammonia assimilation cycle	P	AT5G35630
10716	3	F23 vs. L23	F2-3 up	GO:0019676	Metabolism	Metabolism	ammonia assimilation cycle	P	AT5G53460
10717	3	F23 vs. L23	F2-3 up	GO:0016208	Metabolism	Metabolism	AMP binding	F	AT5G36880
10718	3	F23 vs. L23	F2-3 up	GO:0003876	Metabolism	Metabolism	AMP deaminase activity	F	AT2G38280
10719	3	F23 vs. L23	F2-3 up	GO:0046033	Metabolism	Metabolism	AMP metabolic process	P	AT2G38280
10720	3	F23 vs. L23	F2-3 up	GO:0047668	Metabolism	Metabolism	amygdalin beta-glucosidase activity	F	AT3G18080
10721	3	F23 vs. L23	F2-3 up	GO:0010021	Metabolism	Metabolism	amylopectin biosynthetic process	P	AT3G01180
10722	3	F23 vs. L23	F2-3 up	GO:0102229	Metabolism	Metabolism	amylopectin maltohydrolase activity	F	AT3G23920
10723	3	F23 vs. L23	F2-3 up	GO:0102229	Metabolism	Metabolism	amylopectin maltohydrolase activity	F	AT4G15210
10724	3	F23 vs. L23	F2-3 up	GO:0102229	Metabolism	Metabolism	amylopectin maltohydrolase activity	F	AT4G17090
10725	3	F23 vs. L23	F2-3 up	GO:0009718	Metabolism	Metabolism	anthocyanin-containing compound biosynthetic process	P	AT2G02990
10726	3	F23 vs. L23	F2-3 up	GO:0009718	Metabolism	Metabolism	anthocyanin-containing compound biosynthetic process	P	AT3G21560
10727	3	F23 vs. L23	F2-3 up	GO:0009718	Metabolism	Metabolism	anthocyanin-containing compound biosynthetic process	P	AT5G42800
10728	3	F23 vs. L23	F2-3 up	GO:0004049	Metabolism	Metabolism	anthranilate synthase activity	F	AT5G05730
10729	3	F23 vs. L23	F2-3 up	GO:0005950	Metabolism	Metabolism	anthranilate synthase complex	C	AT5G05730
10730	3	F23 vs. L23	F2-3 up	GO:0019146	Metabolism	Metabolism	arabinose-5-phosphate isomerase activity	F	AT3G54690

10731	3	F23 vs. L23	F2-3 up	GO:0008792	Metabolism	Metabolism	arginine decarboxylase activity	F	AT4G34710
10732	3	F23 vs. L23	F2-3 up	GO:0047769	Metabolism	Metabolism	arogenate dehydratase activity	F	AT5G22630
10733	3	F23 vs. L23	F2-3 up	GO:0070330	Metabolism	Metabolism	aromatase activity	F	AT1G01600
10734	3	F23 vs. L23	F2-3 up	GO:0070330	Metabolism	Metabolism	aromatase activity	F	AT1G63710
10735	3	F23 vs. L23	F2-3 up	GO:0070330	Metabolism	Metabolism	aromatase activity	F	AT4G00360
10736	3	F23 vs. L23	F2-3 up	GO:0009073	Metabolism	Metabolism	aromatic amino acid family biosynthetic process	P	AT1G22410
10737	3	F23 vs. L23	F2-3 up	GO:0009072	Metabolism	Metabolism	aromatic amino acid family metabolic process	P	AT2G02390
10738	3	F23 vs. L23	F2-3 up	GO:0019438	Metabolism	Metabolism	aromatic compound biosynthetic process	P	AT4G35150
10739	3	F23 vs. L23	F2-3 up	GO:0004067	Metabolism	Metabolism	asparaginase activity	F	AT5G08100
10740	3	F23 vs. L23	F2-3 up	GO:0009067	Metabolism	Metabolism	aspartate family amino acid biosynthetic process	P	AT5G14060
10741	3	F23 vs. L23	F2-3 up	GO:0004072	Metabolism	Metabolism	aspartate kinase activity	F	AT5G14060
10742	3	F23 vs. L23	F2-3 up	GO:0004190	Metabolism	Metabolism	aspartic-type endopeptidase activity	F	AT1G03230
10743	3	F23 vs. L23	F2-3 up	GO:0004190	Metabolism	Metabolism	aspartic-type endopeptidase activity	F	AT1G62290
10744	3	F23 vs. L23	F2-3 up	GO:0004190	Metabolism	Metabolism	aspartic-type endopeptidase activity	F	AT1G65240
10745	3	F23 vs. L23	F2-3 up	GO:0004190	Metabolism	Metabolism	aspartic-type endopeptidase activity	F	AT2G03200
10746	3	F23 vs. L23	F2-3 up	GO:0004190	Metabolism	Metabolism	aspartic-type endopeptidase activity	F	AT4G04460
10747	3	F23 vs. L23	F2-3 up	GO:0004190	Metabolism	Metabolism	aspartic-type endopeptidase activity	F	AT5G36260
10748	3	F23 vs. L23	F2-3 up	GO:0045330	Metabolism	Metabolism	aspartyl esterase activity	F	AT2G45220
10749	3	F23 vs. L23	F2-3 up	GO:0045330	Metabolism	Metabolism	aspartyl esterase activity	F	AT3G05620
10750	3	F23 vs. L23	F2-3 up	GO:0045330	Metabolism	Metabolism	aspartyl esterase activity	F	AT3G14310
10751	3	F23 vs. L23	F2-3 up	GO:0045330	Metabolism	Metabolism	aspartyl esterase activity	F	AT3G17060
10752	3	F23 vs. L23	F2-3 up	GO:0045330	Metabolism	Metabolism	aspartyl esterase activity	F	AT3G43270
10753	3	F23 vs. L23	F2-3 up	GO:0045330	Metabolism	Metabolism	aspartyl esterase activity	F	AT3G62170
10754	3	F23 vs. L23	F2-3 up	GO:0045330	Metabolism	Metabolism	aspartyl esterase activity	F	AT4G33220
10755	3	F23 vs. L23	F2-3 up	GO:0045330	Metabolism	Metabolism	aspartyl esterase activity	F	AT5G07430
10756	3	F23 vs. L23	F2-3 up	GO:0045330	Metabolism	Metabolism	aspartyl esterase activity	F	AT5G53370
10757	3	F23 vs. L23	F2-3 up	GO:0005524	Metabolism	Metabolism	ATP binding	F	AT1G03905
10758	3	F23 vs. L23	F2-3 up	GO:0005524	Metabolism	Metabolism	ATP binding	F	AT1G04400
10759	3	F23 vs. L23	F2-3 up	GO:0005524	Metabolism	Metabolism	ATP binding	F	AT1G09430
10760	3	F23 vs. L23	F2-3 up	GO:0005524	Metabolism	Metabolism	ATP binding	F	AT1G14000
10761	3	F23 vs. L23	F2-3 up	GO:0005524	Metabolism	Metabolism	ATP binding	F	AT1G16370
10762	3	F23 vs. L23	F2-3 up	GO:0005524	Metabolism	Metabolism	ATP binding	F	AT1G18150
10763	3	F23 vs. L23	F2-3 up	GO:0005524	Metabolism	Metabolism	ATP binding	F	AT1G21980
10764	3	F23 vs. L23	F2-3 up	GO:0005524	Metabolism	Metabolism	ATP binding	F	AT1G25350
10765	3	F23 vs. L23	F2-3 up	GO:0005524	Metabolism	Metabolism	ATP binding	F	AT1G32060
10766	3	F23 vs. L23	F2-3 up	GO:0005524	Metabolism	Metabolism	ATP binding	F	AT1G35720
10767	3	F23 vs. L23	F2-3 up	GO:0005524	Metabolism	Metabolism	ATP binding	F	AT1G48030
10768	3	F23 vs. L23	F2-3 up	GO:0005524	Metabolism	Metabolism	ATP binding	F	AT1G50250
10769	3	F23 vs. L23	F2-3 up	GO:0005524	Metabolism	Metabolism	ATP binding	F	AT1G50610
10770	3	F23 vs. L23	F2-3 up	GO:0005524	Metabolism	Metabolism	ATP binding	F	AT1G54280
10771	3	F23 vs. L23	F2-3 up	GO:0005524	Metabolism	Metabolism	ATP binding	F	AT1G59870
10772	3	F23 vs. L23	F2-3 up	GO:0005524	Metabolism	Metabolism	ATP binding	F	AT1G60810
10773	3	F23 vs. L23	F2-3 up	GO:0005524	Metabolism	Metabolism	ATP binding	F	AT1G67940
10774	3	F23 vs. L23	F2-3 up	GO:0005524	Metabolism	Metabolism	ATP binding	F	AT1G70610
10775	3	F23 vs. L23	F2-3 up	GO:0005524	Metabolism	Metabolism	ATP binding	F	AT1G71697
10776	3	F23 vs. L23	F2-3 up	GO:0005524	Metabolism	Metabolism	ATP binding	F	AT1G72180
10777	3	F23 vs. L23	F2-3 up	GO:0005524	Metabolism	Metabolism	ATP binding	F	AT1G72730
10778	3	F23 vs. L23	F2-3 up	GO:0005524	Metabolism	Metabolism	ATP binding	F	AT1G76370
10779	3	F23 vs. L23	F2-3 up	GO:0005524	Metabolism	Metabolism	ATP binding	F	AT1G78290
10780	3	F23 vs. L23	F2-3 up	GO:0005524	Metabolism	Metabolism	ATP binding	F	AT1G80460
10781	3	F23 vs. L23	F2-3 up	GO:0005524	Metabolism	Metabolism	ATP binding	F	AT1G80660
10782	3	F23 vs. L23	F2-3 up	GO:0005524	Metabolism	Metabolism	ATP binding	F	AT2G01320
10783	3	F23 vs. L23	F2-3 up	GO:0005524	Metabolism	Metabolism	ATP binding	F	AT2G02800
10784	3	F23 vs. L23	F2-3 up	GO:0005524	Metabolism	Metabolism	ATP binding	F	AT2G07180
10785	3	F23 vs. L23	F2-3 up	GO:0005524	Metabolism	Metabolism	ATP binding	F	AT2G07560
10786	3	F23 vs. L23	F2-3 up	GO:0005524	Metabolism	Metabolism	ATP binding	F	AT2G14750

10787	3	F23 vs. L23	F2-3 up	GO:0005524	Metabolism	Metabolism	ATP binding	F	AT2G16750
10788	3	F23 vs. L23	F2-3 up	GO:0005524	Metabolism	Metabolism	ATP binding	F	AT2G17220
10789	3	F23 vs. L23	F2-3 up	GO:0005524	Metabolism	Metabolism	ATP binding	F	AT2G17290
10790	3	F23 vs. L23	F2-3 up	GO:0005524	Metabolism	Metabolism	ATP binding	F	AT2G18470
10791	3	F23 vs. L23	F2-3 up	GO:0005524	Metabolism	Metabolism	ATP binding	F	AT2G20470
10792	3	F23 vs. L23	F2-3 up	GO:0005524	Metabolism	Metabolism	ATP binding	F	AT2G21960
10793	3	F23 vs. L23	F2-3 up	GO:0005524	Metabolism	Metabolism	ATP binding	F	AT2G22950
10794	3	F23 vs. L23	F2-3 up	GO:0005524	Metabolism	Metabolism	ATP binding	F	AT2G25530
10795	3	F23 vs. L23	F2-3 up	GO:0005524	Metabolism	Metabolism	ATP binding	F	AT2G30360
10796	3	F23 vs. L23	F2-3 up	GO:0005524	Metabolism	Metabolism	ATP binding	F	AT2G31500
10797	3	F23 vs. L23	F2-3 up	GO:0005524	Metabolism	Metabolism	ATP binding	F	AT2G36880
10798	3	F23 vs. L23	F2-3 up	GO:0005524	Metabolism	Metabolism	ATP binding	F	AT2G38280
10799	3	F23 vs. L23	F2-3 up	GO:0005524	Metabolism	Metabolism	ATP binding	F	AT2G39730
10800	3	F23 vs. L23	F2-3 up	GO:0005524	Metabolism	Metabolism	ATP binding	F	AT2G41210
10801	3	F23 vs. L23	F2-3 up	GO:0005524	Metabolism	Metabolism	ATP binding	F	AT3G03770
10802	3	F23 vs. L23	F2-3 up	GO:0005524	Metabolism	Metabolism	ATP binding	F	AT3G07700
10803	3	F23 vs. L23	F2-3 up	GO:0005524	Metabolism	Metabolism	ATP binding	F	AT3G07960
10804	3	F23 vs. L23	F2-3 up	GO:0005524	Metabolism	Metabolism	ATP binding	F	AT3G08720
10805	3	F23 vs. L23	F2-3 up	GO:0005524	Metabolism	Metabolism	ATP binding	F	AT3G08730
10806	3	F23 vs. L23	F2-3 up	GO:0005524	Metabolism	Metabolism	ATP binding	F	AT3G09440
10807	3	F23 vs. L23	F2-3 up	GO:0005524	Metabolism	Metabolism	ATP binding	F	AT3G10420
10808	3	F23 vs. L23	F2-3 up	GO:0005524	Metabolism	Metabolism	ATP binding	F	AT3G10700
10809	3	F23 vs. L23	F2-3 up	GO:0005524	Metabolism	Metabolism	ATP binding	F	AT3G12690
10810	3	F23 vs. L23	F2-3 up	GO:0005524	Metabolism	Metabolism	ATP binding	F	AT3G12780
10811	3	F23 vs. L23	F2-3 up	GO:0005524	Metabolism	Metabolism	ATP binding	F	AT3G13065
10812	3	F23 vs. L23	F2-3 up	GO:0005524	Metabolism	Metabolism	ATP binding	F	AT3G16340
10813	3	F23 vs. L23	F2-3 up	GO:0005524	Metabolism	Metabolism	ATP binding	F	AT3G17390
10814	3	F23 vs. L23	F2-3 up	GO:0005524	Metabolism	Metabolism	ATP binding	F	AT3G18780
10815	3	F23 vs. L23	F2-3 up	GO:0005524	Metabolism	Metabolism	ATP binding	F	AT3G20190
10816	3	F23 vs. L23	F2-3 up	GO:0005524	Metabolism	Metabolism	ATP binding	F	AT3G21180
10817	3	F23 vs. L23	F2-3 up	GO:0005524	Metabolism	Metabolism	ATP binding	F	AT3G21230
10818	3	F23 vs. L23	F2-3 up	GO:0005524	Metabolism	Metabolism	ATP binding	F	AT3G22890
10819	3	F23 vs. L23	F2-3 up	GO:0005524	Metabolism	Metabolism	ATP binding	F	AT3G24190
10820	3	F23 vs. L23	F2-3 up	GO:0005524	Metabolism	Metabolism	ATP binding	F	AT3G42640
10821	3	F23 vs. L23	F2-3 up	GO:0005524	Metabolism	Metabolism	ATP binding	F	AT3G43210
10822	3	F23 vs. L23	F2-3 up	GO:0005524	Metabolism	Metabolism	ATP binding	F	AT3G45640
10823	3	F23 vs. L23	F2-3 up	GO:0005524	Metabolism	Metabolism	ATP binding	F	AT3G45780
10824	3	F23 vs. L23	F2-3 up	GO:0005524	Metabolism	Metabolism	ATP binding	F	AT3G46520
10825	3	F23 vs. L23	F2-3 up	GO:0005524	Metabolism	Metabolism	ATP binding	F	AT3G48990
10826	3	F23 vs. L23	F2-3 up	GO:0005524	Metabolism	Metabolism	ATP binding	F	AT3G50000
10827	3	F23 vs. L23	F2-3 up	GO:0005524	Metabolism	Metabolism	ATP binding	F	AT3G51240
10828	3	F23 vs. L23	F2-3 up	GO:0005524	Metabolism	Metabolism	ATP binding	F	AT3G54660
10829	3	F23 vs. L23	F2-3 up	GO:0005524	Metabolism	Metabolism	ATP binding	F	AT3G54690
10830	3	F23 vs. L23	F2-3 up	GO:0005524	Metabolism	Metabolism	ATP binding	F	AT3G55100
10831	3	F23 vs. L23	F2-3 up	GO:0005524	Metabolism	Metabolism	ATP binding	F	AT3G55610
10832	3	F23 vs. L23	F2-3 up	GO:0005524	Metabolism	Metabolism	ATP binding	F	AT3G56600
10833	3	F23 vs. L23	F2-3 up	GO:0005524	Metabolism	Metabolism	ATP binding	F	AT3G56960
10834	3	F23 vs. L23	F2-3 up	GO:0005524	Metabolism	Metabolism	ATP binding	F	AT3G57330
10835	3	F23 vs. L23	F2-3 up	GO:0005524	Metabolism	Metabolism	ATP binding	F	AT3G60330
10836	3	F23 vs. L23	F2-3 up	GO:0005524	Metabolism	Metabolism	ATP binding	F	AT3G61160
10837	3	F23 vs. L23	F2-3 up	GO:0005524	Metabolism	Metabolism	ATP binding	F	AT4G01850
10838	3	F23 vs. L23	F2-3 up	GO:0005524	Metabolism	Metabolism	ATP binding	F	AT4G11030
10839	3	F23 vs. L23	F2-3 up	GO:0005524	Metabolism	Metabolism	ATP binding	F	AT4G15530
10840	3	F23 vs. L23	F2-3 up	GO:0005524	Metabolism	Metabolism	ATP binding	F	AT4G18050
10841	3	F23 vs. L23	F2-3 up	GO:0005524	Metabolism	Metabolism	ATP binding	F	AT4G18950
10842	3	F23 vs. L23	F2-3 up	GO:0005524	Metabolism	Metabolism	ATP binding	F	AT4G23100

10843	3	F23 vs. L23	F2-3 up	GO:0005524	Metabolism	Metabolism	ATP binding	F	AT4G24450
10844	3	F23 vs. L23	F2-3 up	GO:0005524	Metabolism	Metabolism	ATP binding	F	AT4G26100
10845	3	F23 vs. L23	F2-3 up	GO:0005524	Metabolism	Metabolism	ATP binding	F	AT4G28490
10846	3	F23 vs. L23	F2-3 up	GO:0005524	Metabolism	Metabolism	ATP binding	F	AT4G31230
10847	3	F23 vs. L23	F2-3 up	GO:0005524	Metabolism	Metabolism	ATP binding	F	AT4G35500
10848	3	F23 vs. L23	F2-3 up	GO:0005524	Metabolism	Metabolism	ATP binding	F	AT4G38510
10849	3	F23 vs. L23	F2-3 up	GO:0005524	Metabolism	Metabolism	ATP binding	F	AT4G39110
10850	3	F23 vs. L23	F2-3 up	GO:0005524	Metabolism	Metabolism	ATP binding	F	AT4G39940
10851	3	F23 vs. L23	F2-3 up	GO:0005524	Metabolism	Metabolism	ATP binding	F	AT5G02270
10852	3	F23 vs. L23	F2-3 up	GO:0005524	Metabolism	Metabolism	ATP binding	F	AT5G04720
10853	3	F23 vs. L23	F2-3 up	GO:0005524	Metabolism	Metabolism	ATP binding	F	AT5G05200
10854	3	F23 vs. L23	F2-3 up	GO:0005524	Metabolism	Metabolism	ATP binding	F	AT5G06530
10855	3	F23 vs. L23	F2-3 up	GO:0005524	Metabolism	Metabolism	ATP binding	F	AT5G07440
10856	3	F23 vs. L23	F2-3 up	GO:0005524	Metabolism	Metabolism	ATP binding	F	AT5G08160
10857	3	F23 vs. L23	F2-3 up	GO:0005524	Metabolism	Metabolism	ATP binding	F	AT5G09810
10858	3	F23 vs. L23	F2-3 up	GO:0005524	Metabolism	Metabolism	ATP binding	F	AT5G12000
10859	3	F23 vs. L23	F2-3 up	GO:0005524	Metabolism	Metabolism	ATP binding	F	AT5G13160
10860	3	F23 vs. L23	F2-3 up	GO:0005524	Metabolism	Metabolism	ATP binding	F	AT5G14060
10861	3	F23 vs. L23	F2-3 up	GO:0005524	Metabolism	Metabolism	ATP binding	F	AT5G18280
10862	3	F23 vs. L23	F2-3 up	GO:0005524	Metabolism	Metabolism	ATP binding	F	AT5G19220
10863	3	F23 vs. L23	F2-3 up	GO:0005524	Metabolism	Metabolism	ATP binding	F	AT5G24080
10864	3	F23 vs. L23	F2-3 up	GO:0005524	Metabolism	Metabolism	ATP binding	F	AT5G27000
10865	3	F23 vs. L23	F2-3 up	GO:0005524	Metabolism	Metabolism	ATP binding	F	AT5G35630
10866	3	F23 vs. L23	F2-3 up	GO:0005524	Metabolism	Metabolism	ATP binding	F	AT5G36880
10867	3	F23 vs. L23	F2-3 up	GO:0005524	Metabolism	Metabolism	ATP binding	F	AT5G40010
10868	3	F23 vs. L23	F2-3 up	GO:0005524	Metabolism	Metabolism	ATP binding	F	AT5G44110
10869	3	F23 vs. L23	F2-3 up	GO:0005524	Metabolism	Metabolism	ATP binding	F	AT5G45840
10870	3	F23 vs. L23	F2-3 up	GO:0005524	Metabolism	Metabolism	ATP binding	F	AT5G48300
10871	3	F23 vs. L23	F2-3 up	GO:0005524	Metabolism	Metabolism	ATP binding	F	AT5G53890
10872	3	F23 vs. L23	F2-3 up	GO:0005524	Metabolism	Metabolism	ATP binding	F	AT5G57350
10873	3	F23 vs. L23	F2-3 up	GO:0005524	Metabolism	Metabolism	ATP binding	F	AT5G59370
10874	3	F23 vs. L23	F2-3 up	GO:0005524	Metabolism	Metabolism	ATP binding	F	AT5G60740
10875	3	F23 vs. L23	F2-3 up	GO:0005524	Metabolism	Metabolism	ATP binding	F	AT5G60790
10876	3	F23 vs. L23	F2-3 up	GO:0005524	Metabolism	Metabolism	ATP binding	F	AT5G61700
10877	3	F23 vs. L23	F2-3 up	GO:0005524	Metabolism	Metabolism	ATP binding	F	AT5G63380
10878	3	F23 vs. L23	F2-3 up	GO:0005524	Metabolism	Metabolism	ATP binding	F	AT5G64840
10879	3	F23 vs. L23	F2-3 up	GO:0005524	Metabolism	Metabolism	ATP binding	F	AT5G65460
10880	3	F23 vs. L23	F2-3 up	GO:0005524	Metabolism	Metabolism	ATP binding	F	ATMG00480
10881	3	F23 vs. L23	F2-3 up	GO:0004176	Metabolism	Metabolism	ATP-dependent peptidase activity	F	AT1G50250
10882	3	F23 vs. L23	F2-3 up	GO:0102996	Metabolism	Metabolism	beta,beta digalactosyldiacylglycerol galactosyltransferase activity	F	AT3G06510
10883	3	F23 vs. L23	F2-3 up	GO:0033396	Metabolism	Metabolism	beta-alanine biosynthetic process via 3-ureidopropionate	P	AT5G64370
10884	3	F23 vs. L23	F2-3 up	GO:0019482	Metabolism	Metabolism	beta-alanine metabolic process	P	AT5G64370
10885	3	F23 vs. L23	F2-3 up	GO:0008798	Metabolism	Metabolism	beta-aspartyl-peptidase activity	F	AT5G08100
10886	3	F23 vs. L23	F2-3 up	GO:0004564	Metabolism	Metabolism	beta-fructofuranosidase activity	F	AT1G12240
10887	3	F23 vs. L23	F2-3 up	GO:0004564	Metabolism	Metabolism	beta-fructofuranosidase activity	F	AT3G52600
10888	3	F23 vs. L23	F2-3 up	GO:0004565	Metabolism	Metabolism	beta-galactosidase activity	F	AT1G45130
10889	3	F23 vs. L23	F2-3 up	GO:0004565	Metabolism	Metabolism	beta-galactosidase activity	F	AT4G35010
10890	3	F23 vs. L23	F2-3 up	GO:0080083	Metabolism	Metabolism	beta-gentiobiose beta-glucosidase activity	F	AT3G18080
10891	3	F23 vs. L23	F2-3 up	GO:0008422	Metabolism	Metabolism	beta-glucosidase activity	F	AT1G61820
10892	3	F23 vs. L23	F2-3 up	GO:0008422	Metabolism	Metabolism	beta-glucosidase activity	F	AT3G03640
10893	3	F23 vs. L23	F2-3 up	GO:0008422	Metabolism	Metabolism	beta-glucosidase activity	F	AT3G06510
10894	3	F23 vs. L23	F2-3 up	GO:0008422	Metabolism	Metabolism	beta-glucosidase activity	F	AT3G18080
10895	3	F23 vs. L23	F2-3 up	GO:0008422	Metabolism	Metabolism	beta-glucosidase activity	F	AT4G10060
10896	3	F23 vs. L23	F2-3 up	GO:0008422	Metabolism	Metabolism	beta-glucosidase activity	F	AT5G25980

10897	3	F23 vs. L23	F2-3 up	GO:0008802	Metabolism	Metabolism	betaine-aldehyde dehydrogenase activity	F	AT3G48170
10898	3	F23 vs. L23	F2-3 up	GO:0004567	Metabolism	Metabolism	beta-mannosidase activity	F	AT3G18080
10899	3	F23 vs. L23	F2-3 up	GO:0003837	Metabolism	Metabolism	beta-ureidopropionase activity	F	AT5G64370
10900	3	F23 vs. L23	F2-3 up	GO:0008150	Metabolism	Metabolism	biological_process	P	AT1G04670
10901	3	F23 vs. L23	F2-3 up	GO:0008150	Metabolism	Metabolism	biological_process	P	AT1G09310
10902	3	F23 vs. L23	F2-3 up	GO:0008150	Metabolism	Metabolism	biological_process	P	AT1G12250
10903	3	F23 vs. L23	F2-3 up	GO:0008150	Metabolism	Metabolism	biological_process	P	AT1G12320
10904	3	F23 vs. L23	F2-3 up	GO:0008150	Metabolism	Metabolism	biological_process	P	AT1G15470
10905	3	F23 vs. L23	F2-3 up	GO:0008150	Metabolism	Metabolism	biological_process	P	AT1G16290
10906	3	F23 vs. L23	F2-3 up	GO:0008150	Metabolism	Metabolism	biological_process	P	AT1G17360
10907	3	F23 vs. L23	F2-3 up	GO:0008150	Metabolism	Metabolism	biological_process	P	AT1G19380
10908	3	F23 vs. L23	F2-3 up	GO:0008150	Metabolism	Metabolism	biological_process	P	AT1G21000
10909	3	F23 vs. L23	F2-3 up	GO:0008150	Metabolism	Metabolism	biological_process	P	AT1G21060
10910	3	F23 vs. L23	F2-3 up	GO:0008150	Metabolism	Metabolism	biological_process	P	AT1G22750
10911	3	F23 vs. L23	F2-3 up	GO:0008150	Metabolism	Metabolism	biological_process	P	AT1G23750
10912	3	F23 vs. L23	F2-3 up	GO:0008150	Metabolism	Metabolism	biological_process	P	AT1G27850
10913	3	F23 vs. L23	F2-3 up	GO:0008150	Metabolism	Metabolism	biological_process	P	AT1G29140
10914	3	F23 vs. L23	F2-3 up	GO:0008150	Metabolism	Metabolism	biological_process	P	AT1G31750
10915	3	F23 vs. L23	F2-3 up	GO:0008150	Metabolism	Metabolism	biological_process	P	AT1G47980
10916	3	F23 vs. L23	F2-3 up	GO:0008150	Metabolism	Metabolism	biological_process	P	AT1G63060
10917	3	F23 vs. L23	F2-3 up	GO:0008150	Metabolism	Metabolism	biological_process	P	AT1G64140
10918	3	F23 vs. L23	F2-3 up	GO:0008150	Metabolism	Metabolism	biological_process	P	AT1G67870
10919	3	F23 vs. L23	F2-3 up	GO:0008150	Metabolism	Metabolism	biological_process	P	AT1G68440
10920	3	F23 vs. L23	F2-3 up	GO:0008150	Metabolism	Metabolism	biological_process	P	AT1G70780
10921	3	F23 vs. L23	F2-3 up	GO:0008150	Metabolism	Metabolism	biological_process	P	AT1G72240
10922	3	F23 vs. L23	F2-3 up	GO:0008150	Metabolism	Metabolism	biological_process	P	AT1G74510
10923	3	F23 vs. L23	F2-3 up	GO:0008150	Metabolism	Metabolism	biological_process	P	AT1G75160
10924	3	F23 vs. L23	F2-3 up	GO:0008150	Metabolism	Metabolism	biological_process	P	AT1G78780
10925	3	F23 vs. L23	F2-3 up	GO:0008150	Metabolism	Metabolism	biological_process	P	AT1G79270
10926	3	F23 vs. L23	F2-3 up	GO:0008150	Metabolism	Metabolism	biological_process	P	AT2G05530
10927	3	F23 vs. L23	F2-3 up	GO:0008150	Metabolism	Metabolism	biological_process	P	AT2G07708
10928	3	F23 vs. L23	F2-3 up	GO:0008150	Metabolism	Metabolism	biological_process	P	AT2G13350
10929	3	F23 vs. L23	F2-3 up	GO:0008150	Metabolism	Metabolism	biological_process	P	AT2G15680
10930	3	F23 vs. L23	F2-3 up	GO:0008150	Metabolism	Metabolism	biological_process	P	AT2G19330
10931	3	F23 vs. L23	F2-3 up	GO:0008150	Metabolism	Metabolism	biological_process	P	AT2G19980
10932	3	F23 vs. L23	F2-3 up	GO:0008150	Metabolism	Metabolism	biological_process	P	AT2G20320
10933	3	F23 vs. L23	F2-3 up	GO:0008150	Metabolism	Metabolism	biological_process	P	AT2G20870
10934	3	F23 vs. L23	F2-3 up	GO:0008150	Metabolism	Metabolism	biological_process	P	AT2G25530
10935	3	F23 vs. L23	F2-3 up	GO:0008150	Metabolism	Metabolism	biological_process	P	AT2G26490
10936	3	F23 vs. L23	F2-3 up	GO:0008150	Metabolism	Metabolism	biological_process	P	AT2G36020
10937	3	F23 vs. L23	F2-3 up	GO:0008150	Metabolism	Metabolism	biological_process	P	AT2G39650
10938	3	F23 vs. L23	F2-3 up	GO:0008150	Metabolism	Metabolism	biological_process	P	AT2G44210
10939	3	F23 vs. L23	F2-3 up	GO:0008150	Metabolism	Metabolism	biological_process	P	AT2G46300
10940	3	F23 vs. L23	F2-3 up	GO:0008150	Metabolism	Metabolism	biological_process	P	AT3G03430
10941	3	F23 vs. L23	F2-3 up	GO:0008150	Metabolism	Metabolism	biological_process	P	AT3G04360
10942	3	F23 vs. L23	F2-3 up	GO:0008150	Metabolism	Metabolism	biological_process	P	AT3G06035
10943	3	F23 vs. L23	F2-3 up	GO:0008150	Metabolism	Metabolism	biological_process	P	AT3G06780
10944	3	F23 vs. L23	F2-3 up	GO:0008150	Metabolism	Metabolism	biological_process	P	AT3G08990
10945	3	F23 vs. L23	F2-3 up	GO:0008150	Metabolism	Metabolism	biological_process	P	AT3G13845
10946	3	F23 vs. L23	F2-3 up	GO:0008150	Metabolism	Metabolism	biological_process	P	AT3G15400
10947	3	F23 vs. L23	F2-3 up	GO:0008150	Metabolism	Metabolism	biological_process	P	AT3G19970
10948	3	F23 vs. L23	F2-3 up	GO:0008150	Metabolism	Metabolism	biological_process	P	AT3G20865
10949	3	F23 vs. L23	F2-3 up	GO:0008150	Metabolism	Metabolism	biological_process	P	AT3G24506
10950	3	F23 vs. L23	F2-3 up	GO:0008150	Metabolism	Metabolism	biological_process	P	AT3G27090
10951	3	F23 vs. L23	F2-3 up	GO:0008150	Metabolism	Metabolism	biological_process	P	AT3G50910
10952	3	F23 vs. L23	F2-3 up	GO:0008150	Metabolism	Metabolism	biological_process	P	AT3G56360

10953	3	F23 vs. L23	F2-3 up	GO:0008150	Metabolism	Metabolism	biological_process	P	AT4G16980
10954	3	F23 vs. L23	F2-3 up	GO:0008150	Metabolism	Metabolism	biological_process	P	AT4G18920
10955	3	F23 vs. L23	F2-3 up	GO:0008150	Metabolism	Metabolism	biological_process	P	AT4G24930
10956	3	F23 vs. L23	F2-3 up	GO:0008150	Metabolism	Metabolism	biological_process	P	AT4G27435
10957	3	F23 vs. L23	F2-3 up	GO:0008150	Metabolism	Metabolism	biological_process	P	AT4G27790
10958	3	F23 vs. L23	F2-3 up	GO:0008150	Metabolism	Metabolism	biological_process	P	AT4G29780
10959	3	F23 vs. L23	F2-3 up	GO:0008150	Metabolism	Metabolism	biological_process	P	AT4G30460
10960	3	F23 vs. L23	F2-3 up	GO:0008150	Metabolism	Metabolism	biological_process	P	AT4G31330
10961	3	F23 vs. L23	F2-3 up	GO:0008150	Metabolism	Metabolism	biological_process	P	AT4G32020
10962	3	F23 vs. L23	F2-3 up	GO:0008150	Metabolism	Metabolism	biological_process	P	AT5G02380
10963	3	F23 vs. L23	F2-3 up	GO:0008150	Metabolism	Metabolism	biological_process	P	AT5G11070
10964	3	F23 vs. L23	F2-3 up	GO:0008150	Metabolism	Metabolism	biological_process	P	AT5G11420
10965	3	F23 vs. L23	F2-3 up	GO:0008150	Metabolism	Metabolism	biological_process	P	AT5G17480
10966	3	F23 vs. L23	F2-3 up	GO:0008150	Metabolism	Metabolism	biological_process	P	AT5G22430
10967	3	F23 vs. L23	F2-3 up	GO:0008150	Metabolism	Metabolism	biological_process	P	AT5G22580
10968	3	F23 vs. L23	F2-3 up	GO:0008150	Metabolism	Metabolism	biological_process	P	AT5G38760
10969	3	F23 vs. L23	F2-3 up	GO:0008150	Metabolism	Metabolism	biological_process	P	AT5G41810
10970	3	F23 vs. L23	F2-3 up	GO:0008150	Metabolism	Metabolism	biological_process	P	AT5G45350
10971	3	F23 vs. L23	F2-3 up	GO:0008150	Metabolism	Metabolism	biological_process	P	AT5G46200
10972	3	F23 vs. L23	F2-3 up	GO:0008150	Metabolism	Metabolism	biological_process	P	AT5G47620
10973	3	F23 vs. L23	F2-3 up	GO:0008150	Metabolism	Metabolism	biological_process	P	AT5G53820
10974	3	F23 vs. L23	F2-3 up	GO:0008150	Metabolism	Metabolism	biological_process	P	AT5G55960
10975	3	F23 vs. L23	F2-3 up	GO:0008150	Metabolism	Metabolism	biological_process	P	AT5G56140
10976	3	F23 vs. L23	F2-3 up	GO:0008150	Metabolism	Metabolism	biological_process	P	AT5G61710
10977	3	F23 vs. L23	F2-3 up	GO:0008150	Metabolism	Metabolism	biological_process	P	AT5G62750
10978	3	F23 vs. L23	F2-3 up	GO:0008150	Metabolism	Metabolism	biological_process	P	AT5G64400
10979	3	F23 vs. L23	F2-3 up	GO:0009058	Metabolism	Metabolism	biosynthetic process	P	AT1G70580
10980	3	F23 vs. L23	F2-3 up	GO:0009058	Metabolism	Metabolism	biosynthetic process	P	AT2G30970
10981	3	F23 vs. L23	F2-3 up	GO:0009058	Metabolism	Metabolism	biosynthetic process	P	AT3G57010
10982	3	F23 vs. L23	F2-3 up	GO:0009058	Metabolism	Metabolism	biosynthetic process	P	AT3G57020
10983	3	F23 vs. L23	F2-3 up	GO:0009058	Metabolism	Metabolism	biosynthetic process	P	AT3G59530
10984	3	F23 vs. L23	F2-3 up	GO:0009058	Metabolism	Metabolism	biosynthetic process	P	AT5G11520
10985	3	F23 vs. L23	F2-3 up	GO:0009058	Metabolism	Metabolism	biosynthetic process	P	AT5G36160
10986	3	F23 vs. L23	F2-3 up	GO:0009081	Metabolism	Metabolism	branched-chain amino acid metabolic process	P	AT3G19710
10987	3	F23 vs. L23	F2-3 up	GO:0004084	Metabolism	Metabolism	branched-chain-amino-acid transaminase activity	F	AT3G19710
10988	3	F23 vs. L23	F2-3 up	GO:0010120	Metabolism	Metabolism	camalexin biosynthetic process	P	AT3G45640
10989	3	F23 vs. L23	F2-3 up	GO:0010120	Metabolism	Metabolism	camalexin biosynthetic process	P	AT4G30530
10990	3	F23 vs. L23	F2-3 up	GO:0030552	Metabolism	Metabolism	cAMP binding	F	AT5G54250
10991	3	F23 vs. L23	F2-3 up	GO:0045227	Metabolism	Metabolism	capsule polysaccharide biosynthetic process	P	AT2G34850
10992	3	F23 vs. L23	F2-3 up	GO:0045227	Metabolism	Metabolism	capsule polysaccharide biosynthetic process	P	AT4G20460
10993	3	F23 vs. L23	F2-3 up	GO:0015976	Metabolism	Metabolism	carbon utilization	P	AT1G58180
10994	3	F23 vs. L23	F2-3 up	GO:0015976	Metabolism	Metabolism	carbon utilization	P	AT3G01500
10995	3	F23 vs. L23	F2-3 up	GO:0015976	Metabolism	Metabolism	carbon utilization	P	AT5G14740
10996	3	F23 vs. L23	F2-3 up	GO:0015976	Metabolism	Metabolism	carbon utilization	P	AT5G65810
10997	3	F23 vs. L23	F2-3 up	GO:0016830	Metabolism	Metabolism	carbon-carbon lyase activity	F	AT2G29630
10998	3	F23 vs. L23	F2-3 up	GO:0016846	Metabolism	Metabolism	carbon-sulfur lyase activity	F	AT1G34040
10999	3	F23 vs. L23	F2-3 up	GO:0016846	Metabolism	Metabolism	carbon-sulfur lyase activity	F	AT3G57050
11000	3	F23 vs. L23	F2-3 up	GO:0019752	Metabolism	Metabolism	carboxylic acid metabolic process	P	AT1G27980
11001	3	F23 vs. L23	F2-3 up	GO:0016831	Metabolism	Metabolism	carboxy-lyase activity	F	AT1G27980
11002	3	F23 vs. L23	F2-3 up	GO:0004180	Metabolism	Metabolism	carboxypeptidase activity	F	AT5G65760
11003	3	F23 vs. L23	F2-3 up	GO:0003824	Metabolism	Metabolism	catalytic activity	F	AT1G08940
11004	3	F23 vs. L23	F2-3 up	GO:0016024	Metabolism	Metabolism	CDP-diacylglycerol biosynthetic process	P	AT1G02390
11005	3	F23 vs. L23	F2-3 up	GO:0016024	Metabolism	Metabolism	CDP-diacylglycerol biosynthetic process	P	AT1G75020
11006	3	F23 vs. L23	F2-3 up	GO:0000032	Metabolism	Metabolism	cell wall mannoprotein biosynthetic process	P	AT3G02570
11007	3	F23 vs. L23	F2-3 up	GO:0052325	Metabolism	Metabolism	cell wall pectin biosynthetic process	P	AT3G21190
11008	3	F23 vs. L23	F2-3 up	GO:0052325	Metabolism	Metabolism	cell wall pectin biosynthetic process	P	AT5G15470

11009	3	F23 vs. L23	F2-3 up	GO:0080079	Metabolism	Metabolism	cellobiose glucosidase activity	F	AT3G06510
11010	3	F23 vs. L23	F2-3 up	GO:0080079	Metabolism	Metabolism	cellobiose glucosidase activity	F	AT3G18080
11011	3	F23 vs. L23	F2-3 up	GO:0006081	Metabolism	Metabolism	cellular aldehyde metabolic process	P	AT1G44170
11012	3	F23 vs. L23	F2-3 up	GO:0006081	Metabolism	Metabolism	cellular aldehyde metabolic process	P	AT4G36250
11013	3	F23 vs. L23	F2-3 up	GO:0006520	Metabolism	Metabolism	cellular amino acid metabolic process	P	AT1G44820
11014	3	F23 vs. L23	F2-3 up	GO:0006520	Metabolism	Metabolism	cellular amino acid metabolic process	P	AT5G01410
11015	3	F23 vs. L23	F2-3 up	GO:0006520	Metabolism	Metabolism	cellular amino acid metabolic process	P	AT5G07440
11016	3	F23 vs. L23	F2-3 up	GO:0006520	Metabolism	Metabolism	cellular amino acid metabolic process	P	AT5G36160
11017	3	F23 vs. L23	F2-3 up	GO:0006520	Metabolism	Metabolism	cellular amino acid metabolic process	P	AT5G54080
11018	3	F23 vs. L23	F2-3 up	GO:0044255	Metabolism	Metabolism	cellular lipid metabolic process	P	AT5G49950
11019	3	F23 vs. L23	F2-3 up	GO:0034613	Metabolism	Metabolism	cellular protein localization	P	AT5G06850
11020	3	F23 vs. L23	F2-3 up	GO:0006464	Metabolism	Metabolism	cellular protein modification process	P	AT5G07460
11021	3	F23 vs. L23	F2-3 up	GO:0008810	Metabolism	Metabolism	cellulase activity	F	AT1G64390
11022	3	F23 vs. L23	F2-3 up	GO:0008810	Metabolism	Metabolism	cellulase activity	F	AT2G32990
11023	3	F23 vs. L23	F2-3 up	GO:0008810	Metabolism	Metabolism	cellulase activity	F	AT5G49720
11024	3	F23 vs. L23	F2-3 up	GO:0030244	Metabolism	Metabolism	cellulose biosynthetic process	P	AT1G05850
11025	3	F23 vs. L23	F2-3 up	GO:0030244	Metabolism	Metabolism	cellulose biosynthetic process	P	AT2G22125
11026	3	F23 vs. L23	F2-3 up	GO:0030244	Metabolism	Metabolism	cellulose biosynthetic process	P	AT2G33100
11027	3	F23 vs. L23	F2-3 up	GO:0030244	Metabolism	Metabolism	cellulose biosynthetic process	P	AT2G39770
11028	3	F23 vs. L23	F2-3 up	GO:0030244	Metabolism	Metabolism	cellulose biosynthetic process	P	AT4G32410
11029	3	F23 vs. L23	F2-3 up	GO:0030244	Metabolism	Metabolism	cellulose biosynthetic process	P	AT4G39350
11030	3	F23 vs. L23	F2-3 up	GO:0030244	Metabolism	Metabolism	cellulose biosynthetic process	P	AT5G09870
11031	3	F23 vs. L23	F2-3 up	GO:0030244	Metabolism	Metabolism	cellulose biosynthetic process	P	AT5G16910
11032	3	F23 vs. L23	F2-3 up	GO:0030244	Metabolism	Metabolism	cellulose biosynthetic process	P	AT5G49720
11033	3	F23 vs. L23	F2-3 up	GO:0030244	Metabolism	Metabolism	cellulose biosynthetic process	P	AT5G64740
11034	3	F23 vs. L23	F2-3 up	GO:0010215	Metabolism	Metabolism	cellulose microfibril organization	P	AT3G20580
11035	3	F23 vs. L23	F2-3 up	GO:0016760	Metabolism	Metabolism	cellulose synthase (UDP-forming) activity	F	AT2G33100
11036	3	F23 vs. L23	F2-3 up	GO:0016760	Metabolism	Metabolism	cellulose synthase (UDP-forming) activity	F	AT4G32410
11037	3	F23 vs. L23	F2-3 up	GO:0016760	Metabolism	Metabolism	cellulose synthase (UDP-forming) activity	F	AT4G39350
11038	3	F23 vs. L23	F2-3 up	GO:0016760	Metabolism	Metabolism	cellulose synthase (UDP-forming) activity	F	AT5G09870
11039	3	F23 vs. L23	F2-3 up	GO:0016760	Metabolism	Metabolism	cellulose synthase (UDP-forming) activity	F	AT5G16910
11040	3	F23 vs. L23	F2-3 up	GO:0016760	Metabolism	Metabolism	cellulose synthase (UDP-forming) activity	F	AT5G64740
11041	3	F23 vs. L23	F2-3 up	GO:0016759	Metabolism	Metabolism	cellulose synthase activity	F	AT2G33100
11042	3	F23 vs. L23	F2-3 up	GO:0016759	Metabolism	Metabolism	cellulose synthase activity	F	AT4G16590
11043	3	F23 vs. L23	F2-3 up	GO:0016759	Metabolism	Metabolism	cellulose synthase activity	F	AT4G31590
11044	3	F23 vs. L23	F2-3 up	GO:0016759	Metabolism	Metabolism	cellulose synthase activity	F	AT4G32410
11045	3	F23 vs. L23	F2-3 up	GO:0016759	Metabolism	Metabolism	cellulose synthase activity	F	AT4G39350
11046	3	F23 vs. L23	F2-3 up	GO:0016759	Metabolism	Metabolism	cellulose synthase activity	F	AT5G09870
11047	3	F23 vs. L23	F2-3 up	GO:0016759	Metabolism	Metabolism	cellulose synthase activity	F	AT5G16910
11048	3	F23 vs. L23	F2-3 up	GO:0016759	Metabolism	Metabolism	cellulose synthase activity	F	AT5G64740
11049	3	F23 vs. L23	F2-3 up	GO:0010330	Metabolism	Metabolism	cellulose synthase complex	C	AT5G64740
11050	3	F23 vs. L23	F2-3 up	GO:1902387	Metabolism	Metabolism	ceramide 1-phosphate binding	F	AT3G21260
11051	3	F23 vs. L23	F2-3 up	GO:1902388	Metabolism	Metabolism	ceramide 1-phosphate transfer activity	F	AT3G21260
11052	3	F23 vs. L23	F2-3 up	GO:0030553	Metabolism	Metabolism	cGMP binding	F	AT5G54250
11053	3	F23 vs. L23	F2-3 up	GO:0009715	Metabolism	Metabolism	chalcone biosynthetic process	P	AT5G13930
11054	3	F23 vs. L23	F2-3 up	GO:0008061	Metabolism	Metabolism	chitin binding	F	AT2G43570
11055	3	F23 vs. L23	F2-3 up	GO:0008061	Metabolism	Metabolism	chitin binding	F	AT2G43590
11056	3	F23 vs. L23	F2-3 up	GO:0008061	Metabolism	Metabolism	chitin binding	F	AT3G04720
11057	3	F23 vs. L23	F2-3 up	GO:0004568	Metabolism	Metabolism	chitinase activity	F	AT2G43570
11058	3	F23 vs. L23	F2-3 up	GO:0004568	Metabolism	Metabolism	chitinase activity	F	AT2G43590
11059	3	F23 vs. L23	F2-3 up	GO:0004103	Metabolism	Metabolism	choline kinase activity	F	AT1G71697
11060	3	F23 vs. L23	F2-3 up	GO:0009423	Metabolism	Metabolism	chorismate biosynthetic process	P	AT1G22410
11061	3	F23 vs. L23	F2-3 up	GO:0009800	Metabolism	Metabolism	cinnamic acid biosynthetic process	P	AT2G37040
11062	3	F23 vs. L23	F2-3 up	GO:0009801	Metabolism	Metabolism	cinnamic acid ester metabolic process	P	AT3G21560
11063	3	F23 vs. L23	F2-3 up	GO:0045551	Metabolism	Metabolism	cinnamyl-alcohol dehydrogenase activity	F	AT4G37990
11064	3	F23 vs. L23	F2-3 up	GO:0033961	Metabolism	Metabolism	cis-stilbene-oxide hydrolase activity	F	AT2G26740

11065	3	F23 vs. L23	F2-3 up	GO:0047782	Metabolism	Metabolism	coniferin beta-glucosidase activity	F	AT1G61820
11066	3	F23 vs. L23	F2-3 up	GO:0050269	Metabolism	Metabolism	coniferyl-aldehyde dehydrogenase activity	F	AT3G24503
11067	3	F23 vs. L23	F2-3 up	GO:0016531	Metabolism	Metabolism	copper chaperone activity	F	AT3G56240
11068	3	F23 vs. L23	F2-3 up	GO:0005507	Metabolism	Metabolism	copper ion binding	F	AT1G08830
11069	3	F23 vs. L23	F2-3 up	GO:0005507	Metabolism	Metabolism	copper ion binding	F	AT1G22840
11070	3	F23 vs. L23	F2-3 up	GO:0005507	Metabolism	Metabolism	copper ion binding	F	AT1G31490
11071	3	F23 vs. L23	F2-3 up	GO:0005507	Metabolism	Metabolism	copper ion binding	F	AT1G35720
11072	3	F23 vs. L23	F2-3 up	GO:0005507	Metabolism	Metabolism	copper ion binding	F	AT1G48030
11073	3	F23 vs. L23	F2-3 up	GO:0005507	Metabolism	Metabolism	copper ion binding	F	AT1G50940
11074	3	F23 vs. L23	F2-3 up	GO:0005507	Metabolism	Metabolism	copper ion binding	F	AT1G55570
11075	3	F23 vs. L23	F2-3 up	GO:0005507	Metabolism	Metabolism	copper ion binding	F	AT1G62380
11076	3	F23 vs. L23	F2-3 up	GO:0005507	Metabolism	Metabolism	copper ion binding	F	AT1G79440
11077	3	F23 vs. L23	F2-3 up	GO:0005507	Metabolism	Metabolism	copper ion binding	F	AT2G01520
11078	3	F23 vs. L23	F2-3 up	GO:0005507	Metabolism	Metabolism	copper ion binding	F	AT2G28190
11079	3	F23 vs. L23	F2-3 up	GO:0005507	Metabolism	Metabolism	copper ion binding	F	AT2G30860
11080	3	F23 vs. L23	F2-3 up	GO:0005507	Metabolism	Metabolism	copper ion binding	F	AT2G30970
11081	3	F23 vs. L23	F2-3 up	GO:0005507	Metabolism	Metabolism	copper ion binding	F	AT2G36880
11082	3	F23 vs. L23	F2-3 up	GO:0005507	Metabolism	Metabolism	copper ion binding	F	AT3G04120
11083	3	F23 vs. L23	F2-3 up	GO:0005507	Metabolism	Metabolism	copper ion binding	F	AT3G13390
11084	3	F23 vs. L23	F2-3 up	GO:0005507	Metabolism	Metabolism	copper ion binding	F	AT3G13930
11085	3	F23 vs. L23	F2-3 up	GO:0005507	Metabolism	Metabolism	copper ion binding	F	AT3G15353
11086	3	F23 vs. L23	F2-3 up	GO:0005507	Metabolism	Metabolism	copper ion binding	F	AT3G54660
11087	3	F23 vs. L23	F2-3 up	GO:0005507	Metabolism	Metabolism	copper ion binding	F	AT4G01850
11088	3	F23 vs. L23	F2-3 up	GO:0005507	Metabolism	Metabolism	copper ion binding	F	AT4G10040
11089	3	F23 vs. L23	F2-3 up	GO:0005507	Metabolism	Metabolism	copper ion binding	F	AT4G26970
11090	3	F23 vs. L23	F2-3 up	GO:0005507	Metabolism	Metabolism	copper ion binding	F	AT5G02380
11091	3	F23 vs. L23	F2-3 up	GO:0005507	Metabolism	Metabolism	copper ion binding	F	AT5G07440
11092	3	F23 vs. L23	F2-3 up	GO:0005507	Metabolism	Metabolism	copper ion binding	F	AT5G13490
11093	3	F23 vs. L23	F2-3 up	GO:0005507	Metabolism	Metabolism	copper ion binding	F	AT5G60020
11094	3	F23 vs. L23	F2-3 up	GO:0009805	Metabolism	Metabolism	coumarin biosynthetic process	P	AT2G40890
11095	3	F23 vs. L23	F2-3 up	GO:0009805	Metabolism	Metabolism	coumarin biosynthetic process	P	AT4G34050
11096	3	F23 vs. L23	F2-3 up	GO:0006723	Metabolism	Metabolism	cuticle hydrocarbon biosynthetic process	P	AT5G57800
11097	3	F23 vs. L23	F2-3 up	GO:0010143	Metabolism	Metabolism	cutin biosynthetic process	P	AT1G02390
11098	3	F23 vs. L23	F2-3 up	GO:0010143	Metabolism	Metabolism	cutin biosynthetic process	P	AT2G38110
11099	3	F23 vs. L23	F2-3 up	GO:0010143	Metabolism	Metabolism	cutin biosynthetic process	P	AT3G10570
11100	3	F23 vs. L23	F2-3 up	GO:0016018	Metabolism	Metabolism	cyclosporin A binding	F	AT4G38740
11101	3	F23 vs. L23	F2-3 up	GO:0004121	Metabolism	Metabolism	cystathionine beta-lyase activity	F	AT3G57050
11102	3	F23 vs. L23	F2-3 up	GO:0004123	Metabolism	Metabolism	cystathionine gamma-lyase activity	F	AT3G57050
11103	3	F23 vs. L23	F2-3 up	GO:0019344	Metabolism	Metabolism	cysteine biosynthetic process	P	AT2G14750
11104	3	F23 vs. L23	F2-3 up	GO:0019344	Metabolism	Metabolism	cysteine biosynthetic process	P	AT3G13110
11105	3	F23 vs. L23	F2-3 up	GO:0019344	Metabolism	Metabolism	cysteine biosynthetic process	P	AT4G14880
11106	3	F23 vs. L23	F2-3 up	GO:0019344	Metabolism	Metabolism	cysteine biosynthetic process	P	AT4G21990
11107	3	F23 vs. L23	F2-3 up	GO:0019344	Metabolism	Metabolism	cysteine biosynthetic process	P	AT4G39940
11108	3	F23 vs. L23	F2-3 up	GO:0006535	Metabolism	Metabolism	cysteine biosynthetic process from serine	P	AT3G13110
11109	3	F23 vs. L23	F2-3 up	GO:0006535	Metabolism	Metabolism	cysteine biosynthetic process from serine	P	AT4G14880
11110	3	F23 vs. L23	F2-3 up	GO:0019343	Metabolism	Metabolism	cysteine biosynthetic process via cystathionine	P	AT3G57050
11111	3	F23 vs. L23	F2-3 up	GO:0004124	Metabolism	Metabolism	cysteine synthase activity	F	AT4G14880
11112	3	F23 vs. L23	F2-3 up	GO:0047804	Metabolism	Metabolism	cysteine-S-conjugate beta-lyase activity	F	AT3G57050
11113	3	F23 vs. L23	F2-3 up	GO:0008234	Metabolism	Metabolism	cysteine-type peptidase activity	F	AT5G45890
11114	3	F23 vs. L23	F2-3 up	GO:0008234	Metabolism	Metabolism	cysteine-type peptidase activity	F	AT5G60360
11115	3	F23 vs. L23	F2-3 up	GO:0004126	Metabolism	Metabolism	cytidine deaminase activity	F	AT2G19570
11116	3	F23 vs. L23	F2-3 up	GO:0009972	Metabolism	Metabolism	cytidine deamination	P	AT2G19570
11117	3	F23 vs. L23	F2-3 up	GO:0102485	Metabolism	Metabolism	dATP phosphohydrolase activity	F	AT5G18280
11118	3	F23 vs. L23	F2-3 up	GO:0102486	Metabolism	Metabolism	dCTP phosphohydrolase activity	F	AT5G18280
11119	3	F23 vs. L23	F2-3 up	GO:0009704	Metabolism	Metabolism	de-etiolation	P	AT1G10370
11120	3	F23 vs. L23	F2-3 up	GO:0009704	Metabolism	Metabolism	de-etiolation	P	AT4G38740

11121	3	F23 vs. L23	F2-3 up	GO:0009704	Metabolism	Metabolism	de-etiolation	P	AT5G23060
11122	3	F23 vs. L23	F2-3 up	GO:0045547	Metabolism	Metabolism	dehydrodolichyl diphosphate synthase activity	F	AT5G58770
11123	3	F23 vs. L23	F2-3 up	GO:0052637	Metabolism	Metabolism	delta 3-trans-hexadecenoic acid phosphatidylglycerol desaturase activity	F	AT4G27030
11124	3	F23 vs. L23	F2-3 up	GO:0047844	Metabolism	Metabolism	deoxycytidine deaminase activity	F	AT2G19570
11125	3	F23 vs. L23	F2-3 up	GO:0047364	Metabolism	Metabolism	desulfoglucosinolate sulfotransferase activity	F	AT1G74090
11126	3	F23 vs. L23	F2-3 up	GO:0046396	Metabolism	Metabolism	D-galacturonate metabolic process	P	AT3G10700
11127	3	F23 vs. L23	F2-3 up	GO:0102491	Metabolism	Metabolism	dGTP phosphohydrolase activity	F	AT5G18280
11128	3	F23 vs. L23	F2-3 up	GO:0004144	Metabolism	Metabolism	diacylglycerol O-acyltransferase activity	F	AT1G54570
11129	3	F23 vs. L23	F2-3 up	GO:0004144	Metabolism	Metabolism	diacylglycerol O-acyltransferase activity	F	AT5G37300
11130	3	F23 vs. L23	F2-3 up	GO:0019877	Metabolism	Metabolism	diaminopimelate biosynthetic process	P	AT5G52100
11131	3	F23 vs. L23	F2-3 up	GO:0045552	Metabolism	Metabolism	dihydrokaempferol 4-reductase activity	F	AT5G42800
11132	3	F23 vs. L23	F2-3 up	GO:0004148	Metabolism	Metabolism	dihydrolipoyl dehydrogenase activity	F	AT1G48030
11133	3	F23 vs. L23	F2-3 up	GO:0004742	Metabolism	Metabolism	dihydrolipoyllysine-residue acetyltransferase activity	F	AT3G13930
11134	3	F23 vs. L23	F2-3 up	GO:0050992	Metabolism	Metabolism	dimethylallyl diphosphate biosynthetic process	P	AT3G02780
11135	3	F23 vs. L23	F2-3 up	GO:0051213	Metabolism	Metabolism	dioxygenase activity	F	AT1G05010
11136	3	F23 vs. L23	F2-3 up	GO:0051213	Metabolism	Metabolism	dioxygenase activity	F	AT1G06640
11137	3	F23 vs. L23	F2-3 up	GO:0051213	Metabolism	Metabolism	dioxygenase activity	F	AT1G11840
11138	3	F23 vs. L23	F2-3 up	GO:0051213	Metabolism	Metabolism	dioxygenase activity	F	AT1G35190
11139	3	F23 vs. L23	F2-3 up	GO:0051213	Metabolism	Metabolism	dioxygenase activity	F	AT1G62380
11140	3	F23 vs. L23	F2-3 up	GO:0051213	Metabolism	Metabolism	dioxygenase activity	F	AT2G30830
11141	3	F23 vs. L23	F2-3 up	GO:0051213	Metabolism	Metabolism	dioxygenase activity	F	AT2G38240
11142	3	F23 vs. L23	F2-3 up	GO:0051213	Metabolism	Metabolism	dioxygenase activity	F	AT3G50210
11143	3	F23 vs. L23	F2-3 up	GO:0051213	Metabolism	Metabolism	dioxygenase activity	F	AT3G51240
11144	3	F23 vs. L23	F2-3 up	GO:0051213	Metabolism	Metabolism	dioxygenase activity	F	AT5G05600
11145	3	F23 vs. L23	F2-3 up	GO:0051213	Metabolism	Metabolism	dioxygenase activity	F	AT5G08640
11146	3	F23 vs. L23	F2-3 up	GO:0051213	Metabolism	Metabolism	dioxygenase activity	F	AT5G24530
11147	3	F23 vs. L23	F2-3 up	GO:0008239	Metabolism	Metabolism	dipeptidyl-peptidase activity	F	AT5G65760
11148	3	F23 vs. L23	F2-3 up	GO:0047334	Metabolism	Metabolism	diphosphate-fructose-6-phosphate 1-phosphotransferase activity	F	AT4G04040
11149	3	F23 vs. L23	F2-3 up	GO:0003677	Metabolism	Metabolism	DNA binding	F	AT1G01250
11150	3	F23 vs. L23	F2-3 up	GO:0003677	Metabolism	Metabolism	DNA binding	F	AT1G09250
11151	3	F23 vs. L23	F2-3 up	GO:0003677	Metabolism	Metabolism	DNA binding	F	AT1G09340
11152	3	F23 vs. L23	F2-3 up	GO:0003677	Metabolism	Metabolism	DNA binding	F	AT1G30330
11153	3	F23 vs. L23	F2-3 up	GO:0003677	Metabolism	Metabolism	DNA binding	F	AT1G32640
11154	3	F23 vs. L23	F2-3 up	GO:0003677	Metabolism	Metabolism	DNA binding	F	AT1G63650
11155	3	F23 vs. L23	F2-3 up	GO:0003677	Metabolism	Metabolism	DNA binding	F	AT1G64800
11156	3	F23 vs. L23	F2-3 up	GO:0003677	Metabolism	Metabolism	DNA binding	F	AT1G64860
11157	3	F23 vs. L23	F2-3 up	GO:0003677	Metabolism	Metabolism	DNA binding	F	AT1G69120
11158	3	F23 vs. L23	F2-3 up	GO:0003677	Metabolism	Metabolism	DNA binding	F	AT2G17950
11159	3	F23 vs. L23	F2-3 up	GO:0003677	Metabolism	Metabolism	DNA binding	F	AT2G23760
11160	3	F23 vs. L23	F2-3 up	GO:0003677	Metabolism	Metabolism	DNA binding	F	AT2G35940
11161	3	F23 vs. L23	F2-3 up	GO:0003677	Metabolism	Metabolism	DNA binding	F	AT2G37630
11162	3	F23 vs. L23	F2-3 up	GO:0003677	Metabolism	Metabolism	DNA binding	F	AT2G40140
11163	3	F23 vs. L23	F2-3 up	GO:0003677	Metabolism	Metabolism	DNA binding	F	AT2G42200
11164	3	F23 vs. L23	F2-3 up	GO:0003677	Metabolism	Metabolism	DNA binding	F	AT2G45190
11165	3	F23 vs. L23	F2-3 up	GO:0003677	Metabolism	Metabolism	DNA binding	F	AT2G46820
11166	3	F23 vs. L23	F2-3 up	GO:0003677	Metabolism	Metabolism	DNA binding	F	AT2G46830
11167	3	F23 vs. L23	F2-3 up	GO:0003677	Metabolism	Metabolism	DNA binding	F	AT3G04120
11168	3	F23 vs. L23	F2-3 up	GO:0003677	Metabolism	Metabolism	DNA binding	F	AT3G15030
11169	3	F23 vs. L23	F2-3 up	GO:0003677	Metabolism	Metabolism	DNA binding	F	AT3G17310
11170	3	F23 vs. L23	F2-3 up	GO:0003677	Metabolism	Metabolism	DNA binding	F	AT3G23290
11171	3	F23 vs. L23	F2-3 up	GO:0003677	Metabolism	Metabolism	DNA binding	F	AT3G52800
11172	3	F23 vs. L23	F2-3 up	GO:0003677	Metabolism	Metabolism	DNA binding	F	AT4G00480
11173	3	F23 vs. L23	F2-3 up	GO:0003677	Metabolism	Metabolism	DNA binding	F	AT4G37180
11174	3	F23 vs. L23	F2-3 up	GO:0003677	Metabolism	Metabolism	DNA binding	F	AT5G20240

11175	3	F23 vs. L23	F2-3 up	GO:0003677	Metabolism	Metabolism	DNA binding	F	AT5G24120
11176	3	F23 vs. L23	F2-3 up	GO:0003913	Metabolism	Metabolism	DNA photolyase activity	F	AT2G47590
11177	3	F23 vs. L23	F2-3 up	GO:0008725	Metabolism	Metabolism	DNA-3-methyladenine glycosylase activity	F	AT3G12710
11178	3	F23 vs. L23	F2-3 up	GO:0008725	Metabolism	Metabolism	DNA-3-methyladenine glycosylase activity	F	AT5G44680
11179	3	F23 vs. L23	F2-3 up	GO:0019408	Metabolism	Metabolism	dolichol biosynthetic process	P	AT5G58770
11180	3	F23 vs. L23	F2-3 up	GO:0008830	Metabolism	Metabolism	dTDP-4-dehydrorhamnose 3,5-epimerase activity	F	AT1G63000
11181	3	F23 vs. L23	F2-3 up	GO:0008831	Metabolism	Metabolism	dTDP-4-dehydrorhamnose reductase activity	F	AT1G63000
11182	3	F23 vs. L23	F2-3 up	GO:0008460	Metabolism	Metabolism	dTDP-glucose 4,6-dehydratase activity	F	AT1G53500
11183	3	F23 vs. L23	F2-3 up	GO:0008460	Metabolism	Metabolism	dTDP-glucose 4,6-dehydratase activity	F	AT1G78570
11184	3	F23 vs. L23	F2-3 up	GO:0019305	Metabolism	Metabolism	dTDP-rhamnose biosynthetic process	P	AT1G63000
11185	3	F23 vs. L23	F2-3 up	GO:0102488	Metabolism	Metabolism	dTTP phosphohydrolase activity	F	AT5G18280
11186	3	F23 vs. L23	F2-3 up	GO:0102487	Metabolism	Metabolism	dUTP phosphohydrolase activity	F	AT5G18280
11187	3	F23 vs. L23	F2-3 up	GO:0042732	Metabolism	Metabolism	D-xylose metabolic process	P	AT2G47650
11188	3	F23 vs. L23	F2-3 up	GO:0006897	Metabolism	Metabolism	endocytosis	P	AT1G03050
11189	3	F23 vs. L23	F2-3 up	GO:0006897	Metabolism	Metabolism	endocytosis	P	AT3G56960
11190	3	F23 vs. L23	F2-3 up	GO:0035671	Metabolism	Metabolism	enone reductase activity	F	AT1G23740
11191	3	F23 vs. L23	F2-3 up	GO:0008047	Metabolism	Metabolism	enzyme activator activity	F	AT1G50320
11192	3	F23 vs. L23	F2-3 up	GO:0008047	Metabolism	Metabolism	enzyme activator activity	F	AT3G15360
11193	3	F23 vs. L23	F2-3 up	GO:0019899	Metabolism	Metabolism	enzyme binding	F	AT1G78370
11194	3	F23 vs. L23	F2-3 up	GO:0004857	Metabolism	Metabolism	enzyme inhibitor activity	F	AT2G10970
11195	3	F23 vs. L23	F2-3 up	GO:0004857	Metabolism	Metabolism	enzyme inhibitor activity	F	AT2G47050
11196	3	F23 vs. L23	F2-3 up	GO:0004857	Metabolism	Metabolism	enzyme inhibitor activity	F	AT3G15360
11197	3	F23 vs. L23	F2-3 up	GO:0004857	Metabolism	Metabolism	enzyme inhibitor activity	F	AT4G24640
11198	3	F23 vs. L23	F2-3 up	GO:0004857	Metabolism	Metabolism	enzyme inhibitor activity	F	AT5G50030
11199	3	F23 vs. L23	F2-3 up	GO:0004857	Metabolism	Metabolism	enzyme inhibitor activity	F	AT5G62350
11200	3	F23 vs. L23	F2-3 up	GO:0004857	Metabolism	Metabolism	enzyme inhibitor activity	F	AT5G62360
11201	3	F23 vs. L23	F2-3 up	GO:0004301	Metabolism	Metabolism	epoxide hydrolase activity	F	AT2G26740
11202	3	F23 vs. L23	F2-3 up	GO:0080082	Metabolism	Metabolism	esculin beta-glucosidase activity	F	AT3G18080
11203	3	F23 vs. L23	F2-3 up	GO:0006887	Metabolism	Metabolism	exocytosis	P	AT1G61290
11204	3	F23 vs. L23	F2-3 up	GO:0006887	Metabolism	Metabolism	exocytosis	P	AT3G03800
11205	3	F23 vs. L23	F2-3 up	GO:0006887	Metabolism	Metabolism	exocytosis	P	AT5G13990
11206	3	F23 vs. L23	F2-3 up	GO:0006887	Metabolism	Metabolism	exocytosis	P	AT5G59730
11207	3	F23 vs. L23	F2-3 up	GO:0071949	Metabolism	Metabolism	FAD binding	F	AT1G04400
11208	3	F23 vs. L23	F2-3 up	GO:0071949	Metabolism	Metabolism	FAD binding	F	AT2G34810
11209	3	F23 vs. L23	F2-3 up	GO:0071949	Metabolism	Metabolism	FAD binding	F	AT3G59970
11210	3	F23 vs. L23	F2-3 up	GO:0008199	Metabolism	Metabolism	ferric iron binding	F	AT3G11050
11211	3	F23 vs. L23	F2-3 up	GO:0008199	Metabolism	Metabolism	ferric iron binding	F	AT3G17790
11212	3	F23 vs. L23	F2-3 up	GO:0008199	Metabolism	Metabolism	ferric iron binding	F	AT5G01600
11213	3	F23 vs. L23	F2-3 up	GO:0008198	Metabolism	Metabolism	ferrous iron binding	F	AT3G11050
11214	3	F23 vs. L23	F2-3 up	GO:0008198	Metabolism	Metabolism	ferrous iron binding	F	AT3G17790
11215	3	F23 vs. L23	F2-3 up	GO:0008198	Metabolism	Metabolism	ferrous iron binding	F	AT5G01600
11216	3	F23 vs. L23	F2-3 up	GO:0004322	Metabolism	Metabolism	ferroxidase activity	F	AT1G41830
11217	3	F23 vs. L23	F2-3 up	GO:0004322	Metabolism	Metabolism	ferroxidase activity	F	AT1G55570
11218	3	F23 vs. L23	F2-3 up	GO:0004322	Metabolism	Metabolism	ferroxidase activity	F	AT3G11050
11219	3	F23 vs. L23	F2-3 up	GO:0004322	Metabolism	Metabolism	ferroxidase activity	F	AT3G13390
11220	3	F23 vs. L23	F2-3 up	GO:0004322	Metabolism	Metabolism	ferroxidase activity	F	AT5G01600
11221	3	F23 vs. L23	F2-3 up	GO:0004322	Metabolism	Metabolism	ferroxidase activity	F	AT5G60020
11222	3	F23 vs. L23	F2-3 up	GO:0047890	Metabolism	Metabolism	flavanone 4-reductase activity	F	AT5G42800
11223	3	F23 vs. L23	F2-3 up	GO:0050660	Metabolism	Metabolism	flavin adenine dinucleotide binding	F	AT1G48030
11224	3	F23 vs. L23	F2-3 up	GO:0050660	Metabolism	Metabolism	flavin adenine dinucleotide binding	F	AT1G50940
11225	3	F23 vs. L23	F2-3 up	GO:0050660	Metabolism	Metabolism	flavin adenine dinucleotide binding	F	AT1G62540
11226	3	F23 vs. L23	F2-3 up	GO:0050660	Metabolism	Metabolism	flavin adenine dinucleotide binding	F	AT1G62570
11227	3	F23 vs. L23	F2-3 up	GO:0050660	Metabolism	Metabolism	flavin adenine dinucleotide binding	F	AT3G09940
11228	3	F23 vs. L23	F2-3 up	GO:0050660	Metabolism	Metabolism	flavin adenine dinucleotide binding	F	AT3G44190
11229	3	F23 vs. L23	F2-3 up	GO:0050660	Metabolism	Metabolism	flavin adenine dinucleotide binding	F	AT3G52880
11230	3	F23 vs. L23	F2-3 up	GO:0050660	Metabolism	Metabolism	flavin adenine dinucleotide binding	F	AT3G54660

11231	3	F23 vs. L23	F2-3 up	GO:0050660	Metabolism	Metabolism	flavin adenine dinucleotide binding	F	AT4G37760
11232	3	F23 vs. L23	F2-3 up	GO:0050660	Metabolism	Metabolism	flavin adenine dinucleotide binding	F	AT5G24140
11233	3	F23 vs. L23	F2-3 up	GO:0050660	Metabolism	Metabolism	flavin adenine dinucleotide binding	F	AT5G53460
11234	3	F23 vs. L23	F2-3 up	GO:0072387	Metabolism	Metabolism	flavin adenine dinucleotide metabolic process	P	AT1G04400
11235	3	F23 vs. L23	F2-3 up	GO:0033759	Metabolism	Metabolism	flavone synthase activity	F	AT5G24530
11236	3	F23 vs. L23	F2-3 up	GO:0045431	Metabolism	Metabolism	flavonol synthase activity	F	AT5G08640
11237	3	F23 vs. L23	F2-3 up	GO:0010181	Metabolism	Metabolism	FMN binding	F	AT2G06050
11238	3	F23 vs. L23	F2-3 up	GO:0010181	Metabolism	Metabolism	FMN binding	F	AT3G14420
11239	3	F23 vs. L23	F2-3 up	GO:0010181	Metabolism	Metabolism	FMN binding	F	AT3G45780
11240	3	F23 vs. L23	F2-3 up	GO:0010181	Metabolism	Metabolism	FMN binding	F	AT5G53460
11241	3	F23 vs. L23	F2-3 up	GO:0046655	Metabolism	Metabolism	folic acid metabolic process	P	AT4G13930
11242	3	F23 vs. L23	F2-3 up	GO:0004328	Metabolism	Metabolism	formamidase activity	F	AT4G37550
11243	3	F23 vs. L23	F2-3 up	GO:0006004	Metabolism	Metabolism	fucose metabolic process	P	AT3G21190
11244	3	F23 vs. L23	F2-3 up	GO:0010392	Metabolism	Metabolism	galactoglucomannan metabolic process	P	AT2G22900
11245	3	F23 vs. L23	F2-3 up	GO:0004335	Metabolism	Metabolism	galactokinase activity	F	AT3G10700
11246	3	F23 vs. L23	F2-3 up	GO:0019375	Metabolism	Metabolism	galactolipid biosynthetic process	P	AT3G12780
11247	3	F23 vs. L23	F2-3 up	GO:0046480	Metabolism	Metabolism	galactolipid galactosyltransferase activity	F	AT3G06510
11248	3	F23 vs. L23	F2-3 up	GO:0019374	Metabolism	Metabolism	galactolipid metabolic process	P	AT3G54950
11249	3	F23 vs. L23	F2-3 up	GO:0051070	Metabolism	Metabolism	galactomannan biosynthetic process	P	AT2G22900
11250	3	F23 vs. L23	F2-3 up	GO:0005534	Metabolism	Metabolism	galactose binding	F	AT3G10700
11251	3	F23 vs. L23	F2-3 up	GO:0006012	Metabolism	Metabolism	galactose metabolic process	P	AT2G34850
11252	3	F23 vs. L23	F2-3 up	GO:0006012	Metabolism	Metabolism	galactose metabolic process	P	AT4G20460
11253	3	F23 vs. L23	F2-3 up	GO:0008378	Metabolism	Metabolism	galactosyltransferase activity	F	AT2G22900
11254	3	F23 vs. L23	F2-3 up	GO:0008378	Metabolism	Metabolism	galactosyltransferase activity	F	AT3G06510
11255	3	F23 vs. L23	F2-3 up	GO:0047911	Metabolism	Metabolism	galacturan 1,4-alpha-galacturonidase activity	F	AT1G02790
11256	3	F23 vs. L23	F2-3 up	GO:0047911	Metabolism	Metabolism	galacturan 1,4-alpha-galacturonidase activity	F	AT3G07850
11257	3	F23 vs. L23	F2-3 up	GO:0033481	Metabolism	Metabolism	galacturonate biosynthetic process	P	AT3G23820
11258	3	F23 vs. L23	F2-3 up	GO:0047912	Metabolism	Metabolism	galacturonokinase activity	F	AT3G10700
11259	3	F23 vs. L23	F2-3 up	GO:0003839	Metabolism	Metabolism	gamma-glutamylcyclotransferase activity	F	AT4G31290
11260	3	F23 vs. L23	F2-3 up	GO:0009298	Metabolism	Metabolism	GDP-mannose biosynthetic process	P	AT2G39770
11261	3	F23 vs. L23	F2-3 up	GO:0009298	Metabolism	Metabolism	GDP-mannose biosynthetic process	P	AT3G02570
11262	3	F23 vs. L23	F2-3 up	GO:0047259	Metabolism	Metabolism	glucomannan 4-beta-mannosyltransferase activity	F	AT4G16590
11263	3	F23 vs. L23	F2-3 up	GO:0019761	Metabolism	Metabolism	glucosinolate biosynthetic process	P	AT1G16410
11264	3	F23 vs. L23	F2-3 up	GO:0019761	Metabolism	Metabolism	glucosinolate biosynthetic process	P	AT1G62540
11265	3	F23 vs. L23	F2-3 up	GO:0019761	Metabolism	Metabolism	glucosinolate biosynthetic process	P	AT1G62570
11266	3	F23 vs. L23	F2-3 up	GO:0019761	Metabolism	Metabolism	glucosinolate biosynthetic process	P	AT1G74090
11267	3	F23 vs. L23	F2-3 up	GO:0019761	Metabolism	Metabolism	glucosinolate biosynthetic process	P	AT2G43100
11268	3	F23 vs. L23	F2-3 up	GO:0019761	Metabolism	Metabolism	glucosinolate biosynthetic process	P	AT3G19710
11269	3	F23 vs. L23	F2-3 up	GO:0019761	Metabolism	Metabolism	glucosinolate biosynthetic process	P	AT3G58990
11270	3	F23 vs. L23	F2-3 up	GO:0019761	Metabolism	Metabolism	glucosinolate biosynthetic process	P	AT4G12030
11271	3	F23 vs. L23	F2-3 up	GO:0019761	Metabolism	Metabolism	glucosinolate biosynthetic process	P	AT4G13770
11272	3	F23 vs. L23	F2-3 up	GO:0019761	Metabolism	Metabolism	glucosinolate biosynthetic process	P	AT4G23100
11273	3	F23 vs. L23	F2-3 up	GO:0019761	Metabolism	Metabolism	glucosinolate biosynthetic process	P	AT5G14200
11274	3	F23 vs. L23	F2-3 up	GO:0102799	Metabolism	Metabolism	glucosinolate glucohydrolase activity	F	AT5G25980
11275	3	F23 vs. L23	F2-3 up	GO:0019760	Metabolism	Metabolism	glucosinolate metabolic process	P	AT4G30530
11276	3	F23 vs. L23	F2-3 up	GO:0030775	Metabolism	Metabolism	glucuronoxylan 4-O-methyltransferase activity	F	AT1G27930
11277	3	F23 vs. L23	F2-3 up	GO:0004349	Metabolism	Metabolism	glutamate 5-kinase activity	F	AT3G55610
11278	3	F23 vs. L23	F2-3 up	GO:0006537	Metabolism	Metabolism	glutamate biosynthetic process	P	AT5G04140
11279	3	F23 vs. L23	F2-3 up	GO:0006537	Metabolism	Metabolism	glutamate biosynthetic process	P	AT5G53460
11280	3	F23 vs. L23	F2-3 up	GO:0004351	Metabolism	Metabolism	glutamate decarboxylase activity	F	AT1G65960
11281	3	F23 vs. L23	F2-3 up	GO:0006540	Metabolism	Metabolism	glutamate decarboxylation to succinate	P	AT1G79440
11282	3	F23 vs. L23	F2-3 up	GO:0006536	Metabolism	Metabolism	glutamate metabolic process	P	AT1G65960
11283	3	F23 vs. L23	F2-3 up	GO:0016041	Metabolism	Metabolism	glutamate synthase (ferredoxin) activity	F	AT5G04140
11284	3	F23 vs. L23	F2-3 up	GO:0004350	Metabolism	Metabolism	glutamate-5-semialdehyde dehydrogenase activity	F	AT3G55610
11285	3	F23 vs. L23	F2-3 up	GO:0004356	Metabolism	Metabolism	glutamate-ammonia ligase activity	F	AT5G35630
11286	3	F23 vs. L23	F2-3 up	GO:0004357	Metabolism	Metabolism	glutamate-cysteine ligase activity	F	AT4G23100

11287	3	F23 vs. L23	F2-3 up	GO:0004359	Metabolism	Metabolism	glutaminase activity	F	AT5G01410
11288	3	F23 vs. L23	F2-3 up	GO:0006542	Metabolism	Metabolism	glutamine biosynthetic process	P	AT5G35630
11289	3	F23 vs. L23	F2-3 up	GO:0008465	Metabolism	Metabolism	glycerate dehydrogenase activity	F	AT1G68010
11290	3	F23 vs. L23	F2-3 up	GO:0006662	Metabolism	Metabolism	glycerol ether metabolic process	P	AT1G59730
11291	3	F23 vs. L23	F2-3 up	GO:0006662	Metabolism	Metabolism	glycerol ether metabolic process	P	AT3G15360
11292	3	F23 vs. L23	F2-3 up	GO:0004370	Metabolism	Metabolism	glycerol kinase activity	F	AT1G80460
11293	3	F23 vs. L23	F2-3 up	GO:0006071	Metabolism	Metabolism	glycerol metabolic process	P	AT1G80460
11294	3	F23 vs. L23	F2-3 up	GO:0006071	Metabolism	Metabolism	glycerol metabolic process	P	AT5G58050
11295	3	F23 vs. L23	F2-3 up	GO:0090447	Metabolism	Metabolism	glycerol-3-phosphate 2-O-acyltransferase activity	F	AT1G02390
11296	3	F23 vs. L23	F2-3 up	GO:0090447	Metabolism	Metabolism	glycerol-3-phosphate 2-O-acyltransferase activity	F	AT2G38110
11297	3	F23 vs. L23	F2-3 up	GO:0046167	Metabolism	Metabolism	glycerol-3-phosphate biosynthetic process	P	AT1G80460
11298	3	F23 vs. L23	F2-3 up	GO:0004366	Metabolism	Metabolism	glycerol-3-phosphate O-acyltransferase activity	F	AT1G02390
11299	3	F23 vs. L23	F2-3 up	GO:0008889	Metabolism	Metabolism	glycerophosphodiester phosphodiesterase activity	F	AT5G58050
11300	3	F23 vs. L23	F2-3 up	GO:0016594	Metabolism	Metabolism	glycine binding	F	AT4G33010
11301	3	F23 vs. L23	F2-3 up	GO:0006545	Metabolism	Metabolism	glycine biosynthetic process	P	AT1G23310
11302	3	F23 vs. L23	F2-3 up	GO:0019264	Metabolism	Metabolism	glycine biosynthetic process from serine	P	AT4G13930
11303	3	F23 vs. L23	F2-3 up	GO:0005960	Metabolism	Metabolism	glycine cleavage complex	C	AT2G35370
11304	3	F23 vs. L23	F2-3 up	GO:0005960	Metabolism	Metabolism	glycine cleavage complex	C	AT4G33010
11305	3	F23 vs. L23	F2-3 up	GO:0019464	Metabolism	Metabolism	glycine decarboxylation via glycine cleavage system	P	AT1G11860
11306	3	F23 vs. L23	F2-3 up	GO:0019464	Metabolism	Metabolism	glycine decarboxylation via glycine cleavage system	P	AT2G35370
11307	3	F23 vs. L23	F2-3 up	GO:0019464	Metabolism	Metabolism	glycine decarboxylation via glycine cleavage system	P	AT4G33010
11308	3	F23 vs. L23	F2-3 up	GO:0004375	Metabolism	Metabolism	glycine dehydrogenase (decarboxylating) activity	F	AT2G35370
11309	3	F23 vs. L23	F2-3 up	GO:0004375	Metabolism	Metabolism	glycine dehydrogenase (decarboxylating) activity	F	AT4G33010
11310	3	F23 vs. L23	F2-3 up	GO:0004372	Metabolism	Metabolism	glycine hydroxymethyltransferase activity	F	AT4G13930
11311	3	F23 vs. L23	F2-3 up	GO:0006544	Metabolism	Metabolism	glycine metabolic process	P	AT4G13930
11312	3	F23 vs. L23	F2-3 up	GO:0047958	Metabolism	Metabolism	glycine:2-oxoglutarate aminotransferase activity	F	AT1G23310
11313	3	F23 vs. L23	F2-3 up	GO:0047958	Metabolism	Metabolism	glycine:2-oxoglutarate aminotransferase activity	F	AT1G70580
11314	3	F23 vs. L23	F2-3 up	GO:0005978	Metabolism	Metabolism	glycogen biosynthetic process	P	AT1G70730
11315	3	F23 vs. L23	F2-3 up	GO:0005978	Metabolism	Metabolism	glycogen biosynthetic process	P	AT5G19220
11316	3	F23 vs. L23	F2-3 up	GO:0005978	Metabolism	Metabolism	glycogen biosynthetic process	P	AT5G48300
11317	3	F23 vs. L23	F2-3 up	GO:0005977	Metabolism	Metabolism	glycogen metabolic process	P	AT3G03250
11318	3	F23 vs. L23	F2-3 up	GO:0006024	Metabolism	Metabolism	glycosaminoglycan biosynthetic process	P	AT3G29360
11319	3	F23 vs. L23	F2-3 up	GO:0006024	Metabolism	Metabolism	glycosaminoglycan biosynthetic process	P	AT5G15490
11320	3	F23 vs. L23	F2-3 up	GO:0006024	Metabolism	Metabolism	glycosaminoglycan biosynthetic process	P	AT5G39320
11321	3	F23 vs. L23	F2-3 up	GO:0019172	Metabolism	Metabolism	glyoxalase III activity	F	AT1G53280
11322	3	F23 vs. L23	F2-3 up	GO:0032580	Metabolism	Metabolism	Golgi cisterna membrane	C	AT2G34850
11323	3	F23 vs. L23	F2-3 up	GO:0032580	Metabolism	Metabolism	Golgi cisterna membrane	C	AT2G47650
11324	3	F23 vs. L23	F2-3 up	GO:0032580	Metabolism	Metabolism	Golgi cisterna membrane	C	AT3G23820
11325	3	F23 vs. L23	F2-3 up	GO:0032580	Metabolism	Metabolism	Golgi cisterna membrane	C	AT4G20460
11326	3	F23 vs. L23	F2-3 up	GO:0006506	Metabolism	Metabolism	GPI anchor biosynthetic process	P	AT3G45100
11327	3	F23 vs. L23	F2-3 up	GO:0010597	Metabolism	Metabolism	green leaf volatile biosynthetic process	P	AT3G03480
11328	3	F23 vs. L23	F2-3 up	GO:0010597	Metabolism	Metabolism	green leaf volatile biosynthetic process	P	AT3G45140
11329	3	F23 vs. L23	F2-3 up	GO:0005525	Metabolism	Metabolism	GTP binding	F	AT1G64740
11330	3	F23 vs. L23	F2-3 up	GO:0005525	Metabolism	Metabolism	GTP binding	F	AT2G15310
11331	3	F23 vs. L23	F2-3 up	GO:0005525	Metabolism	Metabolism	GTP binding	F	AT2G18390
11332	3	F23 vs. L23	F2-3 up	GO:0005525	Metabolism	Metabolism	GTP binding	F	AT2G21880
11333	3	F23 vs. L23	F2-3 up	GO:0005525	Metabolism	Metabolism	GTP binding	F	AT2G22450
11334	3	F23 vs. L23	F2-3 up	GO:0005525	Metabolism	Metabolism	GTP binding	F	AT2G39770
11335	3	F23 vs. L23	F2-3 up	GO:0005525	Metabolism	Metabolism	GTP binding	F	AT2G45770
11336	3	F23 vs. L23	F2-3 up	GO:0005525	Metabolism	Metabolism	GTP binding	F	AT3G07050
11337	3	F23 vs. L23	F2-3 up	GO:0005525	Metabolism	Metabolism	GTP binding	F	AT3G21700
11338	3	F23 vs. L23	F2-3 up	GO:0005525	Metabolism	Metabolism	GTP binding	F	AT3G61760
11339	3	F23 vs. L23	F2-3 up	GO:0005525	Metabolism	Metabolism	GTP binding	F	AT3G62290
11340	3	F23 vs. L23	F2-3 up	GO:0005525	Metabolism	Metabolism	GTP binding	F	AT4G17170
11341	3	F23 vs. L23	F2-3 up	GO:0005525	Metabolism	Metabolism	GTP binding	F	AT4G17530
11342	3	F23 vs. L23	F2-3 up	GO:0005525	Metabolism	Metabolism	GTP binding	F	AT4G20360

11343	3	F23 vs. L23	F2-3 up	GO:0005096	Metabolism	Metabolism	GTPase activator activity	F	AT3G07940
11344	3	F23 vs. L23	F2-3 up	GO:0005096	Metabolism	Metabolism	GTPase activator activity	F	AT5G09550
11345	3	F23 vs. L23	F2-3 up	GO:0005096	Metabolism	Metabolism	GTPase activator activity	F	AT5G54780
11346	3	F23 vs. L23	F2-3 up	GO:0003924	Metabolism	Metabolism	GTPase activity	F	AT1G64740
11347	3	F23 vs. L23	F2-3 up	GO:0003924	Metabolism	Metabolism	GTPase activity	F	AT2G18390
11348	3	F23 vs. L23	F2-3 up	GO:0003924	Metabolism	Metabolism	GTPase activity	F	AT2G21880
11349	3	F23 vs. L23	F2-3 up	GO:0003924	Metabolism	Metabolism	GTPase activity	F	AT2G45770
11350	3	F23 vs. L23	F2-3 up	GO:0003924	Metabolism	Metabolism	GTPase activity	F	AT3G21700
11351	3	F23 vs. L23	F2-3 up	GO:0003924	Metabolism	Metabolism	GTPase activity	F	AT3G61760
11352	3	F23 vs. L23	F2-3 up	GO:0003924	Metabolism	Metabolism	GTPase activity	F	AT4G17170
11353	3	F23 vs. L23	F2-3 up	GO:0003924	Metabolism	Metabolism	GTPase activity	F	AT4G17530
11354	3	F23 vs. L23	F2-3 up	GO:0003924	Metabolism	Metabolism	GTPase activity	F	AT4G20360
11355	3	F23 vs. L23	F2-3 up	GO:0106046	Metabolism	Metabolism	guanine deglycation, glyoxal removal	P	AT1G53280
11356	3	F23 vs. L23	F2-3 up	GO:0020037	Metabolism	Metabolism	heme binding	F	AT1G01600
11357	3	F23 vs. L23	F2-3 up	GO:0020037	Metabolism	Metabolism	heme binding	F	AT1G16410
11358	3	F23 vs. L23	F2-3 up	GO:0020037	Metabolism	Metabolism	heme binding	F	AT1G20620
11359	3	F23 vs. L23	F2-3 up	GO:0020037	Metabolism	Metabolism	heme binding	F	AT1G22840
11360	3	F23 vs. L23	F2-3 up	GO:0020037	Metabolism	Metabolism	heme binding	F	AT1G58265
11361	3	F23 vs. L23	F2-3 up	GO:0020037	Metabolism	Metabolism	heme binding	F	AT1G63710
11362	3	F23 vs. L23	F2-3 up	GO:0020037	Metabolism	Metabolism	heme binding	F	AT2G15620
11363	3	F23 vs. L23	F2-3 up	GO:0020037	Metabolism	Metabolism	heme binding	F	AT2G24180
11364	3	F23 vs. L23	F2-3 up	GO:0020037	Metabolism	Metabolism	heme binding	F	AT2G30490
11365	3	F23 vs. L23	F2-3 up	GO:0020037	Metabolism	Metabolism	heme binding	F	AT2G37970
11366	3	F23 vs. L23	F2-3 up	GO:0020037	Metabolism	Metabolism	heme binding	F	AT2G40000
11367	3	F23 vs. L23	F2-3 up	GO:0020037	Metabolism	Metabolism	heme binding	F	AT2G40890
11368	3	F23 vs. L23	F2-3 up	GO:0020037	Metabolism	Metabolism	heme binding	F	AT2G41480
11369	3	F23 vs. L23	F2-3 up	GO:0020037	Metabolism	Metabolism	heme binding	F	AT3G01420
11370	3	F23 vs. L23	F2-3 up	GO:0020037	Metabolism	Metabolism	heme binding	F	AT3G10570
11371	3	F23 vs. L23	F2-3 up	GO:0020037	Metabolism	Metabolism	heme binding	F	AT3G14620
11372	3	F23 vs. L23	F2-3 up	GO:0020037	Metabolism	Metabolism	heme binding	F	AT3G48520
11373	3	F23 vs. L23	F2-3 up	GO:0020037	Metabolism	Metabolism	heme binding	F	AT3G49110
11374	3	F23 vs. L23	F2-3 up	GO:0020037	Metabolism	Metabolism	heme binding	F	AT3G61880
11375	3	F23 vs. L23	F2-3 up	GO:0020037	Metabolism	Metabolism	heme binding	F	AT4G00360
11376	3	F23 vs. L23	F2-3 up	GO:0020037	Metabolism	Metabolism	heme binding	F	AT4G10040
11377	3	F23 vs. L23	F2-3 up	GO:0020037	Metabolism	Metabolism	heme binding	F	AT4G13770
11378	3	F23 vs. L23	F2-3 up	GO:0020037	Metabolism	Metabolism	heme binding	F	AT4G15440
11379	3	F23 vs. L23	F2-3 up	GO:0020037	Metabolism	Metabolism	heme binding	F	AT4G19230
11380	3	F23 vs. L23	F2-3 up	GO:0020037	Metabolism	Metabolism	heme binding	F	AT4G37430
11381	3	F23 vs. L23	F2-3 up	GO:0020037	Metabolism	Metabolism	heme binding	F	AT4G39480
11382	3	F23 vs. L23	F2-3 up	GO:0020037	Metabolism	Metabolism	heme binding	F	AT4G39510
11383	3	F23 vs. L23	F2-3 up	GO:0020037	Metabolism	Metabolism	heme binding	F	AT4G39730
11384	3	F23 vs. L23	F2-3 up	GO:0020037	Metabolism	Metabolism	heme binding	F	AT5G25120
11385	3	F23 vs. L23	F2-3 up	GO:0020037	Metabolism	Metabolism	heme binding	F	AT5G36220
11386	3	F23 vs. L23	F2-3 up	GO:0020037	Metabolism	Metabolism	heme binding	F	AT5G47000
11387	3	F23 vs. L23	F2-3 up	GO:0020037	Metabolism	Metabolism	heme binding	F	AT5G51890
11388	3	F23 vs. L23	F2-3 up	GO:0010297	Metabolism	Metabolism	heteropolysaccharide binding	F	AT1G09340
11389	3	F23 vs. L23	F2-3 up	GO:0010289	Metabolism	Metabolism	homogalacturonan biosynthetic process	P	AT1G78240
11390	3	F23 vs. L23	F2-3 up	GO:0010289	Metabolism	Metabolism	homogalacturonan biosynthetic process	P	AT3G25140
11391	3	F23 vs. L23	F2-3 up	GO:0010394	Metabolism	Metabolism	homogalacturonan metabolic process	P	AT5G65810
11392	3	F23 vs. L23	F2-3 up	GO:0004411	Metabolism	Metabolism	homogentisate 1,2-dioxygenase activity	F	AT5G54080
11393	3	F23 vs. L23	F2-3 up	GO:0009090	Metabolism	Metabolism	homoserine biosynthetic process	P	AT5G14060
11394	3	F23 vs. L23	F2-3 up	GO:0070814	Metabolism	Metabolism	hydrogen sulfide biosynthetic process	P	AT2G14750
11395	3	F23 vs. L23	F2-3 up	GO:0070814	Metabolism	Metabolism	hydrogen sulfide biosynthetic process	P	AT3G22890
11396	3	F23 vs. L23	F2-3 up	GO:0070814	Metabolism	Metabolism	hydrogen sulfide biosynthetic process	P	AT4G39940
11397	3	F23 vs. L23	F2-3 up	GO:0016787	Metabolism	Metabolism	hydrolase activity	F	AT1G33290
11398	3	F23 vs. L23	F2-3 up	GO:0016787	Metabolism	Metabolism	hydrolase activity	F	AT1G56500

11399	3	F23 vs. L23	F2-3 up	GO:0016787	Metabolism	Metabolism	hydrolase activity	F	AT1G80280
11400	3	F23 vs. L23	F2-3 up	GO:0016787	Metabolism	Metabolism	hydrolase activity	F	AT2G16750
11401	3	F23 vs. L23	F2-3 up	GO:0016787	Metabolism	Metabolism	hydrolase activity	F	AT2G32150
11402	3	F23 vs. L23	F2-3 up	GO:0016787	Metabolism	Metabolism	hydrolase activity	F	AT2G41250
11403	3	F23 vs. L23	F2-3 up	GO:0016787	Metabolism	Metabolism	hydrolase activity	F	AT2G45610
11404	3	F23 vs. L23	F2-3 up	GO:0016787	Metabolism	Metabolism	hydrolase activity	F	AT3G02780
11405	3	F23 vs. L23	F2-3 up	GO:0016787	Metabolism	Metabolism	hydrolase activity	F	AT3G10420
11406	3	F23 vs. L23	F2-3 up	GO:0016787	Metabolism	Metabolism	hydrolase activity	F	AT3G17020
11407	3	F23 vs. L23	F2-3 up	GO:0016787	Metabolism	Metabolism	hydrolase activity	F	AT3G19970
11408	3	F23 vs. L23	F2-3 up	GO:0016787	Metabolism	Metabolism	hydrolase activity	F	AT3G48420
11409	3	F23 vs. L23	F2-3 up	GO:0016787	Metabolism	Metabolism	hydrolase activity	F	AT3G50620
11410	3	F23 vs. L23	F2-3 up	GO:0016787	Metabolism	Metabolism	hydrolase activity	F	AT4G31230
11411	3	F23 vs. L23	F2-3 up	GO:0016787	Metabolism	Metabolism	hydrolase activity	F	AT4G32260
11412	3	F23 vs. L23	F2-3 up	GO:0016787	Metabolism	Metabolism	hydrolase activity	F	AT4G38510
11413	3	F23 vs. L23	F2-3 up	GO:0016787	Metabolism	Metabolism	hydrolase activity	F	AT4G39955
11414	3	F23 vs. L23	F2-3 up	GO:0016787	Metabolism	Metabolism	hydrolase activity	F	AT5G12000
11415	3	F23 vs. L23	F2-3 up	GO:0016787	Metabolism	Metabolism	hydrolase activity	F	AT5G49950
11416	3	F23 vs. L23	F2-3 up	GO:0016787	Metabolism	Metabolism	hydrolase activity	F	AT5G59480
11417	3	F23 vs. L23	F2-3 up	GO:0016811	Metabolism	Metabolism	hydrolase activity, acting on carbon-nitrogen (but not peptide) bonds, in linear amides	F	AT4G37550
11418	3	F23 vs. L23	F2-3 up	GO:0016811	Metabolism	Metabolism	hydrolase activity, acting on carbon-nitrogen (but not peptide) bonds, in linear amides	F	AT4G37560
11419	3	F23 vs. L23	F2-3 up	GO:0016788	Metabolism	Metabolism	hydrolase activity, acting on ester bonds	F	AT1G58430
11420	3	F23 vs. L23	F2-3 up	GO:0016788	Metabolism	Metabolism	hydrolase activity, acting on ester bonds	F	AT3G16370
11421	3	F23 vs. L23	F2-3 up	GO:0016788	Metabolism	Metabolism	hydrolase activity, acting on ester bonds	F	AT3G48610
11422	3	F23 vs. L23	F2-3 up	GO:0016788	Metabolism	Metabolism	hydrolase activity, acting on ester bonds	F	AT3G53100
11423	3	F23 vs. L23	F2-3 up	GO:0016788	Metabolism	Metabolism	hydrolase activity, acting on ester bonds	F	AT4G28780
11424	3	F23 vs. L23	F2-3 up	GO:0016788	Metabolism	Metabolism	hydrolase activity, acting on ester bonds	F	AT5G17670
11425	3	F23 vs. L23	F2-3 up	GO:0016788	Metabolism	Metabolism	hydrolase activity, acting on ester bonds	F	AT5G33370
11426	3	F23 vs. L23	F2-3 up	GO:0016799	Metabolism	Metabolism	hydrolase activity, hydrolyzing N-glycosyl compounds	F	AT2G28305
11427	3	F23 vs. L23	F2-3 up	GO:0004553	Metabolism	Metabolism	hydrolase activity, hydrolyzing O-glycosyl compounds	F	AT3G04010
11428	3	F23 vs. L23	F2-3 up	GO:0004553	Metabolism	Metabolism	hydrolase activity, hydrolyzing O-glycosyl compounds	F	AT5G57560
11429	3	F23 vs. L23	F2-3 up	GO:0004553	Metabolism	Metabolism	hydrolase activity, hydrolyzing O-glycosyl compounds	F	AT5G65730
11430	3	F23 vs. L23	F2-3 up	GO:0016618	Metabolism	Metabolism	hydroxypyruvate reductase activity	F	AT1G68010
11431	3	F23 vs. L23	F2-3 up	GO:0102953	Metabolism	Metabolism	hypoglycin A gamma-glutamyl transpeptidase activity	F	AT4G39640
11432	3	F23 vs. L23	F2-3 up	GO:0042802	Metabolism	Metabolism	identical protein binding	F	AT1G04400
11433	3	F23 vs. L23	F2-3 up	GO:0042802	Metabolism	Metabolism	identical protein binding	F	AT2G14750
11434	3	F23 vs. L23	F2-3 up	GO:0042802	Metabolism	Metabolism	identical protein binding	F	AT2G30490
11435	3	F23 vs. L23	F2-3 up	GO:0042802	Metabolism	Metabolism	identical protein binding	F	AT2G40890
11436	3	F23 vs. L23	F2-3 up	GO:0042802	Metabolism	Metabolism	identical protein binding	F	AT3G11050
11437	3	F23 vs. L23	F2-3 up	GO:0042802	Metabolism	Metabolism	identical protein binding	F	AT3G45780
11438	3	F23 vs. L23	F2-3 up	GO:0042802	Metabolism	Metabolism	identical protein binding	F	AT4G11280
11439	3	F23 vs. L23	F2-3 up	GO:0042802	Metabolism	Metabolism	identical protein binding	F	AT4G31800
11440	3	F23 vs. L23	F2-3 up	GO:0042802	Metabolism	Metabolism	identical protein binding	F	AT5G01600
11441	3	F23 vs. L23	F2-3 up	GO:0006188	Metabolism	Metabolism	IMP biosynthetic process	P	AT2G38280
11442	3	F23 vs. L23	F2-3 up	GO:0080071	Metabolism	Metabolism	indol-3-yl-methyl-desulfoglucosinolate sulfotransferase activity	F	AT1G74090
11443	3	F23 vs. L23	F2-3 up	GO:0009759	Metabolism	Metabolism	indole glucosinolate biosynthetic process	P	AT1G32640
11444	3	F23 vs. L23	F2-3 up	GO:0042343	Metabolism	Metabolism	indole glucosinolate metabolic process	P	AT4G37430
11445	3	F23 vs. L23	F2-3 up	GO:0009700	Metabolism	Metabolism	indole phytoalexin biosynthetic process	P	AT4G23100
11446	3	F23 vs. L23	F2-3 up	GO:0052638	Metabolism	Metabolism	indole-3-butyrate beta-glucosyltransferase activity	F	AT2G31750

11447	3	F23 vs. L23	F2-3 up	GO:0042430	Metabolism	Metabolism	indole-containing compound metabolic process	P	AT2G24180
11448	3	F23 vs. L23	F2-3 up	GO:0004427	Metabolism	Metabolism	inorganic diphosphatase activity	F	AT2G46860
11449	3	F23 vs. L23	F2-3 up	GO:0004427	Metabolism	Metabolism	inorganic diphosphatase activity	F	AT4G01480
11450	3	F23 vs. L23	F2-3 up	GO:0004427	Metabolism	Metabolism	inorganic diphosphatase activity	F	AT5G09650
11451	3	F23 vs. L23	F2-3 up	GO:0006021	Metabolism	Metabolism	inositol biosynthetic process	P	AT2G22240
11452	3	F23 vs. L23	F2-3 up	GO:0006021	Metabolism	Metabolism	inositol biosynthetic process	P	AT4G39800
11453	3	F23 vs. L23	F2-3 up	GO:0000822	Metabolism	Metabolism	inositol hexakisphosphate binding	F	AT2G03240
11454	3	F23 vs. L23	F2-3 up	GO:0000822	Metabolism	Metabolism	inositol hexakisphosphate binding	F	AT3G29060
11455	3	F23 vs. L23	F2-3 up	GO:0004512	Metabolism	Metabolism	inositol-3-phosphate synthase activity	F	AT2G22240
11456	3	F23 vs. L23	F2-3 up	GO:0004512	Metabolism	Metabolism	inositol-3-phosphate synthase activity	F	AT4G39800
11457	3	F23 vs. L23	F2-3 up	GO:0006880	Metabolism	Metabolism	intracellular sequestering of iron ion	P	AT3G11050
11458	3	F23 vs. L23	F2-3 up	GO:0006880	Metabolism	Metabolism	intracellular sequestering of iron ion	P	AT5G01600
11459	3	F23 vs. L23	F2-3 up	GO:0005506	Metabolism	Metabolism	iron ion binding	F	AT1G01600
11460	3	F23 vs. L23	F2-3 up	GO:0005506	Metabolism	Metabolism	iron ion binding	F	AT1G16410
11461	3	F23 vs. L23	F2-3 up	GO:0005506	Metabolism	Metabolism	iron ion binding	F	AT1G58265
11462	3	F23 vs. L23	F2-3 up	GO:0005506	Metabolism	Metabolism	iron ion binding	F	AT1G63710
11463	3	F23 vs. L23	F2-3 up	GO:0005506	Metabolism	Metabolism	iron ion binding	F	AT1G69640
11464	3	F23 vs. L23	F2-3 up	GO:0005506	Metabolism	Metabolism	iron ion binding	F	AT2G24180
11465	3	F23 vs. L23	F2-3 up	GO:0005506	Metabolism	Metabolism	iron ion binding	F	AT2G30490
11466	3	F23 vs. L23	F2-3 up	GO:0005506	Metabolism	Metabolism	iron ion binding	F	AT2G40890
11467	3	F23 vs. L23	F2-3 up	GO:0005506	Metabolism	Metabolism	iron ion binding	F	AT2G42750
11468	3	F23 vs. L23	F2-3 up	GO:0005506	Metabolism	Metabolism	iron ion binding	F	AT3G10570
11469	3	F23 vs. L23	F2-3 up	GO:0005506	Metabolism	Metabolism	iron ion binding	F	AT3G11050
11470	3	F23 vs. L23	F2-3 up	GO:0005506	Metabolism	Metabolism	iron ion binding	F	AT3G14620
11471	3	F23 vs. L23	F2-3 up	GO:0005506	Metabolism	Metabolism	iron ion binding	F	AT3G48520
11472	3	F23 vs. L23	F2-3 up	GO:0005506	Metabolism	Metabolism	iron ion binding	F	AT3G61880
11473	3	F23 vs. L23	F2-3 up	GO:0005506	Metabolism	Metabolism	iron ion binding	F	AT4G00360
11474	3	F23 vs. L23	F2-3 up	GO:0005506	Metabolism	Metabolism	iron ion binding	F	AT4G13770
11475	3	F23 vs. L23	F2-3 up	GO:0005506	Metabolism	Metabolism	iron ion binding	F	AT4G15440
11476	3	F23 vs. L23	F2-3 up	GO:0005506	Metabolism	Metabolism	iron ion binding	F	AT4G19230
11477	3	F23 vs. L23	F2-3 up	GO:0005506	Metabolism	Metabolism	iron ion binding	F	AT4G26260
11478	3	F23 vs. L23	F2-3 up	GO:0005506	Metabolism	Metabolism	iron ion binding	F	AT4G37430
11479	3	F23 vs. L23	F2-3 up	GO:0005506	Metabolism	Metabolism	iron ion binding	F	AT4G39480
11480	3	F23 vs. L23	F2-3 up	GO:0005506	Metabolism	Metabolism	iron ion binding	F	AT4G39510
11481	3	F23 vs. L23	F2-3 up	GO:0005506	Metabolism	Metabolism	iron ion binding	F	AT5G01600
11482	3	F23 vs. L23	F2-3 up	GO:0005506	Metabolism	Metabolism	iron ion binding	F	AT5G25120
11483	3	F23 vs. L23	F2-3 up	GO:0005506	Metabolism	Metabolism	iron ion binding	F	AT5G36220
11484	3	F23 vs. L23	F2-3 up	GO:0005506	Metabolism	Metabolism	iron ion binding	F	AT5G53460
11485	3	F23 vs. L23	F2-3 up	GO:0005506	Metabolism	Metabolism	iron ion binding	F	AT5G57800
11486	3	F23 vs. L23	F2-3 up	GO:0005506	Metabolism	Metabolism	iron ion binding	F	AT5G60600
11487	3	F23 vs. L23	F2-3 up	GO:0016226	Metabolism	Metabolism	iron-sulfur cluster assembly	P	AT4G04770
11488	3	F23 vs. L23	F2-3 up	GO:0051536	Metabolism	Metabolism	iron-sulfur cluster binding	F	AT2G29630
11489	3	F23 vs. L23	F2-3 up	GO:0016853	Metabolism	Metabolism	isomerase activity	F	AT1G78570
11490	3	F23 vs. L23	F2-3 up	GO:0009240	Metabolism	Metabolism	isopentenyl diphosphate biosynthetic process	P	AT3G02780
11491	3	F23 vs. L23	F2-3 up	GO:0019288	Metabolism	Metabolism	isopentenyl diphosphate biosynthetic process, methylerythritol 4-phosphate pathway	P	AT5G60600
11492	3	F23 vs. L23	F2-3 up	GO:0019287	Metabolism	Metabolism	isopentenyl diphosphate biosynthetic process, mevalonate pathway	P	AT1G76490
11493	3	F23 vs. L23	F2-3 up	GO:0004452	Metabolism	Metabolism	isopentenyl-diphosphate delta-isomerase activity	F	AT3G02780
11494	3	F23 vs. L23	F2-3 up	GO:0008299	Metabolism	Metabolism	isoprenoid biosynthetic process	P	AT1G76490
11495	3	F23 vs. L23	F2-3 up	GO:0008299	Metabolism	Metabolism	isoprenoid biosynthetic process	P	AT2G17370
11496	3	F23 vs. L23	F2-3 up	GO:0008299	Metabolism	Metabolism	isoprenoid biosynthetic process	P	AT3G02780
11497	3	F23 vs. L23	F2-3 up	GO:0052694	Metabolism	Metabolism	jasmonoyl-isoleucine-12-hydroxylase activity	F	AT3G48520
11498	3	F23 vs. L23	F2-3 up	GO:0008911	Metabolism	Metabolism	lactaldehyde dehydrogenase activity	F	AT2G24270
11499	3	F23 vs. L23	F2-3 up	GO:0019516	Metabolism	Metabolism	lactate oxidation	P	AT3G14420
11500	3	F23 vs. L23	F2-3 up	GO:0004021	Metabolism	Metabolism	L-alanine:2-oxoglutarate aminotransferase activity	F	AT1G23310

11501	3	F23 vs. L23	F2-3 up	GO:0004021	Metabolism	Metabolism	L-alanine:2-oxoglutarate aminotransferase activity	F	AT1G70580
11502	3	F23 vs. L23	F2-3 up	GO:0004069	Metabolism	Metabolism	L-aspartate:2-oxoglutarate aminotransferase activity	F	AT2G30970
11503	3	F23 vs. L23	F2-3 up	GO:0004069	Metabolism	Metabolism	L-aspartate:2-oxoglutarate aminotransferase activity	F	AT5G11520
11504	3	F23 vs. L23	F2-3 up	GO:0009098	Metabolism	Metabolism	leucine biosynthetic process	P	AT2G43100
11505	3	F23 vs. L23	F2-3 up	GO:0009098	Metabolism	Metabolism	leucine biosynthetic process	P	AT3G58990
11506	3	F23 vs. L23	F2-3 up	GO:0009098	Metabolism	Metabolism	leucine biosynthetic process	P	AT5G14200
11507	3	F23 vs. L23	F2-3 up	GO:0103068	Metabolism	Metabolism	leukotriene C4 gamma-glutamyl transferase activity	F	AT4G39640
11508	3	F23 vs. L23	F2-3 up	GO:0097054	Metabolism	Metabolism	L-glutamate biosynthetic process	P	AT5G04140
11509	3	F23 vs. L23	F2-3 up	GO:0097054	Metabolism	Metabolism	L-glutamate biosynthetic process	P	AT5G53460
11510	3	F23 vs. L23	F2-3 up	GO:0016874	Metabolism	Metabolism	ligase activity	F	AT3G01060
11511	3	F23 vs. L23	F2-3 up	GO:0016874	Metabolism	Metabolism	ligase activity	F	AT5G63380
11512	3	F23 vs. L23	F2-3 up	GO:0009809	Metabolism	Metabolism	lignin biosynthetic process	P	AT1G05850
11513	3	F23 vs. L23	F2-3 up	GO:0009809	Metabolism	Metabolism	lignin biosynthetic process	P	AT1G12840
11514	3	F23 vs. L23	F2-3 up	GO:0009809	Metabolism	Metabolism	lignin biosynthetic process	P	AT1G61820
11515	3	F23 vs. L23	F2-3 up	GO:0009809	Metabolism	Metabolism	lignin biosynthetic process	P	AT2G36880
11516	3	F23 vs. L23	F2-3 up	GO:0009809	Metabolism	Metabolism	lignin biosynthetic process	P	AT2G40890
11517	3	F23 vs. L23	F2-3 up	GO:0009809	Metabolism	Metabolism	lignin biosynthetic process	P	AT3G17390
11518	3	F23 vs. L23	F2-3 up	GO:0009809	Metabolism	Metabolism	lignin biosynthetic process	P	AT4G01070
11519	3	F23 vs. L23	F2-3 up	GO:0009809	Metabolism	Metabolism	lignin biosynthetic process	P	AT4G34050
11520	3	F23 vs. L23	F2-3 up	GO:0009809	Metabolism	Metabolism	lignin biosynthetic process	P	AT4G37990
11521	3	F23 vs. L23	F2-3 up	GO:0009809	Metabolism	Metabolism	lignin biosynthetic process	P	AT5G60020
11522	3	F23 vs. L23	F2-3 up	GO:0009808	Metabolism	Metabolism	lignin metabolic process	P	AT2G30490
11523	3	F23 vs. L23	F2-3 up	GO:0009808	Metabolism	Metabolism	lignin metabolic process	P	AT2G41480
11524	3	F23 vs. L23	F2-3 up	GO:0016165	Metabolism	Metabolism	linoleate 13S-lipoxygenase activity	F	AT1G17420
11525	3	F23 vs. L23	F2-3 up	GO:0016165	Metabolism	Metabolism	linoleate 13S-lipoxygenase activity	F	AT1G72520
11526	3	F23 vs. L23	F2-3 up	GO:0016165	Metabolism	Metabolism	linoleate 13S-lipoxygenase activity	F	AT3G45140
11527	3	F23 vs. L23	F2-3 up	GO:1990136	Metabolism	Metabolism	linoleate 9S-lipoxygenase activity	F	AT3G22400
11528	3	F23 vs. L23	F2-3 up	GO:0016298	Metabolism	Metabolism	lipase activity	F	AT1G75880
11529	3	F23 vs. L23	F2-3 up	GO:0008289	Metabolism	Metabolism	lipid binding	F	AT3G21260
11530	3	F23 vs. L23	F2-3 up	GO:0008289	Metabolism	Metabolism	lipid binding	F	AT3G51600
11531	3	F23 vs. L23	F2-3 up	GO:0008289	Metabolism	Metabolism	lipid binding	F	AT3G54800
11532	3	F23 vs. L23	F2-3 up	GO:0008289	Metabolism	Metabolism	lipid binding	F	AT5G07550
11533	3	F23 vs. L23	F2-3 up	GO:0008610	Metabolism	Metabolism	lipid biosynthetic process	P	AT5G57800
11534	3	F23 vs. L23	F2-3 up	GO:0034389	Metabolism	Metabolism	lipid droplet organization	P	AT3G05500
11535	3	F23 vs. L23	F2-3 up	GO:0030259	Metabolism	Metabolism	lipid glycosylation	P	AT5G04500
11536	3	F23 vs. L23	F2-3 up	GO:0006629	Metabolism	Metabolism	lipid metabolic process	P	AT1G09430
11537	3	F23 vs. L23	F2-3 up	GO:0006629	Metabolism	Metabolism	lipid metabolic process	P	AT1G60810
11538	3	F23 vs. L23	F2-3 up	GO:0006629	Metabolism	Metabolism	lipid metabolic process	P	AT1G62290
11539	3	F23 vs. L23	F2-3 up	GO:0006629	Metabolism	Metabolism	lipid metabolic process	P	AT3G01420
11540	3	F23 vs. L23	F2-3 up	GO:0006629	Metabolism	Metabolism	lipid metabolic process	P	AT4G04460
11541	3	F23 vs. L23	F2-3 up	GO:0006629	Metabolism	Metabolism	lipid metabolic process	P	AT5G16010
11542	3	F23 vs. L23	F2-3 up	GO:0006629	Metabolism	Metabolism	lipid metabolic process	P	AT5G55340
11543	3	F23 vs. L23	F2-3 up	GO:0006629	Metabolism	Metabolism	lipid metabolic process	P	AT5G58050
11544	3	F23 vs. L23	F2-3 up	GO:0019915	Metabolism	Metabolism	lipid storage	P	AT3G05500
11545	3	F23 vs. L23	F2-3 up	GO:0019915	Metabolism	Metabolism	lipid storage	P	AT5G07550
11546	3	F23 vs. L23	F2-3 up	GO:0031405	Metabolism	Metabolism	lipoic acid binding	F	AT3G13930
11547	3	F23 vs. L23	F2-3 up	GO:0004459	Metabolism	Metabolism	L-lactate dehydrogenase activity	F	AT3G14420
11548	3	F23 vs. L23	F2-3 up	GO:0062045	Metabolism	Metabolism	L-lysine alpha-aminotransferase	F	AT2G13810
11549	3	F23 vs. L23	F2-3 up	GO:0019279	Metabolism	Metabolism	L-methionine biosynthetic process from L-homoserine via cystathionine	P	AT3G57050
11550	3	F23 vs. L23	F2-3 up	GO:0036456	Metabolism	Metabolism	L-methionine-(S)-S-oxide reductase activity	F	AT5G07460
11551	3	F23 vs. L23	F2-3 up	GO:0033744	Metabolism	Metabolism	L-methionine:thioredoxin-disulfide S-oxidoreductase activity	F	AT5G07460
11552	3	F23 vs. L23	F2-3 up	GO:0009094	Metabolism	Metabolism	L-phenylalanine biosynthetic process	P	AT5G22630

11553	3	F23 vs. L23	F2-3 up	GO:0062034	Metabolism	Metabolism	L-pipecolic acid biosynthetic process	P	AT2G13810
11554	3	F23 vs. L23	F2-3 up	GO:0055129	Metabolism	Metabolism	L-proline biosynthetic process	P	AT3G55610
11555	3	F23 vs. L23	F2-3 up	GO:0004838	Metabolism	Metabolism	L-tyrosine:2-oxoglutarate aminotransferase activity	F	AT5G36160
11556	3	F23 vs. L23	F2-3 up	GO:0016829	Metabolism	Metabolism	lyase activity	F	AT1G44000
11557	3	F23 vs. L23	F2-3 up	GO:0016829	Metabolism	Metabolism	lyase activity	F	AT2G02990
11558	3	F23 vs. L23	F2-3 up	GO:0016829	Metabolism	Metabolism	lyase activity	F	AT3G07820
11559	3	F23 vs. L23	F2-3 up	GO:0016829	Metabolism	Metabolism	lyase activity	F	AT3G07830
11560	3	F23 vs. L23	F2-3 up	GO:0016829	Metabolism	Metabolism	lyase activity	F	AT3G07840
11561	3	F23 vs. L23	F2-3 up	GO:0016829	Metabolism	Metabolism	lyase activity	F	AT5G48140
11562	3	F23 vs. L23	F2-3 up	GO:0016829	Metabolism	Metabolism	lyase activity	F	AT5G55720
11563	3	F23 vs. L23	F2-3 up	GO:0016829	Metabolism	Metabolism	lyase activity	F	AT5G63180
11564	3	F23 vs. L23	F2-3 up	GO:0009089	Metabolism	Metabolism	lysine biosynthetic process via diaminopimelate	P	AT5G14060
11565	3	F23 vs. L23	F2-3 up	GO:0009089	Metabolism	Metabolism	lysine biosynthetic process via diaminopimelate	P	AT5G52100
11566	3	F23 vs. L23	F2-3 up	GO:0000287	Metabolism	Metabolism	magnesium ion binding	F	AT1G51420
11567	3	F23 vs. L23	F2-3 up	GO:0000287	Metabolism	Metabolism	magnesium ion binding	F	AT1G54280
11568	3	F23 vs. L23	F2-3 up	GO:0000287	Metabolism	Metabolism	magnesium ion binding	F	AT1G70730
11569	3	F23 vs. L23	F2-3 up	GO:0000287	Metabolism	Metabolism	magnesium ion binding	F	AT2G24210
11570	3	F23 vs. L23	F2-3 up	GO:0000287	Metabolism	Metabolism	magnesium ion binding	F	AT2G35840
11571	3	F23 vs. L23	F2-3 up	GO:0000287	Metabolism	Metabolism	magnesium ion binding	F	AT2G46860
11572	3	F23 vs. L23	F2-3 up	GO:0000287	Metabolism	Metabolism	magnesium ion binding	F	AT3G09810
11573	3	F23 vs. L23	F2-3 up	GO:0000287	Metabolism	Metabolism	magnesium ion binding	F	AT4G01480
11574	3	F23 vs. L23	F2-3 up	GO:0000287	Metabolism	Metabolism	magnesium ion binding	F	AT5G09650
11575	3	F23 vs. L23	F2-3 up	GO:0000287	Metabolism	Metabolism	magnesium ion binding	F	AT5G14200
11576	3	F23 vs. L23	F2-3 up	GO:0000287	Metabolism	Metabolism	magnesium ion binding	F	AT5G56350
11577	3	F23 vs. L23	F2-3 up	GO:0000287	Metabolism	Metabolism	magnesium ion binding	F	AT5G57350
11578	3	F23 vs. L23	F2-3 up	GO:0000287	Metabolism	Metabolism	magnesium ion binding	F	AT5G63680
11579	3	F23 vs. L23	F2-3 up	GO:0016034	Metabolism	Metabolism	maleylacetoacetate isomerase activity	F	AT2G02390
11580	3	F23 vs. L23	F2-3 up	GO:0102216	Metabolism	Metabolism	maltodextrin water dikinase	F	AT4G24450
11581	3	F23 vs. L23	F2-3 up	GO:0000024	Metabolism	Metabolism	maltose biosynthetic process	P	AT4G17090
11582	3	F23 vs. L23	F2-3 up	GO:0030145	Metabolism	Metabolism	manganese ion binding	F	AT1G72610
11583	3	F23 vs. L23	F2-3 up	GO:0030145	Metabolism	Metabolism	manganese ion binding	F	AT3G05930
11584	3	F23 vs. L23	F2-3 up	GO:0010412	Metabolism	Metabolism	mannan metabolic process	P	AT3G21190
11585	3	F23 vs. L23	F2-3 up	GO:0051753	Metabolism	Metabolism	mannan synthase activity	F	AT2G33100
11586	3	F23 vs. L23	F2-3 up	GO:0051753	Metabolism	Metabolism	mannan synthase activity	F	AT3G21190
11587	3	F23 vs. L23	F2-3 up	GO:0051753	Metabolism	Metabolism	mannan synthase activity	F	AT4G16590
11588	3	F23 vs. L23	F2-3 up	GO:0051753	Metabolism	Metabolism	mannan synthase activity	F	AT5G16910
11589	3	F23 vs. L23	F2-3 up	GO:0004475	Metabolism	Metabolism	mannose-1-phosphate guanylyltransferase activity	F	AT2G39770
11590	3	F23 vs. L23	F2-3 up	GO:0004476	Metabolism	Metabolism	mannose-6-phosphate isomerase activity	F	AT3G02570
11591	3	F23 vs. L23	F2-3 up	GO:0097502	Metabolism	Metabolism	mannosylation	P	AT3G21190
11592	3	F23 vs. L23	F2-3 up	GO:0052854	Metabolism	Metabolism	medium-chain-(S)-2-hydroxy-acid oxidase activity	F	AT3G14420
11593	3	F23 vs. L23	F2-3 up	GO:0046467	Metabolism	Metabolism	membrane lipid biosynthetic process	P	AT3G07700
11594	3	F23 vs. L23	F2-3 up	GO:0046872	Metabolism	Metabolism	metal ion binding	F	AT1G04400
11595	3	F23 vs. L23	F2-3 up	GO:0046872	Metabolism	Metabolism	metal ion binding	F	AT1G05010
11596	3	F23 vs. L23	F2-3 up	GO:0046872	Metabolism	Metabolism	metal ion binding	F	AT1G06640
11597	3	F23 vs. L23	F2-3 up	GO:0046872	Metabolism	Metabolism	metal ion binding	F	AT1G10960
11598	3	F23 vs. L23	F2-3 up	GO:0046872	Metabolism	Metabolism	metal ion binding	F	AT1G11840
11599	3	F23 vs. L23	F2-3 up	GO:0046872	Metabolism	Metabolism	metal ion binding	F	AT1G17420
11600	3	F23 vs. L23	F2-3 up	GO:0046872	Metabolism	Metabolism	metal ion binding	F	AT1G19150
11601	3	F23 vs. L23	F2-3 up	GO:0046872	Metabolism	Metabolism	metal ion binding	F	AT1G20823
11602	3	F23 vs. L23	F2-3 up	GO:0046872	Metabolism	Metabolism	metal ion binding	F	AT1G35190
11603	3	F23 vs. L23	F2-3 up	GO:0046872	Metabolism	Metabolism	metal ion binding	F	AT1G45474
11604	3	F23 vs. L23	F2-3 up	GO:0046872	Metabolism	Metabolism	metal ion binding	F	AT1G47570
11605	3	F23 vs. L23	F2-3 up	GO:0046872	Metabolism	Metabolism	metal ion binding	F	AT1G50250
11606	3	F23 vs. L23	F2-3 up	GO:0046872	Metabolism	Metabolism	metal ion binding	F	AT1G51090
11607	3	F23 vs. L23	F2-3 up	GO:0046872	Metabolism	Metabolism	metal ion binding	F	AT1G56500
11608	3	F23 vs. L23	F2-3 up	GO:0046872	Metabolism	Metabolism	metal ion binding	F	AT1G71050

11609	3	F23 vs. L23	F2-3 up	GO:0046872	Metabolism	Metabolism	metal ion binding	F	AT1G72520
11610	3	F23 vs. L23	F2-3 up	GO:0046872	Metabolism	Metabolism	metal ion binding	F	AT1G75690
11611	3	F23 vs. L23	F2-3 up	GO:0046872	Metabolism	Metabolism	metal ion binding	F	AT1G80660
11612	3	F23 vs. L23	F2-3 up	GO:0046872	Metabolism	Metabolism	metal ion binding	F	AT2G07560
11613	3	F23 vs. L23	F2-3 up	GO:0046872	Metabolism	Metabolism	metal ion binding	F	AT2G15620
11614	3	F23 vs. L23	F2-3 up	GO:0046872	Metabolism	Metabolism	metal ion binding	F	AT2G16430
11615	3	F23 vs. L23	F2-3 up	GO:0046872	Metabolism	Metabolism	metal ion binding	F	AT2G22450
11616	3	F23 vs. L23	F2-3 up	GO:0046872	Metabolism	Metabolism	metal ion binding	F	AT2G22950
11617	3	F23 vs. L23	F2-3 up	GO:0046872	Metabolism	Metabolism	metal ion binding	F	AT2G24820
11618	3	F23 vs. L23	F2-3 up	GO:0046872	Metabolism	Metabolism	metal ion binding	F	AT2G25740
11619	3	F23 vs. L23	F2-3 up	GO:0046872	Metabolism	Metabolism	metal ion binding	F	AT2G29630
11620	3	F23 vs. L23	F2-3 up	GO:0046872	Metabolism	Metabolism	metal ion binding	F	AT2G30020
11621	3	F23 vs. L23	F2-3 up	GO:0046872	Metabolism	Metabolism	metal ion binding	F	AT2G30830
11622	3	F23 vs. L23	F2-3 up	GO:0046872	Metabolism	Metabolism	metal ion binding	F	AT2G31830
11623	3	F23 vs. L23	F2-3 up	GO:0046872	Metabolism	Metabolism	metal ion binding	F	AT2G38240
11624	3	F23 vs. L23	F2-3 up	GO:0046872	Metabolism	Metabolism	metal ion binding	F	AT2G38280
11625	3	F23 vs. L23	F2-3 up	GO:0046872	Metabolism	Metabolism	metal ion binding	F	AT2G40000
11626	3	F23 vs. L23	F2-3 up	GO:0046872	Metabolism	Metabolism	metal ion binding	F	AT2G40140
11627	3	F23 vs. L23	F2-3 up	GO:0046872	Metabolism	Metabolism	metal ion binding	F	AT2G41480
11628	3	F23 vs. L23	F2-3 up	GO:0046872	Metabolism	Metabolism	metal ion binding	F	AT2G41630
11629	3	F23 vs. L23	F2-3 up	GO:0046872	Metabolism	Metabolism	metal ion binding	F	AT2G42200
11630	3	F23 vs. L23	F2-3 up	GO:0046872	Metabolism	Metabolism	metal ion binding	F	AT2G45190
11631	3	F23 vs. L23	F2-3 up	GO:0046872	Metabolism	Metabolism	metal ion binding	F	AT2G45770
11632	3	F23 vs. L23	F2-3 up	GO:0046872	Metabolism	Metabolism	metal ion binding	F	AT2G45800
11633	3	F23 vs. L23	F2-3 up	GO:0046872	Metabolism	Metabolism	metal ion binding	F	AT2G46210
11634	3	F23 vs. L23	F2-3 up	GO:0046872	Metabolism	Metabolism	metal ion binding	F	AT3G01420
11635	3	F23 vs. L23	F2-3 up	GO:0046872	Metabolism	Metabolism	metal ion binding	F	AT3G02750
11636	3	F23 vs. L23	F2-3 up	GO:0046872	Metabolism	Metabolism	metal ion binding	F	AT3G02770
11637	3	F23 vs. L23	F2-3 up	GO:0046872	Metabolism	Metabolism	metal ion binding	F	AT3G02780
11638	3	F23 vs. L23	F2-3 up	GO:0046872	Metabolism	Metabolism	metal ion binding	F	AT3G03420
11639	3	F23 vs. L23	F2-3 up	GO:0046872	Metabolism	Metabolism	metal ion binding	F	AT3G05580
11640	3	F23 vs. L23	F2-3 up	GO:0046872	Metabolism	Metabolism	metal ion binding	F	AT3G07940
11641	3	F23 vs. L23	F2-3 up	GO:0046872	Metabolism	Metabolism	metal ion binding	F	AT3G08990
11642	3	F23 vs. L23	F2-3 up	GO:0046872	Metabolism	Metabolism	metal ion binding	F	AT3G10700
11643	3	F23 vs. L23	F2-3 up	GO:0046872	Metabolism	Metabolism	metal ion binding	F	AT3G11410
11644	3	F23 vs. L23	F2-3 up	GO:0046872	Metabolism	Metabolism	metal ion binding	F	AT3G12620
11645	3	F23 vs. L23	F2-3 up	GO:0046872	Metabolism	Metabolism	metal ion binding	F	AT3G16800
11646	3	F23 vs. L23	F2-3 up	GO:0046872	Metabolism	Metabolism	metal ion binding	F	AT3G17390
11647	3	F23 vs. L23	F2-3 up	GO:0046872	Metabolism	Metabolism	metal ion binding	F	AT3G17611
11648	3	F23 vs. L23	F2-3 up	GO:0046872	Metabolism	Metabolism	metal ion binding	F	AT3G18080
11649	3	F23 vs. L23	F2-3 up	GO:0046872	Metabolism	Metabolism	metal ion binding	F	AT3G19980
11650	3	F23 vs. L23	F2-3 up	GO:0046872	Metabolism	Metabolism	metal ion binding	F	AT3G21180
11651	3	F23 vs. L23	F2-3 up	GO:0046872	Metabolism	Metabolism	metal ion binding	F	AT3G22370
11652	3	F23 vs. L23	F2-3 up	GO:0046872	Metabolism	Metabolism	metal ion binding	F	AT3G22400
11653	3	F23 vs. L23	F2-3 up	GO:0046872	Metabolism	Metabolism	metal ion binding	F	AT3G24670
11654	3	F23 vs. L23	F2-3 up	GO:0046872	Metabolism	Metabolism	metal ion binding	F	AT3G25290
11655	3	F23 vs. L23	F2-3 up	GO:0046872	Metabolism	Metabolism	metal ion binding	F	AT3G42640
11656	3	F23 vs. L23	F2-3 up	GO:0046872	Metabolism	Metabolism	metal ion binding	F	AT3G45140
11657	3	F23 vs. L23	F2-3 up	GO:0046872	Metabolism	Metabolism	metal ion binding	F	AT3G46620
11658	3	F23 vs. L23	F2-3 up	GO:0046872	Metabolism	Metabolism	metal ion binding	F	AT3G48420
11659	3	F23 vs. L23	F2-3 up	GO:0046872	Metabolism	Metabolism	metal ion binding	F	AT3G49110
11660	3	F23 vs. L23	F2-3 up	GO:0046872	Metabolism	Metabolism	metal ion binding	F	AT3G50210
11661	3	F23 vs. L23	F2-3 up	GO:0046872	Metabolism	Metabolism	metal ion binding	F	AT3G51240
11662	3	F23 vs. L23	F2-3 up	GO:0046872	Metabolism	Metabolism	metal ion binding	F	AT3G55800
11663	3	F23 vs. L23	F2-3 up	GO:0046872	Metabolism	Metabolism	metal ion binding	F	AT3G57330
11664	3	F23 vs. L23	F2-3 up	GO:0046872	Metabolism	Metabolism	metal ion binding	F	AT3G60330

11665	3	F23 vs. L23	F2-3 up	GO:0046872	Metabolism	Metabolism	metal ion binding	F	AT3G61230
11666	3	F23 vs. L23	F2-3 up	GO:0046872	Metabolism	Metabolism	metal ion binding	F	AT4G03280
11667	3	F23 vs. L23	F2-3 up	GO:0046872	Metabolism	Metabolism	metal ion binding	F	AT4G15530
11668	3	F23 vs. L23	F2-3 up	GO:0046872	Metabolism	Metabolism	metal ion binding	F	AT4G15700
11669	3	F23 vs. L23	F2-3 up	GO:0046872	Metabolism	Metabolism	metal ion binding	F	AT4G21990
11670	3	F23 vs. L23	F2-3 up	GO:0046872	Metabolism	Metabolism	metal ion binding	F	AT4G24450
11671	3	F23 vs. L23	F2-3 up	GO:0046872	Metabolism	Metabolism	metal ion binding	F	AT4G24890
11672	3	F23 vs. L23	F2-3 up	GO:0046872	Metabolism	Metabolism	metal ion binding	F	AT4G31750
11673	3	F23 vs. L23	F2-3 up	GO:0046872	Metabolism	Metabolism	metal ion binding	F	AT4G32410
11674	3	F23 vs. L23	F2-3 up	GO:0046872	Metabolism	Metabolism	metal ion binding	F	AT4G34050
11675	3	F23 vs. L23	F2-3 up	GO:0046872	Metabolism	Metabolism	metal ion binding	F	AT4G35060
11676	3	F23 vs. L23	F2-3 up	GO:0046872	Metabolism	Metabolism	metal ion binding	F	AT4G39350
11677	3	F23 vs. L23	F2-3 up	GO:0046872	Metabolism	Metabolism	metal ion binding	F	AT5G04140
11678	3	F23 vs. L23	F2-3 up	GO:0046872	Metabolism	Metabolism	metal ion binding	F	AT5G04500
11679	3	F23 vs. L23	F2-3 up	GO:0046872	Metabolism	Metabolism	metal ion binding	F	AT5G05600
11680	3	F23 vs. L23	F2-3 up	GO:0046872	Metabolism	Metabolism	metal ion binding	F	AT5G05730
11681	3	F23 vs. L23	F2-3 up	GO:0046872	Metabolism	Metabolism	metal ion binding	F	AT5G08640
11682	3	F23 vs. L23	F2-3 up	GO:0046872	Metabolism	Metabolism	metal ion binding	F	AT5G09870
11683	3	F23 vs. L23	F2-3 up	GO:0046872	Metabolism	Metabolism	metal ion binding	F	AT5G14660
11684	3	F23 vs. L23	F2-3 up	GO:0046872	Metabolism	Metabolism	metal ion binding	F	AT5G16450
11685	3	F23 vs. L23	F2-3 up	GO:0046872	Metabolism	Metabolism	metal ion binding	F	AT5G17450
11686	3	F23 vs. L23	F2-3 up	GO:0046872	Metabolism	Metabolism	metal ion binding	F	AT5G18600
11687	3	F23 vs. L23	F2-3 up	GO:0046872	Metabolism	Metabolism	metal ion binding	F	AT5G20700
11688	3	F23 vs. L23	F2-3 up	GO:0046872	Metabolism	Metabolism	metal ion binding	F	AT5G22250
11689	3	F23 vs. L23	F2-3 up	GO:0046872	Metabolism	Metabolism	metal ion binding	F	AT5G22580
11690	3	F23 vs. L23	F2-3 up	GO:0046872	Metabolism	Metabolism	metal ion binding	F	AT5G24530
11691	3	F23 vs. L23	F2-3 up	GO:0046872	Metabolism	Metabolism	metal ion binding	F	AT5G35735
11692	3	F23 vs. L23	F2-3 up	GO:0046872	Metabolism	Metabolism	metal ion binding	F	AT5G43380
11693	3	F23 vs. L23	F2-3 up	GO:0046872	Metabolism	Metabolism	metal ion binding	F	AT5G47000
11694	3	F23 vs. L23	F2-3 up	GO:0046872	Metabolism	Metabolism	metal ion binding	F	AT5G47650
11695	3	F23 vs. L23	F2-3 up	GO:0046872	Metabolism	Metabolism	metal ion binding	F	AT5G51720
11696	3	F23 vs. L23	F2-3 up	GO:0046872	Metabolism	Metabolism	metal ion binding	F	AT5G51890
11697	3	F23 vs. L23	F2-3 up	GO:0046872	Metabolism	Metabolism	metal ion binding	F	AT5G54080
11698	3	F23 vs. L23	F2-3 up	GO:0046872	Metabolism	Metabolism	metal ion binding	F	AT5G55720
11699	3	F23 vs. L23	F2-3 up	GO:0046872	Metabolism	Metabolism	metal ion binding	F	AT5G61410
11700	3	F23 vs. L23	F2-3 up	GO:0046872	Metabolism	Metabolism	metal ion binding	F	AT5G63180
11701	3	F23 vs. L23	F2-3 up	GO:0046872	Metabolism	Metabolism	metal ion binding	F	AT5G64740
11702	3	F23 vs. L23	F2-3 up	GO:0046872	Metabolism	Metabolism	metal ion binding	F	AT5G66070
11703	3	F23 vs. L23	F2-3 up	GO:0070006	Metabolism	Metabolism	metalloaminopeptidase activity	F	AT5G04710
11704	3	F23 vs. L23	F2-3 up	GO:0004478	Metabolism	Metabolism	methionine adenosyltransferase activity	F	AT2G36880
11705	3	F23 vs. L23	F2-3 up	GO:0004478	Metabolism	Metabolism	methionine adenosyltransferase activity	F	AT3G17390
11706	3	F23 vs. L23	F2-3 up	GO:0004478	Metabolism	Metabolism	methionine adenosyltransferase activity	F	AT4G01850
11707	3	F23 vs. L23	F2-3 up	GO:0009086	Metabolism	Metabolism	methionine biosynthetic process	P	AT3G59970
11708	3	F23 vs. L23	F2-3 up	GO:0006555	Metabolism	Metabolism	methionine metabolic process	P	AT3G17390
11709	3	F23 vs. L23	F2-3 up	GO:0006555	Metabolism	Metabolism	methionine metabolic process	P	AT4G14710
11710	3	F23 vs. L23	F2-3 up	GO:0010326	Metabolism	Metabolism	methionine-oxo-acid transaminase activity	F	AT3G19710
11711	3	F23 vs. L23	F2-3 up	GO:0008168	Metabolism	Metabolism	methyltransferase activity	F	AT1G04430
11712	3	F23 vs. L23	F2-3 up	GO:0008168	Metabolism	Metabolism	methyltransferase activity	F	AT1G13860
11713	3	F23 vs. L23	F2-3 up	GO:0008168	Metabolism	Metabolism	methyltransferase activity	F	AT1G27930
11714	3	F23 vs. L23	F2-3 up	GO:0008168	Metabolism	Metabolism	methyltransferase activity	F	AT1G31850
11715	3	F23 vs. L23	F2-3 up	GO:0008168	Metabolism	Metabolism	methyltransferase activity	F	AT1G33170
11716	3	F23 vs. L23	F2-3 up	GO:0008168	Metabolism	Metabolism	methyltransferase activity	F	AT1G78240
11717	3	F23 vs. L23	F2-3 up	GO:0008168	Metabolism	Metabolism	methyltransferase activity	F	AT2G27590
11718	3	F23 vs. L23	F2-3 up	GO:0008168	Metabolism	Metabolism	methyltransferase activity	F	AT2G41380
11719	3	F23 vs. L23	F2-3 up	GO:0008168	Metabolism	Metabolism	methyltransferase activity	F	AT3G17310
11720	3	F23 vs. L23	F2-3 up	GO:0008168	Metabolism	Metabolism	methyltransferase activity	F	AT3G23300

11721	3	F23 vs. L23	F2-3 up	GO:0008168	Metabolism	Metabolism	methyltransferase activity	F	AT3G27230
11722	3	F23 vs. L23	F2-3 up	GO:0008168	Metabolism	Metabolism	methyltransferase activity	F	AT3G51070
11723	3	F23 vs. L23	F2-3 up	GO:0008168	Metabolism	Metabolism	methyltransferase activity	F	AT4G10440
11724	3	F23 vs. L23	F2-3 up	GO:0008168	Metabolism	Metabolism	methyltransferase activity	F	AT4G17080
11725	3	F23 vs. L23	F2-3 up	GO:0008168	Metabolism	Metabolism	methyltransferase activity	F	AT4G19120
11726	3	F23 vs. L23	F2-3 up	GO:0008168	Metabolism	Metabolism	methyltransferase activity	F	AT5G65810
11727	3	F23 vs. L23	F2-3 up	GO:0000146	Metabolism	Metabolism	microfilament motor activity	F	AT1G54560
11728	3	F23 vs. L23	F2-3 up	GO:0008017	Metabolism	Metabolism	microtubule binding	F	AT3G43210
11729	3	F23 vs. L23	F2-3 up	GO:0008017	Metabolism	Metabolism	microtubule binding	F	AT3G61760
11730	3	F23 vs. L23	F2-3 up	GO:0008017	Metabolism	Metabolism	microtubule binding	F	AT5G27000
11731	3	F23 vs. L23	F2-3 up	GO:0008017	Metabolism	Metabolism	microtubule binding	F	AT5G65460
11732	3	F23 vs. L23	F2-3 up	GO:0003674	Metabolism	Metabolism	molecular_function	F	AT1G03620
11733	3	F23 vs. L23	F2-3 up	GO:0003674	Metabolism	Metabolism	molecular_function	F	AT1G04670
11734	3	F23 vs. L23	F2-3 up	GO:0003674	Metabolism	Metabolism	molecular_function	F	AT1G12250
11735	3	F23 vs. L23	F2-3 up	GO:0003674	Metabolism	Metabolism	molecular_function	F	AT1G12320
11736	3	F23 vs. L23	F2-3 up	GO:0003674	Metabolism	Metabolism	molecular_function	F	AT1G13970
11737	3	F23 vs. L23	F2-3 up	GO:0003674	Metabolism	Metabolism	molecular_function	F	AT1G16290
11738	3	F23 vs. L23	F2-3 up	GO:0003674	Metabolism	Metabolism	molecular_function	F	AT1G17360
11739	3	F23 vs. L23	F2-3 up	GO:0003674	Metabolism	Metabolism	molecular_function	F	AT1G19610
11740	3	F23 vs. L23	F2-3 up	GO:0003674	Metabolism	Metabolism	molecular_function	F	AT1G21010
11741	3	F23 vs. L23	F2-3 up	GO:0003674	Metabolism	Metabolism	molecular_function	F	AT1G21060
11742	3	F23 vs. L23	F2-3 up	GO:0003674	Metabolism	Metabolism	molecular_function	F	AT1G22750
11743	3	F23 vs. L23	F2-3 up	GO:0003674	Metabolism	Metabolism	molecular_function	F	AT1G23710
11744	3	F23 vs. L23	F2-3 up	GO:0003674	Metabolism	Metabolism	molecular_function	F	AT1G24520
11745	3	F23 vs. L23	F2-3 up	GO:0003674	Metabolism	Metabolism	molecular_function	F	AT1G27850
11746	3	F23 vs. L23	F2-3 up	GO:0003674	Metabolism	Metabolism	molecular_function	F	AT1G29140
11747	3	F23 vs. L23	F2-3 up	GO:0003674	Metabolism	Metabolism	molecular_function	F	AT1G31750
11748	3	F23 vs. L23	F2-3 up	GO:0003674	Metabolism	Metabolism	molecular_function	F	AT1G47980
11749	3	F23 vs. L23	F2-3 up	GO:0003674	Metabolism	Metabolism	molecular_function	F	AT1G49800
11750	3	F23 vs. L23	F2-3 up	GO:0003674	Metabolism	Metabolism	molecular_function	F	AT1G63060
11751	3	F23 vs. L23	F2-3 up	GO:0003674	Metabolism	Metabolism	molecular_function	F	AT1G63930
11752	3	F23 vs. L23	F2-3 up	GO:0003674	Metabolism	Metabolism	molecular_function	F	AT1G64140
11753	3	F23 vs. L23	F2-3 up	GO:0003674	Metabolism	Metabolism	molecular_function	F	AT1G68440
11754	3	F23 vs. L23	F2-3 up	GO:0003674	Metabolism	Metabolism	molecular_function	F	AT1G68610
11755	3	F23 vs. L23	F2-3 up	GO:0003674	Metabolism	Metabolism	molecular_function	F	AT1G70780
11756	3	F23 vs. L23	F2-3 up	GO:0003674	Metabolism	Metabolism	molecular_function	F	AT1G72240
11757	3	F23 vs. L23	F2-3 up	GO:0003674	Metabolism	Metabolism	molecular_function	F	AT1G73390
11758	3	F23 vs. L23	F2-3 up	GO:0003674	Metabolism	Metabolism	molecular_function	F	AT1G74450
11759	3	F23 vs. L23	F2-3 up	GO:0003674	Metabolism	Metabolism	molecular_function	F	AT1G74510
11760	3	F23 vs. L23	F2-3 up	GO:0003674	Metabolism	Metabolism	molecular_function	F	AT1G74670
11761	3	F23 vs. L23	F2-3 up	GO:0003674	Metabolism	Metabolism	molecular_function	F	AT1G75160
11762	3	F23 vs. L23	F2-3 up	GO:0003674	Metabolism	Metabolism	molecular_function	F	AT1G75800
11763	3	F23 vs. L23	F2-3 up	GO:0003674	Metabolism	Metabolism	molecular_function	F	AT1G78780
11764	3	F23 vs. L23	F2-3 up	GO:0003674	Metabolism	Metabolism	molecular_function	F	AT2G04039
11765	3	F23 vs. L23	F2-3 up	GO:0003674	Metabolism	Metabolism	molecular_function	F	AT2G05530
11766	3	F23 vs. L23	F2-3 up	GO:0003674	Metabolism	Metabolism	molecular_function	F	AT2G07708
11767	3	F23 vs. L23	F2-3 up	GO:0003674	Metabolism	Metabolism	molecular_function	F	AT2G13350
11768	3	F23 vs. L23	F2-3 up	GO:0003674	Metabolism	Metabolism	molecular_function	F	AT2G14900
11769	3	F23 vs. L23	F2-3 up	GO:0003674	Metabolism	Metabolism	molecular_function	F	AT2G17840
11770	3	F23 vs. L23	F2-3 up	GO:0003674	Metabolism	Metabolism	molecular_function	F	AT2G19980
11771	3	F23 vs. L23	F2-3 up	GO:0003674	Metabolism	Metabolism	molecular_function	F	AT2G20320
11772	3	F23 vs. L23	F2-3 up	GO:0003674	Metabolism	Metabolism	molecular_function	F	AT2G20870
11773	3	F23 vs. L23	F2-3 up	GO:0003674	Metabolism	Metabolism	molecular_function	F	AT2G24450
11774	3	F23 vs. L23	F2-3 up	GO:0003674	Metabolism	Metabolism	molecular_function	F	AT2G36020
11775	3	F23 vs. L23	F2-3 up	GO:0003674	Metabolism	Metabolism	molecular_function	F	AT2G38640
11776	3	F23 vs. L23	F2-3 up	GO:0003674	Metabolism	Metabolism	molecular_function	F	AT2G39210

11777	3	F23 vs. L23	F2-3 up	GO:0003674	Metabolism	Metabolism	molecular_function	F	AT2G39650
11778	3	F23 vs. L23	F2-3 up	GO:0003674	Metabolism	Metabolism	molecular_function	F	AT2G46140
11779	3	F23 vs. L23	F2-3 up	GO:0003674	Metabolism	Metabolism	molecular_function	F	AT2G46300
11780	3	F23 vs. L23	F2-3 up	GO:0003674	Metabolism	Metabolism	molecular_function	F	AT3G03160
11781	3	F23 vs. L23	F2-3 up	GO:0003674	Metabolism	Metabolism	molecular_function	F	AT3G04360
11782	3	F23 vs. L23	F2-3 up	GO:0003674	Metabolism	Metabolism	molecular_function	F	AT3G06035
11783	3	F23 vs. L23	F2-3 up	GO:0003674	Metabolism	Metabolism	molecular_function	F	AT3G06780
11784	3	F23 vs. L23	F2-3 up	GO:0003674	Metabolism	Metabolism	molecular_function	F	AT3G08610
11785	3	F23 vs. L23	F2-3 up	GO:0003674	Metabolism	Metabolism	molecular_function	F	AT3G10985
11786	3	F23 vs. L23	F2-3 up	GO:0003674	Metabolism	Metabolism	molecular_function	F	AT3G13845
11787	3	F23 vs. L23	F2-3 up	GO:0003674	Metabolism	Metabolism	molecular_function	F	AT3G15400
11788	3	F23 vs. L23	F2-3 up	GO:0003674	Metabolism	Metabolism	molecular_function	F	AT3G17800
11789	3	F23 vs. L23	F2-3 up	GO:0003674	Metabolism	Metabolism	molecular_function	F	AT3G17930
11790	3	F23 vs. L23	F2-3 up	GO:0003674	Metabolism	Metabolism	molecular_function	F	AT3G20220
11791	3	F23 vs. L23	F2-3 up	GO:0003674	Metabolism	Metabolism	molecular_function	F	AT3G20865
11792	3	F23 vs. L23	F2-3 up	GO:0003674	Metabolism	Metabolism	molecular_function	F	AT3G24506
11793	3	F23 vs. L23	F2-3 up	GO:0003674	Metabolism	Metabolism	molecular_function	F	AT3G27090
11794	3	F23 vs. L23	F2-3 up	GO:0003674	Metabolism	Metabolism	molecular_function	F	AT3G50910
11795	3	F23 vs. L23	F2-3 up	GO:0003674	Metabolism	Metabolism	molecular_function	F	AT3G56360
11796	3	F23 vs. L23	F2-3 up	GO:0003674	Metabolism	Metabolism	molecular_function	F	AT4G00880
11797	3	F23 vs. L23	F2-3 up	GO:0003674	Metabolism	Metabolism	molecular_function	F	AT4G02380
11798	3	F23 vs. L23	F2-3 up	GO:0003674	Metabolism	Metabolism	molecular_function	F	AT4G04980
11799	3	F23 vs. L23	F2-3 up	GO:0003674	Metabolism	Metabolism	molecular_function	F	AT4G11760
11800	3	F23 vs. L23	F2-3 up	GO:0003674	Metabolism	Metabolism	molecular_function	F	AT4G12410
11801	3	F23 vs. L23	F2-3 up	GO:0003674	Metabolism	Metabolism	molecular_function	F	AT4G13230
11802	3	F23 vs. L23	F2-3 up	GO:0003674	Metabolism	Metabolism	molecular_function	F	AT4G13560
11803	3	F23 vs. L23	F2-3 up	GO:0003674	Metabolism	Metabolism	molecular_function	F	AT4G14615
11804	3	F23 vs. L23	F2-3 up	GO:0003674	Metabolism	Metabolism	molecular_function	F	AT4G18920
11805	3	F23 vs. L23	F2-3 up	GO:0003674	Metabolism	Metabolism	molecular_function	F	AT4G24930
11806	3	F23 vs. L23	F2-3 up	GO:0003674	Metabolism	Metabolism	molecular_function	F	AT4G27435
11807	3	F23 vs. L23	F2-3 up	GO:0003674	Metabolism	Metabolism	molecular_function	F	AT4G29780
11808	3	F23 vs. L23	F2-3 up	GO:0003674	Metabolism	Metabolism	molecular_function	F	AT4G30460
11809	3	F23 vs. L23	F2-3 up	GO:0003674	Metabolism	Metabolism	molecular_function	F	AT4G30996
11810	3	F23 vs. L23	F2-3 up	GO:0003674	Metabolism	Metabolism	molecular_function	F	AT4G31080
11811	3	F23 vs. L23	F2-3 up	GO:0003674	Metabolism	Metabolism	molecular_function	F	AT4G31330
11812	3	F23 vs. L23	F2-3 up	GO:0003674	Metabolism	Metabolism	molecular_function	F	AT4G32020
11813	3	F23 vs. L23	F2-3 up	GO:0003674	Metabolism	Metabolism	molecular_function	F	AT5G01430
11814	3	F23 vs. L23	F2-3 up	GO:0003674	Metabolism	Metabolism	molecular_function	F	AT5G11070
11815	3	F23 vs. L23	F2-3 up	GO:0003674	Metabolism	Metabolism	molecular_function	F	AT5G11420
11816	3	F23 vs. L23	F2-3 up	GO:0003674	Metabolism	Metabolism	molecular_function	F	AT5G15460
11817	3	F23 vs. L23	F2-3 up	GO:0003674	Metabolism	Metabolism	molecular_function	F	AT5G22430
11818	3	F23 vs. L23	F2-3 up	GO:0003674	Metabolism	Metabolism	molecular_function	F	AT5G26060
11819	3	F23 vs. L23	F2-3 up	GO:0003674	Metabolism	Metabolism	molecular_function	F	AT5G38660
11820	3	F23 vs. L23	F2-3 up	GO:0003674	Metabolism	Metabolism	molecular_function	F	AT5G38760
11821	3	F23 vs. L23	F2-3 up	GO:0003674	Metabolism	Metabolism	molecular_function	F	AT5G39210
11822	3	F23 vs. L23	F2-3 up	GO:0003674	Metabolism	Metabolism	molecular_function	F	AT5G41810
11823	3	F23 vs. L23	F2-3 up	GO:0003674	Metabolism	Metabolism	molecular_function	F	AT5G42680
11824	3	F23 vs. L23	F2-3 up	GO:0003674	Metabolism	Metabolism	molecular_function	F	AT5G44300
11825	3	F23 vs. L23	F2-3 up	GO:0003674	Metabolism	Metabolism	molecular_function	F	AT5G45350
11826	3	F23 vs. L23	F2-3 up	GO:0003674	Metabolism	Metabolism	molecular_function	F	AT5G46200
11827	3	F23 vs. L23	F2-3 up	GO:0003674	Metabolism	Metabolism	molecular_function	F	AT5G50720
11828	3	F23 vs. L23	F2-3 up	GO:0003674	Metabolism	Metabolism	molecular_function	F	AT5G53590
11829	3	F23 vs. L23	F2-3 up	GO:0003674	Metabolism	Metabolism	molecular_function	F	AT5G53820
11830	3	F23 vs. L23	F2-3 up	GO:0003674	Metabolism	Metabolism	molecular_function	F	AT5G55620
11831	3	F23 vs. L23	F2-3 up	GO:0003674	Metabolism	Metabolism	molecular_function	F	AT5G55960
11832	3	F23 vs. L23	F2-3 up	GO:0003674	Metabolism	Metabolism	molecular_function	F	AT5G57785

11833	3	F23 vs. L23	F2-3 up	GO:0003674	Metabolism	Metabolism	molecular_function	F	AT5G61710
11834	3	F23 vs. L23	F2-3 up	GO:0003674	Metabolism	Metabolism	molecular_function	F	AT5G62750
11835	3	F23 vs. L23	F2-3 up	GO:0003674	Metabolism	Metabolism	molecular_function	F	AT5G64400
11836	3	F23 vs. L23	F2-3 up	GO:0003674	Metabolism	Metabolism	molecular_function	F	AT5G65650
11837	3	F23 vs. L23	F2-3 up	GO:0003674	Metabolism	Metabolism	molecular_function	F	AT5G67370
11838	3	F23 vs. L23	F2-3 up	GO:0030366	Metabolism	Metabolism	molybdopterin synthase activity	F	AT2G43760
11839	3	F23 vs. L23	F2-3 up	GO:0004497	Metabolism	Metabolism	monooxygenase activity	F	AT2G24180
11840	3	F23 vs. L23	F2-3 up	GO:0004497	Metabolism	Metabolism	monooxygenase activity	F	AT2G40890
11841	3	F23 vs. L23	F2-3 up	GO:0004497	Metabolism	Metabolism	monooxygenase activity	F	AT3G14620
11842	3	F23 vs. L23	F2-3 up	GO:0004497	Metabolism	Metabolism	monooxygenase activity	F	AT3G61880
11843	3	F23 vs. L23	F2-3 up	GO:0004497	Metabolism	Metabolism	monooxygenase activity	F	AT4G15440
11844	3	F23 vs. L23	F2-3 up	GO:0004497	Metabolism	Metabolism	monooxygenase activity	F	AT4G19230
11845	3	F23 vs. L23	F2-3 up	GO:0004497	Metabolism	Metabolism	monooxygenase activity	F	AT4G37430
11846	3	F23 vs. L23	F2-3 up	GO:0004497	Metabolism	Metabolism	monooxygenase activity	F	AT4G39480
11847	3	F23 vs. L23	F2-3 up	GO:0004497	Metabolism	Metabolism	monooxygenase activity	F	AT4G39510
11848	3	F23 vs. L23	F2-3 up	GO:0004497	Metabolism	Metabolism	monooxygenase activity	F	AT5G38420
11849	3	F23 vs. L23	F2-3 up	GO:0005996	Metabolism	Metabolism	monosaccharide metabolic process	P	AT1G09340
11850	3	F23 vs. L23	F2-3 up	GO:0016099	Metabolism	Metabolism	monoterpenoid biosynthetic process	P	AT2G24210
11851	3	F23 vs. L23	F2-3 up	GO:0005451	Metabolism	Metabolism	monovalent cation:proton antiporter activity	F	AT3G17630
11852	3	F23 vs. L23	F2-3 up	GO:0005451	Metabolism	Metabolism	monovalent cation:proton antiporter activity	F	AT5G01690
11853	3	F23 vs. L23	F2-3 up	GO:0003774	Metabolism	Metabolism	motor activity	F	AT1G54560
11854	3	F23 vs. L23	F2-3 up	GO:0010192	Metabolism	Metabolism	mucilage biosynthetic process	P	AT1G53500
11855	3	F23 vs. L23	F2-3 up	GO:0010192	Metabolism	Metabolism	mucilage biosynthetic process	P	AT2G22900
11856	3	F23 vs. L23	F2-3 up	GO:0010192	Metabolism	Metabolism	mucilage biosynthetic process	P	AT5G09870
11857	3	F23 vs. L23	F2-3 up	GO:0048354	Metabolism	Metabolism	mucilage biosynthetic process involved in seed coat development	P	AT1G21070
11858	3	F23 vs. L23	F2-3 up	GO:0048354	Metabolism	Metabolism	mucilage biosynthetic process involved in seed coat development	P	AT1G63930
11859	3	F23 vs. L23	F2-3 up	GO:0048358	Metabolism	Metabolism	mucilage pectin biosynthetic process	P	AT5G15470
11860	3	F23 vs. L23	F2-3 up	GO:0010264	Metabolism	Metabolism	myo-inositol hexakisphosphate biosynthetic process	P	AT2G22240
11861	3	F23 vs. L23	F2-3 up	GO:0010264	Metabolism	Metabolism	myo-inositol hexakisphosphate biosynthetic process	P	AT4G39800
11862	3	F23 vs. L23	F2-3 up	GO:0050551	Metabolism	Metabolism	myrcene synthase activity	F	AT2G24210
11863	3	F23 vs. L23	F2-3 up	GO:0004499	Metabolism	Metabolism	N,N-dimethylaniline monooxygenase activity	F	AT1G62540
11864	3	F23 vs. L23	F2-3 up	GO:0004499	Metabolism	Metabolism	N,N-dimethylaniline monooxygenase activity	F	AT1G62570
11865	3	F23 vs. L23	F2-3 up	GO:0045486	Metabolism	Metabolism	naringenin 3-dioxygenase activity	F	AT3G51240
11866	3	F23 vs. L23	F2-3 up	GO:0045486	Metabolism	Metabolism	naringenin 3-dioxygenase activity	F	AT5G08640
11867	3	F23 vs. L23	F2-3 up	GO:0016210	Metabolism	Metabolism	naringenin-chalcone synthase activity	F	AT5G13930
11868	3	F23 vs. L23	F2-3 up	GO:0042128	Metabolism	Metabolism	nitrate assimilation	P	AT1G12110
11869	3	F23 vs. L23	F2-3 up	GO:0042128	Metabolism	Metabolism	nitrate assimilation	P	AT1G68570
11870	3	F23 vs. L23	F2-3 up	GO:0042128	Metabolism	Metabolism	nitrate assimilation	P	AT2G15620
11871	3	F23 vs. L23	F2-3 up	GO:0042128	Metabolism	Metabolism	nitrate assimilation	P	AT2G26690
11872	3	F23 vs. L23	F2-3 up	GO:0042128	Metabolism	Metabolism	nitrate assimilation	P	AT3G21670
11873	3	F23 vs. L23	F2-3 up	GO:0042128	Metabolism	Metabolism	nitrate assimilation	P	AT5G53460
11874	3	F23 vs. L23	F2-3 up	GO:0009671	Metabolism	Metabolism	nitrate:proton symporter activity	F	AT5G40890
11875	3	F23 vs. L23	F2-3 up	GO:0006807	Metabolism	Metabolism	nitrogen compound metabolic process	P	AT1G65960
11876	3	F23 vs. L23	F2-3 up	GO:0006807	Metabolism	Metabolism	nitrogen compound metabolic process	P	AT5G11520
11877	3	F23 vs. L23	F2-3 up	GO:0006607	Metabolism	Metabolism	NLS-bearing protein import into nucleus	P	AT4G16143
11878	3	F23 vs. L23	F2-3 up	GO:0045735	Metabolism	Metabolism	nutrient reservoir activity	F	AT1G72610
11879	3	F23 vs. L23	F2-3 up	GO:0045735	Metabolism	Metabolism	nutrient reservoir activity	F	AT3G05930
11880	3	F23 vs. L23	F2-3 up	GO:0045735	Metabolism	Metabolism	nutrient reservoir activity	F	AT5G24780
11881	3	F23 vs. L23	F2-3 up	GO:0045735	Metabolism	Metabolism	nutrient reservoir activity	F	AT5G25610
11882	3	F23 vs. L23	F2-3 up	GO:0016413	Metabolism	Metabolism	O-acetyltransferase activity	F	AT2G30010
11883	3	F23 vs. L23	F2-3 up	GO:0016413	Metabolism	Metabolism	O-acetyltransferase activity	F	AT3G28150
11884	3	F23 vs. L23	F2-3 up	GO:0009924	Metabolism	Metabolism	octadecanal decarbonylase activity	F	AT5G57800

11885	3	F23 vs. L23	F2-3 up	GO:0008171	Metabolism	Metabolism	O-methyltransferase activity	F	AT3G61990
11886	3	F23 vs. L23	F2-3 up	GO:0008171	Metabolism	Metabolism	O-methyltransferase activity	F	AT4G35150
11887	3	F23 vs. L23	F2-3 up	GO:0006730	Metabolism	Metabolism	one-carbon metabolic process	P	AT2G36880
11888	3	F23 vs. L23	F2-3 up	GO:0006730	Metabolism	Metabolism	one-carbon metabolic process	P	AT3G17390
11889	3	F23 vs. L23	F2-3 up	GO:0006730	Metabolism	Metabolism	one-carbon metabolic process	P	AT3G23810
11890	3	F23 vs. L23	F2-3 up	GO:0006730	Metabolism	Metabolism	one-carbon metabolic process	P	AT4G01850
11891	3	F23 vs. L23	F2-3 up	GO:0006730	Metabolism	Metabolism	one-carbon metabolic process	P	AT4G13930
11892	3	F23 vs. L23	F2-3 up	GO:0008948	Metabolism	Metabolism	oxaloacetate decarboxylase activity	F	AT3G02770
11893	3	F23 vs. L23	F2-3 up	GO:0008948	Metabolism	Metabolism	oxaloacetate decarboxylase activity	F	AT5G16450
11894	3	F23 vs. L23	F2-3 up	GO:0006107	Metabolism	Metabolism	oxaloacetate metabolic process	P	AT5G58330
11895	3	F23 vs. L23	F2-3 up	GO:0055114	Metabolism	Metabolism	oxidation-reduction process	P	AT1G01090
11896	3	F23 vs. L23	F2-3 up	GO:0055114	Metabolism	Metabolism	oxidation-reduction process	P	AT1G01600
11897	3	F23 vs. L23	F2-3 up	GO:0055114	Metabolism	Metabolism	oxidation-reduction process	P	AT1G05010
11898	3	F23 vs. L23	F2-3 up	GO:0055114	Metabolism	Metabolism	oxidation-reduction process	P	AT1G06090
11899	3	F23 vs. L23	F2-3 up	GO:0055114	Metabolism	Metabolism	oxidation-reduction process	P	AT1G06640
11900	3	F23 vs. L23	F2-3 up	GO:0055114	Metabolism	Metabolism	oxidation-reduction process	P	AT1G06690
11901	3	F23 vs. L23	F2-3 up	GO:0055114	Metabolism	Metabolism	oxidation-reduction process	P	AT1G08830
11902	3	F23 vs. L23	F2-3 up	GO:0055114	Metabolism	Metabolism	oxidation-reduction process	P	AT1G11840
11903	3	F23 vs. L23	F2-3 up	GO:0055114	Metabolism	Metabolism	oxidation-reduction process	P	AT1G16410
11904	3	F23 vs. L23	F2-3 up	GO:0055114	Metabolism	Metabolism	oxidation-reduction process	P	AT1G19550
11905	3	F23 vs. L23	F2-3 up	GO:0055114	Metabolism	Metabolism	oxidation-reduction process	P	AT1G20020
11906	3	F23 vs. L23	F2-3 up	GO:0055114	Metabolism	Metabolism	oxidation-reduction process	P	AT1G20620
11907	3	F23 vs. L23	F2-3 up	GO:0055114	Metabolism	Metabolism	oxidation-reduction process	P	AT1G23740
11908	3	F23 vs. L23	F2-3 up	GO:0055114	Metabolism	Metabolism	oxidation-reduction process	P	AT1G23800
11909	3	F23 vs. L23	F2-3 up	GO:0055114	Metabolism	Metabolism	oxidation-reduction process	P	AT1G31490
11910	3	F23 vs. L23	F2-3 up	GO:0055114	Metabolism	Metabolism	oxidation-reduction process	P	AT1G35190
11911	3	F23 vs. L23	F2-3 up	GO:0055114	Metabolism	Metabolism	oxidation-reduction process	P	AT1G42970
11912	3	F23 vs. L23	F2-3 up	GO:0055114	Metabolism	Metabolism	oxidation-reduction process	P	AT1G44170
11913	3	F23 vs. L23	F2-3 up	GO:0055114	Metabolism	Metabolism	oxidation-reduction process	P	AT1G50320
11914	3	F23 vs. L23	F2-3 up	GO:0055114	Metabolism	Metabolism	oxidation-reduction process	P	AT1G51410
11915	3	F23 vs. L23	F2-3 up	GO:0055114	Metabolism	Metabolism	oxidation-reduction process	P	AT1G53500
11916	3	F23 vs. L23	F2-3 up	GO:0055114	Metabolism	Metabolism	oxidation-reduction process	P	AT1G55570
11917	3	F23 vs. L23	F2-3 up	GO:0055114	Metabolism	Metabolism	oxidation-reduction process	P	AT1G57770
11918	3	F23 vs. L23	F2-3 up	GO:0055114	Metabolism	Metabolism	oxidation-reduction process	P	AT1G58265
11919	3	F23 vs. L23	F2-3 up	GO:0055114	Metabolism	Metabolism	oxidation-reduction process	P	AT1G58290
11920	3	F23 vs. L23	F2-3 up	GO:0055114	Metabolism	Metabolism	oxidation-reduction process	P	AT1G59730
11921	3	F23 vs. L23	F2-3 up	GO:0055114	Metabolism	Metabolism	oxidation-reduction process	P	AT1G62380
11922	3	F23 vs. L23	F2-3 up	GO:0055114	Metabolism	Metabolism	oxidation-reduction process	P	AT1G62540
11923	3	F23 vs. L23	F2-3 up	GO:0055114	Metabolism	Metabolism	oxidation-reduction process	P	AT1G62570
11924	3	F23 vs. L23	F2-3 up	GO:0055114	Metabolism	Metabolism	oxidation-reduction process	P	AT1G63000
11925	3	F23 vs. L23	F2-3 up	GO:0055114	Metabolism	Metabolism	oxidation-reduction process	P	AT1G63710
11926	3	F23 vs. L23	F2-3 up	GO:0055114	Metabolism	Metabolism	oxidation-reduction process	P	AT1G64060
11927	3	F23 vs. L23	F2-3 up	GO:0055114	Metabolism	Metabolism	oxidation-reduction process	P	AT1G68010
11928	3	F23 vs. L23	F2-3 up	GO:0055114	Metabolism	Metabolism	oxidation-reduction process	P	AT1G69640
11929	3	F23 vs. L23	F2-3 up	GO:0055114	Metabolism	Metabolism	oxidation-reduction process	P	AT1G71500
11930	3	F23 vs. L23	F2-3 up	GO:0055114	Metabolism	Metabolism	oxidation-reduction process	P	AT1G76080
11931	3	F23 vs. L23	F2-3 up	GO:0055114	Metabolism	Metabolism	oxidation-reduction process	P	AT1G76490
11932	3	F23 vs. L23	F2-3 up	GO:0055114	Metabolism	Metabolism	oxidation-reduction process	P	AT1G78570
11933	3	F23 vs. L23	F2-3 up	GO:0055114	Metabolism	Metabolism	oxidation-reduction process	P	AT1G79440
11934	3	F23 vs. L23	F2-3 up	GO:0055114	Metabolism	Metabolism	oxidation-reduction process	P	AT2G02390
11935	3	F23 vs. L23	F2-3 up	GO:0055114	Metabolism	Metabolism	oxidation-reduction process	P	AT2G06050
11936	3	F23 vs. L23	F2-3 up	GO:0055114	Metabolism	Metabolism	oxidation-reduction process	P	AT2G15620
11937	3	F23 vs. L23	F2-3 up	GO:0055114	Metabolism	Metabolism	oxidation-reduction process	P	AT2G17370
11938	3	F23 vs. L23	F2-3 up	GO:0055114	Metabolism	Metabolism	oxidation-reduction process	P	AT2G24180
11939	3	F23 vs. L23	F2-3 up	GO:0055114	Metabolism	Metabolism	oxidation-reduction process	P	AT2G24270
11940	3	F23 vs. L23	F2-3 up	GO:0055114	Metabolism	Metabolism	oxidation-reduction process	P	AT2G24820

11941	3	F23 vs. L23	F2-3 up	GO:0055114	Metabolism	Metabolism	oxidation-reduction process	P	AT2G28190
11942	3	F23 vs. L23	F2-3 up	GO:0055114	Metabolism	Metabolism	oxidation-reduction process	P	AT2G29980
11943	3	F23 vs. L23	F2-3 up	GO:0055114	Metabolism	Metabolism	oxidation-reduction process	P	AT2G30490
11944	3	F23 vs. L23	F2-3 up	GO:0055114	Metabolism	Metabolism	oxidation-reduction process	P	AT2G30830
11945	3	F23 vs. L23	F2-3 up	GO:0055114	Metabolism	Metabolism	oxidation-reduction process	P	AT2G30860
11946	3	F23 vs. L23	F2-3 up	GO:0055114	Metabolism	Metabolism	oxidation-reduction process	P	AT2G31360
11947	3	F23 vs. L23	F2-3 up	GO:0055114	Metabolism	Metabolism	oxidation-reduction process	P	AT2G31570
11948	3	F23 vs. L23	F2-3 up	GO:0055114	Metabolism	Metabolism	oxidation-reduction process	P	AT2G34810
11949	3	F23 vs. L23	F2-3 up	GO:0055114	Metabolism	Metabolism	oxidation-reduction process	P	AT2G37240
11950	3	F23 vs. L23	F2-3 up	GO:0055114	Metabolism	Metabolism	oxidation-reduction process	P	AT2G37760
11951	3	F23 vs. L23	F2-3 up	GO:0055114	Metabolism	Metabolism	oxidation-reduction process	P	AT2G37770
11952	3	F23 vs. L23	F2-3 up	GO:0055114	Metabolism	Metabolism	oxidation-reduction process	P	AT2G38240
11953	3	F23 vs. L23	F2-3 up	GO:0055114	Metabolism	Metabolism	oxidation-reduction process	P	AT2G40890
11954	3	F23 vs. L23	F2-3 up	GO:0055114	Metabolism	Metabolism	oxidation-reduction process	P	AT2G41480
11955	3	F23 vs. L23	F2-3 up	GO:0055114	Metabolism	Metabolism	oxidation-reduction process	P	AT2G46210
11956	3	F23 vs. L23	F2-3 up	GO:0055114	Metabolism	Metabolism	oxidation-reduction process	P	AT3G04120
11957	3	F23 vs. L23	F2-3 up	GO:0055114	Metabolism	Metabolism	oxidation-reduction process	P	AT3G08610
11958	3	F23 vs. L23	F2-3 up	GO:0055114	Metabolism	Metabolism	oxidation-reduction process	P	AT3G09940
11959	3	F23 vs. L23	F2-3 up	GO:0055114	Metabolism	Metabolism	oxidation-reduction process	P	AT3G10570
11960	3	F23 vs. L23	F2-3 up	GO:0055114	Metabolism	Metabolism	oxidation-reduction process	P	AT3G11050
11961	3	F23 vs. L23	F2-3 up	GO:0055114	Metabolism	Metabolism	oxidation-reduction process	P	AT3G11170
11962	3	F23 vs. L23	F2-3 up	GO:0055114	Metabolism	Metabolism	oxidation-reduction process	P	AT3G11630
11963	3	F23 vs. L23	F2-3 up	GO:0055114	Metabolism	Metabolism	oxidation-reduction process	P	AT3G13390
11964	3	F23 vs. L23	F2-3 up	GO:0055114	Metabolism	Metabolism	oxidation-reduction process	P	AT3G14620
11965	3	F23 vs. L23	F2-3 up	GO:0055114	Metabolism	Metabolism	oxidation-reduction process	P	AT3G15360
11966	3	F23 vs. L23	F2-3 up	GO:0055114	Metabolism	Metabolism	oxidation-reduction process	P	AT3G17790
11967	3	F23 vs. L23	F2-3 up	GO:0055114	Metabolism	Metabolism	oxidation-reduction process	P	AT3G22370
11968	3	F23 vs. L23	F2-3 up	GO:0055114	Metabolism	Metabolism	oxidation-reduction process	P	AT3G24503
11969	3	F23 vs. L23	F2-3 up	GO:0055114	Metabolism	Metabolism	oxidation-reduction process	P	AT3G25290
11970	3	F23 vs. L23	F2-3 up	GO:0055114	Metabolism	Metabolism	oxidation-reduction process	P	AT3G26060
11971	3	F23 vs. L23	F2-3 up	GO:0055114	Metabolism	Metabolism	oxidation-reduction process	P	AT3G26650
11972	3	F23 vs. L23	F2-3 up	GO:0055114	Metabolism	Metabolism	oxidation-reduction process	P	AT3G29360
11973	3	F23 vs. L23	F2-3 up	GO:0055114	Metabolism	Metabolism	oxidation-reduction process	P	AT3G48170
11974	3	F23 vs. L23	F2-3 up	GO:0055114	Metabolism	Metabolism	oxidation-reduction process	P	AT3G48520
11975	3	F23 vs. L23	F2-3 up	GO:0055114	Metabolism	Metabolism	oxidation-reduction process	P	AT3G49110
11976	3	F23 vs. L23	F2-3 up	GO:0055114	Metabolism	Metabolism	oxidation-reduction process	P	AT3G50210
11977	3	F23 vs. L23	F2-3 up	GO:0055114	Metabolism	Metabolism	oxidation-reduction process	P	AT3G50560
11978	3	F23 vs. L23	F2-3 up	GO:0055114	Metabolism	Metabolism	oxidation-reduction process	P	AT3G51240
11979	3	F23 vs. L23	F2-3 up	GO:0055114	Metabolism	Metabolism	oxidation-reduction process	P	AT3G52880
11980	3	F23 vs. L23	F2-3 up	GO:0055114	Metabolism	Metabolism	oxidation-reduction process	P	AT3G55610
11981	3	F23 vs. L23	F2-3 up	GO:0055114	Metabolism	Metabolism	oxidation-reduction process	P	AT3G59970
11982	3	F23 vs. L23	F2-3 up	GO:0055114	Metabolism	Metabolism	oxidation-reduction process	P	AT3G61220
11983	3	F23 vs. L23	F2-3 up	GO:0055114	Metabolism	Metabolism	oxidation-reduction process	P	AT3G61880
11984	3	F23 vs. L23	F2-3 up	GO:0055114	Metabolism	Metabolism	oxidation-reduction process	P	AT4G00360
11985	3	F23 vs. L23	F2-3 up	GO:0055114	Metabolism	Metabolism	oxidation-reduction process	P	AT4G11600
11986	3	F23 vs. L23	F2-3 up	GO:0055114	Metabolism	Metabolism	oxidation-reduction process	P	AT4G13770
11987	3	F23 vs. L23	F2-3 up	GO:0055114	Metabolism	Metabolism	oxidation-reduction process	P	AT4G14710
11988	3	F23 vs. L23	F2-3 up	GO:0055114	Metabolism	Metabolism	oxidation-reduction process	P	AT4G15440
11989	3	F23 vs. L23	F2-3 up	GO:0055114	Metabolism	Metabolism	oxidation-reduction process	P	AT4G19230
11990	3	F23 vs. L23	F2-3 up	GO:0055114	Metabolism	Metabolism	oxidation-reduction process	P	AT4G20760
11991	3	F23 vs. L23	F2-3 up	GO:0055114	Metabolism	Metabolism	oxidation-reduction process	P	AT4G21990
11992	3	F23 vs. L23	F2-3 up	GO:0055114	Metabolism	Metabolism	oxidation-reduction process	P	AT4G22870
11993	3	F23 vs. L23	F2-3 up	GO:0055114	Metabolism	Metabolism	oxidation-reduction process	P	AT4G26260
11994	3	F23 vs. L23	F2-3 up	GO:0055114	Metabolism	Metabolism	oxidation-reduction process	P	AT4G27030
11995	3	F23 vs. L23	F2-3 up	GO:0055114	Metabolism	Metabolism	oxidation-reduction process	P	AT4G30470
11996	3	F23 vs. L23	F2-3 up	GO:0055114	Metabolism	Metabolism	oxidation-reduction process	P	AT4G33010

11997	3	F23 vs. L23	F2-3 up	GO:0055114	Metabolism	Metabolism	oxidation-reduction process	P	AT4G36250
11998	3	F23 vs. L23	F2-3 up	GO:0055114	Metabolism	Metabolism	oxidation-reduction process	P	AT4G37430
11999	3	F23 vs. L23	F2-3 up	GO:0055114	Metabolism	Metabolism	oxidation-reduction process	P	AT4G37760
12000	3	F23 vs. L23	F2-3 up	GO:0055114	Metabolism	Metabolism	oxidation-reduction process	P	AT4G37990
12001	3	F23 vs. L23	F2-3 up	GO:0055114	Metabolism	Metabolism	oxidation-reduction process	P	AT4G39480
12002	3	F23 vs. L23	F2-3 up	GO:0055114	Metabolism	Metabolism	oxidation-reduction process	P	AT4G39510
12003	3	F23 vs. L23	F2-3 up	GO:0055114	Metabolism	Metabolism	oxidation-reduction process	P	AT4G39730
12004	3	F23 vs. L23	F2-3 up	GO:0055114	Metabolism	Metabolism	oxidation-reduction process	P	AT5G01600
12005	3	F23 vs. L23	F2-3 up	GO:0055114	Metabolism	Metabolism	oxidation-reduction process	P	AT5G04140
12006	3	F23 vs. L23	F2-3 up	GO:0055114	Metabolism	Metabolism	oxidation-reduction process	P	AT5G05600
12007	3	F23 vs. L23	F2-3 up	GO:0055114	Metabolism	Metabolism	oxidation-reduction process	P	AT5G07440
12008	3	F23 vs. L23	F2-3 up	GO:0055114	Metabolism	Metabolism	oxidation-reduction process	P	AT5G07460
12009	3	F23 vs. L23	F2-3 up	GO:0055114	Metabolism	Metabolism	oxidation-reduction process	P	AT5G08640
12010	3	F23 vs. L23	F2-3 up	GO:0055114	Metabolism	Metabolism	oxidation-reduction process	P	AT5G14200
12011	3	F23 vs. L23	F2-3 up	GO:0055114	Metabolism	Metabolism	oxidation-reduction process	P	AT5G15490
12012	3	F23 vs. L23	F2-3 up	GO:0055114	Metabolism	Metabolism	oxidation-reduction process	P	AT5G24140
12013	3	F23 vs. L23	F2-3 up	GO:0055114	Metabolism	Metabolism	oxidation-reduction process	P	AT5G24530
12014	3	F23 vs. L23	F2-3 up	GO:0055114	Metabolism	Metabolism	oxidation-reduction process	P	AT5G25120
12015	3	F23 vs. L23	F2-3 up	GO:0055114	Metabolism	Metabolism	oxidation-reduction process	P	AT5G35735
12016	3	F23 vs. L23	F2-3 up	GO:0055114	Metabolism	Metabolism	oxidation-reduction process	P	AT5G36220
12017	3	F23 vs. L23	F2-3 up	GO:0055114	Metabolism	Metabolism	oxidation-reduction process	P	AT5G38420
12018	3	F23 vs. L23	F2-3 up	GO:0055114	Metabolism	Metabolism	oxidation-reduction process	P	AT5G39320
12019	3	F23 vs. L23	F2-3 up	GO:0055114	Metabolism	Metabolism	oxidation-reduction process	P	AT5G42800
12020	3	F23 vs. L23	F2-3 up	GO:0055114	Metabolism	Metabolism	oxidation-reduction process	P	AT5G47000
12021	3	F23 vs. L23	F2-3 up	GO:0055114	Metabolism	Metabolism	oxidation-reduction process	P	AT5G47890
12022	3	F23 vs. L23	F2-3 up	GO:0055114	Metabolism	Metabolism	oxidation-reduction process	P	AT5G47910
12023	3	F23 vs. L23	F2-3 up	GO:0055114	Metabolism	Metabolism	oxidation-reduction process	P	AT5G50850
12024	3	F23 vs. L23	F2-3 up	GO:0055114	Metabolism	Metabolism	oxidation-reduction process	P	AT5G51890
12025	3	F23 vs. L23	F2-3 up	GO:0055114	Metabolism	Metabolism	oxidation-reduction process	P	AT5G52100
12026	3	F23 vs. L23	F2-3 up	GO:0055114	Metabolism	Metabolism	oxidation-reduction process	P	AT5G53460
12027	3	F23 vs. L23	F2-3 up	GO:0055114	Metabolism	Metabolism	oxidation-reduction process	P	AT5G54080
12028	3	F23 vs. L23	F2-3 up	GO:0055114	Metabolism	Metabolism	oxidation-reduction process	P	AT5G57800
12029	3	F23 vs. L23	F2-3 up	GO:0055114	Metabolism	Metabolism	oxidation-reduction process	P	AT5G58260
12030	3	F23 vs. L23	F2-3 up	GO:0055114	Metabolism	Metabolism	oxidation-reduction process	P	AT5G60020
12031	3	F23 vs. L23	F2-3 up	GO:0055114	Metabolism	Metabolism	oxidation-reduction process	P	AT5G60600
12032	3	F23 vs. L23	F2-3 up	GO:0055114	Metabolism	Metabolism	oxidation-reduction process	P	ATCG00420
12033	3	F23 vs. L23	F2-3 up	GO:0055114	Metabolism	Metabolism	oxidation-reduction process	P	ATCG01100
12034	3	F23 vs. L23	F2-3 up	GO:0055114	Metabolism	Metabolism	oxidation-reduction process	P	ATMG00650
12035	3	F23 vs. L23	F2-3 up	GO:0031408	Metabolism	Metabolism	oxylipin biosynthetic process	P	AT1G17420
12036	3	F23 vs. L23	F2-3 up	GO:0031408	Metabolism	Metabolism	oxylipin biosynthetic process	P	AT1G72520
12037	3	F23 vs. L23	F2-3 up	GO:0031408	Metabolism	Metabolism	oxylipin biosynthetic process	P	AT2G06050
12038	3	F23 vs. L23	F2-3 up	GO:0031408	Metabolism	Metabolism	oxylipin biosynthetic process	P	AT3G01420
12039	3	F23 vs. L23	F2-3 up	GO:0031408	Metabolism	Metabolism	oxylipin biosynthetic process	P	AT3G22400
12040	3	F23 vs. L23	F2-3 up	GO:0031408	Metabolism	Metabolism	oxylipin biosynthetic process	P	AT3G45140
12041	3	F23 vs. L23	F2-3 up	GO:0031408	Metabolism	Metabolism	oxylipin biosynthetic process	P	AT5G63380
12042	3	F23 vs. L23	F2-3 up	GO:0046409	Metabolism	Metabolism	p-coumarate 3-hydroxylase activity	F	AT2G40890
12043	3	F23 vs. L23	F2-3 up	GO:0030570	Metabolism	Metabolism	pectate lyase activity	F	AT3G24670
12044	3	F23 vs. L23	F2-3 up	GO:0030570	Metabolism	Metabolism	pectate lyase activity	F	AT5G55720
12045	3	F23 vs. L23	F2-3 up	GO:0030570	Metabolism	Metabolism	pectate lyase activity	F	AT5G63180
12046	3	F23 vs. L23	F2-3 up	GO:0052793	Metabolism	Metabolism	pectin acetylesterase activity	F	AT3G05910
12047	3	F23 vs. L23	F2-3 up	GO:0052793	Metabolism	Metabolism	pectin acetylesterase activity	F	AT4G19410
12048	3	F23 vs. L23	F2-3 up	GO:0052793	Metabolism	Metabolism	pectin acetylesterase activity	F	AT4G19420
12049	3	F23 vs. L23	F2-3 up	GO:0052793	Metabolism	Metabolism	pectin acetylesterase activity	F	AT5G45280
12050	3	F23 vs. L23	F2-3 up	GO:0045489	Metabolism	Metabolism	pectin biosynthetic process	P	AT3G25140
12051	3	F23 vs. L23	F2-3 up	GO:0045489	Metabolism	Metabolism	pectin biosynthetic process	P	AT5G65810
12052	3	F23 vs. L23	F2-3 up	GO:0045488	Metabolism	Metabolism	pectin metabolic process	P	AT5G65810

12053	3	F23 vs. L23	F2-3 up	GO:0030599	Metabolism	Metabolism	pectinesterase activity	F	AT2G45220
12054	3	F23 vs. L23	F2-3 up	GO:0030599	Metabolism	Metabolism	pectinesterase activity	F	AT3G05620
12055	3	F23 vs. L23	F2-3 up	GO:0030599	Metabolism	Metabolism	pectinesterase activity	F	AT3G14310
12056	3	F23 vs. L23	F2-3 up	GO:0030599	Metabolism	Metabolism	pectinesterase activity	F	AT3G17060
12057	3	F23 vs. L23	F2-3 up	GO:0030599	Metabolism	Metabolism	pectinesterase activity	F	AT3G43270
12058	3	F23 vs. L23	F2-3 up	GO:0030599	Metabolism	Metabolism	pectinesterase activity	F	AT3G62170
12059	3	F23 vs. L23	F2-3 up	GO:0030599	Metabolism	Metabolism	pectinesterase activity	F	AT4G33220
12060	3	F23 vs. L23	F2-3 up	GO:0030599	Metabolism	Metabolism	pectinesterase activity	F	AT5G07430
12061	3	F23 vs. L23	F2-3 up	GO:0030599	Metabolism	Metabolism	pectinesterase activity	F	AT5G53370
12062	3	F23 vs. L23	F2-3 up	GO:0046910	Metabolism	Metabolism	pectinesterase inhibitor activity	F	AT2G45220
12063	3	F23 vs. L23	F2-3 up	GO:0046910	Metabolism	Metabolism	pectinesterase inhibitor activity	F	AT3G05620
12064	3	F23 vs. L23	F2-3 up	GO:0046910	Metabolism	Metabolism	pectinesterase inhibitor activity	F	AT3G14310
12065	3	F23 vs. L23	F2-3 up	GO:0046910	Metabolism	Metabolism	pectinesterase inhibitor activity	F	AT3G43270
12066	3	F23 vs. L23	F2-3 up	GO:0046910	Metabolism	Metabolism	pectinesterase inhibitor activity	F	AT3G62170
12067	3	F23 vs. L23	F2-3 up	GO:0046910	Metabolism	Metabolism	pectinesterase inhibitor activity	F	AT4G33220
12068	3	F23 vs. L23	F2-3 up	GO:0046910	Metabolism	Metabolism	pectinesterase inhibitor activity	F	AT5G53370
12069	3	F23 vs. L23	F2-3 up	GO:0042277	Metabolism	Metabolism	peptide binding	F	AT1G72180
12070	3	F23 vs. L23	F2-3 up	GO:0000048	Metabolism	Metabolism	peptidyltransferase activity	F	AT4G39640
12071	3	F23 vs. L23	F2-3 up	GO:0045548	Metabolism	Metabolism	phenylalanine ammonia-lyase activity	F	AT2G37040
12072	3	F23 vs. L23	F2-3 up	GO:0009699	Metabolism	Metabolism	phenylpropanoid biosynthetic process	P	AT2G30490
12073	3	F23 vs. L23	F2-3 up	GO:0009699	Metabolism	Metabolism	phenylpropanoid biosynthetic process	P	AT2G37040
12074	3	F23 vs. L23	F2-3 up	GO:0009699	Metabolism	Metabolism	phenylpropanoid biosynthetic process	P	AT2G40890
12075	3	F23 vs. L23	F2-3 up	GO:0009699	Metabolism	Metabolism	phenylpropanoid biosynthetic process	P	AT3G21230
12076	3	F23 vs. L23	F2-3 up	GO:0009699	Metabolism	Metabolism	phenylpropanoid biosynthetic process	P	AT3G24503
12077	3	F23 vs. L23	F2-3 up	GO:0009698	Metabolism	Metabolism	phenylpropanoid metabolic process	P	AT2G30490
12078	3	F23 vs. L23	F2-3 up	GO:0009698	Metabolism	Metabolism	phenylpropanoid metabolic process	P	AT5G60020
12079	3	F23 vs. L23	F2-3 up	GO:0006796	Metabolism	Metabolism	phosphate-containing compound metabolic process	P	AT2G46860
12080	3	F23 vs. L23	F2-3 up	GO:0006796	Metabolism	Metabolism	phosphate-containing compound metabolic process	P	AT4G01480
12081	3	F23 vs. L23	F2-3 up	GO:0006796	Metabolism	Metabolism	phosphate-containing compound metabolic process	P	AT5G09650
12082	3	F23 vs. L23	F2-3 up	GO:0006656	Metabolism	Metabolism	phosphatidylcholine biosynthetic process	P	AT1G48600
12083	3	F23 vs. L23	F2-3 up	GO:0046471	Metabolism	Metabolism	phosphatidylglycerol metabolic process	P	AT4G27030
12084	3	F23 vs. L23	F2-3 up	GO:0035091	Metabolism	Metabolism	phosphatidylinositol binding	F	AT3G54800
12085	3	F23 vs. L23	F2-3 up	GO:0046488	Metabolism	Metabolism	phosphatidylinositol metabolic process	P	AT2G41210
12086	3	F23 vs. L23	F2-3 up	GO:0046488	Metabolism	Metabolism	phosphatidylinositol metabolic process	P	AT3G56960
12087	3	F23 vs. L23	F2-3 up	GO:0017176	Metabolism	Metabolism	phosphatidylinositol N-acetylglucosaminyltransferase activity	F	AT3G45100
12088	3	F23 vs. L23	F2-3 up	GO:0008526	Metabolism	Metabolism	phosphatidylinositol transfer activity	F	AT4G36490
12089	3	F23 vs. L23	F2-3 up	GO:0005546	Metabolism	Metabolism	phosphatidylinositol-4,5-bisphosphate binding	F	AT1G03050
12090	3	F23 vs. L23	F2-3 up	GO:0006659	Metabolism	Metabolism	phosphatidylserine biosynthetic process	P	AT4G39800
12091	3	F23 vs. L23	F2-3 up	GO:0008964	Metabolism	Metabolism	phosphoenolpyruvate carboxylase activity	F	AT3G14940
12092	3	F23 vs. L23	F2-3 up	GO:0000234	Metabolism	Metabolism	phosphoethanolamine N-methyltransferase activity	F	AT1G48600
12093	3	F23 vs. L23	F2-3 up	GO:0004614	Metabolism	Metabolism	phosphoglucomutase activity	F	AT1G70730
12094	3	F23 vs. L23	F2-3 up	GO:0008114	Metabolism	Metabolism	phosphogluconate 2-dehydrogenase activity	F	AT1G64190
12095	3	F23 vs. L23	F2-3 up	GO:0004616	Metabolism	Metabolism	phosphogluconate dehydrogenase (decarboxylating) activity	F	AT1G64190
12096	3	F23 vs. L23	F2-3 up	GO:0008654	Metabolism	Metabolism	phospholipid biosynthetic process	P	AT2G22240
12097	3	F23 vs. L23	F2-3 up	GO:0008654	Metabolism	Metabolism	phospholipid biosynthetic process	P	AT2G38110
12098	3	F23 vs. L23	F2-3 up	GO:0006644	Metabolism	Metabolism	phospholipid metabolic process	P	AT3G54950
12099	3	F23 vs. L23	F2-3 up	GO:0052667	Metabolism	Metabolism	phosphomethylethanolamine N-methyltransferase activity	F	AT1G48600
12100	3	F23 vs. L23	F2-3 up	GO:0016310	Metabolism	Metabolism	phosphorylation	P	AT1G09600
12101	3	F23 vs. L23	F2-3 up	GO:0016310	Metabolism	Metabolism	phosphorylation	P	AT1G32060
12102	3	F23 vs. L23	F2-3 up	GO:0016310	Metabolism	Metabolism	phosphorylation	P	AT1G71697

12103	3	F23 vs. L23	F2-3 up	GO:0016310	Metabolism	Metabolism	phosphorylation	P	AT1G78940
12104	3	F23 vs. L23	F2-3 up	GO:0016310	Metabolism	Metabolism	phosphorylation	P	AT1G80460
12105	3	F23 vs. L23	F2-3 up	GO:0016310	Metabolism	Metabolism	phosphorylation	P	AT2G07180
12106	3	F23 vs. L23	F2-3 up	GO:0016310	Metabolism	Metabolism	phosphorylation	P	AT2G14750
12107	3	F23 vs. L23	F2-3 up	GO:0016310	Metabolism	Metabolism	phosphorylation	P	AT2G20470
12108	3	F23 vs. L23	F2-3 up	GO:0016310	Metabolism	Metabolism	phosphorylation	P	AT3G13065
12109	3	F23 vs. L23	F2-3 up	GO:0016310	Metabolism	Metabolism	phosphorylation	P	AT3G50000
12110	3	F23 vs. L23	F2-3 up	GO:0016310	Metabolism	Metabolism	phosphorylation	P	AT3G55610
12111	3	F23 vs. L23	F2-3 up	GO:0016310	Metabolism	Metabolism	phosphorylation	P	AT4G15530
12112	3	F23 vs. L23	F2-3 up	GO:0016310	Metabolism	Metabolism	phosphorylation	P	AT4G20300
12113	3	F23 vs. L23	F2-3 up	GO:0016310	Metabolism	Metabolism	phosphorylation	P	AT4G24450
12114	3	F23 vs. L23	F2-3 up	GO:0016310	Metabolism	Metabolism	phosphorylation	P	AT4G25030
12115	3	F23 vs. L23	F2-3 up	GO:0016310	Metabolism	Metabolism	phosphorylation	P	AT4G35500
12116	3	F23 vs. L23	F2-3 up	GO:0016310	Metabolism	Metabolism	phosphorylation	P	AT4G39940
12117	3	F23 vs. L23	F2-3 up	GO:0016310	Metabolism	Metabolism	phosphorylation	P	AT5G05200
12118	3	F23 vs. L23	F2-3 up	GO:0016310	Metabolism	Metabolism	phosphorylation	P	AT5G14060
12119	3	F23 vs. L23	F2-3 up	GO:0033306	Metabolism	Metabolism	phytol metabolic process	P	AT1G54570
12120	3	F23 vs. L23	F2-3 up	GO:0031409	Metabolism	Metabolism	pigment binding	F	AT1G45474
12121	3	F23 vs. L23	F2-3 up	GO:0006596	Metabolism	Metabolism	polyamine biosynthetic process	P	AT1G70310
12122	3	F23 vs. L23	F2-3 up	GO:0006596	Metabolism	Metabolism	polyamine biosynthetic process	P	AT3G02470
12123	3	F23 vs. L23	F2-3 up	GO:0006596	Metabolism	Metabolism	polyamine biosynthetic process	P	AT4G34710
12124	3	F23 vs. L23	F2-3 up	GO:0004650	Metabolism	Metabolism	polygalacturonase activity	F	AT1G02790
12125	3	F23 vs. L23	F2-3 up	GO:0004650	Metabolism	Metabolism	polygalacturonase activity	F	AT3G07820
12126	3	F23 vs. L23	F2-3 up	GO:0004650	Metabolism	Metabolism	polygalacturonase activity	F	AT3G07830
12127	3	F23 vs. L23	F2-3 up	GO:0004650	Metabolism	Metabolism	polygalacturonase activity	F	AT3G07840
12128	3	F23 vs. L23	F2-3 up	GO:0004650	Metabolism	Metabolism	polygalacturonase activity	F	AT3G07850
12129	3	F23 vs. L23	F2-3 up	GO:0004650	Metabolism	Metabolism	polygalacturonase activity	F	AT5G48140
12130	3	F23 vs. L23	F2-3 up	GO:0047262	Metabolism	Metabolism	polygalacturonate 4-alpha-galacturonosyltransferase activity	F	AT3G25140
12131	3	F23 vs. L23	F2-3 up	GO:0047262	Metabolism	Metabolism	polygalacturonate 4-alpha-galacturonosyltransferase activity	F	AT5G15470
12132	3	F23 vs. L23	F2-3 up	GO:0016094	Metabolism	Metabolism	polyprenol biosynthetic process	P	AT5G58770
12133	3	F23 vs. L23	F2-3 up	GO:0002094	Metabolism	Metabolism	polyprenyltransferase activity	F	AT5G58770
12134	3	F23 vs. L23	F2-3 up	GO:0030247	Metabolism	Metabolism	polysaccharide binding	F	AT1G05850
12135	3	F23 vs. L23	F2-3 up	GO:0090358	Metabolism	Metabolism	positive regulation of tryptophan metabolic process	P	AT1G75500
12136	3	F23 vs. L23	F2-3 up	GO:0030955	Metabolism	Metabolism	potassium ion binding	F	AT5G56350
12137	3	F23 vs. L23	F2-3 up	GO:0030955	Metabolism	Metabolism	potassium ion binding	F	AT5G63680
12138	3	F23 vs. L23	F2-3 up	GO:0004664	Metabolism	Metabolism	prephenate dehydratase activity	F	AT5G22630
12139	3	F23 vs. L23	F2-3 up	GO:0008131	Metabolism	Metabolism	primary amine oxidase activity	F	AT1G31490
12140	3	F23 vs. L23	F2-3 up	GO:0006561	Metabolism	Metabolism	proline biosynthetic process	P	AT3G55610
12141	3	F23 vs. L23	F2-3 up	GO:0005515	Metabolism	Metabolism	protein binding	F	AT1G04400
12142	3	F23 vs. L23	F2-3 up	GO:0005515	Metabolism	Metabolism	protein binding	F	AT1G08830
12143	3	F23 vs. L23	F2-3 up	GO:0005515	Metabolism	Metabolism	protein binding	F	AT1G09250
12144	3	F23 vs. L23	F2-3 up	GO:0005515	Metabolism	Metabolism	protein binding	F	AT1G09340
12145	3	F23 vs. L23	F2-3 up	GO:0005515	Metabolism	Metabolism	protein binding	F	AT1G12110
12146	3	F23 vs. L23	F2-3 up	GO:0005515	Metabolism	Metabolism	protein binding	F	AT1G12840
12147	3	F23 vs. L23	F2-3 up	GO:0005515	Metabolism	Metabolism	protein binding	F	AT1G14000
12148	3	F23 vs. L23	F2-3 up	GO:0005515	Metabolism	Metabolism	protein binding	F	AT1G14920
12149	3	F23 vs. L23	F2-3 up	GO:0005515	Metabolism	Metabolism	protein binding	F	AT1G15470
12150	3	F23 vs. L23	F2-3 up	GO:0005515	Metabolism	Metabolism	protein binding	F	AT1G16720
12151	3	F23 vs. L23	F2-3 up	GO:0005515	Metabolism	Metabolism	protein binding	F	AT1G17370
12152	3	F23 vs. L23	F2-3 up	GO:0005515	Metabolism	Metabolism	protein binding	F	AT1G17380
12153	3	F23 vs. L23	F2-3 up	GO:0005515	Metabolism	Metabolism	protein binding	F	AT1G18740
12154	3	F23 vs. L23	F2-3 up	GO:0005515	Metabolism	Metabolism	protein binding	F	AT1G19180
12155	3	F23 vs. L23	F2-3 up	GO:0005515	Metabolism	Metabolism	protein binding	F	AT1G20620
12156	3	F23 vs. L23	F2-3 up	GO:0005515	Metabolism	Metabolism	protein binding	F	AT1G20850

12157	3	F23 vs. L23	F2-3 up	GO:0005515	Metabolism	Metabolism	protein binding	F	AT1G22840
12158	3	F23 vs. L23	F2-3 up	GO:0005515	Metabolism	Metabolism	protein binding	F	AT1G23380
12159	3	F23 vs. L23	F2-3 up	GO:0005515	Metabolism	Metabolism	protein binding	F	AT1G27930
12160	3	F23 vs. L23	F2-3 up	GO:0005515	Metabolism	Metabolism	protein binding	F	AT1G28270
12161	3	F23 vs. L23	F2-3 up	GO:0005515	Metabolism	Metabolism	protein binding	F	AT1G28480
12162	3	F23 vs. L23	F2-3 up	GO:0005515	Metabolism	Metabolism	protein binding	F	AT1G30330
12163	3	F23 vs. L23	F2-3 up	GO:0005515	Metabolism	Metabolism	protein binding	F	AT1G31260
12164	3	F23 vs. L23	F2-3 up	GO:0005515	Metabolism	Metabolism	protein binding	F	AT1G32060
12165	3	F23 vs. L23	F2-3 up	GO:0005515	Metabolism	Metabolism	protein binding	F	AT1G32640
12166	3	F23 vs. L23	F2-3 up	GO:0005515	Metabolism	Metabolism	protein binding	F	AT1G35490
12167	3	F23 vs. L23	F2-3 up	GO:0005515	Metabolism	Metabolism	protein binding	F	AT1G45474
12168	3	F23 vs. L23	F2-3 up	GO:0005515	Metabolism	Metabolism	protein binding	F	AT1G50250
12169	3	F23 vs. L23	F2-3 up	GO:0005515	Metabolism	Metabolism	protein binding	F	AT1G50610
12170	3	F23 vs. L23	F2-3 up	GO:0005515	Metabolism	Metabolism	protein binding	F	AT1G55480
12171	3	F23 vs. L23	F2-3 up	GO:0005515	Metabolism	Metabolism	protein binding	F	AT1G58290
12172	3	F23 vs. L23	F2-3 up	GO:0005515	Metabolism	Metabolism	protein binding	F	AT1G61660
12173	3	F23 vs. L23	F2-3 up	GO:0005515	Metabolism	Metabolism	protein binding	F	AT1G62360
12174	3	F23 vs. L23	F2-3 up	GO:0005515	Metabolism	Metabolism	protein binding	F	AT1G63650
12175	3	F23 vs. L23	F2-3 up	GO:0005515	Metabolism	Metabolism	protein binding	F	AT1G64060
12176	3	F23 vs. L23	F2-3 up	GO:0005515	Metabolism	Metabolism	protein binding	F	AT1G64190
12177	3	F23 vs. L23	F2-3 up	GO:0005515	Metabolism	Metabolism	protein binding	F	AT1G64860
12178	3	F23 vs. L23	F2-3 up	GO:0005515	Metabolism	Metabolism	protein binding	F	AT1G69120
12179	3	F23 vs. L23	F2-3 up	GO:0005515	Metabolism	Metabolism	protein binding	F	AT1G70310
12180	3	F23 vs. L23	F2-3 up	GO:0005515	Metabolism	Metabolism	protein binding	F	AT1G70700
12181	3	F23 vs. L23	F2-3 up	GO:0005515	Metabolism	Metabolism	protein binding	F	AT1G72180
12182	3	F23 vs. L23	F2-3 up	GO:0005515	Metabolism	Metabolism	protein binding	F	AT1G74380
12183	3	F23 vs. L23	F2-3 up	GO:0005515	Metabolism	Metabolism	protein binding	F	AT1G76080
12184	3	F23 vs. L23	F2-3 up	GO:0005515	Metabolism	Metabolism	protein binding	F	AT1G76490
12185	3	F23 vs. L23	F2-3 up	GO:0005515	Metabolism	Metabolism	protein binding	F	AT1G77980
12186	3	F23 vs. L23	F2-3 up	GO:0005515	Metabolism	Metabolism	protein binding	F	AT1G80840
12187	3	F23 vs. L23	F2-3 up	GO:0005515	Metabolism	Metabolism	protein binding	F	AT2G01330
12188	3	F23 vs. L23	F2-3 up	GO:0005515	Metabolism	Metabolism	protein binding	F	AT2G05620
12189	3	F23 vs. L23	F2-3 up	GO:0005515	Metabolism	Metabolism	protein binding	F	AT2G07560
12190	3	F23 vs. L23	F2-3 up	GO:0005515	Metabolism	Metabolism	protein binding	F	AT2G13570
12191	3	F23 vs. L23	F2-3 up	GO:0005515	Metabolism	Metabolism	protein binding	F	AT2G17290
12192	3	F23 vs. L23	F2-3 up	GO:0005515	Metabolism	Metabolism	protein binding	F	AT2G17950
12193	3	F23 vs. L23	F2-3 up	GO:0005515	Metabolism	Metabolism	protein binding	F	AT2G22240
12194	3	F23 vs. L23	F2-3 up	GO:0005515	Metabolism	Metabolism	protein binding	F	AT2G23760
12195	3	F23 vs. L23	F2-3 up	GO:0005515	Metabolism	Metabolism	protein binding	F	AT2G24540
12196	3	F23 vs. L23	F2-3 up	GO:0005515	Metabolism	Metabolism	protein binding	F	AT2G26490
12197	3	F23 vs. L23	F2-3 up	GO:0005515	Metabolism	Metabolism	protein binding	F	AT2G30360
12198	3	F23 vs. L23	F2-3 up	GO:0005515	Metabolism	Metabolism	protein binding	F	AT2G30490
12199	3	F23 vs. L23	F2-3 up	GO:0005515	Metabolism	Metabolism	protein binding	F	AT2G31380
12200	3	F23 vs. L23	F2-3 up	GO:0005515	Metabolism	Metabolism	protein binding	F	AT2G31500
12201	3	F23 vs. L23	F2-3 up	GO:0005515	Metabolism	Metabolism	protein binding	F	AT2G31570
12202	3	F23 vs. L23	F2-3 up	GO:0005515	Metabolism	Metabolism	protein binding	F	AT2G35940
12203	3	F23 vs. L23	F2-3 up	GO:0005515	Metabolism	Metabolism	protein binding	F	AT2G36880
12204	3	F23 vs. L23	F2-3 up	GO:0005515	Metabolism	Metabolism	protein binding	F	AT2G37040
12205	3	F23 vs. L23	F2-3 up	GO:0005515	Metabolism	Metabolism	protein binding	F	AT2G37180
12206	3	F23 vs. L23	F2-3 up	GO:0005515	Metabolism	Metabolism	protein binding	F	AT2G37630
12207	3	F23 vs. L23	F2-3 up	GO:0005515	Metabolism	Metabolism	protein binding	F	AT2G38170
12208	3	F23 vs. L23	F2-3 up	GO:0005515	Metabolism	Metabolism	protein binding	F	AT2G38280
12209	3	F23 vs. L23	F2-3 up	GO:0005515	Metabolism	Metabolism	protein binding	F	AT2G38400
12210	3	F23 vs. L23	F2-3 up	GO:0005515	Metabolism	Metabolism	protein binding	F	AT2G39770
12211	3	F23 vs. L23	F2-3 up	GO:0005515	Metabolism	Metabolism	protein binding	F	AT2G40000
12212	3	F23 vs. L23	F2-3 up	GO:0005515	Metabolism	Metabolism	protein binding	F	AT2G40140

12213	3	F23 vs. L23	F2-3 up	GO:0005515	Metabolism	Metabolism	protein binding	F	AT2G40670
12214	3	F23 vs. L23	F2-3 up	GO:0005515	Metabolism	Metabolism	protein binding	F	AT2G40890
12215	3	F23 vs. L23	F2-3 up	GO:0005515	Metabolism	Metabolism	protein binding	F	AT2G41630
12216	3	F23 vs. L23	F2-3 up	GO:0005515	Metabolism	Metabolism	protein binding	F	AT2G45190
12217	3	F23 vs. L23	F2-3 up	GO:0005515	Metabolism	Metabolism	protein binding	F	AT2G45770
12218	3	F23 vs. L23	F2-3 up	GO:0005515	Metabolism	Metabolism	protein binding	F	AT2G46410
12219	3	F23 vs. L23	F2-3 up	GO:0005515	Metabolism	Metabolism	protein binding	F	AT2G46830
12220	3	F23 vs. L23	F2-3 up	GO:0005515	Metabolism	Metabolism	protein binding	F	AT3G02470
12221	3	F23 vs. L23	F2-3 up	GO:0005515	Metabolism	Metabolism	protein binding	F	AT3G03770
12222	3	F23 vs. L23	F2-3 up	GO:0005515	Metabolism	Metabolism	protein binding	F	AT3G04120
12223	3	F23 vs. L23	F2-3 up	GO:0005515	Metabolism	Metabolism	protein binding	F	AT3G05500
12224	3	F23 vs. L23	F2-3 up	GO:0005515	Metabolism	Metabolism	protein binding	F	AT3G07050
12225	3	F23 vs. L23	F2-3 up	GO:0005515	Metabolism	Metabolism	protein binding	F	AT3G07560
12226	3	F23 vs. L23	F2-3 up	GO:0005515	Metabolism	Metabolism	protein binding	F	AT3G08510
12227	3	F23 vs. L23	F2-3 up	GO:0005515	Metabolism	Metabolism	protein binding	F	AT3G08720
12228	3	F23 vs. L23	F2-3 up	GO:0005515	Metabolism	Metabolism	protein binding	F	AT3G08730
12229	3	F23 vs. L23	F2-3 up	GO:0005515	Metabolism	Metabolism	protein binding	F	AT3G09440
12230	3	F23 vs. L23	F2-3 up	GO:0005515	Metabolism	Metabolism	protein binding	F	AT3G11410
12231	3	F23 vs. L23	F2-3 up	GO:0005515	Metabolism	Metabolism	protein binding	F	AT3G11630
12232	3	F23 vs. L23	F2-3 up	GO:0005515	Metabolism	Metabolism	protein binding	F	AT3G12620
12233	3	F23 vs. L23	F2-3 up	GO:0005515	Metabolism	Metabolism	protein binding	F	AT3G12690
12234	3	F23 vs. L23	F2-3 up	GO:0005515	Metabolism	Metabolism	protein binding	F	AT3G13110
12235	3	F23 vs. L23	F2-3 up	GO:0005515	Metabolism	Metabolism	protein binding	F	AT3G14770
12236	3	F23 vs. L23	F2-3 up	GO:0005515	Metabolism	Metabolism	protein binding	F	AT3G15030
12237	3	F23 vs. L23	F2-3 up	GO:0005515	Metabolism	Metabolism	protein binding	F	AT3G15540
12238	3	F23 vs. L23	F2-3 up	GO:0005515	Metabolism	Metabolism	protein binding	F	AT3G16800
12239	3	F23 vs. L23	F2-3 up	GO:0005515	Metabolism	Metabolism	protein binding	F	AT3G18780
12240	3	F23 vs. L23	F2-3 up	GO:0005515	Metabolism	Metabolism	protein binding	F	AT3G19390
12241	3	F23 vs. L23	F2-3 up	GO:0005515	Metabolism	Metabolism	protein binding	F	AT3G19980
12242	3	F23 vs. L23	F2-3 up	GO:0005515	Metabolism	Metabolism	protein binding	F	AT3G20190
12243	3	F23 vs. L23	F2-3 up	GO:0005515	Metabolism	Metabolism	protein binding	F	AT3G23050
12244	3	F23 vs. L23	F2-3 up	GO:0005515	Metabolism	Metabolism	protein binding	F	AT3G23820
12245	3	F23 vs. L23	F2-3 up	GO:0005515	Metabolism	Metabolism	protein binding	F	AT3G26060
12246	3	F23 vs. L23	F2-3 up	GO:0005515	Metabolism	Metabolism	protein binding	F	AT3G26650
12247	3	F23 vs. L23	F2-3 up	GO:0005515	Metabolism	Metabolism	protein binding	F	AT3G45140
12248	3	F23 vs. L23	F2-3 up	GO:0005515	Metabolism	Metabolism	protein binding	F	AT3G45640
12249	3	F23 vs. L23	F2-3 up	GO:0005515	Metabolism	Metabolism	protein binding	F	AT3G45780
12250	3	F23 vs. L23	F2-3 up	GO:0005515	Metabolism	Metabolism	protein binding	F	AT3G48740
12251	3	F23 vs. L23	F2-3 up	GO:0005515	Metabolism	Metabolism	protein binding	F	AT3G50000
12252	3	F23 vs. L23	F2-3 up	GO:0005515	Metabolism	Metabolism	protein binding	F	AT3G51920
12253	3	F23 vs. L23	F2-3 up	GO:0005515	Metabolism	Metabolism	protein binding	F	AT3G56150
12254	3	F23 vs. L23	F2-3 up	GO:0005515	Metabolism	Metabolism	protein binding	F	AT3G57600
12255	3	F23 vs. L23	F2-3 up	GO:0005515	Metabolism	Metabolism	protein binding	F	AT3G61880
12256	3	F23 vs. L23	F2-3 up	GO:0005515	Metabolism	Metabolism	protein binding	F	AT3G62410
12257	3	F23 vs. L23	F2-3 up	GO:0005515	Metabolism	Metabolism	protein binding	F	AT3G62960
12258	3	F23 vs. L23	F2-3 up	GO:0005515	Metabolism	Metabolism	protein binding	F	AT3G63140
12259	3	F23 vs. L23	F2-3 up	GO:0005515	Metabolism	Metabolism	protein binding	F	AT4G00360
12260	3	F23 vs. L23	F2-3 up	GO:0005515	Metabolism	Metabolism	protein binding	F	AT4G00480
12261	3	F23 vs. L23	F2-3 up	GO:0005515	Metabolism	Metabolism	protein binding	F	AT4G01850
12262	3	F23 vs. L23	F2-3 up	GO:0005515	Metabolism	Metabolism	protein binding	F	AT4G03190
12263	3	F23 vs. L23	F2-3 up	GO:0005515	Metabolism	Metabolism	protein binding	F	AT4G03280
12264	3	F23 vs. L23	F2-3 up	GO:0005515	Metabolism	Metabolism	protein binding	F	AT4G04770
12265	3	F23 vs. L23	F2-3 up	GO:0005515	Metabolism	Metabolism	protein binding	F	AT4G05590
12266	3	F23 vs. L23	F2-3 up	GO:0005515	Metabolism	Metabolism	protein binding	F	AT4G08150
12267	3	F23 vs. L23	F2-3 up	GO:0005515	Metabolism	Metabolism	protein binding	F	AT4G11280
12268	3	F23 vs. L23	F2-3 up	GO:0005515	Metabolism	Metabolism	protein binding	F	AT4G13930

12269	3	F23 vs. L23	F2-3 up	GO:0005515	Metabolism	Metabolism	protein binding	F	AT4G14880
12270	3	F23 vs. L23	F2-3 up	GO:0005515	Metabolism	Metabolism	protein binding	F	AT4G15630
12271	3	F23 vs. L23	F2-3 up	GO:0005515	Metabolism	Metabolism	protein binding	F	AT4G18950
12272	3	F23 vs. L23	F2-3 up	GO:0005515	Metabolism	Metabolism	protein binding	F	AT4G20360
12273	3	F23 vs. L23	F2-3 up	GO:0005515	Metabolism	Metabolism	protein binding	F	AT4G25030
12274	3	F23 vs. L23	F2-3 up	GO:0005515	Metabolism	Metabolism	protein binding	F	AT4G28490
12275	3	F23 vs. L23	F2-3 up	GO:0005515	Metabolism	Metabolism	protein binding	F	AT4G29350
12276	3	F23 vs. L23	F2-3 up	GO:0005515	Metabolism	Metabolism	protein binding	F	AT4G31800
12277	3	F23 vs. L23	F2-3 up	GO:0005515	Metabolism	Metabolism	protein binding	F	AT4G32410
12278	3	F23 vs. L23	F2-3 up	GO:0005515	Metabolism	Metabolism	protein binding	F	AT4G33010
12279	3	F23 vs. L23	F2-3 up	GO:0005515	Metabolism	Metabolism	protein binding	F	AT4G34710
12280	3	F23 vs. L23	F2-3 up	GO:0005515	Metabolism	Metabolism	protein binding	F	AT4G35250
12281	3	F23 vs. L23	F2-3 up	GO:0005515	Metabolism	Metabolism	protein binding	F	AT4G36710
12282	3	F23 vs. L23	F2-3 up	GO:0005515	Metabolism	Metabolism	protein binding	F	AT4G39110
12283	3	F23 vs. L23	F2-3 up	GO:0005515	Metabolism	Metabolism	protein binding	F	AT4G39800
12284	3	F23 vs. L23	F2-3 up	GO:0005515	Metabolism	Metabolism	protein binding	F	AT5G01410
12285	3	F23 vs. L23	F2-3 up	GO:0005515	Metabolism	Metabolism	protein binding	F	AT5G04140
12286	3	F23 vs. L23	F2-3 up	GO:0005515	Metabolism	Metabolism	protein binding	F	AT5G06850
12287	3	F23 vs. L23	F2-3 up	GO:0005515	Metabolism	Metabolism	protein binding	F	AT5G08640
12288	3	F23 vs. L23	F2-3 up	GO:0005515	Metabolism	Metabolism	protein binding	F	AT5G09230
12289	3	F23 vs. L23	F2-3 up	GO:0005515	Metabolism	Metabolism	protein binding	F	AT5G09810
12290	3	F23 vs. L23	F2-3 up	GO:0005515	Metabolism	Metabolism	protein binding	F	AT5G11790
12291	3	F23 vs. L23	F2-3 up	GO:0005515	Metabolism	Metabolism	protein binding	F	AT5G13160
12292	3	F23 vs. L23	F2-3 up	GO:0005515	Metabolism	Metabolism	protein binding	F	AT5G13490
12293	3	F23 vs. L23	F2-3 up	GO:0005515	Metabolism	Metabolism	protein binding	F	AT5G13930
12294	3	F23 vs. L23	F2-3 up	GO:0005515	Metabolism	Metabolism	protein binding	F	AT5G13990
12295	3	F23 vs. L23	F2-3 up	GO:0005515	Metabolism	Metabolism	protein binding	F	AT5G18600
12296	3	F23 vs. L23	F2-3 up	GO:0005515	Metabolism	Metabolism	protein binding	F	AT5G20240
12297	3	F23 vs. L23	F2-3 up	GO:0005515	Metabolism	Metabolism	protein binding	F	AT5G22250
12298	3	F23 vs. L23	F2-3 up	GO:0005515	Metabolism	Metabolism	protein binding	F	AT5G24120
12299	3	F23 vs. L23	F2-3 up	GO:0005515	Metabolism	Metabolism	protein binding	F	AT5G24650
12300	3	F23 vs. L23	F2-3 up	GO:0005515	Metabolism	Metabolism	protein binding	F	AT5G27000
12301	3	F23 vs. L23	F2-3 up	GO:0005515	Metabolism	Metabolism	protein binding	F	AT5G40890
12302	3	F23 vs. L23	F2-3 up	GO:0005515	Metabolism	Metabolism	protein binding	F	AT5G41790
12303	3	F23 vs. L23	F2-3 up	GO:0005515	Metabolism	Metabolism	protein binding	F	AT5G42050
12304	3	F23 vs. L23	F2-3 up	GO:0005515	Metabolism	Metabolism	protein binding	F	AT5G42800
12305	3	F23 vs. L23	F2-3 up	GO:0005515	Metabolism	Metabolism	protein binding	F	AT5G45840
12306	3	F23 vs. L23	F2-3 up	GO:0005515	Metabolism	Metabolism	protein binding	F	AT5G47670
12307	3	F23 vs. L23	F2-3 up	GO:0005515	Metabolism	Metabolism	protein binding	F	AT5G47910
12308	3	F23 vs. L23	F2-3 up	GO:0005515	Metabolism	Metabolism	protein binding	F	AT5G50800
12309	3	F23 vs. L23	F2-3 up	GO:0005515	Metabolism	Metabolism	protein binding	F	AT5G53890
12310	3	F23 vs. L23	F2-3 up	GO:0005515	Metabolism	Metabolism	protein binding	F	AT5G54490
12311	3	F23 vs. L23	F2-3 up	GO:0005515	Metabolism	Metabolism	protein binding	F	AT5G56860
12312	3	F23 vs. L23	F2-3 up	GO:0005515	Metabolism	Metabolism	protein binding	F	AT5G60450
12313	3	F23 vs. L23	F2-3 up	GO:0005515	Metabolism	Metabolism	protein binding	F	AT5G60910
12314	3	F23 vs. L23	F2-3 up	GO:0005515	Metabolism	Metabolism	protein binding	F	AT5G61670
12315	3	F23 vs. L23	F2-3 up	GO:0005515	Metabolism	Metabolism	protein binding	F	AT5G64350
12316	3	F23 vs. L23	F2-3 up	GO:0005515	Metabolism	Metabolism	protein binding	F	AT5G64740
12317	3	F23 vs. L23	F2-3 up	GO:0005515	Metabolism	Metabolism	protein binding	F	AT5G65410
12318	3	F23 vs. L23	F2-3 up	GO:0005515	Metabolism	Metabolism	protein binding	F	AT5G65460
12319	3	F23 vs. L23	F2-3 up	GO:0005515	Metabolism	Metabolism	protein binding	F	AT5G67300
12320	3	F23 vs. L23	F2-3 up	GO:0005515	Metabolism	Metabolism	protein binding	F	AT5G67480
12321	3	F23 vs. L23	F2-3 up	GO:0030674	Metabolism	Metabolism	protein binding, bridging	F	AT3G21890
12322	3	F23 vs. L23	F2-3 up	GO:0043424	Metabolism	Metabolism	protein histidine kinase binding	F	AT2G38280
12323	3	F23 vs. L23	F2-3 up	GO:0043424	Metabolism	Metabolism	protein histidine kinase binding	F	AT4G15630
12324	3	F23 vs. L23	F2-3 up	GO:0043394	Metabolism	Metabolism	proteoglycan binding	F	AT4G19840

12325	3	F23 vs. L23	F2-3 up	GO:0015252	Metabolism	Metabolism	proton channel activity	F	ATCG00150
12326	3	F23 vs. L23	F2-3 up	GO:0120029	Metabolism	Metabolism	proton export across plasma membrane	P	AT1G80660
12327	3	F23 vs. L23	F2-3 up	GO:0120029	Metabolism	Metabolism	proton export across plasma membrane	P	AT2G07560
12328	3	F23 vs. L23	F2-3 up	GO:0120029	Metabolism	Metabolism	proton export across plasma membrane	P	AT3G42640
12329	3	F23 vs. L23	F2-3 up	GO:0120029	Metabolism	Metabolism	proton export across plasma membrane	P	AT3G60330
12330	3	F23 vs. L23	F2-3 up	GO:0120029	Metabolism	Metabolism	proton export across plasma membrane	P	AT5G57350
12331	3	F23 vs. L23	F2-3 up	GO:0009446	Metabolism	Metabolism	putrescine biosynthetic process	P	AT4G34710
12332	3	F23 vs. L23	F2-3 up	GO:0033388	Metabolism	Metabolism	putrescine biosynthetic process from arginine	P	AT4G34710
12333	3	F23 vs. L23	F2-3 up	GO:0036381	Metabolism	Metabolism	pyridoxal 5'-phosphate synthase (glutamine hydrolysing) activity	F	AT5G01410
12334	3	F23 vs. L23	F2-3 up	GO:0030170	Metabolism	Metabolism	pyridoxal phosphate binding	F	AT1G23310
12335	3	F23 vs. L23	F2-3 up	GO:0030170	Metabolism	Metabolism	pyridoxal phosphate binding	F	AT1G27980
12336	3	F23 vs. L23	F2-3 up	GO:0030170	Metabolism	Metabolism	pyridoxal phosphate binding	F	AT1G65960
12337	3	F23 vs. L23	F2-3 up	GO:0030170	Metabolism	Metabolism	pyridoxal phosphate binding	F	AT1G70580
12338	3	F23 vs. L23	F2-3 up	GO:0030170	Metabolism	Metabolism	pyridoxal phosphate binding	F	AT2G13810
12339	3	F23 vs. L23	F2-3 up	GO:0030170	Metabolism	Metabolism	pyridoxal phosphate binding	F	AT2G30970
12340	3	F23 vs. L23	F2-3 up	GO:0030170	Metabolism	Metabolism	pyridoxal phosphate binding	F	AT2G38400
12341	3	F23 vs. L23	F2-3 up	GO:0030170	Metabolism	Metabolism	pyridoxal phosphate binding	F	AT3G57050
12342	3	F23 vs. L23	F2-3 up	GO:0030170	Metabolism	Metabolism	pyridoxal phosphate binding	F	AT4G11280
12343	3	F23 vs. L23	F2-3 up	GO:0030170	Metabolism	Metabolism	pyridoxal phosphate binding	F	AT4G13930
12344	3	F23 vs. L23	F2-3 up	GO:0030170	Metabolism	Metabolism	pyridoxal phosphate binding	F	AT4G14880
12345	3	F23 vs. L23	F2-3 up	GO:0030170	Metabolism	Metabolism	pyridoxal phosphate binding	F	AT4G33010
12346	3	F23 vs. L23	F2-3 up	GO:0030170	Metabolism	Metabolism	pyridoxal phosphate binding	F	AT5G11520
12347	3	F23 vs. L23	F2-3 up	GO:0030170	Metabolism	Metabolism	pyridoxal phosphate binding	F	AT5G36160
12348	3	F23 vs. L23	F2-3 up	GO:0042823	Metabolism	Metabolism	pyridoxal phosphate biosynthetic process	P	AT5G01410
12349	3	F23 vs. L23	F2-3 up	GO:0008615	Metabolism	Metabolism	pyridoxine biosynthetic process	P	AT5G01410
12350	3	F23 vs. L23	F2-3 up	GO:0050242	Metabolism	Metabolism	pyruvate, phosphate dikinase activity	F	AT4G15530
12351	3	F23 vs. L23	F2-3 up	GO:0080043	Metabolism	Metabolism	quercetin 3-O-glucosyltransferase activity	F	AT2G31750
12352	3	F23 vs. L23	F2-3 up	GO:0080043	Metabolism	Metabolism	quercetin 3-O-glucosyltransferase activity	F	AT2G31790
12353	3	F23 vs. L23	F2-3 up	GO:0080043	Metabolism	Metabolism	quercetin 3-O-glucosyltransferase activity	F	AT2G36790
12354	3	F23 vs. L23	F2-3 up	GO:0080043	Metabolism	Metabolism	quercetin 3-O-glucosyltransferase activity	F	AT3G21560
12355	3	F23 vs. L23	F2-3 up	GO:0080043	Metabolism	Metabolism	quercetin 3-O-glucosyltransferase activity	F	AT3G46670
12356	3	F23 vs. L23	F2-3 up	GO:0080046	Metabolism	Metabolism	quercetin 4'-O-glucosyltransferase activity	F	AT2G36790
12357	3	F23 vs. L23	F2-3 up	GO:0080044	Metabolism	Metabolism	quercetin 7-O-glucosyltransferase activity	F	AT2G31750
12358	3	F23 vs. L23	F2-3 up	GO:0080044	Metabolism	Metabolism	quercetin 7-O-glucosyltransferase activity	F	AT2G31790
12359	3	F23 vs. L23	F2-3 up	GO:0080044	Metabolism	Metabolism	quercetin 7-O-glucosyltransferase activity	F	AT2G36790
12360	3	F23 vs. L23	F2-3 up	GO:0080044	Metabolism	Metabolism	quercetin 7-O-glucosyltransferase activity	F	AT3G21560
12361	3	F23 vs. L23	F2-3 up	GO:0080044	Metabolism	Metabolism	quercetin 7-O-glucosyltransferase activity	F	AT3G46670
12362	3	F23 vs. L23	F2-3 up	GO:0048038	Metabolism	Metabolism	quinone binding	F	AT1G31490
12363	3	F23 vs. L23	F2-3 up	GO:0048038	Metabolism	Metabolism	quinone binding	F	AT4G23890
12364	3	F23 vs. L23	F2-3 up	GO:0048038	Metabolism	Metabolism	quinone binding	F	AT5G58260
12365	3	F23 vs. L23	F2-3 up	GO:0048038	Metabolism	Metabolism	quinone binding	F	ATCG00420
12366	3	F23 vs. L23	F2-3 up	GO:0048038	Metabolism	Metabolism	quinone binding	F	ATCG00430
12367	3	F23 vs. L23	F2-3 up	GO:0048038	Metabolism	Metabolism	quinone binding	F	ATCG01090
12368	3	F23 vs. L23	F2-3 up	GO:0048038	Metabolism	Metabolism	quinone binding	F	ATCG01100
12369	3	F23 vs. L23	F2-3 up	GO:0009231	Metabolism	Metabolism	riboflavin biosynthetic process	P	AT2G22450
12370	3	F23 vs. L23	F2-3 up	GO:0006557	Metabolism	Metabolism	S-adenosylmethioninamine biosynthetic process	P	AT3G02470
12371	3	F23 vs. L23	F2-3 up	GO:0006557	Metabolism	Metabolism	S-adenosylmethioninamine biosynthetic process	P	AT5G15950
12372	3	F23 vs. L23	F2-3 up	GO:0006556	Metabolism	Metabolism	S-adenosylmethionine biosynthetic process	P	AT2G36880
12373	3	F23 vs. L23	F2-3 up	GO:0006556	Metabolism	Metabolism	S-adenosylmethionine biosynthetic process	P	AT3G17390
12374	3	F23 vs. L23	F2-3 up	GO:0006556	Metabolism	Metabolism	S-adenosylmethionine biosynthetic process	P	AT4G01850
12375	3	F23 vs. L23	F2-3 up	GO:0033353	Metabolism	Metabolism	S-adenosylmethionine cycle	P	AT3G23810
12376	3	F23 vs. L23	F2-3 up	GO:0008757	Metabolism	Metabolism	S-adenosylmethionine-dependent methyltransferase activity	F	AT4G35150
12377	3	F23 vs. L23	F2-3 up	GO:0034785	Metabolism	Metabolism	salicylate 5-hydroxylase activity	F	AT5G24530
12378	3	F23 vs. L23	F2-3 up	GO:0102483	Metabolism	Metabolism	scopolin beta-glucosidase activity	F	AT1G61820

12379	3	F23 vs. L23	F2-3 up	GO:0102483	Metabolism	Metabolism	scopolin beta-glucosidase activity	F	AT3G18080
12380	3	F23 vs. L23	F2-3 up	GO:0050278	Metabolism	Metabolism	sedoheptulose-bisphosphatase activity	F	AT3G55800
12381	3	F23 vs. L23	F2-3 up	GO:0001887	Metabolism	Metabolism	selenium compound metabolic process	P	AT3G22890
12382	3	F23 vs. L23	F2-3 up	GO:0042989	Metabolism	Metabolism	sequestering of actin monomers	P	AT2G19770
12383	3	F23 vs. L23	F2-3 up	GO:0042989	Metabolism	Metabolism	sequestering of actin monomers	P	AT4G29340
12384	3	F23 vs. L23	F2-3 up	GO:0042989	Metabolism	Metabolism	sequestering of actin monomers	P	AT4G29350
12385	3	F23 vs. L23	F2-3 up	GO:0070905	Metabolism	Metabolism	serine binding	F	AT4G13930
12386	3	F23 vs. L23	F2-3 up	GO:0009001	Metabolism	Metabolism	serine O-acetyltransferase activity	F	AT3G13110
12387	3	F23 vs. L23	F2-3 up	GO:0008236	Metabolism	Metabolism	serine-type peptidase activity	F	AT5G65760
12388	3	F23 vs. L23	F2-3 up	GO:0016987	Metabolism	Metabolism	sigma factor activity	F	AT1G64860
12389	3	F23 vs. L23	F2-3 up	GO:0016987	Metabolism	Metabolism	sigma factor activity	F	AT5G24120
12390	3	F23 vs. L23	F2-3 up	GO:0050284	Metabolism	Metabolism	sinapate 1-glucosyltransferase activity	F	AT3G21560
12391	3	F23 vs. L23	F2-3 up	GO:0052747	Metabolism	Metabolism	sinapyl alcohol dehydrogenase activity	F	AT4G37990
12392	3	F23 vs. L23	F2-3 up	GO:0008295	Metabolism	Metabolism	spermidine biosynthetic process	P	AT1G70310
12393	3	F23 vs. L23	F2-3 up	GO:0008295	Metabolism	Metabolism	spermidine biosynthetic process	P	AT3G02470
12394	3	F23 vs. L23	F2-3 up	GO:0008295	Metabolism	Metabolism	spermidine biosynthetic process	P	AT4G34710
12395	3	F23 vs. L23	F2-3 up	GO:0008295	Metabolism	Metabolism	spermidine biosynthetic process	P	AT5G15950
12396	3	F23 vs. L23	F2-3 up	GO:0004766	Metabolism	Metabolism	spermidine synthase activity	F	AT1G70310
12397	3	F23 vs. L23	F2-3 up	GO:0006597	Metabolism	Metabolism	spermine biosynthetic process	P	AT3G02470
12398	3	F23 vs. L23	F2-3 up	GO:0006597	Metabolism	Metabolism	spermine biosynthetic process	P	AT5G15950
12399	3	F23 vs. L23	F2-3 up	GO:0008117	Metabolism	Metabolism	sphinganine-1-phosphate aldolase activity	F	AT1G27980
12400	3	F23 vs. L23	F2-3 up	GO:0046520	Metabolism	Metabolism	sphingoid biosynthetic process	P	AT1G69640
12401	3	F23 vs. L23	F2-3 up	GO:0030148	Metabolism	Metabolism	sphingolipid biosynthetic process	P	AT2G46210
12402	3	F23 vs. L23	F2-3 up	GO:0052631	Metabolism	Metabolism	sphingolipid delta-8 desaturase activity	F	AT2G46210
12403	3	F23 vs. L23	F2-3 up	GO:0006665	Metabolism	Metabolism	sphingolipid metabolic process	P	AT1G27980
12404	3	F23 vs. L23	F2-3 up	GO:0000170	Metabolism	Metabolism	sphingosine hydroxylase activity	F	AT1G69640
12405	3	F23 vs. L23	F2-3 up	GO:0004506	Metabolism	Metabolism	squalene monooxygenase activity	F	AT4G37760
12406	3	F23 vs. L23	F2-3 up	GO:0004506	Metabolism	Metabolism	squalene monooxygenase activity	F	AT5G24140
12407	3	F23 vs. L23	F2-3 up	GO:0000104	Metabolism	Metabolism	succinate dehydrogenase activity	F	AT3G27380
12408	3	F23 vs. L23	F2-3 up	GO:0000104	Metabolism	Metabolism	succinate dehydrogenase activity	F	AT5G40650
12409	3	F23 vs. L23	F2-3 up	GO:0004575	Metabolism	Metabolism	sucrose alpha-glucosidase activity	F	AT1G12240
12410	3	F23 vs. L23	F2-3 up	GO:0050307	Metabolism	Metabolism	sucrose-phosphate phosphatase activity	F	AT1G51420
12411	3	F23 vs. L23	F2-3 up	GO:0050307	Metabolism	Metabolism	sucrose-phosphate phosphatase activity	F	AT2G35840
12412	3	F23 vs. L23	F2-3 up	GO:0004781	Metabolism	Metabolism	sulfate adenylyltransferase (ATP) activity	F	AT3G22890
12413	3	F23 vs. L23	F2-3 up	GO:0019419	Metabolism	Metabolism	sulfate reduction	P	AT4G21990
12414	3	F23 vs. L23	F2-3 up	GO:0008146	Metabolism	Metabolism	sulfotransferase activity	F	AT3G50620
12415	3	F23 vs. L23	F2-3 up	GO:0017025	Metabolism	Metabolism	TBP-class protein binding	F	AT2G41630
12416	3	F23 vs. L23	F2-3 up	GO:0035999	Metabolism	Metabolism	tetrahydrofolate interconversion	P	AT3G59970
12417	3	F23 vs. L23	F2-3 up	GO:0035999	Metabolism	Metabolism	tetrahydrofolate interconversion	P	AT4G13930
12418	3	F23 vs. L23	F2-3 up	GO:0046653	Metabolism	Metabolism	tetrahydrofolate metabolic process	P	AT4G13930
12419	3	F23 vs. L23	F2-3 up	GO:0009228	Metabolism	Metabolism	thiamine biosynthetic process	P	AT2G29630
12420	3	F23 vs. L23	F2-3 up	GO:0009229	Metabolism	Metabolism	thiamine diphosphate biosynthetic process	P	AT2G29630
12421	3	F23 vs. L23	F2-3 up	GO:0019137	Metabolism	Metabolism	thioglucosidase activity	F	AT5G25980
12422	3	F23 vs. L23	F2-3 up	GO:0004791	Metabolism	Metabolism	thioredoxin-disulfide reductase activity	F	AT3G54660
12423	3	F23 vs. L23	F2-3 up	GO:0009088	Metabolism	Metabolism	threonine biosynthetic process	P	AT5G14060
12424	3	F23 vs. L23	F2-3 up	GO:0008483	Metabolism	Metabolism	transaminase activity	F	AT1G11860
12425	3	F23 vs. L23	F2-3 up	GO:0008483	Metabolism	Metabolism	transaminase activity	F	AT1G34040
12426	3	F23 vs. L23	F2-3 up	GO:0008483	Metabolism	Metabolism	transaminase activity	F	AT2G13810
12427	3	F23 vs. L23	F2-3 up	GO:0016710	Metabolism	Metabolism	trans-cinnamate 4-monooxygenase activity	F	AT2G30490
12428	3	F23 vs. L23	F2-3 up	GO:0016740	Metabolism	Metabolism	transferase activity	F	AT1G19550
12429	3	F23 vs. L23	F2-3 up	GO:0016740	Metabolism	Metabolism	transferase activity	F	AT1G64980
12430	3	F23 vs. L23	F2-3 up	GO:0016746	Metabolism	Metabolism	transferase activity, transferring acyl groups	F	AT1G75020
12431	3	F23 vs. L23	F2-3 up	GO:0016746	Metabolism	Metabolism	transferase activity, transferring acyl groups	F	AT1G75880
12432	3	F23 vs. L23	F2-3 up	GO:0016747	Metabolism	Metabolism	transferase activity, transferring acyl groups other than amino-acyl groups	F	AT1G03495

12433	3	F23 vs. L23	F2-3 up	GO:0016747	Metabolism	Metabolism	transferase activity, transferring acyl groups other than amino-acyl groups	F	AT1G28680
12434	3	F23 vs. L23	F2-3 up	GO:0016747	Metabolism	Metabolism	transferase activity, transferring acyl groups other than amino-acyl groups	F	AT1G31490
12435	3	F23 vs. L23	F2-3 up	GO:0016747	Metabolism	Metabolism	transferase activity, transferring acyl groups other than amino-acyl groups	F	AT2G40230
12436	3	F23 vs. L23	F2-3 up	GO:0016747	Metabolism	Metabolism	transferase activity, transferring acyl groups other than amino-acyl groups	F	AT4G24510
12437	3	F23 vs. L23	F2-3 up	GO:0016747	Metabolism	Metabolism	transferase activity, transferring acyl groups other than amino-acyl groups	F	AT5G67160
12438	3	F23 vs. L23	F2-3 up	GO:0016757	Metabolism	Metabolism	transferase activity, transferring glycosyl groups	F	AT1G07850
12439	3	F23 vs. L23	F2-3 up	GO:0016757	Metabolism	Metabolism	transferase activity, transferring glycosyl groups	F	AT1G74380
12440	3	F23 vs. L23	F2-3 up	GO:0016757	Metabolism	Metabolism	transferase activity, transferring glycosyl groups	F	AT2G18570
12441	3	F23 vs. L23	F2-3 up	GO:0016757	Metabolism	Metabolism	transferase activity, transferring glycosyl groups	F	AT2G31750
12442	3	F23 vs. L23	F2-3 up	GO:0016757	Metabolism	Metabolism	transferase activity, transferring glycosyl groups	F	AT2G31790
12443	3	F23 vs. L23	F2-3 up	GO:0016757	Metabolism	Metabolism	transferase activity, transferring glycosyl groups	F	AT2G33100
12444	3	F23 vs. L23	F2-3 up	GO:0016757	Metabolism	Metabolism	transferase activity, transferring glycosyl groups	F	AT2G36790
12445	3	F23 vs. L23	F2-3 up	GO:0016757	Metabolism	Metabolism	transferase activity, transferring glycosyl groups	F	AT3G01180
12446	3	F23 vs. L23	F2-3 up	GO:0016757	Metabolism	Metabolism	transferase activity, transferring glycosyl groups	F	AT3G06510
12447	3	F23 vs. L23	F2-3 up	GO:0016757	Metabolism	Metabolism	transferase activity, transferring glycosyl groups	F	AT3G21190
12448	3	F23 vs. L23	F2-3 up	GO:0016757	Metabolism	Metabolism	transferase activity, transferring glycosyl groups	F	AT3G21760
12449	3	F23 vs. L23	F2-3 up	GO:0016757	Metabolism	Metabolism	transferase activity, transferring glycosyl groups	F	AT3G25140
12450	3	F23 vs. L23	F2-3 up	GO:0016757	Metabolism	Metabolism	transferase activity, transferring glycosyl groups	F	AT3G45100
12451	3	F23 vs. L23	F2-3 up	GO:0016757	Metabolism	Metabolism	transferase activity, transferring glycosyl groups	F	AT3G46670
12452	3	F23 vs. L23	F2-3 up	GO:0016757	Metabolism	Metabolism	transferase activity, transferring glycosyl groups	F	AT4G01070
12453	3	F23 vs. L23	F2-3 up	GO:0016757	Metabolism	Metabolism	transferase activity, transferring glycosyl groups	F	AT4G10120
12454	3	F23 vs. L23	F2-3 up	GO:0016757	Metabolism	Metabolism	transferase activity, transferring glycosyl groups	F	AT4G13990
12455	3	F23 vs. L23	F2-3 up	GO:0016757	Metabolism	Metabolism	transferase activity, transferring glycosyl groups	F	AT4G16590
12456	3	F23 vs. L23	F2-3 up	GO:0016757	Metabolism	Metabolism	transferase activity, transferring glycosyl groups	F	AT4G31590
12457	3	F23 vs. L23	F2-3 up	GO:0016757	Metabolism	Metabolism	transferase activity, transferring glycosyl groups	F	AT4G32410
12458	3	F23 vs. L23	F2-3 up	GO:0016757	Metabolism	Metabolism	transferase activity, transferring glycosyl groups	F	AT4G39350
12459	3	F23 vs. L23	F2-3 up	GO:0016757	Metabolism	Metabolism	transferase activity, transferring glycosyl groups	F	AT5G04500
12460	3	F23 vs. L23	F2-3 up	GO:0016757	Metabolism	Metabolism	transferase activity, transferring glycosyl groups	F	AT5G09870
12461	3	F23 vs. L23	F2-3 up	GO:0016757	Metabolism	Metabolism	transferase activity, transferring glycosyl groups	F	AT5G15470
12462	3	F23 vs. L23	F2-3 up	GO:0016757	Metabolism	Metabolism	transferase activity, transferring glycosyl groups	F	AT5G16910
12463	3	F23 vs. L23	F2-3 up	GO:0016757	Metabolism	Metabolism	transferase activity, transferring glycosyl groups	F	AT5G64740
12464	3	F23 vs. L23	F2-3 up	GO:0016758	Metabolism	Metabolism	transferase activity, transferring hexosyl groups	F	AT2G18570
12465	3	F23 vs. L23	F2-3 up	GO:0016758	Metabolism	Metabolism	transferase activity, transferring hexosyl groups	F	AT3G21760
12466	3	F23 vs. L23	F2-3 up	GO:0019346	Metabolism	Metabolism	transsulfuration	P	AT3G57050
12467	3	F23 vs. L23	F2-3 up	GO:0102701	Metabolism	Metabolism	tricyclene synthase activity	F	AT2G24210
12468	3	F23 vs. L23	F2-3 up	GO:0019432	Metabolism	Metabolism	triglyceride biosynthetic process	P	AT1G54570
12469	3	F23 vs. L23	F2-3 up	GO:0019432	Metabolism	Metabolism	triglyceride biosynthetic process	P	AT5G37300
12470	3	F23 vs. L23	F2-3 up	GO:0006641	Metabolism	Metabolism	triglyceride metabolic process	P	AT1G80460
12471	3	F23 vs. L23	F2-3 up	GO:0016104	Metabolism	Metabolism	triterpenoid biosynthetic process	P	AT2G17370
12472	3	F23 vs. L23	F2-3 up	GO:0000162	Metabolism	Metabolism	tryptophan biosynthetic process	P	AT1G75500
12473	3	F23 vs. L23	F2-3 up	GO:0000162	Metabolism	Metabolism	tryptophan biosynthetic process	P	AT5G05730
12474	3	F23 vs. L23	F2-3 up	GO:0006568	Metabolism	Metabolism	tryptophan metabolic process	P	AT1G32640
12475	3	F23 vs. L23	F2-3 up	GO:0010489	Metabolism	Metabolism	UDP-4-keto-6-deoxy-glucose-3,5-epimerase activity	F	AT1G53500
12476	3	F23 vs. L23	F2-3 up	GO:0010489	Metabolism	Metabolism	UDP-4-keto-6-deoxy-glucose-3,5-epimerase activity	F	AT1G63000
12477	3	F23 vs. L23	F2-3 up	GO:0010490	Metabolism	Metabolism	UDP-4-keto-rhamnose-4-keto-reductase activity	F	AT1G53500
12478	3	F23 vs. L23	F2-3 up	GO:0010490	Metabolism	Metabolism	UDP-4-keto-rhamnose-4-keto-reductase activity	F	AT1G63000
12479	3	F23 vs. L23	F2-3 up	GO:0050373	Metabolism	Metabolism	UDP-arabinose 4-epimerase activity	F	AT2G34850
12480	3	F23 vs. L23	F2-3 up	GO:0050373	Metabolism	Metabolism	UDP-arabinose 4-epimerase activity	F	AT4G20460
12481	3	F23 vs. L23	F2-3 up	GO:0033320	Metabolism	Metabolism	UDP-D-xylose biosynthetic process	P	AT2G47650
12482	3	F23 vs. L23	F2-3 up	GO:0050377	Metabolism	Metabolism	UDP-glucose 4,6-dehydratase activity	F	AT1G53500
12483	3	F23 vs. L23	F2-3 up	GO:0050377	Metabolism	Metabolism	UDP-glucose 4,6-dehydratase activity	F	AT1G78570

12484	3	F23 vs. L23	F2-3 up	GO:0003978	Metabolism	Metabolism	UDP-glucose 4-epimerase activity	F	AT1G09340
12485	3	F23 vs. L23	F2-3 up	GO:0003978	Metabolism	Metabolism	UDP-glucose 4-epimerase activity	F	AT2G34850
12486	3	F23 vs. L23	F2-3 up	GO:0003978	Metabolism	Metabolism	UDP-glucose 4-epimerase activity	F	AT3G63140
12487	3	F23 vs. L23	F2-3 up	GO:0003978	Metabolism	Metabolism	UDP-glucose 4-epimerase activity	F	AT4G20460
12488	3	F23 vs. L23	F2-3 up	GO:0003979	Metabolism	Metabolism	UDP-glucose 6-dehydrogenase activity	F	AT3G29360
12489	3	F23 vs. L23	F2-3 up	GO:0003979	Metabolism	Metabolism	UDP-glucose 6-dehydrogenase activity	F	AT5G15490
12490	3	F23 vs. L23	F2-3 up	GO:0003979	Metabolism	Metabolism	UDP-glucose 6-dehydrogenase activity	F	AT5G39320
12491	3	F23 vs. L23	F2-3 up	GO:0006011	Metabolism	Metabolism	UDP-glucose metabolic process	P	AT3G03250
12492	3	F23 vs. L23	F2-3 up	GO:0035251	Metabolism	Metabolism	UDP-glucosyltransferase activity	F	AT2G36790
12493	3	F23 vs. L23	F2-3 up	GO:0035251	Metabolism	Metabolism	UDP-glucosyltransferase activity	F	AT4G01070
12494	3	F23 vs. L23	F2-3 up	GO:0050378	Metabolism	Metabolism	UDP-glucuronate 4-epimerase activity	F	AT3G23820
12495	3	F23 vs. L23	F2-3 up	GO:0006065	Metabolism	Metabolism	UDP-glucuronate biosynthetic process	P	AT3G29360
12496	3	F23 vs. L23	F2-3 up	GO:0006065	Metabolism	Metabolism	UDP-glucuronate biosynthetic process	P	AT5G15490
12497	3	F23 vs. L23	F2-3 up	GO:0006065	Metabolism	Metabolism	UDP-glucuronate biosynthetic process	P	AT5G39320
12498	3	F23 vs. L23	F2-3 up	GO:0048040	Metabolism	Metabolism	UDP-glucuronate decarboxylase activity	F	AT2G47650
12499	3	F23 vs. L23	F2-3 up	GO:0008194	Metabolism	Metabolism	UDP-glycosyltransferase activity	F	AT2G18570
12500	3	F23 vs. L23	F2-3 up	GO:0008194	Metabolism	Metabolism	UDP-glycosyltransferase activity	F	AT2G31750
12501	3	F23 vs. L23	F2-3 up	GO:0008194	Metabolism	Metabolism	UDP-glycosyltransferase activity	F	AT2G31790
12502	3	F23 vs. L23	F2-3 up	GO:0008194	Metabolism	Metabolism	UDP-glycosyltransferase activity	F	AT2G36790
12503	3	F23 vs. L23	F2-3 up	GO:0008194	Metabolism	Metabolism	UDP-glycosyltransferase activity	F	AT3G21560
12504	3	F23 vs. L23	F2-3 up	GO:0008194	Metabolism	Metabolism	UDP-glycosyltransferase activity	F	AT3G21760
12505	3	F23 vs. L23	F2-3 up	GO:0008194	Metabolism	Metabolism	UDP-glycosyltransferase activity	F	AT3G46670
12506	3	F23 vs. L23	F2-3 up	GO:0008194	Metabolism	Metabolism	UDP-glycosyltransferase activity	F	AT4G01070
12507	3	F23 vs. L23	F2-3 up	GO:0033358	Metabolism	Metabolism	UDP-L-arabinose biosynthetic process	P	AT2G34850
12508	3	F23 vs. L23	F2-3 up	GO:0033358	Metabolism	Metabolism	UDP-L-arabinose biosynthetic process	P	AT4G20460
12509	3	F23 vs. L23	F2-3 up	GO:0010280	Metabolism	Metabolism	UDP-L-rhamnose synthase activity	F	AT1G53500
12510	3	F23 vs. L23	F2-3 up	GO:0010280	Metabolism	Metabolism	UDP-L-rhamnose synthase activity	F	AT1G78570
12511	3	F23 vs. L23	F2-3 up	GO:0010253	Metabolism	Metabolism	UDP-rhamnose biosynthetic process	P	AT1G53500
12512	3	F23 vs. L23	F2-3 up	GO:0010253	Metabolism	Metabolism	UDP-rhamnose biosynthetic process	P	AT1G63000
12513	3	F23 vs. L23	F2-3 up	GO:0010253	Metabolism	Metabolism	UDP-rhamnose biosynthetic process	P	AT1G78570
12514	3	F23 vs. L23	F2-3 up	GO:0003983	Metabolism	Metabolism	UTP:glucose-1-phosphate uridylyltransferase activity	F	AT3G03250
12515	3	F23 vs. L23	F2-3 up	GO:0042819	Metabolism	Metabolism	vitamin B6 biosynthetic process	P	AT5G01410
12516	3	F23 vs. L23	F2-3 up	GO:0010025	Metabolism	Metabolism	wax biosynthetic process	P	AT1G02205
12517	3	F23 vs. L23	F2-3 up	GO:0010025	Metabolism	Metabolism	wax biosynthetic process	P	AT1G68530
12518	3	F23 vs. L23	F2-3 up	GO:0010025	Metabolism	Metabolism	wax biosynthetic process	P	AT4G24510
12519	3	F23 vs. L23	F2-3 up	GO:0010025	Metabolism	Metabolism	wax biosynthetic process	P	AT5G37300
12520	3	F23 vs. L23	F2-3 up	GO:0010025	Metabolism	Metabolism	wax biosynthetic process	P	AT5G57800
12521	3	F23 vs. L23	F2-3 up	GO:0006805	Metabolism	Metabolism	xenobiotic metabolic process	P	AT4G01070
12522	3	F23 vs. L23	F2-3 up	GO:0045492	Metabolism	Metabolism	xylan biosynthetic process	P	AT1G27930
12523	3	F23 vs. L23	F2-3 up	GO:0045492	Metabolism	Metabolism	xylan biosynthetic process	P	AT5G67210
12524	3	F23 vs. L23	F2-3 up	GO:0045491	Metabolism	Metabolism	xylan metabolic process	P	AT1G27930
12525	3	F23 vs. L23	F2-3 up	GO:0045491	Metabolism	Metabolism	xylan metabolic process	P	AT5G67210
12526	3	F23 vs. L23	F2-3 up	GO:0010089	Metabolism	Metabolism	xylem development	P	AT4G08150
12527	3	F23 vs. L23	F2-3 up	GO:0010089	Metabolism	Metabolism	xylem development	P	AT4G32880
12528	3	F23 vs. L23	F2-3 up	GO:0033843	Metabolism	Metabolism	xyloglucan 6-xylosyltransferase activity	F	AT1G74380
12529	3	F23 vs. L23	F2-3 up	GO:0009969	Metabolism	Metabolism	xyloglucan biosynthetic process	P	AT1G74380
12530	3	F23 vs. L23	F2-3 up	GO:0010411	Metabolism	Metabolism	xyloglucan metabolic process	P	AT1G74380
12531	3	F23 vs. L23	F2-3 up	GO:0010411	Metabolism	Metabolism	xyloglucan metabolic process	P	AT3G28150
12532	3	F23 vs. L23	F2-3 up	GO:0010411	Metabolism	Metabolism	xyloglucan metabolic process	P	AT3G44990
12533	3	F23 vs. L23	F2-3 up	GO:0010411	Metabolism	Metabolism	xyloglucan metabolic process	P	AT5G57560
12534	3	F23 vs. L23	F2-3 up	GO:0010411	Metabolism	Metabolism	xyloglucan metabolic process	P	AT5G65730
12535	3	F23 vs. L23	F2-3 up	GO:0016762	Metabolism	Metabolism	xyloglucan:xyloglucosyl transferase activity	F	AT3G44990
12536	3	F23 vs. L23	F2-3 up	GO:0016762	Metabolism	Metabolism	xyloglucan:xyloglucosyl transferase activity	F	AT5G57560
12537	3	F23 vs. L23	F2-3 up	GO:0016762	Metabolism	Metabolism	xyloglucan:xyloglucosyl transferase activity	F	AT5G65730

12538	3	F23 vs. L23	F2-3 up	GO:0033946	Metabolism	Metabolism	xyloglucan-specific endo-beta-1,4-glucanase activity	F	AT3G44990
12539	3	F23 vs. L23	F2-3 up	GO:0008270	Metabolism	Metabolism	zinc ion binding	F	AT1G23740
12540	3	F23 vs. L23	F2-3 up	GO:0008270	Metabolism	Metabolism	zinc ion binding	F	AT1G35720
12541	3	F23 vs. L23	F2-3 up	GO:0008270	Metabolism	Metabolism	zinc ion binding	F	AT1G48030
12542	3	F23 vs. L23	F2-3 up	GO:0008270	Metabolism	Metabolism	zinc ion binding	F	AT1G54280
12543	3	F23 vs. L23	F2-3 up	GO:0008270	Metabolism	Metabolism	zinc ion binding	F	AT1G58180
12544	3	F23 vs. L23	F2-3 up	GO:0008270	Metabolism	Metabolism	zinc ion binding	F	AT2G19570
12545	3	F23 vs. L23	F2-3 up	GO:0008270	Metabolism	Metabolism	zinc ion binding	F	AT2G31380
12546	3	F23 vs. L23	F2-3 up	GO:0008270	Metabolism	Metabolism	zinc ion binding	F	AT3G01500
12547	3	F23 vs. L23	F2-3 up	GO:0008270	Metabolism	Metabolism	zinc ion binding	F	AT3G02010
12548	3	F23 vs. L23	F2-3 up	GO:0008270	Metabolism	Metabolism	zinc ion binding	F	AT3G02570
12549	3	F23 vs. L23	F2-3 up	GO:0008270	Metabolism	Metabolism	zinc ion binding	F	AT3G13110
12550	3	F23 vs. L23	F2-3 up	GO:0008270	Metabolism	Metabolism	zinc ion binding	F	AT3G15353
12551	3	F23 vs. L23	F2-3 up	GO:0008270	Metabolism	Metabolism	zinc ion binding	F	AT3G27380
12552	3	F23 vs. L23	F2-3 up	GO:0008270	Metabolism	Metabolism	zinc ion binding	F	AT3G52720
12553	3	F23 vs. L23	F2-3 up	GO:0008270	Metabolism	Metabolism	zinc ion binding	F	AT3G52800
12554	3	F23 vs. L23	F2-3 up	GO:0008270	Metabolism	Metabolism	zinc ion binding	F	AT4G13930
12555	3	F23 vs. L23	F2-3 up	GO:0008270	Metabolism	Metabolism	zinc ion binding	F	AT4G37990
12556	3	F23 vs. L23	F2-3 up	GO:0008270	Metabolism	Metabolism	zinc ion binding	F	AT5G04710
12557	3	F23 vs. L23	F2-3 up	GO:0008270	Metabolism	Metabolism	zinc ion binding	F	AT5G07440
12558	3	F23 vs. L23	F2-3 up	GO:0008270	Metabolism	Metabolism	zinc ion binding	F	AT5G14740
12559	3	F23 vs. L23	F2-3 up	GO:0008270	Metabolism	Metabolism	zinc ion binding	F	AT5G40650
12560	3	F23 vs. L23	F2-3 up	GO:0008270	Metabolism	Metabolism	zinc ion binding	F	AT5G56860
12561	3	F23 vs. L23	F2-3 up	GO:0008270	Metabolism	Metabolism	zinc ion binding	F	AT5G67480
12562	3	F23 vs. L23	F2-3 up	GO:0008270	Metabolism	Metabolism	zinc ion binding	F	ATCG00120
12563	3	F23 vs. L23	F2-3 up	GO:0009882	Metabolism	Photosynthesis	blue light photoreceptor activity	F	AT1G04400
12564	3	F23 vs. L23	F2-3 up	GO:0009882	Metabolism	Photosynthesis	blue light photoreceptor activity	F	AT3G45780
12565	3	F23 vs. L23	F2-3 up	GO:0015977	Metabolism	Photosynthesis	carbon fixation	P	AT3G14940
12566	3	F23 vs. L23	F2-3 up	GO:0016120	Metabolism	Photosynthesis	carotene biosynthetic process	P	AT5G61670
12567	3	F23 vs. L23	F2-3 up	GO:0016168	Metabolism	Photosynthesis	chlorophyll binding	F	AT1G19150
12568	3	F23 vs. L23	F2-3 up	GO:0016168	Metabolism	Photosynthesis	chlorophyll binding	F	AT1G44575
12569	3	F23 vs. L23	F2-3 up	GO:0016168	Metabolism	Photosynthesis	chlorophyll binding	F	AT1G45474
12570	3	F23 vs. L23	F2-3 up	GO:0015995	Metabolism	Photosynthesis	chlorophyll biosynthetic process	P	AT1G58290
12571	3	F23 vs. L23	F2-3 up	GO:0015995	Metabolism	Photosynthesis	chlorophyll biosynthetic process	P	AT3G02780
12572	3	F23 vs. L23	F2-3 up	GO:0015995	Metabolism	Photosynthesis	chlorophyll biosynthetic process	P	AT3G51820
12573	3	F23 vs. L23	F2-3 up	GO:0015994	Metabolism	Photosynthesis	chlorophyll metabolic process	P	AT5G01410
12574	3	F23 vs. L23	F2-3 up	GO:0046408	Metabolism	Photosynthesis	chlorophyll synthetase activity	F	AT3G51820
12575	3	F23 vs. L23	F2-3 up	GO:0010277	Metabolism	Photosynthesis	chlorophyllide a oxygenase [overall] activity	F	AT2G24820
12576	3	F23 vs. L23	F2-3 up	GO:0009507	Metabolism	Photosynthesis	chloroplast	C	AT1G01090
12577	3	F23 vs. L23	F2-3 up	GO:0009507	Metabolism	Photosynthesis	chloroplast	C	AT1G06690
12578	3	F23 vs. L23	F2-3 up	GO:0009507	Metabolism	Photosynthesis	chloroplast	C	AT1G08510
12579	3	F23 vs. L23	F2-3 up	GO:0009507	Metabolism	Photosynthesis	chloroplast	C	AT1G09340
12580	3	F23 vs. L23	F2-3 up	GO:0009507	Metabolism	Photosynthesis	chloroplast	C	AT1G10370
12581	3	F23 vs. L23	F2-3 up	GO:0009507	Metabolism	Photosynthesis	chloroplast	C	AT1G10960
12582	3	F23 vs. L23	F2-3 up	GO:0009507	Metabolism	Photosynthesis	chloroplast	C	AT1G11840
12583	3	F23 vs. L23	F2-3 up	GO:0009507	Metabolism	Photosynthesis	chloroplast	C	AT1G11860
12584	3	F23 vs. L23	F2-3 up	GO:0009507	Metabolism	Photosynthesis	chloroplast	C	AT1G12250
12585	3	F23 vs. L23	F2-3 up	GO:0009507	Metabolism	Photosynthesis	chloroplast	C	AT1G12840
12586	3	F23 vs. L23	F2-3 up	GO:0009507	Metabolism	Photosynthesis	chloroplast	C	AT1G13270
12587	3	F23 vs. L23	F2-3 up	GO:0009507	Metabolism	Photosynthesis	chloroplast	C	AT1G14560
12588	3	F23 vs. L23	F2-3 up	GO:0009507	Metabolism	Photosynthesis	chloroplast	C	AT1G15980
12589	3	F23 vs. L23	F2-3 up	GO:0009507	Metabolism	Photosynthesis	chloroplast	C	AT1G16410
12590	3	F23 vs. L23	F2-3 up	GO:0009507	Metabolism	Photosynthesis	chloroplast	C	AT1G16720
12591	3	F23 vs. L23	F2-3 up	GO:0009507	Metabolism	Photosynthesis	chloroplast	C	AT1G16880
12592	3	F23 vs. L23	F2-3 up	GO:0009507	Metabolism	Photosynthesis	chloroplast	C	AT1G17380

12593	3	F23 vs. L23	F2-3 up	GO:0009507	Metabolism	Photosynthesis	chloroplast	C	AT1G17420
12594	3	F23 vs. L23	F2-3 up	GO:0009507	Metabolism	Photosynthesis	chloroplast	C	AT1G19150
12595	3	F23 vs. L23	F2-3 up	GO:0009507	Metabolism	Photosynthesis	chloroplast	C	AT1G19550
12596	3	F23 vs. L23	F2-3 up	GO:0009507	Metabolism	Photosynthesis	chloroplast	C	AT1G20020
12597	3	F23 vs. L23	F2-3 up	GO:0009507	Metabolism	Photosynthesis	chloroplast	C	AT1G20620
12598	3	F23 vs. L23	F2-3 up	GO:0009507	Metabolism	Photosynthesis	chloroplast	C	AT1G20823
12599	3	F23 vs. L23	F2-3 up	GO:0009507	Metabolism	Photosynthesis	chloroplast	C	AT1G20850
12600	3	F23 vs. L23	F2-3 up	GO:0009507	Metabolism	Photosynthesis	chloroplast	C	AT1G21060
12601	3	F23 vs. L23	F2-3 up	GO:0009507	Metabolism	Photosynthesis	chloroplast	C	AT1G22410
12602	3	F23 vs. L23	F2-3 up	GO:0009507	Metabolism	Photosynthesis	chloroplast	C	AT1G23310
12603	3	F23 vs. L23	F2-3 up	GO:0009507	Metabolism	Photosynthesis	chloroplast	C	AT1G23740
12604	3	F23 vs. L23	F2-3 up	GO:0009507	Metabolism	Photosynthesis	chloroplast	C	AT1G23800
12605	3	F23 vs. L23	F2-3 up	GO:0009507	Metabolism	Photosynthesis	chloroplast	C	AT1G27980
12606	3	F23 vs. L23	F2-3 up	GO:0009507	Metabolism	Photosynthesis	chloroplast	C	AT1G29340
12607	3	F23 vs. L23	F2-3 up	GO:0009507	Metabolism	Photosynthesis	chloroplast	C	AT1G31260
12608	3	F23 vs. L23	F2-3 up	GO:0009507	Metabolism	Photosynthesis	chloroplast	C	AT1G31850
12609	3	F23 vs. L23	F2-3 up	GO:0009507	Metabolism	Photosynthesis	chloroplast	C	AT1G32060
12610	3	F23 vs. L23	F2-3 up	GO:0009507	Metabolism	Photosynthesis	chloroplast	C	AT1G32080
12611	3	F23 vs. L23	F2-3 up	GO:0009507	Metabolism	Photosynthesis	chloroplast	C	AT1G33290
12612	3	F23 vs. L23	F2-3 up	GO:0009507	Metabolism	Photosynthesis	chloroplast	C	AT1G34040
12613	3	F23 vs. L23	F2-3 up	GO:0009507	Metabolism	Photosynthesis	chloroplast	C	AT1G35720
12614	3	F23 vs. L23	F2-3 up	GO:0009507	Metabolism	Photosynthesis	chloroplast	C	AT1G42970
12615	3	F23 vs. L23	F2-3 up	GO:0009507	Metabolism	Photosynthesis	chloroplast	C	AT1G44575
12616	3	F23 vs. L23	F2-3 up	GO:0009507	Metabolism	Photosynthesis	chloroplast	C	AT1G44820
12617	3	F23 vs. L23	F2-3 up	GO:0009507	Metabolism	Photosynthesis	chloroplast	C	AT1G45474
12618	3	F23 vs. L23	F2-3 up	GO:0009507	Metabolism	Photosynthesis	chloroplast	C	AT1G50250
12619	3	F23 vs. L23	F2-3 up	GO:0009507	Metabolism	Photosynthesis	chloroplast	C	AT1G50310
12620	3	F23 vs. L23	F2-3 up	GO:0009507	Metabolism	Photosynthesis	chloroplast	C	AT1G50320
12621	3	F23 vs. L23	F2-3 up	GO:0009507	Metabolism	Photosynthesis	chloroplast	C	AT1G51110
12622	3	F23 vs. L23	F2-3 up	GO:0009507	Metabolism	Photosynthesis	chloroplast	C	AT1G52580
12623	3	F23 vs. L23	F2-3 up	GO:0009507	Metabolism	Photosynthesis	chloroplast	C	AT1G53280
12624	3	F23 vs. L23	F2-3 up	GO:0009507	Metabolism	Photosynthesis	chloroplast	C	AT1G53500
12625	3	F23 vs. L23	F2-3 up	GO:0009507	Metabolism	Photosynthesis	chloroplast	C	AT1G54570
12626	3	F23 vs. L23	F2-3 up	GO:0009507	Metabolism	Photosynthesis	chloroplast	C	AT1G55480
12627	3	F23 vs. L23	F2-3 up	GO:0009507	Metabolism	Photosynthesis	chloroplast	C	AT1G56500
12628	3	F23 vs. L23	F2-3 up	GO:0009507	Metabolism	Photosynthesis	chloroplast	C	AT1G57770
12629	3	F23 vs. L23	F2-3 up	GO:0009507	Metabolism	Photosynthesis	chloroplast	C	AT1G58180
12630	3	F23 vs. L23	F2-3 up	GO:0009507	Metabolism	Photosynthesis	chloroplast	C	AT1G58290
12631	3	F23 vs. L23	F2-3 up	GO:0009507	Metabolism	Photosynthesis	chloroplast	C	AT1G58430
12632	3	F23 vs. L23	F2-3 up	GO:0009507	Metabolism	Photosynthesis	chloroplast	C	AT1G59870
12633	3	F23 vs. L23	F2-3 up	GO:0009507	Metabolism	Photosynthesis	chloroplast	C	AT1G61740
12634	3	F23 vs. L23	F2-3 up	GO:0009507	Metabolism	Photosynthesis	chloroplast	C	AT1G63000
12635	3	F23 vs. L23	F2-3 up	GO:0009507	Metabolism	Photosynthesis	chloroplast	C	AT1G64190
12636	3	F23 vs. L23	F2-3 up	GO:0009507	Metabolism	Photosynthesis	chloroplast	C	AT1G64510
12637	3	F23 vs. L23	F2-3 up	GO:0009507	Metabolism	Photosynthesis	chloroplast	C	AT1G64860
12638	3	F23 vs. L23	F2-3 up	GO:0009507	Metabolism	Photosynthesis	chloroplast	C	AT1G64980
12639	3	F23 vs. L23	F2-3 up	GO:0009507	Metabolism	Photosynthesis	chloroplast	C	AT1G68010
12640	3	F23 vs. L23	F2-3 up	GO:0009507	Metabolism	Photosynthesis	chloroplast	C	AT1G68590
12641	3	F23 vs. L23	F2-3 up	GO:0009507	Metabolism	Photosynthesis	chloroplast	C	AT1G70480
12642	3	F23 vs. L23	F2-3 up	GO:0009507	Metabolism	Photosynthesis	chloroplast	C	AT1G70580
12643	3	F23 vs. L23	F2-3 up	GO:0009507	Metabolism	Photosynthesis	chloroplast	C	AT1G70610
12644	3	F23 vs. L23	F2-3 up	GO:0009507	Metabolism	Photosynthesis	chloroplast	C	AT1G70730
12645	3	F23 vs. L23	F2-3 up	GO:0009507	Metabolism	Photosynthesis	chloroplast	C	AT1G71500
12646	3	F23 vs. L23	F2-3 up	GO:0009507	Metabolism	Photosynthesis	chloroplast	C	AT1G72520
12647	3	F23 vs. L23	F2-3 up	GO:0009507	Metabolism	Photosynthesis	chloroplast	C	AT1G75690
12648	3	F23 vs. L23	F2-3 up	GO:0009507	Metabolism	Photosynthesis	chloroplast	C	AT1G76080

12649	3	F23 vs. L23	F2-3 up	GO:0009507	Metabolism	Photosynthesis	chloroplast	C	AT1G77090
12650	3	F23 vs. L23	F2-3 up	GO:0009507	Metabolism	Photosynthesis	chloroplast	C	AT1G78160
12651	3	F23 vs. L23	F2-3 up	GO:0009507	Metabolism	Photosynthesis	chloroplast	C	AT1G78370
12652	3	F23 vs. L23	F2-3 up	GO:0009507	Metabolism	Photosynthesis	chloroplast	C	AT1G78570
12653	3	F23 vs. L23	F2-3 up	GO:0009507	Metabolism	Photosynthesis	chloroplast	C	AT1G79440
12654	3	F23 vs. L23	F2-3 up	GO:0009507	Metabolism	Photosynthesis	chloroplast	C	AT2G01290
12655	3	F23 vs. L23	F2-3 up	GO:0009507	Metabolism	Photosynthesis	chloroplast	C	AT2G01320
12656	3	F23 vs. L23	F2-3 up	GO:0009507	Metabolism	Photosynthesis	chloroplast	C	AT2G04039
12657	3	F23 vs. L23	F2-3 up	GO:0009507	Metabolism	Photosynthesis	chloroplast	C	AT2G05620
12658	3	F23 vs. L23	F2-3 up	GO:0009507	Metabolism	Photosynthesis	chloroplast	C	AT2G07180
12659	3	F23 vs. L23	F2-3 up	GO:0009507	Metabolism	Photosynthesis	chloroplast	C	AT2G10940
12660	3	F23 vs. L23	F2-3 up	GO:0009507	Metabolism	Photosynthesis	chloroplast	C	AT2G13680
12661	3	F23 vs. L23	F2-3 up	GO:0009507	Metabolism	Photosynthesis	chloroplast	C	AT2G13810
12662	3	F23 vs. L23	F2-3 up	GO:0009507	Metabolism	Photosynthesis	chloroplast	C	AT2G14750
12663	3	F23 vs. L23	F2-3 up	GO:0009507	Metabolism	Photosynthesis	chloroplast	C	AT2G15620
12664	3	F23 vs. L23	F2-3 up	GO:0009507	Metabolism	Photosynthesis	chloroplast	C	AT2G17840
12665	3	F23 vs. L23	F2-3 up	GO:0009507	Metabolism	Photosynthesis	chloroplast	C	AT2G18400
12666	3	F23 vs. L23	F2-3 up	GO:0009507	Metabolism	Photosynthesis	chloroplast	C	AT2G18570
12667	3	F23 vs. L23	F2-3 up	GO:0009507	Metabolism	Photosynthesis	chloroplast	C	AT2G21330
12668	3	F23 vs. L23	F2-3 up	GO:0009507	Metabolism	Photosynthesis	chloroplast	C	AT2G21960
12669	3	F23 vs. L23	F2-3 up	GO:0009507	Metabolism	Photosynthesis	chloroplast	C	AT2G22450
12670	3	F23 vs. L23	F2-3 up	GO:0009507	Metabolism	Photosynthesis	chloroplast	C	AT2G24180
12671	3	F23 vs. L23	F2-3 up	GO:0009507	Metabolism	Photosynthesis	chloroplast	C	AT2G24210
12672	3	F23 vs. L23	F2-3 up	GO:0009507	Metabolism	Photosynthesis	chloroplast	C	AT2G24270
12673	3	F23 vs. L23	F2-3 up	GO:0009507	Metabolism	Photosynthesis	chloroplast	C	AT2G24450
12674	3	F23 vs. L23	F2-3 up	GO:0009507	Metabolism	Photosynthesis	chloroplast	C	AT2G24820
12675	3	F23 vs. L23	F2-3 up	GO:0009507	Metabolism	Photosynthesis	chloroplast	C	AT2G25530
12676	3	F23 vs. L23	F2-3 up	GO:0009507	Metabolism	Photosynthesis	chloroplast	C	AT2G26500
12677	3	F23 vs. L23	F2-3 up	GO:0009507	Metabolism	Photosynthesis	chloroplast	C	AT2G26690
12678	3	F23 vs. L23	F2-3 up	GO:0009507	Metabolism	Photosynthesis	chloroplast	C	AT2G28190
12679	3	F23 vs. L23	F2-3 up	GO:0009507	Metabolism	Photosynthesis	chloroplast	C	AT2G28305
12680	3	F23 vs. L23	F2-3 up	GO:0009507	Metabolism	Photosynthesis	chloroplast	C	AT2G29630
12681	3	F23 vs. L23	F2-3 up	GO:0009507	Metabolism	Photosynthesis	chloroplast	C	AT2G29980
12682	3	F23 vs. L23	F2-3 up	GO:0009507	Metabolism	Photosynthesis	chloroplast	C	AT2G30020
12683	3	F23 vs. L23	F2-3 up	GO:0009507	Metabolism	Photosynthesis	chloroplast	C	AT2G30860
12684	3	F23 vs. L23	F2-3 up	GO:0009507	Metabolism	Photosynthesis	chloroplast	C	AT2G31750
12685	3	F23 vs. L23	F2-3 up	GO:0009507	Metabolism	Photosynthesis	chloroplast	C	AT2G31790
12686	3	F23 vs. L23	F2-3 up	GO:0009507	Metabolism	Photosynthesis	chloroplast	C	AT2G34460
12687	3	F23 vs. L23	F2-3 up	GO:0009507	Metabolism	Photosynthesis	chloroplast	C	AT2G36790
12688	3	F23 vs. L23	F2-3 up	GO:0009507	Metabolism	Photosynthesis	chloroplast	C	AT2G37240
12689	3	F23 vs. L23	F2-3 up	GO:0009507	Metabolism	Photosynthesis	chloroplast	C	AT2G37770
12690	3	F23 vs. L23	F2-3 up	GO:0009507	Metabolism	Photosynthesis	chloroplast	C	AT2G38170
12691	3	F23 vs. L23	F2-3 up	GO:0009507	Metabolism	Photosynthesis	chloroplast	C	AT2G38280
12692	3	F23 vs. L23	F2-3 up	GO:0009507	Metabolism	Photosynthesis	chloroplast	C	AT2G38400
12693	3	F23 vs. L23	F2-3 up	GO:0009507	Metabolism	Photosynthesis	chloroplast	C	AT2G39210
12694	3	F23 vs. L23	F2-3 up	GO:0009507	Metabolism	Photosynthesis	chloroplast	C	AT2G39470
12695	3	F23 vs. L23	F2-3 up	GO:0009507	Metabolism	Photosynthesis	chloroplast	C	AT2G39510
12696	3	F23 vs. L23	F2-3 up	GO:0009507	Metabolism	Photosynthesis	chloroplast	C	AT2G39730
12697	3	F23 vs. L23	F2-3 up	GO:0009507	Metabolism	Photosynthesis	chloroplast	C	AT2G41380
12698	3	F23 vs. L23	F2-3 up	GO:0009507	Metabolism	Photosynthesis	chloroplast	C	AT2G42750
12699	3	F23 vs. L23	F2-3 up	GO:0009507	Metabolism	Photosynthesis	chloroplast	C	AT2G43100
12700	3	F23 vs. L23	F2-3 up	GO:0009507	Metabolism	Photosynthesis	chloroplast	C	AT2G44120
12701	3	F23 vs. L23	F2-3 up	GO:0009507	Metabolism	Photosynthesis	chloroplast	C	AT2G45770
12702	3	F23 vs. L23	F2-3 up	GO:0009507	Metabolism	Photosynthesis	chloroplast	C	AT2G46820
12703	3	F23 vs. L23	F2-3 up	GO:0009507	Metabolism	Photosynthesis	chloroplast	C	AT2G47050
12704	3	F23 vs. L23	F2-3 up	GO:0009507	Metabolism	Photosynthesis	chloroplast	C	AT3G01060

12705	3	F23 vs. L23	F2-3 up	GO:0009507	Metabolism	Photosynthesis	chloroplast	C	AT3G01180
12706	3	F23 vs. L23	F2-3 up	GO:0009507	Metabolism	Photosynthesis	chloroplast	C	AT3G01500
12707	3	F23 vs. L23	F2-3 up	GO:0009507	Metabolism	Photosynthesis	chloroplast	C	AT3G02750
12708	3	F23 vs. L23	F2-3 up	GO:0009507	Metabolism	Photosynthesis	chloroplast	C	AT3G02780
12709	3	F23 vs. L23	F2-3 up	GO:0009507	Metabolism	Photosynthesis	chloroplast	C	AT3G03420
12710	3	F23 vs. L23	F2-3 up	GO:0009507	Metabolism	Photosynthesis	chloroplast	C	AT3G03640
12711	3	F23 vs. L23	F2-3 up	GO:0009507	Metabolism	Photosynthesis	chloroplast	C	AT3G04120
12712	3	F23 vs. L23	F2-3 up	GO:0009507	Metabolism	Photosynthesis	chloroplast	C	AT3G04790
12713	3	F23 vs. L23	F2-3 up	GO:0009507	Metabolism	Photosynthesis	chloroplast	C	AT3G06510
12714	3	F23 vs. L23	F2-3 up	GO:0009507	Metabolism	Photosynthesis	chloroplast	C	AT3G07700
12715	3	F23 vs. L23	F2-3 up	GO:0009507	Metabolism	Photosynthesis	chloroplast	C	AT3G09440
12716	3	F23 vs. L23	F2-3 up	GO:0009507	Metabolism	Photosynthesis	chloroplast	C	AT3G09810
12717	3	F23 vs. L23	F2-3 up	GO:0009507	Metabolism	Photosynthesis	chloroplast	C	AT3G10420
12718	3	F23 vs. L23	F2-3 up	GO:0009507	Metabolism	Photosynthesis	chloroplast	C	AT3G10570
12719	3	F23 vs. L23	F2-3 up	GO:0009507	Metabolism	Photosynthesis	chloroplast	C	AT3G11050
12720	3	F23 vs. L23	F2-3 up	GO:0009507	Metabolism	Photosynthesis	chloroplast	C	AT3G11170
12721	3	F23 vs. L23	F2-3 up	GO:0009507	Metabolism	Photosynthesis	chloroplast	C	AT3G11630
12722	3	F23 vs. L23	F2-3 up	GO:0009507	Metabolism	Photosynthesis	chloroplast	C	AT3G12610
12723	3	F23 vs. L23	F2-3 up	GO:0009507	Metabolism	Photosynthesis	chloroplast	C	AT3G12780
12724	3	F23 vs. L23	F2-3 up	GO:0009507	Metabolism	Photosynthesis	chloroplast	C	AT3G13110
12725	3	F23 vs. L23	F2-3 up	GO:0009507	Metabolism	Photosynthesis	chloroplast	C	AT3G14420
12726	3	F23 vs. L23	F2-3 up	GO:0009507	Metabolism	Photosynthesis	chloroplast	C	AT3G14940
12727	3	F23 vs. L23	F2-3 up	GO:0009507	Metabolism	Photosynthesis	chloroplast	C	AT3G15360
12728	3	F23 vs. L23	F2-3 up	GO:0009507	Metabolism	Photosynthesis	chloroplast	C	AT3G15400
12729	3	F23 vs. L23	F2-3 up	GO:0009507	Metabolism	Photosynthesis	chloroplast	C	AT3G16170
12730	3	F23 vs. L23	F2-3 up	GO:0009507	Metabolism	Photosynthesis	chloroplast	C	AT3G16370
12731	3	F23 vs. L23	F2-3 up	GO:0009507	Metabolism	Photosynthesis	chloroplast	C	AT3G17630
12732	3	F23 vs. L23	F2-3 up	GO:0009507	Metabolism	Photosynthesis	chloroplast	C	AT3G17800
12733	3	F23 vs. L23	F2-3 up	GO:0009507	Metabolism	Photosynthesis	chloroplast	C	AT3G17930
12734	3	F23 vs. L23	F2-3 up	GO:0009507	Metabolism	Photosynthesis	chloroplast	C	AT3G18080
12735	3	F23 vs. L23	F2-3 up	GO:0009507	Metabolism	Photosynthesis	chloroplast	C	AT3G19800
12736	3	F23 vs. L23	F2-3 up	GO:0009507	Metabolism	Photosynthesis	chloroplast	C	AT3G21230
12737	3	F23 vs. L23	F2-3 up	GO:0009507	Metabolism	Photosynthesis	chloroplast	C	AT3G22840
12738	3	F23 vs. L23	F2-3 up	GO:0009507	Metabolism	Photosynthesis	chloroplast	C	AT3G22890
12739	3	F23 vs. L23	F2-3 up	GO:0009507	Metabolism	Photosynthesis	chloroplast	C	AT3G23700
12740	3	F23 vs. L23	F2-3 up	GO:0009507	Metabolism	Photosynthesis	chloroplast	C	AT3G23920
12741	3	F23 vs. L23	F2-3 up	GO:0009507	Metabolism	Photosynthesis	chloroplast	C	AT3G24190
12742	3	F23 vs. L23	F2-3 up	GO:0009507	Metabolism	Photosynthesis	chloroplast	C	AT3G24506
12743	3	F23 vs. L23	F2-3 up	GO:0009507	Metabolism	Photosynthesis	chloroplast	C	AT3G25290
12744	3	F23 vs. L23	F2-3 up	GO:0009507	Metabolism	Photosynthesis	chloroplast	C	AT3G25690
12745	3	F23 vs. L23	F2-3 up	GO:0009507	Metabolism	Photosynthesis	chloroplast	C	AT3G25760
12746	3	F23 vs. L23	F2-3 up	GO:0009507	Metabolism	Photosynthesis	chloroplast	C	AT3G26060
12747	3	F23 vs. L23	F2-3 up	GO:0009507	Metabolism	Photosynthesis	chloroplast	C	AT3G26520
12748	3	F23 vs. L23	F2-3 up	GO:0009507	Metabolism	Photosynthesis	chloroplast	C	AT3G26650
12749	3	F23 vs. L23	F2-3 up	GO:0009507	Metabolism	Photosynthesis	chloroplast	C	AT3G27300
12750	3	F23 vs. L23	F2-3 up	GO:0009507	Metabolism	Photosynthesis	chloroplast	C	AT3G45140
12751	3	F23 vs. L23	F2-3 up	GO:0009507	Metabolism	Photosynthesis	chloroplast	C	AT3G48420
12752	3	F23 vs. L23	F2-3 up	GO:0009507	Metabolism	Photosynthesis	chloroplast	C	AT3G48460
12753	3	F23 vs. L23	F2-3 up	GO:0009507	Metabolism	Photosynthesis	chloroplast	C	AT3G48740
12754	3	F23 vs. L23	F2-3 up	GO:0009507	Metabolism	Photosynthesis	chloroplast	C	AT3G48990
12755	3	F23 vs. L23	F2-3 up	GO:0009507	Metabolism	Photosynthesis	chloroplast	C	AT3G51820
12756	3	F23 vs. L23	F2-3 up	GO:0009507	Metabolism	Photosynthesis	chloroplast	C	AT3G52880
12757	3	F23 vs. L23	F2-3 up	GO:0009507	Metabolism	Photosynthesis	chloroplast	C	AT3G54510
12758	3	F23 vs. L23	F2-3 up	GO:0009507	Metabolism	Photosynthesis	chloroplast	C	AT3G54660
12759	3	F23 vs. L23	F2-3 up	GO:0009507	Metabolism	Photosynthesis	chloroplast	C	AT3G54690
12760	3	F23 vs. L23	F2-3 up	GO:0009507	Metabolism	Photosynthesis	chloroplast	C	AT3G54960

12761	3	F23 vs. L23	F2-3 up	GO:0009507	Metabolism	Photosynthesis	chloroplast	C	AT3G55610
12762	3	F23 vs. L23	F2-3 up	GO:0009507	Metabolism	Photosynthesis	chloroplast	C	AT3G55640
12763	3	F23 vs. L23	F2-3 up	GO:0009507	Metabolism	Photosynthesis	chloroplast	C	AT3G55800
12764	3	F23 vs. L23	F2-3 up	GO:0009507	Metabolism	Photosynthesis	chloroplast	C	AT3G56240
12765	3	F23 vs. L23	F2-3 up	GO:0009507	Metabolism	Photosynthesis	chloroplast	C	AT3G57010
12766	3	F23 vs. L23	F2-3 up	GO:0009507	Metabolism	Photosynthesis	chloroplast	C	AT3G57020
12767	3	F23 vs. L23	F2-3 up	GO:0009507	Metabolism	Photosynthesis	chloroplast	C	AT3G57050
12768	3	F23 vs. L23	F2-3 up	GO:0009507	Metabolism	Photosynthesis	chloroplast	C	AT3G57330
12769	3	F23 vs. L23	F2-3 up	GO:0009507	Metabolism	Photosynthesis	chloroplast	C	AT3G58990
12770	3	F23 vs. L23	F2-3 up	GO:0009507	Metabolism	Photosynthesis	chloroplast	C	AT3G59360
12771	3	F23 vs. L23	F2-3 up	GO:0009507	Metabolism	Photosynthesis	chloroplast	C	AT3G62410
12772	3	F23 vs. L23	F2-3 up	GO:0009507	Metabolism	Photosynthesis	chloroplast	C	AT3G63140
12773	3	F23 vs. L23	F2-3 up	GO:0009507	Metabolism	Photosynthesis	chloroplast	C	AT4G01070
12774	3	F23 vs. L23	F2-3 up	GO:0009507	Metabolism	Photosynthesis	chloroplast	C	AT4G01470
12775	3	F23 vs. L23	F2-3 up	GO:0009507	Metabolism	Photosynthesis	chloroplast	C	AT4G02380
12776	3	F23 vs. L23	F2-3 up	GO:0009507	Metabolism	Photosynthesis	chloroplast	C	AT4G03200
12777	3	F23 vs. L23	F2-3 up	GO:0009507	Metabolism	Photosynthesis	chloroplast	C	AT4G03280
12778	3	F23 vs. L23	F2-3 up	GO:0009507	Metabolism	Photosynthesis	chloroplast	C	AT4G04770
12779	3	F23 vs. L23	F2-3 up	GO:0009507	Metabolism	Photosynthesis	chloroplast	C	AT4G05590
12780	3	F23 vs. L23	F2-3 up	GO:0009507	Metabolism	Photosynthesis	chloroplast	C	AT4G11030
12781	3	F23 vs. L23	F2-3 up	GO:0009507	Metabolism	Photosynthesis	chloroplast	C	AT4G11600
12782	3	F23 vs. L23	F2-3 up	GO:0009507	Metabolism	Photosynthesis	chloroplast	C	AT4G11960
12783	3	F23 vs. L23	F2-3 up	GO:0009507	Metabolism	Photosynthesis	chloroplast	C	AT4G12030
12784	3	F23 vs. L23	F2-3 up	GO:0009507	Metabolism	Photosynthesis	chloroplast	C	AT4G14690
12785	3	F23 vs. L23	F2-3 up	GO:0009507	Metabolism	Photosynthesis	chloroplast	C	AT4G14880
12786	3	F23 vs. L23	F2-3 up	GO:0009507	Metabolism	Photosynthesis	chloroplast	C	AT4G15530
12787	3	F23 vs. L23	F2-3 up	GO:0009507	Metabolism	Photosynthesis	chloroplast	C	AT4G19230
12788	3	F23 vs. L23	F2-3 up	GO:0009507	Metabolism	Photosynthesis	chloroplast	C	AT4G20360
12789	3	F23 vs. L23	F2-3 up	GO:0009507	Metabolism	Photosynthesis	chloroplast	C	AT4G20760
12790	3	F23 vs. L23	F2-3 up	GO:0009507	Metabolism	Photosynthesis	chloroplast	C	AT4G21990
12791	3	F23 vs. L23	F2-3 up	GO:0009507	Metabolism	Photosynthesis	chloroplast	C	AT4G22750
12792	3	F23 vs. L23	F2-3 up	GO:0009507	Metabolism	Photosynthesis	chloroplast	C	AT4G23100
12793	3	F23 vs. L23	F2-3 up	GO:0009507	Metabolism	Photosynthesis	chloroplast	C	AT4G23890
12794	3	F23 vs. L23	F2-3 up	GO:0009507	Metabolism	Photosynthesis	chloroplast	C	AT4G24380
12795	3	F23 vs. L23	F2-3 up	GO:0009507	Metabolism	Photosynthesis	chloroplast	C	AT4G24450
12796	3	F23 vs. L23	F2-3 up	GO:0009507	Metabolism	Photosynthesis	chloroplast	C	AT4G24640
12797	3	F23 vs. L23	F2-3 up	GO:0009507	Metabolism	Photosynthesis	chloroplast	C	AT4G24890
12798	3	F23 vs. L23	F2-3 up	GO:0009507	Metabolism	Photosynthesis	chloroplast	C	AT4G24930
12799	3	F23 vs. L23	F2-3 up	GO:0009507	Metabolism	Photosynthesis	chloroplast	C	AT4G25030
12800	3	F23 vs. L23	F2-3 up	GO:0009507	Metabolism	Photosynthesis	chloroplast	C	AT4G25640
12801	3	F23 vs. L23	F2-3 up	GO:0009507	Metabolism	Photosynthesis	chloroplast	C	AT4G26970
12802	3	F23 vs. L23	F2-3 up	GO:0009507	Metabolism	Photosynthesis	chloroplast	C	AT4G27030
12803	3	F23 vs. L23	F2-3 up	GO:0009507	Metabolism	Photosynthesis	chloroplast	C	AT4G28780
12804	3	F23 vs. L23	F2-3 up	GO:0009507	Metabolism	Photosynthesis	chloroplast	C	AT4G29350
12805	3	F23 vs. L23	F2-3 up	GO:0009507	Metabolism	Photosynthesis	chloroplast	C	AT4G32260
12806	3	F23 vs. L23	F2-3 up	GO:0009507	Metabolism	Photosynthesis	chloroplast	C	AT4G33010
12807	3	F23 vs. L23	F2-3 up	GO:0009507	Metabolism	Photosynthesis	chloroplast	C	AT4G34710
12808	3	F23 vs. L23	F2-3 up	GO:0009507	Metabolism	Photosynthesis	chloroplast	C	AT4G34720
12809	3	F23 vs. L23	F2-3 up	GO:0009507	Metabolism	Photosynthesis	chloroplast	C	AT4G34950
12810	3	F23 vs. L23	F2-3 up	GO:0009507	Metabolism	Photosynthesis	chloroplast	C	AT4G35250
12811	3	F23 vs. L23	F2-3 up	GO:0009507	Metabolism	Photosynthesis	chloroplast	C	AT4G36490
12812	3	F23 vs. L23	F2-3 up	GO:0009507	Metabolism	Photosynthesis	chloroplast	C	AT4G37550
12813	3	F23 vs. L23	F2-3 up	GO:0009507	Metabolism	Photosynthesis	chloroplast	C	AT4G38100
12814	3	F23 vs. L23	F2-3 up	GO:0009507	Metabolism	Photosynthesis	chloroplast	C	AT4G38740
12815	3	F23 vs. L23	F2-3 up	GO:0009507	Metabolism	Photosynthesis	chloroplast	C	AT4G38970
12816	3	F23 vs. L23	F2-3 up	GO:0009507	Metabolism	Photosynthesis	chloroplast	C	AT4G39030

12817	3	F23 vs. L23	F2-3 up	GO:0009507	Metabolism	Photosynthesis	chloroplast	C	AT4G39110
12818	3	F23 vs. L23	F2-3 up	GO:0009507	Metabolism	Photosynthesis	chloroplast	C	AT4G39940
12819	3	F23 vs. L23	F2-3 up	GO:0009507	Metabolism	Photosynthesis	chloroplast	C	AT5G01600
12820	3	F23 vs. L23	F2-3 up	GO:0009507	Metabolism	Photosynthesis	chloroplast	C	AT5G04140
12821	3	F23 vs. L23	F2-3 up	GO:0009507	Metabolism	Photosynthesis	chloroplast	C	AT5G04710
12822	3	F23 vs. L23	F2-3 up	GO:0009507	Metabolism	Photosynthesis	chloroplast	C	AT5G04980
12823	3	F23 vs. L23	F2-3 up	GO:0009507	Metabolism	Photosynthesis	chloroplast	C	AT5G05200
12824	3	F23 vs. L23	F2-3 up	GO:0009507	Metabolism	Photosynthesis	chloroplast	C	AT5G05730
12825	3	F23 vs. L23	F2-3 up	GO:0009507	Metabolism	Photosynthesis	chloroplast	C	AT5G07550
12826	3	F23 vs. L23	F2-3 up	GO:0009507	Metabolism	Photosynthesis	chloroplast	C	AT5G09650
12827	3	F23 vs. L23	F2-3 up	GO:0009507	Metabolism	Photosynthesis	chloroplast	C	AT5G09660
12828	3	F23 vs. L23	F2-3 up	GO:0009507	Metabolism	Photosynthesis	chloroplast	C	AT5G11520
12829	3	F23 vs. L23	F2-3 up	GO:0009507	Metabolism	Photosynthesis	chloroplast	C	AT5G11790
12830	3	F23 vs. L23	F2-3 up	GO:0009507	Metabolism	Photosynthesis	chloroplast	C	AT5G12170
12831	3	F23 vs. L23	F2-3 up	GO:0009507	Metabolism	Photosynthesis	chloroplast	C	AT5G14060
12832	3	F23 vs. L23	F2-3 up	GO:0009507	Metabolism	Photosynthesis	chloroplast	C	AT5G14200
12833	3	F23 vs. L23	F2-3 up	GO:0009507	Metabolism	Photosynthesis	chloroplast	C	AT5G14660
12834	3	F23 vs. L23	F2-3 up	GO:0009507	Metabolism	Photosynthesis	chloroplast	C	AT5G14740
12835	3	F23 vs. L23	F2-3 up	GO:0009507	Metabolism	Photosynthesis	chloroplast	C	AT5G15390
12836	3	F23 vs. L23	F2-3 up	GO:0009507	Metabolism	Photosynthesis	chloroplast	C	AT5G15470
12837	3	F23 vs. L23	F2-3 up	GO:0009507	Metabolism	Photosynthesis	chloroplast	C	AT5G16010
12838	3	F23 vs. L23	F2-3 up	GO:0009507	Metabolism	Photosynthesis	chloroplast	C	AT5G17670
12839	3	F23 vs. L23	F2-3 up	GO:0009507	Metabolism	Photosynthesis	chloroplast	C	AT5G19220
12840	3	F23 vs. L23	F2-3 up	GO:0009507	Metabolism	Photosynthesis	chloroplast	C	AT5G22580
12841	3	F23 vs. L23	F2-3 up	GO:0009507	Metabolism	Photosynthesis	chloroplast	C	AT5G22630
12842	3	F23 vs. L23	F2-3 up	GO:0009507	Metabolism	Photosynthesis	chloroplast	C	AT5G23060
12843	3	F23 vs. L23	F2-3 up	GO:0009507	Metabolism	Photosynthesis	chloroplast	C	AT5G23120
12844	3	F23 vs. L23	F2-3 up	GO:0009507	Metabolism	Photosynthesis	chloroplast	C	AT5G24120
12845	3	F23 vs. L23	F2-3 up	GO:0009507	Metabolism	Photosynthesis	chloroplast	C	AT5G24650
12846	3	F23 vs. L23	F2-3 up	GO:0009507	Metabolism	Photosynthesis	chloroplast	C	AT5G24780
12847	3	F23 vs. L23	F2-3 up	GO:0009507	Metabolism	Photosynthesis	chloroplast	C	AT5G25120
12848	3	F23 vs. L23	F2-3 up	GO:0009507	Metabolism	Photosynthesis	chloroplast	C	AT5G25980
12849	3	F23 vs. L23	F2-3 up	GO:0009507	Metabolism	Photosynthesis	chloroplast	C	AT5G30510
12850	3	F23 vs. L23	F2-3 up	GO:0009507	Metabolism	Photosynthesis	chloroplast	C	AT5G35630
12851	3	F23 vs. L23	F2-3 up	GO:0009507	Metabolism	Photosynthesis	chloroplast	C	AT5G36220
12852	3	F23 vs. L23	F2-3 up	GO:0009507	Metabolism	Photosynthesis	chloroplast	C	AT5G36700
12853	3	F23 vs. L23	F2-3 up	GO:0009507	Metabolism	Photosynthesis	chloroplast	C	AT5G36880
12854	3	F23 vs. L23	F2-3 up	GO:0009507	Metabolism	Photosynthesis	chloroplast	C	AT5G38420
12855	3	F23 vs. L23	F2-3 up	GO:0009507	Metabolism	Photosynthesis	chloroplast	C	AT5G38660
12856	3	F23 vs. L23	F2-3 up	GO:0009507	Metabolism	Photosynthesis	chloroplast	C	AT5G38900
12857	3	F23 vs. L23	F2-3 up	GO:0009507	Metabolism	Photosynthesis	chloroplast	C	AT5G39210
12858	3	F23 vs. L23	F2-3 up	GO:0009507	Metabolism	Photosynthesis	chloroplast	C	AT5G42680
12859	3	F23 vs. L23	F2-3 up	GO:0009507	Metabolism	Photosynthesis	chloroplast	C	AT5G43745
12860	3	F23 vs. L23	F2-3 up	GO:0009507	Metabolism	Photosynthesis	chloroplast	C	AT5G45840
12861	3	F23 vs. L23	F2-3 up	GO:0009507	Metabolism	Photosynthesis	chloroplast	C	AT5G45890
12862	3	F23 vs. L23	F2-3 up	GO:0009507	Metabolism	Photosynthesis	chloroplast	C	AT5G46800
12863	3	F23 vs. L23	F2-3 up	GO:0009507	Metabolism	Photosynthesis	chloroplast	C	AT5G47620
12864	3	F23 vs. L23	F2-3 up	GO:0009507	Metabolism	Photosynthesis	chloroplast	C	AT5G47650
12865	3	F23 vs. L23	F2-3 up	GO:0009507	Metabolism	Photosynthesis	chloroplast	C	AT5G47890
12866	3	F23 vs. L23	F2-3 up	GO:0009507	Metabolism	Photosynthesis	chloroplast	C	AT5G48300
12867	3	F23 vs. L23	F2-3 up	GO:0009507	Metabolism	Photosynthesis	chloroplast	C	AT5G50030
12868	3	F23 vs. L23	F2-3 up	GO:0009507	Metabolism	Photosynthesis	chloroplast	C	AT5G50720
12869	3	F23 vs. L23	F2-3 up	GO:0009507	Metabolism	Photosynthesis	chloroplast	C	AT5G51720
12870	3	F23 vs. L23	F2-3 up	GO:0009507	Metabolism	Photosynthesis	chloroplast	C	AT5G52100
12871	3	F23 vs. L23	F2-3 up	GO:0009507	Metabolism	Photosynthesis	chloroplast	C	AT5G53460
12872	3	F23 vs. L23	F2-3 up	GO:0009507	Metabolism	Photosynthesis	chloroplast	C	AT5G53820

12873	3	F23 vs. L23	F2-3 up	GO:0009507	Metabolism	Photosynthesis	chloroplast	C	AT5G53890
12874	3	F23 vs. L23	F2-3 up	GO:0009507	Metabolism	Photosynthesis	chloroplast	C	AT5G57350
12875	3	F23 vs. L23	F2-3 up	GO:0009507	Metabolism	Photosynthesis	chloroplast	C	AT5G57560
12876	3	F23 vs. L23	F2-3 up	GO:0009507	Metabolism	Photosynthesis	chloroplast	C	AT5G58050
12877	3	F23 vs. L23	F2-3 up	GO:0009507	Metabolism	Photosynthesis	chloroplast	C	AT5G58260
12878	3	F23 vs. L23	F2-3 up	GO:0009507	Metabolism	Photosynthesis	chloroplast	C	AT5G58330
12879	3	F23 vs. L23	F2-3 up	GO:0009507	Metabolism	Photosynthesis	chloroplast	C	AT5G58770
12880	3	F23 vs. L23	F2-3 up	GO:0009507	Metabolism	Photosynthesis	chloroplast	C	AT5G60600
12881	3	F23 vs. L23	F2-3 up	GO:0009507	Metabolism	Photosynthesis	chloroplast	C	AT5G61410
12882	3	F23 vs. L23	F2-3 up	GO:0009507	Metabolism	Photosynthesis	chloroplast	C	AT5G61670
12883	3	F23 vs. L23	F2-3 up	GO:0009507	Metabolism	Photosynthesis	chloroplast	C	AT5G61700
12884	3	F23 vs. L23	F2-3 up	GO:0009507	Metabolism	Photosynthesis	chloroplast	C	AT5G64040
12885	3	F23 vs. L23	F2-3 up	GO:0009507	Metabolism	Photosynthesis	chloroplast	C	AT5G64840
12886	3	F23 vs. L23	F2-3 up	GO:0009507	Metabolism	Photosynthesis	chloroplast	C	AT5G65460
12887	3	F23 vs. L23	F2-3 up	GO:0009507	Metabolism	Photosynthesis	chloroplast	C	AT5G65760
12888	3	F23 vs. L23	F2-3 up	GO:0009507	Metabolism	Photosynthesis	chloroplast	C	AT5G67370
12889	3	F23 vs. L23	F2-3 up	GO:0009507	Metabolism	Photosynthesis	chloroplast	C	ATCG00120
12890	3	F23 vs. L23	F2-3 up	GO:0009507	Metabolism	Photosynthesis	chloroplast	C	ATCG00150
12891	3	F23 vs. L23	F2-3 up	GO:0009507	Metabolism	Photosynthesis	chloroplast	C	ATCG00220
12892	3	F23 vs. L23	F2-3 up	GO:0009507	Metabolism	Photosynthesis	chloroplast	C	ATCG00420
12893	3	F23 vs. L23	F2-3 up	GO:0009507	Metabolism	Photosynthesis	chloroplast	C	ATCG00430
12894	3	F23 vs. L23	F2-3 up	GO:0009507	Metabolism	Photosynthesis	chloroplast	C	ATCG00560
12895	3	F23 vs. L23	F2-3 up	GO:0009507	Metabolism	Photosynthesis	chloroplast	C	ATCG00820
12896	3	F23 vs. L23	F2-3 up	GO:0009507	Metabolism	Photosynthesis	chloroplast	C	ATCG01090
12897	3	F23 vs. L23	F2-3 up	GO:0009507	Metabolism	Photosynthesis	chloroplast	C	ATCG01100
12898	3	F23 vs. L23	F2-3 up	GO:0009507	Metabolism	Photosynthesis	chloroplast	C	ATMG00480
12899	3	F23 vs. L23	F2-3 up	GO:0009904	Metabolism	Photosynthesis	chloroplast accumulation movement	P	AT3G45780
12900	3	F23 vs. L23	F2-3 up	GO:0009904	Metabolism	Photosynthesis	chloroplast accumulation movement	P	AT5G65460
12901	3	F23 vs. L23	F2-3 up	GO:0009544	Metabolism	Photosynthesis	chloroplast ATP synthase complex	C	ATCG00120
12902	3	F23 vs. L23	F2-3 up	GO:0009544	Metabolism	Photosynthesis	chloroplast ATP synthase complex	C	ATCG00150
12903	3	F23 vs. L23	F2-3 up	GO:0009903	Metabolism	Photosynthesis	chloroplast avoidance movement	P	AT3G45780
12904	3	F23 vs. L23	F2-3 up	GO:0009903	Metabolism	Photosynthesis	chloroplast avoidance movement	P	AT5G65460
12905	3	F23 vs. L23	F2-3 up	GO:0010151	Metabolism	Photosynthesis	chloroplast elongation	P	AT5G56860
12906	3	F23 vs. L23	F2-3 up	GO:0009941	Metabolism	Photosynthesis	chloroplast envelope	C	AT1G01090
12907	3	F23 vs. L23	F2-3 up	GO:0009941	Metabolism	Photosynthesis	chloroplast envelope	C	AT1G06690
12908	3	F23 vs. L23	F2-3 up	GO:0009941	Metabolism	Photosynthesis	chloroplast envelope	C	AT1G09340
12909	3	F23 vs. L23	F2-3 up	GO:0009941	Metabolism	Photosynthesis	chloroplast envelope	C	AT1G11840
12910	3	F23 vs. L23	F2-3 up	GO:0009941	Metabolism	Photosynthesis	chloroplast envelope	C	AT1G11860
12911	3	F23 vs. L23	F2-3 up	GO:0009941	Metabolism	Photosynthesis	chloroplast envelope	C	AT1G16720
12912	3	F23 vs. L23	F2-3 up	GO:0009941	Metabolism	Photosynthesis	chloroplast envelope	C	AT1G16880
12913	3	F23 vs. L23	F2-3 up	GO:0009941	Metabolism	Photosynthesis	chloroplast envelope	C	AT1G20620
12914	3	F23 vs. L23	F2-3 up	GO:0009941	Metabolism	Photosynthesis	chloroplast envelope	C	AT1G23740
12915	3	F23 vs. L23	F2-3 up	GO:0009941	Metabolism	Photosynthesis	chloroplast envelope	C	AT1G32060
12916	3	F23 vs. L23	F2-3 up	GO:0009941	Metabolism	Photosynthesis	chloroplast envelope	C	AT1G32080
12917	3	F23 vs. L23	F2-3 up	GO:0009941	Metabolism	Photosynthesis	chloroplast envelope	C	AT1G42970
12918	3	F23 vs. L23	F2-3 up	GO:0009941	Metabolism	Photosynthesis	chloroplast envelope	C	AT1G50250
12919	3	F23 vs. L23	F2-3 up	GO:0009941	Metabolism	Photosynthesis	chloroplast envelope	C	AT1G51110
12920	3	F23 vs. L23	F2-3 up	GO:0009941	Metabolism	Photosynthesis	chloroplast envelope	C	AT1G55480
12921	3	F23 vs. L23	F2-3 up	GO:0009941	Metabolism	Photosynthesis	chloroplast envelope	C	AT1G59870
12922	3	F23 vs. L23	F2-3 up	GO:0009941	Metabolism	Photosynthesis	chloroplast envelope	C	AT1G70610
12923	3	F23 vs. L23	F2-3 up	GO:0009941	Metabolism	Photosynthesis	chloroplast envelope	C	AT1G71500
12924	3	F23 vs. L23	F2-3 up	GO:0009941	Metabolism	Photosynthesis	chloroplast envelope	C	AT1G76080
12925	3	F23 vs. L23	F2-3 up	GO:0009941	Metabolism	Photosynthesis	chloroplast envelope	C	AT1G76370
12926	3	F23 vs. L23	F2-3 up	GO:0009941	Metabolism	Photosynthesis	chloroplast envelope	C	AT2G01320
12927	3	F23 vs. L23	F2-3 up	GO:0009941	Metabolism	Photosynthesis	chloroplast envelope	C	AT2G21330
12928	3	F23 vs. L23	F2-3 up	GO:0009941	Metabolism	Photosynthesis	chloroplast envelope	C	AT2G24820

12929	3	F23 vs. L23	F2-3 up	GO:0009941	Metabolism	Photosynthesis	chloroplast envelope	C	AT2G34460
12930	3	F23 vs. L23	F2-3 up	GO:0009941	Metabolism	Photosynthesis	chloroplast envelope	C	AT2G36830
12931	3	F23 vs. L23	F2-3 up	GO:0009941	Metabolism	Photosynthesis	chloroplast envelope	C	AT2G39730
12932	3	F23 vs. L23	F2-3 up	GO:0009941	Metabolism	Photosynthesis	chloroplast envelope	C	AT2G46820
12933	3	F23 vs. L23	F2-3 up	GO:0009941	Metabolism	Photosynthesis	chloroplast envelope	C	AT3G01500
12934	3	F23 vs. L23	F2-3 up	GO:0009941	Metabolism	Photosynthesis	chloroplast envelope	C	AT3G04790
12935	3	F23 vs. L23	F2-3 up	GO:0009941	Metabolism	Photosynthesis	chloroplast envelope	C	AT3G06510
12936	3	F23 vs. L23	F2-3 up	GO:0009941	Metabolism	Photosynthesis	chloroplast envelope	C	AT3G11170
12937	3	F23 vs. L23	F2-3 up	GO:0009941	Metabolism	Photosynthesis	chloroplast envelope	C	AT3G11630
12938	3	F23 vs. L23	F2-3 up	GO:0009941	Metabolism	Photosynthesis	chloroplast envelope	C	AT3G12780
12939	3	F23 vs. L23	F2-3 up	GO:0009941	Metabolism	Photosynthesis	chloroplast envelope	C	AT3G13930
12940	3	F23 vs. L23	F2-3 up	GO:0009941	Metabolism	Photosynthesis	chloroplast envelope	C	AT3G15360
12941	3	F23 vs. L23	F2-3 up	GO:0009941	Metabolism	Photosynthesis	chloroplast envelope	C	AT3G25760
12942	3	F23 vs. L23	F2-3 up	GO:0009941	Metabolism	Photosynthesis	chloroplast envelope	C	AT3G26060
12943	3	F23 vs. L23	F2-3 up	GO:0009941	Metabolism	Photosynthesis	chloroplast envelope	C	AT3G26650
12944	3	F23 vs. L23	F2-3 up	GO:0009941	Metabolism	Photosynthesis	chloroplast envelope	C	AT3G45140
12945	3	F23 vs. L23	F2-3 up	GO:0009941	Metabolism	Photosynthesis	chloroplast envelope	C	AT3G46520
12946	3	F23 vs. L23	F2-3 up	GO:0009941	Metabolism	Photosynthesis	chloroplast envelope	C	AT3G48420
12947	3	F23 vs. L23	F2-3 up	GO:0009941	Metabolism	Photosynthesis	chloroplast envelope	C	AT3G55800
12948	3	F23 vs. L23	F2-3 up	GO:0009941	Metabolism	Photosynthesis	chloroplast envelope	C	AT3G63140
12949	3	F23 vs. L23	F2-3 up	GO:0009941	Metabolism	Photosynthesis	chloroplast envelope	C	AT4G03280
12950	3	F23 vs. L23	F2-3 up	GO:0009941	Metabolism	Photosynthesis	chloroplast envelope	C	AT4G15440
12951	3	F23 vs. L23	F2-3 up	GO:0009941	Metabolism	Photosynthesis	chloroplast envelope	C	AT4G20360
12952	3	F23 vs. L23	F2-3 up	GO:0009941	Metabolism	Photosynthesis	chloroplast envelope	C	AT4G24450
12953	3	F23 vs. L23	F2-3 up	GO:0009941	Metabolism	Photosynthesis	chloroplast envelope	C	AT4G32260
12954	3	F23 vs. L23	F2-3 up	GO:0009941	Metabolism	Photosynthesis	chloroplast envelope	C	AT4G33010
12955	3	F23 vs. L23	F2-3 up	GO:0009941	Metabolism	Photosynthesis	chloroplast envelope	C	AT4G38510
12956	3	F23 vs. L23	F2-3 up	GO:0009941	Metabolism	Photosynthesis	chloroplast envelope	C	AT4G38970
12957	3	F23 vs. L23	F2-3 up	GO:0009941	Metabolism	Photosynthesis	chloroplast envelope	C	AT4G39030
12958	3	F23 vs. L23	F2-3 up	GO:0009941	Metabolism	Photosynthesis	chloroplast envelope	C	AT5G04140
12959	3	F23 vs. L23	F2-3 up	GO:0009941	Metabolism	Photosynthesis	chloroplast envelope	C	AT5G09650
12960	3	F23 vs. L23	F2-3 up	GO:0009941	Metabolism	Photosynthesis	chloroplast envelope	C	AT5G09660
12961	3	F23 vs. L23	F2-3 up	GO:0009941	Metabolism	Photosynthesis	chloroplast envelope	C	AT5G09810
12962	3	F23 vs. L23	F2-3 up	GO:0009941	Metabolism	Photosynthesis	chloroplast envelope	C	AT5G13490
12963	3	F23 vs. L23	F2-3 up	GO:0009941	Metabolism	Photosynthesis	chloroplast envelope	C	AT5G14740
12964	3	F23 vs. L23	F2-3 up	GO:0009941	Metabolism	Photosynthesis	chloroplast envelope	C	AT5G16010
12965	3	F23 vs. L23	F2-3 up	GO:0009941	Metabolism	Photosynthesis	chloroplast envelope	C	AT5G17670
12966	3	F23 vs. L23	F2-3 up	GO:0009941	Metabolism	Photosynthesis	chloroplast envelope	C	AT5G19220
12967	3	F23 vs. L23	F2-3 up	GO:0009941	Metabolism	Photosynthesis	chloroplast envelope	C	AT5G23120
12968	3	F23 vs. L23	F2-3 up	GO:0009941	Metabolism	Photosynthesis	chloroplast envelope	C	AT5G24650
12969	3	F23 vs. L23	F2-3 up	GO:0009941	Metabolism	Photosynthesis	chloroplast envelope	C	AT5G35630
12970	3	F23 vs. L23	F2-3 up	GO:0009941	Metabolism	Photosynthesis	chloroplast envelope	C	AT5G38420
12971	3	F23 vs. L23	F2-3 up	GO:0009941	Metabolism	Photosynthesis	chloroplast envelope	C	AT5G38660
12972	3	F23 vs. L23	F2-3 up	GO:0009941	Metabolism	Photosynthesis	chloroplast envelope	C	AT5G43745
12973	3	F23 vs. L23	F2-3 up	GO:0009941	Metabolism	Photosynthesis	chloroplast envelope	C	AT5G58260
12974	3	F23 vs. L23	F2-3 up	GO:0009941	Metabolism	Photosynthesis	chloroplast envelope	C	AT5G58330
12975	3	F23 vs. L23	F2-3 up	GO:0009941	Metabolism	Photosynthesis	chloroplast envelope	C	AT5G60600
12976	3	F23 vs. L23	F2-3 up	GO:0009941	Metabolism	Photosynthesis	chloroplast envelope	C	AT5G61410
12977	3	F23 vs. L23	F2-3 up	GO:0009941	Metabolism	Photosynthesis	chloroplast envelope	C	ATCG00820
12978	3	F23 vs. L23	F2-3 up	GO:0009706	Metabolism	Photosynthesis	chloroplast inner membrane	C	AT1G32080
12979	3	F23 vs. L23	F2-3 up	GO:0009706	Metabolism	Photosynthesis	chloroplast inner membrane	C	AT2G24820
12980	3	F23 vs. L23	F2-3 up	GO:0009706	Metabolism	Photosynthesis	chloroplast inner membrane	C	AT3G11170
12981	3	F23 vs. L23	F2-3 up	GO:0009706	Metabolism	Photosynthesis	chloroplast inner membrane	C	AT5G24650
12982	3	F23 vs. L23	F2-3 up	GO:0009706	Metabolism	Photosynthesis	chloroplast inner membrane	C	AT5G64280
12983	3	F23 vs. L23	F2-3 up	GO:0031969	Metabolism	Photosynthesis	chloroplast membrane	C	AT1G16720
12984	3	F23 vs. L23	F2-3 up	GO:0031969	Metabolism	Photosynthesis	chloroplast membrane	C	AT1G42970

12985	3	F23 vs. L23	F2-3 up	GO:0031969	Metabolism	Photosynthesis	chloroplast membrane	C	AT1G55480
12986	3	F23 vs. L23	F2-3 up	GO:0031969	Metabolism	Photosynthesis	chloroplast membrane	C	AT1G58290
12987	3	F23 vs. L23	F2-3 up	GO:0031969	Metabolism	Photosynthesis	chloroplast membrane	C	AT1G61800
12988	3	F23 vs. L23	F2-3 up	GO:0031969	Metabolism	Photosynthesis	chloroplast membrane	C	AT1G64150
12989	3	F23 vs. L23	F2-3 up	GO:0031969	Metabolism	Photosynthesis	chloroplast membrane	C	AT1G70610
12990	3	F23 vs. L23	F2-3 up	GO:0031969	Metabolism	Photosynthesis	chloroplast membrane	C	AT3G26650
12991	3	F23 vs. L23	F2-3 up	GO:0031969	Metabolism	Photosynthesis	chloroplast membrane	C	AT3G51820
12992	3	F23 vs. L23	F2-3 up	GO:0031969	Metabolism	Photosynthesis	chloroplast membrane	C	AT4G27030
12993	3	F23 vs. L23	F2-3 up	GO:0031969	Metabolism	Photosynthesis	chloroplast membrane	C	AT4G39030
12994	3	F23 vs. L23	F2-3 up	GO:0031969	Metabolism	Photosynthesis	chloroplast membrane	C	AT5G12170
12995	3	F23 vs. L23	F2-3 up	GO:0031969	Metabolism	Photosynthesis	chloroplast membrane	C	AT5G61670
12996	3	F23 vs. L23	F2-3 up	GO:0009658	Metabolism	Photosynthesis	chloroplast organization	P	AT1G09340
12997	3	F23 vs. L23	F2-3 up	GO:0009658	Metabolism	Photosynthesis	chloroplast organization	P	AT1G32080
12998	3	F23 vs. L23	F2-3 up	GO:0009658	Metabolism	Photosynthesis	chloroplast organization	P	AT1G44000
12999	3	F23 vs. L23	F2-3 up	GO:0009658	Metabolism	Photosynthesis	chloroplast organization	P	AT4G20360
13000	3	F23 vs. L23	F2-3 up	GO:0009658	Metabolism	Photosynthesis	chloroplast organization	P	AT5G24120
13001	3	F23 vs. L23	F2-3 up	GO:0009658	Metabolism	Photosynthesis	chloroplast organization	P	AT5G56860
13002	3	F23 vs. L23	F2-3 up	GO:0009707	Metabolism	Photosynthesis	chloroplast outer membrane	C	AT1G70480
13003	3	F23 vs. L23	F2-3 up	GO:0009707	Metabolism	Photosynthesis	chloroplast outer membrane	C	AT3G06510
13004	3	F23 vs. L23	F2-3 up	GO:0009707	Metabolism	Photosynthesis	chloroplast outer membrane	C	AT3G25690
13005	3	F23 vs. L23	F2-3 up	GO:0009707	Metabolism	Photosynthesis	chloroplast outer membrane	C	AT4G04980
13006	3	F23 vs. L23	F2-3 up	GO:0030093	Metabolism	Photosynthesis	chloroplast photosystem I	C	AT2G46820
13007	3	F23 vs. L23	F2-3 up	GO:0030093	Metabolism	Photosynthesis	chloroplast photosystem I	C	AT5G64040
13008	3	F23 vs. L23	F2-3 up	GO:0009902	Metabolism	Photosynthesis	chloroplast relocation	P	AT1G64500
13009	3	F23 vs. L23	F2-3 up	GO:0009902	Metabolism	Photosynthesis	chloroplast relocation	P	AT3G25690
13010	3	F23 vs. L23	F2-3 up	GO:0009570	Metabolism	Photosynthesis	chloroplast stroma	C	AT1G01090
13011	3	F23 vs. L23	F2-3 up	GO:0009570	Metabolism	Photosynthesis	chloroplast stroma	C	AT1G09340
13012	3	F23 vs. L23	F2-3 up	GO:0009570	Metabolism	Photosynthesis	chloroplast stroma	C	AT1G10960
13013	3	F23 vs. L23	F2-3 up	GO:0009570	Metabolism	Photosynthesis	chloroplast stroma	C	AT1G11860
13014	3	F23 vs. L23	F2-3 up	GO:0009570	Metabolism	Photosynthesis	chloroplast stroma	C	AT1G16720
13015	3	F23 vs. L23	F2-3 up	GO:0009570	Metabolism	Photosynthesis	chloroplast stroma	C	AT1G16880
13016	3	F23 vs. L23	F2-3 up	GO:0009570	Metabolism	Photosynthesis	chloroplast stroma	C	AT1G20020
13017	3	F23 vs. L23	F2-3 up	GO:0009570	Metabolism	Photosynthesis	chloroplast stroma	C	AT1G20620
13018	3	F23 vs. L23	F2-3 up	GO:0009570	Metabolism	Photosynthesis	chloroplast stroma	C	AT1G23740
13019	3	F23 vs. L23	F2-3 up	GO:0009570	Metabolism	Photosynthesis	chloroplast stroma	C	AT1G32060
13020	3	F23 vs. L23	F2-3 up	GO:0009570	Metabolism	Photosynthesis	chloroplast stroma	C	AT1G35720
13021	3	F23 vs. L23	F2-3 up	GO:0009570	Metabolism	Photosynthesis	chloroplast stroma	C	AT1G42970
13022	3	F23 vs. L23	F2-3 up	GO:0009570	Metabolism	Photosynthesis	chloroplast stroma	C	AT1G50320
13023	3	F23 vs. L23	F2-3 up	GO:0009570	Metabolism	Photosynthesis	chloroplast stroma	C	AT1G53280
13024	3	F23 vs. L23	F2-3 up	GO:0009570	Metabolism	Photosynthesis	chloroplast stroma	C	AT1G55480
13025	3	F23 vs. L23	F2-3 up	GO:0009570	Metabolism	Photosynthesis	chloroplast stroma	C	AT1G56500
13026	3	F23 vs. L23	F2-3 up	GO:0009570	Metabolism	Photosynthesis	chloroplast stroma	C	AT1G64190
13027	3	F23 vs. L23	F2-3 up	GO:0009570	Metabolism	Photosynthesis	chloroplast stroma	C	AT1G68590
13028	3	F23 vs. L23	F2-3 up	GO:0009570	Metabolism	Photosynthesis	chloroplast stroma	C	AT1G70580
13029	3	F23 vs. L23	F2-3 up	GO:0009570	Metabolism	Photosynthesis	chloroplast stroma	C	AT1G75690
13030	3	F23 vs. L23	F2-3 up	GO:0009570	Metabolism	Photosynthesis	chloroplast stroma	C	AT1G76080
13031	3	F23 vs. L23	F2-3 up	GO:0009570	Metabolism	Photosynthesis	chloroplast stroma	C	AT1G77090
13032	3	F23 vs. L23	F2-3 up	GO:0009570	Metabolism	Photosynthesis	chloroplast stroma	C	AT1G79440
13033	3	F23 vs. L23	F2-3 up	GO:0009570	Metabolism	Photosynthesis	chloroplast stroma	C	AT2G15620
13034	3	F23 vs. L23	F2-3 up	GO:0009570	Metabolism	Photosynthesis	chloroplast stroma	C	AT2G21330
13035	3	F23 vs. L23	F2-3 up	GO:0009570	Metabolism	Photosynthesis	chloroplast stroma	C	AT2G28190
13036	3	F23 vs. L23	F2-3 up	GO:0009570	Metabolism	Photosynthesis	chloroplast stroma	C	AT2G29630
13037	3	F23 vs. L23	F2-3 up	GO:0009570	Metabolism	Photosynthesis	chloroplast stroma	C	AT2G30860
13038	3	F23 vs. L23	F2-3 up	GO:0009570	Metabolism	Photosynthesis	chloroplast stroma	C	AT2G39730
13039	3	F23 vs. L23	F2-3 up	GO:0009570	Metabolism	Photosynthesis	chloroplast stroma	C	AT2G43100
13040	3	F23 vs. L23	F2-3 up	GO:0009570	Metabolism	Photosynthesis	chloroplast stroma	C	AT2G45770

13041	3	F23 vs. L23	F2-3 up	GO:0009570	Metabolism	Photosynthesis	chloroplast stroma	C	AT3G01500
13042	3	F23 vs. L23	F2-3 up	GO:0009570	Metabolism	Photosynthesis	chloroplast stroma	C	AT3G04790
13043	3	F23 vs. L23	F2-3 up	GO:0009570	Metabolism	Photosynthesis	chloroplast stroma	C	AT3G11630
13044	3	F23 vs. L23	F2-3 up	GO:0009570	Metabolism	Photosynthesis	chloroplast stroma	C	AT3G12780
13045	3	F23 vs. L23	F2-3 up	GO:0009570	Metabolism	Photosynthesis	chloroplast stroma	C	AT3G14420
13046	3	F23 vs. L23	F2-3 up	GO:0009570	Metabolism	Photosynthesis	chloroplast stroma	C	AT3G15360
13047	3	F23 vs. L23	F2-3 up	GO:0009570	Metabolism	Photosynthesis	chloroplast stroma	C	AT3G22890
13048	3	F23 vs. L23	F2-3 up	GO:0009570	Metabolism	Photosynthesis	chloroplast stroma	C	AT3G23700
13049	3	F23 vs. L23	F2-3 up	GO:0009570	Metabolism	Photosynthesis	chloroplast stroma	C	AT3G26650
13050	3	F23 vs. L23	F2-3 up	GO:0009570	Metabolism	Photosynthesis	chloroplast stroma	C	AT3G45140
13051	3	F23 vs. L23	F2-3 up	GO:0009570	Metabolism	Photosynthesis	chloroplast stroma	C	AT3G46520
13052	3	F23 vs. L23	F2-3 up	GO:0009570	Metabolism	Photosynthesis	chloroplast stroma	C	AT3G48420
13053	3	F23 vs. L23	F2-3 up	GO:0009570	Metabolism	Photosynthesis	chloroplast stroma	C	AT3G48990
13054	3	F23 vs. L23	F2-3 up	GO:0009570	Metabolism	Photosynthesis	chloroplast stroma	C	AT3G52720
13055	3	F23 vs. L23	F2-3 up	GO:0009570	Metabolism	Photosynthesis	chloroplast stroma	C	AT3G54660
13056	3	F23 vs. L23	F2-3 up	GO:0009570	Metabolism	Photosynthesis	chloroplast stroma	C	AT3G55800
13057	3	F23 vs. L23	F2-3 up	GO:0009570	Metabolism	Photosynthesis	chloroplast stroma	C	AT3G57050
13058	3	F23 vs. L23	F2-3 up	GO:0009570	Metabolism	Photosynthesis	chloroplast stroma	C	AT3G58990
13059	3	F23 vs. L23	F2-3 up	GO:0009570	Metabolism	Photosynthesis	chloroplast stroma	C	AT3G62410
13060	3	F23 vs. L23	F2-3 up	GO:0009570	Metabolism	Photosynthesis	chloroplast stroma	C	AT3G63140
13061	3	F23 vs. L23	F2-3 up	GO:0009570	Metabolism	Photosynthesis	chloroplast stroma	C	AT4G04770
13062	3	F23 vs. L23	F2-3 up	GO:0009570	Metabolism	Photosynthesis	chloroplast stroma	C	AT4G14880
13063	3	F23 vs. L23	F2-3 up	GO:0009570	Metabolism	Photosynthesis	chloroplast stroma	C	AT4G15530
13064	3	F23 vs. L23	F2-3 up	GO:0009570	Metabolism	Photosynthesis	chloroplast stroma	C	AT4G17090
13065	3	F23 vs. L23	F2-3 up	GO:0009570	Metabolism	Photosynthesis	chloroplast stroma	C	AT4G20360
13066	3	F23 vs. L23	F2-3 up	GO:0009570	Metabolism	Photosynthesis	chloroplast stroma	C	AT4G23100
13067	3	F23 vs. L23	F2-3 up	GO:0009570	Metabolism	Photosynthesis	chloroplast stroma	C	AT4G33010
13068	3	F23 vs. L23	F2-3 up	GO:0009570	Metabolism	Photosynthesis	chloroplast stroma	C	AT4G35250
13069	3	F23 vs. L23	F2-3 up	GO:0009570	Metabolism	Photosynthesis	chloroplast stroma	C	AT4G38970
13070	3	F23 vs. L23	F2-3 up	GO:0009570	Metabolism	Photosynthesis	chloroplast stroma	C	AT5G01600
13071	3	F23 vs. L23	F2-3 up	GO:0009570	Metabolism	Photosynthesis	chloroplast stroma	C	AT5G04140
13072	3	F23 vs. L23	F2-3 up	GO:0009570	Metabolism	Photosynthesis	chloroplast stroma	C	AT5G04710
13073	3	F23 vs. L23	F2-3 up	GO:0009570	Metabolism	Photosynthesis	chloroplast stroma	C	AT5G05730
13074	3	F23 vs. L23	F2-3 up	GO:0009570	Metabolism	Photosynthesis	chloroplast stroma	C	AT5G09650
13075	3	F23 vs. L23	F2-3 up	GO:0009570	Metabolism	Photosynthesis	chloroplast stroma	C	AT5G09810
13076	3	F23 vs. L23	F2-3 up	GO:0009570	Metabolism	Photosynthesis	chloroplast stroma	C	AT5G14060
13077	3	F23 vs. L23	F2-3 up	GO:0009570	Metabolism	Photosynthesis	chloroplast stroma	C	AT5G14200
13078	3	F23 vs. L23	F2-3 up	GO:0009570	Metabolism	Photosynthesis	chloroplast stroma	C	AT5G14660
13079	3	F23 vs. L23	F2-3 up	GO:0009570	Metabolism	Photosynthesis	chloroplast stroma	C	AT5G14740
13080	3	F23 vs. L23	F2-3 up	GO:0009570	Metabolism	Photosynthesis	chloroplast stroma	C	AT5G19220
13081	3	F23 vs. L23	F2-3 up	GO:0009570	Metabolism	Photosynthesis	chloroplast stroma	C	AT5G22630
13082	3	F23 vs. L23	F2-3 up	GO:0009570	Metabolism	Photosynthesis	chloroplast stroma	C	AT5G23120
13083	3	F23 vs. L23	F2-3 up	GO:0009570	Metabolism	Photosynthesis	chloroplast stroma	C	AT5G30510
13084	3	F23 vs. L23	F2-3 up	GO:0009570	Metabolism	Photosynthesis	chloroplast stroma	C	AT5G35630
13085	3	F23 vs. L23	F2-3 up	GO:0009570	Metabolism	Photosynthesis	chloroplast stroma	C	AT5G36700
13086	3	F23 vs. L23	F2-3 up	GO:0009570	Metabolism	Photosynthesis	chloroplast stroma	C	AT5G36880
13087	3	F23 vs. L23	F2-3 up	GO:0009570	Metabolism	Photosynthesis	chloroplast stroma	C	AT5G38420
13088	3	F23 vs. L23	F2-3 up	GO:0009570	Metabolism	Photosynthesis	chloroplast stroma	C	AT5G39210
13089	3	F23 vs. L23	F2-3 up	GO:0009570	Metabolism	Photosynthesis	chloroplast stroma	C	AT5G48300
13090	3	F23 vs. L23	F2-3 up	GO:0009570	Metabolism	Photosynthesis	chloroplast stroma	C	AT5G51720
13091	3	F23 vs. L23	F2-3 up	GO:0009570	Metabolism	Photosynthesis	chloroplast stroma	C	AT5G52100
13092	3	F23 vs. L23	F2-3 up	GO:0009570	Metabolism	Photosynthesis	chloroplast stroma	C	AT5G53460
13093	3	F23 vs. L23	F2-3 up	GO:0009570	Metabolism	Photosynthesis	chloroplast stroma	C	AT5G58330
13094	3	F23 vs. L23	F2-3 up	GO:0009570	Metabolism	Photosynthesis	chloroplast stroma	C	AT5G58770
13095	3	F23 vs. L23	F2-3 up	GO:0009570	Metabolism	Photosynthesis	chloroplast stroma	C	AT5G60600
13096	3	F23 vs. L23	F2-3 up	GO:0009570	Metabolism	Photosynthesis	chloroplast stroma	C	AT5G61410

13097	3	F23 vs. L23	F2-3 up	GO:0009570	Metabolism	Photosynthesis	chloroplast stroma	C	ATCG00820
13098	3	F23 vs. L23	F2-3 up	GO:0009533	Metabolism	Photosynthesis	chloroplast stromal thylakoid	C	AT3G26060
13099	3	F23 vs. L23	F2-3 up	GO:0009533	Metabolism	Photosynthesis	chloroplast stromal thylakoid	C	AT5G23120
13100	3	F23 vs. L23	F2-3 up	GO:0009534	Metabolism	Photosynthesis	chloroplast thylakoid	C	AT1G11860
13101	3	F23 vs. L23	F2-3 up	GO:0009534	Metabolism	Photosynthesis	chloroplast thylakoid	C	AT1G12250
13102	3	F23 vs. L23	F2-3 up	GO:0009534	Metabolism	Photosynthesis	chloroplast thylakoid	C	AT1G15980
13103	3	F23 vs. L23	F2-3 up	GO:0009534	Metabolism	Photosynthesis	chloroplast thylakoid	C	AT1G20020
13104	3	F23 vs. L23	F2-3 up	GO:0009534	Metabolism	Photosynthesis	chloroplast thylakoid	C	AT1G44000
13105	3	F23 vs. L23	F2-3 up	GO:0009534	Metabolism	Photosynthesis	chloroplast thylakoid	C	AT1G44575
13106	3	F23 vs. L23	F2-3 up	GO:0009534	Metabolism	Photosynthesis	chloroplast thylakoid	C	AT1G50250
13107	3	F23 vs. L23	F2-3 up	GO:0009534	Metabolism	Photosynthesis	chloroplast thylakoid	C	AT1G51110
13108	3	F23 vs. L23	F2-3 up	GO:0009534	Metabolism	Photosynthesis	chloroplast thylakoid	C	AT1G55480
13109	3	F23 vs. L23	F2-3 up	GO:0009534	Metabolism	Photosynthesis	chloroplast thylakoid	C	AT1G56500
13110	3	F23 vs. L23	F2-3 up	GO:0009534	Metabolism	Photosynthesis	chloroplast thylakoid	C	AT1G71500
13111	3	F23 vs. L23	F2-3 up	GO:0009534	Metabolism	Photosynthesis	chloroplast thylakoid	C	AT1G75690
13112	3	F23 vs. L23	F2-3 up	GO:0009534	Metabolism	Photosynthesis	chloroplast thylakoid	C	AT1G77090
13113	3	F23 vs. L23	F2-3 up	GO:0009534	Metabolism	Photosynthesis	chloroplast thylakoid	C	AT2G05620
13114	3	F23 vs. L23	F2-3 up	GO:0009534	Metabolism	Photosynthesis	chloroplast thylakoid	C	AT2G21330
13115	3	F23 vs. L23	F2-3 up	GO:0009534	Metabolism	Photosynthesis	chloroplast thylakoid	C	AT2G21960
13116	3	F23 vs. L23	F2-3 up	GO:0009534	Metabolism	Photosynthesis	chloroplast thylakoid	C	AT2G30440
13117	3	F23 vs. L23	F2-3 up	GO:0009534	Metabolism	Photosynthesis	chloroplast thylakoid	C	AT2G34460
13118	3	F23 vs. L23	F2-3 up	GO:0009534	Metabolism	Photosynthesis	chloroplast thylakoid	C	AT2G45770
13119	3	F23 vs. L23	F2-3 up	GO:0009534	Metabolism	Photosynthesis	chloroplast thylakoid	C	AT2G46820
13120	3	F23 vs. L23	F2-3 up	GO:0009534	Metabolism	Photosynthesis	chloroplast thylakoid	C	AT3G26060
13121	3	F23 vs. L23	F2-3 up	GO:0009534	Metabolism	Photosynthesis	chloroplast thylakoid	C	AT3G51820
13122	3	F23 vs. L23	F2-3 up	GO:0009534	Metabolism	Photosynthesis	chloroplast thylakoid	C	AT3G63140
13123	3	F23 vs. L23	F2-3 up	GO:0009534	Metabolism	Photosynthesis	chloroplast thylakoid	C	AT4G03280
13124	3	F23 vs. L23	F2-3 up	GO:0009534	Metabolism	Photosynthesis	chloroplast thylakoid	C	AT4G20360
13125	3	F23 vs. L23	F2-3 up	GO:0009534	Metabolism	Photosynthesis	chloroplast thylakoid	C	AT4G23890
13126	3	F23 vs. L23	F2-3 up	GO:0009534	Metabolism	Photosynthesis	chloroplast thylakoid	C	AT4G32260
13127	3	F23 vs. L23	F2-3 up	GO:0009534	Metabolism	Photosynthesis	chloroplast thylakoid	C	AT4G33010
13128	3	F23 vs. L23	F2-3 up	GO:0009534	Metabolism	Photosynthesis	chloroplast thylakoid	C	AT4G35250
13129	3	F23 vs. L23	F2-3 up	GO:0009534	Metabolism	Photosynthesis	chloroplast thylakoid	C	AT5G23060
13130	3	F23 vs. L23	F2-3 up	GO:0009534	Metabolism	Photosynthesis	chloroplast thylakoid	C	AT5G23120
13131	3	F23 vs. L23	F2-3 up	GO:0009534	Metabolism	Photosynthesis	chloroplast thylakoid	C	AT5G38660
13132	3	F23 vs. L23	F2-3 up	GO:0009534	Metabolism	Photosynthesis	chloroplast thylakoid	C	AT5G64040
13133	3	F23 vs. L23	F2-3 up	GO:0009534	Metabolism	Photosynthesis	chloroplast thylakoid	C	ATCG00120
13134	3	F23 vs. L23	F2-3 up	GO:0009534	Metabolism	Photosynthesis	chloroplast thylakoid	C	ATCG00150
13135	3	F23 vs. L23	F2-3 up	GO:0009534	Metabolism	Photosynthesis	chloroplast thylakoid	C	ATCG00560
13136	3	F23 vs. L23	F2-3 up	GO:0009543	Metabolism	Photosynthesis	chloroplast thylakoid lumen	C	AT1G12250
13137	3	F23 vs. L23	F2-3 up	GO:0009543	Metabolism	Photosynthesis	chloroplast thylakoid lumen	C	AT1G77090
13138	3	F23 vs. L23	F2-3 up	GO:0009543	Metabolism	Photosynthesis	chloroplast thylakoid lumen	C	AT2G39470
13139	3	F23 vs. L23	F2-3 up	GO:0009543	Metabolism	Photosynthesis	chloroplast thylakoid lumen	C	AT3G26060
13140	3	F23 vs. L23	F2-3 up	GO:0009543	Metabolism	Photosynthesis	chloroplast thylakoid lumen	C	AT4G24930
13141	3	F23 vs. L23	F2-3 up	GO:0009543	Metabolism	Photosynthesis	chloroplast thylakoid lumen	C	AT5G23120
13142	3	F23 vs. L23	F2-3 up	GO:0009543	Metabolism	Photosynthesis	chloroplast thylakoid lumen	C	AT5G64040
13143	3	F23 vs. L23	F2-3 up	GO:0009535	Metabolism	Photosynthesis	chloroplast thylakoid membrane	C	AT1G06690
13144	3	F23 vs. L23	F2-3 up	GO:0009535	Metabolism	Photosynthesis	chloroplast thylakoid membrane	C	AT1G12250
13145	3	F23 vs. L23	F2-3 up	GO:0009535	Metabolism	Photosynthesis	chloroplast thylakoid membrane	C	AT1G15980
13146	3	F23 vs. L23	F2-3 up	GO:0009535	Metabolism	Photosynthesis	chloroplast thylakoid membrane	C	AT1G16720
13147	3	F23 vs. L23	F2-3 up	GO:0009535	Metabolism	Photosynthesis	chloroplast thylakoid membrane	C	AT1G16880
13148	3	F23 vs. L23	F2-3 up	GO:0009535	Metabolism	Photosynthesis	chloroplast thylakoid membrane	C	AT1G19150
13149	3	F23 vs. L23	F2-3 up	GO:0009535	Metabolism	Photosynthesis	chloroplast thylakoid membrane	C	AT1G20020
13150	3	F23 vs. L23	F2-3 up	GO:0009535	Metabolism	Photosynthesis	chloroplast thylakoid membrane	C	AT1G32060
13151	3	F23 vs. L23	F2-3 up	GO:0009535	Metabolism	Photosynthesis	chloroplast thylakoid membrane	C	AT1G42970
13152	3	F23 vs. L23	F2-3 up	GO:0009535	Metabolism	Photosynthesis	chloroplast thylakoid membrane	C	AT1G44575

13153	3	F23 vs. L23	F2-3 up	GO:0009535	Metabolism	Photosynthesis	chloroplast thylakoid membrane	C	AT1G45474
13154	3	F23 vs. L23	F2-3 up	GO:0009535	Metabolism	Photosynthesis	chloroplast thylakoid membrane	C	AT1G50250
13155	3	F23 vs. L23	F2-3 up	GO:0009535	Metabolism	Photosynthesis	chloroplast thylakoid membrane	C	AT1G51110
13156	3	F23 vs. L23	F2-3 up	GO:0009535	Metabolism	Photosynthesis	chloroplast thylakoid membrane	C	AT1G55480
13157	3	F23 vs. L23	F2-3 up	GO:0009535	Metabolism	Photosynthesis	chloroplast thylakoid membrane	C	AT1G56500
13158	3	F23 vs. L23	F2-3 up	GO:0009535	Metabolism	Photosynthesis	chloroplast thylakoid membrane	C	AT1G64150
13159	3	F23 vs. L23	F2-3 up	GO:0009535	Metabolism	Photosynthesis	chloroplast thylakoid membrane	C	AT1G64510
13160	3	F23 vs. L23	F2-3 up	GO:0009535	Metabolism	Photosynthesis	chloroplast thylakoid membrane	C	AT1G68590
13161	3	F23 vs. L23	F2-3 up	GO:0009535	Metabolism	Photosynthesis	chloroplast thylakoid membrane	C	AT1G71500
13162	3	F23 vs. L23	F2-3 up	GO:0009535	Metabolism	Photosynthesis	chloroplast thylakoid membrane	C	AT1G75690
13163	3	F23 vs. L23	F2-3 up	GO:0009535	Metabolism	Photosynthesis	chloroplast thylakoid membrane	C	AT2G05620
13164	3	F23 vs. L23	F2-3 up	GO:0009535	Metabolism	Photosynthesis	chloroplast thylakoid membrane	C	AT2G10940
13165	3	F23 vs. L23	F2-3 up	GO:0009535	Metabolism	Photosynthesis	chloroplast thylakoid membrane	C	AT2G26500
13166	3	F23 vs. L23	F2-3 up	GO:0009535	Metabolism	Photosynthesis	chloroplast thylakoid membrane	C	AT2G30440
13167	3	F23 vs. L23	F2-3 up	GO:0009535	Metabolism	Photosynthesis	chloroplast thylakoid membrane	C	AT2G34460
13168	3	F23 vs. L23	F2-3 up	GO:0009535	Metabolism	Photosynthesis	chloroplast thylakoid membrane	C	AT2G39470
13169	3	F23 vs. L23	F2-3 up	GO:0009535	Metabolism	Photosynthesis	chloroplast thylakoid membrane	C	AT2G39730
13170	3	F23 vs. L23	F2-3 up	GO:0009535	Metabolism	Photosynthesis	chloroplast thylakoid membrane	C	AT2G45770
13171	3	F23 vs. L23	F2-3 up	GO:0009535	Metabolism	Photosynthesis	chloroplast thylakoid membrane	C	AT2G46820
13172	3	F23 vs. L23	F2-3 up	GO:0009535	Metabolism	Photosynthesis	chloroplast thylakoid membrane	C	AT3G01500
13173	3	F23 vs. L23	F2-3 up	GO:0009535	Metabolism	Photosynthesis	chloroplast thylakoid membrane	C	AT3G04790
13174	3	F23 vs. L23	F2-3 up	GO:0009535	Metabolism	Photosynthesis	chloroplast thylakoid membrane	C	AT3G07700
13175	3	F23 vs. L23	F2-3 up	GO:0009535	Metabolism	Photosynthesis	chloroplast thylakoid membrane	C	AT3G15360
13176	3	F23 vs. L23	F2-3 up	GO:0009535	Metabolism	Photosynthesis	chloroplast thylakoid membrane	C	AT3G17930
13177	3	F23 vs. L23	F2-3 up	GO:0009535	Metabolism	Photosynthesis	chloroplast thylakoid membrane	C	AT3G22840
13178	3	F23 vs. L23	F2-3 up	GO:0009535	Metabolism	Photosynthesis	chloroplast thylakoid membrane	C	AT3G25760
13179	3	F23 vs. L23	F2-3 up	GO:0009535	Metabolism	Photosynthesis	chloroplast thylakoid membrane	C	AT3G26060
13180	3	F23 vs. L23	F2-3 up	GO:0009535	Metabolism	Photosynthesis	chloroplast thylakoid membrane	C	AT3G26650
13181	3	F23 vs. L23	F2-3 up	GO:0009535	Metabolism	Photosynthesis	chloroplast thylakoid membrane	C	AT3G45140
13182	3	F23 vs. L23	F2-3 up	GO:0009535	Metabolism	Photosynthesis	chloroplast thylakoid membrane	C	AT3G51820
13183	3	F23 vs. L23	F2-3 up	GO:0009535	Metabolism	Photosynthesis	chloroplast thylakoid membrane	C	AT4G03280
13184	3	F23 vs. L23	F2-3 up	GO:0009535	Metabolism	Photosynthesis	chloroplast thylakoid membrane	C	AT4G11960
13185	3	F23 vs. L23	F2-3 up	GO:0009535	Metabolism	Photosynthesis	chloroplast thylakoid membrane	C	AT4G14690
13186	3	F23 vs. L23	F2-3 up	GO:0009535	Metabolism	Photosynthesis	chloroplast thylakoid membrane	C	AT4G20360
13187	3	F23 vs. L23	F2-3 up	GO:0009535	Metabolism	Photosynthesis	chloroplast thylakoid membrane	C	AT4G23890
13188	3	F23 vs. L23	F2-3 up	GO:0009535	Metabolism	Photosynthesis	chloroplast thylakoid membrane	C	AT4G32260
13189	3	F23 vs. L23	F2-3 up	GO:0009535	Metabolism	Photosynthesis	chloroplast thylakoid membrane	C	AT4G35250
13190	3	F23 vs. L23	F2-3 up	GO:0009535	Metabolism	Photosynthesis	chloroplast thylakoid membrane	C	AT4G38100
13191	3	F23 vs. L23	F2-3 up	GO:0009535	Metabolism	Photosynthesis	chloroplast thylakoid membrane	C	AT4G39730
13192	3	F23 vs. L23	F2-3 up	GO:0009535	Metabolism	Photosynthesis	chloroplast thylakoid membrane	C	AT5G01600
13193	3	F23 vs. L23	F2-3 up	GO:0009535	Metabolism	Photosynthesis	chloroplast thylakoid membrane	C	AT5G14740
13194	3	F23 vs. L23	F2-3 up	GO:0009535	Metabolism	Photosynthesis	chloroplast thylakoid membrane	C	AT5G23060
13195	3	F23 vs. L23	F2-3 up	GO:0009535	Metabolism	Photosynthesis	chloroplast thylakoid membrane	C	AT5G23120
13196	3	F23 vs. L23	F2-3 up	GO:0009535	Metabolism	Photosynthesis	chloroplast thylakoid membrane	C	AT5G35630
13197	3	F23 vs. L23	F2-3 up	GO:0009535	Metabolism	Photosynthesis	chloroplast thylakoid membrane	C	AT5G38660
13198	3	F23 vs. L23	F2-3 up	GO:0009535	Metabolism	Photosynthesis	chloroplast thylakoid membrane	C	AT5G58260
13199	3	F23 vs. L23	F2-3 up	GO:0009535	Metabolism	Photosynthesis	chloroplast thylakoid membrane	C	AT5G64040
13200	3	F23 vs. L23	F2-3 up	GO:0009535	Metabolism	Photosynthesis	chloroplast thylakoid membrane	C	ATCG00120
13201	3	F23 vs. L23	F2-3 up	GO:0009535	Metabolism	Photosynthesis	chloroplast thylakoid membrane	C	ATCG00150
13202	3	F23 vs. L23	F2-3 up	GO:0009535	Metabolism	Photosynthesis	chloroplast thylakoid membrane	C	ATCG00220
13203	3	F23 vs. L23	F2-3 up	GO:0009535	Metabolism	Photosynthesis	chloroplast thylakoid membrane	C	ATCG00420
13204	3	F23 vs. L23	F2-3 up	GO:0009535	Metabolism	Photosynthesis	chloroplast thylakoid membrane	C	ATCG00430
13205	3	F23 vs. L23	F2-3 up	GO:0009535	Metabolism	Photosynthesis	chloroplast thylakoid membrane	C	ATCG00560
13206	3	F23 vs. L23	F2-3 up	GO:0009535	Metabolism	Photosynthesis	chloroplast thylakoid membrane	C	ATCG01090
13207	3	F23 vs. L23	F2-3 up	GO:0009535	Metabolism	Photosynthesis	chloroplast thylakoid membrane	C	ATCG01100
13208	3	F23 vs. L23	F2-3 up	GO:0009575	Metabolism	Photosynthesis	chromoplast stroma	C	AT1G55480

13209	3	F23 vs. L23	F2-3 up	GO:0009512	Metabolism	Photosynthesis	cytochrome b6f complex	C	AT2G26500
13210	3	F23 vs. L23	F2-3 up	GO:0009512	Metabolism	Photosynthesis	cytochrome b6f complex	C	AT4G03280
13211	3	F23 vs. L23	F2-3 up	GO:0010190	Metabolism	Photosynthesis	cytochrome b6f complex assembly	P	AT3G17930
13212	3	F23 vs. L23	F2-3 up	GO:0046028	Metabolism	Photosynthesis	electron transporter, transferring electrons from cytochrome b6/f complex of photosystem II activity	F	AT4G03280
13213	3	F23 vs. L23	F2-3 up	GO:0045158	Metabolism	Photosynthesis	electron transporter, transferring electrons within cytochrome b6/f complex of photosystem II activity	F	AT4G03280
13214	3	F23 vs. L23	F2-3 up	GO:0035448	Metabolism	Photosynthesis	extrinsic component of thylakoid membrane	C	AT1G55480
13215	3	F23 vs. L23	F2-3 up	GO:0004324	Metabolism	Photosynthesis	ferredoxin-NADP+ reductase activity	F	AT1G20020
13216	3	F23 vs. L23	F2-3 up	GO:0042132	Metabolism	Photosynthesis	fructose 1,6-bisphosphate 1-phosphatase activity	F	AT3G55800
13217	3	F23 vs. L23	F2-3 up	GO:0030388	Metabolism	Photosynthesis	fructose 1,6-bisphosphate metabolic process	P	AT2G21330
13218	3	F23 vs. L23	F2-3 up	GO:0030388	Metabolism	Photosynthesis	fructose 1,6-bisphosphate metabolic process	P	AT3G55800
13219	3	F23 vs. L23	F2-3 up	GO:0030388	Metabolism	Photosynthesis	fructose 1,6-bisphosphate metabolic process	P	AT4G38970
13220	3	F23 vs. L23	F2-3 up	GO:0006002	Metabolism	Photosynthesis	fructose 6-phosphate metabolic process	P	AT3G55800
13221	3	F23 vs. L23	F2-3 up	GO:0004332	Metabolism	Photosynthesis	fructose-bisphosphate aldolase activity	F	AT2G21330
13222	3	F23 vs. L23	F2-3 up	GO:0004332	Metabolism	Photosynthesis	fructose-bisphosphate aldolase activity	F	AT4G38970
13223	3	F23 vs. L23	F2-3 up	GO:0006783	Metabolism	Photosynthesis	heme biosynthetic process	P	AT1G58290
13224	3	F23 vs. L23	F2-3 up	GO:0080153	Metabolism	Photosynthesis	negative regulation of reductive pentose-phosphate cycle	P	AT3G62410
13225	3	F23 vs. L23	F2-3 up	GO:0009854	Metabolism	Photosynthesis	oxidative photosynthetic carbon pathway	P	AT1G68010
13226	3	F23 vs. L23	F2-3 up	GO:0009854	Metabolism	Photosynthesis	oxidative photosynthetic carbon pathway	P	AT3G14420
13227	3	F23 vs. L23	F2-3 up	GO:0009915	Metabolism	Photosynthesis	phloem sucrose loading	P	AT1G22710
13228	3	F23 vs. L23	F2-3 up	GO:0110128	Metabolism	Photosynthesis	phloem sucrose unloading	P	AT1G35720
13229	3	F23 vs. L23	F2-3 up	GO:0015979	Metabolism	Photosynthesis	photosynthesis	P	AT1G20020
13230	3	F23 vs. L23	F2-3 up	GO:0015979	Metabolism	Photosynthesis	photosynthesis	P	AT1G27730
13231	3	F23 vs. L23	F2-3 up	GO:0015979	Metabolism	Photosynthesis	photosynthesis	P	AT1G44575
13232	3	F23 vs. L23	F2-3 up	GO:0015979	Metabolism	Photosynthesis	photosynthesis	P	AT1G55480
13233	3	F23 vs. L23	F2-3 up	GO:0015979	Metabolism	Photosynthesis	photosynthesis	P	AT1G61800
13234	3	F23 vs. L23	F2-3 up	GO:0015979	Metabolism	Photosynthesis	photosynthesis	P	AT1G77090
13235	3	F23 vs. L23	F2-3 up	GO:0015979	Metabolism	Photosynthesis	photosynthesis	P	AT2G39470
13236	3	F23 vs. L23	F2-3 up	GO:0015979	Metabolism	Photosynthesis	photosynthesis	P	AT3G01500
13237	3	F23 vs. L23	F2-3 up	GO:0015979	Metabolism	Photosynthesis	photosynthesis	P	AT3G02780
13238	3	F23 vs. L23	F2-3 up	GO:0015979	Metabolism	Photosynthesis	photosynthesis	P	AT3G11050
13239	3	F23 vs. L23	F2-3 up	GO:0015979	Metabolism	Photosynthesis	photosynthesis	P	AT3G14940
13240	3	F23 vs. L23	F2-3 up	GO:0015979	Metabolism	Photosynthesis	photosynthesis	P	AT3G22840
13241	3	F23 vs. L23	F2-3 up	GO:0015979	Metabolism	Photosynthesis	photosynthesis	P	AT4G04040
13242	3	F23 vs. L23	F2-3 up	GO:0015979	Metabolism	Photosynthesis	photosynthesis	P	AT4G11960
13243	3	F23 vs. L23	F2-3 up	GO:0015979	Metabolism	Photosynthesis	photosynthesis	P	AT4G14690
13244	3	F23 vs. L23	F2-3 up	GO:0015979	Metabolism	Photosynthesis	photosynthesis	P	AT4G15530
13245	3	F23 vs. L23	F2-3 up	GO:0015979	Metabolism	Photosynthesis	photosynthesis	P	AT5G01600
13246	3	F23 vs. L23	F2-3 up	GO:0015979	Metabolism	Photosynthesis	photosynthesis	P	AT5G23120
13247	3	F23 vs. L23	F2-3 up	GO:0015979	Metabolism	Photosynthesis	photosynthesis	P	AT5G64040
13248	3	F23 vs. L23	F2-3 up	GO:0015979	Metabolism	Photosynthesis	photosynthesis	P	ATCG01090
13249	3	F23 vs. L23	F2-3 up	GO:0015979	Metabolism	Photosynthesis	photosynthesis	P	ATCG01100
13250	3	F23 vs. L23	F2-3 up	GO:0009768	Metabolism	Photosynthesis	photosynthesis, light harvesting in photosystem I	P	AT1G19150
13251	3	F23 vs. L23	F2-3 up	GO:0009768	Metabolism	Photosynthesis	photosynthesis, light harvesting in photosystem I	P	AT1G45474
13252	3	F23 vs. L23	F2-3 up	GO:0019684	Metabolism	Photosynthesis	photosynthesis, light reaction	P	AT5G52100
13253	3	F23 vs. L23	F2-3 up	GO:0019684	Metabolism	Photosynthesis	photosynthesis, light reaction	P	ATCG00220
13254	3	F23 vs. L23	F2-3 up	GO:0009643	Metabolism	Photosynthesis	photosynthetic acclimation	P	AT1G61800
13255	3	F23 vs. L23	F2-3 up	GO:0009767	Metabolism	Photosynthesis	photosynthetic electron transport chain	P	AT4G03280
13256	3	F23 vs. L23	F2-3 up	GO:0009767	Metabolism	Photosynthesis	photosynthetic electron transport chain	P	AT4G23890
13257	3	F23 vs. L23	F2-3 up	GO:0009773	Metabolism	Photosynthesis	photosynthetic electron transport in photosystem I	P	AT1G15980
13258	3	F23 vs. L23	F2-3 up	GO:0009773	Metabolism	Photosynthesis	photosynthetic electron transport in photosystem I	P	AT2G05620
13259	3	F23 vs. L23	F2-3 up	GO:0009773	Metabolism	Photosynthesis	photosynthetic electron transport in photosystem I	P	AT2G46820
13260	3	F23 vs. L23	F2-3 up	GO:0009773	Metabolism	Photosynthesis	photosynthetic electron transport in photosystem I	P	AT4G11960
13261	3	F23 vs. L23	F2-3 up	GO:0009773	Metabolism	Photosynthesis	photosynthetic electron transport in photosystem I	P	AT5G64040

13262	3	F23 vs. L23	F2-3 up	GO:0009772	Metabolism	Photosynthesis	photosynthetic electron transport in photosystem II	P	AT2G45770
13263	3	F23 vs. L23	F2-3 up	GO:0009522	Metabolism	Photosynthesis	photosystem I	C	AT1G19150
13264	3	F23 vs. L23	F2-3 up	GO:0009522	Metabolism	Photosynthesis	photosystem I	C	AT3G22840
13265	3	F23 vs. L23	F2-3 up	GO:0009522	Metabolism	Photosynthesis	photosystem I	C	AT4G14690
13266	3	F23 vs. L23	F2-3 up	GO:0009522	Metabolism	Photosynthesis	photosystem I	C	AT5G64040
13267	3	F23 vs. L23	F2-3 up	GO:0009782	Metabolism	Photosynthesis	photosystem I antenna complex	C	AT1G45474
13268	3	F23 vs. L23	F2-3 up	GO:0009523	Metabolism	Photosynthesis	photosystem II	C	AT1G16720
13269	3	F23 vs. L23	F2-3 up	GO:0009523	Metabolism	Photosynthesis	photosystem II	C	AT1G44575
13270	3	F23 vs. L23	F2-3 up	GO:0009523	Metabolism	Photosynthesis	photosystem II	C	AT1G55480
13271	3	F23 vs. L23	F2-3 up	GO:0009523	Metabolism	Photosynthesis	photosystem II	C	AT1G71500
13272	3	F23 vs. L23	F2-3 up	GO:0009523	Metabolism	Photosynthesis	photosystem II	C	AT3G22840
13273	3	F23 vs. L23	F2-3 up	GO:0009523	Metabolism	Photosynthesis	photosystem II	C	AT4G14690
13274	3	F23 vs. L23	F2-3 up	GO:0009523	Metabolism	Photosynthesis	photosystem II	C	AT4G35250
13275	3	F23 vs. L23	F2-3 up	GO:0009523	Metabolism	Photosynthesis	photosystem II	C	AT5G23120
13276	3	F23 vs. L23	F2-3 up	GO:0009523	Metabolism	Photosynthesis	photosystem II	C	ATCG00220
13277	3	F23 vs. L23	F2-3 up	GO:0010207	Metabolism	Photosynthesis	photosystem II assembly	P	AT1G16720
13278	3	F23 vs. L23	F2-3 up	GO:0010207	Metabolism	Photosynthesis	photosystem II assembly	P	AT1G55480
13279	3	F23 vs. L23	F2-3 up	GO:0010207	Metabolism	Photosynthesis	photosystem II assembly	P	AT1G71500
13280	3	F23 vs. L23	F2-3 up	GO:0010207	Metabolism	Photosynthesis	photosystem II assembly	P	AT4G35250
13281	3	F23 vs. L23	F2-3 up	GO:0010207	Metabolism	Photosynthesis	photosystem II assembly	P	AT5G24120
13282	3	F23 vs. L23	F2-3 up	GO:0009654	Metabolism	Photosynthesis	photosystem II oxygen evolving complex	C	AT1G77090
13283	3	F23 vs. L23	F2-3 up	GO:0009654	Metabolism	Photosynthesis	photosystem II oxygen evolving complex	C	AT2G39470
13284	3	F23 vs. L23	F2-3 up	GO:0010270	Metabolism	Photosynthesis	photosystem II oxygen evolving complex assembly	P	AT1G64150
13285	3	F23 vs. L23	F2-3 up	GO:0009539	Metabolism	Photosynthesis	photosystem II reaction center	C	ATCG00560
13286	3	F23 vs. L23	F2-3 up	GO:0010206	Metabolism	Photosynthesis	photosystem II repair	P	AT1G50250
13287	3	F23 vs. L23	F2-3 up	GO:0010206	Metabolism	Photosynthesis	photosystem II repair	P	AT1G55480
13288	3	F23 vs. L23	F2-3 up	GO:0010206	Metabolism	Photosynthesis	photosystem II repair	P	AT1G75690
13289	3	F23 vs. L23	F2-3 up	GO:0042549	Metabolism	Photosynthesis	photosystem II stabilization	P	AT1G71500
13290	3	F23 vs. L23	F2-3 up	GO:0080005	Metabolism	Photosynthesis	photosystem stoichiometry adjustment	P	AT1G64860
13291	3	F23 vs. L23	F2-3 up	GO:0009536	Metabolism	Photosynthesis	plastid	C	AT1G01090
13292	3	F23 vs. L23	F2-3 up	GO:0009536	Metabolism	Photosynthesis	plastid	C	AT1G08510
13293	3	F23 vs. L23	F2-3 up	GO:0009536	Metabolism	Photosynthesis	plastid	C	AT1G22410
13294	3	F23 vs. L23	F2-3 up	GO:0009536	Metabolism	Photosynthesis	plastid	C	AT1G44170
13295	3	F23 vs. L23	F2-3 up	GO:0009536	Metabolism	Photosynthesis	plastid	C	AT1G64190
13296	3	F23 vs. L23	F2-3 up	GO:0009536	Metabolism	Photosynthesis	plastid	C	AT1G68590
13297	3	F23 vs. L23	F2-3 up	GO:0009536	Metabolism	Photosynthesis	plastid	C	AT2G13810
13298	3	F23 vs. L23	F2-3 up	GO:0009536	Metabolism	Photosynthesis	plastid	C	AT2G14750
13299	3	F23 vs. L23	F2-3 up	GO:0009536	Metabolism	Photosynthesis	plastid	C	AT2G29630
13300	3	F23 vs. L23	F2-3 up	GO:0009536	Metabolism	Photosynthesis	plastid	C	AT2G30020
13301	3	F23 vs. L23	F2-3 up	GO:0009536	Metabolism	Photosynthesis	plastid	C	AT2G43100
13302	3	F23 vs. L23	F2-3 up	GO:0009536	Metabolism	Photosynthesis	plastid	C	AT3G06510
13303	3	F23 vs. L23	F2-3 up	GO:0009536	Metabolism	Photosynthesis	plastid	C	AT3G24506
13304	3	F23 vs. L23	F2-3 up	GO:0009536	Metabolism	Photosynthesis	plastid	C	AT3G56360
13305	3	F23 vs. L23	F2-3 up	GO:0009536	Metabolism	Photosynthesis	plastid	C	AT3G58990
13306	3	F23 vs. L23	F2-3 up	GO:0009536	Metabolism	Photosynthesis	plastid	C	AT4G12030
13307	3	F23 vs. L23	F2-3 up	GO:0009536	Metabolism	Photosynthesis	plastid	C	AT4G20360
13308	3	F23 vs. L23	F2-3 up	GO:0009536	Metabolism	Photosynthesis	plastid	C	AT4G23100
13309	3	F23 vs. L23	F2-3 up	GO:0009536	Metabolism	Photosynthesis	plastid	C	AT4G39030
13310	3	F23 vs. L23	F2-3 up	GO:0009536	Metabolism	Photosynthesis	plastid	C	AT4G39940
13311	3	F23 vs. L23	F2-3 up	GO:0009536	Metabolism	Photosynthesis	plastid	C	AT5G09650
13312	3	F23 vs. L23	F2-3 up	GO:0009536	Metabolism	Photosynthesis	plastid	C	AT5G11520
13313	3	F23 vs. L23	F2-3 up	GO:0009536	Metabolism	Photosynthesis	plastid	C	AT5G12170
13314	3	F23 vs. L23	F2-3 up	GO:0009536	Metabolism	Photosynthesis	plastid	C	AT5G13490
13315	3	F23 vs. L23	F2-3 up	GO:0009536	Metabolism	Photosynthesis	plastid	C	AT5G13710
13316	3	F23 vs. L23	F2-3 up	GO:0009536	Metabolism	Photosynthesis	plastid	C	AT5G14200
13317	3	F23 vs. L23	F2-3 up	GO:0009536	Metabolism	Photosynthesis	plastid	C	AT5G14660

13318	3	F23 vs. L23	F2-3 up	GO:0009536	Metabolism	Photosynthesis	plastid	C	AT5G14740
13319	3	F23 vs. L23	F2-3 up	GO:0009536	Metabolism	Photosynthesis	plastid	C	AT5G15910
13320	3	F23 vs. L23	F2-3 up	GO:0009536	Metabolism	Photosynthesis	plastid	C	AT5G16010
13321	3	F23 vs. L23	F2-3 up	GO:0009536	Metabolism	Photosynthesis	plastid	C	AT5G16450
13322	3	F23 vs. L23	F2-3 up	GO:0009536	Metabolism	Photosynthesis	plastid	C	AT5G22580
13323	3	F23 vs. L23	F2-3 up	GO:0009536	Metabolism	Photosynthesis	plastid	C	AT5G23060
13324	3	F23 vs. L23	F2-3 up	GO:0009536	Metabolism	Photosynthesis	plastid	C	AT5G23120
13325	3	F23 vs. L23	F2-3 up	GO:0009536	Metabolism	Photosynthesis	plastid	C	AT5G24650
13326	3	F23 vs. L23	F2-3 up	GO:0009536	Metabolism	Photosynthesis	plastid	C	AT5G25980
13327	3	F23 vs. L23	F2-3 up	GO:0009536	Metabolism	Photosynthesis	plastid	C	AT5G30510
13328	3	F23 vs. L23	F2-3 up	GO:0009536	Metabolism	Photosynthesis	plastid	C	AT5G35630
13329	3	F23 vs. L23	F2-3 up	GO:0009536	Metabolism	Photosynthesis	plastid	C	AT5G36160
13330	3	F23 vs. L23	F2-3 up	GO:0009536	Metabolism	Photosynthesis	plastid	C	AT5G36700
13331	3	F23 vs. L23	F2-3 up	GO:0009536	Metabolism	Photosynthesis	plastid	C	AT5G38420
13332	3	F23 vs. L23	F2-3 up	GO:0009536	Metabolism	Photosynthesis	plastid	C	AT5G38660
13333	3	F23 vs. L23	F2-3 up	GO:0009536	Metabolism	Photosynthesis	plastid	C	AT5G40650
13334	3	F23 vs. L23	F2-3 up	GO:0009536	Metabolism	Photosynthesis	plastid	C	AT5G41790
13335	3	F23 vs. L23	F2-3 up	GO:0009536	Metabolism	Photosynthesis	plastid	C	AT5G43745
13336	3	F23 vs. L23	F2-3 up	GO:0009536	Metabolism	Photosynthesis	plastid	C	AT5G46800
13337	3	F23 vs. L23	F2-3 up	GO:0009536	Metabolism	Photosynthesis	plastid	C	AT5G47890
13338	3	F23 vs. L23	F2-3 up	GO:0009536	Metabolism	Photosynthesis	plastid	C	AT5G47910
13339	3	F23 vs. L23	F2-3 up	GO:0009536	Metabolism	Photosynthesis	plastid	C	AT5G48300
13340	3	F23 vs. L23	F2-3 up	GO:0009536	Metabolism	Photosynthesis	plastid	C	AT5G49360
13341	3	F23 vs. L23	F2-3 up	GO:0009536	Metabolism	Photosynthesis	plastid	C	AT5G50850
13342	3	F23 vs. L23	F2-3 up	GO:0009536	Metabolism	Photosynthesis	plastid	C	AT5G51720
13343	3	F23 vs. L23	F2-3 up	GO:0009536	Metabolism	Photosynthesis	plastid	C	AT5G52450
13344	3	F23 vs. L23	F2-3 up	GO:0009536	Metabolism	Photosynthesis	plastid	C	AT5G53460
13345	3	F23 vs. L23	F2-3 up	GO:0009536	Metabolism	Photosynthesis	plastid	C	AT5G54080
13346	3	F23 vs. L23	F2-3 up	GO:0009536	Metabolism	Photosynthesis	plastid	C	AT5G54760
13347	3	F23 vs. L23	F2-3 up	GO:0009536	Metabolism	Photosynthesis	plastid	C	AT5G54940
13348	3	F23 vs. L23	F2-3 up	GO:0009536	Metabolism	Photosynthesis	plastid	C	AT5G56350
13349	3	F23 vs. L23	F2-3 up	GO:0009536	Metabolism	Photosynthesis	plastid	C	AT5G57350
13350	3	F23 vs. L23	F2-3 up	GO:0009536	Metabolism	Photosynthesis	plastid	C	AT5G58260
13351	3	F23 vs. L23	F2-3 up	GO:0009536	Metabolism	Photosynthesis	plastid	C	AT5G58330
13352	3	F23 vs. L23	F2-3 up	GO:0009536	Metabolism	Photosynthesis	plastid	C	AT5G59370
13353	3	F23 vs. L23	F2-3 up	GO:0009536	Metabolism	Photosynthesis	plastid	C	AT5G60360
13354	3	F23 vs. L23	F2-3 up	GO:0009536	Metabolism	Photosynthesis	plastid	C	AT5G60600
13355	3	F23 vs. L23	F2-3 up	GO:0009536	Metabolism	Photosynthesis	plastid	C	AT5G60790
13356	3	F23 vs. L23	F2-3 up	GO:0009536	Metabolism	Photosynthesis	plastid	C	AT5G61410
13357	3	F23 vs. L23	F2-3 up	GO:0009536	Metabolism	Photosynthesis	plastid	C	AT5G61670
13358	3	F23 vs. L23	F2-3 up	GO:0009536	Metabolism	Photosynthesis	plastid	C	AT5G62350
13359	3	F23 vs. L23	F2-3 up	GO:0009536	Metabolism	Photosynthesis	plastid	C	AT5G63180
13360	3	F23 vs. L23	F2-3 up	GO:0009536	Metabolism	Photosynthesis	plastid	C	AT5G63680
13361	3	F23 vs. L23	F2-3 up	GO:0009536	Metabolism	Photosynthesis	plastid	C	AT5G64040
13362	3	F23 vs. L23	F2-3 up	GO:0009536	Metabolism	Photosynthesis	plastid	C	AT5G64280
13363	3	F23 vs. L23	F2-3 up	GO:0009536	Metabolism	Photosynthesis	plastid	C	AT5G64350
13364	3	F23 vs. L23	F2-3 up	GO:0009536	Metabolism	Photosynthesis	plastid	C	ATCG00120
13365	3	F23 vs. L23	F2-3 up	GO:0009536	Metabolism	Photosynthesis	plastid	C	ATCG00150
13366	3	F23 vs. L23	F2-3 up	GO:0009536	Metabolism	Photosynthesis	plastid	C	ATCG00430
13367	3	F23 vs. L23	F2-3 up	GO:0009536	Metabolism	Photosynthesis	plastid	C	ATCG00820
13368	3	F23 vs. L23	F2-3 up	GO:0009536	Metabolism	Photosynthesis	plastid	C	ATCG01090
13369	3	F23 vs. L23	F2-3 up	GO:0009508	Metabolism	Photosynthesis	plastid chromosome	C	AT2G46820
13370	3	F23 vs. L23	F2-3 up	GO:0042170	Metabolism	Photosynthesis	plastid membrane	C	AT3G11170
13371	3	F23 vs. L23	F2-3 up	GO:0009668	Metabolism	Photosynthesis	plastid membrane organization	P	AT5G58770
13372	3	F23 vs. L23	F2-3 up	GO:0009657	Metabolism	Photosynthesis	plastid organization	P	AT5G23120
13373	3	F23 vs. L23	F2-3 up	GO:0009547	Metabolism	Photosynthesis	plastid ribosome	C	ATCG00820

13374	3	F23 vs. L23	F2-3 up	GO:0000312	Metabolism	Photosynthesis	plastid small ribosomal subunit	C	ATCG00820
13375	3	F23 vs. L23	F2-3 up	GO:0032544	Metabolism	Photosynthesis	plastid translation	P	AT1G09340
13376	3	F23 vs. L23	F2-3 up	GO:0032544	Metabolism	Photosynthesis	plastid translation	P	AT1G68590
13377	3	F23 vs. L23	F2-3 up	GO:0032544	Metabolism	Photosynthesis	plastid translation	P	AT3G63140
13378	3	F23 vs. L23	F2-3 up	GO:0010287	Metabolism	Photosynthesis	plastoglobule	C	AT1G06690
13379	3	F23 vs. L23	F2-3 up	GO:0010287	Metabolism	Photosynthesis	plastoglobule	C	AT1G09340
13380	3	F23 vs. L23	F2-3 up	GO:0010287	Metabolism	Photosynthesis	plastoglobule	C	AT1G54570
13381	3	F23 vs. L23	F2-3 up	GO:0010287	Metabolism	Photosynthesis	plastoglobule	C	AT2G21330
13382	3	F23 vs. L23	F2-3 up	GO:0010287	Metabolism	Photosynthesis	plastoglobule	C	AT2G34460
13383	3	F23 vs. L23	F2-3 up	GO:0010287	Metabolism	Photosynthesis	plastoglobule	C	AT2G39730
13384	3	F23 vs. L23	F2-3 up	GO:0010287	Metabolism	Photosynthesis	plastoglobule	C	AT3G07700
13385	3	F23 vs. L23	F2-3 up	GO:0010287	Metabolism	Photosynthesis	plastoglobule	C	AT3G26060
13386	3	F23 vs. L23	F2-3 up	GO:0010287	Metabolism	Photosynthesis	plastoglobule	C	AT3G63140
13387	3	F23 vs. L23	F2-3 up	GO:0010287	Metabolism	Photosynthesis	plastoglobule	C	AT4G38970
13388	3	F23 vs. L23	F2-3 up	GO:0010287	Metabolism	Photosynthesis	plastoglobule	C	AT4G39730
13389	3	F23 vs. L23	F2-3 up	GO:0010287	Metabolism	Photosynthesis	plastoglobule	C	AT5G05200
13390	3	F23 vs. L23	F2-3 up	GO:0010287	Metabolism	Photosynthesis	plastoglobule	C	ATCG00120
13391	3	F23 vs. L23	F2-3 up	GO:0080177	Metabolism	Photosynthesis	plastoglobule organization	P	AT3G07700
13392	3	F23 vs. L23	F2-3 up	GO:0009496	Metabolism	Photosynthesis	plastoquinol--plastocyanin reductase activity	F	AT4G03280
13393	3	F23 vs. L23	F2-3 up	GO:0006779	Metabolism	Photosynthesis	porphyrin-containing compound biosynthetic process	P	AT1G58290
13394	3	F23 vs. L23	F2-3 up	GO:1902326	Metabolism	Photosynthesis	positive regulation of chlorophyll biosynthetic process	P	AT5G56860
13395	3	F23 vs. L23	F2-3 up	GO:1905157	Metabolism	Photosynthesis	positive regulation of photosynthesis	P	AT5G65810
13396	3	F23 vs. L23	F2-3 up	GO:0045038	Metabolism	Photosynthesis	protein import into chloroplast thylakoid membrane	P	AT2G45770
13397	3	F23 vs. L23	F2-3 up	GO:0045036	Metabolism	Photosynthesis	protein targeting to chloroplast	P	AT2G24820
13398	3	F23 vs. L23	F2-3 up	GO:0045036	Metabolism	Photosynthesis	protein targeting to chloroplast	P	AT5G24650
13399	3	F23 vs. L23	F2-3 up	GO:0018298	Metabolism	Photosynthesis	protein-chromophore linkage	P	AT1G04400
13400	3	F23 vs. L23	F2-3 up	GO:0018298	Metabolism	Photosynthesis	protein-chromophore linkage	P	AT1G19150
13401	3	F23 vs. L23	F2-3 up	GO:0018298	Metabolism	Photosynthesis	protein-chromophore linkage	P	AT1G45474
13402	3	F23 vs. L23	F2-3 up	GO:0018298	Metabolism	Photosynthesis	protein-chromophore linkage	P	AT2G47590
13403	3	F23 vs. L23	F2-3 up	GO:0018298	Metabolism	Photosynthesis	protein-chromophore linkage	P	AT3G45780
13404	3	F23 vs. L23	F2-3 up	GO:0006782	Metabolism	Photosynthesis	protoporphyrinogen IX biosynthetic process	P	AT1G58290
13405	3	F23 vs. L23	F2-3 up	GO:0009517	Metabolism	Photosynthesis	PSII associated light-harvesting complex II	C	AT1G44575
13406	3	F23 vs. L23	F2-3 up	GO:0031540	Metabolism	Photosynthesis	regulation of anthocyanin biosynthetic process	P	AT5G13930
13407	3	F23 vs. L23	F2-3 up	GO:0010380	Metabolism	Photosynthesis	regulation of chlorophyll biosynthetic process	P	AT3G22840
13408	3	F23 vs. L23	F2-3 up	GO:0010380	Metabolism	Photosynthesis	regulation of chlorophyll biosynthetic process	P	AT4G14690
13409	3	F23 vs. L23	F2-3 up	GO:0010380	Metabolism	Photosynthesis	regulation of chlorophyll biosynthetic process	P	AT5G56860
13410	3	F23 vs. L23	F2-3 up	GO:0010109	Metabolism	Photosynthesis	regulation of photosynthesis	P	AT1G61800
13411	3	F23 vs. L23	F2-3 up	GO:0046863	Metabolism	Photosynthesis	ribulose-1,5-bisphosphate carboxylase/oxygenase activator activity	F	AT2G39730
13412	3	F23 vs. L23	F2-3 up	GO:0016984	Metabolism	Photosynthesis	ribulose-bisphosphate carboxylase activity	F	AT5G38420
13413	3	F23 vs. L23	F2-3 up	GO:0016114	Metabolism	Photosynthesis	terpenoid biosynthetic process	P	AT5G60600
13414	3	F23 vs. L23	F2-3 up	GO:0009579	Metabolism	Photosynthesis	thylakoid	C	AT1G12250
13415	3	F23 vs. L23	F2-3 up	GO:0009579	Metabolism	Photosynthesis	thylakoid	C	AT1G20020
13416	3	F23 vs. L23	F2-3 up	GO:0009579	Metabolism	Photosynthesis	thylakoid	C	AT1G23740
13417	3	F23 vs. L23	F2-3 up	GO:0009579	Metabolism	Photosynthesis	thylakoid	C	AT1G32060
13418	3	F23 vs. L23	F2-3 up	GO:0009579	Metabolism	Photosynthesis	thylakoid	C	AT1G35720
13419	3	F23 vs. L23	F2-3 up	GO:0009579	Metabolism	Photosynthesis	thylakoid	C	AT1G44575
13420	3	F23 vs. L23	F2-3 up	GO:0009579	Metabolism	Photosynthesis	thylakoid	C	AT1G55480
13421	3	F23 vs. L23	F2-3 up	GO:0009579	Metabolism	Photosynthesis	thylakoid	C	AT1G64510
13422	3	F23 vs. L23	F2-3 up	GO:0009579	Metabolism	Photosynthesis	thylakoid	C	AT1G68590
13423	3	F23 vs. L23	F2-3 up	GO:0009579	Metabolism	Photosynthesis	thylakoid	C	AT1G77090
13424	3	F23 vs. L23	F2-3 up	GO:0009579	Metabolism	Photosynthesis	thylakoid	C	AT2G04039
13425	3	F23 vs. L23	F2-3 up	GO:0009579	Metabolism	Photosynthesis	thylakoid	C	AT2G21330
13426	3	F23 vs. L23	F2-3 up	GO:0009579	Metabolism	Photosynthesis	thylakoid	C	AT2G28190

13427	3	F23 vs. L23	F2-3 up	GO:0009579	Metabolism	Photosynthesis	thylakoid	C	AT2G30860
13428	3	F23 vs. L23	F2-3 up	GO:0009579	Metabolism	Photosynthesis	thylakoid	C	AT2G35370
13429	3	F23 vs. L23	F2-3 up	GO:0009579	Metabolism	Photosynthesis	thylakoid	C	AT2G39470
13430	3	F23 vs. L23	F2-3 up	GO:0009579	Metabolism	Photosynthesis	thylakoid	C	AT2G39730
13431	3	F23 vs. L23	F2-3 up	GO:0009579	Metabolism	Photosynthesis	thylakoid	C	AT2G46820
13432	3	F23 vs. L23	F2-3 up	GO:0009579	Metabolism	Photosynthesis	thylakoid	C	AT3G01500
13433	3	F23 vs. L23	F2-3 up	GO:0009579	Metabolism	Photosynthesis	thylakoid	C	AT3G04790
13434	3	F23 vs. L23	F2-3 up	GO:0009579	Metabolism	Photosynthesis	thylakoid	C	AT3G11630
13435	3	F23 vs. L23	F2-3 up	GO:0009579	Metabolism	Photosynthesis	thylakoid	C	AT3G12780
13436	3	F23 vs. L23	F2-3 up	GO:0009579	Metabolism	Photosynthesis	thylakoid	C	AT3G15360
13437	3	F23 vs. L23	F2-3 up	GO:0009579	Metabolism	Photosynthesis	thylakoid	C	AT3G55800
13438	3	F23 vs. L23	F2-3 up	GO:0009579	Metabolism	Photosynthesis	thylakoid	C	AT3G63140
13439	3	F23 vs. L23	F2-3 up	GO:0009579	Metabolism	Photosynthesis	thylakoid	C	AT4G03280
13440	3	F23 vs. L23	F2-3 up	GO:0009579	Metabolism	Photosynthesis	thylakoid	C	AT4G24930
13441	3	F23 vs. L23	F2-3 up	GO:0009579	Metabolism	Photosynthesis	thylakoid	C	AT4G32260
13442	3	F23 vs. L23	F2-3 up	GO:0009579	Metabolism	Photosynthesis	thylakoid	C	AT4G38970
13443	3	F23 vs. L23	F2-3 up	GO:0009579	Metabolism	Photosynthesis	thylakoid	C	AT4G39730
13444	3	F23 vs. L23	F2-3 up	GO:0009579	Metabolism	Photosynthesis	thylakoid	C	AT5G01600
13445	3	F23 vs. L23	F2-3 up	GO:0009579	Metabolism	Photosynthesis	thylakoid	C	AT5G09650
13446	3	F23 vs. L23	F2-3 up	GO:0009579	Metabolism	Photosynthesis	thylakoid	C	AT5G23060
13447	3	F23 vs. L23	F2-3 up	GO:0009579	Metabolism	Photosynthesis	thylakoid	C	AT5G23120
13448	3	F23 vs. L23	F2-3 up	GO:0009579	Metabolism	Photosynthesis	thylakoid	C	AT5G30510
13449	3	F23 vs. L23	F2-3 up	GO:0009579	Metabolism	Photosynthesis	thylakoid	C	AT5G35630
13450	3	F23 vs. L23	F2-3 up	GO:0009579	Metabolism	Photosynthesis	thylakoid	C	AT5G38420
13451	3	F23 vs. L23	F2-3 up	GO:0009579	Metabolism	Photosynthesis	thylakoid	C	AT5G58330
13452	3	F23 vs. L23	F2-3 up	GO:0009579	Metabolism	Photosynthesis	thylakoid	C	AT5G61410
13453	3	F23 vs. L23	F2-3 up	GO:0009579	Metabolism	Photosynthesis	thylakoid	C	AT5G64040
13454	3	F23 vs. L23	F2-3 up	GO:0009579	Metabolism	Photosynthesis	thylakoid	C	ATCG00120
13455	3	F23 vs. L23	F2-3 up	GO:0009579	Metabolism	Photosynthesis	thylakoid	C	ATCG00560
13456	3	F23 vs. L23	F2-3 up	GO:0031977	Metabolism	Photosynthesis	thylakoid lumen	C	AT1G50250
13457	3	F23 vs. L23	F2-3 up	GO:0031977	Metabolism	Photosynthesis	thylakoid lumen	C	AT1G77090
13458	3	F23 vs. L23	F2-3 up	GO:0031977	Metabolism	Photosynthesis	thylakoid lumen	C	AT2G21330
13459	3	F23 vs. L23	F2-3 up	GO:0031977	Metabolism	Photosynthesis	thylakoid lumen	C	AT4G24930
13460	3	F23 vs. L23	F2-3 up	GO:0031977	Metabolism	Photosynthesis	thylakoid lumen	C	AT5G23120
13461	3	F23 vs. L23	F2-3 up	GO:0031977	Metabolism	Photosynthesis	thylakoid lumen	C	ATCG00120
13462	3	F23 vs. L23	F2-3 up	GO:0042651	Metabolism	Photosynthesis	thylakoid membrane	C	AT1G16720
13463	3	F23 vs. L23	F2-3 up	GO:0042651	Metabolism	Photosynthesis	thylakoid membrane	C	AT1G56500
13464	3	F23 vs. L23	F2-3 up	GO:0042651	Metabolism	Photosynthesis	thylakoid membrane	C	AT4G35250
13465	3	F23 vs. L23	F2-3 up	GO:0010027	Metabolism	Photosynthesis	thylakoid membrane organization	P	AT1G44575
13466	3	F23 vs. L23	F2-3 up	GO:0010027	Metabolism	Photosynthesis	thylakoid membrane organization	P	AT1G50250
13467	3	F23 vs. L23	F2-3 up	GO:0010027	Metabolism	Photosynthesis	thylakoid membrane organization	P	AT2G30440
13468	3	F23 vs. L23	F2-3 up	GO:0010027	Metabolism	Photosynthesis	thylakoid membrane organization	P	AT2G45770
13469	3	F23 vs. L23	F2-3 up	GO:0010027	Metabolism	Photosynthesis	thylakoid membrane organization	P	AT3G12780
13470	3	F23 vs. L23	F2-3 up	GO:0051738	Metabolism	Photosynthesis	xanthophyll binding	F	AT1G44575
13471	3	F23 vs. L23	F2-3 up	GO:0015172	Metabolism	Transporters	acidic amino acid transmembrane transporter activity	F	AT1G24400
13472	3	F23 vs. L23	F2-3 up	GO:0015800	Metabolism	Transporters	acidic amino acid transport	P	AT5G09220
13473	3	F23 vs. L23	F2-3 up	GO:0046715	Metabolism	Transporters	active borate transmembrane transporter activity	F	AT1G80760
13474	3	F23 vs. L23	F2-3 up	GO:0046715	Metabolism	Transporters	active borate transmembrane transporter activity	F	AT2G47160
13475	3	F23 vs. L23	F2-3 up	GO:0003333	Metabolism	Transporters	amino acid transmembrane transport	P	AT1G24400
13476	3	F23 vs. L23	F2-3 up	GO:0003333	Metabolism	Transporters	amino acid transmembrane transport	P	AT2G39130
13477	3	F23 vs. L23	F2-3 up	GO:0003333	Metabolism	Transporters	amino acid transmembrane transport	P	AT3G11900
13478	3	F23 vs. L23	F2-3 up	GO:0003333	Metabolism	Transporters	amino acid transmembrane transport	P	AT3G30390
13479	3	F23 vs. L23	F2-3 up	GO:0003333	Metabolism	Transporters	amino acid transmembrane transport	P	AT5G09220
13480	3	F23 vs. L23	F2-3 up	GO:0015171	Metabolism	Transporters	amino acid transmembrane transporter activity	F	AT1G24400
13481	3	F23 vs. L23	F2-3 up	GO:0015171	Metabolism	Transporters	amino acid transmembrane transporter activity	F	AT2G39130

13482	3	F23 vs. L23	F2-3 up	GO:0015171	Metabolism	Transporters	amino acid transmembrane transporter activity	F	AT3G11900
13483	3	F23 vs. L23	F2-3 up	GO:0015171	Metabolism	Transporters	amino acid transmembrane transporter activity	F	AT3G30390
13484	3	F23 vs. L23	F2-3 up	GO:0015171	Metabolism	Transporters	amino acid transmembrane transporter activity	F	AT5G09220
13485	3	F23 vs. L23	F2-3 up	GO:0015171	Metabolism	Transporters	amino acid transmembrane transporter activity	F	AT5G14940
13486	3	F23 vs. L23	F2-3 up	GO:0006865	Metabolism	Transporters	amino acid transport	P	AT5G09220
13487	3	F23 vs. L23	F2-3 up	GO:0005253	Metabolism	Transporters	anion channel activity	F	AT5G40890
13488	3	F23 vs. L23	F2-3 up	GO:0015301	Metabolism	Transporters	anion:anion antiporter activity	F	AT2G47160
13489	3	F23 vs. L23	F2-3 up	GO:0015297	Metabolism	Transporters	antiporter activity	F	AT1G21070
13490	3	F23 vs. L23	F2-3 up	GO:0015297	Metabolism	Transporters	antiporter activity	F	AT1G47530
13491	3	F23 vs. L23	F2-3 up	GO:0015297	Metabolism	Transporters	antiporter activity	F	AT1G61800
13492	3	F23 vs. L23	F2-3 up	GO:0015297	Metabolism	Transporters	antiporter activity	F	AT1G76670
13493	3	F23 vs. L23	F2-3 up	GO:0015297	Metabolism	Transporters	antiporter activity	F	AT4G24570
13494	3	F23 vs. L23	F2-3 up	GO:0015297	Metabolism	Transporters	antiporter activity	F	AT4G39030
13495	3	F23 vs. L23	F2-3 up	GO:0015297	Metabolism	Transporters	antiporter activity	F	AT5G01690
13496	3	F23 vs. L23	F2-3 up	GO:0015297	Metabolism	Transporters	antiporter activity	F	AT5G17860
13497	3	F23 vs. L23	F2-3 up	GO:0015297	Metabolism	Transporters	antiporter activity	F	AT5G49130
13498	3	F23 vs. L23	F2-3 up	GO:0015297	Metabolism	Transporters	antiporter activity	F	AT5G52450
13499	3	F23 vs. L23	F2-3 up	GO:0015173	Metabolism	Transporters	aromatic amino acid transmembrane transporter activity	F	AT3G11900
13500	3	F23 vs. L23	F2-3 up	GO:1901684	Metabolism	Transporters	arsenate ion transmembrane transport	P	AT3G54700
13501	3	F23 vs. L23	F2-3 up	GO:0005471	Metabolism	Transporters	ATP:ADP antiporter activity	F	AT5G13490
13502	3	F23 vs. L23	F2-3 up	GO:0140326	Metabolism	Transporters	ATPase-coupled intramembrane lipid transporter activity	F	AT1G54280
13503	3	F23 vs. L23	F2-3 up	GO:0042626	Metabolism	Transporters	ATPase-coupled transmembrane transporter activity	F	AT1G59870
13504	3	F23 vs. L23	F2-3 up	GO:0042626	Metabolism	Transporters	ATPase-coupled transmembrane transporter activity	F	AT1G70610
13505	3	F23 vs. L23	F2-3 up	GO:0042626	Metabolism	Transporters	ATPase-coupled transmembrane transporter activity	F	AT2G01320
13506	3	F23 vs. L23	F2-3 up	GO:0042626	Metabolism	Transporters	ATPase-coupled transmembrane transporter activity	F	AT3G16340
13507	3	F23 vs. L23	F2-3 up	GO:0042626	Metabolism	Transporters	ATPase-coupled transmembrane transporter activity	F	AT3G55100
13508	3	F23 vs. L23	F2-3 up	GO:0042626	Metabolism	Transporters	ATPase-coupled transmembrane transporter activity	F	AT4G04770
13509	3	F23 vs. L23	F2-3 up	GO:0042626	Metabolism	Transporters	ATPase-coupled transmembrane transporter activity	F	AT4G18050
13510	3	F23 vs. L23	F2-3 up	GO:0042626	Metabolism	Transporters	ATPase-coupled transmembrane transporter activity	F	AT5G06530
13511	3	F23 vs. L23	F2-3 up	GO:0042626	Metabolism	Transporters	ATPase-coupled transmembrane transporter activity	F	AT5G60740
13512	3	F23 vs. L23	F2-3 up	GO:0042626	Metabolism	Transporters	ATPase-coupled transmembrane transporter activity	F	AT5G61690
13513	3	F23 vs. L23	F2-3 up	GO:0042626	Metabolism	Transporters	ATPase-coupled transmembrane transporter activity	F	AT5G61700
13514	3	F23 vs. L23	F2-3 up	GO:0080139	Metabolism	Transporters	borate efflux transmembrane transporter activity	F	AT2G47160
13515	3	F23 vs. L23	F2-3 up	GO:0046713	Metabolism	Transporters	borate transport	P	AT1G80760
13516	3	F23 vs. L23	F2-3 up	GO:0046713	Metabolism	Transporters	borate transport	P	AT2G47160
13517	3	F23 vs. L23	F2-3 up	GO:0015086	Metabolism	Transporters	cadmium ion transmembrane transporter activity	F	AT1G16310
13518	3	F23 vs. L23	F2-3 up	GO:0015086	Metabolism	Transporters	cadmium ion transmembrane transporter activity	F	AT1G59870
13519	3	F23 vs. L23	F2-3 up	GO:0015691	Metabolism	Transporters	cadmium ion transport	P	AT1G59870
13520	3	F23 vs. L23	F2-3 up	GO:0070588	Metabolism	Transporters	calcium ion transmembrane transport	P	AT1G35720
13521	3	F23 vs. L23	F2-3 up	GO:0070588	Metabolism	Transporters	calcium ion transmembrane transport	P	AT2G22950
13522	3	F23 vs. L23	F2-3 up	GO:0070588	Metabolism	Transporters	calcium ion transmembrane transport	P	AT2G38170
13523	3	F23 vs. L23	F2-3 up	GO:0070588	Metabolism	Transporters	calcium ion transmembrane transport	P	AT3G21180
13524	3	F23 vs. L23	F2-3 up	GO:0070588	Metabolism	Transporters	calcium ion transmembrane transport	P	AT3G57330

13525	3	F23 vs. L23	F2-3 up	GO:0015085	Metabolism	Transporters	calcium ion transmembrane transporter activity	F	AT2G38170
13526	3	F23 vs. L23	F2-3 up	GO:0006816	Metabolism	Transporters	calcium ion transport	P	AT1G64150
13527	3	F23 vs. L23	F2-3 up	GO:0006816	Metabolism	Transporters	calcium ion transport	P	AT2G38170
13528	3	F23 vs. L23	F2-3 up	GO:0005388	Metabolism	Transporters	calcium transmembrane transporter activity, phosphorylative mechanism	F	AT2G22950
13529	3	F23 vs. L23	F2-3 up	GO:0005388	Metabolism	Transporters	calcium transmembrane transporter activity, phosphorylative mechanism	F	AT3G21180
13530	3	F23 vs. L23	F2-3 up	GO:0005388	Metabolism	Transporters	calcium transmembrane transporter activity, phosphorylative mechanism	F	AT3G57330
13531	3	F23 vs. L23	F2-3 up	GO:0034219	Metabolism	Transporters	carbohydrate transmembrane transport	P	AT3G05960
13532	3	F23 vs. L23	F2-3 up	GO:0015144	Metabolism	Transporters	carbohydrate transmembrane transporter activity	F	AT1G16370
13533	3	F23 vs. L23	F2-3 up	GO:0015144	Metabolism	Transporters	carbohydrate transmembrane transporter activity	F	AT1G50310
13534	3	F23 vs. L23	F2-3 up	GO:0015144	Metabolism	Transporters	carbohydrate transmembrane transporter activity	F	AT2G48020
13535	3	F23 vs. L23	F2-3 up	GO:0015144	Metabolism	Transporters	carbohydrate transmembrane transporter activity	F	AT3G05960
13536	3	F23 vs. L23	F2-3 up	GO:0008643	Metabolism	Transporters	carbohydrate transport	P	AT1G21070
13537	3	F23 vs. L23	F2-3 up	GO:0008643	Metabolism	Transporters	carbohydrate transport	P	AT1G76670
13538	3	F23 vs. L23	F2-3 up	GO:0008643	Metabolism	Transporters	carbohydrate transport	P	AT3G14770
13539	3	F23 vs. L23	F2-3 up	GO:0008643	Metabolism	Transporters	carbohydrate transport	P	AT3G48740
13540	3	F23 vs. L23	F2-3 up	GO:0008643	Metabolism	Transporters	carbohydrate transport	P	AT3G59360
13541	3	F23 vs. L23	F2-3 up	GO:0008643	Metabolism	Transporters	carbohydrate transport	P	AT5G50800
13542	3	F23 vs. L23	F2-3 up	GO:0008643	Metabolism	Transporters	carbohydrate transport	P	AT5G65650
13543	3	F23 vs. L23	F2-3 up	GO:0005351	Metabolism	Transporters	carbohydrate:proton symporter activity	F	AT1G50310
13544	3	F23 vs. L23	F2-3 up	GO:0005351	Metabolism	Transporters	carbohydrate:proton symporter activity	F	AT2G48020
13545	3	F23 vs. L23	F2-3 up	GO:0005351	Metabolism	Transporters	carbohydrate:proton symporter activity	F	AT3G05960
13546	3	F23 vs. L23	F2-3 up	GO:0005351	Metabolism	Transporters	carbohydrate:proton symporter activity	F	AT3G51490
13547	3	F23 vs. L23	F2-3 up	GO:1905039	Metabolism	Transporters	carboxylic acid transmembrane transport	P	AT5G50800
13548	3	F23 vs. L23	F2-3 up	GO:0005261	Metabolism	Transporters	cation channel activity	F	AT1G70480
13549	3	F23 vs. L23	F2-3 up	GO:0008324	Metabolism	Transporters	cation transmembrane transporter activity	F	AT1G16310
13550	3	F23 vs. L23	F2-3 up	GO:0008324	Metabolism	Transporters	cation transmembrane transporter activity	F	AT5G54250
13551	3	F23 vs. L23	F2-3 up	GO:0006812	Metabolism	Transporters	cation transport	P	AT3G17630
13552	3	F23 vs. L23	F2-3 up	GO:0006812	Metabolism	Transporters	cation transport	P	AT5G01690
13553	3	F23 vs. L23	F2-3 up	GO:0015491	Metabolism	Transporters	cation:cation antiporter activity	F	AT5G17860
13554	3	F23 vs. L23	F2-3 up	GO:0035627	Metabolism	Transporters	ceramide transport	P	AT3G21260
13555	3	F23 vs. L23	F2-3 up	GO:0015267	Metabolism	Transporters	channel activity	F	AT1G80760
13556	3	F23 vs. L23	F2-3 up	GO:0015267	Metabolism	Transporters	channel activity	F	AT3G56950
13557	3	F23 vs. L23	F2-3 up	GO:0015267	Metabolism	Transporters	channel activity	F	AT5G37810
13558	3	F23 vs. L23	F2-3 up	GO:0034707	Metabolism	Transporters	chloride channel complex	C	AT5G40890
13559	3	F23 vs. L23	F2-3 up	GO:0006821	Metabolism	Transporters	chloride transport	P	AT5G40890
13560	3	F23 vs. L23	F2-3 up	GO:0006824	Metabolism	Transporters	cobalt ion transport	P	AT2G38460
13561	3	F23 vs. L23	F2-3 up	GO:0015228	Metabolism	Transporters	coenzyme A transmembrane transporter activity	F	AT1G14560
13562	3	F23 vs. L23	F2-3 up	GO:0006825	Metabolism	Transporters	copper ion transport	P	AT3G56240
13563	3	F23 vs. L23	F2-3 up	GO:0005310	Metabolism	Transporters	dicarboxylic acid transmembrane transporter activity	F	AT4G24570
13564	3	F23 vs. L23	F2-3 up	GO:0005769	Metabolism	Transporters	early endosome	C	AT5G49720
13565	3	F23 vs. L23	F2-3 up	GO:0031901	Metabolism	Transporters	early endosome membrane	C	AT1G04760
13566	3	F23 vs. L23	F2-3 up	GO:0006888	Metabolism	Transporters	endoplasmic reticulum to Golgi vesicle-mediated transport	P	AT3G03160
13567	3	F23 vs. L23	F2-3 up	GO:0006888	Metabolism	Transporters	endoplasmic reticulum to Golgi vesicle-mediated transport	P	AT4G17170
13568	3	F23 vs. L23	F2-3 up	GO:0005768	Metabolism	Transporters	endosome	C	AT1G04430
13569	3	F23 vs. L23	F2-3 up	GO:0005768	Metabolism	Transporters	endosome	C	AT1G04760
13570	3	F23 vs. L23	F2-3 up	GO:0005768	Metabolism	Transporters	endosome	C	AT1G13860
13571	3	F23 vs. L23	F2-3 up	GO:0005768	Metabolism	Transporters	endosome	C	AT1G27930
13572	3	F23 vs. L23	F2-3 up	GO:0005768	Metabolism	Transporters	endosome	C	AT1G31850
13573	3	F23 vs. L23	F2-3 up	GO:0005768	Metabolism	Transporters	endosome	C	AT1G33170
13574	3	F23 vs. L23	F2-3 up	GO:0005768	Metabolism	Transporters	endosome	C	AT1G62360

13575	3	F23 vs. L23	F2-3 up	GO:0005768	Metabolism	Transporters	endosome	C	AT1G74380
13576	3	F23 vs. L23	F2-3 up	GO:0005768	Metabolism	Transporters	endosome	C	AT1G78240
13577	3	F23 vs. L23	F2-3 up	GO:0005768	Metabolism	Transporters	endosome	C	AT2G03240
13578	3	F23 vs. L23	F2-3 up	GO:0005768	Metabolism	Transporters	endosome	C	AT2G22900
13579	3	F23 vs. L23	F2-3 up	GO:0005768	Metabolism	Transporters	endosome	C	AT2G47160
13580	3	F23 vs. L23	F2-3 up	GO:0005768	Metabolism	Transporters	endosome	C	AT2G47650
13581	3	F23 vs. L23	F2-3 up	GO:0005768	Metabolism	Transporters	endosome	C	AT3G21190
13582	3	F23 vs. L23	F2-3 up	GO:0005768	Metabolism	Transporters	endosome	C	AT3G23300
13583	3	F23 vs. L23	F2-3 up	GO:0005768	Metabolism	Transporters	endosome	C	AT3G23820
13584	3	F23 vs. L23	F2-3 up	GO:0005768	Metabolism	Transporters	endosome	C	AT3G25140
13585	3	F23 vs. L23	F2-3 up	GO:0005768	Metabolism	Transporters	endosome	C	AT3G27230
13586	3	F23 vs. L23	F2-3 up	GO:0005768	Metabolism	Transporters	endosome	C	AT3G51070
13587	3	F23 vs. L23	F2-3 up	GO:0005768	Metabolism	Transporters	endosome	C	AT4G10440
13588	3	F23 vs. L23	F2-3 up	GO:0005768	Metabolism	Transporters	endosome	C	AT4G19120
13589	3	F23 vs. L23	F2-3 up	GO:0005768	Metabolism	Transporters	endosome	C	AT4G27720
13590	3	F23 vs. L23	F2-3 up	GO:0005768	Metabolism	Transporters	endosome	C	AT4G30996
13591	3	F23 vs. L23	F2-3 up	GO:0005768	Metabolism	Transporters	endosome	C	AT4G32410
13592	3	F23 vs. L23	F2-3 up	GO:0005768	Metabolism	Transporters	endosome	C	AT5G16910
13593	3	F23 vs. L23	F2-3 up	GO:0005768	Metabolism	Transporters	endosome	C	AT5G18280
13594	3	F23 vs. L23	F2-3 up	GO:0005768	Metabolism	Transporters	endosome	C	AT5G25610
13595	3	F23 vs. L23	F2-3 up	GO:0005768	Metabolism	Transporters	endosome	C	AT5G42050
13596	3	F23 vs. L23	F2-3 up	GO:0005768	Metabolism	Transporters	endosome	C	AT5G49720
13597	3	F23 vs. L23	F2-3 up	GO:0005768	Metabolism	Transporters	endosome	C	AT5G65810
13598	3	F23 vs. L23	F2-3 up	GO:0015093	Metabolism	Transporters	ferrous iron transmembrane transporter activity	F	AT1G16310
13599	3	F23 vs. L23	F2-3 up	GO:0015783	Metabolism	Transporters	GDP-fucose transmembrane transport	P	AT1G76670
13600	3	F23 vs. L23	F2-3 up	GO:0005457	Metabolism	Transporters	GDP-fucose transmembrane transporter activity	F	AT1G76670
13601	3	F23 vs. L23	F2-3 up	GO:0015152	Metabolism	Transporters	glucose-6-phosphate transmembrane transporter activity	F	AT1G61800
13602	3	F23 vs. L23	F2-3 up	GO:0015760	Metabolism	Transporters	glucose-6-phosphate transport	P	AT1G61800
13603	3	F23 vs. L23	F2-3 up	GO:1901349	Metabolism	Transporters	glucosinolate transport	P	AT3G47960
13604	3	F23 vs. L23	F2-3 up	GO:1901349	Metabolism	Transporters	glucosinolate transport	P	AT5G62680
13605	3	F23 vs. L23	F2-3 up	GO:0090448	Metabolism	Transporters	glucosinolate:proton symporter activity	F	AT3G47960
13606	3	F23 vs. L23	F2-3 up	GO:0090448	Metabolism	Transporters	glucosinolate:proton symporter activity	F	AT5G62680
13607	3	F23 vs. L23	F2-3 up	GO:0015168	Metabolism	Transporters	glycerol transmembrane transporter activity	F	AT1G80760
13608	3	F23 vs. L23	F2-3 up	GO:0005452	Metabolism	Transporters	inorganic anion exchanger activity	F	AT2G47160
13609	3	F23 vs. L23	F2-3 up	GO:0005315	Metabolism	Transporters	inorganic phosphate transmembrane transporter activity	F	AT1G61800
13610	3	F23 vs. L23	F2-3 up	GO:0005315	Metabolism	Transporters	inorganic phosphate transmembrane transporter activity	F	AT1G67940
13611	3	F23 vs. L23	F2-3 up	GO:0005315	Metabolism	Transporters	inorganic phosphate transmembrane transporter activity	F	AT3G54700
13612	3	F23 vs. L23	F2-3 up	GO:0031359	Metabolism	Transporters	integral component of chloroplast outer membrane	C	AT3G06510
13613	3	F23 vs. L23	F2-3 up	GO:0120009	Metabolism	Transporters	intermembrane lipid transfer	P	AT3G21260
13614	3	F23 vs. L23	F2-3 up	GO:0006886	Metabolism	Transporters	intracellular protein transport	P	AT1G61290
13615	3	F23 vs. L23	F2-3 up	GO:0006886	Metabolism	Transporters	intracellular protein transport	P	AT2G15310
13616	3	F23 vs. L23	F2-3 up	GO:0006886	Metabolism	Transporters	intracellular protein transport	P	AT2G21880
13617	3	F23 vs. L23	F2-3 up	GO:0006886	Metabolism	Transporters	intracellular protein transport	P	AT3G03160
13618	3	F23 vs. L23	F2-3 up	GO:0006886	Metabolism	Transporters	intracellular protein transport	P	AT3G03800
13619	3	F23 vs. L23	F2-3 up	GO:0006886	Metabolism	Transporters	intracellular protein transport	P	AT3G21700
13620	3	F23 vs. L23	F2-3 up	GO:0006886	Metabolism	Transporters	intracellular protein transport	P	AT3G62290
13621	3	F23 vs. L23	F2-3 up	GO:0006886	Metabolism	Transporters	intracellular protein transport	P	AT4G17170
13622	3	F23 vs. L23	F2-3 up	GO:0006886	Metabolism	Transporters	intracellular protein transport	P	AT4G17530
13623	3	F23 vs. L23	F2-3 up	GO:0006886	Metabolism	Transporters	intracellular protein transport	P	AT5G54780
13624	3	F23 vs. L23	F2-3 up	GO:0005216	Metabolism	Transporters	ion channel activity	F	AT5G54250
13625	3	F23 vs. L23	F2-3 up	GO:0015662	Metabolism	Transporters	ion transmembrane transporter activity, phosphorylative mechanism	F	AT1G54280

13626	3	F23 vs. L23	F2-3 up	GO:0006811	Metabolism	Transporters	ion transport	P	AT1G16370
13627	3	F23 vs. L23	F2-3 up	GO:0006811	Metabolism	Transporters	ion transport	P	ATMG00480
13628	3	F23 vs. L23	F2-3 up	GO:0034755	Metabolism	Transporters	iron ion transmembrane transport	P	AT2G38460
13629	3	F23 vs. L23	F2-3 up	GO:0005381	Metabolism	Transporters	iron ion transmembrane transporter activity	F	AT2G38460
13630	3	F23 vs. L23	F2-3 up	GO:0006826	Metabolism	Transporters	iron ion transport	P	AT1G41830
13631	3	F23 vs. L23	F2-3 up	GO:0006826	Metabolism	Transporters	iron ion transport	P	AT1G55570
13632	3	F23 vs. L23	F2-3 up	GO:0006826	Metabolism	Transporters	iron ion transport	P	AT2G38460
13633	3	F23 vs. L23	F2-3 up	GO:0006826	Metabolism	Transporters	iron ion transport	P	AT3G11050
13634	3	F23 vs. L23	F2-3 up	GO:0006826	Metabolism	Transporters	iron ion transport	P	AT3G13390
13635	3	F23 vs. L23	F2-3 up	GO:0006826	Metabolism	Transporters	iron ion transport	P	AT5G01600
13636	3	F23 vs. L23	F2-3 up	GO:0006826	Metabolism	Transporters	iron ion transport	P	AT5G60020
13637	3	F23 vs. L23	F2-3 up	GO:0005871	Metabolism	Transporters	kinesin complex	C	AT3G43210
13638	3	F23 vs. L23	F2-3 up	GO:0005871	Metabolism	Transporters	kinesin complex	C	AT5G27000
13639	3	F23 vs. L23	F2-3 up	GO:0005871	Metabolism	Transporters	kinesin complex	C	AT5G65460
13640	3	F23 vs. L23	F2-3 up	GO:0098712	Metabolism	Transporters	L-glutamate import across plasma membrane	P	AT2G39510
13641	3	F23 vs. L23	F2-3 up	GO:0015186	Metabolism	Transporters	L-glutamine transmembrane transporter activity	F	AT2G39510
13642	3	F23 vs. L23	F2-3 up	GO:0006869	Metabolism	Transporters	lipid transport	P	AT3G18280
13643	3	F23 vs. L23	F2-3 up	GO:0006869	Metabolism	Transporters	lipid transport	P	AT3G51600
13644	3	F23 vs. L23	F2-3 up	GO:0006869	Metabolism	Transporters	lipid transport	P	AT5G50800
13645	3	F23 vs. L23	F2-3 up	GO:0006869	Metabolism	Transporters	lipid transport	P	AT5G61690
13646	3	F23 vs. L23	F2-3 up	GO:0006869	Metabolism	Transporters	lipid transport	P	AT5G61700
13647	3	F23 vs. L23	F2-3 up	GO:0005319	Metabolism	Transporters	lipid transporter activity	F	AT3G51600
13648	3	F23 vs. L23	F2-3 up	GO:0005319	Metabolism	Transporters	lipid transporter activity	F	AT5G61690
13649	3	F23 vs. L23	F2-3 up	GO:0005319	Metabolism	Transporters	lipid transporter activity	F	AT5G61700
13650	3	F23 vs. L23	F2-3 up	GO:0000064	Metabolism	Transporters	L-ornithine transmembrane transporter activity	F	AT5G46800
13651	3	F23 vs. L23	F2-3 up	GO:0080054	Metabolism	Transporters	low-affinity nitrate transmembrane transporter activity	F	AT1G22550
13652	3	F23 vs. L23	F2-3 up	GO:0080054	Metabolism	Transporters	low-affinity nitrate transmembrane transporter activity	F	AT1G52190
13653	3	F23 vs. L23	F2-3 up	GO:0015095	Metabolism	Transporters	magnesium ion transmembrane transporter activity	F	AT1G64150
13654	3	F23 vs. L23	F2-3 up	GO:0015095	Metabolism	Transporters	magnesium ion transmembrane transporter activity	F	AT4G13800
13655	3	F23 vs. L23	F2-3 up	GO:0015095	Metabolism	Transporters	magnesium ion transmembrane transporter activity	F	AT5G11960
13656	3	F23 vs. L23	F2-3 up	GO:0015693	Metabolism	Transporters	magnesium ion transport	P	AT4G13800
13657	3	F23 vs. L23	F2-3 up	GO:0071423	Metabolism	Transporters	malate transmembrane transport	P	AT4G24570
13658	3	F23 vs. L23	F2-3 up	GO:0015140	Metabolism	Transporters	malate transmembrane transporter activity	F	AT4G24570
13659	3	F23 vs. L23	F2-3 up	GO:0005384	Metabolism	Transporters	manganese ion transmembrane transporter activity	F	AT1G64150
13660	3	F23 vs. L23	F2-3 up	GO:0006828	Metabolism	Transporters	manganese ion transport	P	AT1G64150
13661	3	F23 vs. L23	F2-3 up	GO:0046873	Metabolism	Transporters	metal ion transmembrane transporter activity	F	AT1G31260
13662	3	F23 vs. L23	F2-3 up	GO:0030001	Metabolism	Transporters	metal ion transport	P	AT1G51090
13663	3	F23 vs. L23	F2-3 up	GO:0030001	Metabolism	Transporters	metal ion transport	P	AT1G71050
13664	3	F23 vs. L23	F2-3 up	GO:0030001	Metabolism	Transporters	metal ion transport	P	AT4G35060
13665	3	F23 vs. L23	F2-3 up	GO:0030001	Metabolism	Transporters	metal ion transport	P	AT5G17450
13666	3	F23 vs. L23	F2-3 up	GO:1990575	Metabolism	Transporters	mitochondrial L-ornithine transmembrane transport	P	AT5G46800
13667	3	F23 vs. L23	F2-3 up	GO:0006839	Metabolism	Transporters	mitochondrial transport	P	AT4G24570
13668	3	F23 vs. L23	F2-3 up	GO:0006839	Metabolism	Transporters	mitochondrial transport	P	AT5G46800
13669	3	F23 vs. L23	F2-3 up	GO:0015098	Metabolism	Transporters	molybdate ion transmembrane transporter activity	F	AT4G27720
13670	3	F23 vs. L23	F2-3 up	GO:0015689	Metabolism	Transporters	molybdate ion transport	P	AT4G27720
13671	3	F23 vs. L23	F2-3 up	GO:0008028	Metabolism	Transporters	monocarboxylic acid transmembrane transporter activity	F	AT4G12030
13672	3	F23 vs. L23	F2-3 up	GO:0015145	Metabolism	Transporters	monosaccharide transmembrane transporter activity	F	AT3G05960
13673	3	F23 vs. L23	F2-3 up	GO:0010360	Metabolism	Transporters	negative regulation of anion channel activity	P	AT4G05590
13674	3	F23 vs. L23	F2-3 up	GO:0010362	Metabolism	Transporters	negative regulation of anion channel activity by blue light	P	AT3G45780
13675	3	F23 vs. L23	F2-3 up	GO:1901017	Metabolism	Transporters	negative regulation of potassium ion transmembrane transporter activity	P	AT4G05590

13676	3	F23 vs. L23	F2-3 up	GO:0015175	Metabolism	Transporters	neutral amino acid transmembrane transporter activity	F	AT1G24400
13677	3	F23 vs. L23	F2-3 up	GO:0015175	Metabolism	Transporters	neutral amino acid transmembrane transporter activity	F	AT3G11900
13678	3	F23 vs. L23	F2-3 up	GO:0015804	Metabolism	Transporters	neutral amino acid transport	P	AT1G24400
13679	3	F23 vs. L23	F2-3 up	GO:0015804	Metabolism	Transporters	neutral amino acid transport	P	AT5G09220
13680	3	F23 vs. L23	F2-3 up	GO:1902025	Metabolism	Transporters	nitrate import	P	AT1G72180
13681	3	F23 vs. L23	F2-3 up	GO:0015112	Metabolism	Transporters	nitrate transmembrane transporter activity	F	AT1G12110
13682	3	F23 vs. L23	F2-3 up	GO:0015112	Metabolism	Transporters	nitrate transmembrane transporter activity	F	AT1G32450
13683	3	F23 vs. L23	F2-3 up	GO:0015112	Metabolism	Transporters	nitrate transmembrane transporter activity	F	AT3G21670
13684	3	F23 vs. L23	F2-3 up	GO:0015112	Metabolism	Transporters	nitrate transmembrane transporter activity	F	AT5G40890
13685	3	F23 vs. L23	F2-3 up	GO:0015706	Metabolism	Transporters	nitrate transport	P	AT1G12110
13686	3	F23 vs. L23	F2-3 up	GO:0015706	Metabolism	Transporters	nitrate transport	P	AT1G32450
13687	3	F23 vs. L23	F2-3 up	GO:0015706	Metabolism	Transporters	nitrate transport	P	AT5G40890
13688	3	F23 vs. L23	F2-3 up	GO:0008139	Metabolism	Transporters	nuclear localization sequence binding	F	AT4G16143
13689	3	F23 vs. L23	F2-3 up	GO:0005337	Metabolism	Transporters	nucleoside transmembrane transporter activity	F	AT3G51490
13690	3	F23 vs. L23	F2-3 up	GO:0005338	Metabolism	Transporters	nucleotide-sugar transmembrane transporter activity	F	AT1G21070
13691	3	F23 vs. L23	F2-3 up	GO:0005338	Metabolism	Transporters	nucleotide-sugar transmembrane transporter activity	F	AT1G76670
13692	3	F23 vs. L23	F2-3 up	GO:0035673	Metabolism	Transporters	oligopeptide transmembrane transporter activity	F	AT1G22550
13693	3	F23 vs. L23	F2-3 up	GO:0035673	Metabolism	Transporters	oligopeptide transmembrane transporter activity	F	AT1G52190
13694	3	F23 vs. L23	F2-3 up	GO:0035673	Metabolism	Transporters	oligopeptide transmembrane transporter activity	F	AT1G68570
13695	3	F23 vs. L23	F2-3 up	GO:0035673	Metabolism	Transporters	oligopeptide transmembrane transporter activity	F	AT2G26690
13696	3	F23 vs. L23	F2-3 up	GO:0035673	Metabolism	Transporters	oligopeptide transmembrane transporter activity	F	AT2G40460
13697	3	F23 vs. L23	F2-3 up	GO:0035673	Metabolism	Transporters	oligopeptide transmembrane transporter activity	F	AT3G53960
13698	3	F23 vs. L23	F2-3 up	GO:0035673	Metabolism	Transporters	oligopeptide transmembrane transporter activity	F	AT5G14940
13699	3	F23 vs. L23	F2-3 up	GO:0006857	Metabolism	Transporters	oligopeptide transport	P	AT1G12110
13700	3	F23 vs. L23	F2-3 up	GO:0015850	Metabolism	Transporters	organic hydroxy compound transport	P	AT3G16340
13701	3	F23 vs. L23	F2-3 up	GO:0015822	Metabolism	Transporters	ornithine transport	P	AT5G46800
13702	3	F23 vs. L23	F2-3 up	GO:0015131	Metabolism	Transporters	oxaloacetate transmembrane transporter activity	F	AT4G24570
13703	3	F23 vs. L23	F2-3 up	GO:0015729	Metabolism	Transporters	oxaloacetate transport	P	AT4G24570
13704	3	F23 vs. L23	F2-3 up	GO:0015367	Metabolism	Transporters	oxoglutarate:malate antiporter activity	F	AT5G64280
13705	3	F23 vs. L23	F2-3 up	GO:1901140	Metabolism	Transporters	p-coumaryl alcohol transport	P	AT3G16340
13706	3	F23 vs. L23	F2-3 up	GO:1904680	Metabolism	Transporters	peptide transmembrane transporter activity	F	AT1G22550
13707	3	F23 vs. L23	F2-3 up	GO:1904680	Metabolism	Transporters	peptide transmembrane transporter activity	F	AT1G52190
13708	3	F23 vs. L23	F2-3 up	GO:1904680	Metabolism	Transporters	peptide transmembrane transporter activity	F	AT1G68570
13709	3	F23 vs. L23	F2-3 up	GO:1904680	Metabolism	Transporters	peptide transmembrane transporter activity	F	AT2G26690
13710	3	F23 vs. L23	F2-3 up	GO:1904680	Metabolism	Transporters	peptide transmembrane transporter activity	F	AT2G40460
13711	3	F23 vs. L23	F2-3 up	GO:1904680	Metabolism	Transporters	peptide transmembrane transporter activity	F	AT3G53960
13712	3	F23 vs. L23	F2-3 up	GO:1904680	Metabolism	Transporters	peptide transmembrane transporter activity	F	AT5G14940
13713	3	F23 vs. L23	F2-3 up	GO:0015333	Metabolism	Transporters	peptide:proton symporter activity	F	AT1G22550
13714	3	F23 vs. L23	F2-3 up	GO:0015333	Metabolism	Transporters	peptide:proton symporter activity	F	AT1G52190
13715	3	F23 vs. L23	F2-3 up	GO:0015333	Metabolism	Transporters	peptide:proton symporter activity	F	AT1G68570
13716	3	F23 vs. L23	F2-3 up	GO:0015333	Metabolism	Transporters	peptide:proton symporter activity	F	AT2G26690
13717	3	F23 vs. L23	F2-3 up	GO:0015333	Metabolism	Transporters	peptide:proton symporter activity	F	AT2G40460
13718	3	F23 vs. L23	F2-3 up	GO:0015333	Metabolism	Transporters	peptide:proton symporter activity	F	AT3G53960
13719	3	F23 vs. L23	F2-3 up	GO:0015333	Metabolism	Transporters	peptide:proton symporter activity	F	AT5G14940
13720	3	F23 vs. L23	F2-3 up	GO:1990429	Metabolism	Transporters	peroxisomal importomer complex	C	AT3G07560
13721	3	F23 vs. L23	F2-3 up	GO:0010233	Metabolism	Transporters	phloem transport	P	AT1G14920
13722	3	F23 vs. L23	F2-3 up	GO:0035435	Metabolism	Transporters	phosphate ion transmembrane transport	P	AT1G67940
13723	3	F23 vs. L23	F2-3 up	GO:0035435	Metabolism	Transporters	phosphate ion transmembrane transport	P	AT4G24570
13724	3	F23 vs. L23	F2-3 up	GO:0015114	Metabolism	Transporters	phosphate ion transmembrane transporter activity	F	AT2G03240
13725	3	F23 vs. L23	F2-3 up	GO:0015114	Metabolism	Transporters	phosphate ion transmembrane transporter activity	F	AT3G29060
13726	3	F23 vs. L23	F2-3 up	GO:0015114	Metabolism	Transporters	phosphate ion transmembrane transporter activity	F	AT3G54700
13727	3	F23 vs. L23	F2-3 up	GO:0006817	Metabolism	Transporters	phosphate ion transport	P	AT2G03240

13728	3	F23 vs. L23	F2-3 up	GO:0006817	Metabolism	Transporters	phosphate ion transport	P	AT3G29060
13729	3	F23 vs. L23	F2-3 up	GO:0006817	Metabolism	Transporters	phosphate ion transport	P	AT3G54700
13730	3	F23 vs. L23	F2-3 up	GO:0015714	Metabolism	Transporters	phosphoenolpyruvate transport	P	AT1G61800
13731	3	F23 vs. L23	F2-3 up	GO:0015713	Metabolism	Transporters	phosphoglycerate transmembrane transport	P	AT1G61800
13732	3	F23 vs. L23	F2-3 up	GO:0015120	Metabolism	Transporters	phosphoglycerate transmembrane transporter activity	F	AT1G61800
13733	3	F23 vs. L23	F2-3 up	GO:0045332	Metabolism	Transporters	phospholipid translocation	P	AT1G54280
13734	3	F23 vs. L23	F2-3 up	GO:0015914	Metabolism	Transporters	phospholipid transport	P	AT4G36490
13735	3	F23 vs. L23	F2-3 up	GO:0009506	Metabolism	Transporters	plasmodesma	C	AT1G09340
13736	3	F23 vs. L23	F2-3 up	GO:0009506	Metabolism	Transporters	plasmodesma	C	AT1G20620
13737	3	F23 vs. L23	F2-3 up	GO:0009506	Metabolism	Transporters	plasmodesma	C	AT1G28270
13738	3	F23 vs. L23	F2-3 up	GO:0009506	Metabolism	Transporters	plasmodesma	C	AT1G35720
13739	3	F23 vs. L23	F2-3 up	GO:0009506	Metabolism	Transporters	plasmodesma	C	AT1G41830
13740	3	F23 vs. L23	F2-3 up	GO:0009506	Metabolism	Transporters	plasmodesma	C	AT1G42470
13741	3	F23 vs. L23	F2-3 up	GO:0009506	Metabolism	Transporters	plasmodesma	C	AT1G44170
13742	3	F23 vs. L23	F2-3 up	GO:0009506	Metabolism	Transporters	plasmodesma	C	AT1G60030
13743	3	F23 vs. L23	F2-3 up	GO:0009506	Metabolism	Transporters	plasmodesma	C	AT1G62360
13744	3	F23 vs. L23	F2-3 up	GO:0009506	Metabolism	Transporters	plasmodesma	C	AT1G62380
13745	3	F23 vs. L23	F2-3 up	GO:0009506	Metabolism	Transporters	plasmodesma	C	AT1G63000
13746	3	F23 vs. L23	F2-3 up	GO:0009506	Metabolism	Transporters	plasmodesma	C	AT1G78570
13747	3	F23 vs. L23	F2-3 up	GO:0009506	Metabolism	Transporters	plasmodesma	C	AT2G10940
13748	3	F23 vs. L23	F2-3 up	GO:0009506	Metabolism	Transporters	plasmodesma	C	AT2G16430
13749	3	F23 vs. L23	F2-3 up	GO:0009506	Metabolism	Transporters	plasmodesma	C	AT2G22125
13750	3	F23 vs. L23	F2-3 up	GO:0009506	Metabolism	Transporters	plasmodesma	C	AT2G30490
13751	3	F23 vs. L23	F2-3 up	GO:0009506	Metabolism	Transporters	plasmodesma	C	AT2G30860
13752	3	F23 vs. L23	F2-3 up	GO:0009506	Metabolism	Transporters	plasmodesma	C	AT2G35840
13753	3	F23 vs. L23	F2-3 up	GO:0009506	Metabolism	Transporters	plasmodesma	C	AT2G36880
13754	3	F23 vs. L23	F2-3 up	GO:0009506	Metabolism	Transporters	plasmodesma	C	AT2G39210
13755	3	F23 vs. L23	F2-3 up	GO:0009506	Metabolism	Transporters	plasmodesma	C	AT2G39330
13756	3	F23 vs. L23	F2-3 up	GO:0009506	Metabolism	Transporters	plasmodesma	C	AT2G39700
13757	3	F23 vs. L23	F2-3 up	GO:0009506	Metabolism	Transporters	plasmodesma	C	AT2G41480
13758	3	F23 vs. L23	F2-3 up	GO:0009506	Metabolism	Transporters	plasmodesma	C	AT2G44120
13759	3	F23 vs. L23	F2-3 up	GO:0009506	Metabolism	Transporters	plasmodesma	C	AT3G03770
13760	3	F23 vs. L23	F2-3 up	GO:0009506	Metabolism	Transporters	plasmodesma	C	AT3G04840
13761	3	F23 vs. L23	F2-3 up	GO:0009506	Metabolism	Transporters	plasmodesma	C	AT3G05930
13762	3	F23 vs. L23	F2-3 up	GO:0009506	Metabolism	Transporters	plasmodesma	C	AT3G07050
13763	3	F23 vs. L23	F2-3 up	GO:0009506	Metabolism	Transporters	plasmodesma	C	AT3G09440
13764	3	F23 vs. L23	F2-3 up	GO:0009506	Metabolism	Transporters	plasmodesma	C	AT3G14420
13765	3	F23 vs. L23	F2-3 up	GO:0009506	Metabolism	Transporters	plasmodesma	C	AT3G17390
13766	3	F23 vs. L23	F2-3 up	GO:0009506	Metabolism	Transporters	plasmodesma	C	AT3G18780
13767	3	F23 vs. L23	F2-3 up	GO:0009506	Metabolism	Transporters	plasmodesma	C	AT3G25170
13768	3	F23 vs. L23	F2-3 up	GO:0009506	Metabolism	Transporters	plasmodesma	C	AT3G25290
13769	3	F23 vs. L23	F2-3 up	GO:0009506	Metabolism	Transporters	plasmodesma	C	AT3G43210
13770	3	F23 vs. L23	F2-3 up	GO:0009506	Metabolism	Transporters	plasmodesma	C	AT3G47960
13771	3	F23 vs. L23	F2-3 up	GO:0009506	Metabolism	Transporters	plasmodesma	C	AT3G48990
13772	3	F23 vs. L23	F2-3 up	GO:0009506	Metabolism	Transporters	plasmodesma	C	AT3G51600
13773	3	F23 vs. L23	F2-3 up	GO:0009506	Metabolism	Transporters	plasmodesma	C	AT3G52470
13774	3	F23 vs. L23	F2-3 up	GO:0009506	Metabolism	Transporters	plasmodesma	C	AT3G53420
13775	3	F23 vs. L23	F2-3 up	GO:0009506	Metabolism	Transporters	plasmodesma	C	AT3G55610
13776	3	F23 vs. L23	F2-3 up	GO:0009506	Metabolism	Transporters	plasmodesma	C	AT3G62290
13777	3	F23 vs. L23	F2-3 up	GO:0009506	Metabolism	Transporters	plasmodesma	C	AT4G01850
13778	3	F23 vs. L23	F2-3 up	GO:0009506	Metabolism	Transporters	plasmodesma	C	AT4G13930
13779	3	F23 vs. L23	F2-3 up	GO:0009506	Metabolism	Transporters	plasmodesma	C	AT4G15630
13780	3	F23 vs. L23	F2-3 up	GO:0009506	Metabolism	Transporters	plasmodesma	C	AT4G16143
13781	3	F23 vs. L23	F2-3 up	GO:0009506	Metabolism	Transporters	plasmodesma	C	AT4G17530
13782	3	F23 vs. L23	F2-3 up	GO:0009506	Metabolism	Transporters	plasmodesma	C	AT4G19410

13783	3	F23 vs. L23	F2-3 up	GO:0009506	Metabolism	Transporters	plasmodesma	C	AT4G26100
13784	3	F23 vs. L23	F2-3 up	GO:0009506	Metabolism	Transporters	plasmodesma	C	AT4G35060
13785	3	F23 vs. L23	F2-3 up	GO:0009506	Metabolism	Transporters	plasmodesma	C	AT4G38400
13786	3	F23 vs. L23	F2-3 up	GO:0009506	Metabolism	Transporters	plasmodesma	C	AT4G39110
13787	3	F23 vs. L23	F2-3 up	GO:0009506	Metabolism	Transporters	plasmodesma	C	AT4G39640
13788	3	F23 vs. L23	F2-3 up	GO:0009506	Metabolism	Transporters	plasmodesma	C	AT5G06320
13789	3	F23 vs. L23	F2-3 up	GO:0009506	Metabolism	Transporters	plasmodesma	C	AT5G06850
13790	3	F23 vs. L23	F2-3 up	GO:0009506	Metabolism	Transporters	plasmodesma	C	AT5G09810
13791	3	F23 vs. L23	F2-3 up	GO:0009506	Metabolism	Transporters	plasmodesma	C	AT5G13710
13792	3	F23 vs. L23	F2-3 up	GO:0009506	Metabolism	Transporters	plasmodesma	C	AT5G25980
13793	3	F23 vs. L23	F2-3 up	GO:0009506	Metabolism	Transporters	plasmodesma	C	AT5G45280
13794	3	F23 vs. L23	F2-3 up	GO:0009506	Metabolism	Transporters	plasmodesma	C	AT5G51890
13795	3	F23 vs. L23	F2-3 up	GO:0009506	Metabolism	Transporters	plasmodesma	C	AT5G57350
13796	3	F23 vs. L23	F2-3 up	GO:0009506	Metabolism	Transporters	plasmodesma	C	AT5G62680
13797	3	F23 vs. L23	F2-3 up	GO:0010497	Metabolism	Transporters	plasmodesmata-mediated intercellular transport	P	AT3G05930
13798	3	F23 vs. L23	F2-3 up	GO:0071805	Metabolism	Transporters	potassium ion transmembrane transport	P	AT4G33530
13799	3	F23 vs. L23	F2-3 up	GO:0015079	Metabolism	Transporters	potassium ion transmembrane transporter activity	F	AT4G33530
13800	3	F23 vs. L23	F2-3 up	GO:0006813	Metabolism	Transporters	potassium ion transport	P	AT3G17630
13801	3	F23 vs. L23	F2-3 up	GO:0006813	Metabolism	Transporters	potassium ion transport	P	AT4G33530
13802	3	F23 vs. L23	F2-3 up	GO:0006813	Metabolism	Transporters	potassium ion transport	P	AT5G01690
13803	3	F23 vs. L23	F2-3 up	GO:0006813	Metabolism	Transporters	potassium ion transport	P	AT5G17860
13804	3	F23 vs. L23	F2-3 up	GO:0015386	Metabolism	Transporters	potassium:proton antiporter activity	F	AT1G32450
13805	3	F23 vs. L23	F2-3 up	GO:0016560	Metabolism	Transporters	protein import into peroxisome matrix, docking	P	AT3G07560
13806	3	F23 vs. L23	F2-3 up	GO:0045039	Metabolism	Transporters	protein insertion into mitochondrial inner membrane	P	AT5G24650
13807	3	F23 vs. L23	F2-3 up	GO:0070973	Metabolism	Transporters	protein localization to endoplasmic reticulum exit site	P	AT3G03160
13808	3	F23 vs. L23	F2-3 up	GO:0072659	Metabolism	Transporters	protein localization to plasma membrane	P	AT1G03050
13809	3	F23 vs. L23	F2-3 up	GO:0008320	Metabolism	Transporters	protein transmembrane transporter activity	F	AT5G24650
13810	3	F23 vs. L23	F2-3 up	GO:0015031	Metabolism	Transporters	protein transport	P	AT1G03550
13811	3	F23 vs. L23	F2-3 up	GO:0015031	Metabolism	Transporters	protein transport	P	AT1G04760
13812	3	F23 vs. L23	F2-3 up	GO:0015031	Metabolism	Transporters	protein transport	P	AT1G13890
13813	3	F23 vs. L23	F2-3 up	GO:0015031	Metabolism	Transporters	protein transport	P	AT2G20840
13814	3	F23 vs. L23	F2-3 up	GO:0015031	Metabolism	Transporters	protein transport	P	AT4G36490
13815	3	F23 vs. L23	F2-3 up	GO:0015031	Metabolism	Transporters	protein transport	P	AT5G09550
13816	3	F23 vs. L23	F2-3 up	GO:0015031	Metabolism	Transporters	protein transport	P	AT5G13990
13817	3	F23 vs. L23	F2-3 up	GO:0015031	Metabolism	Transporters	protein transport	P	AT5G59730
13818	3	F23 vs. L23	F2-3 up	GO:0005345	Metabolism	Transporters	purine nucleobase transmembrane transporter activity	F	AT4G18220
13819	3	F23 vs. L23	F2-3 up	GO:0015211	Metabolism	Transporters	purine nucleoside transmembrane transporter activity	F	AT4G18220
13820	3	F23 vs. L23	F2-3 up	GO:0015865	Metabolism	Transporters	purine nucleotide transport	P	AT5G13490
13821	3	F23 vs. L23	F2-3 up	GO:0090481	Metabolism	Transporters	pyrimidine nucleotide-sugar transmembrane transport	P	AT3G59360
13822	3	F23 vs. L23	F2-3 up	GO:0015165	Metabolism	Transporters	pyrimidine nucleotide-sugar transmembrane transporter activity	F	AT3G59360
13823	3	F23 vs. L23	F2-3 up	GO:0050833	Metabolism	Transporters	pyruvate transmembrane transporter activity	F	AT4G05590
13824	3	F23 vs. L23	F2-3 up	GO:1901979	Metabolism	Transporters	regulation of inward rectifier potassium channel activity	P	AT2G31500
13825	3	F23 vs. L23	F2-3 up	GO:0034765	Metabolism	Transporters	regulation of ion transmembrane transport	P	AT5G40890
13826	3	F23 vs. L23	F2-3 up	GO:0010966	Metabolism	Transporters	regulation of phosphate transport	P	AT1G20823
13827	3	F23 vs. L23	F2-3 up	GO:0042306	Metabolism	Transporters	regulation of protein import into nucleus	P	AT5G41790
13828	3	F23 vs. L23	F2-3 up	GO:0010155	Metabolism	Transporters	regulation of proton transport	P	AT3G45780
13829	3	F23 vs. L23	F2-3 up	GO:0099503	Metabolism	Transporters	secretory vesicle	C	AT1G11840
13830	3	F23 vs. L23	F2-3 up	GO:0099503	Metabolism	Transporters	secretory vesicle	C	AT1G41830
13831	3	F23 vs. L23	F2-3 up	GO:0099503	Metabolism	Transporters	secretory vesicle	C	AT1G62290

13832	3	F23 vs. L23	F2-3 up	GO:0099503	Metabolism	Transporters	secretory vesicle	C	AT1G64190
13833	3	F23 vs. L23	F2-3 up	GO:0099503	Metabolism	Transporters	secretory vesicle	C	AT2G10940
13834	3	F23 vs. L23	F2-3 up	GO:0099503	Metabolism	Transporters	secretory vesicle	C	AT2G43570
13835	3	F23 vs. L23	F2-3 up	GO:0099503	Metabolism	Transporters	secretory vesicle	C	AT2G43590
13836	3	F23 vs. L23	F2-3 up	GO:0099503	Metabolism	Transporters	secretory vesicle	C	AT3G04720
13837	3	F23 vs. L23	F2-3 up	GO:0099503	Metabolism	Transporters	secretory vesicle	C	AT3G29360
13838	3	F23 vs. L23	F2-3 up	GO:0099503	Metabolism	Transporters	secretory vesicle	C	AT3G49110
13839	3	F23 vs. L23	F2-3 up	GO:0099503	Metabolism	Transporters	secretory vesicle	C	AT3G53980
13840	3	F23 vs. L23	F2-3 up	GO:0099503	Metabolism	Transporters	secretory vesicle	C	AT3G54960
13841	3	F23 vs. L23	F2-3 up	GO:0099503	Metabolism	Transporters	secretory vesicle	C	AT4G01610
13842	3	F23 vs. L23	F2-3 up	GO:0099503	Metabolism	Transporters	secretory vesicle	C	AT4G15210
13843	3	F23 vs. L23	F2-3 up	GO:0099503	Metabolism	Transporters	secretory vesicle	C	AT4G16660
13844	3	F23 vs. L23	F2-3 up	GO:0099503	Metabolism	Transporters	secretory vesicle	C	AT4G30530
13845	3	F23 vs. L23	F2-3 up	GO:0099503	Metabolism	Transporters	secretory vesicle	C	AT4G39730
13846	3	F23 vs. L23	F2-3 up	GO:0099503	Metabolism	Transporters	secretory vesicle	C	AT5G08370
13847	3	F23 vs. L23	F2-3 up	GO:0099503	Metabolism	Transporters	secretory vesicle	C	AT5G25980
13848	3	F23 vs. L23	F2-3 up	GO:0099503	Metabolism	Transporters	secretory vesicle	C	AT5G57560
13849	3	F23 vs. L23	F2-3 up	GO:0099503	Metabolism	Transporters	secretory vesicle	C	AT5G60360
13850	3	F23 vs. L23	F2-3 up	GO:0099503	Metabolism	Transporters	secretory vesicle	C	AT5G65760
13851	3	F23 vs. L23	F2-3 up	GO:0005484	Metabolism	Transporters	SNAP receptor activity	F	AT1G13890
13852	3	F23 vs. L23	F2-3 up	GO:0005484	Metabolism	Transporters	SNAP receptor activity	F	AT1G61290
13853	3	F23 vs. L23	F2-3 up	GO:0005484	Metabolism	Transporters	SNAP receptor activity	F	AT3G03800
13854	3	F23 vs. L23	F2-3 up	GO:0000149	Metabolism	Transporters	SNARE binding	F	AT1G03050
13855	3	F23 vs. L23	F2-3 up	GO:0000149	Metabolism	Transporters	SNARE binding	F	AT1G61290
13856	3	F23 vs. L23	F2-3 up	GO:0000149	Metabolism	Transporters	SNARE binding	F	AT3G03800
13857	3	F23 vs. L23	F2-3 up	GO:0031201	Metabolism	Transporters	SNARE complex	C	AT1G61290
13858	3	F23 vs. L23	F2-3 up	GO:0031201	Metabolism	Transporters	SNARE complex	C	AT3G03800
13859	3	F23 vs. L23	F2-3 up	GO:0006814	Metabolism	Transporters	sodium ion transport	P	AT5G17860
13860	3	F23 vs. L23	F2-3 up	GO:0015385	Metabolism	Transporters	sodium:proton antiporter activity	F	AT3G17630
13861	3	F23 vs. L23	F2-3 up	GO:0015385	Metabolism	Transporters	sodium:proton antiporter activity	F	AT5G01690
13862	3	F23 vs. L23	F2-3 up	GO:0015299	Metabolism	Transporters	solute:proton antiporter activity	F	AT3G17630
13863	3	F23 vs. L23	F2-3 up	GO:0071422	Metabolism	Transporters	succinate transmembrane transport	P	AT4G24570
13864	3	F23 vs. L23	F2-3 up	GO:0015141	Metabolism	Transporters	succinate transmembrane transporter activity	F	AT4G24570
13865	3	F23 vs. L23	F2-3 up	GO:0008515	Metabolism	Transporters	sucrose transmembrane transporter activity	F	AT1G22710
13866	3	F23 vs. L23	F2-3 up	GO:0008515	Metabolism	Transporters	sucrose transmembrane transporter activity	F	AT3G48740
13867	3	F23 vs. L23	F2-3 up	GO:0008515	Metabolism	Transporters	sucrose transmembrane transporter activity	F	AT5G50800
13868	3	F23 vs. L23	F2-3 up	GO:0015770	Metabolism	Transporters	sucrose transport	P	AT1G22710
13869	3	F23 vs. L23	F2-3 up	GO:0015770	Metabolism	Transporters	sucrose transport	P	AT3G48740
13870	3	F23 vs. L23	F2-3 up	GO:0015770	Metabolism	Transporters	sucrose transport	P	AT5G50800
13871	3	F23 vs. L23	F2-3 up	GO:0008506	Metabolism	Transporters	sucrose:proton symporter activity	F	AT1G22710
13872	3	F23 vs. L23	F2-3 up	GO:0051119	Metabolism	Transporters	sugar transmembrane transporter activity	F	AT3G14770
13873	3	F23 vs. L23	F2-3 up	GO:0051119	Metabolism	Transporters	sugar transmembrane transporter activity	F	AT3G48740
13874	3	F23 vs. L23	F2-3 up	GO:0051119	Metabolism	Transporters	sugar transmembrane transporter activity	F	AT5G50800
13875	3	F23 vs. L23	F2-3 up	GO:0000103	Metabolism	Transporters	sulfate assimilation	P	AT2G14750
13876	3	F23 vs. L23	F2-3 up	GO:0000103	Metabolism	Transporters	sulfate assimilation	P	AT3G22890
13877	3	F23 vs. L23	F2-3 up	GO:0000103	Metabolism	Transporters	sulfate assimilation	P	AT4G21990
13878	3	F23 vs. L23	F2-3 up	GO:0000103	Metabolism	Transporters	sulfate assimilation	P	AT4G39940
13879	3	F23 vs. L23	F2-3 up	GO:0015116	Metabolism	Transporters	sulfate transmembrane transporter activity	F	AT4G24570
13880	3	F23 vs. L23	F2-3 up	GO:0008272	Metabolism	Transporters	sulfate transport	P	AT4G24570
13881	3	F23 vs. L23	F2-3 up	GO:0015293	Metabolism	Transporters	symporter activity	F	AT1G12110
13882	3	F23 vs. L23	F2-3 up	GO:0015293	Metabolism	Transporters	symporter activity	F	AT1G24400
13883	3	F23 vs. L23	F2-3 up	GO:0015293	Metabolism	Transporters	symporter activity	F	AT1G32450
13884	3	F23 vs. L23	F2-3 up	GO:0015293	Metabolism	Transporters	symporter activity	F	AT3G21670
13885	3	F23 vs. L23	F2-3 up	GO:0015293	Metabolism	Transporters	symporter activity	F	AT3G54700
13886	3	F23 vs. L23	F2-3 up	GO:0015293	Metabolism	Transporters	symporter activity	F	AT5G09220
13887	3	F23 vs. L23	F2-3 up	GO:0015117	Metabolism	Transporters	thiosulfate transmembrane transporter activity	F	AT4G24570

13888	3	F23 vs. L23	F2-3 up	GO:0015709	Metabolism	Transporters	thiosulfate transport	P	AT4G24570
13889	3	F23 vs. L23	F2-3 up	GO:0005802	Metabolism	Transporters	trans-Golgi network	C	AT1G04430
13890	3	F23 vs. L23	F2-3 up	GO:0005802	Metabolism	Transporters	trans-Golgi network	C	AT1G13860
13891	3	F23 vs. L23	F2-3 up	GO:0005802	Metabolism	Transporters	trans-Golgi network	C	AT1G27930
13892	3	F23 vs. L23	F2-3 up	GO:0005802	Metabolism	Transporters	trans-Golgi network	C	AT1G31850
13893	3	F23 vs. L23	F2-3 up	GO:0005802	Metabolism	Transporters	trans-Golgi network	C	AT1G33170
13894	3	F23 vs. L23	F2-3 up	GO:0005802	Metabolism	Transporters	trans-Golgi network	C	AT1G74380
13895	3	F23 vs. L23	F2-3 up	GO:0005802	Metabolism	Transporters	trans-Golgi network	C	AT1G78240
13896	3	F23 vs. L23	F2-3 up	GO:0005802	Metabolism	Transporters	trans-Golgi network	C	AT2G03240
13897	3	F23 vs. L23	F2-3 up	GO:0005802	Metabolism	Transporters	trans-Golgi network	C	AT2G22900
13898	3	F23 vs. L23	F2-3 up	GO:0005802	Metabolism	Transporters	trans-Golgi network	C	AT2G33100
13899	3	F23 vs. L23	F2-3 up	GO:0005802	Metabolism	Transporters	trans-Golgi network	C	AT2G47650
13900	3	F23 vs. L23	F2-3 up	GO:0005802	Metabolism	Transporters	trans-Golgi network	C	AT3G21190
13901	3	F23 vs. L23	F2-3 up	GO:0005802	Metabolism	Transporters	trans-Golgi network	C	AT3G23300
13902	3	F23 vs. L23	F2-3 up	GO:0005802	Metabolism	Transporters	trans-Golgi network	C	AT3G23820
13903	3	F23 vs. L23	F2-3 up	GO:0005802	Metabolism	Transporters	trans-Golgi network	C	AT3G25140
13904	3	F23 vs. L23	F2-3 up	GO:0005802	Metabolism	Transporters	trans-Golgi network	C	AT3G27230
13905	3	F23 vs. L23	F2-3 up	GO:0005802	Metabolism	Transporters	trans-Golgi network	C	AT3G29060
13906	3	F23 vs. L23	F2-3 up	GO:0005802	Metabolism	Transporters	trans-Golgi network	C	AT3G51070
13907	3	F23 vs. L23	F2-3 up	GO:0005802	Metabolism	Transporters	trans-Golgi network	C	AT4G10440
13908	3	F23 vs. L23	F2-3 up	GO:0005802	Metabolism	Transporters	trans-Golgi network	C	AT4G19120
13909	3	F23 vs. L23	F2-3 up	GO:0005802	Metabolism	Transporters	trans-Golgi network	C	AT4G27720
13910	3	F23 vs. L23	F2-3 up	GO:0005802	Metabolism	Transporters	trans-Golgi network	C	AT4G30996
13911	3	F23 vs. L23	F2-3 up	GO:0005802	Metabolism	Transporters	trans-Golgi network	C	AT4G32410
13912	3	F23 vs. L23	F2-3 up	GO:0005802	Metabolism	Transporters	trans-Golgi network	C	AT4G39350
13913	3	F23 vs. L23	F2-3 up	GO:0005802	Metabolism	Transporters	trans-Golgi network	C	AT5G09870
13914	3	F23 vs. L23	F2-3 up	GO:0005802	Metabolism	Transporters	trans-Golgi network	C	AT5G16910
13915	3	F23 vs. L23	F2-3 up	GO:0005802	Metabolism	Transporters	trans-Golgi network	C	AT5G18280
13916	3	F23 vs. L23	F2-3 up	GO:0005802	Metabolism	Transporters	trans-Golgi network	C	AT5G25610
13917	3	F23 vs. L23	F2-3 up	GO:0005802	Metabolism	Transporters	trans-Golgi network	C	AT5G49720
13918	3	F23 vs. L23	F2-3 up	GO:0005802	Metabolism	Transporters	trans-Golgi network	C	AT5G64740
13919	3	F23 vs. L23	F2-3 up	GO:0005802	Metabolism	Transporters	trans-Golgi network	C	AT5G65810
13920	3	F23 vs. L23	F2-3 up	GO:0055085	Metabolism	Transporters	transmembrane transport	P	AT1G12110
13921	3	F23 vs. L23	F2-3 up	GO:0055085	Metabolism	Transporters	transmembrane transport	P	AT1G16370
13922	3	F23 vs. L23	F2-3 up	GO:0055085	Metabolism	Transporters	transmembrane transport	P	AT1G59870
13923	3	F23 vs. L23	F2-3 up	GO:0055085	Metabolism	Transporters	transmembrane transport	P	AT1G60030
13924	3	F23 vs. L23	F2-3 up	GO:0055085	Metabolism	Transporters	transmembrane transport	P	AT1G70610
13925	3	F23 vs. L23	F2-3 up	GO:0055085	Metabolism	Transporters	transmembrane transport	P	AT1G70940
13926	3	F23 vs. L23	F2-3 up	GO:0055085	Metabolism	Transporters	transmembrane transport	P	AT1G76670
13927	3	F23 vs. L23	F2-3 up	GO:0055085	Metabolism	Transporters	transmembrane transport	P	AT2G01320
13928	3	F23 vs. L23	F2-3 up	GO:0055085	Metabolism	Transporters	transmembrane transport	P	AT2G01420
13929	3	F23 vs. L23	F2-3 up	GO:0055085	Metabolism	Transporters	transmembrane transport	P	AT2G36830
13930	3	F23 vs. L23	F2-3 up	GO:0055085	Metabolism	Transporters	transmembrane transport	P	AT2G37180
13931	3	F23 vs. L23	F2-3 up	GO:0055085	Metabolism	Transporters	transmembrane transport	P	AT2G39210
13932	3	F23 vs. L23	F2-3 up	GO:0055085	Metabolism	Transporters	transmembrane transport	P	AT3G16340
13933	3	F23 vs. L23	F2-3 up	GO:0055085	Metabolism	Transporters	transmembrane transport	P	AT3G21670
13934	3	F23 vs. L23	F2-3 up	GO:0055085	Metabolism	Transporters	transmembrane transport	P	AT3G47960
13935	3	F23 vs. L23	F2-3 up	GO:0055085	Metabolism	Transporters	transmembrane transport	P	AT3G53420
13936	3	F23 vs. L23	F2-3 up	GO:0055085	Metabolism	Transporters	transmembrane transport	P	AT3G55100
13937	3	F23 vs. L23	F2-3 up	GO:0055085	Metabolism	Transporters	transmembrane transport	P	AT3G55640
13938	3	F23 vs. L23	F2-3 up	GO:0055085	Metabolism	Transporters	transmembrane transport	P	AT3G56950
13939	3	F23 vs. L23	F2-3 up	GO:0055085	Metabolism	Transporters	transmembrane transport	P	AT4G18050
13940	3	F23 vs. L23	F2-3 up	GO:0055085	Metabolism	Transporters	transmembrane transport	P	AT4G39030
13941	3	F23 vs. L23	F2-3 up	GO:0055085	Metabolism	Transporters	transmembrane transport	P	AT5G06530
13942	3	F23 vs. L23	F2-3 up	GO:0055085	Metabolism	Transporters	transmembrane transport	P	AT5G13490
13943	3	F23 vs. L23	F2-3 up	GO:0055085	Metabolism	Transporters	transmembrane transport	P	AT5G17860

13944	3	F23 vs. L23	F2-3 up	GO:0055085	Metabolism	Transporters	transmembrane transport	P	AT5G37810
13945	3	F23 vs. L23	F2-3 up	GO:0055085	Metabolism	Transporters	transmembrane transport	P	AT5G60740
13946	3	F23 vs. L23	F2-3 up	GO:0055085	Metabolism	Transporters	transmembrane transport	P	AT5G61700
13947	3	F23 vs. L23	F2-3 up	GO:0055085	Metabolism	Transporters	transmembrane transport	P	AT5G62680
13948	3	F23 vs. L23	F2-3 up	GO:0055085	Metabolism	Transporters	transmembrane transport	P	AT5G64280
13949	3	F23 vs. L23	F2-3 up	GO:0022857	Metabolism	Transporters	transmembrane transporter activity	F	AT1G16370
13950	3	F23 vs. L23	F2-3 up	GO:0022857	Metabolism	Transporters	transmembrane transporter activity	F	AT1G21070
13951	3	F23 vs. L23	F2-3 up	GO:0022857	Metabolism	Transporters	transmembrane transporter activity	F	AT1G47530
13952	3	F23 vs. L23	F2-3 up	GO:0022857	Metabolism	Transporters	transmembrane transporter activity	F	AT1G60030
13953	3	F23 vs. L23	F2-3 up	GO:0022857	Metabolism	Transporters	transmembrane transporter activity	F	AT1G61800
13954	3	F23 vs. L23	F2-3 up	GO:0022857	Metabolism	Transporters	transmembrane transporter activity	F	AT1G75500
13955	3	F23 vs. L23	F2-3 up	GO:0022857	Metabolism	Transporters	transmembrane transporter activity	F	AT1G76670
13956	3	F23 vs. L23	F2-3 up	GO:0022857	Metabolism	Transporters	transmembrane transporter activity	F	AT3G55640
13957	3	F23 vs. L23	F2-3 up	GO:0022857	Metabolism	Transporters	transmembrane transporter activity	F	AT5G40890
13958	3	F23 vs. L23	F2-3 up	GO:0022857	Metabolism	Transporters	transmembrane transporter activity	F	AT5G49130
13959	3	F23 vs. L23	F2-3 up	GO:0022857	Metabolism	Transporters	transmembrane transporter activity	F	AT5G52450
13960	3	F23 vs. L23	F2-3 up	GO:0022857	Metabolism	Transporters	transmembrane transporter activity	F	AT5G64280
13961	3	F23 vs. L23	F2-3 up	GO:0030133	Metabolism	Transporters	transport vesicle	C	AT1G61290
13962	3	F23 vs. L23	F2-3 up	GO:0030658	Metabolism	Transporters	transport vesicle membrane	C	AT1G03550
13963	3	F23 vs. L23	F2-3 up	GO:0030658	Metabolism	Transporters	transport vesicle membrane	C	AT2G20840
13964	3	F23 vs. L23	F2-3 up	GO:0005215	Metabolism	Transporters	transporter activity	F	AT1G60030
13965	3	F23 vs. L23	F2-3 up	GO:0005215	Metabolism	Transporters	transporter activity	F	AT1G67940
13966	3	F23 vs. L23	F2-3 up	GO:0005215	Metabolism	Transporters	transporter activity	F	AT1G70610
13967	3	F23 vs. L23	F2-3 up	GO:0005215	Metabolism	Transporters	transporter activity	F	AT2G40090
13968	3	F23 vs. L23	F2-3 up	GO:0005215	Metabolism	Transporters	transporter activity	F	AT4G04770
13969	3	F23 vs. L23	F2-3 up	GO:0005215	Metabolism	Transporters	transporter activity	F	AT5G02270
13970	3	F23 vs. L23	F2-3 up	GO:0005215	Metabolism	Transporters	transporter activity	F	AT5G44110
13971	3	F23 vs. L23	F2-3 up	GO:0005215	Metabolism	Transporters	transporter activity	F	AT5G60790
13972	3	F23 vs. L23	F2-3 up	GO:0005215	Metabolism	Transporters	transporter activity	F	AT5G61690
13973	3	F23 vs. L23	F2-3 up	GO:0005215	Metabolism	Transporters	transporter activity	F	AT5G61700
13974	3	F23 vs. L23	F2-3 up	GO:0005215	Metabolism	Transporters	transporter activity	F	AT5G64280
13975	3	F23 vs. L23	F2-3 up	GO:0005215	Metabolism	Transporters	transporter activity	F	AT5G64840
13976	3	F23 vs. L23	F2-3 up	GO:0071917	Metabolism	Transporters	triose-phosphate transmembrane transporter activity	F	AT1G61800
13977	3	F23 vs. L23	F2-3 up	GO:0072334	Metabolism	Transporters	UDP-galactose transmembrane transport	P	AT1G76670
13978	3	F23 vs. L23	F2-3 up	GO:0005459	Metabolism	Transporters	UDP-galactose transmembrane transporter activity	F	AT1G21070
13979	3	F23 vs. L23	F2-3 up	GO:0005459	Metabolism	Transporters	UDP-galactose transmembrane transporter activity	F	AT1G76670
13980	3	F23 vs. L23	F2-3 up	GO:0015786	Metabolism	Transporters	UDP-glucose transmembrane transport	P	AT1G76670
13981	3	F23 vs. L23	F2-3 up	GO:0005460	Metabolism	Transporters	UDP-glucose transmembrane transporter activity	F	AT1G76670
13982	3	F23 vs. L23	F2-3 up	GO:0015204	Metabolism	Transporters	urea transmembrane transporter activity	F	AT1G80760
13983	3	F23 vs. L23	F2-3 up	GO:0015204	Metabolism	Transporters	urea transmembrane transporter activity	F	AT2G36830
13984	3	F23 vs. L23	F2-3 up	GO:0015204	Metabolism	Transporters	urea transmembrane transporter activity	F	AT4G01470
13985	3	F23 vs. L23	F2-3 up	GO:0015840	Metabolism	Transporters	urea transport	P	AT2G36830
13986	3	F23 vs. L23	F2-3 up	GO:0015840	Metabolism	Transporters	urea transport	P	AT4G01470
13987	3	F23 vs. L23	F2-3 up	GO:0043182	Metabolism	Transporters	vacuolar sequestering of sodium ion	P	AT4G30996
13988	3	F23 vs. L23	F2-3 up	GO:0016192	Metabolism	Transporters	vesicle-mediated transport	P	AT1G04760
13989	3	F23 vs. L23	F2-3 up	GO:0016192	Metabolism	Transporters	vesicle-mediated transport	P	AT1G13890
13990	3	F23 vs. L23	F2-3 up	GO:0016192	Metabolism	Transporters	vesicle-mediated transport	P	AT2G15310
13991	3	F23 vs. L23	F2-3 up	GO:0016192	Metabolism	Transporters	vesicle-mediated transport	P	AT3G62290
13992	3	F23 vs. L23	F2-3 up	GO:0016192	Metabolism	Transporters	vesicle-mediated transport	P	AT4G17530
13993	3	F23 vs. L23	F2-3 up	GO:0016192	Metabolism	Transporters	vesicle-mediated transport	P	AT5G01430
13994	3	F23 vs. L23	F2-3 up	GO:0016192	Metabolism	Transporters	vesicle-mediated transport	P	AT5G09550
13995	3	F23 vs. L23	F2-3 up	GO:0005247	Metabolism	Transporters	voltage-gated chloride channel activity	F	AT5G40890
13996	3	F23 vs. L23	F2-3 up	GO:0015250	Metabolism	Transporters	water channel activity	F	AT1G80760
13997	3	F23 vs. L23	F2-3 up	GO:0015250	Metabolism	Transporters	water channel activity	F	AT2G36830
13998	3	F23 vs. L23	F2-3 up	GO:0015250	Metabolism	Transporters	water channel activity	F	AT2G37180

13999	3	F23 vs. L23	F2-3 up	GO:0015250	Metabolism	Transporters	water channel activity	F	AT3G26520
14000	3	F23 vs. L23	F2-3 up	GO:0015250	Metabolism	Transporters	water channel activity	F	AT3G53420
14001	3	F23 vs. L23	F2-3 up	GO:0015250	Metabolism	Transporters	water channel activity	F	AT4G01470
14002	3	F23 vs. L23	F2-3 up	GO:0015250	Metabolism	Transporters	water channel activity	F	AT5G37810
14003	3	F23 vs. L23	F2-3 up	GO:0006833	Metabolism	Transporters	water transport	P	AT2G36830
14004	3	F23 vs. L23	F2-3 up	GO:0006833	Metabolism	Transporters	water transport	P	AT2G37180
14005	3	F23 vs. L23	F2-3 up	GO:0006833	Metabolism	Transporters	water transport	P	AT3G53420
14006	3	F23 vs. L23	F2-3 up	GO:0006833	Metabolism	Transporters	water transport	P	AT4G01470
14007	3	F23 vs. L23	F2-3 up	GO:0042910	Metabolism	Transporters	xenobiotic transmembrane transporter activity	F	AT1G47530
14008	3	F23 vs. L23	F2-3 up	GO:0042910	Metabolism	Transporters	xenobiotic transmembrane transporter activity	F	AT4G39030
14009	3	F23 vs. L23	F2-3 up	GO:0042910	Metabolism	Transporters	xenobiotic transmembrane transporter activity	F	AT5G49130
14010	3	F23 vs. L23	F2-3 up	GO:0042910	Metabolism	Transporters	xenobiotic transmembrane transporter activity	F	AT5G52450
14011	3	F23 vs. L23	F2-3 up	GO:0015341	Metabolism	Transporters	zinc efflux active transmembrane transporter activity	F	AT1G16310
14012	3	F23 vs. L23	F2-3 up	GO:0022883	Metabolism	Transporters	zinc efflux transmembrane transporter activity	F	AT2G48020
14013	3	F23 vs. L23	F2-3 up	GO:0071577	Metabolism	Transporters	zinc ion transmembrane transport	P	AT1G31260
14014	3	F23 vs. L23	F2-3 up	GO:0005385	Metabolism	Transporters	zinc ion transmembrane transporter activity	F	AT1G31260
14015	3	F23 vs. L23	F2-3 up	GO:0006829	Metabolism	Transporters	zinc ion transport	P	AT2G48020
14016	3	F23 vs. L23	F2-3 up	GO:0051087	Regulation	Protein modification	chaperone binding	F	AT1G44160
14017	3	F23 vs. L23	F2-3 up	GO:0051087	Regulation	Protein modification	chaperone binding	F	AT3G47940
14018	3	F23 vs. L23	F2-3 up	GO:0061077	Regulation	Protein modification	chaperone-mediated protein folding	P	AT5G64350
14019	3	F23 vs. L23	F2-3 up	GO:0043686	Regulation	Protein modification	co-translational protein modification	P	AT5G14660
14020	3	F23 vs. L23	F2-3 up	GO:0005783	Regulation	Protein modification	endoplasmic reticulum	C	AT1G16410
14021	3	F23 vs. L23	F2-3 up	GO:0005783	Regulation	Protein modification	endoplasmic reticulum	C	AT1G27980
14022	3	F23 vs. L23	F2-3 up	GO:0005783	Regulation	Protein modification	endoplasmic reticulum	C	AT1G44170
14023	3	F23 vs. L23	F2-3 up	GO:0005783	Regulation	Protein modification	endoplasmic reticulum	C	AT1G44820
14024	3	F23 vs. L23	F2-3 up	GO:0005783	Regulation	Protein modification	endoplasmic reticulum	C	AT1G62380
14025	3	F23 vs. L23	F2-3 up	GO:0005783	Regulation	Protein modification	endoplasmic reticulum	C	AT1G68530
14026	3	F23 vs. L23	F2-3 up	GO:0005783	Regulation	Protein modification	endoplasmic reticulum	C	AT1G69640
14027	3	F23 vs. L23	F2-3 up	GO:0005783	Regulation	Protein modification	endoplasmic reticulum	C	AT1G70940
14028	3	F23 vs. L23	F2-3 up	GO:0005783	Regulation	Protein modification	endoplasmic reticulum	C	AT1G75020
14029	3	F23 vs. L23	F2-3 up	GO:0005783	Regulation	Protein modification	endoplasmic reticulum	C	AT1G76090
14030	3	F23 vs. L23	F2-3 up	GO:0005783	Regulation	Protein modification	endoplasmic reticulum	C	AT1G76490
14031	3	F23 vs. L23	F2-3 up	GO:0005783	Regulation	Protein modification	endoplasmic reticulum	C	AT2G01420
14032	3	F23 vs. L23	F2-3 up	GO:0005783	Regulation	Protein modification	endoplasmic reticulum	C	AT2G24180
14033	3	F23 vs. L23	F2-3 up	GO:0005783	Regulation	Protein modification	endoplasmic reticulum	C	AT2G29980
14034	3	F23 vs. L23	F2-3 up	GO:0005783	Regulation	Protein modification	endoplasmic reticulum	C	AT2G30490
14035	3	F23 vs. L23	F2-3 up	GO:0005783	Regulation	Protein modification	endoplasmic reticulum	C	AT2G31360
14036	3	F23 vs. L23	F2-3 up	GO:0005783	Regulation	Protein modification	endoplasmic reticulum	C	AT2G34460
14037	3	F23 vs. L23	F2-3 up	GO:0005783	Regulation	Protein modification	endoplasmic reticulum	C	AT2G35370
14038	3	F23 vs. L23	F2-3 up	GO:0005783	Regulation	Protein modification	endoplasmic reticulum	C	AT2G35840
14039	3	F23 vs. L23	F2-3 up	GO:0005783	Regulation	Protein modification	endoplasmic reticulum	C	AT2G36790
14040	3	F23 vs. L23	F2-3 up	GO:0005783	Regulation	Protein modification	endoplasmic reticulum	C	AT2G36880
14041	3	F23 vs. L23	F2-3 up	GO:0005783	Regulation	Protein modification	endoplasmic reticulum	C	AT2G37180
14042	3	F23 vs. L23	F2-3 up	GO:0005783	Regulation	Protein modification	endoplasmic reticulum	C	AT2G37760
14043	3	F23 vs. L23	F2-3 up	GO:0005783	Regulation	Protein modification	endoplasmic reticulum	C	AT2G37770
14044	3	F23 vs. L23	F2-3 up	GO:0005783	Regulation	Protein modification	endoplasmic reticulum	C	AT2G37970
14045	3	F23 vs. L23	F2-3 up	GO:0005783	Regulation	Protein modification	endoplasmic reticulum	C	AT2G38280
14046	3	F23 vs. L23	F2-3 up	GO:0005783	Regulation	Protein modification	endoplasmic reticulum	C	AT2G39420
14047	3	F23 vs. L23	F2-3 up	GO:0005783	Regulation	Protein modification	endoplasmic reticulum	C	AT2G40890
14048	3	F23 vs. L23	F2-3 up	GO:0005783	Regulation	Protein modification	endoplasmic reticulum	C	AT2G46210
14049	3	F23 vs. L23	F2-3 up	GO:0005783	Regulation	Protein modification	endoplasmic reticulum	C	AT3G03640
14050	3	F23 vs. L23	F2-3 up	GO:0005783	Regulation	Protein modification	endoplasmic reticulum	C	AT3G26520
14051	3	F23 vs. L23	F2-3 up	GO:0005783	Regulation	Protein modification	endoplasmic reticulum	C	AT3G52720
14052	3	F23 vs. L23	F2-3 up	GO:0005783	Regulation	Protein modification	endoplasmic reticulum	C	AT3G54960
14053	3	F23 vs. L23	F2-3 up	GO:0005783	Regulation	Protein modification	endoplasmic reticulum	C	AT3G56950
14054	3	F23 vs. L23	F2-3 up	GO:0005783	Regulation	Protein modification	endoplasmic reticulum	C	AT3G57010

14055	3	F23 vs. L23	F2-3 up	GO:0005783	Regulation	Protein modification	endoplasmic reticulum	C	AT3G57020
14056	3	F23 vs. L23	F2-3 up	GO:0005783	Regulation	Protein modification	endoplasmic reticulum	C	AT3G59530
14057	3	F23 vs. L23	F2-3 up	GO:0005783	Regulation	Protein modification	endoplasmic reticulum	C	AT3G61990
14058	3	F23 vs. L23	F2-3 up	GO:0005783	Regulation	Protein modification	endoplasmic reticulum	C	AT4G16660
14059	3	F23 vs. L23	F2-3 up	GO:0005783	Regulation	Protein modification	endoplasmic reticulum	C	AT4G22750
14060	3	F23 vs. L23	F2-3 up	GO:0005783	Regulation	Protein modification	endoplasmic reticulum	C	AT4G24510
14061	3	F23 vs. L23	F2-3 up	GO:0005783	Regulation	Protein modification	endoplasmic reticulum	C	AT4G27790
14062	3	F23 vs. L23	F2-3 up	GO:0005783	Regulation	Protein modification	endoplasmic reticulum	C	AT4G30996
14063	3	F23 vs. L23	F2-3 up	GO:0005783	Regulation	Protein modification	endoplasmic reticulum	C	AT4G31080
14064	3	F23 vs. L23	F2-3 up	GO:0005783	Regulation	Protein modification	endoplasmic reticulum	C	AT4G36250
14065	3	F23 vs. L23	F2-3 up	GO:0005783	Regulation	Protein modification	endoplasmic reticulum	C	AT4G37430
14066	3	F23 vs. L23	F2-3 up	GO:0005783	Regulation	Protein modification	endoplasmic reticulum	C	AT4G37760
14067	3	F23 vs. L23	F2-3 up	GO:0005783	Regulation	Protein modification	endoplasmic reticulum	C	AT4G39730
14068	3	F23 vs. L23	F2-3 up	GO:0005783	Regulation	Protein modification	endoplasmic reticulum	C	AT5G06850
14069	3	F23 vs. L23	F2-3 up	GO:0005783	Regulation	Protein modification	endoplasmic reticulum	C	AT5G13710
14070	3	F23 vs. L23	F2-3 up	GO:0005783	Regulation	Protein modification	endoplasmic reticulum	C	AT5G13930
14071	3	F23 vs. L23	F2-3 up	GO:0005783	Regulation	Protein modification	endoplasmic reticulum	C	AT5G24140
14072	3	F23 vs. L23	F2-3 up	GO:0005783	Regulation	Protein modification	endoplasmic reticulum	C	AT5G37300
14073	3	F23 vs. L23	F2-3 up	GO:0005783	Regulation	Protein modification	endoplasmic reticulum	C	AT5G40010
14074	3	F23 vs. L23	F2-3 up	GO:0034663	Regulation	Protein modification	endoplasmic reticulum chaperone complex	C	AT4G16660
14075	3	F23 vs. L23	F2-3 up	GO:0005789	Regulation	Protein modification	endoplasmic reticulum membrane	C	AT1G06090
14076	3	F23 vs. L23	F2-3 up	GO:0005789	Regulation	Protein modification	endoplasmic reticulum membrane	C	AT1G12240
14077	3	F23 vs. L23	F2-3 up	GO:0005789	Regulation	Protein modification	endoplasmic reticulum membrane	C	AT1G13860
14078	3	F23 vs. L23	F2-3 up	GO:0005789	Regulation	Protein modification	endoplasmic reticulum membrane	C	AT1G16410
14079	3	F23 vs. L23	F2-3 up	GO:0005789	Regulation	Protein modification	endoplasmic reticulum membrane	C	AT1G27980
14080	3	F23 vs. L23	F2-3 up	GO:0005789	Regulation	Protein modification	endoplasmic reticulum membrane	C	AT1G33170
14081	3	F23 vs. L23	F2-3 up	GO:0005789	Regulation	Protein modification	endoplasmic reticulum membrane	C	AT1G68530
14082	3	F23 vs. L23	F2-3 up	GO:0005789	Regulation	Protein modification	endoplasmic reticulum membrane	C	AT1G69640
14083	3	F23 vs. L23	F2-3 up	GO:0005789	Regulation	Protein modification	endoplasmic reticulum membrane	C	AT1G76490
14084	3	F23 vs. L23	F2-3 up	GO:0005789	Regulation	Protein modification	endoplasmic reticulum membrane	C	AT2G17370
14085	3	F23 vs. L23	F2-3 up	GO:0005789	Regulation	Protein modification	endoplasmic reticulum membrane	C	AT2G29980
14086	3	F23 vs. L23	F2-3 up	GO:0005789	Regulation	Protein modification	endoplasmic reticulum membrane	C	AT2G31360
14087	3	F23 vs. L23	F2-3 up	GO:0005789	Regulation	Protein modification	endoplasmic reticulum membrane	C	AT2G46210
14088	3	F23 vs. L23	F2-3 up	GO:0005789	Regulation	Protein modification	endoplasmic reticulum membrane	C	AT3G03160
14089	3	F23 vs. L23	F2-3 up	GO:0005789	Regulation	Protein modification	endoplasmic reticulum membrane	C	AT3G11900
14090	3	F23 vs. L23	F2-3 up	GO:0005789	Regulation	Protein modification	endoplasmic reticulum membrane	C	AT3G45100
14091	3	F23 vs. L23	F2-3 up	GO:0005789	Regulation	Protein modification	endoplasmic reticulum membrane	C	AT3G48520
14092	3	F23 vs. L23	F2-3 up	GO:0005789	Regulation	Protein modification	endoplasmic reticulum membrane	C	AT3G51070
14093	3	F23 vs. L23	F2-3 up	GO:0005789	Regulation	Protein modification	endoplasmic reticulum membrane	C	AT3G56950
14094	3	F23 vs. L23	F2-3 up	GO:0005789	Regulation	Protein modification	endoplasmic reticulum membrane	C	AT4G10440
14095	3	F23 vs. L23	F2-3 up	GO:0005789	Regulation	Protein modification	endoplasmic reticulum membrane	C	AT4G13770
14096	3	F23 vs. L23	F2-3 up	GO:0005789	Regulation	Protein modification	endoplasmic reticulum membrane	C	AT4G19120
14097	3	F23 vs. L23	F2-3 up	GO:0005789	Regulation	Protein modification	endoplasmic reticulum membrane	C	AT5G06850
14098	3	F23 vs. L23	F2-3 up	GO:0005789	Regulation	Protein modification	endoplasmic reticulum membrane	C	AT5G37300
14099	3	F23 vs. L23	F2-3 up	GO:0005789	Regulation	Protein modification	endoplasmic reticulum membrane	C	AT5G57800
14100	3	F23 vs. L23	F2-3 up	GO:0030234	Regulation	Protein modification	enzyme regulator activity	F	AT2G39730
14101	3	F23 vs. L23	F2-3 up	GO:0005794	Regulation	Protein modification	Golgi apparatus	C	AT1G03050
14102	3	F23 vs. L23	F2-3 up	GO:0005794	Regulation	Protein modification	Golgi apparatus	C	AT1G03230
14103	3	F23 vs. L23	F2-3 up	GO:0005794	Regulation	Protein modification	Golgi apparatus	C	AT1G03550
14104	3	F23 vs. L23	F2-3 up	GO:0005794	Regulation	Protein modification	Golgi apparatus	C	AT1G04430
14105	3	F23 vs. L23	F2-3 up	GO:0005794	Regulation	Protein modification	Golgi apparatus	C	AT1G05850
14106	3	F23 vs. L23	F2-3 up	GO:0005794	Regulation	Protein modification	Golgi apparatus	C	AT1G07850
14107	3	F23 vs. L23	F2-3 up	GO:0005794	Regulation	Protein modification	Golgi apparatus	C	AT1G09340
14108	3	F23 vs. L23	F2-3 up	GO:0005794	Regulation	Protein modification	Golgi apparatus	C	AT1G12840
14109	3	F23 vs. L23	F2-3 up	GO:0005794	Regulation	Protein modification	Golgi apparatus	C	AT1G13860
14110	3	F23 vs. L23	F2-3 up	GO:0005794	Regulation	Protein modification	Golgi apparatus	C	AT1G21070

14111	3	F23 vs. L23	F2-3 up	GO:0005794	Regulation	Protein modification	Golgi apparatus	C	AT1G27930
14112	3	F23 vs. L23	F2-3 up	GO:0005794	Regulation	Protein modification	Golgi apparatus	C	AT1G31850
14113	3	F23 vs. L23	F2-3 up	GO:0005794	Regulation	Protein modification	Golgi apparatus	C	AT1G33170
14114	3	F23 vs. L23	F2-3 up	GO:0005794	Regulation	Protein modification	Golgi apparatus	C	AT1G44170
14115	3	F23 vs. L23	F2-3 up	GO:0005794	Regulation	Protein modification	Golgi apparatus	C	AT1G61800
14116	3	F23 vs. L23	F2-3 up	GO:0005794	Regulation	Protein modification	Golgi apparatus	C	AT1G62380
14117	3	F23 vs. L23	F2-3 up	GO:0005794	Regulation	Protein modification	Golgi apparatus	C	AT1G69640
14118	3	F23 vs. L23	F2-3 up	GO:0005794	Regulation	Protein modification	Golgi apparatus	C	AT1G74380
14119	3	F23 vs. L23	F2-3 up	GO:0005794	Regulation	Protein modification	Golgi apparatus	C	AT1G76670
14120	3	F23 vs. L23	F2-3 up	GO:0005794	Regulation	Protein modification	Golgi apparatus	C	AT1G78240
14121	3	F23 vs. L23	F2-3 up	GO:0005794	Regulation	Protein modification	Golgi apparatus	C	AT2G03240
14122	3	F23 vs. L23	F2-3 up	GO:0005794	Regulation	Protein modification	Golgi apparatus	C	AT2G15310
14123	3	F23 vs. L23	F2-3 up	GO:0005794	Regulation	Protein modification	Golgi apparatus	C	AT2G20840
14124	3	F23 vs. L23	F2-3 up	GO:0005794	Regulation	Protein modification	Golgi apparatus	C	AT2G22900
14125	3	F23 vs. L23	F2-3 up	GO:0005794	Regulation	Protein modification	Golgi apparatus	C	AT2G24180
14126	3	F23 vs. L23	F2-3 up	GO:0005794	Regulation	Protein modification	Golgi apparatus	C	AT2G30010
14127	3	F23 vs. L23	F2-3 up	GO:0005794	Regulation	Protein modification	Golgi apparatus	C	AT2G30490
14128	3	F23 vs. L23	F2-3 up	GO:0005794	Regulation	Protein modification	Golgi apparatus	C	AT2G33100
14129	3	F23 vs. L23	F2-3 up	GO:0005794	Regulation	Protein modification	Golgi apparatus	C	AT2G34850
14130	3	F23 vs. L23	F2-3 up	GO:0005794	Regulation	Protein modification	Golgi apparatus	C	AT2G37970
14131	3	F23 vs. L23	F2-3 up	GO:0005794	Regulation	Protein modification	Golgi apparatus	C	AT2G39420
14132	3	F23 vs. L23	F2-3 up	GO:0005794	Regulation	Protein modification	Golgi apparatus	C	AT2G39730
14133	3	F23 vs. L23	F2-3 up	GO:0005794	Regulation	Protein modification	Golgi apparatus	C	AT2G47650
14134	3	F23 vs. L23	F2-3 up	GO:0005794	Regulation	Protein modification	Golgi apparatus	C	AT3G09440
14135	3	F23 vs. L23	F2-3 up	GO:0005794	Regulation	Protein modification	Golgi apparatus	C	AT3G21190
14136	3	F23 vs. L23	F2-3 up	GO:0005794	Regulation	Protein modification	Golgi apparatus	C	AT3G23300
14137	3	F23 vs. L23	F2-3 up	GO:0005794	Regulation	Protein modification	Golgi apparatus	C	AT3G23820
14138	3	F23 vs. L23	F2-3 up	GO:0005794	Regulation	Protein modification	Golgi apparatus	C	AT3G25140
14139	3	F23 vs. L23	F2-3 up	GO:0005794	Regulation	Protein modification	Golgi apparatus	C	AT3G26520
14140	3	F23 vs. L23	F2-3 up	GO:0005794	Regulation	Protein modification	Golgi apparatus	C	AT3G27230
14141	3	F23 vs. L23	F2-3 up	GO:0005794	Regulation	Protein modification	Golgi apparatus	C	AT3G28150
14142	3	F23 vs. L23	F2-3 up	GO:0005794	Regulation	Protein modification	Golgi apparatus	C	AT3G29060
14143	3	F23 vs. L23	F2-3 up	GO:0005794	Regulation	Protein modification	Golgi apparatus	C	AT3G44190
14144	3	F23 vs. L23	F2-3 up	GO:0005794	Regulation	Protein modification	Golgi apparatus	C	AT3G47960
14145	3	F23 vs. L23	F2-3 up	GO:0005794	Regulation	Protein modification	Golgi apparatus	C	AT3G57330
14146	3	F23 vs. L23	F2-3 up	GO:0005794	Regulation	Protein modification	Golgi apparatus	C	AT3G58730
14147	3	F23 vs. L23	F2-3 up	GO:0005794	Regulation	Protein modification	Golgi apparatus	C	AT3G59360
14148	3	F23 vs. L23	F2-3 up	GO:0005794	Regulation	Protein modification	Golgi apparatus	C	AT3G62290
14149	3	F23 vs. L23	F2-3 up	GO:0005794	Regulation	Protein modification	Golgi apparatus	C	AT4G10440
14150	3	F23 vs. L23	F2-3 up	GO:0005794	Regulation	Protein modification	Golgi apparatus	C	AT4G13990
14151	3	F23 vs. L23	F2-3 up	GO:0005794	Regulation	Protein modification	Golgi apparatus	C	AT4G16590
14152	3	F23 vs. L23	F2-3 up	GO:0005794	Regulation	Protein modification	Golgi apparatus	C	AT4G16660
14153	3	F23 vs. L23	F2-3 up	GO:0005794	Regulation	Protein modification	Golgi apparatus	C	AT4G17170
14154	3	F23 vs. L23	F2-3 up	GO:0005794	Regulation	Protein modification	Golgi apparatus	C	AT4G17530
14155	3	F23 vs. L23	F2-3 up	GO:0005794	Regulation	Protein modification	Golgi apparatus	C	AT4G19120
14156	3	F23 vs. L23	F2-3 up	GO:0005794	Regulation	Protein modification	Golgi apparatus	C	AT4G20460
14157	3	F23 vs. L23	F2-3 up	GO:0005794	Regulation	Protein modification	Golgi apparatus	C	AT4G22750
14158	3	F23 vs. L23	F2-3 up	GO:0005794	Regulation	Protein modification	Golgi apparatus	C	AT4G27720
14159	3	F23 vs. L23	F2-3 up	GO:0005794	Regulation	Protein modification	Golgi apparatus	C	AT4G30996
14160	3	F23 vs. L23	F2-3 up	GO:0005794	Regulation	Protein modification	Golgi apparatus	C	AT4G31590
14161	3	F23 vs. L23	F2-3 up	GO:0005794	Regulation	Protein modification	Golgi apparatus	C	AT4G32410
14162	3	F23 vs. L23	F2-3 up	GO:0005794	Regulation	Protein modification	Golgi apparatus	C	AT4G38510
14163	3	F23 vs. L23	F2-3 up	GO:0005794	Regulation	Protein modification	Golgi apparatus	C	AT4G38740
14164	3	F23 vs. L23	F2-3 up	GO:0005794	Regulation	Protein modification	Golgi apparatus	C	AT4G39350
14165	3	F23 vs. L23	F2-3 up	GO:0005794	Regulation	Protein modification	Golgi apparatus	C	AT5G06320
14166	3	F23 vs. L23	F2-3 up	GO:0005794	Regulation	Protein modification	Golgi apparatus	C	AT5G09870

14167	3	F23 vs. L23	F2-3 up	GO:0005794	Regulation	Protein modification	Golgi apparatus	C	AT5G15470
14168	3	F23 vs. L23	F2-3 up	GO:0005794	Regulation	Protein modification	Golgi apparatus	C	AT5G16910
14169	3	F23 vs. L23	F2-3 up	GO:0005794	Regulation	Protein modification	Golgi apparatus	C	AT5G18280
14170	3	F23 vs. L23	F2-3 up	GO:0005794	Regulation	Protein modification	Golgi apparatus	C	AT5G25610
14171	3	F23 vs. L23	F2-3 up	GO:0005794	Regulation	Protein modification	Golgi apparatus	C	AT5G47910
14172	3	F23 vs. L23	F2-3 up	GO:0005794	Regulation	Protein modification	Golgi apparatus	C	AT5G49720
14173	3	F23 vs. L23	F2-3 up	GO:0005794	Regulation	Protein modification	Golgi apparatus	C	AT5G57560
14174	3	F23 vs. L23	F2-3 up	GO:0005794	Regulation	Protein modification	Golgi apparatus	C	AT5G64740
14175	3	F23 vs. L23	F2-3 up	GO:0005794	Regulation	Protein modification	Golgi apparatus	C	AT5G65810
14176	3	F23 vs. L23	F2-3 up	GO:0005794	Regulation	Protein modification	Golgi apparatus	C	AT5G67210
14177	3	F23 vs. L23	F2-3 up	GO:0000139	Regulation	Protein modification	Golgi membrane	C	AT1G03550
14178	3	F23 vs. L23	F2-3 up	GO:0000139	Regulation	Protein modification	Golgi membrane	C	AT1G04430
14179	3	F23 vs. L23	F2-3 up	GO:0000139	Regulation	Protein modification	Golgi membrane	C	AT1G12240
14180	3	F23 vs. L23	F2-3 up	GO:0000139	Regulation	Protein modification	Golgi membrane	C	AT1G21070
14181	3	F23 vs. L23	F2-3 up	GO:0000139	Regulation	Protein modification	Golgi membrane	C	AT1G27930
14182	3	F23 vs. L23	F2-3 up	GO:0000139	Regulation	Protein modification	Golgi membrane	C	AT1G31850
14183	3	F23 vs. L23	F2-3 up	GO:0000139	Regulation	Protein modification	Golgi membrane	C	AT1G74380
14184	3	F23 vs. L23	F2-3 up	GO:0000139	Regulation	Protein modification	Golgi membrane	C	AT1G76670
14185	3	F23 vs. L23	F2-3 up	GO:0000139	Regulation	Protein modification	Golgi membrane	C	AT1G78240
14186	3	F23 vs. L23	F2-3 up	GO:0000139	Regulation	Protein modification	Golgi membrane	C	AT2G20840
14187	3	F23 vs. L23	F2-3 up	GO:0000139	Regulation	Protein modification	Golgi membrane	C	AT2G22900
14188	3	F23 vs. L23	F2-3 up	GO:0000139	Regulation	Protein modification	Golgi membrane	C	AT2G33100
14189	3	F23 vs. L23	F2-3 up	GO:0000139	Regulation	Protein modification	Golgi membrane	C	AT3G21190
14190	3	F23 vs. L23	F2-3 up	GO:0000139	Regulation	Protein modification	Golgi membrane	C	AT3G23300
14191	3	F23 vs. L23	F2-3 up	GO:0000139	Regulation	Protein modification	Golgi membrane	C	AT3G25140
14192	3	F23 vs. L23	F2-3 up	GO:0000139	Regulation	Protein modification	Golgi membrane	C	AT4G13990
14193	3	F23 vs. L23	F2-3 up	GO:0000139	Regulation	Protein modification	Golgi membrane	C	AT4G16590
14194	3	F23 vs. L23	F2-3 up	GO:0000139	Regulation	Protein modification	Golgi membrane	C	AT4G17530
14195	3	F23 vs. L23	F2-3 up	GO:0000139	Regulation	Protein modification	Golgi membrane	C	AT4G31590
14196	3	F23 vs. L23	F2-3 up	GO:0000139	Regulation	Protein modification	Golgi membrane	C	AT4G36490
14197	3	F23 vs. L23	F2-3 up	GO:0000139	Regulation	Protein modification	Golgi membrane	C	AT5G15470
14198	3	F23 vs. L23	F2-3 up	GO:0000139	Regulation	Protein modification	Golgi membrane	C	AT5G18280
14199	3	F23 vs. L23	F2-3 up	GO:0000139	Regulation	Protein modification	Golgi membrane	C	AT5G65810
14200	3	F23 vs. L23	F2-3 up	GO:0000139	Regulation	Protein modification	Golgi membrane	C	AT5G67210
14201	3	F23 vs. L23	F2-3 up	GO:0030173	Regulation	Protein modification	integral component of Golgi membrane	C	AT3G59360
14202	3	F23 vs. L23	F2-3 up	GO:0030173	Regulation	Protein modification	integral component of Golgi membrane	C	AT5G16910
14203	3	F23 vs. L23	F2-3 up	GO:0032259	Regulation	Protein modification	methylation	P	AT1G04430
14204	3	F23 vs. L23	F2-3 up	GO:0032259	Regulation	Protein modification	methylation	P	AT1G13860
14205	3	F23 vs. L23	F2-3 up	GO:0032259	Regulation	Protein modification	methylation	P	AT1G27930
14206	3	F23 vs. L23	F2-3 up	GO:0032259	Regulation	Protein modification	methylation	P	AT1G31850
14207	3	F23 vs. L23	F2-3 up	GO:0032259	Regulation	Protein modification	methylation	P	AT1G33170
14208	3	F23 vs. L23	F2-3 up	GO:0032259	Regulation	Protein modification	methylation	P	AT1G48600
14209	3	F23 vs. L23	F2-3 up	GO:0032259	Regulation	Protein modification	methylation	P	AT1G76090
14210	3	F23 vs. L23	F2-3 up	GO:0032259	Regulation	Protein modification	methylation	P	AT1G78240
14211	3	F23 vs. L23	F2-3 up	GO:0032259	Regulation	Protein modification	methylation	P	AT2G27590
14212	3	F23 vs. L23	F2-3 up	GO:0032259	Regulation	Protein modification	methylation	P	AT2G41380
14213	3	F23 vs. L23	F2-3 up	GO:0032259	Regulation	Protein modification	methylation	P	AT3G23300
14214	3	F23 vs. L23	F2-3 up	GO:0032259	Regulation	Protein modification	methylation	P	AT3G27230
14215	3	F23 vs. L23	F2-3 up	GO:0032259	Regulation	Protein modification	methylation	P	AT3G51070
14216	3	F23 vs. L23	F2-3 up	GO:0032259	Regulation	Protein modification	methylation	P	AT4G10440
14217	3	F23 vs. L23	F2-3 up	GO:0032259	Regulation	Protein modification	methylation	P	AT4G17080
14218	3	F23 vs. L23	F2-3 up	GO:0032259	Regulation	Protein modification	methylation	P	AT4G19120
14219	3	F23 vs. L23	F2-3 up	GO:0032259	Regulation	Protein modification	methylation	P	AT4G34050
14220	3	F23 vs. L23	F2-3 up	GO:0032259	Regulation	Protein modification	methylation	P	AT4G35150
14221	3	F23 vs. L23	F2-3 up	GO:0032259	Regulation	Protein modification	methylation	P	AT5G13710
14222	3	F23 vs. L23	F2-3 up	GO:0032259	Regulation	Protein modification	methylation	P	AT5G65810

14223	3	F23 vs. L23	F2-3 up	GO:0031365	Regulation	Protein modification	N-terminal protein amino acid modification	P	AT1G13270
14224	3	F23 vs. L23	F2-3 up	GO:0031365	Regulation	Protein modification	N-terminal protein amino acid modification	P	AT5G14660
14225	3	F23 vs. L23	F2-3 up	GO:0006499	Regulation	Protein modification	N-terminal protein myristoylation	P	AT2G15310
14226	3	F23 vs. L23	F2-3 up	GO:0006499	Regulation	Protein modification	N-terminal protein myristoylation	P	AT3G62290
14227	3	F23 vs. L23	F2-3 up	GO:0018119	Regulation	Protein modification	peptidyl-cysteine S-nitrosylation	P	AT1G09340
14228	3	F23 vs. L23	F2-3 up	GO:0018119	Regulation	Protein modification	peptidyl-cysteine S-nitrosylation	P	AT1G32060
14229	3	F23 vs. L23	F2-3 up	GO:0018119	Regulation	Protein modification	peptidyl-cysteine S-nitrosylation	P	AT3G12780
14230	3	F23 vs. L23	F2-3 up	GO:0018119	Regulation	Protein modification	peptidyl-cysteine S-nitrosylation	P	AT3G25760
14231	3	F23 vs. L23	F2-3 up	GO:0018119	Regulation	Protein modification	peptidyl-cysteine S-nitrosylation	P	AT3G26650
14232	3	F23 vs. L23	F2-3 up	GO:0018119	Regulation	Protein modification	peptidyl-cysteine S-nitrosylation	P	AT3G52880
14233	3	F23 vs. L23	F2-3 up	GO:0018119	Regulation	Protein modification	peptidyl-cysteine S-nitrosylation	P	AT4G20360
14234	3	F23 vs. L23	F2-3 up	GO:0018230	Regulation	Protein modification	peptidyl-L-cysteine S-palmitoylation	P	AT4G22750
14235	3	F23 vs. L23	F2-3 up	GO:0003755	Regulation	Protein modification	peptidyl-prolyl cis-trans isomerase activity	F	AT4G38740
14236	3	F23 vs. L23	F2-3 up	GO:0003755	Regulation	Protein modification	peptidyl-prolyl cis-trans isomerase activity	F	AT5G64350
14237	3	F23 vs. L23	F2-3 up	GO:0016540	Regulation	Protein modification	protein autoprocessing	P	AT5G08100
14238	3	F23 vs. L23	F2-3 up	GO:0036524	Regulation	Protein modification	protein deglycase activity	F	AT1G53280
14239	3	F23 vs. L23	F2-3 up	GO:0036529	Regulation	Protein modification	protein deglycation, glyoxal removal	P	AT1G53280
14240	3	F23 vs. L23	F2-3 up	GO:0016579	Regulation	Protein modification	protein deubiquitination	P	AT5G22030
14241	3	F23 vs. L23	F2-3 up	GO:0046983	Regulation	Protein modification	protein dimerization activity	F	AT1G09250
14242	3	F23 vs. L23	F2-3 up	GO:0046983	Regulation	Protein modification	protein dimerization activity	F	AT1G59640
14243	3	F23 vs. L23	F2-3 up	GO:0046983	Regulation	Protein modification	protein dimerization activity	F	AT1G61660
14244	3	F23 vs. L23	F2-3 up	GO:0046983	Regulation	Protein modification	protein dimerization activity	F	AT1G63650
14245	3	F23 vs. L23	F2-3 up	GO:0046983	Regulation	Protein modification	protein dimerization activity	F	AT1G77980
14246	3	F23 vs. L23	F2-3 up	GO:0046983	Regulation	Protein modification	protein dimerization activity	F	AT3G54340
14247	3	F23 vs. L23	F2-3 up	GO:0046983	Regulation	Protein modification	protein dimerization activity	F	AT4G00480
14248	3	F23 vs. L23	F2-3 up	GO:0046983	Regulation	Protein modification	protein dimerization activity	F	AT4G35150
14249	3	F23 vs. L23	F2-3 up	GO:0003756	Regulation	Protein modification	protein disulfide isomerase activity	F	AT1G75690
14250	3	F23 vs. L23	F2-3 up	GO:0003756	Regulation	Protein modification	protein disulfide isomerase activity	F	AT3G54960
14251	3	F23 vs. L23	F2-3 up	GO:0015035	Regulation	Protein modification	protein disulfide oxidoreductase activity	F	AT1G28480
14252	3	F23 vs. L23	F2-3 up	GO:0015035	Regulation	Protein modification	protein disulfide oxidoreductase activity	F	AT1G50320
14253	3	F23 vs. L23	F2-3 up	GO:0015035	Regulation	Protein modification	protein disulfide oxidoreductase activity	F	AT1G59730
14254	3	F23 vs. L23	F2-3 up	GO:0015035	Regulation	Protein modification	protein disulfide oxidoreductase activity	F	AT1G64500
14255	3	F23 vs. L23	F2-3 up	GO:0015035	Regulation	Protein modification	protein disulfide oxidoreductase activity	F	AT3G15360
14256	3	F23 vs. L23	F2-3 up	GO:0015035	Regulation	Protein modification	protein disulfide oxidoreductase activity	F	AT3G62960
14257	3	F23 vs. L23	F2-3 up	GO:0015035	Regulation	Protein modification	protein disulfide oxidoreductase activity	F	AT4G15700
14258	3	F23 vs. L23	F2-3 up	GO:0015035	Regulation	Protein modification	protein disulfide oxidoreductase activity	F	AT5G18600
14259	3	F23 vs. L23	F2-3 up	GO:0015035	Regulation	Protein modification	protein disulfide oxidoreductase activity	F	AT5G38900
14260	3	F23 vs. L23	F2-3 up	GO:0019904	Regulation	Protein modification	protein domain specific binding	F	AT1G26480
14261	3	F23 vs. L23	F2-3 up	GO:0019904	Regulation	Protein modification	protein domain specific binding	F	AT2G31380
14262	3	F23 vs. L23	F2-3 up	GO:0019904	Regulation	Protein modification	protein domain specific binding	F	AT2G46830
14263	3	F23 vs. L23	F2-3 up	GO:0006457	Regulation	Protein modification	protein folding	P	AT3G09440
14264	3	F23 vs. L23	F2-3 up	GO:0006457	Regulation	Protein modification	protein folding	P	AT3G54960
14265	3	F23 vs. L23	F2-3 up	GO:0010731	Regulation	Protein modification	protein glutathionylation	P	AT1G19550
14266	3	F23 vs. L23	F2-3 up	GO:0010731	Regulation	Protein modification	protein glutathionylation	P	AT5G02790
14267	3	F23 vs. L23	F2-3 up	GO:0006486	Regulation	Protein modification	protein glycosylation	P	AT3G02570
14268	3	F23 vs. L23	F2-3 up	GO:0006486	Regulation	Protein modification	protein glycosylation	P	AT4G13990
14269	3	F23 vs. L23	F2-3 up	GO:0006486	Regulation	Protein modification	protein glycosylation	P	AT5G04500
14270	3	F23 vs. L23	F2-3 up	GO:0006486	Regulation	Protein modification	protein glycosylation	P	AT5G58770
14271	3	F23 vs. L23	F2-3 up	GO:0046982	Regulation	Protein modification	protein heterodimerization activity	F	AT1G69120
14272	3	F23 vs. L23	F2-3 up	GO:0046982	Regulation	Protein modification	protein heterodimerization activity	F	AT2G13570
14273	3	F23 vs. L23	F2-3 up	GO:0046982	Regulation	Protein modification	protein heterodimerization activity	F	AT2G35940
14274	3	F23 vs. L23	F2-3 up	GO:0046982	Regulation	Protein modification	protein heterodimerization activity	F	AT5G01410
14275	3	F23 vs. L23	F2-3 up	GO:0046982	Regulation	Protein modification	protein heterodimerization activity	F	AT5G47670
14276	3	F23 vs. L23	F2-3 up	GO:0042803	Regulation	Protein modification	protein homodimerization activity	F	AT1G04400
14277	3	F23 vs. L23	F2-3 up	GO:0042803	Regulation	Protein modification	protein homodimerization activity	F	AT1G35720
14278	3	F23 vs. L23	F2-3 up	GO:0042803	Regulation	Protein modification	protein homodimerization activity	F	AT1G45474

14279	3	F23 vs. L23	F2-3 up	GO:0042803	Regulation	Protein modification	protein homodimerization activity	F	AT1G62360
14280	3	F23 vs. L23	F2-3 up	GO:0042803	Regulation	Protein modification	protein homodimerization activity	F	AT2G19570
14281	3	F23 vs. L23	F2-3 up	GO:0042803	Regulation	Protein modification	protein homodimerization activity	F	AT2G35940
14282	3	F23 vs. L23	F2-3 up	GO:0042803	Regulation	Protein modification	protein homodimerization activity	F	AT2G37630
14283	3	F23 vs. L23	F2-3 up	GO:0042803	Regulation	Protein modification	protein homodimerization activity	F	AT3G01680
14284	3	F23 vs. L23	F2-3 up	GO:0042803	Regulation	Protein modification	protein homodimerization activity	F	AT4G34710
14285	3	F23 vs. L23	F2-3 up	GO:0042803	Regulation	Protein modification	protein homodimerization activity	F	AT5G01410
14286	3	F23 vs. L23	F2-3 up	GO:0051260	Regulation	Protein modification	protein homooligomerization	P	AT3G48740
14287	3	F23 vs. L23	F2-3 up	GO:0051289	Regulation	Protein modification	protein homotetramerization	P	AT1G32640
14288	3	F23 vs. L23	F2-3 up	GO:0005956	Regulation	Protein modification	protein kinase CK2 complex	C	AT3G50000
14289	3	F23 vs. L23	F2-3 up	GO:0006479	Regulation	Protein modification	protein methylation	P	AT1G27930
14290	3	F23 vs. L23	F2-3 up	GO:0008276	Regulation	Protein modification	protein methyltransferase activity	F	AT3G61990
14291	3	F23 vs. L23	F2-3 up	GO:0000413	Regulation	Protein modification	protein peptidyl-prolyl isomerization	P	AT4G38740
14292	3	F23 vs. L23	F2-3 up	GO:0000413	Regulation	Protein modification	protein peptidyl-prolyl isomerization	P	AT5G64350
14293	3	F23 vs. L23	F2-3 up	GO:0043621	Regulation	Protein modification	protein self-association	F	AT3G01680
14294	3	F23 vs. L23	F2-3 up	GO:0043621	Regulation	Protein modification	protein self-association	F	AT5G24650
14295	3	F23 vs. L23	F2-3 up	GO:0050821	Regulation	Protein modification	protein stabilization	P	AT5G61670
14296	3	F23 vs. L23	F2-3 up	GO:0031386	Regulation	Protein modification	protein tag	F	AT4G02890
14297	3	F23 vs. L23	F2-3 up	GO:0006605	Regulation	Protein modification	protein targeting	P	AT2G45770
14298	3	F23 vs. L23	F2-3 up	GO:0006612	Regulation	Protein modification	protein targeting to membrane	P	AT4G22750
14299	3	F23 vs. L23	F2-3 up	GO:0019707	Regulation	Protein modification	protein-cysteine S-acyltransferase activity	F	AT4G22750
14300	3	F23 vs. L23	F2-3 up	GO:0019706	Regulation	Protein modification	protein-cysteine S-palmitoyltransferase activity	F	AT4G22750
14301	3	F23 vs. L23	F2-3 up	GO:0050790	Regulation	Protein modification	regulation of catalytic activity	P	AT4G01610
14302	3	F23 vs. L23	F2-3 up	GO:0043496	Regulation	Protein modification	regulation of protein homodimerization activity	P	AT2G46830
14303	3	F23 vs. L23	F2-3 up	GO:0006614	Regulation	Protein modification	SRP-dependent cotranslational protein targeting to membrane	P	AT2G45770
14304	3	F23 vs. L23	F2-3 up	GO:0018316	Regulation	Protein modification	peptide cross-linking via L-cystine	P	AT3G62410
14305	3	F23 vs. L23	F2-3 up	GO:0042586	Regulation	Protein modification	peptide deformylase activity	F	AT5G14660
14306	3	F23 vs. L23	F2-3 up	GO:0008113	Regulation	Protein modification	peptide-methionine (S)-S-oxide reductase activity	F	AT5G07460
14307	3	F23 vs. L23	F2-3 up	GO:0030835	Regulation	Regulation	negative regulation of actin filament depolymerization	P	AT4G38510
14308	3	F23 vs. L23	F2-3 up	GO:0043086	Regulation	Regulation	negative regulation of catalytic activity	P	AT2G10970
14309	3	F23 vs. L23	F2-3 up	GO:0043086	Regulation	Regulation	negative regulation of catalytic activity	P	AT2G47050
14310	3	F23 vs. L23	F2-3 up	GO:0043086	Regulation	Regulation	negative regulation of catalytic activity	P	AT3G15360
14311	3	F23 vs. L23	F2-3 up	GO:0043086	Regulation	Regulation	negative regulation of catalytic activity	P	AT5G50030
14312	3	F23 vs. L23	F2-3 up	GO:0043086	Regulation	Regulation	negative regulation of catalytic activity	P	AT5G62350
14313	3	F23 vs. L23	F2-3 up	GO:0043086	Regulation	Regulation	negative regulation of catalytic activity	P	AT5G62360
14314	3	F23 vs. L23	F2-3 up	GO:0009910	Regulation	Regulation	negative regulation of flower development	P	AT3G19980
14315	3	F23 vs. L23	F2-3 up	GO:0009910	Regulation	Regulation	negative regulation of flower development	P	AT5G56860
14316	3	F23 vs. L23	F2-3 up	GO:0045926	Regulation	Regulation	negative regulation of growth	P	AT3G16800
14317	3	F23 vs. L23	F2-3 up	GO:2000122	Regulation	Regulation	negative regulation of stomatal complex development	P	AT3G01500
14318	3	F23 vs. L23	F2-3 up	GO:0030836	Regulation	Regulation	positive regulation of actin filament depolymerization	P	AT2G01330
14319	3	F23 vs. L23	F2-3 up	GO:1904143	Regulation	Regulation	positive regulation of carotenoid biosynthetic process	P	AT5G61670
14320	3	F23 vs. L23	F2-3 up	GO:0045597	Regulation	Regulation	positive regulation of cell differentiation	P	AT4G32880
14321	3	F23 vs. L23	F2-3 up	GO:0045962	Regulation	Regulation	positive regulation of development, heterochronic	P	AT3G15030
14322	3	F23 vs. L23	F2-3 up	GO:0048639	Regulation	Regulation	positive regulation of developmental growth	P	AT5G65810
14323	3	F23 vs. L23	F2-3 up	GO:0009911	Regulation	Regulation	positive regulation of flower development	P	AT1G04400
14324	3	F23 vs. L23	F2-3 up	GO:0009911	Regulation	Regulation	positive regulation of flower development	P	AT5G06850
14325	3	F23 vs. L23	F2-3 up	GO:0009911	Regulation	Regulation	positive regulation of flower development	P	AT5G08370
14326	3	F23 vs. L23	F2-3 up	GO:0009911	Regulation	Regulation	positive regulation of flower development	P	AT5G60910
14327	3	F23 vs. L23	F2-3 up	GO:0080114	Regulation	Regulation	positive regulation of glycine hydroxymethyltransferase activity	P	AT5G04140
14328	3	F23 vs. L23	F2-3 up	GO:0045927	Regulation	Regulation	positive regulation of growth	P	AT3G05500

14329	3	F23 vs. L23	F2-3 up	GO:0031116	Regulation	Regulation	positive regulation of microtubule polymerization	P	AT2G18390
14330	3	F23 vs. L23	F2-3 up	GO:0010030	Regulation	Regulation	positive regulation of seed germination	P	AT1G62380
14331	3	F23 vs. L23	F2-3 up	GO:0010030	Regulation	Regulation	positive regulation of seed germination	P	AT3G22840
14332	3	F23 vs. L23	F2-3 up	GO:0010030	Regulation	Regulation	positive regulation of seed germination	P	AT3G48990
14333	3	F23 vs. L23	F2-3 up	GO:0010030	Regulation	Regulation	positive regulation of seed germination	P	AT4G14690
14334	3	F23 vs. L23	F2-3 up	GO:0010063	Regulation	Regulation	positive regulation of trichoblast fate specification	P	AT2G46410
14335	3	F23 vs. L23	F2-3 up	GO:0051512	Regulation	Regulation	positive regulation of unidimensional cell growth	P	AT5G65810
14336	3	F23 vs. L23	F2-3 up	GO:0008360	Regulation	Regulation	regulation of cell shape	P	AT2G13680
14337	3	F23 vs. L23	F2-3 up	GO:0045995	Regulation	Regulation	regulation of embryonic development	P	AT3G07050
14338	3	F23 vs. L23	F2-3 up	GO:0045604	Regulation	Regulation	regulation of epidermal cell differentiation	P	AT3G07050
14339	3	F23 vs. L23	F2-3 up	GO:0009909	Regulation	Regulation	regulation of flower development	P	AT1G04400
14340	3	F23 vs. L23	F2-3 up	GO:0009909	Regulation	Regulation	regulation of flower development	P	AT1G20823
14341	3	F23 vs. L23	F2-3 up	GO:0009909	Regulation	Regulation	regulation of flower development	P	AT2G45190
14342	3	F23 vs. L23	F2-3 up	GO:0009909	Regulation	Regulation	regulation of flower development	P	AT3G21890
14343	3	F23 vs. L23	F2-3 up	GO:0010439	Regulation	Regulation	regulation of glucosinolate biosynthetic process	P	AT2G14750
14344	3	F23 vs. L23	F2-3 up	GO:0010439	Regulation	Regulation	regulation of glucosinolate biosynthetic process	P	AT4G39940
14345	3	F23 vs. L23	F2-3 up	GO:0040008	Regulation	Regulation	regulation of growth	P	AT1G10370
14346	3	F23 vs. L23	F2-3 up	GO:0040008	Regulation	Regulation	regulation of growth	P	AT1G78370
14347	3	F23 vs. L23	F2-3 up	GO:0040008	Regulation	Regulation	regulation of growth	P	AT3G23050
14348	3	F23 vs. L23	F2-3 up	GO:0040008	Regulation	Regulation	regulation of growth	P	AT3G54950
14349	3	F23 vs. L23	F2-3 up	GO:0040008	Regulation	Regulation	regulation of growth	P	AT3G61880
14350	3	F23 vs. L23	F2-3 up	GO:0040008	Regulation	Regulation	regulation of growth	P	AT4G39730
14351	3	F23 vs. L23	F2-3 up	GO:0040008	Regulation	Regulation	regulation of growth	P	AT5G11790
14352	3	F23 vs. L23	F2-3 up	GO:2000024	Regulation	Regulation	regulation of leaf development	P	AT2G45190
14353	3	F23 vs. L23	F2-3 up	GO:2000024	Regulation	Regulation	regulation of leaf development	P	AT3G07050
14354	3	F23 vs. L23	F2-3 up	GO:2000025	Regulation	Regulation	regulation of leaf formation	P	AT2G42200
14355	3	F23 vs. L23	F2-3 up	GO:1901371	Regulation	Regulation	regulation of leaf morphogenesis	P	AT1G04400
14356	3	F23 vs. L23	F2-3 up	GO:1901371	Regulation	Regulation	regulation of leaf morphogenesis	P	AT2G32460
14357	3	F23 vs. L23	F2-3 up	GO:1901141	Regulation	Regulation	regulation of lignin biosynthetic process	P	AT3G16340
14358	3	F23 vs. L23	F2-3 up	GO:1905038	Regulation	Regulation	regulation of membrane lipid metabolic process	P	AT1G54280
14359	3	F23 vs. L23	F2-3 up	GO:0010075	Regulation	Regulation	regulation of meristem growth	P	AT1G04400
14360	3	F23 vs. L23	F2-3 up	GO:0009934	Regulation	Regulation	regulation of meristem structural organization	P	AT1G62360
14361	3	F23 vs. L23	F2-3 up	GO:0051171	Regulation	Regulation	regulation of nitrogen compound metabolic process	P	AT5G56860
14362	3	F23 vs. L23	F2-3 up	GO:0006808	Regulation	Regulation	regulation of nitrogen utilization	P	AT1G14920
14363	3	F23 vs. L23	F2-3 up	GO:0080092	Regulation	Regulation	regulation of pollen tube growth	P	AT1G28270
14364	3	F23 vs. L23	F2-3 up	GO:0080092	Regulation	Regulation	regulation of pollen tube growth	P	AT1G50610
14365	3	F23 vs. L23	F2-3 up	GO:0080092	Regulation	Regulation	regulation of pollen tube growth	P	AT1G77980
14366	3	F23 vs. L23	F2-3 up	GO:0080092	Regulation	Regulation	regulation of pollen tube growth	P	AT2G13680
14367	3	F23 vs. L23	F2-3 up	GO:0080092	Regulation	Regulation	regulation of pollen tube growth	P	AT2G31500
14368	3	F23 vs. L23	F2-3 up	GO:0080092	Regulation	Regulation	regulation of pollen tube growth	P	AT2G32460
14369	3	F23 vs. L23	F2-3 up	GO:0080092	Regulation	Regulation	regulation of pollen tube growth	P	AT2G41630
14370	3	F23 vs. L23	F2-3 up	GO:0080092	Regulation	Regulation	regulation of pollen tube growth	P	AT3G20190
14371	3	F23 vs. L23	F2-3 up	GO:0080092	Regulation	Regulation	regulation of pollen tube growth	P	AT4G39110
14372	3	F23 vs. L23	F2-3 up	GO:0080092	Regulation	Regulation	regulation of pollen tube growth	P	AT5G13990
14373	3	F23 vs. L23	F2-3 up	GO:2000214	Regulation	Regulation	regulation of proline metabolic process	P	AT1G61660
14374	3	F23 vs. L23	F2-3 up	GO:0010082	Regulation	Regulation	regulation of root meristem growth	P	AT1G70940
14375	3	F23 vs. L23	F2-3 up	GO:0010337	Regulation	Regulation	regulation of salicylic acid metabolic process	P	AT1G05850
14376	3	F23 vs. L23	F2-3 up	GO:2000652	Regulation	Regulation	regulation of secondary cell wall biogenesis	P	AT1G32640
14377	3	F23 vs. L23	F2-3 up	GO:2000652	Regulation	Regulation	regulation of secondary cell wall biogenesis	P	AT5G16600
14378	3	F23 vs. L23	F2-3 up	GO:2000032	Regulation	Regulation	regulation of secondary shoot formation	P	AT3G15030
14379	3	F23 vs. L23	F2-3 up	GO:0080050	Regulation	Regulation	regulation of seed development	P	AT5G56860
14380	3	F23 vs. L23	F2-3 up	GO:1902183	Regulation	Regulation	regulation of shoot apical meristem development	P	AT2G45190
14381	3	F23 vs. L23	F2-3 up	GO:0048831	Regulation	Regulation	regulation of shoot system development	P	AT1G72180
14382	3	F23 vs. L23	F2-3 up	GO:0048831	Regulation	Regulation	regulation of shoot system development	P	AT3G15030
14383	3	F23 vs. L23	F2-3 up	GO:0048831	Regulation	Regulation	regulation of shoot system development	P	AT4G36710

14384	3	F23 vs. L23	F2-3 up	GO:0090153	Regulation	Regulation	regulation of sphingolipid biosynthetic process	P	AT1G42470
14385	3	F23 vs. L23	F2-3 up	GO:2000038	Regulation	Regulation	regulation of stomatal complex development	P	AT3G45640
14386	3	F23 vs. L23	F2-3 up	GO:2000037	Regulation	Regulation	regulation of stomatal complex patterning	P	AT3G45640
14387	3	F23 vs. L23	F2-3 up	GO:0033613	Regulation	Transcription	activating transcription factor binding	F	AT5G47670
14388	3	F23 vs. L23	F2-3 up	GO:0045014	Regulation	Transcription	carbon catabolite repression of transcription by glucose	P	AT4G03190
14389	3	F23 vs. L23	F2-3 up	GO:0031490	Regulation	Transcription	chromatin DNA binding	F	AT1G11950
14390	3	F23 vs. L23	F2-3 up	GO:0006338	Regulation	Transcription	chromatin remodeling	P	AT1G04400
14391	3	F23 vs. L23	F2-3 up	GO:0006338	Regulation	Transcription	chromatin remodeling	P	AT1G30810
14392	3	F23 vs. L23	F2-3 up	GO:0006306	Regulation	Transcription	DNA methylation	P	AT3G17310
14393	3	F23 vs. L23	F2-3 up	GO:0001216	Regulation	Transcription	DNA-binding transcription activator activity	F	AT3G15030
14394	3	F23 vs. L23	F2-3 up	GO:0001228	Regulation	Transcription	DNA-binding transcription activator activity, RNA polymerase II-specific	F	AT1G61660
14395	3	F23 vs. L23	F2-3 up	GO:0003700	Regulation	Transcription	DNA-binding transcription factor activity	F	AT1G01250
14396	3	F23 vs. L23	F2-3 up	GO:0003700	Regulation	Transcription	DNA-binding transcription factor activity	F	AT1G01720
14397	3	F23 vs. L23	F2-3 up	GO:0003700	Regulation	Transcription	DNA-binding transcription factor activity	F	AT1G09250
14398	3	F23 vs. L23	F2-3 up	GO:0003700	Regulation	Transcription	DNA-binding transcription factor activity	F	AT1G14920
14399	3	F23 vs. L23	F2-3 up	GO:0003700	Regulation	Transcription	DNA-binding transcription factor activity	F	AT1G23380
14400	3	F23 vs. L23	F2-3 up	GO:0003700	Regulation	Transcription	DNA-binding transcription factor activity	F	AT1G26610
14401	3	F23 vs. L23	F2-3 up	GO:0003700	Regulation	Transcription	DNA-binding transcription factor activity	F	AT1G27730
14402	3	F23 vs. L23	F2-3 up	GO:0003700	Regulation	Transcription	DNA-binding transcription factor activity	F	AT1G30330
14403	3	F23 vs. L23	F2-3 up	GO:0003700	Regulation	Transcription	DNA-binding transcription factor activity	F	AT1G32640
14404	3	F23 vs. L23	F2-3 up	GO:0003700	Regulation	Transcription	DNA-binding transcription factor activity	F	AT1G35490
14405	3	F23 vs. L23	F2-3 up	GO:0003700	Regulation	Transcription	DNA-binding transcription factor activity	F	AT1G59640
14406	3	F23 vs. L23	F2-3 up	GO:0003700	Regulation	Transcription	DNA-binding transcription factor activity	F	AT1G61660
14407	3	F23 vs. L23	F2-3 up	GO:0003700	Regulation	Transcription	DNA-binding transcription factor activity	F	AT1G62360
14408	3	F23 vs. L23	F2-3 up	GO:0003700	Regulation	Transcription	DNA-binding transcription factor activity	F	AT1G63650
14409	3	F23 vs. L23	F2-3 up	GO:0003700	Regulation	Transcription	DNA-binding transcription factor activity	F	AT1G64800
14410	3	F23 vs. L23	F2-3 up	GO:0003700	Regulation	Transcription	DNA-binding transcription factor activity	F	AT1G64860
14411	3	F23 vs. L23	F2-3 up	GO:0003700	Regulation	Transcription	DNA-binding transcription factor activity	F	AT1G69120
14412	3	F23 vs. L23	F2-3 up	GO:0003700	Regulation	Transcription	DNA-binding transcription factor activity	F	AT1G77980
14413	3	F23 vs. L23	F2-3 up	GO:0003700	Regulation	Transcription	DNA-binding transcription factor activity	F	AT1G80840
14414	3	F23 vs. L23	F2-3 up	GO:0003700	Regulation	Transcription	DNA-binding transcription factor activity	F	AT2G13570
14415	3	F23 vs. L23	F2-3 up	GO:0003700	Regulation	Transcription	DNA-binding transcription factor activity	F	AT2G17950
14416	3	F23 vs. L23	F2-3 up	GO:0003700	Regulation	Transcription	DNA-binding transcription factor activity	F	AT2G23760
14417	3	F23 vs. L23	F2-3 up	GO:0003700	Regulation	Transcription	DNA-binding transcription factor activity	F	AT2G31380
14418	3	F23 vs. L23	F2-3 up	GO:0003700	Regulation	Transcription	DNA-binding transcription factor activity	F	AT2G32460
14419	3	F23 vs. L23	F2-3 up	GO:0003700	Regulation	Transcription	DNA-binding transcription factor activity	F	AT2G35940
14420	3	F23 vs. L23	F2-3 up	GO:0003700	Regulation	Transcription	DNA-binding transcription factor activity	F	AT2G37630
14421	3	F23 vs. L23	F2-3 up	GO:0003700	Regulation	Transcription	DNA-binding transcription factor activity	F	AT2G40140
14422	3	F23 vs. L23	F2-3 up	GO:0003700	Regulation	Transcription	DNA-binding transcription factor activity	F	AT2G42200
14423	3	F23 vs. L23	F2-3 up	GO:0003700	Regulation	Transcription	DNA-binding transcription factor activity	F	AT2G45190
14424	3	F23 vs. L23	F2-3 up	GO:0003700	Regulation	Transcription	DNA-binding transcription factor activity	F	AT2G46410
14425	3	F23 vs. L23	F2-3 up	GO:0003700	Regulation	Transcription	DNA-binding transcription factor activity	F	AT2G46830
14426	3	F23 vs. L23	F2-3 up	GO:0003700	Regulation	Transcription	DNA-binding transcription factor activity	F	AT3G06490
14427	3	F23 vs. L23	F2-3 up	GO:0003700	Regulation	Transcription	DNA-binding transcription factor activity	F	AT3G15030
14428	3	F23 vs. L23	F2-3 up	GO:0003700	Regulation	Transcription	DNA-binding transcription factor activity	F	AT3G15540
14429	3	F23 vs. L23	F2-3 up	GO:0003700	Regulation	Transcription	DNA-binding transcription factor activity	F	AT3G21890
14430	3	F23 vs. L23	F2-3 up	GO:0003700	Regulation	Transcription	DNA-binding transcription factor activity	F	AT3G23050
14431	3	F23 vs. L23	F2-3 up	GO:0003700	Regulation	Transcription	DNA-binding transcription factor activity	F	AT3G46600
14432	3	F23 vs. L23	F2-3 up	GO:0003700	Regulation	Transcription	DNA-binding transcription factor activity	F	AT3G54340
14433	3	F23 vs. L23	F2-3 up	GO:0003700	Regulation	Transcription	DNA-binding transcription factor activity	F	AT3G57600
14434	3	F23 vs. L23	F2-3 up	GO:0003700	Regulation	Transcription	DNA-binding transcription factor activity	F	AT4G00480
14435	3	F23 vs. L23	F2-3 up	GO:0003700	Regulation	Transcription	DNA-binding transcription factor activity	F	AT4G08150
14436	3	F23 vs. L23	F2-3 up	GO:0003700	Regulation	Transcription	DNA-binding transcription factor activity	F	AT4G17230
14437	3	F23 vs. L23	F2-3 up	GO:0003700	Regulation	Transcription	DNA-binding transcription factor activity	F	AT4G31800

14438	3	F23 vs. L23	F2-3 up	GO:0003700	Regulation	Transcription	DNA-binding transcription factor activity	F	AT4G32800
14439	3	F23 vs. L23	F2-3 up	GO:0003700	Regulation	Transcription	DNA-binding transcription factor activity	F	AT4G32880
14440	3	F23 vs. L23	F2-3 up	GO:0003700	Regulation	Transcription	DNA-binding transcription factor activity	F	AT4G36710
14441	3	F23 vs. L23	F2-3 up	GO:0003700	Regulation	Transcription	DNA-binding transcription factor activity	F	AT4G37180
14442	3	F23 vs. L23	F2-3 up	GO:0003700	Regulation	Transcription	DNA-binding transcription factor activity	F	AT5G16600
14443	3	F23 vs. L23	F2-3 up	GO:0003700	Regulation	Transcription	DNA-binding transcription factor activity	F	AT5G20240
14444	3	F23 vs. L23	F2-3 up	GO:0003700	Regulation	Transcription	DNA-binding transcription factor activity	F	AT5G24120
14445	3	F23 vs. L23	F2-3 up	GO:0003700	Regulation	Transcription	DNA-binding transcription factor activity	F	AT5G47670
14446	3	F23 vs. L23	F2-3 up	GO:0003700	Regulation	Transcription	DNA-binding transcription factor activity	F	AT5G56860
14447	3	F23 vs. L23	F2-3 up	GO:0003700	Regulation	Transcription	DNA-binding transcription factor activity	F	AT5G60450
14448	3	F23 vs. L23	F2-3 up	GO:0003700	Regulation	Transcription	DNA-binding transcription factor activity	F	AT5G60910
14449	3	F23 vs. L23	F2-3 up	GO:0003700	Regulation	Transcription	DNA-binding transcription factor activity	F	AT5G62020
14450	3	F23 vs. L23	F2-3 up	GO:0003700	Regulation	Transcription	DNA-binding transcription factor activity	F	AT5G62570
14451	3	F23 vs. L23	F2-3 up	GO:0003700	Regulation	Transcription	DNA-binding transcription factor activity	F	AT5G67300
14452	3	F23 vs. L23	F2-3 up	GO:0006352	Regulation	Transcription	DNA-templated transcription, initiation	P	AT1G64860
14453	3	F23 vs. L23	F2-3 up	GO:0006352	Regulation	Transcription	DNA-templated transcription, initiation	P	AT2G41630
14454	3	F23 vs. L23	F2-3 up	GO:0006352	Regulation	Transcription	DNA-templated transcription, initiation	P	AT5G24120
14455	3	F23 vs. L23	F2-3 up	GO:0016458	Regulation	Transcription	gene silencing	P	AT3G02470
14456	3	F23 vs. L23	F2-3 up	GO:0035195	Regulation	Transcription	gene silencing by miRNA	P	AT1G08830
14457	3	F23 vs. L23	F2-3 up	GO:0035195	Regulation	Transcription	gene silencing by miRNA	P	AT2G28190
14458	3	F23 vs. L23	F2-3 up	GO:0008883	Regulation	Transcription	glutamyl-tRNA reductase activity	F	AT1G58290
14459	3	F23 vs. L23	F2-3 up	GO:0004402	Regulation	Transcription	histone acetyltransferase activity	F	AT5G67480
14460	3	F23 vs. L23	F2-3 up	GO:0032452	Regulation	Transcription	histone demethylase activity	F	AT1G30810
14461	3	F23 vs. L23	F2-3 up	GO:0032453	Regulation	Transcription	histone demethylase activity (H3-K4 specific)	F	AT1G30810
14462	3	F23 vs. L23	F2-3 up	GO:0032454	Regulation	Transcription	histone demethylase activity (H3-K9 specific)	F	AT1G11950
14463	3	F23 vs. L23	F2-3 up	GO:0034647	Regulation	Transcription	histone demethylase activity (H3-trimethyl-K4 specific)	F	AT1G30810
14464	3	F23 vs. L23	F2-3 up	GO:0043970	Regulation	Transcription	histone H3-K9 acetylation	P	AT5G09230
14465	3	F23 vs. L23	F2-3 up	GO:0033169	Regulation	Transcription	histone H3-K9 demethylation	P	AT1G11950
14466	3	F23 vs. L23	F2-3 up	GO:0035097	Regulation	Transcription	histone methyltransferase complex	C	AT1G30810
14467	3	F23 vs. L23	F2-3 up	GO:0006397	Regulation	Transcription	mRNA processing	P	AT1G17370
14468	3	F23 vs. L23	F2-3 up	GO:0006397	Regulation	Transcription	mRNA processing	P	AT1G22760
14469	3	F23 vs. L23	F2-3 up	GO:0010629	Regulation	Transcription	negative regulation of gene expression	P	AT4G37180
14470	3	F23 vs. L23	F2-3 up	GO:0000122	Regulation	Transcription	negative regulation of transcription by RNA polymerase II	P	AT1G28480
14471	3	F23 vs. L23	F2-3 up	GO:0000122	Regulation	Transcription	negative regulation of transcription by RNA polymerase II	P	AT5G18600
14472	3	F23 vs. L23	F2-3 up	GO:0045892	Regulation	Transcription	negative regulation of transcription, DNA-templated	P	AT1G27730
14473	3	F23 vs. L23	F2-3 up	GO:0045892	Regulation	Transcription	negative regulation of transcription, DNA-templated	P	AT1G80840
14474	3	F23 vs. L23	F2-3 up	GO:0045892	Regulation	Transcription	negative regulation of transcription, DNA-templated	P	AT2G37630
14475	3	F23 vs. L23	F2-3 up	GO:0045892	Regulation	Transcription	negative regulation of transcription, DNA-templated	P	AT2G46830
14476	3	F23 vs. L23	F2-3 up	GO:0045892	Regulation	Transcription	negative regulation of transcription, DNA-templated	P	AT3G07050
14477	3	F23 vs. L23	F2-3 up	GO:0000289	Regulation	Transcription	nuclear-transcribed mRNA poly(A) tail shortening	P	AT1G71770
14478	3	F23 vs. L23	F2-3 up	GO:0000289	Regulation	Transcription	nuclear-transcribed mRNA poly(A) tail shortening	P	AT5G22250
14479	3	F23 vs. L23	F2-3 up	GO:0005730	Regulation	Transcription	nucleolus	C	AT2G37630
14480	3	F23 vs. L23	F2-3 up	GO:0005730	Regulation	Transcription	nucleolus	C	AT2G44120
14481	3	F23 vs. L23	F2-3 up	GO:0005730	Regulation	Transcription	nucleolus	C	AT3G07050
14482	3	F23 vs. L23	F2-3 up	GO:0005730	Regulation	Transcription	nucleolus	C	AT3G17390
14483	3	F23 vs. L23	F2-3 up	GO:0005730	Regulation	Transcription	nucleolus	C	AT3G50000
14484	3	F23 vs. L23	F2-3 up	GO:0005730	Regulation	Transcription	nucleolus	C	AT4G01850
14485	3	F23 vs. L23	F2-3 up	GO:0005730	Regulation	Transcription	nucleolus	C	AT4G16143
14486	3	F23 vs. L23	F2-3 up	GO:0005730	Regulation	Transcription	nucleolus	C	AT4G20360
14487	3	F23 vs. L23	F2-3 up	GO:0005730	Regulation	Transcription	nucleolus	C	AT5G09810
14488	3	F23 vs. L23	F2-3 up	GO:0005730	Regulation	Transcription	nucleolus	C	AT5G50850
14489	3	F23 vs. L23	F2-3 up	GO:0005730	Regulation	Transcription	nucleolus	C	AT5G67470
14490	3	F23 vs. L23	F2-3 up	GO:0000932	Regulation	Transcription	P-body	C	AT5G22250

14491	3	F23 vs. L23	F2-3 up	GO:0070816	Regulation	Transcription	phosphorylation of RNA polymerase II C-terminal domain	P	AT1G09600
14492	3	F23 vs. L23	F2-3 up	GO:0000427	Regulation	Transcription	plastid-encoded plastid RNA polymerase complex	C	AT1G09340
14493	3	F23 vs. L23	F2-3 up	GO:0008143	Regulation	Transcription	poly(A) binding	F	AT1G22760
14494	3	F23 vs. L23	F2-3 up	GO:0008143	Regulation	Transcription	poly(A) binding	F	AT1G71770
14495	3	F23 vs. L23	F2-3 up	GO:1903508	Regulation	Transcription	positive regulation of nucleic acid-templated transcription	P	AT1G14920
14496	3	F23 vs. L23	F2-3 up	GO:0045944	Regulation	Transcription	positive regulation of transcription by RNA polymerase II	P	AT1G69120
14497	3	F23 vs. L23	F2-3 up	GO:0045944	Regulation	Transcription	positive regulation of transcription by RNA polymerase II	P	AT1G77980
14498	3	F23 vs. L23	F2-3 up	GO:0045944	Regulation	Transcription	positive regulation of transcription by RNA polymerase II	P	AT3G54340
14499	3	F23 vs. L23	F2-3 up	GO:0045944	Regulation	Transcription	positive regulation of transcription by RNA polymerase II	P	AT5G20240
14500	3	F23 vs. L23	F2-3 up	GO:0045944	Regulation	Transcription	positive regulation of transcription by RNA polymerase II	P	AT5G60910
14501	3	F23 vs. L23	F2-3 up	GO:0032968	Regulation	Transcription	positive regulation of transcription elongation from RNA polymerase II promoter	P	AT1G09600
14502	3	F23 vs. L23	F2-3 up	GO:0061408	Regulation	Transcription	positive regulation of transcription from RNA polymerase II promoter in response to heat stress	P	AT5G62020
14503	3	F23 vs. L23	F2-3 up	GO:0045893	Regulation	Transcription	positive regulation of transcription, DNA-templated	P	AT1G09340
14504	3	F23 vs. L23	F2-3 up	GO:0045893	Regulation	Transcription	positive regulation of transcription, DNA-templated	P	AT1G32640
14505	3	F23 vs. L23	F2-3 up	GO:0045893	Regulation	Transcription	positive regulation of transcription, DNA-templated	P	AT1G61660
14506	3	F23 vs. L23	F2-3 up	GO:0045893	Regulation	Transcription	positive regulation of transcription, DNA-templated	P	AT1G69120
14507	3	F23 vs. L23	F2-3 up	GO:0045893	Regulation	Transcription	positive regulation of transcription, DNA-templated	P	AT2G13570
14508	3	F23 vs. L23	F2-3 up	GO:0045893	Regulation	Transcription	positive regulation of transcription, DNA-templated	P	AT2G32460
14509	3	F23 vs. L23	F2-3 up	GO:0045893	Regulation	Transcription	positive regulation of transcription, DNA-templated	P	AT2G46830
14510	3	F23 vs. L23	F2-3 up	GO:0045893	Regulation	Transcription	positive regulation of transcription, DNA-templated	P	AT3G57600
14511	3	F23 vs. L23	F2-3 up	GO:0045893	Regulation	Transcription	positive regulation of transcription, DNA-templated	P	AT3G63140
14512	3	F23 vs. L23	F2-3 up	GO:0045893	Regulation	Transcription	positive regulation of transcription, DNA-templated	P	AT5G24120
14513	3	F23 vs. L23	F2-3 up	GO:0045893	Regulation	Transcription	positive regulation of transcription, DNA-templated	P	AT5G47670
14514	3	F23 vs. L23	F2-3 up	GO:0051090	Regulation	Transcription	regulation of DNA-binding transcription factor activity	P	AT1G32640
14515	3	F23 vs. L23	F2-3 up	GO:0010468	Regulation	Transcription	regulation of gene expression	P	AT1G09340
14516	3	F23 vs. L23	F2-3 up	GO:0010468	Regulation	Transcription	regulation of gene expression	P	AT1G18150
14517	3	F23 vs. L23	F2-3 up	GO:0010468	Regulation	Transcription	regulation of gene expression	P	AT2G32460
14518	3	F23 vs. L23	F2-3 up	GO:0010468	Regulation	Transcription	regulation of gene expression	P	AT2G46830
14519	3	F23 vs. L23	F2-3 up	GO:0010468	Regulation	Transcription	regulation of gene expression	P	AT3G45640
14520	3	F23 vs. L23	F2-3 up	GO:0010468	Regulation	Transcription	regulation of gene expression	P	AT4G28490
14521	3	F23 vs. L23	F2-3 up	GO:0010468	Regulation	Transcription	regulation of gene expression	P	AT5G56860
14522	3	F23 vs. L23	F2-3 up	GO:2001141	Regulation	Transcription	regulation of RNA biosynthetic process	P	AT1G64860
14523	3	F23 vs. L23	F2-3 up	GO:2001141	Regulation	Transcription	regulation of RNA biosynthetic process	P	AT5G24120
14524	3	F23 vs. L23	F2-3 up	GO:0043619	Regulation	Transcription	regulation of transcription from RNA polymerase II promoter in response to oxidative stress	P	AT1G32640
14525	3	F23 vs. L23	F2-3 up	GO:0006355	Regulation	Transcription	regulation of transcription, DNA-templated	P	AT1G01250
14526	3	F23 vs. L23	F2-3 up	GO:0006355	Regulation	Transcription	regulation of transcription, DNA-templated	P	AT1G01720
14527	3	F23 vs. L23	F2-3 up	GO:0006355	Regulation	Transcription	regulation of transcription, DNA-templated	P	AT1G23380
14528	3	F23 vs. L23	F2-3 up	GO:0006355	Regulation	Transcription	regulation of transcription, DNA-templated	P	AT1G26610
14529	3	F23 vs. L23	F2-3 up	GO:0006355	Regulation	Transcription	regulation of transcription, DNA-templated	P	AT1G30330
14530	3	F23 vs. L23	F2-3 up	GO:0006355	Regulation	Transcription	regulation of transcription, DNA-templated	P	AT1G30810
14531	3	F23 vs. L23	F2-3 up	GO:0006355	Regulation	Transcription	regulation of transcription, DNA-templated	P	AT1G59640
14532	3	F23 vs. L23	F2-3 up	GO:0006355	Regulation	Transcription	regulation of transcription, DNA-templated	P	AT1G62360
14533	3	F23 vs. L23	F2-3 up	GO:0006355	Regulation	Transcription	regulation of transcription, DNA-templated	P	AT1G63650
14534	3	F23 vs. L23	F2-3 up	GO:0006355	Regulation	Transcription	regulation of transcription, DNA-templated	P	AT1G64800
14535	3	F23 vs. L23	F2-3 up	GO:0006355	Regulation	Transcription	regulation of transcription, DNA-templated	P	AT1G64860

14536	3	F23 vs. L23	F2-3 up	GO:0006355	Regulation	Transcription	regulation of transcription, DNA-templated	P	AT1G77980
14537	3	F23 vs. L23	F2-3 up	GO:0006355	Regulation	Transcription	regulation of transcription, DNA-templated	P	AT2G17950
14538	3	F23 vs. L23	F2-3 up	GO:0006355	Regulation	Transcription	regulation of transcription, DNA-templated	P	AT2G23760
14539	3	F23 vs. L23	F2-3 up	GO:0006355	Regulation	Transcription	regulation of transcription, DNA-templated	P	AT2G31380
14540	3	F23 vs. L23	F2-3 up	GO:0006355	Regulation	Transcription	regulation of transcription, DNA-templated	P	AT2G35940
14541	3	F23 vs. L23	F2-3 up	GO:0006355	Regulation	Transcription	regulation of transcription, DNA-templated	P	AT2G40140
14542	3	F23 vs. L23	F2-3 up	GO:0006355	Regulation	Transcription	regulation of transcription, DNA-templated	P	AT2G40670
14543	3	F23 vs. L23	F2-3 up	GO:0006355	Regulation	Transcription	regulation of transcription, DNA-templated	P	AT2G41630
14544	3	F23 vs. L23	F2-3 up	GO:0006355	Regulation	Transcription	regulation of transcription, DNA-templated	P	AT2G42200
14545	3	F23 vs. L23	F2-3 up	GO:0006355	Regulation	Transcription	regulation of transcription, DNA-templated	P	AT3G15030
14546	3	F23 vs. L23	F2-3 up	GO:0006355	Regulation	Transcription	regulation of transcription, DNA-templated	P	AT3G15540
14547	3	F23 vs. L23	F2-3 up	GO:0006355	Regulation	Transcription	regulation of transcription, DNA-templated	P	AT3G21890
14548	3	F23 vs. L23	F2-3 up	GO:0006355	Regulation	Transcription	regulation of transcription, DNA-templated	P	AT3G23050
14549	3	F23 vs. L23	F2-3 up	GO:0006355	Regulation	Transcription	regulation of transcription, DNA-templated	P	AT3G46600
14550	3	F23 vs. L23	F2-3 up	GO:0006355	Regulation	Transcription	regulation of transcription, DNA-templated	P	AT4G00480
14551	3	F23 vs. L23	F2-3 up	GO:0006355	Regulation	Transcription	regulation of transcription, DNA-templated	P	AT4G08150
14552	3	F23 vs. L23	F2-3 up	GO:0006355	Regulation	Transcription	regulation of transcription, DNA-templated	P	AT4G17230
14553	3	F23 vs. L23	F2-3 up	GO:0006355	Regulation	Transcription	regulation of transcription, DNA-templated	P	AT4G31800
14554	3	F23 vs. L23	F2-3 up	GO:0006355	Regulation	Transcription	regulation of transcription, DNA-templated	P	AT4G32800
14555	3	F23 vs. L23	F2-3 up	GO:0006355	Regulation	Transcription	regulation of transcription, DNA-templated	P	AT4G36710
14556	3	F23 vs. L23	F2-3 up	GO:0006355	Regulation	Transcription	regulation of transcription, DNA-templated	P	AT4G37180
14557	3	F23 vs. L23	F2-3 up	GO:0006355	Regulation	Transcription	regulation of transcription, DNA-templated	P	AT5G47670
14558	3	F23 vs. L23	F2-3 up	GO:0006355	Regulation	Transcription	regulation of transcription, DNA-templated	P	AT5G56860
14559	3	F23 vs. L23	F2-3 up	GO:0006355	Regulation	Transcription	regulation of transcription, DNA-templated	P	AT5G60450
14560	3	F23 vs. L23	F2-3 up	GO:0006355	Regulation	Transcription	regulation of transcription, DNA-templated	P	AT5G65410
14561	3	F23 vs. L23	F2-3 up	GO:0006355	Regulation	Transcription	regulation of transcription, DNA-templated	P	AT5G67300
14562	3	F23 vs. L23	F2-3 up	GO:0006355	Regulation	Transcription	regulation of transcription, DNA-templated	P	AT5G67480
14563	3	F23 vs. L23	F2-3 up	GO:0003723	Regulation	Transcription	RNA binding	F	AT1G09340
14564	3	F23 vs. L23	F2-3 up	GO:0003723	Regulation	Transcription	RNA binding	F	AT1G22760
14565	3	F23 vs. L23	F2-3 up	GO:0003723	Regulation	Transcription	RNA binding	F	AT1G54290
14566	3	F23 vs. L23	F2-3 up	GO:0003723	Regulation	Transcription	RNA binding	F	AT1G62360
14567	3	F23 vs. L23	F2-3 up	GO:0003723	Regulation	Transcription	RNA binding	F	AT1G71770
14568	3	F23 vs. L23	F2-3 up	GO:0003723	Regulation	Transcription	RNA binding	F	AT1G72730
14569	3	F23 vs. L23	F2-3 up	GO:0003723	Regulation	Transcription	RNA binding	F	AT1G78160
14570	3	F23 vs. L23	F2-3 up	GO:0003723	Regulation	Transcription	RNA binding	F	AT2G02990
14571	3	F23 vs. L23	F2-3 up	GO:0003723	Regulation	Transcription	RNA binding	F	AT3G19090
14572	3	F23 vs. L23	F2-3 up	GO:0003723	Regulation	Transcription	RNA binding	F	AT5G15390
14573	3	F23 vs. L23	F2-3 up	GO:0003723	Regulation	Transcription	RNA binding	F	AT5G22250
14574	3	F23 vs. L23	F2-3 up	GO:0003723	Regulation	Transcription	RNA binding	F	AT5G54760
14575	3	F23 vs. L23	F2-3 up	GO:0003723	Regulation	Transcription	RNA binding	F	AT5G54940
14576	3	F23 vs. L23	F2-3 up	GO:0003723	Regulation	Transcription	RNA binding	F	AT5G56140
14577	3	F23 vs. L23	F2-3 up	GO:0034337	Regulation	Transcription	RNA folding	P	AT3G23700
14578	3	F23 vs. L23	F2-3 up	GO:0009451	Regulation	Transcription	RNA modification	P	AT3G02010
14579	3	F23 vs. L23	F2-3 up	GO:0001139	Regulation	Transcription	RNA polymerase II complex recruiting activity	F	AT2G41630
14580	3	F23 vs. L23	F2-3 up	GO:0000979	Regulation	Transcription	RNA polymerase II core promoter sequence-specific DNA binding	F	AT2G41630
14581	3	F23 vs. L23	F2-3 up	GO:0008353	Regulation	Transcription	RNA polymerase II CTD heptapeptide repeat kinase activity	F	AT1G09600
14582	3	F23 vs. L23	F2-3 up	GO:0000978	Regulation	Transcription	RNA polymerase II proximal promoter sequence-specific DNA binding	F	AT1G61660
14583	3	F23 vs. L23	F2-3 up	GO:0000978	Regulation	Transcription	RNA polymerase II proximal promoter sequence-specific DNA binding	F	AT5G62020
14584	3	F23 vs. L23	F2-3 up	GO:0000977	Regulation	Transcription	RNA polymerase II regulatory region sequence-specific DNA binding	F	AT1G69120
14585	3	F23 vs. L23	F2-3 up	GO:0000977	Regulation	Transcription	RNA polymerase II regulatory region sequence-specific DNA binding	F	AT1G77980

14586	3	F23 vs. L23	F2-3 up	GO:0000977	Regulation	Transcription	RNA polymerase II regulatory region sequence-specific DNA binding	F	AT3G54340
14587	3	F23 vs. L23	F2-3 up	GO:0000977	Regulation	Transcription	RNA polymerase II regulatory region sequence-specific DNA binding	F	AT5G60910
14588	3	F23 vs. L23	F2-3 up	GO:0043565	Regulation	Transcription	sequence-specific DNA binding	F	AT1G14920
14589	3	F23 vs. L23	F2-3 up	GO:0043565	Regulation	Transcription	sequence-specific DNA binding	F	AT1G27730
14590	3	F23 vs. L23	F2-3 up	GO:0043565	Regulation	Transcription	sequence-specific DNA binding	F	AT1G30810
14591	3	F23 vs. L23	F2-3 up	GO:0043565	Regulation	Transcription	sequence-specific DNA binding	F	AT1G32640
14592	3	F23 vs. L23	F2-3 up	GO:0043565	Regulation	Transcription	sequence-specific DNA binding	F	AT1G59640
14593	3	F23 vs. L23	F2-3 up	GO:0043565	Regulation	Transcription	sequence-specific DNA binding	F	AT1G61660
14594	3	F23 vs. L23	F2-3 up	GO:0043565	Regulation	Transcription	sequence-specific DNA binding	F	AT1G62360
14595	3	F23 vs. L23	F2-3 up	GO:0043565	Regulation	Transcription	sequence-specific DNA binding	F	AT1G69120
14596	3	F23 vs. L23	F2-3 up	GO:0043565	Regulation	Transcription	sequence-specific DNA binding	F	AT1G77980
14597	3	F23 vs. L23	F2-3 up	GO:0043565	Regulation	Transcription	sequence-specific DNA binding	F	AT2G13570
14598	3	F23 vs. L23	F2-3 up	GO:0043565	Regulation	Transcription	sequence-specific DNA binding	F	AT2G32460
14599	3	F23 vs. L23	F2-3 up	GO:0043565	Regulation	Transcription	sequence-specific DNA binding	F	AT2G37630
14600	3	F23 vs. L23	F2-3 up	GO:0043565	Regulation	Transcription	sequence-specific DNA binding	F	AT2G46410
14601	3	F23 vs. L23	F2-3 up	GO:0043565	Regulation	Transcription	sequence-specific DNA binding	F	AT2G46830
14602	3	F23 vs. L23	F2-3 up	GO:0043565	Regulation	Transcription	sequence-specific DNA binding	F	AT3G06490
14603	3	F23 vs. L23	F2-3 up	GO:0043565	Regulation	Transcription	sequence-specific DNA binding	F	AT3G15030
14604	3	F23 vs. L23	F2-3 up	GO:0043565	Regulation	Transcription	sequence-specific DNA binding	F	AT3G46600
14605	3	F23 vs. L23	F2-3 up	GO:0043565	Regulation	Transcription	sequence-specific DNA binding	F	AT3G54340
14606	3	F23 vs. L23	F2-3 up	GO:0043565	Regulation	Transcription	sequence-specific DNA binding	F	AT3G57600
14607	3	F23 vs. L23	F2-3 up	GO:0043565	Regulation	Transcription	sequence-specific DNA binding	F	AT4G08150
14608	3	F23 vs. L23	F2-3 up	GO:0043565	Regulation	Transcription	sequence-specific DNA binding	F	AT4G17230
14609	3	F23 vs. L23	F2-3 up	GO:0043565	Regulation	Transcription	sequence-specific DNA binding	F	AT4G31800
14610	3	F23 vs. L23	F2-3 up	GO:0043565	Regulation	Transcription	sequence-specific DNA binding	F	AT4G32800
14611	3	F23 vs. L23	F2-3 up	GO:0043565	Regulation	Transcription	sequence-specific DNA binding	F	AT4G36710
14612	3	F23 vs. L23	F2-3 up	GO:0043565	Regulation	Transcription	sequence-specific DNA binding	F	AT5G16600
14613	3	F23 vs. L23	F2-3 up	GO:0043565	Regulation	Transcription	sequence-specific DNA binding	F	AT5G47670
14614	3	F23 vs. L23	F2-3 up	GO:0043565	Regulation	Transcription	sequence-specific DNA binding	F	AT5G56860
14615	3	F23 vs. L23	F2-3 up	GO:0043565	Regulation	Transcription	sequence-specific DNA binding	F	AT5G60910
14616	3	F23 vs. L23	F2-3 up	GO:0043565	Regulation	Transcription	sequence-specific DNA binding	F	AT5G62020
14617	3	F23 vs. L23	F2-3 up	GO:0043565	Regulation	Transcription	sequence-specific DNA binding	F	AT5G62570
14618	3	F23 vs. L23	F2-3 up	GO:0043565	Regulation	Transcription	sequence-specific DNA binding	F	AT5G67300
14619	3	F23 vs. L23	F2-3 up	GO:1990837	Regulation	Transcription	sequence-specific double-stranded DNA binding	F	AT3G15030
14620	3	F23 vs. L23	F2-3 up	GO:0003713	Regulation	Transcription	transcription coactivator activity	F	AT1G14920
14621	3	F23 vs. L23	F2-3 up	GO:0003712	Regulation	Transcription	transcription coregulator activity	F	AT5G67480
14622	3	F23 vs. L23	F2-3 up	GO:0008134	Regulation	Transcription	transcription factor binding	F	AT1G69120
14623	3	F23 vs. L23	F2-3 up	GO:0008134	Regulation	Transcription	transcription factor binding	F	AT1G77980
14624	3	F23 vs. L23	F2-3 up	GO:0008134	Regulation	Transcription	transcription factor binding	F	AT2G41630
14625	3	F23 vs. L23	F2-3 up	GO:0008134	Regulation	Transcription	transcription factor binding	F	AT3G54340
14626	3	F23 vs. L23	F2-3 up	GO:0008134	Regulation	Transcription	transcription factor binding	F	AT5G24780
14627	3	F23 vs. L23	F2-3 up	GO:0008134	Regulation	Transcription	transcription factor binding	F	AT5G60910
14628	3	F23 vs. L23	F2-3 up	GO:0044212	Regulation	Transcription	transcription regulatory region DNA binding	F	AT1G01720
14629	3	F23 vs. L23	F2-3 up	GO:0044212	Regulation	Transcription	transcription regulatory region DNA binding	F	AT1G01780
14630	3	F23 vs. L23	F2-3 up	GO:0044212	Regulation	Transcription	transcription regulatory region DNA binding	F	AT1G14920
14631	3	F23 vs. L23	F2-3 up	GO:0044212	Regulation	Transcription	transcription regulatory region DNA binding	F	AT1G23380
14632	3	F23 vs. L23	F2-3 up	GO:0044212	Regulation	Transcription	transcription regulatory region DNA binding	F	AT1G26610
14633	3	F23 vs. L23	F2-3 up	GO:0044212	Regulation	Transcription	transcription regulatory region DNA binding	F	AT1G27730
14634	3	F23 vs. L23	F2-3 up	GO:0044212	Regulation	Transcription	transcription regulatory region DNA binding	F	AT1G30810
14635	3	F23 vs. L23	F2-3 up	GO:0044212	Regulation	Transcription	transcription regulatory region DNA binding	F	AT1G32640
14636	3	F23 vs. L23	F2-3 up	GO:0044212	Regulation	Transcription	transcription regulatory region DNA binding	F	AT1G69120
14637	3	F23 vs. L23	F2-3 up	GO:0044212	Regulation	Transcription	transcription regulatory region DNA binding	F	AT1G77980
14638	3	F23 vs. L23	F2-3 up	GO:0044212	Regulation	Transcription	transcription regulatory region DNA binding	F	AT1G80840
14639	3	F23 vs. L23	F2-3 up	GO:0044212	Regulation	Transcription	transcription regulatory region DNA binding	F	AT2G22900

14640	3	F23 vs. L23	F2-3 up	GO:0044212	Regulation	Transcription	transcription regulatory region DNA binding	F	AT2G32460
14641	3	F23 vs. L23	F2-3 up	GO:0044212	Regulation	Transcription	transcription regulatory region DNA binding	F	AT2G37630
14642	3	F23 vs. L23	F2-3 up	GO:0044212	Regulation	Transcription	transcription regulatory region DNA binding	F	AT2G46410
14643	3	F23 vs. L23	F2-3 up	GO:0044212	Regulation	Transcription	transcription regulatory region DNA binding	F	AT3G06490
14644	3	F23 vs. L23	F2-3 up	GO:0044212	Regulation	Transcription	transcription regulatory region DNA binding	F	AT3G21890
14645	3	F23 vs. L23	F2-3 up	GO:0044212	Regulation	Transcription	transcription regulatory region DNA binding	F	AT3G23050
14646	3	F23 vs. L23	F2-3 up	GO:0044212	Regulation	Transcription	transcription regulatory region DNA binding	F	AT3G46600
14647	3	F23 vs. L23	F2-3 up	GO:0044212	Regulation	Transcription	transcription regulatory region DNA binding	F	AT3G54340
14648	3	F23 vs. L23	F2-3 up	GO:0044212	Regulation	Transcription	transcription regulatory region DNA binding	F	AT3G57600
14649	3	F23 vs. L23	F2-3 up	GO:0044212	Regulation	Transcription	transcription regulatory region DNA binding	F	AT4G32800
14650	3	F23 vs. L23	F2-3 up	GO:0044212	Regulation	Transcription	transcription regulatory region DNA binding	F	AT4G32880
14651	3	F23 vs. L23	F2-3 up	GO:0044212	Regulation	Transcription	transcription regulatory region DNA binding	F	AT4G37180
14652	3	F23 vs. L23	F2-3 up	GO:0044212	Regulation	Transcription	transcription regulatory region DNA binding	F	AT5G16600
14653	3	F23 vs. L23	F2-3 up	GO:0044212	Regulation	Transcription	transcription regulatory region DNA binding	F	AT5G20240
14654	3	F23 vs. L23	F2-3 up	GO:0044212	Regulation	Transcription	transcription regulatory region DNA binding	F	AT5G56860
14655	3	F23 vs. L23	F2-3 up	GO:0044212	Regulation	Transcription	transcription regulatory region DNA binding	F	AT5G60450
14656	3	F23 vs. L23	F2-3 up	GO:0044212	Regulation	Transcription	transcription regulatory region DNA binding	F	AT5G60910
14657	3	F23 vs. L23	F2-3 up	GO:0044212	Regulation	Transcription	transcription regulatory region DNA binding	F	AT5G62020
14658	3	F23 vs. L23	F2-3 up	GO:0044212	Regulation	Transcription	transcription regulatory region DNA binding	F	AT5G65410
14659	3	F23 vs. L23	F2-3 up	GO:0044212	Regulation	Transcription	transcription regulatory region DNA binding	F	AT5G67300
14660	3	F23 vs. L23	F2-3 up	GO:0000976	Regulation	Transcription	transcription regulatory region sequence-specific DNA binding	F	AT1G11950
14661	3	F23 vs. L23	F2-3 up	GO:0006351	Regulation	Transcription	transcription, DNA-templated	P	AT2G37630
14662	3	F23 vs. L23	F2-3 up	GO:0097550	Regulation	Transcription	transcriptional preinitiation complex	C	AT2G41630
14663	3	F23 vs. L23	F2-3 up	GO:0001174	Regulation	Transcription	transcriptional start site selection at RNA polymerase II promoter	P	AT2G41630
14664	3	F23 vs. L23	F2-3 up	GO:0003746	Regulation	Transcription	translation elongation factor activity	F	AT4G20360
14665	3	F23 vs. L23	F2-3 up	GO:0003743	Regulation	Transcription	translation initiation factor activity	F	AT1G22760
14666	3	F23 vs. L23	F2-3 up	GO:0003743	Regulation	Transcription	translation initiation factor activity	F	AT1G54290
14667	3	F23 vs. L23	F2-3 up	GO:0003743	Regulation	Transcription	translation initiation factor activity	F	AT1G71770
14668	3	F23 vs. L23	F2-3 up	GO:0003743	Regulation	Transcription	translation initiation factor activity	F	AT1G72730
14669	3	F23 vs. L23	F2-3 up	GO:0003743	Regulation	Transcription	translation initiation factor activity	F	AT3G19030
14670	3	F23 vs. L23	F2-3 up	GO:0003743	Regulation	Transcription	translation initiation factor activity	F	AT3G56150
14671	3	F23 vs. L23	F2-3 up	GO:0003743	Regulation	Transcription	translation initiation factor activity	F	AT4G35250
14672	3	F23 vs. L23	F2-3 up	GO:0003743	Regulation	Transcription	translation initiation factor activity	F	AT5G54760
14673	3	F23 vs. L23	F2-3 up	GO:0003743	Regulation	Transcription	translation initiation factor activity	F	AT5G54940
14674	3	F23 vs. L23	F2-3 up	GO:0030015	Regulation	Translation	CCR4-NOT core complex	C	AT5G22250
14675	3	F23 vs. L23	F2-3 up	GO:0002183	Regulation	Translation	cytoplasmic translational initiation	P	AT1G72730
14676	3	F23 vs. L23	F2-3 up	GO:0022625	Regulation	Translation	cytosolic large ribosomal subunit	C	AT2G44120
14677	3	F23 vs. L23	F2-3 up	GO:0022626	Regulation	Translation	cytosolic ribosome	C	AT1G11860
14678	3	F23 vs. L23	F2-3 up	GO:0022626	Regulation	Translation	cytosolic ribosome	C	AT1G20620
14679	3	F23 vs. L23	F2-3 up	GO:0022626	Regulation	Translation	cytosolic ribosome	C	AT2G21330
14680	3	F23 vs. L23	F2-3 up	GO:0022626	Regulation	Translation	cytosolic ribosome	C	AT2G44120
14681	3	F23 vs. L23	F2-3 up	GO:0022626	Regulation	Translation	cytosolic ribosome	C	AT3G04840
14682	3	F23 vs. L23	F2-3 up	GO:0022626	Regulation	Translation	cytosolic ribosome	C	AT3G09440
14683	3	F23 vs. L23	F2-3 up	GO:0022626	Regulation	Translation	cytosolic ribosome	C	AT3G14420
14684	3	F23 vs. L23	F2-3 up	GO:0022626	Regulation	Translation	cytosolic ribosome	C	AT3G18080
14685	3	F23 vs. L23	F2-3 up	GO:0022626	Regulation	Translation	cytosolic ribosome	C	AT5G25980
14686	3	F23 vs. L23	F2-3 up	GO:0022626	Regulation	Translation	cytosolic ribosome	C	AT5G35630
14687	3	F23 vs. L23	F2-3 up	GO:0022626	Regulation	Translation	cytosolic ribosome	C	AT5G38420
14688	3	F23 vs. L23	F2-3 up	GO:0022627	Regulation	Translation	cytosolic small ribosomal subunit	C	AT1G64510
14689	3	F23 vs. L23	F2-3 up	GO:0022627	Regulation	Translation	cytosolic small ribosomal subunit	C	AT3G04840
14690	3	F23 vs. L23	F2-3 up	GO:0071784	Regulation	Translation	endoplasmic reticulum cisternal network assembly	P	AT4G31080
14691	3	F23 vs. L23	F2-3 up	GO:0005852	Regulation	Translation	eukaryotic translation initiation factor 3 complex	C	AT3G56150
14692	3	F23 vs. L23	F2-3 up	GO:0004819	Regulation	Translation	glutamine-tRNA ligase activity	F	AT1G25350
14693	3	F23 vs. L23	F2-3 up	GO:0006425	Regulation	Translation	glutaminyl-tRNA aminoacylation	P	AT1G25350

14694	3	F23 vs. L23	F2-3 up	GO:0006424	Regulation	Translation	glutamyl-tRNA aminoacylation	P	AT1G25350
14695	3	F23 vs. L23	F2-3 up	GO:0000481	Regulation	Translation	maturation of 5S rRNA	P	AT3G23700
14696	3	F23 vs. L23	F2-3 up	GO:0000463	Regulation	Translation	maturation of LSU-rRNA from tricistronic rRNA transcript (SSU-rRNA, 5.8S rRNA, LSU-rRNA)	P	AT2G44120
14697	3	F23 vs. L23	F2-3 up	GO:0005763	Regulation	Translation	mitochondrial small ribosomal subunit	C	ATCG00820
14698	3	F23 vs. L23	F2-3 up	GO:0003730	Regulation	Translation	mRNA 3'-UTR binding	F	AT1G22760
14699	3	F23 vs. L23	F2-3 up	GO:0003730	Regulation	Translation	mRNA 3'-UTR binding	F	AT1G71770
14700	3	F23 vs. L23	F2-3 up	GO:0003729	Regulation	Translation	mRNA binding	F	AT1G17370
14701	3	F23 vs. L23	F2-3 up	GO:0003729	Regulation	Translation	mRNA binding	F	AT1G20620
14702	3	F23 vs. L23	F2-3 up	GO:0003729	Regulation	Translation	mRNA binding	F	AT1G78160
14703	3	F23 vs. L23	F2-3 up	GO:0003729	Regulation	Translation	mRNA binding	F	AT1G79270
14704	3	F23 vs. L23	F2-3 up	GO:0003729	Regulation	Translation	mRNA binding	F	AT2G44120
14705	3	F23 vs. L23	F2-3 up	GO:0003729	Regulation	Translation	mRNA binding	F	AT3G04840
14706	3	F23 vs. L23	F2-3 up	GO:0003729	Regulation	Translation	mRNA binding	F	AT3G17390
14707	3	F23 vs. L23	F2-3 up	GO:0003729	Regulation	Translation	mRNA binding	F	AT3G23700
14708	3	F23 vs. L23	F2-3 up	GO:0003729	Regulation	Translation	mRNA binding	F	AT3G45780
14709	3	F23 vs. L23	F2-3 up	GO:0003729	Regulation	Translation	mRNA binding	F	AT3G53420
14710	3	F23 vs. L23	F2-3 up	GO:0003729	Regulation	Translation	mRNA binding	F	AT3G56150
14711	3	F23 vs. L23	F2-3 up	GO:0003729	Regulation	Translation	mRNA binding	F	AT5G30510
14712	3	F23 vs. L23	F2-3 up	GO:0000184	Regulation	Translation	nuclear-transcribed mRNA catabolic process, nonsense-mediated decay	P	AT1G22760
14713	3	F23 vs. L23	F2-3 up	GO:0004535	Regulation	Translation	poly(A)-specific ribonuclease activity	F	AT5G22250
14714	3	F23 vs. L23	F2-3 up	GO:0008266	Regulation	Translation	poly(U) RNA binding	F	AT1G20020
14715	3	F23 vs. L23	F2-3 up	GO:0008266	Regulation	Translation	poly(U) RNA binding	F	AT1G22760
14716	3	F23 vs. L23	F2-3 up	GO:0008266	Regulation	Translation	poly(U) RNA binding	F	AT1G68010
14717	3	F23 vs. L23	F2-3 up	GO:0008266	Regulation	Translation	poly(U) RNA binding	F	AT1G71770
14718	3	F23 vs. L23	F2-3 up	GO:0008266	Regulation	Translation	poly(U) RNA binding	F	AT3G63140
14719	3	F23 vs. L23	F2-3 up	GO:0045727	Regulation	Translation	positive regulation of translation	P	AT1G09340
14720	3	F23 vs. L23	F2-3 up	GO:0045727	Regulation	Translation	positive regulation of translation	P	AT3G08720
14721	3	F23 vs. L23	F2-3 up	GO:0045727	Regulation	Translation	positive regulation of translation	P	AT3G08730
14722	3	F23 vs. L23	F2-3 up	GO:0045727	Regulation	Translation	positive regulation of translation	P	AT3G63140
14723	3	F23 vs. L23	F2-3 up	GO:0060211	Regulation	Translation	regulation of nuclear-transcribed mRNA poly(A) tail shortening	P	AT1G22760
14724	3	F23 vs. L23	F2-3 up	GO:0051252	Regulation	Translation	regulation of RNA metabolic process	P	AT3G02770
14725	3	F23 vs. L23	F2-3 up	GO:0051252	Regulation	Translation	regulation of RNA metabolic process	P	AT5G16450
14726	3	F23 vs. L23	F2-3 up	GO:0006417	Regulation	Translation	regulation of translation	P	AT1G21760
14727	3	F23 vs. L23	F2-3 up	GO:0006417	Regulation	Translation	regulation of translation	P	AT1G22760
14728	3	F23 vs. L23	F2-3 up	GO:0006417	Regulation	Translation	regulation of translation	P	AT1G54290
14729	3	F23 vs. L23	F2-3 up	GO:0006417	Regulation	Translation	regulation of translation	P	AT1G71770
14730	3	F23 vs. L23	F2-3 up	GO:0006417	Regulation	Translation	regulation of translation	P	AT1G78160
14731	3	F23 vs. L23	F2-3 up	GO:0004540	Regulation	Translation	ribonuclease activity	F	AT2G02990
14732	3	F23 vs. L23	F2-3 up	GO:0004540	Regulation	Translation	ribonuclease activity	F	AT3G04720
14733	3	F23 vs. L23	F2-3 up	GO:0008428	Regulation	Translation	ribonuclease inhibitor activity	F	AT3G02770
14734	3	F23 vs. L23	F2-3 up	GO:0008428	Regulation	Translation	ribonuclease inhibitor activity	F	AT5G16450
14735	3	F23 vs. L23	F2-3 up	GO:0033897	Regulation	Translation	ribonuclease T2 activity	F	AT2G02990
14736	3	F23 vs. L23	F2-3 up	GO:1990904	Regulation	Translation	ribonucleoprotein complex	C	AT1G22760
14737	3	F23 vs. L23	F2-3 up	GO:1990904	Regulation	Translation	ribonucleoprotein complex	C	AT1G71770
14738	3	F23 vs. L23	F2-3 up	GO:1990904	Regulation	Translation	ribonucleoprotein complex	C	AT3G19090
14739	3	F23 vs. L23	F2-3 up	GO:0000028	Regulation	Translation	ribosomal small subunit assembly	P	ATCG00820
14740	3	F23 vs. L23	F2-3 up	GO:0005840	Regulation	Translation	ribosome	C	AT1G09340
14741	3	F23 vs. L23	F2-3 up	GO:0005840	Regulation	Translation	ribosome	C	AT1G68590
14742	3	F23 vs. L23	F2-3 up	GO:0005840	Regulation	Translation	ribosome	C	AT2G18400
14743	3	F23 vs. L23	F2-3 up	GO:0005840	Regulation	Translation	ribosome	C	AT5G30510
14744	3	F23 vs. L23	F2-3 up	GO:0042254	Regulation	Translation	ribosome biogenesis	P	AT3G19800
14745	3	F23 vs. L23	F2-3 up	GO:0003724	Regulation	Translation	RNA helicase activity	F	AT1G72730
14746	3	F23 vs. L23	F2-3 up	GO:0006396	Regulation	Translation	RNA processing	P	AT1G04530

14747	3	F23 vs. L23	F2-3 up	GO:0006396	Regulation	Translation	RNA processing	P	AT3G19090
14748	3	F23 vs. L23	F2-3 up	GO:0019843	Regulation	Translation	rRNA binding	F	AT1G09340
14749	3	F23 vs. L23	F2-3 up	GO:0019843	Regulation	Translation	rRNA binding	F	AT2G18400
14750	3	F23 vs. L23	F2-3 up	GO:0019843	Regulation	Translation	rRNA binding	F	AT3G63140
14751	3	F23 vs. L23	F2-3 up	GO:0006364	Regulation	Translation	rRNA processing	P	AT1G09340
14752	3	F23 vs. L23	F2-3 up	GO:0006364	Regulation	Translation	rRNA processing	P	AT3G63140
14753	3	F23 vs. L23	F2-3 up	GO:0015935	Regulation	Translation	small ribosomal subunit	C	ATCG00820
14754	3	F23 vs. L23	F2-3 up	GO:0070181	Regulation	Translation	small ribosomal subunit rRNA binding	F	AT1G64510
14755	3	F23 vs. L23	F2-3 up	GO:0003735	Regulation	Translation	structural constituent of ribosome	F	AT1G64510
14756	3	F23 vs. L23	F2-3 up	GO:0003735	Regulation	Translation	structural constituent of ribosome	F	AT1G68590
14757	3	F23 vs. L23	F2-3 up	GO:0003735	Regulation	Translation	structural constituent of ribosome	F	AT2G18400
14758	3	F23 vs. L23	F2-3 up	GO:0003735	Regulation	Translation	structural constituent of ribosome	F	AT2G44120
14759	3	F23 vs. L23	F2-3 up	GO:0003735	Regulation	Translation	structural constituent of ribosome	F	AT5G30510
14760	3	F23 vs. L23	F2-3 up	GO:0003735	Regulation	Translation	structural constituent of ribosome	F	ATCG00820
14761	3	F23 vs. L23	F2-3 up	GO:0006412	Regulation	Translation	translation	P	AT1G25350
14762	3	F23 vs. L23	F2-3 up	GO:0006412	Regulation	Translation	translation	P	AT1G64510
14763	3	F23 vs. L23	F2-3 up	GO:0006412	Regulation	Translation	translation	P	AT2G18400
14764	3	F23 vs. L23	F2-3 up	GO:0006412	Regulation	Translation	translation	P	AT5G14660
14765	3	F23 vs. L23	F2-3 up	GO:0006412	Regulation	Translation	translation	P	AT5G30510
14766	3	F23 vs. L23	F2-3 up	GO:0006412	Regulation	Translation	translation	P	ATCG00820
14767	3	F23 vs. L23	F2-3 up	GO:0031369	Regulation	Translation	translation initiation factor binding	F	AT3G56150
14768	3	F23 vs. L23	F2-3 up	GO:0006414	Regulation	Translation	translational elongation	P	AT4G20360
14769	3	F23 vs. L23	F2-3 up	GO:0006413	Regulation	Translation	translational initiation	P	AT1G16720
14770	3	F23 vs. L23	F2-3 up	GO:0006413	Regulation	Translation	translational initiation	P	AT1G54290
14771	3	F23 vs. L23	F2-3 up	GO:0006413	Regulation	Translation	translational initiation	P	AT1G71770
14772	3	F23 vs. L23	F2-3 up	GO:0006413	Regulation	Translation	translational initiation	P	AT3G19030
14773	3	F23 vs. L23	F2-3 up	GO:0006413	Regulation	Translation	translational initiation	P	AT3G56150
14774	3	F23 vs. L23	F2-3 up	GO:0006413	Regulation	Translation	translational initiation	P	AT5G54760
14775	3	F23 vs. L23	F2-3 up	GO:0006413	Regulation	Translation	translational initiation	P	AT5G54940
14776	3	F23 vs. L23	F2-3 up	GO:0009020	Regulation	Translation	tRNA (guanosine-2'-O-)-methyltransferase activity	F	AT5G15390
14777	3	F23 vs. L23	F2-3 up	GO:0002938	Regulation	Translation	tRNA guanine ribose methylation	P	AT5G15390
14778	3	F23 vs. L23	F2-3 up	GO:0016031	Regulation	Translation	tRNA import into mitochondrion	P	AT5G24650
14779	3	F23 vs. L23	F2-3 up	GO:0004430	Signaling and response	Signaling	1-phosphatidylinositol 4-kinase activity	F	AT3G56600
14780	3	F23 vs. L23	F2-3 up	GO:0016308	Signaling and response	Signaling	1-phosphatidylinositol-4-phosphate 5-kinase activity	F	AT1G21980
14781	3	F23 vs. L23	F2-3 up	GO:0016308	Signaling and response	Signaling	1-phosphatidylinositol-4-phosphate 5-kinase activity	F	AT2G41210
14782	3	F23 vs. L23	F2-3 up	GO:0016308	Signaling and response	Signaling	1-phosphatidylinositol-4-phosphate 5-kinase activity	F	AT3G07960
14783	3	F23 vs. L23	F2-3 up	GO:0016308	Signaling and response	Signaling	1-phosphatidylinositol-4-phosphate 5-kinase activity	F	AT3G56960
14784	3	F23 vs. L23	F2-3 up	GO:0030797	Signaling and response	Signaling	24-methylenesterol C-methyltransferase activity	F	AT1G76090
14785	3	F23 vs. L23	F2-3 up	GO:0003993	Signaling and response	Signaling	acid phosphatase activity	F	AT2G16430
14786	3	F23 vs. L23	F2-3 up	GO:0003993	Signaling and response	Signaling	acid phosphatase activity	F	AT3G17790
14787	3	F23 vs. L23	F2-3 up	GO:0003993	Signaling and response	Signaling	acid phosphatase activity	F	AT4G24890
14788	3	F23 vs. L23	F2-3 up	GO:0003993	Signaling and response	Signaling	acid phosphatase activity	F	AT5G24780
14789	3	F23 vs. L23	F2-3 up	GO:0090630	Signaling and response	Signaling	activation of GTPase activity	P	AT5G54780
14790	3	F23 vs. L23	F2-3 up	GO:0010011	Signaling and response	Signaling	auxin binding	F	AT4G03190
14791	3	F23 vs. L23	F2-3 up	GO:0009851	Signaling and response	Signaling	auxin biosynthetic process	P	AT1G75500
14792	3	F23 vs. L23	F2-3 up	GO:0009851	Signaling and response	Signaling	auxin biosynthetic process	P	AT5G05730
14793	3	F23 vs. L23	F2-3 up	GO:0010315	Signaling and response	Signaling	auxin efflux	P	AT1G70940
14794	3	F23 vs. L23	F2-3 up	GO:0010315	Signaling and response	Signaling	auxin efflux	P	AT1G75500
14795	3	F23 vs. L23	F2-3 up	GO:0010315	Signaling and response	Signaling	auxin efflux	P	AT1G78570
14796	3	F23 vs. L23	F2-3 up	GO:0010315	Signaling and response	Signaling	auxin efflux	P	AT2G01420
14797	3	F23 vs. L23	F2-3 up	GO:0010329	Signaling and response	Signaling	auxin efflux transmembrane transporter activity	F	AT1G70940
14798	3	F23 vs. L23	F2-3 up	GO:0010329	Signaling and response	Signaling	auxin efflux transmembrane transporter activity	F	AT2G01420

14799	3	F23 vs. L23	F2-3 up	GO:0009850	Signaling and response	Signaling	auxin metabolic process	P	AT2G31750
14800	3	F23 vs. L23	F2-3 up	GO:0009926	Signaling and response	Signaling	auxin polar transport	P	AT1G70940
14801	3	F23 vs. L23	F2-3 up	GO:0009926	Signaling and response	Signaling	auxin polar transport	P	AT2G01420
14802	3	F23 vs. L23	F2-3 up	GO:0009926	Signaling and response	Signaling	auxin polar transport	P	AT5G13930
14803	3	F23 vs. L23	F2-3 up	GO:0009734	Signaling and response	Signaling	auxin-activated signaling pathway	P	AT1G12110
14804	3	F23 vs. L23	F2-3 up	GO:0009734	Signaling and response	Signaling	auxin-activated signaling pathway	P	AT1G14000
14805	3	F23 vs. L23	F2-3 up	GO:0009734	Signaling and response	Signaling	auxin-activated signaling pathway	P	AT1G30330
14806	3	F23 vs. L23	F2-3 up	GO:0009734	Signaling and response	Signaling	auxin-activated signaling pathway	P	AT1G70940
14807	3	F23 vs. L23	F2-3 up	GO:0009734	Signaling and response	Signaling	auxin-activated signaling pathway	P	AT1G75500
14808	3	F23 vs. L23	F2-3 up	GO:0009734	Signaling and response	Signaling	auxin-activated signaling pathway	P	AT2G01420
14809	3	F23 vs. L23	F2-3 up	GO:0009734	Signaling and response	Signaling	auxin-activated signaling pathway	P	AT3G15540
14810	3	F23 vs. L23	F2-3 up	GO:0009734	Signaling and response	Signaling	auxin-activated signaling pathway	P	AT3G23050
14811	3	F23 vs. L23	F2-3 up	GO:0009734	Signaling and response	Signaling	auxin-activated signaling pathway	P	AT5G54510
14812	3	F23 vs. L23	F2-3 up	GO:0009734	Signaling and response	Signaling	auxin-activated signaling pathway	P	AT5G60450
14813	3	F23 vs. L23	F2-3 up	GO:0009785	Signaling and response	Signaling	blue light signaling pathway	P	AT1G04400
14814	3	F23 vs. L23	F2-3 up	GO:0009785	Signaling and response	Signaling	blue light signaling pathway	P	AT3G45780
14815	3	F23 vs. L23	F2-3 up	GO:0009785	Signaling and response	Signaling	blue light signaling pathway	P	AT4G38740
14816	3	F23 vs. L23	F2-3 up	GO:0009742	Signaling and response	Signaling	brassinosteroid mediated signaling pathway	P	AT1G14000
14817	3	F23 vs. L23	F2-3 up	GO:0009742	Signaling and response	Signaling	brassinosteroid mediated signaling pathway	P	AT1G50990
14818	3	F23 vs. L23	F2-3 up	GO:0009742	Signaling and response	Signaling	brassinosteroid mediated signaling pathway	P	AT4G38740
14819	3	F23 vs. L23	F2-3 up	GO:0005509	Signaling and response	Signaling	calcium ion binding	F	AT1G35720
14820	3	F23 vs. L23	F2-3 up	GO:0005509	Signaling and response	Signaling	calcium ion binding	F	AT1G64060
14821	3	F23 vs. L23	F2-3 up	GO:0005509	Signaling and response	Signaling	calcium ion binding	F	AT1G76130
14822	3	F23 vs. L23	F2-3 up	GO:0005509	Signaling and response	Signaling	calcium ion binding	F	AT1G77090
14823	3	F23 vs. L23	F2-3 up	GO:0005509	Signaling and response	Signaling	calcium ion binding	F	AT2G15680
14824	3	F23 vs. L23	F2-3 up	GO:0005509	Signaling and response	Signaling	calcium ion binding	F	AT2G17290
14825	3	F23 vs. L23	F2-3 up	GO:0005509	Signaling and response	Signaling	calcium ion binding	F	AT2G31500
14826	3	F23 vs. L23	F2-3 up	GO:0005509	Signaling and response	Signaling	calcium ion binding	F	AT2G39470
14827	3	F23 vs. L23	F2-3 up	GO:0005509	Signaling and response	Signaling	calcium ion binding	F	AT3G03430
14828	3	F23 vs. L23	F2-3 up	GO:0005509	Signaling and response	Signaling	calcium ion binding	F	AT3G51920
14829	3	F23 vs. L23	F2-3 up	GO:0005509	Signaling and response	Signaling	calcium ion binding	F	AT4G05020
14830	3	F23 vs. L23	F2-3 up	GO:0005509	Signaling and response	Signaling	calcium ion binding	F	AT4G26470
14831	3	F23 vs. L23	F2-3 up	GO:0005509	Signaling and response	Signaling	calcium ion binding	F	AT4G27790
14832	3	F23 vs. L23	F2-3 up	GO:0005509	Signaling and response	Signaling	calcium ion binding	F	AT5G17480
14833	3	F23 vs. L23	F2-3 up	GO:0005509	Signaling and response	Signaling	calcium ion binding	F	AT5G47910
14834	3	F23 vs. L23	F2-3 up	GO:0005509	Signaling and response	Signaling	calcium ion binding	F	AT5G54130
14835	3	F23 vs. L23	F2-3 up	GO:0005509	Signaling and response	Signaling	calcium ion binding	F	AT5G54490
14836	3	F23 vs. L23	F2-3 up	GO:0009931	Signaling and response	Signaling	calcium-dependent protein serine/threonine kinase activity	F	AT2G17290
14837	3	F23 vs. L23	F2-3 up	GO:0009931	Signaling and response	Signaling	calcium-dependent protein serine/threonine kinase activity	F	AT2G31500
14838	3	F23 vs. L23	F2-3 up	GO:0019722	Signaling and response	Signaling	calcium-mediated signaling	P	AT1G28270
14839	3	F23 vs. L23	F2-3 up	GO:0019722	Signaling and response	Signaling	calcium-mediated signaling	P	AT2G18470
14840	3	F23 vs. L23	F2-3 up	GO:0019722	Signaling and response	Signaling	calcium-mediated signaling	P	AT3G25170
14841	3	F23 vs. L23	F2-3 up	GO:0019722	Signaling and response	Signaling	calcium-mediated signaling	P	AT3G51920
14842	3	F23 vs. L23	F2-3 up	GO:0019722	Signaling and response	Signaling	calcium-mediated signaling	P	AT4G26470
14843	3	F23 vs. L23	F2-3 up	GO:0005516	Signaling and response	Signaling	calmodulin binding	F	AT1G18150
14844	3	F23 vs. L23	F2-3 up	GO:0005516	Signaling and response	Signaling	calmodulin binding	F	AT1G65960
14845	3	F23 vs. L23	F2-3 up	GO:0005516	Signaling and response	Signaling	calmodulin binding	F	AT2G17290
14846	3	F23 vs. L23	F2-3 up	GO:0005516	Signaling and response	Signaling	calmodulin binding	F	AT2G22950
14847	3	F23 vs. L23	F2-3 up	GO:0005516	Signaling and response	Signaling	calmodulin binding	F	AT2G31500
14848	3	F23 vs. L23	F2-3 up	GO:0005516	Signaling and response	Signaling	calmodulin binding	F	AT2G33670
14849	3	F23 vs. L23	F2-3 up	GO:0005516	Signaling and response	Signaling	calmodulin binding	F	AT3G21180
14850	3	F23 vs. L23	F2-3 up	GO:0005516	Signaling and response	Signaling	calmodulin binding	F	AT3G43120
14851	3	F23 vs. L23	F2-3 up	GO:0005516	Signaling and response	Signaling	calmodulin binding	F	AT3G57330
14852	3	F23 vs. L23	F2-3 up	GO:0005516	Signaling and response	Signaling	calmodulin binding	F	AT5G54250

14853	3	F23 vs. L23	F2-3 up	GO:0005516	Signaling and response	Signaling	calmodulin binding	F	AT5G62570
14854	3	F23 vs. L23	F2-3 up	GO:0005516	Signaling and response	Signaling	calmodulin binding	F	AT5G64040
14855	3	F23 vs. L23	F2-3 up	GO:0005516	Signaling and response	Signaling	calmodulin binding	F	AT5G67480
14856	3	F23 vs. L23	F2-3 up	GO:0004683	Signaling and response	Signaling	calmodulin-dependent protein kinase activity	F	AT2G17290
14857	3	F23 vs. L23	F2-3 up	GO:0004683	Signaling and response	Signaling	calmodulin-dependent protein kinase activity	F	AT2G31500
14858	3	F23 vs. L23	F2-3 up	GO:0007166	Signaling and response	Signaling	cell surface receptor signaling pathway	P	AT5G45840
14859	3	F23 vs. L23	F2-3 up	GO:0007267	Signaling and response	Signaling	cell-cell signaling	P	AT1G28270
14860	3	F23 vs. L23	F2-3 up	GO:0007267	Signaling and response	Signaling	cell-cell signaling	P	AT3G25170
14861	3	F23 vs. L23	F2-3 up	GO:0071483	Signaling and response	Signaling	cellular response to blue light	P	AT3G22840
14862	3	F23 vs. L23	F2-3 up	GO:0071483	Signaling and response	Signaling	cellular response to blue light	P	AT4G14690
14863	3	F23 vs. L23	F2-3 up	GO:0071483	Signaling and response	Signaling	cellular response to blue light	P	AT5G24120
14864	3	F23 vs. L23	F2-3 up	GO:0080029	Signaling and response	Signaling	cellular response to boron-containing substance levels	P	AT1G80760
14865	3	F23 vs. L23	F2-3 up	GO:0071277	Signaling and response	Signaling	cellular response to calcium ion	P	AT5G23060
14866	3	F23 vs. L23	F2-3 up	GO:0070417	Signaling and response	Signaling	cellular response to cold	P	AT1G20823
14867	3	F23 vs. L23	F2-3 up	GO:0070417	Signaling and response	Signaling	cellular response to cold	P	AT2G46210
14868	3	F23 vs. L23	F2-3 up	GO:0070417	Signaling and response	Signaling	cellular response to cold	P	AT3G62410
14869	3	F23 vs. L23	F2-3 up	GO:0071280	Signaling and response	Signaling	cellular response to copper ion	P	AT1G08830
14870	3	F23 vs. L23	F2-3 up	GO:0071490	Signaling and response	Signaling	cellular response to far red light	P	AT3G22840
14871	3	F23 vs. L23	F2-3 up	GO:0071490	Signaling and response	Signaling	cellular response to far red light	P	AT4G14690
14872	3	F23 vs. L23	F2-3 up	GO:0071398	Signaling and response	Signaling	cellular response to fatty acid	P	AT1G05010
14873	3	F23 vs. L23	F2-3 up	GO:0071398	Signaling and response	Signaling	cellular response to fatty acid	P	AT1G62380
14874	3	F23 vs. L23	F2-3 up	GO:0071370	Signaling and response	Signaling	cellular response to gibberellin stimulus	P	AT1G12240
14875	3	F23 vs. L23	F2-3 up	GO:0071366	Signaling and response	Signaling	cellular response to indolebutyric acid stimulus	P	AT1G59870
14876	3	F23 vs. L23	F2-3 up	GO:0071482	Signaling and response	Signaling	cellular response to light stimulus	P	AT1G07180
14877	3	F23 vs. L23	F2-3 up	GO:0071482	Signaling and response	Signaling	cellular response to light stimulus	P	AT1G64860
14878	3	F23 vs. L23	F2-3 up	GO:0071482	Signaling and response	Signaling	cellular response to light stimulus	P	AT1G68010
14879	3	F23 vs. L23	F2-3 up	GO:0043562	Signaling and response	Signaling	cellular response to nitrogen levels	P	AT5G64370
14880	3	F23 vs. L23	F2-3 up	GO:0071491	Signaling and response	Signaling	cellular response to red light	P	AT3G22840
14881	3	F23 vs. L23	F2-3 up	GO:0071491	Signaling and response	Signaling	cellular response to red light	P	AT4G14690
14882	3	F23 vs. L23	F2-3 up	GO:0071446	Signaling and response	Signaling	cellular response to salicylic acid stimulus	P	AT3G01420
14883	3	F23 vs. L23	F2-3 up	GO:0071329	Signaling and response	Signaling	cellular response to sucrose stimulus	P	AT1G08830
14884	3	F23 vs. L23	F2-3 up	GO:0071329	Signaling and response	Signaling	cellular response to sucrose stimulus	P	AT2G28190
14885	3	F23 vs. L23	F2-3 up	GO:0071329	Signaling and response	Signaling	cellular response to sucrose stimulus	P	AT5G44110
14886	3	F23 vs. L23	F2-3 up	GO:1904482	Signaling and response	Signaling	cellular response to tetrahydrofolate	P	AT4G13930
14887	3	F23 vs. L23	F2-3 up	GO:0010617	Signaling and response	Signaling	circadian regulation of calcium ion oscillation	P	AT1G04400
14888	3	F23 vs. L23	F2-3 up	GO:0007623	Signaling and response	Signaling	circadian rhythm	P	AT1G04400
14889	3	F23 vs. L23	F2-3 up	GO:0007623	Signaling and response	Signaling	circadian rhythm	P	AT1G09340
14890	3	F23 vs. L23	F2-3 up	GO:0007623	Signaling and response	Signaling	circadian rhythm	P	AT2G17840
14891	3	F23 vs. L23	F2-3 up	GO:0007623	Signaling and response	Signaling	circadian rhythm	P	AT2G46830
14892	3	F23 vs. L23	F2-3 up	GO:0007623	Signaling and response	Signaling	circadian rhythm	P	AT3G01060
14893	3	F23 vs. L23	F2-3 up	GO:0007623	Signaling and response	Signaling	circadian rhythm	P	AT3G14620
14894	3	F23 vs. L23	F2-3 up	GO:0007623	Signaling and response	Signaling	circadian rhythm	P	AT3G21890
14895	3	F23 vs. L23	F2-3 up	GO:0007623	Signaling and response	Signaling	circadian rhythm	P	AT3G45780
14896	3	F23 vs. L23	F2-3 up	GO:0007623	Signaling and response	Signaling	circadian rhythm	P	AT3G50000
14897	3	F23 vs. L23	F2-3 up	GO:0007623	Signaling and response	Signaling	circadian rhythm	P	AT4G13930
14898	3	F23 vs. L23	F2-3 up	GO:0007623	Signaling and response	Signaling	circadian rhythm	P	AT5G56860
14899	3	F23 vs. L23	F2-3 up	GO:0050897	Signaling and response	Signaling	cobalt ion binding	F	AT1G20620
14900	3	F23 vs. L23	F2-3 up	GO:0050897	Signaling and response	Signaling	cobalt ion binding	F	AT1G48030
14901	3	F23 vs. L23	F2-3 up	GO:0050897	Signaling and response	Signaling	cobalt ion binding	F	AT4G13930
14902	3	F23 vs. L23	F2-3 up	GO:0050897	Signaling and response	Signaling	cobalt ion binding	F	AT5G07440
14903	3	F23 vs. L23	F2-3 up	GO:0008180	Signaling and response	Signaling	COP9 signalosome	C	AT3G23050
14904	3	F23 vs. L23	F2-3 up	GO:0009691	Signaling and response	Signaling	cytokinin biosynthetic process	P	AT1G62360
14905	3	F23 vs. L23	F2-3 up	GO:0009691	Signaling and response	Signaling	cytokinin biosynthetic process	P	AT2G28305
14906	3	F23 vs. L23	F2-3 up	GO:0009736	Signaling and response	Signaling	cytokinin-activated signaling pathway	P	AT2G40670
14907	3	F23 vs. L23	F2-3 up	GO:0009736	Signaling and response	Signaling	cytokinin-activated signaling pathway	P	AT5G56860

14908	3	F23 vs. L23	F2-3 up	GO:0016311	Signaling and response	Signaling	dephosphorylation	P	AT2G31830
14909	3	F23 vs. L23	F2-3 up	GO:0016311	Signaling and response	Signaling	dephosphorylation	P	AT3G17790
14910	3	F23 vs. L23	F2-3 up	GO:0016311	Signaling and response	Signaling	dephosphorylation	P	AT4G24890
14911	3	F23 vs. L23	F2-3 up	GO:0016311	Signaling and response	Signaling	dephosphorylation	P	AT5G24780
14912	3	F23 vs. L23	F2-3 up	GO:0005513	Signaling and response	Signaling	detection of calcium ion	P	AT3G51920
14913	3	F23 vs. L23	F2-3 up	GO:0106167	Signaling and response	Signaling	extracellular ATP signaling	P	AT1G32640
14914	3	F23 vs. L23	F2-3 up	GO:0009740	Signaling and response	Signaling	gibberellic acid mediated signaling pathway	P	AT1G14920
14915	3	F23 vs. L23	F2-3 up	GO:0009740	Signaling and response	Signaling	gibberellic acid mediated signaling pathway	P	AT1G74670
14916	3	F23 vs. L23	F2-3 up	GO:0009740	Signaling and response	Signaling	gibberellic acid mediated signaling pathway	P	AT2G14900
14917	3	F23 vs. L23	F2-3 up	GO:0009740	Signaling and response	Signaling	gibberellic acid mediated signaling pathway	P	AT2G32460
14918	3	F23 vs. L23	F2-3 up	GO:0009740	Signaling and response	Signaling	gibberellic acid mediated signaling pathway	P	AT2G36830
14919	3	F23 vs. L23	F2-3 up	GO:0009740	Signaling and response	Signaling	gibberellic acid mediated signaling pathway	P	AT5G56860
14920	3	F23 vs. L23	F2-3 up	GO:1905200	Signaling and response	Signaling	gibberellic acid transmembrane transport	P	AT5G50800
14921	3	F23 vs. L23	F2-3 up	GO:1905201	Signaling and response	Signaling	gibberellin transmembrane transporter activity	F	AT5G50800
14922	3	F23 vs. L23	F2-3 up	GO:0010255	Signaling and response	Signaling	glucose mediated signaling pathway	P	AT5G56860
14923	3	F23 vs. L23	F2-3 up	GO:0009630	Signaling and response	Signaling	gravitropism	P	AT1G70940
14924	3	F23 vs. L23	F2-3 up	GO:0009630	Signaling and response	Signaling	gravitropism	P	AT2G18390
14925	3	F23 vs. L23	F2-3 up	GO:0009630	Signaling and response	Signaling	gravitropism	P	AT3G15540
14926	3	F23 vs. L23	F2-3 up	GO:0009630	Signaling and response	Signaling	gravitropism	P	AT3G23050
14927	3	F23 vs. L23	F2-3 up	GO:0008083	Signaling and response	Signaling	growth factor activity	F	AT5G65870
14928	3	F23 vs. L23	F2-3 up	GO:0102489	Signaling and response	Signaling	GTP phosphohydrolase activity	F	AT5G18280
14929	3	F23 vs. L23	F2-3 up	GO:0010274	Signaling and response	Signaling	hydrotropism	P	AT5G42680
14930	3	F23 vs. L23	F2-3 up	GO:0010279	Signaling and response	Signaling	indole-3-acetic acid amido synthetase activity	F	AT2G23170
14931	3	F23 vs. L23	F2-3 up	GO:0010279	Signaling and response	Signaling	indole-3-acetic acid amido synthetase activity	F	AT5G54510
14932	3	F23 vs. L23	F2-3 up	GO:0046855	Signaling and response	Signaling	inositol phosphate dephosphorylation	P	AT2G31830
14933	3	F23 vs. L23	F2-3 up	GO:0007229	Signaling and response	Signaling	integrin-mediated signaling pathway	P	AT4G18950
14934	3	F23 vs. L23	F2-3 up	GO:0035556	Signaling and response	Signaling	intracellular signal transduction	P	AT1G18150
14935	3	F23 vs. L23	F2-3 up	GO:0035556	Signaling and response	Signaling	intracellular signal transduction	P	AT1G78290
14936	3	F23 vs. L23	F2-3 up	GO:0035556	Signaling and response	Signaling	intracellular signal transduction	P	AT2G17290
14937	3	F23 vs. L23	F2-3 up	GO:0035556	Signaling and response	Signaling	intracellular signal transduction	P	AT2G20470
14938	3	F23 vs. L23	F2-3 up	GO:0035556	Signaling and response	Signaling	intracellular signal transduction	P	AT2G31500
14939	3	F23 vs. L23	F2-3 up	GO:0035556	Signaling and response	Signaling	intracellular signal transduction	P	AT3G45640
14940	3	F23 vs. L23	F2-3 up	GO:0009695	Signaling and response	Signaling	jasmonic acid biosynthetic process	P	AT1G17420
14941	3	F23 vs. L23	F2-3 up	GO:0009695	Signaling and response	Signaling	jasmonic acid biosynthetic process	P	AT1G70480
14942	3	F23 vs. L23	F2-3 up	GO:0009695	Signaling and response	Signaling	jasmonic acid biosynthetic process	P	AT1G72520
14943	3	F23 vs. L23	F2-3 up	GO:0009695	Signaling and response	Signaling	jasmonic acid biosynthetic process	P	AT2G06050
14944	3	F23 vs. L23	F2-3 up	GO:0009695	Signaling and response	Signaling	jasmonic acid biosynthetic process	P	AT3G25760
14945	3	F23 vs. L23	F2-3 up	GO:0009695	Signaling and response	Signaling	jasmonic acid biosynthetic process	P	AT3G45140
14946	3	F23 vs. L23	F2-3 up	GO:0009695	Signaling and response	Signaling	jasmonic acid biosynthetic process	P	AT5G63380
14947	3	F23 vs. L23	F2-3 up	GO:0120091	Signaling and response	Signaling	jasmonic acid hydrolase	F	AT2G38240
14948	3	F23 vs. L23	F2-3 up	GO:0120091	Signaling and response	Signaling	jasmonic acid hydrolase	F	AT5G05600
14949	3	F23 vs. L23	F2-3 up	GO:0009867	Signaling and response	Signaling	jasmonic acid mediated signaling pathway	P	AT1G14920
14950	3	F23 vs. L23	F2-3 up	GO:0009867	Signaling and response	Signaling	jasmonic acid mediated signaling pathway	P	AT1G19180
14951	3	F23 vs. L23	F2-3 up	GO:0009867	Signaling and response	Signaling	jasmonic acid mediated signaling pathway	P	AT1G28480
14952	3	F23 vs. L23	F2-3 up	GO:0009867	Signaling and response	Signaling	jasmonic acid mediated signaling pathway	P	AT1G32640
14953	3	F23 vs. L23	F2-3 up	GO:0009867	Signaling and response	Signaling	jasmonic acid mediated signaling pathway	P	AT1G63650
14954	3	F23 vs. L23	F2-3 up	GO:0009694	Signaling and response	Signaling	jasmonic acid metabolic process	P	AT3G48520
14955	3	F23 vs. L23	F2-3 up	GO:0016301	Signaling and response	Signaling	kinase activity	F	AT1G50610
14956	3	F23 vs. L23	F2-3 up	GO:0016301	Signaling and response	Signaling	kinase activity	F	AT1G72180
14957	3	F23 vs. L23	F2-3 up	GO:0016301	Signaling and response	Signaling	kinase activity	F	AT1G78940
14958	3	F23 vs. L23	F2-3 up	GO:0016301	Signaling and response	Signaling	kinase activity	F	AT2G20470
14959	3	F23 vs. L23	F2-3 up	GO:0016301	Signaling and response	Signaling	kinase activity	F	AT3G08720
14960	3	F23 vs. L23	F2-3 up	GO:0016301	Signaling and response	Signaling	kinase activity	F	AT3G08730
14961	3	F23 vs. L23	F2-3 up	GO:0016301	Signaling and response	Signaling	kinase activity	F	AT3G12690
14962	3	F23 vs. L23	F2-3 up	GO:0016301	Signaling and response	Signaling	kinase activity	F	AT3G13065
14963	3	F23 vs. L23	F2-3 up	GO:0016301	Signaling and response	Signaling	kinase activity	F	AT3G20190

14964	3	F23 vs. L23	F2-3 up	GO:0016301	Signaling and response	Signaling	kinase activity	F	AT3G45780
14965	3	F23 vs. L23	F2-3 up	GO:0016301	Signaling and response	Signaling	kinase activity	F	AT3G50000
14966	3	F23 vs. L23	F2-3 up	GO:0016301	Signaling and response	Signaling	kinase activity	F	AT4G15530
14967	3	F23 vs. L23	F2-3 up	GO:0016301	Signaling and response	Signaling	kinase activity	F	AT4G20300
14968	3	F23 vs. L23	F2-3 up	GO:0016301	Signaling and response	Signaling	kinase activity	F	AT4G25030
14969	3	F23 vs. L23	F2-3 up	GO:0016301	Signaling and response	Signaling	kinase activity	F	AT4G28490
14970	3	F23 vs. L23	F2-3 up	GO:0016301	Signaling and response	Signaling	kinase activity	F	AT4G39110
14971	3	F23 vs. L23	F2-3 up	GO:0016301	Signaling and response	Signaling	kinase activity	F	AT5G05200
14972	3	F23 vs. L23	F2-3 up	GO:0016301	Signaling and response	Signaling	kinase activity	F	AT5G12000
14973	3	F23 vs. L23	F2-3 up	GO:0016301	Signaling and response	Signaling	kinase activity	F	AT5G56350
14974	3	F23 vs. L23	F2-3 up	GO:0016301	Signaling and response	Signaling	kinase activity	F	AT5G58050
14975	3	F23 vs. L23	F2-3 up	GO:0016301	Signaling and response	Signaling	kinase activity	F	AT5G63680
14976	3	F23 vs. L23	F2-3 up	GO:0019900	Signaling and response	Signaling	kinase binding	F	AT1G32640
14977	3	F23 vs. L23	F2-3 up	GO:0019900	Signaling and response	Signaling	kinase binding	F	AT3G11410
14978	3	F23 vs. L23	F2-3 up	GO:0048574	Signaling and response	Signaling	long-day photoperiodism, flowering	P	AT1G04400
14979	3	F23 vs. L23	F2-3 up	GO:0048574	Signaling and response	Signaling	long-day photoperiodism, flowering	P	AT2G46830
14980	3	F23 vs. L23	F2-3 up	GO:0048574	Signaling and response	Signaling	long-day photoperiodism, flowering	P	AT5G06850
14981	3	F23 vs. L23	F2-3 up	GO:0004724	Signaling and response	Signaling	magnesium-dependent protein serine/threonine phosphatase activity	F	AT2G30020
14982	3	F23 vs. L23	F2-3 up	GO:0004724	Signaling and response	Signaling	magnesium-dependent protein serine/threonine phosphatase activity	F	AT3G02750
14983	3	F23 vs. L23	F2-3 up	GO:0004724	Signaling and response	Signaling	magnesium-dependent protein serine/threonine phosphatase activity	F	AT3G11410
14984	3	F23 vs. L23	F2-3 up	GO:0004724	Signaling and response	Signaling	magnesium-dependent protein serine/threonine phosphatase activity	F	AT3G12620
14985	3	F23 vs. L23	F2-3 up	GO:0004724	Signaling and response	Signaling	magnesium-dependent protein serine/threonine phosphatase activity	F	AT3G16800
14986	3	F23 vs. L23	F2-3 up	GO:0004724	Signaling and response	Signaling	magnesium-dependent protein serine/threonine phosphatase activity	F	AT4G31750
14987	3	F23 vs. L23	F2-3 up	GO:0004707	Signaling and response	Signaling	MAP kinase activity	F	AT1G18150
14988	3	F23 vs. L23	F2-3 up	GO:0004707	Signaling and response	Signaling	MAP kinase activity	F	AT3G45640
14989	3	F23 vs. L23	F2-3 up	GO:0080030	Signaling and response	Signaling	methyl indole-3-acetate esterase activity	F	AT2G45610
14990	3	F23 vs. L23	F2-3 up	GO:0031930	Signaling and response	Signaling	mitochondria-nucleus signaling pathway	P	AT3G22370
14991	3	F23 vs. L23	F2-3 up	GO:0042754	Signaling and response	Signaling	negative regulation of circadian rhythm	P	AT2G46830
14992	3	F23 vs. L23	F2-3 up	GO:0009938	Signaling and response	Signaling	negative regulation of gibberellic acid mediated signaling pathway	P	AT1G14920
14993	3	F23 vs. L23	F2-3 up	GO:0009938	Signaling and response	Signaling	negative regulation of gibberellic acid mediated signaling pathway	P	AT5G56860
14994	3	F23 vs. L23	F2-3 up	GO:0010100	Signaling and response	Signaling	negative regulation of photomorphogenesis	P	AT2G31380
14995	3	F23 vs. L23	F2-3 up	GO:0007231	Signaling and response	Signaling	osmosensory signaling pathway	P	AT1G64060
14996	3	F23 vs. L23	F2-3 up	GO:0007231	Signaling and response	Signaling	osmosensory signaling pathway	P	AT5G47910
14997	3	F23 vs. L23	F2-3 up	GO:0002221	Signaling and response	Signaling	pattern recognition receptor signaling pathway	P	AT3G49110
14998	3	F23 vs. L23	F2-3 up	GO:0002221	Signaling and response	Signaling	pattern recognition receptor signaling pathway	P	AT5G13160
14999	3	F23 vs. L23	F2-3 up	GO:0017046	Signaling and response	Signaling	peptide hormone binding	F	AT1G72180
15000	3	F23 vs. L23	F2-3 up	GO:0001653	Signaling and response	Signaling	peptide receptor activity	F	AT1G72180
15001	3	F23 vs. L23	F2-3 up	GO:0001653	Signaling and response	Signaling	peptide receptor activity	F	AT5G53890
15002	3	F23 vs. L23	F2-3 up	GO:0018105	Signaling and response	Signaling	peptidyl-serine phosphorylation	P	AT2G17290
15003	3	F23 vs. L23	F2-3 up	GO:0018105	Signaling and response	Signaling	peptidyl-serine phosphorylation	P	AT2G20470
15004	3	F23 vs. L23	F2-3 up	GO:0018105	Signaling and response	Signaling	peptidyl-serine phosphorylation	P	AT2G31500
15005	3	F23 vs. L23	F2-3 up	GO:0018105	Signaling and response	Signaling	peptidyl-serine phosphorylation	P	AT3G50000
15006	3	F23 vs. L23	F2-3 up	GO:0018105	Signaling and response	Signaling	peptidyl-serine phosphorylation	P	AT3G61160
15007	3	F23 vs. L23	F2-3 up	GO:0018105	Signaling and response	Signaling	peptidyl-serine phosphorylation	P	AT4G26100
15008	3	F23 vs. L23	F2-3 up	GO:0018107	Signaling and response	Signaling	peptidyl-threonine phosphorylation	P	AT3G50000
15009	3	F23 vs. L23	F2-3 up	GO:0018107	Signaling and response	Signaling	peptidyl-threonine phosphorylation	P	AT3G61160
15010	3	F23 vs. L23	F2-3 up	GO:0016791	Signaling and response	Signaling	phosphatase activity	F	AT1G02390
15011	3	F23 vs. L23	F2-3 up	GO:0016791	Signaling and response	Signaling	phosphatase activity	F	AT2G38110

15012	3	F23 vs. L23	F2-3 up	GO:0016791	Signaling and response	Signaling	phosphatase activity	F	AT3G11410
15013	3	F23 vs. L23	F2-3 up	GO:0016791	Signaling and response	Signaling	phosphatase activity	F	AT3G17790
15014	3	F23 vs. L23	F2-3 up	GO:0036149	Signaling and response	Signaling	phosphatidylinositol acyl-chain remodeling	P	AT1G75020
15015	3	F23 vs. L23	F2-3 up	GO:0046856	Signaling and response	Signaling	phosphatidylinositol dephosphorylation	P	AT2G31830
15016	3	F23 vs. L23	F2-3 up	GO:0046856	Signaling and response	Signaling	phosphatidylinositol dephosphorylation	P	AT5G04980
15017	3	F23 vs. L23	F2-3 up	GO:0016307	Signaling and response	Signaling	phosphatidylinositol phosphate kinase activity	F	AT1G21980
15018	3	F23 vs. L23	F2-3 up	GO:0004435	Signaling and response	Signaling	phosphatidylinositol phospholipase C activity	F	AT3G08510
15019	3	F23 vs. L23	F2-3 up	GO:0004435	Signaling and response	Signaling	phosphatidylinositol phospholipase C activity	F	AT5G58690
15020	3	F23 vs. L23	F2-3 up	GO:0004435	Signaling and response	Signaling	phosphatidylinositol phospholipase C activity	F	AT5G58700
15021	3	F23 vs. L23	F2-3 up	GO:0034485	Signaling and response	Signaling	phosphatidylinositol-3,4,5-trisphosphate 5-phosphatase activity	F	AT2G31830
15022	3	F23 vs. L23	F2-3 up	GO:0034485	Signaling and response	Signaling	phosphatidylinositol-3,4,5-trisphosphate 5-phosphatase activity	F	AT5G04980
15023	3	F23 vs. L23	F2-3 up	GO:0004439	Signaling and response	Signaling	phosphatidylinositol-4,5-bisphosphate 5-phosphatase activity	F	AT2G31830
15024	3	F23 vs. L23	F2-3 up	GO:0004439	Signaling and response	Signaling	phosphatidylinositol-4,5-bisphosphate 5-phosphatase activity	F	AT5G04980
15025	3	F23 vs. L23	F2-3 up	GO:0004618	Signaling and response	Signaling	phosphoglycerate kinase activity	F	AT3G12780
15026	3	F23 vs. L23	F2-3 up	GO:0008967	Signaling and response	Signaling	phosphoglycolate phosphatase activity	F	AT5G36700
15027	3	F23 vs. L23	F2-3 up	GO:0016004	Signaling and response	Signaling	phospholipase activator activity	F	AT3G62290
15028	3	F23 vs. L23	F2-3 up	GO:0004620	Signaling and response	Signaling	phospholipase activity	F	AT3G54950
15029	3	F23 vs. L23	F2-3 up	GO:0004629	Signaling and response	Signaling	phospholipase C activity	F	AT3G08510
15030	3	F23 vs. L23	F2-3 up	GO:0004721	Signaling and response	Signaling	phosphoprotein phosphatase activity	F	AT3G11410
15031	3	F23 vs. L23	F2-3 up	GO:0004721	Signaling and response	Signaling	phosphoprotein phosphatase activity	F	AT3G19980
15032	3	F23 vs. L23	F2-3 up	GO:0000156	Signaling and response	Signaling	phosphorelay response regulator activity	F	AT2G40670
15033	3	F23 vs. L23	F2-3 up	GO:0000160	Signaling and response	Signaling	phosphorelay signal transduction system	P	AT2G40670
15034	3	F23 vs. L23	F2-3 up	GO:0010205	Signaling and response	Signaling	photoinhibition	P	AT1G50250
15035	3	F23 vs. L23	F2-3 up	GO:0009640	Signaling and response	Signaling	photomorphogenesis	P	AT1G69640
15036	3	F23 vs. L23	F2-3 up	GO:0009640	Signaling and response	Signaling	photomorphogenesis	P	AT2G31380
15037	3	F23 vs. L23	F2-3 up	GO:0048573	Signaling and response	Signaling	photoperiodism, flowering	P	AT1G12110
15038	3	F23 vs. L23	F2-3 up	GO:0048573	Signaling and response	Signaling	photoperiodism, flowering	P	AT1G30810
15039	3	F23 vs. L23	F2-3 up	GO:0048573	Signaling and response	Signaling	photoperiodism, flowering	P	AT5G48300
15040	3	F23 vs. L23	F2-3 up	GO:0009881	Signaling and response	Signaling	photoreceptor activity	F	AT2G47590
15041	3	F23 vs. L23	F2-3 up	GO:0009638	Signaling and response	Signaling	phototropism	P	AT1G04400
15042	3	F23 vs. L23	F2-3 up	GO:0009638	Signaling and response	Signaling	phototropism	P	AT2G30520
15043	3	F23 vs. L23	F2-3 up	GO:0009638	Signaling and response	Signaling	phototropism	P	AT3G15540
15044	3	F23 vs. L23	F2-3 up	GO:0009638	Signaling and response	Signaling	phototropism	P	AT3G45780
15045	3	F23 vs. L23	F2-3 up	GO:0009958	Signaling and response	Signaling	positive gravitropism	P	AT1G70940
15046	3	F23 vs. L23	F2-3 up	GO:0010601	Signaling and response	Signaling	positive regulation of auxin biosynthetic process	P	AT3G08510
15047	3	F23 vs. L23	F2-3 up	GO:0010929	Signaling and response	Signaling	positive regulation of auxin mediated signaling pathway	P	AT5G67300
15048	3	F23 vs. L23	F2-3 up	GO:0090355	Signaling and response	Signaling	positive regulation of auxin metabolic process	P	AT1G75500
15049	3	F23 vs. L23	F2-3 up	GO:0009939	Signaling and response	Signaling	positive regulation of gibberellic acid mediated signaling pathway	P	AT1G14920
15050	3	F23 vs. L23	F2-3 up	GO:0046777	Signaling and response	Signaling	protein autophosphorylation	P	AT2G17290
15051	3	F23 vs. L23	F2-3 up	GO:0046777	Signaling and response	Signaling	protein autophosphorylation	P	AT2G31500
15052	3	F23 vs. L23	F2-3 up	GO:0046777	Signaling and response	Signaling	protein autophosphorylation	P	AT3G03770
15053	3	F23 vs. L23	F2-3 up	GO:0046777	Signaling and response	Signaling	protein autophosphorylation	P	AT3G45780
15054	3	F23 vs. L23	F2-3 up	GO:0046777	Signaling and response	Signaling	protein autophosphorylation	P	AT3G61160
15055	3	F23 vs. L23	F2-3 up	GO:0046777	Signaling and response	Signaling	protein autophosphorylation	P	AT4G28490
15056	3	F23 vs. L23	F2-3 up	GO:0046777	Signaling and response	Signaling	protein autophosphorylation	P	AT4G39110
15057	3	F23 vs. L23	F2-3 up	GO:0046777	Signaling and response	Signaling	protein autophosphorylation	P	AT5G13160
15058	3	F23 vs. L23	F2-3 up	GO:0046777	Signaling and response	Signaling	protein autophosphorylation	P	AT5G53890
15059	3	F23 vs. L23	F2-3 up	GO:0006470	Signaling and response	Signaling	protein dephosphorylation	P	AT2G30020
15060	3	F23 vs. L23	F2-3 up	GO:0006470	Signaling and response	Signaling	protein dephosphorylation	P	AT3G02750
15061	3	F23 vs. L23	F2-3 up	GO:0006470	Signaling and response	Signaling	protein dephosphorylation	P	AT3G05580

15062	3	F23 vs. L23	F2-3 up	GO:0006470	Signaling and response	Signaling	protein dephosphorylation	P	AT3G11410
15063	3	F23 vs. L23	F2-3 up	GO:0006470	Signaling and response	Signaling	protein dephosphorylation	P	AT3G12620
15064	3	F23 vs. L23	F2-3 up	GO:0006470	Signaling and response	Signaling	protein dephosphorylation	P	AT3G16800
15065	3	F23 vs. L23	F2-3 up	GO:0006470	Signaling and response	Signaling	protein dephosphorylation	P	AT3G19980
15066	3	F23 vs. L23	F2-3 up	GO:0006470	Signaling and response	Signaling	protein dephosphorylation	P	AT4G31750
15067	3	F23 vs. L23	F2-3 up	GO:0006470	Signaling and response	Signaling	protein dephosphorylation	P	AT5G43380
15068	3	F23 vs. L23	F2-3 up	GO:0004672	Signaling and response	Signaling	protein kinase activity	F	AT2G18470
15069	3	F23 vs. L23	F2-3 up	GO:0004672	Signaling and response	Signaling	protein kinase activity	F	AT2G30360
15070	3	F23 vs. L23	F2-3 up	GO:0004672	Signaling and response	Signaling	protein kinase activity	F	AT2G31500
15071	3	F23 vs. L23	F2-3 up	GO:0004672	Signaling and response	Signaling	protein kinase activity	F	AT3G03770
15072	3	F23 vs. L23	F2-3 up	GO:0004672	Signaling and response	Signaling	protein kinase activity	F	AT3G07700
15073	3	F23 vs. L23	F2-3 up	GO:0004672	Signaling and response	Signaling	protein kinase activity	F	AT3G08720
15074	3	F23 vs. L23	F2-3 up	GO:0004672	Signaling and response	Signaling	protein kinase activity	F	AT3G08730
15075	3	F23 vs. L23	F2-3 up	GO:0004672	Signaling and response	Signaling	protein kinase activity	F	AT3G12780
15076	3	F23 vs. L23	F2-3 up	GO:0004672	Signaling and response	Signaling	protein kinase activity	F	AT3G20190
15077	3	F23 vs. L23	F2-3 up	GO:0004672	Signaling and response	Signaling	protein kinase activity	F	AT3G24190
15078	3	F23 vs. L23	F2-3 up	GO:0004672	Signaling and response	Signaling	protein kinase activity	F	AT3G45640
15079	3	F23 vs. L23	F2-3 up	GO:0004672	Signaling and response	Signaling	protein kinase activity	F	AT3G45780
15080	3	F23 vs. L23	F2-3 up	GO:0004672	Signaling and response	Signaling	protein kinase activity	F	AT4G18950
15081	3	F23 vs. L23	F2-3 up	GO:0004672	Signaling and response	Signaling	protein kinase activity	F	AT4G28490
15082	3	F23 vs. L23	F2-3 up	GO:0004672	Signaling and response	Signaling	protein kinase activity	F	AT4G31230
15083	3	F23 vs. L23	F2-3 up	GO:0004672	Signaling and response	Signaling	protein kinase activity	F	AT4G39110
15084	3	F23 vs. L23	F2-3 up	GO:0004672	Signaling and response	Signaling	protein kinase activity	F	AT5G12000
15085	3	F23 vs. L23	F2-3 up	GO:0004672	Signaling and response	Signaling	protein kinase activity	F	AT5G13160
15086	3	F23 vs. L23	F2-3 up	GO:0000159	Signaling and response	Signaling	protein phosphatase type 2A complex	C	AT3G19980
15087	3	F23 vs. L23	F2-3 up	GO:0045309	Signaling and response	Signaling	protein phosphorylated amino acid binding	F	AT1G26480
15088	3	F23 vs. L23	F2-3 up	GO:0006468	Signaling and response	Signaling	protein phosphorylation	P	AT1G09600
15089	3	F23 vs. L23	F2-3 up	GO:0006468	Signaling and response	Signaling	protein phosphorylation	P	AT1G14000
15090	3	F23 vs. L23	F2-3 up	GO:0006468	Signaling and response	Signaling	protein phosphorylation	P	AT1G50610
15091	3	F23 vs. L23	F2-3 up	GO:0006468	Signaling and response	Signaling	protein phosphorylation	P	AT1G76370
15092	3	F23 vs. L23	F2-3 up	GO:0006468	Signaling and response	Signaling	protein phosphorylation	P	AT1G78290
15093	3	F23 vs. L23	F2-3 up	GO:0006468	Signaling and response	Signaling	protein phosphorylation	P	AT2G02800
15094	3	F23 vs. L23	F2-3 up	GO:0006468	Signaling and response	Signaling	protein phosphorylation	P	AT2G07180
15095	3	F23 vs. L23	F2-3 up	GO:0006468	Signaling and response	Signaling	protein phosphorylation	P	AT2G16750
15096	3	F23 vs. L23	F2-3 up	GO:0006468	Signaling and response	Signaling	protein phosphorylation	P	AT2G17220
15097	3	F23 vs. L23	F2-3 up	GO:0006468	Signaling and response	Signaling	protein phosphorylation	P	AT2G17290
15098	3	F23 vs. L23	F2-3 up	GO:0006468	Signaling and response	Signaling	protein phosphorylation	P	AT2G18470
15099	3	F23 vs. L23	F2-3 up	GO:0006468	Signaling and response	Signaling	protein phosphorylation	P	AT2G30360
15100	3	F23 vs. L23	F2-3 up	GO:0006468	Signaling and response	Signaling	protein phosphorylation	P	AT3G08720
15101	3	F23 vs. L23	F2-3 up	GO:0006468	Signaling and response	Signaling	protein phosphorylation	P	AT3G08730
15102	3	F23 vs. L23	F2-3 up	GO:0006468	Signaling and response	Signaling	protein phosphorylation	P	AT3G12690
15103	3	F23 vs. L23	F2-3 up	GO:0006468	Signaling and response	Signaling	protein phosphorylation	P	AT3G13065
15104	3	F23 vs. L23	F2-3 up	GO:0006468	Signaling and response	Signaling	protein phosphorylation	P	AT3G20190
15105	3	F23 vs. L23	F2-3 up	GO:0006468	Signaling and response	Signaling	protein phosphorylation	P	AT3G20530
15106	3	F23 vs. L23	F2-3 up	GO:0006468	Signaling and response	Signaling	protein phosphorylation	P	AT3G24190
15107	3	F23 vs. L23	F2-3 up	GO:0006468	Signaling and response	Signaling	protein phosphorylation	P	AT3G45640
15108	3	F23 vs. L23	F2-3 up	GO:0006468	Signaling and response	Signaling	protein phosphorylation	P	AT3G45780
15109	3	F23 vs. L23	F2-3 up	GO:0006468	Signaling and response	Signaling	protein phosphorylation	P	AT3G50000
15110	3	F23 vs. L23	F2-3 up	GO:0006468	Signaling and response	Signaling	protein phosphorylation	P	AT3G59830
15111	3	F23 vs. L23	F2-3 up	GO:0006468	Signaling and response	Signaling	protein phosphorylation	P	AT4G18950
15112	3	F23 vs. L23	F2-3 up	GO:0006468	Signaling and response	Signaling	protein phosphorylation	P	AT4G31230
15113	3	F23 vs. L23	F2-3 up	GO:0006468	Signaling and response	Signaling	protein phosphorylation	P	AT4G35500
15114	3	F23 vs. L23	F2-3 up	GO:0006468	Signaling and response	Signaling	protein phosphorylation	P	AT5G08160
15115	3	F23 vs. L23	F2-3 up	GO:0006468	Signaling and response	Signaling	protein phosphorylation	P	AT5G12000
15116	3	F23 vs. L23	F2-3 up	GO:0006468	Signaling and response	Signaling	protein phosphorylation	P	AT5G13160
15117	3	F23 vs. L23	F2-3 up	GO:0006468	Signaling and response	Signaling	protein phosphorylation	P	AT5G24080

15118	3	F23 vs. L23	F2-3 up	GO:0006468	Signaling and response	Signaling	protein phosphorylation	P	AT5G45840
15119	3	F23 vs. L23	F2-3 up	GO:0004674	Signaling and response	Signaling	protein serine/threonine kinase activity	F	AT1G14000
15120	3	F23 vs. L23	F2-3 up	GO:0004674	Signaling and response	Signaling	protein serine/threonine kinase activity	F	AT1G50610
15121	3	F23 vs. L23	F2-3 up	GO:0004674	Signaling and response	Signaling	protein serine/threonine kinase activity	F	AT1G72180
15122	3	F23 vs. L23	F2-3 up	GO:0004674	Signaling and response	Signaling	protein serine/threonine kinase activity	F	AT1G78290
15123	3	F23 vs. L23	F2-3 up	GO:0004674	Signaling and response	Signaling	protein serine/threonine kinase activity	F	AT2G02800
15124	3	F23 vs. L23	F2-3 up	GO:0004674	Signaling and response	Signaling	protein serine/threonine kinase activity	F	AT2G03410
15125	3	F23 vs. L23	F2-3 up	GO:0004674	Signaling and response	Signaling	protein serine/threonine kinase activity	F	AT2G07180
15126	3	F23 vs. L23	F2-3 up	GO:0004674	Signaling and response	Signaling	protein serine/threonine kinase activity	F	AT2G17220
15127	3	F23 vs. L23	F2-3 up	GO:0004674	Signaling and response	Signaling	protein serine/threonine kinase activity	F	AT2G17290
15128	3	F23 vs. L23	F2-3 up	GO:0004674	Signaling and response	Signaling	protein serine/threonine kinase activity	F	AT2G20470
15129	3	F23 vs. L23	F2-3 up	GO:0004674	Signaling and response	Signaling	protein serine/threonine kinase activity	F	AT2G30360
15130	3	F23 vs. L23	F2-3 up	GO:0004674	Signaling and response	Signaling	protein serine/threonine kinase activity	F	AT3G08720
15131	3	F23 vs. L23	F2-3 up	GO:0004674	Signaling and response	Signaling	protein serine/threonine kinase activity	F	AT3G08730
15132	3	F23 vs. L23	F2-3 up	GO:0004674	Signaling and response	Signaling	protein serine/threonine kinase activity	F	AT3G12690
15133	3	F23 vs. L23	F2-3 up	GO:0004674	Signaling and response	Signaling	protein serine/threonine kinase activity	F	AT3G19980
15134	3	F23 vs. L23	F2-3 up	GO:0004674	Signaling and response	Signaling	protein serine/threonine kinase activity	F	AT3G45780
15135	3	F23 vs. L23	F2-3 up	GO:0004674	Signaling and response	Signaling	protein serine/threonine kinase activity	F	AT3G50000
15136	3	F23 vs. L23	F2-3 up	GO:0004674	Signaling and response	Signaling	protein serine/threonine kinase activity	F	AT3G59830
15137	3	F23 vs. L23	F2-3 up	GO:0004674	Signaling and response	Signaling	protein serine/threonine kinase activity	F	AT3G61160
15138	3	F23 vs. L23	F2-3 up	GO:0004674	Signaling and response	Signaling	protein serine/threonine kinase activity	F	AT4G18950
15139	3	F23 vs. L23	F2-3 up	GO:0004674	Signaling and response	Signaling	protein serine/threonine kinase activity	F	AT4G26100
15140	3	F23 vs. L23	F2-3 up	GO:0004674	Signaling and response	Signaling	protein serine/threonine kinase activity	F	AT4G28490
15141	3	F23 vs. L23	F2-3 up	GO:0004674	Signaling and response	Signaling	protein serine/threonine kinase activity	F	AT4G35500
15142	3	F23 vs. L23	F2-3 up	GO:0004674	Signaling and response	Signaling	protein serine/threonine kinase activity	F	AT4G39110
15143	3	F23 vs. L23	F2-3 up	GO:0004674	Signaling and response	Signaling	protein serine/threonine kinase activity	F	AT5G08160
15144	3	F23 vs. L23	F2-3 up	GO:0004674	Signaling and response	Signaling	protein serine/threonine kinase activity	F	AT5G13160
15145	3	F23 vs. L23	F2-3 up	GO:0004674	Signaling and response	Signaling	protein serine/threonine kinase activity	F	AT5G45840
15146	3	F23 vs. L23	F2-3 up	GO:0004674	Signaling and response	Signaling	protein serine/threonine kinase activity	F	AT5G53890
15147	3	F23 vs. L23	F2-3 up	GO:0004722	Signaling and response	Signaling	protein serine/threonine phosphatase activity	F	AT2G30020
15148	3	F23 vs. L23	F2-3 up	GO:0004722	Signaling and response	Signaling	protein serine/threonine phosphatase activity	F	AT3G02750
15149	3	F23 vs. L23	F2-3 up	GO:0004722	Signaling and response	Signaling	protein serine/threonine phosphatase activity	F	AT3G05580
15150	3	F23 vs. L23	F2-3 up	GO:0004722	Signaling and response	Signaling	protein serine/threonine phosphatase activity	F	AT3G11410
15151	3	F23 vs. L23	F2-3 up	GO:0004722	Signaling and response	Signaling	protein serine/threonine phosphatase activity	F	AT3G12620
15152	3	F23 vs. L23	F2-3 up	GO:0004722	Signaling and response	Signaling	protein serine/threonine phosphatase activity	F	AT3G16800
15153	3	F23 vs. L23	F2-3 up	GO:0004722	Signaling and response	Signaling	protein serine/threonine phosphatase activity	F	AT4G31750
15154	3	F23 vs. L23	F2-3 up	GO:0004722	Signaling and response	Signaling	protein serine/threonine phosphatase activity	F	AT5G43380
15155	3	F23 vs. L23	F2-3 up	GO:0004712	Signaling and response	Signaling	protein serine/threonine/tyrosine kinase activity	F	AT1G14000
15156	3	F23 vs. L23	F2-3 up	GO:0004712	Signaling and response	Signaling	protein serine/threonine/tyrosine kinase activity	F	AT3G59830
15157	3	F23 vs. L23	F2-3 up	GO:0004712	Signaling and response	Signaling	protein serine/threonine/tyrosine kinase activity	F	AT4G18950
15158	3	F23 vs. L23	F2-3 up	GO:0047134	Signaling and response	Signaling	protein-disulfide reductase activity	F	AT1G50320
15159	3	F23 vs. L23	F2-3 up	GO:0047134	Signaling and response	Signaling	protein-disulfide reductase activity	F	AT3G15360
15160	3	F23 vs. L23	F2-3 up	GO:0005093	Signaling and response	Signaling	Rab GDP-dissociation inhibitor activity	F	AT5G09550
15161	3	F23 vs. L23	F2-3 up	GO:0017137	Signaling and response	Signaling	Rab GTPase binding	F	AT5G09550
15162	3	F23 vs. L23	F2-3 up	GO:0017137	Signaling and response	Signaling	Rab GTPase binding	F	AT5G54780
15163	3	F23 vs. L23	F2-3 up	GO:0032482	Signaling and response	Signaling	Rab protein signal transduction	P	AT2G21880
15164	3	F23 vs. L23	F2-3 up	GO:0032482	Signaling and response	Signaling	Rab protein signal transduction	P	AT3G21700
15165	3	F23 vs. L23	F2-3 up	GO:0032482	Signaling and response	Signaling	Rab protein signal transduction	P	AT4G17170
15166	3	F23 vs. L23	F2-3 up	GO:0032482	Signaling and response	Signaling	Rab protein signal transduction	P	AT4G17530
15167	3	F23 vs. L23	F2-3 up	GO:0048365	Signaling and response	Signaling	Rac GTPase binding	F	AT1G12070
15168	3	F23 vs. L23	F2-3 up	GO:0048365	Signaling and response	Signaling	Rac GTPase binding	F	AT1G62450
15169	3	F23 vs. L23	F2-3 up	GO:0010017	Signaling and response	Signaling	red or far-red light signaling pathway	P	AT2G24540
15170	3	F23 vs. L23	F2-3 up	GO:0010017	Signaling and response	Signaling	red or far-red light signaling pathway	P	AT2G37970
15171	3	F23 vs. L23	F2-3 up	GO:0009585	Signaling and response	Signaling	red, far-red light phototransduction	P	AT2G24540
15172	3	F23 vs. L23	F2-3 up	GO:0009585	Signaling and response	Signaling	red, far-red light phototransduction	P	AT4G38740
15173	3	F23 vs. L23	F2-3 up	GO:0010600	Signaling and response	Signaling	regulation of auxin biosynthetic process	P	AT5G05730

15174	3	F23 vs. L23	F2-3 up	GO:0010928	Signaling and response	Signaling	regulation of auxin mediated signaling pathway	P	AT1G27340
15175	3	F23 vs. L23	F2-3 up	GO:2000012	Signaling and response	Signaling	regulation of auxin polar transport	P	AT5G11790
15176	3	F23 vs. L23	F2-3 up	GO:0042752	Signaling and response	Signaling	regulation of circadian rhythm	P	AT1G04400
15177	3	F23 vs. L23	F2-3 up	GO:0042752	Signaling and response	Signaling	regulation of circadian rhythm	P	AT3G50000
15178	3	F23 vs. L23	F2-3 up	GO:0010371	Signaling and response	Signaling	regulation of gibberellin biosynthetic process	P	AT5G65410
15179	3	F23 vs. L23	F2-3 up	GO:2000022	Signaling and response	Signaling	regulation of jasmonic acid mediated signaling pathway	P	AT1G16310
15180	3	F23 vs. L23	F2-3 up	GO:2000022	Signaling and response	Signaling	regulation of jasmonic acid mediated signaling pathway	P	AT1G17380
15181	3	F23 vs. L23	F2-3 up	GO:2000022	Signaling and response	Signaling	regulation of jasmonic acid mediated signaling pathway	P	AT1G19180
15182	3	F23 vs. L23	F2-3 up	GO:2000022	Signaling and response	Signaling	regulation of jasmonic acid mediated signaling pathway	P	AT1G70700
15183	3	F23 vs. L23	F2-3 up	GO:2000022	Signaling and response	Signaling	regulation of jasmonic acid mediated signaling pathway	P	AT2G34600
15184	3	F23 vs. L23	F2-3 up	GO:2000022	Signaling and response	Signaling	regulation of jasmonic acid mediated signaling pathway	P	AT2G38240
15185	3	F23 vs. L23	F2-3 up	GO:2000022	Signaling and response	Signaling	regulation of jasmonic acid mediated signaling pathway	P	AT5G05600
15186	3	F23 vs. L23	F2-3 up	GO:2000022	Signaling and response	Signaling	regulation of jasmonic acid mediated signaling pathway	P	AT5G67300
15187	3	F23 vs. L23	F2-3 up	GO:2000028	Signaling and response	Signaling	regulation of photoperiodism, flowering	P	AT1G04400
15188	3	F23 vs. L23	F2-3 up	GO:2000028	Signaling and response	Signaling	regulation of photoperiodism, flowering	P	AT5G56860
15189	3	F23 vs. L23	F2-3 up	GO:0001932	Signaling and response	Signaling	regulation of protein phosphorylation	P	AT4G38740
15190	3	F23 vs. L23	F2-3 up	GO:2000030	Signaling and response	Signaling	regulation of response to red or far red light	P	AT1G78370
15191	3	F23 vs. L23	F2-3 up	GO:2000030	Signaling and response	Signaling	regulation of response to red or far red light	P	AT4G04770
15192	3	F23 vs. L23	F2-3 up	GO:2000280	Signaling and response	Signaling	regulation of root development	P	AT1G72180
15193	3	F23 vs. L23	F2-3 up	GO:2000280	Signaling and response	Signaling	regulation of root development	P	AT3G05930
15194	3	F23 vs. L23	F2-3 up	GO:0080142	Signaling and response	Signaling	regulation of salicylic acid biosynthetic process	P	AT5G62570
15195	3	F23 vs. L23	F2-3 up	GO:2000031	Signaling and response	Signaling	regulation of salicylic acid mediated signaling pathway	P	AT1G16310
15196	3	F23 vs. L23	F2-3 up	GO:2000031	Signaling and response	Signaling	regulation of salicylic acid mediated signaling pathway	P	AT5G67300
15197	3	F23 vs. L23	F2-3 up	GO:0010029	Signaling and response	Signaling	regulation of seed germination	P	AT1G14920
15198	3	F23 vs. L23	F2-3 up	GO:0010029	Signaling and response	Signaling	regulation of seed germination	P	AT5G56860
15199	3	F23 vs. L23	F2-3 up	GO:0060320	Signaling and response	Signaling	rejection of self pollen	P	AT5G26060
15200	3	F23 vs. L23	F2-3 up	GO:0048838	Signaling and response	Signaling	release of seed from dormancy	P	AT4G19230
15201	3	F23 vs. L23	F2-3 up	GO:0072722	Signaling and response	Signaling	response to amitrole	P	AT2G43570
15202	3	F23 vs. L23	F2-3 up	GO:0060359	Signaling and response	Signaling	response to ammonium ion	P	AT2G39770
15203	3	F23 vs. L23	F2-3 up	GO:0009733	Signaling and response	Signaling	response to auxin	P	AT1G30330
15204	3	F23 vs. L23	F2-3 up	GO:0009733	Signaling and response	Signaling	response to auxin	P	AT2G21220
15205	3	F23 vs. L23	F2-3 up	GO:0009733	Signaling and response	Signaling	response to auxin	P	AT2G22240
15206	3	F23 vs. L23	F2-3 up	GO:0009733	Signaling and response	Signaling	response to auxin	P	AT2G26740
15207	3	F23 vs. L23	F2-3 up	GO:0009733	Signaling and response	Signaling	response to auxin	P	AT2G37630
15208	3	F23 vs. L23	F2-3 up	GO:0009733	Signaling and response	Signaling	response to auxin	P	AT2G46830
15209	3	F23 vs. L23	F2-3 up	GO:0009733	Signaling and response	Signaling	response to auxin	P	AT3G15540
15210	3	F23 vs. L23	F2-3 up	GO:0009733	Signaling and response	Signaling	response to auxin	P	AT3G20220
15211	3	F23 vs. L23	F2-3 up	GO:0009733	Signaling and response	Signaling	response to auxin	P	AT3G23050
15212	3	F23 vs. L23	F2-3 up	GO:0009733	Signaling and response	Signaling	response to auxin	P	AT3G43120
15213	3	F23 vs. L23	F2-3 up	GO:0009733	Signaling and response	Signaling	response to auxin	P	AT4G00880
15214	3	F23 vs. L23	F2-3 up	GO:0009733	Signaling and response	Signaling	response to auxin	P	AT4G03190
15215	3	F23 vs. L23	F2-3 up	GO:0009733	Signaling and response	Signaling	response to auxin	P	AT4G11280
15216	3	F23 vs. L23	F2-3 up	GO:0009733	Signaling and response	Signaling	response to auxin	P	AT4G12410
15217	3	F23 vs. L23	F2-3 up	GO:0009733	Signaling and response	Signaling	response to auxin	P	AT4G32880
15218	3	F23 vs. L23	F2-3 up	GO:0009733	Signaling and response	Signaling	response to auxin	P	AT5G08640
15219	3	F23 vs. L23	F2-3 up	GO:0009733	Signaling and response	Signaling	response to auxin	P	AT5G09810

15220	3	F23 vs. L23	F2-3 up	GO:0009733	Signaling and response	Signaling	response to auxin	P	AT5G13930
15221	3	F23 vs. L23	F2-3 up	GO:0009733	Signaling and response	Signaling	response to auxin	P	AT5G45890
15222	3	F23 vs. L23	F2-3 up	GO:0009733	Signaling and response	Signaling	response to auxin	P	AT5G53590
15223	3	F23 vs. L23	F2-3 up	GO:0009733	Signaling and response	Signaling	response to auxin	P	AT5G54490
15224	3	F23 vs. L23	F2-3 up	GO:0009733	Signaling and response	Signaling	response to auxin	P	AT5G54510
15225	3	F23 vs. L23	F2-3 up	GO:0009733	Signaling and response	Signaling	response to auxin	P	AT5G56860
15226	3	F23 vs. L23	F2-3 up	GO:0009733	Signaling and response	Signaling	response to auxin	P	AT5G57560
15227	3	F23 vs. L23	F2-3 up	GO:0009733	Signaling and response	Signaling	response to auxin	P	AT5G67300
15228	3	F23 vs. L23	F2-3 up	GO:0009733	Signaling and response	Signaling	response to auxin	P	AT5G67480
15229	3	F23 vs. L23	F2-3 up	GO:0009617	Signaling and response	Signaling	response to bacterium	P	AT1G12240
15230	3	F23 vs. L23	F2-3 up	GO:0009617	Signaling and response	Signaling	response to bacterium	P	AT1G72520
15231	3	F23 vs. L23	F2-3 up	GO:0009617	Signaling and response	Signaling	response to bacterium	P	AT1G80460
15232	3	F23 vs. L23	F2-3 up	GO:0009617	Signaling and response	Signaling	response to bacterium	P	AT2G45220
15233	3	F23 vs. L23	F2-3 up	GO:0009617	Signaling and response	Signaling	response to bacterium	P	AT3G45140
15234	3	F23 vs. L23	F2-3 up	GO:0009617	Signaling and response	Signaling	response to bacterium	P	AT3G45640
15235	3	F23 vs. L23	F2-3 up	GO:0009617	Signaling and response	Signaling	response to bacterium	P	AT4G37990
15236	3	F23 vs. L23	F2-3 up	GO:0009617	Signaling and response	Signaling	response to bacterium	P	AT5G01600
15237	3	F23 vs. L23	F2-3 up	GO:0009617	Signaling and response	Signaling	response to bacterium	P	AT5G05730
15238	3	F23 vs. L23	F2-3 up	GO:0009617	Signaling and response	Signaling	response to bacterium	P	AT5G06320
15239	3	F23 vs. L23	F2-3 up	GO:0009617	Signaling and response	Signaling	response to bacterium	P	AT5G24530
15240	3	F23 vs. L23	F2-3 up	GO:0009617	Signaling and response	Signaling	response to bacterium	P	AT5G60600
15241	3	F23 vs. L23	F2-3 up	GO:0009617	Signaling and response	Signaling	response to bacterium	P	AT5G67160
15242	3	F23 vs. L23	F2-3 up	GO:0009607	Signaling and response	Signaling	response to biotic stimulus	P	AT2G33670
15243	3	F23 vs. L23	F2-3 up	GO:0009637	Signaling and response	Signaling	response to blue light	P	AT1G04400
15244	3	F23 vs. L23	F2-3 up	GO:0009637	Signaling and response	Signaling	response to blue light	P	AT1G19150
15245	3	F23 vs. L23	F2-3 up	GO:0009637	Signaling and response	Signaling	response to blue light	P	AT3G45780
15246	3	F23 vs. L23	F2-3 up	GO:0009637	Signaling and response	Signaling	response to blue light	P	AT5G24120
15247	3	F23 vs. L23	F2-3 up	GO:0009637	Signaling and response	Signaling	response to blue light	P	AT5G38420
15248	3	F23 vs. L23	F2-3 up	GO:0010036	Signaling and response	Signaling	response to boron-containing substance	P	AT2G47160
15249	3	F23 vs. L23	F2-3 up	GO:0009741	Signaling and response	Signaling	response to brassinosteroid	P	AT5G57560
15250	3	F23 vs. L23	F2-3 up	GO:0046686	Signaling and response	Signaling	response to cadmium ion	P	AT1G07440
15251	3	F23 vs. L23	F2-3 up	GO:0046686	Signaling and response	Signaling	response to cadmium ion	P	AT1G11840
15252	3	F23 vs. L23	F2-3 up	GO:0046686	Signaling and response	Signaling	response to cadmium ion	P	AT1G11860
15253	3	F23 vs. L23	F2-3 up	GO:0046686	Signaling and response	Signaling	response to cadmium ion	P	AT1G20620
15254	3	F23 vs. L23	F2-3 up	GO:0046686	Signaling and response	Signaling	response to cadmium ion	P	AT1G35720
15255	3	F23 vs. L23	F2-3 up	GO:0046686	Signaling and response	Signaling	response to cadmium ion	P	AT1G42970
15256	3	F23 vs. L23	F2-3 up	GO:0046686	Signaling and response	Signaling	response to cadmium ion	P	AT1G48030
15257	3	F23 vs. L23	F2-3 up	GO:0046686	Signaling and response	Signaling	response to cadmium ion	P	AT1G70730
15258	3	F23 vs. L23	F2-3 up	GO:0046686	Signaling and response	Signaling	response to cadmium ion	P	AT2G21330
15259	3	F23 vs. L23	F2-3 up	GO:0046686	Signaling and response	Signaling	response to cadmium ion	P	AT2G30860
15260	3	F23 vs. L23	F2-3 up	GO:0046686	Signaling and response	Signaling	response to cadmium ion	P	AT2G30970
15261	3	F23 vs. L23	F2-3 up	GO:0046686	Signaling and response	Signaling	response to cadmium ion	P	AT2G35840
15262	3	F23 vs. L23	F2-3 up	GO:0046686	Signaling and response	Signaling	response to cadmium ion	P	AT2G37630
15263	3	F23 vs. L23	F2-3 up	GO:0046686	Signaling and response	Signaling	response to cadmium ion	P	AT2G37760
15264	3	F23 vs. L23	F2-3 up	GO:0046686	Signaling and response	Signaling	response to cadmium ion	P	AT2G41380
15265	3	F23 vs. L23	F2-3 up	GO:0046686	Signaling and response	Signaling	response to cadmium ion	P	AT2G46830
15266	3	F23 vs. L23	F2-3 up	GO:0046686	Signaling and response	Signaling	response to cadmium ion	P	AT3G02570
15267	3	F23 vs. L23	F2-3 up	GO:0046686	Signaling and response	Signaling	response to cadmium ion	P	AT3G04120
15268	3	F23 vs. L23	F2-3 up	GO:0046686	Signaling and response	Signaling	response to cadmium ion	P	AT3G09440
15269	3	F23 vs. L23	F2-3 up	GO:0046686	Signaling and response	Signaling	response to cadmium ion	P	AT3G12780
15270	3	F23 vs. L23	F2-3 up	GO:0046686	Signaling and response	Signaling	response to cadmium ion	P	AT3G13110
15271	3	F23 vs. L23	F2-3 up	GO:0046686	Signaling and response	Signaling	response to cadmium ion	P	AT3G22890
15272	3	F23 vs. L23	F2-3 up	GO:0046686	Signaling and response	Signaling	response to cadmium ion	P	AT3G27300
15273	3	F23 vs. L23	F2-3 up	GO:0046686	Signaling and response	Signaling	response to cadmium ion	P	AT3G48990
15274	3	F23 vs. L23	F2-3 up	GO:0046686	Signaling and response	Signaling	response to cadmium ion	P	AT3G52880
15275	3	F23 vs. L23	F2-3 up	GO:0046686	Signaling and response	Signaling	response to cadmium ion	P	AT3G56240

15276	3	F23 vs. L23	F2-3 up	GO:0046686	Signaling and response	Signaling	response to cadmium ion	P	AT4G04460
15277	3	F23 vs. L23	F2-3 up	GO:0046686	Signaling and response	Signaling	response to cadmium ion	P	AT4G11600
15278	3	F23 vs. L23	F2-3 up	GO:0046686	Signaling and response	Signaling	response to cadmium ion	P	AT4G13930
15279	3	F23 vs. L23	F2-3 up	GO:0046686	Signaling and response	Signaling	response to cadmium ion	P	AT4G14880
15280	3	F23 vs. L23	F2-3 up	GO:0046686	Signaling and response	Signaling	response to cadmium ion	P	AT4G23100
15281	3	F23 vs. L23	F2-3 up	GO:0046686	Signaling and response	Signaling	response to cadmium ion	P	AT4G26970
15282	3	F23 vs. L23	F2-3 up	GO:0046686	Signaling and response	Signaling	response to cadmium ion	P	AT4G33010
15283	3	F23 vs. L23	F2-3 up	GO:0046686	Signaling and response	Signaling	response to cadmium ion	P	AT4G34050
15284	3	F23 vs. L23	F2-3 up	GO:0046686	Signaling and response	Signaling	response to cadmium ion	P	AT4G38740
15285	3	F23 vs. L23	F2-3 up	GO:0046686	Signaling and response	Signaling	response to cadmium ion	P	AT4G38970
15286	3	F23 vs. L23	F2-3 up	GO:0046686	Signaling and response	Signaling	response to cadmium ion	P	AT5G02790
15287	3	F23 vs. L23	F2-3 up	GO:0046686	Signaling and response	Signaling	response to cadmium ion	P	AT5G07440
15288	3	F23 vs. L23	F2-3 up	GO:0046686	Signaling and response	Signaling	response to cadmium ion	P	AT5G09650
15289	3	F23 vs. L23	F2-3 up	GO:0046686	Signaling and response	Signaling	response to cadmium ion	P	AT5G12170
15290	3	F23 vs. L23	F2-3 up	GO:0046686	Signaling and response	Signaling	response to cadmium ion	P	AT5G23120
15291	3	F23 vs. L23	F2-3 up	GO:0046686	Signaling and response	Signaling	response to cadmium ion	P	AT5G35630
15292	3	F23 vs. L23	F2-3 up	GO:0046686	Signaling and response	Signaling	response to cadmium ion	P	AT5G53460
15293	3	F23 vs. L23	F2-3 up	GO:0046686	Signaling and response	Signaling	response to cadmium ion	P	AT5G63680
15294	3	F23 vs. L23	F2-3 up	GO:0046686	Signaling and response	Signaling	response to cadmium ion	P	AT5G67300
15295	3	F23 vs. L23	F2-3 up	GO:0010037	Signaling and response	Signaling	response to carbon dioxide	P	AT3G01500
15296	3	F23 vs. L23	F2-3 up	GO:0010200	Signaling and response	Signaling	response to chitin	P	AT1G20823
15297	3	F23 vs. L23	F2-3 up	GO:0010200	Signaling and response	Signaling	response to chitin	P	AT1G27730
15298	3	F23 vs. L23	F2-3 up	GO:0010200	Signaling and response	Signaling	response to chitin	P	AT1G32640
15299	3	F23 vs. L23	F2-3 up	GO:0010200	Signaling and response	Signaling	response to chitin	P	AT1G80840
15300	3	F23 vs. L23	F2-3 up	GO:0010200	Signaling and response	Signaling	response to chitin	P	AT2G34600
15301	3	F23 vs. L23	F2-3 up	GO:0010200	Signaling and response	Signaling	response to chitin	P	AT2G40140
15302	3	F23 vs. L23	F2-3 up	GO:0010200	Signaling and response	Signaling	response to chitin	P	AT3G45640
15303	3	F23 vs. L23	F2-3 up	GO:0010200	Signaling and response	Signaling	response to chitin	P	AT3G46600
15304	3	F23 vs. L23	F2-3 up	GO:0010200	Signaling and response	Signaling	response to chitin	P	AT3G46620
15305	3	F23 vs. L23	F2-3 up	GO:0010200	Signaling and response	Signaling	response to chitin	P	AT3G52800
15306	3	F23 vs. L23	F2-3 up	GO:0010200	Signaling and response	Signaling	response to chitin	P	AT4G17230
15307	3	F23 vs. L23	F2-3 up	GO:0010200	Signaling and response	Signaling	response to chitin	P	AT4G31800
15308	3	F23 vs. L23	F2-3 up	GO:0010200	Signaling and response	Signaling	response to chitin	P	AT5G62020
15309	3	F23 vs. L23	F2-3 up	GO:0010200	Signaling and response	Signaling	response to chitin	P	AT5G64660
15310	3	F23 vs. L23	F2-3 up	GO:0010200	Signaling and response	Signaling	response to chitin	P	AT5G66070
15311	3	F23 vs. L23	F2-3 up	GO:0010200	Signaling and response	Signaling	response to chitin	P	AT5G67300
15312	3	F23 vs. L23	F2-3 up	GO:0032025	Signaling and response	Signaling	response to cobalt ion	P	AT3G02570
15313	3	F23 vs. L23	F2-3 up	GO:0046688	Signaling and response	Signaling	response to copper ion	P	AT1G08830
15314	3	F23 vs. L23	F2-3 up	GO:0046688	Signaling and response	Signaling	response to copper ion	P	AT2G28190
15315	3	F23 vs. L23	F2-3 up	GO:0009735	Signaling and response	Signaling	response to cytokinin	P	AT1G05850
15316	3	F23 vs. L23	F2-3 up	GO:0009735	Signaling and response	Signaling	response to cytokinin	P	AT1G78240
15317	3	F23 vs. L23	F2-3 up	GO:0009735	Signaling and response	Signaling	response to cytokinin	P	AT2G40670
15318	3	F23 vs. L23	F2-3 up	GO:0009735	Signaling and response	Signaling	response to cytokinin	P	AT3G18780
15319	3	F23 vs. L23	F2-3 up	GO:0009735	Signaling and response	Signaling	response to cytokinin	P	AT5G45890
15320	3	F23 vs. L23	F2-3 up	GO:0009735	Signaling and response	Signaling	response to cytokinin	P	AT5G49720
15321	3	F23 vs. L23	F2-3 up	GO:0009735	Signaling and response	Signaling	response to cytokinin	P	AT5G56860
15322	3	F23 vs. L23	F2-3 up	GO:0010218	Signaling and response	Signaling	response to far red light	P	AT1G14920
15323	3	F23 vs. L23	F2-3 up	GO:0010218	Signaling and response	Signaling	response to far red light	P	AT1G19150
15324	3	F23 vs. L23	F2-3 up	GO:0010218	Signaling and response	Signaling	response to far red light	P	AT3G18780
15325	3	F23 vs. L23	F2-3 up	GO:0010218	Signaling and response	Signaling	response to far red light	P	AT4G14690
15326	3	F23 vs. L23	F2-3 up	GO:0010218	Signaling and response	Signaling	response to far red light	P	AT5G24120
15327	3	F23 vs. L23	F2-3 up	GO:0010218	Signaling and response	Signaling	response to far red light	P	AT5G38420
15328	3	F23 vs. L23	F2-3 up	GO:0010218	Signaling and response	Signaling	response to far red light	P	AT5G44110
15329	3	F23 vs. L23	F2-3 up	GO:0009750	Signaling and response	Signaling	response to fructose	P	AT1G74670
15330	3	F23 vs. L23	F2-3 up	GO:0009750	Signaling and response	Signaling	response to fructose	P	AT5G45890
15331	3	F23 vs. L23	F2-3 up	GO:0009739	Signaling and response	Signaling	response to gibberellin	P	AT1G14920

15332	3	F23 vs. L23	F2-3 up	GO:0009739	Signaling and response	Signaling	response to gibberellin	P	AT1G69530
15333	3	F23 vs. L23	F2-3 up	GO:0009739	Signaling and response	Signaling	response to gibberellin	P	AT2G32460
15334	3	F23 vs. L23	F2-3 up	GO:0009739	Signaling and response	Signaling	response to gibberellin	P	AT2G37630
15335	3	F23 vs. L23	F2-3 up	GO:0009739	Signaling and response	Signaling	response to gibberellin	P	AT2G46830
15336	3	F23 vs. L23	F2-3 up	GO:0009739	Signaling and response	Signaling	response to gibberellin	P	AT5G56860
15337	3	F23 vs. L23	F2-3 up	GO:0009739	Signaling and response	Signaling	response to gibberellin	P	AT5G67300
15338	3	F23 vs. L23	F2-3 up	GO:0009739	Signaling and response	Signaling	response to gibberellin	P	AT5G67480
15339	3	F23 vs. L23	F2-3 up	GO:0009749	Signaling and response	Signaling	response to glucose	P	AT1G61800
15340	3	F23 vs. L23	F2-3 up	GO:0009749	Signaling and response	Signaling	response to glucose	P	AT1G74670
15341	3	F23 vs. L23	F2-3 up	GO:0009749	Signaling and response	Signaling	response to glucose	P	AT4G04040
15342	3	F23 vs. L23	F2-3 up	GO:0009749	Signaling and response	Signaling	response to glucose	P	AT5G20700
15343	3	F23 vs. L23	F2-3 up	GO:0009749	Signaling and response	Signaling	response to glucose	P	AT5G45890
15344	3	F23 vs. L23	F2-3 up	GO:0009629	Signaling and response	Signaling	response to gravity	P	AT1G78370
15345	3	F23 vs. L23	F2-3 up	GO:0009629	Signaling and response	Signaling	response to gravity	P	AT5G13930
15346	3	F23 vs. L23	F2-3 up	GO:0060416	Signaling and response	Signaling	response to growth hormone	P	AT1G10370
15347	3	F23 vs. L23	F2-3 up	GO:0009725	Signaling and response	Signaling	response to hormone	P	AT2G40710
15348	3	F23 vs. L23	F2-3 up	GO:0010039	Signaling and response	Signaling	response to iron ion	P	AT1G08830
15349	3	F23 vs. L23	F2-3 up	GO:0010039	Signaling and response	Signaling	response to iron ion	P	AT2G28190
15350	3	F23 vs. L23	F2-3 up	GO:0010039	Signaling and response	Signaling	response to iron ion	P	AT3G11050
15351	3	F23 vs. L23	F2-3 up	GO:0010039	Signaling and response	Signaling	response to iron ion	P	AT5G01600
15352	3	F23 vs. L23	F2-3 up	GO:0009753	Signaling and response	Signaling	response to jasmonic acid	P	AT1G17380
15353	3	F23 vs. L23	F2-3 up	GO:0009753	Signaling and response	Signaling	response to jasmonic acid	P	AT1G17420
15354	3	F23 vs. L23	F2-3 up	GO:0009753	Signaling and response	Signaling	response to jasmonic acid	P	AT1G18150
15355	3	F23 vs. L23	F2-3 up	GO:0009753	Signaling and response	Signaling	response to jasmonic acid	P	AT1G19180
15356	3	F23 vs. L23	F2-3 up	GO:0009753	Signaling and response	Signaling	response to jasmonic acid	P	AT1G32640
15357	3	F23 vs. L23	F2-3 up	GO:0009753	Signaling and response	Signaling	response to jasmonic acid	P	AT1G70700
15358	3	F23 vs. L23	F2-3 up	GO:0009753	Signaling and response	Signaling	response to jasmonic acid	P	AT2G24210
15359	3	F23 vs. L23	F2-3 up	GO:0009753	Signaling and response	Signaling	response to jasmonic acid	P	AT2G26690
15360	3	F23 vs. L23	F2-3 up	GO:0009753	Signaling and response	Signaling	response to jasmonic acid	P	AT2G34600
15361	3	F23 vs. L23	F2-3 up	GO:0009753	Signaling and response	Signaling	response to jasmonic acid	P	AT2G34810
15362	3	F23 vs. L23	F2-3 up	GO:0009753	Signaling and response	Signaling	response to jasmonic acid	P	AT2G37630
15363	3	F23 vs. L23	F2-3 up	GO:0009753	Signaling and response	Signaling	response to jasmonic acid	P	AT2G39730
15364	3	F23 vs. L23	F2-3 up	GO:0009753	Signaling and response	Signaling	response to jasmonic acid	P	AT2G39770
15365	3	F23 vs. L23	F2-3 up	GO:0009753	Signaling and response	Signaling	response to jasmonic acid	P	AT2G46410
15366	3	F23 vs. L23	F2-3 up	GO:0009753	Signaling and response	Signaling	response to jasmonic acid	P	AT2G46830
15367	3	F23 vs. L23	F2-3 up	GO:0009753	Signaling and response	Signaling	response to jasmonic acid	P	AT3G06490
15368	3	F23 vs. L23	F2-3 up	GO:0009753	Signaling and response	Signaling	response to jasmonic acid	P	AT3G09940
15369	3	F23 vs. L23	F2-3 up	GO:0009753	Signaling and response	Signaling	response to jasmonic acid	P	AT3G23050
15370	3	F23 vs. L23	F2-3 up	GO:0009753	Signaling and response	Signaling	response to jasmonic acid	P	AT3G45140
15371	3	F23 vs. L23	F2-3 up	GO:0009753	Signaling and response	Signaling	response to jasmonic acid	P	AT4G11280
15372	3	F23 vs. L23	F2-3 up	GO:0009753	Signaling and response	Signaling	response to jasmonic acid	P	AT4G12030
15373	3	F23 vs. L23	F2-3 up	GO:0009753	Signaling and response	Signaling	response to jasmonic acid	P	AT4G23100
15374	3	F23 vs. L23	F2-3 up	GO:0009753	Signaling and response	Signaling	response to jasmonic acid	P	AT4G34710
15375	3	F23 vs. L23	F2-3 up	GO:0009753	Signaling and response	Signaling	response to jasmonic acid	P	AT4G37760
15376	3	F23 vs. L23	F2-3 up	GO:0009753	Signaling and response	Signaling	response to jasmonic acid	P	AT5G13930
15377	3	F23 vs. L23	F2-3 up	GO:0009753	Signaling and response	Signaling	response to jasmonic acid	P	AT5G14200
15378	3	F23 vs. L23	F2-3 up	GO:0009753	Signaling and response	Signaling	response to jasmonic acid	P	AT5G24780
15379	3	F23 vs. L23	F2-3 up	GO:0009753	Signaling and response	Signaling	response to jasmonic acid	P	AT5G67160
15380	3	F23 vs. L23	F2-3 up	GO:0009753	Signaling and response	Signaling	response to jasmonic acid	P	AT5G67300
15381	3	F23 vs. L23	F2-3 up	GO:0009753	Signaling and response	Signaling	response to jasmonic acid	P	AT5G67480
15382	3	F23 vs. L23	F2-3 up	GO:0080167	Signaling and response	Signaling	response to karrikin	P	AT1G07440
15383	3	F23 vs. L23	F2-3 up	GO:0080167	Signaling and response	Signaling	response to karrikin	P	AT1G10370
15384	3	F23 vs. L23	F2-3 up	GO:0080167	Signaling and response	Signaling	response to karrikin	P	AT1G10960
15385	3	F23 vs. L23	F2-3 up	GO:0080167	Signaling and response	Signaling	response to karrikin	P	AT1G44575
15386	3	F23 vs. L23	F2-3 up	GO:0080167	Signaling and response	Signaling	response to karrikin	P	AT1G61800
15387	3	F23 vs. L23	F2-3 up	GO:0080167	Signaling and response	Signaling	response to karrikin	P	AT1G74670

15388	3	F23 vs. L23	F2-3 up	GO:0080167	Signaling and response	Signaling	response to karrikin	P	AT1G78290
15389	3	F23 vs. L23	F2-3 up	GO:0080167	Signaling and response	Signaling	response to karrikin	P	AT1G80460
15390	3	F23 vs. L23	F2-3 up	GO:0080167	Signaling and response	Signaling	response to karrikin	P	AT2G23910
15391	3	F23 vs. L23	F2-3 up	GO:0080167	Signaling and response	Signaling	response to karrikin	P	AT2G29980
15392	3	F23 vs. L23	F2-3 up	GO:0080167	Signaling and response	Signaling	response to karrikin	P	AT2G30490
15393	3	F23 vs. L23	F2-3 up	GO:0080167	Signaling and response	Signaling	response to karrikin	P	AT2G37040
15394	3	F23 vs. L23	F2-3 up	GO:0080167	Signaling and response	Signaling	response to karrikin	P	AT3G09440
15395	3	F23 vs. L23	F2-3 up	GO:0080167	Signaling and response	Signaling	response to karrikin	P	AT3G21560
15396	3	F23 vs. L23	F2-3 up	GO:0080167	Signaling and response	Signaling	response to karrikin	P	AT3G22840
15397	3	F23 vs. L23	F2-3 up	GO:0080167	Signaling and response	Signaling	response to karrikin	P	AT3G61220
15398	3	F23 vs. L23	F2-3 up	GO:0080167	Signaling and response	Signaling	response to karrikin	P	AT4G03280
15399	3	F23 vs. L23	F2-3 up	GO:0080167	Signaling and response	Signaling	response to karrikin	P	AT4G27030
15400	3	F23 vs. L23	F2-3 up	GO:0080167	Signaling and response	Signaling	response to karrikin	P	AT4G34710
15401	3	F23 vs. L23	F2-3 up	GO:0080167	Signaling and response	Signaling	response to karrikin	P	AT5G02270
15402	3	F23 vs. L23	F2-3 up	GO:0080167	Signaling and response	Signaling	response to karrikin	P	AT5G05600
15403	3	F23 vs. L23	F2-3 up	GO:0080167	Signaling and response	Signaling	response to karrikin	P	AT5G07460
15404	3	F23 vs. L23	F2-3 up	GO:0080167	Signaling and response	Signaling	response to karrikin	P	AT5G08640
15405	3	F23 vs. L23	F2-3 up	GO:0080167	Signaling and response	Signaling	response to karrikin	P	AT5G57785
15406	3	F23 vs. L23	F2-3 up	GO:0080167	Signaling and response	Signaling	response to karrikin	P	AT5G62680
15407	3	F23 vs. L23	F2-3 up	GO:1902065	Signaling and response	Signaling	response to L-glutamate	P	AT3G45640
15408	3	F23 vs. L23	F2-3 up	GO:0009416	Signaling and response	Signaling	response to light stimulus	P	AT1G04400
15409	3	F23 vs. L23	F2-3 up	GO:0009416	Signaling and response	Signaling	response to light stimulus	P	AT1G16880
15410	3	F23 vs. L23	F2-3 up	GO:0009416	Signaling and response	Signaling	response to light stimulus	P	AT1G19150
15411	3	F23 vs. L23	F2-3 up	GO:0009416	Signaling and response	Signaling	response to light stimulus	P	AT1G20620
15412	3	F23 vs. L23	F2-3 up	GO:0009416	Signaling and response	Signaling	response to light stimulus	P	AT1G42970
15413	3	F23 vs. L23	F2-3 up	GO:0009416	Signaling and response	Signaling	response to light stimulus	P	AT1G45474
15414	3	F23 vs. L23	F2-3 up	GO:0009416	Signaling and response	Signaling	response to light stimulus	P	AT1G48030
15415	3	F23 vs. L23	F2-3 up	GO:0009416	Signaling and response	Signaling	response to light stimulus	P	AT1G55480
15416	3	F23 vs. L23	F2-3 up	GO:0009416	Signaling and response	Signaling	response to light stimulus	P	AT1G58290
15417	3	F23 vs. L23	F2-3 up	GO:0009416	Signaling and response	Signaling	response to light stimulus	P	AT1G68530
15418	3	F23 vs. L23	F2-3 up	GO:0009416	Signaling and response	Signaling	response to light stimulus	P	AT1G70940
15419	3	F23 vs. L23	F2-3 up	GO:0009416	Signaling and response	Signaling	response to light stimulus	P	AT1G79440
15420	3	F23 vs. L23	F2-3 up	GO:0009416	Signaling and response	Signaling	response to light stimulus	P	AT2G28190
15421	3	F23 vs. L23	F2-3 up	GO:0009416	Signaling and response	Signaling	response to light stimulus	P	AT2G30490
15422	3	F23 vs. L23	F2-3 up	GO:0009416	Signaling and response	Signaling	response to light stimulus	P	AT2G39730
15423	3	F23 vs. L23	F2-3 up	GO:0009416	Signaling and response	Signaling	response to light stimulus	P	AT3G02570
15424	3	F23 vs. L23	F2-3 up	GO:0009416	Signaling and response	Signaling	response to light stimulus	P	AT3G19710
15425	3	F23 vs. L23	F2-3 up	GO:0009416	Signaling and response	Signaling	response to light stimulus	P	AT3G23290
15426	3	F23 vs. L23	F2-3 up	GO:0009416	Signaling and response	Signaling	response to light stimulus	P	AT3G26650
15427	3	F23 vs. L23	F2-3 up	GO:0009416	Signaling and response	Signaling	response to light stimulus	P	AT3G49110
15428	3	F23 vs. L23	F2-3 up	GO:0009416	Signaling and response	Signaling	response to light stimulus	P	AT3G62410
15429	3	F23 vs. L23	F2-3 up	GO:0009416	Signaling and response	Signaling	response to light stimulus	P	AT4G02380
15430	3	F23 vs. L23	F2-3 up	GO:0009416	Signaling and response	Signaling	response to light stimulus	P	AT4G38740
15431	3	F23 vs. L23	F2-3 up	GO:0009416	Signaling and response	Signaling	response to light stimulus	P	AT5G04140
15432	3	F23 vs. L23	F2-3 up	GO:0009416	Signaling and response	Signaling	response to light stimulus	P	AT5G08640
15433	3	F23 vs. L23	F2-3 up	GO:0009416	Signaling and response	Signaling	response to light stimulus	P	AT5G09810
15434	3	F23 vs. L23	F2-3 up	GO:0009416	Signaling and response	Signaling	response to light stimulus	P	AT5G56860
15435	3	F23 vs. L23	F2-3 up	GO:0010555	Signaling and response	Signaling	response to mannitol	P	AT1G72180
15436	3	F23 vs. L23	F2-3 up	GO:1905582	Signaling and response	Signaling	response to mannose	P	AT5G20700
15437	3	F23 vs. L23	F2-3 up	GO:0010167	Signaling and response	Signaling	response to nitrate	P	AT1G05850
15438	3	F23 vs. L23	F2-3 up	GO:0010167	Signaling and response	Signaling	response to nitrate	P	AT1G12110
15439	3	F23 vs. L23	F2-3 up	GO:0010167	Signaling and response	Signaling	response to nitrate	P	AT1G32450
15440	3	F23 vs. L23	F2-3 up	GO:0010167	Signaling and response	Signaling	response to nitrate	P	AT2G15620
15441	3	F23 vs. L23	F2-3 up	GO:0010167	Signaling and response	Signaling	response to nitrate	P	AT3G21670
15442	3	F23 vs. L23	F2-3 up	GO:0010167	Signaling and response	Signaling	response to nitrate	P	AT5G40890
15443	3	F23 vs. L23	F2-3 up	GO:0010243	Signaling and response	Signaling	response to organonitrogen compound	P	AT2G46830

15444	3	F23 vs. L23	F2-3 up	GO:0080184	Signaling and response	Signaling	response to phenylpropanoid	P	AT2G01520
15445	3	F23 vs. L23	F2-3 up	GO:0010114	Signaling and response	Signaling	response to red light	P	AT1G19150
15446	3	F23 vs. L23	F2-3 up	GO:0010114	Signaling and response	Signaling	response to red light	P	AT1G69530
15447	3	F23 vs. L23	F2-3 up	GO:0010114	Signaling and response	Signaling	response to red light	P	AT3G18780
15448	3	F23 vs. L23	F2-3 up	GO:0010114	Signaling and response	Signaling	response to red light	P	AT4G14690
15449	3	F23 vs. L23	F2-3 up	GO:0010114	Signaling and response	Signaling	response to red light	P	AT5G24120
15450	3	F23 vs. L23	F2-3 up	GO:0010114	Signaling and response	Signaling	response to red light	P	AT5G38420
15451	3	F23 vs. L23	F2-3 up	GO:0010114	Signaling and response	Signaling	response to red light	P	AT5G44110
15452	3	F23 vs. L23	F2-3 up	GO:0009639	Signaling and response	Signaling	response to red or far red light	P	AT4G19230
15453	3	F23 vs. L23	F2-3 up	GO:0009751	Signaling and response	Signaling	response to salicylic acid	P	AT1G28480
15454	3	F23 vs. L23	F2-3 up	GO:0009751	Signaling and response	Signaling	response to salicylic acid	P	AT1G80840
15455	3	F23 vs. L23	F2-3 up	GO:0009751	Signaling and response	Signaling	response to salicylic acid	P	AT2G37630
15456	3	F23 vs. L23	F2-3 up	GO:0009751	Signaling and response	Signaling	response to salicylic acid	P	AT2G40000
15457	3	F23 vs. L23	F2-3 up	GO:0009751	Signaling and response	Signaling	response to salicylic acid	P	AT2G46410
15458	3	F23 vs. L23	F2-3 up	GO:0009751	Signaling and response	Signaling	response to salicylic acid	P	AT2G46830
15459	3	F23 vs. L23	F2-3 up	GO:0009751	Signaling and response	Signaling	response to salicylic acid	P	AT3G01420
15460	3	F23 vs. L23	F2-3 up	GO:0009751	Signaling and response	Signaling	response to salicylic acid	P	AT4G31800
15461	3	F23 vs. L23	F2-3 up	GO:0009751	Signaling and response	Signaling	response to salicylic acid	P	AT4G39030
15462	3	F23 vs. L23	F2-3 up	GO:0009751	Signaling and response	Signaling	response to salicylic acid	P	AT5G06320
15463	3	F23 vs. L23	F2-3 up	GO:0009751	Signaling and response	Signaling	response to salicylic acid	P	AT5G24530
15464	3	F23 vs. L23	F2-3 up	GO:0009751	Signaling and response	Signaling	response to salicylic acid	P	AT5G67300
15465	3	F23 vs. L23	F2-3 up	GO:0009751	Signaling and response	Signaling	response to salicylic acid	P	AT5G67480
15466	3	F23 vs. L23	F2-3 up	GO:0010272	Signaling and response	Signaling	response to silver ion	P	AT2G43570
15467	3	F23 vs. L23	F2-3 up	GO:0050896	Signaling and response	Signaling	response to stimulus	P	AT2G47590
15468	3	F23 vs. L23	F2-3 up	GO:1902347	Signaling and response	Signaling	response to strigolactone	P	AT1G04400
15469	3	F23 vs. L23	F2-3 up	GO:0009744	Signaling and response	Signaling	response to sucrose	P	AT1G16880
15470	3	F23 vs. L23	F2-3 up	GO:0009744	Signaling and response	Signaling	response to sucrose	P	AT1G42970
15471	3	F23 vs. L23	F2-3 up	GO:0009744	Signaling and response	Signaling	response to sucrose	P	AT1G61800
15472	3	F23 vs. L23	F2-3 up	GO:0009744	Signaling and response	Signaling	response to sucrose	P	AT1G74670
15473	3	F23 vs. L23	F2-3 up	GO:0009744	Signaling and response	Signaling	response to sucrose	P	AT3G04120
15474	3	F23 vs. L23	F2-3 up	GO:0009744	Signaling and response	Signaling	response to sucrose	P	AT3G26650
15475	3	F23 vs. L23	F2-3 up	GO:0009744	Signaling and response	Signaling	response to sucrose	P	AT3G62410
15476	3	F23 vs. L23	F2-3 up	GO:0009744	Signaling and response	Signaling	response to sucrose	P	AT5G04140
15477	3	F23 vs. L23	F2-3 up	GO:0009744	Signaling and response	Signaling	response to sucrose	P	AT5G20700
15478	3	F23 vs. L23	F2-3 up	GO:0009744	Signaling and response	Signaling	response to sucrose	P	AT5G45890
15479	3	F23 vs. L23	F2-3 up	GO:0009411	Signaling and response	Signaling	response to UV	P	AT3G12610
15480	3	F23 vs. L23	F2-3 up	GO:0009411	Signaling and response	Signaling	response to UV	P	AT4G13770
15481	3	F23 vs. L23	F2-3 up	GO:0010225	Signaling and response	Signaling	response to UV-C	P	AT3G50000
15482	3	F23 vs. L23	F2-3 up	GO:0010266	Signaling and response	Signaling	response to vitamin B1	P	AT2G29630
15483	3	F23 vs. L23	F2-3 up	GO:0010043	Signaling and response	Signaling	response to zinc ion	P	AT2G01520
15484	3	F23 vs. L23	F2-3 up	GO:0010043	Signaling and response	Signaling	response to zinc ion	P	AT2G30860
15485	3	F23 vs. L23	F2-3 up	GO:0010043	Signaling and response	Signaling	response to zinc ion	P	AT3G02570
15486	3	F23 vs. L23	F2-3 up	GO:0010043	Signaling and response	Signaling	response to zinc ion	P	AT5G01600
15487	3	F23 vs. L23	F2-3 up	GO:0007266	Signaling and response	Signaling	Rho protein signal transduction	P	AT1G12070
15488	3	F23 vs. L23	F2-3 up	GO:0007266	Signaling and response	Signaling	Rho protein signal transduction	P	AT1G62450
15489	3	F23 vs. L23	F2-3 up	GO:0009697	Signaling and response	Signaling	salicylic acid biosynthetic process	P	AT4G39030
15490	3	F23 vs. L23	F2-3 up	GO:0009697	Signaling and response	Signaling	salicylic acid biosynthetic process	P	AT5G67160
15491	3	F23 vs. L23	F2-3 up	GO:0009863	Signaling and response	Signaling	salicylic acid mediated signaling pathway	P	AT1G14920
15492	3	F23 vs. L23	F2-3 up	GO:0009863	Signaling and response	Signaling	salicylic acid mediated signaling pathway	P	AT1G28480
15493	3	F23 vs. L23	F2-3 up	GO:0006465	Signaling and response	Signaling	signal peptide processing	P	AT2G30440
15494	3	F23 vs. L23	F2-3 up	GO:0005047	Signaling and response	Signaling	signal recognition particle binding	F	AT2G45770
15495	3	F23 vs. L23	F2-3 up	GO:0007165	Signaling and response	Signaling	signal transduction	P	AT1G18150
15496	3	F23 vs. L23	F2-3 up	GO:0007165	Signaling and response	Signaling	signal transduction	P	AT2G30360
15497	3	F23 vs. L23	F2-3 up	GO:0007165	Signaling and response	Signaling	signal transduction	P	AT2G30520
15498	3	F23 vs. L23	F2-3 up	GO:0007165	Signaling and response	Signaling	signal transduction	P	AT3G12610
15499	3	F23 vs. L23	F2-3 up	GO:0007165	Signaling and response	Signaling	signal transduction	P	AT3G45640

15500	3	F23 vs. L23	F2-3 up	GO:0007165	Signaling and response	Signaling	signal transduction	P	AT3G54800
15501	3	F23 vs. L23	F2-3 up	GO:0007165	Signaling and response	Signaling	signal transduction	P	AT3G61160
15502	3	F23 vs. L23	F2-3 up	GO:0007165	Signaling and response	Signaling	signal transduction	P	AT4G38740
15503	3	F23 vs. L23	F2-3 up	GO:0023014	Signaling and response	Signaling	signal transduction by protein phosphorylation	P	AT2G03410
15504	3	F23 vs. L23	F2-3 up	GO:0038023	Signaling and response	Signaling	signaling receptor activity	F	AT2G40710
15505	3	F23 vs. L23	F2-3 up	GO:0007264	Signaling and response	Signaling	small GTPase mediated signal transduction	P	AT5G09550
15506	3	F23 vs. L23	F2-3 up	GO:0016229	Signaling and response	Signaling	steroid dehydrogenase activity	F	AT2G37760
15507	3	F23 vs. L23	F2-3 up	GO:0016229	Signaling and response	Signaling	steroid dehydrogenase activity	F	AT2G37770
15508	3	F23 vs. L23	F2-3 up	GO:0003838	Signaling and response	Signaling	sterol 24-C-methyltransferase activity	F	AT1G76090
15509	3	F23 vs. L23	F2-3 up	GO:0003838	Signaling and response	Signaling	sterol 24-C-methyltransferase activity	F	AT5G13710
15510	3	F23 vs. L23	F2-3 up	GO:0016126	Signaling and response	Signaling	sterol biosynthetic process	P	AT1G76090
15511	3	F23 vs. L23	F2-3 up	GO:0016126	Signaling and response	Signaling	sterol biosynthetic process	P	AT1G76490
15512	3	F23 vs. L23	F2-3 up	GO:0016126	Signaling and response	Signaling	sterol biosynthetic process	P	AT2G17370
15513	3	F23 vs. L23	F2-3 up	GO:0016126	Signaling and response	Signaling	sterol biosynthetic process	P	AT4G37760
15514	3	F23 vs. L23	F2-3 up	GO:0016126	Signaling and response	Signaling	sterol biosynthetic process	P	AT5G13710
15515	3	F23 vs. L23	F2-3 up	GO:0016126	Signaling and response	Signaling	sterol biosynthetic process	P	AT5G24140
15516	3	F23 vs. L23	F2-3 up	GO:0016125	Signaling and response	Signaling	sterol metabolic process	P	AT4G15440
15517	3	F23 vs. L23	F2-3 up	GO:0016125	Signaling and response	Signaling	sterol metabolic process	P	AT4G19230
15518	3	F23 vs. L23	F2-3 up	GO:0010118	Signaling and response	Signaling	stomatal movement	P	AT1G04400
15519	3	F23 vs. L23	F2-3 up	GO:0010118	Signaling and response	Signaling	stomatal movement	P	AT1G32080
15520	3	F23 vs. L23	F2-3 up	GO:0010118	Signaling and response	Signaling	stomatal movement	P	AT3G56960
15521	3	F23 vs. L23	F2-3 up	GO:0004714	Signaling and response	Signaling	transmembrane receptor protein tyrosine kinase activity	F	AT1G72180
15522	3	F23 vs. L23	F2-3 up	GO:0004714	Signaling and response	Signaling	transmembrane receptor protein tyrosine kinase activity	F	AT4G28490
15523	3	F23 vs. L23	F2-3 up	GO:0009606	Signaling and response	Signaling	tropism	P	AT1G70940
15524	3	F23 vs. L23	F2-3 up	GO:0010050	Signaling and response	Signaling	vegetative phase change	P	AT5G60450
15525	3	F23 vs. L23	F2-3 up	GO:0010295	Signaling and response	Stress	(+)-abscisic acid 8'-hydroxylase activity	F	AT4G19230
15526	3	F23 vs. L23	F2-3 up	GO:0006075	Signaling and response	Stress	(1->3)-beta-D-glucan biosynthetic process	P	AT2G13680
15527	3	F23 vs. L23	F2-3 up	GO:0003843	Signaling and response	Stress	1,3-beta-D-glucan synthase activity	F	AT2G13680
15528	3	F23 vs. L23	F2-3 up	GO:0000148	Signaling and response	Stress	1,3-beta-D-glucan synthase complex	C	AT2G13680
15529	3	F23 vs. L23	F2-3 up	GO:0046345	Signaling and response	Stress	abscisic acid catabolic process	P	AT4G19230
15530	3	F23 vs. L23	F2-3 up	GO:0009687	Signaling and response	Stress	abscisic acid metabolic process	P	AT4G19230
15531	3	F23 vs. L23	F2-3 up	GO:0009738	Signaling and response	Stress	abscisic acid-activated signaling pathway	P	AT1G12240
15532	3	F23 vs. L23	F2-3 up	GO:0009738	Signaling and response	Stress	abscisic acid-activated signaling pathway	P	AT1G32640
15533	3	F23 vs. L23	F2-3 up	GO:0009738	Signaling and response	Stress	abscisic acid-activated signaling pathway	P	AT1G64060
15534	3	F23 vs. L23	F2-3 up	GO:0009738	Signaling and response	Stress	abscisic acid-activated signaling pathway	P	AT2G17290
15535	3	F23 vs. L23	F2-3 up	GO:0009738	Signaling and response	Stress	abscisic acid-activated signaling pathway	P	AT2G18470
15536	3	F23 vs. L23	F2-3 up	GO:0009738	Signaling and response	Stress	abscisic acid-activated signaling pathway	P	AT2G30020
15537	3	F23 vs. L23	F2-3 up	GO:0009738	Signaling and response	Stress	abscisic acid-activated signaling pathway	P	AT3G11410
15538	3	F23 vs. L23	F2-3 up	GO:0009738	Signaling and response	Stress	abscisic acid-activated signaling pathway	P	AT3G45640
15539	3	F23 vs. L23	F2-3 up	GO:0009738	Signaling and response	Stress	abscisic acid-activated signaling pathway	P	AT3G46620
15540	3	F23 vs. L23	F2-3 up	GO:0009738	Signaling and response	Stress	abscisic acid-activated signaling pathway	P	AT5G25980
15541	3	F23 vs. L23	F2-3 up	GO:0009738	Signaling and response	Stress	abscisic acid-activated signaling pathway	P	AT5G47670
15542	3	F23 vs. L23	F2-3 up	GO:0033741	Signaling and response	Stress	adenylyl-sulfate reductase (glutathione) activity	F	AT4G21990
15543	3	F23 vs. L23	F2-3 up	GO:0007568	Signaling and response	Stress	aging	P	AT2G02990
15544	3	F23 vs. L23	F2-3 up	GO:0007568	Signaling and response	Stress	aging	P	AT3G10985
15545	3	F23 vs. L23	F2-3 up	GO:0007568	Signaling and response	Stress	aging	P	AT3G50210
15546	3	F23 vs. L23	F2-3 up	GO:0007568	Signaling and response	Stress	aging	P	AT3G56240
15547	3	F23 vs. L23	F2-3 up	GO:0007568	Signaling and response	Stress	aging	P	AT4G14880
15548	3	F23 vs. L23	F2-3 up	GO:0007568	Signaling and response	Stress	aging	P	AT5G35630
15549	3	F23 vs. L23	F2-3 up	GO:0007568	Signaling and response	Stress	aging	P	AT5G45890
15550	3	F23 vs. L23	F2-3 up	GO:0007568	Signaling and response	Stress	aging	P	AT5G60360
15551	3	F23 vs. L23	F2-3 up	GO:0009916	Signaling and response	Stress	alternative oxidase activity	F	AT3G22370
15552	3	F23 vs. L23	F2-3 up	GO:0010230	Signaling and response	Stress	alternative respiration	P	AT2G18660
15553	3	F23 vs. L23	F2-3 up	GO:0010230	Signaling and response	Stress	alternative respiration	P	AT3G22370

15554	3	F23 vs. L23	F2-3 up	GO:0055081	Signaling and response	Stress	anion homeostasis	P	AT3G57330
15555	3	F23 vs. L23	F2-3 up	GO:0016209	Signaling and response	Stress	antioxidant activity	F	AT2G37240
15556	3	F23 vs. L23	F2-3 up	GO:1901683	Signaling and response	Stress	arsenate ion transmembrane transporter activity	F	AT3G54700
15557	3	F23 vs. L23	F2-3 up	GO:0010252	Signaling and response	Stress	auxin homeostasis	P	AT1G70940
15558	3	F23 vs. L23	F2-3 up	GO:0010252	Signaling and response	Stress	auxin homeostasis	P	AT2G01420
15559	3	F23 vs. L23	F2-3 up	GO:0010252	Signaling and response	Stress	auxin homeostasis	P	AT2G23170
15560	3	F23 vs. L23	F2-3 up	GO:0010252	Signaling and response	Stress	auxin homeostasis	P	AT5G54510
15561	3	F23 vs. L23	F2-3 up	GO:0055073	Signaling and response	Stress	cadmium ion homeostasis	P	AT1G71050
15562	3	F23 vs. L23	F2-3 up	GO:0055073	Signaling and response	Stress	cadmium ion homeostasis	P	AT5G17450
15563	3	F23 vs. L23	F2-3 up	GO:0015368	Signaling and response	Stress	calcium:cation antiporter activity	F	AT2G38170
15564	3	F23 vs. L23	F2-3 up	GO:0015369	Signaling and response	Stress	calcium:proton antiporter activity	F	AT2G38170
15565	3	F23 vs. L23	F2-3 up	GO:0005432	Signaling and response	Stress	calcium:sodium antiporter activity	F	AT5G17860
15566	3	F23 vs. L23	F2-3 up	GO:0005544	Signaling and response	Stress	calcium-dependent phospholipid binding	F	AT1G35720
15567	3	F23 vs. L23	F2-3 up	GO:0052543	Signaling and response	Stress	callose deposition in cell wall	P	AT3G03250
15568	3	F23 vs. L23	F2-3 up	GO:0052543	Signaling and response	Stress	callose deposition in cell wall	P	AT5G50800
15569	3	F23 vs. L23	F2-3 up	GO:0033500	Signaling and response	Stress	carbohydrate homeostasis	P	AT1G64060
15570	3	F23 vs. L23	F2-3 up	GO:0033500	Signaling and response	Stress	carbohydrate homeostasis	P	AT5G47910
15571	3	F23 vs. L23	F2-3 up	GO:0004096	Signaling and response	Stress	catalase activity	F	AT1G20620
15572	3	F23 vs. L23	F2-3 up	GO:0004096	Signaling and response	Stress	catalase activity	F	AT4G39730
15573	3	F23 vs. L23	F2-3 up	GO:0045454	Signaling and response	Stress	cell redox homeostasis	P	AT1G28480
15574	3	F23 vs. L23	F2-3 up	GO:0045454	Signaling and response	Stress	cell redox homeostasis	P	AT1G48030
15575	3	F23 vs. L23	F2-3 up	GO:0045454	Signaling and response	Stress	cell redox homeostasis	P	AT1G50320
15576	3	F23 vs. L23	F2-3 up	GO:0045454	Signaling and response	Stress	cell redox homeostasis	P	AT1G56500
15577	3	F23 vs. L23	F2-3 up	GO:0045454	Signaling and response	Stress	cell redox homeostasis	P	AT1G59730
15578	3	F23 vs. L23	F2-3 up	GO:0045454	Signaling and response	Stress	cell redox homeostasis	P	AT1G64500
15579	3	F23 vs. L23	F2-3 up	GO:0045454	Signaling and response	Stress	cell redox homeostasis	P	AT1G76080
15580	3	F23 vs. L23	F2-3 up	GO:0045454	Signaling and response	Stress	cell redox homeostasis	P	AT3G11630
15581	3	F23 vs. L23	F2-3 up	GO:0045454	Signaling and response	Stress	cell redox homeostasis	P	AT3G15360
15582	3	F23 vs. L23	F2-3 up	GO:0045454	Signaling and response	Stress	cell redox homeostasis	P	AT3G26060
15583	3	F23 vs. L23	F2-3 up	GO:0045454	Signaling and response	Stress	cell redox homeostasis	P	AT3G54660
15584	3	F23 vs. L23	F2-3 up	GO:0045454	Signaling and response	Stress	cell redox homeostasis	P	AT3G54960
15585	3	F23 vs. L23	F2-3 up	GO:0045454	Signaling and response	Stress	cell redox homeostasis	P	AT3G62960
15586	3	F23 vs. L23	F2-3 up	GO:0045454	Signaling and response	Stress	cell redox homeostasis	P	AT4G15700
15587	3	F23 vs. L23	F2-3 up	GO:0045454	Signaling and response	Stress	cell redox homeostasis	P	AT4G21990
15588	3	F23 vs. L23	F2-3 up	GO:0045454	Signaling and response	Stress	cell redox homeostasis	P	AT5G18600
15589	3	F23 vs. L23	F2-3 up	GO:0006876	Signaling and response	Stress	cellular cadmium ion homeostasis	P	AT1G16310
15590	3	F23 vs. L23	F2-3 up	GO:0006874	Signaling and response	Stress	cellular calcium ion homeostasis	P	AT2G38170
15591	3	F23 vs. L23	F2-3 up	GO:0006878	Signaling and response	Stress	cellular copper ion homeostasis	P	AT3G15353
15592	3	F23 vs. L23	F2-3 up	GO:0006878	Signaling and response	Stress	cellular copper ion homeostasis	P	AT3G56240
15593	3	F23 vs. L23	F2-3 up	GO:0006879	Signaling and response	Stress	cellular iron ion homeostasis	P	AT1G16310
15594	3	F23 vs. L23	F2-3 up	GO:0006879	Signaling and response	Stress	cellular iron ion homeostasis	P	AT4G04770
15595	3	F23 vs. L23	F2-3 up	GO:0030026	Signaling and response	Stress	cellular manganese ion homeostasis	P	AT2G38170
15596	3	F23 vs. L23	F2-3 up	GO:0098869	Signaling and response	Stress	cellular oxidant detoxification	P	AT1G19550
15597	3	F23 vs. L23	F2-3 up	GO:0098869	Signaling and response	Stress	cellular oxidant detoxification	P	AT2G41480
15598	3	F23 vs. L23	F2-3 up	GO:0098869	Signaling and response	Stress	cellular oxidant detoxification	P	AT4G11600
15599	3	F23 vs. L23	F2-3 up	GO:0098869	Signaling and response	Stress	cellular oxidant detoxification	P	AT5G51890
15600	3	F23 vs. L23	F2-3 up	GO:0030643	Signaling and response	Stress	cellular phosphate ion homeostasis	P	AT3G17790
15601	3	F23 vs. L23	F2-3 up	GO:0071215	Signaling and response	Stress	cellular response to abscisic acid stimulus	P	AT1G61660
15602	3	F23 vs. L23	F2-3 up	GO:0071215	Signaling and response	Stress	cellular response to abscisic acid stimulus	P	AT5G41790
15603	3	F23 vs. L23	F2-3 up	GO:0071454	Signaling and response	Stress	cellular response to anoxia	P	AT3G62410
15604	3	F23 vs. L23	F2-3 up	GO:0034605	Signaling and response	Stress	cellular response to heat	P	AT3G09440
15605	3	F23 vs. L23	F2-3 up	GO:0034605	Signaling and response	Stress	cellular response to heat	P	AT3G22840
15606	3	F23 vs. L23	F2-3 up	GO:0034605	Signaling and response	Stress	cellular response to heat	P	AT3G62410
15607	3	F23 vs. L23	F2-3 up	GO:0034605	Signaling and response	Stress	cellular response to heat	P	AT4G14690
15608	3	F23 vs. L23	F2-3 up	GO:0034605	Signaling and response	Stress	cellular response to heat	P	AT5G62020
15609	3	F23 vs. L23	F2-3 up	GO:0071486	Signaling and response	Stress	cellular response to high light intensity	P	AT3G22840

15610	3	F23 vs. L23	F2-3 up	GO:0071486	Signaling and response	Stress	cellular response to high light intensity	P	AT4G14690
15611	3	F23 vs. L23	F2-3 up	GO:0071456	Signaling and response	Stress	cellular response to hypoxia	P	AT4G16660
15612	3	F23 vs. L23	F2-3 up	GO:0071484	Signaling and response	Stress	cellular response to light intensity	P	AT1G08830
15613	3	F23 vs. L23	F2-3 up	GO:0071484	Signaling and response	Stress	cellular response to light intensity	P	AT2G05620
15614	3	F23 vs. L23	F2-3 up	GO:0071484	Signaling and response	Stress	cellular response to light intensity	P	AT2G28190
15615	3	F23 vs. L23	F2-3 up	GO:0071732	Signaling and response	Stress	cellular response to nitric oxide	P	AT1G62380
15616	3	F23 vs. L23	F2-3 up	GO:0071732	Signaling and response	Stress	cellular response to nitric oxide	P	AT3G01420
15617	3	F23 vs. L23	F2-3 up	GO:0006995	Signaling and response	Stress	cellular response to nitrogen starvation	P	AT1G20620
15618	3	F23 vs. L23	F2-3 up	GO:0034599	Signaling and response	Stress	cellular response to oxidative stress	P	AT1G08830
15619	3	F23 vs. L23	F2-3 up	GO:0034599	Signaling and response	Stress	cellular response to oxidative stress	P	AT2G28190
15620	3	F23 vs. L23	F2-3 up	GO:0034599	Signaling and response	Stress	cellular response to oxidative stress	P	AT3G07700
15621	3	F23 vs. L23	F2-3 up	GO:0034599	Signaling and response	Stress	cellular response to oxidative stress	P	AT3G26060
15622	3	F23 vs. L23	F2-3 up	GO:0034599	Signaling and response	Stress	cellular response to oxidative stress	P	AT5G07460
15623	3	F23 vs. L23	F2-3 up	GO:0071457	Signaling and response	Stress	cellular response to ozone	P	AT1G08830
15624	3	F23 vs. L23	F2-3 up	GO:0071457	Signaling and response	Stress	cellular response to ozone	P	AT2G28190
15625	3	F23 vs. L23	F2-3 up	GO:0016036	Signaling and response	Stress	cellular response to phosphate starvation	P	AT1G20620
15626	3	F23 vs. L23	F2-3 up	GO:0016036	Signaling and response	Stress	cellular response to phosphate starvation	P	AT2G02990
15627	3	F23 vs. L23	F2-3 up	GO:0016036	Signaling and response	Stress	cellular response to phosphate starvation	P	AT2G03240
15628	3	F23 vs. L23	F2-3 up	GO:0016036	Signaling and response	Stress	cellular response to phosphate starvation	P	AT2G16430
15629	3	F23 vs. L23	F2-3 up	GO:0016036	Signaling and response	Stress	cellular response to phosphate starvation	P	AT2G22240
15630	3	F23 vs. L23	F2-3 up	GO:0016036	Signaling and response	Stress	cellular response to phosphate starvation	P	AT3G03250
15631	3	F23 vs. L23	F2-3 up	GO:0016036	Signaling and response	Stress	cellular response to phosphate starvation	P	AT3G29060
15632	3	F23 vs. L23	F2-3 up	GO:0034614	Signaling and response	Stress	cellular response to reactive oxygen species	P	AT3G01420
15633	3	F23 vs. L23	F2-3 up	GO:0071461	Signaling and response	Stress	cellular response to redox state	P	AT1G64860
15634	3	F23 vs. L23	F2-3 up	GO:0071472	Signaling and response	Stress	cellular response to salt stress	P	AT1G08830
15635	3	F23 vs. L23	F2-3 up	GO:0071472	Signaling and response	Stress	cellular response to salt stress	P	AT1G16370
15636	3	F23 vs. L23	F2-3 up	GO:0071472	Signaling and response	Stress	cellular response to salt stress	P	AT1G61660
15637	3	F23 vs. L23	F2-3 up	GO:0071472	Signaling and response	Stress	cellular response to salt stress	P	AT2G28190
15638	3	F23 vs. L23	F2-3 up	GO:0071472	Signaling and response	Stress	cellular response to salt stress	P	AT5G24120
15639	3	F23 vs. L23	F2-3 up	GO:0009267	Signaling and response	Stress	cellular response to starvation	P	AT3G50210
15640	3	F23 vs. L23	F2-3 up	GO:0009970	Signaling and response	Stress	cellular response to sulfate starvation	P	AT1G20620
15641	3	F23 vs. L23	F2-3 up	GO:0009970	Signaling and response	Stress	cellular response to sulfate starvation	P	ATCG00420
15642	3	F23 vs. L23	F2-3 up	GO:0097237	Signaling and response	Stress	cellular response to toxic substance	P	AT5G05600
15643	3	F23 vs. L23	F2-3 up	GO:0034620	Signaling and response	Stress	cellular response to unfolded protein	P	AT3G09440
15644	3	F23 vs. L23	F2-3 up	GO:0071492	Signaling and response	Stress	cellular response to UV-A	P	AT3G22840
15645	3	F23 vs. L23	F2-3 up	GO:0071492	Signaling and response	Stress	cellular response to UV-A	P	AT4G14690
15646	3	F23 vs. L23	F2-3 up	GO:0071493	Signaling and response	Stress	cellular response to UV-B	P	AT1G08830
15647	3	F23 vs. L23	F2-3 up	GO:0071493	Signaling and response	Stress	cellular response to UV-B	P	AT2G28190
15648	3	F23 vs. L23	F2-3 up	GO:0042631	Signaling and response	Stress	cellular response to water deprivation	P	AT1G09340
15649	3	F23 vs. L23	F2-3 up	GO:0042631	Signaling and response	Stress	cellular response to water deprivation	P	AT1G16370
15650	3	F23 vs. L23	F2-3 up	GO:0042631	Signaling and response	Stress	cellular response to water deprivation	P	AT1G32080
15651	3	F23 vs. L23	F2-3 up	GO:0042631	Signaling and response	Stress	cellular response to water deprivation	P	AT1G61660
15652	3	F23 vs. L23	F2-3 up	GO:0042631	Signaling and response	Stress	cellular response to water deprivation	P	AT1G68010
15653	3	F23 vs. L23	F2-3 up	GO:0042631	Signaling and response	Stress	cellular response to water deprivation	P	AT4G02380
15654	3	F23 vs. L23	F2-3 up	GO:0006882	Signaling and response	Stress	cellular zinc ion homeostasis	P	AT1G16310
15655	3	F23 vs. L23	F2-3 up	GO:0006882	Signaling and response	Stress	cellular zinc ion homeostasis	P	AT2G38170
15656	3	F23 vs. L23	F2-3 up	GO:0051085	Signaling and response	Stress	chaperone cofactor-dependent protein refolding	P	AT1G44160
15657	3	F23 vs. L23	F2-3 up	GO:0051085	Signaling and response	Stress	chaperone cofactor-dependent protein refolding	P	AT3G09440
15658	3	F23 vs. L23	F2-3 up	GO:0051085	Signaling and response	Stress	chaperone cofactor-dependent protein refolding	P	AT3G47940
15659	3	F23 vs. L23	F2-3 up	GO:0009631	Signaling and response	Stress	cold acclimation	P	AT1G18740
15660	3	F23 vs. L23	F2-3 up	GO:0009631	Signaling and response	Stress	cold acclimation	P	AT2G38170
15661	3	F23 vs. L23	F2-3 up	GO:0010494	Signaling and response	Stress	cytoplasmic stress granule	C	AT1G72730
15662	3	F23 vs. L23	F2-3 up	GO:0006952	Signaling and response	Stress	defense response	P	AT1G05010
15663	3	F23 vs. L23	F2-3 up	GO:0006952	Signaling and response	Stress	defense response	P	AT1G17380
15664	3	F23 vs. L23	F2-3 up	GO:0006952	Signaling and response	Stress	defense response	P	AT1G17420
15665	3	F23 vs. L23	F2-3 up	GO:0006952	Signaling and response	Stress	defense response	P	AT1G19610

15666	3	F23 vs. L23	F2-3 up	GO:0006952	Signaling and response	Stress	defense response	P	AT1G20823
15667	3	F23 vs. L23	F2-3 up	GO:0006952	Signaling and response	Stress	defense response	P	AT1G62380
15668	3	F23 vs. L23	F2-3 up	GO:0006952	Signaling and response	Stress	defense response	P	AT1G64060
15669	3	F23 vs. L23	F2-3 up	GO:0006952	Signaling and response	Stress	defense response	P	AT1G70700
15670	3	F23 vs. L23	F2-3 up	GO:0006952	Signaling and response	Stress	defense response	P	AT1G72520
15671	3	F23 vs. L23	F2-3 up	GO:0006952	Signaling and response	Stress	defense response	P	AT1G75500
15672	3	F23 vs. L23	F2-3 up	GO:0006952	Signaling and response	Stress	defense response	P	AT1G75800
15673	3	F23 vs. L23	F2-3 up	GO:0006952	Signaling and response	Stress	defense response	P	AT1G76370
15674	3	F23 vs. L23	F2-3 up	GO:0006952	Signaling and response	Stress	defense response	P	AT2G01520
15675	3	F23 vs. L23	F2-3 up	GO:0006952	Signaling and response	Stress	defense response	P	AT2G02140
15676	3	F23 vs. L23	F2-3 up	GO:0006952	Signaling and response	Stress	defense response	P	AT2G02800
15677	3	F23 vs. L23	F2-3 up	GO:0006952	Signaling and response	Stress	defense response	P	AT2G07180
15678	3	F23 vs. L23	F2-3 up	GO:0006952	Signaling and response	Stress	defense response	P	AT2G17220
15679	3	F23 vs. L23	F2-3 up	GO:0006952	Signaling and response	Stress	defense response	P	AT2G30860
15680	3	F23 vs. L23	F2-3 up	GO:0006952	Signaling and response	Stress	defense response	P	AT2G33670
15681	3	F23 vs. L23	F2-3 up	GO:0006952	Signaling and response	Stress	defense response	P	AT2G34600
15682	3	F23 vs. L23	F2-3 up	GO:0006952	Signaling and response	Stress	defense response	P	AT2G37040
15683	3	F23 vs. L23	F2-3 up	GO:0006952	Signaling and response	Stress	defense response	P	AT2G43590
15684	3	F23 vs. L23	F2-3 up	GO:0006952	Signaling and response	Stress	defense response	P	AT3G06490
15685	3	F23 vs. L23	F2-3 up	GO:0006952	Signaling and response	Stress	defense response	P	AT3G49110
15686	3	F23 vs. L23	F2-3 up	GO:0006952	Signaling and response	Stress	defense response	P	AT3G61220
15687	3	F23 vs. L23	F2-3 up	GO:0006952	Signaling and response	Stress	defense response	P	AT4G00360
15688	3	F23 vs. L23	F2-3 up	GO:0006952	Signaling and response	Stress	defense response	P	AT4G01610
15689	3	F23 vs. L23	F2-3 up	GO:0006952	Signaling and response	Stress	defense response	P	AT4G02380
15690	3	F23 vs. L23	F2-3 up	GO:0006952	Signaling and response	Stress	defense response	P	AT4G03190
15691	3	F23 vs. L23	F2-3 up	GO:0006952	Signaling and response	Stress	defense response	P	AT4G11280
15692	3	F23 vs. L23	F2-3 up	GO:0006952	Signaling and response	Stress	defense response	P	AT4G39030
15693	3	F23 vs. L23	F2-3 up	GO:0006952	Signaling and response	Stress	defense response	P	AT5G13160
15694	3	F23 vs. L23	F2-3 up	GO:0006952	Signaling and response	Stress	defense response	P	AT5G24780
15695	3	F23 vs. L23	F2-3 up	GO:0006952	Signaling and response	Stress	defense response	P	AT5G47910
15696	3	F23 vs. L23	F2-3 up	GO:0006952	Signaling and response	Stress	defense response	P	AT5G66070
15697	3	F23 vs. L23	F2-3 up	GO:0006952	Signaling and response	Stress	defense response	P	AT5G67160
15698	3	F23 vs. L23	F2-3 up	GO:0052542	Signaling and response	Stress	defense response by callose deposition	P	AT1G64060
15699	3	F23 vs. L23	F2-3 up	GO:0052544	Signaling and response	Stress	defense response by callose deposition in cell wall	P	AT1G59870
15700	3	F23 vs. L23	F2-3 up	GO:0052544	Signaling and response	Stress	defense response by callose deposition in cell wall	P	AT4G23100
15701	3	F23 vs. L23	F2-3 up	GO:0042742	Signaling and response	Stress	defense response to bacterium	P	AT1G02205
15702	3	F23 vs. L23	F2-3 up	GO:0042742	Signaling and response	Stress	defense response to bacterium	P	AT1G08830
15703	3	F23 vs. L23	F2-3 up	GO:0042742	Signaling and response	Stress	defense response to bacterium	P	AT1G09340
15704	3	F23 vs. L23	F2-3 up	GO:0042742	Signaling and response	Stress	defense response to bacterium	P	AT1G19180
15705	3	F23 vs. L23	F2-3 up	GO:0042742	Signaling and response	Stress	defense response to bacterium	P	AT1G20020
15706	3	F23 vs. L23	F2-3 up	GO:0042742	Signaling and response	Stress	defense response to bacterium	P	AT1G20850
15707	3	F23 vs. L23	F2-3 up	GO:0042742	Signaling and response	Stress	defense response to bacterium	P	AT1G32060
15708	3	F23 vs. L23	F2-3 up	GO:0042742	Signaling and response	Stress	defense response to bacterium	P	AT1G59870
15709	3	F23 vs. L23	F2-3 up	GO:0042742	Signaling and response	Stress	defense response to bacterium	P	AT1G80460
15710	3	F23 vs. L23	F2-3 up	GO:0042742	Signaling and response	Stress	defense response to bacterium	P	AT1G80840
15711	3	F23 vs. L23	F2-3 up	GO:0042742	Signaling and response	Stress	defense response to bacterium	P	AT2G13810
15712	3	F23 vs. L23	F2-3 up	GO:0042742	Signaling and response	Stress	defense response to bacterium	P	AT2G22240
15713	3	F23 vs. L23	F2-3 up	GO:0042742	Signaling and response	Stress	defense response to bacterium	P	AT2G30860
15714	3	F23 vs. L23	F2-3 up	GO:0042742	Signaling and response	Stress	defense response to bacterium	P	AT2G37630
15715	3	F23 vs. L23	F2-3 up	GO:0042742	Signaling and response	Stress	defense response to bacterium	P	AT2G39730
15716	3	F23 vs. L23	F2-3 up	GO:0042742	Signaling and response	Stress	defense response to bacterium	P	AT2G39770
15717	3	F23 vs. L23	F2-3 up	GO:0042742	Signaling and response	Stress	defense response to bacterium	P	AT3G01420
15718	3	F23 vs. L23	F2-3 up	GO:0042742	Signaling and response	Stress	defense response to bacterium	P	AT3G01500
15719	3	F23 vs. L23	F2-3 up	GO:0042742	Signaling and response	Stress	defense response to bacterium	P	AT3G04720
15720	3	F23 vs. L23	F2-3 up	GO:0042742	Signaling and response	Stress	defense response to bacterium	P	AT3G04790
15721	3	F23 vs. L23	F2-3 up	GO:0042742	Signaling and response	Stress	defense response to bacterium	P	AT3G08510

15722	3	F23 vs. L23	F2-3 up	GO:0042742	Signaling and response	Stress	defense response to bacterium	P	AT3G11630
15723	3	F23 vs. L23	F2-3 up	GO:0042742	Signaling and response	Stress	defense response to bacterium	P	AT3G14420
15724	3	F23 vs. L23	F2-3 up	GO:0042742	Signaling and response	Stress	defense response to bacterium	P	AT3G26520
15725	3	F23 vs. L23	F2-3 up	GO:0042742	Signaling and response	Stress	defense response to bacterium	P	AT3G49110
15726	3	F23 vs. L23	F2-3 up	GO:0042742	Signaling and response	Stress	defense response to bacterium	P	AT3G55800
15727	3	F23 vs. L23	F2-3 up	GO:0042742	Signaling and response	Stress	defense response to bacterium	P	AT3G57330
15728	3	F23 vs. L23	F2-3 up	GO:0042742	Signaling and response	Stress	defense response to bacterium	P	AT4G03280
15729	3	F23 vs. L23	F2-3 up	GO:0042742	Signaling and response	Stress	defense response to bacterium	P	AT4G23100
15730	3	F23 vs. L23	F2-3 up	GO:0042742	Signaling and response	Stress	defense response to bacterium	P	AT4G31750
15731	3	F23 vs. L23	F2-3 up	GO:0042742	Signaling and response	Stress	defense response to bacterium	P	AT4G31800
15732	3	F23 vs. L23	F2-3 up	GO:0042742	Signaling and response	Stress	defense response to bacterium	P	AT4G32260
15733	3	F23 vs. L23	F2-3 up	GO:0042742	Signaling and response	Stress	defense response to bacterium	P	AT4G39030
15734	3	F23 vs. L23	F2-3 up	GO:0042742	Signaling and response	Stress	defense response to bacterium	P	AT5G04720
15735	3	F23 vs. L23	F2-3 up	GO:0042742	Signaling and response	Stress	defense response to bacterium	P	AT5G06320
15736	3	F23 vs. L23	F2-3 up	GO:0042742	Signaling and response	Stress	defense response to bacterium	P	AT5G09230
15737	3	F23 vs. L23	F2-3 up	GO:0042742	Signaling and response	Stress	defense response to bacterium	P	AT5G09650
15738	3	F23 vs. L23	F2-3 up	GO:0042742	Signaling and response	Stress	defense response to bacterium	P	AT5G13160
15739	3	F23 vs. L23	F2-3 up	GO:0042742	Signaling and response	Stress	defense response to bacterium	P	AT5G14740
15740	3	F23 vs. L23	F2-3 up	GO:0042742	Signaling and response	Stress	defense response to bacterium	P	AT5G22250
15741	3	F23 vs. L23	F2-3 up	GO:0042742	Signaling and response	Stress	defense response to bacterium	P	AT5G24530
15742	3	F23 vs. L23	F2-3 up	GO:0042742	Signaling and response	Stress	defense response to bacterium	P	AT5G50850
15743	3	F23 vs. L23	F2-3 up	GO:0042742	Signaling and response	Stress	defense response to bacterium	P	AT5G67300
15744	3	F23 vs. L23	F2-3 up	GO:0042742	Signaling and response	Stress	defense response to bacterium	P	ATCG00120
15745	3	F23 vs. L23	F2-3 up	GO:0009816	Signaling and response	Stress	defense response to bacterium, incompatible interaction	P	AT2G40000
15746	3	F23 vs. L23	F2-3 up	GO:0009816	Signaling and response	Stress	defense response to bacterium, incompatible interaction	P	AT4G23100
15747	3	F23 vs. L23	F2-3 up	GO:0009816	Signaling and response	Stress	defense response to bacterium, incompatible interaction	P	AT4G25030
15748	3	F23 vs. L23	F2-3 up	GO:0009816	Signaling and response	Stress	defense response to bacterium, incompatible interaction	P	AT5G06320
15749	3	F23 vs. L23	F2-3 up	GO:0009816	Signaling and response	Stress	defense response to bacterium, incompatible interaction	P	AT5G13160
15750	3	F23 vs. L23	F2-3 up	GO:0050832	Signaling and response	Stress	defense response to fungus	P	AT1G02205
15751	3	F23 vs. L23	F2-3 up	GO:0050832	Signaling and response	Stress	defense response to fungus	P	AT1G19610
15752	3	F23 vs. L23	F2-3 up	GO:0050832	Signaling and response	Stress	defense response to fungus	P	AT1G51090
15753	3	F23 vs. L23	F2-3 up	GO:0050832	Signaling and response	Stress	defense response to fungus	P	AT1G80840
15754	3	F23 vs. L23	F2-3 up	GO:0050832	Signaling and response	Stress	defense response to fungus	P	AT2G02140
15755	3	F23 vs. L23	F2-3 up	GO:0050832	Signaling and response	Stress	defense response to fungus	P	AT2G22240
15756	3	F23 vs. L23	F2-3 up	GO:0050832	Signaling and response	Stress	defense response to fungus	P	AT2G30020
15757	3	F23 vs. L23	F2-3 up	GO:0050832	Signaling and response	Stress	defense response to fungus	P	AT2G37630
15758	3	F23 vs. L23	F2-3 up	GO:0050832	Signaling and response	Stress	defense response to fungus	P	AT2G38870
15759	3	F23 vs. L23	F2-3 up	GO:0050832	Signaling and response	Stress	defense response to fungus	P	AT2G39210
15760	3	F23 vs. L23	F2-3 up	GO:0050832	Signaling and response	Stress	defense response to fungus	P	AT2G40140
15761	3	F23 vs. L23	F2-3 up	GO:0050832	Signaling and response	Stress	defense response to fungus	P	AT3G01420
15762	3	F23 vs. L23	F2-3 up	GO:0050832	Signaling and response	Stress	defense response to fungus	P	AT3G23820
15763	3	F23 vs. L23	F2-3 up	GO:0050832	Signaling and response	Stress	defense response to fungus	P	AT3G48990
15764	3	F23 vs. L23	F2-3 up	GO:0050832	Signaling and response	Stress	defense response to fungus	P	AT3G49110
15765	3	F23 vs. L23	F2-3 up	GO:0050832	Signaling and response	Stress	defense response to fungus	P	AT4G11760
15766	3	F23 vs. L23	F2-3 up	GO:0050832	Signaling and response	Stress	defense response to fungus	P	AT4G19230
15767	3	F23 vs. L23	F2-3 up	GO:0050832	Signaling and response	Stress	defense response to fungus	P	AT4G23100
15768	3	F23 vs. L23	F2-3 up	GO:0050832	Signaling and response	Stress	defense response to fungus	P	AT4G31800
15769	3	F23 vs. L23	F2-3 up	GO:0050832	Signaling and response	Stress	defense response to fungus	P	AT5G47910
15770	3	F23 vs. L23	F2-3 up	GO:0050832	Signaling and response	Stress	defense response to fungus	P	AT5G67300
15771	3	F23 vs. L23	F2-3 up	GO:0009817	Signaling and response	Stress	defense response to fungus, incompatible interaction	P	AT1G20020

15772	3	F23 vs. L23	F2-3 up	GO:0009817	Signaling and response	Stress	defense response to fungus, incompatible interaction	P	AT1G29340
15773	3	F23 vs. L23	F2-3 up	GO:0009817	Signaling and response	Stress	defense response to fungus, incompatible interaction	P	AT1G59870
15774	3	F23 vs. L23	F2-3 up	GO:0009817	Signaling and response	Stress	defense response to fungus, incompatible interaction	P	AT3G01500
15775	3	F23 vs. L23	F2-3 up	GO:0009817	Signaling and response	Stress	defense response to fungus, incompatible interaction	P	AT3G04720
15776	3	F23 vs. L23	F2-3 up	GO:0009817	Signaling and response	Stress	defense response to fungus, incompatible interaction	P	AT5G38900
15777	3	F23 vs. L23	F2-3 up	GO:0009817	Signaling and response	Stress	defense response to fungus, incompatible interaction	P	AT5G45890
15778	3	F23 vs. L23	F2-3 up	GO:0009817	Signaling and response	Stress	defense response to fungus, incompatible interaction	P	AT5G58260
15779	3	F23 vs. L23	F2-3 up	GO:0050829	Signaling and response	Stress	defense response to Gram-negative bacterium	P	AT2G45220
15780	3	F23 vs. L23	F2-3 up	GO:0050829	Signaling and response	Stress	defense response to Gram-negative bacterium	P	AT3G14310
15781	3	F23 vs. L23	F2-3 up	GO:0050829	Signaling and response	Stress	defense response to Gram-negative bacterium	P	AT3G23820
15782	3	F23 vs. L23	F2-3 up	GO:0050829	Signaling and response	Stress	defense response to Gram-negative bacterium	P	AT4G28490
15783	3	F23 vs. L23	F2-3 up	GO:0050829	Signaling and response	Stress	defense response to Gram-negative bacterium	P	AT4G33220
15784	3	F23 vs. L23	F2-3 up	GO:0002213	Signaling and response	Stress	defense response to insect	P	AT3G48520
15785	3	F23 vs. L23	F2-3 up	GO:0002213	Signaling and response	Stress	defense response to insect	P	AT4G23100
15786	3	F23 vs. L23	F2-3 up	GO:0002213	Signaling and response	Stress	defense response to insect	P	AT5G25980
15787	3	F23 vs. L23	F2-3 up	GO:0002229	Signaling and response	Stress	defense response to oomycetes	P	AT5G12170
15788	3	F23 vs. L23	F2-3 up	GO:0002229	Signaling and response	Stress	defense response to oomycetes	P	AT5G24530
15789	3	F23 vs. L23	F2-3 up	GO:0051607	Signaling and response	Stress	defense response to virus	P	AT1G04400
15790	3	F23 vs. L23	F2-3 up	GO:0051607	Signaling and response	Stress	defense response to virus	P	AT2G22240
15791	3	F23 vs. L23	F2-3 up	GO:0051607	Signaling and response	Stress	defense response to virus	P	AT5G06320
15792	3	F23 vs. L23	F2-3 up	GO:0009814	Signaling and response	Stress	defense response, incompatible interaction	P	AT1G29340
15793	3	F23 vs. L23	F2-3 up	GO:0016045	Signaling and response	Stress	detection of bacterium	P	AT2G29630
15794	3	F23 vs. L23	F2-3 up	GO:0009727	Signaling and response	Stress	detection of ethylene stimulus	P	AT1G62380
15795	3	F23 vs. L23	F2-3 up	GO:0071585	Signaling and response	Stress	detoxification of cadmium ion	P	AT1G71050
15796	3	F23 vs. L23	F2-3 up	GO:0071585	Signaling and response	Stress	detoxification of cadmium ion	P	AT5G17450
15797	3	F23 vs. L23	F2-3 up	GO:0009819	Signaling and response	Stress	drought recovery	P	AT2G37040
15798	3	F23 vs. L23	F2-3 up	GO:0009819	Signaling and response	Stress	drought recovery	P	AT3G16800
15799	3	F23 vs. L23	F2-3 up	GO:0009693	Signaling and response	Stress	ethylene biosynthetic process	P	AT1G05010
15800	3	F23 vs. L23	F2-3 up	GO:0009693	Signaling and response	Stress	ethylene biosynthetic process	P	AT1G62380
15801	3	F23 vs. L23	F2-3 up	GO:0009693	Signaling and response	Stress	ethylene biosynthetic process	P	AT4G11280
15802	3	F23 vs. L23	F2-3 up	GO:0009873	Signaling and response	Stress	ethylene-activated signaling pathway	P	AT1G01250
15803	3	F23 vs. L23	F2-3 up	GO:0009873	Signaling and response	Stress	ethylene-activated signaling pathway	P	AT1G05850
15804	3	F23 vs. L23	F2-3 up	GO:0009873	Signaling and response	Stress	ethylene-activated signaling pathway	P	AT1G64060
15805	3	F23 vs. L23	F2-3 up	GO:0009873	Signaling and response	Stress	ethylene-activated signaling pathway	P	AT4G32800
15806	3	F23 vs. L23	F2-3 up	GO:0009873	Signaling and response	Stress	ethylene-activated signaling pathway	P	AT5G09230
15807	3	F23 vs. L23	F2-3 up	GO:0048307	Signaling and response	Stress	ferredoxin-nitrite reductase activity	F	AT2G15620
15808	3	F23 vs. L23	F2-3 up	GO:0009813	Signaling and response	Stress	flavonoid biosynthetic process	P	AT2G38240
15809	3	F23 vs. L23	F2-3 up	GO:0009813	Signaling and response	Stress	flavonoid biosynthetic process	P	AT2G40890
15810	3	F23 vs. L23	F2-3 up	GO:0009813	Signaling and response	Stress	flavonoid biosynthetic process	P	AT3G51240
15811	3	F23 vs. L23	F2-3 up	GO:0009813	Signaling and response	Stress	flavonoid biosynthetic process	P	AT5G05600
15812	3	F23 vs. L23	F2-3 up	GO:0009813	Signaling and response	Stress	flavonoid biosynthetic process	P	AT5G08640
15813	3	F23 vs. L23	F2-3 up	GO:0009813	Signaling and response	Stress	flavonoid biosynthetic process	P	AT5G13930
15814	3	F23 vs. L23	F2-3 up	GO:0009813	Signaling and response	Stress	flavonoid biosynthetic process	P	AT5G24530
15815	3	F23 vs. L23	F2-3 up	GO:0009812	Signaling and response	Stress	flavonoid metabolic process	P	AT4G25640
15816	3	F23 vs. L23	F2-3 up	GO:0051555	Signaling and response	Stress	flavonol biosynthetic process	P	AT1G78570
15817	3	F23 vs. L23	F2-3 up	GO:0051555	Signaling and response	Stress	flavonol biosynthetic process	P	AT2G36790
15818	3	F23 vs. L23	F2-3 up	GO:0010336	Signaling and response	Stress	gibberellic acid homeostasis	P	AT1G14920
15819	3	F23 vs. L23	F2-3 up	GO:0010336	Signaling and response	Stress	gibberellic acid homeostasis	P	AT1G22840
15820	3	F23 vs. L23	F2-3 up	GO:0010336	Signaling and response	Stress	gibberellic acid homeostasis	P	AT1G68570

15821	3	F23 vs. L23	F2-3 up	GO:0010336	Signaling and response	Stress	gibberellic acid homeostasis	P	AT4G10040
15822	3	F23 vs. L23	F2-3 up	GO:0043295	Signaling and response	Stress	glutathione binding	F	AT1G78370
15823	3	F23 vs. L23	F2-3 up	GO:0043295	Signaling and response	Stress	glutathione binding	F	AT2G30860
15824	3	F23 vs. L23	F2-3 up	GO:0043295	Signaling and response	Stress	glutathione binding	F	AT3G03190
15825	3	F23 vs. L23	F2-3 up	GO:0006750	Signaling and response	Stress	glutathione biosynthetic process	P	AT4G23100
15826	3	F23 vs. L23	F2-3 up	GO:0045174	Signaling and response	Stress	glutathione dehydrogenase (ascorbate) activity	F	AT1G19550
15827	3	F23 vs. L23	F2-3 up	GO:0016756	Signaling and response	Stress	glutathione gamma-glutamylcysteinyltransferase activity	F	AT4G39640
15828	3	F23 vs. L23	F2-3 up	GO:0036374	Signaling and response	Stress	glutathione hydrolase activity	F	AT4G39640
15829	3	F23 vs. L23	F2-3 up	GO:0006749	Signaling and response	Stress	glutathione metabolic process	P	AT1G10370
15830	3	F23 vs. L23	F2-3 up	GO:0006749	Signaling and response	Stress	glutathione metabolic process	P	AT1G78320
15831	3	F23 vs. L23	F2-3 up	GO:0006749	Signaling and response	Stress	glutathione metabolic process	P	AT1G78370
15832	3	F23 vs. L23	F2-3 up	GO:0006749	Signaling and response	Stress	glutathione metabolic process	P	AT2G02390
15833	3	F23 vs. L23	F2-3 up	GO:0006749	Signaling and response	Stress	glutathione metabolic process	P	AT2G30860
15834	3	F23 vs. L23	F2-3 up	GO:0006749	Signaling and response	Stress	glutathione metabolic process	P	AT3G03190
15835	3	F23 vs. L23	F2-3 up	GO:0006749	Signaling and response	Stress	glutathione metabolic process	P	AT3G54660
15836	3	F23 vs. L23	F2-3 up	GO:0004602	Signaling and response	Stress	glutathione peroxidase activity	F	AT2G30860
15837	3	F23 vs. L23	F2-3 up	GO:0004602	Signaling and response	Stress	glutathione peroxidase activity	F	AT2G31570
15838	3	F23 vs. L23	F2-3 up	GO:0004602	Signaling and response	Stress	glutathione peroxidase activity	F	AT4G11600
15839	3	F23 vs. L23	F2-3 up	GO:0004364	Signaling and response	Stress	glutathione transferase activity	F	AT1G10370
15840	3	F23 vs. L23	F2-3 up	GO:0004364	Signaling and response	Stress	glutathione transferase activity	F	AT1G78320
15841	3	F23 vs. L23	F2-3 up	GO:0004364	Signaling and response	Stress	glutathione transferase activity	F	AT1G78370
15842	3	F23 vs. L23	F2-3 up	GO:0004364	Signaling and response	Stress	glutathione transferase activity	F	AT2G02390
15843	3	F23 vs. L23	F2-3 up	GO:0004364	Signaling and response	Stress	glutathione transferase activity	F	AT2G30860
15844	3	F23 vs. L23	F2-3 up	GO:0004364	Signaling and response	Stress	glutathione transferase activity	F	AT3G03190
15845	3	F23 vs. L23	F2-3 up	GO:0004364	Signaling and response	Stress	glutathione transferase activity	F	AT5G02790
15846	3	F23 vs. L23	F2-3 up	GO:0034635	Signaling and response	Stress	glutathione transport	P	AT5G12170
15847	3	F23 vs. L23	F2-3 up	GO:0004362	Signaling and response	Stress	glutathione-disulfide reductase activity	F	AT3G54660
15848	3	F23 vs. L23	F2-3 up	GO:0010286	Signaling and response	Stress	heat acclimation	P	AT1G54280
15849	3	F23 vs. L23	F2-3 up	GO:0031072	Signaling and response	Stress	heat shock protein binding	F	AT3G09440
15850	3	F23 vs. L23	F2-3 up	GO:0050665	Signaling and response	Stress	hydrogen peroxide biosynthetic process	P	AT1G64060
15851	3	F23 vs. L23	F2-3 up	GO:0050665	Signaling and response	Stress	hydrogen peroxide biosynthetic process	P	AT3G14420
15852	3	F23 vs. L23	F2-3 up	GO:0042744	Signaling and response	Stress	hydrogen peroxide catabolic process	P	AT1G20620
15853	3	F23 vs. L23	F2-3 up	GO:0042744	Signaling and response	Stress	hydrogen peroxide catabolic process	P	AT2G41480
15854	3	F23 vs. L23	F2-3 up	GO:0042744	Signaling and response	Stress	hydrogen peroxide catabolic process	P	AT3G11630
15855	3	F23 vs. L23	F2-3 up	GO:0042744	Signaling and response	Stress	hydrogen peroxide catabolic process	P	AT3G49110
15856	3	F23 vs. L23	F2-3 up	GO:0042744	Signaling and response	Stress	hydrogen peroxide catabolic process	P	AT3G52880
15857	3	F23 vs. L23	F2-3 up	GO:0042744	Signaling and response	Stress	hydrogen peroxide catabolic process	P	AT5G47000
15858	3	F23 vs. L23	F2-3 up	GO:0042744	Signaling and response	Stress	hydrogen peroxide catabolic process	P	AT5G51890
15859	3	F23 vs. L23	F2-3 up	GO:0080170	Signaling and response	Stress	hydrogen peroxide transmembrane transport	P	AT2G36830
15860	3	F23 vs. L23	F2-3 up	GO:0080170	Signaling and response	Stress	hydrogen peroxide transmembrane transport	P	AT3G26520
15861	3	F23 vs. L23	F2-3 up	GO:0080170	Signaling and response	Stress	hydrogen peroxide transmembrane transport	P	AT3G53420
15862	3	F23 vs. L23	F2-3 up	GO:0042538	Signaling and response	Stress	hyperosmotic salinity response	P	AT1G14920
15863	3	F23 vs. L23	F2-3 up	GO:0042538	Signaling and response	Stress	hyperosmotic salinity response	P	AT3G55610
15864	3	F23 vs. L23	F2-3 up	GO:0042538	Signaling and response	Stress	hyperosmotic salinity response	P	AT4G30996
15865	3	F23 vs. L23	F2-3 up	GO:0042538	Signaling and response	Stress	hyperosmotic salinity response	P	AT4G32410
15866	3	F23 vs. L23	F2-3 up	GO:0042538	Signaling and response	Stress	hyperosmotic salinity response	P	AT5G01410
15867	3	F23 vs. L23	F2-3 up	GO:0042538	Signaling and response	Stress	hyperosmotic salinity response	P	AT5G49720
15868	3	F23 vs. L23	F2-3 up	GO:0042538	Signaling and response	Stress	hyperosmotic salinity response	P	AT5G50720
15869	3	F23 vs. L23	F2-3 up	GO:0045087	Signaling and response	Stress	innate immune response	P	AT4G39030
15870	3	F23 vs. L23	F2-3 up	GO:0045087	Signaling and response	Stress	innate immune response	P	AT5G13160
15871	3	F23 vs. L23	F2-3 up	GO:0050113	Signaling and response	Stress	inositol oxygenase activity	F	AT4G26260
15872	3	F23 vs. L23	F2-3 up	GO:0052658	Signaling and response	Stress	inositol-1,4,5-trisphosphate 5-phosphatase activity	F	AT2G31830
15873	3	F23 vs. L23	F2-3 up	GO:0044419	Signaling and response	Stress	interspecies interaction between organisms	P	AT4G31750
15874	3	F23 vs. L23	F2-3 up	GO:0055072	Signaling and response	Stress	iron ion homeostasis	P	AT1G41830
15875	3	F23 vs. L23	F2-3 up	GO:0055072	Signaling and response	Stress	iron ion homeostasis	P	AT1G55570

15876	3	F23 vs. L23	F2-3 up	GO:0055072	Signaling and response	Stress	iron ion homeostasis	P	AT3G07700
15877	3	F23 vs. L23	F2-3 up	GO:0055072	Signaling and response	Stress	iron ion homeostasis	P	AT3G13390
15878	3	F23 vs. L23	F2-3 up	GO:0055072	Signaling and response	Stress	iron ion homeostasis	P	AT5G01600
15879	3	F23 vs. L23	F2-3 up	GO:0055072	Signaling and response	Stress	iron ion homeostasis	P	AT5G51720
15880	3	F23 vs. L23	F2-3 up	GO:0055072	Signaling and response	Stress	iron ion homeostasis	P	AT5G60020
15881	3	F23 vs. L23	F2-3 up	GO:0031640	Signaling and response	Stress	killing of cells of other organism	P	AT1G19610
15882	3	F23 vs. L23	F2-3 up	GO:0031640	Signaling and response	Stress	killing of cells of other organism	P	AT2G02140
15883	3	F23 vs. L23	F2-3 up	GO:0031640	Signaling and response	Stress	killing of cells of other organism	P	AT3G04720
15884	3	F23 vs. L23	F2-3 up	GO:0031640	Signaling and response	Stress	killing of cells of other organism	P	AT4G11760
15885	3	F23 vs. L23	F2-3 up	GO:0004462	Signaling and response	Stress	lactoylglutathione lyase activity	F	AT1G11840
15886	3	F23 vs. L23	F2-3 up	GO:0031418	Signaling and response	Stress	L-ascorbic acid binding	F	AT1G05010
15887	3	F23 vs. L23	F2-3 up	GO:0031418	Signaling and response	Stress	L-ascorbic acid binding	F	AT1G62380
15888	3	F23 vs. L23	F2-3 up	GO:0031418	Signaling and response	Stress	L-ascorbic acid binding	F	AT3G51240
15889	3	F23 vs. L23	F2-3 up	GO:0031418	Signaling and response	Stress	L-ascorbic acid binding	F	AT5G08640
15890	3	F23 vs. L23	F2-3 up	GO:0019853	Signaling and response	Stress	L-ascorbic acid biosynthetic process	P	AT2G39770
15891	3	F23 vs. L23	F2-3 up	GO:0019853	Signaling and response	Stress	L-ascorbic acid biosynthetic process	P	AT4G26260
15892	3	F23 vs. L23	F2-3 up	GO:0019243	Signaling and response	Stress	methylglyoxal catabolic process to D-lactate via S-lactoyl-glutathione	P	AT1G11840
15893	3	F23 vs. L23	F2-3 up	GO:0051787	Signaling and response	Stress	misfolded protein binding	F	AT3G09440
15894	3	F23 vs. L23	F2-3 up	GO:0016656	Signaling and response	Stress	monodehydroascorbate reductase (NADH) activity	F	AT3G09940
15895	3	F23 vs. L23	F2-3 up	GO:0016656	Signaling and response	Stress	monodehydroascorbate reductase (NADH) activity	F	AT3G52880
15896	3	F23 vs. L23	F2-3 up	GO:0009788	Signaling and response	Stress	negative regulation of abscisic acid-activated signaling pathway	P	AT1G01720
15897	3	F23 vs. L23	F2-3 up	GO:0009788	Signaling and response	Stress	negative regulation of abscisic acid-activated signaling pathway	P	AT3G11410
15898	3	F23 vs. L23	F2-3 up	GO:0009788	Signaling and response	Stress	negative regulation of abscisic acid-activated signaling pathway	P	AT5G66070
15899	3	F23 vs. L23	F2-3 up	GO:0031348	Signaling and response	Stress	negative regulation of defense response	P	AT1G59870
15900	3	F23 vs. L23	F2-3 up	GO:0031348	Signaling and response	Stress	negative regulation of defense response	P	AT4G39030
15901	3	F23 vs. L23	F2-3 up	GO:0031348	Signaling and response	Stress	negative regulation of defense response	P	AT5G09230
15902	3	F23 vs. L23	F2-3 up	GO:1900425	Signaling and response	Stress	negative regulation of defense response to bacterium	P	AT1G51090
15903	3	F23 vs. L23	F2-3 up	GO:1900425	Signaling and response	Stress	negative regulation of defense response to bacterium	P	AT3G12620
15904	3	F23 vs. L23	F2-3 up	GO:1902478	Signaling and response	Stress	negative regulation of defense response to bacterium, incompatible interaction	P	AT5G62570
15905	3	F23 vs. L23	F2-3 up	GO:1900366	Signaling and response	Stress	negative regulation of defense response to insect	P	AT3G22400
15906	3	F23 vs. L23	F2-3 up	GO:0080148	Signaling and response	Stress	negative regulation of response to water deprivation	P	AT1G10370
15907	3	F23 vs. L23	F2-3 up	GO:0010187	Signaling and response	Stress	negative regulation of seed germination	P	AT1G14920
15908	3	F23 vs. L23	F2-3 up	GO:0010187	Signaling and response	Stress	negative regulation of seed germination	P	AT5G56860
15909	3	F23 vs. L23	F2-3 up	GO:0017148	Signaling and response	Stress	negative regulation of translation	P	AT5G22250
15910	3	F23 vs. L23	F2-3 up	GO:0050421	Signaling and response	Stress	nitrite reductase (NO-forming) activity	F	AT2G15620
15911	3	F23 vs. L23	F2-3 up	GO:0010196	Signaling and response	Stress	nonphotochemical quenching	P	AT1G44575
15912	3	F23 vs. L23	F2-3 up	GO:0010196	Signaling and response	Stress	nonphotochemical quenching	P	AT1G56500
15913	3	F23 vs. L23	F2-3 up	GO:0010196	Signaling and response	Stress	nonphotochemical quenching	P	AT4G03280
15914	3	F23 vs. L23	F2-3 up	GO:0051920	Signaling and response	Stress	peroxiredoxin activity	F	AT3G11630
15915	3	F23 vs. L23	F2-3 up	GO:0051920	Signaling and response	Stress	peroxiredoxin activity	F	AT3G26060
15916	3	F23 vs. L23	F2-3 up	GO:0055062	Signaling and response	Stress	phosphate ion homeostasis	P	AT2G38170
15917	3	F23 vs. L23	F2-3 up	GO:0046854	Signaling and response	Stress	phosphatidylinositol phosphorylation	P	AT1G21980
15918	3	F23 vs. L23	F2-3 up	GO:0048015	Signaling and response	Stress	phosphatidylinositol-mediated signaling	P	AT3G08510
15919	3	F23 vs. L23	F2-3 up	GO:0048015	Signaling and response	Stress	phosphatidylinositol-mediated signaling	P	AT5G58690
15920	3	F23 vs. L23	F2-3 up	GO:0048015	Signaling and response	Stress	phosphatidylinositol-mediated signaling	P	AT5G58700
15921	3	F23 vs. L23	F2-3 up	GO:0047066	Signaling and response	Stress	phospholipid-hydroperoxide glutathione peroxidase activity	F	AT4G11600
15922	3	F23 vs. L23	F2-3 up	GO:0010117	Signaling and response	Stress	photoprotection	P	AT1G27730

15923	3	F23 vs. L23	F2-3 up	GO:0010117	Signaling and response	Stress	photoprotection	P	AT2G05620
15924	3	F23 vs. L23	F2-3 up	GO:0010117	Signaling and response	Stress	photoprotection	P	AT3G22840
15925	3	F23 vs. L23	F2-3 up	GO:0010117	Signaling and response	Stress	photoprotection	P	AT4G14690
15926	3	F23 vs. L23	F2-3 up	GO:0009626	Signaling and response	Stress	plant-type hypersensitive response	P	AT1G29340
15927	3	F23 vs. L23	F2-3 up	GO:0009626	Signaling and response	Stress	plant-type hypersensitive response	P	AT3G01420
15928	3	F23 vs. L23	F2-3 up	GO:0009626	Signaling and response	Stress	plant-type hypersensitive response	P	AT3G45640
15929	3	F23 vs. L23	F2-3 up	GO:0009626	Signaling and response	Stress	plant-type hypersensitive response	P	AT4G37990
15930	3	F23 vs. L23	F2-3 up	GO:0009626	Signaling and response	Stress	plant-type hypersensitive response	P	AT4G38740
15931	3	F23 vs. L23	F2-3 up	GO:0009626	Signaling and response	Stress	plant-type hypersensitive response	P	AT5G54250
15932	3	F23 vs. L23	F2-3 up	GO:0009789	Signaling and response	Stress	positive regulation of abscisic acid-activated signaling pathway	P	AT2G32460
15933	3	F23 vs. L23	F2-3 up	GO:0009963	Signaling and response	Stress	positive regulation of flavonoid biosynthetic process	P	AT1G32640
15934	3	F23 vs. L23	F2-3 up	GO:0043068	Signaling and response	Stress	positive regulation of programmed cell death	P	AT2G32460
15935	3	F23 vs. L23	F2-3 up	GO:2000379	Signaling and response	Stress	positive regulation of reactive oxygen species metabolic process	P	AT1G04400
15936	3	F23 vs. L23	F2-3 up	GO:1902584	Signaling and response	Stress	positive regulation of response to water deprivation	P	AT3G05500
15937	3	F23 vs. L23	F2-3 up	GO:0055075	Signaling and response	Stress	potassium ion homeostasis	P	AT1G32450
15938	3	F23 vs. L23	F2-3 up	GO:0080136	Signaling and response	Stress	priming of cellular response to stress	P	AT3G45640
15939	3	F23 vs. L23	F2-3 up	GO:0002020	Signaling and response	Stress	protease binding	F	AT3G09440
15940	3	F23 vs. L23	F2-3 up	GO:0044183	Signaling and response	Stress	protein folding chaperone	F	AT3G09440
15941	3	F23 vs. L23	F2-3 up	GO:0042026	Signaling and response	Stress	protein refolding	P	AT3G09440
15942	3	F23 vs. L23	F2-3 up	GO:0042026	Signaling and response	Stress	protein refolding	P	AT4G38740
15943	3	F23 vs. L23	F2-3 up	GO:0072593	Signaling and response	Stress	reactive oxygen species metabolic process	P	AT1G64060
15944	3	F23 vs. L23	F2-3 up	GO:0072593	Signaling and response	Stress	reactive oxygen species metabolic process	P	AT1G79440
15945	3	F23 vs. L23	F2-3 up	GO:0072593	Signaling and response	Stress	reactive oxygen species metabolic process	P	AT3G49110
15946	3	F23 vs. L23	F2-3 up	GO:0072593	Signaling and response	Stress	reactive oxygen species metabolic process	P	AT5G47910
15947	3	F23 vs. L23	F2-3 up	GO:0072593	Signaling and response	Stress	reactive oxygen species metabolic process	P	AT5G51720
15948	3	F23 vs. L23	F2-3 up	GO:0072593	Signaling and response	Stress	reactive oxygen species metabolic process	P	AT5G62520
15949	3	F23 vs. L23	F2-3 up	GO:0009787	Signaling and response	Stress	regulation of abscisic acid-activated signaling pathway	P	AT1G16310
15950	3	F23 vs. L23	F2-3 up	GO:0010359	Signaling and response	Stress	regulation of anion channel activity	P	AT2G17290
15951	3	F23 vs. L23	F2-3 up	GO:1900067	Signaling and response	Stress	regulation of cellular response to alkaline pH	P	AT1G19180
15952	3	F23 vs. L23	F2-3 up	GO:0031347	Signaling and response	Stress	regulation of defense response	P	AT1G17380
15953	3	F23 vs. L23	F2-3 up	GO:0031347	Signaling and response	Stress	regulation of defense response	P	AT1G19180
15954	3	F23 vs. L23	F2-3 up	GO:0031347	Signaling and response	Stress	regulation of defense response	P	AT1G70700
15955	3	F23 vs. L23	F2-3 up	GO:0031347	Signaling and response	Stress	regulation of defense response	P	AT1G80840
15956	3	F23 vs. L23	F2-3 up	GO:0031347	Signaling and response	Stress	regulation of defense response	P	AT2G34600
15957	3	F23 vs. L23	F2-3 up	GO:0031347	Signaling and response	Stress	regulation of defense response	P	AT4G31800
15958	3	F23 vs. L23	F2-3 up	GO:1900424	Signaling and response	Stress	regulation of defense response to bacterium	P	AT5G67160
15959	3	F23 vs. L23	F2-3 up	GO:1900150	Signaling and response	Stress	regulation of defense response to fungus	P	AT5G67160
15960	3	F23 vs. L23	F2-3 up	GO:1900150	Signaling and response	Stress	regulation of defense response to fungus	P	AT5G67300
15961	3	F23 vs. L23	F2-3 up	GO:2000068	Signaling and response	Stress	regulation of defense response to insect	P	AT1G32640
15962	3	F23 vs. L23	F2-3 up	GO:0050691	Signaling and response	Stress	regulation of defense response to virus by host	P	AT1G80840
15963	3	F23 vs. L23	F2-3 up	GO:0050691	Signaling and response	Stress	regulation of defense response to virus by host	P	AT4G31800
15964	3	F23 vs. L23	F2-3 up	GO:0045088	Signaling and response	Stress	regulation of innate immune response	P	AT2G37630
15965	3	F23 vs. L23	F2-3 up	GO:0051453	Signaling and response	Stress	regulation of intracellular pH	P	AT1G80660
15966	3	F23 vs. L23	F2-3 up	GO:0051453	Signaling and response	Stress	regulation of intracellular pH	P	AT2G07560
15967	3	F23 vs. L23	F2-3 up	GO:0051453	Signaling and response	Stress	regulation of intracellular pH	P	AT3G42640
15968	3	F23 vs. L23	F2-3 up	GO:0051453	Signaling and response	Stress	regulation of intracellular pH	P	AT3G60330
15969	3	F23 vs. L23	F2-3 up	GO:0051453	Signaling and response	Stress	regulation of intracellular pH	P	AT5G57350
15970	3	F23 vs. L23	F2-3 up	GO:0006885	Signaling and response	Stress	regulation of pH	P	AT3G17630
15971	3	F23 vs. L23	F2-3 up	GO:0006885	Signaling and response	Stress	regulation of pH	P	AT5G01690
15972	3	F23 vs. L23	F2-3 up	GO:2000377	Signaling and response	Stress	regulation of reactive oxygen species metabolic process	P	AT1G14920
15973	3	F23 vs. L23	F2-3 up	GO:2000377	Signaling and response	Stress	regulation of reactive oxygen species metabolic process	P	AT1G61660

15974	3	F23 vs. L23	F2-3 up	GO:2000377	Signaling and response	Stress	regulation of reactive oxygen species metabolic process	P	AT4G22750
15975	3	F23 vs. L23	F2-3 up	GO:2001020	Signaling and response	Stress	regulation of response to DNA damage stimulus	P	AT3G50000
15976	3	F23 vs. L23	F2-3 up	GO:1901031	Signaling and response	Stress	regulation of response to reactive oxygen species	P	AT3G07700
15977	3	F23 vs. L23	F2-3 up	GO:2000070	Signaling and response	Stress	regulation of response to water deprivation	P	AT4G05590
15978	3	F23 vs. L23	F2-3 up	GO:2000033	Signaling and response	Stress	regulation of seed dormancy process	P	AT1G14920
15979	3	F23 vs. L23	F2-3 up	GO:0090333	Signaling and response	Stress	regulation of stomatal closure	P	AT5G23060
15980	3	F23 vs. L23	F2-3 up	GO:0010119	Signaling and response	Stress	regulation of stomatal movement	P	AT1G64060
15981	3	F23 vs. L23	F2-3 up	GO:0010119	Signaling and response	Stress	regulation of stomatal movement	P	AT1G69530
15982	3	F23 vs. L23	F2-3 up	GO:0010119	Signaling and response	Stress	regulation of stomatal movement	P	AT2G17290
15983	3	F23 vs. L23	F2-3 up	GO:0010119	Signaling and response	Stress	regulation of stomatal movement	P	AT3G01500
15984	3	F23 vs. L23	F2-3 up	GO:0010119	Signaling and response	Stress	regulation of stomatal movement	P	AT3G11410
15985	3	F23 vs. L23	F2-3 up	GO:0010119	Signaling and response	Stress	regulation of stomatal movement	P	AT3G45780
15986	3	F23 vs. L23	F2-3 up	GO:0010119	Signaling and response	Stress	regulation of stomatal movement	P	AT4G18950
15987	3	F23 vs. L23	F2-3 up	GO:0010119	Signaling and response	Stress	regulation of stomatal movement	P	AT5G25980
15988	3	F23 vs. L23	F2-3 up	GO:1902456	Signaling and response	Stress	regulation of stomatal opening	P	AT2G17290
15989	3	F23 vs. L23	F2-3 up	GO:0043903	Signaling and response	Stress	regulation of symbiosis, encompassing mutualism through parasitism	P	AT3G09940
15990	3	F23 vs. L23	F2-3 up	GO:0051209	Signaling and response	Stress	release of sequestered calcium ion into cytosol	P	AT3G08510
15991	3	F23 vs. L23	F2-3 up	GO:0051209	Signaling and response	Stress	release of sequestered calcium ion into cytosol	P	AT5G58690
15992	3	F23 vs. L23	F2-3 up	GO:0051209	Signaling and response	Stress	release of sequestered calcium ion into cytosol	P	AT5G58700
15993	3	F23 vs. L23	F2-3 up	GO:0019430	Signaling and response	Stress	removal of superoxide radicals	P	AT1G08830
15994	3	F23 vs. L23	F2-3 up	GO:0019430	Signaling and response	Stress	removal of superoxide radicals	P	AT2G28190
15995	3	F23 vs. L23	F2-3 up	GO:0002679	Signaling and response	Stress	respiratory burst involved in defense response	P	AT1G64060
15996	3	F23 vs. L23	F2-3 up	GO:0009737	Signaling and response	Stress	response to abscisic acid	P	AT1G14920
15997	3	F23 vs. L23	F2-3 up	GO:0009737	Signaling and response	Stress	response to abscisic acid	P	AT1G27730
15998	3	F23 vs. L23	F2-3 up	GO:0009737	Signaling and response	Stress	response to abscisic acid	P	AT1G32080
15999	3	F23 vs. L23	F2-3 up	GO:0009737	Signaling and response	Stress	response to abscisic acid	P	AT1G32640
16000	3	F23 vs. L23	F2-3 up	GO:0009737	Signaling and response	Stress	response to abscisic acid	P	AT1G35720
16001	3	F23 vs. L23	F2-3 up	GO:0009737	Signaling and response	Stress	response to abscisic acid	P	AT1G44170
16002	3	F23 vs. L23	F2-3 up	GO:0009737	Signaling and response	Stress	response to abscisic acid	P	AT1G59870
16003	3	F23 vs. L23	F2-3 up	GO:0009737	Signaling and response	Stress	response to abscisic acid	P	AT2G17290
16004	3	F23 vs. L23	F2-3 up	GO:0009737	Signaling and response	Stress	response to abscisic acid	P	AT2G17840
16005	3	F23 vs. L23	F2-3 up	GO:0009737	Signaling and response	Stress	response to abscisic acid	P	AT2G35940
16006	3	F23 vs. L23	F2-3 up	GO:0009737	Signaling and response	Stress	response to abscisic acid	P	AT2G38280
16007	3	F23 vs. L23	F2-3 up	GO:0009737	Signaling and response	Stress	response to abscisic acid	P	AT2G46820
16008	3	F23 vs. L23	F2-3 up	GO:0009737	Signaling and response	Stress	response to abscisic acid	P	AT2G46830
16009	3	F23 vs. L23	F2-3 up	GO:0009737	Signaling and response	Stress	response to abscisic acid	P	AT3G01420
16010	3	F23 vs. L23	F2-3 up	GO:0009737	Signaling and response	Stress	response to abscisic acid	P	AT3G06490
16011	3	F23 vs. L23	F2-3 up	GO:0009737	Signaling and response	Stress	response to abscisic acid	P	AT3G11050
16012	3	F23 vs. L23	F2-3 up	GO:0009737	Signaling and response	Stress	response to abscisic acid	P	AT3G11410
16013	3	F23 vs. L23	F2-3 up	GO:0009737	Signaling and response	Stress	response to abscisic acid	P	AT3G23700
16014	3	F23 vs. L23	F2-3 up	GO:0009737	Signaling and response	Stress	response to abscisic acid	P	AT3G46620
16015	3	F23 vs. L23	F2-3 up	GO:0009737	Signaling and response	Stress	response to abscisic acid	P	AT3G48170
16016	3	F23 vs. L23	F2-3 up	GO:0009737	Signaling and response	Stress	response to abscisic acid	P	AT3G51920
16017	3	F23 vs. L23	F2-3 up	GO:0009737	Signaling and response	Stress	response to abscisic acid	P	AT3G53420
16018	3	F23 vs. L23	F2-3 up	GO:0009737	Signaling and response	Stress	response to abscisic acid	P	AT3G55610
16019	3	F23 vs. L23	F2-3 up	GO:0009737	Signaling and response	Stress	response to abscisic acid	P	AT4G02380
16020	3	F23 vs. L23	F2-3 up	GO:0009737	Signaling and response	Stress	response to abscisic acid	P	AT4G34710
16021	3	F23 vs. L23	F2-3 up	GO:0009737	Signaling and response	Stress	response to abscisic acid	P	AT4G38970
16022	3	F23 vs. L23	F2-3 up	GO:0009737	Signaling and response	Stress	response to abscisic acid	P	AT5G04500
16023	3	F23 vs. L23	F2-3 up	GO:0009737	Signaling and response	Stress	response to abscisic acid	P	AT5G16600
16024	3	F23 vs. L23	F2-3 up	GO:0009737	Signaling and response	Stress	response to abscisic acid	P	AT5G25610
16025	3	F23 vs. L23	F2-3 up	GO:0009737	Signaling and response	Stress	response to abscisic acid	P	AT5G25980
16026	3	F23 vs. L23	F2-3 up	GO:0009737	Signaling and response	Stress	response to abscisic acid	P	AT5G40010
16027	3	F23 vs. L23	F2-3 up	GO:0009737	Signaling and response	Stress	response to abscisic acid	P	AT5G50720

16028	3	F23 vs. L23	F2-3 up	GO:0009737	Signaling and response	Stress	response to abscisic acid	P	AT5G67300
16029	3	F23 vs. L23	F2-3 up	GO:0009646	Signaling and response	Stress	response to absence of light	P	AT1G04400
16030	3	F23 vs. L23	F2-3 up	GO:0009646	Signaling and response	Stress	response to absence of light	P	AT4G02380
16031	3	F23 vs. L23	F2-3 up	GO:0009646	Signaling and response	Stress	response to absence of light	P	AT5G01410
16032	3	F23 vs. L23	F2-3 up	GO:0046685	Signaling and response	Stress	response to arsenic-containing substance	P	AT4G33540
16033	3	F23 vs. L23	F2-3 up	GO:0009409	Signaling and response	Stress	response to cold	P	AT1G09340
16034	3	F23 vs. L23	F2-3 up	GO:0009409	Signaling and response	Stress	response to cold	P	AT1G16880
16035	3	F23 vs. L23	F2-3 up	GO:0009409	Signaling and response	Stress	response to cold	P	AT1G20620
16036	3	F23 vs. L23	F2-3 up	GO:0009409	Signaling and response	Stress	response to cold	P	AT1G21760
16037	3	F23 vs. L23	F2-3 up	GO:0009409	Signaling and response	Stress	response to cold	P	AT1G23740
16038	3	F23 vs. L23	F2-3 up	GO:0009409	Signaling and response	Stress	response to cold	P	AT1G27730
16039	3	F23 vs. L23	F2-3 up	GO:0009409	Signaling and response	Stress	response to cold	P	AT1G32060
16040	3	F23 vs. L23	F2-3 up	GO:0009409	Signaling and response	Stress	response to cold	P	AT1G35720
16041	3	F23 vs. L23	F2-3 up	GO:0009409	Signaling and response	Stress	response to cold	P	AT1G42970
16042	3	F23 vs. L23	F2-3 up	GO:0009409	Signaling and response	Stress	response to cold	P	AT1G45474
16043	3	F23 vs. L23	F2-3 up	GO:0009409	Signaling and response	Stress	response to cold	P	AT1G68530
16044	3	F23 vs. L23	F2-3 up	GO:0009409	Signaling and response	Stress	response to cold	P	AT1G79440
16045	3	F23 vs. L23	F2-3 up	GO:0009409	Signaling and response	Stress	response to cold	P	AT2G17840
16046	3	F23 vs. L23	F2-3 up	GO:0009409	Signaling and response	Stress	response to cold	P	AT2G37760
16047	3	F23 vs. L23	F2-3 up	GO:0009409	Signaling and response	Stress	response to cold	P	AT2G37770
16048	3	F23 vs. L23	F2-3 up	GO:0009409	Signaling and response	Stress	response to cold	P	AT2G39730
16049	3	F23 vs. L23	F2-3 up	GO:0009409	Signaling and response	Stress	response to cold	P	AT2G40140
16050	3	F23 vs. L23	F2-3 up	GO:0009409	Signaling and response	Stress	response to cold	P	AT2G46830
16051	3	F23 vs. L23	F2-3 up	GO:0009409	Signaling and response	Stress	response to cold	P	AT3G01500
16052	3	F23 vs. L23	F2-3 up	GO:0009409	Signaling and response	Stress	response to cold	P	AT3G05890
16053	3	F23 vs. L23	F2-3 up	GO:0009409	Signaling and response	Stress	response to cold	P	AT3G06510
16054	3	F23 vs. L23	F2-3 up	GO:0009409	Signaling and response	Stress	response to cold	P	AT3G08720
16055	3	F23 vs. L23	F2-3 up	GO:0009409	Signaling and response	Stress	response to cold	P	AT3G08730
16056	3	F23 vs. L23	F2-3 up	GO:0009409	Signaling and response	Stress	response to cold	P	AT3G11170
16057	3	F23 vs. L23	F2-3 up	GO:0009409	Signaling and response	Stress	response to cold	P	AT3G11410
16058	3	F23 vs. L23	F2-3 up	GO:0009409	Signaling and response	Stress	response to cold	P	AT3G11630
16059	3	F23 vs. L23	F2-3 up	GO:0009409	Signaling and response	Stress	response to cold	P	AT3G12780
16060	3	F23 vs. L23	F2-3 up	GO:0009409	Signaling and response	Stress	response to cold	P	AT3G17020
16061	3	F23 vs. L23	F2-3 up	GO:0009409	Signaling and response	Stress	response to cold	P	AT3G17390
16062	3	F23 vs. L23	F2-3 up	GO:0009409	Signaling and response	Stress	response to cold	P	AT3G22370
16063	3	F23 vs. L23	F2-3 up	GO:0009409	Signaling and response	Stress	response to cold	P	AT3G22840
16064	3	F23 vs. L23	F2-3 up	GO:0009409	Signaling and response	Stress	response to cold	P	AT3G23700
16065	3	F23 vs. L23	F2-3 up	GO:0009409	Signaling and response	Stress	response to cold	P	AT3G26650
16066	3	F23 vs. L23	F2-3 up	GO:0009409	Signaling and response	Stress	response to cold	P	AT3G45640
16067	3	F23 vs. L23	F2-3 up	GO:0009409	Signaling and response	Stress	response to cold	P	AT4G02380
16068	3	F23 vs. L23	F2-3 up	GO:0009409	Signaling and response	Stress	response to cold	P	AT4G17090
16069	3	F23 vs. L23	F2-3 up	GO:0009409	Signaling and response	Stress	response to cold	P	AT4G34710
16070	3	F23 vs. L23	F2-3 up	GO:0009409	Signaling and response	Stress	response to cold	P	AT4G39730
16071	3	F23 vs. L23	F2-3 up	GO:0009409	Signaling and response	Stress	response to cold	P	AT5G01600
16072	3	F23 vs. L23	F2-3 up	GO:0009409	Signaling and response	Stress	response to cold	P	AT5G16910
16073	3	F23 vs. L23	F2-3 up	GO:0009409	Signaling and response	Stress	response to cold	P	AT5G40010
16074	3	F23 vs. L23	F2-3 up	GO:0009409	Signaling and response	Stress	response to cold	P	AT5G50720
16075	3	F23 vs. L23	F2-3 up	GO:0009409	Signaling and response	Stress	response to cold	P	AT5G57560
16076	3	F23 vs. L23	F2-3 up	GO:0009409	Signaling and response	Stress	response to cold	P	AT5G58770
16077	3	F23 vs. L23	F2-3 up	GO:0009409	Signaling and response	Stress	response to cold	P	AT5G61410
16078	3	F23 vs. L23	F2-3 up	GO:0009409	Signaling and response	Stress	response to cold	P	ATCG00120
16079	3	F23 vs. L23	F2-3 up	GO:0010201	Signaling and response	Stress	response to continuous far red light stimulus by the high-irradiance response system	P	AT2G35940
16080	3	F23 vs. L23	F2-3 up	GO:0010583	Signaling and response	Stress	response to cyclopentenone	P	AT4G38400
16081	3	F23 vs. L23	F2-3 up	GO:0010583	Signaling and response	Stress	response to cyclopentenone	P	AT5G09870
16082	3	F23 vs. L23	F2-3 up	GO:0010583	Signaling and response	Stress	response to cyclopentenone	P	AT5G57560

16083	3	F23 vs. L23	F2-3 up	GO:0010583	Signaling and response	Stress	response to cyclopentenone	P	AT5G64740
16084	3	F23 vs. L23	F2-3 up	GO:0009269	Signaling and response	Stress	response to desiccation	P	AT1G32640
16085	3	F23 vs. L23	F2-3 up	GO:0009269	Signaling and response	Stress	response to desiccation	P	AT1G44170
16086	3	F23 vs. L23	F2-3 up	GO:0009269	Signaling and response	Stress	response to desiccation	P	AT2G37180
16087	3	F23 vs. L23	F2-3 up	GO:0009269	Signaling and response	Stress	response to desiccation	P	AT2G46140
16088	3	F23 vs. L23	F2-3 up	GO:0009269	Signaling and response	Stress	response to desiccation	P	AT3G25760
16089	3	F23 vs. L23	F2-3 up	GO:0009269	Signaling and response	Stress	response to desiccation	P	AT5G25610
16090	3	F23 vs. L23	F2-3 up	GO:0042493	Signaling and response	Stress	response to drug	P	AT3G12610
16091	3	F23 vs. L23	F2-3 up	GO:0034976	Signaling and response	Stress	response to endoplasmic reticulum stress	P	AT3G54960
16092	3	F23 vs. L23	F2-3 up	GO:0045471	Signaling and response	Stress	response to ethanol	P	AT2G17950
16093	3	F23 vs. L23	F2-3 up	GO:0009723	Signaling and response	Stress	response to ethylene	P	AT1G14920
16094	3	F23 vs. L23	F2-3 up	GO:0009723	Signaling and response	Stress	response to ethylene	P	AT1G64060
16095	3	F23 vs. L23	F2-3 up	GO:0009723	Signaling and response	Stress	response to ethylene	P	AT2G46830
16096	3	F23 vs. L23	F2-3 up	GO:0009723	Signaling and response	Stress	response to ethylene	P	AT3G04720
16097	3	F23 vs. L23	F2-3 up	GO:0009723	Signaling and response	Stress	response to ethylene	P	AT3G06490
16098	3	F23 vs. L23	F2-3 up	GO:0009723	Signaling and response	Stress	response to ethylene	P	AT4G02380
16099	3	F23 vs. L23	F2-3 up	GO:0009723	Signaling and response	Stress	response to ethylene	P	AT4G11280
16100	3	F23 vs. L23	F2-3 up	GO:0009723	Signaling and response	Stress	response to ethylene	P	AT5G05730
16101	3	F23 vs. L23	F2-3 up	GO:0009723	Signaling and response	Stress	response to ethylene	P	AT5G45890
16102	3	F23 vs. L23	F2-3 up	GO:0009723	Signaling and response	Stress	response to ethylene	P	AT5G60360
16103	3	F23 vs. L23	F2-3 up	GO:0009723	Signaling and response	Stress	response to ethylene	P	AT5G67300
16104	3	F23 vs. L23	F2-3 up	GO:0050826	Signaling and response	Stress	response to freezing	P	AT3G06510
16105	3	F23 vs. L23	F2-3 up	GO:0050826	Signaling and response	Stress	response to freezing	P	AT3G45640
16106	3	F23 vs. L23	F2-3 up	GO:0009620	Signaling and response	Stress	response to fungus	P	AT1G05010
16107	3	F23 vs. L23	F2-3 up	GO:0009620	Signaling and response	Stress	response to fungus	P	AT1G17420
16108	3	F23 vs. L23	F2-3 up	GO:0009620	Signaling and response	Stress	response to fungus	P	AT2G06050
16109	3	F23 vs. L23	F2-3 up	GO:0009620	Signaling and response	Stress	response to fungus	P	AT2G30020
16110	3	F23 vs. L23	F2-3 up	GO:0009620	Signaling and response	Stress	response to fungus	P	AT2G45220
16111	3	F23 vs. L23	F2-3 up	GO:0009620	Signaling and response	Stress	response to fungus	P	AT3G06490
16112	3	F23 vs. L23	F2-3 up	GO:0009620	Signaling and response	Stress	response to fungus	P	AT3G10985
16113	3	F23 vs. L23	F2-3 up	GO:0009620	Signaling and response	Stress	response to fungus	P	AT3G14310
16114	3	F23 vs. L23	F2-3 up	GO:0009620	Signaling and response	Stress	response to fungus	P	AT3G45140
16115	3	F23 vs. L23	F2-3 up	GO:0009620	Signaling and response	Stress	response to fungus	P	AT5G08370
16116	3	F23 vs. L23	F2-3 up	GO:0009620	Signaling and response	Stress	response to fungus	P	AT5G24530
16117	3	F23 vs. L23	F2-3 up	GO:0010332	Signaling and response	Stress	response to gamma radiation	P	AT3G50000
16118	3	F23 vs. L23	F2-3 up	GO:0009408	Signaling and response	Stress	response to heat	P	AT1G05850
16119	3	F23 vs. L23	F2-3 up	GO:0009408	Signaling and response	Stress	response to heat	P	AT1G21760
16120	3	F23 vs. L23	F2-3 up	GO:0009408	Signaling and response	Stress	response to heat	P	AT1G35720
16121	3	F23 vs. L23	F2-3 up	GO:0009408	Signaling and response	Stress	response to heat	P	AT1G59870
16122	3	F23 vs. L23	F2-3 up	GO:0009408	Signaling and response	Stress	response to heat	P	AT1G79440
16123	3	F23 vs. L23	F2-3 up	GO:0009408	Signaling and response	Stress	response to heat	P	AT2G39770
16124	3	F23 vs. L23	F2-3 up	GO:0009408	Signaling and response	Stress	response to heat	P	AT3G04120
16125	3	F23 vs. L23	F2-3 up	GO:0009408	Signaling and response	Stress	response to heat	P	AT3G08720
16126	3	F23 vs. L23	F2-3 up	GO:0009408	Signaling and response	Stress	response to heat	P	AT3G09440
16127	3	F23 vs. L23	F2-3 up	GO:0009408	Signaling and response	Stress	response to heat	P	AT4G17230
16128	3	F23 vs. L23	F2-3 up	GO:0009408	Signaling and response	Stress	response to heat	P	AT4G23100
16129	3	F23 vs. L23	F2-3 up	GO:0009408	Signaling and response	Stress	response to heat	P	AT5G47910
16130	3	F23 vs. L23	F2-3 up	GO:0009408	Signaling and response	Stress	response to heat	P	AT5G57560
16131	3	F23 vs. L23	F2-3 up	GO:0009408	Signaling and response	Stress	response to heat	P	AT5G67300
16132	3	F23 vs. L23	F2-3 up	GO:0009635	Signaling and response	Stress	response to herbicide	P	AT1G12110
16133	3	F23 vs. L23	F2-3 up	GO:0080027	Signaling and response	Stress	response to herbivore	P	AT2G24210
16134	3	F23 vs. L23	F2-3 up	GO:0080027	Signaling and response	Stress	response to herbivore	P	AT3G04720
16135	3	F23 vs. L23	F2-3 up	GO:0080027	Signaling and response	Stress	response to herbivore	P	AT3G45140
16136	3	F23 vs. L23	F2-3 up	GO:0080027	Signaling and response	Stress	response to herbivore	P	AT4G15210
16137	3	F23 vs. L23	F2-3 up	GO:0009644	Signaling and response	Stress	response to high light intensity	P	AT1G17420
16138	3	F23 vs. L23	F2-3 up	GO:0009644	Signaling and response	Stress	response to high light intensity	P	AT1G27730

16139	3	F23 vs. L23	F2-3 up	GO:0009644	Signaling and response	Stress	response to high light intensity	P	AT1G45474
16140	3	F23 vs. L23	F2-3 up	GO:0009644	Signaling and response	Stress	response to high light intensity	P	AT1G55480
16141	3	F23 vs. L23	F2-3 up	GO:0009644	Signaling and response	Stress	response to high light intensity	P	AT2G05620
16142	3	F23 vs. L23	F2-3 up	GO:0009644	Signaling and response	Stress	response to high light intensity	P	AT2G17840
16143	3	F23 vs. L23	F2-3 up	GO:0009644	Signaling and response	Stress	response to high light intensity	P	AT3G18780
16144	3	F23 vs. L23	F2-3 up	GO:0042542	Signaling and response	Stress	response to hydrogen peroxide	P	AT1G18150
16145	3	F23 vs. L23	F2-3 up	GO:0042542	Signaling and response	Stress	response to hydrogen peroxide	P	AT1G20620
16146	3	F23 vs. L23	F2-3 up	GO:0042542	Signaling and response	Stress	response to hydrogen peroxide	P	AT3G04120
16147	3	F23 vs. L23	F2-3 up	GO:0042542	Signaling and response	Stress	response to hydrogen peroxide	P	AT3G17790
16148	3	F23 vs. L23	F2-3 up	GO:0042542	Signaling and response	Stress	response to hydrogen peroxide	P	AT5G01600
16149	3	F23 vs. L23	F2-3 up	GO:0042542	Signaling and response	Stress	response to hydrogen peroxide	P	AT5G67480
16150	3	F23 vs. L23	F2-3 up	GO:0001666	Signaling and response	Stress	response to hypoxia	P	AT1G23310
16151	3	F23 vs. L23	F2-3 up	GO:0009625	Signaling and response	Stress	response to insect	P	AT1G16410
16152	3	F23 vs. L23	F2-3 up	GO:0009625	Signaling and response	Stress	response to insect	P	AT4G02380
16153	3	F23 vs. L23	F2-3 up	GO:0009625	Signaling and response	Stress	response to insect	P	AT4G13770
16154	3	F23 vs. L23	F2-3 up	GO:0009625	Signaling and response	Stress	response to insect	P	AT4G19840
16155	3	F23 vs. L23	F2-3 up	GO:1990641	Signaling and response	Stress	response to iron ion starvation	P	AT3G07700
16156	3	F23 vs. L23	F2-3 up	GO:1990641	Signaling and response	Stress	response to iron ion starvation	P	AT4G26970
16157	3	F23 vs. L23	F2-3 up	GO:1990641	Signaling and response	Stress	response to iron ion starvation	P	AT5G67370
16158	3	F23 vs. L23	F2-3 up	GO:0033591	Signaling and response	Stress	response to L-ascorbic acid	P	AT3G02570
16159	3	F23 vs. L23	F2-3 up	GO:0006982	Signaling and response	Stress	response to lipid hydroperoxide	P	AT5G01410
16160	3	F23 vs. L23	F2-3 up	GO:0010244	Signaling and response	Stress	response to low fluence blue light stimulus by blue low-fluence system	P	AT1G04400
16161	3	F23 vs. L23	F2-3 up	GO:0009645	Signaling and response	Stress	response to low light intensity stimulus	P	AT1G45474
16162	3	F23 vs. L23	F2-3 up	GO:0009612	Signaling and response	Stress	response to mechanical stimulus	P	AT4G11280
16163	3	F23 vs. L23	F2-3 up	GO:0009612	Signaling and response	Stress	response to mechanical stimulus	P	AT5G57560
16164	3	F23 vs. L23	F2-3 up	GO:0010188	Signaling and response	Stress	response to microbial phytotoxin	P	AT1G80460
16165	3	F23 vs. L23	F2-3 up	GO:0002237	Signaling and response	Stress	response to molecule of bacterial origin	P	AT1G80460
16166	3	F23 vs. L23	F2-3 up	GO:0002237	Signaling and response	Stress	response to molecule of bacterial origin	P	AT1G80840
16167	3	F23 vs. L23	F2-3 up	GO:0002237	Signaling and response	Stress	response to molecule of bacterial origin	P	AT4G31800
16168	3	F23 vs. L23	F2-3 up	GO:0002238	Signaling and response	Stress	response to molecule of fungal origin	P	AT3G10985
16169	3	F23 vs. L23	F2-3 up	GO:0009624	Signaling and response	Stress	response to nematode	P	AT1G61800
16170	3	F23 vs. L23	F2-3 up	GO:0009624	Signaling and response	Stress	response to nematode	P	AT1G76670
16171	3	F23 vs. L23	F2-3 up	GO:0009624	Signaling and response	Stress	response to nematode	P	AT2G40460
16172	3	F23 vs. L23	F2-3 up	GO:0009624	Signaling and response	Stress	response to nematode	P	AT3G10985
16173	3	F23 vs. L23	F2-3 up	GO:0009624	Signaling and response	Stress	response to nematode	P	AT3G11900
16174	3	F23 vs. L23	F2-3 up	GO:0009624	Signaling and response	Stress	response to nematode	P	AT3G14310
16175	3	F23 vs. L23	F2-3 up	GO:0009624	Signaling and response	Stress	response to nematode	P	AT4G39030
16176	3	F23 vs. L23	F2-3 up	GO:0009624	Signaling and response	Stress	response to nematode	P	AT5G14940
16177	3	F23 vs. L23	F2-3 up	GO:0009624	Signaling and response	Stress	response to nematode	P	AT5G52450
16178	3	F23 vs. L23	F2-3 up	GO:0009624	Signaling and response	Stress	response to nematode	P	AT5G61410
16179	3	F23 vs. L23	F2-3 up	GO:0009624	Signaling and response	Stress	response to nematode	P	AT5G64840
16180	3	F23 vs. L23	F2-3 up	GO:0090548	Signaling and response	Stress	response to nitrate starvation	P	AT1G72180
16181	3	F23 vs. L23	F2-3 up	GO:0010335	Signaling and response	Stress	response to non-ionic osmotic stress	P	AT5G01410
16182	3	F23 vs. L23	F2-3 up	GO:0002239	Signaling and response	Stress	response to oomycetes	P	AT5G24530
16183	3	F23 vs. L23	F2-3 up	GO:0006970	Signaling and response	Stress	response to osmotic stress	P	AT1G35720
16184	3	F23 vs. L23	F2-3 up	GO:0006970	Signaling and response	Stress	response to osmotic stress	P	AT1G72180
16185	3	F23 vs. L23	F2-3 up	GO:0006970	Signaling and response	Stress	response to osmotic stress	P	AT1G78290
16186	3	F23 vs. L23	F2-3 up	GO:0006970	Signaling and response	Stress	response to osmotic stress	P	AT2G37180
16187	3	F23 vs. L23	F2-3 up	GO:0006970	Signaling and response	Stress	response to osmotic stress	P	AT3G08730
16188	3	F23 vs. L23	F2-3 up	GO:0006970	Signaling and response	Stress	response to osmotic stress	P	AT3G45640
16189	3	F23 vs. L23	F2-3 up	GO:0006970	Signaling and response	Stress	response to osmotic stress	P	AT4G34710
16190	3	F23 vs. L23	F2-3 up	GO:0051707	Signaling and response	Stress	response to other organism	P	AT3G45140
16191	3	F23 vs. L23	F2-3 up	GO:0051707	Signaling and response	Stress	response to other organism	P	AT3G54950
16192	3	F23 vs. L23	F2-3 up	GO:0006979	Signaling and response	Stress	response to oxidative stress	P	AT1G08830
16193	3	F23 vs. L23	F2-3 up	GO:0006979	Signaling and response	Stress	response to oxidative stress	P	AT1G27730

16194	3	F23 vs. L23	F2-3 up	GO:0006979	Signaling and response	Stress	response to oxidative stress	P	AT1G35720
16195	3	F23 vs. L23	F2-3 up	GO:0006979	Signaling and response	Stress	response to oxidative stress	P	AT1G76080
16196	3	F23 vs. L23	F2-3 up	GO:0006979	Signaling and response	Stress	response to oxidative stress	P	AT2G28190
16197	3	F23 vs. L23	F2-3 up	GO:0006979	Signaling and response	Stress	response to oxidative stress	P	AT2G31570
16198	3	F23 vs. L23	F2-3 up	GO:0006979	Signaling and response	Stress	response to oxidative stress	P	AT2G37040
16199	3	F23 vs. L23	F2-3 up	GO:0006979	Signaling and response	Stress	response to oxidative stress	P	AT2G40000
16200	3	F23 vs. L23	F2-3 up	GO:0006979	Signaling and response	Stress	response to oxidative stress	P	AT2G41480
16201	3	F23 vs. L23	F2-3 up	GO:0006979	Signaling and response	Stress	response to oxidative stress	P	AT3G01420
16202	3	F23 vs. L23	F2-3 up	GO:0006979	Signaling and response	Stress	response to oxidative stress	P	AT3G03190
16203	3	F23 vs. L23	F2-3 up	GO:0006979	Signaling and response	Stress	response to oxidative stress	P	AT3G04120
16204	3	F23 vs. L23	F2-3 up	GO:0006979	Signaling and response	Stress	response to oxidative stress	P	AT3G07700
16205	3	F23 vs. L23	F2-3 up	GO:0006979	Signaling and response	Stress	response to oxidative stress	P	AT3G11050
16206	3	F23 vs. L23	F2-3 up	GO:0006979	Signaling and response	Stress	response to oxidative stress	P	AT3G11630
16207	3	F23 vs. L23	F2-3 up	GO:0006979	Signaling and response	Stress	response to oxidative stress	P	AT3G15360
16208	3	F23 vs. L23	F2-3 up	GO:0006979	Signaling and response	Stress	response to oxidative stress	P	AT3G45640
16209	3	F23 vs. L23	F2-3 up	GO:0006979	Signaling and response	Stress	response to oxidative stress	P	AT3G49110
16210	3	F23 vs. L23	F2-3 up	GO:0006979	Signaling and response	Stress	response to oxidative stress	P	AT4G02380
16211	3	F23 vs. L23	F2-3 up	GO:0006979	Signaling and response	Stress	response to oxidative stress	P	AT4G11280
16212	3	F23 vs. L23	F2-3 up	GO:0006979	Signaling and response	Stress	response to oxidative stress	P	AT4G11600
16213	3	F23 vs. L23	F2-3 up	GO:0006979	Signaling and response	Stress	response to oxidative stress	P	AT4G26970
16214	3	F23 vs. L23	F2-3 up	GO:0006979	Signaling and response	Stress	response to oxidative stress	P	AT4G34710
16215	3	F23 vs. L23	F2-3 up	GO:0006979	Signaling and response	Stress	response to oxidative stress	P	AT4G39640
16216	3	F23 vs. L23	F2-3 up	GO:0006979	Signaling and response	Stress	response to oxidative stress	P	AT5G01410
16217	3	F23 vs. L23	F2-3 up	GO:0006979	Signaling and response	Stress	response to oxidative stress	P	AT5G07460
16218	3	F23 vs. L23	F2-3 up	GO:0006979	Signaling and response	Stress	response to oxidative stress	P	AT5G13930
16219	3	F23 vs. L23	F2-3 up	GO:0006979	Signaling and response	Stress	response to oxidative stress	P	AT5G47000
16220	3	F23 vs. L23	F2-3 up	GO:0006979	Signaling and response	Stress	response to oxidative stress	P	AT5G47650
16221	3	F23 vs. L23	F2-3 up	GO:0006979	Signaling and response	Stress	response to oxidative stress	P	AT5G51890
16222	3	F23 vs. L23	F2-3 up	GO:0010193	Signaling and response	Stress	response to ozone	P	AT1G08830
16223	3	F23 vs. L23	F2-3 up	GO:0010193	Signaling and response	Stress	response to ozone	P	AT1G72520
16224	3	F23 vs. L23	F2-3 up	GO:0010193	Signaling and response	Stress	response to ozone	P	AT2G06050
16225	3	F23 vs. L23	F2-3 up	GO:0010193	Signaling and response	Stress	response to ozone	P	AT2G39770
16226	3	F23 vs. L23	F2-3 up	GO:0010193	Signaling and response	Stress	response to ozone	P	AT3G10985
16227	3	F23 vs. L23	F2-3 up	GO:0010193	Signaling and response	Stress	response to ozone	P	AT3G17800
16228	3	F23 vs. L23	F2-3 up	GO:0010193	Signaling and response	Stress	response to ozone	P	AT4G23100
16229	3	F23 vs. L23	F2-3 up	GO:0009268	Signaling and response	Stress	response to pH	P	AT2G30360
16230	3	F23 vs. L23	F2-3 up	GO:0000302	Signaling and response	Stress	response to reactive oxygen species	P	AT1G18150
16231	3	F23 vs. L23	F2-3 up	GO:0000302	Signaling and response	Stress	response to reactive oxygen species	P	AT3G11050
16232	3	F23 vs. L23	F2-3 up	GO:0000302	Signaling and response	Stress	response to reactive oxygen species	P	AT3G56240
16233	3	F23 vs. L23	F2-3 up	GO:0000302	Signaling and response	Stress	response to reactive oxygen species	P	AT4G02380
16234	3	F23 vs. L23	F2-3 up	GO:0000302	Signaling and response	Stress	response to reactive oxygen species	P	AT5G01600
16235	3	F23 vs. L23	F2-3 up	GO:0051775	Signaling and response	Stress	response to redox state	P	AT3G04120
16236	3	F23 vs. L23	F2-3 up	GO:0051775	Signaling and response	Stress	response to redox state	P	AT5G58330
16237	3	F23 vs. L23	F2-3 up	GO:1902074	Signaling and response	Stress	response to salt	P	AT1G79440
16238	3	F23 vs. L23	F2-3 up	GO:0009651	Signaling and response	Stress	response to salt stress	P	AT1G05850
16239	3	F23 vs. L23	F2-3 up	GO:0009651	Signaling and response	Stress	response to salt stress	P	AT1G08830
16240	3	F23 vs. L23	F2-3 up	GO:0009651	Signaling and response	Stress	response to salt stress	P	AT1G10370
16241	3	F23 vs. L23	F2-3 up	GO:0009651	Signaling and response	Stress	response to salt stress	P	AT1G14920
16242	3	F23 vs. L23	F2-3 up	GO:0009651	Signaling and response	Stress	response to salt stress	P	AT1G27730
16243	3	F23 vs. L23	F2-3 up	GO:0009651	Signaling and response	Stress	response to salt stress	P	AT1G35720
16244	3	F23 vs. L23	F2-3 up	GO:0009651	Signaling and response	Stress	response to salt stress	P	AT1G44170
16245	3	F23 vs. L23	F2-3 up	GO:0009651	Signaling and response	Stress	response to salt stress	P	AT2G17840
16246	3	F23 vs. L23	F2-3 up	GO:0009651	Signaling and response	Stress	response to salt stress	P	AT2G36830
16247	3	F23 vs. L23	F2-3 up	GO:0009651	Signaling and response	Stress	response to salt stress	P	AT2G37630
16248	3	F23 vs. L23	F2-3 up	GO:0009651	Signaling and response	Stress	response to salt stress	P	AT2G37760
16249	3	F23 vs. L23	F2-3 up	GO:0009651	Signaling and response	Stress	response to salt stress	P	AT2G37770

16250	3	F23 vs. L23	F2-3 up	GO:0009651	Signaling and response	Stress	response to salt stress	P	AT2G38170
16251	3	F23 vs. L23	F2-3 up	GO:0009651	Signaling and response	Stress	response to salt stress	P	AT2G39770
16252	3	F23 vs. L23	F2-3 up	GO:0009651	Signaling and response	Stress	response to salt stress	P	AT2G46830
16253	3	F23 vs. L23	F2-3 up	GO:0009651	Signaling and response	Stress	response to salt stress	P	AT3G03640
16254	3	F23 vs. L23	F2-3 up	GO:0009651	Signaling and response	Stress	response to salt stress	P	AT3G06490
16255	3	F23 vs. L23	F2-3 up	GO:0009651	Signaling and response	Stress	response to salt stress	P	AT3G08720
16256	3	F23 vs. L23	F2-3 up	GO:0009651	Signaling and response	Stress	response to salt stress	P	AT3G08730
16257	3	F23 vs. L23	F2-3 up	GO:0009651	Signaling and response	Stress	response to salt stress	P	AT3G26520
16258	3	F23 vs. L23	F2-3 up	GO:0009651	Signaling and response	Stress	response to salt stress	P	AT3G51920
16259	3	F23 vs. L23	F2-3 up	GO:0009651	Signaling and response	Stress	response to salt stress	P	AT3G53420
16260	3	F23 vs. L23	F2-3 up	GO:0009651	Signaling and response	Stress	response to salt stress	P	AT4G34710
16261	3	F23 vs. L23	F2-3 up	GO:0009651	Signaling and response	Stress	response to salt stress	P	AT4G39730
16262	3	F23 vs. L23	F2-3 up	GO:0009651	Signaling and response	Stress	response to salt stress	P	AT5G01410
16263	3	F23 vs. L23	F2-3 up	GO:0009651	Signaling and response	Stress	response to salt stress	P	AT5G04500
16264	3	F23 vs. L23	F2-3 up	GO:0009651	Signaling and response	Stress	response to salt stress	P	AT5G25610
16265	3	F23 vs. L23	F2-3 up	GO:0009651	Signaling and response	Stress	response to salt stress	P	AT5G40010
16266	3	F23 vs. L23	F2-3 up	GO:0009651	Signaling and response	Stress	response to salt stress	P	AT5G62520
16267	3	F23 vs. L23	F2-3 up	GO:0009651	Signaling and response	Stress	response to salt stress	P	AT5G67300
16268	3	F23 vs. L23	F2-3 up	GO:0009651	Signaling and response	Stress	response to salt stress	P	AT5G67480
16269	3	F23 vs. L23	F2-3 up	GO:0042594	Signaling and response	Stress	response to starvation	P	AT5G20700
16270	3	F23 vs. L23	F2-3 up	GO:0009610	Signaling and response	Stress	response to symbiotic fungus	P	AT2G35940
16271	3	F23 vs. L23	F2-3 up	GO:0009610	Signaling and response	Stress	response to symbiotic fungus	P	AT3G09940
16272	3	F23 vs. L23	F2-3 up	GO:0009636	Signaling and response	Stress	response to toxic substance	P	AT2G37760
16273	3	F23 vs. L23	F2-3 up	GO:0009636	Signaling and response	Stress	response to toxic substance	P	AT2G37770
16274	3	F23 vs. L23	F2-3 up	GO:0009636	Signaling and response	Stress	response to toxic substance	P	AT3G21560
16275	3	F23 vs. L23	F2-3 up	GO:0009636	Signaling and response	Stress	response to toxic substance	P	AT4G01070
16276	3	F23 vs. L23	F2-3 up	GO:0009636	Signaling and response	Stress	response to toxic substance	P	AT5G02790
16277	3	F23 vs. L23	F2-3 up	GO:0006986	Signaling and response	Stress	response to unfolded protein	P	AT3G09440
16278	3	F23 vs. L23	F2-3 up	GO:0010224	Signaling and response	Stress	response to UV-B	P	AT2G37040
16279	3	F23 vs. L23	F2-3 up	GO:0010224	Signaling and response	Stress	response to UV-B	P	AT3G17800
16280	3	F23 vs. L23	F2-3 up	GO:0010224	Signaling and response	Stress	response to UV-B	P	AT3G21890
16281	3	F23 vs. L23	F2-3 up	GO:0010224	Signaling and response	Stress	response to UV-B	P	AT3G45640
16282	3	F23 vs. L23	F2-3 up	GO:0010224	Signaling and response	Stress	response to UV-B	P	AT3G51240
16283	3	F23 vs. L23	F2-3 up	GO:0010224	Signaling and response	Stress	response to UV-B	P	AT4G14690
16284	3	F23 vs. L23	F2-3 up	GO:0010224	Signaling and response	Stress	response to UV-B	P	AT5G01410
16285	3	F23 vs. L23	F2-3 up	GO:0010224	Signaling and response	Stress	response to UV-B	P	AT5G13930
16286	3	F23 vs. L23	F2-3 up	GO:0010224	Signaling and response	Stress	response to UV-B	P	AT5G45890
16287	3	F23 vs. L23	F2-3 up	GO:0009615	Signaling and response	Stress	response to virus	P	AT2G37630
16288	3	F23 vs. L23	F2-3 up	GO:0009615	Signaling and response	Stress	response to virus	P	AT2G43570
16289	3	F23 vs. L23	F2-3 up	GO:0009615	Signaling and response	Stress	response to virus	P	AT3G04720
16290	3	F23 vs. L23	F2-3 up	GO:0009615	Signaling and response	Stress	response to virus	P	AT3G09440
16291	3	F23 vs. L23	F2-3 up	GO:0009414	Signaling and response	Stress	response to water deprivation	P	AT1G02205
16292	3	F23 vs. L23	F2-3 up	GO:0009414	Signaling and response	Stress	response to water deprivation	P	AT1G04400
16293	3	F23 vs. L23	F2-3 up	GO:0009414	Signaling and response	Stress	response to water deprivation	P	AT1G05850
16294	3	F23 vs. L23	F2-3 up	GO:0009414	Signaling and response	Stress	response to water deprivation	P	AT1G12110
16295	3	F23 vs. L23	F2-3 up	GO:0009414	Signaling and response	Stress	response to water deprivation	P	AT1G27730
16296	3	F23 vs. L23	F2-3 up	GO:0009414	Signaling and response	Stress	response to water deprivation	P	AT1G35720
16297	3	F23 vs. L23	F2-3 up	GO:0009414	Signaling and response	Stress	response to water deprivation	P	AT1G76080
16298	3	F23 vs. L23	F2-3 up	GO:0009414	Signaling and response	Stress	response to water deprivation	P	AT2G05620
16299	3	F23 vs. L23	F2-3 up	GO:0009414	Signaling and response	Stress	response to water deprivation	P	AT2G17840
16300	3	F23 vs. L23	F2-3 up	GO:0009414	Signaling and response	Stress	response to water deprivation	P	AT2G26740
16301	3	F23 vs. L23	F2-3 up	GO:0009414	Signaling and response	Stress	response to water deprivation	P	AT2G37180
16302	3	F23 vs. L23	F2-3 up	GO:0009414	Signaling and response	Stress	response to water deprivation	P	AT2G37760
16303	3	F23 vs. L23	F2-3 up	GO:0009414	Signaling and response	Stress	response to water deprivation	P	AT2G37770
16304	3	F23 vs. L23	F2-3 up	GO:0009414	Signaling and response	Stress	response to water deprivation	P	AT3G09940
16305	3	F23 vs. L23	F2-3 up	GO:0009414	Signaling and response	Stress	response to water deprivation	P	AT3G11410

16306	3	F23 vs. L23	F2-3 up	GO:0009414	Signaling and response	Stress	response to water deprivation	P	AT3G23050
16307	3	F23 vs. L23	F2-3 up	GO:0009414	Signaling and response	Stress	response to water deprivation	P	AT3G23920
16308	3	F23 vs. L23	F2-3 up	GO:0009414	Signaling and response	Stress	response to water deprivation	P	AT3G45140
16309	3	F23 vs. L23	F2-3 up	GO:0009414	Signaling and response	Stress	response to water deprivation	P	AT3G46620
16310	3	F23 vs. L23	F2-3 up	GO:0009414	Signaling and response	Stress	response to water deprivation	P	AT3G48170
16311	3	F23 vs. L23	F2-3 up	GO:0009414	Signaling and response	Stress	response to water deprivation	P	AT3G51920
16312	3	F23 vs. L23	F2-3 up	GO:0009414	Signaling and response	Stress	response to water deprivation	P	AT3G53420
16313	3	F23 vs. L23	F2-3 up	GO:0009414	Signaling and response	Stress	response to water deprivation	P	AT4G02380
16314	3	F23 vs. L23	F2-3 up	GO:0009414	Signaling and response	Stress	response to water deprivation	P	AT4G39730
16315	3	F23 vs. L23	F2-3 up	GO:0009414	Signaling and response	Stress	response to water deprivation	P	AT5G06530
16316	3	F23 vs. L23	F2-3 up	GO:0009414	Signaling and response	Stress	response to water deprivation	P	AT5G40010
16317	3	F23 vs. L23	F2-3 up	GO:0009414	Signaling and response	Stress	response to water deprivation	P	AT5G50720
16318	3	F23 vs. L23	F2-3 up	GO:0009414	Signaling and response	Stress	response to water deprivation	P	AT5G65730
16319	3	F23 vs. L23	F2-3 up	GO:0009414	Signaling and response	Stress	response to water deprivation	P	AT5G67300
16320	3	F23 vs. L23	F2-3 up	GO:0009611	Signaling and response	Stress	response to wounding	P	AT1G01720
16321	3	F23 vs. L23	F2-3 up	GO:0009611	Signaling and response	Stress	response to wounding	P	AT1G09340
16322	3	F23 vs. L23	F2-3 up	GO:0009611	Signaling and response	Stress	response to wounding	P	AT1G17380
16323	3	F23 vs. L23	F2-3 up	GO:0009611	Signaling and response	Stress	response to wounding	P	AT1G17420
16324	3	F23 vs. L23	F2-3 up	GO:0009611	Signaling and response	Stress	response to wounding	P	AT1G18150
16325	3	F23 vs. L23	F2-3 up	GO:0009611	Signaling and response	Stress	response to wounding	P	AT1G19180
16326	3	F23 vs. L23	F2-3 up	GO:0009611	Signaling and response	Stress	response to wounding	P	AT1G27730
16327	3	F23 vs. L23	F2-3 up	GO:0009611	Signaling and response	Stress	response to wounding	P	AT1G32640
16328	3	F23 vs. L23	F2-3 up	GO:0009611	Signaling and response	Stress	response to wounding	P	AT1G55480
16329	3	F23 vs. L23	F2-3 up	GO:0009611	Signaling and response	Stress	response to wounding	P	AT1G70700
16330	3	F23 vs. L23	F2-3 up	GO:0009611	Signaling and response	Stress	response to wounding	P	AT1G71697
16331	3	F23 vs. L23	F2-3 up	GO:0009611	Signaling and response	Stress	response to wounding	P	AT1G72520
16332	3	F23 vs. L23	F2-3 up	GO:0009611	Signaling and response	Stress	response to wounding	P	AT1G80840
16333	3	F23 vs. L23	F2-3 up	GO:0009611	Signaling and response	Stress	response to wounding	P	AT2G02990
16334	3	F23 vs. L23	F2-3 up	GO:0009611	Signaling and response	Stress	response to wounding	P	AT2G06050
16335	3	F23 vs. L23	F2-3 up	GO:0009611	Signaling and response	Stress	response to wounding	P	AT2G24210
16336	3	F23 vs. L23	F2-3 up	GO:0009611	Signaling and response	Stress	response to wounding	P	AT2G26690
16337	3	F23 vs. L23	F2-3 up	GO:0009611	Signaling and response	Stress	response to wounding	P	AT2G30020
16338	3	F23 vs. L23	F2-3 up	GO:0009611	Signaling and response	Stress	response to wounding	P	AT2G30490
16339	3	F23 vs. L23	F2-3 up	GO:0009611	Signaling and response	Stress	response to wounding	P	AT2G34600
16340	3	F23 vs. L23	F2-3 up	GO:0009611	Signaling and response	Stress	response to wounding	P	AT2G34810
16341	3	F23 vs. L23	F2-3 up	GO:0009611	Signaling and response	Stress	response to wounding	P	AT2G37040
16342	3	F23 vs. L23	F2-3 up	GO:0009611	Signaling and response	Stress	response to wounding	P	AT2G38870
16343	3	F23 vs. L23	F2-3 up	GO:0009611	Signaling and response	Stress	response to wounding	P	AT3G17800
16344	3	F23 vs. L23	F2-3 up	GO:0009611	Signaling and response	Stress	response to wounding	P	AT3G19710
16345	3	F23 vs. L23	F2-3 up	GO:0009611	Signaling and response	Stress	response to wounding	P	AT3G23050
16346	3	F23 vs. L23	F2-3 up	GO:0009611	Signaling and response	Stress	response to wounding	P	AT3G45140
16347	3	F23 vs. L23	F2-3 up	GO:0009611	Signaling and response	Stress	response to wounding	P	AT3G45640
16348	3	F23 vs. L23	F2-3 up	GO:0009611	Signaling and response	Stress	response to wounding	P	AT3G48520
16349	3	F23 vs. L23	F2-3 up	GO:0009611	Signaling and response	Stress	response to wounding	P	AT4G11280
16350	3	F23 vs. L23	F2-3 up	GO:0009611	Signaling and response	Stress	response to wounding	P	AT4G12030
16351	3	F23 vs. L23	F2-3 up	GO:0009611	Signaling and response	Stress	response to wounding	P	AT4G15440
16352	3	F23 vs. L23	F2-3 up	GO:0009611	Signaling and response	Stress	response to wounding	P	AT4G34710
16353	3	F23 vs. L23	F2-3 up	GO:0009611	Signaling and response	Stress	response to wounding	P	AT4G37760
16354	3	F23 vs. L23	F2-3 up	GO:0009611	Signaling and response	Stress	response to wounding	P	AT5G05730
16355	3	F23 vs. L23	F2-3 up	GO:0009611	Signaling and response	Stress	response to wounding	P	AT5G06320
16356	3	F23 vs. L23	F2-3 up	GO:0009611	Signaling and response	Stress	response to wounding	P	AT5G09810
16357	3	F23 vs. L23	F2-3 up	GO:0009611	Signaling and response	Stress	response to wounding	P	AT5G13930
[truncated: 618,582 more chars]
